# Induction of the Erythroid Differentiation of K562 Cells Is Coupled with Changes in the Inter-Chromosomal Contacts of rDNA Clusters

**DOI:** 10.3390/ijms24129842

**Published:** 2023-06-07

**Authors:** Nickolai A. Tchurikov, Elena S. Klushevskaya, Ildar R. Alembekov, Antonina N. Kretova, Vladimir R. Chechetkin, Galina I. Kravatskaya, Yuri V. Kravatsky

**Affiliations:** 1 Department of Epigenetic Mechanisms of Gene Expression Regulation, Engelhardt Institute of Molecular Biology Russian Academy of Sciences, 119334 Moscow, Russia; giedre@inbox.ru (E.S.K.); alembeki@gmail.com (I.R.A.); tonya_kretova@mail.ru (A.N.K.); vladimir_chechet@mail.ru (V.R.C.); galina.kravatskaya@gmail.com (G.I.K.); jiri@eimb.ru (Y.V.K.); 2 Center for Precision Genome Editing and Genetic Technologies for Biomedicine, Engelhardt Institute of Molecular Biology Russian Academy of Sciences, 119334 Moscow, Russia

**Keywords:** rDNA clusters, differentiation, inter-chromosomal contacts, 4C, gene expression, K562

## Abstract

The expression of clusters of rDNA genes influences pluripotency; however, the underlying mechanisms are not yet known. These clusters shape inter-chromosomal contacts with numerous genes controlling differentiation in human and *Drosophila* cells. This suggests a possible role of these contacts in the formation of 3D chromosomal structures and the regulation of gene expression in development. However, it has not yet been demonstrated whether inter-chromosomal rDNA contacts are changed during differentiation. In this study, we used human leukemia K562 cells and induced their erythroid differentiation in order to study both the changes in rDNA contacts and the expression of genes. We observed that approximately 200 sets of rDNA-contacting genes are co-expressed in different combinations in both untreated and differentiated K562 cells. rDNA contacts are changed during differentiation and coupled with the upregulation of genes whose products are mainly located in the nucleus and are highly associated with DNA- and RNA-binding, along with the downregulation of genes whose products mainly reside in the cytoplasm or intra- or extracellular vesicles. The most downregulated gene is *ID3*, which is known as an inhibitor of differentiation, and thus should be switched off to allow for differentiation. Our data suggest that the differentiation of K562 cells leads to alterations in the inter-chromosomal contacts of rDNA clusters and 3D structures in particular chromosomal regions as well as to changes in the expression of genes located in the corresponding chromosomal domains. We conclude that approximately half of the rDNA-contacting genes are co-expressed in human cells and that rDNA clusters are involved in the global regulation of gene expression.

## 1. Introduction

Nucleoli play important roles not only in ribosome biogenesis but also in a number of other functions, including genome stability, autophagy regulation, the regulation of gene expression, the positioning of chromosomes, the regulation of cellular stresses, and aging [1,2,3,4,5]. In embryonic stem cells, the formation of heterochromatin isles inside rDNA clusters leads to the appearance of repressed chromatin structures in different genomic regions, coupled with the transcriptional activation of differentiation genes and the loss of pluripotency [6,7]. The underlying mechanisms are not yet clear. One hint comes from the observation that rDNA clusters in human and *Drosophila* cells form dynamic inter-chromosomal contacts with numerous genes controlling development [8,9,10]. The heat-shock treatment induces dramatic but reversible changes in these contacts, highlighting their physiological role [9,10]. It has been shown that the changes occurring in such long-range rDNA–genomic interactions are associated with changes in gene expression patterns during tumorigenesis [11]. rDNA clusters often shape numerous contacts in chromosomal regions possessing the long stretches of H3K27ac marks that are associated with super-enhancers and active chromatin regions [9,12,13]. However, it has been observed that rDNA contact sites can exactly correspond to repressed chromatin areas [14] and that rDNA contacts are enriched in segments of closed, repressed, late-replicating chromatin, CTCF-binding sites, and with the regions possessing the most frequent DNA DSBs inside active and silenced genes [15,16]. Taken together, these facts support the view on the role of rDNA genes in the global epigenetic regulation of gene expression by the shaping of physiological 3D chromosomal structures [17,18]. However, to date, it has not been demonstrated if rDNA contacts are changed during differentiation and if these changes are associated with alterations in the expression of developmental genes.

In this study, we used the hemin-induced differentiation of human leukemia K562 cells and compared both the changes in the inter-chromosomal contacts of rDNA genes and the expression of genes. In our study, we observed approximately 200 sets of rDNA-contacting genes that are co-expressed in both untreated and differentiated K562 cells. We also observed that differentiation is coupled with alterations in rDNA contacts with developmental genes. Upon differentiation, the downregulation of more than 1000 genes whose products are mainly located in the cytoplasm was detected, along with the upregulation of approximately the same number of genes whose products bind with DNA or RNA and mainly reside in the nucleoplasm. We found that upon differentiation, the *ID3* gene, known as an inhibitor of differentiation, is extremely downregulated. We also detected that about half of the rDNA-contacting genes are co-expressed in human cells. The results of our study suggest that the inter-chromosomal contacts of rDNA clusters are involved in the formation of a 3D net of chromosomal structures that are associated with changes occurring in the expression of developmental genes.

## 2. Results

### 2.1. rDNA Clusters Contact with the Numerous Genes in Initial K562 Cells

For the identification of the contact sites of rDNA genes in initial K562 cells, we used the circular chromatin conformation capture (4C-rDNA) approach and paired-end sequencing of the corresponding libraries (see Section 4). In these experiments, we detected 3699 genes, which formed the most frequent contacts (≥100 contacts under the *p*-adjusted value ≤ 0.05) with rDNA clusters in the population of K562 cells (Table S1). Among these genes, there were 86 *ZNF* genes that could regulate gene expression occurring at the transcriptional and translational levels [19], 280 genes for long intergenic non-coding RNAs (lincRNAs) that affect gene expression [20], *Ago1-3* and *PIWI* genes that are involved in RNAi mechanisms, and 13 interleukin genes that modulate growth and differentiation.

**Figure 1:**
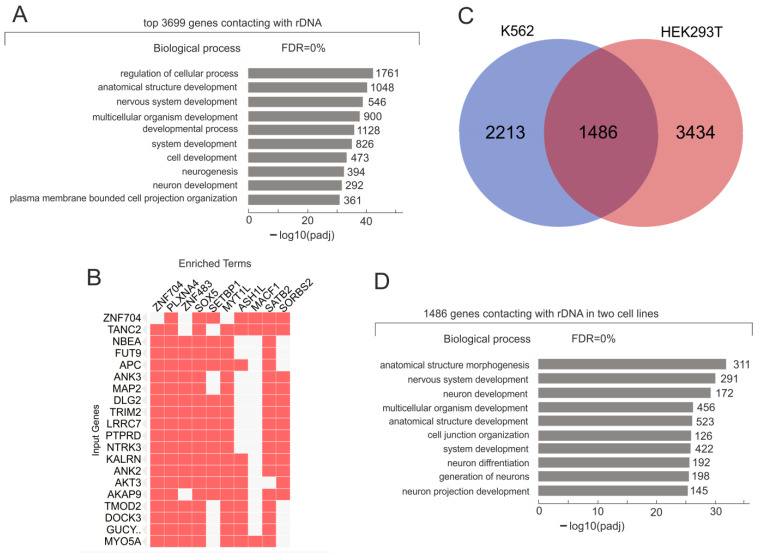
Search of rDNA-contacting genes in K562 cells for enriched Gene Ontology (GO) terms using g:Profiler (https://biit.cs.ut.ee/gprofiler/gost, accessed on 4 June 2023). (**A**) The top-10 detected terms of biological processes associated with rDNA-contacting genes. The values to the right of the bars show the number of rDNA-contacting genes associated with a term. The list of corresponding genes is shown in Table S2. (**B**) rDNA-contacting genes detected in K562 cells (indicated as “Input Genes”) are co-expressed with different transcription factors (indicated as “Enriched Terms”) across different human cell lines and tissues. Top-20 input genes are also shown. The search was performed in https://maayanlab.cloud/Enrichr/enrich# (accessed on 4 June 2023) for ARCHS4 TFs Coexp. The complete list of 177 sets of co-expressing rDNA-contacting genes and corresponding transcription factors are shown in Table S3. (**C**) The Venn diagram shows the intersections among rDNA-contacting genes in K562 and HEK293T cells (the list of corresponding genes is shown in Table S4). (**D**) The top-10 detected terms of biological processes associated with 1486 rDNA-contacting genes common for K562 and HEK293T cells. The values to the right of the bars show the number of rDNA-contacting genes associated with a term. The list of corresponding genes is shown in Table S5.

A Gene Ontology (GO) search revealed that hundreds of these rDNA-contacting genes are highly associated with the regulation of cellular processes and development (Figure 1A, Table S2). These results were anticipated because it has previously been demonstrated that rDNA genes mostly shape contacts with developmental genes in human and *Drosophila* cells [9,10]. It is known that erythroleukemia-type K562 cells have been isolated from bone marrow [21]. For this reason, we can expect that some level of differentiation in this direction can be preserved in the cells, and different agents (hemin, guanine, guanosine, guanine ribonucleotides, thymidine, deoxyguanosine, sodium butyrate, and 5-aminolevulinic acid) to some extent activated the erythroid differentiation [22]. Our GO data were in agreement with our supposition and strongly suggest that rDNA clusters often shape inter-chromosomal contacts with developmental genes in untreated K562 cells.

However, the following questions arise: what does this fact mean, and what are rDNA clusters doing via these contacts with the specific sets of genes in different cell types and even different organisms? Could this be some mechanism that is required for the concerted expression of the specific pattern of genes necessary for differentiation? Some hint for answering the questions comes from a search of the detected rDNA-contacting genes in the ARCHS4 TFs Coexp database [23]. Surprisingly, we observed that, in untreated K562 cells, approximately half of the detected 3699 rDNA-contacting genes (namely, 1985 genes) formed the 177 sets of co-expressed genes in different combinations. (Figure 1B, Table S3). K562 cells are not unique in this respect. These genes are also co-expressed in different combinations across different human cell lines and tissues [23]. The data suggest the important role of the inter-chromosomal contacts of rDNA clusters in the global regulation of gene expression in human cells.

These data prompted us to compare whether the sets of rDNA-contacting genes in human cells of different origins essentially overlap. Figure 1C shows that 40% of the rDNA-contacting genes detected in K562 cells also shape frequent contacts in HEK293T cells. HEK293T cells originated from human embryonic kidney neurons [24], while K562 cells were obtained from bone marrow and were of the erythroleukemia type. These data are clearly consistent with the idea that different sets of rDNA-contacting genes in multiple combinations are co-expressed to direct differentiation in various cell types.

We performed a search of 1486 overlapping rDNA-contacting genes between two cell lines in GO databases and observed that they were highly associated with development and morphogenesis and nervous system development (Figure 1D). The relations between neuronal and myeloid cells are discussed in ref. [25]. Our data at least indicate that myeloid cells share inter-chromosomal rDNA contacts with a set of co-expressed developmental genes controlling the differentiation of neurons.

### 2.2. Induced Differentiation of K562 Cells Leads to Essential Changes in the Pattern of rDNA Inter-Chromosomal Contacts

**Figure 2:**
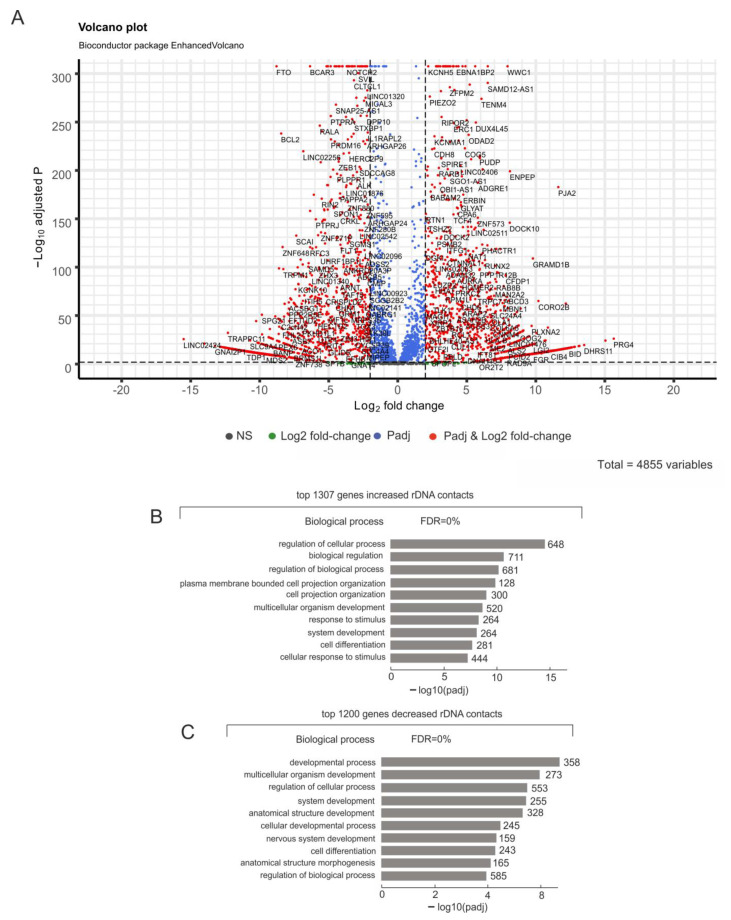
Analysis of changes in the contacts of rDNA clusters with genes following induced differentiation in K562 cells. (**A**) The volcano plot presents statistically significant changes (padj < 0.05) in contacts of genes with rDNA clusters determined in 4C-rDNA experiments. (**B**) The top 10 detected terms of biological processes associated with genes that increased the number of contacts with rDNA clusters. The search was performed using g:Profiler (https://biit.cs.ut.ee/gprofiler/gost, 4 June 2023). The values to the right of the bars show the number of rDNA-contacting genes associated with a term. The list of corresponding genes is shown in Table S6. (**C**) The top 10 detected terms of biological processes associated with genes that decreased the number of contacts with rDNA clusters. The values to the right of the bars show the number of rDNA-contacting genes associated with a term. The list of corresponding genes is shown in Table S7.

We attempted to search rDNA inter-chromosomal contacts in K562 cells following the induction of differentiation by hemin (see Section 4). We observed that, upon differentiation, 1307 genes increased the number of contacts with rDNA genes, while 1200 genes decreased the number of contacts (Figure 2A, Table S1). Some genes changed the number of contacts by up to 50-fold. This result indicates that hemin serves as a strong signaling molecule for K562 cells and creates essential changes in the inter-chromosomal contacts of rDNA genes.

The analysis of these two sets of genes, either increasing or decreasing the numbers of contacts with rDNA genes, revealed that they corresponded to GO items containing genes that were involved in differentiation and development (Figure 2B,C). Interestingly, there was evidence of a few of the same GO items (regulation of the biological process, system development, and cell differentiation) possessing large groups of non-overlapping lists of genes (Tables S6 and S7). Our results suggest that members of the same GO item can behave differently during the differentiation of K562 cells. We detected that a set of genes, which increased the number of contacts with rDNA genes, included the genes *AGO1* (whose product binds to miRNAs and siRNAs and is involved in transcriptional gene-silencing), 36 *ZNF*, and 85 *LINC* genes. However, the list of genes that decreased the number of contacts with rDNA genes included *AGO2* (whose product is an important component of the RNA-induced silencing complex), another set of 30 *ZNF* genes, and 89 *LINC* genes. We assumed that these two sets of genes, located on opposite sides of the volcano plot in Figure 2A, play important roles in the differentiation of K562 cells by distinctive mechanisms.

When differentiating K562 cells, it can be observed that there are 2566 genes possessing at least 100 contacts with rDNA clusters (Table S1). The analysis of these genes in the ARCHS4 TFs Coexp database [23] revealed that 1485 genes were co-expressed in different combinations shaping 184 groups (Table S8). The data independently confirmed the previous conclusion (Section 2.1) that approximately half of the rDNA-contacting genes are co-expressed and that the inter-chromosomal contacts of rDNA clusters are involved in the co-regulation of large groups of genes.

### 2.3. Induced Differentiation of K562 Cells Leads to Extreme Downregulation of the ID3 Gene

**Figure 3:**
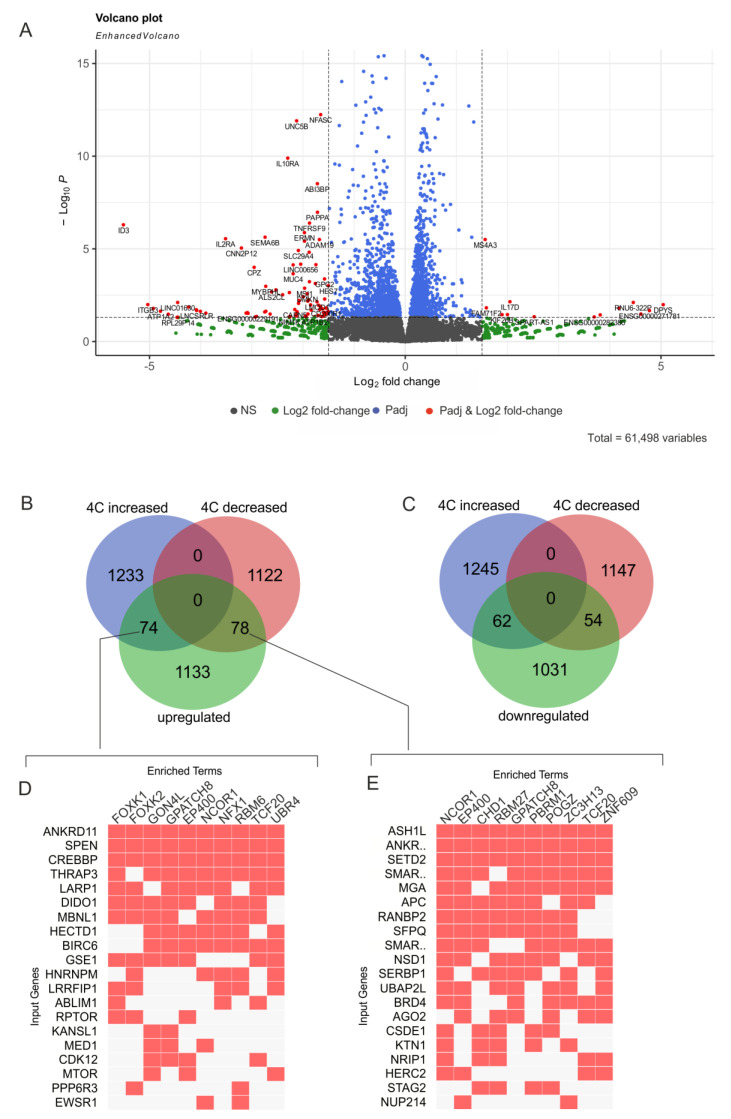
Differential expressions of genes in K562 cells following the induction of differentiation by hemin. (**A**) The volcano plot presents the statistically significant log2-fold changes in the expression of genes determined in RNA-Seq experiments. The results of the RNA-Seq experiments are presented in Table S9. (**B**) The Venn diagram shows the intersections of upregulated genes (*p* value < 0.05) with the lists of genes that reveal an increase or decrease in rDNA contacts. The list of corresponding genes is presented in Table S10. (**C**) The Venn diagram shows the intersections of downregulated genes (*p*-value < 0.05) with lists of genes that reveal an increase or decrease in rDNA contacts. The list of corresponding genes is presented in Table S11. (**D**) The top 20 upregulated genes that increased their contacts with rDNA upon differentiation (indicated as “input genes”) are simultaneously regulated by different transcription factors (indicated as “enriched terms”). The data were obtained via a search of corresponding genes in Enrichr Submissions TF-Gene Co-occurrence (https://maayanlab.cloud/Enrichr/, accessed on 4 June 2023). (**E**) The top 20 upregulated genes that decreased their contacts with rDNA upon differentiation (indicated as “input genes”) are simultaneously regulated by different transcription factors (indicated as “enriched terms”). The data were obtained via a search of corresponding genes in Enrichr Submissions TF-Gene Co-occurrence (https://maayanlab.cloud/Enrichr/, accessed on 4 June 2023).

The volcano plot presented in Figure 3A shows the statistically significant log2-fold changes in the expression of genes following hemin treatment. The results of the RNA-Seq experiments we conducted are presented in Table S9. The expressions of some genes changed to a considerable degree—up to log2 > 5. Interestingly, the *ID3* gene was extremely downregulated (more than 45-fold). This gene belongs to the family of inhibitors of differentiation (*ID*) genes. The corresponding helix–loop–helix (HLH) proteins in ID genes shape heterodimers with numerous members of the basic HLH family of transcription factors possessing basic DNA-binding domains. *ID3* genes encode proteins that lack this basic DNA-binding domain and, therefore, inhibit the DNA-binding behavior of different transcription factors and change the expression of numerous genes controlling cell growth and differentiation processes [26]. It is likely that the extreme repression of the *ID3* gene, which is considered a dominant negative regulator of differentiation [27], is required for instigating K562 cell differentiation. We also observed approximately 2-fold downregulation of the *ID1* gene, another member of the negative regulators of differentiation (Table S9).

### 2.4. Hemin-Induced Differentiation Leads to the Up- or Downregulation of Thousands of Genes, Including More Than 200 rDNA-Contacting Genes

We expected that the induction of differentiation should lead to changes in the expression of numerous genes in K562 cells and that some of these genes could correspond to rDNA-contacting genes. In fact, we detected that, in the course of differentiation, more than 1000 genes are either up- or downregulated (*p*-value < 0.05) (Table S9). The GO search revealed that 1285 upregulated genes were highly associated with DNA- and RNA-binding (padj up to 8 × 10^−57^), and their products mainly reside in the nucleoplasm (padj up to 8 × 10^−141^) (Table S12). However, 1147 downregulated genes (*p*-value < 0.05) were mainly associated with differentiation and development, and their products mainly reside in the cytoplasm or intra- or extracellular vesicles (Table S13).

Figure 3B shows the intersections of upregulated genes and genes that reveal a decrease or increase in rDNA contacts. It can be observed that approximately 12% of upregulated genes are associated with rDNA. Similarly, approximately 10% of downregulated genes correspond to rDNA-contacting genes that changed their number of contacts with rDNA (Figure 3C). The lists of these genes are presented in Tables S14 and S15, respectively.

The analysis of these genes revealed that they were simultaneously regulated by different transcription factors (Figure 3D,E). Interestingly, some of the factors were common to upregulated rDNA-contacting genes, which increased or decreased the number of contacts. These data are clearly consistent with the previous conclusion suggesting that rDNA-contacting genes are highly associated with numerous transcription factors and are also co-expressed.

### 2.5. Differentiation Is Coupled with Changes in rDNA Contacts in Areas Located around Globin-Gene Clusters

**Figure 4:**
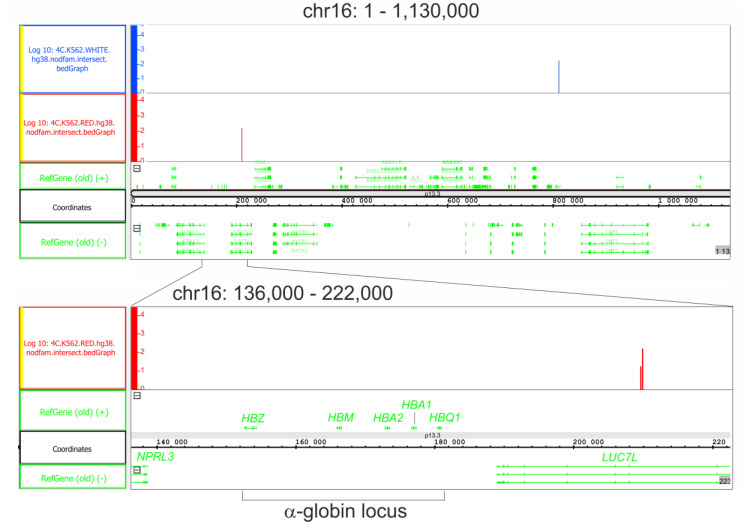
rDNA contacts inside the region of chr16 possessing an α-globin locus. The upper panel shows the tip of the chromosome, approximately 1 Mb in length (coordinates as in hg38). The contacts detected in initial K562 cells are presented in blue. The contacts detected in the cells following the induction of differentiation are presented in red. Prior to the mapping, 4C-rDNA reads were filtered to remove the reads that entirely corresponded to repetitive sequences (Dfam filtration, nodfam). The Y-axis presents the log10 value of the number of mapped 4C reads. The lower panel presents the contacts in the cells detected following the induction of differentiation in the lower scale.

In our study, we expected that the hemin-induced differentiation of K562 cells may lead to changes in the expression of genes controlling globin genes and that these events were not associated with the direct contacts of rDNA clusters with the regions in which globin genes reside. Surprisingly, we observed changes in rDNA contacts on the tip of chromosome 16, where the α-globin gene cluster resides. There was an rDNA-contacting region located approximately 600 kb downstream of the cluster inside the *PRR25* gene in the initial K562 cells. Following the induction of differentiation, these contacts completely disappeared, while new rDNA-contacting regions located inside the *LUC7L* gene appeared much closer to the α-globin gene cluster, approximately 29 kb downstream from the locus (Figure 4).

**Figure 5:**
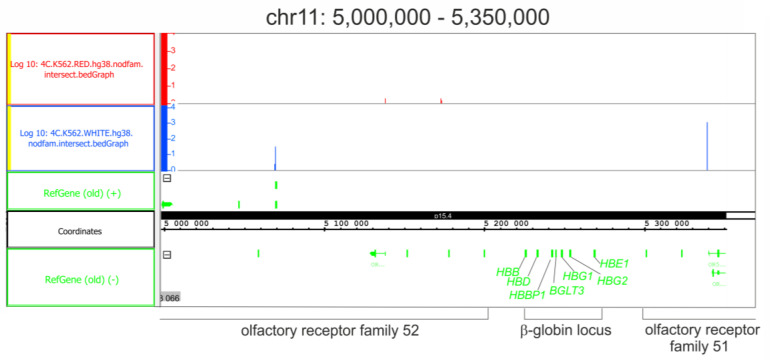
rDNA contacts located inside the chr11 region possessing the β-globin locus. The contacts detected in initial K562 cells are presented in blue. Coordinates are shown as in hg38. The contacts detected in the cells following the induction of differentiation are presented in red. Prior to the mapping, the 4C-rDNA reads were filtered to remove the reads that entirely corresponded to repetitive sequences (Dfam filtration, nodfam). The Y-axis shows the log10 value of the number of mapped 4C reads.

We also detected the changes occurring in rDNA contacts in chromosome 11, where the β-globin gene cluster resides (Figure 5). Again, the rDNA contacts were detected around the β-globin gene cluster in initial K562 cells located inside olfactory receptor gene families that are positioned around the cluster. These contacts completely disappeared following the induction of differentiation. Only a few rDNA contacts were observed following differentiation approximately 40 kb upstream of the cluster.

**Figure 6:**
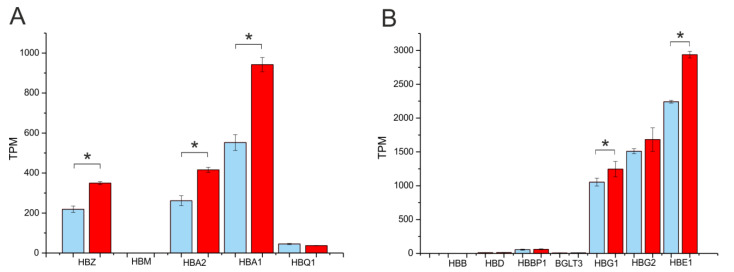
Expression values of α- and β-globin genes prior to and following the initial step of the differentiation of K562 cells. The expression detected in initial K562 cells is shown in blue; the expression detected following hemin treatment is presented in red. The Y-axis shows the values in TPM. (**A**)—expression in α-globin cluster; (**B**)—expression in β-globin cluster. The genes are presented in the order in which they occur in chr16 (α-globin gene cluster) or chr11 (β-globin gene cluster). * *p*-value < 0.05.

The initial step of differentiation that we observed upon the action of hemin also caused the slight activation of the transcription of globin genes. Figure 6 shows that a statistically significant increase in expression values was detected in *HBZ*, *HBA2*, *HBA*1, *HBG1*, *HBG2*, and *HBE1* genes. The basic transcription level for different globin genes was observed in initial K562 cells (Table S16). The data we obtained indicate that, during the early differentiation stage of K562 cells, both the 3D structures of the chromosomal regions possessing globin genes and the expression levels of the genes are changed.

### 2.6. rDNA Contact Appearance at the Region of the ID3 Gene Is Coupled with Its Strong Repression

**Figure 7:**
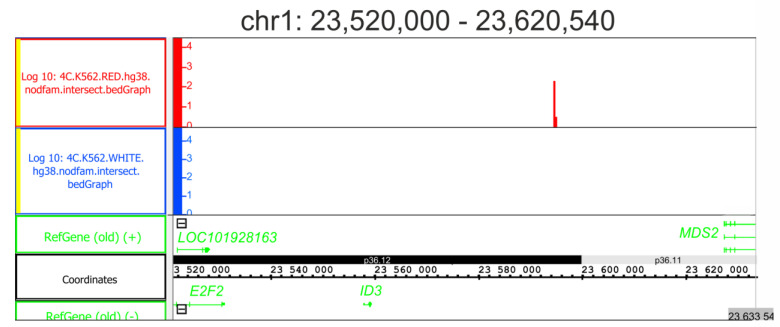
rDNA contacts appeared inside the region of chr1 possessing the *ID3* gene. No rDNA contacts were detected inside the approximate 100 kb region in initial K562 cells (scale presented in blue). The contacts detected in the cells following the induction of differentiation are presented in red. Coordinates are shown as in hg38. Prior to the mapping, the 4C-rDNA reads were filtered to remove the reads that entirely corresponded to repetitive sequences (Dfam filtration, nodfam). The Y-axis presents the log10 value of the number of mapped 4C reads.

In our study, we detected that, upon differentiation, the *ID3* gene was subjected to the most severe downregulation activity (Figure 3A). Thus, it was of interest to verify whether rDNA contacts appeared in the region of chromosome 1, where the gene resides. Figure 7 shows that the contacts emerged approximately 34 kb downstream from the gene rDNA-contacting region. Prior to the hemin-induced differentiation, no rDNA contacts were detected in the region. The cause–effect relationship between these contacts and the observed repression of the *ID3* gene remains unclear. However, the data indicate that during the differentiation, the 3D structures in the region were changed.

### 2.7. Differentiation Does Not Affect Either the Frequency of rDNA Contacts or the Expression of DUX4 Genes

**Figure 8:**
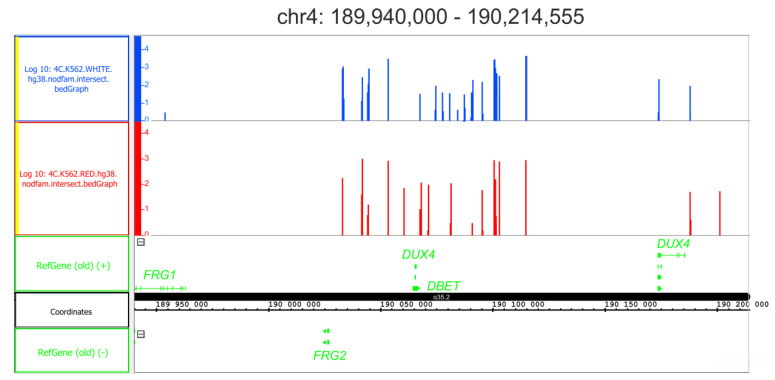
rDNA contacts inside the telomere region of chr4 possessing *DUX4* genes. The contacts detected in initial K562 cells are presented in blue. The contacts detected in the cells following the induction of differentiation are presented in red. Coordinates are shown as in hg38. Prior to the mapping, 4C-rDNA reads were filtered to remove the reads that entirely corresponded to repetitive sequences (Dfam filtration, nodfam). The Y-axis presents the log10 values of the number of mapped 4C reads.

Upon the differentiation of K562 cells, many regions preserved their inter-chromosomal contacts with rDNA genes. It was observed that rDNA contacts located inside *DUX4* genes were characteristic of different human cell lines and were very sensitive to heat-shock treatment, mostly disappearing following a brief treatment [17,28]. This was why we were interested in verifying whether the initial differentiation step of K562 cells affected the contacts of rDNA clusters with *DUX4* genes. Figure 8 presents the contacts present at the very tip of chromosome 4 possessing *DUX4* genes. We did not observe any changes in either rDNA contacts or expression levels of the gene in initial cells or the cells following differentiation (Tables S1 and S16). The genes were completely silenced. The result indicates that some inter-chromosomal contacts of rDNA clusters remain unchanged during the initial differentiation stage of K562 cells.

Our data strongly suggest that the differentiation of K562 cells leads to alterations in the inter-chromosomal contacts of rDNA clusters located in particular chromosomal regions. These alterations are coupled with changes in the expression of genes located in the corresponding chromosomal domains.

## 3. Discussion

### 3.1. How Does Hemin Affect rDNA Inter-Chromosomal Contacts?

Hemin is a normal substrate that is physiologically produced during the destruction of senescent erythrocytes, and a constitutive mechanism exists for the degradation of toxic heme by HMOX1 (Heme oxygenase 1) into biliverdin, carbon monoxide, and free iron, which have potent anti-oxidative stress and anti-inflammatory functions [29]. It was determined that hemin induces the expression of *HMOX1* genes and the JNK/Nrf2 signaling pathway [30]. At present, the molecular mechanisms by which Nrf2 and NF-κB signaling pathways cooperate to maintain homeostasis of the cellular redox status and regulate the cellular response to stress and inflammation are not yet known [31]. In our experiments, we observed that *HMOX1* expression increased three-fold upon the action of hemin (Table S16) and that the gene involved in the formation of the JNK cascade makes contact with rDNA clusters (Table S2).

It is well-known that nucleoli play an important role in the regulation of cellular stress [30] and are very sensitive to physiological heat-shock treatment [9,10,28]. One of the consequences of hemin-induced stress is erythroid differentiation, in which the genes involved in RNA- and DNA-binding are upregulated. The GO data (Table S12) suggest that the products of these genes are highly associated with nucleoplasm (padj up to 8 ×·10^−141^), strongly suggesting that the upregulated genes play important roles in the regulation of chromosomal structures and gene expression. The molecular functions of these genes include controlling chromatin-binding, DNA and RNA helicase activity, transcription-factor-binding, and transcription coregulator activity (Table S12).

Different types of stress induce different effects on rDNA interactions. The response to this stress also depends on both the genomic region and the cell type. The stress induced by hemin does not affect rDNA contacts located inside *DUX4* in K562 cells (Figure 8), while the physiological heat-shock treatment of HEK293T cells almost completely removes the contacts present inside these genes [28]. It is well-known that the induction of nucleolar stress leads to the rapid large-scale re-organization of rDNA clusters and the formation of closed chromatin regions called nucleolar caps [32]. Moreover, it seems that rDNA units that are subjected to heterochromatization lose their inter-chromosomal contacts. Nevertheless, the heat-shock treatment of *Drosophila* S2 cells, which leads to the essential downregulation of rDNA genes, induces the appearance of novel and frequent rDNA contacts inside the cluster of *Ste* genes [10]. It is probable that the nucleoli that are very sensitive to different external and internal signals play complex roles in the nucleus via dynamic inter-chromosomal contacts.

At present, we do not know the detailed mechanisms by which hemin-induced stress induces erythroid differentiation. However, a search of KEGG pathways revealed that upregulated genes were mainly involved in the RNA splicing pathway (Figure S1). Another clue comes from the observation of the extreme downregulation of the *ID3* gene (Figure 3A). The gene product prevents the function of cognate helix–loop–helix (HLH) proteins as transcription factors by arresting their action and preventing their function as transcription factors driving the differentiation. HLH proteins include a large superfamily of transcriptional regulators with more than 100 members, which control cell lineage development in human cells [33]. The arrest of these proteins can prevent the development of K562 cells toward a more mature erythroid differentiation state. We believe that the severe hemin-induced downregulation of the *ID3* gene should promote this development. Recently, we detected that, during the malignization of cancer cells, the opposite effect occurred—the simultaneous extreme upregulation of three *ID* genes: *ID1*, *ID2*, and *ID3* (paper in preparation). Thus, we suggest that the upregulation of *ID* genes caused de-differentiation, while their downregulation led to the acquisition of an increasingly differentiated state of cells.

### 3.2. rDNA Inter-Chromosomal Contacts and Mechanisms of Gene Expression Regulation

The main aim of this study was to highlight the possible link between differentiation and rDNA inter-chromosomal contacts and the supposed nature of these contacts. The data we obtained strongly suggest that the differentiation of K562 cells is coupled with the ordered changes of the contacts, which were mainly observed in the regions where the hundreds of genes involved in the regulation of biological processes, system development, and cell differentiation reside (Figure 2). Additionally, there was differentiation clearly accompanied by the contacts with genes controlling different aspects of differentiation. It is clear from the results that the rDNA contact itself does not directly regulate a gene. There was a higher number of genes that changed the number of contacts with rDNA (4855 genes, Figure 2A) than rDNA-contacting genes, which changed the overall expression (268 in total, as shown in Figure 2B,D). Approximately 90% of the upregulated (1285 genes) and downregulated genes (1147 genes) revealed no close contacts (≤2.5 kb distances, see Section 4) with rDNA. However, changes in expression were observed when the contact sites were located at a distance (Figure 4, Figure 5 and Figure 7). In addition, there were examples of direct contact with *DUX* (Figure 8) or *Ste* genes [10]. It may follow that the influence on the expression of rDNA contacts can occur at 50–100 kb distances. We have now developed a bioinformatics approach by which to perform the accurate genome-wide correlation analysis of supposed distance-dependent effects on rDNA contacts on gene expression upon the differentiation of K562 cells. The analysis performed on several regions presented in Figure 4, Figure 5 and Figure 7 suggests that the effects were obtained from 40–100 kb distances.

We speculate that rDNA contacts, on the one hand, could change 3D structures around the contact sites and/or, on the other hand, could direct the corresponding chromosomal regions into particular liquid–liquid-phase-separation condensates. There are several lines of evidence supporting this supposition. First, we observed that numerous sets of rDNA-contacting genes are co-regulated (Figure 3C,E) and co-expressed (Tables S3 and S8), suggesting the involvement of rDNA clusters in these events. Second, the existence of 50–100 kb chromosomal domains possessing coordinately expressed genes was detected [34]. The sizes of these domains more or less overlapped with the postulated sizes of nuclear condensates (approximately 0.3–0.4 μm), corresponding to a 30-nm-like fiber structure of 50 kb DNA stretches [35,36]. Third, the analysis of rDNA-contacting regions in genome browsers revealed that these regions very often corresponded to long stretches of up to 50 kb, decorated with H3K27ac marks, which are known as super-enhancer sites that can shape condensates [10,13,16,37]. Nucleoli, as the largest membrane-less organelles in nuclei, are good candidates for the organization of condensates, possessing either set of activators or repressors of transcription. Thus, the co-expression and co-regulation of numerous rDNA-contacting genes that are located in different chromosomal regions could be achieved (Tables S3 and S8). This function of nucleoli can explain the numerous roles of rDNA clusters beyond ribosome biogenesis. Currently, we are addressing several questions raised in this study, especially regarding the formation of condensates around the nucleoli and epigenetic states around rDNA-contacting regions.

## 4. Materials and Methods

### 4.1. Cell Culture Growth and the Induction of Differentiation by Hemin

Human leukemia K562 cells were obtained from the American Type Culture Collection (ATCC, Manassas, VA, USA). The cells were grown in RPMI 1640 media (PanEco, Moscow, Russia) supplemented with heat-inactivated fetal calf serum (HyClone, Logan, UT, USA), 2 mM glutamine, 250 u/mL penicillin, and 250 μg/mL streptomycin (PanEco, Russia) at a temperature of 37 °C in a humidified atmosphere containing 5% CO_2_. Hemin (50 μM, neoFroxx, Einhausen, Germany) was added to the medium to induce erythroid differentiation, as previously described [38], and cells were incubated further for 108 h. At this concentration, the drug did not affect cell proliferation.

### 4.2. 4C-rDNA Procedure

The DNA samples used for 4C experiments were isolated according to the procedures previously described [16]. The cells were then fixed in 1.5% formaldehyde, and the nuclei were isolated. Then, digestion with a 6-cutter *EcoR*I enzyme and the ligation of extensively diluted DNA to favor intramolecular ligations were performed. To shorten the ligated DNA fragments, digestion with a 4-cutter *Fae*I endonuclease was performed, followed by the ligation of diluted DNA samples to favor circularization and to minimize dimerization. The primers 5′ TCTTTGAAAAAAATCCCAGAAGTGGT 3′ and 5′ AAGTCCAGAAATCAACTCGCCAGT 3′ for 4C-rDNA were selected inside the IGS (intergenic spacer in rDNA genes), as previously described [12]. The final DNA samples were used for the preparation of DNA libraries that were subjected to deep sequencing using Illumina HiSeq 2500 Rapid v2 (Illumina, San Diego, CA, USA), using 150 nt long reads. The 4C-rDNA raw data corresponding to two pairs of biological replicates, corresponding to initial or differentiated K562 cells, were deposited under accession number GSE232392.

### 4.3. Mapping and Processing of 4C-rDNA 4C Data

Both raw reads and processed mappings were uploaded to the GEO database under accession no. GSE232392. The number of reads we obtained was replicate 1: 17831199, replicate 2: 18964663 for K562 white/untreated cells, and replicate 1: 11110278, replicate 2: 11603232 for K562 red/treated-by-hemin cells.

K562 4C-rDNA-associated contacting region mappings were obtained in the following way. Adapters were removed separately from direct (R1) and reverse (R2) reads by cutadapt [39] 3.5 using the following multi-step procedure:

1. 4C full-length adapters (both direct and reverse complement (RC)):
  A1D TTCACTTCTGACATCCCAGATTTGATCTCCCTACAGAATGCTGTACAGAACTGGCGAGTTGATTTCTGGACTT,
  A1RC AAGTCCAGAAATCAACTCGCCAGTTCTGTACAGCATTCTGTAGGGAGATCAAATCTGGGATGTCAGAAGTGAA,
  A2D TCTTTGAAAAAAATCCCAGAAGTGGTTTTGGCTTTTTGGCTAGGAGGCCTAAGCCTGCTGAGAACTTTCCTGCCCAGGATCCT,
  A2RC AGGATCCTGGGCAGGAAAGTTCTCAGCAGGCTTAGGCCTCCTAGCCAAAAAGCCAAAACCACTTCTGGGATTTTTTTCAAAGA
  were removed at 5′ ends by the options -O 10 (minimal overlap adapter with the read) --trim-n (omit N’s at the ends of reads) --times = 4 (search for the adapter up to 4 times in the read consequently) --minimum-length = 20 (minimum acceptable read length after trimming) -q 24 (minimal acceptable quality). All untrimmed reads were collected in a separate file.
2. Illumina 3′ adapter arrays from AGATCGGAAGAGC to AGATCGGAA-GAGCNNNNNNNNNN and from GATCGGAAGAGC to GATCGGAA-GAGCNNNNNNNNNN anchored to 3′ ends of reads were removed by cutadapt with the following options: -O 10 (minimal overlap adapter with the read) --times = 4 (search for the adapter up to 4 times in the read consequently) --minimum-length = 20 (minimum acceptable read length after trimming) -q 24 (minimal acceptable quality).
3. Incomplete from 5′ 4C full-length adapter arrays: from TTCACTTCTGACATCCCAGATTTGATCTCCCTACAGAATGCTGTACAGAACTGGCGAGTTGATTTCTGGACTT to TTCACTTCTGACATCCCAGA (minimal length = 20) and from TCTTTGAAAAAAATCCCAGAAGTGGTTTTGGCTTTTTGGCTAGGAGGCCTAAGCCTGCTGAGAACTTTCCTGCCCAGGATCCT to TCTTTGAAAAAAATCCCAGA (minimal length = 20) both direct and reverse complement reads, anchored to 5′ ends of reads were removed by cutadapt with the same options as mentioned above.
4. Illumina adapter GATCGGAAGAGC and IlluminaPE adapter AGATCGGAA-GAGC were removed by cutadapt from 3′ ends of reads with the following options: -O 5 (minimal overlap adapter with the read) --times = 4 --minimum-length = 20 (minimum acceptable read length after trimming) -q 24 (minimal acceptable quality)
5. Incomplete from 3′ 4C full-length adapter arrays: from TTCACTTCTGACATCCCAGATTTGATCTCCCTACAGAATGCTGTACAGAACTGGCGAGTTGATTTCTGGACTT to GCGAGTTGATTTCTGGACTT (minimal length = 20) and from TCTTTGAAAAAAATCCCAGAAGTGGTTTTGGCTTTTTGGCTAGGAGGCCTAAGCCTGCTGAGAACTTTCCTGCCCAGGATCCT to ACTTTCCTGCCCAGGATCCT (minimal length = 20) both direct and reverse complement reads were removed by cutadapt from the 3′ ends of reads with the same options as in points 2 and 3 of this protocol.
6. All untrimmed reads collected during step 1 of the described procedure (i.e., the reads without at least a 10-nucleotides overlap of the adapter and read) were trimmed again by the previously described procedure (points 1–5) with the only difference: in the first step, the changed set of cudatapt options was applied: -O 15 (instead of -O 10, thus requiring additional adapter nucleotides to overlap with the read); -e 0.2 (this option fixed the error rate at 0.2, thus enabling us to find adapters that were read with a reduced quality).

Only the reads that were trimmed from 4C adapters were preserved for further analysis, thus ensuring that they were 4C-associated. Direct and reverse reads were repaired using BBTools [40] 38.62 repair.sh script with the option “repair”. Only paired-end reads were preserved to perform an additional analysis: 16848786 (replicate 1, white/untreated), 17951920 (replicate 2, white/untreated), 10259396 (replicate 1, red/treated by hemin), and 10762480 (replicate 2, red/treated by hemin); all singleton reads were omitted.

The alignment to the genome was performed by the bwa 0.7.17-r1188 [41] mem method with the GRCh38/hg38 p13 genome; samtools 1.14 [42] were used to filter the aligned reads from the unaligned reads in alignment BAM files (-F4 option), re-sorting them by their coordinates (samtools sort). In-house bash and Perl scripts were used to obtain the final mappings with genome coordinates, number of reads, coverage, and sequence per mapping.

Replicate quality control was performed using deepTools2 [43]: BAM files obtained from the different replicates were RPKM-normalized (bamCoverage --effectiveGenomeSize 2913022398 --normalizeUsing RPKM --ignoreForNormalization chr14 --exactScaling), and the plotCorrelation tool with options --removeOutliers --skipZeros -p scatterplot was applied to calculate both Pearson’s and Spearman’s correlation coefficients. The Pearson’s correlation coefficients between replicates were r = 0.99 both for untreated- and treated-by-hemin 4C-rDNA K562 datasets. The Spearman’s correlation coefficients were *ρ* = 0.74 for untreated- and *ρ* = 0.83 for treated-by-hemin 4C-rDNA K562 datasets. The calculated correlation coefficients indicate that replicates obtained during 4C-rDNA experiments both for untreated- and treated-by-hemin K562 cells were in good mutual agreement. The derived scatterplots and correlation coefficients imply that the results we obtained are reliable.

An intersection of K562 4C-rDNA-mapped replicates was created by an in-house bash script that utilizes intersect and merge methods from bedtools v.2.29.1 [44] and partition, bedmap, and map-id-uniq methods from bedops v.2.4.40 [45].

The scripts and data files that performed the described procedure are deposited in the public GitHub repository (https://github.com/lokapal/K562.hemin/tree/main/4C, accessed on 4 June 2023).

### 4.4. 4C-rDNA-Associated Gene Quantifications and Differential 4C Analysis

As described in Section 4.2, we used the 6-cutter *EcoR*I enzyme in the 4C-rDNA experiments. This implies that 4C-rDNA contacts were cut at a resolution of ±2.5 kb and, therefore, each dataset was projected onto hg19/hg38 genes lists, with each contact size/length being extended to 5 kb by applying in-house Perl scripts that utilize bedtools. The GRCh38/hg38 genome genes’ coordinates were obtained from Ensemble, GTF annotation v. 97 for the GRch38/hg38 genome build. Each 4C-rDNA-associated gene could be featured by the corresponding count of the associated 4C contact reads; therefore, the resulting set of genes could be ranked according to these counts. We selected the most prominent 4C-rDNA-associated genes with the counts of corresponding reads exceeding 100. Thus, we finally obtained lists of 3699 and 2566 4C-rDNA-contacting genes for untreated or hemin-induced K562 cells, respectively.

As previously mentioned, our molecular biology procedures implied that 4C contacts were mapped at a resolution of ±2.5 kb. Therefore, we quantified 4C-rDNA-associated reads to the hg38 genome by featureCounts [46] with the options appropriately changed: -a hg38.97.gtf -t gene -g gene_id -M --readExtension5 2500 --readExtension3 2500. The differential 4C analysis was performed by the DESeq2 1.30.1 [47] R library using two 4C untreated replicates as the “control” and two 4C treated-by-hemin replicates as “experiment” and parameter fitType = “local” that was optimal to process 4C-generated data. An in-house R script was applied to associate ISO gene names with gene IDs for further genetic and GO analyses. Volcano plots were created by EnhancedVolcano 1.8.0 R library [48].

The scripts and data files that performed the described procedure were deposited in the public GitHub repository (https://github.com/lokapal/K562.hemin/tree/main/4C, accessed on 4 June 2023).

### 4.5. RNA-Seq Analysis

Total RNA was extracted from the cells lysed with Trizol using a PureLink RNA Micro Kit (Invitrogen, Waltham, MA, USA) in accordance with the manufacturer’s instructions. RNA quality was checked using a Bioanalyzer and RNA 6000 Nano Kit (Agilent, Santa Clara, CA, USA). Poly(A)+RNA was purified using a Dynabeads^®^ mRNA Purification Kit (Ambion, CT, USA). The Illumina library was prepared from poly(A)+RNA with a NEBNext^®^ Ultra™II RNA Library Prep Kit for Illumina^®^ (NEB, Ipswich, MA, USA), according to the manual. Sequencing was performed on a 50 bp read length. At least 10 million reads were generated for each sample. Both raw RNA-Seq reads and processed gene expression values for the initial and differentiated K562 cells were deposited in the Gene Expression Omnibus (GEO) repository under accession number GSE232390. The number of reads we obtained was replicate 1: 10899858, replicate 2: 10110489 for K562 white/untreated cells and replicate 1: 10376685, replicate 2: 11214611 for K562 red/treated-by-hemin cells.

The processing of RNA-Seq expression data for K562 cells was performed in the following way: Trimmomatic [49] 0.39 was applied to remove low-quality reads (Q < 22), too-short reads (length < 20 bp), and remaining Illumina TruSeq 3′ SE adapters with the following options: LEADING:18, TRAILING:18, SLIDINGWINDOW:4:22, MINLEN:20 and ILLUMINACLIP:TruSeq3-SE:2:30:10.

Trimmed reads were accurately quantified by RSEM [50] 1.3.1 to *H.sapiens* Ensembl v.106 genome/annotation with the following options: --fragment-length-mean 255 --star --calc-ci --ci-memory 30720 for both replicates separately. The obtained expression values (TPM per gene) were averaged between replicates by an in-house R script.

Differential RNA-Seq analysis was performed as follows: to assure compatibility with the derived expression values, STAR [51] 2.7.5c was applied to align the filtered reads to the same genome/annotation (Ensembl v.106 genome and annotation) with the options that RSEM employs for STAR: --outSAMunmapped Within --outFilterType BySJout --outSAMattributes NH HI AS NM MD --outFilterMultimapNmax 20 --outFilterMismatchNmax 999 --outFilterMismatchNoverLmax 0.04 --alignIntronMin 20 --alignIntronMax 1000000 --alignMatesGapMax 1000000 --alignSJoverhangMin 8 --alignSJDBoverhangMin 1 --sjdbScore 1 --quantMode TranscriptomeSAM. featureCounts 2.0.3 [44] was applied with the recommended options (-t exon -g gene_id) to obtain absolute reads values per gene. DESeq2 [45] 1.30.1 R library was applied to create a differential expression table for white/untreated- and red/treated-by-hemin K562 cells.

Differential expression figures were plotted by EnhancedVolcano [46] 1.8.0 R library. The scatterplots of quantified-to-genome datasets were created by DESeq2 to check the consistency between replicates. Additional consistency checks between replicates were performed by deepTools2. Pearson’s and Spearman’s correlation coefficients for white/untreated- (*r* = 0.99, *ρ* = 0.80) and red/treated-by- hemin (*r* = 0.99, *ρ* = 0.80) RNA-Seq datasets proved the high degree of consistency between replicates. The scripts and data files used to perform the described procedures were deposited in the public GitHub repository (https://github.com/lokapal/K562.hemin/tree/main/RNASeq, accessed on 4 June 2023).

## Data Availability

Not applicable.

## Supplementary Materials

The supporting information can be downloaded at: https://www.mdpi.com/article/10.3390/ijms24129842/s1.
