# Supplementary material for: Induction of the Erythroid Differentiation of K562 Cells Is Coupled with Changes in the Inter-Chromosomal Contacts of rDNA Clusters
Source: Int J Mol Sci. 2023 Jun 7;24(12):9842. doi: 10.3390/ijms24129842 (PMC10298330; doi:10.3390/ijms24129842)
Supplement: Supplementary file 1 [file ijms-24-09842-s001.zip › Supplemental Information-22-5-23.pdf]

Supplemental Information for:

Induction of erythroid differentiation of K562 cells is coupled with the changes in inter-chromosomal contacts of rDNA clusters

Nickolai A. Tchurikov,[1*] Elena S. Klushevskaya[1], Ildar R. Alembekov[1], Antonina N. Kretova[1], Vladimir R. Chechetkin[1], Galina I. Kravatskaya[1], Yuri V. Kravatsky[1,2]

[1] Department of Epigenetic Mechanisms of Gene Expression Regulation, Engelhardt Institute of Molecular Biology Russian Academy of Sciences, Moscow, 119334, Russia

[2] Center for Precision Genome Editing and Genetic Technologies for Biomedicine, Engelhardt Institute of Molecular Biology Russian Academy of Sciences, Moscow, 119334, Russia

Tables S1–S16

Table S1. Lists of rDNA-contacting genes in untreated K562 cells (white) and cells treated with hemin (red), and differential analysis of the contacts of rDNA clusters with genes upon the induced differentiation of K562 cells. 4C-rDNA reads were processed as described in the Materials and Methods Section. Excel file attached separately. Related to Figure 2.

Table S2. GO associations with molecular functions (MF) and biological processes (BPs) (GO Profiler) of the top-3699 genes possessing ≥ 100 contacts with rDNA clusters. Related to Figure 1A.

GO.ID	Description	padj	Genes
**MF**			
GO:0005515	protein binding	1.155841429996664e-32	*EBNA1BP2,NOTCH2,BCAR3,MTOR,CNTN4,SPOCK1,NSG1,WWC1,IMMP2L,LRP12,PTPRD,FREM1,TRAPPC9,BNC2,NEBL,LRRC4C,TMTC1,KCNH5,MICU2,ANKS1B,POTEG,SMOC1,MYO9A,ULK2,NLK,LONP2,UNC13C,LRRC49,SCAPER,FTO,KSR1,MGA,RFX7,AGBL1,ZNF236,PLCB1,TTC3,MX2,TMPRSS2,TAFA5,SVIL,TLN2,CLTCL1,ZFPM2,ARL15,MICAL3,POTEM,TENM4,NUBPL,L3MBTL4,DLC1,TNRC6B,MGAM,DPP10,ZDHHC21,PTPRA,ITPR2,RIPOR2,PDE4D,RDX,RP1,STXBP1,ERC1,RALA,NME7,SLC44A5,EPS15L1,BCL2,MYO5A,ODAD2,KCNMA1,SYT16,ARPP21,PRDM16,ARHGAP26,FBN1,LRFN2,LPCAT2,F13A1,LRRTM4,SETBP1,GPHN,COG5,CDH8,CHRNA7,DCDC1,ROBO2,PUDP,RIMS1,PIK3C3,SPIRE1,TENM3,GABRB3,ZEB1,TMEM132D,CNTLN,SDCCAG8,RARB,FGD4,EXOC6B,SPRED1,NAV2,SPAG16,MYO1E,TRAPPC8,PLPPR1,USH2A,CEP192,MINAR1,CDC42EP3,LAIR1,TTC33,RIMS2,PCMTD1,ALK,MICOS10,AUTS2,FOXJ2,CDYL2,CARMIL1,PJA2,BABAM2,SV2C,PAPPA2,GLIS3,FANK1,ERBIN,ERCC6L2,RHPN2,HACD2,ASTN1,UNC79,HLCS,FCHO2,RIN2,PARVB,ANO6,CACNG2,DLGAP1,NEGR1,GLYAT,NAALADL2,MLLT3,EGLN3,GPC6,SUSD4,CNTNAP2,MAP4,MAP3K9,MYO3B,MOCOS,SPON1,APC,ZMYM4,ZNF595,HHLA2,TSHZ3,DSCAM,MYO5C,RTN1,TCF4,CRKL,SOX5,SETD2,ERG,ARHGAP24,TNIK,PTPRJ,EFCAB2,OCA2,KDM4C,DOCK10,TSHZ2,EGFR,RFX3,DENND1A,USP14,ANGPT1,CDK12,BACH1,MACF1,CTNNA3,PRKACB,NEK7,RGS3,NCOR1,RNF220,DOCK2,DIAPH3,CCDC138,NEDD4,MYOF,MAML2,SPATA17,SND1,SCAI,GNPTAB,CRB1,NSMCE2,BTBD9,BCL11A,SOX6,FAM83F,TMEM182,PSMB2,SGMS1,CECR2,ARMC2,FLI1,RPRD1A,PTPN4,CDH4,B3GALT5,ATP2B2,NTRK3,LARGE1,RXFP1,C5,PDE1C,FBXL7,ZFAND6,PHACTR1,DKK2,FLT1,DNAJC13,ZNF648,RFC3,RABEP1,GK,TASP1,FNDC3B,CNTN3,THRAP3,MAPKBP1,AOAH,GABRB1,DGKI,INVS,C12ORF4,EDAR,GRIA1,CRACD,CAST,TTC39B,NUP214,NEO1,CNTN6,CABLES1,SLC8A3,UHRF1BP1L,MALRD1,TOM1L2,NELL2,SEZ6L,PRKD1,TBC1D19,PAK1,GMDS,EPHA7,CTNNAL1,NCOA7,KHDRBS2,CHRM3,ADSS2,RALGPS1,SPEN,RAPGEF2,PELI2,LRP2,ADGRB3,DEUP1,RUNX2,FGF12,CPS1,TAOK3,LDLRAD3,CPEB4,TMEM38B,AGK,CSTF3,BCKDHB,RANBP17,UBE2L3,LDB2,TAFA4,SAMD5,PPP2R2B,BTBD11,PUM3,SYN2,CCL28,SMYD3,PATJ,HERC2,LRGUK,TMEM241,GRM7,SEPTIN9,RETREG1,RPTOR,DNAH6,GHR,WDSUB1,EPB41L3,KIF4A,THADA,DHX32,COL4A2,AIG1,SSBP3,TMEM74,RALGAPA1,RAPGEF5,TBCD,NEDD4L,ADAM32,PPP1R12B,TRPM1,ADAM10,HDAC9,ZHX3,ATF7IP,UBE2G1,IL1R1,APBB2,PHACTR2,APP,RPS6KA2,SAMSN1,KYNU,CACNA1C,KDM1B,CACNB2,KLHL13,MTUS1,PHKB,DCLK1,STAU2,GABRG2,DOCK8,MAPRE2,ZNF600,USP18,SEMA5A,SYT1,VCL,ARHGAP44,NTF3,PARP15,NDUFAF2,CD2AP,AURKA,PARN,TTC29,PYGO1,SLC8A1,HERPUD2,CCDC116,SSBP2,PTPRR,SRGAP2C,ANKRD31,FIG4,DUX4,TAFA2,MARCHF1,CMIP,ABCG8,PLGRKT,FRMD3,UPP2,CCSER2,LOXHD1,ECPAS,SRGAP2B,KANK1,KCNE4,MAP4K4,ABCD2,BMPR1B,FMN2,THSD7A,PCSK6,AKAP6,HOMER2,CTNNA2,HADHB,POTEH,ARNT,RAB8B,PDZRN4,PA*

			K3,PDE1A,TTLL7,DIP2B,RANBP2,LARP1,ITPKB,TRPC5,RGS20,PDE10A,UBE2E2,RAP1GDS1,HHAT,RNLS,CLIC6,CHST8,KICS2,ERC2,TMEM236,DNM3,NBN,CUBN,SCP2,IFT57,PHF21B,INTS7,RBM47,SUSD6,PRKCZ,CALD1,SNTG2,KLHL1,SPOP,BTLA,GRB10,RALYL,RYR3,TAF15,DIP2A,MSH6,MCPH1,ARHGAP32,RAB27B,ST8SIA5,CNST,HEATR4,HECW1,DEFA3,LRRC7,MBNL2,C7ORF31,PHF19,MRTFA,TAF4B,ANKRD33B,COBL,SENP6,DUSP22,PDXDC1,EBF2,UBN1,SV2B,YAP1,ESS2,SEM1,NFIA,WDR70,RIPK4,ZKSCAN5,SHC4,VPS35L,BRINP1,SCGB2B2,MAPK1,ABCD3,RABGAP1L,SGTB,DNAH14,TRPC7,ADAM22,USP25,KMT2E,ALCAM,PLG,PAPPA,PCGF5,PDGFD,C2ORF88,COPB1,SYT10,ZNRF3,DNAJC21,CCDC150,MTUS2,PPP1R1C,ABLIM1,CCDC172,ITGBL1,ARHGEF17,NRG3,UBE2O,SFMBT2,ANKFY1,NCAM1,GFRA1,NIPBL,RNF17,SLC16A1,SPIDR,RNGTT,IPO11,EWSR1,GABPA,MICU1,CORO2B,CARD18,CHD6,STK38,LCE1F,PTPN13,TBC1D22A,CHN1,SORCS3,KANSL1,LIMCH1,FMN1,ECT2L,MBNL1,PAFAH1B1,ATF6,EFEMP1,DCAF1,ITGB8,STON2,ZFAND3,VPS13D,TLK1,TPM1,NF2,LRRC38,WDR25,CNKSR2,RBFOX1,WDFY4,C1ORF21,HIVEP1,CORIN,CTNNA1,PPP1R9A,ANKRD11,EFCAB8,CDH7,MOB3B,BIRC6,AKAP9,KLF15,RASGRF2,PPARA,MEIS2,SNX30,LCLAT1,NFIB,KCNS3,ERMP1,MRTFB,PPP6R3,PRTG,RGL1,SYNJ1,NR5A2,ADAMTS3,TIAM1,MPRIP,ARAP2,GRM1,FOXJ3,UBE3D,KAZN,RSRC1,PTPRK,ARHGEF12,GABRG1,ENAH,PAK5,ST6GALNAC3,TRERF1,SF3B6,PARD3B,PPP2R5E,PDZRN3,KIAA1958,PLA2R1,GIPC2,EIF3D,TMCO4,SEMA3C,DAPK1,NAV3,SLC24A4,SEC14L1,TMEM108,ACSM2B,AGO2,WDHD1,PHC3,MAGI1,DNAH11,JARID2,SCN2A,RIC8B,TBC1D9,RAB22A,SORCS1,DNAJC15,AMPH,GATAD2B,CPE,PALS2,DYSF,IL34,ANK2,STAG2,BRWD1,TANC1,THUMPD2,ADGRV1,ZNF846,MELK,BCAS3,RYR2,SYNE2,BBS2,PEBP4,WNT9B,MSANTD4,CLPX,RANBP3L,NKG7,SEMA6D,AIF1L,NBEA,DUSP16,ANKS6,SMARCA4,CDH11,USP8,LDB3,FABP7,NOL4,PARD3,SLC36A1,MAPKAP1,EFTUD2,TNRC6C,PIAS1,TBC1D5,SPG21,UBE2R2,BLK,COL23A1,RBM6,EBF1,TNR,OLA1,DST,CXADR,DOCK4,MBD5,ATRX,NUAK1,PTPRT,XIRP2,ELAVL4,ABL1,KLHL32,AGPS,MXI1,PTPN12,HDAC4,OXR1,SLC1A1,PRKAA1,SDC2,GAS2,SLC12A8,KCNH1,ITGB3BP,MRPS27,LRFN5,RIMBP2,CRTAC1,DROSHA,TTLL5,APBB1IP,ANO4,L3MBTL3,DMXL2,EIPR1,APLF,NFAT5,MAST4,DNAH5,GUCY1A2,NBAS,CDH18,PSMF1,ATE1,SLFN11,RAP1A,GLIS1,TMCO5A,ACSS3,LYRM4,MYO10,SLC46A3,GPC5,TOX3,ZNHIT6,CAMK4,BAZ2A,INPP5A,CPSF3,FGF10,FBXL13,C2ORF42,ZC3HAV1,UQCC1,GRID2,CDHR3,TGM1,PEAK1,LATS2,NRG1,GSG1L,KLHL33,CLIP1,ASPM,AP3B1,DENND2B,COL6A5,EFCAB6,RASGRF1,ATP11C,GSE1,ZNF438,DHX40,ABCB7,SYNE1,ZBTB16,MUSK,KIR3DL2,ZNF675,ACTR3C,GNG7,SMARCAD1,SH3GL3,SETDB2,RPF1,PRKCE,FOXK2,SLCO3A1,ASAP2,MED15,SLMAP,ESRRG,C12ORF40,USP33,DENND4C,CEP83,CERS6,FBN2,CD44,RGS12,PTPRO,EGF,PRRC1,ABCC9,TRIO,PDE3A,EXT1,STXBP6,COL5A3,NSMAF,NLRP13,LNPEP,LIMD1,PEX14,SPRED2,RPS6KA3,CTNND2,MARCHF8,IFT43,ATP8A2,SCG5,MTMR3,PTPN2,TRIM5,LHFPL3,PLXNA2,POC5,MCF2L,ATXN3,RFC1,HTR2C,RIC3,SLC2A3,ARHGEF7,ALG10B,ATP8A1,ZCCHC7,AMBRA1,RFTN2,LTBP1,STK38L,ZFYVE9,GALNT10,GUCD1,KDM7A,OPRM1,ABCC4,PRMT8,HTR2A,BIN2,PLCXD3,FANCM,FANCA,CYBRD1,DAZL,INPP4B,MATN2,FARS2,GTF2F2,PPP2R2C,CNNM4,KREMEN1,STAC,ANKRD28,SEMA3E,TAF3,RPRD1B,MARK2,GCSAML,TMEM67,RCL1,EBF3,ALPL,ZNF33B,LPP,C10ORF90,FHL2,ABHD17C,ADGRA3,CNIH3,PUM1,TMOD2,HERC1,MSH2,IGF2BP3,MPPED2,GNAL,CDIN1,EPHA6,ANKRD17,APBA2,MAIP1,LINGO2,ZNF397,SH3KBP1,ATL1,SLC2A13,LUC7L,RELL1,CDKN2C,EPN2,KCND2,TNPO3,SNRPN,GRK3,CD163,SPSB4,CLSPN,NOS2,BICRAL,AFG3L2,CPNE4,STK10,TTC7B,MNAT1,RBBP8,MDFIC,SGCZ,TMTC2,ADAM12,MYLK2,ANK3,SNTG1,EMILIN2,HMGA2,MYOM2,COG2,CCND3,BCL11B,VPS41,FOLH1,DOCK5,F5,ECE1,KLHL4,ZIM3,STK32A,CREM,LYPLA1,MBP,AK8,TRPS1,TRAPPC11,HHIPL1,PLCE1,TGFA,IL17RA,ANKFN1,HIP1,CRIM1,XPNPEP1,FUT9,PRR5L,VPS37A,ATP6V1E1,UTP4,VAV1,CDH20,MSRA,RUFY2,FBXO32,TJP1,LDLRAD4,NPHP4,EGFLAM

			,PACSIN2,CNTN1,HLA-B,TARS3,FKBP5,IQSEC1,MTHFD1L,SNX3,CACNA1I,NAA35,PDLIM5,KCNJ15,CEPT1,BRCA2,AQR,DISC1,ZBTB2,EXD3,DNER,BLM,ASB7,WDPCP,SEMA3A,MAGI3,HSF2BP,INTS8,NAP1L4,LIN54,LRP1B,ADCY10,PSG8,STRN,AGL,ZNF121,ANKRD30BL,STX12,PHACTR3,BMP2,MYLK4,UNC5D,ATP9A,FAM217B,TRAK1,WDR26,PSG9,CDC42BPB,EVI5,PTCD2,MSR1,VRK1,GNAI1,RALGAPA2,SGSM1,ZC3H14,NCAM2,GFI1B,TBC1D4,RANBP9,RESF1,MYRIP,TTR,HPCAL1,RIN3,MSI2,TPGS2,DNAL1,SLC15A5,RNF38,TTC6,PGPEP1,TMEM161A,SEMA3D,ASXL3,NETO2,PDE6C,ANKRD7,CABIN1,LEMD3,RELN,ARHGAP42,HMGB1,GNAQ,TUBGCP3,NUDCD3,CDS2,AP4E1,FGF9,NFATC2,TDRD7,SH3BP5,CPAMD8,RTTN,MDM1,ZNF106,C19ORF18,MYOM1,ZNF567,CLVS1,TRAF3,ZNF462,ANKRD26,ESRP1,UNC13B,TTC21B,ETS2,UBAP2L,GEMIN5,UIMC1,DOCK1,LRRFIP1,TSPAN2,PFKFB4,RAP1GAP,PLS1,SRGAP2,IKZF2,SNX8,SEC23B,ENOX1,SLC39A6,NIN,HAUS6,DRAXIN,DNAH8,TRIT1,ATF1,CCDC186,SLAMF1,SMARCA2,ETS1,FAM83B,GLI3,CGAS,MEGF11,SMARCC1,SNX6,GABRR2,SMOC2,PACS1,PCP4,CNKSR3,CASP5,VENTX,IDE,WDR12,KIF21A,KIF15,PRDM10,CUL1,ZFYVE26,RERE,PSD3,MAP2,ANKMY1,BTAF1,GAREM1,DAW1,FBXO47,PEX6,ZNF618,FARP1,MOB1B,ATF2,NDUFAF6,GOLGA8B,HIRA,CYLD,UMODL1,BBS4,LRRC8B,MAPK8IP1,MX1,TMEM171,ZMAT4,CLVS2,PSG6,HIVEP3,COL5A1,GABBR2,PSIP1,ITGA9,KIAA0753,CFTR,MYEOV,KPNA1,CSE1L,NELL1,DOP1B,TBC1D13,UBASH3A,AHDC1,FAM214A,COL14A1,RGMB,NEU3,PHAF1,CEP44,MRPL13,KITLG,ZZEF1,DNAJC7,ATP10B,CAMTA1,UBR1,DCC,SMPDL3A,CHRM5,MAP4K3,YLPM1,SLC30A10,RCAN1,GTF2I,RORB,CHAF1A,TADA2A,DAB1,MED27,SELENON,RB1CC1,MYO3A,AKAP10,UBE2E1,PTPRE,REPS1,PRKN,AGMO,MTMR2,SH3PXD2A,ZFAND4,SPSB1,CDC42BPA,TBX20,SP110,CCDC102B,DLGAP2,AFAP1,MAPK10,DACH1,ZNF541,FBXO3,RWDD2B,DPF3,LGI2,LYST,NGEF,GRIN2A,ARID5B,H2BC15,JPH1,TXNRD2,ATXN1,WSB1,TRPM6,CDH23,PRKCH,PKP1,HUNK,TG,IL6R,FRMPD4,PEPD,ALS2,RACGAP1,NLRC5,ZNF627,OR51E1,ACO1,ANKRD30A,TFDP1,DHRS11,CNOT6L,MKNK1,HEMGN,KANK4,DOCK9,DMC1,FBLN5,LCE3B,KCNQ3,TOX,POLR1D,SHISA9,SLC4A4,PTPRB,ZFP90,TRMT61B,PDE6A,COPS8,TSPAN33,TBATA,ZNF124,SCN10A,LRBA,RBMX2,ANKRD55,SHANK2,ST8SIA1,ANKRD18A,MAP7,USP7,VAV3,PSMA1,MON2,LRRC37A3,TASOR2,PLAGL1,KCND3,HAAO,FAH,MESD,ITSN2,SOX30,PTGFRN,KIAA0825,SYBU,KIR2DL4,ARHGEF28,RALB,NPAS2,ADGRG6,YIPF6,KCNN3,MYO1D,SEC24D,PPA2,FAR1,CA1,ROCK1,LYN,VCAM1,SEL1L,ARHGAP31,CTSB,EIF2B3,LRIG1,TTC37,SUMO3,SLC15A2,ZNF169,PLEKHB2,KIF11,DTX1,BZW1,TENM2,OVOL2,PIWIL3,ZBTB33,ADA2,NTN1,CHKA,PLCB4,ZFHX3,FANCL,DPYSL5,SLC13A5,ZNF44,RRAGD,BANP,SUPT16H,ARID1B,HOXC13,CRACR2A,FAM81A,RNF152,BAZ1A,OTUD7A,INSR,CUL5,DMBT1,OR7A17,BMF,YTHDF3,TFF1,DEDD2,NEK6,HECTD1,GRID1,SHROOM3,XRCC4,COLQ,FAM118A,SLC52A1,HDAC11,NMU,LYPLAL1,DDHD1,PBX3,SUMO2,HS1BP3,DPYD,ARFGEF1,PDE4DIP,GAST,POGK,SNAI2,ASH1L,UBL3,IGHV3-74,HOXC4,BID,SIAH2,PIGK,OSBPL10,RPH3A,TANC2,ZBTB8OS,COX5A,TRABD2B,UFD1,RXRG,SP3,DRAM1,ERN2,FNDC1,MBTPS2,FLNB,TRIM58,TIAL1,TOM1,ELF2,IFI44,PLPP4,NREP,ZDHHC17,NSD2,FYCO1,CERS3,ESYT2,SH3GLB1,SLC22A14,CD9,CARD10,LTN1,KRT6B,RALGPS2,TWIST2,CTIF,SAMHD1,HSD17B14,IFT81,ENPP1,ENTPD5,UTRN,MOCS2,RASGRP1,IGSF11,SNX9,CDH26,DZANK1,PXDNL,UCK2,NDRG2,CSNK2A1,BMP5,PWWP3A,WDR72,KCNC1,CSF1,GHRH,PPIL6,POTEB3,EOGT,CCDC34,HDGFL3,NUP37,BCL2L1,SERPINB9,SCAF4,KRT25,CTDP1,HCN1,PRKG1,LAMA3,ASB4,GRIN2B,ST13,ANKRD66,GRB14,TMEM71,INO80,FANCB,IGHV2-70D,CLNS1A,CNMD,KIF21B,SMAD5,CELF4,SYNJ2,TCERG1,ABCG1,FOXN3,KCNK5,DCUN1D4,PLXDC2,VSTM4,SLC40A1,PRAME,HADHA,MYCL,TNN,FAM149B1,CABYR,CIDEC,KLHL7,PSAP,PSMA5,CFHR4,MICALL2,MED1,IPCEF1,POTEB2,ATG4B,CDC14B,PCNT,KDM6A,ATRN,IL33,AJAP1,GPRC5C,TLNRD1,ROR2,CFH,PPP2R

			2A,ZNF521,NPL,KL,BANK1,CSDE1,FAT1,HGD,OTOG,LMX1A,IL10,ACTR2,SFPQ,SCML2,CALN1,RIOK1,CLSTN2,TTC39C,PTH,SOSTDC1,TOP3B,PRKAA2,CSF2RB,DIRAS2,SKA1,NDC80,GOLGA8G,RNF182,SOHLH1,LARP6,PACRG,ERO1B,PHF20L1,ITPRIP,VSTM2A,FAM204A,MAP6,VASP,ETV6,TACC2,SCFD2,SHISAL1,PALMD,SNRPC,KIFC1,IQGAP1,RPS12,PRB3,CAMLG,ZBTB7C,TEAD1,MORC2,SREBF2,ANP32B,YBX3,AIMP1,LASP1,THNSL2,FYB2,NRXN1,EPHX4,PCID2,HIPK1,ENTHD1,CISD1,CIB4,SNAP91,CD70,CYP4F22,CIBAR1,PBLD,FICD,ERICH5,CACYBP,CADM1,CENPE,PEG10,LMX1B,NET1,SIPA1L2,TUBB6,NGDN,ELOC,ANLN,TWIST1,AKT3,ALKAL2,JAK2,ADAM28,VSX1,RPF2,FSTL1,CHCHD6,ZBTB38,MPPE1,ISX,MADD,PTGS1,PATL1,ZNF449,PRSS2,FH,TDP1,CREBBP,MELTF,MRM1,TNKS,ARL11,SGO1,GORAB,PCNA,SIAH3,TRPV5,UFL1,ADAMTS5,NFKBIA,PRKCB,ANKRD24,FBXW2,CFAP299,NTM,KIF6,ABCC8,ANXA4,MT1HL1,ZC3H15,ANP32A,OTULINL,RFC2,SMTN,ST6GAL2,ALX4,RTRAF,USH1C,BRD4,ZBTB21,SERBP1,NEDD9,OLFM4,NRBP1,ITGA6,ATP2B1,GAP43,SLC14A2,DGLUCY,ASS1,MTCL1,GRIP1,IGHV1OR15-9,CTNNBL1,GTSF1L,SAR1A,EML1,CNIH1,MAST2,HPSE2,BTG3,ERLIN2,GOLGA8J,TRAPPC3,MAPK1IP1L,UBAP2,ADAMTSL3,EFHD2,CIDEA,PCMTD2,ZBTB49,BBS9,EXT2,EXOC1,KRT6A,AGO1,FRA10AC1,DIPK1A,MEOX2,SLC6A1,GID8,ELL2,GRXCR1,SDS,LINGO1,SNAPC3,STAT1,ZCCHC14,BRMS1L,FAM189A2,NDFIP2,NR2C1,MAP2K6,S100PBP,CMTM7,VAT1L,ERICH3,SHROOM2,KCNJ18,MARCHF6,MTPN,ABI1,MYO18B,NECTIN4,ARMC6,CEMIP,POU6F2,IMPACT,CBLIF,CCBE1,SLX4IP,PARK7,MAPK8,ITGA4,TOP3A,OAZ2,EIF3F,PPME1,MED12L,ZSCAN30,FBXL17,UBL7,POU1F1,UBE2J2,ADCYAP1R1,MTF2,CSMD1,NCAPG2,TM9SF4,RAPGEF4,SCGB1D1,FOXP2,ASB2,MYOCD,HMCN1,CEP120,MYH13,DHTKD1,CYFIP2,UBE2QL1,HNRNPM,ACACA,KRT85,ASCC2,ARL4C,EFHB,ARID3B,MEF2C,STOML1,ZNF613,ADGRB1,RXRA,WNT7A,RBPMS2,ECHDC1,OXNAD1,MAP3K5,NDFIP1,IKBIP,MAP3K4,TRIM43B,WASF3,S100B,SERPINI2,PRDM13,TRIM43,FOXO6,ERI1,SUMF1,CD82,ATP6V1C2,C9ORF43,CHAMP1,C16ORF72,BTF3L4,MAGEL2,PKN2,RAD51AP1,SLC10A6,FAM25C,PDE2A,RAB38,LRRC2,KRTAP21-2,SFI1,DBF4B,FBXW8,SDCBP,NECTIN1,DSG1,JPT2,SPPL2B,NSMCE1,C3ORF52,COMMD8,ERICH1,WWOX,ZBTB25,FAM72A,PASK,MLLT1,MS4A4A,NCK1,FLVCR1,SCAF8,FGR,CWC22,CCDC106,DRC7,CDCA8,PPP2R3A,DNMBP,TRIM23,ATP6V1B2,CXCL2,TOP1,FAM72B,SNAP29,FAM72D,MLLT10,C2,IFNAR1,RNF8,GNG12,LCE3D,KLHL29,EPHA4,PPIP5K2,TEX29,CYTH4,EMP1,INTS13,GABRA5,KIAA0319L,MECOM,DNMT3L,NTRK2,ANKRD20A1,IL1RAPL1,FNDC3A,RSPH1,KHDC4,NUMB,LHX9,WNT2B,COLEC12,ZBTB10,TNNI1,PLEKHA3,OCLN,CCDC152,POSTN,FAM110A,CREB5,SNRPD1,SHISA6,MEGF10,FBXO31,EXTL3,AKAP11,TRPM7,KTN1,KRTAP26-1,PRKAB1,DTHD1,IREB2,MVB12B,HS6ST1,PTK2,ERP27,MARK4,CDH5,ANKRD6,APOL2,SCGN,NFKBID,ARHGAP12,CLDN18,ASCL3,MPP7,DIAPH1,FBXO41,FEZ2,INIP,LAMB1,SCAMP1,APIP,CYFIP1,UBE3A,TMEM54,SCG3,APOL1,SEMA4D,JAM2,DNAH10,PITPNC1,FRMD6,MC2R,ZBTB20,FAT4,IMPA2,FAM102A,LRMDA,AP2B1,RUNX1,AKR1B1,C9,KIRREL1,WNT5B,RASGEF1B,AMFR,SAXO1,SCARA5,CTSE,NENF,SH2D1B,HEATR5A,PSTPIP2,ZFYVE1,SANBR,ASAP1,SAMD13,ICA1,EDIL3,NOS1AP,MTTP,TPTE,SORBS2,PDCL3,SRP9,CNKSR1,CCDC88A,UBAP1L,CHCHD2,SPAG6,SLC5A1,MDN1,CDC45,BICD1,TNFSF11,FYN,BUB1,KDM5A,PCBP3,MYL12B,NLRP14,PPM1F,GOLGA8F,UNC45B,ARL13B,XPO7,SDE2,UHRF2,SCN8A,HDAC2,SNTB1,AVEN,SLF1,SACM1L,TBX15,SH2D3C,PSME3IP1,DOCK3,TRNAU1AP,NCS1,COL18A1,CDH9,LHFPL2,LYSMD2,ATP5PF,ALB,DOK5,NALCN,UGP2,MTMR7,EHBP1,ZFYVE28,MAPK9,RSPH14,PABPC1,CRTAM,APELA,MDGA2,STT3A,DEFB108B,ROR1,SLC16A9,GALNT2,FUT8,ARNT2,ASB3,HECW2,POTEJ,CDH2,CNTN5,ITGA8,SEL1L2,FBXL20,NTN4,RAD9A,XRN2,PHLPP1,PLEKHA2,GPR137B,EPHB1,GRM5,RAI14,SPOPL,ZNF705D,RPS6KA5,SPTB,TBC1D1,LRRC69,PTPRG,ANKRD36C,PID1,NRP

			1,FCHSD2,SDK1,PRKCA,GBP4,IFT46,NLRP4,ANKRD36B,ATPSCKMT,SPHKAP,FAIM,SAMD12,USP24,FAAP24,MOGAT3,FHIT,ITGA1,PCCA,CROT,KLF12,RNF138,RC3H1,NRIP1,CHODL,POR,SLC14A1,MCC,GOLGA8S,SUPT3H,BCR,TUT4,NRXN3,ELMO1,RGS6,RERG,NLRP8,TCERG1L,KIF16B,CDH12,PRIM2,ARMC3,MIPOL1,C14ORF39,ARFGAP3,SENP8,USP49,ELP2,CFAP70,FBLN1,STK36,NSG2,PAQR5,RAG1,KCNJ6,B9D1,ZMYM1,DGCR2,DNPEP,RRAS2,GNA14,BMPER,RABL2A,KIAA1328,PRDM15,SRGAP3,SLC35F1,ZNF420,MACROH2A1,MITF,NBPF1,EPHB2,TOGARAM1,CSNK1G1,SACS,BCL2L13,RNF11,SGCG,CD38,EYA4,CHCHD3,MYO5B,RGPD4,PPIL2,CDK14,RSRP1,AKAIN1,MET,MUC16,SPPL3,DLG2,CDH17,COMMD10,ATP6V0D2,SPECC1,CAMK1G,IBA57,METTL15,PPFIA2,CDH13,STXBP4,POTED,MAB21L3,KRTAP19-7,CACNG3,ATG5,USP32,NRAP,MAGI2,KIAA1217,PRDM11,VMP1,FAM171A1,MLIP,FLRT2,MYB,KALRN,ZNF704,SLC1A2,GNAS,LAMA1,MFHAS1,CPQ,NUP43,TRIM9,ATRNL1,TIAM2,DHX29,BMP7,TTC28,TMPRSS15,ASTN2,DLG5,TNFAIP8,ZMYND8,GAPVD1,RNF217,KIRREL3,PRSS23,KCTD1,GOLGA6A,DNAH3,BPTF,BTBD10,CCSER1,AK3,ZMYND11,TMEM25,NUDT21,TRAPPC10,GRM3,KMT2C,DDX6,ADGRF5,UPRT,PDGFC,WDR41,DNAH17,PLIN2,PPP1R13B,ELOVL7,FOCAD,EPB41L4A,ABL2,TRAPPC6B,BACE2,RFX2,PARPBP,NECAB1,PKNOX2,EYA1,FHOD3,PDZD2,TTLL11,GOLGA8T,PRPF18,SLIT2,CMPK1,TMPRSS3,EXOC4,CNOT7,FAM126A,KCNIP4,ESCO1,KCTD8,CCDC141,PLCL1,ERBB4,ANKRD30B,IL20RB,FAM3B,FAM126B,GSAP,SYNDIG1,ROBO1,SAMD4A,PBX1,SPATS2L,IRAG1,NPAS3,NUF2,PRKCQ,RGPD2,IPP,SAMM50,ANTXR1,NDRG1,MYH15,SORCS2,SIPA1L3,TRDN,ZNF679,NLGN1,SYNPR,CTTNBP2,SHLD2,NOS1,SLC6A3,GLDC,CHD9,PRR16,ASIC2,TXNDC16,EFNA5,TCF12,LRRC9,GAS2L1,ARHGEF11,MTREX,VCAN,RAB27A,NSD1,EHMT1,SLIT3,DTNA,KIF13A,AP5M1,FRMD5,ESR1,DNAH9,SLC25A48,MYO9B,NTNG1,CYP2C8,KCNQ5,LOXL2,NYAP2,IGLC3,ANKRD36,C16ORF74,CEP57L1,IQCJ-SCHIP1,MPDZ,FAM153A,IRAG2,ADGRG7,ORC4,SKAP2,PRLR,AGO3,HTT,FOXB1,RAD51B,CAMK1D,PIK3R3,SLC25A18,CFAP44,POTEC,CDKAL1,EML6,OPCML,AK2,HLA-F,FER,ZNF302,EVA1A,EYA2,CCR2,RPGRIP1,STARD13,PITPNM3,SNTB2,WDR64,INTS12,A2M,WDFY3,CHFR,PCMT1,EPS8,OSBPL6,JAZF1,ZNF578,OARD1,SPOCK3,SEMA4B,NRF1,IGHV1OR21-1,ANO2,PHC2,GRIA4,AGAP1,ROCK2,PRDM1,RORA,STMP1,IL16,TERB2,DMRT1,EIF4G3,CDCA5,PPP1CB,CATSPER2,RGS8,RAB31,PDK1,HSPG2,PSMD2,PTPRQ,HERPUD1,NCOA6,TRIM2,COL4A3,WASHC1,RGS7,HOOK3,KIF7,GNG2,PCSK2,FSTL4,CLDN10,BARD1,PALD1,CLCN5,HSPA12A,STK3,DEPTOR,ZNF423,SLC13A4,C1QL3,RSU1,HNRNPU,VTI1A,CEP72,RAB3GAP2,TULP4,CADPS,APCDD1,IGF1R,KCNAB1,PRKAG2,GLI2,ANKRD10,MFSD11,APMAP,IQCM,THRB,LSAMP,AKAP13,MORC3,ATP10A,SEPTIN6,DNM1L
GO:0043167	ion binding	9.230719696092971e-26	NOTCH2,MTOR,CACNA2D3,SPOCK1,EXOC1L,ABCA13,FREM1,BNC2,MICU2,SMOC1,MYO9A,ULK2,NLK,LONP2,UNC13C,SCAPER,FTO,KSR1,AGBL1,ZNF236,PLCB1,ZNF536,TTC3,MX2,LIPI,SVIL,ZFPM2,ARL15,MICAL3,NUBPL,L3MBTL4,ITPR2,PDE4D,RALA,NME7,EPS15L1,MYO5A,KCNMA1,PRDM16,FBN1,LPCAT2,F13A1,GPHN,CDH8,CHRNA7,PUDP,RIMS1,PIK3C3,ZEB1,AKR1C3,RARB,FGD4,GALNT1,NAV2,ENPEP,MYO1E,RIMS2,ALK,ADGRE1,PCDH7,MCTP1,PJA2,PAPPA2,GLIS3,ERCC6L2,HLCS,FCHO2,ANO6,ZNF880,EGLN3,MAP3K9,MYO3B,MOCOS,SPON1,CPA6,ZMYM4,ZNF595,TSHZ3,MYO5C,SETD2,ZNF573,TNIK,EFCAB2,KDM4C,NEK4,TSHZ2,EGFR,ZNF280B,CDK12,MACF1,PRKACB,NEK7,RNF220,HMCN2,ZNF407,MYOF,GNPTAB,CRB1,NSMCE2,BCL11A,CHSY1,CDH4,ATP2B2,NTRK3,LARGE1,RXFP1,PDE1C,ZFAND6,CYP2C9,FLT1,ZNF648,ADAMTS6,ZNF382,GK,THRAP3,AOAH,DGKI,SLC8A3,NELL2,PRKD1,PAK1,GMDS,EPHA7,CHRM3,ADSS2,GRAMD1B,CHSY3,RAPGEF2,LRP2,RUNX2,ARSB,CPS1,TAOK3,CPEB4,AGK,PRICKLE2,RANBP17,UBE2L3,SYN2,SMYD3,TYW1,HERC2,LRGUK,GRM7,SEPTIN9,DNAH6,KIF4A,DHX32,ADAM10,HDAC9,ZHX3,UBE2G1,APP,ABCB5,ADK,RPS6KA2,KYNU,CACNA1C,KDM1B,DCLK1,ZNF600,SYT1,ACER2,PARP15,ZNF723,AURKA,PARN,ST18,PY

			GO1,SLC8A1,MARCHF1,ABCG8,MAP4K4,HIVEP2,ABCD2,BMPR1B,ZNF717,RAB8B,PDZRN4,PAK3,PDE1A,ZNF257,TTLL7,RANBP2,ITPKB,TRPC5,PDE10A,UBE2E2,HHAT,RNLS,DNM3,CUBN,SCP2,SYN3,PHF21B,PRKCZ,MAN2A2,RYR3,TAF15,MSH6,RAB27B,COL27A1,ZSWIM6,FER1L6,MBNL2,ADAMTS17,ABCA5,PHF19,GALNT14,PDXDC1,EBF2,PPM1L,RIPK4,ZKSCAN5,MAPK1,MGAT5,CADPS2,KCNJ1,ABCD3,DNAH14,TRPC7,KMT2E,PAPPA,PCGF5,SYT10,ZNRF3,DNAJC21,CA5A,XXYLT1,ABLIM1,UBE2O,ANKFY1,RNF17,GALNT16,PI4K2B,RNGTT,EWSR1,FAT3,MICU1,ZNF735,CHD6,STK38,CHN1,MYLK3,ACSBG1,LIMCH1,MBNL1,EFEMP1,TLL1,ZNF684,DCAF1,ZFAND3,TLK1,HIVEP1,EFCAB8,CDH7,MOB3B,KLF15,PPARA,PPIP5K1,ERMP1,NR5A2,ADAMTS3,ARAP2,PAK5,TRERF1,PCDH11Y,PDZRN3,DAPK1,NAV3,ACSM2B,AGO2,STK32B,PHC3,MAGI1,ALPK2,DNAH11,TBC1D9,RAB22A,GATAD2B,CPE,DYSF,ADGRV1,ZNF846,MELK,RYR2,ZNF606,CLPX,AIF1L,SMARCA4,CDH11,LDB3,FABP7,PARD3,MAPKAP1,EFTUD2,PIAS1,UBE2R2,BLK,EBF1,OLA1,DST,ATRX,NUAK1,XIRP2,ABL1,AGPS,HDAC4,SLC1A1,PRKAA1,CRTAC1,DROSHA,TTLL5,L3MBTL3,APLF,ADAMTS14,MAST4,DNAH5,GUCY1A2,CDH18,SLFN11,RAP1A,GLIS1,ACSS3,MORC1,MYO10,ZNHIT6,CAMK4,BAZ2A,PLEKHA8,CPSF3,ZC3HAV1,CDHR3,TGM1,PEAK1,LATS2,CLIP1,EFCAB6,PAH,ATP11C,ZNF438,DHX40,ABCB7,ZBTB16,MUSK,GALNTL6,ZNF675,ACTR3C,SMARCAD1,ABCC12,SETDB2,PRKCE,FOXK2,PGM5,ASAP2,METAP1D,WNK2,ESRRG,ZNF718,DGKB,USP33,FBN2,ZNF831,EGF,ALPK3,ABCC9,P2RX6,TRIO,PDE3A,EXT1,STXBP6,NLRP13,LNPEP,LIMD1,ADAMTS2,RPS6KA3,MARCHF8,ATP8A2,SCG5,MTMR3,TRIM5,RFC1,HTR2C,ATP8A1,ZCCHC7,LTBP1,STK38L,ZFYVE9,GALNT10,KDM7A,ABCC4,PRMT8,HTR2A,KIAA0232,FANCM,CYBRD1,CYP4A11,MATN2,FARS2,GTF2F2,STAC,TAF3,MARK2,GMPR,EBF3,ALPL,ZNF33B,LPP,FHL2,MSH2,MPPED2,GNAL,EPHA6,ZNF397,ATL1,HIPK3,KCND2,ABCA10,GRK3,CPXM2,NOS2,AFG3L2,CPNE4,STK10,MNAT1,ADAM12,MYLK2,XYLT1,GBP6,BCL11B,VPS41,FOLH1,F5,ECE1,ZIM3,STK32A,AK8,TRPS1,TMEM163,PLCE1,HIP1,XPNPEP1,GSR,PCDH9,VAV1,CYP4Z1,CDH20,RUFY2,MYT1L,ZNF160,EGFLAM,PACSIN2,TARS3,MTHFD1L,SNX3,ZNF367,PDLIM5,CEPT1,AQR,ZBTB2,GALNT13,EXD3,DNER,BLM,NRK,MAGI3,LIN54,LRP1B,ADCY10,ZNF121,RC3H2,MYLK4,ATP9A,CDC42BPB,DSE,VRK1,GNAI1,ZC3H14,GFI1B,MYRIP,HPCAL1,BMP2K,RNF38,ASXL3,PDE6C,POLR3A,RELN,GNAQ,ZNF106,ZNF567,CLVS1,TRAF3,ZNF462,UNC13B,ZNF875,DSTYK,UIMC1,B4GALT6,PFKFB4,PLS1,IKZF2,SEC23B,NIN,DNAH8,TRIT1,GADL1,SMARCA2,ARSJ,GLI3,CGAS,SMOC2,PCP4,IDE,MCTP2,KIF21A,KIF15,PRDM10,RERGL,ZFYVE26,ZNF431,RERE,ANKMY1,BTAF1,MYL1,PEX6,ZNF618,NEK10,MOB1B,ATF2,CYLD,UMODL1,ADARB2,MX1,ZMAT4,CLVS2,ANTXRL,HIVEP3,COL5A1,ITGA9,CFTR,NELL1,ME2,ZZEF1,ATP10B,UBR1,MYT1,SMPDL3A,MAP4K3,YPEL1,RORB,TADA2A,ZNF208,SELENON,MYO3A,UBE2E1,REPS1,PRKN,AGMO,ZNF608,SH3PXD2A,ZFAND4,CDC42BPA,SP110,MAPK10,PCDH15,ZNF541,DPF3,HEPHL1,GRIN2A,TXNRD2,TRPM6,CDH23,LALBA,PRKCH,HUNK,FRMPD4,PEPD,RACGAP1,NLRC5,ZNF627,ACO1,CNOT6L,MKNK1,DMC1,FBLN5,ZFP90,AOPEP,PDE6A,ZNF124,VAV3,ENPP3,PLAGL1,KCND3,HAAO,FAH,ITSN2,MOK,ARHGEF28,RALB,NPAS2,MYO1D,SEC24D,PPA2,CA1,ROCK1,LYN,ZNF780B,ZNF169,PLEKHB2,KIF11,DTX1,TENM2,OVOL2,ZBTB33,ADA2,CHKA,PLCB4,MMP16,PRUNE2,ZFHX3,FANCL,ZNF44,RRAGD,CRACR2A,RNF152,BAZ1A,CASZ1,OTUD7A,INSR,NEK6,HECTD1,DDHD1,ZNF292,ADAMTS19,DPYD,SNAI2,ASH1L,SIAH2,RPH3A,ZBTB8OS,COX5A,ABCA4,TRABD2B,RXRG,SP3,ERN2,ZNF879,MBTPS2,TRIM58,ZNF804B,NSD2,FYCO1,ESYT2,LTN1,SAMHD1,ENPP1,ENTPD5,UTRN,RASGRP1,PAMR1,CDH26,DZANK1,PXDNL,UCK2,CSNK2A1,ZNF385D,HCN1,PRKG1,GRIN2B,INO80,ETNPPL,KIF21B,SMAD5,ABCG1,MARCHF11,SLC40A1,HADHA,CABYR,PSAP,MICALL2,KDM6A,ROR2,ZNF521,FAT1,HGD,LMX1A,ACTR2,CALN1,RIOK1,CLSTN2,SDF4,PRKAA2,DIRAS2,RNF182,ERO1B,PHF20L1,PLA2G4A,RAB12,SNRPC,KIFC1,IQGAP1,ZBTB7C,MORC2,LASP1,THNSL2,NRXN1,HIPK1,ZNF234,CISD1,CIB4,

			ZNF518A,DGKK,SNAP91,CYP4F22,FICD,CENPE,PEG10,LMX1B,TUBB6,AKT3,JAK2,ADAM28,FSTL1,ZBTB38,MPPE1,BPNT1,SVEP1,PTGS1,ZNF287,CELSR2,ZNF449,PRSS2,CREBBP,MELTF,TNKS,ARL11,SIAH3,TRPV5,ADAMTS5,PRKCB,GOT2,KIF6,ABCC8,MIPEP,PCDH11X,ANXA4,OVCH1,MT1HL1,CACNA1E,ZC3H15,RFC2,ZNF354C,ZBTB21,SMPD4,NRBP1,ITGA6,ATP2B1,GAP43,IARS2,CLCA4,ASS1,CNDP2,GTSF1L,AGAP9,ADGRE3,SAR1A,ADCY9,EML1,MAST2,ZNF528,ZNF611,EFHD2,ZBTB49,EXT2,EXOC1,PDP2,SLC6A1,SDS,ZCCHC14,NR2C1,MAP2K6,DGKG,VAT1L,MARCHF6,GATAD1,MYO18B,CCBE1,PARK7,ADAMTS18,MAPK8,ITGA4,TOP3A,ZSCAN30,UBE2J2,PLA2G12B,MTF2,RAPGEF4,FOXP2,HFM1,HMCN1,MYH13,ATP13A3,DHTKD1,ZSCAN5C,UBE2QL1,ACACA,ST8SIA4,ARL4C,EFHB,ZNF613,RXRA,MAP3K5,MAP3K4,TRIM43B,S100B,PRDM13,TRIM43,ERI1,SUMF1,EFCAB14,ZNF112,CHAMP1,PKN2,PDE2A,RAB38,DBF4B,SDCBP,DSG1,NSMCE1,ZNF813,ZBTB25,PASK,FGR,PPP2R3A,TRIM23,ATP6V1B2,TOP1,MLLT10,C2,RNF8,EPHA4,PPIP5K2,MECOM,DNMT3L,NTRK2,ACSM2A,LHX9,ADAMTS9,COLEC12,FRRS1,ZBTB10,TNNI1,PLEKHA3,POSTN,CREB5,EXTL3,TRPM7,IREB2,PTK2,MARK4,CDH5,TPH2,SCGN,APIP,SUSD1,UBE3A,PCDH8,DNAH10,PITPNC1,ZBTB20,FAT4,IMPA2,ZNF66,RUNX1,AMFR,NENF,POMT2,ZNF845,ZFYVE1,ASAP1,PLCZ1,EDIL3,DIDO1,GALNT18,HKDC1,ADAMTS16,ACOXL,MDN1,FYN,BUB1,KDM5A,MYL12B,NLRP14,ZNF705G,PPM1F,ARL13B,UHRF2,SCN8A,NCS1,COL18A1,GALNT17,CDH9,ALB,ATP9B,UGP2,ZFYVE28,MAPK9,STT3A,ROR1,GALNT2,TET1,CDH2,ITGA8,XRN2,PHLPP1,PLEKHA2,EPHB1,EYS,DDX10,ADCK1,ZNF705D,RPS6KA5,NRP1,FCHSD2,PRKCA,GBP4,NLRP4,RNF215,ITGA1,ZNF615,PCCA,KLF12,RNF138,RC3H1,POR,ZNF850,ZNF235,ABCA6,EFL1,MCC,ZNF738,BCR,TUT4,NRXN3,RERG,ZNF215,NLRP8,KIF16B,CDH12,PRIM2,SNRK,ARFGAP3,USP49,PGM2L1,FBLN1,STK36,MB,RAG1,ZMYM1,DNPEP,CYP4B1,RRAS2,GNA14,ZNF678,RABL2A,PRDM15,ZNF420,EPHB2,CSNK1G1,RNF11,EYA4,DPH6,MYO5B,CDK14,MET,CDH17,ZNF705B,CAMK1G,CDH13,USP32,NRAP,UNK,AIFM3,KALRN,ME3,ZNF704,SLC1A2,GNAS,MFHAS1,CA10,CPQ,TRIM9,DHX29,ASTN2,ZMYND8,RNF217,DNAH3,ZNF74,BPTF,AK3,ZMYND11,KMT2C,DDX6,UPRT,DNAH17,ABL2,MMP26,NECAB1,EYA1,TTLL11,SLIT2,CMPK1,CNOT7,KCNIP4,ESCO1,PLCL1,ERBB4,TRHDE,PRKCQ,ANTXR1,MYH15,MGMT,ZNF679,AK9,NOS1,SLC6A3,GLDC,CHD9,MTREX,VCAN,RAB27A,NSD1,EHMT1,SLIT3,DTNA,KIF13A,ESR1,DNAH9,MYO9B,KDM4B,CYP2C8,LOXL2,CACNA2D1,ORC4,PRLR,AGO3,LARS2,RAD51B,CAMK1D,CDKAL1,AK2,FER,ZNF302,EYA2,PITPNM3,INTS12,WDFY3,CHFR,ZNF721,JAZF1,ZNF578,ZNF891,SPOCK3,ZNF14,PHC2,AGAP1,ROCK2,PRDM1,RORA,NARS2,DMRT1,PPP1CB,RAB31,PDK1,HSPG2,TRIM2,ZFP30,KIF7,FSTL4,BARD1,CLCN5,PNPLA3,HSPA12A,STK3,ZNF423,PNPLA8,ZNF568,HNRNPU,CADPS,IGF1R,KCNAB1,PRKAG2,GLI2,THRB,AKAP13,MORC3,ATP10A,SEPTIN6,DNM1L
GO:0046872	metal ion binding	2.0254286122048157e-18	*NOTCH2,CACNA2D3,SPOCK1,FREM1,BNC2,MICU2,SMOC1,MYO9A,NLK,UNC13C,SCAPER,FTO,KSR1,AGBL1,ZNF236,PLCB1,ZNF536,TTC3,LIPI,ZFPM2,MICAL3,NUBPL,L3MBTL4,ITPR2,PDE4D,NME7,EPS15L1,KCNMA1,PRDM16,FBN1,LPCAT2,F13A1,GPHN,CDH8,PUDP,RIMS1,ZEB1,RARB,FGD4,GALNT1,ENPEP,RIMS2,ADGRE1,PCDH7,MCTP1,PJA2,PAPPA2,GLIS3,ANO6,ZNF880,EGLN3,MOCOS,SPON1,CPA6,ZMYM4,ZNF595,TSHZ3,SETD2,ZNF573,EFCAB2,KDM4C,NEK4,TSHZ2,ZNF280B,MACF1,PRKACB,NEK7,RNF220,HMCN2,ZNF407,MYOF,GNPTAB,CRB1,NSMCE2,BCL11A,CHSY1,CDH4,ATP2B2,LARGE1,RXFP1,PDE1C,ZFAND6,CYP2C9,ZNF648,ADAMTS6,ZNF382,AOAH,DGKI,SLC8A3,NELL2,PRKD1,ADSS2,CHSY3,RAPGEF2,LRP2,ARSB,CPS1,CPEB4,PRICKLE2,SMYD3,TYW1,HERC2,GRM7,KIF4A,ADAM10,HDAC9,ZHX3,APP,ADK,RPS6KA2,CACNA1C,KDM1B,ZNF600,SYT1,ACER2,ZNF723,PARN,ST18,PYGO1,SLC8A1,MARCHF1,ABCG8,HIVEP2,BMPR1B,ZNF717,PDZRN4,PAK3,PDE1A,ZNF257,TTLL7,RANBP2,PDE10A,CUBN,PHF21B,PRKCZ,MAN2A2,RYR3,TAF15,MSH6,COL27A1,ZSWIM6,FER1L6,MBNL2,ADAMTS17,PHF19,GALNT14,EBF2,PPM1L,*

			ZKSCAN5,MGAT5,CADPS2,KMT2E,PAPPA,PCGF5,SYT10,ZNRF3,DNAJC21,CA5A,XXYLT1,ABLIM1,ANKFY1,RNF17,GALNT16,EWSR1,FAT3,MICU1,ZNF735,STK38,CHN1,LIMCH1,MBNL1,EFEMP1,TLL1,ZNF684,ZFAND3,HIVEP1,EFCAB8,CDH7,MOB3B,KLF15,PPARA,ERMP1,NR5A2,ADAMTS3,ARAP2,TRERF1,PCDH11Y,PDZRN3,ACSM2B,AGO2,STK32B,PHC3,TBC1D9,GATAD2B,CPE,DYSF,ADGRV1,ZNF846,MELK,RYR2,ZNF606,CLPX,AIF1L,CDH11,LDB3,PIAS1,EBF1,OLA1,DST,ATRX,NUAK1,XIRP2,ABL1,HDAC4,SLC1A1,PRKAA1,CRTAC1,DROSHA,TTLL5,L3MBTL3,APLF,ADAMTS14,MAST4,CDH18,GLIS1,MORC1,ZNHIT6,BAZ2A,CPSF3,ZC3HAV1,CDHR3,TGM1,LATS2,CLIP1,EFCAB6,PAH,ATP11C,ZNF438,ZBTB16,MUSK,GALNTL6,ZNF675,SETDB2,PRKCE,FOXK2,PGM5,ASAP2,METAP1D,ESRRG,ZNF718,DGKB,USP33,FBN2,ZNF831,EGF,PDE3A,EXT1,LNPEP,LIMD1,ADAMTS2,RPS6KA3,MARCHF8,ATP8A2,MTMR3,TRIM5,ATP8A1,ZCCHC7,LTBP1,STK38L,ZFYVE9,GALNT10,KDM7A,CYBRD1,CYP4A11,MATN2,STAC,TAF3,MARK2,GMPR,EBF3,ALPL,ZNF33B,LPP,FHL2,MSH2,MPPED2,GNAL,ZNF397,KCND2,CPXM2,NOS2,AFG3L2,CPNE4,MNAT1,ADAM12,XYLT1,BCL11B,VPS41,FOLH1,F5,ECE1,ZIM3,STK32A,TRPS1,TMEM163,PLCE1,XPNPEP1,PCDH9,VAV1,CYP4Z1,CDH20,RUFY2,MYT1L,ZNF160,EGFLAM,ZNF367,PDLIM5,CEPT1,ZBTB2,GALNT13,EXD3,DNER,BLM,LIN54,LRP1B,ADCY10,ZNF121,RC3H2,ATP9A,CDC42BPB,DSE,GNAI1,ZC3H14,GFI1B,MYRIP,HPCAL1,RNF38,ASXL3,PDE6C,POLR3A,RELN,GNAQ,ZNF106,ZNF567,TRAF3,ZNF462,UNC13B,ZNF875,UIMC1,B4GALT6,PLS1,IKZF2,SEC23B,NIN,TRIT1,ARSJ,GLI3,CGAS,SMOC2,PCP4,IDE,MCTP2,PRDM10,ZFYVE26,ZNF431,RERE,ANKMY1,MYL1,ZNF618,NEK10,MOB1B,ATF2,CYLD,UMODL1,ADARB2,ZMAT4,ANTXRL,HIVEP3,COL5A1,ITGA9,NELL1,ME2,ZZEF1,ATP10B,UBR1,MYT1,SMPDL3A,YPEL1,RORB,TADA2A,ZNF208,SELENON,REPS1,PRKN,AGMO,ZNF608,ZFAND4,CDC42BPA,SP110,PCDH15,ZNF541,DPF3,HEPHL1,GRIN2A,TRPM6,CDH23,LALBA,PRKCH,PEPD,RACGAP1,ZNF627,ACO1,CNOT6L,MKNK1,FBLN5,ZFP90,AOPEP,PDE6A,ZNF124,VAV3,ENPP3,PLAGL1,KCND3,HAAO,FAH,ITSN2,MOK,ARHGEF28,NPAS2,SEC24D,PPA2,CA1,ROCK1,ZNF780B,ZNF169,DTX1,TENM2,OVOL2,ZBTB33,ADA2,PLCB4,MMP16,PRUNE2,ZFHX3,FANCL,ZNF44,CRACR2A,RNF152,BAZ1A,CASZ1,OTUD7A,NEK6,HECTD1,DDHD1,ZNF292,ADAMTS19,DPYD,SNAI2,ASH1L,SIAH2,RPH3A,ZBTB8OS,COX5A,TRABD2B,RXRG,SP3,ERN2,ZNF879,MBTPS2,TRIM58,ZNF804B,NSD2,FYCO1,ESYT2,LTN1,SAMHD1,ENPP1,UTRN,RASGRP1,PAMR1,CDH26,DZANK1,PXDNL,ZNF385D,GRIN2B,SMAD5,MARCHF11,SLC40A1,CABYR,MICALL2,KDM6A,ROR2,ZNF521,FAT1,HGD,LMX1A,CALN1,RIOK1,CLSTN2,SDF4,PRKAA2,RNF182,PHF20L1,PLA2G4A,SNRPC,IQGAP1,ZBTB7C,MORC2,LASP1,NRXN1,ZNF234,CISD1,CIB4,ZNF518A,DGKK,CYP4F22,PEG10,LMX1B,JAK2,ADAM28,FSTL1,ZBTB38,MPPE1,BPNT1,SVEP1,PTGS1,ZNF287,CELSR2,ZNF449,PRSS2,CREBBP,MELTF,TNKS,SIAH3,TRPV5,ADAMTS5,PRKCB,MIPEP,PCDH11X,ANXA4,OVCH1,MT1HL1,CACNA1E,ZC3H15,ZNF354C,ZBTB21,SMPD4,ITGA6,ATP2B1,CLCA4,CNDP2,GTSF1L,AGAP9,ADGRE3,ADCY9,EML1,MAST2,ZNF528,ZNF611,EFHD2,ZBTB49,EXT2,PDP2,SLC6A1,ZCCHC14,NR2C1,DGKG,VAT1L,MARCHF6,GATAD1,CCBE1,PARK7,ADAMTS18,ITGA4,TOP3A,ZSCAN30,PLA2G12B,MTF2,FOXP2,HMCN1,ATP13A3,ZSCAN5C,ACACA,EFHB,ZNF613,RXRA,MAP3K5,MAP3K4,TRIM43B,S100B,PRDM13,TRIM43,ERI1,SUMF1,EFCAB14,ZNF112,CHAMP1,PDE2A,DBF4B,DSG1,NSMCE1,ZNF813,ZBTB25,PPP2R3A,TRIM23,MLLT10,C2,RNF8,MECOM,DNMT3L,ACSM2A,LHX9,ADAMTS9,COLEC12,FRRS1,ZBTB10,TNNI1,POSTN,CREB5,EXTL3,TRPM7,IREB2,CDH5,TPH2,SCGN,APIP,SUSD1,UBE3A,PCDH8,ZBTB20,FAT4,IMPA2,ZNF66,RUNX1,AMFR,NENF,POMT2,ZNF845,ZFYVE1,ASAP1,PLCZ1,EDIL3,DIDO1,GALNT18,ADAMTS16,FYN,KDM5A,MYL12B,ZNF705G,PPM1F,UHRF2,NCS1,COL18A1,GALNT17,CDH9,ALB,ATP9B,UGP2,ZFYVE28,STT3A,GALNT2,TET1,CDH2,ITGA8,XRN2,PHLPP1,EYS,ZNF705D,RPS6KA5,NRP1,PRKCA,RNF215,ITGA1,ZNF615,PCCA,KLF12,RNF138,RC3H1,ZNF850,ZNF235,MCC,ZNF738,TUT4,NRXN3,ZNF215,

			CDH12,PRIM2,SNRK,ARFGAP3,USP49,PGM2L1,FBLN1,STK36,MB,RAG1,ZMYM1,DNPEP,CYP4B1,GNA14,ZNF678,PRDM15,ZNF420,RNF11,EYA4,CDH17,ZNF705B,CDH13,USP32,NRAP,UNK,AIFM3,KALRN,ME3,ZNF704,SLC1A2,GNAS,CA10,CPQ,TRIM9,ASTN2,ZMYND8,RNF217,ZNF74,BPTF,ZMYND11,KMT2C,ABL2,MMP26,NECAB1,EYA1,TTLL11,SLIT2,CNOT7,KCNIP4,ESCO1,TRHDE,PRKCQ,ANTXR1,MGMT,ZNF679,NOS1,SLC6A3,VCAN,NSD1,EHMT1,SLIT3,DTNA,ESR1,MYO9B,KDM4B,CYP2C8,LOXL2,CACNA2D1,PRLR,AGO3,CDKAL1,ZNF302,EYA2,PITPNM3,INTS12,WDFY3,CHFR,ZNF721,JAZF1,ZNF578,ZNF891,SPOCK3,ZNF14,PHC2,AGAP1,ROCK2,PRDM1,RORA,DMRT1,PPP1CB,HSPG2,TRIM2,ZFP30,FSTL4,BARD1,STK3,ZNF423,ZNF568,CADPS,GLI2,THRB,AKAP13,MORC3,ATP10A
GO:0043169	cation binding	1.364741464091 8938e-17	NOTCH2,CACNA2D3,SPOCK1,FREM1,BNC2,MICU2,SMOC1,MYO9A,NLK,UNC13C,SCAPER,FTO,KSR1,AGBL1,ZNF236,PLCB1,ZNF536,TTC3,LIPI,ZFPM2,MICAL3,NUBPL,L3MBTL4,ITPR2,PDE4D,NME7,EPS15L1,KCNMA1,PRDM16,FBN1,LPCAT2,F13A1,GPHN,CDH8,CHRNA7,PUDP,RIMS1,ZEB1,RARB,FGD4,GALNT1,ENPEP,RIMS2,ADGRE1,PCDH7,MCTP1,PJA2,PAPPA2,GLIS3,ANO6,ZNF880,EGLN3,MOCOS,SPON1,CPA6,ZMYM4,ZNF595,TSHZ3,SETD2,ZNF573,EFCAB2,KDM4C,NEK4,TSHZ2,ZNF280B,MACF1,PRKACB,NEK7,RNF220,HMCN2,ZNF407,MYOF,GNPTAB,CRB1,NSMCE2,BCL11A,CHSY1,CDH4,ATP2B2,LARGE1,RXFP1,PDE1C,ZFAND6,CYP2C9,ZNF648,ADAMTS6,ZNF382,AOAH,DGKI,SLC8A3,NELL2,PRKD1,CHRM3,ADSS2,CHSY3,RAPGEF2,LRP2,ARSB,CPS1,CPEB4,PRICKLE2,SMYD3,TYW1,HERC2,GRM7,KIF4A,ADAM10,HDAC9,ZHX3,APP,ADK,RPS6KA2,CACNA1C,KDM1B,ZNF600,SYT1,ACER2,ZNF723,PARN,ST18,PYGO1,SLC8A1,MARCHF1,ABCG8,HIVEP2,BMPR1B,ZNF717,PDZRN4,PAK3,PDE1A,ZNF257,TTLL7,RANBP2,PDE10A,CUBN,PHF21B,PRKCZ,MAN2A2,RYR3,TAF15,MSH6,COL27A1,ZSWIM6,FER1L6,MBNL2,ADAMTS17,PHF19,GALNT14,EBF2,PPM1L,ZKSCAN5,MGAT5,CADPS2,KMT2E,PAPPA,PCGF5,SYT10,ZNRF3,DNAJC21,CA5A,XXYLT1,ABLIM1,ANKFY1,RNF17,GALNT16,EWSR1,FAT3,MICU1,ZNF735,STK38,CHN1,LIMCH1,MBNL1,EFEMP1,TLL1,ZNF684,ZFAND3,HIVEP1,EFCAB8,CDH7,MOB3B,KLF15,PPARA,ERMP1,NR5A2,ADAMTS3,ARAP2,TRERF1,PCDH11Y,PDZRN3,ACSM2B,AGO2,STK32B,PHC3,TBC1D9,GATAD2B,CPE,DYSF,ADGRV1,ZNF846,MELK,RYR2,ZNF606,CLPX,AIF1L,CDH11,LDB3,PIAS1,EBF1,OLA1,DST,ATRX,NUAK1,XIRP2,ABL1,HDAC4,SLC1A1,PRKAA1,CRTAC1,DROSHA,TTLL5,L3MBTL3,APLF,ADAMTS14,MAST4,CDH18,GLIS1,MORC1,ZNHIT6,BAZ2A,CPSF3,ZC3HAV1,CDHR3,TGM1,LATS2,CLIP1,EFCAB6,PAH,ATP11C,ZNF438,ZBTB16,MUSK,GALNTL6,ZNF675,SETDB2,PRKCE,FOXK2,PGM5,ASAP2,METAP1D,ESRRG,ZNF718,DGKB,USP33,FBN2,ZNF831,EGF,PDE3A,EXT1,LNPEP,LIMD1,ADAMTS2,RPS6KA3,MARCHF8,ATP8A2,MTMR3,TRIM5,HTR2C,ATP8A1,ZCCHC7,LTBP1,STK38L,ZFYVE9,GALNT10,KDM7A,PRMT8,HTR2A,CYBRD1,CYP4A11,MATN2,STAC,TAF3,MARK2,GMPR,EBF3,ALPL,ZNF33B,LPP,FHL2,MSH2,MPPED2,GNAL,ZNF397,KCND2,CPXM2,NOS2,AFG3L2,CPNE4,MNAT1,ADAM12,XYLT1,BCL11B,VPS41,FOLH1,F5,ECE1,ZIM3,STK32A,AK8,TRPS1,TMEM163,PLCE1,XPNPEP1,PCDH9,VAV1,CYP4Z1,CDH20,RUFY2,MYT1L,ZNF160,EGFLAM,ZNF367,PDLIM5,CEPT1,ZBTB2,GALNT13,EXD3,DNER,BLM,LIN54,LRP1B,ADCY10,ZNF121,RC3H2,ATP9A,CDC42BPB,DSE,GNAI1,ZC3H14,GFI1B,MYRIP,HPCAL1,RNF38,ASXL3,PDE6C,POLR3A,RELN,GNAQ,ZNF106,ZNF567,TRAF3,ZNF462,UNC13B,ZNF875,UIMC1,B4GALT6,PLS1,IKZF2,SEC23B,NIN,TRIT1,ARSJ,GLI3,CGAS,SMOC2,PCP4,IDE,MCTP2,PRDM10,ZFYVE26,ZNF431,RERE,ANKMY1,MYL1,ZNF618,NEK10,MOB1B,ATF2,CYLD,UMODL1,ADARB2,ZMAT4,ANTXRL,HIVEP3,COL5A1,ITGA9,NELL1,ME2,ZZEF1,ATP10B,UBR1,MYT1,SMPDL3A,YPEL1,RORB,TADA2A,ZNF208,SELENON,REPS1,PRKN,AGMO,ZNF608,ZFAND4,CDC42BPA,SP110,PCDH15,ZNF541,DPF3,HEPHL1,GRIN2A,TRPM6,CDH23,LALBA,PRKCH,PEPD,RACGAP1,ZNF627,ACO1,CNOT6L,MKNK1,FBLN5,ZFP90,AOPEP,PDE6A,ZNF124,VAV3,ENPP3,PLAGL1,KCND3,HAAO,FAH,ITSN2,MOK,ARHGEF28,NPAS2,SEC24D,P

			PA2,CA1,ROCK1,ZNF780B,ZNF169,DTX1,TENM2,OVOL2,ZBTB33,ADA2,PLCB4,MMP16,PRUNE2,ZFHX3,FANCL,ZNF44,CRACR2A,RNF152,BAZ1A,CASZ1,OTUD7A,NEK6,HECTD1,DDHD1,ZNF292,ADAMTS19,DPYD,SNAI2,ASH1L,SIAH2,RPH3A,ZBTB8OS,COX5A,TRABD2B,RXRG,SP3,ERN2,ZNF879,MBTPS2,TRIM58,ZNF804B,NSD2,FYCO1,ESYT2,LTN1,SAMHD1,ENPP1,UTRN,RASGRP1,PAMR1,CDH26,DZANK1,PXDNL,ZNF385D,GRIN2B,SMAD5,MARCHF11,SLC40A1,CABYR,MICALL2,KDM6A,ROR2,ZNF521,FAT1,HGD,LMX1A,CALN1,RIOK1,CLSTN2,SDF4,PRKAA2,RNF182,PHF20L1,PLA2G4A,SNRPC,IQGAP1,ZBTB7C,MORC2,LASP1,THNSL2,NRXN1,ZNF234,CISD1,CIB4,ZNF518A,DGKK,CYP4F22,PEG10,LMX1B,JAK2,ADAM28,FSTL1,ZBTB38,MPPE1,BPNT1,SVEP1,PTGS1,ZNF287,CELSR2,ZNF449,PRSS2,CREBBP,MELTF,TNKS,SIAH3,TRPV5,ADAMTS5,PRKCB,MIPEP,PCDH11X,ANXA4,OVCH1,MT1HL1,CACNA1E,ZC3H15,ZNF354C,ZBTB21,SMPD4,ITGA6,ATP2B1,CLCA4,CNDP2,GTSF1L,AGAP9,ADGRE3,ADCY9,EML1,MAST2,ZNF528,ZNF611,EFHD2,ZBTB49,EXT2,PDP2,SLC6A1,ZCCHC14,NR2C1,DGKG,VAT1L,MARCHF6,GATAD1,CCBE1,PARK7,ADAMTS18,ITGA4,TOP3A,ZSCAN30,PLA2G12B,MTF2,FOXP2,HMCN1,ATP13A3,DHTKD1,ZSCAN5C,ACACA,EFHB,ZNF613,RXRA,MAP3K5,MAP3K4,TRIM43B,S100B,PRDM13,TRIM43,ERI1,SUMF1,EFCAB14,ZNF112,CHAMP1,PDE2A,DBF4B,DSG1,NSMCE1,ZNF813,ZBTB25,PPP2R3A,TRIM23,MLLT10,C2,RNF8,MECOM,DNMT3L,ACSM2A,LHX9,ADAMTS9,COLEC12,FRRS1,ZBTB10,TNNI1,POSTN,CREB5,EXTL3,TRPM7,IREB2,CDH5,TPH2,SCGN,APIP,SUSD1,UBE3A,PCDH8,ZBTB20,FAT4,IMPA2,ZNF66,RUNX1,AMFR,NENF,POMT2,ZNF845,ZFYVE1,ASAP1,PLCZ1,EDIL3,DIDO1,GALNT18,ADAMTS16,FYN,KDM5A,MYL12B,ZNF705G,PPM1F,UHRF2,NCS1,COL18A1,GALNT17,CDH9,ALB,ATP9B,UGP2,ZFYVE28,STT3A,GALNT2,TET1,CDH2,ITGA8,XRN2,PHLPP1,EYS,ZNF705D,RPS6KA5,NRP1,PRKCA,RNF215,ITGA1,ZNF615,PCCA,KLF12,RNF138,RC3H1,ZNF850,ZNF235,MCC,ZNF738,TUT4,NRXN3,ZNF215,CDH12,PRIM2,SNRK,ARFGAP3,USP49,PGM2L1,FBLN1,STK36,MB,RAG1,ZMYM1,DNPEP,CYP4B1,GNA14,ZNF678,PRDM15,ZNF420,RNF11,EYA4,CDH17,ZNF705B,CDH13,USP32,NRAP,UNK,AIFM3,KALRN,ME3,ZNF704,SLC1A2,GNAS,CA10,CPQ,TRIM9,ASTN2,ZMYND8,RNF217,ZNF74,BPTF,ZMYND11,KMT2C,ABL2,MMP26,NECAB1,EYA1,TTLL11,SLIT2,CNOT7,KCNIP4,ESCO1,TRHDE,PRKCQ,ANTXR1,MGMT,ZNF679,NOS1,SLC6A3,GLDC,VCAN,NSD1,EHMT1,SLIT3,DTNA,ESR1,MYO9B,KDM4B,CYP2C8,LOXL2,CACNA2D1,PRLR,AGO3,CDKAL1,ZNF302,EYA2,PITPNM3,INTS12,WDFY3,CHFR,ZNF721,JAZF1,ZNF578,ZNF891,SPOCK3,ZNF14,PHC2,AGAP1,ROCK2,PRDM1,RORA,DMRT1,PPP1CB,HSPG2,TRIM2,ZFP30,FSTL4,BARD1,STK3,ZNF423,ZNF568,CADPS,PRKAG2,GLI2,THRB,AKAP13,MORC3,ATP10A
GO:0008092	cytoskeletal protein binding	2.290700930554225e-12	NEBL,MYO9A,AGBL1,MX2,SVIL,TLN2,MICAL3,RDX,RP1,RALA,MYO5A,KCNMA1,DCDC1,SPIRE1,FGD4,MYO1E,USH2A,CDC42EP3,PARVB,MAP4,MYO3B,APC,MYO5C,SETD2,EGFR,MACF1,CTNNA3,DIAPH3,PTPN4,PHACTR1,PAK1,CTNNAL1,EPB41L3,KIF4A,TBCD,PHACTR2,CACNA1C,CACNB2,MTUS1,DCLK1,STAU2,MAPRE2,VCL,SLC8A1,FRMD3,CCSER2,MAP4K4,FMN2,HOMER2,CTNNA2,TTLL7,DIP2B,TRPC5,DNM3,CALD1,SNTG2,KLHL1,RAB27B,MRTFA,COBL,MTUS2,ABLIM1,CORO2B,LIMCH1,FMN1,PAFAH1B1,TPM1,NF2,CTNNA1,PPP1R9A,MRTFB,MPRIP,ENAH,NAV3,MAGI1,ANK2,BCAS3,SYNE2,AIF1L,LDB3,DST,PTPRT,XIRP2,ABL1,PRKAA1,GAS2,TTLL5,MYO10,CLIP1,SYNE1,ACTR3C,PRKCE,PEX14,ARHGEF7,STK38L,MARK2,TMEM67,C10ORF90,TMOD2,MYLK2,ANK3,SNTG1,MYOM2,VPS41,KLHL4,HIP1,PACSIN2,PDLIM5,BRCA2,DISC1,PHACTR3,TRAK1,MYRIP,DNAL1,TUBGCP3,MDM1,MYOM1,PLS1,NIN,HAUS6,SNX6,KIF21A,KIF15,MAP2,FARP1,BBS4,MAPK8IP1,MX1,CEP44,MYO3A,PRKN,AFAP1,RACGAP1,SYBU,MYO1D,ROCK1,LYN,KIF11,DPYSL5,NEK6,SHROOM3,ARFGEF1,FLNB,IFT81,UTRN,KCNC1,HDGFL3,INO80,KIF21B,MICALL2,TLNRD1,ACTR2,CLSTN2,SKA1,LARP6,PACRG,MAP6,VASP,KIFC1,IQGAP1,LASP1,CACYBP,CENPE,ANLN,KIF6,SMTN,USH1C,MTCL1,EML1,MAST2,SHROOM2,ABI1,MYO18B,IMPACT,MYH13,ARL4C,W

			ASF3,S100B,NCK1,TNNI1,TRPM7,KTN1,PTK2,MARK4,DIAPH1,CYFIP1,KIRREL1,SAXO1,PSTPIP2,CCDC88A,SPAG6,BICD1,FYN,MYL12B,SNTB1,RAI14,SPTB,KIF16B,TOGARAM1,MYO5B,NRAP,DLG5,EPB41L4A,ABL2,PKNOX2,FHOD3,TTLL11,IPP,ANTXR1,NDRG1,MYH15,GAS2L1,RAB27A,KIF13A,FRMD5,MYO9B,CEP57L1,IRAG2,HTT,EML6,FER,SNTB2,EPS8,ROCK2,WASHC1,HOOK3,KIF7,HNRNPU,DNM1L
GO:0030695	GTPase regulator activity	5.937633417364246e-11	*BCAR3,GARNL3,MYO9A,PLCB1,DLC1,ARHGAP26,RIMS1,FGD4,RIN2,ARHGAP24,DOCK10,DENND1A,RGS3,DOCK2,RABEP1,DGKI,TBC1D19,RALGPS1,RAPGEF2,ADGRB3,HERC2,RALGAPA1,RAPGEF5,TBCD,DOCK8,ARHGAP44,RANBP2,RGS20,RAP1GDS1,ARHGAP32,RGS9,RABGAP1L,ARHGEF17,TBC1D22A,CHN1,ECT2L,RASGRF2,RGL1,TIAM1,ARAP2,ARHGEF12,RIC8B,TBC1D9,RANBP3L,TBC1D5,DOCK4,RAP1A,DENND2B,RASGRF1,ASAP2,DENND4C,RGS12,EGF,TRIO,MCF2L,ARHGEF7,HERC1,KNDC1,DOCK5,PLCE1,VAV1,IQSEC1,EVI5,RALGAPA2,SGSM1,TBC1D4,RIN3,ARHGAP42,GNAQ,SH3BP5,DOCK1,RAP1GAP,SRGAP2,SEC23B,PSD3,FARP1,TBC1D13,NGEF,ALS2,RACGAP1,DOCK9,VAV3,ITSN2,ARHGEF28,DENND2C,ARHGAP28,ARHGAP31,EIF2B3,ARFGEF1,RALGPS2,RASGRP1,RASGEF1C,IQGAP1,NET1,SIPA1L2,MADD,AGAP9,ARFGEF3,RAPGEF4,DNMBP,CYTH4,ARHGAP12,RASGEF1B,ASAP1,CCDC88A,SH2D3C,DOCK3,TBC1D1,NRP1,BCR,ELMO1,RGS6,ARFGAP3,SRGAP3,RGPD4,KALRN,TIAM2,GAPVD1,WDR41,SLIT2,RGPD2,SIPA1L3,ARHGEF11,MYO9B,STARD13,AGAP1,RGS8,RGS7,RAB3GAP2,AKAP13,DNM1L*
GO:0060589	nucleoside-triphosphatase regulator activity	5.937633417364246e-11	*BCAR3,GARNL3,MYO9A,PLCB1,DLC1,ARHGAP26,RIMS1,FGD4,RIN2,ARHGAP24,DOCK10,DENND1A,RGS3,DOCK2,RABEP1,DGKI,TBC1D19,RALGPS1,RAPGEF2,ADGRB3,HERC2,RALGAPA1,RAPGEF5,TBCD,DOCK8,ARHGAP44,RANBP2,RGS20,RAP1GDS1,ARHGAP32,RGS9,RABGAP1L,ARHGEF17,TBC1D22A,CHN1,ECT2L,RASGRF2,RGL1,TIAM1,ARAP2,ARHGEF12,RIC8B,TBC1D9,RANBP3L,TBC1D5,DOCK4,RAP1A,DENND2B,RASGRF1,ASAP2,DENND4C,RGS12,EGF,TRIO,MCF2L,ARHGEF7,HERC1,KNDC1,DOCK5,PLCE1,VAV1,IQSEC1,EVI5,RALGAPA2,SGSM1,TBC1D4,RIN3,ARHGAP42,GNAQ,SH3BP5,DOCK1,RAP1GAP,SRGAP2,SEC23B,PSD3,FARP1,TBC1D13,NGEF,ALS2,RACGAP1,DOCK9,VAV3,ITSN2,ARHGEF28,DENND2C,ARHGAP28,ARHGAP31,EIF2B3,ARFGEF1,RALGPS2,RASGRP1,RASGEF1C,IQGAP1,NET1,SIPA1L2,MADD,AGAP9,ARFGEF3,RAPGEF4,DNMBP,CYTH4,ARHGAP12,RASGEF1B,ASAP1,CCDC88A,SH2D3C,DOCK3,TBC1D1,NRP1,BCR,ELMO1,RGS6,ARFGAP3,SRGAP3,RGPD4,KALRN,TIAM2,GAPVD1,WDR41,SLIT2,RGPD2,SIPA1L3,ARHGEF11,MYO9B,STARD13,AGAP1,RGS8,RGS7,RAB3GAP2,AKAP13,DNM1L*
GO:0005524	ATP binding	6.32110237942662e-11	*MTOR,ABCA13,MYO9A,ULK2,NLK,LONP2,KSR1,NUBPL,NME7,MYO5A,GPHN,PIK3C3,NAV2,MYO1E,ALK,ERCC6L2,HLCS,MAP3K9,MYO3B,MYO5C,TNIK,NEK4,EGFR,CDK12,PRKACB,NEK7,ATP2B2,NTRK3,FLT1,GK,THRAP3,DGKI,PRKD1,PAK1,EPHA7,RUNX2,CPS1,TAOK3,AGK,UBE2L3,SYN2,LRGUK,DNAH6,KIF4A,DHX32,UBE2G1,ABCB5,ADK,RPS6KA2,DCLK1,AURKA,ABCG8,MAP4K4,ABCD2,BMPR1B,PAK3,TTLL7,ITPKB,UBE2E2,SYN3,PRKCZ,MSH6,ABCA5,RIPK4,MAPK1,KCNJ1,ABCD3,DNAH14,UBE2O,PI4K2B,RNGTT,CHD6,STK38,MYLK3,ACSBG1,DCAF1,TLK1,PPIP5K1,PAK5,DAPK1,NAV3,ACSM2B,STK32B,MAGI1,ALPK2,DNAH11,MELK,CLPX,SMARCA4,UBE2R2,BLK,OLA1,ATRX,NUAK1,ABL1,PRKAA1,TTLL5,MAST4,DNAH5,SLFN11,ACSS3,MYO10,CAMK4,PEAK1,LATS2,ATP11C,DHX40,ABCB7,MUSK,ACTR3C,SMARCAD1,ABCC12,PRKCE,WNK2,DGKB,ALPK3,ABCC9,P2RX6,TRIO,NLRP13,RPS6KA3,ATP8A2,RFC1,ATP8A1,STK38L,ABCC4,KIAA0232,FANCM,FARS2,GTF2F2,MARK2,MSH2,EPHA6,HIPK3,ABCA10,GRK3,AFG3L2,STK10,MYLK2,STK32A,AK8,TARS3,MTHFD1L,AQR,BLM,NRK,MAGI3,ADCY10,MYLK4,ATP9A,CDC42BPB,VRK1,BMP2K,DSTYK,PFKFB4,DNAH8,TRIT1,SMARCA2,CGAS,IDE,KIF21A,KIF15,BTAF1,PEX6,NEK10,CFTR,ATP10B,MAP4K3,MYO3A,UBE2E1,CDC42BPA,MAPK10,TRPM6,PRKCH,HUNK,NLRC5,MKNK1,DMC1,MOK,MYO1D,ROCK1,LYN,KIF11,CHKA,INSR,NEK6,ABCA4,ERN2,ENPP1,ENTPD5,UCK2,CSNK2A1,PRKG1,INO80,KIF21B,ABCG1,RO*

			R2,ACTR2,RIOK1,PRKAA2,KIFC1,MORC2,HIPK1,DGKK,FICD,CENPE,AKT3,JAK2,PRKCB,KIF6,ABCC8,RFC2,NRBP1,ATP2B1,IARS2,ASS1,ADCY9,MAST2,MAP2K6,DGKG,MYO18B,MAPK8,UBE2J2,HFM1,MYH13,ATP13A3,UBE2QL1,ACACA,MAP3K5,MAP3K4,PKN2,PASK,FGR,ATP6V1B2,TOP1,EPHA4,PPIP5K2,NTRK2,ACSM2A,TRPM7,PTK2,MARK4,DNAH10,RUNX1,HKDC1,MDN1,FYN,BUB1,NLRP14,SCN8A,ATP9B,MAPK9,ROR1,EPHB1,DDX10,ADCK1,RPS6KA5,PRKCA,NLRP4,PCCA,ABCA6,BCR,NLRP8,KIF16B,SNRK,STK36,EPHB2,CSNK1G1,DPH6,MYO5B,CDK14,MET,CAMK1G,KALRN,DHX29,DNAH3,AK3,DDX6,DNAH17,ABL2,TTLL11,CMPK1,ERBB4,PRKCQ,MYH15,AK9,CHD9,MTREX,KIF13A,DNAH9,MYO9B,ORC4,LARS2,RAD51B,CAMK1D,AK2,FER,ROCK2,NARS2,PDK1,KIF7,CLCN5,HSPA12A,STK3,PNPLA8,HNRNPU,IGF1R,PRKAG2,ATP10A
GO:0004712	protein serine/threonine/tyrosine kinase activity	7.104580306930085e-11	*MTOR,ULK2,NLK,KSR1,ALK,MAP3K9,MYO3B,TNIK,NEK4,EGFR,CDK12,PRKACB,NEK7,NTRK3,FLT1,PRKD1,PAK1,EPHA7,TAOK3,RPS6KA2,DCLK1,AURKA,MAP4K4,PAK3,PRKCZ,RIPK4,MAPK1,STK38,DCAF1,TLK1,PAK5,DAPK1,STK32B,ALPK2,MELK,BLK,NUAK1,ABL1,PRKAA1,MAST4,CAMK4,LATS2,MUSK,PRKCE,WNK2,ALPK3,TRIO,RPS6KA3,STK38L,MARK2,EPHA6,HIPK3,STK10,STK32A,NRK,MYLK4,CDC42BPB,VRK1,BMP2K,DSTYK,NEK10,MAP4K3,MYO3A,CDC42BPA,MAPK10,TRPM6,PRKCH,HUNK,MKNK1,MOK,ROCK1,LYN,CHKA,INSR,NEK6,ERN2,CSNK2A1,PRKG1,ROR2,RIOK1,PRKAA2,HIPK1,AKT3,JAK2,PRKCB,MAST2,MAP2K6,MAPK8,MAP3K5,MAP3K4,PKN2,PASK,FGR,EPHA4,NTRK2,TRPM7,PTK2,MARK4,FYN,BUB1,MAPK9,EPHB1,RPS6KA5,PRKCA,BCR,SNRK,STK36,EPHB2,CSNK1G1,CDK14,MET,CAMK1G,KALRN,ABL2,ERBB4,PRKCQ,CAMK1D,FER,ROCK2,STK3,IGF1R*
GO:0050839	cell adhesion molecule binding	7.598244062334822e-11	*CNTN4,PTPRD,LRRC4C,TLN2,TENM4,RDX,ERC1,EPS15L1,FBN1,CDH8,ROBO2,TENM3,DSCAM,CRKL,PTPRJ,EGFR,MACF1,CTNNA3,DIAPH3,SND1,CDH4,CAST,CNTN6,CTNNAL1,SEPTIN9,ADAM10,VCL,CD2AP,CTNNA2,LARP1,CALD1,PDXDC1,ADAM22,ITGBL1,GFRA1,STK38,ITGB8,NF2,CTNNA1,CDH7,MRTFB,MPRIP,CPE,CDH11,USP8,OLA1,DST,CXADR,PTPRT,CDH18,ZC3HAV1,CDHR3,NRG1,PTPRO,STXBP6,CTNND2,PTPN2,MARK2,EPN2,ANK3,CDH20,TJP1,PACSIN2,CNTN1,PDLIM5,HMGB1,LRRFIP1,COL5A1,ITGA9,CDH23,PKP1,DOCK9,FBLN5,PTPRB,LYN,VCAM1,BZW1,TENM2,FLNB,ESYT2,SH3GLB1,CD9,UTRN,SNX9,CDH26,LAMA3,TNN,VASP,IQGAP1,LASP1,NRXN1,CADM1,ANLN,ADAMTS5,ZC3H15,SERBP1,OLFM4,ITGA6,SLC14A2,UBAP2,EFHD2,STAT1,ABI1,PARK7,ITGA4,PPME1,PKN2,SDCBP,NECTIN1,NCK1,NUMB,POSTN,KTN1,PTK2,CDH5,MPP7,LAMB1,JAM2,KIRREL1,ASAP1,EDIL3,CDH9,CDH2,CNTN5,ITGA8,PRKCA,ITGA1,NRXN3,CDH12,FBLN1,CDH17,CDH13,DHX29,GAPVD1,KIRREL3,DDX6,NDRG1,NLGN1,FRMD5,NTNG1,FER,PCMT1,COL4A3*
GO:0032559	adenyl ribonucleotide binding	8.376655929969694e-11	*MTOR,ABCA13,MYO9A,ULK2,NLK,LONP2,KSR1,NUBPL,PDE4D,NME7,MYO5A,GPHN,PIK3C3,NAV2,MYO1E,ALK,ERCC6L2,HLCS,MAP3K9,MYO3B,MYO5C,TNIK,NEK4,EGFR,CDK12,PRKACB,NEK7,ATP2B2,NTRK3,FLT1,GK,THRAP3,DGKI,PRKD1,PAK1,EPHA7,RAPGEF2,RUNX2,CPS1,TAOK3,AGK,UBE2L3,SYN2,LRGUK,DNAH6,KIF4A,DHX32,UBE2G1,ABCB5,ADK,RPS6KA2,DCLK1,AURKA,ABCG8,MAP4K4,ABCD2,BMPR1B,PAK3,TTLL7,ITPKB,PDE10A,UBE2E2,SCP2,SYN3,PRKCZ,MSH6,ABCA5,RIPK4,MAPK1,KCNJ1,ABCD3,DNAH14,UBE2O,PI4K2B,RNGTT,CHD6,STK38,MYLK3,ACSBG1,DCAF1,TLK1,PPIP5K1,PAK5,DAPK1,NAV3,ACSM2B,STK32B,MAGI1,ALPK2,DNAH11,MELK,CLPX,SMARCA4,UBE2R2,BLK,OLA1,ATRX,NUAK1,ABL1,PRKAA1,TTLL5,MAST4,DNAH5,SLFN11,ACSS3,MYO10,CAMK4,PEAK1,LATS2,ATP11C,DHX40,ABCB7,MUSK,ACTR3C,SMARCAD1,ABCC12,PRKCE,WNK2,DGKB,ALPK3,ABCC9,P2RX6,TRIO,NLRP13,RPS6KA3,ATP8A2,RFC1,ATP8A1,STK38L,ABCC4,KIAA0232,FANCM,FARS2,GTF2F2,MARK2,MSH2,MPPED2,EPHA6,HIPK3,ABCA10,GRK3,AFG3L2,STK10,MYLK2,STK32A,AK8,TARS3,MTHFD1L,AQR,BLM,NRK,MAGI3,ADCY10,MYLK4,ATP9A,CDC42BPB,VRK1,BMP2K,DSTYK,PFKFB4,DNAH8,TRIT1,SMARCA2,CGAS,IDE,KIF21A,KIF15,BTAF1,PEX6,NEK10,CFTR,ATP10B,MAP4K3,MYO3A,UBE2E1,CDC42BPA,MAPK10,TRPM*

			6,PRKCH,HUNK,NLRC5,MKNK1,DMC1,MOK,MYO1D,ROCK1,LYN,KIF11,CHKA,INSR,NEK6,ABCA4,ERN2,ENPP1,ENTPD5,UCK2,CSNK2A1,HCN1,PRKG1,INO80,KIF21B,ABCG1,HADHA,ROR2,ACTR2,RIOK1,PRKAA2,KIFC1,MORC2,HIPK1,DGKK,FICD,CENPE,AKT3,JAK2,PRKCB,KIF6,ABCC8,RFC2,NRBP1,ATP2B1,IARS2,ASS1,ADCY9,MAST2,MAP2K6,DGKG,MYO18B,MAPK8,UBE2J2,RAPGEF4,HFM1,MYH13,ATP13A3,UBE2QL1,ACACA,MAP3K5,MAP3K4,PKN2,PDE2A,PASK,FGR,ATP6V1B2,TOP1,EPHA4,PPIP5K2,NTRK2,ACSM2A,TRPM7,PTK2,MARK4,DNAH10,RUNX1,HKDC1,MDN1,FYN,BUB1,NLRP14,SCN8A,ATP9B,MAPK9,ROR1,EPHB1,DDX10,ADCK1,RPS6KA5,PRKCA,NLRP4,PCCA,ABCA6,BCR,NLRP8,KIF16B,SNRK,STK36,EPHB2,CSNK1G1,DPH6,MYO5B,CDK14,MET,CAMK1G,KALRN,DHX29,DNAH3,AK3,DDX6,DNAH17,ABL2,TTLL11,CMPK1,ERBB4,PRKCQ,MYH15,AK9,CHD9,MTREX,KIF13A,DNAH9,MYO9B,ORC4,LARS2,RAD51B,CAMK1D,AK2,FER,ROCK2,NARS2,PDK1,KIF7,CLCN5,PNPLA3,HSPA12A,STK3,PNPLA8,HNRNPU,IGF1R,PRKAG2,ATP10A
GO:0140096	catalytic activity, acting on a protein	9.011985489618256e-11	*MTOR,IMMP2L,PTPRD,TMTC1,ULK2,NLK,LONP2,KSR1,AGBL1,TTC3,TMPRSS2,DPP10,ZDHHC21,PTPRA,PRDM16,F13A1,PIK3C3,GALNT1,ENPEP,PCMTD1,ALK,PJA2,PAPPA2,HLCS,EGLN3,MAP3K9,MYO3B,CPA6,SETD2,TNIK,PTPRJ,KDM4C,NEK4,EGFR,USP14,CDK12,PRKACB,NEK7,RNF220,NEDD4,NSMCE2,PSMB2,PTPN4,NTRK3,FLT1,ADAMTS6,TASP1,PRKD1,TPTE2,PAK1,EPHA7,PELI2,CPS1,TAOK3,ADAMTSL1,UBE2L3,PTPRN2,SMYD3,HERC2,WDSUB1,NEDD4L,ADAM32,ADAM10,HDAC9,UBE2G1,RPS6KA2,KDM1B,KLHL13,DCLK1,USP18,PARP15,AURKA,PTPRR,FIG4,MARCHF1,MAP4K4,BMPR1B,PCSK6,PAK3,TTLL7,RANBP2,UBE2E2,PRKCZ,HECW1,ADAMTS17,SENP6,DUSP22,GALNT14,PPM1L,RIPK4,MAPK1,MGAT5,ADAM22,USP25,PLG,PAPPA,ZNRF3,UBE2O,GALNT16,RNGTT,STK38,PTPN13,MYLK3,EFEMP1,TLL1,DCAF1,TLK1,ZDHHC14,CORIN,BIRC6,ERMP1,ADAMTS3,UBE3D,PTPRK,PAK5,PDZRN3,DAPK1,STK32B,ALPK2,JARID2,CPE,MELK,HECTD4,CLPX,DUSP16,USP8,PIAS1,UBE2R2,BLK,NUAK1,PTPRT,ABL1,PTPN12,HDAC4,PRKAA1,TTLL5,ADAMTS14,MAST4,ATE1,HECTD2,CAMK4,TGM1,PEAK1,LATS2,MUSK,GALNTL6,SETDB2,PRKCE,METAP1D,NXN,WNK2,B4GALNT3,USP33,PTPRO,ALPK3,TRIO,LNPEP,ADAMTS2,RPS6KA3,MARCHF8,MTMR3,PTPN2,TRIM5,ATXN3,LTBP1,STK38L,GALNT10,KDM7A,PRMT8,MARK2,C10ORF90,ABHD17C,HERC1,EPHA6,HIPK3,GRK3,CPXM2,AFG3L2,STK10,TMTC2,ADAM12,MYLK2,XYLT1,CCND3,FOLH1,ECE1,STK32A,LYPLA1,CWC27,CRIM1,XPNPEP1,GSR,CAPN5,FKBP5,BRCA2,GALNT13,NRK,RC3H2,MYLK4,CDC42BPB,VRK1,BMP2K,RNF38,PGPEP1,RELN,TRAF3,DSTYK,ENOX1,CASP5,IDE,NEK10,ATF2,CYLD,ZZEF1,UBR1,MAP4K3,MED27,MYO3A,UBE2E1,PTPRE,PRKN,MTMR2,CDC42BPA,MAPK10,FBXO3,TXNRD2,WSB1,USP43,TRPM6,PRKCH,HUNK,PEPD,CPVL,MKNK1,PTPRB,AOPEP,USP7,MOK,ADGRG6,PPA2,ROCK1,LYN,CTSB,DTX1,CHKA,MMP16,FANCL,RNF152,OTUD7A,INSR,CUL5,NEK6,HECTD1,HDAC11,LYPLAL1,ADAMTS19,ASH1L,SIAH2,PIGK,TRABD2B,UFD1,ERN2,MBTPS2,TRIM58,ZDHHC17,NSD2,PTAR1,LTN1,PAMR1,CSNK2A1,PPIL6,EOGT,CTDP1,PRKG1,ASB4,MARCHF11,ATG4B,CDC14B,KDM6A,ROR2,RIOK1,SOSTDC1,PRKAA2,QSOX2,RNF182,ERO1B,HIPK1,FICD,AKT3,JAK2,ADAM28,PRSS2,CREBBP,TNKS,SIAH3,UFL1,ADAMTS5,PRKCB,FBXW2,MIPEP,OVCH1,BRD4,NRBP1,CLCA4,CNDP2,MAST2,PCMTD2,PDP2,GRXCR1,MAP2K6,MARCHF6,PARK7,ADAMTS18,MAPK8,EIF3F,PPME1,UBE2J2,ASB2,UBE2QL1,MAP3K5,MAP3K4,TRIM43B,PRDM13,TRIM43,MAGEL2,PKN2,LRRC2,FBXW8,SPPL2B,NSMCE1,PASK,FGR,TRIM23,TOP1,TINAG,C2,RNF8,EPHA4,MECOM,NTRK2,ADAMTS9,TRPM7,PRKAB1,PTK2,MARK4,CD5L,UBE3A,AMFR,CTSE,POMT2,TPTE,GALNT18,ADAMTS16,FYN,BUB1,KDM5A,PPM1F,UHRF2,HDAC2,GALNT17,MTMR7,MAPK9,ROR1,GALNT2,FUT8,HECW2,OVCH2,PHLPP1,EPHB1,ZDHHC18,ADCK1,RPS6KA5,PTPRG,NRP1,PRKCA,ATPSCKMT,RNF215,USP24,RNF138,RC3H1,ZNF738,BCR,SNRK,SENP8,USP49,STK36,RAG1,DNPEP,DPP6,EPHB2,CSNK1G1,RNF11,EYA4,PPIL2,PRSS51,CDK14,MET,SPPL3,CAMK1G,USP32,ADAM29,KALRN,CPQ,TRIM9,TMPRSS15,*

			RNF217,PRSS23,KMT2C,ABL2,MMP26,BACE2,EYA1,TTLL11,PARP8,TMPRSS3,ESCO1,ERBB4,TRHDE,PRKCQ,NSD1,EHMT1,USP31,KDM4B,LOXL2,ZDHHC11B,CAMK1D,CFAP44,FER,EYA2,CHFR,PCMT1,ROCK2,ATAT1,PPP1CB,PDK1,PTPRQ,TRIM2,PCSK2,BARD1,PALD1,STK3,IGF1R,PRKAG2,AKAP13
GO:0030554	adenyl nucleotide binding	2.31766355359874e-10	*MTOR,ABCA13,MYO9A,ULK2,NLK,LONP2,KSR1,NUBPL,PDE4D,NME7,MYO5A,GPHN,PIK3C3,NAV2,MYO1E,ALK,ERCC6L2,HLCS,MAP3K9,MYO3B,MYO5C,TNIK,NEK4,EGFR,CDK12,PRKACB,NEK7,ATP2B2,NTRK3,FLT1,GK,THRAP3,DGKI,PRKD1,PAK1,EPHA7,RAPGEF2,RUNX2,CPS1,TAOK3,AGK,UBE2L3,SYN2,LRGUK,DNAH6,KIF4A,DHX32,UBE2G1,ABCB5,ADK,RPS6KA2,DCLK1,AURKA,ABCG8,MAP4K4,ABCD2,BMPR1B,PAK3,TTLL7,ITPKB,PDE10A,UBE2E2,SCP2,SYN3,PRKCZ,MSH6,ABCA5,RIPK4,MAPK1,KCNJ1,ABCD3,DNAH14,UBE2O,PI4K2B,RNGTT,CHD6,STK38,MYLK3,ACSBG1,DCAF1,TLK1,PPIP5K1,PAK5,DAPK1,NAV3,ACSM2B,STK32B,MAGI1,ALPK2,DNAH11,MELK,CLPX,SMARCA4,UBE2R2,BLK,OLA1,ATRX,NUAK1,ABL1,PRKAA1,TTLL5,MAST4,DNAH5,SLFN11,ACSS3,MYO10,CAMK4,PEAK1,LATS2,ATP11C,DHX40,ABCB7,MUSK,ACTR3C,SMARCAD1,ABCC12,PRKCE,WNK2,DGKB,ALPK3,ABCC9,P2RX6,TRIO,NLRP13,RPS6KA3,ATP8A2,RFC1,ATP8A1,STK38L,ABCC4,KIAA0232,FANCM,FARS2,GTF2F2,MARK2,MSH2,MPPED2,EPHA6,HIPK3,ABCA10,GRK3,AFG3L2,STK10,MYLK2,STK32A,AK8,TARS3,MTHFD1L,AQR,BLM,NRK,MAGI3,ADCY10,MYLK4,ATP9A,CDC42BPB,VRK1,BMP2K,DSTYK,PFKFB4,DNAH8,TRIT1,SMARCA2,CGAS,IDE,KIF21A,KIF15,BTAF1,PEX6,NEK10,CFTR,ATP10B,MAP4K3,MYO3A,UBE2E1,CDC42BPA,MAPK10,TRPM6,PRKCH,HUNK,NLRC5,MKNK1,DMC1,MOK,MYO1D,ROCK1,LYN,KIF11,CHKA,INSR,NEK6,ABCA4,ERN2,ENPP1,ENTPD5,UCK2,CSNK2A1,HCN1,PRKG1,INO80,KIF21B,ABCG1,HADHA,ROR2,ACTR2,RIOK1,PRKAA2,KIFC1,MORC2,HIPK1,DGKK,FICD,CENPE,AKT3,JAK2,PRKCB,KIF6,ABCC8,RFC2,NRBP1,ATP2B1,IARS2,ASS1,ADCY9,MAST2,MAP2K6,DGKG,MYO18B,MAPK8,UBE2J2,RAPGEF4,HFM1,MYH13,ATP13A3,UBE2QL1,ACACA,MAP3K5,MAP3K4,PKN2,PDE2A,PASK,FGR,ATP6V1B2,TOP1,EPHA4,PPIP5K2,NTRK2,ACSM2A,TRPM7,PTK2,MARK4,DNAH10,RUNX1,HKDC1,MDN1,FYN,BUB1,NLRP14,SCN8A,ATP9B,MAPK9,ROR1,EPHB1,DDX10,ADCK1,RPS6KA5,PRKCA,NLRP4,PCCA,ABCA6,BCR,NLRP8,KIF16B,SNRK,STK36,EPHB2,CSNK1G1,DPH6,MYO5B,CDK14,MET,CAMK1G,KALRN,DHX29,DNAH3,AK3,DDX6,DNAH17,ABL2,TTLL11,CMPK1,ERBB4,PRKCQ,MYH15,AK9,CHD9,MTREX,KIF13A,DNAH9,MYO9B,ORC4,LARS2,RAD51B,CAMK1D,AK2,FER,ROCK2,NARS2,PDK1,KIF7,CLCN5,PNPLA3,HSPA12A,STK3,PNPLA8,HNRNPU,IGF1R,PRKAG2,ATP10A*
GO:0032553	ribonucleotide binding	8.584607347321197e-10	*MTOR,ABCA13,MYO9A,ULK2,NLK,LONP2,KSR1,MX2,ARL15,NUBPL,PDE4D,RALA,NME7,MYO5A,GPHN,PIK3C3,NAV2,MYO1E,ALK,ERCC6L2,HLCS,MAP3K9,MYO3B,MYO5C,TNIK,NEK4,EGFR,CDK12,PRKACB,NEK7,ATP2B2,NTRK3,FLT1,GK,THRAP3,DGKI,PRKD1,PAK1,EPHA7,ADSS2,RAPGEF2,RUNX2,CPS1,TAOK3,AGK,RANBP17,UBE2L3,SYN2,TYW1,LRGUK,SEPTIN9,DNAH6,KIF4A,DHX32,UBE2G1,ABCB5,ADK,RPS6KA2,DCLK1,AURKA,ABCG8,MAP4K4,ABCD2,BMPR1B,RAB8B,PAK3,TTLL7,ITPKB,PDE10A,UBE2E2,HHAT,DNM3,SCP2,SYN3,PRKCZ,MSH6,RAB27B,ABCA5,RIPK4,MAPK1,KCNJ1,ABCD3,DNAH14,UBE2O,PI4K2B,RNGTT,CHD6,STK38,MYLK3,ACSBG1,DCAF1,TLK1,PPIP5K1,PAK5,DAPK1,NAV3,ACSM2B,STK32B,MAGI1,ALPK2,DNAH11,RAB22A,MELK,CLPX,SMARCA4,EFTUD2,UBE2R2,BLK,OLA1,ATRX,NUAK1,ABL1,PRKAA1,TTLL5,MAST4,DNAH5,GUCY1A2,SLFN11,RAP1A,ACSS3,MYO10,CAMK4,PEAK1,LATS2,ATP11C,DHX40,ABCB7,MUSK,ACTR3C,SMARCAD1,ABCC12,PRKCE,WNK2,DGKB,ALPK3,ABCC9,P2RX6,TRIO,NLRP13,RPS6KA3,ATP8A2,SCG5,RFC1,ATP8A1,STK38L,ABCC4,KIAA0232,FANCM,FARS2,GTF2F2,MARK2,MSH2,MPPED2,GNAL,EPHA6,ATL1,HIPK3,ABCA10,GRK3,NOS2,AFG3L2,STK10,MYLK2,GBP6,STK32A,AK8,TARS3,MTHFD1L,AQR,BLM,NRK,MAGI3,ADCY10,MYLK4,ATP9A,CDC42BPB,VRK1,GNAI1,BMP2K,PDE6C,GNAQ,DSTYK,PFKFB4,NIN,DNAH8,TRIT1,SMARCA2,CGAS,IDE,KIF21A,KIF15,RERGL,BTAF1,PEX6,NEK10,MX1,CFTR,*

			ATP10B,MAP4K3,MYO3A,UBE2E1,CDC42BPA,MAPK10,TRPM6,PRKCH,HUNK,NLRC5,MKNK1,DMC1,MOK,RALB,MYO1D,ROCK1,LYN,KIF11,CHKA,RRAGD,CRACR2A,INSR,NEK6,ABCA4,ERN2,SAMHD1,ENPP1,ENTPD5,UCK2,CSNK2A1,HCN1,PRKG1,INO80,KIF21B,ABCG1,HADHA,ROR2,ACTR2,RIOK1,PRKAA2,DIRAS2,RAB12,KIFC1,MORC2,HIPK1,DGKK,FICD,CENPE,TUBB6,AKT3,JAK2,ARL11,PRKCB,KIF6,ABCC8,RFC2,NRBP1,ATP2B1,IARS2,ASS1,SAR1A,ADCY9,MAST2,MAP2K6,DGKG,MYO18B,MAPK8,UBE2J2,RAPGEF4,HFM1,MYH13,ATP13A3,UBE2QL1,ACACA,ARL4C,MAP3K5,MAP3K4,PKN2,PDE2A,RAB38,PASK,FGR,TRIM23,ATP6V1B2,TOP1,EPHA4,PPIP5K2,NTRK2,ACSM2A,TRPM7,PTK2,MARK4,DNAH10,RUNX1,HKDC1,MDN1,FYN,BUB1,NLRP14,ARL13B,SCN8A,ATP9B,UGP2,MAPK9,ROR1,EPHB1,DDX10,ADCK1,RPS6KA5,PRKCA,GBP4,NLRP4,PCCA,POR,ABCA6,EFL1,BCR,RERG,NLRP8,KIF16B,SNRK,STK36,RRAS2,GNA14,RABL2A,EPHB2,CSNK1G1,DPH6,MYO5B,CDK14,MET,CAMK1G,KALRN,GNAS,MFHAS1,DHX29,DNAH3,AK3,DDX6,UPRT,DNAH17,ABL2,TTLL11,CMPK1,ERBB4,PRKCQ,MYH15,AK9,NOS1,CHD9,MTREX,RAB27A,KIF13A,DNAH9,MYO9B,ORC4,LARS2,RAD51B,CAMK1D,AK2,FER,AGAP1,ROCK2,NARS2,RAB31,PDK1,KIF7,CLCN5,PNPLA3,HSPA12A,STK3,PNPLA8,HNRNPU,IGF1R,PRKAG2,ATP10A,SEPTIN6,DNM1L
GO:0035639	purine ribonucleoside triphosphate binding	2.1719537909004807e-9	*MTOR,ABCA13,MYO9A,ULK2,NLK,LONP2,KSR1,MX2,ARL15,NUBPL,RALA,NME7,MYO5A,GPHN,PIK3C3,NAV2,MYO1E,ALK,ERCC6L2,HLCS,MAP3K9,MYO3B,MYO5C,TNIK,NEK4,EGFR,CDK12,PRKACB,NEK7,ATP2B2,NTRK3,FLT1,GK,THRAP3,DGKI,PRKD1,PAK1,EPHA7,ADSS2,RUNX2,CPS1,TAOK3,AGK,RANBP17,UBE2L3,SYN2,LRGUK,SEPTIN9,DNAH6,KIF4A,DHX32,UBE2G1,ABCB5,ADK,RPS6KA2,DCLK1,AURKA,ABCG8,MAP4K4,ABCD2,BMPR1B,RAB8B,PAK3,TTLL7,ITPKB,UBE2E2,HHAT,DNM3,SYN3,PRKCZ,MSH6,RAB27B,ABCA5,RIPK4,MAPK1,KCNJ1,ABCD3,DNAH14,UBE2O,PI4K2B,RNGTT,CHD6,STK38,MYLK3,ACSBG1,DCAF1,TLK1,PPIP5K1,PAK5,DAPK1,NAV3,ACSM2B,STK32B,MAGI1,ALPK2,DNAH11,RAB22A,MELK,CLPX,SMARCA4,EFTUD2,UBE2R2,BLK,OLA1,ATRX,NUAK1,ABL1,PRKAA1,TTLL5,MAST4,DNAH5,GUCY1A2,SLFN11,RAP1A,ACSS3,MYO10,CAMK4,PEAK1,LATS2,ATP11C,DHX40,ABCB7,MUSK,ACTR3C,SMARCAD1,ABCC12,PRKCE,WNK2,DGKB,ALPK3,ABCC9,P2RX6,TRIO,NLRP13,RPS6KA3,ATP8A2,SCG5,RFC1,ATP8A1,STK38L,ABCC4,KIAA0232,FANCM,FARS2,GTF2F2,MARK2,MSH2,GNAL,EPHA6,ATL1,HIPK3,ABCA10,GRK3,AFG3L2,STK10,MYLK2,GBP6,STK32A,AK8,TARS3,MTHFD1L,AQR,BLM,NRK,MAGI3,ADCY10,MYLK4,ATP9A,CDC42BPB,VRK1,GNAI1,BMP2K,GNAQ,DSTYK,PFKFB4,NIN,DNAH8,TRIT1,SMARCA2,CGAS,IDE,KIF21A,KIF15,RERGL,BTAF1,PEX6,NEK10,MX1,CFTR,ATP10B,MAP4K3,MYO3A,UBE2E1,CDC42BPA,MAPK10,TRPM6,PRKCH,HUNK,NLRC5,MKNK1,DMC1,MOK,RALB,MYO1D,ROCK1,LYN,KIF11,CHKA,RRAGD,CRACR2A,INSR,NEK6,ABCA4,ERN2,SAMHD1,ENPP1,ENTPD5,UCK2,CSNK2A1,PRKG1,INO80,KIF21B,ABCG1,ROR2,ACTR2,RIOK1,PRKAA2,DIRAS2,RAB12,KIFC1,MORC2,HIPK1,DGKK,FICD,CENPE,TUBB6,AKT3,JAK2,ARL11,PRKCB,KIF6,ABCC8,RFC2,NRBP1,ATP2B1,IARS2,ASS1,SAR1A,ADCY9,MAST2,MAP2K6,DGKG,MYO18B,MAPK8,UBE2J2,HFM1,MYH13,ATP13A3,UBE2QL1,ACACA,ARL4C,MAP3K5,MAP3K4,PKN2,RAB38,PASK,FGR,TRIM23,ATP6V1B2,TOP1,EPHA4,PPIP5K2,NTRK2,ACSM2A,TRPM7,PTK2,MARK4,DNAH10,RUNX1,HKDC1,MDN1,FYN,BUB1,NLRP14,ARL13B,SCN8A,ATP9B,MAPK9,ROR1,EPHB1,DDX10,ADCK1,RPS6KA5,PRKCA,GBP4,NLRP4,PCCA,ABCA6,EFL1,BCR,RERG,NLRP8,KIF16B,SNRK,STK36,RRAS2,GNA14,RABL2A,EPHB2,CSNK1G1,DPH6,MYO5B,CDK14,MET,CAMK1G,KALRN,GNAS,MFHAS1,DHX29,DNAH3,AK3,DDX6,UPRT,DNAH17,ABL2,TTLL11,CMPK1,ERBB4,PRKCQ,MYH15,AK9,CHD9,MTREX,RAB27A,KIF13A,DNAH9,MYO9B,ORC4,LARS2,RAD51B,CAMK1D,AK2,FER,AGAP1,ROCK2,NARS2,RAB31,PDK1,KIF7,CLCN5,HSPA12A,STK3,PNPLA8,HNRNPU,IGF1R,PRKAG2,ATP10A,SEPTIN6,DNM1L*
GO:0032555	purine ribonucleo	2.361950701799	*MTOR,ABCA13,MYO9A,ULK2,NLK,LONP2,KSR1,MX2,ARL15,NUBPL,PDE4D,RALA,NME7,MYO5A,GPHN,PIK3C3,NAV2,MYO1E,ALK,ERCC6L2,HLCS,MAP3K9,MYO3B,MYO5C,TNIK,NEK4,EGFR,CDK*

	tide binding	8428e-9	*12,PRKACB,NEK7,ATP2B2,NTRK3,FLT1,GK,THRAP3,DGKI,PRKD1,PAK1,EPHA7,ADSS2,RAPGEF2,RUNX2,CPS1,TAOK3,AGK,RANBP17,UBE2L3,SYN2,LRGUK,SEPTIN9,DNAH6,KIF4A,DHX32,UBE2G1,ABCB5,ADK,RPS6KA2,DCLK1,AURKA,ABCG8,MAP4K4,ABCD2,BMPR1B,RAB8B,PAK3,TTLL7,ITPKB,PDE10A,UBE2E2,HHAT,DNM3,SCP2,SYN3,PRKCZ,MSH6,RAB27B,ABCA5,RIPK4,MAPK1,KCNJ1,ABCD3,DNAH14,UBE2O,PI4K2B,RNGTT,CHD6,STK38,MYLK3,ACSBG1,DCAF1,TLK1,PPIP5K1,PAK5,DAPK1,NAV3,ACSM2B,STK32B,MAGI1,ALPK2,DNAH11,RAB22A,MELK,CLPX,SMARCA4,EFTUD2,UBE2R2,BLK,OLA1,ATRX,NUAK1,ABL1,PRKAA1,TTLL5,MAST4,DNAH5,GUCY1A2,SLFN11,RAP1A,ACSS3,MYO10,CAMK4,PEAK1,LATS2,ATP11C,DHX40,ABCB7,MUSK,ACTR3C,SMARCAD1,ABCC12,PRKCE,WNK2,DGKB,ALPK3,ABCC9,P2RX6,TRIO,NLRP13,RPS6KA3,ATP8A2,SCG5,RFC1,ATP8A1,STK38L,ABCC4,KIAA0232,FANCM,FARS2,GTF2F2,MARK2,MSH2,MPPED2,GNAL,EPHA6,ATL1,HIPK3,ABCA10,GRK3,AFG3L2,STK10,MYLK2,GBP6,STK32A,AK8,TARS3,MTHFD1L,AQR,BLM,NRK,MAGI3,ADCY10,MYLK4,ATP9A,CDC42BPB,VRK1,GNAI1,BMP2K,PDE6C,GNAQ,DSTYK,PFKFB4,NIN,DNAH8,TRIT1,SMARCA2,CGAS,IDE,KIF21A,KIF15,RERGL,BTAF1,PEX6,NEK10,MX1,CFTR,ATP10B,MAP4K3,MYO3A,UBE2E1,CDC42BPA,MAPK10,TRPM6,PRKCH,HUNK,NLRC5,MKNK1,DMC1,MOK,RALB,MYO1D,ROCK1,LYN,KIF11,CHKA,RRAGD,CRACR2A,INSR,NEK6,ABCA4,ERN2,SAMHD1,ENPP1,ENTPD5,UCK2,CSNK2A1,HCN1,PRKG1,INO80,KIF21B,ABCG1,HADHA,ROR2,ACTR2,RIOK1,PRKAA2,DIRAS2,RAB12,KIFC1,MORC2,HIPK1,DGKK,FICD,CENPE,TUBB6,AKT3,JAK2,ARL11,PRKCB,KIF6,ABCC8,RFC2,NRBP1,ATP2B1,IARS2,ASS1,SAR1A,ADCY9,MAST2,MAP2K6,DGKG,MYO18B,MAPK8,UBE2J2,RAPGEF4,HFM1,MYH13,ATP13A3,UBE2QL1,ACACA,ARL4C,MAP3K5,MAP3K4,PKN2,PDE2A,RAB38,PASK,FGR,TRIM23,ATP6V1B2,TOP1,EPHA4,PPIP5K2,NTRK2,ACSM2A,TRPM7,PTK2,MARK4,DNAH10,RUNX1,HKDC1,MDN1,FYN,BUB1,NLRP14,ARL13B,SCN8A,ATP9B,MAPK9,ROR1,EPHB1,DDX10,ADCK1,RPS6KA5,PRKCA,GBP4,NLRP4,PCCA,ABCA6,EFL1,BCR,RERG,NLRP8,KIF16B,SNRK,STK36,RRAS2,GNA14,RABL2A,EPHB2,CSNK1G1,DPH6,MYO5B,CDK14,MET,CAMK1G,KALRN,GNAS,MFHAS1,DHX29,DNAH3,AK3,DDX6,UPRT,DNAH17,ABL2,TTLL11,CMPK1,ERBB4,PRKCQ,MYH15,AK9,CHD9,MTREX,RAB27A,KIF13A,DNAH9,MYO9B,ORC4,LARS2,RAD51B,CAMK1D,AK2,FER,AGAP1,ROCK2,NARS2,RAB31,PDK1,KIF7,CLCN5,PNPLA3,HSPA12A,STK3,PNPLA8,HNRNPU,IGF1R,PRKAG2,ATP10A,SEPTIN6,DNM1L*
GO:0043168	anion binding	3.440312112254 6087e-9	*MTOR,EXOC1L,ABCA13,MYO9A,ULK2,NLK,LONP2,KSR1,PLCB1,MX2,SVIL,ARL15,MICAL3,NUBPL,ITPR2,PDE4D,RALA,NME7,MYO5A,GPHN,PIK3C3,AKR1C3,NAV2,MYO1E,ALK,ERCC6L2,HLCS,FCHO2,EGLN3,MAP3K9,MYO3B,MOCOS,MYO5C,TNIK,NEK4,EGFR,CDK12,PRKACB,NEK7,ATP2B2,NTRK3,FLT1,GK,THRAP3,DGKI,PRKD1,PAK1,GMDS,EPHA7,ADSS2,GRAMD1B,RAPGEF2,RUNX2,CPS1,TAOK3,AGK,RANBP17,UBE2L3,SYN2,TYW1,LRGUK,GRM7,SEPTIN9,DNAH6,KIF4A,DHX32,UBE2G1,ABCB5,ADK,RPS6KA2,KYNU,KDM1B,DCLK1,SYT1,PARP15,AURKA,ABCG8,MAP4K4,ABCD2,BMPR1B,RAB8B,PAK3,TTLL7,ITPKB,TRPC5,PDE10A,UBE2E2,HHAT,RNLS,DNM3,SCP2,SYN3,PRKCZ,MSH6,RAB27B,ABCA5,PDXDC1,RIPK4,MAPK1,KCNJ1,ABCD3,DNAH14,TRPC7,SYT10,UBE2O,PI4K2B,RNGTT,CHD6,STK38,MYLK3,ACSBG1,DCAF1,TLK1,PPIP5K1,ARAP2,PAK5,DAPK1,NAV3,ACSM2B,STK32B,MAGI1,ALPK2,DNAH11,RAB22A,MELK,RYR2,CLPX,SMARCA4,FABP7,PARD3,MAPKAP1,EFTUD2,UBE2R2,BLK,OLA1,ATRX,NUAK1,ABL1,AGPS,SLC1A1,PRKAA1,TTLL5,MAST4,DNAH5,GUCY1A2,SLFN11,RAP1A,ACSS3,MYO10,CAMK4,PLEKHA8,PEAK1,LATS2,ATP11C,DHX40,ABCB7,MUSK,ACTR3C,SMARCAD1,ABCC12,PRKCE,WNK2,DGKB,ALPK3,ABCC9,P2RX6,TRIO,STXBP6,NLRP13,RPS6KA3,ATP8A2,SCG5,RFC1,ATP8A1,STK38L,ABCC4,KIAA0232,FANCM,FARS2,GTF2F2,MARK2,MSH2,MPPED2,GNAL,EPHA6,ATL1,HIPK3,ABCA10,GRK3,NOS2,AFG3L2,STK10,MYLK2,GBP6,FOLH1,STK32A,AK8,HIP1,GSR,PACSIN2,TARS3,MTHFD1L,SNX3,AQR,BLM,NRK,MAGI3,ADCY10,MYLK4,ATP9A,CDC42BPB,VRK1,GNA*

			I1,BMP2K,PDE6C,GNAQ,CLVS1,DSTYK,PFKFB4,NIN,DNAH8,TRIT1,GADL1,SMARCA2,CGAS,IDE,KIF21A,KIF15,RERGL,BTAF1,PEX6,NEK10,MX1,CLVS2,CFTR,ATP10B,MAP4K3,MYO3A,UBE2E1,SH3PXD2A,CDC42BPA,MAPK10,TXNRD2,TRPM6,PRKCH,HUNK,FRMPD4,RACGAP1,NLRC5,MKNK1,DMC1,MOK,RALB,MYO1D,ROCK1,LYN,PLEKHB2,KIF11,CHKA,RRAGD,CRACR2A,INSR,NEK6,DPYD,RPH3A,ABCA4,ERN2,SAMHD1,ENPP1,ENTPD5,UCK2,CSNK2A1,HCN1,PRKG1,GRIN2B,INO80,ETNPPL,KIF21B,ABCG1,HADHA,PSAP,ROR2,ACTR2,RIOK1,PRKAA2,DIRAS2,ERO1B,PLA2G4A,RAB12,KIFC1,IQGAP1,MORC2,THNSL2,HIPK1,DGKK,SNAP91,FICD,CENPE,TUBB6,AKT3,JAK2,ARL11,PRKCB,GOT2,KIF6,ABCC8,RFC2,NRBP1,ATP2B1,GAP43,IARS2,ASS1,SAR1A,ADCY9,MAST2,EXOC1,SDS,MAP2K6,DGKG,MYO18B,MAPK8,UBE2J2,RAPGEF4,HFM1,MYH13,ATP13A3,DHTKD1,UBE2QL1,ACACA,ST8SIA4,ARL4C,RXRA,MAP3K5,MAP3K4,PKN2,PDE2A,RAB38,SDCBP,PASK,FGR,TRIM23,ATP6V1B2,TOP1,EPHA4,PPIP5K2,NTRK2,ACSM2A,PLEKHA3,TRPM7,PTK2,MARK4,DNAH10,PITPNC1,RUNX1,ZFYVE1,ASAP1,PLCZ1,HKDC1,ACOXL,MDN1,FYN,BUB1,NLRP14,ARL13B,SCN8A,ALB,ATP9B,MAPK9,ROR1,PLEKHA2,EPHB1,DDX10,ADCK1,RPS6KA5,FCHSD2,PRKCA,GBP4,NLRP4,PCCA,POR,ABCA6,EFL1,BCR,RERG,NLRP8,KIF16B,SNRK,STK36,RRAS2,GNA14,RABL2A,EPHB2,CSNK1G1,DPH6,MYO5B,CDK14,MET,CAMK1G,AIFM3,KALRN,ME3,GNAS,MFHAS1,DHX29,ASTN2,DNAH3,AK3,DDX6,UPRT,DNAH17,ABL2,TTLL11,CMPK1,PLCL1,ERBB4,PRKCQ,MYH15,AK9,NOS1,GLDC,CHD9,MTREX,RAB27A,KIF13A,DNAH9,MYO9B,ORC4,LARS2,RAD51B,CAMK1D,AK2,FER,AGAP1,ROCK2,NARS2,RAB31,PDK1,KIF7,CLCN5,PNPLA3,HSPA12A,STK3,PNPLA8,HNRNPU,IGF1R,KCNAB1,PRKAG2,ATP10A,SEPTIN6,DNM1L
GO:0004672	protein kinase activity	5.513301307526684e-9	*MTOR,ULK2,NLK,KSR1,PIK3C3,ALK,MAP3K9,MYO3B,TNIK,NEK4,EGFR,CDK12,PRKACB,NEK7,NTRK3,FLT1,PRKD1,PAK1,EPHA7,TAOK3,RPS6KA2,DCLK1,AURKA,MAP4K4,BMPR1B,PAK3,PRKCZ,RIPK4,MAPK1,STK38,MYLK3,EFEMP1,DCAF1,TLK1,PAK5,DAPK1,STK32B,ALPK2,MELK,BLK,NUAK1,ABL1,PRKAA1,MAST4,CAMK4,PEAK1,LATS2,MUSK,PRKCE,WNK2,ALPK3,TRIO,RPS6KA3,LTBP1,STK38L,MARK2,EPHA6,HIPK3,GRK3,STK10,MYLK2,CCND3,STK32A,CRIM1,NRK,MYLK4,CDC42BPB,VRK1,BMP2K,DSTYK,NEK10,MAP4K3,MYO3A,CDC42BPA,MAPK10,TRPM6,PRKCH,HUNK,MKNK1,MOK,ROCK1,LYN,CHKA,INSR,NEK6,ERN2,CSNK2A1,PRKG1,ROR2,RIOK1,SOSTDC1,PRKAA2,HIPK1,AKT3,JAK2,PRKCB,BRD4,NRBP1,MAST2,MAP2K6,MAPK8,MAP3K5,MAP3K4,PKN2,PASK,FGR,TOP1,EPHA4,NTRK2,TRPM7,PRKAB1,PTK2,MARK4,FYN,BUB1,MAPK9,ROR1,EPHB1,ADCK1,RPS6KA5,NRP1,PRKCA,BCR,SNRK,STK36,EPHB2,CSNK1G1,CDK14,MET,CAMK1G,KALRN,ABL2,ERBB4,PRKCQ,CAMK1D,FER,ROCK2,PDK1,STK3,IGF1R,PRKAG2,AKAP13*
GO:0017076	purine nucleotide binding	6.420590105487593e-9	*MTOR,ABCA13,MYO9A,ULK2,NLK,LONP2,KSR1,MX2,ARL15,NUBPL,PDE4D,RALA,NME7,MYO5A,GPHN,PIK3C3,NAV2,MYO1E,ALK,ERCC6L2,HLCS,MAP3K9,MYO3B,MYO5C,TNIK,NEK4,EGFR,CDK12,PRKACB,NEK7,ATP2B2,NTRK3,FLT1,GK,THRAP3,DGKI,PRKD1,PAK1,EPHA7,ADSS2,RAPGEF2,RUNX2,CPS1,TAOK3,AGK,RANBP17,UBE2L3,SYN2,LRGUK,SEPTIN9,DNAH6,KIF4A,DHX32,UBE2G1,ABCB5,ADK,RPS6KA2,DCLK1,AURKA,ABCG8,MAP4K4,ABCD2,BMPR1B,RAB8B,PAK3,TTLL7,ITPKB,PDE10A,UBE2E2,HHAT,DNM3,SCP2,SYN3,PRKCZ,MSH6,RAB27B,ABCA5,RIPK4,MAPK1,KCNJ1,ABCD3,DNAH14,UBE2O,PI4K2B,RNGTT,CHD6,STK38,MYLK3,ACSBG1,DCAF1,TLK1,PPIP5K1,PAK5,DAPK1,NAV3,ACSM2B,STK32B,MAGI1,ALPK2,DNAH11,RAB22A,MELK,CLPX,SMARCA4,EFTUD2,UBE2R2,BLK,OLA1,ATRX,NUAK1,ABL1,PRKAA1,TTLL5,MAST4,DNAH5,GUCY1A2,SLFN11,RAP1A,ACSS3,MYO10,CAMK4,PEAK1,LATS2,ATP11C,DHX40,ABCB7,MUSK,ACTR3C,SMARCAD1,ABCC12,PRKCE,WNK2,DGKB,ALPK3,ABCC9,P2RX6,TRIO,NLRP13,RPS6KA3,ATP8A2,SCG5,RFC1,ATP8A1,STK38L,ABCC4,KIAA0232,FANCM,FARS2,GTF2F2,MARK2,MSH2,MPPED2,GNAL,EPHA6,ATL1,HIPK3,ABCA10,GRK3,AFG3L2,STK10,MYLK2,GBP6,STK32A,AK8,TARS3,MTHFD1L,AQR,BLM,NRK,MAGI3,ADCY*

			10,MYLK4,ATP9A,CDC42BPB,VRK1,GNAI1,BMP2K,PDE6C,GNAQ,DSTYK,PFKFB4,NIN,DNAH8,TRIT1,SMARCA2,CGAS,IDE,KIF21A,KIF15,RERGL,BTAF1,PEX6,NEK10,MX1,CFTR,ATP10B,MAP4K3,MYO3A,UBE2E1,CDC42BPA,MAPK10,TRPM6,PRKCH,HUNK,NLRC5,MKNK1,DMC1,MOK,RALB,MYO1D,ROCK1,LYN,KIF11,CHKA,RRAGD,CRACR2A,INSR,NEK6,ABCA4,ERN2,SAMHD1,ENPP1,ENTPD5,UCK2,CSNK2A1,HCN1,PRKG1,INO80,KIF21B,ABCG1,HADHA,ROR2,ACTR2,RIOK1,PRKAA2,DIRAS2,RAB12,KIFC1,MORC2,HIPK1,DGKK,FICD,CENPE,TUBB6,AKT3,JAK2,ARL11,PRKCB,KIF6,ABCC8,RFC2,NRBP1,ATP2B1,IARS2,ASS1,SAR1A,ADCY9,MAST2,MAP2K6,DGKG,MYO18B,MAPK8,UBE2J2,RAPGEF4,HFM1,MYH13,ATP13A3,UBE2QL1,ACACA,ARL4C,MAP3K5,MAP3K4,PKN2,PDE2A,RAB38,PASK,FGR,TRIM23,ATP6V1B2,TOP1,EPHA4,PPIP5K2,NTRK2,ACSM2A,TRPM7,PTK2,MARK4,DNAH10,RUNX1,HKDC1,MDN1,FYN,BUB1,NLRP14,ARL13B,SCN8A,ATP9B,MAPK9,ROR1,EPHB1,DDX10,ADCK1,RPS6KA5,PRKCA,GBP4,NLRP4,PCCA,ABCA6,EFL1,BCR,RERG,NLRP8,KIF16B,SNRK,STK36,RRAS2,GNA14,RABL2A,EPHB2,CSNK1G1,DPH6,MYO5B,CDK14,MET,CAMK1G,KALRN,GNAS,MFHAS1,DHX29,DNAH3,AK3,DDX6,UPRT,DNAH17,ABL2,TTLL11,CMPK1,ERBB4,PRKCQ,MYH15,AK9,CHD9,MTREX,RAB27A,KIF13A,DNAH9,MYO9B,ORC4,LARS2,RAD51B,CAMK1D,AK2,FER,AGAP1,ROCK2,NARS2,RAB31,PDK1,KIF7,CLCN5,PNPLA3,HSPA12A,STK3,PNPLA8,HNRNPU,IGF1R,PRKAG2,ATP10A,SEPTIN6,DNM1L
GO:0097367	carbohydrate derivative binding	1.044298773101908e-8	*MTOR,ABCA13,SMOC1,MYO9A,ULK2,NLK,LONP2,KSR1,MX2,LIPI,ARL15,NUBPL,PDE4D,RALA,NME7,MYO5A,FBN1,GPHN,PIK3C3,NAV2,MYO1E,ALK,COL25A1,ERCC6L2,HLCS,MAP3K9,MYO3B,MYO5C,TNIK,NEK4,EGFR,CDK12,PRKACB,NEK7,ATP2B2,NTRK3,FLT1,GK,THRAP3,EVA1C,DGKI,NELL2,PRKD1,PAK1,EPHA7,ADSS2,RAPGEF2,RUNX2,FGF12,CPS1,TAOK3,AGK,RANBP17,UBE2L3,SYN2,TYW1,LRGUK,SEPTIN9,DNAH6,KIF4A,DHX32,UBE2G1,APP,ABCB5,ADK,RPS6KA2,DCLK1,SEMA5A,AURKA,ABCG8,MAP4K4,ABCD2,BMPR1B,PCSK6,RAB8B,PAK3,TTLL7,ITPKB,PDE10A,UBE2E2,HHAT,DNM3,SCP2,SYN3,PRKCZ,MSH6,RAB27B,ABCA5,RIPK4,MAPK1,KCNJ1,ABCD3,DNAH14,CRISPLD2,UBE2O,PI4K2B,RNGTT,CHD6,STK38,MYLK3,ACSBG1,PAFAH1B1,DCAF1,TLK1,PPIP5K1,ADAMTS3,PAK5,DAPK1,NAV3,ACSM2B,STK32B,MAGI1,ALPK2,DNAH11,RAB22A,MELK,CLPX,SMARCA4,EFTUD2,UBE2R2,BLK,COL23A1,OLA1,ATRX,NUAK1,ABL1,PRKAA1,DROSHA,TTLL5,MAST4,DNAH5,GUCY1A2,SLFN11,RAP1A,ACSS3,MYO10,CAMK4,PLEKHA8,FGF10,PEAK1,LATS2,ATP11C,DHX40,ABCB7,MUSK,ACTR3C,SMARCAD1,ABCC12,PRKCE,WNK2,DGKB,CD44,ALPK3,ABCC9,P2RX6,TRIO,COL5A3,NLRP13,RPS6KA3,ATP8A2,SCG5,RFC1,ATP8A1,STK38L,ABCC4,KIAA0232,FANCM,FARS2,GTF2F2,MARK2,MSH2,MPPED2,GNAL,EPHA6,ATL1,HIPK3,ABCA10,GRK3,NOS2,AFG3L2,STK10,MYLK2,GBP6,STK32A,AK8,EGFLAM,TARS3,MTHFD1L,AQR,BLM,NRK,MAGI3,ADCY10,MYLK4,ATP9A,CDC42BPB,VRK1,GNAI1,BMP2K,PDE6C,HMGB1,GNAQ,FGF9,DSTYK,PFKFB4,NIN,DNAH8,TRIT1,SMARCA2,CGAS,SMOC2,IDE,KIF21A,KIF15,RERGL,BTAF1,PEX6,NEK10,MX1,COL5A1,CFTR,NELL1,ATP10B,MAP4K3,MYO3A,UBE2E1,CDC42BPA,MAPK10,TRPM6,PRKCH,HUNK,NLRC5,MKNK1,DMC1,PSMA1,MOK,RALB,MYO1D,ROCK1,LYN,CTSB,KIF11,ADA2,CHKA,RRAGD,CRACR2A,INSR,NEK6,COLQ,PIGK,ABCA4,ERN2,SAMHD1,ENPP1,ENTPD5,UCK2,CSNK2A1,HCN1,PRKG1,INO80,KIF21B,ABCG1,HADHA,PSAP,ROR2,CFH,ACTR2,RIOK1,PRKAA2,DIRAS2,RAB12,KIFC1,REG4,MORC2,HIPK1,DGKK,FICD,CENPE,TUBB6,AKT3,JAK2,FSTL1,MPPE1,ARL11,ADAMTS5,PRKCB,KIF6,ABCC8,RFC2,NRBP1,ATP2B1,GAP43,IARS2,ASS1,SAR1A,ADCY9,MAST2,HPSE2,MAP2K6,DGKG,MYO18B,CEMIP,MAPK8,UBE2J2,RAPGEF4,HFM1,MYH13,ATP13A3,UBE2QL1,ACACA,ST8SIA4,ARL4C,ADGRB1,MAP3K5,MAP3K4,PKN2,PDE2A,RAB38,SDCBP,PASK,FGR,TRIM23,ATP6V1B2,TOP1,EPHA4,PPIP5K2,NTRK2,ACSM2A,POSTN,TRPM7,PTK2,MARK4,DNAH10,RUNX1,HKDC1,MDN1,FYN,BUB1,NLRP14,ARL13B,SCN8A,ATP9B,UGP2,MAPK9,ROR1,EPHB1,DDX10,ADCK1,RPS6KA5,NRP1,PRKCA,GBP4,NLRP4,PCCA,POR,ABCA6,EFL1,*

			BCR,RERG,NLRP8,KIF16B,SNRK,STK36,RRAS2,GNA14,RABL2A,EPHB2,CSNK1G1,DPH6,MYO5B,CDK14,MET,CAMK1G,KALRN,GNAS,LAMA1,MFHAS1,DHX29,BMP7,CHIT1,DNAH3,AK3,DDX6,UPRT,DNAH17,ABL2,TTLL11,SLIT2,CMPK1,ERBB4,PRKCQ,MYH15,AK9,NOS1,CHD9,MTREX,VCAN,RAB27A,SLIT3,KIF13A,DNAH9,MYO9B,ORC4,LARS2,RAD51B,CAMK1D,AK2,FER,OARD1,SPOCK3,AGAP1,ROCK2,NARS2,RAB31,PDK1,KIF7,CLCN5,PNPLA3,HSPA12A,STK3,PNPLA8,HNRNPU,IGF1R,PRKAG2,ATP10A,SEPTIN6,DNM1L
GO:0003779	actin binding	1.213499175922 5245e-8	*NEBL,MYO9A,SVIL,TLN2,MICAL3,RDX,MYO5A,KCNMA1,SPIRE1,FGD4,MYO1E,PARVB,MYO3B,MYO5C,EGFR,MACF1,CTNNA3,DIAPH3,PHACTR1,CTNNAL1,EPB41L3,PHACTR2,CACNB2,VCL,FMN2,HOMER2,CTNNA2,TRPC5,CALD1,SNTG2,KLHL1,MRTFA,COBL,ABLIM1,CORO2B,LIMCH1,FMN1,TPM1,NF2,CTNNA1,PPP1R9A,MRTFB,MPRIP,ENAH,SYNE2,AIF1L,LDB3,DST,XIRP2,ABL1,GAS2,MYO10,SYNE1,ACTR3C,PRKCE,STK38L,TMOD2,SNTG1,MYOM2,KLHL4,HIP1,PDLIM5,PHACTR3,MYRIP,MYOM1,PLS1,MYO3A,PRKN,AFAP1,MYO1D,SHROOM3,FLNB,UTRN,INO80,MICALL2,TLNRD1,ACTR2,PACRG,VASP,IQGAP1,LASP1,ANLN,SMTN,USH1C,SHROOM2,MYO18B,IMPACT,MYH13,WASF3,NCK1,TNNI1,TRPM7,PTK2,DIAPH1,CYFIP1,PSTPIP2,CCDC88A,SNTB1,RAI14,SPTB,MYO5B,NRAP,ABL2,PKNOX2,FHOD3,IPP,ANTXR1,MYH15,GAS2L1,MYO9B,FER,SNTB2,EPS8,WASHC1,HNRNPU*
GO:0005085	guanyl-nucleotide exchange factor activity	4.134771020392 312e-8	*BCAR3,FGD4,RIN2,DOCK10,DENND1A,DOCK2,RALGPS1,RAPGEF2,HERC2,RAPGEF5,DOCK8,RAP1GDS1,ARHGEF17,ECT2L,RASGRF2,RGL1,TIAM1,ARHGEF12,RIC8B,DOCK4,RAP1A,DENND2B,RASGRF1,DENND4C,EGF,TRIO,MCF2L,ARHGEF7,HERC1,KNDC1,DOCK5,PLCE1,VAV1,IQSEC1,RIN3,SH3BP5,DOCK1,PSD3,FARP1,NGEF,ALS2,DOCK9,VAV3,ITSN2,ARHGEF28,DENND2C,EIF2B3,ARFGEF1,RALGPS2,RASGRP1,RASGEF1C,NET1,MADD,ARFGEF3,RAPGEF4,DNMBP,CYTH4,RASGEF1B,CCDC88A,SH2D3C,DOCK3,BCR,ELMO1,KALRN,TIAM2,GAPVD1,WDR41,ARHGEF11,RAB3GAP2,AKAP13*
GO:0016301	kinase activity	9.794797760977 386e-8	*MTOR,ULK2,NLK,KSR1,NME7,PIK3C3,ALK,MAP3K9,MYO3B,TNIK,NEK4,EGFR,CDK12,PRKACB,NEK7,SGMS1,NTRK3,FLT1,GK,DGKI,PRKD1,PAK1,EPHA7,TAOK3,AGK,LRGUK,ADK,RPS6KA2,DCLK1,AURKA,MAP4K4,BMPR1B,PAK3,ITPKB,PRKCZ,RIPK4,MAPK1,PI4K2B,STK38,MYLK3,EFEMP1,DCAF1,TLK1,PPIP5K1,PAK5,DAPK1,STK32B,ALPK2,MELK,MAPKAP1,BLK,NUAK1,ABL1,PRKAA1,MAST4,CAMK4,PEAK1,LATS2,MUSK,PRKCE,WNK2,DGKB,ALPK3,TRIO,RPS6KA3,LTBP1,STK38L,MARK2,EPHA6,SH3KBP1,HIPK3,CDKN2C,GRK3,STK10,MYLK2,CCND3,STK32A,AK8,CRIM1,NRK,MAGI3,MYLK4,CDC42BPB,VRK1,BMP2K,DSTYK,PFKFB4,NEK10,MAP4K3,MYO3A,AKAP10,CDC42BPA,MAPK10,TRPM6,PRKCH,HUNK,MKNK1,MOK,ROCK1,LYN,CHKA,INSR,NEK6,ERN2,UCK2,CSNK2A1,PRKG1,ROR2,RIOK1,SOSTDC1,PRKAA2,HIPK1,DGKK,AKT3,JAK2,PRKCB,BRD4,NRBP1,MAST2,MAP2K6,DGKG,MAPK8,MAP3K5,MAP3K4,PKN2,PASK,FGR,TOP1,EPHA4,PPIP5K2,NTRK2,TRPM7,PRKAB1,PTK2,MARK4,CNKSR1,HKDC1,FYN,BUB1,MAPK9,ROR1,EPHB1,ADCK1,RPS6KA5,NRP1,PRKCA,BCR,SNRK,PGM2L1,STK36,EPHB2,CSNK1G1,CDK14,MET,DLG2,CAMK1G,MAGI2,KALRN,AK3,ABL2,CMPK1,ERBB4,PRKCQ,AK9,CAMK1D,PIK3R3,AK2,FER,FGGY,ROCK2,PDK1,STK3,IGF1R,PRKAG2,AKAP13*
GO:0000166	nucleotide binding	1.297024524827 3205e-7	*MTOR,ABCA13,MYO9A,ULK2,NLK,LONP2,KSR1,MX2,ARL15,MICAL3,NUBPL,PDE4D,RALA,NME7,MYO5A,GPHN,PIK3C3,NAV2,MYO1E,ALK,ERCC6L2,HLCS,MAP3K9,MYO3B,MYO5C,TNIK,NEK4,EGFR,CDK12,PRKACB,NEK7,ATP2B2,NTRK3,FLT1,GK,THRAP3,DGKI,PRKD1,PAK1,GMDS,EPHA7,ADSS2,RAPGEF2,RUNX2,CPS1,TAOK3,AGK,RANBP17,UBE2L3,SYN2,TYW1,LRGUK,SEPTIN9,DNAH6,KIF4A,DHX32,UBE2G1,ABCB5,ADK,RPS6KA2,KDM1B,DCLK1,PARP15,AURKA,ABCG8,MAP4K4,ABCD2,BMPR1B,RAB8B,PAK3,TTLL7,ITPKB,PDE10A,UBE2E2,HHAT,RNLS,DNM3,SCP2,SYN3,PRKCZ,MSH6,RAB27B,ABCA5,RIPK4,MAPK1,KCNJ1,ABCD3,DNAH14,UBE2O,PI4K2B,RNGTT,CHD6,STK38,MYLK3,ACSBG1,DCAF1,TLK1,PPIP5K1,PAK5,DAPK1,NAV3,ACSM2B,STK32B,MAGI1,ALPK2,DNAH11,RAB22A,MELK,CLPX,SMARCA4,EFTUD2,UBE2R*

			2,BLK,OLA1,ATRX,NUAK1,ABL1,AGPS,PRKAA1,TTLL5,APLF,MAST4,DNAH5,GUCY1A2,SLFN11,RAP1A,ACSS3,MYO10,CAMK4,PEAK1,LATS2,ATP11C,DHX40,ABCB7,MUSK,ACTR3C,SMARCAD1,ABCC12,PRKCE,WNK2,DGKB,ALPK3,ABCC9,P2RX6,TRIO,NLRP13,RPS6KA3,ATP8A2,SCG5,RFC1,ATP8A1,STK38L,ABCC4,KIAA0232,FANCM,FARS2,GTF2F2,MARK2,MSH2,MPPED2,GNAL,EPHA6,ATL1,HIPK3,ABCA10,GRK3,NOS2,AFG3L2,STK10,MYLK2,GBP6,STK32A,AK8,GSR,TARS3,MTHFD1L,AQR,BLM,NRK,MAGI3,ADCY10,MYLK4,ATP9A,CDC42BPB,VRK1,GNAI1,BMP2K,PDE6C,GNAQ,DSTYK,PFKFB4,NIN,DNAH8,TRIT1,SMARCA2,CGAS,IDE,KIF21A,KIF15,RERGL,BTAF1,PEX6,NEK10,MX1,CFTR,ME2,ATP10B,MAP4K3,MYO3A,UBE2E1,CDC42BPA,MAPK10,TXNRD2,TRPM6,PRKCH,HUNK,NLRC5,DHRS11,MKNK1,DMC1,MOK,RALB,MYO1D,ROCK1,LYN,KIF11,ZBTB33,CHKA,RRAGD,CRACR2A,INSR,NEK6,DPYD,ABCA4,ERN2,SAMHD1,ENPP1,ENTPD5,MOCS2,UCK2,CSNK2A1,HCN1,PRKG1,INO80,DHRS3,KIF21B,ABCG1,HADHA,ROR2,ACTR2,RIOK1,PRKAA2,DIRAS2,ERO1B,RAB12,KIFC1,MORC2,HIPK1,DGKK,FICD,CENPE,TUBB6,AKT3,JAK2,ZBTB38,ARL11,PRKCB,KIF6,ABCC8,RFC2,ZBTB21,NRBP1,ATP2B1,IARS2,ASS1,SAR1A,ADCY9,MAST2,MAP2K6,DGKG,MYO18B,MAPK8,UBE2J2,RAPGEF4,HFM1,MYH13,ATP13A3,UBE2QL1,ACACA,ARL4C,MAP3K5,MAP3K4,PKN2,PDE2A,RAB38,PASK,FGR,TRIM23,ATP6V1B2,TOP1,TINAG,EPHA4,PPIP5K2,NTRK2,ACSM2A,TRPM7,PTK2,MARK4,DNAH10,RUNX1,SLC27A6,HKDC1,ACOXL,MDN1,FYN,BUB1,NLRP14,ARL13B,SCN8A,ATP9B,UGP2,MAPK9,ROR1,TET1,EPHB1,DDX10,ADCK1,RPS6KA5,PRKCA,GBP4,NLRP4,FHIT,PCCA,POR,ABCA6,EFL1,BCR,RERG,NLRP8,KIF16B,SNRK,STK36,RRAS2,GNA14,RABL2A,EPHB2,CSNK1G1,DPH6,MYO5B,CDK14,MET,CAMK1G,AIFM3,KALRN,ME3,GNAS,MFHAS1,DHX29,DNAH3,AK3,DDX6,UPRT,DNAH17,ABL2,TTLL11,CMPK1,ERBB4,PRKCQ,MYH15,AK9,NOS1,CHD9,MTREX,RAB27A,KIF13A,DNAH9,MYO9B,ORC4,LARS2,RAD51B,CAMK1D,AK2,FER,CHFR,AGAP1,ROCK2,NARS2,RAB31,PDK1,KIF7,CLCN5,PNPLA3,HSPA12A,STK3,PNPLA8,HNRNPU,IGF1R,KCNAB1,PRKAG2,ATP10A,SEPTIN6,DNM1L
GO:1901265	nucleoside phosphate binding	1.379783838549978e-7	*MTOR,ABCA13,MYO9A,ULK2,NLK,LONP2,KSR1,MX2,ARL15,MICAL3,NUBPL,PDE4D,RALA,NME7,MYO5A,GPHN,PIK3C3,NAV2,MYO1E,ALK,ERCC6L2,HLCS,MAP3K9,MYO3B,MYO5C,TNIK,NEK4,EGFR,CDK12,PRKACB,NEK7,ATP2B2,NTRK3,FLT1,GK,THRAP3,DGKI,PRKD1,PAK1,GMDS,EPHA7,ADSS2,RAPGEF2,RUNX2,CPS1,TAOK3,AGK,RANBP17,UBE2L3,SYN2,TYW1,LRGUK,SEPTIN9,DNAH6,KIF4A,DHX32,UBE2G1,ABCB5,ADK,RPS6KA2,KDM1B,DCLK1,PARP15,AURKA,ABCG8,MAP4K4,ABCD2,BMPR1B,RAB8B,PAK3,TTLL7,ITPKB,PDE10A,UBE2E2,HHAT,RNLS,DNM3,SCP2,SYN3,PRKCZ,MSH6,RAB27B,ABCA5,RIPK4,MAPK1,KCNJ1,ABCD3,DNAH14,UBE2O,PI4K2B,RNGTT,CHD6,STK38,MYLK3,ACSBG1,DCAF1,TLK1,PPIP5K1,PAK5,DAPK1,NAV3,ACSM2B,STK32B,MAGI1,ALPK2,DNAH11,RAB22A,MELK,CLPX,SMARCA4,EFTUD2,UBE2R2,BLK,OLA1,ATRX,NUAK1,ABL1,AGPS,PRKAA1,TTLL5,APLF,MAST4,DNAH5,GUCY1A2,SLFN11,RAP1A,ACSS3,MYO10,CAMK4,PEAK1,LATS2,ATP11C,DHX40,ABCB7,MUSK,ACTR3C,SMARCAD1,ABCC12,PRKCE,WNK2,DGKB,ALPK3,ABCC9,P2RX6,TRIO,NLRP13,RPS6KA3,ATP8A2,SCG5,RFC1,ATP8A1,STK38L,ABCC4,KIAA0232,FANCM,FARS2,GTF2F2,MARK2,MSH2,MPPED2,GNAL,EPHA6,ATL1,HIPK3,ABCA10,GRK3,NOS2,AFG3L2,STK10,MYLK2,GBP6,STK32A,AK8,GSR,TARS3,MTHFD1L,AQR,BLM,NRK,MAGI3,ADCY10,MYLK4,ATP9A,CDC42BPB,VRK1,GNAI1,BMP2K,PDE6C,GNAQ,DSTYK,PFKFB4,NIN,DNAH8,TRIT1,SMARCA2,CGAS,IDE,KIF21A,KIF15,RERGL,BTAF1,PEX6,NEK10,MX1,CFTR,ME2,ATP10B,MAP4K3,MYO3A,UBE2E1,CDC42BPA,MAPK10,TXNRD2,TRPM6,PRKCH,HUNK,NLRC5,DHRS11,MKNK1,DMC1,MOK,RALB,MYO1D,ROCK1,LYN,KIF11,ZBTB33,CHKA,RRAGD,CRACR2A,INSR,NEK6,DPYD,ABCA4,ERN2,SAMHD1,ENPP1,ENTPD5,MOCS2,UCK2,CSNK2A1,HCN1,PRKG1,INO80,DHRS3,KIF21B,ABCG1,HADHA,ROR2,ACTR2,RIOK1,PRKAA2,DIRAS2,ERO1B,RAB12,KIFC1,MORC2,HIPK1,DGKK,FICD,CENPE,TUBB6,AKT3,JAK2,ZBTB38,ARL11,PRKCB,KIF6,ABCC8,RFC2,ZBTB21,NRBP1,ATP2B1,IARS2,AS*

			S1,SAR1A,ADCY9,MAST2,MAP2K6,DGKG,MYO18B,MAPK8,UBE2J2,RAPGEF4,HFM1,MYH13,ATP13A3,UBE2QL1,ACACA,ARL4C,MAP3K5,MAP3K4,PKN2,PDE2A,RAB38,PASK,FGR,TRIM23,ATP6V1B2,TOP1,TINAG,EPHA4,PPIP5K2,NTRK2,ACSM2A,TRPM7,PTK2,MARK4,DNAH10,RUNX1,SLC27A6,HKDC1,ACOXL,MDN1,FYN,BUB1,NLRP14,ARL13B,SCN8A,ATP9B,UGP2,MAPK9,ROR1,TET1,EPHB1,DDX10,ADCK1,RPS6KA5,PRKCA,GBP4,NLRP4,FHIT,PCCA,POR,ABCA6,EFL1,BCR,RERG,NLRP8,KIF16B,SNRK,STK36,RRAS2,GNA14,RABL2A,EPHB2,CSNK1G1,DPH6,MYO5B,CDK14,MET,CAMK1G,AIFM3,KALRN,ME3,GNAS,MFHAS1,DHX29,DNAH3,AK3,DDX6,UPRT,DNAH17,ABL2,TTLL11,CMPK1,ERBB4,PRKCQ,MYH15,AK9,NOS1,CHD9,MTREX,RAB27A,KIF13A,DNAH9,MYO9B,ORC4,LARS2,RAD51B,CAMK1D,AK2,FER,CHFR,AGAP1,ROCK2,NARS2,RAB31,PDK1,KIF7,CLCN5,PNPLA3,HSPA12A,STK3,PNPLA8,HNRNPU,IGF1R,KCNAB1,PRKAG2,ATP10A,SEPTIN6,DNM1L
GO:0106310	protein serine kinase activity	1.391067658976 6532e-7	*MTOR,ULK2,NLK,KSR1,MAP3K9,MYO3B,TNIK,NEK4,CDK12,PRKACB,NEK7,PRKD1,PAK1,TAOK3,RPS6KA2,DCLK1,AURKA,MAP4K4,PAK3,PRKCZ,RIPK4,MAPK1,STK38,DCAF1,TLK1,PAK5,DAPK1,STK32B,ALPK2,MELK,NUAK1,PRKAA1,MAST4,CAMK4,LATS2,PRKCE,WNK2,ALPK3,TRIO,RPS6KA3,STK38L,MARK2,HIPK3,STK10,STK32A,NRK,MYLK4,CDC42BPB,VRK1,BMP2K,DSTYK,NEK10,MAP4K3,MYO3A,CDC42BPA,MAPK10,TRPM6,PRKCH,HUNK,MKNK1,MOK,ROCK1,NEK6,ERN2,CSNK2A1,PRKG1,RIOK1,PRKAA2,HIPK1,AKT3,PRKCB,MAST2,MAP2K6,MAPK8,MAP3K5,MAP3K4,PKN2,PASK,TRPM7,MARK4,BUB1,MAPK9,RPS6KA5,PRKCA,BCR,SNRK,STK36,CSNK1G1,CDK14,CAMK1G,KALRN,PRKCQ,CAMK1D,ROCK2,STK3*
GO:0016773	phosphotransferase activity, alcohol group as acceptor	1.508339820510 8067e-7	*MTOR,ULK2,NLK,KSR1,PIK3C3,ALK,MAP3K9,MYO3B,TNIK,NEK4,EGFR,CDK12,PRKACB,NEK7,NTRK3,FLT1,GK,DGKI,PRKD1,PAK1,EPHA7,TAOK3,AGK,ADK,RPS6KA2,DCLK1,AURKA,MAP4K4,BMPR1B,PAK3,ITPKB,PRKCZ,RIPK4,MAPK1,PI4K2B,STK38,MYLK3,EFEMP1,DCAF1,TLK1,PAK5,DAPK1,STK32B,ALPK2,MELK,BLK,NUAK1,ABL1,PRKAA1,MAST4,CAMK4,PEAK1,LATS2,MUSK,PRKCE,WNK2,DGKB,ALPK3,TRIO,RPS6KA3,LTBP1,STK38L,MARK2,EPHA6,HIPK3,GRK3,STK10,MYLK2,CCND3,STK32A,CRIM1,NRK,MYLK4,CDC42BPB,VRK1,BMP2K,DSTYK,PFKFB4,NEK10,MAP4K3,MYO3A,CDC42BPA,MAPK10,TRPM6,PRKCH,HUNK,MKNK1,MOK,ROCK1,LYN,CHKA,INSR,NEK6,ERN2,UCK2,CSNK2A1,PRKG1,ROR2,RIOK1,SOSTDC1,PRKAA2,HIPK1,DGKK,AKT3,JAK2,PRKCB,BRD4,NRBP1,MAST2,MAP2K6,DGKG,MAPK8,MAP3K5,MAP3K4,PKN2,PASK,FGR,TOP1,EPHA4,NTRK2,TRPM7,PRKAB1,PTK2,MARK4,HKDC1,FYN,BUB1,MAPK9,ROR1,EPHB1,ADCK1,RPS6KA5,NRP1,PRKCA,BCR,SNRK,PGM2L1,STK36,EPHB2,CSNK1G1,CDK14,MET,CAMK1G,KALRN,ABL2,ERBB4,PRKCQ,CAMK1D,PIK3R3,FER,FGGY,ROCK2,PDK1,STK3,IGF1R,PRKAG2,AKAP13*
GO:0004674	protein serine/threonine kinase activity	5.283088762016 135e-7	*MTOR,ULK2,NLK,KSR1,MAP3K9,MYO3B,TNIK,NEK4,EGFR,CDK12,PRKACB,NEK7,PRKD1,PAK1,TAOK3,RPS6KA2,DCLK1,AURKA,MAP4K4,BMPR1B,PAK3,PRKCZ,RIPK4,MAPK1,STK38,MYLK3,DCAF1,TLK1,PAK5,DAPK1,STK32B,ALPK2,MELK,NUAK1,PRKAA1,MAST4,CAMK4,LATS2,PRKCE,WNK2,ALPK3,TRIO,RPS6KA3,LTBP1,STK38L,MARK2,HIPK3,GRK3,STK10,MYLK2,CCND3,STK32A,NRK,MYLK4,CDC42BPB,VRK1,BMP2K,DSTYK,NEK10,MAP4K3,MYO3A,CDC42BPA,MAPK10,TRPM6,PRKCH,HUNK,MKNK1,MOK,ROCK1,NEK6,ERN2,CSNK2A1,PRKG1,RIOK1,SOSTDC1,PRKAA2,HIPK1,AKT3,PRKCB,BRD4,MAST2,MAP2K6,MAPK8,MAP3K5,MAP3K4,PKN2,PASK,TOP1,TRPM7,MARK4,BUB1,MAPK9,ADCK1,RPS6KA5,PRKCA,BCR,SNRK,STK36,CSNK1G1,CDK14,CAMK1G,KALRN,PRKCQ,CAMK1D,ROCK2,STK3,PRKAG2,AKAP13*
GO:0045296	cadherin binding	0.000003500060 7915707286	*RDX,ERC1,EPS15L1,CDH8,CRKL,PTPRJ,EGFR,MACF1,CTNNA3,DIAPH3,SND1,CDH4,CAST,CTNNAL1,SEPTIN9,VCL,CD2AP,CTNNA2,LARP1,CALD1,PDXDC1,STK38,CTNNA1,CDH7,MRTFB,MPRIP,CDH11,USP8,OLA1,PTPRT,CDH18,ZC3HAV1,CDHR3,PTPRO,STXBP6,CTNND2,MARK2,EPN2,ANK3,CDH20,TJP1,PACSIN2,PDLIM5,LRRFIP1,CDH23,PKP1,DOCK9,PTPRB,BZW1,FLNB,ESYT2,SH3GLB1,SNX9,CDH26,VASP,IQGAP1,LASP1,ANLN,ZC3H15,SERBP1,OLFM4,ITGA6,UBAP2,EFHD2,STAT1,ABI1,PARK7,PPME1*

			,PKN2,NCK1,NUMB,KTN1,CDH5,MPP7,ASAP1,CDH9,CDH2,CDH12,CDH17,CDH13,DHX29,GAPVD1,DDX6,NDRG1,FER,PCMT1
GO:0005096	GTPase activator activity	0.000007523794305872622 5	*GARNL3,MYO9A,PLCB1,DLC1,ARHGAP26,RIN2,ARHGAP24,RGS3,DOCK2,RABEP1,TBC1D19,RAPGEF2,ADGRB3,RALGAPA1,TBCD,ARHGAP44,RANBP2,RGS20,RAP1GDS1,ARHGAP32,RGS9,RABGAP1L,TBC1D22A,CHN1,ARAP2,ARHGEF12,TBC1D9,RANBP3L,TBC1D5,DOCK4,ASAP2,RGS12,DOCK5,EVI5,RALGAPA2,SGSM1,TBC1D4,RIN3,ARHGAP42,GNAQ,DOCK1,RAP1GAP,SRGAP2,SEC23B,TBC1D13,ALS2,RACGAP1,VAV3,ARHGAP28,ARHGAP31,IQGAP1,SIPA1L2,AGAP9,ARHGAP12,ASAP1,TBC1D1,NRP1,BCR,RGS6,ARFGAP3,SRGAP3,RGPD4,TIAM2,GAPVD1,RGPD2,SIPA1L3,ARHGEF11,MYO9B,STARD13,AGAP1,RGS8,RGS7,RAB3GAP2,DNM1L*
GO:0005516	calmodulin binding	0.00002180126627878591 7	*KCNH5,UNC13C,PLCB1,MYO5A,ARPP21,MYO1E,MYO5C,SPATA17,ATP2B2,PDE1C,INVS,SLC8A3,CACNA1C,PHKB,SYT1,SLC8A1,PDE1A,ITPKB,CALD1,RYR3,EWSR1,RASGRF2,DAPK1,SLC24A4,RYR2,KCNH1,MYO10,CAMK4,ASPM,NOS2,MYLK2,MBP,TJP1,STRN,UNC13B,PCP4,MAP2,MYO3A,MKNK1,KCNQ3,KCNN3,MYO1D,PCNT,MAP6,IQGAP1,TRPV5,ATP2B1,GAP43,MYH13,SNTB1,MYO5B,CAMK1G,MYH15,NOS1,MYO9B,KCNQ5,CAMK1D,SNTB2*
GO:0036094	small molecule binding	0.00002252169275821980 4	*MTOR,ABCA13,MYO9A,ULK2,NLK,LONP2,KSR1,MX2,ARL15,MICAL3,NUBPL,ITPR2,PDE4D,RALA,NME7,MYO5A,ALDH1A2,GPHN,PIK3C3,NAV2,MYO1E,ALK,ERCC6L2,HLCS,EGLN3,MAP3K9,MYO3B,MOCOS,MYO5C,TNIK,NEK4,EGFR,CDK12,PRKACB,NEK7,ATP2B2,NTRK3,FLT1,GK,THRAP3,DGKI,PRKD1,PAK1,GMDS,EPHA7,ADSS2,GRAMD1B,RAPGEF2,RUNX2,CPS1,TAOK3,AGK,RANBP17,UBE2L3,SYN2,TYW1,LRGUK,GRM7,SEPTIN9,DNAH6,KIF4A,DHX32,UBE2G1,ABCB5,ADK,RPS6KA2,KYNU,KDM1B,DCLK1,PARP15,AURKA,ABCG8,MAP4K4,ABCD2,BMPR1B,RAB8B,PAK3,TTLL7,ITPKB,TRPC5,PDE10A,UBE2E2,HHAT,RNLS,DNM3,CUBN,SCP2,SYN3,PRKCZ,MSH6,RAB27B,ABCA5,PDXDC1,RIPK4,MAPK1,KCNJ1,ABCD3,DNAH14,TRPC7,UBE2O,PI4K2B,RNGTT,CHD6,STK38,MYLK3,ACSBG1,DCAF1,TLK1,PPIP5K1,PAK5,DAPK1,NAV3,ACSM2B,STK32B,MAGI1,ALPK2,DNAH11,RAB22A,MELK,CLPX,SMARCA4,EFTUD2,UBE2R2,BLK,OLA1,ATRX,NUAK1,ABL1,AGPS,SLC1A1,PRKAA1,TTLL5,APLF,MAST4,DNAH5,GUCY1A2,SLFN11,RAP1A,ACSS3,MYO10,CAMK4,MANBA,PEAK1,LATS2,ATP11C,DHX40,ABCB7,MUSK,ACTR3C,SMARCAD1,ABCC12,PRKCE,WNK2,DGKB,ALPK3,ABCC9,P2RX6,TRIO,NLRP13,RPS6KA3,ATP8A2,SCG5,RFC1,SLC2A3,ATP8A1,STK38L,ABCC4,KIAA0232,FANCM,FARS2,GTF2F2,MARK2,MSH2,MPPED2,GNAL,EPHA6,ATL1,HIPK3,ABCA10,GRK3,NOS2,AFG3L2,STK10,MYLK2,GBP6,STK32A,AK8,GSR,TARS3,MTHFD1L,AQR,BLM,NRK,MAGI3,ADCY10,MYLK4,ATP9A,CDC42BPB,VRK1,GNAI1,BMP2K,PDE6C,GNAQ,DSTYK,PFKFB4,NIN,DNAH8,TRIT1,GADL1,SMARCA2,CGAS,IDE,KIF21A,KIF15,RERGL,BTAF1,PEX6,NEK10,MX1,CFTR,ME2,ATP10B,UBR1,MAP4K3,MYO3A,UBE2E1,CDC42BPA,MAPK10,TXNRD2,TRPM6,PRKCH,HUNK,NLRC5,DHRS11,MKNK1,DMC1,MOK,RALB,MYO1D,ROCK1,LYN,KIF11,ZBTB33,CHKA,RRAGD,CRACR2A,INSR,NEK6,DPYD,OSBPL10,RPH3A,ABCA4,ERN2,SAMHD1,ENPP1,ENTPD5,MOCS2,UCK2,CSNK2A1,HCN1,PRKG1,GRIN2B,INO80,ETNPPL,DHRS3,KIF21B,ABCG1,HADHA,ROR2,KL,ACTR2,RIOK1,PRKAA2,DIRAS2,ERO1B,RAB12,KIFC1,MORC2,THNSL2,HIPK1,DGKK,FICD,CENPE,TUBB6,AKT3,JAK2,ZBTB38,ARL11,PRKCB,GOT2,KIF6,ABCC8,RFC2,ZBTB21,NRBP1,ATP2B1,IARS2,ASS1,SAR1A,ADCY9,MAST2,ERLIN2,SDS,MAP2K6,DGKG,MYO18B,CBLIF,PARK7,MAPK8,UBE2J2,RAPGEF4,HFM1,MYH13,ATP13A3,DHTKD1,UBE2QL1,ACACA,ARL4C,RXRA,MAP3K5,MAP3K4,PKN2,PDE2A,RAB38,PASK,FGR,TRIM23,ATP6V1B2,TOP1,TINAG,EPHA4,PPIP5K2,NTRK2,ACSM2A,COLEC12,TRPM7,PTK2,MARK4,DNAH10,RUNX1,SLC27A6,HKDC1,ACOXL,MDN1,FYN,BUB1,NLRP14,ARL13B,SCN8A,ALB,ATP9B,UGP2,MAPK9,ROR1,TET1,EPHB1,DDX10,ADCK1,RPS6KA5,PRKCA,GBP4,NLRP4,FHIT,PCCA,POR,ABCA6,EFL1,BCR,RERG,NLRP8,KIF16B,SNRK,STK36,MB,CYP4B1,RRAS2,GNA14,RABL2A,EPHB2,CSNK1G1,DPH6,MYO5B,CDK14,MET,CAMK1G,AIFM3,KALRN,ME3,GNAS,MFHAS1,DHX29,ASTN2,DNAH3,AK3,DDX6,UPRT,DNAH17,ABL2,TTLL11,CMPK1,PLCL1,ERBB4,PR*

			KCQ,MYH15,AK9,NOS1,GLDC,CHD9,MTREX,RAB27A,KIF13A,DNAH9,MYO9B,ORC4,LARS2,RAD51B,CAMK1D,AK2,FER,OSBPL5,CHFR,OSBPL6,OARD1,AGAP1,ROCK2,NARS2,RAB31,PDK1,KIF7,CLCN5,PNPLA3,HSPA12A,STK3,PNPLA8,HNRNPU,IGF1R,KCNAB1,PRKAG2,ATP10A,SEPTIN6,DNM1L
GO:0016772	transferase activity, transferring phosphorus-containing groups	0.00004803378832631001	*MTOR,ULK2,NLK,KSR1,NME7,GPHN,PIK3C3,ALK,MAP3K9,MYO3B,TNIK,NEK4,EGFR,CDK12,PRKACB,NEK7,GNPTAB,SGMS1,NTRK3,FLT1,GK,DGKI,PRKD1,PAK1,EPHA7,TAOK3,AGK,LRGUK,ADK,RPS6KA2,DCLK1,AURKA,MAP4K4,BMPR1B,PAK3,ITPKB,PRKCZ,RIPK4,MAPK1,PI4K2B,RNGTT,STK38,MYLK3,EFEMP1,DCAF1,TLK1,PPIP5K1,PAK5,DAPK1,STK32B,ALPK2,MELK,MAPKAP1,BLK,NUAK1,ABL1,PRKAA1,MAST4,CAMK4,PEAK1,LATS2,MUSK,PRKCE,WNK2,DGKB,ALPK3,TRIO,RPS6KA3,LTBP1,STK38L,MARK2,EPHA6,SH3KBP1,HIPK3,CDKN2C,GRK3,STK10,MYLK2,CCND3,STK32A,AK8,CRIM1,CEPT1,NRK,MAGI3,MYLK4,CDC42BPB,VRK1,BMP2K,POLR3A,CDS2,DSTYK,PFKFB4,CGAS,NEK10,PIGN,MAP4K3,MYO3A,AKAP10,CDC42BPA,MAPK10,TRPM6,PRKCH,HUNK,MKNK1,POLR1D,MOK,ROCK1,LYN,EIF2B3,CHKA,INSR,NEK6,ERN2,UCK2,CSNK2A1,PRKG1,ROR2,RIOK1,SOSTDC1,PRKAA2,HIPK1,DGKK,FICD,AKT3,JAK2,PRKCB,BRD4,NRBP1,MAST2,MAP2K6,DGKG,MAPK8,MAP3K5,MAP3K4,PKN2,PASK,FGR,TOP1,EPHA4,PPIP5K2,NTRK2,TRPM7,PRKAB1,PTK2,MARK4,CNKSR1,HKDC1,FYN,BUB1,UGP2,MAPK9,ROR1,EPHB1,ADCK1,RPS6KA5,NRP1,PRKCA,FHIT,BCR,TUT4,SNRK,PGM2L1,STK36,EPHB2,CSNK1G1,CDK14,MET,DLG2,CAMK1G,MAGI2,KALRN,AK3,ABL2,CMPK1,ERBB4,PRKCQ,AK9,CAMK1D,PIK3R3,AK2,FER,FGGY,ROCK2,PDK1,STK3,IGF1R,PRKAG2,AKAP13*
GO:0046873	metal ion transmembrane transporter activity	0.00007868769891244554	*CACNA2D3,SLC17A1,SLC24A2,KCNH5,ITPR2,KCNMA1,CHRNA7,CACNG2,SLC4A10,GRIK3,ATP2B2,TUSC3,SLC39A12,SLC8A3,TMEM38B,SLC24A3,TRPM1,SLC39A11,CACNA1C,CACNB2,TMC1,SLC8A1,KCNE4,KCNK10,TRPC5,RYR3,KCNJ1,TRPC7,NIPAL2,LRRC38,GRIK4,KCNS3,SLC24A4,SCN2A,RYR2,SLC9C1,SLC1A1,SLC12A8,KCNH1,ABCC9,OPRM1,CNNM4,KCND2,NIPA2,CACNA1I,KCNJ15,SCN11A,SLC23A2,SLC39A6,KCNH8,SLC9A4,GRIK2,SLC30A10,GRIN2A,TRPM6,SLC12A1,KCNQ3,SLC4A4,SCN10A,KCND3,KCNN3,SLC13A5,CUL5,ZDHHC17,KCNC1,HCN1,GRIN2B,KCNK5,SLC40A1,SLC5A12,SLC1A7,TRPV5,ABCC8,CACNA1E,ATP2B1,SLC6A1,SHROOM2,SLC6A11,KCNJ18,SLC5A9,SLC10A6,TRPM7,GRIK1,SLC9A5,SLC5A1,SCN8A,NCS1,NALCN,TRPM3,SLC39A8,KCNJ6,CACNG3,SLC1A2,KCNIP4,SLC6A3,ASIC2,KCNQ5,CACNA2D1,CATSPER2,SLC13A4,KCNAB1*
GO:0030234	enzyme regulator activity	0.00008504886562065322	*BCAR3,SPOCK1,GARNL3,MYO9A,PLCB1,DLC1,ARHGAP26,RIMS1,FGD4,SPRED1,ALK,RIN2,APC,ARHGAP24,DOCK10,EGFR,DENND1A,USP14,RGS3,DOCK2,C5,PHACTR1,RABEP1,DGKI,CAST,TBC1D19,RALGPS1,RAPGEF2,ADGRB3,TAOK3,UBE2L3,PPP2R2B,HERC2,GRM7,RPTOR,RALGAPA1,RAPGEF5,TBCD,PPP1R12B,PHACTR2,APP,DOCK8,ARHGAP44,SERPINA6,BMPR1B,RANBP2,RGS20,RAP1GDS1,ARHGAP32,RGS9,MRTFA,MGAT5,RABGAP1L,ITIH5,PPP1R1C,ARHGEF17,NRG3,UBE2O,CARD18,TBC1D22A,CHN1,ECT2L,CCNG2,MOB3B,BIRC6,RASGRF2,MRTFB,PPP6R3,RGL1,TIAM1,ARAP2,ARHGEF12,PPP2R5E,RIC8B,TBC1D9,ADGRV1,BCAS3,CLPX,RANBP3L,TBC1D5,DOCK4,PSMF1,RAP1A,NRG1,DENND2B,RASGRF1,PRKCE,ASAP2,DENND4C,RGS12,EGF,TRIO,NSMAF,SPRED2,RPS6KA3,SCG5,MCF2L,RFC1,ARHGEF7,PPP2R2C,MARK2,HERC1,CDKN2C,KNDC1,MNAT1,CCND3,DOCK5,PLCE1,TGFA,CRIM1,VAV1,IQSEC1,PHACTR3,BMP2,EVI5,RALGAPA2,SGSM1,TBC1D4,RIN3,BMP2K,CABIN1,ARHGAP42,GNAQ,SH3BP5,CPAMD8,DOCK1,RAP1GAP,SRGAP2,SEC23B,PSD3,FARP1,MOB1B,UMODL1,MAPK8IP1,TBC1D13,RCAN1,SH3PXD2A,NGEF,ALS2,RACGAP1,DOCK9,VAV3,ITSN2,ARHGEF28,DENND2C,ROCK1,ARHGAP28,ARHGAP31,EIF2B3,MMP16,ARFGEF1,RALGPS2,RASGRP1,SERPINB9,PSAP,GPRC5C,PPP2R2A,RASGEF1C,ITPRIP,IQGAP1,FRY,NET1,SIPA1L2,ALKAL2,MADD,PCNA,ANXA4,AGAP9,PPP1R17,ARFGEF3,MAP2K6,ABI1,PARK7,MAPK8,OAZ2,PPME1,NCAPG2,RAPGEF4,SERPINI2,DBF4B,NCK1,PPP2R3A,DNMBP,TRIM23,CYTH4,DNMT3L,RCAN2,ARHGAP12,SERPINB10,RASGEF1B,ASAP1,CCDC*

			88A,FYN,SH2D3C,DOCK3,NCS1,GRM5,TBC1D1,NRP1,BCR,ELMO1,RGS6,ARFGAP3,FBLN1,SRGAP3,MACROH2A1,BCL2L13,RGPD4,SERPINB2,KALRN,GNAS,SERPINB7,TIAM2,TNFAIP8,GAPVD1,WDR41,SLIT2,ERBB4,SERPINB11,RGPD2,SIPA1L3,EFNA5,ARHGEF11,ESR1,MYO9B,PIK3R3,STARD13,A2M,SPOCK3,AGAP1,RGS8,PSMD2,COL4A3,RGS7,STK3,RAB3GAP2,PRKAG2,AKAP13,DNM1L
GO:0005488	binding	0.000153739643158278	*EBNA1BP2,NOTCH2,BCAR3,MTOR,CNTN4,CACNA2D3,SPOCK1,NSG1,EXOC1L,WWC1,ABCA13,IMMP2L,LRP12,PTPRD,FREM1,TRAPPC9,BNC2,PVT1,NEBL,LRRC4C,TMTC1,KCNH5,MICU2,ANKS1B,POTEG,SMOC1,MYO9A,ULK2,NLK,LONP2,UNC13C,LRRC49,SCAPER,FTO,KSR1,MGA,RFX7,AGBL1,ZNF236,PLCB1,ZNF536,TTC3,MX2,TMPRSS2,LIPI,TAFA5,SVIL,TLN2,CLTCL1,ZFPM2,ARL15,MICAL3,POTEM,TENM4,NUBPL,L3MBTL4,DLC1,TNRC6B,MGAM,DPP10,ZDHHC21,PTPRA,ITPR2,RIPOR2,PDE4D,RDX,RP1,STXBP1,ERC1,RALA,NME7,SLC44A5,EPS15L1,BCL2,MYO5A,ODAD2,KCNMA1,SYT16,ARPP21,PRDM16,ALDH1A2,ARHGAP26,FBN1,LRFN2,LPCAT2,F13A1,LRRTM4,SETBP1,GPHN,COG5,CDH8,CHRNA7,DCDC1,ROBO2,PUDP,RIMS1,PIK3C3,SPIRE1,TENM3,GABRB3,ZEB1,TMEM132D,AKR1C3,CNTLN,SDCCAG8,RARB,FGD4,EXOC6B,SPRED1,GALNT1,NAV2,ENPEP,SPAG16,MYO1E,TRAPPC8,PLPPR1,USH2A,CEP192,MINAR1,CDC42EP3,LAIR1,TTC33,RIMS2,PCMTD1,ALK,MICOS10,AUTS2,ADGRE1,PCDH7,FOXJ2,CDYL2,CARMIL1,MCTP1,PJA2,COL25A1,BABAM2,SV2C,PAPPA2,GLIS3,FANK1,ERBIN,ERCC6L2,RHPN2,HACD2,ASTN1,UNC79,HLCS,FCHO2,RIN2,PARVB,ANO6,CACNG2,DLGAP1,NEGR1,ZNF880,GLYAT,NAALADL2,MLLT3,EGLN3,GPC6,SUSD4,CNTNAP2,MAP4,MAP3K9,MYO3B,MOCOS,SPON1,CPA6,APC,ZMYM4,ZNF595,HHLA2,TSHZ3,RBFOX3,DSCAM,MYO5C,RTN1,TCF4,CRKL,SOX5,SETD2,ERG,ARHGAP24,ZNF573,TNIK,URB1,PTPRJ,EFCAB2,OCA2,KDM4C,NEK4,DOCK10,TSHZ2,EGFR,ZNF280B,RFX3,DENND1A,USP14,ANGPT1,CDK12,BACH1,MACF1,CTNNA3,PRKACB,NEK7,RGS3,NCOR1,RNF220,HMCN2,DOCK2,DIAPH3,ZNF407,CCDC138,NEDD4,MYOF,MAML2,MTRF1,SPATA17,SND1,SCAI,GNPTAB,CRB1,NSMCE2,BTBD9,BCL11A,SOX6,FAM83F,TMEM182,PSMB2,SGMS1,CECR2,ARMC2,CHSY1,FLI1,RPRD1A,PTPN4,CDH4,B3GALT5,ATP2B2,NTRK3,LARGE1,RXFP1,C5,PDE1C,FBXL7,ZFAND6,CYP2C9,PHACTR1,DKK2,FLT1,DNAJC13,ZNF648,RFC3,RABEP1,ADAMTS6,ZNF382,GK,TASP1,FNDC3B,CNTN3,THRAP3,MAPKBP1,AOAH,EVA1C,GABRB1,DGKI,INVS,C12ORF4,EDAR,GRIA1,CRACD,CAST,TTC39B,NUP214,NEO1,CNTN6,CABLES1,SLC8A3,UHRF1BP1L,MALRD1,TOM1L2,NELL2,SEZ6L,PRKD1,TBC1D19,PAK1,GMDS,EPHA7,CTNNAL1,NCOA7,KHDRBS2,CHRM3,ADSS2,GRAMD1B,RALGPS1,SPEN,CHSY3,RAPGEF2,PELI2,LRP2,ADGRB3,DEUP1,RUNX2,ARSB,FGF12,CPS1,TAOK3,ONECUT1,LDLRAD3,CPEB4,TMEM38B,AGK,CSTF3,BCKDHB,PRICKLE2,RANBP17,UBE2L3,LDB2,TAFA4,SAMD5,PPP2R2B,BTBD11,PUM3,SYN2,CCL28,SMYD3,PATJ,TYW1,HERC2,LRGUK,TMEM241,GRM7,SEPTIN9,RETREG1,RPTOR,DNAH6,GHR,WDSUB1,EPB41L3,KIF4A,THADA,DHX32,COL4A2,AIG1,SSBP3,TMEM74,RALGAPA1,CELF2,RAPGEF5,TBCD,NEDD4L,ADAM32,PPP1R12B,TRPM1,ADAM10,HDAC9,ZHX3,ATF7IP,UBE2G1,IL1R1,APBB2,PHACTR2,APP,ABCB5,ADK,RPS6KA2,SAMSN1,KYNU,CACNA1C,KDM1B,CACNB2,KLHL13,MTUS1,PHKB,DCLK1,STAU2,GABRG2,DOCK8,MAPRE2,ZNF600,USP18,SEMA5A,SYT1,VCL,ARHGAP44,NTF3,ACER2,PARP15,NDUFAF2,CD2AP,ZNF723,AURKA,PARN,TTC29,ST18,PYGO1,SLC8A1,HERPUD2,CCDC116,SSBP2,PTPRR,SRGAP2C,ANKRD31,FIG4,DUX4,TAFA2,MARCHF1,CMIP,ABCG8,SERPINA6,PLGRKT,FRMD3,UPP2,CCSER2,LOXHD1,ECPAS,SRGAP2B,KANK1,KCNE4,MAP4K4,HIVEP2,ABCD2,BMPR1B,FMN2,THSD7A,PCSK6,AKAP6,HOMER2,ZNF717,CTNNA2,HADHB,POTEH,ARNT,RAB8B,PDZRN4,PAK3,RFTN1,PDE1A,ZNF257,TTLL7,DIP2B,RANBP2,LARP1,ITPKB,TRPC5,RGS20,PDE10A,UBE2E2,RAP1GDS1,HHAT,RNLS,CLIC6,CHST8,KICS2,ERC2,TMEM236,DNM3,NBN,CUBN,SCP2,SYN3,IFT57,PHF21B,INTS7,RBM47,SUSD6,PRKCZ,CALD1,SNTG2,KLHL1,SPOP,BTLA,MAN2A2,GRB10,RALYL,RYR3,TAF15,DIP2A,MSH6,MCPH1,ARHGAP*

			32,RAB27B,COL27A1,ZSWIM6,FER1L6,ST8SIA5,CNST,HEATR4,HECW1,DEFA3,LRRC7,MBNL2,ADAMTS17,C7ORF31,ABCA5,PHF19,MRTFA,TAF4B,ANKRD33B,COBL,SENP6,DUSP22,GALNT14,PDXDC1,EBF2,UBN1,SV2B,YAP1,ESS2,SEM1,NFIA,WDR70,PPM1L,RIPK4,ZKSCAN5,SHC4,VPS35L,BRINP1,SCGB2B2,MAPK1,MGAT5,CADPS2,KCNJ1,ABCD3,RABGAP1L,SGTB,DNAH14,TRPC7,ADAM22,USP25,CRISPLD2,KMT2E,ALCAM,PLG,PAPPA,PCGF5,PDGFD,C2ORF88,COPB1,SYT10,ZNRF3,DNAJC21,CCDC150,CA5A,XXYLT1,MTUS2,PPP1R1C,ABLIM1,CCDC172,ITGBL1,ARHGEF17,NRG3,UBE2O,SFMBT2,ANKFY1,NCAM1,GFRA1,SYCP1,NIPBL,RNF17,SLC16A1,SPIDR,GALNT16,PI4K2B,RNGTT,IPO11,EWSR1,GABPA,FAT3,MICU1,ZNF735,CORO2B,CARD18,CHD6,STK38,LCE1F,PTPN13,TBC1D22A,CHN1,SORCS3,MYLK3,ACSBG1,KANSL1,GLP2R,LIMCH1,FMN1,ECT2L,MBNL1,PAFAH1B1,ATF6,EFEMP1,TLL1,ZNF684,DCAF1,ITGB8,STON2,ZFAND3,VPS13D,TLK1,TPM1,NF2,LRRC38,WDR25,CNKSR2,RBFOX1,WDFY4,C1ORF21,HIVEP1,CORIN,CTNNA1,PPP1R9A,ANKRD11,EFCAB8,CDH7,MOB3B,BIRC6,AKAP9,KLF15,RASGRF2,PPARA,PPIP5K1,MEIS2,SNX30,LCLAT1,NFIB,KCNS3,SNX29,ERMP1,MRTFB,PPP6R3,PRTG,RGL1,SYNJ1,NR5A2,ADAMTS3,TIAM1,MPRIP,ARAP2,GRM1,FOXJ3,UBE3D,KAZN,RSRC1,PTPRK,ARHGEF12,GABRG1,ENAH,PAK5,ST6GALNAC3,TRERF1,SF3B6,PARD3B,PCDH11Y,PPP2R5E,PDZRN3,KIAA1958,PLA2R1,GIPC2,EIF3D,TMCO4,SEMA3C,DAPK1,NAV3,SLC24A4,SEC14L1,TMEM108,ACSM2B,AGO2,WDHD1,STK32B,PHC3,MAGI1,ALPK2,DNAH11,JARID2,SCN2A,RIC8B,TBC1D9,RAB22A,SORCS1,DNAJC15,AMPH,GATAD2B,CPE,PALS2,DYSF,IL34,ANK2,STAG2,BRWD1,TANC1,THUMPD2,ADGRV1,ZNF846,MELK,BCAS3,RYR2,SYNE2,BBS2,PEBP4,WNT9B,ZNF606,MSANTD4,CLPX,RANBP3L,NKG7,SEMA6D,AIF1L,NBEA,SHOC1,DUSP16,ANKS6,SRFBP1,SMARCA4,MRPS35,CDH11,USP8,LDB3,FABP7,NOL4,PARD3,SLC36A1,MAPKAP1,EFTUD2,TNRC6C,PIAS1,TBC1D5,SPG21,UBE2R2,BLK,COL23A1,RBM6,EBF1,TNR,OLA1,DST,CXADR,DOCK4,MBD5,ATRX,NUAK1,PTPRT,XIRP2,ELAVL4,ABL1,KLHL32,AGPS,MXI1,PTPN12,HDAC4,OXR1,SLC1A1,PRKAA1,SDC2,GAS2,SLC12A8,KCNH1,ITGB3BP,MRPS27,LRFN5,RIMBP2,CRTAC1,CRYBG1,DROSHA,TTLL5,APBB1IP,ANO4,L3MBTL3,DMXL2,EIPR1,APLF,NFAT5,ADAMTS14,MAST4,DNAH5,GUCY1A2,NBAS,CDH18,PSMF1,ATE1,SLFN11,RAP1A,GLIS1,TMCO5A,ACSS3,MORC1,LYRM4,MYO10,SLC46A3,GPC5,TOX3,ZNHIT6,CAMK4,BAZ2A,MANBA,PLEKHA8,INPP5A,CPSF3,FGF10,FBXL13,C2ORF42,ZC3HAV1,UQCC1,GRID2,CDHR3,TGM1,PEAK1,LATS2,NRG1,GSG1L,KLHL33,CLIP1,ASPM,AP3B1,DENND2B,COL6A5,EFCAB6,RASGRF1,PAH,ATP11C,GSE1,ZNF438,DHX40,ABCB7,SYNE1,ZBTB16,MUSK,GALNTL6,KIR3DL2,ZNF675,ACTR3C,GNG7,SMARCAD1,SH3GL3,ABCC12,SETDB2,RPF1,PRKCE,FOXK2,SLCO3A1,PGM5,ASAP2,MED15,SLMAP,METAP1D,WNK2,ESRRG,ZNF718,C12ORF40,DGKB,USP33,DENND4C,CEP83,CERS6,FBN2,CD44,RGS12,ZNF831,PTPRO,EGF,ALPK3,PRRC1,ABCC9,P2RX6,TRIO,PDE3A,EXT1,STXBP6,COL5A3,NSMAF,NLRP13,LNPEP,LIMD1,PEX14,SPRED2,ADAMTS2,RPS6KA3,CTNND2,MARCHF8,IFT43,ATP8A2,SCG5,MTMR3,PTPN2,TRIM5,LHFPL3,PLXNA2,POC5,MCF2L,ATXN3,RFC1,HTR2C,RIC3,SLC2A3,ARHGEF7,ALG10B,ATP8A1,ZCCHC7,AMBRA1,RFTN2,LTBP1,STK38L,ZFYVE9,GALNT10,GUCD1,KDM7A,OPRM1,ABCC4,PRMT8,HTR2A,KIAA0232,BIN2,PLCXD3,FANCM,FANCA,CYBRD1,CYP4A11,DAZL,INPP4B,MATN2,FARS2,GTF2F2,PPP2R2C,CNNM4,KREMEN1,STAC,ANKRD28,SEMA3E,TAF3,RPRD1B,MARK2,GCSAML,GMPR,TMEM67,RCL1,EBF3,ALPL,ZNF33B,LPP,C10ORF90,FHL2,ABHD17C,ADGRA3,CNIH3,PUM1,TMOD2,HERC1,MSH2,IGF2BP3,MPPED2,IGLV2-14,GNAL,CDIN1,EPHA6,ANKRD17,APBA2,MAIP1,LINGO2,ZNF397,SH3KBP1,ATL1,SLC2A13,LUC7L,RELL1,HIPK3,CDKN2C,EPN2,KCND2,TNPO3,SNRPN,ABCA10,GRK3,CPXM2,CD163,SPSB4,CLSPN,NOS2,BICRAL,AFG3L2,CPNE4,STK10,TTC7B,MNAT1,RBBP8,MDFIC,SGCZ,TMTC2,ADAM12,MYLK2,ANK3,SNTG1,EMILIN2,XYLT1,HMGA2,MYOM2,COG2,GBP6,CCND3,BCL11B,VPS41,FOLH1,DOCK5,F5,ECE1,KLHL4,ZIM3,STK32A,IGLV3-

			27,CREM,LYPLA1,MBP,AK8,TRPS1,TRAPPC11,TMEM163,HHIPL1,PLCE1,TGFA,IL17RA,ANKFN1,HIP1,CRIM1,XPNPEP1,FUT9,PRR5L,VPS37A,GSR,PCDH9,ATP6V1E1,UTP4,VAV1,CYP4Z1,CDH20,MSRA,RUFY2,MYT1L,FBXO32,ZNF160,TJP1,LDLRAD4,NPHP4,EGFLAM,PACSIN2,CNTN1,HLA-B,TARS3,FKBP5,IQSEC1,HSF5,MTHFD1L,SNX3,CACNA1I,NAA35,ZNF367,PDLIM5,KCNJ15,CEPT1,BRCA2,AQR,DISC1,ZBTB2,GALNT13,EXD3,DNER,BLM,ASB7,WDPCP,NRK,SEMA3A,MAGI3,HSF2BP,INTS8,NAP1L4,LIN54,LRP1B,ADCY10,PSG8,STRN,AGL,OR9Q1,ZNF121,ANKRD30BL,STX12,PHACTR3,BMP2,RC3H2,MYLK4,UNC5D,ATP9A,FAM217B,TRAK1,WDR26,PSG9,CDC42BPB,EVI5,DSE,PTCD2,MSR1,VRK1,GNAI1,RALGAPA2,SGSM1,ZC3H14,NCAM2,GFI1B,TBC1D4,RANBP9,RESF1,MYRIP,TTR,HPCAL1,RIN3,MSI2,TPGS2,BMP2K,DNAL1,SLC15A5,RNF38,TTC6,PGPEP1,TMEM161A,SEMA3D,ASXL3,NETO2,PDE6C,ANKRD7,CABIN1,POLR3A,LEMD3,RELN,ARHGAP42,HMGB1,GNAQ,TUBGCP3,NUDCD3,CDS2,AP4E1,FGF9,NFATC2,TDRD7,SH3BP5,CPAMD8,RTTN,MDM1,ZNF106,C19ORF18,MYOM1,ZNF567,CLVS1,TRAF3,ZNF462,PRG4,ANKRD26,ESRP1,UNC13B,TTC21B,ETS2,UBAP2L,GEMIN5,ZNF875,DSTYK,UIMC1,DOCK1,B4GALT6,LRRFIP1,TSPAN2,PFKFB4,RAP1GAP,PLS1,SRGAP2,IKZF2,SNX8,SEC23B,ENOX1,SLC39A6,NIN,HAUS6,DRAXIN,DNAH8,TRIT1,ATF1,GADL1,CCDC186,SLAMF1,SMARCA2,ETS1,FAM83B,ARSJ,GLI3,CGAS,MEGF11,SMARCC1,SNX6,AFF3,GABRR2,SMOC2,PACS1,PCP4,CNKSR3,CASP5,VENTX,IDE,WDR12,MCTP2,KIF21A,KIF15,PRDM10,RERGL,CUL1,MYEF2,ZFYVE26,ZNF431,RERE,PSD3,MAP2,ANKMY1,BTAF1,GAREM1,DAW1,MYL1,FBXO47,PEX6,ZNF618,NEK10,RRBP1,FARP1,MOB1B,ATF2,NDUFAF6,GOLGA8B,HIRA,CYLD,UMODL1,BBS4,ADARB2,LRRC8B,MAPK8IP1,MX1,TMEM171,ZMAT4,CLVS2,ANTXRL,PSG6,HIVEP3,COL5A1,GABBR2,PSIP1,ITGA9,KIAA0753,CFTR,MYEOV,KPNA1,CSE1L,NELL1,DOP1B,ME2,TBC1D13,UBASH3A,AHDC1,FAM214A,COL14A1,RGMB,NEU3,PHAF1,CEP44,MRPL13,KITLG,ZZEF1,DNAJC7,ATP10B,CAMTA1,UBR1,DCC,MYT1,RNU2-47P,SMPDL3A,CHRM5,MAP4K3,YLPM1,YPEL1,SLC30A10,RCAN1,GTF2I,RORB,CHAF1A,TADA2A,DAB1,MED27,ZNF208,SELENON,RB1CC1,NMD3,MYO3A,AKAP10,UBE2E1,PTPRE,REPS1,PRKN,AGMO,MTMR2,ZNF608,SH3PXD2A,ZFAND4,SPSB1,CDC42BPA,TBX20,SP110,CCDC102B,DLGAP2,AFAP1,MAPK10,DACH1,PCDH15,ZNF541,FBXO3,RWDD2B,DPF3,LGI2,LYST,NGEF,HEPHL1,GRIN2A,ARID5B,ZBED9,H2BC15,JPH1,TXNRD2,ATXN1,WSB1,TRPM6,CDH23,LALBA,PRKCH,PKP1,HUNK,TG,IL6R,FRMPD4,PEPD,ALS2,RACGAP1,NLRC5,ZNF627,OR51E1,ACO1,ANKRD30A,TFDP1,DHRS11,CNOT6L,MKNK1,HEMGN,KANK4,DOCK9,SNX25,DMC1,FBLN5,LCE3B,KCNQ3,TOX,POLR1D,SHISA9,SLC4A4,PTPRB,ZFP90,AOPEP,TRMT61B,PDE6A,COPS8,TSPAN33,TBATA,ZNF124,SCN10A,LRBA,RBMX2,ANKRD55,SHANK2,ST8SIA1,ANKRD18A,MAP7,USP7,VAV3,PSMA1,MON2,LRRC37A3,ENPP3,TASOR2,PLAGL1,KCND3,HAAO,FAH,MESD,ITSN2,SOX30,PTGFRN,KIAA0825,MOK,SYBU,KIR2DL4,ARHGEF28,RALB,NPAS2,ADGRG6,YIPF6,KCNN3,MYO1D,SEC24D,PPA2,FAR1,CA1,ROCK1,LYN,VCAM1,SEL1L,ARHGAP31,ZNF780B,CTSB,EIF2B3,LRIG1,TTC37,SUMO3,SLC15A2,ZNF169,PLEKHB2,KIF11,DTX1,BZW1,TENM2,OVOL2,PIWIL3,ZBTB33,ADA2,NTN1,CHKA,PLCB4,MMP16,PRUNE2,ZFHX3,FANCL,DPYSL5,SLC13A5,ZNF44,RRAGD,BANP,SUPT16H,ARID1B,HOXC13,CRACR2A,FAM81A,RNF152,BAZ1A,CASZ1,OTUD7A,INSR,CUL5,DMBT1,OR7A17,BMF,YTHDF3,TFF1,DEDD2,NEK6,HECTD1,GRID1,SHROOM3,XRCC4,COLQ,FAM118A,SLC52A1,HDAC11,NMU,LYPLAL1,DDHD1,PBX3,SUMO2,HS1BP3,ZNF292,ADAMTS19,DPYD,ARFGEF1,PDE4DIP,GAST,POGK,SNAI2,ASH1L,UBL3,IGHV3-74,HOXC4,BID,SIAH2,PIGK,OSBPL10,RPH3A,TANC2,ZBTB8OS,COX5A,ABCA4,TRABD2B,UFD1,RXRG,SP3,DRAM1,ERN2,FNDC1,ZNF879,MBTPS2,FLNB,TRIM58,TIAL1,TOM1,ELF2,ZNF804B,IFI44,PLPP4,NREP,ZDHHC17,NSD2,FYCO1,CERS3,ESYT2,SH3GLB1,SLC22A14,CD9,CARD10,LTN1,KRT6B,RALGPS2,TWIST2,

			CTIF,SAMHD1,HSD17B14,IFT81,ENPP1,ENTPD5,UTRN,MOCS2,RASGRP1,IGSF11,SNX9,PAMR1,CDH26,DZANK1,PXDNL,UCK2,NDRG2,CSNK2A1,BMP5,PWWP3A,WDR72,KCNC1,CSF1,GHRH,PPIL6,POTEB3,EOGT,CCDC34,HDGFL3,ZNF385D,NUP37,BCL2L1,SERPINB9,SCAF4,KRT25,CTDP1,HCN1,PRKG1,LAMA3,ASB4,GRIN2B,ST13,ANKRD66,GRB14,TMEM71,INO80,FANCB,IGHV2-70D,CLNS1A,CNMD,ETNPPL,DHRS3,KIF21B,SMAD5,CELF4,SYNJ2,TCERG1,HEATR6,ABCG1,MARCHF11,FOXN3,KCNK5,DCUN1D4,PLXDC2,VSTM4,SLC40A1,PRAME,HADHA,MYCL,TNN,FAM149B1,CABYR,CIDEC,KLHL7,PSAP,PSMA5,CFHR4,MICALL2,MED1,IPCEF1,POTEB2,NSUN6,ATG4B,CDC14B,PCNT,KDM6A,ATRN,IL33,AJAP1,GPRC5C,TLNRD1,ROR2,CFH,PPP2R2A,ZNF521,NPL,KL,BANK1,CSDE1,FAT1,HGD,OTOG,LMX1A,IL10,ACTR2,SFPQ,SCML2,CALN1,RIOK1,CLSTN2,TTC39C,PTH,SDF4,SOSTDC1,TOP3B,PRKAA2,CSF2RB,DIRAS2,SKA1,NDC80,GOLGA8G,RNF182,SOHLH1,LARP6,PACRG,ERO1B,PHF20L1,ABHD2,ITPRIP,VSTM2A,FAM204A,MAP6,VASP,PLA2G4A,ETV6,TACC2,SCFD2,SHISAL1,PALMD,RAB12,SNRPC,KIFC1,IQGAP1,RPS12,REG4,PRB3,CAMLG,ZBTB7C,TEAD1,MORC2,SREBF2,ANP32B,YBX3,AIMP1,LASP1,THNSL2,FYB2,NRXN1,EPHX4,PCID2,HIPK1,ZNF234,ENTHD1,CISD1,CIB4,ZNF518A,DGKK,SNAP91,CD70,CYP4F22,CIBAR1,PBLD,FICD,ERICH5,CACYBP,CADM1,CENPE,PEG10,LMX1B,NET1,SIPA1L2,TUBB6,NGDN,ELOC,ANLN,TWIST1,RNU6-1150P,AKT3,ALKAL2,RNU1-51P,JAK2,ADAM28,VSX1,RPF2,FSTL1,CHCHD6,ZBTB38,MPPE1,ISX,BPNT1,SEC14L3,SVEP1,MADD,HCRTR1,RBM19,PTGS1,PATL1,ZNF287,CELSR2,ZNF449,PRSS2,FH,TDP1,CREBBP,MELTF,MRM1,TNKS,ARL11,SGO1,GORAB,PCNA,SIAH3,TRPV5,UFL1,ADAMTS5,NFKBIA,PRKCB,ANKRD24,FBXW2,CFAP299,GOT2,NTM,KIF6,ABCC8,MIPEP,PCDH11X,ANXA4,OVCH1,MT1HL1,CACNA1E,ZC3H15,ANP32A,OTULINL,RFC2,SMTN,ZNF354C,ST6GAL2,ALX4,RNU6-113P,RTRAF,USH1C,BRD4,ZBTB21,SERBP1,SMPD4,NEDD9,OLFM4,NRBP1,ITGA6,ATP2B1,GAP43,SLC14A2,IARS2,CLCA4,DGLUCY,ATP1A1-AS1,ASS1,CNDP2,MTCL1,GRIP1,IGHV1OR15-9,CTNNBL1,GTSF1L,AGAP9,ADGRE3,SAR1A,ADCY9,EML1,CNIH1,MAST2,HPSE2,BTG3,ZNF528,ERLIN2,GOLGA8J,ZNF611,TRAPPC3,MAPK1IP1L,UBAP2,ADAMTSL3,EFHD2,CIDEA,PCMTD2,ZBTB49,BBS9,EXT2,EXOC1,KRT6A,STOX2,AGO1,FRA10AC1,PDP2,DIPK1A,MEOX2,SLC6A1,GID8,ELL2,GRXCR1,SDS,LINGO1,SNAPC3,STAT1,ZCCHC14,BRMS1L,FAM189A2,NDFIP2,NR2C1,MAP2K6,S100PBP,CMTM7,DGKG,VAT1L,ERICH3,SHROOM2,SLC6A11,KCNJ18,MARCHF6,GATAD1,MTPN,ABI1,MYO18B,NECTIN4,ARMC6,CEMIP,POU6F2,IMPACT,CBLIF,CCBE1,SLX4IP,PARK7,ADAMTS18,MAPK8,ITGA4,TOP3A,OAZ2,EIF3F,PPME1,MED12L,ZSCAN30,FBXL17,UBL7,POU1F1,UBE2J2,ADCYAP1R1,PLA2G12B,MTF2,CSMD1,NCAPG2,TM9SF4,RAPGEF4,SCGB1D1,FOXP2,ASB2,MYOCD,HFM1,HMCN1,CEP120,MYH13,ATP13A3,DHTKD1,ZSCAN5C,CYFIP2,UBE2QL1,HNRNPM,ACACA,KRT85,ASCC2,ST8SIA4,ARL4C,EFHB,ARID3B,MEF2C,STOML1,ZNF613,ADGRB1,RXRA,WNT7A,RBPMS2,ECHDC1,OXNAD1,MAP3K5,NDFIP1,IKBIP,MAP3K4,TRIM43B,WASF3,S100B,SERPINI2,PRDM13,TRIM43,FOXO6,ERI1,SUMF1,EFCAB14,ZNF112,CD82,ATP6V1C2,C9ORF43,CHAMP1,C16ORF72,BTF3L4,MAGEL2,PKN2,RAD51AP1,SLC10A6,FAM25C,PDE2A,RAB38,LRRC2,KRTAP21-2,SFI1,DBF4B,FBXW8,SDCBP,NECTIN1,DSG1,JPT2,SPPL2B,NSMCE1,C3ORF52,ZNF813,COMMD8,ERICH1,WWOX,ZBTB25,FAM72A,PASK,MLLT1,MS4A4A,NCK1,FLVCR1,SCAF8,FGR,CWC22,CCDC106,DRC7,CDCA8,PPP2R3A,DNMBP,RNU6-1007P,TRIM23,ATP6V1B2,CXCL2,TOP1,TINAG,FAM72B,SNAP29,FAM72D,MLLT10,C2,IFNAR1,RNF8,GNG12,LCE3D,KLHL29,EPHA4,PPIP5K2,TEX29,CYTH4,EMP1,INTS13,GABRA5,KIAA0319L,MECOM,DNMT3L,NTRK2,ANKRD20A1,IL1RAPL1,MGAM2,FNDC3A,ACSM2A,RSPH1,KHDC4,NUMB,LHX9,ADAMTS9,WNT2B,COLEC12,FRRS1,ZBTB10,TNNI1,PLEKHA3,OCLN,CCDC152,POSTN,FAM110A,CREB5,SNRPD1,SHISA6,MEGF10,FBXO31,EXTL3,AKAP1

			1,TRPM7,KTN1,KRTAP26-1,PRKAB1,DTHD1,IREB2,MVB12B,HS6ST1,PTK2,ERP27,MARK4,CDH5,TPH2,RCAN2,ANKRD6,APOL2,SCGN,NFKBID,ARHGAP12,CLDN18,ASCL3,MPP7,DIAPH1,FBXO41,FEZ2,INIP,LAMB1,SCAMP1,APIP,CYFIP1,SUSD1,UBE3A,TMEM54,SCG3,APOL1,PCDH8,SEMA4D,JAM2,DNAH10,PITPNC1,FRMD6,MC2R,ZBTB20,FAT4,IMPA2,FAM102A,LRMDA,ZNF66,AP2B1,RUNX1,AKR1B1,C9,KIRREL1,WNT5B,RASGEF1B,AMFR,SAXO1,SCARA5,CTSE,NENF,SH2D1B,POMT2,HEATR5A,ZNF845,PSTPIP2,ZFYVE1,SANBR,ASAP1,SAMD13,ICA1,PLCZ1,EDIL3,NOS1AP,MTTP,DIDO1,TPTE,SORBS2,PDCL3,SRP9,CNKSR1,CCDC88A,NSUN2,SLC27A6,UBAP1L,CHCHD2,GALNT18,HKDC1,ADAMTS16,SPAG6,ACOXL,SLC5A1,MDN1,CDC45,BICD1,TNFSF11,FYN,BUB1,KDM5A,PCBP3,MYL12B,NLRP14,ZNF705G,PPM1F,GOLGA8F,ADGRL2,UNC45B,ARL13B,XPO7,SDE2,RBMS3,UHRF2,SCN8A,HDAC2,SNTB1,AVEN,SLF1,SACM1L,TBX15,SH2D3C,PSME3IP1,DOCK3,TRNAU1AP,NCS1,COL18A1,GALNT17,CDH9,LHFPL2,LYSMD2,ATP5PF,ALB,DOK5,ATP9B,NALCN,UGP2,MTMR7,EHBP1,ZFYVE28,MAPK9,RSPH14,PABPC1,CRTAM,APELA,MDGA2,STT3A,DEFB108B,ROR1,SLC16A9,GALNT2,FUT8,TET1,ARNT2,ASB3,HECW2,POTEJ,CDH2,CNTN5,ITGA8,SEL1L2,FBXL20,NTN4,RAD9A,XRN2,PHLPP1,PLEKHA2,GPR137B,RNU6-929P,EPHB1,EYS,GRM5,DDX10,ADCK1,RAI14,SPOPL,ZNF705D,RPS6KA5,SPTB,TBC1D1,LRRC69,PTPRG,ANKRD36C,PID1,NRP1,MIDEAS,FCHSD2,SDK1,PRKCA,GBP4,IFT46,MRPL58,NLRP4,ANKRD36B,ATPSCKMT,SPHKAP,FAIM,RNF215,SAMD12,USP24,FAAP24,MOGAT3,FHIT,ITGA1,ZNF615,PCCA,CROT,KLF12,RNF138,RC3H1,NRIP1,CHODL,POR,ZNF850,ZNF235,ABCA6,SLC14A1,CLEC20A,EFL1,MCC,GOLGA8S,ZNF738,SUPT3H,BCR,TUT4,NRXN3,ELMO1,RGS6,RERG,ZNF215,NLRP8,TCERG1L,KIF16B,CDH12,PRIM2,SNRK,ARMC3,MIPOL1,C14ORF39,ARFGAP3,SENP8,USP49,ELP2,CFAP70,PGM2L1,FBLN1,STK36,NSG2,PAQR5,MB,RAG1,KCNJ6,B9D1,ZMYM1,DGCR2,DNPEP,CYP4B1,RRAS2,GNA14,ZNF678,BMPER,RABL2A,KIAA1328,PRDM15,CUX1,SRGAP3,SLC35F1,ZNF420,MACROH2A1,MITF,NBPF1,EPHB2,TOGARAM1,CSNK1G1,SACS,BCL2L13,RNF11,SGCG,CD38,EYA4,CHCHD3,DPH6,MYO5B,RGPD4,PPIL2,CDK14,RSRP1,AKAIN1,MET,MUC16,SPPL3,DLG2,CDH17,COMMD10,ZNF705B,ATP6V0D2,SPECC1,CAMK1G,IBA57,METTL15,PPFIA2,CDH13,STXBP4,POTED,MAB21L3,KRTAP19-7,CACNG3,ATG5,USP32,NRAP,MAGI2,KIAA1217,PRDM11,VMP1,UNK,AIFM3,FAM171A1,MLIP,FLRT2,MYB,KALRN,ME3,ZNF704,SLC1A2,GNAS,LAMA1,MFHAS1,CA10,CPQ,NUP43,TRIM9,ATRNL1,TIAM2,DHX29,BMP7,TTC28,CHIT1,TMPRSS15,ASTN2,DLG5,TNFAIP8,ZMYND8,GAPVD1,RNF217,KIRREL3,PRSS23,KCTD1,GOLGA6A,DNAH3,ZNF74,BPTF,BTBD10,CCSER1,AK3,ZMYND11,TMEM25,NUDT21,TRAPPC10,GRM3,KMT2C,DDX6,ADGRF5,UPRT,PDGFC,WDR41,DNAH17,PLIN2,PPP1R13B,ELOVL7,FOCAD,EPB41L4A,ABL2,MMP26,MRPL37,TRAPPC6B,BACE2,RFX2,PARPBP,NECAB1,PKNOX2,EYA1,FHOD3,PDZD2,TTLL11,GOLGA8T,PRPF18,SLIT2,CMPK1,TMPRSS3,EXOC4,RNU6-835P,CNOT7,FAM126A,KCNIP4,ESCO1,KCTD8,CCDC141,PLCL1,ERBB4,ANKRD30B,IL20RB,FAM3B,FAM126B,GSAP,TRHDE,SYNDIG1,ROBO1,SAMD4A,PBX1,SPATS2L,IRAG1,NPAS3,NUF2,PRKCQ,RGPD2,IPP,SAMM50,ANTXR1,NDRG1,MYH15,SORCS2,SIPA1L3,TRDN,MGMT,ZNF679,NLGN1,SYNPR,CTTNBP2,AK9,SHLD2,NOS1,SLC6A3,GLDC,CHD9,PRR16,ASIC2,TXNDC16,EFNA5,TCF12,LRRC9,GAS2L1,ARHGEF11,MTREX,VCAN,RAB27A,NSD1,EHMT1,SLIT3,DTNA,KIF13A,AP5M1,FRMD5,ESR1,DNAH9,SLC25A48,MYO9B,NTNG1,KDM4B,CYP2C8,KCNQ5,LOXL2,CACNA2D1,NYAP2,IGLC3,ANKRD36,C16ORF74,CEP57L1,IQCJ-SCHIP1,MPDZ,FAM153A,IRAG2,ADGRG7,ORC4,SKAP2,PRLR,AGO3,HTT,LARS2,FOXB1,RAD51B,CAMK1D,PIK3R3,SLC25A18,CFAP44,POTEC,CDKAL1,EML6,OPCML,AK2,HLA-F,FER,ZNF302,EVA1A,EYA2,CCR2,RPGRIP1,STARD13,PITPNM3,SNTB2,WDR64,OSBPL5,INTS12,IGLV3-

			1,A2M,WDFY3,CHFR,ZNF721,PCMT1,EPS8,OSBPL6,AUH,JAZF1,ZNF578,OARD1,ZNF891,SPOCK3,SEMA4B,NRF1,IGHV1OR21-1,ZNF14,ANO2,PHC2,GRIA4,AGAP1,ROCK2,PRDM1,RORA,STMP1,IL16,TERB2,NARS2,DMRT1,EIF4G3,CDCA5,PPP1CB,CATSPER2,RGS8,RAB31,PDK1,HSPG2,PSMD2,PTPRQ,HERPUD1,NCOA6,TRIM2,COL4A3,WASHC1,ZFP30,RGS7,HOOK3,KIF7,GNG2,PCSK2,FSTL4,CLDN10,BARD1,PALD1,CLCN5,PNPLA3,HSPA12A,STK3,DEPTOR,ZNF423,SLC13A4,C1QL3,RSU1,PNPLA8,ZNF568,HNRNPU,LINC00240,VTI1A,CEP72,RAB3GAP2,TULP4,CADPS,APCDD1,IGF1R,KCNAB1,PRKAG2,GLI2,ANKRD10,MFSD11,APMAP,IQCM,THRB,LSAMP,AKAP13,MORC3,ATP10A,SEPTIN6,DNM1L
GO:0031267	small GTPase binding	0.00022933543613750437	*MICAL3,ERC1,MYO5A,RIMS1,FGD4,RIMS2,RIN2,DENND1A,DOCK2,DIAPH3,DGKI,PAK1,RANBP17,ARHGAP44,PAK3,RANBP2,RABGAP1L,ANKFY1,IPO11,MAPKAP1,DOCK4,DMXL2,RAP1A,USP33,TNPO3,DOCK5,PLCE1,CDC42BPB,EVI5,SGSM1,RANBP9,MYRIP,RIN3,UNC13B,DOCK1,RAP1GAP,SRGAP2,CCDC186,FARP1,CSE1L,TBC1D13,PRKCH,ALS2,ROCK1,RNF152,RPH3A,MICALL2,IQGAP1,YBX3,ADCYAP1R1,RAPGEF4,CYFIP2,PKN2,DIAPH1,CYFIP1,BICD1,XPO7,DOCK3,MYO5B,GAPVD1,EXOC4,NDRG1,MYO9B,FER,EPS8,ROCK2,RAB3GAP2,AKAP13,DNM1L*
GO:0005509	calcium ion binding	0.0002599763236418613	*NOTCH2,SPOCK1,MICU2,SMOC1,UNC13C,PLCB1,ITPR2,EPS15L1,FBN1,LPCAT2,CDH8,ADGRE1,PCDH7,MCTP1,EFCAB2,MACF1,HMCN2,GNPTAB,CRB1,CDH4,ATP2B2,AOAH,NELL2,RAPGEF2,LRP2,CPS1,GRM7,SYT1,SLC8A1,CUBN,RYR3,SYT10,FAT3,MICU1,EFEMP1,TLL1,EFCAB8,CDH7,PCDH11Y,TBC1D9,DYSF,ADGRV1,MELK,RYR2,AIF1L,CDH11,DST,CRTAC1,CDH18,CDHR3,EFCAB6,DGKB,FBN2,EGF,LTBP1,MATN2,ALPL,PCDH9,CDH20,EGFLAM,DNER,LRP1B,HPCAL1,UNC13B,PLS1,NIN,SMOC2,PCP4,MCTP2,MYL1,UMODL1,NELL1,ZZEF1,SELENON,REPS1,PCDH15,CDH23,LALBA,FBLN5,ENPP3,ITSN2,TENM2,PLCB4,CRACR2A,RPH3A,ESYT2,ENPP1,RASGRP1,PAMR1,CDH26,CABYR,FAT1,CALN1,CLSTN2,SDF4,PLA2G4A,IQGAP1,NRXN1,CIB4,FSTL1,SVEP1,CELSR2,PRSS2,PCDH11X,ANXA4,CACNA1E,ADGRE3,EML1,EFHD2,DGKG,CCBE1,PLA2G12B,HMCN1,EFHB,S100B,EFCAB14,DSG1,PPP2R3A,CDH5,SCGN,SUSD1,PCDH8,FAT4,RUNX1,PLCZ1,EDIL3,MYL12B,NCS1,CDH9,CDH2,EYS,MCC,CDH12,FBLN1,CDH17,CDH13,USP32,ASTN2,NECAB1,SLIT2,KCNIP4,MGMT,VCAN,SLIT3,LOXL2,PITPNM3,SPOCK3,HSPG2,FSTL4*
GO:0016740	transferase activity	0.00026602777488331307	*MTOR,TMTC1,ULK2,NLK,FTO,KSR1,TTC3,ZDHHC21,NME7,PRDM16,LPCAT2,F13A1,GPHN,PIK3C3,GALNT1,PCMTD1,ALK,PJA2,GLYAT,MAP3K9,MYO3B,MOCOS,SETD2,TNIK,NEK4,EGFR,CDK12,PRKACB,NEK7,RNF220,UGT3A2,NEDD4,GNPTAB,NSMCE2,SGMS1,CHSY1,B3GALT5,NTRK3,LARGE1,FLT1,GK,NAT1,DGKI,PRKD1,PAK1,EPHA7,CHSY3,PELI2,TAOK3,AGK,UBE2L3,SMYD3,HERC2,LRGUK,WDSUB1,NEDD4L,UBE2G1,ADK,RPS6KA2,KLHL13,DCLK1,PARP15,AURKA,DTWD2,MARCHF1,UPP2,MAP4K4,BMPR1B,HADHB,PAK3,RANBP2,ITPKB,UBE2E2,HHAT,CHST8,SCP2,PRKCZ,ST8SIA5,HECW1,GALNT14,RIPK4,MAPK1,MGAT5,ZNRF3,XXYLT1,UBE2O,GALNT16,PI4K2B,RNGTT,STK38,MYLK3,EFEMP1,DCAF1,TLK1,ZDHHC14,BIRC6,PPIP5K1,HS3ST2,LCLAT1,UBE3D,PAK5,ST6GALNAC3,PDZRN3,DAPK1,ACSM2B,STK32B,ALPK2,THUMPD2,MELK,HECTD4,SUGCT,MAPKAP1,PIAS1,UBE2R2,BLK,NUAK1,ABL1,AGPS,HDAC4,PRKAA1,MAST4,ATE1,HECTD2,CAMK4,ZC3HAV1,TGM1,PEAK1,LATS2,MUSK,GALNTL6,SETDB2,PRKCE,WNK2,B4GALNT3,DGKB,CERS6,GLT1D1,ALPK3,TRIO,EXT1,RPS6KA3,MARCHF8,TRIM5,ST8SIA6,ALG10B,LTBP1,STK38L,GALNT10,PRMT8,MARK2,C10ORF90,HERC1,EPHA6,SH3KBP1,HIPK3,CDKN2C,GRK3,STK10,TMTC2,MYLK2,XYLT1,CCND3,STK32A,AK8,CRIM1,FUT9,GXYLT2,CEPT1,BRCA2,GALNT13,NRK,MAGI3,AGL,RC3H2,MYLK4,CDC42BPB,VRK1,BMP2K,RNF38,POLR3A,CDS2,UST,TRAF3,DSTYK,B4GALT6,PFKFB4,TRIT1,CGAS,PRDM10,NEK10,PIGN,ATF2,ZZEF1,UBR1,MAP4K3,HS3ST4,MED27,MYO3A,AKAP10,UBE2E1,PRKN,CDC42BPA,MAPK10,FBXO3,WSB1,TRPM6,LALBA,PRKCH,HUNK,MKNK1,POLR1D,TRMT61B,ST8SIA1,MOK,ROCK1,LYN,EIF2B3,GSTA3,DTX1,CHKA,FANCL,RNF152,INSR,CUL5,NEK6,HECTD1,ASH1L,SIAH2,PGAP4,ERN2,TRIM58,ZD*

			HHC17,NSD2,CERS3,PTAR1,LTN1,MOCS2,UCK2,CSNK2A1,EOGT,PRKG1,HS6ST3,ASB4,ETNPPL,MARCHF11,HADHA,LPGAT1,NSUN6,ROR2,RIOK1,SOSTDC1,PRKAA2,GLYATL1,RNF182,PLA2G4A,HIPK1,DGKK,FICD,AKT3,INMT-MINDY4,JAK2,CREBBP,MRM1,TNKS,SIAH3,UFL1,GLYATL2,PRKCB,FBXW2,GOT2,ST6GAL2,BRD4,NRBP1,MAST2,PCMTD2,EXT2,MAP2K6,DGKG,MARCHF6,MAPK8,UBE2J2,ASB2,UBE2QL1,ST8SIA4,MAP3K5,MAP3K4,TRIM43B,PRDM13,TRIM43,MAGEL2,PKN2,FBXW8,NSMCE1,PASK,FGR,TRIM23,TOP1,RNF8,EPHA4,PPIP5K2,MECOM,GLYATL3,NTRK2,ACSM2A,EXTL3,TRPM7,PRKAB1,HS6ST1,PTK2,MARK4,UBE3A,AMFR,POMT2,DPY19L2,CNKSR1,NSUN2,GALNT18,HKDC1,FYN,BUB1,DPY19L1,UHRF2,GALNT17,UGP2,MAPK9,STT3A,ROR1,GALNT2,FUT8,HECW2,EPHB1,ZDHHC18,ADCK1,RPS6KA5,NRP1,PRKCA,COX10,ATPSCKMT,RNF215,MOGAT3,FHIT,CROT,RNF138,RC3H1,ZNF738,BCR,TUT4,SNRK,PGM2L1,STK36,RAG1,PRDM15,EPHB2,CSNK1G1,RNF11,CD38,PPIL2,CDK14,MET,DLG2,CAMK1G,IBA57,METTL15,MAGI2,PRDM11,KALRN,CHST3,TRIM9,RNF217,AK3,KMT2C,WSCD1,ELOVL7,ABL2,PARP8,CMPK1,ESCO1,ERBB4,PRKCQ,MGMT,AK9,NSD1,EHMT1,PIGB,ZDHHC11B,CAMK1D,PIK3R3,CDKAL1,AK2,FER,FGGY,CHFR,PCMT1,ROCK2,PRDM1,ATAT1,PDK1,TRIM2,BARD1,PNPLA3,STK3,IGF1R,PRKAG2,AKAP13
GO:0008066	glutamate receptor activity	0.000268770624 4589073	*GRIK3,GRIA1,GRM7,GRIK4,GRM1,GRM8,GRID2,GRIK2,GRIN2A,GRID1,GRIN2B,GRIK1,GRM5,GRM3,GRIA4*
GO:0008047	enzyme activator activity	0.000583422634 1997858	*GARNL3,MYO9A,PLCB1,DLC1,ARHGAP26,ALK,RIN2,ARHGAP24,EGFR,RGS3,DOCK2,RABEP1,TBC1D19,RAPGEF2,ADGRB3,UBE2L3,RPTOR,RALGAPA1,TBCD,PPP1R12B,APP,ARHGAP44,BMPR1B,RANBP2,RGS20,RAP1GDS1,ARHGAP32,RGS9,RABGAP1L,NRG3,TBC1D22A,CHN1,MOB3B,ARAP2,ARHGEF12,PPP2R5E,TBC1D9,BCAS3,CLPX,RANBP3L,TBC1D5,DOCK4,NRG1,PRKCE,ASAP2,RGS12,EGF,NSMAF,RFC1,MARK2,MNAT1,DOCK5,TGFA,BMP2,EVI5,RALGAPA2,SGSM1,TBC1D4,RIN3,ARHGAP42,GNAQ,DOCK1,RAP1GAP,SRGAP2,SEC23B,MOB1B,TBC1D13,SH3PXD2A,ALS2,RACGAP1,VAV3,ARHGAP28,ARHGAP31,MMP16,PSAP,GPRC5C,IQGAP1,SIPA1L2,ALKAL2,MADD,PCNA,AGAP9,MAP2K6,ABI1,PARK7,NCAPG2,DBF4B,TRIM23,DNMT3L,ARHGAP12,ASAP1,FYN,NCS1,GRM5,TBC1D1,NRP1,BCR,RGS6,ARFGAP3,FBLN1,SRGAP3,BCL2L13,RGPD4,GNAS,TIAM2,GAPVD1,ERBB4,RGPD2,SIPA1L3,EFNA5,ARHGEF11,MYO9B,STARD13,AGAP1,RGS8,RGS7,STK3,RAB3GAP2,PRKAG2,DNM1L*
GO:0015318	inorganic molecular entity transmembrane transporter activity	0.001049851469 1585723	*UNC80,CACNA2D3,SLC17A1,SLC24A2,KCNH5,SLC37A1,PIEZO2,ITPR2,KCNMA1,CHRNA7,GABRB3,ANO6,CACNG2,SLC4A10,GRIK3,ATP2B2,TUSC3,GABRB1,GRIA1,SLC39A12,SLC8A3,GABRA6,TMEM38B,SLC24A3,TRPM1,SLC39A11,CACNA1C,CACNB2,GABRG2,TMC1,SLC8A1,KCNE4,KCNK10,TRPC5,CLIC6,RYR3,KCNJ1,TRPC7,SLC45A4,NIPAL2,LRRC38,GRIK4,KCNS3,GABRG1,SLC24A4,SCN2A,RYR2,SLC9C1,SLC36A1,SLC1A1,SLC12A8,KCNH1,ANO4,GRID2,ABCC9,P2RX6,ATP8A1,OPRM1,ABCC4,CNNM4,SLC2A13,KCND2,NIPA2,TMC7,ATP6V1E1,CACNA1I,KCNJ15,SCN11A,SLC23A2,SLC39A6,KCNH8,SLC37A2,SLC9A4,GABRR2,GRIK2,LRRC8B,CFTR,SLC30A10,GRIN2A,TRPM6,SLC12A1,KCNQ3,SLC4A4,SCN10A,KCND3,KCNN3,SLC15A2,SLC13A5,CUL5,GRID1,COX5A,GABRG3,ZDHHC17,KCNC1,HCN1,GRIN2B,KCNK5,SLC40A1,SLC5A12,COX7A2L,SLC1A7,TRPV5,ABCC8,CACNA1E,ATP2B1,CLCA4,OTOP1,SLC6A1,SHROOM2,SLC6A11,KCNJ18,ATP13A3,SLC5A9,ATP6V1C2,SLC10A6,ATP6V1B2,GABRA5,TRPM7,GRIK1,APOL1,SLC26A2,SLC9A5,SLC5A1,ANO10,SCN8A,TMEM63C,NCS1,ATP5PF,NALCN,TRPM3,SLC39A8,SLC14A1,KCNJ6,ATP6V0D2,CACNG3,SLC1A2,GABRA2,KCNIP4,SLC6A3,ASIC2,KCNQ5,CACNA2D1,SLC25A18,ANO2,GRIA4,CATSPER2,CLCN5,SLC13A4,KCNAB1*
GO:0022836	gated channel activity	0.001374358721 7693615	*CACNA2D3,KCNH5,PIEZO2,ITPR2,KCNMA1,CHRNA7,GABRB3,ANO6,CACNG2,GRIK3,GABRB1,GRIA1,GABRA6,TRPM1,CACNA1C,CACNB2,GABRG2,TMC1,KCNE4,KCNK10,CLIC6,RYR3,KCNJ1,LRR*

			C38,GRIK4,KCNS3,GABRG1,SCN2A,RYR2,KCNH1,ANO4,GRID2,ABCC9,P2RX6,OPRM1,KCND2,TMC7,CACNA1I,KCNJ15,SCN11A,KCNH8,GABRR2,GRIK2,CFTR,GRIN2A,KCNQ3,SCN10A,KCND3,KCNN3,GRID1,GABRG3,KCNC1,HCN1,GRIN2B,KCNK5,ABCC8,CACNA1E,CLCA4,SHROOM2,KCNJ18,GABRA5,GRIK1,ANO10,SCN8A,TMEM63C,NCS1,NALCN,TRPM3,KCNJ6,CACNG3,GABRA2,KCNIP4,ASIC2,KCNQ5,CACNA2D1,ANO2,GRIA4,CATSPER2,CLCN5,KCNAB1
GO:0003824	catalytic activity	0.0016781687582752816	*MTOR,IMMP2L,PTPRD,TMTC1,ULK2,NLK,LONP2,FTO,KSR1,AGBL1,PLCB1,TTC3,MX2,TMPRSS2,LIPI,ARL15,MICAL3,MGAM,DPP10,ZDHHC21,PTPRA,PDE4D,RALA,NME7,IL1RAPL2,PRDM16,ALDH1A2,LPCAT2,F13A1,GPHN,PUDP,PIK3C3,AKR1C3,GALNT1,NAV2,ENPEP,MYO1E,PLPPR1,PCMTD1,ALK,CDYL2,PJA2,THSD4,PAPPA2,ERCC6L2,HACD2,HLCS,GLYAT,EGLN3,MAP3K9,MYO3B,PGBD5,MOCOS,CPA6,PLPPR5,SETD2,TNIK,PTPRJ,KDM4C,NEK4,EGFR,USP14,CDK12,PRKACB,NEK7,RGS3,RNF220,UGT3A2,NEDD4,SND1,GNPTAB,NSMCE2,PSMB2,SGMS1,CECR2,CHSY1,PTPN4,B3GALT5,ATP2B2,NTRK3,LARGE1,PDE1C,CYP2C9,FLT1,RFC3,ADAMTS6,GK,TASP1,AOAH,NAT1,DGKI,PRKD1,TPTE2,PAK1,GMDS,EPHA7,CHRM3,ADSS2,CHSY3,PELI2,ARSB,CPS1,TAOK3,AGK,ADAMTSL1,BCKDHB,UBE2L3,PTPRN2,SMYD3,MPPED1,TYW1,HERC2,LRGUK,SEPTIN9,DNAH6,WDSUB1,DHX32,AIG1,NEDD4L,ADAM32,ADAM10,HDAC9,ATF7IP,UBE2G1,IL1R1,ADK,RPS6KA2,KYNU,KDM1B,KLHL13,DCLK1,USP18,ACER2,PARP15,AURKA,PARN,PTPRR,DTWD2,FIG4,MARCHF1,ABCG8,UPP2,MAP4K4,ABCD2,BMPR1B,PCSK6,HADHB,RAB8B,PAK3,PDE1A,TTLL7,RANBP2,ITPKB,RGS20,PDE10A,UBE2E2,HHAT,RNLS,CHST8,DNM3,SCP2,PRKCZ,MAN2A2,DIP2A,MSH6,RAB27B,ST8SIA5,HEATR4,RGS9,HECW1,ADAMTS17,SENP6,DUSP22,GALNT14,PDXDC1,PPM1L,RIPK4,MAPK1,MGAT5,ABCD3,DNAH14,ADAM22,USP25,PLG,PAPPA,ZNRF3,CA5A,XXYLT1,UBE2O,GALNT16,PI4K2B,RNGTT,MTMR10,CHD6,STK38,PTPN13,MYLK3,ACSBG1,EFEMP1,TLL1,DCAF1,TLK1,ZDHHC14,CORIN,BIRC6,PPIP5K1,HS3ST2,LCLAT1,ERMP1,SYNJ1,ADAMTS3,UBE3D,PTPRK,PAK5,ST6GALNAC3,PDZRN3,DAPK1,NAV3,FAR2,ACSM2B,AGO2,STK32B,ALPK2,JARID2,RAB22A,CPE,THUMPD2,ADGRV1,MELK,HECTD4,CLPX,ASAH2B,SHOC1,DUSP16,SUGCT,SMARCA4,USP8,MAPKAP1,EFTUD2,PIAS1,UBE2R2,BLK,OLA1,ATRX,NUAK1,PTPRT,ABL1,AGPS,PTPN12,HDAC4,OXR1,PRKAA1,DROSHA,TTLL5,APLF,ADAMTS14,MAST4,GUCY1A2,ATE1,SLFN11,RAP1A,ACSS3,HECTD2,CAMK4,MANBA,INPP5A,CPSF3,ZC3HAV1,GALC,TGM1,PEAK1,LATS2,PAH,ATP11C,DHX40,MUSK,GALNTL6,SMARCAD1,SETDB2,PRKCE,PGM5,METAP1D,NXN,WNK2,B4GALNT3,DGKB,USP33,CERS6,RGS12,PTPRO,GLT1D1,ALPK3,TRIO,PDE3A,EXT1,LNPEP,ADAMTS2,RPS6KA3,MARCHF8,ATP8A2,MTMR3,PTPN2,TRIM5,ATXN3,RFC1,ST8SIA6,ALG10B,ATP8A1,LTBP1,STK38L,GALNT10,KDM7A,PLD5,ABCC4,PRMT8,PLCXD3,FANCM,CYBRD1,CYP4A11,INPP4B,FARS2,GTF2F2,MARK2,GMPR,RCL1,ALPL,C10ORF90,ABHD17C,HERC1,MSH2,MPPED2,GNAL,EPHA6,SH3KBP1,ATL1,HIPK3,CDKN2C,GRK3,CPXM2,NOS2,AFG3L2,STK10,RBBP8,TMTC2,ADAM12,MYLK2,XYLT1,HMGA2,GBP6,CCND3,FOLH1,ECE1,STK32A,LYPLA1,AK8,CWC27,PLCE1,CRIM1,XPNPEP1,FUT9,GXYLT2,GSR,CAPN5,CYP4Z1,MSRA,TARS3,FKBP5,MTHFD1L,CEPT1,BRCA2,AQR,GALNT13,EXD3,BLM,NRK,MAGI3,ADCY10,AGL,RC3H2,MYLK4,ATP9A,CDC42BPB,DSE,VRK1,GNAI1,BMP2K,RNF38,PGPEP1,PDE6C,POLR3A,RELN,HMGB1,GNAQ,CDS2,UST,TRAF3,DSTYK,B4GALT6,PFKFB4,RAP1GAP,ENOX1,TRIT1,GADL1,SMARCA2,ARSJ,CGAS,CASP5,IDE,KIF21A,KIF15,PRDM10,RERGL,BTAF1,PEX6,NEK10,PIGN,ATF2,CYLD,ADARB2,MX1,CFTR,ME2,NEU3,ZZEF1,ATP10B,UBR1,SMPDL3A,CHRM5,MAP4K3,HS3ST4,MED27,SELENON,MYO3A,AKAP10,UBE2E1,PTPRE,PRKN,AGMO,MTMR2,CDC42BPA,MAPK10,PNPLA7,FBXO3,HEPHL1,TXNRD2,WSB1,USP43,TRPM6,LALBA,PRKCH,HUNK,PEPD,CPVL,ACO1,DHRS11,CNOT6L,MKNK1,DMC1,POLR1D,PTPRB,AOPEP,TRMT61B,PDE6A,ST8SIA1,USP7,GLB1L3,ENPP3,HAAO,FAH,MOK,RALB,ADGRG6,PPA2,FAR1,CA1,ROCK1,LYN,VCAM1,CTSB,EIF2B3,GSTA3,DTX1,ADA2,CHKA,*

			PLCB4,MMP16,PRUNE2,FANCL,DPYSL5,RRAGD,CRACR2A,RNF152,OTUD7A,INSR,CUL5,NEK6,HECTD1,HDAC11,LYPLAL1,DDHD1,ADAMTS19,DPYD,ASH1L,SIAH2,PIGK,PGAP4,COX5A,ABCA4,TRABD2B,UFD1,ERN2,MBTPS2,TRIM58,PLPP4,ZDHHC17,NSD2,CERS3,PTAR1,LTN1,SAMHD1,HSD17B14,ENPP1,ENTPD5,MOCS2,SDR42E2,PAMR1,PXDNL,UCK2,CSNK2A1,PPIL6,EOGT,CTDP1,PRKG1,HS6ST3,ASB4,INO80,ETNPPL,DHRS3,KIF21B,SYNJ2,MARCHF11,HADHA,LPGAT1,IPCEF1,NSUN6,ATG4B,CDC14B,KDM6A,ROR2,NPL,KL,HGD,OTOG,RIOK1,SOSTDC1,TOP3B,PRKAA2,DIRAS2,GLYATL1,QSOX2,RNF182,ERO1B,ABHD2,PLA2G4A,RAB12,KIFC1,COX7A2L,MORC2,THNSL2,EPHX4,HIPK1,DGKK,CYP4F22,PBLD,FICD,TUBB6,AKT3,INMT-MINDY4,JAK2,ADAM28,MPPE1,BPNT1,PTGS1,PRSS2,FH,TDP1,CREBBP,MRM1,TNKS,ARL11,PCNA,SIAH3,UFL1,ADAMTS5,GLYATL2,PRKCB,FBXW2,GOT2,KIF6,MIPEP,OVCH1,RFC2,ST6GAL2,BRD4,SMPD4,NRBP1,ATP2B1,IARS2,CLCA4,DGLUCY,NOXRED1,ASS1,CNDP2,SAR1A,ADCY9,MAST2,HPSE2,PCMTD2,EXT2,FRA10AC1,PDP2,GRXCR1,SDS,MAP2K6,DGKG,VAT1L,MARCHF6,CEMIP,PARK7,ADAMTS18,MAPK8,TOP3A,EIF3F,PPME1,UBE2J2,PLA2G12B,ASB2,HFM1,ATP13A3,DHTKD1,UBE2QL1,ACACA,ST8SIA4,NDUFA10,ARL4C,ECHDC1,OXNAD1,MAP3K5,MAP3K4,TRIM43B,PRDM13,TRIM43,HSDL2,ERI1,SUMF1,MAGEL2,PKN2,PDE2A,RAB38,LRRC2,FBXW8,SPPL2B,NSMCE1,WWOX,PASK,FGR,TRIM23,TOP1,TINAG,C2,RNF8,EPHA4,PPIP5K2,MECOM,GLYATL3,NTRK2,IL1RAPL1,MGAM2,ACSM2A,ADAMTS9,FRRS1,CD101,EXTL3,TRPM7,PRKAB1,IREB2,HS6ST1,PTK2,MARK4,CD5L,TPH2,APIP,UBE3A,IMPA2,AKR1B1,AMFR,CTSE,POMT2,PLCZ1,DPY19L2,TPTE,CNKSR1,NSUN2,SLC27A6,GALNT18,HKDC1,ADAMTS16,ACOXL,MDN1,FYN,BUB1,KDM5A,DPY19L1,PPM1F,ARL13B,UHRF2,HDAC2,SACM1L,GALNT17,ATP5PF,ATP9B,UGP2,MTMR7,MAPK9,STT3A,ROR1,GALNT2,FUT8,TET1,HECW2,OVCH2,RAD9A,XRN2,PHLPP1,EPHB1,ZDHHC18,DDX10,ADCK1,RPS6KA5,PTPRG,NRP1,PRKCA,GBP4,MRPL58,COX10,ATPSCKMT,RNF215,USP24,MOGAT3,FHIT,PCCA,CROT,RNF138,RC3H1,POR,EFL1,ZNF738,BCR,TUT4,RGS6,RERG,KIF16B,SNRK,SENP8,USP49,PGM2L1,STK36,RAG1,DNPEP,CYP4B1,RRAS2,GNA14,RABL2A,PRDM15,DPP6,EPHB2,CSNK1G1,RNF11,CD38,EYA4,DPH6,PPIL2,PRSS51,CDK14,MET,SPPL3,DLG2,CAMK1G,IBA57,METTL15,USP32,MAGI2,PRDM11,AIFM3,ADAM29,KALRN,ME3,CHST3,GNAS,CA10,CPQ,TRIM9,DHX29,CHIT1,TMPRSS15,RNF217,PRSS23,BTD,BPTF,AK3,KMT2C,WSCD1,DDX6,ELOVL7,ABL2,MMP26,BACE2,EYA1,TTLL11,PARP8,CMPK1,TMPRSS3,CNOT7,ESCO1,PLCL1,ERBB4,TRHDE,PRKCQ,MGMT,AK9,NOS1,GLDC,CHD9,MTREX,RAB27A,NSD1,EHMT1,USP31,KIF13A,SDR42E1,MYO9B,KDM4B,CYP2C8,LOXL2,ORC4,PIGB,AGO3,LARS2,ZDHHC11B,RAD51B,CAMK1D,PIK3R3,MACROD2,CFAP44,CDKAL1,AK2,FER,EYA2,PITPNM3,FGGY,CHFR,PCMT1,AUH,OARD1,AGAP1,ROCK2,PRDM1,ATAT1,NARS2,PPP1CB,RGS8,RAB31,PDK1,PTPRQ,TRIM2,HSD17B2,RGS7,KIF7,PCSK2,BARD1,PALD1,PNPLA3,STK3,PNPLA8,IGF1R,KCNAB1,PRKAG2,APMAP,AKAP13,MORC3,ATP10A,SEPTIN6,DNM1L
GO:0051020	GTPase binding	0.001743249940952738	MICAL3,ERC1,MYO5A,RIMS1,FGD4,RIMS2,RIN2,DENND1A,DOCK2,DIAPH3,DGKI,PAK1,RANBP17,ARHGAP44,PAK3,RANBP2,RABGAP1L,ANKFY1,IPO11,MAPKAP1,DOCK4,DMXL2,RAP1A,USP33,AMBRA1,TNPO3,DOCK5,PLCE1,CDC42BPB,EVI5,SGSM1,RANBP9,MYRIP,RIN3,UNC13B,DOCK1,RAP1GAP,SRGAP2,CCDC186,FARP1,CSE1L,TBC1D13,PRKCH,ALS2,ROCK1,RRAGD,RNF152,RPH3A,MICALL2,IQGAP1,YBX3,AIMP1,ADCYAP1R1,RAPGEF4,CYFIP2,PKN2,DIAPH1,CYFIP1,BICD1,XPO7,DOCK3,MYO5B,GAPVD1,EXOC4,NDRG1,MYO9B,FER,EPS8,ROCK2,RAB3GAP2,AKAP13,DNM1L
GO:0022890	inorganic cation transmembrane transporte	0.0018332682329047021	UNC80,CACNA2D3,SLC17A1,SLC24A2,KCNH5,PIEZO2,ITPR2,KCNMA1,CHRNA7,ANO6,CACNG2,SLC4A10,GRIK3,ATP2B2,TUSC3,GRIA1,SLC39A12,SLC8A3,TMEM38B,SLC24A3,TRPM1,SLC39A11,CACNA1C,CACNB2,TMC1,SLC8A1,KCNE4,KCNK10,TRPC5,RYR3,KCNJ1,TRPC7,SLC45A4,NIPAL2,LRRC38,GRIK4,KCNS3,SLC24A4,SCN2A,RYR2,SLC9C1,SLC36A1,SLC1A1,SLC12A8,KCNH

	r activity		*1,ABCC9,P2RX6,ATP8A1,OPRM1,CNNM4,SLC2A13,KCND2,NIPA2,ATP6V1E1,CACNA1I,KCNJ15,SCN11A,SLC23A2,SLC39A6,KCNH8,SLC9A4,GRIK2,SLC30A10,GRIN2A,TRPM6,SLC12A1,KCNQ3,SLC4A4,SCN10A,KCND3,KCNN3,SLC15A2,SLC13A5,CUL5,COX5A,ZDHHC17,KCNC1,HCN1,GRIN2B,KCNK5,SLC40A1,SLC5A12,COX7A2L,SLC1A7,TRPV5,ABCC8,CACNA1E,ATP2B1,OTOP1,SLC6A1,SHROOM2,SLC6A11,KCNJ18,ATP13A3,SLC5A9,ATP6V1C2,SLC10A6,ATP6V1B2,TRPM7,GRIK1,SLC9A5,SLC5A1,ANO10,SCN8A,TMEM63C,NCS1,ATP5PF,NALCN,TRPM3,SLC39A8,KCNJ6,ATP6V0D2,CACNG3,SLC1A2,KCNIP4,SLC6A3,ASIC2,KCNQ5,CACNA2D1,SLC25A18,GRIA4,CATSPER2,CLCN5,SLC13A4,KCNAB1*
GO:0015631	tubulin binding	0.0025577797014176553	*AGBL1,MX2,RP1,DCDC1,MAP4,APC,SETD2,MACF1,DIAPH3,PAK1,KIF4A,TBCD,MTUS1,DCLK1,MAPRE2,CCSER2,MAP4K4,FMN2,TTLL7,DIP2B,DNM3,MTUS2,FMN1,PAFAH1B1,NAV3,BCAS3,DST,GAS2,TTLL5,CLIP1,PEX14,ARHGEF7,C10ORF90,VPS41,BRCA2,DNAL1,TUBGCP3,MDM1,NIN,HAUS6,KIF21A,KIF15,MAP2,BBS4,MX1,CEP44,PRKN,RACGAP1,LYN,KIF11,DPYSL5,IFT81,HDGFL3,INO80,KIF21B,SKA1,PACRG,MAP6,KIFC1,CACYBP,CENPE,KIF6,MTCL1,EML1,MAST2,ARL4C,MARK4,DIAPH1,SAXO1,CCDC88A,SPAG6,FYN,KIF16B,TOGARAM1,TTLL11,NDRG1,GAS2L1,KIF13A,CEP57L1,IRAG2,HTT,EML6,WASHC1,HOOK3,KIF7,DNM1L*
GO:0005216	ion channel activity	0.003254780822118313	*UNC80,CACNA2D3,SLC24A2,KCNH5,PIEZO2,ITPR2,KCNMA1,CHRNA7,GABRB3,ANO6,CACNG2,GRIK3,GABRB1,GRIA1,GABRA6,TMEM38B,SLC24A3,TRPM1,CACNA1C,CACNB2,GABRG2,TMC1,KCNE4,KCNK10,TRPC5,CLIC6,RYR3,KCNJ1,TRPC7,LRRC38,GRIK4,KCNS3,GABRG1,SLC24A4,SCN2A,RYR2,SLC9C1,SLC1A1,KCNH1,ANO4,GRID2,ABCC9,P2RX6,OPRM1,KCND2,TMC7,CACNA1I,KCNJ15,SCN11A,KCNH8,GABRR2,GRIK2,LRRC8B,CFTR,GRIN2A,TRPM6,KCNQ3,SCN10A,KCND3,KCNN3,CUL5,GRID1,GABRG3,KCNC1,HCN1,GRIN2B,KCNK5,TRPV5,ABCC8,CACNA1E,CLCA4,OTOP1,SHROOM2,KCNJ18,GABRA5,TRPM7,GRIK1,APOL1,ANO10,SCN8A,TMEM63C,NCS1,ATP5PF,NALCN,TRPM3,KCNJ6,CACNG3,GABRA2,KCNIP4,ASIC2,KCNQ5,CACNA2D1,ANO2,GRIA4,CATSPER2,CLCN5,KCNAB1*
GO:0051015	actin filament binding	0.003522468749321158	*NEBL,SVIL,TLN2,MICAL3,MYO5A,MYO1E,MYO5C,EGFR,MACF1,CTNNA3,CTNNAL1,CACNB2,VCL,CTNNA2,ABLIM1,CORO2B,TPM1,CTNNA1,PPP1R9A,MPRIP,SYNE2,AIF1L,XIRP2,ABL1,GAS2,MYO10,SYNE1,ACTR3C,MYOM2,HIP1,MYOM1,PLS1,MYO1D,SHROOM3,FLNB,UTRN,MICALL2,ACTR2,IQGAP1,LASP1,USH1C,SHROOM2,MYH13,CYFIP1,PSTPIP2,SPTB,MYO5B,NRAP,ABL2,PKNOX2,FHOD3,ANTXR1,MYH15,GAS2L1,MYO9B*
GO:0035091	phosphatidylinositol binding	0.004883516932728665	*EXOC1L,PLCB1,SVIL,ITPR2,MYO1E,FCHO2,DENND1A,TOM1L2,SYT1,ARHGAP32,KCNJ1,SYT10,ANKFY1,SNX30,SNX29,ARAP2,PARD3B,BCAS3,PARD3,MAPKAP1,KCNH1,MYO10,PLEKHA8,STXBP6,MCF2L,ZFYVE9,HIP1,SNX3,CLVS1,SNX8,CGAS,SNX6,ZFYVE26,CLVS2,SH3PXD2A,FRMPD4,RACGAP1,SNX25,PLEKHB2,HS1BP3,PIGK,RPH3A,TOM1,ESYT2,SNX9,PLA2G4A,IQGAP1,SNAP91,MPPE1,GAP43,EXOC1,ZCCHC14,SDCBP,PASK,PLEKHA3,PITPNC1,ZFYVE1,ASAP1,PLCZ1,CCDC88A,ZFYVE28,FCHSD2,KIF16B,OSBPL5,WDFY3*
GO:0008324	cation transmembrane transporter activity	0.005446281502606583	*UNC80,CACNA2D3,SLC17A1,SLC24A2,KCNH5,PIEZO2,ITPR2,SLC44A5,KCNMA1,CHRNA7,ANO6,CACNG2,SLC4A10,OCA2,GRIK3,ATP2B2,TUSC3,GRIA1,SLC39A12,SLC8A3,TMEM38B,SLC24A3,SLC44A1,TRPM1,SLC39A11,SLC7A2,CACNA1C,CACNB2,TMC1,SLC8A1,KCNE4,KCNK10,TRPC5,RYR3,KCNJ1,TRPC7,SLC45A4,NIPAL2,LRRC38,GRIK4,KCNS3,SLC24A4,SCN2A,RYR2,SLC9C1,SLC36A1,SLC1A1,SLC12A8,KCNH1,ABCC9,P2RX6,ATP8A1,OPRM1,CNNM4,SLC2A13,KCND2,NIPA2,ATP6V1E1,CACNA1I,KCNJ15,SCN11A,SLC23A2,SLC39A6,KCNH8,SLC9A4,GRIK2,SLC30A10,GRIN2A,TRPM6,SLC12A1,KCNQ3,SLC4A4,SCN10A,KCND3,KCNN3,SLC44A2,SLC15A2,SLC13A5,CUL5,COX5A,ZDHHC17,KCNC1,HCN1,GRIN2B,KCNK5,SLC40A1,SLC5A12,COX7A2L,SLC1A7,TRPV5,ABCC8,CACNA1E,ATP2B1,OTOP1,SLC6A1,SHROOM2,SLC6A11,KCNJ18,ATP13A3,SLC5A9,ATP6V1C2,SLC10A6,ATP6V1B2,TRPM7,GRIK1,SLC9A5,SLC5A1,ANO10,SCN8A,TMEM63C,NC*

			S1,ATP5PF,NALCN,TRPM3,SLC39A8,SLC16A9,KCNJ6,ATP6V0D2,CACNG3,SLC1A2,KCNIP4,SLC6A3,ASIC2,KCNQ5,CACNA2D1,SLC25A18,GRIA4,CATSPER2,CLCN5,SLC13A4,KCNAB1
GO:0019899	enzyme binding	0.008361968679786093	*NOTCH2,BCAR3,WWC1,NLK,LONP2,KSR1,PLCB1,MICAL3,PDE4D,RDX,STXBP1,ERC1,RALA,BCL2,MYO5A,RIMS1,CNTLN,FGD4,SPRED1,CEP192,RIMS2,PJA2,ERCC6L2,HACD2,HLCS,RIN2,CNTNAP2,APC,DSCAM,CRKL,PTPRJ,KDM4C,EGFR,DENND1A,ANGPT1,CDK12,PRKACB,NCOR1,DOCK2,DIAPH3,NEDD4,BCL11A,FAM83F,RPRD1A,PHACTR1,DGKI,TOM1L2,NELL2,PAK1,RANBP17,UBE2L3,LDB2,SMYD3,HERC2,RPTOR,GHR,PPP1R12B,ADAM10,HDAC9,UBE2G1,IL1R1,APP,STAU2,MAPRE2,VCL,ARHGAP44,AURKA,PARN,PTPRR,AKAP6,PAK3,RANBP2,TRPC5,DNM3,PRKCZ,SPOP,MSH6,CNST,DUSP22,WDR70,SHC4,MAPK1,RABGAP1L,USP25,KMT2E,PLG,NRG3,ANKFY1,NIPBL,IPO11,CARD18,STK38,KANSL1,PAFAH1B1,ATF6,CNKSR2,PPARA,PPP6R3,TIAM1,UBE3D,PTPRK,PLA2R1,AGO2,ANK2,BCAS3,RYR2,NBEA,DUSP16,SMARCA4,LDB3,MAPKAP1,PIAS1,DOCK4,PTPRT,ABL1,HDAC4,KCNH1,DROSHA,DMXL2,RAP1A,ZNHIT6,NRG1,AP3B1,SYNE1,ZNF675,PRKCE,USP33,SPRED2,RPS6KA3,MTMR3,PTPN2,TRIM5,ATXN3,ARHGEF7,AMBRA1,PRMT8,RPRD1B,MSH2,SH3KBP1,SLC2A13,CDKN2C,TNPO3,MYOM2,CCND3,DOCK5,MBP,PLCE1,PRR5L,ATP6V1E1,SNX3,PDLIM5,BRCA2,ADCY10,PSG8,STRN,PHACTR3,ATP9A,PSG9,CDC42BPB,EVI5,VRK1,SGSM1,RANBP9,RESF1,MYRIP,RIN3,HMGB1,NFATC2,MYOM1,TRAF3,UNC13B,DOCK1,RAP1GAP,SRGAP2,NIN,CCDC186,FAM83B,GLI3,CUL1,ZFYVE26,FARP1,MOB1B,ATF2,CYLD,MAPK8IP1,PSG6,CFTR,CSE1L,NELL1,TBC1D13,RB1CC1,PRKN,SH3PXD2A,LYST,PRKCH,IL6R,ALS2,RACGAP1,TSPAN33,LRBA,USP7,RALB,ROCK1,LYN,SUMO3,KIF11,DTX1,ZFHX3,FANCL,RRAGD,RNF152,CUL5,DMBT1,NEK6,SUMO2,PDE4DIP,BID,SIAH2,RPH3A,UTRN,SNX9,BCL2L1,SERPINB9,SCAF4,ASB4,GRB14,SMAD5,TCERG1,DCUN1D4,CABYR,PSAP,MICALL2,ROR2,BANK1,SFPQ,PACRG,IQGAP1,CAMLG,ANP32B,YBX3,AIMP1,SNAP91,CD70,CACYBP,ALKAL2,JAK2,SGO1,PCNA,SIAH3,UFL1,NFKBIA,PRKCB,RFC2,RTRAF,BRD4,NEDD9,CTNNBL1,MAST2,ERLIN2,AGO1,STAT1,BRMS1L,NR2C1,MAP2K6,MARCHF6,CCBE1,PARK7,MAPK8,PPME1,UBE2J2,ADCYAP1R1,RAPGEF4,MYOCD,CYFIP2,MEF2C,RXRA,MAP3K5,PKN2,SFI1,DBF4B,WWOX,NCK1,SCAF8,FGR,RNF8,EPHA4,DNMT3L,NTRK2,AKAP11,PRKAB1,PTK2,CDH5,DIAPH1,CYFIP1,AMFR,NOS1AP,CCDC88A,BICD1,FYN,XPO7,HDAC2,SLF1,DOCK3,CDH2,RAD9A,GRM5,SPOPL,NRP1,PRKCA,FHIT,ITGA1,PCCA,RNF138,NRIP1,POR,TCERG1L,ELP2,MACROH2A1,CHCHD3,MYO5B,MET,DLG2,MAGI2,MFHAS1,TTC28,GAPVD1,RNF217,NUDT21,EXOC4,NDRG1,SLC6A3,FRMD5,ESR1,MYO9B,PRLR,HTT,FER,PITPNM3,A2M,EPS8,ROCK2,PRDM1,PPP1CB,NCOA6,WASHC1,BARD1,HNRNPU,RAB3GAP2,CADPS,PRKAG2,THRB,AKAP13,DNM1L*
GO:0019904	protein domain specific binding	0.009866126708524941	*NLK,DLC1,STXBP1,ERC1,BCL2,CNTLN,SPON1,DENND1A,NEDD4,ATP2B2,RABEP1,GRIA1,KHDRBS2,RAPGEF2,LRP2,RUNX2,LDB2,GRM7,GHR,ADAM10,APP,CD2AP,RAB8B,PAK3,SNTG2,RAB27B,MRTFA,SHC4,PLG,NIPBL,CARD18,FMN1,PPARA,SYNJ1,ENAH,USP8,EBF1,CXADR,DOCK4,ATRX,ABL1,PTPN12,SDC2,INPP5A,GRID2,ZBTB16,ESRRG,RFC1,ZFYVE9,OPRM1,SH3KBP1,LUC7L,ADAM12,HMGA2,TRPS1,CRIM1,RUFY2,TJP1,STRN,SH3BP5,ZNF106,ETS2,DOCK1,SLAMF1,GABRR2,KIF21A,ATF2,CFTR,CHAF1A,REPS1,PRKN,AFAP1,TFDP1,SHISA9,SHANK2,MYO1D,LYN,ARHGAP31,DTX1,INSR,XRCC4,HOXC4,CARD10,BCL2L1,SYNJ2,CABYR,MED1,ZNF521,VASP,ETV6,IQGAP1,CACYBP,CADM1,TWIST1,JAK2,ALX4,ZBTB21,ATP2B1,NDFIP2,ABI1,HNRNPM,ADGRB1,RXRA,MAP3K5,NDFIP1,PDE2A,NCK1,TOP1,GNG12,EPHA4,OCLN,SHISA6,PTK2,MPP7,SCAMP1,ICA1,CCDC88A,FYN,SNTB1,DOCK3,FUT8,RAD9A,PLEKHA2,ELMO1,CACNG3,TRIM9,ZMYND8,DDX6,EXOC4,ROBO1,NLGN1,CTTNBP2,TCF12,RAB27A,EHMT1,KCNAB1*
GO:0005001	transmembrane	0.012069061837	*PTPRD,PTPRA,PTPRN2,PTPRR,PTPRK,PTPRT,PTPRO,PTPRE,PTPRB,PTPRG*

	receptor protein tyrosine phosphatase activity	718154	
GO:0019198	transmembrane receptor protein phosphatase activity	0.012069061837718154	*PTPRD,PTPRA,PTPRN2,PTPRR,PTPRK,PTPRT,PTPRO,PTPRE,PTPRB,PTPRG*
GO:0008013	beta-catenin binding	0.012754743283072537	*APC,TCF4,PTPRJ,CTNNA3,VCL,CD2AP,KANK1,CTNNA2,CTNNA1,PTPRK,CXADR,PTPRT,CTNND2,TJP1,GLI3,PRKN,SOX30,CDH26,AJAP1,GRIP1,SHROOM2,MED12L,NUMB,CDH5,CDH2,DLG5,ESR1,RORA*
GO:0015075	ion transmembrane transporter activity	0.01277379943724249	*UNC80,CACNA2D3,SLC17A1,SLC24A2,KCNH5,SLC25A21,SLC37A1,PIEZO2,ITPR2,SLC44A5,KCNMA1,CHRNA7,GABRB3,ANO6,CACNG2,SLC4A10,OCA2,GRIK3,ATP2B2,TUSC3,GABRB1,GRIA1,SLC39A12,SLC8A3,GABRA6,TMEM38B,SLC24A3,SLC44A1,TRPM1,SLC39A11,SLC7A2,CACNA1C,CACNB2,GABRG2,TMC1,SLC8A1,KCNE4,KCNK10,TRPC5,CLIC6,RYR3,KCNJ1,TRPC7,SLC45A4,SLC16A1,NIPAL2,LRRC38,GRIK4,KCNS3,GABRG1,SLC24A4,SCN2A,RYR2,SLC9C1,SLC36A1,SLC1A1,SLC12A8,KCNH1,ANO4,GRID2,SLCO3A1,ABCC9,P2RX6,SLC2A3,ATP8A1,OPRM1,ABCC4,CNNM4,SLC2A13,KCND2,NIPA2,TMC7,ATP6V1E1,CACNA1I,KCNJ15,SCN11A,SLC23A2,SLC39A6,KCNH8,SLC37A2,SLC9A4,GABRR2,GRIK2,LRRC8B,CFTR,SLC30A10,GRIN2A,TRPM6,SLC12A1,KCNQ3,SLC4A4,SCN10A,KCND3,KCNN3,SLC44A2,SLC15A2,SLC13A5,CUL5,GRID1,SLC52A1,COX5A,GABRG3,ZDHHC17,SLC22A14,KCNC1,HCN1,GRIN2B,KCNK5,SLC40A1,SLC5A12,COX7A2L,LASP1,SLC1A7,TRPV5,ABCC8,CACNA1E,ATP2B1,CLCA4,OTOP1,SLC6A1,SHROOM2,SLC6A11,KCNJ18,ATP13A3,SLC5A9,ATP6V1C2,SLC10A6,ATP6V1B2,GABRA5,TRPM7,GRIK1,APOL1,SLC26A2,SLC9A5,SLC27A6,SLC5A1,ANO10,SCN8A,TMEM63C,NCS1,ATP5PF,NALCN,TRPM3,SLC39A8,SLC16A9,KCNJ6,ATP6V0D2,CACNG3,SLC1A2,GABRA2,KCNIP4,SLC6A3,ASIC2,KCNQ5,CACNA2D1,SLC25A18,ANO2,GRIA4,CATSPER2,CLCN5,SLC13A4,KCNAB1*
GO:0017124	SH3 domain binding	0.013778847673907614	*DENND1A,KHDRBS2,LRP2,ADAM10,CD2AP,PAK3,FMN1,SYNJ1,ENAH,USP8,DOCK4,ABL1,PTPN12,SH3KBP1,ADAM12,RUFY2,SH3BP5,ZNF106,DOCK1,REPS1,PRKN,AFAP1,SHANK2,LYN,ARHGAP31,DTX1,SYNJ2,CABYR,VASP,ABI1,DOCK3,FUT8,RAD9A,ELMO1,CTTNBP2*
GO:0003774	cytoskeletal motor activity	0.01612403247762649	*MYO9A,MYO5A,MYO1E,MYO3B,MYO5C,DNAH6,KIF4A,DNAH14,DNAH11,DNAH5,MYO10,DNAH8,KIF21A,KIF15,MYO3A,MYO1D,KIF11,KIF21B,KIFC1,CENPE,KIF6,MYO18B,MYH13,DNAH10,KIF16B,MYO5B,DNAH3,DNAH17,MYH15,KIF13A,DNAH9,MYO9B,KIF7*
GO:0004713	protein tyrosine kinase activity	0.022924847299007296	*ALK,MAP3K9,EGFR,NTRK3,FLT1,EPHA7,EFEMP1,MELK,BLK,ABL1,PEAK1,MUSK,EPHA6,HIPK3,CRIM1,DSTYK,LYN,CHKA,INSR,ROR2,HIPK1,JAK2,MAP2K6,FGR,EPHA4,NTRK2,PTK2,FYN,ROR1,EPHB1,RPS6KA5,NRP1,BCR,EPHB2,MET,ABL2,ERBB4,FER,IGF1R*
GO:0008017	microtubule binding	0.02351079469108689	*MX2,RP1,DCDC1,MAP4,APC,MACF1,DIAPH3,KIF4A,MTUS1,DCLK1,MAPRE2,CCSER2,MAP4K4,FMN2,DNM3,MTUS2,FMN1,PAFAH1B1,NAV3,DST,GAS2,CLIP1,PEX14,C10ORF90,VPS41,TUBGCP3,MDM1,NIN,HAUS6,KIF21A,KIF15,MAP2,MX1,CEP44,RACGAP1,KIF11,DPYSL5,HDGFL3,KIF21B,SKA1,MAP6,KIFC1,CENPE,KIF6,MTCL1,EML1,MAST2,MARK4,DIAPH1,SAXO1,CCDC88A,SPAG6,KIF16B,TOGARAM1,NDRG1,GAS2L1,KIF13A,CEP57L1,IRAG2,EML6,HOOK3,KIF7,DNM1L*
GO:0008237	metallopeptidase activity	0.02393772072991509	*AGBL1,ENPEP,PAPPA2,CPA6,ADAMTS6,ADAMTSL1,ADAM32,ADAM10,ADAMTS17,ADAM22,PAPPA,TLL1,ERMP1,ADAMTS3,CPE,ADAMTS14,METAP1D,LNPEP,ADAMTS2,CPXM2,AFG3L2,ADAM12,FOLH1,ECE1,XPNPEP1,IDE,PEPD,AOPEP,MMP16,ADAMTS19,TRAB*

			D2B,MBTPS2,ADAM28,PRSS2,ADAMTS5,MIPEP,CLCA4,CNDP2,ADAMTS18,EIF3F,ADAMTS9,ADAMTS16,DNPEP,ADAM29,CPQ,MMP26,TRHDE
GO:0045499	chemorepellent activity	0.032203382347647136	*EPHA7,SEMA5A,NRG3,SEMA3C,SEMA6D,NRG1,SEMA3E,SEMA3A,SEMA3D,SEMA4D,FLRT2,EFNA5,SEMA4B*
GO:0016917	GABA receptor activity	0.03514243867570173	*GABRB3,GABRB1,GABRA6,GABRG2,GABRG1,GABRR2,GABBR2,GABRG3,GPR156,GABRA5,GABRA2*
GO:0005261	cation channel activity	0.0399206720204274	*UNC80,CACNA2D3,SLC24A2,KCNH5,PIEZO2,ITPR2,KCNMA1,CHRNA7,ANO6,CACNG2,GRIK3,GRIA1,TMEM38B,SLC24A3,TRPM1,CACNA1C,CACNB2,TMC1,KCNE4,KCNK10,TRPC5,RYR3,KCNJ1,TRPC7,LRRC38,GRIK4,KCNS3,SLC24A4,SCN2A,RYR2,KCNH1,ABCC9,P2RX6,OPRM1,KCND2,CACNA1I,KCNJ15,SCN11A,KCNH8,GRIK2,GRIN2A,TRPM6,KCNQ3,SCN10A,KCND3,KCNN3,CUL5,KCNC1,HCN1,GRIN2B,KCNK5,TRPV5,ABCC8,CACNA1E,OTOP1,SHROOM2,KCNJ18,TRPM7,GRIK1,ANO10,SCN8A,TMEM63C,NCS1,ATP5PF,NALCN,TRPM3,KCNJ6,CACNG3,KCNIP4,ASIC2,KCNQ5,CACNA2D1,GRIA4,CATSPER2,KCNAB1*
GO:0004970	ionotropic glutamate receptor activity	0.043889434261199387	*GRIK3,GRIA1,GRIK4,GRID2,GRIK2,GRIN2A,GRID1,GRIN2B,GRIK1,GRIA4*
BP			
GO:0050794	regulation of cellular process	4.0433960957366594e-38	*NOTCH2,BCAR3,BRINP3,MTOR,CNTN4,CACNA2D3,SPOCK1,NSG1,SGCD,WWC1,ABCA13,GARNL3,LRP12,PTPRD,SLC24A2,TRAPPC9,BNC2,LRRC4C,KCNH5,ANKS1B,SMOC1,MYO9A,ULK2,NLK,LONP2,UNC13C,FTO,KSR1,MGA,RFX7,ZNF236,PLCB1,ZNF536,MX2,TAFA5,SVIL,CLTCL1,ZFPM2,TENM4,L3MBTL4,DLC1,TNRC6B,DPP10,ZDHHC21,PTPRA,ITPR2,RIPOR2,PDE4D,RDX,RP1,STXBP1,ERC1,RALA,IL1RAPL2,BCL2,ODAD2,KCNMA1,PRDM16,ALDH1A2,ARHGAP26,FBN1,LRFN2,CDH8,CHRNA7,DCDC1,GPR158,ROBO2,RIMS1,PIK3C3,EPC2,SPIRE1,TENM3,GABRB3,ZEB1,AKR1C3,SDCCAG8,RARB,FGD4,SPRED1,ENPEP,MYO1E,PLPPR1,USH2A,MINAR1,CDC42EP3,RIMS2,ALK,AUTS2,ADGRE1,FOXJ2,CDYL2,CARMIL1,MCTP1,PJA2,BABAM2,PAPPA2,GLIS3,FANK1,ERBIN,RHPN2,RIN2,ANO6,CACNG2,DLGAP1,NEGR1,ZNF880,MLLT3,EGLN3,GPC6,CNTNAP2,MAP4,MAP3K9,SPON1,APC,ZNF595,HHLA2,TSHZ3,RBFOX3,PLPPR5,DSCAM,RTN1,TCF4,CRKL,ILDR2,SOX5,SETD2,ERG,ARHGAP24,ZNF573,TNIK,SLC4A10,PTPRJ,KDM4C,NEK4,DOCK10,TSHZ2,EGFR,ZNF280B,RFX3,DENND1A,USP14,ANGPT1,CDK12,BACH1,MACF1,CTNNA3,PRKACB,NEK7,RGS3,NCOR1,RNF220,DOCK2,ZNF407,NEDD4,MAML2,MTRF1,SND1,SCAI,NSMCE2,BTBD9,BCL11A,SOX6,FAM83F,TMEM182,SGMS1,GRIK3,CHSY1,FLI1,RPRD1A,CDH4,NTRK3,RXFP1,C5,PDE1C,ZFAND6,PHACTR1,DKK2,FLT1,DNAJC13,ZNF648,RFC3,RABEP1,ZNF382,TASP1,THRAP3,MAPKBP1,GABRB1,PSMA8,DGKI,INVS,C12ORF4,EDAR,GRIA1,CRACD,CAST,NUP214,NEO1,CNTN6,SLC39A12,CABLES1,SLC8A3,MALRD1,TOM1L2,PRKD1,TPTE2,PAK1,GMDS,EPHA7,CTNNAL1,NCOA7,KHDRBS2,CHRM3,RALGPS1,SPEN,RAPGEF2,PELI2,LRP2,ADGRB3,RUNX2,ARSB,FGF12,GABRA6,TAOK3,ONECUT1,CPEB4,TMEM38B,PRICKLE2,UBE2L3,LDB2,TAFA4,BTBD11,PUM3,CCL28,SMYD3,PATJ,GRM7,SEPTIN9,RETREG1,RPTOR,TMEM117,GHR,EPB41L3,THADA,COL4A2,SSBP3,RALGAPA1,CELF2,RAPGEF5,TBCD,NEDD4L,PPP1R12B,TRPM1,ADAM10,HDAC9,ZHX3,ATF7IP,IL1R1,APBB2,APP,RPS6KA2,SAMSN1,CACNA1C,KDM1B,CACNB2,KLHL13,MTUS1,DCLK1,STAU2,GABRG2,DOCK8,TMC1,MAPRE2,ZNF600,USP18,SEMA5A,SYT1,VCL,ARHGAP44,NTF3,ACER2,PARP15,NDUFAF2,CD2AP,ZNF723,AURKA,PARN,CFDP1,ST18,PYGO1,SLC8A1,HERPUD2,SSBP2,PTPRR,SRGAP2C,ANKRD31,FIG4,DUX4,TAFA2,ABCG8,SRGAP2B,KANK1,KCNE4,MAP4K4,HIVEP2,ABCD2,BMPR1B,FMN2,PCSK6,AKAP6,HOMER2,ZNF717,CTNNA2,ARNT,RAB8B,PAK3,RFTN1,PDE1A,ZNF257,DIP2B,KCNK10,RANBP2,LARP1,ITPKB,TRPC5,RGS20,PD*

			E10A,UBE2E2,RAP1GDS1,HHAT,CLIC6,KICS2,ERC2,DNM3,NBN,SCP2,SYN3,IFT57,INTS7,PRKCZ,BTLA,GRB10,RYR3,TAF15,MSH6,MCPH1,ARHGAP32,RAB27B,CNST,RGS9,HECW1,DEFA3,MBNL2,ABCA5,PHF19,MRTFA,TAF4B,COBL,SENP6,DUSP22,EBF2,YAP1,NFIA,WDR70,PPM1L,RIPK4,ZKSCAN5,SHC4,BRINP1,MAPK1,MGAT5,CADPS2,KCNJ1,HRH2,RABGAP1L,ADAM22,USP25,KMT2E,ALCAM,PLG,PCGF5,PDGFD,SYT10,ZNRF3,PPP1R1C,ITGBL1,ARHGEF17,NRG3,UBE2O,SFMBT2,ANKFY1,NCAM1,GFRA1,SYCP1,NIPBL,SLC16A1,SPIDR,EWSR1,GABPA,FAT3,MICU1,ZNF735,CORO2B,CARD18,CHD6,STK38,PTPN13,CHN1,HRH4,SORCS3,MYLK3,KANSL1,GLP2R,LIMCH1,FMN1,MBNL1,PAFAH1B1,ATF6,EFEMP1,ZNF684,TM7SF3,DCAF1,ITGB8,STON2,VPS13D,CCNG2,TLK1,TPM1,NF2,LRRC38,CNKSR2,GRIK4,RBFOX1,HIVEP1,CTNNA1,PPP1R9A,MOB3B,BIRC6,AKAP9,KLF15,RASGRF2,PPARA,MEIS2,SNX30,NFIB,KCNS3,ERMP1,MRTFB,PPP6R3,PRTG,RGL1,SYNJ1,NR5A2,ADAMTS3,TIAM1,ARAP2,GRM1,FOXJ3,PTPRK,ARHGEF12,GABRG1,PAK5,TRERF1,PCDH11Y,PPP2R5E,PLA2R1,EIF3D,SEMA3C,DAPK1,NAV3,SLC24A4,SEC14L1,VPS13C,TMEM108,AGO2,STK32B,PHC3,MAGI1,ALPK2,DNAH11,JARID2,SCN2A,RIC8B,SORCS1,DNAJC15,GATAD2B,CPE,EVC2,DYSF,IL34,ANK2,BRWD1,TANC1,ADGRV1,ZNF846,MELK,BCAS3,RYR2,SYNE2,BBS2,WNT9B,ZNF606,RANBP3L,OR4F6,NKG7,SEMA6D,DUSP16,SMARCA4,CDH11,USP8,FABP7,PARD3,MAPKAP1,TNRC6C,PIAS1,TBC1D5,SPG21,BLK,EBF1,TNR,GRM8,DST,CXADR,DOCK4,MBD5,ATRX,NUAK1,PTPRT,ELAVL4,ABL1,MXI1,PTPN12,HDAC4,OXR1,SLC1A1,PRKAA1,SDC2,GAS2,KCNH1,ITGB3BP,MRPS27,LRFN5,CREG1,DROSHA,APBB1IP,L3MBTL3,EIPR1,APLF,NFAT5,MAST4,GUCY1A2,NBAS,SLFN11,RAP1A,GLIS1,MORC1,MYO10,GPC5,TOX3,CAMK4,BAZ2A,INPP5A,CPSF3,FGF10,ZC3HAV1,GRID2,TGM1,PEAK1,LATS2,NRG1,INO80D,GSG1L,CLIP1,ASPM,AP3B1,DENND2B,RASGRF1,ATP11C,ZNF438,ABCB7,ZBTB16,MUSK,KIR3DL2,ZNF675,GNG7,SMARCAD1,SH3GL3,SETDB2,PRKCE,FOXK2,SLCO3A1,MED15,SLMAP,NXN,WNK2,ESRRG,ZNF718,DGKB,USP33,DENND4C,FBN2,CD44,RGS12,PTPRO,EGF,PRRC1,ABCC9,P2RX6,TRIO,PDE3A,EXT1,STXBP6,NSMAF,LNPEP,LIMD1,PEX14,SPRED2,RPS6KA3,CTNND2,ATP8A2,SCG5,MTMR3,PTPN2,TRIM5,PLXNA2,MCF2L,OR4F15,ATXN3,RFC1,HTR2C,RIC3,CLEC16A,ARHGEF7,ALG10B,ATP8A1,AMBRA1,LTBP1,STK38L,ZFYVE9,KDM7A,OPRM1,HTR2A,PLCXD3,FANCA,DAZL,INPP4B,GTF2F2,KREMEN1,STAC,SEMA3E,TAF3,RPRD1B,MARK2,GCSAML,TMEM67,EBF3,ZNF33B,C10ORF90,FHL2,ABHD17C,ADGRA3,CNIH3,PUM1,TMOD2,HERC1,MSH2,IGF2BP3,GNAL,EPHA6,ANKRD17,APBA2,LINGO2,ZNF397,SH3KBP1,SLC2A13,RELL1,HIPK3,CDKN2C,EPN2,KCND2,EVC,GRK3,KNDC1,SPSB4,CLSPN,NOS2,BICRAL,STK10,MOSMO,GFRA2,MNAT1,TMEM116,RBBP8,MDFIC,ADAM12,MYLK2,ANK3,EMILIN2,HMGA2,CCND3,BCL11B,VPS41,DOCK5,ECE1,ZIM3,STK32A,CREM,LYPLA1,MBP,TRPS1,PLCE1,TGFA,IL17RA,ANKFN1,HIP1,CRIM1,FUT9,PRR5L,GSR,ATP6V1E1,UTP4,CAPN5,VAV1,RUFY2,MYT1L,ZNF160,TJP1,LDLRAD4,NPHP4,EGFLAM,PACSIN2,CNTN1,HLA-B,IQSEC1,HSF5,SNX3,CACNA1I,NAA35,ZNF367,PDLIM5,KCNJ15,BRCA2,DISC1,ZBTB2,DNER,BLM,ASB7,WDPCP,NRK,SEMA3A,MAGI3,INTS8,LIN54,ADCY10,PSG8,STRN,OR9Q1,ZNF121,BMP2,RC3H2,UNC5D,ATP9A,TRAK1,PSG9,CDC42BPB,SOGA1,PTCD2,SCN11A,MSR1,VRK1,GNAI1,RALGAPA2,ZC3H14,GFI1B,TBC1D4,RANBP9,RESF1,MYRIP,TTR,RIN3,BMP2K,TMEM161A,SEMA3D,ASXL3,NETO2,PDE6C,CABIN1,LEMD3,RELN,ARHGAP42,HMGB1,GNAQ,FGF9,NFATC2,SH3BP5,UST,MDM1,SLC23A2,POLR2M,ZNF106,MYOM1,ZNF567,TRAF3,ZNF462,ANKRD26,ESRP1,UNC13B,TTC21B,ETS2,GEMIN5,ZNF875,DSTYK,UIMC1,DOCK1,LRRFIP1,RAP1GAP,PLS1,SRGAP2,IKZF2,NIN,DRAXIN,ATF1,SLAMF1,KCNH8,SMARCA2,ETS1,FAM83B,GLI3,CGAS,SMARCC1,SNX6,AFF3,GABRR2,SMOC2,PCP4,CNKSR3,CASP5,VENTX,GRIK2,IDE,WDR12,MCTP2,KIF15,PRDM10,CUL1,MYEF2,ZFYVE26,ZNF431,RERE,PSD3,MAP2,BTAF1,GAREM1,LAMC1,ZNF618,NEK10,FARP1,MOB1B,ATF2,HIRA,CYLD,UMODL1,BBS4,MAPK8IP1,MX1,PSG

			6,HIVEP3,COL5A1,GABBR2,PSIP1,ITGA9,CFTR,KPNA1,NELL1,ME2,UBASH3A,RGMB,NEU3,MRPL13,KITLG,ZZEF1,DNAJC7,CAMTA1,UBR1,DCC,MYT1,CHRM5,MAP4K3,YLPM1,SLC30A10,RCAN1,GTF2I,RORB,TADA2A,DAB1,MED27,ZNF208,SELENON,RB1CC1,NMD3,AKAP10,PTPRE,PRKN,MTMR2,ZNF608,TBX20,SP110,DLGAP2,AFAP1,MAPK10,DACH1,ZNF541,DPF3,NGEF,GRIN2A,ARID5B,ZBED9,JPH1,TXNRD2,ATXN1,WSB1,LALBA,PRKCH,PKP1,HUNK,FRMD4A,TG,IL6R,FRMPD4,ALS2,RACGAP1,NLRC5,ZNF627,OR51E1,ACO1,TFDP1,CNOT6L,MKNK1,HEMGN,KANK4,DOCK9,SNX25,FBLN5,KCNQ3,TOX,SHISA9,SLC4A4,PTPRB,ZFP90,PDE6A,COPS8,ZNF124,SCN10A,SHANK2,ST8SIA1,USP7,VAV3,ENPP3,PLAGL1,KCND3,MESD,ITSN2,SOX30,MOK,KIR2DL4,ARHGEF28,RALB,NPAS2,ADGRG6,ROCK1,LYN,VCAM1,SEL1L,ARHGAP28,ARHGAP31,ZNF780B,CTSB,EIF2B3,SLC44A2,SUMO3,SLC15A2,ZNF169,PLEKHB2,KIF11,DTX1,BZW1,TENM2,OVOL2,PIWIL3,ZBTB33,ADA2,NTN1,PLCB4,ZFHX3,FANCL,DPYSL5,ZNF44,RRAGD,BANP,SUPT16H,ARID1B,HOXC13,CRACR2A,RNF152,BAZ1A,CASZ1,OTUD7A,INSR,CUL5,OR7A17,BMF,YTHDF3,TFF1,DEDD2,NEK6,HECTD1,GRID1,COLQ,NMU,DDHD1,PBX3,SUMO2,HS1BP3,ZNF292,ARFGEF1,PDE4DIP,GAST,POGK,SNAI2,ASH1L,IGHV3-74,HOXC4,BID,SIAH2,TANC2,ABCA4,TRABD2B,UFD1,RXRG,SP3,DRAM1,ERN2,GABRG3,ZNF879,MBTPS2,FLNB,TRIM58,TIAL1,TOM1,ELF2,PLPP4,NREP,ZDHHC17,NSD2,FYCO1,SH3GLB1,CD9,CARD10,RALGPS2,JCAD,TWIST2,OR4K2,CTIF,SAMHD1,IFT81,ENPP1,UTRN,RASGRP1,IGSF11,SNX9,TP53I11,TMEM225,ANAPC1,NDRG2,CSNK2A1,BMP5,KCNC1,CSF1,GHRH,HDGFL3,BCL2L1,SERPINB9,SCAF4,CTDP1,HCN1,PRKG1,LAMA3,ASB4,GRIN2B,GRB14,INO80,FANCB,GPR156,IGHV2-70D,CLNS1A,CNMD,DHRS3,SMAD5,CELF4,TCERG1,ABCG1,OR4C46,FOXN3,KCNK5,SLC40A1,PRAME,MYCL,TNN,CIDEC,PSAP,LPGAT1,MICALL2,MED1,CDC14B,PCNT,IL33,AJAP1,GPRC5C,ROR2,CFH,ZNF521,KL,RASGEF1C,BANK1,CSDE1,LMX1A,TMEM178A,IL10,ACTR2,OR1L6,SFPQ,SCML2,PRAMEF25,RIOK1,CLSTN2,PTH,SOSTDC1,PRKAA2,CSF2RB,DIRAS2,SKA1,NDC80,SOHLH1,LARP6,PACRG,PHF20L1,ABHD2,ITPRIP,VSTM2A,MAP6,VASP,PLA2G4A,ETV6,RAB12,IQGAP1,RPS12,CAMLG,COX7A2L,ZBTB7C,TEAD1,MORC2,SREBF2,ANP32B,YBX3,AIMP1,THNSL2,FYB2,NRXN1,PCID2,HIPK1,ZNF234,CISD1,ZNF518A,DGKK,SNAP91,CD70,CIBAR1,PBLD,FICD,CADM1,CENPE,PEG10,LMX1B,NET1,SIPA1L2,NGDN,ELOC,ANLN,TWIST1,AKT3,ALKAL2,JAK2,VSX1,RPF2,FSTL1,ZBTB38,ISX,SVEP1,MADD,HCRTR1,PTGS1,PATL1,ZNF287,CELSR2,ZNF449,PRSS2,CREBBP,MELTF,TNKS,GORAB,PCNA,SIAH3,UFL1,NFKBIA,PRKCB,OR2T3,ABCC8,ANXA4,CACNA1E,ZC3H15,ANP32A,RFC2,ZNF354C,ALX4,RTRAF,USH1C,BRD4,ZBTB21,SERBP1,NEDD9,OLFM4,NRBP1,ITGA6,ATP2B1,GAP43,ASS1,MTCL1,GRIP1,IGHV1OR15-9,CTNNBL1,ADGRE3,SAR1A,ADCY9,PPP1R17,CNIH1,MAST2,HPSE2,BTG3,ZNF528,ERLIN2,ZNF611,OTOP1,CIDEA,ARFGEF3,ZBTB49,EXT2,EXOC1,KRT6A,STOX2,AGO1,MEOX2,SLC6A1,GID8,ELL2,STAT1,BRMS1L,NDFIP2,NR2C1,MAP2K6,CMTM7,DGKG,KCNJ18,GATAD1,MTPN,ABI1,CEMIP,PRAMEF2,POU6F2,IMPACT,CCBE1,PARK7,ADAMTS18,MAPK8,ITGA4,OAZ2,MED12L,ZSCAN30,FBXL17,POU1F1,UBE2J2,ADCYAP1R1,MTF2,NCAPG2,TM9SF4,RAPGEF4,OR6C75,FOXP2,ASB2,MYOCD,CEP120,ZSCAN5C,CYFIP2,HNRNPM,ASCC2,EFHB,OR13C9,ARID3B,MEF2C,ZNF613,ADGRB1,RXRA,WNT7A,RBPMS2,MAP3K5,NDFIP1,MAP3K4,WASF3,S100B,SERPINI2,PRDM13,FOXO6,ZNF112,ATP6V1C2,C16ORF72,MAGEL2,PKN2,RAD51AP1,OR10H2,PDE2A,RAB38,LRRC2,DBF4B,FBXW8,SDCBP,NECTIN1,JPT2,SPPL2B,NSMCE1,ZNF813,WWOX,ZBTB25,PASK,MLLT1,NCK1,SCAF8,FGR,CWC22,CDCA8,PPP2R3A,DNMBP,ATP6V1B2,CXCL2,MLLT10,C2,IFNAR1,RNF8,GNG12,EPHA4,CYTH4,INTS13,GABRA5,MECOM,DNMT3L,NTRK2,IL1RAPL1,NUMB,LHX9,ADAMTS9,WNT2B,COLEC12,ZBTB10,OCLN,POSTN,CREB5,CD101,SHISA6,MEGF10,IL17RD,FBXO31,EXTL3,AKAP11,GRIK1,PRKAB1,DTHD1,IREB2,MVB12B,PTK2,MARK4,CD

			H5,CD5L,RCAN2,ANKRD6,SCGN,NFKBID,ARHGAP12,CLDN18,ASCL3,MPP7,DIAPH1,FEZ2,INIP,LAMB1,APIP,CYFIP1,UBE3A,PCDH8,SEMA4D,JAM2,PITPNC1,FRMD6,MC2R,ZBTB20,FAT4,IMPA2,ZNF66,RUNX1,AKR1B1,KIRREL1,WNT5B,RASGEF1B,AMFR,SAXO1,NENF,POMT2,PTGFR,ZNF845,OR4L1,ASAP1,SAMD13,ICA1,PLCZ1,EDIL3,NOS1AP,FCRLA,DIDO1,TPTE,SORBS2,PDCL3,SRP9,CNKSR1,CCDC88A,GPR55,NSUN2,CHCHD2,ADAMTS16,CDC45,OR11G2,BICD1,TNFSF11,FYN,BUB1,KDM5A,PCBP3,ZNF705G,PPM1F,ADGRL2,ARL13B,SDE2,RBMS3,UHRF2,SCN8A,HDAC2,AVEN,SLF1,GON4L,TBX15,SH2D3C,DOCK3,TRNAU1AP,NCS1,COL18A1,ALB,DOK5,NALCN,ZFYVE28,MAPK9,PABPC1,CRTAM,APELA,SLC39A8,ROR1,FUT8,TET1,ARNT2,ASB3,HECW2,CDH2,ITGA8,FBXL20,RAD9A,PHLPP1,GPR137B,EPHB1,RP1L1,GRM5,ADCK1,RAI14,ZNF705D,RPS6KA5,SPTB,TBC1D1,LRRC69,PTPRG,PID1,NRP1,MIDEAS,FCHSD2,PRKCA,ATPSCKMT,FAIM,SAMD12,FHIT,ITGA1,ZNF615,KLF12,RNF138,RC3H1,NRIP1,CHODL,POR,ZNF850,ZNF235,MCC,ZNF738,SUPT3H,BCR,TUT4,NRXN3,ELMO1,RGS6,RERG,ZNF215,TCERG1L,KIF16B,PRIM2,SNRK,C14ORF39,TM9SF2,ELP2,FBLN1,STK36,NSG2,RAG1,KCNJ6,B9D1,RRAS2,GNA14,ZNF678,BMPER,PRDM15,CUX1,DPP6,SRGAP3,ZNF420,MACROH2A1,MITF,EPHB2,TSPAN13,TOGARAM1,CSNK1G1,SACS,BCL2L13,CD38,EYA4,DPH6,CDK14,AKAIN1,MET,SPPL3,CDH17,ZNF705B,ATP6V0D2,PPFIA2,CDH13,MED13L,STXBP4,SERPINB2,CACNG3,ATG5,MAGI2,PRDM11,VMP1,UNK,MLIP,FLRT2,MYB,KALRN,ZNF704,SLC1A2,GNAS,LAMA1,MFHAS1,SERPINB7,ATRNL1,TIAM2,DHX29,BMP7,TTC28,ASTN2,DLG5,TNFAIP8,ZMYND8,GAPVD1,GABRA2,RNF217,KCTD1,OR2T2,ZNF74,BPTF,BTBD10,ZMYND11,TMEM25,NUDT21,GRM3,KMT2C,DDX6,ADGRF5,OR4N2,PDGFC,WDR41,PPP1R13B,ABL2,BACE2,RFX2,PARPBP,NECAB1,PKNOX2,EYA1,FHOD3,SLIT2,EXOC4,CNOT7,KCNIP4,ESCO1,KCTD8,PLCL1,ERBB4,IL20RB,FAM3B,GSAP,TRHDE,SYNDIG1,ROBO1,SAMD4A,PBX1,IRAG1,NPAS3,NUF2,PRKCQ,ANTXR1,NDRG1,SORCS2,SIPA1L3,TRDN,MGMT,ZNF679,NLGN1,CTTNBP2,SHLD2,NOS1,SLC6A3,PRR16,ASIC2,EFNA5,TCF12,GAS2L1,ARHGEF11,RAB27A,NSD1,EHMT1,SLIT3,DTNA,KIF13A,FRMD5,ESR1,MYO9B,NTNG1,KDM4B,KCNQ5,LOXL2,CACNA2D1,NYAP2,IGLC3,IQCJ-SCHIP1,ADGRG7,SKAP2,PRLR,AGO3,HTT,FOXB1,RAD51B,CAMK1D,PIK3R3,HLA-F,FER,ZNF302,EYA2,CCR2,STARD13,INTS12,CHFR,ZNF721,EPS8,JAZF1,ZNF578,ZNF891,SPOCK3,SEMA4B,NRF1,IGHV1OR21-1,ZNF14,HRH1,PHC2,GRIA4,ROCK2,PRDM1,RORA,STMP1,IL16,ATAT1,DMRT1,EIF4G3,CDCA5,PPP1CB,CATSPER2,RGS8,RAB31,PDK1,CSMD3,HERPUD1,NCOA6,TRIM2,COL4A3,WASHC1,ZFP30,RGS7,HOOK3,KIF7,GNG2,FSTL4,BARD1,STK3,DEPTOR,ZNF423,C1QL3,RSU1,PNPLA8,ZNF568,HNRNPU,CEP72,RAB3GAP2,CADPS,APCDD1,IGF1R,KCNAB1,PRKAG2,GLI2,THRB,AKAP13,MORC3,ATP10A,DNM1L
GO:0048856	anatomical structure development	2.2437523791736848e-36	*NOTCH2,BCAR3,BRINP3,MTOR,CNTN4,SPOCK1,SGCD,IMMP2L,LRP12,PTPRD,FREM1,TRAPPC9,BNC2,NEBL,LRRC4C,SMOC1,MYO9A,ULK2,SCAPER,FTO,PLCB1,ZNF536,TAFA5,SVIL,CLTCL1,ZFPM2,TENM4,NUBPL,DLC1,ZDHHC21,RIPOR2,PDE4D,RDX,RP1,STXBP1,RALA,IL1RAPL2,BCL2,ODAD2,ALDH1A2,ARHGAP26,FBN1,CDH8,CHRNA7,ROBO2,RIMS1,SPIRE1,TENM3,GABRB3,ZEB1,AKR1C3,SDCCAG8,RARB,FGD4,SPRED1,NAV2,ENPEP,SPAG16,MYO1E,PLPPR1,USH2A,MINAR1,CDC42EP3,RIMS2,ALK,AUTS2,FOXJ2,CARMIL1,PAPPA2,ASTN1,RIN2,PARVB,ANO6,NEGR1,MLLT3,GPC6,CNTNAP2,MAP4,MYO3B,APC,ZMYM4,RBFOX3,PLPPR5,DSCAM,RTN1,TCF4,CRKL,ILDR2,SOX5,SETD2,ARHGAP24,TNIK,SLC4A10,PTPRJ,OCA2,KDM4C,DOCK10,EGFR,RFX3,ANGPT1,MACF1,PRKACB,RNF220,DOCK2,NEDD4,MYOF,CRB1,BCL11A,SOX6,TMEM182,CECR2,ARMC2,CHSY1,FLI1,CDH4,ATP2B2,NTRK3,LARGE1,RXFP1,C5,PHACTR1,DKK2,FLT1,ADAMTS6,GABRB1,EDAR,NEO1,CNTN6,SLC39A12,CABLES1,SLC8A3,PRKD1,PAK1,EPHA7,CHRM3,SPEN,RAPGEF2,LRP2,ADGRB3,DEUP1,RUNX2,ARS*

			B,FGF12,CPS1,TAOK3,ONECUT1,TMEM38B,ADAMTSL1,PRICKLE2,SLC24A3,LDB2,SMYD3,GRM7,GHR,LUZP1,EPB41L3,COL4A2,SSBP3,RAPGEF5,TBCD,NEDD4L,ADAM10,HDAC9,APP,ABCB5,RPS6KA2,CACNA1C,DCLK1,STAU2,GABRG2,TMC1,SEMA5A,SYT1,VCL,ARHGAP44,NTF3,AURKA,CFDP1,PYGO1,SLC8A1,PTPRR,SRGAP2C,FIG4,CMIP,SRGAP2B,KANK1,MAP4K4,ABCD2,BMPR1B,FMN2,THSD7A,PCSK6,AKAP6,CTNNA2,ARNT,PAK3,TTLL7,DIP2B,ITPKB,TRPC5,CHST8,DNM3,NBN,IFT57,RBM47,PRKCZ,CALD1,SNTG2,KLHL1,DIP2A,MSH6,MCPH1,COL27A1,ZSWIM6,HECW1,MRTFA,TAF4B,COBL,EBF2,YAP1,ESS2,FRYL,NFIA,RIPK4,BRINP1,MAPK1,ADAM22,CRISPLD2,KMT2E,ALCAM,PLG,PDGFD,ZNRF3,ABLIM1,NRG3,NCAM1,GFRA1,SYCP1,NIPBL,RNF17,GABPA,FAT3,LCE1F,CHN1,MYLK3,ACSBG1,FMN1,MBNL1,PAFAH1B1,ATF6,EFEMP1,TLL1,DCAF1,ITGB8,TPM1,NF2,RBFOX1,CTNNA1,PPP1R9A,ANKRD11,CDH7,BIRC6,KLF15,PPARA,MEIS2,NFIB,MRTFB,PRTG,SYNJ1,NR5A2,ADAMTS3,TIAM1,KAZN,ENAH,SF3B6,SEMA3C,NAV3,SLC24A4,TMEM108,AGO2,ALPK2,DNAH11,JARID2,SCN2A,CPE,DYSF,IL34,ANK2,BRWD1,TANC1,ADGRV1,MELK,BCAS3,RYR2,SYNE2,BBS2,WNT9B,RANBP3L,SEMA6D,ANKS6,SMARCA4,CDH11,LDB3,FABP7,PARD3,MAPKAP1,BLK,TNR,COL22A1,CXADR,MBD5,ATRX,XIRP2,ELAVL4,ABL1,PTPN12,HDAC4,SLC1A1,PRKAA1,SDC2,GAS2,KCNH1,LRFN5,CRTAC1,DROSHA,TTLL5,L3MBTL3,APLF,DNAH5,CDH18,RAP1A,MYO10,CAMK4,FGF10,GRID2,CDHR3,GALC,TGM1,PEAK1,LATS2,NRG1,INO80D,ASPM,AP3B1,RASGRF1,ATP11C,SYNE1,ZBTB16,MUSK,ZNF675,SH3GL3,SETDB2,PGM5,NXN,USP33,FBN2,CD44,PTPRO,EGF,ALPK3,TRIO,PDE3A,EXT1,COL5A3,LIMD1,SPRED2,ADAMTS2,RPS6KA3,CTNND2,NHS,ATP8A2,PTPN2,PLXNA2,ATXN3,ST8SIA6,ARHGEF7,AMBRA1,KDM7A,OPRM1,FANCA,CYP4A11,DAZL,CNNM4,KREMEN1,SEMA3E,MARK2,ALPL,FHL2,TMOD2,HERC1,MSH2,IGF2BP3,CDIN1,EPHA6,ANKRD17,APBA2,LINGO2,SH3KBP1,ATL1,LUC7L,CDKN2C,EPN2,EVC,KNDC1,AFG3L2,MOSMO,GFRA2,MNAT1,RBBP8,SGCZ,ADAM12,MYLK2,ANK3,EMILIN2,XYLT1,HMGA2,MYOM2,BCL11B,DOCK5,ECE1,MBP,AK8,TRPS1,PLCE1,TGFA,CRIM1,FUT9,VAV1,CDH20,MYT1L,ZNF160,TJP1,LDLRAD4,NPHP4,EGFLAM,PACSIN2,CNTN1,HLA-B,IQSEC1,MTHFD1L,SNX3,PDLIM5,BRCA2,DISC1,DNER,WDPCP,NRK,SLC10A7,SEMA3A,STRN,BMP2,RC3H2,UNC5D,TRAK1,PSG9,PTCD2,NCAM2,GFI1B,MSI2,BMP2K,RNF38,SEMA3D,ASXL3,PDE6C,RELN,HMGB1,FGF9,NFATC2,TDRD7,UST,CPAMD8,RTTN,MDM1,SLC23A2,ESRP1,TTC21B,ETS2,DOCK1,B4GALT6,TSPAN2,RAP1GAP,PLS1,SRGAP2,NIN,DRAXIN,ATF1,SLAMF1,SMARCA2,ETS1,GLI3,MEGF11,SMARCC1,AFF3,SLC9A4,SMOC2,PCP4,CASP5,CUL1,MYEF2,RERE,MAP2,DAW1,LAMC1,FARP1,TDRD5,ATF2,HIRA,UMODL1,BBS4,LAMC3,HIVEP3,COL5A1,CFTR,KPNA1,NELL1,DOP1B,KITLG,DCC,MYT1,RCAN1,GTF2I,RORB,TADA2A,DAB1,SELENON,RB1CC1,MYO3A,PRKN,MTMR2,SH3PXD2A,TBX20,DACH1,PCDH15,DPF3,LGI2,NGEF,GRIN2A,ARID5B,JPH1,ATXN1,CDH23,PRKCH,TG,IL6R,ALS2,RACGAP1,ACO1,TFDP1,DMC1,LCE3B,TOX,PTPRB,PDE6A,SCN10A,SHANK2,VAV3,MESD,ITSN2,SOX30,PTGFRN,SYBU,ARHGEF28,NPAS2,ADGRG6,YIPF6,SEC24D,ROCK1,LYN,VCAM1,CTSB,EIF2B3,LRIG1,DTX1,TENM2,OVOL2,NTN1,MMP16,ZFHX3,DPYSL5,ARID1B,HOXC13,CRACR2A,CASZ1,INSR,DMBT1,YTHDF3,HECTD1,SHROOM3,XRCC4,COLQ,HDAC11,DDHD1,PBX3,SNAI2,ASH1L,HOXC4,SIAH2,TANC2,UFD1,RXRG,SP3,MBTPS2,FLNB,TRIM58,TIAL1,PLPP4,NREP,ZDHHC17,NSD2,CERS3,SLC22A14,CD9,CARD10,KRT6B,XKR5,JCAD,SAMHD1,IFT81,ENPP1,UTRN,RASGRP1,DZANK1,NDRG2,BMP5,WDR72,KCNC1,CSF1,GHRH,HDGFL3,BCL2L1,KRT25,CTDP1,HCN1,PRKG1,LAMA3,ASB4,GRIN2B,INO80,CNMD,DHRS3,SMAD5,CELF4,SYNJ2,FOXN3,VSTM4,SLC40A1,MYCL,TNN,CABYR,PSAP,MICALL2,MED1,ATG4B,KDM6A,ATRN,IL33,AJAP1,ROR2,ZNF521,KL,CSDE1,FAT1,LMX1A,TMEM178A,IL10,ACTR2,SCML2,CLSTN2,TTC39C,PTH,SOSTDC1,SOHLH1,PACRG,ABHD2,MAP6,VASP,ETV6,TACC2,PALMD,IQGAP1,TEAD1,ANP32B,YBX3,AIMP1,NRXN1,PCID2,HIPK1,FRY,CIBAR1,CACYBP,CADM1,LMX1B,ANLN,TWIST1

			,AKT3,ALKAL2,JAK2,VSX1,FSTL1,ISX,BPNT1,SVEP1,RBM19,CELSR2,CREBBP,MELTF,ARL11,GORAB,PCNA,SIAH3,UFL1,ADAMTS5,NFKBIA,PRKCB,NTM,ABCC8,ANXA4,SMTN,ALX4,USH1C,SMPD4,NEDD9,OLFM4,ITGA6,ATP2B1,GAP43,ASS1,GRIP1,CTNNBL1,ADCY9,EML1,PPP1R17,XKR6,OTOP1,EXT2,KRT6A,STOX2,AGO1,MEOX2,GRXCR1,STAT1,NR2C1,MAP2K6,CMTM7,DGKG,SHROOM2,SLC6A11,MTPN,ABI1,MYO18B,ARMC6,POU6F2,IMPACT,CCBE1,ADAMTS18,ITGA4,FBXL17,POU1F1,ADCYAP1R1,MTF2,CSMD1,NCAPG2,FOXP2,ASB2,MYOCD,CEP120,DHTKD1,CYFIP2,KRT85,ST8SIA4,MEF2C,ADGRB1,RXRA,WNT7A,RBPMS2,MAP3K5,NDFIP1,MAP3K4,WASF3,S100B,PRDM13,FOXO6,PDE2A,FBXW8,SDCBP,NECTIN1,WWOX,NCK1,FLVCR1,FGR,DRC7,PPP2R3A,DNMBP,TOP1,SPRR2D,RNF8,LCE3D,EPHA4,EMP1,GABRA5,MECOM,DNMT3L,NTRK2,IL1RAPL1,FNDC3A,RSPH1,NUMB,LHX9,ADAMTS9,WNT2B,TNNI1,POSTN,CD101,MEGF10,IL17RD,FBXO31,GRIK1,PRKAB1,IREB2,HS6ST1,PTK2,MARK4,CDH5,ANKRD6,NFKBID,ARHGAP12,CLDN18,DIAPH1,FEZ2,LAMB1,CYFIP1,UBE3A,PCDH8,SEMA4D,JAM2,FRMD6,FAT4,AP2B1,RUNX1,AKR1B1,WNT5B,SANBR,ASAP1,DPY19L2,SORBS2,PDCL3,CCDC88A,GPR55,NSUN2,ADAMTS16,SPAG6,BICD1,TNFSF11,FYN,MYL12B,ADGRL2,UNC45B,ARL13B,HYDIN,SCN8A,HDAC2,GON4L,TBX15,NCS1,COL18A1,CDH9,LHFPL2,ATP5PF,DOK5,UGP2,CRTAM,COL19A1,APELA,MDGA2,ROR1,FUT8,TET1,ARNT2,HECW2,CDH2,CNTN5,ITGA8,NTN4,XRN2,PHLPP1,GPR137B,EPHB1,EYS,RP1L1,GRM5,DDX10,ADCK1,RPS6KA5,PTPRG,PID1,NRP1,SDK1,PRKCA,FAIM,ITGA1,RC3H1,NRIP1,CHODL,POR,BCR,TUT4,NRXN3,KIF16B,CDH12,SNRK,C14ORF39,FBLN1,STK36,PAQR5,MB,RAG1,B9D1,DGCR2,RRAS2,BMPER,CUX1,MACROH2A1,MITF,EPHB2,IGSF3,SGCG,CD38,EYA4,MET,CDH17,SPECC1,PPFIA2,CDH13,ATG5,NRAP,MAGI2,KIAA1217,VMP1,UNK,FAM171A1,ADAM29,FLRT2,MYB,KALRN,SLC1A2,GNAS,LAMA1,MFHAS1,GREB1L,SERPINB7,CA10,CPQ,ATRNL1,TIAM2,IGSF21,BMP7,ASTN2,DLG5,GABRA2,KIRREL3,BTD,BPTF,NUDT21,DDX6,ADGRF5,PDGFC,ABL2,TRAPPC6B,RFX2,NECAB1,EYA1,FHOD3,SLIT2,EXOC4,FAM126A,CCDC141,ERBB4,SYNDIG1,ROBO1,PBX1,PRKCQ,ANTXR1,NDRG1,MYH15,SIPA1L3,MGMT,NLGN1,CTTNBP2,SHLD2,NOS1,SLC6A3,ASIC2,EFNA5,TCF12,VCAN,EHMT1,SLIT3,ESR1,NTNG1,KDM4B,LOXL2,NYAP2,PRLR,FOXB1,RAD51B,CAMK1D,PIK3R3,MACROD2,CFAP44,OPCML,CATSPERE,FER,EYA2,CCR2,RPGRIP1,STARD13,A2M,EPS8,SEMA4B,ROCK2,PRDM1,RORA,ATAT1,DMRT1,CATSPER2,HSPG2,PTPRQ,CSMD3,NCOA6,HSD17B2,COL4A3,RGS7,HOOK3,PCSK2,FSTL4,STK3,ZNF423,ZNF568,HNRNPU,APCDD1,IGF1R,GLI2,THRB,LSAMP,AKAP13,MORC3,ATP10A,DNM1L
GO:0007399	nervous system development	4.125901321282941e-36	*NOTCH2,BRINP3,MTOR,CNTN4,SPOCK1,IMMP2L,LRP12,PTPRD,TRAPPC9,LRRC4C,MYO9A,ULK2,PLCB1,ZNF536,TENM4,DLC1,RIPOR2,RP1,STXBP1,RALA,IL1RAPL2,BCL2,ODAD2,ALDH1A2,ARHGAP26,CHRNA7,ROBO2,RIMS1,TENM3,GABRB3,ZEB1,SDCCAG8,RARB,NAV2,PLPPR1,USH2A,MINAR1,RIMS2,ALK,AUTS2,ASTN1,NEGR1,GPC6,CNTNAP2,MAP4,APC,RBFOX3,PLPPR5,DSCAM,RTN1,TCF4,CRKL,SOX5,SETD2,TNIK,SLC4A10,DOCK10,EGFR,MACF1,PRKACB,RNF220,NEDD4,CRB1,BCL11A,SOX6,CECR2,CDH4,ATP2B2,NTRK3,PHACTR1,GABRB1,NEO1,CNTN6,SLC39A12,CABLES1,SLC8A3,PRKD1,PAK1,EPHA7,CHRM3,SPEN,RAPGEF2,LRP2,ADGRB3,RUNX2,ARSB,FGF12,TAOK3,ADAMTSL1,LDB2,GRM7,LUZP1,EPB41L3,SSBP3,RAPGEF5,TBCD,NEDD4L,HDAC9,APP,DCLK1,STAU2,GABRG2,TMC1,SEMA5A,SYT1,VCL,ARHGAP44,NTF3,AURKA,SLC8A1,SRGAP2C,FIG4,SRGAP2B,KANK1,MAP4K4,ABCD2,BMPR1B,CTNNA2,PAK3,TTLL7,DIP2B,TRPC5,CHST8,DNM3,IFT57,PRKCZ,SNTG2,KLHL1,DIP2A,MCPH1,ZSWIM6,HECW1,COBL,YAP1,ESS2,FRYL,NFIA,BRINP1,MAPK1,ADAM22,ALCAM,NRG3,NCAM1,GFRA1,NIPBL,FAT3,CHN1,ACSBG1,MBNL1,PAFAH1B1,NF2,RBFOX1,CTNNA1,PPP1R9A,KLF15,MEIS2,NFIB,PRTG,SYNJ1,TIAM1,ENAH,SEMA3C,NAV3,SLC24A4,TMEM108,DNAH11,JARID2,SCN2A,IL34,ANK2,ADGRV1,SYNE2,BBS2,WNT9B,SEMA6D,SMARCA4,CDH11,FABP7,PARD3,MAPKAP1,BLK,TNR,MBD5,ATRX,ELAVL4,ABL1,HDAC4,SLC1A1,SDC2,LRFN5,CRTAC1,*

			DNAH5,RAP1A,FGF10,GRID2,GALC,NRG1,ASPM,RASGRF1,ZBTB16,MUSK,SH3GL3,USP33,PTPRO,EGF,TRIO,EXT1,RPS6KA3,CTNND2,ATP8A2,PLXNA2,ATXN3,ARHGEF7,AMBRA1,KDM7A,OPRM1,KREMEN1,SEMA3E,MARK2,TMOD2,HERC1,IGF2BP3,EPHA6,APBA2,LINGO2,ATL1,CDKN2C,KNDC1,AFG3L2,MOSMO,GFRA2,MNAT1,ANK3,BCL11B,ECE1,MBP,AK8,CRIM1,FUT9,MYT1L,NPHP4,CNTN1,IQSEC1,MTHFD1L,SNX3,PDLIM5,BRCA2,DISC1,DNER,WDPCP,NRK,SEMA3A,STRN,BMP2,UNC5D,TRAK1,NCAM2,SEMA3D,PDE6C,RELN,HMGB1,FGF9,UST,SLC23A2,ESRP1,TTC21B,B4GALT6,TSPAN2,RAP1GAP,PLS1,SRGAP2,NIN,DRAXIN,ATF1,SMARCA2,GLI3,SMARCC1,PCP4,CASP5,MYEF2,RERE,MAP2,FARP1,ATF2,BBS4,LAMC3,NELL1,DCC,MYT1,RORB,DAB1,PRKN,MTMR2,TBX20,PCDH15,DPF3,LGI2,NGEF,GRIN2A,ATXN1,CDH23,PRKCH,TG,ALS2,RACGAP1,TOX,PTPRB,SHANK2,ITSN2,SYBU,NPAS2,ADGRG6,ROCK1,LYN,VCAM1,EIF2B3,LRIG1,DTX1,TENM2,OVOL2,NTN1,ZFHX3,DPYSL5,ARID1B,CASZ1,HECTD1,SHROOM3,COLQ,HDAC11,PBX3,TANC2,RXRG,NREP,ZDHHC17,CD9,DZANK1,NDRG2,BMP5,KCNC1,CSF1,GHRH,HDGFL3,HCN1,PRKG1,LAMA3,GRIN2B,SYNJ2,MYCL,TNN,MICALL2,MED1,KDM6A,ATRN,IL33,ROR2,ZNF521,LMX1A,ACTR2,CLSTN2,MAP6,VASP,ETV6,TACC2,IQGAP1,ANP32B,NRXN1,HIPK1,FRY,CADM1,LMX1B,TWIST1,AKT3,ALKAL2,JAK2,VSX1,ISX,BPNT1,CELSR2,GORAB,UFL1,NTM,ABCC8,USH1C,ITGA6,ATP2B1,GAP43,GRIP1,EML1,PPP1R17,GRXCR1,DGKG,SHROOM2,SLC6A11,MTPN,ABI1,POU6F2,IMPACT,ITGA4,FBXL17,POU1F1,FOXP2,CEP120,CYFIP2,ST8SIA4,MEF2C,ADGRB1,WNT7A,WASF3,S100B,PRDM13,FOXO6,FBXW8,SDCBP,NECTIN1,NCK1,PPP2R3A,EPHA4,GABRA5,NTRK2,IL1RAPL1,NUMB,LHX9,WNT2B,FBXO31,GRIK1,HS6ST1,PTK2,MARK4,FEZ2,LAMB1,CYFIP1,UBE3A,SEMA4D,JAM2,FAT4,RUNX1,WNT5B,ASAP1,CCDC88A,SPAG6,FYN,ADGRL2,ARL13B,HYDIN,SCN8A,HDAC2,NCS1,ATP5PF,DOK5,UGP2,MDGA2,ROR1,ARNT2,HECW2,CDH2,CNTN5,ITGA8,NTN4,XRN2,EPHB1,RP1L1,GRM5,RPS6KA5,PTPRG,NRP1,SDK1,FAIM,ITGA1,CHODL,BCR,NRXN3,STK36,RRAS2,CUX1,EPHB2,CD38,MET,PPFIA2,MAGI2,UNK,FLRT2,KALRN,SLC1A2,LAMA1,CA10,TIAM2,IGSF21,BMP7,ASTN2,DLG5,GABRA2,KIRREL3,BTD,BPTF,DDX6,PDGFC,ABL2,TRAPPC6B,EYA1,SLIT2,FAM126A,CCDC141,ERBB4,SYNDIG1,ROBO1,PBX1,PRKCQ,NDRG1,NLGN1,CTTNBP2,SLC6A3,ASIC2,EFNA5,TCF12,VCAN,SLIT3,NTNG1,KDM4B,NYAP2,FOXB1,CAMK1D,MACROD2,OPCML,CCR2,RPGRIP1,SEMA4B,PRDM1,RORA,ATAT1,HSPG2,PTPRQ,CSMD3,NCOA6,RGS7,HOOK3,PCSK2,FSTL4,STK3,ZNF423,APCDD1,IGF1R,GLI2,THRB,LSAMP
GO:0007275	multicellular organism development	6.175511944125696e-36	*NOTCH2,BCAR3,BRINP3,MTOR,CNTN4,SPOCK1,SGCD,IMMP2L,LRP12,PTPRD,FREM1,TRAPPC9,BNC2,NEBL,LRRC4C,SMOC1,MYO9A,ULK2,SCAPER,PLCB1,ZNF536,TAFA5,ZFPM2,TENM4,DLC1,ZDHHC21,RIPOR2,RP1,STXBP1,RALA,IL1RAPL2,BCL2,ODAD2,ALDH1A2,ARHGAP26,FBN1,CHRNA7,ROBO2,RIMS1,TENM3,GABRB3,ZEB1,AKR1C3,SDCCAG8,RARB,SPRED1,NAV2,ENPEP,MYO1E,PLPPR1,USH2A,MINAR1,RIMS2,ALK,AUTS2,FOXJ2,PAPPA2,ASTN1,RIN2,ANO6,NEGR1,MLLT3,GPC6,CNTNAP2,MAP4,MYO3B,APC,RBFOX3,PLPPR5,DSCAM,RTN1,TCF4,CRKL,SOX5,SETD2,ARHGAP24,TNIK,SLC4A10,PTPRJ,KDM4C,DOCK10,EGFR,RFX3,ANGPT1,MACF1,PRKACB,RNF220,DOCK2,NEDD4,CRB1,BCL11A,SOX6,CECR2,CHSY1,FLI1,CDH4,ATP2B2,NTRK3,RXFP1,C5,PHACTR1,DKK2,FLT1,ADAMTS6,GABRB1,EDAR,NEO1,CNTN6,SLC39A12,CABLES1,SLC8A3,PRKD1,PAK1,EPHA7,CHRM3,SPEN,RAPGEF2,LRP2,ADGRB3,RUNX2,ARSB,FGF12,CPS1,TAOK3,ONECUT1,TMEM38B,ADAMTSL1,LDB2,GRM7,GHR,LUZP1,EPB41L3,COL4A2,SSBP3,RAPGEF5,TBCD,NEDD4L,ADAM10,HDAC9,APP,ABCB5,RPS6KA2,CACNA1C,DCLK1,STAU2,GABRG2,TMC1,SEMA5A,SYT1,VCL,ARHGAP44,NTF3,AURKA,PYGO1,SLC8A1,PTPRR,SRGAP2C,FIG4,CMIP,SRGAP2B,KANK1,MAP4K4,ABCD2,BMPR1B,THSD7A,PCSK6,AKAP6,CTNNA2,ARNT,PAK3,TTLL7,DIP2B,ITPKB,TRPC5,CHST8,DNM3,NBN,IFT57,RBM47,PRKCZ,CALD1,SNTG2,KLHL1,DIP2A,MSH6,MCPH1,COL27A1,ZSWIM6,HECW1,COBL,YAP1,ESS2,FRYL,NFIA,BRINP1,MAPK1,ADAM22,CRISPLD2,KMT2E,A*

			LCAM,PLG,PDGFD,ZNRF3,NRG3,NCAM1,GFRA1,NIPBL,GABPA,FAT3,CHN1,MYLK3,ACSBG1,FMN1,MBNL1,PAFAH1B1,ATF6,EFEMP1,TLL1,DCAF1,ITGB8,TPM1,NF2,RBFOX1,CTNNA1,PPP1R9A,ANKRD11,BIRC6,KLF15,PPARA,MEIS2,NFIB,PRTG,SYNJ1,NR5A2,ADAMTS3,TIAM1,ENAH,SF3B6,SEMA3C,NAV3,SLC24A4,TMEM108,AGO2,ALPK2,DNAH11,JARID2,SCN2A,CPE,IL34,ANK2,ADGRV1,MELK,BCAS3,RYR2,SYNE2,BBS2,WNT9B,RANBP3L,SEMA6D,ANKS6,SMARCA4,CDH11,LDB3,FABP7,PARD3,MAPKAP1,BLK,TNR,COL22A1,CXADR,MBD5,ATRX,XIRP2,ELAVL4,ABL1,HDAC4,SLC1A1,SDC2,GAS2,LRFN5,CRTAC1,DROSHA,TTLL5,L3MBTL3,APLF,DNAH5,RAP1A,CAMK4,FGF10,GRID2,GALC,LATS2,NRG1,INO80D,ASPM,AP3B1,RASGRF1,ATP11C,ZBTB16,MUSK,ZNF675,SH3GL3,SETDB2,NXN,USP33,FBN2,CD44,PTPRO,EGF,ALPK3,TRIO,EXT1,SPRED2,ADAMTS2,RPS6KA3,CTNND2,NHS,ATP8A2,PTPN2,PLXNA2,ATXN3,ST8SIA6,ARHGEF7,AMBRA1,KDM7A,OPRM1,FANCA,CYP4A11,KREMEN1,SEMA3E,MARK2,ALPL,FHL2,TMOD2,HERC1,MSH2,IGF2BP3,CDIN1,EPHA6,ANKRD17,APBA2,LINGO2,ATL1,CDKN2C,EPN2,EVC,KNDC1,AFG3L2,MOSMO,GFRA2,MNAT1,RBBP8,SGCZ,ADAM12,MYLK2,ANK3,EMILIN2,XYLT1,HMGA2,BCL11B,ECE1,MBP,AK8,TRPS1,PLCE1,TGFA,CRIM1,FUT9,VAV1,MYT1L,ZNF160,TJP1,NPHP4,CNTN1,HLA-B,IQSEC1,MTHFD1L,SNX3,PDLIM5,BRCA2,DISC1,DNER,WDPCP,NRK,SLC10A7,SEMA3A,STRN,BMP2,RC3H2,UNC5D,TRAK1,PSG9,PTCD2,NCAM2,GFI1B,BMP2K,RNF38,SEMA3D,PDE6C,RELN,HMGB1,FGF9,NFATC2,TDRD7,UST,CPAMD8,RTTN,MDM1,SLC23A2,ESRP1,TTC21B,ETS2,DOCK1,B4GALT6,TSPAN2,RAP1GAP,PLS1,SRGAP2,NIN,DRAXIN,ATF1,SLAMF1,SMARCA2,ETS1,GLI3,MEGF11,SMARCC1,AFF3,SMOC2,PCP4,CASP5,MYEF2,RERE,MAP2,DAW1,FARP1,TDRD5,ATF2,HIRA,UMODL1,BBS4,LAMC3,COL5A1,CFTR,NELL1,DOP1B,KITLG,DCC,MYT1,GTF2I,RORB,TADA2A,DAB1,SELENON,RB1CC1,MYO3A,PRKN,MTMR2,SH3PXD2A,TBX20,DACH1,PCDH15,DPF3,LGI2,NGEF,GRIN2A,ARID5B,ATXN1,CDH23,PRKCH,TG,IL6R,ALS2,RACGAP1,ACO1,DMC1,TOX,PTPRB,PDE6A,SHANK2,VAV3,ITSN2,SYBU,NPAS2,ADGRG6,YIPF6,SEC24D,ROCK1,LYN,VCAM1,EIF2B3,LRIG1,DTX1,TENM2,OVOL2,NTN1,MMP16,ZFHX3,DPYSL5,ARID1B,HOXC13,CRACR2A,CASZ1,INSR,YTHDF3,HECTD1,SHROOM3,XRCC4,COLQ,HDAC11,PBX3,SNAI2,ASH1L,HOXC4,SIAH2,TANC2,UFD1,RXRG,SP3,MBTPS2,TRIM58,PLPP4,NREP,ZDHHC17,NSD2,CD9,CARD10,JCAD,SAMHD1,ENPP1,RASGRP1,DZANK1,NDRG2,BMP5,KCNC1,CSF1,GHRH,HDGFL3,BCL2L1,CTDP1,HCN1,PRKG1,LAMA3,ASB4,GRIN2B,INO80,CNMD,DHRS3,SMAD5,CELF4,SYNJ2,FOXN3,VSTM4,SLC40A1,MYCL,TNN,PSAP,MICALL2,MED1,KDM6A,ATRN,IL33,AJAP1,ROR2,ZNF521,KL,CSDE1,FAT1,LMX1A,TMEM178A,IL10,ACTR2,CLSTN2,TTC39C,PTH,SOSTDC1,MAP6,VASP,ETV6,TACC2,IQGAP1,TEAD1,ANP32B,YBX3,AIMP1,NRXN1,PCID2,HIPK1,FRY,CIBAR1,CACYBP,CADM1,LMX1B,ANLN,TWIST1,AKT3,ALKAL2,JAK2,VSX1,ISX,BPNT1,SVEP1,RBM19,CELSR2,CREBBP,ARL11,GORAB,PCNA,SIAH3,UFL1,NFKBIA,PRKCB,NTM,ABCC8,ALX4,USH1C,SMPD4,NEDD9,ITGA6,ATP2B1,GAP43,ASS1,GRIP1,CTNNBL1,ADCY9,EML1,PPP1R17,OTOP1,EXT2,STOX2,AGO1,MEOX2,GRXCR1,STAT1,MAP2K6,CMTM7,DGKG,SHROOM2,SLC6A11,MTPN,ABI1,MYO18B,ARMC6,POU6F2,IMPACT,CCBE1,ADAMTS18,ITGA4,FBXL17,POU1F1,ADCYAP1R1,MTF2,CSMD1,NCAPG2,FOXP2,ASB2,MYOCD,CEP120,DHTKD1,CYFIP2,ST8SIA4,MEF2C,ADGRB1,RXRA,WNT7A,RBPMS2,NDFIP1,MAP3K4,WASF3,S100B,PRDM13,FOXO6,PDE2A,FBXW8,SDCBP,NECTIN1,WWOX,NCK1,FLVCR1,FGR,PPP2R3A,TOP1,RNF8,EPHA4,GABRA5,MECOM,DNMT3L,NTRK2,IL1RAPL1,FNDC3A,NUMB,LHX9,ADAMTS9,WNT2B,TNNI1,CD101,FBXO31,GRIK1,PRKAB1,IREB2,HS6ST1,PTK2,MARK4,CDH5,NFKBID,CLDN18,FEZ2,LAMB1,CYFIP1,UBE3A,PCDH8,SEMA4D,JAM2,FAT4,AP2B1,RUNX1,AKR1B1,WNT5B,SANBR,ASAP1,SORBS2,PDCL3,CCDC88A,GPR55,NSUN2,ADAMTS16,SPAG6,TNFSF11,FYN,ADGRL2,UNC45B,ARL13B,HYDIN,SCN8A,HDAC2,GON4L,TBX15,NCS1,COL18A1,LHFPL2,ATP5PF,DOK5,UGP2,CRTAM,COL19A1,APELA,MDGA2,ROR1,FUT8,TET1,ARNT2,HECW2,CDH2,C

			NTN5,ITGA8,NTN4,XRN2,PHLPP1,GPR137B,EPHB1,RP1L1,GRM5,RPS6KA5,PTPRG,NRP1,SDK1,PRKCA,FAIM,ITGA1,RC3H1,NRIP1,CHODL,POR,BCR,NRXN3,KIF16B,SNRK,FBLN1,STK36,MB,RAG1,B9D1,RRAS2,BMPER,CUX1,MACROH2A1,MITF,EPHB2,IGSF3,SGCG,CD38,EYA4,MET,CDH17,SPECC1,PPFIA2,CDH13,ATG5,NRAP,MAGI2,KIAA1217,VMP1,UNK,ADAM29,FLRT2,MYB,KALRN,SLC1A2,GNAS,LAMA1,MFHAS1,GREB1L,SERPINB7,CA10,TIAM2,IGSF21,BMP7,ASTN2,DLG5,GABRA2,KIRREL3,BTD,BPTF,NUDT21,DDX6,ADGRF5,PDGFC,ABL2,TRAPPC6B,NECAB1,EYA1,FHOD3,SLIT2,EXOC4,FAM126A,CCDC141,ERBB4,SYNDIG1,ROBO1,PBX1,PRKCQ,ANTXR1,NDRG1,MYH15,SIPA1L3,NLGN1,CTTNBP2,SHLD2,SLC6A3,ASIC2,EFNA5,TCF12,VCAN,EHMT1,SLIT3,ESR1,NTNG1,KDM4B,LOXL2,NYAP2,PRLR,FOXB1,RAD51B,CAMK1D,PIK3R3,MACROD2,OPCML,FER,EYA2,CCR2,RPGRIP1,STARD13,A2M,SEMA4B,ROCK2,PRDM1,RORA,ATAT1,DMRT1,HSPG2,PTPRQ,CSMD3,NCOA6,HSD17B2,COL4A3,RGS7,HOOK3,PCSK2,FSTL4,STK3,ZNF423,ZNF568,HNRNPU,APCDD1,IGF1R,GLI2,THRB,LSAMP,AKAP13,MORC3
GO:0032502	developmental process	1.195574565495 0131e-35	*NOTCH2,BCAR3,BRINP3,MTOR,CNTN4,SPOCK1,SGCD,WWC1,IMMP2L,LRP12,PTPRD,FREM1,TRAPPC9,BNC2,NEBL,LRRC4C,SMOC1,MYO9A,ULK2,UNC13C,SCAPER,FTO,MGA,PLCB1,ZNF536,TAFA5,SVIL,CLTCL1,ZFPM2,TENM4,NUBPL,DLC1,ZDHHC21,RIPOR2,PDE4D,RDX,RP1,STXBP1,RALA,IL1RAPL2,BCL2,ODAD2,PRDM16,ALDH1A2,ARHGAP26,FBN1,CDH8,CHRNA7,ROBO2,RIMS1,SPIRE1,TENM3,GABRB3,ZEB1,AKR1C3,SDCCAG8,RARB,FGD4,SPRED1,NAV2,ENPEP,SPAG16,MYO1E,PLPPR1,USH2A,MINAR1,CDC42EP3,RIMS2,ALK,AUTS2,FOXJ2,CARMIL1,PAPPA2,ASTN1,RIN2,PARVB,ANO6,NEGR1,MLLT3,GPC6,CNTNAP2,MAP4,MYO3B,APC,ZMYM4,RBFOX3,PLPPR5,DSCAM,RTN1,TCF4,CRKL,ILDR2,SOX5,SETD2,ERG,ARHGAP24,TNIK,SLC4A10,PTPRJ,OCA2,KDM4C,DOCK10,EGFR,RFX3,ANGPT1,CDK12,MACF1,PRKACB,RNF220,DOCK2,NEDD4,MYOF,SND1,CRB1,BCL11A,SOX6,TMEM182,CECR2,ARMC2,CHSY1,FLI1,CDH4,ATP2B2,NTRK3,LARGE1,RXFP1,C5,PHACTR1,DKK2,FLT1,DNAJC13,ADAMTS6,GABRB1,PSMA8,EDAR,NEO1,CNTN6,SLC39A12,CABLES1,SLC8A3,PRKD1,PAK1,EPHA7,CHRM3,SPEN,RAPGEF2,LRP2,ADGRB3,DEUP1,RUNX2,ARSB,FGF12,CPS1,TAOK3,ONECUT1,TMEM38B,ADAMTSL1,PRICKLE2,SLC24A3,LDB2,SMYD3,HERC2,LRGUK,GRM7,RETREG1,GHR,LUZP1,EPB41L3,COL4A2,SSBP3,RAPGEF5,TBCD,NEDD4L,ADAM10,HDAC9,ZHX3,APP,ABCB5,RPS6KA2,CACNA1C,DCLK1,STAU2,GABRG2,TMC1,SEMA5A,SYT1,VCL,ARHGAP44,NTF3,AURKA,CFDP1,PYGO1,SLC8A1,HERPUD2,PTPRR,SRGAP2C,FIG4,CMIP,SRGAP2B,KANK1,MAP4K4,ABCD2,BMPR1B,FMN2,THSD7A,PCSK6,AKAP6,CTNNA2,ARNT,PAK3,TTLL7,DIP2B,ITPKB,TRPC5,CHST8,DNM3,NBN,IFT57,RBM47,PRKCZ,CALD1,SNTG2,KLHL1,DIP2A,MSH6,MCPH1,COL27A1,ZSWIM6,HECW1,ABCA5,PHF19,MRTFA,TAF4B,COBL,EBF2,YAP1,ESS2,FRYL,NFIA,RIPK4,SHC4,BRINP1,MAPK1,ADAM22,CRISPLD2,KMT2E,ALCAM,PLG,PDGFD,ZNRF3,ABLIM1,NRG3,NCAM1,GFRA1,SYCP1,NIPBL,RNF17,GABPA,FAT3,LCE1F,CHN1,MYLK3,ACSBG1,FMN1,MBNL1,PAFAH1B1,ATF6,EFEMP1,TLL1,DCAF1,ITGB8,TPM1,NF2,RBFOX1,CTNNA1,PPP1R9A,ANKRD11,CDH7,BIRC6,KLF15,PPARA,MEIS2,NFIB,MRTFB,PRTG,SYNJ1,NR5A2,ADAMTS3,TIAM1,FOXJ3,KAZN,ENAH,SF3B6,PCDH11Y,SEMA3C,NAV3,SLC24A4,TMEM108,AGO2,ALPK2,DNAH11,JARID2,SCN2A,GATAD2B,CPE,DYSF,IL34,ANK2,BRWD1,TANC1,ADGRV1,MELK,BCAS3,RYR2,SYNE2,BBS2,WNT9B,SLC9C1,RANBP3L,SEMA6D,ANKS6,SMARCA4,CDH11,LDB3,FABP7,PARD3,MAPKAP1,PIAS1,BLK,TNR,COL22A1,CXADR,MBD5,ATRX,XIRP2,ELAVL4,ABL1,PTPN12,HDAC4,SLC1A1,PRKAA1,SDC2,GAS2,KCNH1,LRFN5,CRTAC1,DROSHA,TTLL5,L3MBTL3,APLF,DNAH5,CDH18,RAP1A,GLIS1,MORC1,MYO10,CAMK4,FGF10,GRID2,CDHR3,GALC,TGM1,PEAK1,LATS2,NRG1,INO80D,ASPM,AP3B1,RASGRF1,ATP11C,SYNE1,ZBTB16,MUSK,ZNF675,SH3GL3,SETDB2,PGM5,MED15,NXN,USP33,FBN2,CD44,PTPRO,EGF,ALPK3,TRIO,PDE3A,EXT1,COL5A3,LIMD1,SPRED2,ADAMTS2,RPS6KA3,CTNND2,NHS,ATP8A2,PTPN2,PLXNA2,ATXN3,ST8SIA6,HTR2*

			C,ARHGEF7,AMBRA1,KDM7A,OPRM1,HTR2A,FANCA,CYP4A11,DAZL,CNNM4,KREMEN1,SEMA3E,MARK2,ALPL,FHL2,PUM1,TMOD2,HERC1,MSH2,IGF2BP3,CDIN1,EPHA6,ANKRD17,APBA2,LINGO2,SH3KBP1,ATL1,LUC7L,CDKN2C,EPN2,EVC,KNDC1,BICRAL,AFG3L2,MOSMO,GFRA2,MNAT1,RBBP8,SGCZ,ADAM12,MYLK2,ANK3,EMILIN2,XYLT1,HMGA2,MYOM2,BCL11B,DOCK5,ECE1,CREM,MBP,AK8,TRPS1,PLCE1,TGFA,HIP1,CRIM1,FUT9,VAV1,CDH20,MYT1L,ZNF160,TJP1,LDLRAD4,NPHP4,EGFLAM,PACSIN2,CNTN1,HLA-B,IQSEC1,MTHFD1L,SNX3,PDLIM5,BRCA2,DISC1,DNER,WDPCP,NRK,SLC10A7,SEMA3A,HSF2BP,CFAP97,ADCY10,STRN,BMP2,RC3H2,UNC5D,TRAK1,PSG9,PTCD2,MSR1,NCAM2,GFI1B,MSI2,BMP2K,RNF38,SEMA3D,ASXL3,PDE6C,RELN,HMGB1,FGF9,NFATC2,TDRD7,UST,CPAMD8,RTTN,MDM1,SLC23A2,ANKRD26,ESRP1,UNC13B,TTC21B,ETS2,DOCK1,B4GALT6,TSPAN2,RAP1GAP,PLS1,SRGAP2,NIN,DRAXIN,ATF1,SLAMF1,SMARCA2,ETS1,GLI3,MEGF11,SMARCC1,NHSL1,AFF3,SLC9A4,SMOC2,PCP4,CASP5,CUL1,MYEF2,ZNF431,RERE,MAP2,DAW1,LAMC1,RRBP1,FARP1,TDRD5,ATF2,HIRA,UMODL1,BBS4,LAMC3,HIVEP3,COL5A1,CFTR,KPNA1,NELL1,DOP1B,KITLG,DCC,MYT1,RCAN1,GTF2I,RORB,TADA2A,DAB1,MED27,SELENON,RB1CC1,MYO3A,PRKN,MTMR2,SH3PXD2A,TBX20,DACH1,PCDH15,ZNF541,DPF3,LGI2,NGEF,GRIN2A,ARID5B,JPH1,ATXN1,CDH23,PRKCH,TG,IL6R,ALS2,RACGAP1,ACO1,TFDP1,HEMGN,DMC1,LCE3B,TOX,PTPRB,CATSPERG,PDE6A,TBATA,SCN10A,SHANK2,VAV3,MESD,ITSN2,SOX30,PTGFRN,SYBU,ARHGEF28,NPAS2,ADGRG6,YIPF6,SEC24D,ROCK1,LYN,VCAM1,CTSB,EIF2B3,LRIG1,PLEKHB2,DTX1,TENM2,OVOL2,PIWIL3,NTN1,MMP16,ZFHX3,DPYSL5,ARID1B,HOXC13,CRACR2A,CASZ1,INSR,DMBT1,YTHDF3,TFF1,HECTD1,SHROOM3,XRCC4,COLQ,HDAC11,DDHD1,PBX3,SNAI2,ASH1L,HOXC4,SIAH2,TANC2,UFD1,RXRG,SP3,MBTPS2,FLNB,TRIM58,TIAL1,ELF2,PLPP4,NREP,ZDHHC17,NSD2,CERS3,SLC22A14,CD9,CARD10,KRT6B,XKR5,JCAD,TWIST2,SAMHD1,IFT81,ENPP1,UTRN,RASGRP1,DZANK1,NDRG2,BMP5,WDR72,KCNC1,CSF1,GHRH,HDGFL3,BCL2L1,SPATA48,KRT25,CTDP1,HCN1,PRKG1,LAMA3,ASB4,GRIN2B,INO80,CNMD,DHRS3,SMAD5,CELF4,SYNJ2,ABCG1,FOXN3,VSTM4,SLC40A1,PRAME,MYCL,TNN,CABYR,PSAP,MICALL2,MED1,ATG4B,KDM6A,ATRN,IL33,AJAP1,ROR2,ZNF521,KL,CSDE1,FAT1,LMX1A,TMEM178A,IL10,ACTR2,SCML2,PRAMEF25,CLSTN2,TTC39C,PTH,SDF4,SOSTDC1,SOHLH1,PACRG,ABHD2,VSTM2A,MAP6,VASP,ETV6,TACC2,PALMD,IQGAP1,ZBTB7C,TEAD1,ANP32B,YBX3,AIMP1,NRXN1,PCID2,HIPK1,FRY,CIBAR1,CACYBP,CADM1,PEG10,LMX1B,ANLN,TWIST1,AKT3,ALKAL2,JAK2,ADAM28,VSX1,FSTL1,ISX,BPNT1,SVEP1,RBM19,CELSR2,ZNF449,CREBBP,MELTF,ARL11,GORAB,PCNA,SIAH3,UFL1,ADAMTS5,NFKBIA,PRKCB,NTM,ABCC8,ANXA4,SMTN,ALX4,USH1C,SMPD4,NEDD9,OLFM4,ITGA6,ATP2B1,GAP43,ASS1,GRIP1,CTNNBL1,ADCY9,EML1,PPP1R17,MAST2,XKR6,OTOP1,BBS9,EXT2,KRT6A,STOX2,AGO1,MEOX2,GRXCR1,STAT1,BRMS1L,NR2C1,MAP2K6,CMTM7,DGKG,SHROOM2,SLC6A11,MTPN,ABI1,MYO18B,ARMC6,PRAMEF2,POU6F2,IMPACT,CCBE1,ADAMTS18,ITGA4,BCAP29,FBXL17,POU1F1,ADCYAP1R1,MTF2,CSMD1,NCAPG2,NDC1,FOXP2,ASB2,MYOCD,CEP120,DHTKD1,CYFIP2,KRT85,ST8SIA4,MEF2C,ADGRB1,RXRA,WNT7A,RBPMS2,MAP3K5,NDFIP1,MAP3K4,WASF3,S100B,PRDM13,FOXO6,PDE2A,RAB38,FBXW8,SDCBP,NECTIN1,WWOX,NCK1,FLVCR1,FGR,DRC7,PPP2R3A,DNMBP,TOP1,SPRR2D,RNF8,LCE3D,EPHA4,EMP1,GABRA5,MECOM,DNMT3L,NTRK2,IL1RAPL1,FNDC3A,RSPH1,NUMB,LHX9,ADAMTS9,WNT2B,TNNI1,POSTN,CD101,SHISA6,MEGF10,IL17RD,FBXO31,GRIK1,PRKAB1,IREB2,HS6ST1,PTK2,MARK4,CDH5,ANKRD6,NFKBID,ARHGAP12,CLDN18,DIAPH1,FEZ2,LAMB1,CYFIP1,UBE3A,HOATZ,PCDH8,SEMA4D,JAM2,FRMD6,FAT4,LRMDA,AP2B1,RUNX1,AKR1B1,WNT5B,AMFR,SANBR,ASAP1,FCRLA,DPY19L2,SORBS2,PDCL3,CCDC88A,GPR55,NSUN2,ADAMTS16,SPAG6,BICD1,TNFSF11,FYN,MYL12B,NLRP14,ADGRL2,UNC45B,ARL13B,HYDIN,UHRF2,SCN8A,HDAC2,GON4L,TBX15,NCS1,COL18A1,CDH9,LHFPL2,ATP5PF,DOK5

			,UGP2,MAPK9,CRTAM,COL19A1,APELA,MDGA2,ROR1,FUT8,TET1,ARNT2,HECW2,CDH2,CNTN5,ITGA8,NTN4,XRN2,PHLPP1,GPR137B,EPHB1,EYS,RP1L1,GRM5,DDX10,ADCK1,RAI14,RPS6KA5,PTPRG,PID1,NRP1,SDK1,PRKCA,FAIM,ITGA1,RC3H1,NRIP1,CHODL,POR,BCR,TUT4,NRXN3,KIF16B,CDH12,SNRK,C14ORF39,FBLN1,STK36,PAQR5,MB,RAG1,B9D1,DGCR2,RRAS2,BMPER,CUX1,MACROH2A1,MITF,EPHB2,IGSF3,SGCG,CD38,EYA4,MET,CDH17,SPECC1,PPFIA2,CDH13,ATG5,NRAP,MAGI2,KIAA1217,VMP1,UNK,FAM171A1,ADAM29,FLRT2,MYB,KALRN,SLC1A2,GNAS,LAMA1,MFHAS1,GREB1L,SERPINB7,CA10,CPQ,ATRNL1,TIAM2,IGSF21,BMP7,ASTN2,DLG5,GABRA2,KIRREL3,BTD,BPTF,NUDT21,DDX6,ADGRF5,PDGFC,ABL2,TRAPPC6B,RFX2,NECAB1,EYA1,FHOD3,SLIT2,EXOC4,FAM126A,CCDC141,ERBB4,SYNDIG1,ROBO1,PBX1,PRKCQ,ANTXR1,NDRG1,MYH15,SIPA1L3,MGMT,NLGN1,CTTNBP2,SHLD2,NOS1,SLC6A3,ASIC2,EFNA5,TCF12,VCAN,RAB27A,EHMT1,SLIT3,ESR1,NTNG1,KDM4B,LOXL2,NYAP2,PRLR,FOXB1,RAD51B,CAMK1D,PIK3R3,MACROD2,CFAP44,OPCML,CATSPERE,FER,EYA2,CCR2,RPGRIP1,STARD13,A2M,EPS8,SEMA4B,PHC2,ROCK2,PRDM1,RORA,ATAT1,DMRT1,CATSPER2,HSPG2,PTPRQ,CSMD3,NCOA6,HSD17B2,COL4A3,RGS7,HOOK3,PCSK2,FSTL4,PNPLA3,STK3,ZNF423,ZNF568,HNRNPU,APCDD1,IGF1R,GLI2,THRB,LSAMP,AKAP13,MORC3,ATP10A,SEPTIN6,DNM1L
GO:0048731	system development	4.14682928730004e-34	NOTCH2,BCAR3,BRINP3,MTOR,CNTN4,SPOCK1,SGCD,IMMP2L,LRP12,PTPRD,FREM1,TRAPPC9,BNC2,NEBL,LRRC4C,SMOC1,MYO9A,ULK2,SCAPER,PLCB1,ZNF536,TAFA5,ZFPM2,TENM4,DLC1,RIPOR2,RP1,STXBP1,RALA,IL1RAPL2,BCL2,ODAD2,ALDH1A2,ARHGAP26,FBN1,CHRNA7,ROBO2,RIMS1,TENM3,GABRB3,ZEB1,AKR1C3,SDCCAG8,RARB,SPRED1,NAV2,ENPEP,MYO1E,PLPPR1,USH2A,MINAR1,RIMS2,ALK,AUTS2,FOXJ2,PAPPA2,ASTN1,RIN2,NEGR1,MLLT3,GPC6,CNTNAP2,MAP4,APC,RBFOX3,PLPPR5,DSCAM,RTN1,TCF4,CRKL,SOX5,SETD2,ARHGAP24,TNIK,SLC4A10,PTPRJ,DOCK10,EGFR,RFX3,ANGPT1,MACF1,PRKACB,RNF220,DOCK2,NEDD4,CRB1,BCL11A,SOX6,CECR2,CHSY1,FLI1,CDH4,ATP2B2,NTRK3,RXFP1,C5,PHACTR1,FLT1,ADAMTS6,GABRB1,EDAR,NEO1,CNTN6,SLC39A12,CABLES1,SLC8A3,PRKD1,PAK1,EPHA7,CHRM3,SPEN,RAPGEF2,LRP2,ADGRB3,RUNX2,ARSB,FGF12,CPS1,TAOK3,ONECUT1,TMEM38B,ADAMTSL1,LDB2,GRM7,GHR,LUZP1,EPB41L3,COL4A2,SSBP3,RAPGEF5,TBCD,NEDD4L,ADAM10,HDAC9,APP,ABCB5,RPS6KA2,CACNA1C,DCLK1,STAU2,GABRG2,TMC1,SEMA5A,SYT1,VCL,ARHGAP44,NTF3,AURKA,PYGO1,SLC8A1,SRGAP2C,FIG4,SRGAP2B,KANK1,MAP4K4,ABCD2,BMPR1B,THSD7A,AKAP6,CTNNA2,ARNT,PAK3,TTLL7,DIP2B,ITPKB,TRPC5,CHST8,DNM3,NBN,IFT57,RBM47,PRKCZ,CALD1,SNTG2,KLHL1,DIP2A,MSH6,MCPH1,COL27A1,ZSWIM6,HECW1,COBL,YAP1,ESS2,FRYL,NFIA,BRINP1,MAPK1,ADAM22,CRISPLD2,KMT2E,ALCAM,PLG,PDGFD,NRG3,NCAM1,GFRA1,NIPBL,GABPA,FAT3,CHN1,MYLK3,ACSBG1,FMN1,MBNL1,PAFAH1B1,ATF6,EFEMP1,TLL1,DCAF1,ITGB8,TPM1,NF2,RBFOX1,CTNNA1,PPP1R9A,ANKRD11,KLF15,PPARA,MEIS2,NFIB,PRTG,SYNJ1,TIAM1,ENAH,SEMA3C,NAV3,SLC24A4,TMEM108,AGO2,ALPK2,DNAH11,JARID2,SCN2A,CPE,IL34,ANK2,ADGRV1,MELK,BCAS3,RYR2,SYNE2,BBS2,WNT9B,RANBP3L,SEMA6D,ANKS6,SMARCA4,CDH11,LDB3,FABP7,PARD3,MAPKAP1,BLK,TNR,COL22A1,CXADR,MBD5,ATRX,XIRP2,ELAVL4,ABL1,HDAC4,SLC1A1,SDC2,GAS2,LRFN5,CRTAC1,DROSHA,TTLL5,L3MBTL3,APLF,DNAH5,RAP1A,CAMK4,FGF10,GRID2,GALC,NRG1,ASPM,AP3B1,RASGRF1,ATP11C,ZBTB16,MUSK,ZNF675,SH3GL3,SETDB2,NXN,USP33,FBN2,CD44,PTPRO,EGF,ALPK3,TRIO,EXT1,SPRED2,ADAMTS2,RPS6KA3,CTNND2,NHS,ATP8A2,PTPN2,PLXNA2,ATXN3,ARHGEF7,AMBRA1,KDM7A,OPRM1,FANCA,CYP4A11,KREMEN1,SEMA3E,MARK2,ALPL,FHL2,TMOD2,HERC1,MSH2,IGF2BP3,CDIN1,EPHA6,ANKRD17,APBA2,LINGO2,ATL1,CDKN2C,EPN2,EVC,KNDC1,AFG3L2,MOSMO,GFRA2,MNAT1,SGCZ,ADAM12,MYLK2,ANK3,EMILIN2,XYLT1,HMGA2,BCL11B,ECE1,MBP,AK8,TRPS1,PLCE1,TGFA,CRIM1,FUT9,VAV1,MYT1L,ZNF160,TJP1,NPHP4,CNTN1,HLA-B,IQSEC1,MTHFD1L,SNX3,PDLIM5,BRCA2,DISC1,DNER,WDPCP

			,NRK,SLC10A7,SEMA3A,STRN,BMP2,RC3H2,UNC5D,TRAK1,PSG9,PTCD2,NCAM2,GFI1B,RNF38,SEMA3D,PDE6C,RELN,HMGB1,FGF9,NFATC2,TDRD7,UST,CPAMD8,MDM1,SLC23A2,ESRP1,TTC21B,ETS2,DOCK1,B4GALT6,TSPAN2,RAP1GAP,PLS1,SRGAP2,NIN,DRAXIN,ATF1,SLAMF1,SMARCA2,ETS1,GLI3,MEGF11,SMARCC1,SMOC2,PCP4,CASP5,MYEF2,RERE,MAP2,DAW1,FARP1,ATF2,UMODL1,BBS4,LAMC3,COL5A1,NELL1,KITLG,DCC,MYT1,GTF2I,RORB,DAB1,SELENON,RB1CC1,PRKN,MTMR2,SH3PXD2A,TBX20,PCDH15,DPF3,LGI2,NGEF,GRIN2A,ARID5B,ATXN1,CDH23,PRKCH,TG,IL6R,ALS2,RACGAP1,DMC1,TOX,PTPRB,PDE6A,SHANK2,VAV3,ITSN2,SYBU,NPAS2,ADGRG6,YIPF6,ROCK1,LYN,VCAM1,EIF2B3,LRIG1,DTX1,TENM2,OVOL2,NTN1,MMP16,ZFHX3,DPYSL5,ARID1B,CRACR2A,CASZ1,INSR,HECTD1,SHROOM3,XRCC4,COLQ,HDAC11,PBX3,SNAI2,ASH1L,HOXC4,TANC2,UFD1,RXRG,SP3,MBTPS2,TRIM58,NREP,ZDHHC17,NSD2,CD9,CARD10,JCAD,SAMHD1,RASGRP1,DZANK1,NDRG2,BMP5,KCNC1,CSF1,GHRH,HDGFL3,BCL2L1,CTDP1,HCN1,PRKG1,LAMA3,ASB4,GRIN2B,CNMD,DHRS3,SMAD5,CELF4,SYNJ2,FOXN3,VSTM4,SLC40A1,MYCL,TNN,PSAP,MICALL2,MED1,KDM6A,ATRN,IL33,ROR2,ZNF521,CSDE1,FAT1,LMX1A,TMEM178A,IL10,ACTR2,CLSTN2,PTH,MAP6,VASP,ETV6,TACC2,IQGAP1,ANP32B,YBX3,AIMP1,NRXN1,PCID2,HIPK1,FRY,CACYBP,CADM1,LMX1B,ANLN,TWIST1,AKT3,ALKAL2,JAK2,VSX1,ISX,BPNT1,SVEP1,CELSR2,ARL11,GORAB,PCNA,UFL1,NFKBIA,PRKCB,NTM,ABCC8,ALX4,USH1C,NEDD9,ITGA6,ATP2B1,GAP43,ASS1,GRIP1,CTNNBL1,EML1,PPP1R17,EXT2,AGO1,MEOX2,GRXCR1,STAT1,MAP2K6,CMTM7,DGKG,SHROOM2,SLC6A11,MTPN,ABI1,MYO18B,ARMC6,POU6F2,IMPACT,CCBE1,ADAMTS18,ITGA4,FBXL17,POU1F1,CSMD1,NCAPG2,FOXP2,ASB2,MYOCD,CEP120,DHTKD1,CYFIP2,ST8SIA4,MEF2C,ADGRB1,WNT7A,RBPMS2,NDFIP1,WASF3,S100B,PRDM13,FOXO6,PDE2A,FBXW8,SDCBP,NECTIN1,WWOX,NCK1,FLVCR1,FGR,PPP2R3A,RNF8,EPHA4,GABRA5,MECOM,NTRK2,IL1RAPL1,FNDC3A,NUMB,LHX9,ADAMTS9,WNT2B,TNNI1,CD101,FBXO31,GRIK1,IREB2,HS6ST1,PTK2,MARK4,CDH5,NFKBID,CLDN18,FEZ2,LAMB1,CYFIP1,UBE3A,SEMA4D,JAM2,FAT4,AP2B1,RUNX1,AKR1B1,WNT5B,SANBR,ASAP1,SORBS2,PDCL3,CCDC88A,GPR55,ADAMTS16,SPAG6,TNFSF11,FYN,ADGRL2,UNC45B,ARL13B,HYDIN,SCN8A,HDAC2,GON4L,TBX15,NCS1,COL18A1,ATP5PF,DOK5,UGP2,CRTAM,COL19A1,APELA,MDGA2,ROR1,ARNT2,HECW2,CDH2,CNTN5,ITGA8,NTN4,XRN2,PHLPP1,GPR137B,EPHB1,RP1L1,GRM5,RPS6KA5,PTPRG,NRP1,SDK1,PRKCA,FAIM,ITGA1,RC3H1,NRIP1,CHODL,POR,BCR,NRXN3,SNRK,STK36,MB,RAG1,B9D1,RRAS2,BMPER,CUX1,MITF,EPHB2,IGSF3,SGCG,CD38,MET,CDH17,PPFIA2,CDH13,ATG5,NRAP,MAGI2,KIAA1217,UNK,ADAM29,FLRT2,MYB,KALRN,SLC1A2,GNAS,LAMA1,MFHAS1,GREB1L,SERPINB7,CA10,TIAM2,IGSF21,BMP7,ASTN2,DLG5,GABRA2,KIRREL3,BTD,BPTF,NUDT21,DDX6,ADGRF5,PDGFC,ABL2,TRAPPC6B,EYA1,FHOD3,SLIT2,FAM126A,CCDC141,ERBB4,SYNDIG1,ROBO1,PBX1,PRKCQ,ANTXR1,NDRG1,MYH15,SIPA1L3,NLGN1,CTTNBP2,SHLD2,SLC6A3,ASIC2,EFNA5,TCF12,VCAN,SLIT3,ESR1,NTNG1,KDM4B,LOXL2,NYAP2,PRLR,FOXB1,CAMK1D,PIK3R3,MACROD2,OPCML,FER,CCR2,RPGRIP1,STARD13,A2M,SEMA4B,ROCK2,PRDM1,RORA,ATAT1,DMRT1,HSPG2,PTPRQ,CSMD3,NCOA6,COL4A3,RGS7,HOOK3,PCSK2,FSTL4,STK3,ZNF423,HNRNPU,APCDD1,IGF1R,GLI2,THRB,LSAMP,AKAP13
GO:0048468	cell development	2.211142408365 6914e-32	NOTCH2,MTOR,CNTN4,SPOCK1,SGCD,LRP12,PTPRD,NEBL,LRRC4C,MYO9A,ULK2,PLCB1,TENM4,ZDHHC21,RIPOR2,PDE4D,RDX,RP1,STXBP1,BCL2,ALDH1A2,FBN1,CHRNA7,ROBO2,RIMS1,TENM3,RARB,SPAG16,MYO1E,USH2A,MINAR1,RIMS2,ALK,AUTS2,CARMIL1,PARVB,NEGR1,CNTNAP2,MAP4,PLPPR5,DSCAM,CRKL,SETD2,TNIK,SLC4A10,OCA2,DOCK10,RFX3,MACF1,RNF220,NEDD4,MYOF,CRB1,BCL11A,TMEM182,CECR2,ARMC2,CHSY1,FLI1,CDH4,NTRK3,PHACTR1,GABRB1,NEO1,CNTN6,SLC39A12,PRKD1,PAK1,EPHA7,SPEN,RAPGEF2,LRP2,ADGRB3,RUNX2,ARSB,TAOK3,ONECUT1,ADAMTSL1,SMYD3,GRM7,EPB41L3,TBCD,NEDD4L,HDAC9,APP,RPS6KA2,DCLK1,STAU2,TMC1,SEMA5A,SYT1,VCL,

			ARHGAP44,NTF3,AURKA,PYGO1,SLC8A1,SRGAP2C,FIG4,KANK1,MAP4K4,BMPR1B,FMN2,AKAP6,CTNNA2,PAK3,DIP2B,TRPC5,DNM3,PRKCZ,KLHL1,DIP2A,COL27A1,HECW1,TAF4B,COBL,YAP1,FRYL,BRINP1,MAPK1,ADAM22,ALCAM,NCAM1,GFRA1,SYCP1,RNF17,FAT3,CHN1,MYLK3,PAFAH1B1,TPM1,NF2,CTNNA1,PPP1R9A,PPARA,NFIB,PRTG,SYNJ1,TIAM1,ENAH,SEMA3C,TMEM108,ALPK2,DYSF,IL34,ANK2,ADGRV1,BBS2,SEMA6D,CDH11,LDB3,PARD3,BLK,TNR,COL22A1,CXADR,ATRX,ELAVL4,ABL1,HDAC4,SDC2,CRTAC1,L3MBTL3,RAP1A,GRID2,PEAK1,LATS2,NRG1,ASPM,RASGRF1,ZBTB16,PGM5,USP33,PTPRO,ALPK3,TRIO,PDE3A,EXT1,LIMD1,CTNND2,ATP8A2,PLXNA2,ARHGEF7,OPRM1,DAZL,KREMEN1,SEMA3E,MARK2,FHL2,TMOD2,HERC1,MSH2,EPHA6,ATL1,KNDC1,AFG3L2,SGCZ,ANK3,MYOM2,BCL11B,DOCK5,ECE1,MBP,FUT9,MYT1L,TJP1,NPHP4,CNTN1,IQSEC1,SNX3,PDLIM5,BRCA2,DISC1,DNER,WDPCP,NRK,SEMA3A,STRN,BMP2,UNC5D,PTCD2,NCAM2,MSI2,SEMA3D,PDE6C,RELN,HMGB1,NFATC2,TDRD7,UST,SLC23A2,DOCK1,B4GALT6,TSPAN2,PLS1,SRGAP2,NIN,DRAXIN,ATF1,SMARCA2,GLI3,SLC9A4,RERE,MAP2,LAMC1,FARP1,TDRD5,BBS4,LAMC3,CFTR,KITLG,DCC,RCAN1,RORB,DAB1,SELENON,PRKN,MTMR2,TBX20,PCDH15,NGEF,ARID5B,CDH23,PRKCH,ALS2,DMC1,TOX,ITSN2,SOX30,ADGRG6,YIPF6,ROCK1,LYN,EIF2B3,TENM2,OVOL2,NTN1,DPYSL5,SHROOM3,HDAC11,PBX3,SNAI2,TANC2,FLNB,TRIM58,TIAL1,NREP,ZDHHC17,SLC22A14,IFT81,DZANK1,BMP5,HDGFL3,BCL2L1,CTDP1,HCN1,PRKG1,LAMA3,SMAD5,CELF4,TNN,CABYR,MICALL2,MED1,ATRN,IL33,ROR2,FAT1,LMX1A,ACTR2,SOHLH1,PACRG,ABHD2,MAP6,VASP,IQGAP1,YBX3,NRXN1,FRY,TWIST1,ALKAL2,JAK2,VSX1,CELSR2,MELTF,UFL1,NTM,ABCC8,USH1C,NEDD9,OLFM4,ITGA6,GAP43,GRIP1,GRXCR1,DGKG,ABI1,MYO18B,IMPACT,ITGA4,ASB2,CYFIP2,MEF2C,ADGRB1,WNT7A,WASF3,S100B,FOXO6,PDE2A,FBXW8,NECTIN1,NCK1,FLVCR1,DRC7,RNF8,EPHA4,GABRA5,NTRK2,IL1RAPL1,FNDC3A,RSPH1,NUMB,LHX9,MEGF10,FBXO31,HS6ST1,PTK2,CDH5,CLDN18,FEZ2,LAMB1,CYFIP1,UBE3A,SEMA4D,JAM2,FRMD6,FAT4,RUNX1,AKR1B1,WNT5B,ASAP1,DPY19L2,SORBS2,CCDC88A,NSUN2,SPAG6,TNFSF11,FYN,ARL13B,HYDIN,HDAC2,NCS1,COL18A1,ROR1,HECW2,CDH2,CNTN5,ITGA8,NTN4,EPHB1,RP1L1,GRM5,RPS6KA5,PTPRG,NRP1,SDK1,FAIM,ITGA1,CHODL,TUT4,NRXN3,C14ORF39,FBLN1,PAQR5,CUX1,EPHB2,CD38,MET,PPFIA2,ATG5,NRAP,MAGI2,UNK,FLRT2,MYB,KALRN,LAMA1,ATRNL1,TIAM2,BMP7,DLG5,KIRREL3,ADGRF5,ABL2,RFX2,FHOD3,SLIT2,CCDC141,ERBB4,ROBO1,PBX1,PRKCQ,ANTXR1,NDRG1,SIPA1L3,NLGN1,EFNA5,SLIT3,ESR1,NTNG1,NYAP2,FOXB1,CAMK1D,CFAP44,OPCML,CATSPERE,FER,RPGRIP1,SEMA4B,ROCK2,PRDM1,ATAT1,DMRT1,CATSPER2,PTPRQ,CSMD3,HOOK3,FSTL4,HNRNPU,IGF1R,GLI2,THRB,AKAP13
GO:0022008	neurogenesis	5.200148498154413e-32	*NOTCH2,BRINP3,MTOR,CNTN4,SPOCK1,LRP12,PTPRD,TRAPPC9,LRRC4C,MYO9A,ULK2,ZNF536,TENM4,RIPOR2,RP1,STXBP1,BCL2,ALDH1A2,CHRNA7,ROBO2,RIMS1,TENM3,ZEB1,SDCCAG8,RARB,NAV2,USH2A,MINAR1,RIMS2,ALK,AUTS2,ASTN1,NEGR1,CNTNAP2,MAP4,PLPPR5,DSCAM,RTN1,TCF4,CRKL,SOX5,TNIK,SLC4A10,DOCK10,MACF1,RNF220,NEDD4,CRB1,BCL11A,SOX6,CECR2,CDH4,ATP2B2,NTRK3,PHACTR1,GABRB1,NEO1,CNTN6,SLC39A12,SLC8A3,PRKD1,PAK1,EPHA7,SPEN,RAPGEF2,LRP2,ADGRB3,RUNX2,ARSB,TAOK3,ADAMTSL1,GRM7,EPB41L3,TBCD,NEDD4L,HDAC9,APP,DCLK1,STAU2,TMC1,SEMA5A,SYT1,VCL,ARHGAP44,NTF3,AURKA,SRGAP2C,FIG4,KANK1,MAP4K4,BMPR1B,CTNNA2,PAK3,DIP2B,TRPC5,DNM3,PRKCZ,KLHL1,DIP2A,ZSWIM6,HECW1,COBL,YAP1,FRYL,BRINP1,MAPK1,ADAM22,ALCAM,NRG3,NCAM1,GFRA1,NIPBL,FAT3,CHN1,PAFAH1B1,NF2,CTNNA1,PPP1R9A,KLF15,NFIB,PRTG,SYNJ1,TIAM1,ENAH,SEMA3C,NAV3,TMEM108,IL34,ADGRV1,SYNE2,WNT9B,SEMA6D,CDH11,PARD3,BLK,TNR,ELAVL4,ABL1,SLC1A1,SDC2,CRTAC1,RAP1A,FGF10,GRID2,NRG1,ASPM,RASGRF1,SH3GL3,USP33,PTPRO,TRIO,EXT1,CTNND2,ATP8A2,PLXNA2,OPRM1,KREMEN1,SEMA3E,MARK2,HERC1,EPHA6,ATL1,CDKN2C,KNDC1,AFG3L2,MOSMO,ANK3,BCL11B,ECE1,MBP,FUT9,MYT1L,NPHP4,CNTN1,IQSEC1,SNX3,PD*

			LIM5,DISC1,DNER,WDPCP,NRK,SEMA3A,STRN,BMP2,UNC5D,TRAK1,NCAM2,SEMA3D,PDE6C,RELN,HMGB1,UST,SLC23A2,ESRP1,TTC21B,B4GALT6,TSPAN2,RAP1GAP,PLS1,SRGAP2,NIN,DRAXIN,ATF1,GLI3,PCP4,MYEF2,RERE,MAP2,FARP1,BBS4,LAMC3,DCC,RORB,DAB1,PRKN,MTMR2,TBX20,PCDH15,NGEF,GRIN2A,CDH23,PRKCH,ALS2,RACGAP1,TOX,PTPRB,ITSN2,ADGRG6,ROCK1,LYN,VCAM1,EIF2B3,DTX1,TENM2,NTN1,ZFHX3,DPYSL5,CASZ1,HDAC11,PBX3,TANC2,RXRG,NREP,ZDHHC17,CD9,DZANK1,BMP5,CSF1,HDGFL3,HCN1,PRKG1,LAMA3,MYCL,TNN,MICALL2,MED1,IL33,ROR2,ZNF521,LMX1A,ACTR2,MAP6,VASP,ETV6,IQGAP1,NRXN1,HIPK1,FRY,LMX1B,TWIST1,ALKAL2,JAK2,VSX1,CELSR2,UFL1,NTM,ABCC8,USH1C,ITGA6,GAP43,GRIP1,EML1,GRXCR1,DGKG,MTPN,ABI1,IMPACT,ITGA4,CEP120,CYFIP2,MEF2C,ADGRB1,WNT7A,WASF3,S100B,PRDM13,FOXO6,FBXW8,NECTIN1,NCK1,PPP2R3A,EPHA4,GABRA5,NTRK2,IL1RAPL1,NUMB,LHX9,WNT2B,FBXO31,HS6ST1,PTK2,FEZ2,LAMB1,CYFIP1,UBE3A,SEMA4D,FAT4,RUNX1,WNT5B,ASAP1,CCDC88A,SPAG6,FYN,HDAC2,NCS1,DOK5,MDGA2,ROR1,HECW2,CDH2,CNTN5,NTN4,XRN2,EPHB1,RP1L1,GRM5,RPS6KA5,PTPRG,NRP1,SDK1,FAIM,ITGA1,CHODL,NRXN3,RRAS2,CUX1,EPHB2,CD38,MET,PPFIA2,MAGI2,UNK,FLRT2,KALRN,LAMA1,TIAM2,BMP7,ASTN2,DLG5,KIRREL3,DDX6,ABL2,EYA1,SLIT2,CCDC141,ERBB4,ROBO1,PBX1,PRKCQ,NDRG1,NLGN1,EFNA5,TCF12,VCAN,SLIT3,NTNG1,NYAP2,FOXB1,CAMK1D,OPCML,CCR2,RPGRIP1,SEMA4B,PRDM1,RORA,ATAT1,PTPRQ,CSMD3,HOOK3,FSTL4,APCDD1,IGF1R,GLI2,THRB
GO:0048666	neuron development	6.614732768681943e-31	*NOTCH2,CNTN4,SPOCK1,LRP12,PTPRD,LRRC4C,MYO9A,ULK2,TENM4,RIPOR2,RP1,STXBP1,BCL2,CHRNA7,ROBO2,RIMS1,TENM3,MINAR1,RIMS2,ALK,AUTS2,NEGR1,CNTNAP2,MAP4,PLPPR5,DSCAM,CRKL,TNIK,SLC4A10,DOCK10,MACF1,RNF220,NEDD4,CRB1,BCL11A,CECR2,CDH4,NTRK3,PHACTR1,GABRB1,NEO1,CNTN6,SLC39A12,PRKD1,PAK1,EPHA7,RAPGEF2,LRP2,ADGRB3,ARSB,TAOK3,ADAMTSL1,GRM7,EPB41L3,TBCD,NEDD4L,APP,DCLK1,STAU2,TMC1,SEMA5A,SYT1,VCL,ARHGAP44,NTF3,AURKA,SRGAP2C,FIG4,KANK1,MAP4K4,BMPR1B,CTNNA2,PAK3,DIP2B,TRPC5,DNM3,PRKCZ,KLHL1,DIP2A,HECW1,COBL,FRYL,ALCAM,NCAM1,GFRA1,FAT3,CHN1,PAFAH1B1,CTNNA1,PPP1R9A,NFIB,PRTG,TIAM1,ENAH,SEMA3C,TMEM108,ADGRV1,SEMA6D,CDH11,PARD3,BLK,TNR,ELAVL4,ABL1,SDC2,CRTAC1,RAP1A,GRID2,RASGRF1,USP33,PTPRO,TRIO,EXT1,CTNND2,ATP8A2,PLXNA2,KREMEN1,SEMA3E,MARK2,HERC1,EPHA6,ATL1,KNDC1,AFG3L2,ANK3,BCL11B,ECE1,MBP,FUT9,MYT1L,NPHP4,CNTN1,IQSEC1,SNX3,PDLIM5,DISC1,WDPCP,NRK,SEMA3A,STRN,UNC5D,NCAM2,SEMA3D,PDE6C,RELN,HMGB1,UST,SLC23A2,B4GALT6,TSPAN2,PLS1,SRGAP2,NIN,DRAXIN,ATF1,GLI3,RERE,MAP2,FARP1,BBS4,DCC,RORB,DAB1,PRKN,MTMR2,PCDH15,NGEF,CDH23,ALS2,TOX,ITSN2,ROCK1,LYN,TENM2,NTN1,DPYSL5,PBX3,TANC2,NREP,ZDHHC17,DZANK1,BMP5,HDGFL3,HCN1,PRKG1,LAMA3,TNN,MICALL2,ROR2,LMX1A,ACTR2,MAP6,VASP,IQGAP1,NRXN1,FRY,ALKAL2,JAK2,VSX1,CELSR2,NTM,USH1C,ITGA6,GAP43,GRIP1,GRXCR1,DGKG,ABI1,IMPACT,ITGA4,CYFIP2,MEF2C,ADGRB1,WNT7A,S100B,FOXO6,FBXW8,NECTIN1,NCK1,EPHA4,GABRA5,NTRK2,IL1RAPL1,NUMB,LHX9,FBXO31,HS6ST1,PTK2,FEZ2,LAMB1,CYFIP1,UBE3A,SEMA4D,FAT4,RUNX1,ASAP1,CCDC88A,SPAG6,FYN,HDAC2,NCS1,ROR1,HECW2,CDH2,CNTN5,NTN4,EPHB1,RP1L1,RPS6KA5,PTPRG,NRP1,SDK1,ITGA1,CHODL,NRXN3,CUX1,EPHB2,CD38,PPFIA2,MAGI2,UNK,FLRT2,KALRN,LAMA1,TIAM2,BMP7,DLG5,KIRREL3,ABL2,SLIT2,CCDC141,ROBO1,PBX1,PRKCQ,NLGN1,EFNA5,SLIT3,NTNG1,NYAP2,FOXB1,CAMK1D,OPCML,RPGRIP1,SEMA4B,PRDM1,ATAT1,PTPRQ,CSMD3,FSTL4,IGF1R,GLI2,THRB*
GO:0120036	plasma membrane bounded cell	8.334462447501137e-31	*NOTCH2,MTOR,CNTN4,SPOCK1,LRP12,PTPRD,LRRC4C,MYO9A,ULK2,LRRC49,RIPOR2,RDX,RP1,STXBP1,RALA,BCL2,ODAD2,CHRNA7,ROBO2,RIMS1,TENM3,SDCCAG8,FGD4,SPAG16,MINAR1,CDC42EP3,RIMS2,ALK,AUTS2,CARMIL1,PARVB,ANO6,NEGR1,CNTNAP2,MAP4,APC,PLPPR5,DSCAM,CRKL,ARHGAP24,TNIK,DOCK*

	projection organization		10,RFX3,MACF1,NEDD4,BCL11A,CECR2,ARMC2,CDH4,NTRK3,PHACTR1,NEO1,CNTN6,SLC39A12,PRKD1,PAK1,EPHA7,RAPGEF2,LRP2,ADGRB3,ARSB,TAOK3,ONECUT1,ADAMTSL1,LRGUK,GRM7,SEPTIN9,EPB41L3,NEDD4L,APP,DCLK1,STAU2,SEMA5A,SYT1,VCL,ARHGAP44,NTF3,CD2AP,AURKA,TTC29,SRGAP2C,FIG4,KANK1,MAP4K4,ABCD2,BMPR1B,CTNNA2,PAK3,DIP2B,TRPC5,DNM3,IFT57,PRKCZ,KLHL1,DIP2A,HECW1,COBL,YAP1,FRYL,ALCAM,ABLIM1,NCAM1,GFRA1,FAT3,CHN1,PAFAH1B1,TPM1,CTNNA1,PPP1R9A,NFIB,PRTG,TIAM1,ENAH,SEMA3C,TMEM108,TANC1,ADGRV1,BCAS3,SYNE2,BBS2,SEMA6D,AIF1L,CDH11,PARD3,BLK,TNR,ELAVL4,ABL1,HDAC4,SDC2,CRTAC1,DNAH5,RAP1A,MYO10,GRID2,RASGRF1,USP33,CEP83,CD44,PTPRO,TRIO,EXT1,CTNND2,IFT43,ATP8A2,PLXNA2,ARHGEF7,ABCC4,KREMEN1,SEMA3E,MARK2,TMEM67,HERC1,EPHA6,ATL1,KNDC1,AFG3L2,CFAP61,ANK3,BCL11B,ECE1,MBP,PLCE1,FUT9,CNTN1,IQSEC1,SNX3,PDLIM5,DISC1,WDPCP,NRK,SEMA3A,STRN,UNC5D,NCAM2,DNAL1,SEMA3D,RELN,HMGB1,NUDCD3,UST,RTTN,SLC23A2,TTC21B,B4GALT6,TSPAN2,RAP1GAP,PLS1,SRGAP2,NIN,DRAXIN,DNAH8,ATF1,GLI3,RERE,MAP2,DAW1,FARP1,CYLD,BBS4,KIAA0753,DCC,DAB1,PRKN,MTMR2,PCDH15,NGEF,CDH23,ALS2,TOX,VAV3,ITSN2,CFAP74,ROCK1,LYN,TENM2,NTN1,DPYSL5,INSR,TANC2,NREP,ZDHHC17,IFT81,BMP5,HDGFL3,PRKG1,LAMA3,GRIN2B,TNN,FAM149B1,MICALL2,CDC14B,PCNT,ROR2,LMX1A,ACTR2,TTC39C,MAP6,VASP,IQGAP1,NRXN1,FRY,CIBAR1,ANLN,ALKAL2,JAK2,CELSR2,GORAB,USH1C,NEDD9,ITGA6,GAP43,GRIP1,BBS9,GRXCR1,DGKG,ABI1,IMPACT,ITGA4,CEP120,CYFIP2,MEF2C,ADGRB1,WNT7A,WASF3,S100B,FOXO6,FBXW8,SDCBP,NECTIN1,NCK1,DRC7,SNAP29,EPHA4,EMP1,NTRK2,IL1RAPL1,RSPH1,NUMB,LHX9,OCLN,FBXO31,PTK2,MARK4,FEZ2,LAMB1,CYFIP1,UBE3A,HOATZ,SEMA4D,FAT4,SAXO1,ASAP1,CCDC88A,ADAMTS16,SPAG6,FYN,ARL13B,HYDIN,HDAC2,NCS1,ROR1,HECW2,CDH2,CNTN5,EPHB1,RP1L1,RPS6KA5,PTPRG,NRP1,SDK1,IFT46,ITGA1,CHODL,NRXN3,CFAP70,STK36,B9D1,CUX1,EPHB2,TOGARAM1,CD38,PPFIA2,CDH13,ATG5,MAGI2,FLRT2,KALRN,LAMA1,TIAM2,BMP7,DLG5,KIRREL3,DNAH17,ABL2,RFX2,SLIT2,CCDC141,ROBO1,PRKCQ,NLGN1,EFNA5,SLIT3,NTNG1,NYAP2,HTT,FOXB1,CAMK1D,CFAP44,FER,RPGRIP1,EPS8,SEMA4B,ATAT1,CSMD3,WASHC1,FSTL4,ZNF423,IGF1R,GLI2,SEPTIN6*
GO:0030182	neuron differentiation	1.416103534242001e-30	*NOTCH2,BRINP3,CNTN4,SPOCK1,LRP12,PTPRD,TRAPPC9,LRRC4C,MYO9A,ULK2,ZNF536,TENM4,RIPOR2,RP1,STXBP1,BCL2,ALDH1A2,CHRNA7,ROBO2,RIMS1,TENM3,ZEB1,USH2A,MINAR1,RIMS2,ALK,AUTS2,NEGR1,CNTNAP2,MAP4,PLPPR5,DSCAM,RTN1,TCF4,CRKL,TNIK,SLC4A10,DOCK10,MACF1,RNF220,NEDD4,CRB1,BCL11A,CECR2,CDH4,ATP2B2,NTRK3,PHACTR1,GABRB1,NEO1,CNTN6,SLC39A12,PRKD1,PAK1,EPHA7,RAPGEF2,LRP2,ADGRB3,RUNX2,ARSB,TAOK3,ADAMTSL1,GRM7,EPB41L3,TBCD,NEDD4L,HDAC9,APP,DCLK1,STAU2,TMC1,SEMA5A,SYT1,VCL,ARHGAP44,NTF3,AURKA,SRGAP2C,FIG4,KANK1,MAP4K4,BMPR1B,CTNNA2,PAK3,DIP2B,TRPC5,DNM3,PRKCZ,KLHL1,DIP2A,ZSWIM6,HECW1,COBL,FRYL,BRINP1,ALCAM,NCAM1,GFRA1,FAT3,CHN1,PAFAH1B1,CTNNA1,PPP1R9A,NFIB,PRTG,TIAM1,ENAH,SEMA3C,TMEM108,ADGRV1,WNT9B,SEMA6D,CDH11,PARD3,BLK,TNR,ELAVL4,ABL1,SDC2,CRTAC1,RAP1A,GRID2,NRG1,ASPM,RASGRF1,SH3GL3,USP33,PTPRO,TRIO,EXT1,CTNND2,ATP8A2,PLXNA2,KREMEN1,SEMA3E,MARK2,HERC1,EPHA6,ATL1,KNDC1,AFG3L2,MOSMO,ANK3,BCL11B,ECE1,MBP,FUT9,MYT1L,NPHP4,CNTN1,IQSEC1,SNX3,PDLIM5,DISC1,WDPCP,NRK,SEMA3A,STRN,BMP2,UNC5D,NCAM2,SEMA3D,PDE6C,RELN,HMGB1,UST,SLC23A2,ESRP1,TTC21B,B4GALT6,TSPAN2,RAP1GAP,PLS1,SRGAP2,NIN,DRAXIN,ATF1,GLI3,PCP4,MYEF2,RERE,MAP2,FARP1,BBS4,DCC,RORB,DAB1,PRKN,MTMR2,TBX20,PCDH15,NGEF,CDH23,ALS2,TOX,ITSN2,ROCK1,LYN,VCAM1,DTX1,TENM2,NTN1,ZFHX3,DPYSL5,CASZ1,PBX3,TANC2,RXRG,NREP,ZDHHC17,DZANK1,BMP5,HDGFL3,HCN1,PRKG1,LAMA3,MYCL,TNN,MICALL2,MED1,ROR2,ZNF521,LMX1A,ACTR2,MAP6,VASP,IQGAP1,NRXN1,HIPK1,FRY,LMX1B,ALKAL2,JAK2,VSX1,CELSR2,NTM,USH1C,ITGA6,GAP*

			43,GRIP1,GRXCR1,DGKG,MTPN,ABI1,IMPACT,ITGA4,CYFIP2,MEF2C,ADGRB1,WNT7A,S100B,FOXO6,FBXW8,NECTIN1,NCK1,PPP2R3A,EPHA4,GABRA5,NTRK2,IL1RAPL1,NUMB,LHX9,WNT2B,FBXO31,HS6ST1,PTK2,FEZ2,LAMB1,CYFIP1,UBE3A,SEMA4D,FAT4,RUNX1,WNT5B,ASAP1,CCDC88A,SPAG6,FYN,HDAC2,NCS1,DOK5,MDGA2,ROR1,HECW2,CDH2,CNTN5,NTN4,XRN2,EPHB1,RP1L1,RPS6KA5,PTPRG,NRP1,SDK1,ITGA1,CHODL,NRXN3,CUX1,EPHB2,CD38,MET,PPFIA2,MAGI2,UNK,FLRT2,KALRN,LAMA1,TIAM2,BMP7,DLG5,KIRREL3,DDX6,ABL2,EYA1,SLIT2,CCDC141,ERBB4,ROBO1,PBX1,PRKCQ,NLGN1,EFNA5,TCF12,SLIT3,NTNG1,NYAP2,FOXB1,CAMK1D,OPCML,RPGRIP1,SEMA4B,PRDM1,RORA,ATAT1,PTPRQ,CSMD3,FSTL4,IGF1R,GLI2,THRB
GO:0030030	cell projection organization	2.127764766826225e-30	NOTCH2,MTOR,CNTN4,SPOCK1,LRP12,PTPRD,LRRC4C,MYO9A,ULK2,LRRC49,RIPOR2,RDX,RP1,STXBP1,RALA,BCL2,ODAD2,CHRNA7,ROBO2,RIMS1,TENM3,SDCCAG8,FGD4,SPAG16,MINAR1,CDC42EP3,RIMS2,ALK,AUTS2,CARMIL1,PARVB,ANO6,NEGR1,CNTNAP2,MAP4,APC,PLPPR5,DSCAM,CRKL,ARHGAP24,TNIK,DOCK10,RFX3,MACF1,NEDD4,BCL11A,CECR2,ARMC2,CDH4,NTRK3,PHACTR1,NEO1,CNTN6,SLC39A12,PRKD1,PAK1,EPHA7,RAPGEF2,LRP2,ADGRB3,DEUP1,ARSB,TAOK3,ONECUT1,ADAMTSL1,LRGUK,GRM7,SEPTIN9,EPB41L3,NEDD4L,APP,DCLK1,STAU2,SEMA5A,SYT1,VCL,ARHGAP44,NTF3,CD2AP,AURKA,TTC29,SRGAP2C,FIG4,KANK1,MAP4K4,ABCD2,BMPR1B,CTNNA2,RAB8B,PAK3,DIP2B,TRPC5,DNM3,IFT57,PRKCZ,KLHL1,DIP2A,HECW1,COBL,YAP1,FRYL,ALCAM,ABLIM1,NCAM1,GFRA1,FAT3,CHN1,PAFAH1B1,TPM1,CTNNA1,PPP1R9A,NFIB,PRTG,TIAM1,ENAH,SEMA3C,TMEM108,TANC1,ADGRV1,BCAS3,SYNE2,BBS2,SEMA6D,AIF1L,CDH11,PARD3,BLK,TNR,ELAVL4,ABL1,HDAC4,SDC2,CRTAC1,DNAH5,RAP1A,MYO10,GRID2,RASGRF1,USP33,CEP83,CD44,PTPRO,TRIO,EXT1,CTNND2,IFT43,ATP8A2,PLXNA2,ARHGEF7,ABCC4,KREMEN1,SEMA3E,MARK2,TMEM67,HERC1,EPHA6,ATL1,KNDC1,AFG3L2,CFAP61,ANK3,BCL11B,ECE1,MBP,PLCE1,FUT9,PACSIN2,CNTN1,IQSEC1,SNX3,PDLIM5,DISC1,WDPCP,NRK,SEMA3A,STRN,UNC5D,NCAM2,DNAL1,SEMA3D,RELN,HMGB1,NUDCD3,UST,RTTN,SLC23A2,TTC21B,B4GALT6,TSPAN2,RAP1GAP,PLS1,SRGAP2,NIN,DRAXIN,DNAH8,ATF1,GLI3,RERE,MAP2,DAW1,FARP1,CYLD,BBS4,KIAA0753,DCC,DAB1,PRKN,MTMR2,PCDH15,NGEF,CDH23,ALS2,TOX,VAV3,ITSN2,CFAP74,ROCK1,LYN,TENM2,NTN1,DPYSL5,INSR,TANC2,NREP,ZDHHC17,IFT81,BMP5,HDGFL3,PRKG1,LAMA3,GRIN2B,TNN,FAM149B1,MICALL2,CDC14B,PCNT,ROR2,LMX1A,ACTR2,TTC39C,MAP6,VASP,IQGAP1,NRXN1,FRY,CIBAR1,ANLN,ALKAL2,JAK2,CELSR2,GORAB,USH1C,NEDD9,ITGA6,GAP43,GRIP1,BBS9,GRXCR1,DGKG,ABI1,IMPACT,ITGA4,CEP120,CYFIP2,MEF2C,ADGRB1,WNT7A,WASF3,S100B,FOXO6,PKN2,FBXW8,SDCBP,NECTIN1,NCK1,DRC7,SNAP29,EPHA4,EMP1,NTRK2,IL1RAPL1,RSPH1,NUMB,LHX9,OCLN,FBXO31,PTK2,MARK4,FEZ2,LAMB1,CYFIP1,UBE3A,HOATZ,SEMA4D,FAT4,SAXO1,ASAP1,CCDC88A,ADAMTS16,SPAG6,FYN,ARL13B,HYDIN,HDAC2,NCS1,ROR1,HECW2,CDH2,CNTN5,ITGA8,EPHB1,RP1L1,RPS6KA5,PTPRG,NRP1,SDK1,IFT46,ITGA1,CHODL,NRXN3,CFAP70,STK36,B9D1,CUX1,EPHB2,TOGARAM1,CD38,PPFIA2,CDH13,ATG5,MAGI2,FLRT2,KALRN,LAMA1,TIAM2,BMP7,DLG5,KIRREL3,DNAH17,ABL2,RFX2,SLIT2,CCDC141,ROBO1,PRKCQ,NLGN1,EFNA5,SLIT3,DNAH9,NTNG1,NYAP2,HTT,FOXB1,CAMK1D,CFAP44,FER,RPGRIP1,EPS8,SEMA4B,ATAT1,CSMD3,WASHC1,FSTL4,ZNF423,IGF1R,GLI2,SEPTIN6
GO:0048869	cellular developmental process	4.547450955755203e-30	NOTCH2,BRINP3,MTOR,CNTN4,SPOCK1,SGCD,LRP12,PTPRD,TRAPPC9,NEBL,LRRC4C,SMOC1,MYO9A,ULK2,FTO,MGA,PLCB1,ZNF536,ZFPM2,TENM4,NUBPL,ZDHHC21,RIPOR2,PDE4D,RDX,RP1,STXBP1,BCL2,PRDM16,ALDH1A2,FBN1,CHRNA7,ROBO2,RIMS1,TENM3,ZEB1,AKR1C3,SDCCAG8,RARB,SPRED1,NAV2,SPAG16,MYO1E,USH2A,MINAR1,RIMS2,ALK,AUTS2,FOXJ2,CARMIL1,ASTN1,RIN2,PARVB,NEGR1,MLLT3,CNTNAP2,MAP4,APC,PLPPR5,DSCAM,RTN1,TCF4,CRKL,ILDR2,SOX5,SETD2,ERG,ARHGAP24,TNIK,SLC4A10,PTPRJ,OCA2,KDM4C,DOCK10,EGFR,RFX3,ANGPT1,CDK12,MACF1,RNF220,DOCK2,NEDD4,MYOF,SND1,CRB1,BC

			L11A,SOX6,TMEM182,CECR2,ARMC2,CHSY1,FLI1,CDH4,ATP2B2,NTRK3,RXFP1,PHACTR1,FLT1,DNAJC13,GABRB1,PSMA8,EDAR,NEO1,CNTN6,SLC39A12,SLC8A3,PRKD1,PAK1,EPHA7,SPEN,RAPGEF2,LRP2,ADGRB3,DEUP1,RUNX2,ARSB,CPS1,TAOK3,ONECUT1,TMEM38B,ADAMTSL1,SMYD3,LRGUK,GRM7,RETREG1,GHR,EPB41L3,COL4A2,SSBP3,TBCD,NEDD4L,HDAC9,ZHX3,APP,ABCB5,RPS6KA2,DCLK1,STAU2,TMC1,SEMA5A,SYT1,VCL,ARHGAP44,NTF3,AURKA,PYGO1,SLC8A1,SRGAP2C,FIG4,KANK1,MAP4K4,BMPR1B,FMN2,THSD7A,AKAP6,CTNNA2,ARNT,PAK3,TTLL7,DIP2B,ITPKB,TRPC5,DNM3,RBM47,PRKCZ,KLHL1,DIP2A,COL27A1,ZSWIM6,HECW1,ABCA5,PHF19,MRTFA,TAF4B,COBL,EBF2,YAP1,FRYL,SHC4,BRINP1,MAPK1,ADAM22,KMT2E,ALCAM,PLG,NRG3,NCAM1,GFRA1,SYCP1,NIPBL,RNF17,GABPA,FAT3,LCE1F,CHN1,MYLK3,MBNL1,PAFAH1B1,EFEMP1,TLL1,DCAF1,ITGB8,TPM1,NF2,RBFOX1,CTNNA1,PPP1R9A,KLF15,PPARA,MEIS2,NFIB,MRTFB,PRTG,SYNJ1,NR5A2,TIAM1,FOXJ3,KAZN,ENAH,SEMA3C,NAV3,TMEM108,ALPK2,JARID2,GATAD2B,DYSF,IL34,ANK2,TANC1,ADGRV1,SYNE2,BBS2,WNT9B,SLC9C1,RANBP3L,SEMA6D,SMARCA4,CDH11,LDB3,PARD3,PIAS1,BLK,TNR,COL22A1,CXADR,ATRX,ELAVL4,ABL1,HDAC4,SLC1A1,SDC2,KCNH1,CRTAC1,DROSHA,L3MBTL3,RAP1A,GLIS1,MORC1,CAMK4,FGF10,GRID2,TGM1,PEAK1,LATS2,NRG1,ASPM,AP3B1,RASGRF1,ATP11C,SYNE1,ZBTB16,MUSK,ZNF675,SH3GL3,PGM5,NXN,USP33,FBN2,CD44,PTPRO,ALPK3,TRIO,PDE3A,EXT1,LIMD1,SPRED2,RPS6KA3,CTNND2,NHS,ATP8A2,PTPN2,PLXNA2,HTR2C,ARHGEF7,AMBRA1,OPRM1,HTR2A,FANCA,DAZL,KREMEN1,SEMA3E,MARK2,ALPL,FHL2,PUM1,TMOD2,HERC1,MSH2,CDIN1,EPHA6,ANKRD17,ATL1,CDKN2C,KNDC1,BICRAL,AFG3L2,MOSMO,SGCZ,ADAM12,MYLK2,ANK3,HMGA2,MYOM2,BCL11B,DOCK5,ECE1,CREM,MBP,TRPS1,HIP1,CRIM1,FUT9,VAV1,MYT1L,TJP1,LDLRAD4,NPHP4,PACSIN2,CNTN1,HLA-B,IQSEC1,SNX3,PDLIM5,BRCA2,DISC1,DNER,WDPCP,NRK,SEMA3A,STRN,BMP2,RC3H2,UNC5D,TRAK1,PSG9,PTCD2,MSR1,NCAM2,MSI2,SEMA3D,PDE6C,RELN,HMGB1,FGF9,NFATC2,TDRD7,UST,SLC23A2,ANKRD26,ESRP1,TTC21B,ETS2,DOCK1,B4GALT6,TSPAN2,RAP1GAP,PLS1,SRGAP2,NIN,DRAXIN,ATF1,SLAMF1,SMARCA2,ETS1,GLI3,SMARCC1,NHSL1,SLC9A4,PCP4,MYEF2,ZNF431,RERE,MAP2,LAMC1,RRBP1,FARP1,TDRD5,ATF2,HIRA,BBS4,LAMC3,HIVEP3,COL5A1,CFTR,NELL1,KITLG,DCC,MYT1,RCAN1,RORB,DAB1,SELENON,PRKN,MTMR2,SH3PXD2A,TBX20,PCDH15,ZNF541,DPF3,NGEF,GRIN2A,ARID5B,CDH23,PRKCH,IL6R,ALS2,RACGAP1,HEMGN,DMC1,LCE3B,TOX,PTPRB,CATSPERG,TBATA,ITSN2,SOX30,PTGFRN,ARHGEF28,ADGRG6,YIPF6,ROCK1,LYN,VCAM1,CTSB,EIF2B3,PLEKHB2,DTX1,TENM2,OVOL2,PIWIL3,NTN1,ZFHX3,DPYSL5,ARID1B,CRACR2A,CASZ1,DMBT1,TFF1,HECTD1,SHROOM3,HDAC11,PBX3,SNAI2,TANC2,RXRG,SP3,FLNB,TRIM58,TIAL1,ELF2,NREP,ZDHHC17,CERS3,SLC22A14,CD9,KRT6B,TWIST2,IFT81,ENPP1,RASGRP1,DZANK1,NDRG2,BMP5,CSF1,HDGFL3,BCL2L1,SPATA48,CTDP1,HCN1,PRKG1,LAMA3,ASB4,CNMD,SMAD5,CELF4,ABCG1,PRAME,MYCL,TNN,CABYR,PSAP,MICALL2,MED1,KDM6A,ATRN,IL33,AJAP1,ROR2,ZNF521,FAT1,LMX1A,TMEM178A,IL10,ACTR2,PRAMEF25,PTH,SDF4,SOSTDC1,SOHLH1,PACRG,ABHD2,VSTM2A,MAP6,VASP,ETV6,IQGAP1,ZBTB7C,ANP32B,YBX3,NRXN1,PCID2,HIPK1,FRY,CADM1,PEG10,LMX1B,ANLN,TWIST1,ALKAL2,JAK2,VSX1,FSTL1,CELSR2,MELTF,ARL11,PCNA,UFL1,ADAMTS5,NFKBIA,NTM,ABCC8,ANXA4,USH1C,NEDD9,OLFM4,ITGA6,GAP43,GRIP1,EML1,MAST2,BBS9,EXT2,KRT6A,GRXCR1,STAT1,NR2C1,MAP2K6,CMTM7,DGKG,MTPN,ABI1,MYO18B,ARMC6,PRAMEF2,POU6F2,IMPACT,ITGA4,BCAP29,FBXL17,ADCYAP1R1,MTF2,NCAPG2,ASB2,MYOCD,CEP120,DHTKD1,CYFIP2,KRT85,MEF2C,ADGRB1,RXRA,WNT7A,RBPMS2,MAP3K5,NDFIP1,MAP3K4,WASF3,S100B,PRDM13,FOXO6,PDE2A,RAB38,FBXW8,SDCBP,NECTIN1,WWOX,NCK1,FLVCR1,FGR,DRC7,PPP2R3A,SPRR2D,RNF8,LCE3D,EPHA4,GABRA5,MECOM,DNMT3L,NTRK2,IL1RAPL1,FNDC3A,RSPH1,NUMB,LHX9,ADAMTS9,WNT2B,CD101,MEGF10,IL17RD,FBXO31,IREB2,HS6ST1,

			PTK2,CDH5,NFKBID,CLDN18,FEZ2,LAMB1,CYFIP1,UBE3A,SEMA4D,JAM2,FRMD6,FAT4,LRMDA,RUNX1,AKR1B1,WNT5B,ASAP1,FCRLA,DPY19L2,SORBS2,CCDC88A,GPR55,NSUN2,SPAG6,TNFSF11,FYN,NLRP14,UNC45B,ARL13B,HYDIN,UHRF2,HDAC2,GON4L,TBX15,NCS1,COL18A1,DOK5,MAPK9,CRTAM,COL19A1,APELA,MDGA2,ROR1,TET1,HECW2,CDH2,CNTN5,ITGA8,NTN4,XRN2,GPR137B,EPHB1,RP1L1,GRM5,RAI14,RPS6KA5,PTPRG,PID1,NRP1,SDK1,PRKCA,FAIM,ITGA1,RC3H1,CHODL,POR,BCR,TUT4,NRXN3,SNRK,C14ORF39,FBLN1,PAQR5,MB,RAG1,B9D1,RRAS2,CUX1,MACROH2A1,MITF,EPHB2,CD38,EYA4,MET,CDH17,PPFIA2,ATG5,NRAP,MAGI2,UNK,FLRT2,MYB,KALRN,LAMA1,MFHAS1,ATRNL1,TIAM2,BMP7,ASTN2,DLG5,KIRREL3,NUDT21,DDX6,ADGRF5,ABL2,RFX2,EYA1,FHOD3,SLIT2,CCDC141,ERBB4,ROBO1,PBX1,PRKCQ,ANTXR1,NDRG1,SIPA1L3,MGMT,NLGN1,NOS1,EFNA5,TCF12,VCAN,RAB27A,SLIT3,ESR1,NTNG1,LOXL2,NYAP2,PRLR,FOXB1,CAMK1D,PIK3R3,CFAP44,OPCML,CATSPERE,FER,EYA2,CCR2,RPGRIP1,A2M,SEMA4B,ROCK2,PRDM1,RORA,ATAT1,DMRT1,CATSPER2,HSPG2,PTPRQ,CSMD3,NCOA6,HOOK3,FSTL4,PNPLA3,STK3,ZNF423,HNRNPU,APCDD1,IGF1R,GLI2,THRB,AKAP13,SEPTIN6,DNM1L
GO:0009653	anatomical structure morphogenesis	1.0077130133980986e-29	*NOTCH2,BCAR3,MTOR,CNTN4,SGCD,PTPRD,FREM1,NEBL,LRRC4C,MYO9A,ULK2,TAFA5,CLTCL1,ZFPM2,TENM4,NUBPL,DLC1,RIPOR2,RDX,RP1,STXBP1,RALA,BCL2,ALDH1A2,FBN1,CDH8,CHRNA7,ROBO2,RIMS1,SPIRE1,TENM3,ZEB1,SDCCAG8,RARB,FGD4,SPRED1,ENPEP,MYO1E,USH2A,MINAR1,CDC42EP3,RIMS2,AUTS2,FOXJ2,CARMIL1,PAPPA2,RIN2,PARVB,MLLT3,GPC6,CNTNAP2,MYO3B,ZMYM4,DSCAM,CRKL,SOX5,SETD2,ARHGAP24,TNIK,SLC4A10,KDM4C,DOCK10,EGFR,ANGPT1,MACF1,PRKACB,NEDD4,MYOF,CRB1,BCL11A,SOX6,TMEM182,CECR2,CHSY1,FLI1,CDH4,RXFP1,C5,PHACTR1,FLT1,EDAR,NEO1,CNTN6,SLC39A12,PRKD1,PAK1,EPHA7,RAPGEF2,LRP2,ADGRB3,RUNX2,TAOK3,ONECUT1,ADAMTSL1,PRICKLE2,GHR,LUZP1,EPB41L3,COL4A2,SSBP3,TBCD,NEDD4L,HDAC9,APP,CACNA1C,DCLK1,STAU2,SEMA5A,SYT1,VCL,ARHGAP44,NTF3,AURKA,CFDP1,FIG4,KANK1,MAP4K4,BMPR1B,THSD7A,CTNNA2,PAK3,DIP2B,TRPC5,DNM3,IFT57,PRKCZ,CALD1,DIP2A,COL27A1,HECW1,MRTFA,COBL,YAP1,FRYL,RIPK4,MAPK1,CRISPLD2,ALCAM,ZNRF3,ABLIM1,NRG3,NCAM1,NIPBL,GABPA,FAT3,CHN1,MYLK3,FMN1,MBNL1,PAFAH1B1,EFEMP1,ITGB8,TPM1,NF2,CTNNA1,ANKRD11,CDH7,PPARA,MEIS2,NFIB,PRTG,NR5A2,TIAM1,ENAH,SF3B6,SEMA3C,SLC24A4,TMEM108,AGO2,ALPK2,DNAH11,CPE,ANK2,BRWD1,TANC1,BCAS3,RYR2,BBS2,WNT9B,SEMA6D,CDH11,LDB3,PARD3,TNR,COL22A1,ATRX,XIRP2,ELAVL4,ABL1,SLC1A1,SDC2,GAS2,KCNH1,CDH18,RAP1A,MYO10,FGF10,GRID2,CDHR3,PEAK1,LATS2,NRG1,AP3B1,ZBTB16,SETDB2,PGM5,USP33,FBN2,CD44,PTPRO,EGF,TRIO,EXT1,LIMD1,CTNND2,ATP8A2,PLXNA2,ARHGEF7,CNNM4,SEMA3E,MARK2,ALPL,FHL2,TMOD2,HERC1,IGF2BP3,EPHA6,SH3KBP1,ATL1,EPN2,KNDC1,AFG3L2,ADAM12,MYLK2,ANK3,EMILIN2,HMGA2,MYOM2,BCL11B,DOCK5,ECE1,MBP,TGFA,CDH20,TJP1,EGFLAM,PACSIN2,CNTN1,MTHFD1L,PDLIM5,DISC1,WDPCP,NRK,SEMA3A,BMP2,UNC5D,PTCD2,SEMA3D,ASXL3,PDE6C,RELN,FGF9,NFATC2,TDRD7,UST,SLC23A2,TTC21B,ETS2,DOCK1,B4GALT6,PLS1,SRGAP2,NIN,DRAXIN,ETS1,GLI3,MEGF11,SMARCC1,AFF3,SMOC2,CUL1,RERE,MAP2,LAMC1,FARP1,ATF2,HIRA,BBS4,LAMC3,COL5A1,CFTR,DCC,GTF2I,RORB,DAB1,MYO3A,PRKN,MTMR2,SH3PXD2A,TBX20,PCDH15,NGEF,ARID5B,CDH23,ALS2,PTPRB,SCN10A,VAV3,ITSN2,SOX30,PTGFRN,ARHGEF28,ADGRG6,ROCK1,LRIG1,OVOL2,NTN1,MMP16,DPYSL5,HOXC13,INSR,HECTD1,SHROOM3,DDHD1,PBX3,SNAI2,ASH1L,HOXC4,TANC2,SP3,FLNB,ZDHHC17,NSD2,CD9,CARD10,JCAD,DZANK1,BMP5,WDR72,CSF1,BCL2L1,KRT25,HCN1,LAMA3,ASB4,CNMD,DHRS3,SMAD5,FOXN3,VSTM4,SLC40A1,TNN,MICALL2,MED1,KDM6A,ATRN,AJAP1,FAT1,LMX1A,IL10,ACTR2,SCML2,TTC39C,SOSTDC1,MAP6,VASP,PALMD,IQGAP1,AIMP1,NRXN1,HIPK1,FRY,CIBAR1,TWIST1,AKT3,VSX1,SVEP1,CELSR2,CREBBP,MELTF,GORAB,ADAMTS5,PRKCB,ABCC8,ALX4,USH1C,NEDD9,OLFM4,ITGA6,GAP*

			43,GRIP1,OTOP1,EXT2,KRT6A,AGO1,MEOX2,GRXCR1,STAT1,SHROOM2,MTPN,ABI1,MYO18B,IMPACT,CCBE1,ITGA4,CSMD1,ASB2,MYOCD,CYFIP2,MEF2C,ADGRB1,WNT7A,RBPMS2,WASF3,S100B,FBXW8,NECTIN1,WWOX,FLVCR1,FGR,PPP2R3A,DNMBP,EPHA4,NTRK2,IL1RAPL1,NUMB,LHX9,ADAMTS9,WNT2B,TNNI1,FBXO31,HS6ST1,PTK2,CDH5,ANKRD6,ARHGAP12,DIAPH1,FEZ2,LAMB1,CYFIP1,UBE3A,PCDH8,SEMA4D,JAM2,FRMD6,FAT4,RUNX1,WNT5B,PDCL3,ADAMTS16,SPAG6,BICD1,TNFSF11,FYN,MYL12B,ARL13B,HDAC2,TBX15,COL18A1,CDH9,APELA,ROR1,TET1,HECW2,CDH2,CNTN5,ITGA8,NTN4,EPHB1,ADCK1,RPS6KA5,PID1,NRP1,SDK1,PRKCA,ITGA1,RC3H1,CHODL,POR,BCR,NRXN3,KIF16B,CDH12,FBLN1,B9D1,DGCR2,BMPER,CUX1,MACROH2A1,EPHB2,EYA4,MET,PPFIA2,CDH13,NRAP,MAGI2,UNK,FAM171A1,FLRT2,KALRN,GNAS,LAMA1,GREB1L,ATRNL1,TIAM2,BMP7,ASTN2,DLG5,KIRREL3,ADGRF5,PDGFC,RFX2,EYA1,FHOD3,SLIT2,EXOC4,CCDC141,ERBB4,ROBO1,PBX1,PRKCQ,ANTXR1,SIPA1L3,NLGN1,NOS1,SLC6A3,EFNA5,SLIT3,ESR1,NTNG1,LOXL2,NYAP2,FOXB1,PIK3R3,FER,EYA2,CCR2,RPGRIP1,STARD13,EPS8,SEMA4B,ROCK2,PRDM1,RORA,DMRT1,HSPG2,PTPRQ,COL4A3,FSTL4,STK3,ZNF568,APCDD1,IGF1R,GLI2,THRB,AKAP13,ATP10A,DNM1L
GO:0030154	cell differentiation	1.042667622961 3627e-29	*NOTCH2,BRINP3,MTOR,CNTN4,SPOCK1,SGCD,LRP12,PTPRD,TRAPPC9,NEBL,LRRC4C,SMOC1,MYO9A,ULK2,FTO,MGA,PLCB1,ZNF536,ZFPM2,TENM4,ZDHHC21,RIPOR2,PDE4D,RDX,RP1,STXBP1,BCL2,PRDM16,ALDH1A2,FBN1,CHRNA7,ROBO2,RIMS1,TENM3,ZEB1,AKR1C3,SDCCAG8,RARB,SPRED1,NAV2,SPAG16,MYO1E,USH2A,MINAR1,RIMS2,ALK,AUTS2,FOXJ2,CARMIL1,ASTN1,RIN2,PARVB,NEGR1,MLLT3,CNTNAP2,MAP4,APC,PLPPR5,DSCAM,RTN1,TCF4,CRKL,ILDR2,SOX5,SETD2,ERG,ARHGAP24,TNIK,SLC4A10,PTPRJ,OCA2,KDM4C,DOCK10,EGFR,RFX3,ANGPT1,CDK12,MACF1,RNF220,DOCK2,NEDD4,MYOF,SND1,CRB1,BCL11A,SOX6,TMEM182,CECR2,ARMC2,CHSY1,FLI1,CDH4,ATP2B2,NTRK3,RXFP1,PHACTR1,FLT1,DNAJC13,GABRB1,PSMA8,EDAR,NEO1,CNTN6,SLC39A12,SLC8A3,PRKD1,PAK1,EPHA7,SPEN,RAPGEF2,LRP2,ADGRB3,DEUP1,RUNX2,ARSB,CPS1,TAOK3,ONECUT1,TMEM38B,ADAMTSL1,SMYD3,LRGUK,GRM7,RETREG1,GHR,EPB41L3,COL4A2,SSBP3,TBCD,NEDD4L,HDAC9,ZHX3,APP,ABCB5,RPS6KA2,DCLK1,STAU2,TMC1,SEMA5A,SYT1,VCL,ARHGAP44,NTF3,AURKA,PYGO1,SLC8A1,SRGAP2C,FIG4,KANK1,MAP4K4,BMPR1B,FMN2,THSD7A,AKAP6,CTNNA2,ARNT,PAK3,TTLL7,DIP2B,ITPKB,TRPC5,DNM3,RBM47,PRKCZ,KLHL1,DIP2A,COL27A1,ZSWIM6,HECW1,ABCA5,PHF19,MRTFA,TAF4B,COBL,EBF2,YAP1,FRYL,SHC4,BRINP1,MAPK1,ADAM22,KMT2E,ALCAM,PLG,NRG3,NCAM1,GFRA1,SYCP1,NIPBL,RNF17,GABPA,FAT3,LCE1F,CHN1,MYLK3,MBNL1,PAFAH1B1,EFEMP1,TLL1,DCAF1,ITGB8,TPM1,NF2,RBFOX1,CTNNA1,PPP1R9A,KLF15,PPARA,MEIS2,NFIB,MRTFB,PRTG,SYNJ1,NR5A2,TIAM1,FOXJ3,KAZN,ENAH,SEMA3C,NAV3,TMEM108,ALPK2,JARID2,GATAD2B,DYSF,IL34,ANK2,TANC1,ADGRV1,SYNE2,BBS2,WNT9B,SLC9C1,RANBP3L,SEMA6D,SMARCA4,CDH11,LDB3,PARD3,PIAS1,BLK,TNR,COL22A1,CXADR,ATRX,ELAVL4,ABL1,HDAC4,SLC1A1,SDC2,KCNH1,CRTAC1,DROSHA,L3MBTL3,RAP1A,GLIS1,MORC1,CAMK4,FGF10,GRID2,TGM1,PEAK1,LATS2,NRG1,ASPM,AP3B1,RASGRF1,ATP11C,SYNE1,ZBTB16,MUSK,ZNF675,SH3GL3,PGM5,NXN,USP33,FBN2,PTPRO,ALPK3,TRIO,PDE3A,EXT1,LIMD1,SPRED2,RPS6KA3,CTNND2,NHS,ATP8A2,PTPN2,PLXNA2,HTR2C,ARHGEF7,AMBRA1,OPRM1,HTR2A,FANCA,DAZL,KREMEN1,SEMA3E,MARK2,ALPL,FHL2,PUM1,TMOD2,HERC1,MSH2,CDIN1,EPHA6,ANKRD17,ATL1,CDKN2C,KNDC1,BICRAL,AFG3L2,MOSMO,SGCZ,ADAM12,MYLK2,ANK3,HMGA2,MYOM2,BCL11B,DOCK5,ECE1,CREM,MBP,TRPS1,HIP1,CRIM1,FUT9,VAV1,MYT1L,TJP1,LDLRAD4,NPHP4,CNTN1,HLA-B,IQSEC1,SNX3,PDLIM5,BRCA2,DISC1,DNER,WDPCP,NRK,SEMA3A,STRN,BMP2,RC3H2,UNC5D,TRAK1,PSG9,PTCD2,MSR1,NCAM2,MSI2,SEMA3D,PDE6C,RELN,HMGB1,FGF9,NFATC2,TDRD7,UST,SLC23A2,ANKRD26,ESRP1,TTC21B,ETS2,DOCK1,B4GALT6,TSPAN2,RAP1GAP,PLS1,SRGAP2,NIN,DRAXIN,ATF1,SLAMF1,S*

			MARCA2,ETS1,GLI3,SMARCC1,NHSL1,SLC9A4,PCP4,MYEF2,ZNF431,RERE,MAP2,LAMC1,RRBP1,FARP1,TDRD5,ATF2,HIRA,BBS4,LAMC3,HIVEP3,COL5A1,CFTR,NELL1,KITLG,DCC,MYT1,RCAN1,RORB,DAB1,SELENON,PRKN,MTMR2,SH3PXD2A,TBX20,PCDH15,ZNF541,DPF3,NGEF,GRIN2A,ARID5B,CDH23,PRKCH,IL6R,ALS2,RACGAP1,HEMGN,DMC1,LCE3B,TOX,PTPRB,CATSPERG,TBATA,ITSN2,SOX30,PTGFRN,ARHGEF28,ADGRG6,YIPF6,ROCK1,LYN,VCAM1,CTSB,EIF2B3,PLEKHB2,DTX1,TENM2,OVOL2,PIWIL3,NTN1,ZFHX3,DPYSL5,ARID1B,CRACR2A,CASZ1,DMBT1,TFF1,HECTD1,SHROOM3,HDAC11,PBX3,SNAI2,TANC2,RXRG,SP3,FLNB,TRIM58,TIAL1,ELF2,NREP,ZDHHC17,CERS3,SLC22A14,CD9,KRT6B,TWIST2,IFT81,ENPP1,RASGRP1,DZANK1,NDRG2,BMP5,CSF1,HDGFL3,BCL2L1,SPATA48,CTDP1,HCN1,PRKG1,LAMA3,ASB4,CNMD,SMAD5,CELF4,ABCG1,PRAME,MYCL,TNN,CABYR,PSAP,MICALL2,MED1,KDM6A,ATRN,IL33,AJAP1,ROR2,ZNF521,FAT1,LMX1A,TMEM178A,IL10,ACTR2,PRAMEF25,PTH,SDF4,SOSTDC1,SOHLH1,PACRG,ABHD2,VSTM2A,MAP6,VASP,ETV6,IQGAP1,ZBTB7C,ANP32B,YBX3,NRXN1,PCID2,HIPK1,FRY,CADM1,PEG10,LMX1B,ANLN,TWIST1,ALKAL2,JAK2,VSX1,FSTL1,CELSR2,MELTF,ARL11,PCNA,UFL1,ADAMTS5,NFKBIA,NTM,ABCC8,ANXA4,USH1C,NEDD9,OLFM4,ITGA6,GAP43,GRIP1,EML1,MAST2,BBS9,EXT2,KRT6A,GRXCR1,STAT1,NR2C1,MAP2K6,CMTM7,DGKG,MTPN,ABI1,MYO18B,ARMC6,PRAMEF2,POU6F2,IMPACT,ITGA4,BCAP29,FBXL17,ADCYAP1R1,MTF2,NCAPG2,ASB2,MYOCD,CEP120,DHTKD1,CYFIP2,KRT85,MEF2C,ADGRB1,RXRA,WNT7A,RBPMS2,MAP3K5,NDFIP1,MAP3K4,WASF3,S100B,PRDM13,FOXO6,PDE2A,RAB38,FBXW8,SDCBP,NECTIN1,WWOX,NCK1,FLVCR1,FGR,DRC7,PPP2R3A,SPRR2D,RNF8,LCE3D,EPHA4,GABRA5,MECOM,DNMT3L,NTRK2,IL1RAPL1,FNDC3A,RSPH1,NUMB,LHX9,ADAMTS9,WNT2B,CD101,MEGF10,IL17RD,FBXO31,IREB2,HS6ST1,PTK2,CDH5,NFKBID,CLDN18,FEZ2,LAMB1,CYFIP1,UBE3A,SEMA4D,JAM2,FRMD6,FAT4,LRMDA,RUNX1,AKR1B1,WNT5B,ASAP1,FCRLA,DPY19L2,SORBS2,CCDC88A,GPR55,NSUN2,SPAG6,TNFSF11,FYN,NLRP14,UNC45B,ARL13B,HYDIN,UHRF2,HDAC2,GON4L,TBX15,NCS1,COL18A1,DOK5,MAPK9,CRTAM,COL19A1,APELA,MDGA2,ROR1,TET1,HECW2,CDH2,CNTN5,ITGA8,NTN4,XRN2,GPR137B,EPHB1,RP1L1,GRM5,RAI14,RPS6KA5,PTPRG,NRP1,SDK1,PRKCA,FAIM,ITGA1,RC3H1,CHODL,POR,BCR,TUT4,NRXN3,SNRK,C14ORF39,FBLN1,PAQR5,MB,RAG1,B9D1,RRAS2,CUX1,MACROH2A1,MITF,EPHB2,CD38,EYA4,MET,CDH17,PPFIA2,ATG5,NRAP,MAGI2,UNK,FLRT2,MYB,KALRN,LAMA1,MFHAS1,ATRNL1,TIAM2,BMP7,ASTN2,DLG5,KIRREL3,NUDT21,DDX6,ADGRF5,ABL2,RFX2,EYA1,FHOD3,SLIT2,CCDC141,ERBB4,ROBO1,PBX1,PRKCQ,ANTXR1,NDRG1,SIPA1L3,MGMT,NLGN1,NOS1,EFNA5,TCF12,VCAN,RAB27A,SLIT3,ESR1,NTNG1,LOXL2,NYAP2,PRLR,FOXB1,CAMK1D,PIK3R3,CFAP44,OPCML,CATSPERE,FER,EYA2,CCR2,RPGRIP1,A2M,SEMA4B,ROCK2,PRDM1,RORA,ATAT1,DMRT1,CATSPER2,HSPG2,PTPRQ,CSMD3,NCOA6,HOOK3,FSTL4,PNPLA3,STK3,ZNF423,HNRNPU,APCDD1,IGF1R,GLI2,THRB,AKAP13,SEPTIN6
GO:0048699	generation of neurons	1.320217355247 3385e-29	*NOTCH2,BRINP3,CNTN4,SPOCK1,LRP12,PTPRD,TRAPPC9,LRRC4C,MYO9A,ULK2,ZNF536,TENM4,RIPOR2,RP1,STXBP1,BCL2,ALDH1A2,CHRNA7,ROBO2,RIMS1,TENM3,ZEB1,SDCCAG8,USH2A,MINAR1,RIMS2,ALK,AUTS2,ASTN1,NEGR1,CNTNAP2,MAP4,PLPPR5,DSCAM,RTN1,TCF4,CRKL,SOX5,TNIK,SLC4A10,DOCK10,MACF1,RNF220,NEDD4,CRB1,BCL11A,CECR2,CDH4,ATP2B2,NTRK3,PHACTR1,GABRB1,NEO1,CNTN6,SLC39A12,PRKD1,PAK1,EPHA7,RAPGEF2,LRP2,ADGRB3,RUNX2,ARSB,TAOK3,ADAMTSL1,GRM7,EPB41L3,TBCD,NEDD4L,HDAC9,APP,DCLK1,STAU2,TMC1,SEMA5A,SYT1,VCL,ARHGAP44,NTF3,AURKA,SRGAP2C,FIG4,KANK1,MAP4K4,BMPR1B,CTNNA2,PAK3,DIP2B,TRPC5,DNM3,PRKCZ,KLHL1,DIP2A,ZSWIM6,HECW1,COBL,FRYL,BRINP1,ALCAM,NRG3,NCAM1,GFRA1,NIPBL,FAT3,CHN1,PAFAH1B1,CTNNA1,PPP1R9A,NFIB,PRTG,TIAM1,ENAH,SEMA3C,TMEM108,ADGRV1,WNT9B,SEMA6D,CDH11,PARD3,BLK,TNR,ELAVL4,ABL1,SDC2,CRTAC1,RAP1A,GRID2,NRG1,ASPM,RASGRF1,SH3GL3,USP33,PTPRO*

			,TRIO,EXT1,CTNND2,ATP8A2,PLXNA2,KREMEN1,SEMA3E,MARK2,HERC1,EPHA6,ATL1,KNDC1,AFG3L2,MOSMO,ANK3,BCL11B,ECE1,MBP,FUT9,MYT1L,NPHP4,CNTN1,IQSEC1,SNX3,PDLIM5,DISC1,DNER,WDPCP,NRK,SEMA3A,STRN,BMP2,UNC5D,NCAM2,SEMA3D,PDE6C,RELN,HMGB1,UST,SLC23A2,ESRP1,TTC21B,B4GALT6,TSPAN2,RAP1GAP,PLS1,SRGAP2,NIN,DRAXIN,ATF1,GLI3,PCP4,MYEF2,RERE,MAP2,FARP1,BBS4,DCC,RORB,DAB1,PRKN,MTMR2,TBX20,PCDH15,NGEF,CDH23,ALS2,RACGAP1,TOX,ITSN2,ROCK1,LYN,VCAM1,DTX1,TENM2,NTN1,ZFHX3,DPYSL5,CASZ1,PBX3,TANC2,RXRG,NREP,ZDHHC17,DZANK1,BMP5,HDGFL3,HCN1,PRKG1,LAMA3,MYCL,TNN,MICALL2,MED1,ROR2,ZNF521,LMX1A,ACTR2,MAP6,VASP,IQGAP1,NRXN1,HIPK1,FRY,LMX1B,TWIST1,ALKAL2,JAK2,VSX1,CELSR2,NTM,USH1C,ITGA6,GAP43,GRIP1,EML1,GRXCR1,DGKG,MTPN,ABI1,IMPACT,ITGA4,CYFIP2,MEF2C,ADGRB1,WNT7A,S100B,FOXO6,FBXW8,NECTIN1,NCK1,PPP2R3A,EPHA4,GABRA5,NTRK2,IL1RAPL1,NUMB,LHX9,WNT2B,FBXO31,HS6ST1,PTK2,FEZ2,LAMB1,CYFIP1,UBE3A,SEMA4D,FAT4,RUNX1,WNT5B,ASAP1,CCDC88A,SPAG6,FYN,HDAC2,NCS1,DOK5,MDGA2,ROR1,HECW2,CDH2,CNTN5,NTN4,XRN2,EPHB1,RP1L1,RPS6KA5,PTPRG,NRP1,SDK1,ITGA1,CHODL,NRXN3,CUX1,EPHB2,CD38,MET,PPFIA2,MAGI2,UNK,FLRT2,KALRN,LAMA1,TIAM2,BMP7,ASTN2,DLG5,KIRREL3,DDX6,ABL2,EYA1,SLIT2,CCDC141,ERBB4,ROBO1,PBX1,PRKCQ,NLGN1,EFNA5,TCF12,SLIT3,NTNG1,NYAP2,FOXB1,CAMK1D,OPCML,RPGRIP1,SEMA4B,PRDM1,RORA,ATAT1,PTPRQ,CSMD3,FSTL4,IGF1R,GLI2,THRB
GO:0031175	neuron projection development	6.151076946157889e-29	NOTCH2,CNTN4,SPOCK1,LRP12,PTPRD,LRRC4C,MYO9A,ULK2,RIPOR2,STXBP1,BCL2,CHRNA7,ROBO2,RIMS1,TENM3,MINAR1,RIMS2,ALK,AUTS2,NEGR1,CNTNAP2,MAP4,PLPPR5,DSCAM,CRKL,TNIK,DOCK10,MACF1,NEDD4,BCL11A,CECR2,CDH4,NTRK3,PHACTR1,NEO1,CNTN6,SLC39A12,PRKD1,PAK1,EPHA7,RAPGEF2,LRP2,ADGRB3,ARSB,TAOK3,ADAMTSL1,GRM7,EPB41L3,NEDD4L,APP,DCLK1,STAU2,SEMA5A,SYT1,VCL,ARHGAP44,NTF3,AURKA,SRGAP2C,FIG4,KANK1,MAP4K4,BMPR1B,CTNNA2,PAK3,DIP2B,TRPC5,DNM3,PRKCZ,KLHL1,DIP2A,HECW1,COBL,FRYL,ALCAM,NCAM1,GFRA1,FAT3,CHN1,PAFAH1B1,CTNNA1,PPP1R9A,NFIB,PRTG,TIAM1,ENAH,SEMA3C,TMEM108,ADGRV1,SEMA6D,CDH11,PARD3,BLK,TNR,ELAVL4,ABL1,SDC2,CRTAC1,RAP1A,GRID2,RASGRF1,USP33,PTPRO,TRIO,EXT1,CTNND2,ATP8A2,PLXNA2,KREMEN1,SEMA3E,MARK2,HERC1,EPHA6,ATL1,KNDC1,AFG3L2,ANK3,BCL11B,ECE1,MBP,FUT9,CNTN1,IQSEC1,SNX3,PDLIM5,DISC1,NRK,SEMA3A,STRN,UNC5D,NCAM2,SEMA3D,RELN,HMGB1,UST,SLC23A2,B4GALT6,TSPAN2,PLS1,SRGAP2,NIN,DRAXIN,ATF1,GLI3,RERE,MAP2,FARP1,BBS4,DCC,DAB1,PRKN,PCDH15,NGEF,CDH23,ALS2,TOX,ITSN2,ROCK1,LYN,NTN1,DPYSL5,TANC2,NREP,ZDHHC17,BMP5,HDGFL3,PRKG1,LAMA3,TNN,MICALL2,ROR2,LMX1A,ACTR2,MAP6,VASP,IQGAP1,NRXN1,FRY,ALKAL2,JAK2,CELSR2,USH1C,ITGA6,GAP43,GRIP1,GRXCR1,DGKG,ABI1,IMPACT,ITGA4,CYFIP2,MEF2C,ADGRB1,WNT7A,S100B,FOXO6,FBXW8,NECTIN1,NCK1,EPHA4,NTRK2,IL1RAPL1,NUMB,LHX9,FBXO31,PTK2,FEZ2,LAMB1,CYFIP1,UBE3A,SEMA4D,FAT4,ASAP1,CCDC88A,SPAG6,FYN,HDAC2,NCS1,ROR1,HECW2,CDH2,CNTN5,EPHB1,RPS6KA5,PTPRG,NRP1,SDK1,ITGA1,CHODL,NRXN3,CUX1,EPHB2,CD38,PPFIA2,MAGI2,FLRT2,KALRN,LAMA1,TIAM2,BMP7,DLG5,KIRREL3,ABL2,SLIT2,CCDC141,ROBO1,PRKCQ,NLGN1,EFNA5,SLIT3,NTNG1,NYAP2,FOXB1,CAMK1D,SEMA4B,CSMD3,FSTL4,IGF1R,GLI2
GO:0032501	multicellular organismal process	2.487961724690268e-28	NOTCH2,BCAR3,BRINP3,MTOR,CNTN4,SPOCK1,SGCD,WWC1,IMMP2L,LRP12,PTPRD,SLC24A2,FREM1,TRAPPC9,BNC2,NEBL,LRRC4C,SMOC1,MYO9A,ULK2,SCAPER,FTO,PLCB1,ZNF536,TAFA5,ZFPM2,PIEZO2,TENM4,DLC1,ZDHHC21,RIPOR2,PDE4D,RP1,STXBP1,RALA,IL1RAPL2,BCL2,ODAD2,KCNMA1,PRDM16,ALDH1A2,ARHGAP26,FBN1,F13A1,CHRNA7,ROBO2,RIMS1,SPIRE1,TENM3,GABRB3,ZEB1,AKR1C3,SDCCAG8,RARB,SPRED1,NAV2,ENPEP,SPAG16,MYO1E,PLPPR1,USH2A,MINAR1,RIMS2,ALK,AUTS2,FOXJ2,PJA2,PAPPA2,ERBIN,RHPN2,ASTN1,RIN2,ANO6,CACNG2

			,DLGAP1,NEGR1,MLLT3,GPC6,CNTNAP2,MAP4,MYO3B,APC,HHLA2,TSHZ3,RBFOX3,PLPPR5,DSCAM,RTN1,TCF4,CRKL,ILDR2,SOX5,SETD2,ARHGAP24,TNIK,SLC4A10,PTPRJ,OCA2,KDM4C,DOCK10,EGFR,RFX3,ANGPT1,MACF1,CTNNA3,PRKACB,NCOR1,RNF220,DOCK2,NEDD4,MYOF,SND1,CRB1,BTBD9,BCL11A,SOX6,CECR2,ARMC2,CHSY1,FLI1,CDH4,ATP2B2,NTRK3,RXFP1,C5,TUSC3,PHACTR1,DKK2,FLT1,DNAJC13,ADAMTS6,MAPKBP1,GABRB1,PSMA8,DGKI,EDAR,GRIA1,CRACD,NEO1,CNTN6,SLC39A12,CABLES1,SLC8A3,PRKD1,PAK1,EPHA7,CHRM3,SPEN,RAPGEF2,LRP2,ADGRB3,RUNX2,ARSB,FGF12,GABRA6,CPS1,TAOK3,ONECUT1,TMEM38B,ADAMTSL1,SLC24A3,SLC44A1,LDB2,TAFA4,HERC2,LRGUK,GRM7,RETREG1,RPTOR,GHR,LUZP1,EPB41L3,COL4A2,SSBP3,CELF2,RAPGEF5,TBCD,NEDD4L,PPP1R12B,TRPM1,ADAM10,HDAC9,ZHX3,IL1R1,APBB2,APP,SLC7A2,ABCB5,RPS6KA2,CACNA1C,KDM1B,CACNB2,DCLK1,STAU2,GABRG2,TMC1,MAPRE2,SEMA5A,SYT1,VCL,ARHGAP44,NTF3,CD2AP,AURKA,PARN,PYGO1,SLC8A1,HERPUD2,PTPRR,SRGAP2C,FIG4,TAFA2,CMIP,ABCG8,LOXHD1,SRGAP2B,KANK1,KCNE4,MAP4K4,ABCD2,BMPR1B,FMN2,THSD7A,PCSK6,AKAP6,HOMER2,CTNNA2,ARNT,RAB8B,PAK3,RFTN1,TTLL7,DIP2B,KCNK10,ITPKB,TRPC5,RAP1GDS1,RNLS,CHST8,DNM3,NBN,CUBN,IFT57,RBM47,PRKCZ,CALD1,SNTG2,KLHL1,GRB10,DIP2A,MSH6,MCPH1,COL27A1,ZSWIM6,RGS9,HECW1,ABCA5,PHF19,TAF4B,COBL,EBF2,YAP1,ESS2,FRYL,NFIA,BRINP1,MAPK1,HRH2,ADAM22,CRISPLD2,KMT2E,ALCAM,PLG,PAPPA,PDGFD,SYT10,ZNRF3,ABLIM1,NRG3,NCAM1,GFRA1,SYCP1,NIPBL,RNF17,SLC16A1,GABPA,FAT3,CORO2B,CARD18,LCE1F,CHN1,SORCS3,MYLK3,ACSBG1,FMN1,MBNL1,PAFAH1B1,ATF6,EFEMP1,TLL1,DCAF1,ITGB8,TPM1,NF2,RBFOX1,CORIN,CTNNA1,PPP1R9A,ANKRD11,BIRC6,AKAP9,KLF15,PPARA,MEIS2,NFIB,MRTFB,PRTG,SYNJ1,NR5A2,ADAMTS3,TIAM1,GRM1,FOXJ3,KAZN,GABRG1,ENAH,PAK5,SF3B6,PCDH11Y,PLA2R1,SEMA3C,NAV3,SLC24A4,TMEM108,AGO2,ALPK2,DNAH11,JARID2,SCN2A,CPE,IL34,ANK2,TANC1,ADGRV1,MELK,BCAS3,RYR2,SYNE2,BBS2,WNT9B,SLC9C1,RANBP3L,OR4F6,SEMA6D,ANKS6,SMARCA4,CDH11,LDB3,FABP7,PARD3,MAPKAP1,BLK,TNR,COL22A1,GRM8,CXADR,DOCK4,MBD5,ATRX,XIRP2,ELAVL4,ABL1,HDAC4,OXR1,SLC1A1,PRKAA1,SDC2,GAS2,KCNH1,LRFN5,CRTAC1,DROSHA,TTLL5,L3MBTL3,APLF,DNAH5,RAP1A,GLIS1,MORC1,CAMK4,FGF10,ZC3HAV1,GRID2,GALC,TGM1,LATS2,NRG1,INO80D,ASPM,AP3B1,RASGRF1,ATP11C,SYNE1,ZBTB16,MUSK,ZNF675,SH3GL3,SETDB2,PRKCE,SLCO3A1,MED15,SLMAP,NXN,ESRRG,DGKB,USP33,FBN2,CD44,PTPRO,EGF,ALPK3,ABCC9,P2RX6,TRIO,PDE3A,EXT1,LNPEP,LIMD1,SPRED2,ADAMTS2,RPS6KA3,CTNND2,NHS,ATP8A2,PTPN2,LHFPL3,PLXNA2,OR4F15,ATXN3,ST8SIA6,HTR2C,SLC2A3,ARHGEF7,CD96,ATP8A1,AMBRA1,LTBP1,KDM7A,PKHD1L1,OPRM1,ABCC4,HTR2A,FANCA,CYP4A11,DAZL,CNNM4,KREMEN1,STAC,SEMA3E,MARK2,ALPL,FHL2,PUM1,TMOD2,HERC1,MSH2,IGF2BP3,GNAL,CDIN1,EPHA6,ANKRD17,APBA2,LINGO2,ATL1,SLC2A13,LUC7L,CDKN2C,EPN2,KCND2,EVC,KNDC1,NOS2,BICRAL,AFG3L2,MOSMO,GFRA2,MNAT1,RBBP8,SGCZ,ADAM12,MYLK2,ANK3,EMILIN2,XYLT1,HMGA2,MYOM2,BCL11B,DOCK5,F5,ECE1,CREM,MBP,AK8,TRPS1,PLCE1,TGFA,IL17RA,ANKFN1,CRIM1,FUT9,VAV1,MYT1L,FBXO32,ZNF160,TJP1,LDLRAD4,NPHP4,CNTN1,HLA-B,IQSEC1,MTHFD1L,SNX3,CACNA1I,PDLIM5,BRCA2,DISC1,DNER,WDPCP,NRK,SLC10A7,SEMA3A,HSF2BP,CFAP97,ADCY10,STRN,OR9Q1,BMP2,RC3H2,UNC5D,TRAK1,PSG9,PTCD2,SCN11A,MSR1,NCAM2,GFI1B,BMP2K,RNF38,SEMA3D,PDE6C,POLR3A,RELN,ARHGAP42,HMGB1,GNAQ,FGF9,NFATC2,TDRD7,UST,CPAMD8,RTTN,MDM1,SLC23A2,MYOM1,TRAF3,ESRP1,UNC13B,TTC21B,ETS2,DOCK1,B4GALT6,TSPAN2,RAP1GAP,PLS1,SRGAP2,NIN,DRAXIN,ATF1,SLAMF1,SMARCA2,ETS1,GLI3,CGAS,MEGF11,SMARCC1,AFF3,SLC9A4,GABRR2,SMOC2,PCP4,CASP5,GRIK2,IDE,MYEF2,RERE,MAP2,DAW1,MYL1,LAMC1,NEK10,RRBP1,FARP1,TDRD5,ATF2,HIRA,CYLD,UMODL1,BBS4,LAMC3,PSG6,COL5A1,CFTR,NELL1,DOP1B,UBASH3A,KITLG,CAMTA1,DCC,MYT1,CHRM5,

			RCAN1,GTF2I,RORB,TADA2A,DAB1,MED27,SELENON,RB1CC1,MYO3A,PRKN,MTMR2,SH3PXD2A,TBX20,DLGAP2,DACH1,PCDH15,ZNF541,DPF3,LGI2,SMPX,NGEF,GRIN2A,ARID5B,ATXN1,CDH23,PRKCH,TG,IL6R,ALS2,RACGAP1,OR51E1,ACO1,HEMGN,DMC1,LCE3B,TOX,SHISA9,SLC4A4,PTPRB,CATSPERG,PDE6A,TBATA,SCN10A,SHANK2,VAV3,KCND3,MESD,ITSN2,SOX30,SYBU,KIR2DL4,NPAS2,ADGRG6,YIPF6,SEC24D,ROCK1,LYN,VCAM1,CTSB,EIF2B3,LRIG1,SLC15A2,DTX1,TENM2,OVOL2,PIWIL3,NTN1,MMP16,ZFHX3,FANCL,DPYSL5,ARID1B,HOXC13,CRACR2A,CASZ1,INSR,OR7A17,YTHDF3,TFF1,HECTD1,GRID1,SHROOM3,XRCC4,COLQ,HDAC11,NMU,PBX3,SNAI2,ASH1L,HOXC4,SIAH2,TANC2,ABCA4,UFD1,RXRG,SP3,GABRG3,MBTPS2,TRIM58,TIAL1,PLPP4,NREP,ZDHHC17,NSD2,CERS3,SLC22A14,CD9,CARD10,KRT6B,JCAD,TWIST2,OR4K2,SAMHD1,IFT81,ENPP1,UTRN,RASGRP1,IGSF11,DZANK1,NDRG2,BMP5,KCNC1,CSF1,GHRH,HDGFL3,BCL2L1,SPATA48,KRT25,CTDP1,HCN1,PRKG1,LAMA3,ASB4,GRIN2B,INO80,CNMD,DHRS3,SMAD5,CELF4,SYNJ2,ABCG1,OR4C46,FOXN3,VSTM4,SLC40A1,MYCL,TNN,CABYR,PSAP,MICALL2,MED1,KDM6A,ATRN,IL33,AJAP1,ROR2,ZNF521,KL,BANK1,CSDE1,FAT1,OTOG,LMX1A,TMEM178A,IL10,ACTR2,OR1L6,CLSTN2,TTC39C,PTH,SOSTDC1,PRKAA2,CSF2RB,NDC80,SOHLH1,PACRG,ABHD2,MAP6,VASP,PLA2G4A,ETV6,TACC2,IQGAP1,TEAD1,ANP32B,YBX3,AIMP1,NRXN1,PCID2,HIPK1,DGKK,FRY,CIBAR1,PBLD,CACYBP,CADM1,SSPN,LMX1B,ANLN,TWIST1,AKT3,ALKAL2,JAK2,ADAM28,VSX1,FSTL1,ISX,BPNT1,SVEP1,HCRTR1,RBM19,PTGS1,ZNF287,CELSR2,ZNF449,PRSS2,FH,CREBBP,ARL11,GORAB,PCNA,SIAH3,TRPV5,UFL1,ADAMTS5,NFKBIA,PRKCB,OR2T3,NTM,ABCC8,ANXA4,SMTN,ALX4,USH1C,SMPD4,NEDD9,ITGA6,ATP2B1,GAP43,ASS1,GRIP1,CTNNBL1,ADCY9,EML1,PPP1R17,MAST2,OTOP1,CIDEA,BBS9,EXT2,KRT6A,STOX2,AGO1,MEOX2,SLC6A1,GRXCR1,STAT1,BRMS1L,MAP2K6,CMTM7,DGKG,SHROOM2,SLC6A11,MTPN,ABI1,MYO18B,ARMC6,CEMIP,POU6F2,IMPACT,CCBE1,PARK7,ADAMTS18,ITGA4,BCAP29,FBXL17,POU1F1,ADCYAP1R1,MTF2,CSMD1,NCAPG2,NDC1,OR6C75,FOXP2,ASB2,MYOCD,HMCN1,CEP120,MYH13,DHTKD1,CYFIP2,ACACA,KRT85,ST8SIA4,OR13C9,MEF2C,ADGRB1,RXRA,WNT7A,RBPMS2,NDFIP1,MAP3K4,WASF3,S100B,PRDM13,FOXO6,PKN2,OR10H2,PDE2A,FBXW8,SDCBP,NECTIN1,DSG1,WWOX,PASK,NCK1,FLVCR1,FGR,DRC7,PPP2R3A,TOP1,SPRR2D,RNF8,LCE3D,EPHA4,PPIP5K2,GABRA5,MECOM,DNMT3L,NTRK2,IL1RAPL1,FNDC3A,RSPH1,NUMB,LHX9,ADAMTS9,WNT2B,TNNI1,OCLN,CD101,SHISA6,IL17RD,FBXO31,AKAP11,GRIK1,PRKAB1,IREB2,HS6ST1,PTK2,MARK4,CDH5,APOL2,NFKBID,CLDN18,DIAPH1,FEZ2,LAMB1,CYFIP1,UBE3A,HOATZ,PCDH8,SEMA4D,JAM2,ZBTB20,FAT4,AP2B1,RUNX1,AKR1B1,KIRREL1,WNT5B,AMFR,SLC26A2,PTGFR,OR4L1,SANBR,ASAP1,NOS1AP,MTTP,DPY19L2,SORBS2,PDCL3,CCDC88A,GPR55,NSUN2,ADAMTS16,SPAG6,SLC5A1,OR11G2,TNFSF11,FYN,KDM5A,NLRP14,PPM1F,ADGRL2,UNC45B,ARL13B,HYDIN,SCN8A,HDAC2,SNTB1,GON4L,TBX15,TMEM63C,NCS1,COL18A1,LHFPL2,ATP5PF,ALB,DOK5,UGP2,CRTAM,COL19A1,APELA,MDGA2,TRPM3,SLC39A8,ROR1,OPA3,FUT8,TET1,ARNT2,ASB3,HECW2,POTEJ,CDH2,CNTN5,ITGA8,FBXL20,NTN4,XRN2,PHLPP1,GPR137B,EPHB1,EYS,RP1L1,GRM5,RAI14,RPS6KA5,PTPRG,NRP1,SDK1,PRKCA,ATPSCKMT,FAIM,ITGA1,RC3H1,NRIP1,CHODL,POR,MCC,BCR,TUT4,NRXN3,KIF16B,SNRK,C14ORF39,FBLN1,STK36,PAQR5,MB,RAG1,B9D1,DGCR2,RRAS2,BMPER,CUX1,MACROH2A1,MITF,EPHB2,IGSF3,SGCG,CD38,EYA4,MYO5B,MET,CDH17,SPECC1,PPFIA2,CDH13,SERPINB2,CACNG3,ATG5,NRAP,MAGI2,KIAA1217,VMP1,UNK,ADAM29,MLIP,FLRT2,MYB,KALRN,SLC1A2,GNAS,LAMA1,MFHAS1,GREB1L,SERPINB7,CA10,TIAM2,IGSF21,BMP7,CHIT1,ASTN2,DLG5,GABRA2,KIRREL3,BTD,OR2T2,BPTF,AK3,TMEM25,NUDT21,DDX6,ADGRF5,OR4N2,PDGFC,ABL2,TRAPPC6B,RFX2,NECAB1,EYA1,FHOD3,SLIT2,TMPRSS3,EXOC4,FAM126A,CCDC141,PLCL1,ERBB4,IL20RB,TRHDE,SYNDIG1,ROBO1,PBX1,PRKCQ,ANTXR1,NDRG1,MYH15,SIPA1L3,TRDN,NLGN1,CTTNBP2,SHLD2,NOS1,SLC6A3,ASIC2,EFNA5,TCF12,AR

			HGEF11,VCAN,RAB27A,EHMT1,SLIT3,DTNA,ESR1,DNAH9,NTNG1,KDM4B,LOXL2,CACNA2D1,NYAP2,PRLR,HTT,FOXB1,RAD51B,CAMK1D,PIK3R3,MACROD2,CFAP44,OPCML,CATSPERE,HLA-F,FER,EYA2,KATNIP,CCR2,RPGRIP1,STARD13,A2M,EPS8,SEMA4B,HRH1,PHC2,ROCK2,PRDM1,RORA,STMP1,IL16,ATAT1,DMRT1,PPP1CB,CATSPER2,HSPG2,PTPRQ,CSMD3,NCOA6,HSD17B2,COL4A3,RGS7,HOOK3,PCSK2,FSTL4,BARD1,CLCN5,STK3,ZNF423,ZNF568,HNRNPU,VTI1A,APCDD1,IGF1R,GLI2,THRB,LSAMP,AKAP13,MORC3,SEPTIN6,DNM1L
GO:0065007	biological regulation	5.290972093177678e-27	NOTCH2,BCAR3,BRINP3,MTOR,UNC80,CNTN4,CACNA2D3,SPOCK1,NSG1,SGCD,WWC1,ABCA13,GARNL3,LRP12,PTPRD,SLC24A2,TRAPPC9,BNC2,PVT1,LRRC4C,KCNH5,MICU2,ANKS1B,SMOC1,MYO9A,ULK2,NLK,LONP2,UNC13C,FTO,KSR1,MGA,RFX7,ZNF236,PLCB1,ZNF536,MX2,TMPRSS2,TAFA5,SVIL,CLTCL1,ZFPM2,PIEZO2,TENM4,L3MBTL4,DLC1,TNRC6B,DPP10,ZDHHC21,PTPRA,ITPR2,RIPOR2,PDE4D,RDX,RP1,STXBP1,ERC1,RALA,IL1RAPL2,BCL2,ODAD2,KCNMA1,PRDM16,ALDH1A2,ARHGAP26,FBN1,LRFN2,F13A1,GPHN,CDH8,CHRNA7,DCDC1,GPR158,ROBO2,RIMS1,PIK3C3,EPC2,SPIRE1,TENM3,GABRB3,ZEB1,AKR1C3,SDCCAG8,RARB,FGD4,SPRED1,NAV2,ENPEP,MYO1E,PLPPR1,USH2A,MINAR1,CDC42EP3,RIMS2,ALK,AUTS2,ADGRE1,FOXJ2,CDYL2,CARMIL1,MCTP1,PJA2,BABAM2,PAPPA2,GLIS3,FANK1,ERBIN,RHPN2,RIN2,PARVB,ANO6,CACNG2,DLGAP1,NEGR1,ZNF880,MLLT3,EGLN3,GPC6,SUSD4,CNTNAP2,MAP4,MAP3K9,SPON1,APC,ZMYM4,ZNF595,HHLA2,TSHZ3,RBFOX3,PLPPR5,DSCAM,RTN1,TCF4,CRKL,ILDR2,SOX5,SETD2,ERG,ARHGAP24,ZNF573,TNIK,SLC4A10,PTPRJ,KDM4C,NEK4,DOCK10,TSHZ2,EGFR,ZNF280B,RFX3,DENND1A,USP14,ANGPT1,CDK12,BACH1,MACF1,CTNNA3,PRKACB,NEK7,RGS3,NCOR1,RNF220,DOCK2,ZNF407,NEDD4,MAML2,MTRF1,SND1,SCAI,CRB1,NSMCE2,BTBD9,BCL11A,SOX6,FAM83F,TMEM182,SGMS1,GRIK3,CHSY1,FLI1,RPRD1A,CDH4,ATP2B2,NTRK3,LARGE1,RXFP1,C5,PDE1C,ZFAND6,CYP2C9,PHACTR1,DKK2,FLT1,DNAJC13,ZNF648,RFC3,RABEP1,ZNF382,TASP1,THRAP3,MAPKBP1,AOAH,GABRB1,PSMA8,DGKI,INVS,C12ORF4,EDAR,GRIA1,CRACD,CAST,TTC39B,NUP214,NEO1,CNTN6,SLC39A12,CABLES1,SLC8A3,MALRD1,TOM1L2,NELL2,PRKD1,TBC1D19,TPTE2,PAK1,GMDS,EPHA7,CTNNAL1,NCOA7,KHDRBS2,CHRM3,GRAMD1B,RALGPS1,SPEN,RAPGEF2,PELI2,LRP2,ADGRB3,RUNX2,ARSB,FGF12,GABRA6,CPS1,TAOK3,ONECUT1,LDLRAD3,CPEB4,TMEM38B,PRICKLE2,SLC24A3,UBE2L3,LDB2,TAFA4,PPP2R2B,BTBD11,PUM3,PTPRN2,SYN2,CCL28,SMYD3,PATJ,HERC2,GRM7,SEPTIN9,RETREG1,RPTOR,TMEM117,GHR,EPB41L3,THADA,COL4A2,SSBP3,RALGAPA1,CELF2,RAPGEF5,TBCD,NEDD4L,PPP1R12B,TRPM1,ADAM10,HDAC9,ZHX3,ATF7IP,IL1R1,APBB2,PHACTR2,APP,ABCB5,RPS6KA2,SAMSN1,CACNA1C,KDM1B,CACNB2,KLHL13,MTUS1,DCLK1,STAU2,GABRG2,DOCK8,TMC1,MAPRE2,ZNF600,USP18,SEMA5A,SYT1,VCL,ARHGAP44,NTF3,ACER2,PARP15,NDUFAF2,CD2AP,ZNF723,AURKA,PARN,CFDP1,ST18,PYGO1,SLC8A1,HERPUD2,SSBP2,PTPRR,SRGAP2C,ANKRD31,FIG4,DUX4,TAFA2,ABCG8,SERPINA6,PLGRKT,SRGAP2B,KANK1,KCNE4,MAP4K4,HIVEP2,ABCD2,BMPR1B,FMN2,PCSK6,AKAP6,HOMER2,ZNF717,CTNNA2,ARNT,RAB8B,PAK3,RFTN1,PDE1A,ZNF257,DIP2B,KCNK10,RANBP2,LARP1,ITPKB,TRPC5,RGS20,PDE10A,UBE2E2,RAP1GDS1,HHAT,RNLS,CLIC6,CHST8,KICS2,ERC2,DNM3,NBN,CUBN,SCP2,SYN3,IFT57,INTS7,PRKCZ,SPOP,BTLA,GRB10,RYR3,TAF15,DIP2A,MSH6,MCPH1,ARHGAP32,RAB27B,CNST,RGS9,HECW1,DEFA3,MBNL2,ABCA5,PHF19,MRTFA,TAF4B,COBL,SENP6,DUSP22,EBF2,YAP1,NFIA,WDR70,PPM1L,RIPK4,ZKSCAN5,SHC4,BRINP1,MAPK1,MGAT5,CADPS2,KCNJ1,HRH2,RABGAP1L,ITIH5,TRPC7,ADAM22,USP25,KMT2E,ALCAM,PLG,PCGF5,PDGFD,SYT10,ZNRF3,PPP1R1C,ITGBL1,ARHGEF17,NRG3,UBE2O,SFMBT2,MIR663AHG,ANKFY1,NCAM1,GFRA1,SYCP1,NIPBL,SLC16A1,SPIDR,EWSR1,GABPA,FAT3,MICU1,ZNF735,CORO2B,CARD18,CHD6,STK38,PTPN13,TBC1D22A,CHN1,HRH4,SORCS3,MYLK3,KANSL1,GLP2R,LIMCH1,FMN1,ECT2L,MBNL1,PAFAH1B1,ATF6,EFEMP1,ZNF684,TM7SF3,DCAF1,ITGB8,STON2,

			VPS13D,CCNG2,TLK1,TPM1,NF2,LRRC38,CNKSR2,GRIK4,RBFOX1,HIVEP1,CORIN,CTNNA1,PPP1R9A,MOB3B,BIRC6,AKAP9,KLF15,RASGRF2,PPARA,MEIS2,SNX30,NFIB,KCNS3,ERMP1,MRTFB,PPP6R3,PRTG,RGL1,SYNJ1,NR5A2,ADAMTS3,TIAM1,ARAP2,GRM1,FOXJ3,PTPRK,ARHGEF12,GABRG1,PAK5,TRERF1,PCDH11Y,PPP2R5E,PLA2R1,EIF3D,SEMA3C,DAPK1,NAV3,SLC24A4,SEC14L1,VPS13C,TMEM108,AGO2,STK32B,PHC3,MAGI1,ALPK2,DNAH11,JARID2,SCN2A,RIC8B,TBC1D9,RAB22A,SORCS1,DNAJC15,GATAD2B,CPE,EVC2,DYSF,IL34,ANK2,BRWD1,TANC1,ADGRV1,ZNF846,MELK,BCAS3,RYR2,SYNE2,BBS2,WNT9B,ZNF606,SLC9C1,CLPX,RANBP3L,OR4F6,NKAIN3,NKG7,SEMA6D,NBEA,DUSP16,SMARCA4,CDH11,USP8,FABP7,PARD3,MAPKAP1,TNRC6C,PIAS1,TBC1D5,SPG21,BLK,EBF1,TNR,GRM8,DST,CXADR,DOCK4,MBD5,ATRX,NUAK1,PTPRT,ELAVL4,ABL1,MXI1,PTPN12,HDAC4,OXR1,SLC1A1,PRKAA1,SDC2,GAS2,SLC12A8,KCNH1,ITGB3BP,MRPS27,LRFN5,CREG1,DROSHA,APBB1IP,ANO4,L3MBTL3,DMXL2,EIPR1,APLF,NFAT5,MAST4,GUCY1A2,NBAS,PSMF1,SLFN11,RAP1A,NKAIN2,GLIS1,MORC1,MYO10,GPC5,TOX3,CAMK4,BAZ2A,INPP5A,CPSF3,FGF10,ZC3HAV1,GRID2,TGM1,PEAK1,LATS2,NRG1,INO80D,GSG1L,CLIP1,ASPM,AP3B1,DENND2B,RASGRF1,PAH,ATP11C,ZNF438,ABCB7,ZBTB16,MUSK,KIR3DL2,ZNF675,GNG7,SMARCAD1,SH3GL3,SETDB2,PRKCE,FOXK2,SLCO3A1,ASAP2,MED15,SLMAP,NXN,WNK2,ESRRG,ZNF718,DGKB,USP33,DENND4C,FBN2,CD44,RGS12,PTPRO,EGF,PRRC1,ABCC9,P2RX6,TRIO,PDE3A,EXT1,STXBP6,NSMAF,LNPEP,LIMD1,PEX14,SPRED2,RPS6KA3,CTNND2,ATP8A2,SCG5,MTMR3,PTPN2,TRIM5,PLXNA2,MCF2L,OR4F15,ATXN3,RFC1,HTR2C,RIC3,CLEC16A,ARHGEF7,CD96,ALG10B,ATP8A1,AMBRA1,LTBP1,STK38L,ZFYVE9,KDM7A,OPRM1,PRMT8,HTR2A,PLCXD3,FANCM,FANCA,CYBRD1,CYP4A11,DAZL,INPP4B,GTF2F2,PPP2R2C,CNNM4,KREMEN1,STAC,SEMA3E,TAF3,RPRD1B,MARK2,GCSAML,TMEM67,EBF3,ALPL,ZNF33B,C10ORF90,FHL2,ABHD17C,ADGRA3,CNIH3,PUM1,TMOD2,HERC1,MSH2,IGF2BP3,GNAL,CDIN1,EPHA6,ANKRD17,APBA2,MAIP1,LINGO2,ZNF397,SH3KBP1,SLC2A13,LUC7L,RELL1,HIPK3,CDKN2C,EPN2,KCND2,EVC,GRK3,KNDC1,SPSB4,CLSPN,NOS2,BICRAL,AFG3L2,STK10,MOSMO,GFRA2,MNAT1,TMEM116,RBBP8,MDFIC,SGCZ,TMTC2,ADAM12,MYLK2,ANK3,EMILIN2,HMGA2,CCND3,BCL11B,VPS41,DOCK5,F5,ECE1,ZIM3,STK32A,CREM,LYPLA1,MBP,LINC01151,TRPS1,TRAPPC11,PLCE1,TGFA,IL17RA,ANKFN1,HIP1,CRIM1,FUT9,PRR5L,GSR,ATP6V1E1,UTP4,CAPN5,VAV1,RUFY2,MYT1L,FBXO32,ZNF160,TJP1,LDLRAD4,NPHP4,EGFLAM,PACSIN2,CNTN1,HLA-B,IQSEC1,HSF5,SNX3,CACNA1I,NAA35,ZNF367,PDLIM5,KCNJ15,BRCA2,DISC1,ZBTB2,DNER,BLM,ASB7,WDPCP,NRK,SLC10A7,SEMA3A,MAGI3,INTS8,LIN54,ADCY10,PSG8,STRN,OR9Q1,ZNF121,STX12,PHACTR3,BMP2,RC3H2,UNC5D,ATP9A,TRAK1,PSG9,CDC42BPB,SOGA1,EVI5,PTCD2,SCN11A,MSR1,VRK1,GNAI1,RALGAPA2,SGSM1,ZC3H14,GFI1B,TBC1D4,RANBP9,RESF1,MYRIP,TTR,RIN3,BMP2K,TMEM161A,SEMA3D,ASXL3,NETO2,PDE6C,CABIN1,POLR3A,LEMD3,RELN,ARHGAP42,HMGB1,GNAQ,FGF9,NFATC2,TDRD7,SH3BP5,UST,CPAMD8,MDM1,SLC23A2,POLR2M,ZNF106,MYOM1,ZNF567,TRAF3,ZNF462,ANKRD26,ESRP1,UNC13B,TTC21B,ETS2,UBAP2L,GEMIN5,ZNF875,DSTYK,UIMC1,DOCK1,LRRFIP1,RAP1GAP,PLS1,SRGAP2,IKZF2,SEC23B,SLC39A6,NIN,DRAXIN,ATF1,CCDC186,SLAMF1,KCNH8,SMARCA2,ETS1,FAM83B,GLI3,CGAS,SMARCC1,SNX6,AFF3,SLC9A4,GABRR2,SMOC2,PCP4,CNKSR3,CASP5,VENTX,GRIK2,IDE,WDR12,MCTP2,KIF15,PRDM10,CUL1,MYEF2,ZFYVE26,ZNF431,RERE,PSD3,MAP2,BTAF1,GAREM1,PEX6,LAMC1,ZNF618,NEK10,FARP1,MOB1B,ATF2,HIRA,CYLD,UMODL1,BBS4,MAPK8IP1,MX1,PSG6,HIVEP3,COL5A1,GABBR2,PSIP1,ITGA9,CFTR,KPNA1,NELL1,ME2,TBC1D13,UBASH3A,RGMB,NEU3,MRPL13,KITLG,ZZEF1,DNAJC7,ATP10B,CAMTA1,UBR1,DCC,MYT1,CHRM5,MAP4K3,YLPM1,SLC30A10,RCAN1,GTF2I,RORB,TADA2A,DAB1,MED27,ZNF208,SELENON,RB1CC1,NMD3,AKAP10,PTPRE,PRKN,MTMR2,ZNF608,SH3PXD2A,TBX20,SP110,DLGAP2,AFAP1,MAPK10,DACH1,PCDH15,ZN

			F541,DPF3,NGEF,HEPHL1,GRIN2A,ARID5B,ZBED9,JPH1,TXNRD2,ATXN1,WSB1,CDH23,LALBA,PRKCH,PKP1,HUNK,SLC12A1,FRMD4A,TG,IL6R,FRMPD4,ALS2,RACGAP1,NLRC5,ZNF627,OR51E1,ACO1,TFDP1,DHRS11,CNOT6L,MKNK1,HEMGN,KANK4,DOCK9,SNX25,HULC,FBLN5,KCNQ3,TOX,SHISA9,SLC4A4,PTPRB,ZFP90,PDE6A,COPS8,ZNF124,SCN10A,SHANK2,ST8SIA1,USP7,VAV3,PSMA1,ENPP3,PLAGL1,KCND3,HAAO,MESD,ITSN2,SOX30,MOK,KIR2DL4,ARHGEF28,RALB,NPAS2,ADGRG6,DENND2C,PPA2,ROCK1,LYN,VCAM1,SEL1L,ARHGAP28,ARHGAP31,ZNF780B,CTSB,EIF2B3,TTC37,SLC44A2,SUMO3,SLC15A2,ZNF169,PLEKHB2,KIF11,DTX1,BZW1,TENM2,OVOL2,PIWIL3,ZBTB33,ADA2,NTN1,PLCB4,MMP16,ZFHX3,FANCL,DPYSL5,ZNF44,RRAGD,BANP,SUPT16H,ARID1B,HOXC13,CRACR2A,RNF152,BAZ1A,CASZ1,OTUD7A,INSR,CUL5,OR7A17,BMF,YTHDF3,TFF1,DEDD2,NEK6,HECTD1,GRID1,SHROOM3,XRCC4,COLQ,NMU,DDHD1,PBX3,SUMO2,HS1BP3,ZNF292,ARFGEF1,PDE4DIP,GAST,POGK,SNAI2,ASH1L,IGHV3-74,HOXC4,BID,SIAH2,RPH3A,TANC2,ABCA4,TRABD2B,UFD1,RXRG,SP3,DRAM1,ERN2,GABRG3,ZNF879,MBTPS2,FLNB,TRIM58,TIAL1,TOM1,ELF2,PLPP4,NREP,ZDHHC17,NSD2,FYCO1,SH3GLB1,CD9,CARD10,XKR5,RALGPS2,JCAD,TWIST2,OR4K2,CTIF,SAMHD1,IFT81,ENPP1,UTRN,RASGRP1,IGSF11,SNX9,TP53I11,TMEM225,ANAPC1,NDRG2,CSNK2A1,BMP5,KCNC1,CSF1,GHRH,HDGFL3,BCL2L1,SERPINB9,SCAF4,MIR3142HG,CTDP1,HCN1,PRKG1,LAMA3,ASB4,GRIN2B,GRB14,INO80,FANCB,GPR156,IGHV2-70D,CLNS1A,CNMD,DHRS3,SMAD5,CELF4,TCERG1,ABCG1,OR4C46,FOXN3,KCNK5,DCUN1D4,VSTM4,SLC40A1,PRAME,MYCL,TNN,CIDEC,PSAP,LPGAT1,MICALL2,MED1,CDC14B,PCNT,KDM6A,ATRN,IL33,AJAP1,GPRC5C,ROR2,CFH,PPP2R2A,ZNF521,KL,RASGEF1C,BANK1,CSDE1,LMX1A,TMEM178A,IL10,ACTR2,OR1L6,SFPQ,SCML2,PRAMEF25,RIOK1,CLSTN2,PTH,SOSTDC1,PRKAA2,CSF2RB,DIRAS2,SKA1,NDC80,SOHLH1,LARP6,PACRG,PHF20L1,ABHD2,ITPRIP,VSTM2A,MAP6,VASP,PLA2G4A,ETV6,PALMD,RAB12,IQGAP1,RPS12,CAMLG,COX7A2L,ZBTB7C,TEAD1,MORC2,SREBF2,ANP32B,YBX3,AIMP1,THNSL2,FYB2,NRXN1,PCID2,HIPK1,ZNF234,CISD1,ZNF518A,DGKK,FRY,SNAP91,CD70,CIBAR1,PBLD,FICD,CADM1,CENPE,PEG10,LMX1B,NET1,SIPA1L2,NGDN,ELOC,ANLN,TWIST1,AKT3,ALKAL2,JAK2,SLC1A7,VSX1,RPF2,FSTL1,ZBTB38,ISX,SVEP1,MADD,HCRTR1,RBM19,PTGS1,PATL1,ZNF287,CELSR2,ZNF449,PRSS2,FH,CREBBP,MELTF,TNKS,GORAB,PCNA,SIAH3,TRPV5,UFL1,ADAMTS5,NFKBIA,PRKCB,OR2T3,ABCC8,ANXA4,MT1HL1,CACNA1E,ZC3H15,ANP32A,RFC2,ZNF354C,ALX4,RTRAF,USH1C,BRD4,ZBTB21,SERBP1,NEDD9,OLFM4,NRBP1,ITGA6,ATP2B1,GAP43,IARS2,ASS1,MTCL1,GRIP1,IGHV1OR15-9,CTNNBL1,AGAP9,ADGRE3,SAR1A,ADCY9,PPP1R17,CNIH1,MAST2,HPSE2,BTG3,ZNF528,ERLIN2,ZNF611,UBAP2,XKR6,OTOP1,CIDEA,ARFGEF3,ZBTB49,EXT2,EXOC1,HEPACAM,KRT6A,STOX2,AGO1,PDP2,MEOX2,SLC6A1,GID8,ELL2,STAT1,BRMS1L,NDFIP2,NR2C1,MAP2K6,CMTM7,DGKG,SLC6A11,KCNJ18,GATAD1,MTPN,ABI1,CEMIP,PRAMEF2,POU6F2,IMPACT,CCBE1,PARK7,ADAMTS18,MAPK8,ITGA4,OAZ2,PPME1,MED12L,ZSCAN30,FBXL17,UBL7,POU1F1,UBE2J2,ADCYAP1R1,PLA2G12B,MTF2,CSMD1,NCAPG2,TM9SF4,RAPGEF4,OR6C75,FOXP2,ASB2,MYOCD,CEP120,ATP13A3,ZSCAN5C,CYFIP2,HNRNPM,ACACA,ASCC2,EFHB,OR13C9,ARID3B,MEF2C,ZNF613,ADGRB1,RXRA,WNT7A,RBPMS2,MAP3K5,NDFIP1,MAP3K4,TRIM43B,WASF3,S100B,SERPINI2,PRDM13,TRIM43,FOXO6,ERI1,ZNF112,ATP6V1C2,C16ORF72,MAGEL2,PKN2,RAD51AP1,OR10H2,PDE2A,RAB38,LRRC2,DBF4B,FBXW8,SDCBP,NECTIN1,DSG1,JPT2,SPPL2B,NSMCE1,ZNF813,WWOX,ZBTB25,PASK,MLLT1,NCK1,FLVCR1,SCAF8,FGR,CWC22,CDCA8,PPP2R3A,DNMBP,TRIM23,ATP6V1B2,CXCL2,TOP1,SNAP29,MLLT10,C2,IFNAR1,RNF8,GNG12,EPHA4,CYTH4,INTS13,GABRA5,MECOM,DNMT3L,NTRK2,IL1RAPL1,ACSM2A,NUMB,LHX9,ADAMTS9,WNT2B,COLEC12,FRRS1,ZBTB10,TNNI1,OCLN,POSTN,C

			REB5,MIR548H4,CD101,SHISA6,MEGF10,IL17RD,FBXO31,EXTL3,AKAP11,TRPM7,GRIK1,PRKAB1,DTHD1,IREB2,MVB12B,PTK2,MARK4,CDH5,CD5L,RCAN2,ANKRD6,SCGN,NFKBID,ARHGAP12,CLDN18,ASCL3,MPP7,DIAPH1,FEZ2,INIP,LAMB1,MIR17HG,APIP,CYFIP1,UBE3A,PCDH8,SEMA4D,JAM2,SERPINB10,PITPNC1,FRMD6,MC2R,ZBTB20,FAT4,IMPA2,ZNF66,AP2B1,RUNX1,AKR1B1,C9,KIRREL1,WNT5B,RASGEF1B,AMFR,SAXO1,SCARA5,NENF,SH2D1B,POMT2,PTGFR,ZNF845,OR4L1,ASAP1,SAMD13,ICA1,PLCZ1,EDIL3,NOS1AP,MTTP,SLC9A5,FCRLA,DIDO1,TPTE,SORBS2,PDCL3,SRP9,CNKSR1,CCDC88A,GPR55,NSUN2,CHCHD2,HKDC1,ADAMTS16,ACOXL,CDC45,OR11G2,BICD1,TNFSF11,FYN,BUB1,KDM5A,PCBP3,MYL12B,ZNF705G,PPM1F,ADGRL2,ARL13B,SDE2,RBMS3,UHRF2,SCN8A,HDAC2,AVEN,SLF1,GON4L,TBX15,SH2D3C,PSME3IP1,DOCK3,TRNAU1AP,NCS1,COL18A1,LHFPL2,ALB,DOK5,ATP9B,NALCN,ZFYVE28,MAPK9,PABPC1,CRTAM,APELA,SLC39A8,ROR1,OPA3,FUT8,TET1,ARNT2,ASB3,HECW2,POTEJ,CDH2,ITGA8,FBXL20,NTN4,RAD9A,XRN2,PHLPP1,GPR137B,EPHB1,RP1L1,GRM5,ADCK1,RAI14,SPOPL,ZNF705D,RPS6KA5,SPTB,TBC1D1,LRRC69,PTPRG,PID1,NRP1,MIDEAS,FCHSD2,SDK1,PRKCA,IFT46,ATPSCKMT,FAIM,SAMD12,FHIT,ITGA1,ZNF615,KLF12,RNF138,RC3H1,NRIP1,CHODL,POR,ZNF850,ZNF235,MCC,ZNF738,SUPT3H,BCR,TUT4,NRXN3,ELMO1,RGS6,RERG,ZNF215,TCERG1L,KIF16B,PRIM2,SNRK,C14ORF39,ARFGAP3,TM9SF2,ELP2,FBLN1,STK36,NSG2,MB,RAG1,KCNJ6,B9D1,RRAS2,GNA14,ZNF678,BMPER,PRDM15,CUX1,DPP6,SRGAP3,ZNF420,MACROH2A1,MITF,EPHB2,TSPAN13,TOGARAM1,CSNK1G1,SACS,BCL2L13,CD38,EYA4,DPH6,MYO5B,RGPD4,CDK14,AKAIN1,MET,SPPL3,CDH17,ZNF705B,ATP6V0D2,PPFIA2,CDH13,MED13L,STXBP4,SERPINB2,CACNG3,ATG5,MAGI2,PRDM11,VMP1,UNK,FAM171A1,MLIP,FLRT2,MYB,KALRN,ZNF704,SLC1A2,GNAS,LAMA1,MFHAS1,SERPINB7,CPQ,ATRNL1,TIAM2,DHX29,BMP7,TTC28,ASTN2,DLG5,TNFAIP8,ZMYND8,GAPVD1,GABRA2,RNF217,KCTD1,OR2T2,ZNF74,BPTF,BTBD10,AK3,ZMYND11,TMEM25,NUDT21,GRM3,KMT2C,DDX6,ADGRF5,OR4N2,PDGFC,WDR41,PLIN2,PPP1R13B,FOCAD,ABL2,MMP26,TRAPPC6B,BACE2,RFX2,PARPBP,NECAB1,PKNOX2,EYA1,FHOD3,PRPF18,SLIT2,TMPRSS3,EXOC4,CNOT7,KCNIP4,ESCO1,KCTD8,PLCL1,ERBB4,IL20RB,SERPINB11,FAM3B,GSAP,TRHDE,SYNDIG1,ROBO1,SAMD4A,PBX1,IRAG1,NPAS3,NUF2,PRKCQ,RGPD2,ANTXR1,NDRG1,SORCS2,SIPA1L3,TRDN,MGMT,ZNF679,NLGN1,CTTNBP2,SHLD2,NOS1,SLC6A3,PRR16,ASIC2,EFNA5,TCF12,GAS2L1,ARHGEF11,RAB27A,NSD1,EHMT1,SLIT3,DTNA,KIF13A,FRMD5,ESR1,MYO9B,NTNG1,KDM4B,CYP2C8,KCNQ5,LOXL2,CACNA2D1,NYAP2,IGLC3,IQCJ-SCHIP1,ADGRG7,SKAP2,PRLR,AGO3,HTT,LARS2,FOXB1,RAD51B,CAMK1D,PIK3R3,HLA-F,FER,ZNF302,EYA2,CCR2,STARD13,INTS12,A2M,FGGY,CHFR,ZNF721,EPS8,OSBPL6,JAZF1,ZNF578,ZNF891,SPOCK3,SEMA4B,NRF1,IGHV1OR21-1,ZNF14,HRH1,PHC2,GRIA4,AGAP1,ROCK2,PRDM1,RORA,STMP1,IL16,ATAT1,DMRT1,EIF4G3,CDCA5,PPP1CB,CATSPER2,RGS8,RAB31,PDK1,HSPG2,PSMD2,CSMD3,HERPUD1,NCOA6,TRIM2,HSD17B2,COL4A3,WASHC1,ZFP30,RGS7,HOOK3,KIF7,GNG2,PCSK2,FSTL4,CLDN10,BARD1,PNPLA3,STK3,DEPTOR,ZNF423,C1QL3,RSU1,PNPLA8,ZNF568,HNRNPU,CEP72,RAB3GAP2,CADPS,APCDD1,IGF1R,KCNAB1,PRKAG2,GLI2,THRB,AKAP13,MORC3,ATP10A,DNM1L
GO:0000902	cell morphogenesis	7.530316488123854e-27	*NOTCH2,CNTN4,PTPRD,LRRC4C,MYO9A,ULK2,DLC1,RIPOR2,RDX,STXBP1,BCL2,CDH8,CHRNA7,ROBO2,RIMS1,FGD4,USH2A,CDC42EP3,RIMS2,AUTS2,CARMIL1,PARVB,CNTNAP2,ZMYM4,DSCAM,CRKL,TNIK,DOCK10,EGFR,MACF1,NEDD4,CRB1,BCL11A,CDH4,PHACTR1,NEO1,CNTN6,PAK1,EPHA7,RAPGEF2,LRP2,ADGRB3,TAOK3,ADAMTSL1,EPB41L3,TBCD,NEDD4L,APP,DCLK1,STAU2,SEMA5A,SYT1,VCL,ARHGAP44,NTF3,AURKA,CFDP1,KANK1,MAP4K4,BMPR1B,CTNNA2,PAK3,DIP2B,TRPC5,DNM3,PRKCZ,DIP2A,HECW1,COBL,YAP1,FRYL,ALCAM,NCAM1,FAT3,CHN1,PAFAH1B1,TPM1,CDH7,NFIB,PRTG,TIAM1,ENAH,SEMA3C,TMEM108,BR*

			WD1,SEMA6D,CDH11,PARD3,TNR,COL22A1,ELAVL4,ABL1,SDC2,GAS2,CDH18,MYO10,CDHR3,PEAK1,LATS2,NRG1,AP3B1,USP33,CD44,PTPRO,TRIO,EXT1,LIMD1,CTNND2,ATP8A2,PLXNA2,ARHGEF7,SEMA3E,MARK2,EPHA6,SH3KBP1,ATL1,KNDC1,AFG3L2,ANK3,BCL11B,DOCK5,ECE1,MBP,CDH20,PACSIN2,CNTN1,PDLIM5,DISC1,WDPCP,NRK,SEMA3A,UNC5D,SEMA3D,RELN,UST,SLC23A2,DOCK1,B4GALT6,PLS1,SRGAP2,NIN,DRAXIN,GLI3,RERE,MAP2,LAMC1,FARP1,LAMC3,DCC,DAB1,PRKN,PCDH15,NGEF,CDH23,ALS2,ITSN2,ARHGEF28,ROCK1,NTN1,DPYSL5,SHROOM3,TANC2,FLNB,ZDHHC17,LAMA3,TNN,MICALL2,MED1,ATRN,FAT1,LMX1A,ACTR2,MAP6,VASP,PALMD,IQGAP1,NRXN1,FRY,CELSR2,MELTF,NEDD9,OLFM4,GAP43,GRIP1,ABI1,IMPACT,ITGA4,CYFIP2,MEF2C,ADGRB1,WNT7A,WASF3,S100B,FBXW8,NECTIN1,FGR,DNMBP,EPHA4,NTRK2,IL1RAPL1,NUMB,LHX9,FBXO31,PTK2,CDH5,DIAPH1,FEZ2,LAMB1,CYFIP1,UBE3A,SEMA4D,FRMD6,SPAG6,FYN,MYL12B,ARL13B,COL18A1,CDH9,HECW2,CDH2,CNTN5,ITGA8,NTN4,EPHB1,RPS6KA5,NRP1,ITGA1,CHODL,NRXN3,CDH12,FBLN1,CUX1,EPHB2,MET,PPFIA2,UNK,FAM171A1,FLRT2,KALRN,LAMA1,ATRNL1,TIAM2,BMP7,KIRREL3,SLIT2,CCDC141,ROBO1,PRKCQ,ANTXR1,SIPA1L3,NLGN1,EFNA5,SLIT3,NTNG1,NYAP2,FOXB1,FER,EPS8,SEMA4B,DMRT1,PTPRQ,FSTL4,IGF1R,GLI2,ATP10A
GO:0034330	cell junction organization	1.748731818729 3132e-25	*PTPRD,LRRC4C,MYO9A,UNC13C,TLN2,DLC1,PTPRA,RDX,ERC1,IL1RAPL2,BCL2,LRFN2,GPHN,CDH8,CHRNA7,ROBO2,GABRB3,CACNG2,NEGR1,GPC6,CNTNAP2,APC,DSCAM,CRKL,PTPRJ,DOCK10,MACF1,NEDD4,NTRK3,CAST,SLC8A3,EPHA7,RAPGEF2,ADGRB3,PATJ,EPB41L3,TBCD,ADAM10,APBB2,APP,CACNB2,STAU2,GABRG2,MAPRE2,VCL,ARHGAP44,SRGAP2C,MAP4K4,CTNNA2,RAB8B,PAK3,ERC2,DNM3,DIP2A,DUSP22,NFIA,CORO2B,LIMCH1,FMN1,PAFAH1B1,NF2,CNKSR2,CTNNA1,CDH7,PTPRK,PDZRN3,TMEM108,ANK2,TANC1,BCAS3,CDH11,PARD3,TNR,DST,CXADR,XIRP2,ABL1,SLC1A1,LRFN5,CDH18,RAP1A,GRID2,CDHR3,PEAK1,NRG1,MUSK,DGKB,PTPRO,EXT1,CTNND2,ARHGEF7,SEMA3E,ABHD17C,LINGO2,AFG3L2,ANK3,CDH20,TJP1,NPHP4,IQSEC1,PDLIM5,DISC1,DNER,WDPCP,STRN,RELN,UNC13B,SRGAP2,LAMC1,FARP1,MTMR2,LGI2,NGEF,PRKCH,PKP1,FRMPD4,ALS2,SHANK2,MESD,SYBU,ROCK1,NTN1,INSR,COLQ,SNAI2,TANC2,CD9,GRIN2B,MICALL2,LMX1A,IL10,ACTR2,CLSTN2,NRXN1,HIPK1,SVEP1,ABCC8,NEDD9,ITGA6,GAP43,SLC6A1,MEF2C,ADGRB1,WNT7A,WASF3,PKN2,SDCBP,NECTIN1,DSG1,EPHA4,NTRK2,IL1RAPL1,NUMB,OCLN,SHISA6,PTK2,CDH5,CLDN18,MPP7,CYFIP1,UBE3A,PCDH8,SEMA4D,KIRREL1,NOS1AP,FYN,PPM1F,ADGRL2,CDH9,CDH2,CNTN5,EPHB1,GRM5,NRP1,SDK1,PRKCA,BCR,NRXN3,CDH12,EPHB2,PPFIA2,VMP1,FLRT2,KALRN,IGSF21,DLG5,GABRA2,KIRREL3,ERBB4,SYNDIG1,NLGN1,CTTNBP2,ASIC2,EFNA5,NTNG1,FER,ROCK2,CLDN10,C1QL3,IGF1R*
GO:0032990	cell part morphogenesis	2.380734267217 6264e-23	*NOTCH2,CNTN4,PTPRD,LRRC4C,MYO9A,ULK2,NUBPL,STXBP1,BCL2,CHRNA7,ROBO2,RIMS1,RIMS2,AUTS2,CNTNAP2,DSCAM,TNIK,DOCK10,MACF1,NEDD4,BCL11A,CDH4,PHACTR1,NEO1,CNTN6,PAK1,EPHA7,RAPGEF2,LRP2,ADGRB3,TAOK3,ADAMTSL1,EPB41L3,NEDD4L,APP,DCLK1,STAU2,SEMA5A,SYT1,VCL,ARHGAP44,NTF3,AURKA,KANK1,MAP4K4,BMPR1B,CTNNA2,PAK3,DIP2B,TRPC5,DNM3,PRKCZ,DIP2A,HECW1,COBL,ALCAM,NCAM1,CHN1,PAFAH1B1,NFIB,PRTG,TIAM1,ENAH,SEMA3C,TMEM108,SEMA6D,CDH11,PARD3,TNR,ELAVL4,ABL1,SDC2,USP33,CD44,PTPRO,TRIO,EXT1,CTNND2,ATP8A2,PLXNA2,ARHGEF7,SEMA3E,MARK2,EPHA6,ATL1,KNDC1,AFG3L2,ANK3,BCL11B,ECE1,MBP,PACSIN2,CNTN1,PDLIM5,DISC1,NRK,SEMA3A,UNC5D,SEMA3D,RELN,UST,SLC23A2,B4GALT6,SRGAP2,NIN,DRAXIN,GLI3,RERE,MAP2,FARP1,DCC,DAB1,PRKN,NGEF,ALS2,ITSN2,ROCK1,NTN1,DPYSL5,TANC2,ZDHHC17,BCL2L1,LAMA3,TNN,LMX1A,ACTR2,MAP6,VASP,IQGAP1,NRXN1,CELSR2,GAP43,GRIP1,ABI1,IMPACT,ITGA4,CYFIP2,ADGRB1,WNT7A,S100B,FBXW8,NECTIN1,EPHA4,NTRK2,IL1RAPL1,NUMB,LHX9,FBXO31,PTK2,FEZ2,CYFIP1,UBE3A,SEMA4D,SPAG6,FYN,HECW2,CDH2,CNTN5,EPHB1,RPS6KA5,PID1,NRP1,ITGA1,CHODL,NRXN3,CUX1,EPHB2,PPFIA2,FLR*

			T2,KALRN,LAMA1,TIAM2,BMP7,KIRREL3,SLIT2,CCDC141,ROBO1,PRKCQ,NLGN1,EFNA5,SLIT3,NTNG1,NYAP2,FOXB1,SEMA4B,FSTL4,IGF1R,GLI2,DNM1L
GO:0048858	cell projection morphogenesis	3.224019629916792e-23	*NOTCH2,CNTN4,PTPRD,LRRC4C,MYO9A,ULK2,STXBP1,BCL2,CHRNA7,ROBO2,RIMS1,RIMS2,AUTS2,CNTNAP2,DSCAM,TNIK,DOCK10,MACF1,NEDD4,BCL11A,CDH4,PHACTR1,NEO1,CNTN6,PAK1,EPHA7,RAPGEF2,LRP2,ADGRB3,TAOK3,ADAMTSL1,EPB41L3,NEDD4L,APP,DCLK1,STAU2,SEMA5A,SYT1,VCL,ARHGAP44,NTF3,AURKA,KANK1,MAP4K4,BMPR1B,CTNNA2,PAK3,DIP2B,TRPC5,DNM3,PRKCZ,DIP2A,HECW1,COBL,ALCAM,NCAM1,CHN1,PAFAH1B1,NFIB,PRTG,TIAM1,ENAH,SEMA3C,TMEM108,SEMA6D,CDH11,PARD3,TNR,ELAVL4,ABL1,SDC2,USP33,CD44,PTPRO,TRIO,EXT1,CTNND2,ATP8A2,PLXNA2,ARHGEF7,SEMA3E,MARK2,EPHA6,ATL1,KNDC1,AFG3L2,ANK3,BCL11B,ECE1,MBP,PACSIN2,CNTN1,PDLIM5,DISC1,NRK,SEMA3A,UNC5D,SEMA3D,RELN,UST,SLC23A2,B4GALT6,SRGAP2,NIN,DRAXIN,GLI3,RERE,MAP2,FARP1,DCC,DAB1,PRKN,NGEF,ALS2,ITSN2,ROCK1,NTN1,DPYSL5,TANC2,ZDHHC17,LAMA3,TNN,LMX1A,ACTR2,MAP6,VASP,IQGAP1,NRXN1,CELSR2,GAP43,GRIP1,ABI1,IMPACT,ITGA4,CYFIP2,ADGRB1,WNT7A,S100B,FBXW8,NECTIN1,EPHA4,NTRK2,IL1RAPL1,NUMB,LHX9,FBXO31,PTK2,FEZ2,CYFIP1,UBE3A,SEMA4D,SPAG6,FYN,HECW2,CDH2,CNTN5,EPHB1,RPS6KA5,NRP1,ITGA1,CHODL,NRXN3,CUX1,EPHB2,PPFIA2,FLRT2,KALRN,LAMA1,TIAM2,BMP7,KIRREL3,SLIT2,CCDC141,ROBO1,PRKCQ,NLGN1,EFNA5,SLIT3,NTNG1,NYAP2,FOXB1,SEMA4B,FSTL4,IGF1R,GLI2*
GO:0120039	plasma membrane bounded cell projection morphogenesis	3.2979439515516833e-23	*NOTCH2,CNTN4,PTPRD,LRRC4C,MYO9A,ULK2,STXBP1,BCL2,CHRNA7,ROBO2,RIMS1,RIMS2,AUTS2,CNTNAP2,DSCAM,TNIK,DOCK10,MACF1,NEDD4,BCL11A,CDH4,PHACTR1,NEO1,CNTN6,PAK1,EPHA7,RAPGEF2,LRP2,ADGRB3,TAOK3,ADAMTSL1,EPB41L3,NEDD4L,APP,DCLK1,STAU2,SEMA5A,SYT1,VCL,ARHGAP44,NTF3,AURKA,KANK1,MAP4K4,BMPR1B,CTNNA2,PAK3,DIP2B,TRPC5,DNM3,PRKCZ,DIP2A,HECW1,COBL,ALCAM,NCAM1,CHN1,PAFAH1B1,NFIB,PRTG,TIAM1,ENAH,SEMA3C,TMEM108,SEMA6D,CDH11,PARD3,TNR,ELAVL4,ABL1,SDC2,USP33,CD44,PTPRO,TRIO,EXT1,CTNND2,ATP8A2,PLXNA2,ARHGEF7,SEMA3E,MARK2,EPHA6,ATL1,KNDC1,AFG3L2,ANK3,BCL11B,ECE1,MBP,CNTN1,PDLIM5,DISC1,NRK,SEMA3A,UNC5D,SEMA3D,RELN,UST,SLC23A2,B4GALT6,SRGAP2,NIN,DRAXIN,GLI3,RERE,MAP2,FARP1,DCC,DAB1,PRKN,NGEF,ALS2,ITSN2,ROCK1,NTN1,DPYSL5,TANC2,ZDHHC17,LAMA3,TNN,LMX1A,ACTR2,MAP6,VASP,IQGAP1,NRXN1,CELSR2,GAP43,GRIP1,ABI1,IMPACT,ITGA4,CYFIP2,ADGRB1,WNT7A,S100B,FBXW8,NECTIN1,EPHA4,NTRK2,IL1RAPL1,NUMB,LHX9,FBXO31,PTK2,FEZ2,CYFIP1,UBE3A,SEMA4D,SPAG6,FYN,HECW2,CDH2,CNTN5,EPHB1,RPS6KA5,NRP1,ITGA1,CHODL,NRXN3,CUX1,EPHB2,PPFIA2,FLRT2,KALRN,LAMA1,TIAM2,BMP7,KIRREL3,SLIT2,CCDC141,ROBO1,PRKCQ,NLGN1,EFNA5,SLIT3,NTNG1,NYAP2,FOXB1,SEMA4B,FSTL4,IGF1R,GLI2*
GO:0048812	neuron projection morphogenesis	3.4998531859256274e-23	*NOTCH2,CNTN4,PTPRD,LRRC4C,MYO9A,ULK2,STXBP1,BCL2,CHRNA7,ROBO2,RIMS1,RIMS2,AUTS2,CNTNAP2,DSCAM,TNIK,DOCK10,MACF1,NEDD4,BCL11A,CDH4,PHACTR1,NEO1,CNTN6,PAK1,EPHA7,RAPGEF2,LRP2,ADGRB3,TAOK3,ADAMTSL1,EPB41L3,NEDD4L,APP,DCLK1,STAU2,SEMA5A,SYT1,VCL,ARHGAP44,NTF3,AURKA,MAP4K4,BMPR1B,CTNNA2,PAK3,DIP2B,TRPC5,DNM3,PRKCZ,DIP2A,HECW1,COBL,ALCAM,NCAM1,CHN1,PAFAH1B1,NFIB,PRTG,TIAM1,ENAH,SEMA3C,TMEM108,SEMA6D,CDH11,PARD3,TNR,ELAVL4,ABL1,SDC2,USP33,PTPRO,TRIO,EXT1,CTNND2,ATP8A2,PLXNA2,SEMA3E,MARK2,EPHA6,ATL1,KNDC1,AFG3L2,ANK3,BCL11B,ECE1,MBP,CNTN1,PDLIM5,DISC1,NRK,SEMA3A,UNC5D,SEMA3D,RELN,UST,SLC23A2,B4GALT6,SRGAP2,NIN,DRAXIN,GLI3,RERE,MAP2,FARP1,DCC,DAB1,PRKN,NGEF,ALS2,ITSN2,ROCK1,NTN1,DPYSL5,TANC2,ZDHHC17,LAMA3,TNN,LMX1A,ACTR2,MAP6,VASP,IQGAP1,NRXN1,CELSR2,GAP43,GRIP1,ABI1,IMPACT,ITGA4,CYFIP2,ADGRB1,WNT7A,S100B,FBXW8,NECTIN1,EPHA4,NTRK2,IL1RAPL1,NUMB,LHX9,FBXO31,PTK2,FEZ2,CYFIP1,UBE3A,SEMA4D,SPAG6,FYN,HECW2,CDH2,CNTN5,EPHB1,RPS6KA5,NRP1,ITGA1,CHODL,NRXN3,CUX1,EPHB2,PPFI*

			A2,FLRT2,KALRN,LAMA1,TIAM2,BMP7,KIRREL3,SLIT2,CCDC141,ROBO1,PRKCQ,NLGN1,EFNA5,SLIT3,NTNG1,NYAP2,FOXB1,SEMA4B,FSTL4,IGF1R,GLI2
GO:0032989	cellular component morphogenesis	1.636357796410 2115e-22	*NOTCH2,CNTN4,PTPRD,NEBL,LRRC4C,MYO9A,ULK2,TENM4,NUBPL,STXBP1,BCL2,CHRNA7,ROBO2,RIMS1,RIMS2,AUTS2,CNTNAP2,DSCAM,TNIK,DOCK10,MACF1,NEDD4,BCL11A,CDH4,PHACTR1,NEO1,CNTN6,PAK1,EPHA7,RAPGEF2,LRP2,ADGRB3,TAOK3,ADAMTSL1,EPB41L3,NEDD4L,APP,DCLK1,STAU2,SEMA5A,SYT1,VCL,ARHGAP44,NTF3,AURKA,FIG4,KANK1,MAP4K4,BMPR1B,CTNNA2,PAK3,DIP2B,TRPC5,DNM3,PRKCZ,DIP2A,HECW1,COBL,ALCAM,NCAM1,CHN1,MYLK3,PAFAH1B1,TPM1,NFIB,PRTG,TIAM1,ENAH,SEMA3C,TMEM108,ANK2,SEMA6D,CDH11,LDB3,PARD3,TNR,ELAVL4,ABL1,SDC2,PGM5,USP33,CD44,PTPRO,TRIO,EXT1,CTNND2,ATP8A2,PLXNA2,ARHGEF7,SEMA3E,MARK2,TMOD2,EPHA6,ATL1,KNDC1,AFG3L2,ANK3,MYOM2,BCL11B,ECE1,MBP,PACSIN2,CNTN1,PDLIM5,DISC1,NRK,SEMA3A,UNC5D,SEMA3D,RELN,UST,SLC23A2,B4GALT6,SRGAP2,NIN,DRAXIN,GLI3,RERE,MAP2,FARP1,DCC,DAB1,PRKN,MTMR2,NGEF,ALS2,ITSN2,SOX30,ROCK1,NTN1,DPYSL5,TANC2,ZDHHC17,CD9,BCL2L1,LAMA3,TNN,LMX1A,ACTR2,MAP6,VASP,IQGAP1,NRXN1,CELSR2,GAP43,GRIP1,ABI1,IMPACT,ITGA4,CYFIP2,ADGRB1,WNT7A,S100B,FBXW8,NECTIN1,EPHA4,NTRK2,IL1RAPL1,NUMB,LHX9,FBXO31,PTK2,FEZ2,CYFIP1,UBE3A,SEMA4D,SPAG6,FYN,HECW2,CDH2,CNTN5,EPHB1,RPS6KA5,PID1,NRP1,ITGA1,CHODL,NRXN3,CUX1,EPHB2,PPFIA2,NRAP,FLRT2,KALRN,LAMA1,TIAM2,BMP7,KIRREL3,RFX2,FHOD3,SLIT2,CCDC141,ROBO1,PRKCQ,NLGN1,EFNA5,SLIT3,NTNG1,NYAP2,FOXB1,SEMA4B,FSTL4,IGF1R,GLI2,AKAP13,DNM1L*
GO:0000904	cell morphogenesis involved in differentiation	4.298754718018 709e-22	*NOTCH2,CNTN4,PTPRD,LRRC4C,ULK2,RIPOR2,STXBP1,BCL2,CHRNA7,ROBO2,USH2A,AUTS2,CARMIL1,PARVB,DSCAM,CRKL,TNIK,DOCK10,MACF1,NEDD4,BCL11A,CDH4,PHACTR1,NEO1,CNTN6,PAK1,EPHA7,RAPGEF2,ADGRB3,ADAMTSL1,TBCD,NEDD4L,APP,DCLK1,STAU2,SEMA5A,VCL,ARHGAP44,KANK1,BMPR1B,CTNNA2,PAK3,DIP2B,TRPC5,DNM3,DIP2A,HECW1,COBL,ALCAM,NCAM1,FAT3,CHN1,PAFAH1B1,NFIB,PRTG,TIAM1,ENAH,SEMA3C,SEMA6D,CDH11,PARD3,TNR,COL22A1,ELAVL4,ABL1,SDC2,PEAK1,LATS2,USP33,PTPRO,TRIO,EXT1,CTNND2,ATP8A2,PLXNA2,ARHGEF7,SEMA3E,MARK2,EPHA6,ATL1,KNDC1,AFG3L2,ANK3,BCL11B,DOCK5,ECE1,MBP,CNTN1,PDLIM5,DISC1,WDPCP,SEMA3A,UNC5D,SEMA3D,RELN,UST,DOCK1,B4GALT6,PLS1,SRGAP2,NIN,DRAXIN,GLI3,RERE,MAP2,LAMC1,FARP1,LAMC3,DCC,DAB1,PCDH15,NGEF,CDH23,ALS2,ROCK1,NTN1,DPYSL5,TANC2,FLNB,ZDHHC17,LAMA3,TNN,MICALL2,ATRN,FAT1,LMX1A,ACTR2,MAP6,VASP,NRXN1,CELSR2,MELTF,NEDD9,OLFM4,GAP43,ABI1,ITGA4,MEF2C,ADGRB1,WNT7A,S100B,FBXW8,NECTIN1,EPHA4,NTRK2,IL1RAPL1,NUMB,LHX9,FBXO31,PTK2,FEZ2,LAMB1,CYFIP1,UBE3A,SEMA4D,FRMD6,FYN,ARL13B,COL18A1,HECW2,CDH2,CNTN5,ITGA8,NTN4,EPHB1,RPS6KA5,NRP1,CHODL,NRXN3,FBLN1,CUX1,EPHB2,MET,PPFIA2,UNK,FLRT2,KALRN,LAMA1,ATRNL1,TIAM2,BMP7,SLIT2,CCDC141,ROBO1,PRKCQ,ANTXR1,SIPA1L3,NLGN1,EFNA5,SLIT3,NTNG1,FOXB1,FER,SEMA4B,PTPRQ,FSTL4,IGF1R,GLI2*
GO:0050789	regulation of biological process	5.049953940328 589e-22	*NOTCH2,BCAR3,BRINP3,MTOR,CNTN4,CACNA2D3,SPOCK1,NSG1,SGCD,WWC1,ABCA13,GARNL3,LRP12,PTPRD,SLC24A2,TRAPPC9,BNC2,PVT1,LRRC4C,KCNH5,ANKS1B,SMOC1,MYO9A,ULK2,NLK,LONP2,UNC13C,FTO,KSR1,MGA,RFX7,ZNF236,PLCB1,ZNF536,MX2,TMPRSS2,TAFA5,SVIL,CLTCL1,ZFPM2,TENM4,L3MBTL4,DLC1,TNRC6B,DPP10,ZDHHC21,PTPRA,ITPR2,RIPOR2,PDE4D,RDX,RP1,STXBP1,ERC1,RALA,IL1RAPL2,BCL2,ODAD2,KCNMA1,PRDM16,ALDH1A2,ARHGAP26,FBN1,LRFN2,CDH8,CHRNA7,DCDC1,GPR158,ROBO2,RIMS1,PIK3C3,EPC2,SPIRE1,TENM3,GABRB3,ZEB1,AKR1C3,SDCCAG8,RARB,FGD4,SPRED1,ENPEP,MYO1E,PLPPR1,USH2A,MINAR1,CDC42EP3,RIMS2,ALK,AUTS2,ADGRE1,FOXJ2,CDYL2,CARMIL1,MCTP1,PJA2,BABAM2,PAPPA2,GLIS3,FANK1,ERBIN,RHPN2,RIN2,PARVB,ANO6,CACNG2,DLGAP1,NEGR1,ZNF880,MLLT3,EGLN3,GPC6,SUSD4,CNTNAP2,MAP4,MA*

			P3K9,SPON1,APC,ZMYM4,ZNF595,HHLA2,TSHZ3,RBFOX3,PLPPR5,DSCAM,RTN1,TCF4,CRKL,ILDR2,SOX5,SETD2,ERG,ARHGAP24,ZNF573,TNIK,SLC4A10,PTPRJ,KDM4C,NEK4,DOCK10,TSHZ2,EGFR,ZNF280B,RFX3,DENND1A,USP14,ANGPT1,CDK12,BACH1,MACF1,CTNNA3,PRKACB,NEK7,RGS3,NCOR1,RNF220,DOCK2,ZNF407,NEDD4,MAML2,MTRF1,SND1,SCAI,NSMCE2,BTBD9,BCL11A,SOX6,FAM83F,TMEM182,SGMS1,GRIK3,CHSY1,FLI1,RPRD1A,CDH4,ATP2B2,NTRK3,RXFP1,C5,PDE1C,ZFAND6,PHACTR1,DKK2,FLT1,DNAJC13,ZNF648,RFC3,RABEP1,ZNF382,TASP1,THRAP3,MAPKBP1,AOAH,GABRB1,PSMA8,DGKI,INVS,C12ORF4,EDAR,GRIA1,CRACD,CAST,TTC39B,NUP214,NEO1,CNTN6,SLC39A12,CABLES1,SLC8A3,MALRD1,TOM1L2,PRKD1,TPTE2,PAK1,GMDS,EPHA7,CTNNAL1,NCOA7,KHDRBS2,CHRM3,RALGPS1,SPEN,RAPGEF2,PELI2,LRP2,ADGRB3,RUNX2,ARSB,FGF12,GABRA6,TAOK3,ONECUT1,LDLRAD3,CPEB4,TMEM38B,PRICKLE2,SLC24A3,UBE2L3,LDB2,TAFA4,BTBD11,PUM3,CCL28,SMYD3,PATJ,GRM7,SEPTIN9,RETREG1,RPTOR,TMEM117,GHR,EPB41L3,THADA,COL4A2,SSBP3,RALGAPA1,CELF2,RAPGEF5,TBCD,NEDD4L,PPP1R12B,TRPM1,ADAM10,HDAC9,ZHX3,ATF7IP,IL1R1,APBB2,APP,RPS6KA2,SAMSN1,CACNA1C,KDM1B,CACNB2,KLHL13,MTUS1,DCLK1,STAU2,GABRG2,DOCK8,TMC1,MAPRE2,ZNF600,USP18,SEMA5A,SYT1,VCL,ARHGAP44,NTF3,ACER2,PARP15,NDUFAF2,CD2AP,ZNF723,AURKA,PARN,CFDP1,ST18,PYGO1,SLC8A1,HERPUD2,SSBP2,PTPRR,SRGAP2C,ANKRD31,FIG4,DUX4,TAFA2,ABCG8,SERPINA6,PLGRKT,SRGAP2B,KANK1,KCNE4,MAP4K4,HIVEP2,ABCD2,BMPR1B,FMN2,PCSK6,AKAP6,HOMER2,ZNF717,CTNNA2,ARNT,RAB8B,PAK3,RFTN1,PDE1A,ZNF257,DIP2B,KCNK10,RANBP2,LARP1,ITPKB,TRPC5,RGS20,PDE10A,UBE2E2,RAP1GDS1,HHAT,RNLS,CLIC6,KICS2,ERC2,DNM3,NBN,SCP2,SYN3,IFT57,INTS7,PRKCZ,SPOP,BTLA,GRB10,RYR3,TAF15,DIP2A,MSH6,MCPH1,ARHGAP32,RAB27B,CNST,RGS9,HECW1,DEFA3,MBNL2,ABCA5,PHF19,MRTFA,TAF4B,COBL,SENP6,DUSP22,EBF2,YAP1,NFIA,WDR70,PPM1L,RIPK4,ZKSCAN5,SHC4,BRINP1,MAPK1,MGAT5,CADPS2,KCNJ1,HRH2,RABGAP1L,ITIH5,ADAM22,USP25,KMT2E,ALCAM,PLG,PCGF5,PDGFD,SYT10,ZNRF3,PPP1R1C,ITGBL1,ARHGEF17,NRG3,UBE2O,SFMBT2,MIR663AHG,ANKFY1,NCAM1,GFRA1,SYCP1,NIPBL,SLC16A1,SPIDR,EWSR1,GABPA,FAT3,MICU1,ZNF735,CORO2B,CARD18,CHD6,STK38,PTPN13,CHN1,HRH4,SORCS3,MYLK3,KANSL1,GLP2R,LIMCH1,FMN1,MBNL1,PAFAH1B1,ATF6,EFEMP1,ZNF684,TM7SF3,DCAF1,ITGB8,STON2,VPS13D,CCNG2,TLK1,TPM1,NF2,LRRC38,CNKSR2,GRIK4,RBFOX1,HIVEP1,CORIN,CTNNA1,PPP1R9A,MOB3B,BIRC6,AKAP9,KLF15,RASGRF2,PPARA,MEIS2,SNX30,NFIB,KCNS3,ERMP1,MRTFB,PPP6R3,PRTG,RGL1,SYNJ1,NR5A2,ADAMTS3,TIAM1,ARAP2,GRM1,FOXJ3,PTPRK,ARHGEF12,GABRG1,PAK5,TRERF1,PCDH11Y,PPP2R5E,PLA2R1,EIF3D,SEMA3C,DAPK1,NAV3,SLC24A4,SEC14L1,VPS13C,TMEM108,AGO2,STK32B,PHC3,MAGI1,ALPK2,DNAH11,JARID2,SCN2A,RIC8B,SORCS1,DNAJC15,GATAD2B,CPE,EVC2,DYSF,IL34,ANK2,BRWD1,TANC1,ADGRV1,ZNF846,MELK,BCAS3,RYR2,SYNE2,BBS2,WNT9B,ZNF606,CLPX,RANBP3L,OR4F6,NKAIN3,NKG7,SEMA6D,DUSP16,SMARCA4,CDH11,USP8,FABP7,PARD3,MAPKAP1,TNRC6C,PIAS1,TBC1D5,SPG21,BLK,EBF1,TNR,GRM8,DST,CXADR,DOCK4,MBD5,ATRX,NUAK1,PTPRT,ELAVL4,ABL1,MXI1,PTPN12,HDAC4,OXR1,SLC1A1,PRKAA1,SDC2,GAS2,KCNH1,ITGB3BP,MRPS27,LRFN5,CREG1,DROSHA,APBB1IP,L3MBTL3,EIPR1,APLF,NFAT5,MAST4,GUCY1A2,NBAS,PSMF1,SLFN11,RAP1A,NKAIN2,GLIS1,MORC1,MYO10,GPC5,TOX3,CAMK4,BAZ2A,INPP5A,CPSF3,FGF10,ZC3HAV1,GRID2,TGM1,PEAK1,LATS2,NRG1,INO80D,GSG1L,CLIP1,ASPM,AP3B1,DENND2B,RASGRF1,ATP11C,ZNF438,ABCB7,ZBTB16,MUSK,KIR3DL2,ZNF675,GNG7,SMARCAD1,SH3GL3,SETDB2,PRKCE,FOXK2,SLCO3A1,MED15,SLMAP,NXN,WNK2,ESRRG,ZNF718,DGKB,USP33,DENND4C,FBN2,CD44,RGS12,PTPRO,EGF,PRRC1,ABCC9,P2RX6,TRIO,PDE3A,EXT1,STXBP6,NSMAF,LNPEP,LIMD1,PEX14,SPRED2,RPS6KA3,CTNND2,ATP8A2,SCG5,MTMR3,PTPN2,TRIM5,PLXNA2,MCF2L,OR4F15,ATXN3,RFC1,HTR2C,RIC3,CLEC16A,ARHGEF

			7,CD96,ALG10B,ATP8A1,AMBRA1,LTBP1,STK38L,ZFYVE9,KDM7A,OPRM1,HTR2A,PLCXD3,FANCM,FANCA,CYP4A11,DAZL,INPP4B,GTF2F2,KREMEN1,STAC,SEMA3E,TAF3,RPRD1B,MARK2,GCSAML,TMEM67,EBF3,ALPL,ZNF33B,C10ORF90,FHL2,ABHD17C,ADGRA3,CNIH3,PUM1,TMOD2,HERC1,MSH2,IGF2BP3,GNAL,EPHA6,ANKRD17,APBA2,LINGO2,ZNF397,SH3KBP1,SLC2A13,LUC7L,RELL1,HIPK3,CDKN2C,EPN2,KCND2,EVC,GRK3,KNDC1,SPSB4,CLSPN,NOS2,BICRAL,AFG3L2,STK10,MOSMO,GFRA2,MNAT1,TMEM116,RBBP8,MDFIC,ADAM12,MYLK2,ANK3,EMILIN2,HMGA2,CCND3,BCL11B,VPS41,DOCK5,ECE1,ZIM3,STK32A,CREM,LYPLA1,MBP,LINC01151,TRPS1,PLCE1,TGFA,IL17RA,ANKFN1,HIP1,CRIM1,FUT9,PRR5L,GSR,ATP6V1E1,UTP4,CAPN5,VAV1,RUFY2,MYT1L,FBXO32,ZNF160,TJP1,LDLRAD4,NPHP4,EGFLAM,PACSIN2,CNTN1,HLA-B,IQSEC1,HSF5,SNX3,CACNA1I,NAA35,ZNF367,PDLIM5,KCNJ15,BRCA2,DISC1,ZBTB2,DNER,BLM,ASB7,WDPCP,NRK,SEMA3A,MAGI3,INTS8,LIN54,ADCY10,PSG8,STRN,OR9Q1,ZNF121,BMP2,RC3H2,UNC5D,ATP9A,TRAK1,PSG9,CDC42BPB,SOGA1,PTCD2,SCN11A,MSR1,VRK1,GNAI1,RALGAPA2,ZC3H14,GFI1B,TBC1D4,RANBP9,RESF1,MYRIP,TTR,RIN3,BMP2K,TMEM161A,SEMA3D,ASXL3,NETO2,PDE6C,CABIN1,POLR3A,LEMD3,RELN,ARHGAP42,HMGB1,GNAQ,FGF9,NFATC2,TDRD7,SH3BP5,UST,CPAMD8,MDM1,SLC23A2,POLR2M,ZNF106,MYOM1,ZNF567,TRAF3,ZNF462,ANKRD26,ESRP1,UNC13B,TTC21B,ETS2,GEMIN5,ZNF875,DSTYK,UIMC1,DOCK1,LRRFIP1,RAP1GAP,PLS1,SRGAP2,IKZF2,NIN,DRAXIN,ATF1,SLAMF1,KCNH8,SMARCA2,ETS1,FAM83B,GLI3,CGAS,SMARCC1,SNX6,AFF3,GABRR2,SMOC2,PCP4,CNKSR3,CASP5,VENTX,GRIK2,IDE,WDR12,MCTP2,KIF15,PRDM10,CUL1,MYEF2,ZFYVE26,ZNF431,RERE,PSD3,MAP2,BTAF1,GAREM1,LAMC1,ZNF618,NEK10,FARP1,MOB1B,ATF2,HIRA,CYLD,UMODL1,BBS4,MAPK8IP1,MX1,PSG6,HIVEP3,COL5A1,GABBR2,PSIP1,ITGA9,CFTR,KPNA1,NELL1,ME2,UBASH3A,RGMB,NEU3,MRPL13,KITLG,ZZEF1,DNAJC7,CAMTA1,UBR1,DCC,MYT1,CHRM5,MAP4K3,YLPM1,SLC30A10,RCAN1,GTF2I,RORB,TADA2A,DAB1,MED27,ZNF208,SELENON,RB1CC1,NMD3,AKAP10,PTPRE,PRKN,MTMR2,ZNF608,TBX20,SP110,DLGAP2,AFAP1,MAPK10,DACH1,ZNF541,DPF3,NGEF,GRIN2A,ARID5B,ZBED9,JPH1,TXNRD2,ATXN1,WSB1,LALBA,PRKCH,PKP1,HUNK,FRMD4A,TG,IL6R,FRMPD4,ALS2,RACGAP1,NLRC5,ZNF627,OR51E1,ACO1,TFDP1,CNOT6L,MKNK1,HEMGN,KANK4,DOCK9,SNX25,HULC,FBLN5,KCNQ3,TOX,SHISA9,SLC4A4,PTPRB,ZFP90,PDE6A,COPS8,ZNF124,SCN10A,SHANK2,ST8SIA1,USP7,VAV3,PSMA1,ENPP3,PLAGL1,KCND3,MESD,ITSN2,SOX30,MOK,KIR2DL4,ARHGEF28,RALB,NPAS2,ADGRG6,ROCK1,LYN,VCAM1,SEL1L,ARHGAP28,ARHGAP31,ZNF780B,CTSB,EIF2B3,TTC37,SLC44A2,SUMO3,SLC15A2,ZNF169,PLEKHB2,KIF11,DTX1,BZW1,TENM2,OVOL2,PIWIL3,ZBTB33,ADA2,NTN1,PLCB4,ZFHX3,FANCL,DPYSL5,ZNF44,RRAGD,BANP,SUPT16H,ARID1B,HOXC13,CRACR2A,RNF152,BAZ1A,CASZ1,OTUD7A,INSR,CUL5,OR7A17,BMF,YTHDF3,TFF1,DEDD2,NEK6,HECTD1,GRID1,SHROOM3,COLQ,NMU,DDHD1,PBX3,SUMO2,HS1BP3,ZNF292,ARFGEF1,PDE4DIP,GAST,POGK,SNAI2,ASH1L,IGHV3-74,HOXC4,BID,SIAH2,TANC2,ABCA4,TRABD2B,UFD1,RXRG,SP3,DRAM1,ERN2,GABRG3,ZNF879,MBTPS2,FLNB,TRIM58,TIAL1,TOM1,ELF2,PLPP4,NREP,ZDHHC17,NSD2,FYCO1,SH3GLB1,CD9,CARD10,RALGPS2,JCAD,TWIST2,OR4K2,CTIF,SAMHD1,IFT81,ENPP1,UTRN,RASGRP1,IGSF11,SNX9,TP53I11,TMEM225,ANAPC1,NDRG2,CSNK2A1,BMP5,KCNC1,CSF1,GHRH,HDGFL3,BCL2L1,SERPINB9,SCAF4,MIR3142HG,CTDP1,HCN1,PRKG1,LAMA3,ASB4,GRIN2B,GRB14,INO80,FANCB,GPR156,IGHV2-70D,CLNS1A,CNMD,DHRS3,SMAD5,CELF4,TCERG1,ABCG1,OR4C46,FOXN3,KCNK5,DCUN1D4,SLC40A1,PRAME,MYCL,TNN,CIDEC,PSAP,LPGAT1,MICALL2,MED1,CDC14B,PCNT,KDM6A,ATRN,IL33,AJAP1,GPRC5C,ROR2,CFH,ZNF521,KL,RASGEF1C,BANK1,CSDE1,LMX1A,TMEM178A,IL10,ACTR2,OR1L6,SFPQ,SCML2,PRAMEF25,RIOK1,CLSTN2,PTH,SOSTDC1,PRKAA2,CSF2RB,DIRAS2,SKA1,NDC80,SOHLH1,LARP6,PACRG,PHF20L1,ABHD2,ITPRIP

			,VSTM2A,MAP6,VASP,PLA2G4A,ETV6,PALMD,RAB12,IQGAP1,RPS12,CAMLG,COX7A2L,ZBTB7C,TEAD1,MORC2,SREBF2,ANP32B,YBX3,AIMP1,THNSL2,FYB2,NRXN1,PCID2,HIPK1,ZNF234,CISD1,ZNF518A,DGKK,FRY,SNAP91,CD70,CIBAR1,PBLD,FICD,CADM1,CENPE,PEG10,LMX1B,NET1,SIPA1L2,NGDN,ELOC,ANLN,TWIST1,AKT3,ALKAL2,JAK2,VSX1,RPF2,FSTL1,ZBTB38,ISX,SVEP1,MADD,HCRTR1,RBM19,PTGS1,PATL1,ZNF287,CELSR2,ZNF449,PRSS2,FH,CREBBP,MELTF,TNKS,GORAB,PCNA,SIAH3,UFL1,ADAMTS5,NFKBIA,PRKCB,OR2T3,ABCC8,ANXA4,CACNA1E,ZC3H15,ANP32A,RFC2,ZNF354C,ALX4,RTRAF,USH1C,BRD4,ZBTB21,SERBP1,NEDD9,OLFM4,NRBP1,ITGA6,ATP2B1,GAP43,ASS1,MTCL1,GRIP1,IGHV1OR15-9,CTNNBL1,ADGRE3,SAR1A,ADCY9,PPP1R17,CNIH1,MAST2,HPSE2,BTG3,ZNF528,ERLIN2,ZNF611,UBAP2,OTOP1,CIDEA,ARFGEF3,ZBTB49,EXT2,EXOC1,HEPACAM,KRT6A,STOX2,AGO1,MEOX2,SLC6A1,GID8,ELL2,STAT1,BRMS1L,NDFIP2,NR2C1,MAP2K6,CMTM7,DGKG,KCNJ18,GATAD1,MTPN,ABI1,CEMIP,PRAMEF2,POU6F2,IMPACT,CCBE1,PARK7,ADAMTS18,MAPK8,ITGA4,OAZ2,MED12L,ZSCAN30,FBXL17,UBL7,POU1F1,UBE2J2,ADCYAP1R1,MTF2,NCAPG2,TM9SF4,RAPGEF4,OR6C75,FOXP2,ASB2,MYOCD,CEP120,ZSCAN5C,CYFIP2,HNRNPM,ASCC2,EFHB,OR13C9,ARID3B,MEF2C,ZNF613,ADGRB1,RXRA,WNT7A,RBPMS2,MAP3K5,NDFIP1,MAP3K4,TRIM43B,WASF3,S100B,SERPINI2,PRDM13,TRIM43,FOXO6,ERI1,ZNF112,ATP6V1C2,C16ORF72,MAGEL2,PKN2,RAD51AP1,OR10H2,PDE2A,RAB38,LRRC2,DBF4B,FBXW8,SDCBP,NECTIN1,JPT2,SPPL2B,NSMCE1,ZNF813,WWOX,ZBTB25,PASK,MLLT1,NCK1,FLVCR1,SCAF8,FGR,CWC22,CDCA8,PPP2R3A,DNMBP,ATP6V1B2,CXCL2,TOP1,MLLT10,C2,IFNAR1,RNF8,GNG12,EPHA4,CYTH4,INTS13,GABRA5,MECOM,DNMT3L,NTRK2,IL1RAPL1,NUMB,LHX9,ADAMTS9,WNT2B,COLEC12,ZBTB10,TNNI1,OCLN,POSTN,CREB5,MIR548H4,CD101,SHISA6,MEGF10,IL17RD,FBXO31,EXTL3,AKAP11,GRIK1,PRKAB1,DTHD1,IREB2,MVB12B,PTK2,MARK4,CDH5,CD5L,RCAN2,ANKRD6,SCGN,NFKBID,ARHGAP12,CLDN18,ASCL3,MPP7,DIAPH1,FEZ2,INIP,LAMB1,MIR17HG,APIP,CYFIP1,UBE3A,PCDH8,SEMA4D,JAM2,SERPINB10,PITPNC1,FRMD6,MC2R,ZBTB20,FAT4,IMPA2,ZNF66,RUNX1,AKR1B1,C9,KIRREL1,WNT5B,RASGEF1B,AMFR,SAXO1,NENF,SH2D1B,POMT2,PTGFR,ZNF845,OR4L1,ASAP1,SAMD13,ICA1,PLCZ1,EDIL3,NOS1AP,MTTP,FCRLA,DIDO1,TPTE,SORBS2,PDCL3,SRP9,CNKSR1,CCDC88A,GPR55,NSUN2,CHCHD2,ADAMTS16,CDC45,OR11G2,BICD1,TNFSF11,FYN,BUB1,KDM5A,PCBP3,MYL12B,ZNF705G,PPM1F,ADGRL2,ARL13B,SDE2,RBMS3,UHRF2,SCN8A,HDAC2,AVEN,SLF1,GON4L,TBX15,SH2D3C,PSME3IP1,DOCK3,TRNAU1AP,NCS1,COL18A1,LHFPL2,ALB,DOK5,NALCN,ZFYVE28,MAPK9,PABPC1,CRTAM,APELA,SLC39A8,ROR1,OPA3,FUT8,TET1,ARNT2,ASB3,HECW2,CDH2,ITGA8,FBXL20,NTN4,RAD9A,XRN2,PHLPP1,GPR137B,EPHB1,RP1L1,GRM5,ADCK1,RAI14,SPOPL,ZNF705D,RPS6KA5,SPTB,TBC1D1,LRRC69,PTPRG,PID1,NRP1,MIDEAS,FCHSD2,SDK1,PRKCA,ATPSCKMT,FAIM,SAMD12,FHIT,ITGA1,ZNF615,KLF12,RNF138,RC3H1,NRIP1,CHODL,POR,ZNF850,ZNF235,MCC,ZNF738,SUPT3H,BCR,TUT4,NRXN3,ELMO1,RGS6,RERG,ZNF215,TCERG1L,KIF16B,PRIM2,SNRK,C14ORF39,TM9SF2,ELP2,FBLN1,STK36,NSG2,RAG1,KCNJ6,B9D1,RRAS2,GNA14,ZNF678,BMPER,PRDM15,CUX1,DPP6,SRGAP3,ZNF420,MACROH2A1,MITF,EPHB2,TSPAN13,TOGARAM1,CSNK1G1,SACS,BCL2L13,CD38,EYA4,DPH6,CDK14,AKAIN1,MET,SPPL3,CDH17,ZNF705B,ATP6V0D2,PPFIA2,CDH13,MED13L,STXBP4,SERPINB2,CACNG3,ATG5,MAGI2,PRDM11,VMP1,UNK,FAM171A1,MLIP,FLRT2,MYB,KALRN,ZNF704,SLC1A2,GNAS,LAMA1,MFHAS1,SERPINB7,ATRNL1,TIAM2,DHX29,BMP7,TTC28,ASTN2,DLG5,TNFAIP8,ZMYND8,GAPVD1,GABRA2,RNF217,KCTD1,OR2T2,ZNF74,BPTF,BTBD10,ZMYND11,TMEM25,NUDT21,GRM3,KMT2C,DDX6,ADGRF5,OR4N2,PDGFC,WDR41,PLIN2,PPP1R13B,FOCAD,ABL2,MMP26,BACE2,RFX2,PARPBP,NECAB1,PKNOX2,EYA1,FHOD3,PRPF18,SLIT2,EXOC4,CNOT7,KCNIP4,ESCO1,KCTD8,PLCL1,ERBB4,IL20RB,SERPINB11,FAM3B,GSAP,TRHDE,SYNDIG1,ROBO1,SAMD

			4A,PBX1,IRAG1,NPAS3,NUF2,PRKCQ,ANTXR1,NDRG1,SORCS2,SIPA1L3,TRDN,MGMT,ZNF679,NLGN1,CTTNBP2,SHLD2,NOS1,SLC6A3,PRR16,ASIC2,EFNA5,TCF12,GAS2L1,ARHGEF11,RAB27A,NSD1,EHMT1,SLIT3,DTNA,KIF13A,FRMD5,ESR1,MYO9B,NTNG1,KDM4B,KCNQ5,LOXL2,CACNA2D1,NYAP2,IGLC3,IQCJ-SCHIP1,ADGRG7,SKAP2,PRLR,AGO3,HTT,FOXB1,RAD51B,CAMK1D,PIK3R3,HLA-F,FER,ZNF302,EYA2,CCR2,STARD13,INTS12,A2M,CHFR,ZNF721,EPS8,OSBPL6,JAZF1,ZNF578,ZNF891,SPOCK3,SEMA4B,NRF1,IGHV1OR21-1,ZNF14,HRH1,PHC2,GRIA4,ROCK2,PRDM1,RORA,STMP1,IL16,ATAT1,DMRT1,EIF4G3,CDCA5,PPP1CB,CATSPER2,RGS8,RAB31,PDK1,HSPG2,PSMD2,CSMD3,HERPUD1,NCOA6,TRIM2,COL4A3,WASHC1,ZFP30,RGS7,HOOK3,KIF7,GNG2,FSTL4,CLDN10,BARD1,STK3,DEPTOR,ZNF423,C1QL3,RSU1,PNPLA8,ZNF568,HNRNPU,CEP72,RAB3GAP2,CADPS,APCDD1,IGF1R,KCNAB1,PRKAG2,GLI2,THRB,AKAP13,MORC3,ATP10A,DNM1L
GO:0007154	cell communication	6.028348848483416e-22	*NOTCH2,BCAR3,MTOR,CNTN4,NSG1,SGCD,WWC1,GARNL3,LRP12,PTPRD,SLC24A2,FREM1,LRRC4C,ANKS1B,MYO9A,ULK2,NLK,UNC13C,KSR1,PLCB1,ZNF536,TAFA5,TENM4,DLC1,ZDHHC21,PTPRA,ITPR2,RIPOR2,PDE4D,RDX,RP1,STXBP1,ERC1,RALA,IL1RAPL2,BCL2,PRDM16,ALDH1A2,ARHGAP26,FBN1,LRFN2,CDH8,CHRNA7,DCDC1,GPR158,ROBO2,RIMS1,PIK3C3,TENM3,GABRB3,ZEB1,AKR1C3,RARB,FGD4,SPRED1,ENPEP,MYO1E,PLPPR1,MINAR1,CDC42EP3,RIMS2,ALK,AUTS2,ADGRE1,MCTP1,PJA2,BABAM2,SV2C,ERBIN,RHPN2,RIN2,ANO6,CACNG2,DLGAP1,MLLT3,GPC6,CNTNAP2,MAP3K9,APC,HHLA2,TSHZ3,PLPPR5,DSCAM,CRKL,ILDR2,ERG,ARHGAP24,TNIK,SLC4A10,PTPRJ,KDM4C,DOCK10,EGFR,RFX3,DENND1A,USP14,ANGPT1,MACF1,CTNNA3,PRKACB,RGS3,NCOR1,RNF220,DOCK2,NEDD4,MAML2,SCAI,CRB1,BTBD9,FAM83F,SGMS1,GRIK3,CHSY1,NTRK3,RXFP1,C5,PDE1C,ZFAND6,DKK2,FLT1,RABEP1,MAPKBP1,GABRB1,DGKI,INVS,EDAR,GRIA1,NEO1,CNTN6,SLC39A12,SLC8A3,TOM1L2,PRKD1,TPTE2,PAK1,GMDS,EPHA7,CTNNAL1,CHRM3,RALGPS1,SPEN,RAPGEF2,PELI2,LRP2,ADGRB3,RUNX2,FGF12,GABRA6,TAOK3,ONECUT1,CPEB4,TMEM38B,PRICKLE2,TAFA4,BTBD11,PTPRN2,SYN2,CCL28,PATJ,GRM7,RPTOR,TMEM117,GHR,COL4A2,RALGAPA1,RAPGEF5,PPP1R12B,TRPM1,ADAM10,IL1R1,APBB2,APP,RPS6KA2,CACNA1C,CACNB2,DCLK1,STAU2,GABRG2,DOCK8,MAPRE2,USP18,SEMA5A,SYT1,ARHGAP44,NTF3,ACER2,NDUFAF2,CD2AP,AURKA,ST18,PYGO1,SLC8A1,HERPUD2,PTPRR,TAFA2,ABCG8,KANK1,MAP4K4,BMPR1B,FMN2,PCSK6,AKAP6,HOMER2,ARNT,RAB8B,PAK3,RFTN1,PDE1A,KCNK10,LARP1,ITPKB,RGS20,PDE10A,RAP1GDS1,HHAT,KICS2,ERC2,NBN,SYN3,IFT57,INTS7,PRKCZ,BTLA,GRB10,MSH6,ARHGAP32,RGS9,HECW1,DEFA3,DUSP22,SV2B,YAP1,PPM1L,SHC4,MAPK1,MGAT5,CADPS2,HRH2,ALCAM,PLG,PDGFD,SYT10,ZNRF3,PPP1R1C,ITGBL1,ARHGEF17,NRG3,UBE2O,NCAM1,GFRA1,SLC16A1,STK38,PTPN13,CHN1,HRH4,SORCS3,GLP2R,PAFAH1B1,ATF6,EFEMP1,TM7SF3,ITGB8,TLK1,NF2,CNKSR2,GRIK4,HIVEP1,CTNNA1,PPP1R9A,MOB3B,BIRC6,AKAP9,KLF15,RASGRF2,PPARA,ERMP1,RGL1,SYNJ1,NR5A2,ADAMTS3,TIAM1,ARAP2,GRM1,PTPRK,ARHGEF12,GABRG1,PAK5,TRERF1,PCDH11Y,PPP2R5E,PLA2R1,SEMA3C,DAPK1,SLC24A4,SEC14L1,TMEM108,STK32B,MAGI1,ALPK2,SCN2A,RIC8B,SORCS1,DNAJC15,AMPH,CPE,EVC2,IL34,ANK2,ADGRV1,MELK,RYR2,BBS2,WNT9B,OR4F6,NKG7,SEMA6D,DUSP16,SMARCA4,CDH11,USP8,PARD3,MAPKAP1,PIAS1,SPG21,BLK,TNR,GRM8,DST,CXADR,DOCK4,MBD5,ATRX,NUAK1,PTPRT,ELAVL4,ABL1,PTPN12,HDAC4,SLC1A1,PRKAA1,GAS2,KCNH1,ITGB3BP,RIMBP2,APBB1IP,EIPR1,APLF,NFAT5,MAST4,GUCY1A2,RAP1A,MYO10,GPC5,CAMK4,INPP5A,FGF10,ZC3HAV1,GRID2,LATS2,NRG1,GSG1L,ASPM,AP3B1,DENND2B,RASGRF1,MUSK,ZNF675,GNG7,SH3GL3,PRKCE,NXN,WNK2,ESRRG,DGKB,USP33,DENND4C,FBN2,CD44,RGS12,PTPRO,EGF,P2RX6,TRIO,PDE3A,EXT1,NSMAF,LNPEP,LIMD1,SPRED2,RPS6KA3,CTNND2,SCG5,MTMR3,PTPN2,TRIM5,PLXNA2,MCF2L,OR4F15,ATXN3,HTR2C,RIC3,CLEC16A,ARHGEF7,AMBRA*

			1,LTBP1,STK38L,ZFYVE9,OPRM1,ABCC4,HTR2A,PLCXD3,FANCA,INPP4B,KREMEN1,STAC,SEMA3E,MARK2,GCSAML,FHL2,ADGRA3,CNIH3,PUM1,TMOD2,MSH2,GNAL,EPHA6,ANKRD17,APBA2,SH3KBP1,RELL1,HIPK3,EPN2,KCND2,EVC,GRK3,KNDC1,SPSB4,CLSPN,NOS2,MOSMO,GFRA2,TMEM116,RBBP8,MDFIC,ADAM12,MYLK2,ANK3,SNTG1,HMGA2,CCND3,VPS41,DOCK5,ECE1,STK32A,CREM,MBP,PLCE1,TGFA,IL17RA,HIP1,CRIM1,PRR5L,CAPN5,VAV1,LDLRAD4,NPHP4,PACSIN2,CNTN1,IQSEC1,SNX3,CACNA1I,BRCA2,DISC1,DNER,BLM,ASB7,WDPCP,NRK,SEMA3A,MAGI3,ADCY10,PSG8,STRN,OR9Q1,BMP2,RC3H2,UNC5D,PSG9,CDC42BPB,SOGA1,SCN11A,VRK1,GNAI1,RALGAPA2,RANBP9,MYRIP,TTR,RIN3,BMP2K,TMEM161A,SEMA3D,NETO2,PDE6C,CABIN1,LEMD3,RELN,ARHGAP42,HMGB1,GNAQ,FGF9,NFATC2,SH3BP5,POLR2M,ZNF106,MYOM1,TRAF3,UNC13B,TTC21B,DSTYK,UIMC1,DOCK1,RAP1GAP,SRGAP2,DRAXIN,ATF1,CCDC186,SLAMF1,FAM83B,GLI3,CGAS,SMARCC1,SNX6,GABRR2,SMOC2,PCP4,CNKSR3,CASP5,GRIK2,IDE,WDR12,MCTP2,CUL1,PSD3,GAREM1,LAMC1,NEK10,FARP1,MOB1B,ATF2,CYLD,BBS4,MAPK8IP1,MX1,PSG6,GABBR2,ITGA9,CFTR,KPNA1,UBASH3A,RGMB,NEU3,KITLG,ZZEF1,CAMTA1,UBR1,DCC,CHRM5,MAP4K3,SLC30A10,RCAN1,RORB,DAB1,RB1CC1,AKAP10,PTPRE,PRKN,MTMR2,TBX20,DLGAP2,AFAP1,MAPK10,NGEF,GRIN2A,ARID5B,WSB1,LALBA,PRKCH,PKP1,HUNK,TG,IL6R,ALS2,RACGAP1,NLRC5,OR51E1,MKNK1,DOCK9,SNX25,KCNQ3,SHISA9,PDE6A,COPS8,SCN10A,SHANK2,USP7,VAV3,MESD,SOX30,MOK,KIR2DL4,ARHGEF28,RALB,ADGRG6,ROCK1,LYN,VCAM1,SEL1L,ARHGAP28,ARHGAP31,EIF2B3,SLC44A2,SLC15A2,DTX1,TENM2,OVOL2,ZBTB33,ADA2,NTN1,CHKA,PLCB4,DPYSL5,RRAGD,CRACR2A,RNF152,OTUD7A,INSR,CUL5,OR7A17,BMF,YTHDF3,TFF1,DEDD2,NEK6,GRID1,COLQ,NMU,ARFGEF1,GAST,SNAI2,ASH1L,IGHV3-74,BID,SIAH2,RPH3A,ABCA4,TRABD2B,UFD1,RXRG,ERN2,GABRG3,MBTPS2,FLNB,TIAL1,TOM1,PLPP4,NREP,ZDHHC17,SH3GLB1,CARD10,RALGPS2,JCAD,OR4K2,SAMHD1,IFT81,ENPP1,RASGRP1,IGSF11,NDRG2,CSNK2A1,BMP5,CSF1,GHRH,HDGFL3,BCL2L1,HCN1,PRKG1,LAMA3,ASB4,GRIN2B,GRB14,GPR156,IGHV2-70D,DHRS3,SMAD5,CELF4,OR4C46,FOXN3,PRAME,TNN,PSAP,MED1,CDC14B,PCNT,IL33,GPRC5C,ROR2,KL,RASGEF1C,BANK1,FAT1,IL10,OR1L6,SFPQ,CLSTN2,PTH,SOSTDC1,PRKAA2,CSF2RB,DIRAS2,NDC80,ABHD2,ITPRIP,PLA2G4A,RAB12,IQGAP1,RPS12,CAMLG,TEAD1,SREBF2,YBX3,AIMP1,THNSL2,FYB2,NRXN1,PCID2,HIPK1,DGKK,CD70,CIBAR1,PBLD,FICD,PEG10,NET1,SIPA1L2,TWIST1,AKT3,ALKAL2,JAK2,RPF2,FSTL1,SVEP1,MADD,HCRTR1,CELSR2,CREBBP,TNKS,GORAB,UFL1,NFKBIA,PRKCB,OR2T3,ABCC8,ANXA4,CACNA1E,ZC3H15,ANP32A,BRD4,NEDD9,OLFM4,NRBP1,ITGA6,ATP2B1,GAP43,GRIP1,IGHV1OR15-9,ADGRE3,ADCY9,PPP1R17,CNIH1,MAST2,ERLIN2,OTOP1,CIDEA,ARFGEF3,EXT2,EXOC1,SLC6A1,GID8,STAT1,BRMS1L,NDFIP2,NR2C1,MAP2K6,CMTM7,DGKG,ABI1,IMPACT,CCBE1,PARK7,ADAMTS18,MAPK8,ITGA4,FBXL17,ADCYAP1R1,NCAPG2,RAPGEF4,OR6C75,ASB2,MYOCD,MYH13,CYFIP2,EFHB,OR13C9,MEF2C,ADGRB1,RXRA,WNT7A,RBPMS2,MAP3K5,NDFIP1,MAP3K4,S100B,ATP6V1C2,C16ORF72,PKN2,OR10H2,PDE2A,RAB38,LRRC2,SDCBP,JPT2,SPPL2B,WWOX,PASK,NCK1,FGR,CDCA8,PPP2R3A,DNMBP,CXCL2,SNAP29,IFNAR1,GNG12,EPHA4,CYTH4,GABRA5,MECOM,NTRK2,IL1RAPL1,WNT2B,COLEC12,POSTN,CD101,SHISA6,IL17RD,FBXO31,AKAP11,GRIK1,PRKAB1,DTHD1,MVB12B,PTK2,MARK4,CDH5,RCAN2,ANKRD6,SCGN,NFKBID,ARHGAP12,CLDN18,FEZ2,INIP,LAMB1,APIP,CYFIP1,UBE3A,PCDH8,SEMA4D,PITPNC1,MC2R,FAT4,IMPA2,WNT5B,RASGEF1B,AMFR,NENF,PTGFR,ZFYVE1,OR4L1,ICA1,PLCZ1,NOS1AP,FCRLA,DIDO1,TPTE,SORBS2,CNKSR1,CCDC88A,GPR55,NSUN2,CDC45,OR11G2,BICD1,TNFSF11,FYN,BUB1,PPM1F,ADGRL2,ARL13B,SDE2,RBMS3,SCN8A,HDAC2,SH2D3C,DOCK3,ALB,DOK5,ZFYVE28,MAPK9,APELA,ROR1,FUT8,TET1,ASB3,CDH2,ITGA8,FBXL20,NTN4,RAD9A,PHLPP1,GPR137B,EPHB1,RP1L1,GRM5,RAI14,RPS6KA5,TBC1D

			1,LRRC69,PTPRG,PID1,NRP1,FCHSD2,PRKCA,FAIM,SAMD12,FHIT,ITGA1,RNF138,RC3H1,POR,MCC,BCR,NRXN3,ELMO1,RGS6,RERG,KIF16B,SNRK,ELP2,FBLN1,STK36,NSG2,B9D1,RRAS2,GNA14,BMPER,PRDM15,SRGAP3,MITF,EPHB2,CSNK1G1,CD38,EYA4,CDK14,MET,SPPL3,DLG2,CDH17,CDH13,STXBP4,CACNG3,ATG5,MAGI2,PRDM11,FLRT2,KALRN,SLC1A2,GNAS,LAMA1,MFHAS1,ATRNL1,TIAM2,BMP7,DLG5,GAPVD1,GABRA2,OR2T2,ZMYND11,TMEM25,GRM3,ADGRF5,OR4N2,PDGFC,PLIN2,PPP1R13B,ABL2,EYA1,SLIT2,EXOC4,CNOT7,KCTD8,PLCL1,ERBB4,IL20RB,FAM3B,TRHDE,ROBO1,IRAG1,NUF2,PRKCQ,NDRG1,SORCS2,SIPA1L3,TRDN,NLGN1,NOS1,SLC6A3,ASIC2,EFNA5,GAS2L1,ARHGEF11,SLIT3,DTNA,ESR1,MYO9B,NTNG1,CACNA2D1,NYAP2,IGLC3,IQCJ-SCHIP1,ADGRG7,SKAP2,PRLR,AGO3,HTT,FOXB1,PIK3R3,FER,EYA2,CCR2,STARD13,CHFR,EPS8,SEMA4B,IGHV1OR21-1,HRH1,GRIA4,ROCK2,RORA,IL16,DMRT1,PPP1CB,RGS8,PDK1,HERPUD1,COL4A3,RGS7,KIF7,GNG2,FSTL4,BARD1,STK3,DEPTOR,ZNF423,RSU1,CADPS,APCDD1,IGF1R,PRKAG2,GLI2,THRB,AKAP13,DNM1L
GO:0023052	signaling	2.171776249778723e-21	NOTCH2,BCAR3,MTOR,CNTN4,NSG1,SGCD,WWC1,GARNL3,LRP12,PTPRD,SLC24A2,LRRC4C,ANKS1B,MYO9A,ULK2,NLK,UNC13C,KSR1,PLCB1,ZNF536,TAFA5,TENM4,DLC1,ZDHHC21,PTPRA,ITPR2,RIPOR2,PDE4D,RDX,RP1,STXBP1,ERC1,RALA,IL1RAPL2,BCL2,PRDM16,ALDH1A2,ARHGAP26,FBN1,LRFN2,CDH8,CHRNA7,DCDC1,GPR158,ROBO2,RIMS1,PIK3C3,TENM3,GABRB3,ZEB1,AKR1C3,RARB,FGD4,SPRED1,ENPEP,MYO1E,PLPPR1,MINAR1,CDC42EP3,RIMS2,ALK,AUTS2,ADGRE1,MCTP1,PJA2,BABAM2,SV2C,ERBIN,RHPN2,RIN2,ANO6,CACNG2,DLGAP1,MLLT3,GPC6,CNTNAP2,MAP3K9,APC,HHLA2,TSHZ3,PLPPR5,DSCAM,CRKL,ILDR2,ERG,ARHGAP24,TNIK,SLC4A10,PTPRJ,KDM4C,DOCK10,EGFR,RFX3,DENND1A,USP14,ANGPT1,MACF1,CTNNA3,PRKACB,RGS3,NCOR1,RNF220,DOCK2,NEDD4,MAML2,SCAI,CRB1,BTBD9,FAM83F,SGMS1,GRIK3,CHSY1,ATP2B2,NTRK3,RXFP1,C5,PDE1C,ZFAND6,DKK2,FLT1,RABEP1,MAPKBP1,GABRB1,DGKI,INVS,EDAR,GRIA1,NEO1,CNTN6,SLC39A12,SLC8A3,TOM1L2,PRKD1,TPTE2,PAK1,GMDS,EPHA7,CTNNAL1,CHRM3,RALGPS1,SPEN,RAPGEF2,PELI2,LRP2,ADGRB3,RUNX2,FGF12,GABRA6,TAOK3,ONECUT1,CPEB4,TMEM38B,PRICKLE2,TAFA4,BTBD11,PTPRN2,SYN2,CCL28,PATJ,GRM7,RPTOR,TMEM117,GHR,COL4A2,RALGAPA1,RAPGEF5,PPP1R12B,TRPM1,ADAM10,IL1R1,APBB2,APP,RPS6KA2,CACNA1C,CACNB2,DCLK1,STAU2,GABRG2,DOCK8,MAPRE2,USP18,SEMA5A,SYT1,ARHGAP44,NTF3,ACER2,NDUFAF2,CD2AP,AURKA,ST18,PYGO1,SLC8A1,HERPUD2,PTPRR,TAFA2,ABCG8,KANK1,KCNE4,MAP4K4,BMPR1B,FMN2,PCSK6,AKAP6,HOMER2,ARNT,RAB8B,PAK3,RFTN1,PDE1A,KCNK10,LARP1,ITPKB,RGS20,PDE10A,RAP1GDS1,HHAT,KICS2,ERC2,NBN,SYN3,IFT57,INTS7,PRKCZ,BTLA,GRB10,MSH6,ARHGAP32,RGS9,HECW1,DEFA3,DUSP22,SV2B,YAP1,PPM1L,SHC4,MAPK1,MGAT5,CADPS2,HRH2,ALCAM,PLG,PDGFD,SYT10,ZNRF3,PPP1R1C,ITGBL1,ARHGEF17,NRG3,UBE2O,NCAM1,GFRA1,SLC16A1,STK38,PTPN13,CHN1,HRH4,SORCS3,GLP2R,PAFAH1B1,ATF6,EFEMP1,TM7SF3,ITGB8,TLK1,NF2,CNKSR2,GRIK4,HIVEP1,CORIN,CTNNA1,PPP1R9A,MOB3B,BIRC6,AKAP9,KLF15,RASGRF2,PPARA,ERMP1,RGL1,SYNJ1,NR5A2,ADAMTS3,TIAM1,ARAP2,GRM1,PTPRK,ARHGEF12,GABRG1,PAK5,TRERF1,PCDH11Y,PPP2R5E,PLA2R1,SEMA3C,DAPK1,SLC24A4,SEC14L1,TMEM108,STK32B,MAGI1,ALPK2,SCN2A,RIC8B,SORCS1,AMPH,CPE,EVC2,IL34,ANK2,ADGRV1,MELK,RYR2,BBS2,WNT9B,OR4F6,NKG7,SEMA6D,DUSP16,SMARCA4,CDH11,USP8,PARD3,MAPKAP1,PIAS1,SPG21,BLK,TNR,GRM8,DST,CXADR,DOCK4,MBD5,ATRX,NUAK1,PTPRT,ELAVL4,ABL1,PTPN12,HDAC4,SLC1A1,PRKAA1,GAS2,KCNH1,ITGB3BP,RIMBP2,APBB1IP,EIPR1,APLF,NFAT5,MAST4,GUCY1A2,RAP1A,MYO10,GPC5,CAMK4,INPP5A,FGF10,ZC3HAV1,GRID2,LATS2,NRG1,GSG1L,ASPM,AP3B1,DENND2B,RASGRF1,MUSK,ZNF675,GNG7,SH3GL3,PRKCE,NXN,WNK2,ESRRG,DGKB,USP33,DENND4C,FBN2,CD44,RGS12,PTPRO,EGF,ABCC9,P2RX6,TRIO,PDE3A,EXT1,NSMAF,LN

			PEP,LIMD1,SPRED2,RPS6KA3,CTNND2,SCG5,PTPN2,TRIM5,PLXNA2,MCF2L,OR4F15,ATXN3,HTR2C,RIC3,CLEC16A,ARHGEF7,LTBP1,STK38L,ZFYVE9,OPRM1,ABCC4,HTR2A,PLCXD3,FANCA,INPP4B,KREMEN1,STAC,SEMA3E,MARK2,GCSAML,FHL2,ADGRA3,CNIH3,PUM1,TMOD2,MSH2,GNAL,EPHA6,ANKRD17,APBA2,SH3KBP1,RELL1,HIPK3,EPN2,KCND2,EVC,GRK3,KNDC1,SPSB4,CLSPN,NOS2,MOSMO,GFRA2,TMEM116,RBBP8,MDFIC,ADAM12,MYLK2,ANK3,HMGA2,CCND3,DOCK5,ECE1,STK32A,CREM,MBP,PLCE1,TGFA,IL17RA,HIP1,CRIM1,PRR5L,CAPN5,VAV1,LDLRAD4,NPHP4,PACSIN2,CNTN1,IQSEC1,SNX3,CACNA1I,BRCA2,DISC1,DNER,BLM,ASB7,WDPCP,NRK,SEMA3A,MAGI3,ADCY10,PSG8,STRN,OR9Q1,BMP2,RC3H2,UNC5D,PSG9,CDC42BPB,SOGA1,SCN11A,VRK1,GNAI1,RALGAPA2,RANBP9,MYRIP,TTR,RIN3,BMP2K,TMEM161A,SEMA3D,NETO2,PDE6C,CABIN1,LEMD3,RELN,ARHGAP42,HMGB1,GNAQ,FGF9,NFATC2,SH3BP5,POLR2M,ZNF106,MYOM1,TRAF3,UNC13B,TTC21B,DSTYK,UIMC1,DOCK1,RAP1GAP,SRGAP2,DRAXIN,ATF1,CCDC186,SLAMF1,FAM83B,GLI3,CGAS,SMARCC1,SNX6,GABRR2,SMOC2,PCP4,CNKSR3,CASP5,GRIK2,IDE,WDR12,MCTP2,CUL1,PSD3,GAREM1,LAMC1,NEK10,FARP1,MOB1B,ATF2,CYLD,BBS4,MAPK8IP1,MX1,PSG6,GABBR2,ITGA9,CFTR,KPNA1,UBASH3A,RGMB,NEU3,KITLG,ZZEF1,CAMTA1,UBR1,DCC,CHRM5,MAP4K3,SLC30A10,RCAN1,RORB,DAB1,RB1CC1,AKAP10,PTPRE,PRKN,MTMR2,TBX20,DLGAP2,AFAP1,MAPK10,NGEF,GRIN2A,ARID5B,WSB1,LALBA,PRKCH,PKP1,HUNK,TG,IL6R,ALS2,RACGAP1,NLRC5,OR51E1,MKNK1,DOCK9,SNX25,KCNQ3,SHISA9,PDE6A,COPS8,SCN10A,SHANK2,USP7,VAV3,KCND3,MESD,SOX30,MOK,KIR2DL4,ARHGEF28,RALB,ADGRG6,ROCK1,LYN,VCAM1,SEL1L,ARHGAP28,ARHGAP31,EIF2B3,SLC44A2,SLC15A2,DTX1,TENM2,OVOL2,ZBTB33,ADA2,NTN1,PLCB4,DPYSL5,RRAGD,CRACR2A,RNF152,OTUD7A,INSR,CUL5,OR7A17,BMF,YTHDF3,TFF1,DEDD2,NEK6,GRID1,COLQ,NMU,ARFGEF1,GAST,SNAI2,ASH1L,IGHV3-74,BID,SIAH2,RPH3A,ABCA4,TRABD2B,UFD1,RXRG,ERN2,GABRG3,MBTPS2,FLNB,TIAL1,TOM1,PLPP4,NREP,ZDHHC17,CARD10,RALGPS2,JCAD,OR4K2,SAMHD1,IFT81,ENPP1,RASGRP1,IGSF11,NDRG2,CSNK2A1,BMP5,CSF1,GHRH,HDGFL3,BCL2L1,HCN1,PRKG1,LAMA3,ASB4,GRIN2B,GRB14,GPR156,IGHV2-70D,DHRS3,SMAD5,CELF4,OR4C46,FOXN3,PRAME,TNN,PSAP,MED1,CDC14B,PCNT,IL33,GPRC5C,ROR2,KL,RASGEF1C,BANK1,FAT1,IL10,OR1L6,SFPQ,CLSTN2,PTH,SOSTDC1,PRKAA2,CSF2RB,DIRAS2,NDC80,ABHD2,ITPRIP,PLA2G4A,RAB12,IQGAP1,RPS12,CAMLG,TEAD1,SREBF2,YBX3,AIMP1,THNSL2,FYB2,NRXN1,PCID2,HIPK1,DGKK,CD70,CIBAR1,PBLD,FICD,PEG10,NET1,SIPA1L2,TWIST1,AKT3,ALKAL2,JAK2,RPF2,FSTL1,SVEP1,MADD,HCRTR1,CELSR2,CREBBP,TNKS,GORAB,UFL1,NFKBIA,PRKCB,OR2T3,ABCC8,ANXA4,CACNA1E,ZC3H15,ANP32A,BRD4,NEDD9,OLFM4,NRBP1,ITGA6,ATP2B1,GAP43,GRIP1,IGHV1OR15-9,ADGRE3,ADCY9,PPP1R17,CNIH1,MAST2,ERLIN2,OTOP1,CIDEA,ARFGEF3,EXT2,EXOC1,SLC6A1,GID8,STAT1,BRMS1L,NDFIP2,NR2C1,MAP2K6,CMTM7,DGKG,ABI1,CCBE1,PARK7,ADAMTS18,MAPK8,ITGA4,FBXL17,ADCYAP1R1,NCAPG2,RAPGEF4,OR6C75,ASB2,MYOCD,CYFIP2,EFHB,OR13C9,MEF2C,ADGRB1,RXRA,WNT7A,RBPMS2,MAP3K5,NDFIP1,MAP3K4,S100B,ATP6V1C2,C16ORF72,PKN2,OR10H2,PDE2A,RAB38,LRRC2,SDCBP,JPT2,SPPL2B,WWOX,PASK,NCK1,FGR,CDCA8,PPP2R3A,DNMBP,CXCL2,SNAP29,IFNAR1,GNG12,EPHA4,CYTH4,GABRA5,MECOM,NTRK2,IL1RAPL1,WNT2B,COLEC12,POSTN,CD101,SHISA6,IL17RD,FBXO31,AKAP11,GRIK1,PRKAB1,DTHD1,MVB12B,PTK2,MARK4,CDH5,RCAN2,ANKRD6,SCGN,NFKBID,ARHGAP12,CLDN18,FEZ2,INIP,LAMB1,APIP,CYFIP1,UBE3A,PCDH8,SEMA4D,PITPNC1,MC2R,FAT4,IMPA2,WNT5B,RASGEF1B,AMFR,NENF,PTGFR,OR4L1,ICA1,PLCZ1,NOS1AP,FCRLA,DIDO1,TPTE,SORBS2,CNKSR1,CCDC88A,GPR55,NSUN2,CDC45,OR11G2,BICD1,TNFSF11,FYN,BUB1,PPM1F,ADGRL2,ARL13B,SDE2,RBMS3,SCN8A,HDAC2,SH2D3C,DOCK3,DOK5,ZFYVE28,MAPK9,APELA,ROR1,FUT8,TET1,ASB3,CDH2,ITGA8,FBXL20,NTN4,RAD9A,PHLPP1,GPR137B,EPHB1,RP1

			L1,GRM5,RAI14,RPS6KA5,TBC1D1,LRRC69,PTPRG,PID1,NRP1,FCHSD2,PRKCA,FAIM,SAMD12,FHIT,ITGA1,RNF138,RC3H1,POR,MCC,BCR,NRXN3,ELMO1,RGS6,RERG,KIF16B,SNRK,ELP2,FBLN1,STK36,NSG2,B9D1,RRAS2,GNA14,BMPER,PRDM15,SRGAP3,MITF,EPHB2,CSNK1G1,CD38,EYA4,CDK14,MET,SPPL3,DLG2,CDH17,CDH13,STXBP4,CACNG3,MAGI2,PRDM11,FLRT2,KALRN,SLC1A2,GNAS,LAMA1,MFHAS1,ATRNL1,TIAM2,BMP7,DLG5,GAPVD1,GABRA2,OR2T2,ZMYND11,TMEM25,GRM3,ADGRF5,OR4N2,PDGFC,PPP1R13B,ABL2,EYA1,SLIT2,EXOC4,CNOT7,KCTD8,PLCL1,ERBB4,IL20RB,FAM3B,TRHDE,ROBO1,IRAG1,NUF2,PRKCQ,NDRG1,SORCS2,SIPA1L3,TRDN,NLGN1,NOS1,SLC6A3,ASIC2,EFNA5,ARHGEF11,SLIT3,DTNA,ESR1,MYO9B,NTNG1,CACNA2D1,NYAP2,IGLC3,IQCJ-SCHIP1,ADGRG7,SKAP2,PRLR,AGO3,HTT,PIK3R3,FER,EYA2,CCR2,STARD13,CHFR,EPS8,SEMA4B,IGHV1OR21-1,HRH1,GRIA4,ROCK2,RORA,IL16,DMRT1,PPP1CB,RGS8,PDK1,HERPUD1,COL4A3,RGS7,KIF7,GNG2,FSTL4,BARD1,STK3,DEPTOR,ZNF423,RSU1,CADPS,APCDD1,IGF1R,PRKAG2,GLI2,THRB,AKAP13,DNM1L
GO:0035556	intracellular signal transduction	8.181691505147024e-21	*NOTCH2,BCAR3,MTOR,SGCD,WWC1,GARNL3,MYO9A,NLK,KSR1,PLCB1,DLC1,ITPR2,RIPOR2,PDE4D,RDX,RP1,ERC1,RALA,BCL2,ARHGAP26,CHRNA7,DCDC1,PIK3C3,AKR1C3,FGD4,SPRED1,MINAR1,CDC42EP3,ALK,AUTS2,MCTP1,PJA2,BABAM2,ERBIN,RIN2,MAP3K9,APC,CRKL,ARHGAP24,TNIK,PTPRJ,DOCK10,EGFR,DENND1A,ANGPT1,PRKACB,NCOR1,DOCK2,NEDD4,SCAI,SGMS1,NTRK3,ZFAND6,FLT1,MAPKBP1,DGKI,EDAR,PRKD1,TPTE2,PAK1,EPHA7,CTNNAL1,CHRM3,RALGPS1,RAPGEF2,PELI2,LRP2,FGF12,TAOK3,TMEM38B,PATJ,RPTOR,TMEM117,GHR,RALGAPA1,RAPGEF5,APBB2,APP,RPS6KA2,CACNA1C,DCLK1,DOCK8,MAPRE2,SEMA5A,ARHGAP44,NTF3,ACER2,CD2AP,AURKA,SLC8A1,PTPRR,KANK1,MAP4K4,FMN2,AKAP6,HOMER2,PAK3,LARP1,ITPKB,PDE10A,RAP1GDS1,KICS2,NBN,INTS7,PRKCZ,GRB10,MSH6,ARHGAP32,RGS9,DUSP22,YAP1,PPM1L,SHC4,MAPK1,PDGFD,PPP1R1C,ARHGEF17,NRG3,STK38,PTPN13,CHN1,HRH4,PAFAH1B1,TLK1,NF2,CNKSR2,PPP1R9A,MOB3B,RASGRF2,PPARA,RGL1,NR5A2,TIAM1,GRM1,ARHGEF12,PAK5,PLA2R1,DAPK1,SLC24A4,SEC14L1,STK32B,SCN2A,IL34,ANK2,ADGRV1,MELK,RYR2,DUSP16,USP8,MAPKAP1,BLK,DOCK4,ATRX,NUAK1,ABL1,HDAC4,PRKAA1,KCNH1,NFAT5,MAST4,GUCY1A2,RAP1A,CAMK4,INPP5A,FGF10,ZC3HAV1,LATS2,NRG1,DENND2B,RASGRF1,ZNF675,PRKCE,WNK2,DGKB,DENND4C,CD44,EGF,TRIO,PDE3A,LIMD1,SPRED2,RPS6KA3,PTPN2,TRIM5,MCF2L,HTR2C,CLEC16A,ARHGEF7,STK38L,OPRM1,HTR2A,SEMA3E,MARK2,FHL2,PUM1,MSH2,ANKRD17,RELL1,HIPK3,KNDC1,SPSB4,CLSPN,NOS2,RBBP8,MDFIC,HMGA2,DOCK5,STK32A,MBP,PLCE1,TGFA,HIP1,PRR5L,VAV1,IQSEC1,BRCA2,DISC1,BLM,ASB7,NRK,SEMA3A,MAGI3,ADCY10,BMP2,RC3H2,GNAI1,RALGAPA2,RANBP9,TMEM161A,LEMD3,RELN,ARHGAP42,HMGB1,NFATC2,SH3BP5,POLR2M,MYOM1,TRAF3,DSTYK,UIMC1,DOCK1,RAP1GAP,SRGAP2,ATF1,SLAMF1,CGAS,CNKSR3,MCTP2,CUL1,PSD3,GAREM1,NEK10,MOB1B,ATF2,CYLD,MAPK8IP1,KITLG,CAMTA1,UBR1,MAP4K3,SLC30A10,RCAN1,DAB1,RB1CC1,PRKN,MAPK10,NGEF,GRIN2A,WSB1,PRKCH,HUNK,IL6R,ALS2,RACGAP1,NLRC5,MKNK1,DOCK9,COPS8,SHANK2,USP7,VAV3,MOK,ARHGEF28,RALB,ADGRG6,ROCK1,LYN,VCAM1,ARHGAP28,ARHGAP31,SLC44A2,SLC15A2,ZBTB33,NTN1,PLCB4,RRAGD,CRACR2A,RNF152,OTUD7A,INSR,CUL5,DEDD2,NEK6,ARFGEF1,SNAI2,ASH1L,BID,SIAH2,UFD1,ERN2,TIAL1,ZDHHC17,CARD10,RALGPS2,JCAD,RASGRP1,NDRG2,CSF1,BCL2L1,PRKG1,ASB4,GRIN2B,FOXN3,CDC14B,ROR2,KL,RASGEF1C,BANK1,SFPQ,PTH,PRKAA2,NDC80,RAB12,IQGAP1,TEAD1,YBX3,NRXN1,PCID2,HIPK1,DGKK,NET1,SIPA1L2,TWIST1,AKT3,ALKAL2,JAK2,RPF2,MADD,HCRTR1,UFL1,NFKBIA,PRKCB,ANP32A,BRD4,NRBP1,GRIP1,ADCY9,PPP1R17,MAST2,ARFGEF3,EXOC1,STAT1,NDFIP2,MAP2K6,DGKG,PARK7,MAPK8,ADCYAP1R1,RAPGEF4,ASB2,EFHB,MEF2C,WNT7A,MAP3K5,NDFIP1,MAP3K4,S100B,C16ORF72,PKN2,PDE2A,RAB38,SDCBP,JPT2,WWOX,PASK,NCK1,FGR,CDCA8,D*

			NMBP,EPHA4,CYTH4,MECOM,NTRK2,FBXO31,AKAP11,PTK2,MARK4,RCAN2,ANKRD6,ARHGAP12,INIP,APIP,CYFIP1,UBE3A,SEMA4D,FAT4,RASGEF1B,NENF,PTGFR,PLCZ1,NOS1AP,TPTE,CNKSR1,CCDC88A,GPR55,NSUN2,CDC45,TNFSF11,FYN,BUB1,PPM1F,SDE2,SH2D3C,DOCK3,DOK5,MAPK9,APELA,ROR1,ASB3,CDH2,RAD9A,PHLPP1,GPR137B,EPHB1,RP1L1,GRM5,RAI14,RPS6KA5,NRP1,PRKCA,FAIM,FHIT,ITGA1,RC3H1,BCR,ELMO1,RGS6,RERG,SNRK,FBLN1,STK36,RRAS2,BMPER,PRDM15,SRGAP3,EPHB2,MET,SPPL3,CDH13,MAGI2,PRDM11,KALRN,GNAS,MFHAS1,TIAM2,BMP7,DLG5,ZMYND11,PDGFC,PPP1R13B,ABL2,SLIT2,PLCL1,ERBB4,ROBO1,IRAG1,NUF2,PRKCQ,NDRG1,SIPA1L3,NLGN1,NOS1,ARHGEF11,ESR1,MYO9B,NYAP2,IQCJ-SCHIP1,AGO3,HTT,PIK3R3,FER,CCR2,STARD13,CHFR,EPS8,HRH1,ROCK2,RORA,DMRT1,PPP1CB,PDK1,HERPUD1,RGS7,BARD1,STK3,DEPTOR,IGF1R,PRKAG2,AKAP13,DNM1L
GO:0023051	regulation of signaling	1.767283726543 5958e-20	*NOTCH2,BCAR3,MTOR,CNTN4,NSG1,WWC1,GARNL3,PTPRD,SLC24A2,LRRC4C,MYO9A,NLK,UNC13C,KSR1,PLCB1,ZNF536,DLC1,PTPRA,RIPOR2,PDE4D,RDX,STXBP1,ERC1,BCL2,PRDM16,ARHGAP26,FBN1,LRFN2,CHRNA7,ROBO2,RIMS1,ZEB1,AKR1C3,FGD4,SPRED1,MINAR1,RIMS2,ALK,AUTS2,MCTP1,PJA2,BABAM2,ERBIN,CACNG2,DLGAP1,MLLT3,GPC6,APC,TSHZ3,CRKL,ARHGAP24,TNIK,SLC4A10,PTPRJ,KDM4C,EGFR,RFX3,DENND1A,ANGPT1,MACF1,PRKACB,RGS3,NCOR1,RNF220,DOCK2,NEDD4,SCAI,BTBD9,SGMS1,GRIK3,CHSY1,ATP2B2,NTRK3,ZFAND6,DKK2,FLT1,MAPKBP1,DGKI,INVS,EDAR,GRIA1,NEO1,CNTN6,SLC8A3,PRKD1,TPTE2,PAK1,EPHA7,RALGPS1,RAPGEF2,PELI2,LRP2,RUNX2,FGF12,TAOK3,ONECUT1,TAFA4,GRM7,RPTOR,GHR,RALGAPA1,ADAM10,IL1R1,APP,CACNB2,STAU2,DOCK8,MAPRE2,USP18,SEMA5A,SYT1,ARHGAP44,NTF3,NDUFAF2,CD2AP,AURKA,SLC8A1,PTPRR,KANK1,MAP4K4,BMPR1B,PCSK6,AKAP6,HOMER2,ARNT,RAB8B,PAK3,ITPKB,RGS20,PDE10A,RAP1GDS1,KICS2,ERC2,SYN3,PRKCZ,GRB10,ARHGAP32,RGS9,HECW1,DUSP22,YAP1,MAPK1,MGAT5,PDGFD,ZNRF3,ARHGEF17,NRG3,UBE2O,NCAM1,SLC16A1,STK38,PTPN13,CHN1,HRH4,SORCS3,PAFAH1B1,ATF6,TM7SF3,ITGB8,NF2,CNKSR2,GRIK4,CORIN,CTNNA1,PPP1R9A,MOB3B,BIRC6,AKAP9,KLF15,RASGRF2,PPARA,ADAMTS3,TIAM1,GRM1,ARHGEF12,PAK5,PCDH11Y,PLA2R1,DAPK1,SLC24A4,SEC14L1,TMEM108,ALPK2,RIC8B,IL34,ANK2,ADGRV1,RYR2,DUSP16,SMARCA4,CDH11,USP8,MAPKAP1,BLK,TNR,GRM8,MBD5,NUAK1,PTPRT,ELAVL4,ABL1,PTPN12,SLC1A1,PRKAA1,GAS2,EIPR1,NFAT5,GUCY1A2,RAP1A,GPC5,FGF10,ZC3HAV1,GRID2,LATS2,NRG1,GSG1L,ASPM,DENND2B,RASGRF1,ZNF675,GNG7,PRKCE,NXN,WNK2,DGKB,USP33,DENND4C,FBN2,CD44,RGS12,PTPRO,EGF,TRIO,PDE3A,LIMD1,SPRED2,CTNND2,SCG5,PTPN2,TRIM5,MCF2L,HTR2C,CLEC16A,ARHGEF7,LTBP1,OPRM1,HTR2A,FANCA,KREMEN1,SEMA3E,GCSAML,FHL2,CNIH3,PUM1,TMOD2,ANKRD17,APBA2,RELL1,HIPK3,EPN2,EVC,GRK3,NOS2,MOSMO,MDFIC,CCND3,ECE1,PLCE1,TGFA,HIP1,CRIM1,PRR5L,VAV1,LDLRAD4,NPHP4,PACSIN2,IQSEC1,SNX3,BRCA2,DISC1,NRK,SEMA3A,MAGI3,BMP2,RC3H2,GNAI1,RALGAPA2,RANBP9,MYRIP,BMP2K,TMEM161A,NETO2,LEMD3,RELN,ARHGAP42,HMGB1,GNAQ,FGF9,TRAF3,UNC13B,TTC21B,DSTYK,RAP1GAP,SRGAP2,DRAXIN,SLAMF1,GLI3,SNX6,SMOC2,CNKSR3,GRIK2,MCTP2,PSD3,GAREM1,LAMC1,NEK10,CYLD,MAPK8IP1,CFTR,KPNA1,UBASH3A,NEU3,KITLG,ZZEF1,CAMTA1,UBR1,DCC,SLC30A10,RCAN1,DAB1,RB1CC1,PTPRE,PRKN,MTMR2,TBX20,DLGAP2,AFAP1,NGEF,GRIN2A,PRKCH,IL6R,ALS2,RACGAP1,NLRC5,SNX25,SHISA9,SHANK2,USP7,VAV3,SOX30,ARHGEF28,ROCK1,LYN,ARHGAP28,ARHGAP31,SLC44A2,SLC15A2,DTX1,OVOL2,PLCB4,RRAGD,CRACR2A,RNF152,OTUD7A,INSR,YTHDF3,DEDD2,NEK6,GRID1,NMU,ARFGEF1,SNAI2,ASH1L,BID,SIAH2,TRABD2B,UFD1,ERN2,TIAL1,NREP,ZDHHC17,RALGPS2,JCAD,SAMHD1,IFT81,ENPP1,RASGRP1,IGSF11,NDRG2,CSNK2A1,BMP5,CSF1,GHRH,BCL2L1,HCN1,GRIN2B,GRB14,DHRS3,CELF4,PRAME,TNN,MED1,ROR2,KL,BANK1,IL10,SFPQ,CLSTN2,PTH,SOSTDC1,PRKAA2,NDC80,ITPRIP,IQGAP1,RPS12,SREBF2,YBX3,AIMP1,NRXN1,PCID2,HIPK1,CIBAR1,P*

			BLD,FICD,PEG10,NET1,SIPA1L2,TWIST1,AKT3,ALKAL2,JAK2,RPF2,FSTL1,MADD,HCRTR1,CREBBP,TNKS,GORAB,UFL1,NFKBIA,PRKCB,ABCC8,BRD4,ITGA6,ATP2B1,OTOP1,CIDEA,ARFGEF3,SLC6A1,GID8,STAT1,BRMS1L,NDFIP2,NR2C1,MAP2K6,DGKG,CCBE1,PARK7,FBXL17,ADCYAP1R1,NCAPG2,MYOCD,CYFIP2,EFHB,MEF2C,ADGRB1,RXRA,WNT7A,RBPMS2,MAP3K5,NDFIP1,MAP3K4,S100B,ATP6V1C2,C16ORF72,PDE2A,SDCBP,JPT2,SPPL2B,WWOX,PASK,NCK1,FGR,CDCA8,PPP2R3A,DNMBP,EPHA4,CYTH4,MECOM,NTRK2,POSTN,SHISA6,IL17RD,GRIK1,MVB12B,PTK2,CDH5,ANKRD6,SCGN,ARHGAP12,LAMB1,APIP,CYFIP1,UBE3A,SEMA4D,WNT5B,AMFR,NENF,ICA1,NOS1AP,TPTE,CCDC88A,GPR55,BICD1,TNFSF11,FYN,RBMS3,HDAC2,DOCK3,DOK5,ZFYVE28,MAPK9,APELA,ROR1,TET1,CDH2,ITGA8,FBXL20,RAD9A,PHLPP1,GPR137B,EPHB1,GRM5,RAI14,TBC1D1,PID1,NRP1,PRKCA,FAIM,ITGA1,RC3H1,POR,MCC,BCR,NRXN3,RGS6,KIF16B,ELP2,FBLN1,STK36,BMPER,PRDM15,SRGAP3,EPHB2,CSNK1G1,CD38,EYA4,CDK14,MET,SPPL3,CDH13,STXBP4,CACNG3,MAGI2,PRDM11,KALRN,GNAS,LAMA1,MFHAS1,TIAM2,BMP7,DLG5,ZMYND11,TMEM25,GRM3,PDGFC,ABL2,EYA1,SLIT2,CNOT7,KCTD8,PLCL1,ERBB4,ROBO1,PRKCQ,SORCS2,SIPA1L3,NLGN1,EFNA5,ARHGEF11,SLIT3,ESR1,MYO9B,NTNG1,IQCJ-SCHIP1,PRLR,AGO3,HTT,FER,EYA2,CCR2,STARD13,EPS8,HRH1,ROCK2,RORA,DMRT1,RGS8,HERPUD1,RGS7,KIF7,FSTL4,BARD1,STK3,DEPTOR,ZNF423,APCDD1,IGF1R,GLI2,THRB,AKAP13,DNM1L
GO:0010646	regulation of cell communication	2.129415699285 3608e-20	*NOTCH2,BCAR3,MTOR,CNTN4,NSG1,WWC1,GARNL3,PTPRD,SLC24A2,LRRC4C,MYO9A,NLK,UNC13C,KSR1,PLCB1,ZNF536,DLC1,PTPRA,RIPOR2,PDE4D,RDX,STXBP1,ERC1,BCL2,PRDM16,ARHGAP26,FBN1,LRFN2,CHRNA7,ROBO2,RIMS1,ZEB1,AKR1C3,FGD4,SPRED1,MINAR1,RIMS2,ALK,AUTS2,MCTP1,PJA2,BABAM2,ERBIN,CACNG2,DLGAP1,MLLT3,GPC6,APC,TSHZ3,CRKL,ARHGAP24,TNIK,SLC4A10,PTPRJ,KDM4C,EGFR,RFX3,DENND1A,ANGPT1,MACF1,PRKACB,RGS3,NCOR1,RNF220,DOCK2,NEDD4,SCAI,BTBD9,SGMS1,GRIK3,CHSY1,NTRK3,ZFAND6,DKK2,FLT1,MAPKBP1,DGKI,INVS,EDAR,GRIA1,NEO1,CNTN6,SLC8A3,PRKD1,TPTE2,PAK1,EPHA7,RALGPS1,RAPGEF2,PELI2,LRP2,RUNX2,FGF12,TAOK3,ONECUT1,TAFA4,GRM7,RPTOR,GHR,RALGAPA1,ADAM10,IL1R1,APP,CACNB2,STAU2,DOCK8,MAPRE2,USP18,SEMA5A,SYT1,ARHGAP44,NTF3,NDUFAF2,CD2AP,AURKA,SLC8A1,PTPRR,KANK1,MAP4K4,BMPR1B,PCSK6,AKAP6,HOMER2,ARNT,RAB8B,PAK3,ITPKB,RGS20,PDE10A,RAP1GDS1,KICS2,ERC2,SYN3,PRKCZ,GRB10,ARHGAP32,RGS9,HECW1,DUSP22,YAP1,MAPK1,MGAT5,PDGFD,ZNRF3,ARHGEF17,NRG3,UBE2O,NCAM1,SLC16A1,STK38,PTPN13,CHN1,HRH4,SORCS3,PAFAH1B1,ATF6,TM7SF3,ITGB8,NF2,CNKSR2,GRIK4,CTNNA1,PPP1R9A,MOB3B,BIRC6,AKAP9,KLF15,RASGRF2,PPARA,ADAMTS3,TIAM1,GRM1,ARHGEF12,PAK5,PCDH11Y,PLA2R1,DAPK1,SLC24A4,SEC14L1,TMEM108,ALPK2,RIC8B,IL34,ANK2,ADGRV1,RYR2,DUSP16,SMARCA4,CDH11,USP8,MAPKAP1,BLK,TNR,GRM8,CXADR,MBD5,NUAK1,PTPRT,ELAVL4,ABL1,PTPN12,SLC1A1,PRKAA1,GAS2,EIPR1,NFAT5,GUCY1A2,RAP1A,GPC5,FGF10,ZC3HAV1,GRID2,LATS2,NRG1,GSG1L,ASPM,DENND2B,RASGRF1,ZNF675,GNG7,PRKCE,NXN,WNK2,DGKB,USP33,DENND4C,FBN2,CD44,RGS12,PTPRO,EGF,TRIO,PDE3A,LIMD1,SPRED2,CTNND2,SCG5,PTPN2,TRIM5,MCF2L,HTR2C,CLEC16A,ARHGEF7,LTBP1,OPRM1,HTR2A,FANCA,KREMEN1,SEMA3E,GCSAML,FHL2,CNIH3,PUM1,TMOD2,ANKRD17,APBA2,RELL1,HIPK3,EPN2,EVC,GRK3,NOS2,MOSMO,MDFIC,ANK3,CCND3,PLCE1,TGFA,HIP1,CRIM1,PRR5L,VAV1,LDLRAD4,NPHP4,PACSIN2,IQSEC1,SNX3,BRCA2,DISC1,NRK,SEMA3A,MAGI3,BMP2,RC3H2,GNAI1,RALGAPA2,RANBP9,MYRIP,BMP2K,TMEM161A,NETO2,LEMD3,RELN,ARHGAP42,HMGB1,GNAQ,FGF9,TRAF3,UNC13B,TTC21B,DSTYK,RAP1GAP,SRGAP2,DRAXIN,SLAMF1,GLI3,SNX6,SMOC2,CNKSR3,GRIK2,MCTP2,PSD3,GAREM1,LAMC1,NEK10,CYLD,MAPK8IP1,CFTR,KPNA1,UBASH3A,NEU3,KITLG,ZZEF1,CAMTA1,UBR1,DCC,SLC30A10,RCAN1,DAB1,RB1CC1,PTPRE,PRKN,MTMR2,TBX20,DLGAP2,AFAP1,NGEF,GRIN2A,PRKCH,IL6*

			R,ALS2,RACGAP1,NLRC5,SNX25,SHISA9,SHANK2,USP7,VAV3,SOX30,ARHGEF28,ROCK1,LYN,ARHGAP28,ARHGAP31,SLC44A2,SLC15A2,DTX1,OVOL2,PLCB4,RRAGD,CRACR2A,RNF152,OTUD7A,INSR,YTHDF3,DEDD2,NEK6,GRID1,NMU,ARFGEF1,SNAI2,ASH1L,BID,SIAH2,TRABD2B,UFD1,ERN2,TIAL1,NREP,ZDHHC17,RALGPS2,JCAD,SAMHD1,IFT81,ENPP1,RASGRP1,IGSF11,NDRG2,CSNK2A1,BMP5,CSF1,GHRH,BCL2L1,HCN1,GRIN2B,GRB14,DHRS3,CELF4,PRAME,TNN,MED1,ROR2,KL,BANK1,IL10,SFPQ,CLSTN2,PTH,SOSTDC1,PRKAA2,NDC80,ITPRIP,IQGAP1,RPS12,SREBF2,YBX3,AIMP1,NRXN1,PCID2,HIPK1,CIBAR1,PBLD,FICD,PEG10,NET1,SIPA1L2,TWIST1,AKT3,ALKAL2,JAK2,RPF2,FSTL1,MADD,HCRTR1,CREBBP,TNKS,GORAB,UFL1,NFKBIA,PRKCB,ABCC8,BRD4,ITGA6,OTOP1,CIDEA,ARFGEF3,SLC6A1,GID8,STAT1,BRMS1L,NDFIP2,NR2C1,MAP2K6,DGKG,CCBE1,PARK7,FBXL17,ADCYAP1R1,NCAPG2,MYOCD,CYFIP2,EFHB,MEF2C,ADGRB1,RXRA,WNT7A,RBPMS2,MAP3K5,NDFIP1,MAP3K4,S100B,ATP6V1C2,C16ORF72,PDE2A,SDCBP,JPT2,SPPL2B,WWOX,PASK,NCK1,FGR,CDCA8,PPP2R3A,DNMBP,EPHA4,CYTH4,MECOM,NTRK2,POSTN,SHISA6,IL17RD,GRIK1,MVB12B,PTK2,CDH5,ANKRD6,SCGN,ARHGAP12,LAMB1,APIP,CYFIP1,UBE3A,SEMA4D,WNT5B,AMFR,NENF,ICA1,NOS1AP,TPTE,CCDC88A,GPR55,BICD1,TNFSF11,FYN,RBMS3,HDAC2,DOCK3,DOK5,ZFYVE28,MAPK9,APELA,ROR1,TET1,CDH2,ITGA8,FBXL20,RAD9A,PHLPP1,GPR137B,EPHB1,GRM5,RAI14,TBC1D1,PID1,NRP1,PRKCA,FAIM,ITGA1,RC3H1,POR,MCC,BCR,NRXN3,RGS6,ELP2,FBLN1,STK36,BMPER,PRDM15,SRGAP3,EPHB2,CSNK1G1,CD38,EYA4,CDK14,MET,SPPL3,CDH13,STXBP4,CACNG3,MAGI2,PRDM11,KALRN,GNAS,LAMA1,MFHAS1,TIAM2,BMP7,DLG5,ZMYND11,TMEM25,GRM3,PDGFC,ABL2,EYA1,SLIT2,CNOT7,KCTD8,PLCL1,ERBB4,ROBO1,PRKCQ,SORCS2,SIPA1L3,TRDN,NLGN1,EFNA5,ARHGEF11,SLIT3,ESR1,MYO9B,NTNG1,IQCJ-SCHIP1,PRLR,AGO3,HTT,FER,EYA2,CCR2,STARD13,EPS8,HRH1,ROCK2,RORA,DMRT1,RGS8,HERPUD1,RGS7,KIF7,FSTL4,BARD1,STK3,DEPTOR,ZNF423,APCDD1,IGF1R,GLI2,THRB,AKAP13,DNM1L
GO:0048667	cell morphogenesis involved in neuron differentiation	2.748980471249 4596e-20	NOTCH2,CNTN4,PTPRD,LRRC4C,ULK2,RIPOR2,STXBP1,BCL2,CHRNA7,ROBO2,AUTS2,DSCAM,TNIK,DOCK10,MACF1,NEDD4,BCL11A,CDH4,PHACTR1,NEO1,CNTN6,PAK1,EPHA7,RAPGEF2,ADGRB3,ADAMTSL1,TBCD,NEDD4L,APP,DCLK1,STAU2,SEMA5A,VCL,ARHGAP44,BMPR1B,CTNNA2,PAK3,DIP2B,TRPC5,DNM3,DIP2A,HECW1,COBL,ALCAM,NCAM1,CHN1,PAFAH1B1,NFIB,PRTG,TIAM1,ENAH,SEMA3C,SEMA6D,CDH11,PARD3,TNR,ELAVL4,ABL1,SDC2,USP33,PTPRO,TRIO,EXT1,CTNND2,ATP8A2,PLXNA2,SEMA3E,MARK2,EPHA6,ATL1,KNDC1,AFG3L2,ANK3,BCL11B,ECE1,MBP,CNTN1,PDLIM5,DISC1,WDPCP,SEMA3A,UNC5D,SEMA3D,RELN,UST,B4GALT6,PLS1,NIN,DRAXIN,GLI3,RERE,MAP2,FARP1,DCC,DAB1,PCDH15,NGEF,CDH23,ALS2,NTN1,DPYSL5,TANC2,ZDHHC17,LAMA3,TNN,LMX1A,ACTR2,MAP6,VASP,NRXN1,CELSR2,GAP43,ABI1,ITGA4,MEF2C,ADGRB1,WNT7A,S100B,FBXW8,NECTIN1,EPHA4,NTRK2,IL1RAPL1,NUMB,LHX9,FBXO31,PTK2,FEZ2,CYFIP1,UBE3A,SEMA4D,FYN,HECW2,CDH2,CNTN5,EPHB1,RPS6KA5,NRP1,CHODL,NRXN3,CUX1,EPHB2,PPFIA2,UNK,FLRT2,KALRN,LAMA1,TIAM2,BMP7,SLIT2,CCDC141,ROBO1,PRKCQ,NLGN1,EFNA5,SLIT3,NTNG1,FOXB1,SEMA4B,PTPRQ,FSTL4,IGF1R,GLI2
GO:0051179	localization	4.263569762047 5034e-20	MTOR,UNC80,CACNA2D3,NSG1,SGCD,EXOC1L,WWC1,SLC17A1,ABCA13,IMMP2L,LRP12,SLC24A2,TRAPPC9,KCNH5,MICU2,SLC25A21,ANKS1B,LONP2,UNC13C,FTO,MX2,TMPRSS2,CLTCL1,SLC37A1,PIEZO2,MICAL3,SNAP25-AS1,DPP10,ZDHHC21,ITPR2,RIPOR2,PDE4D,RDX,STXBP1,ERC1,RALA,SLC44A5,EPS15L1,BCL2,MYO5A,KCNMA1,SYT16,FBN1,GPHN,COG5,CHRNA7,GPR158,RIMS1,PIK3C3,SPIRE1,GABRB3,CNTLN,EXOC6B,SPAG16,MYO1E,TRAPPC8,USH2A,CEP192,RIMS2,CARMIL1,MCTP1,SV2C,ERBIN,FCHO2,RIN2,ANO6,CACNG2,GPC6,CNTNAP2,MAP4,CEP112,APC,MYO5C,CRKL,ILDR2,SETD2,TANGO6,TNIK,SLC4A10,PTPRJ,OCA2,EGFR,RFX3,DENND1A,A

			NGPT1,MACF1,DOCK2,NEDD4,GNPTAB,CRB1,BTBD9,CECR2,GRIK3,ATP2B2,TUSC3,ZFAND6,DNAJC13,RABEP1,GABRB1,DGKI,C12ORF4,GRIA1,TTC39B,NUP214,SLC39A12,SLC8A3,TOM1L2,CEP128,PRKD1,PAK1,CHRM3,GRAMD1B,RAPGEF2,LRP2,ARSB,FGF12,GABRA6,LDLRAD3,TMEM38B,AGK,RANBP17,SLC24A3,SLC44A1,UBE2L3,TAFA4,PTPRN2,SYN2,SMYD3,HERC2,TMEM241,GRM7,SEPTIN9,GHR,EPB41L3,KIF4A,THADA,NEDD4L,TRPM1,ADAM10,SLC39A11,APP,SLC7A2,ABCB5,CACNA1C,CACNB2,DCLK1,STAU2,GABRG2,TMC1,MAPRE2,SYT1,VCL,ARHGAP44,NTF3,NDUFAF2,CD2AP,AURKA,PARN,PYGO1,SLC8A1,ABCG8,KCNE4,ABCD2,FMN2,PCSK6,AKAP6,HOMER2,RAB8B,RFTN1,KCNK10,RANBP2,TRPC5,RAP1GDS1,CLIC6,KICS2,ERC2,DNM3,CUBN,SCP2,SYN3,IFT57,PRKCZ,GRB10,RYR3,MCPH1,RAB27B,CNST,HECW1,ABCA5,SV2B,YAP1,SEM1,VPS35L,MAPK1,CADPS2,KCNJ1,HRH2,ABCD3,RABGAP1L,SGTB,TRPC7,ADAM22,SLC45A4,COPB1,SYT10,UBE2O,ANKFY1,SYCP1,NIPBL,SLC16A1,SPIDR,NIPAL2,IPO11,MICU1,CORO2B,PAFAH1B1,TM7SF3,STON2,VPS13D,TLK1,NF2,LRRC38,GRIK4,RBFOX1,ZDHHC14,CORIN,CTNNA1,AKAP9,KLF15,RASGRF2,PPARA,SNX30,KCNS3,SYNJ1,GRM1,RSRC1,PTPRK,GABRG1,PARD3B,PLA2R1,DAPK1,SLC24A4,SEC14L1,VPS13C,TMEM108,DNAH11,JARID2,SCN2A,RAB22A,SORCS1,DNAJC15,AMPH,CPE,DYSF,ANK2,ADGRV1,BCAS3,RYR2,SYNE2,BBS2,SLC9C1,RANBP3L,NKAIN3,NKG7,NBEA,DUSP16,USP8,FABP7,PARD3,SLC36A1,TBC1D5,BLK,DST,CXADR,ATRX,ABL1,SLC1A1,PRKAA1,SLC12A8,KCNH1,FHIP1A,PRELID2,ANO4,CCDC91,EIPR1,DNAH5,NBAS,RAP1A,NKAIN2,MYO10,SLC46A3,GPC5,ZNHIT6,PLEKHA8,FGF10,GRID2,LATS2,NRG1,GSG1L,ASPM,AP3B1,RASGRF1,ATP11C,ABCB7,SYNE1,ZBTB16,MUSK,SH3GL3,ABCC12,PRKCE,SLCO3A1,SLMAP,WNK2,USP33,DENND4C,CEP83,FBN2,EGF,ABCC9,P2RX6,EXT1,STXBP6,PEX14,IFT43,ATP8A2,SCG5,PTPN2,TRIM5,PLXNA2,ATXN3,HTR2C,RIC3,CLEC16A,SLC2A3,ARHGEF7,ALG10B,ATP8A1,RFTN2,LTBP1,ZFYVE9,OPRM1,ABCC4,HTR2A,BIN2,CYBRD1,CYP4A11,CNNM4,STAC,TAF3,MARK2,ABHD17C,CNIH3,MSH2,IGF2BP3,APBA2,MAIP1,SH3KBP1,SLC2A13,EPN2,KCND2,TNPO3,ABCA10,GRK3,CD163,NOS2,AFG3L2,TTC7B,MDFIC,MYLK2,ANK3,NIPA2,TMC7,COG2,VPS41,LYPLA1,TRAPPC11,TMEM163,HHIPL1,ANKFN1,HIP1,PRR5L,VPS37A,ATP6V1E1,VAV1,EFR3A,RUFY2,TJP1,NPHP4,PACSIN2,CNTN1,SNX3,CACNA1I,BHLHE40-AS1,KCNJ15,BRCA2,DISC1,DNER,WDPCP,SLC10A7,LRP1B,ADCY10,STX12,BMP2,ATP9A,TRAK1,EVI5,SCN11A,MSR1,VRK1,GNAI1,TBC1D4,MYRIP,TTR,RIN3,BMP2K,SLC15A5,NETO2,RELN,HMGB1,NUDCD3,CDS2,AP4E1,FGF9,SLC23A2,POLR2M,MYOM1,PRG4,UNC13B,TTC21B,DOCK1,PLS1,SNX8,SEC23B,SLC39A6,NIN,CCDC186,SLAMF1,KCNH8,GLI3,SNX6,SLC37A2,SLC9A4,GABRR2,PACS1,CNKSR3,GRIK2,MCTP2,MAP2,DAW1,PEX6,NEK10,RRBP1,ATF2,BBS4,LRRC8B,MAPK8IP1,ANTXRL,KIAA0753,CFTR,KPNA1,CSE1L,DOP1B,TBC1D13,NEU3,PHAF1,ATP10B,CHRM5,SLC30A10,DAB1,SELENON,NMD3,AKAP10,REPS1,PRKN,MTMR2,LYST,HEPHL1,GRIN2A,JPH1,ATXN1,TRPM6,CDH23,PRKCH,SLC12A1,FRMD4A,TG,ALS2,RACGAP1,ACO1,SNX25,FBLN5,OSCP1,KCNQ3,SHISA9,SLC4A4,TSPAN33,SCN10A,LRBA,MAP7,USP7,VAV3,MON2,KCND3,MESD,ITSN2,SOX30,SYBU,RALB,YIPF6,KCNN3,MYO1D,SEC24D,ROCK1,LYN,SEL1L,SLC44A2,SUMO3,SLC15A2,NTN1,CHKA,SLC13A5,RRAGD,BANP,CRACR2A,INSR,NPIPA1,CUL5,DMBT1,HECTD1,GRID1,SHROOM3,XRCC4,COLQ,SLC52A1,ARFGEF1,IGHV3-74,BID,PIGK,OSBPL10,RPH3A,TANC2,COX5A,ABCA4,UFD1,GABRG3,TRIM58,TOM1,PLPP4,ZDHHC17,NSD2,FYCO1,ESYT2,SH3GLB1,SLC22A14,CD9,CARD10,TMED3,XKR5,IFT81,ENPP1,UTRN,RASGRP1,IGSF11,SNX9,WDR72,KCNC1,GHRH,NUP37,BCL2L1,HCN1,GRIN2B,IGHV2-70D,CLNS1A,SYNJ2,ABCG1,KCNK5,SLC40A1,FAM149B1,CABYR,CIDEC,PSAP,CFHR4,MICALL2,MED1,IPCEF1,ATG4B,PCNT,SLC5A12,IL10,ACTR2,SFPQ,PTH,PRKAA2,NDC80,PACRG,VSTM2A,MAP6,PLA2G4A,SCFD2,KIFC1,SLC25A52,CAMLG,COX7A2L,SR

			EBF2,ANP32B,AIMP1,LASP1,FYB2,NRXN1,PCID2,ENTHD1,SNAP91,CENPE,PEG10,TWIST1,JAK2,SLC1A7,RPF2,MPPE1,CELSR2,MELTF,TNKS,ARL11,SIAH3,TRPV5,UFL1,NFKBIA,PRKCB,GOT2,ABCC8,MIPEP,CACNA1E,ANP32A,RTRAF,USH1C,NEDD9,NRBP1,ATP2B1,SLC14A2,CLCA4,MTCL1,GRIP1,IGHV1OR15-9,TM9SF3,SAR1A,CNIH1,TRAPPC3,XKR6,OTOP1,CIDEA,BBS9,EXT2,EXOC1,HEPACAM,SLC6A1,NDFIP2,MAP2K6,SHROOM2,RN7SL483P,SLC6A11,KCNJ18,CEMIP,CBLIF,PARK7,MAPK8,ITGA4,OAZ2,BCAP29,UBE2J2,ADCYAP1R1,PLA2G12B,NDC1,TM9SF4,RAPGEF4,CEP120,ATP13A3,ARL4C,EFHB,MEF2C,STOML1,ADGRB1,RXRA,WNT7A,NDFIP1,SLC5A9,ATP6V1C2,CHAMP1,MAGEL2,SLC10A6,RAB38,SDCBP,NECTIN1,JPT2,PASK,FLVCR1,FGR,CDCA8,TRIM23,ATP6V1B2,SNAP29,C2,INTS13,GABRA5,NTRK2,IL1RAPL1,NUMB,ADAMTS9,RN7SL767P,COLEC12,PLEKHA3,OCLN,STON1-GTF2A1L,SHISA6,MEGF10,AKAP11,TRPM7,KTN1,GRIK1,IREB2,MFSD9,MVB12B,PTK2,MARK4,CDH5,CD5L,APOL2,AP4S1,ARHGAP12,CLDN18,MPP7,DIAPH1,SCAMP1,CYFIP1,UBE3A,SCG3,APOL1,PITPNC1,FRMD6,AP2B1,SCARA5,SLC26A2,HEATR5A,ZFYVE1,ICA1,PLCZ1,NOS1AP,MTTP,SLC9A5,SRP9,CCDC88A,NSUN2,SLC27A6,SPAG6,SLC5A1,BICD1,ANO10,TNFSF11,FYN,PPM1F,ARL13B,XPO7,ODR4,SCN8A,SLF1,TMEM63C,NCS1,ATP5PF,ALB,ATP9B,NALCN,EHBP1,MAPK9,APELA,TRPM3,SLC39A8,SLC16A9,ASB3,HECW2,CDH2,ITGA8,FBXL20,GPR137B,ZDHHC18,GRM5,TBC1D1,PID1,NRP1,FCHSD2,IFT46,ATPSCKMT,RNF215,CROT,NRIP1,ABCA6,SLC14A1,MCC,BCR,NRXN3,ELMO1,KIF16B,ARFGAP3,TM9SF2,STK36,NSG2,MB,KCNJ6,B9D1,BMPER,RABL2A,CUX1,DPP6,SLC35F1,MACROH2A1,EPHB2,TSPAN13,CSNK1G1,CD38,MYO5B,RGPD4,PPIL2,AKAIN1,MET,DLG2,CDH17,ATP6V0D2,PPFIA2,CDH13,STXBP4,CACNG3,ATG5,MAGI2,SLC35F4,VMP1,KALRN,SLC1A2,GNAS,MFHAS1,NUP43,BMP7,TMPRSS15,ASTN2,DLG5,GAPVD1,GABRA2,TRAPPC10,DDX6,WDR41,PLIN2,ABL2,VPS13B,TRAPPC6B,TMPRSS3,EXOC4,FAM126A,KCNIP4,CCDC141,ERBB4,FAM3B,FAM126B,SYNDIG1,NUF2,RGPD2,SAMM50,ANTXR1,SORCS2,TRDN,NLGN1,NOS1,SLC6A3,ASIC2,EFNA5,GAS2L1,RAB27A,KIF13A,AP5M1,ESR1,DNAH9,SLC25A48,KCNQ5,LOXL2,CACNA2D1,IGLC3,IRAG2,PRLR,HTT,ZDHHC11B,FOXB1,CAMK1D,SLC25A18,HLA-F,FER,EYA2,KATNIP,CCR2,PITPNM3,OSBPL5,OSBPL6,IGHV1OR21-1,ANO2,GRIA4,AGAP1,ROCK2,IL16,TERB2,CDCA5,CATSPER2,RAB31,HSPG2,HERPUD1,WASHC1,RGS7,HOOK3,CLDN10,BARD1,CLCN5,STK3,ZNF423,SLC13A4,PNPLA8,HNRNPU,VTI1A,CEP72,RAB3GAP2,CADPS,IGF1R,KCNAB1,PRKAG2,TANGO2,AKAP13,MORC3,ATP10A,SEPTIN6,DNM1L
GO:0051716	cellular response to stimulus	1.9101105817948162e-19	NOTCH2,BCAR3,BRINP3,MTOR,NSG1,SGCD,WWC1,IMMP2L,GARNL3,LRP12,PTPRD,SLC24A2,ANKS1B,MYO9A,ULK2,NLK,FTO,KSR1,ZNF236,PLCB1,ZNF536,TAFA5,PIEZO2,TENM4,DLC1,ZDHHC21,PTPRA,ITPR2,RIPOR2,PDE4D,RDX,RP1,STXBP1,ERC1,RALA,IL1RAPL2,BCL2,MYO5A,ARPP21,PRDM16,ALDH1A2,ARHGAP26,FBN1,CHRNA7,DCDC1,GPR158,ROBO2,RIMS1,PIK3C3,EPC2,SPIRE1,TENM3,GABRB3,ZEB1,AKR1C3,RARB,FGD4,SPRED1,ENPEP,MYO1E,PLPPR1,MINAR1,CDC42EP3,RIMS2,ALK,AUTS2,ADGRE1,MCTP1,PJA2,BABAM2,ERBIN,ERCC6L2,RHPN2,RIN2,ANO6,CACNG2,DLGAP1,GLYAT,MLLT3,EGLN3,GPC6,MAP3K9,APC,HHLA2,PLPPR5,DSCAM,CRKL,SOX5,SETD2,ERG,ARHGAP24,TNIK,PTPRJ,KDM4C,NEK4,DOCK10,EGFR,DENND1A,USP14,ANGPT1,BACH1,MACF1,PRKACB,NEK7,RGS3,NCOR1,RNF220,DOCK2,UGT3A2,NEDD4,MAML2,SCAI,CRB1,NSMCE2,BCL11A,SOX6,FAM83F,SGMS1,GRIK3,CHSY1,NTRK3,RXFP1,C5,PDE1C,ZFAND6,CYP2C9,DKK2,FLT1,RFC3,RABEP1,MAPKBP1,NAT1,GABRB1,DGKI,INVS,EDAR,GRIA1,NEO1,CNTN6,SLC39A12,SLC8A3,TOM1L2,PRKD1,TPTE2,PAK1,GMDS,EPHA7,CTNNAL1,NCOA7,CHRM3,ADSS2,GRAMD1B,RALGPS1,SPEN,RAPGEF2,PELI2,LRP2,ADGRB3,RUNX2,FGF12,GABRA6,CPS1,TAOK3,ONECUT1,CPEB4,TMEM38B,PRICKLE2,UBE2L3,TAFA4,BTBD11,PTPRN2,CCL28,SMYD3,PATJ

			,HERC2,GRM7,RPTOR,TMEM117,GHR,COL4A2,RALGAPA1,RAPGEF5,PPP1R12B,TRPM1,ADAM10,HDAC9,IL1R1,APBB2,APP,RPS6KA2,CACNA1C,MTUS1,DCLK1,STAU2,GABRG2,DOCK8,MAPRE2,USP18,SEMA5A,SYT1,ARHGAP44,NTF3,ACER2,NDUFAF2,CD2AP,AURKA,ST18,PYGO1,SLC8A1,HERPUD2,PTPRR,TAFA2,ABCG8,ECPAS,KANK1,MAP4K4,BMPR1B,FMN2,PCSK6,AKAP6,HOMER2,HADHB,ARNT,RAB8B,PAK3,RFTN1,PDE1A,KCNK10,LARP1,ITPKB,RGS20,PDE10A,UBE2E2,RAP1GDS1,HHAT,KICS2,NBN,IFT57,INTS7,SUSD6,PRKCZ,BTLA,GRB10,RYR3,MSH6,ARHGAP32,RGS9,HECW1,DEFA3,DUSP22,YAP1,SEM1,WDR70,PPM1L,SHC4,BRINP1,MAPK1,MGAT5,HRH2,SGTB,USP25,ALCAM,PDGFD,SYT10,ZNRF3,PPP1R1C,ITGBL1,ARHGEF17,NRG3,UBE2O,NCAM1,GFRA1,SYCP1,NIPBL,SLC16A1,SPIDR,GABPA,MICU1,CORO2B,CHD6,STK38,PTPN13,CHN1,HRH4,SORCS3,MYLK3,GLP2R,PAFAH1B1,ATF6,EFEMP1,TM7SF3,ITGB8,TLK1,TPM1,NF2,CNKSR2,GRIK4,HIVEP1,CTNNA1,PPP1R9A,MOB3B,BIRC6,AKAP9,KLF15,RASGRF2,PPARA,ERMP1,RGL1,NR5A2,ADAMTS3,TIAM1,ARAP2,GRM1,PTPRK,ARHGEF12,GABRG1,PAK5,TRERF1,PCDH11Y,PPP2R5E,PLA2R1,SEMA3C,DAPK1,SLC24A4,SEC14L1,VPS13C,TMEM108,ACSM2B,WDHD1,STK32B,MAGI1,ALPK2,JARID2,SCN2A,RIC8B,SORCS1,DNAJC15,CPE,EVC2,IL34,ANK2,ADGRV1,MELK,BCAS3,RYR2,BBS2,WNT9B,OR4F6,NKG7,SEMA6D,DUSP16,SMARCA4,USP8,PARD3,MAPKAP1,EFTUD2,PIAS1,SPG21,BLK,TNR,GRM8,DST,CXADR,DOCK4,MBD5,ATRX,NUAK1,PTPRT,ELAVL4,ABL1,PTPN12,HDAC4,OXR1,SLC1A1,PRKAA1,GAS2,KCNH1,ITGB3BP,APBB1IP,APLF,NFAT5,MAST4,GUCY1A2,SLFN11,RAP1A,MYO10,GPC5,CAMK4,INPP5A,FGF10,ZC3HAV1,GRID2,LATS2,NRG1,INO80D,GSG1L,ASPM,AP3B1,DENND2B,RASGRF1,MUSK,ZNF675,GNG7,SMARCAD1,SH3GL3,PRKCE,NXN,WNK2,ESRRG,DGKB,USP33,DENND4C,FBN2,CD44,RGS12,PTPRO,EGF,P2RX6,TRIO,PDE3A,EXT1,NSMAF,LNPEP,LIMD1,SPRED2,RPS6KA3,CTNND2,SCG5,MTMR3,PTPN2,TRIM5,PLXNA2,MCF2L,OR4F15,ATXN3,RFC1,HTR2C,CLEC16A,ARHGEF7,AMBRA1,LTBP1,STK38L,ZFYVE9,OPRM1,ABCC4,HTR2A,BIN2,PLCXD3,FANCM,FANCA,INPP4B,KREMEN1,STAC,SEMA3E,MARK2,GCSAML,TMEM67,ALPL,FHL2,ADGRA3,CNIH3,PUM1,TMOD2,MSH2,GNAL,EPHA6,ANKRD17,RELL1,HIPK3,EPN2,KCND2,EVC,GRK3,KNDC1,SPSB4,CLSPN,NOS2,CPNE4,MOSMO,GFRA2,MNAT1,TMEM116,RBBP8,MDFIC,ADAM12,ANK3,HMGA2,GBP6,CCND3,VPS41,DOCK5,ECE1,STK32A,CREM,MBP,PLCE1,TGFA,IL17RA,HIP1,CRIM1,PRR5L,GSR,CAPN5,VAV1,MSRA,FBXO32,TJP1,LDLRAD4,NPHP4,CNTN1,IQSEC1,SNX3,CACNA1I,BRCA2,DISC1,DNER,BLM,ASB7,WDPCP,NRK,SEMA3A,MAGI3,HSF2BP,ADCY10,PSG8,STRN,OR9Q1,BMP2,RC3H2,UNC5D,PSG9,CDC42BPB,SOGA1,MSR1,VRK1,GNAI1,RALGAPA2,TBC1D4,RANBP9,TTR,RIN3,BMP2K,TMEM161A,SEMA3D,NETO2,PDE6C,CABIN1,LEMD3,RELN,ARHGAP42,HMGB1,GNAQ,FGF9,NFATC2,SH3BP5,SLC23A2,POLR2M,ZNF106,MYOM1,TRAF3,UNC13B,TTC21B,DSTYK,UIMC1,DOCK1,RAP1GAP,SRGAP2,DRAXIN,ATF1,CCDC186,SLAMF1,SMARCA2,FAM83B,GLI3,CGAS,SMARCC1,SNX6,GABRR2,SMOC2,PCP4,CNKSR3,CASP5,GRIK2,IDE,WDR12,MCTP2,CUL1,MYEF2,ZFYVE26,PSD3,GAREM1,LAMC1,NEK10,MOB1B,ATF2,CYLD,UMODL1,BBS4,MAPK8IP1,MX1,PSG6,GABBR2,ITGA9,CFTR,KPNA1,UBASH3A,RGMB,NEU3,KITLG,DNAJC7,CAMTA1,UBR1,DCC,CHRM5,MAP4K3,SLC30A10,RCAN1,RORB,CHAF1A,DAB1,SELENON,RB1CC1,AKAP10,PTPRE,PRKN,MTMR2,TBX20,DLGAP2,AFAP1,MAPK10,DPF3,LYST,NGEF,GRIN2A,ARID5B,TXNRD2,WSB1,USP43,LALBA,PRKCH,PKP1,HUNK,TG,IL6R,ALS2,RACGAP1,NLRC5,OR51E1,MKNK1,DOCK9,SNX25,DMC1,FBLN5,SHISA9,PDE6A,COPS8,SHANK2,ST8SIA1,USP7,VAV3,MESD,SOX30,MOK,KIR2DL4,ARHGEF28,RALB,NPAS2,ADGRG6,ROCK1,LYN,VCAM1,SEL1L,ARHGAP28,ARHGAP31,CTSB,EIF2B3,SLC44A2,GSTA3,SLC15A2,DTX1,TENM2,OVOL2,ZBTB33,ADA2,NTN1,CHKA,PLCB4,FANCL,DPYSL5,SLC13A5,RRAGD,SUPT16H,ARID1B,CRACR2A,RNF152,OTUD7A,INSR,CUL5,OR7A17,BMF,YTHDF3,TFF1,DEDD2,NEK6,GRID1,SHROOM3,XRCC4,NMU,ARFGEF1,GAST,SNAI2,ASH1L,IGHV3-

			74,BID,SIAH2,ABCA4,TRABD2B,UFD1,RXRG,ERN2,GABRG3,MBTPS2,FLNB,TIAL1,TOM1,PLPP4,NREP,ZDHHC17,NSD2,SH3GLB1,CD9,CARD10,RALGPS2,JCAD,OR4K2,SAMHD1,IFT81,ENPP1,RASGRP1,IGSF11,PXDNL,NDRG2,CSNK2A1,BMP5,PWWP3A,KCNC1,CSF1,GHRH,HDGFL3,BCL2L1,SERPINB9,HCN1,PRKG1,LAMA3,ASB4,GRIN2B,GRB14,INO80,FANCB,GPR156,IGHV2-70D,DHRS3,SMAD5,CELF4,ABCG1,OR4C46,FOXN3,SLC40A1,PRAME,TNN,PSAP,MED1,IPCEF1,CDC14B,PCNT,IL33,GPRC5C,ROR2,KL,RASGEF1C,BANK1,IL10,ACTR2,OR1L6,SFPQ,PTH,SOSTDC1,PRKAA2,CSF2RB,DIRAS2,NDC80,PACRG,ABHD2,ITPRIP,VSTM2A,PLA2G4A,RAB12,IQGAP1,RPS12,CAMLG,TEAD1,MORC2,SREBF2,YBX3,AIMP1,THNSL2,FYB2,NRXN1,PCID2,HIPK1,DGKK,CD70,CIBAR1,PBLD,FICD,CACYBP,PEG10,NET1,SIPA1L2,TWIST1,AKT3,ALKAL2,JAK2,RPF2,FSTL1,CHCHD6,ZBTB38,SVEP1,MADD,HCRTR1,PTGS1,CELSR2,FH,TDP1,CREBBP,TNKS,GORAB,PCNA,UFL1,NFKBIA,PRKCB,OR2T3,ABCC8,ANXA4,MT1HL1,ZC3H15,ANP32A,RFC2,BRD4,SMPD4,NEDD9,OLFM4,NRBP1,ITGA6,ATP2B1,GAP43,ASS1,GRIP1,IGHV1OR15-9,ADGRE3,ADCY9,PPP1R17,CNIH1,MAST2,ERLIN2,OTOP1,CIDEA,ARFGEF3,EXT2,EXOC1,GID8,STAT1,BRMS1L,NDFIP2,NR2C1,MAP2K6,CMTM7,DGKG,SHROOM2,MARCHF6,MTPN,ABI1,IMPACT,CCBE1,PARK7,ADAMTS18,MAPK8,ITGA4,TOP3A,FBXL17,UBL7,UBE2J2,ADCYAP1R1,MTF2,NCAPG2,RAPGEF4,OR6C75,ASB2,MYOCD,MYH13,CYFIP2,HNRNPM,ACACA,ASCC2,EFHB,OR13C9,MEF2C,ADGRB1,RXRA,WNT7A,RBPMS2,MAP3K5,NDFIP1,MAP3K4,S100B,ATP6V1C2,C16ORF72,PKN2,RAD51AP1,OR10H2,PDE2A,RAB38,LRRC2,SDCBP,JPT2,SPPL2B,NSMCE1,WWOX,PASK,NCK1,FGR,CDCA8,PPP2R3A,DNMBP,CXCL2,IFNAR1,RNF8,GNG12,EPHA4,CYTH4,GABRA5,MECOM,NTRK2,IL1RAPL1,WNT2B,COLEC12,POSTN,CD101,SHISA6,IL17RD,FBXO31,AKAP11,GRIK1,PRKAB1,DTHD1,MVB12B,PTK2,ERP27,MARK4,CDH5,TPH2,RCAN2,ANKRD6,NFKBID,ARHGAP12,CLDN18,DIAPH1,FEZ2,INIP,LAMB1,APIP,CYFIP1,UBE3A,SEMA4D,PITPNC1,MC2R,ZBTB20,FAT4,IMPA2,AKR1B1,WNT5B,RASGEF1B,AMFR,SAXO1,SCARA5,NENF,PTGFR,ZFYVE1,OR4L1,PLCZ1,NOS1AP,FCRLA,DIDO1,TPTE,SORBS2,CNKSR1,CCDC88A,GPR55,NSUN2,CHCHD2,CDC45,OR11G2,BICD1,TNFSF11,FYN,BUB1,PPM1F,ADGRL2,ARL13B,SDE2,RBMS3,HDAC2,SLF1,SH2D3C,DOCK3,ALB,DOK5,ZFYVE28,MAPK9,APELA,ROR1,FUT8,TET1,ASB3,CDH2,ITGA8,SEL1L2,RAD9A,PHLPP1,GPR137B,EPHB1,RP1L1,GRM5,RAI14,RPS6KA5,TBC1D1,LRRC69,PTPRG,PID1,NRP1,PRKCA,GBP4,FAIM,SAMD12,FAAP24,FHIT,ITGA1,RNF138,RC3H1,NRIP1,POR,MCC,SUPT3H,BCR,NRXN3,ELMO1,RGS6,RERG,KIF16B,SNRK,C14ORF39,ELP2,FBLN1,STK36,NSG2,B9D1,RRAS2,GNA14,BMPER,PRDM15,SRGAP3,MITF,EPHB2,CSNK1G1,CD38,EYA4,CDK14,MET,SPPL3,DLG2,CDH17,CDH13,STXBP4,CACNG3,ATG5,MAGI2,PRDM11,FLRT2,KALRN,SLC1A2,GNAS,LAMA1,MFHAS1,ATRNL1,TIAM2,BMP7,DLG5,GAPVD1,GABRA2,OR2T2,ZMYND11,TMEM25,GRM3,ADGRF5,OR4N2,PDGFC,PLIN2,PPP1R13B,ABL2,RFX2,PARPBP,EYA1,SLIT2,CNOT7,KCTD8,PLCL1,ERBB4,IL20RB,FAM3B,TRHDE,ROBO1,IRAG1,NUF2,PRKCQ,NDRG1,SORCS2,SIPA1L3,MGMT,NLGN1,SHLD2,NOS1,GLDC,ASIC2,EFNA5,GAS2L1,ARHGEF11,MTREX,SLIT3,DTNA,ESR1,MYO9B,CYP2C8,CACNA2D1,NYAP2,IGLC3,IQCJ-SCHIP1,ADGRG7,SKAP2,PRLR,AGO3,HTT,RAD51B,CAMK1D,PIK3R3,MACROD2,FER,EYA2,CCR2,STARD13,CHFR,EPS8,OARD1,SEMA4B,IGHV1OR21-1,HRH1,GRIA4,ROCK2,RORA,IL16,DMRT1,CDCA5,PPP1CB,RGS8,RAB31,PDK1,HERPUD1,NCOA6,COL4A3,RGS7,KIF7,GNG2,FSTL4,BARD1,PNPLA3,STK3,DEPTOR,ZNF423,RSU1,PNPLA8,HNRNPU,APCDD1,IGF1R,PRKAG2,GLI2,THRB,AKAP13,MORC3,DNM1L
GO:0050896	response to stimulus	1.6136615129673651e-18	*NOTCH2,BCAR3,BRINP3,MTOR,CNTN4,NSG1,SGCD,WWC1,IMMP2L,GARNL3,LRP12,PTPRD,SLC24A2,ANKS1B,MYO9A,ULK2,NLK,LONP2,FTO,KSR1,ZNF236,PLCB1,ZNF536,MX2,TAFA5,PIEZO2,TENM4,DLC1,ZDHHC21,PTPRA,ITPR2,RIPOR2,PDE4D,RDX,RP1,STXBP1,ERC1,RALA,IL1RAPL2,BCL2,MYO5A,KCNMA1,ARPP2*

			1,PRDM16,ALDH1A2,ARHGAP26,FBN1,F13A1,GPHN,CDH8,CHRNA7,DCDC1,GPR158,ROBO2,RIMS1,PIK3C3,EPC2,SPIRE1,TENM3,GABRB3,ZEB1,AKR1C3,RARB,FGD4,SPRED1,ENPEP,MYO1E,PLPPR1,USH2A,CEP192,MINAR1,CDC42EP3,LAIR1,RIMS2,ALK,AUTS2,ADGRE1,MCTP1,PJA2,BABAM2,PAPPA2,ERBIN,ERCC6L2,RHPN2,HLCS,RIN2,ANO6,CACNG2,DLGAP1,GLYAT,NAALADL2,MLLT3,EGLN3,GPC6,SUSD4,CNTNAP2,MAP3K9,MYO3B,APC,HHLA2,PLPPR5,DSCAM,CRKL,ILDR2,SOX5,SETD2,ERG,ARHGAP24,TNIK,SLC4A10,PTPRJ,KDM4C,NEK4,DOCK10,EGFR,DENND1A,USP14,ANGPT1,BACH1,MACF1,PRKACB,NEK7,RGS3,NCOR1,RNF220,HMCN2,DOCK2,UGT3A2,NEDD4,MYOF,MAML2,SCAI,CRB1,NSMCE2,BCL11A,SOX6,FAM83F,PSMB2,SGMS1,GRIK3,CHSY1,CDH4,B3GALT5,NTRK3,RXFP1,C5,PDE1C,ZFAND6,CYP2C9,DKK2,FLT1,RFC3,RABEP1,MAPKBP1,AOAH,NAT1,GABRB1,DGKI,INVS,C12ORF4,EDAR,GRIA1,NEO1,CNTN6,SLC39A12,SLC8A3,TOM1L2,PRKD1,TPTE2,PAK1,GMDS,EPHA7,CTNNAL1,NCOA7,CHRM3,ADSS2,GRAMD1B,RALGPS1,SPEN,RAPGEF2,PELI2,LRP2,ADGRB3,RUNX2,ARSB,FGF12,GABRA6,CPS1,TAOK3,ONECUT1,CPEB4,TMEM38B,ADAMTSL1,BCKDHB,PRICKLE2,UBE2L3,TAFA4,BTBD11,PTPRN2,CCL28,SMYD3,IGLV10-54,PATJ,HERC2,GRM7,RPTOR,TMEM117,GHR,COL4A2,RALGAPA1,RAPGEF5,PPP1R12B,TRPM1,ADAM10,HDAC9,IL1R1,APBB2,APP,RPS6KA2,SAMSN1,KYNU,CACNA1C,MTUS1,DCLK1,STAU2,GABRG2,DOCK8,TMC1,MAPRE2,USP18,SEMA5A,SYT1,VCL,ARHGAP44,NTF3,ACER2,NDUFAF2,CD2AP,AURKA,ST18,PYGO1,SLC8A1,HERPUD2,PTPRR,TAFA2,MARCHF1,ABCG8,PLGRKT,ECPAS,KANK1,MAP4K4,ABCD2,BMPR1B,FMN2,PCSK6,AKAP6,HOMER2,CTNNA2,HADHB,ARNT,RAB8B,PAK3,RFTN1,PDE1A,KCNK10,LARP1,ITPKB,RGS20,PDE10A,UBE2E2,RAP1GDS1,HHAT,RNLS,KICS2,NBN,CUBN,IFT57,INTS7,SUSD6,PRKCZ,BTLA,GRB10,RYR3,MSH6,MCPH1,ARHGAP32,FER1L6,RGS9,HECW1,DEFA3,MRTFA,DUSP22,YAP1,SEM1,WDR70,PPM1L,SHC4,BRINP1,MAPK1,MGAT5,HRH2,ABCD3,SGTB,USP25,ALCAM,PLG,PAPPA,PDGFD,SYT10,ZNRF3,PPP1R1C,ITGBL1,ARHGEF17,NRG3,UBE2O,NCAM1,GFRA1,SYCP1,NIPBL,SLC16A1,SPIDR,GABPA,MICU1,CORO2B,CARD18,CHD6,STK38,PTPN13,CHN1,HRH4,SORCS3,MYLK3,ACSBG1,GLP2R,PAFAH1B1,ATF6,EFEMP1,TM7SF3,ITGB8,TLK1,TPM1,NF2,CNKSR2,GRIK4,WDFY4,HIVEP1,CTNNA1,PPP1R9A,MOB3B,BIRC6,AKAP9,KLF15,RASGRF2,PPARA,MEIS2,NFIB,ERMP1,PRTG,RGL1,SYNJ1,NR5A2,ADAMTS3,TIAM1,ARAP2,GRM1,RSRC1,PTPRK,ARHGEF12,GABRG1,ENAH,PAK5,TRERF1,PCDH11Y,PPP2R5E,PLA2R1,SEMA3C,DAPK1,SLC24A4,SEC14L1,VPS13C,TMEM108,ACSM2B,WDHD1,STK32B,MAGI1,ALPK2,JARID2,SCN2A,RIC8B,SORCS1,DNAJC15,CPE,EVC2,DYSF,IL34,ANK2,TANC1,ADGRV1,MELK,BCAS3,RYR2,BBS2,WNT9B,OR4F6,NKG7,SEMA6D,DUSP16,SMARCA4,USP8,PARD3,MAPKAP1,EFTUD2,PIAS1,TBC1D5,SPG21,BLK,TNR,GRM8,DST,CXADR,DOCK4,MBD5,ATRX,NUAK1,PTPRT,ELAVL4,ABL1,PTPN12,HDAC4,OXR1,SLC1A1,PRKAA1,GAS2,KCNH1,ITGB3BP,LRFN5,DROSHA,APBB1IP,APLF,NFAT5,MAST4,GUCY1A2,SLFN11,RAP1A,MORC1,MYO10,GPC5,CAMK4,INPP5A,FGF10,ZC3HAV1,GRID2,LATS2,NRG1,INO80D,GSG1L,ASPM,AP3B1,DENND2B,RASGRF1,MUSK,KIR3DL2,ZNF675,GNG7,SMARCAD1,SH3GL3,PRKCE,FOXK2,NXN,WNK2,ESRRG,DGKB,USP33,DENND4C,CERS6,FBN2,CD44,RGS12,PTPRO,EGF,ABCC9,P2RX6,TRIO,PDE3A,EXT1,NSMAF,LNPEP,LIMD1,SPRED2,RPS6KA3,CTNND2,MARCHF8,ATP8A2,SCG5,MTMR3,PTPN2,TRIM5,PLXNA2,MCF2L,OR4F15,ATXN3,RFC1,HTR2C,CLEC16A,ARHGEF7,CD96,AMBRA1,RFTN2,LTBP1,STK38L,ZFYVE9,PKHD1L1,OPRM1,ABCC4,HTR2A,BIN2,PLCXD3,FANCM,FANCA,CYBRD1,INPP4B,CNNM4,KREMEN1,STAC,SEMA3E,MARK2,GCSAML,GMPR,TMEM67,ALPL,FHL2,ADGRA3,CNIH3,PUM1,TMOD2,MSH2,IGLV2-14,GNAL,EPHA6,ANKRD17,RELL1,HIPK3,EPN2,KCND2,EVC,GRK3,CD163,KNDC1,SPSB4,CLSPN,NOS2,AFG3L2,CPNE4,MOSMO,GFRA2,MNAT1,TMEM116,RBBP8,MDFIC,ADAM12,ANK3,EMILIN2,HMGA2,GBP6,CCND3,BCL11B,VPS41,DOCK5,F5,ECE1,STK32A,IGLV3-

			27,CREM,MBP,PLCE1,TGFA,IL17RA,ANKFN1,HIP1,CRIM1,PRR5L,GSR,CAPN5,VAV1,MSRA,FBXO32,TJP1,LDLRAD4,NPHP4,CNTN1,HLA-B,FKBP5,IQSEC1,SNX3,CACNA1I,BRCA2,DISC1,DNER,BLM,ASB7,WDPCP,NRK,SEMA3A,MAGI3,HSF2BP,ADCY10,PSG8,STRN,AGL,OR9Q1,BMP2,RC3H2,UNC5D,PSG9,CDC42BPB,SOGA1,SCN11A,MSR1,VRK1,GNAI1,RALGAPA2,TBC1D4,RANBP9,TTR,RIN3,BMP2K,TMEM161A,SEMA3D,NETO2,PDE6C,CABIN1,POLR3A,LEMD3,RELN,ARHGAP42,HMGB1,GNAQ,FGF9,NFATC2,SH3BP5,SLC23A2,POLR2M,ZNF106,MYOM1,TRAF3,PRG4,UNC13B,TTC21B,DSTYK,UIMC1,DOCK1,TSPAN2,RAP1GAP,SRGAP2,DRAXIN,ATF1,CCDC186,SLAMF1,SMARCA2,ETS1,FAM83B,GLI3,CGAS,SMARCC1,SNX6,AFF3,SLC9A4,GABRR2,SMOC2,PCP4,CNKSR3,CASP5,GRIK2,IDE,WDR12,MCTP2,CUL1,MYEF2,ZFYVE26,PSD3,GAREM1,LAMC1,NEK10,MOB1B,ATF2,CYLD,UMODL1,BBS4,MAPK8IP1,MX1,PSG6,COL5A1,GABBR2,PSIP1,ITGA9,CFTR,KPNA1,UBASH3A,RGMB,NEU3,KITLG,DNAJC7,CAMTA1,UBR1,DCC,CHRM5,MAP4K3,SLC30A10,RCAN1,RORB,CHAF1A,DAB1,SELENON,RB1CC1,MYO3A,AKAP10,PTPRE,PRKN,MTMR2,TBX20,DLGAP2,AFAP1,MAPK10,DACH1,PCDH15,DPF3,LYST,NGEF,GRIN2A,ARID5B,TXNRD2,WSB1,USP43,TRPM6,CDH23,LALBA,PRKCH,PKP1,HUNK,TG,IL6R,ALS2,RACGAP1,NLRC5,OR51E1,ACO1,MKNK1,DOCK9,SNX25,DMC1,FBLN5,OSCP1,LCE3B,SHISA9,PDE6A,COPS8,SHANK2,ST8SIA1,MAP7,USP7,VAV3,PSMA1,ENPP3,HAAO,IGLV5-45,MESD,SOX30,MOK,KIR2DL4,ARHGEF28,RALB,NPAS2,ADGRG6,ROCK1,LYN,VCAM1,SEL1L,ARHGAP28,ARHGAP31,CTSB,EIF2B3,TLDC2,SLC44A2,GSTA3,SLC15A2,DTX1,TENM2,OVOL2,ZBTB33,ADA2,NTN1,CHKA,PLCB4,ZFHX3,FANCL,DPYSL5,SLC13A5,RRAGD,SUPT16H,ARID1B,CRACR2A,RNF152,OTUD7A,INSR,CUL5,DMBT1,OR7A17,BMF,YTHDF3,TFF1,DEDD2,NEK6,GRID1,SHROOM3,XRCC4,NMU,ARFGEF1,GAST,SNAI2,ASH1L,IGHV3-74,BID,SIAH2,ABCA4,TRABD2B,UFD1,RXRG,ERN2,GABRG3,MBTPS2,FLNB,TRIM58,TIAL1,TOM1,IFI44,PLPP4,NREP,ZDHHC17,NSD2,SH3GLB1,CD9,CARD10,RALGPS2,JCAD,OR4K2,SAMHD1,IFT81,ENPP1,RASGRP1,IGSF11,PXDNL,NDRG2,CSNK2A1,BMP5,PWWP3A,KCNC1,CSF1,GHRH,HDGFL3,BCL2L1,SERPINB9,CTDP1,HCN1,PRKG1,LAMA3,ASB4,GRIN2B,ST13,GRB14,INO80,FANCB,GPR156,IGHV2-70D,DHRS3,SMAD5,CELF4,ABCG1,OR4C46,FOXN3,SLC40A1,PRAME,HADHA,TNN,PSAP,MED1,IPCEF1,CDC14B,PCNT,ATRN,IL33,AJAP1,GPRC5C,ROR2,CFH,PPP2R2A,KL,RASGEF1C,BANK1,LMX1A,IL10,ACTR2,OR1L6,SFPQ,PTH,SOSTDC1,PRKAA2,CSF2RB,DIRAS2,TRAV8-6,NDC80,PACRG,ABHD2,ITPRIP,VSTM2A,VASP,PLA2G4A,RAB12,IQGAP1,RPS12,REG4,PRB3,CAMLG,TEAD1,MORC2,SREBF2,YBX3,AIMP1,THNSL2,FYB2,NRXN1,PCID2,HIPK1,DGKK,CD70,CIBAR1,PBLD,FICD,CACYBP,CADM1,PEG10,NET1,SIPA1L2,TWIST1,AKT3,ALKAL2,JAK2,VSX1,RPF2,FSTL1,CHCHD6,ZBTB38,SVEP1,MADD,HCRTR1,PTGS1,CELSR2,PRSS2,FH,TDP1,CREBBP,TNKS,GORAB,PCNA,UFL1,ADAMTS5,NFKBIA,PRKCB,OR2T3,GOT2,ABCC8,TRGJ1,ANXA4,MT1HL1,ZC3H15,ANP32A,RFC2,BRD4,SMPD4,NEDD9,OLFM4,NRBP1,ITGA6,ATP2B1,GAP43,ASS1,GRIP1,IGHV1OR15-9,ADGRE3,ADCY9,PPP1R17,CNIH1,MAST2,ERLIN2,OTOP1,CIDEA,ARFGEF3,BBS9,EXT2,EXOC1,KRT6A,SLC6A1,GID8,STAT1,BRMS1L,NDFIP2,NR2C1,MAP2K6,CMTM7,DGKG,SHROOM2,SLC6A11,MARCHF6,MTPN,ABI1,IMPACT,CCBE1,PARK7,ADAMTS18,MAPK8,ITGA4,TOP3A,FBXL17,UBL7,UBE2J2,ADCYAP1R1,MTF2,CSMD1,NCAPG2,TM9SF4,RAPGEF4,OR6C75,ASB2,MYOCD,HMCN1,MYH13,CYFIP2,HNRNPM,ACACA,ASCC2,EFHB,OR13C9,MEF2C,ADGRB1,RXRA,WNT7A,RBPMS2,MAP3K5,NDFIP1,IKBIP,MAP3K4,TRIM43B,S100B,TRIM43,ATP6V1C2,C16ORF72,PKN2,RAD51AP1,OR10H2,PDE2A,RAB38,LRRC2,SDCBP,NECTIN1,DSG1,JPT2,SPPL2B,NSMCE1,TRAV8-1,WWOX,PASK,NCK1,FGR,CDCA8,PPP2R3A,DNMBP,TRIM23,CXCL2,TOP1,C2,IFNAR1,RNF8,GNG12,EPHA4,CYTH4,GABRA5,MEC

			OM,NTRK2,IL1RAPL1,LHX9,ADAMTS9,WNT2B,COLEC12,TNNI1,OCLN,POSTN,CD101,SHISA6,IL17RD,FBXO31,AKAP11,GRIK1,PRKAB1,DTHD1,MVB12B,PTK2,ERP27,MARK4,CDH5,CD5L,TPH2,RCAN2,ANKRD6,APOL2,NFKBID,ARHGAP12,CLDN18,DIAPH1,FEZ2,INIP,LAMB1,DEFB116,APIP,CYFIP1,UBE3A,APOL1,SEMA4D,PITPNC1,MC2R,ZBTB20,FAT4,IMPA2,AKR1B1,C9,WNT5B,RASGEF1B,AMFR,SAXO1,SCARA5,NENF,SH2D1B,PTGFR,ZFYVE1,OR4L1,SANBR,PLCZ1,NOS1AP,MTTP,FCRLA,DIDO1,TPTE,SORBS2,CNKSR1,CCDC88A,GPR55,NSUN2,CHCHD2,SLC5A1,CDC45,OR11G2,BICD1,TNFSF11,FYN,BUB1,PPM1F,ADGRL2,ARL13B,SDE2,RBMS3,HDAC2,SLF1,SH2D3C,DOCK3,ALB,DOK5,IGLV4-3,ZFYVE28,MAPK9,CRTAM,APELA,TRPM3,DEFB108B,ROR1,OPA3,FUT8,TET1,ARNT2,ASB3,CDH2,CNTN5,ITGA8,SEL1L2,FBXL20,RAD9A,PHLPP1,GPR137B,EPHB1,EYS,RP1L1,GRM5,RAI14,RPS6KA5,TBC1D1,LRRC69,PTPRG,PID1,NRP1,SDK1,PRKCA,GBP4,NLRP4,FAIM,SAMD12,FAAP24,FHIT,ITGA1,RNF138,RC3H1,NRIP1,POR,MCC,SUPT3H,BCR,NRXN3,ELMO1,RGS6,RERG,KIF16B,SNRK,C14ORF39,ELP2,FBLN1,STK36,NSG2,MB,RAG1,B9D1,RRAS2,GNA14,BMPER,PRDM15,SRGAP3,MACROH2A1,MITF,EPHB2,CSNK1G1,CD38,EYA4,CDK14,MET,SPPL3,DLG2,CDH17,CDH13,STXBP4,SERPINB2,CACNG3,ATG5,MAGI2,PRDM11,MLIP,FLRT2,MYB,KALRN,SLC1A2,GNAS,LAMA1,MFHAS1,ATRNL1,TIAM2,BMP7,CHIT1,DLG5,GAPVD1,GABRA2,OR2T2,AK3,ZMYND11,TMEM25,GRM3,ADGRF5,OR4N2,PDGFC,WDR41,PLIN2,PPP1R13B,ABL2,MMP26,RFX2,PARPBP,EYA1,SLIT2,CNOT7,KCTD8,CCDC141,PLCL1,ERBB4,IL20RB,FAM3B,TRHDE,ROBO1,IRAG1,NUF2,PRKCQ,NDRG1,SORCS2,SIPA1L3,TRDN,MGMT,NLGN1,SHLD2,NOS1,SLC6A3,GLDC,ASIC2,EFNA5,TCF12,GAS2L1,ARHGEF11,MTREX,RAB27A,SLIT3,DTNA,ESR1,MYO9B,CYP2C8,LOXL2,CACNA2D1,NYAP2,IGLC3,IQCJ-SCHIP1,ADGRG7,SKAP2,PRLR,AGO3,HTT,FOXB1,RAD51B,CAMK1D,PIK3R3,MACROD2,HLA-F,FER,EYA2,CCR2,RPGRIP1,STARD13,IGLV3-1,A2M,CHFR,EPS8,OARD1,SEMA4B,IGHV1OR21-1,HRH1,GRIA4,IGLV2-18,ROCK2,PRDM1,RORA,STMP1,IL16,DMRT1,CDCA5,PPP1CB,RGS8,RAB31,PDK1,HSPG2,PTPRQ,HERPUD1,NCOA6,HSD17B2,COL4A3,RGS7,KIF7,GNG2,FSTL4,BARD1,PNPLA3,STK3,DEPTOR,ZNF423,RSU1,PNPLA8,HNRNPU,APCDD1,IGF1R,PRKAG2,GLI2,THRB,AKAP13,MORC3,DNM1L
GO:0120035	regulation of plasma membrane bounded cell projection organization	3.642566335414852e-18	MTOR,SPOCK1,PTPRD,LRRC4C,ULK2,RIPOR2,RDX,RP1,RALA,ROBO2,TENM3,SDCCAG8,MINAR1,CDC42EP3,ALK,AUTS2,CARMIL1,NEGR1,MAP4,APC,PLPPR5,DSCAM,CRKL,ARHGAP24,TNIK,MACF1,NEDD4,BCL11A,CDH4,NTRK3,SLC39A12,PRKD1,PAK1,EPHA7,RAPGEF2,ADGRB3,ARSB,SEPTIN9,NEDD4L,STAU2,SEMA5A,ARHGAP44,SRGAP2C,FIG4,KANK1,CTNNA2,PAK3,DIP2B,TRPC5,DNM3,HECW1,COBL,YAP1,FAT3,CHN1,PAFAH1B1,TIAM1,SEMA3C,BCAS3,SYNE2,SEMA6D,TNR,ELAVL4,ABL1,HDAC4,SDC2,RAP1A,MYO10,GRID2,CD44,PTPRO,ATP8A2,PLXNA2,ARHGEF7,KREMEN1,SEMA3E,MARK2,KNDC1,MBP,PLCE1,FUT9,CNTN1,SNX3,PDLIM5,DISC1,WDPCP,SEMA3A,SEMA3D,RELN,UST,RAP1GAP,PLS1,NIN,DRAXIN,ATF1,MAP2,CYLD,BBS4,DCC,DAB1,NGEF,TOX,LYN,TENM2,NTN1,DPYSL5,TANC2,BMP5,GRIN2B,TNN,ROR2,ACTR2,MAP6,NRXN1,ANLN,ALKAL2,USH1C,NEDD9,ITGA6,GAP43,DGKG,CEP120,WNT7A,FBXW8,NCK1,EPHA4,NTRK2,IL1RAPL1,OCLN,FBXO31,MARK4,CYFIP1,UBE3A,SEMA4D,SAXO1,CCDC88A,ADAMTS16,FYN,HDAC2,NCS1,ROR1,HECW2,CDH2,PTPRG,NRP1,CHODL,CUX1,EPHB2,CD38,PPFIA2,ATG5,MAGI2,KALRN,TIAM2,BMP7,ABL2,SLIT2,ROBO1,NLGN1,EFNA5,NTNG1,HTT,CAMK1D,FER,EPS8,SEMA4B,CSMD3,WASHC1,FSTL4,IGF1R
GO:0031344	regulation of cell projection organization	3.707032494821488e-18	MTOR,SPOCK1,PTPRD,LRRC4C,MYO9A,ULK2,RIPOR2,RDX,RP1,RALA,ROBO2,TENM3,SDCCAG8,MINAR1,CDC42EP3,ALK,AUTS2,CARMIL1,NEGR1,MAP4,APC,PLPPR5,DSCAM,CRKL,ARHGAP24,TNIK,MACF1,NEDD4,BCL11A,CDH4,NTRK3,SLC39A12,PRKD1,PAK1,EPHA7,RAPGEF2,ADGRB3,ARSB,SEPTIN9,NEDD4L,STAU2,SEMA5A,ARHGAP44,SRGAP2C,FIG4,KANK1,CTNNA2,RAB8B,PAK3

			,DIP2B,TRPC5,DNM3,HECW1,COBL,YAP1,FAT3,CHN1,PAFAH1B1,TIAM1,SEMA3C,BCAS3,SYNE2,SEMA6D,TNR,ELAVL4,ABL1,HDAC4,SDC2,RAP1A,MYO10,GRID2,CD44,PTPRO,ATP8A2,PLXNA2,ARHGEF7,KREMEN1,SEMA3E,MARK2,KNDC1,MBP,PLCE1,FUT9,CNTN1,SNX3,PDLIM5,DISC1,WDPCP,SEMA3A,SEMA3D,RELN,UST,RAP1GAP,PLS1,NIN,DRAXIN,ATF1,MAP2,CYLD,BBS4,DCC,DAB1,NGEF,TOX,LYN,TENM2,NTN1,DPYSL5,TANC2,BMP5,GRIN2B,TNN,ROR2,ACTR2,MAP6,NRXN1,ANLN,ALKAL2,USH1C,NEDD9,ITGA6,GAP43,GRIP1,DGKG,CEP120,WNT7A,FBXW8,NCK1,EPHA4,NTRK2,IL1RAPL1,OCLN,FBXO31,MARK4,CYFIP1,UBE3A,SEMA4D,SAXO1,CCDC88A,ADAMTS16,FYN,HDAC2,NCS1,ROR1,HECW2,CDH2,PTPRG,NRP1,CHODL,CUX1,EPHB2,CD38,PPFIA2,ATG5,MAGI2,KALRN,TIAM2,BMP7,ABL2,SLIT2,ROBO1,NLGN1,EFNA5,NTNG1,HTT,CAMK1D,FER,EPS8,SEMA4B,CSMD3,WASHC1,FSTL4,IGF1R
GO:0007165	signal transduction	5.235674510414 1425e-18	*NOTCH2,BCAR3,MTOR,NSG1,SGCD,WWC1,GARNL3,LRP12,PTPRD,SLC24A2,ANKS1B,MYO9A,ULK2,NLK,KSR1,PLCB1,ZNF536,TAFA5,TENM4,DLC1,ZDHHC21,PTPRA,ITPR2,RIPOR2,PDE4D,RDX,RP1,ERC1,RALA,IL1RAPL2,BCL2,PRDM16,ALDH1A2,ARHGAP26,FBN1,CHRNA7,DCDC1,GPR158,ROBO2,RIMS1,PIK3C3,TENM3,GABRB3,ZEB1,AKR1C3,RARB,FGD4,SPRED1,ENPEP,MYO1E,PLPPR1,MINAR1,CDC42EP3,RIMS2,ALK,AUTS2,ADGRE1,MCTP1,PJA2,BABAM2,ERBIN,RHPN2,RIN2,ANO6,CACNG2,DLGAP1,MLLT3,GPC6,MAP3K9,APC,HHLA2,PLPPR5,DSCAM,CRKL,ERG,ARHGAP24,TNIK,PTPRJ,KDM4C,DOCK10,EGFR,DENND1A,ANGPT1,MACF1,PRKACB,RGS3,NCOR1,RNF220,DOCK2,NEDD4,MAML2,SCAI,FAM83F,SGMS1,GRIK3,CHSY1,NTRK3,RXFP1,C5,PDE1C,ZFAND6,DKK2,FLT1,RABEP1,MAPKBP1,GABRB1,DGKI,INVS,EDAR,GRIA1,NEO1,CNTN6,SLC39A12,SLC8A3,TOM1L2,PRKD1,TPTE2,PAK1,GMDS,EPHA7,CTNNAL1,CHRM3,RALGPS1,SPEN,RAPGEF2,PELI2,LRP2,ADGRB3,RUNX2,FGF12,GABRA6,TAOK3,ONECUT1,CPEB4,TMEM38B,PRICKLE2,TAFA4,BTBD11,CCL28,PATJ,GRM7,RPTOR,TMEM117,GHR,COL4A2,RALGAPA1,RAPGEF5,PPP1R12B,TRPM1,ADAM10,IL1R1,APBB2,APP,RPS6KA2,CACNA1C,DCLK1,GABRG2,DOCK8,MAPRE2,USP18,SEMA5A,ARHGAP44,NTF3,ACER2,CD2AP,AURKA,ST18,PYGO1,SLC8A1,HERPUD2,PTPRR,TAFA2,ABCG8,KANK1,MAP4K4,BMPR1B,FMN2,PCSK6,AKAP6,HOMER2,ARNT,PAK3,RFTN1,PDE1A,KCNK10,LARP1,ITPKB,RGS20,PDE10A,RAP1GDS1,HHAT,KICS2,NBN,IFT57,INTS7,PRKCZ,BTLA,GRB10,MSH6,ARHGAP32,RGS9,HECW1,DEFA3,DUSP22,YAP1,PPM1L,SHC4,MAPK1,MGAT5,HRH2,ALCAM,PDGFD,ZNRF3,PPP1R1C,ITGBL1,ARHGEF17,NRG3,UBE2O,NCAM1,GFRA1,STK38,PTPN13,CHN1,HRH4,SORCS3,GLP2R,PAFAH1B1,ATF6,EFEMP1,ITGB8,TLK1,NF2,CNKSR2,GRIK4,HIVEP1,CTNNA1,PPP1R9A,MOB3B,BIRC6,AKAP9,KLF15,RASGRF2,PPARA,ERMP1,RGL1,NR5A2,ADAMTS3,TIAM1,ARAP2,GRM1,PTPRK,ARHGEF12,GABRG1,PAK5,TRERF1,PCDH11Y,PPP2R5E,PLA2R1,SEMA3C,DAPK1,SLC24A4,SEC14L1,TMEM108,STK32B,MAGI1,ALPK2,SCN2A,RIC8B,SORCS1,CPE,EVC2,IL34,ANK2,ADGRV1,MELK,RYR2,BBS2,WNT9B,OR4F6,NKG7,SEMA6D,DUSP16,SMARCA4,USP8,PARD3,MAPKAP1,PIAS1,SPG21,BLK,GRM8,DST,DOCK4,MBD5,ATRX,NUAK1,PTPRT,ABL1,PTPN12,HDAC4,SLC1A1,PRKAA1,GAS2,KCNH1,ITGB3BP,APBB1IP,APLF,NFAT5,MAST4,GUCY1A2,RAP1A,MYO10,GPC5,CAMK4,INPP5A,FGF10,ZC3HAV1,GRID2,LATS2,NRG1,GSG1L,ASPM,AP3B1,DENND2B,RASGRF1,MUSK,ZNF675,GNG7,SH3GL3,PRKCE,NXN,WNK2,ESRRG,DGKB,USP33,DENND4C,FBN2,CD44,RGS12,PTPRO,EGF,P2RX6,TRIO,PDE3A,EXT1,NSMAF,LNPEP,LIMD1,SPRED2,RPS6KA3,CTNND2,SCG5,PTPN2,TRIM5,PLXNA2,MCF2L,OR4F15,HTR2C,CLEC16A,ARHGEF7,LTBP1,STK38L,ZFYVE9,OPRM1,HTR2A,PLCXD3,FANCA,INPP4B,KREMEN1,STAC,SEMA3E,MARK2,GCSAML,FHL2,ADGRA3,CNIH3,PUM1,TMOD2,MSH2,GNAL,EPHA6,ANKRD17,RELL1,HIPK3,EPN2,EVC,GRK3,KNDC1,SPSB4,CLSPN,NOS2,MOSMO,GFRA2,TMEM116,RBBP8,MDFIC,ADAM12,ANK3,HMGA2,CCND3,DOCK5,ECE1,STK32A,CREM,MBP,PLCE1,TGFA,IL17RA,HIP1,CRIM1,PRR5L,CAPN5,VAV1,LDLRAD4,NPHP4,CNTN1,IQSEC1,SNX3,CACNA1I,BRCA2,DISC1,DNER,BLM*

			,ASB7,WDPCP,NRK,SEMA3A,MAGI3,ADCY10,PSG8,STRN,OR9Q1,BMP2,RC3H2,UNC5D,PSG9,CDC42BPB,SOGA1,VRK1,GNAI1,RALGAPA2,RANBP9,TTR,RIN3,BMP2K,TMEM161A,SEMA3D,NETO2,PDE6C,CABIN1,LEMD3,RELN,ARHGAP42,HMGB1,GNAQ,FGF9,NFATC2,SH3BP5,POLR2M,ZNF106,MYOM1,TRAF3,UNC13B,TTC21B,DSTYK,UIMC1,DOCK1,RAP1GAP,SRGAP2,DRAXIN,ATF1,SLAMF1,FAM83B,GLI3,CGAS,SMARCC1,SNX6,GABRR2,SMOC2,PCP4,CNKSR3,CASP5,GRIK2,IDE,WDR12,MCTP2,CUL1,PSD3,GAREM1,LAMC1,NEK10,MOB1B,ATF2,CYLD,BBS4,MAPK8IP1,MX1,PSG6,GABBR2,ITGA9,KPNA1,UBASH3A,RGMB,NEU3,KITLG,CAMTA1,UBR1,DCC,CHRM5,MAP4K3,SLC30A10,RCAN1,RORB,DAB1,RB1CC1,AKAP10,PTPRE,PRKN,MTMR2,TBX20,DLGAP2,AFAP1,MAPK10,NGEF,GRIN2A,ARID5B,WSB1,LALBA,PRKCH,PKP1,HUNK,TG,IL6R,ALS2,RACGAP1,NLRC5,OR51E1,MKNK1,DOCK9,SNX25,SHISA9,PDE6A,COPS8,SHANK2,USP7,VAV3,MESD,SOX30,MOK,KIR2DL4,ARHGEF28,RALB,ADGRG6,ROCK1,LYN,VCAM1,SEL1L,ARHGAP28,ARHGAP31,EIF2B3,SLC44A2,SLC15A2,DTX1,TENM2,OVOL2,ZBTB33,ADA2,NTN1,PLCB4,DPYSL5,RRAGD,CRACR2A,RNF152,OTUD7A,INSR,CUL5,OR7A17,BMF,YTHDF3,TFF1,DEDD2,NEK6,GRID1,NMU,ARFGEF1,GAST,SNAI2,ASH1L,IGHV3-74,BID,SIAH2,ABCA4,TRABD2B,UFD1,RXRG,ERN2,GABRG3,MBTPS2,FLNB,TIAL1,TOM1,PLPP4,NREP,ZDHHC17,CARD10,RALGPS2,JCAD,OR4K2,SAMHD1,IFT81,ENPP1,RASGRP1,IGSF11,NDRG2,CSNK2A1,BMP5,CSF1,GHRH,HDGFL3,BCL2L1,PRKG1,LAMA3,ASB4,GRIN2B,GRB14,GPR156,IGHV2-70D,DHRS3,SMAD5,CELF4,OR4C46,FOXN3,PRAME,TNN,PSAP,MED1,CDC14B,PCNT,IL33,GPRC5C,ROR2,KL,RASGEF1C,BANK1,IL10,OR1L6,SFPQ,PTH,SOSTDC1,PRKAA2,CSF2RB,DIRAS2,NDC80,ABHD2,ITPRIP,RAB12,IQGAP1,RPS12,CAMLG,TEAD1,SREBF2,YBX3,AIMP1,THNSL2,FYB2,NRXN1,PCID2,HIPK1,DGKK,CD70,CIBAR1,PBLD,FICD,PEG10,NET1,SIPA1L2,TWIST1,AKT3,ALKAL2,JAK2,RPF2,FSTL1,SVEP1,MADD,HCRTR1,CELSR2,CREBBP,TNKS,GORAB,UFL1,NFKBIA,PRKCB,OR2T3,ANXA4,ZC3H15,ANP32A,BRD4,NEDD9,OLFM4,NRBP1,ITGA6,GAP43,GRIP1,IGHV1OR15-9,ADGRE3,ADCY9,PPP1R17,CNIH1,MAST2,ERLIN2,OTOP1,CIDEA,ARFGEF3,EXT2,EXOC1,GID8,STAT1,BRMS1L,NDFIP2,NR2C1,MAP2K6,CMTM7,DGKG,ABI1,CCBE1,PARK7,ADAMTS18,MAPK8,ITGA4,FBXL17,ADCYAP1R1,NCAPG2,RAPGEF4,OR6C75,ASB2,MYOCD,CYFIP2,EFHB,OR13C9,MEF2C,ADGRB1,RXRA,WNT7A,RBPMS2,MAP3K5,NDFIP1,MAP3K4,S100B,ATP6V1C2,C16ORF72,PKN2,OR10H2,PDE2A,RAB38,LRRC2,SDCBP,JPT2,SPPL2B,WWOX,PASK,NCK1,FGR,CDCA8,PPP2R3A,DNMBP,CXCL2,IFNAR1,GNG12,EPHA4,CYTH4,GABRA5,MECOM,NTRK2,IL1RAPL1,WNT2B,COLEC12,POSTN,CD101,SHISA6,IL17RD,FBXO31,AKAP11,GRIK1,PRKAB1,DTHD1,MVB12B,PTK2,MARK4,CDH5,RCAN2,ANKRD6,NFKBID,ARHGAP12,CLDN18,FEZ2,INIP,LAMB1,APIP,CYFIP1,UBE3A,SEMA4D,PITPNC1,MC2R,FAT4,IMPA2,WNT5B,RASGEF1B,AMFR,NENF,PTGFR,OR4L1,PLCZ1,NOS1AP,FCRLA,DIDO1,TPTE,SORBS2,CNKSR1,CCDC88A,GPR55,NSUN2,CDC45,OR11G2,BICD1,TNFSF11,FYN,BUB1,PPM1F,ADGRL2,ARL13B,SDE2,RBMS3,HDAC2,SH2D3C,DOCK3,DOK5,ZFYVE28,MAPK9,APELA,ROR1,FUT8,TET1,ASB3,CDH2,ITGA8,RAD9A,PHLPP1,GPR137B,EPHB1,RP1L1,GRM5,RAI14,RPS6KA5,LRRC69,PTPRG,PID1,NRP1,PRKCA,FAIM,SAMD12,FHIT,ITGA1,RNF138,RC3H1,POR,MCC,BCR,NRXN3,ELMO1,RGS6,RERG,KIF16B,SNRK,ELP2,FBLN1,STK36,NSG2,B9D1,RRAS2,GNA14,BMPER,PRDM15,SRGAP3,MITF,EPHB2,CSNK1G1,CD38,EYA4,CDK14,MET,SPPL3,CDH17,CDH13,STXBP4,CACNG3,MAGI2,PRDM11,FLRT2,KALRN,GNAS,LAMA1,MFHAS1,ATRNL1,TIAM2,BMP7,DLG5,GAPVD1,GABRA2,OR2T2,ZMYND11,TMEM25,GRM3,ADGRF5,OR4N2,PDGFC,PPP1R13B,ABL2,EYA1,SLIT2,CNOT7,KCTD8,PLCL1,ERBB4,IL20RB,FAM3B,TRHDE,ROBO1,IRAG1,NUF2,PRKCQ,NDRG1,SORCS2,SIPA1L3,NLGN1,NOS1,ASIC2,EFNA5,ARHGEF11,SLIT3,DTNA,ESR1,MYO9B,NYAP2,IGLC3,IQCJ-SCHIP1,ADGRG7,SKAP2,PRLR,AGO3,HTT,PIK3R3,FER,EYA2,C

			CR2,STARD13,CHFR,EPS8,SEMA4B,IGHV1OR21-1,HRH1,GRIA4,ROCK2,RORA,IL16,DMRT1,PPP1CB,RGS8,PDK1,HERPUD1,COL4A3,RGS7,KIF7,GNG2,FSTL4,BARD1,STK3,DEPTOR,ZNF423,RSU1,APCDD1,IGF1R,PRKAG2,GLI2,THRB,AKAP13,DNM1L
GO:0050793	regulation of developmental process	1.404110495602 0207e-17	*NOTCH2,BRINP3,MTOR,CNTN4,WWC1,PTPRD,LRRC4C,SMOC1,MYO9A,ULK2,FTO,PLCB1,ZNF536,TAFA5,ZFPM2,TENM4,DLC1,ZDHHC21,RIPOR2,RDX,RALA,BCL2,FBN1,CHRNA7,ROBO2,RIMS1,SPIRE1,ZEB1,RARB,FGD4,SPRED1,USH2A,MINAR1,CDC42EP3,RIMS2,ALK,FOXJ2,CARMIL1,RIN2,PARVB,ANO6,MLLT3,GPC6,APC,ZMYM4,DSCAM,TCF4,CRKL,SOX5,TNIK,KDM4C,EGFR,RFX3,CDK12,MACF1,NEDD4,BCL11A,SOX6,TMEM182,CDH4,NTRK3,C5,FLT1,SLC39A12,PRKD1,PAK1,EPHA7,SPEN,RAPGEF2,LRP2,ADGRB3,RUNX2,PRICKLE2,GHR,EPB41L3,COL4A2,SSBP3,NEDD4L,ADAM10,HDAC9,ZHX3,APP,STAU2,SEMA5A,SYT1,VCL,ARHGAP44,NTF3,AURKA,CFDP1,SLC8A1,SRGAP2C,FIG4,KANK1,BMPR1B,AKAP6,ARNT,PAK3,DIP2B,ITPKB,TRPC5,DNM3,PRKCZ,HECW1,ABCA5,COBL,YAP1,BRINP1,MAPK1,ZNRF3,NIPBL,GABPA,FAT3,CHN1,MYLK3,PAFAH1B1,EFEMP1,ITGB8,TPM1,NF2,RBFOX1,CTNNA1,PPARA,MEIS2,NFIB,MRTFB,PRTG,SYNJ1,TIAM1,SEMA3C,AGO2,ALPK2,JARID2,GATAD2B,IL34,BRWD1,ADGRV1,BBS2,WNT9B,RANBP3L,SEMA6D,SMARCA4,PARD3,TNR,CXADR,MBD5,ELAVL4,ABL1,HDAC4,PRKAA1,SDC2,GAS2,DROSHA,RAP1A,GLIS1,MYO10,CAMK4,FGF10,GRID2,LATS2,NRG1,INO80D,ASPM,AP3B1,ATP11C,ZBTB16,MUSK,ZNF675,SH3GL3,FBN2,CD44,EGF,TRIO,PDE3A,LIMD1,SPRED2,RPS6KA3,ATP8A2,PTPN2,PLXNA2,HTR2C,ARHGEF7,AMBRA1,OPRM1,HTR2A,FANCA,KREMEN1,SEMA3E,MARK2,MSH2,ANKRD17,LINGO2,SH3KBP1,LUC7L,EPN2,KNDC1,BICRAL,AFG3L2,MOSMO,ADAM12,EMILIN2,HMGA2,BCL11B,DOCK5,MBP,TRPS1,CRIM1,TJP1,LDLRAD4,HLA-B,PDLIM5,DISC1,WDPCP,SEMA3A,BMP2,RC3H2,PSG9,MSR1,GFI1B,BMP2K,SEMA3D,RELN,HMGB1,FGF9,NFATC2,UST,SLC23A2,ANKRD26,ESRP1,DOCK1,RAP1GAP,PLS1,NIN,DRAXIN,SMARCA2,ETS1,GLI3,SMARCC1,SMOC2,PCP4,MAP2,LAMC1,ATF2,UMODL1,BBS4,COL5A1,CFTR,NELL1,KITLG,DCC,GTF2I,RORB,TADA2A,DAB1,PRKN,MTMR2,TBX20,DPF3,NGEF,PRKCH,TG,IL6R,RACGAP1,HEMGN,TOX,ITSN2,ROCK1,LYN,PLEKHB2,DTX1,OVOL2,NTN1,ZFHX3,DPYSL5,ARID1B,CASZ1,INSR,SHROOM3,COLQ,DDHD1,SNAI2,TANC2,RXRG,TRIM58,NREP,NSD2,JCAD,TWIST2,ENPP1,RASGRP1,BMP5,CSF1,GHRH,BCL2L1,CTDP1,LAMA3,ASB4,INO80,CNMD,SMAD5,CELF4,ABCG1,PRAME,MYCL,TNN,MED1,ATRN,IL33,AJAP1,ROR2,KL,LMX1A,TMEM178A,IL10,ACTR2,PRAMEF25,CLSTN2,PTH,SOSTDC1,VSTM2A,MAP6,PALMD,ZBTB7C,ANP32B,YBX3,NRXN1,PCID2,HIPK1,TWIST1,AKT3,JAK2,RBM19,CELSR2,MELTF,UFL1,NFKBIA,PRKCB,ABCC8,NEDD9,OLFM4,ATP2B1,GRIP1,AGO1,STAT1,BRMS1L,DGKG,MTPN,PRAMEF2,IMPACT,CCBE1,MYOCD,MEF2C,ADGRB1,RXRA,WNT7A,RBPMS2,MAP3K5,NDFIP1,WASF3,FOXO6,FBXW8,SDCBP,FLVCR1,FGR,DNMBP,EPHA4,NTRK2,IL1RAPL1,NUMB,ADAMTS9,WNT2B,CD101,MEGF10,IL17RD,FBXO31,PTK2,CDH5,ANKRD6,NFKBID,CLDN18,DIAPH1,LAMB1,CYFIP1,UBE3A,SEMA4D,JAM2,FAT4,RUNX1,WNT5B,ASAP1,PDCL3,GPR55,NSUN2,TNFSF11,FYN,MYL12B,ADGRL2,HDAC2,MAPK9,CRTAM,APELA,ROR1,TET1,HECW2,CDH2,NTN4,GPR137B,EPHB1,GRM5,ADCK1,PID1,NRP1,SDK1,PRKCA,FAIM,RC3H1,CHODL,POR,BCR,FBLN1,RAG1,BMPER,CUX1,MACROH2A1,MITF,EPHB2,PPFIA2,MAGI2,FAM171A1,FLRT2,MYB,KALRN,GNAS,LAMA1,TIAM2,BMP7,DLG5,NUDT21,DDX6,EYA1,SLIT2,ERBB4,SYNDIG1,ROBO1,PBX1,NLGN1,SHLD2,SLC6A3,ASIC2,EFNA5,TCF12,EHMT1,ESR1,NTNG1,LOXL2,PRLR,CAMK1D,CCR2,EPS8,SEMA4B,ROCK2,PRDM1,RORA,ATAT1,DMRT1,HSPG2,CSMD3,COL4A3,HOOK3,FSTL4,STK3,HNRNPU,APCDD1,IGF1R,GLI2,ATP10A,DNM1L*
GO:0048523	negative regulation of	1.514164393451 4607e-	*NOTCH2,BRINP3,MTOR,CNTN4,SPOCK1,WWC1,PTPRD,SLC24A2,ULK2,NLK,FTO,PLCB1,ZNF536,TAFA5,SVIL,ZFPM2,L3MBTL4,DLC1,TNRC6B,ITPR2,RIPOR2,PDE4D,RDX,STXBP1,BCL2,PRDM16,ALDH1A2,FBN1,CHRNA7,ROBO2,ZEB1,AKR1C3,RARB,SPRED*

	cellular process	17	*1,USH2A,MINAR1,FOXJ2,CDYL2,CARMIL1,MCTP1,BABAM2,GLIS3,FANK1,ERBIN,RHPN2,MLLT3,MAP4,SPON1,APC,TSHZ3,DSCAM,RTN1,CRKL,ILDR2,ARHGAP24,PTPRJ,KDM4C,EGFR,RFX3,USP14,ANGPT1,CDK12,BACH1,PRKACB,RGS3,NCOR1,NEDD4,SCAI,BCL11A,SOX6,TMEM182,GRIK3,NTRK3,C5,ZFAND6,DKK2,FLT1,THRAP3,MAPKBP1,DGKI,INVS,GRIA1,CRACD,CAST,SLC8A3,MALRD1,TOM1L2,PRKD1,TPTE2,PAK1,EPHA7,NCOA7,SPEN,RAPGEF2,LRP2,RUNX2,TAOK3,ONECUT1,CPEB4,TMEM38B,LDB2,CCL28,SMYD3,GRM7,RETREG1,RPTOR,GHR,THADA,TBCD,NEDD4L,ADAM10,HDAC9,ZHX3,ATF7IP,APBB2,APP,RPS6KA2,SAMSN1,CACNA1C,KDM1B,DCLK1,STAU2,DOCK8,USP18,SEMA5A,VCL,ARHGAP44,NTF3,ACER2,PARP15,NDUFAF2,CD2AP,AURKA,PARN,CFDP1,ST18,SLC8A1,PTPRR,SRGAP2C,FIG4,DUX4,SRGAP2B,KANK1,KCNE4,MAP4K4,ABCD2,BMPR1B,FMN2,AKAP6,HOMER2,CTNNA2,DIP2B,LARP1,ITPKB,TRPC5,RGS20,PDE10A,KICS2,DNM3,NBN,IFT57,INTS7,PRKCZ,GRB10,RYR3,TAF15,MSH6,MCPH1,RGS9,HECW1,ABCA5,PHF19,DUSP22,YAP1,BRINP1,MAPK1,MGAT5,ADAM22,USP25,PLG,ZNRF3,NRG3,UBE2O,SFMBT2,NIPBL,GABPA,FAT3,CORO2B,STK38,PTPN13,SORCS3,LIMCH1,PAFAH1B1,EFEMP1,ZNF684,TM7SF3,DCAF1,TPM1,NF2,HIVEP1,CTNNA1,BIRC6,PPARA,MEIS2,NFIB,PRTG,PTPRK,PAK5,TRERF1,SEMA3C,DAPK1,NAV3,SLC24A4,SEC14L1,VPS13C,AGO2,PHC3,ALPK2,JARID2,DNAJC15,GATAD2B,ANK2,ADGRV1,ZNF846,BCAS3,RYR2,RANBP3L,SEMA6D,DUSP16,SMARCA4,FABP7,PARD3,MAPKAP1,TNRC6C,PIAS1,BLK,TNR,CXADR,ATRX,PTPRT,ELAVL4,ABL1,MXI1,PTPN12,HDAC4,OXR1,SLC1A1,PRKAA1,LRFN5,CREG1,L3MBTL3,NBAS,SLFN11,RAP1A,GLIS1,MORC1,TOX3,INPP5A,FGF10,GRID2,LATS2,NRG1,ASPM,ZNF438,ABCB7,ZBTB16,KIR3DL2,ZNF675,SH3GL3,SETDB2,PRKCE,FOXK2,NXN,WNK2,FBN2,CD44,RGS12,PTPRO,TRIO,PDE3A,STXBP6,LIMD1,PEX14,SPRED2,RPS6KA3,ATP8A2,PTPN2,PLXNA2,RFC1,HTR2C,CLEC16A,ARHGEF7,AMBRA1,LTBP1,OPRM1,HTR2A,DAZL,KREMEN1,SEMA3E,TAF3,TMEM67,FHL2,ABHD17C,PUM1,TMOD2,HERC1,MSH2,IGF2BP3,ANKRD17,ZNF397,HIPK3,CDKN2C,EPN2,GRK3,CLSPN,BICRAL,MOSMO,MNAT1,RBBP8,MDFIC,ANK3,EMILIN2,HMGA2,CCND3,BCL11B,CREM,LYPLA1,MBP,TRPS1,TGFA,CRIM1,PRR5L,MYT1L,TJP1,LDLRAD4,NPHP4,PACSIN2,HLA-B,SNX3,NAA35,BRCA2,ZBTB2,BLM,SEMA3A,STRN,BMP2,RC3H2,ATP9A,SOGA1,ZC3H14,GFI1B,TBC1D4,RANBP9,RIN3,TMEM161A,SEMA3D,LEMD3,ARHGAP42,HMGB1,GNAQ,FGF9,NFATC2,SH3BP5,MDM1,SLC23A2,ANKRD26,ETS2,ZNF875,DSTYK,UIMC1,LRRFIP1,RAP1GAP,SRGAP2,DRAXIN,SLAMF1,SMARCA2,ETS1,GLI3,CGAS,SMARCC1,SNX6,CNKSR3,GRIK2,ZNF431,RERE,MAP2,BTAF1,ATF2,HIRA,CYLD,BBS4,MAPK8IP1,COL5A1,NELL1,UBASH3A,NEU3,MRPL13,KITLG,UBR1,DCC,SLC30A10,RCAN1,RORB,DAB1,SELENON,RB1CC1,PTPRE,PRKN,MTMR2,ZNF608,TBX20,DACH1,ZNF541,DPF3,NGEF,ARID5B,JPH1,ATXN1,PRKCH,PKP1,FRMD4A,NLRC5,TFDP1,CNOT6L,KANK4,SNX25,PTPRB,ZFP90,COPS8,ZNF124,SHANK2,USP7,ENPP3,PLAGL1,SOX30,KIR2DL4,NPAS2,ROCK1,LYN,ARHGAP28,ZNF169,DTX1,TENM2,OVOL2,ZBTB33,NTN1,ZFHX3,DPYSL5,RNF152,OTUD7A,BMF,YTHDF3,TFF1,DEDD2,HECTD1,ARFGEF1,SNAI2,ASH1L,BID,SIAH2,TRABD2B,UFD1,SP3,ERN2,TIAL1,ELF2,NSD2,CD9,CARD10,TWIST2,SAMHD1,ENPP1,TP53I11,TMEM225,NDRG2,CSNK2A1,BMP5,CSF1,HDGFL3,BCL2L1,SERPINB9,SCAF4,CTDP1,HCN1,PRKG1,GRIN2B,GRB14,FANCB,CNMD,DHRS3,SMAD5,CELF4,TCERG1,ABCG1,FOXN3,SLC40A1,PRAME,TNN,MED1,CDC14B,IL33,AJAP1,BANK1,CSDE1,LMX1A,TMEM178A,IL10,SFPQ,SCML2,PRAMEF25,PTH,SOSTDC1,PRKAA2,NDC80,PACRG,ABHD2,ITPRIP,ETV6,IQGAP1,CAMLG,ZBTB7C,SREBF2,ANP32B,YBX3,AIMP1,NRXN1,PCID2,PBLD,PEG10,TWIST1,AKT3,JAK2,FSTL1,ZBTB38,PATL1,CREBBP,MELTF,TNKS,PCNA,SIAH3,UFL1,NFKBIA,PRKCB,ABCC8,ANXA4,RTRAF,BRD4,ZBTB21,NEDD9,ITGA6,ASS1,BTG3,ERLIN2,OTOP1,CIDEA,ZBTB49,AGO1,MEOX2,SLC6A1,STAT1,BRMS1L,NDFIP2,NR2C1,DGKG,MTPN,ABI1,CEMIP,PRAMEF2,IMPACT,PARK7,ADAMTS18,MAPK8,OAZ2,POU1F1,ADCYAP1R1,MTF2,NCAPG*

			2,FOXP2,MYOCD,MEF2C,ADGRB1,RXRA,WNT7A,RBPMS2,NDFIP1,PRDM13,C16ORF72,MAGEL2,PDE2A,SDCBP,WWOX,ZBTB25,PASK,MLLT1,NCK1,SCAF8,FGR,CDCA8,PPP2R3A,RNF8,EPHA4,GABRA5,MECOM,DNMT3L,NTRK2,IL1RAPL1,NUMB,LHX9,ADAMTS9,ZBTB10,OCLN,SHISA6,IL17RD,FBXO31,IREB2,PTK2,CDH5,ANKRD6,ARHGAP12,CLDN18,ASCL3,DIAPH1,FEZ2,INIP,APIP,UBE3A,SEMA4D,JAM2,ZBTB20,RUNX1,AKR1B1,KIRREL1,AMFR,NENF,PTGFR,SAMD13,TPTE,PDCL3,SRP9,GPR55,NSUN2,CDC45,BICD1,TNFSF11,FYN,BUB1,KDM5A,PCBP3,ZNF705G,PPM1F,SDE2,RBMS3,HDAC2,AVEN,GON4L,TBX15,COL18A1,ALB,ZFYVE28,PABPC1,CRTAM,APELA,TET1,HECW2,CDH2,RAD9A,PHLPP1,GPR137B,EPHB1,GRM5,ADCK1,ZNF705D,RPS6KA5,SPTB,TBC1D1,PTPRG,PID1,NRP1,MIDEAS,PRKCA,FAIM,FHIT,ITGA1,KLF12,RC3H1,NRIP1,POR,MCC,BCR,TUT4,RGS6,RERG,FBLN1,RAG1,BMPER,PRDM15,CUX1,SRGAP3,MACROH2A1,MITF,EPHB2,SACS,CD38,EYA4,AKAIN1,MET,ZNF705B,CDH13,SERPINB2,ATG5,MAGI2,PRDM11,UNK,MLIP,MYB,KALRN,MFHAS1,BMP7,ASTN2,DLG5,TNFAIP8,ZMYND8,KCTD1,BPTF,BTBD10,ZMYND11,TMEM25,DDX6,ADGRF5,WDR41,PPP1R13B,ABL2,BACE2,PARPBP,EYA1,FHOD3,SLIT2,CNOT7,ERBB4,IL20RB,ROBO1,SAMD4A,PBX1,NUF2,PRKCQ,ANTXR1,NDRG1,SORCS2,TRDN,MGMT,NLGN1,SHLD2,NOS1,ASIC2,EFNA5,NSD1,EHMT1,SLIT3,FRMD5,ESR1,KDM4B,LOXL2,IQCJ-SCHIP1,SKAP2,PRLR,AGO3,HTT,FOXB1,CAMK1D,PIK3R3,HLA-F,FER,EYA2,CCR2,STARD13,CHFR,EPS8,JAZF1,ZNF891,SPOCK3,SEMA4B,PHC2,GRIA4,ROCK2,PRDM1,RORA,DMRT1,RGS8,HERPUD1,COL4A3,WASHC1,RGS7,HOOK3,KIF7,FSTL4,BARD1,STK3,DEPTOR,ZNF423,ZNF568,HNRNPU,APCDD1,IGF1R,KCNAB1,PRKAG2,GLI2,THRB,MORC3
GO:0051128	regulation of cellular component organization	2.1959357350615172e-17	*NOTCH2,MTOR,SPOCK1,ABCA13,PTPRD,LRRC4C,MYO9A,ULK2,PLCB1,SVIL,DLC1,PTPRA,RIPOR2,RDX,RP1,STXBP1,RALA,IL1RAPL2,BCL2,LRFN2,CDH8,CHRNA7,ROBO2,RIMS1,SPIRE1,TENM3,SDCCAG8,MINAR1,CDC42EP3,RIMS2,ALK,AUTS2,CARMIL1,MCTP1,PAPPA2,RHPN2,ANO6,NEGR1,MLLT3,GPC6,CNTNAP2,MAP4,APC,PLPPR5,DSCAM,CRKL,ARHGAP24,TNIK,PTPRJ,EGFR,ANGPT1,MACF1,NEK7,NEDD4,MTRF1,NSMCE2,BTBD9,BCL11A,TMEM182,CDH4,NTRK3,PSMA8,CRACD,SLC39A12,TOM1L2,PRKD1,PAK1,EPHA7,RAPGEF2,ADGRB3,ARSB,SEPTIN9,RPTOR,EPB41L3,TBCD,NEDD4L,ADAM10,ATF7IP,APP,RPS6KA2,STAU2,MAPRE2,SEMA5A,SYT1,VCL,ARHGAP44,NTF3,CD2AP,AURKA,PARN,SRGAP2C,FIG4,KANK1,MAP4K4,AKAP6,CTNNA2,RAB8B,PAK3,DIP2B,TRPC5,RAP1GDS1,DNM3,NBN,PRKCZ,MCPH1,HECW1,COBL,SENP6,DUSP22,YAP1,MAPK1,NRG3,ANKFY1,SPIDR,FAT3,CORO2B,CHN1,MYLK3,LIMCH1,FMN1,PAFAH1B1,STON2,VPS13D,TLK1,TPM1,NF2,AKAP9,PPARA,SNX30,SYNJ1,TIAM1,PAK5,SEMA3C,NAV3,VPS13C,DNAJC15,TANC1,BCAS3,SYNE2,SEMA6D,SMARCA4,MAPKAP1,TBC1D5,TNR,ATRX,ELAVL4,ABL1,HDAC4,PRKAA1,SDC2,LRFN5,L3MBTL3,RAP1A,MYO10,GRID2,PEAK1,NRG1,INO80D,GSG1L,CLIP1,ABCB7,MUSK,SH3GL3,SETDB2,PRKCE,DGKB,CD44,PTPRO,EGF,PDE3A,STXBP6,RPS6KA3,ATP8A2,MTMR3,PLXNA2,CLEC16A,ARHGEF7,ATP8A1,AMBRA1,STK38L,KREMEN1,SEMA3E,MARK2,TMEM67,C10ORF90,ABHD17C,TMOD2,LINGO2,CDKN2C,KNDC1,MNAT1,VPS41,MBP,PLCE1,TGFA,HIP1,CRIM1,FUT9,RUFY2,TJP1,LDLRAD4,NPHP4,PACSIN2,CNTN1,IQSEC1,SNX3,PDLIM5,DISC1,WDPCP,SEMA3A,BMP2,GNAI1,TBC1D4,RESF1,RIN3,BMP2K,SEMA3D,RELN,HMGB1,NFATC2,UST,MDM1,SLC23A2,UNC13B,RAP1GAP,PLS1,NIN,DRAXIN,ATF1,SLAMF1,SMARCA2,SMARCC1,KIF15,MAP2,LAMC1,FARP1,CYLD,BBS4,COL5A1,NEU3,DCC,YLPM1,DAB1,PRKN,MTMR2,TBX20,AFAP1,DPF3,NGEF,PRKCH,FRMPD4,CNOT6L,KANK4,FBLN5,TOX,SHANK2,USP7,ITSN2,RALB,ROCK1,LYN,ARHGAP28,TENM2,NTN1,DPYSL5,ARID1B,INSR,BMF,NEK6,COLQ,DDHD1,ARFGEF1,PDE4DIP,SNAI2,BID,TANC2,TRABD2B,TRIM58,FYCO1,SH3GLB1,ENPP1,SNX9,ANAPC1,CSNK2A1,BMP5,HDGFL3,BCL2L1,SCAF4,CTDP1,GRIN2B,INO80,TNN,ROR2,LMX1A,IL10,ACTR2,SFPQ,CLSTN2,PRKAA2,SKA1,NDC80,MAP6,VASP,IQGAP1,TEAD1,MORC2,SREBF2,N*

			RXN1,PCID2,SNAP91,CENPE,NET1,ANLN,ALKAL2,PRSS2,MELTF,TNKS,ABCC8,USH1C,NEDD9,ITGA6,GAP43,GRIP1,SAR1A,DGKG,MTPN,IMPACT,PARK7,MAPK8,MYOCD,CEP120,CYFIP2,MEF2C,ADGRB1,WNT7A,MAP3K4,WASF3,MAGEL2,RAD51AP1,PDE2A,FBXW8,SDCBP,NECTIN1,NSMCE1,NCK1,SCAF8,CDCA8,EPHA4,NTRK2,IL1RAPL1,NUMB,OCLN,FBXO31,EXTL3,PTK2,MARK4,CDH5,MPP7,DIAPH1,FEZ2,LAMB1,CYFIP1,UBE3A,PCDH8,SEMA4D,RUNX1,KIRREL1,SAXO1,ASAP1,CCDC88A,ADAMTS16,BICD1,FYN,BUB1,PPM1F,ADGRL2,HDAC2,SLF1,NCS1,MAPK9,APELA,ROR1,TET1,HECW2,CDH2,EPHB1,ADCK1,SPTB,PTPRG,PID1,NRP1,FCHSD2,CHODL,RERG,CUX1,MACROH2A1,EPHB2,TOGARAM1,SACS,CD38,AKAIN1,MET,CDH17,PPFIA2,CDH13,ATG5,MAGI2,PRDM11,FLRT2,KALRN,LAMA1,TIAM2,BMP7,DLG5,ABL2,FHOD3,SLIT2,SYNDIG1,ROBO1,NUF2,PRKCQ,ANTXR1,NLGN1,CTTNBP2,ASIC2,EFNA5,GAS2L1,ARHGEF11,SLIT3,ESR1,NTNG1,IQCJ-SCHIP1,HTT,CAMK1D,FER,CHFR,EPS8,SEMA4B,ROCK2,STMP1,ATAT1,DMRT1,CDCA5,RAB31,CSMD3,WASHC1,FSTL4,C1QL3,HNRNPU,RAB3GAP2,IGF1R,AKAP13,ATP10A,DNM1L
GO:0007155	cell adhesion	6.378908581003301e-17	CNTN4,SPOCK1,PTPRD,FREM1,LRRC4C,TLN2,TENM4,DLC1,ZDHHC21,PTPRA,RIPOR2,RDX,STXBP1,BCL2,FBN1,CDH8,ROBO2,TENM3,CNTNAP5,USH2A,ADGRE1,PCDH7,CARMIL1,ERBIN,ASTN1,RIN2,PARVB,NEGR1,GPC6,CNTNAP2,SPON1,APC,HHLA2,DSCAM,CRKL,ILDR2,PTPRJ,EGFR,ANGPT1,MACF1,CTNNA3,HMCN2,CRB1,CDH4,CNTN3,NEO1,CNTN6,EPHA7,CTNNAL1,ONECUT1,ADAMTSL1,CCL28,TBCD,ADAM32,ADAM10,APP,DOCK8,SEMA5A,VCL,ACER2,CD2AP,IGSF5,CFDP1,KANK1,MAP4K4,CTNNA2,ITPKB,PRKCZ,DUSP22,CADM2,ADAM22,ALCAM,PLG,ITGBL1,NCAM1,FAT3,CORO2B,LIMCH1,FMN1,ITGB8,TPM1,NF2,CTNNA1,CDH7,PPARA,PRTG,TIAM1,PTPRK,PARD3B,PCDH11Y,MAGI1,ADGRV1,BCAS3,SMARCA4,CDH11,PARD3,BLK,TNR,DST,CXADR,NUAK1,PTPRT,ABL1,ITGB3BP,LRFN5,APBB1IP,NFAT5,CDH18,MYO10,GRID2,CDHR3,PEAK1,NRG1,AP3B1,COL6A5,ZBTB16,PRKCE,PGM5,CD44,PTPRO,EXT1,STXBP6,COL5A3,CTNND2,PTPN2,PLXNA2,ATXN3,ARHGEF7,CD96,AMBRA1,SEMA3E,LPP,COL6A6,STK10,ADAM12,ANK3,EMILIN2,DOCK5,MBP,FUT9,PCDH9,VAV1,CDH20,TJP1,NPHP4,EGFLAM,CNTN1,PDLIM5,DISC1,WDPCP,BMP2,RC3H2,UNC5D,NCAM2,RELN,HMGB1,DOCK1,SRGAP2,SLAMF1,SMARCA2,ETS1,GLI3,MEGF11,SMARCC1,LAMC1,LAMC3,COL5A1,ITGA9,COL14A1,RGMB,KITLG,DCC,DAB1,PCDH15,CDH23,PKP1,FBLN5,VAV3,ROCK1,LYN,VCAM1,DTX1,TENM2,NTN1,ZFHX3,ARID1B,SNAI2,CD9,JCAD,UTRN,RASGRP1,IGSF11,CDH26,BMP5,CSF1,PRKG1,LAMA3,TNN,MICALL2,ATRN,AJAP1,FAT1,IL10,CLSTN2,FYB2,NRXN1,CD70,CADM1,SSPN,JAK2,SVEP1,CELSR2,PRSS2,MELTF,NTM,PCDH11X,NEDD9,OLFM4,ITGA6,ASS1,HEPACAM,NECTIN4,ADAMTS18,ITGA4,TM9SF4,HMCN1,CYFIP2,ADGRB1,NDFIP1,SERPINI2,PKN2,NECTIN1,DSG1,NCK1,TINAG,EPHA4,IL1RAPL1,FNDC3A,ADAMTS9,POSTN,MEGF10,TRPM7,PTK2,CDH5,NFKBID,CLDN18,LAMB1,PCDH8,SEMA4D,JAM2,FAT4,RUNX1,KIRREL1,EDIL3,TNFSF11,FYN,PPM1F,CNTNAP3,COL18A1,CDH9,CRTAM,COL19A1,SLC39A8,CDH2,CNTN5,ITGA8,NTN4,EPHB1,NRP1,SDK1,PRKCA,ITGA1,RC3H1,BCR,NRXN3,CDH12,FBLN1,RAG1,DGCR2,EPHB2,MUC16,DLG2,CDH17,PPFIA2,CDH13,VMP1,FLRT2,MYB,GNAS,LAMA1,ATRNL1,IGSF21,BMP7,ASTN2,DLG5,KIRREL3,ABL2,PDZD2,CCDC141,IL20RB,ROBO1,PRKCQ,ANTXR1,NLGN1,EFNA5,VCAN,FRMD5,NTNG1,LOXL2,PRLR,OPCML,FER,CCR2,ROCK2,PPP1CB,COL4A3,CLDN10,RSU1,GLI2,LSAMP
GO:0050808	synapse organization	4.650376056439441e-16	PTPRD,LRRC4C,UNC13C,ERC1,IL1RAPL2,LRFN2,GPHN,CDH8,CHRNA7,ROBO2,GABRB3,CACNG2,NEGR1,GPC6,DSCAM,CRKL,DOCK10,NEDD4,NTRK3,CAST,SLC8A3,EPHA7,ADGRB3,ADAM10,APBB2,APP,CACNB2,STAU2,GABRG2,ARHGAP44,SRGAP2C,CTNNA2,PAK3,ERC2,DNM3,DIP2A,NFIA,PAFAH1B1,CNKSR2,PDZRN3,TMEM108,TANC1,TNR,ABL1,SLC1A1,LRFN5,GRID2,NRG1,MUSK,DGKB,PTPRO,CTNND2,SEMA3E,ABHD17C,LINGO2,AFG3L2,ANK3,PDLIM5,DISC1,DNER,RELN,UNC13B,SRGAP2,FARP1,MTMR2,LGI2,NGEF,FRMPD4,ALS2,SHANK2,MESD,SYBU,NTN1,INSR,COLQ,TANC2,GRIN2B,LMX1A,IL10,ACTR2,CLSTN2,NRXN1,NEDD9,G

			AP43,SLC6A1,MEF2C,ADGRB1,WNT7A,WASF3,SDCBP,NECTIN1,EPHA4,NTRK2,IL1RAPL1,SHISA6,CYFIP1,UBE3A,PCDH8,SEMA4D,NOS1AP,FYN,ADGRL2,CDH2,CNTN5,EPHB1,GRM5,NRP1,SDK1,NRXN3,EPHB2,PPFIA2,FLRT2,KALRN,IGSF21,DLG5,GABRA2,KIRREL3,ERBB4,SYNDIG1,NLGN1,CTTNBP2,ASIC2,EFNA5,NTNG1,C1QL3,IGF1R
GO:0016358	dendrite development	9.24587095345252e-16	*PTPRD,CHRNA7,ALK,DSCAM,CRKL,TNIK,DOCK10,NEDD4,BCL11A,PHACTR1,RAPGEF2,ADGRB3,NEDD4L,APP,DCLK1,STAU2,ARHGAP44,SRGAP2C,CTNNA2,PAK3,TRPC5,DNM3,KLHL1,DIP2A,HECW1,COBL,FAT3,PAFAH1B1,ELAVL4,ABL1,SDC2,CTNND2,KNDC1,IQSEC1,PDLIM5,DISC1,SEMA3A,STRN,RELN,SRGAP2,RERE,MAP2,FARP1,BBS4,DCC,DAB1,NGEF,NTN1,DPYSL5,TANC2,BMP5,PRKG1,ACTR2,MAP6,CELSR2,GRIP1,DGKG,ABI1,MEF2C,WNT7A,FOXO6,FBXW8,EPHA4,IL1RAPL1,FBXO31,CYFIP1,UBE3A,SEMA4D,ASAP1,FYN,HDAC2,HECW2,EPHB1,NRP1,SDK1,CUX1,EPHB2,PPFIA2,KALRN,BMP7,DLG5,NLGN1,CAMK1D,CSMD3,FSTL4*
GO:0009966	regulation of signal transduction	1.4360535308943907e-15	*NOTCH2,BCAR3,MTOR,WWC1,GARNL3,PTPRD,MYO9A,NLK,KSR1,PLCB1,ZNF536,DLC1,RIPOR2,PDE4D,RDX,BCL2,PRDM16,ARHGAP26,FBN1,CHRNA7,ROBO2,RIMS1,ZEB1,AKR1C3,FGD4,SPRED1,MINAR1,RIMS2,ALK,AUTS2,PJA2,BABAM2,ERBIN,CACNG2,DLGAP1,MLLT3,GPC6,APC,CRKL,ARHGAP24,TNIK,PTPRJ,KDM4C,EGFR,DENND1A,ANGPT1,MACF1,PRKACB,RGS3,NCOR1,RNF220,DOCK2,NEDD4,SCAI,SGMS1,CHSY1,NTRK3,ZFAND6,DKK2,FLT1,MAPKBP1,DGKI,INVS,EDAR,NEO1,CNTN6,SLC8A3,PRKD1,TPTE2,PAK1,EPHA7,RALGPS1,RAPGEF2,PELI2,LRP2,RUNX2,TAOK3,ONECUT1,TAFA4,RPTOR,GHR,RALGAPA1,ADAM10,IL1R1,APP,DOCK8,MAPRE2,USP18,SEMA5A,ARHGAP44,NTF3,CD2AP,AURKA,PTPRR,KANK1,MAP4K4,BMPR1B,PCSK6,AKAP6,HOMER2,ARNT,PAK3,ITPKB,RGS20,PDE10A,RAP1GDS1,KICS2,PRKCZ,GRB10,ARHGAP32,RGS9,HECW1,DUSP22,YAP1,MAPK1,MGAT5,PDGFD,ZNRF3,ARHGEF17,UBE2O,NCAM1,STK38,PTPN13,CHN1,HRH4,PAFAH1B1,ATF6,NF2,CNKSR2,CTNNA1,MOB3B,BIRC6,AKAP9,KLF15,RASGRF2,PPARA,ADAMTS3,TIAM1,GRM1,ARHGEF12,PAK5,PCDH11Y,PLA2R1,DAPK1,SLC24A4,SEC14L1,TMEM108,ALPK2,RIC8B,IL34,ADGRV1,DUSP16,SMARCA4,USP8,MAPKAP1,BLK,MBD5,NUAK1,PTPRT,ABL1,PTPN12,PRKAA1,GAS2,NFAT5,GUCY1A2,RAP1A,GPC5,FGF10,ZC3HAV1,LATS2,NRG1,GSG1L,ASPM,DENND2B,RASGRF1,ZNF675,GNG7,PRKCE,NXN,WNK2,USP33,DENND4C,FBN2,CD44,RGS12,PTPRO,EGF,TRIO,PDE3A,LIMD1,SPRED2,CTNND2,PTPN2,TRIM5,MCF2L,HTR2C,CLEC16A,LTBP1,OPRM1,HTR2A,FANCA,KREMEN1,SEMA3E,GCSAML,FHL2,CNIH3,PUM1,TMOD2,ANKRD17,RELL1,HIPK3,EPN2,EVC,GRK3,MOSMO,MDFIC,CCND3,PLCE1,TGFA,HIP1,CRIM1,PRR5L,VAV1,LDLRAD4,NPHP4,IQSEC1,SNX3,BRCA2,DISC1,NRK,SEMA3A,MAGI3,BMP2,RC3H2,GNAI1,RALGAPA2,RANBP9,BMP2K,TMEM161A,NETO2,LEMD3,RELN,ARHGAP42,HMGB1,GNAQ,FGF9,TRAF3,UNC13B,TTC21B,DSTYK,RAP1GAP,SRGAP2,DRAXIN,SLAMF1,GLI3,SNX6,SMOC2,CNKSR3,PSD3,GAREM1,LAMC1,NEK10,CYLD,MAPK8IP1,KPNA1,UBASH3A,NEU3,KITLG,CAMTA1,UBR1,SLC30A10,RCAN1,DAB1,RB1CC1,PTPRE,PRKN,MTMR2,TBX20,DLGAP2,AFAP1,NGEF,GRIN2A,PRKCH,IL6R,ALS2,RACGAP1,NLRC5,SNX25,SHISA9,SHANK2,USP7,VAV3,SOX30,ARHGEF28,ROCK1,LYN,ARHGAP28,ARHGAP31,SLC44A2,SLC15A2,DTX1,OVOL2,RRAGD,CRACR2A,RNF152,OTUD7A,INSR,YTHDF3,DEDD2,NEK6,ARFGEF1,SNAI2,ASH1L,BID,SIAH2,TRABD2B,UFD1,ERN2,TIAL1,NREP,ZDHHC17,RALGPS2,JCAD,SAMHD1,IFT81,ENPP1,RASGRP1,IGSF11,NDRG2,CSNK2A1,BMP5,CSF1,GHRH,BCL2L1,GRIN2B,GRB14,DHRS3,CELF4,PRAME,TNN,MED1,ROR2,KL,BANK1,IL10,SFPQ,PTH,SOSTDC1,PRKAA2,NDC80,ITPRIP,IQGAP1,RPS12,SREBF2,YBX3,NRXN1,PCID2,HIPK1,CIBAR1,PBLD,FICD,PEG10,NET1,SIPA1L2,TWIST1,AKT3,ALKAL2,JAK2,RPF2,FSTL1,MADD,HCRTR1,CREBBP,TNKS,GORAB,UFL1,NFKBIA,PRKCB,BRD4,ITGA6,OTOP1,CIDEA,ARFGEF3,GID8,STAT1,BRMS1L,NDFIP2,NR2C1,MAP2K6,DGKG,CCBE1,PARK7,FBXL17,ADCYAP1R1,NCAPG2,MYOCD,CYFIP2,EFHB,MEF2C,RXRA,WNT7A,RBPMS2,MAP3K5,NDFIP1,MAP3K4,S100B,ATP6V1C2,C16ORF72,PDE2A,SDCBP,JPT2,SPPL*

			2B,WWOX,NCK1,FGR,CDCA8,PPP2R3A,DNMBP,EPHA4,CYTH4,MECOM,NTRK2,POSTN,SHISA6,IL17RD,MVB12B,PTK2,CDH5,ANKRD6,ARHGAP12,LAMB1,APIP,CYFIP1,UBE3A,SEMA4D,WNT5B,AMFR,NENF,NOS1AP,TPTE,CCDC88A,GPR55,BICD1,TNFSF11,FYN,RBMS3,HDAC2,DOCK3,DOK5,ZFYVE28,MAPK9,APELA,ROR1,TET1,CDH2,ITGA8,RAD9A,PHLPP1,GPR137B,EPHB1,GRM5,RAI14,PID1,NRP1,PRKCA,FAIM,ITGA1,RC3H1,POR,MCC,BCR,RGS6,ELP2,FBLN1,STK36,BMPER,PRDM15,SRGAP3,EPHB2,CSNK1G1,EYA4,CDK14,MET,SPPL3,CDH13,CACNG3,MAGI2,PRDM11,KALRN,GNAS,LAMA1,MFHAS1,TIAM2,BMP7,DLG5,ZMYND11,TMEM25,PDGFC,ABL2,EYA1,SLIT2,CNOT7,KCTD8,ERBB4,ROBO1,PRKCQ,SIPA1L3,NLGN1,ARHGEF11,SLIT3,ESR1,MYO9B,IQCJ-SCHIP1,PRLR,AGO3,HTT,FER,EYA2,CCR2,STARD13,EPS8,ROCK2,RORA,DMRT1,RGS8,HERPUD1,RGS7,KIF7,FSTL4,BARD1,STK3,DEPTOR,ZNF423,APCDD1,IGF1R,GLI2,THRB,AKAP13,DNM1L
GO:0010975	regulation of neuron projection development	1.599878890814 9386e-15	*SPOCK1,PTPRD,LRRC4C,ULK2,ROBO2,TENM3,MINAR1,ALK,NEGR1,PLPPR5,DSCAM,CRKL,TNIK,MACF1,NEDD4,BCL11A,CDH4,NTRK3,SLC39A12,PRKD1,PAK1,EPHA7,RAPGEF2,ADGRB3,ARSB,NEDD4L,STAU2,SEMA5A,ARHGAP44,FIG4,KANK1,CTNNA2,PAK3,DIP2B,TRPC5,DNM3,HECW1,COBL,FAT3,CHN1,PAFAH1B1,TIAM1,SEMA3C,SEMA6D,TNR,ELAVL4,ABL1,SDC2,RAP1A,GRID2,PTPRO,ATP8A2,PLXNA2,KREMEN1,SEMA3E,MARK2,KNDC1,MBP,FUT9,CNTN1,SNX3,PDLIM5,DISC1,SEMA3A,SEMA3D,RELN,UST,NIN,DRAXIN,ATF1,MAP2,DCC,DAB1,NGEF,TOX,LYN,NTN1,DPYSL5,TANC2,BMP5,TNN,ROR2,ACTR2,MAP6,NRXN1,ALKAL2,ITGA6,DGKG,WNT7A,FBXW8,NCK1,EPHA4,NTRK2,IL1RAPL1,FBXO31,CYFIP1,UBE3A,SEMA4D,CCDC88A,FYN,HDAC2,NCS1,ROR1,HECW2,CDH2,PTPRG,NRP1,CHODL,CUX1,EPHB2,CD38,PPFIA2,MAGI2,KALRN,TIAM2,BMP7,ABL2,SLIT2,ROBO1,NLGN1,EFNA5,NTNG1,CAMK1D,SEMA4B,CSMD3,FSTL4,IGF1R*
GO:0034329	cell junction assembly	7.036877948408 7355e-15	*PTPRD,MYO9A,TLN2,DLC1,PTPRA,IL1RAPL2,BCL2,CDH8,ROBO2,GABRB3,NEGR1,GPC6,CNTNAP2,APC,DSCAM,CRKL,PTPRJ,MACF1,NTRK3,EPHA7,RAPGEF2,ADGRB3,PATJ,EPB41L3,TBCD,APP,STAU2,GABRG2,VCL,SRGAP2C,MAP4K4,DNM3,DUSP22,CORO2B,LIMCH1,FMN1,CTNNA1,CDH7,PTPRK,ANK2,BCAS3,CDH11,PARD3,DST,ABL1,LRFN5,CDH18,RAP1A,GRID2,CDHR3,PEAK1,NRG1,MUSK,PTPRO,CTNND2,ARHGEF7,LINGO2,CDH20,TJP1,NPHP4,PDLIM5,DNER,WDPCP,STRN,SRGAP2,LAMC1,FARP1,LGI2,PRKCH,PKP1,SHANK2,ROCK1,NTN1,COLQ,SNAI2,CD9,MICALL2,CLSTN2,NRXN1,HIPK1,ITGA6,GAP43,MEF2C,ADGRB1,WNT7A,PKN2,SDCBP,NECTIN1,DSG1,NTRK2,IL1RAPL1,OCLN,PTK2,CDH5,CLDN18,MPP7,SEMA4D,PPM1F,ADGRL2,CDH9,CDH2,CNTN5,EPHB1,NRP1,SDK1,PRKCA,BCR,NRXN3,CDH12,EPHB2,VMP1,FLRT2,DLG5,GABRA2,KIRREL3,ERBB4,SYNDIG1,NLGN1,ASIC2,EFNA5,FER,ROCK2,CLDN10*
GO:0065008	regulation of biological quality	7.283938180043 852e-15	*MTOR,UNC80,CNTN4,NSG1,SGCD,PTPRD,SLC24A2,KCNH5,MICU2,ULK2,NLK,UNC13C,FTO,ZNF236,SVIL,PIEZO2,TNRC6B,ZDHHC21,ITPR2,PDE4D,RDX,RP1,STXBP1,ERC1,IL1RAPL2,BCL2,KCNMA1,PRDM16,ALDH1A2,LRFN2,F13A1,GPHN,CDH8,CHRNA7,ROBO2,RIMS1,GABRB3,AKR1C3,NAV2,ENPEP,USH2A,CDC42EP3,RIMS2,ALK,CARMIL1,MCTP1,ERBIN,ANO6,CACNG2,NEGR1,GPC6,APC,TSHZ3,DSCAM,ILDR2,SLC4A10,PTPRJ,DOCK10,RFX3,ANGPT1,MACF1,CTNNA3,PRKACB,NEDD4,CRB1,BTBD9,BCL11A,GRIK3,FLI1,CDH4,ATP2B2,NTRK3,LARGE1,CYP2C9,THRAP3,GABRB1,DGKI,GRIA1,CRACD,TTC39B,NEO1,SLC39A12,SLC8A3,MALRD1,NELL2,PRKD1,EPHA7,CHRM3,GRAMD1B,RAPGEF2,ADGRB3,FGF12,GABRA6,CPS1,TMEM38B,SLC24A3,LDB2,TAFA4,PTPRN2,SYN2,CCL28,RPTOR,GHR,THADA,NEDD4L,TRPM1,ADAM10,HDAC9,ATF7IP,APBB2,APP,ABCB5,RPS6KA2,CACNA1C,CACNB2,STAU2,GABRG2,SEMA5A,SYT1,VCL,ARHGAP44,NDUFAF2,AURKA,PARN,SLC8A1,ABCG8,KANK1,KCNE4,PCSK6,AKAP6,HOMER2,CTNNA2,ARNT,RAB8B,PAK3,DIP2B,KCNK10,LARP1,ITPKB,TRPC5,RAP1GDS1,RNLS,CHST8,ERC2,DNM3,CUBN,SCP2,SYN3,PRKCZ,GRB10,RYR3,TAF15,ABCA5,EBF2,YAP1,MAPK1,CADPS2,HRH2,TRPC7,ADAM22,KMT2E,PLG,SLC16A1,MICU1,CORO2B,HRH4*

			,SORCS3,MYLK3,FMN1,PAFAH1B1,TM7SF3,TPM1,NF2,GRIK4,CORIN,AKAP9,KLF15,RASGRF2,PPARA,SYNJ1,NR5A2,GRM1,GABRG1,SEMA3C,SLC24A4,TMEM108,AGO2,SCN2A,RAB22A,CPE,ANK2,TANC1,ADGRV1,RYR2,BBS2,WNT9B,SLC9C1,SEMA6D,NBEA,SMARCA4,USP8,TNRC6C,BLK,TNR,CXADR,DOCK4,MBD5,ELAVL4,ABL1,SLC1A1,PRKAA1,SLC12A8,KCNH1,LRFN5,ANO4,L3MBTL3,DMXL2,EIPR1,RAP1A,FGF10,GRID2,NRG1,GSG1L,AP3B1,RASGRF1,PAH,ATP11C,ABCB7,MUSK,ZNF675,PRKCE,FOXK2,SLMAP,WNK2,ESRRG,DGKB,USP33,PTPRO,ABCC9,P2RX6,PDE3A,EXT1,LNPEP,ATP8A2,SCG5,PTPN2,HTR2C,RIC3,ARHGEF7,ATP8A1,AMBRA1,OPRM1,HTR2A,CYBRD1,CYP4A11,DAZL,CNNM4,SEMA3E,ALPL,C10ORF90,ABHD17C,PUM1,TMOD2,IGF2BP3,CDIN1,APBA2,MAIP1,LINGO2,KCND2,NOS2,AFG3L2,MOSMO,SGCZ,TMTC2,ANK3,EMILIN2,DOCK5,F5,ECE1,MBP,TRAPPC11,PLCE1,HIP1,GSR,VAV1,TJP1,NPHP4,CACNA1I,PDLIM5,DISC1,SLC10A7,SEMA3A,STX12,BMP2,RC3H2,ATP9A,SCN11A,ZC3H14,MYRIP,TTR,RIN3,SEMA3D,RELN,ARHGAP42,HMGB1,GNAQ,UNC13B,UBAP2L,PLS1,SLC39A6,DRAXIN,CCDC186,KCNH8,ETS1,SLC9A4,GABRR2,GRIK2,IDE,MCTP2,MYEF2,MAP2,PEX6,LAMC1,FARP1,ATF2,BBS4,CFTR,KITLG,ZZEF1,ATP10B,DCC,SLC30A10,TADA2A,SELENON,RB1CC1,PRKN,MTMR2,PCDH15,NGEF,HEPHL1,GRIN2A,JPH1,TXNRD2,CDH23,SLC12A1,TG,FRMPD4,ALS2,ACO1,DHRS11,CNOT6L,KANK4,KCNQ3,SHISA9,SLC4A4,SCN10A,SHANK2,USP7,VAV3,ENPP3,KCND3,HAAO,PPA2,ROCK1,LYN,SEL1L,ARHGAP28,CTSB,NTN1,INSR,YTHDF3,TFF1,DEDD2,GRID1,COLQ,NMU,ARFGEF1,BID,RPH3A,TANC2,ABCA4,SP3,GABRG3,TRIM58,SH3GLB1,CD9,XKR5,ENPP1,IGSF11,SNX9,BMP5,KCNC1,CSF1,GHRH,BCL2L1,HCN1,PRKG1,GRIN2B,CLNS1A,DHRS3,SMAD5,CELF4,ABCG1,KCNK5,VSTM4,SLC40A1,MED1,KL,CSDE1,TMEM178A,IL10,ACTR2,CLSTN2,PTH,PRKAA2,NDC80,VASP,PLA2G4A,CAMLG,SREBF2,YBX3,AIMP1,NRXN1,PCID2,DGKK,PBLD,AKT3,JAK2,SLC1A7,FSTL1,HCRTR1,PTGS1,PATL1,CELSR2,FH,CREBBP,MELTF,SIAH3,TRPV5,UFL1,ADAMTS5,PRKCB,ABCC8,MT1HL1,USH1C,SERBP1,NEDD9,ATP2B1,IARS2,GRIP1,XKR6,CIDEA,EXT2,SLC6A1,STAT1,MAP2K6,DGKG,SLC6A11,MTPN,CEMIP,PARK7,ADAMTS18,MAPK8,ADCYAP1R1,PLA2G12B,CSMD1,NCAPG2,TM9SF4,RAPGEF4,ATP13A3,CYFIP2,HNRNPM,ACACA,MEF2C,ADGRB1,WNT7A,NDFIP1,S100B,PDE2A,RAB38,NECTIN1,DSG1,PASK,NCK1,FLVCR1,SNAP29,EPHA4,GABRA5,NTRK2,IL1RAPL1,ACSM2A,NUMB,FRRS1,OCLN,SHISA6,AKAP11,TRPM7,GRIK1,PRKAB1,IREB2,PTK2,CDH5,SCGN,CLDN18,DIAPH1,CYFIP1,UBE3A,PCDH8,SEMA4D,JAM2,ZBTB20,AP2B1,AKR1B1,KIRREL1,SAXO1,SCARA5,NENF,PTGFR,ICA1,PLCZ1,NOS1AP,MTTP,SLC9A5,PDCL3,GPR55,NSUN2,HKDC1,ADAMTS16,ACOXL,TNFSF11,FYN,ADGRL2,SCN8A,ALB,ATP9B,NALCN,PABPC1,SLC39A8,POTEJ,CDH2,FBXL20,GPR137B,EPHB1,RP1L1,GRM5,ADCK1,SPTB,TBC1D1,PID1,NRP1,FCHSD2,PRKCA,IFT46,ITGA1,RC3H1,POR,BCR,TUT4,NRXN3,FBLN1,MB,RAG1,GNA14,EPHB2,CD38,MYO5B,MET,SPPL3,ATP6V0D2,PPFIA2,STXBP4,SERPINB2,CACNG3,ATG5,VMP1,FLRT2,KALRN,SLC1A2,GNAS,MFHAS1,CPQ,DLG5,GABRA2,AK3,TMEM25,ADGRF5,PDGFC,ABL2,BACE2,FHOD3,SLIT2,TMPRSS3,CNOT7,PLCL1,ERBB4,IL20RB,FAM3B,TRHDE,SYNDIG1,SAMD4A,PRKCQ,SORCS2,TRDN,NLGN1,CTTNBP2,NOS1,SLC6A3,PRR16,ASIC2,EFNA5,RAB27A,EHMT1,ESR1,CYP2C8,CACNA2D1,PRLR,AGO3,HTT,LARS2,FOXB1,FER,EYA2,CCR2,FGGY,CHFR,EPS8,SEMA4B,HRH1,ROCK2,RORA,HERPUD1,HSD17B2,PCSK2,FSTL4,BARD1,PNPLA3,STK3,DEPTOR,ZNF423,C1QL3,PNPLA8,HNRNPU,CADPS,IGF1R,THRB,MORC3,ATP10A
GO:0048583	regulation of response to stimulus	8.258946726337699e-15	*NOTCH2,BCAR3,MTOR,WWC1,GARNL3,PTPRD,MYO9A,NLK,KSR1,PLCB1,ZNF536,TAFA5,DLC1,RIPOR2,PDE4D,RDX,STXBP1,BCL2,PRDM16,ARHGAP26,FBN1,CHRNA7,ROBO2,RIMS1,EPC2,SPIRE1,ZEB1,AKR1C3,FGD4,SPRED1,MINAR1,RIMS2,ALK,AUTS2,MCTP1,PJA2,BABAM2,ERBIN,ANO6,CACNG2,DLGAP1,MLLT3,GPC6,SUSD4,APC,HHLA2,DSCAM,CRKL,SETD2,ARHGAP24,TNIK,PTPRJ,KDM4C,NEK4,EGFR,DENND1A,USP14,ANGPT1,MACF1,PRKACB,RGS3,NCOR1,RNF220,DOCK2,NEDD4,SCAI,SGMS1,CHSY1,N*

			TRK3,C5,ZFAND6,DKK2,FLT1,MAPKBP1,AOAH,DGKI,INVS,C12ORF4,EDAR,NEO1,CNTN6,SLC8A3,PRKD1,TPTE2,PAK1,EPHA7,NCOA7,RALGPS1,RAPGEF2,PELI2,LRP2,RUNX2,TAOK3,ONECUT1,TAFA4,RPTOR,GHR,RALGAPA1,ADAM10,IL1R1,APP,SAMSN1,MTUS1,DOCK8,MAPRE2,USP18,SEMA5A,ARHGAP44,NTF3,NDUFAF2,CD2AP,AURKA,PTPRR,KANK1,MAP4K4,BMPR1B,FMN2,PCSK6,AKAP6,HOMER2,CTNNA2,ARNT,PAK3,RFTN1,ITPKB,RGS20,PDE10A,RAP1GDS1,KICS2,PRKCZ,BTLA,GRB10,MCPH1,ARHGAP32,RGS9,HECW1,DUSP22,YAP1,MAPK1,MGAT5,USP25,PLG,PDGFD,ZNRF3,ARHGEF17,UBE2O,NCAM1,SPIDR,MICU1,CORO2B,STK38,PTPN13,CHN1,HRH4,PAFAH1B1,ATF6,NF2,CNKSR2,CTNNA1,MOB3B,BIRC6,AKAP9,KLF15,RASGRF2,PPARA,ADAMTS3,TIAM1,GRM1,ARHGEF12,PAK5,PCDH11Y,PLA2R1,SEMA3C,DAPK1,SLC24A4,SEC14L1,VPS13C,TMEM108,ALPK2,JARID2,RIC8B,IL34,ADGRV1,BBS2,NKG7,SEMA6D,DUSP16,SMARCA4,USP8,MAPKAP1,SPG21,BLK,TNR,MBD5,NUAK1,PTPRT,ABL1,PTPN12,HDAC4,OXR1,PRKAA1,GAS2,LRFN5,DROSHA,APLF,NFAT5,GUCY1A2,RAP1A,GPC5,FGF10,ZC3HAV1,GRID2,LATS2,NRG1,INO80D,GSG1L,ASPM,AP3B1,DENND2B,RASGRF1,ZNF675,GNG7,PRKCE,NXN,WNK2,USP33,DENND4C,FBN2,CD44,RGS12,PTPRO,EGF,TRIO,PDE3A,LIMD1,SPRED2,RPS6KA3,CTNND2,PTPN2,TRIM5,MCF2L,ATXN3,HTR2C,CLEC16A,CD96,LTBP1,OPRM1,HTR2A,FANCA,KREMEN1,SEMA3E,GCSAML,FHL2,CNIH3,PUM1,TMOD2,MSH2,ANKRD17,RELL1,HIPK3,EPN2,EVC,GRK3,MOSMO,RBBP8,MDFIC,EMILIN2,HMGA2,CCND3,PLCE1,TGFA,IL17RA,HIP1,CRIM1,PRR5L,VAV1,FBXO32,LDLRAD4,NPHP4,HLA-B,IQSEC1,SNX3,BRCA2,DISC1,NRK,SEMA3A,MAGI3,BMP2,RC3H2,PSG9,GNAI1,RALGAPA2,RANBP9,RIN3,BMP2K,TMEM161A,SEMA3D,NETO2,LEMD3,RELN,ARHGAP42,HMGB1,GNAQ,FGF9,NFATC2,TRAF3,UNC13B,TTC21B,DSTYK,UIMC1,RAP1GAP,SRGAP2,DRAXIN,SLAMF1,SMARCA2,ETS1,GLI3,CGAS,SMARCC1,SNX6,SMOC2,CNKSR3,CASP5,PSD3,GAREM1,LAMC1,NEK10,CYLD,BBS4,MAPK8IP1,KPNA1,UBASH3A,NEU3,KITLG,DNAJC7,CAMTA1,UBR1,SLC30A10,RCAN1,DAB1,SELENON,RB1CC1,PTPRE,PRKN,MTMR2,TBX20,DLGAP2,AFAP1,MAPK10,DPF3,NGEF,GRIN2A,PRKCH,IL6R,ALS2,RACGAP1,NLRC5,SNX25,FBLN5,SHISA9,SHANK2,USP7,VAV3,PSMA1,ENPP3,SOX30,KIR2DL4,ARHGEF28,NPAS2,ROCK1,LYN,ARHGAP28,ARHGAP31,EIF2B3,SLC44A2,SLC15A2,DTX1,OVOL2,RRAGD,ARID1B,CRACR2A,RNF152,OTUD7A,INSR,YTHDF3,DEDD2,NEK6,ARFGEF1,SNAI2,ASH1L,IGHV3-74,BID,SIAH2,TRABD2B,UFD1,ERN2,MBTPS2,TIAL1,PLPP4,NREP,ZDHHC17,NSD2,CD9,RALGPS2,JCAD,SAMHD1,IFT81,ENPP1,RASGRP1,IGSF11,NDRG2,CSNK2A1,BMP5,CSF1,GHRH,BCL2L1,SERPINB9,CTDP1,PRKG1,GRIN2B,GRB14,INO80,FANCB,IGHV2-70D,DHRS3,CELF4,PRAME,TNN,MED1,IL33,AJAP1,ROR2,CFH,KL,BANK1,IL10,ACTR2,SFPQ,PTH,SOSTDC1,PRKAA2,NDC80,ITPRIP,PLA2G4A,IQGAP1,RPS12,SREBF2,YBX3,FYB2,NRXN1,PCID2,HIPK1,CIBAR1,PBLD,FICD,CADM1,PEG10,NET1,SIPA1L2,TWIST1,AKT3,ALKAL2,JAK2,RPF2,FSTL1,MADD,HCRTR1,CREBBP,TNKS,GORAB,PCNA,UFL1,NFKBIA,PRKCB,ABCC8,BRD4,NEDD9,ITGA6,ATP2B1,IGHV1OR15-9,OTOP1,CIDEA,ARFGEF3,GID8,STAT1,BRMS1L,NDFIP2,NR2C1,MAP2K6,DGKG,MTPN,CCBE1,PARK7,ADAMTS18,MAPK8,FBXL17,ADCYAP1R1,NCAPG2,MYOCD,CYFIP2,EFHB,MEF2C,RXRA,WNT7A,RBPMS2,MAP3K5,NDFIP1,MAP3K4,S100B,ATP6V1C2,C16ORF72,RAD51AP1,PDE2A,SDCBP,JPT2,SPPL2B,NSMCE1,WWOX,NCK1,FGR,CDCA8,PPP2R3A,DNMBP,C2,RNF8,EPHA4,CYTH4,MECOM,NTRK2,COLEC12,TNNI1,OCLN,POSTN,SHISA6,IL17RD,MVB12B,PTK2,CDH5,CD5L,ANKRD6,NFKBID,ARHGAP12,LAMB1,APIP,CYFIP1,UBE3A,SEMA4D,C9,WNT5B,AMFR,NENF,SH2D1B,NOS1AP,TPTE,CCDC88A,GPR55,CHCHD2,BICD1,TNFSF11,FYN,PPM1F,RBMS3,HDAC2,SLF1,DOCK3,DOK5,ZFYVE28,MAPK9,CRTAM,APELA,ROR1,FUT8,TET1,CDH2,ITGA8,RAD9A,PHLPP1,GPR137B,EPHB1,GRM5,RAI14,TBC1D1,PID1,NRP1,PRKCA,FAIM,ITGA1,RC3H1,POR,MCC,SUPT3H,BCR,RGS6,ELP2,FBLN1,STK36,RAG

			1,BMPER,PRDM15,SRGAP3,MACROH2A1,EPHB2,CSNK1G1,CD38,EYA4,CDK14,MET,SPPL3,CDH13,SERPINB2,CACNG3,ATG5,MAGI2,PRDM11,MLIP,MYB,KALRN,GNAS,LAMA1,MFHAS1,TIAM2,BMP7,DLG5,ZMYND11,TMEM25,PDGFC,WDR41,ABL2,MMP26,PARPBP,EYA1,SLIT2,CNOT7,KCTD8,ERBB4,IL20RB,ROBO1,PRKCQ,SIPA1L3,MGMT,NLGN1,SHLD2,SLC6A3,ARHGEF11,SLIT3,ESR1,MYO9B,IGLC3,IQCJ-SCHIP1,PRLR,AGO3,HTT,CAMK1D,HLA-F,FER,EYA2,CCR2,STARD13,A2M,EPS8,SEMA4B,IGHV1OR21-1,ROCK2,RORA,IL16,DMRT1,RGS8,HERPUD1,RGS7,KIF7,FSTL4,BARD1,STK3,DEPTOR,ZNF423,PNPLA8,APCDD1,IGF1R,GLI2,THRB,AKAP13,DNM1L*
GO:0048522	positive regulation of cellular process	1.7817562744196112e-14	*NOTCH2,BCAR3,BRINP3,MTOR,NSG1,WWC1,ABCA13,PTPRD,SLC24A2,ULK2,FTO,KSR1,MGA,PLCB1,SVIL,CLTCL1,ZFPM2,TENM4,DLC1,TNRC6B,DPP10,RIPOR2,RDX,RP1,STXBP1,RALA,BCL2,KCNMA1,PRDM16,ALDH1A2,CHRNA7,ROBO2,RIMS1,EPC2,SPIRE1,TENM3,ZEB1,AKR1C3,RARB,SPRED1,CDC42EP3,RIMS2,ALK,AUTS2,FOXJ2,CARMIL1,PJA2,BABAM2,GLIS3,FANK1,ERBIN,RIN2,ANO6,CACNG2,NEGR1,MLLT3,CNTNAP2,MAP3K9,APC,HHLA2,TSHZ3,PLPPR5,DSCAM,TCF4,CRKL,SOX5,ERG,TNIK,PTPRJ,KDM4C,NEK4,EGFR,RFX3,ANGPT1,CDK12,BACH1,MACF1,NEK7,RNF220,ZNF407,NEDD4,MAML2,NSMCE2,BCL11A,SOX6,CHSY1,FLI1,CDH4,NTRK3,DKK2,FLT1,RFC3,TASP1,THRAP3,MAPKBP1,DGKI,EDAR,CRACD,NEO1,CNTN6,SLC8A3,PRKD1,PAK1,EPHA7,NCOA7,SPEN,RAPGEF2,PELI2,LRP2,ADGRB3,RUNX2,ARSB,TAOK3,ONECUT1,UBE2L3,LDB2,CCL28,SMYD3,SEPTIN9,RPTOR,GHR,SSBP3,NEDD4L,ADAM10,HDAC9,ZHX3,ATF7IP,IL1R1,APBB2,APP,RPS6KA2,KDM1B,CACNB2,STAU2,DOCK8,MAPRE2,ZNF600,SEMA5A,SYT1,NTF3,ACER2,CD2AP,AURKA,PARN,ST18,PYGO1,SLC8A1,SSBP2,SRGAP2C,ANKRD31,FIG4,DUX4,KANK1,MAP4K4,ABCD2,BMPR1B,FMN2,AKAP6,ZNF717,ARNT,RAB8B,PAK3,RANBP2,LARP1,ITPKB,TRPC5,UBE2E2,DNM3,NBN,SCP2,PRKCZ,GRB10,TAF15,RAB27B,CNST,PHF19,MRTFA,TAF4B,COBL,DUSP22,EBF2,YAP1,NFIA,SHC4,BRINP1,MAPK1,MGAT5,CADPS2,KMT2E,PCGF5,PDGFD,SYT10,NRG3,UBE2O,ANKFY1,GFRA1,NIPBL,SPIDR,GABPA,CORO2B,CHD6,MYLK3,KANSL1,GLP2R,LIMCH1,FMN1,PAFAH1B1,ATF6,TM7SF3,VPS13D,TPM1,NF2,LRRC38,HIVEP1,CTNNA1,MOB3B,BIRC6,AKAP9,KLF15,RASGRF2,PPARA,MEIS2,SNX30,NFIB,MRTFB,SYNJ1,NR5A2,ADAMTS3,TIAM1,GRM1,FOXJ3,TRERF1,PCDH11Y,PLA2R1,EIF3D,SEMA3C,DAPK1,NAV3,TMEM108,AGO2,MAGI1,JARID2,GATAD2B,IL34,ANK2,ADGRV1,MELK,BCAS3,RYR2,SYNE2,ZNF606,RANBP3L,SEMA6D,SMARCA4,USP8,PARD3,MAPKAP1,TNRC6C,PIAS1,TBC1D5,BLK,TNR,DOCK4,MBD5,ATRX,ELAVL4,ABL1,HDAC4,SLC1A1,PRKAA1,MRPS27,APBB1IP,EIPR1,APLF,NFAT5,GUCY1A2,SLFN11,RAP1A,GLIS1,MYO10,GPC5,TOX3,CAMK4,CPSF3,FGF10,ZC3HAV1,GRID2,TGM1,LATS2,NRG1,INO80D,CLIP1,ASPM,AP3B1,DENND2B,RASGRF1,ATP11C,ZNF438,ABCB7,ZBTB16,MUSK,SH3GL3,SETDB2,PRKCE,FOXK2,SLCO3A1,MED15,WNK2,ESRRG,FBN2,CD44,EGF,PRRC1,PDE3A,NSMAF,SPRED2,RPS6KA3,ATP8A2,PTPN2,TRIM5,PLXNA2,MCF2L,ATXN3,RFC1,HTR2C,RIC3,CLEC16A,ARHGEF7,ALG10B,ATP8A1,AMBRA1,KDM7A,OPRM1,HTR2A,DAZL,GTF2F2,STAC,SEMA3E,TAF3,RPRD1B,MARK2,EBF3,ZNF33B,ABHD17C,PUM1,TMOD2,MSH2,EPHA6,ANKRD17,LINGO2,SH3KBP1,SLC2A13,RELL1,EPN2,EVC,KNDC1,CLSPN,NOS2,BICRAL,MNAT1,RBBP8,MDFIC,ANK3,EMILIN2,HMGA2,CCND3,BCL11B,DOCK5,ECE1,CREM,PLCE1,TGFA,HIP1,FUT9,PRR5L,VAV1,TJP1,NPHP4,EGFLAM,CNTN1,HLA-B,IQSEC1,SNX3,CACNA1I,BRCA2,DISC1,BLM,SEMA3A,ADCY10,BMP2,RC3H2,MSR1,VRK1,GNAI1,GFI1B,RESF1,MYRIP,BMP2K,TMEM161A,SEMA3D,ASXL3,RELN,HMGB1,FGF9,NFATC2,SLC23A2,MYOM1,TRAF3,ZNF462,UNC13B,TTC21B,ETS2,DSTYK,UIMC1,DOCK1,PLS1,NIN,ATF1,SLAMF1,SMARCA2,ETS1,GLI3,CGAS,SMARCC1,SMOC2,PCP4,CNKSR3,VENTX,GRIK2,PRDM10,RERE,MAP2,GAREM1,LAMC1,NEK10,MOB1B,ATF2,CYLD,BBS4,MAPK8IP1,HIVEP3,PSIP1,CFTR,NELL1,RGMB,NEU3,KITLG,CAMTA1,SLC30A10,GTF2I,RORB,TADA2A,DAB1,MED27,ZNF208,SELENON*

			,*RB1CC1,NMD3,PRKN,MTMR2,TBX20,DPF3,GRIN2A,ARID5B,ZBED9,PRKCH,FRMD4A,IL6R,FRMPD4,ALS2,RACGAP1,NLRC5,TFDP1,CNOT6L,TOX,SLC4A4,ZFP90,COPS8,SHANK2,ST8SIA1,USP7,VAV3,PLAGL1,MESD,ITSN2,SOX30,KIR2DL4,RALB,NPAS2,ROCK1,LYN,VCAM1,ZNF780B,SLC44A2,DTX1,TENM2,OVOL2,NTN1,ZFHX3,RRAGD,BANP,SUPT16H,ARID1B,HOXC13,CRACR2A,RNF152,BAZ1A,CASZ1,INSR,BMF,YTHDF3,DEDD2,NEK6,HECTD1,NMU,DDHD1,PBX3,SUMO2,ZNF292,PDE4DIP,SNAI2,ASH1L,IGHV3-74,HOXC4,BID,TRABD2B,RXRG,SP3,ERN2,MBTPS2,TRIM58,TIAL1,ELF2,ZDHHC17,NSD2,FYCO1,SH3GLB1,CARD10,JCAD,TWIST2,CTIF,UTRN,RASGRP1,IGSF11,SNX9,CSNK2A1,BMP5,KCNC1,CSF1,GHRH,BCL2L1,ASB4,GRIN2B,INO80,FANCB,IGHV2-70D,CLNS1A,SMAD5,CELF4,TCERG1,ABCG1,SLC40A1,PRAME,TNN,LPGAT1,MICALL2,MED1,CDC14B,PCNT,IL33,GPRC5C,ROR2,ZNF521,KL,BANK1,CSDE1,LMX1A,IL10,ACTR2,SFPQ,PRAMEF25,RIOK1,CLSTN2,PTH,PRKAA2,CSF2RB,NDC80,LARP6,VSTM2A,MAP6,VASP,PLA2G4A,ETV6,IQGAP1,RPS12,ZBTB7C,TEAD1,MORC2,SREBF2,ANP32B,YBX3,AIMP1,NRXN1,PCID2,HIPK1,CD70,CIBAR1,CADM1,CENPE,LMX1B,NET1,ANLN,TWIST1,AKT3,ALKAL2,JAK2,ZBTB38,MADD,HCRTR1,PATL1,ZNF287,PRSS2,CREBBP,MELTF,TNKS,GORAB,PCNA,UFL1,NFKBIA,PRKCB,ABCC8,RFC2,ALX4,RTRAF,BRD4,NEDD9,OLFM4,ITGA6,ASS1,MTCL1,GRIP1,IGHV1OR15-9,CTNNBL1,SAR1A,HPSE2,ZBTB49,EXOC1,KRT6A,STOX2,AGO1,MEOX2,GID8,ELL2,STAT1,NDFIP2,NR2C1,MAP2K6,MTPN,ABI1,CEMIP,PRAMEF2,IMPACT,CCBE1,PARK7,MAPK8,ITGA4,OAZ2,MED12L,POU1F1,UBE2J2,ADCYAP1R1,MTF2,NCAPG2,TM9SF4,MYOCD,CEP120,CYFIP2,ARID3B,MEF2C,ADGRB1,RXRA,WNT7A,RBPMS2,MAP3K5,NDFIP1,MAP3K4,WASF3,S100B,ZNF112,ATP6V1C2,MAGEL2,PKN2,RAD51AP1,RAB38,DBF4B,FBXW8,SDCBP,NSMCE1,WWOX,PASK,NCK1,SCAF8,FGR,CDCA8,PPP2R3A,MLLT10,IFNAR1,RNF8,EPHA4,MECOM,DNMT3L,NTRK2,IL1RAPL1,NUMB,ADAMTS9,OCLN,CREB5,CD101,MEGF10,FBXO31,EXTL3,PTK2,MARK4,CDH5,CD5L,ANKRD6,SCGN,NFKBID,ASCL3,MPP7,DIAPH1,LAMB1,CYFIP1,UBE3A,SEMA4D,JAM2,ZBTB20,RUNX1,KIRREL1,WNT5B,SAXO1,NENF,POMT2,PTGFR,ZNF845,ASAP1,EDIL3,NOS1AP,PDCL3,CCDC88A,GPR55,CHCHD2,BICD1,TNFSF11,FYN,KDM5A,PPM1F,ADGRL2,HDAC2,SLF1,TBX15,SH2D3C,DOCK3,NCS1,DOK5,MAPK9,PABPC1,CRTAM,APELA,ROR1,TET1,ARNT2,CDH2,ITGA8,RAD9A,GPR137B,EPHB1,GRM5,ADCK1,RPS6KA5,PID1,NRP1,FCHSD2,PRKCA,ATPSCKMT,FAIM,ITGA1,ZNF615,KLF12,RC3H1,NRIP1,CHODL,POR,ZNF850,SUPT3H,TUT4,PRIM2,FBLN1,STK36,RAG1,RRAS2,BMPER,PRDM15,CUX1,MACROH2A1,MITF,EPHB2,TOGARAM1,CSNK1G1,CD38,EYA4,MET,SPPL3,CDH17,CDH13,STXBP4,CACNG3,MAGI2,PRDM11,VMP1,MLIP,FLRT2,MYB,KALRN,SLC1A2,GNAS,LAMA1,MFHAS1,SERPINB7,TIAM2,DHX29,BMP7,DLG5,TNFAIP8,RNF217,BPTF,BTBD10,NUDT21,KMT2C,ADGRF5,PDGFC,ABL2,RFX2,EYA1,SLIT2,CNOT7,ERBB4,GSAP,SYNDIG1,ROBO1,SAMD4A,PBX1,NPAS3,PRKCQ,TRDN,MGMT,NLGN1,SHLD2,NOS1,PRR16,ASIC2,EFNA5,TCF12,ARHGEF11,RAB27A,NSD1,FRMD5,ESR1,LOXL2,CACNA2D1,IGLC3,IQCJ-SCHIP1,PRLR,AGO3,HTT,RAD51B,CAMK1D,PIK3R3,HLA-F,FER,EYA2,CCR2,CHFR,ZNF721,EPS8,SEMA4B,NRF1,IGHV1OR21-1,HRH1,ROCK2,RORA,STMP1,ATAT1,DMRT1,CDCA5,RAB31,HERPUD1,NCOA6,WASHC1,RGS7,KIF7,BARD1,STK3,ZNF423,RSU1,HNRNPU,RAB3GAP2,CADPS,IGF1R,PRKAG2,GLI2,THRB,AKAP13,MORC3,ATP10A,DNM1L*
GO:0007010	cytoskeleton organization	2.049360590256 0283e-14	*NOTCH2,MTOR,NEBL,LRRC49,SVIL,TLN2,MICAL3,DLC1,RIPOR2,RDX,RP1,RALA,BCL2,MYO5A,ODAD2,ARHGAP26,SPIRE1,CNTLN,SDCCAG8,FGD4,SPAG16,MYO1E,CEP192,CDC42EP3,AUTS2,CARMIL1,ERBIN,RHPN2,PARVB,MAP4,APC,ZMYM4,MYO5C,SETD2,TNIK,MACF1,CTNNA3,NEK7,NCOR1,DOCK2,DIAPH3,CECR2,ARMC2,NTRK3,PHACTR1,CRACD,SLC39A12,PAK1,DEUP1,LRGUK,EPB41L3,KIF4A,TBCD,PHACTR2,DCLK1,STAU2,MAPRE2,SEMA5*

			A,ARHGAP44,NTF3,CD2AP,AURKA,SRGAP2C,FRMD3,CCSER2,KANK1,FMN2,THSD7A,CTNNA2,PAK3,TTLL7,RAP1GDS1,PRKCZ,CALD1,KLHL1,MCPH1,MRTFA,COBL,SENP6,MAPK1,ABLIM1,ARHGEF17,SLC16A1,CORO2B,MYLK3,LIMCH1,FMN1,PAFAH1B1,TPM1,NF2,CTNNA1,PPP1R9A,AKAP9,MPRIP,ENAH,PAK5,PARD3B,NAV3,ANK2,STAG2,BRWD1,BCAS3,SYNE2,BBS2,AIF1L,LDB3,PARD3,MAPKAP1,DST,CXADR,ATRX,XIRP2,ABL1,PRKAA1,GAS2,TTLL5,MAST4,DNAH5,FGF10,CLIP1,ASPM,PRKCE,PGM5,USP33,LIMD1,PEX14,ATP8A2,ATXN3,ARHGEF7,SEMA3E,MARK2,TMEM67,C10ORF90,TMOD2,SH3KBP1,ANK3,MYOM2,PLCE1,ANKFN1,HIP1,TJP1,NPHP4,PACSIN2,IQSEC1,PDLIM5,BRCA2,DISC1,WDPCP,NRK,PHACTR3,CDC42BPB,GNAI1,RANBP9,DNAL1,TUBGCP3,RTTN,MDM1,PLS1,SRGAP2,NIN,HAUS6,DNAH8,KIF15,MAP2,DAW1,FARP1,ATF2,GOLGA8B,CYLD,BBS4,THSD7B,KIAA0753,CEP44,PRKN,CDC42BPA,AFAP1,PCDH15,PKP1,FRMPD4,RACGAP1,KANK4,MAP7,CFAP74,MYO1D,ROCK1,ARHGAP28,KIF11,NEK6,SHROOM3,ARFGEF1,PDE4DIP,FLNB,KRT6B,UTRN,SNX9,HDGFL3,KRT25,PRKG1,INO80,MICALL2,CDC14B,PCNT,FAT1,ACTR2,PRKAA2,SKA1,NDC80,MAP6,VASP,TACC2,KIFC1,IQGAP1,CENPE,TUBB6,ANLN,JAK2,TNKS,SGO1,SMTN,USH1C,NEDD9,MTCL1,EML1,MAST2,ARFGEF3,KRT6A,SHROOM2,MTPN,ABI1,ASB2,HMCN1,CEP120,CYFIP2,KRT85,WASF3,MAGEL2,SDCBP,NCK1,DRC7,CDCA8,INTS13,RSPH1,OCLN,AKAP11,TRPM7,PTK2,MARK4,CDH5,ARHGAP12,DIAPH1,CYFIP1,HOATZ,FRMD6,KIRREL1,SAXO1,PSTPIP2,NOS1AP,SORBS2,PDCL3,CCDC88A,SPAG6,BICD1,PPM1F,HYDIN,EHBP1,RP1L1,SPTB,NRP1,FCHSD2,BCR,ELMO1,STK36,TOGARAM1,MYO5B,MET,SPECC1,NRAP,FAM171A1,DNAH17,EPB41L4A,ABL2,FHOD3,TTLL11,SLIT2,NUF2,ANTXR1,SIPA1L3,TRDN,NLGN1,EFNA5,GAS2L1,ARHGEF11,FRMD5,IQCJ-SCHIP1,HTT,CFAP44,FER,STARD13,EPS8,ROCK2,ATAT1,WASHC1,HOOK3,HNRNPU,CEP72,AKAP13
GO:0048513	animal organ development	5.020880141050604e-14	*NOTCH2,BCAR3,MTOR,CNTN4,SGCD,IMMP2L,FREM1,TRAPPC9,BNC2,NEBL,SMOC1,SCAPER,FTO,PLCB1,SVIL,ZFPM2,TENM4,DLC1,ZDHHC21,RIPOR2,RP1,BCL2,ODAD2,ALDH1A2,FBN1,ROBO2,TENM3,ZEB1,AKR1C3,RARB,SPRED1,ENPEP,MYO1E,USH2A,ALK,PAPPA2,ANO6,NEGR1,MLLT3,GPC6,CNTNAP2,MYO3B,DSCAM,CRKL,ILDR2,SOX5,SETD2,SLC4A10,PTPRJ,DOCK10,EGFR,RFX3,ANGPT1,DOCK2,CRB1,SOX6,TMEM182,CECR2,CHSY1,FLI1,ATP2B2,NTRK3,LARGE1,RXFP1,PHACTR1,FLT1,ADAMTS6,EDAR,SLC8A3,EPHA7,RAPGEF2,LRP2,RUNX2,FGF12,CPS1,ONECUT1,TMEM38B,PRICKLE2,SLC24A3,LDB2,GHR,LUZP1,SSBP3,ADAM10,HDAC9,APP,ABCB5,RPS6KA2,CACNA1C,DCLK1,STAU2,TMC1,SEMA5A,SYT1,AURKA,PYGO1,SLC8A1,SRGAP2C,BMPR1B,AKAP6,CTNNA2,ARNT,ITPKB,IFT57,RBM47,PRKCZ,KLHL1,MCPH1,COL27A1,ZSWIM6,COBL,EBF2,YAP1,NFIA,MAPK1,CRISPLD2,KMT2E,PLG,PDGFD,ZNRF3,ABLIM1,NRG3,GFRA1,NIPBL,GABPA,FAT3,LCE1F,MYLK3,ACSBG1,FMN1,PAFAH1B1,ATF6,EFEMP1,DCAF1,ITGB8,TPM1,NF2,RBFOX1,CTNNA1,ANKRD11,BIRC6,KLF15,PPARA,MEIS2,NFIB,MRTFB,SYNJ1,NR5A2,TIAM1,KAZN,SEMA3C,SLC24A4,TMEM108,ALPK2,DNAH11,JARID2,CPE,IL34,ANK2,ADGRV1,MELK,RYR2,SYNE2,BBS2,WNT9B,RANBP3L,SEMA6D,ANKS6,SMARCA4,LDB3,MAPKAP1,BLK,TNR,CXADR,ATRX,XIRP2,ELAVL4,ABL1,HDAC4,SLC1A1,PRKAA1,GAS2,DROSHA,TTLL5,L3MBTL3,DNAH5,CAMK4,FGF10,GRID2,TGM1,LATS2,NRG1,ASPM,AP3B1,ATP11C,ZBTB16,ZNF675,SETDB2,FBN2,CD44,PTPRO,EGF,ALPK3,EXT1,COL5A3,SPRED2,ADAMTS2,NHS,ATP8A2,PTPN2,PLXNA2,ARHGEF7,AMBRA1,KDM7A,FANCA,CYP4A11,CNNM4,SEMA3E,ALPL,FHL2,HERC1,MSH2,CDIN1,LUC7L,EVC,KNDC1,MNAT1,SGCZ,MYLK2,XYLT1,HMGA2,BCL11B,ECE1,MBP,AK8,TRPS1,PLCE1,TGFA,VAV1,ZNF160,LDLRAD4,NPHP4,EGFLAM,CNTN1,HLA-B,MTHFD1L,PDLIM5,BRCA2,DISC1,DNER,WDPCP,SLC10A7,SEMA3A,BMP2,RC3H2,PSG9,PTCD2,GFI1B,BMP2K,RNF38,SEMA3D,ASXL3,PDE6C,RELN,HMGB1,FGF9,TDRD7,CPAMD8,MDM1,ESRP1,TTC21B,DOCK1,TSPAN2,RAP1GAP,PLS1,SRGAP2,NIN,DRAXIN,SLAMF1,SMARCA2,ETS1,GLI3,MEGF11,SMARCC1,CASP5,CUL1*

			,RERE,DAW1,LAMC1,ATF2,UMODL1,BBS4,LAMC3,HIVEP3,COL5A1,CFTR,NELL1,KITLG,DCC,RCAN1,RORB,DAB1,SELENON,RB1CC1,MYO3A,SH3PXD2A,TBX20,PCDH15,DPF3,GRIN2A,ARID5B,JPH1,ATXN1,CDH23,PRKCH,TG,IL6R,DMC1,LCE3B,TOX,PDE6A,SCN10A,SHANK2,ADGRG6,ROCK1,LYN,VCAM1,CTSB,LRIG1,DTX1,OVOL2,NTN1,MMP16,ZFHX3,ARID1B,HOXC13,CRACR2A,INSR,HECTD1,PBX3,SNAI2,ASH1L,HOXC4,RXRG,SP3,MBTPS2,FLNB,TRIM58,NSD2,CERS3,KRT6B,ENPP1,UTRN,RASGRP1,DZANK1,NDRG2,BMP5,WDR72,KCNC1,CSF1,GHRH,BCL2L1,KRT25,CTDP1,HCN1,PRKG1,LAMA3,GRIN2B,CNMD,DHRS3,SMAD5,CELF4,SYNJ2,FOXN3,VSTM4,SLC40A1,MYCL,TNN,PSAP,MED1,ATG4B,KDM6A,ATRN,AJAP1,ROR2,KL,CSDE1,FAT1,LMX1A,TMEM178A,IL10,TTC39C,PTH,SOSTDC1,ETV6,TACC2,IQGAP1,TEAD1,ANP32B,YBX3,NRXN1,PCID2,HIPK1,CACYBP,CADM1,ANLN,TWIST1,AKT3,JAK2,VSX1,CELSR2,ARL11,GORAB,PCNA,UFL1,ADAMTS5,NFKBIA,SMTN,ALX4,USH1C,NEDD9,ITGA6,ATP2B1,ASS1,EML1,OTOP1,EXT2,KRT6A,STOX2,MEOX2,GRXCR1,STAT1,MAP2K6,CMTM7,SHROOM2,SLC6A11,MTPN,ABI1,MYO18B,ARMC6,CCBE1,ADAMTS18,ITGA4,FBXL17,POU1F1,CSMD1,NCAPG2,FOXP2,ASB2,MYOCD,CEP120,DHTKD1,KRT85,MEF2C,ADGRB1,RXRA,WNT7A,RBPMS2,NDFIP1,MAP3K4,PDE2A,FBXW8,SDCBP,NECTIN1,WWOX,FLVCR1,FGR,PPP2R3A,SPRR2D,LCE3D,EPHA4,GABRA5,MECOM,DNMT3L,NTRK2,FNDC3A,NUMB,LHX9,ADAMTS9,WNT2B,TNNI1,CD101,MEGF10,IL17RD,IREB2,HS6ST1,PTK2,ANKRD6,NFKBID,CLDN18,LAMB1,UBE3A,SEMA4D,FAT4,AP2B1,RUNX1,AKR1B1,WNT5B,SORBS2,GPR55,NSUN2,ADAMTS16,TNFSF11,FYN,ADGRL2,UNC45B,ARL13B,HYDIN,HDAC2,GON4L,TBX15,COL18A1,ATP5PF,UGP2,CRTAM,COL19A1,APELA,MDGA2,ROR1,ARNT2,CDH2,CNTN5,ITGA8,NTN4,XRN2,GPR137B,EPHB1,RP1L1,PTPRG,NRP1,SDK1,PRKCA,RC3H1,NRIP1,CHODL,POR,BCR,SNRK,STK36,MB,RAG1,B9D1,DGCR2,BMPER,MACROH2A1,MITF,EPHB2,IGSF3,SGCG,EYA4,MET,CDH17,ATG5,NRAP,MAGI2,ADAM29,FLRT2,MYB,KALRN,SLC1A2,GNAS,LAMA1,MFHAS1,GREB1L,SERPINB7,CA10,ATRNL1,BMP7,DLG5,KIRREL3,BPTF,NUDT21,ADGRF5,PDGFC,EYA1,FHOD3,SLIT2,EXOC4,CCDC141,ERBB4,ROBO1,PBX1,MYH15,SIPA1L3,MGMT,CTTNBP2,SLC6A3,TCF12,SLIT3,ESR1,NTNG1,KDM4B,LOXL2,PRLR,FOXB1,PIK3R3,MACROD2,FER,CCR2,RPGRIP1,A2M,SEMA4B,ROCK2,PRDM1,RORA,ATAT1,DMRT1,HSPG2,PTPRQ,NCOA6,HSD17B2,COL4A3,RGS7,HOOK3,STK3,ZNF423,ZNF568,HNRNPU,APCDD1,IGF1R,GLI2,THRB,AKAP13
GO:0016477	cell migration	1.3656437665311091e-13	MTOR,SPOCK1,WWC1,LRP12,PLCB1,TAFA5,DLC1,RIPOR2,RDX,BCL2,SDCCAG8,SPRED1,ENPEP,USH2A,AUTS2,CARMIL1,MCTP1,ASTN1,RIN2,ANO6,GPC6,APC,CRKL,SETD2,ARHGAP24,PTPRJ,DOCK10,EGFR,ANGPT1,MACF1,CTNNA3,DOCK2,SCAI,NTRK3,C5,PHACTR1,FLT1,NEO1,PRKD1,PAK1,RAPGEF2,ARSB,ONECUT1,LDB2,TAFA4,CCL28,ADAM10,HDAC9,IL1R1,APP,MTUS1,DCLK1,DOCK8,MAPRE2,SEMA5A,VCL,NTF3,CD2AP,SLC8A1,PTPRR,SRGAP2C,SRGAP2B,KANK1,MAP4K4,CTNNA2,PAK3,PRKCZ,GRB10,MRTFA,DUSP22,MAPK1,MGAT5,PLG,PDGFD,ITGBL1,NRG3,GFRA1,NIPBL,FAT3,LIMCH1,PAFAH1B1,ITGB8,TPM1,NF2,AVL9,CTNNA1,TIAM1,PTPRK,PAK5,SEMA3C,NAV3,AGO2,IL34,BCAS3,SYNE2,SEMA6D,TNR,CXADR,DOCK4,PTPRT,ABL1,HDAC4,SDC2,GPC5,FGF10,PEAK1,NRG1,ASPM,PRKCE,USP33,CD44,PTPRO,EGF,EXT1,LIMD1,PLXNA2,ARHGEF7,ATP8A1,BIN2,SEMA3E,MARK2,GCSAML,SH3KBP1,STK10,EMILIN2,BCL11B,DOCK5,IL17RA,FUT9,PRR5L,VAV1,TJP1,LDLRAD4,IQSEC1,DISC1,DNER,WDPCP,SEMA3A,BMP2,UNC5D,CDC42BPB,RIN3,SEMA3D,RELN,HMGB1,FGF9,NFATC2,DOCK1,SRGAP2,SLAMF1,ETS1,GLI3,SMOC2,RERE,LAMC1,UMODL1,BBS4,LAMC3,COL5A1,ITGA9,KITLG,DCC,DAB1,CDC42BPA,TBX20,DACH1,LYST,ARID5B,IL6R,PTPRB,VAV3,ROCK1,LYN,VCAM1,OVOL2,NTN1,INSR,YTHDF3,SNAI2,CD9,CARD10,JCAD,TWIST2,BMP5,CSF1,PRKG1,LAMA3,VSTM4,TNN,ATRN,IL33,ROR2,FAT1,IL10,TSPAN11,ABHD2,IQGAP1,AIMP1,NET1,ANLN,TWIST1,AKT3,JAK2,FSTL1,CELSR2,ABCC8,NEDD9,ITGA6,MEOX2,BRMS1L,SHROOM2,CEMIP,CCBE1,ITGA4,ASB2,MYOCD,MEF2C,ADGRB1,WNT7A,PKN2,SDCBP,NCK1,FGR,PPP2

			R3A,CXCL2,EPHA4,NTRK2,NUMB,ADAMTS9,MEGF10,FBXO31,PTK2,CDH5,DIAPH1,LAMB1,SEMA4D,JAM2,WNT5B,PSTPIP2,CCDC88A,TNFSF11,FYN,PPM1F,ARL13B,HDAC2,CRTAM,APELA,FUT8,TET1,CDH2,NTN4,EPHB1,PTPRG,NRP1,PRKCA,ITGA1,MCC,BCR,ELMO1,FBLN1,RRAS2,BMPER,SRGAP3,MITF,EPHB2,MET,CDH13,MAGI2,UNK,FLRT2,LAMA1,ATRNL1,BMP7,ASTN2,DLG5,ZMYND8,KIRREL3,PDGFC,ABL2,SLIT2,CCDC141,ERBB4,ROBO1,PRKCQ,VCAN,FRMD5,NTNG1,LOXL2,FOXB1,CAMK1D,PIK3R3,FER,CCR2,STARD13,EPS8,SEMA4B,HRH1,ROCK2,IL16,DMRT1,WASHC1,APCDD1,IGF1R,DNM1L
GO:0099536	synaptic signaling	1.894053170759 9639e-13	*CNTN4,NSG1,PTPRD,SLC24A2,LRRC4C,UNC13C,PLCB1,PTPRA,STXBP1,ERC1,LRFN2,CDH8,CHRNA7,RIMS1,GABRB3,RIMS2,MCTP1,SV2C,CACNG2,DLGAP1,TSHZ3,CRKL,SLC4A10,USP14,BTBD9,GRIK3,GABRB1,DGKI,GRIA1,SLC8A3,CHRM3,RAPGEF2,FGF12,GABRA6,PTPRN2,SYN2,GRM7,APP,RPS6KA2,CACNB2,STAU2,GABRG2,SYT1,NTF3,ERC2,SYN3,PRKCZ,SV2B,MAPK1,CADPS2,HRH2,PLG,SYT10,NRG3,HRH4,SORCS3,PAFAH1B1,GRIK4,PPP1R9A,AKAP9,RASGRF2,SYNJ1,GRM1,GABRG1,TMEM108,AMPH,CDH11,USP8,TNR,GRM8,ELAVL4,ABL1,SLC1A1,RIMBP2,RAP1A,GRID2,NRG1,RASGRF1,PRKCE,DGKB,P2RX6,EXT1,RPS6KA3,ATXN3,HTR2C,RIC3,OPRM1,HTR2A,TMOD2,APBA2,KCND2,MYLK2,MBP,PACSIN2,DISC1,RELN,GNAQ,UNC13B,GABRR2,GRIK2,MCTP2,FARP1,GABBR2,KPNA1,ZZEF1,DCC,CHRM5,PRKN,MTMR2,DLGAP2,GRIN2A,ALS2,KCNQ3,SHISA9,SHANK2,PLCB4,GRID1,COLQ,NMU,RPH3A,GABRG3,IGSF11,HCN1,GRIN2B,CELF4,ROR2,CLSTN2,NRXN1,JAK2,HCRTR1,PRKCB,CACNA1E,SLC6A1,PARK7,MEF2C,ADGRB1,WNT7A,S100B,OR10H2,SDCBP,SNAP29,EPHA4,GABRA5,NTRK2,IL1RAPL1,SHISA6,GRIK1,SCGN,CYFIP1,PCDH8,FYN,CDH2,FBXL20,EPHB1,GRM5,FCHSD2,BCR,NRXN3,EPHB2,CD38,DLG2,CACNG3,SLC1A2,GABRA2,TMEM25,GRM3,EXOC4,PLCL1,SORCS2,NLGN1,NOS1,SLC6A3,ASIC2,DTNA,NTNG1,CCR2,HRH1,RGS8,CADPS*
GO:0048518	positive regulation of biological process	2.354684695722 861e-13	*NOTCH2,BCAR3,BRINP3,MTOR,NSG1,WWC1,ABCA13,PTPRD,SLC24A2,PVT1,ULK2,FTO,KSR1,MGA,PLCB1,TMPRSS2,SVIL,CLTCL1,ZFPM2,TENM4,DLC1,TNRC6B,DPP10,RIPOR2,PDE4D,RDX,RP1,STXBP1,RALA,BCL2,KCNMA1,PRDM16,ALDH1A2,CHRNA7,ROBO2,RIMS1,EPC2,SPIRE1,TENM3,ZEB1,AKR1C3,RARB,SPRED1,CDC42EP3,RIMS2,ALK,AUTS2,FOXJ2,CARMIL1,PJA2,BABAM2,GLIS3,FANK1,ERBIN,RIN2,ANO6,CACNG2,NEGR1,MLLT3,EGLN3,SUSD4,CNTNAP2,MAP3K9,SPON1,APC,HHLA2,TSHZ3,PLPPR5,DSCAM,TCF4,CRKL,SOX5,SETD2,ERG,TNIK,PTPRJ,KDM4C,NEK4,EGFR,RFX3,ANGPT1,CDK12,BACH1,MACF1,NEK7,NCOR1,RNF220,DOCK2,ZNF407,NEDD4,MAML2,NSMCE2,BCL11A,SOX6,CHSY1,FLI1,CDH4,NTRK3,C5,DKK2,FLT1,RFC3,TASP1,THRAP3,MAPKBP1,DGKI,C12ORF4,EDAR,CRACD,NEO1,CNTN6,SLC39A12,SLC8A3,PRKD1,PAK1,EPHA7,NCOA7,CHRM3,SPEN,RAPGEF2,PELI2,LRP2,ADGRB3,RUNX2,ARSB,FGF12,TAOK3,ONECUT1,SLC24A3,UBE2L3,LDB2,CCL28,SMYD3,SEPTIN9,RPTOR,GHR,SSBP3,NEDD4L,ADAM10,HDAC9,ZHX3,ATF7IP,IL1R1,APBB2,APP,RPS6KA2,KDM1B,CACNB2,STAU2,DOCK8,MAPRE2,ZNF600,SEMA5A,SYT1,NTF3,ACER2,CD2AP,AURKA,PARN,ST18,PYGO1,SLC8A1,SSBP2,SRGAP2C,ANKRD31,FIG4,DUX4,PLGRKT,KANK1,MAP4K4,ABCD2,BMPR1B,FMN2,AKAP6,ZNF717,ARNT,RAB8B,PAK3,RFTN1,DIP2B,RANBP2,LARP1,ITPKB,TRPC5,UBE2E2,DNM3,NBN,SCP2,IFT57,PRKCZ,GRB10,TAF15,DIP2A,RAB27B,CNST,HECW1,ABCA5,PHF19,MRTFA,TAF4B,COBL,DUSP22,EBF2,YAP1,NFIA,SHC4,BRINP1,MAPK1,MGAT5,CADPS2,HRH2,KMT2E,PLG,PCGF5,PDGFD,SYT10,NRG3,UBE2O,ANKFY1,GFRA1,NIPBL,SPIDR,GABPA,CORO2B,CHD6,MYLK3,KANSL1,GLP2R,LIMCH1,FMN1,PAFAH1B1,ATF6,TM7SF3,ITGB8,VPS13D,TPM1,NF2,LRRC38,HIVEP1,CTNNA1,MOB3B,BIRC6,AKAP9,KLF15,RASGRF2,PPARA,MEIS2,SNX30,NFIB,MRTFB,SYNJ1,NR5A2,ADAMTS3,TIAM1,GRM1,FOXJ3,TRERF1,PCDH11Y,PLA2R1,EIF3D,SEMA3C,DAPK1,NAV3,SLC24A4,TMEM108,AGO2,MAGI1,JARID2,GATAD2B,IL34,ANK2,ADGRV1,MELK,BCAS3,RYR2,SYNE2,BBS2,ZNF606,CLPX,RANBP3L,NKG7,SEMA6D,SMARCA4,USP8,PARD3,MAPKAP1,TNRC*

			6C,PIAS1,TBC1D5,SPG21,BLK,TNR,DOCK4,MBD5,ATRX,ELAVL4,ABL1,HDAC4,SLC1A1,PRKAA1,MRPS27,DROSHA,APBB1IP,EIPR1,APLF,NFAT5,GUCY1A2,NBAS,SLFN11,RAP1A,GLIS1,MYO10,GPC5,TOX3,CAMK4,CPSF3,FGF10,ZC3HAV1,GRID2,TGM1,LATS2,NRG1,INO80D,CLIP1,ASPM,AP3B1,DENND2B,RASGRF1,ATP11C,ZNF438,ABCB7,ZBTB16,MUSK,SH3GL3,SETDB2,PRKCE,FOXK2,SLCO3A1,MED15,WNK2,ESRRG,FBN2,CD44,EGF,PRRC1,PDE3A,NSMAF,LIMD1,SPRED2,RPS6KA3,ATP8A2,PTPN2,TRIM5,PLXNA2,MCF2L,ATXN3,RFC1,HTR2C,RIC3,CLEC16A,ARHGEF7,ALG10B,ATP8A1,AMBRA1,KDM7A,OPRM1,HTR2A,FANCM,CYP4A11,DAZL,GTF2F2,STAC,SEMA3E,TAF3,RPRD1B,MARK2,GCSAML,EBF3,ALPL,ZNF33B,ABHD17C,PUM1,TMOD2,MSH2,IGF2BP3,EPHA6,ANKRD17,LINGO2,SH3KBP1,SLC2A13,RELL1,EPN2,EVC,KNDC1,SPSB4,CLSPN,NOS2,BICRAL,MNAT1,RBBP8,MDFIC,ADAM12,MYLK2,ANK3,EMILIN2,HMGA2,CCND3,BCL11B,DOCK5,ECE1,CREM,MBP,PLCE1,TGFA,IL17RA,HIP1,FUT9,PRR5L,VAV1,TJP1,NPHP4,EGFLAM,CNTN1,HLA-B,IQSEC1,SNX3,CACNA1I,BRCA2,DISC1,BLM,SEMA3A,ADCY10,BMP2,RC3H2,PSG9,MSR1,VRK1,GNAI1,GFI1B,RANBP9,RESF1,MYRIP,BMP2K,TMEM161A,SEMA3D,ASXL3,POLR3A,RELN,HMGB1,FGF9,NFATC2,SLC23A2,MYOM1,TRAF3,ZNF462,UNC13B,TTC21B,ETS2,DSTYK,UIMC1,DOCK1,PLS1,NIN,ATF1,SLAMF1,SMARCA2,ETS1,GLI3,CGAS,SMARCC1,SMOC2,PCP4,CNKSR3,VENTX,GRIK2,IDE,PRDM10,RERE,MAP2,GAREM1,LAMC1,NEK10,MOB1B,ATF2,CYLD,BBS4,MAPK8IP1,HIVEP3,PSIP1,CFTR,NELL1,UBASH3A,RGMB,NEU3,KITLG,CAMTA1,SLC30A10,GTF2I,RORB,TADA2A,DAB1,MED27,ZNF208,SELENON,RB1CC1,NMD3,PRKN,MTMR2,TBX20,DPF3,GRIN2A,ARID5B,ZBED9,PRKCH,PKP1,FRMD4A,IL6R,FRMPD4,ALS2,RACGAP1,NLRC5,TFDP1,CNOT6L,TOX,SLC4A4,ZFP90,COPS8,SHANK2,ST8SIA1,USP7,VAV3,ENPP3,PLAGL1,MESD,ITSN2,SOX30,KIR2DL4,RALB,NPAS2,ROCK1,LYN,VCAM1,ZNF780B,EIF2B3,SLC44A2,DTX1,TENM2,OVOL2,NTN1,ZFHX3,RRAGD,BANP,SUPT16H,ARID1B,HOXC13,CRACR2A,RNF152,BAZ1A,CASZ1,INSR,BMF,YTHDF3,DEDD2,NEK6,HECTD1,NMU,DDHD1,PBX3,SUMO2,ZNF292,ARFGEF1,PDE4DIP,SNAI2,ASH1L,IGHV3-74,HOXC4,BID,TRABD2B,RXRG,SP3,ERN2,MBTPS2,TRIM58,TIAL1,ELF2,PLPP4,ZDHHC17,NSD2,FYCO1,SH3GLB1,CARD10,JCAD,TWIST2,CTIF,UTRN,RASGRP1,IGSF11,SNX9,CSNK2A1,BMP5,KCNC1,CSF1,GHRH,BCL2L1,ASB4,GRIN2B,INO80,FANCB,IGHV2-70D,CLNS1A,SMAD5,CELF4,TCERG1,ABCG1,DCUN1D4,SLC40A1,PRAME,TNN,LPGAT1,MICALL2,MED1,CDC14B,PCNT,KDM6A,IL33,GPRC5C,ROR2,CFH,ZNF521,KL,BANK1,CSDE1,LMX1A,IL10,ACTR2,SFPQ,PRAMEF25,RIOK1,CLSTN2,PTH,PRKAA2,CSF2RB,NDC80,LARP6,VSTM2A,MAP6,VASP,PLA2G4A,ETV6,IQGAP1,RPS12,ZBTB7C,TEAD1,MORC2,SREBF2,ANP32B,YBX3,AIMP1,FYB2,NRXN1,PCID2,HIPK1,CD70,CIBAR1,CADM1,CENPE,LMX1B,NET1,ANLN,TWIST1,AKT3,ALKAL2,JAK2,ZBTB38,MADD,HCRTR1,RBM19,PATL1,ZNF287,PRSS2,FH,CREBBP,MELTF,TNKS,GORAB,PCNA,UFL1,NFKBIA,PRKCB,ABCC8,RFC2,ALX4,RTRAF,BRD4,NEDD9,OLFM4,ITGA6,ATP2B1,ASS1,MTCL1,GRIP1,IGHV1OR15-9,CTNNBL1,SAR1A,HPSE2,CIDEA,ZBTB49,EXOC1,KRT6A,STOX2,AGO1,MEOX2,SLC6A1,GID8,ELL2,STAT1,BRMS1L,NDFIP2,NR2C1,MAP2K6,MTPN,ABI1,CEMIP,PRAMEF2,IMPACT,CCBE1,PARK7,MAPK8,ITGA4,OAZ2,MED12L,POU1F1,UBE2J2,ADCYAP1R1,MTF2,NCAPG2,TM9SF4,MYOCD,CEP120,CYFIP2,ARID3B,MEF2C,ADGRB1,RXRA,WNT7A,RBPMS2,MAP3K5,NDFIP1,MAP3K4,WASF3,S100B,FOXO6,ZNF112,ATP6V1C2,MAGEL2,PKN2,RAD51AP1,PDE2A,RAB38,DBF4B,FBXW8,SDCBP,NSMCE1,WWOX,PASK,NCK1,SCAF8,FGR,CDCA8,PPP2R3A,MLLT10,C2,IFNAR1,RNF8,EPHA4,MECOM,DNMT3L,NTRK2,IL1RAPL1,NUMB,ADAMTS9,WNT2B,OCLN,CREB5,CD101,MEGF10,FBXO31,EXTL3,PRKAB1,PTK2,MARK4,CDH5,CD5L,ANKRD6,SCGN,NFKBID,ASCL3,MPP7,DIAPH1,LAMB1,CYFIP1,UBE3A,SEMA4D,JAM2,ZBTB20,RUNX1,C9,KIRRE

			L1,WNT5B,SAXO1,NENF,SH2D1B,POMT2,PTGFR,ZNF845,ASAP1,EDIL3,NOS1AP,PDCL3,CCDC88A,GPR55,CHCHD2,BICD1,TNFSF11,FYN,KDM5A,PPM1F,ADGRL2,RBMS3,HDAC2,SLF1,TBX15,SH2D3C,DOCK3,NCS1,LHFPL2,DOK5,MAPK9,PABPC1,CRTAM,APELA,ROR1,TET1,ARNT2,HECW2,CDH2,ITGA8,RAD9A,GPR137B,EPHB1,GRM5,ADCK1,RPS6KA5,PID1,NRP1,FCHSD2,PRKCA,ATPSCKMT,FAIM,ITGA1,ZNF615,KLF12,RC3H1,NRIP1,CHODL,POR,ZNF850,SUPT3H,BCR,TUT4,PRIM2,FBLN1,STK36,RAG1,RRAS2,BMPER,PRDM15,CUX1,MACROH2A1,MITF,EPHB2,TOGARAM1,CSNK1G1,BCL2L13,CD38,EYA4,MET,SPPL3,CDH17,CDH13,STXBP4,CACNG3,ATG5,MAGI2,PRDM11,VMP1,MLIP,FLRT2,MYB,KALRN,SLC1A2,GNAS,LAMA1,MFHAS1,SERPINB7,TIAM2,DHX29,BMP7,DLG5,TNFAIP8,RNF217,BPTF,BTBD10,NUDT21,KMT2C,ADGRF5,PDGFC,PLIN2,ABL2,RFX2,EYA1,SLIT2,CNOT7,ERBB4,IL20RB,GSAP,SYNDIG1,ROBO1,SAMD4A,PBX1,NPAS3,PRKCQ,ANTXR1,TRDN,MGMT,NLGN1,SHLD2,NOS1,SLC6A3,PRR16,ASIC2,EFNA5,TCF12,ARHGEF11,RAB27A,NSD1,EHMT1,FRMD5,ESR1,LOXL2,CACNA2D1,IGLC3,IQCJ-SCHIP1,PRLR,AGO3,HTT,RAD51B,CAMK1D,PIK3R3,HLA-F,FER,EYA2,CCR2,A2M,CHFR,ZNF721,EPS8,SEMA4B,NRF1,IGHV1OR21-1,HRH1,ROCK2,PRDM1,RORA,STMP1,IL16,ATAT1,DMRT1,CDCA5,RAB31,HERPUD1,NCOA6,COL4A3,WASHC1,RGS7,KIF7,BARD1,STK3,ZNF423,RSU1,HNRNPU,RAB3GAP2,CADPS,IGF1R,PRKAG2,GLI2,THRB,AKAP13,MORC3,ATP10A,DNM1L
GO:0099537	trans-synaptic signaling	3.4521031773406745e-13	CNTN4,NSG1,PTPRD,SLC24A2,LRRC4C,UNC13C,PLCB1,PTPRA,STXBP1,ERC1,LRFN2,CDH8,CHRNA7,RIMS1,GABRB3,RIMS2,MCTP1,SV2C,CACNG2,DLGAP1,TSHZ3,SLC4A10,USP14,BTBD9,GRIK3,GABRB1,DGKI,GRIA1,SLC8A3,CHRM3,RAPGEF2,FGF12,GABRA6,PTPRN2,SYN2,GRM7,APP,RPS6KA2,CACNB2,STAU2,GABRG2,SYT1,NTF3,ERC2,SYN3,PRKCZ,SV2B,MAPK1,CADPS2,HRH2,PLG,SYT10,NRG3,HRH4,SORCS3,PAFAH1B1,GRIK4,PPP1R9A,AKAP9,RASGRF2,SYNJ1,GRM1,GABRG1,TMEM108,AMPH,CDH11,USP8,TNR,GRM8,ELAVL4,ABL1,SLC1A1,RIMBP2,RAP1A,GRID2,RASGRF1,PRKCE,DGKB,P2RX6,EXT1,RPS6KA3,ATXN3,HTR2C,RIC3,OPRM1,HTR2A,TMOD2,APBA2,KCND2,MYLK2,MBP,PACSIN2,DISC1,RELN,UNC13B,GABRR2,GRIK2,MCTP2,FARP1,GABBR2,ZZEF1,DCC,CHRM5,PRKN,MTMR2,DLGAP2,GRIN2A,ALS2,KCNQ3,SHISA9,SHANK2,PLCB4,GRID1,COLQ,NMU,RPH3A,GABRG3,IGSF11,HCN1,GRIN2B,CELF4,ROR2,CLSTN2,NRXN1,JAK2,HCRTR1,PRKCB,CACNA1E,SLC6A1,PARK7,MEF2C,ADGRB1,WNT7A,S100B,OR10H2,SDCBP,SNAP29,EPHA4,GABRA5,NTRK2,IL1RAPL1,SHISA6,GRIK1,SCGN,CYFIP1,PCDH8,FYN,CDH2,FBXL20,EPHB1,GRM5,FCHSD2,BCR,NRXN3,EPHB2,CD38,DLG2,CACNG3,SLC1A2,GABRA2,TMEM25,GRM3,EXOC4,PLCL1,SORCS2,NLGN1,NOS1,SLC6A3,ASIC2,DTNA,NTNG1,CCR2,HRH1,CADPS
GO:0048870	cell motility	6.736532327464382e-13	MTOR,SPOCK1,WWC1,LRP12,PLCB1,TAFA5,DLC1,RIPOR2,RDX,BCL2,SDCCAG8,SPRED1,ENPEP,SPAG16,USH2A,AUTS2,CARMIL1,MCTP1,ASTN1,RIN2,ANO6,GPC6,APC,CRKL,SETD2,ARHGAP24,PTPRJ,DOCK10,EGFR,RFX3,ANGPT1,MACF1,CTNNA3,DOCK2,SCAI,ARMC2,NTRK3,C5,PHACTR1,FLT1,NEO1,PRKD1,TPTE2,PAK1,RAPGEF2,ARSB,ONECUT1,LDB2,TAFA4,CCL28,DNAH6,ADAM10,HDAC9,IL1R1,APP,MTUS1,DCLK1,DOCK8,MAPRE2,SEMA5A,VCL,NTF3,CD2AP,SLC8A1,PTPRR,SRGAP2C,SRGAP2B,KANK1,MAP4K4,CTNNA2,PAK3,PRKCZ,GRB10,MRTFA,DUSP22,MAPK1,MGAT5,DNAH14,PLG,PDGFD,ITGBL1,NRG3,GFRA1,NIPBL,FAT3,LIMCH1,PAFAH1B1,ITGB8,TPM1,NF2,AVL9,CTNNA1,TIAM1,PTPRK,ENAH,PAK5,SEMA3C,NAV3,AGO2,DNAH11,IL34,BCAS3,SYNE2,BBS2,SLC9C1,SEMA6D,TNR,DST,CXADR,DOCK4,PTPRT,ABL1,HDAC4,SDC2,DNAH5,GPC5,FGF10,PEAK1,NRG1,ASPM,PRKCE,USP33,CD44,PTPRO,EGF,EXT1,LIMD1,PLXNA2,ARHGEF7,ATP8A1,BIN2,SEMA3E,MARK2,GCSAML,SH3KBP1,STK10,EMILIN2,BCL11B,DOCK5,IL17RA,FUT9,PRR5L,VAV1,TJP1,LDLRAD4,NPHP4,IQSEC1,CACNA1I,DISC1,DNER,WDPCP,SEMA3A,BMP2,UNC5D,CDC42BPB,RIN3,SEMA3D,RELN,HMGB1,FGF9,NFATC2,DOCK1,SRGAP2,DNAH8,SLAMF1,ETS1,GLI3,SMOC2,RERE,LAMC1,U

			MODL1,BBS4,LAMC3,COL5A1,ITGA9,KITLG,DCC,DAB1,CDC42BPA,TBX20,DACH1,LYST,ARID5B,IL6R,PTPRB,VAV3,ROCK1,LYN,VCAM1,OVOL2,NTN1,INSR,YTHDF3,SNAI2,ASH1L,SLC22A14,CD9,CARD10,JCAD,TWIST2,IFT81,BMP5,CSF1,PRKG1,LAMA3,VSTM4,TNN,ATRN,IL33,ROR2,FAT1,IL10,TSPAN11,ABHD2,IQGAP1,AIMP1,NET1,ANLN,TWIST1,AKT3,JAK2,FSTL1,CELSR2,ABCC8,NEDD9,ITGA6,MEOX2,BRMS1L,SHROOM2,CEMIP,CCBE1,ITGA4,ASB2,MYOCD,MEF2C,ADGRB1,WNT7A,PKN2,SDCBP,NCK1,FGR,DRC7,PPP2R3A,CXCL2,EPHA4,INTS13,NTRK2,NUMB,ADAMTS9,MEGF10,FBXO31,PTK2,CDH5,DIAPH1,LAMB1,HOATZ,SEMA4D,JAM2,WNT5B,PSTPIP2,TPTE,CCDC88A,SPAG6,TNFSF11,FYN,PPM1F,ARL13B,HDAC2,CRTAM,APELA,FUT8,TET1,CDH2,NTN4,EPHB1,PTPRG,NRP1,PRKCA,ITGA1,MCC,BCR,ELMO1,FBLN1,RRAS2,BMPER,SRGAP3,MITF,EPHB2,MET,CDH13,MAGI2,UNK,FLRT2,LAMA1,ATRNL1,BMP7,ASTN2,DLG5,ZMYND8,KIRREL3,DNAH3,PDGFC,DNAH17,ABL2,SLIT2,CCDC141,ERBB4,ROBO1,PRKCQ,VCAN,FRMD5,NTNG1,LOXL2,FOXB1,CAMK1D,PIK3R3,CFAP44,CATSPERE,FER,CCR2,STARD13,EPS8,SPOCK3,SEMA4B,HRH1,ROCK2,IL16,DMRT1,CATSPER2,WASHC1,APCDD1,IGF1R,DNM1L
GO:0007409	axonogenesis	1.1626209056584975e-12	*NOTCH2,CNTN4,LRRC4C,ULK2,STXBP1,BCL2,ROBO2,AUTS2,DSCAM,MACF1,BCL11A,CDH4,NEO1,CNTN6,PAK1,EPHA7,ADAMTSL1,APP,DCLK1,SEMA5A,VCL,BMPR1B,CTNNA2,PAK3,DIP2B,TRPC5,COBL,ALCAM,NCAM1,CHN1,PAFAH1B1,NFIB,PRTG,TIAM1,ENAH,SEMA3C,SEMA6D,CDH11,PARD3,TNR,ABL1,USP33,PTPRO,TRIO,EXT1,ATP8A2,PLXNA2,SEMA3E,MARK2,EPHA6,ATL1,AFG3L2,ANK3,BCL11B,ECE1,MBP,CNTN1,DISC1,SEMA3A,UNC5D,SEMA3D,RELN,UST,B4GALT6,NIN,DRAXIN,GLI3,MAP2,DCC,DAB1,ALS2,NTN1,DPYSL5,ZDHHC17,LAMA3,TNN,LMX1A,MAP6,VASP,NRXN1,GAP43,ITGA4,ADGRB1,WNT7A,S100B,NECTIN1,EPHA4,NTRK2,NUMB,LHX9,PTK2,FEZ2,CYFIP1,SEMA4D,FYN,CDH2,CNTN5,EPHB1,RPS6KA5,NRP1,CHODL,NRXN3,EPHB2,FLRT2,KALRN,LAMA1,TIAM2,BMP7,SLIT2,CCDC141,ROBO1,PRKCQ,EFNA5,SLIT3,NTNG1,FOXB1,SEMA4B,FSTL4,IGF1R,GLI2*
GO:0007268	chemical synaptic transmission	1.2065444169235557e-12	*CNTN4,NSG1,PTPRD,SLC24A2,LRRC4C,UNC13C,PLCB1,PTPRA,STXBP1,ERC1,LRFN2,CDH8,CHRNA7,RIMS1,GABRB3,RIMS2,MCTP1,SV2C,CACNG2,DLGAP1,TSHZ3,SLC4A10,USP14,BTBD9,GRIK3,GABRB1,DGKI,GRIA1,SLC8A3,CHRM3,RAPGEF2,FGF12,GABRA6,PTPRN2,SYN2,GRM7,APP,RPS6KA2,CACNB2,STAU2,GABRG2,SYT1,NTF3,ERC2,SYN3,PRKCZ,SV2B,MAPK1,CADPS2,HRH2,PLG,SYT10,NRG3,HRH4,SORCS3,PAFAH1B1,GRIK4,PPP1R9A,AKAP9,RASGRF2,SYNJ1,GRM1,GABRG1,TMEM108,AMPH,CDH11,USP8,TNR,GRM8,ELAVL4,ABL1,SLC1A1,RIMBP2,RAP1A,GRID2,RASGRF1,PRKCE,DGKB,P2RX6,EXT1,RPS6KA3,ATXN3,HTR2C,RIC3,OPRM1,HTR2A,TMOD2,APBA2,KCND2,MYLK2,MBP,PACSIN2,DISC1,RELN,UNC13B,GABRR2,GRIK2,MCTP2,GABBR2,ZZEF1,DCC,CHRM5,PRKN,MTMR2,DLGAP2,GRIN2A,ALS2,KCNQ3,SHISA9,SHANK2,PLCB4,GRID1,COLQ,NMU,RPH3A,GABRG3,IGSF11,HCN1,GRIN2B,CELF4,ROR2,CLSTN2,NRXN1,JAK2,HCRTR1,PRKCB,CACNA1E,SLC6A1,PARK7,MEF2C,ADGRB1,WNT7A,S100B,OR10H2,SDCBP,SNAP29,EPHA4,GABRA5,NTRK2,SHISA6,GRIK1,SCGN,CYFIP1,PCDH8,FYN,CDH2,FBXL20,EPHB1,GRM5,FCHSD2,BCR,NRXN3,EPHB2,CD38,DLG2,CACNG3,SLC1A2,GABRA2,TMEM25,GRM3,EXOC4,PLCL1,SORCS2,NLGN1,SLC6A3,ASIC2,DTNA,NTNG1,CCR2,HRH1,CADPS*
GO:0098916	anterograde trans-synaptic signaling	1.2065444169235557e-12	*CNTN4,NSG1,PTPRD,SLC24A2,LRRC4C,UNC13C,PLCB1,PTPRA,STXBP1,ERC1,LRFN2,CDH8,CHRNA7,RIMS1,GABRB3,RIMS2,MCTP1,SV2C,CACNG2,DLGAP1,TSHZ3,SLC4A10,USP14,BTBD9,GRIK3,GABRB1,DGKI,GRIA1,SLC8A3,CHRM3,RAPGEF2,FGF12,GABRA6,PTPRN2,SYN2,GRM7,APP,RPS6KA2,CACNB2,STAU2,GABRG2,SYT1,NTF3,ERC2,SYN3,PRKCZ,SV2B,MAPK1,CADPS2,HRH2,PLG,SYT10,NRG3,HRH4,SORCS3,PAFAH1B1,GRIK4,PPP1R9A,AKAP9,RASGRF2,SYNJ1,GRM1,GABRG1,TMEM108,AMPH,CDH11,USP8,TNR,GRM8,ELAVL4,ABL1,SLC1A1,RIMBP2,RAP1A,GRID2,RASGRF1,PRKCE,DGKB,P2RX6,EXT1,RPS6KA3,ATXN3,HTR2C,RIC3,OPRM1,HTR2A,TMOD2,APBA2,KCND2,MYLK2,MBP,PACSIN*

			2,DISC1,RELN,UNC13B,GABRR2,GRIK2,MCTP2,GABBR2,ZZEF1,DCC,CHRM5,PRKN,MTMR2,DLGAP2,GRIN2A,ALS2,KCNQ3,SHISA9,SHANK2,PLCB4,GRID1,COLQ,NMU,RPH3A,GABRG3,IGSF11,HCN1,GRIN2B,CELF4,ROR2,CLSTN2,NRXN1,JAK2,HCRTR1,PRKCB,CACNA1E,SLC6A1,PARK7,MEF2C,ADGRB1,WNT7A,S100B,OR10H2,SDCBP,SNAP29,EPHA4,GABRA5,NTRK2,SHISA6,GRIK1,SCGN,CYFIP1,PCDH8,FYN,CDH2,FBXL20,EPHB1,GRM5,FCHSD2,BCR,NRXN3,EPHB2,CD38,DLG2,CACNG3,SLC1A2,GABRA2,TMEM25,GRM3,EXOC4,PLCL1,SORCS2,NLGN1,SLC6A3,ASIC2,DTNA,NTNG1,CCR2,HRH1,CADPS
GO:0036211	protein modification process	1.787807744756 3017e-12	*BCAR3,MTOR,PTPRD,TMTC1,ULK2,NLK,KSR1,AGBL1,TTC3,DLC1,ZDHHC21,PTPRA,PDE4D,ERC1,BCL2,PRDM16,F13A1,GPHN,CHRNA7,PIK3C3,EPC2,SPRED1,GALNT1,MINAR1,PCMTD1,ALK,AUTS2,PJA2,BABAM2,HLCS,MLLT3,EGLN3,MAP3K9,MYO3B,APC,CRKL,SETD2,ERG,TNIK,PTPRJ,KDM4C,NEK4,EGFR,USP14,ANGPT1,CDK12,PRKACB,NEK7,RNF220,NEDD4,NSMCE2,BCL11A,RPRD1A,PTPN4,B3GALT5,NTRK3,LARGE1,TUSC3,FBXL7,FLT1,SLC8A3,PRKD1,TPTE2,PAK1,EPHA7,NCOA7,CHRM3,RAPGEF2,PELI2,TAOK3,UBE2L3,PPP2R2B,PUM3,PTPRN2,SMYD3,HERC2,RPTOR,GHR,WDSUB1,NEDD4L,ADAM10,HDAC9,UBE2G1,APP,RPS6KA2,SAMSN1,KDM1B,KLHL13,PHKB,DCLK1,USP18,NTF3,ACER2,PARP15,AURKA,SLC8A1,PTPRR,MARCHF1,MAP4K4,BMPR1B,ARNT,PAK3,TTLL7,DIP2B,RANBP2,ITPKB,TRPC5,UBE2E2,HHAT,NBN,PRKCZ,SPOP,MAN2A2,DIP2A,ST8SIA5,HECW1,PHF19,TAF4B,SENP6,DUSP22,GALNT14,WDR70,PPM1L,RIPK4,MAPK1,MGAT5,USP25,KMT2E,PCGF5,PDGFD,ZNRF3,XXYLT1,NRG3,UBE2O,GFRA1,NIPBL,GALNT16,RNGTT,STK38,PTPN13,MYLK3,KANSL1,LIMCH1,EFEMP1,DCAF1,CCNG2,TLK1,NF2,ZDHHC14,MOB3B,BIRC6,AKAP9,KLF15,PPP6R3,UBE3D,RSRC1,PTPRK,PAK5,ST6GALNAC3,TRERF1,PPP2R5E,PDZRN3,DAPK1,STK32B,ALPK2,JARID2,GATAD2B,CPE,IL34,MELK,WNT9B,HECTD4,DUSP16,USP8,PARD3,MAPKAP1,PIAS1,UBE2R2,BLK,ATRX,NUAK1,PTPRT,ABL1,PTPN12,HDAC4,OXR1,SLC1A1,PRKAA1,TTLL5,EIPR1,MAST4,ATE1,RAP1A,HECTD2,CAMK4,BAZ2A,MANBA,FGF10,TGM1,PEAK1,LATS2,NRG1,AP3B1,ZBTB16,MUSK,GALNTL6,ZNF675,SMARCAD1,SETDB2,PRKCE,SLCO3A1,METAP1D,NXN,WNK2,USP33,CD44,PTPRO,EGF,ALPK3,PRRC1,TRIO,EXT1,LNPEP,SPRED2,RPS6KA3,MARCHF8,MTMR3,PTPN2,TRIM5,ATXN3,ST8SIA6,ALG10B,AMBRA1,STK38L,GALNT10,KDM7A,PRMT8,HTR2A,FANCM,FANCA,PPP2R2C,TAF3,RPRD1B,MARK2,C10ORF90,ABHD17C,HERC1,EPHA6,HIPK3,CDKN2C,GRK3,KNDC1,SPSB4,CLSPN,NOS2,STK10,MNAT1,TMTC2,MYLK2,HMGA2,CCND3,FOLH1,STK32A,LYPLA1,CWC27,PLCE1,TGFA,FUT9,PRR5L,GXYLT2,MSRA,FBXO32,LDLRAD4,EGFLAM,CNTN1,FKBP5,NAA35,BRCA2,GALNT13,BLM,ASB7,NRK,BMP2,RC3H2,MYLK4,TRAK1,CDC42BPB,VRK1,TPGS2,BMP2K,RNF38,RELN,GNAQ,FGF9,SH3BP5,UST,TRAF3,DSTYK,UIMC1,B4GALT6,SNX6,CNKSR3,CUL1,DAW1,NEK10,MOB1B,PIGN,ATF2,CYLD,MAPK8IP1,KITLG,ZZEF1,CAMTA1,UBR1,MAP4K3,RCAN1,TADA2A,DAB1,MED27,RB1CC1,MYO3A,UBE2E1,PTPRE,PRKN,MTMR2,SPSB1,CDC42BPA,MAPK10,ZNF541,FBXO3,WSB1,USP43,TRPM6,PRKCH,HUNK,IL6R,ALS2,MKNK1,SNX25,PTPRB,COPS8,ST8SIA1,USP7,MOK,RALB,ROCK1,LYN,SUMO3,DTX1,CHKA,FANCL,RNF152,OTUD7A,INSR,CUL5,NEK6,HECTD1,HDAC11,LYPLAL1,SUMO2,ARFGEF1,SNAI2,ASH1L,SIAH2,PIGK,PGAP4,TRABD2B,ERN2,TRIM58,ZDHHC17,NSD2,PTAR1,CARD10,LTN1,ENPP1,ENTPD5,RASGRP1,SNX9,ANAPC1,CSNK2A1,BMP5,CSF1,PPIL6,EOGT,CTDP1,PRKG1,ASB4,FANCB,CLNS1A,SMAD5,MARCHF11,DCUN1D4,PRAME,KLHL7,ATG4B,CDC14B,KDM6A,GPRC5C,ROR2,PPP2R2A,BANK1,SFPQ,PRKAA2,CSF2RB,RNF182,PHF20L1,IQGAP1,CAMLG,NRXN1,HIPK1,FRY,FICD,CENPE,ELOC,TWIST1,AKT3,ALKAL2,JAK2,MPPE1,MADD,CREBBP,TNKS,SIAH3,UFL1,PRKCB,FBXW2,ST6GAL2,RTRAF,BRD4,NEDD9,NRBP1,MAST2,PCMTD2,EXT2,PDP2,BRMS1L,NDFIP2,MAP2K6,MARCHF6,ABI1,CEMIP,IMPACT,PARK7,MAPK8,EIF3F,PPME1,FBXL17,UBE2J2,MTF2,NCAPG2,ASB2,MYOCD,UBE2QL1,ST8SIA4,MEF2C,ADGRB1,MAP3K5,NDFIP1,MAP3K4,TRIM43B,PRDM13,TRIM43,SUMF1,MAGE*

			L2,PKN2,DBF4B,FBXW8,SDCBP,NSMCE1,PASK,MLLT1,NCK1,FGR,CDCA8,PPP2R3A,TRIM23,TOP1,RNF8,EPHA4,MECOM,NTRK2,OCLN,FBXO31,EXTL3,TRPM7,PRKAB1,PTK2,MARK4,CDH5,UBE3A,SEMA4D,KIRREL1,AMFR,POMT2,NOS1AP,MTTP,DPY19L2,TPTE,PDCL3,CCDC88A,GALNT18,TNFSF11,FYN,BUB1,KDM5A,DPY19L1,PPM1F,SDE2,UHRF2,HDAC2,SLF1,SH2D3C,DOCK3,GALNT17,MTMR7,ZFYVE28,MAPK9,STT3A,SLC39A8,ROR1,GALNT2,FUT8,TET1,ASB3,HECW2,EPHB1,ZDHHC18,GRM5,SPOPL,RPS6KA5,PTPRG,PID1,NRP1,MIDEAS,PRKCA,ATPSCKMT,RNF215,USP24,ITGA1,RNF138,RC3H1,POR,ZNF738,SUPT3H,BCR,SNRK,SENP8,USP49,FBLN1,STK36,RAG1,BMPER,MACROH2A1,EPHB2,CSNK1G1,RNF11,DPH6,PPIL2,CDK14,MET,SPPL3,CAMK1G,ATG5,USP32,MAGI2,MYB,KALRN,LAMA1,MFHAS1,TRIM9,BMP7,RNF217,KMT2C,PDGFC,ABL2,EYA1,TTLL11,SLIT2,PARP8,CNOT7,ESCO1,ERBB4,ROBO1,PRKCQ,NOS1,EFNA5,NSD1,EHMT1,USP31,KDM4B,LOXL2,PRLR,PIGB,HTT,ZDHHC11B,CAMK1D,PIK3R3,MACROD2,FER,EYA2,CHFR,PCMT1,OARD1,SPOCK3,ROCK2,PRDM1,ATAT1,PPP1CB,PDK1,PTPRQ,HERPUD1,NCOA6,TRIM2,WASHC1,BARD1,STK3,DEPTOR,RAB3GAP2,TULP4,IGF1R,PRKAG2,AKAP13,MORC3
GO:0051234	establishment of localization	3.146155296810111e-12	*UNC80,CACNA2D3,NSG1,EXOC1L,WWC1,SLC17A1,ABCA13,IMMP2L,LRP12,SLC24A2,TRAPPC9,KCNH5,MICU2,SLC25A21,LONP2,UNC13C,MX2,TMPRSS2,CLTCL1,SLC37A1,PIEZO2,MICAL3,SNAP25-AS1,DPP10,ZDHHC21,ITPR2,RIPOR2,PDE4D,RDX,STXBP1,ERC1,RALA,SLC44A5,EPS15L1,BCL2,MYO5A,KCNMA1,SYT16,COG5,CHRNA7,RIMS1,PIK3C3,SPIRE1,GABRB3,EXOC6B,SPAG16,MYO1E,TRAPPC8,USH2A,RIMS2,CARMIL1,MCTP1,SV2C,ERBIN,FCHO2,RIN2,ANO6,CACNG2,MAP4,MYO5C,ILDR2,SETD2,TANGO6,SLC4A10,PTPRJ,OCA2,EGFR,RFX3,DENND1A,ANGPT1,MACF1,DOCK2,NEDD4,GNPTAB,BTBD9,CECR2,GRIK3,ATP2B2,TUSC3,ZFAND6,DNAJC13,RABEP1,GABRB1,DGKI,C12ORF4,GRIA1,TTC39B,NUP214,SLC39A12,SLC8A3,TOM1L2,PRKD1,PAK1,CHRM3,GRAMD1B,LRP2,ARSB,FGF12,GABRA6,LDLRAD3,TMEM38B,AGK,RANBP17,SLC24A3,SLC44A1,UBE2L3,TAFA4,PTPRN2,SYN2,SMYD3,HERC2,TMEM241,GRM7,GHR,KIF4A,THADA,NEDD4L,TRPM1,ADAM10,SLC39A11,APP,SLC7A2,ABCB5,CACNA1C,CACNB2,DCLK1,STAU2,GABRG2,TMC1,SYT1,ARHGAP44,NTF3,NDUFAF2,CD2AP,SLC8A1,ABCG8,KCNE4,ABCD2,FMN2,PCSK6,AKAP6,HOMER2,RAB8B,RFTN1,KCNK10,RANBP2,TRPC5,RAP1GDS1,CLIC6,ERC2,DNM3,CUBN,SCP2,SYN3,IFT57,PRKCZ,GRB10,RYR3,MCPH1,RAB27B,CNST,HECW1,ABCA5,SV2B,SEM1,VPS35L,MAPK1,CADPS2,KCNJ1,HRH2,ABCD3,RABGAP1L,SGTB,TRPC7,SLC45A4,COPB1,SYT10,UBE2O,ANKFY1,NIPBL,SLC16A1,SPIDR,NIPAL2,IPO11,MICU1,CORO2B,PAFAH1B1,TM7SF3,STON2,VPS13D,TLK1,LRRC38,GRIK4,RBFOX1,ZDHHC14,CORIN,AKAP9,KLF15,RASGRF2,PPARA,SNX30,KCNS3,SYNJ1,GRM1,RSRC1,GABRG1,PARD3B,PLA2R1,DAPK1,SLC24A4,SEC14L1,VPS13C,TMEM108,DNAH11,SCN2A,RAB22A,SORCS1,DNAJC15,AMPH,CPE,DYSF,ANK2,ADGRV1,BCAS3,RYR2,SYNE2,BBS2,SLC9C1,RANBP3L,NKAIN3,NKG7,DUSP16,FABP7,PARD3,SLC36A1,TBC1D5,BLK,DST,CXADR,ABL1,SLC1A1,PRKAA1,SLC12A8,KCNH1,PRELID2,ANO4,CCDC91,EIPR1,DNAH5,NBAS,RAP1A,NKAIN2,MYO10,SLC46A3,PLEKHA8,FGF10,GRID2,NRG1,GSG1L,AP3B1,RASGRF1,ATP11C,ABCB7,SH3GL3,ABCC12,PRKCE,SLCO3A1,SLMAP,WNK2,USP33,DENND4C,CEP83,EGF,ABCC9,P2RX6,EXT1,STXBP6,PEX14,IFT43,ATP8A2,SCG5,HTR2C,CLEC16A,SLC2A3,ARHGEF7,ALG10B,ATP8A1,RFTN2,ZFYVE9,OPRM1,ABCC4,HTR2A,BIN2,CYBRD1,CYP4A11,CNNM4,STAC,CNIH3,IGF2BP3,APBA2,MAIP1,SH3KBP1,SLC2A13,EPN2,KCND2,TNPO3,ABCA10,GRK3,CD163,NOS2,AFG3L2,MDFIC,MYLK2,ANK3,NIPA2,TMC7,COG2,VPS41,LYPLA1,TRAPPC11,TMEM163,HHIPL1,ANKFN1,HIP1,PRR5L,VPS37A,ATP6V1E1,VAV1,RUFY2,PACSIN2,CNTN1,SNX3,CACNA1I,BHLHE40-AS1,KCNJ15,BRCA2,DNER,WDPCP,SLC10A7,LRP1B,ADCY10,STX12,BMP2,ATP9A,TRAK1,EVI5,SCN11A,MSR1,TBC1D4,MYRIP,TTR,RIN3,BMP2K,SLC15A5,NETO2,RELN,HMGB1,AP4E1,FGF9,*

			SLC23A2,MYOM1,PRG4,UNC13B,TTC21B,DOCK1,PLS1,SNX8,SEC23B,SLC39A6,CCDC186,SLAMF1,KCNH8,GLI3,SNX6,SLC37A2,SLC9A4,GABRR2,CNKSR3,GRIK2,MCTP2,MAP2,DAW1,PEX6,NEK10,RRBP1,ATF2,BBS4,LRRC8B,MAPK8IP1,ANTXRL,CFTR,KPNA1,CSE1L,DOP1B,TBC1D13,NEU3,PHAF1,ATP10B,CHRM5,SLC30A10,SELENON,NMD3,REPS1,PRKN,MTMR2,LYST,HEPHL1,GRIN2A,JPH1,ATXN1,TRPM6,CDH23,SLC12A1,FRMD4A,TG,ALS2,RACGAP1,ACO1,SNX25,FBLN5,OSCP1,KCNQ3,SHISA9,SLC4A4,SCN10A,USP7,VAV3,MON2,KCND3,MESD,ITSN2,SOX30,SYBU,RALB,YIPF6,KCNN3,MYO1D,SEC24D,ROCK1,LYN,SEL1L,SLC44A2,SLC15A2,NTN1,CHKA,SLC13A5,CRACR2A,INSR,NPIPA1,CUL5,DMBT1,GRID1,COLQ,SLC52A1,ARFGEF1,IGHV3-74,BID,OSBPL10,RPH3A,TANC2,COX5A,ABCA4,UFD1,GABRG3,TRIM58,TOM1,PLPP4,ZDHHC17,NSD2,FYCO1,ESYT2,SH3GLB1,SLC22A14,CD9,TMED3,XKR5,IFT81,ENPP1,UTRN,RASGRP1,SNX9,KCNC1,GHRH,NUP37,BCL2L1,HCN1,GRIN2B,IGHV2-70D,CLNS1A,SYNJ2,ABCG1,KCNK5,SLC40A1,CABYR,CIDEC,PSAP,CFHR4,MICALL2,MED1,IPCEF1,ATG4B,PCNT,SLC5A12,ACTR2,SFPQ,PTH,NDC80,MAP6,PLA2G4A,SCFD2,KIFC1,SLC25A52,CAMLG,COX7A2L,SREBF2,ANP32B,AIMP1,LASP1,NRXN1,PCID2,ENTHD1,SNAP91,CENPE,PEG10,TWIST1,JAK2,SLC1A7,MPPE1,CELSR2,MELTF,TNKS,ARL11,SIAH3,TRPV5,NFKBIA,PRKCB,GOT2,ABCC8,MIPEP,CACNA1E,ANP32A,RTRAF,NRBP1,ATP2B1,SLC14A2,CLCA4,MTCL1,GRIP1,IGHV1OR15-9,SAR1A,CNIH1,TRAPPC3,XKR6,OTOP1,BBS9,EXT2,EXOC1,SLC6A1,NDFIP2,MAP2K6,SHROOM2,RN7SL483P,SLC6A11,KCNJ18,CEMIP,CBLIF,PARK7,MAPK8,ITGA4,OAZ2,BCAP29,UBE2J2,ADCYAP1R1,PLA2G12B,NDC1,TM9SF4,RAPGEF4,CEP120,ATP13A3,ARL4C,EFHB,MEF2C,STOML1,ADGRB1,RXRA,WNT7A,NDFIP1,SLC5A9,ATP6V1C2,CHAMP1,MAGEL2,SLC10A6,RAB38,SDCBP,NECTIN1,JPT2,PASK,FLVCR1,FGR,CDCA8,TRIM23,ATP6V1B2,SNAP29,C2,GABRA5,NTRK2,IL1RAPL1,NUMB,ADAMTS9,RN7SL767P,COLEC12,PLEKHA3,OCLN,STON1-GTF2A1L,SHISA6,MEGF10,TRPM7,KTN1,GRIK1,IREB2,MFSD9,MVB12B,PTK2,CD5L,APOL2,AP4S1,ARHGAP12,DIAPH1,SCAMP1,CYFIP1,UBE3A,APOL1,PITPNC1,AP2B1,SCARA5,SLC26A2,HEATR5A,ICA1,PLCZ1,NOS1AP,MTTP,SLC9A5,SRP9,CCDC88A,NSUN2,SLC27A6,SPAG6,SLC5A1,BICD1,ANO10,TNFSF11,FYN,PPM1F,XPO7,SCN8A,TMEM63C,NCS1,ATP5PF,ATP9B,NALCN,EHBP1,APELA,TRPM3,SLC39A8,SLC16A9,HECW2,CDH2,ITGA8,FBXL20,ZDHHC18,GRM5,TBC1D1,PID1,NRP1,FCHSD2,IFT46,ATPSCKMT,RNF215,CROT,ABCA6,SLC14A1,MCC,BCR,NRXN3,ELMO1,KIF16B,ARFGAP3,STK36,NSG2,MB,KCNJ6,RABL2A,CUX1,DPP6,SLC35F1,MACROH2A1,EPHB2,TSPAN13,CSNK1G1,CD38,MYO5B,RGPD4,MET,DLG2,CDH17,ATP6V0D2,PPFIA2,CDH13,STXBP4,CACNG3,ATG5,MAGI2,SLC35F4,VMP1,KALRN,SLC1A2,GNAS,NUP43,TMPRSS15,ASTN2,GAPVD1,GABRA2,TRAPPC10,DDX6,WDR41,PLIN2,ABL2,VPS13B,TRAPPC6B,TMPRSS3,EXOC4,KCNIP4,ERBB4,FAM3B,SYNDIG1,NUF2,RGPD2,SAMM50,ANTXR1,SORCS2,TRDN,NLGN1,NOS1,SLC6A3,ASIC2,EFNA5,RAB27A,KIF13A,AP5M1,DNAH9,SLC25A48,KCNQ5,LOXL2,CACNA2D1,IGLC3,IRAG2,PRLR,HTT,ZDHHC11B,FOXB1,CAMK1D,SLC25A18,HLA-F,FER,EYA2,KATNIP,CCR2,PITPNM3,OSBPL5,OSBPL6,IGHV1OR21-1,ANO2,GRIA4,AGAP1,IL16,TERB2,CDCA5,CATSPER2,RAB31,HSPG2,HERPUD1,WASHC1,RGS7,HOOK3,CLDN10,BARD1,CLCN5,STK3,SLC13A4,PNPLA8,HNRNPU,VTI1A,RAB3GAP2,CADPS,IGF1R,KCNAB1,PRKAG2,TANGO2,AKAP13,ATP10A,DNM1L
GO:0061564	axon development	3.1775160542857953e-12	*NOTCH2,CNTN4,LRRC4C,ULK2,STXBP1,BCL2,ROBO2,AUTS2,DSCAM,MACF1,BCL11A,CDH4,NEO1,CNTN6,PAK1,EPHA7,ADAMTSL1,GRM7,APP,DCLK1,SEMA5A,VCL,BMPR1B,CTNNA2,PAK3,DIP2B,TRPC5,COBL,ALCAM,NCAM1,CHN1,PAFAH1B1,CTNNA1,NFIB,PRTG,TIAM1,ENAH,SEMA3C,SEMA6D,CDH11,PARD3,TNR,ABL1,CRTAC1,USP33,PTPRO,TRIO,EXT1,ATP8A2,PLXNA2,KREMEN1,SEMA3E,MARK2,EPHA6,ATL1,AFG3L2,ANK3,BCL11B,ECE1,MBP,CNTN1,DISC1,SEMA3A,UNC5D,NCAM2,SEMA3D,RELN,UST,B4G*

			ALT6,TSPAN2,NIN,DRAXIN,GLI3,MAP2,DCC,DAB1,ALS2,NTN1,DPYSL5,NREP,ZDHHC17,LAMA3,TNN,LMX1A,MAP6,VASP,NRXN1,JAK2,GAP43,ITGA4,ADGRB1,WNT7A,S100B,NECTIN1,EPHA4,NTRK2,NUMB,LHX9,PTK2,FEZ2,CYFIP1,SEMA4D,FYN,CDH2,CNTN5,EPHB1,RPS6KA5,NRP1,CHODL,NRXN3,EPHB2,FLRT2,KALRN,LAMA1,TIAM2,BMP7,SLIT2,CCDC141,ROBO1,PRKCQ,EFNA5,SLIT3,NTNG1,FOXB1,SEMA4B,FSTL4,IGF1R,GLI2
GO:0022603	regulation of anatomical structure morphogenesis	5.912535988550438e-12	*PTPRD,LRRC4C,MYO9A,ULK2,TAFA5,TENM4,DLC1,RDX,RALA,CHRNA7,ROBO2,RIMS1,SPIRE1,FGD4,SPRED1,MINAR1,CDC42EP3,RIMS2,FOXJ2,CARMIL1,PARVB,MLLT3,GPC6,ZMYM4,DSCAM,CRKL,TNIK,MACF1,NEDD4,BCL11A,CDH4,C5,FLT1,SLC39A12,PRKD1,PAK1,EPHA7,RAPGEF2,ADGRB3,RUNX2,PRICKLE2,EPB41L3,COL4A2,NEDD4L,STAU2,SEMA5A,SYT1,ARHGAP44,AURKA,CFDP1,KANK1,PAK3,DIP2B,TRPC5,DNM3,HECW1,ZNRF3,CHN1,PAFAH1B1,ITGB8,TPM1,TIAM1,SEMA3C,AGO2,BRWD1,WNT9B,SEMA6D,TNR,ABL1,SDC2,GAS2,MYO10,FGF10,CD44,EGF,LIMD1,PLXNA2,ARHGEF7,SEMA3E,MARK2,SH3KBP1,EPN2,KNDC1,ADAM12,EMILIN2,HMGA2,DOCK5,MBP,TJP1,PDLIM5,DISC1,WDPCP,SEMA3A,BMP2,SEMA3D,RELN,UST,SLC23A2,DOCK1,NIN,DRAXIN,ETS1,SMOC2,MAP2,ATF2,DCC,GTF2I,DAB1,PRKN,NGEF,ITSN2,ROCK1,NTN1,DPYSL5,SHROOM3,DDHD1,SNAI2,TANC2,JCAD,CSF1,CNMD,TNN,AJAP1,IL10,ACTR2,MAP6,PALMD,HIPK1,TWIST1,AKT3,CELSR2,MELTF,PRKCB,ABCC8,NEDD9,OLFM4,GRIP1,AGO1,STAT1,CCBE1,MEF2C,ADGRB1,WNT7A,WASF3,FBXW8,FGR,DNMBP,EPHA4,NTRK2,IL1RAPL1,ADAMTS9,WNT2B,FBXO31,PTK2,CDH5,ANKRD6,DIAPH1,CYFIP1,UBE3A,SEMA4D,RUNX1,WNT5B,PDCL3,FYN,MYL12B,APELA,ROR1,HECW2,CDH2,NTN4,ADCK1,PID1,NRP1,PRKCA,RC3H1,CHODL,BCR,FBLN1,BMPER,CUX1,EPHB2,PPFIA2,MAGI2,FAM171A1,KALRN,TIAM2,BMP7,SLIT2,ROBO1,NLGN1,EFNA5,ESR1,NTNG1,CCR2,EPS8,SEMA4B,ROCK2,HSPG2,COL4A3,FSTL4,APCDD1,ATP10A,DNM1L*
GO:0006810	transport	9.173857206111255e-12	*UNC80,CACNA2D3,NSG1,EXOC1L,WWC1,SLC17A1,ABCA13,IMMP2L,LRP12,SLC24A2,TRAPPC9,KCNH5,MICU2,SLC25A21,LONP2,UNC13C,MX2,TMPRSS2,CLTCL1,SLC37A1,PIEZO2,MICAL3,SNAP25-AS1,DPP10,ZDHHC21,ITPR2,PDE4D,RDX,STXBP1,ERC1,RALA,SLC44A5,EPS15L1,BCL2,MYO5A,KCNMA1,SYT16,COG5,CHRNA7,RIMS1,PIK3C3,SPIRE1,GABRB3,EXOC6B,SPAG16,MYO1E,TRAPPC8,RIMS2,CARMIL1,MCTP1,SV2C,ERBIN,FCHO2,RIN2,ANO6,CACNG2,MYO5C,ILDR2,SETD2,TANGO6,SLC4A10,PTPRJ,OCA2,EGFR,RFX3,DENND1A,ANGPT1,MACF1,DOCK2,NEDD4,GNPTAB,BTBD9,CECR2,GRIK3,ATP2B2,TUSC3,ZFAND6,DNAJC13,RABEP1,GABRB1,DGKI,C12ORF4,GRIA1,TTC39B,NUP214,SLC39A12,SLC8A3,TOM1L2,PRKD1,PAK1,CHRM3,GRAMD1B,LRP2,ARSB,FGF12,GABRA6,LDLRAD3,TMEM38B,AGK,RANBP17,SLC24A3,SLC44A1,UBE2L3,TAFA4,PTPRN2,SYN2,HERC2,TMEM241,GRM7,GHR,KIF4A,THADA,NEDD4L,TRPM1,ADAM10,SLC39A11,APP,SLC7A2,ABCB5,CACNA1C,CACNB2,DCLK1,STAU2,GABRG2,TMC1,SYT1,ARHGAP44,NTF3,NDUFAF2,CD2AP,SLC8A1,ABCG8,KCNE4,ABCD2,FMN2,PCSK6,AKAP6,HOMER2,RAB8B,RFTN1,KCNK10,RANBP2,TRPC5,RAP1GDS1,CLIC6,ERC2,DNM3,CUBN,SCP2,SYN3,IFT57,PRKCZ,GRB10,RYR3,RAB27B,CNST,HECW1,ABCA5,SV2B,SEM1,VPS35L,MAPK1,CADPS2,KCNJ1,HRH2,ABCD3,RABGAP1L,SGTB,TRPC7,SLC45A4,COPB1,SYT10,UBE2O,ANKFY1,SLC16A1,NIPAL2,IPO11,MICU1,PAFAH1B1,TM7SF3,STON2,VPS13D,TLK1,LRRC38,GRIK4,RBFOX1,ZDHHC14,CORIN,AKAP9,KLF15,RASGRF2,PPARA,SNX30,KCNS3,SYNJ1,GRM1,RSRC1,GABRG1,PLA2R1,DAPK1,SLC24A4,SEC14L1,VPS13C,TMEM108,DNAH11,SCN2A,RAB22A,SORCS1,DNAJC15,AMPH,CPE,DYSF,ANK2,BCAS3,RYR2,SYNE2,BBS2,SLC9C1,RANBP3L,NKAIN3,NKG7,DUSP16,FABP7,PARD3,SLC36A1,TBC1D5,BLK,DST,CXADR,ABL1,SLC1A1,PRKAA1,SLC12A8,KCNH1,PRELID2,ANO4,CCDC91,EIPR1,DNAH5,NBAS,RAP1A,NKAIN2,MYO10,SLC46A3,PLEKHA8,FGF10,GRID2,NRG1,GSG1L,AP3B1,RASGRF1,ATP11C,ABCB7,SH3GL3,ABCC12,PRKCE,SLCO3A1,SLMAP,WNK2,USP33,DENND4C,CEP83,EGF,ABCC9,P2RX6,EXT1,STXBP6,PEX14,IFT43,ATP8A2,SCG5,HTR*

			2C,CLEC16A,SLC2A3,ARHGEF7,ALG10B,ATP8A1,RFTN2,ZFYVE9,OPRM1,ABCC4,HTR2A,BIN2,CYBRD1,CYP4A11,CNNM4,STAC,CNIH3,IGF2BP3,APBA2,MAIP1,SH3KBP1,SLC2A13,EPN2,KCND2,TNPO3,ABCA10,GRK3,CD163,NOS2,AFG3L2,MDFIC,MYLK2,ANK3,NIPA2,TMC7,COG2,VPS41,LYPLA1,TRAPPC11,TMEM163,HHIPL1,HIP1,PRR5L,VPS37A,ATP6V1E1,VAV1,RUFY2,PACSIN2,CNTN1,SNX3,CACNA1I,BHLHE40-AS1,KCNJ15,DNER,WDPCP,SLC10A7,LRP1B,ADCY10,STX12,BMP2,ATP9A,TRAK1,EVI5,SCN11A,MSR1,TBC1D4,MYRIP,TTR,RIN3,BMP2K,SLC15A5,NETO2,RELN,HMGB1,AP4E1,FGF9,SLC23A2,MYOM1,PRG4,UNC13B,TTC21B,DOCK1,PLS1,SNX8,SEC23B,SLC39A6,CCDC186,SLAMF1,KCNH8,GLI3,SNX6,SLC37A2,SLC9A4,GABRR2,CNKSR3,GRIK2,MCTP2,MAP2,DAW1,PEX6,NEK10,RRBP1,ATF2,BBS4,LRRC8B,MAPK8IP1,ANTXRL,CFTR,KPNA1,CSE1L,DOP1B,TBC1D13,NEU3,PHAF1,ATP10B,CHRM5,SLC30A10,SELENON,NMD3,REPS1,PRKN,MTMR2,LYST,HEPHL1,GRIN2A,JPH1,ATXN1,TRPM6,CDH23,SLC12A1,FRMD4A,TG,ALS2,RACGAP1,ACO1,SNX25,FBLN5,OSCP1,KCNQ3,SHISA9,SLC4A4,SCN10A,USP7,VAV3,MON2,KCND3,MESD,ITSN2,SOX30,SYBU,RALB,YIPF6,KCNN3,MYO1D,SEC24D,ROCK1,LYN,SEL1L,SLC44A2,SLC15A2,NTN1,CHKA,SLC13A5,CRACR2A,INSR,NPIPA1,CUL5,DMBT1,GRID1,SLC52A1,ARFGEF1,IGHV3-74,BID,OSBPL10,RPH3A,TANC2,COX5A,ABCA4,UFD1,GABRG3,TRIM58,TOM1,PLPP4,ZDHHC17,FYCO1,ESYT2,SH3GLB1,SLC22A14,CD9,TMED3,XKR5,IFT81,ENPP1,UTRN,RASGRP1,SNX9,KCNC1,GHRH,NUP37,BCL2L1,HCN1,GRIN2B,IGHV2-70D,CLNS1A,SYNJ2,ABCG1,KCNK5,SLC40A1,CABYR,CIDEC,PSAP,CFHR4,MICALL2,MED1,IPCEF1,ATG4B,PCNT,SLC5A12,ACTR2,SFPQ,PTH,MAP6,PLA2G4A,SCFD2,SLC25A52,CAMLG,COX7A2L,SREBF2,ANP32B,AIMP1,LASP1,NRXN1,PCID2,ENTHD1,SNAP91,PEG10,TWIST1,JAK2,SLC1A7,MPPE1,CELSR2,MELTF,TNKS,ARL11,SIAH3,TRPV5,NFKBIA,PRKCB,GOT2,ABCC8,MIPEP,CACNA1E,ANP32A,RTRAF,NRBP1,ATP2B1,SLC14A2,CLCA4,MTCL1,GRIP1,IGHV1OR15-9,SAR1A,CNIH1,TRAPPC3,XKR6,OTOP1,BBS9,EXT2,EXOC1,SLC6A1,NDFIP2,MAP2K6,SHROOM2,RN7SL483P,SLC6A11,KCNJ18,CEMIP,CBLIF,PARK7,MAPK8,ITGA4,OAZ2,BCAP29,UBE2J2,ADCYAP1R1,PLA2G12B,NDC1,TM9SF4,RAPGEF4,CEP120,ATP13A3,ARL4C,EFHB,MEF2C,STOML1,ADGRB1,RXRA,WNT7A,NDFIP1,SLC5A9,ATP6V1C2,MAGEL2,SLC10A6,RAB38,SDCBP,NECTIN1,JPT2,PASK,FLVCR1,FGR,TRIM23,ATP6V1B2,SNAP29,C2,GABRA5,NTRK2,IL1RAPL1,NUMB,ADAMTS9,RN7SL767P,COLEC12,PLEKHA3,OCLN,STON1-GTF2A1L,SHISA6,MEGF10,TRPM7,KTN1,GRIK1,IREB2,MFSD9,MVB12B,PTK2,CD5L,APOL2,AP4S1,ARHGAP12,DIAPH1,SCAMP1,CYFIP1,UBE3A,APOL1,PITPNC1,AP2B1,SCARA5,SLC26A2,HEATR5A,ICA1,PLCZ1,NOS1AP,MTTP,SLC9A5,SRP9,CCDC88A,NSUN2,SLC27A6,SPAG6,SLC5A1,BICD1,ANO10,TNFSF11,FYN,PPM1F,XPO7,SCN8A,TMEM63C,NCS1,ATP5PF,ATP9B,NALCN,EHBP1,APELA,TRPM3,SLC39A8,SLC16A9,HECW2,CDH2,FBXL20,ZDHHC18,GRM5,TBC1D1,PID1,NRP1,FCHSD2,IFT46,ATPSCKMT,RNF215,CROT,ABCA6,SLC14A1,BCR,NRXN3,ELMO1,KIF16B,ARFGAP3,STK36,NSG2,MB,KCNJ6,RABL2A,CUX1,DPP6,SLC35F1,EPHB2,TSPAN13,CSNK1G1,CD38,MYO5B,RGPD4,MET,DLG2,CDH17,ATP6V0D2,PPFIA2,CDH13,STXBP4,CACNG3,ATG5,MAGI2,SLC35F4,VMP1,KALRN,SLC1A2,GNAS,NUP43,TMPRSS15,ASTN2,GAPVD1,GABRA2,TRAPPC10,DDX6,WDR41,PLIN2,ABL2,VPS13B,TRAPPC6B,TMPRSS3,EXOC4,KCNIP4,ERBB4,FAM3B,SYNDIG1,RGPD2,SAMM50,ANTXR1,SORCS2,TRDN,NLGN1,NOS1,SLC6A3,ASIC2,EFNA5,RAB27A,KIF13A,AP5M1,DNAH9,SLC25A48,KCNQ5,LOXL2,CACNA2D1,IGLC3,IRAG2,PRLR,HTT,ZDHHC11B,FOXB1,CAMK1D,SLC25A18,HLA-F,FER,EYA2,KATNIP,CCR2,PITPNM3,OSBPL5,OSBPL6,IGHV1OR21-1,ANO2,GRIA4,AGAP1,IL16,CATSPER2,RAB31,HSPG2,HERPUD1,WASHC1,RGS7,HOOK3,CLDN10,BARD1,CLCN5,STK3,SLC13A4

			,PNPLA8,HNRNPU,VTI1A,RAB3GAP2,CADPS,IGF1R,KCNAB1,PRKAG2,TANGO2,AKAP13,ATP10A,DNM1L
GO:0031346	positive regulation of cell projection organization	1.4527605720822816e-11	MTOR,PTPRD,RIPOR2,RP1,RALA,ROBO2,TENM3,CDC42EP3,ALK,AUTS2,CARMIL1,NEGR1,APC,PLPPR5,DSCAM,MACF1,BCL11A,CDH4,NTRK3,PRKD1,RAPGEF2,ARSB,SEPTIN9,STAU2,SEMA5A,FIG4,RAB8B,PAK3,TRPC5,DNM3,COBL,PAFAH1B1,TIAM1,BCAS3,ELAVL4,ABL1,HDAC4,RAP1A,ATP8A2,PLXNA2,ARHGEF7,MARK2,PLCE1,FUT9,CNTN1,SNX3,DISC1,RELN,NIN,ATF1,BBS4,TOX,LYN,TENM2,NTN1,BMP5,TNN,ROR2,ACTR2,MAP6,NRXN1,ANLN,ALKAL2,NEDD9,ITGA6,GRIP1,CEP120,FBXW8,NCK1,EPHA4,NTRK2,IL1RAPL1,OCLN,FBXO31,MARK4,CYFIP1,SEMA4D,SAXO1,CCDC88A,FYN,ROR1,NRP1,CHODL,CUX1,EPHB2,MAGI2,KALRN,TIAM2,BMP7,ABL2,SLIT2,ROBO1,NLGN1,EFNA5,HTT,CAMK1D,EPS8,WASHC1,IGF1R
GO:0065009	regulation of molecular function	1.9569897049398207e-11	BCAR3,MTOR,SPOCK1,GARNL3,TRAPPC9,MYO9A,KSR1,PLCB1,DLC1,ZDHHC21,RIPOR2,PDE4D,RDX,ERC1,BCL2,ARHGAP26,CHRNA7,RIMS1,FGD4,SPRED1,ALK,FANK1,ERBIN,RIN2,CACNG2,DLGAP1,EGLN3,SPON1,APC,CRKL,ARHGAP24,PTPRJ,DOCK10,EGFR,DENND1A,USP14,ANGPT1,CDK12,NEK7,RGS3,RNF220,DOCK2,NEDD4,NTRK3,RXFP1,C5,PHACTR1,DKK2,FLT1,RFC3,RABEP1,DGKI,CAST,SLC8A3,PRKD1,TBC1D19,PAK1,EPHA7,CHRM3,RALGPS1,RAPGEF2,ADGRB3,FGF12,TAOK3,UBE2L3,LDB2,TAFA4,PPP2R2B,SMYD3,HERC2,GRM7,RPTOR,GHR,THADA,RALGAPA1,RAPGEF5,TBCD,NEDD4L,PPP1R12B,HDAC9,PHACTR2,APP,CACNA1C,CACNB2,DOCK8,MAPRE2,ARHGAP44,NTF3,ACER2,AURKA,PARN,ST18,SLC8A1,SERPINA6,KCNE4,MAP4K4,BMPR1B,PCSK6,AKAP6,RANBP2,RGS20,RAP1GDS1,NBN,IFT57,PRKCZ,MSH6,MCPH1,ARHGAP32,RGS9,HECW1,MRTFA,DUSP22,EBF2,RIPK4,MAPK1,MGAT5,RABGAP1L,ITIH5,PDGFD,PPP1R1C,ARHGEF17,NRG3,UBE2O,CARD18,STK38,TBC1D22A,CHN1,ECT2L,PAFAH1B1,CCNG2,TPM1,NF2,LRRC38,MOB3B,BIRC6,AKAP9,RASGRF2,PPARA,MRTFB,PPP6R3,RGL1,TIAM1,ARAP2,ARHGEF12,PPP2R5E,PLA2R1,EIF3D,DAPK1,SLC24A4,RIC8B,TBC1D9,IL34,ANK2,ADGRV1,BCAS3,RYR2,WNT9B,CLPX,RANBP3L,DUSP16,SMARCA4,TBC1D5,BLK,DOCK4,NUAK1,PTPRT,ABL1,HDAC4,SLC1A1,PSMF1,RAP1A,FGF10,LATS2,NRG1,GSG1L,AP3B1,DENND2B,RASGRF1,MUSK,ZNF675,PRKCE,SLCO3A1,ASAP2,SLMAP,WNK2,USP33,DENND4C,CD44,RGS12,PTPRO,EGF,PRRC1,ABCC9,TRIO,PDE3A,NSMAF,PEX14,SPRED2,RPS6KA3,SCG5,PTPN2,TRIM5,PLXNA2,MCF2L,RFC1,ARHGEF7,ALG10B,AMBRA1,OPRM1,PRMT8,HTR2A,FANCA,PPP2R2C,STAC,TAF3,MARK2,CNIH3,HERC1,MSH2,EPHA6,HIPK3,CDKN2C,KNDC1,CLSPN,NOS2,MNAT1,ANK3,HMGA2,CCND3,DOCK5,MBP,PLCE1,TGFA,HIP1,CRIM1,VAV1,IQSEC1,DISC1,BLM,PHACTR3,BMP2,EVI5,RALGAPA2,SGSM1,TBC1D4,RIN3,BMP2K,NETO2,CABIN1,RELN,ARHGAP42,HMGB1,GNAQ,SH3BP5,CPAMD8,TRAF3,ZNF462,DSTYK,DOCK1,RAP1GAP,SRGAP2,SEC23B,SLAMF1,GLI3,SNX6,CNKSR3,IDE,ZNF431,PSD3,MAP2,BTAF1,ZNF618,NEK10,FARP1,MOB1B,ATF2,CYLD,UMODL1,BBS4,MAPK8IP1,GABBR2,CFTR,TBC1D13,KITLG,RCAN1,DAB1,SELENON,NMD3,PRKN,SH3PXD2A,DLGAP2,MAPK10,NGEF,GRIN2A,ARID5B,JPH1,PRKCH,IL6R,ALS2,RACGAP1,NLRC5,TFDP1,DOCK9,SHISA9,PTPRB,ZFP90,COPS8,USP7,VAV3,ITSN2,ARHGEF28,RALB,DENND2C,ROCK1,LYN,ARHGAP28,ARHGAP31,CTSB,EIF2B3,SUMO3,MMP16,CRACR2A,INSR,XRCC4,ARFGEF1,BID,SIAH2,ERN2,MBTPS2,CARD10,RALGPS2,ENPP1,UTRN,RASGRP1,SNX9,TMEM225,CSNK2A1,KCNC1,CSF1,SERPINB9,HCN1,PRKG1,GRIN2B,DCUN1D4,PSAP,CDC14B,GPRC5C,ROR2,PPP2R2A,RASGEF1C,IL10,PTH,LARP6,ITPRIP,IQGAP1,ZBTB7C,ANP32B,NRXN1,PCID2,FRY,FICD,CENPE,NET1,SIPA1L2,TWIST1,ALKAL2,JAK2,MADD,TNKS,PCNA,UFL1,NFKBIA,PRKCB,ABCC8,ANXA4,ZC3H15,RFC2,RTRAF,NEDD9,ITGA6,AGAP9,PPP1R17,ARFGEF3,PDP2,NDFIP2,MAP2K6,MTPN,ABI1,CEMIP,PARK7,MAPK8,ITGA4,OAZ2,PPME1,ADCYAP1R1,NCAPG2,RAPGEF4,MYOCD,CYFIP2,EFHB,MEF2C,RXRA,MAP3K5,NDFIP1,MAP3K4,SERPINI2,DBF4B,MLLT1,NCK1,FGR,PPP2R3A,DNMBP,TRIM23,EPHA4,CYTH4,DNMT3L,NTRK2,SHISA6,PRKAB1,PTK2,RCAN2,ARHGAP12,SEMA4D,SERPIN

			B10,RASGEF1B,AMFR,ASAP1,NOS1AP,CCDC88A,GPR55,BICD1,TNFSF11,FYN,KDM5A,PPM1F,HDAC2,SH2D3C,PSME3IP1,DOCK3,NCS1,ZFYVE28,MAPK9,ROR1,HECW2,GPR137B,EPHB1,GRM5,RPS6KA5,TBC1D1,NRP1,ATPSCKMT,ITGA1,POR,BCR,ELMO1,RGS6,PRIM2,C14ORF39,ARFGAP3,FBLN1,STK36,RAG1,SRGAP3,MACROH2A1,EPHB2,BCL2L13,RGPD4,MET,SPPL3,SERPINB2,CACNG3,MAGI2,VMP1,KALRN,GNAS,SERPINB7,TIAM2,BMP7,TNFAIP8,GAPVD1,GRM3,PDGFC,WDR41,ABL2,TRAPPC6B,SLIT2,PLCL1,ERBB4,SERPINB11,ROBO1,PBX1,PRKCQ,RGPD2,ANTXR1,SIPA1L3,TRDN,MGMT,NLGN1,NOS1,EFNA5,ARHGEF11,NSD1,ESR1,MYO9B,CACNA2D1,PRLR,HTT,CAMK1D,PIK3R3,FER,CCR2,STARD13,A2M,SPOCK3,AGAP1,ROCK2,RGS8,PSMD2,COL4A3,WASHC1,RGS7,STK3,DEPTOR,RSU1,HNRNPU,RAB3GAP2,IGF1R,KCNAB1,PRKAG2,AKAP13,DNM1L
GO:0040011	locomotion	2.984860064457008e-11	NOTCH2,MTOR,CNTN4,LRP12,PLCB1,TAFA5,DLC1,RIPOR2,RDX,RALA,BCL2,ROBO2,SPRED1,CARMIL1,MCTP1,RIN2,ANO6,APC,DSCAM,CRKL,PTPRJ,DOCK10,EGFR,USP14,ANGPT1,MACF1,DOCK2,SCAI,CDH4,NTRK3,C5,PHACTR1,FLT1,NEO1,CNTN6,PRKD1,PAK1,EPHA7,RAPGEF2,ARSB,ONECUT1,ADAMTSL1,LDB2,TAFA4,CCL28,ADAM10,HDAC9,IL1R1,APP,MTUS1,DOCK8,MAPRE2,SEMA5A,VCL,NTF3,SLC8A1,PTPRR,SRGAP2C,PLGRKT,SRGAP2B,KANK1,MAP4K4,BMPR1B,CTNNA2,PAK3,DUSP22,MAPK1,MGAT5,ALCAM,PDGFD,NRG3,NCAM1,NIPBL,CHN1,LIMCH1,TPM1,NF2,CTNNA1,NFIB,PRTG,TIAM1,PTPRK,ENAH,SEMA3C,NAV3,AGO2,IL34,BCAS3,SYNE2,BBS2,SEMA6D,TNR,CXADR,DOCK4,PTPRT,ABL1,HDAC4,FGF10,NRG1,PRKCE,USP33,PTPRO,EGF,TRIO,EXT1,PTPN2,PLXNA2,ARHGEF7,ATP8A1,BIN2,SEMA3E,GCSAML,EPHA6,STK10,EMILIN2,BCL11B,DOCK5,ECE1,IL17RA,FUT9,PRR5L,VAV1,TJP1,LDLRAD4,CNTN1,IQSEC1,WDPCP,SEMA3A,BMP2,UNC5D,RIN3,SEMA3D,RELN,HMGB1,FGF9,DOCK1,SRGAP2,DRAXIN,SLAMF1,ETS1,GLI3,SMOC2,BBS4,ITGA9,KITLG,DCC,RCAN1,DACH1,LYST,GRIN2A,IL6R,VAV3,ROCK1,LYN,VCAM1,NTN1,DPYSL5,INSR,YTHDF3,SNAI2,CD9,CARD10,JCAD,TWIST2,BMP5,CSF1,PRKG1,LAMA3,TNN,IL33,ROR2,LMX1A,IL10,ABHD2,VASP,NRXN1,TWIST1,AKT3,JAK2,ABCC8,NEDD9,ITGA6,GAP43,MEOX2,BRMS1L,CMTM7,CEMIP,CCBE1,ITGA4,MYOCD,MEF2C,ADGRB1,WNT7A,PKN2,SDCBP,NECTIN1,NCK1,FGR,PPP2R3A,CXCL2,EPHA4,NUMB,LHX9,ADAMTS9,FBXO31,PTK2,CDH5,DIAPH1,FEZ2,LAMB1,CYFIP1,SEMA4D,JAM2,WNT5B,TNFSF11,FYN,PPM1F,HDAC2,APELA,TET1,CNTN5,EPHB1,GRM5,RPS6KA5,PTPRG,NRP1,PRKCA,ITGA1,MCC,BCR,NRXN3,FBLN1,RRAS2,BMPER,SRGAP3,MITF,EPHB2,MET,CDH13,MAGI2,FLRT2,KALRN,LAMA1,BMP7,DLG5,ZMYND8,PDGFC,ABL2,SLIT2,CCDC141,ERBB4,ROBO1,PRKCQ,EFNA5,SLIT3,FRMD5,NTNG1,CAMK1D,PIK3R3,FER,CCR2,STARD13,SPOCK3,SEMA4B,HRH1,ROCK2,IL16,WASHC1,IGF1R,GLI2,DNM1L
GO:0043412	macromolecule modification	4.902367270983254e-11	BCAR3,MTOR,PTPRD,TMTC1,ULK2,NLK,FTO,KSR1,AGBL1,TTC3,DLC1,ZDHHC21,PTPRA,PDE4D,ERC1,BCL2,PRDM16,F13A1,GPHN,CHRNA7,PIK3C3,EPC2,SPRED1,GALNT1,MINAR1,PCMTD1,ALK,AUTS2,PJA2,BABAM2,HLCS,MLLT3,EGLN3,MAP3K9,MYO3B,APC,CRKL,SETD2,ERG,TNIK,PTPRJ,KDM4C,NEK4,EGFR,USP14,ANGPT1,CDK12,PRKACB,NEK7,RNF220,NEDD4,NSMCE2,BCL11A,RPRD1A,PTPN4,B3GALT5,NTRK3,LARGE1,TUSC3,FBXL7,FLT1,SLC8A3,PRKD1,TPTE2,PAK1,EPHA7,NCOA7,CHRM3,RAPGEF2,PELI2,TAOK3,UBE2L3,PPP2R2B,PUM3,PTPRN2,SMYD3,TYW1,HERC2,RPTOR,GHR,WDSUB1,THADA,NEDD4L,ADAM10,HDAC9,ATF7IP,UBE2G1,APP,RPS6KA2,SAMSN1,KDM1B,KLHL13,PHKB,DCLK1,USP18,NTF3,ACER2,PARP15,AURKA,PARN,SLC8A1,PTPRR,DTWD2,MARCHF1,MAP4K4,BMPR1B,ARNT,PAK3,TTLL7,DIP2B,RANBP2,ITPKB,TRPC5,UBE2E2,HHAT,NBN,RBM47,PRKCZ,SPOP,MAN2A2,DIP2A,ST8SIA5,HECW1,PHF19,TAF4B,SENP6,DUSP22,GALNT14,WDR70,PPM1L,RIPK4,MAPK1,MGAT5,USP25,KMT2E,PCGF5,PDGFD,ZNRF3,XXYLT1,NRG3,UBE2O,GFRA1,NIPBL,GALNT16,RNGTT,STK38,PTPN13,MYLK3,KANSL1,LIMCH1,EFEMP1,DCAF1,CCNG2,TLK1,NF2,ZDHHC14,MOB3B,BIRC6,AKAP9,KLF15,PPP6R3,UBE3D,RSRC1,PTPRK,PAK5,ST6GALNAC3,TRERF1,

			PPP2R5E,PDZRN3,DAPK1,STK32B,ALPK2,JARID2,GATAD2B,CPE,IL34,THUMPD2,MELK,WNT9B,HECTD4,DUSP16,USP8,PARD3,MAPKAP1,PIAS1,UBE2R2,BLK,ATRX,NUAK1,PTPRT,ABL1,PTPN12,HDAC4,OXR1,SLC1A1,PRKAA1,TTLL5,EIPR1,MAST4,ATE1,RAP1A,HECTD2,MORC1,CAMK4,BAZ2A,MANBA,FGF10,TGM1,PEAK1,LATS2,NRG1,AP3B1,ZBTB16,MUSK,GALNTL6,ZNF675,SMARCAD1,SETDB2,PRKCE,SLCO3A1,METAP1D,NXN,WNK2,USP33,CD44,PTPRO,EGF,ALPK3,PRRC1,TRIO,EXT1,LNPEP,SPRED2,RPS6KA3,MARCHF8,MTMR3,PTPN2,TRIM5,ATXN3,ST8SIA6,ALG10B,AMBRA1,STK38L,GALNT10,KDM7A,PRMT8,HTR2A,FANCM,FANCA,PPP2R2C,TAF3,RPRD1B,MARK2,C10ORF90,ABHD17C,HERC1,EPHA6,HIPK3,CDKN2C,GRK3,KNDC1,SPSB4,CLSPN,NOS2,STK10,MNAT1,TMTC2,MYLK2,HMGA2,CCND3,FOLH1,STK32A,LYPLA1,CWC27,PLCE1,TGFA,FUT9,PRR5L,GXYLT2,MSRA,FBXO32,LDLRAD4,EGFLAM,CNTN1,FKBP5,NAA35,BRCA2,GALNT13,BLM,ASB7,NRK,BMP2,RC3H2,MYLK4,TRAK1,CDC42BPB,VRK1,TPGS2,BMP2K,RNF38,RELN,GNAQ,FGF9,SH3BP5,UST,TRAF3,DSTYK,UIMC1,B4GALT6,TRIT1,SNX6,CNKSR3,CUL1,DAW1,NEK10,TDRD5,MOB1B,PIGN,ATF2,CYLD,ADARB2,MAPK8IP1,KITLG,ZZEF1,CAMTA1,UBR1,MAP4K3,RCAN1,TADA2A,DAB1,MED27,RB1CC1,MYO3A,UBE2E1,PTPRE,PRKN,MTMR2,SPSB1,CDC42BPA,MAPK10,ZNF541,FBXO3,WSB1,USP43,TRPM6,PRKCH,HUNK,IL6R,ALS2,MKNK1,SNX25,TOX,PTPRB,TRMT61B,COPS8,ST8SIA1,USP7,MOK,RALB,ROCK1,LYN,SUMO3,DTX1,CHKA,FANCL,RNF152,OTUD7A,INSR,CUL5,NEK6,HECTD1,HDAC11,LYPLAL1,SUMO2,ARFGEF1,SNAI2,ASH1L,SIAH2,PIGK,PGAP4,TRABD2B,ERN2,TRIM58,ZDHHC17,NSD2,PTAR1,CARD10,LTN1,ENPP1,ENTPD5,RASGRP1,SNX9,ANAPC1,CSNK2A1,BMP5,CSF1,PPIL6,EOGT,CTDP1,PRKG1,ASB4,FANCB,CLNS1A,SMAD5,MARCHF11,DCUN1D4,PRAME,KLHL7,NSUN6,ATG4B,CDC14B,KDM6A,GPRC5C,ROR2,PPP2R2A,BANK1,SFPQ,PRKAA2,CSF2RB,RNF182,PHF20L1,IQGAP1,CAMLG,NRXN1,HIPK1,FRY,FICD,CENPE,ELOC,TWIST1,AKT3,ALKAL2,JAK2,MPPE1,MADD,CREBBP,MRM1,TNKS,SIAH3,UFL1,PRKCB,FBXW2,ST6GAL2,RTRAF,BRD4,NEDD9,NRBP1,MAST2,PCMTD2,EXT2,PDP2,BRMS1L,NDFIP2,MAP2K6,MARCHF6,ABI1,CEMIP,IMPACT,PARK7,MAPK8,EIF3F,PPME1,FBXL17,UBE2J2,MTF2,NCAPG2,ASB2,MYOCD,UBE2QL1,ASCC2,ST8SIA4,MEF2C,ADGRB1,MAP3K5,NDFIP1,MAP3K4,TRIM43B,PRDM13,TRIM43,SUMF1,MAGEL2,PKN2,DBF4B,FBXW8,SDCBP,NSMCE1,PASK,MLLT1,NCK1,FGR,CDCA8,PPP2R3A,TRIM23,TOP1,RNF8,EPHA4,MECOM,DNMT3L,NTRK2,OCLN,SNRPD1,FBXO31,EXTL3,TRPM7,PRKAB1,PTK2,MARK4,CDH5,UBE3A,SEMA4D,KIRREL1,AMFR,POMT2,NOS1AP,MTTP,DPY19L2,TPTE,PDCL3,CCDC88A,NSUN2,GALNT18,TNFSF11,FYN,BUB1,KDM5A,DPY19L1,PPM1F,SDE2,UHRF2,HDAC2,SLF1,SH2D3C,DOCK3,GALNT17,MTMR7,ZFYVE28,MAPK9,STT3A,SLC39A8,ROR1,GALNT2,FUT8,TET1,ASB3,HECW2,EPHB1,ZDHHC18,GRM5,SPOPL,RPS6KA5,PTPRG,PID1,NRP1,MIDEAS,PRKCA,ATPSCKMT,RNF215,USP24,ITGA1,RNF138,RC3H1,POR,ZNF738,SUPT3H,BCR,SNRK,SENP8,USP49,ELP2,FBLN1,STK36,RAG1,BMPER,MACROH2A1,EPHB2,CSNK1G1,RNF11,DPH6,PPIL2,CDK14,MET,SPPL3,CAMK1G,METTL15,ATG5,USP32,MAGI2,MYB,KALRN,LAMA1,MFHAS1,TRIM9,BMP7,RNF217,KMT2C,PDGFC,ABL2,EYA1,TTLL11,SLIT2,PARP8,CNOT7,ESCO1,ERBB4,ROBO1,PRKCQ,MGMT,NOS1,EFNA5,NSD1,EHMT1,USP31,KDM4B,LOXL2,PRLR,PIGB,HTT,ZDHHC11B,CAMK1D,PIK3R3,MACROD2,CDKAL1,FER,EYA2,CHFR,PCMT1,OARD1,SPOCK3,ROCK2,PRDM1,ATAT1,PPP1CB,PDK1,PTPRQ,HERPUD1,NCOA6,TRIM2,WASHC1,BARD1,STK3,DEPTOR,RAB3GAP2,TULP4,IGF1R,PRKAG2,AKAP13,MORC3
GO:0051130	positive regulation of cellular component organization	1.422307576373 8924e-10	MTOR,ABCA13,PTPRD,PLCB1,RIPOR2,RP1,RALA,ROBO2,SPIRE1,TENM3,CDC42EP3,ALK,AUTS2,CARMIL1,ANO6,NEGR1,CNTNAP2,APC,PLPPR5,DSCAM,PTPRJ,ANGPT1,MACF1,NEK7,NSMCE2,BCL11A,CDH4,NTRK3,CRACD,PRKD1,PAK1,RAPGEF2,ADGRB3,ARSB,SEPTIN9,NEDD4L,ATF7IP,APP,STAU2,MAPRE2,SEMA5A,SYT1,NTF3,AURKA,PARN,FIG4,MAP4K4,RAB8B,PAK3,TRPC5,DNM3,NBN,COBL,MAPK1,ANKFY1,SPIDR,MYLK3,LIMCH1,FMN1,PAFAH1B1,VPS13D,TPM1,NF2,AKAP9,SNX30,SYNJ1,TIAM1,NAV3

			,BCAS3,TBC1D5,ATRX,ELAVL4,ABL1,HDAC4,RAP1A,GRID2,NRG1,INO80D,CLIP1,ABCB7,SETDB2,PRKCE,EGF,ATP8A2,PLXNA2,ARHGEF7,ATP8A1,AMBRA1,MARK2,LINGO2,PLCE1,TGFA,HIP1,FUT9,NPHP4,CNTN1,IQSEC1,SNX3,DISC1,BMP2,RESF1,RELN,NFATC2,UNC13B,NIN,ATF1,BBS4,PRKN,FRMPD4,CNOT6L,TOX,RALB,ROCK1,LYN,TENM2,NTN1,INSR,BMF,DDHD1,PDE4DIP,BID,TRABD2B,TRIM58,FYCO1,SH3GLB1,SNX9,BMP5,INO80,TNN,ROR2,ACTR2,SFPQ,CLSTN2,MAP6,VASP,MORC2,NRXN1,ANLN,ALKAL2,MELTF,TNKS,ABCC8,NEDD9,ITGA6,GRIP1,PARK7,MAPK8,CEP120,ADGRB1,WNT7A,MAP3K4,WASF3,MAGEL2,RAD51AP1,FBXW8,SDCBP,NCK1,EPHA4,NTRK2,IL1RAPL1,OCLN,FBXO31,MARK4,CDH5,MPP7,CYFIP1,SEMA4D,RUNX1,KIRREL1,SAXO1,ASAP1,CCDC88A,BICD1,FYN,PPM1F,ADGRL2,SLF1,MAPK9,APELA,ROR1,EPHB1,ADCK1,NRP1,FCHSD2,CHODL,CUX1,MACROH2A1,EPHB2,TOGARAM1,MET,CDH17,MAGI2,FLRT2,KALRN,TIAM2,BMP7,DLG5,ABL2,SLIT2,SYNDIG1,ROBO1,PRKCQ,NLGN1,ASIC2,EFNA5,ESR1,HTT,CAMK1D,FER,EPS8,ROCK2,STMP1,ATAT1,DMRT1,RAB31,WASHC1,RAB3GAP2,IGF1R,ATP10A,DNM1L
GO:0050807	regulation of synapse organization	1.502939495876 7202e-10	PTPRD,IL1RAPL2,LRFN2,CDH8,ROBO2,NEGR1,GPC6,NEDD4,NTRK3,EPHA7,ADGRB3,APP,STAU2,ARHGAP44,CTNNA2,PAK3,DNM3,PAFAH1B1,TANC1,ABL1,LRFN5,GRID2,MUSK,DGKB,PTPRO,ABHD17C,LINGO2,PDLIM5,DISC1,RELN,FARP1,NGEF,FRMPD4,SHANK2,NTN1,COLQ,TANC2,GRIN2B,IL10,ACTR2,CLSTN2,NRXN1,NEDD9,MEF2C,ADGRB1,WNT7A,NECTIN1,EPHA4,NTRK2,IL1RAPL1,CYFIP1,UBE3A,PCDH8,SEMA4D,FYN,ADGRL2,CDH2,EPHB1,EPHB2,PPFIA2,FLRT2,KALRN,DLG5,SYNDIG1,NLGN1,CTTNBP2,ASIC2,EFNA5,C1QL3
GO:0030029	actin filament-based process	3.115429395493 357e-10	NOTCH2,MTOR,SGCD,NEBL,SVIL,MICAL3,DLC1,PDE4D,RDX,RALA,BCL2,MYO5A,ARHGAP26,SPIRE1,FGD4,MYO1E,CDC42EP3,AUTS2,CARMIL1,RHPN2,PARVB,MYO5C,TNIK,CTNNA3,DOCK2,DIAPH3,NTRK3,PHACTR1,CRACD,PAK1,FGF12,EPB41L3,NEDD4L,PHACTR2,CACNA1C,CACNB2,STAU2,SEMA5A,ARHGAP44,NTF3,CD2AP,FRMD3,KANK1,KCNE4,FMN2,THSD7A,CTNNA2,PAK3,RAP1GDS1,CALD1,KLHL1,MRTFA,COBL,ARHGEF17,CORO2B,MYLK3,LIMCH1,FMN1,PAFAH1B1,TPM1,NF2,CTNNA1,PPP1R9A,AKAP9,MPRIP,ENAH,ANK2,BCAS3,RYR2,SYNE2,AIF1L,LDB3,CXADR,XIRP2,ABL1,GAS2,FGF10,PRKCE,PGM5,ABCC9,ATXN3,SEMA3E,TMOD2,SH3KBP1,MYLK2,MYOM2,HIP1,TJP1,NPHP4,PACSIN2,IQSEC1,PDLIM5,NRK,PHACTR3,CDC42BPB,PLS1,SRGAP2,MYL1,FARP1,BBS4,THSD7B,PRKN,CDC42BPA,PCDH15,FRMPD4,RACGAP1,KANK4,KCND3,MYO1D,ROCK1,ARHGAP28,SHROOM3,ARFGEF1,FLNB,UTRN,SNX9,PRKG1,MICALL2,FAT1,ACTR2,VASP,IQGAP1,ANLN,JAK2,SMTN,USH1C,NEDD9,ARFGEF3,SHROOM2,MTPN,ABI1,ASB2,HMCN1,CYFIP2,WASF3,MAGEL2,SDCBP,NCK1,AKAP11,TRPM7,ARHGAP12,DIAPH1,CYFIP1,FRMD6,KIRREL1,PSTPIP2,NOS1AP,SORBS2,PDCL3,CCDC88A,PPM1F,EHBP1,SPTB,NRP1,FCHSD2,BCR,ELMO1,MYO5B,MET,SPECC1,NRAP,FAM171A1,EPB41L4A,ABL2,FHOD3,SLIT2,ANTXR1,EFNA5,GAS2L1,ARHGEF11,FRMD5,MYO9B,CACNA2D1,FER,STARD13,EPS8,ROCK2,WASHC1,AKAP13
GO:0007267	cell-cell signaling	3.278886830060 6663e-10	CNTN4,NSG1,PTPRD,SLC24A2,LRRC4C,NLK,UNC13C,PLCB1,PTPRA,STXBP1,ERC1,LRFN2,CDH8,CHRNA7,RIMS1,GABRB3,ENPEP,RIMS2,MCTP1,SV2C,CACNG2,DLGAP1,MLLT3,GPC6,APC,TSHZ3,CRKL,ILDR2,TNIK,SLC4A10,EGFR,RFX3,USP14,MACF1,RNF220,CRB1,BTBD9,GRIK3,DKK2,GABRB1,DGKI,INVS,GRIA1,SLC8A3,CHRM3,RAPGEF2,FGF12,GABRA6,PRICKLE2,PTPRN2,SYN2,GRM7,ADAM10,APP,RPS6KA2,CACNA1C,CACNB2,STAU2,GABRG2,SEMA5A,SYT1,NTF3,NDUFAF2,PYGO1,KANK1,RAB8B,ERC2,SYN3,PRKCZ,GRB10,HECW1,SV2B,YAP1,MAPK1,CADPS2,HRH2,PLG,SYT10,ZNRF3,NRG3,SLC16A1,HRH4,SORCS3,PAFAH1B1,TM7SF3,ITGB8,GRIK4,PPP1R9A,AKAP9,KLF15,RASGRF2,SYNJ1,TIAM1,GRM1,GABRG1,PCDH11Y,TMEM108,ALPK2,AMPH,CPE,ANK2,RYR2,WNT9B,SMARCA4,CDH11,USP8,BLK,TNR,GRM8,CXADR,ELAVL4,ABL1,SLC1A1,PRKAA1,RIMBP2,EIPR1,RAP1A,GPC5,FGF10,GRID2,LATS2,NRG1,ASPM,RASGRF1,PRKCE,NXN,WNK2,DGKB,PTPRO,EGF,P2RX6,EXT1,LNPEP,LIMD1,RPS6KA3,CTN

			ND2,SCG5,ATXN3,HTR2C,RIC3,ARHGEF7,LTBP1,OPRM1,ABCC4,HTR2A,KREMEN1,MARK2,TMOD2,APBA2,SH3KBP1,KCND2,NOS2,MDFIC,MYLK2,HMGA2,MBP,NPHP4,PACSIN2,SNX3,DISC1,STRN,BMP2,PSG9,MYRIP,RELN,GNAQ,FGF9,UNC13B,TTC21B,DRAXIN,CCDC186,GLI3,CGAS,GABRR2,GRIK2,MCTP2,FARP1,CYLD,GABBR2,CFTR,KPNA1,ZZEF1,DCC,CHRM5,PRKN,MTMR2,DLGAP2,GRIN2A,LALBA,ALS2,KCNQ3,SHISA9,SCN10A,SHANK2,MESD,SOX30,LYN,ZBTB33,PLCB4,GRID1,COLQ,NMU,SNAI2,SIAH2,RPH3A,TRABD2B,GABRG3,IGSF11,NDRG2,CSNK2A1,GHRH,HCN1,GRIN2B,GPR156,CELF4,TNN,ROR2,FAT1,CLSTN2,PTH,SOSTDC1,PRKAA2,PLA2G4A,RPS12,AIMP1,NRXN1,CD70,JAK2,HCRTR1,CELSR2,TNKS,PRKCB,ABCC8,CACNA1E,SLC6A1,GID8,MAP2K6,PARK7,RAPGEF4,MEF2C,ADGRB1,WNT7A,S100B,ATP6V1C2,OR10H2,SDCBP,WWOX,PASK,PPP2R3A,SNAP29,EPHA4,GABRA5,NTRK2,IL1RAPL1,WNT2B,SHISA6,GRIK1,ANKRD6,SCGN,CYFIP1,PCDH8,WNT5B,AMFR,ICA1,CCDC88A,TNFSF11,FYN,RBMS3,ROR1,CDH2,FBXL20,EPHB1,GRM5,TBC1D1,NRP1,FCHSD2,RNF138,MCC,BCR,NRXN3,PRDM15,MITF,EPHB2,CSNK1G1,CD38,CDK14,DLG2,STXBP4,CACNG3,MAGI2,KALRN,SLC1A2,GABRA2,TMEM25,GRM3,EXOC4,PLCL1,FAM3B,TRHDE,SORCS2,NLGN1,NOS1,SLC6A3,ASIC2,EFNA5,DTNA,NTNG1,CACNA2D1,CCR2,HRH1,RGS8,STK3,ZNF423,CADPS,APCDD1,GLI2
GO:0019538	protein metabolic process	3.380785071000752e-10	BCAR3,MTOR,SPOCK1,NSG1,IMMP2L,PTPRD,TMTC1,ULK2,NLK,LONP2,FTO,KSR1,AGBL1,PLCB1,TTC3,TMPRSS2,DLC1,TNRC6B,DPP10,ZDHHC21,PTPRA,PDE4D,RDX,ERC1,BCL2,PRDM16,F13A1,GPHN,CHRNA7,PIK3C3,EPC2,SPRED1,GALNT1,ENPEP,MINAR1,PCMTD1,ALK,AUTS2,PJA2,BABAM2,PAPPA2,HLCS,MLLT3,EGLN3,MAP3K9,MYO3B,MOCOS,SPON1,CPA6,APC,RTN1,CRKL,SETD2,ERG,TNIK,PTPRJ,KDM4C,NEK4,EGFR,USP14,ANGPT1,CDK12,PRKACB,NEK7,RNF220,NEDD4,MTRF1,GNPTAB,NSMCE2,BCL11A,PSMB2,CHSY1,RPRD1A,PTPN4,B3GALT5,NTRK3,LARGE1,C5,TUSC3,FBXL7,FLT1,ADAMTS6,TASP1,PSMA8,CAST,SLC8A3,PRKD1,TPTE2,PAK1,EPHA7,NCOA7,CHRM3,CHSY3,RAPGEF2,PELI2,LRP2,TAOK3,LDLRAD3,CPEB4,UBE2L3,PPP2R2B,PUM3,PTPRN2,SMYD3,HERC2,RPTOR,GHR,WDSUB1,NEDD4L,ADAM32,ADAM10,HDAC9,UBE2G1,APP,RPS6KA2,SAMSN1,KDM1B,KLHL13,PHKB,DCLK1,USP18,NTF3,ACER2,PARP15,CD2AP,AURKA,PARN,ST18,SLC8A1,PTPRR,MARCHF1,SERPINA6,PLGRKT,ECPAS,MAP4K4,BMPR1B,FMN2,PCSK6,ARNT,PAK3,TTLL7,DIP2B,RANBP2,LARP1,ITPKB,TRPC5,UBE2E2,RAP1GDS1,HHAT,CHST8,NBN,IFT57,PRKCZ,SPOP,MAN2A2,DIP2A,ST8SIA5,HECW1,ADAMTS17,PHF19,TAF4B,SENP6,DUSP22,GALNT14,SEM1,WDR70,PPM1L,RIPK4,MAPK1,MGAT5,ITIH5,SGTB,ADAM22,USP25,KMT2E,PLG,PAPPA,PCGF5,PDGFD,ZNRF3,XXYLT1,NRG3,UBE2O,GFRA1,NIPBL,GALNT16,RNGTT,CARD18,STK38,PTPN13,MYLK3,KANSL1,LIMCH1,ATF6,EFEMP1,TLL1,DCAF1,CCNG2,TLK1,NF2,MRPS22,ZDHHC14,CORIN,MOB3B,BIRC6,AKAP9,KLF15,PPARA,ERMP1,PPP6R3,ADAMTS3,UBE3D,RSRC1,PTPRK,PAK5,ST6GALNAC3,TRERF1,PPP2R5E,PDZRN3,EIF3D,DAPK1,AGO2,STK32B,ALPK2,JARID2,GATAD2B,CPE,IL34,ADGRV1,MELK,WNT9B,HECTD4,CLPX,DUSP16,MRPS35,USP8,PARD3,MAPKAP1,TNRC6C,PIAS1,UBE2R2,BLK,ATRX,NUAK1,PTPRT,ELAVL4,ABL1,PTPN12,HDAC4,OXR1,SLC1A1,PRKAA1,MRPS27,TTLL5,EIPR1,ADAMTS14,MAST4,PSMF1,ATE1,RAP1A,HECTD2,CAMK4,BAZ2A,MANBA,FGF10,FBXL13,TGM1,PEAK1,LATS2,NRG1,AP3B1,ZBTB16,MUSK,GALNTL6,ZNF675,SMARCAD1,SETDB2,PRKCE,SLCO3A1,METAP1D,NXN,WNK2,USP33,CD44,PTPRO,EGF,ALPK3,PRRC1,TRIO,EXT1,LNPEP,SPRED2,ADAMTS2,RPS6KA3,MARCHF8,SCG5,MTMR3,PTPN2,TRIM5,ATXN3,ST8SIA6,ALG10B,AMBRA1,STK38L,GALNT10,KDM7A,PRMT8,HTR2A,FANCM,FANCA,DAZL,FARS2,PPP2R2C,TAF3,RPRD1B,MARK2,TMEM67,C10ORF90,ABHD17C,PUM1,HERC1,IGF2BP3,EPHA6,SLC2A13,HIPK3,CDKN2C,GRK3,CPXM2,KNDC1,SPSB4,CLSPN,NOS2,AFG3L2,STK10,MNAT1,TMTC2,ADAM12,MYLK2,XYLT1,HMGA2,CCND3,FOLH1,ECE1,STK32A,LYPLA1,MBP,CWC27,PLCE1,TGFA,IL17RA,HIP1,CRIM1,XPNPEP1,FUT9,PRR5L,GXYLT2,VPS37A,CAPN5,MSRA,FBXO32,LDLRAD4,EGFLAM,CNTN

			1,TARS3,FKBP5,SNX3,NAA35,BRCA2,DISC1,GALNT13,DNER,BLM,ASB7,NRK,BMP2,RC3H2,MYLK4,TRAK1,WDR26,CDC42BPB,DSE,VRK1,RANBP9,TPGS2,BMP2K,RNF38,PGPEP1,RELN,HMGB1,GNAQ,FGF9,SH3BP5,UST,CPAMD8,TRAF3,GEMIN5,DSTYK,UIMC1,B4GALT6,GLI3,SMARCC1,SNX6,CNKSR3,CASP5,IDE,CUL1,DAW1,NEK10,RRBP1,MOB1B,PIGN,ATF2,CYLD,UMODL1,MAPK8IP1,NELL1,MRPL13,KITLG,ZZEF1,CAMTA1,UBR1,MAP4K3,HS3ST4,RCAN1,TADA2A,DAB1,MED27,RB1CC1,MYO3A,UBE2E1,PTPRE,PRKN,MTMR2,SPSB1,CDC42BPA,MAPK10,ZNF541,FBXO3,GRIN2A,WSB1,USP43,TRPM6,PRKCH,HUNK,IL6R,PEPD,ALS2,CPVL,ACO1,CNOT6L,MKNK1,SNX25,PTPRB,AOPEP,COPS8,TSPAN33,ST8SIA1,USP7,PSMA1,MOK,RALB,ROCK1,LYN,SEL1L,CTSB,EIF2B3,SUMO3,DTX1,BZW1,PIWIL3,CHKA,MMP16,FANCL,BANP,RNF152,OTUD7A,INSR,CUL5,YTHDF3,NEK6,HECTD1,HDAC11,LYPLAL1,SUMO2,ADAMTS19,ARFGEF1,SNAI2,ASH1L,BID,SIAH2,PIGK,PGAP4,TRABD2B,UFD1,ERN2,MBTPS2,TRIM58,ZDHHC17,NSD2,PTAR1,CARD10,LTN1,CTIF,ENPP1,ENTPD5,MOCS2,RASGRP1,SNX9,PAMR1,ANAPC1,CSNK2A1,BMP5,CSF1,PPIL6,EOGT,SERPINB9,CTDP1,PRKG1,HS6ST3,ASB4,GRIN2B,FANCB,CLNS1A,CNMD,SMAD5,CELF4,ABCG1,MARCHF11,DCUN1D4,PRAME,KLHL7,PSMA5,ATG4B,CDC14B,KDM6A,IL33,GPRC5C,ROR2,CFH,PPP2R2A,BANK1,CSDE1,IL10,SFPQ,PRKAA2,CSF2RB,RNF182,LARP6,PHF20L1,IQGAP1,RPS12,CAMLG,ANP32B,YBX3,AIMP1,NRXN1,PCID2,HIPK1,FRY,FICD,CENPE,NGDN,ELOC,TWIST1,AKT3,ALKAL2,JAK2,ADAM28,MPPE1,MADD,PATL1,PRSS2,CREBBP,MELTF,TNKS,SIAH3,UFL1,ADAMTS5,NFKBIA,PRKCB,FBXW2,MIPEP,OVCH1,ZC3H15,ST6GAL2,RTRAF,BRD4,NEDD9,NRBP1,IARS2,CLCA4,CNDP2,MAST2,ERLIN2,PCMTD2,EXT2,AGO1,PDP2,GID8,BRMS1L,NDFIP2,MAP2K6,MARCHF6,MTPN,ABI1,CEMIP,IMPACT,CCBE1,PARK7,ADAMTS18,MAPK8,OAZ2,EIF3F,PPME1,FBXL17,UBL7,UBE2J2,MTF2,NCAPG2,ASB2,MYOCD,CYFIP2,UBE2QL1,ACACA,ASCC2,ST8SIA4,MEF2C,ADGRB1,WNT7A,MAP3K5,NDFIP1,MAP3K4,TRIM43B,SERPINI2,PRDM13,TRIM43,SUMF1,MAGEL2,PKN2,DBF4B,FBXW8,SDCBP,SPPL2B,NSMCE1,PASK,MLLT1,NCK1,FGR,CDCA8,PPP2R3A,TRIM23,TOP1,TINAG,C2,RNF8,EPHA4,MECOM,NTRK2,ADAMTS9,OCLN,FBXO31,EXTL3,TRPM7,PRKAB1,IREB2,MVB12B,HS6ST1,PTK2,MARK4,CDH5,CD5L,APOL2,CYFIP1,UBE3A,APOL1,SEMA4D,SERPINB10,RUNX1,KIRREL1,AMFR,CTSE,POMT2,NOS1AP,MTTP,DPY19L2,TPTE,PDCL3,SRP9,CCDC88A,UBAP1L,GALNT18,ADAMTS16,TNFSF11,FYN,BUB1,KDM5A,DPY19L1,PPM1F,SDE2,UHRF2,HDAC2,SLF1,SH2D3C,PSME3IP1,DOCK3,TRNAU1AP,GALNT17,MTMR7,ZFYVE28,MAPK9,PABPC1,STT3A,SLC39A8,ROR1,GALNT2,FUT8,TET1,ASB3,HECW2,OVCH2,SEL1L2,FBXL20,EPHB1,ZDHHC18,GRM5,SPOPL,RPS6KA5,PTPRG,PID1,NRP1,MIDEAS,PRKCA,MRPL58,ATPSCKMT,RNF215,USP24,FHIT,ITGA1,RNF138,RC3H1,POR,EFL1,ZNF738,SUPT3H,BCR,TUT4,SNRK,TM9SF2,SENP8,USP49,ELP2,FBLN1,STK36,RAG1,DNPEP,BMPER,DPP6,MACROH2A1,EPHB2,CSNK1G1,BCL2L13,RNF11,DPH6,PPIL2,PRSS51,CDK14,MET,SPPL3,CAMK1G,SERPINB2,ATG5,USP32,MAGI2,UNK,ADAM29,MYB,KALRN,CHST3,LAMA1,MFHAS1,SERPINB7,CPQ,TRIM9,DHX29,BMP7,TMPRSS15,TNFAIP8,RNF217,PRSS23,KMT2C,DDX6,PDGFC,ABL2,MMP26,MRPL37,BACE2,NECAB1,EYA1,TTLL11,SLIT2,PARP8,TMPRSS3,CNOT7,ESCO1,ERBB4,SERPINB11,GSAP,TRHDE,ROBO1,SAMD4A,PRKCQ,ANTXR1,MGMT,NOS1,PRR16,EFNA5,NSD1,EHMT1,USP31,KDM4B,LOXL2,PRLR,PIGB,AGO3,HTT,LARS2,ZDHHC11B,CAMK1D,PIK3R3,MACROD2,CDKAL1,FER,EYA2,A2M,CHFR,PCMT1,OARD1,SPOCK3,ROCK2,PRDM1,ATAT1,NARS2,EIF4G3,PPP1CB,PDK1,PSMD2,PTPRQ,HERPUD1,NCOA6,TRIM2,COL4A3,WASHC1,PCSK2,BARD1,STK3,DEPTOR,HNRNPU,LINC00240,RAB3GAP2,TULP4,IGF1R,PRKAG2,AKAP13,MORC3
GO:1902531	regulation of intracellular signal	5.138487690619894e-10	NOTCH2,BCAR3,MTOR,WWC1,GARNL3,MYO9A,KSR1,PLCB1,DLC1,RIPOR2,PDE4D,RDX,BCL2,ARHGAP26,CHRNA7,AKR1C3,FGD4,SPRED1,MINAR1,ALK,AUTS2,PJA2,BABAM2,ERBIN,CRKL,ARHGAP24,TNIK,PTPRJ,EGFR,DENND1A,ANGPT1,NCOR1,DOCK2,NEDD4,SCAI,SGMS1,NTRK3,ZFAND6,FLT1,MAPKBP1,DGKI,EDAR,P

	transduction		*RKD1,TPTE2,PAK1,EPHA7,RALGPS1,RAPGEF2,PELI2,LRP2,TAOK3,RPTOR,GHR,RALGAPA1,APP,DOCK8,MAPRE2,SEMA5A,ARHGAP44,NTF3,CD2AP,AURKA,PTPRR,KANK1,MAP4K4,AKAP6,HOMER2,PAK3,ITPKB,PDE10A,RAP1GDS1,KICS2,PRKCZ,ARHGAP32,DUSP22,MAPK1,PDGFD,ARHGEF17,STK38,PTPN13,CHN1,HRH4,PAFAH1B1,NF2,MOB3B,RASGRF2,PPARA,TIAM1,GRM1,ARHGEF12,PAK5,PLA2R1,SLC24A4,SEC14L1,IL34,ADGRV1,DUSP16,MAPKAP1,NUAK1,ABL1,PRKAA1,NFAT5,GUCY1A2,RAP1A,FGF10,ZC3HAV1,NRG1,DENND2B,RASGRF1,ZNF675,PRKCE,WNK2,DENND4C,CD44,EGF,TRIO,PDE3A,LIMD1,SPRED2,PTPN2,TRIM5,MCF2L,HTR2C,CLEC16A,OPRM1,HTR2A,SEMA3E,FHL2,PUM1,ANKRD17,RELL1,HIPK3,MDFIC,PLCE1,TGFA,HIP1,PRR5L,VAV1,IQSEC1,BRCA2,NRK,SEMA3A,MAGI3,BMP2,RC3H2,GNAI1,RALGAPA2,RANBP9,TMEM161A,LEMD3,RELN,ARHGAP42,HMGB1,TRAF3,DSTYK,RAP1GAP,SRGAP2,SLAMF1,CNKSR3,PSD3,GAREM1,NEK10,CYLD,MAPK8IP1,KITLG,CAMTA1,UBR1,SLC30A10,RCAN1,RB1CC1,PRKN,NGEF,IL6R,ALS2,RACGAP1,SHANK2,USP7,VAV3,ARHGEF28,ROCK1,LYN,ARHGAP28,ARHGAP31,SLC44A2,SLC15A2,RRAGD,CRACR2A,RNF152,OTUD7A,INSR,NEK6,ARFGEF1,SNAI2,ASH1L,BID,UFD1,ERN2,TIAL1,ZDHHC17,RALGPS2,JCAD,RASGRP1,NDRG2,CSF1,BCL2L1,ROR2,KL,BANK1,SFPQ,PRKAA2,NDC80,IQGAP1,YBX3,NRXN1,PCID2,NET1,SIPA1L2,TWIST1,AKT3,ALKAL2,JAK2,RPF2,MADD,HCRTR1,UFL1,NFKBIA,PRKCB,BRD4,ARFGEF3,STAT1,NDFIP2,MAP2K6,DGKG,PARK7,ADCYAP1R1,EFHB,MEF2C,WNT7A,MAP3K5,NDFIP1,MAP3K4,S100B,C16ORF72,PDE2A,SDCBP,JPT2,NCK1,FGR,CDCA8,DNMBP,EPHA4,CYTH4,MECOM,NTRK2,PTK2,ANKRD6,ARHGAP12,APIP,UBE3A,SEMA4D,NENF,NOS1AP,TPTE,GPR55,TNFSF11,FYN,DOCK3,DOK5,APELA,ROR1,CDH2,RAD9A,PHLPP1,GPR137B,EPHB1,GRM5,RAI14,NRP1,PRKCA,ITGA1,RC3H1,BCR,FBLN1,BMPER,PRDM15,SRGAP3,EPHB2,MET,SPPL3,CDH13,MAGI2,PRDM11,KALRN,GNAS,MFHAS1,TIAM2,BMP7,DLG5,ZMYND11,PDGFC,ABL2,SLIT2,ERBB4,ROBO1,SIPA1L3,NLGN1,ARHGEF11,ESR1,MYO9B,IQCJ-SCHIP1,AGO3,HTT,STARD13,EPS8,ROCK2,RORA,HERPUD1,BARD1,STK3,DEPTOR,IGF1R,AKAP13,DNM1L*
GO:0007264	small GTPase mediated signal transduction	5.990016607076961e-10	*NOTCH2,BCAR3,GARNL3,MYO9A,KSR1,DLC1,RIPOR2,RDX,RALA,ARHGAP26,FGD4,CDC42EP3,AUTS2,ERBIN,RIN2,CRKL,ARHGAP24,DOCK10,DENND1A,DOCK2,SCAI,DGKI,PRKD1,CTNNAL1,RALGPS1,RAPGEF2,RALGAPA1,RAPGEF5,DOCK8,MAPRE2,ARHGAP44,CD2AP,KANK1,MAP4K4,ITPKB,ARHGAP32,ARHGEF17,CHN1,RASGRF2,RGL1,TIAM1,ARHGEF12,USP8,MAPKAP1,DOCK4,ABL1,RAP1A,FGF10,NRG1,RASGRF1,DENND4C,TRIO,MCF2L,KNDC1,DOCK5,PLCE1,VAV1,IQSEC1,RALGAPA2,RELN,ARHGAP42,DOCK1,RAP1GAP,SRGAP2,PSD3,KITLG,DAB1,NGEF,ALS2,RACGAP1,DOCK9,VAV3,ARHGEF28,RALB,ROCK1,ARHGAP28,ARHGAP31,NTN1,ARFGEF1,SIAH2,RALGPS2,RASGRP1,CSF1,RASGEF1C,PTH,RAB12,NET1,SIPA1L2,MADD,ARFGEF3,PARK7,ADCYAP1R1,RAPGEF4,RAB38,SDCBP,DNMBP,CYTH4,ARHGAP12,CYFIP1,RASGEF1B,CNKSR1,CCDC88A,GPR55,SH2D3C,DOCK3,NRP1,BCR,ELMO1,RERG,RRAS2,SRGAP3,EPHB2,MET,CDH13,KALRN,TIAM2,ABL2,SLIT2,ROBO1,SIPA1L3,ARHGEF11,MYO9B,STARD13,EPS8,ROCK2,AKAP13*
GO:0048813	dendrite morphogenesis	6.092293932042417e-10	*PTPRD,CHRNA7,DSCAM,TNIK,DOCK10,NEDD4,PHACTR1,RAPGEF2,ADGRB3,NEDD4L,DCLK1,STAU2,ARHGAP44,CTNNA2,PAK3,TRPC5,DNM3,DIP2A,HECW1,PAFAH1B1,ELAVL4,SDC2,CTNND2,KNDC1,PDLIM5,SEMA3A,RELN,RERE,MAP2,FARP1,NGEF,DPYSL5,TANC2,ACTR2,MAP6,CELSR2,ABI1,WNT7A,FBXW8,EPHA4,IL1RAPL1,FBXO31,UBE3A,SEMA4D,FYN,HECW2,EPHB1,NRP1,CUX1,EPHB2,PPFIA2,KALRN,NLGN1*
GO:0007417	central nervous system development	6.486077425849245e-10	*MTOR,CNTN4,SPOCK1,IMMP2L,TRAPPC9,PLCB1,TENM4,DLC1,IL1RAPL2,BCL2,ODAD2,ALDH1A2,ROBO2,ZEB1,RARB,NAV2,ALK,NEGR1,CNTNAP2,CRKL,SETD2,SLC4A10,EGFR,SOX6,ATP2B2,NTRK3,PHACTR1,GABRB1,CNTN6,SLC8A3,EPHA7,RAPGEF2,LRP2,ARSB,SSBP3,APP,DCLK1,SEMA5A,SYT1,SLC8A1,SRGAP2C,BMPR1B,CTNNA2,CHST8,SNTG2,KLHL1,MCPH1,ZSWIM6,MAPK1,ADAM22,NRG3,NIPBL,PAFAH1B1,NF2,CTNNA1,MEIS2,NFIB,SYN*

			J1,TMEM108,JARID2,IL34,SYNE2,BBS2,WNT9B,SEMA6D,CDH11,MAPKAP1,TNR,ATRX,ELAVL4,ABL1,SLC1A1,DNAH5,FGF10,GRID2,NRG1,ASPM,ZBTB16,SH3GL3,EGF,TRIO,EXT1,RPS6KA3,PLXNA2,KDM7A,SEMA3E,HERC1,CDKN2C,KNDC1,MNAT1,BCL11B,MBP,AK8,CNTN1,BRCA2,DISC1,DNER,SEMA3A,BMP2,RELN,FGF9,TTC21B,B4GALT6,TSPAN2,SRGAP2,NIN,DRAXIN,GLI3,CASP5,RERE,MAP2,ATF2,BBS4,LAMC3,DCC,DAB1,PRKN,TBX20,GRIN2A,ATXN1,TOX,SHANK2,NPAS2,LYN,EIF2B3,ZFHX3,HDAC11,PBX3,NDRG2,BMP5,KCNC1,GHRH,PRKG1,GRIN2B,SYNJ2,MED1,ATRN,IL33,ROR2,LMX1A,TACC2,ANP32B,NRXN1,CADM1,AKT3,CELSR2,ATP2B1,GAP43,EML1,PPP1R17,SHROOM2,SLC6A11,MTPN,POU6F2,POU1F1,FOXP2,CEP120,WNT7A,WASF3,S100B,EPHA4,GABRA5,NTRK2,NUMB,WNT2B,GRIK1,LAMB1,UBE3A,FAT4,FYN,ADGRL2,ARL13B,HYDIN,HDAC2,ATP5PF,UGP2,MDGA2,ROR1,ARNT2,CDH2,CNTN5,ITGA8,XRN2,EPHB1,PTPRG,NRP1,BCR,STK36,EPHB2,KALRN,SLC1A2,CA10,BMP7,DLG5,KIRREL3,BTD,BPTF,PDGFC,SLIT2,CCDC141,ERBB4,ROBO1,PBX1,CTTNBP2,SLC6A3,ASIC2,VCAN,KDM4B,FOXB1,MACROD2,RORA,ATAT1,HSPG2,NCOA6,RGS7,HOOK3,STK3,ZNF423,IGF1R,GLI2
GO:0050803	regulation of synapse structure or activity	6.949480550521 53e-10	*PTPRD,IL1RAPL2,LRFN2,CDH8,ROBO2,NEGR1,GPC6,NEDD4,NTRK3,EPHA7,ADGRB3,APP,STAU2,ARHGAP44,CTNNA2,PAK3,DNM3,PAFAH1B1,TANC1,ABL1,LRFN5,GRID2,MUSK,DGKB,PTPRO,ABHD17C,LINGO2,PDLIM5,DISC1,RELN,FARP1,NGEF,FRMPD4,SHANK2,NTN1,COLQ,TANC2,GRIN2B,IL10,ACTR2,CLSTN2,NRXN1,NEDD9,MEF2C,ADGRB1,WNT7A,NECTIN1,EPHA4,NTRK2,IL1RAPL1,CYFIP1,UBE3A,PCDH8,SEMA4D,FYN,ADGRL2,CDH2,EPHB1,EPHB2,PPFIA2,FLRT2,KALRN,DLG5,SYNDIG1,NLGN1,CTTNBP2,ASIC2,EFNA5,C1QL3*
GO:0007166	cell surface receptor signaling pathway	8.575824079632 514e-10	*NOTCH2,BCAR3,PTPRD,ANKS1B,NLK,PLCB1,PTPRA,PDE4D,IL1RAPL2,BCL2,PRDM16,FBN1,CHRNA7,ROBO2,RIMS1,ZEB1,SPRED1,MYO1E,RIMS2,ALK,ADGRE1,ERBIN,ANO6,MLLT3,GPC6,APC,HHLA2,DSCAM,CRKL,TNIK,PTPRJ,EGFR,ANGPT1,MACF1,PRKACB,RNF220,NEDD4,MAML2,GRIK3,CHSY1,NTRK3,C5,DKK2,FLT1,DGKI,INVS,GRIA1,NEO1,CNTN6,SLC8A3,PRKD1,PAK1,GMDS,EPHA7,SPEN,RAPGEF2,PELI2,LRP2,ADGRB3,RUNX2,FGF12,ONECUT1,CPEB4,PRICKLE2,BTBD11,GRM7,GHR,COL4A2,TRPM1,ADAM10,IL1R1,APP,USP18,SEMA5A,NTF3,ST18,PYGO1,PTPRR,KANK1,BMPR1B,PCSK6,HOMER2,ARNT,PAK3,RFTN1,ITPKB,HHAT,IFT57,PRKCZ,BTLA,GRB10,HECW1,DUSP22,YAP1,PPM1L,SHC4,MAPK1,MGAT5,PDGFD,ZNRF3,ITGBL1,NRG3,UBE2O,NCAM1,GFRA1,CHN1,GLP2R,PAFAH1B1,EFEMP1,ITGB8,NF2,GRIK4,HIVEP1,CTNNA1,BIRC6,KLF15,PPARA,ADAMTS3,TIAM1,GRM1,PTPRK,PAK5,PCDH11Y,SEMA3C,DAPK1,TMEM108,MAGI1,ALPK2,CPE,EVC2,IL34,ADGRV1,BBS2,WNT9B,SEMA6D,SMARCA4,USP8,PIAS1,SPG21,BLK,GRM8,DST,MBD5,PTPRT,ABL1,PTPN12,SLC1A1,PRKAA1,GAS2,RAP1A,GPC5,FGF10,GRID2,LATS2,NRG1,ASPM,MUSK,ZNF675,PRKCE,NXN,WNK2,FBN2,CD44,PTPRO,EGF,P2RX6,TRIO,EXT1,LNPEP,LIMD1,SPRED2,CTNND2,PTPN2,TRIM5,PLXNA2,ARHGEF7,LTBP1,ZFYVE9,OPRM1,FANCA,KREMEN1,SEMA3E,MARK2,GCSAML,ADGRA3,EPHA6,EPN2,EVC,MOSMO,GFRA2,MDFIC,ADAM12,CCND3,ECE1,PLCE1,TGFA,IL17RA,HIP1,CRIM1,VAV1,LDLRAD4,NPHP4,CNTN1,SNX3,DISC1,DNER,WDPCP,SEMA3A,STRN,BMP2,RC3H2,UNC5D,PSG9,SOGA1,RANBP9,BMP2K,SEMA3D,CABIN1,LEMD3,RELN,GNAQ,FGF9,NFATC2,ZNF106,TRAF3,UNC13B,TTC21B,DSTYK,DOCK1,DRAXIN,SLAMF1,FAM83B,GLI3,SMARCC1,SNX6,SMOC2,GRIK2,IDE,WDR12,GAREM1,LAMC1,ATF2,CYLD,BBS4,MX1,ITGA9,KPNA1,UBASH3A,RGMB,NEU3,KITLG,DCC,SLC30A10,DAB1,RB1CC1,PTPRE,PRKN,MTMR2,TBX20,MAPK10,NGEF,GRIN2A,ARID5B,PRKCH,IL6R,NLRC5,SNX25,VAV3,MESD,SOX30,ARHGEF28,ADGRG6,LYN,SEL1L,EIF2B3,DTX1,OVOL2,ZBTB33,INSR,CUL5,YTHDF3,DEDD2,GRID1,SNAI2,IGHV3-74,BID,SIAH2,TRABD2B,PLPP4,NREP,ZDHHC17,JCAD,SAMHD1,IFT81,ENPP1,IGSF11,NDRG2,CSNK2A1,BMP5,CSF1,GHRH,BCL2L1,LAMA3,GRIN2B,GRB14,IGHV2-70D,SMAD5,CELF4,TNN,MED1,IL33,ROR2,KL,BANK1,IL10,SO*

			STDC1,PRKAA2,CSF2RB,ITPRIP,IQGAP1,RPS12,CAMLG,SREBF2,FYB2,NRXN1,HIPK1,CD70,CIBAR1,PBLD,PEG10,JAK2,FSTL1,SVEP1,MADD,CELSR2,CREBBP,TNKS,GORAB,NFKBIA,PRKCB,ANXA4,ZC3H15,NEDD9,ITGA6,IGHV1OR15-9,ADGRE3,OTOP1,CIDEA,GID8,STAT1,BRMS1L,ABI1,CCBE1,PARK7,ADAMTS18,MAPK8,ITGA4,FBXL17,ADCYAP1R1,MYOCD,CYFIP2,MEF2C,ADGRB1,WNT7A,RBPMS2,ATP6V1C2,SDCBP,SPPL2B,WWOX,NCK1,FGR,PPP2R3A,CXCL2,IFNAR1,EPHA4,NTRK2,WNT2B,POSTN,CD101,SHISA6,IL17RD,GRIK1,MVB12B,PTK2,CDH5,ANKRD6,NFKBID,CLDN18,LAMB1,CYFIP1,SEMA4D,FAT4,WNT5B,AMFR,FCRLA,SORBS2,CNKSR1,CCDC88A,TNFSF11,FYN,ADGRL2,ARL13B,RBMS3,HDAC2,DOK5,ZFYVE28,MAPK9,ROR1,FUT8,TET1,CDH2,ITGA8,EPHB1,GRM5,RPS6KA5,PTPRG,PID1,NRP1,PRKCA,FAIM,SAMD12,ITGA1,RNF138,RC3H1,POR,MCC,KIF16B,ELP2,STK36,B9D1,BMPER,PRDM15,MITF,EPHB2,CSNK1G1,CD38,EYA4,CDK14,MET,SPPL3,CDH17,CDH13,STXBP4,MAGI2,FLRT2,KALRN,LAMA1,BMP7,DLG5,ZMYND11,TMEM25,GRM3,ADGRF5,PDGFC,EYA1,SLIT2,CNOT7,ERBB4,IL20RB,ROBO1,PRKCQ,NLGN1,EFNA5,SLIT3,MYO9B,IGLC3,ADGRG7,PRLR,HTT,PIK3R3,FER,EYA2,CCR2,SEMA4B,IGHV1OR21-1,GRIA4,RORA,DMRT1,COL4A3,KIF7,FSTL4,STK3,DEPTOR,ZNF423,APCDD1,IGF1R,GLI2
GO:0006796	phosphate-containing compound metabolic process	9.108933562388092e-10	*BCAR3,MTOR,PTPRD,ULK2,NLK,KSR1,PLCB1,LIPI,DLC1,PTPRA,PDE4D,ERC1,NME7,SLC44A5,BCL2,LPCAT2,GPHN,CHRNA7,PUDP,PIK3C3,SPRED1,PLPPR1,ALK,HACD2,GLYAT,MAP3K9,MYO3B,MOCOS,APC,PLPPR5,DSCAM,CRKL,ERG,TNIK,PTPRJ,NEK4,EGFR,ANGPT1,CDK12,PRKACB,NEK7,NCOR1,GNPTAB,SGMS1,RPRD1A,PTPN4,NTRK3,FLT1,GK,DGKI,SLC8A3,PRKD1,TPTE2,PAK1,EPHA7,ADSS2,RAPGEF2,PELI2,CPS1,TAOK3,TMEM38B,AGK,SLC44A1,LDB2,PPP2R2B,PTPRN2,SMYD3,LRGUK,RPTOR,GHR,ADAM10,APP,ADK,RPS6KA2,SAMSN1,KYNU,PHKB,DCLK1,NTF3,AURKA,SLC8A1,PTPRR,FIG4,UPP2,MAP4K4,BMPR1B,ARNT,PAK3,RANBP2,ITPKB,TRPC5,PDE10A,NBN,SCP2,PRKCZ,GRB10,DIP2A,MCPH1,TAF4B,DUSP22,PPM1L,RIPK4,MAPK1,MGAT5,PDGFD,NRG3,GFRA1,PI4K2B,RNGTT,MTMR10,STK38,PTPN13,MYLK3,ACSBG1,LIMCH1,PAFAH1B1,EFEMP1,DCAF1,CCNG2,TLK1,NF2,MOB3B,BIRC6,AKAP9,PPARA,PPIP5K1,LCLAT1,PPP6R3,SYNJ1,RSRC1,PTPRK,PAK5,PPP2R5E,DAPK1,FAR2,ACSM2B,STK32B,ALPK2,IL34,MELK,WNT9B,CLPX,DUSP16,PARD3,MAPKAP1,BLK,OLA1,NUAK1,PTPRT,ABL1,PTPN12,HDAC4,SLC1A1,PRKAA1,MAST4,GUCY1A2,RAP1A,CAMK4,INPP5A,FGF10,PEAK1,LATS2,NRG1,MUSK,ZNF675,PRKCE,FOXK2,SLCO3A1,WNK2,DGKB,CD44,PTPRO,EGF,ALPK3,PRRC1,TRIO,LIMD1,SPRED2,RPS6KA3,MTMR3,PTPN2,HTR2C,AMBRA1,STK38L,HTR2A,INPP4B,PPP2R2C,TAF3,RPRD1B,MARK2,GMPR,ALPL,EPHA6,SH3KBP1,HIPK3,CDKN2C,GRK3,KNDC1,CLSPN,NOS2,STK10,TTC7B,MNAT1,MYLK2,HMGA2,CCND3,STK32A,AK8,PLCE1,TGFA,PRR5L,EFR3A,LDLRAD4,CNTN1,CEPT1,BLM,NRK,MAGI3,ADCY10,BMP2,MYLK4,CDC42BPB,VRK1,BMP2K,RELN,GNAQ,CDS2,FGF9,SH3BP5,DSTYK,PFKFB4,SNX6,CNKSR3,NEK10,MOB1B,PIGN,ATF2,MAPK8IP1,ME2,KITLG,CAMTA1,SMPDL3A,CHRM5,MAP4K3,RCAN1,TADA2A,DAB1,RB1CC1,MYO3A,AKAP10,PTPRE,PRKN,MTMR2,CDC42BPA,MAPK10,PNPLA7,TRPM6,PRKCH,HUNK,IL6R,ALS2,NLRC5,MKNK1,SNX25,SLC4A4,PTPRB,COPS8,VAV3,ENPP3,HAAO,MOK,RALB,PPA2,FAR1,ROCK1,LYN,SLC44A2,CHKA,INSR,NEK6,DPYD,PIGK,OSBPL10,PGAP4,ERN2,PLPP4,CARD10,SAMHD1,ENPP1,ENTPD5,MOCS2,RASGRP1,SNX9,TMEM225,UCK2,CSNK2A1,BMP5,CSF1,CTDP1,PRKG1,SMAD5,SYNJ2,HADHA,LPGAT1,CDC14B,GPRC5C,ROR2,PPP2R2A,BANK1,RIOK1,HDHD5,PTH,PRKAA2,CSF2RB,PLA2G4A,IQGAP1,THNSL2,NRXN1,HIPK1,DGKK,CENPE,AKT3,ALKAL2,JAK2,MPPE1,BPNT1,MADD,TNKS,PRKCB,RTRAF,BRD4,SMPD4,NEDD9,NRBP1,ITGA6,ADCY9,PPP1R17,MAST2,ZBTB49,FRA10AC1,PDP2,MAP2K6,DGKG,ABI1,CEMIP,IMPACT,PARK7,MAPK8,PPME1,ADCYAP1R1,PLA2G12B,NCAPG2,MYOCD,DHTKD1,ACACA,NDUFA10,MEF2C,MAP3K5,MAP3K4,PKN2,PDE2A,RAB38,DBF4B,SDCBP,PASK,MLLT1,NCK1,FGR,CDCA8,PPP2R3A,ATP6V1B2,T*

			OP1,EPHA4,PPIP5K2,NTRK2,ACSM2A,OCLN,TRPM7,PRKAB1,PTK2,MARK4,CDH5,SEMA4D,ZBTB20,IMPA2,KIRREL1,SLC26A2,TPTE,PDCL3,CNKSR1,CCDC88A,HKDC1,TNFSF11,FYN,BUB1,PPM1F,HDAC2,SACM1L,SH2D3C,DOCK3,NCS1,ATP5PF,UGP2,MTMR7,ZFYVE28,MAPK9,ROR1,EPHB1,GRM5,ADCK1,RPS6KA5,PTPRG,PID1,NRP1,PRKCA,ATPSCKMT,FHIT,ITGA1,CROT,EFL1,BCR,SNRK,FBLN1,STK36,BMPER,MACROH2A1,EPHB2,CSNK1G1,CD38,CDK14,MET,SPPL3,DLG2,CAMK1G,MAGI2,KALRN,LAMA1,MFHAS1,BMP7,BTBD10,AK3,ADGRF5,PDGFC,ELOVL7,ABL2,EYA1,SLIT2,CMPK1,CNOT7,FAM126A,ERBB4,FAM126B,ROBO1,PRKCQ,AK9,NOS1,EFNA5,NSD1,PRLR,PIGB,HTT,CAMK1D,PIK3R3,AK2,FER,EYA2,PITPNM3,OSBPL5,FGGY,HRH1,ROCK2,RORA,PPP1CB,PDK1,PTPRQ,WASHC1,BARD1,PNPLA3,STK3,DEPTOR,PNPLA8,HNRNPU,IGF1R,PRKAG2,AKAP13,MORC3,DNM1L
GO:0006793	phosphorus metabolic process	1.688017325227711e-9	*BCAR3,MTOR,PTPRD,ULK2,NLK,KSR1,PLCB1,LIPI,DLC1,PTPRA,PDE4D,ERC1,NME7,SLC44A5,BCL2,LPCAT2,GPHN,CHRNA7,PUDP,PIK3C3,SPRED1,PLPPR1,ALK,HACD2,GLYAT,MAP3K9,MYO3B,MOCOS,APC,PLPPR5,DSCAM,CRKL,ERG,TNIK,PTPRJ,NEK4,EGFR,ANGPT1,CDK12,PRKACB,NEK7,NCOR1,GNPTAB,SGMS1,RPRD1A,PTPN4,NTRK3,FLT1,GK,DGKI,SLC8A3,PRKD1,TPTE2,PAK1,GMDS,EPHA7,ADSS2,RAPGEF2,PELI2,CPS1,TAOK3,TMEM38B,AGK,SLC44A1,LDB2,PPP2R2B,PTPRN2,SMYD3,LRGUK,RPTOR,GHR,ADAM10,APP,ADK,RPS6KA2,SAMSN1,KYNU,PHKB,DCLK1,NTF3,AURKA,SLC8A1,PTPRR,FIG4,UPP2,MAP4K4,BMPR1B,ARNT,PAK3,RANBP2,ITPKB,TRPC5,PDE10A,NBN,SCP2,PRKCZ,GRB10,DIP2A,MCPH1,TAF4B,DUSP22,PPM1L,RIPK4,MAPK1,MGAT5,PDGFD,NRG3,GFRA1,PI4K2B,RNGTT,MTMR10,STK38,PTPN13,MYLK3,ACSBG1,LIMCH1,PAFAH1B1,EFEMP1,DCAF1,CCNG2,TLK1,NF2,MOB3B,BIRC6,AKAP9,PPARA,PPIP5K1,LCLAT1,PPP6R3,SYNJ1,RSRC1,PTPRK,PAK5,PPP2R5E,DAPK1,FAR2,ACSM2B,STK32B,ALPK2,IL34,MELK,WNT9B,CLPX,DUSP16,PARD3,MAPKAP1,BLK,OLA1,NUAK1,PTPRT,ABL1,PTPN12,HDAC4,SLC1A1,PRKAA1,MAST4,GUCY1A2,RAP1A,CAMK4,INPP5A,FGF10,PEAK1,LATS2,NRG1,MUSK,ZNF675,PRKCE,FOXK2,SLCO3A1,WNK2,DGKB,CD44,PTPRO,EGF,ALPK3,PRRC1,TRIO,LIMD1,SPRED2,RPS6KA3,MTMR3,PTPN2,HTR2C,AMBRA1,STK38L,HTR2A,INPP4B,PPP2R2C,TAF3,RPRD1B,MARK2,GMPR,ALPL,EPHA6,SH3KBP1,HIPK3,CDKN2C,GRK3,KNDC1,CLSPN,NOS2,STK10,TTC7B,MNAT1,MYLK2,HMGA2,CCND3,STK32A,AK8,PLCE1,TGFA,PRR5L,EFR3A,LDLRAD4,CNTN1,CEPT1,BLM,NRK,MAGI3,ADCY10,BMP2,MYLK4,CDC42BPB,VRK1,BMP2K,RELN,GNAQ,CDS2,FGF9,SH3BP5,DSTYK,PFKFB4,SNX6,CNKSR3,NEK10,MOB1B,PIGN,ATF2,MAPK8IP1,ME2,KITLG,CAMTA1,SMPDL3A,CHRM5,MAP4K3,RCAN1,TADA2A,DAB1,RB1CC1,MYO3A,AKAP10,PTPRE,PRKN,MTMR2,CDC42BPA,MAPK10,PNPLA7,TRPM6,PRKCH,HUNK,IL6R,ALS2,NLRC5,MKNK1,SNX25,SLC4A4,PTPRB,COPS8,VAV3,ENPP3,HAAO,MOK,RALB,PPA2,FAR1,ROCK1,LYN,SLC44A2,CHKA,INSR,NEK6,DPYD,PIGK,OSBPL10,PGAP4,ERN2,PLPP4,CARD10,SAMHD1,ENPP1,ENTPD5,MOCS2,RASGRP1,SNX9,TMEM225,UCK2,CSNK2A1,BMP5,CSF1,CTDP1,PRKG1,SMAD5,SYNJ2,HADHA,LPGAT1,CDC14B,GPRC5C,ROR2,PPP2R2A,BANK1,RIOK1,HDHD5,PTH,PRKAA2,CSF2RB,PLA2G4A,IQGAP1,THNSL2,NRXN1,HIPK1,DGKK,CENPE,AKT3,ALKAL2,JAK2,MPPE1,BPNT1,MADD,TNKS,PRKCB,RTRAF,BRD4,SMPD4,NEDD9,NRBP1,ITGA6,ADCY9,PPP1R17,MAST2,ZBTB49,FRA10AC1,PDP2,MAP2K6,DGKG,ABI1,CEMIP,IMPACT,PARK7,MAPK8,PPME1,ADCYAP1R1,PLA2G12B,NCAPG2,MYOCD,DHTKD1,ACACA,NDUFA10,MEF2C,MAP3K5,MAP3K4,PKN2,PDE2A,RAB38,DBF4B,SDCBP,PASK,MLLT1,NCK1,FGR,CDCA8,PPP2R3A,ATP6V1B2,TOP1,EPHA4,PPIP5K2,NTRK2,ACSM2A,OCLN,TRPM7,PRKAB1,PTK2,MARK4,CDH5,SEMA4D,ZBTB20,IMPA2,KIRREL1,SLC26A2,TPTE,PDCL3,CNKSR1,CCDC88A,HKDC1,TNFSF11,FYN,BUB1,PPM1F,HDAC2,SACM1L,SH2D3C,DOCK3,NCS1,ATP5PF,UGP2,MTMR7,ZFYVE28,MAPK9,ROR1,FUT8,EPHB1,GRM5,ADCK1,RPS6KA5,PTPRG,PID1,NRP1,PRKCA,ATPSCKMT,FHIT,ITGA1,CROT,EFL1,BCR,SNRK,FBLN1,STK36,BMPER,MACROH2A1,EPHB2,CSNK1G1,CD38,CDK14,MET,SPPL3,DLG2,CAMK1G,MAGI2,KALRN,L*

			AMA1,MFHAS1,BMP7,BTBD10,AK3,ADGRF5,PDGFC,ELOVL7,ABL2,EYA1,SLIT2,CMPK1,CNOT7,FAM126A,ERBB4,FAM126B,ROBO1,PRKCQ,AK9,NOS1,EFNA5,NSD1,PRLR,PIGB,HTT,CAMK1D,PIK3R3,AK2,FER,EYA2,PITPNM3,OSBPL5,FGGY,HRH1,ROCK2,RORA,PPP1CB,PDK1,PTPRQ,WASHC1,BARD1,PNPLA3,STK3,DEPTOR,PNPLA8,HNRNPU,IGF1R,PRKAG2,AKAP13,MORC3,DNM1L
GO:0016310	phosphorylation	1.906854711025 1563e-9	*BCAR3,MTOR,ULK2,NLK,KSR1,PTPRA,PDE4D,ERC1,NME7,BCL2,CHRNA7,PIK3C3,SPRED1,ALK,MAP3K9,MYO3B,APC,DSCAM,CRKL,ERG,TNIK,PTPRJ,NEK4,EGFR,ANGPT1,CDK12,PRKACB,NEK7,NCOR1,GNPTAB,SGMS1,PTPN4,NTRK3,FLT1,GK,DGKI,SLC8A3,PRKD1,PAK1,EPHA7,RAPGEF2,PELI2,TAOK3,AGK,LDB2,SMYD3,LRGUK,RPTOR,GHR,ADAM10,APP,ADK,RPS6KA2,SAMSN1,PHKB,DCLK1,NTF3,AURKA,SLC8A1,MAP4K4,BMPR1B,ARNT,PAK3,RANBP2,ITPKB,TRPC5,NBN,PRKCZ,GRB10,MCPH1,TAF4B,DUSP22,RIPK4,MAPK1,PDGFD,NRG3,GFRA1,PI4K2B,STK38,PTPN13,MYLK3,LIMCH1,EFEMP1,DCAF1,CCNG2,TLK1,NF2,MOB3B,BIRC6,AKAP9,PPARA,PPIP5K1,RSRC1,PAK5,DAPK1,STK32B,ALPK2,IL34,MELK,WNT9B,DUSP16,PARD3,MAPKAP1,BLK,NUAK1,PTPRT,ABL1,HDAC4,SLC1A1,PRKAA1,MAST4,RAP1A,CAMK4,FGF10,PEAK1,LATS2,NRG1,MUSK,ZNF675,PRKCE,FOXK2,SLCO3A1,WNK2,DGKB,CD44,PTPRO,EGF,ALPK3,PRRC1,TRIO,LIMD1,SPRED2,RPS6KA3,PTPN2,AMBRA1,STK38L,HTR2A,TAF3,MARK2,EPHA6,SH3KBP1,HIPK3,CDKN2C,GRK3,KNDC1,CLSPN,STK10,MNAT1,MYLK2,HMGA2,CCND3,STK32A,AK8,PLCE1,TGFA,PRR5L,LDLRAD4,CNTN1,BLM,NRK,BMP2,MYLK4,CDC42BPB,VRK1,BMP2K,RELN,GNAQ,FGF9,SH3BP5,DSTYK,PFKFB4,SNX6,CNKSR3,NEK10,MOB1B,ATF2,MAPK8IP1,KITLG,MAP4K3,TADA2A,DAB1,RB1CC1,MYO3A,AKAP10,PRKN,CDC42BPA,MAPK10,TRPM6,PRKCH,HUNK,IL6R,ALS2,NLRC5,MKNK1,SNX25,SLC4A4,PTPRB,COPS8,VAV3,MOK,RALB,ROCK1,LYN,CHKA,INSR,NEK6,ERN2,CARD10,ENPP1,RASGRP1,SNX9,UCK2,CSNK2A1,BMP5,CSF1,PRKG1,SMAD5,GPRC5C,ROR2,BANK1,RIOK1,PRKAA2,CSF2RB,IQGAP1,NRXN1,HIPK1,DGKK,CENPE,AKT3,ALKAL2,JAK2,MADD,TNKS,PRKCB,RTRAF,BRD4,NEDD9,NRBP1,ITGA6,MAST2,MAP2K6,DGKG,ABI1,CEMIP,IMPACT,PARK7,MAPK8,PPME1,NCAPG2,MYOCD,DHTKD1,MEF2C,MAP3K5,MAP3K4,PKN2,DBF4B,SDCBP,PASK,MLLT1,NCK1,FGR,CDCA8,TOP1,EPHA4,PPIP5K2,NTRK2,OCLN,TRPM7,PRKAB1,PTK2,MARK4,CDH5,SEMA4D,ZBTB20,KIRREL1,PDCL3,CNKSR1,CCDC88A,HKDC1,TNFSF11,FYN,BUB1,PPM1F,HDAC2,SH2D3C,DOCK3,ZFYVE28,MAPK9,ROR1,EPHB1,GRM5,ADCK1,RPS6KA5,PID1,NRP1,PRKCA,BCR,SNRK,FBLN1,STK36,BMPER,MACROH2A1,EPHB2,CSNK1G1,CDK14,MET,CAMK1G,MAGI2,KALRN,LAMA1,BMP7,BTBD10,AK3,PDGFC,ABL2,SLIT2,CMPK1,CNOT7,ERBB4,ROBO1,PRKCQ,AK9,NOS1,EFNA5,NSD1,PRLR,HTT,CAMK1D,PIK3R3,AK2,FER,FGGY,ROCK2,PDK1,WASHC1,BARD1,STK3,DEPTOR,HNRNPU,IGF1R,PRKAG2,AKAP13,MORC3*
GO:0006996	organelle organization	2.191118052480 8768e-9	*NOTCH2,MTOR,IMMP2L,PTPRD,TRAPPC9,NEBL,ULK2,LONP2,UNC13C,LRRC49,PLCB1,SVIL,TLN2,MICAL3,NUBPL,DLC1,RIPOR2,RDX,RP1,STXBP1,RALA,BCL2,MYO5A,ODAD2,ARHGAP26,COG5,PIK3C3,SPIRE1,CNTLN,SDCCAG8,FGD4,NAV2,SPAG16,MYO1E,TRAPPC8,CEP192,CDC42EP3,MICOS10,AUTS2,FOXJ2,CARMIL1,ERBIN,RHPN2,PARVB,MAP4,APC,ZMYM4,MYO5C,SETD2,TNIK,RFX3,MACF1,CTNNA3,NEK7,NCOR1,DOCK2,DIAPH3,GNPTAB,NSMCE2,CECR2,ARMC2,NTRK3,ZFAND6,PHACTR1,DNAJC13,RFC3,PSMA8,CRACD,SLC39A12,TOM1L2,PRKD1,PAK1,DEUP1,ARSB,ONECUT1,TMEM38B,AGK,UBE2L3,LRGUK,SEPTIN9,RETREG1,EPB41L3,KIF4A,TBCD,ATF7IP,PHACTR2,RPS6KA2,DCLK1,STAU2,MAPRE2,SEMA5A,SYT1,ARHGAP44,NTF3,NDUFAF2,CD2AP,AURKA,PARN,TTC29,PYGO1,SRGAP2C,ANKRD31,FIG4,FRMD3,CCSER2,KANK1,ABCD2,FMN2,THSD7A,CTNNA2,RAB8B,PAK3,TTLL7,RAP1GDS1,NBN,IFT57,PRKCZ,CALD1,KLHL1,MCPH1,MRTFA,COBL,SENP6,YAP1,MAPK1,ABCD3,ABLIM1,ARHGEF17,ANKFY1,SYCP1,NIPBL,SLC16A1,PI4K2B,CORO2B,CHD6,MYLK3,LIMCH1,FMN1,PAFAH1B1,VPS13D,TPM1,NF2,CTNNA1,PPP1R9A,AKAP9,SNX30,SYNJ1,MPRIP,FOXJ3,ENAH,PAK5,PARD3B,NAV3,VPS13C,TMEM108,RAB22A,DNAJC15,DYSF,ANK2,STAG2,BRWD1,BCAS*

			3,SYNE2,BBS2,AIF1L,SHOC1,SMARCA4,USP8,LDB3,PARD3,MAPKAP1,DST,CXADR,ATRX,XIRP2,ABL1,PRKAA1,GAS2,ITGB3BP,TTLL5,MAST4,DNAH5,BAZ2A,FGF10,UQCC1,GRID2,INO80D,CLIP1,ASPM,AP3B1,SYNE1,SMARCAD1,SETDB2,PRKCE,PGM5,USP33,CEP83,EGF,DMAC1,PDE3A,EXT1,STXBP6,LIMD1,PEX14,IFT43,ATP8A2,MTMR3,ATXN3,RFC1,CLEC16A,ARHGEF7,AMBRA1,ABCC4,BIN2,FANCM,FANCA,GTF2F2,SEMA3E,MARK2,TMEM67,C10ORF90,TMOD2,MSH2,MAIP1,SH3KBP1,ATL1,AFG3L2,MNAT1,CFAP61,ANK3,HMGA2,MYOM2,COG2,VPS41,TRAPPC11,PLCE1,TGFA,ANKFN1,HIP1,VPS37A,TJP1,NPHP4,PACSIN2,IQSEC1,SNX3,PDLIM5,BRCA2,DISC1,BLM,WDPCP,NRK,HSF2BP,STX12,PHACTR3,TRAK1,CDC42BPB,PTCD2,VRK1,GNAI1,TBC1D4,RANBP9,RESF1,DNAL1,LEMD3,RELN,HMGB1,TUBGCP3,NUDCD3,CDS2,TDRD7,RTTN,MDM1,POLR2M,CLVS1,UNC13B,TTC21B,UBAP2L,PLS1,SRGAP2,SEC23B,NIN,HAUS6,DNAH8,SLAMF1,SMARCA2,ETS1,SMARCC1,KIF15,ZFYVE26,MAP2,DAW1,PEX6,FARP1,TDRD5,ATF2,NDUFAF6,GOLGA8B,CYLD,BBS4,GOLGA6B,CLVS2,THSD7B,KIAA0753,DOP1B,CEP44,ATP10B,GOLGA6D,YLPM1,SELENON,RB1CC1,PRKN,CDC42BPA,AFAP1,PCDH15,DPF3,LYST,PKP1,FRMPD4,ALS2,RACGAP1,CNOT6L,KANK4,DMC1,GOLGA6C,LRBA,SHANK2,MAP7,USP7,VAV3,SOX30,PTGFRN,RALB,CFAP74,MYO1D,SEC24D,ROCK1,ARHGAP28,KIF11,CHKA,ARID1B,INSR,BMF,YTHDF3,NEK6,SHROOM3,DDHD1,ARFGEF1,PDE4DIP,BID,ERN2,FLNB,FYCO1,ESYT2,SH3GLB1,TMED3,KRT6B,IFT81,UTRN,SNX9,ANAPC1,HDGFL3,BCL2L1,KRT25,CTDP1,PRKG1,INO80,FAM149B1,CIDEC,MICALL2,ATG4B,CDC14B,PCNT,CSDE1,FAT1,ACTR2,SFPQ,TTC39C,TOP3B,PRKAA2,SKA1,NDC80,MAP6,VASP,TACC2,KIFC1,IQGAP1,CAMLG,COX7A2L,MORC2,SREBF2,NRXN1,PCID2,SNAP91,CIBAR1,CENPE,TUBB6,ANLN,AKT3,JAK2,RPF2,CHCHD6,PATL1,CELSR2,TNKS,SGO1,GORAB,PCNA,SIAH3,UFL1,PRKCB,MIPEP,RFC2,SMTN,USH1C,SERBP1,SMPD4,NEDD9,GAP43,MTCL1,SAR1A,EML1,MAST2,GOLGA8J,TRAPPC3,ARFGEF3,BBS9,KRT6A,SHROOM2,MTPN,ABI1,PARK7,MAPK8,TOP3A,UBE2J2,NCAPG2,NDC1,ASB2,HFM1,HMCN1,CEP120,CYFIP2,KRT85,ASCC2,NDUFA10,MAP3K4,WASF3,CHAMP1,MAGEL2,RAD51AP1,PDE2A,RAB38,FBXW8,SDCBP,NSMCE1,NCK1,DRC7,CDCA8,TOP1,SNAP29,INTS13,DNMT3L,RSPH1,OCLN,AKAP11,TRPM7,MVB12B,PTK2,MARK4,CDH5,ARHGAP12,DIAPH1,FEZ2,CYFIP1,HOATZ,FRMD6,KIRREL1,SAXO1,PSTPIP2,ZFYVE1,ASAP1,NOS1AP,SORBS2,PDCL3,CCDC88A,CHCHD2,ADAMTS16,SPAG6,MDN1,CDC45,BICD1,BUB1,PPM1F,GOLGA8F,ARL13B,HYDIN,SLF1,EHBP1,MAPK9,HECW2,CDH2,RP1L1,ADCK1,SPTB,PID1,NRP1,FCHSD2,PRKCA,IFT46,COX10,RC3H1,EFL1,BCR,ELMO1,C14ORF39,ARFGAP3,CFAP70,STK36,B9D1,MACROH2A1,TOGARAM1,CHCHD3,MYO5B,MET,SPECC1,ATG5,NRAP,VMP1,FAM171A1,DHX29,BMP7,GOLGA6A,TRAPPC10,DDX6,DNAH17,PLIN2,EPB41L4A,ABL2,TRAPPC6B,RFX2,FHOD3,TTLL11,GOLGA8T,SLIT2,CNOT7,ESCO1,ERBB4,NUF2,PRKCQ,SAMM50,ANTXR1,SIPA1L3,TRDN,NLGN1,SHLD2,CHD9,EFNA5,GAS2L1,ARHGEF11,RAB27A,KIF13A,FRMD5,IQCJ-SCHIP1,IRAG2,HTT,RAD51B,CFAP44,FER,EYA2,RPGRIP1,STARD13,CHFR,EPS8,ROCK2,ATAT1,TERB2,DMRT1,CDCA5,RAB31,WASHC1,HOOK3,PNPLA3,ZNF423,HNRNPU,VTI1A,CEP72,RAB3GAP2,TANGO2,AKAP13,SEPTIN6,DNM1L
GO:0030036	actin cytoskeleton organization	2.930490141329686e-9	NOTCH2,MTOR,NEBL,SVIL,MICAL3,DLC1,RDX,RALA,BCL2,MYO5A,ARHGAP26,SPIRE1,FGD4,MYO1E,CDC42EP3,AUTS2,CARMIL1,RHPN2,PARVB,MYO5C,TNIK,CTNNA3,DOCK2,DIAPH3,NTRK3,PHACTR1,CRACD,PAK1,EPB41L3,PHACTR2,STAU2,SEMA5A,ARHGAP44,NTF3,CD2AP,FRMD3,KANK1,FMN2,THSD7A,CTNNA2,PAK3,RAP1GDS1,CALD1,KLHL1,MRTFA,COBL,ARHGEF17,CORO2B,MYLK3,LIMCH1,FMN1,PAFAH1B1,TPM1,NF2,CTNNA1,PPP1R9A,MPRIP,ENAH,BCAS3,AIF1L,LDB3,CXADR,XIRP2,ABL1,GAS2,FGF10,PRKCE,PGM5,ATXN3,SEMA3E,TMOD2,SH3KBP1,MYOM2,HIP1,TJP1,NPHP4,PACSIN2,IQSEC1,PDLIM5,NRK,PHACTR3,CDC42BPB,PLS1,FARP1,BBS4,THSD7B,PRKN,CDC42BPA,PCDH15,FRMPD4,RACGAP1,KANK4,MYO1D,ROCK1,ARHGAP28,SHROOM3,ARF

			GEF1,FLNB,UTRN,SNX9,PRKG1,MICALL2,FAT1,ACTR2,VASP,IQGAP1,ANLN,JAK2,SMTN,USH1C,NEDD9,ARFGEF3,SHROOM2,MTPN,ABI1,ASB2,HMCN1,CYFIP2,WASF3,MAGEL2,SDCBP,NCK1,AKAP11,TRPM7,ARHGAP12,DIAPH1,CYFIP1,FRMD6,KIRREL1,PSTPIP2,NOS1AP,SORBS2,PDCL3,CCDC88A,PPM1F,EHBP1,SPTB,NRP1,FCHSD2,BCR,ELMO1,MYO5B,MET,SPECC1,NRAP,FAM171A1,EPB41L4A,ABL2,FHOD3,SLIT2,ANTXR1,EFNA5,GAS2L1,ARHGEF11,FRMD5,FER,STARD13,EPS8,ROCK2,WASHC1,AKAP13
GO:0030334	regulation of cell migration	6.631650067360577e-9	*MTOR,PLCB1,TAFA5,DLC1,RIPOR2,RDX,BCL2,SPRED1,CARMIL1,MCTP1,RIN2,ANO6,APC,CRKL,PTPRJ,DOCK10,EGFR,ANGPT1,MACF1,SCAI,NTRK3,C5,PHACTR1,FLT1,PRKD1,PAK1,RAPGEF2,ARSB,ONECUT1,LDB2,CCL28,ADAM10,HDAC9,IL1R1,APP,MTUS1,DOCK8,MAPRE2,SEMA5A,VCL,NTF3,SLC8A1,PTPRR,SRGAP2C,SRGAP2B,KANK1,MAP4K4,CTNNA2,PAK3,DUSP22,MAPK1,MGAT5,PDGFD,NRG3,NIPBL,LIMCH1,TPM1,NF2,TIAM1,PTPRK,SEMA3C,NAV3,AGO2,IL34,BCAS3,SYNE2,SEMA6D,TNR,DOCK4,PTPRT,ABL1,HDAC4,FGF10,NRG1,PRKCE,EGF,PLXNA2,ARHGEF7,ATP8A1,SEMA3E,GCSAML,STK10,EMILIN2,DOCK5,FUT9,PRR5L,TJP1,LDLRAD4,IQSEC1,WDPCP,SEMA3A,BMP2,UNC5D,RIN3,SEMA3D,RELN,HMGB1,FGF9,DOCK1,SRGAP2,SLAMF1,ETS1,SMOC2,KITLG,DACH1,IL6R,ROCK1,LYN,NTN1,INSR,YTHDF3,SNAI2,CD9,CARD10,JCAD,TWIST2,BMP5,CSF1,PRKG1,LAMA3,TNN,IL33,ROR2,ABHD2,AKT3,JAK2,ABCC8,NEDD9,ITGA6,MEOX2,BRMS1L,CEMIP,CCBE1,ITGA4,MYOCD,MEF2C,ADGRB1,WNT7A,SDCBP,NCK1,FGR,PPP2R3A,EPHA4,NUMB,ADAMTS9,FBXO31,PTK2,CDH5,DIAPH1,LAMB1,SEMA4D,JAM2,WNT5B,PPM1F,HDAC2,APELA,TET1,PTPRG,NRP1,PRKCA,MCC,BCR,FBLN1,RRAS2,BMPER,SRGAP3,MITF,EPHB2,MET,CDH13,MAGI2,FLRT2,LAMA1,BMP7,DLG5,ZMYND8,PDGFC,ABL2,SLIT2,ERBB4,ROBO1,FRMD5,NTNG1,CAMK1D,PIK3R3,FER,CCR2,STARD13,SEMA4B,ROCK2,WASHC1,IGF1R,DNM1L*
GO:0050790	regulation of catalytic activity	6.867803008308252e-9	*BCAR3,MTOR,SPOCK1,GARNL3,MYO9A,KSR1,PLCB1,DLC1,ZDHHC21,RIPOR2,RDX,BCL2,ARHGAP26,CHRNA7,RIMS1,FGD4,SPRED1,ALK,RIN2,EGLN3,APC,CRKL,ARHGAP24,PTPRJ,DOCK10,EGFR,DENND1A,USP14,ANGPT1,CDK12,NEK7,RGS3,DOCK2,NTRK3,RXFP1,C5,PHACTR1,FLT1,RFC3,RABEP1,DGKI,CAST,SLC8A3,PRKD1,TBC1D19,PAK1,EPHA7,RALGPS1,RAPGEF2,ADGRB3,TAOK3,UBE2L3,LDB2,PPP2R2B,SMYD3,HERC2,GRM7,RPTOR,GHR,RALGAPA1,RAPGEF5,TBCD,PPP1R12B,HDAC9,PHACTR2,APP,CACNA1C,DOCK8,MAPRE2,ARHGAP44,NTF3,ACER2,PARN,ST18,SLC8A1,SERPINA6,MAP4K4,BMPR1B,PCSK6,RANBP2,RGS20,RAP1GDS1,NBN,IFT57,PRKCZ,MSH6,MCPH1,ARHGAP32,RGS9,MRTFA,DUSP22,MAPK1,MGAT5,RABGAP1L,ITIH5,PDGFD,PPP1R1C,ARHGEF17,NRG3,UBE2O,CARD18,STK38,TBC1D22A,CHN1,ECT2L,PAFAH1B1,CCNG2,NF2,MOB3B,BIRC6,AKAP9,RASGRF2,MRTFB,PPP6R3,RGL1,TIAM1,ARAP2,ARHGEF12,PPP2R5E,PLA2R1,DAPK1,RIC8B,TBC1D9,IL34,ADGRV1,BCAS3,WNT9B,CLPX,RANBP3L,DUSP16,TBC1D5,DOCK4,NUAK1,PTPRT,ABL1,SLC1A1,PSMF1,RAP1A,LATS2,NRG1,AP3B1,DENND2B,RASGRF1,MUSK,ZNF675,PRKCE,ASAP2,DENND4C,CD44,RGS12,PTPRO,EGF,PRRC1,TRIO,PDE3A,NSMAF,SPRED2,RPS6KA3,SCG5,PTPN2,PLXNA2,MCF2L,RFC1,ARHGEF7,AMBRA1,HTR2A,PPP2R2C,MARK2,HERC1,MSH2,EPHA6,HIPK3,CDKN2C,KNDC1,CLSPN,NOS2,MNAT1,HMGA2,CCND3,DOCK5,MBP,PLCE1,TGFA,HIP1,CRIM1,VAV1,IQSEC1,BLM,PHACTR3,BMP2,EVI5,RALGAPA2,SGSM1,TBC1D4,RIN3,BMP2K,CABIN1,RELN,ARHGAP42,HMGB1,GNAQ,SH3BP5,CPAMD8,DSTYK,DOCK1,RAP1GAP,SRGAP2,SEC23B,SLAMF1,SNX6,PSD3,NEK10,FARP1,MOB1B,UMODL1,BBS4,MAPK8IP1,GABBR2,TBC1D13,KITLG,RCAN1,DAB1,PRKN,SH3PXD2A,NGEF,GRIN2A,IL6R,ALS2,RACGAP1,NLRC5,DOCK9,PTPRB,COPS8,VAV3,ITSN2,ARHGEF28,RALB,DENND2C,ROCK1,LYN,ARHGAP28,ARHGAP31,CTSB,EIF2B3,MMP16,INSR,XRCC4,ARFGEF1,BID,SIAH2,ERN2,CARD10,RALGPS2,RASGRP1,SNX9,TMEM225,CSNK2A1,CSF1,SERPINB9,PRKG1,GRIN2B,DCUN1D4,PSAP,CDC14B,GPRC5C,ROR2,PPP2R2A,RASGEF1C,PTH,ITPRIP,IQGAP1,ANP32B,NRXN1,PCID2,FRY,FICD,CENPE,NET1,SIPA1L2,ALKAL2,JAK2,MADD,TNKS,PCN*

			A,ANXA4,ZC3H15,RFC2,RTRAF,NEDD9,ITGA6,AGAP9,PPP1R17,ARFGEF3,PDP2,MAP2K6,ABI1,CEMIP,PARK7,MAPK8,OAZ2,PPME1,ADCYAP1R1,NCAPG2,RAPGEF4,MYOCD,CYFIP2,MEF2C,MAP3K5,MAP3K4,SERPINI2,DBF4B,MLLT1,NCK1,FGR,PPP2R3A,DNMBP,TRIM23,EPHA4,CYTH4,DNMT3L,NTRK2,PRKAB1,PTK2,RCAN2,ARHGAP12,SEMA4D,SERPINB10,RASGEF1B,ASAP1,NOS1AP,CCDC88A,GPR55,BICD1,TNFSF11,FYN,KDM5A,PPM1F,SH2D3C,DOCK3,NCS1,ZFYVE28,ROR1,GPR137B,EPHB1,GRM5,TBC1D1,NRP1,ATPSCKMT,ITGA1,POR,BCR,ELMO1,RGS6,PRIM2,ARFGAP3,FBLN1,RAG1,SRGAP3,MACROH2A1,EPHB2,BCL2L13,RGPD4,MET,SERPINB2,MAGI2,KALRN,GNAS,SERPINB7,TIAM2,BMP7,TNFAIP8,GAPVD1,GRM3,PDGFC,WDR41,ABL2,TRAPPC6B,SLIT2,ERBB4,SERPINB11,ROBO1,PRKCQ,RGPD2,ANTXR1,SIPA1L3,MGMT,NOS1,EFNA5,ARHGEF11,ESR1,MYO9B,PRLR,HTT,PIK3R3,CCR2,STARD13,A2M,SPOCK3,AGAP1,ROCK2,RGS8,PSMD2,COL4A3,WASHC1,RGS7,STK3,DEPTOR,RSU1,HNRNPU,RAB3GAP2,IGF1R,PRKAG2,AKAP13,DNM1L
GO:0051056	regulation of small GTPase mediated signal transduction	8.613067322772321e-9	*NOTCH2,GARNL3,MYO9A,DLC1,RIPOR2,RDX,ARHGAP26,FGD4,AUTS2,ERBIN,ARHGAP24,DENND1A,DOCK2,SCAI,DGKI,RALGPS1,RALGAPA1,DOCK8,MAPRE2,ARHGAP44,CD2AP,KANK1,MAP4K4,ITPKB,ARHGAP32,ARHGEF17,CHN1,RASGRF2,TIAM1,ARHGEF12,MAPKAP1,ABL1,FGF10,NRG1,RASGRF1,DENND4C,TRIO,MCF2L,PLCE1,VAV1,IQSEC1,RALGAPA2,RELN,ARHGAP42,RAP1GAP,SRGAP2,PSD3,KITLG,NGEF,ALS2,RACGAP1,VAV3,ARHGEF28,ARHGAP28,ARHGAP31,ARFGEF1,RALGPS2,RASGRP1,CSF1,NET1,SIPA1L2,MADD,ARFGEF3,ADCYAP1R1,DNMBP,CYTH4,ARHGAP12,GPR55,DOCK3,NRP1,BCR,SRGAP3,EPHB2,MET,KALRN,TIAM2,ABL2,SLIT2,ROBO1,SIPA1L3,ARHGEF11,MYO9B,STARD13,EPS8,AKAP13*
GO:0007411	axon guidance	1.2314372031808234e-8	*NOTCH2,CNTN4,ROBO2,DSCAM,CDH4,NEO1,CNTN6,EPHA7,ADAMTSL1,APP,SEMA5A,BMPR1B,ALCAM,NCAM1,CHN1,NFIB,PRTG,ENAH,SEMA3C,SEMA6D,TNR,USP33,PTPRO,TRIO,EXT1,PLXNA2,SEMA3E,EPHA6,BCL11B,ECE1,CNTN1,SEMA3A,UNC5D,SEMA3D,RELN,DRAXIN,GLI3,DCC,NTN1,DPYSL5,LAMA3,LMX1A,VASP,NRXN1,GAP43,NECTIN1,EPHA4,LHX9,PTK2,FEZ2,CYFIP1,SEMA4D,FYN,CNTN5,EPHB1,RPS6KA5,NRP1,NRXN3,EPHB2,FLRT2,KALRN,LAMA1,BMP7,SLIT2,CCDC141,ROBO1,PRKCQ,EFNA5,SLIT3,SEMA4B,GLI2*
GO:0051239	regulation of multicellular organismal process	1.336318962861873e-8	*NOTCH2,MTOR,WWC1,PTPRD,ULK2,FTO,PLCB1,TAFA5,ZFPM2,TENM4,ZDHHC21,PDE4D,BCL2,KCNMA1,PRDM16,FBN1,CHRNA7,ROBO2,RIMS1,ZEB1,RARB,SPRED1,MINAR1,RIMS2,FOXJ2,ERBIN,RIN2,ANO6,DLGAP1,APC,HHLA2,TSHZ3,DSCAM,SOX5,SETD2,PTPRJ,EGFR,RFX3,ANGPT1,MACF1,CTNNA3,BCL11A,SOX6,CHSY1,CDH4,ATP2B2,NTRK3,C5,FLT1,MAPKBP1,SLC39A12,SLC8A3,PRKD1,EPHA7,CHRM3,SPEN,RAPGEF2,LRP2,ADGRB3,RUNX2,ARSB,FGF12,TMEM38B,TAFA4,GHR,COL4A2,CELF2,PPP1R12B,ADAM10,HDAC9,IL1R1,APP,CACNA1C,CACNB2,STAU2,MAPRE2,SEMA5A,VCL,CD2AP,SLC8A1,PTPRR,FIG4,ABCG8,KCNE4,MAP4K4,ABCD2,BMPR1B,AKAP6,HOMER2,ARNT,RAB8B,PAK3,RFTN1,DIP2B,ITPKB,TRPC5,RNLS,PRKCZ,GRB10,EBF2,YAP1,BRINP1,MAPK1,HRH2,PLG,NIPBL,GABPA,CORO2B,CARD18,PAFAH1B1,EFEMP1,ITGB8,TPM1,NF2,CORIN,CTNNA1,AKAP9,PPARA,MEIS2,NFIB,MRTFB,PRTG,SYNJ1,TIAM1,GRM1,PLA2R1,SEMA3C,NAV3,SLC24A4,TMEM108,AGO2,ALPK2,JARID2,IL34,ANK2,ADGRV1,BCAS3,RYR2,BBS2,WNT9B,SEMA6D,SMARCA4,PARD3,TNR,CXADR,DOCK4,MBD5,ABL1,HDAC4,SLC1A1,DROSHA,CAMK4,FGF10,ZC3HAV1,GRID2,LATS2,NRG1,INO80D,ASPM,AP3B1,ATP11C,ZBTB16,ZNF675,PRKCE,ESRRG,FBN2,PTPRO,EGF,ABCC9,PDE3A,LNPEP,SPRED2,ATP8A2,PTPN2,PLXNA2,HTR2C,CD96,AMBRA1,LTBP1,OPRM1,HTR2A,FANCA,KREMEN1,SEMA3E,ALPL,MSH2,IGF2BP3,LINGO2,LUC7L,EPN2,NOS2,BICRAL,AFG3L2,ADAM12,MYLK2,EMILIN2,HMGA2,DOCK5,ECE1,MBP,TRPS1,PLCE1,IL17RA,FBXO32,TJP1,LDLRAD4,HLA-B,IQSEC1,BRCA2,DISC1,WDPCP,SEMA3A,BMP2,RC3H2,PSG9,SCN11A,GFI1B,BMP2K,SEMA3D,POLR3A,RELN,ARHGAP42,HMGB1,FGF9,TRAF3,ESRP1,UNC13B,TTC21B,DOCK1,RAP1GAP,PLS1,*

			NIN,DRAXIN,SLAMF1,SMARCA2,ETS1,GLI3,CGAS,SMARCC1,SMOC2,MAP2,ATF2,CYLD,BBS4,CFTR,NELL1,UBASH3A,KITLG,DCC,GTF2I,TADA2A,DAB1,SELENON,MTMR2,TBX20,DLGAP2,GRIN2A,PRKCH,TG,IL6R,RACGAP1,TOX,SHISA9,SCN10A,KCND3,KIR2DL4,NPAS2,ROCK1,LYN,DTX1,OVOL2,NTN1,ZFHX3,DPYSL5,ARID1B,INSR,NMU,PBX3,SNAI2,UFD1,RXRG,NSD2,CD9,CARD10,JCAD,ENPP1,RASGRP1,IGSF11,NDRG2,BMP5,CSF1,GHRH,CTDP1,HCN1,PRKG1,LAMA3,GRIN2B,INO80,CNMD,DHRS3,CELF4,MYCL,TNN,MED1,ATRN,IL33,AJAP1,ROR2,KL,BANK1,TMEM178A,IL10,ACTR2,CLSTN2,PTH,SOSTDC1,MAP6,IQGAP1,YBX3,NRXN1,PCID2,HIPK1,CADM1,TWIST1,AKT3,JAK2,RBM19,ZNF287,FH,UFL1,ADAMTS5,NFKBIA,PRKCB,ABCC8,ANXA4,NEDD9,ATP2B1,MAST2,CIDEA,AGO1,MEOX2,STAT1,BRMS1L,MAP2K6,MTPN,CCBE1,PARK7,ADAMTS18,MYOCD,MEF2C,ADGRB1,RXRA,WNT7A,NDFIP1,WASF3,FBXW8,SDCBP,PASK,FLVCR1,FGR,EPHA4,NTRK2,IL1RAPL1,NUMB,ADAMTS9,WNT2B,TNNI1,CD101,SHISA6,IL17RD,FBXO31,PRKAB1,PTK2,CDH5,NFKBID,CLDN18,CYFIP1,SEMA4D,JAM2,ZBTB20,FAT4,RUNX1,NOS1AP,PDCL3,GPR55,TNFSF11,PPM1F,ADGRL2,HDAC2,CRTAM,APELA,TET1,ASB3,NTN4,GPR137B,EPHB1,GRM5,RPS6KA5,PTPRG,NRP1,PRKCA,ATPSCKMT,FAIM,RC3H1,CHODL,POR,MCC,BCR,FBLN1,RAG1,BMPER,CUX1,MACROH2A1,MITF,EPHB2,CD38,MET,SERPINB2,ATG5,MLIP,FLRT2,MYB,KALRN,GNAS,LAMA1,SERPINB7,TIAM2,BMP7,DLG5,TMEM25,PDGFC,SLIT2,PLCL1,ERBB4,IL20RB,SYNDIG1,ROBO1,PBX1,PRKCQ,TRDN,NLGN1,SHLD2,NOS1,SLC6A3,ASIC2,EFNA5,EHMT1,ESR1,LOXL2,CACNA2D1,PRLR,HLA-F,CCR2,STARD13,SEMA4B,HRH1,ROCK2,PRDM1,RORA,STMP1,IL16,DMRT1,HSPG2,COL4A3,HOOK3,FSTL4,STK3,ZNF423,IGF1R,GLI2,THRB
GO:0097485	neuron projection guidance	1.531076770303552e-8	*NOTCH2,CNTN4,ROBO2,DSCAM,CDH4,NEO1,CNTN6,EPHA7,ADAMTSL1,APP,SEMA5A,BMPR1B,ALCAM,NCAM1,CHN1,NFIB,PRTG,ENAH,SEMA3C,SEMA6D,TNR,USP33,PTPRO,TRIO,EXT1,PLXNA2,SEMA3E,EPHA6,BCL11B,ECE1,CNTN1,SEMA3A,UNC5D,SEMA3D,RELN,DRAXIN,GLI3,DCC,NTN1,DPYSL5,LAMA3,LMX1A,VASP,NRXN1,GAP43,NECTIN1,EPHA4,LHX9,PTK2,FEZ2,CYFIP1,SEMA4D,FYN,CNTN5,EPHB1,RPS6KA5,NRP1,NRXN3,EPHB2,FLRT2,KALRN,LAMA1,BMP7,SLIT2,CCDC141,ROBO1,PRKCQ,EFNA5,SLIT3,SEMA4B,GLI2*
GO:0007420	brain development	1.533557565019834e-8	*CNTN4,IMMP2L,TRAPPC9,PLCB1,DLC1,BCL2,ODAD2,ALDH1A2,ROBO2,ZEB1,RARB,ALK,NEGR1,CNTNAP2,CRKL,SETD2,SLC4A10,EGFR,SOX6,ATP2B2,PHACTR1,EPHA7,RAPGEF2,LRP2,SSBP3,APP,DCLK1,SEMA5A,SYT1,SLC8A1,SRGAP2C,CTNNA2,KLHL1,MCPH1,ZSWIM6,NRG3,NIPBL,PAFAH1B1,NF2,CTNNA1,MEIS2,NFIB,SYNJ1,TMEM108,SYNE2,BBS2,WNT9B,SEMA6D,MAPKAP1,TNR,ATRX,ELAVL4,ABL1,SLC1A1,DNAH5,FGF10,GRID2,NRG1,ASPM,EGF,EXT1,PLXNA2,KDM7A,SEMA3E,HERC1,KNDC1,MNAT1,BCL11B,MBP,AK8,CNTN1,BRCA2,DISC1,SEMA3A,BMP2,RELN,FGF9,TTC21B,TSPAN2,SRGAP2,NIN,DRAXIN,GLI3,CASP5,RERE,ATF2,BBS4,DAB1,GRIN2A,ATXN1,TOX,SHANK2,ZFHX3,PBX3,NDRG2,BMP5,KCNC1,GHRH,PRKG1,GRIN2B,SYNJ2,MED1,ATRN,LMX1A,TACC2,ANP32B,NRXN1,CADM1,AKT3,CELSR2,ATP2B1,EML1,SHROOM2,SLC6A11,MTPN,POU1F1,FOXP2,CEP120,WNT7A,GABRA5,NTRK2,NUMB,WNT2B,LAMB1,UBE3A,FAT4,FYN,ADGRL2,ARL13B,HYDIN,ATP5PF,UGP2,ARNT2,CDH2,CNTN5,ITGA8,XRN2,EPHB1,PTPRG,NRP1,BCR,STK36,EPHB2,SLC1A2,CA10,BMP7,DLG5,KIRREL3,BPTF,SLIT2,CCDC141,ERBB4,ROBO1,PBX1,CTTNBP2,SLC6A3,KDM4B,FOXB1,MACROD2,RORA,ATAT1,HSPG2,NCOA6,RGS7,HOOK3,ZNF423,IGF1R,GLI2*
GO:0050804	modulation of chemical synaptic transmission	1.851677784471103e-8	*CNTN4,NSG1,PTPRD,SLC24A2,LRRC4C,UNC13C,PLCB1,PTPRA,STXBP1,ERC1,LRFN2,CHRNA7,RIMS1,RIMS2,MCTP1,CACNG2,DLGAP1,TSHZ3,SLC4A10,BTBD9,GRIK3,DGKI,GRIA1,SLC8A3,RAPGEF2,GRM7,APP,CACNB2,STAU2,SYT1,NTF3,ERC2,SYN3,PRKCZ,MAPK1,NRG3,SORCS3,GRIK4,PPP1R9A,AKAP9,RASGRF2,GRM1,TMEM108,CDH11,USP8,TNR,GRM8,ELAVL4,ABL1,SLC1A1,RAP1A,GRID2,RASGRF1,PRKCE,DGKB,HTR2A,APBA2,PACSIN2,DISC1,RELN,GRIK2,MCTP2,ZZEF1,DCC,PRKN,MTMR2,DLGAP2,*

			GRIN2A,SHISA9,SHANK2,PLCB4,GRID1,NMU,IGSF11,HCN1,GRIN2B,CELF4,ROR2,CLSTN2,NRXN1,JAK2,PRKCB,SLC6A1,MEF2C,ADGRB1,WNT7A,S100B,EPHA4,NTRK2,SHISA6,GRIK1,SCGN,CYFIP1,FYN,CDH2,FBXL20,EPHB1,GRM5,BCR,NRXN3,EPHB2,CD38,CACNG3,TMEM25,GRM3,PLCL1,SORCS2,NLGN1,NTNG1,CCR2,HRH1
GO:0099177	regulation of trans-synaptic signaling	2.147304317530614e-8	*CNTN4,NSG1,PTPRD,SLC24A2,LRRC4C,UNC13C,PLCB1,PTPRA,STXBP1,ERC1,LRFN2,CHRNA7,RIMS1,RIMS2,MCTP1,CACNG2,DLGAP1,TSHZ3,SLC4A10,BTBD9,GRIK3,DGKI,GRIA1,SLC8A3,RAPGEF2,GRM7,APP,CACNB2,STAU2,SYT1,NTF3,ERC2,SYN3,PRKCZ,MAPK1,NRG3,SORCS3,GRIK4,PPP1R9A,AKAP9,RASGRF2,GRM1,TMEM108,CDH11,USP8,TNR,GRM8,ELAVL4,ABL1,SLC1A1,RAP1A,GRID2,RASGRF1,PRKCE,DGKB,HTR2A,APBA2,PACSIN2,DISC1,RELN,GRIK2,MCTP2,ZZEF1,DCC,PRKN,MTMR2,DLGAP2,GRIN2A,SHISA9,SHANK2,PLCB4,GRID1,NMU,IGSF11,HCN1,GRIN2B,CELF4,ROR2,CLSTN2,NRXN1,JAK2,PRKCB,SLC6A1,MEF2C,ADGRB1,WNT7A,S100B,EPHA4,NTRK2,SHISA6,GRIK1,SCGN,CYFIP1,FYN,CDH2,FBXL20,EPHB1,GRM5,BCR,NRXN3,EPHB2,CD38,CACNG3,TMEM25,GRM3,PLCL1,SORCS2,NLGN1,NTNG1,CCR2,HRH1*
GO:0007169	transmembrane receptor protein tyrosine kinase signaling pathway	2.500754923438 7142e-8	*BCAR3,ANKS1B,PLCB1,PTPRA,MYO1E,ALK,ERBIN,APC,CRKL,PTPRJ,EGFR,ANGPT1,NEDD4,NTRK3,FLT1,PRKD1,PAK1,EPHA7,RAPGEF2,RUNX2,FGF12,GHR,COL4A2,NTF3,PTPRR,KANK1,ARNT,PAK3,PRKCZ,GRB10,SHC4,MAPK1,PDGFD,NRG3,GFRA1,CHN1,EFEMP1,ADAMTS3,TIAM1,TMEM108,BLK,MBD5,PTPRT,ABL1,PTPN12,PRKAA1,FGF10,NRG1,MUSK,EGF,EXT1,PTPN2,ARHGEF7,EPHA6,EPN2,GFRA2,CCND3,PLCE1,TGFA,HIP1,CRIM1,VAV1,BMP2,SOGA1,FGF9,ZNF106,DSTYK,FAM83B,SMARCC1,SNX6,SMOC2,IDE,GAREM1,NEU3,SLC30A10,PTPRE,NGEF,ARID5B,ARHGEF28,LYN,INSR,CUL5,ZDHHC17,JCAD,ENPP1,BMP5,CSF1,GHRH,GRB14,ROR2,KL,IQGAP1,CAMLG,NRXN1,JAK2,SVEP1,PRKCB,NEDD9,ABI1,CCBE1,MYOCD,CYFIP2,NCK1,FGR,EPHA4,NTRK2,MVB12B,PTK2,CYFIP1,FAT4,CNKSR1,CCDC88A,FYN,DOK5,ZFYVE28,ROR1,EPHB1,PTPRG,PID1,NRP1,SAMD12,ITGA1,KIF16B,EPHB2,MET,CDH13,STXBP4,FLRT2,KALRN,PDGFC,ERBB4,ROBO1,PRKCQ,EFNA5,PRLR,PIK3R3,FER,COL4A3,FSTL4,IGF1R*
GO:0048589	developmental growth	3.895050962230821e-8	*NOTCH2,MTOR,WWC1,BNC2,ULK2,SCAPER,FTO,PLCB1,ZFPM2,TENM4,BCL2,RIMS1,RARB,RIMS2,AUTS2,DSCAM,SLC4A10,MACF1,BCL11A,TMEM182,CDH4,LARGE1,EPHA7,TMEM38B,GHR,NEDD4L,APP,DCLK1,SEMA5A,SYT1,VCL,AURKA,BMPR1B,AKAP6,DIP2B,TRPC5,NBN,PRKCZ,COL27A1,COBL,YAP1,ALCAM,PLG,NIPBL,FMN1,PAFAH1B1,PPARA,SEMA3C,TMEM108,JARID2,BBS2,SEMA6D,TNR,CXADR,MBD5,ATRX,ABL1,PTPN12,GAS2,FGF10,LATS2,NRG1,ASPM,MUSK,EXT1,ATP8A2,SEMA3E,APBA2,EVC,AFG3L2,PDLIM5,BRCA2,DISC1,SEMA3A,RC3H2,SEMA3D,FGF9,SLC23A2,PLS1,NIN,DRAXIN,GLI3,MAP2,ATF2,BBS4,KPNA1,DCC,SELENON,PRKN,TBX20,PCDH15,ARID5B,ITSN2,PTGFRN,NTN1,INSR,COLQ,CD9,CSF1,GHRH,CTDP1,TNN,PSAP,MED1,KDM6A,ATRN,IQGAP1,YBX3,GAP43,MTPN,IMPACT,ITGA4,NCAPG2,CYFIP2,MEF2C,WNT7A,FLVCR1,PPP2R3A,CYFIP1,UBE3A,SEMA4D,RUNX1,SORBS2,SPAG6,EYS,NRP1,POR,MAGI2,SLC1A2,GNAS,CPQ,SLIT2,ERBB4,SLC6A3,EFNA5,SLIT3,ESR1,PRLR,RAD51B,SEMA4B,FSTL4,STK3,GLI2,AKAP13*
GO:0009887	animal organ morphogenesis	4.358782473292 5886e-8	*NOTCH2,BCAR3,MTOR,FREM1,ZFPM2,DLC1,RIPOR2,RP1,BCL2,ALDH1A2,FBN1,ROBO2,TENM3,ZEB1,RARB,USH2A,PAPPA2,MLLT3,GPC6,MYO3B,DSCAM,CRKL,SOX5,SETD2,SLC4A10,EGFR,CRB1,SOX6,CHSY1,FLI1,RXFP1,EDAR,LRP2,RUNX2,PRICKLE2,GHR,STAU2,BMPR1B,CTNNA2,IFT57,COL27A1,YAP1,MAPK1,ZNRF3,ABLIM1,NRG3,NIPBL,FAT3,FMN1,PAFAH1B1,EFEMP1,TPM1,NF2,CTNNA1,ANKRD11,PPARA,MEIS2,NFIB,NR5A2,TIAM1,SEMA3C,SLC24A4,ALPK2,DNAH11,CPE,RYR2,BBS2,WNT9B,XIRP2,ABL1,SLC1A1,FGF10,NRG1,AP3B1,SETDB2,FBN2,EXT1,ATP8A2,CNNM4,ALPL,FHL2,MYLK2,BCL11B,TGFA,EGFLAM,MTHFD1L,WDPCP,BMP2,PTCD2,ASXL3,PDE6C,FGF9,TDRD7,PLS1,GLI3,MEGF11,SMARCC1,CUL1,LAMC1,ATF2,BBS4,LAMC3,COL5A1,CFTR,RORB,MYO3A,TBX20,PCDH15,ARID5B,CDH23,SCN10A,ADGR*

			G6,LRIG1,OVOL2,NTN1,MMP16,HOXC13,INSR,PBX3,SNAI2,ASH1L,HOXC4,SP3,NSD2,DZANK1,BMP5,WDR72,CSF1,HCN1,LAMA3,DHRS3,FOXN3,SLC40A1,MED1,KDM6A,ATRN,AJAP1,FAT1,TTC39C,SOSTDC1,HIPK1,TWIST1,AKT3,VSX1,CELSR2,ADAMTS5,ALX4,USH1C,ITGA6,OTOP1,EXT2,GRXCR1,STAT1,SHROOM2,CSMD1,ASB2,MEF2C,WNT7A,RBPMS2,NECTIN1,WWOX,FLVCR1,FGR,PPP2R3A,NTRK2,LHX9,WNT2B,TNNI1,PTK2,ANKRD6,LAMB1,FAT4,ADAMTS16,TNFSF11,ARL13B,HDAC2,TBX15,COL18A1,ROR1,CDH2,ITGA8,NTN4,EPHB1,NRP1,SDK1,POR,BCR,DGCR2,EPHB2,MAGI2,FLRT2,LAMA1,GREB1L,ATRNL1,BMP7,DLG5,PDGFC,EYA1,SLIT2,EXOC4,ERBB4,ROBO1,PBX1,SLIT3,ESR1,NTNG1,RPGRIP1,PRDM1,HSPG2,PTPRQ,APCDD1,GLI2,THRB
GO:0006468	protein phosphorylation	5.254943632760095e-8	BCAR3,MTOR,ULK2,NLK,KSR1,PTPRA,PDE4D,ERC1,BCL2,CHRNA7,PIK3C3,SPRED1,ALK,MAP3K9,MYO3B,APC,CRKL,ERG,TNIK,PTPRJ,NEK4,EGFR,ANGPT1,CDK12,PRKACB,NEK7,PTPN4,NTRK3,FLT1,SLC8A3,PRKD1,PAK1,EPHA7,RAPGEF2,PELI2,TAOK3,SMYD3,RPTOR,GHR,ADAM10,APP,RPS6KA2,SAMSN1,PHKB,DCLK1,NTF3,AURKA,SLC8A1,MAP4K4,BMPR1B,PAK3,ITPKB,TRPC5,NBN,PRKCZ,TAF4B,DUSP22,RIPK4,MAPK1,PDGFD,NRG3,GFRA1,STK38,PTPN13,MYLK3,LIMCH1,EFEMP1,CCNG2,TLK1,NF2,MOB3B,BIRC6,AKAP9,RSRC1,PAK5,DAPK1,STK32B,ALPK2,IL34,MELK,WNT9B,DUSP16,PARD3,MAPKAP1,BLK,NUAK1,PTPRT,ABL1,SLC1A1,PRKAA1,MAST4,RAP1A,CAMK4,FGF10,PEAK1,LATS2,NRG1,MUSK,ZNF675,PRKCE,SLCO3A1,WNK2,CD44,PTPRO,EGF,ALPK3,PRRC1,TRIO,SPRED2,RPS6KA3,PTPN2,STK38L,HTR2A,TAF3,MARK2,EPHA6,HIPK3,CDKN2C,GRK3,KNDC1,CLSPN,STK10,MNAT1,MYLK2,HMGA2,CCND3,STK32A,PLCE1,TGFA,PRR5L,LDLRAD4,CNTN1,BLM,NRK,BMP2,MYLK4,CDC42BPB,VRK1,BMP2K,RELN,GNAQ,FGF9,SH3BP5,DSTYK,SNX6,CNKSR3,NEK10,MOB1B,ATF2,MAPK8IP1,KITLG,MAP4K3,TADA2A,DAB1,RB1CC1,MYO3A,PRKN,CDC42BPA,MAPK10,TRPM6,PRKCH,HUNK,IL6R,ALS2,MKNK1,SNX25,PTPRB,COPS8,MOK,RALB,ROCK1,LYN,CHKA,INSR,NEK6,ERN2,CARD10,ENPP1,RASGRP1,SNX9,CSNK2A1,BMP5,CSF1,PRKG1,SMAD5,GPRC5C,ROR2,BANK1,PRKAA2,CSF2RB,IQGAP1,NRXN1,HIPK1,CENPE,AKT3,ALKAL2,JAK2,MADD,TNKS,PRKCB,RTRAF,BRD4,NEDD9,NRBP1,MAST2,MAP2K6,ABI1,CEMIP,IMPACT,PARK7,MAPK8,PPME1,NCAPG2,MYOCD,MEF2C,MAP3K5,MAP3K4,PKN2,DBF4B,SDCBP,PASK,MLLT1,NCK1,FGR,CDCA8,TOP1,EPHA4,NTRK2,OCLN,TRPM7,PRKAB1,PTK2,MARK4,CDH5,SEMA4D,KIRREL1,PDCL3,CCDC88A,TNFSF11,FYN,BUB1,PPM1F,HDAC2,SH2D3C,DOCK3,ZFYVE28,MAPK9,ROR1,EPHB1,GRM5,RPS6KA5,PID1,NRP1,PRKCA,BCR,SNRK,FBLN1,STK36,BMPER,MACROH2A1,EPHB2,CSNK1G1,CDK14,MET,CAMK1G,KALRN,LAMA1,BMP7,PDGFC,ABL2,SLIT2,CNOT7,ERBB4,ROBO1,PRKCQ,NOS1,EFNA5,NSD1,PRLR,HTT,CAMK1D,PIK3R3,FER,ROCK2,PDK1,STK3,DEPTOR,IGF1R,PRKAG2,AKAP13,MORC3
GO:0060322	head development	5.415562675149977e-8	CNTN4,IMMP2L,TRAPPC9,PLCB1,DLC1,BCL2,ODAD2,ALDH1A2,ROBO2,ZEB1,RARB,ALK,NEGR1,CNTNAP2,CRKL,SETD2,SLC4A10,EGFR,SOX6,ATP2B2,PHACTR1,EPHA7,RAPGEF2,LRP2,SSBP3,APP,DCLK1,SEMA5A,SYT1,SLC8A1,SRGAP2C,CTNNA2,KLHL1,MCPH1,ZSWIM6,MAPK1,CRISPLD2,NRG3,NIPBL,PAFAH1B1,NF2,CTNNA1,ANKRD11,MEIS2,NFIB,SYNJ1,TMEM108,SYNE2,BBS2,WNT9B,SEMA6D,MAPKAP1,TNR,ATRX,ELAVL4,ABL1,SLC1A1,DNAH5,FGF10,GRID2,NRG1,ASPM,EGF,EXT1,PLXNA2,KDM7A,SEMA3E,HERC1,KNDC1,MNAT1,BCL11B,MBP,AK8,CNTN1,BRCA2,DISC1,SEMA3A,BMP2,RELN,FGF9,TTC21B,TSPAN2,SRGAP2,NIN,DRAXIN,GLI3,CASP5,RERE,ATF2,BBS4,DAB1,GRIN2A,ARID5B,ATXN1,TOX,SHANK2,ZFHX3,PBX3,NDRG2,BMP5,KCNC1,GHRH,PRKG1,GRIN2B,SYNJ2,MED1,ATRN,LMX1A,TACC2,ANP32B,NRXN1,CADM1,AKT3,CELSR2,ATP2B1,EML1,SHROOM2,SLC6A11,MTPN,POU1F1,FOXP2,CEP120,WNT7A,FLVCR1,GABRA5,NTRK2,NUMB,WNT2B,LAMB1,UBE3A,FAT4,FYN,ADGRL2,ARL13B,HYDIN,ATP5PF,UGP2,ARNT2,CDH2,CNTN5,ITGA8,XRN2,EPHB1,DDX10,PTPRG,NRP1,BCR,STK36,EPHB2,SLC1A2,CA10,BMP7,DLG5,KIRREL3,BPTF,SLIT2,CCDC141,ERBB4,ROBO1,PBX1,CTTNBP2,SLC6A3,KDM4B,FOXB1,MACROD2,RORA,ATAT1,HSPG2,NCOA6,R

			GS7,HOOK3,ZNF423,IGF1R,GLI2
GO:1901564	organonitrogen compound metabolic process	5.735515533067498e-8	BCAR3,MTOR,SPOCK1,NSG1,SLC17A1,IMMP2L,PTPRD,TMTC1,SLC25A21,ULK2,NLK,LONP2,FTO,KSR1,AGBL1,PLCB1,TTC3,TMPRSS2,DLC1,TNRC6B,DPP10,ZDHHC21,PTPRA,PDE4D,RDX,ERC1,NME7,SLC44A5,BCL2,PRDM16,LPCAT2,F13A1,GPHN,CHRNA7,PIK3C3,EPC2,SPRED1,GALNT1,ENPEP,MINAR1,PCMTD1,ALK,AUTS2,CARMIL1,PJA2,BABAM2,PAPPA2,HACD2,HLCS,GLYAT,MLLT3,EGLN3,MAP3K9,MYO3B,MOCOS,SPON1,CPA6,APC,RTN1,CRKL,SETD2,ERG,TNIK,PTPRJ,KDM4C,NEK4,EGFR,USP14,ANGPT1,CDK12,PRKACB,NEK7,NCOR1,RNF220,NEDD4,MTRF1,GNPTAB,NSMCE2,BTBD9,BCL11A,PSMB2,SGMS1,CHSY1,RPRD1A,PTPN4,B3GALT5,NTRK3,LARGE1,C5,TUSC3,FBXL7,CYP2C9,FLT1,ADAMTS6,TASP1,PSMA8,CAST,SLC8A3,PRKD1,TPTE2,PAK1,EPHA7,NCOA7,CHRM3,ADSS2,CHSY3,RAPGEF2,PELI2,LRP2,CPS1,TAOK3,LDLRAD3,CPEB4,AGK,BCKDHB,SLC44A1,UBE2L3,PPP2R2B,PUM3,PTPRN2,SMYD3,TYW1,HERC2,LRGUK,RPTOR,GHR,WDSUB1,NEDD4L,ADAM32,ADAM10,HDAC9,UBE2G1,APP,ADK,RPS6KA2,SAMSN1,KYNU,KDM1B,KLHL13,PHKB,DCLK1,USP18,NTF3,ACER2,PARP15,CD2AP,AURKA,PARN,ST18,SLC8A1,PTPRR,MARCHF1,SERPINA6,PLGRKT,UPP2,ECPAS,MAP4K4,BMPR1B,FMN2,PCSK6,ARNT,PAK3,TTLL7,DIP2B,RANBP2,LARP1,ITPKB,TRPC5,PDE10A,UBE2E2,RAP1GDS1,HHAT,CHST8,NBN,CUBN,IFT57,PRKCZ,SPOP,MAN2A2,DIP2A,ST8SIA5,HECW1,ADAMTS17,PHF19,TAF4B,SENP6,DUSP22,GALNT14,SEM1,WDR70,PPM1L,RIPK4,MAPK1,MGAT5,ITIH5,SGTB,ADAM22,USP25,KMT2E,PLG,PAPPA,PCGF5,PDGFD,ZNRF3,XXYLT1,NRG3,UBE2O,GFRA1,NIPBL,GALNT16,RNGTT,CARD18,STK38,PTPN13,MYLK3,ACSBG1,KANSL1,LIMCH1,PAFAH1B1,ATF6,EFEMP1,TLL1,DCAF1,ITGB8,CCNG2,TLK1,NF2,MRPS22,ZDHHC14,CORIN,MOB3B,BIRC6,AKAP9,KLF15,PPARA,HS3ST2,ERMP1,PPP6R3,ADAMTS3,UBE3D,RSRC1,PTPRK,PAK5,ST6GALNAC3,TRERF1,PPP2R5E,PDZRN3,EIF3D,DAPK1,FAR2,ACSM2B,AGO2,STK32B,ALPK2,JARID2,GATAD2B,CPE,IL34,ADGRV1,MELK,WNT9B,HECTD4,CLPX,ASAH2B,DUSP16,MRPS35,USP8,PARD3,MAPKAP1,TNRC6C,PIAS1,UBE2R2,BLK,OLA1,ATRX,NUAK1,PTPRT,ELAVL4,ABL1,PTPN12,HDAC4,OXR1,SLC1A1,PRKAA1,MRPS27,TTLL5,EIPR1,ADAMTS14,MAST4,GUCY1A2,PSMF1,ATE1,RAP1A,HECTD2,CAMK4,BAZ2A,MANBA,FGF10,FBXL13,GALC,TGM1,PEAK1,LATS2,NRG1,AP3B1,PAH,ABCB7,ZBTB16,MUSK,GALNTL6,ZNF675,SMARCAD1,SETDB2,PRKCE,FOXK2,SLCO3A1,METAP1D,NXN,WNK2,USP33,CERS6,CD44,PTPRO,EGF,ALPK3,PRRC1,TRIO,EXT1,NSMAF,LNPEP,SPRED2,ADAMTS2,RPS6KA3,MARCHF8,SCG5,MTMR3,PTPN2,TRIM5,ATXN3,ST8SIA6,ALG10B,AMBRA1,STK38L,GALNT10,KDM7A,PRMT8,HTR2A,FANCM,FANCA,DAZL,FARS2,PPP2R2C,TAF3,RPRD1B,MARK2,GMPR,TMEM67,ALPL,C10ORF90,ABHD17C,PUM1,HERC1,IGF2BP3,EPHA6,SLC2A13,HIPK3,CDKN2C,GRK3,CPXM2,KNDC1,SPSB4,CLSPN,NOS2,AFG3L2,STK10,MNAT1,TMTC2,ADAM12,MYLK2,XYLT1,HMGA2,CCND3,FOLH1,ECE1,STK32A,CREM,LYPLA1,MBP,CWC27,PLCE1,TGFA,IL17RA,HIP1,CRIM1,XPNPEP1,FUT9,PRR5L,GXYLT2,VPS37A,GSR,CAPN5,MSRA,FBXO32,LDLRAD4,EGFLAM,CNTN1,TARS3,FKBP5,MTHFD1L,SNX3,NAA35,CEPT1,BRCA2,DISC1,GALNT13,DNER,BLM,ASB7,NRK,SLC10A7,MAGI3,ADCY10,BMP2,RC3H2,MYLK4,TRAK1,WDR26,CDC42BPB,DSE,VRK1,RANBP9,TTR,TPGS2,BMP2K,RNF38,PGPEP1,RELN,HMGB1,GNAQ,FGF9,SH3BP5,UST,CPAMD8,TRAF3,GEMIN5,DSTYK,UIMC1,B4GALT6,GLI3,SMARCC1,SNX6,CNKSR3,CASP5,IDE,CUL1,DAW1,NEK10,RRBP1,MOB1B,PIGN,ATF2,CYLD,UMODL1,MAPK8IP1,NELL1,ME2,NEU3,MRPL13,KITLG,ZZEF1,CAMTA1,UBR1,SMPDL3A,MAP4K3,HS3ST4,RCAN1,TADA2A,DAB1,MED27,RB1CC1,MYO3A,UBE2E1,PTPRE,PRKN,MTMR2,SPSB1,CDC42BPA,MAPK10,PNPLA7,ZNF541,FBXO3,GRIN2A,WSB1,USP43,TRPM6,PRKCH,HUNK,TG,IL6R,PEPD,ALS2,CPVL,ACO1,CNOT6L,MKNK1,SNX25,SLC4A4,PTPRB,AOPEP,COPS8,TSPAN33,ST8SIA1,USP7,PSMA1,ENPP3,HAAO,FAH,MOK,RALB,FAR1,ROCK1,LYN,VCAM1,SEL1L,CTSB,EIF2B3,SLC44A2,GSTA3,SUMO3,DTX1,BZW1,PIWIL3,ADA2,CHKA,MMP16,FANCL,BANP,RNF152,OTUD7A,INSR,CUL5,YTHDF3

			,NEK6,HECTD1,SLC52A1,HDAC11,LYPLAL1,SUMO2,ADAMTS19,DPYD,ARFGEF1,SNAI2,ASH1L,BID,SIAH2,PIGK,OSBPL10,PGAP4,TRABD2B,UFD1,ERN2,MBTPS2,TRIM58,ZDHHC17,NSD2,CERS3,PTAR1,CARD10,LTN1,CTIF,SAMHD1,ENPP1,ENTPD5,MOCS2,RASGRP1,SNX9,PAMR1,ANAPC1,UCK2,CSNK2A1,BMP5,CSF1,PPIL6,EOGT,SERPINB9,CTDP1,PRKG1,HS6ST3,ASB4,GRIN2B,FANCB,CLNS1A,CNMD,SMAD5,CELF4,ABCG1,MARCHF11,DCUN1D4,PRAME,KLHL7,PSAP,LPGAT1,PSMA5,MED1,ATG4B,CDC14B,KDM6A,IL33,GPRC5C,ROR2,CFH,PPP2R2A,NPL,KL,BANK1,CSDE1,HGD,IL10,SFPQ,PTH,PRKAA2,CSF2RB,GLYATL1,RNF182,LARP6,PHF20L1,PLA2G4A,IQGAP1,RPS12,CAMLG,ANP32B,YBX3,AIMP1,THNSL2,NRXN1,PCID2,HIPK1,FRY,CYP4F22,FICD,CENPE,NGDN,ELOC,TWIST1,AKT3,ALKAL2,JAK2,ADAM28,MPPE1,BPNT1,MADD,PATL1,PRSS2,CREBBP,MELTF,TNKS,SIAH3,UFL1,ADAMTS5,NFKBIA,PRKCB,FBXW2,GOT2,MIPEP,OVCH1,ZC3H15,ST6GAL2,RTRAF,BRD4,SMPD4,NEDD9,NRBP1,IARS2,CLCA4,DGLUCY,NOXRED1,ASS1,CNDP2,ADCY9,MAST2,HPSE2,ERLIN2,PCMTD2,ZBTB49,EXT2,AGO1,PDP2,GID8,SDS,BRMS1L,NDFIP2,MAP2K6,MARCHF6,MTPN,ABI1,CEMIP,IMPACT,CBLIF,CCBE1,PARK7,ADAMTS18,MAPK8,OAZ2,EIF3F,PPME1,FBXL17,UBL7,UBE2J2,MTF2,NCAPG2,ASB2,MYOCD,DHTKD1,CYFIP2,UBE2QL1,ACACA,ASCC2,ST8SIA4,NDUFA10,MEF2C,ADGRB1,WNT7A,MAP3K5,NDFIP1,MAP3K4,TRIM43B,SERPINI2,PRDM13,TRIM43,SUMF1,MAGEL2,PKN2,PDE2A,RAB38,DBF4B,FBXW8,SDCBP,SPPL2B,NSMCE1,PASK,MLLT1,NCK1,FLVCR1,FGR,CDCA8,PPP2R3A,TRIM23,ATP6V1B2,TOP1,TINAG,C2,RNF8,EPHA4,MECOM,NTRK2,ACSM2A,ADAMTS9,OCLN,FBXO31,EXTL3,TRPM7,PRKAB1,IREB2,MVB12B,HS6ST1,PTK2,MARK4,CDH5,CD5L,TPH2,APOL2,APIP,CYFIP1,UBE3A,APOL1,SEMA4D,SERPINB10,ZBTB20,RUNX1,KIRREL1,AMFR,CTSE,SLC26A2,POMT2,NOS1AP,MTTP,DPY19L2,TPTE,PDCL3,SRP9,CCDC88A,UBAP1L,GALNT18,HKDC1,ADAMTS16,TNFSF11,FYN,BUB1,KDM5A,DPY19L1,PPM1F,SDE2,UHRF2,HDAC2,SLF1,SH2D3C,PSME3IP1,DOCK3,TRNAU1AP,NCS1,GALNT17,ATP5PF,MTMR7,ZFYVE28,MAPK9,PABPC1,STT3A,SLC39A8,ROR1,SLC16A9,GALNT2,FUT8,TET1,ASB3,HECW2,OVCH2,SEL1L2,FBXL20,EPHB1,ZDHHC18,GRM5,GDAP1L1,SPOPL,RPS6KA5,PTPRG,PID1,NRP1,MIDEAS,PRKCA,MRPL58,COX10,ATPSCKMT,RNF215,USP24,FHIT,ITGA1,PCCA,CROT,RNF138,RC3H1,POR,EFL1,ZNF738,SUPT3H,BCR,TUT4,SNRK,TM9SF2,SENP8,USP49,ELP2,FBLN1,STK36,RAG1,DNPEP,BMPER,DPP6,MACROH2A1,EPHB2,CSNK1G1,BCL2L13,RNF11,CD38,DPH6,PPIL2,PRSS51,CDK14,MET,SPPL3,DLG2,CAMK1G,IBA57,SERPINB2,ATG5,USP32,MAGI2,UNK,ADAM29,MYB,KALRN,SLC1A2,CHST3,LAMA1,MFHAS1,SERPINB7,CPQ,TRIM9,DHX29,BMP7,CHIT1,TMPRSS15,TNFAIP8,RNF217,PRSS23,BTD,AK3,KMT2C,DDX6,PDGFC,ELOVL7,ABL2,MMP26,MRPL37,BACE2,NECAB1,EYA1,TTLL11,SLIT2,PARP8,CMPK1,TMPRSS3,CNOT7,ESCO1,ERBB4,SERPINB11,GSAP,TRHDE,ROBO1,SAMD4A,PRKCQ,ANTXR1,MGMT,AK9,NOS1,SLC6A3,GLDC,PRR16,EFNA5,NSD1,EHMT1,USP31,KDM4B,LOXL2,PRLR,PIGB,AGO3,HTT,LARS2,ZDHHC11B,CAMK1D,PIK3R3,MACROD2,CDKAL1,AK2,FER,EYA2,OSBPL5,A2M,CHFR,PCMT1,AUH,OARD1,SPOCK3,ROCK2,PRDM1,RORA,ATAT1,NARS2,EIF4G3,PPP1CB,PDK1,PSMD2,PTPRQ,HERPUD1,NCOA6,TRIM2,COL4A3,WASHC1,PCSK2,BARD1,STK3,DEPTOR,PNPLA8,HNRNPU,LINC00240,RAB3GAP2,TULP4,IGF1R,PRKAG2,AKAP13,MORC3,DNM1L
GO:2000145	regulation of cell motility	7.679421596483845e-8	MTOR,PLCB1,TAFA5,DLC1,RIPOR2,RDX,BCL2,SPRED1,CARMIL1,MCTP1,RIN2,ANO6,APC,CRKL,PTPRJ,DOCK10,EGFR,ANGPT1,MACF1,SCAI,NTRK3,C5,PHACTR1,FLT1,PRKD1,PAK1,RAPGEF2,ARSB,ONECUT1,LDB2,CCL28,ADAM10,HDAC9,IL1R1,APP,MTUS1,DOCK8,MAPRE2,SEMA5A,VCL,NTF3,SLC8A1,PTPRR,SRGAP2C,SRGAP2B,KANK1,MAP4K4,CTNNA2,PAK3,DUSP22,MAPK1,MGAT5,PDGFD,NRG3,NIPBL,LIMCH1,TPM1,NF2,CTNNA1,TIAM1,PTPRK,SEMA3C,NAV3,AGO2,IL34,BCAS3,SYNE2,BBS2,SEMA6D,TNR,DOCK4,PTPRT,ABL1,HDAC4,FGF10,NRG1,PRKCE,EGF,PLXNA2,ARHGEF7,ATP8A1,SEMA3E,GCSAML,STK10,EMILIN2,DOCK5,FUT9,PRR5L,TJP1,LDLRAD4,IQSEC1,WDPCP,SEMA3A,BMP2,

			UNC5D,RIN3,SEMA3D,RELN,HMGB1,FGF9,DOCK1,SRGAP2,SLAMF1,ETS1,SMOC2,BBS4,KITLG,DACH1,IL6R,ROCK1,LYN,NTN1,INSR,YTHDF3,SNAI2,CD9,CARD10,JCAD,TWIST2,BMP5,CSF1,PRKG1,LAMA3,TNN,IL33,ROR2,ABHD2,TWIST1,AKT3,JAK2,ABCC8,NEDD9,ITGA6,MEOX2,BRMS1L,CEMIP,CCBE1,ITGA4,MYOCD,MEF2C,ADGRB1,WNT7A,PKN2,SDCBP,NCK1,FGR,PPP2R3A,EPHA4,NUMB,ADAMTS9,FBXO31,PTK2,CDH5,DIAPH1,LAMB1,SEMA4D,JAM2,WNT5B,PPM1F,HDAC2,APELA,TET1,PTPRG,NRP1,PRKCA,MCC,BCR,FBLN1,RRAS2,BMPER,SRGAP3,MITF,EPHB2,MET,CDH13,MAGI2,FLRT2,LAMA1,BMP7,DLG5,ZMYND8,PDGFC,ABL2,SLIT2,ERBB4,ROBO1,FRMD5,NTNG1,CAMK1D,PIK3R3,FER,CCR2,STARD13,SPOCK3,SEMA4B,ROCK2,WASHC1,IGF1R,DNM1L
GO:0040012	regulation of locomotion	8.763117442391262e-8	*MTOR,PLCB1,TAFA5,DLC1,RIPOR2,RDX,BCL2,ROBO2,SPRED1,CARMIL1,MCTP1,RIN2,ANO6,APC,DSCAM,CRKL,PTPRJ,DOCK10,EGFR,USP14,ANGPT1,MACF1,SCAI,NTRK3,C5,PHACTR1,FLT1,PRKD1,PAK1,RAPGEF2,ARSB,ONECUT1,LDB2,CCL28,ADAM10,HDAC9,IL1R1,APP,MTUS1,DOCK8,MAPRE2,SEMA5A,VCL,NTF3,SLC8A1,PTPRR,SRGAP2C,SRGAP2B,KANK1,MAP4K4,CTNNA2,PAK3,DUSP22,MAPK1,MGAT5,PDGFD,NRG3,NIPBL,LIMCH1,TPM1,NF2,CTNNA1,TIAM1,PTPRK,SEMA3C,NAV3,AGO2,IL34,BCAS3,SYNE2,BBS2,SEMA6D,TNR,DOCK4,PTPRT,ABL1,HDAC4,FGF10,NRG1,PRKCE,PTPRO,EGF,PTPN2,PLXNA2,ARHGEF7,ATP8A1,SEMA3E,GCSAML,STK10,EMILIN2,DOCK5,FUT9,PRR5L,TJP1,LDLRAD4,IQSEC1,WDPCP,SEMA3A,BMP2,UNC5D,RIN3,SEMA3D,RELN,HMGB1,FGF9,DOCK1,SRGAP2,SLAMF1,ETS1,SMOC2,BBS4,KITLG,DACH1,IL6R,ROCK1,LYN,NTN1,INSR,YTHDF3,SNAI2,CD9,CARD10,JCAD,TWIST2,BMP5,CSF1,PRKG1,LAMA3,TNN,IL33,ROR2,ABHD2,TWIST1,AKT3,JAK2,ABCC8,NEDD9,ITGA6,MEOX2,BRMS1L,CEMIP,CCBE1,ITGA4,MYOCD,MEF2C,ADGRB1,WNT7A,PKN2,SDCBP,NCK1,FGR,PPP2R3A,EPHA4,NUMB,ADAMTS9,FBXO31,PTK2,CDH5,DIAPH1,LAMB1,SEMA4D,JAM2,WNT5B,PPM1F,HDAC2,APELA,TET1,GRM5,PTPRG,NRP1,PRKCA,MCC,BCR,FBLN1,RRAS2,BMPER,SRGAP3,MITF,EPHB2,MET,CDH13,MAGI2,FLRT2,LAMA1,BMP7,DLG5,ZMYND8,PDGFC,ABL2,SLIT2,ERBB4,ROBO1,FRMD5,NTNG1,CAMK1D,PIK3R3,FER,CCR2,STARD13,SPOCK3,SEMA4B,ROCK2,IL16,WASHC1,IGF1R,DNM1L*
GO:0044087	regulation of cellular component biogenesis	9.411867144620245e-8	*MTOR,PTPRD,SVIL,DLC1,PTPRA,RIPOR2,RDX,RP1,STXBP1,RALA,IL1RAPL2,ROBO2,SDCCAG8,CDC42EP3,AUTS2,CARMIL1,RHPN2,NEGR1,GPC6,CNTNAP2,MAP4,APC,PLPPR5,ARHGAP24,PTPRJ,MACF1,NEK7,BCL11A,NTRK3,CRACD,SLC39A12,PAK1,EPHA7,RAPGEF2,ADGRB3,LDB2,SEPTIN9,TBCD,ATF7IP,APP,STAU2,VCL,ARHGAP44,SRGAP2C,KANK1,MAP4K4,PAK3,DNM3,PRKCZ,COBL,SENP6,DUSP22,YAP1,SPIDR,CORO2B,LIMCH1,FMN1,TPM1,NF2,AKAP9,SNX30,NAV3,DNAJC15,BCAS3,SYNE2,ABL1,HDAC4,PRKAA1,LRFN5,RAP1A,MYO10,GRID2,PEAK1,NRG1,CLIP1,ABCB7,MUSK,SETDB2,PRKCE,STXBP6,MTMR3,ARHGEF7,AMBRA1,C10ORF90,TMOD2,LINGO2,VPS41,PLCE1,TJP1,LDLRAD4,NPHP4,PDLIM5,WDPCP,RESF1,HMGB1,MDM1,UNC13B,RAP1GAP,KIF15,MAP2,FARP1,CYLD,BBS4,PRKN,TBX20,PRKCH,CNOT6L,KANK4,RALB,ROCK1,ARHGAP28,TENM2,NTN1,BMF,COLQ,ARFGEF1,PDE4DIP,SNAI2,BID,TRABD2B,SH3GLB1,SNX9,ACTR2,CLSTN2,PRKAA2,VASP,MORC2,NRXN1,ANLN,GAP43,SAR1A,MTPN,IMPACT,PARK7,CEP120,CYFIP2,MEF2C,ADGRB1,WNT7A,SDCBP,NECTIN1,NCK1,NTRK2,IL1RAPL1,OCLN,PTK2,MARK4,CDH5,MPP7,FEZ2,CYFIP1,SEMA4D,KIRREL1,SAXO1,ASAP1,CCDC88A,ADAMTS16,PPM1F,ADGRL2,SLF1,MAPK9,EPHB1,SPTB,NRP1,FCHSD2,PRKCA,EPHB2,TOGARAM1,SACS,AKAIN1,MET,CDH17,ATG5,FLRT2,BMP7,DLG5,FHOD3,SLIT2,SYNDIG1,ANTXR1,NLGN1,ASIC2,EFNA5,ESR1,HTT,FER,EPS8,ROCK2,STMP1,ATAT1,WASHC1,HNRNPU,RAB3GAP2*
GO:0007167	enzyme-linked receptor protein signaling	1.0547889810093185e-7	*NOTCH2,BCAR3,PTPRD,ANKS1B,NLK,PLCB1,PTPRA,PRDM16,FBN1,ZEB1,SPRED1,MYO1E,ALK,ERBIN,APC,CRKL,PTPRJ,EGFR,ANGPT1,NEDD4,NTRK3,FLT1,NEO1,PRKD1,PAK1,EPHA7,RAPGEF2,LRP2,RUNX2,FGF12,ONECUT1,BTBD11,GHR,COL4A2,NTF3,PTPRR,KANK1,BMPR1B,PCSK6,ARNT,PAK3,PRKCZ,GRB10,DUSP22,PPM1L,SHC4,MAPK1,PDGFD,NRG3,UBE2O,GFRA1,CHN1,EFE*

	pathway		*MP1,ITGB8,HIVEP1,PPARA,ADAMTS3,TIAM1,PTPRK,TMEM108,BLK,MBD5,PTPRT,ABL1,PTPN12,PRKAA1,FGF10,LATS2,NRG1,MUSK,FBN2,EGF,TRIO,EXT1,LNPEP,SPRED2,PTPN2,ARHGEF7,LTBP1,ZFYVE9,EPHA6,EPN2,GFRA2,CCND3,PLCE1,TGFA,HIP1,CRIM1,VAV1,LDLRAD4,BMP2,PSG9,SOGA1,LEMD3,FGF9,ZNF106,DSTYK,FAM83B,SMARCC1,SNX6,SMOC2,IDE,GAREM1,ATF2,RGMB,NEU3,SLC30A10,PTPRE,TBX20,NGEF,ARID5B,SNX25,ARHGEF28,LYN,OVOL2,INSR,CUL5,NREP,ZDHHC17,JCAD,ENPP1,BMP5,CSF1,GHRH,GRB14,SMAD5,ROR2,KL,SOSTDC1,IQGAP1,CAMLG,NRXN1,PBLD,PEG10,JAK2,FSTL1,SVEP1,CREBBP,PRKCB,NEDD9,CIDEA,BRMS1L,ABI1,CCBE1,MYOCD,CYFIP2,RBPMS2,SDCBP,NCK1,FGR,EPHA4,NTRK2,IL17RD,MVB12B,PTK2,CDH5,CYFIP1,FAT4,CNKSR1,CCDC88A,FYN,HDAC2,DOK5,ZFYVE28,ROR1,FUT8,TET1,ITGA8,EPHB1,PTPRG,PID1,NRP1,SAMD12,ITGA1,KIF16B,BMPER,EPHB2,MET,CDH13,STXBP4,MAGI2,FLRT2,KALRN,BMP7,PDGFC,ERBB4,ROBO1,PRKCQ,EFNA5,PRLR,PIK3R3,FER,DMRT1,COL4A3,FSTL4,ZNF423,IGF1R*
GO:0009888	tissue development	1.562805956929967e-7	*NOTCH2,MTOR,SGCD,BNC2,NEBL,MYO9A,FTO,PLCB1,SVIL,ZFPM2,TENM4,DLC1,ZDHHC21,RIPOR2,PDE4D,RDX,RALA,BCL2,ALDH1A2,ROBO2,ZEB1,AKR1C3,RARB,SPRED1,MYO1E,USH2A,FOXJ2,ANO6,MLLT3,GPC6,SOX5,SETD2,ARHGAP24,EGFR,RFX3,PRKACB,RNF220,SOX6,CECR2,CHSY1,RXFP1,EDAR,PAK1,EPHA7,RAPGEF2,LRP2,DEUP1,RUNX2,CPS1,ONECUT1,TMEM38B,PRICKLE2,SLC24A3,LDB2,GHR,LUZP1,COL4A2,SSBP3,HDAC9,TMC1,SEMA5A,VCL,SLC8A1,BMPR1B,AKAP6,IFT57,COL27A1,MRTFA,COBL,EBF2,YAP1,RIPK4,MAPK1,PDGFD,ZNRF3,LCE1F,MYLK3,FMN1,PAFAH1B1,EFEMP1,ITGB8,TPM1,NF2,RBFOX1,BIRC6,KLF15,PPARA,NFIB,MRTFB,NR5A2,TIAM1,KAZN,SEMA3C,SLC24A4,ALPK2,JARID2,ADGRV1,RYR2,BBS2,WNT9B,RANBP3L,SEMA6D,COL22A1,CXADR,ATRX,XIRP2,ABL1,HDAC4,PRKAA1,RAP1A,FGF10,TGM1,LATS2,NRG1,AP3B1,ZBTB16,SETDB2,PGM5,FBN2,CD44,PTPRO,EGF,ALPK3,EXT1,SPRED2,PLXNA2,AMBRA1,CNNM4,SEMA3E,ALPL,FHL2,LUC7L,EVC,SGCZ,MYLK2,HMGA2,BCL11B,ECE1,TRPS1,TJP1,LDLRAD4,EGFLAM,MTHFD1L,PDLIM5,BRCA2,DNER,WDPCP,SEMA3A,BMP2,PTCD2,BMP2K,SEMA3D,FGF9,TDRD7,ESRP1,ETS2,PLS1,GLI3,SLC9A4,LAMC1,ATF2,UMODL1,BBS4,LAMC3,HIVEP3,COL5A1,CFTR,NELL1,KITLG,RCAN1,SELENON,TBX20,PCDH15,ARID5B,CDH23,PRKCH,IL6R,TFDP1,LCE3B,TOX,MESD,YIPF6,ROCK1,CTSB,OVOL2,NTN1,HOXC13,INSR,DMBT1,HECTD1,SHROOM3,SNAI2,ASH1L,HOXC4,RXRG,FLNB,CERS3,KRT6B,ENPP1,BMP5,WDR72,CSF1,KRT25,CTDP1,LAMA3,CNMD,SMAD5,SLC40A1,MYCL,TNN,PSAP,MED1,ATG4B,KDM6A,ATRN,AJAP1,ROR2,KL,FAT1,IL10,PTH,SOSTDC1,VASP,IQGAP1,YBX3,TWIST1,JAK2,FSTL1,SVEP1,CELSR2,GORAB,PCNA,ANXA4,ALX4,USH1C,ITGA6,ATP2B1,OTOP1,EXT2,KRT6A,MEOX2,GRXCR1,STAT1,MTPN,ABI1,MYO18B,ITGA4,FBXL17,CSMD1,ASB2,MYOCD,KRT85,MEF2C,RXRA,WNT7A,PDE2A,FBXW8,SDCBP,NECTIN1,FGR,PPP2R3A,SPRR2D,LCE3D,EPHA4,EMP1,FNDC3A,ADAMTS9,WNT2B,TNNI1,POSTN,MEGF10,IL17RD,CDH5,ANKRD6,ARHGAP12,LAMB1,PCDH8,SEMA4D,FRMD6,FAT4,RUNX1,AKR1B1,WNT5B,SORBS2,NSUN2,ADAMTS16,TNFSF11,ARL13B,HYDIN,HDAC2,COL18A1,COL19A1,APELA,ROR1,CDH2,ITGA8,NTN4,EPHB1,NRP1,POR,BCR,KIF16B,B9D1,BMPER,MACROH2A1,SGCG,MET,ATG5,NRAP,MAGI2,GNAS,LAMA1,GREB1L,SERPINB7,ATRNL1,BMP7,ASTN2,DLG5,BPTF,EYA1,FHOD3,SLIT2,EXOC4,ERBB4,ROBO1,PBX1,MYH15,SIPA1L3,MGMT,ESR1,NTNG1,LOXL2,PRLR,FOXB1,RAD51B,FER,EYA2,STARD13,SEMA4B,ROCK2,PRDM1,DMRT1,HSPG2,PTPRQ,STK3,HNRNPU,APCDD1,GLI2,THRB,AKAP13*
GO:0018193	peptidyl-amino acid modification	1.876185873421855e-7	*BCAR3,MTOR,ULK2,NLK,AGBL1,ZDHHC21,PDE4D,BCL2,EPC2,SPRED1,GALNT1,ALK,AUTS2,MLLT3,EGLN3,MAP3K9,SETD2,PTPRJ,KDM4C,EGFR,ANGPT1,NSMCE2,BCL11A,PTPN4,NTRK3,TUSC3,FLT1,PRKD1,PAK1,EPHA7,NCOA7,SMYD3,RPTOR,GHR,HDAC9,APP,RPS6KA2,SAMSN1,DCLK1,NTF3,AURKA,ARNT,TTLL7,DIP2B,RANBP2,TRPC5,HHAT,PRKCZ,DIP2A,PHF19,SENP6,DUSP22,MAPK1,MGAT5,KMT2E,PDGFD,GFRA1,GALNT16,STK38,KANSL1*

			,EFEMP1,TLK1,NF2,ZDHHC14,AKAP9,KLF15,STK32B,JARID2,IL34,MELK,PARD3,MAPKAP1,PIAS1,BLK,ATRX,ABL1,HDAC4,OXR1,SLC1A1,PRKAA1,TTLL5,MAST4,CAMK4,FGF10,PEAK1,LATS2,NRG1,MUSK,GALNTL6,SETDB2,PRKCE,METAP1D,CD44,EGF,SPRED2,RPS6KA3,PTPN2,STK38L,PRMT8,HTR2A,MARK2,EPHA6,HIPK3,CLSPN,NOS2,MYLK2,FOLH1,STK32A,CWC27,TGFA,EGFLAM,CNTN1,FKBP5,NAA35,BRCA2,GALNT13,CDC42BPB,VRK1,TPGS2,RELN,SH3BP5,DSTYK,SNX6,CNKSR3,ATF2,KITLG,TADA2A,CDC42BPA,PRKCH,IL6R,MKNK1,ROCK1,LYN,SUMO3,CHKA,INSR,NEK6,SUMO2,SNAI2,ASH1L,ZDHHC17,NSD2,CSNK2A1,PPIL6,EOGT,CLNS1A,KDM6A,ROR2,BANK1,PRKAA2,PHF20L1,NRXN1,HIPK1,TWIST1,AKT3,ALKAL2,JAK2,CREBBP,TNKS,PRKCB,BRD4,NEDD9,MAST2,MAP2K6,ABI1,CEMIP,PARK7,MAPK8,MTF2,NCAPG2,MYOCD,PKN2,NSMCE1,MLLT1,NCK1,FGR,TOP1,EPHA4,NTRK2,PTK2,SEMA4D,NOS1AP,DPY19L2,PDCL3,FYN,DPY19L1,PPM1F,UHRF2,HDAC2,SLF1,SH2D3C,DOCK3,ZFYVE28,MAPK9,ST3A,ROR1,GALNT2,FUT8,EPHB1,ZDHHC18,GRM5,RPS6KA5,NRP1,PRKCA,ATPSCKMT,POR,SUPT3H,BCR,MACROH2A1,EPHB2,CSNK1G1,DPH6,PPIL2,MET,CAMK1G,ATG5,MYB,BMP7,KMT2C,PDGFC,ABL2,EYA1,TTLL11,CNOT7,ESCO1,ERBB4,PRKCQ,NOS1,EFNA5,NSD1,EHMT1,KDM4B,LOXL2,PRLR,ZDHHC11B,CAMK1D,FER,SPOCK3,ROCK2,PRDM1,ATAT1,NCOA6,IGF1R,PRKAG2,MORC3
GO:0072359	circulatory system development	2.095757102982 9237e-7	*NOTCH2,MTOR,SGCD,IMMP2L,NEBL,TAFA5,ZFPM2,TENM4,DLC1,ODAD2,ALDH1A2,FBN1,CHRNA7,ROBO2,RARB,SPRED1,ENPEP,MYO1E,MINAR1,FOXJ2,RIN2,CRKL,SETD2,ARHGAP24,EGFR,ANGPT1,SOX6,NTRK3,C5,FLT1,ADAMTS6,SLC39A12,PRKD1,RAPGEF2,LRP2,ADGRB3,FGF12,LUZP1,COL4A2,ADAM10,HDAC9,RPS6KA2,CACNA1C,SEMA5A,SLC8A1,THSD7A,AKAP6,IFT57,CALD1,YAP1,MAPK1,PLG,PDGFD,NIPBL,MYLK3,ITGB8,TPM1,PPARA,SEMA3C,AGO2,ALPK2,DNAH11,JARID2,CPE,ANK2,BCAS3,RYR2,ANKS6,LDB3,COL22A1,CXADR,XIRP2,ABL1,SLC1A1,DNAH5,RAP1A,FGF10,NRG1,SETDB2,NXN,EGF,ALPK3,EXT1,SEMA3E,FHL2,ANKRD17,EPN2,MNAT1,SGCZ,ADAM12,MYLK2,EMILIN2,HMGA2,ECE1,PLCE1,TGFA,TJP1,PDLIM5,WDPCP,BMP2,PTCD2,FGF9,NFATC2,ETS1,GLI3,SMOC2,DAW1,ATF2,BBS4,COL5A1,GTF2I,RB1CC1,TBX20,IL6R,PTPRB,VAV3,ADGRG6,ROCK1,VCAM1,OVOL2,INSR,HECTD1,SNAI2,RXRG,NSD2,CARD10,JCAD,BMP5,CTDP1,ASB4,CNMD,DHRS3,SMAD5,VSTM4,TNN,MED1,KDM6A,IL10,ANP32B,AIMP1,NRXN1,HIPK1,CACYBP,TWIST1,AKT3,SVEP1,PCNA,PRKCB,ABCC8,AGO1,MEOX2,STAT1,MYO18B,CCBE1,ASB2,MYOCD,MEF2C,ADGRB1,WNT7A,PDE2A,FBXW8,FLVCR1,NTRK2,ADAMTS9,TNNI1,HS6ST1,PTK2,CDH5,FAT4,AP2B1,RUNX1,SORBS2,PDCL3,ARL13B,COL18A1,APELA,CDH2,EPHB1,NRP1,PRKCA,NRXN3,MB,B9D1,BMPER,EPHB2,SGCG,CDH13,ATG5,NRAP,FLRT2,LAMA1,GREB1L,SERPINB7,BMP7,ADGRF5,EYA1,FHOD3,SLIT2,ERBB4,ROBO1,ANTXR1,SLIT3,LOXL2,PIK3R3,CCR2,STARD13,ROCK2,PRDM1,RORA,HSPG2,NCOA6,COL4A3,STK3,HNRNPU,IGF1R,GLI2,AKAP13*
GO:0099173	postsynapse organization	2.100997571602 7933e-7	*PTPRD,LRFN2,GPHN,CHRNA7,CRKL,DOCK10,NTRK3,EPHA7,ADAM10,STAU2,ARHGAP44,PAK3,DNM3,DIP2A,PAFAH1B1,CNKSR2,TMEM108,TANC1,GRID2,MUSK,DGKB,CTNND2,ABHD17C,PDLIM5,RELN,FARP1,MTMR2,NGEF,FRMPD4,SHANK2,MESD,INSR,COLQ,TANC2,GRIN2B,ACTR2,NRXN1,NEDD9,GAP43,WNT7A,EPHA4,IL1RAPL1,SHISA6,UBE3A,NOS1AP,FYN,CDH2,EPHB1,NRP1,EPHB2,PPFIA2,KALRN,NLGN1,IGF1R*
GO:0051641	cellular localization	2.230512802532 9082e-7	*NSG1,EXOC1L,WWC1,ABCA13,IMMP2L,TRAPPC9,LONP2,UNC13C,MX2,CLTCL1,SNAP25-AS1,DPP10,ZDHHC21,ITPR2,RIPOR2,PDE4D,RDX,STXBP1,ERC1,RALA,EPS15L1,BCL2,MYO5A,FBN1,GPHN,COG5,GPR158,RIMS1,PIK3C3,SPIRE1,CNTLN,EXOC6B,MYO1E,TRAPPC8,USH2A,CEP192,RIMS2,MCTP1,ERBIN,FCHO2,CACNG2,GPC6,CNTNAP2,MAP4,APC,MYO5C,CRKL,ILDR2,SETD2,TANGO6,TNIK,EGFR,RFX3,DENND1A,ANGPT1,MACF1,DOCK2,NEDD4,GNPTAB,CRB1,BTBD9,ZFAND6,DNAJC13,RABEP1,DGKI,C12ORF4,NUP214,TOM1L2,CEP128,PRKD1,GRAMD1B,RAPGEF2,LRP2,ARSB,TMEM38B,AGK,RANBP17,UBE2L3,PTPRN2,SYN2,SMYD3,HERC2,SEPTIN9,EPB4*

			1L3,KIF4A,NEDD4L,ADAM10,APP,CACNA1C,CACNB2,DCLK1,STAU2,MAPRE2,SYT1,VCL,ARHGAP44,NDUFAF2,CD2AP,AURKA,PYGO1,SLC8A1,ABCD2,FMN2,AKAP6,RAB8B,RFTN1,RANBP2,RAP1GDS1,KICS2,ERC2,DNM3,CUBN,SCP2,SYN3,IFT57,PRKCZ,RYR3,MCPH1,RAB27B,CNST,YAP1,SEM1,VPS35L,MAPK1,CADPS2,ABCD3,RABGAP1L,SGTB,ADAM22,COPB1,UBE2O,ANKFY1,SYCP1,NIPBL,SLC16A1,SPIDR,IPO11,CORO2B,PAFAH1B1,TM7SF3,STON2,VPS13D,TLK1,NF2,ZDHHC14,CTNNA1,AKAP9,SNX30,SYNJ1,RSRC1,PTPRK,PARD3B,VPS13C,TMEM108,DNAH11,JARID2,RAB22A,DNAJC15,AMPH,CPE,ANK2,ADGRV1,BCAS3,RYR2,SYNE2,BBS2,RANBP3L,NKG7,NBEA,DUSP16,USP8,PARD3,TBC1D5,BLK,DST,ATRX,ABL1,SLC1A1,PRKAA1,FHIP1A,ANO4,CCDC91,EIPR1,DNAH5,NBAS,RAP1A,MYO10,GPC5,PLEKHA8,FGF10,GRID2,LATS2,NRG1,GSG1L,ASPM,AP3B1,SYNE1,ZBTB16,MUSK,SH3GL3,PRKCE,SLMAP,DENND4C,CEP83,FBN2,EGF,STXBP6,PEX14,IFT43,SCG5,TRIM5,ATXN3,HTR2C,RIC3,CLEC16A,LTBP1,ZFYVE9,OPRM1,ABCC4,HTR2A,CYBRD1,STAC,TAF3,ABHD17C,MSH2,APBA2,MAIP1,TNPO3,NOS2,TTC7B,MDFIC,MYLK2,ANK3,COG2,VPS41,LYPLA1,TRAPPC11,ANKFN1,HIP1,PRR5L,VPS37A,EFR3A,TJP1,NPHP4,PACSIN2,SNX3,BHLHE40-AS1,BRCA2,DISC1,WDPCP,SLC10A7,LRP1B,STX12,ATP9A,TRAK1,EVI5,SCN11A,MSR1,VRK1,GNAI1,MYRIP,SLC15A5,RELN,NUDCD3,AP4E1,FGF9,POLR2M,MYOM1,UNC13B,TTC21B,PLS1,SNX8,SEC23B,NIN,CCDC186,GLI3,SNX6,PACS1,MCTP2,MAP2,DAW1,PEX6,RRBP1,ATF2,BBS4,KIAA0753,CFTR,KPNA1,CSE1L,DOP1B,TBC1D13,PHAF1,SELENON,NMD3,AKAP10,REPS1,PRKN,MTMR2,LYST,GRIN2A,JPH1,ATXN1,PRKCH,FRMD4A,ALS2,SNX25,FBLN5,KCNQ3,TSPAN33,LRBA,MAP7,USP7,MON2,MESD,ITSN2,SYBU,YIPF6,MYO1D,SEC24D,ROCK1,LYN,SEL1L,SUMO3,SLC15A2,NTN1,RRAGD,BANP,CRACR2A,NPIPA1,DMBT1,HECTD1,SHROOM3,XRCC4,COLQ,ARFGEF1,BID,PIGK,RPH3A,TANC2,UFD1,TRIM58,TOM1,ZDHHC17,NSD2,FYCO1,SH3GLB1,CARD10,TMED3,IFT81,RASGRP1,IGSF11,SNX9,WDR72,NUP37,BCL2L1,HCN1,SYNJ2,ABCG1,SLC40A1,FAM149B1,PSAP,MICALL2,MED1,ATG4B,PCNT,IL10,ACTR2,SFPQ,PRKAA2,NDC80,PACRG,MAP6,PLA2G4A,SCFD2,KIFC1,CAMLG,SREBF2,ANP32B,FYB2,NRXN1,PCID2,SNAP91,CENPE,JAK2,SLC1A7,RPF2,MPPE1,CELSR2,TNKS,ARL11,SIAH3,UFL1,NFKBIA,PRKCB,ABCC8,MIPEP,ANP32A,USH1C,NEDD9,NRBP1,MTCL1,GRIP1,TM9SF3,SAR1A,TRAPPC3,BBS9,EXOC1,HEPACAM,SLC6A1,NDFIP2,SHROOM2,RN7SL483P,SLC6A11,CEMIP,PARK7,MAPK8,ITGA4,OAZ2,BCAP29,UBE2J2,ADCYAP1R1,NDC1,TM9SF4,RAPGEF4,CEP120,ARL4C,MEF2C,WNT7A,NDFIP1,CHAMP1,MAGEL2,RAB38,SDCBP,NECTIN1,FLVCR1,FGR,CDCA8,TRIM23,SNAP29,INTS13,NUMB,ADAMTS9,RN7SL767P,OCLN,SHISA6,AKAP11,KTN1,IREB2,MVB12B,MARK4,CDH5,AP4S1,CLDN18,MPP7,DIAPH1,SCAMP1,CYFIP1,SCG3,FRMD6,AP2B1,HEATR5A,ICA1,MTTP,SRP9,CCDC88A,NSUN2,BICD1,FYN,PPM1F,ARL13B,XPO7,ODR4,SLF1,ALB,EHBP1,MAPK9,SLC39A8,ASB3,CDH2,ITGA8,FBXL20,GPR137B,ZDHHC18,TBC1D1,PID1,NRP1,FCHSD2,IFT46,RNF215,SLC14A1,MCC,BCR,NRXN3,KIF16B,ARFGAP3,TM9SF2,NSG2,B9D1,BMPER,RABL2A,DPP6,MACROH2A1,EPHB2,CD38,MYO5B,RGPD4,PPIL2,AKAIN1,DLG2,ATP6V0D2,PPFIA2,CDH13,STXBP4,CACNG3,ATG5,MAGI2,VMP1,SLC1A2,GNAS,MFHAS1,NUP43,BMP7,ASTN2,DLG5,GAPVD1,TRAPPC10,DDX6,VPS13B,TRAPPC6B,EXOC4,FAM126A,KCNIP4,ERBB4,FAM3B,FAM126B,SYNDIG1,NUF2,RGPD2,SAMM50,SORCS2,TRDN,NLGN1,NOS1,SLC6A3,ASIC2,EFNA5,GAS2L1,RAB27A,KIF13A,AP5M1,ESR1,DNAH9,IRAG2,HTT,ZDHHC11B,HLA-F,FER,EYA2,CCR2,OSBPL5,ANO2,AGAP1,ROCK2,TERB2,CDCA5,RAB31,HERPUD1,WASHC1,HOOK3,BARD1,STK3,ZNF423,HNRNPU,VTI1A,CEP72,RAB3GAP2,CADPS,TANGO2,AKAP13,MORC3,SEPTIN6,DNM1L
GO:1901888	regulation of cell junction	2.268446356211928e-7	PTPRD,DLC1,PTPRA,IL1RAPL2,ROBO2,NEGR1,GPC6,CNTNAP2,PTPRJ,MACF1,NTRK3,EPHA7,RAPGEF2,ADGRB3,APP,STAU2,VCL,MAP4K4,DUSP22,LIMCH1,FMN1,BCAS3,ABL1,LRFN5,RAP1A,GRID2,PEAK1,MUSK,LINGO2,TJP1,NPHP4,PDLIM5,WDPCP,FAR

	assembly		*P1,PRKCH,ROCK1,NTN1,COLQ,SNAI2,CLSTN2,NRXN1,MEF2C,ADGRB1,WNT7A,NECTIN1,NTRK2,IL1RAPL1,PTK2,SEMA4D,PPM1F,ADGRL2,EPHB1,NRP1,EPHB2,FLRT2,DLG5,SYNDIG1,NLGN1,ASIC2,EFNA5,ROCK2*
GO:0034220	ion transmembrane transport	2.553805575865543e-7	*UNC80,CACNA2D3,SLC17A1,SLC24A2,KCNH5,MICU2,SLC25A21,SLC37A1,PIEZO2,DPP10,ITPR2,PDE4D,BCL2,KCNMA1,CHRNA7,GABRB3,ANO6,CACNG2,SLC4A10,NEDD4,GRIK3,ATP2B2,TUSC3,GABRB1,GRIA1,SLC39A12,SLC8A3,PRKD1,CHRM3,LRP2,FGF12,GABRA6,TMEM38B,SLC24A3,THADA,NEDD4L,TRPM1,SLC39A11,APP,SLC7A2,CACNA1C,CACNB2,GABRG2,TMC1,SLC8A1,KCNE4,AKAP6,KCNK10,TRPC5,CLIC6,RYR3,HECW1,KCNJ1,TRPC7,SLC16A1,NIPAL2,MICU1,LRRC38,GRIK4,AKAP9,RASGRF2,KCNS3,GRM1,GABRG1,DAPK1,SLC24A4,SCN2A,ANK2,RYR2,SLC9C1,SLC36A1,ABL1,SLC1A1,SLC12A8,KCNH1,ANO4,GRID2,GSG1L,RASGRF1,ATP11C,ABCB7,PRKCE,SLMAP,WNK2,ABCC9,P2RX6,HTR2C,ALG10B,ATP8A1,OPRM1,ABCC4,HTR2A,CNNM4,STAC,CNIH3,MAIP1,KCND2,AFG3L2,ANK3,NIPA2,TMC7,TMEM163,ATP6V1E1,CACNA1I,KCNJ15,SCN11A,NETO2,RELN,SLC23A2,SLC39A6,KCNH8,SLC37A2,SLC9A4,GABRR2,CNKSR3,GRIK2,LRRC8B,CFTR,SLC30A10,SELENON,GRIN2A,JPH1,TRPM6,SLC12A1,KCNQ3,SHISA9,SLC4A4,SCN10A,KCND3,KCNN3,LYN,SLC15A2,SLC13A5,CRACR2A,CUL5,GRID1,COX5A,GABRG3,ZDHHC17,UTRN,KCNC1,HCN1,GRIN2B,KCNK5,SLC40A1,SLC5A12,COX7A2L,LASP1,NRXN1,TWIST1,SLC1A7,TRPV5,ABCC8,CACNA1E,ATP2B1,CLCA4,OTOP1,SLC6A1,SHROOM2,SLC6A11,KCNJ18,CEMIP,CBLIF,PARK7,ADCYAP1R1,ATP13A3,MEF2C,ATP6V1C2,ATP6V1B2,GABRA5,SHISA6,TRPM7,GRIK1,DIAPH1,APOL1,SCARA5,SLC26A2,NOS1AP,SLC9A5,SLC5A1,ANO10,FYN,SCN8A,TMEM63C,NCS1,ATP5PF,NALCN,TRPM3,SLC39A8,SLC16A9,HECW2,GRM5,ATPSCKMT,KCNJ6,DPP6,EPHB2,TSPAN13,ATP6V0D2,CACNG3,ATG5,VMP1,SLC1A2,GABRA2,KCNIP4,TRDN,NLGN1,NOS1,SLC6A3,ASIC2,KCNQ5,CACNA2D1,HTT,SLC25A18,CCR2,ANO2,GRIA4,CATSPER2,RGS7,CLCN5,SLC13A4,KCNAB1,ATP10A*
GO:0071495	cellular response to endogenous stimulus	2.572959194240459e-7	*NOTCH2,BCAR3,MTOR,NSG1,NLK,PLCB1,PTPRA,ITPR2,PDE4D,RDX,MYO5A,PRDM16,FBN1,ROBO2,GABRB3,ZEB1,AKR1C3,RARB,SPRED1,ALK,APC,CRKL,SOX5,KDM4C,EGFR,NCOR1,NEDD4,BCL11A,SOX6,NTRK3,RXFP1,GABRB1,NEO1,PAK1,CHRM3,RAPGEF2,LRP2,RUNX2,FGF12,CPS1,ONECUT1,CPEB4,TMEM38B,UBE2L3,SMYD3,RPTOR,GHR,COL4A2,HDAC9,APP,GABRG2,NTF3,SLC8A1,KANK1,BMPR1B,PCSK6,AKAP6,RAB8B,LARP1,RAP1GDS1,PRKCZ,GRB10,RYR3,DEFA3,DUSP22,YAP1,MAPK1,HRH2,PDGFD,UBE2O,SPIDR,GABPA,HRH4,GLP2R,ITGB8,HIVEP1,CTNNA1,AKAP9,KLF15,PPARA,NR5A2,PTPRK,TRERF1,TMEM108,BCAS3,RYR2,BBS2,SMARCA4,USP8,MBD5,ELAVL4,ABL1,PTPN12,HDAC4,SLC1A1,PRKAA1,RAP1A,FGF10,LATS2,PRKCE,ESRRG,DENND4C,FBN2,CD44,PDE3A,EXT1,SPRED2,PTPN2,HTR2C,LTBP1,ZFYVE9,OPRM1,HTR2A,GNAL,CCND3,CRIM1,FBXO32,LDLRAD4,BLM,BMP2,PSG9,SOGA1,TBC1D4,LEMD3,FGF9,ZNF106,DSTYK,RAP1GAP,SMARCC1,SNX6,SMOC2,IDE,GAREM1,ATF2,UMODL1,BBS4,CFTR,RGMB,UBR1,CHRM5,SLC30A10,SELENON,PTPRE,PRKN,TBX20,SNX25,ROCK1,LYN,VCAM1,CTSB,OVOL2,RRAGD,ARID1B,INSR,SNAI2,RXRG,NREP,ENPP1,BMP5,BCL2L1,SERPINB9,HCN1,GRB14,SMAD5,MED1,KL,ACTR2,PTH,SOSTDC1,PRKAA2,ABHD2,VSTM2A,IQGAP1,NRXN1,PBLD,PEG10,JAK2,FSTL1,CREBBP,UFL1,PRKCB,ATP2B1,ASS1,OTOP1,CIDEA,EXT2,STAT1,BRMS1L,NR2C1,IMPACT,PARK7,ITGA4,MYOCD,ACACA,MEF2C,RXRA,WNT7A,RBPMS2,OR10H2,PDE2A,SDCBP,WWOX,NCK1,EPHA4,NTRK2,IL17RD,PTK2,CDH5,DIAPH1,CYFIP1,UBE3A,FAT4,PTGFR,FYN,HDAC2,FUT8,TET1,ITGA8,GRM5,PID1,POR,KIF16B,NSG2,GNA14,BMPER,EPHB2,STXBP4,MAGI2,FLRT2,SLC1A2,GNAS,BMP7,PDGFC,SLIT2,ERBB4,PRKCQ,EFNA5,GAS2L1,SLIT3,ESR1,CACNA2D1,PRLR,PIK3R3,FER,HRH1,ROCK2,RGS8,RAB31,GNG2,PNPLA3,ZNF423,HNRNPU,IGF1R,THRB*
GO:0016192	vesicle-mediated	3.189940367705	*NSG1,EXOC1L,ABCA13,LRP12,TRAPPC9,UNC13C,TMPRSS2,CLTCL1,MICAL3,RDX,STXBP1,ERC1,RALA,EPS15L1,MYO5A,SYT16,COG5,RIMS1,PIK3C3,SPIRE1,EXOC6B,MYO1E,TRAPPC8,RIMS*

	transport	4265e-7	2,CARMIL1,MCTP1,FCHO2,RIN2,ANO6,CACNG2,PTPRJ,EGFR,DENND1A,ANGPT1,MACF1,DOCK2,NEDD4,BTBD9,CECR2,DNAJC13,RABEP1,DGKI,C12ORF4,GRIA1,PRKD1,PAK1,LRP2,LDLRAD3,TAFA4,SYN2,GHR,NEDD4L,APP,CACNB2,DCLK1,SYT1,ARHGAP44,NTF3,CD2AP,FMN2,RAB8B,ERC2,DNM3,CUBN,SYN3,RAB27B,CNST,VPS35L,MAPK1,CADPS2,RABGAP1L,COPB1,SYT10,UBE2O,ANKFY1,STON2,SYNJ1,PLA2R1,VPS13C,TMEM108,RAB22A,SORCS1,AMPH,DYSF,ANK2,BBS2,NKG7,TBC1D5,BLK,ABL1,SLC1A1,PRKAA1,CCDC91,EIPR1,NBAS,RAP1A,NRG1,GSG1L,AP3B1,SH3GL3,PRKCE,USP33,CEP83,EGF,STXBP6,CLEC16A,ZFYVE9,ABCC4,HTR2A,BIN2,CNIH3,APBA2,SH3KBP1,EPN2,GRK3,CD163,ANK3,COG2,VPS41,LYPLA1,TRAPPC11,HHIPL1,HIP1,VPS37A,VAV1,RUFY2,PACSIN2,SNX3,CACNA1I,DNER,SLC10A7,LRP1B,STX12,ATP9A,TRAK1,EVI5,MSR1,TBC1D4,RIN3,BMP2K,HMGB1,AP4E1,PRG4,UNC13B,DOCK1,SNX8,SEC23B,SLAMF1,SNX6,MAPK8IP1,CFTR,DOP1B,NEU3,PHAF1,REPS1,PRKN,MTMR2,LYST,ALS2,USP7,VAV3,MON2,MESD,ITSN2,SOX30,RALB,YIPF6,MYO1D,SEC24D,ROCK1,LYN,CRACR2A,INSR,DMBT1,ARFGEF1,IGHV3-74,TOM1,PLPP4,ESYT2,CD9,TMED3,XKR5,ENPP1,RASGRP1,SNX9,BCL2L1,IGHV2-70D,SYNJ2,MICALL2,SCFD2,CAMLG,NRXN1,ENTHD1,SNAP91,PEG10,MPPE1,ARL11,PRKCB,NRBP1,GRIP1,IGHV1OR15-9,SAR1A,CNIH1,TRAPPC3,XKR6,EXOC1,ITGA4,BCAP29,TM9SF4,RAPGEF4,ARL4C,ADGRB1,WNT7A,MAGEL2,RAB38,SDCBP,JPT2,FGR,TRIM23,SNAP29,C2,IL1RAPL1,NUMB,ADAMTS9,COLEC12,STON1-GTF2A1L,MEGF10,MVB12B,PTK2,CD5L,AP4S1,ARHGAP12,SCAMP1,CYFIP1,UBE3A,AP2B1,SCARA5,HEATR5A,BICD1,FYN,ATP9B,EHBP1,APELA,CDH2,FBXL20,NRP1,FCHSD2,RNF215,BCR,NRXN3,ELMO1,KIF16B,ARFGAP3,NSG2,CUX1,EPHB2,CSNK1G1,MYO5B,MET,CDH13,CACNG3,ATG5,MAGI2,KALRN,TMPRSS15,GAPVD1,TRAPPC10,WDR41,ABL2,TRAPPC6B,TMPRSS3,EXOC4,SYNDIG1,NLGN1,RAB27A,KIF13A,AP5M1,LOXL2,IGLC3,IRAG2,HTT,CAMK1D,HLA-F,FER,CCR2,OSBPL5,IGHV1OR21-1,RAB31,HSPG2,WASHC1,HOOK3,CLCN5,VTI1A,CADPS,IGF1R,DNM1L
GO:0045595	regulation of cell differentiation	3.537199777647 1997e-7	NOTCH2,BRINP3,MTOR,CNTN4,PTPRD,SMOC1,ULK2,FTO,PLCB1,ZNF536,ZFPM2,TENM4,ZDHHC21,RIPOR2,BCL2,FBN1,ROBO2,ZEB1,RARB,SPRED1,USH2A,ALK,FOXJ2,CARMIL1,RIN2,APC,DSCAM,TCF4,CRKL,SOX5,KDM4C,EGFR,RFX3,CDK12,MACF1,BCL11A,SOX6,TMEM182,CDH4,NTRK3,PRKD1,EPHA7,SPEN,RAPGEF2,LRP2,RUNX2,GHR,HDAC9,ZHX3,APP,STAU2,SEMA5A,VCL,NTF3,AURKA,KANK1,BMPR1B,AKAP6,ARNT,PAK3,DIP2B,ITPKB,TRPC5,PRKCZ,ABCA5,YAP1,BRINP1,MAPK1,GABPA,MYLK3,PAFAH1B1,EFEMP1,NF2,RBFOX1,CTNNA1,PPARA,MEIS2,PRTG,SYNJ1,TIAM1,SEMA3C,GATAD2B,IL34,ADGRV1,WNT9B,RANBP3L,SEMA6D,SMARCA4,TNR,ABL1,HDAC4,DROSHA,RAP1A,GLIS1,CAMK4,FGF10,NRG1,ASPM,AP3B1,ATP11C,ZBTB16,ZNF675,SH3GL3,FBN2,TRIO,PDE3A,LIMD1,SPRED2,RPS6KA3,PTPN2,PLXNA2,HTR2C,ARHGEF7,AMBRA1,OPRM1,HTR2A,FANCA,SEMA3E,ANKRD17,BICRAL,MOSMO,BCL11B,DOCK5,MBP,TRPS1,CRIM1,LDLRAD4,HLA-B,DISC1,SEMA3A,BMP2,RC3H2,PSG9,MSR1,SEMA3D,RELN,HMGB1,FGF9,NFATC2,ANKRD26,ESRP1,DOCK1,RAP1GAP,NIN,DRAXIN,SMARCA2,ETS1,GLI3,SMARCC1,PCP4,MAP2,LAMC1,COL5A1,NELL1,KITLG,DCC,RORB,DAB1,TBX20,DPF3,PRKCH,IL6R,HEMGN,TOX,ROCK1,LYN,PLEKHB2,DTX1,OVOL2,NTN1,ZFHX3,DPYSL5,ARID1B,CASZ1,SNAI2,TRIM58,NREP,TWIST2,ENPP1,RASGRP1,BMP5,CSF1,CTDP1,ASB4,SMAD5,ABCG1,PRAME,MYCL,TNN,MED1,IL33,AJAP1,ROR2,LMX1A,TMEM178A,IL10,ACTR2,PRAMEF25,PTH,SOSTDC1,VSTM2A,MAP6,ZBTB7C,ANP32B,PCID2,TWIST1,JAK2,MELTF,UFL1,NFKBIA,ABCC8,NEDD9,OLFM4,STAT1,PRAMEF2,IMPACT,MYOCD,MEF2C,WNT7A,RBPMS2,MAP3K5,NDFIP1,FBXW8,SDCBP,EPHA4,NTRK2,IL1RAPL1,NUMB,ADAMTS9,CD101,MEGF10,IL17RD,FBXO31,PTK2,CDH5,NFKBID,CLDN18

			,LAMB1,CYFIP1,SEMA4D,FAT4,RUNX1,GPR55,NSUN2,TNFSF11,HDAC2,MAPK9,CRTAM,GPR137B,EPHB1,GRM5,NRP1,PRKCA,FAIM,RC3H1,CHODL,POR,FBLN1,RAG1,CUX1,MACROH2A1,MITF,EPHB2,MYB,KALRN,LAMA1,TIAM2,BMP7,NUDT21,DDX6,EYA1,SLIT2,ROBO1,PBX1,NLGN1,EFNA5,TCF12,LOXL2,PRLR,CCR2,SEMA4B,ROCK2,PRDM1,RORA,ATAT1,HOOK3,FSTL4,STK3,HNRNPU,GLI2
GO:0043087	regulation of GTPase activity	3.587646404531 0197e-7	*BCAR3,MTOR,GARNL3,MYO9A,RIPOR2,RDX,FGD4,CRKL,ARHGAP24,DOCK10,DENND1A,NTRK3,DGKI,RALGPS1,RAPGEF2,RALGAPA1,RAPGEF5,DOCK8,MAPRE2,ARHGAP44,NTF3,MAP4K4,RAP1GDS1,RABGAP1L,TBC1D22A,CHN1,PAFAH1B1,RASGRF2,RGL1,TIAM1,ARAP2,TBC1D9,BCAS3,TBC1D5,RAP1A,RASGRF1,ASAP2,PLXNA2,ARHGEF7,VAV1,IQSEC1,EVI5,RALGAPA2,SGSM1,TBC1D4,ARHGAP42,RAP1GAP,SRGAP2,BBS4,TBC1D13,NGEF,ALS2,DOCK9,VAV3,ARFGEF1,RALGPS2,RASGRP1,SNX9,PRKG1,RASGEF1C,IQGAP1,FICD,NET1,SIPA1L2,ZC3H15,NEDD9,ITGA6,RAPGEF4,EPHA4,NTRK2,PTK2,ARHGAP12,SEMA4D,RASGEF1B,ASAP1,GPR137B,TBC1D1,BCR,RGS6,MET,KALRN,GNAS,TIAM2,GAPVD1,WDR41,TRAPPC6B,SIPA1L3,EFNA5,RGS8,RGS7,RSU1,RAB3GAP2*
GO:0007423	sensory organ development	3.872429963251 375e-7	*NOTCH2,BCAR3,BNC2,SMOC1,SCAPER,RIPOR2,RP1,BCL2,ALDH1A2,FBN1,TENM3,ZEB1,RARB,SPRED1,USH2A,MYO3B,DSCAM,EGFR,CRB1,CECR2,ATP2B2,NTRK3,FLT1,ADAM10,ABCB5,CACNA1C,DCLK1,STAU2,TMC1,BMPR1B,MAPK1,NIPBL,FAT3,PAFAH1B1,ATF6,EFEMP1,NF2,MEIS2,ADGRV1,WNT9B,SMARCA4,SLC1A1,TTLL5,FGF10,FBN2,SPRED2,NHS,ATP8A2,BCL11B,ECE1,NPHP4,WDPCP,BMP2,PDE6C,FGF9,TDRD7,CPAMD8,MDM1,ESRP1,PLS1,GLI3,MEGF11,BBS4,LAMC3,COL5A1,RORB,MYO3A,PCDH15,CDH23,PDE6A,LRIG1,NTN1,HOXC13,PBX3,SP3,DZANK1,BMP5,HCN1,CELF4,VSTM4,MYCL,MED1,ATG4B,FAT1,TTC39C,ANP32B,HIPK1,TWIST1,VSX1,USH1C,ATP2B1,OTOP1,GRXCR1,SHROOM2,ADAMTS18,WNT7A,NECTIN1,PPP2R3A,EPHA4,GABRA5,NTRK2,WNT2B,FAT4,WNT5B,UNC45B,HDAC2,ROR1,ITGA8,XRN2,EPHB1,RP1L1,NRP1,SDK1,BCR,B9D1,BMPER,MITF,EPHB2,EYA4,ATG5,LAMA1,BMP7,EYA1,PBX1,MYH15,SIPA1L3,SLC6A3,RPGRIP1,PRDM1,PTPRQ,GLI2,THRB*
GO:0050773	regulation of dendrite development	4.386173813276 32e-7	*PTPRD,ALK,CRKL,TNIK,NEDD4,BCL11A,RAPGEF2,ADGRB3,NEDD4L,STAU2,PAK3,TRPC5,HECW1,COBL,FAT3,PAFAH1B1,ELAVL4,ABL1,SDC2,KNDC1,RELN,DCC,DPYSL5,BMP5,ACTR2,DGKG,FBXW8,EPHA4,IL1RAPL1,FBXO31,CYFIP1,SEMA4D,HECW2,CUX1,EPHB2,KALRN,BMP7,CAMK1D,CSMD3*
GO:0009987	cellular process	5.951912435029 6e-7	*EBNA1BP2,NOTCH2,BCAR3,BRINP3,MTOR,UNC80,CNTN4,CACNA2D3,SPOCK1,NSG1,SGCD,EXOC1L,WWC1,SLC17A1,ABCA13,IMMP2L,GARNL3,LRP12,PTPRD,SLC24A2,FREM1,TRAPPC9,BNC2,PVT1,NEBL,LRRC4C,TMTC1,KCNH5,MICU2,SLC25A21,ANKS1B,SMOC1,MYO9A,ULK2,NLK,LONP2,UNC13C,LRRC49,FTO,KSR1,MGA,RFX7,SNHG14,ZNF236,PLCB1,ZNF536,TTC3,MX2,LIPI,TAFA5,SVIL,TLN2,CLTCL1,SLC37A1,ZFPM2,PIEZO2,MICAL3,TENM4,NUBPL,L3MBTL4,SNAP25-AS1,DLC1,TNRC6B,MGAM,DPP10,ZDHHC21,PTPRA,ITPR2,RIPOR2,PDE4D,RDX,RP1,STXBP1,ERC1,RALA,NME7,SLC44A5,EPS15L1,IL1RAPL2,BCL2,MYO5A,ODAD2,KCNMA1,SYT16,ARPP21,PRDM16,ALDH1A2,ARHGAP26,FBN1,LRFN2,LPCAT2,GPHN,COG5,CDH8,CHRNA7,DCDC1,GPR158,ROBO2,PUDP,RIMS1,PIK3C3,EPC2,SPIRE1,TENM3,GABRB3,ZEB1,AKR1C3,CNTLN,CNTNAP5,SDCCAG8,RARB,FGD4,EXOC6B,SPRED1,GALNT1,NAV2,ENPEP,SPAG16,MYO1E,TRAPPC8,PLPPR1,USH2A,CEP192,MINAR1,CDC42EP3,RIMS2,PCMTD1,ALK,MICOS10,AUTS2,ADGRE1,PCDH7,FOXJ2,CDYL2,CARMIL1,MCTP1,PJA2,FAM135B,THSD4,BABAM2,SV2C,PAPPA2,GLIS3,FANK1,ERBIN,ERCC6L2,RHPN2,HACD2,ASTN1,HLCS,FCHO2,RIN2,PARVB,ANO6,CACNG2,DLGAP1,NEGR1,ZNF880,GLYAT,MLLT3,EGLN3,GPC6,CNTNAP2,MAP4,MAP3K9,MYO3B,PGBD5,MOCOS,SPON1,APC,ZMYM4,ZNF595,HHLA2,TSHZ3,RBFOX3,PLPPR5,DSCAM,MYO5C,RTN1,TCF4,CRKL,ILDR2,SOX5,SETD2,TANGO6,ERG,ARHGAP24,ZNF573,TNIK,SLC4A10,URB1,*

			PTPRJ,OCA2,KDM4C,NEK4,DOCK10,TSHZ2,EGFR,ZNF280B,RFX3,DENND1A,USP14,ANGPT1,CDK12,BACH1,MACF1,CTNNA3,PRKACB,NEK7,RGS3,NCOR1,RNF220,HMCN2,DOCK2,DIAPH3,ZNF407,UGT3A2,NEDD4,MYOF,MAML2,MTRF1,SND1,SCAI,GNPTAB,CRB1,NSMCE2,BTBD9,BCL11A,SOX6,FAM83F,TMEM182,PSMB2,SGMS1,CECR2,GRIK3,ARMC2,CHSY1,FLI1,RPRD1A,PTPN4,CDH4,B3GALT5,ATP2B2,NTRK3,LARGE1,RXFP1,C5,PDE1C,TUSC3,FBXL7,ZFAND6,CYP2C9,PHACTR1,DKK2,FLT1,DNAJC13,ZNF648,RFC3,RABEP1,ADAMTS6,ZNF382,GK,TASP1,CNTN3,THRAP3,MAPKBP1,AOAH,NAT1,GABRB1,PSMA8,DGKI,INVS,C12ORF4,EDAR,GRIA1,CRACD,CAST,NUP214,NEO1,CNTN6,SLC39A12,CABLES1,SLC8A3,MALRD1,TOM1L2,CEP128,NELL2,PRKD1,TPTE2,PAK1,GMDS,EPHA7,CTNNAL1,NCOA7,KHDRBS2,CHRM3,ADSS2,GRAMD1B,RALGPS1,SPEN,CHSY3,RAPGEF2,PELI2,LRP2,ADGRB3,DEUP1,RUNX2,ARSB,FGF12,GABRA6,CPS1,TAOK3,ONECUT1,CPEB4,TMEM38B,AGK,ADAMTSL1,CSTF3,BCKDHB,PRICKLE2,RANBP17,SLC24A3,SLC44A1,UBE2L3,LDB2,TAFA4,PPP2R2B,BTBD11,PUM3,PTPRN2,SYN2,CCL28,SMYD3,PATJ,TYW1,HERC2,LRGUK,TMEM241,GRM7,SEPTIN9,RETREG1,RPTOR,DNAH6,TMEM117,GHR,EPB41L3,KIF4A,THADA,COL4A2,AIG1,SSBP3,TMEM74,RALGAPA1,CELF2,RAPGEF5,TBCD,NEDD4L,ADAM32,PPP1R12B,TRPM1,ADAM10,HDAC9,ZHX3,UBA6-DT,ATF7IP,SLC39A11,UBE2G1,IL1R1,APBB2,PHACTR2,APP,SLC7A2,ABCB5,ADK,RPS6KA2,SAMSN1,KYNU,CACNA1C,KDM1B,CACNB2,KLHL13,MTUS1,PHKB,DCLK1,STAU2,GABRG2,DOCK8,TMC1,MAPRE2,ZNF600,USP18,SEMA5A,SYT1,VCL,ARHGAP44,NTF3,ACER2,PARP15,NDUFAF2,CD2AP,ZNF723,AURKA,PARN,IGSF5,TTC29,CFDP1,ST18,PYGO1,SLC8A1,HERPUD2,SSBP2,PTPRR,SRGAP2C,DTWD2,ANKRD31,FIG4,DUX4,TAFA2,ABCG8,FRMD3,UPP2,CCSER2,ECPAS,SRGAP2B,KANK1,KCNE4,MAP4K4,HIVEP2,ABCD2,BMPR1B,FMN2,THSD7A,PCSK6,AKAP6,HOMER2,ZNF717,CTNNA2,HADHB,ARNT,RAB8B,PAK3,RFTN1,PDE1A,ZNF257,TTLL7,DIP2B,KCNK10,RANBP2,LARP1,ITPKB,TRPC5,RGS20,PDE10A,UBE2E2,RAP1GDS1,HHAT,CLIC6,CHST8,KICS2,ERC2,DNM3,NBN,CUBN,SCP2,SYN3,IFT57,INTS7,RBM47,SUSD6,PRKCZ,CALD1,KLHL1,SPOP,BTLA,MAN2A2,GRB10,RYR3,TAF15,DIP2A,MSH6,MCPH1,ARHGAP32,RAB27B,COL27A1,ZSWIM6,FER1L6,ST8SIA5,CNST,RGS9,HECW1,DEFA3,MBNL2,ADAMTS17,ABCA5,PHF19,MRTFA,TAF4B,COBL,SENP6,DUSP22,GALNT14,LMNTD1,PDXDC1,EBF2,UBN1,SV2B,YAP1,ESS2,FRYL,SEM1,NFIA,WDR70,PPM1L,RIPK4,ZKSCAN5,SHC4,VPS35L,BRINP1,MAPK1,MGAT5,CADPS2,KCNJ1,CADM2,HRH2,ABCD3,RABGAP1L,SGTB,DNAH14,TRPC7,ADAM22,USP25,CRISPLD2,KMT2E,ALCAM,PLG,PCGF5,PDGFD,COPB1,SYT10,ZNRF3,DNAJC21,CA5A,XXYLT1,PPP1R1C,ABLIM1,ITGBL1,ARHGEF17,NRG3,UBE2O,SFMBT2,ANKFY1,NCAM1,GFRA1,SYCP1,NIPBL,RNF17,SLC16A1,SPIDR,GALNT16,NIPAL2,PI4K2B,RNGTT,IPO11,EWSR1,MTMR10,GABPA,FAT3,MICU1,ZNF735,CORO2B,CARD18,CHD6,STK38,LCE1F,PTPN13,CHN1,HRH4,SORCS3,MYLK3,ACSBG1,KANSL1,GLP2R,LIMCH1,FMN1,MBNL1,PAFAH1B1,ATF6,EFEMP1,TLL1,ZNF684,TM7SF3,DCAF1,ITGB8,STON2,VPS13D,CCNG2,TLK1,TPM1,NF2,LRRC38,AVL9,CNKSR2,MRPS22,GRIK4,RBFOX1,WDFY4,ZDHHC14,HIVEP1,CORIN,CTNNA1,PPP1R9A,CDH7,MOB3B,BIRC6,AKAP9,KLF15,RASGRF2,PPARA,PPIP5K1,MEIS2,SNX30,LCLAT1,NFIB,KCNS3,ERMP1,MRTFB,PPP6R3,PRTG,RGL1,SYNJ1,NR5A2,ADAMTS3,TIAM1,MPRIP,ARAP2,GRM1,FOXJ3,UBE3D,KAZN,RSRC1,PTPRK,ARHGEF12,GABRG1,ENAH,PAK5,ST6GALNAC3,TRERF1,SF3B6,PARD3B,PCDH11Y,PPP2R5E,PDZRN3,PLA2R1,EIF3D,SEMA3C,DAPK1,NAV3,SLC24A4,SEC14L1,FAR2,VPS13C,TMEM108,ACSM2B,AGO2,WDHD1,STK32B,PHC3,MAGI1,ALPK2,DNAH11,JARID2,SCN2A,RIC8B,RAB22A,SORCS1,DNAJC15,AMPH,GATAD2B,CPE,PALS2,EVC2,DYSF,IL34,ANK2,STAG2,BRWD1,TANC1,THUMPD2,ADGRV1,ZNF846,MELK,BCAS3,RYR2,SYNE2,BBS2,WNT9B,ZNF606,SLC9C1,CLPX,RANBP3L,OR4F6,NKG7,SEMA6D,AIF1L,NBEA,ASAH2B,SHOC1,DUSP16,SRFBP1,SMARCA4,MRPS35,CDH11,USP8,LDB3,FABP7,PARD3,SLC36A1,MAPKAP1,EFTUD2,TNRC6C,PI

			AS1,TBC1D5,SPG21,UBE2R2,BLK,COL23A1,RBM6,EBF1,TNR,COL22A1,GRM8,OLA1,DST,CXADR,DOCK4,MBD5,ATRX,NUAK1,PTPRT,XIRP2,ELAVL4,ABL1,AGPS,MXI1,PTPN12,HDAC4,OXR1,SLC1A1,PRKAA1,SDC2,GAS2,SLC12A8,KCNH1,ITGB3BP,MRPS27,LRFN5,RIMBP2,CRTAC1,FHIP1A,CREG1,DROSHA,PRELID2,TTLL5,APBB1IP,ANO4,L3MBTL3,DMXL2,CCDC91,EIPR1,APLF,NFAT5,ADAMTS14,MAST4,DNAH5,GUCY1A2,NBAS,CDH18,PSMF1,ATE1,SLFN11,RAP1A,GLIS1,ACSS3,MORC1,LYRM4,MYO10,SLC46A3,GPC5,TOX3,ZNHIT6,CAMK4,BAZ2A,MANBA,PLEKHA8,INPP5A,CPSF3,FGF10,FBXL13,ZC3HAV1,UQCC1,GRID2,CDHR3,GALC,TGM1,PEAK1,LATS2,NRG1,INO80D,GSG1L,CLIP1,ASPM,AP3B1,DENND2B,COL6A5,RASGRF1,PAH,ATP11C,ZNF438,ABCB7,SYNE1,ZBTB16,MUSK,GALNTL6,KIR3DL2,ZNF675,GNG7,SMARCAD1,SH3GL3,ABCC12,SETDB2,RPF1,PRKCE,FOXK2,SLCO3A1,PGM5,MED15,SLMAP,NXN,WNK2,ESRRG,ZNF718,DGKB,USP33,DENND4C,CEP83,CERS6,FBN2,CD44,RGS12,PTPRO,EGF,ALPK3,PRRC1,DMAC1,ABCC9,P2RX6,TRIO,PDE3A,EXT1,STXBP6,COL5A3,NSMAF,LNPEP,LIMD1,PEX14,SPRED2,ADAMTS2,RPS6KA3,CTNND2,NHS,IFT43,ATP8A2,SCG5,MTMR3,PTPN2,TRIM5,PLXNA2,POC5,MCF2L,OR4F15,ATXN3,RFC1,ST8SIA6,HTR2C,RIC3,CLEC16A,SLC2A3,ARHGEF7,CD96,ALG10B,ATP8A1,AMBRA1,LTBP1,STK38L,ZFYVE9,GALNT10,KDM7A,OPRM1,ABCC4,PRMT8,HTR2A,BIN2,PLCXD3,FANCM,FANCA,CYBRD1,CYP4A11,DAZL,INPP4B,MATN2,FARS2,GTF2F2,PPP2R2C,CNNM4,KREMEN1,STAC,SEMA3E,TAF3,RPRD1B,MARK2,GCSAML,GMPR,TMEM67,RCL1,EBF3,ALPL,ZNF33B,LPP,C10ORF90,FHL2,ABHD17C,ADGRA3,CNIH3,PUM1,TMOD2,HERC1,MSH2,IGF2BP3,GNAL,CDIN1,EPHA6,ANKRD17,APBA2,COL6A6,MAIP1,LINGO2,ZNF397,SH3KBP1,ATL1,SLC2A13,LUC7L,RELL1,HIPK3,CDKN2C,EPN2,KCND2,TNPO3,EVC,SNRPN,ABCA10,GRK3,CPXM2,KNDC1,SPSB4,CLSPN,NOS2,BICRAL,AFG3L2,CPNE4,STK10,MOSMO,GFRA2,TTC7B,MNAT1,TMEM116,RBBP8,MDFIC,SGCZ,TMTC2,CFAP61,ADAM12,MYLK2,ANK3,SNTG1,NIPA2,TMC7,EMILIN2,XYLT1,HMGA2,MYOM2,COG2,GBP6,CCND3,BCL11B,VPS41,FOLH1,DOCK5,ECE1,ZIM3,STK32A,CREM,LYPLA1,MBP,AK8,LINC01151,TRPS1,TRAPPC11,TMEM163,CWC27,PLCE1,TGFA,IL17RA,ANKFN1,HIP1,CRIM1,XPNPEP1,FUT9,PRR5L,GXYLT2,VPS37A,GSR,PCDH9,ATP6V1E1,UTP4,CAPN5,VAV1,CYP4Z1,CDH20,EFR3A,MSRA,RUFY2,MYT1L,FBXO32,ZNF160,TJP1,LDLRAD4,NPHP4,EGFLAM,PACSIN2,CNTN1,HLA-B,TARS3,FKBP5,IQSEC1,HSF5,MTHFD1L,SNX3,CACNA1I,NAA35,ZNF367,PDLIM5,BHLHE40-AS1,KCNJ15,CEPT1,BRCA2,AQR,DISC1,ZBTB2,GALNT13,EXD3,DNER,BLM,ASB7,WDPCP,NRK,SLC10A7,SEMA3A,MAGI3,HSF2BP,INTS8,NAP1L4,LIN54,LRP1B,ADCY10,PSG8,STRN,AGL,OR9Q1,ZNF121,ANKRD30BL,STX12,PHACTR3,BMP2,RC3H2,MYLK4,UNC5D,ATP9A,TRAK1,WDR26,PSG9,CDC42BPB,SOGA1,EVI5,DSE,PTCD2,SCN11A,MSR1,VRK1,GNAI1,RALGAPA2,ZC3H14,NCAM2,GFI1B,TBC1D4,RANBP9,RESF1,MYRIP,TTR,RIN3,MSI2,BMP2K,DNAL1,SLC15A5,TMEM161A,SEMA3D,ASXL3,NETO2,PDE6C,CABIN1,POLR3A,LEMD3,RELN,ARHGAP42,HMGB1,GNAQ,TUBGCP3,NUDCD3,CDS2,AP4E1,FGF9,NFATC2,TDRD7,SH3BP5,UST,RTTN,MDM1,SLC23A2,POLR2M,ZNF106,MYOM1,ZNF567,CLVS1,TRAF3,ZNF462,ANKRD26,ESRP1,UNC13B,TTC21B,ETS2,UBAP2L,GEMIN5,ZNF875,DSTYK,UIMC1,DOCK1,B4GALT6,LRRFIP1,TSPAN2,PFKFB4,RAP1GAP,PLS1,SRGAP2,IKZF2,SNX8,SEC23B,SLC39A6,NIN,HAUS6,DRAXIN,DNAH8,TRIT1,ATF1,GADL1,CCDC186,SLAMF1,KCNH8,SMARCA2,ETS1,FAM83B,GLI3,CGAS,MEGF11,SMARCC1,NHSL1,SNX6,AFF3,SLC37A2,SLC9A4,GABRR2,SMOC2,PACS1,PCP4,CNKSR3,CASP5,VENTX,GRIK2,IDE,WDR12,MCTP2,KIF21A,KIF15,PRDM10,CUL1,MYEF2,ZFYVE26,ZNF431,RERE,PSD3,MAP2,BTAF1,GAREM1,DAW1,MYL1,PEX6,LAMC1,ZNF618,NEK10,RRBP1,FARP1,TDRD5,MOB1B,PIGN,ATF2,NDUFAF6,GOLGA8B,HIRA,CYLD,UMODL1,BBS4,ADARB2,LRRC8B,MAPK8IP1,GOLGA6B,MX1,CLVS2,THSD7B,LAMC3,PSG6,HIVEP3,COL5A1,GABBR2,PSIP1,ITGA9,KIAA0753,CFTR,KPNA1,CSE1L,NELL

			1,DOP1B,ME2,TBC1D13,UBASH3A,COL14A1,RGMB,NEU3,PHAF1,CEP44,MRPL13,KITLG,ZZEF1,DNAJC7,ATP10B,CAMTA1,UBR1,DCC,MYT1,RNU2-47P,SMPDL3A,CHRM5,GOLGA6D,MAP4K3,HS3ST4,YLPM1,SLC30A10,RCAN1,GTF2I,RORB,CHAF1A,TADA2A,DAB1,MED27,ZNF208,SELENON,RB1CC1,NMD3,MYO3A,AKAP10,UBE2E1,PTPRE,REPS1,PRKN,AGMO,MTMR2,ZNF608,SH3PXD2A,SPSB1,CDC42BPA,TBX20,SP110,DLGAP2,AFAP1,MAPK10,DACH1,PCDH15,PNPLA7,ZNF541,DPF3,LGI2,LYST,NGEF,HEPHL1,GRIN2A,ARID5B,ZBED9,H2BC15,JPH1,TXNRD2,ATXN1,WSB1,USP43,TRPM6,CDH23,LALBA,PRKCH,PKP1,HUNK,SLC12A1,FRMD4A,TG,IL6R,FRMPD4,PEPD,ALS2,RACGAP1,NLRC5,ZNF627,OR51E1,ACO1,TFDP1,DHRS11,CNOT6L,MKNK1,HEMGN,KANK4,DOCK9,SNX25,DMC1,FBLN5,OSCP1,LCE3B,KCNQ3,TOX,POLR1D,SHISA9,SLC4A4,PTPRB,GOLGA6C,ZFP90,TRMT61B,CATSPERG,PDE6A,COPS8,TSPAN33,TBATA,ZNF124,SCN10A,LRBA,RBMX2,SHANK2,ST8SIA1,MAP7,USP7,VAV3,PSMA1,MON2,ENPP3,PLAGL1,KCND3,HAAO,FAH,MESD,ITSN2,SOX30,PTGFRN,MOK,SYBU,KIR2DL4,ARHGEF28,RALB,NPAS2,ADGRG6,YIPF6,CFAP74,KCNN3,MYO1D,SEC24D,PPA2,FAR1,CA1,ROCK1,LYN,VCAM1,SEL1L,ARHGAP28,ARHGAP31,ZNF780B,CTSB,EIF2B3,TTC37,SLC44A2,GSTA3,SUMO3,SLC15A2,ZNF169,PLEKHB2,KIF11,DTX1,BZW1,TENM2,OVOL2,PIWIL3,ZBTB33,ADA2,NTN1,CHKA,PLCB4,MMP16,PRUNE2,ZFHX3,FANCL,DPYSL5,SLC13A5,ZNF44,RRAGD,BANP,SUPT16H,ARID1B,HOXC13,CRACR2A,RNF152,BAZ1A,CASZ1,OTUD7A,INSR,NPIPA1,CUL5,DMBT1,OR7A17,BMF,YTHDF3,TFF1,DEDD2,NEK6,HECTD1,GRID1,SHROOM3,XRCC4,COLQ,SLC52A1,HDAC11,NMU,DDHD1,PBX3,SUMO2,HS1BP3,ZNF292,ADAMTS19,DPYD,ARFGEF1,PDE4DIP,GAST,POGK,SNAI2,ASH1L,IGHV3-74,HOXC4,BID,SIAH2,PIGK,OSBPL10,RPH3A,TANC2,PGAP4,ZBTB8OS,COX5A,ABCA4,TRABD2B,UFD1,RXRG,SP3,DRAM1,ERN2,GABRG3,ZNF879,MBTPS2,FLNB,TRIM58,TIAL1,TOM1,ELF2,PLPP4,NREP,ZDHHC17,NSD2,FYCO1,CERS3,ESYT2,SH3GLB1,PTAR1,SLC22A14,CD9,CARD10,LTN1,TMED3,KRT6B,XKR5,RALGPS2,JCAD,TWIST2,OR4K2,CTIF,SAMHD1,IFT81,ENPP1,ENTPD5,UTRN,MOCS2,RASGRP1,IGSF11,SNX9,TP53I11,CDH26,TMEM225,DZANK1,ANAPC1,PXDNL,UCK2,NDRG2,CSNK2A1,BMP5,PWWP3A,WDR72,KCNC1,CSF1,GHRH,PPIL6,EOGT,HDGFL3,NUP37,BCL2L1,SERPINB9,SCAF4,SPATA48,KRT25,CTDP1,HCN1,PRKG1,LAMA3,HS6ST3,ASB4,GRIN2B,ST13,GRB14,INO80,FANCB,GPR156,IGHV2-70D,CLNS1A,CNMD,DHRS3,KIF21B,SMAD5,CELF4,SYNJ2,TCERG1,ABCG1,OR4C46,FOXN3,KCNK5,VSTM4,SLC40A1,PRAME,HADHA,MYCL,TNN,FAM149B1,CABYR,CIDEC,PSAP,LPGAT1,PSMA5,MICALL2,MED1,IPCEF1,NSUN6,ATG4B,CDC14B,PCNT,SLC5A12,KDM6A,ATRN,IL33,AJAP1,GPRC5C,ROR2,CFH,PPP2R2A,ZNF521,NPL,KL,RASGEF1C,BANK1,CSDE1,FAT1,HGD,LMX1A,TMEM178A,IL10,TSPAN11,ACTR2,OR1L6,SFPQ,SCML2,PRAMEF25,RIOK1,CLSTN2,HDHD5,TTC39C,PTH,SDF4,SOSTDC1,TOP3B,PRKAA2,CSF2RB,DIRAS2,SKA1,GLYATL1,NDC80,QSOX2,SOHLH1,LARP6,PACRG,ERO1B,PHF20L1,ABHD2,ITPRIP,VSTM2A,MAP6,VASP,PLA2G4A,ETV6,TACC2,SCFD2,RAB12,SNRPC,KIFC1,SLC25A52,IQGAP1,RPS12,CAMLG,COX7A2L,ZBTB7C,TEAD1,MORC2,SREBF2,ANP32B,YBX3,AIMP1,LASP1,THNSL2,FYB2,NRXN1,PCID2,HIPK1,ZNF234,CISD1,ZNF518A,DGKK,FRY,SNAP91,CD70,CYP4F22,CIBAR1,PBLD,FICD,CACYBP,CADM1,SSPN,CENPE,PEG10,LMX1B,NET1,SIPA1L2,TUBB6,NGDN,ELOC,ANLN,TWIST1,RNU6-1150P,AKT3,ALKAL2,RNU1-51P,JAK2,SLC1A7,VSX1,RPF2,FSTL1,CHCHD6,ZBTB38,MPPE1,ISX,BPNT1,SVEP1,MADD,HCRTR1,RBM19,PTGS1,PATL1,ZNF287,CELSR2,ZNF449,PRSS2,FH,TDP1,CREBBP,MELTF,MRM1,TNKS,ARL11,SGO1,GORAB,PCNA,SIAH3,TRPV5,UFL1,ADAMTS5,GLYATL2,NFKBIA,PRKCB,OR2T3,GOT2,NTM,KIF6,ABCC8,MIPEP,PCDH11X,ANXA4,MT1HL1,CACNA1E,ZC3H15,ANP32A,RFC2,SMTN,ZNF354C,ST6GAL2,ALX4,RNU6-113P,RTRAF,USH1C,BRD4,ZBTB21,SERBP1,SMPD4,NEDD9,OLF

			M4,NRBP1,ITGA6,ATP2B1,GAP43,SLC14A2,IARS2,CLCA4,DGLUCY,ATP1A1-AS1,NOXRED1,ASS1,MTCL1,GRIP1,IGHV1OR15-9,CTNNBL1,TM9SF3,ADGRE3,SAR1A,ADCY9,EML1,PPP1R17,CNIH1,MAST2,HPSE2,BTG3,ZNF528,ERLIN2,GOLGA8J,ZNF611,TRAPPC3,XKR6,OTOP1,ADAMTSL3,CIDEA,PCMTD2,ARFGEF3,ZBTB49,BBS9,EXT2,EXOC1,HEPACAM,KRT6A,STOX2,AGO1,FRA10AC1,PDP2,MEOX2,SLC6A1,GID8,ELL2,GRXCR1,SDS,SNAPC3,STAT1,BRMS1L,NDFIP2,NR2C1,MAP2K6,CMTM7,DGKG,SHROOM2,RN7SL483P,SLC6A11,KCNJ18,MARCHF6,GATAD1,MTPN,ABI1,MYO18B,NECTIN4,ARMC6,CEMIP,PRAMEF2,POU6F2,IMPACT,CBLIF,CCBE1,PARK7,ADAMTS18,MAPK8,ITGA4,TOP3A,OAZ2,EIF3F,BCAP29,PPME1,MED12L,ZSCAN30,FBXL17,UBL7,POU1F1,UBE2J2,ADCYAP1R1,PLA2G12B,MTF2,NCAPG2,NDC1,TM9SF4,RAPGEF4,OR6C75,FOXP2,ASB2,MYOCD,HFM1,HMCN1,CEP120,MYH13,ATP13A3,DHTKD1,ZSCAN5C,CYFIP2,HNRNPM,ACACA,KRT85,ASCC2,ST8SIA4,NDUFA10,ARL4C,EFHB,OR13C9,ARID3B,MEF2C,ZNF613,ADGRB1,RXRA,WNT7A,RBPMS2,ECHDC1,MAP3K5,NDFIP1,SLC5A9,MAP3K4,WASF3,S100B,SERPINI2,PRDM13,FOXO6,ERI1,SUMF1,ZNF112,ATP6V1C2,CHAMP1,C16ORF72,MAGEL2,PKN2,RAD51AP1,SLC10A6,OR10H2,PDE2A,RAB38,LRRC2,DBF4B,FBXW8,SDCBP,NECTIN1,DSG1,JPT2,SPPL2B,NSMCE1,ZNF813,WWOX,ZBTB25,PASK,MLLT1,NCK1,FLVCR1,SCAF8,FGR,CWC22,DRC7,CDCA8,PPP2R3A,DNMBP,RNU6-1007P,TRIM23,ATP6V1B2,CXCL2,TOP1,TINAG,SNAP29,MLLT10,C2,SPRR2D,IFNAR1,RNF8,GNG12,LCE3D,EPHA4,PPIP5K2,CYTH4,EMP1,INTS13,GABRA5,MECOM,DNMT3L,NTRK2,IL1RAPL1,FNDC3A,ACSM2A,RSPH1,KHDC4,NUMB,LHX9,ADAMTS9,RN7SL767P,WNT2B,COLEC12,FRRS1,ZBTB10,PLEKHA3,OCLN,POSTN,CREB5,SNRPD1,CD101,STON1-GTF2A1L,SHISA6,MEGF10,IL17RD,FBXO31,EXTL3,AKAP11,TRPM7,KTN1,GRIK1,PRKAB1,DTHD1,IREB2,MFSD9,MVB12B,HS6ST1,PTK2,ERP27,MARK4,CDH5,CD5L,TPH2,RCAN2,ANKRD6,AP4S1,SCGN,NFKBID,ARHGAP12,CLDN18,ASCL3,MPP7,DIAPH1,FEZ2,INIP,LAMB1,SCAMP1,APIP,CYFIP1,UBE3A,SCG3,HOATZ,APOL1,PCDH8,SEMA4D,JAM2,DNAH10,PITPNC1,FRMD6,MC2R,ZBTB20,FAT4,IMPA2,LRMDA,CCDC162P,ZNF66,AP2B1,RUNX1,AKR1B1,C9,KIRREL1,WNT5B,RASGEF1B,AMFR,SAXO1,SCARA5,NENF,SH2D1B,SLC26A2,POMT2,HEATR5A,PTGFR,ZNF845,PSTPIP2,ZFYVE1,OR4L1,SANBR,ASAP1,SAMD13,ICA1,PLCZ1,EDIL3,NOS1AP,MTTP,SLC9A5,FCRLA,DIDO1,DPY19L2,TPTE,SORBS2,PDCL3,SRP9,CNKSR1,CCDC88A,GPR55,NSUN2,SLC27A6,UBAP1L,CHCHD2,GALNT18,HKDC1,ADAMTS16,SPAG6,ACOXL,SLC5A1,MDN1,CDC45,OR11G2,BICD1,ANO10,TNFSF11,FYN,BUB1,KDM5A,PCBP3,NLRP14,DPY19L1,ZNF705G,PPM1F,GOLGA8F,ADGRL2,UNC45B,ARL13B,XPO7,SDE2,ODR4,RBMS3,HYDIN,UHRF2,SCN8A,HDAC2,AVEN,SLF1,SACM1L,GON4L,TBX15,CNTNAP3,TMEM63C,SH2D3C,DOCK3,TRNAU1AP,NCS1,COL18A1,GALNT17,CDH9,ATP5PF,ALB,DOK5,ATP9B,NALCN,UGP2,MTMR7,EHBP1,ZFYVE28,MAPK9,PABPC1,CRTAM,COL19A1,APELA,MDGA2,STT3A,TRPM3,SLC39A8,ROR1,SLC16A9,GALNT2,FUT8,TET1,ARNT2,ASB3,HECW2,CDH2,CNTN5,ITGA8,SEL1L2,FBXL20,NTN4,RAD9A,XRN2,PHLPP1,GPR137B,RNU6-929P,EPHB1,RP1L1,ZDHHC18,GRM5,DDX10,ADCK1,RAI14,GDAP1L1,SPOPL,ZNF705D,RPS6KA5,SPTB,TBC1D1,LRRC69,PTPRG,PID1,NRP1,MIDEAS,FCHSD2,SDK1,PRKCA,GBP4,IFT46,MRPL58,COX10,ATPSCKMT,FAIM,RNF215,SAMD12,USP24,FAAP24,MOGAT3,FHIT,ITGA1,ZNF615,PCCA,CROT,KLF12,RNF138,RC3H1,NRIP1,CHODL,POR,ZNF850,ZNF235,ABCA6,SLC14A1,EFL1,MCC,ZNF738,SUPT3H,BCR,TUT4,NRXN3,ELMO1,RGS6,RERG,ZNF215,TCERG1L,KIF16B,CDH12,PRIM2,SNRK,C14ORF39,ARFGAP3,TM9SF2,USP49,ELP2,CFAP70,FBLN1,STK36,NSG2,PAQR5,MB,RAG1,KCNJ6,B9D1,DGCR2,DNPEP,CYP4B1,RRAS2,GNA14,ZNF678,BMPER,RABL2A,PRDM15,CUX1,DPP6,SRGAP3,SLC35F1,ZNF420,MACROH2A1,MITF,EPHB2,TSPAN13,TOGARAM1,CSNK1G1,SACS,BCL2L13,RNF11,CD38,EYA4,CHCHD3,DPH6,MYO5B,RGPD4,PPIL2,CDK14,AKAIN1,MET,MUC16,SPPL3,DLG2,CDH17,Z

			NF705B,ATP6V0D2,SPECC1,CAMK1G,IBA57,METTL15,PPFIA2,CDH13,MED13L,STXBP4,SERPINB2,CACNG3,ATG5,USP32,NRAP,MAGI2,SLC35F4,PRDM11,VMP1,UNK,AIFM3,FAM171A1,MLIP,FLRT2,MYB,KALRN,ME3,ZNF704,SLC1A2,CHST3,GNAS,LAMA1,MFHAS1,SERPINB7,CA10,CPQ,NUP43,TRIM9,ATRNL1,TIAM2,DHX29,IGSF21,BMP7,TTC28,ASTN2,DLG5,TNFAIP8,ZMYND8,GAPVD1,GABRA2,RNF217,KIRREL3,KCTD1,BTD,GOLGA6A,OR2T2,DNAH3,ZNF74,BPTF,BTBD10,AK3,ZMYND11,TMEM25,NUDT21,TRAPPC10,GRM3,KMT2C,DDX6,ADGRF5,OR4N2,PDGFC,WDR41,DNAH17,PLIN2,PPP1R13B,ELOVL7,EPB41L4A,ABL2,MMP26,MRPL37,VPS13B,TRAPPC6B,BACE2,RFX2,PARPBP,NECAB1,PKNOX2,EYA1,FHOD3,PDZD2,TTLL11,GOLGA8T,PRPF18,SLIT2,CMPK1,TMPRSS3,EXOC4,RNU6-835P,CNOT7,FAM126A,KCNIP4,ESCO1,KCTD8,CCDC141,PLCL1,ERBB4,IL20RB,FAM3B,FAM126B,GSAP,TRHDE,SYNDIG1,ROBO1,SAMD4A,PBX1,IRAG1,NPAS3,NUF2,PRKCQ,RGPD2,SAMM50,ANTXR1,NDRG1,SORCS2,SIPA1L3,TRDN,MGMT,ZNF679,NLGN1,CTTNBP2,AK9,SHLD2,NOS1,SLC6A3,GLDC,CHD9,PRR16,ASIC2,EFNA5,TCF12,GAS2L1,ARHGEF11,MTREX,VCAN,RAB27A,NSD1,EHMT1,SLIT3,USP31,DTNA,KIF13A,AP5M1,FRMD5,ESR1,DNAH9,SLC25A48,MYO9B,NTNG1,KDM4B,CYP2C8,KCNQ5,LOXL2,CACNA2D1,NYAP2,IGLC3,IQCJ-SCHIP1,FRMD4B,IRAG2,ADGRG7,ORC4,SKAP2,PRLR,PIGB,AGO3,HTT,LARS2,ZDHHC11B,FOXB1,RAD51B,CAMK1D,PIK3R3,SLC25A18,MACROD2,CFAP44,CDKAL1,OPCML,CATSPERE,AK2,HLA-F,FER,ZNF302,EVA1A,EYA2,KATNIP,CCR2,RPGRIP1,STARD13,PITPNM3,OSBPL5,INTS12,A2M,FGGY,WDFY3,CHFR,ZNF721,PCMT1,EPS8,OSBPL6,AUH,JAZF1,ZNF578,OARD1,ZNF891,SPOCK3,SEMA4B,NRF1,IGHV1OR21-1,ZNF14,ANO2,HRH1,PHC2,GRIA4,AGAP1,ROCK2,PRDM1,RORA,STMP1,IL16,ATAT1,TERB2,NARS2,DMRT1,EIF4G3,CDCA5,PPP1CB,CATSPER2,RGS8,RAB31,PDK1,HSPG2,PSMD2,PTPRQ,CSMD3,HERPUD1,NCOA6,TRIM2,HSD17B2,COL4A3,WASHC1,ZFP30,RGS7,HOOK3,KIF7,GNG2,PCSK2,FSTL4,CLDN10,BARD1,CLCN5,PNPLA3,STK3,DEPTOR,ZNF423,SLC13A4,C1QL3,RSU1,PNPLA8,ZNF568,HNRNPU,LINC00240,VTI1A,CEP72,RAB3GAP2,CADPS,APCDD1,IGF1R,KCNAB1,MEG8,PRKAG2,GLI2,THRB,TANGO2,LSAMP,AKAP13,MORC3,ATP10A,SEPTIN6,DNM1L
GO:2000026	regulation of multicellular organismal development	0.0000011461631116736055	*NOTCH2,MTOR,PTPRD,ULK2,PLCB1,TAFA5,ZFPM2,TENM4,ZDHHC21,FBN1,CHRNA7,ROBO2,ZEB1,RARB,SPRED1,MINAR1,FOXJ2,ANO6,DSCAM,SOX5,RFX3,MACF1,BCL11A,SOX6,CDH4,NTRK3,C5,FLT1,SLC39A12,PRKD1,EPHA7,SPEN,RAPGEF2,LRP2,ADGRB3,RUNX2,COL4A2,ADAM10,STAU2,SEMA5A,VCL,SLC8A1,FIG4,BMPR1B,AKAP6,ARNT,PAK3,DIP2B,ITPKB,TRPC5,PRKCZ,YAP1,BRINP1,NIPBL,GABPA,PAFAH1B1,EFEMP1,ITGB8,NF2,CTNNA1,PPARA,MEIS2,PRTG,SYNJ1,TIAM1,SEMA3C,AGO2,JARID2,IL34,ADGRV1,WNT9B,SEMA6D,SMARCA4,PARD3,TNR,CXADR,ABL1,DROSHA,CAMK4,FGF10,GRID2,NRG1,INO80D,ASPM,AP3B1,ATP11C,ZBTB16,ZNF675,FBN2,EGF,SPRED2,PTPN2,PLXNA2,AMBRA1,OPRM1,FANCA,SEMA3E,LINGO2,EPN2,ADAM12,EMILIN2,HMGA2,MBP,TRPS1,TJP1,HLA-B,DISC1,WDPCP,SEMA3A,BMP2,RC3H2,PSG9,GFI1B,BMP2K,SEMA3D,RELN,HMGB1,FGF9,ESRP1,NIN,DRAXIN,SMARCA2,ETS1,GLI3,SMARCC1,SMOC2,MAP2,ATF2,CFTR,NELL1,KITLG,DCC,GTF2I,TADA2A,DAB1,MTMR2,TBX20,PRKCH,TG,RACGAP1,TOX,ROCK1,LYN,DTX1,OVOL2,NTN1,DPYSL5,ARID1B,INSR,SNAI2,RXRG,JCAD,ENPP1,RASGRP1,CSF1,CTDP1,LAMA3,INO80,CNMD,MYCL,TNN,MED1,IL33,AJAP1,ROR2,KL,TMEM178A,IL10,ACTR2,CLSTN2,PTH,MAP6,NRXN1,PCID2,HIPK1,TWIST1,AKT3,RBM19,UFL1,NFKBIA,PRKCB,ABCC8,NEDD9,ATP2B1,AGO1,STAT1,CCBE1,MEF2C,ADGRB1,RXRA,WNT7A,NDFIP1,WASF3,FBXW8,EPHA4,NTRK2,IL1RAPL1,NUMB,ADAMTS9,WNT2B,CD101,FBXO31,CDH5,NFKBID,CLDN18,CYFIP1,SEMA4D,JAM2,FAT4,RUNX1,PDCL3,GPR55,TNFSF11,ADGRL2,HDAC2,CRTAM,APELA,GPR137B,EPHB1,GRM5,NRP1,PRKCA,FAIM,RC3H1,CHODL,POR,RAG1,BMPER,CUX1,MACROH2A1,MITF,EPHB2,FLRT2,MYB,KALRN,LAMA1,*

			TIAM2,BMP7,DLG5,SLIT2,ERBB4,SYNDIG1,ROBO1,NLGN1,ASIC2,EFNA5,EHMT1,LOXL2,PRLR,CCR2,SEMA4B,ROCK2,PRDM1,HSPG2,COL4A3,HOOK3,FSTL4,GLI2
GO:0009719	response to endogenous stimulus	0.000001234838 1768130318	*NOTCH2,BCAR3,MTOR,NSG1,NLK,PLCB1,PTPRA,ITPR2,PDE4D,RDX,BCL2,MYO5A,PRDM16,FBN1,ROBO2,GABRB3,ZEB1,AKR1C3,RARB,SPRED1,ALK,APC,CRKL,SOX5,KDM4C,EGFR,NCOR1,NEDD4,BCL11A,SOX6,NTRK3,RXFP1,GABRB1,NEO1,PAK1,CHRM3,RAPGEF2,LRP2,RUNX2,ARSB,FGF12,CPS1,ONECUT1,CPEB4,TMEM38B,BCKDHB,UBE2L3,SMYD3,RPTOR,GHR,COL4A2,HDAC9,APP,GABRG2,NTF3,SLC8A1,KANK1,BMPR1B,PCSK6,AKAP6,RAB8B,LARP1,RAP1GDS1,PRKCZ,GRB10,RYR3,DEFA3,DUSP22,YAP1,MAPK1,HRH2,PAPPA,PDGFD,UBE2O,SPIDR,GABPA,HRH4,ACSBG1,GLP2R,ITGB8,HIVEP1,CTNNA1,AKAP9,KLF15,PPARA,NR5A2,PTPRK,TRERF1,SLC24A4,VPS13C,TMEM108,BCAS3,RYR2,BBS2,SMARCA4,USP8,MBD5,ELAVL4,ABL1,PTPN12,HDAC4,SLC1A1,PRKAA1,RAP1A,FGF10,LATS2,PRKCE,ESRRG,DENND4C,FBN2,CD44,PDE3A,EXT1,SPRED2,PTPN2,HTR2C,LTBP1,ZFYVE9,OPRM1,HTR2A,ALPL,FHL2,GNAL,NOS2,CCND3,MBP,CRIM1,FBXO32,LDLRAD4,BLM,AGL,BMP2,PSG9,SOGA1,GNAI1,TBC1D4,LEMD3,FGF9,ZNF106,DSTYK,RAP1GAP,GLI3,SMARCC1,SNX6,SMOC2,IDE,GAREM1,ATF2,UMODL1,BBS4,CFTR,RGMB,UBR1,CHRM5,SLC30A10,SELENON,PTPRE,PRKN,TBX20,SNX25,SOX30,ROCK1,LYN,VCAM1,CTSB,EIF2B3,OVOL2,ZFHX3,RRAGD,ARID1B,INSR,TFF1,SNAI2,RXRG,NREP,CD9,ENPP1,BMP5,KCNC1,BCL2L1,SERPINB9,HCN1,GRB14,SMAD5,HADHA,MED1,KL,IL10,ACTR2,PTH,SOSTDC1,PRKAA2,ABHD2,VSTM2A,IQGAP1,SREBF2,NRXN1,PBLD,PEG10,JAK2,FSTL1,CREBBP,PCNA,UFL1,PRKCB,ABCC8,ATP2B1,ASS1,OTOP1,CIDEA,EXT2,STAT1,BRMS1L,NR2C1,IMPACT,PARK7,ITGA4,MYOCD,ACACA,MEF2C,RXRA,WNT7A,RBPMS2,OR10H2,PDE2A,SDCBP,DSG1,WWOX,NCK1,C2,EPHA4,NTRK2,IL17RD,PTK2,CDH5,TPH2,DIAPH1,CYFIP1,UBE3A,FAT4,PTGFR,FYN,HDAC2,FUT8,TET1,ITGA8,GRM5,PID1,POR,RERG,KIF16B,NSG2,GNA14,BMPER,EPHB2,CD38,CDH13,STXBP4,MAGI2,FLRT2,SLC1A2,GNAS,BMP7,PDGFC,SLIT2,ERBB4,PRKCQ,NOS1,EFNA5,GAS2L1,SLIT3,ESR1,CACNA2D1,PRLR,PIK3R3,FER,A2M,HRH1,ROCK2,RGS8,RAB31,NCOA6,GNG2,PNPLA3,ZNF423,HNRNPU,IGF1R,THRB*
GO:0120031	plasma membrane bounded cell projection assembly	0.000001287975 1542594827	*MTOR,LRRC49,RIPOR2,RDX,RP1,RALA,ODAD2,SDCCAG8,FGD4,SPAG16,CDC42EP3,AUTS2,CARMIL1,PARVB,ANO6,MAP4,APC,PLPPR5,ARHGAP24,TNIK,RFX3,ARMC2,RAPGEF2,ONECUT1,LRGUK,SEPTIN9,DCLK1,STAU2,VCL,ARHGAP44,CD2AP,SRGAP2C,KANK1,DNM3,IFT57,COBL,YAP1,ABLIM1,BCAS3,SYNE2,BBS2,AIF1L,ABL1,HDAC4,DNAH5,MYO10,CEP83,PTPRO,IFT43,ARHGEF7,ABCC4,TMEM67,PLCE1,DISC1,WDPCP,DNAL1,NUDCD3,TTC21B,RAP1GAP,SRGAP2,DNAH8,DAW1,CYLD,BBS4,KIAA0753,PCDH15,VAV3,CFAP74,ROCK1,TENM2,NTN1,IFT81,FAM149B1,CDC14B,PCNT,ACTR2,TTC39C,NRXN1,CIBAR1,ANLN,CELSR2,GORAB,GAP43,BBS9,CEP120,WASF3,SDCBP,NCK1,DRC7,SNAP29,EMP1,RSPH1,OCLN,MARK4,CYFIP1,HOATZ,SAXO1,ASAP1,CCDC88A,ADAMTS16,SPAG6,ARL13B,HYDIN,RP1L1,NRP1,IFT46,CFAP70,STK36,B9D1,EPHB2,TOGARAM1,CDH13,ATG5,DNAH17,RFX2,SLIT2,NLGN1,HTT,CFAP44,FER,RPGRIP1,EPS8,ATAT1,WASHC1,ZNF423,SEPTIN6*
GO:0007416	synapse assembly	0.000001515464 4759790474	*PTPRD,IL1RAPL2,ROBO2,GABRB3,NEGR1,GPC6,DSCAM,CRKL,NTRK3,EPHA7,ADGRB3,APP,STAU2,GABRG2,SRGAP2C,DNM3,LRFN5,GRID2,NRG1,MUSK,LINGO2,PDLIM5,DNER,SRGAP2,FARP1,LGI2,SHANK2,NTN1,COLQ,CLSTN2,NRXN1,GAP43,MEF2C,ADGRB1,WNT7A,SDCBP,NECTIN1,NTRK2,IL1RAPL1,SEMA4D,ADGRL2,CDH2,CNTN5,EPHB1,SDK1,NRXN3,EPHB2,FLRT2,DLG5,GABRA2,KIRREL3,ERBB4,SYNDIG1,NLGN1,ASIC2,EFNA5*
GO:0098609	cell-cell adhesion	0.000001958913 5883742 22	*CNTN4,PTPRD,LRRC4C,TLN2,TENM4,ZDHHC21,RIPOR2,RDX,STXBP1,BCL2,CDH8,ROBO2,TENM3,PCDH7,ASTN1,NEGR1,GPC6,HHLA2,DSCAM,ILDR2,EGFR,CTNNA3,CRB1,CDH4,NEO1,CNTN6,EPHA7,CCL28,DOCK8,VCL,CD2AP,IGSF5,CTNNA2,ITPKB,PRKCZ,DUSP22,ALCAM,PLG,FAT3,NF2,CTNNA1,CDH7,PPARA,PCDH11Y,MAGI1,ADGRV1,SMARCA4,CDH11,BLK,TNR,CXADR,PTPRT,AB*

			L1,LRFN5,NFAT5,CDH18,MYO10,GRID2,CDHR3,NRG1,AP3B1,ZBTB16,CD44,EXT1,STXBP6,CTNND2,PTPN2,AMBRA1,LPP,STK10,ANK3,EMILIN2,MBP,FUT9,PCDH9,VAV1,CDH20,TJP1,NPHP4,CNTN1,PDLIM5,BMP2,RC3H2,UNC5D,NCAM2,HMGB1,SLAMF1,SMARCA2,ETS1,GLI3,MEGF11,SMARCC1,ITGA9,COL14A1,KITLG,DCC,DAB1,PCDH15,CDH23,PKP1,ROCK1,LYN,VCAM1,DTX1,TENM2,NTN1,ARID1B,CD9,RASGRP1,IGSF11,CDH26,BMP5,PRKG1,LAMA3,FAT1,IL10,CLSTN2,NRXN1,CD70,CADM1,JAK2,CELSR2,PCDH11X,ITGA6,ASS1,NECTIN4,ADAMTS18,ITGA4,HMCN1,CYFIP2,NDFIP1,NECTIN1,DSG1,NCK1,IL1RAPL1,FNDC3A,MEGF10,PTK2,CDH5,NFKBID,CLDN18,LAMB1,PCDH8,SEMA4D,JAM2,FAT4,RUNX1,KIRREL1,TNFSF11,FYN,PPM1F,CDH9,CRTAM,COL19A1,SLC39A8,CDH2,CNTN5,ITGA8,SDK1,PRKCA,ITGA1,RC3H1,NRXN3,CDH12,RAG1,DLG2,CDH17,CDH13,VMP1,MYB,GNAS,IGSF21,BMP7,ASTN2,DLG5,KIRREL3,ABL2,IL20RB,ROBO1,PRKCQ,NLGN1,EFNA5,NTNG1,FER,CCR2,CLDN10,GLI2
GO:0050770	regulation of axonogenesis	0.0000023179188937657035	*LRRC4C,ULK2,ROBO2,DSCAM,MACF1,BCL11A,CDH4,PAK1,EPHA7,SEMA5A,PAK3,DIP2B,TRPC5,CHN1,PAFAH1B1,TIAM1,SEMA3C,SEMA6D,TNR,PLXNA2,SEMA3E,MARK2,MBP,DISC1,SEMA3A,SEMA3D,UST,NIN,DRAXIN,MAP2,DCC,DAB1,NTN1,MAP6,WNT7A,EPHA4,NTRK2,CYFIP1,SEMA4D,CDH2,NRP1,CHODL,EPHB2,TIAM2,SLIT2,ROBO1,EFNA5,SEMA4B,FSTL4*
GO:0048585	negative regulation of response to stimulus	0.0000027503548345930766	*MTOR,WWC1,PTPRD,NLK,ZNF536,TAFA5,DLC1,RIPOR2,PDE4D,BCL2,PRDM16,FBN1,ROBO2,SPRED1,MINAR1,MCTP1,ERBIN,MLLT3,SUSD4,APC,ARHGAP24,PTPRJ,EGFR,USP14,PRKACB,RGS3,NCOR1,NEDD4,SCAI,C5,DKK2,MAPKBP1,AOAH,INVS,NCOA7,LRP2,RUNX2,TAOK3,ONECUT1,SAMSN1,USP18,SEMA5A,ARHGAP44,NDUFAF2,CD2AP,PTPRR,KANK1,HOMER2,CTNNA2,RGS20,PDE10A,KICS2,PRKCZ,GRB10,RGS9,HECW1,DUSP22,YAP1,USP25,PLG,ZNRF3,STK38,PAFAH1B1,NF2,CTNNA1,BIRC6,PPARA,PAK5,SEMA3C,SLC24A4,SEC14L1,VPS13C,ALPK2,BBS2,SEMA6D,DUSP16,SMARCA4,MAPKAP1,TNR,PTPRT,ABL1,PTPN12,OXR1,PRKAA1,LRFN5,FGF10,GRID2,LATS2,NRG1,ZNF675,NXN,WNK2,FBN2,CD44,RGS12,PTPRO,PDE3A,LIMD1,SPRED2,PTPN2,CD96,LTBP1,OPRM1,KREMEN1,SEMA3E,FHL2,HIPK3,EPN2,GRK3,MOSMO,HMGA2,CRIM1,PRR5L,LDLRAD4,NPHP4,HLA-B,SEMA3A,BMP2,RC3H2,RANBP9,RIN3,TMEM161A,SEMA3D,LEMD3,ARHGAP42,FGF9,DRAXIN,SLAMF1,GLI3,CGAS,SNX6,CNKSR3,CYLD,BBS4,MAPK8IP1,UBASH3A,UBR1,RCAN1,DAB1,RB1CC1,PTPRE,PRKN,MTMR2,TBX20,NLRC5,SNX25,SHANK2,PSMA1,ENPP3,SOX30,KIR2DL4,LYN,OVOL2,RNF152,OTUD7A,YTHDF3,SNAI2,ASH1L,BID,SIAH2,TRABD2B,UFD1,CD9,SAMHD1,ENPP1,NDRG2,CSNK2A1,BMP5,BCL2L1,SERPINB9,PRKG1,GRB14,FANCB,DHRS3,CELF4,PRAME,TNN,IL33,AJAP1,BANK1,IL10,SOSTDC1,PRKAA2,ITPRIP,YBX3,NRXN1,PBLD,PEG10,TWIST1,UFL1,NFKBIA,PRKCB,ABCC8,BRD4,ITGA6,OTOP1,CIDEA,STAT1,BRMS1L,DGKG,PARK7,ADAMTS18,MYOCD,RBPMS2,NDFIP1,C16ORF72,PDE2A,WWOX,NCK1,FGR,PPP2R3A,EPHA4,MECOM,SHISA6,IL17RD,CDH5,ANKRD6,ARHGAP12,SEMA4D,AMFR,NENF,BICD1,FYN,RBMS3,HDAC2,ZFYVE28,APELA,TET1,CDH2,PHLPP1,GRM5,TBC1D1,PID1,NRP1,FAIM,ITGA1,RC3H1,MCC,BCR,RGS6,FBLN1,BMPER,PRDM15,MACROH2A1,EPHB2,EYA4,MET,SERPINB2,ATG5,MAGI2,MLIP,MFHAS1,BMP7,DLG5,ZMYND11,TMEM25,WDR41,ABL2,MMP26,PARPBP,EYA1,SLIT2,CNOT7,IL20RB,ROBO1,PRKCQ,SHLD2,SLC6A3,SLIT3,ESR1,HTT,HLA-F,FER,EYA2,CCR2,A2M,SEMA4B,RORA,RGS8,HERPUD1,RGS7,KIF7,FSTL4,STK3,DEPTOR,APCDD1,IGF1R,GLI2*
GO:0007610	behavior	0.000003004718054374706	*MTOR,PLCB1,BCL2,CHRNA7,NAV2,ALK,PJA2,ASTN1,NEGR1,CNTNAP2,DSCAM,SLC4A10,EGFR,NCOR1,BTBD9,DGKI,GRIA1,SLC8A3,ADGRB3,FGF12,RPTOR,APP,GABRG2,NTF3,FIG4,TAFA2,HOMER2,KCNK10,PRKCZ,KLHL1,BRINP1,MAPK1,ADAM22,SLC16A1,SORCS3,PAFAH1B1,PPARA,MEIS2,SYNJ1,GRM1,PAK5,SLC24A4,DNAH11,SCN2A,TANC1,BBS2,TNR,MBD5,ELAVL4,ABL1,HDAC4,OXR1,SLC1A1,PRKAA1,MORC1,CAMK4,NRG1,RASGRF1,MUSK,PRKCE,EXT1,ATP8A2,HTR2C,ATP8A1,OPRM1,HTR2A,PUM1,TMOD2,APBA2,KCND2,ANKFN1,NPHP4,CNTN1,STRN,RELN,TTC21B*

			,GLI3,GRIK2,BBS4,RCAN1,DAB1,PRKN,DACH1,PCDH15,GRIN2A,ATXN1,CDH23,ALS2,SHANK2,NPAS2,ZFHX3,INSR,GRID1,NMU,PBX3,GHRH,GRIN2B,OTOG,LMX1A,ACTR2,NRXN1,HCRTR1,ABCC8,NEDD9,SLC6A1,PARK7,CSMD1,MEF2C,S100B,FOXO6,EPHA4,GABRA5,NTRK2,UBE3A,AMFR,FYN,HDAC2,ITGA8,FBXL20,GRM5,SDK1,NRXN3,RAG1,EPHB2,SPECC1,KALRN,SLC1A2,KIRREL3,ABL2,NLGN1,SLC6A3,HTT,FOXB1,EPS8,HRH1,THRB
GO:0040007	growth	0.00000361647709441838 9	NOTCH2,MTOR,SPOCK1,WWC1,LRP12,BNC2,ULK2,SCAPER,FTO,PLCB1,ZFPM2,TENM4,BCL2,RIMS1,RARB,MINAR1,RIMS2,AUTS2,PAPPA2,DSCAM,CRKL,SLC4A10,PTPRJ,EGFR,MACF1,BCL11A,TMEM182,CDH4,LARGE1,EPHA7,TMEM38B,RPTOR,GHR,EPB41L3,NEDD4L,ADAM10,APP,DCLK1,SEMA5A,SYT1,VCL,AURKA,BMPR1B,AKAP6,RFTN1,DIP2B,TRPC5,NBN,PRKCZ,COL27A1,COBL,YAP1,ALCAM,PLG,NRG3,NIPBL,FMN1,PAFAH1B1,DCAF1,PPARA,PAK5,SEMA3C,TMEM108,JARID2,BBS2,SEMA6D,SMARCA4,MAPKAP1,TNR,CXADR,MBD5,ATRX,ABL1,PTPN12,GAS2,CREG1,FGF10,LATS2,NRG1,ASPM,MUSK,EXT1,RPS6KA3,ATP8A2,SEMA3E,APBA2,CDKN2C,EVC,AFG3L2,HMGA2,PLCE1,CRIM1,PDLIM5,BRCA2,DISC1,SEMA3A,RC3H2,SEMA3D,FGF9,SLC23A2,PLS1,NIN,DRAXIN,SMARCA2,GLI3,MAP2,ATF2,BBS4,KPNA1,DCC,SELENON,PRKN,TBX20,PCDH15,ARID5B,FBLN5,ITSN2,PTGFRN,NTN1,INSR,COLQ,CD9,ENPP1,IGSF11,CSNK2A1,CSF1,GHRH,BCL2L1,CTDP1,INO80,PRAME,TNN,PSAP,MED1,KDM6A,ATRN,LMX1A,IQGAP1,TEAD1,YBX3,NET1,PRSS2,NEDD9,GAP43,HEPACAM,BRMS1L,MTPN,IMPACT,ITGA4,NCAPG2,MYOCD,CYFIP2,MEF2C,WNT7A,SDCBP,FLVCR1,PPP2R3A,EXTL3,CYFIP1,UBE3A,SEMA4D,RUNX1,SORBS2,SPAG6,PPM1F,EYS,NRP1,POR,RERG,CD38,MAGI2,PRDM11,SLC1A2,GNAS,CPQ,SLIT2,ERBB4,PRKCQ,SLC6A3,EFNA5,ARHGEF11,SLIT3,ESR1,PRLR,RAD51B,SEMA4B,FSTL4,STK3,GLI2,AKAP13
GO:0055085	transmembrane transport	0.00000433451324529610 1	UNC80,CACNA2D3,SLC17A1,ABCA13,SLC24A2,KCNH5,MICU2,SLC25A21,LONP2,CLTCL1,SLC37A1,PIEZO2,DPP10,ITPR2,PDE4D,SLC44A5,BCL2,KCNMA1,CHRNA7,GABRB3,SV2C,ANO6,CACNG2,SLC4A10,OCA2,NEDD4,GRIK3,ATP2B2,TUSC3,GABRB1,GRIA1,SLC39A12,SLC8A3,PRKD1,CHRM3,LRP2,FGF12,GABRA6,TMEM38B,SLC24A3,SLC44A1,TMEM241,THADA,NEDD4L,TRPM1,SLC39A11,APP,SLC7A2,ABCB5,CACNA1C,CACNB2,GABRG2,TMC1,SLC8A1,ABCG8,KCNE4,ABCD2,AKAP6,KCNK10,TRPC5,CLIC6,GRB10,RYR3,HECW1,ABCA5,SV2B,KCNJ1,ABCD3,TRPC7,SLC16A1,NIPAL2,MICU1,LRRC38,GRIK4,AKAP9,KLF15,RASGRF2,KCNS3,GRM1,GABRG1,DAPK1,SLC24A4,SCN2A,DNAJC15,ANK2,RYR2,SLC9C1,SLC36A1,ABL1,SLC1A1,SLC12A8,KCNH1,ANO4,SLC46A3,GRID2,GSG1L,RASGRF1,ATP11C,ABCB7,ABCC12,PRKCE,SLCO3A1,SLMAP,WNK2,ABCC9,P2RX6,PEX14,HTR2C,SLC2A3,ALG10B,ATP8A1,OPRM1,ABCC4,HTR2A,CYBRD1,CNNM4,STAC,CNIH3,MAIP1,SLC2A13,KCND2,ABCA10,AFG3L2,ANK3,NIPA2,TMC7,TMEM163,ATP6V1E1,CACNA1I,KCNJ15,SLC10A7,SCN11A,SLC15A5,NETO2,RELN,SLC23A2,SLC39A6,KCNH8,SLC37A2,SLC9A4,GABRR2,CNKSR3,GRIK2,PEX6,LRRC8B,CFTR,SLC30A10,SELENON,GRIN2A,JPH1,TRPM6,SLC12A1,OSCP1,KCNQ3,SHISA9,SLC4A4,SCN10A,KCND3,KCNN3,LYN,SLC44A2,SLC15A2,SLC13A5,CRACR2A,INSR,CUL5,GRID1,COX5A,ABCA4,GABRG3,ZDHHC17,SLC22A14,ENPP1,UTRN,KCNC1,HCN1,GRIN2B,ABCG1,KCNK5,SLC40A1,SLC5A12,PTH,SLC25A52,COX7A2L,LASP1,NRXN1,TWIST1,SLC1A7,TRPV5,PRKCB,ABCC8,CACNA1E,ATP2B1,SLC14A2,CLCA4,OTOP1,SLC6A1,SHROOM2,SLC6A11,KCNJ18,CEMIP,CBLIF,PARK7,OAZ2,ADCYAP1R1,ATP13A3,MEF2C,SLC5A9,ATP6V1C2,SLC10A6,FLVCR1,ATP6V1B2,GABRA5,OCLN,SHISA6,TRPM7,GRIK1,MFSD9,DIAPH1,APOL1,SCARA5,SLC26A2,NOS1AP,SLC9A5,SLC5A1,ANO10,FYN,SCN8A,TMEM63C,NCS1,ATP5PF,NALCN,TRPM3,SLC39A8,SLC16A9,HECW2,GRM5,PID1,ATPSCKMT,ABCA6,SLC14A1,BCR,KCNJ6,DPP6,SLC35F1,EPHB2,TSPAN13,CDH17,ATP6V0D2,STXBP4,CACNG3,ATG5,SLC35F4,VMP1,SLC1A2,GABRA2,KCNIP4,TRDN,NLGN1,NOS1,SLC6A3,ASIC2,SLC25A48,KCNQ5,CACNA2D1,HTT,SLC25A18,CCR2,ANO2,GRIA4,CATSPER2,RGS7,CLCN5,SLC13A4,KCNAB1,PRKAG2,ATP10A

GO:0007017	microtubule-based process	0.000004715262652334339	*LRRC49,RIPOR2,RP1,ODAD2,SPIRE1,CNTLN,SDCCAG8,SPAG16,CEP192,MAP4,APC,SETD2,RFX3,MACF1,NEK7,NCOR1,ARMC2,SLC39A12,PAK1,DEUP1,LRGUK,DNAH6,KIF4A,TBCD,APP,DCLK1,STAU2,MAPRE2,AURKA,TTC29,SRGAP2C,CCSER2,FMN2,TTLL7,IFT57,PRKCZ,MCPH1,RAB27B,SENP6,DNAH14,SLC16A1,PAFAH1B1,AKAP9,PARD3B,NAV3,TMEM108,DNAH11,STAG2,BCAS3,SYNE2,BBS2,SLC9C1,PARD3,DST,ATRX,ABL1,PRKAA1,TTLL5,DNAH5,FGF10,CLIP1,ASPM,AP3B1,USP33,PEX14,IFT43,ATXN3,ARHGEF7,MARK2,TMEM67,C10ORF90,CFAP61,ANKFN1,NPHP4,CACNA1I,BRCA2,DISC1,WDPCP,ADCY10,TRAK1,GNAI1,RANBP9,DNAL1,TUBGCP3,RTTN,MDM1,TTC21B,SRGAP2,NIN,HAUS6,DNAH8,KIF21A,KIF15,MAP2,DAW1,NEK10,GOLGA8B,CYLD,BBS4,KIAA0753,CEP44,RACGAP1,MAP7,SYBU,CFAP74,ROCK1,KIF11,NEK6,PDE4DIP,ASH1L,TRIM58,FYCO1,SLC22A14,IFT81,RASGRP1,HDGFL3,INO80,KIF21B,CABYR,CDC14B,PCNT,ACTR2,SFPQ,PRKAA2,SKA1,NDC80,MAP6,TACC2,KIFC1,CENPE,TUBB6,CELSR2,TNKS,SGO1,KIF6,MTCL1,EML1,CEP120,DRC7,CDCA8,INTS13,RSPH1,OCLN,KTN1,MARK4,CDH5,DIAPH1,HOATZ,DNAH10,SAXO1,CCDC88A,SPAG6,BICD1,HYDIN,RP1L1,IFT46,KIF16B,CFAP70,STK36,TOGARAM1,MET,DLG2,DNAH3,DNAH17,TTLL11,NUF2,TRDN,EFNA5,GAS2L1,KIF13A,DNAH9,HTT,CFAP44,CATSPERE,FER,KATNIP,ROCK2,ATAT1,CATSPER2,HOOK3,KIF7,HNRNPU,CEP72*
GO:0044093	positive regulation of molecular function	0.000004902414368826 78	*BCAR3,MTOR,GARNL3,TRAPPC9,MYO9A,DLC1,RIPOR2,ERC1,BCL2,CHRNA7,ALK,FANK1,CACNG2,EGLN3,SPON1,CRKL,ARHGAP24,DOCK10,EGFR,DENND1A,ANGPT1,NEK7,RNF220,NTRK3,RXFP1,FLT1,RFC3,PRKD1,PAK1,EPHA7,RALGPS1,RAPGEF2,TAOK3,UBE2L3,RPTOR,GHR,RALGAPA1,RAPGEF5,APP,CACNA1C,CACNB2,DOCK8,MAPRE2,NTF3,ACER2,PARN,ST18,MAP4K4,BMPR1B,AKAP6,RANBP2,RAP1GDS1,NBN,IFT57,PRKCZ,MSH6,EBF2,RIPK4,MAPK1,RABGAP1L,PDGFD,NRG3,TBC1D22A,CHN1,TPM1,LRRC38,MOB3B,AKAP9,RASGRF2,RGL1,TIAM1,ARAP2,EIF3D,DAPK1,TBC1D9,IL34,ANK2,BCAS3,RYR2,WNT9B,CLPX,SMARCA4,TBC1D5,BLK,ABL1,HDAC4,SLC1A1,RAP1A,FGF10,NRG1,RASGRF1,MUSK,SLCO3A1,ASAP2,WNK2,USP33,CD44,EGF,PRRC1,TRIM5,ARHGEF7,ALG10B,AMBRA1,HTR2A,STAC,MARK2,MSH2,EPHA6,CLSPN,NOS2,MNAT1,ANK3,HMGA2,CCND3,MBP,TGFA,HIP1,VAV1,IQSEC1,BMP2,EVI5,RALGAPA2,SGSM1,TBC1D4,RELN,ARHGAP42,HMGB1,GNAQ,DSTYK,RAP1GAP,SRGAP2,CNKSR3,IDE,ZNF618,NEK10,MOB1B,ATF2,CFTR,TBC1D13,KITLG,DAB1,NMD3,PRKN,NGEF,GRIN2A,ARID5B,PRKCH,IL6R,ALS2,TFDP1,DOCK9,COPS8,VAV3,RALB,ROCK1,LYN,CRACR2A,INSR,XRCC4,BID,ERN2,MBTPS2,CARD10,RALGPS2,RASGRP1,SNX9,KCNC1,CSF1,GRIN2B,DCUN1D4,PSAP,CDC14B,GPRC5C,ROR2,RASGEF1C,IL10,PTH,LARP6,IQGAP1,ANP32B,NRXN1,CENPE,NET1,SIPA1L2,TWIST1,ALKAL2,JAK2,MADD,TNKS,PCNA,PRKCB,ABCC8,ZC3H15,RFC2,NEDD9,ITGA6,PDP2,MAP2K6,MTPN,ABI1,CEMIP,PARK7,MAPK8,ADCYAP1R1,RAPGEF4,MYOCD,CYFIP2,MEF2C,RXRA,MAP3K5,MAP3K4,DBF4B,FGR,EPHA4,NTRK2,PTK2,SEMA4D,RASGEF1B,AMFR,ASAP1,NOS1AP,CCDC88A,GPR55,TNFSF11,FYN,PPM1F,HDAC2,DOCK3,NCS1,ROR1,EPHB1,GRM5,RPS6KA5,TBC1D1,NRP1,ATPSCKMT,ITGA1,POR,BCR,RGS6,PRIM2,FBLN1,STK36,EPHB2,BCL2L13,MET,SPPL3,CACNG3,MAGI2,VMP1,KALRN,GNAS,TIAM2,PDGFC,WDR41,ABL2,PLCL1,ERBB4,ROBO1,PRKCQ,ANTXR1,SIPA1L3,TRDN,NOS1,EFNA5,ESR1,CACNA2D1,PRLR,HTT,CAMK1D,FER,CCR2,ROCK2,RGS8,COL4A3,RGS7,STK3,RSU1,HNRNPU,IGF1R,PRKAG2,AKAP13*
GO:0051094	positive regulation of developmental process	0.000005289866595831649	*NOTCH2,BRINP3,MTOR,PTPRD,PLCB1,ZFPM2,TENM4,RIPOR2,RALA,BCL2,CHRNA7,ROBO2,RIMS1,SPIRE1,ZEB1,RIMS2,ALK,CARMIL1,RIN2,ANO6,DSCAM,TCF4,CRKL,SOX5,RFX3,MACF1,BCL11A,SOX6,CDH4,C5,FLT1,SLC39A12,PRKD1,SPEN,RAPGEF2,LRP2,ADGRB3,RUNX2,GHR,SSBP3,NEDD4L,ZHX3,STAU2,SEMA5A,SYT1,AURKA,SLC8A1,BMPR1B,AKAP6,ARNT,PAK3,ITPKB,TRPC5,PRKCZ,COBL,YAP1,BRINP1,NIPBL,MYLK3,PAFAH1B1,ITGB8,NF2,MRTFB,SYNJ1,TIAM1,AGO2,IL34,ADGRV1,BBS2,RANBP3L,SMARCA4,ELAVL4,ABL1,PRKAA1,RAP1A,FGF10,GRID2,NR*

			G1,ASPM,AP3B1,ATP11C,ZBTB16,SH3GL3,FBN2,EGF,PDE3A,RPS6KA3,ATP8A2,PLXNA2,HTR2C,ARHGEF7,AMBRA1,OPRM1,HTR2A,MSH2,LINGO2,BICRAL,ADAM12,EMILIN2,HMGA2,DOCK5,TJP1,DISC1,BMP2,MSR1,RELN,HMGB1,FGF9,NFATC2,SLC23A2,DOCK1,PLS1,NIN,SMARCA2,ETS1,GLI3,SMARCC1,SMOC2,PCP4,LAMC1,BBS4,CFTR,NELL1,KITLG,DAB1,PRKN,TBX20,DPF3,PRKCH,IL6R,TOX,ITSN2,LYN,OVOL2,NTN1,ZFHX3,ARID1B,INSR,DDHD1,SNAI2,NSD2,JCAD,RASGRP1,BMP5,CSF1,GHRH,ASB4,SMAD5,TNN,MED1,IL33,ROR2,KL,IL10,ACTR2,CLSTN2,PTH,VSTM2A,MAP6,ZBTB7C,YBX3,NRXN1,PCID2,HIPK1,TWIST1,AKT3,JAK2,RBM19,UFL1,PRKCB,NEDD9,OLFM4,ATP2B1,GRIP1,STAT1,BRMS1L,IMPACT,CCBE1,MYOCD,MEF2C,ADGRB1,RXRA,WNT7A,MAP3K5,FOXO6,FBXW8,SDCBP,EPHA4,NTRK2,IL1RAPL1,NUMB,ADAMTS9,WNT2B,CD101,FBXO31,CDH5,NFKBID,LAMB1,CYFIP1,SEMA4D,RUNX1,WNT5B,PDCL3,TNFSF11,ADGRL2,HDAC2,MAPK9,APELA,TET1,EPHB1,GRM5,NRP1,PRKCA,FAIM,CHODL,POR,RAG1,BMPER,CUX1,MACROH2A1,EPHB2,FLRT2,MYB,KALRN,LAMA1,TIAM2,BMP7,DLG5,NUDT21,SLIT2,ERBB4,SYNDIG1,ROBO1,NLGN1,SHLD2,SLC6A3,ASIC2,EFNA5,TCF12,LOXL2,DMRT1,STK3,HNRNPU,IGF1R,GLI2,DNM1L
GO:0048519	negative regulation of biological process	0.000005680694652414687	*NOTCH2,BRINP3,MTOR,CNTN4,SPOCK1,WWC1,PTPRD,SLC24A2,PVT1,ULK2,NLK,FTO,PLCB1,ZNF536,TAFA5,SVIL,ZFPM2,L3MBTL4,DLC1,TNRC6B,ITPR2,RIPOR2,PDE4D,RDX,STXBP1,BCL2,KCNMA1,PRDM16,ALDH1A2,FBN1,CHRNA7,ROBO2,ZEB1,AKR1C3,RARB,SPRED1,USH2A,MINAR1,ALK,FOXJ2,CDYL2,CARMIL1,MCTP1,BABAM2,GLIS3,FANK1,ERBIN,RHPN2,MLLT3,SUSD4,MAP4,SPON1,APC,TSHZ3,DSCAM,RTN1,CRKL,ILDR2,ARHGAP24,PTPRJ,KDM4C,EGFR,RFX3,USP14,ANGPT1,CDK12,BACH1,PRKACB,RGS3,NCOR1,NEDD4,SND1,SCAI,BCL11A,SOX6,TMEM182,GRIK3,CHSY1,NTRK3,C5,ZFAND6,DKK2,FLT1,THRAP3,MAPKBP1,AOAH,DGKI,INVS,GRIA1,CRACD,CAST,TTC39B,SLC8A3,MALRD1,TOM1L2,PRKD1,TPTE2,PAK1,EPHA7,NCOA7,SPEN,RAPGEF2,LRP2,ADGRB3,RUNX2,TAOK3,ONECUT1,CPEB4,TMEM38B,SLC24A3,LDB2,CCL28,SMYD3,GRM7,RETREG1,RPTOR,GHR,THADA,COL4A2,TBCD,NEDD4L,ADAM10,HDAC9,ZHX3,ATF7IP,APBB2,APP,RPS6KA2,SAMSN1,CACNA1C,KDM1B,DCLK1,STAU2,DOCK8,USP18,SEMA5A,VCL,ARHGAP44,NTF3,ACER2,PARP15,NDUFAF2,CD2AP,AURKA,PARN,CFDP1,ST18,SLC8A1,PTPRR,SRGAP2C,FIG4,DUX4,ABCG8,SERPINA6,SRGAP2B,KANK1,KCNE4,MAP4K4,ABCD2,BMPR1B,FMN2,AKAP6,HOMER2,CTNNA2,DIP2B,LARP1,ITPKB,TRPC5,RGS20,PDE10A,RAP1GDS1,RNLS,KICS2,DNM3,NBN,IFT57,INTS7,PRKCZ,GRB10,RYR3,TAF15,DIP2A,MSH6,MCPH1,RGS9,HECW1,ABCA5,PHF19,DUSP22,YAP1,BRINP1,MAPK1,MGAT5,ITIH5,ADAM22,USP25,PLG,ZNRF3,NRG3,UBE2O,SFMBT2,MIR663AHG,NIPBL,GABPA,FAT3,CORO2B,CARD18,STK38,PTPN13,SORCS3,LIMCH1,PAFAH1B1,EFEMP1,ZNF684,TM7SF3,DCAF1,ITGB8,TPM1,NF2,HIVEP1,CTNNA1,BIRC6,KLF15,PPARA,MEIS2,NFIB,PRTG,PTPRK,PAK5,TRERF1,PLA2R1,SEMA3C,DAPK1,NAV3,SLC24A4,SEC14L1,VPS13C,AGO2,PHC3,ALPK2,JARID2,DNAJC15,GATAD2B,DYSF,ANK2,ADGRV1,ZNF846,BCAS3,RYR2,BBS2,WNT9B,RANBP3L,SEMA6D,DUSP16,SMARCA4,FABP7,PARD3,MAPKAP1,TNRC6C,PIAS1,BLK,TNR,CXADR,DOCK4,ATRX,PTPRT,ELAVL4,ABL1,MXI1,PTPN12,HDAC4,OXR1,SLC1A1,PRKAA1,LRFN5,CREG1,DROSHA,L3MBTL3,NBAS,PSMF1,SLFN11,RAP1A,GLIS1,MORC1,TOX3,BAZ2A,INPP5A,FGF10,ZC3HAV1,GRID2,LATS2,NRG1,ASPM,ZNF438,ABCB7,ZBTB16,KIR3DL2,ZNF675,SH3GL3,SETDB2,PRKCE,FOXK2,NXN,WNK2,FBN2,CD44,RGS12,PTPRO,EGF,TRIO,PDE3A,STXBP6,LNPEP,LIMD1,PEX14,SPRED2,RPS6KA3,ATP8A2,PTPN2,TRIM5,PLXNA2,RFC1,HTR2C,CLEC16A,ARHGEF7,CD96,AMBRA1,LTBP1,OPRM1,HTR2A,DAZL,KREMEN1,SEMA3E,TAF3,TMEM67,FHL2,ABHD17C,PUM1,TMOD2,HERC1,MSH2,IGF2BP3,ANKRD17,ZNF397,LUC7L,HIPK3,CDKN2C,EPN2,GRK3,CLSPN,NOS2,BICRAL,MOSMO,MNAT1,RBBP8,MDFIC,ANK3,EMILIN2,HMGA2,CCND3,BCL11B,DOCK5,CREM,LYPLA1,MBP,LINC01151,TRPS1,TGFA,CRIM1,PRR5L,MYT1L,TJP1,LDLRAD4,NPHP4,PACSIN2,HLA-*

			B,SNX3,NAA35,BRCA2,ZBTB2,BLM,SEMA3A,STRN,BMP2,RC3H2,ATP9A,PSG9,SOGA1,MSR1,ZC3H14,GFI1B,TBC1D4,RANBP9,RESF1,RIN3,TMEM161A,SEMA3D,ASXL3,LEMD3,ARHGAP42,HMGB1,GNAQ,FGF9,NFATC2,SH3BP5,CPAMD8,MDM1,SLC23A2,ANKRD26,TTC21B,ETS2,ZNF875,DSTYK,UIMC1,LRRFIP1,RAP1GAP,SRGAP2,DRAXIN,SLAMF1,SMARCA2,ETS1,GLI3,CGAS,SMARCC1,SNX6,CNKSR3,GRIK2,MYEF2,ZNF431,RERE,MAP2,BTAF1,ATF2,HIRA,CYLD,BBS4,MAPK8IP1,MX1,COL5A1,NELL1,UBASH3A,NEU3,MRPL13,KITLG,UBR1,DCC,SLC30A10,RCAN1,GTF2I,RORB,DAB1,SELENON,RB1CC1,PTPRE,PRKN,MTMR2,ZNF608,TBX20,DACH1,ZNF541,DPF3,NGEF,GRIN2A,ARID5B,JPH1,ATXN1,PRKCH,PKP1,FRMD4A,IL6R,NLRC5,TFDP1,CNOT6L,KANK4,SNX25,PTPRB,ZFP90,COPS8,ZNF124,SHANK2,USP7,PSMA1,ENPP3,PLAGL1,SOX30,KIR2DL4,NPAS2,ROCK1,LYN,ARHGAP28,CTSB,TTC37,ZNF169,DTX1,TENM2,OVOL2,PIWIL3,ZBTB33,NTN1,ZFHX3,DPYSL5,BANP,RNF152,OTUD7A,BMF,YTHDF3,TFF1,DEDD2,HECTD1,ARFGEF1,SNAI2,ASH1L,BID,SIAH2,TRABD2B,UFD1,SP3,ERN2,TIAL1,ELF2,NSD2,CD9,CARD10,TWIST2,CTIF,SAMHD1,ENPP1,TP53I11,TMEM225,NDRG2,CSNK2A1,BMP5,CSF1,HDGFL3,BCL2L1,SERPINB9,SCAF4,MIR3142HG,CTDP1,HCN1,PRKG1,GRIN2B,GRB14,FANCB,CNMD,DHRS3,SMAD5,CELF4,TCERG1,ABCG1,FOXN3,SLC40A1,PRAME,TNN,MED1,CDC14B,IL33,AJAP1,BANK1,CSDE1,LMX1A,TMEM178A,IL10,SFPQ,SCML2,PRAMEF25,PTH,SOSTDC1,PRKAA2,NDC80,PACRG,ABHD2,ITPRIP,ETV6,IQGAP1,CAMLG,ZBTB7C,MORC2,SREBF2,ANP32B,YBX3,AIMP1,NRXN1,PCID2,FRY,PBLD,FICD,PEG10,TWIST1,AKT3,JAK2,FSTL1,ZBTB38,PATL1,CREBBP,MELTF,TNKS,PCNA,SIAH3,UFL1,ADAMTS5,NFKBIA,PRKCB,ABCC8,ANXA4,RTRAF,BRD4,ZBTB21,SERBP1,NEDD9,ITGA6,ATP2B1,ASS1,BTG3,ERLIN2,OTOP1,CIDEA,ZBTB49,KRT6A,AGO1,MEOX2,SLC6A1,STAT1,BRMS1L,NDFIP2,NR2C1,MAP2K6,DGKG,MTPN,ABI1,CEMIP,PRAMEF2,IMPACT,PARK7,ADAMTS18,MAPK8,OAZ2,POU1F1,ADCYAP1R1,MTF2,NCAPG2,FOXP2,MYOCD,HNRNPM,MEF2C,ADGRB1,RXRA,WNT7A,RBPMS2,NDFIP1,SERPINI2,PRDM13,ERI1,C16ORF72,MAGEL2,PDE2A,SDCBP,WWOX,ZBTB25,PASK,MLLT1,NCK1,SCAF8,FGR,CDCA8,PPP2R3A,RNF8,EPHA4,GABRA5,MECOM,DNMT3L,NTRK2,IL1RAPL1,NUMB,LHX9,ADAMTS9,ZBTB10,OCLN,MIR548H4,SHISA6,IL17RD,FBXO31,IREB2,PTK2,CDH5,ANKRD6,ARHGAP12,CLDN18,ASCL3,DIAPH1,FEZ2,INIP,MIR17HG,APIP,CYFIP1,UBE3A,SEMA4D,JAM2,SERPINB10,ZBTB20,RUNX1,AKR1B1,KIRREL1,AMFR,NENF,PTGFR,ASAP1,SAMD13,TPTE,PDCL3,SRP9,GPR55,NSUN2,CDC45,BICD1,TNFSF11,FYN,BUB1,KDM5A,PCBP3,ZNF705G,PPM1F,SDE2,RBMS3,HDAC2,AVEN,GON4L,TBX15,PSME3IP1,COL18A1,ALB,ZFYVE28,PABPC1,CRTAM,APELA,TET1,HECW2,CDH2,RAD9A,XRN2,PHLPP1,GPR137B,EPHB1,GRM5,ADCK1,SPOPL,ZNF705D,RPS6KA5,SPTB,TBC1D1,PTPRG,PID1,NRP1,MIDEAS,PRKCA,FAIM,FHIT,ITGA1,KLF12,RC3H1,NRIP1,POR,MCC,BCR,TUT4,RGS6,RERG,FBLN1,RAG1,BMPER,PRDM15,CUX1,SRGAP3,MACROH2A1,MITF,EPHB2,SACS,CD38,EYA4,AKAIN1,MET,ZNF705B,CDH13,SERPINB2,ATG5,MAGI2,PRDM11,UNK,MLIP,MYB,KALRN,GNAS,MFHAS1,SERPINB7,BMP7,ASTN2,DLG5,TNFAIP8,ZMYND8,KCTD1,BPTF,BTBD10,ZMYND11,TMEM25,DDX6,ADGRF5,WDR41,PPP1R13B,FOCAD,ABL2,MMP26,BACE2,PARPBP,EYA1,FHOD3,PRPF18,SLIT2,CNOT7,PLCL1,ERBB4,IL20RB,SERPINB11,ROBO1,SAMD4A,PBX1,NUF2,PRKCQ,ANTXR1,NDRG1,SORCS2,TRDN,MGMT,NLGN1,SHLD2,NOS1,SLC6A3,ASIC2,EFNA5,GAS2L1,NSD1,EHMT1,SLIT3,FRMD5,ESR1,KDM4B,LOXL2,IQCJ-SCHIP1,SKAP2,PRLR,AGO3,HTT,FOXB1,CAMK1D,PIK3R3,HLA-F,FER,EYA2,CCR2,STARD13,A2M,CHFR,EPS8,JAZF1,ZNF891,SPOCK3,SEMA4B,PHC2,GRIA4,ROCK2,PRDM1,RORA,DMRT1,RGS8,HSPG2,HERPUD1,COL4A3,WASHC1,RGS7,HOOK3,KIF7,FSTL4,BARD1,STK3,DEPTOR,ZNF423,ZNF568,HNRNPU,APCDD1,IGF1R,KCNAB1,PRKAG2,GLI2,THRB,MORC3
GO:00	regulation	0.00000	*NOTCH2,BCAR3,WWC1,KSR1,PLCB1,CHRNA7,SPRED1,ALK,PJA2,CRKL,TNIK,PTPRJ,EGFR,ANGPT1,NCOR1,NTRK3,FLT1,MAPKB*

43408	of MAPK cascade	6164994 1254514 6	*P1,EDAR,PAK1,EPHA7,RAPGEF2,PELI2,TAOK3,GHR,APP,NTF3,PTPRR,MAP4K4,PAK3,RAP1GDS1,PRKCZ,DUSP22,MAPK1,PDGFD,STK38,HRH4,PAFAH1B1,NF2,GRM1,PAK5,IL34,DUSP16,ABL1,RAP1A,FGF10,NRG1,DENND2B,ZNF675,PRKCE,WNK2,CD44,EGF,SPRED2,PTPN2,TRIM5,HTR2C,OPRM1,HTR2A,RELL1,HIPK3,MDFIC,PLCE1,TGFA,NRK,SEMA3A,MAGI3,BMP2,RANBP9,HMGB1,TRAF3,DSTYK,SLAMF1,CNKSR3,GAREM1,NEK10,CYLD,MAPK8IP1,KITLG,SLC30A10,RB1CC1,PRKN,IL6R,ROCK1,LYN,CRACR2A,INSR,ASH1L,ERN2,ZDHHC17,JCAD,RASGRP1,NDRG2,ROR2,KL,BANK1,IQGAP1,NRXN1,ALKAL2,JAK2,MADD,HCRTR1,MAP2K6,MEF2C,WNT7A,MAP3K5,MAP3K4,SDCBP,EPHA4,MECOM,NTRK2,ANKRD6,APIP,NENF,GPR55,TNFSF11,DOK5,APELA,CDH2,PHLPP1,EPHB1,GRM5,NRP1,PRKCA,ITGA1,FBLN1,BMPER,PRDM15,EPHB2,PRDM11,MFHAS1,BMP7,ZMYND11,PDGFC,ERBB4,ROBO1,ROCK2,STK3,IGF1R,AKAP13*
GO:00 30031	cell projection assembly	0.00000 6280679 7749516 43	*MTOR,LRRC49,RIPOR2,RDX,RP1,RALA,ODAD2,SDCCAG8,FGD4,SPAG16,CDC42EP3,AUTS2,CARMIL1,PARVB,ANO6,MAP4,APC,PLPPR5,ARHGAP24,TNIK,RFX3,ARMC2,RAPGEF2,ONECUT1,LRGUK,SEPTIN9,DCLK1,STAU2,VCL,ARHGAP44,CD2AP,SRGAP2C,KANK1,DNM3,IFT57,COBL,YAP1,ABLIM1,BCAS3,SYNE2,BBS2,AIF1L,ABL1,HDAC4,DNAH5,MYO10,CEP83,PTPRO,IFT43,ARHGEF7,ABCC4,TMEM67,PLCE1,DISC1,WDPCP,DNAL1,NUDCD3,TTC21B,RAP1GAP,SRGAP2,DNAH8,DAW1,CYLD,BBS4,KIAA0753,PCDH15,VAV3,CFAP74,ROCK1,TENM2,NTN1,IFT81,FAM149B1,CDC14B,PCNT,ACTR2,TTC39C,NRXN1,CIBAR1,ANLN,CELSR2,GORAB,GAP43,BBS9,CEP120,WASF3,SDCBP,NCK1,DRC7,SNAP29,EMP1,RSPH1,OCLN,MARK4,CYFIP1,HOATZ,SAXO1,ASAP1,CCDC88A,ADAMTS16,SPAG6,ARL13B,HYDIN,RP1L1,NRP1,IFT46,CFAP70,STK36,B9D1,EPHB2,TOGARAM1,CDH13,ATG5,DNAH17,RFX2,SLIT2,NLGN1,HTT,CFAP44,FER,RPGRIP1,EPS8,ATAT1,WASHC1,ZNF423,SEPTIN6*
GO:00 31323	regulation of cellular metabolic process	0.00000 6587506 6588402 93	*NOTCH2,BCAR3,MTOR,NSG1,WWC1,TRAPPC9,BNC2,ULK2,NLK,LONP2,FTO,KSR1,MGA,RFX7,ZNF236,PLCB1,ZNF536,ZFPM2,L3MBTL4,DLC1,TNRC6B,PDE4D,ERC1,BCL2,PRDM16,CHRNA7,PIK3C3,EPC2,SPIRE1,ZEB1,AKR1C3,RARB,SPRED1,ALK,AUTS2,FOXJ2,CDYL2,BABAM2,GLIS3,FANK1,ERBIN,ZNF880,MLLT3,SPON1,APC,ZNF595,TSHZ3,RBFOX3,DSCAM,RTN1,TCF4,CRKL,ILDR2,SOX5,SETD2,ERG,ZNF573,TNIK,PTPRJ,KDM4C,NEK4,TSHZ2,EGFR,ZNF280B,RFX3,USP14,ANGPT1,CDK12,BACH1,NEK7,NCOR1,RNF220,ZNF407,NEDD4,MAML2,MTRF1,SND1,SCAI,NSMCE2,BCL11A,SOX6,FLI1,RPRD1A,NTRK3,FLT1,ZNF648,RFC3,ZNF382,TASP1,THRAP3,SLC8A3,MALRD1,PRKD1,PAK1,EPHA7,NCOA7,KHDRBS2,SPEN,RAPGEF2,PELI2,RUNX2,TAOK3,ONECUT1,CPEB4,UBE2L3,LDB2,PUM3,SMYD3,RPTOR,GHR,SSBP3,CELF2,HDAC9,ZHX3,ATF7IP,APBB2,APP,SAMSN1,KDM1B,ZNF600,NTF3,ACER2,PARP15,ZNF723,AURKA,PARN,ST18,PYGO1,SLC8A1,SSBP2,ANKRD31,DUX4,HIVEP2,ABCD2,BMPR1B,FMN2,ZNF717,ARNT,PAK3,ZNF257,RANBP2,LARP1,ITPKB,TRPC5,NBN,SCP2,INTS7,PRKCZ,GRB10,TAF15,MSH6,MCPH1,MBNL2,PHF19,MRTFA,TAF4B,DUSP22,EBF2,YAP1,NFIA,WDR70,RIPK4,ZKSCAN5,MAPK1,MGAT5,KMT2E,PCGF5,PDGFD,NRG3,SFMBT2,GFRA1,NIPBL,SPIDR,EWSR1,GABPA,ZNF735,CHD6,STK38,PTPN13,KANSL1,LIMCH1,MBNL1,ATF6,EFEMP1,ZNF684,DCAF1,VPS13D,CCNG2,NF2,RBFOX1,HIVEP1,MOB3B,AKAP9,KLF15,PPARA,MEIS2,SNX30,NFIB,MRTFB,PPP6R3,NR5A2,FOXJ3,PTPRK,TRERF1,EIF3D,DAPK1,VPS13C,AGO2,PHC3,JARID2,DNAJC15,GATAD2B,IL34,BRWD1,ZNF846,BCAS3,WNT9B,ZNF606,DUSP16,SMARCA4,PARD3,MAPKAP1,TNRC6C,PIAS1,EBF1,ATRX,NUAK1,PTPRT,ELAVL4,ABL1,MXI1,HDAC4,SLC1A1,PRKAA1,ITGB3BP,MRPS27,CREG1,DROSHA,L3MBTL3,APLF,NFAT5,NBAS,SLFN11,RAP1A,GLIS1,MORC1,TOX3,CAMK4,BAZ2A,FGF10,ZC3HAV1,LATS2,NRG1,INO80D,AP3B1,ZNF438,ABCB7,ZBTB16,MUSK,ZNF675,SMARCAD1,SETDB2,PRKCE,FOXK2,SLCO3A1,MED15,ESRRG,ZNF718,USP33,CD44,PTPRO,EGF,PRRC1,PDE3A,LIMD1,PEX14,SPRED2,RPS6KA3,MTMR3,PTPN2,TRIM5,RFC1,HTR2C,CLEC16A,ALG10B,AMBRA1,KDM7A,OPRM1,HTR2A,FANCA,DAZL,GTF2F2,TAF3,RP*

			RD1B,MARK2,EBF3,ZNF33B,FHL2,PUM1,HERC1,MSH2,IGF2BP3,EPHA6,ANKRD17,ZNF397,SLC2A13,HIPK3,CDKN2C,KNDC1,CLSPN,NOS2,BICRAL,MNAT1,RBBP8,MDFIC,HMGA2,CCND3,BCL11B,ECE1,ZIM3,CREM,TRPS1,PLCE1,TGFA,PRR5L,ATP6V1E1,UTP4,MYT1L,ZNF160,LDLRAD4,CNTN1,HSF5,ZNF367,BRCA2,DISC1,ZBTB2,BLM,INTS8,LIN54,ZNF121,BMP2,RC3H2,TRAK1,SOGA1,PTCD2,ZC3H14,GFI1B,TMEM161A,ASXL3,RELN,HMGB1,GNAQ,FGF9,NFATC2,SH3BP5,ZNF567,TRAF3,ZNF462,ESRP1,TTC21B,ETS2,GEMIN5,ZNF875,DSTYK,UIMC1,LRRFIP1,IKZF2,ATF1,SMARCA2,ETS1,GLI3,CGAS,SMARCC1,SNX6,AFF3,SMOC2,CNKSR3,VENTX,IDE,PRDM10,MYEF2,ZNF431,RERE,BTAF1,ZNF618,NEK10,MOB1B,ATF2,HIRA,CYLD,MAPK8IP1,HIVEP3,PSIP1,KPNA1,ME2,RGMB,MRPL13,KITLG,CAMTA1,MYT1,CHRM5,YLPM1,SLC30A10,RCAN1,GTF2I,RORB,TADA2A,DAB1,MED27,ZNF208,RB1CC1,NMD3,PRKN,MTMR2,ZNF608,TBX20,SP110,MAPK10,DACH1,ZNF541,DPF3,ARID5B,ATXN1,PRKCH,PKP1,IL6R,ALS2,NLRC5,ZNF627,ACO1,TFDP1,CNOT6L,MKNK1,SNX25,FBLN5,TOX,SLC4A4,PTPRB,ZFP90,COPS8,ZNF124,USP7,VAV3,PLAGL1,SOX30,KIR2DL4,RALB,NPAS2,ROCK1,LYN,ZNF780B,ZNF169,DTX1,BZW1,TENM2,OVOL2,PIWIL3,ZBTB33,ZFHX3,ZNF44,RRAGD,BANP,SUPT16H,ARID1B,HOXC13,RNF152,BAZ1A,CASZ1,INSR,BMF,YTHDF3,DEDD2,NEK6,HECTD1,PBX3,SUMO2,ZNF292,POGK,SNAI2,ASH1L,HOXC4,SIAH2,RXRG,SP3,DRAM1,ERN2,ZNF879,MBTPS2,TIAL1,ELF2,NSD2,FYCO1,SH3GLB1,CARD10,TWIST2,CTIF,ENPP1,RASGRP1,SNX9,TMEM225,CSNK2A1,BMP5,CSF1,HDGFL3,BCL2L1,SCAF4,CTDP1,INO80,FANCB,CLNS1A,SMAD5,CELF4,TCERG1,ABCG1,FOXN3,SLC40A1,PRAME,MYCL,PSAP,LPGAT1,MED1,IL33,GPRC5C,ROR2,ZNF521,BANK1,CSDE1,LMX1A,IL10,ACTR2,SFPQ,SCML2,PRAMEF25,RIOK1,PTH,PRKAA2,SOHLH1,LARP6,PHF20L1,ETV6,IQGAP1,CAMLG,COX7A2L,ZBTB7C,TEAD1,SREBF2,YBX3,NRXN1,PCID2,ZNF234,CISD1,ZNF518A,CENPE,LMX1B,NGDN,ELOC,TWIST1,AKT3,ALKAL2,JAK2,VSX1,ZBTB38,ISX,MADD,PATL1,ZNF287,CELSR2,ZNF449,CREBBP,TNKS,PCNA,UFL1,NFKBIA,PRKCB,ANXA4,RFC2,ZNF354C,ALX4,RTRAF,BRD4,ZBTB21,SERBP1,NEDD9,ITGA6,ASS1,MTCL1,PPP1R17,ZNF528,ERLIN2,ZNF611,CIDEA,ZBTB49,EXOC1,STOX2,AGO1,MEOX2,ELL2,STAT1,BRMS1L,NR2C1,MAP2K6,GATAD1,MTPN,ABI1,CEMIP,PRAMEF2,POU6F2,IMPACT,PARK7,MAPK8,MED12L,ZSCAN30,POU1F1,ADCYAP1R1,MTF2,NCAPG2,FOXP2,MYOCD,ZSCAN5C,HNRNPM,ASCC2,ARID3B,MEF2C,ZNF613,ADGRB1,RXRA,WNT7A,MAP3K5,NDFIP1,MAP3K4,PRDM13,FOXO6,ZNF112,ATP6V1C2,MAGEL2,RAD51AP1,PDE2A,RAB38,DBF4B,FBXW8,SDCBP,NSMCE1,ZNF813,WWOX,ZBTB25,PASK,MLLT1,NCK1,SCAF8,FGR,CWC22,CDCA8,ATP6V1B2,MLLT10,IFNAR1,RNF8,EPHA4,INTS13,MECOM,DNMT3L,NTRK2,LHX9,ZBTB10,OCLN,CREB5,IREB2,PTK2,CDH5,ASCL3,FEZ2,CYFIP1,UBE3A,SEMA4D,ZBTB20,ZNF66,RUNX1,KIRREL1,POMT2,ZNF845,SAMD13,NOS1AP,PDCL3,SRP9,CCDC88A,NSUN2,CHCHD2,TNFSF11,FYN,KDM5A,PCBP3,ZNF705G,PPM1F,HDAC2,SLF1,GON4L,TBX15,SH2D3C,DOCK3,TRNAU1AP,NCS1,ZFYVE28,MAPK9,PABPC1,SLC39A8,ROR1,TET1,ARNT2,ITGA8,GPR137B,EPHB1,GRM5,ZNF705D,RPS6KA5,PID1,NRP1,MIDEAS,PRKCA,ATPSCKMT,FHIT,ITGA1,ZNF615,KLF12,RC3H1,NRIP1,POR,ZNF850,ZNF235,ZNF738,SUPT3H,BCR,TUT4,ZNF215,TCERG1L,KIF16B,PRIM2,C14ORF39,TM9SF2,ELP2,FBLN1,STK36,ZNF678,BMPER,PRDM15,CUX1,ZNF420,MACROH2A1,MITF,EPHB2,CD38,EYA4,DPH6,CDK14,MET,SPPL3,ZNF705B,ATP6V0D2,CDH13,MED13L,ATG5,MAGI2,PRDM11,UNK,MLIP,MYB,ZNF704,MFHAS1,DHX29,BMP7,ZMYND8,RNF217,KCTD1,ZNF74,BPTF,BTBD10,ZMYND11,NUDT21,KMT2C,DDX6,ADGRF5,PDGFC,WDR41,ABL2,BACE2,RFX2,PARPBP,NECAB1,PKNOX2,EYA1,SLIT2,EXOC4,CNOT7,ESCO1,ERBB4,GSAP,ROBO1,SAMD4A,PBX1,NPAS3,PRKCQ,MGMT,ZNF679,SHLD2,NOS1,SLC6A3,PRR16,EFNA5,TCF12,ARHGEF11,RAB27A,NSD1,EHMT1,ESR1,KDM4B,LOXL2,PRLR,AGO3,HTT,FOXB1,CAMK1D,PIK3R3,FER,ZNF302,EYA2,INTS12,CHFR,ZNF721,JAZF1,ZNF578,ZNF891,NRF1,ZNF14,HRH1,PHC2,ROCK2,PRDM1,RORA,DMRT1,EIF4G3,

			PDK1,HERPUD1,NCOA6,WASHC1,ZFP30,BARD1,STK3,DEPTOR,ZNF423,ZNF568,HNRNPU,RAB3GAP2,IGF1R,PRKAG2,GLI2,THRB,AKAP13,MORC3,DNM1L
GO:0003008	system process	0.000006996733231261712	*MTOR,SGCD,IMMP2L,SLC24A2,MYO9A,FTO,PLCB1,PIEZO2,TENM4,ZDHHC21,RIPOR2,PDE4D,RP1,BCL2,KCNMA1,CHRNA7,RIMS1,GABRB3,AKR1C3,NAV2,ENPEP,SPAG16,MYO1E,USH2A,RIMS2,PJA2,RHPN2,CACNG2,DLGAP1,CNTNAP2,MYO3B,TSHZ3,SLC4A10,PTPRJ,EGFR,ANGPT1,CTNNA3,PRKACB,MYOF,CRB1,BTBD9,FLI1,ATP2B2,TUSC3,GABRB1,DGKI,GRIA1,CRACD,SLC8A3,CHRM3,LRP2,ADGRB3,FGF12,GABRA6,CPS1,TMEM38B,SLC24A3,SLC44A1,TAFA4,GRM7,RETREG1,CELF2,NEDD4L,PPP1R12B,TRPM1,APBB2,APP,SLC7A2,RPS6KA2,CACNA1C,CACNB2,GABRG2,TMC1,NTF3,SLC8A1,FIG4,TAFA2,ABCG8,LOXHD1,KCNE4,AKAP6,HOMER2,CTNNA2,RAB8B,KCNK10,RAP1GDS1,RNLS,NBN,PRKCZ,CALD1,RGS9,YAP1,BRINP1,MAPK1,HRH2,SYT10,ABLIM1,NIPBL,SLC16A1,CORO2B,SORCS3,MYLK3,PAFAH1B1,ATF6,EFEMP1,TPM1,RBFOX1,CORIN,AKAP9,KLF15,PPARA,MEIS2,SYNJ1,GRM1,GABRG1,PAK5,SLC24A4,TMEM108,DNAH11,JARID2,SCN2A,ANK2,TANC1,ADGRV1,RYR2,BBS2,OR4F6,PARD3,TNR,GRM8,CXADR,DOCK4,ELAVL4,ABL1,HDAC4,SLC1A1,KCNH1,CAMK4,FGF10,GRID2,AP3B1,RASGRF1,MUSK,SLCO3A1,SLMAP,PTPRO,ABCC9,P2RX6,PDE3A,EXT1,LNPEP,ATP8A2,LHFPL3,OR4F15,HTR2C,SLC2A3,ATP8A1,PKHD1L1,OPRM1,ABCC4,HTR2A,CYP4A11,CNNM4,STAC,TMOD2,HERC1,GNAL,SLC2A13,KCND2,NOS2,SGCZ,MYLK2,ANK3,EMILIN2,MYOM2,DOCK5,F5,ECE1,MBP,PLCE1,ANKFN1,FBXO32,TJP1,CACNA1I,PDLIM5,OR9Q1,SCN11A,NCAM2,PDE6C,RELN,ARHGAP42,MYOM1,UNC13B,PLS1,SLC9A4,GABRR2,GRIK2,DAW1,MYL1,NEK10,BBS4,LAMC3,DOP1B,CAMTA1,CHRM5,RCAN1,RORB,SELENON,MYO3A,PRKN,MTMR2,TBX20,DLGAP2,PCDH15,SMPX,GRIN2A,ATXN1,CDH23,OR51E1,ACO1,SHISA9,SLC4A4,PDE6A,SCN10A,SHANK2,KCND3,ROCK1,LRIG1,SLC15A2,INSR,OR7A17,TFF1,NMU,PBX3,SNAI2,ABCA4,GABRG3,OR4K2,UTRN,IGSF11,CTDP1,HCN1,PRKG1,GRIN2B,DHRS3,SMAD5,CELF4,OR4C46,VSTM4,KDM6A,IL33,KL,OTOG,LMX1A,IL10,ACTR2,OR1L6,NRXN1,PBLD,SSPN,JAK2,VSX1,SVEP1,PTGS1,TRPV5,OR2T3,ABCC8,SMTN,USH1C,NEDD9,ATP2B1,BBS9,EXT2,SLC6A1,GRXCR1,STAT1,MAP2K6,MTPN,CEMIP,POU6F2,CCBE1,CSMD1,OR6C75,ASB2,MYOCD,HMCN1,MYH13,OR13C9,MEF2C,WNT7A,WASF3,S100B,FOXO6,OR10H2,PDE2A,PPIP5K2,GABRA5,NTRK2,TNNI1,OCLN,SHISA6,AKAP11,IREB2,CDH5,DIAPH1,CYFIP1,UBE3A,JAM2,AKR1B1,KIRREL1,AMFR,OR4L1,NOS1AP,SORBS2,ADAMTS16,SLC5A1,OR11G2,FYN,SCN8A,HDAC2,SNTB1,TMEM63C,COL18A1,APELA,TRPM3,ROR1,OPA3,ASB3,CNTN5,ITGA8,EPHB1,EYS,RP1L1,GRM5,PRKCA,ATPSCKMT,ITGA1,BCR,NRXN3,RAG1,DGCR2,EPHB2,SGCG,CD38,EYA4,MYO5B,SPECC1,CACNG3,ATG5,MLIP,KALRN,SLC1A2,GNAS,GABRA2,OR2T2,TMEM25,ADGRF5,OR4N2,EYA1,SLIT2,TMPRSS3,TRHDE,TRDN,NLGN1,NOS1,SLC6A3,ASIC2,ARHGEF11,DTNA,DNAH9,CACNA2D1,HTT,FOXB1,KATNIP,CCR2,RPGRIP1,HRH1,ROCK2,PTPRQ,COL4A3,CLCN5,VTI1A,THRB,AKAP13,DNM1L*
GO:0031589	cell-substrate adhesion	0.000007140672253730093	*SPOCK1,FREM1,DLC1,PTPRA,BCL2,USH2A,CARMIL1,RIN2,PARVB,CRKL,PTPRJ,ANGPT1,MACF1,ONECUT1,CCL28,TBCD,VCL,ACER2,KANK1,MAP4K4,PRKCZ,DUSP22,PLG,ITGBL1,CORO2B,LIMCH1,FMN1,ITGB8,NF2,TIAM1,PTPRK,BCAS3,ABL1,PEAK1,PRKCE,CD44,PTPRO,COL5A3,ATXN3,ARHGEF7,CD96,SEMA3E,DOCK5,EGFLAM,DISC1,WDPCP,DOCK1,SRGAP2,LAMC1,LAMC3,ITGA9,FBLN5,ROCK1,VCAM1,UTRN,CSF1,TNN,MICALL2,ATRN,AJAP1,JAK2,MELTF,NEDD9,OLFM4,ITGA6,ITGA4,ADAMTS9,TRPM7,PTK2,LAMB1,EDIL3,PPM1F,ITGA8,NTN4,EPHB1,NRP1,ITGA1,BCR,FBLN1,PPFIA2,CDH13,ATRNL1,ANTXR1,EFNA5,NTNG1,FER,ROCK2,RSU1*
GO:0010976	positive regulation of neuron projection	0.000007346806458500755	*TENM3,ALK,NEGR1,PLPPR5,BCL11A,NTRK3,PRKD1,RAPGEF2,ARSB,STAU2,FIG4,PAK3,COBL,PAFAH1B1,ELAVL4,ABL1,RAP1A,ATP8A2,MARK2,FUT9,CNTN1,SNX3,DISC1,RELN,ATF1,TOX,LYN,BMP5,TNN,ROR2,ACTR2,NRXN1,ALKAL2,ITGA6,NCK1,NTRK2,CYFIP1,FYN,ROR1,NRP1,EPHB2,MAGI2,KALRN,BMP7,ABL2,*

	development		*NLGN1,CAMK1D,IGF1R*
GO:0035249	synaptic transmission, glutamatergic	0.000007916535454463932	*UNC13C,STXBP1,CDH8,CACNG2,TSHZ3,GRIK3,DGKI,GRM7,SYT1,GRIK4,GRM1,TNR,GRM8,GRID2,EXT1,HTR2A,DISC1,RELN,UNC13B,GRIK2,PRKN,GRIN2A,ALS2,GRID1,HCN1,GRIN2B,ROR2,NRXN1,MEF2C,GRIK1,CDH2,GRM5,CACNG3,GRM3,NLGN1,CCR2*
GO:0051171	regulation of nitrogen compound metabolic process	0.000008024549655026 88	*NOTCH2,BCAR3,MTOR,SPOCK1,WWC1,TRAPPC9,BNC2,NLK,FTO,KSR1,MGA,RFX7,ZNF236,PLCB1,ZNF536,ZFPM2,L3MBTL4,DLC1,TNRC6B,PDE4D,RDX,ERC1,BCL2,PRDM16,CHRNA7,EPC2,SPIRE1,ZEB1,RARB,SPRED1,MINAR1,ALK,AUTS2,FOXJ2,CDYL2,BABAM2,GLIS3,FANK1,ERBIN,ZNF880,MLLT3,EGLN3,SPON1,APC,ZNF595,TSHZ3,RBFOX3,RTN1,TCF4,CRKL,ILDR2,SOX5,SETD2,ERG,ZNF573,TNIK,PTPRJ,KDM4C,NEK4,TSHZ2,EGFR,ZNF280B,RFX3,USP14,ANGPT1,CDK12,BACH1,PRKACB,NEK7,NCOR1,RNF220,ZNF407,NEDD4,MAML2,MTRF1,SND1,SCAI,NSMCE2,BCL11A,SOX6,FLI1,RPRD1A,NTRK3,C5,FLT1,ZNF648,RFC3,ZNF382,TASP1,THRAP3,CAST,SLC8A3,PRKD1,PAK1,EPHA7,NCOA7,KHDRBS2,SPEN,RAPGEF2,PELI2,LRP2,RUNX2,TAOK3,ONECUT1,LDLRAD3,CPEB4,UBE2L3,LDB2,PUM3,SMYD3,RPTOR,GHR,SSBP3,CELF2,NEDD4L,HDAC9,ZHX3,ATF7IP,APBB2,APP,RPS6KA2,SAMSN1,KDM1B,ZNF600,NTF3,ACER2,PARP15,ZNF723,AURKA,PARN,ST18,PYGO1,SLC8A1,SSBP2,ANKRD31,DUX4,SERPINA6,PLGRKT,HIVEP2,BMPR1B,FMN2,ZNF717,ARNT,PAK3,ZNF257,DIP2B,LARP1,ITPKB,TRPC5,NBN,IFT57,INTS7,PRKCZ,SPOP,TAF15,DIP2A,MSH6,MCPH1,HECW1,MBNL2,PHF19,MRTFA,TAF4B,DUSP22,EBF2,YAP1,NFIA,WDR70,RIPK4,ZKSCAN5,MAPK1,MGAT5,ITIH5,USP25,KMT2E,PCGF5,PDGFD,NRG3,UBE2O,SFMBT2,GFRA1,NIPBL,SPIDR,EWSR1,GABPA,ZNF735,CARD18,CHD6,STK38,PTPN13,KANSL1,LIMCH1,MBNL1,ATF6,EFEMP1,ZNF684,DCAF1,CCNG2,NF2,RBFOX1,HIVEP1,MOB3B,BIRC6,AKAP9,KLF15,PPARA,MEIS2,NFIB,MRTFB,PPP6R3,NR5A2,FOXJ3,PTPRK,TRERF1,EIF3D,DAPK1,AGO2,PHC3,JARID2,GATAD2B,IL34,BRWD1,ZNF846,BCAS3,WNT9B,ZNF606,CLPX,DUSP16,SMARCA4,USP8,PARD3,MAPKAP1,TNRC6C,PIAS1,EBF1,ATRX,NUAK1,PTPRT,ELAVL4,ABL1,MXI1,HDAC4,OXR1,SLC1A1,PRKAA1,ITGB3BP,MRPS27,CREG1,DROSHA,L3MBTL3,APLF,NFAT5,NBAS,PSMF1,SLFN11,RAP1A,GLIS1,MORC1,TOX3,CAMK4,BAZ2A,FGF10,ZC3HAV1,LATS2,NRG1,INO80D,AP3B1,ZNF438,ABCB7,ZBTB16,MUSK,ZNF675,SMARCAD1,SETDB2,PRKCE,FOXK2,SLCO3A1,MED15,NXN,ESRRG,ZNF718,CD44,PTPRO,EGF,PRRC1,PDE3A,LIMD1,PEX14,SPRED2,RPS6KA3,PTPN2,TRIM5,ATXN3,RFC1,ALG10B,AMBRA1,KDM7A,OPRM1,HTR2A,FANCM,FANCA,DAZL,GTF2F2,TAF3,RPRD1B,MARK2,EBF3,ZNF33B,FHL2,PUM1,MSH2,IGF2BP3,ANKRD17,ZNF397,SLC2A13,HIPK3,CDKN2C,KNDC1,SPSB4,CLSPN,NOS2,BICRAL,MNAT1,RBBP8,MDFIC,HMGA2,CCND3,BCL11B,ZIM3,CREM,MBP,TRPS1,PLCE1,TGFA,HIP1,CRIM1,PRR5L,UTP4,MYT1L,ZNF160,LDLRAD4,CNTN1,HSF5,SNX3,ZNF367,BRCA2,DISC1,ZBTB2,BLM,INTS8,LIN54,ZNF121,BMP2,RC3H2,TRAK1,PTCD2,ZC3H14,GFI1B,RANBP9,TMEM161A,ASXL3,RELN,HMGB1,GNAQ,FGF9,NFATC2,SH3BP5,CPAMD8,ZNF567,TRAF3,ZNF462,ESRP1,TTC21B,ETS2,GEMIN5,ZNF875,UIMC1,LRRFIP1,IKZF2,ATF1,SMARCA2,ETS1,GLI3,CGAS,SMARCC1,SNX6,AFF3,SMOC2,CNKSR3,VENTX,IDE,PRDM10,MYEF2,ZNF431,RERE,BTAF1,ZNF618,NEK10,MOB1B,ATF2,HIRA,CYLD,UMODL1,MAPK8IP1,HIVEP3,PSIP1,KPNA1,NELL1,ME2,RGMB,MRPL13,KITLG,CAMTA1,MYT1,YLPM1,RCAN1,GTF2I,RORB,TADA2A,DAB1,MED27,ZNF208,RB1CC1,NMD3,PRKN,ZNF608,TBX20,SP110,MAPK10,DACH1,ZNF541,DPF3,GRIN2A,ARID5B,ATXN1,PRKCH,PKP1,IL6R,ALS2,NLRC5,ZNF627,ACO1,TFDP1,CNOT6L,MKNK1,SNX25,TOX,SLC4A4,PTPRB,ZFP90,COPS8,ZNF124,USP7,PLAGL1,SOX30,RALB,NPAS2,ROCK1,LYN,ZNF780B,CTSB,ZNF169,DTX1,BZW1,TENM2,OVOL2,PIWIL3,ZBTB33,ZFHX3,ZNF44,BANP,SUPT16H,ARID1B,HOXC13,BAZ1A,CASZ1,INSR,YTHDF3,DEDD2,HECTD1,PBX3,SUMO2,ZNF292,POGK,SNAI2,ASH1L,HOXC4,BID,S*

			IAH2,TRABD2B,RXRG,SP3,ERN2,ZNF879,MBTPS2,TIAL1,ELF2,NSD2,CARD10,TWIST2,CTIF,ENPP1,RASGRP1,SNX9,CSNK2A1,BMP5,CSF1,HDGFL3,SERPINB9,SCAF4,CTDP1,GRIN2B,INO80,FANCB,CLNS1A,SMAD5,CELF4,TCERG1,ABCG1,FOXN3,DCUN1D4,SLC40A1,PRAME,MYCL,MED1,CDC14B,IL33,GPRC5C,ROR2,ZNF521,BANK1,CSDE1,LMX1A,IL10,ACTR2,SFPQ,SCML2,PRAMEF25,RIOK1,PTH,PRKAA2,SOHLH1,LARP6,PHF20L1,ETV6,IQGAP1,CAMLG,ZBTB7C,TEAD1,SREBF2,ANP32B,YBX3,NRXN1,PCID2,ZNF234,ZNF518A,FRY,CENPE,LMX1B,NGDN,ELOC,TWIST1,ALKAL2,JAK2,VSX1,ZBTB38,ISX,MADD,PATL1,ZNF287,CELSR2,ZNF449,CREBBP,MELTF,TNKS,PCNA,UFL1,NFKBIA,PRKCB,ANXA4,RFC2,ZNF354C,ALX4,RTRAF,BRD4,ZBTB21,SERBP1,NEDD9,ITGA6,ASS1,ZNF528,ZNF611,CIDEA,ZBTB49,STOX2,AGO1,MEOX2,ELL2,STAT1,BRMS1L,NDFIP2,NR2C1,MAP2K6,GATAD1,MTPN,ABI1,CEMIP,PRAMEF2,POU6F2,IMPACT,CCBE1,PARK7,MAPK8,OAZ2,MED12L,ZSCAN30,UBL7,POU1F1,MTF2,NCAPG2,FOXP2,MYOCD,ZSCAN5C,CYFIP2,HNRNPM,ASCC2,ARID3B,MEF2C,ZNF613,ADGRB1,RXRA,WNT7A,MAP3K5,NDFIP1,MAP3K4,SERPINI2,PRDM13,FOXO6,ZNF112,MAGEL2,RAD51AP1,PDE2A,RAB38,DBF4B,FBXW8,SDCBP,NSMCE1,ZNF813,WWOX,ZBTB25,PASK,MLLT1,NCK1,SCAF8,FGR,CWC22,CDCA8,PPP2R3A,MLLT10,RNF8,EPHA4,INTS13,MECOM,DNMT3L,NTRK2,LHX9,ZBTB10,OCLN,CREB5,IREB2,PTK2,CDH5,ASCL3,CYFIP1,UBE3A,SEMA4D,SERPINB10,ZBTB20,ZNF66,RUNX1,KIRREL1,POMT2,ZNF845,SAMD13,NOS1AP,PDCL3,SRP9,CCDC88A,NSUN2,CHCHD2,TNFSF11,FYN,KDM5A,PCBP3,ZNF705G,PPM1F,HDAC2,SLF1,GON4L,TBX15,SH2D3C,PSME3IP1,DOCK3,TRNAU1AP,NCS1,ZFYVE28,MAPK9,PABPC1,SLC39A8,ROR1,TET1,ARNT2,HECW2,ITGA8,FBXL20,GRM5,SPOPL,ZNF705D,RPS6KA5,PID1,NRP1,MIDEAS,PRKCA,ATPSCKMT,FHIT,ITGA1,ZNF615,KLF12,RC3H1,NRIP1,POR,ZNF850,ZNF235,ZNF738,SUPT3H,BCR,TUT4,ZNF215,TCERG1L,PRIM2,C14ORF39,TM9SF2,ELP2,FBLN1,STK36,RAG1,ZNF678,BMPER,PRDM15,CUX1,ZNF420,MACROH2A1,MITF,EPHB2,BCL2L13,CD38,EYA4,DPH6,CDK14,MET,SPPL3,ZNF705B,CDH13,MED13L,SERPINB2,ATG5,MAGI2,PRDM11,UNK,MLIP,MYB,ZNF704,MFHAS1,SERPINB7,DHX29,BMP7,TNFAIP8,ZMYND8,RNF217,KCTD1,ZNF74,BPTF,ZMYND11,NUDT21,KMT2C,DDX6,PDGFC,BACE2,RFX2,PARPBP,NECAB1,PKNOX2,EYA1,SLIT2,CNOT7,ESCO1,ERBB4,SERPINB11,GSAP,ROBO1,SAMD4A,PBX1,NPAS3,PRKCQ,ANTXR1,MGMT,ZNF679,SHLD2,NOS1,SLC6A3,PRR16,EFNA5,TCF12,ARHGEF11,NSD1,EHMT1,ESR1,KDM4B,LOXL2,PRLR,AGO3,HTT,FOXB1,CAMK1D,PIK3R3,FER,ZNF302,EYA2,INTS12,A2M,CHFR,ZNF721,JAZF1,ZNF578,ZNF891,SPOCK3,NRF1,ZNF14,PHC2,ROCK2,PRDM1,RORA,DMRT1,EIF4G3,PDK1,PSMD2,HERPUD1,NCOA6,COL4A3,WASHC1,ZFP30,BARD1,STK3,DEPTOR,ZNF423,ZNF568,HNRNPU,RAB3GAP2,IGF1R,PRKAG2,GLI2,THRB,AKAP13,DNM1L
GO:0006811	ion transport	0.0000108600456871982 6	*UNC80,CACNA2D3,SLC17A1,SLC24A2,KCNH5,MICU2,SLC25A21,SLC37A1,PIEZO2,DPP10,ITPR2,PDE4D,STXBP1,SLC44A5,BCL2,KCNMA1,CHRNA7,GABRB3,ANO6,CACNG2,SLC4A10,NEDD4,GRIK3,ATP2B2,TUSC3,GABRB1,GRIA1,SLC39A12,SLC8A3,PRKD1,CHRM3,LRP2,FGF12,GABRA6,TMEM38B,SLC24A3,SLC44A1,GRM7,THADA,NEDD4L,TRPM1,SLC39A11,APP,SLC7A2,CACNA1C,CACNB2,GABRG2,TMC1,SYT1,SLC8A1,KCNE4,AKAP6,HOMER2,KCNK10,TRPC5,CLIC6,RYR3,HECW1,KCNJ1,ABCD3,TRPC7,SYT10,SLC16A1,NIPAL2,MICU1,LRRC38,GRIK4,AKAP9,RASGRF2,KCNS3,GRM1,GABRG1,PLA2R1,DAPK1,SLC24A4,SEC14L1,SCN2A,ANK2,RYR2,SLC9C1,NKAIN3,SLC36A1,ABL1,SLC1A1,PRKAA1,SLC12A8,KCNH1,ANO4,NKAIN2,PLEKHA8,GRID2,GSG1L,RASGRF1,ATP11C,ABCB7,PRKCE,SLCO3A1,SLMAP,WNK2,EGF,ABCC9,P2RX6,HTR2C,SLC2A3,ALG10B,ATP8A1,OPRM1,ABCC4,HTR2A,CYBRD1,CYP4A11,CNNM4,STAC,CNIH3,MAIP1,KCND2,NOS2,AFG3L2,ANK3,NIPA2,TMC7,TMEM163,ATP6V1E1,CNTN1,CACNA1I,KCNJ15,SLC10A7,SCN11A,NETO2,RELN,SLC23A2,SLC39A6,KCNH8,SLC37A2,SLC9A4,GABRR2,CNKSR3,GRIK2,LRRC8B,CFTR,CHRM5,SLC30A10,SELENON,PRKN,HEPHL1,GRIN2A,JPH1,TR*

			PM6,CDH23,SLC12A1,TG,RACGAP1,KCNQ3,SHISA9,SLC4A4,SCN10A,KCND3,KCNN3,LYN,SLC44A2,SLC15A2,SLC13A5,CRACR2A,CUL5,GRID1,SLC52A1,COX5A,GABRG3,PLPP4,ZDHHC17,SLC22A14,ENPP1,UTRN,KCNC1,HCN1,GRIN2B,CLNS1A,KCNK5,SLC40A1,PSAP,SLC5A12,PLA2G4A,COX7A2L,LASP1,NRXN1,TWIST1,SLC1A7,MELTF,TRPV5,PRKCB,ABCC8,CACNA1E,ATP2B1,CLCA4,OTOP1,SLC6A1,NDFIP2,MAP2K6,SHROOM2,SLC6A11,KCNJ18,CEMIP,CBLIF,PARK7,ADCYAP1R1,PLA2G12B,ATP13A3,EFHB,MEF2C,NDFIP1,SLC5A9,ATP6V1C2,SLC10A6,NECTIN1,FLVCR1,ATP6V1B2,GABRA5,NTRK2,PLEKHA3,SHISA6,TRPM7,GRIK1,IREB2,DIAPH1,APOL1,SCARA5,SLC26A2,PLCZ1,NOS1AP,MTTP,SLC9A5,SLC27A6,SLC5A1,ANO10,TNFSF11,FYN,SCN8A,TMEM63C,NCS1,ATP5PF,NALCN,TRPM3,SLC39A8,SLC16A9,HECW2,GRM5,ATPSCKMT,KCNJ6,DPP6,EPHB2,TSPAN13,ATP6V0D2,CACNG3,ATG5,VMP1,SLC1A2,GABRA2,KCNIP4,TRDN,NLGN1,NOS1,SLC6A3,ASIC2,KCNQ5,CACNA2D1,HTT,SLC25A18,CCR2,ANO2,GRIA4,IL16,CATSPER2,RGS7,CLDN10,CLCN5,SLC13A4,PNPLA8,KCNAB1,ATP10A,DNM1L
GO:0098660	inorganic ion transmembrane transport	0.000017052823401408904	*CACNA2D3,SLC17A1,SLC24A2,KCNH5,MICU2,SLC37A1,DPP10,ITPR2,PDE4D,BCL2,KCNMA1,CHRNA7,GABRB3,ANO6,CACNG2,SLC4A10,NEDD4,ATP2B2,TUSC3,GABRB1,SLC39A12,SLC8A3,PRKD1,FGF12,GABRA6,TMEM38B,SLC24A3,THADA,NEDD4L,TRPM1,SLC39A11,CACNA1C,CACNB2,GABRG2,TMC1,SLC8A1,KCNE4,AKAP6,KCNK10,TRPC5,CLIC6,RYR3,HECW1,KCNJ1,TRPC7,NIPAL2,MICU1,LRRC38,AKAP9,KCNS3,GABRG1,SLC24A4,SCN2A,ANK2,RYR2,SLC9C1,SLC36A1,ABL1,SLC1A1,SLC12A8,KCNH1,ANO4,ABCB7,PRKCE,SLMAP,WNK2,ABCC9,HTR2C,ALG10B,OPRM1,HTR2A,CNNM4,STAC,MAIP1,KCND2,AFG3L2,ANK3,NIPA2,TMEM163,ATP6V1E1,CACNA1I,KCNJ15,SCN11A,NETO2,SLC39A6,KCNH8,SLC37A2,SLC9A4,GABRR2,CNKSR3,CFTR,SLC30A10,SELENON,GRIN2A,JPH1,TRPM6,SLC12A1,KCNQ3,SLC4A4,SCN10A,KCND3,KCNN3,LYN,SLC15A2,CRACR2A,CUL5,COX5A,GABRG3,ZDHHC17,UTRN,KCNC1,HCN1,GRIN2B,KCNK5,SLC40A1,COX7A2L,TWIST1,TRPV5,ABCC8,CACNA1E,ATP2B1,CLCA4,OTOP1,SLC6A1,SHROOM2,SLC6A11,KCNJ18,CEMIP,CBLIF,PARK7,ADCYAP1R1,ATP6V1C2,ATP6V1B2,GABRA5,TRPM7,DIAPH1,APOL1,SCARA5,SLC26A2,NOS1AP,SLC9A5,SLC5A1,ANO10,FYN,SCN8A,NCS1,ATP5PF,NALCN,TRPM3,SLC39A8,HECW2,GRM5,ATPSCKMT,KCNJ6,DPP6,TSPAN13,ATP6V0D2,CACNG3,ATG5,VMP1,GABRA2,KCNIP4,TRDN,NOS1,SLC6A3,ASIC2,KCNQ5,CACNA2D1,HTT,SLC25A18,ANO2,CATSPER2,RGS7,CLCN5,SLC13A4,KCNAB1*
GO:0033036	macromolecule localization	0.000017503758514173714	*NSG1,ABCA13,IMMP2L,LONP2,FTO,MX2,CLTCL1,SNAP25-AS1,DPP10,ZDHHC21,RIPOR2,RDX,STXBP1,ERC1,RALA,BCL2,MYO5A,FBN1,GPHN,COG5,GPR158,RIMS1,PIK3C3,SPIRE1,CNTLN,EXOC6B,TRAPPC8,USH2A,CEP192,RIMS2,ERBIN,FCHO2,ANO6,CACNG2,GPC6,CNTNAP2,APC,CRKL,ILDR2,SETD2,TANGO6,TNIK,EGFR,RFX3,DENND1A,ANGPT1,MACF1,NEDD4,GNPTAB,CRB1,ZFAND6,DNAJC13,RABEP1,TTC39B,NUP214,TOM1L2,CEP128,PRKD1,GRAMD1B,RAPGEF2,LRP2,AGK,RANBP17,UBE2L3,PTPRN2,SMYD3,HERC2,SEPTIN9,EPB41L3,NEDD4L,ADAM10,CACNB2,DCLK1,MAPRE2,VCL,ARHGAP44,NDUFAF2,CD2AP,AURKA,PARN,PYGO1,ABCG8,ABCD2,FMN2,AKAP6,RAB8B,RFTN1,RANBP2,TRPC5,RAP1GDS1,KICS2,CUBN,SCP2,PRKCZ,MCPH1,RAB27B,CNST,ABCA5,YAP1,SEM1,VPS35L,CADPS2,ABCD3,RABGAP1L,SGTB,ADAM22,COPB1,SYCP1,NIPBL,SLC16A1,SPIDR,IPO11,CORO2B,PAFAH1B1,TM7SF3,VPS13D,TLK1,NF2,RBFOX1,ZDHHC14,CTNNA1,PPARA,SNX30,PTPRK,PARD3B,PLA2R1,VPS13C,DNAH11,JARID2,RAB22A,DNAJC15,CPE,ANK2,ADGRV1,BCAS3,RYR2,BBS2,RANBP3L,NBEA,DUSP16,USP8,FABP7,PARD3,TBC1D5,BLK,ATRX,ABL1,SLC1A1,PRKAA1,FHIP1A,PRELID2,ANO4,CCDC91,EIPR1,NBAS,RAP1A,GPC5,ZNHIT6,PLEKHA8,FGF10,GRID2,LATS2,AP3B1,ATP11C,SYNE1,ZBTB16,MUSK,PRKCE,SLCO3A1,SLMAP,DENND4C,CEP83,FBN2,EGF,PEX14,ATP8A2,SCG5,PTPN2,TRIM5,ATXN3,RIC3,ATP8A1,RFTN2,LTBP1,OPRM1,ABCC4,HTR2A,CYP4A11,STAC,TAF3,ABHD17C,MSH2,IGF2BP3,APBA2,MAIP1,TNPO3,ABCA10,NOS2,TTC7B,MDFIC,ANK3,COG2,VPS41,L*

			YPLA1,HIP1,PRR5L,VPS37A,EFR3A,TJP1,NPHP4,PACSIN2,SNX3,BHLHE40-AS1,BRCA2,DISC1,WDPCP,SLC10A7,LRP1B,STX12,ATP9A,TRAK1,MSR1,VRK1,GNAI1,MYRIP,SLC15A5,RELN,NUDCD3,CDS2,AP4E1,FGF9,MYOM1,UNC13B,TTC21B,PLS1,SNX8,SEC23B,NIN,CCDC186,GLI3,SNX6,PACS1,MAP2,PEX6,RRBP1,ATF2,BBS4,KIAA0753,CFTR,KPNA1,CSE1L,DOP1B,TBC1D13,PHAF1,ATP10B,NMD3,AKAP10,PRKN,LYST,GRIN2A,PRKCH,FRMD4A,ALS2,SNX25,FBLN5,TSPAN33,LRBA,MAP7,MON2,MESD,MYO1D,SEC24D,ROCK1,SEL1L,SUMO3,SLC15A2,CHKA,RRAGD,BANP,NPIPA1,DMBT1,HECTD1,SHROOM3,XRCC4,COLQ,ARFGEF1,BID,PIGK,OSBPL10,RPH3A,ABCA4,UFD1,TOM1,ZDHHC17,NSD2,ESYT2,SH3GLB1,CARD10,TMED3,XKR5,ENPP1,IGSF11,SNX9,WDR72,NUP37,BCL2L1,HCN1,ABCG1,FAM149B1,CIDEC,PSAP,CFHR4,MICALL2,MED1,ATG4B,PCNT,IL10,PRKAA2,NDC80,PACRG,VSTM2A,PLA2G4A,SCFD2,CAMLG,SREBF2,ANP32B,FYB2,NRXN1,PCID2,SNAP91,PEG10,JAK2,RPF2,CELSR2,TNKS,ARL11,SIAH3,UFL1,NFKBIA,PRKCB,GOT2,ABCC8,MIPEP,RTRAF,USH1C,NEDD9,MTCL1,GRIP1,TM9SF3,SAR1A,XKR6,CIDEA,BBS9,EXOC1,HEPACAM,NDFIP2,MAP2K6,SHROOM2,RN7SL483P,CEMIP,PARK7,MAPK8,ITGA4,OAZ2,BCAP29,UBE2J2,PLA2G12B,NDC1,TM9SF4,RAPGEF4,CEP120,ARL4C,STOML1,RXRA,WNT7A,NDFIP1,CHAMP1,SLC10A6,RAB38,SDCBP,NECTIN1,TRIM23,SNAP29,INTS13,NUMB,ADAMTS9,RN7SL767P,PLEKHA3,OCLN,SHISA6,AKAP11,KTN1,MVB12B,MARK4,CDH5,APOL2,AP4S1,CLDN18,MPP7,DIAPH1,SCAMP1,SCG3,APOL1,PITPNC1,FRMD6,AP2B1,HEATR5A,ZFYVE1,ICA1,MTTP,SRP9,CCDC88A,NSUN2,SLC27A6,BICD1,TNFSF11,FYN,PPM1F,ARL13B,XPO7,ODR4,SLF1,ATP9B,EHBP1,MAPK9,ASB3,CDH2,ITGA8,GPR137B,ZDHHC18,TBC1D1,PID1,NRP1,FCHSD2,RNF215,CROT,NRIP1,ABCA6,MCC,BCR,ARFGAP3,TM9SF2,B9D1,BMPER,RABL2A,DPP6,MACROH2A1,EPHB2,CD38,MYO5B,RGPD4,PPIL2,AKAIN1,DLG2,STXBP4,CACNG3,MAGI2,VMP1,GNAS,MFHAS1,NUP43,BMP7,ASTN2,DLG5,GAPVD1,DDX6,PLIN2,VPS13B,EXOC4,FAM126A,KCNIP4,ERBB4,FAM3B,FAM126B,SYNDIG1,RGPD2,SAMM50,SORCS2,NLGN1,ASIC2,EFNA5,GAS2L1,KIF13A,AP5M1,ESR1,ZDHHC11B,PITPNM3,OSBPL5,OSBPL6,AGAP1,ROCK2,CDCA5,RAB31,HERPUD1,WASHC1,HOOK3,BARD1,STK3,ZNF423,PNPLA8,HNRNPU,VTI1A,CEP72,RAB3GAP2,CADPS,TANGO2,MORC3,ATP10A,SEPTIN6,DNM1L
GO:0080090	regulation of primary metabolic process	0.000018044265034162777	*NOTCH2,BCAR3,MTOR,SPOCK1,WWC1,TRAPPC9,BNC2,NLK,LONP2,FTO,KSR1,MGA,RFX7,ZNF236,PLCB1,ZNF536,ZFPM2,L3MBTL4,DLC1,TNRC6B,PDE4D,RDX,ERC1,BCL2,PRDM16,CHRNA7,EPC2,SPIRE1,ZEB1,AKR1C3,RARB,SPRED1,MINAR1,ALK,AUTS2,FOXJ2,CDYL2,BABAM2,GLIS3,FANK1,ERBIN,ZNF880,MLLT3,EGLN3,SPON1,APC,ZNF595,TSHZ3,RBFOX3,RTN1,TCF4,CRKL,ILDR2,SOX5,SETD2,ERG,ZNF573,TNIK,PTPRJ,KDM4C,NEK4,TSHZ2,EGFR,ZNF280B,RFX3,USP14,ANGPT1,CDK12,BACH1,PRKACB,NEK7,NCOR1,RNF220,ZNF407,NEDD4,MAML2,MTRF1,SND1,SCAI,NSMCE2,BCL11A,SOX6,FLI1,RPRD1A,NTRK3,C5,FLT1,ZNF648,RFC3,ZNF382,TASP1,THRAP3,CAST,TTC39B,SLC8A3,MALRD1,PRKD1,PAK1,EPHA7,NCOA7,KHDRBS2,SPEN,RAPGEF2,PELI2,LRP2,RUNX2,TAOK3,ONECUT1,LDLRAD3,CPEB4,UBE2L3,LDB2,PUM3,SMYD3,RPTOR,GHR,SSBP3,CELF2,NEDD4L,HDAC9,ZHX3,ATF7IP,APBB2,APP,RPS6KA2,SAMSN1,KDM1B,ZNF600,NTF3,ACER2,PARP15,ZNF723,AURKA,PARN,ST18,PYGO1,SLC8A1,SSBP2,ANKRD31,DUX4,SERPINA6,PLGRKT,HIVEP2,ABCD2,BMPR1B,FMN2,ZNF717,ARNT,PAK3,ZNF257,DIP2B,RANBP2,LARP1,ITPKB,TRPC5,NBN,SCP2,IFT57,INTS7,PRKCZ,SPOP,GRB10,TAF15,DIP2A,MSH6,MCPH1,HECW1,MBNL2,PHF19,MRTFA,TAF4B,DUSP22,EBF2,YAP1,NFIA,WDR70,RIPK4,ZKSCAN5,MAPK1,MGAT5,ITIH5,USP25,KMT2E,PCGF5,PDGFD,NRG3,UBE2O,SFMBT2,GFRA1,NIPBL,SPIDR,EWSR1,GABPA,ZNF735,CARD18,CHD6,STK38,PTPN13,KANSL1,LIMCH1,MBNL1,ATF6,EFEMP1,ZNF684,DCAF1,CCNG2,NF2,RBFOX1,HIVEP1,MOB3B,BIRC6,AKAP9,KLF15,PPARA,MEIS2,NFIB,MRTFB,PPP6R3,NR5A2,FOXJ3,PTPRK,TRERF1,EIF3D,DAPK1,AGO2,PHC3,JARID2,DNAJC15,GATA*

			D2B,IL34,BRWD1,ZNF846,BCAS3,WNT9B,ZNF606,CLPX,DUSP16,SMARCA4,USP8,PARD3,MAPKAP1,TNRC6C,PIAS1,EBF1,ATRX,NUAK1,PTPRT,ELAVL4,ABL1,MXI1,HDAC4,OXR1,SLC1A1,PRKAA1,ITGB3BP,MRPS27,CREG1,DROSHA,L3MBTL3,APLF,NFAT5,NBAS,PSMF1,SLFN11,RAP1A,GLIS1,MORC1,TOX3,CAMK4,BAZ2A,FGF10,ZC3HAV1,LATS2,NRG1,INO80D,AP3B1,ZNF438,ZBTB16,MUSK,ZNF675,SMARCAD1,SETDB2,PRKCE,FOXK2,SLCO3A1,MED15,NXN,ESRRG,ZNF718,CD44,PTPRO,EGF,PRRC1,PDE3A,LIMD1,PEX14,SPRED2,RPS6KA3,MTMR3,PTPN2,TRIM5,ATXN3,RFC1,HTR2C,ALG10B,AMBRA1,KDM7A,HTR2A,FANCM,FANCA,DAZL,GTF2F2,TAF3,RPRD1B,MARK2,EBF3,ZNF33B,FHL2,PUM1,MSH2,IGF2BP3,ANKRD17,ZNF397,SLC2A13,HIPK3,CDKN2C,KNDC1,SPSB4,CLSPN,NOS2,BICRAL,MNAT1,RBBP8,MDFIC,HMGA2,CCND3,BCL11B,ZIM3,CREM,MBP,TRPS1,PLCE1,TGFA,HIP1,CRIM1,PRR5L,UTP4,MYT1L,ZNF160,LDLRAD4,CNTN1,HSF5,SNX3,ZNF367,BRCA2,DISC1,ZBTB2,BLM,INTS8,LIN54,ZNF121,BMP2,RC3H2,TRAK1,SOGA1,PTCD2,ZC3H14,GFI1B,RANBP9,TMEM161A,ASXL3,RELN,HMGB1,GNAQ,FGF9,NFATC2,SH3BP5,CPAMD8,ZNF567,TRAF3,ZNF462,ESRP1,TTC21B,ETS2,GEMIN5,ZNF875,UIMC1,LRRFIP1,IKZF2,ATF1,SMARCA2,ETS1,GLI3,CGAS,SMARCC1,SNX6,AFF3,SMOC2,CNKSR3,VENTX,IDE,PRDM10,MYEF2,ZNF431,RERE,BTAF1,ZNF618,NEK10,MOB1B,ATF2,HIRA,CYLD,UMODL1,BBS4,MAPK8IP1,HIVEP3,PSIP1,KPNA1,NELL1,ME2,RGMB,MRPL13,KITLG,CAMTA1,MYT1,CHRM5,YLPM1,RCAN1,GTF2I,RORB,TADA2A,DAB1,MED27,ZNF208,RB1CC1,NMD3,PRKN,MTMR2,ZNF608,TBX20,SP110,MAPK10,DACH1,ZNF541,DPF3,GRIN2A,ARID5B,ATXN1,PRKCH,PKP1,IL6R,ALS2,NLRC5,ZNF627,ACO1,TFDP1,CNOT6L,MKNK1,SNX25,TOX,SLC4A4,PTPRB,ZFP90,COPS8,ZNF124,USP7,VAV3,PLAGL1,SOX30,RALB,NPAS2,ROCK1,LYN,ZNF780B,CTSB,ZNF169,DTX1,BZW1,TENM2,OVOL2,PIWIL3,ZBTB33,ZFHX3,ZNF44,BANP,SUPT16H,ARID1B,HOXC13,BAZ1A,CASZ1,INSR,YTHDF3,DEDD2,HECTD1,PBX3,SUMO2,ZNF292,POGK,SNAI2,ASH1L,HOXC4,BID,SIAH2,TRABD2B,RXRG,SP3,ERN2,ZNF879,MBTPS2,TIAL1,ELF2,NSD2,CARD10,TWIST2,CTIF,ENPP1,RASGRP1,SNX9,CSNK2A1,BMP5,CSF1,HDGFL3,SERPINB9,SCAF4,CTDP1,GRIN2B,INO80,FANCB,CLNS1A,SMAD5,CELF4,TCERG1,ABCG1,FOXN3,DCUN1D4,SLC40A1,PRAME,MYCL,PSAP,LPGAT1,MED1,CDC14B,IL33,GPRC5C,ROR2,ZNF521,BANK1,CSDE1,LMX1A,IL10,ACTR2,SFPQ,SCML2,PRAMEF25,RIOK1,PTH,PRKAA2,SOHLH1,LARP6,PHF20L1,ETV6,IQGAP1,CAMLG,ZBTB7C,TEAD1,SREBF2,ANP32B,YBX3,NRXN1,PCID2,ZNF234,ZNF518A,FRY,CENPE,LMX1B,NGDN,ELOC,TWIST1,ALKAL2,JAK2,VSX1,ZBTB38,ISX,MADD,PATL1,ZNF287,CELSR2,ZNF449,CREBBP,MELTF,TNKS,PCNA,UFL1,NFKBIA,PRKCB,ANXA4,RFC2,ZNF354C,ALX4,RTRAF,BRD4,ZBTB21,SERBP1,NEDD9,ITGA6,ZNF528,ERLIN2,ZNF611,CIDEA,ZBTB49,STOX2,AGO1,MEOX2,ELL2,STAT1,BRMS1L,NDFIP2,NR2C1,MAP2K6,GATAD1,MTPN,ABI1,CEMIP,PRAMEF2,POU6F2,IMPACT,CCBE1,PARK7,MAPK8,OAZ2,MED12L,ZSCAN30,UBL7,POU1F1,ADCYAP1R1,MTF2,NCAPG2,FOXP2,MYOCD,ZSCAN5C,CYFIP2,HNRNPM,ASCC2,ARID3B,MEF2C,ZNF613,ADGRB1,RXRA,WNT7A,MAP3K5,NDFIP1,MAP3K4,SERPINI2,PRDM13,FOXO6,ZNF112,MAGEL2,RAD51AP1,PDE2A,RAB38,DBF4B,FBXW8,SDCBP,NSMCE1,ZNF813,WWOX,ZBTB25,PASK,MLLT1,NCK1,SCAF8,FGR,CWC22,CDCA8,PPP2R3A,MLLT10,RNF8,EPHA4,INTS13,MECOM,DNMT3L,NTRK2,LHX9,ZBTB10,OCLN,CREB5,IREB2,PTK2,CDH5,ASCL3,CYFIP1,UBE3A,SEMA4D,SERPINB10,ZBTB20,ZNF66,RUNX1,KIRREL1,POMT2,ZNF845,SAMD13,NOS1AP,PDCL3,SRP9,CCDC88A,NSUN2,CHCHD2,TNFSF11,FYN,KDM5A,PCBP3,ZNF705G,PPM1F,HDAC2,SLF1,GON4L,TBX15,SH2D3C,PSME3IP1,DOCK3,TRNAU1AP,NCS1,ZFYVE28,MAPK9,PABPC1,SLC39A8,ROR1,OPA3,TET1,ARNT2,HECW2,ITGA8,FBXL20,GRM5,SPOPL,ZNF705D,RPS6KA5,PID1,NRP1,MIDEAS,PRKCA,ATPSCKMT,FHIT,ITGA1,ZNF615,KLF12,RC3H1,NRIP1,POR,ZNF850,ZNF235,ZNF738,SUPT3H,TUT4,ZNF215,TCERG1L,PRIM2,C14ORF39,TM9SF2,ELP2,FBLN1,STK36,RAG1,ZNF678,BMPER,PRDM15,CUX1,ZNF420,MACROH2A1,MITF,EPHB2

			,BCL2L13,CD38,EYA4,DPH6,CDK14,MET,SPPL3,ZNF705B,CDH13,MED13L,SERPINB2,ATG5,MAGI2,PRDM11,UNK,MLIP,MYB,ZNF704,MFHAS1,SERPINB7,DHX29,BMP7,TNFAIP8,ZMYND8,RNF217,KCTD1,ZNF74,BPTF,ZMYND11,NUDT21,KMT2C,DDX6,ADGRF5,PDGFC,BACE2,RFX2,PARPBP,NECAB1,PKNOX2,EYA1,SLIT2,CNOT7,ESCO1,ERBB4,SERPINB11,GSAP,ROBO1,SAMD4A,PBX1,NPAS3,PRKCQ,ANTXR1,MGMT,ZNF679,SHLD2,NOS1,SLC6A3,PRR16,EFNA5,TCF12,ARHGEF11,NSD1,EHMT1,ESR1,KDM4B,LOXL2,PRLR,AGO3,HTT,FOXB1,CAMK1D,PIK3R3,FER,ZNF302,EYA2,INTS12,A2M,CHFR,ZNF721,JAZF1,ZNF578,ZNF891,SPOCK3,NRF1,ZNF14,HRH1,PHC2,ROCK2,PRDM1,RORA,DMRT1,EIF4G3,PDK1,PSMD2,HERPUD1,NCOA6,COL4A3,WASHC1,ZFP30,BARD1,STK3,DEPTOR,ZNF423,ZNF568,HNRNPU,RAB3GAP2,IGF1R,PRKAG2,GLI2,THRB,AKAP13,DNM1L
GO:0051173	positive regulation of nitrogen compound metabolic process	0.00001841992416122705	BCAR3,MTOR,WWC1,FTO,MGA,PLCB1,ZFPM2,DLC1,TNRC6B,RDX,BCL2,PRDM16,CHRNA7,EPC2,SPIRE1,ZEB1,RARB,ALK,AUTS2,FOXJ2,BABAM2,GLIS3,FANK1,MLLT3,EGLN3,SPON1,APC,TCF4,CRKL,ERG,TNIK,PTPRJ,NEK4,EGFR,RFX3,ANGPT1,CDK12,BACH1,NEK7,ZNF407,NEDD4,MAML2,BCL11A,FLI1,NTRK3,FLT1,RFC3,TASP1,THRAP3,PRKD1,PAK1,EPHA7,NCOA7,RAPGEF2,PELI2,LRP2,RUNX2,TAOK3,ONECUT1,UBE2L3,LDB2,SMYD3,RPTOR,GHR,SSBP3,NEDD4L,ATF7IP,APBB2,APP,KDM1B,ZNF600,NTF3,ACER2,AURKA,PARN,ST18,PYGO1,SSBP2,ANKRD31,DUX4,PLGRKT,BMPR1B,FMN2,ZNF717,ARNT,PAK3,DIP2B,LARP1,TRPC5,NBN,IFT57,PRKCZ,TAF15,DIP2A,HECW1,PHF19,MRTFA,TAF4B,EBF2,YAP1,NFIA,MAPK1,KMT2E,PCGF5,PDGFD,NRG3,GFRA1,NIPBL,SPIDR,GABPA,CHD6,KANSL1,LIMCH1,ATF6,HIVEP1,MOB3B,AKAP9,KLF15,PPARA,MEIS2,NFIB,MRTFB,NR5A2,FOXJ3,TRERF1,EIF3D,DAPK1,AGO2,JARID2,GATAD2B,IL34,BCAS3,ZNF606,CLPX,SMARCA4,MAPKAP1,TNRC6C,PIAS1,ATRX,ABL1,HDAC4,SLC1A1,PRKAA1,MRPS27,APLF,NFAT5,RAP1A,GLIS1,TOX3,CAMK4,FGF10,ZC3HAV1,NRG1,INO80D,AP3B1,ZNF438,ABCB7,ZBTB16,MUSK,FOXK2,SLCO3A1,MED15,ESRRG,CD44,EGF,PRRC1,RPS6KA3,TRIM5,ATXN3,RFC1,ALG10B,AMBRA1,KDM7A,OPRM1,HTR2A,FANCM,DAZL,GTF2F2,TAF3,RPRD1B,MARK2,EBF3,ZNF33B,PUM1,MSH2,SLC2A13,KNDC1,SPSB4,CLSPN,NOS2,BICRAL,MNAT1,RBBP8,MDFIC,HMGA2,CCND3,BCL11B,CREM,MBP,TGFA,HIP1,PRR5L,CNTN1,BRCA2,DISC1,BLM,BMP2,RC3H2,GFI1B,RANBP9,TMEM161A,ASXL3,RELN,HMGB1,FGF9,NFATC2,ZNF462,ETS2,UIMC1,ATF1,SMARCA2,ETS1,GLI3,SMARCC1,SMOC2,VENTX,IDE,PRDM10,RERE,NEK10,MOB1B,ATF2,HIVEP3,PSIP1,RGMB,KITLG,CAMTA1,GTF2I,RORB,TADA2A,DAB1,MED27,ZNF208,RB1CC1,NMD3,PRKN,TBX20,DPF3,GRIN2A,ARID5B,IL6R,ALS2,NLRC5,TFDP1,CNOT6L,TOX,SLC4A4,ZFP90,COPS8,USP7,PLAGL1,SOX30,RALB,NPAS2,ROCK1,LYN,ZNF780B,DTX1,OVOL2,ZFHX3,BANP,SUPT16H,ARID1B,HOXC13,BAZ1A,CASZ1,INSR,YTHDF3,HECTD1,PBX3,SUMO2,ZNF292,SNAI2,ASH1L,HOXC4,BID,TRABD2B,RXRG,SP3,ERN2,MBTPS2,ELF2,NSD2,CARD10,CTIF,RASGRP1,SNX9,CSNK2A1,BMP5,CSF1,GRIN2B,INO80,FANCB,CLNS1A,SMAD5,CELF4,TCERG1,ABCG1,DCUN1D4,SLC40A1,MED1,CDC14B,IL33,GPRC5C,ROR2,ZNF521,BANK1,CSDE1,LMX1A,IL10,ACTR2,SFPQ,RIOK1,PTH,PRKAA2,LARP6,ETV6,IQGAP1,ZBTB7C,TEAD1,SREBF2,ANP32B,YBX3,NRXN1,PCID2,CENPE,LMX1B,TWIST1,ALKAL2,JAK2,ZBTB38,MADD,PATL1,ZNF287,CREBBP,MELTF,TNKS,PCNA,NFKBIA,PRKCB,RFC2,ALX4,RTRAF,BRD4,NEDD9,ITGA6,ASS1,ZBTB49,STOX2,AGO1,MEOX2,ELL2,STAT1,NDFIP2,MAP2K6,MTPN,ABI1,CEMIP,IMPACT,CCBE1,PARK7,MAPK8,OAZ2,MED12L,POU1F1,MTF2,MYOCD,CYFIP2,ARID3B,MEF2C,RXRA,WNT7A,MAP3K5,NDFIP1,MAP3K4,ZNF112,RAD51AP1,RAB38,DBF4B,FBXW8,SDCBP,WWOX,PASK,NCK1,SCAF8,CDCA8,PPP2R3A,MLLT10,RNF8,EPHA4,MECOM,DNMT3L,NTRK2,CREB5,PTK2,CDH5,ASCL3,UBE3A,SEMA4D,ZBTB20,RUNX1,POMT2,ZNF845,NOS1AP,CCDC88A,CHCHD2,TNFSF11,FYN,KDM5A,PPM1F,HDAC2,SLF1,TBX15,SH2D3C,DOCK3,NCS1,MAPK9,PABPC1,TET1,ARNT2,HECW2,ITGA8,GRM5,RPS6KA5,PID1,NRP1,ITGA1,ZNF615,KLF12,RC3H1,NRIP1,ZNF850,SUPT3H,T

			UT4,PRIM2,FBLN1,PRDM15,MITF,BCL2L13,CD38,EYA4,MET,SPPL3,CDH13,MAGI2,PRDM11,MLIP,MYB,DHX29,BMP7,RNF217,BPTF,NUDT21,KMT2C,PDGFC,RFX2,EYA1,CNOT7,ERBB4,GSAP,ROBO1,SAMD4A,PBX1,NPAS3,PRKCQ,ANTXR1,MGMT,SHLD2,NOS1,PRR16,EFNA5,TCF12,ARHGEF11,NSD1,ESR1,PRLR,AGO3,PIK3R3,EYA2,CHFR,ZNF721,NRF1,ROCK2,RORA,DMRT1,HERPUD1,NCOA6,COL4A3,BARD1,STK3,ZNF423,HNRNPU,RAB3GAP2,IGF1R,PRKAG2,GLI2,THRB,AKAP13
GO:0032879	regulation of localization	0.000020217450808842 65	*CACNA2D3,WWC1,ABCA13,KCNH5,FTO,MX2,CLTCL1,DPP10,ITPR2,RIPOR2,PDE4D,RDX,STXBP1,RALA,BCL2,KCNMA1,RIMS1,PIK3C3,RIMS2,MCTP1,ANO6,CACNG2,GPC6,APC,CRKL,SETD2,PTPRJ,EGFR,RFX3,ANGPT1,DOCK2,NEDD4,BTBD9,DNAJC13,DGKI,C12ORF4,TTC39B,NUP214,PRKD1,CHRM3,FGF12,TMEM38B,UBE2L3,GRM7,THADA,NEDD4L,ADAM10,APP,CACNA1C,CACNB2,DCLK1,TMC1,SYT1,VCL,ARHGAP44,NTF3,NDUFAF2,CD2AP,PARN,SLC8A1,ABCG8,KCNE4,AKAP6,HOMER2,RAB8B,KCNK10,RAP1GDS1,CLIC6,DNM3,SCP2,PRKCZ,GRB10,RYR3,RAB27B,CNST,HECW1,ABCA5,YAP1,MAPK1,CADPS2,KCNJ1,RABGAP1L,SYT10,ANKFY1,SYCP1,SLC16A1,SPIDR,CORO2B,TM7SF3,STON2,NF2,LRRC38,CORIN,CTNNA1,AKAP9,KLF15,RASGRF2,PPARA,KCNS3,SYNJ1,PLA2R1,DAPK1,SCN2A,DYSF,ANK2,BCAS3,RYR2,NKAIN3,USP8,TBC1D5,BLK,ABL1,SLC1A1,PRKAA1,KCNH1,EIPR1,RAP1A,NKAIN2,GPC5,FGF10,LATS2,NRG1,GSG1L,RASGRF1,SH3GL3,PRKCE,SLMAP,WNK2,EGF,ABCC9,STXBP6,ATP8A2,SCG5,PTPN2,TRIM5,HTR2C,RIC3,ARHGEF7,ALG10B,ATP8A1,OPRM1,HTR2A,CYP4A11,STAC,ABHD17C,CNIH3,APBA2,KCND2,NOS2,MDFIC,MYLK2,ANK3,LYPLA1,HIP1,PRR5L,RUFY2,PACSIN2,CNTN1,SNX3,CACNA1I,KCNJ15,WDPCP,BMP2,ATP9A,SCN11A,MSR1,VRK1,GNAI1,TBC1D4,MYRIP,RIN3,BMP2K,NETO2,RELN,HMGB1,MYOM1,UNC13B,TTC21B,PLS1,SLAMF1,KCNH8,GLI3,CNKSR3,MCTP2,MAP2,CFTR,NEU3,SLC30A10,SELENON,NMD3,PRKN,MTMR2,GRIN2A,JPH1,PRKCH,FRMD4A,KCNQ3,SHISA9,SCN10A,USP7,KCND3,MESD,RALB,ROCK1,LYN,SUMO3,CRACR2A,INSR,HECTD1,TRIM58,PLPP4,NSD2,SH3GLB1,CARD10,ENPP1,UTRN,KCNC1,GHRH,BCL2L1,HCN1,GRIN2B,ABCG1,KCNK5,MICALL2,PCNT,PTH,PRKAA2,NDC80,VSTM2A,PLA2G4A,SREBF2,ANP32B,AIMP1,NRXN1,SNAP91,TWIST1,JAK2,CELSR2,SIAH3,UFL1,NFKBIA,PRKCB,ABCC8,CACNA1E,NEDD9,ATP2B1,MTCL1,SAR1A,CIDEA,EXOC1,SLC6A1,NDFIP2,MAP2K6,KCNJ18,CEMIP,PARK7,MAPK8,OAZ2,UBE2J2,ADCYAP1R1,TM9SF4,RAPGEF4,CEP120,EFHB,MEF2C,RXRA,WNT7A,NDFIP1,SDCBP,PASK,FGR,CDCA8,C2,IL1RAPL1,NUMB,OCLN,SHISA6,MARK4,CLDN18,DIAPH1,CYFIP1,ICA1,NOS1AP,CCDC88A,NSUN2,BICD1,TNFSF11,FYN,PPM1F,SCN8A,NALCN,APELA,HECW2,CDH2,FBXL20,GPR137B,GRM5,TBC1D1,PID1,NRP1,ATPSCKMT,BCR,NRXN3,KCNJ6,B9D1,BMPER,DPP6,MACROH2A1,EPHB2,TSPAN13,CD38,CDH13,STXBP4,CACNG3,ATG5,MAGI2,VMP1,KALRN,SLC1A2,MFHAS1,ASTN2,GAPVD1,WDR41,PLIN2,ABL2,KCNIP4,ERBB4,TRDN,NLGN1,NOS1,ASIC2,EFNA5,RAB27A,KCNQ5,CACNA2D1,HTT,CAMK1D,HLA-F,FER,CCR2,OSBPL6,ROCK2,IL16,CDCA5,CATSPER2,RAB31,RGS7,CLDN10,BARD1,HNRNPU,CEP72,CADPS,KCNAB1,PRKAG2,DNM1L*
GO:0042391	regulation of membrane potential	0.000020630266425093342	*MTOR,KCNH5,PIEZO2,BCL2,KCNMA1,CHRNA7,RIMS1,GABRB3,RIMS2,CACNG2,CTNNA3,NEDD4,GRIK3,GABRB1,DGKI,GRIA1,SLC8A3,FGF12,GABRA6,TAFA4,NEDD4L,APP,ABCB5,CACNA1C,CACNB2,GABRG2,SLC8A1,KCNE4,AKAP6,KCNK10,TRPC5,PRKCZ,GRIK4,AKAP9,GRM1,GABRG1,SLC24A4,TMEM108,SCN2A,ANK2,RYR2,CXADR,ABL1,KCNH1,GRID2,SLMAP,P2RX6,OPRM1,KCND2,ANK3,CACNA1I,SCN11A,RELN,GNAQ,UNC13B,KCNH8,GABRR2,GRIK2,CFTR,PRKN,MTMR2,GRIN2A,KCNQ3,SLC4A4,SCN10A,KCND3,PPA2,GRID1,BID,GABRG3,IGSF11,KCNC1,BCL2L1,HCN1,GRIN2B,CELF4,KCNK5,NRXN1,PARK7,MEF2C,WNT7A,GABRA5,NTRK2,GRIK1,NOS1AP,SCN8A,NALCN,SLC39A8,GRM5,PID1,GNA14,GABRA2,TMEM25,TRDN,NLGN1,ASIC2,CACNA2D1*
GO:00	positive	0.00002	*BCAR3,MTOR,NSG1,WWC1,ULK2,FTO,MGA,PLCB1,ZFPM2,DLC1,TNRC6B,BCL2,PRDM16,CHRNA7,EPC2,SPIRE1,ZEB1,AKR1C3,R*

31325	regulation of cellular metabolic process	3673876814076056	ARB,ALK,AUTS2,FOXJ2,BABAM2,GLIS3,FANK1,MLLT3,DSCAM,TCF4,CRKL,ERG,TNIK,PTPRJ,NEK4,EGFR,RFX3,ANGPT1,CDK12,BACH1,NEK7,ZNF407,MAML2,BCL11A,FLI1,NTRK3,FLT1,RFC3,TASP1,THRAP3,PRKD1,PAK1,EPHA7,NCOA7,RAPGEF2,PELI2,RUNX2,TAOK3,ONECUT1,UBE2L3,LDB2,SMYD3,RPTOR,GHR,SSBP3,ATF7IP,APBB2,APP,KDM1B,ZNF600,NTF3,AURKA,PARN,ST18,PYGO1,SSBP2,ANKRD31,DUX4,ABCD2,BMPR1B,FMN2,ZNF717,ARNT,PAK3,RANBP2,LARP1,TRPC5,NBN,PRKCZ,GRB10,TAF15,PHF19,MRTFA,TAF4B,EBF2,YAP1,NFIA,MAPK1,KMT2E,PCGF5,PDGFD,NRG3,GFRA1,NIPBL,SPIDR,GABPA,CHD6,KANSL1,LIMCH1,ATF6,VPS13D,HIVEP1,MOB3B,AKAP9,KLF15,PPARA,MEIS2,SNX30,NFIB,MRTFB,NR5A2,FOXJ3,TRERF1,EIF3D,DAPK1,AGO2,JARID2,GATAD2B,IL34,BCAS3,ZNF606,SMARCA4,MAPKAP1,TNRC6C,PIAS1,ATRX,ABL1,HDAC4,SLC1A1,PRKAA1,MRPS27,APLF,NFAT5,RAP1A,GLIS1,TOX3,CAMK4,FGF10,ZC3HAV1,NRG1,INO80D,AP3B1,ZNF438,ABCB7,ZBTB16,MUSK,PRKCE,FOXK2,SLCO3A1,MED15,ESRRG,CD44,EGF,PRRC1,RPS6KA3,PTPN2,TRIM5,RFC1,HTR2C,ALG10B,AMBRA1,KDM7A,OPRM1,HTR2A,DAZL,GTF2F2,TAF3,RPRD1B,MARK2,EBF3,ZNF33B,PUM1,MSH2,EPHA6,SLC2A13,KNDC1,CLSPN,NOS2,BICRAL,MNAT1,RBBP8,MDFIC,HMGA2,CCND3,BCL11B,ECE1,CREM,TGFA,PRR5L,CNTN1,BRCA2,DISC1,BLM,BMP2,RC3H2,GFI1B,TMEM161A,ASXL3,RELN,HMGB1,FGF9,NFATC2,ZNF462,ETS2,DSTYK,UIMC1,ATF1,SMARCA2,ETS1,GLI3,CGAS,SMARCC1,SMOC2,VENTX,PRDM10,RERE,NEK10,MOB1B,ATF2,HIVEP3,PSIP1,RGMB,KITLG,CAMTA1,GTF2I,RORB,TADA2A,DAB1,MED27,ZNF208,RB1CC1,NMD3,PRKN,TBX20,DPF3,ARID5B,IL6R,ALS2,NLRC5,TFDP1,CNOT6L,TOX,SLC4A4,ZFP90,COPS8,USP7,VAV3,PLAGL1,SOX30,KIR2DL4,RALB,NPAS2,ROCK1,LYN,ZNF780B,DTX1,OVOL2,ZFHX3,BANP,SUPT16H,ARID1B,HOXC13,RNF152,BAZ1A,CASZ1,INSR,YTHDF3,HECTD1,PBX3,SUMO2,ZNF292,ASH1L,HOXC4,RXRG,SP3,ERN2,MBTPS2,ELF2,NSD2,FYCO1,SH3GLB1,CARD10,CTIF,RASGRP1,SNX9,BMP5,CSF1,INO80,FANCB,CLNS1A,SMAD5,CELF4,TCERG1,ABCG1,SLC40A1,LPGAT1,MED1,IL33,GPRC5C,ROR2,ZNF521,BANK1,CSDE1,LMX1A,IL10,ACTR2,SFPQ,RIOK1,PTH,PRKAA2,LARP6,ETV6,IQGAP1,ZBTB7C,TEAD1,SREBF2,YBX3,NRXN1,PCID2,CENPE,LMX1B,TWIST1,ALKAL2,JAK2,ZBTB38,MADD,PATL1,ZNF287,CREBBP,TNKS,PCNA,UFL1,NFKBIA,PRKCB,RFC2,ALX4,RTRAF,BRD4,NEDD9,ITGA6,ASS1,ZBTB49,STOX2,AGO1,MEOX2,ELL2,STAT1,MAP2K6,ABI1,CEMIP,IMPACT,PARK7,MED12L,POU1F1,ADCYAP1R1,MTF2,MYOCD,ARID3B,MEF2C,ADGRB1,RXRA,WNT7A,MAP3K5,MAP3K4,ZNF112,RAD51AP1,RAB38,DBF4B,FBXW8,SDCBP,WWOX,PASK,NCK1,SCAF8,FGR,CDCA8,MLLT10,IFNAR1,RNF8,EPHA4,MECOM,DNMT3L,NTRK2,CREB5,PTK2,CDH5,ASCL3,UBE3A,SEMA4D,ZBTB20,RUNX1,POMT2,ZNF845,NOS1AP,CCDC88A,CHCHD2,TNFSF11,FYN,KDM5A,HDAC2,SLF1,TBX15,SH2D3C,DOCK3,NCS1,MAPK9,PABPC1,ROR1,TET1,ARNT2,ITGA8,EPHB1,GRM5,RPS6KA5,PID1,NRP1,ITGA1,ZNF615,KLF12,RC3H1,NRIP1,POR,ZNF850,SUPT3H,TUT4,PRIM2,PRDM15,MITF,EPHB2,CD38,EYA4,MET,SPPL3,CDH13,MAGI2,PRDM11,MLIP,MYB,DHX29,BMP7,RNF217,BPTF,BTBD10,NUDT21,KMT2C,ADGRF5,PDGFC,RFX2,EYA1,CNOT7,ERBB4,GSAP,ROBO1,SAMD4A,PBX1,NPAS3,PRKCQ,MGMT,SHLD2,NOS1,PRR16,EFNA5,TCF12,ARHGEF11,RAB27A,NSD1,ESR1,PRLR,AGO3,HTT,PIK3R3,EYA2,CHFR,ZNF721,NRF1,HRH1,ROCK2,RORA,DMRT1,HERPUD1,NCOA6,STK3,ZNF423,HNRNPU,RAB3GAP2,IGF1R,PRKAG2,GLI2,THRB,AKAP13,MORC3
GO:0098655	cation transmembrane transport	0.00002449118276569826 3	UNC80,CACNA2D3,SLC17A1,SLC24A2,KCNH5,MICU2,PIEZO2,DPP10,ITPR2,PDE4D,BCL2,KCNMA1,CHRNA7,ANO6,CACNG2,SLC4A10,NEDD4,ATP2B2,TUSC3,SLC39A12,SLC8A3,PRKD1,FGF12,TMEM38B,SLC24A3,THADA,NEDD4L,TRPM1,SLC39A11,APP,SLC7A2,CACNA1C,CACNB2,TMC1,SLC8A1,KCNE4,AKAP6,KCNK10,TRPC5,RYR3,HECW1,KCNJ1,TRPC7,NIPAL2,MICU1,LRRC38,AKAP9,RASGRF2,KCNS3,DAPK1,SLC24A4,SCN2A,ANK2,RYR2,SLC9C1,SLC36A1,ABL1,SLC1A1,SLC12A8,KCNH1,GSG1L,RASGRF1,ABCB7,PRKCE,SLMAP,WNK2,ABCC9,P2RX6,HTR2C,ALG10B,AT

			P8A1,OPRM1,HTR2A,CNNM4,STAC,CNIH3,MAIP1,KCND2,AFG3L2,ANK3,NIPA2,TMEM163,ATP6V1E1,CACNA1I,KCNJ15,SCN11A,NETO2,RELN,SLC39A6,KCNH8,SLC9A4,CNKSR3,SLC30A10,SELENON,GRIN2A,JPH1,TRPM6,SLC12A1,KCNQ3,SHISA9,SLC4A4,SCN10A,KCND3,KCNN3,LYN,SLC15A2,CRACR2A,CUL5,COX5A,ZDHHC17,UTRN,KCNC1,HCN1,GRIN2B,KCNK5,SLC40A1,COX7A2L,NRXN1,TWIST1,TRPV5,ABCC8,CACNA1E,ATP2B1,OTOP1,SLC6A1,SHROOM2,SLC6A11,KCNJ18,CEMIP,CBLIF,PARK7,ADCYAP1R1,ATP13A3,MEF2C,ATP6V1C2,ATP6V1B2,SHISA6,TRPM7,DIAPH1,SCARA5,NOS1AP,SLC9A5,SLC5A1,ANO10,FYN,SCN8A,TMEM63C,NCS1,ATP5PF,NALCN,TRPM3,SLC39A8,SLC16A9,HECW2,ATPSCKMT,KCNJ6,DPP6,EPHB2,TSPAN13,ATP6V0D2,CACNG3,ATG5,VMP1,SLC1A2,KCNIP4,TRDN,NLGN1,NOS1,SLC6A3,ASIC2,KCNQ5,CACNA2D1,HTT,SLC25A18,CCR2,CATSPER2,RGS7,KCNAB1
GO:0043085	positive regulation of catalytic activity	0.00002466489453047691	*BCAR3,MTOR,GARNL3,MYO9A,DLC1,BCL2,CHRNA7,ALK,EGLN3,CRKL,ARHGAP24,DOCK10,EGFR,DENND1A,ANGPT1,NEK7,NTRK3,RXFP1,FLT1,RFC3,PRKD1,PAK1,EPHA7,RALGPS1,RAPGEF2,TAOK3,UBE2L3,RPTOR,GHR,RALGAPA1,RAPGEF5,APP,CACNA1C,DOCK8,MAPRE2,NTF3,ACER2,PARN,ST18,MAP4K4,BMPR1B,RANBP2,RAP1GDS1,NBN,IFT57,PRKCZ,MSH6,MAPK1,RABGAP1L,PDGFD,NRG3,TBC1D22A,CHN1,MOB3B,RASGRF2,RGL1,TIAM1,ARAP2,DAPK1,TBC1D9,IL34,BCAS3,WNT9B,CLPX,TBC1D5,ABL1,SLC1A1,RAP1A,NRG1,RASGRF1,MUSK,ASAP2,CD44,EGF,PRRC1,ARHGEF7,AMBRA1,HTR2A,MARK2,MSH2,EPHA6,CLSPN,NOS2,MNAT1,HMGA2,CCND3,MBP,TGFA,HIP1,VAV1,IQSEC1,BMP2,EVI5,RALGAPA2,SGSM1,TBC1D4,RELN,ARHGAP42,HMGB1,GNAQ,DSTYK,RAP1GAP,SRGAP2,NEK10,MOB1B,TBC1D13,KITLG,DAB1,NGEF,GRIN2A,IL6R,ALS2,DOCK9,COPS8,VAV3,RALB,ROCK1,LYN,INSR,XRCC4,BID,ERN2,CARD10,RALGPS2,RASGRP1,SNX9,CSF1,GRIN2B,DCUN1D4,PSAP,CDC14B,GPRC5C,ROR2,RASGEF1C,PTH,IQGAP1,ANP32B,NRXN1,CENPE,NET1,SIPA1L2,ALKAL2,JAK2,MADD,TNKS,PCNA,ZC3H15,RFC2,NEDD9,ITGA6,PDP2,MAP2K6,ABI1,CEMIP,PARK7,MAPK8,ADCYAP1R1,RAPGEF4,CYFIP2,MEF2C,MAP3K5,MAP3K4,DBF4B,FGR,EPHA4,NTRK2,PTK2,SEMA4D,RASGEF1B,ASAP1,NOS1AP,CCDC88A,GPR55,TNFSF11,FYN,PPM1F,DOCK3,NCS1,ROR1,EPHB1,GRM5,TBC1D1,ATPSCKMT,ITGA1,POR,BCR,RGS6,PRIM2,FBLN1,EPHB2,BCL2L13,MET,MAGI2,KALRN,GNAS,TIAM2,PDGFC,WDR41,ABL2,ERBB4,ROBO1,PRKCQ,ANTXR1,SIPA1L3,NOS1,EFNA5,ESR1,PRLR,ROCK2,RGS8,COL4A3,RGS7,STK3,RSU1,HNRNPU,IGF1R,PRKAG2,AKAP13*
GO:0000165	MAPK cascade	0.000026058251205588595	*NOTCH2,BCAR3,WWC1,NLK,KSR1,PLCB1,CHRNA7,SPRED1,ALK,PJA2,MAP3K9,CRKL,TNIK,PTPRJ,EGFR,ANGPT1,NCOR1,NTRK3,FLT1,MAPKBP1,EDAR,PAK1,EPHA7,RAPGEF2,PELI2,FGF12,TAOK3,GHR,APP,NTF3,PTPRR,MAP4K4,PAK3,ITPKB,RAP1GDS1,PRKCZ,GRB10,DUSP22,PPM1L,MAPK1,PDGFD,STK38,HRH4,PAFAH1B1,NF2,GRM1,PAK5,IL34,DUSP16,ABL1,RAP1A,FGF10,NRG1,DENND2B,ZNF675,PRKCE,WNK2,CD44,EGF,SPRED2,PTPN2,TRIM5,HTR2C,OPRM1,HTR2A,RELL1,HIPK3,MDFIC,MBP,PLCE1,TGFA,NRK,SEMA3A,MAGI3,BMP2,RANBP9,HMGB1,TRAF3,DSTYK,SLAMF1,CNKSR3,GAREM1,NEK10,ATF2,CYLD,MAPK8IP1,KITLG,MAP4K3,SLC30A10,RB1CC1,PRKN,MAPK10,IL6R,ROCK1,LYN,CRACR2A,INSR,ASH1L,ERN2,ZDHHC17,JCAD,RASGRP1,NDRG2,ROR2,KL,BANK1,IQGAP1,NRXN1,ALKAL2,JAK2,MADD,HCRTR1,MAP2K6,MAPK8,MEF2C,WNT7A,MAP3K5,MAP3K4,SDCBP,EPHA4,MECOM,NTRK2,ANKRD6,APIP,NENF,GPR55,TNFSF11,SH2D3C,DOK5,MAPK9,APELA,CDH2,PHLPP1,EPHB1,GRM5,NRP1,PRKCA,ITGA1,FBLN1,BMPER,PRDM15,EPHB2,PRDM11,MFHAS1,BMP7,ZMYND11,PDGFC,ERBB4,ROBO1,ROCK2,PPP1CB,STK3,IGF1R,AKAP13*
GO:0051963	regulation of synapse assembly	0.000026393394584697964	*PTPRD,IL1RAPL2,ROBO2,NEGR1,GPC6,NTRK3,EPHA7,ADGRB3,APP,STAU2,LRFN5,GRID2,MUSK,LINGO2,PDLIM5,FARP1,NTN1,COLQ,CLSTN2,NRXN1,MEF2C,ADGRB1,WNT7A,NECTIN1,NTRK2,IL1RAPL1,SEMA4D,ADGRL2,EPHB1,EPHB2,FLRT2,DLG5,SYNDIG1,NLGN1,ASIC2,EFNA5*

GO:0048588	developmental cell growth	0.0000291018368950213	*ULK2,RIMS1,RIMS2,AUTS2,DSCAM,MACF1,BCL11A,CDH4,EPHA7,NEDD4L,APP,DCLK1,SEMA5A,SYT1,VCL,AURKA,AKAP6,DIP2B,TRPC5,PRKCZ,COBL,ALCAM,PAFAH1B1,PPARA,SEMA3C,TMEM108,SEMA6D,TNR,ABL1,EXT1,SEMA3E,PDLIM5,DISC1,SEMA3A,SEMA3D,SLC23A2,NIN,DRAXIN,MAP2,DCC,PRKN,ITSN2,NTN1,CTDP1,TNN,IQGAP1,IMPACT,ITGA4,CYFIP2,CYFIP1,SEMA4D,SORBS2,SPAG6,NRP1,SLIT2,EFNA5,SLIT3,SEMA4B,FSTL4,AKAP13*
GO:1905114	cell surface receptor signaling pathway involved in cell-cell signaling	0.0000313289440822076 7	*NLK,CHRNA7,RIMS1,RIMS2,MLLT3,GPC6,APC,TNIK,EGFR,MACF1,RNF220,DKK2,DGKI,INVS,SLC8A3,PRICKLE2,APP,SEMA5A,PYGO1,KANK1,PRKCZ,GRB10,HECW1,YAP1,ZNRF3,KLF15,TIAM1,PCDH11Y,TMEM108,ALPK2,CPE,WNT9B,SMARCA4,USP8,ABL1,PRKAA1,GPC5,FGF10,GRID2,LATS2,ASPM,NXN,WNK2,PTPRO,EGF,P2RX6,EXT1,LIMD1,CTNND2,OPRM1,KREMEN1,MARK2,MDFIC,NPHP4,SNX3,DISC1,STRN,BMP2,RELN,GNAQ,FGF9,UNC13B,TTC21B,DRAXIN,GLI3,GRIK2,CYLD,KPNA1,PRKN,MTMR2,GRIN2A,MESD,SOX30,ZBTB33,SNAI2,SIAH2,TRABD2B,IGSF11,NDRG2,CSNK2A1,GRIN2B,CELF4,TNN,ROR2,SOSTDC1,PRKAA2,RPS12,NRXN1,CELSR2,TNKS,GID8,MEF2C,WNT7A,ATP6V1C2,WWOX,PPP2R3A,WNT2B,SHISA6,ANKRD6,WNT5B,AMFR,CCDC88A,RBMS3,ROR1,CDH2,RNF138,MCC,PRDM15,MITF,CSNK1G1,CDK14,MAGI2,TMEM25,NLGN1,STK3,ZNF423,APCDD1,GLI2*
GO:0051960	regulation of nervous system development	0.0000330214236117588 85	*MTOR,PTPRD,ULK2,TENM4,ROBO2,DSCAM,MACF1,BCL11A,CDH4,NTRK3,EPHA7,SPEN,RAPGEF2,LRP2,ADGRB3,STAU2,SEMA5A,FIG4,PAK3,DIP2B,TRPC5,YAP1,BRINP1,PAFAH1B1,NF2,CTNNA1,PRTG,SYNJ1,TIAM1,SEMA3C,IL34,SEMA6D,PARD3,TNR,GRID2,ASPM,PLXNA2,OPRM1,SEMA3E,LINGO2,MBP,DISC1,SEMA3A,BMP2,SEMA3D,RELN,NIN,DRAXIN,GLI3,MAP2,DCC,DAB1,MTMR2,PRKCH,TG,LYN,NTN1,DPYSL5,RXRG,IL33,ACTR2,CLSTN2,MAP6,NRXN1,UFL1,ABCC8,ADGRB1,WNT7A,WASF3,FBXW8,EPHA4,NTRK2,IL1RAPL1,NUMB,FBXO31,CYFIP1,SEMA4D,JAM2,ADGRL2,HDAC2,EPHB1,GRM5,NRP1,FAIM,CHODL,CUX1,EPHB2,FLRT2,KALRN,TIAM2,BMP7,DLG5,SLIT2,SYNDIG1,ROBO1,NLGN1,ASIC2,EFNA5,SEMA4B,HOOK3,FSTL4*
GO:0001654	eye development	0.0000332194120137957 6	*NOTCH2,BCAR3,SMOC1,SCAPER,RP1,BCL2,ALDH1A2,FBN1,TENM3,ZEB1,RARB,SPRED1,DSCAM,EGFR,CRB1,ATP2B2,FLT1,ABCB5,CACNA1C,DCLK1,STAU2,BMPR1B,NIPBL,FAT3,ATF6,EFEMP1,NF2,MEIS2,WNT9B,SMARCA4,SLC1A1,TTLL5,FGF10,FBN2,SPRED2,NHS,ATP8A2,BCL11B,NPHP4,WDPCP,PDE6C,FGF9,TDRD7,CPAMD8,MDM1,GLI3,MEGF11,BBS4,LAMC3,COL5A1,RORB,PDE6A,PBX3,SP3,DZANK1,HCN1,CELF4,VSTM4,MED1,FAT1,HIPK1,TWIST1,VSX1,USH1C,ATP2B1,SHROOM2,ADAMTS18,WNT7A,NECTIN1,PPP2R3A,NTRK2,WNT2B,WNT5B,UNC45B,HDAC2,XRN2,EPHB1,RP1L1,NRP1,SDK1,B9D1,MITF,EPHB2,LAMA1,BMP7,PBX1,MYH15,SIPA1L3,SLC6A3,RPGRIP1,PRDM1,THRB*
GO:0000226	microtubule cytoskeleton organization	0.0000396446285197589 66	*LRRC49,RIPOR2,RP1,ODAD2,SPIRE1,CNTLN,SDCCAG8,SPAG16,CEP192,MAP4,APC,SETD2,NEK7,NCOR1,ARMC2,SLC39A12,PAK1,DEUP1,LRGUK,KIF4A,TBCD,DCLK1,MAPRE2,AURKA,SRGAP2C,CCSER2,FMN2,TTLL7,PRKCZ,MCPH1,SENP6,SLC16A1,PAFAH1B1,AKAP9,PARD3B,NAV3,STAG2,BCAS3,SYNE2,BBS2,PARD3,DST,ATRX,ABL1,PRKAA1,TTLL5,DNAH5,FGF10,CLIP1,ASPM,USP33,PEX14,ATXN3,ARHGEF7,MARK2,TMEM67,C10ORF90,ANKFN1,BRCA2,DISC1,GNAI1,RANBP9,DNAL1,TUBGCP3,RTTN,MDM1,SRGAP2,NIN,HAUS6,DNAH8,KIF15,MAP2,DAW1,GOLGA8B,CYLD,BBS4,KIAA0753,CEP44,RACGAP1,MAP7,CFAP74,ROCK1,KIF11,NEK6,PDE4DIP,HDGFL3,INO80,CDC14B,PCNT,PRKAA2,SKA1,NDC80,MAP6,TACC2,KIFC1,CENPE,TUBB6,TNKS,SGO1,MTCL1,EML1,CEP120,DRC7,CDCA8,INTS13,RSPH1,OCLN,MARK4,CDH5,HOATZ,SAXO1,CCDC88A,SPAG6,BICD1,HYDIN,RP1L1,STK36,TOGARAM1,MET,DNAH17,TTLL11,NUF2,TRDN,EFNA5,GAS2L1,HTT,CFAP44,FER,ROCK2,ATAT1,HOOK3,HNRNPU,CEP72*
GO:0150063	visual system development	0.0000552675000193349 5	*NOTCH2,BCAR3,SMOC1,SCAPER,RP1,BCL2,ALDH1A2,FBN1,TENM3,ZEB1,RARB,SPRED1,DSCAM,EGFR,CRB1,ATP2B2,FLT1,ABCB5,CACNA1C,DCLK1,STAU2,BMPR1B,NIPBL,FAT3,ATF6,EFEMP1,NF2,MEIS2,WNT9B,SMARCA4,SLC1A1,TTLL5,FGF10,FBN2,SPRED2,NHS,ATP8A2,BCL11B,NPHP4,WDPCP,PDE6C,FGF9,TDRD*

			7,CPAMD8,MDM1,GLI3,MEGF11,BBS4,LAMC3,COL5A1,RORB,PDE6A,PBX3,SP3,DZANK1,HCN1,CELF4,VSTM4,MED1,FAT1,HIPK1,TWIST1,VSX1,USH1C,ATP2B1,SHROOM2,ADAMTS18,WNT7A,NECTIN1,PPP2R3A,NTRK2,WNT2B,WNT5B,UNC45B,HDAC2,XRN2,EPHB1,RP1L1,NRP1,SDK1,B9D1,MITF,EPHB2,LAMA1,BMP7,PBX1,MYH15,SIPA1L3,SLC6A3,RPGRIP1,PRDM1,THRB
GO:0048880	sensory system development	0.00005686595255634882	*NOTCH2,BCAR3,SMOC1,SCAPER,RP1,BCL2,ALDH1A2,FBN1,TENM3,ZEB1,RARB,SPRED1,DSCAM,EGFR,CRB1,ATP2B2,FLT1,ABCB5,CACNA1C,DCLK1,STAU2,BMPR1B,NIPBL,FAT3,ATF6,EFEMP1,NF2,MEIS2,WNT9B,SMARCA4,SLC1A1,TTLL5,FGF10,FBN2,SPRED2,NHS,ATP8A2,BCL11B,NPHP4,WDPCP,SEMA3A,PDE6C,FGF9,TDRD7,CPAMD8,MDM1,GLI3,MEGF11,BBS4,LAMC3,COL5A1,RORB,PDE6A,PBX3,SP3,DZANK1,HCN1,CELF4,VSTM4,MED1,FAT1,HIPK1,TWIST1,VSX1,USH1C,ATP2B1,SHROOM2,ADAMTS18,WNT7A,NECTIN1,PPP2R3A,NTRK2,WNT2B,WNT5B,UNC45B,HDAC2,XRN2,EPHB1,RP1L1,NRP1,SDK1,B9D1,MITF,EPHB2,LAMA1,BMP7,PBX1,MYH15,SIPA1L3,SLC6A3,RPGRIP1,PRDM1,THRB*
GO:0010604	positive regulation of macromolecule metabolic process	0.000060875283851310745	*BCAR3,MTOR,NSG1,WWC1,PVT1,FTO,MGA,PLCB1,ZFPM2,DLC1,TNRC6B,PDE4D,RDX,BCL2,PRDM16,ALDH1A2,CHRNA7,RIMS1,EPC2,SPIRE1,ZEB1,RARB,ALK,AUTS2,FOXJ2,BABAM2,GLIS3,FANK1,MLLT3,EGLN3,SPON1,APC,HHLA2,TCF4,CRKL,SETD2,ERG,TNIK,PTPRJ,NEK4,EGFR,RFX3,ANGPT1,CDK12,BACH1,NEK7,NCOR1,ZNF407,NEDD4,MAML2,BCL11A,FLI1,NTRK3,C5,FLT1,RFC3,TASP1,THRAP3,EDAR,PRKD1,PAK1,EPHA7,NCOA7,RAPGEF2,PELI2,LRP2,RUNX2,TAOK3,ONECUT1,SLC24A3,UBE2L3,LDB2,SMYD3,RPTOR,GHR,SSBP3,NEDD4L,ATF7IP,IL1R1,APBB2,APP,RPS6KA2,KDM1B,ZNF600,NTF3,ACER2,AURKA,PARN,ST18,PYGO1,SSBP2,ANKRD31,DUX4,PLGRKT,BMPR1B,FMN2,ZNF717,ARNT,PAK3,RFTN1,DIP2B,LARP1,TRPC5,NBN,IFT57,PRKCZ,TAF15,DIP2A,HECW1,PHF19,MRTFA,TAF4B,EBF2,YAP1,NFIA,MAPK1,KMT2E,PCGF5,PDGFD,NRG3,GFRA1,NIPBL,SPIDR,GABPA,CHD6,KANSL1,LIMCH1,ATF6,ITGB8,HIVEP1,MOB3B,AKAP9,KLF15,PPARA,MEIS2,NFIB,MRTFB,NR5A2,FOXJ3,TRERF1,PLA2R1,EIF3D,DAPK1,SLC24A4,AGO2,JARID2,GATAD2B,IL34,ANK2,BCAS3,ZNF606,CLPX,SMARCA4,MAPKAP1,TNRC6C,PIAS1,ATRX,ELAVL4,ABL1,HDAC4,SLC1A1,PRKAA1,MRPS27,DROSHA,APLF,NFAT5,NBAS,RAP1A,GLIS1,TOX3,CAMK4,FGF10,ZC3HAV1,NRG1,INO80D,AP3B1,ZNF438,ZBTB16,MUSK,FOXK2,SLCO3A1,MED15,ESRRG,CD44,EGF,PRRC1,RPS6KA3,TRIM5,ATXN3,RFC1,ALG10B,AMBRA1,KDM7A,HTR2A,FANCM,DAZL,GTF2F2,TAF3,RPRD1B,MARK2,EBF3,ZNF33B,PUM1,MSH2,IGF2BP3,SLC2A13,KNDC1,SPSB4,CLSPN,NOS2,BICRAL,MNAT1,RBBP8,MDFIC,MYLK2,ANK3,HMGA2,CCND3,BCL11B,ECE1,CREM,MBP,TGFA,IL17RA,HIP1,PRR5L,CNTN1,BRCA2,DISC1,BLM,BMP2,RC3H2,PSG9,GFI1B,RANBP9,TMEM161A,ASXL3,POLR3A,RELN,HMGB1,FGF9,NFATC2,MYOM1,ZNF462,TTC21B,ETS2,UIMC1,ATF1,SLAMF1,SMARCA2,ETS1,GLI3,CGAS,SMARCC1,SMOC2,VENTX,IDE,PRDM10,RERE,NEK10,MOB1B,ATF2,HIVEP3,PSIP1,RGMB,KITLG,CAMTA1,GTF2I,RORB,TADA2A,DAB1,MED27,ZNF208,RB1CC1,NMD3,PRKN,TBX20,DPF3,GRIN2A,ARID5B,PKP1,IL6R,ALS2,NLRC5,TFDP1,CNOT6L,TOX,ZFP90,COPS8,USP7,PLAGL1,SOX30,KIR2DL4,RALB,NPAS2,ROCK1,LYN,ZNF780B,DTX1,OVOL2,ZFHX3,BANP,SUPT16H,ARID1B,HOXC13,BAZ1A,CASZ1,INSR,YTHDF3,HECTD1,PBX3,SUMO2,ZNF292,SNAI2,ASH1L,HOXC4,BID,TRABD2B,RXRG,SP3,ERN2,MBTPS2,ELF2,NSD2,CARD10,CTIF,RASGRP1,SNX9,CSNK2A1,BMP5,CSF1,GRIN2B,INO80,FANCB,CLNS1A,SMAD5,CELF4,TCERG1,ABCG1,DCUN1D4,SLC40A1,MED1,CDC14B,KDM6A,IL33,GPRC5C,ROR2,ZNF521,BANK1,CSDE1,LMX1A,IL10,ACTR2,SFPQ,RIOK1,PTH,PRKAA2,LARP6,VSTM2A,ETV6,IQGAP1,ZBTB7C,TEAD1,SREBF2,ANP32B,YBX3,NRXN1,PCID2,CADM1,CENPE,LMX1B,TWIST1,ALKAL2,JAK2,ZBTB38,MADD,PATL1,ZNF287,CREBBP,MELTF,TNKS,PCNA,NFKBIA,PRKCB,ABCC8,RFC2,ALX4,RTRAF,BRD4,NEDD9,ITGA6,ZBTB49,STOX2,AGO1,MEOX2,ELL2,STAT1,NDFIP2,MAP2K6,MTPN,ABI1,CEMIP,IMPACT,CCBE1,PARK7,MAPK8,OAZ2,MED12L,POU1F1,MTF2,MYOCD,CYFIP2,ARID3B,MEF2C,RXRA,WNT7A,MAP3K5,NDFIP1,MA*

			P3K4,ZNF112,RAD51AP1,PDE2A,DBF4B,FBXW8,SDCBP,WWOX,PASK,NCK1,SCAF8,FGR,CDCA8,PPP2R3A,MLLT10,RNF8,EPHA4,MECOM,DNMT3L,NTRK2,OCLN,CREB5,PRKAB1,PTK2,CDH5,ASCL3,UBE3A,SEMA4D,ZBTB20,RUNX1,POMT2,PTGFR,ZNF845,NOS1AP,PDCL3,CCDC88A,CHCHD2,TNFSF11,FYN,KDM5A,PPM1F,RBMS3,HDAC2,SLF1,TBX15,SH2D3C,DOCK3,MAPK9,PABPC1,CRTAM,TET1,ARNT2,HECW2,ITGA8,GRM5,RPS6KA5,PID1,NRP1,ITGA1,ZNF615,KLF12,RC3H1,NRIP1,ZNF850,SUPT3H,TUT4,PRIM2,FBLN1,PRDM15,MACROH2A1,MITF,EPHB2,BCL2L13,CD38,EYA4,MET,SPPL3,CDH13,MAGI2,PRDM11,MLIP,MYB,SERPINB7,DHX29,BMP7,RNF217,BPTF,NUDT21,KMT2C,PDGFC,RFX2,EYA1,CNOT7,ERBB4,IL20RB,GSAP,ROBO1,SAMD4A,PBX1,NPAS3,PRKCQ,ANTXR1,MGMT,SHLD2,NOS1,PRR16,EFNA5,TCF12,ARHGEF11,RAB27A,NSD1,ESR1,PRLR,AGO3,PIK3R3,HLA-F,EYA2,CCR2,CHFR,ZNF721,NRF1,ROCK2,PRDM1,RORA,STMP1,IL16,DMRT1,HERPUD1,NCOA6,COL4A3,BARD1,STK3,ZNF423,HNRNPU,RAB3GAP2,IGF1R,PRKAG2,GLI2,THRB,AKAP13
GO:0060560	developmental growth involved in morphogenesis	0.000063594238824150007	*ULK2,RIMS1,RIMS2,AUTS2,DSCAM,MACF1,BCL11A,CDH4,EPHA7,NEDD4L,APP,DCLK1,SEMA5A,SYT1,VCL,AURKA,DIP2B,TRPC5,PRKCZ,COBL,YAP1,ALCAM,FMN1,PAFAH1B1,SEMA3C,TMEM108,SEMA6D,TNR,ABL1,FGF10,EXT1,SEMA3E,DISC1,SEMA3A,SEMA3D,SLC23A2,NIN,DRAXIN,MAP2,DCC,PRKN,ITSN2,NTN1,CSF1,TNN,MED1,IQGAP1,IMPACT,ITGA4,CYFIP2,PPP2R3A,CYFIP1,SEMA4D,SPAG6,NRP1,MAGI2,SLIT2,EFNA5,SLIT3,ESR1,SEMA4B,FSTL4*
GO:0044089	positive regulation of cellular component biogenesis	0.000080541208522715 29	*MTOR,PTPRD,RIPOR2,RP1,RALA,CDC42EP3,AUTS2,CARMIL1,CNTNAP2,APC,PLPPR5,PTPRJ,NEK7,CRACD,PAK1,ADGRB3,LDB2,SEPTIN9,ATF7IP,STAU2,MAP4K4,DNM3,COBL,SPIDR,LIMCH1,FMN1,TPM1,NF2,AKAP9,SNX30,NAV3,BCAS3,ABL1,HDAC4,GRID2,NRG1,CLIP1,SETDB2,PRKCE,AMBRA1,LINGO2,PLCE1,NPHP4,RESF1,UNC13B,BBS4,CNOT6L,RALB,ROCK1,TENM2,BMF,PDE4DIP,BID,TRABD2B,SH3GLB1,SNX9,ACTR2,CLSTN2,VASP,MORC2,NRXN1,ANLN,PARK7,CEP120,ADGRB1,WNT7A,SDCBP,NCK1,NTRK2,IL1RAPL1,OCLN,MARK4,CDH5,MPP7,CYFIP1,SEMA4D,KIRREL1,SAXO1,ASAP1,CCDC88A,PPM1F,ADGRL2,SLF1,MAPK9,EPHB1,NRP1,FCHSD2,PRKCA,EPHB2,TOGARAM1,MET,CDH17,FLRT2,BMP7,DLG5,SYNDIG1,NLGN1,ASIC2,EFNA5,ESR1,HTT,FER,EPS8,ROCK2,STMP1,ATAT1,WASHC1,RAB3GAP2*
GO:0106027	neuron projection organization	0.000081828513428484 19	*CHRNA7,DOCK10,APP,STAU2,ARHGAP44,ABCD2,PAK3,DNM3,DIP2A,PAFAH1B1,TANC1,CTNND2,PDLIM5,RELN,PLS1,MTMR2,NGEF,INSR,TANC2,GRIN2B,ACTR2,NEDD9,WNT7A,EPHA4,UBE3A,FYN,EPHB1,EPHB2,PPFIA2,KALRN,NLGN1,IGF1R*
GO:0007215	glutamate receptor signaling pathway	0.000096245000172861 38	*PLCB1,GRIK3,GRIA1,CPEB4,GRM7,TRPM1,APP,HOMER2,GRIK4,GRM1,GRM8,SLC1A1,GRID2,GNAQ,GRIK2,GRIN2A,GRID1,GRIN2B,GRIK1,FYN,GRM5,GRM3,GRIA4*
GO:0009893	positive regulation of metabolic process	0.000101615731403975 34	*BCAR3,MTOR,NSG1,WWC1,PVT1,ULK2,FTO,MGA,PLCB1,ZFPM2,DLC1,TNRC6B,PDE4D,RDX,BCL2,PRDM16,ALDH1A2,CHRNA7,RIMS1,EPC2,SPIRE1,ZEB1,AKR1C3,RARB,ALK,AUTS2,FOXJ2,BABAM2,GLIS3,FANK1,MLLT3,EGLN3,SPON1,APC,HHLA2,DSCAM,TCF4,CRKL,SETD2,ERG,TNIK,PTPRJ,NEK4,EGFR,RFX3,ANGPT1,CDK12,BACH1,NEK7,NCOR1,ZNF407,NEDD4,MAML2,BCL11A,FLI1,NTRK3,C5,FLT1,RFC3,TASP1,THRAP3,EDAR,PRKD1,PAK1,EPHA7,NCOA7,RAPGEF2,PELI2,LRP2,RUNX2,TAOK3,ONECUT1,SLC24A3,UBE2L3,LDB2,SMYD3,RPTOR,GHR,SSBP3,NEDD4L,ATF7IP,IL1R1,APBB2,APP,RPS6KA2,KDM1B,ZNF600,NTF3,ACER2,AURKA,PARN,ST18,PYGO1,SSBP2,ANKRD31,DUX4,PLGRKT,ABCD2,BMPR1B,FMN2,ZNF717,ARNT,PAK3,RFTN1,DIP2B,RANBP2,LARP1,TRPC5,NBN,SCP2,IFT57,PRKCZ,GRB10,TAF15,DIP2A,HECW1,PHF19,MRTFA,TAF4B,EBF2,YAP1,NFIA,MAPK1,KMT2E,PCGF5,PDGFD,NRG3,GFRA1,NIPBL,SPIDR,GABPA,CHD6,KANSL1,LIMCH1,ATF6,ITGB8,VPS13D,HIVEP1,MOB3B,AKAP9,KLF15,PPARA,MEIS2,SNX30,NFIB,MRTFB,NR5A2,FOXJ3,TRERF1,PLA2R1,EIF3D,DAPK1,SLC24A4,AGO2,JARID2,GATAD2B,IL*

			34,ANK2,BCAS3,ZNF606,CLPX,SMARCA4,MAPKAP1,TNRC6C,PIAS1,ATRX,ELAVL4,ABL1,HDAC4,SLC1A1,PRKAA1,MRPS27,DROSHA,APLF,NFAT5,NBAS,RAP1A,GLIS1,TOX3,CAMK4,FGF10,ZC3HAV1,NRG1,INO80D,AP3B1,ZNF438,ABCB7,ZBTB16,MUSK,PRKCE,FOXK2,SLCO3A1,MED15,ESRRG,CD44,EGF,PRRC1,RPS6KA3,PTPN2,TRIM5,ATXN3,RFC1,HTR2C,ALG10B,AMBRA1,KDM7A,OPRM1,HTR2A,FANCM,DAZL,GTF2F2,TAF3,RPRD1B,MARK2,EBF3,ALPL,ZNF33B,PUM1,MSH2,IGF2BP3,EPHA6,SLC2A13,KNDC1,SPSB4,CLSPN,NOS2,BICRAL,MNAT1,RBBP8,MDFIC,MYLK2,ANK3,HMGA2,CCND3,BCL11B,ECE1,CREM,MBP,TGFA,IL17RA,HIP1,PRR5L,CNTN1,BRCA2,DISC1,BLM,BMP2,RC3H2,PSG9,GFI1B,RANBP9,TMEM161A,ASXL3,POLR3A,RELN,HMGB1,FGF9,NFATC2,MYOM1,ZNF462,TTC21B,ETS2,DSTYK,UIMC1,ATF1,SLAMF1,SMARCA2,ETS1,GLI3,CGAS,SMARCC1,SMOC2,VENTX,IDE,PRDM10,RERE,NEK10,MOB1B,ATF2,HIVEP3,PSIP1,RGMB,KITLG,CAMTA1,GTF2I,RORB,TADA2A,DAB1,MED27,ZNF208,RB1CC1,NMD3,PRKN,TBX20,DPF3,GRIN2A,ARID5B,PKP1,IL6R,ALS2,NLRC5,TFDP1,CNOT6L,TOX,SLC4A4,ZFP90,COPS8,USP7,VAV3,PLAGL1,SOX30,KIR2DL4,RALB,NPAS2,ROCK1,LYN,ZNF780B,DTX1,OVOL2,ZFHX3,BANP,SUPT16H,ARID1B,HOXC13,RNF152,BAZ1A,CASZ1,INSR,YTHDF3,HECTD1,PBX3,SUMO2,ZNF292,SNAI2,ASH1L,HOXC4,BID,TRABD2B,RXRG,SP3,ERN2,MBTPS2,ELF2,NSD2,FYCO1,SH3GLB1,CARD10,CTIF,RASGRP1,SNX9,CSNK2A1,BMP5,CSF1,GRIN2B,INO80,FANCB,CLNS1A,SMAD5,CELF4,TCERG1,ABCG1,DCUN1D4,SLC40A1,LPGAT1,MED1,CDC14B,KDM6A,IL33,GPRC5C,ROR2,ZNF521,BANK1,CSDE1,LMX1A,IL10,ACTR2,SFPQ,RIOK1,PTH,PRKAA2,LARP6,VSTM2A,ETV6,IQGAP1,ZBTB7C,TEAD1,SREBF2,ANP32B,YBX3,NRXN1,PCID2,CADM1,CENPE,LMX1B,TWIST1,ALKAL2,JAK2,ZBTB38,MADD,PATL1,ZNF287,FH,CREBBP,MELTF,TNKS,PCNA,UFL1,NFKBIA,PRKCB,ABCC8,RFC2,ALX4,RTRAF,BRD4,NEDD9,ITGA6,ASS1,ZBTB49,STOX2,AGO1,MEOX2,ELL2,STAT1,NDFIP2,MAP2K6,MTPN,ABI1,CEMIP,IMPACT,CCBE1,PARK7,MAPK8,OAZ2,MED12L,POU1F1,ADCYAP1R1,MTF2,MYOCD,CYFIP2,ARID3B,MEF2C,ADGRB1,RXRA,WNT7A,MAP3K5,NDFIP1,MAP3K4,ZNF112,RAD51AP1,PDE2A,RAB38,DBF4B,FBXW8,SDCBP,WWOX,PASK,NCK1,SCAF8,FGR,CDCA8,PPP2R3A,MLLT10,IFNAR1,RNF8,EPHA4,MECOM,DNMT3L,NTRK2,OCLN,CREB5,PRKAB1,PTK2,CDH5,ASCL3,UBE3A,SEMA4D,ZBTB20,RUNX1,POMT2,PTGFR,ZNF845,NOS1AP,PDCL3,CCDC88A,CHCHD2,TNFSF11,FYN,KDM5A,PPM1F,RBMS3,HDAC2,SLF1,TBX15,SH2D3C,DOCK3,NCS1,MAPK9,PABPC1,CRTAM,ROR1,TET1,ARNT2,HECW2,ITGA8,EPHB1,GRM5,RPS6KA5,PID1,NRP1,ITGA1,ZNF615,KLF12,RC3H1,NRIP1,POR,ZNF850,SUPT3H,TUT4,PRIM2,FBLN1,PRDM15,MACROH2A1,MITF,EPHB2,BCL2L13,CD38,EYA4,MET,SPPL3,CDH13,MAGI2,PRDM11,MLIP,MYB,GNAS,SERPINB7,DHX29,BMP7,RNF217,BPTF,BTBD10,NUDT21,KMT2C,ADGRF5,PDGFC,RFX2,EYA1,CNOT7,ERBB4,IL20RB,GSAP,ROBO1,SAMD4A,PBX1,NPAS3,PRKCQ,ANTXR1,MGMT,SHLD2,NOS1,PRR16,EFNA5,TCF12,ARHGEF11,RAB27A,NSD1,EHMT1,ESR1,PRLR,AGO3,HTT,CAMK1D,PIK3R3,HLA-F,EYA2,CCR2,CHFR,ZNF721,NRF1,HRH1,ROCK2,PRDM1,RORA,STMP1,IL16,DMRT1,HERPUD1,NCOA6,COL4A3,BARD1,STK3,ZNF423,HNRNPU,RAB3GAP2,IGF1R,PRKAG2,GLI2,THRB,AKAP13,MORC3*
GO:0008104	protein localization	0.00010298635197089249	*NSG1,IMMP2L,LONP2,MX2,CLTCL1,SNAP25-AS1,DPP10,ZDHHC21,RIPOR2,RDX,STXBP1,ERC1,RALA,BCL2,MYO5A,FBN1,GPHN,COG5,GPR158,RIMS1,PIK3C3,SPIRE1,CNTLN,EXOC6B,TRAPPC8,USH2A,CEP192,RIMS2,ERBIN,FCHO2,CACNG2,GPC6,CNTNAP2,APC,CRKL,ILDR2,SETD2,TANGO6,TNIK,EGFR,RFX3,DENND1A,ANGPT1,MACF1,NEDD4,GNPTAB,CRB1,ZFAND6,DNAJC13,RABEP1,NUP214,TOM1L2,CEP128,PRKD1,RAPGEF2,LRP2,AGK,RANBP17,UBE2L3,PTPRN2,SMYD3,HERC2,SEPTIN9,EPB41L3,NEDD4L,ADAM10,CACNB2,DCLK1,MAPRE2,VCL,ARHGAP44,NDUFAF2,CD2AP,AURKA,PYGO1,FMN2,AKAP6,RAB8B,RFTN1,RANBP2,RAP1GDS1,KICS2,CUBN,SCP2,PRKCZ,MCPH1,RAB27B,CNST,YAP1,VPS35L,CADPS2,RABGAP1L,SGTB,ADAM22,*

			COPB1,SYCP1,NIPBL,SLC16A1,SPIDR,IPO11,CORO2B,PAFAH1B1,TM7SF3,VPS13D,TLK1,NF2,ZDHHC14,CTNNA1,SNX30,PTPRK,PARD3B,VPS13C,DNAH11,JARID2,RAB22A,DNAJC15,CPE,ANK2,ADGRV1,BCAS3,RYR2,BBS2,RANBP3L,NBEA,DUSP16,USP8,PARD3,TBC1D5,BLK,ATRX,ABL1,SLC1A1,PRKAA1,FHIP1A,CCDC91,EIPR1,NBAS,RAP1A,GPC5,PLEKHA8,FGF10,GRID2,LATS2,AP3B1,SYNE1,ZBTB16,MUSK,PRKCE,SLMAP,DENND4C,CEP83,FBN2,EGF,PEX14,SCG5,TRIM5,ATXN3,RIC3,LTBP1,OPRM1,HTR2A,STAC,TAF3,ABHD17C,MSH2,APBA2,MAIP1,TNPO3,NOS2,TTC7B,MDFIC,ANK3,COG2,VPS41,LYPLA1,HIP1,PRR5L,VPS37A,EFR3A,TJP1,NPHP4,PACSIN2,SNX3,BHLHE40-AS1,BRCA2,DISC1,WDPCP,SLC10A7,LRP1B,STX12,TRAK1,MSR1,VRK1,GNAI1,MYRIP,SLC15A5,RELN,NUDCD3,AP4E1,FGF9,MYOM1,UNC13B,TTC21B,PLS1,SNX8,SEC23B,NIN,CCDC186,GLI3,SNX6,PACS1,MAP2,PEX6,RRBP1,ATF2,BBS4,KIAA0753,CFTR,KPNA1,CSE1L,DOP1B,TBC1D13,PHAF1,NMD3,AKAP10,PRKN,LYST,GRIN2A,PRKCH,FRMD4A,ALS2,SNX25,FBLN5,TSPAN33,LRBA,MAP7,MON2,MESD,MYO1D,SEC24D,ROCK1,SEL1L,SUMO3,SLC15A2,RRAGD,BANP,NPIPA1,DMBT1,HECTD1,SHROOM3,XRCC4,COLQ,ARFGEF1,BID,PIGK,RPH3A,UFD1,TOM1,ZDHHC17,NSD2,SH3GLB1,CARD10,TMED3,IGSF11,SNX9,WDR72,NUP37,BCL2L1,HCN1,ABCG1,FAM149B1,MICALL2,MED1,ATG4B,PCNT,IL10,PRKAA2,NDC80,PACRG,SCFD2,CAMLG,SREBF2,ANP32B,FYB2,NRXN1,PCID2,SNAP91,JAK2,RPF2,CELSR2,TNKS,ARL11,SIAH3,UFL1,NFKBIA,PRKCB,ABCC8,MIPEP,USH1C,NEDD9,MTCL1,GRIP1,TM9SF3,SAR1A,BBS9,EXOC1,HEPACAM,NDFIP2,SHROOM2,RN7SL483P,CEMIP,PARK7,MAPK8,ITGA4,OAZ2,BCAP29,UBE2J2,NDC1,TM9SF4,RAPGEF4,CEP120,ARL4C,WNT7A,NDFIP1,CHAMP1,RAB38,SDCBP,NECTIN1,TRIM23,SNAP29,INTS13,NUMB,ADAMTS9,RN7SL767P,OCLN,SHISA6,AKAP11,KTN1,MVB12B,MARK4,CDH5,AP4S1,CLDN18,MPP7,DIAPH1,SCAMP1,SCG3,FRMD6,AP2B1,HEATR5A,ICA1,MTTP,SRP9,CCDC88A,BICD1,FYN,PPM1F,ARL13B,XPO7,ODR4,SLF1,EHBP1,MAPK9,ASB3,CDH2,ITGA8,GPR137B,ZDHHC18,TBC1D1,PID1,NRP1,FCHSD2,RNF215,MCC,BCR,ARFGAP3,TM9SF2,B9D1,BMPER,RABL2A,DPP6,MACROH2A1,EPHB2,CD38,MYO5B,RGPD4,PPIL2,AKAIN1,DLG2,STXBP4,CACNG3,MAGI2,VMP1,GNAS,MFHAS1,NUP43,BMP7,ASTN2,DLG5,GAPVD1,DDX6,VPS13B,EXOC4,FAM126A,KCNIP4,ERBB4,FAM3B,FAM126B,SYNDIG1,RGPD2,SAMM50,SORCS2,NLGN1,ASIC2,EFNA5,GAS2L1,KIF13A,AP5M1,ESR1,ZDHHC11B,AGAP1,ROCK2,CDCA5,RAB31,HERPUD1,WASHC1,HOOK3,BARD1,STK3,ZNF423,HNRNPU,VTI1A,CEP72,RAB3GAP2,CADPS,TANGO2,MORC3,SEPTIN6,DNM1L
GO:0034765	regulation of ion transmembrane transport	0.000111363822158694227	*CACNA2D3,KCNH5,DPP10,PDE4D,BCL2,KCNMA1,ANO6,CACNG2,NEDD4,PRKD1,CHRM3,FGF12,TMEM38B,THADA,NEDD4L,APP,CACNA1C,CACNB2,TMC1,SLC8A1,KCNE4,AKAP6,KCNK10,CLIC6,HECW1,KCNJ1,LRRC38,AKAP9,RASGRF2,KCNS3,DAPK1,SCN2A,ANK2,RYR2,ABL1,KCNH1,GSG1L,RASGRF1,PRKCE,SLMAP,WNK2,ABCC9,ALG10B,OPRM1,STAC,CNIH3,KCND2,ANK3,CACNA1I,KCNJ15,SCN11A,NETO2,RELN,KCNH8,CNKSR3,CFTR,SELENON,GRIN2A,JPH1,KCNQ3,SHISA9,SCN10A,KCND3,LYN,CRACR2A,UTRN,KCNC1,HCN1,GRIN2B,KCNK5,NRXN1,TWIST1,ABCC8,CACNA1E,KCNJ18,CEMIP,PARK7,ADCYAP1R1,MEF2C,SHISA6,DIAPH1,NOS1AP,FYN,SCN8A,NALCN,HECW2,GRM5,ATPSCKMT,KCNJ6,DPP6,EPHB2,TSPAN13,CACNG3,ATG5,VMP1,KCNIP4,TRDN,NLGN1,NOS1,ASIC2,KCNQ5,CACNA2D1,HTT,CCR2,CATSPER2,RGS7,KCNAB1*
GO:0030335	positive regulation of cell migration	0.000112304931901969899	*MTOR,RIPOR2,RDX,BCL2,CARMIL1,RIN2,ANO6,APC,CRKL,EGFR,ANGPT1,NTRK3,FLT1,PRKD1,PAK1,RAPGEF2,ONECUT1,ADAM10,HDAC9,IL1R1,APP,DOCK8,MAPRE2,SEMA5A,NTF3,SLC8A1,SRGAP2C,MAP4K4,PAK3,MAPK1,MGAT5,PDGFD,NIPBL,TIAM1,SEMA3C,AGO2,IL34,BCAS3,SYNE2,SEMA6D,DOCK4,ABL1,HDAC4,FGF10,PRKCE,EGF,ARHGEF7,ATP8A1,SEMA3E,DOCK5,TJP1,IQSEC1,SEMA3A,BMP2,SEMA3D,RELN,HMGB1,FGF9,DOCK1,SLAMF1,ETS1,SMOC2,KITLG,IL6R,LYN,INSR,SNAI2,JCAD,TWIST2,CSF1,ROR2,AKT3,JAK2,NEDD9,ITGA6,CEMIP,CCBE1,ITGA4,*

			WNT7A,SDCBP,FGR,EPHA4,NUMB,FBXO31,PTK2,CDH5,DIAPH1,LAMB1,SEMA4D,JAM2,WNT5B,PPM1F,APELA,NRP1,PRKCA,FBLN1,RRAS2,EPHB2,MET,CDH13,BMP7,PDGFC,ABL2,CAMK1D,PIK3R3,FER,CCR2,SEMA4B,ROCK2,WASHC1,IGF1R,DNM1L
GO:0051336	regulation of hydrolase activity	0.000113080369772578	*BCAR3,MTOR,SPOCK1,GARNL3,MYO9A,DLC1,RIPOR2,RDX,FGD4,EGLN3,CRKL,ARHGAP24,DOCK10,EGFR,DENND1A,USP14,NTRK3,C5,FLT1,DGKI,CAST,PRKD1,EPHA7,RALGPS1,RAPGEF2,RALGAPA1,RAPGEF5,HDAC9,APP,DOCK8,MAPRE2,ARHGAP44,NTF3,ACER2,ST18,SERPINA6,MAP4K4,PCSK6,RAP1GDS1,IFT57,PRKCZ,MGAT5,RABGAP1L,ITIH5,UBE2O,CARD18,TBC1D22A,CHN1,PAFAH1B1,BIRC6,RASGRF2,PPP6R3,RGL1,TIAM1,ARAP2,PLA2R1,DAPK1,TBC1D9,BCAS3,CLPX,TBC1D5,NUAK1,ABL1,SLC1A1,PSMF1,RAP1A,RASGRF1,ASAP2,CD44,PDE3A,RPS6KA3,PLXNA2,ARHGEF7,HTR2A,MBP,HIP1,CRIM1,VAV1,IQSEC1,BMP2,EVI5,RALGAPA2,SGSM1,TBC1D4,ARHGAP42,HMGB1,GNAQ,CPAMD8,RAP1GAP,SRGAP2,UMODL1,BBS4,TBC1D13,RCAN1,NGEF,GRIN2A,ALS2,DOCK9,VAV3,ROCK1,LYN,CTSB,ARFGEF1,BID,SIAH2,RALGPS2,RASGRP1,SNX9,TMEM225,CSNK2A1,SERPINB9,PRKG1,GRIN2B,PSAP,RASGEF1C,PTH,IQGAP1,ANP32B,PCID2,FICD,NET1,SIPA1L2,JAK2,PCNA,ZC3H15,NEDD9,ITGA6,PPP1R17,PARK7,MAPK8,ADCYAP1R1,RAPGEF4,CYFIP2,MEF2C,MAP3K5,SERPINI2,EPHA4,NTRK2,PTK2,ARHGAP12,SEMA4D,SERPINB10,RASGEF1B,ASAP1,GPR55,BICD1,FYN,KDM5A,PPM1F,GPR137B,TBC1D1,ITGA1,POR,BCR,RGS6,FBLN1,RAG1,BCL2L13,MET,SERPINB2,MAGI2,KALRN,GNAS,SERPINB7,TIAM2,TNFAIP8,GAPVD1,WDR41,ABL2,TRAPPC6B,SERPINB11,ROBO1,ANTXR1,SIPA1L3,MGMT,NOS1,EFNA5,ESR1,HTT,A2M,SPOCK3,ROCK2,RGS8,COL4A3,RGS7,RSU1,RAB3GAP2*
GO:0045216	cell-cell junction organization	0.000127045011154362 32	*TLN2,RDX,CDH8,CNTNAP2,APC,PATJ,EPB41L3,TBCD,ADAM10,VCL,NF2,CTNNA1,CDH7,ANK2,CDH11,PARD3,CXADR,XIRP2,CDH18,CDHR3,PTPRO,EXT1,CTNND2,CDH20,TJP1,NPHP4,STRN,PRKCH,PKP1,ROCK1,SNAI2,CD9,MICALL2,HIPK1,SVEP1,ABCC8,PKN2,NECTIN1,DSG1,EPHA4,NUMB,OCLN,CDH5,CLDN18,MPP7,KIRREL1,CDH9,CDH2,PRKCA,CDH12,EPHB2,DLG5,FER,ROCK2,CLDN10*
GO:0007611	learning or memory	0.000131778787022769 56	*PLCB1,CHRNA7,PJA2,CNTNAP2,EGFR,BTBD9,DGKI,GRIA1,SLC8A3,ADGRB3,APP,NTF3,TAFA2,KCNK10,PRKCZ,BRINP1,MAPK1,SORCS3,PAFAH1B1,MEIS2,SYNJ1,PAK5,DNAH11,SCN2A,TANC1,TNR,ELAVL4,ABL1,SLC1A1,CAMK4,RASGRF1,MUSK,ATP8A1,HTR2A,TMOD2,RELN,RCAN1,PRKN,GRIN2A,ATXN1,SHANK2,INSR,GRIN2B,LMX1A,ACTR2,NRXN1,ABCC8,NEDD9,SLC6A1,CSMD1,MEF2C,S100B,FOXO6,GABRA5,NTRK2,UBE3A,AMFR,FYN,ITGA8,GRM5,NRXN3,RAG1,EPHB2,SPECC1,KALRN,HTT,FOXB1,HRH1*
GO:0007507	heart development	0.000131906351978647 48	*NOTCH2,MTOR,SGCD,NEBL,ZFPM2,TENM4,DLC1,ODAD2,ALDH1A2,FBN1,ROBO2,RARB,SPRED1,CRKL,SETD2,EGFR,SOX6,NTRK3,ADAMTS6,LRP2,FGF12,LUZP1,HDAC9,RPS6KA2,CACNA1C,SLC8A1,AKAP6,IFT57,YAP1,MAPK1,NIPBL,MYLK3,TPM1,PPARA,SEMA3C,ALPK2,DNAH11,JARID2,CPE,ANK2,RYR2,ANKS6,LDB3,CXADR,XIRP2,DNAH5,NRG1,SETDB2,ALPK3,EXT1,FHL2,MNAT1,SGCZ,MYLK2,ECE1,PLCE1,PDLIM5,BMP2,PTCD2,FGF9,GLI3,DAW1,ATF2,BBS4,COL5A1,RB1CC1,TBX20,ADGRG6,ROCK1,VCAM1,OVOL2,INSR,HECTD1,SNAI2,RXRG,NSD2,BMP5,CTDP1,DHRS3,SMAD5,MED1,KDM6A,CACYBP,TWIST1,PCNA,MYO18B,ASB2,MYOCD,MEF2C,PDE2A,ADAMTS9,TNNI1,PTK2,FAT4,AP2B1,RUNX1,SORBS2,ARL13B,APELA,NRP1,MB,SGCG,ATG5,NRAP,FLRT2,GREB1L,BMP7,EYA1,FHOD3,SLIT2,ERBB4,ROBO1,SLIT3,ROCK2,PRDM1,NCOA6,STK3,HNRNPU,IGF1R,GLI2,AKAP13*
GO:0006935	chemotaxis	0.000136299247697899 4	*NOTCH2,CNTN4,RIPOR2,RALA,ROBO2,ANO6,DSCAM,CRKL,PTPRJ,USP14,ANGPT1,DOCK2,CDH4,NTRK3,C5,FLT1,NEO1,CNTN6,PRKD1,EPHA7,ADAMTSL1,TAFA4,CCL28,ADAM10,APP,MTUS1,SEMA5A,NTF3,PLGRKT,BMPR1B,MAPK1,ALCAM,PDGFD,NRG3,NCAM1,CHN1,NFIB,PRTG,ENAH,SEMA3C,IL34,SEMA6D,TNR,CXADR,DOCK4,FGF10,NRG1,USP33,PTPRO,TRIO,EXT1,PTPN2,PLXNA2,BIN2,SEMA3E,EPHA6,BCL11B,ECE1,IL17RA,VAV1,CNTN1,SEMA3A,UNC5D,RIN3,SEMA3D,RELN,HMGB1,DRAXIN,SLAMF1,GLI3,SMOC2,ITGA9,DCC,LYST,IL6R,VAV3,LYN,VCAM1,NTN1,DP*

			YSL5,CSF1,LAMA3,LMX1A,IL10,VASP,NRXN1,NEDD9,GAP43,CMTM7,NECTIN1,CXCL2,EPHA4,LHX9,PTK2,FEZ2,CYFIP1,SEMA4D,TNFSF11,FYN,PPM1F,CNTN5,EPHB1,RPS6KA5,NRP1,ITGA1,NRXN3,EPHB2,MET,CDH13,FLRT2,KALRN,LAMA1,BMP7,SLIT2,CCDC141,ROBO1,PRKCQ,EFNA5,SLIT3,CAMK1D,FER,CCR2,SEMA4B,HRH1,IL16,GLI2,DNM1L
GO:0060284	regulation of cell development	0.0001544301392777993	*NOTCH2,MTOR,PTPRD,ULK2,PLCB1,TENM4,ZDHHC21,BCL2,FBN1,ROBO2,CARMIL1,DSCAM,CRKL,RFX3,MACF1,BCL11A,CDH4,NTRK3,EPHA7,SPEN,RAPGEF2,LRP2,HDAC9,STAU2,SEMA5A,VCL,AURKA,KANK1,PAK3,DIP2B,TRPC5,YAP1,BRINP1,PAFAH1B1,NF2,CTNNA1,PRTG,SYNJ1,TIAM1,SEMA3C,IL34,SEMA6D,TNR,ABL1,HDAC4,ASPM,PDE3A,PLXNA2,ARHGEF7,OPRM1,SEMA3E,DOCK5,MBP,DISC1,SEMA3A,BMP2,SEMA3D,RELN,DOCK1,NIN,DRAXIN,GLI3,MAP2,DCC,DAB1,PRKCH,ROCK1,LYN,NTN1,DPYSL5,TRIM58,IL33,ACTR2,MAP6,MELTF,UFL1,ABCC8,NEDD9,OLFM4,WNT7A,FBXW8,EPHA4,NTRK2,IL1RAPL1,NUMB,FBXO31,CDH5,CLDN18,CYFIP1,SEMA4D,TNFSF11,HDAC2,GRM5,NRP1,FAIM,CHODL,FBLN1,CUX1,EPHB2,KALRN,TIAM2,BMP7,SLIT2,ROBO1,EFNA5,SEMA4B,ROCK2,HOOK3,FSTL4*
GO:0070727	cellular macromolecule localization	0.00015509464846155885	*NSG1,IMMP2L,LONP2,MX2,CLTCL1,SNAP25-AS1,DPP10,ZDHHC21,RIPOR2,RDX,STXBP1,ERC1,RALA,BCL2,MYO5A,FBN1,GPHN,COG5,GPR158,RIMS1,PIK3C3,SPIRE1,CNTLN,EXOC6B,TRAPPC8,USH2A,CEP192,RIMS2,ERBIN,FCHO2,CACNG2,GPC6,CNTNAP2,APC,CRKL,ILDR2,SETD2,TANGO6,TNIK,EGFR,RFX3,DENND1A,ANGPT1,MACF1,NEDD4,GNPTAB,CRB1,ZFAND6,DNAJC13,RABEP1,NUP214,TOM1L2,CEP128,PRKD1,RAPGEF2,LRP2,AGK,RANBP17,UBE2L3,PTPRN2,SMYD3,HERC2,SEPTIN9,EPB41L3,NEDD4L,ADAM10,CACNB2,DCLK1,MAPRE2,VCL,ARHGAP44,NDUFAF2,CD2AP,AURKA,PYGO1,FMN2,AKAP6,RAB8B,RFTN1,RANBP2,RAP1GDS1,KICS2,CUBN,SCP2,PRKCZ,MCPH1,RAB27B,CNST,YAP1,VPS35L,CADPS2,RABGAP1L,SGTB,ADAM22,COPB1,SYCP1,NIPBL,SLC16A1,SPIDR,IPO11,CORO2B,PAFAH1B1,TM7SF3,VPS13D,TLK1,NF2,ZDHHC14,CTNNA1,SNX30,PTPRK,PARD3B,VPS13C,DNAH11,JARID2,RAB22A,DNAJC15,CPE,ANK2,ADGRV1,BCAS3,RYR2,BBS2,RANBP3L,NBEA,DUSP16,USP8,PARD3,TBC1D5,BLK,ATRX,ABL1,SLC1A1,PRKAA1,FHIP1A,CCDC91,EIPR1,NBAS,RAP1A,GPC5,PLEKHA8,FGF10,GRID2,LATS2,AP3B1,SYNE1,ZBTB16,MUSK,PRKCE,SLMAP,DENND4C,CEP83,FBN2,EGF,PEX14,SCG5,TRIM5,ATXN3,RIC3,LTBP1,OPRM1,HTR2A,STAC,TAF3,ABHD17C,MSH2,APBA2,MAIP1,TNPO3,NOS2,TTC7B,MDFIC,ANK3,COG2,VPS41,LYPLA1,HIP1,PRR5L,VPS37A,EFR3A,TJP1,NPHP4,PACSIN2,SNX3,BHLHE40-AS1,BRCA2,DISC1,WDPCP,SLC10A7,LRP1B,STX12,TRAK1,MSR1,VRK1,GNAI1,MYRIP,SLC15A5,RELN,NUDCD3,AP4E1,FGF9,MYOM1,UNC13B,TTC21B,PLS1,SNX8,SEC23B,NIN,CCDC186,GLI3,SNX6,PACS1,MAP2,PEX6,RRBP1,ATF2,BBS4,KIAA0753,CFTR,KPNA1,CSE1L,DOP1B,TBC1D13,PHAF1,NMD3,AKAP10,PRKN,LYST,GRIN2A,PRKCH,FRMD4A,ALS2,SNX25,FBLN5,TSPAN33,LRBA,MAP7,MON2,MESD,MYO1D,SEC24D,ROCK1,SEL1L,SUMO3,SLC15A2,RRAGD,BANP,NPIPA1,DMBT1,HECTD1,SHROOM3,XRCC4,COLQ,ARFGEF1,BID,PIGK,RPH3A,UFD1,TOM1,ZDHHC17,NSD2,SH3GLB1,CARD10,TMED3,IGSF11,SNX9,WDR72,NUP37,BCL2L1,HCN1,ABCG1,FAM149B1,MICALL2,MED1,ATG4B,PCNT,IL10,PRKAA2,NDC80,PACRG,SCFD2,CAMLG,SREBF2,ANP32B,FYB2,NRXN1,PCID2,SNAP91,JAK2,RPF2,CELSR2,TNKS,ARL11,SIAH3,UFL1,NFKBIA,PRKCB,ABCC8,MIPEP,USH1C,NEDD9,MTCL1,GRIP1,TM9SF3,SAR1A,BBS9,EXOC1,HEPACAM,NDFIP2,SHROOM2,RN7SL483P,CEMIP,PARK7,MAPK8,ITGA4,OAZ2,BCAP29,UBE2J2,NDC1,TM9SF4,RAPGEF4,CEP120,ARL4C,WNT7A,NDFIP1,CHAMP1,RAB38,SDCBP,NECTIN1,TRIM23,SNAP29,INTS13,NUMB,ADAMTS9,RN7SL767P,OCLN,SHISA6,AKAP11,KTN1,MVB12B,MARK4,CDH5,AP4S1,CLDN18,MPP7,DIAPH1,SCAMP1,SCG3,FRMD6,AP2B1,HEATR5A,ICA1,MTTP,SRP9,CCDC88A,BICD1,FYN,PPM1F,ARL13B,XPO7,ODR4,SLF1,EHBP1,MAPK9,ASB3,CDH2,ITGA8,GPR137B,ZDHHC18,TBC1D1,PID1,NRP1,FCHSD2,RNF215,MCC,BCR,ARFGAP3,TM9SF2,B9D1,BMPER,RABL2A,DPP6,MACROH2A*

			1,EPHB2,CD38,MYO5B,RGPD4,PPIL2,AKAIN1,DLG2,STXBP4,CACNG3,MAGI2,VMP1,GNAS,MFHAS1,NUP43,BMP7,ASTN2,DLG5,GAPVD1,DDX6,VPS13B,EXOC4,FAM126A,KCNIP4,ERBB4,FAM3B,FAM126B,SYNDIG1,RGPD2,SAMM50,SORCS2,NLGN1,ASIC2,EFNA5,GAS2L1,KIF13A,AP5M1,ESR1,ZDHHC11B,AGAP1,ROCK2,CDCA5,RAB31,HERPUD1,WASHC1,HOOK3,BARD1,STK3,ZNF423,HNRNPU,VTI1A,CEP72,RAB3GAP2,CADPS,TANGO2,MORC3,SEPTIN6,DNM1L
GO:0042330	taxis	0.000165004793700342 25	*NOTCH2,CNTN4,RIPOR2,RALA,ROBO2,ANO6,DSCAM,CRKL,PTPRJ,USP14,ANGPT1,DOCK2,CDH4,NTRK3,C5,FLT1,NEO1,CNTN6,PRKD1,EPHA7,ADAMTSL1,TAFA4,CCL28,ADAM10,APP,MTUS1,SEMA5A,NTF3,PLGRKT,BMPR1B,MAPK1,ALCAM,PDGFD,NRG3,NCAM1,CHN1,NFIB,PRTG,ENAH,SEMA3C,IL34,SEMA6D,TNR,CXADR,DOCK4,FGF10,NRG1,USP33,PTPRO,TRIO,EXT1,PTPN2,PLXNA2,BIN2,SEMA3E,EPHA6,BCL11B,ECE1,IL17RA,VAV1,CNTN1,SEMA3A,UNC5D,RIN3,SEMA3D,RELN,HMGB1,DRAXIN,SLAMF1,GLI3,SMOC2,ITGA9,DCC,LYST,IL6R,VAV3,LYN,VCAM1,NTN1,DPYSL5,CSF1,LAMA3,LMX1A,IL10,VASP,NRXN1,NEDD9,GAP43,CMTM7,NECTIN1,CXCL2,EPHA4,LHX9,PTK2,FEZ2,CYFIP1,SEMA4D,TNFSF11,FYN,PPM1F,CNTN5,EPHB1,RPS6KA5,NRP1,ITGA1,NRXN3,EPHB2,MET,CDH13,FLRT2,KALRN,LAMA1,BMP7,SLIT2,CCDC141,ROBO1,PRKCQ,EFNA5,SLIT3,CAMK1D,FER,CCR2,SEMA4B,HRH1,IL16,GLI2,DNM1L*
GO:0043547	positive regulation of GTPase activity	0.000178139264997237 46	*BCAR3,GARNL3,MYO9A,CRKL,ARHGAP24,DOCK10,DENND1A,NTRK3,RALGPS1,RAPGEF2,RALGAPA1,RAPGEF5,DOCK8,MAPRE2,NTF3,MAP4K4,RAP1GDS1,RABGAP1L,TBC1D22A,CHN1,RASGRF2,RGL1,TIAM1,ARAP2,TBC1D9,BCAS3,TBC1D5,RAP1A,RASGRF1,ASAP2,ARHGEF7,VAV1,IQSEC1,EVI5,RALGAPA2,SGSM1,TBC1D4,ARHGAP42,RAP1GAP,SRGAP2,TBC1D13,NGEF,ALS2,DOCK9,RALGPS2,RASGRP1,SNX9,RASGEF1C,NET1,SIPA1L2,ZC3H15,NEDD9,ITGA6,RAPGEF4,SEMA4D,RASGEF1B,ASAP1,TBC1D1,BCR,RGS6,KALRN,GNAS,TIAM2,WDR41,SIPA1L3,RGS8,RGS7,RSU1*
GO:0034762	regulation of transmembrane transport	0.000179133388003588 64	*CACNA2D3,KCNH5,CLTCL1,DPP10,PDE4D,BCL2,KCNMA1,ANO6,CACNG2,NEDD4,PRKD1,CHRM3,FGF12,TMEM38B,THADA,NEDD4L,APP,CACNA1C,CACNB2,TMC1,SLC8A1,KCNE4,AKAP6,KCNK10,CLIC6,GRB10,HECW1,KCNJ1,LRRC38,AKAP9,KLF15,RASGRF2,KCNS3,DAPK1,SCN2A,ANK2,RYR2,ABL1,KCNH1,GSG1L,RASGRF1,PRKCE,SLMAP,WNK2,ABCC9,ALG10B,OPRM1,STAC,CNIH3,KCND2,ANK3,CACNA1I,KCNJ15,SCN11A,NETO2,RELN,KCNH8,CNKSR3,CFTR,SELENON,GRIN2A,JPH1,KCNQ3,SHISA9,SCN10A,KCND3,LYN,CRACR2A,INSR,ENPP1,UTRN,KCNC1,HCN1,GRIN2B,KCNK5,PTH,NRXN1,TWIST1,PRKCB,ABCC8,CACNA1E,KCNJ18,CEMIP,PARK7,OAZ2,ADCYAP1R1,MEF2C,OCLN,SHISA6,DIAPH1,NOS1AP,FYN,SCN8A,NALCN,HECW2,GRM5,PID1,ATPSCKMT,KCNJ6,DPP6,EPHB2,TSPAN13,STXBP4,CACNG3,ATG5,VMP1,SLC1A2,KCNIP4,TRDN,NLGN1,NOS1,ASIC2,KCNQ5,CACNA2D1,HTT,CCR2,CATSPER2,RGS7,KCNAB1,PRKAG2*
GO:0010648	negative regulation of cell communication	0.000202736951796225 6	*MTOR,WWC1,PTPRD,SLC24A2,NLK,ZNF536,DLC1,RIPOR2,PDE4D,STXBP1,BCL2,PRDM16,FBN1,SPRED1,MINAR1,ERBIN,MLLT3,APC,ARHGAP24,PTPRJ,EGFR,PRKACB,RGS3,NCOR1,NEDD4,SCAI,GRIK3,DKK2,MAPKBP1,DGKI,INVS,GRIA1,LRP2,RUNX2,TAOK3,ONECUT1,STAU2,USP18,ARHGAP44,NDUFAF2,CD2AP,PTPRR,KANK1,HOMER2,RGS20,PDE10A,KICS2,PRKCZ,GRB10,RGS9,HECW1,DUSP22,YAP1,ZNRF3,STK38,SORCS3,PAFAH1B1,NF2,CTNNA1,BIRC6,PPARA,PAK5,SLC24A4,SEC14L1,ALPK2,DUSP16,SMARCA4,MAPKAP1,TNR,PTPRT,ABL1,PTPN12,PRKAA1,RAP1A,FGF10,GRID2,LATS2,NRG1,ZNF675,NXN,WNK2,FBN2,CD44,RGS12,PTPRO,PDE3A,LIMD1,SPRED2,PTPN2,LTBP1,OPRM1,HTR2A,KREMEN1,FHL2,HIPK3,EPN2,GRK3,MOSMO,CRIM1,PRR5L,LDLRAD4,NPHP4,BMP2,RANBP9,TMEM161A,LEMD3,ARHGAP42,FGF9,DRAXIN,SLAMF1,GLI3,SNX6,CNKSR3,GRIK2,CYLD,MAPK8IP1,UBASH3A,UBR1,RCAN1,DAB1,RB1CC1,PTPRE,PRKN,MTMR2,TBX20,NLRC5,SNX25,SHANK2,SOX30,LYN,OVOL2,RNF152,OTUD7A,YTHDF3,SNAI2,ASH1L,BID,SIAH2,TRABD2B,UFD1,SAMHD1,ENPP1,NDRG2,CSNK2A1,BMP5,BCL2L1,HCN1,GRB14,DHRS3,CELF4,PRAME,TNN,BANK1,IL10,SOSTDC1,PRKAA2,ITPRIP,YB*

			X3,PBLD,PEG10,TWIST1,UFL1,NFKBIA,PRKCB,ABCC8,BRD4,ITGA6,OTOP1,CIDEA,SLC6A1,STAT1,BRMS1L,DGKG,PARK7,MYOCD,RBPMS2,C16ORF72,PDE2A,WWOX,NCK1,PPP2R3A,EPHA4,MECOM,SHISA6,IL17RD,ANKRD6,ARHGAP12,AMFR,BICD1,RBMS3,HDAC2,ZFYVE28,APELA,TET1,CDH2,PHLPP1,GRM5,TBC1D1,PID1,NRP1,FAIM,ITGA1,MCC,RGS6,FBLN1,BMPER,PRDM15,EPHB2,CD38,EYA4,MET,MAGI2,KALRN,MFHAS1,BMP7,DLG5,ZMYND11,TMEM25,ABL2,EYA1,SLIT2,CNOT7,ROBO1,PRKCQ,SORCS2,SLIT3,ESR1,HTT,EYA2,RORA,RGS8,HERPUD1,RGS7,KIF7,FSTL4,STK3,DEPTOR,APCDD1,IGF1R,GLI2
GO:0050890	cognition	0.0002045093337383042	*PLCB1,CHRNA7,PJA2,CNTNAP2,EGFR,BTBD9,TUSC3,DGKI,GRIA1,SLC8A3,ADGRB3,APP,NTF3,TAFA2,KCNK10,PRKCZ,BRINP1,MAPK1,NIPBL,SORCS3,PAFAH1B1,MEIS2,SYNJ1,PAK5,DNAH11,SCN2A,TANC1,TNR,ELAVL4,ABL1,SLC1A1,CAMK4,RASGRF1,MUSK,ATP8A1,HTR2A,TMOD2,RELN,BBS4,DOP1B,RCAN1,PRKN,GRIN2A,ATXN1,SHANK2,INSR,GRIN2B,LMX1A,ACTR2,NRXN1,ABCC8,NEDD9,SLC6A1,CSMD1,MEF2C,S100B,FOXO6,GABRA5,NTRK2,CYFIP1,UBE3A,AMFR,FYN,ITGA8,GRM5,NRXN3,RAG1,DGCR2,EPHB2,SPECC1,KALRN,GNAS,HTT,FOXB1,HRH1*
GO:0060996	dendritic spine development	0.00026467118589628774	*DOCK10,STAU2,ARHGAP44,SRGAP2C,PAK3,DNM3,DIP2A,PAFAH1B1,CTNND2,IQSEC1,PDLIM5,DISC1,RELN,SRGAP2,NGEF,TANC2,ACTR2,MEF2C,WNT7A,FOXO6,EPHA4,UBE3A,ASAP1,HDAC2,EPHB1,SDK1,EPHB2,PPFIA2,KALRN,DLG5,NLGN1,FSTL4*
GO:0023057	negative regulation of signaling	0.0002769072908582638	*MTOR,WWC1,PTPRD,SLC24A2,NLK,ZNF536,DLC1,RIPOR2,PDE4D,STXBP1,BCL2,PRDM16,FBN1,SPRED1,MINAR1,ERBIN,MLLT3,APC,ARHGAP24,PTPRJ,EGFR,PRKACB,RGS3,NCOR1,NEDD4,SCAI,GRIK3,DKK2,MAPKBP1,DGKI,INVS,GRIA1,LRP2,RUNX2,TAOK3,ONECUT1,STAU2,USP18,ARHGAP44,NDUFAF2,CD2AP,PTPRR,KANK1,HOMER2,RGS20,PDE10A,KICS2,PRKCZ,GRB10,RGS9,HECW1,DUSP22,YAP1,ZNRF3,STK38,SORCS3,PAFAH1B1,NF2,CTNNA1,BIRC6,PPARA,PAK5,SLC24A4,SEC14L1,ALPK2,DUSP16,SMARCA4,MAPKAP1,TNR,PTPRT,ABL1,PTPN12,PRKAA1,RAP1A,FGF10,GRID2,LATS2,NRG1,ZNF675,NXN,WNK2,FBN2,CD44,RGS12,PTPRO,PDE3A,LIMD1,SPRED2,PTPN2,LTBP1,OPRM1,HTR2A,KREMEN1,FHL2,HIPK3,EPN2,GRK3,MOSMO,CRIM1,PRR5L,LDLRAD4,NPHP4,BMP2,RANBP9,TMEM161A,LEMD3,ARHGAP42,FGF9,DRAXIN,SLAMF1,GLI3,SNX6,CNKSR3,GRIK2,CYLD,MAPK8IP1,UBASH3A,UBR1,RCAN1,DAB1,RB1CC1,PTPRE,PRKN,MTMR2,TBX20,NLRC5,SNX25,SHANK2,SOX30,LYN,OVOL2,RNF152,OTUD7A,YTHDF3,SNAI2,ASH1L,BID,SIAH2,TRABD2B,UFD1,SAMHD1,ENPP1,NDRG2,CSNK2A1,BMP5,BCL2L1,HCN1,GRB14,DHRS3,CELF4,PRAME,TNN,BANK1,IL10,SOSTDC1,PRKAA2,ITPRIP,YBX3,PBLD,PEG10,TWIST1,UFL1,NFKBIA,PRKCB,ABCC8,BRD4,ITGA6,OTOP1,CIDEA,SLC6A1,STAT1,BRMS1L,DGKG,PARK7,MYOCD,RBPMS2,C16ORF72,PDE2A,WWOX,NCK1,PPP2R3A,EPHA4,MECOM,SHISA6,IL17RD,ANKRD6,ARHGAP12,AMFR,BICD1,RBMS3,HDAC2,ZFYVE28,APELA,TET1,CDH2,PHLPP1,GRM5,TBC1D1,PID1,NRP1,FAIM,ITGA1,MCC,RGS6,FBLN1,BMPER,PRDM15,EPHB2,CD38,EYA4,MET,MAGI2,KALRN,MFHAS1,BMP7,DLG5,ZMYND11,TMEM25,ABL2,EYA1,SLIT2,CNOT7,ROBO1,PRKCQ,SORCS2,SLIT3,ESR1,HTT,EYA2,RORA,RGS8,HERPUD1,RGS7,KIF7,FSTL4,STK3,DEPTOR,APCDD1,IGF1R,GLI2*
GO:0097061	dendritic spine organization	0.0002981108447178989	*CHRNA7,DOCK10,STAU2,ARHGAP44,PAK3,DNM3,DIP2A,PAFAH1B1,TANC1,CTNND2,PDLIM5,RELN,MTMR2,NGEF,INSR,TANC2,GRIN2B,ACTR2,NEDD9,WNT7A,EPHA4,UBE3A,FYN,EPHB1,EPHB2,PPFIA2,KALRN,NLGN1,IGF1R*
GO:0007265	Ras protein signal transduction	0.0002981206310161855	*NOTCH2,KSR1,DLC1,RIPOR2,RDX,RALA,CDC42EP3,AUTS2,ERBIN,CRKL,ARHGAP24,DENND1A,SCAI,DGKI,PRKD1,CTNNAL1,RALGPS1,RAPGEF2,RAPGEF5,MAPRE2,ARHGAP44,KANK1,MAP4K4,ITPKB,RASGRF2,RGL1,TIAM1,ARHGEF12,USP8,MAPKAP1,ABL1,RAP1A,FGF10,NRG1,RASGRF1,DENND4C,MCF2L,PLCE1,IQSEC1,ARHGAP42,PSD3,KITLG,ALS2,ARHGEF28,RALB,ROCK1,NTN1,ARFGEF1,RALGPS2,RASGRP1,CSF1,RASGEF1C,PTH,RAB12,NET1,MADD,ARFGEF3,PARK7,RAPGEF4,SDCBP,CYTH4,CYFIP1,RA*

			SGEF1B,CNKSR1,GPR55,NRP1,BCR,ELMO1,RERG,RRAS2,EPHB2,MET,CDH13,ABL2,ROBO1,ARHGEF11,MYO9B,STARD13,EPS8,ROCK2,AKAP13
GO:0099175	regulation of postsynapse organization	0.000307062178346611	*PTPRD,LRFN2,NTRK3,EPHA7,STAU2,ARHGAP44,PAK3,DNM3,PAFAH1B1,TANC1,GRID2,DGKB,ABHD17C,PDLIM5,RELN,NGEF,TANC2,GRIN2B,ACTR2,NRXN1,NEDD9,WNT7A,EPHA4,IL1RAPL1,UBE3A,FYN,CDH2,EPHB2,PPFIA2,KALRN,NLGN1*
GO:0051093	negative regulation of developmental process	0.000397152737297468 7	*CNTN4,WWC1,ULK2,ZNF536,TAFA5,ZFPM2,BCL2,FBN1,ROBO2,ZEB1,RARB,SPRED1,USH2A,MINAR1,FOXJ2,EGFR,CDK12,BCL11A,SOX6,TMEM182,NTRK3,EPHA7,RAPGEF2,ADGRB3,COL4A2,APP,SEMA5A,SRGAP2C,KANK1,DIP2B,ITPKB,TRPC5,DNM3,ABCA5,YAP1,BRINP1,MAPK1,GABPA,FAT3,EFEMP1,NF2,CTNNA1,PPARA,MEIS2,NFIB,PRTG,SEMA3C,ALPK2,JARID2,ADGRV1,BBS2,WNT9B,RANBP3L,SEMA6D,SMARCA4,TNR,CXADR,HDAC4,GLIS1,FGF10,ASPM,ZBTB16,ZNF675,TRIO,LIMD1,SPRED2,PTPN2,KREMEN1,SEMA3E,ANKRD17,LUC7L,EPN2,BICRAL,MBP,CRIM1,LDLRAD4,SEMA3A,BMP2,RC3H2,SEMA3D,HMGB1,FGF9,NFATC2,ANKRD26,RAP1GAP,DRAXIN,SMARCA2,GLI3,SMARCC1,MAP2,ATF2,COL5A1,DCC,GTF2I,RORB,DAB1,PRKN,NGEF,ROCK1,LYN,DTX1,OVOL2,NTN1,ZFHX3,DPYSL5,SNAI2,TWIST2,ENPP1,BMP5,BCL2L1,CTDP1,CNMD,ABCG1,PRAME,TNN,MED1,LMX1A,TMEM178A,PRAMEF25,PTH,SOSTDC1,ANP32B,YBX3,TWIST1,MELTF,NFKBIA,ABCC8,AGO1,STAT1,BRMS1L,PRAMEF2,MYOCD,ADGRB1,WNT7A,RBPMS2,NDFIP1,EPHA4,ADAMTS9,IL17RD,CLDN18,UBE3A,SEMA4D,RUNX1,ASAP1,GPR55,HDAC2,TET1,GPR137B,EPHB1,ADCK1,NRP1,RC3H1,BCR,FBLN1,EPHB2,MYB,GNAS,BMP7,DDX6,PBX1,NLGN1,EFNA5,LOXL2,CCR2,SEMA4B,ROCK2,RORA,HSPG2,COL4A3,HOOK3,FSTL4,STK3,HNRNPU,GLI2*
GO:0040017	positive regulation of locomotion	0.000403657098465345 66	*MTOR,RIPOR2,RDX,BCL2,CARMIL1,RIN2,ANO6,APC,DSCAM,CRKL,EGFR,ANGPT1,NTRK3,FLT1,PRKD1,PAK1,RAPGEF2,ONECUT1,ADAM10,HDAC9,IL1R1,APP,DOCK8,MAPRE2,SEMA5A,NTF3,SLC8A1,SRGAP2C,MAP4K4,PAK3,MAPK1,MGAT5,PDGFD,NIPBL,TIAM1,SEMA3C,AGO2,IL34,BCAS3,SYNE2,SEMA6D,DOCK4,ABL1,HDAC4,FGF10,PRKCE,EGF,ARHGEF7,ATP8A1,SEMA3E,DOCK5,TJP1,IQSEC1,SEMA3A,BMP2,SEMA3D,RELN,HMGB1,FGF9,DOCK1,SLAMF1,ETS1,SMOC2,KITLG,IL6R,LYN,NTN1,INSR,SNAI2,JCAD,TWIST2,CSF1,ROR2,TWIST1,AKT3,JAK2,NEDD9,ITGA6,CEMIP,CCBE1,ITGA4,WNT7A,SDCBP,FGR,EPHA4,NUMB,FBXO31,PTK2,CDH5,DIAPH1,LAMB1,SEMA4D,JAM2,WNT5B,PPM1F,APELA,NRP1,PRKCA,FBLN1,RRAS2,EPHB2,MET,CDH13,BMP7,PDGFC,ABL2,SLIT2,CAMK1D,PIK3R3,FER,CCR2,SEMA4B,ROCK2,IL16,WASHC1,IGF1R,DNM1L*
GO:1901699	cellular response to nitrogen compound	0.000407860915599444 53	*BCAR3,MTOR,NSG1,PLCB1,PTPRA,ITPR2,PDE4D,MYO5A,FBN1,CHRNA7,GABRB3,ZEB1,ALK,APC,CRKL,EGFR,BCL11A,GABRB1,GRIA1,CHRM3,RAPGEF2,CPS1,CPEB4,TMEM38B,RPTOR,GHR,HDAC9,APP,GABRG2,SLC8A1,KANK1,AKAP6,RAB8B,LARP1,RAP1GDS1,PRKCZ,GRB10,RYR3,MAPK1,HRH2,PDGFD,SPIDR,GABPA,HRH4,GLP2R,CTNNA1,AKAP9,KLF15,RYR2,MBD5,ATRX,ABL1,SLC1A1,PRKAA1,RAP1A,DENND4C,PDE3A,PTPN2,HTR2C,OPRM1,HTR2A,GNAL,CCND3,BLM,SOGA1,TBC1D4,GNAQ,ZNF106,CGAS,SMARCC1,SNX6,IDE,ATF2,UMODL1,CFTR,UBR1,CHRM5,SLC30A10,SELENON,PTPRE,PRKN,RALB,ROCK1,LYN,VCAM1,RRAGD,ARID1B,INSR,ENPP1,BCL2L1,HCN1,GRB14,KL,ACTR2,JAK2,PRKCB,ATP2B1,ASS1,OTOP1,STAT1,IMPACT,ITGA4,MEF2C,MAP3K5,OR10H2,PDE2A,NCK1,EPHA4,NTRK2,COLEC12,PTK2,DIAPH1,CYFIP1,FYN,HDAC2,GRM5,PID1,POR,NSG2,GNA14,EPHB2,STXBP4,SLC1A2,GNAS,PDGFC,SLIT2,PRKCQ,CACNA2D1,PIK3R3,FER,HRH1,ROCK2,RGS8,RAB31,GNG2,PNPLA3,IGF1R*
GO:0071417	cellular response to organonitr	0.000411109278273778 64	*BCAR3,MTOR,NSG1,PLCB1,PTPRA,ITPR2,PDE4D,MYO5A,FBN1,GABRB3,ZEB1,ALK,APC,EGFR,BCL11A,GABRB1,CHRM3,RAPGEF2,CPS1,CPEB4,TMEM38B,RPTOR,GHR,HDAC9,APP,GABRG2,SLC8A1,KANK1,AKAP6,RAB8B,LARP1,RAP1GDS1,PRKCZ,GRB10,RYR3,MAPK1,HRH2,PDGFD,SPIDR,GABPA,HRH4,GLP2R,CTNNA1,A*

	ogen compound		*KAP9,KLF15,RYR2,MBD5,ABL1,SLC1A1,PRKAA1,RAP1A,DENND4C,PDE3A,PTPN2,HTR2C,OPRM1,HTR2A,GNAL,CCND3,BLM,SOGA1,TBC1D4,ZNF106,SMARCC1,SNX6,IDE,ATF2,UMODL1,CFTR,UBR1,CHRM5,SLC30A10,SELENON,PTPRE,PRKN,ROCK1,LYN,VCAM1,RRAGD,ARID1B,INSR,ENPP1,BCL2L1,HCN1,GRB14,KL,ACTR2,JAK2,PRKCB,ATP2B1,ASS1,OTOP1,STAT1,IMPACT,ITGA4,MEF2C,OR10H2,PDE2A,NCK1,EPHA4,NTRK2,PTK2,DIAPH1,CYFIP1,FYN,HDAC2,GRM5,PID1,POR,NSG2,GNA14,EPHB2,STXBP4,SLC1A2,GNAS,PDGFC,SLIT2,PRKCQ,CACNA2D1,PIK3R3,FER,HRH1,ROCK2,RGS8,RAB31,GNG2,PNPLA3,IGF1R*
GO:0051345	positive regulation of hydrolase activity	0.000444443359 0193902	*BCAR3,MTOR,GARNL3,MYO9A,DLC1,EGLN3,CRKL,ARHGAP24,DOCK10,EGFR,DENND1A,NTRK3,FLT1,PRKD1,RALGPS1,RAPGEF2,RALGAPA1,RAPGEF5,APP,DOCK8,MAPRE2,NTF3,ACER2,ST18,MAP4K4,RAP1GDS1,IFT57,PRKCZ,RABGAP1L,TBC1D22A,CHN1,RASGRF2,RGL1,TIAM1,ARAP2,DAPK1,TBC1D9,BCAS3,CLPX,TBC1D5,SLC1A1,RAP1A,RASGRF1,ASAP2,ARHGEF7,HTR2A,MBP,HIP1,VAV1,IQSEC1,BMP2,EVI5,RALGAPA2,SGSM1,TBC1D4,ARHGAP42,HMGB1,GNAQ,RAP1GAP,SRGAP2,TBC1D13,NGEF,GRIN2A,ALS2,DOCK9,ROCK1,LYN,BID,RALGPS2,RASGRP1,SNX9,GRIN2B,PSAP,RASGEF1C,PTH,ANP32B,NET1,SIPA1L2,JAK2,PCNA,ZC3H15,NEDD9,ITGA6,MAPK8,ADCYAP1R1,RAPGEF4,CYFIP2,MEF2C,MAP3K5,EPHA4,SEMA4D,RASGEF1B,ASAP1,GPR55,FYN,PPM1F,TBC1D1,ITGA1,BCR,RGS6,FBLN1,BCL2L13,MAGI2,KALRN,GNAS,TIAM2,WDR41,ABL2,ROBO1,ANTXR1,SIPA1L3,ESR1,ROCK2,RGS8,COL4A3,RGS7,RSU1*
GO:0010243	response to organonitrogen compound	0.000450097815 5581418	*BCAR3,MTOR,NSG1,PLCB1,PTPRA,ITPR2,PDE4D,MYO5A,FBN1,CHRNA7,PIK3C3,GABRB3,ZEB1,ALK,ERBIN,HLCS,APC,TNIK,EGFR,USP14,BCL11A,PSMB2,GABRB1,CHRM3,ADSS2,RAPGEF2,CPS1,CPEB4,TMEM38B,BCKDHB,RPTOR,GHR,HDAC9,APP,KYNU,GABRG2,SLC8A1,ECPAS,KANK1,AKAP6,HOMER2,RAB8B,LARP1,RAP1GDS1,RNLS,PRKCZ,GRB10,RYR3,MAPK1,HRH2,SGTB,USP25,PDGFD,SPIDR,GABPA,HRH4,GLP2R,ATF6,CTNNA1,AKAP9,KLF15,PPARA,SLC24A4,VPS13C,RYR2,EFTUD2,MBD5,ELAVL4,ABL1,SLC1A1,PRKAA1,RAP1A,PRKCE,DENND4C,ABCC9,P2RX6,PDE3A,EXT1,PTPN2,ATXN3,HTR2C,OPRM1,HTR2A,TMEM67,ALPL,GNAL,CCND3,BLM,SOGA1,GNAI1,TBC1D4,ZNF106,ATF1,SMARCC1,SNX6,IDE,ATF2,UMODL1,CFTR,UBR1,CHRM5,SLC30A10,SELENON,PTPRE,PRKN,GRIN2A,ROCK1,LYN,VCAM1,SEL1L,EIF2B3,RRAGD,ARID1B,CRACR2A,INSR,TFF1,UFD1,CD9,ENPP1,KCNC1,BCL2L1,HCN1,GRB14,HADHA,PPP2R2A,KL,IL10,ACTR2,SREBF2,JAK2,PCNA,UFL1,NFKBIA,PRKCB,ABCC8,ATP2B1,ASS1,ERLIN2,OTOP1,SLC6A1,STAT1,MARCHF6,IMPACT,ITGA4,UBE2J2,MEF2C,OR10H2,PDE2A,NCK1,EPHA4,NTRK2,PTK2,DIAPH1,CYFIP1,UBE3A,AMFR,FYN,HDAC2,SEL1L2,GRM5,PID1,SDK1,POR,NSG2,GNA14,EPHB2,CDH13,STXBP4,SLC1A2,GNAS,BMP7,PDGFC,SLIT2,PRKCQ,MGMT,SLC6A3,GLDC,CACNA2D1,PIK3R3,FER,HRH1,ROCK2,RGS8,RAB31,HERPUD1,RGS7,GNG2,PNPLA3,IGF1R*
GO:0007043	cell-cell junction assembly	0.000452629147 69690605	*TLN2,CDH8,CNTNAP2,APC,PATJ,EPB41L3,TBCD,VCL,CTNNA1,CDH7,ANK2,CDH11,PARD3,CDH18,CDHR3,PTPRO,CTNND2,CDH20,TJP1,NPHP4,STRN,PRKCH,PKP1,ROCK1,SNAI2,CD9,MICALL2,HIPK1,PKN2,DSG1,OCLN,CDH5,CLDN18,MPP7,CDH9,CDH2,PRKCA,CDH12,EPHB2,DLG5,FER,ROCK2,CLDN10*
GO:0030001	metal ion transport	0.000484202163 92044155	*CACNA2D3,SLC17A1,SLC24A2,KCNH5,MICU2,DPP10,ITPR2,PDE4D,BCL2,KCNMA1,CHRNA7,ANO6,CACNG2,SLC4A10,NEDD4,ATP2B2,TUSC3,SLC39A12,SLC8A3,PRKD1,LRP2,FGF12,TMEM38B,SLC24A3,THADA,NEDD4L,TRPM1,SLC39A11,CACNA1C,CACNB2,TMC1,SLC8A1,KCNE4,AKAP6,HOMER2,KCNK10,TRPC5,RYR3,HECW1,KCNJ1,TRPC7,NIPAL2,MICU1,LRRC38,AKAP9,KCNS3,SLC24A4,SCN2A,ANK2,RYR2,SLC9C1,NKAIN3,ABL1,SLC1A1,SLC12A8,KCNH1,NKAIN2,ABCB7,PRKCE,SLMAP,WNK2,EGF,ABCC9,HTR2C,ALG10B,OPRM1,HTR2A,CYBRD1,CNNM4,STAC,MAIP1,KCND2,AFG3L2,ANK3,NIPA2,TMEM163,CNTN1,CACNA1I,KCNJ15,SLC10A7,SCN11A,NETO2,SLC23A2,SLC39A6,KCNH8,SLC9A4,CNKSR3,SLC30A10,SELENON,HEPHL1,GRIN2A,JPH1,TRPM6,CDH23,SLC12A1,KCNQ3,SLC4A4,SCN10A,KCND3,KCNN3,LYN,SLC1*

			3A5,CRACR2A,CUL5,PLPP4,ZDHHC17,UTRN,KCNC1,HCN1,GRIN2B,KCNK5,SLC40A1,SLC5A12,MELTF,TRPV5,PRKCB,ABCC8,CACNA1E,ATP2B1,SLC6A1,NDFIP2,SHROOM2,SLC6A11,KCNJ18,CEMIP,CBLIF,ADCYAP1R1,EFHB,NDFIP1,SLC5A9,SLC10A6,NECTIN1,FLVCR1,TRPM7,IREB2,DIAPH1,SCARA5,PLCZ1,NOS1AP,SLC9A5,SLC5A1,FYN,SCN8A,NCS1,NALCN,TRPM3,SLC39A8,HECW2,KCNJ6,DPP6,TSPAN13,CACNG3,ATG5,VMP1,KCNIP4,TRDN,NOS1,SLC6A3,ASIC2,KCNQ5,CACNA2D1,HTT,IL16,CATSPER2,RGS7,SLC13A4,KCNAB1,DNM1L
GO:2000147	positive regulation of cell motility	0.0005070603850326129	*MTOR,RIPOR2,RDX,BCL2,CARMIL1,RIN2,ANO6,APC,CRKL,EGFR,ANGPT1,NTRK3,FLT1,PRKD1,PAK1,RAPGEF2,ONECUT1,ADAM10,HDAC9,IL1R1,APP,DOCK8,MAPRE2,SEMA5A,NTF3,SLC8A1,SRGAP2C,MAP4K4,PAK3,MAPK1,MGAT5,PDGFD,NIPBL,TIAM1,SEMA3C,AGO2,IL34,BCAS3,SYNE2,SEMA6D,DOCK4,ABL1,HDAC4,FGF10,PRKCE,EGF,ARHGEF7,ATP8A1,SEMA3E,DOCK5,TJP1,IQSEC1,SEMA3A,BMP2,SEMA3D,RELN,HMGB1,FGF9,DOCK1,SLAMF1,ETS1,SMOC2,KITLG,IL6R,LYN,NTN1,INSR,SNAI2,JCAD,TWIST2,CSF1,ROR2,TWIST1,AKT3,JAK2,NEDD9,ITGA6,CEMIP,CCBE1,ITGA4,WNT7A,SDCBP,FGR,EPHA4,NUMB,FBXO31,PTK2,CDH5,DIAPH1,LAMB1,SEMA4D,JAM2,WNT5B,PPM1F,APELA,NRP1,PRKCA,FBLN1,RRAS2,EPHB2,MET,CDH13,BMP7,PDGFC,ABL2,CAMK1D,PIK3R3,FER,CCR2,SEMA4B,ROCK2,WASHC1,IGF1R,DNM1L*
GO:0060078	regulation of postsynaptic membrane potential	0.000553932763618564	*CHRNA7,RIMS1,RIMS2,GRIK3,GABRB1,DGKI,GRIA1,SLC8A3,GABRA6,APP,GABRG2,PRKCZ,GRIK4,GRM1,GABRG1,TMEM108,GRID2,P2RX6,OPRM1,KCND2,RELN,UNC13B,GABRR2,GRIK2,MTMR2,GRIN2A,GRID1,GABRG3,IGSF11,GRIN2B,CELF4,NRXN1,MEF2C,WNT7A,GABRA5,GRIK1,GRM5,GABRA2,TMEM25,NLGN1*
GO:0018209	peptidyl-serine modification	0.000618030685211050З	*BCAR3,MTOR,ULK2,NLK,PDE4D,BCL2,GALNT1,EGFR,ANGPT1,NTRK3,PRKD1,PAK1,SMYD3,RPTOR,APP,RPS6KA2,DCLK1,NTF3,AURKA,PRKCZ,MAPK1,GALNT16,STK38,TLK1,AKAP9,STK32B,MAPKAP1,SLC1A1,PRKAA1,MAST4,CAMK4,LATS2,PRKCE,CD44,RPS6KA3,STK38L,MARK2,HIPK3,CLSPN,STK32A,EGFLAM,GALNT13,VRK1,CNKSR3,PRKCH,MKNK1,ROCK1,NEK6,CSNK2A1,NRXN1,HIPK1,AKT3,TNKS,PRKCB,MAST2,PARK7,MAPK8,PKN2,NCK1,TOP1,NTRK2,PPM1F,SH2D3C,MAPK9,GALNT2,RPS6KA5,PRKCA,CSNK1G1,CAMK1G,PRKCQ,NOS1,NSD1,CAMK1D,SPOCK3,ROCK2,MORC3*
GO:0051962	positive regulation of nervous system development	0.000618258218764З645	*MTOR,PTPRD,TENM4,ROBO2,DSCAM,MACF1,BCL11A,CDH4,SPEN,LRP2,ADGRB3,STAU2,SEMA5A,PAK3,TRPC5,PAFAH1B1,SYNJ1,TIAM1,IL34,GRID2,ASPM,PLXNA2,OPRM1,LINGO2,DISC1,BMP2,RELN,NIN,GLI3,PRKCH,LYN,NTN1,IL33,ACTR2,CLSTN2,MAP6,NRXN1,UFL1,ADGRB1,WNT7A,FBXW8,EPHA4,NTRK2,IL1RAPL1,NUMB,FBXO31,CYFIP1,SEMA4D,ADGRL2,HDAC2,EPHB1,GRM5,NRP1,FAIM,CHODL,CUX1,EPHB2,FLRT2,KALRN,TIAM2,DLG5,SLIT2,SYNDIG1,ROBO1,NLGN1,ASIC2,EFNA5*
GO:0030155	regulation of cell adhesion	0.0006350488405771256	*SPOCK1,DLC1,ZDHHC21,PTPRA,RIPOR2,RDX,BCL2,TENM3,CARMIL1,RIN2,HHLA2,DSCAM,CRKL,ILDR2,PTPRJ,ANGPT1,MACF1,EPHA7,ONECUT1,CCL28,TBCD,ADAM10,DOCK8,SEMA5A,VCL,ACER2,KANK1,MAP4K4,ITPKB,PRKCZ,DUSP22,ADAM22,PLG,CORO2B,LIMCH1,FMN1,TPM1,NF2,PPARA,MAGI1,BCAS3,SMARCA4,BLK,TNR,NUAK1,ABL1,APBB1IP,NFAT5,MYO10,PEAK1,NRG1,AP3B1,ZBTB16,PRKCE,CD44,PTPRO,PTPN2,PLXNA2,ATXN3,ARHGEF7,AMBRA1,SEMA3E,ANK3,EMILIN2,DOCK5,MBP,FUT9,VAV1,TJP1,EGFLAM,DISC1,WDPCP,BMP2,RC3H2,HMGB1,DOCK1,SLAMF1,SMARCA2,ETS1,GLI3,SMARCC1,LAMC1,KITLG,DAB1,VAV3,ROCK1,LYN,VCAM1,DTX1,ZFHX3,ARID1B,SNAI2,CD9,UTRN,RASGRP1,CSF1,PRKG1,LAMA3,TNN,AJAP1,IL10,CD70,JAK2,CELSR2,PRSS2,MELTF,NEDD9,OLFM4,ASS1,ADAMTS18,ITGA4,NDFIP1,SERPINI2,NCK1,EPHA4,MEGF10,PTK2,NFKBID,LAMB1,PCDH8,SEMA4D,RUNX1,EDIL3,TNFSF11,FYN,PPM1F,CRTAM,NRP1,PRKCA,RC3H1,FBLN1,RAG1,EPHB2,CDH13,MYB,LAMA1,BMP7,DLG5,ABL2,IL20RB,PRKCQ,EFNA5,FRMD5,PRLR,CCR2,ROCK2,PPP1CB,RSU1,GLI2*

GO:0014706	striated muscle tissue development	0.000638695379491689	*NOTCH2,MTOR,SGCD,NEBL,ZFPM2,TENM4,ALDH1A2,RARB,SOX6,LRP2,SLC8A1,AKAP6,YAP1,MYLK3,TPM1,PPARA,MRTFB,SEMA3C,ALPK2,JARID2,RYR2,CXADR,XIRP2,NRG1,PGM5,ALPK3,FHL2,LUC7L,SGCZ,MYLK2,PDLIM5,BMP2,PTCD2,FGF9,TBX20,BMP5,CTDP1,SMAD5,MED1,TWIST1,MTPN,MYO18B,ASB2,MYOCD,MEF2C,ADAMTS9,TNNI1,RUNX1,SORBS2,SGCG,ATG5,NRAP,BMP7,EYA1,FHOD3,ERBB4,EYA2,HNRNPU,AKAP13*
GO:0098662	inorganic cation transmembrane transport	0.0006434937532816875	*CACNA2D3,SLC17A1,SLC24A2,KCNH5,MICU2,DPP10,ITPR2,PDE4D,BCL2,KCNMA1,CHRNA7,ANO6,CACNG2,SLC4A10,NEDD4,ATP2B2,TUSC3,SLC39A12,SLC8A3,PRKD1,FGF12,TMEM38B,SLC24A3,THADA,NEDD4L,TRPM1,SLC39A11,CACNA1C,CACNB2,TMC1,SLC8A1,KCNE4,AKAP6,KCNK10,TRPC5,RYR3,HECW1,KCNJ1,TRPC7,NIPAL2,MICU1,LRRC38,AKAP9,KCNS3,SLC24A4,SCN2A,ANK2,RYR2,SLC9C1,SLC36A1,ABL1,SLC1A1,SLC12A8,KCNH1,ABCB7,PRKCE,SLMAP,WNK2,ABCC9,HTR2C,ALG10B,OPRM1,HTR2A,CNNM4,STAC,MAIP1,KCND2,AFG3L2,ANK3,NIPA2,TMEM163,ATP6V1E1,CACNA1I,KCNJ15,SCN11A,NETO2,SLC39A6,KCNH8,SLC9A4,CNKSR3,SLC30A10,SELENON,GRIN2A,JPH1,TRPM6,SLC12A1,KCNQ3,SLC4A4,SCN10A,KCND3,KCNN3,LYN,SLC15A2,CRACR2A,CUL5,COX5A,ZDHHC17,UTRN,KCNC1,HCN1,GRIN2B,KCNK5,SLC40A1,COX7A2L,TWIST1,TRPV5,ABCC8,CACNA1E,ATP2B1,OTOP1,SLC6A1,SHROOM2,SLC6A11,KCNJ18,CEMIP,CBLIF,PARK7,ADCYAP1R1,ATP6V1C2,ATP6V1B2,TRPM7,DIAPH1,SCARA5,NOS1AP,SLC9A5,SLC5A1,FYN,SCN8A,NCS1,ATP5PF,NALCN,TRPM3,SLC39A8,HECW2,ATPSCKMT,KCNJ6,DPP6,TSPAN13,ATP6V0D2,CACNG3,ATG5,VMP1,KCNIP4,TRDN,NOS1,SLC6A3,ASIC2,KCNQ5,CACNA2D1,HTT,SLC25A18,CATSPER2,RGS7,KCNAB1*
GO:0030010	establishment of cell polarity	0.0006814627405619193	*WWC1,MYO9A,RIPOR2,SDCCAG8,MAP4,CRKL,DOCK2,CRB1,PATJ,DOCK8,KANK1,PRKCZ,MCPH1,PAFAH1B1,PARD3B,ALPK2,BCAS3,PARD3,ABL1,FGF10,MARK2,ANKFN1,WDPCP,UST,FRMD4A,ROCK1,ARFGEF1,FAT1,NDC80,WNT7A,PTK2,CDH5,MPP7,CRTAM,EPHB1,LAMA1,ABL2,SIPA1L3,ARHGEF11,FRMD4B,HTT,ROCK2,IGF1R*
GO:0003013	circulatory system process	0.0006816317124228458	*MTOR,SGCD,IMMP2L,ZDHHC21,PDE4D,KCNMA1,NAV2,ENPEP,PTPRJ,ANGPT1,CTNNA3,MYOF,FLI1,ATP2B2,SLC8A3,CHRM3,LRP2,FGF12,CPS1,TMEM38B,SLC24A3,SLC44A1,CELF2,NEDD4L,SLC7A2,RPS6KA2,CACNA1C,CACNB2,SLC8A1,KCNE4,RAP1GDS1,RNLS,YAP1,HRH2,SLC16A1,CORO2B,MYLK3,TPM1,CORIN,AKAP9,PPARA,ANK2,RYR2,BBS2,CXADR,DOCK4,ABL1,HDAC4,SLC1A1,SLCO3A1,PTPRO,ABCC9,PDE3A,EXT1,LNPEP,SLC2A3,ATP8A1,ABCC4,HTR2A,CYP4A11,SLC2A13,NOS2,SGCZ,MYLK2,EMILIN2,DOCK5,F5,ECE1,TJP1,ARHGAP42,BBS4,TBX20,SLC4A4,SCN10A,KCND3,ROCK1,SLC15A2,INSR,HCN1,PRKG1,SMAD5,VSTM4,KL,JAK2,SVEP1,PTGS1,ABCC8,ATP2B1,EXT2,SLC6A1,STAT1,MAP2K6,PDE2A,TNNI1,OCLN,CDH5,NOS1AP,ADAMTS16,SLC5A1,FYN,APELA,ASB3,ITGA1,BCR,SGCG,CD38,ATG5,SLC1A2,SLIT2,TRHDE,TRDN,NOS1,ASIC2,CACNA2D1,HRH1,ROCK2,COL4A3,THRB,AKAP13,DNM1L*
GO:0006812	cation transport	0.0007021715742743854	*UNC80,CACNA2D3,SLC17A1,SLC24A2,KCNH5,MICU2,PIEZO2,DPP10,ITPR2,PDE4D,SLC44A5,BCL2,KCNMA1,CHRNA7,ANO6,CACNG2,SLC4A10,NEDD4,ATP2B2,TUSC3,SLC39A12,SLC8A3,PRKD1,LRP2,FGF12,TMEM38B,SLC24A3,SLC44A1,THADA,NEDD4L,TRPM1,SLC39A11,APP,SLC7A2,CACNA1C,CACNB2,TMC1,SYT1,SLC8A1,KCNE4,AKAP6,HOMER2,KCNK10,TRPC5,RYR3,HECW1,KCNJ1,TRPC7,SYT10,NIPAL2,MICU1,LRRC38,AKAP9,RASGRF2,KCNS3,DAPK1,SLC24A4,SEC14L1,SCN2A,ANK2,RYR2,SLC9C1,NKAIN3,SLC36A1,ABL1,SLC1A1,SLC12A8,KCNH1,NKAIN2,GSG1L,RASGRF1,ABCB7,PRKCE,SLMAP,WNK2,EGF,ABCC9,P2RX6,HTR2C,ALG10B,ATP8A1,OPRM1,HTR2A,CYBRD1,CNNM4,STAC,CNIH3,MAIP1,KCND2,AFG3L2,ANK3,NIPA2,TMEM163,ATP6V1E1,CNTN1,CACNA1I,KCNJ15,SLC10A7,SCN11A,NETO2,RELN,SLC23A2,SLC39A6,KCNH8,SLC9A4,CNKSR3,CHRM5,SLC30A10,SELENON,PRKN,HEPHL1,GRIN2A,JPH1,TRPM6,CDH23,SLC12A1,KCNQ3,SHISA9,SLC4A4,SCN10A,KCND3,KCNN3,LYN,SLC44A2,SLC15A2,SLC13A5,CRACR2A,CUL5,COX5A,PLPP4,ZDHHC17,UTRN,*

			KCNC1,HCN1,GRIN2B,KCNK5,SLC40A1,SLC5A12,COX7A2L,NRXN1,TWIST1,MELTF,TRPV5,PRKCB,ABCC8,CACNA1E,ATP2B1,OTOP1,SLC6A1,NDFIP2,SHROOM2,SLC6A11,KCNJ18,CEMIP,CBLIF,PARK7,ADCYAP1R1,ATP13A3,EFHB,MEF2C,NDFIP1,SLC5A9,ATP6V1C2,SLC10A6,NECTIN1,FLVCR1,ATP6V1B2,SHISA6,TRPM7,IREB2,DIAPH1,SCARA5,PLCZ1,NOS1AP,SLC9A5,SLC5A1,ANO10,FYN,SCN8A,TMEM63C,NCS1,ATP5PF,NALCN,TRPM3,SLC39A8,SLC16A9,HECW2,ATPSCKMT,KCNJ6,DPP6,EPHB2,TSPAN13,ATP6V0D2,CACNG3,ATG5,VMP1,SLC1A2,KCNIP4,TRDN,NLGN1,NOS1,SLC6A3,ASIC2,KCNQ5,CACNA2D1,HTT,SLC25A18,CCR2,IL16,CATSPER2,RGS7,SLC13A4,KCNAB1,DNM1L
GO:0010647	positive regulation of cell communication	0.000720065371 9779469	*NOTCH2,BCAR3,NSG1,WWC1,SLC24A2,KSR1,PLCB1,STXBP1,CHRNA7,ROBO2,RIMS1,AKR1C3,SPRED1,RIMS2,ALK,AUTS2,PJA2,ERBIN,CACNG2,MLLT3,TSHZ3,CRKL,TNIK,PTPRJ,EGFR,ANGPT1,MACF1,RNF220,NEDD4,CHSY1,NTRK3,DKK2,FLT1,MAPKBP1,DGKI,EDAR,NEO1,CNTN6,SLC8A3,PRKD1,PAK1,RAPGEF2,PELI2,LRP2,TAOK3,RPTOR,GHR,IL1R1,APP,CACNB2,MAPRE2,SEMA5A,SYT1,NTF3,KANK1,MAP4K4,BMPR1B,AKAP6,ARNT,RAB8B,ITPKB,PRKCZ,GRB10,DUSP22,YAP1,MAPK1,MGAT5,PDGFD,UBE2O,PAFAH1B1,ATF6,TM7SF3,CTNNA1,RASGRF2,ADAMTS3,GRM1,PCDH11Y,PLA2R1,TMEM108,IL34,ADGRV1,SMARCA4,USP8,BLK,TNR,MBD5,ABL1,SLC1A1,NFAT5,GUCY1A2,RAP1A,GPC5,FGF10,ZC3HAV1,NRG1,ASPM,DENND2B,RASGRF1,PRKCE,WNK2,CD44,EGF,SPRED2,PTPN2,TRIM5,MCF2L,HTR2C,CLEC16A,ARHGEF7,OPRM1,HTR2A,SEMA3E,PUM1,TMOD2,ANKRD17,RELL1,EPN2,EVC,ANK3,PLCE1,TGFA,HIP1,PRR5L,DISC1,SEMA3A,BMP2,RC3H2,MYRIP,BMP2K,RELN,HMGB1,FGF9,TRAF3,TTC21B,DSTYK,SLAMF1,SMOC2,GRIK2,GAREM1,LAMC1,NEK10,CYLD,MAPK8IP1,CFTR,NEU3,KITLG,CAMTA1,SLC30A10,RB1CC1,PRKN,TBX20,GRIN2A,PRKCH,IL6R,ALS2,NLRC5,SHANK2,ROCK1,LYN,SLC44A2,RRAGD,CRACR2A,INSR,DEDD2,NEK6,NMU,BID,ERN2,TIAL1,ZDHHC17,JCAD,RASGRP1,IGSF11,CSNK2A1,BMP5,CSF1,GHRH,GRIN2B,MED1,ROR2,KL,BANK1,IL10,SFPQ,CLSTN2,PTH,NDC80,IQGAP1,RPS12,AIMP1,NRXN1,PCID2,CIBAR1,NET1,AKT3,ALKAL2,JAK2,MADD,HCRTR1,CREBBP,TNKS,GORAB,PRKCB,BRD4,GID8,NDFIP2,NR2C1,MAP2K6,CCBE1,PARK7,ADCYAP1R1,MYOCD,CYFIP2,MEF2C,RXRA,WNT7A,MAP3K5,NDFIP1,MAP3K4,S100B,ATP6V1C2,SDCBP,WWOX,NCK1,FGR,CDCA8,PPP2R3A,EPHA4,NTRK2,PTK2,CDH5,ANKRD6,SCGN,LAMB1,CYFIP1,UBE3A,SEMA4D,WNT5B,NENF,NOS1AP,CCDC88A,GPR55,TNFSF11,FYN,DOK5,MAPK9,APELA,ROR1,CDH2,ITGA8,RAD9A,GPR137B,GRM5,NRP1,PRKCA,ITGA1,RC3H1,POR,STK36,BMPER,PRDM15,EPHB2,CSNK1G1,CD38,MET,SPPL3,CDH13,CACNG3,GNAS,LAMA1,MFHAS1,BMP7,DLG5,PDGFC,ERBB4,ROBO1,TRDN,NLGN1,IQCJ-SCHIP1,PRLR,AGO3,HTT,CCR2,ROCK2,KIF7,STK3,ZNF423,IGF1R,THRB,AKAP13,DNM1L*
GO:0070848	response to growth factor	0.000806964472 9910724	*NOTCH2,NLK,RDX,PRDM16,FBN1,ZEB1,SPRED1,CRKL,SOX5,EGFR,NEDD4,SOX6,NTRK3,FLT1,NEO1,PRKD1,RAPGEF2,LRP2,RUNX2,FGF12,CPS1,ONECUT1,COL4A2,APP,NTF3,BMPR1B,PCSK6,ARNT,GRB10,DUSP22,MAPK1,PDGFD,UBE2O,GFRA1,ITGB8,HIVEP1,PPARA,MEIS2,ADAMTS3,PTPRK,TMEM108,USP8,ELAVL4,ABL1,PTPN12,RAP1A,FGF10,LATS2,FBN2,CD44,PDE3A,EXT1,SPRED2,LTBP1,ZFYVE9,OPRM1,EPN2,CRIM1,LDLRAD4,BMP2,PSG9,LEMD3,FGF9,DSTYK,RAP1GAP,SNX6,SMOC2,GAREM1,ATF2,RGMB,TBX20,SNX25,ROCK1,VCAM1,OVOL2,ZFHX3,INSR,SNAI2,NREP,ZDHHC17,JCAD,BMP5,KCNC1,SMAD5,MED1,KL,IL10,PTH,SOSTDC1,VSTM2A,IQGAP1,NRXN1,PBLD,PEG10,TWIST1,FSTL1,CREBBP,PRKCB,CIDEA,EXT2,BRMS1L,CCBE1,MYOCD,CYFIP2,MEF2C,WNT7A,RBPMS2,PDE2A,SDCBP,WWOX,NTRK2,IL17RD,PTK2,CDH5,CYFIP1,UBE3A,FAT4,FYN,HDAC2,DOK5,FUT8,TET1,ITGA8,NRP1,KIF16B,BMPER,MAGI2,FLRT2,BMP7,SLIT2,ERBB4,ROBO1,NOS1,FER,ROCK2,ZNF423,IGF1R*
GO:0048814	regulation of dendrite	0.000874892427 6724662	*PTPRD,TNIK,NEDD4,RAPGEF2,ADGRB3,NEDD4L,STAU2,PAK3,TRPC5,HECW1,PAFAH1B1,SDC2,KNDC1,RELN,DPYSL5,ACTR2,FBXW8,EPHA4,IL1RAPL1,FBXO31,SEMA4D,HECW2,CUX1,EPHB2,KALRN*

	morphogenesis		
GO:0009968	negative regulation of signal transduction	0.0009112099758380914	*MTOR,WWC1,PTPRD,NLK,ZNF536,DLC1,RIPOR2,PDE4D,BCL2,PRDM16,FBN1,SPRED1,MINAR1,ERBIN,MLLT3,APC,ARHGAP24,PTPRJ,EGFR,PRKACB,RGS3,NCOR1,NEDD4,SCAI,DKK2,MAPKBP1,INVS,LRP2,RUNX2,TAOK3,ONECUT1,USP18,ARHGAP44,CD2AP,PTPRR,KANK1,HOMER2,RGS20,PDE10A,KICS2,PRKCZ,GRB10,RGS9,HECW1,DUSP22,YAP1,ZNRF3,STK38,PAFAH1B1,NF2,CTNNA1,BIRC6,PPARA,PAK5,SLC24A4,SEC14L1,ALPK2,DUSP16,SMARCA4,MAPKAP1,PTPRT,ABL1,PTPN12,PRKAA1,FGF10,LATS2,NRG1,ZNF675,NXN,WNK2,FBN2,CD44,RGS12,PTPRO,PDE3A,LIMD1,SPRED2,PTPN2,LTBP1,OPRM1,KREMEN1,FHL2,HIPK3,EPN2,GRK3,MOSMO,CRIM1,PRR5L,LDLRAD4,NPHP4,BMP2,RANBP9,TMEM161A,LEMD3,ARHGAP42,FGF9,DRAXIN,SLAMF1,GLI3,SNX6,CNKSR3,CYLD,MAPK8IP1,UBASH3A,UBR1,RCAN1,DAB1,RB1CC1,PTPRE,PRKN,MTMR2,TBX20,NLRC5,SNX25,SHANK2,SOX30,LYN,OVOL2,RNF152,OTUD7A,YTHDF3,SNAI2,ASH1L,BID,SIAH2,TRABD2B,UFD1,SAMHD1,ENPP1,NDRG2,CSNK2A1,BMP5,BCL2L1,GRB14,DHRS3,CELF4,PRAME,TNN,BANK1,IL10,SOSTDC1,PRKAA2,ITPRIP,YBX3,PBLD,PEG10,TWIST1,UFL1,NFKBIA,PRKCB,BRD4,ITGA6,OTOP1,CIDEA,STAT1,BRMS1L,DGKG,PARK7,MYOCD,RBPMS2,C16ORF72,PDE2A,WWOX,NCK1,PPP2R3A,EPHA4,MECOM,SHISA6,IL17RD,ANKRD6,ARHGAP12,AMFR,BICD1,RBMS3,HDAC2,ZFYVE28,APELA,TET1,CDH2,PHLPP1,GRM5,PID1,NRP1,FAIM,ITGA1,MCC,RGS6,FBLN1,BMPER,PRDM15,EPHB2,EYA4,MET,MAGI2,MFHAS1,BMP7,DLG5,ZMYND11,TMEM25,ABL2,EYA1,SLIT2,CNOT7,ROBO1,PRKCQ,SLIT3,ESR1,HTT,EYA2,RORA,RGS8,HERPUD1,RGS7,KIF7,FSTL4,STK3,DEPTOR,APCDD1,IGF1R,GLI2*
GO:0031345	negative regulation of cell projection organization	0.0009436334271976512	*SPOCK1,ULK2,MINAR1,MAP4,ARHGAP24,BCL11A,EPHA7,RAPGEF2,SEMA5A,ARHGAP44,SRGAP2C,KANK1,DIP2B,TRPC5,DNM3,YAP1,FAT3,PAFAH1B1,SEMA3C,SEMA6D,TNR,PTPRO,KREMEN1,SEMA3E,MBP,SEMA3A,SEMA3D,RAP1GAP,DRAXIN,MAP2,DCC,DAB1,NGEF,NTN1,DPYSL5,GRIN2B,NRXN1,EPHA4,UBE3A,SEMA4D,FYN,HDAC2,PTPRG,NRP1,EPHB2,CD38,SLIT2,NLGN1,SEMA4B,FSTL4*
GO:0051966	regulation of synaptic transmission, glutamatergic	0.0009611726618432697	*STXBP1,CACNG2,TSHZ3,GRIK3,DGKI,GRM7,SYT1,GRM1,TNR,GRM8,HTR2A,DISC1,RELN,GRIK2,GRIN2A,HCN1,GRIN2B,ROR2,NRXN1,MEF2C,GRIK1,CDH2,GRM5,CACNG3,GRM3,NLGN1,CCR2*
GO:0098742	cell-cell adhesion via plasma-membrane adhesion molecules	0.0010788173963851438	*CNTN4,PTPRD,LRRC4C,TENM4,CDH8,ROBO2,TENM3,PCDH7,GPC6,DSCAM,CRB1,CDH4,CNTN6,ALCAM,FAT3,CDH7,PCDH11Y,CDH11,CXADR,PTPRT,LRFN5,CDH18,GRID2,CDHR3,NRG1,MBP,PCDH9,CDH20,BMP2,UNC5D,DAB1,PCDH15,CDH23,VCAM1,TENM2,IGSF11,CDH26,FAT1,IL10,CLSTN2,NRXN1,CADM1,CELSR2,PCDH11X,NECTIN4,HMCN1,NECTIN1,DSG1,IL1RAPL1,CDH5,CLDN18,PCDH8,FAT4,CDH9,CRTAM,CDH2,SDK1,CDH12,CDH17,CDH13,IGSF21,KIRREL3,ROBO1,NLGN1,EFNA5,NTNG1,CLDN10*
GO:0016311	dephosphorylation	0.0011299266424761381	*MTOR,PTPRD,DLC1,PTPRA,BCL2,PLPPR1,PLPPR5,PTPRJ,RPRD1A,PTPN4,TPTE2,PPP2R2B,PTPRN2,PTPRR,FIG4,DUSP22,PPM1L,MGAT5,RNGTT,MTMR10,PTPN13,PPP6R3,SYNJ1,PTPRK,PPP2R5E,DUSP16,NUAK1,PTPRT,PTPN12,INPP5A,PTPRO,MTMR3,PTPN2,AMBRA1,INPP4B,PPP2R2C,RPRD1B,ALPL,BMP2,CAMTA1,CHRM5,RCAN1,PTPRE,MTMR2,PTPRB,ROCK1,PLPP4,TMEM225,CTDP1,SYNJ2,CDC14B,PPP2R2A,IQGAP1,THNSL2,JAK2,BPNT1,PPP1R17,FRA10AC1,PDP2,MEF2C,NCK1,PPP2R3A,CDH5,SEMA4D,IMPA2,TPTE,PPM1F,SACM1L,MTMR7,PTPRG,ITGA1,SPPL3,MAGI2,MFHAS1,EYA1,HTT,EYA2,ROCK2,PPP1CB,PTPRQ*
GO:0009967	positive regulation	0.001181766271	*NOTCH2,BCAR3,WWC1,KSR1,PLCB1,CHRNA7,ROBO2,RIMS1,AKR1C3,SPRED1,RIMS2,ALK,AUTS2,PJA2,ERBIN,MLLT3,CRKL,TNIK,PTPRJ,EGFR,ANGPT1,MACF1,RNF220,NEDD4,CHSY1,NTRK3*

	of signal transduction	442579	,DKK2,FLT1,MAPKBP1,DGKI,EDAR,NEO1,CNTN6,PRKD1,PAK1,RAPGEF2,PELI2,LRP2,TAOK3,RPTOR,GHR,IL1R1,APP,MAPRE2,SEMA5A,NTF3,KANK1,MAP4K4,BMPR1B,AKAP6,ARNT,ITPKB,PRKCZ,GRB10,DUSP22,YAP1,MGAT5,PDGFD,UBE2O,PAFAH1B1,ATF6,CTNNA1,ADAMTS3,GRM1,PCDH11Y,PLA2R1,TMEM108,IL34,ADGRV1,SMARCA4,USP8,MBD5,ABL1,NFAT5,GUCY1A2,RAP1A,GPC5,FGF10,ZC3HAV1,NRG1,ASPM,DENND2B,RASGRF1,PRKCE,WNK2,CD44,EGF,SPRED2,PTPN2,TRIM5,MCF2L,HTR2C,CLEC16A,OPRM1,HTR2A,SEMA3E,PUM1,TMOD2,ANKRD17,RELL1,EPN2,EVC,PLCE1,TGFA,HIP1,PRR5L,DISC1,SEMA3A,BMP2,RC3H2,BMP2K,RELN,HMGB1,FGF9,TRAF3,TTC21B,DSTYK,SLAMF1,SMOC2,GAREM1,LAMC1,NEK10,CYLD,MAPK8IP1,NEU3,KITLG,CAMTA1,SLC30A10,RB1CC1,PRKN,TBX20,GRIN2A,PRKCH,IL6R,ALS2,NLRC5,ROCK1,LYN,SLC44A2,RRAGD,CRACR2A,INSR,DEDD2,NEK6,BID,ERN2,TIAL1,ZDHHC17,JCAD,RASGRP1,IGSF11,CSNK2A1,BMP5,CSF1,GHRH,GRIN2B,MED1,ROR2,KL,BANK1,IL10,SFPQ,PTH,NDC80,IQGAP1,RPS12,NRXN1,PCID2,CIBAR1,NET1,AKT3,ALKAL2,JAK2,MADD,HCRTR1,CREBBP,TNKS,GORAB,PRKCB,BRD4,GID8,NDFIP2,NR2C1,MAP2K6,CCBE1,PARK7,ADCYAP1R1,MYOCD,CYFIP2,MEF2C,RXRA,WNT7A,MAP3K5,NDFIP1,MAP3K4,S100B,ATP6V1C2,SDCBP,WWOX,NCK1,FGR,CDCA8,PPP2R3A,EPHA4,NTRK2,PTK2,CDH5,ANKRD6,LAMB1,CYFIP1,UBE3A,SEMA4D,WNT5B,NENF,NOS1AP,CCDC88A,GPR55,TNFSF11,FYN,DOK5,MAPK9,APELA,ROR1,CDH2,ITGA8,RAD9A,GPR137B,GRM5,NRP1,PRKCA,ITGA1,RC3H1,POR,STK36,BMPER,PRDM15,CSNK1G1,MET,SPPL3,CDH13,GNAS,LAMA1,MFHAS1,BMP7,DLG5,PDGFC,ERBB4,ROBO1,NLGN1,IQCJ-SCHIP1,PRLR,AGO3,HTT,ROCK2,KIF7,STK3,ZNF423,IGF1R,THRB,AKAP13,DNM1L*
GO:0023056	positive regulation of signaling	0.0012722146807059092	*NOTCH2,BCAR3,NSG1,WWC1,SLC24A2,KSR1,PLCB1,STXBP1,CHRNA7,ROBO2,RIMS1,AKR1C3,SPRED1,RIMS2,ALK,AUTS2,PJA2,ERBIN,CACNG2,MLLT3,TSHZ3,CRKL,TNIK,PTPRJ,EGFR,ANGPT1,MACF1,RNF220,NEDD4,CHSY1,NTRK3,DKK2,FLT1,MAPKBP1,DGKI,EDAR,NEO1,CNTN6,SLC8A3,PRKD1,PAK1,RAPGEF2,PELI2,LRP2,TAOK3,RPTOR,GHR,IL1R1,APP,CACNB2,MAPRE2,SEMA5A,SYT1,NTF3,KANK1,MAP4K4,BMPR1B,AKAP6,ARNT,RAB8B,ITPKB,PRKCZ,GRB10,DUSP22,YAP1,MAPK1,MGAT5,PDGFD,UBE2O,PAFAH1B1,ATF6,TM7SF3,CTNNA1,RASGRF2,ADAMTS3,GRM1,PCDH11Y,PLA2R1,TMEM108,IL34,ADGRV1,SMARCA4,USP8,BLK,TNR,MBD5,ABL1,SLC1A1,NFAT5,GUCY1A2,RAP1A,GPC5,FGF10,ZC3HAV1,NRG1,ASPM,DENND2B,RASGRF1,PRKCE,WNK2,CD44,EGF,SPRED2,PTPN2,TRIM5,MCF2L,HTR2C,CLEC16A,ARHGEF7,OPRM1,HTR2A,SEMA3E,PUM1,TMOD2,ANKRD17,RELL1,EPN2,EVC,ECE1,PLCE1,TGFA,HIP1,PRR5L,DISC1,SEMA3A,BMP2,RC3H2,MYRIP,BMP2K,RELN,HMGB1,FGF9,TRAF3,TTC21B,DSTYK,SLAMF1,SMOC2,GRIK2,GAREM1,LAMC1,NEK10,CYLD,MAPK8IP1,CFTR,NEU3,KITLG,CAMTA1,SLC30A10,RB1CC1,PRKN,TBX20,GRIN2A,PRKCH,IL6R,ALS2,NLRC5,SHANK2,ROCK1,LYN,SLC44A2,RRAGD,CRACR2A,INSR,DEDD2,NEK6,NMU,BID,ERN2,TIAL1,ZDHHC17,JCAD,RASGRP1,IGSF11,CSNK2A1,BMP5,CSF1,GHRH,GRIN2B,MED1,ROR2,KL,BANK1,IL10,SFPQ,CLSTN2,PTH,NDC80,IQGAP1,RPS12,AIMP1,NRXN1,PCID2,CIBAR1,NET1,AKT3,ALKAL2,JAK2,MADD,HCRTR1,CREBBP,TNKS,GORAB,PRKCB,BRD4,GID8,NDFIP2,NR2C1,MAP2K6,CCBE1,PARK7,ADCYAP1R1,MYOCD,CYFIP2,MEF2C,RXRA,WNT7A,MAP3K5,NDFIP1,MAP3K4,S100B,ATP6V1C2,SDCBP,WWOX,NCK1,FGR,CDCA8,PPP2R3A,EPHA4,NTRK2,PTK2,CDH5,ANKRD6,SCGN,LAMB1,CYFIP1,UBE3A,SEMA4D,WNT5B,NENF,NOS1AP,CCDC88A,GPR55,TNFSF11,FYN,DOK5,MAPK9,APELA,ROR1,CDH2,ITGA8,RAD9A,GPR137B,GRM5,NRP1,PRKCA,ITGA1,RC3H1,POR,STK36,BMPER,PRDM15,EPHB2,CSNK1G1,CD38,MET,SPPL3,CDH13,CACNG3,GNAS,LAMA1,MFHAS1,BMP7,DLG5,PDGFC,ERBB4,ROBO1,NLGN1,IQCJ-SCHIP1,PRLR,AGO3,HTT,CCR2,ROCK2,KIF7,STK3,ZNF423,IGF1R,THRB,AKAP13,DNM1L*
GO:00	JNK	0.00129	*PLCB1,PJA2,CRKL,TNIK,EGFR,NCOR1,MAPKBP1,EDAR,FGF12,TAOK3,APP,MAP4K4,DUSP22,PAFAH1B1,DUSP16,ZNF675,HIPK*

07254	cascade	7046862 5333584	*3,MDFIC,SEMA3A,MAGI3,HMGB1,TRAF3,SLAMF1,ATF2,CYLD,MAPK8IP1,MAP4K3,RB1CC1,PRKN,MAPK10,CRACR2A,RASGRP1,MAPK8,WNT7A,MAP3K5,SDCBP,MECOM,ANKRD6,TNFSF11,SH2D3C,MAPK9,PHLPP1,EPHB1,MFHAS1,ZMYND11,STK3,IGF1R*
GO:0019220	regulation of phosphate metabolic process	0.001412715859 5690595	*BCAR3,MTOR,KSR1,DLC1,PDE4D,BCL2,CHRNA7,SPRED1,ALK,APC,DSCAM,CRKL,TNIK,PTPRJ,EGFR,ANGPT1,CDK12,NCOR1,NTRK3,FLT1,SLC8A3,PRKD1,PAK1,EPHA7,RAPGEF2,PELI2,TAOK3,LDB2,SMYD3,RPTOR,GHR,APP,SAMSN1,NTF3,SLC8A1,BMPR1B,ARNT,RANBP2,ITPKB,TRPC5,NBN,SCP2,PRKCZ,GRB10,MCPH1,DUSP22,MAPK1,MGAT5,PDGFD,NRG3,GFRA1,STK38,PTPN13,LIMCH1,CCNG2,NF2,MOB3B,AKAP9,PPARA,PPP6R3,IL34,WNT9B,DUSP16,PARD3,MAPKAP1,NUAK1,PTPRT,ABL1,HDAC4,SLC1A1,PRKAA1,RAP1A,FGF10,LATS2,NRG1,MUSK,ZNF675,PRKCE,SLCO3A1,CD44,PTPRO,EGF,PRRC1,SPRED2,MTMR3,PTPN2,HTR2C,AMBRA1,HTR2A,MARK2,EPHA6,HIPK3,CDKN2C,KNDC1,CLSPN,NOS2,MNAT1,HMGA2,CCND3,PLCE1,TGFA,PRR5L,LDLRAD4,CNTN1,BLM,BMP2,RELN,GNAQ,FGF9,SH3BP5,DSTYK,SNX6,CNKSR3,NEK10,MOB1B,ATF2,MAPK8IP1,ME2,KITLG,CAMTA1,CHRM5,RCAN1,TADA2A,DAB1,RB1CC1,PRKN,MTMR2,IL6R,ALS2,NLRC5,SNX25,SLC4A4,PTPRB,COPS8,VAV3,RALB,ROCK1,LYN,INSR,ERN2,CARD10,ENPP1,RASGRP1,SNX9,TMEM225,BMP5,CSF1,GPRC5C,ROR2,BANK1,PTH,PRKAA2,IQGAP1,NRXN1,CENPE,ALKAL2,JAK2,MADD,RTRAF,BRD4,NEDD9,ITGA6,PPP1R17,MAP2K6,ABI1,CEMIP,IMPACT,PARK7,ADCYAP1R1,NCAPG2,MYOCD,MEF2C,MAP3K5,MAP3K4,RAB38,DBF4B,SDCBP,MLLT1,NCK1,FGR,CDCA8,EPHA4,NTRK2,OCLN,PTK2,CDH5,SEMA4D,ZBTB20,KIRREL1,PDCL3,CCDC88A,TNFSF11,FYN,PPM1F,HDAC2,SH2D3C,DOCK3,NCS1,ZFYVE28,ROR1,EPHB1,GRM5,PID1,NRP1,ATPSCKMT,ITGA1,FBLN1,BMPER,MACROH2A1,EPHB2,MET,SPPL3,MAGI2,MFHAS1,BMP7,BTBD10,ADGRF5,PDGFC,SLIT2,CNOT7,ERBB4,ROBO1,NOS1,EFNA5,NSD1,PRLR,HTT,PIK3R3,FER,HRH1,ROCK2,PDK1,WASHC1,BARD1,STK3,DEPTOR,HNRNPU,IGF1R,PRKAG2,AKAP13,DNM1L*
GO:0048646	anatomical structure formation involved in morphogenesis	0.001413820554 7156474	*NOTCH2,NEBL,TAFA5,TENM4,DLC1,RIPOR2,RALA,ALDH1A2,CHRNA7,ROBO2,SDCCAG8,SPRED1,ENPEP,MINAR1,FOXJ2,DSCAM,SETD2,ARHGAP24,KDM4C,ANGPT1,PRKACB,MYOF,CRB1,TMEM182,CECR2,C5,FLT1,EDAR,SLC39A12,PRKD1,LRP2,ADGRB3,LUZP1,EPB41L3,COL4A2,SSBP3,HDAC9,SEMA5A,FIG4,THSD7A,IFT57,CALD1,COBL,YAP1,MAPK1,NRG3,GABPA,FAT3,MYLK3,FMN1,PAFAH1B1,ITGB8,TPM1,NF2,PPARA,NFIB,SF3B6,SEMA3C,SLC24A4,AGO2,ANK2,TANC1,BCAS3,WNT9B,LDB3,COL22A1,ABL1,SLC1A1,KCNH1,FGF10,GRID2,LATS2,PGM5,FBN2,EGF,EXT1,ATP8A2,PLXNA2,CNNM4,SEMA3E,FHL2,TMOD2,HERC1,EPN2,KNDC1,ADAM12,EMILIN2,HMGA2,MYOM2,TGFA,TJP1,CNTN1,MTHFD1L,BMP2,RELN,FGF9,NFATC2,TTC21B,ETS2,ETS1,GLI3,MEGF11,SMOC2,ATF2,BBS4,COL5A1,CFTR,GTF2I,MTMR2,SH3PXD2A,TBX20,PTPRB,VAV3,SOX30,PTGFRN,ADGRG6,ROCK1,OVOL2,HECTD1,SHROOM3,SNAI2,SP3,CD9,CARD10,JCAD,BMP5,WDR72,LAMA3,CNMD,VSTM4,SLC40A1,TNN,MED1,KDM6A,IL10,VASP,AIMP1,NRXN1,HIPK1,TWIST1,AKT3,ADAMTS5,PRKCB,ABCC8,EXT2,AGO1,MEOX2,STAT1,MTPN,ABI1,CCBE1,ITGA4,MEF2C,ADGRB1,WNT7A,NECTIN1,PPP2R3A,ADAMTS9,WNT2B,HS6ST1,PTK2,CDH5,LAMB1,PCDH8,JAM2,RUNX1,PDCL3,HDAC2,COL18A1,APELA,TET1,ITGA8,EPHB1,NRP1,SDK1,PRKCA,RC3H1,NRXN3,KIF16B,BMPER,MACROH2A1,EPHB2,CDH13,NRAP,GNAS,BMP7,RFX2,EYA1,FHOD3,SLIT2,EXOC4,ROBO1,NOS1,LOXL2,FOXB1,PIK3R3,EYA2,CCR2,STARD13,ROCK2,RORA,HSPG2,COL4A3,STK3,GLI2,AKAP13*
GO:0051174	regulation of phosphorus metabolic process	0.001496182430 6183986	*BCAR3,MTOR,KSR1,DLC1,PDE4D,BCL2,CHRNA7,SPRED1,ALK,APC,DSCAM,CRKL,TNIK,PTPRJ,EGFR,ANGPT1,CDK12,NCOR1,NTRK3,FLT1,SLC8A3,PRKD1,PAK1,EPHA7,RAPGEF2,PELI2,TAOK3,LDB2,SMYD3,RPTOR,GHR,APP,SAMSN1,NTF3,SLC8A1,BMPR1B,ARNT,RANBP2,ITPKB,TRPC5,NBN,SCP2,PRKCZ,GRB10,MCPH1,DUSP22,MAPK1,MGAT5,PDGFD,NRG3,GFRA1,STK38,PTPN13,LIMCH1,CCNG2,NF2,MOB3B,AKAP9,PPARA,PPP6R3,IL34,WNT9B,DUSP16,PARD3,MAPKAP1,NUAK1,PTPRT,ABL1,HDAC4,SLC1A*

			1,PRKAA1,RAP1A,FGF10,LATS2,NRG1,MUSK,ZNF675,PRKCE,SLCO3A1,CD44,PTPRO,EGF,PRRC1,SPRED2,MTMR3,PTPN2,HTR2C,AMBRA1,HTR2A,MARK2,EPHA6,HIPK3,CDKN2C,KNDC1,CLSPN,NOS2,MNAT1,HMGA2,CCND3,PLCE1,TGFA,PRR5L,LDLRAD4,CNTN1,BLM,BMP2,RELN,GNAQ,FGF9,SH3BP5,DSTYK,SNX6,CNKSR3,NEK10,MOB1B,ATF2,MAPK8IP1,ME2,KITLG,CAMTA1,CHRM5,RCAN1,TADA2A,DAB1,RB1CC1,PRKN,MTMR2,IL6R,ALS2,NLRC5,SNX25,SLC4A4,PTPRB,COPS8,VAV3,RALB,ROCK1,LYN,INSR,ERN2,CARD10,ENPP1,RASGRP1,SNX9,TMEM225,BMP5,CSF1,GPRC5C,ROR2,BANK1,PTH,PRKAA2,IQGAP1,NRXN1,CENPE,ALKAL2,JAK2,MADD,RTRAF,BRD4,NEDD9,ITGA6,PPP1R17,MAP2K6,ABI1,CEMIP,IMPACT,PARK7,ADCYAP1R1,NCAPG2,MYOCD,MEF2C,MAP3K5,MAP3K4,RAB38,DBF4B,SDCBP,MLLT1,NCK1,FGR,CDCA8,EPHA4,NTRK2,OCLN,PTK2,CDH5,SEMA4D,ZBTB20,KIRREL1,PDCL3,CCDC88A,TNFSF11,FYN,PPM1F,HDAC2,SH2D3C,DOCK3,NCS1,ZFYVE28,ROR1,EPHB1,GRM5,PID1,NRP1,ATPSCKMT,ITGA1,FBLN1,BMPER,MACROH2A1,EPHB2,MET,SPPL3,MAGI2,MFHAS1,BMP7,BTBD10,ADGRF5,PDGFC,SLIT2,CNOT7,ERBB4,ROBO1,NOS1,EFNA5,NSD1,PRLR,HTT,PIK3R3,FER,HRH1,ROCK2,PDK1,WASHC1,BARD1,STK3,DEPTOR,HNRNPU,IGF1R,PRKAG2,AKAP13,DNM1L
GO:0060998	regulation of dendritic spine development	0.001549728015425211	*STAU2,SRGAP2C,PAK3,DNM3,PAFAH1B1,DISC1,RELN,NGEF,TANC2,ACTR2,MEF2C,FOXO6,UBE3A,ASAP1,HDAC2,SDK1,EPHB2,PPFIA2,KALRN,DLG5,NLGN1,FSTL4*
GO:0048638	regulation of developmental growth	0.0015524453576872522	*WWC1,ULK2,FTO,PLCB1,ZFPM2,BCL2,RIMS1,RIMS2,DSCAM,MACF1,BCL11A,CDH4,EPHA7,GHR,NEDD4L,APP,SEMA5A,SYT1,AKAP6,DIP2B,TRPC5,YAP1,NIPBL,PAFAH1B1,PPARA,SEMA3C,JARID2,BBS2,SEMA6D,TNR,CXADR,MBD5,ABL1,LATS2,NRG1,MUSK,ATP8A2,SEMA3E,AFG3L2,DISC1,SEMA3A,SEMA3D,FGF9,SLC23A2,PLS1,DRAXIN,MAP2,BBS4,DCC,PRKN,TBX20,ITSN2,NTN1,INSR,COLQ,CSF1,GHRH,CTDP1,ATRN,YBX3,MEF2C,FLVCR1,CYFIP1,SEMA4D,RUNX1,NRP1,GNAS,ERBB4,SLC6A3,EFNA5,SEMA4B,FSTL4,STK3*
GO:0198738	cell-cell signaling by wnt	0.0015817458733880737	*NLK,MLLT3,GPC6,APC,TNIK,EGFR,MACF1,RNF220,DKK2,INVS,PRICKLE2,APP,SEMA5A,PYGO1,KANK1,GRB10,HECW1,YAP1,ZNRF3,KLF15,TIAM1,PCDH11Y,ALPK2,CPE,WNT9B,SMARCA4,USP8,ABL1,PRKAA1,GPC5,FGF10,LATS2,ASPM,NXN,WNK2,PTPRO,EGF,EXT1,LIMD1,CTNND2,OPRM1,KREMEN1,MARK2,MDFIC,NPHP4,SNX3,DISC1,STRN,BMP2,GNAQ,FGF9,TTC21B,DRAXIN,GLI3,CYLD,KPNA1,PRKN,MESD,SOX30,ZBTB33,SNAI2,SIAH2,TRABD2B,NDRG2,CSNK2A1,TNN,ROR2,SOSTDC1,PRKAA2,RPS12,CELSR2,TNKS,GID8,WNT7A,ATP6V1C2,WWOX,PPP2R3A,WNT2B,SHISA6,ANKRD6,WNT5B,AMFR,CCDC88A,RBMS3,ROR1,CDH2,RNF138,MCC,PRDM15,MITF,CSNK1G1,CDK14,MAGI2,STK3,APCDD1*
GO:0006897	endocytosis	0.0016699632123134294	*ABCA13,LRP12,TMPRSS2,CLTCL1,EPS15L1,PIK3C3,MYO1E,CARMIL1,MCTP1,FCHO2,RIN2,ANO6,CACNG2,EGFR,DENND1A,ANGPT1,DOCK2,NEDD4,BTBD9,DNAJC13,RABEP1,GRIA1,PRKD1,LRP2,LDLRAD3,GHR,NEDD4L,APP,SYT1,NTF3,CD2AP,DNM3,CUBN,RAB27B,MAPK1,RABGAP1L,ANKFY1,STON2,SYNJ1,PLA2R1,TMEM108,RAB22A,AMPH,ANK2,TBC1D5,ABL1,NRG1,GSG1L,SH3GL3,USP33,EGF,ZFYVE9,BIN2,SH3KBP1,EPN2,GRK3,CD163,HHIPL1,HIP1,RUFY2,PACSIN2,DNER,LRP1B,ATP9A,MSR1,RIN3,BMP2K,AP4E1,PRG4,DOCK1,NEU3,REPS1,MTMR2,ITSN2,ROCK1,INSR,DMBT1,IGHV3-74,TOM1,ESYT2,CD9,XKR5,ENPP1,SNX9,BCL2L1,IGHV2-70D,SYNJ2,ENTHD1,SNAP91,IGHV1OR15-9,XKR6,ITGA4,ADGRB1,SDCBP,JPT2,NUMB,COLEC12,STON1-GTF2A1L,MEGF10,CD5L,ARHGAP12,SCAMP1,UBE3A,AP2B1,SCARA5,HEATR5A,BICD1,ATP9B,EHBP1,APELA,FCHSD2,ELMO1,CSNK1G1,CDH13,CACNG3,MAGI2,TMPRSS15,GAPVD1,ABL2,TMPRSS3,NLGN1,LOXL2,IGLC3,IGHV1OR21-1,RAB31,HSPG2,CLCN5,DNM1L*

GO:0022604	regulation of cell morphogenesis	0.0018796811074577808	*PTPRD,MYO9A,DLC1,RDX,RIMS1,FGD4,CDC42EP3,RIMS2,CARMIL1,PARVB,ZMYM4,CRKL,MACF1,BCL11A,EPB41L3,NEDD4L,STAU2,SYT1,CFDP1,KANK1,PAK3,PAFAH1B1,TPM1,BRWD1,ABL1,GAS2,MYO10,CD44,LIMD1,PLXNA2,ARHGEF7,SEMA3E,MARK2,SH3KBP1,DOCK5,WDPCP,RELN,SLC23A2,DOCK1,PRKN,ITSN2,SHROOM3,ACTR2,PALMD,MELTF,NEDD9,OLFM4,GRIP1,WASF3,FBXW8,FGR,DNMBP,EPHA4,IL1RAPL1,FBXO31,PTK2,DIAPH1,CYFIP1,SEMA4D,FYN,MYL12B,NRP1,FBLN1,CUX1,EPHB2,FAM171A1,KALRN,EFNA5,NTNG1,EPS8,ATP10A*
GO:0040013	negative regulation of locomotion	0.0018958513665066296	*PLCB1,TAFA5,DLC1,RIPOR2,BCL2,ROBO2,SPRED1,MCTP1,PTPRJ,SCAI,C5,CCL28,SEMA5A,VCL,PTPRR,SRGAP2C,SRGAP2B,KANK1,DUSP22,NRG3,LIMCH1,TPM1,NF2,CTNNA1,PTPRK,SEMA3C,NAV3,SEMA6D,PTPRT,NRG1,PTPRO,PTPN2,SEMA3E,EMILIN2,LDLRAD4,SEMA3A,RIN3,SEMA3D,HMGB1,SRGAP2,DACH1,CARD10,BMP5,PRKG1,TNN,IL33,ABHD2,ABCC8,NEDD9,MEOX2,BRMS1L,MYOCD,MEF2C,ADGRB1,EPHA4,ADAMTS9,SEMA4D,HDAC2,TET1,GRM5,PTPRG,NRP1,MCC,BCR,FBLN1,SRGAP3,MITF,MAGI2,DLG5,ZMYND8,SLIT2,ROBO1,FRMD5,STARD13,SPOCK3,SEMA4B*
GO:0043410	positive regulation of MAPK cascade	0.002082349620827072	*NOTCH2,BCAR3,WWC1,KSR1,PLCB1,CHRNA7,ALK,PJA2,CRKL,TNIK,PTPRJ,EGFR,ANGPT1,NTRK3,FLT1,MAPKBP1,EDAR,PAK1,RAPGEF2,PELI2,TAOK3,GHR,APP,NTF3,PRKCZ,DUSP22,PDGFD,GRM1,IL34,ABL1,RAP1A,FGF10,NRG1,DENND2B,PRKCE,CD44,EGF,TRIM5,HTR2C,OPRM1,HTR2A,RELL1,PLCE1,TGFA,SEMA3A,BMP2,HMGB1,TRAF3,DSTYK,SLAMF1,GAREM1,NEK10,MAPK8IP1,KITLG,SLC30A10,RB1CC1,IL6R,ROCK1,CRACR2A,INSR,ERN2,JCAD,RASGRP1,ROR2,KL,BANK1,IQGAP1,NRXN1,ALKAL2,JAK2,MADD,HCRTR1,MAP2K6,MEF2C,WNT7A,MAP3K5,MAP3K4,SDCBP,EPHA4,NTRK2,ANKRD6,NENF,GPR55,TNFSF11,DOK5,APELA,CDH2,GRM5,NRP1,PRKCA,ITGA1,BMPER,MFHAS1,PDGFC,ERBB4,ROBO1,ROCK2,STK3,IGF1R,AKAP13*
GO:1901698	response to nitrogen compound	0.002119541676264687	*BCAR3,MTOR,NSG1,PLCB1,PTPRA,ITPR2,PDE4D,MYO5A,FBN1,CHRNA7,PIK3C3,GABRB3,ZEB1,ALK,ERBIN,HLCS,APC,CRKL,TNIK,EGFR,USP14,BCL11A,PSMB2,GABRB1,GRIA1,CHRM3,ADSS2,RAPGEF2,CPS1,CPEB4,TMEM38B,BCKDHB,RPTOR,GHR,HDAC9,APP,KYNU,GABRG2,SLC8A1,ECPAS,KANK1,AKAP6,HOMER2,RAB8B,RFTN1,LARP1,RAP1GDS1,RNLS,PRKCZ,GRB10,RYR3,MAPK1,HRH2,SGTB,USP25,PDGFD,SPIDR,GABPA,HRH4,GLP2R,ATF6,CTNNA1,AKAP9,KLF15,PPARA,SLC24A4,VPS13C,RYR2,EFTUD2,MBD5,ATRX,ELAVL4,ABL1,SLC1A1,PRKAA1,RAP1A,PRKCE,DENND4C,ABCC9,P2RX6,PDE3A,EXT1,PTPN2,ATXN3,HTR2C,RFTN2,OPRM1,HTR2A,TMEM67,ALPL,GNAL,CCND3,BLM,SOGA1,GNAI1,TBC1D4,GNAQ,ZNF106,ATF1,CGAS,SMARCC1,SNX6,IDE,ATF2,UMODL1,CFTR,UBR1,CHRM5,SLC30A10,SELENON,PTPRE,PRKN,GRIN2A,RALB,ROCK1,LYN,VCAM1,SEL1L,EIF2B3,RRAGD,ARID1B,CRACR2A,INSR,TFF1,UFD1,CD9,ENPP1,KCNC1,BCL2L1,HCN1,GRB14,HADHA,PPP2R2A,KL,IL10,ACTR2,SREBF2,JAK2,PCNA,UFL1,NFKBIA,PRKCB,ABCC8,ATP2B1,ASS1,ERLIN2,OTOP1,SLC6A1,STAT1,MARCHF6,IMPACT,ITGA4,UBE2J2,MEF2C,MAP3K5,OR10H2,PDE2A,NCK1,EPHA4,NTRK2,COLEC12,PTK2,DIAPH1,CYFIP1,UBE3A,AMFR,FYN,HDAC2,SEL1L2,GRM5,PID1,SDK1,POR,NSG2,GNA14,EPHB2,CDH13,STXBP4,SLC1A2,GNAS,BMP7,PDGFC,SLIT2,PRKCQ,MGMT,SLC6A3,GLDC,CACNA2D1,PIK3R3,FER,HRH1,ROCK2,RGS8,RAB31,HERPUD1,RGS7,GNG2,PNPLA3,IGF1R*
GO:0050767	regulation of neurogenesis	0.002312388420581863	*MTOR,PTPRD,ULK2,TENM4,ROBO2,DSCAM,MACF1,BCL11A,CDH4,NTRK3,EPHA7,SPEN,RAPGEF2,LRP2,STAU2,SEMA5A,PAK3,DIP2B,TRPC5,YAP1,BRINP1,PAFAH1B1,NF2,CTNNA1,PRTG,SYNJ1,TIAM1,SEMA3C,IL34,SEMA6D,TNR,ASPM,PLXNA2,OPRM1,SEMA3E,MBP,DISC1,SEMA3A,BMP2,SEMA3D,RELN,NIN,DRAXIN,GLI3,MAP2,DCC,DAB1,PRKCH,LYN,NTN1,DPYSL5,IL33,ACTR2,MAP6,UFL1,ABCC8,WNT7A,FBXW8,EPHA4,NTRK2,IL1RAPL1,NUMB,FBXO31,CYFIP1,SEMA4D,HDAC2,GRM5,NRP1,FAIM,CHODL,CUX1,EPHB2,KALRN,TIAM2,BMP7,SLIT2,ROBO1,EFNA5,SEMA4B,HOOK3,FSTL4*
GO:19	neuron	0.00231	*ULK2,RIMS1,RIMS2,AUTS2,DSCAM,MACF1,BCL11A,CDH4,NEDD4L,DCLK1,SEMA5A,SYT1,VCL,AURKA,DIP2B,TRPC5,PRKCZ,AL*

90138	projection extension	5742168 6287086	*CAM,PAFAH1B1,SEMA3C,TMEM108,SEMA6D,TNR,ABL1,SEMA3E,DISC1,SEMA3A,SEMA3D,SLC23A2,DRAXIN,MAP2,PRKN,ITSN2,NTN1,TNN,IQGAP1,IMPACT,ITGA4,CYFIP2,CYFIP1,SEMA4D,SPAG6,NRP1,SLIT2,SLIT3,SEMA4B*
GO:0016055	Wnt signaling pathway	0.002397166632 0501584	*NLK,MLLT3,GPC6,APC,TNIK,EGFR,MACF1,RNF220,DKK2,INVS,PRICKLE2,APP,SEMA5A,PYGO1,KANK1,GRB10,HECW1,YAP1,ZNRF3,KLF15,TIAM1,PCDH11Y,ALPK2,CPE,WNT9B,SMARCA4,USP8,ABL1,PRKAA1,GPC5,FGF10,LATS2,ASPM,NXN,WNK2,PTPRO,EGF,EXT1,LIMD1,CTNND2,KREMEN1,MARK2,MDFIC,NPHP4,SNX3,DISC1,STRN,BMP2,GNAQ,FGF9,TTC21B,DRAXIN,GLI3,CYLD,KPNA1,PRKN,MESD,SOX30,ZBTB33,SNAI2,SIAH2,TRABD2B,NDRG2,CSNK2A1,TNN,ROR2,SOSTDC1,PRKAA2,RPS12,CELSR2,TNKS,GID8,WNT7A,ATP6V1C2,WWOX,PPP2R3A,WNT2B,SHISA6,ANKRD6,WNT5B,AMFR,CCDC88A,RBMS3,ROR1,CDH2,RNF138,MCC,PRDM15,MITF,CSNK1G1,CDK14,MAGI2,STK3,APCDD1*
GO:0046578	regulation of Ras protein signal transduction	0.002595762893 245768	*NOTCH2,DLC1,RIPOR2,RDX,AUTS2,ERBIN,ARHGAP24,DENND1A,SCAI,DGKI,RALGPS1,MAPRE2,ARHGAP44,KANK1,MAP4K4,ITPKB,RASGRF2,MAPKAP1,ABL1,FGF10,NRG1,RASGRF1,DENND4C,MCF2L,PLCE1,IQSEC1,ARHGAP42,PSD3,KITLG,ALS2,ARHGEF28,ARFGEF1,RALGPS2,RASGRP1,CSF1,NET1,MADD,ARFGEF3,CYTH4,GPR55,NRP1,BCR,EPHB2,MET,ABL2,ROBO1,MYO9B,STARD13,EPS8,AKAP13*
GO:0051049	regulation of transport	0.002632112656 6239686	*CACNA2D3,WWC1,ABCA13,KCNH5,MX2,CLTCL1,DPP10,PDE4D,RDX,STXBP1,RALA,BCL2,KCNMA1,RIMS1,PIK3C3,RIMS2,MCTP1,ANO6,CACNG2,SETD2,PTPRJ,RFX3,ANGPT1,DOCK2,NEDD4,BTBD9,DNAJC13,DGKI,C12ORF4,TTC39B,NUP214,PRKD1,CHRM3,FGF12,TMEM38B,UBE2L3,GRM7,THADA,NEDD4L,APP,CACNA1C,CACNB2,TMC1,SYT1,ARHGAP44,NTF3,NDUFAF2,CD2AP,SLC8A1,ABCG8,KCNE4,AKAP6,HOMER2,RAB8B,KCNK10,RAP1GDS1,CLIC6,DNM3,SCP2,PRKCZ,GRB10,RAB27B,CNST,HECW1,ABCA5,MAPK1,CADPS2,KCNJ1,SYT10,ANKFY1,SLC16A1,TM7SF3,STON2,LRRC38,CORIN,AKAP9,KLF15,RASGRF2,PPARA,KCNS3,SYNJ1,PLA2R1,DAPK1,SCN2A,DYSF,ANK2,BCAS3,RYR2,NKAIN3,TBC1D5,BLK,ABL1,SLC1A1,PRKAA1,KCNH1,EIPR1,RAP1A,NKAIN2,FGF10,NRG1,GSG1L,RASGRF1,SH3GL3,PRKCE,SLMAP,WNK2,EGF,ABCC9,STXBP6,ATP8A2,SCG5,HTR2C,ARHGEF7,ALG10B,ATP8A1,OPRM1,HTR2A,CYP4A11,STAC,CNIH3,APBA2,KCND2,NOS2,MDFIC,MYLK2,ANK3,LYPLA1,HIP1,PRR5L,RUFY2,PACSIN2,CNTN1,SNX3,CACNA1I,KCNJ15,BMP2,ATP9A,SCN11A,TBC1D4,MYRIP,RIN3,BMP2K,NETO2,RELN,HMGB1,MYOM1,UNC13B,TTC21B,SLAMF1,KCNH8,GLI3,CNKSR3,MCTP2,MAP2,CFTR,NEU3,SLC30A10,SELENON,PRKN,MTMR2,GRIN2A,JPH1,FRMD4A,KCNQ3,SHISA9,SCN10A,USP7,KCND3,ROCK1,LYN,CRACR2A,INSR,TRIM58,PLPP4,SH3GLB1,ENPP1,UTRN,KCNC1,GHRH,BCL2L1,HCN1,GRIN2B,ABCG1,KCNK5,MICALL2,PCNT,PTH,PLA2G4A,SREBF2,ANP32B,AIMP1,NRXN1,SNAP91,TWIST1,JAK2,SIAH3,NFKBIA,PRKCB,ABCC8,CACNA1E,ATP2B1,MTCL1,SAR1A,EXOC1,SLC6A1,NDFIP2,MAP2K6,KCNJ18,CEMIP,PARK7,MAPK8,OAZ2,UBE2J2,ADCYAP1R1,TM9SF4,RAPGEF4,EFHB,MEF2C,RXRA,WNT7A,NDFIP1,SDCBP,PASK,FGR,C2,IL1RAPL1,NUMB,OCLN,SHISA6,DIAPH1,CYFIP1,ICA1,NOS1AP,NSUN2,BICD1,TNFSF11,FYN,PPM1F,SCN8A,NALCN,APELA,HECW2,CDH2,FBXL20,GRM5,TBC1D1,PID1,NRP1,ATPSCKMT,BCR,NRXN3,KCNJ6,DPP6,EPHB2,TSPAN13,CD38,CDH13,STXBP4,CACNG3,ATG5,MAGI2,VMP1,KALRN,SLC1A2,GAPVD1,WDR41,ABL2,KCNIP4,TRDN,NLGN1,NOS1,ASIC2,EFNA5,RAB27A,KCNQ5,CACNA2D1,HTT,CAMK1D,HLA-F,FER,CCR2,OSBPL6,IL16,CATSPER2,RAB31,RGS7,CLDN10,BARD1,CADPS,KCNAB1,PRKAG2,DNM1L*
GO:0018105	peptidyl-serine phosphorylation	0.002662809662 4353188	*BCAR3,MTOR,ULK2,NLK,PDE4D,BCL2,EGFR,ANGPT1,NTRK3,PRKD1,PAK1,SMYD3,RPTOR,APP,RPS6KA2,DCLK1,NTF3,AURKA,PRKCZ,MAPK1,STK38,TLK1,AKAP9,STK32B,MAPKAP1,SLC1A1,PRKAA1,MAST4,CAMK4,LATS2,PRKCE,CD44,RPS6KA3,STK38L,MARK2,HIPK3,CLSPN,STK32A,VRK1,CNKSR3,PRKCH,MKNK1,ROCK1,NEK6,CSNK2A1,NRXN1,HIPK1,AKT3,TNKS,PRKCB,MAST2,PARK7,MAPK8,PKN2,NCK1,TOP1,NTRK2,PPM1F,SH2D3C,MAPK9,RPS6KA5,PRKCA,CSNK1G1,CAMK1G,PRKCQ,NOS1,NSD1,CAMK1D*

			,ROCK2,MORC3
GO:0007157	heterophilic cell-cell adhesion via plasma membrane cell adhesion molecules	0.003375973804313724	*PTPRD,TENM4,TENM3,CRB1,CDH4,ALCAM,CXADR,GRID2,VCAM1,TENM2,NRXN1,CADM1,NECTIN4,HMCN1,NECTIN1,IL1RAPL1,FAT4,CRTAM,CDH2,IGSF21,NLGN1*
GO:0120032	regulation of plasma membrane bounded cell projection assembly	0.0035858313976942645	*MTOR,RIPOR2,RDX,RP1,RALA,SDCCAG8,CDC42EP3,AUTS2,MAP4,APC,PLPPR5,ARHGAP24,SEPTIN9,STAU2,ARHGAP44,SRGAP2C,KANK1,DNM3,COBL,YAP1,BCAS3,SYNE2,HDAC4,MYO10,PLCE1,WDPCP,RAP1GAP,CYLD,BBS4,TENM2,ACTR2,NRXN1,ANLN,GAP43,CEP120,OCLN,MARK4,CYFIP1,SAXO1,CCDC88A,ADAMTS16,NRP1,EPHB2,ATG5,SLIT2,NLGN1,HTT,FER,EPS8,WASHC1*
GO:0007015	actin filament organization	0.0039683189535357965	*MTOR,NEBL,SVIL,MICAL3,DLC1,RDX,BCL2,MYO5A,SPIRE1,MYO1E,CDC42EP3,CARMIL1,RHPN2,MYO5C,CTNNA3,DIAPH3,PHACTR1,CRACD,PAK1,SEMA5A,CD2AP,KANK1,FMN2,CTNNA2,PAK3,CALD1,COBL,CORO2B,LIMCH1,FMN1,TPM1,NF2,CTNNA1,PPP1R9A,MPRIP,ENAH,AIF1L,XIRP2,ABL1,GAS2,PRKCE,TMOD2,SH3KBP1,HIP1,TJP1,PLS1,BBS4,PRKN,PCDH15,KANK4,MYO1D,ROCK1,ARHGAP28,SHROOM3,ARFGEF1,SNX9,MICALL2,FAT1,ACTR2,VASP,JAK2,USH1C,NEDD9,SHROOM2,MTPN,ABI1,HMCN1,CYFIP2,WASF3,MAGEL2,NCK1,ARHGAP12,DIAPH1,CYFIP1,KIRREL1,PSTPIP2,SORBS2,CCDC88A,PPM1F,SPTB,NRP1,FCHSD2,ELMO1,MYO5B,MET,NRAP,FAM171A1,FHOD3,SLIT2,GAS2L1,FER,EPS8,ROCK2,WASHC1*
GO:0035295	tube development	0.004519911542017463	*NOTCH2,SGCD,SCAPER,TAFA5,ZFPM2,DLC1,RALA,BCL2,ALDH1A2,CHRNA7,ROBO2,ZEB1,SDCCAG8,RARB,SPRED1,ENPEP,MYO1E,MINAR1,FOXJ2,RIN2,CRKL,SETD2,ARHGAP24,EGFR,ANGPT1,PRKACB,RNF220,CECR2,RXFP1,C5,FLT1,EDAR,SLC39A12,PRKD1,PAK1,EPHA7,RAPGEF2,LRP2,ADGRB3,CPS1,TMEM38B,LUZP1,COL4A2,SSBP3,HDAC9,SEMA5A,THSD7A,IFT57,CALD1,COBL,YAP1,NFIA,MAPK1,CRISPLD2,NIPBL,FMN1,ITGB8,NFIB,SEMA3C,AGO2,BCAS3,RYR2,WNT9B,COL22A1,ATRX,ABL1,SLC1A1,RAP1A,FGF10,AP3B1,SETDB2,EGF,EXT1,ADAMTS2,PLXNA2,AMBRA1,SEMA3E,EPN2,ADAM12,EMILIN2,HMGA2,ECE1,TGFA,TJP1,MTHFD1L,WDPCP,BMP2,RC3H2,FGF9,ETS1,GLI3,SMOC2,ATF2,BBS4,GTF2I,SELENON,TBX20,PTPRB,VAV3,YIPF6,ROCK1,OVOL2,NTN1,HECTD1,SHROOM3,SP3,CARD10,JCAD,BMP5,CSF1,ASB4,CNMD,SMAD5,VSTM4,TNN,MED1,KDM6A,IL10,SOSTDC1,VASP,AIMP1,NRXN1,HIPK1,TWIST1,AKT3,GORAB,PRKCB,ABCC8,ALX4,ASS1,AGO1,MEOX2,STAT1,MYO18B,CCBE1,CSMD1,ASB2,MYOCD,MEF2C,ADGRB1,WNT7A,RBPMS2,EPHA4,NTRK2,ADAMTS9,WNT2B,HS6ST1,PTK2,CDH5,CLDN18,FAT4,RUNX1,AKR1B1,PDCL3,ADAMTS16,ARL13B,COL18A1,APELA,CDH2,EPHB1,NRP1,PRKCA,NRXN3,BMPER,EPHB2,MET,CDH13,LAMA1,GREB1L,BMP7,DLG5,ADGRF5,PDGFC,EYA1,SLIT2,ROBO1,PBX1,ESR1,LOXL2,FOXB1,PIK3R3,CCR2,STARD13,ROCK2,PRDM1,RORA,HSPG2,COL4A3,STK3,GLI2,THRB*
GO:0030900	forebrain development	0.00456189180896792	*TRAPPC9,PLCB1,DLC1,ALDH1A2,ROBO2,ZEB1,RARB,ALK,CNTNAP2,CRKL,SETD2,SLC4A10,EGFR,PHACTR1,RAPGEF2,LRP2,APP,DCLK1,SEMA5A,SLC8A1,SRGAP2C,MCPH1,ZSWIM6,NRG3,PAFAH1B1,NF2,NFIB,TMEM108,SYNE2,BBS2,TNR,ATRX,ELAVL4,DNAH5,FGF10,NRG1,ASPM,EXT1,SEMA3E,BCL11B,DISC1,SEMA3A,BMP2,RELN,TTC21B,SRGAP2,NIN,DRAXIN,GLI3,BBS4,DAB1,TOX,KCNC1,GHRH,PRKG1,LMX1A,TACC2,POU1F1,FOXP2,CEP120,WNT7A,NTRK2,NUMB,WNT2B,LAMB1,FAT4,FYN,ARL13B,CDH2,XRN2,NRP1,EPHB2,SLC1A2,KIRREL3,SLIT2,CCDC141,ERBB4,ROBO1,SLC6A3,FOXB1,ATAT1,HOOK3,IGF1R,GLI2*
GO:00	regulation	0.00459	*SPOCK1,DLC1,PTPRA,BCL2,CARMIL1,RIN2,CRKL,PTPRJ,MACF*

10810	of cell-substrate adhesion	4919075054831	*1,ONECUT1,CCL28,TBCD,VCL,ACER2,KANK1,MAP4K4,PRKCZ,DUSP22,PLG,CORO2B,LIMCH1,FMN1,NF2,BCAS3,ABL1,PEAK1,PRKCE,PTPRO,ATXN3,ARHGEF7,SEMA3E,DOCK5,EGFLAM,DISC1,WDPCP,DOCK1,ROCK1,UTRN,CSF1,AJAP1,JAK2,MELTF,NEDD9,OLFM4,PTK2,EDIL3,PPM1F,NRP1,FBLN1,CDH13,EFNA5,ROCK2,RSU1*
GO:0001764	neuron migration	0.004609110068319701	*SPOCK1,LRP12,SDCCAG8,AUTS2,ASTN1,CRKL,NTRK3,PHACTR1,NEO1,RAPGEF2,DCLK1,SRGAP2C,CTNNA2,NRG3,NIPBL,FAT3,PAFAH1B1,NRG1,ASPM,SEMA3E,MARK2,DISC1,DNER,SEMA3A,UNC5D,RELN,SRGAP2,BBS4,DCC,DAB1,TBX20,NTN1,PRKG1,TNN,TWIST1,CELSR2,MEF2C,NTRK2,FBXO31,FYN,NRP1,UNK,FLRT2,ASTN2,KIRREL3,NTNG1*
GO:0060491	regulation of cell projection assembly	0.004919902619861064	*MTOR,RIPOR2,RDX,RP1,RALA,SDCCAG8,CDC42EP3,AUTS2,MAP4,APC,PLPPR5,ARHGAP24,SEPTIN9,STAU2,ARHGAP44,SRGAP2C,KANK1,DNM3,COBL,YAP1,BCAS3,SYNE2,HDAC4,MYO10,PLCE1,WDPCP,RAP1GAP,CYLD,BBS4,TENM2,ACTR2,NRXN1,ANLN,GAP43,CEP120,OCLN,MARK4,CYFIP1,SAXO1,CCDC88A,ADAMTS16,NRP1,EPHB2,ATG5,SLIT2,NLGN1,HTT,FER,EPS8,WASHC1*
GO:0048592	eye morphogenesis	0.005147889214197348	*NOTCH2,BCAR3,RP1,BCL2,FBN1,TENM3,ZEB1,RARB,DSCAM,CRB1,STAU2,NIPBL,FAT3,EFEMP1,SLC1A1,FBN2,ATP8A2,PDE6C,TDRD7,GLI3,MEGF11,BBS4,COL5A1,RORB,SP3,DZANK1,HCN1,FAT1,HIPK1,TWIST1,VSX1,USH1C,SHROOM2,NECTIN1,PPP2R3A,NTRK2,WNT2B,EPHB1,SDK1,EPHB2,BMP7,RPGRIP1,PRDM1,THRB*
GO:0046328	regulation of JNK cascade	0.005290547474640238	*PLCB1,PJA2,TNIK,EGFR,NCOR1,MAPKBP1,EDAR,TAOK3,APP,MAP4K4,DUSP22,PAFAH1B1,DUSP16,ZNF675,HIPK3,MDFIC,SEMA3A,MAGI3,HMGB1,TRAF3,SLAMF1,CYLD,MAPK8IP1,RB1CC1,PRKN,CRACR2A,RASGRP1,WNT7A,MAP3K5,SDCBP,MECOM,ANKRD6,TNFSF11,PHLPP1,EPHB1,MFHAS1,ZMYND11,STK3,IGF1R*
GO:0070887	cellular response to chemical stimulus	0.005651452101491875	*NOTCH2,BCAR3,BRINP3,MTOR,NSG1,NLK,ZNF236,PLCB1,PTPRA,ITPR2,RIPOR2,PDE4D,RDX,STXBP1,IL1RAPL2,BCL2,MYO5A,PRDM16,ALDH1A2,FBN1,CHRNA7,ROBO2,GABRB3,ZEB1,AKR1C3,RARB,SPRED1,ALK,ERBIN,ERCC6L2,ANO6,GLYAT,EGLN3,APC,CRKL,SOX5,PTPRJ,KDM4C,EGFR,ANGPT1,BACH1,NEK7,NCOR1,UGT3A2,NEDD4,BCL11A,SOX6,NTRK3,RXFP1,C5,ZFAND6,CYP2C9,FLT1,NAT1,GABRB1,GRIA1,NEO1,SLC8A3,PRKD1,PAK1,NCOA7,CHRM3,GRAMD1B,RAPGEF2,LRP2,RUNX2,FGF12,CPS1,ONECUT1,CPEB4,TMEM38B,UBE2L3,TAFA4,PTPRN2,CCL28,SMYD3,RPTOR,GHR,COL4A2,ADAM10,HDAC9,IL1R1,APP,RPS6KA2,MTUS1,STAU2,GABRG2,DOCK8,USP18,SEMA5A,SYT1,NTF3,ACER2,NDUFAF2,ST18,SLC8A1,HERPUD2,KANK1,BMPR1B,FMN2,PCSK6,AKAP6,HADHB,ARNT,RAB8B,PAK3,LARP1,ITPKB,RAP1GDS1,PRKCZ,GRB10,RYR3,DEFA3,DUSP22,YAP1,BRINP1,MAPK1,HRH2,PDGFD,SYT10,UBE2O,GFRA1,SLC16A1,SPIDR,GABPA,MICU1,CHD6,HRH4,MYLK3,GLP2R,PAFAH1B1,ATF6,TM7SF3,ITGB8,TPM1,HIVEP1,CTNNA1,AKAP9,KLF15,PPARA,ERMP1,NR5A2,ADAMTS3,PTPRK,TRERF1,PLA2R1,DAPK1,TMEM108,ACSM2B,JARID2,SCN2A,IL34,ADGRV1,MELK,BCAS3,RYR2,BBS2,WNT9B,SMARCA4,USP8,MAPKAP1,EFTUD2,CXADR,DOCK4,MBD5,ATRX,PTPRT,ELAVL4,ABL1,PTPN12,HDAC4,OXR1,SLC1A1,PRKAA1,KCNH1,NFAT5,RAP1A,FGF10,LATS2,ZNF675,PRKCE,NXN,ESRRG,DENND4C,FBN2,CD44,PTPRO,PDE3A,EXT1,SPRED2,PTPN2,TRIM5,ATXN3,HTR2C,LTBP1,ZFYVE9,OPRM1,ABCC4,HTR2A,BIN2,ALPL,GNAL,EPN2,KCND2,NOS2,CPNE4,ANK3,GBP6,CCND3,IL17RA,CRIM1,PRR5L,GSR,VAV1,MSRA,FBXO32,TJP1,LDLRAD4,BLM,ADCY10,BMP2,PSG9,SOGA1,MSR1,GNAI1,TBC1D4,RIN3,TMEM161A,LEMD3,HMGB1,GNAQ,FGF9,SLC23A2,ZNF106,TRAF3,UNC13B,DSTYK,RAP1GAP,CCDC186,SLAMF1,CGAS,SMARCC1,SNX6,SMOC2,IDE,GAREM1,ATF2,CYLD,UMODL1,BBS4,MX1,ITGA9,CFTR,RGMB,UBR1,CHRM5,SLC30A10,RORB,SELENON,PTPRE,PRKN,TBX20,LYST,ARID5B,TXNRD2,IL6R,NLRC5,SNX25,FBLN5,VAV3,RALB,ROCK1,LYN,VCAM1,CTSB,GSTA3,DTX1,OVOL2,NTN1,SLC13A5,RRAGD,ARID1B,INSR,YTHDF3,SNAI2,UFD1,RXRG,ERN2,MBTPS2,FLNB,NREP,ZDHHC17,JCAD,SAMHD1,ENPP1,PXDNL,BMP5,KCNC1,CSF1,BCL2L1,SERPINB9,HCN1,GRB14,SMAD5,SLC40A1,MED1,IPCEF1,IL33,KL,IL10,ACTR2,SFPQ,PTH,SOSTD*

			C1,PRKAA2,CSF2RB,PACRG,ABHD2,VSTM2A,PLA2G4A,IQGAP1,YBX3,NRXN1,HIPK1,CD70,PBLD,FICD,CACYBP,PEG10,NET1,TWIST1,JAK2,FSTL1,PTGS1,CREBBP,PCNA,UFL1,NFKBIA,PRKCB,ABCC8,MT1HL1,ZC3H15,SMPD4,NEDD9,ATP2B1,ASS1,OTOP1,CIDEA,EXT2,STAT1,BRMS1L,NR2C1,MAP2K6,IMPACT,CCBE1,PARK7,MAPK8,ITGA4,MTF2,MYOCD,CYFIP2,ACACA,MEF2C,RXRA,WNT7A,RBPMS2,MAP3K5,OR10H2,PDE2A,SDCBP,SPPL2B,WWOX,NCK1,CXCL2,IFNAR1,EPHA4,NTRK2,COLEC12,POSTN,IL17RD,PTK2,CDH5,TPH2,CLDN18,DIAPH1,CYFIP1,UBE3A,ZBTB20,FAT4,AKR1B1,WNT5B,AMFR,PTGFR,CHCHD2,TNFSF11,FYN,PPM1F,HDAC2,ALB,DOK5,MAPK9,FUT8,TET1,ITGA8,EPHB1,GRM5,RPS6KA5,TBC1D1,PID1,NRP1,PRKCA,GBP4,ITGA1,RC3H1,NRIP1,POR,BCR,KIF16B,NSG2,GNA14,BMPER,EPHB2,MET,DLG2,STXBP4,ATG5,MAGI2,FLRT2,SLC1A2,GNAS,BMP7,PDGFC,ABL2,RFX2,SLIT2,CNOT7,ERBB4,IL20RB,ROBO1,PRKCQ,NDRG1,MGMT,NLGN1,NOS1,GLDC,EFNA5,GAS2L1,SLIT3,ESR1,CYP2C8,CACNA2D1,PRLR,CAMK1D,PIK3R3,FER,CCR2,EPS8,HRH1,ROCK2,RORA,IL16,RGS8,RAB31,PDK1,HERPUD1,GNG2,PNPLA3,ZNF423,PNPLA8,HNRNPU,IGF1R,GLI2,THRB,DNM1L
GO:0007612	learning	0.005933037886143992	PLCB1,CNTNAP2,DGKI,SLC8A3,ADGRB3,APP,TAFA2,SORCS3,MEIS2,SYNJ1,PAK5,TANC1,TNR,ELAVL4,ABL1,SLC1A1,ATP8A1,RELN,PRKN,GRIN2A,ATXN1,SHANK2,INSR,ACTR2,NRXN1,ABCC8,SLC6A1,CSMD1,GABRA5,NTRK2,UBE3A,FYN,GRM5,NRXN3,RAG1,EPHB2,SPECC1,KALRN,HTT,FOXB1,HRH1
GO:0090596	sensory organ morphogenesis	0.006277173620124004	NOTCH2,BCAR3,RIPOR2,RP1,BCL2,FBN1,TENM3,ZEB1,RARB,MYO3B,DSCAM,CRB1,STAU2,MAPK1,NIPBL,FAT3,EFEMP1,SLC1A1,FGF10,FBN2,ATP8A2,WDPCP,PDE6C,FGF9,TDRD7,PLS1,GLI3,MEGF11,BBS4,COL5A1,RORB,MYO3A,PCDH15,CDH23,LRIG1,NTN1,HOXC13,SP3,DZANK1,HCN1,FAT1,TTC39C,HIPK1,TWIST1,VSX1,USH1C,OTOP1,SHROOM2,NECTIN1,PPP2R3A,NTRK2,WNT2B,HDAC2,ITGA8,EPHB1,SDK1,BCR,EPHB2,BMP7,EYA1,RPGRIP1,PRDM1,PTPRQ,GLI2,THRB
GO:0048584	positive regulation of response to stimulus	0.00630982081599716	NOTCH2,BCAR3,MTOR,WWC1,KSR1,PLCB1,RIPOR2,PDE4D,STXBP1,BCL2,CHRNA7,ROBO2,RIMS1,EPC2,SPIRE1,AKR1C3,SPRED1,RIMS2,ALK,AUTS2,PJA2,BABAM2,ERBIN,ANO6,MLLT3,SUSD4,HHLA2,DSCAM,CRKL,TNIK,PTPRJ,EGFR,ANGPT1,MACF1,RNF220,NEDD4,CHSY1,NTRK3,C5,DKK2,FLT1,MAPKBP1,DGKI,EDAR,NEO1,CNTN6,PRKD1,PAK1,RAPGEF2,PELI2,LRP2,TAOK3,RPTOR,GHR,ADAM10,IL1R1,APP,MAPRE2,SEMA5A,NTF3,KANK1,MAP4K4,BMPR1B,FMN2,AKAP6,ARNT,PAK3,RFTN1,ITPKB,PRKCZ,GRB10,DUSP22,YAP1,MAPK1,MGAT5,PLG,PDGFD,UBE2O,SPIDR,PAFAH1B1,ATF6,CTNNA1,ADAMTS3,GRM1,PCDH11Y,PLA2R1,TMEM108,IL34,ADGRV1,NKG7,SMARCA4,USP8,SPG21,BLK,MBD5,ABL1,NFAT5,GUCY1A2,RAP1A,GPC5,FGF10,ZC3HAV1,NRG1,INO80D,ASPM,DENND2B,RASGRF1,PRKCE,WNK2,CD44,EGF,SPRED2,PTPN2,TRIM5,MCF2L,ATXN3,HTR2C,CLEC16A,OPRM1,HTR2A,SEMA3E,GCSAML,PUM1,TMOD2,MSH2,ANKRD17,RELL1,EPN2,EVC,RBBP8,EMILIN2,PLCE1,TGFA,IL17RA,HIP1,PRR5L,VAV1,HLA-B,DISC1,SEMA3A,BMP2,RC3H2,BMP2K,TMEM161A,RELN,HMGB1,FGF9,NFATC2,TRAF3,TTC21B,DSTYK,UIMC1,SLAMF1,SMARCA2,ETS1,CGAS,SMARCC1,SMOC2,GAREM1,LAMC1,NEK10,CYLD,MAPK8IP1,UBASH3A,NEU3,KITLG,CAMTA1,SLC30A10,SELENON,RB1CC1,PRKN,TBX20,DPF3,GRIN2A,PRKCH,IL6R,ALS2,NLRC5,VAV3,KIR2DL4,NPAS2,ROCK1,LYN,EIF2B3,SLC44A2,RRAGD,ARID1B,CRACR2A,INSR,DEDD2,NEK6,ARFGEF1,IGHV3-74,BID,ERN2,TIAL1,PLPP4,ZDHHC17,NSD2,JCAD,RASGRP1,IGSF11,CSNK2A1,BMP5,CSF1,GHRH,GRIN2B,INO80,FANCB,IGHV2-70D,MED1,IL33,ROR2,CFH,KL,BANK1,IL10,ACTR2,SFPQ,PTH,NDC80,PLA2G4A,IQGAP1,RPS12,FYB2,NRXN1,PCID2,CIBAR1,CADM1,NET1,AKT3,ALKAL2,JAK2,MADD,HCRTR1,CREBBP,TNKS,GORAB,PCNA,NFKBIA,PRKCB,BRD4,NEDD9,IGHV1OR15-9,GID8,NDFIP2,NR2C1,MAP2K6,CCBE1,PARK7,ADCYAP1R1,MYOCD,CYFIP2,MEF2C,RXRA,WNT7A,MAP3K5,NDFIP1,MAP3K4,S100B,ATP6V1C2,RAD51AP1,PDE2A,SDCBP,NSMCE1,WWOX,NCK1,

			FGR,CDCA8,PPP2R3A,C2,RNF8,EPHA4,NTRK2,OCLN,PTK2,CDH5,CD5L,ANKRD6,NFKBID,LAMB1,CYFIP1,UBE3A,SEMA4D,C9,WNT5B,NENF,SH2D1B,NOS1AP,CCDC88A,GPR55,TNFSF11,FYN,PPM1F,SLF1,DOK5,MAPK9,CRTAM,APELA,ROR1,CDH2,ITGA8,RAD9A,GPR137B,GRM5,NRP1,PRKCA,ITGA1,RC3H1,POR,STK36,BMPER,PRDM15,MACROH2A1,CSNK1G1,CD38,EYA4,MET,SPPL3,CDH13,MYB,GNAS,LAMA1,MFHAS1,BMP7,DLG5,PDGFC,EYA1,SLIT2,ERBB4,ROBO1,PRKCQ,MGMT,NLGN1,SHLD2,IGLC3,IQCJ-SCHIP1,PRLR,AGO3,HTT,CAMK1D,HLA-F,EYA2,CCR2,A2M,IGHV1OR21-1,ROCK2,IL16,HERPUD1,KIF7,STK3,ZNF423,IGF1R,THRB,AKAP13,DNM1L
GO:0045935	positive regulation of nucleobase-containing compound metabolic process	0.006930479175590292	*BCAR3,MTOR,WWC1,FTO,MGA,PLCB1,ZFPM2,TNRC6B,PRDM16,EPC2,SPIRE1,ZEB1,RARB,AUTS2,FOXJ2,BABAM2,GLIS3,FANK1,MLLT3,TCF4,ERG,NEK4,EGFR,RFX3,CDK12,BACH1,NEK7,ZNF407,MAML2,BCL11A,FLI1,RFC3,TASP1,THRAP3,PRKD1,NCOA7,RUNX2,ONECUT1,UBE2L3,LDB2,SMYD3,RPTOR,SSBP3,ATF7IP,APBB2,APP,KDM1B,ZNF600,PARN,ST18,PYGO1,SSBP2,ANKRD31,DUX4,BMPR1B,FMN2,ZNF717,ARNT,PAK3,NBN,TAF15,MRTFA,TAF4B,EBF2,YAP1,NFIA,MAPK1,KMT2E,PCGF5,NIPBL,SPIDR,GABPA,CHD6,KANSL1,ATF6,HIVEP1,KLF15,PPARA,MEIS2,NFIB,MRTFB,NR5A2,FOXJ3,TRERF1,AGO2,GATAD2B,BCAS3,ZNF606,SMARCA4,TNRC6C,ATRX,ABL1,HDAC4,PRKAA1,APLF,NFAT5,GLIS1,TOX3,CAMK4,FGF10,ZC3HAV1,INO80D,AP3B1,ZNF438,ZBTB16,FOXK2,MED15,ESRRG,EGF,RPS6KA3,TRIM5,RFC1,KDM7A,HTR2A,GTF2F2,TAF3,RPRD1B,EBF3,ZNF33B,PUM1,MSH2,NOS2,BICRAL,RBBP8,MDFIC,HMGA2,BCL11B,CREM,PRR5L,BRCA2,BLM,BMP2,RC3H2,GFI1B,TMEM161A,ASXL3,HMGB1,NFATC2,ZNF462,ETS2,UIMC1,ATF1,SMARCA2,ETS1,GLI3,SMARCC1,SMOC2,VENTX,PRDM10,RERE,ATF2,HIVEP3,PSIP1,RGMB,CAMTA1,GTF2I,RORB,TADA2A,MED27,ZNF208,NMD3,PRKN,TBX20,DPF3,ARID5B,NLRC5,TFDP1,CNOT6L,TOX,SLC4A4,ZFP90,USP7,PLAGL1,SOX30,NPAS2,ROCK1,ZNF780B,DTX1,OVOL2,ZFHX3,BANP,SUPT16H,ARID1B,HOXC13,BAZ1A,CASZ1,INSR,YTHDF3,PBX3,ZNF292,ASH1L,HOXC4,RXRG,SP3,MBTPS2,ELF2,NSD2,BMP5,INO80,FANCB,CLNS1A,SMAD5,CELF4,TCERG1,SLC40A1,MED1,IL33,ZNF521,CSDE1,LMX1A,IL10,ACTR2,SFPQ,RIOK1,PTH,PRKAA2,ETV6,ZBTB7C,TEAD1,SREBF2,PCID2,LMX1B,TWIST1,JAK2,ZBTB38,PATL1,ZNF287,CREBBP,TNKS,PCNA,NFKBIA,PRKCB,RFC2,ALX4,RTRAF,BRD4,ITGA6,ZBTB49,STOX2,AGO1,MEOX2,ELL2,STAT1,PARK7,MED12L,POU1F1,MTF2,MYOCD,ARID3B,MEF2C,RXRA,WNT7A,MAP3K5,MAP3K4,ZNF112,RAD51AP1,DBF4B,WWOX,NCK1,SCAF8,MLLT10,RNF8,MECOM,DNMT3L,CREB5,ASCL3,UBE3A,ZBTB20,RUNX1,ZNF845,CHCHD2,TNFSF11,KDM5A,HDAC2,SLF1,TBX15,NCS1,PABPC1,TET1,ARNT2,ITGA8,RPS6KA5,PID1,ZNF615,KLF12,RC3H1,NRIP1,ZNF850,SUPT3H,TUT4,PRIM2,PRDM15,MITF,CD38,EYA4,MET,CDH13,PRDM11,MLIP,MYB,BMP7,BPTF,NUDT21,KMT2C,RFX2,EYA1,CNOT7,ERBB4,SAMD4A,PBX1,NPAS3,PRKCQ,MGMT,SHLD2,NOS1,TCF12,ARHGEF11,NSD1,ESR1,AGO3,EYA2,ZNF721,NRF1,ROCK2,RORA,DMRT1,NCOA6,ZNF423,HNRNPU,IGF1R,GLI2,THRB*
GO:0048013	ephrin receptor signaling pathway	0.007333486930732641	*ANKS1B,NTRK3,PAK1,EPHA7,PAK3,CHN1,TIAM1,ARHGEF7,EPHA6,NGEF,ARHGEF28,LYN,NCK1,EPHA4,PTK2,FYN,EPHB1,EPHB2,KALRN,EFNA5*
GO:0018108	peptidyl-tyrosine phosphorylation	0.007734320056 7419265	*MTOR,ALK,MAP3K9,PTPRJ,EGFR,ANGPT1,PTPN4,NTRK3,FLT1,EPHA7,GHR,APP,SAMSN1,NTF3,PRKCZ,DUSP22,PDGFD,GFRA1,EFEMP1,NF2,IL34,MELK,BLK,ABL1,FGF10,PEAK1,NRG1,MUSK,PRKCE,CD44,EGF,PTPN2,HTR2A,EPHA6,HIPK3,TGFA,CNTN1,RELN,SH3BP5,DSTYK,SNX6,KITLG,IL6R,LYN,CHKA,INSR,ROR2,BANK1,HIPK1,ALKAL2,JAK2,NEDD9,MAP2K6,ABI1,NCAPG2,FGR,EPHA4,NTRK2,PTK2,SEMA4D,PDCL3,FYN,HDAC2,DOCK3,ZFYVE28,ROR1,EPHB1,GRM5,NRP1,BCR,EPHB2,MET,PDGFC,ABL2,CNOT7,ERBB4,EFNA5,PRLR,FER,IGF1R*
GO:00	cellular	0.00779	*NOTCH2,NLK,RDX,PRDM16,FBN1,ZEB1,SPRED1,CRKL,SOX5,EG*

71363	response to growth factor stimulus	98275996693355	*FR,NEDD4,SOX6,NTRK3,FLT1,NEO1,PRKD1,RAPGEF2,LRP2,RUNX2,FGF12,CPS1,ONECUT1,COL4A2,APP,NTF3,BMPR1B,PCSK6,ARNT,GRB10,DUSP22,PDGFD,UBE2O,GFRA1,ITGB8,HIVEP1,PPARA,ADAMTS3,PTPRK,TMEM108,USP8,ELAVL4,ABL1,PTPN12,RAP1A,FGF10,LATS2,FBN2,CD44,PDE3A,EXT1,SPRED2,LTBP1,ZFYVE9,EPN2,CRIM1,LDLRAD4,BMP2,PSG9,LEMD3,FGF9,DSTYK,RAP1GAP,SNX6,SMOC2,GAREM1,ATF2,RGMB,TBX20,SNX25,VCAM1,OVOL2,INSR,SNAI2,NREP,ZDHHC17,JCAD,BMP5,SMAD5,MED1,KL,IL10,SOSTDC1,VSTM2A,IQGAP1,NRXN1,PBLD,PEG10,TWIST1,FSTL1,CREBBP,PRKCB,CIDEA,EXT2,BRMS1L,CCBE1,MYOCD,CYFIP2,MEF2C,WNT7A,RBPMS2,PDE2A,SDCBP,WWOX,NTRK2,IL17RD,PTK2,CDH5,CYFIP1,UBE3A,FAT4,FYN,HDAC2,DOK5,FUT8,TET1,ITGA8,NRP1,KIF16B,BMPER,MAGI2,FLRT2,BMP7,SLIT2,ERBB4,ROBO1,NOS1,ZNF423,IGF1R*
GO:0035239	tube morphogenesis	0.009311962672062924	*NOTCH2,SGCD,TAFA5,ZFPM2,DLC1,RALA,BCL2,CHRNA7,ZEB1,SDCCAG8,SPRED1,ENPEP,MYO1E,MINAR1,FOXJ2,RIN2,SETD2,ARHGAP24,EGFR,ANGPT1,PRKACB,CECR2,C5,FLT1,EDAR,SLC39A12,PRKD1,PAK1,EPHA7,RAPGEF2,LRP2,ADGRB3,LUZP1,COL4A2,HDAC9,SEMA5A,THSD7A,IFT57,CALD1,COBL,YAP1,NIPBL,FMN1,ITGB8,NFIB,AGO2,BCAS3,RYR2,WNT9B,COL22A1,ABL1,SLC1A1,RAP1A,FGF10,SETDB2,EGF,EXT1,SEMA3E,EPN2,ADAM12,EMILIN2,HMGA2,TGFA,TJP1,MTHFD1L,BMP2,FGF9,ETS1,GLI3,SMOC2,ATF2,BBS4,GTF2I,TBX20,PTPRB,VAV3,ROCK1,OVOL2,NTN1,HECTD1,SHROOM3,CARD10,JCAD,BMP5,CSF1,ASB4,CNMD,VSTM4,TNN,MED1,KDM6A,IL10,SOSTDC1,VASP,AIMP1,NRXN1,HIPK1,TWIST1,AKT3,PRKCB,ABCC8,AGO1,MEOX2,STAT1,MYO18B,CCBE1,CSMD1,ASB2,MYOCD,MEF2C,ADGRB1,WNT7A,RBPMS2,EPHA4,NTRK2,ADAMTS9,WNT2B,HS6ST1,PTK2,CDH5,FAT4,RUNX1,PDCL3,ADAMTS16,ARL13B,COL18A1,APELA,CDH2,EPHB1,NRP1,PRKCA,NRXN3,BMPER,EPHB2,MET,CDH13,LAMA1,GREB1L,BMP7,DLG5,ADGRF5,EYA1,SLIT2,ROBO1,PBX1,ESR1,LOXL2,PIK3R3,CCR2,STARD13,ROCK2,PRDM1,RORA,HSPG2,COL4A3,STK3,GLI2*
GO:0009790	embryo development	0.009519360203306902	*NOTCH2,PLCB1,ZFPM2,TENM4,DLC1,RIPOR2,RALA,ALDH1A2,FBN1,ZEB1,RARB,MYO1E,MYO3B,SETD2,KDM4C,EGFR,ANGPT1,PRKACB,RNF220,CECR2,C5,FLT1,LRP2,RUNX2,LUZP1,COL4A2,SSBP3,ADAM10,CACNA1C,PTPRR,CMIP,PCSK6,ARNT,NBN,IFT57,COBL,YAP1,MAPK1,PLG,NIPBL,GABPA,MBNL1,PAFAH1B1,EFEMP1,NF2,BIRC6,MEIS2,NR5A2,ADAMTS3,SF3B6,SEMA3C,RYR2,WNT9B,ANKS6,ABL1,FGF10,LATS2,INO80D,ZBTB16,SETDB2,NXN,FBN2,EXT1,ATP8A2,PLXNA2,ST8SIA6,AMBRA1,MSH2,APBA2,RBBP8,XYLT1,HMGA2,ECE1,MBP,TJP1,MTHFD1L,BRCA2,WDPCP,BMP2,FGF9,TDRD7,ETS2,PLS1,GLI3,AFF3,TDRD5,ATF2,HIRA,BBS4,COL5A1,DOP1B,KITLG,TADA2A,MYO3A,SH3PXD2A,TBX20,PCDH15,CDH23,ALS2,RACGAP1,SEC24D,ROCK1,LRIG1,OVOL2,NTN1,MMP16,INSR,HECTD1,SHROOM3,PBX3,HOXC4,TANC2,SP3,PLPP4,BMP5,BCL2L1,LAMA3,INO80,SMAD5,CELF4,MED1,KDM6A,IL10,TTC39C,VASP,TEAD1,YBX3,HIPK1,TWIST1,RBM19,CREBBP,GORAB,ALX4,USH1C,SMPD4,ADCY9,OTOP1,EXT2,STOX2,MEOX2,ABI1,MYO18B,ITGA4,NCAPG2,ASB2,MEF2C,WNT7A,RBPMS2,FBXW8,FLVCR1,PPP2R3A,TOP1,DNMT3L,WNT2B,HS6ST1,LAMB1,PCDH8,WNT5B,NSUN2,ARL13B,HDAC2,TBX15,APELA,FUT8,TET1,ARNT2,ITGA8,NRP1,BCR,KIF16B,B9D1,MACROH2A1,EPHB2,SPECC1,KIAA1217,UNK,LAMA1,BMP7,BPTF,ADGRF5,PDGFC,NECAB1,EYA1,EXOC4,ERBB4,PBX1,EHMT1,FOXB1,RAD51B,EYA2,A2M,ROCK2,PRDM1,PTPRQ,HSD17B2,STK3,ZNF568,GLI2*
GO:0018212	peptidyl-tyrosine modification	0.009563279105495684	*MTOR,ALK,MAP3K9,PTPRJ,EGFR,ANGPT1,PTPN4,NTRK3,FLT1,EPHA7,GHR,APP,SAMSN1,NTF3,PRKCZ,DUSP22,PDGFD,GFRA1,EFEMP1,NF2,IL34,MELK,BLK,ABL1,FGF10,PEAK1,NRG1,MUSK,PRKCE,CD44,EGF,PTPN2,HTR2A,EPHA6,HIPK3,TGFA,CNTN1,RELN,SH3BP5,DSTYK,SNX6,KITLG,IL6R,LYN,CHKA,INSR,ROR2,BANK1,HIPK1,ALKAL2,JAK2,NEDD9,MAP2K6,ABI1,NCAPG2,FGR,EPHA4,NTRK2,PTK2,SEMA4D,PDCL3,FYN,HDAC2,DOCK3,ZFYVE28,ROR1,EPHB1,GRM5,NRP1,BCR,EPHB2,MET,PDGFC,ABL2,CNOT7,ERBB4,EFNA5,PRLR,FER,IGF1R*

GO:0042325	regulation of phosphorylation	0.010158690253331423	*BCAR3,MTOR,KSR1,PDE4D,BCL2,CHRNA7,SPRED1,ALK,APC,DSCAM,CRKL,TNIK,PTPRJ,EGFR,ANGPT1,CDK12,NCOR1,NTRK3,FLT1,SLC8A3,PRKD1,PAK1,EPHA7,RAPGEF2,PELI2,TAOK3,LDB2,SMYD3,RPTOR,GHR,APP,SAMSN1,NTF3,SLC8A1,BMPR1B,ARNT,RANBP2,ITPKB,TRPC5,NBN,PRKCZ,GRB10,MCPH1,DUSP22,MAPK1,PDGFD,NRG3,GFRA1,STK38,PTPN13,LIMCH1,CCNG2,NF2,MOB3B,AKAP9,PPARA,IL34,WNT9B,DUSP16,PARD3,MAPKAP1,PTPRT,ABL1,HDAC4,SLC1A1,PRKAA1,RAP1A,FGF10,LATS2,NRG1,MUSK,ZNF675,PRKCE,SLCO3A1,CD44,PTPRO,EGF,PRRC1,SPRED2,PTPN2,AMBRA1,HTR2A,MARK2,EPHA6,HIPK3,CDKN2C,KNDC1,CLSPN,MNAT1,HMGA2,CCND3,PLCE1,TGFA,PRR5L,LDLRAD4,CNTN1,BLM,BMP2,RELN,GNAQ,FGF9,SH3BP5,DSTYK,SNX6,CNKSR3,NEK10,MOB1B,ATF2,MAPK8IP1,KITLG,TADA2A,DAB1,RB1CC1,PRKN,IL6R,ALS2,NLRC5,SNX25,SLC4A4,PTPRB,COPS8,VAV3,RALB,ROCK1,LYN,INSR,ERN2,CARD10,ENPP1,RASGRP1,SNX9,BMP5,CSF1,GPRC5C,ROR2,BANK1,PRKAA2,IQGAP1,NRXN1,CENPE,ALKAL2,JAK2,MADD,RTRAF,BRD4,NEDD9,ITGA6,MAP2K6,ABI1,CEMIP,IMPACT,PARK7,NCAPG2,MYOCD,MEF2C,MAP3K5,MAP3K4,DBF4B,SDCBP,MLLT1,NCK1,FGR,CDCA8,EPHA4,NTRK2,OCLN,PTK2,CDH5,SEMA4D,ZBTB20,KIRREL1,PDCL3,CCDC88A,TNFSF11,FYN,PPM1F,HDAC2,SH2D3C,DOCK3,ZFYVE28,ROR1,EPHB1,GRM5,PID1,NRP1,FBLN1,BMPER,MACROH2A1,EPHB2,MET,BMP7,BTBD10,PDGFC,SLIT2,CNOT7,ERBB4,ROBO1,NOS1,EFNA5,NSD1,PRLR,HTT,PIK3R3,FER,ROCK2,WASHC1,BARD1,STK3,DEPTOR,HNRNPU,IGF1R,PRKAG2,AKAP13*
GO:0030111	regulation of Wnt signaling pathway	0.011387336604000744	*NLK,MLLT3,APC,EGFR,MACF1,RNF220,DKK2,INVS,APP,SEMA5A,KANK1,GRB10,HECW1,YAP1,ZNRF3,KLF15,TIAM1,PCDH11Y,ALPK2,SMARCA4,USP8,ABL1,GPC5,FGF10,LATS2,ASPM,NXN,WNK2,PTPRO,EGF,LIMD1,CTNND2,KREMEN1,MDFIC,NPHP4,SNX3,DISC1,BMP2,GNAQ,FGF9,TTC21B,DRAXIN,GLI3,CYLD,KPNA1,PRKN,SOX30,SNAI2,SIAH2,TRABD2B,CSNK2A1,TNN,SOSTDC1,RPS12,TNKS,GID8,ATP6V1C2,WWOX,PPP2R3A,SHISA6,ANKRD6,WNT5B,AMFR,RBMS3,CDH2,MCC,PRDM15,CSNK1G1,CDK14,STK3,APCDD1*
GO:0010977	negative regulation of neuron projection development	0.011591125781495962	*SPOCK1,ULK2,MINAR1,BCL11A,EPHA7,SEMA5A,KANK1,DIP2B,DNM3,FAT3,PAFAH1B1,SEMA3C,SEMA6D,TNR,PTPRO,KREMEN1,SEMA3E,MBP,SEMA3A,SEMA3D,DRAXIN,MAP2,DCC,DAB1,NGEF,NTN1,EPHA4,UBE3A,SEMA4D,HDAC2,PTPRG,NRP1,EPHB2,CD38,SLIT2,NLGN1,SEMA4B,FSTL4*
GO:0033554	cellular response to stress	0.011665787700067768	*MTOR,IMMP2L,FTO,PLCB1,BCL2,ARPP21,PIK3C3,EPC2,SPIRE1,AKR1C3,SPRED1,PJA2,BABAM2,ERCC6L2,EGLN3,APC,CRKL,SETD2,TNIK,NEK4,EGFR,USP14,BACH1,NCOR1,NEDD4,NSMCE2,RFC3,MAPKBP1,EDAR,SLC8A3,PRKD1,PAK1,NCOA7,FGF12,TAOK3,CPEB4,HERC2,RPTOR,TMEM117,APP,STAU2,ACER2,SLC8A1,HERPUD2,ECPAS,MAP4K4,FMN2,ARNT,UBE2E2,KICS2,NBN,INTS7,SUSD6,MSH6,DUSP22,YAP1,SEM1,WDR70,MAPK1,SGTB,USP25,PDGFD,SYCP1,NIPBL,SPIDR,MICU1,CORO2B,CHD6,PAFAH1B1,ATF6,TM7SF3,TLK1,TPM1,CTNNA1,RASGRF2,PPARA,ERMP1,PTPRK,PLA2R1,DAPK1,VPS13C,WDHD1,SCN2A,DNAJC15,MELK,WNT9B,DUSP16,SMARCA4,MAPKAP1,BLK,TNR,ATRX,NUAK1,ELAVL4,ABL1,OXR1,SLC1A1,PRKAA1,APLF,NFAT5,SLFN11,FGF10,INO80D,RASGRF1,ZNF675,SMARCAD1,PRKCE,USP33,CD44,SPRED2,RPS6KA3,MTMR3,PTPN2,ATXN3,RFC1,CLEC16A,AMBRA1,OPRM1,FANCM,FANCA,KREMEN1,STAC,TMEM67,MSH2,RELL1,HIPK3,KCND2,CLSPN,MNAT1,RBBP8,MDFIC,HMGA2,VPS41,PRR5L,GSR,MSRA,BRCA2,BLM,SEMA3A,MAGI3,HSF2BP,BMP2,VRK1,TMEM161A,HMGB1,NFATC2,TRAF3,UIMC1,SLAMF1,SMARCA2,CGAS,SMARCC1,MYEF2,ZFYVE26,ATF2,CYLD,MAPK8IP1,CFTR,DNAJC7,MAP4K3,SLC30A10,CHAF1A,SELENON,RB1CC1,PRKN,MAPK10,DPF3,USP43,DMC1,FBLN5,ST8SIA1,USP7,VAV3,KIR2DL4,RALB,NPAS2,LYN,SEL1L,CHKA,FANCL,RRAGD,SUPT16H,ARID1B,CRACR2A,RNF152,NEK6,XRCC4,SNAI2,BID,UFD1,ERN2,MBTPS2,NREP,NSD2,SH3GLB1,SAMHD1,RASGRP1,PWWP3A,BCL2L1,INO80,FANCB,FOXN3,CDC14B,IL10,ACTR2,SFPQ,PRKAA2,*

			PACRG,MORC2,SREBF2,YBX3,HIPK1,FICD,NET1,TWIST1,AKT3,JAK2,CHCHD6,ZBTB38,FH,TDP1,CREBBP,PCNA,UFL1,NFKBIA,RFC2,BRD4,GAP43,ASS1,ERLIN2,CIDEA,MAP2K6,MARCHF6,IMPACT,PARK7,MAPK8,TOP3A,UBL7,UBE2J2,MYH13,HNRNPM,ASCC2,MEF2C,WNT7A,MAP3K5,MAP3K4,RAD51AP1,SDCBP,NSMCE1,NCK1,RNF8,EPHA4,MECOM,WNT2B,FBXO31,ERP27,ANKRD6,INIP,AKR1B1,AMFR,SAXO1,SCARA5,ZFYVE1,CHCHD2,CDC45,TNFSF11,FYN,SDE2,HDAC2,SLF1,SH2D3C,ALB,MAPK9,FUT8,SEL1L2,RAD9A,PHLPP1,EPHB1,FAAP24,RNF138,SUPT3H,C14ORF39,EYA4,MET,STXBP4,ATG5,MFHAS1,BMP7,ZMYND11,PLIN2,PARPBP,EYA1,NDRG1,MGMT,SHLD2,GAS2L1,MTREX,RAD51B,MACROD2,FER,EYA2,OARD1,ROCK2,RORA,CDCA5,PDK1,HERPUD1,NCOA6,BARD1,STK3,PNPLA8,IGF1R,PRKAG2,MORC3
GO:0010033	response to organic substance	0.012159616646323444	*NOTCH2,BCAR3,BRINP3,MTOR,NSG1,NLK,LONP2,ZNF236,PLCB1,MX2,PTPRA,ITPR2,RIPOR2,PDE4D,RDX,STXBP1,IL1RAPL2,BCL2,MYO5A,KCNMA1,PRDM16,ALDH1A2,FBN1,CHRNA7,ROBO2,PIK3C3,GABRB3,ZEB1,AKR1C3,RARB,SPRED1,ALK,ERBIN,HLCS,APC,CRKL,ILDR2,SOX5,SETD2,TNIK,PTPRJ,KDM4C,EGFR,USP14,ANGPT1,NCOR1,UGT3A2,NEDD4,BCL11A,SOX6,PSMB2,NTRK3,RXFP1,ZFAND6,FLT1,RFC3,GABRB1,NEO1,PRKD1,PAK1,CHRM3,ADSS2,GRAMD1B,RAPGEF2,LRP2,RUNX2,ARSB,FGF12,CPS1,ONECUT1,CPEB4,TMEM38B,BCKDHB,UBE2L3,PTPRN2,SMYD3,RPTOR,GHR,COL4A2,ADAM10,HDAC9,IL1R1,APP,RPS6KA2,KYNU,GABRG2,DOCK8,USP18,NTF3,ACER2,NDUFAF2,ST18,SLC8A1,HERPUD2,ECPAS,KANK1,BMPR1B,PCSK6,AKAP6,HOMER2,HADHB,ARNT,RAB8B,PAK3,RFTN1,LARP1,RAP1GDS1,RNLS,PRKCZ,GRB10,RYR3,DEFA3,DUSP22,YAP1,BRINP1,MAPK1,HRH2,ABCD3,SGTB,USP25,PAPPA,PDGFD,UBE2O,GFRA1,SLC16A1,SPIDR,GABPA,HRH4,MYLK3,ACSBG1,GLP2R,PAFAH1B1,ATF6,TM7SF3,ITGB8,HIVEP1,CTNNA1,AKAP9,KLF15,PPARA,MEIS2,ERMP1,SYNJ1,NR5A2,ADAMTS3,PTPRK,TRERF1,DAPK1,SLC24A4,VPS13C,TMEM108,JARID2,IL34,ANK2,BCAS3,RYR2,BBS2,WNT9B,SMARCA4,USP8,EFTUD2,MBD5,ATRX,PTPRT,ELAVL4,ABL1,PTPN12,HDAC4,SLC1A1,PRKAA1,NFAT5,RAP1A,FGF10,LATS2,ZNF675,PRKCE,ESRRG,DENND4C,FBN2,CD44,ABCC9,P2RX6,PDE3A,EXT1,SPRED2,RPS6KA3,PTPN2,TRIM5,ATXN3,HTR2C,CD96,RFTN2,LTBP1,ZFYVE9,OPRM1,HTR2A,TMEM67,ALPL,FHL2,GNAL,EPN2,NOS2,GBP6,CCND3,F5,MBP,IL17RA,CRIM1,FBXO32,TJP1,LDLRAD4,BLM,AGL,BMP2,PSG9,SOGA1,MSR1,GNAI1,TBC1D4,TMEM161A,LEMD3,HMGB1,GNAQ,FGF9,SLC23A2,ZNF106,TRAF3,UNC13B,DSTYK,RAP1GAP,ATF1,CCDC186,GLI3,CGAS,SMARCC1,SNX6,AFF3,SMOC2,IDE,GAREM1,ATF2,CYLD,UMODL1,BBS4,MX1,CFTR,RGMB,UBR1,CHRM5,MAP4K3,SLC30A10,RORB,SELENON,PTPRE,PRKN,TBX20,GRIN2A,ARID5B,IL6R,NLRC5,SNX25,SOX30,RALB,ROCK1,LYN,VCAM1,SEL1L,CTSB,EIF2B3,DTX1,OVOL2,ZFHX3,RRAGD,ARID1B,CRACR2A,INSR,DMBT1,YTHDF3,TFF1,SNAI2,UFD1,RXRG,ERN2,MBTPS2,FLNB,NREP,ZDHHC17,CD9,JCAD,SAMHD1,ENPP1,BMP5,KCNC1,CSF1,BCL2L1,SERPINB9,HCN1,GRIN2B,GRB14,SMAD5,ABCG1,HADHA,MED1,IL33,PPP2R2A,KL,IL10,ACTR2,PTH,SOSTDC1,PRKAA2,CSF2RB,PACRG,ABHD2,VSTM2A,IQGAP1,SREBF2,YBX3,NRXN1,HIPK1,CD70,PBLD,FICD,CACYBP,PEG10,TWIST1,JAK2,FSTL1,CREBBP,PCNA,UFL1,NFKBIA,PRKCB,GOT2,ABCC8,ZC3H15,SMPD4,ATP2B1,ASS1,ERLIN2,OTOP1,CIDEA,EXT2,SLC6A1,STAT1,BRMS1L,NR2C1,MAP2K6,MARCHF6,IMPACT,CCBE1,PARK7,MAPK8,ITGA4,UBE2J2,ADCYAP1R1,MTF2,MYOCD,CYFIP2,ACACA,MEF2C,RXRA,WNT7A,RBPMS2,MAP3K5,OR10H2,PDE2A,SDCBP,DSG1,SPPL2B,WWOX,NCK1,CXCL2,C2,IFNAR1,EPHA4,NTRK2,COLEC12,POSTN,IL17RD,PTK2,ERP27,CDH5,TPH2,CLDN18,DIAPH1,CYFIP1,UBE3A,ZBTB20,FAT4,WNT5B,AMFR,PTGFR,TNFSF11,FYN,HDAC2,DOK5,FUT8,TET1,ITGA8,SEL1L2,GRM5,RPS6KA5,TBC1D1,PID1,NRP1,SDK1,PRKCA,GBP4,RC3H1,NRIP1,POR,BCR,RERG,KIF16B,NSG2,GNA14,BMPER,EPHB2,CD38,CDH13,STXBP4,MAGI2,FLRT2,SLC1A2,GNAS,BMP7,PDGFC,PLIN2,ABL2,RFX2,SLIT2,CNOT7,ERBB4,IL20RB,ROBO1,PRKCQ,MGMT,NOS1,SLC6A3,GLDC,EFNA5,GAS2L1,SLIT3,ESR1,CACNA2D1,PRLR,PIK3R3,FER,C*

			CR2,A2M,EPS8,HRH1,ROCK2,RORA,RGS8,RAB31,HERPUD1,NCOA6,HSD17B2,COL4A3,RGS7,GNG2,PNPLA3,ZNF423,HNRNPU,IGF1R,GLI2,THRB
GO:0032409	regulation of transporter activity	0.0128388895034 1599	*PDE4D,BCL2,CACNG2,NEDD4,CHRM3,FGF12,THADA,NEDD4L,APP,CACNB2,KCNE4,AKAP6,HECW1,LRRC38,AKAP9,RASGRF2,DAPK1,ANK2,RYR2,GSG1L,RASGRF1,PRKCE,SLMAP,WNK2,ABCC9,ALG10B,OPRM1,STAC,CNIH3,ANK3,NETO2,RELN,CNKSR3,CFTR,SELENON,JPH1,SHISA9,CRACR2A,UTRN,KCNC1,HCN1,NRXN1,TWIST1,ABCC8,NDFIP2,PARK7,MEF2C,RXRA,NDFIP1,SHISA6,NOS1AP,HECW2,GRM5,ATPSCKMT,EPHB2,CACNG3,VMP1,TRDN,NLGN1,NOS1,CACNA2D1,HTT,CCR2,KCNAB1*
GO:0044380	protein localization to cytoskeleton	0.012943239011543465	*CEP192,APC,MAPRE2,AURKA,MCPH1,ABL1,CEP83,HTR2A,ABHD17C,DISC1,NUDCD3,BBS4,KIAA0753,CHAMP1,MARK4,DIAPH1,BICD1,GAS2L1,HOOK3,HNRNPU,CEP72*
GO:0018210	peptidyl-threonine modification	0.012991390706947499	*MTOR,NLK,BCL2,SPRED1,GALNT1,PRKD1,RPTOR,APP,TRPC5,MAPK1,GALNT16,PARD3,GALNTL6,EGF,SPRED2,MARK2,HIPK3,MYLK2,GALNT13,CDC42BPB,ATF2,CDC42BPA,ROCK1,CSNK2A1,EOGT,HIPK1,TNKS,CEMIP,MAPK8,GALNT2,PRKCA,BMP7,ROCK2,PRKAG2*
GO:0060429	epithelium development	0.013242172129204888	*NOTCH2,MTOR,MYO9A,PLCB1,DLC1,ZDHHC21,RIPOR2,PDE4D,RDX,RALA,BCL2,ALDH1A2,ROBO2,ZEB1,AKR1C3,RARB,SPRED1,MYO1E,USH2A,FOXJ2,MLLT3,GPC6,SETD2,ARHGAP24,EGFR,RFX3,PRKACB,RNF220,CECR2,EDAR,PAK1,EPHA7,RAPGEF2,LRP2,DEUP1,CPS1,ONECUT1,TMEM38B,PRICKLE2,LDB2,LUZP1,SSBP3,TMC1,VCL,IFT57,MRTFA,COBL,YAP1,RIPK4,MAPK1,ZNRF3,LCE1F,FMN1,PAFAH1B1,NF2,KLF15,NFIB,NR5A2,TIAM1,KAZN,SEMA3C,RYR2,WNT9B,COL22A1,ATRX,ABL1,RAP1A,FGF10,TGM1,LATS2,NRG1,AP3B1,SETDB2,CD44,PTPRO,EGF,EXT1,SPRED2,PLXNA2,AMBRA1,SEMA3E,BCL11B,ECE1,TJP1,MTHFD1L,BRCA2,WDPCP,BMP2,TDRD7,ESRP1,PLS1,GLI3,SLC9A4,BBS4,COL5A1,TBX20,PCDH15,CDH23,PRKCH,LCE3B,YIPF6,ROCK1,CTSB,OVOL2,NTN1,HOXC13,DMBT1,HECTD1,SHROOM3,SNAI2,FLNB,CERS3,KRT6B,BMP5,CSF1,KRT25,LAMA3,SMAD5,SLC40A1,MYCL,PSAP,MED1,KDM6A,AJAP1,FAT1,IL10,SOSTDC1,VASP,IQGAP1,TWIST1,JAK2,FSTL1,CELSR2,GORAB,PCNA,ANXA4,ALX4,USH1C,KRT6A,MEOX2,GRXCR1,STAT1,ABI1,CSMD1,ASB2,KRT85,MEF2C,WNT7A,PDE2A,PPP2R3A,SPRR2D,LCE3D,EPHA4,FNDC3A,WNT2B,CDH5,ANKRD6,ARHGAP12,PCDH8,FRMD6,FAT4,AKR1B1,WNT5B,NSUN2,ADAMTS16,TNFSF11,ARL13B,HYDIN,HDAC2,COL18A1,APELA,ROR1,CDH2,NTN4,NRP1,BCR,B9D1,BMPER,MACROH2A1,MET,MAGI2,GNAS,LAMA1,GREB1L,BMP7,ASTN2,DLG5,EYA1,SLIT2,ERBB4,ROBO1,PBX1,SIPA1L3,MGMT,ESR1,PRLR,FOXB1,RAD51B,FER,STARD13,ROCK2,PRDM1,DMRT1,PTPRQ,STK3,APCDD1,GLI2,THRB*
GO:0031644	regulation of nervous system process	0.013309562453316465	*TENM4,CHRNA7,RIMS1,RIMS2,DLGAP1,SLC8A3,FGF12,TAFA4,APP,FIG4,PRKCZ,AKAP9,GRM1,TMEM108,PARD3,TNR,HTR2C,OPRM1,SCN11A,RELN,UNC13B,MTMR2,DLGAP2,GRIN2A,SHISA9,IGSF11,GRIN2B,CELF4,IL33,IL10,NRXN1,WNT7A,WASF3,SHISA6,JAM2,ATPSCKMT,TMEM25,NLGN1,ROCK2*
GO:0022898	regulation of transmembrane transporter activity	0.013446735273550205	*PDE4D,BCL2,CACNG2,NEDD4,CHRM3,FGF12,THADA,NEDD4L,APP,CACNB2,KCNE4,AKAP6,HECW1,LRRC38,AKAP9,RASGRF2,DAPK1,ANK2,RYR2,GSG1L,RASGRF1,PRKCE,SLMAP,WNK2,ABCC9,ALG10B,OPRM1,STAC,CNIH3,ANK3,NETO2,RELN,CNKSR3,CFTR,SELENON,JPH1,SHISA9,CRACR2A,UTRN,KCNC1,HCN1,NRXN1,TWIST1,ABCC8,PARK7,MEF2C,SHISA6,NOS1AP,HECW2,GRM5,ATPSCKMT,EPHB2,CACNG3,VMP1,TRDN,NLGN1,NOS1,CACNA2D1,HTT,CCR2,KCNAB1*
GO:0016049	cell growth	0.013789261819505967	*MTOR,SPOCK1,ULK2,BCL2,RIMS1,MINAR1,RIMS2,AUTS2,PAPPA2,DSCAM,CRKL,PTPRJ,EGFR,MACF1,BCL11A,CDH4,EPHA7,RPTOR,EPB41L3,NEDD4L,ADAM10,APP,DCLK1,SEMA5A,SYT1,VCL,AURKA,AKAP6,DIP2B,TRPC5,PRKCZ,COBL,YAP1,ALCAM,NRG3,PAFAH1B1,PPARA,PAK5,SEMA3C,TMEM108,SEMA6D,SMARCA4,MAPKAP1,TNR,ABL1,NRG1,EXT1,RPS6KA3,SEMA3E,CDKN2C,PL*

			CE1,CRIM1,PDLIM5,DISC1,SEMA3A,SEMA3D,SLC23A2,NIN,DRAXIN,SMARCA2,MAP2,DCC,PRKN,FBLN5,ITSN2,NTN1,ENPP1,CSNK2A1,CTDP1,INO80,TNN,LMX1A,IQGAP1,TEAD1,NET1,PRSS2,MTPN,IMPACT,ITGA4,MYOCD,CYFIP2,SDCBP,EXTL3,CYFIP1,SEMA4D,SORBS2,SPAG6,NRP1,RERG,CD38,PRDM11,SLIT2,PRKCQ,EFNA5,ARHGEF11,SLIT3,SEMA4B,FSTL4,AKAP13
GO:0072698	protein localization to microtubule cytoskeleton	0.014460724337194197	*CEP192,APC,MAPRE2,AURKA,MCPH1,ABL1,CEP83,ABHD17C,DISC1,NUDCD3,BBS4,KIAA0753,CHAMP1,MARK4,DIAPH1,BICD1,GAS2L1,HOOK3,HNRNPU,CEP72*
GO:0045597	positive regulation of cell differentiation	0.015276223195639498	*NOTCH2,BRINP3,MTOR,PTPRD,PLCB1,TENM4,RIPOR2,BCL2,ROBO2,ZEB1,CARMIL1,RIN2,DSCAM,TCF4,CRKL,SOX5,RFX3,MACF1,BCL11A,SOX6,CDH4,PRKD1,SPEN,RAPGEF2,LRP2,RUNX2,GHR,ZHX3,STAU2,SEMA5A,AURKA,BMPR1B,AKAP6,ARNT,PAK3,ITPKB,TRPC5,PRKCZ,YAP1,BRINP1,MYLK3,PAFAH1B1,NF2,SYNJ1,TIAM1,IL34,RANBP3L,SMARCA4,ABL1,RAP1A,NRG1,ASPM,AP3B1,ATP11C,ZBTB16,SH3GL3,FBN2,PDE3A,RPS6KA3,PLXNA2,HTR2C,ARHGEF7,AMBRA1,OPRM1,HTR2A,DOCK5,DISC1,BMP2,MSR1,RELN,HMGB1,DOCK1,NIN,SMARCA2,ETS1,GLI3,SMARCC1,PCP4,LAMC1,NELL1,KITLG,DAB1,TBX20,DPF3,PRKCH,IL6R,TOX,LYN,OVOL2,NTN1,ZFHX3,ARID1B,SNAI2,RASGRP1,CSF1,ASB4,SMAD5,MED1,IL33,ROR2,IL10,ACTR2,VSTM2A,MAP6,ZBTB7C,PCID2,TWIST1,JAK2,UFL1,NEDD9,OLFM4,STAT1,IMPACT,MYOCD,MEF2C,MAP3K5,FBXW8,SDCBP,EPHA4,NTRK2,IL1RAPL1,NUMB,ADAMTS9,CD101,FBXO31,CDH5,NFKBID,LAMB1,CYFIP1,SEMA4D,RUNX1,TNFSF11,HDAC2,MAPK9,GRM5,NRP1,PRKCA,FAIM,CHODL,POR,RAG1,CUX1,MACROH2A1,EPHB2,MYB,KALRN,LAMA1,TIAM2,BMP7,NUDT21,SLIT2,ROBO1,EFNA5,TCF12,LOXL2,STK3,HNRNPU,GLI2*
GO:0071310	cellular response to organic substance	0.015428538346456477	*NOTCH2,BCAR3,BRINP3,MTOR,NSG1,NLK,ZNF236,PLCB1,PTPRA,ITPR2,RIPOR2,PDE4D,RDX,STXBP1,IL1RAPL2,BCL2,MYO5A,PRDM16,ALDH1A2,FBN1,ROBO2,GABRB3,ZEB1,AKR1C3,RARB,SPRED1,ALK,ERBIN,APC,CRKL,SOX5,PTPRJ,KDM4C,EGFR,ANGPT1,NCOR1,UGT3A2,NEDD4,BCL11A,SOX6,NTRK3,RXFP1,ZFAND6,FLT1,GABRB1,NEO1,PRKD1,PAK1,CHRM3,GRAMD1B,RAPGEF2,LRP2,RUNX2,FGF12,CPS1,ONECUT1,CPEB4,TMEM38B,UBE2L3,PTPRN2,SMYD3,RPTOR,GHR,COL4A2,HDAC9,IL1R1,APP,RPS6KA2,GABRG2,DOCK8,USP18,NTF3,NDUFAF2,ST18,SLC8A1,HERPUD2,KANK1,BMPR1B,PCSK6,AKAP6,HADHB,ARNT,RAB8B,PAK3,LARP1,RAP1GDS1,PRKCZ,GRB10,RYR3,DEFA3,DUSP22,YAP1,BRINP1,MAPK1,HRH2,PDGFD,UBE2O,GFRA1,SLC16A1,SPIDR,GABPA,HRH4,MYLK3,GLP2R,PAFAH1B1,ATF6,TM7SF3,ITGB8,HIVEP1,CTNNA1,AKAP9,KLF15,PPARA,ERMP1,NR5A2,ADAMTS3,PTPRK,TRERF1,DAPK1,TMEM108,JARID2,IL34,BCAS3,RYR2,BBS2,WNT9B,SMARCA4,USP8,MBD5,PTPRT,ELAVL4,ABL1,PTPN12,HDAC4,SLC1A1,PRKAA1,NFAT5,RAP1A,FGF10,LATS2,ZNF675,PRKCE,ESRRG,DENND4C,FBN2,CD44,PDE3A,EXT1,SPRED2,PTPN2,TRIM5,ATXN3,HTR2C,LTBP1,ZFYVE9,OPRM1,HTR2A,ALPL,GNAL,EPN2,NOS2,GBP6,CCND3,IL17RA,CRIM1,FBXO32,TJP1,LDLRAD4,BLM,BMP2,PSG9,SOGA1,MSR1,GNAI1,TBC1D4,LEMD3,HMGB1,FGF9,SLC23A2,ZNF106,TRAF3,UNC13B,DSTYK,RAP1GAP,CCDC186,CGAS,SMARCC1,SNX6,SMOC2,IDE,GAREM1,ATF2,CYLD,UMODL1,BBS4,MX1,CFTR,RGMB,UBR1,CHRM5,SLC30A10,RORB,SELENON,PTPRE,PRKN,TBX20,ARID5B,IL6R,NLRC5,SNX25,RALB,ROCK1,LYN,VCAM1,CTSB,DTX1,OVOL2,RRAGD,ARID1B,INSR,YTHDF3,SNAI2,UFD1,RXRG,ERN2,MBTPS2,FLNB,NREP,ZDHHC17,JCAD,SAMHD1,ENPP1,BMP5,CSF1,BCL2L1,SERPINB9,HCN1,GRB14,SMAD5,MED1,IL33,KL,IL10,ACTR2,PTH,SOSTDC1,PRKAA2,CSF2RB,PACRG,ABHD2,VSTM2A,IQGAP1,YBX3,NRXN1,HIPK1,CD70,PBLD,FICD,CACYBP,PEG10,TWIST1,JAK2,FSTL1,CREBBP,UFL1,NFKBIA,PRKCB,ABCC8,ZC3H15,SMPD4,ATP2B1,ASS1,OTOP1,CIDEA,EXT2,STAT1,BRMS1L,NR2C1,MAP*

			2K6,IMPACT,CCBE1,PARK7,MAPK8,ITGA4,MTF2,MYOCD,CYFIP2,ACACA,MEF2C,RXRA,WNT7A,RBPMS2,MAP3K5,OR10H2,PDE2A,SDCBP,SPPL2B,WWOX,NCK1,CXCL2,IFNAR1,EPHA4,NTRK2,COLEC12,IL17RD,PTK2,CDH5,CLDN18,DIAPH1,CYFIP1,UBE3A,ZBTB20,FAT4,WNT5B,AMFR,PTGFR,TNFSF11,FYN,HDAC2,DOK5,FUT8,TET1,ITGA8,GRM5,RPS6KA5,TBC1D1,PID1,NRP1,PRKCA,GBP4,RC3H1,NRIP1,POR,BCR,KIF16B,NSG2,GNA14,BMPER,EPHB2,STXBP4,MAGI2,FLRT2,SLC1A2,GNAS,BMP7,PDGFC,ABL2,RFX2,SLIT2,CNOT7,ERBB4,IL20RB,ROBO1,PRKCQ,MGMT,NOS1,GLDC,EFNA5,GAS2L1,SLIT3,ESR1,CACNA2D1,PRLR,PIK3R3,FER,CCR2,EPS8,HRH1,ROCK2,RORA,RGS8,RAB31,HERPUD1,GNG2,PNPLA3,ZNF423,HNRNPU,IGF1R,GLI2,THRB
GO:0045596	negative regulation of cell differentiation	0.015484228103370301	*CNTN4,ULK2,ZNF536,ZFPM2,FBN1,ZEB1,RARB,SPRED1,USH2A,FOXJ2,EGFR,CDK12,BCL11A,SOX6,TMEM182,NTRK3,EPHA7,RAPGEF2,APP,SEMA5A,KANK1,DIP2B,ITPKB,TRPC5,ABCA5,YAP1,BRINP1,MAPK1,GABPA,EFEMP1,NF2,CTNNA1,PPARA,MEIS2,PRTG,SEMA3C,ADGRV1,RANBP3L,SEMA6D,SMARCA4,TNR,HDAC4,GLIS1,FGF10,ASPM,ZBTB16,ZNF675,TRIO,LIMD1,SPRED2,PTPN2,SEMA3E,ANKRD17,BICRAL,MBP,CRIM1,LDLRAD4,SEMA3A,BMP2,RC3H2,SEMA3D,HMGB1,FGF9,NFATC2,ANKRD26,RAP1GAP,DRAXIN,SMARCA2,GLI3,SMARCC1,MAP2,COL5A1,DCC,RORB,DAB1,LYN,DTX1,OVOL2,NTN1,ZFHX3,DPYSL5,SNAI2,TWIST2,ENPP1,BMP5,CTDP1,ABCG1,PRAME,TNN,MED1,LMX1A,TMEM178A,PRAMEF25,PTH,SOSTDC1,ANP32B,TWIST1,MELTF,NFKBIA,ABCC8,STAT1,PRAMEF2,MYOCD,WNT7A,RBPMS2,EPHA4,IL17RD,CLDN18,SEMA4D,RUNX1,GPR55,GPR137B,EPHB1,NRP1,RC3H1,FBLN1,EPHB2,MYB,BMP7,DDX6,PBX1,EFNA5,SEMA4B,RORA,HOOK3,FSTL4,HNRNPU,GLI2*
GO:0051172	negative regulation of nitrogen compound metabolic process	0.016164841520431364	*NOTCH2,SPOCK1,WWC1,FTO,PLCB1,ZNF536,ZFPM2,L3MBTL4,TNRC6B,PDE4D,PRDM16,CHRNA7,ZEB1,RARB,SPRED1,MINAR1,CDYL2,GLIS3,SPON1,APC,TSHZ3,RTN1,CRKL,PTPRJ,KDM4C,EGFR,RFX3,USP14,ANGPT1,BACH1,NCOR1,NEDD4,SCAI,BCL11A,SOX6,NTRK3,C5,THRAP3,CAST,SLC8A3,NCOA7,SPEN,RUNX2,CPEB4,LDB2,SMYD3,HDAC9,ZHX3,ATF7IP,APBB2,APP,SAMSN1,KDM1B,NTF3,PARP15,PARN,SLC8A1,SERPINA6,FMN2,LARP1,NBN,PRKCZ,TAF15,MSH6,MCPH1,PHF19,DUSP22,YAP1,MGAT5,ITIH5,USP25,UBE2O,SFMBT2,NIPBL,GABPA,CARD18,STK38,PTPN13,ZNF684,DCAF1,NF2,HIVEP1,BIRC6,KLF15,PPARA,MEIS2,NFIB,PTPRK,TRERF1,DAPK1,AGO2,PHC3,JARID2,GATAD2B,ZNF846,DUSP16,SMARCA4,PARD3,TNRC6C,PIAS1,ATRX,PTPRT,ELAVL4,ABL1,MXI1,HDAC4,OXR1,PRKAA1,CREG1,L3MBTL3,NBAS,PSMF1,SLFN11,GLIS1,MORC1,LATS2,NRG1,ZNF438,ZBTB16,ZNF675,SETDB2,PRKCE,FOXK2,NXN,CD44,PTPRO,LIMD1,PEX14,SPRED2,RPS6KA3,PTPN2,RFC1,OPRM1,DAZL,TAF3,FHL2,PUM1,MSH2,IGF2BP3,ZNF397,HIPK3,CDKN2C,NOS2,RBBP8,MDFIC,HMGA2,CCND3,CREM,TRPS1,CRIM1,PRR5L,MYT1L,LDLRAD4,SNX3,BRCA2,ZBTB2,BLM,BMP2,RC3H2,ZC3H14,GFI1B,HMGB1,GNAQ,FGF9,NFATC2,SH3BP5,CPAMD8,ETS2,ZNF875,UIMC1,LRRFIP1,SMARCA2,GLI3,CGAS,SMARCC1,SNX6,CNKSR3,ZNF431,RERE,BTAF1,ATF2,HIRA,MAPK8IP1,NELL1,MRPL13,RORB,PRKN,ZNF608,TBX20,DACH1,ZNF541,DPF3,GRIN2A,ARID5B,ATXN1,PKP1,CNOT6L,SNX25,PTPRB,ZFP90,ZNF124,USP7,PLAGL1,SOX30,ROCK1,LYN,CTSB,ZNF169,TENM2,OVOL2,ZBTB33,ZFHX3,BANP,YTHDF3,DEDD2,SNAI2,SIAH2,SP3,ERN2,ELF2,NSD2,TWIST2,ENPP1,CSNK2A1,SERPINB9,SCAF4,FANCB,SMAD5,CELF4,TCERG1,FOXN3,PRAME,MED1,IL33,BANK1,CSDE1,IL10,SFPQ,SCML2,PRAMEF25,PRKAA2,ETV6,CAMLG,SREBF2,YBX3,PCID2,FRY,TWIST1,ZBTB38,PATL1,CREBBP,TNKS,PCNA,UFL1,RTRAF,ZBTB21,ZBTB49,AGO1,STAT1,BRMS1L,NR2C1,PRAMEF2,IMPACT,PARK7,POU1F1,MTF2,NCAPG2,FOXP2,MYOCD,MEF2C,ADGRB1,RXRA,NDFIP1,SERPINI2,PRDM13,MAGEL2,PDE2A,SDCBP,ZBTB25,MLLT1,NCK1,SCAF8,RNF8,EPHA4,MECOM,DNMT3L,NTRK2,LHX9,ZBTB10,OCLN,IREB2,ASCL3,SEMA4D,SERPINB10,ZBTB20,RUNX1,KIRREL1,SAMD13,PDCL3,SRP9,NSUN2,TNFSF11,FYN,KDM5A,PCBP3,ZNF705G,PPM1F,HDAC2,GON4L,TBX15,PSME3IP1,ZFYVE28,PABPC1,TET1,SPOPL,ZNF705D,RPS6KA5*

			,PID1,MIDEAS,FHIT,KLF12,RC3H1,NRIP1,POR,TUT4,FBLN1,RAG1,CUX1,MACROH2A1,MITF,EPHB2,CD38,ZNF705B,SERPINB2,ATG5,PRDM11,UNK,MLIP,MYB,MFHAS1,SERPINB7,BMP7,TNFAIP8,ZMYND8,KCTD1,BPTF,ZMYND11,DDX6,BACE2,PARPBP,SLIT2,CNOT7,SERPINB11,SAMD4A,SHLD2,NSD1,EHMT1,ESR1,KDM4B,LOXL2,AGO3,A2M,JAZF1,ZNF891,SPOCK3,PHC2,ROCK2,PRDM1,DMRT1,COL4A3,BARD1,DEPTOR,ZNF423,ZNF568,HNRNPU,PRKAG2,GLI2,THRB
GO:0043010	camera-type eye development	0.016178719428165577	*NOTCH2,BCAR3,SCAPER,RP1,ALDH1A2,FBN1,TENM3,ZEB1,SPRED1,DSCAM,EGFR,CRB1,ATP2B2,FLT1,CACNA1C,DCLK1,BMPR1B,FAT3,EFEMP1,NF2,WNT9B,SMARCA4,SLC1A1,TTLL5,FGF10,FBN2,SPRED2,NHS,ATP8A2,BCL11B,NPHP4,WDPCP,PDE6C,TDRD7,MDM1,GLI3,MEGF11,BBS4,LAMC3,RORB,PDE6A,SP3,HCN1,CELF4,VSTM4,MED1,FAT1,HIPK1,TWIST1,VSX1,USH1C,ATP2B1,SHROOM2,WNT7A,NECTIN1,NTRK2,WNT2B,WNT5B,UNC45B,HDAC2,XRN2,EPHB1,RP1L1,NRP1,SDK1,B9D1,MITF,EPHB2,LAMA1,BMP7,MYH15,SLC6A3,RPGRIP1,THRB*
GO:0150115	cell-substrate junction organization	0.01620772359194752	*DLC1,PTPRA,BCL2,PTPRJ,MACF1,MAPRE2,VCL,MAP4K4,RAB8B,DUSP22,CORO2B,LIMCH1,FMN1,PTPRK,BCAS3,DST,ABL1,PEAK1,ARHGEF7,IQSEC1,WDPCP,LAMC1,ROCK1,ITGA6,PTK2,PPM1F,NRP1,BCR,EFNA5,ROCK2*
GO:0043269	regulation of ion transport	0.0162875824324 1906	*CACNA2D3,KCNH5,DPP10,PDE4D,STXBP1,BCL2,KCNMA1,ANO6,CACNG2,NEDD4,PRKD1,CHRM3,FGF12,TMEM38B,GRM7,THADA,NEDD4L,APP,CACNA1C,CACNB2,TMC1,SYT1,SLC8A1,KCNE4,AKAP6,HOMER2,KCNK10,CLIC6,HECW1,KCNJ1,SYT10,LRRC38,AKAP9,RASGRF2,KCNS3,PLA2R1,DAPK1,SCN2A,ANK2,RYR2,NKAIN3,ABL1,PRKAA1,KCNH1,NKAIN2,GSG1L,RASGRF1,PRKCE,SLMAP,WNK2,EGF,ABCC9,ALG10B,OPRM1,HTR2A,CYP4A11,STAC,CNIH3,KCND2,ANK3,CNTN1,CACNA1I,KCNJ15,SCN11A,NETO2,RELN,KCNH8,CNKSR3,CFTR,SLC30A10,SELENON,PRKN,GRIN2A,JPH1,KCNQ3,SHISA9,SCN10A,KCND3,LYN,CRACR2A,PLPP4,UTRN,KCNC1,HCN1,GRIN2B,KCNK5,PLA2G4A,NRXN1,TWIST1,ABCC8,CACNA1E,ATP2B1,SLC6A1,MAP2K6,KCNJ18,CEMIP,PARK7,ADCYAP1R1,EFHB,MEF2C,SHISA6,DIAPH1,NOS1AP,TNFSF11,FYN,SCN8A,NALCN,HECW2,GRM5,ATPSCKMT,KCNJ6,DPP6,EPHB2,TSPAN13,CACNG3,ATG5,VMP1,KCNIP4,TRDN,NLGN1,NOS1,ASIC2,KCNQ5,CACNA2D1,HTT,CCR2,IL16,CATSPER2,RGS7,CLDN10,KCNAB1*
GO:0010720	positive regulation of cell development	0.017149413872875444	*MTOR,PTPRD,TENM4,BCL2,ROBO2,CARMIL1,DSCAM,CRKL,RFX3,MACF1,BCL11A,CDH4,SPEN,LRP2,STAU2,SEMA5A,AURKA,PAK3,TRPC5,PAFAH1B1,SYNJ1,TIAM1,IL34,ABL1,ASPM,PDE3A,PLXNA2,ARHGEF7,OPRM1,DOCK5,DISC1,BMP2,RELN,DOCK1,NIN,GLI3,PRKCH,LYN,NTN1,IL33,ACTR2,MAP6,UFL1,NEDD9,OLFM4,FBXW8,EPHA4,NTRK2,IL1RAPL1,NUMB,FBXO31,CDH5,CYFIP1,SEMA4D,TNFSF11,HDAC2,GRM5,NRP1,FAIM,CHODL,CUX1,EPHB2,KALRN,TIAM2,SLIT2,ROBO1,EFNA5*
GO:0060997	dendritic spine morphogenesis	0.0176657115640 0133	*DOCK10,STAU2,ARHGAP44,PAK3,DNM3,DIP2A,PAFAH1B1,CTNND2,PDLIM5,RELN,NGEF,TANC2,ACTR2,WNT7A,EPHA4,UBE3A,EPHB1,EPHB2,PPFIA2,KALRN,NLGN1*
GO:1904062	regulation of cation transmembrane transport	0.017938562260802596	*DPP10,PDE4D,BCL2,ANO6,CACNG2,NEDD4,PRKD1,FGF12,TMEM38B,THADA,NEDD4L,APP,CACNA1C,CACNB2,TMC1,SLC8A1,KCNE4,AKAP6,HECW1,LRRC38,AKAP9,RASGRF2,DAPK1,ANK2,RYR2,ABL1,GSG1L,RASGRF1,PRKCE,SLMAP,WNK2,ABCC9,ALG10B,OPRM1,STAC,CNIH3,ANK3,NETO2,RELN,CNKSR3,SELENON,GRIN2A,JPH1,SHISA9,LYN,CRACR2A,UTRN,KCNC1,HCN1,GRIN2B,NRXN1,TWIST1,ABCC8,CEMIP,PARK7,ADCYAP1R1,MEF2C,SHISA6,DIAPH1,NOS1AP,FYN,HECW2,ATPSCKMT,DPP6,EPHB2,TSPAN13,CACNG3,ATG5,VMP1,KCNIP4,TRDN,NLGN1,NOS1,CACNA2D1,HTT,CCR2,RGS7,KCNAB1*
GO:0033043	regulation of organelle	0.018241915291361422	*NOTCH2,MTOR,PTPRD,PLCB1,SVIL,DLC1,RIPOR2,RDX,RP1,STXBP1,RALA,SPIRE1,SDCCAG8,CDC42EP3,CARMIL1,RHPN2,MAP4,APC,NEK7,NSMCE2,NTRK3,PSMA8,CRACD,SLC39A12,TOM1L2*

	organization		,PAK1,SEPTIN9,TBCD,ATF7IP,RPS6KA2,STAU2,MAPRE2,SEMA5A,SYT1,ARHGAP44,NTF3,CD2AP,AURKA,PARN,KANK1,CTNNA2,PAK3,RAP1GDS1,NBN,MCPH1,SENP6,YAP1,MAPK1,CORO2B,MYLK3,LIMCH1,FMN1,PAFAH1B1,VPS13D,TPM1,NF2,AKAP9,SNX30,SYNJ1,NAV3,VPS13C,BCAS3,SYNE2,SMARCA4,ATRX,ABL1,PRKAA1,GRID2,INO80D,CLIP1,SETDB2,PRKCE,EGF,PDE3A,MTMR3,CLEC16A,ARHGEF7,AMBRA1,SEMA3E,MARK2,TMEM67,C10ORF90,TMOD2,MNAT1,TGFA,TJP1,WDPCP,GNAI1,TBC1D4,RESF1,MDM1,SLAMF1,SMARCA2,SMARCC1,KIF15,MAP2,CYLD,BBS4,YLPM1,PRKN,AFAP1,DPF3,CNOT6L,KANK4,USP7,RALB,ROCK1,ARHGAP28,ARID1B,INSR,BMF,NEK6,DDHD1,ARFGEF1,PDE4DIP,BID,FYCO1,SH3GLB1,SNX9,ANAPC1,HDGFL3,BCL2L1,INO80,SFPQ,PRKAA2,SKA1,NDC80,MAP6,VASP,IQGAP1,MORC2,SREBF2,NRXN1,PCID2,CENPE,TNKS,NEDD9,GAP43,SAR1A,MTPN,PARK7,MAPK8,CEP120,CYFIP2,MAP3K4,WASF3,MAGEL2,RAD51AP1,PDE2A,SDCBP,NSMCE1,NCK1,CDCA8,OCLN,PTK2,MARK4,CDH5,DIAPH1,FEZ2,CYFIP1,KIRREL1,SAXO1,ASAP1,CCDC88A,ADAMTS16,BICD1,BUB1,PPM1F,SLF1,MAPK9,HECW2,CDH2,ADCK1,SPTB,PID1,NRP1,FCHSD2,MACROH2A1,TOGARAM1,MET,ATG5,BMP7,ABL2,FHOD3,SLIT2,NUF2,PRKCQ,EFNA5,GAS2L1,IQCJ-SCHIP1,HTT,FER,CHFR,EPS8,ROCK2,ATAT1,DMRT1,CDCA5,WASHC1,HNRNPU,RAB3GAP2,AKAP13,DNM1L*
GO:0051246	regulation of protein metabolic process	0.0192314039093310 8	*BCAR3,MTOR,SPOCK1,FTO,KSR1,PLCB1,DLC1,TNRC6B,PDE4D,RDX,BCL2,CHRNA7,SPRED1,MINAR1,ALK,AUTS2,EGLN3,SPON1,APC,RTN1,CRKL,TNIK,PTPRJ,KDM4C,EGFR,USP14,ANGPT1,CDK12,PRKACB,NEDD4,MTRF1,NTRK3,C5,FLT1,CAST,SLC8A3,PRKD1,PAK1,EPHA7,NCOA7,RAPGEF2,PELI2,LRP2,TAOK3,LDLRAD3,CPEB4,UBE2L3,PUM3,SMYD3,RPTOR,GHR,NEDD4L,APP,RPS6KA2,SAMSN1,NTF3,ACER2,AURKA,PARN,ST18,SLC8A1,SERPINA6,PLGRKT,BMPR1B,FMN2,ARNT,DIP2B,LARP1,ITPKB,TRPC5,NBN,IFT57,PRKCZ,SPOP,DIP2A,HECW1,PHF19,DUSP22,WDR70,MAPK1,MGAT5,ITIH5,USP25,KMT2E,PDGFD,NRG3,UBE2O,GFRA1,NIPBL,CARD18,STK38,PTPN13,KANSL1,LIMCH1,CCNG2,NF2,MOB3B,BIRC6,AKAP9,KLF15,PPP6R3,EIF3D,DAPK1,AGO2,JARID2,IL34,WNT9B,CLPX,DUSP16,USP8,PARD3,MAPKAP1,TNRC6C,PIAS1,ATRX,NUAK1,PTPRT,ELAVL4,ABL1,HDAC4,OXR1,SLC1A1,PRKAA1,MRPS27,PSMF1,RAP1A,FGF10,LATS2,NRG1,MUSK,ZNF675,PRKCE,SLCO3A1,NXN,CD44,PTPRO,EGF,PRRC1,SPRED2,RPS6KA3,PTPN2,ATXN3,ALG10B,AMBRA1,HTR2A,FANCM,DAZL,MARK2,PUM1,IGF2BP3,SLC2A13,HIPK3,CDKN2C,KNDC1,SPSB4,CLSPN,NOS2,MNAT1,HMGA2,CCND3,MBP,PLCE1,TGFA,HIP1,CRIM1,PRR5L,LDLRAD4,CNTN1,SNX3,DISC1,BLM,BMP2,RC3H2,RANBP9,RELN,HMGB1,GNAQ,FGF9,SH3BP5,CPAMD8,TRAF3,GEMIN5,SMARCC1,SNX6,CNKSR3,IDE,NEK10,MOB1B,ATF2,UMODL1,MAPK8IP1,NELL1,MRPL13,KITLG,CAMTA1,RCAN1,TADA2A,DAB1,RB1CC1,PRKN,GRIN2A,IL6R,ALS2,ACO1,CNOT6L,MKNK1,SNX25,PTPRB,COPS8,USP7,RALB,ROCK1,LYN,CTSB,BZW1,PIWIL3,BANP,INSR,YTHDF3,HECTD1,SUMO2,SNAI2,BID,SIAH2,TRABD2B,ERN2,CARD10,CTIF,ENPP1,RASGRP1,SNX9,CSNK2A1,BMP5,CSF1,SERPINB9,GRIN2B,CELF4,ABCG1,DCUN1D4,CDC14B,IL33,GPRC5C,ROR2,BANK1,CSDE1,IL10,PRKAA2,LARP6,IQGAP1,CAMLG,ANP32B,YBX3,NRXN1,PCID2,FRY,CENPE,NGDN,TWIST1,ALKAL2,JAK2,MADD,PATL1,MELTF,UFL1,NFKBIA,RTRAF,BRD4,NEDD9,AGO1,NDFIP2,MAP2K6,MTPN,ABI1,CEMIP,IMPACT,CCBE1,PARK7,MAPK8,OAZ2,UBL7,MTF2,NCAPG2,MYOCD,CYFIP2,MEF2C,ADGRB1,WNT7A,MAP3K5,NDFIP1,MAP3K4,SERPINI2,DBF4B,FBXW8,SDCBP,PASK,MLLT1,NCK1,FGR,CDCA8,PPP2R3A,EPHA4,NTRK2,OCLN,IREB2,PTK2,CDH5,CYFIP1,UBE3A,SEMA4D,SERPINB10,RUNX1,KIRREL1,POMT2,NOS1AP,PDCL3,SRP9,CCDC88A,TNFSF11,FYN,KDM5A,PPM1F,HDAC2,SH2D3C,PSME3IP1,DOCK3,TRNAU1AP,ZFYVE28,MAPK9,PABPC1,TET1,HECW2,FBXL20,GRM5,SPOPL,RPS6KA5,PID1,NRP1,FHIT,ITGA1,RC3H1,POR,TUT4,TM9SF2,ELP2,FBLN1,RAG1,BMPER,MACROH2A1,EPHB2,BCL2L13,DPH6,SPPL3,SERPINB2,ATG5,MAGI2,UNK,MYB,MFHAS1,SERPINB7,DHX29,BMP7,TNFAIP8,RNF217,DDX6,PDGFC,BACE2,NECAB1,SLIT2,CNOT7,ERBB4,SERPINB1*

			1,GSAP,ROBO1,SAMD4A,ANTXR1,MGMT,NOS1,PRR16,EFNA5,NSD1,KDM4B,PRLR,AGO3,HTT,PIK3R3,FER,A2M,CHFR,SPOCK3,ROCK2,EIF4G3,PSMD2,HERPUD1,COL4A3,WASHC1,BARD1,STK3,DEPTOR,HNRNPU,RAB3GAP2,PRKAG2,AKAP13
GO:1902533	positive regulation of intracellular signal transduction	0.021479761270144067	NOTCH2,BCAR3,WWC1,KSR1,PLCB1,CHRNA7,AKR1C3,SPRED1,ALK,AUTS2,PJA2,ERBIN,CRKL,TNIK,PTPRJ,EGFR,ANGPT1,NEDD4,NTRK3,FLT1,MAPKBP1,DGKI,EDAR,PRKD1,PAK1,RAPGEF2,PELI2,LRP2,TAOK3,RPTOR,GHR,APP,MAPRE2,SEMA5A,NTF3,MAP4K4,AKAP6,ITPKB,PRKCZ,DUSP22,PDGFD,GRM1,PLA2R1,IL34,ADGRV1,ABL1,NFAT5,GUCY1A2,RAP1A,FGF10,ZC3HAV1,NRG1,DENND2B,RASGRF1,PRKCE,CD44,EGF,SPRED2,PTPN2,TRIM5,MCF2L,HTR2C,CLEC16A,OPRM1,HTR2A,SEMA3E,PUM1,ANKRD17,RELL1,PLCE1,TGFA,HIP1,PRR5L,SEMA3A,BMP2,RC3H2,RELN,HMGB1,TRAF3,DSTYK,SLAMF1,GAREM1,NEK10,MAPK8IP1,KITLG,CAMTA1,SLC30A10,RB1CC1,PRKN,IL6R,ALS2,ROCK1,LYN,SLC44A2,RRAGD,CRACR2A,INSR,NEK6,BID,ERN2,TIAL1,ZDHHC17,JCAD,RASGRP1,CSF1,ROR2,KL,BANK1,SFPQ,NDC80,IQGAP1,NRXN1,PCID2,NET1,AKT3,ALKAL2,JAK2,MADD,HCRTR1,PRKCB,BRD4,NDFIP2,MAP2K6,PARK7,ADCYAP1R1,MEF2C,WNT7A,MAP3K5,NDFIP1,MAP3K4,S100B,SDCBP,NCK1,FGR,CDCA8,EPHA4,NTRK2,PTK2,ANKRD6,UBE3A,SEMA4D,NENF,NOS1AP,GPR55,TNFSF11,FYN,DOK5,APELA,ROR1,CDH2,RAD9A,GPR137B,GRM5,NRP1,PRKCA,ITGA1,RC3H1,BMPER,MET,SPPL3,CDH13,GNAS,MFHAS1,DLG5,PDGFC,ERBB4,ROBO1,NLGN1,IQCJ-SCHIP1,AGO3,ROCK2,STK3,IGF1R,AKAP13,DNM1L
GO:0034332	adherens junction organization	0.022260478980711047	RDX,CDH8,TBCD,ADAM10,VCL,CDH7,CDH11,CDH18,CDHR3,CDH20,TJP1,HIPK1,EPHA4,NUMB,CDH5,CDH9,CDH12,DLG5,FER
GO:0008015	blood circulation	0.022790994865161562	MTOR,SGCD,IMMP2L,ZDHHC21,PDE4D,KCNMA1,NAV2,ENPEP,PTPRJ,ANGPT1,CTNNA3,MYOF,FLI1,ATP2B2,SLC8A3,CHRM3,FGF12,CPS1,TMEM38B,CELF2,NEDD4L,RPS6KA2,CACNA1C,CACNB2,SLC8A1,KCNE4,RAP1GDS1,RNLS,HRH2,CORO2B,MYLK3,TPM1,CORIN,AKAP9,PPARA,ANK2,RYR2,BBS2,CXADR,DOCK4,ABL1,HDAC4,SLC1A1,PTPRO,ABCC9,PDE3A,EXT1,LNPEP,HTR2A,CYP4A11,NOS2,SGCZ,MYLK2,EMILIN2,DOCK5,F5,ECE1,TJP1,ARHGAP42,BBS4,TBX20,SCN10A,KCND3,ROCK1,HCN1,PRKG1,SMAD5,VSTM4,KL,JAK2,PTGS1,ABCC8,ATP2B1,EXT2,STAT1,MAP2K6,PDE2A,TNNI1,OCLN,CDH5,NOS1AP,ADAMTS16,APELA,ASB3,ITGA1,BCR,SGCG,CD38,ATG5,SLIT2,TRHDE,TRDN,NOS1,ASIC2,CACNA2D1,HRH1,ROCK2,COL4A3,THRB,DNM1L
GO:0061061	muscle structure development	0.024486282252023407	MTOR,SGCD,NEBL,PLCB1,SVIL,ZFPM2,RIPOR2,BCL2,ZEB1,RARB,MYOF,SOX6,TMEM182,LARGE1,LRP2,ADGRB3,SMYD3,HDAC9,SLC8A1,AKAP6,MRTFA,PLG,MYLK3,MBNL1,TPM1,RBFOX1,PPARA,MRTFB,ALPK2,DYSF,ANK2,TANC1,RYR2,RANBP3L,SMARCA4,LDB3,CXADR,XIRP2,HDAC4,PRKAA1,KCNH1,FGF10,NRG1,SYNE1,PGM5,ALPK3,FHL2,TMOD2,ANKRD17,LUC7L,EVC,AFG3L2,SGCZ,ADAM12,MYLK2,MYOM2,PDLIM5,DNER,BMP2,PTCD2,FGF9,NFATC2,SMARCA2,SMARCC1,MYEF2,LAMC1,HIRA,HIVEP3,RCAN1,SELENON,TBX20,DPF3,ARID5B,JPH1,PTGFRN,ZFHX3,ARID1B,RXRG,FLNB,CD9,UTRN,CTDP1,MED1,SOSTDC1,YBX3,TWIST1,ADAMTS5,SMTN,ALX4,ASS1,MEOX2,MTPN,MYO18B,ASB2,MYOCD,MEF2C,ADGRB1,RBPMS2,MAP3K5,PPP2R3A,TNNI1,MEGF10,LAMB1,JAM2,WNT5B,SORBS2,UNC45B,COL19A1,CDH2,ITGA8,EPHB1,CHODL,SGCG,ATG5,NRAP,LAMA1,FHOD3,MYH15,NOS1,TCF12,RORA,HNRNPU,AKAP13
GO:0001944	vasculature development	0.0252225415576 6406	NOTCH2,SGCD,IMMP2L,TAFA5,ZFPM2,ALDH1A2,CHRNA7,ROBO2,SPRED1,ENPEP,MYO1E,MINAR1,FOXJ2,RIN2,CRKL,SETD2,ARHGAP24,ANGPT1,C5,FLT1,ADAMTS6,SLC39A12,PRKD1,RAPGEF2,LRP2,ADGRB3,LUZP1,COL4A2,ADAM10,HDAC9,SEMA5A,THSD7A,CALD1,YAP1,MAPK1,PLG,PDGFD,ITGB8,SEMA3C,AGO2,BCAS3,COL22A1,ABL1,SLC1A1,RAP1A,FGF10,EGF,SEMA3E,ANKRD17,EPN2,ADAM12,EMILIN2,HMGA2,TGFA,TJP1,FGF9,NFATC2,ETS1,GLI3,SMOC2,ATF2,COL5A1,GTF2I,TBX20,IL6R,PTPRB,VAV3,ROCK1,OVOL2,HECTD1,CARD10,JCAD,ASB4,CNMD,VSTM4

			,TNN,MED1,IL10,ANP32B,AIMP1,NRXN1,HIPK1,TWIST1,AKT3,SVEP1,PRKCB,ABCC8,AGO1,MEOX2,STAT1,MYO18B,CCBE1,MYOCD,MEF2C,ADGRB1,WNT7A,PDE2A,FBXW8,FLVCR1,NTRK2,ADAMTS9,HS6ST1,PTK2,CDH5,AP2B1,RUNX1,PDCL3,COL18A1,APELA,CDH2,EPHB1,NRP1,PRKCA,NRXN3,B9D1,BMPER,EPHB2,CDH13,LAMA1,SERPINB7,BMP7,ADGRF5,EYA1,SLIT2,ROBO1,ANTXR1,LOXL2,PIK3R3,CCR2,STARD13,ROCK2,PRDM1,RORA,HSPG2,COL4A3
GO:0014070	response to organic cyclic compound	0.025437976649548846	*NSG1,LONP2,PLCB1,ITPR2,PDE4D,STXBP1,BCL2,ALDH1A2,GABRB3,AKR1C3,ALK,HLCS,KDM4C,EGFR,NCOR1,UGT3A2,NEDD4,PSMB2,NTRK3,GABRB1,PAK1,CHRM3,ADSS2,GRAMD1B,RAPGEF2,RUNX2,CPS1,TMEM38B,BCKDHB,UBE2L3,SMYD3,GHR,APP,KYNU,GABRG2,USP18,SLC8A1,AKAP6,HOMER2,PAK3,RFTN1,LARP1,RNLS,RYR3,DEFA3,YAP1,MAPK1,HRH2,ABCD3,PAPPA,SLC16A1,SPIDR,GABPA,HRH4,ACSBG1,CTNNA1,AKAP9,PPARA,TRERF1,RYR2,SMARCA4,USP8,EFTUD2,ELAVL4,ABL1,SLC1A1,PRKAA1,RAP1A,FGF10,PRKCE,ESRRG,ABCC9,P2RX6,PDE3A,HTR2C,RFTN2,OPRM1,HTR2A,ALPL,GNAL,MBP,FBXO32,BLM,AGL,BMP2,MSR1,GNAI1,ATF1,CGAS,ATF2,CFTR,CHRM5,SELENON,PRKN,SOX30,RALB,LYN,CRACR2A,SNAI2,RXRG,ENPP1,BCL2L1,HCN1,SMAD5,MED1,PPP2R2A,KL,IL10,ACTR2,PTH,ABHD2,JAK2,PCNA,UFL1,NFKBIA,ATP2B1,ASS1,CIDEA,SLC6A1,STAT1,NR2C1,IMPACT,PARK7,ADCYAP1R1,MEF2C,RXRA,WNT7A,OR10H2,PDE2A,DSG1,COLEC12,TPH2,DIAPH1,UBE3A,PTGFR,HDAC2,SDK1,NRIP1,POR,NSG2,GNA14,CD38,SLC1A2,GNAS,BMP7,PLIN2,SLIT2,MGMT,SLC6A3,GLDC,EFNA5,SLIT3,ESR1,A2M,HRH1,ROCK2,RORA,RGS8,GNG2,PNPLA3,HNRNPU,IGF1R,GLI2*
GO:0031324	negative regulation of cellular metabolic process	0.025820840500465316	*NOTCH2,MTOR,WWC1,FTO,PLCB1,ZNF536,ZFPM2,L3MBTL4,TNRC6B,PDE4D,BCL2,PRDM16,CHRNA7,ZEB1,AKR1C3,RARB,SPRED1,CDYL2,GLIS3,SPON1,APC,TSHZ3,RTN1,CRKL,PTPRJ,KDM4C,RFX3,USP14,ANGPT1,BACH1,NCOR1,NEDD4,SCAI,BCL11A,SOX6,NTRK3,THRAP3,SLC8A3,MALRD1,SPEN,RAPGEF2,RUNX2,CPEB4,LDB2,SMYD3,RPTOR,HDAC9,ZHX3,ATF7IP,APBB2,SAMSN1,KDM1B,NTF3,PARP15,PARN,SLC8A1,ABCD2,LARP1,NBN,PRKCZ,GRB10,TAF15,MSH6,MCPH1,PHF19,DUSP22,YAP1,MGAT5,SFMBT2,NIPBL,GABPA,STK38,PTPN13,ZNF684,DCAF1,NF2,HIVEP1,PPARA,MEIS2,NFIB,PTPRK,TRERF1,DAPK1,AGO2,PHC3,JARID2,DNAJC15,GATAD2B,ZNF846,DUSP16,SMARCA4,PARD3,TNRC6C,PIAS1,ATRX,PTPRT,ELAVL4,ABL1,MXI1,HDAC4,PRKAA1,CREG1,L3MBTL3,NBAS,SLFN11,GLIS1,MORC1,LATS2,NRG1,ZNF438,ABCB7,ZBTB16,ZNF675,SETDB2,FOXK2,PTPRO,LIMD1,PEX14,SPRED2,PTPN2,RFC1,CLEC16A,OPRM1,DAZL,TAF3,FHL2,PUM1,HERC1,MSH2,IGF2BP3,ZNF397,HIPK3,CDKN2C,RBBP8,MDFIC,HMGA2,CCND3,CREM,TRPS1,PRR5L,MYT1L,LDLRAD4,BRCA2,ZBTB2,BLM,BMP2,RC3H2,SOGA1,ZC3H14,GFI1B,HMGB1,GNAQ,FGF9,NFATC2,SH3BP5,ETS2,ZNF875,UIMC1,LRRFIP1,SMARCA2,GLI3,CGAS,SMARCC1,SNX6,CNKSR3,ZNF431,RERE,BTAF1,ATF2,HIRA,MAPK8IP1,MRPL13,SLC30A10,RORB,PRKN,MTMR2,ZNF608,TBX20,DACH1,ZNF541,DPF3,ARID5B,ATXN1,PKP1,CNOT6L,SNX25,PTPRB,ZFP90,ZNF124,USP7,PLAGL1,SOX30,ROCK1,LYN,ZNF169,TENM2,OVOL2,ZBTB33,ZFHX3,BMF,YTHDF3,DEDD2,SNAI2,SIAH2,SP3,ERN2,ELF2,NSD2,TWIST2,ENPP1,TMEM225,CSNK2A1,BMP5,BCL2L1,SCAF4,FANCB,SMAD5,CELF4,TCERG1,FOXN3,PRAME,MED1,IL33,BANK1,CSDE1,IL10,SFPQ,SCML2,PRAMEF25,ETV6,IQGAP1,CAMLG,SREBF2,YBX3,TWIST1,AKT3,ZBTB38,PATL1,CREBBP,TNKS,PCNA,RTRAF,ZBTB21,ERLIN2,ZBTB49,AGO1,STAT1,BRMS1L,NR2C1,PRAMEF2,IMPACT,PARK7,POU1F1,MTF2,NCAPG2,FOXP2,MYOCD,MEF2C,RXRA,NDFIP1,PRDM13,MAGEL2,PDE2A,SDCBP,ZBTB25,PASK,MLLT1,NCK1,SCAF8,RNF8,MECOM,DNMT3L,NTRK2,LHX9,ZBTB10,OCLN,IREB2,ASCL3,FEZ2,SEMA4D,ZBTB20,RUNX1,KIRREL1,SAMD13,PDCL3,SRP9,NSUN2,TNFSF11,FYN,KDM5A,PCBP3,ZNF705G,PPM1F,HDAC2,GON4L,TBX15,ZFYVE28,PABPC1,TET1,ZNF705D,RPS6KA5,PID1,MIDEAS,FHIT,KLF12,RC3H1,NRIP1,BCR,TUT4,FBLN1,CUX1,MACROH2A1,MITF,EPHB2,CD38,MET,ZNF705B,ATG5,PRDM11,UNK,MLIP,MYB,MFHAS1,BMP7,ZMYND8,KCTD1,B*

			PTF,ZMYND11,DDX6,BACE2,PARPBP,SLIT2,CNOT7,SAMD4A,SHLD2,NSD1,EHMT1,ESR1,KDM4B,LOXL2,AGO3,JAZF1,ZNF891,PHC2,ROCK2,PRDM1,DMRT1,WASHC1,BARD1,DEPTOR,ZNF423,ZNF568,HNRNPU,PRKAG2,GLI2,THRB
GO:0050771	negative regulation of axonogenesis	0.027448591467411992	*ULK2,BCL11A,EPHA7,SEMA5A,DIP2B,SEMA3C,SEMA6D,TNR,SEMA3E,MBP,SEMA3A,SEMA3D,DRAXIN,MAP2,DCC,DAB1,NTN1,SEMA4D,NRP1,EPHB2,SEMA4B,FSTL4*
GO:0090066	regulation of anatomical structure size	0.0276606257828 8965	*MTOR,ULK2,SVIL,ZDHHC21,RDX,KCNMA1,CDC42EP3,CARMIL1,ANO6,DSCAM,MACF1,BCL11A,CDH4,CRACD,EPHA7,CHRM3,CPS1,RPTOR,SEMA5A,SLC8A1,KANK1,PAK3,DIP2B,TRPC5,RAP1GDS1,HRH2,FMN1,PAFAH1B1,SEMA3C,RAB22A,BBS2,WNT9B,SEMA6D,TNR,DOCK4,ABL1,SLC12A8,PRKCE,EXT1,HTR2A,SEMA3E,TMOD2,DOCK5,ECE1,VAV1,DISC1,SEMA3A,RIN3,SEMA3D,ARHGAP42,PLS1,DRAXIN,MAP2,BBS4,DCC,RB1CC1,SLC12A1,ALS2,KANK4,VAV3,ROCK1,ARHGAP28,NTN1,ARFGEF1,SNX9,PRKG1,CLNS1A,VSTM4,VASP,AKT3,USH1C,ATP2B1,EXT2,MTPN,CYFIP2,WNT7A,NCK1,CYFIP1,SEMA4D,KIRREL1,SPTB,NRP1,FCHSD2,ITGA1,CD38,ATG5,FHOD3,SLIT2,NOS1,PRR16,ASIC2,EFNA5,FER,EPS8,SEMA4B,HRH1,ROCK2,FSTL4,DEPTOR*
GO:0043549	regulation of kinase activity	0.030718755027 61471	*MTOR,KSR1,SPRED1,ALK,APC,PTPRJ,EGFR,ANGPT1,CDK12,NTRK3,FLT1,SLC8A3,PRKD1,PAK1,EPHA7,RAPGEF2,TAOK3,LDB2,SMYD3,RPTOR,GHR,APP,NTF3,SLC8A1,BMPR1B,RANBP2,NBN,PRKCZ,MCPH1,DUSP22,PDGFD,NRG3,STK38,CCNG2,NF2,MOB3B,IL34,DUSP16,PTPRT,ABL1,SLC1A1,RAP1A,LATS2,NRG1,MUSK,ZNF675,CD44,PTPRO,EGF,PRRC1,PTPN2,AMBRA1,HTR2A,MARK2,EPHA6,HIPK3,CDKN2C,CLSPN,MNAT1,HMGA2,CCND3,PLCE1,TGFA,BLM,BMP2,RELN,GNAQ,SH3BP5,DSTYK,SNX6,NEK10,MOB1B,MAPK8IP1,KITLG,DAB1,PRKN,IL6R,ALS2,NLRC5,PTPRB,COPS8,VAV3,RALB,LYN,INSR,ERN2,CARD10,RASGRP1,SNX9,CSF1,GPRC5C,ROR2,IQGAP1,NRXN1,CENPE,ALKAL2,JAK2,MADD,RTRAF,NEDD9,MAP2K6,ABI1,CEMIP,PARK7,NCAPG2,MYOCD,MEF2C,MAP3K5,MAP3K4,DBF4B,MLLT1,FGR,EPHA4,NTRK2,PTK2,CCDC88A,TNFSF11,PPM1F,DOCK3,ZFYVE28,ROR1,EPHB1,GRM5,MACROH2A1,EPHB2,MET,BMP7,PDGFC,ERBB4,ROBO1,EFNA5,PRLR,HTT,PIK3R3,WASHC1,STK3,DEPTOR,HNRNPU,IGF1R,PRKAG2,AKAP13*
GO:0048639	positive regulation of developmental growth	0.032547062482 80808	*PLCB1,ZFPM2,BCL2,RIMS1,RIMS2,DSCAM,MACF1,BCL11A,CDH4,GHR,NEDD4L,SEMA5A,SYT1,AKAP6,TRPC5,YAP1,NIPBL,PAFAH1B1,BBS2,NRG1,ATP8A2,DISC1,FGF9,SLC23A2,PLS1,BBS4,PRKN,TBX20,ITSN2,NTN1,INSR,CSF1,GHRH,YBX3,MEF2C,CYFIP1,SEMA4D,NRP1,ERBB4,SLC6A3,EFNA5*
GO:0050769	positive regulation of neurogenesis	0.032675100243 81528	*MTOR,PTPRD,TENM4,ROBO2,DSCAM,MACF1,BCL11A,CDH4,SPEN,LRP2,STAU2,SEMA5A,PAK3,TRPC5,PAFAH1B1,SYNJ1,TIAM1,IL34,ASPM,PLXNA2,OPRM1,DISC1,BMP2,RELN,NIN,GLI3,PRKCH,LYN,NTN1,IL33,ACTR2,MAP6,UFL1,FBXW8,EPHA4,NTRK2,IL1RAPL1,NUMB,FBXO31,CYFIP1,SEMA4D,HDAC2,GRM5,NRP1,FAIM,CHODL,CUX1,EPHB2,KALRN,TIAM2,SLIT2,ROBO1,EFNA5*
GO:0032412	regulation of ion transmembrane transporter activity	0.033443073762 28895	*PDE4D,CACNG2,NEDD4,CHRM3,FGF12,THADA,NEDD4L,APP,CACNB2,KCNE4,AKAP6,HECW1,LRRC38,AKAP9,RASGRF2,DAPK1,ANK2,RYR2,GSG1L,RASGRF1,PRKCE,SLMAP,WNK2,ABCC9,ALG10B,OPRM1,STAC,CNIH3,ANK3,NETO2,RELN,CNKSR3,CFTR,SELENON,JPH1,SHISA9,CRACR2A,UTRN,KCNC1,HCN1,NRXN1,ABCC8,MEF2C,SHISA6,NOS1AP,HECW2,GRM5,ATPSCKMT,EPHB2,CACNG3,VMP1,TRDN,NLGN1,NOS1,CACNA2D1,HTT,CCR2,KCNAB1*
GO:0050919	negative chemotaxis	0.034263154843 67749	*ROBO2,EPHA7,SEMA5A,NRG3,SEMA3C,SEMA6D,NRG1,SEMA3E,SEMA3A,SEMA3D,NTN1,SEMA4D,FLRT2,SLIT2,ROBO1,EFNA5,SLIT3,SEMA4B*
GO:0008038	neuron recognition	0.034263154843 67749	*CNTN4,ROBO2,CNTNAP2,DSCAM,CNTN6,APP,SEMA5A,CRTAC1,EXT1,NCAM2,TNN,NTM,GAP43,EPHA4,NRP1,EPHB2,ROBO1,OPCML*
GO:00	homophilic	0.03549	*CNTN4,CDH8,ROBO2,TENM3,PCDH7,DSCAM,CDH4,CNTN6,FAT3,*

07156	cell adhesion via plasma membrane adhesion molecules	2840766518687	*CDH7,PCDH11Y,CDH11,PTPRT,CDH18,CDHR3,PCDH9,CDH20,PCDH15,CDH23,IGSF11,CDH26,FAT1,CLSTN2,CADM1,CELSR2,PCDH11X,NECTIN4,HMCN1,NECTIN1,DSG1,CDH5,PCDH8,FAT4,CDH9,CDH2,SDK1,CDH12,CDH17,CDH13,IGSF21,KIRREL3,ROBO1*
GO:0098815	modulation of excitatory postsynaptic potential	0.0372684386674 2529	*CHRNA7,RIMS1,RIMS2,SLC8A3,APP,PRKCZ,TMEM108,RELN,MTMR2,GRIN2A,IGSF11,GRIN2B,CELF4,NRXN1,WNT7A,TMEM25,NLGN1*
GO:0048738	cardiac muscle tissue development	0.03933306239926026	*NOTCH2,MTOR,SGCD,NEBL,ZFPM2,TENM4,ALDH1A2,RARB,SOX6,LRP2,SLC8A1,AKAP6,YAP1,MYLK3,TPM1,PPARA,ALPK2,JARID2,RYR2,CXADR,XIRP2,NRG1,ALPK3,FHL2,SGCZ,MYLK2,PDLIM5,BMP2,PTCD2,FGF9,TBX20,BMP5,CTDP1,SMAD5,MED1,MYO18B,ASB2,MYOCD,MEF2C,ADAMTS9,TNNI1,RUNX1,SORBS2,SGCG,ATG5,NRAP,BMP7,FHOD3,ERBB4,HNRNPU,AKAP13*
GO:0007626	locomotory behavior	0.04124088273627785	*MTOR,NAV2,ALK,ASTN1,NEGR1,DSCAM,SLC4A10,NCOR1,BTBD9,FGF12,APP,FIG4,KLHL1,ADAM22,PAFAH1B1,GRM1,PAK5,TNR,ELAVL4,OXR1,SLC1A1,NRG1,PRKCE,HTR2C,PUM1,APBA2,KCND2,ANKFN1,CNTN1,STRN,RELN,RCAN1,DAB1,PRKN,PCDH15,CDH23,ALS2,SHANK2,ZFHX3,PBX3,OTOG,LMX1A,PARK7,EPHA4,UBE3A,GRM5,KALRN,SLC6A3,EPS8*
GO:0045944	positive regulation of transcription by RNA polymerase II	0.04208086625129514	*ZFPM2,PRDM16,ZEB1,RARB,AUTS2,FOXJ2,GLIS3,TCF4,ERG,EGFR,RFX3,CDK12,BACH1,ZNF407,MAML2,BCL11A,FLI1,THRAP3,PRKD1,NCOA7,RUNX2,ONECUT1,LDB2,SMYD3,SSBP3,APBB2,APP,KDM1B,ZNF600,ST18,PYGO1,SSBP2,DUX4,BMPR1B,ZNF717,ARNT,MRTFA,TAF4B,EBF2,YAP1,NFIA,PCGF5,NIPBL,GABPA,CHD6,ATF6,HIVEP1,KLF15,PPARA,MEIS2,NFIB,MRTFB,NR5A2,FOXJ3,TRERF1,AGO2,BCAS3,ZNF606,SMARCA4,ATRX,ABL1,HDAC4,NFAT5,GLIS1,TOX3,FGF10,AP3B1,ZNF438,ZBTB16,FOXK2,MED15,ESRRG,RPS6KA3,GTF2F2,TAF3,RPRD1B,EBF3,ZNF33B,HMGA2,BCL11B,CREM,BMP2,GFI1B,ASXL3,HMGB1,NFATC2,ZNF462,ETS2,ATF1,SMARCA2,ETS1,GLI3,SMARCC1,VENTX,PRDM10,ATF2,PSIP1,CAMTA1,GTF2I,RORB,MED27,ZNF208,PRKN,TBX20,DPF3,NLRC5,TFDP1,PLAGL1,SOX30,NPAS2,ZNF780B,OVOL2,ZFHX3,SUPT16H,HOXC13,CASZ1,PBX3,ZNF292,ASH1L,HOXC4,RXRG,SP3,MBTPS2,BMP5,INO80,SMAD5,TCERG1,SLC40A1,MED1,IL33,ZNF521,LMX1A,IL10,ACTR2,SFPQ,PTH,ETV6,ZBTB7C,TEAD1,SREBF2,LMX1B,TWIST1,JAK2,ZBTB38,CREBBP,TNKS,NFKBIA,ALX4,RTRAF,BRD4,ITGA6,ZBTB49,STOX2,AGO1,MEOX2,ELL2,STAT1,PARK7,POU1F1,MTF2,MYOCD,ARID3B,MEF2C,RXRA,WNT7A,ZNF112,WWOX,NCK1,MLLT10,MECOM,ASCL3,UBE3A,RUNX1,ZNF845,CHCHD2,TNFSF11,HDAC2,TET1,ARNT2,ITGA8,RPS6KA5,PID1,ZNF615,KLF12,NRIP1,ZNF850,PRDM15,MITF,MET,CDH13,MLIP,MYB,BMP7,BPTF,KMT2C,RFX2,EYA1,CNOT7,PBX1,NOS1,TCF12,ESR1,ZNF721,NRF1,RORA,DMRT1,NCOA6,HNRNPU,GLI2,THRB*
GO:0038127	ERBB signaling pathway	0.04259115998586117	*BCAR3,ERBIN,PTPRJ,EGFR,PTPRR,MAPK1,NRG3,EFEMP1,ABL1,PTPN12,NRG1,EGF,PTPN2,PLCE1,TGFA,HIP1,FAM83B,SNX6,GAREM1,NEU3,SLC30A10,CUL5,IQGAP1,CAMLG,MVB12B,PTK2,CCDC88A,ZFYVE28,ITGA1,KIF16B,CDH13,ERBB4,FER*
GO:0007613	memory	0.04259115998586117	*PLCB1,CHRNA7,PJA2,BTBD9,GRIA1,SLC8A3,NTF3,TAFA2,KCNK10,PRKCZ,BRINP1,SORCS3,PAK5,SCN2A,SLC1A1,CAMK4,RASGRF1,MUSK,HTR2A,RELN,RCAN1,GRIN2A,ATXN1,INSR,LMX1A,ABCC8,SLC6A1,CSMD1,S100B,FOXO6,ITGA8,KALRN,HRH1*
GO:0007160	cell-matrix adhesion	0.043660876976674916	*FREM1,DLC1,PTPRA,BCL2,RIN2,PTPRJ,MACF1,ONECUT1,CCL28,VCL,ACER2,MAP4K4,PRKCZ,DUSP22,ITGBL1,CORO2B,LIMCH1,FMN1,ITGB8,NF2,TIAM1,PTPRK,BCAS3,ABL1,PEAK1,CD44,COL5A3,ARHGEF7,CD96,SEMA3E,DISC1,WDPCP,ITGA9,FBLN5,ROCK1,VCAM1,UTRN,CSF1,TNN,AJAP1,ITGA6,ITGA4,ADAMTS9,TRPM7,PTK2,PPM1F,ITGA8,NRP1,ITGA1,BCR,PPFIA2,CDH13,EFNA5,ROCK2*

GO:0001568	blood vessel development	0.044133197752 10206	*NOTCH2,SGCD,TAFA5,ZFPM2,ALDH1A2,CHRNA7,ROBO2,SPRED1,ENPEP,MYO1E,MINAR1,FOXJ2,RIN2,CRKL,SETD2,ARHGAP24,ANGPT1,C5,FLT1,ADAMTS6,SLC39A12,PRKD1,RAPGEF2,LRP2,ADGRB3,LUZP1,COL4A2,HDAC9,SEMA5A,THSD7A,CALD1,YAP1,MAPK1,PLG,PDGFD,ITGB8,SEMA3C,AGO2,BCAS3,COL22A1,ABL1,SLC1A1,RAP1A,FGF10,EGF,SEMA3E,ANKRD17,EPN2,ADAM12,EMILIN2,HMGA2,TGFA,TJP1,FGF9,ETS1,GLI3,SMOC2,ATF2,COL5A1,GTF2I,TBX20,IL6R,PTPRB,VAV3,ROCK1,OVOL2,HECTD1,CARD10,JCAD,ASB4,CNMD,VSTM4,TNN,MED1,IL10,AIMP1,NRXN1,HIPK1,TWIST1,AKT3,PRKCB,ABCC8,AGO1,MEOX2,STAT1,MYO18B,CCBE1,MYOCD,MEF2C,ADGRB1,WNT7A,PDE2A,FBXW8,FLVCR1,NTRK2,ADAMTS9,HS6ST1,PTK2,CDH5,AP2B1,RUNX1,PDCL3,COL18A1,APELA,CDH2,EPHB1,NRP1,PRKCA,NRXN3,BMPER,EPHB2,CDH13,LAMA1,SERPINB7,BMP7,ADGRF5,EYA1,SLIT2,ROBO1,ANTXR1,LOXL2,PIK3R3,CCR2,STARD13,ROCK2,PRDM1,RORA,HSPG2,COL4A3*
GO:0040008	regulation of growth	0.045603544688 21034	*MTOR,SPOCK1,WWC1,LRP12,ULK2,FTO,PLCB1,ZFPM2,BCL2,RIMS1,MINAR1,RIMS2,PAPPA2,DSCAM,CRKL,PTPRJ,EGFR,MACF1,BCL11A,CDH4,EPHA7,RPTOR,GHR,EPB41L3,NEDD4L,ADAM10,APP,SEMA5A,SYT1,AKAP6,RFTN1,DIP2B,TRPC5,YAP1,NRG3,NIPBL,PAFAH1B1,DCAF1,PPARA,PAK5,SEMA3C,JARID2,BBS2,SEMA6D,SMARCA4,MAPKAP1,TNR,CXADR,MBD5,ABL1,CREG1,LATS2,NRG1,MUSK,RPS6KA3,ATP8A2,SEMA3E,CDKN2C,AFG3L2,HMGA2,PLCE1,CRIM1,DISC1,SEMA3A,SEMA3D,FGF9,SLC23A2,PLS1,DRAXIN,SMARCA2,MAP2,BBS4,DCC,PRKN,TBX20,FBLN5,ITSN2,NTN1,INSR,COLQ,ENPP1,IGSF11,CSNK2A1,CSF1,GHRH,BCL2L1,CTDP1,INO80,PRAME,ATRN,LMX1A,TEAD1,YBX3,NET1,PRSS2,NEDD9,GAP43,HEPACAM,BRMS1L,MTPN,MYOCD,MEF2C,SDCBP,FLVCR1,EXTL3,CYFIP1,SEMA4D,RUNX1,PPM1F,NRP1,RERG,CD38,PRDM11,GNAS,SLIT2,ERBB4,PRKCQ,SLC6A3,EFNA5,ARHGEF11,SLIT3,SEMA4B,FSTL4,STK3*
GO:0044057	regulation of system process	0.048913705743 196936	*FTO,TENM4,ZDHHC21,PDE4D,KCNMA1,CHRNA7,RIMS1,RIMS2,DLGAP1,TSHZ3,CTNNA3,ATP2B2,SLC8A3,CHRM3,FGF12,TMEM38B,TAFA4,CELF2,PPP1R12B,APP,CACNA1C,CACNB2,SLC8A1,FIG4,ABCG8,KCNE4,AKAP6,RAB8B,RNLS,PRKCZ,HRH2,CORO2B,TPM1,CORIN,AKAP9,PPARA,GRM1,TMEM108,JARID2,ANK2,RYR2,PARD3,TNR,CXADR,DOCK4,ABL1,HDAC4,SLC1A1,FGF10,PTPRO,ABCC9,HTR2C,OPRM1,HTR2A,MYLK2,DOCK5,ECE1,PLCE1,FBXO32,SCN11A,RELN,ARHGAP42,UNC13B,SELENON,MTMR2,DLGAP2,GRIN2A,SHISA9,SCN10A,KCND3,ROCK1,NMU,PBX3,IGSF11,CTDP1,HCN1,PRKG1,GRIN2B,CELF4,IL33,IL10,NRXN1,JAK2,ABCC8,ATP2B1,MTPN,MYOCD,WNT7A,WASF3,TNNI1,SHISA6,JAM2,NOS1AP,APELA,ASB3,PRKCA,ATPSCKMT,CD38,MLIP,TMEM25,TRDN,NLGN1,NOS1,ASIC2,CACNA2D1,HRH1,ROCK2,THRB*
GO:0042995	cell projection	5.051871066663 704e-42	*NOTCH2,BRINP3,MTOR,UNC80,CNTN4,SPOCK1,NSG1,WWC1,ANKS1B,MYO9A,UNC13C,KSR1,SVIL,TLN2,MICAL3,TENM4,DLC1,RIPOR2,RDX,RP1,STXBP1,ERC1,MYO5A,ODAD2,GPHN,CDH8,CHRNA7,ROBO2,RIMS1,PIK3C3,TENM3,GABRB3,SDCCAG8,FGD4,SPAG16,MYO1E,PLPPR1,USH2A,RIMS2,AUTS2,CARMIL1,SV2C,FANK1,PARVB,CACNG2,NEGR1,CNTNAP2,MAP4,MYO3B,APC,TSHZ3,DSCAM,RTN1,ARHGAP24,SLC4A10,PTPRJ,EFCAB2,NEK4,DOCK10,EGFR,DENND1A,ANGPT1,MACF1,CTNNA3,PRKACB,NEDD4,CRB1,GRIK3,ATP2B2,NTRK3,CNTN3,GABRB1,DGKI,INVS,GRIA1,NEO1,CNTN6,SLC8A3,CEP128,TPTE2,PAK1,EPHA7,CHRM3,RAPGEF2,LRP2,GABRA6,CPEB4,LRGUK,GRM7,SEPTIN9,RPTOR,DNAH6,EPB41L3,KIF4A,TRPM1,ADAM10,APBB2,APP,SAMSN1,CACNA1C,DCLK1,STAU2,GABRG2,DOCK8,TMC1,SYT1,ARHGAP44,NTF3,CD2AP,AURKA,TTC29,SLC8A1,LOXHD1,KANK1,FMN2,THSD7A,HOMER2,CTNNA2,TTLL7,DIP2B,TRPC5,ERC2,DNM3,CUBN,IFT57,PRKCZ,KLHL1,DIP2A,ARHGAP32,RAB27B,COBL,DUSP22,SV2B,BRINP1,MAPK1,CADM2,HRH2,RABGAP1L,DNAH14,ADAM22,ALCAM,ABLIM1,CCDC172,NCAM1,GFRA1,FAT3,PTPN13,HRH4,PAFAH1B1,STON2,TPM1,NF2,CNKSR2,CTNNA1,PPP1R9A,AKAP9,NFIB,SYNJ1,GRM1,GABRG1,ENAH,PCDH11Y,SLC24A4,TMEM108,AGO2,MAGI1,DNAH11,SCN2A,RAB22A,EVC2,ANK2,TANC1,ADGRV1,SYNE2,BBS2,SLC9C1,AIF1L,ANKS6,USP8,LDB3,PARD3,DS*

			T,CXADR,DOCK4,ELAVL4,ABL1,PTPN12,SLC1A1,PRKAA1,KCNH1,TTLL5,APBB1IP,DNAH5,RAP1A,MYO10,INPP5A,FBXL13,GRID2,NRG1,CLIP1,AP3B1,RASGRF1,CEP83,CD44,RGS12,PTPRO,P2RX6,TRIO,CTNND2,NHS,IFT43,ATP8A2,HTR2C,RIC3,SLC2A3,ARHGEF7,AMBRA1,PKHD1L1,OPRM1,HTR2A,BIN2,CYBRD1,MARK2,TMEM67,ABHD17C,CNIH3,EPHA6,APBA2,SH3KBP1,ATL1,SLC2A13,KCND2,EVC,GRK3,KNDC1,MOSMO,CFAP61,ANK3,SNTG1,BCL11B,MBP,AK8,PLCE1,PCDH9,ATP6V1E1,TJP1,NPHP4,EGFLAM,PACSIN2,CNTN1,CACNA1I,PDLIM5,DISC1,DNER,WDPCP,SEMA3A,ADCY10,STRN,TRAK1,CDC42BPB,SCN11A,ZC3H14,NCAM2,MYRIP,RIN3,DNAL1,RNF38,RELN,GNAQ,RTTN,UNC13B,TTC21B,RAP1GAP,PLS1,SRGAP2,SLC39A6,NIN,DNAH8,GLI3,GABRR2,KIF21A,PSD3,MAP2,DAW1,PEX6,FARP1,CYLD,BBS4,MAPK8IP1,GABBR2,KPNA1,PHAF1,KITLG,DCC,CHRM5,MYO3A,PRKN,MTMR2,SH3PXD2A,CDC42BPA,PCDH15,NGEF,GRIN2A,TXNRD2,TRPM6,CDH23,FRMPD4,ALS2,KCNQ3,SHISA9,CATSPERG,PDE6A,TBATA,SCN10A,SHANK2,MAP7,KCND3,MOK,SYBU,CFAP74,KCNN3,MYO1D,ROCK1,VCAM1,ARHGAP31,TENM2,PLCB4,DPYSL5,INSR,NMU,SIAH2,RPH3A,TANC2,ABCA4,GABRG3,FAM183A,ZDHHC17,SLC22A14,JCAD,IFT81,UTRN,SNX9,NDRG2,KCNC1,GHRH,HCN1,GRIN2B,DHRS3,KIF21B,SYNJ2,TNN,FAM149B1,CABYR,MICALL2,ROR2,FAT1,ACTR2,SFPQ,CLSTN2,PRKAA2,PACRG,ABHD2,MAP6,VASP,PALMD,IQGAP1,NRXN1,CIBAR1,CADM1,NGDN,ANLN,MADD,PTGS1,UFL1,PRKCB,USH1C,NEDD9,NRBP1,ATP2B1,GAP43,ASS1,GRIP1,ADCY9,BBS9,HEPACAM,SLC6A1,GRXCR1,STAT1,SLC6A11,MTPN,ABI1,CBLIF,PARK7,MAPK8,ITGA4,ADCYAP1R1,CYFIP2,ARL4C,ADGRB1,NDFIP1,WASF3,S100B,PKN2,OR10H2,NECTIN1,WWOX,FGR,DRC7,ATP6V1B2,SNAP29,EPHA4,GABRA5,NTRK2,IL1RAPL1,RSPH1,RSPH3,SHISA6,MEGF10,TRPM7,PTK2,MARK4,TPH2,SCGN,DIAPH1,FEZ2,CYFIP1,HOATZ,PCDH8,DNAH10,KIRREL1,AMFR,SAXO1,SLC26A2,ASAP1,NOS1AP,MTTP,TPTE,SORBS2,CCDC88A,SPAG6,SLC5A1,CDC45,FYN,ADGRL2,ARL13B,HYDIN,SCN8A,SH2D3C,NCS1,PABPC1,ROR1,CDH2,CNTN5,ITGA8,EPHB1,EYS,RP1L1,GRM5,SPTB,NRP1,FCHSD2,IFT46,ITGA1,MCC,BCR,CFAP70,NSG2,B9D1,EPHB2,TOGARAM1,SACS,DLG2,CAMK1G,PPFIA2,CDH13,CACNG3,ATG5,MAGI2,FLRT2,SLC1A2,GNAS,TRIM9,TIAM2,DLG5,GABRA2,KIRREL3,DNAH3,GRM3,DNAH17,TTLL11,EXOC4,FAM126A,KCNIP4,KCTD8,CCDC141,SYNDIG1,ROBO1,SAMD4A,ANTXR1,SORCS2,NLGN1,SYNPR,CTTNBP2,NOS1,SLC6A3,ASIC2,VCAN,RAB27A,DNAH9,MYO9B,IQCJ-SCHIP1,MPDZ,CCDC178,FRMD4B,HTT,CFAP44,CATSPERE,AK2,FER,KATNIP,CCR2,RPGRIP1,PITPNM3,WDFY3,EPS8,ANO2,HRH1,GRIA4,ATAT1,CATSPER2,RGS8,KIF7,PCSK2,HNRNPU,VTI1A,IGF1R,KCNAB1,GLI2,SEPTIN6
GO:0120025	plasma membrane bounded cell projection	7.725853532806657e-39	NOTCH2,BRINP3,MTOR,UNC80,CNTN4,SPOCK1,NSG1,WWC1,ANKS1B,MYO9A,UNC13C,KSR1,TLN2,TENM4,DLC1,RIPOR2,RDX,RP1,STXBP1,ERC1,MYO5A,ODAD2,GPHN,CDH8,CHRNA7,ROBO2,PIK3C3,TENM3,GABRB3,SDCCAG8,FGD4,SPAG16,MYO1E,PLPPR1,USH2A,AUTS2,CARMIL1,SV2C,FANK1,PARVB,CACNG2,NEGR1,CNTNAP2,MAP4,MYO3B,APC,TSHZ3,DSCAM,RTN1,SLC4A10,PTPRJ,EFCAB2,NEK4,DOCK10,EGFR,DENND1A,ANGPT1,MACF1,CTNNA3,PRKACB,NEDD4,CRB1,GRIK3,ATP2B2,NTRK3,CNTN3,GABRB1,DGKI,INVS,GRIA1,NEO1,CNTN6,SLC8A3,CEP128,PAK1,EPHA7,CHRM3,RAPGEF2,LRP2,GABRA6,CPEB4,GRM7,SEPTIN9,RPTOR,DNAH6,EPB41L3,KIF4A,TRPM1,ADAM10,APBB2,APP,SAMSN1,CACNA1C,DCLK1,STAU2,GABRG2,DOCK8,TMC1,SYT1,ARHGAP44,NTF3,CD2AP,AURKA,TTC29,SLC8A1,LOXHD1,KANK1,FMN2,HOMER2,CTNNA2,TTLL7,DIP2B,TRPC5,ERC2,DNM3,CUBN,IFT57,PRKCZ,KLHL1,DIP2A,ARHGAP32,RAB27B,COBL,DUSP22,SV2B,BRINP1,MAPK1,CADM2,HRH2,RABGAP1L,DNAH14,ADAM22,ALCAM,ABLIM1,CCDC172,NCAM1,GFRA1,FAT3,PTPN13,HRH4,PAFAH1B1,STON2,TPM1,NF2,CNKSR2,CTNNA1,PPP1R9A,AKAP9,NFIB,SYNJ1,GRM1,GABRG1,ENAH,PCDH11Y,SLC24A4,TMEM108,AGO2,DNAH11,SCN2A,RAB22A,EVC2,ANK2,TANC1,ADGRV1,SYNE2,BBS2,SLC9C1,AIF1L,ANKS6,USP8,LDB3,PARD3,DST,CXADR

			,DOCK4,ELAVL4,ABL1,SLC1A1,PRKAA1,KCNH1,TTLL5,APBB1IP,DNAH5,RAP1A,MYO10,INPP5A,FBXL13,GRID2,NRG1,CLIP1,AP3B1,RASGRF1,CEP83,CD44,RGS12,PTPRO,P2RX6,CTNND2,NHS,IFT43,HTR2C,RIC3,ARHGEF7,AMBRA1,PKHD1L1,OPRM1,HTR2A,CYBRD1,MARK2,TMEM67,ABHD17C,CNIH3,EPHA6,APBA2,SH3KBP1,ATL1,SLC2A13,KCND2,EVC,KNDC1,MOSMO,CFAP61,ANK3,SNTG1,BCL11B,MBP,AK8,PLCE1,PCDH9,ATP6V1E1,NPHP4,PACSIN2,CNTN1,CACNA1I,DISC1,DNER,WDPCP,SEMA3A,ADCY10,STRN,TRAK1,CDC42BPB,SCN11A,ZC3H14,NCAM2,MYRIP,RIN3,DNAL1,RNF38,RELN,GNAQ,RTTN,UNC13B,TTC21B,RAP1GAP,PLS1,SRGAP2,SLC39A6,NIN,DNAH8,GLI3,GABRR2,KIF21A,PSD3,MAP2,DAW1,PEX6,FARP1,CYLD,BBS4,MAPK8IP1,GABBR2,KPNA1,PHAF1,KITLG,DCC,CHRM5,MYO3A,PRKN,MTMR2,CDC42BPA,PCDH15,NGEF,GRIN2A,TXNRD2,TRPM6,CDH23,FRMPD4,ALS2,KCNQ3,SHISA9,CATSPERG,PDE6A,TBATA,SCN10A,SHANK2,MAP7,KCND3,MOK,SYBU,CFAP74,KCNN3,MYO1D,ROCK1,VCAM1,ARHGAP31,TENM2,PLCB4,DPYSL5,INSR,NMU,SIAH2,RPH3A,TANC2,ABCA4,GABRG3,FAM183A,SLC22A14,JCAD,IFT81,UTRN,SNX9,NDRG2,KCNC1,GHRH,HCN1,GRIN2B,DHRS3,KIF21B,TNN,FAM149B1,CABYR,MICALL2,ROR2,FAT1,ACTR2,SFPQ,CLSTN2,PRKAA2,PACRG,ABHD2,MAP6,VASP,PALMD,IQGAP1,NRXN1,CIBAR1,CADM1,NGDN,ANLN,MADD,PTGS1,UFL1,PRKCB,USH1C,NEDD9,NRBP1,ATP2B1,GAP43,ASS1,GRIP1,ADCY9,BBS9,HEPACAM,SLC6A1,GRXCR1,STAT1,SLC6A11,MTPN,ABI1,CBLIF,PARK7,MAPK8,ITGA4,ADCYAP1R1,CYFIP2,ARL4C,ADGRB1,NDFIP1,WASF3,S100B,PKN2,OR10H2,NECTIN1,WWOX,FGR,DRC7,ATP6V1B2,SNAP29,EPHA4,GABRA5,NTRK2,IL1RAPL1,RSPH1,SHISA6,TRPM7,PTK2,MARK4,TPH2,SCGN,DIAPH1,FEZ2,CYFIP1,HOATZ,PCDH8,DNAH10,KIRREL1,AMFR,SAXO1,SLC26A2,ASAP1,NOS1AP,MTTP,SORBS2,CCDC88A,SPAG6,SLC5A1,CDC45,FYN,ADGRL2,ARL13B,HYDIN,SCN8A,SH2D3C,NCS1,PABPC1,ROR1,CDH2,CNTN5,ITGA8,EPHB1,EYS,RP1L1,GRM5,NRP1,FCHSD2,IFT46,ITGA1,MCC,BCR,CFAP70,NSG2,B9D1,EPHB2,TOGARAM1,SACS,DLG2,CAMK1G,PPFIA2,CDH13,CACNG3,ATG5,MAGI2,FLRT2,SLC1A2,GNAS,TRIM9,TIAM2,DLG5,GABRA2,KIRREL3,DNAH3,GRM3,DNAH17,TTLL11,EXOC4,FAM126A,KCNIP4,CCDC141,SYNDIG1,ROBO1,SAMD4A,ANTXR1,SORCS2,NLGN1,SYNPR,CTTNBP2,NOS1,SLC6A3,ASIC2,VCAN,RAB27A,DNAH9,MYO9B,IQCJ-SCHIP1,MPDZ,CCDC178,FRMD4B,HTT,CFAP44,CATSPERE,AK2,FER,CCR2,RPGRIP1,WDFY3,EPS8,ANO2,HRH1,GRIA4,ATAT1,CATSPER2,RGS8,KIF7,PCSK2,HNRNPU,VTI1A,IGF1R,KCNAB1,GLI2,SEPTIN6

Table S3. The 177 sets of co-expressed rDNA-contacting genes in untreated K562 cells. The search was performed in https://maayanlab.cloud/Enrichr/enrich# for ARCHS4 TFs Coexp. The database presents the top-300 genes that are co-expressed with transcription factors. All 1985 genes specify the transcription factors and are co-expressed in different combinations.

Term	Overlap	Adjusted P-value	Genes
ZNF704 human tf ARCHS4 coexpression	144/299	1.1308931274681654E-28	ATP8A2;CTNND2;ZBTB20;MSI2;SOGA1;SLC4A4;CELSR2;SLC8A1;MYLK3;HERC2;ZNF608;HERC1;AKT3;KIF21A;DIP2C;SCAPER;MAGI1;GUCY1A2;RBFOX1;TMEM178B;RALGAPA1;CACNA2D1;TMOD2;MTUS1;ANK2;FRMD4A;ANK3;TANC2;PYGO1;TANC1;AKAP9;WDFY3;ASTN2;DGKI;ASTN1;MACF1;IGSF3;TNKS;KMT2C;PCDH15;NEDD4L;TMTC2;PIK3R3;AGAP1;ILDR2;CACNA1C;KALRN;NPAS3;FLRT2;MAP2;FUT9;PLXNA2;SRGAP3;MPDZ;BPTF;AUTS2;CADM2;NEBL;MICAL3;ZBTB10;MYO5A;GRIN2B;CORO2B;DCLK1;PBX1;PTPRD;CCDC88A;ARHGAP32;SDK1;DLG2;NBEA;WNK2;SPIRE1;FAT3;TCF4;FAT4;BRWD1;ROBO2;RERE;DOCK3;TENM4;MAST2;GRIK3;ZCCHC14;ROBO1;AKAP11;TRIM2;MCF2L;PSD3;TMEM108;HYDIN2;DLGAP1;NPIPA1;N

			EO1;ADAMTS9;ARHGEF12;DST;VPS13D;ASH1L;NAV2;SEZ6L;TOX3;MPPED1;SETBP1;LRRC7;MPPED2;ARHGEF7;RAPGEF5;PLCB1;SHANK2;PAFAH1B1;PPM1L;LUZP2;ADAM22;NRXN3;AKAP6;MIPOL1;STOX2;KIAA1328;HECTD2;HECTD4;CLVS2;CTNNA3;ATP9A;MAP4K4;ARNT2;ZNF462;FARP1;MYEF2;NTRK3;PCDH7;LSAMP;YLPM1;KIAA1549L;PDE4DIP;SORBS2;MYO9A;TTLL7;TJP1;PDE10A;APC;ASXL3;TTC3;RGS12
PLXNA4 human tf ARCHS4 coexpression	142/299	9.883127277665778E-28	*ATP8A2;FRMPD4;MYT1L;ANKRD36;CTNND2;RORB;FRY;SOGA1;SLC8A1;HS6ST3;RIMS1;CDH4;ZNF608;DPYSL5;AKT3;TNR;KIF21A;KIF21B;PKNOX2;ANKS1B;SOX5;PPFIA2;GUCY1A2;ANKRD36C;RBFOX1;MEF2C;EPHA6;KCNH5;TMEM178B;CACNA2D1;PRKCE;TMOD2;ANK2;FRMD4A;ANK3;TANC2;MAPK8IP1;SCN8A;AKAP9;HECW1;WDFY3;DGKI;ASTN1;MACF1;CTTNBP2;STXBP1;AGAP1;ILDR2;KALRN;CACNA1E;NKAIN2;CTIF;DPP6;FLRT2;MAP2;FUT9;ZNF704;PLXNA2;ST8SIA5;HIVEP2;SRGAP3;NDFIP1;ARPP21;SYT1;CADM2;MYO5A;CORO2B;DCLK1;PBX1;SNAP91;PTPRD;CCDC88A;LRFN2;DLG2;DAB1;NBEA;SYNJ1;GNAQ;CNTN1;SPIRE1;FAT3;CCSER1;TCF4;FAT4;SCN2A;SLC44A5;ROBO2;DOCK3;TENM4;CELF2;PTPRO;AFF3;ROBO1;C4ORF50;GRM5;AKAP11;GRM7;TRIM2;NCS1;PSD3;DLGAP1;ZNF385D;NEO1;TRPC5;FAM219A;SORCS3;AJAP1;CNKSR2;MPPED1;NAV3;SETBP1;LRRC7;ARHGEF7;PLCB1;PAFAH1B1;PPM1L;PLPPR5;ADAM22;NRXN3;PRSS51;MAPK8;HECTD4;CLVS2;NCAM1;CTNNA2;CSMD2;PAK3;CSMD1;ATP9A;OPCML;ARNT2;NTRK3;LSAMP;SYT16;KIAA1549L;MEIS2;TTLL7;APC;PPP2R2B;ASXL3;TTC3;RIMBP2*
ZNF483 human tf ARCHS4 coexpression	139/299	4.346528433386478E-26	*ATP8A1;FRMPD4;MYT1L;DGKB;ZBTB20;RORB;LCLAT1;SLC8A1;RIMS2;RIMS1;AKT3;SAMD12;PRKACB;ANKS1B;KCNH1;UNC13C;GUCY1A2;EPHA6;KCNH5;RALGAPA1;TMOD2;MAGI2;TTC7B;ANK2;ANK3;SHISA9;GABRG1;EML6;ADGRB3;SCN8A;HECW1;SCG5;WDPCP;WDFY3;SCG3;ASTN2;DGKI;FTO;NECAB1;SLC1A2;KALRN;NALCN;GLB1L3;MTMR7;GRIN2A;DPP6;PDZD2;MAP2;SNTG1;FUT9;AP4S1;SCAI;DTNA;ZNF382;SYT1;CADM2;SLC4A10;MYO5A;GRIN2B;LRP1B;PTPRD;FAM135B;DLG2;NBEA;TMEM116;CNTN1;CPE;FAT3;SCN2A;CDS2;GABRB3;SPAG16;CCDC122;ZNF891;DOCK3;RASGRF2;DIRAS2;EFCAB6;PPP1R9A;RYR3;SYNE1;C4ORF50;UNC80;GRM5;AKAP11;LRRTM4;CA5A;TRIM2;ADAMTSL3;PSD3;HYDIN2;DLGAP1;DLGAP2;SLC2A13;OPRM1;KIF6;AJAP1;CNKSR2;DNM3;LRRC7;KCNMA1;RAPGEF5;PLCB1;SLC24A2;PPM1L;RGPD6;ADAM22;TULP4;RGPD5;AKAP6;MIPOL1;FBXL20;RANBP3L;KIAA1328;SV2B;CHN1;CLVS2;PAK3;CSMD1;ATP9B;GPR158;ATP9A;OPCML;RIC3;PCDH9;NEGR1;NTRK3;CADPS;SYT16;KIAA1549L;TTLL7;MAPK10;APC;PPP2R2B;PTPN4;ASB3;ZNF850;TNRC6B;HCN1*
SOX5 human tf ARCHS4 coexpression	138/299	1.281717654919808E-25	*ATP8A2;MYT1L;ANKRD36;ZNF292;CTNND2;UBE3A;RORB;SLC8A1;CDH7;SRGAP2C;GRIP1;AKT3;KIF21A;DIP2C;SRGAP2B;POTEB;SYBU;PKNOX2;PPFIA2;MAGI1;EPHA4;GUCY1A2;MEF2C;EPHA6;CXADR;KCNH5;TMEM178B;RALGAPA1;CACNA2D1;TMOD2;MAGI2;RFX3;KAZN;ANK2;FRMD4A;ANK3;TANC2;TIAM2;ADGRB3;RUFY2;AKAP9;HECW1;KCNQ3;DGKI;GRIA1;CTTNBP2;NEDD4L;ILDR2;NREP;KALRN;CACNA1E;FLRT2;MAP2;FUT9;ZNF704;SRGAP3;MPDZ;BPTF;BCL11B;ZNF382;BCL11A;SIAH3;SLC4A10;MYO5A;CORO2B;DCLK1;ST18;PBX1;LRP1B;PTPRD;CCDC88A;FER;DLG2;DAB1;NBEA;SPIRE1;FAT3;TCF4;FAT4;CNTN4;FGF12;LRP12;SLC44A5;ROBO2;CNTNAP2;DOCK3;TENM4;CELF2;PTPRO;GRIK3;ELAVL4;GRIK2;FMN2;ROBO1;SLC22A14;NHSL1;ADAMTS3;GRM7;LRRTM4;TRIM2;TMEM108;PHACTR3;ITGB8;DLGAP1;ZNF385D;ERC1;NEO1;TTC37;LRRC49;ATRX;MPPED1;NAV3;SETBP1;LRRC7;MPPED2;SLC24A2;PPM1L;TULP4;NOL4;FGD4;MAPK8;HECTD2;NCAM1;PAK3;CSMD1;MYEF2;NTRK3;LSAMP;SYT16;KI*

			AA1549L;MEIS2;PDE10A;NFIA;APC;NFIB;PPP2R2B;TTC3;SSBP2
SETBP1 human tf ARCHS4 coexpression	125/299	1.5776077320992477E-18	CPNE4;MYT1L;ANKRD36;ZNF292;CTNND2;ZBTB20;SOGA1;GRIP1;ZNF608;DACH1;CDH2;DPYSL5;AKT3;KIF21A;SOX6;SRGAP2B;SOX5;PPFIA2;MAGI1;ANKRD36C;WSB1;KCNK10;TMEM178B;CACNA2D1;MAGI2;RFX3;ANK2;FRMD4A;MYT1;PYGO1;IFT81;ADGRB3;DOK5;RUFY2;AKAP9;ASTN2;TOX;ASTN1;KMT2E;GRIA1;TNKS;PDE1A;NEDD4L;ILDR2;BAZ2B;NREP;KALRN;NPAS3;NKAIN3;SNTG1;FUT9;ZNF704;PLXNA2;SRGAP3;LRRC4C;MPDZ;AUTS2;ST8SIA1;CORO2B;DCLK1;PBX1;PTPRD;CCDC88A;LRFN5;DLG2;NBEA;RNF182;ZNF536;FAT3;TCF4;FAT4;SLC44A5;ROBO2;DRAXIN;TENM4;ELAVL4;BICD1;SYNE2;ROBO1;GRM7;LRRTM4;TRIM2;GDAP1L1;ITGB8;RALGPS1;GARNL3;KLF12;ADGRV1;KCND3;LRRC49;TCF12;ATRX;TOX3;LRRC7;MPPED2;NRXN3;NOL4;MIPOL1;FGD4;STOX2;GNG2;NCAM1;CTNNA2;CSMD2;PAK3;PAK5;ZNF462;FARP1;NTRK2;MBD5;MYEF2;NTRK3;LSAMP;YLPM1;MEIS2;ATAT1;MAPK10;NFIA;APC;FABP7;ASXL3;TTC3;YPEL1;SSBP2;TNRC6B
MYT1L human tf ARCHS4 coexpression	124/299	4.26243007682826E-18	ATP8A2;FRMPD4;ANKRD36;CTNND2;ZBTB20;RORB;RIMS2;RPH3A;RIMS1;AKT3;ANKS1B;PPFIA2;RGS7;GUCY1A2;ANKRD36C;RBFOX1;MEF2C;RBFOX3;TMEM178B;TMOD2;MAGI2;ANK2;ANK3;TANC2;ADGRB3;SCN8A;AKAP9;HECW1;KCNQ3;ASTN1;GRIA1;RTN1;CTTNBP2;STXBP1;NTM;SLC1A2;NREP;NYAP2;KALRN;NALCN;CACNA1E;GRIN2A;DPP6;PGBD5;MAP2;FUT9;CAMTA1;SRGAP3;PTPRN2;DTNA;SYT1;CADM2;SLC4A10;MYO5A;GRIN2B;DCLK1;SNAP91;PTPRD;CCDC88A;RALYL;DLG2;NBEA;CNTN1;FAT3;TCF4;C8ORF34;GABRB3;DPP10;DOCK3;PTPRO;DIRAS2;CELF4;ELAVL4;GRIK2;UNC80;GRM5;GRM7;TRIM2;MCF2L;PSD3;PHACTR3;DLGAP1;DLGAP2;GARNL3;TRPC5;LRRC49;UBE2QL1;SYN2;PGM2L1;AJAP1;CNKSR2;DNM3;MPPED1;LRRC7;PLCB1;SLC24A2;PPM1L;ADAM22;NRXN3;AKAP6;STOX2;SV2B;CHN1;CLVS2;NCAM1;CTNNA2;PAK3;CACNG3;PAK5;ATP9A;OPCML;GABBR2;ARNT2;KCNJ6;NTRK3;SYT16;KIAA1549L;ATP2B2;TTLL7;MAPK10;APC;PPP2R2C;PPP2R2B;TTC3
ASH1L human tf ARCHS4 coexpression	122/299	3.222755136962082E-17	PATJ;TRIO;ATP8A1;ANKRD36;RORA;FRY;SOGA1;ZFYVE26;DNM1P47;HERC2;HERC1;AKT3;DIP2B;GUCY1A2;RALGAPA1;RALGAPA2;TMOD2;ANK2;TANC2;CLIP1;AKAP9;WDPCP;WDFY3;ASTN2;UTRN;ANKRD17;MACF1;DDX6;KMT2C;ITPR2;CACNA1C;KALRN;CACNA1E;LPP;PCNX1;PDZD2;ZNF704;HIVEP1;TRPM7;CEP192;HIVEP2;BPTF;CREBBP;ZNF382;LRBA;DENND4C;MICAL3;MYO5A;LNPEP;ARHGAP26;SMARCA2;PHC3;GRIN2B;ZZEF1;MED13L;ARHGAP32;AGO3;DMXL2;STRN;CDK12;BRWD1;SETD2;DOCK3;PPP1R13B;DOCK9;RASGRF2;LYST;SYNE2;SYNE1;UNC80;AKAP13;NIPBL;C16ORF72;AKAP11;ADAMTSL3;ZNF407;PSD3;ERC1;ARHGEF12;KCND2;DST;VPS13C;ARAP2;VPS13B;ARID1B;NCOR1;PEAK1;BIRC6;RAPGEF5;PLCB1;NFAT5;ROCK2;PRUNE2;MIPOL1;BTAF1;KIAA1328;HECTD1;NSD1;HECTD4;PCNT;ATP9B;RANBP2;ARFGEF1;USP24;SPEN;MBD5;MON2;MGA;ZNF804B;ERBIN;YLPM1;ADAM32;PDE4DIP;DDHD1;MYO9A;GATAD2B;TTLL7;APC;KANSL1;NEDD4;SLMAP;FRYL
MACF1 human tf ARCHS4 coexpression	122/299	3.222755136962082E-17	ITSN2;TRIO;MAML2;ATP8A1;RORA;FRY;SOGA1;ETS1;DOCK10;ZFYVE26;PTAR1;DNM1P47;HERC2;HERC1;MPRIP;PIEZO2;SACS;ANKFY1;DIP2B;MAP3K5;MBNL1;RALGAPA1;RALGAPA2;FNDC3B;TANC2;TANC1;WDFY3;UTRN;NOTCH2;ANKRD17;DDX6;KMT2C;ITPR2;IQGAP1;CACNA1C;LPP;PCNX1;ATXN1;ZNF704;CEP192;HIVEP2;CREBBP;LRBA;DENND4C;EXOC6B;MICAL3;MYO5A;LNPEP;DNAJC13;ARHGAP26;SMARCA2;ZZEF1;MED13L;CYLD;PTPRB;DMXL2;FAT1;BCL2;CCSER2;STRN;FAT4;DOCK4;DOCK9;DOCK8;RASGRF2;PTPRJ;LIMD1;LYST;SYNE2;SYNE1;AKAP13

			;NIPBL;C16ORF72;AKAP11;ZNF407;KIF13A;MYOCD;ARHGEF12;ITGA4;DST;VPS13C;ARAP2;VPS13B;ASH1L;URB1;ARID1B;VCAN;NCOR1;PEAK1;BIRC6;PPP1R12B;DOCK2;DOCK1;KDM7A;NFAT5;WDR26;ROCK2;HERC2P2;HTT;BTAF1;KIAA1328;HECTD1;NSD1;HECTD4;ZNF106;RANBP2;ARFGEF1;USP24;SPEN;MON2;TRAPPC10;MGA;ERBIN;YLPM1;ADAM32;DDHD1;MYO9A;MTOR;TJP1;SLMAP;ESYT2;FRYL
SATB2 human tf ARCHS4 coexpression	121/299	8.906749700651784E-17	*ZFYVE9;FRMPD4;MYT1L;ANKRD36;SLC8A1;EPS8;CDH4;KIF21A;PKNOX2;ANKS1B;SOX5;PPFIA2;RGS7;GUCY1A2;ANKRD36C;MEF2C;KCNH5;TMEM178B;CACNA2D1;TMOD2;MAGI2;ZNF271P;ANK2;FRMD4A;ANK3;SHISA9;EML1;TANC2;TIAM2;GAREM1;AKAP9;ULK2;TTLL11;ASTN2;DGKI;CTTNBP2;TNKS;KALRN;NKAIN2;FLRT2;MAP2;FUT9;ZNF704;PLXNA2;ANKRD20A5P;ST8SIA5;ARPP21;CADM2;MYO5A;GRIN2B;CORO2B;DCLK1;PTPRD;CCDC88A;DLG2;DAB1;NBEA;SYNJ1;SPIRE1;FAT3;CCSER1;CNTN3;TCF4;FAT4;SCN2A;RGL1;BRWD1;SLC44A5;ROBO2;SETD2;DOCK3;TENM4;CELF2;PTPRO;NHSL1;GRM7;PPP2R5E;TRIM2;ZNF648;TMEM108;PHACTR3;HYDIN2;DLGAP1;DLGAP2;NEO1;KDM6A;SLC2A13;ATRX;COBL;FAM126B;ARID1B;ITFG1;DNM3;VCAN;MMP16;MPPED1;NAV3;LRRC7;SHANK2;LUZP2;PLPPR5;RGPD6;ATP10B;MIPOL1;PRSS51;MAPK8;NCAM1;PAK5;ATP9A;NTRK3;KIAA1549L;LHFPL3;C1ORF21;MEIS2;TTLL7;MAB21L3;APC;CAMK4;ASXL3;PTPN4;CCDC171*
SORBS2 human tf ARCHS4 coexpression	120/299	2.457624343872832E-16	*FHOD3;MYT1L;SLC8A1;MYLK3;CDH2;DPYSL5;ZSCAN30;AKT3;LONP2;KIF21A;LARGE1;MAGI1;EPHA7;MLIP;ST6GAL2;CACNA2D1;MAGI2;MTUS2;ANK2;FRMD4A;ANK3;UNC5D;TANC2;TIAM2;TOM1L2;AKAP9;KCNQ3;SLC27A6;ALPK3;WDFY3;MXRA7;ALPK2;DGKI;MACF1;BAZ2B;CACNA1C;NREP;KALRN;LPP;MAP2;ZNF704;PLXNA2;SRGAP3;CCDC141;MPDZ;CADM1;SPHKAP;ZNF382;AUTS2;NEBL;ST18;SNAP91;PTPRD;DLG2;WNK2;RCAN2;PDE3A;TCF4;ROBO2;RYR2;MYOM1;TENM4;ELAVL4;FHL2;LDB3;SIPA1L2;PPP1R9A;MYOM2;PTPRG;RASGEF1B;TRIM2;MYO18B;PSD3;PGM5;RALGPS1;UNC45B;MYOCD;DST;DCC;LRRC49;ATRX;ENAH;SETBP1;LRRC7;NOS1AP;SHANK2;PRKAA2;TULP4;AKAP6;STOX2;TBX20;NHS;CTNNA3;NCAM1;CSMD3;PAK3;PDLIM5;CORIN;CLVS1;ZNF462;FARP1;MYEF2;NTRK3;CADPS;SYT16;PDE4DIP;PPP2R3A;KIAA0232;CDC42BPA;MAPK10;PDE10A;NFIA;NFIB;KLHL7;ASXL3;TTC3;YPEL1;TACC2;FRYL;HCN1*
PBX1 human tf ARCHS4 coexpression	117/299	5.916974092828944E-15	*ATP8A2;MYT1L;CTNND2;KLHL32;ZBTB20;ADARB2;SOGA1;RPS6KA5;ZNF608;CDH2;DPYSL5;PEG10;KIF21A;KIF21B;EPHB2;LARGE1;MAGI1;GUCY1A2;KCNK10;TMEM178B;CACNA2D1;MAGI2;EBF3;ANK2;FRMD4A;ANK3;SHISA9;FOXP2;TANC2;PYGO1;WDPCP;WDFY3;ASTN2;DGKI;FTO;IGSF3;CHRNA7;NTM;PIK3R3;AGAP1;CACNA1C;NREP;KALRN;NPAS3;ORC4;PGBD5;MAP2;ZNF704;MAP6;CAMTA1;SRGAP3;ASIC2;ZNF423;MPDZ;CADM1;AUTS2;NETO2;HOOK3;GRIN2B;DCLK1;PTPRD;CCDC88A;NBEA;GNAQ;FAT3;FAT4;GABRB3;ROBO2;DRAXIN;DOCK3;TENM4;PTPRO;CELF4;ELAVL4;TSPAN11;ROBO1;TRIM2;ADAMTSL3;ZNF385D;NEO1;LRRC49;UBE2QL1;GFRA1;NAV2;SORCS3;PGM2L1;NAALADL2;AJAP1;TOX3;GAP43;SETBP1;LRRC7;PPP1R12B;PPM1L;MIPOL1;STOX2;CECR2;GTF2IP1;KIAA1328;CTNNA2;PAK3;CSMD1;PAK5;ATP9A;ZNF462;FARP1;MBD5;KCNJ6;MYEF2;NTRK3;PCDH8;LSAMP;MAPK10;NEDD4;TTC3;ASB4;ADGRL2*
TCF4 human tf ARCHS4 coexpression	116/299	1.3471390640769162E-14	*ATF2;MYT1L;ANKRD36;ZNF292;SLC8A1;SRGAP2C;ZNF608;ZSCAN30;AKT3;KIF21A;SCAPER;SRGAP2B;ANKS1B;PPFIA2;MAGI1;ANKRD36C;MEF2C;MAGI2;RFX3;ANK2;SHISA9;TIAM2;ADGRB3;DOK5;RUFY2;AKAP9;KCNQ3;ASTN2;KMT2E;GRIA1;CTTNBP2;NTM;PCDH15;NEDD4L;BAZ2B;NREP;NYAP2;KALRN;NPAS3;NKAIN2;FLRT2;MAP2;SN*

			TG1;FUT9;ZNF704;ST8SIA5;SRGAP3;GRIA4;ZNF382;BCL11A;CORO2B;DCLK1;PTPRD;CCDC88A;FER;DLG2;NBEA;ZNF536;FAT3;CCSER1;SCN2A;ROBO2;TENM4;CHD9;CELF2;PTPRO;ELAVL4;SLC35F1;AFF3;BICD1;NHSL1;GRM7;LRRTM4;TRIM2;RB1CC1;DLGAP1;TCF12;ATRX;FAM126B;ZDHHC17;ARID1B;TOX3;MMP16;MPPED1;NAV3;SETBP1;IL1RAPL2;MPPED2;VSTM2A;CRB1;PLPPR5;NRXN3;MIPOL1;PRSS51;STOX2;HECTD2;CLVS2;NCAM1;PAK5;RFTN2;ZNF462;MBD5;MYEF2;NTRK3;KIAA1549L;DDHD1;TTLL7;NFIA;APC;NFIB;KLHL7;ASXL3;TTC3;PTPN4;SSBP2;ASB3
POGZ human tf ARCHS4 coexpression	116/299	1.3471390640769162E-14	ANKRD36;SOGA1;CELSR2;IGF1R;TIAL1;HERC2;ZNF608;LIPI;AKT3;KIF21A;SRGAP2B;POTEC;MAGI1;ANKRD36C;HFM1;RALGAPA1;TTC7B;SHISA9;FOXP2;TANC2;AKAP9;WDPCP;ASTN2;FTO;MACF1;INO80D;TNKS;KMT2C;AGAP1;BAZ2A;BAZ2B;KALRN;ATXN3;MAP2;ZNF704;GSE1;ANKRD10;TRPM7;CEP192;MPDZ;BPTF;CREBBP;ZNF382;AUTS2;RANBP17;MICAL3;PHC3;PTPRD;MLLT10;NBEA;WNK2;AGO1;DLG5;AGO2;TCF4;EIF4G3;GABRB3;RERE;CNTNAP2;CHD9;GADL1;PPP1R9A;AFF3;SYNE2;ZCCHC14;DTWD2;UNC80;EPB41L4A;TRIM2;ADAMTSL3;MCF2L;TLK1;NPIPA1;NEO1;ARFGEF3;RBM6;USP49;NCOA6;VPS13B;ASH1L;NAV2;SEZ6L;ARID1B;SFPQ;NAV3;SETBP1;PEAK1;FAM193A;SHANK2;NFAT5;NRXN1;RGPD6;TULP4;RGPD5;FAM214A;MIPOL1;KIAA1328;HECTD4;SPEN;ZNF462;FARP1;MYEF2;MGA;CADPS;YLPM1;ADAM32;CDC42BPA;GATAD2B;NFIA;KANSL1;NFIB;TTC3;RIMBP2;ASB4;ASB3;TNRC6B
FAM171B human tf ARCHS4 coexpression	116/299	1.3471390640769162E-14	SEMA5A;ERO1B;FRMPD4;DGKB;LCLAT1;TIAL1;PPP1CB;SRGAP2C;SYNPR;KIF21A;ZNF568;SAMD12;PRKACB;SRGAP2B;ANKS1B;PPFIA2;EPHA7;WSB1;CXADR;LIMCH1;SLX4IP;MAGI2;RFX3;ZNF271P;ANK2;GABRG2;ADGRB3;RUFY2;KCNQ3;SCG5;SCG3;RIN2;NECAB1;SAR1A;CTTNBP2;BTF3L4;EFNA5;NALCN;SDCBP;GRIN2A;MAP2;SNTG1;FUT9;CADM1;SYT1;CADM2;INSR;SLC4A10;SNAP91;PTPRD;IGSF11;NBEA;CNTN1;CPE;TCF4;SCN2A;FGF12;CPEB4;GABRB3;SPAG16;ROBO2;DPP10;SLC35F1;PPP1R9A;PTPRG;PCMTD2;TRIM9;GRM5;NHSL1;LRRTM4;TRIM2;RB1CC1;TLK1;ITGB8;DLGAP2;EDIL3;PCMTD1;MAPK1IP1L;SLC2A13;ATRX;SEZ6L;SRP9;PJA2;CNKSR2;SETBP1;LRRC7;MPPED2;PLCB1;SLC24A2;NLGN1;ATL1;NRXN1;PLPPR5;RAP1GDS1;NOL4;CHN1;NCAM2;GPR158;AKAIN1;RFTN2;MYEF2;GABRA5;NTRK3;CADPS;SYT16;CDC42BPA;OXR1;TTLL7;NFIA;APC;NFIB;PPP2R2B;TTC3;ASB4;PTPN4;SSBP2
TRIM23 human tf ARCHS4 coexpression	114/299	1.0116600222967192E-13	ATP8A2;ATP8A1;FRMPD4;MYT1L;DGKB;CTNND2;RPH3A;SYNPR;AKT3;KIF21A;PRKACB;SYBU;ANKS1B;PPFIA2;KCNH1;GUCY1A2;RBFOX1;CACNA2D1;TMOD2;CACNA2D3;ANK2;SCAMP1;GABRG2;GABRG1;ADGRB3;SCN8A;RUFY2;FAR1;KCNQ5;WDFY3;ASTN1;NECAB1;RTN1;STXBP1;SLC1A2;NALCN;GRIN2A;MAP2;FUT9;NDFIP1;DTNA;SYT1;CADM2;SLC4A10;MYO5A;HSPA12A;BTBD10;CORO2B;DCLK1;SNAP91;PTPRD;DLG2;NBEA;SYNJ1;RCAN2;CNTN1;SCN2A;BRWD1;CDK14;FGF12;CPEB4;GABRB3;DPP10;RNF11;DOCK3;DIRAS2;PPP1R9A;SYNE1;TRIM9;GRM5;AKAP11;TRIM2;RB1CC1;PSD3;DLGAP1;EDIL3;KCND2;PDE4D;KCNAB1;SYN2;PJA2;CNKSR2;DNM3;RAPGEF2;RAPGEF5;PLCB1;PAFAH1B1;RAPGEF4;SLC24A2;PPM1L;ADAM22;NRXN3;AKAP6;KIAA0513;SV2B;CHN1;CLVS2;SPOCK1;NCAM1;CTNNA2;NCAM2;GPR158;ATP9A;OPCML;ARNT2;CA10;ATRNL1;SYT16;KIAA1549L;ATP2B2;TTLL7;APC;PPP2R2C;PPP2R2B
NFAT5 human tf ARCHS4	113/299	2.2175635187573056E-13	ITSN2;TRIO;MAML2;RORA;SOGA1;ZFYVE26;HERC2;HERC1;MPRIP;MBNL1;MBNL2;RALGAPA1;FNDC3B;TANC2;TANC1;CLIP1;WDPCP;WDFY3;ASTN2;UTRN;FTO;NOTCH2;ANKRD17;MACF1;DDX6;KMT2C;ITPR2;BAZ2A;IQGAP1;NL

coexpression			K;LPP;ATXN3;PCNX1;SEC14L1;ATXN1;HIVEP1;HIVEP2;CREBBP;LRBA;DENND4C;MICAL3;LNPEP;HOOK3;ARHGAP26;SMARCA2;PHC3;ZZEF1;MED13L;AGO3;AGO2;DMXL2;FAT1;STRN;CDK12;DIDO1;DOCK5;SETD2;DOCK9;KIAA1671;PTPRJ;LIMD1;SYNE2;SYNE1;AKAP13;NIPBL;C16ORF72;AKAP11;SH3PXD2A;ADAMTSL3;ZNF407;KYNU;KIF13A;ARHGEF11;ARHGEF12;DST;VPS13C;VPS13D;ARAP2;VPS13B;ASH1L;ARID1B;PATL1;NCOR1;PEAK1;BIRC6;KDM7A;WDR26;ROCK2;HTT;RRBP1;MIPOL1;KIAA1328;HECTD1;NSD1;HECTD4;ATP9B;RANBP2;ARFGEF1;USP24;SPEN;FARP1;MON2;TRAPPC10;ERBIN;YLPM1;ADAM32;MYO9A;GATAD2B;TJP1;KANSL1;NEDD4;SLMAP;ESYT2
MEIS2 human tf ARCHS4 coexpression	113/299	2.2175635187573056E-13	ERO1B;MYT1L;CTNND2;ZBTB20;SOGA1;SLC8A1;HS6ST3;SCGN;CDH4;ZNF608;DACH1;KIF21A;KIF21B;RGS8;SOX5;MAGI1;KCNH5;TMEM178B;CACNA2D1;TMOD2;MAGI2;MTUS2;ANK2;FRMD4A;ANK3;SHISA9;EML1;MYT1;TANC2;CLIP1;RRAGD;SCG5;WDPCP;SCG3;ASTN2;RIN2;DGKI;FTO;CTTNBP2;BAZ2B;NREP;NYAP2;KALRN;FLRT2;MAP2;FUT9;ZNF704;PLXNA2;STXBP6;SRGAP3;ZFHX3;PTPRN2;CADM1;ZNF382;AUTS2;NEBL;BTBD9;GRIN2B;LRP1B;PTPRD;RALYL;PLCXD3;NBEA;WNK2;RNF182;SPIRE1;CPE;FAT4;LHX9;GABRB3;ROBO2;DRAXIN;TENM4;ELAVL4;GLI3;ROBO1;UNC80;GRM7;TRIM2;MCF2L;PHACTR3;HYDIN2;TRPC5;ABCC8;NAV3;SETBP1;LRRC7;CNKSR3;MPPED2;MYO3A;SAMD5;RGPD5;NOL4;MIPOL1;GNG2;NCAM1;CSMD3;PAK3;MBD5;MYEF2;ANKRD30BL;NTRK3;CADPS;LSAMP;SYT16;MAPK10;PDE10A;APC;PPP2R2B;NEDD4;ASXL3;TTC3;ASB3
RORB human tf ARCHS4 coexpression	113/299	2.2175635187573056E-13	ATP8A2;FRMPD4;MYT1L;CTNND2;SLC8A1;RIMS2;RPH3A;RIMS1;PRKACB;ANKS1B;PPFIA2;KCNH1;RGS7;UNC13C;GUCY1A2;RBFOX1;KCNH5;TMOD2;ANK2;ANK3;GABRG2;GABRG1;ADGRB3;SCN8A;HECW1;DGKI;ASTN1;NECAB1;STXBP1;SLC1A2;KALRN;NALCN;GRIN2A;DPP6;PGBD5;MAP2;FUT9;ST8SIA5;CAMTA1;MBP;SRGAP3;NDFIP1;DTNA;SYT1;CADM2;SLC4A10;MYO5A;HSPA12A;GRIN2B;DCLK1;SNAP91;PTPRD;DLG2;SYNJ1;CNTN1;SCN2A;GABRB3;PTPRT;DOCK3;KCNC1;DIRAS2;RASGRF1;OTUD7A;UNC80;TRIM9;GRM5;AKAP11;TRIM2;NCS1;PSD3;DLGAP1;DLGAP2;MYRIP;SYN2;AJAP1;CNKSR2;DNM3;MPPED1;LRRC7;RAPGEF5;PLCB1;PAFAH1B1;RAPGEF4;SLC24A2;PPM1L;ADAM22;KIAA0513;SV2B;CHN1;CLVS2;NCAM1;CTNNA2;PDE6A;PAK3;CACNG3;ATP9A;WASF3;OPCML;GABBR2;ARNT2;GABRA5;ATRNL1;NTRK3;CADPS;LSAMP;SYT16;KIAA1549L;ATP2B2;TTLL7;APC;PPP2R2C;PPP2R2B;HCN1
SLC4A10 human tf ARCHS4 coexpression	113/299	2.2175635187573056E-13	ATP8A1;FRMPD4;MYT1L;DGKB;KNDC1;LDLRAD4;SLC8A1;HS6ST3;RPH3A;SYNPR;SAMD12;PRKACB;SYBU;ANKS1B;PPFIA2;KCNH1;RGS7;UNC13C;RBFOX1;PRKCE;TMOD2;ANK2;GABRG2;GABRG1;PCP4;ADGRB3;SCN8A;KCNQ3;SCG3;NGEF;NECAB1;STXBP1;SLC1A2;KALRN;NALCN;GRIN2A;DPP6;PGBD5;MAP2;PTPRN2;NDFIP1;SYT1;CADM2;MYO5A;HSPA12A;GRIN2B;CORO2B;DCLK1;SNAP91;LRP1B;DLG2;SYNJ1;RCAN2;CNTN1;CPE;SCN2A;FGF12;GABRB3;DOCK3;RASGRF2;DIRAS2;RASGRF1;GRM1;UNC80;TRIM9;GRM5;AKAP11;TRIM2;NCS1;PSD3;DLGAP1;KCND3;SLC2A13;KCNAB1;MYRIP;SYN2;C1QL3;PJA2;AJAP1;CNKSR2;DNM3;LRRC7;KCNMA1;RAPGEF5;PLCB1;RAPGEF4;SLC24A2;ADAM22;KIAA0513;SV2B;CHN1;CLVS2;CTNNA2;NCAM2;CACNG3;GPR158;ATP9A;OPCML;GABBR2;ARNT2;CA10;GABRA5;ATRNL1;NTRK3;KCNIP4;SYT16;KIAA1549L;ATP2B2;TTLL7;MAPK10;PPP2R2C;PPP2R2B;HCN1
KIAA1549 human tf ARCHS4 coexpression	111/299	1.4816734962619541E-12	ATP8A2;MYT1L;ZNF292;CTNND2;MSI2;SOGA1;CDH4;ZNF608;CDH2;DPYSL5;AKT3;KIF21A;RGS8;ANKS1B;PPFIA2;MAGI1;TMEM178B;CACNA2D1;MAGI2;RFX3;ANK2;FRMD4A;ANK3;SHISA9;MYT1;TANC2;IFT81;AKAP9;HECW1;KCNQ3;IGSF3;CHRNA7;NTM;NEDD4L;PIK3R3;AGAP1;NREP;KALRN;CACNA1E;NPAS3;PHF21B;MAP2;FUT9;ZNF7

			04;PLXNA2;MAP6;CAMTA1;SRGAP3;MPDZ;AUTS2;NETO2;GRIN2B;DCLK1;PBX1;PTPRD;CCDC88A;DLG2;NBEA;DLG5;FAT3;TCF4;SCN2A;GABRB3;PTPRT;ROBO2;DRAXIN;TENM3;TENM4;PTPRO;CELF4;ELAVL4;GRIK2;ROBO1;GRM7;TRIM2;ADAMTSL3;GDAP1L1;TMEM108;NCOA6;DCC;LRRC49;HUNK;UBE2QL1;IL17RD;PGM2L1;GAP43;NAV3;SETBP1;LRRC7;ARHGEF7;AKAP6;STOX2;MAPK8;GNG2;NCAM1;CSMD2;PAK3;PAK5;ARNT2;ZNF462;FARP1;MBD5;MYEF2;NTRK3;LSAMP;KIAA1549L;ATAT1;TNRC6C;APC;ZNF618;ASXL3
NPAS3 human tf ARCHS4 coexpression	111/299	1.4816734962619541E-12	APP;SPON1;CTNND2;ZBTB20;CDH4;DACH1;CDH2;DPYSL5;PEG10;AKT3;KIF21A;SOX6;EPHB1;SRGAP2B;ANKS1B;MAGI1;GUCY1A2;TMEM178B;TMOD2;MAGI2;RFX3;KAZN;ANK2;FRMD4A;PYGO1;IFT81;ADGRB3;TNIK;ASTN1;GRIA1;ADCYAP1R1;NTM;AGAP1;ILDR2;NREP;DPP6;NKAIN3;MAP2;CDH20;FUT9;CAMTA1;KCNN3;SRGAP3;MPDZ;NDFIP1;DTNA;CADM1;WSCD1;AUTS2;CADM2;ST8SIA1;PXDNL;NETO2;CORO2B;DCLK1;PBX1;PTPRD;CCDC88A;SDK1;CNTN1;FAT3;TCF4;SPAG16;DPP10;DRAXIN;TENM3;MEGF10;GRIK4;SLC35F1;FMN2;SIPA1L2;GRM3;TRIM9;GRM5;TRIM2;TMEM108;ITGB8;ADGRV1;KCND3;LRRC49;IL17RD;SEZ6L;PARD3B;TOX3;NRG3;SETBP1;MPPED2;LUZP2;NTN1;STOX2;GNG2;TMEM67;LRIG1;NCAM1;CTNNA2;PAK3;JAM2;ATP9A;ARNT2;ZNF462;NTRK2;MYEF2;NTRK3;KIAA1549L;TTLL7;MAPK10;APC;PPP2R2B;FABP7;SMOC1;TTC3
ZNF236 human tf ARCHS4 coexpression	110/299	3.597870346084322E-12	ZBTB21;RORA;PTAR1;DNM1P47;HERC1;PPIP5K2;RTTN;POTEC;MBNL1;USP7;RALGAPA1;CLIP1;AKAP9;WDFY3;ASTN2;UTRN;CFAP61;ANKRD17;MACF1;KMT2C;CLEC16A;BAZ2B;LPP;PCNX1;GRK3;ATXN1;RNF217;ADAMTS17;HIVEP1;CEP192;HIVEP2;BPTF;LRRC37A3;CREBBP;LRBA;DENND4C;LNPEP;DNAJC13;ARHGAP26;SMARCA2;ZZEF1;MED13L;CYLD;ARHGAP32;GON4L;DMXL2;STRN;CDK12;BRWD1;EIF4G3;RERE;DIDO1;SETD2;USP33;SMG1P5;LYST;SYNE2;SYNE1;AKAP13;NIPBL;C16ORF72;AKAP11;ADAMTSL3;ZNF407;ERC1;DST;NCOA6;VPS13C;VPS13D;ARAP2;VPS13B;ASH1L;ARID1B;FAM153A;BIRC6;KDM7A;NFAT5;WDR26;ROCK1;RNF38;RGPD6;RGPD8;MIPOL1;FGD4;SCAF8;KIAA1328;HECTD1;HECTD4;ANKRD36BP2;PCNT;ATP9B;RANBP2;ARFGEF1;SPEN;USP25;MBD5;MON2;PNPLA7;TRAPPC10;MGA;ERBIN;YLPM1;ADAM32;DDHD1;HIPK1;MYO9A;TTLL5;APLF;FRYL;TNRC6B
PKNOX2 human tf ARCHS4 coexpression	110/299	3.597870346084322E-12	CTNND2;RORB;ADARB2;SOGA1;MYLK3;RIMS1;RPS6KA5;ZNF608;TNR;KIF21B;SOX5;PPFIA2;GUCY1A2;RBFOX1;MEF2C;EPHA6;KCNH5;TMEM178B;CACNA2D1;TMOD2;TTC7B;ANK2;FRMD4A;ANK3;SHISA9;TANC2;CLIP1;WDPCP;DGKI;FTO;STXBP1;AGAP1;ILDR2;KALRN;ORC4;NUAK1;DPP6;FLRT2;MAP2;FUT9;ZNF704;SRGAP3;KLHL29;ZFHX3;AUTS2;MICAL3;MYO5A;HOOK3;GRIN2B;DCLK1;PBX1;PTPRD;CCDC88A;DLG2;DAB1;NBEA;WNK2;FAT3;FAT4;FBXL7;ROBO2;ZNF891;DOCK3;TENM4;PTPRO;GRIK3;ROBO1;GRM7;CA5A;TRIM2;ADAMTSL3;MCF2L;RASGEF1C;OPA3;PSD3;DLGAP1;ZNF385D;NEO1;TRPC5;ABCC9;SORCS3;KLF15;AJAP1;MPPED1;NAV3;LRRC7;PEAK1;CNKSR3;KIAA1328;HECTD4;NCAM1;PAK3;CSMD1;ATP9B;ATP9A;ARNT2;FARP1;NEGR1;ANKRD30BL;NTRK3;LSAMP;YLPM1;KIAA1549L;MEIS2;PDE10A;APC;PPP2R2B;NEDD4;RGS12;ASB3
NFIA human tf ARCHS4 coexpression	109/299	9.038405770908416E-12	MYT1L;ANKRD36;CDH7;SRGAP2C;AKT3;KIF21A;SRGAP2B;SOX5;PPFIA2;RGS7;GUCY1A2;EPHA7;CXADR;ZNF160;MAGI2;RFX3;KAZN;ANK2;CDKAL1;FRMD4B;ADGRB3;AKAP9;HECW1;KCNQ3;ZFPM2;GRIA1;INO80D;CTTNBP2;BTF3L4;NEDD4L;BAZ2B;NYAP2;NPAS3;MAP2;SNTG1;PLXNA2;ANKRD10;SRGAP3;BCL11B;ST8SIA1;BCL11A;INSR;CORO2B;ST18;PTPRD;CCDC88A;DAB1;NBEA;AGO1;FAT3;CNTN3;TCF4;FAT4;FGF12;LRP12;ROBO2;CNTNAP2;TE

			NM4;CLCN3P1;PTPRO;GRIK3;ELAVL4;SLC35F1;GRIK2;EFCAB6;BICD1;SYNE2;PTPRG;NHSL1;ADAMTS3;RASGEF1B;LRRTM4;RB1CC1;TMEM108;PHACTR3;ITGB8;GARNL3;MAPK1IP1L;LRRC49;ATRX;ENAH;TOX3;SETBP1;LRRC7;MPPED2;RAPGEF2;ZNF234;SHANK2;SLC24A2;CRB1;TULP4;NOL4;JAM2;PAK5;OPCML;RFTN2;ZNF462;MYEF2;NTRK3;SYT16;SORBS2;PPP2R3A;KIAA0232;NBEAP1;APC;PPP2R2B;TTC3;ASB4;HCN1
AFF3 human tf ARCHS4 coexpression	108/299	2.1555984452683744E-11	*MYT1L;ANKRD36;CTNND2;DPY19L2P2;LDLRAD4;FRY;SLC8A1;CEP128;POTEKP;CCDC91;ZSCAN30;PPIP5K2;AKT3;PRKACB;ANKS1B;PPFIA2;POTEC;GUCY1A2;ANKRD36C;MEF2C;PRKCB;PRKCE;TMOD2;MAGI2;ANK2;FRMD4B;SHISA9;GAREM1;AKAP9;HECW1;KCNQ3;WDPCP;ASTN2;ANKRD36B;BLK;KMT2E;GRIA1;TPH2;NREP;KALRN;NPAS3;MAP2;SRGAP3;SCAI;CEP112;ZNF382;AUTS2;SYT1;CADM2;SETDB2;RABL2A;MYO5A;PARP15;GRIN2B;CORO2B;DCLK1;PTPRD;CCDC88A;DLG2;SYNJ1;TCF4;CDK14;PTPRT;DPP10;CCDC122;ZNF891;CELF2;EFCAB6;UNC80;GRM7;TRIM2;ADAMTSL3;RALGPS2;KCND2;NCOA6;VPS13B;TBC1D9;ASH1L;ARID1B;MPPED1;SETBP1;BANK1;LRRC7;ZMYND11;AKAP6;LRP2;MIPOL1;KIAA1328;GNG7;HECTD4;NRIP1;CLVS2;ANKRD36BP2;NCAM1;ATP9B;NTRK2;MBD5;NTRK3;ADAM32;DDHD1;TTLL7;TTC3;NEK10;YPEL1;RAB3GAP2;SSBP2;ASB3;TNRC6B*
PBRM1 human tf ARCHS4 coexpression	108/299	2.1555984452683744E-11	*ANKRD36;ZNF292;LTN1;UBE3A;GRIP1;ZNF608;HERC1;SACS;SMARCAD1;EPC2;THSD7A;GTF2I;SMARCC1;FRMD4A;RFX7;AKAP9;WDFY3;UTRN;MACF1;DDX6;TNKS;KMT2C;IREB2;PIK3R3;BAZ2B;NREP;PHF21B;MAP2;ZNF704;BPTF;BCL11B;AUTS2;BCL11A;ZBTB10;PBX1;MED13L;PTPRD;FER;AGO3;TCF4;UBAP1L;FAT4;EIF4G3;SLC44A5;SETD2;TENM4;CHD9;ELAVL4;BICD1;SYNE2;PTPRG;ROBO1;VN1R7P;NIPBL;ZMYM4;PPP2R5E;ZNF407;SPIN1;DNM1L;RBM6;NCOA6;DCC;LRRC49;VPS13C;ATRX;VPS13B;NSUN6;ZDHHC17;ARID1B;PARD3B;ASPM;SFPQ;NCOR1;MMP16;DNAJC7;SETBP1;MTF2;BIRC6;PIK3C3;HDAC2;ROCK1;STAU2;MAPK8;KIAA1328;NSD1;ZSWIM6;NCAM1;PAK3;MAP4K4;RANBP2;ARFGEF1;USP24;ATF7IP;ZNF462;MBD5;MYEF2;MGA;YLPM1;SYT14;PPP2R3A;MYO9A;GATAD2B;PLEKHA8;APC;KANSL1;ASXL3;TTC3;FRYL*
ZNF638 human tf ARCHS4 coexpression	107/299	5.285713566693269E-11	*ATP8A1;CTNND2;DPY19L2P2;LTN1;PTAR1;HERC2;HERC1;ZSCAN30;PPIP5K2;SACS;DIP2B;SAMD13;TRIM23;PPFIA2;KCNH1;UNC13C;EPHA4;RALGAPA1;TMOD2;WDR72;ANK2;ANK3;UNC5D;ADCY9;HECW2;WDFY3;ASTN1;FTO;MACF1;ZBED9;TNKS;KMT2C;BAZ2B;KALRN;PCNX1;MAP2;FUT9;TRPM7;CEP192;BPTF;CADM2;ST8SIA1;LRBA;PHC3;LRP1B;DLG2;AGO3;FAT3;BRWD1;GABRB1;DOCK3;DOCK9;PPP1R9A;OSBPL10;SYNE1;UNC80;C16ORF72;AKAP11;TRIM2;RB1CC1;EDIL3;VWFP1;ZYG11A;ARHGEF12;DST;VPS13C;VPS13D;VPS13B;ASH1L;ZDHHC17;C1QL3;ARID1B;DNM3;HEATR5A;BIRC6;RAPGEF5;PLCB1;SLC24A2;NFAT5;ROCK2;HERC2P3;TULP4;AKAP6;ZDHHC21;MIPOL1;MAPK9;RANBP3L;KIAA1328;HECTD1;HECTD4;SPOCK3;ATP9B;RANBP2;MBD5;MON2;PCDH9;ATRNL1;MGA;NTRK3;SYT16;MYO9A;TJP1;POLR3A;APC;PTPN4;ASB3;FRYL*
SOX6 human tf ARCHS4 coexpression	106/299	1.2790945942033093E-10	*SEMA5A;ZNF292;ZBTB20;MYLK3;SRGAP2C;DPYSL5;ZSCAN30;ZNF606;TNR;WSB1;ST6GAL2;TMEM178B;MAGI2;RFX3;FRMD4A;IFT81;ADGRB3;RUFY2;AKAP9;SCG3;ASTN2;ZFPM2;ASTN1;KMT2E;TNKS;PDE1A;PCDH15;TPTE2P2;PIK3R3;ILDR2;BAZ2B;NPAS3;MAP2;SNTG1;FUT9;SRGAP3;GRIA4;SPECC1;ANKRD26;SLC14A2;ZNF382;ARHGAP28;DCDC1;CCDC88A;AGO3;TMEM236;TCF4;CPEB4;SLC44A5;SPAG16;ROBO2;OSCP1;CCDC122;ZNF891;CHD9;GADL1;SLC35F1;EFCAB6;PPP1R9A;RYR3;UNC80;NHSL1;TRIM2;ADAMTSL3;SNAPC3;ITGB8;NOS1;DENND2C;LRRC49;TCF12;ABCC9;SPTB;EPN2;MMP16;SETBP1;ZNF234;*

			CRB1;PLPPR1;LUZP2;NRXN3;NOL4;MIPOL1;FGD4;GNG2;KIAA1328;HECTD2;ANKRD36BP2;JAM2;PAK5;GABRA2;RFTN2;ATF7IP;RIC3;ZNF462;MBD5;NTRK3;LSAMP;PDE4DIP;LHFPL3;MAPK10;APC;KLHL7;TTC3;ASB4;ZNF611;ASB3
ZNF407 human tf ARCHS4 coexpression	105/299	3.0542293385940733E-10	*ITSN2;ATP8A1;FRY;BACH1;DOCK10;ZFYVE26;DNM1P47;HERC2;PPIP5K2;DIP2B;MAP3K5;MBNL1;MORC3;RALGAPA1;RALGAPA2;AKAP9;RELL1;WDFY3;ZFPM2;UTRN;NOTCH2;MACF1;DDX6;MTMR3;KMT2C;ITPR2;IQGAP1;PCNX1;HIVEP1;CEP192;HIVEP2;CREBBP;LRBA;DENND4C;LNPEP;DNAJC13;ARHGAP26;SMARCA2;ZZEF1;MED13L;CYLD;DMXL2;SP3;STRN;CCSER1;CDK12;BRWD1;KIAA0825;KDM5A;DOCK5;SETD2;DOCK8;SMG1P2;SMG1P5;PTPRJ;GRIK2;LYST;SYNE2;SYNE1;AKAP13;NIPBL;C16ORF72;JAK2;PCMTD1;ITGA4;DST;VPS13C;VPS13D;ARAP2;VPS13B;ASH1L;PARP8;PHF20L1;NCOR1;DPYD;SPOPL;BIRC6;DOCK2;MCTP2;KDM7A;NFAT5;WDR26;ROCK1;HTT;SCAF8;KIAA1328;HECTD1;NSD1;HECTD4;PCNT;ATP9B;RANBP2;ARFGEF1;USP24;USP25;MON2;KDM4C;TRAPPC10;MGA;ERBIN;DDHD1;MYO9A;SNRK;KANSL1;FRYL*
AFF4 human tf ARCHS4 coexpression	104/299	6.745469857686141E-10	*APP;TRIO;ZFYVE9;SOGA1;CMIP;ALCAM;HERC1;MPRIP;SACS;ANKFY1;DIP2B;GTF2I;UNC13B;IL1R1;RALGAPA1;ZBTB38;FNDC3B;AFAP1;FNDC3A;RC3H2;TANC2;TANC1;WDFY3;UTRN;NOTCH2;ANKRD17;MACF1;DDX6;ABHD2;KMT2C;LPP;ACACA;PCNX1;ABL1;HIVEP2;MAP4;CREBBP;EXOC6B;DNAJC13;SYNJ2;PUM1;GNG12;ZZEF1;MED13L;EXT1;DLG5;FAT1;STRN;CDK12;PTPRK;LAMC1;ZCCHC14;AKAP11;SH3PXD2A;KIF13A;ERC1;TEAD1;NEO1;ARHGEF11;ZHX3;ARHGEF12;DST;VPS13C;ARHGEF17;MYOF;VPS13D;ASH1L;ATRN;SFPQ;BIRC6;DOCK1;PAFAH1B1;YAP1;NFAT5;RABGAP1;WDR26;ROCK2;HTT;TM9SF3;SCAF8;MAPK8;HECTD1;NSD1;HECTD4;CTNNA1;FLNB;ATP9A;MAP4K4;RANBP2;USP24;SPEN;TRAPPC10;SAMD4A;ERBIN;LAMB1;AP2B1;CDC42BPB;MTOR;PLEKHA8;TJP1;VMP1;SLMAP;ATP13A3;SEC24D*
MBNL2 human tf ARCHS4 coexpression	104/299	6.745469857686141E-10	*ATP8A1;FRMPD4;RORA;RPH3A;SYNPR;RPS6KA3;EFR3A;PRKACB;KCNH1;CAST;RBFOX1;LIMCH1;RALGAPA1;PRKCE;TMOD2;MTUS1;GABRG2;GABRG1;TOM1L2;TANC1;WDFY3;NGEF;NECAB1;STXBP1;SEL1L;SLC1A2;NDRG2;NALCN;RAP1GAP;GRIN2A;PDZD2;MBP;MAP4;NDFIP2;ABCA5;CADM2;CRIM1;SLC4A10;MYO5A;SYNJ2;HSPA12A;GNG12;ELL2;SNAP91;ARHGAP32;PTPRB;DLG2;SYNJ1;RCAN2;CNTN1;CPE;STRN;SCN2A;CPEB4;RNF11;DOCK9;DIRAS2;RASGRF1;PTPRK;SYNE1;AKAP11;UBL3;TRIM2;KIF13A;PSD3;EDIL3;PCMTD1;ARHGEF12;DST;SLC2A13;VPS13D;ARAP2;COBL;KCNAB1;MYRIP;SYN2;DNM3;KCNMA1;PLCB1;RAPGEF4;SLC24A2;NFAT5;ROCK2;ADAM22;KIAA0513;ABLIM1;SV2B;CHN1;SPOCK3;SPOCK1;CTNNA3;GPR158;ATP9A;OPCML;PCGF5;ATRNL1;SAMD4A;SYT16;KIAA1549L;PDE4DIP;ATP2B2;TTLL7;TJP1;PPP2R2C*
HELZ human tf ARCHS4 coexpression	104/299	6.745469857686141E-10	*RORA;ETS1;DOCK10;PTAR1;HERC1;THSD7A;MBNL1;MORC3;PRKCH;RALGAPA1;RALGAPA2;ADAM10;INPP4B;WDFY3;UTRN;KMT2E;ANKRD17;MACF1;DDX6;KMT2C;ITPR2;LPP;RASGRP1;PCNX1;HIVEP1;TRPM7;CEP192;HIVEP2;BPTF;MAP2K6;CREBBP;RABGAP1L;ARPP21;LRBA;DENND4C;LNPEP;DNAJC13;SMARCA2;PHC3;ZZEF1;MED13L;CYLD;SP3;BCL2;STRN;CDK12;BRWD1;SETD2;USP32;DOCK8;SMG1P2;LYST;SYNE2;SYNE1;AKAP13;NIPBL;C16ORF72;ZNF407;KDM6A;KLF12;ARHGEF12;ITGA4;DST;VPS13C;VPS13D;ARAP2;VPS13B;ASH1L;ARID1B;PARP8;BIRC6;PIK3C3;DOCK2;MCTP2;KDM7A;NFAT5;WDR26;ROCK1;HTT;SCAF8;BTAF1;KIAA1328;HECTD1;NSD1;HECTD4;RANBP2;ARFGEF1;USP24;SPEN;MBD5;MON2;KDM4C;TRAPPC10;MGA;ERBIN;YLPM1;ADAM32;DDHD1;HIPK1;MYO9A;GATAD2B;KANSL1;SLMAP;UNC79*

ZNF91 human tf ARCHS4 coexpression	103/299	1.5707670061403296E-9	*MYT1L;ANKRD36;ZNF292;ZBTB20;UBE3A;PPIP5K2;AKT3;KIF21A;SOX6;GUCY1A2;EPHA6;WSB1;CXADR;RALGAPA1;SEMA6D;TMOD2;MAGI2;RFX3;ANK2;ANK3;TANC2;RUFY2;AKAP9;ASTN2;CFAP97;ANKRD36B;TNKS;BAZ2B;NREP;NYAP2;KALRN;ORC4;MAP2;SNTG1;FUT9;SRGAP3;SCAI;ZNF382;RANBP17;GRIN2B;PTPRD;CCDC88A;CNOT7;DLG2;NBEA;FAT3;CCSER1;TCF4;BRWD1;COPS8;CPEB4;SLC44A5;ROBO2;ZNF891;SETD2;CHD9;PTPRO;ELAVL4;SMG1P5;EFCAB6;PPP1R9A;UNC80;GRM7;TRIM2;RB1CC1;TMEM108;TLK1;PCMTD1;DST;LRRC49;ATRX;ASH1L;FAM126B;PJA2;NAV3;SETBP1;LRRC7;NRXN3;TULP4;AKAP6;NOL4;MIPOL1;FBXL20;FGD4;MAPK8;GNG2;HECTD2;ZNF627;PAK3;MBD5;NTRK3;SYT16;DDHD1;DEFB108B;MAPK10;APC;KLHL7;ASXL3;TTC3;PTPN4;SSBP2;ASB3;TNRC6B*
ZKSCAN1 human tf ARCHS4 coexpression	102/299	3.6073487754967736E-9	*ERO1B;ANKRD36;TUSC3;ZNF292;ZBTB20;MSI2;RPS6KA5;ZNF606;PPIP5K2;LONP2;LUC7L;KIF21A;SOX6;MAGI1;ANKRD36C;WSB1;RALGAPA1;MAGI2;RFX3;TANC2;IFT81;AKAP9;SCG5;WDPCP;SCG3;ASTN2;ANKRD36B;ZNF397;FTO;STXBP4;PIK3R3;BAZ2B;NALCN;LPP;NPAS3;ORC4;FAM83F;MAP2;SRGAP3;SLC14A2;PTPRN2;CADM1;ZNF382;PUM1;ST18;DCDC1;CCDC88A;AGO3;TMEM236;TMEM116;CPE;XKR6;PCSK2;SPAG16;CCDC122;ZNF891;CHD9;ELAVL4;PPP1R9A;PCMTD2;UNC80;CA5A;TRIM2;ADAMTSL3;GATAD1;GDAP1L1;ITGB8;ARFGEF3;TCF12;HUNK;COBL;ARID1B;ZFP90;EPN2;PLCB4;EVC;SETBP1;PEAK1;CNKSR3;RGPD5;HERC2P9;NOL4;MIPOL1;FBXL20;FGD4;KIAA1328;PAK3;ZNF462;FARP1;MBD5;MYEF2;CADPS;ELP2;C1ORF21;SORBS2;ZDHHC11B;NEDD4;TTC3;ASB4;RGS12;ASB3;TNRC6B*
ST18 human tf ARCHS4 coexpression	101/299	7.727886902866618E-9	*ERO1B;MYT1L;DGKB;CDH7;RIMS2;DPYSL5;AKT3;KIF21A;ANKS1B;EPHA7;MAGI2;MTUS2;EBF2;CDKAL1;MYT1;SHISA6;RUFY2;AKAP9;HECW1;KCNQ3;SCG5;SCG3;RIN2;TNKS;STXBP1;BTF3L4;BAZ2B;NREP;NYAP2;KALRN;NALCN;MTMR7;DPP6;MAP2;SNTG1;SRGAP3;PTPRN2;CADM1;ZNF382;BCL11A;SLC4A10;SNAP91;PTPRD;CCDC88A;PLCXD3;CNTN1;CPE;CPEB4;PCSK2;GABRB3;ROBO2;ZNF891;ELAVL4;GRIK2;EFCAB6;PPP1R9A;BICD1;UNC80;RASGEF1B;TRIM2;MCF2L;RB1CC1;HYDIN2;EDIL3;ARFGEF3;KCND3;DCC;LRRC49;SEZ6L;SETBP1;SAMD5;SLC24A2;CRB1;PPM1L;ATL1;NRXN3;TULP4;AKAP6;TMEM163;NOL4;CLVS2;NCAM1;CTNNA2;CSMD3;PAK3;MBD5;MYEF2;PCDH9;NTRK3;CADPS;SYT16;SORBS2;TTLL7;MAPK10;NFIB;ASXL3;TTC3;RIMBP2;APBA2;ASB3;HCN1*
ZNF462 human tf ARCHS4 coexpression	101/299	7.727886902866618E-9	*ZNF292;ZBTB20;IGF1R;RIMS2;ZNF608;CDH2;DPYSL5;SNRPD1;KIF21A;PPFIA2;MAGI1;EPHA4;SMARCC1;CXADR;RFX3;ANK3;MYT1;PYGO1;IFT81;AKAP9;ZNF431;ZNF397;BTF3L4;NEDD4L;ILDR2;BAZ2B;NREP;KALRN;ACACA;PHF21B;APELA;MAP2;ZNF704;CAMTA1;SRGAP3;ZNF423;MPDZ;BPTF;CADM1;ZNF382;AUTS2;PBX1;PTPRD;CCDC88A;WNK2;AGO1;ZNF536;FAT3;TCF4;GABRB3;ROBO2;ZNF891;DRAXIN;TENM4;CHD6;PSIP1;PPP1R9A;BICD1;TTC28;TRIM2;ADAMTSL3;SUMO2;JARID2;ARFGEF3;USP49;ADGRV1;LRRC49;ATRX;SHROOM2;HUNK;TET1;NAV2;IL17RD;ARID1B;NAALADL2;ENAH;NCOR1;SETBP1;HDAC2;PRUNE2;TULP4;STOX2;CECR2;HECTD2;APBB2;NCAM1;PAK5;FARP1;MYEF2;SLC16A1;YLPM1;TJP1;ATAT1;ZNF738;KLHL7;TTC3;YPEL1;RGS12;KIAA1958;ADGRL2;TNRC6B*
NCOA1 human tf ARCHS4 coexpression	101/299	7.727886902866618E-9	*ITSN2;ATP8A1;RORA;FRY;HERC1;KIF21B;DIP2B;ANKS1B;MAP3K5;RBFOX1;MBNL1;PRKCB;RALGAPA1;TMOD2;ANK2;ANK3;IPCEF1;MADD;WDFY3;UTRN;MACF1;DDX6;MTMR3;KMT2C;KALRN;MED12L;GRIN2A;PCNX1;ATXN1;HIVEP2;LYN;CREBBP;IQSEC1;MYO5A;LNPEP;FOXN3;ARHGAP26;SMARCA2;PHC3;GRIN2B;ZZEF1;MED13L;CYLD;DLG2;SYNJ1;GNAQ;DMXL2;FAT3;STRN;MTMR10;CPQ;DOCK8*

			;CELF2;DYSF;PTPRJ;HTR2A;LYST;RYR3;SYNE2;GRM1;SYNE1;UNC80;SENP6;AKAP13;GLT1D1;AKAP11;ADGRE3;CNOT6L;VPS13C;VPS13D;ARAP2;VPS13B;ASH1L;FAM126B;IL17RA;PARP8;CNKSR2;ITPKB;RAPGEF2;PLCB1;DOCK2;MCTP2;KDM7A;SLC24A2;ADAM22;AOAH;KIAA0513;HECTD4;MAPK1;GABBR2;SEMA4D;PCGF5;PLCL1;ERBIN;PDE4DIP;ATP2B2;MYO9A;SNRK;APC;CAMK4;PTPN4
POU3F2 human tf ARCHS4 coexpression	100/299	1.6839264537274902E-8	MYT1L;CTNND2;LDLRAD3;CELSR2;CDH4;DACH1;CDH2;DPYSL5;KIF21A;EPHB2;EPHB1;MAGI1;ANKRD36C;WSB1;ST6GAL2;TMEM178B;RFX3;EBF3;ANK2;FRMD4A;ADGRB3;RUFY2;TNKS;CHRNA7;NTM;PIK3R3;ILDR2;NREP;KALRN;NPAS3;PHF21B;NKAIN3;MAP2;ZNF704;PLXNA2;MAP6;CAMTA1;SRGAP3;WSCD1;ST8SIA1;NETO2;CORO2B;DCLK1;PBX1;PTPRD;CCDC88A;RNF182;FAT3;TCF4;SLC44A5;ROBO2;DRAXIN;TENM4;ELAVL4;TSPAN11;ROBO1;GRM3;TRIM2;DNER;ITGB8;ADGRV1;DCC;LRRC49;TCF12;HUNK;TOX3;GAP43;ZEB1;MPPED1;SETBP1;LRRC7;ZNF780B;HDAC2;STOX2;GTF2IP1;GNG2;ZSWIM6;NCAM1;CTNNA2;PAK3;JAM2;PAK5;ATP9A;MAP4K4;ARNT2;MYEF2;NTRK3;ENOX1;TTLL7;ATAT1;MAPK10;NELL2;FABP7;ASXL3;TTC3;YPEL1;RGS12;APBA2;ADGRL2;SPSB4
CHD9 human tf ARCHS4 coexpression	100/299	1.6839264537274902E-8	ZBTB25;ANKRD36;ZNF292;FRG1HP;ZBTB20;UBE3A;SRGAP2C;CNST;PPIP5K2;AKT3;DLEU1;KIF21A;SOX6;SCAPER;SRGAP2B;ANKS1B;ANKRD36C;WSB1;RALGAPA1;KCNH8;MAGI2;ANK2;IFT81;AKAP9;ZNF438;ASTN2;ZNF675;ANKRD36B;KMT2E;SHC4;STXBP4;KMT2C;BAZ2B;FGGY;MAP2;SNTG1;TTC21B;ANKRD10;LRRC4C;BPTF;PRELID2;SLC14A2;DCDC1;CCDC88A;FER;AGO3;CEP83;TCF4;BRWD1;DPP10;ZNF891;USP33;EFCAB6;PPP1R9A;SYNE2;SENP6;EPB41L4A;LRRTM4;TRIM2;ADAMTSL3;GABPA;DST;GRID1;LRRC49;VPS13C;ATRX;VPS13B;ARID1B;ZFP90;PCCA;SETBP1;PEAK1;MPPED2;USP41;SDCCAG8;CRB1;CATSPER2;RGPD6;RGPD5;MIPOL1;ATP6AP1L;FBXL20;KIAA1328;ANKRD36BP2;PAK3;ATP9B;JAM2;S100PBP;MBD5;MYEF2;CDC42BPA;MAPK10;MAB21L3;APC;NEDD4;TTC3;SSBP2;ASB3;ZNF354C;TNRC6B
PLXNA2 human tf ARCHS4 coexpression	99/299	3.6243832076160924E-8	APP;MYT1L;CTNND2;SOGA1;CELSR2;CDH5;CDH4;MPRIP;DPYSL5;PEG10;AKT3;KIF21B;EPHB2;PPFIA2;MAGI1;RBFOX1;TMEM178B;CACNA2D1;TMOD2;AFAP1;KAZN;ANK2;FRMD4A;HSPG2;TANC2;MAPK8IP1;COL4A2;HECW2;SCN8A;KCNQ3;WDFY3;TNIK;GRIA1;IGSF3;STXBP1;AGAP1;NREP;KALRN;FAM171A1;CTIF;MAP2;ZNF704;EPB41L3;MAP4;SRGAP3;NDFIP1;AUTS2;SYT1;NETO2;CORO2B;DCLK1;SNAP91;PTPRD;SDK1;PTPRB;WNK2;DLG5;SPIRE1;FAT4;DOCK4;TENM4;DOCK9;MAST2;GRIK3;ECE1;SIPA1L2;ROBO1;TRIM2;MCF2L;NCS1;PHACTR3;NEO1;FAM219A;NAV2;RGMB;MPPED1;SETBP1;LRRC7;PAFAH1B1;STOX2;GNG2;HECTD4;GTF2IP4;MVB12B;NCAM1;CTNNA2;PAK5;ATP9A;MAP4K4;ARNT2;NTRK3;KIAA1549L;CDC42BPB;MEIS2;SGSM1;APC;NFIB;TTC3;APBA2
BDP1 human tf ARCHS4 coexpression	99/299	3.6243832076160924E-8	TCERG1;ZFYVE9;ANKRD36;ZNF292;HNRNPU;UBE3A;CWC27;HERC2;HERC1;SACS;PPFIA2;POTEC;ANKRD36C;ESCO1;RALGAPA1;ZNF271P;ANK2;AKAP9;WDPCP;WDFY3;ZNF678;ASTN2;UTRN;ANKRD36B;ANKRD17;MACF1;DDX6;TBC1D19;KMT2C;BAZ2B;TTC21B;ANKRD20A5P;CEP192;BPTF;LRBA;ZBTB10;CCDC88A;AGO3;NBEA;FAT3;CCSER1;TCF4;CDK12;BRWD1;SETD2;USP33;SYNE2;AKAP13;NIPBL;NHSL1;ADAMTSL3;KDM6A;TTC37;ARHGEF12;DST;VPS13C;VPS13D;ATRX;VPS13B;ASH1L;ARID1B;SUPT3H;SFPQ;NCOR1;LRRC7;PEAK1;BIRC6;ZNF236;NFAT5;ROCK1;ROCK2;RGPD6;MIPOL1;KIAA1328;NSD1;ANKRD36BP2;PCNT;ATP9B;RANBP2;ARFGEF1;USP24;SPEN;USP25;MBD5;MGA;ERBIN;YLPM1;ADAM32;DDHD1;MYO9A;KTN1;MAB21L3;APC;CAMK4;NEDD4;PTPN4;FRYL;TNRC6B;CCDC171

SMAD4 human tf ARCHS4 coexpression	98/299	7.527923016959694E-8	*PATJ;ZFYVE9;XYLT1;HNRNPU;PTAR1;HERC2;ZNF608;HERC1;SACS;DIP2B;GTF2I;UNC13B;CADPS2;SMARCC1;RALGAPA1;RALGAPA2;ADAM10;WDFY3;UTRN;NOTCH2;MACF1;DDX6;KMT2C;IREB2;LPP;ARHGAP12;PCNX1;CEP192;MPDZ;BPTF;CREBBP;LRBA;DENND4C;LNPEP;DNAJC13;FOXN3;PUM1;ZZEF1;MED13L;AGO2;SP3;FAT1;STRN;BRWD1;FGF10;TENM4;SYNE2;PTPRG;AKAP13;NIPBL;C16ORF72;AKAP11;ZMYM4;KIF13A;TEAD1;NEO1;ARHGEF12;DST;VPS13C;VPS13D;VPS13B;ASH1L;VCAN;SFPQ;ELF2;SPOPL;BIRC6;DOCK1;KDM7A;NFAT5;WDR26;ROCK2;HTT;ZDHHC21;TM9SF3;CECR2;SCAF8;BTAF1;HECTD1;NSD1;HECTD4;RANBP2;ARFGEF1;USP24;SVIL;MON2;TRAPPC10;MGA;ERBIN;PTPN13;MYO9A;MTOR;PLEKHA8;TJP1;ITCH;SLMAP;PKN2;ADGRL2*
ELK4 human tf ARCHS4 coexpression	98/299	7.527923016959694E-8	*ITSN2;PATJ;MAML2;CEP120;RORA;ETS1;DOCK10;POTEM;PTAR1;HERC1;CAST;MBNL1;PRKCH;ENTPD5;RALGAPA2;TC2N;RUNX1;INPP4B;ZNF831;NOTCH2;MACF1;DDX6;CASZ1;ABHD2;KMT2C;ITPR2;BAZ2A;IQGAP1;LPP;RASGRP1;PCNX1;TRPM7;HIVEP2;CREBBP;LRBA;DENND4C;LNPEP;FOXN3;SMARCA2;PHC3;PARP15;FLI1;ZZEF1;MED13L;CYLD;AGO2;SP3;BCL2;STRN;CDK12;DOCK9;DOCK8;EFCAB14;PTPRJ;LIMD1;SYNE2;AKAP13;NIPBL;LRRFIP1;ABCC4;CNOT6L;ARHGEF12;ITGA4;VPS13C;VPS13D;ARAP2;VPS13B;ASH1L;ARID1B;PARP8;PACS1;BIRC6;DOCK2;MCTP2;KDM7A;NFAT5;WDR26;DNAH8;NLRC5;KIAA1328;HECTD1;NSD1;HECTD4;PDLIM5;RANBP2;ARFGEF1;USP24;OR51E1;SEMA4D;PLEKHA2;STAT1;TRAPPC10;ERBIN;HIPK1;GATAD2B;SNRK;SLMAP;ESYT2*
GLIS3 human tf ARCHS4 coexpression	98/299	7.527923016959694E-8	*APP;ERO1B;TRIO;MAML2;TUSC3;WWC1;ZBTB20;SLC4A4;IGF1R;SCGN;C1ORF127;ALCAM;CDH2;PEG10;IL1R1;FNDC3B;AFAP1;RFX3;ARID5B;FNDC3A;STON1-GTF2A1L;TANC2;RRAGD;SCG5;SCG3;MXRA7;RIN2;RAI14;SDC2;ARHGEF28;SEL1L;FBLN5;NPAS3;CALD1;ARSJ;SNX9;TSPAN3;MPDZ;ARSB;STARD13;VCAM1;NEK6;PXDNL;GNG12;ELL2;IGSF11;MOB1B;SDK1;RCAN1;PLCXD3;COL5A1;PTPRA;DLG5;GNAS;STT3A;CPE;SPAG16;NRP1;TENM3;ECE1;FMN2;LAMC1;HYDIN;ITGB8;TEAD1;NEO1;DST;NCOA6;ABCC8;ANXA4;MYOF;NAV2;PARD3B;EVC;HEATR5A;KCNMA1;MYO3A;DOCK1;CD44;CHST3;DNAH5;DNAH6;RGPD6;FSTL1;SLC7A2;THSD4;ERICH5;CTNNA1;MAP4K4;NTRK2;LAMB1;CDC42BPB;CDC42BPA;TJP1;SPSB1;TTC6;SEC24D;CCL28*
FOXG1 human tf ARCHS4 coexpression	97/299	1.6161399679560872E-7	*MYT1L;ANKRD36;ZNF292;CTNND2;RORB;CDH4;CDH2;DPYSL5;AKT3;LUC7L;KIF21A;ZNF721;SOX6;SOX5;PPFIA2;RGS7;EPHA4;ANKRD36C;CXADR;MAGI2;RFX3;ANK2;EML1;GAREM1;IFT81;RUFY2;AKAP9;HECW1;KCNQ3;AMPH;ASTN1;GRIA1;CTTNBP2;TNKS;NTM;NEDD4L;BAZ2B;NREP;KALRN;PHF21B;MAP2;FUT9;ZNF704;PRDM16;SRGAP3;MPDZ;ZNF382;ST8SIA1;CORO2B;ST18;PTPRD;CCDC88A;DLG2;DAB1;NBEA;RNF182;TCF4;SLC44A5;ROBO2;TENM4;GRIK3;ELAVL4;BICD1;GLI3;SYNE2;GLI2;TTC28;NHSL1;LRRTM4;TRIM2;MAPK1IP1L;LRRC49;ATRX;IL17RD;ENAH;C12ORF4;MPPED1;SETBP1;LRRC7;MPPED2;CRB1;PHLPP1;STOX2;NCAM1;CTNNA2;PAK5;MYEF2;NTRK3;TTLL7;ATAT1;NELL2;MAB21L3;NFIA;APC;NFIB;KLHL7;TTC3*
ZMAT3 human tf ARCHS4 coexpression	96/299	3.2098623590077647E-7	*SEMA5A;APP;RNF11;TRIO;ANKRD33B;ECE1;LAMC1;ANTXR1;SLC8A1;GALNT10;LOXL2;ADAMTSL1;TRIM9;SPRED1;ADAMTS2;AKAP11;SH3PXD2A;RPS6KA2;NCS1;PSD3;SACS;VSTM4;TEAD1;PAMR1;EDIL3;ZNF124;KCNH1;ZHX3;ARHGEF12;DST;ZBTB38;ARHGEF17;MYOF;TMOD2;FNDC3B;AFAP1;FRMD6;EVC;COL4A2;HECW2;KCNMA1;CDC42EP3;CDH11;WDFY3;MXRA7;DOCK1;DGKI;CHST3;PAFAH1B1;MACF1;NFAT5;ROCK1;TWIST2;TMTC1;NLK;FSTL1;TRHDE;LTBP1;FBLN5;CTIF;ATXN1;CALD1;ARSJ;SNX25;MGAT5;CHN1;CLVS2;SPOCK1;KCNN3;MAP4;ATP9A;MAP4K*

			4;NTRK2;STARD13;EGLN3;CADM2;NEK6;RFTN1;NEK7;SAMD4A;CRIM1;SYT16;MYO5A;CYBRD1;LAMB1;GNG12;DCLK1;EXT1;ARHGAP31;COL5A1;FAT1;ITGBL1;SPIRE1;RGL1;SEC24D;FBN1
ZNF827 human tf ARCHS4 coexpression	96/299	3.2098623590077647E-7	DPP10;DRAXIN;MYT1L;CHD9;SYCP1;ZNF292;CTNND2;ZBTB20;MSI2;PPP1R9A;BICD1;TSPAN11;PTPRG;IGF1R;ZNF608;NHSL1;DACH1;CDH2;SCN11A;ADAMTSL3;ITGB8;KIF21A;STK32A;PPFIA2;CFAP44;MAGI1;USP49;TMEM178B;CACNA2D1;LRRC49;ATRX;HUNK;RFX3;FRMD4A;IL17RD;RGMB;ZDHHC17;MYT1;ARID1B;ENAH;SETBP1;PEAK1;AKAP9;SCG3;ASTN2;ZNF236;ARHGEF7;ZNF431;ANKRD17;TNKS;TSSC2;CHRNA7;NEDD4L;AGAP1;ILDR2;BAZ2B;NREP;MIPOL1;NPAS3;PHF21B;STOX2;PDCD6IPP2;MAP2;SNTG1;FUT9;MAP6;NCAM1;SRGAP3;JAM2;S100PBP;BPTF;ZNF541;ZNF462;FARP1;MYEF2;AUTS2;NTRK3;RANBP17;CADPS;SORBS2;HOOK3;DCLK1;PBX1;PTPRD;MAB21L3;CCDC88A;AGO3;NFIA;PPP2R2B;ASXL3;TTC3;RIMBP2;RNF182;TCF4;UBAP1L;TNRC6B
BAZ2B human tf ARCHS4 coexpression	96/299	3.2098623590077647E-7	DPP10;KDM5A;CCDC122;ZNF891;ANKRD36;CHD9;ZNF292;USP33;GADL1;ELAVL4;FRG1HP;ZBTB20;EFCAB6;PPP1R9A;MYLK3;SYNE2;UNC80;EPB41L4A;DACH1;ZSCAN30;PPIP5K2;CA5A;ADAMTSL3;GATAD1;SCAPER;POTEC;MAGI1;ANKRD36C;WSB1;LRRC49;KCNH8;MAGI2;ATRX;RFX3;VPS13B;FAM126B;SHISA9;ARID1B;SLC9A4;PCCA;SETBP1;IFT81;PEAK1;CATSPERG;RUFY2;AKAP9;WDPCP;WDFY3;ASTN2;ANKRD36B;SDCCAG8;ZNF431;FTO;KMT2E;CRB1;KCNE4;KMT2C;RGPD5;MIPOL1;STK3;FBXL20;FGD4;ORC4;FGGY;KIAA1328;MAP2;SNTG1;MAP6;ANKRD36BP2;AP4S1;CSMD3;SRGAP3;LRRC4C;ATP9B;BPTF;RFTN2;MBD5;MYEF2;ZNF382;RANBP17;ADAM32;ARHGAP28;DDHD1;ST18;DCDC1;MAPK10;MAB21L3;CCDC88A;FER;AGO3;NEDD4;TTC3;BRWD1;ASB3;FRYL;TNRC6B
NCOR1 human tf ARCHS4 coexpression	96/299	3.2098623590077647E-7	ZNF891;SETD2;ZBTB25;DOCK8;DPY19L2P2;GADL1;MSI2;SYNE2;TTC28;SYNE1;UNC80;AKAP13;ZFYVE26;NIPBL;HERC2;HERC1;CA5A;ADAMTSL3;ZNF407;ERC1;GTF2I;MAP3K5;USP8;MBNL1;ARHGEF12;RALGAPA1;RALGAPA2;VPS13C;VPS13D;TTC7B;ANK3;ASH1L;NBPF1;ARID1B;ZDHHC14;PEAK1;AKAP9;CNKSR3;WDPCP;WDFY3;BIRC6;ASTN2;UTRN;ANAPC1;FTO;KMT2E;ANKRD17;MACF1;NFAT5;KMT2C;ITPR2;ATP10B;BAZ2B;ZC3HAV1;LPP;MIPOL1;FBXL20;ATXN3;PCNX1;KIAA1328;NSD1;HECTD4;ANKRD36BP2;PCNT;ATP9B;BPTF;SPECC1;ARFGEF1;USP24;SPEN;ZNF462;CREBBP;RABGAP1L;MBD5;LRBA;MGA;DENND4C;YLPM1;ADAM32;LNPEP;HOOK3;DDHD1;PHC3;PARP15;MYO9A;ZZEF1;MED13L;ARHGAP32;AGO3;KANSL1;NEDD4;CDK12;BRWD1;ASB3;FRYL;TNRC6B
CUX2 human tf ARCHS4 coexpression	95/299	6.204120332203394E-7	SLC44A5;RERE;DOCK3;TENM4;FRMPD4;MYT1L;ANKRD36;CTNND2;SLC8A1;RPH3A;CDH4;ZNF608;GRM7;DPYSL5;TRIM2;MCF2L;NCS1;PHACTR3;KIF21A;DLGAP1;KIF21B;NEO1;PPFIA2;ANKRD36C;RBFOX1;MEF2C;KCNH5;TMEM178B;CACNA2D1;TMOD2;CYP4A11;ANK2;ANK3;MYRIP;SYN2;MAPK8IP1;MPPED1;SETBP1;LRRC7;SCN8A;ARHGEF7;NGEF;SHANK2;ASTN1;CTTNBP2;STXBP1;PLPPR5;ADAM22;KALRN;PRSS51;CTIF;SV2B;MAP2;FUT9;ZNF704;HECTD4;CHN1;PLXNA2;CLVS2;NCAM1;CTNNA2;SRGAP3;CACNG3;PAK5;ATP9A;GABBR2;ARNT2;LINGO1;ANKRD24;AUTS2;SYT1;CADM2;NTRK3;KIAA1549L;MYO5A;ATP2B2;HSPA12A;GRIN2B;CORO2B;DCLK1;PBX1;SNAP91;PTPRD;CYP2C9;DLG2;NBEA;APC;PPP2R2C;PPP2R2B;WNK2;ASXL3;FAT3;TCF4;SCN2A;APBA2
SOX9 human tf ARCHS4 coexpression	95/299	6.204120332203394E-7	APP;SPON1;TENM4;MEGF10;CTNND2;WWC1;ZBTB20;SLC35F1;PTPRK;SLC4A4;CELSR2;GLI3;CMIP;KIF15;SPRED2;GRM5;CDH2;TRIM2;TRPS1;ITGB8;KIF21A;PTGFRN;EPHB2;NEO1;SRGAP2B;ANKRD6;MAGI1;COL27A1;ADGRV1;KCNK10;TMEM178B;TCF12;RFX3;ADGRA3;SLC6A11;SEZ6L;SORCS2;PARD3B;EPN2;PYGO1;TOX3;ASPM;LMX1A

			;SETBP1;ADGRB3;PARD3;MPPED2;CHST3;CREB5;FTO;NFAT5;ADCYAP1R1;PHLPP1;NXN;NTM;AGAP1;ILDR2;ASAP2;NTN1;NPAS2;NPAS3;STOX2;RELN;ZNRF3;ZNF704;LRIG1;APBB2;SRGAP3;MPDZ;JAM2;MAP4K4;RFTN2;ARNT2;ZNF462;FARP1;NTRK2;DTNA;MYEF2;WSCD1;AUTS2;SGTB;PCDH8;HMGA2;BTBD9;CORO2B;PTK2;ARHGAP32;NFIA;FABP7;SMOC1;DLG5;FAT1;RNF182;SPIRE1;NECTIN4
ZNF510 human tf ARCHS4 coexpression	95/299	6.204120332203394E-7	DIDO1;LPGAT1;TRIO;ATP8A1;ZFYVE9;DOCK9;USP32;RASGRF2;CEP120;ZBTB21;HNRNPU;SYNE1;NIPBL;PTAR1;GRM5;HERC2;ZNF608;AKAP11;HERC1;RB1CC1;SACS;DIP2B;GTF2I;MED1;WDHD1;CD96;KLF12;ARHGEF12;DST;RALGAPA1;PRKCE;VPS13C;TMOD2;VPS13D;VPS13B;ANK2;ASH1L;SFPQ;RABEP1;CSDE1;WDFY3;BIRC6;TNIK;RAPGEF5;UTRN;DGKI;ANAPC1;PAFAH1B1;ANKRD17;MACF1;DDX6;MTPN;KMT2C;ADAM22;HTT;ACACA;TM9SF3;PCNX1;SCAF8;BTAF1;HECTD1;ZNF704;NSD1;HECTD4;GTF2IP4;HIVEP2;ATP9A;RANBP2;ARFGEF1;USP24;SPEN;KDM4C;LRBA;TRAPPC10;MGA;ERBIN;MYO5A;LNPEP;DNAJC13;ESRRG;AP2B1;SMARCA2;ZZEF1;MTOR;MED13L;KTN1;APC;CAMK4;SP3;BCL2;FAT3;STRN;CDK12;BRWD1;TAF3
MGA human tf ARCHS4 coexpression	95/299	6.204120332203394E-7	LIN54;ZNF891;PATJ;SETD2;SMG1P2;LTN1;ZBTB20;SYNE2;OSBPL10;IGF1R;SYNE1;AKAP13;NIPBL;PTAR1;HERC2;C16ORF72;HERC1;ADAMTSL3;PPP6R3;SACS;ERC1;ZNF121;USP8;AQR;ARHGEF12;ESCO1;DST;RALGAPA1;VPS13C;VPS13D;VPS13B;ASH1L;ARID1B;NCOR1;PEAK1;AKAP9;WDPCP;WDFY3;PIK3C3;ASTN2;UTRN;ANAPC1;FTO;ANKRD17;MACF1;DDX6;NFAT5;RABGAP1;KMT2C;IREB2;TULP4;RGPD5;ITPR2;BAZ2B;THADA;UBR1;MIPOL1;PCNX1;SCAF8;BTAF1;KIAA1328;HECTD1;NSD1;HIVEP1;TRPM7;CEP192;NUP43;MPDZ;ATP9B;BPTF;RANBP2;SPECC1;ARFGEF1;USP24;MBD5;LRBA;DENND4C;ERBIN;ADAM32;LNPEP;HOOK3;PHC3;MYO9A;PTK2;MED13L;TJP1;AGO3;KANSL1;AGO1;NEDD4;CDK12;BRWD1;ASB3;ZNF850;TNRC6B
NCOA2 human tf ARCHS4 coexpression	95/299	6.204120332203394E-7	ITSN2;RERE;DIDO1;RNF11;LPGAT1;ATP8A1;USP32;DOCK8;EFCAB14;PTPRJ;FRY;BACH1;SIPA1L3;LYST;SYNE2;SYNE1;AKAP13;EFR3A;GLT1D1;NIPBL;C16ORF72;HERC1;ZNF407;ADGRE3;DIP2B;JAK2;IL6R;MAP3K5;MBNL1;ARHGEF12;RALGAPA1;RALGAPA2;VPS13C;VPS13D;ARAP2;VPS13B;ASH1L;IL17RA;PARP8;DPYD;SPOPL;WDFY3;BIRC6;UTRN;DOCK2;MCTP2;KDM7A;NOTCH2;MACF1;DDX6;NFAT5;MTMR3;WDR26;ABHD2;KMT2C;RNF38;HTT;ITPR2;BAZ2A;CSF2RB;IQGAP1;PCNX1;SCAF8;HECTD1;NSD1;HECTD4;HIVEP2;MARK2;LYN;RANBP2;ARFGEF1;USP24;CREBBP;MGAM;SEMA4D;IQSEC1;PCGF5;LRBA;TRAPPC10;DENND4C;ERBIN;LNPEP;DNAJC13;HIPK1;ARHGAP26;SMARCA2;ZZEF1;MED13L;CYLD;SNRK;DMXL2;SP3;STRN;CDK12;FRYL
FOXP2 human tf ARCHS4 coexpression	94/299	1.1912051206656543E-6	DPP10;ZNF891;DRAXIN;MYT1L;TUSC3;CELF4;ELAVL4;ZBTB20;GRIK1;GRIK2;EFCAB6;PPP1R9A;TSPAN11;PTPRG;PCMT1;RIMS1;DACH1;ZNF606;ZNF280B;DNER;KIF21A;RALGPS1;ANKS1B;ZNF684;GUCY1A2;CXADR;USP49;TMEM178B;DCC;LRRC49;EBF1;MTUS2;ANK3;MYT1;ARID1B;PGM2L1;SHISA6;ADGRB3;DOK5;AKAP9;AMPH;ALG10B;CDH18;KHDRBS2;RTN1;TSHZ3;CHRNA7;TSHZ2;PLPPR5;TULP4;ZNF66;KLHL13;NREP;NYAP2;FBXL20;ADAM29;DPP6;PDCD6IPP2;GNG2;MAP2;SMIM11B;ZMAT4;MAP6;CAMTA1;ZKSCAN5;NCAM1;CTNNA2;CSMD3;PAK3;MORC2;VAT1L;CLVS1;ZNF382;CNTN5;AUTS2;NEGR1;ST8SIA1;RANBP17;CADPS;PBX3;KLHL1;PBX1;ENOX1;ATAT1;PTPRD;CCDC88A;RALYL;FER;ASXL3;TTC3;ZNF74;BRWD1;LRP12;LHX9
NFIB	94/299	1.1912051206	SLC44A5;CNTNAP3;CNTNAP2;ATP8A2;USP31;MYT1L;GRIK3;UBE3A;GRIK2;BICD1;CDH7;SRGAP2C;NHSL1;ADAM

human tf ARCHS4 coexpression		656543E-6	*TS3;LRRTM4;RB1CC1;AKT3;TMEM108;PHACTR3;HYDIN2;THSD7A;DNM1L;SRGAP2B;SOX5;GARNL3;PPFIA2;RGS7;GUCY1A2;EPHA7;MEF2C;CXADR;LIMCH1;ZNF160;ATRX;KAZN;PRKCA;EML1;TIAM2;SETBP1;ADGRB3;LRRC7;AKAP9;MPPED2;MTF2;KCNQ3;RAPGEF2;ZFPM2;ZNF234;SHANK2;ZNF431;GRIA1;SLC24A2;INO80D;CTTNBP2;TNKS;TULP4;NEDD4L;EFNA5;NOL4;CACNA1E;MAP2;PLXNA2;ANKRD20A5P;PAK3;OPCML;CA10;MYEF2;BCL11B;ARPP21;BCL11A;INSR;NTRK3;SLC4A10;SORBS2;SYT14;PPP2R3A;CORO2B;ST18;SNAP91;SGSM1;CCDC88A;NELL1;DLG2;DAB1;SLCO3A1;NBEA;ASXL3;TTC3;TCF4;ASB4;FAT4;FGF12;LRP12;UNC79*
ZBTB38 human tf ARCHS4 coexpression	94/299	1.1912051206656543E-6	*BCAR3;LPGAT1;TRIO;ANKRD33B;DOCK9;EFCAB14;PTPRK;LAMC1;IKZF2;LIMD1;GALNT10;SYNE1;EPS8;AKAP13;EFR3A;AKAP11;SH3PXD2A;KIF13A;C3ORF52;SAMD12;TEAD1;CAST;MBNL1;MBNL2;ZHX3;ARHGEF12;DST;IL1R1;ANXA4;VPS13C;MYOF;VPS13D;FNDC3B;ANO6;ASH1L;KIAA1217;FRMD6;EVC;TRAF3;KCNMA1;CMPK1;ITGA8;MET;MACF1;NFAT5;ROCK2;ARHGEF28;SEL1L;TGFA;TMTC1;RRBP1;IQGAP1;LPP;TM9SF3;ATXN1;IGLV2-14;ARSJ;SNX25;MGAT5;BTLA;STK38L;SPOCK1;SNX9;HIVEP2;MAP4;STARD13;PCGF5;STAT1;NEK6;RFTN1;NEK7;SAMD4A;ERBIN;CRIM1;CYBRD1;CABLES1;LNPEP;DNAJC13;LAMB1;SYNJ2;GNG12;SMARCA2;ELL2;ZZEF1;EXT1;MYO1D;MYO1E;DPY19L1;SLMAP;FAT1;BCL2;ATP13A3;SEC24D;FBN1*
ZNF532 human tf ARCHS4 coexpression	94/299	1.1912051206656543E-6	*GABRB3;DRAXIN;TENM3;HIP1;TENM4;MEGF10;CTNND2;GRIK3;ELAVL4;PSIP1;SOGA1;CELSR2;BICD1;PTPRG;GLI2;ROBO1;IGF1R;ZNF608;CDH2;DPYSL5;TRIM2;AKT3;RBPMS2;DIP2C;LARGE1;NEO1;MAGI1;WSB1;CXADR;SNRPN;TMEM178B;DCC;CACNA2D1;RFX3;TET1;ANK2;FRMD4A;NAV2;IL17RD;ENAH;SETBP1;ADGRB3;LRRC7;ASTN1;HDAC2;CTTNBP2;TNKS;NXN;AGAP1;ILDR2;AKAP6;NREP;KALRN;NPAS3;FAM171A1;STOX2;CECR2;APELA;MAP2;ZNF704;LRIG1;NCAM1;IGF2BP3;CTNNA2;SRGAP3;CSMD2;ZNF423;EXTL3;MPDZ;WASF3;ARNT2;ZNF462;FARP1;MYEF2;WSCD1;AUTS2;NTRK3;YLPM1;PTPN13;CORO2B;DCLK1;PBX1;PTK2;TJP1;PTPRD;ZNF738;WNK2;KLHL7;DLG5;TTC3;FAT3;TCF4;FAT4;ADGRL2*
ZNF536 human tf ARCHS4 coexpression	94/299	1.1912051206656543E-6	*MYT1L;DGKB;CTNND2;PTPRO;GRIK3;ELAVL4;SLC6A1;GPHN;ROBO1;GRM3;DPYSL5;TRIM2;DNER;GDAP1L1;PSD3;PHACTR3;KIF21A;DLGAP1;TUBB2BP1;SYBU;WSB1;KCND3;TMEM178A;LRRC49;TMOD2;RFX3;EBF3;ANK2;ANK3;DNM3;GAP43;NRG3;SETBP1;ADGRB3;LRRC7;RUFY2;HECW1;MPPED2;AMPH;RAPGEF5;TOX;ASTN1;GRIA1;RTN1;STXBP1;PDE1A;ATL1;BTF3L4;NRXN3;PIK3R3;AGAP1;ILDR2;AKAP6;NREP;KALRN;NOL4;HDAC9;STOX2;MAPK8;GNG2;MAP2;CLVS2;APBB2;NCAM1;CTNNA2;SRGAP3;PAK5;GRIA4;GABRA2;ATF7IP;ARNT2;ZNF462;MYEF2;AUTS2;SYT1;PCDH9;PCDH8;LSAMP;DCLK1;PBX1;ENOX1;TTLL7;ATAT1;PTPRD;MAPK10;NELL2;CCDC88A;LRFN5;APC;KLHL7;TTC3;FAT3;TCF4;APBA2*
SOX4 human tf ARCHS4 coexpression	93/299	2.268655035672371E-6	*SLC44A5;ROBO2;ZNF891;CNTNAP2;DRAXIN;MYT1L;ZNF292;CLCN3P1;PTPRO;ELAVL4;SLC35F1;GRIK2;GPHN;SRGAP2C;NHSL1;DPYSL5;LRRTM4;GDAP1L1;SUMO2;TMEM108;ITGB8;KIF21A;SOX6;SRGAP2B;MAGI1;MAPK1IP1L;EPHA7;WSB1;CXADR;LRRC49;TCF12;ATRX;ANK2;ENAH;TOX3;SETBP1;IFT81;LRRC7;RUFY2;AKAP9;MPPED2;ZNF234;GRIA1;CRB1;HDAC2;RTN1;CTTNBP2;ATL1;CHRNA7;BTF3L4;NRXN3;NEDD4L;PIK3R3;BAZ2B;NREP;FGD4;GNG2;HECTD2;MAP2;SNTG1;ANKRD10;SRGAP3;PAK3;JAM2;PAK5;GRIA4;RFTN2;ZNF462;CADM1;ZNF382;AUTS2;BCL11A;SIAH3;INSR;RANBP17;CORO2B;ST18;PBX1;ATAT1;MAPK10;CCDC88A;NFIA;NBEA;APC;NFIB;ZNF738;KLHL7;ASXL3;TTC3;ZNF536;TCF4;SSBP2;LRP12*

ZFHX3 human tf ARCHS4 coexpression	93/299	2.268655035672371E-6	*RYR2;PATJ;DOCK4;TRIO;DOCK9;CELF4;KIAA1671;SOGA1;SIPA1L3;RYR3;SYNE1;RPTOR;C4ORF50;UNC80;CDH5;HERC2;RPS6KA5;HERC1;MPRIP;ADAMTSL3;MCF2L;PSD3;GAST;ERC1;PKNOX2;GUCY1A2;VWFP1;ARHGEF12;DST;VPS13D;ABCC9;ANK3;ASH1L;URB1;SHISA9;HSPG2;KLF15;TANC2;TANC1;CLIP1;GAP43;NAV3;PEAK1;HECW2;WDPCP;WDFY3;BIRC6;RAPGEF5;PPARA;DGKI;FTO;NOTCH2;MACF1;NFAT5;KMT2C;AGAP1;CACNA1C;CACNA1E;LPP;PDZD2;KIAA1328;HECTD4;PLXNA2;GSG1L;ABL2;FLNB;NCAM1;CSMD1;ATP9B;ATP9A;ARNT2;FARP1;ANKRD30BL;NTRK3;MICAL3;NSG1;HOOK3;CDC42BPB;GRIN2B;MYO9A;HS3ST4;MEIS2;PBX1;ZZEF1;TJP1;ARHGAP32;GNAL;PDE10A;AGO3;WNK2;NEDD4;FAT3;FAT4*
ZFPM2 human tf ARCHS4 coexpression	93/299	2.268655035672371E-6	*SLC44A5;OSCP1;CCDC122;CNTNAP2;MYT1L;DPY19L2P2;DIRAS2;LTN1;GADL1;TMEM182;GRIK2;EFCAB6;MYLK3;GPHN;RPH3A;UNC80;SYNPR;ADAMTS3;LRRTM4;TRIM2;ADAMTSL3;ZNF407;AKT3;PSD3;DPF3;DLGAP1;NOS1;ERC1;SOX6;DIP2C;SRGAP2B;PPFIA2;POTEC;MAPK1IP1L;UNC13C;RBFOX1;KCND3;CACNA2D1;TMOD2;TTC7B;KAZN;ANK2;SHISA9;FAM126A;SYN2;CNKSR2;SETBP1;LRRC7;HECW1;KCNQ3;PLCB1;SHANK2;GRIA1;SHC4;NECAB1;TPH2;NLGN1;STXBP1;NDRG2;KALRN;MIPOL1;STOX2;GRIN2A;HECTD1;SV2B;MAP2;CLVS2;RFPL3S;OPCML;SLC14A2;SPHKAP;GABRA6;SYT1;CADM2;ST8SIA1;INSR;NTRK3;KIAA1549L;ADAM32;PDE4DIP;ATP2B2;MYO9A;SNAP91;TTLL7;FER;NFIA;NFIB;NEDD4;TTC3;SPIRE1;SCN2A;LRP12;CPEB4*
ZNF385D human tf ARCHS4 coexpression	93/299	2.268655035672371E-6	*ROBO2;DPP10;CCDC122;ZNF891;ATP8A2;MYT1L;ANKRD36;C2ORF88;CELF4;SLC8A1;MYLK3;GRM1;RIMS2;UNC80;EPB41L4A;GRM5;GRM7;CA5A;TRIM2;ADAMTSL3;PHACTR3;DLGAP1;ANKS1B;SOX5;GARNL3;KCNH5;LRRC49;TMOD2;MAGI2;TTC7B;UBE2QL1;ANK2;ABCC9;ANK3;SHISA9;AJAP1;CNKSR2;FRMD3;DNM3;LRRC7;HECW1;ASTN2;PLCB1;ZNF397;SLC24A2;TPH2;PPM1L;KCNE4;ATL1;NTM;KALRN;CACNA1E;MIPOL1;GRIN2A;DPP6;KIAA1328;FLRT2;PGBD5;MAP2;FUT9;CHN1;CLVS2;CAMTA1;NCAM1;CSMD3;PAK3;ASIC2;MORC1;CSMD1;GABBR2;RIC3;ZNF382;SYT1;NEGR1;CADM2;NTRK3;LSAMP;SLC4A10;SYT16;ADAM32;GRIN2B;DCLK1;PBX1;SNAP91;TTLL7;CCDC88A;GFI1B;DLG2;PDE10A;PPP2R2B;GNAQ;SCN2A;ASB3*
AFF1 human tf ARCHS4 coexpression	93/299	2.268655035672371E-6	*ITSN2;RERE;DIDO1;ATP8A1;KDM1B;DOCK9;DOCK8;CHD6;KIAA1671;EFCAB14;PTPRJ;LIMD1;SIPA1L3;YBX3;AKAP13;FYCO1;NIPBL;HERC1;ZNF407;KIF13A;LRRFIP1;ARFGEF3;CAST;MBNL1;ARHGEF12;ITGA4;RALGAPA2;VPS13C;VPS13D;VPS13B;ASH1L;RUNX1;KIAA1217;BMP2K;WDFY3;BIRC6;UTRN;DOCK2;MCTP2;KDM7A;FKBP5;ANKRD17;MACF1;NFAT5;CASZ1;MTMR3;WDR26;ABHD2;KMT2C;DNAH5;PRUNE2;HTT;ITPR2;BAZ2A;IQGAP1;AMBRA1;ACACA;PCNX1;SCAF8;LARP1;HECTD1;NSD1;HECTD4;FLNB;HIVEP2;PCNT;MARK2;RANBP2;ARFGEF1;USP24;SPEN;CREBBP;LRBA;TRAPPC10;DENND4C;ERBIN;XPO7;LNPEP;MYO9B;DNAJC13;FOXN3;HIPK1;SMARCA2;FLI1;ZZEF1;MTOR;MED13L;DIAPH1;SP3;BCL2;STRN;ESYT2;CDK12*
ETV1 human tf ARCHS4 coexpression	92/299	4.3437660445467605E-6	*GABRB3;PPP1R17;HIP1;GALNT13;DOCK3;MTCL1;MEGF11;CTNND2;DIRAS2;RASGRF1;LDLRAD3;KNDC1;RORB;PCMTD2;RPH3A;SRGAP2C;TRIM9;TRIM2;MCF2L;DNER;PSD3;TNR;ITGB8;DLGAP1;SRGAP2B;MAGI1;CADPS2;RBFOX3;KCND2;KCND3;TMEM178A;DCC;SEMA6D;TCF12;TMOD2;RFX3;ANK2;SEZ6L;SYN2;MAPK8IP1;EPN2;CNKSR2;TIAM1;TOX3;ADGRB3;ADGRB1;SCG3;ASTN1;SAMD5;ADCYAP1R1;NLGN1;PHLPP1;STXBP1;BTF3L4;ADAM22;ILDR2;NDRG2;DPP6;SV2B;MAP2;SNTG1;FUT9;LRIG1;CLVS2;SPOCK1;GPR158;SLC15A5;WASF3;GRIA4;BBS2;RFTN2;ARNT2;LINGO1;PTPRN2;DTNA;MYEF2;GABRA6;WSCD1;SYT1;CADM2;ATP2B2;KLHL4;S100B;CORO2B;SNAP91;IGS*

			F11;MAPK10;APC;SMOC1;CNTN1;SPIRE1;APBA2
RFX3 human tf ARCHS4 coexpression	92/299	4.3437660445467605E-6	*FANK1;SPAG16;OSCP1;ZNF292;ELAVL4;EFCAB6;TTC29;TRIM9;ZNF608;DACH1;CDH2;DPYSL5;ZNF606;LRRTM4;TRIM2;SNAPC3;HYDIN;KIF21A;EPHB1;CFAP44;MAGI1;EPHA4;WSB1;CXADR;ADGRV1;DCC;LRRC49;TCF12;ANK2;IL17RD;SRP9;TOX3;SETBP1;IFT81;LRRC7;ARMC3;RUFY2;CDHR3;ASTN1;CFAP61;GRIA1;DNAH3;HDAC2;TNKS;DNAH6;GREB1L;ILDR2;AKAP6;DNAH9;BAZ2B;NREP;NOL4;NPAS3;STOX2;GNG2;HECTD2;MAP2;FUT9;TMEM67;MAP6;TSPAN3;CTNNA2;CSMD3;SRGAP3;SLIT2;PAK3;ZNF462;NTRK2;DNAH10;MYEF2;AUTS2;SPAG6;CFAP70;RABL2A;VWA3B;DCLK1;ATAT1;PTPRD;TMEM232;MAPK10;NELL2;CCDC88A;RSPH1;FAM183A;APC;ZNF738;FABP7;TTC3;FAT3;C6ORF118;SSBP2;PPIL6*
ZNF540 human tf ARCHS4 coexpression	92/299	4.3437660445467605E-6	*PCSK2;ZNF891;ZNRF2P2;MYT1L;DGKB;ZNF292;ELAVL4;FRG1HP;CDH8;UNC80;SCGN;DPYSL5;TRIM2;GDAP1L1;RNF17;TRAPPC11;KIF21A;RALGPS1;ANKS1B;RGS7;ZNF287;EPHA7;TANGO6;WSB1;TET1P1;LRRC49;NGDN;TMOD2;MAGI2;RFX3;SEZ6L;GABRG2;ITFG1;PJA2;ADGRB3;LRRC7;TRAPPC6B;RUFY2;HECW1;SCG5;SCG3;MAPRE2;ZNF675;CDH18;ASTN1;ZNF112;GRIA1;STX12;MAGEL2;RTN1;STOML1;STXBP1;ATL1;NRXN3;RGPD5;NREP;NYAP2;NOL4;MTMR7;RIC8B;GNG2;HECTD2;MAP2;SNTG1;SPOCK3;CTNNA2;CSMD3;SRGAP3;PAK3;GRIA4;CCDC178;PTPRN2;CADM1;ZNF382;CADM2;LSAMP;SLC4A10;SYT16;MEIS2;SGCZ;ATAT1;MAPK10;LRFN5;APC;ASXL3;TTC3;CNTN1;PARGP1;CNTN4;SCN2A;FSIP1;SSBP2*
ZBTB37 human tf ARCHS4 coexpression	92/299	4.3437660445467605E-6	*CCDC122;ZNF891;ANKRD36;ZNF292;DOCK8;USP33;GADL1;TMEM182;FRY;LYST;MYLK3;SYNE2;SYNE1;AKAP13;NIPBL;PTAR1;RPS6KA5;C16ORF72;HERC1;ZSCAN30;CA5A;ADAMTSL3;KYNU;LONP2;POTEC;RALGAPA1;RALGAPA2;VPS13C;VPS13D;TTC7B;VPS13B;ABCC9;ASH1L;SHISA9;ARID1B;PARP8;PHF20L1;PEAK1;AKAP9;CNKSR3;WDPCP;WDFY3;BIRC6;PIK3C3;ASTN2;UTRN;MCTP2;KDM7A;FTO;MACF1;TPH2;DDX6;NFAT5;CATSPER2;KMT2C;RGPD6;RGPD5;BAZ2B;LPP;MIPOL1;FBXL20;ATXN3;ORC4;PCNX1;KIAA1328;ANKRD36BP2;TRPM7;ATP9B;SCAI;BPTF;RABGAP1L;MBD5;ZNF382;INSR;MGA;ERBIN;ADAM32;LNPEP;ARHGAP28;HOOK3;DDHD1;PHC3;PARP15;CYLD;AGO3;KANSL1;NEDD4;DMXL2;BRWD1;PTPN4;ASB3;TNRC6B*
PAX8 human tf ARCHS4 coexpression	91/299	8.452643855222726E-6	*COL18A1;SPON1;PATJ;PPP1R13B;ATP8A1;DOCK9;CPQ;KIAA1671;ZBTB20;LAMC1;SIPA1L3;SYNE2;PTPRG;SYNE1;AKAP13;DEPTOR;MPRIP;MECOM;ENPP3;VSTM4;ADAMTS9;GTF2I;CUBN;ZHX3;ARHGEF12;RALGAPA2;ARHGEF17;COL23A1;VPS13D;ARAP2;WDR72;KIAA1217;TANC1;GOLGA8B;POR;FNDC1;COL4A3;WDFY3;BIRC6;PKHD1L1;PLA2R1;UTRN;GPRC5C;DOCK1;MET;DGKI;MATN2;FKBP5;ITGA9;YAP1;NOTCH2;MACF1;NFAT5;RBM47;SEMA3D;SDC2;ARHGEF28;GLIS3;TGFA;RRBP1;LRP2;PRDM11;RAP1GAP;THSD4;HIRA;HECTD1;FLNB;APOL1;KCNJ1;ZBTB7C;FARP1;STARD13;HOMER2;LRBA;ZBTB16;SLC12A1;ZNF804B;FAM189A2;CYBRD1;SORBS2;LAMB1;MYO9A;AIF1L;PTPRB;ARHGAP31;MYO5B;LPCAT2;FAT4;GGT3P;ATP13A3;ADGRL2*
ZMAT4 human tf ARCHS4 coexpression	91/299	8.452643855222726E-6	*GABRB3;DPP10;DOCK3;ATP8A1;FRMPD4;MYT1L;DGKB;CTNND2;OTUD7A;CELF4;ZBTB20;GRIK1;C4ORF50;GRM5;CDH2;PEG10;TRIM2;DNER;SUMO2;KIF21A;DLGAP1;KCNH1;GUCY1A2;POU1F1;CXADR;SNRPN;TMEM178B;TMEM178A;KCNH8;TMOD2;ANK2;PGM2L1;FOXP2;PJA2;AJAP1;IFT43;ADGRB3;DOK5;SCN8A;HECW1;TNIK;DGKI;CDH18;MAGEL2;KHDRBS2;NLGN1;HDAC2;RTN1;PLPPR5;BTF3L4;RNF8;KLHL13;NREP;MTMR7;CECR2;GRIN2A;DPP6;MAP2;FUT9;CLVS2;CAMTA1;DPH6;NCAM1;STXBP6;PAK3;ATP9A;GRIA4;ARNT2;ZNF462;SYT1;PCDH9;CADM2;ATRNL1;NTRK3;PCDH7;PBX3;LSAMP;KLHL1;ESRRG;DCLK1;TT*

			LL7;PTPRD;IGSF11;RALYL;ZNF738;PPP2R2B;CAMK4;SCN2A;COPS8;TAF3;ADGRL2
KLF12 human tf ARCHS4 coexpression	90/299	1.5355064310228337E-5	*CYFIP2;OXNAD1;ABCD2;MAML2;DOCK8;CELF2;CEP120;RORA;LYST;ETS1;SYNE2;SYNE1;DOCK10;AKAP13;HERC1;TRIM2;SACS;UBASH3A;KIF21B;TRIM23;CD96;MBNL1;PRKCH;CNOT6L;ITGA4;CACNA2D1;VPS13C;TCF12;ARAP2;RFX3;VPS13B;ANK2;ASH1L;TC2N;IPCEF1;ZDHHC17;ZFP90;PARP8;PHF20L1;ITPKB;ARL4C;GOLGA8B;SETBP1;DPYD;ZNF780B;BIRC6;ZNF831;TNIK;UTRN;DOCK2;KDM7A;KMT2E;MACF1;DDX6;TNKS;KMT2C;NLRC5;BAZ2B;NREP;RASGRP1;CACNA1I;STOX2;PCNX1;GNG2;MAP2;NCAM1;FYN;HIVEP2;SCAI;CREBBP;BCL11B;SEMA4D;TRAPPC10;ERBIN;MYO5A;LNPEP;DCLK1;CCDC88A;CYLD;SNRK;APC;KANSL1;CAMK4;TTC3;BCL2;CCSER2;TCF4;BRWD1;PTPN4;FRYL*
SOX11 human tf ARCHS4 coexpression	90/299	1.5355064310228337E-5	*SLC44A5;DRAXIN;MYT1L;ANKRD36;ZNF292;PTPRO;ELAVL4;MSANTD4;GPHN;NHSL1;CDH2;DPYSL5;TRIM2;SNAPC3;GDAP1L1;LUC7L;KIF21A;MAPK1IP1L;EPHA4;WSB1;CXADR;DCC;LRRC49;MAGI2;ATRX;RFX3;MYT1;SRP9;ENAH;SETBP1;IFT81;LRRC7;RUFY2;AKAP9;MPPED2;ZNF675;KMT2E;GRIA1;CRB1;HDAC2;RTN1;STAU2;ATL1;NTM;BTF3L4;NRXN3;NEDD4L;PIK3R3;AKAP6;BAZ2B;NREP;NOL4;PHF21B;FGD4;GNG2;MAP2;ZNF627;MAP6;NCAM1;CTNNA2;SRGAP3;PAK3;PAK5;CLVS1;ATF7IP;RIC3;ZNF462;MON2;MYEF2;CADM1;ZNF382;AUTS2;BCL11A;ST18;ATAT1;MAPK10;NELL2;CCDC88A;NFIA;NFIB;ZNF738;AGO1;KLHL7;ASXL3;TTC3;YPEL1;ZNF536;TCF4;SSBP2;LRP12*
POU3F3 human tf ARCHS4 coexpression	90/299	1.5355064310228337E-5	*DRAXIN;TENM4;MYT1L;CTNND2;ELAVL4;ZBTB20;CELSR2;TSPAN11;ROBO1;TRIM9;CDH4;ZNF608;CDH2;DPYSL5;PEG10;TRIM2;AKT3;DNER;GDAP1L1;KIF21A;KIF21B;ERC2;PPFIA2;TMEM178B;TMOD2;RFX3;KAZN;ANK2;FRMD4A;ANK3;IL17RD;SORCS3;MYT1;MPPED1;SETBP1;LRRC7;ADGRB1;TNIK;ASTN1;GRIA1;STAU2;AGAP1;ILDR2;NREP;KALRN;NPAS3;PHF21B;FAM171A1;STOX2;GTF2IP1;GNG2;MAP2;CDH20;FUT9;ZNF704;PLXNA2;MAP6;CAMTA1;NCAM1;CTNNA2;SRGAP3;ZNF423;PAK5;ATP9A;ARNT2;NTRK2;NDFIP1;MYEF2;WSCD1;AUTS2;NTRK3;PCDH8;GRIN2B;CORO2B;DCLK1;TTLL7;ATAT1;PTPRD;MAPK10;MAB21L3;CCDC88A;DLG2;APC;SMOC1;TTC3;ZNF536;FAT3;TCF4;RGS12;APBA2*
TBX20 human tf ARCHS4 coexpression	90/299	1.5355064310228337E-5	*FHOD3;COL18A1;RYR2;MYOM1;PPP1R13B;DPY19L2P1;FHL2;LDB3;LAMC1;SIPA1L2;MYOM2;SLC8A1;MYLK3;AKAP13;ARHGAP42;CDH2;KIF13A;MYO18B;DPF3;PGM5;TEAD1;ADAMTS9;PDK1;UNC45B;MLIP;MYOCD;CERS6;ARHGEF17;TPM1;MTUS2;TOM1L2;TANC1;VCAN;COL4A2;COL21A1;SLC27A6;ALPK3;MXRA7;ALPK2;TLN2;PPP1R12B;DGKI;MATN2;ITGA9;YAP1;FBN2;PRKAA2;SMPX;TMTC1;CACNA1C;LTBP1;THSD4;ABLIM1;INPP5A;MGAT5;TNNI1;CTNNA3;SLIT3;MAP4;B4GALNT3;CCDC141;PDLIM5;MPDZ;CORIN;SVIL;SPHKAP;CNTN5;NEBL;PCDH7;NEK7;EXOC6B;MICAL3;SAMD4A;FAM189A2;PDE4DIP;SORBS2;PXDNL;LAMB1;MCC;PTPN13;BMP5;TJP1;PLCXD3;BMP2;COL5A1;DLC1;SLMAP;PDE3A;STRN;TACC2*
ZNF292 human tf ARCHS4 coexpression	90/299	1.5355064310228337E-5	*SLC44A5;TCERG1;CCDC122;ZNF891;ZBTB25;ANKRD36;CHD9;ELAVL4;EFCAB6;PPP1R9A;SYNE2;GRM7;ZSCAN30;PPIP5K2;TRIM2;ADAMTSL3;KIF21A;ANKRD36C;WSB1;CXADR;HFM1;DCC;LRRC49;VPS13C;MAGI2;ATRX;RFX2;RFX3;VPS13B;FAM126B;ARID1B;SETBP1;IFT81;LRRC7;RUFY2;HECW1;MPPED2;WDPCP;PIK3C3;ASTN2;ANKRD36B;ZNF431;KMT2E;HDAC2;CHRNA7;KMT2C;RGPD6;RGPD5;BAZ2B;NREP;CACNA1E;MTMR7;MIPOL1;ORC4;MAPK8;GNG2;KIAA1328;MAP2;SNTG1;ANKRD36BP2;SRGAP3;CSMD2;PAK3;ATP9B;SCAI;PAK5;BPTF;RIC3;ZNF462;MBD5;MYEF2;ZNF382;ERCC6L2;ADAM32;DDHD1;DCDC1;MAB21L3;CCDC88A;AGO3;NBEA;KLHL7;NEDD4;ASXL3;TTC3*

			;TCF4;BRWD1;SSBP2;ASB3;FRYL;TNRC6B
ZFP28 human tf ARCHS4 coexpression	90/299	1.5355064310228337E-5	CYFIP2;GABRB3;SPAG16;POMT2;DOCK3;PLEKHB2;FRMPD4;ANKRD36;CELF4;ZBTB20;ZNF44;HTR2A;PPP1R9A;UNC80;TRIM9;DACH1;GRB14;DPYSL5;PEG10;AKT3;HYDIN2;BBS9;DNM1L;TRIM23;RGS7;GUSBP1;EPHA6;RALGAPA1;ABCC8;PDE4D;PRKCA;ANK2;ABCC9;SCAMP1;ARID1B;FOXP2;PJA2;ZFP90;CACNB2;LRRC7;RRAGD;AKAP9;MYO3A;SCG5;SCG3;AMPH;PRKD1;CNTNAP5;SAMD5;STX12;PPM1L;TNKS;NTM;NOL4;NALCN;SLC7A2;MTMR7;SDCBP;GRIN2A;MAP2;FUT9;ZMAT4;TYW1;NCAM1;PTCHD4;CSMD2;ATP9A;OPCML;PTPRN2;NTRK3;LSAMP;FAM189A2;SLC4A10;MYO5A;CDC42BPA;GRIN2B;MEIS2;PBX1;PTK2;PTPRD;TMEM232;FER;NBEA;APC;GSTA3;RCAN2;TTC3;BRWD1;COPS8;CPEB4
HIVEP2 human tf ARCHS4 coexpression	89/299	2.6349441037109094E-5	CYFIP2;ITSN2;MAML2;ATP8A1;FRMPD4;DOCK9;DOCK8;RASGRF2;RASGRF1;PTPRJ;RORA;ETS1;SYNE1;DOCK10;RPH3A;AKAP13;FAM102A;AKAP11;HERC1;MPRIP;PSD3;DLGAP1;MAP3K5;KCNH1;ARHGEF11;RBFOX1;MBNL1;MEF2C;PRKCH;PRKCB;PRKCE;VPS13C;TMOD2;VPS13D;ARAP2;ANK2;ASH1L;IPCEF1;SYN2;ITPKB;PACS1;MADD;KCNQ5;WDFY3;ZNF831;RAPGEF5;UTRN;DOCK2;NGEF;KDM7A;MACF1;NFAT5;ADAM22;SLC1A2;NLRC5;HTT;IQGAP1;RASGRP1;KIAA0513;CACNA1I;GRIN2A;PCNX1;ABLIM1;ATXN1;SV2B;HECTD4;CHN1;CREBBP;BCL11B;SEMA4D;IQSEC1;PLEKHA2;PCGF5;ERBIN;MYO5A;PDE4DIP;LNPEP;MYO9B;ATP2B2;ARHGAP26;SMARCA2;GRIN2B;ZZEF1;CYLD;SYNJ1;PPP2R2C;CAMK4;BCL2;STRN
ZHX1 human tf ARCHS4 coexpression	89/299	2.6349441037109094E-5	PCSK2;LIN54;ERO1B;ABCD3;ZFYVE9;CPQ;USP32;LAMC3;POGK;RAB22A;SCGN;EFR3A;AKAP11;PPIP5K1;TRIM2;RB1CC1;PSD3;ANKRD31;LONP2;BCAP29;KIF21A;DIP2B;SOX6;DIP2C;SCAPER;PCMTD1;PPFIA2;MBNL2;ZHX3;ARHGEF12;MUSK;DST;ARL15;LIMCH1;RALGAPA1;ZNF160;VPS13D;ANK2;FNDC3A;ASH1L;ITFG1;TANC2;PJA2;RABEP1;CSDE1;AKAP9;CMPK1;SCG5;WDFY3;SCG3;ARHGEF7;MICU1;ZMYND11;ZNF235;STX12;PRKAA1;ROCK2;PIK3R3;ZDHHC21;SLC30A10;HACD2;NOL4;SNTG2;HECTD1;FCHO2;GSE1;MAPK1;FAM83B;ATP9A;MON2;NDFIP1;ABCA5;ERBIN;SYT16;RANBP9;CDC42BPA;ELL2;MEIS2;FIG4;MOB1B;ARHGAP32;SYNJ1;APC;TTC3;CNTN1;CCSER2;SPIRE1;CPE;STRN
PBX3 human tf ARCHS4 coexpression	89/299	2.6349441037109094E-5	DPP10;DRAXIN;TENM3;ATP8A1;TUSC3;GRIK1;GRIK2;BICD1;CDH8;PTPRG;ROBO1;DACH1;CDH2;DPYSL5;PEG10;TRIM2;DNER;GDAP1L1;DIRC3;ZNF521;RALGPS1;MAGI1;CXADR;TMEM178B;DCC;CACNA2D1;LRRC49;MAGI2;EBF1;EBF2;EBF3;GFRA1;RGMB;MYT1;PGM2L1;FOXP2;PJA2;ADGRB3;NME7;LRRC7;DOK5;LMX1B;TOX;CNTNAP5;KHDRBS2;HDAC2;RTN1;PLPPR5;GREB1L;PIK3R3;BAZ2B;KLHL13;NREP;KALRN;TMEM163;STOX2;MAPK8;GNG2;MAP2;ZMAT4;MVB12B;MAP6;NCAM1;CTNNA2;CSMD3;PAK3;PAK5;ATF7IP;CA10;NDFIP1;MYEF2;ZNF382;CNTN5;AUTS2;KLHL1;DCLK1;ARHGAP24;ENOX1;ATAT1;PTPRD;MAPK10;RALYL;FER;KLHL7;ASXL3;TTC3;KIAA1958;SSBP2;APBA2
ZEB2 human tf ARCHS4 coexpression	89/299	2.6349441037109094E-5	DOCK4;PID1;MCTP1;ANKRD36;MEGF11;DOCK8;CELF2;ANKRD20A1;FRY;BACH1;LYST;SLC8A1;FAM107B;SYNE1;DOCK10;AKAP13;GRM5;HERC1;GRM7;TRIM2;TMEM108;LUC7L;TRAPPC11;EMILIN2;KIF21B;GUCY1A2;MEF2C;LIMCH1;DAPK1;GRID1;VPS13C;PDE4D;TMOD2;ARAP2;VPS13B;ANK2;FAM126B;ZDHHC17;RUNX1;PHF20L1;DNM3;AGAP9;VCAN;MPPED1;LRRC7;DPYD;MTF2;KCNQ3;RAPGEF2;WDFY3;COL6A6;ECT2L;DOCK2;MACF1;RABGAP1;WDR26;CTTNBP2;KMT2C;AOAH;NR2C1;FBXL20;GNG2;MAP2;NCAM1;MYEF2;NTRK3;SYT16;MYO5A;RAB27A;SYT14;FOXN3;DDHD1;PPP2R3A;ATP2B1;PLXDC2;ARHGAP26;SMARCA2;GRIN2B;MED13L;PTPRD;CCDC88A;PTPRE;DLG2;DAB1;APC;NFIB;DMXL2;CCSER1;TCF4

DZIP1 human tf ARCHS4 coexpression	89/299	2.6349441037109094E-5	*APP;SH3GL3;TENM3;TENM4;CPNE4;CTNND2;FMN2;SIPA1L2;PTPRG;ROBO1;ZNF608;CDH2;TRIM2;AKT3;PSD3;SACS;KIF21A;TEAD1;NEO1;MAGI1;GUCY1A2;TMEM178B;DST;TMOD2;ANK2;IL17RD;EML1;ZDHHC17;MAPK8IP1;ENAH;PYGO1;SETBP1;DOK5;PEAK1;CDH11;MXRA7;DOCK1;PAFAH1B1;RAI14;RABGAP1;NXN;RGPD6;ADAM22;AGAP1;ILDR2;FSTL1;EHBP1;NPAS3;FAM171A1;CTIF;STOX2;FLRT2;MAP2;STK36;ZNF704;NCAM1;CTNNA2;SRGAP3;ZNF423;GPC6;MPDZ;ATP9A;WASF3;ARNT2;FARP1;MYEF2;WSCD1;AUTS2;NTRK3;RDX;KIAA1549L;HMGA2;HSPA12A;PTPN13;CDC42BPA;MYO9A;CORO2B;DCLK1;PLEKHA8;TTLL7;PTPRD;CCDC88A;TTLL5;FER;SPIRE1;FAT3;EIF4G3;ADGRL2;TBATA*
RAPGEF5 human tf ARCHS4 coexpression	89/299	2.6349441037109094E-5	*DOCK4;DOCK3;ATP8A1;FRMPD4;DOCK9;CTNND2;RASGRF2;DIRAS2;RASGRF1;OTUD7A;KNDC1;SYNE1;RPH3A;SYNPR;GRM5;AKAP11;TRIM2;MCF2L;NCS1;PSD3;DLGAP1;EDIL3;CALN1;KCNH1;CADPS2;RBFOX1;MBNL2;DST;PRKCE;TMOD2;MTUS1;COBL;ANK2;MYRIP;SYN2;MAPK8IP1;GABRG1;AJAP1;CNKSR2;DNM3;PITPNM3;SCN8A;WDFY3;PLCB1;NGEF;PAFAH1B1;RAPGEF4;SLC24A2;NECAB1;STXBP1;ADAM22;SLC1A2;NDRG2;PRKCZ;KALRN;NALCN;RAP1GAP;KIAA0513;GRIN2A;SV2B;EPB41L3;CHN1;SPOCK3;CLVS2;SPOCK1;MBP;GPR158;ATP9A;OPCML;ARNT2;DTNA;SYT1;CADM2;ATRNL1;KIAA1549L;MYO5A;ATP2B2;HSPA12A;SNAP91;LRP1B;ETNPPL;TTLL7;PTPRD;ARHGAP32;DLG2;SYNJ1;PPP2R2C;CNTN1;SCN2A*
GATAD2B human tf ARCHS4 coexpression	89/299	2.6349441037109094E-5	*CYFIP2;RERE;SETD2;DOCK8;CELF2;LDLRAD4;MSI2;SOGA1;CMIP;SYNE2;IGF1R;AKAP13;NIPBL;AKAP11;HERC1;AKT3;UBAP2L;KIF21B;ERC1;USP49;CAMK1D;TMEM178B;PRKCB;NCOA6;SFMBT2;ANK3;ASH1L;ARID1B;TANC2;AJAP1;SFPQ;UBE2R2;SETBP1;RFX7;PACS1;MADD;FAM193A;TNIK;ARHGEF7;PAFAH1B1;KMT2E;MACF1;DDX6;NFAT5;WDR26;ANP32A;KMT2C;ANKRD11;AGAP1;HTT;BAZ2A;KALRN;KIAA1328;NSD1;HECTD4;HIVEP1;GSE1;DROSHA;TRPM7;NCAM1;HIVEP2;PAK5;MARK2;BRD4;BPTF;SPEN;CREBBP;KDM4B;AUTS2;SEMA4D;IQSEC1;TRAPPC10;FOXJ3;MICAL3;LSAMP;YLPM1;LNPEP;SMARCA2;SCAF4;MED13L;TNRC6C;DLG2;AGO3;NBEA;KANSL1;AGO2;CDK12;SSBP3;TNRC6B*
CHD7 human tf ARCHS4 coexpression	89/299	2.6349441037109094E-5	*TCERG1;ZNF891;DRAXIN;HIP1;TENM4;ZNF292;ZFAND6;ELAVL4;PSIP1;GLI3;SYNE2;KIF15;PTPRG;ZNF608;NHSL1;DACH1;CDH2;DPYSL5;ADAMTSL3;SMARCAD1;KIF21A;SOX6;SRGAP2B;MAGI1;EPHA4;SMARCC1;WSB1;USP49;DCC;LRRC49;MAGI3;TCF12;ATRX;RFX3;TET1;IL17RD;MYT1;ENAH;TOX3;MYCL;MMP16;SETBP1;IFT81;CCDC150;ZNF431;UBE2Q2P1;CRB1;HDAC2;PPM1L;TNKS;BTF3L4;GREB1L;PIK3R3;ILDR2;BAZ2B;NREP;KALRN;PHF21B;PRTG;FGD4;STOX2;CECR2;KIAA1328;MAP2;MAP6;IGF2BP3;SRGAP3;MPDZ;BPTF;ATF7IP;ZNF462;FARP1;MYEF2;AUTS2;RANBP17;ST18;MAPK10;CCDC88A;AGO3;NFIA;KANSL1;ZNF738;AGO1;KLHL7;TTC3;YPEL1;TCF4;KIAA1958;ASB3*
CHD2 human tf ARCHS4 coexpression	89/299	2.6349441037109094E-5	*ITSN2;KDM5A;RERE;SETD2;ZNF292;DOCK8;SMG1P2;RORA;LYST;ETS1;SYNE2;SYNE1;DOCK10;AKAP13;STK10;NIPBL;C16ORF72;HERC1;ADAMTSL3;ZNF407;LRRFIP1;MBNL1;MORC3;PRKCH;ITGA4;VPS13C;VPS13D;ARAP2;VPS13B;ASH1L;ARID1B;PARP8;GOLGA8B;NCOR1;PEAK1;FAM153A;BIRC6;UTRN;DOCK2;MCTP2;KDM7A;KMT2E;MACF1;DDX6;NFAT5;WDR26;KMT2C;RGPD6;NLRC5;RGPD5;BAZ2A;RGPD8;BAZ2B;PCNX1;BTAF1;KIAA1328;NSD1;HECTD4;HIVEP1;TRPM7;CEP192;HIVEP2;ATP9B;BPTF;LRRC37A3;RANBP2;USP24;SPEN;CREBBP;MBD5;KDM4C;LRBA;TRAPPC10;MGA;YLPM1;LNPEP;HIPK1;ARHGAP26;SMARCA2;PHC3;PARP15;ZZEF1;MED13L;CYLD;SNRK;KANSL1;DMXL2;BCL2;CDK12*

RGS7 human tf ARCHS4 coexpression	89/299	2.6349441037109094E-5	*PCSK2;DOCK3;KCNC1;FRMPD4;MYT1L;CTNND2;DIRAS2;RASGRF1;KNDC1;HS6ST3;RPH3A;UNC80;SYNPR;GRM5;TRIM2;MCF2L;NCS1;PSD3;CKMT1B;DLGAP1;PRKACB;PPFIA2;KCNH1;UNC13C;RBFOX1;RBFOX3;PRKCE;TMOD2;ANK2;MYRIP;SEZ6L;SYN2;GABRG2;MAPK8IP1;AJAP1;CNKSR2;DNM3;MPPED1;SCN8A;KCNMA1;SCG3;PLCB1;NGEF;RAPGEF4;SLC24A2;NECAB1;RTN1;STXBP1;ADAM22;SLC1A2;NDRG2;PRKCZ;KALRN;NALCN;RAP1GAP;KIAA0513;GRIN2A;DPP6;PGBD5;SV2B;MAP2;CHN1;SPOCK3;CLVS2;CACNG3;GPR158;ATP9A;OPCML;GABBR2;ARNT2;PTPRN2;NDFIP1;SYT1;CADM2;CADPS;SLC4A10;KIAA1549L;MYO5A;ATP2B2;HSPA12A;CORO2B;SNAP91;DLG2;SYNJ1;PPP2R2C;RCAN2;CNTN1;CPE;SCN2A*
DACH2 human tf ARCHS4 coexpression	88/299	4.8131828448290184E-5	*GABRB3;PCDH11Y;MYT1L;PTPRO;RASGRF1;CELF4;ELAVL4;CDH8;SYNPR;C1ORF127;FGF9;GRM7;DPYSL5;TRIM2;KIF21A;ZNF385D;SYBU;ANKS1B;PPFIA2;EPHA6;CXADR;CACNA2D1;LRRC49;TMOD2;MAGI2;ATRX;EBF1;MTUS2;SEZ6L;PGM2L1;GAP43;NAV3;NRG3;IFT81;ADGRB3;LRRC7;AKAP9;SCG3;CDH18;ASTN1;ZNF112;FTO;NECAB1;RTN1;NRXN3;ZNF66;NREP;NOL4;NALCN;CACNA1E;FGD4;STOX2;DPP6;GNG2;MAP2;SUSD4;MAP6;CAMTA1;SUSD6;NCAM1;CTNNA2;SRGAP3;PAK3;PAK5;CADM1;SYT1;PCDH9;CADM2;LSAMP;SLC4A10;GRIN2B;USH2A;SNAP91;DCDC1;MAPK10;NELL2;RALYL;DLG2;NBEA;PPP2R2B;ASXL3;TTC3;RIMBP2;CNTN1;FAT3;GALNTL6;SCN2A;SSBP2*
FOXE1 human tf ARCHS4 coexpression	88/299	4.8131828448290184E-5	*DOCK5;PATJ;PPP1R13B;ATP8A1;DOCK9;CPQ;GALNT18;KIAA1671;LAMC1;SYNE2;SYNE1;AKAP13;MPRIP;KIF13A;VSTM4;GTF2I;KRT6A;GBP6;CAST;ARHGEF12;RALGAPA2;ARHGEF17;COL23A1;RIPK4;MYOF;VPS13D;ARAP2;EMP1;WDR72;HSPG2;KIAA1217;TOM1L2;TANC1;GOLGA8B;ATRN;POR;FNDC1;COL4A3;PKP1;BIRC6;MXRA7;PKHD1L1;PLA2R1;UTRN;DOCK1;MET;DGKI;MATN2;FKBP5;ITGA9;YAP1;NOTCH2;MACF1;RBM47;SEMA3D;SDC2;ARHGEF28;GLIS3;NTN4;RRBP1;LRP2;RAP1GAP;THSD4;HIRA;HECTD1;CTNNA1;ABL1;FLNB;CDH26;CTNNAL1;ZBTB7C;STARD13;ZBTB16;ZNF804B;FAM189A2;CYBRD1;SORBS2;HOOK3;MYO9A;LRP1B;ARHGAP31;TG;MYO5B;LPCAT2;CD9;GGT3P;ATP13A3;FBN1*
TOX3 human tf ARCHS4 coexpression	88/299	4.8131828448290184E-5	*ROBO2;DRAXIN;TENM4;ELAVL4;SLC35F1;TSPAN11;SRGAP2C;ZNF608;NHSL1;DACH1;CDH2;DPYSL5;TRIM2;SUMO2;TMEM108;ITGB8;KIF21A;SOX6;EPHB2;SRGAP2B;MAGI1;WSB1;TMEM178B;NCOA6;LRRC49;MAGI3;TCF12;MAGI2;ATRX;RFX3;ANK2;NSUN6;GAREM1;CFDP1;LMX1A;SETBP1;IFT81;MPPED2;PDZRN3;ZNF234;ZNF431;CRB1;CHRNA7;NEDD4L;PIK3R3;ILDR2;BAZ2B;NREP;NPAS3;FGD4;ORC4;STOX2;GTF2IP1;NKAIN3;ZNRF3;MAP2;SNTG1;ZNF704;ZNF627;MAP6;SRGAP3;PAK3;JAM2;BPTF;RFTN2;FOXB1;ZNF462;FARP1;PTPRN2;MYEF2;AUTS2;RANBP17;LSAMP;ST18;PBX1;PTPRD;IGSF11;CCDC88A;CENPE;NFIA;ZNF738;TTC3;YPEL1;FAT3;TCF4;KIAA1958;BMPR1B;TNRC6B*
ID4 human tf ARCHS4 coexpression	88/299	4.8131828448290184E-5	*SPON1;TENM4;CTNND2;GRIK4;ZBTB20;FMN2;SIPA1L2;SYNE2;GRM3;TRIM9;UBL3;DACH1;CDH2;TRIM2;KIF21A;EPHB1;NEO1;MAGI1;SLC15A2;ADGRV1;KCNK10;TMEM178B;KCND3;LRRC49;ATRX;RFX3;KAZN;IL17RD;SEZ6L;PARD3B;PYGO1;TOX3;ANKFN1;SETBP1;IFT81;ADGRB3;PARD3;LRRC9;ZNF678;DGKI;CHST3;ADCYAP1R1;MAGEL2;PHLPP1;NTM;ILDR2;APCDD1;NTN1;NPAS3;STOX2;GTF2IP1;MAP2;ZNF704;LRIG1;GPC5;NCAM1;KCNN3;TSPAN3;SRGAP3;SLIT2;MPDZ;JAM2;ARNT2;NTRK2;MBD5;NDFIP1;DTNA;MYEF2;VCAM1;AUTS2;ST8SIA1;PCDH7;FAM189A2;ZBTB10;HOOK3;BMP7;DCLK1;PBX1;PTPRD;NIN;TTC6;ZNF738;FABP7;SMOC1;SPIRE1;FAT3;HCG22;BRWD1*
THRB	87/299	8.5807811114	*PTPRT;DOCK3;MAST4;FRMPD4;DIRAS2;CHRM5;RASGRF1;KNDC1;C12ORF42;RPH3A;UNC80;SYNPR;SLC25A48;GR*

human tf ARCHS4 coexpression		18924E-5	*M5;ADAMTSL3;PSD3;DLGAP1;ANKS1B;KCNH1;UNC13C;RBFOX1;MEF2C;KCNH5;ARHGEF12;RBFOX3;KCND3;PRKCE;TMOD2;KCTD1;ANK3;ELOVL7;SYN2;CNKSR2;DNM3;SCN8A;PPARA;PLCB1;VSTM2A;NGEF;RAPGEF4;SLC24A2;STOML1;HEPACAM;STXBP1;ADAM22;SLC1A2;AKAP6;NDRG2;KALRN;NALCN;ATP6AP1L;KIAA0513;GRIN2A;DPP6;RIC8B;PGBD5;SV2B;CHN1;ST8SIA5;CAMTA1;GPC5;CACNG3;ATP9A;OPCML;GABBR2;ARNT2;OSBPL6;SYT1;NEGR1;CADM2;ATRNL1;SLC4A10;KIAA1549L;FBXL17;ATP2B2;SNAP91;ETNPPL;TTC39B;TTLL7;RPGRIP1;ARHGAP32;DLG2;PPP2R2C;RCAN2;RAB12;SCN2A;HCN1*
DACH1 human tf ARCHS4 coexpression	87/299	8.580781111418924E-5	*ROBO2;DRAXIN;TENM4;ZNF292;CTNND2;CELF4;ELAVL4;PPP1R9A;TSPAN11;RIMS2;GRIP1;CDH4;ZNF608;CDH2;DPYSL5;GDAP1L1;EPHB2;ZNF521;PPFIA2;KCNK10;DCC;LRRC49;KCNH8;MAGI3;ATRX;EBF2;RFX3;EBF3;IL17RD;MYT1;PGM2L1;TOX3;SLC9A4;GAP43;LMX1A;SETBP1;AKAP9;HECW1;PRR16;LMX1B;CNTNAP5;PPM1L;CHRNA7;TSHZ2;GREB1L;BAZ2B;NREP;KALRN;TMEM163;NPAS3;PHF21B;STOX2;GTF2IP1;PDCD6IPP2;MAP2;MAP6;NCAM1;CTNNA2;SRGAP3;PAK3;ZNF423;LRRC4C;JAM2;CLVS1;ZNF462;PTPRN2;MYEF2;ZNF382;ST8SIA1;CADPS;PBX3;HOOK3;MEIS2;ST18;PBX1;PTPRD;MAPK10;CCDC88A;ZNF618;ZNF738;AGO1;ASXL3;TTC3;KIAA1958;SSBP3;VSX1;LHX9*
ZKSCAN2 human tf ARCHS4 coexpression	87/299	8.580781111418924E-5	*TENM4;ZFYVE9;ZBTB21;ELAVL4;HNRNPU;PSIP1;UBE3A;KIF11;BRCA2;BICD1;TXNDC16;GLI3;ZCCHC14;PTPRG;ROBO1;ZNF608;CDH2;DPYSL5;ZNF606;ZMYM4;TRIM2;AKT3;DNER;CHAMP1;SACS;SMARCAD1;KIF21A;NEO1;MAGI1;SMARCC1;SUPT16H;WSB1;CSNK2A1;DCC;CACNA2D1;RFX3;RGMB;NBPF1;MSH6;SETBP1;ADGRB3;DOK5;MPPED2;ZNF678;ST6GALNAC3;WDR25;HDAC2;GREB1L;PIK3R3;AGAP1;NREP;EHBP1;PHF21B;STOX2;MAP2;FUT9;ZNF704;CLVS2;DROSHA;NCAM1;RPRD1A;SRGAP3;MPDZ;S100PBP;VAT1L;BPTF;ZNF462;FARP1;MYEF2;WSCD1;AUTS2;ERCC6L2;YLPM1;ZBTB10;PUM1;BTBD10;PBX1;SMARCA4;PLEKHA8;ATAT1;PTPRD;CCDC88A;ZNF738;FAT3;UBAP1L;FAT4;ADGRL2*
BNC2 human tf ARCHS4 coexpression	87/299	8.580781111418924E-5	*NRP1;PTPRQ;COL14A1;CHRM5;EFCAB6;GXYLT2;LAMC1;C12ORF42;ANTXR1;PTPRG;EPS8;ADAMTSL1;ADAMTS5;TUBB6;ARHGAP42;ADAMTS2;SGCD;HMCN2;PHACTR2;VSTM4;TEAD1;ZNF124;PRKG1;ADAMTS6;POSTN;MYOCD;MUSK;DST;MYOF;FNDC3B;ARID5B;NAV2;EML1;FAM126A;TBC1D1;FRMD6;ELF2;EVC;HEATR5A;NAV3;CRISPLD2;PEAK1;CDH11;CDH13;ALPK2;TLN2;PPP1R12B;DOCK1;VCL;RAI14;RBMS3;MACF1;SAR1A;TWIST1;FSTL1;LPP;LTBP1;FBLN5;GLIS1;CALD1;ARSJ;PDGFC;RNF217;CHSY3;PDLIM5;STARD13;EYA4;CRIM1;CYBRD1;ARHGAP28;LAMB1;GNG12;FBXO32;CDC42BPA;DAZL;TJP1;TBX15;COL5A1;DLC1;SLMAP;SSPN;ITGBL1;SPATS2L;SNAI2;FBXL7;FBN1;SNTB2*
SCAPER human tf ARCHS4 coexpression	87/299	8.580781111418924E-5	*PCSK2;ROBO2;DPP10;ERO1B;ZNF891;MYT1L;ANKRD36;CHD9;ZNF292;FRG1HP;EFCAB6;PPP1R9A;LCLAT1;RIMS2;UNC80;DSTYK;EPB41L4A;GRM7;ZSCAN30;LRRTM4;ADAMTSL3;KIF21A;ZNF568;ANKS1B;RBM6;ANKRD36C;USP49;TMEM178B;SLX4IP;MAGI2;ATRX;ANK2;OPRM1;SEZ6L;ARID1B;DNM3;LRRC7;RUFY2;AKAP9;SCG5;SCG3;ASTN2;ANKRD36B;SDCCAG8;KMT2E;TPH2;LUZP2;PCDH15;RGPD6;ADAM22;TULP4;RGPD5;ILDR2;BAZ2B;MTMR7;MIPOL1;AK9;SNTG2;TTR;MAP2;SNTG1;ZNF704;HSD17B2;ANKRD20A5P;ANKRD36BP2;NCAM1;SRGAP3;PAK3;CORIN;BPTF;RIC3;ZNF382;ZBTB10;DCDC1;TTLL7;MAPK10;CCDC88A;FER;NBEA;TMEM236;ASXL3;TTC3;TCF4;CNTN4;PTPN4;ASB3;CCDC171*
RREB1 human tf	86/299	1.5252159931210862E-4	*ITSN2;DIDO1;PATJ;DOCK8;KIAA1671;LIMD1;SIPA1L3;ETS2;CABIN1;RPTOR;AKAP13;ZFYVE26;HERC2;HERC1;ZNF407;KYNU;MYB;LRRFIP1;PGPEP1;MAP3K5;NUP214*

ARCHS4 coexpression			;VAV3;MBNL1;ITGA4;SFMBT2;RALGAPA2;VPS13C;VPS13D;FNDC3B;GOLGA8J;VPS13B;ASH1L;URB1;ARID1B;RUNX1;ETV6;NCOR1;BMP2K;WDPCP;BIRC6;PPARA;UTRN;DOCK2;WDFY4;MACF1;NFAT5;CASZ1;RBM47;CTDP1;KMT2C;HTT;ITPR2;BAZ2A;RRBP1;LPP;PCNX1;KIAA1328;HECTD1;CUX1;NSD1;TRPM7;CEP192;PCNT;ATP9B;MDN1;RANBP2;USP24;SPEN;CREBBP;LRBA;TRAPPC10;MGA;LNPEP;MYO9B;HIPK1;SMARCA2;PHC3;PARP15;ZZEF1;MED13L;DIAPH1;AGO2;BCL2;ESYT2;CDK12;AVL9
TCF12 human tf ARCHS4 coexpression	86/299	1.5252159931210862E-4	MAML2;MEGF10;LDLRAD3;SLC35F1;TSPAN11;KIF15;ROBO1;SRGAP2C;TRIM9;CDH2;SACS;TNR;SMARCAD1;ITGB8;SOX6;ZNF521;SRGAP2B;GTF2I;MAGI1;KLF12;WSB1;ADGRV1;DST;DCC;SEMA6D;RFX3;ANK2;RGMB;MOB3B;ENAH;TOX3;ASPM;ZEB1;SETBP1;IFT81;ADGRB3;TOX;ZNF112;CREB5;CRB1;ADCYAP1R1;HDAC2;STXBP4;TNKS;BTF3L4;PIK3R3;ILDR2;BAZ2B;KLHL13;NREP;NPAS3;STOX2;NKAIN3;HECTD2;MAP2;SNTG1;ZNF704;LRIG1;APBB2;TSPAN3;SRGAP3;MPDZ;JAM2;SLC15A5;GRIA4;BBS2;RFTN2;ATF7IP;NTRK2;MYEF2;WSCD1;RANBP17;PUM1;CORO2B;ST18;PTPRD;IGSF11;MAPK10;CCDC88A;APC;FABP7;SMOC1;FAT3;TCF4;KIAA1958;FRYL
ZFHX4 human tf ARCHS4 coexpression	86/299	1.5252159931210862E-4	ROBO2;DRAXIN;PTPRO;CELF4;ELAVL4;SLC35F1;GRIK2;PPP1R9A;ZNF608;DACH1;CDH2;DPYSL5;DNER;GDAP1L1;SUMO2;KIF21A;ZNF521;ANKS1B;GARNL3;PPFIA2;KIRREL3;MAGI1;EPHA4;WSB1;CXADR;TMEM178B;DCC;LRRC49;KCNH8;MAGI2;ATRX;EBF1;RFX3;ANK2;FRMD4A;ANK3;IL17RD;MYT1;PGM2L1;FOXP2;TOX3;GAP43;SETBP1;IFT81;AKAP9;AMPH;ZNF397;CHRNA7;PLPPR5;GREB1L;TPTE2P2;BAZ2B;NREP;EFNA5;PHF21B;FGD4;STOX2;GTF2IP1;MAP2;MAP6;NCAM1;CTNNA2;SRGAP3;PAK3;ZNF423;SCAI;LINGO2;ZNF462;LINGO1;MYEF2;AUTS2;NEGR1;ST8SIA1;NTRK3;PBX3;LSAMP;PBX1;ATAT1;MAPK10;CCDC88A;AGO3;PPP2R2B;TTC3;FAT3;KIAA1958;LHX9
MYSM1 human tf ARCHS4 coexpression	86/299	1.5252159931210862E-4	ITSN2;TCERG1;DIDO1;SETD2;LPGAT1;SMG1P2;LTN1;HNRNPU;RORA;SYNE2;AKAP13;NIPBL;PTAR1;C16ORF72;HERC1;ZMYM4;JARID2;KDM6A;MBNL1;SMARCC1;USP7;RALGAPA1;VPS13C;VPS13D;ARAP2;TET1;VPS13B;ASH1L;TC2N;ARID1B;PARP8;MSH6;GOLGA8B;SFPQ;FAR1;WDFY3;BIRC6;UTRN;ANAPC1;KDM7A;MACF1;DDX6;NFAT5;WDR26;ROCK1;KMT2C;IREB2;BAZ2A;IQGAP1;LPP;PCNX1;SCAF8;LARP1;BTAF1;HECTD1;NSD1;HECTD4;TRPM7;MDN1;BPTF;RANBP2;ARFGEF1;USP24;SPEN;SLC16A1;KDM4C;LRBA;TRAPPC10;MGA;ERBIN;LNPEP;PHC3;SCAF4;MTOR;MED13L;TJP1;MLLT10;KANSL1;AGO2;SERBP1;DMXL2;SP3;STRN;PKN2;CDK12;BRWD1
MEF2C human tf ARCHS4 coexpression	85/299	2.4801055876356824E-4	MYOM1;MYLK2;ATP8A1;MYT1L;CELF2;LDB3;SLC6A1;JPH1;LRRC2;MYOM2;SLC8A1;CRKL;GRM5;AKAP11;SGCD;HERC1;PPP2R5E;MB;TRIM2;PEBP4;MYO18B;DLGAP1;PRKACB;SGCG;GUCY1A2;UNC45B;RBFOX1;DSCAM;PRKCB;CACNA2D1;PRKCE;TMOD2;ANK2;TRDN;DNM3;MMP16;MPPED1;MYL1;LRRC7;SCN8A;ALPK3;KCNQ5;DGKI;PAFAH1B1;PRKAA2;SMPX;PPM1L;ADAM22;TRAK1;KALRN;GRIN2A;SV2B;FUT9;CHN1;NRAP;SYNDIG1;TNNI1;XIRP2;ZNF106;CTNNA3;NCAM1;HIVEP2;ATP9A;OPCML;SVIL;SYT1;CADM2;AGL;SYT16;KIAA1549L;MYO5A;PDE4DIP;ATP2B2;GRIN2B;DCLK1;SNAP91;TTLL7;PTPRD;DLG2;AGBL1;APC;TCF4;SCN2A;ASB2;FGF12
PGR human tf ARCHS4 coexpression	85/299	2.4801055876356824E-4	PCSK2;ERO1B;PATJ;ICA1;ZBTB20;ECE1;RORB;SERPINA6;AFF3;IGF1R;POTEKP;CDH5;SCGN;EFEMP1;TRPS1;PPP6R3;TMED3;SLC39A6;LONP2;PLCE1;PAMR1;ADAMTS9;ARFGEF3;VAV3;GUCY1A2;VWFP1;ARHGEF12;DST;RALGAPA1;ZBTB38;VPS13C;MYOF;MAGI2;VPS13B;TBC1D9;HSPG2;PRLR;CACNB2;ALDH1A2;SCG5;SCG3;PPP1R12B;RIN2;FREM1;DOCK1;MACF1;SEMA3C;KMT2C;PRICKLE2;LRP2;FAM214A;LPP;SLC7A2;THSD4;ERMP1;TTR;ZNF704;GSE1;FLNB;AKAIN1;ARFGEF1;STARD13;ABCA5;ANKRD

			30A;LRBA;GREB1;LAMB1;TDRD5;CDC42BPA;ELL2;ESR1;MPP7;MED13L;MOB1B;SDK1;PLCXD3;TSPAN13;TMEM236;DLG5;MYO5C;VPS41;NEK10;CPE;CNTN4;SEC24D
HIVEP1 human tf ARCHS4 coexpression	85/299	2.4801055876356824E-4	*ITSN2;UHRF1BP1L;ANKRD33B;PPP1R13B;MAML2;KDM1B;IKZF2;BACH1;DOCK10;AKAP13;ZFYVE26;NIPBL;RPS6KA5;C16ORF72;HERC1;ADAMTSL3;ZNF407;KYNU;STPG2;KSR1;NCOA6;SFMBT2;VPS13C;VPS13D;FNDC3B;ARAP2;VPS13B;ASH1L;ARID1B;TRAF3;PEAK1;WDPCP;ZNF780B;WDFY3;BIRC6;UTRN;DOCK2;WDFY4;KDM7A;MACF1;DDX6;NFAT5;MTMR3;WDR26;KMT2C;ITPR2;BAZ2A;IQGAP1;AMBRA1;PCNX1;SCAF8;BTAF1;ATXN1;KIAA1328;NSD1;NLRP4;BTLA;TRPM7;SUSD6;HIVEP2;ATP9B;LYN;RANBP2;USP24;SPEN;CREBBP;PLEKHA2;LRBA;TRAPPC10;MGA;GPR55;LNPEP;DNAJC13;DDHD1;SMARCA2;PARP15;GATAD2B;ZZEF1;MED13L;CYLD;NLRP13;AGO3;DMXL2;BCL2;CDK12*
ZNF25 human tf ARCHS4 coexpression	85/299	2.4801055876356824E-4	*GABRB1;ATP8A1;MYT1L;CTNND2;DIRAS2;ELAVL4;TRIM9;DPYSL5;TRIM2;DNER;PSD3;DLGAP1;PRKACB;RALGPS1;SYBU;PPFIA2;RBFOX1;WSB1;TMEM178B;TMEM178A;CACNA2D1;TMOD2;ANK2;FRMD4B;SYN2;GABRG2;PGM2L1;PJA2;DNM3;ADGRB3;RUFY2;ULK2;AMPH;ZMYND11;VSTM2A;ASTN1;GRIA1;RTN1;STXBP1;ATL1;ADAM22;NRXN3;AKAP6;NREP;PRKCZ;KALRN;NALCN;DPP6;GNG2;SV2B;MAP2;CDH20;FUT9;SMIM11B;CHN1;CLVS2;NCAM1;CTNNA2;PAK3;ATP9A;OPCML;GABRA2;ARNT2;NDFIP1;SYT1;CADM2;PBX3;SLC4A10;SYT16;ATP2B2;DCLK1;SNAP91;TTLL7;ATAT1;MAPK10;DLG2;APC;PPP2R2B;RCAN2;TTC3;YPEL1;CNTN1;SCN2A;SSBP2;APBA2*
CAMTA1 human tf ARCHS4 coexpression	85/299	2.4801055876356824E-4	*GABRB3;PTPRT;ATP8A2;DOCK3;MYT1L;CTNND2;DIRAS2;CELF4;ELAVL4;RPH3A;RIMS1;GRM5;TRIM2;PSD3;KIF21A;DLGAP1;ZNF385D;ANKS1B;PPFIA2;RBFOX1;TMEM178B;TMEM178A;TMOD2;KAZN;UBE2QL1;ANK2;ANK3;SYN2;GABRG2;PGM2L1;AJAP1;CNKSR2;DNM3;GAP43;SCN8A;HECW1;NOS1AP;ASTN1;SLC24A2;RTN1;STXBP1;ATL1;NTM;ADAM22;NRXN3;KALRN;STOX2;GRIN2A;DPP6;PGBD5;SV2B;MAP2;FUT9;CHN1;CLVS2;MAP6;NCAM1;CTNNA2;SRGAP3;PAK3;PAK5;ATP9A;WASF3;OPCML;GABBR2;ARNT2;SYT1;CADM2;NTRK3;LSAMP;KIAA1549L;ATP2B2;HSPA12A;DCLK1;PBX1;SNAP91;TTLL7;PTPRD;MAPK10;RALYL;DLG2;PPP2R2C;PPP2R2B;RCAN2;SCN2A*
ZBTB41 human tf ARCHS4 coexpression	85/299	2.4801055876356824E-4	*PCSK2;ROBO2;ERO1B;TUSC3;ZNF292;PPP1R9A;LCLAT1;PCMTD2;PPP1CB;TRIM9;SCGN;ZSCAN30;TRIM2;PPP6R3;LONP2;ARFGEF3;PCMTD1;TTC37;WSB1;CXADR;DST;LIMCH1;RALGAPA1;CACNA2D1;SLC2A13;LRRC49;VPS13C;RFX3;ANK2;FNDC3A;SEZ6L;SRP9;PJA2;CACNB2;MMP16;MYO3A;PIGK;SCG3;RIN2;SAMD5;INO80D;FICD;SAR1A;STXBP4;TNKS;BTF3L4;TULP4;UBR1;NOL4;NALCN;NR2C1;TTR;MAP2;TTC21B;SYNDIG1;PAK3;EVI5;MON2;PTPRN2;NDFIP1;ABCA5;PNPLA8;CADPS;SYT16;CDC42BPA;ELL2;OXR1;ST18;MOB1B;MAPK10;ITCH;CNOT7;NBEA;APC;ASXL3;TTC3;VPS41;CNTN1;CPE;CNTN4;SCN2A;BRWD1;PTPN4;LRP12;CPEB4*
ZNF248 human tf ARCHS4 coexpression	85/299	2.4801055876356824E-4	*ATF2;MYT1L;ZNF292;PTPRO;ELAVL4;UBE3A;GRM3;DPYSL5;TRIM2;AKT3;DNER;GDAP1L1;HMCN2;KIF21A;RALGPS1;ANKS1B;PPFIA2;EPHA4;PRMT8;MYOCD;WSB1;LRRC49;TMOD2;OPRM1;SEZ6L;PJA2;SETBP1;ADGRB3;LRRC7;RUFY2;PPP1R12B;MAPRE2;ANKRD36B;CAMK1G;ASTN1;ZNF112;GRIA1;KHDRBS2;RTN1;TNKS;STXBP1;NRXN3;NEDD4L;PIK3R3;AKAP6;CACNA1C;NREP;NYAP2;CACNA1E;LPP;TMEM25;STOX2;MAPK8;GNG2;MAP2;CLVS2;MAP6;NCAM1;CTNNA2;SRGAP3;PAK3;GRIA4;GABRA2;CADM1;SYT1;LSAMP;DCLK1;PBX1;ENOX1;TTLL7;ATAT1;PTPRD;MAPK10;CCDC88A;DLG2;APC;KLHL7;SLMAP;TTC3;UBAP1L;SCN2A;KIAA1958;BRWD1;SSBP2;APBA2*
TUB	85/299	2.4801055876	*GABRB3;APP;CNTNAP2;GALNT13;ATP8A2;DOCK3;MYT1L;CTNND2;DIRAS2;KNDC1;RPH3A;TRIM9;GRM5;DPYSL5;*

human tf ARCHS4 coexpression		356824E-4	*TRIM2;MCF2L;AKT3;NCS1;PSD3;TNR;KIF21A;DLGAP1;PPFIA2;CXADR;TMEM178B;TMEM178A;TMOD2;KAZN;ANK2;FAM219A;SEZ6L;SYN2;MAPK8IP1;LRRC7;SCN8A;SHANK2;ASTN1;PAFAH1B1;ADCYAP1R1;RTN1;CTTNBP2;TNKS;STXBP1;NTM;ADAM22;AGAP1;NREP;KALRN;NOL4;CTIF;STOX2;SV2B;MAP2;CHN1;CLVS2;CAMTA1;NCAM1;CTNNA2;ATP9A;WASF3;OPCML;ARNT2;NDFIP1;MYEF2;SYT1;CADM2;NTRK3;LSAMP;KIAA1549L;ATP2B2;CORO2B;DCLK1;SNAP91;PTPRD;MAPK10;DLG2;NBEA;APC;PPP2R2C;PPP2R2B;RCAN2;TTC3;CNTN1;SCN2A;APBA2*
ZNF804A human tf ARCHS4 coexpression	85/299	2.4801055876356824E-4	*ROBO2;DRAXIN;PPP1R17;TENM4;MYT1L;ANKRD36;CHD9;CTNND2;CELF2;PTPRO;ELAVL4;CDH9;CMIP;RPH3A;CDH4;GRM7;DPYSL5;ZSCAN30;TRIM2;DNER;DLGAP1;ZNF385D;PRKACB;ZNF521;PKNOX2;ANKS1B;CALN1;SOX5;PPFIA2;TRPC5;ST6GAL2;KCNH5;TMEM178B;DCC;CACNA2D1;LRRC49;TMOD2;ANK2;SORCS1;ANK3;UNC5D;GFRA2;ZDHHC17;TIAM2;LRRC7;IL1RAPL2;CDC42EP3;IL1RAPL1;NLGN1;AKAP6;NREP;KALRN;NKAIN2;NEU3;STOX2;GRK3;MAPK8;SAMM50;MAP2;STK36;FUT9;PLXNA2;CLVS2;ZSWIM6;NCAM1;CTNNA2;SRGAP3;PAK5;CLVS1;NTRK3;GRIN2B;DCLK1;SNAP91;PTPRD;NELL2;CCDC88A;FER;DLG2;NFIA;APC;PPP2R2B;COL5A3;TTC3;FAT4;LRP12*
CHD6 human tf ARCHS4 coexpression	85/299	2.4801055876356824E-4	*RERE;DIDO1;PATJ;TRIO;ZMYND8;CISD1;MSI2;SOGA1;SIPA1L3;SYNE2;IGF1R;SYNE1;HERC2;ZNF608;C16ORF72;HERC1;MPRIP;FBXO3;DIP2A;ARFGEF3;RPS12;GTF2I;ARHGEF12;RALGAPA2;VPS13C;VPS13D;VPS13B;ASH1L;ARID1B;NAALADL2;INIP;KIAA1217;ZEB1;PEAK1;AKAP9;WDFY3;BIRC6;ARHGEF7;UTRN;SFI1;MATN2;ANKRD17;MACF1;DDX6;NFAT5;CASZ1;CCDC186;UHRF2;KMT2C;DNAH5;PRUNE2;HTT;KALRN;ACACA;LARP1;KIAA1328;HECTD1;ZNF704;NSD1;HECTD4;STK38;GSE1;HS1BP3;FLNB;PCNT;MARK2;BPTF;SPEN;ZNF462;CREBBP;GLYATL2;LRBA;MGA;GREB1;YLPM1;ZBTB10;SLC52A1;MED13L;TJP1;ARHGAP32;PDP2;KANSL1;WNK2;CDK12;BRWD1*
FMNL2 human tf ARCHS4 coexpression	85/299	2.4801055876356824E-4	*APP;DOCK4;DOCK3;ATP8A1;MEGF10;CTNND2;DIRAS2;KNDC1;SOGA1;CELSR2;TRIM9;SPRED1;AKAP11;MPRIP;TRIM2;MCF2L;NCS1;PSD3;TNR;DLGAP1;DIP2B;EDIL3;KCNH1;RBFOX1;DST;TMOD2;MTUS1;KAZN;ANK2;FAM219A;SYN2;MAPK8IP1;GABRG1;DNM3;ATRN;SCN8A;WDFY3;TLN2;RAPGEF5;NGEF;ASTN1;PAFAH1B1;RAPGEF4;SLC24A2;PDE1C;STXBP1;ADAM22;SLC1A2;AGAP1;NDRG2;FAM171A1;CTIF;SV2B;CHN1;SPOCK3;CLVS2;SPOCK1;MBP;NCAM1;MAP4;GPR158;ATP9A;WASF3;MAP4K4;OPCML;ARNT2;DTNA;SYT1;CADM2;EXOC6B;KIAA1549L;MYO5A;ATP2B2;CDC42BPB;HSPA12A;S100B;CORO2B;SNAP91;ETNPPL;TTLL7;TJP1;PTPRD;DLG2;PPP2R2C;SCN2A*
ZC3H6 human tf ARCHS4 coexpression	85/299	2.4801055876356824E-4	*CCDC122;ZNF891;CRYBB2P1;ZBTB25;PCDH11Y;MYT1L;ANKRD36;DPY19L2P2;GADL1;ZBTB20;TMEM182;EFCAB6;MYLK3;RIMS2;UNC80;EPB41L4A;CCDC91;RPS6KA5;HERC1;ZSCAN30;CA5A;ADAMTSL3;EFCAB2;MACROD2;NOS1;ANKS1B;PCMTD1;POTEC;USP8;ANKRD36C;MAGI2;TTC7B;VPS13B;ANK3;FAM126B;SHISA9;ARID1B;SPATA17;NCOR1;LRRC7;PEAK1;CATSPERG;AKAP9;CNKSR3;WDPCP;ASTN2;ANKRD36B;SDCCAG8;FTO;TPH2;KCNE4;RGPD5;BAZ2B;MIPOL1;STK3;FGD4;ORC4;DPP6;KIAA1328;ANKRD36BP2;AP4S1;SRGAP3;ATP9B;SCAI;LRRC37A3;MBD5;SLC14A2;ZNF382;NTRK3;ADAM32;ARHGAP28;HOOK3;MYO9A;DCDC1;TTC39B;TTLL7;MAB21L3;MFSD14C;AGO3;TMEM116;NEDD4;TTC3;BRWD1;ASB3;TNRC6B*
TEAD1 human tf ARCHS4 coexpression	84/299	4.2583582371160024E-4	*BCAR3;TRIO;TENM3;MAST2;PTPRK;ANTXR1;LOXL2;FYCO1;TUBB6;SGCD;MPRIP;SH3PXD2A;KIF13A;MYO18B;SACS;PRKG1;ARHGEF12;DST;ARHGEF17;MYOF;FNDC3B;AFAP1;ANO6;NAV2;FRMD6;EVC;COL4A2;CDH11;ALPK3;MXRA7;ALPK2;DOCK1;MET;VCL;CHST3;RAI14;YAP1;NOTCH2;SMPX;SEMA3C;ROCK2;NXN;NTN4;TMTC1;FSTL1;LTBP1;HECTD1;CALD1;ARSJ;NRAP;CTNNA1;ABL1;TNNI1;X*

			IRP2;ZNF106;FLNB;MAP4;PDLIM5;MPDZ;CTNNAL1;SVIL;GALNT2;NEK7;EYA4;SAMD4A;CRIM1;LAMB1;AP2B1;CDC42BPB;GNG12;FBXO32;KTN1;EXT1;TJP1;EXT2;ANLN;SMTN;DPY19L1;COL5A1;DLC1;DLG5;FAT1;SNAI2;FBN1
ZNF300 human tf ARCHS4 coexpression	84/299	4.2583582371160024E-4	SLC44A5;ATF2;DRAXIN;USP33;PTPRO;ELAVL4;GRIK2;TXNDC16;CDH8;TPGS2;ROBO1;GRIP1;NHSL1;CDH2;GRM7;DPYSL5;ZNF606;PPIP5K2;TRIM2;GDAP1L1;SUMO2;SPIN1;PHACTR3;SMARCAD1;KIF21A;TUBB2BP1;EPHB1;PPFIA2;WSB1;CXADR;ARL15;LRRC49;TCF12;MAGI2;ATRX;RFX3;TET1;ZNF271P;SRP9;ITFG1;PGM2L1;SETBP1;IFT81;LRRC7;RUFY2;AKAP9;PDZRN4;KMT2E;GRIA1;HDAC2;RTN1;STAU2;BTF3L4;NRXN3;PIK3R3;BAZ2B;NREP;NOL4;STOX2;MAPK8;GNG2;HECTD2;MAP2;ZNF627;NCAM1;CTNNA2;PAK5;MYEF2;CADM1;ZNF804B;PBX3;LHFPL3;ATAT1;PTPRD;MAPK10;CCDC88A;RALYL;APC;ZNF738;KLHL7;TTC3;CCSER1;TCF4;SSBP2
ZNF33A human tf ARCHS4 coexpression	84/299	4.2583582371160024E-4	SPAG16;ZNF891;PATJ;UHRF1BP1L;ANKRD36;ZNF292;USP33;ELAVL4;UBE3A;TMEM260;RORB;PPP1R9A;BICD1;SYNE2;RIMS2;PPIP5K2;LRRTM4;ADAMTSL3;DNER;GDAP1L1;SCAPER;BBS4;ANKRD36C;CXADR;RALGAPA1;DCC;LRRC49;RALGAPA2;KCNH8;VPS13C;ATRX;VPS13B;ZNF271P;ANK3;ZDHHC17;ARID1B;PGM2L1;ZFP90;PARP8;AKAP9;SCG5;ZNF675;ANKRD36B;KDM7A;ZNF431;SAMD5;KMT2E;HDAC2;TNKS;KMT2C;TULP4;BAZ2B;NREP;NOL4;GLB1L3;MTMR7;GTF2IP1;MAP6;ANKRD36BP2;PAK3;EVI5;BPTF;CLVS1;ATF7IP;RIC3;MBD5;MYEF2;ZNF382;PBX3;PHC3;MAPK10;MAB21L3;CCDC88A;CLCN5;PPP2R2B;NEDD4;TTC3;CCSER1;KIAA1958;BRWD1;SSBP2;KIAA0825;FRYL;TNRC6B
FOXJ3 human tf ARCHS4 coexpression	84/299	4.2583582371160024E-4	RERE;DIDO1;SETD2;DPY19L2P3;KDM1B;CELF2;MAST2;CEP120;HNRNPU;EFCAB14;MSI2;SOGA1;ETS1;RPS6KA3;AKAP13;NIPBL;PTAR1;HERC2;HERC1;MPRIP;ZMYM4;UBAP2L;EPC2;QSOX2;NEO1;TRPC7;MBNL1;KLF12;ZHX3;CACNA2D1;VPS13D;ASH1L;TANC2;PHF20L1;SFPQ;RFX7;BIRC6;PABPC1;PAFAH1B1;ANKRD17;MACF1;DDX6;NFAT5;MTMR3;WDR26;KMT2C;HERC2P2;HTT;BAZ2A;AMBRA1;MAPK8;LARP1;BTAF1;HECTD1;NSD1;HECTD4;HIVEP2;MAP4;RANBP2;USP24;SPEN;CREBBP;TRAPPC10;ERBIN;YLPM1;LNPEP;AP2B1;HIPK1;FBXO32;SMARCA2;GATAD2B;SCAF4;ZZEF1;MTOR;MED13L;EXT2;CYLD;DIAPH1;KANSL1;AGO2;STRN;PKN2;ESYT2;CDK12
ZCCHC11 human tf ARCHS4 coexpression	84/299	4.2583582371160024E-4	CCDC122;ZNF891;DRAXIN;CRYBB2P1;ZBTB25;PCDH11Y;ANKRD36;CHD9;ZNF292;USP33;ELAVL4;EFCAB6;SYNE2;NIPBL;ZNF608;ZSCAN30;PPIP5K2;CA5A;ADAMTSL3;GDAP1L1;SCAPER;ZNF521;RBM6;ANKRD36C;WSB1;CXADR;USP49;DCC;ZNF160;LRRC49;SLX4IP;MAGI2;ATRX;RFX3;VPS13B;SETBP1;IFT81;NME7;RUFY2;WDPCP;ASTN2;ANKRD36B;ZNF431;ZNF397;KMT2E;CATSPER2;GREB1L;TULP4;TPTE2P2;RGPD5;BAZ2B;NREP;NR2C1;MIPOL1;AK9;STK3;FBXL20;ORC4;KIAA1328;DZANK1;ANKRD36BP2;ANKRD10;AP4S1;SRGAP3;ATP9B;BPTF;RIC3;MBD5;ZNF382;ST8SIA1;MGA;ARHGAP28;DDHD1;MAB21L3;CCDC88A;AGO3;KANSL1;TMEM116;KLHL7;NEDD4;TTC3;BRWD1;ASB3;TNRC6B
MEF2A human tf ARCHS4 coexpression	83/299	7.091736420908814E-4	ATF1;RYR2;MYOM1;DOCK4;DOCK9;EFCAB14;LDB3;FRY;MYOM2;SLC8A1;SYNE1;AKAP13;FYCO1;ZFYVE26;EFR3A;ALCAM;SGCD;HERC1;MB;PEBP4;KIF13A;MYO18B;PGM5;SGCG;SH3GLB1;UNC45B;MBNL1;MEF2C;MLIP;MBNL2;ZHX3;SFMBT2;VPS13C;VPS13D;TRDN;TOM1L2;ZNF717;MYL1;KCNMA1;BMP2K;ALPK3;KCNQ5;WDFY3;ALPK2;TLN2;PPP1R12B;MACF1;RABGAP1;SMPX;PRUNE2;ITPR2;CACNA1C;TRAK1;BCL2L13;ABLIM1;SCFD2;NRAP;TBX20;TNNI1;XIRP2;ZNF106;CTNNA3;HIVEP2;MAP4;PDLIM5;SVIL;NEBL;EFL1;NEK7;SAMD4A;PDE4DIP;LNPEP;MYO9B;LHFPL2;PLXDC2;FBXO32;SMARCA2;MOB1B;CYLD;ARHGA

			P31;ANKRD18A;RGL1;ASB2
HIF1A human tf ARCHS4 coexpression	83/299	7.091736420908814E-4	*BCAR3;NRP1;DOCK5;TRIO;ITPRIP;C2CD2;ECE1;PTPRK;LAMC1;BACH1;BZW1;CXCL2;GALNT10;LOXL2;TUBB6;ADAMTS2;ALCAM;C16ORF72;DSE;TEAD1;ADAMTS9;IKBIP;CAST;IL1R1;MYOF;FNDC3B;AFAP1;ADAM10;ANO6;HSPG2;VCAN;FRMD6;LATS2;DPYD;CDC42EP3;CDH11;DOCK1;MET;CD44;VCL;CHST3;RAI14;YAP1;NOTCH2;GRAMD1B;WDR26;SEMA3C;NTN4;IQGAP1;FSTL1;LTBP1;PCNX1;DRAM1;CALD1;ARSJ;FAM180A;ABL2;SNX9;FLNB;CTNNAL1;RANBP2;GALNT2;GALNT1;NEK7;CRIM1;DNAJC13;LAMB1;SYNJ2;ATP2B1;GNG12;KTN1;EXT1;EXT2;MYO1E;COL5A1;FAT1;ITGBL1;SNAI2;ESYT2;ACO1;ATP13A3;SEC24D;FBN1*
NR3C1 human tf ARCHS4 coexpression	83/299	7.091736420908814E-4	*ITSN2;PI4K2B;TNFAIP8;TRIO;ANKRD33B;ATP8A1;SH3KBP1;DOCK8;EFCAB14;PTPRJ;IKZF2;BACH1;LYST;ETS1;FAM107B;DOCK10;AKAP13;ZFYVE26;EFR3A;HERC1;KIF13A;DSE;DIP2B;LRRFIP1;MAP3K5;CAST;CD96;MBNL1;ITGA4;PRKCB;ZBTB38;VPS13C;VPS13D;ARAP2;ANO6;APBB1IP;TRAF3;DPYD;AGPS;SPOPL;BIRC6;UTRN;DOCK2;WDFY4;KDM7A;NOTCH2;WDR26;HTT;ITPR2;IQGAP1;SAMSN1;PCNX1;BTAF1;RAP1A;ATXN1;BTLA;SUSD6;HIVEP2;LYN;USP24;PLEKHA2;PCGF5;STAT1;TRAPPC10;NEK6;RFTN1;GPR55;NEK7;ERBIN;LNPEP;MYO9B;DNAJC13;SMARCA2;FLI1;ZZEF1;MED13L;CYLD;MYO1E;ARHGAP31;SP3;BCL2;ESYT2;ATP13A3*
LCORL human tf ARCHS4 coexpression	83/299	7.091736420908814E-4	*SPAG16;CNTNAP2;ANKRD36;ZNF292;USP33;ELAVL4;UBE3A;PPP1R9A;NHSL1;ZNF606;PPIP5K2;LRRTM4;TRIM2;SNAPC3;SMARCAD1;KIF21A;SOX6;ANKRD36C;WSB1;CXADR;LRRC49;TCF12;MAGI2;ATRX;RFX3;ZDHHC17;SRP9;PYGO1;SETBP1;IFT81;NME7;LRRC7;RUFY2;AKAP9;ZNF678;ASTN2;ANKRD36B;KMT2E;HDAC2;STXBP4;STAU2;ATL1;BTF3L4;PIK3R3;ZNF518A;BAZ2B;NREP;NOL4;MTMR7;MIPOL1;ORC4;MAPK8;GNG2;HECTD2;MAP2;ATP9B;SCAI;ATF7IP;MBD5;MYEF2;ZNF382;ERCC6L2;RANBP17;DDHD1;ST18;ATAT1;PTPRD;CCDC88A;LRFN5;FER;CNOT7;NBEA;TMEM236;APC;ZNF738;KLHL7;ASXL3;TTC3;PARGP1;CNTN4;BRWD1;ASB3;FRYL*
KLF7 human tf ARCHS4 coexpression	83/299	7.091736420908814E-4	*ROBO2;DRAXIN;MYT1L;ZNF292;PTPRO;CELF4;ELAVL4;GRIK1;GRIK2;BICD1;GPHN;GRIP1;ZNF608;DPYSL5;TRIM2;AKT3;DNER;GDAP1L1;ZNF449;KIF21A;C19ORF18;TUBB2BP1;RALGPS1;PPFIA2;KLF12;WSB1;CXADR;DCC;RC3H1;RFX3;ANK3;RGMB;ZDHHC17;MYT1;PGM2L1;CLIP1;SETBP1;LRRC7;RUFY2;AKAP9;ALPK3;EXOC1;DEFB103A;KMT2E;GRIA1;RTN1;ATL1;NEDD4L;NREP;KALRN;STOX2;GNG2;HECTD2;MAP2;ZNF627;MYH13;TNNI1;MAP6;APBB2;NPHP4;NCAM1;CTNNA2;SRGAP3;PAK3;PAK5;MBD5;OSBPL6;CADM1;ZNF382;AUTS2;PNPLA8;MYO5A;UBE2G1;SYNJ2;DCLK1;ST18;ATAT1;CCDC88A;TTC3;YPEL1;TCF4;FSIP1;FBXL7*
ZNF365 human tf ARCHS4 coexpression	83/299	7.091736420908814E-4	*DOCK3;KCNC1;FRMPD4;DIRAS2;RASGRF1;OTUD7A;KNDC1;RPH3A;UNC80;SYNPR;GRM5;AKAP11;MCF2L;NCS1;PSD3;DLGAP1;PRKACB;CALN1;KCNH1;RBFOX1;RBFOX3;TMEM178A;PRKCE;TMOD2;ANK2;MYRIP;SYN2;GABRG2;MAPK8IP1;GABRG1;AJAP1;CNKSR2;DNM3;MPPED1;PITPNM3;SCN8A;KCNMA1;RAPGEF5;PLCB1;NGEF;ASTN1;RAPGEF4;SLC24A2;NECAB1;STXBP1;ADAM22;SLC1A2;NDRG2;PRKCZ;NALCN;RAP1GAP;KIAA0513;GRIN2A;CHN1;CLVS2;SPOCK1;MBP;CACNG3;GPR158;ATP9A;OPCML;GABBR2;ARNT2;GABRA6;ANKRD24;SYT1;CADM2;ATRNL1;SLC4A10;SYT16;KIAA1549L;MYO5A;ATP2B2;HSPA12A;SNAP91;TTLL7;DLG2;SYNJ1;PPP2R2C;RCAN2;CNTN1;SCN2A;HCN1*
ZNF84 human tf ARCHS4	83/299	7.091736420908814E-4	*ZNF891;ANKRD36;ZNF292;USP33;MSANTD4;GPHN;CDH2;DPYSL5;TRIM2;KIF21A;ANKS1B;PDK1;MAGI1;ANKRD36C;WSB1;CXADR;HFM1;DST;LRRC49;ATRX;RFX3;TET1;ZNF271P;ANK2;ZNF14;SRP9;ITFG1;ENAH;GAP43;SETB*

coexpression			P1;IFT81;ADGRB3;LRRC7;RUFY2;AKAP9;ZNF234;ANKRD36B;ZNF397;CRB1;HDAC2;STAU2;TNKS;PLPPR5;BTF3L4;PIK3R3;BAZ2B;NREP;CACNA1E;NR2C1;STOX2;GNG2;HECTD2;MAP2;CTNNA2;SRGAP3;PAK3;PAK5;BBS2;ZNF462;MBD5;MYEF2;ZNF382;ERCC6L2;RANBP17;LSAMP;ZNF33B;ENOX1;ATAT1;PTPRD;MAPK10;TNRC6C;CCDC88A;AGO3;NBEA;APC;ZNF738;KLHL7;TTC3;TCF4;BRWD1;PTPN4;SSBP2;CCDC171
ETV5 human tf ARCHS4 coexpression	82/299	0.0011750227071781077	SEMA5A;PLEKHB2;GLDC;CSE1L;LDLRAD3;FMN1;KIF11;LAMC1;BZW1;KIF15;PTPRG;EPS8;GRM3;SRGAP2C;SPRED2;TUBB6;ARHGAP42;SPRED1;NHSL1;PSMD2;NUF2;CWC22;BCAP29;HMCN1;PTGFRN;STK32A;TEAD1;SRGAP2B;MAPK1IP1L;SUPT16H;CSNK2A1;KCND3;TCF12;FNDC3B;RC3H1;ADGRA3;MITF;SEZ6L;EPN2;ENAH;TANC1;MELK;FRMD6;RAB38;DOCK1;CHST3;EXOC1;RAI14;MTMR2;ROCK2;CDCA5;ILDR2;ASAP2;TRPM1;C10ORF90;SDCBP;GNA14;NUAK1;MGAT5;RNF217;EXTL3;BUB1;ARNT2;STARD13;WSCD1;EFL1;MYO5A;HMGA2;UBE2G1;NETO2;SYNJ2;PTPN12;GNG12;CDC42BPA;S100B;CORO2B;NDC80;ANLN;DPY19L1;FABP7;SPIRE1;FBXL7
LCOR human tf ARCHS4 coexpression	82/299	0.0011750227071781077	USP31;MYT1L;ANKRD36;ZNF292;GALNT18;CLCN3P1;ELAVL4;FRG1HP;C12ORF40;C16ORF72;NHSL1;ZNF606;LRRTM4;TRIM2;PDK1;MAPK1IP1L;ANKRD36C;KLF12;WSB1;CXADR;SLX4IP;MAGI2;ATRX;FAM118A;RFX3;PRKCA;ASH1L;OPRM1;GFRA2;ARID1B;ENAH;FRMD5;IFT81;LRRC7;RUFY2;AKAP9;BIRC6;ASTN2;ALG10B;ANKRD36B;MCPH1;UBE2Q2P1;KMT2E;TPH2;INO80D;CUL5;KMT2C;BTF3L4;RGPD6;TULP4;TPTE2P2;RGPD5;BAZ2B;NREP;NOL4;MIPOL1;NKAIN2;FBXL20;CHSY1;MAP2;ANKRD10;PAK5;MBD5;MYEF2;ZNF382;ERCC6L2;INSR;MGA;PHC3;KITLG;MFSD14C;CNOT7;DAB1;NBEA;KLHL7;TTC3;TCF4;ASB4;BRWD1;ASB3;XKR5;TNRC6B
ZNF436 human tf ARCHS4 coexpression	82/299	0.0011750227071781077	SEMA5A;DRAXIN;THSD7B;MEGF10;SLC35F1;PPP1R9A;BICD1;PTPRG;ROBO1;SRGAP2C;TRIM9;ADAMTS3;CDH2;DPYSL5;TRIM2;AKT3;DNER;SPIN1;SACS;ITGB8;KIF21A;PTGFRN;ERC1;SOX6;SRGAP2B;MAGI1;EPHA4;DST;TET1P1;SEMA6D;ANK2;FOXP2;TANC2;PJA2;IMPACT;SETBP1;ADGRB3;RUFY2;COL21A1;ZMYND11;VSTM2A;CHST3;EXOC1;GRIA1;MACF1;MAGEL2;IGSF3;TNKS;ZBTB49;NREP;NPAS3;GNG2;MAP2;ZNF704;MVB12B;APBB2;NCAM1;TSPAN3;MAP4;JAM2;PAK5;VAT1L;RFTN2;NDFIP1;WSCD1;NTRK3;SORBS2;NETO2;AP2B1;CORO2B;DCLK1;ATAT1;PTPRD;DLG2;APC;RCAN2;TTC3;TRPV5;SPIRE1;TCF4;FBXL7;LRP12
ZNF460 human tf ARCHS4 coexpression	82/299	0.0011750227071781077	ZNF292;SMG1P2;LTN1;SMG1P5;CDH8;SYNE1;C16ORF72;HERC1;CDH2;ZSCAN30;TRIM2;ZNF407;PPP6R3;SACS;ITGB8;WSB1;CXADR;ESCO1;DST;RALGAPA1;VPS13C;PDE4D;TTC3P1;VPS13D;RFX3;VPS13B;ANK2;ASH1L;GAPVD1;ZDHHC17;SRP9;TANC2;PJA2;SETBP1;LRRC7;RUFY2;WDFY3;ZNF678;BIRC6;PIK3C3;ZNF236;UTRN;ZNF112;MACF1;NFAT5;FOCAD;INO80D;TNKS;KMT2C;BTF3L4;RGPD5;RGPD2;ARHGAP12;FGD4;BTAF1;HECTD1;HECTD2;MAP2;FUT9;ZNF704;NSD1;ZNF627;KIAA0753;CEP192;ZC3H14;ATP9B;GRIA4;GABRA2;RANBP2;MBD5;MON2;ERCC6L2;LRBA;MGA;RANBP17;LRFN5;APC;TTC3;PARGP1;BRWD1;FRYL;TNRC6B
ZNF521 human tf ARCHS4 coexpression	82/299	0.0011750227071781077	ZNF573;CHRM3;DRAXIN;TENM3;WNT2B;TENM4;GALNT16;ZNF292;CTNND2;CELF4;LDLRAD3;MSI2;CDH9;CDH8;TTC28;ZNF608;DACH1;CDH2;DPYSL5;PSD3;SUMO2;CALN1;EPHA4;WSB1;DCC;LRRC49;TCF12;ATRX;RFX3;IL17RD;EML1;SRP9;PYGO1;IFT81;ST6GALNAC3;ASTN1;ZNF397;KHDRBS2;HDAC2;CHRNA7;GREB1L;ILDR2;NREP;HERC2P9;MED12L;NPAS3;PRTG;STOX2;MAP2;PRDM16;MAP6;CAMTA1;NCAM1;TSPAN3;CTNNA2;SRGAP3;SLIT2;ZNF423;MPDZ;TRPM3;JAM2;WASF3;TMEM132C;MYEF2;WSCD1;AUTS2;ANGPT1;PBX3;C9ORF43;PBX1;PTPRD;MAPK10;

			NELL2;ZNF618;ZNF738;COL5A3;TTC3;YPEL1;KIAA1958;SSBP2;ADGRL2;SPSB4
RC3H2 human tf ARCHS4 coexpression	82/299	0.0011750227071781077	*TRIO;FRY;PITPNC1;SYNE2;CEP128;C16ORF72;AKAP11;HERC1;ZMYM4;TRIM2;PSD3;CHAMP1;SACS;DIP2B;ZNF600;JAK2;GUCY1A2;ACTR2;USP7;ARHGEF12;SLC13A4;KSR1;DST;NSUN2;VPS13C;PDE4D;VPS13D;ASH1L;FRMD4B;TANC2;HADHB;SFPQ;BTD;TBC1D5;SPOPL;FAR1;WDFY3;BIRC6;ZMYND11;DGKI;DTHD1;MACF1;DDX6;MTPN;NFAT5;RABGAP1;WDR26;ROCK2;ASAP2;AMBRA1;FAM214A;TM9SF3;PCNX1;MAPK8;LARP1;ATXN1;HECTD1;NSD1;HIVEP1;HIVEP2;PTCHD4;ATP9A;RANBP2;ARFGEF1;USP24;LRBA;TRAPPC10;MGA;ERBIN;MYO5A;LNPEP;TRAPPC8;DNAJC13;KIAA0232;MED13L;APC;SP3;FAT1;STRN;FAT4;BRWD1;FRYL*
ARX human tf ARCHS4 coexpression	81/299	0.001978492206006498	*PCSK2;ROBO2;ERO1B;TUSC3;ZNF292;PTPRO;ELAVL4;HS6ST3;SCGN;GRM7;DPYSL5;TRIM2;ZNF846;PLCE1;SOX6;ARFGEF3;RGS7;ANKRD36C;PACRG;KCND3;LRRC49;MAGI2;HUNK;UBE2QL1;TOX3;CACNB2;MDS2;MMP16;SETBP1;AKAP9;MPPED2;SCG5;SCG3;VSTM2A;RIN2;SAMD5;PDE1A;BTF3L4;NRXN3;RGPD5;BAZ2B;NREP;NOL4;SLC7A2;MTMR7;NPAS3;GNG2;TTR;MAP2;SNTG1;NCAM1;PAK3;CORIN;AKAIN1;PRELID2;RIC3;ANKRD26;PTPRN2;MYEF2;ZNF382;CADPS;POU6F2;EPHX4;ELL2;ST18;DCDC1;MOB1B;MAPK10;CCDC88A;TMEM236;KLHL7;ASXL3;TTC3;RIMBP2;CNTN1;ZNF536;CPE;TCF4;CNTN4;FGF12;XKR5*
ZNF608 human tf ARCHS4 coexpression	81/299	0.001978492206006498	*GABRB3;RERE;SH3GL3;DRAXIN;TENM3;HIP1;TENM4;CTNND2;ELAVL4;PPP1R9A;TSPAN11;ROBO1;CDH4;DACH1;CDH2;DPYSL5;PEG10;TRIM2;HYDIN2;NEO1;PPFIA2;MAGI1;SMARCC1;WSB1;TMEM178B;ATRX;RFX3;TET1;ANK2;FRMD4A;IL17RD;SETBP1;CATSPERG;AKAP9;TNKS;GREB1L;PIK3R3;AGAP1;ILDR2;BAZ2B;NREP;KALRN;PHF21B;STOX2;CECR2;FLRT2;MAP2;ZNF704;HECTD4;MAP6;NCAM1;CTNNA2;SRGAP3;PAK3;ZNF423;MPDZ;MAP4K4;BPTF;LRRC37A3;ZNF462;AUTS2;YLPM1;ESRRG;PTPN13;DCLK1;PBX1;PTPRD;CCDC88A;SDK1;NBEA;APC;ZNF618;WNK2;ASXL3;TTC3;TCF4;KIAA1958;EIF4G3;ADGRL2;TBATA;SPSB4*
EBF3 human tf ARCHS4 coexpression	80/299	0.003182858220596114	*APP;DRAXIN;MYT1L;CTNND2;CELF4;ELAVL4;XYLT1;TSPAN11;SLC6A3;ROBO1;ZNF608;DACH1;DPYSL5;PEG10;DNER;GDAP1L1;KIF21A;KIF21B;EPHB2;NEO1;RPL23AP87;CHST8;DCC;CACNA2D1;EBF1;AFAP1;EBF2;UBE2QL1;GFRA1;RGMB;MYT1;AJAP1;SFPQ;GAP43;ELF2;PDZRN4;PPM1L;NTM;TMTC2;AGAP1;NREP;NYAP2;KALRN;TMEM163;NPAS3;PHF21B;STOX2;GTF2IP1;GNG2;MAP2;MAP6;NCAM1;CTNNA2;SRGAP3;ZNF423;ATP9A;MAP4K4;KLHL29;LINGO1;KCNJ6;NDFIP1;MYEF2;AUTS2;PLCL1;PCDH8;PBX3;LSAMP;CALM1P2;HSPA12A;DCLK1;PBX1;ENOX1;ATAT1;PTPRD;CCDC88A;ZNF618;ZNF536;FAT3;APBA2;SSBP3*
CLOCK human tf ARCHS4 coexpression	80/299	0.003182858220596114	*PATJ;RNF11;ZFYVE9;KDM1B;LTN1;UBE3A;LCLAT1;OSBPL10;PPP1CB;SRGAP2C;EFR3A;PPP2R5E;PPP6R3;LONP2;TEAD1;UNC13C;ZHX3;ARHGEF12;DST;LIMCH1;RALGAPA1;SLC2A13;FNDC3B;VPS13B;ASH1L;FBXW2;TANC2;KIAA1217;PEAK1;ESRP1;FEZ2;AKAP9;NUMB;GEMIN5;PLIN2;WDFY3;CD44;CSNK1G1;ANKRD17;NFAT5;INO80D;PRKAA2;ROCK2;KMT2C;RGPD6;PRICKLE2;TULP4;ASAP1;FAM214A;LPP;AURKA;GNA14;NLRP8;HECTD1;ARSJ;NLRP4;HIVEP1;TRPM7;MAP4K3;RANBP2;ARFGEF1;ABCA5;MGA;DENND4C;KIAA1549L;DNAJC13;CDC42BPA;ELL2;MYO9A;PTK2;KTN1;EXT1;TJP1;MOB1B;NLRP13;SYNJ1;SLMAP;SPIRE1;CPEB4;SNTB2*
POU2F1 human tf ARCHS4 coexpres	80/299	0.003182858220596114	*GABRB3;DRAXIN;HIP1;ZMYND8;ZNF292;PSIP1;GLI2;ZNF608;SNRPD1;ADAMTSL3;FLVCR1;RBPMS2;JARID2;MAGI1;WDHD1;ZNF121;SMARCC1;ADGRV1;LRRC49;TET1;IL17RD;ARID1B;SGO1;MSH6;SFPQ;MSH2;MTF2;ROR1;CCDC150;FAM72B;ZNF431;ZNF397;DDX6;HDAC2;TNKS;IR*

sion			EB2;GREB1L;BAZ2B;GLB1L3;MIPOL1;PHF21B;FGD4;ATXN3;STOX2;APELA;GTF2IP1;RIC8B;KIAA1328;ADAMTS19;NSD1;MAP6;IGF2BP3;CLSPN;SRGAP3;ZNF423;SCAI;BPTF;ZNF462;MYEF2;SLC16A1;AUTS2;MGA;PCDH8;YLPM1;ARHGAP28;ARID3B;PBX1;GATAD2B;MAB21L3;MLLT10;CCDC88A;CENPE;KANSL1;ZNF738;NEDD4;FAT3;KIAA1958;ASB3;ZNF850;TNRC6B
RCOR3 human tf ARCHS4 coexpression	80/299	0.003182858220596114	PCSK2;SPAG16;ERO1B;LPGAT1;ATP8A1;CHD9;ICA1;ZBTB20;TMEM182;ZNF44;EFCAB6;FRY;MYLK3;IGF1R;PCMTD2;PPP1CB;SENP6;SCGN;CNST;HERC1;PPIP5K2;FBXO3;TLK1;PCMTD1;RBM6;MAPK1IP1L;LIMCH1;RALGAPA1;ABCC8;SLX4IP;VPS13C;COMMD10;VPS13B;OPRM1;ARID1B;ETV6;AKAP9;MYO3A;SCG5;SCG3;USP41;ASTN2;ZNF236;KMT2E;KMT2C;RGPD6;TULP4;RGPD5;RGPD8;ZDHHC21;FAM214A;FBXL20;GRK3;TTR;RNF217;ANKRD36BP2;USP24;RABGAP1L;ABCA5;PHKB;PARVB;DDHD1;SMARCA2;PHC3;ELL2;MOB1B;OCLN;PLCXD3;DPY19L2;AGO3;ZNF615;CCSER2;CPE;ASB4;BRWD1;ZNF354C;CPEB4;CDS2;TNRC6B;CCDC171
ZFP1 human tf ARCHS4 coexpression	80/299	0.003182858220596114	ATF2;ELAVL4;GRIK2;BICD1;TPGS2;ROBO1;PCMT1;CDH2;DPYSL5;ZNF606;LRRTM4;TRIM2;DNER;SUMO2;PHACTR3;KIF21A;TUBB2BP1;DNM1L;EPHB1;PPFIA2;RGS7;EPHA4;WSB1;CXADR;ATRX;RFX3;ANK2;SRP9;FOXP2;PYGO1;FRMD5;SETBP1;IFT81;ADGRB3;DOK5;MPPED2;IL1RAPL1;KMT2E;MAGEL2;NLGN1;HDAC2;RTN1;STAU2;TNKS;BTF3L4;NRXN3;NREP;NOL4;MAPK8;GNG2;MAP2;FUT9;ZMAT4;CLVS2;CTNNA2;SRGAP3;PAK5;OPCML;CLVS1;ATF7IP;CADM2;RANBP17;PBX3;LSAMP;KLHL1;ESRRG;BTBD10;ATAT1;PTPRD;MAPK10;CCDC88A;CNOT7;NFIA;NBEA;APC;NFIB;KLHL7;TTC3;ZNF536;MDGA2
ZC3H11A human tf ARCHS4 coexpression	80/299	0.003182858220596114	ITSN2;DIDO1;SETD2;LPGAT1;USP32;HNRNPU;RPS6KA3;AKAP13;EFR3A;NIPBL;PTAR1;C16ORF72;HERC1;KIF13A;DIP2B;ZNF124;GABPA;PCMTD1;GTF2I;CAST;MBNL1;USP7;ARHGEF12;RALGAPA1;VPS13C;VPS13D;VPS13B;ANO6;ASH1L;FBXW2;SFPQ;CSDE1;SPOPL;WDFY3;BIRC6;UTRN;DOCK1;KDM7A;NOTCH2;MACF1;DDX6;MTPN;NFAT5;MTMR3;WDR26;ROCK1;UHRF2;ROCK2;BAZ2A;IQGAP1;TM9SF3;PCNX1;SCAF8;ABLIM1;BTAF1;HECTD1;NSD1;TRPM7;RANBP2;USP24;SPEN;CREBBP;EGLN3;HOMER2;LRBA;SIAH2;TRAPPC10;ERBIN;LNPEP;DNAJC13;HIPK1;SMARCA2;ZZEF1;MTOR;MED13L;SLMAP;SP3;STRN;PKN2;ESYT2
RORA human tf ARCHS4 coexpression	79/299	0.0050474083731396864	ITSN2;OXNAD1;DOCK9;DOCK8;CELF2;TMEM182;FRY;LYST;ETS1;SYNE2;SYNE1;DOCK10;AKAP13;HERC1;KIF13A;FAM25G;PCMTD1;CERS3;MBNL1;KLF12;GRID2;PRKCH;MBNL2;CNOT6L;ITGA4;RALGAPA1;VPS13C;VPS13D;ARAP2;VPS13B;KCNAB1;ASH1L;TC2N;IPCEF1;PARP8;INPP4B;CLIP1;PCP4;CARD18;PRKCQ;BIRC6;ZNF831;ASTN2;UTRN;KDM7A;KMT2E;MACF1;NFAT5;NLRC5;RASGRP1;CACNA1I;PCNX1;ABLIM1;ATXN1;HIVEP2;KDM4C;BCL11B;ABCA5;SEMA4D;KCNIP4;ERBIN;ADAM32;LNPEP;FOXN3;DDHD1;ARHGAP26;SMARCA2;PHC3;PARP15;MPP7;ZZEF1;TTC39B;CYLD;SNRK;CAMK4;NEDD4;CCSER2;PTPN4;CPEB4
PRDM2 human tf ARCHS4 coexpression	79/299	0.0050474083731396864	CYFIP2;ITSN2;RERE;SETD2;ANKRD33B;DOCK8;CELF2;AFF3;ETS1;SYNE2;FAM107B;SYNE1;FCRLA;DOCK10;AKAP13;NIPBL;C16ORF72;AKAP11;HERC1;EPC2;MBNL1;PRKCB;NCOA6;VPS13C;VPS13D;ARAP2;VPS13B;ASH1L;IPCEF1;FCHSD2;BANK1;PACS1;SPOPL;BIRC6;UTRN;DOCK2;WDFY4;KDM7A;BLK;KMT2E;NFAT5;WDR26;ROCK1;UHRF2;KMT2C;NLRC5;HTT;BAZ2A;RASGRP1;PCNX1;HECTD4;HIVEP1;BTLA;BMF;HIVEP2;BPTF;LYN;SPEN;CREBBP;RABGAP1L;BCL11B;SEMA4D;IQSEC1;PLEKHA2;TRAPPC10;YLPM1;LNPEP;MYO9B;SMARCA2;PARP15;GATAD2B;SCAF4;ZZEF1;MED13L;CYLD;ARHGAP32;KANSL1;BCL2;NFKBID

EBF1 human tf ARCHS4 coexpression	79/299	0.0050474083731396864	*ZNF891;DRAXIN;PTPRO;ELAVL4;BICD1;CCDC102B;GRM1;PTPRG;ROBO1;ZNF608;DACH1;DPYSL5;DNER;GDAP1L1;ANKS1B;PPFIA2;TRPC7;USP49;DCC;CACNA2D1;LRRC49;EBF2;UBE2E2;EBF3;ANK3;RGMB;MYT1;ARID1B;PGM2L1;FOXP2;GAP43;MMP16;NAV3;SETBP1;RUFY2;AKAP9;HECW1;ARHGEF7;CNTNAP5;FTO;KMT2E;RTN1;TPTE2P5;KCNE4;CHRNA7;GREB1L;AGAP1;NREP;KALRN;TMEM163;PHF21B;FGD4;STOX2;NKAIN3;GNG2;MAP2;MAP6;NCAM1;SRGAP3;KLHL29;CADM1;AUTS2;PCDH9;PLCL1;PBX3;POU6F2;KLHL1;YLPM1;ST18;PBX1;ATAT1;NELL2;CCDC88A;RALYL;TTC3;YPEL1;GALNTL6;CDK14;LHX9*
ZNF81 human tf ARCHS4 coexpression	79/299	0.0050474083731396864	*KDM5A;MCTP1;THSD7B;ATP8A1;DOCK8;CELF2;USP33;PTPRJ;TMEM260;LYST;RAB22A;SYNE1;AKAP13;NIPBL;C16ORF72;HERC1;TRAPPC11;JAK2;TRPC7;MBNL1;RALGAPA1;VPS13C;VPS13D;VPS13B;ASH1L;FAM126B;PJA2;PHF20L1;KCNQ3;SPOPL;WDFY3;BIRC6;UTRN;DOCK2;MCTP2;KDM7A;CNTNAP5;MACF1;NFAT5;NLGN1;KMT2C;BAZ2B;THADA;UBR1;FGD4;PCNX1;MAPK8;BTAF1;NSD1;FCHO2;ANKRD36BP2;TRPM7;CEP192;ST8SIA6;BPTF;AKAIN1;ARFGEF1;USP24;RABGAP1L;MBD5;FANCM;ERCC6L2;LRBA;MGA;PBX3;KLHL1;ERBIN;SYT16;LNPEP;DDHD1;SMARCA2;PHC3;CYLD;SNRK;DMXL2;STRN;FAT4;BRWD1;CCDC171*
AHCTF1 human tf ARCHS4 coexpression	79/299	0.0050474083731396864	*DIDO1;USP32;CSE1L;NCAPG2;LTN1;HNRNPU;KIF11;NIPBL;PTAR1;SACS;UBAP2L;DIP2B;SMARCC1;SUPT16H;USP7;NSUN2;VPS13D;URB1;PATL1;ASPM;SFPQ;TFDP1;KIFC1;CSDE1;SPOPL;WDFY3;BIRC6;PABPC1;UTRN;MET;VCL;ANAPC1;NOTCH2;MACF1;MTPN;NFAT5;WDR26;ANP32A;ROCK2;HTT;BAZ2A;IQGAP1;ACACA;TM9SF3;HIRA;PCNX1;SCAF8;LARP1;HECTD1;RACGAP1;NSD1;MAPK1;CEP192;IARS2;BUB1;RANBP2;ARFGEF1;USP24;LRBA;TRAPPC10;MGA;ERBIN;XPO7;DNAJC13;HIPK1;MTOR;MED13L;KTN1;ANLN;DIAPH1;KIF4A;SERBP1;SP3;FAT1;STRN;PKN2;ESYT2;CDK12;ATP13A3*
SHPRH human tf ARCHS4 coexpression	79/299	0.0050474083731396864	*CCDC122;ZNF891;ANKRD36;ZNF292;USP33;DPY19L2P2;GADL1;TMEM182;EFCAB6;AFF3;LCLAT1;MYLK3;CEP128;UNC80;EPB41L4A;CCDC91;HERC1;ZSCAN30;PPIP5K2;LRRTM4;ADAMTSL3;KYNU;PCMTD1;POTEC;RBM6;ANKRD36C;MAGI2;ATRX;TTC7B;VPS13B;FRMD4B;OPRM1;SHISA9;ARID1B;GAREM1;PEAK1;RUFY2;AKAP9;WDPCP;ASTN2;ZMYND11;L3MBTL4;ANKRD36B;ZNF431;FTO;SHC4;TPH2;INO80D;KCNE4;RGPD5;ATP10B;BAZ2B;GLB1L3;MIPOL1;STK3;ATXN3;ORC4;KIAA1328;ANKRD36BP2;CSMD3;ATP9B;MBD5;CEP112;SLC14A2;RANBP17;ADAM32;ARHGAP28;DDHD1;PARP15;MAB21L3;CCDC88A;NEDD4;RAB3GAP2;TCF4;ASB4;BRWD1;ASB3;TNRC6B;CCDC171*
RLF human tf ARCHS4 coexpression	78/299	0.007941755863418297	*KDM5A;ATF2;TCERG1;SETD2;UHRF1BP1L;ZNF292;SMG1P2;ZBTB21;LTN1;UBE3A;BACH1;SYNE2;NIPBL;C16ORF72;HERC1;ZNF407;RB1CC1;RAB8B;KPNA1;KDM6A;EPHA4;MORC3;WSB1;CXADR;ESCO1;COG5;ZNF160;ATRX;RFX3;VPS13B;ASH1L;PJA2;SDE2;BIRC6;TOX;PDZRN4;KDM7A;KMT2E;DDX6;NFAT5;HDAC2;WDR26;TNKS;KMT2C;TULP4;RGPD8;RGPD2;NOL4;SCAF8;MAPK8;BTAF1;HECTD2;MAP2;SLIT2;ZC3H15;RANBP2;USP24;ATF7IP;SPEN;CA10;KDM4C;ERCC6L2;MGA;ZBTB10;PUM1;BTBD10;MED13L;CCDC88A;NIN;NBEA;APC;TTC3;DMXL2;SP3;BRWD1;FGF12;FRYL;CPEB4*
ZNF385B human tf ARCHS4 coexpression	78/299	0.007941755863418297	*RERE;DOCK3;ATP8A1;DIRAS2;RASGRF1;CHD6;KNDC1;SIPA1L3;IGF1R;RPH3A;UNC80;GRM5;ZNF608;MCF2L;NCS1;PSD3;DLGAP1;SYBU;ARFGEF3;PPFIA2;UNC13B;CADPS2;RBFOX1;ARHGEF12;PRKCE;RALGAPA2;TMOD2;COBL;MYRIP;SYN2;KIAA1217;TOM1L2;SCN8A;AKAP9;ADGRF5;ARHGEF7;RAPGEF5;UTRN;NGEF;RAPGEF4;ANKRD17;CASZ1;STXBP1;DNAH5;PRUNE2;ADAM22;TMPRSS2;NLK;NDRG2;PRKCZ;ACACA;RAP1GAP;KIAA0513;SV2B;HECTD4;CHN1;CLVS2;STXBP6;MAP4;CACNG3;GPR158;MARK2;A*

			TP9A;WASF3;OPCML;ZNF462;GABRA6;SYT1;HOMER2;CADM2;IQSEC1;ATP2B2;SNAP91;ARHGAP32;DLG2;PPP2R2C;WNK2;HCN1
ZNF117 human tf ARCHS4 coexpression	78/299	0.007941755863418297	CCDC122;ZNF891;ANKRD36;CHD9;ZNF292;USP33;ONECUT1;ELAVL4;TMEM182;SYNE2;EPB41L4A;ZSCAN30;PPIP5K2;ADAMTSL3;ZNF568;SOX6;PCMTD1;KLF12;WSB1;CXADR;USP49;DCC;LRRC49;ATRX;RFX3;TET1;VPS13B;ZNF271P;FAM126B;TOX3;SETBP1;IFT81;RUFY2;AKAP9;WDPCP;ZNF780B;ASTN2;MCTP2;ZNF431;KMT2E;HDAC2;STXBP4;BTF3L4;TULP4;PIK3R3;ZNF518A;ILDR2;BAZ2B;NREP;MIPOL1;FBXL20;ORC4;MAPK8;GNG2;MAP2;ANKRD20A5P;ANKRD10;RPRD1A;SRGAP3;DISC1;EVI5;ATF7IP;MBD5;RANBP17;DDHD1;CCDC88A;FER;AGO3;APC;ZNF738;KLHL7;TTC3;CCSER1;TCF4;BRWD1;SSBP2;ASB3;XKR6
ZBTB10 human tf ARCHS4 coexpression	78/299	0.007941755863418297	SETD2;ZNF292;SMG1P2;ZBTB21;SMG1P4;CHD6;MSI2;SYNE2;IGF1R;NIPBL;ZNF608;C16ORF72;ZMYM4;RB1CC1;SMARCAD1;SCAPER;ARFGEF3;ANKRD36C;RALGAPA1;ATRX;VPS13B;NSUN6;ASH1L;ZDHHC17;ARID1B;MSH6;ASPM;SFPQ;SETBP1;AKAP9;MTF2;WDFY3;BIRC6;ASTN2;ANKRD17;DDX6;INO80D;TNKS;KMT2C;IREB2;RGPD6;NEDD4L;RGPD5;TMPRSS2;BAZ2B;LPP;MIPOL1;SCAF8;ZNF704;HECTD4;TRPM7;ZSWIM6;BPTF;RANBP2;ARFGEF1;ZNF462;MYEF2;HOMER2;MGA;DNAH14;PPP2R3A;MYO9A;PBX1;MED13L;MLLT10;CCDC88A;CENPE;FER;AGO3;KANSL1;CCNG2;ZNF615;PARGP1;TCF4;BRWD1;CEP44;FRYL;TNRC6B
TOX human tf ARCHS4 coexpression	78/299	0.007941755863418297	ROBO2;ATF2;DRAXIN;ZBTB25;ZNF292;ELAVL4;GRIK2;CDH8;DACH1;DPYSL5;ZNF606;TRIM2;ITGB8;GARNL3;WSB1;ST6GAL2;CXADR;KCNK10;LRRC49;TCF12;MAGI2;RFX3;ZDHHC17;TOX3;SETBP1;IFT81;ADGRB3;RUFY2;MAPRE2;ANKRD36B;ASTN1;KMT2E;CRB1;MAGEL2;KHDRBS2;RTN1;PDE1A;BTF3L4;NRXN3;BAZ2B;NREP;NOL4;NKAIN3;PDCD6IPP2;GNG2;MAP2;SNTG1;FUT9;DPH6;NCAM1;TSPAN3;CTNNA2;SRGAP3;PAK3;SCAI;JAM2;GRIA4;GABRA2;ATF7IP;CA10;MYEF2;ZNF382;ST8SIA1;ERCC6L2;PBX3;SLC4A10;ATAT1;MAPK10;NELL2;LRFN5;RALYL;APC;KLHL7;TTC3;ZNF536;SSBP2;FGF12;LRP12
NEUROD6 human tf ARCHS4 coexpression	77/299	0.012387665035661253	TCERG1;CNTNAP2;ZNRF2P2;MYT1L;PTPRO;GRIK3;RORB;BICD1;SRGAP2C;SLC22A14;NHSL1;ADAMTS3;TRIM2;RB1CC1;AKT3;TMEM108;PHACTR3;SOX5;RGS7;GUCY1A2;EPHA7;CXADR;USP49;CAMLG;KAZN;EML1;MPPED1;LRRC7;AKAP9;HECW1;KCNQ3;RAPGEF2;ZFPM2;SHANK2;LINC00643;GRIA1;SLC24A2;PLPPR1;INO80D;CTTNBP2;NEDD4L;NREP;NYAP2;NOL4;CACNA1E;AP5M1;PCBP3;MAP2;PAK5;OPCML;CLVS1;OSBPL6;BCL11B;BCL11A;SIAH3;ERCC6L2;NTRK3;SORBS2;CORO2B;ST18;PTK2;SNAP91;ATAT1;NELL2;DAB1;NFIA;NBEA;APC;NFIB;PPP2R2B;TTC3;TCF4;FAT4;COPS8;SSBP2;LRP12;SPG21
ZNF354B human tf ARCHS4 coexpression	77/299	0.012387665035661253	DIDO1;DOCK5;TRIO;DPY19L2P2;BACH1;ANTXR1;RPTOR;AKAP13;POTEM;ZFYVE26;PTAR1;DNM1P47;HERC2;C16ORF72;HERC1;ZNF407;KYNU;SACS;ARHGEF12;RALGAPA2;VPS13C;PDE4D;VPS13D;GLP2R;VPS13B;ASH1L;URB1;PEAK1;SPOPL;WDPCP;RELL1;WDFY3;BIRC6;UTRN;MCTP2;NOTCH2;MACF1;NFAT5;NBAS;KMT2C;ITPR2;LPP;ATXN3;MAPK9;PCNX1;KIAA1328;HECTD1;NSD1;HECTD4;TRPM7;CEP192;ATP9B;RANBP2;USP24;ZFHX3;ST8SIA1;LRBA;TRAPPC10;MGA;MOCOS;MICAL3;ERBIN;FANCA;ZZEF1;MTOR;MED13L;TMPRSS15;MYO1E;NEDD4;SLMAP;AGO2;DMXL2;SP3;FAT1;ESYT2;CDK12;KIAA0825
ZNF781 human tf ARCHS4 coexpression	77/299	0.012387665035661253	SLC44A5;ROBO2;MYT1L;ZNF292;PTPRO;CHRM5;ELAVL4;SLC35F1;C12ORF40;EFCAB6;PTPRG;SRGAP2C;SYNPR;GRM7;ZNF606;PPP2R5E;TRIM2;ITGB8;KIF21A;SOX6;SRGAP2B;BBS4;ANKS1B;ANKRD36C;MEF2C;WSB1;LRRC49;TMOD2;MAGI2;ATRX;ZNF271P;SEZ6L;TIAM2;MMP16;LRRC7;RUFY2;AKAP9;ZNF780B;VSTM2A;KMT2E;CRB1;IN

			O80D;ATL1;BTF3L4;ZNF518A;BAZ2B;NREP;NOL4;GNG2;MAP2;SNTG1;SRGAP3;JAM2;PAK5;GRIA4;CLVS1;RFTN2;ATF7IP;SIAH3;RANBP17;SORBS2;NBEA;APC;NFIB;TMEM116;KLHL7;ASXL3;TTC3;YPEL1;RSPH14;ZNF536;ZNF613;TCF4;SSBP2;KIAA0825;ASB3;XKR5
ZNF808 human tf ARCHS4 coexpression	77/299	0.012387665035661253	*CCDC122;ZNF891;ZBTB25;CHD9;ZNF292;SMG1P2;UBE3A;LYST;FAM204A;MYLK3;SYNE2;ABCC13;AKAP13;NIPBL;ACOXL;RPS6KA5;C16ORF72;HERC1;ZFP30;PPIP5K2;ADAMTSL3;PPP6R3;DLEU1;ZNF600;MAPK1IP1L;DENND2C;TANGO6;ITGA4;RALGAPA1;VPS13C;VPS13B;ASH1L;ARID1B;CACNB2;AKAP9;WDPCP;BIRC6;ZNF236;UTRN;ZNF675;KDM7A;ZNF431;FTO;MACF1;CHRNA7;KMT2C;TPTE2P2;RGPD5;BAZ2B;FBXL20;SCAF8;PDCD6IPP2;KIAA1328;ANKRD36BP2;TRPM7;CEP192;AP4S1;S100PBP;USP25;MBD5;MGA;LNPEP;DDHD1;PHC3;PARP15;MYO9A;CHFR;PDP2;AGO3;ZNF813;PARGP1;ZNF611;ASB3;FRA10AC1;ZNF850;TNRC6B;CCDC171*
SALL1 human tf ARCHS4 coexpression	76/299	0.018661520499443168	*GABRB3;HIP1;TENM4;MEGF10;GLDC;CTNND2;PSIP1;LDLRAD3;SLC35F1;HMGB1;HS6ST1;SOGA1;CELSR2;KIF15;PTPRG;GLI2;ROBO1;SPRED1;CDH2;NUF2;RBPMS2;NEO1;SRGAP2B;MAGI1;WDHD1;SMARCC1;SUPT16H;ADGRV1;SEMA6D;TCF12;ADGRA3;HAUS6;NAV2;SGO1;TOX3;ASPM;SETBP1;ADCYAP1R1;PHLPP1;ILDR2;NTN1;PRTG;STOX2;CECR2;APELA;GTF2IP4;LRIG1;IGF2BP3;ZNF423;SRGAP2;EXTL3;MPDZ;BUB1;JAM2;WASF3;ZNF462;FARP1;NTRK2;MYEF2;SLC16A1;WSCD1;AUTS2;HMGA2;ARID3B;PTPN13;CORO2B;AIF1L;TJP1;PTPRD;CENPE;FABP7;KIF4A;FAT3;TCF4;ADGRL2;SPSB4*
ARNT2 human tf ARCHS4 coexpression	76/299	0.018661520499443168	*DOCK3;FRMPD4;MYT1L;CTNND2;DIRAS2;RASGRF1;KNDC1;RPH3A;TRIM9;GRM5;TRIM2;MCF2L;NCS1;PSD3;DLGAP1;KCNH1;RBFOX1;TMEM178A;TMOD2;ANK2;FAM219A;SYN2;MAPK8IP1;GABRG1;AJAP1;CNKSR2;MPPED1;PITPNM3;SCN8A;ADGRB1;RAPGEF5;NGEF;ASTN1;RAPGEF4;STXBP1;ADAM22;SLC1A2;AGAP1;NDRG2;KALRN;RAP1GAP;KIAA0513;CTIF;GRIN2A;PGBD5;SV2B;MAP2;GNG7;CHN1;NCAM1;CTNNA2;CACNG3;GPR158;ATP9A;WASF3;OPCML;GABBR2;NDFIP1;DTNA;ANKRD24;SYT1;CADM2;NTRK3;KIAA1549L;MYO5A;ATP2B2;HSPA12A;CORO2B;DCLK1;SNAP91;DLG2;PPP2R2C;RCAN2;CNTN1;SCN2A;APBA2*
NR1D2 human tf ARCHS4 coexpression	76/299	0.018661520499443168	*MYOM1;MYLK2;RNF11;ATP8A1;ZFYVE9;DOCK9;RORA;LDB3;JPH1;MYOM2;SYNE1;FYCO1;EFR3A;AKAP11;UBL3;SGCD;HERC1;MPRIP;MB;PEBP4;KIF13A;MYO18B;PSD3;TEAD1;CAST;UNC45B;RBFOX1;MBNL2;ARHGEF12;DST;RALGAPA1;CACNA2D1;ZBTB38;VPS13C;VPS13D;MTUS1;TRDN;INPP4B;MYL1;CSDE1;ALPK3;KCNQ5;WDFY3;PRKAA2;SMPX;ROCK2;RGPD6;NDRG2;ABLIM1;HECTD1;NRAP;XIRP2;MAPK1;ZNF106;CTNNA3;HIVEP2;MAP4;RANBP2;USP24;SVIL;ABCA5;AGL;NEK7;SAMD4A;ERBIN;FBXL17;PDE4DIP;LNPEP;ATP2B2;ATP2B1;FBXO32;CYLD;KCNS3;SLMAP;STRN;ESYT2*
NR2C2 human tf ARCHS4 coexpression	76/299	0.018661520499443168	*RERE;DIDO1;TRIO;DOCK8;HNRNPU;PTPRJ;LIMD1;ETS1;CABIN1;SYNE1;RPTOR;AKAP13;NIPBL;PTAR1;HERC2;HERC1;MPRIP;NPIPA1;DIP2B;GTF2I;ARHGEF11;MBNL1;ARHGEF12;PRKCB;VPS13C;VPS13D;VPS13B;ASH1L;URB1;SFPQ;PACS1;MADD;WDFY3;BIRC6;UTRN;DOCK2;ANAPC1;KDM7A;NOTCH2;MACF1;NFAT5;WDR26;KMT2C;HERC2P2;HTT;BAZ2A;PCNX1;BTAF1;HECTD1;MAN2A2;NSD1;HECTD4;MAPK1;CEP192;HIVEP2;RANBP2;USP24;SPEN;CREBBP;MON2;LRBA;TRAPPC10;MICAL3;ERBIN;LNPEP;MYO9B;HIPK1;SMARCA2;ZZEF1;MTOR;MED13L;DIAPH1;SP3;STRN;ESYT2;CDK12*
ZSCAN23 human tf ARCHS4 coexpres	76/299	0.018661520499443168	*SPAG16;ATF2;LTN1;ELAVL4;CWC27;PTPRG;ANKRD30BP2;CDH2;DPYSL5;TRIM2;SUMO2;SOX6;ZNF521;MAGI1;WDHD1;EPHA4;ANKRD36C;ZNF880;EPHA7;GUSBP1;WSB1;CXADR;KCNK10;DCC;LRRC49;TCF12;ATRX;RFX3;TET1;IL17RD;FAM126A;SRP9;SETBP1;IFT81;ADGRB3;RUFY2*

sion			;AKAP9;MPPED2;ANKRD36B;KHDRBS2;HDAC2;STXBP4;TNKS;BTF3L4;GREB1L;ZNF66;BAZ2B;NREP;NR2C1;HDAC9;GNG2;MAP2;ARSJ;ZNF627;CSMD3;SRGAP3;SLIT2;MPDZ;JAM2;PAK5;RFTN2;ATF7IP;ZNF462;FANCM;RANBP17;POU6F2;PTK2;ATAT1;MAPK10;APC;ZNF738;KLHL7;BRMS1L;TTC3;TCF4;SSBP2
ATMIN human tf ARCHS4 coexpression	76/299	0.018661520499443168	ZFYVE9;USP32;CSE1L;PTPRK;BZW1;ZCCHC14;CDC14B;EFR3A;AKAP11;ZMYM4;KIF13A;SPIN1;SACS;RNF17;DIP2B;TEAD1;NEO1;NDC1;USP7;ZHX3;ARHGEF12;RRAS2;ATRN;PARD3;CSDE1;ESRP1;ZMYND11;DOCK1;ANAPC1;PAFAH1B1;YAP1;RABGAP1;IGSF3;SEMA3C;ROCK2;MAPKAP1;HACD2;ACACA;TM9SF3;LARP1;ERMP1;HECTD1;RACGAP1;MGAT5;NSD1;GTF2IP4;CTNNA1;MAPK1;MAP4K3;FAM83B;ATP9A;RANBP2;ARFGEF1;TRAPPC10;GALNT1;GSR;ERBIN;DNAJC13;AP2B1;CDC42BPB;GNG12;HIPK1;MTOR;MED13L;PLEKHA8;KTN1;DAZL;TJP1;DPY19L1;DLG5;GID8;SP3;FAT1;GNAS;SPIRE1;STRN
LGR4 human tf ARCHS4 coexpression	76/299	0.018661520499443168	KCNK5;COL18A1;ABCD3;GLDC;KIAA1671;PTPRK;LAMC1;SIPA1L2;ARHGAP42;TEAD1;NEO1;PLS1;UNC13B;SMARCC1;MYOCD;ARHGEF12;ENTPD5;RRAS2;SHROOM3;ADGRA3;ANO6;ATRN;PARD3;ESRP1;HKDC1;ALPK2;FREM1;CDH17;DOCK1;MET;VCL;YAP1;FBN2;ACSS3;ROCK2;LAMA1;MTTP;RRBP1;ACACA;TM9SF3;C5;APELA;UGP2;HECTD1;ADGRG7;TBX20;MAP7;CTNNA1;ABL1;FLNB;IARS2;IGF2BP3;PDLIM5;RANBP2;SLC16A1;NEBL;GALNT1;GSR;AKR1C3;LAMB1;B3GALT5;CDC42BPB;GNG12;PTPN13;ASS1;ISX;KTN1;TJP1;MYO1D;MYO1E;BMP2;CPS1;MYO5B;SERBP1;FAT1;ADGRL2
RARB human tf ARCHS4 coexpression	75/299	0.027054509331880916	SEMA5A;MIPEP;RYR2;BNC2;ZFYVE9;SYCP1;DPY19L2P1;SLC2A3;LAMC1;LDB2;MEOX2;SLC8A1;ROBO1;ARHGAP42;CDH2;SPIN1;TUBB2BP1;RGS8;DIRC3;RNF152;TEAD1;ADAMTS9;PRKG1;KCNH1;KPNA1;POSTN;MUSK;TPM1;WDR72;DKK2;NRBP1;CNKSR2;TANC1;VCAN;AGPS;CDH11;ALPK2;TLN2;ST6GALNAC3;FREM1;DOCK1;KANK4;TRABD2B;DGKI;RAI14;FBN2;FOCAD;SAR1A;SEMA3D;TMTC2;SEMA3E;RANBP3L;FLRT2;CALD1;ERBB4;CTNNA1;ALX4;CCDC141;ZNF423;RXRG;MPDZ;STARD13;CNTN5;NEBL;TXNRD2;PCDH7;LAMB1;GNG12;MCC;PTPN13;ARHGAP24;TJP1;DLC1;SNAI2;NF2
DMTF1 human tf ARCHS4 coexpression	75/299	0.027054509331880916	KDM5A;ANKRD36;CHD9;DPY19L2P1;CELF2;USP33;SMG1P5;C12ORF40;CROT;BICD1;RYR3;SYNE2;PCMTD2;SENP6;AKAP13;DSTYK;NIPBL;C16ORF72;HERC1;ZSCAN30;PPIP5K2;LUC7L;RBM6;ANKRD36C;RALGAPA1;VPS13C;VPS13B;FAM126B;ARID1B;GOLGA8B;AKAP9;BIRC6;ASTN2;ZMYND11;MCTP2;ALG10B;CREB5;KMT2E;MACF1;TPH2;TNKS;KMT2C;RGPD5;BAZ2B;HERC2P9;MIPOL1;SCAF8;BTAF1;KIAA1328;ANKRD10;ATP9B;S100PBP;BPTF;USP24;ATF7IP;MBD5;RANBP17;ADAM32;LNPEP;DDHD1;PHC3;PARP15;MLLT10;FER;APC;KANSL1;BCL2;CEP83;PKN2;TCF4;BRWD1;PTPN4;ASB3;FRYL;TNRC6B
PAX3 human tf ARCHS4 coexpression	75/299	0.027054509331880916	TENM3;WNT2B;MEGF10;DENND1A;CCDC102B;ROBO1;ZNF608;NHSL1;DACH1;CDH2;PEG10;TRAPPC11;ITGB8;EPHB2;ZNF521;ANKRD6;WSB1;ADGRV1;KCNK10;DST;TCF12;RFX3;TET1;PLA2G4A;PARD3B;ENAH;TOX3;MYCL;ANKFN1;SETBP1;IFT81;ARMC6;ADGRB3;ATF6;SMPDL3A;GRIA1;MTMR2;STXBP4;GREB1L;GLIS3;PRDM13;NR2C1;OAZ2;NPAS3;PRTG;STOX2;GNG2;HECTD2;CALD1;PLXNA2;NHS;TSPAN3;CSMD3;SRGAP3;ZNF423;LRRC4C;MPDZ;BBS2;NTRK2;MYEF2;WSCD1;AUTS2;NOS2;ST8SIA1;RANBP17;PXDNL;PHKB;PTPN13;ZNF33B;TMEM232;MAPK10;TTC3;FAT3;FAT4;BMPR1B
IRX1 human tf ARCHS4 coexpres	75/299	0.027054509331880916	APP;DRAXIN;TENM2;TENM3;MEGF10;CTNND2;MSI2;SIPA1L2;HS6ST1;CELSR2;TSPAN11;CDH4;ZNF608;DACH1;CDH2;DPYSL5;PEG10;TRIM2;ITGB8;KIF21A;ANKRD20A7P;EPHB2;TMEM178B;EBF2;RFX3;KAZN;EBF3;FRMD4A;FAM219A;IL17RD;RGMB;SORCS2;CFDP1;TNIK;CHST3;G

sion			RIA1;IGSF3;NXN;GREB1L;AGAP1;MLLT1;NPAS3;PHF21B;STOX2;GTF2IP1;GNG2;MAP2;CDH20;PLXNA2;MAP6;NCAM1;SRGAP3;ZNF423;EXTL3;BRD4;MAP4K4;TMEM132C;NTRK2;NDFIP1;MYEF2;WSCD1;AUTS2;PCDH8;YLPM1;PXDNL;ESRRG;NETO2;ZDHHC11B;DCLK1;PTPRD;SDK1;ZNF618;PTPRA;TTC3;SPSB4
ZBTB20 human tf ARCHS4 coexpression	75/299	0.027054509331880916	CCDC122;ZNF891;PATJ;ZBTB25;MYT1L;CELF4;EHMT1;KLHL33;TMEM182;MYLK3;SYNE1;UNC80;EPB41L4A;RPS6KA5;CA5A;ADAMTSL3;OPA3;ERC2;USP8;ANKRD36C;MAGI2;TTC7B;ABCC9;SORCS3;SHISA9;ARID1B;NAALADL2;SLC9A4;SPATA17;MYO3B;SETBP1;PEAK1;CNKSR3;WDPCP;ZNF780B;ASTN2;PPARA;ANKRD36B;LINC00643;SDCCAG8;ZNF397;FTO;KCNE4;NMD3;BAZ2B;LPP;MIPOL1;FBXL20;ORC4;DPP6;KIAA1328;FLRT2;ZNF704;AP4S1;PAK3;SCAI;MBD5;KCNJ6;CADM1;NEGR1;HOMER2;NUBPL;PCDH8;KCNJ15;HOOK3;BTBD9;PHC3;CNOT7;AGO3;NEDD4;RGS12;CEACAM22P;ASB3;C8ORF34;TNRC6B
RFX7 human tf ARCHS4 coexpression	75/299	0.027054509331880916	LIN54;DIDO1;SETD2;ATP8A1;CELF2;HNRNPU;PSIP1;MSI2;NIPBL;HERC1;GNPTAB;ZMYM4;MYB;SACS;SMARCAD1;EPC2;QSOX2;GTF2I;KDM6A;AQR;MEF2C;SMARCC1;ITGA4;TANC2;RUNX1;SFPQ;FCHSD2;AGPS;GCSAML;BIRC6;PABPC1;MCTP2;ANAPC1;ANKRD17;MACF1;DDX6;NFAT5;ANP32A;TNKS;HTT;ITPR2;BAZ2A;SCAF8;LARP1;BTAF1;HECTD1;FUT9;NSD1;HECTD4;CEP192;BPTF;RANBP2;USP24;SPEN;CREBBP;TRAPPC10;YLPM1;RANBP9;XPO7;LNPEP;FOXN3;PUM1;GATAD2B;SCAF4;MED13L;PLEKHA8;MLLT10;APC;KANSL1;AGO2;SP3;STRN;CDK12;PTPN4;EIF4G3
ZBTB44 human tf ARCHS4 coexpression	75/299	0.027054509331880916	PATJ;ZFAND6;CSE1L;CELF2;PCDH11X;PSIP1;BRCA2;JPH1;LRRC2;PCMTD2;PPP1CB;PTAR1;C16ORF72;HERC1;ZMYM4;MYB;MYO18B;PPP6R3;PHACTR2;TLK1;PRKG1;PCMTD1;ZNF121;AQR;LYPLA1;CNOT6L;SNRPN;ESCO1;RALGAPA2;VPS13C;ATP11C;GOLGA8J;HAUS6;ARID1B;TRDN;ENAH;MSH6;MSH2;THRAP3;PRKCQ;BIRC6;B4GALT6;CCDC150;UBE2Q2P1;INO80D;PRKAA2;IREB2;ZNF518A;GLB1L3;APELA;SCAF8;UGP2;ADAMTS19;NRAP;XIRP2;ZNF106;NUP43;BARD1;RANBP2;ARFGEF1;USP24;USP25;SLC16A1;AGL;MGA;LNPEP;UBE2G1;FOXN3;TTC39C;PHC3;CENPE;SLMAP;GNAQ;BRWD1;PTPN4
SIM1 human tf ARCHS4 coexpression	75/299	0.027054509331880916	PCSK2;SEMA5A;MYOM1;ERO1B;ZFAND4;PTPRQ;SLC35F4;ZBTB20;LCLAT1;LRRC2;SCGN;FYCO1;C1ORF127;SGCD;MYO18B;SLC16A9;ARFGEF3;PCMTD1;MLIP;ZNF705D;IL1R1;RALGAPA1;ABCC8;SLC2A13;ABCC9;TRDN;ABCA10;PLCB4;NAV3;RRAGD;COL4A3;MYO3A;GAS2;SCG5;ALPK3;SCG3;RIN2;RWDD2B;INO80D;PRKAA2;SMPX;STXBP4;GLIS3;NOL4;NALCN;SLC7A2;TTR;NRAP;SUSD4;XIRP2;TSPAN2;STXBP6;AKAIN1;PTPRN2;ABCA5;CNTN5;CADPS;PDE4DIP;PARVB;BTBD9;CDC42BPA;ELL2;MEIS2;BMP5;MOB1B;TBX15;OCLN;PLCXD3;AGBL1;TMEM236;ASXL3;RIMBP2;CPE;CNTN4;CPEB4
ARID5B human tf ARCHS4 coexpression	75/299	0.027054509331880916	SEMA5A;DOCK5;BNC2;PTPRQ;FHL2;ZBTB20;SLC35F1;GXYLT2;LCLAT1;EPS8;PPP1CB;SRGAP2C;SLC25A48;ADAMTS5;NHSL1;LONP2;PHACTR2;ITGB8;ZNF568;PHACTR1;SRGAP2B;ADAMTS6;MAPK1IP1L;CCBE1;DST;LIMCH1;SLX4IP;PRKCA;ENAH;INPP4B;SPATA17;FRMD6;CRISPLD2;CDH11;ALPK2;RIN2;ATF6;CD44;RBMS3;RWDD2B;CEMIP;FICD;SAR1A;COPB1;PRICKLE2;GLIS3;ANKRD19P;NEDD9;CARM1P1;LPP;FBLN5;MYL12B;ATXN1;CALD1;ARSJ;PDGFC;RNF217;EVI5;LRRC69;STARD13;CRIM1;TTC39C;CDC42BPA;RCAN1;TBX15;DLC1;TMEM117;STT3A;ITGBL1;SNAI2;NF2;SEC24D;LRP12;TNRC6B;SNTB2
ZNF780B human tf ARCHS4 coexpres	75/299	0.027054509331880916	CCDC122;ZNF891;ZBTB25;MYT1L;CHD9;ZNF292;USP33;DPY19L2P2;IPP;EHMT1;ZBTB20;TMEM182;MYLK3;SYNE2;UNC80;SYNPR;RPS6KA5;HERC1;CA5A;ADAMTSL3;SOX6;KLF12;SEMA6D;VPS13C;VPS13B;ABCC9;SHISA9;ZNF14;ARID1B;ADGRB3;CATSPERG;RUFY2;CNKSR3;WDPCP

sion			;BIRC6;ASTN2;ZNF234;ANKRD36B;CDH18;ZNF431;FTO;MACF1;TPTE2P2;ZDHHC21;BAZ2B;MTMR7;MIPOL1;FBXL20;FGD4;ORC4;KIAA1328;HIVEP1;AP4S1;SRGAP3;SCAI;MBD5;SLC14A2;MYEF2;ZNF382;MGA;AMFR;ADAM32;ARHGAP28;DDHD1;PARP15;CCDC88A;AGO3;TMEM116;YPEL1;CEACAM22P;BRWD1;ASB3;ZNF850;C8ORF34;TNRC6B
CHD1 human tf ARCHS4 coexpression	75/299	0.027054509331880916	ITSN2;KDM5A;TCERG1;SETD2;DOCK8;SMG1P2;HNRNPU;BRCA2;BACH1;LYST;SYNE2;KIF15;AKAP13;NIPBL;PTAR1;C16ORF72;HERC1;RB1CC1;PPP6R3;LRRFIP1;JARID2;ZNF367;ANKRD36C;AQR;MBNL1;SMARCC1;USP7;MORC3;ESCO1;ITGA4;VPS13C;VPS13B;HAUS6;BAZ1A;ASH1L;PARP8;PHF20L1;ASPM;SFPQ;SDE2;BIRC6;UTRN;DOCK2;KDM7A;KMT2E;DDX6;WDR26;ROCK1;KMT2C;RGPD8;PCNX1;SCAF8;BTAF1;NSD1;CEP192;BPTF;ARFGEF1;USP24;SPEN;TRAPPC10;MGA;ERBIN;ZBTB10;DDHD1;HIPK1;MED13L;CYLD;CENPE;SNRK;KANSL1;DMXL2;SP3;PKN2;CDK12;BRWD1
ISL1 human tf ARCHS4 coexpression	74/299	0.03961614031621126	PCSK2;SPAG16;ERO1B;TUSC3;SLC35F4;ICA1;LCLAT1;HS6ST3;RIMS2;SCGN;C1ORF127;ACOXL;FAM3B;PLCE1;TLK1;SAMD12;SCAPER;RGS9;ARFGEF3;RGS7;ABCC8;SLC2A13;SLX4IP;MTUS2;FNDC3A;ZDHHC14;CACNB2;RRAGD;CDC42EP3;MYO3A;GAS2;SCG3;USP41;RIN2;LINC00643;SAMD5;RWDD2B;INO80D;RGPD6;USH1C;RGPD5;HSD17B14;NOL4;SLC7A2;MTMR7;TTR;SUSD4;TSPAN2;SUSD6;AKAIN1;KCNJ6;PTPRN2;ABCA5;WSCD2;CADPS;POU6F2;PARVB;CDC42BPA;ELL2;USH2A;ST18;BMP5;MOB1B;OCLN;PLCXD3;TMEM236;ASXL3;RIMBP2;CNTN1;GNAS;RSPH14;CPE;CNTN4;CPEB4
SP4 human tf ARCHS4 coexpression	74/299	0.03961614031621126	ZNF891;UHRF1BP1L;TUSC3;ZNF292;SMG1P4;UBE3A;SLC6A1;CRKL;ZSCAN30;ZNF606;PPP2R5E;PPP6R3;SUMO2;LUC7L;TMEM38B;ZNF521;SRGAP2B;ZNF287;MEF2C;LYPLA1;WSB1;ST6GAL2;CXADR;DSCAM;LRRC49;TTC3P1;MAGI2;HUNK;UNC5D;KIF6;SRP9;MMP16;ZNF675;ZNF234;MCPH1;ZNF397;KMT2E;HDAC2;TNKS;BTF3L4;NRXN3;TULP4;TPTE2P2;RGPD5;BAZ2B;NREP;NYAP2;HERC2P9;NOL4;GLB1L3;ATP6AP1L;FBXL20;GNG2;DZANK1;SNTG1;SYNDIG1;CLVS1;MYEF2;RNGTT;BCL11B;ZNF382;CNOT7;TMEM116;ZNF738;TDP1;KLHL7;ZNF813;ABI1;CCSER1;TCF4;PTPN4;TRMT61B;XKR5;TNRC6B
ATF2 human tf ARCHS4 coexpression	74/299	0.03961614031621126	GABRB3;ZNF292;ELAVL4;BACH1;PCMTD2;DPYSL5;TRIM2;SPIN1;FBXO3;KIF21A;TUBB2BP1;PRKACB;TRIM23;PCMTD1;MAPK1IP1L;WSB1;CXADR;DCC;VPS13C;RFX3;ANK2;ZDHHC17;RUNX2;SRP9;PGM2L1;PJA2;PIAS1;PYGO1;LATS2;MDFIC;LRRC7;RUFY2;NAA35;ZNF678;TOX;KMT2E;GRIA1;STX12;RTN1;STAU2;TNKS;ATL1;BTF3L4;TULP4;ZNF518A;NREP;NOL4;GNAI1;MAPK8;GNG2;HECTD2;MAP2;SNTG1;CTNNA2;PAK3;CADM1;SYT1;SYT16;PUM1;OXR1;FAM217B;ATAT1;PTPRD;MAPK10;CNOT7;SYNJ1;APC;KLHL7;TTC3;CNTN1;TCF4;BRWD1;SSBP2;CDS2
NR3C2 human tf ARCHS4 coexpression	74/299	0.03961614031621126	SLC46A3;PATJ;LPGAT1;DHRS11;PPP1R13B;ATP8A1;KDM1B;DOCK9;KIAA1671;SLC4A4;SYNE1;FYCO1;HERC1;DMBT1;KIF13A;CAPN5;FAM3B;MALRD1;TINAG;NEO1;PLS1;UNC13B;CAST;ZHX3;ARHGEF12;RALGAPA1;ENTPD5;SLC2A13;VPS13C;VPS13D;MTUS1;ARAP2;OLFM4;CEACAM7;CDH17;SMPDL3A;ENPEP;SLC26A2;RBM47;MTMR3;HHLA2;MTTP;SLC1A1;USH1C;TMPRSS2;ATP10B;SLC5A12;SLC5A1;TRAK1;PBLD;ABLIM1;ADGRG7;NRAP;XIRP2;HIVEP2;CLCA4;CNNM4;SVIL;MGAM;ABCA5;REG4;ERBIN;CYBRD1;PDE4DIP;LNPEP;B3GALT5;ZZEF1;ISX;MYO1D;NLRP13;ARHGAP32;TMEM54;MYO5B;GIPC2
POU3F4 human tf ARCHS4 coexpres	74/299	0.03961614031621126	DRAXIN;CHD9;CTNND2;ELAVL4;MSANTD4;TSPAN11;DACH1;CDH2;DPYSL5;GDAP1L1;KIF21A;EPHB2;EPHB1;WSB1;ADGRV1;CAMK1D;KCNK10;LRRC49;KCNH8;TCF12;RFX3;KCTD1;EBF3;RGMB;GAB4;TOX3;GAP43;PCCA;SETBP1;ADGRB3;MPPED2;GRIA1;HDAC2;CHRNA7;NTM;GREB1L;

sion			*BAZ2B;NREP;NPAS3;PHF21B;STOX2;MAPK8;NKAIN3;GNG2;MAP2;MVB12B;MAP6;CAMTA1;NCAM1;CTNNA2;SRGAP3;ZNF423;LRRC4C;JAM2;FOXB1;ZNF462;NTRK2;MYEF2;ZNF382;AUTS2;PLCL1;PCDH8;PBX3;PBX1;ENOX1;ATAT1;PTPRD;MAPK10;CCDC88A;ZNF738;FABP7;TTC3;FAT3;TCF4*
NPAS4 human tf ARCHS4 coexpression	74/299	0.03961614031621126	*PCSK2;GABRB3;PTPRT;INTS12;ERO1B;DOCK3;MYT1L;TUSC3;CISD1;CELF4;PPP1R9A;RIMS2;IQCJ-SCHIP1;UNC80;SCGN;HYDIN2;ERC1;RGS9;ARFGEF3;ANKS1B;ABCC8;LRRC49;MAGI2;FOXP2;CACNB2;EML6;LRRC7;SCG5;SCG3;ZNF236;RIN2;LINC00643;SAMD5;FTO;STOML1;CTTNBP2;STXBP1;ATL1;PLPPR5;HSD17B14;CACNA1C;NREP;KALRN;NOL4;SLC7A2;MTMR7;DPP6;UNK;PDZD2;TTR;HECTD4;SUSD4;PAK3;ATP9A;LINGO2;ZNF462;PTPRN2;GABRA5;CADPS;POU6F2;SYT16;ELL2;GRIN2B;MEIS2;ST18;ATAT1;MOB1B;NELL1;PPP2R2B;ASXL3;RIMBP2;RSPH14;CPE;CNIH3*
YEATS2 human tf ARCHS4 coexpression	74/299	0.03961614031621126	*APP;DIDO1;MAST2;HNRNPU;MSI2;LAMC1;IKZF2;SOGA1;ROBO1;CABIN1;RPTOR;HERC2;SMPD4;HERC1;MPRIP;ZMYM4;PIEZO2;SACS;ANKFY1;UBAP2L;DIP2B;QSOX2;GTF2I;SMARCC1;KSR1;DST;VPS13D;UBE2E1;NAV2;URB1;SND1;SREBF2;BCR;SFPQ;KIFC1;BIRC6;ARHGEF7;DOCK1;ANAPC1;MACF1;HERC2P2;CLEC16A;HTT;FOXK2;BAZ2A;ACACA;LARP1;BTAF1;HECTD1;MTHFD1L;MGAT5;NSD1;HECTD4;ABL1;GSE1;IGF2BP3;MAP4K4;SPEN;FARP1;TAF15;LRBA;MICAL3;YLPM1;FANCA;DNAJC13;AP2B1;CDC42BPB;MTOR;SMARCA4;AGO2;FAT1;BCL2;STRN;CDK12*

Table S4. The overlap between the top-rDNA-contacting genes obtained from untreated K562 cells (3699 genes) and for HEK293T cells (4920 genes). Related to the Venn diagram presented in Figure 1C.

Names	total	elements
HEK293T K562	1486	*CD44 SAMD4A KCNMA1 C10orf90 PKNOX2 RALYL ZHX3 APBB2 GLT1D1 GGT3P PTPRR SCAF8 CPXM2 RTN1 ERG PARN PDE1C SEMA4D INIP WSCD1 SLC12A8 IGSF3 MED13L ZFYVE1 EVC TEAD1 NFIA SYN3 RPS6KA5 POTED SUGCT DHRS11 CCDC34 TAOK3 GADL1 PRKCB EIF4G3 A2M TRPM6 CPNE4 SLC8A1 ARHGEF26-AS1 ANKRD6 KCNC1 GPR55 DNAJC15 SIAH3 CD38 LINC00159 ZNF257 VAT1L FBXO31 C12orf40 PIWIL3 TRAPPC9 FAM182B FNDC1 BCL2 MMP16 CAMTA1 SAMSN1 CHFR THADA COL18A1 TOX3 RSRC1 THRB FGF7P2 DNAH11 ZSCAN5C CDC42BPA SLC13A4 B3GALT5 RAG1 SAMM50 CDC42EP3 FSIP1 GRIK2 IGSF11 SNX25 DPYD UNC13B MAPK10 NCAM1 OTX2-AS1 KDM4B GNG12 IL6R KCNQ5 CDH8 ZBTB20 HEPHL1 IGHVIII-82 SCN11A ATP10A SORBS2 SKAP2 HS1BP3 GOT2 KCNJ6 CASP5 PCAT1 KCNK10 BLOC1S5-TXNDC5 RNF182 BRD4 ZDHHC11B GPR158 ZNF568 NDRG2 TMEM241 GRIP1 APBA2 TTC3 CPVL MIR3118-2 TLK1 ASTN2 TANC2 KIF4A CSMD3 DLGAP1 DUX4L15 RCAN1 FAM193A NTN4 GTF2IP1 JAK2 LINC00348 TM9SF4 BICD1 LRP1B ZBTB8OS MIR17HG ABCG8 KRTAP26-1 OTUD7A TPTE2 ANKRD20A5P KALRN SUMF1 USH2A NEGR1 FGF12 CACNG2 BTBD9 CASC9 NFAT5 FLI1 SLC44A5 MEGF11 SPATS2L LINC00882 TRHDE ZNF536 EPB41L3 LAMA1 PARVB CDH11 SETBP1 ZBTB7C CDS2 GRIN2A MAST4 NRXN1 WDR26 DTWD2 AGBL1 NELL2 ARID1B DEFT1P2 DPY19L2P2 IL1RAPL1 WDPCP NIPA2 MAGI1 LAMA3 SLC14A2 ADAMTS17 GBP4 PTCSC3 ADCYAP1R1 ST6GALNAC3 CTDP1 RERGL ADAMTS6 DPP6 PRELID2 GOLGA8J GRID1 XKR3 RPS6KA2 TDP1 PTGER4P2 LINC00504 SMOC2 ZDHHC17 KCNH1 HLCS ACSS3 CNBD1 DCDC1 CACNA1C CCSER2 AMPH BMF EXOC4 HEATR5A WDR70 CKMT1B PNPLA3 ANO4 GIPC2 BBS9 FAM83B CTNNA1 NCOR1P1 MYO9A NTRK2 FOXN3 ENTHD1 WDFY3 C19orf18 OCLN AK8 NLK ITGBL1 THSD7A ABCA6 NBEA POTEC ANKRD30BP2 RASGEF1B AFAP1 NCAM2 DPY19L2P1 TIAM2 MYT1L TMPRSS3 SRGAP2B IQCJ-SCHIP1 CARM1P1 LRRC49 LINC00273 SMARCA4 HERC2P3 RNF152 CNTN1 ZNRF3 PALMD MEOX2 TTC39B ENPEP PLEKHB2 GLIS3 OR4K6P ANKRD36 NRG3 PTPRG MC2R BTBD11 ELMO1 SLC24A4 CABIN1 SYNE1 ABCD1P4 FBXO47 FBXL17 SLC9B1P4 PDZD2 ANKRD20A9P DNAH10 GAS2 GRIK3 CACNB2 PDE10A NUMB STXBP4 MED15 MTPN MT1HL1 ESYT2 SOX6 MECOM POTEM SYBU TBC1D22A PDE4DIP TRPM3 STK32B LINC01020 VCAN POTEB2 DNAH8 NHS CNTNAP5 RGS12 SHANK2 RAPGEF5 UBE2E2 PTGFRN NBEAP1 KCTD8 CHCHD6 UNC5D HS3ST2 EVA1A ZNF567 NREP GABRA5 DOK5 AGMO DLG5 CFDP1 PGM5 SMARCAD1 MIR3118-3 FNDC3B ZIM3 ASTN1 ADAMTS9-AS2 AIFM3 GNG12-AS1 ATRNL1 DUX4L2 CHRM3 CPE CALD1 AIG1 ERICH1 ABCC13 ARHGAP24 TMEM132D EFCAB8 SPRED2 N4BP2L1 IGHV1OR15-9 LINC00907 SLIT2 PITPNC1 TPTE2P6 MYLK3 C2orf88 CEP128 ABCA5 POTEB ROR1 GLP2R CCDC178 SLC4A4 ADAMTSL1 KAZN ZNF675 CSNK2A1 DTNA AKT3 CRB1 PHKB KMT2C KCNE4 TRIM5 KCNS3 RPL23AP82 CYP4B1 PSD3 ALPK2 ABCA13 HECW1 RAP1GDS1 AFF3 LCE1F ERBB4 KANK1 STT3A GPHN LPP VWFP1 ATRX DMRT1 SLC9B1P3 CHST8 BID MACF1 MEF2C-AS1 MNAT1 TAF4B RAP1A TRIO ZNF385D SLC15A5 CTNNBL1 RAD51B TRMT61B PTPRE TSPAN3 EPDR1 MYO3B DUSP22 CHSY1 MYOM1 PSG8 EXT2 URB1*

		ZSCAN30 OR4C46 ABL2 DIP2C PSMA1 MAP3K5 NOS1 ARPP21 ACACA ABCG1 RGS3 MAML2 SPAG16 EML1 RERG HTR2C CCDC141 NEK4 CACNA1E SPATA17 CTIF CNTN4 TBC1D5 MUC16 CSTF3 CEACAM22P SAMD13 LINC00511 RNF17 PRAMEF26 SLC40A1 SLCO3A1 GABRR2 PIK3C3 SLC9C1 TRAF3 SND1 MPPED1 SNAP25-AS1 CHD6 HMCN1 ZFAND4 FGD4 ETS2 GOLGA6D ITGA1 TCF12 ZNF721 VN1R7P KTN1-AS1 HIRA CORO2B POC5 ITGA8 GRIK4 RBM19 RUNX1 KIR3DL2 FEM1AP1 ALDH1A2 GABRG1 TSHZ2 MAPK9 ESRRG PTGFR IGHV1OR21-1 FAR2 FAM171A1 CNN2P12 ZNF595 CDH17 NLRP13 SV2B PEAK1 EYA1 ADAMTS19 KIAA1217 MORC3 ANKS1B CDH18 P2RX6 HSF2BP AKAP10 SPOCK3 FRMD6 PLS1 UNC79 SPON1 ANK2 PLA2G4A SLC1A2 ZBTB16 STXBP5-AS1 ANO2 SUPT3H SLIT3 BAGE2 GRIN2B ZNF518A IGLV3-31 PHC2 ROBO1 ZNF578 ANKRD30BL ANKRD11 EGFLAM PLD5 RABGAP1 PAK3 DGKB GARNL3 PWRN1 DPH6 EBF1 TNKS KLF12 NDFIP2 AQR GABRA2 ANKRD36BP2 MDM1 SMPX OVOL2 CSE1L C1orf87 PRUNE2 FCHSD2 SGMS1 HERC2P4 LINC01122 ITPR2 BRINP1 C8orf34 LINC00158 MLLT3 BCL2L13 IGSF5 DRG1 SOX9-AS1 TRAPPC10 LEMD3 KHDRBS2 CPQ RNF138 CHODL EHBP1 GABPA PRICKLE2 PSTPIP2 LINC00861 ITGB3BP CACNA2D3 ZNF831 DGKK TMEM67 PRKCE CNTN3 MGAM GLIS1 PSG9 ARHGEF11 PRKAA2 CD163 PACRG BBS2 IL1RAPL2 CHCHD3 MUC19 EDIL3 KLHL33 CDYL2 LRRTM4 PTPRN2 MYOM2 MYO3A USP31 UBE2R2 HIVEP2 KCNH8 GRIK1 NUBPL SOX5 PRAMENP KIF6 DSCAM DGKI RIN3 REG4 UBL3 TBC1D30 ANKFN1 NME7 DNMBP EFHB TRAPPC8 KDM4C ACSM2A DCUN1D4 SDK1 SLC9B1P2 SLC1A1 SLC12A1 MAPKAPK5P1 GRM5 PAMR1 EPHA6 NTN1 CA10 ARAP2 LINC00937 NR5A2 LDB2 IGF1R CYP4Z2P FAR1 SPTB WDR72 SNX30 NLGN1 DNAH9 TMEM260 SHISA9 OR9Q1 SHROOM3 JAM2 ZNF355P SNRPN ALX4 CORIN MSI2 LRRC7 TEX41 LINC00221 PDZRN4 MON2 CNTNAP2 LINC00930 MAP2 LIPE-AS1 AQP4-AS1 KCNIP4 CFTR CAMK1D FLRT2 NALCN-AS1 EFCAB6 ZNF402P MLLT10 C2CD2 NOS1AP PTPRO LINC00840 ZDHHC14 MSRA SYNPR PCDH9 GLB1L3 NKAIN2 CD96 RBMS3 OFCC1 MATN2 NHSL1 INSR IGHVIII-13-1 COBL MDN1 CTNNAL1 CLEC16A PHF20L1 TTC6 ME3 ITGA9 CYP4A22-AS1 KIAA1328 LINC00298 MTRF1 OVCH1 CATSPER2 CRTAC1 HS6ST3 EGLN3 CUX1 ANK3 CDH12 ZMAT4 MORC2 FRMD6-AS2 SV2C GMDS LHFPL3 CNIH3 DOCK3 THSD4 TBCD GPC6 RELN RASGRF2 TRPS1 HS3ST4 MFSD9 ADAMTS5 STK38 ADAMTSL3 EVA1C SNHG14 AOAH FBXO32 PHF2P2 MYO18B CDH4 TNR ADCY9 DPP10 OCA2 CELF4 CDKAL1 DAPK1 VAV3 INPP5A ZNF600 SUSD1 VRK1 ZNF678 CNTN6 CLIC6 ACSM2B ZNF420 TTC7B APP GTF2IP2 FBLN5 PUM1 CCDC88A ARNT2 KCTD1 RNLS SPOCK1 NF1P6 ANKRD20A7P HPSE2 PLCE1 TACC2 ANKRD36B ADAM12 FAM214A PAK1 ATP9B GNAL MITF IGF2BP3 CACNA2D1 ADCK1 HCN1 PPP1R13B TOP3A CHRM5 NSMCE2 ZNF208 FRY LINC00670 CXADR EPS8 GGT2 LRFN5 UTRN GPC5 TENM4 CECR2 PRR16 TSPAN13 GHR DUX4 RIPK4 RASGRF1 RIN2 PRDM16 FRMD5 RNF217 LINC00323 USP7 RBFOX1 MEIS2 KIR2DL4 STARD13 PCDH7 SCP2 KL LRRC4C ALCAM PPP1R9A PDZRN3 AVEN TMEM117 TPTE2P2 ADAMTS16 TASP1 POTEG LINC01090 MICU1 ZZEF1 LTBP1 SLAMF1 RGL1 NKAIN3 BACE2 INO80D SLC25A21 CLSTN2 MEG8 TTLL11 NEBL RARB DIDO1 CCDC18 MYH13 WDR12 TCF4 NAALADL2 FRYL TIAM1 FOCAD PBX1 FAM126B PHACTR1 MLIP SORCS2 PRIM2 PGM2L1 SLC39A12 LIPI ASAP2 DISC1 OSBPL10 FMN1 ANKRD20A1 RALGPS1 ARHGAP42 SLC16A1-AS1 CHAF1A PHF21B GUSBP1 ZFPM2 PIEZO2 SLC35F1 VSTM4 FAM66A SVEP1 LINC00113 NTM VTI1A ASAP1 PCBP3 FRMPD4 LMCD1-AS1 COL23A1 EDAR EGF LINC00960 PDGFD FYN FAM3B KCND3 RIMBP2 PRMT8 IFT43 XRCC4 LRBA GAB4 EPHA7 MAP7 FHIT SENP8 NSG2 AGGF1P2 GRIA1 ZNF627 TRABD2B SPIDR NAV2 STK3 ANO10 CNOT7 POTEKP COL19A1 MSR1 PSIP1 AP2B1 USP18 S100B NET1 TOX PCDH15 ESR1 ARHGAP12 GABRG3 KCNN3 SGCZ SEL1L2 PLCXD3 LUZP2 KCNAB1 GRM1 PDE4D CNTN5 LRIG1 ERC2 PRKACB GNG2 PDE3A PCDH11X RIMS1 POR L3MBTL4 DOCK4 ATP6V1E1 FRMD4A MCTP2 CERS3 PIGK SLC25A15P4 WWOX PCSK2 HUNK KRTAP19-10P CNKSR2 FUT8 SNX29 SAMD5 EPHB1 SSBP2 CREM LSAMP CTTNBP2 FHOD3 GREB1L PARP8 EFEMP1 ARMC2 TNRC6B PIGB AJAP1 IGSF21 SLC9B1P1 MIPEP ABCC9 SNAP29 GSG1L HERC1 DOCK1 MTUS2 DIAPH3 TMEM178B PARP15 FAM126A TRPM7 FLT1 EXT1 EFNA5 NOL4 LINC00536 NXN CDC14B ABCA10 TLN2 C14orf39 HDAC4 ZNF717 STK36 KLHL1 TRPC5 NF1P4 AMFR PLCB4 MRM1 ATP9A FTO PPFIA2 MYEOV ADAM28 ENOX1 B4GALNT3 SH3BP5 AKAP6 ACSBG1 ANKRD36C CSMD2 SORCS3 VPS37A LINC00571 LINC00299 EML6 POU6F2 TENM3 LINGO2 OPCML MARK2 DUX4L19 SFI1 ATF2 TUSC3 PHACTR2 ZNF72P ZNF880 RBBP8 CCDC91 GRID2 CALN1 ZNF423 LRP2 SEMA6D ZNF573 C2 RALGPS2 NTF3 FER SNRK GLDC TTC29 SUSD4 CAMK4 GALNT14 LINC00466 ANKRD20A17P CELF2 TP53I11 PDXDC1 NTNG1 DDX10 ZNF804B FBXL7 MAPRE2 ARFGAP3 MICU2 ISX RAD51AP1 SGCD TMEM108 RIC8B GABRB3 TPTE SEZ6L GRM7 SLC39A8 RAPGEF2 NAV3 MX1 PLGRKT PPA2 IMMP2L ZNF615 MIPOL1 GTF2I DNAH3 ATXN1 PRKCQ SSPN KIRREL3 GABRG2 NUDCD3 CPS1 PRKCH NRXN3 RHPN2 RABGAP1L KRT25 DLC1 PNPLA7 NSG1 ANKRD20A8P GABBR2 KCND2 LY86-AS1 ATP8A2 SNTB1 SLC24A3 UBE3A ORC4 MPRIP GRIA4 IDE CERS6 TPH2 LOXHD1 APC ZBTB25 MACROD2 TTLL5 INO80 TMTC1 MOCS2 AUTS2 TFF1 EPHB2 STON1-GTF2A1L SCAF4 SYT16 ADARB2 ERC1 ZNF850 COX10-AS1 PDLIM5 XYLT1 AGO3 C9 TMTC2 MCTP1 RNU1-51P MOB3B RYR3 NBAS MTND1P17 PRTG NBN C12orf42 ADAMTS18 RGMB CTNND2 FRMD3 COL22A1 TC2N SETD2 PACSIN2 PKP1 MIR181A1HG DOCK2 NUP214 TRIM23 SDCCAG8 FLVCR1 NRP1 CDH13 MDGA2 RFC3 PHACTR3 ADAM5 ANKRD26P1 ZNF879 RPGRIP1 DACH1 TRDN DEFB116 SLC2A13 ZNF397 DAB1 LINC00623 RFTN1 RSU1P1 TEKT4P2 CHODL-AS1 SNTG1 ALK EXOC6B EVC2 GREB1 LDLRAD4 SEMA3A CCDC26 SEMA3E DNAH6 MGAT5 ATP13A3 STOML1 CADM2 MALRD1 MYEF2 DCLK1 MAGI3 IFNG-AS1 ANKRD30BP1 CCDC122 COL21A1 FAM135B MORN1 KIF16B NRIP1 PAXIP1-AS2 CDH2 ARID5B SIPA1L2 CCNG2 RCAN2 LRRC69 TENM2 TANC1 PAPPA SERPINB7 EVI5 IGHVII-65-1 VPS41 SYCP1 ZNF407 MIR3118-4 ASB3 HDAC9 C1orf21 ELOVL7 PIK3R3 MAP2K6 FSTL4 ARHGAP28 MTOR STK38L KSR1 RALGAPA2 RORB GABRB1 FBLN1 SGSM1 TPTEP1 ST8SIA1 BLM SH3KBP1 FHL2 PSMB2 CADPS NEU3 NCAPG2 RGS7 TPTE2P5 KYNU STK32A CD2AP ZFP30 TTC39C CLVS2 DIO2-AS1 USP25 SLC44A1 SPRED1 AP5M1 SIPA1L3 ADAM10 GALC MRPS22 DRAM1 TSPAN33 PPP2R2C KANSL1 CES1P2 LRFN2 FLNB WDFY4 SCAI TULP4 PAPPA2 NCOR1P3 ABCB5 HEATR4 SPECC1 DPY19L2 MTMR10 LINC00559 NEK2P2 PTPRT FAM118A TRIM9 CSMD1 TRERF1 SLC24A2 CENPBD1P1 GLI3 COL25A1 NTRK3 RXFP1 FBN1 SGCG HYDIN CHKA RAB31 CTNNA3 TXNDC16 VPS13D ABHD17C ZNF292 TBX15 PRB3 RAPGEF4 BMPER LINC00922 ANKRD31 ZNF521 PDE1A TMPRSS2 LINC01036 CMAHP ATF7IP DUXAP10 HMGA2 TMPRSS15 MX2 CREB5 THSD7B

		CPA6 NSUN2 DEFA3 PTPRK KIR2DS4 SORCS1 TBC1D4 DNM3 SYT1 APIP SYNDIG1 ASXL3 DPF3 DHX32 SCAPER CRYBB2P1 NPHP4 DOCK9 DUX4L17 DLG2 PPP1R12B SACS PPARA PLXNA2 SCFD2 PTPRD RORA MYH15 SHISA6 SCARA5 IGKV2OR22-4 LINC00923 PLCB1 LOXL2 AGGF1P1 BPTF RPS10P7 PRKG1 CTBP2P1 RASGRP1 PLXDC2 ELAVL4 CDH9 NLRC5 STXBP6 CYP4Z1 WDR25 DMBT1 MXI1 TTC28 MAGI2 NELL1 ANKRD30B STX12 PLCL1 ABI1 HPCAL1 GALNTL6 KIAA1671 PXDNL TSHZ3 ASIC2 RALA DOCK10 GNPTAB TRPM1 CACNG3 CNTNAP3 FNDC3A NECAB1 PRKD1 ATP8A1 TNFAIP8 POT1-AS1 BCL2L1 MICAL3 ADK HDAC2 RANBP17 ETS1 MRPS27 TNN MIR155HG ST6GAL2 RYR2 SEMA3D LDLRAD3 SLX4IP BANP TGFA YME1L1P1 PRLR TBX20 PTPRA FAT3 OR11G2 MTMR2 KCNH5 FAM189A2 TMEM163 MPDZ ATF6 EYS IPO11 IL16 FAM189A1 VCL DEPTOR BACH1 ATAT1 POTEH ROBO2 EWSR1 IFT81 OSCP1 ZMYND11 CDH23 RGS6 SRGAP3 EFCAB2 AKAP13 WDR41 NEDD9 MYRIP SLC39A11 ENPP1 UNC13C PCP4 RIMS2 CCDC171 SLC5A1 C16orf95 STAC SCN8A RAB27A EYA4 RALGAPA1 L3MBTL3 DLGAP2 IGKV2OR22-3 ARL15 POMT2 HIVEP3 CLIP1 SEMA5A CABLES1 PRDM15 OR4N2 BCL11A AGAP1 FREM1 DCC ZNF112 CTNNA2 ATE1 CEP44 PVT1 PARD3B CHN1 ETV6 VPS13C KCNJ15 CEP112 PARD3 NRG1 NPL CAST TBC1D9 FANK1 ZNF845 DLEU1 SLC25A48 ATP10B SLC35F4 NPAS3 PRKCA PGPEP1 FMN2 SEMA3C FANCB DPY19L1 CSF2RB KIAA0825 PCNT BCKDHB PAH ST18 FRMD4B OSBPL5 TOP1
K562	2213	*FSTL1 SLMAP FAM219A FARP1 LRRC37A5P FAM153A CCDC116 KLHL13 RPS4XP6 ABCB7 PDCL3 SLC10A7 LDB3 TPI1P1 SLC6A11 MYO9B NOS2 LINC02645 ZEB1 LINC01708 CDK14 LINC01685 FAT4 LRMDA CHD9 FGR EPN2 SLC15A2 MET THRAP3 BANK1 KNDC1 PRSS51 SLC25A52 LINC02355 HERC2P2 DMC1 GLRA4 OR7A17 LHFPL2 C9orf43 DIP2A NBPF21P PCAT19 TMC3-AS1 UNC45B LUZP1 LINC01346 ROCK2 SCYGR8 LINC02249 LRGUK ZNF723 FAAHP1 LINC01479 LINC02476 FOXK2 FGGY LINC01138 LINC01620 LINC01915 SMIM35 LINC01782 SMYD3 ANKRD20A4P LGI2 LINC02267 DNAH17 TAFA2 STAU2 PGAP4 LINC01387 PGBD5 TIAL1 ROR2 USP14 LINC01163 ST8SIA4 GMPR HYDIN2 GAS2L1 RFX3 CD82 ARL13B LINC02224 TMEM156 SEC14L1 ATP11C SF3B6 LIMD1 XRN2 ADAM32 ASS1 IPP SNX6 CEP120 RN7SL52P FAM204A NCF4-AS1 LINC02122 ATP5PBP3 MYO5C LINC00841 SDC2 USP32 KDM6A MED27 PDYN-AS1 SLC22A14 RBPJP6 UFL1 COX10 CROCC2 EPHA4 PKNOX2-DT LINC01967 AGPS TARID H1-9P KLHL7 ECM1P1 GACAT3 WARS2-AS1 PCSK6 RPRD1B CCDC172 LINC01828 PTPN2 PBLD LINC00486 GORAB GPR1-AS ARMC6 LINC02675 RSU1 MYLK2 ZFHX4-AS1 IRAG2 RBM6 PSMF1 GLYATL2 SH3PXD2A NRF1 IGHV2-70D OPA3 MOB1B UIMC1 LINC02607 RAB38 RIC3 ZNF738 EXOC1 UPP2 VMP1 NENF HRH2 ATP6AP1L RDX SVIL DEUP1 CADPS2 C5 ANKRD26 ATP2B2 RGS20 HCRTR1 RPL37P3 RELL1 ABCC12 LINC02176 EIF3D ADAMTS19-AS1 SNRPC OVCH2 FBXL20 GOLGA8EP COP1P1 POTEJ SHISAL1 SUSD6 LINC02133 LINC01427 MYCL RGPD2 LINC01727 SPAG6 IFNAR1 KIAA0319L OPN3 NRAP MAP4K3 XXYLT1 KIAA0753 SPATA48 HHIPL1 NOTCH2 FAM72D FRG1-DT TTC28-AS1 ANP32A CLDN10 USP43 FKBP5 KIAA0232 SRGAP2C FBXO3 TMOD2 BTBD10 MELK YPEL1 ARHGAP26 PTPN4 WSB1 MAP6 LINC00896 MORC1 APOL1 NDC80 AIF1L SCGB1D5P KANK4 CEP72 SGO1 DUX4L20 FAM230C SLC37A1 SUPT16H GAS1RR LINC01213 LINC02439 LINC02668 AUH RALB LINC02234 MOSMO MRPL37 BLK PPP1R17 PSMA5 RESF1 MAPK1IP1L FARS2 DPH6-DT PIAS1 CDH20 MAGI2-AS3 ZNF611 NCOA6 LRRFIP1 ZC3H14 MROH5 LINC02180 LINC01483 LINC00662 GALNT10 FHIP2A CFAP74 ZNF846 UBE2QL1 PLPPR5 RNF217-AS1 ZNF287 AVL9 ZFYVE9 FER1L6 WBP2P1 LINC02542 SYN2 ATL1 PTCD2 ADGRF5 SERPINB11 QSOX2 NCSTNP1 HFM1 SMG1P5 MCF2L FH STK10 CFAP70 ABCD3 MTF2 GSR CCDC162P FIG4 INTS7 ASB4 GRM3 PRKN GRAMD1B FOXP2 SIAH2 RB1CC1 POLR3A LALBA LINC00375 F13A1 BRCA2 RNU6-1007P ANKMY1 KDM7A APELA UBN1 PLA2G12B ADAM29 CDC27P2 FAR1P1 TMEM182 SP110 KIAA0513 LINC01906 DUX4L34 IL10 LINC02305 LCLAT1 LIFR-AS1 EFL1 BIRC6 OLFM4 SLC6A1 SAMHD1 EPC2 TUT4 SDF4 EFHD2 CXCL2 GNAI1 EGFR GOLGA6B LINC00334 NDRG1 SLC10A6 PTPRQ CARD10 MRPL13 PACS1 BCR SLC49A4 DNPEP TRAPPC11 HOXC4 FANCM NEO1 MELTF MXRA7 LINC01443 NECTIN4 CNMD LINC01309 PDP2 UFD1 ERP27 POTEB3 ZBTB21 NGF-AS1 AGL BBS4 MIR99AHG HS6ST1 ITPKB LINC01829 ZBED9 RTRAF IPCEF1 NF1P9 BIN2 TCERG1 FAIM UBE2O RPRD1A LINC01608 PPP2R2A ZNF541 LINC00869 CPEB4 CKMT1A CDC27P3 ABL1 NCOR1 MOCOS LINC02213 PRDM10 CDC45 CWC22 C16orf72 PPP1CB USP33 ERBIN LINC02087 ZNF121 HERC2P9 MBTPS2 MTHFD1L KHDC4 HOATZ LINC00598 IRAG1 HRH1 LINC02223 ZNF705G LINC01684 KRT89P USP41 RBMX2 ZBTB10 PKHD1L1 SOX1-OT MCC CEP192 SLC26A2 PTH GUSBP11 RAB22A FAM66C ROCR ZNF160 HDAC11 SLC9A4 DHX29 HADHB GRXCR1 STPG2 MIDEAS TM9SF2 MAP3K4 LINC02646 LINC02466 INTS13 VSTM2A HEATR6 GNG7 RUNX2 LRP12 FGF10 TAPT1-AS1 LINC01331 JAZF1-AS1 LINC01035 PLEKHA2 LINC01492 ESRP1 RAC1P3 NLRP8 ZMYM4 FRG1JP PLG DUX4L37 SLFN11 MED12L ZDHHC21 BRMS1L ERO1B TM9SF3 CABYR LINC02505 ANK3-DT DDHD1 PPP6R3 CDH7 MFSD14C ACOXL CYCSP39 GUCD1 ILDR2 LINC02492 NCK1 SOHLH1 FRG1FP CDV3P1 LINC01192 DRAIC PUDP FAT1 PATJ ITCH MBP TANGO6 SOSTDC1 CELSR2 CFAP97 EBNA1BP2 SLC46A3 PTPRJ TET1 SEC24B-AS1 KCNQ3 ANKRD33B SNX8 HADHA MRPL58 CCBE1 SELENON CNIH1 FRG1BP TMEM232 CLCA4-AS1 MAPKBP1 UST LINC02613 BTD NSMAF PYGO1 CDIN1 NFKBIA TYW1 HSF5 ALB CD101 JPH1 ANKRD20A3P GSE1 EFR3A FHIP1A UBAP2 SCN10A NCOA7 ANKRD18A MDS2 ANKRD7 LINC01622 NARS2 MARCHF1 OR4L1 PTCSC2 ABCC8 LINC00539 NOXRED1 BNC2 TTC33 C9orf92 MCPH1 MINAR1 MYO1D HSD17B14 EIPR1 BMP2K LINC01707 LINC02543 HNRNPU CHCHD2 CCDC126 LINC02366 GOLGA6L17P BABAM2 TWIST1 LINC02653 GEMIN5 IL1R1 RPL15P3 TRIM77BP KTN1 PASK HEPACAM USP8 MRTFB XPO7 ARSJ LINC02006 VWA3B LINC01801 LINC01320 LYPLAL1 ALPL STEAP2-AS1 CHST3 MAP3K9 ABLIM1 BTAF1 COLQ PDCD6IPP2 NYAP2 LINC02660 HNRNPCP9 RFX2 MAPK8IP1 CHRNA7 SLC66A1L LYPLAL1-DT ACTR2 HMGB1 MEF2C FOXO1B MYB ARPC3P2 ST8SIA5 TBC1D19 RPTOR CLCN3P1 MAP4 ZNRF2P2 HIVEP1 TNIK COX5A LINC02252 SEC24D MPPED2 MDFIC EPB41L4A WNK2 PCMTD2 MBNL2 LINC02226 STARD4-AS1 TUBGCP3 KIF7 SDAD1P2 ITIH5 LGALS9DP HOXC13 HCP5 ECHDC1 SMARCC1 AMBRA1 STX18-AS1 DOP1B FAM66D LINC02063 LINC00355 FBXW8 SLC9A5 LINC02465 MUSK KCNJ18 ECPAS LINC00583 SFPQ IL21-AS1 RNU6-835P PTPN12 GPR137B LINC00434 LINC02424 RXRA TOP3B LINC01649 STAG2 HTR5BP ARL11 UBA6-DT ARHGEF12 LYPLA1 LNPEP DDX39BP1 UNC93B3 RPS3AP6 CDK12 ANKRD10 GALNT13 NEDD4 YTHDF3 SYT10 PEX14 SEPTIN6 ZBTB38 PAFAH1B1 CFAP61 LINC02380 FYCO1 HRH4 DOCK8 LRRC38 CNKSR3 LINC01340 ZNF648 DRAXIN LINC02058 TRNAU1AP LINC02145*

		ARFGEF3 KIF11 LINC02400 PHC3 TMCO5A CCSER1 FANCL SH3GLB1 LINC02237 OTULINL SCML2 ANKRD28 LINC00701 GRK3 ZBTB2 ZMYND8 CCDC186 GSAP EFTUD2 LINC01695 ZNF382 ACTR3BP1 NDUFAF6 LINC01412 INTS8 ERMP1 ARL4C CIBAR1 ATG4B ADGRL2 GAGE13 OSER1-DT KIFC1 TRPC7 NPSR1-AS1 COL4A3 MYO10 LINC02693 TMEM74 PRKAA1 ENAH TENM3-AS1 GAGE12J TAFA4 POGK CROT LINC00862 STON2 MIR3681HG IREB2 STOX2 LINC02458 ABCC4 PLAGL1 FRG1HP ABCD2 DNAJC7 RRAS2 RPL5P35 ERN2 HECW2 NRBP1 CYP2C58P ZNF679 TLNRD1 SEC14L3 SERPINB2 GTF2F2 AOX3P SOX30 TMEM132C KRTAP19-7 TTLL7 EFCAB14 PLEKHA3 WWC1 CPSF3 NF1P7 SH3GL3 SENP6 MIR100HG LARP1 INVS SUMO3 LNP1 KIF21A UNK LINC01938 PHF19 XKR5 ADAMTS14 ZNF875 LINC02191 DCAF1 SYNJ2 ARSB PARPBP IL34 SIGLEC29P DPYSL5 AGK EBF3 CEP83 NFKBID ATXN3 CIDEA CFAP299 LINC01924 OXR1 LINC01033 SFMBT2 RNU6-1150P NPIPA1 OBI1-AS1 OR2T2 MADD PCID2 LTN1 LINC00667 TINAG AXDND1 ZSWIM6 MYL1 KLHL4 MTREX CD9 EIF3F TOM1 CAMK1G LINC02327 FAM30A PDZPH1P RNU6-113P CFAP418-AS1 WDR64 GOLGA8S PRKAG2 IGLV2-34 LINC02540 MTND2P8 RPL23AP7 JAZF1 WASF3 MTUS1 GABRA6 CCDC192 PTPRVP MGMT BUB1 BNIP3P41 PANTR1 KRTAP21-2 LASP1 KRT6A VPS35L GGT4P LCE3B SKA1 PALS2 ADCY10 SCGB2B2 LINC01692 LINC02165 PTAR1 DSG1-AS1 LINC02091 TRIM58 NAA35 ATP5PF KLHL32 ZMYM1 UBAP1L MVB12B ZCCHC14 AGGF1P10 C4orf50 LINC02253 UBASH3A ELOC LINC01588 PRAMEF2 ENTPD5 CUL1 SLC7A2 PKN2 C16orf74 AGO1 GLYATL1 AGAP14P PRDM11 ODAD2 ETNPPL ARMC3 IGLV3-27 LINC01128 GRB10 PARGP1 LATS2 LINC02141 AOPEP APCDD1L-DT DNMT3L CBLIF ATP6V1B2 TERB2 LINC01602 TTR PSAP NCS1 RANBP3L ONECUT1 MARK2P12 LYN ADGRB3 INPP4B PCDH11Y HGD SUMO2 SDCBP TASOR2 CUL5 MICOS10 LINC01221 LINC00363 DIP2B LINC01151 CD5L IGLV2-14 LINC01189 TFDP1 LINC00383 ZNF876P LINC02464 LCE3D MIR9-1HG PPME1 TNPO3 LARGE1 HTT MBNL1 TPGS2 DAZL FUT9 LINC02291 MOK TBC1D1 IQGAP1 TUBB6 CCDC195 TMCO4 LINC02098 BAZ2A FAM27C RPS12 MED1 DDX6 LINC02328 ANKRD20A21P DIAPH1 COMMD10 COG5 COPS8 SACM1L AKAP9 MIR3197 LINC02008 IQSEC1 PIGN PAQR5 DBF4B LINC02236 CCL28 MRTFA FAM241A LINC00838 PRSS23 WNT2B PPM1L CPHL1P POLR1D CASZ1 LRRC37A3 PAK5 SETDB2 EXD3 FAH OR51E1 RABEP1 TMEM116 NR2F1-AS1 ZNF705B NUTM2HP FRG1CP ALKAL2 VSX1 ZNF280B GOLGA8F NFATC2 TNRC6C LINC02663 PRAMEF25 LINC02451 KRTAP20-4 ARHGAP44 ZNF970P MTCL1 RPL23AP87 GOLGA6C BAZ1A ARID3B ZFAND3 TCERG1L C6orf118 FAM83F RAB12 ITFG1 LINC02235 C21orf62-AS1 EXOC1L XKR6 CYP2C8 PKN2-AS1 LINC02649 DOCK5 ABCA9-AS1 C7orf31 MAGEL2 SRFBP1 COPB1 MIR646HG IL33 SGO1-AS1 DTHD1 MARK4 CRACR2A CFAP20DC HAAO CIBAR1-DT LINC01901 ATP6V0D2 OR2T3 SYNJ1 SERPINB10 ACTN1-DT KLF15 HLA-B PPP2R2B ARHGEF17 LINC02196 ZNF431 CACYBP IL12A-AS1 CATSPERE ANKRD66 ULK2 LYRM4 RPL23AP49 LINC02099 ZNF462 HNRNPH3P1 UGP2 TMEM44-AS1 FAM25G SHROOM2 PABPC1 RP1L1 PPM1F OR4K8P ZFHX3 EFCAB6-AS1 RRAGD RPSAP68 ST13 GPR156 MARCHF8 RGPD8 SHC4 LINC02112 FCRLA THEM7P LINC01937 ZNF613 DEFA8P PSMA8 GOLGA8T KITLG KRT6B AKR1C3 PPP2R5E ASB7 COL5A1 LINC01426 IFT57 IL20RB ADAM22 RABL2A LINC02582 MAP4K4 FICD FEZ2 KIF21B WNT5B PEG10 INHBA-AS1 PRAME LARS2 HULC LINC01414 APLF HIP1 OLA1 WNT9B RFX7 ADGRV1 MFHAS1 LINC02662 ANKRD55 SERBP1 SKINT1L EOGT LINC02254 ADD3-AS1 NF2 CRISPLD2 DMAC1 ANTXRLP1 MTMR3 FAM25C CRKL ANP32B FAM90A28P AP4E1 ITPRIP H2BC15 SCG5 NDFIP1 LINC00581 NDC1 TUBB2BP1 PTPN13 SNAP91 LINC02074 ST8SIA6 LINC01908 CDH5 TRAV8-6 LINC01362 A2MP1 LINC02406 UBBP4 BHLHE40-AS1 LINC00895 UBE2Q2P1 BTG3 ATP2B1 CMTM7 SMAD5 PPIL6 PATL1 UBE2J2 ATRN OR4F6 CNOT6L UGT3A2 TRAPPC6B ZNHIT6 RNGTT CDCA8 WDSUB1 PITX1-AS1 ARHGAP5-AS1 FGF7P3 ZNF684 APOL2 COX7A2L AKAIN1 RNF38 FYB2 AHDC1 LINC02073 HERPUD2 CUBN CRTAM SLC52A1 HSD17B2 UBE2G1 PELI2 B9D1 KIRREL1 LINC01467 PEBP4 MIR548H4 CRACDL LINC01491 MRPL45 LINC02664 TTC37 RN7SL483P RCL1 MIR4435-2HG OAZ2 ZNF718 DKK2 SPPL2B TANGO2 OR7E19P HERC2 RIOK1 DNAJC27-AS1 PUM3 ZNF66 HOOK3 BVES-AS1 LINC02563 CCDC106 ANLN SLC1A7 GNAS MKNK1 ADGRE3 DYSF NPM1P2 SERPINB9 LINC01876 TMEM63C LAIR1 GTF2IP4 MSH6 HECTD2 LINC01410 KATNIP PDE6C FRRS1 ADGRE4P IQCM PTK2 TRIM2 DGLUCY MFSD11 PLIN2 IGLV3-2 SPPL3 CARMIL1 FAM167B LINC02558 DENND1A ABHD2 RACGAP1 MIR3667HG NUP37 ERLIN2 SAA3P LINC02250 KCNK15-AS1 TDRD5 NIPBL GOLGA6L3 ATPSCKMT FRA10AC1 FOXB1 HHLA2 UQCC1 C3orf52 SHOC1 MBD5 FAAP24 KDM5A ATF1 MIR548XHG SAMD12-AS1 RGPD5 GDAP1L1 LINC02096 LINC01358 C12orf4 LINC01579 PLA2R1 LYSMD2 NGDN ADAMTS2 PJA2 TRPV5 SMG1P2 GALNT18 GTSF1L AURKA LINC01145 GPRC5C COLCA1 AP3B1 CPAMD8 RNU6-929P STMP1 UBL7 ERICH5 ASH1L CALM1P2 BCAP29 TTC21B SLC5A4-AS1 NEK6 MEGF10 ECE1 OR13C9 LINC01445 YIPF6 SEMA3F-AS1 TMEM25 DZANK1 CLTCL1 NUAK1 ZNF891 SLC25A18 RNU2-47P SNTG2 CTSB BCRP2 CCDC77 IGLC3 FANCA LINC02306 CHAMP1 LINC02325 ZNF354C GALNT17 LRRC2 NSD1 NEDD4L HDGFL3 CNDP2 CCNYL3 AGO2 CREBBP CFAP44 PARK7 DSTYK BRINP3 LINC01237 ZNF271P C2orf69P4 LINC01498 DRC7 DISC1FP1 LINC00240 DSE LINC02346 ANTXR1 BARD1 LINC02256 RGS8 PDE4DIPP1 BMP7 LHFPL6 GALNT2 ANKRD17 CYP2C9 EPHX4 IMPACT ITGA6 CA5A IMPA2 PSMD2 ZFP90 LINC02641 BMP2 LAMC3 LINC02011 FAM107B ANAPC1 SH2D3C LINC02240 IBA57 LINC02147 RBPJP2 ITGB8 GAP43 LINC02422 OR4K3 PRDM13 LINC01226 GXYLT2 HIPK3 BCAR3 TMEM225 SREBF2 MAIP1 OR10H2 ZNF780B LINC01900 GAST UBE2L3 SAR1A BCL11B LINC01814 LINC01993 LMX1A RSPH3 CHIT1 TSPAN2 ZFAND6 TMEM178A NUF2 CKMT2-AS1 OR52B3P ASAH2B INTS12 GFRA2 NEK10 ZNF74 STAT1 POLR2M SLC44A3-AS1 COLEC12 CDCA5 ZNF705CP MYOCD RPH3A PRDM1 HAGLR LINC01088 ZNF215 RERE ALS2 ZNF33B ZNF608 COMMD8 CYTH4 TBATA MYO1E LINC01681 HDAC2-AS2 SAXO1 LINC01098 RWDD2B BBOX1-AS1 PLPP4 PWRN4 CCDC102B NPAS2 SDS LINC01571 UBR1 COL5A3 SOGA1 COL4A2 ME2 ARHGAP32 SLC27A6 NECTIN1 TMED3 GAREM1 ZNF528 LINC01222 ZNF44 ADA2 PRKCZ FOXJ3 CENPE CNTLN BPNT1 MYL12B RSPH14 IL17RD ALG10B HCG22 SSBP3 HECTD1 CYP4F22 LINC01182 NGEF GNAI2P1 FAM102A LINC01566 PRSS2 MAPK1 ZNF705D OARD1 POSTN LINC00476 SEM1 DMXL2 SEMA6A-AS2 MOGAT3 TMEM236 MS4A4A TNFSF11 SPOP CASC15 LINC01473 RFPL3S SPRY4-AS1 SNX9 GCSAML ARHGEF28 RAB3GAP2 IGHV3-62 SLC37A2 ATP1A1-AS1 CRYZL2P-SEC16B BAZ2B CRACD CEMIP CTSE LINC00877 VENTX DIRC3 MAPK8 ESS2 PHAF1 AK6P2 PLCZ1 ESCO1 PID1 RFC1 COL6A5 ZFYVE28

		NSUN6 LINC02174 EBF2 TMEM38B DNAJC21 AKR1B1 MMP26 RNY4 PCMTD1 OR6C75 EXTL3 EHMT1 PI4K2B ZNF735 FAM72A IL17RA PLPPR1 CLPX H2ACP1 OR7H1P NSMCE1 REPS1 CD70 SH2D1B ZNF606 LRRC8B CSNK1G1 ZNF169 KIF13A KRT18P59 NLRP4 RC3H2 SMTN TWIST2 RNU6-374P SCG3 TNNI1 TAF3 SP3 ZNF106 NR2C1 ADAMTS3 LINC01476 GLI2 CLSPN ICA1 PLEKHD1 EMILIN2 TLDC2 ZNF438 HORMAD2-AS1 VASP UNC80 SDE2 UPRT SPSB4 PPIP5K2 FRG1GP PALD1 C15orf32 KDM1B SPSB1 F5 NUP50-DT S100PBP MESD CDKN2C TRAK1 STXBP1 FAM66B LINC00836 ANKRD30A NMU DENND2B LINC00603 LINC00944 RPS3AP4 LINC00114 C2orf69P3 LINC01500 MANBA GFRA1 SHLD2 SPHKAP RMST ECT2L TDRD7 ELP2 RNF8 MIR663AHG LINC01543 DEDD2 DNM1L LINC01723 DNAL1 TEX29 TAF15 TRAPPC3 TARS3 PFKFB4 LINC02910 RNF11 HMCN2 HTR2A IGLV10-54 LINC01204 HSDL2 EIF4BP3 ZNF350-AS1 ZC3H15 CCDC152 LINC01135 GOLGA8G GALNT1 SNAPC3 MARCHF11 CIB4 AKAP11 RBM15-AS1 NRK NAT1 KICS2 CYBRD1 PPP2R3A KRT16P6 COL27A1 CYFIP2 BMP5 APOOP5 CRYBG1 HACD2 MSANTD4 LINC02061 BCAS3 TMC7 FBXL13 OTOG ERCC6L2 RUFY2 SLC16A1 RANBP9 FAM245A NUDT21 LINC01344 ENPP3 ZNF235 LINC02228 HSPA12A KRTAP21-3 RGPD6 BRWD1 THNSL2 LINC01674 SLC6A3 TMEM54 PPIP5K1 EYA2 ADGRB1 ADGRE1 LINC01837 UCK2 TET1P1 ACTR3C TSPAN11 PDE6A LINC00643 KRT18P55 OR1L6 MGAM2 SLC17A1 OR2T7 KMT2E DNAH14 MAST2 ERI1 DELEC1 MRPS35 RBFOX3 PWWP3A UTP4 BTLA SEC23B TRGJ1 SYNE2 CLEC20A NETO2 GTF2IP6 LINC01416 SERPINA6 ASCL3 FABP7 IFT46 NUP210L GK AK3 ALPK3 LMNTD1 LINC02343 RAB8B RC3H1 LUCAT1 MAB21L3 NSD2 SMPD4 MTCO2P3 LINC00469 SVIL2P ZYG11A CGAS GRM8 SLC23A2 ARHGEF7 LIN54 MPPE1 SLC44A2 LINC01592 ZNF704 TRIM43B SAMD12 MAN2A2 CMIP LINC02198 LINC01117 C1QL3 MPP7 POU1F1 FBN2 KIAA1958 CARD18 LPGAT1 FOLH1 MAPKAP1 SPRR2D CYFIP1 LINC00906 PCMT1 GON4L LINC01019 AFG3L2 ARNT LINC00929 USP49 EIF2B3 CSF1 LINC02109 SLC8A3 HAS2-AS1 SLC2A3 FAM72B MYO5B USP24 UHRF2 SDR42E1 EPCAM-DT CSDE1 LINC01581 RAP1GAP DEFT1P RAD9A RNF215 SPOPL ADAMTS9 RAI14 LINC01664 TMEM273 APMAP HAUS6 CFH LINC00649 PTPRB MARCHF6 CATSPERG ITSN2 LINC01917 ODR4 DHX40 ANKS6 DTX1 GFI1B RASGEF1C TRIM43 FOXO6 MTTP FBXW2 TOM1L2 METTL15 LINC02652 LINC01229 FOXJ2 AGAP9 KIAA1549L MYT1 HECTD4 BICRAL NEK7 NKG7 EMP1 TMEM171 ACTR3BP7 COG2 ZC3HAV1 LNCAROD CCDC138 FAM110A NBPF1 LINC00314 TXNRD2 FAM245B LINC01322 RAB27B ZNF236 LINC01337 MYOF RP1 LUC7L LINC01266 LYST DEFB103A SLC16A9 RSRP1 CCR2 RSPH1 NALCN CFHR4 GATAD1 SNRPD1 LPCAT2 BZW1 DDX39AP1 LINC01477 PCNA B4GALT6 ATG5 TRIM60P19 SGTB SCGN SRGAP2 ZCCHC7 AK9 MYLK4 ZNF234 LINC02680 CWC27 ZNF124 TAFA5 ASCC2 CNNM4 MYO5A SPEN NAP1L4 TMC1 CAMLG WASHC1 CDH26 TPM1 LINGO1 LINC01667 CIDEC CRIM1 SEPTIN9 LINC01706 DHTKD1 SMPDL3A ACO1 EIF1B-AS1 DIRAS2 DUX4L45 SLC5A9 RRBP1 CDHR3 TJP1 DSG1 ERICH3 LINC00354 JCAD ANKRD24 GID8 TADA2A RPL15P2 RBPMS2 AK2 CYP4A11 LRRC9 OSBPL6 GRB14 PNPLA8 LINC01425 LARP6 SEMA4B NIPAL2 LINC01807 LONP2 PEX6 DHRS3 INTS4P1 PRRC1 SPIN1 C21orf91-OT1 DROSHA ZNF813 SNTB2 KPNA1 TTC3P1 FAM149B1 LAMC1 CCND3 SRP9 PBX3 SMIM11B ANGPT1 PCNX1 RPF2 HHAT CHASERR MTCO1P1 SEL1L IGLV5-45 ADGRG6 SLC4A10 PHLPP1 GPR89B BTF3L4 LINC00844 MIR3936HG ZNF618 ITGA4 LIX1-AS1 TSSC2 CEP57L1 ROCK1 RBPJP5 TGM1 MIR3142HG FTLP13 GAPVD1 LINC01310 ZDHHC18 ARHGAP31 HLA-F RXRG FAM183A RGPD4 UHRF1BP1L FRG1DP ADSS2 OR4F15 IKBIP RNF220 IKZF2 OR8B9P PDE2A ASPM RFC2 LINC01643 DENND2C TBC1D13 KCNJ1 PRG4 IGLV3-1 CLCA4 SCGB1D1 LINC01877 SLC5A12 MLLT1 TG HERPUD1 DGKG ATP8A2P3 METAP1D SLC36A1 ATP6V0CP3 LAMB1 ANKRD19P OR5AQ1P XPNPEP1 HOMER2 GLYATL3 PCGF5 FGF7P1 TUBBP9 IGLV2-18 NFIB NDUFAF2 GUCY1A2 LINC01524 UMODL1 KREMEN1 MB FAM66E ZBTB33 SOD1P2 MGA MIR924HG LINC02549 CLCN5 MIR4300HG OR4R3P RTTN FGF9 NIN RPL23AP53 SPG21 ZNF302 GARS1-DT ANKFY1 CREG1 LINC02488 LIMCH1 CMPK1 SCN2A ZKSCAN5 WNT7A GMDS-DT GNAQ ZNF449 MIR548A1HG MTMR7 HNRNPM SPIRE1 TMEM71 ZNF14 C1QTNF3-AMACR UBE2E1 SLC30A10 AIMP1 PPIL2 AP4S1 LINC02149 E2F6P2 COL14A1 TRAV8-1 RBM47 CASC17 GBP6 JARID2 SANBR ZNF367 FBXO41 LINC01666 PRR5L MACROH2A1 UBAP2L TRIM43CP PCDH8 LINC01673 PRPF18 SMOC1 GSTA3 WDHD1 MAMDC2-AS1 TOGARAM1 SLC45A4 ELF2 SCAMP1 PTCHD4 ANTXRL LINC02615 GLYAT KIF15 JPT2 ZFYVE26 SNX3 LINC01422 CEACAM7 DST TMEM161A MSH2 CYLD FAM242A DEFB108B WSCD2 UBE3D VLDLR-AS1 GLIDR CERS3-AS1 CAPN5 CADM1 KLHL29 ABCA4 LINC01718 OPRM1 ACER2 THUMPD2 APCDD1 RGS9 PCCA CNKSR1 CLVS1 YLPM1 DIPK1A SLF1 SLC13A5 VCAM1 LINC02511 LINC01818 ATP6V1C2 LINC01992 INMT-MINDY4 OXNAD1 GNA14 TM7SF3 PLEKHA8 LINC01721 PDK1 LINC02055 CYP2A7P1 PROX1-AS1 DENND4C PSG6 ARFGEF1 HDHD5 PITPNM3 PPP1R1C SDR42E2 IARS2 CCDC150 DNM1P47 HEMGN CDC42BPB FAM217B CISD1 SYT14 LINC02241 ZNF295-AS1 CHSY3 PRKAB1 YAP1 YBX3 LINC01748 LMX1B MICALL2 LINC02245 BMPR1B LINC01173 C2orf42 SMARCA2 C2orf83 TLL1 LINC01297 C1orf127 DUSP16 ELL2 DAW1 SAMMSON IGHV3-74 RIPOR2 PTGS1 LINC01811 LINC02899 RPF1 TRIT1 NDUFA10 CEPT1 KCNK5 HSPD1P3 HKDC1 CLNS1A DUX4L33 NUP43 EPS15L1 NMD3 DNER GOLGA6A GATAD2B XIRP2 KRT85 ANXA4 SLC14A1 CA1 C5orf52 FAM72C RANBP2 IGKV3OR22-2 SERPINI2 APBB1IP ADGRG7 STRN TSBP1-AS1 NPM1P1 FAM81A SMG1P4 SNAI2 ZBTB49 DGCR2 RN7SL250P RNA5SP99 ADGRA3 CLDN18 HSPG2 FAM180A LHX9 GALNT16 MSANTD2 FCHO2 RFTN2 ANO6 COL6A6 OR4K2 RN7SL767P USH1C DNAH5 HIPK1 PTPRD-AS2 SATB1-AS1 VAV1 CACNA1I PDGFC PEPD IFI44 RPS6KA3 RETREG1 LINC02307 LINC02269 NLRP14 DNAJC13 NPHP3-AS1 IGLV3-30 CNST VPS13B FAM138E ASB2 OTOP1 PSME3IP1 GHRH GOLGA8B DPY19L2P3 IGLV4-3 LINC02315 SLC39A6
HEK293T	3434	DDC8 ELMO2 USP17L28 RIT2 CASC20 TRAF3IP2-AS1 DPY30 MIR4677 PAX7 CMTR2 GPR98 GNL3 BBOX1 IFNA20P ZNF799 FRG2 NCF4 LCORL MIR4461 ALDOAP2 BSN-AS2 FGFR1OP2P1 DDB1 DLEU7 CNGB1 EPB41L4B EP300 HS3ST3A1 RDH10 GPSM2 KLHL14 PRKCG KCNA6 DTNBP1 RGN MIR3156-3 C4orf22 BCRP6 MSRB3 GDA MREG AK7 MAP3K3 NINL MIR489 RNF185 TNS3 NR4A2 PIR RRP7A CYP4F59P KIAA1199 FIBCD1 SORL1 C15orf41 SNORA46 SLC22A2 LINC00698 ACTG1P4 CNTN4-AS2 MAPKAPK2 ATXN10 ACTR8 KIAA1210 ITGA3 ZNF510 STX8 EFNB1 ACSM3 C5AR1 ZNF623 HIGD1B SLCO2B1 RN7SL872P PHF20 GTPBP1 GRK5 DGUOK C1QTNF3 MANEA SEL1L3 RNU6-749P SRRM4 FAM73A RAPGEF6 RRN3P2 OR8J3 PYY STARD5 GBP7 RSU1P2 TLK1P1 LINC01121 C2CD3 RPL26P9 GPR78 GSTA2

		ISM1 GRAMD2 ANKRD20A3 CORO2A ARHGAP42P4 MIR4499 AP4B1-AS1 RNF4 CDK8 KIAA1211L HDAC7 ABCD1P3 FAM19A4 CHST15 FPGT CDK1 NLGN1-AS1 N4BP2L2 ZNF232 OPHN1 RPS11P6 RPS15AP6 BANF2 FAM90A6P KIAA0100 MYH6 MAP3K13 CLUHP4 LCMT1 LINC00924 SSXP1 SAA2-SAA4 SLC24A5 MIR654 LINC01047 OR7E25P KIAA1324 OR4M2 PLCL2 BDKRB1 C4orf29 WDFY2 NME8 TP53TG3C PXDN GXYLT1P1 PRAMEF7 FAXC TOMM34 TF COG1 CHST11 PWP2 DNAL4 SLC9A9 GSTM4 ANHX RCSD1 EXOC2 OR10J6P TSPAN7 MIR1185-1 HPRTP4 HERC2P8 PAQR8 MIR4768 RNU6-966P CDK17 GNRHR HDAC1P2 SUZ12P SARS QKI GAGE2B LAMB4 MAN2B1 RN7SL318P PRDX4 AGT LRRTM3 FGF1 CHRM1 FUT4 OR4A42P DBH RPLP0P7 OR5G1P GPATCH2 TMEM11 RPL7P55 QRICH1 KRT222 UBE2L6 PCCB IPO13 LTBR ARHGAP15 TDRKH CCR3 GNB4 AGTPBP1 HBG2 PRR5 SPRR2B SMTNL2 HNRNPA1P74 SALL4P7 PALM2-AKAP2 AHI1 BDH2P1 ACTR5 HEATR5B FHL1 LRRC53 TMEM161B-AS1 EMR1 FAM225B NMNAT2 UBE2N IL23R ACAD11 NEGR1-IT1 SYTL5 PIGL GDI2P2 C1orf112 HUS1 DYRK4 ZNF667 TFEC GRIK1-AS1 PLSCR4 PPARGC1A STRIP1 MSNP1 GGN ZNF709 ETFA AHRR CYP3A5 PCDHGB4 GTSF1 LINC00313 GPX1P2 VRK2 ITSN1 DUX4L3 EP400NL ELMO1-AS1 STOML3 PLK1S1 C3orf22 ZNF962P CMA1 TBC1D3B UPK3B PREX2 PI15 FAM177A1 SNORD11 ELAVL2 RPS20P22 FAM9B TNFAIP8L3 WDR16 ZNF863P SNCAIP DEFA1B KLHL28 DLGAP1-AS4 OR11L1 DNAJB4 TMEM189 PECR PCDHGA10 SVOPL EIF2S1 EDDM3B POLR1A LGR5 TMPRSS4 TSHZ1 LINC01028 LRRC16B RAB5A AKAP2 SMCO2 JRK H2AFZP1 FAM131C ARHGAP25 SNX18P9 RNA5SP470 EMCN-IT2 MIR491 CCDC60 OR8K3 SMAD1-AS2 RAB11FIP4 NF1 LPPR4 RPL39P36 PPFIBP1 USP24P1 FAM49A BCL7A PCDHGA5 IL20RA ELF1 MIR4439 IGLL5 OR9I3P FPGT-TNNI3K SAFB2 VPS16 SLC9A2 SULF2 SOST CCDC144A OR52T1P RRAGC TAF1D RN7SL345P IGHVIII-47-1 WIF1 STRN3 CMKLR1 PGBD4P7 PLCB1-IT1 GPRIN2 SDK2 ANKRD40 CCT6B KRT74 FAM90A19P ZFHX2 FBXL5 RNU6-1241P RNF43 NR2F2-AS1 SNX18P25 ZNF677 MAP2K4 CSRP2BP CD72 KAT5 MIR361 SRRD PROS1 MAS1 OR2L13 PPP6R2 GIMAP6 RPS7P5 ZBTB41 CDK2AP2P1 ITGA2 SHANK2-AS1 MME FAM213A RNU1-142P ZNF713 SNORD116-27 DSCAM-IT1 MBL1P MALL IL10RB PARK2 MAPK8IP2 VWA3A ARHGAP10 ALPK1 FLJ00273 IGHVIII-2-1 C21orf2 RNU6-554P BZRAP1-AS1 KLK9 RN7SKP80 RN7SKP16 GSK3B PDXP KIF9-AS1 VPS37B KIR2DL1 PCDHGA4 FOXO1 PCDH9-AS2 IGLV5-37 PANK3 FAM211A WDR82 MTND6P3 PKP2 AMPD3 NPHP3 SFXN3 SNORA71 CHDC2 GCLC TUBAP SNORD113 SERPINA4 MTCO3P2 IL18RAP TBPL2 RNU6-164P TRIM16 CDC40 SETD3 MIR539 PQLC3 MIR4713 GPR110 ACOX3 CD109 CR1 SLC22A3 LINC00161 NUP210P1 SNORD116-17 DAAM2 ARHGAP8 ZNF571-AS1 PLXNA4 SLC22A5 GRIN3A OR2T4 RN7SL678P KIF23 GRAMD1C ANAPC1P1 NDE1 IGHVIII-26-1 STK24 ZNF695 VPS26B PARVG RNU1-106P SEPT7P4 FEM1AP4 RNU6-438P PDCD6 BRI3BP BACH1-AS1 LMO7 NUTF2 COX16 CDRT4 KRTAP10-5 MAN1A1 CD177P1 PCED1B NLGN4X RNU6-1021P KLF3P1 IGHV4-28 FSD2 EIF4E LINC01162 ATP5A1 SPRR2C BPIFB1 HTR4 PPP1R26P3 EMB RNU6-890P C14orf183 LINC00320 FAM19A5 C12orf55 RNU6-1003P KRT72 OGN PIK3C2B FMR1 GPR21 PA2G4P3 C5orf64 RN7SL542P FAM184B ERCC4 OR4K15 LINC00595 RNASEH2C C9orf153 IRAK2 COMMD1 CCDC3 RNU6-258P ANXA10 MIR765 WDR33 RN7SKP233 OR5K4 PCMTD1P2 RN7SL92P MTCO1P3 PAK7 CHD7 SLC30A8 RNF126 DPM1 PSMB7 RXFP4 C5orf51 ANKH MOV10L1 RN7SL179P KRT4 MEP1AP1 IGKC ANKRD20A14P MDM2 LINC00630 BCRP7 TDRD3 MIR544A OVCH1-AS1 NDST3 RN7SL686P FAM19A2 CDHR4 GBE1 OR5L2 ZNF285 CLASP1 FARP2 SNX5 CYSLTR1 PAPD7 MIR154 SNORD115-35 OR51B2 NR4A1 TMEM175 TIGD4 SLX1B-SULT1A4 C14orf37 GLT8D1 UPB1 CLDN14 RGS17P1 SKIV2L2 SCARNA15 GUCY1B2 SERBP1P6 ARMC9 MCOLN3 LRP8 TAGLN3 IGHV1-69 NFE2L3 MINPP1 KCNG3 PSMD6 TMC5 DUX4L12 KIAA1257 LINC00365 SKI HIGD1AP13 ZNF544 LRRC70 TMEM50B KRTAP13-5P SNORA1 SP140 MTATP8P1 TNFSF8 PDE5A FBLN2 HBE1 TEKT4 OR5G5P TFP1 BCKDHA RPS2P48 RNA5SP265 SCLT1 TMEM52B LINC00970 RNU6-141P EIF4ENIF1 UBE2Q2P10 ZNF433 RPL7L1P12 SRGAP1 ABCA11P SLC16A4 PLAC1 PWAR6 LINC01094 CALCR DUSP27 TBX3 OR8K4P NANOGNBP2 MIR620 MIR4753 DIO2 UNC13A RNU6-837P SLC5A10 TRIOBP TUBA3FP NCOA1 MRPS31P5 CHEK2 DOCK9-AS2 OIT3 ZBBX LRMP HTRA1 RAD1P2 ZNF585A IFT88 SIGLEC30P CHST12 ARHGAP5 DDX21 KIAA1731 P2RX1 ILDR1 DGCR14 TNIP1 PAN3 FSIP2 TOX2 ZBED4 ZNF720 METTL24 RHOJ ZER1 CLUHP5 FCRL5 MRPS31P4 BRE RNU1-60P FAM9A ZNF474 OGFOD1 OR52X1P ZBTB8B BACH1-IT2 PHEX ZNF730 QRSL1P3 FAM182A POLE TSPEAR FILIP1L COL4A6 CATSPERB ZNF559-ZNF177 DET1 FECH SLC7A7 TACR3 RSPRY1 MPTX1 ACTBP8 IGLC2 SLC1A3 MIR3173 PCDHGA11 C14orf119 DNAJA1P1 MT1F FGF12-AS1 RNA5SP438 ASNSP5 LRCH1 LRRC18 FAM124A TCL1B FGF13 SULF1 SLC39A10 CDCA2 DTD2 IGHVIV-44-1 PDIA6 RNU7-119P PRKG1-AS1 SNORA76 SDIM1 FTCDNL1 RNU6-631P SPIN3 HCN4 OR11I1P CD8B CD84 MRPS21 ZNF830 MAGEA11 SH3BP1 ISLR2 B3GNTL1 ERMN MIR605 IGKV1D-12 SIM2 C12orf36 TRIM48 KIRREL GPC3 RNA5-8SP3 TLK2P1 DMD C22orf39 NANOGP4 RNU4-56P TMEM50A STAMBPL1 ADCY2 ZRANB2-AS2 SH3BP5-AS1 TCF20 STAG3L2 CRYL1 OTC MRPS6 NOVA1 KCNC2 IGHV3-64 PER1 ANKRD20A4 RNU6ATAC31P RN7SL736P RNMT MYH4 MIR1324 RNU6-449P SH3BGR PCDHGB3 NF1P8 RN7SL714P SLC25A1P5 KIR3DL3 DTX2P1 TMEM135 ARHGAP35 PRDX2P3 PREX1 WARS2 TBX22 C16orf3 RNA5SP385 LINC00587 UBE2V1 POLD1 RFFL MBOAT7 IGLJ2 POMC EIF3E TRDV1 RPL21P41 NLRP12 MIR4681 TLE4 CNNM3 TLK2 SH3GL2 COL24A1 SNX18P8 ANO3 MIR759 GAK PSPC1 COL11A1 TREM1 SLC13A3 CCP110 ITGAE LRRC37A17P ODF3B CACNG6 RNU2-33P MCM3AP-AS1 FAR1-IT1 ZNF804A WDR83 MIR1254-2 DOCK7 N6AMT2 WRB OR4N3P TTC4 ZNF734P XKR4 C15orf54 OR10AK1P KCNJ16 PTGER4P3 PCOLCE2 CDC20B IGLVI-68 LINC01143 LINC00347 SLC26A7 GPC5-IT1 ZNF57 C9orf3 SYNM RAPGEF4-AS1 MAPK4 RNASEH2B-AS1 FAM3D OMG ZNF486 RN7SL83P EZH2P1 BRAFP1 CWF19L2 NRG3-AS1 VN2R17P EXOSC3P1 OR4F16 MRPS18B AOX1 IGKV1-5 USP17L27 JRKL SERPINA5 ANKRD34C ZNF697 PRED60 RBAK LINC00472 TYMP DIO3OS GULP1 IFNWP4 GPR75 TMEM110 PRAMEF8 ARHGAP11B PCDHGA3 THEMIS SLC5A8 FLVCR2 COL12A1 MIR4529 MTND2P25 ZNF280D GAGE2C FAM230B RHBDD1 OR8K2P DRG2 IGHV3-76 HDLBP MLLT4 RNF6P1 MAST4-AS1 CBFA2T3 COG7 IGHVII-31-1 KLF3 DPH1 BAIAP2L1 SLC6A6P1 EFTUD1P1 PTGER3 IGKJ5 USP29 LINC01013 KIF26B UGCG MIR3936 SLC6A2 SNORA56 ORC3 TVP23C ELP4 RYK IER2 SNORD115-48 LINC00839 TGFB2 CHRDL1 TRAF3IP2 HSPB8 LAMA2 PXT1 ATP6V0D1 RLBP1 LZTR1 ZNF85 C8A TEX36 CAPN3 IGHV3-72 TFDP2 CCDC12 IGLVIV-64 REXO1L10P KCNE1 ITLN1 KLF8P1 FAM20A ZSCAN5A LINC00645 SOX4 UGT3A1 IFI27L1 SLX1B KRT223P FAM95B1 SH3RF3 C18orf8 TPP2 NPM1P25 GTF2E1 ST6GALNAC5 RGS16 C2orf91 TCEB3CL DIAPH2 RTL1 TUBAL3

		RNU6-58P CRYZL1 MECP2 CSN3 BMPR1A CLDN11 TIMP3 MIR4742 RPL23AP12 SLC39A12-AS1 ZNF299P RARRES2P6 DSCAML1 FAM207CP DUXAP8 PLN DUX4L8 PRDM9 FBXO27 SMAD3 CTDSPL2 C14orf182 NAPG MRVI1 PABPC1P12 CCDC39 C1orf101 USP17L6P SLC16A7 FAM192A RNU6-196P MDH1 ZRANB3 MRPL19 DCBLD2 PRKAR2A TMEM185AP1 THYN1 KATNAL1 MAGI2-IT1 MYO6 OR9G2P AKIRIN2 MRPL33 VCAN-AS1 KIFAP3 MIR127 C1orf63 GLRA2 BTBD17 SPRN BORA PI4KA POU2AF1 VWA8 ACBD5 ALDH4A1 TMC2 FAM105B DHRS7C OR51B8P PCDHGA7 TRIM72 INSC SYNJ2BP-COX16 TNFRSF11B PIBF1 ARF4 SNORD115-20 MIR3667 LMF1 TMPRSS11E NCF2 MRPL48 CAPN14 RNA5SP453 USP16 SNORA40 IGHV4OR15-8 GALNT5 RIPPLY3 COMMD7 PLXNB1 C9orf171 CNGB3 SLC25A15P2 MIRLET7C STAT4 DPYD-AS1 LY86 CYCSP51 OR4C16 PDXK MTND1P12 CDC27P1 LRRC4 ZNF670 NDNL2 IFFO2 IGFBP7 POTEE UFD1L CTBP2P4 EPHA5 FAM24B MACROD2-IT1 OVGP1 LINC01136 FAM27B SGIP1 MERTK CEACAM18 RN7SL801P SNORD115-36 SLC47A1 RNF144A CST1 USP17L30 RN7SL864P RPL37P4 CCL7 TP73 IGKV1-12 RBMS2 TMPRSS6 SERPINA1 LINC00376 TAPBP IL1RL1 RN7SL77P ZNF765 IGHV1-14 GPR64 LINC00535 TAC4 MYO16 CYP4F31P ALG12 ZNF366 RPL30P13 CHIAP3 MIR548U MTND4P12 ZNF540 LOH12CR1 ISPD CD58 SNX31 GRHL2 RNU6-1178P CDR2 IGHVII-30-1 SELE BPIFC PSMC6 MAGT1 KLF6 PCNX ITGB7 CHKB SCUBE1 ARHGAP42P5 RNU6-1171P TMPO SNORD69 GPR112 FAM230A MIR663A NOTCH2NL ABCA8 PDE9A LINC00894 SLCO1B7 KCNQ1OT1 MAPK14 PRR14L DYNLL2 RNU2-53P FBXW12 MYPN RPL29P30 LINC00669 SATB2 RNF216P1 ABTB2 MOCS3 MIR4477A GPR126 PPHLN1 RNU6-185P SNORD115-21 CHL1 RAPGEF1 VANGL2 DGKZ INTS4L2 REXO1L8P RAB23 ROCK1P1 FAM8A2P ZNF516 VLDLR LINC00092 HSPD1P7 KRTAP12-2 ELN SAMD8 REG1B CST9L HPS3 KCNK17 ARHGEF3 SNORD115 RHBDL3 MAML3 GABRB2 EDNRB MAN1C1 CFTRP1 ATP5S RCVRN NLRP7 CD226 RBM14-RBM4 STAT3 RNU6-513P CYCSP17 TPCN2 CNN2P4 DMBT1P1 LRRIQ3 GPR141 PRR5-ARHGAP8 TPH1 MAD1L1 SNORA51 MARK2P5 GPR176 RNU6-1266P MYH11 MATN3 IFNGR2 PKP4 RNASE9 SHPRH FAM155A-IT1 UOX SIPA1L1 RNU6-538P DPYS TGFBR1 GBP2 WDR4 DUX4L13 TTLL2 RN7SL607P UACA LRPPRC HEXB RPL34P3 ZNF209P RPS4XP22 ABI3BP LAMTOR5-AS1 TSNAX C12orf60 TAS2R1 C5orf34 IGHV1OR15-6 PRRC2C TUB ARHGEF18 RUNDC3B TRIM51FP SCAMP5 ADAMTS20 LINC00473 WBSCR17 GNAS-AS1 CDH6 RNA5SP35 PCDHGB2 IPMK CAMP KRT126P TRAV8-5 DENND5B TNS1 SSUH2 RBL2 MS4A15 CASP7 LENG8 DUX4L9 USP53 IGHV3-30-2 AATF OR10K2 MRPL50 CHEK2P4 ACSL5 RNU6-352P DOK6 RN7SL449P DKC1 hsa-mir-4528 ADCY8 PCDHGA12 GRAMD4 PSG1 RNU6-978P TPST2P1 DNAJB6 NFIC ELP3 PPIAP1 GABRR3 SUN2 SNRPGP9 HIAT1 SEPT7P9 FRMPD2 KEAP1 NPM1P31 OMA1 RPL30 CCDC30 SULT4A1 SHC3 DYNC2H1 WDR7-OT1 ARRDC4 MIR1299 SRP19 UROS KLHL25 IGLVIVOR22-1 DYNLRB2 SLITRK6 STX16-NPEPL1 ABHD17AP5 TCAIM FOXD4L4 MYO7B OASL KCTD9 TM4SF2 OSGIN2 ACTN2 DARC PET112 CRISP3 NEB SRSF4 POU5F1P3 CAMK2G STAG3L5P TPTE2P4 C6orf3 HNRNPA1P7 FGF14 BRD1 KIF3B SNX7 MIR1290 CCL15-CCL14 GLRA3 SPRED3 VPS8 CEP164P1 BCL7C TMEM100 SNORD115-12 NPM1P48 RNF165 ANP32C CASC4P1 FRG2C ZNF429 CUBNP1 HDHD1 CTBP2P5 DRD5P1 RNF213 LYPD6 DHX9 PDIA5 SUCLG1 OR51I1 KCTD20 WDR47 VDR MIR4452 SLC25A1P2 KRTAP13-6P SPESP1 snR65 DTD1 MIR4695 DUSP11 DUX4L7 RNU6-1061P OR51L1 SH3RF2 RPS3AP46 OR4A5 FOXP4 DUX4L10 RN7SKP168 CACNA1D ASGR2 ELOVL2-AS1 DPT RGS5 ACSS1 ABCG2 LINC00111 TBL1X CLSTN1 LANCL3 SPAG17 ZDHHC11 EPHA3 MOV10 RPL39P40 TMEM150C SEMA5B TMBIM7P MIR1280 ZNF337-AS1 SELK MS4A1 RPS6KA2-IT1 MYH14 IGHV3-16 SKAP1 PILRB CC2D2A DSC3 WDR11 MIR487B TPRG1 RNA5SP269 SNX29P2 SNORA22 ST13P15 CCR1 CACNB4 LINC00710 CHM MTND1P2 SDAD1P1 LINC00395 USP6 ZNF732 MIR655 KLF7 AHSA1 AKR1B1P1 MGAT2 LARGE-IT1 RN7SL673P PYGL FAM65B TXK NOL12 CHTF8 FPGS LINC00446 OSBPL10-AS1 ADH5P3 GXYLT1P2 ZNF354B IL12RB2 USP17L29 IQCA1 NLN C8B OR4A12P TCEB3C RNU6-49P RREB1 PABPC1L MTND1P23 MND1 GTF2A1L PABPC1P5 MIR4519 ANKRD30BP3 TAF1 SPNS2 HERC2P5 RNU6ATAC33P AP1G2 ATP6V1E2 NBPF10 ITIH6 CCDC53 FGF14-IT1 CYCSP41 ATP6V1H C5orf66 DHX57 ACSM1 PTPN21 PLAG1 MIR1267 RNA5SP366 SNORA8 PPP6R2P1 ENTPD4 NPEPL1 THAP7 WDR35 CTBP2P7 RNF144B ZNF841 CCDC33 ATG4C SNTN RBM11 FEM1AP2 ABCB10 LHFPL1 RPL31P40 FBXL21 KRTAP20-3 C15orf57 LGI1 U6 IGLC1 IGHV7-34-1 ENTPD3-AS1 SIL1 TATDN2P3 SNORD112 PGAP3 EFNB2 C21orf62 PKD1L1 ALDH1A1 TSNARE1 HELLS FAM69A OR11H1 BTF3P14 ARHGAP42P3 CNGA4 CYCSP32 GCFC2 ABCD1 OR4K17 IGHV4-4 SNHG17 RNA5SP125 LINC00458 FAM172A GLYR1 ANKRD29 EPM2A COL18A1-AS1 DEFB127 SGMS2 MXD3 TNFRSF10B PPAP2B HIGD1AP8 S100A7L2 SNORD45 SPECC1L-ADORA2A SHFM1 QTRTD1 LCE6A KIF18A LARP1B C1DP1 RN7SL743P CBLN4 ADH1A CDC73 SOX2-OT OR4C15 MIR96 RNU2-27P SDF2 KMT2A CCDC144NL RN7SL435P XBP1P1 FAM210A TCEB1P32 RAD51L3-RFFL FBXW11 TDGF1 ERCC6 EFTUD1 IGKV2OR2-2 HSPE1P25 USP17L5 RPS20P5 DPP8 CDC16 ZFP64 EMCN-IT3 DDX51 MKRN3 LINC00349 SLC30A5 LINC01029 IMPG1 C22orf34 SUFU ARHGEF33 RN7SKP99 SNORD114-20 DSCR4 WDR5 RANBP10 LINC01053 CST13P FAR2P4 FAM90A17P CSNK1G3 TMOD1 RNF219 SMG6 ARHGEF6 SNORD115-41 CIZ1 ITGA11 WWTR1 GAS7 SNORD17 CIT AMOTL1 ZNF607 HTR4-IT1 PEMT ARHGAP6 MCM3AP KCTD7 RNVU1-18 ZNF98 SLC2A3P1 ST3GAL3 PLCD3 MIR4307 TCF7L2 APCDD1L-AS1 CHD5 JAK1 SETD7 HAUS8P1 CHAF1B RN7SKP96 PARP4P2 Y_RNA RNU6-286P ZMIZ1 DAP RNU6ATAC5P GBA3 RN7SKP238 IGKV2D-10 RPGR COL6A3 GPR125 DNMBP-AS1 STARD9 LYSMD1 RN7SL766P PCSK5 LINC00537 FKSG68 WBP1LP1 MUC5AC GRM4 PLB1 CLEC4A CKAP5 PRR12 MAP2K1 AIM2 RNU6-724P TIMP2 LINC00911 CARF UBR2 GTF3C6 CCL15 GP5 SNX24 ZNF19 OR11A1 TMEM132B SAE1 ANKRD44-IT1 CPSF1P1 SLC25A15P5 ENPP7P10 PTPLAD2 SAMD9 FLG-AS1 HNRNPKP3 ZNF490 ARID4B CACNA2D4 CTSH RGPD1 C14orf144 FBXL18 RCC2P8 MFNG PLEKHM2 ANKRD62P1-PARP4P3 STX16 RNU1-33P LINC00702 CDC5L SLC9B1 EFCAB1 CASQ2 LRRC37A7P KPNB1 TAS2R38 FOXO3 MIR431 TRIM59 NF1P3 TTLL8 TTN-AS1 AHNAK2 ACTR3BP3 MRPS31P2 LINC00284 ST7 TM4SF19 RNU6-1193P MTUS2-AS2 KRBOX4 PCDHGA6 NXPH1 AR DDX3X RASA3 LINC00871 IGLV4-69 CDKN3 ARHGEF10 RNU6-410P SERPINB12 GABRA3 E2F3 FRG2B TNFAIP8L2 NUDCD1 KLHL3 FGD6 MIR603 LINC00687 SSX5 PPP4R4 DAPL1 RMI2 DAAM1 MROH1 NPC1 PCDHGB1 RPL12L3 C17orf51 SMARCE1 STK17B MLLT10P1 CXorf30 MBOAT2 CDKL1 SMEK2 ATMIN CENPM SCN9A CDK2AP2P2 RBL1 ATF7IP2 CENPF BNIP3P2 C6 KCTD9P2 MTND2P2 MDM4 KRT23 SERHL FAM21C MAMLD1 SLC25A53 RNU7-174P HAPLN1 MIR3152 RAB7A MIR551B CHKB-CPT1B C10orf11 C5orf17 WDR63 SPEF2 MIR3118-1 CXorf22

		RN7SL247P H2AFY ACTN4 RAB2A SNORD115-11 CEP250 CEACAMP6 TFIP11 GSKIP KRTAP4-12 GRAP2 WASF1 HNRNPA1P71 U2SURP RNU6-1280P ITPK1 G3BP1 NUP155 CYP39A1 DNAJA1P5 NT5DC1 RPL7L1P11 CUBNP3 AMZ2 FRAS1 PPP1R14A SNORD103B SIDT1 TECTA SNORD19B ARL2BPP8 TPRG1L BTN2A1 ASNSP1 STK32C DHX35 DEC1 LINC00520 SP140L G2E3 RPS15AP34 CCDC88B TMCC3 RN7SL143P NFU1P1 TCL6 SAMSN1-AS1 PAPOLA FGFR1 CERK SPECC1L TET3 TMEM110-MUSTN1 CASP6 LINC00693 MAGEB3 LRRN2 ZNF346-IT1 NOC4L MSANTD2P1 PTPN20B ATG2B FAM209A DAD1 ENTPD3 NASP PIK3R5 CDC14A CTBP2P8 PALLD TRAV24 ENOX2 AGBL4 PARP16 DUX4L18 A1CF MACROD1 ASB13 USP32P2 hsa-mir-490 TMCO3 KPNA3 CASC16 IPO7P2 RHOC PPP3CA CHRNA5 RNU6-1291P FAM63B SPIRE2 SPTSSA FTH1P27 MIR3118-6 BCLAF1 GOLGA3 NKD1 SCUBE3 ARHGAP39 AKR1CL1 C1orf174 MTRR ZNF345 MYL12A WDR59 CCDC57 C10orf120 LRRC8D TRUB2 CEP170 LINC00656 RPS15AP3 CASC8 TRIM41 PIN1 SLC38A10 MIR99A SNORD67 ULK4 RNU6-1132P GPR75-ASB3 FAM90A9P DLX6-AS1 SORT1 LLGL2 SERPING1 TMEM120B CDH10 USP17L25 ASUN FAM211A-AS1 MZT1 ZNF605 INTS4L1 OBP2A TBC1D3P5 C1orf168 ZNF41 HLA-DRA DNMT3B GUCY2C OR10T1P ZNF331 SLC17A3 RAD18 EBPL CNN2P7 ZNF114P1 DEPDC5 RYR1 VN1R33P DUX4L6 MIR3198-1 LRRK2 UBTFL8 CCDC181 MIR548AX KIAA0355 PCDHGA1 ZNF728 SLC38A4 PALM2 DYNC1H1 LSM12 PTGES RNU6-316P CD300A SLC35A5 PKHD1 KIF2A MTND2P28 MIR1281 SNORD114-17 FGF14-AS1 CBFA2T2 KRTAP29-1 AGGF1 DUX4L11 ITPRIPL2 C1orf141 ABCC11 NR2C2 MYH1 MGST1 SRD5A2 ZNF432 SLC16A14P1 SPG11 RARRES2P4 FUBP1 STON1 AGGF1P3 PCA3 MMP20 DPH6-AS1 PRG3 GANC KRT43P MIR4533 DDX24 SERTAD2 FAM90A10P OR5H8P ATP1A1 LINC01058 MIR487A OR4C9P LILRP2 GNAO1 SCN1A RNU4-59P RPS6KA6 STRC IL1RL2 SNN BCL2L15 OR4H6P PSMD6-AS2 BMP6 MAP3K7CL DNM1P51 MIR485 TMEM212 PMS2P11 ANKRD62P1 TRAV6 RRP7B MRPS28 SEC63 TDRD10 GC TOR1AIP1 ADC POC1A TMEM170A TNF C21orf49 USP17L24 PHYKPL PDSS1 LINC00971 ATP8B4 GLDN NHLH2 CYP1D1P LINC00639 OR7E89P HIATL2 DSCR10 OR10V3P LRRC42 CCL14 KIR3DP1 ABCC2 TTC12 SELP CSPP1 RNF185-AS1 RABGEF1 KIT MEMO1 RNU6-617P OR4A9P OSBPL3 PPOX ZNF439 MARVELD3 MIR1911 MIR3687 OR8A2P LINC00842 ZNF177 MIP IGKV2-36 FAM19A1 POM121L9P MTUS2-AS1 RNU2-42P FAM81B LINC00229 LRP5 ARR3 RNU6-55P UBA6-AS1 ANKHD1-EIF4EBP3 SNORD115-23 MIR183 EFCAB5 TERF1P5 CHN2 ZNF781 ESRP2 FSHR GAS8 NBEAP3 LCE2B MARCH3 GAD2 BMP15 C1GALT1 RUNX1T1 SGK2 PIK3C2G SNORD115-40 NOL11 TRPM2 SPRR4 SLC30A7 SMCHD1 LINC00507 MED15P7 NRG1-IT1 SLCO1B1 DPY19L3 CILP2 CCDC176 ICT1 EDA2R TNFRSF19 NAP1L6 MIR889 DENND2A CLN6 DMXL1 SIK2 PTPN14 MXRA5 SCFD1 TRPC4 MTND1P31 THEM4 ZNF705A DMGDH PCBD2 TEX14 RRH TSPY26P DRP2 LINC00387 RNU7-176P PDZD7 ENPP2 PIK3CD FHL5 KCNMB3P1 FHAD1 GLULP5 VPS53 SLC5A3 DHRS7B TRIM51 RUFY1 LINC00382 TMPRSS4-AS1 IGHV3-41 RPS3AP41 BLOC1S5 ZNF965P SNORD115-38 ART4 DCAF8L1 RNPC3 EGFEM1P PTPN9 ABCA12 FIGN PI3 OR4C14P ARID2 BROX MARK2P8 GNA13 FAM228B RNU6-78P LINC01043 DNM3-IT1 TRMT11 HMGB3P20 SNURF RPS23P5 BEND5 COL8A1 ISCA1P3 ATXN3L BMS1P18 FAM85B GABRA1 LPHN3 REEP1 LINC00478 FAM90A22P CHMP4C MIR548F1 KCNQ1 NR2F2 PSG7 IGHVIII-67-3 BSG TEAD4 PDE7B RNU6-725P DTX4 SRRM1 RN7SL321P SLC38A6 KIAA1109 USP40 IGLVI-38 RASSF8-AS1 DUSP23 HRNR IGKJ4 IPO8P1 BPIFB4 MRPL42P4 RAB24 NR3C2 SERPINA2P MIR134 RNU6-1233P PRSS3P2 SNORD115-47 CRADD SHC2 NCOA5 EMCN RNA5SP478 BLNK VASH2 LINC00704 OR8U1 TPTE2P1 PTTG1IP ADARB1 GGT1 VPS35 LINC01134 ZNF664 GPC4 HAS3 SOD1P3 LRRC63 BPGM UBASH3B RPL18P13 TTC40 RASA3-IT1 PAGE1 SERPINB8 PPP2R4 REXO1L9P LINC00276 CDRT1 RN7SKP6 LINC00032 VSTM2B TMEM68 GTF2IP3 SNORD103A ENPP7P5 AAR2 ACTR3B NEK5 ASL C1orf173 PM20D1 KLHDC7A CMSS1 PHC1 SLC22A23 PLCH1 CACNA1C-IT3 NAMPT PCNXL4 PCDHGB7 ZNF493 BPESC1 RNASE13 GLRA1 MIR376A1 RNU4-24P CTNNBIP1 TEP1 YAF2 WDR95P KIDINS220 KIAA1644 SMPD4P2 TBCE CT49 FNIP2 G3BP2 HNRNPA2B1 GADD45A MIR214 MGC4294 UGGT2 STIM2 POTEF SNORD23 RNU6-1327P NF1P1 PLAT FAM201B SNORD54 ADORA2A-AS1 RNA5SP492 EIF3A C10ORF68 HS1BP3-IT1 PTPRF SEC24B DNM3OS RNU6-618P IGSF11-AS1 ZNF268 MYLK RN7SKP60 UNC5C PRIMA1 CHI3L2 MTMR4 MIR648 NKAIN1P1 TTC39A POLR2F KRTAP5-8 CSGALNACT1 LYVE1 RNU6-1005P PTPN20A KRTAP20-1 SMAD1 SLC22A15 LST3 LINC00523 ATP1A4 RPL21P6 ANKHD1 LINC00189 C2orf43 TTC8 BRD7 FCF1P10 SNORD6 WDR7 SNORD114-16 DIAPH2-AS1 OR52N5 RBMX2P1 MGAT4C ZNF501 TSPEAR-AS1 HMG20A CEP85L ARL6IP5 TCEB3CL2 ARFIP1 RNU4-60P PAPPA-AS1 PLSCR1 CD163L1 RNA5SP79 TRPV4 NOX5 C1orf167 EEF1A1P1 KCNJ12 ATP5J HDAC8 OR51AB1P SRP54 OSBPL1A STARD4 NLRP11 DNAAF2 ADORA3 LINC00563 PIK3R2 SH2D7 ANKRD44 HYAL4 SP2 CYP3A43 SMPD3 PDE7A KAL1 CYTIP CYBB SPTA1 ZNF483 ZCCHC17 KCNJ3 TBX18 PCDHGA8 DPP9 RGL2 NID2 DHRS4L2 SUZ12 PDE6B SPA17 ZNF729 MIR548AS LHFP TSPY5P TMEM189-UBE2V1 PJA1 IGLVIV-65 FAM76A RNU6-721P DKFZP761J1410 ZFP2 VWC2 BZW2 GUCY2F IGHVII-40-1 ERLIN1 KRTAP6-1 SMIM2-AS1 CACNA1C-IT1 KRTAP9-2 VWA8P1 ADTRP RBFOX2 CRMP1 PDE4B ZNF507 OR4C2P SNORD116-18 ZNF26 PCDH17 PHF8 TFPI USP17L9P MIR323B TDRD6 CA3 PQLC1 DDX59 CYB5R2 GOSR2 LRRC20 DSCR9 ANKRD20A11P LPPR1 LCP2 RMRPP4 ISOC1 RARRES2P9 PET117 IL4R TMEM123 MIR653 SHROOM4 SCO2 C13orf35 TM4SF1 IGHV3-29 SMG7 CT64 PRPSAP2 PAXBP1 ANKRD13A ZCCHC11 RNU6-713P KIAA1211 RPS27L TGFBR3 MCUR1 UMOD MAP3K7 SNORA31 CENPP C16orf80 SLC7A1 PTPRM ACTR3BP5 ADAM17 USP36 CHRNA3 TMEM186 AK5 NCOA2 EGFLAM-AS1 N4BP1 SLC35F2 BNIP3L LCP1 GALNT8 PCDHGB6 RFPL4AP7 RASSF8 OR7D1P USP12 WBP11P1 OR6N1 IMPDH1 KATNAL2 C9orf40 KCNH7 ASXL1 GNG5P5 KRTAP15-1 ELTD1 LCE2C KMO RNU7-114P NUDT4 SNORA70C RNU1-104P CLDN1 PCTP UBE2U TSNAX-DISC1 ABCC1 NXPE2P1 IGHV7-56 SPRR2G C14orf64 RBAK-RBAKDN WDR83OS CDKL5 BCRP1 NSUN7 INPP4A PIP5K1B IGF2R C21orf90 PTPRU AFTPH NHSL2 ADPGK SBF2-AS1 SIAE RPL3P9 TGFB1I1 SNRNP200 SOBP TRBV7-5 TESK2 SHOX2 NPY4R MIR381 ZNF252P ZNF207 KLF13 RN7SKP100 NMNAT1P4 ANKRD32 SCMH1 MARK2P9 KIRREL3-AS1 POLD3 RHEBP3 USP32P1 ZNF91 SNORD115-10 BAALC KIAA1598 JDP2 MORF4L1 POU2F1 PMP22 FAM108A8P ELK3 OLFM3 INADL GPR139 P2RX7 COX5BP6 ADNP2 PLEKHA5 OR5AK4P GAPDHP67 IL18R1 SPTBN4 FAM90A21P MARCH11 AMOT ARMCX4 LINC00317 CCDC7 HGFAC RGS7BP SNORA57 LEPREL1 FSD1L PPARGC1B LPHN2 MTND6P4 PLEKHA6 KDSR BMS1P17 TMEM56 ESR2 LACTB ENPP7P6 MTND5P14 RALBP1 IGHV3-47 RNU6-16P ZCWPW2 KAT7 HNRNPA1L2 HGF MIR376C C8orf87 MYBPC2 REEP2

		FAM220BP ACSL4 RNA5SP303 CAPZB SATB1 IGHV4-31 VPS45 OR10N1P ST20-MTHFS COMT ZNF440 ROS1 EFCAB11 SLC5A4 NRG1-IT2 SNX19P1 KRT75 SLC6A17 RNU1-39P RNA5SP405 ZBED5 TMEM63A MAGEB2 DUX4L16 S1PR3 CKAP4 SLFN12L L3MBTL1 SNORA16 PGK1 RNASEH1 WLS ERVK3-1 RLF IGLV1-36 MIR656 ANKS1A RPL10P3 PRCP SPIB RN7SKP86 MYO1B ZPLD1 HPN PANX1 FGL2 DCHS2 SOS1 MIR767 EHF EEF1E1-BLOC1S5 ZNF189 KLRF2 SLC28A3 RPS15AP1 RNU6-1320P RASSF3 KCNQ5-IT1 CYCSP6 KRTAP9-3 ACOT1 SNORD116-19 LGR6 ZBTB34 RNU6-954P SQRDL BAI3 DPYSL2 SYNPO2 EXOC5 MIR381HG NGF OR4Q1P SLCO1B3 DSCR8 PTPDC1 CYLC2 PPM1J TRIM22 UBE2E3 SAMD3 VEPH1 FAM129A OOSP1P2 BCAN AKAP7 TGM2 BASP1 MIR4535 RPL21P1 ZNF202 ACYP2 MARCH10 BDNF-AS GRK6 FAM46B LINC00492 MIR382 C7orf69 ZNF277 RNA5-8SP7 7SK LRRC8C SNX18P4 RNU6-1066P CALML4 KRTAP4-3 KRT39 MIR5704 MIR1276 SYT2 LINC00442 DPP4 GUSBP3 DICER1 ZNF148 ZNF141 SLC26A8 RNY4P23 MYH16 AQP10 SULT1A4 SEC16B GGT8P C5orf38 SLC16A12 THOC2 TAF1B LINC00898 COL15A1 CACHD1 HDAC5 TAF7L CREG2 FAM27E3 NUP62CL REEP3 TCAM1P MARK1 MGST3 BDNF MIR320B2 BTRC EIF4EBP3 IQCH ACBD6 RBM4 C15orf43 MIR433 FNIP1 DSCR4-IT1 TSPAN8 IGHVII-44-2 MRPS11 MAEL MRPS17 NUP153 MYH9 LINC01057 RNU6-127P RNU6-469P TMEM233 RNA5SP465 SNORD109B BDKRB2 RNF180 ZNF665 MEF2A DUX4L5 NDST4 MIR543 KATNA1 ADORA2A FAM90A23P MRVI1-AS1 MIR556 STIM1 TUBA3C FAM216A GPATCH2L BACH1-IT3 TSC22D3 SWT1 PTCD2P1 IGLJ1 EEFSEC MROH7 ARF1 FLNC PLCH2 TMEM261 PPIAP22 JAG1 CNN3 MIR105-2 LINC01065 AKNAD1 FAM27A IFT80 BMS1P13 RNA5SP61 LINC00879 COX6CP10 AKR1C2 GPALPP1 RN7SL373P HSPA4L ZNF527 RPL21P11 NUPL1 FAM208B LITAF DYM OR7A5 OR5J7P LRRC3-AS1 CUZD1 RNF219-AS1 LINC00351 CHST13 ANKRD20A18P GOLGA1 LINC00857 VDAC2P1 SEPT7P5 GUCY1B3 SNORD114-19 NR3C1 PPIAP6 MIR300 BRF1 FAM108A10P JRKL-AS1 ACTR3BP6 FAM222B KCNB2 MCM3 LINC00658 C17orf75 ZNF585B TENM1 THTPA OR4C5 CYP3A54P CCDC170 HNRNPA1P40 CBLB KIAA1407 LARGE VPS39 CFL1P6 SNAP25 MTFMT ANO1 REG1P JAKMIP1 MIR5582 IGHV1-46 PCMTD1P1 RIMS3 KRT76 RTFDC1 MIR4290 MIR410 CDC42BPG IGHV1-18 ADRA1B LTB LINC01101 OR4A41P PCAT6 ZFPM1 RNU7-145P GDAP1 PRKAR1A RPL8P2 RNA5SP222 ZRANB1 XKR7 DISP1 PRB2 HS6ST2 MYCT1 CPT1B MS4A5 DCST2 GPN3 TFAP2D TNMD SPATA6 CYP7B1 FAM212B STXBP5 TBXAS1 OSBPL9P4 KXD1 PRKX RN7SL674P ARMC7 FCRL2 FAM196B SULT1C2P1 H3F3C BMS1P9 PCK1 KBTBD11 NPR3 HBD MIR4743 REV1 FAM220A RNU6-1269P TTLL11-IT1 GNG4 CNTNAP4 TTC32 LINC00393 RGCC NOX4 IGKV1-37 VWF CYP2E1 RNU6-1049P UBE2Q2P11 LINC01154 RNU6-157P MEGF9 MIR5190 OR4C12 SNORA32 RNU1-11P CYB5D2 STK33 PGM5P2 SNUPN QRFP TRIM51DP PCDHGA9 JPH4 NAPEPLD MIR3648 CISD2 GCNT7 HRASLS5 RN7SL484P FAM155A snoZ6 FCF1P9 OR4Q2 GACAT1 C20orf196 NCOR1P2 CYYR1 IL1RAP UBXN2B KIR3DL1 TXNDC5 RALGDS HNRNPA1P58 SETD5-AS1 DEF6 MTAPP2 WRAP73 RPH3AL ERI2 LINC01076 MANEAL SNORD115-24 LRRTM1 MTND5P11 EIF4E2P1 FAM90A7P SCEL BCOR GRIA3 RNA5SP488 NSUN3 BHLHB9 ILF3 GOPC OR5E1P LINC00457 MIAT POLR3C SLC35F3 DCDC2 NANP ANKRD20A12P DYNC1I2 RN7SKP285 AGGF1P4 LGALS14 ZFP82 LINC00418 CPPED1 GAB2 CEP89 DIS3L2 FREM2 hsa-mir-6723 NOS2P3 SLC7A8 MOGAT2 MYO5BP3 S100A11 CLASP2 EFHC2 DOT1L LHFPL3-AS1 SNORD113-2 SMYD2 PVRL2 OR10R2 TMEM194B ONECUT3 ZMAT3 OR4H12P LIMK2 MIR1185-2 MTATP6P1 TRPM4 ZNF558 LINC00632 MLLT10P2 C11orf30 ICK FAM227B LAMP5 EFCAB4B PSPC1P2 KIAA0040 RN7SL683P HLCS-IT1 KRT2 C1orf95 RBKS FERMT2 DNAJC6 MIR105-1 DSG4 KATNBL1 TPD52 U3 CPNE8 DACH2 PGM2 LINC00521 STX3 M1AP AGBL4-IT1 ZC3H13 ZNF610 TAX1BP1 WBP1L DNAH10OS H2BFM KCNK2 KDR BRD9 PCDHGA2 OR2M5 OGT MSL2 RNF207 FAM13C HNRNPA1P68 KCNT2 CCDC36 GZMH DENND5A CDK2AP2P3 OR4K13 SHCBP1 PNPT1P1 TNC LINC00856 IGHV3-32 PML EBAG9 ADAM6 RN7SKP5 DAB2 FAM101A TTLL12 AFF2 RFC3P1 NCKAP5 DNM1P46 THAP7-AS1 RN7SL552P MGLL FAM27E2 FAM83G LINC00609 RNA5-8SP5 TRAPPC12 FAP NSF EBF4 ANKRD20A2 NFASC RPS4XP15 OR52U1P TPT1P5 LINC01141 TMSB15B ERICH2 OPTN RN7SKP85 METTL9 ATP5O RN7SL50P MAP2K5 ZNF663P PCDHGB8P RAI1 RPS20 SEPP1 CPEB2 LINC01146 TMEM56-RWDD3 ZNF229 LRRK1 DYNAP GRIK1-AS2 RPL7AP28 DNMT1 MTND1P32 PIWIL4 RAP1B MRGPRG ZNF571 BRIP1 WIPF2 UBFD1P1 SNORD116-16 SBF2 DIAPH3-AS2 CADM3 PPM1B PIP5K1P2 KRTAP9-8 KIR2DL3 FOXRED2 IGHV3-65 FGF7 SLC15A1 DGUOK-AS1 SNORD116-26 FMNL3 SNAP23 ANXA8L1 PPAPDC1A CHST9 ZDHHC9 USP17L26 NLRP2 FLT3 GALK2 CEP97 SNX18P15 PCDH19 JMY ZNF451 SRIP1 ARL2BPP5 MIR514A1 MRPL39 OR11H13P SRPK2 CACNA1A MEG9 RNU6-368P MCM9 RNU6-405P ST7-OT4 EDA CCDC144CP UPK3A SPCS2P4 IGDCC4 RN7SKP126 PRB1 MGME1 CCDC144B EDDM3A LINC00491 CTXN2 RN7SL327P RPS26P30 OR11K2P ABCC10 RARRES2P1 EPG5 TTC34 COL2A1 PTH2R RPA1 EEF1E1 PPIAP14 MIR4760 NAP1L4P1 AKR1B10P1 ZNF346 KHDRBS3 TPK1 MYO5BP1 SOX8 KIAA0195 ATXN8OS MTHFS CALCRL ABAT SLC22A25 EGFL6 ZNF674 LGALS9 MIR136 MIR495 CYP1B1-AS1 CELSR1 TNNI3K THBS2 LINC00271 TPO GBAS TAS1R1 TVP23C-CDRT4 RNU6-466P RPS2P44 TMEM138 TRBV6-8 C9orf131 TMEM131 GTDC1 RNU6-230P TRIM51CP MYH7 OR8K5 RNA5SP497 RNASE11 MIR4480 PAPSS1 BICC1 CRNN NBPF11 POLN DISC1-IT1 KCNK13 MTND2P4 SERHL2 DUX4L4 DDC MIR548AL RNU6-614P MS4A4E SNORD114-18 HNRNPA1P53 FLNB-AS1 ENPP7P1 SNORA25 POTEH-AS1 SERTM1 SNORA70 STK35 LINC00707 YWHAQP9 RRM1 MFSD12 PDE11A TSPAN9 ARID4A SYCP2 CLYBL KCNC4 TSPAN1 USP32P3 ACIN1 LECT2 OR4C6 RAB30 CEP41 GNB5 PRKAR1B LINC00972 UBTD2 IQCJ ZSWIM7 CCDC88C SPATA5 B4GALT3 POU2F3 IGHV3-60 RNA5SP490 NVL PPFIBP2 LINC01030 CENPV SMIM20 DTX2P1-UPK3BP1-PMS2P11 CTBP2 MYH8 SNORD113-1 TTN ZNF525 NCALD RNF144A-AS1 PGM5P1 ZFYVE21 CACNA1C-IT2 ZNF93 ZNF337 RAD51D ADH1B MIR369 SARDH EMR4P CPA5 AMY2B PDS5A NCAPH2 CUL2 KIAA0319 RNASET2 C6orf106 CCDC146 MIR4273 FAM227A CLRN1-AS1 FAM221A CDK19 NCMAP SCNN1A KCNE2 PPP2R3C CAB39L LINC00378 CFB QSOX1 ZNF443 DUX4L14 OR5V1 TMEM55A IGHVII-28-1 SYT17 ACTA2-AS1 FOXP1 MAS1LP1 SNORA80 SNRPD3 RBMX2P3 OR4M1 FRG1B ADCK3 FAM114A1 SNORD19 FDXR PAX3 PNLIPRP1 DEFB122 PARP4P3 LRP4 KSR2 TMTC4 NOVA1-AS1 MROH7-TTC4 IGHV3OR16-12 SPATA13 ZNF736 RNA5SP518 RARRES2P2 GUSBP6 TUBGCP6 MORN2 GAREM CST2 PLAC4 HEPACAM2 BLOC1S6 MTND4P14 F11R MIR432 RFX4 RPL18AP14 CEACAMP10 CACNA1C-AS2 EIF3FP1 RNU1-117P OR5B19P LRRC16A LARP4P SNORD115-45 MKL1 SAA2 ATP6V1D ADPGK-AS1 LAMA4 SNORD115-19 PMEPA1 C1orf94 RN7SL659P IGHV4-55 TMEM185A ZZZ3

		UBE3C CCDC92 RPSAP55 MYO5BP2 PEX5L TERF1P1 OR8L1P CYB561A3 RNU6-602P SLC2A9 UCHL1-AS1 PRAMEF12 RNU6-249P LARP4B HGSNAT RAG2 KIAA0754 LCE2A LRCH3 FMNL2 CAPN7 CCL3 RNU6-540P RNU6-458P KDM3A snoU13 MYOM3 CCDC73 SYNPR-AS1 FAM110B CES1P1 SNORD115-34 LGR4 SYT9 SULT1B1 DHRS4-AS1 LINC00534 RFPL4AP5 VSIG10 TRDC RPS20P1 SLC38A7 CYP4F29P SPINT2 SLC22A10 CASK RNF128 PXK SLCO4C1 DENND2D CP LINC00973 FAM160A1 OLFML1 STAG3L5P-PVRIG2P-PILRB SCNM1 AMD1 LCE4A ZNF562 SPRR2E LRRC3B DNAJA1P4 KRTAP19-3 CCNYL2 MARCH1 TUBB1 GRAMD3 TP63 ARMCX2 GRIA2 ANXA11 NUSAP1 ANKRD23 CCDC11P1 MIR3713 IFNA8 TRMU OR4K11P TP53TG3B USP3 LINC00940 HBG1 C3orf67 ADRBK2 VSTM1 LINC00353 ZNF622 SELO DHRS2 SNORD115-39 RXFP2 TRDV3 EXOC6 SNORD115-25

Table S5. GO associations with biological processes (GO Profiler) of 1486 rDNA-contacting genes common to K562 and HEK293T cells. Related to Figure 1D.

.

GO.ID	Description	padj	Genes
**BP**			
GO:0009653	anatomical structure morphogenesis	2.2643101764575657e-30	CD44,SEMA4D,RPS6KA5,TAOK3,PRKCB,ANKRD6,FBXO31,BCL2,MMP16,COL18A1,THRB,DNAH11,CDC42EP3,NCAM1,CDH8,ATP10A,ZNF568,GRIP1,ASTN2,TANC2,NTN4,BICD1,KALRN,USH2A,FLI1,MEGF11,EPB41L3,LAMA1,PARVB,CDH11,NRXN1,IL1RAPL1,WDPCP,LAMA3,SMOC2,ZDHHC17,KCNH1,CACNA1C,EXOC4,CTNNA1,MYO9A,NTRK2,FOXN3,THSD7A,TIAM2,CNTN1,ZNRF3,PALMD,MEOX2,ENPEP,NRG3,SLC24A4,GAS2,NUMB,MTPN,SOX6,PTGFRN,UNC5D,DLG5,CFDP1,PGM5,ATRNL1,CPE,CALD1,ARHGAP24,SLIT2,MYLK3,ROR1,ADAMTSL1,AKT3,CRB1,ALPK2,HECW1,AFF3,ERBB4,KANK1,ATRX,DMRT1,MACF1,RAP1A,TRIO,MYO3B,CHSY1,EXT2,NOS1,CCDC141,CNTN4,SLC40A1,FGD4,ETS2,ITGA1,HIRA,ITGA8,RUNX1,ALDH1A2,FAM171A1,PEAK1,EYA1,CDH18,FRMD6,PLS1,ANK2,ZBTB16,SLIT3,ROBO1,ANKRD11,EGFLAM,PAK3,OVOL2,MLLT3,CHODL,GABPA,PRICKLE2,BBS2,MYOM2,MYO3A,NUBPL,SOX5,DSCAM,DNMBP,KDM4C,SDK1,SLC1A1,EPHA6,NTN1,NR5A2,IGF1R,WDR72,NLGN1,SHROOM3,JAM2,ALX4,CNTNAP2,MAP2,CFTR,FLRT2,PTPRO,INSR,COBL,CUX1,ANK3,CDH12,TBCD,GPC6,RELN,ADAMTS5,MYO18B,CDH4,TNR,VAV3,CNTN6,APP,ADAM12,PAK1,IGF2BP3,ADCK1,HCN1,FRY,EPS8,TENM4,CECR2,GHR,RIPK4,RIN2,MEIS2,STARD13,LRRC4C,ALCAM,ADAMTS16,NEBL,RARB,FRYL,TIAM1,PBX1,PHACTR1,SLC39A12,DISC1,FMN1,ZFPM2,VSTM4,SVEP1,EDAR,EGF,FYN,EPHA7,STK3,S100B,PCDH15,ESR1,ARHGAP12,CNTN5,LRIG1,PRKACB,RIMS1,POR,WWOX,EPHB1,FHOD3,GREB1L,EFEMP1,AJAP1,HERC1,DOCK1,FLT1,EXT1,EFNA5,TRPC5,PPFIA2,TENM3,MARK2,ATF2,GRID2,LRP2,SEMA6D,NTF3,FER,NTNG1,SGCD,TMEM108,RAPGEF2,GTF2I,PRKCQ,KIRREL3,NRXN3,KRT25,DLC1,ATP8A2,UBE3A,AUTS2,EPHB2,PDLIM5,PRTG,CTNND2,COL22A1,SETD2,PACSIN2,SDCCAG8,FLVCR1,NRP1,CDH13,RPGRIP1,DAB1,SEMA3A,SEMA3E,DCLK1,KIF16B,CDH2,ARID5B,TANC1,HDAC9,PIK3R3,FSTL4,MTOR,RORB,FBLN1,SH3KBP1,FHL2,TTC39C,SPRED1,SIPA1L3,FLNB,PAPPA2,CSMD1,GLI3,RXFP1,FBN1,TBX15,BMPER,HMGA2,DNM3,SYT1,ASXL3,PPARA,PLXNA2,PTPRD,RORA,LOXL2,ELAVL4,CDH9,MAGI2,ABI1,RALA,DOCK10,PRKD1,BCL2L1,HDAC2,ETS1,TNN,RYR2,SEMA3D,TGFA,TBX20,FAT3,MTMR2,VCL,ROBO2,CDH23,AKAP13,NEDD9,RIMS2,EYA4,SEMA5A,BCL11A,FREM1,DCC,CTNNA2,CHN1,PARD3,NRG1,PRKCA,SEMA3C
GO:0007399	nervous system development	2.8981794910728524e-29	RTN1,SEMA4D,NFIA,RPS6KA5,TAOK3,SLC8A1,KCNC1,CD38,FBXO31,TRAPPC9,BCL2,THRB,DNAH11,NCAM1,KDM4B,CASP5,NDRG2,GRIP1,APBA2,ASTN2,TANC2,CSMD3,NTN4,JAK2,KALRN,USH2A,NEGR1,FGF12,ZNF536,EPB41L3,LAMA1,CDH11,GRIN2A,NRXN1,ARID1B,IL1RAPL1,WDPCP,LAMA3,ZDHHC17,CTNNA1,MYO9A,NTRK2,AK8,NCAM2,TIAM2,MYT1L,SRGAP2B,SMARCA4,CNTN1,NRG3,PTPRG,SLC24A4,FBXL17,NUMB,MTPN,SOX6,SYBU,VCAN,SHANK2,RAPGEF5,UNC5D,NREP,GABRA5,DOK5,DLG5,ASTN1,CHRM3,SLIT2,ROR1

			,ADAMTSL1,AKT3,CRB1,HECW1,ERBB4,KANK1,ATRX,CHST8,MACF1,MNAT1,RAP1A,TRIO,ABL2,EML1,CCDC141,CNTN4,ITGA1,TCF12,ITGA8,RUNX1,ALDH1A2,EYA1,PLS1,ANK2,SLC1A2,ZBTB16,SLIT3,GRIN2B,ROBO1,PAK3,GABRA2,OVOL2,BRINP1,CHODL,BBS2,IL1RAPL2,GRIK1,SOX5,DSCAM,SDK1,SLC1A1,GRM5,EPHA6,NTN1,CA10,LDB2,IGF1R,NLGN1,SHROOM3,JAM2,CNTNAP2,MAP2,CAMK1D,FLRT2,PTPRO,COBL,CRTAC1,CUX1,ANK3,TBCD,GPC6,RELN,CDH4,TNR,CNTN6,APP,CCDC88A,ARNT2,SPOCK1,TACC2,PAK1,IGF2BP3,HCN1,FRY,LRFN5,TENM4,CECR2,RASGRF1,RBFOX1,MEIS2,LRRC4C,ALCAM,PPP1R9A,CLSTN2,RARB,TCF4,FRYL,TIAM1,PBX1,PHACTR1,SLC39A12,DISC1,NTM,ASAP1,EGF,FYN,EPHA7,NAV2,STK3,S100B,TOX,PCDH15,CNTN5,LRIG1,PRKACB,RIMS1,PCSK2,EPHB1,LSAMP,CTTNBP2,IGSF21,HERC1,FAM126A,EXT1,EFNA5,HDAC4,STK36,KLHL1,TRPC5,PPFIA2,ACSBG1,POU6F2,TENM3,LINGO2,OPCML,MARK2,ATF2,GRID2,ZNF423,LRP2,SEMA6D,NTF3,NTNG1,ISX,TMEM108,GABRB3,GRM7,RAPGEF2,NAV3,IMMP2L,ATXN1,PRKCQ,KIRREL3,GABRG2,PRKCH,NRXN3,DLC1,ATP8A2,UBE3A,APC,MACROD2,AUTS2,EPHB2,PDLIM5,PRTG,CTNND2,SETD2,SDCCAG8,NRP1,MDGA2,RPGRIP1,DAB1,ALK,SEMA3A,SEMA3E,MYEF2,DCLK1,CDH2,TENM2,HDAC9,FSTL4,MTOR,RORB,GABRB1,RGS7,GALC,GLI3,NTRK3,HYDIN,ZNF521,DNM3,SYT1,SYNDIG1,DPF3,NPHP4,PLXNA2,PTPRD,RORA,PLCB1,BPTF,PRKG1,ELAVL4,MAGI2,NELL1,ABI1,ASIC2,RALA,DOCK10,PRKD1,HDAC2,TNN,SEMA3D,TBX20,FAT3,MTMR2,VCL,ATAT1,ROBO2,CDH23,PCP4,RIMS2,SCN8A,SEMA5A,CABLES1,BCL11A,DCC,CTNNA2,CHN1,ETV6,PARD3,NRG1,SEMA3C
GO:0048666	neuron development	4.1199301133787966e-29	SEMA4D,RPS6KA5,TAOK3,CD38,FBXO31,BCL2,THRB,NCAM1,GRIP1,TANC2,CSMD3,NTN4,JAK2,KALRN,NEGR1,EPB41L3,LAMA1,CDH11,NRXN1,IL1RAPL1,WDPCP,LAMA3,ZDHHC17,CTNNA1,MYO9A,NTRK2,NCAM2,TIAM2,MYT1L,CNTN1,PTPRG,NUMB,UNC5D,NREP,GABRA5,DLG5,SLIT2,ROR1,ADAMTSL1,CRB1,HECW1,KANK1,MACF1,RAP1A,TRIO,ABL2,CCDC141,CNTN4,ITGA1,RUNX1,PLS1,SLIT3,ROBO1,PAK3,CHODL,DSCAM,SDK1,EPHA6,NTN1,IGF1R,NLGN1,CNTNAP2,MAP2,CAMK1D,FLRT2,PTPRO,COBL,CRTAC1,CUX1,ANK3,TBCD,RELN,CDH4,TNR,CNTN6,APP,CCDC88A,SPOCK1,PAK1,HCN1,FRY,TENM4,CECR2,RASGRF1,LRRC4C,ALCAM,PPP1R9A,FRYL,TIAM1,PBX1,PHACTR1,SLC39A12,DISC1,NTM,ASAP1,FYN,EPHA7,S100B,TOX,PCDH15,CNTN5,RIMS1,EPHB1,HERC1,EXT1,EFNA5,KLHL1,TRPC5,PPFIA2,TENM3,OPCML,MARK2,GRID2,LRP2,SEMA6D,NTF3,NTNG1,TMEM108,GRM7,RAPGEF2,PRKCQ,KIRREL3,NRXN3,ATP8A2,UBE3A,AUTS2,EPHB2,PDLIM5,PRTG,CTNND2,NRP1,RPGRIP1,DAB1,ALK,SEMA3A,SEMA3E,DCLK1,CDH2,TENM2,FSTL4,RORB,GABRB1,GLI3,NTRK3,DNM3,SYT1,NPHP4,PLXNA2,PTPRD,PRKG1,ELAVL4,MAGI2,ABI1,DOCK10,PRKD1,HDAC2,TNN,SEMA3D,FAT3,MTMR2,VCL,ATAT1,ROBO2,CDH23,RIMS2,SEMA5A,BCL11A,DCC,CTNNA2,CHN1,PARD3,SEMA3C
GO:0007275	multicellular organism development	1.5586519351291916e-27	CD44,PTPRR,RTN1,SEMA4D,IGSF3,EVC,TEAD1,NFIA,RPS6KA5,TAOK3,PRKCB,A2M,SLC8A1,KCNC1,GPR55,SIAH3,CD38,FBXO31,TRAPPC9,BCL2,MMP16,COL18A1,THRB,DNAH11,RAG1,NCAM1,KDM4B,IL6R,SORBS2,CASP5,ZNF568,NDRG2,GRIP1,APBA2,ASTN2,TANC2,CSMD3,NTN4,JAK2,KALRN,USH2A,NEGR1,FGF12,FLI1,MEGF11,ZNF536,EPB41L3,LAMA1,CDH11,GRIN2A,NRXN1,ARID1B,IL1RAPL1,WDPCP,LAMA3,ADCYAP1R1,CTDP1,ADAMTS6,RPS6KA2,SMOC2,ZDHHC17,CACNA1C,EXOC4,CTNNA1,MYO9A,NTRK2,FOXN3,AK8,THSD7A,NCAM2,TIAM2,MYT1L,SRGAP2B,SMARCA4,CNTN1,ZNRF3,MEOX2,ENPEP,NRG3,PTPRG,SLC24A4,FBXL17,GAS2,NUMB,MTPN,SOX6,MECOM,SYBU,VCAN,NHS,SHANK2,RAPGEF5,UNC5D,NREP,GABRA5,DOK5,DLG5,ASTN1,CHRM3,CPE,CALD1,ARHGAP24,SPRED2,SLIT2,MYLK3,ROR1,ADAMTSL1,ZNF675,AKT3,CRB1,ALPK2,HECW1,AFF3,ERBB4,KANK1,ATRX,DMRT1,CHST8,MACF1,MNAT1,RAP1A,TRIO,CTNNBL1,RAD51B,MYO3B,CHSY1,EXT2,ABL2,EML1,CCDC141,CNTN4,SLC40A1,ETS2,ITGA1,TCF12,HIRA,ITGA8,RBM19,RUNX1,ALDH1A2,CDH17,EYA1,KIAA1217,MORC3,PLS1,ANK2,SLC1A2,ZBTB16,SLIT3,GRIN2B,ROBO1,ANKRD11,PAK3,GABRA2,MDM1,OVOL2,BRINP1,MLLT3,CHODL,GABPA,PSG9,BBS2,IL1RAPL2,MYO3A,GRIK1,SOX5,DSCAM,KDM4C,SDK1,SLC1A1,GRM5,EPHA6,NTN

			1,CA10,NR5A2,LDB2,IGF1R,NLGN1,SHROOM3,JAM2,ALX4,CNTNAP2,MAP2,CFTR,CAMK1D,FLRT2,PTPRO,INSR,COBL,CRTAC1,CUX1,ANK3,TBCD,GPC6,RELN,TRPS1,MYO18B,CDH4,TNR,ADCY9,CELF4,VAV3,CNTN6,APP,CCDC88A,ARNT2,SPOCK1,PLCE1,TACC2,ADAM12,PAK1,MITF,IGF2BP3,HCN1,FRY,CXADR,LRFN5,TENM4,CECR2,GHR,RASGRF1,RIN2,RBFOX1,MEIS2,STARD13,KL,LRRC4C,ALCAM,PPP1R9A,ADAMTS16,SLAMF1,INO80D,CLSTN2,NEBL,RARB,TCF4,FRYL,TIAM1,PBX1,PHACTR1,SLC39A12,DISC1,FMN1,ZFPM2,VSTM4,SVEP1,NTM,ASAP1,EDAR,EGF,PDGFD,FYN,XRCC4,EPHA7,NAV2,STK3,COL19A1,AP2B1,S100B,TOX,PCDH15,ESR1,SGCZ,CNTN5,LRIG1,PRKACB,RIMS1,POR,WWOX,PCSK2,FUT8,EPHB1,LSAMP,CTTNBP2,FHOD3,GREB1L,EFEMP1,AJAP1,IGSF21,HERC1,DOCK1,FAM126A,FLT1,EXT1,EFNA5,NXN,HDAC4,STK36,KLHL1,TRPC5,PPFIA2,AKAP6,ACSBG1,POU6F2,TENM3,LINGO2,OPCML,MARK2,ATF2,RBBP8,GRID2,ZNF423,LRP2,SEMA6D,NTF3,FER,SNRK,CAMK4,NTNG1,ISX,SGCD,TMEM108,GABRB3,GRM7,RAPGEF2,NAV3,IMMP2L,GTF2I,ATXN1,PRKCQ,KIRREL3,GABRG2,CPS1,PRKCH,NRXN3,DLC1,ATP8A2,UBE3A,APC,MACROD2,TTLL5,INO80,AUTS2,EPHB2,PDLIM5,XYLT1,PRTG,NBN,ADAMTS18,CTNND2,COL22A1,SETD2,DOCK2,SDCCAG8,FLVCR1,NRP1,CDH13,MDGA2,RPGRIP1,DACH1,DAB1,ALK,SEMA3A,SEMA3E,MYEF2,DCLK1,KIF16B,NRIP1,CDH2,ARID5B,TENM2,SERPINB7,HDAC9,PIK3R3,MAP2K6,FSTL4,MTOR,RORB,GABRB1,FBLN1,FHL2,NCAPG2,RGS7,TTC39C,SPRED1,SIPA1L3,ADAM10,GALC,PAPPA2,ABCB5,SPECC1,CSMD1,GLI3,NTRK3,RXFP1,FBN1,SGCG,HYDIN,TBX15,BMPER,ZNF521,HMGA2,NSUN2,DNM3,SYT1,SYNDIG1,DPF3,SCAPER,NPHP4,PPARA,PLXNA2,PTPRD,RORA,MYH15,PLCB1,LOXL2,BPTF,PRKG1,RASGRP1,ELAVL4,MAGI2,NELL1,ABI1,ASIC2,RALA,DOCK10,FNDC3A,NECAB1,PRKD1,BCL2L1,HDAC2,ETS1,TNN,RYR2,SEMA3D,TGFA,PRLR,TBX20,FAT3,MTMR2,ATF6,VCL,ATAT1,ROBO2,CDH23,AKAP13,NEDD9,ENPP1,PCP4,RIMS2,SCN8A,EYA4,L3MBTL3,SEMA5A,CABLES1,BCL11A,FREM1,DCC,CTNNA2,CHN1,ETV6,PARD3,NRG1,PRKCA,SEMA3C,TOP1
GO:0048856	anatomical structure development	3.523510932749443e-27	CD44,PTPRR,RTN1,SEMA4D,IGSF3,EVC,TEAD1,NFIA,RPS6KA5,TAOK3,PRKCB,A2M,SLC8A1,ANKRD6,KCNC1,GPR55,SIAH3,CD38,FBXO31,TRAPPC9,BCL2,MMP16,COL18A1,THRB,DNAH11,RAG1,CDC42EP3,NCAM1,KDM4B,IL6R,CDH8,ATP10A,SORBS2,CASP5,ZNF568,NDRG2,GRIP1,APBA2,ASTN2,TANC2,CSMD3,RCAN1,NTN4,JAK2,BICD1,KALRN,USH2A,NEGR1,FGF12,FLI1,MEGF11,ZNF536,EPB41L3,LAMA1,PARVB,CDH11,GRIN2A,NRXN1,ARID1B,IL1RAPL1,WDPCP,LAMA3,ADCYAP1R1,CTDP1,ADAMTS6,RPS6KA2,SMOC2,ZDHHC17,KCNH1,CACNA1C,EXOC4,CTNNA1,MYO9A,NTRK2,FOXN3,AK8,THSD7A,NCAM2,TIAM2,MYT1L,SRGAP2B,SMARCA4,CNTN1,ZNRF3,PALMD,MEOX2,ENPEP,NRG3,PTPRG,SLC24A4,SYNE1,FBXL17,GAS2,NUMB,MTPN,SOX6,MECOM,SYBU,VCAN,NHS,SHANK2,RAPGEF5,PTGFRN,UNC5D,NREP,GABRA5,DOK5,DLG5,CFDP1,PGM5,ASTN1,ATRNL1,CHRM3,CPE,CALD1,ARHGAP24,SPRED2,SLIT2,MYLK3,ROR1,ADAMTSL1,KAZN,ZNF675,AKT3,CRB1,ALPK2,HECW1,AFF3,LCE1F,ERBB4,KANK1,ATRX,DMRT1,CHST8,MACF1,MNAT1,TAF4B,RAP1A,TRIO,CTNNBL1,RAD51B,MYO3B,CHSY1,EXT2,ABL2,MAP3K5,NOS1,SPAG16,EML1,CCDC141,CNTN4,RNF17,SLC40A1,FGD4,ETS2,ITGA1,TCF12,HIRA,ITGA8,RBM19,RUNX1,ALDH1A2,FAM171A1,CDH17,PEAK1,EYA1,KIAA1217,MORC3,CDH18,FRMD6,PLS1,ANK2,SLC1A2,ZBTB16,SLIT3,GRIN2B,ROBO1,ANKRD11,EGFLAM,PAK3,GABRA2,MDM1,OVOL2,BRINP1,MLLT3,CPQ,CHODL,GABPA,PRICKLE2,PSG9,PACRG,BBS2,IL1RAPL2,MYOM2,MYO3A,GRIK1,NUBPL,SOX5,DSCAM,DNMBP,KDM4C,SDK1,SLC1A1,GRM5,EPHA6,NTN1,CA10,NR5A2,LDB2,IGF1R,WDR72,NLGN1,SHROOM3,JAM2,ALX4,MSI2,CNTNAP2,MAP2,CFTR,CAMK1D,FLRT2,PTPRO,INSR,COBL,CATSPER2,CRTAC1,CUX1,ANK3,CDH12,TBCD,GPC6,RELN,TRPS1,ADAMTS5,MYO18B,CDH4,TNR,ADCY9,OCA2,CELF4,VAV3,CNTN6,APP,CCDC88A,ARNT2,SPOCK1,PLCE1,TACC2,ADAM12,PAK1,MITF,IGF2BP3,ADCK1,HCN1,FRY,CXADR,EPS8,LRFN5,UTRN,TENM4,CECR2,GHR,RIPK4,RASGRF1,RIN2,RBFOX1,MEIS2,STARD13,KL,LRRC4C,ALCAM,PPP1R9A,ADAMTS16,SLAMF1,INO80D,CLSTN2,NEBL,RARB,TCF4,FRYL,TIAM1,PBX1,PHACTR1,SLC39A12,DISC1,FMN1,ZFP

			M2,VSTM4,SVEP1,NTM,ASAP1,EDAR,EGF,PDGFD,FYN,XRCC4,EPHA7,NAV2,STK3,COL19A1,AP2B1,S100B,TOX,PCDH15,ESR1,ARHGAP12,SGCZ,PDE4D,CNTN5,LRIG1,PRKACB,PDE3A,RIMS1,POR,CERS3,WWOX,PCSK2,FUT8,EPHB1,LSAMP,CTTNBP2,FHOD3,GREB1L,EFEMP1,ARMC2,AJAP1,IGSF21,HERC1,DOCK1,FAM126A,FLT1,EXT1,EFNA5,NXN,C14ORF39,HDAC4,STK36,KLHL1,TRPC5,FTO,PPFIA2,AKAP6,ACSBG1,POU6F2,TENM3,LINGO2,OPCML,MARK2,ATF2,RBBP8,GRID2,ZNF423,LRP2,SEMA6D,NTF3,FER,SNRK,CAMK4,NTNG1,DDX10,ISX,SGCD,TMEM108,GABRB3,GRM7,RAPGEF2,NAV3,IMMP2L,GTF2I,ATXN1,PRKCQ,KIRREL3,GABRG2,CPS1,PRKCH,NRXN3,KRT25,DLC1,ATP8A2,SLC24A3,UBE3A,APC,MACROD2,TTLL5,INO80,AUTS2,EPHB2,PDLIM5,XYLT1,PRTG,NBN,ADAMTS18,CTNND2,COL22A1,SETD2,PACSIN2,DOCK2,SDCCAG8,FLVCR1,NRP1,CDH13,MDGA2,RPGRIP1,DACH1,DAB1,ALK,LDLRAD4,SEMA3A,SEMA3E,MYEF2,DCLK1,KIF16B,NRIP1,CDH2,ARID5B,TENM2,TANC1,SERPINB7,SYCP1,HDAC9,PIK3R3,MAP2K6,FSTL4,MTOR,RORB,GABRB1,FBLN1,SH3KBP1,FHL2,NCAPG2,RGS7,TTC39C,SPRED1,SIPA1L3,ADAM10,GALC,FLNB,PAPPA2,ABCB5,SPECC1,DPY19L2,CSMD1,GLI3,NTRK3,RXFP1,FBN1,SGCG,HYDIN,TBX15,BMPER,ZNF521,HMGA2,NSUN2,DNM3,SYT1,SYNDIG1,ASXL3,DPF3,SCAPER,NPHP4,PPARA,PLXNA2,PTPRD,RORA,MYH15,PLCB1,LOXL2,BPTF,PRKG1,RASGRP1,ELAVL4,CDH9,DMBT1,MAGI2,NELL1,ABI1,ASIC2,RALA,DOCK10,FNDC3A,NECAB1,PRKD1,BCL2L1,HDAC2,ETS1,TNN,RYR2,SEMA3D,TGFA,PRLR,TBX20,FAT3,MTMR2,ATF6,EYS,VCL,ATAT1,ROBO2,IFT81,CDH23,AKAP13,NEDD9,ENPP1,PCP4,RIMS2,SCN8A,EYA4,L3MBTL3,HIVEP3,SEMA5A,CABLES1,BCL11A,FREM1,DCC,CTNNA2,CHN1,ETV6,PARD3,NRG1,PRKCA,FMN2,SEMA3C,TOP1
GO:0034330	cell junction organization	6.792358375283443e-27	APBB2,SEMA4D,NFIA,BCL2,UNC13B,CDH8,TANC2,KALRN,NEGR1,CACNG2,EPB41L3,CDH11,NRXN1,IL1RAPL1,WDPCP,CTNNA1,MYO9A,NTRK2,OCLN,CACNB2,NUMB,SYBU,SHANK2,DLG5,ERBB4,GPHN,MACF1,RAP1A,DUSP22,CORO2B,PEAK1,CDH18,ANK2,GRIN2B,PAK3,DGKB,GABRA2,IL1RAPL2,DSCAM,SDK1,SLC1A1,GRM5,NTN1,IGF1R,NLGN1,CNTNAP2,FLRT2,NOS1AP,PTPRO,INSR,ANK3,CDH12,TBCD,GPC6,RELN,TNR,APP,CXADR,LRFN5,LRRC4C,PDZRN3,CLSTN2,DISC1,FMN1,SVEP1,FRMPD4,FYN,EPHA7,CNTN5,ERC2,CNKSR2,EPHB1,CTTNBP2,IGSF21,EXT1,EFNA5,TLN2,PPFIA2,LINGO2,GRID2,FER,NTNG1,MAPRE2,TMEM108,GABRB3,RAPGEF2,KIRREL3,GABRG2,PRKCH,NRXN3,DLC1,UBE3A,APC,EPHB2,ERC1,PDLIM5,CTNND2,PKP1,NRP1,SEMA3E,CDH2,TANC1,ADAM10,LRFN2,NTRK3,ABHD17C,PTPRK,DNM3,SYNDIG1,NPHP4,PTPRD,SHISA6,CDH9,ASIC2,DOCK10,PTPRA,MTMR2,VCL,ROBO2,NEDD9,UNC13C,CTNNA2,PARD3,NRG1,CAST,PRKCA
GO:0048731	system development	1.0066545972324792e-26	CD44,RTN1,SEMA4D,IGSF3,EVC,NFIA,RPS6KA5,TAOK3,PRKCB,A2M,SLC8A1,KCNC1,GPR55,CD38,FBXO31,TRAPPC9,BCL2,MMP16,COL18A1,THRB,DNAH11,RAG1,NCAM1,KDM4B,IL6R,SORBS2,CASP5,NDRG2,GRIP1,APBA2,ASTN2,TANC2,CSMD3,NTN4,JAK2,KALRN,USH2A,NEGR1,FGF12,FLI1,MEGF11,ZNF536,EPB41L3,LAMA1,CDH11,GRIN2A,NRXN1,ARID1B,IL1RAPL1,WDPCP,LAMA3,CTDP1,ADAMTS6,RPS6KA2,SMOC2,ZDHHC17,CACNA1C,CTNNA1,MYO9A,NTRK2,FOXN3,AK8,THSD7A,NCAM2,TIAM2,MYT1L,SRGAP2B,SMARCA4,CNTN1,MEOX2,ENPEP,NRG3,PTPRG,SLC24A4,FBXL17,GAS2,NUMB,MTPN,SOX6,MECOM,SYBU,VCAN,NHS,SHANK2,RAPGEF5,UNC5D,NREP,GABRA5,DOK5,DLG5,ASTN1,CHRM3,CPE,CALD1,ARHGAP24,SPRED2,SLIT2,MYLK3,ROR1,ADAMTSL1,ZNF675,AKT3,CRB1,ALPK2,HECW1,ERBB4,KANK1,ATRX,DMRT1,CHST8,MACF1,MNAT1,RAP1A,TRIO,CTNNBL1,CHSY1,EXT2,ABL2,EML1,CCDC141,CNTN4,SLC40A1,ETS2,ITGA1,TCF12,ITGA8,RUNX1,ALDH1A2,CDH17,EYA1,KIAA1217,PLS1,ANK2,SLC1A2,ZBTB16,SLIT3,GRIN2B,ROBO1,ANKRD11,PAK3,GABRA2,MDM1,OVOL2,BRINP1,MLLT3,CHODL,GABPA,PSG9,BBS2,IL1RAPL2,GRIK1,SOX5,DSCAM,SDK1,SLC1A1,GRM5,EPHA6,NTN1,CA10,LDB2,IGF1R,NLGN1,SHROOM3,JAM2,ALX4,CNTNAP2,MAP2,CAMK1D,FLRT2,PTPRO,INSR,COBL,CRTAC1,CUX1,ANK3,TBCD,GPC6,RELN,TRPS1,MYO18B,CDH4,TNR,CELF4,VAV3,CNTN6,APP,CCDC88A,ARNT2,SPOCK1,PLCE1,TACC2,ADAM12,PAK1,MITF,IGF2BP3,HCN1,FRY,CXADR,LRFN5,TENM4,CECR2,GHR

			,RASGRF1,RIN2,RBFOX1,MEIS2,STARD13,LRRC4C,ALCAM,PPP1R9A,ADAMTS16,SLAMF1,CLSTN2,NEBL,RARB,TCF4,FRYL,TIAM1,PBX1,PHACTR1,SLC39A12,DISC1,FMN1,ZFPM2,VSTM4,SVEP1,NTM,ASAP1,EDAR,EGF,PDGFD,FYN,XRCC4,EPHA7,NAV2,STK3,COL19A1,AP2B1,S100B,TOX,PCDH15,ESR1,SGCZ,CNTN5,LRIG1,PRKACB,RIMS1,POR,WWOX,PCSK2,EPHB1,LSAMP,CTTNBP2,FHOD3,GREB1L,EFEMP1,IGSF21,HERC1,DOCK1,FAM126A,FLT1,EXT1,EFNA5,NXN,HDAC4,STK36,KLHL1,TRPC5,PPFIA2,AKAP6,ACSBG1,POU6F2,TENM3,LINGO2,OPCML,MARK2,ATF2,GRID2,ZNF423,LRP2,SEMA6D,NTF3,FER,SNRK,CAMK4,NTNG1,ISX,SGCD,TMEM108,GABRB3,GRM7,RAPGEF2,NAV3,IMMP2L,GTF2I,ATXN1,PRKCQ,KIRREL3,GABRG2,CPS1,PRKCH,NRXN3,DLC1,ATP8A2,UBE3A,APC,MACROD2,TTLL5,AUTS2,EPHB2,PDLIM5,XYLT1,PRTG,NBN,ADAMTS18,CTNND2,COL22A1,SETD2,DOCK2,SDCCAG8,FLVCR1,NRP1,CDH13,MDGA2,RPGRIP1,DAB1,ALK,SEMA3A,SEMA3E,MYEF2,DCLK1,NRIP1,CDH2,ARID5B,TENM2,SERPINB7,HDAC9,PIK3R3,MAP2K6,FSTL4,MTOR,RORB,GABRB1,FHL2,NCAPG2,RGS7,SPRED1,SIPA1L3,ADAM10,GALC,PAPPA2,ABCB5,CSMD1,GLI3,NTRK3,RXFP1,FBN1,SGCG,HYDIN,TBX15,BMPER,ZNF521,HMGA2,DNM3,SYT1,SYNDIG1,DPF3,SCAPER,NPHP4,PPARA,PLXNA2,PTPRD,RORA,MYH15,PLCB1,LOXL2,BPTF,PRKG1,RASGRP1,ELAVL4,MAGI2,NELL1,ABI1,ASIC2,RALA,DOCK10,FNDC3A,PRKD1,BCL2L1,HDAC2,ETS1,TNN,RYR2,SEMA3D,TGFA,PRLR,TBX20,FAT3,MTMR2,ATF6,VCL,ATAT1,ROBO2,CDH23,AKAP13,NEDD9,PCP4,RIMS2,SCN8A,L3MBTL3,SEMA5A,CABLES1,BCL11A,FREM1,DCC,CTNNA2,CHN1,ETV6,PARD3,NRG1,PRKCA,SEMA3C
GO:0030182	neuron differentiation	1.0477175510684315e-26	RTN1,SEMA4D,RPS6KA5,TAOK3,CD38,FBXO31,TRAPPC9,BCL2,THRB,NCAM1,GRIP1,TANC2,CSMD3,NTN4,JAK2,KALRN,USH2A,NEGR1,ZNF536,EPB41L3,LAMA1,CDH11,NRXN1,IL1RAPL1,WDPCP,LAMA3,ZDHHC17,CTNNA1,MYO9A,NTRK2,NCAM2,TIAM2,MYT1L,CNTN1,PTPRG,NUMB,MTPN,UNC5D,NREP,GABRA5,DOK5,DLG5,SLIT2,ROR1,ADAMTSL1,CRB1,HECW1,ERBB4,KANK1,MACF1,RAP1A,TRIO,ABL2,CCDC141,CNTN4,ITGA1,TCF12,RUNX1,ALDH1A2,EYA1,PLS1,SLIT3,ROBO1,PAK3,BRINP1,CHODL,DSCAM,SDK1,EPHA6,NTN1,IGF1R,NLGN1,CNTNAP2,MAP2,CAMK1D,FLRT2,PTPRO,COBL,CRTAC1,CUX1,ANK3,TBCD,RELN,CDH4,TNR,CNTN6,APP,CCDC88A,SPOCK1,PAK1,HCN1,FRY,TENM4,CECR2,RASGRF1,LRRC4C,ALCAM,PPP1R9A,TCF4,FRYL,TIAM1,PBX1,PHACTR1,SLC39A12,DISC1,NTM,ASAP1,FYN,EPHA7,S100B,TOX,PCDH15,CNTN5,RIMS1,EPHB1,HERC1,EXT1,EFNA5,KLHL1,TRPC5,PPFIA2,TENM3,OPCML,MARK2,GRID2,LRP2,SEMA6D,NTF3,NTNG1,TMEM108,GRM7,RAPGEF2,PRKCQ,KIRREL3,NRXN3,ATP8A2,UBE3A,AUTS2,EPHB2,PDLIM5,PRTG,CTNND2,NRP1,MDGA2,RPGRIP1,DAB1,ALK,SEMA3A,SEMA3E,MYEF2,DCLK1,CDH2,TENM2,HDAC9,FSTL4,RORB,GABRB1,GLI3,NTRK3,ZNF521,DNM3,SYT1,NPHP4,PLXNA2,PTPRD,RORA,PRKG1,ELAVL4,MAGI2,ABI1,DOCK10,PRKD1,HDAC2,TNN,SEMA3D,TBX20,FAT3,MTMR2,VCL,ATAT1,ROBO2,CDH23,PCP4,RIMS2,SEMA5A,BCL11A,DCC,CTNNA2,CHN1,PARD3,NRG1,SEMA3C
GO:0048699	generation of neurons	1.5675297942235246e-26	RTN1,SEMA4D,RPS6KA5,TAOK3,CD38,FBXO31,TRAPPC9,BCL2,THRB,NCAM1,GRIP1,ASTN2,TANC2,CSMD3,NTN4,JAK2,KALRN,USH2A,NEGR1,ZNF536,EPB41L3,LAMA1,CDH11,NRXN1,IL1RAPL1,WDPCP,LAMA3,ZDHHC17,CTNNA1,MYO9A,NTRK2,NCAM2,TIAM2,MYT1L,CNTN1,NRG3,PTPRG,NUMB,MTPN,UNC5D,NREP,GABRA5,DOK5,DLG5,ASTN1,SLIT2,ROR1,ADAMTSL1,CRB1,HECW1,ERBB4,KANK1,MACF1,RAP1A,TRIO,ABL2,EML1,CCDC141,CNTN4,ITGA1,TCF12,RUNX1,ALDH1A2,EYA1,PLS1,SLIT3,ROBO1,PAK3,BRINP1,CHODL,SOX5,DSCAM,SDK1,EPHA6,NTN1,IGF1R,NLGN1,CNTNAP2,MAP2,CAMK1D,FLRT2,PTPRO,COBL,CRTAC1,CUX1,ANK3,TBCD,RELN,CDH4,TNR,CNTN6,APP,CCDC88A,SPOCK1,PAK1,HCN1,FRY,TENM4,CECR2,RASGRF1,LRRC4C,ALCAM,PPP1R9A,TCF4,FRYL,TIAM1,PBX1,PHACTR1,SLC39A12,DISC1,NTM,ASAP1,FYN,EPHA7,S100B,TOX,PCDH15,CNTN5,RIMS1,EPHB1,HERC1,EXT1,EFNA5,KLHL1,TRPC5,PPFIA2,TENM3,OPCML,MARK2,GRID2,LRP2,SEMA6D,NTF3,NTNG1,TMEM108,GRM7,RAPGEF2,PRKCQ,KIRREL3,NRXN3,ATP8A2,UBE3A,AUTS2,EPHB2,PDLIM5,PRTG,CTNND2,SDCCAG8,NRP1,MDGA2,RPGRIP1,DAB1,ALK,SEMA3A,SEMA3E,MYEF2,DCLK1,CDH2,TENM

			2,HDAC9,FSTL4,RORB,GABRB1,GLI3,NTRK3,ZNF521,DNM3,SYT1,NPHP4,PLXNA2,PTPRD,RORA,PRKG1,ELAVL4,MAGI2,ABI1,DOCK10,PRKD1,HDAC2,TNN,SEMA3D,TBX20,FAT3,MTMR2,VCL,ATAT1,ROBO2,CDH23,PCP4,RIMS2,SEMA5A,BCL11A,DCC,CTNNA2,CHN1,PARD3,NRG1,SEMA3C
GO:0031175	neuron projection development	2.4029273483665926e-26	SEMA4D,RPS6KA5,TAOK3,CD38,FBXO31,BCL2,NCAM1,GRIP1,TANC2,CSMD3,JAK2,KALRN,NEGR1,EPB41L3,LAMA1,CDH11,NRXN1,IL1RAPL1,LAMA3,ZDHHC17,CTNNA1,MYO9A,NTRK2,NCAM2,TIAM2,CNTN1,PTPRG,NUMB,UNC5D,NREP,DLG5,SLIT2,ROR1,ADAMTSL1,HECW1,KANK1,MACF1,RAP1A,TRIO,ABL2,CCDC141,CNTN4,ITGA1,PLS1,SLIT3,ROBO1,PAK3,CHODL,DSCAM,SDK1,EPHA6,NTN1,IGF1R,NLGN1,CNTNAP2,MAP2,CAMK1D,FLRT2,PTPRO,COBL,CRTAC1,CUX1,ANK3,RELN,CDH4,TNR,CNTN6,APP,CCDC88A,SPOCK1,PAK1,FRY,CECR2,RASGRF1,LRRC4C,ALCAM,PPP1R9A,FRYL,TIAM1,PHACTR1,SLC39A12,DISC1,ASAP1,FYN,EPHA7,S100B,TOX,PCDH15,CNTN5,RIMS1,EPHB1,HERC1,EXT1,EFNA5,KLHL1,TRPC5,PPFIA2,TENM3,MARK2,GRID2,LRP2,SEMA6D,NTF3,NTNG1,TMEM108,GRM7,RAPGEF2,PRKCQ,KIRREL3,NRXN3,ATP8A2,UBE3A,AUTS2,EPHB2,PDLIM5,PRTG,CTNND2,NRP1,DAB1,ALK,SEMA3A,SEMA3E,DCLK1,CDH2,FSTL4,GLI3,NTRK3,DNM3,SYT1,PLXNA2,PTPRD,PRKG1,ELAVL4,MAGI2,ABI1,DOCK10,PRKD1,HDAC2,TNN,SEMA3D,FAT3,VCL,ROBO2,CDH23,RIMS2,SEMA5A,BCL11A,DCC,CTNNA2,CHN1,PARD3,SEMA3C
GO:0048468	cell development	4.122501271812563e-26	SEMA4D,RPS6KA5,TAOK3,SLC8A1,CD38,FBXO31,BCL2,COL18A1,THRB,NCAM1,SORBS2,GRIP1,TANC2,CSMD3,RCAN1,NTN4,JAK2,KALRN,USH2A,NEGR1,FLI1,EPB41L3,LAMA1,PARVB,CDH11,NRXN1,IL1RAPL1,WDPCP,LAMA3,CTDP1,RPS6KA2,ZDHHC17,CTNNA1,MYO9A,NTRK2,NCAM2,TIAM2,MYT1L,CNTN1,PTPRG,NUMB,UNC5D,NREP,GABRA5,DLG5,PGM5,ATRNL1,SLIT2,MYLK3,ROR1,ADAMTSL1,CRB1,ALPK2,HECW1,ERBB4,KANK1,ATRX,DMRT1,MACF1,TAF4B,RAP1A,TRIO,CHSY1,ABL2,SPAG16,CCDC141,CNTN4,RNF17,ITGA1,ITGA8,RUNX1,ALDH1A2,PEAK1,FRMD6,PLS1,ANK2,ZBTB16,SLIT3,ROBO1,PAK3,OVOL2,BRINP1,CHODL,PACRG,BBS2,MYOM2,DSCAM,SDK1,GRM5,EPHA6,NTN1,IGF1R,NLGN1,SHROOM3,JAM2,MSI2,CNTNAP2,MAP2,CFTR,CAMK1D,FLRT2,PTPRO,COBL,CATSPER2,CRTAC1,CUX1,ANK3,TBCD,RELN,MYO18B,CDH4,TNR,OCA2,CELF4,CNTN6,APP,CCDC88A,SPOCK1,PAK1,HCN1,FRY,CXADR,TENM4,CECR2,RASGRF1,LRRC4C,ALCAM,PPP1R9A,NEBL,RARB,FRYL,TIAM1,PBX1,PHACTR1,SLC39A12,DISC1,NTM,ASAP1,FYN,EPHA7,S100B,TOX,PCDH15,ESR1,SGCZ,PDE4D,CNTN5,PDE3A,RIMS1,EPHB1,FHOD3,ARMC2,HERC1,DOCK1,EXT1,EFNA5,C14ORF39,HDAC4,KLHL1,TRPC5,PPFIA2,AKAP6,TENM3,OPCML,MARK2,GRID2,LRP2,SEMA6D,NTF3,FER,NTNG1,SGCD,TMEM108,GRM7,RAPGEF2,PRKCQ,KIRREL3,PRKCH,NRXN3,ATP8A2,UBE3A,AUTS2,EPHB2,PDLIM5,PRTG,CTNND2,COL22A1,SETD2,FLVCR1,NRP1,RPGRIP1,DAB1,ALK,SEMA3A,SEMA3E,DCLK1,CDH2,ARID5B,TENM2,SYCP1,HDAC9,FSTL4,MTOR,RORB,GABRB1,FBLN1,FHL2,SIPA1L3,FLNB,DPY19L2,GLI3,NTRK3,FBN1,HYDIN,NSUN2,DNM3,SYT1,NPHP4,PPARA,PLXNA2,PTPRD,PLCB1,PRKG1,ELAVL4,MAGI2,ABI1,DOCK10,FNDC3A,PRKD1,BCL2L1,HDAC2,TNN,SEMA3D,TBX20,FAT3,MTMR2,VCL,ATAT1,ROBO2,IFT81,CDH23,AKAP13,NEDD9,RIMS2,L3MBTL3,SEMA5A,BCL11A,DCC,CTNNA2,CHN1,PARD3,NRG1,FMN2,SEMA3C
GO:0000902	cell morphogenesis	1.894545623454407e-25	CD44,SEMA4D,RPS6KA5,TAOK3,FBXO31,BCL2,COL18A1,CDC42EP3,NCAM1,CDH8,ATP10A,GRIP1,TANC2,NTN4,KALRN,USH2A,EPB41L3,LAMA1,PARVB,CDH11,NRXN1,IL1RAPL1,WDPCP,LAMA3,ZDHHC17,MYO9A,NTRK2,TIAM2,CNTN1,PALMD,GAS2,NUMB,UNC5D,CFDP1,ATRNL1,SLIT2,ADAMTSL1,CRB1,HECW1,KANK1,DMRT1,MACF1,TRIO,CCDC141,CNTN4,FGD4,ITGA1,ITGA8,FAM171A1,PEAK1,CDH18,FRMD6,PLS1,SLIT3,ROBO1,PAK3,CHODL,DSCAM,DNMBP,EPHA6,NTN1,IGF1R,NLGN1,SHROOM3,CNTNAP2,MAP2,FLRT2,PTPRO,COBL,CUX1,ANK3,CDH12,TBCD,RELN,CDH4,TNR,CNTN6,APP,PAK1,FRY,EPS8,LRRC4C,ALCAM,FRYL,TIAM1,PHACTR1,DISC1,FYN,EPHA7,S100B,PCDH15,CNTN5,RIMS1,EPHB1,DOCK1,EXT1,EFNA5,TRPC5,PPFIA2,MARK2,LRP2,SEMA6D,NTF3,FER,NTNG1,TMEM108,RAPGEF2,PRKCQ,KIRREL3,NRXN3,DLC1,ATP8A2,UBE3A,AUTS2,EPHB2,PDLIM5,PRTG,CTNND2,COL22A1,PACSIN2,NRP1,DAB1,

			SEMA3A,SEMA3E,DCLK1,CDH2,FSTL4,FBLN1,SH3KBP1,SIPA1L3,FLNB,GLI3,DNM3,SYT1,PLXNA2,PTPRD,ELAVL4,CDH9,ABI1,DOCK10,TNN,SEMA3D,FAT3,VCL,ROBO2,CDH23,NEDD9,RIMS2,SEMA5A,BCL11A,DCC,CTNNA2,CHN1,PARD3,NRG1,SEMA3C
GO:0032502	developmental process	1.983572016498455e-24	CD44,ZHX3,PTPRR,RTN1,ERG,SEMA4D,IGSF3,EVC,TEAD1,NFIA,RPS6KA5,TAOK3,PRKCB,A2M,SLC8A1,ANKRD6,KCNC1,GPR55,SIAH3,CD38,FBXO31,PIWIL3,TRAPPC9,BCL2,MMP16,COL18A1,THRB,DNAH11,RAG1,CDC42EP3,UNC13B,NCAM1,KDM4B,IL6R,CDH8,ATP10A,SORBS2,CASP5,ZNF568,NDRG2,GRIP1,APBA2,ASTN2,TANC2,CSMD3,RCAN1,NTN4,JAK2,BICD1,KALRN,USH2A,NEGR1,FGF12,FLI1,MEGF11,ZNF536,EPB41L3,LAMA1,PARVB,CDH11,ZBTB7C,GRIN2A,NRXN1,ARID1B,IL1RAPL1,WDPCP,LAMA3,ADCYAP1R1,CTDP1,ADAMTS6,RPS6KA2,SMOC2,ZDHHC17,KCNH1,CACNA1C,EXOC4,PNPLA3,BBS9,CTNNA1,MYO9A,NTRK2,FOXN3,AK8,THSD7A,NCAM2,TIAM2,MYT1L,SRGAP2B,SMARCA4,CNTN1,ZNRF3,PALMD,MEOX2,ENPEP,PLEKHB2,NRG3,PTPRG,SLC24A4,SYNE1,FBXL17,GAS2,NUMB,MED15,MTPN,SOX6,MECOM,SYBU,VCAN,NHS,SHANK2,RAPGEF5,PTGFRN,UNC5D,NREP,GABRA5,DOK5,DLG5,CFDP1,PGM5,ASTN1,ATRNL1,CHRM3,CPE,CALD1,ARHGAP24,SPRED2,SLIT2,MYLK3,ABCA5,ROR1,ADAMTSL1,KAZN,ZNF675,AKT3,CRB1,ALPK2,HECW1,AFF3,LCE1F,ERBB4,KANK1,ATRX,DMRT1,CHST8,MACF1,MNAT1,TAF4B,RAP1A,TRIO,CTNNBL1,RAD51B,MYO3B,CHSY1,EXT2,ABL2,MAP3K5,NOS1,ABCG1,SPAG16,EML1,HTR2C,CCDC141,CNTN4,RNF17,SLC40A1,SLC9C1,SND1,FGD4,ETS2,ITGA1,TCF12,HIRA,ITGA8,RBM19,RUNX1,ALDH1A2,MAPK9,FAM171A1,CDH17,PEAK1,EYA1,KIAA1217,MORC3,CDH18,HSF2BP,FRMD6,PLS1,ANK2,SLC1A2,ZBTB16,SLIT3,GRIN2B,PHC2,ROBO1,ANKRD11,EGFLAM,PAK3,GABRA2,MDM1,OVOL2,BRINP1,MLLT3,CPQ,CHODL,GABPA,PRICKLE2,GLIS1,PSG9,PACRG,BBS2,IL1RAPL2,MYOM2,MYO3A,GRIK1,NUBPL,SOX5,DSCAM,DNMBP,KDM4C,SDK1,SLC1A1,GRM5,EPHA6,NTN1,CA10,NR5A2,LDB2,IGF1R,WDR72,NLGN1,SHROOM3,JAM2,ALX4,MSI2,CNTNAP2,MAP2,CFTR,CAMK1D,FLRT2,PTPRO,NHSL1,INSR,COBL,CATSPER2,CRTAC1,CUX1,ANK3,CDH12,TBCD,GPC6,RELN,TRPS1,ADAMTS5,MYO18B,CDH4,TNR,ADCY9,OCA2,CELF4,VAV3,CNTN6,APP,PUM1,CCDC88A,ARNT2,SPOCK1,PLCE1,TACC2,ADAM12,PAK1,MITF,IGF2BP3,ADCK1,HCN1,FRY,CXADR,EPS8,LRFN5,UTRN,TENM4,CECR2,GHR,RIPK4,RASGRF1,RIN2,PRDM16,RBFOX1,MEIS2,STARD13,KL,LRRC4C,ALCAM,PPP1R9A,ADAMTS16,SLAMF1,INO80D,CLSTN2,NEBL,RARB,TCF4,FRYL,TIAM1,PBX1,PHACTR1,SLC39A12,DISC1,FMN1,ZFPM2,VSTM4,SVEP1,NTM,ASAP1,EDAR,EGF,PDGFD,FYN,XRCC4,EPHA7,NAV2,STK3,COL19A1,MSR1,AP2B1,S100B,TOX,PCDH15,ESR1,ARHGAP12,SGCZ,PDE4D,CNTN5,LRIG1,PRKACB,PDE3A,RIMS1,POR,CERS3,WWOX,PCSK2,FUT8,EPHB1,CREM,LSAMP,CTTNBP2,FHOD3,GREB1L,EFEMP1,ARMC2,AJAP1,IGSF21,HERC1,DOCK1,FAM126A,FLT1,EXT1,EFNA5,NXN,C14ORF39,HDAC4,STK36,KLHL1,TRPC5,AMFR,FTO,PPFIA2,ADAM28,AKAP6,ACSBG1,POU6F2,TENM3,LINGO2,OPCML,MARK2,ATF2,RBBP8,GRID2,ZNF423,LRP2,SEMA6D,NTF3,FER,SNRK,CAMK4,NTNG1,DDX10,ISX,SGCD,TMEM108,GABRB3,GRM7,RAPGEF2,NAV3,IMMP2L,GTF2I,ATXN1,PRKCQ,KIRREL3,GABRG2,CPS1,PRKCH,NRXN3,KRT25,DLC1,ATP8A2,SLC24A3,UBE3A,APC,MACROD2,TTLL5,INO80,AUTS2,TFF1,EPHB2,PDLIM5,XYLT1,PRTG,NBN,ADAMTS18,CTNND2,COL22A1,SETD2,PACSIN2,DOCK2,SDCCAG8,FLVCR1,NRP1,CDH13,MDGA2,RPGRIP1,DACH1,DAB1,ALK,LDLRAD4,SEMA3A,SEMA3E,MYEF2,DCLK1,KIF16B,NRIP1,CDH2,ARID5B,TENM2,TANC1,SERPINB7,SYCP1,HDAC9,PIK3R3,MAP2K6,FSTL4,MTOR,RORB,GABRB1,FBLN1,SH3KBP1,FHL2,NCAPG2,RGS7,TTC39C,SPRED1,SIPA1L3,ADAM10,GALC,FLNB,PAPPA2,ABCB5,SPECC1,DPY19L2,CSMD1,GLI3,NTRK3,RXFP1,FBN1,SGCG,HYDIN,TBX15,BMPER,ZNF521,HMGA2,NSUN2,DNM3,SYT1,SYNDIG1,ASXL3,DPF3,SCAPER,NPHP4,PPARA,PLXNA2,PTPRD,RORA,MYH15,SHISA6,PLCB1,LOXL2,BPTF,PRKG1,RASGRP1,ELAVL4,CDH9,DMBT1,MAGI2,NELL1,ABI1,ASIC2,RALA,DOCK10,FNDC3A,NECAB1,PRKD1,BCL2L1,HDAC2,ETS1,TNN,RYR2,SEMA3D,TGFA,PRLR,TBX20,FAT3,MTMR2,ATF6,EYS,VCL,ATAT1,ROBO2,IFT81,CDH23,AKAP13,NEDD9,ENPP1,UNC13C,PCP4,RIMS2,SCN8A,RAB27A,EYA4,L3MBTL3,HIVEP3,SEMA5A,CABL

			ES1,BCL11A,FREM1,DCC,CTNNA2,CHN1,ETV6,PARD3,NRG1,PRKCA,FMN2,SEMA3C,TOP1
GO:0120036	plasma membrane bounded cell projection organization	2.1421707586527715e-24	CD44,SEMA4D,RPS6KA5,TAOK3,CD38,FBXO31,BCL2,CDC42EP3,NCAM1,GRIP1,TANC2,CSMD3,JAK2,KALRN,NEGR1,EPB41L3,LAMA1,PARVB,CDH11,NRXN1,IL1RAPL1,WDPCP,LAMA3,ZDHHC17,BBS9,CTNNA1,MYO9A,NTRK2,OCLN,NCAM2,TIAM2,LRRC49,CNTN1,PTPRG,NUMB,DNAH8,UNC5D,NREP,DLG5,ARHGAP24,SLIT2,ROR1,ADAMTSL1,HECW1,KANK1,MACF1,RAP1A,TRIO,ABL2,SPAG16,CCDC141,CNTN4,FGD4,ITGA1,PLS1,SLIT3,GRIN2B,ROBO1,PAK3,CHODL,TMEM67,BBS2,DSCAM,SDK1,EPHA6,NTN1,IGF1R,NLGN1,CNTNAP2,MAP2,CAMK1D,FLRT2,PTPRO,INSR,COBL,CRTAC1,CUX1,ANK3,RELN,CDH4,TNR,VAV3,CNTN6,APP,CCDC88A,SPOCK1,PLCE1,PAK1,FRY,EPS8,CECR2,RASGRF1,LRRC4C,ALCAM,PPP1R9A,ADAMTS16,FRYL,TIAM1,PHACTR1,SLC39A12,DISC1,ASAP1,FYN,IFT43,EPHA7,S100B,TOX,PCDH15,CNTN5,RIMS1,EPHB1,ARMC2,SNAP29,HERC1,EXT1,EFNA5,CDC14B,HDAC4,STK36,KLHL1,TRPC5,PPFIA2,TENM3,MARK2,GRID2,ZNF423,LRP2,SEMA6D,NTF3,FER,TTC29,NTNG1,TMEM108,GRM7,RAPGEF2,PRKCQ,KIRREL3,NUDCD3,NRXN3,ATP8A2,UBE3A,APC,AUTS2,EPHB2,PDLIM5,PRTG,CTNND2,SDCCAG8,NRP1,CDH13,RPGRIP1,DAB1,ALK,SEMA3A,SEMA3E,DCLK1,CDH2,TENM2,TANC1,FSTL4,MTOR,CD2AP,TTC39C,GLI3,NTRK3,HYDIN,DNM3,SYT1,PLXNA2,PTPRD,PRKG1,ELAVL4,MAGI2,ABI1,RALA,DOCK10,PRKD1,HDAC2,TNN,SEMA3D,FAT3,MTMR2,VCL,ATAT1,ROBO2,IFT81,CDH23,NEDD9,RIMS2,SEMA5A,BCL11A,DCC,CTNNA2,CHN1,PARD3,SEMA3C,PCNT
GO:0030030	cell projection organization	3.621199812300097e-24	CD44,SEMA4D,RPS6KA5,TAOK3,CD38,FBXO31,BCL2,CDC42EP3,NCAM1,GRIP1,TANC2,CSMD3,JAK2,KALRN,NEGR1,EPB41L3,LAMA1,PARVB,CDH11,NRXN1,IL1RAPL1,WDPCP,LAMA3,ZDHHC17,BBS9,CTNNA1,MYO9A,NTRK2,OCLN,NCAM2,TIAM2,LRRC49,CNTN1,PTPRG,NUMB,DNAH8,UNC5D,NREP,DLG5,ARHGAP24,SLIT2,ROR1,ADAMTSL1,HECW1,KANK1,MACF1,RAP1A,TRIO,ABL2,SPAG16,CCDC141,CNTN4,FGD4,ITGA1,ITGA8,PLS1,SLIT3,GRIN2B,ROBO1,PAK3,CHODL,TMEM67,BBS2,DSCAM,SDK1,EPHA6,NTN1,IGF1R,NLGN1,DNAH9,CNTNAP2,MAP2,CAMK1D,FLRT2,PTPRO,INSR,COBL,CRTAC1,CUX1,ANK3,RELN,CDH4,TNR,VAV3,CNTN6,APP,CCDC88A,SPOCK1,PLCE1,PAK1,FRY,EPS8,CECR2,RASGRF1,LRRC4C,ALCAM,PPP1R9A,ADAMTS16,FRYL,TIAM1,PHACTR1,SLC39A12,DISC1,ASAP1,FYN,IFT43,EPHA7,S100B,TOX,PCDH15,CNTN5,RIMS1,EPHB1,ARMC2,SNAP29,HERC1,EXT1,EFNA5,CDC14B,HDAC4,STK36,KLHL1,TRPC5,PPFIA2,TENM3,MARK2,GRID2,ZNF423,LRP2,SEMA6D,NTF3,FER,TTC29,NTNG1,TMEM108,GRM7,RAPGEF2,PRKCQ,KIRREL3,NUDCD3,NRXN3,ATP8A2,UBE3A,APC,AUTS2,EPHB2,PDLIM5,PRTG,CTNND2,PACSIN2,SDCCAG8,NRP1,CDH13,RPGRIP1,DAB1,ALK,SEMA3A,SEMA3E,DCLK1,CDH2,TENM2,TANC1,FSTL4,MTOR,CD2AP,TTC39C,GLI3,NTRK3,HYDIN,DNM3,SYT1,PLXNA2,PTPRD,PRKG1,ELAVL4,MAGI2,ABI1,RALA,DOCK10,PRKD1,HDAC2,TNN,SEMA3D,FAT3,MTMR2,VCL,ATAT1,ROBO2,IFT81,CDH23,NEDD9,RIMS2,SEMA5A,BCL11A,DCC,CTNNA2,CHN1,PARD3,SEMA3C,PCNT
GO:0022008	neurogenesis	2.553501793363751e-23	RTN1,SEMA4D,RPS6KA5,TAOK3,CD38,FBXO31,TRAPPC9,BCL2,THRB,NCAM1,GRIP1,ASTN2,TANC2,CSMD3,NTN4,JAK2,KALRN,USH2A,NEGR1,ZNF536,EPB41L3,LAMA1,CDH11,GRIN2A,NRXN1,IL1RAPL1,WDPCP,LAMA3,ZDHHC17,CTNNA1,MYO9A,NTRK2,NCAM2,TIAM2,MYT1L,CNTN1,NRG3,PTPRG,NUMB,MTPN,SOX6,VCAN,UNC5D,NREP,GABRA5,DOK5,DLG5,ASTN1,SLIT2,ROR1,ADAMTSL1,CRB1,HECW1,ERBB4,KANK1,MACF1,RAP1A,TRIO,ABL2,EML1,CCDC141,CNTN4,ITGA1,TCF12,RUNX1,ALDH1A2,EYA1,PLS1,SLIT3,ROBO1,PAK3,BRINP1,CHODL,SOX5,DSCAM,SDK1,SLC1A1,GRM5,EPHA6,NTN1,IGF1R,NLGN1,CNTNAP2,MAP2,CAMK1D,FLRT2,PTPRO,COBL,CRTAC1,CUX1,ANK3,TBCD,RELN,CDH4,TNR,CNTN6,APP,CCDC88A,SPOCK1,PAK1,HCN1,FRY,TENM4,CECR2,RASGRF1,LRRC4C,ALCAM,PPP1R9A,RARB,TCF4,FRYL,TIAM1,PBX1,PHACTR1,SLC39A12,DISC1,NTM,ASAP1,FYN,EPHA7,NAV2,S100B,TOX,PCDH15,CNTN5,RIMS1,EPHB1,HERC1,EXT1,EFNA5,KLHL1,TRPC5,PPFIA2,TENM3,OPCML,MARK2,GRID2,LRP2,SEMA6D,NTF3,NTNG1,TMEM108,GRM7,RAPGEF2,NAV3,PRKCQ,KIRREL3,PRKCH,NRXN3,ATP8A2,UBE3A,AUTS2,EPHB2,PDLIM5,PRTG,CTNND2,SDCCAG8,NRP1,MDGA2,RP

			GRIP1,DAB1,ALK,SEMA3A,SEMA3E,MYEF2,DCLK1,CDH2,TENM2,HDAC9,FSTL4,MTOR,RORB,GABRB1,GLI3,NTRK3,ZNF521,DNM3,SYT1,NPHP4,PLXNA2,PTPRD,RORA,PRKG1,ELAVL4,MAGI2,ABI1,DOCK10,PRKD1,HDAC2,TNN,SEMA3D,TBX20,FAT3,MTMR2,VCL,ATAT1,ROBO2,CDH23,PCP4,RIMS2,SEMA5A,BCL11A,DCC,CTNNA2,CHN1,ETV6,PARD3,NRG1,SEMA3C
GO:0032990	cell part morphogenesis	4.292831504561165e-23	CD44,SEMA4D,RPS6KA5,TAOK3,FBXO31,BCL2,NCAM1,GRIP1,TANC2,KALRN,EPB41L3,LAMA1,CDH11,NRXN1,IL1RAPL1,LAMA3,ZDHHC17,MYO9A,NTRK2,TIAM2,CNTN1,NUMB,UNC5D,SLIT2,ADAMTSL1,HECW1,KANK1,MACF1,TRIO,CCDC141,CNTN4,ITGA1,SLIT3,ROBO1,PAK3,CHODL,NUBPL,DSCAM,EPHA6,NTN1,IGF1R,NLGN1,CNTNAP2,MAP2,FLRT2,PTPRO,COBL,CUX1,ANK3,RELN,CDH4,TNR,CNTN6,APP,PAK1,LRRC4C,ALCAM,TIAM1,PHACTR1,DISC1,FYN,EPHA7,S100B,CNTN5,RIMS1,EPHB1,EXT1,EFNA5,TRPC5,PPFIA2,MARK2,LRP2,SEMA6D,NTF3,NTNG1,TMEM108,RAPGEF2,PRKCQ,KIRREL3,NRXN3,ATP8A2,UBE3A,AUTS2,EPHB2,PDLIM5,PRTG,CTNND2,PACSIN2,NRP1,DAB1,SEMA3A,SEMA3E,DCLK1,CDH2,FSTL4,GLI3,DNM3,SYT1,PLXNA2,PTPRD,ELAVL4,ABI1,DOCK10,BCL2L1,TNN,SEMA3D,VCL,ROBO2,RIMS2,SEMA5A,BCL11A,DCC,CTNNA2,CHN1,PARD3,SEMA3C
GO:0048858	cell projection morphogenesis	4.4061159722917124e-23	CD44,SEMA4D,RPS6KA5,TAOK3,FBXO31,BCL2,NCAM1,GRIP1,TANC2,KALRN,EPB41L3,LAMA1,CDH11,NRXN1,IL1RAPL1,LAMA3,ZDHHC17,MYO9A,NTRK2,TIAM2,CNTN1,NUMB,UNC5D,SLIT2,ADAMTSL1,HECW1,KANK1,MACF1,TRIO,CCDC141,CNTN4,ITGA1,SLIT3,ROBO1,PAK3,CHODL,DSCAM,EPHA6,NTN1,IGF1R,NLGN1,CNTNAP2,MAP2,FLRT2,PTPRO,COBL,CUX1,ANK3,RELN,CDH4,TNR,CNTN6,APP,PAK1,LRRC4C,ALCAM,TIAM1,PHACTR1,DISC1,FYN,EPHA7,S100B,CNTN5,RIMS1,EPHB1,EXT1,EFNA5,TRPC5,PPFIA2,MARK2,LRP2,SEMA6D,NTF3,NTNG1,TMEM108,RAPGEF2,PRKCQ,KIRREL3,NRXN3,ATP8A2,UBE3A,AUTS2,EPHB2,PDLIM5,PRTG,CTNND2,PACSIN2,NRP1,DAB1,SEMA3A,SEMA3E,DCLK1,CDH2,FSTL4,GLI3,DNM3,SYT1,PLXNA2,PTPRD,ELAVL4,ABI1,DOCK10,TNN,SEMA3D,VCL,ROBO2,RIMS2,SEMA5A,BCL11A,DCC,CTNNA2,CHN1,PARD3,SEMA3C
GO:0050794	regulation of cellular process	4.7838800311472634e-23	CD44,SAMD4A,KCNMA1,C10ORF90,PKNOX2,ZHX3,APBB2,PTPRR,SCAF8,RTN1,ERG,PARN,PDE1C,SEMA4D,INIP,MED13L,EVC,TEAD1,NFIA,SYN3,RPS6KA5,TAOK3,PRKCB,EIF4G3,SLC8A1,ANKRD6,KCNC1,GPR55,DNAJC15,SIAH3,CD38,ZNF257,FBXO31,PIWIL3,TRAPPC9,BCL2,CAMTA1,SAMSN1,CHFR,THADA,COL18A1,TOX3,THRB,DNAH11,ZSCAN5C,RAG1,CDC42EP3,GRIK2,IGSF11,SNX25,UNC13B,MAPK10,NCAM1,KDM4B,GNG12,IL6R,KCNQ5,CDH8,ZBTB20,SCN11A,ATP10A,SORBS2,SKAP2,HS1BP3,KCNJ6,CASP5,KCNK10,BRD4,GPR158,ZNF568,NDRG2,GRIP1,APBA2,TLK1,ASTN2,TANC2,CSMD3,DLGAP1,RCAN1,JAK2,TM9SF4,BICD1,ABCG8,OTUD7A,TPTE2,KALRN,USH2A,NEGR1,FGF12,CACNG2,BTBD9,NFAT5,FLI1,TRHDE,ZNF536,EPB41L3,LAMA1,CDH11,ZBTB7C,GRIN2A,MAST4,NRXN1,ARID1B,IL1RAPL1,WDPCP,MAGI1,LAMA3,ADCYAP1R1,CTDP1,DPP6,GRID1,RPS6KA2,SMOC2,ZDHHC17,KCNH1,DCDC1,CACNA1C,BMF,EXOC4,WDR70,FAM83B,CTNNA1,MYO9A,NTRK2,FOXN3,OCLN,NLK,ITGBL1,RASGEF1B,AFAP1,TIAM2,MYT1L,SRGAP2B,IQCJ-SCHIP1,SMARCA4,RNF152,CNTN1,ZNRF3,MEOX2,ENPEP,PLEKHB2,GLIS3,NRG3,PTPRG,MC2R,BTBD11,ELMO1,SLC24A4,CABIN1,FBXL17,GAS2,GRIK3,CACNB2,PDE10A,NUMB,STXBP4,MED15,MTPN,SOX6,MECOM,PDE4DIP,STK32B,RGS12,SHANK2,RAPGEF5,UBE2E2,KCTD8,UNC5D,ZNF567,NREP,GABRA5,DOK5,DLG5,CFDP1,SMARCAD1,ZIM3,ATRNL1,CHRM3,CPE,ARHGAP24,SPRED2,IGHV1OR15-9,SLIT2,PITPNC1,MYLK3,ABCA5,ROR1,GLP2R,SLC4A4,ZNF675,CSNK2A1,DTNA,AKT3,KMT2C,KCNE4,TRIM5,KCNS3,PSD3,ALPK2,ABCA13,HECW1,RAP1GDS1,AFF3,ERBB4,KANK1,ATRX,DMRT1,BID,MACF1,MNAT1,TAF4B,RAP1A,TRIO,CTNNBL1,RAD51B,PTPRE,DUSP22,CHSY1,MYOM1,PSG8,EXT2,ZSCAN30,OR4C46,ABL2,MAP3K5,NOS1,ABCG1,RGS3,MAML2,RERG,HTR2C,NEK4,CACNA1E,CTIF,CNTN4,TBC1D5,SAMD13,SLC40A1,SLCO3A1,GABRR2,PIK3C3,TRAF3,SND1,CHD6,FGD4,ETS2,ITGA1,TCF12,ZNF721,HIRA,CORO2B,ITGA8,GRIK4,RUNX1,KIR3DL2,ALDH1A2,GABRG1,TSHZ2,MAPK9,ESRRG,PTGFR,IGHV1OR21-

			1,ZNF595,CDH17,PEAK1,EYA1,MORC3,ANKS1B,P2RX6,AKAP10,SPOCK3,FRMD6,PLS1,SPON1,ANK2,PLA2G4A,SLC1A2,ZBTB16,SUPT3H,SLIT3,GRIN2B,ZNF518A,PHC2,ROBO1,ZNF578,EGFLAM,PAK3,DGKB,GARNL3,DPH6,EBF1,TNKS,KLF12,NDFIP2,GABRA2,MDM1,OVOL2,FCHSD2,SGMS1,ITPR2,BRINP1,MLLT3,BCL2L13,LEMD3,KHDRBS2,RNF138,CHODL,GABPA,PRICKLE2,ITGB3BP,CACNA2D3,DGKK,TMEM67,PRKCE,GLIS1,PSG9,ARHGEF11,PRKAA2,PACRG,BBS2,IL1RAPL2,EDIL3,CDYL2,HIVEP2,KCNH8,GRIK1,SOX5,DSCAM,DGKI,RIN3,ANKFN1,DNMBP,EFHB,KDM4C,SLC1A1,GRM5,EPHA6,NTN1,ARAP2,NR5A2,LDB2,IGF1R,SPTB,SNX30,NLGN1,SHISA9,OR9Q1,JAM2,ALX4,CNTNAP2,MAP2,KCNIP4,CFTR,CAMK1D,FLRT2,MLLT10,NOS1AP,PTPRO,RBMS3,INSR,COBL,CTNNAL1,CLEC16A,PHF20L1,ITGA9,MTRF1,CATSPER2,EGLN3,CUX1,ANK3,MORC2,GMDS,CNIH3,DOCK3,TBCD,GPC6,RELN,RASGRF2,TRPS1,STK38,CDH4,TNR,ADCY9,DPP10,CELF4,DAPK1,VAV3,INPP5A,ZNF600,VRK1,ZNF678,CNTN6,CLIC6,ZNF420,APP,FBLN5,PUM1,CCDC88A,ARNT2,KCTD1,SPOCK1,HPSE2,PLCE1,ADAM12,PAK1,GNAL,MITF,IGF2BP3,CACNA2D1,ADCK1,HCN1,PPP1R13B,CHRM5,NSMCE2,ZNF208,CXADR,EPS8,LRFN5,UTRN,GPC5,TENM4,PRR16,TSPAN13,GHR,DUX4,RIPK4,RASGRF1,RIN2,PRDM16,FRMD5,RNF217,USP7,RBFOX1,MEIS2,KIR2DL4,STARD13,SCP2,KL,LRRC4C,ALCAM,PPP1R9A,AVEN,TMEM117,ADAMTS16,TASP1,MICU1,ZZEF1,LTBP1,SLAMF1,RGL1,BACE2,INO80D,CLSTN2,RARB,DIDO1,WDR12,TCF4,TIAM1,PBX1,PHACTR1,MLIP,SORCS2,PRIM2,SLC39A12,DISC1,FMN1,RALGPS1,ARHGAP42,ZFPM2,SVEP1,ASAP1,PCBP3,FRMPD4,EDAR,EGF,PDGFD,FYN,FAM3B,KCND3,EPHA7,FHIT,NSG2,GRIA1,ZNF627,TRABD2B,SPIDR,STK3,CNOT7,MSR1,PSIP1,USP18,S100B,NET1,TOX,ESR1,ARHGAP12,GABRG3,PLCXD3,KCNAB1,GRM1,PDE4D,ERC2,PRKACB,GNG2,PDE3A,RIMS1,POR,L3MBTL4,DOCK4,ATP6V1E1,FRMD4A,MCTP2,WWOX,HUNK,CNKSR2,FUT8,EPHB1,SSBP2,CREM,CTNBP2,FHOD3,EFEMP1,TNRC6B,AJAP1,ABCC9,GSG1L,HERC1,DOCK1,PARP15,FLT1,EXT1,EFNA5,NXN,CDC14B,C14ORF39,HDAC4,ZNF717,STK36,TRPC5,AMFR,PLCB4,ATP9A,FTO,PPFIA2,SH3BP5,AKAP6,SORCS3,POU6F2,TENM3,LINGO2,MARK2,ATF2,ZNF880,RBBP8,GRID2,ZNF423,LRP2,SEMA6D,ZNF573,C2,RALGPS2,NTF3,FER,SNRK,CAMK4,CELF2,TP53I11,NTNG1,MAPRE2,ISX,RAD51AP1,SGCD,TMEM108,RIC8B,GABRB3,TPTE,GRM7,SLC39A8,RAPGEF2,NAV3,MX1,ZNF615,GTF2I,ATXN1,PRKCQ,GABRG2,PRKCH,NRXN3,RHPN2,RABGAP1L,DLC1,NSG1,GABBR2,KCND2,ATP8A2,UBE3A,GRIA4,IDE,APC,ZBTB25,INO80,AUTS2,TFF1,EPHB2,SCAF4,ERC1,ZNF850,PDLIM5,AGO3,MCTP1,MOB3B,RYR3,NBAS,PRTG,NBN,ADAMTS18,RGMB,CTNND2,SETD2,PACSIN2,PKP1,DOCK2,NUP214,SDCCAG8,NRP1,CDH13,RFC3,ZNF879,DACH1,TRDN,SLC2A13,ZNF397,DAB1,RFTN1,ALK,EVC2,LDLRAD4,SEMA3A,SEMA3E,MGAT5,MALRD1,MYEF2,DCLK1,MAGI3,KIF16B,NRIP1,CDH2,ARID5B,SIPA1L2,CCNG2,RCAN2,LRRC69,TENM2,TANC1,SERPINB7,VPS41,SYCP1,ZNF407,ASB3,HDAC9,PIK3R3,MAP2K6,FSTL4,ARHGAP28,MTOR,STK38L,KSR1,RALGAPA2,RORB,GABRB1,FBLN1,ST8SIA1,BLM,SH3KBP1,FHL2,CADPS,NEU3,NCAPG2,RGS7,STK32A,CD2AP,ZFP30,USP25,SPRED1,SIPA1L3,ADAM10,DRAM1,KANSL1,LRFN2,FLNB,SCAI,PAPPA2,PTPRT,TRERF1,SLC24A2,GLI3,NTRK3,RXFP1,FBN1,RAB31,CTNNA3,VPS13D,ABHD17C,ZNF292,TBX15,RAPGEF4,BMPER,ANKRD31,ZNF521,PDE1A,ATF7IP,HMGA2,MX2,CREB5,NSUN2,DEFA3,PTPRK,SORCS1,TBC1D4,DNM3,SYT1,APIP,SYNDIG1,ASXL3,DPF3,NPHP4,DOCK9,PPP1R12B,SACS,PPARA,PLXNA2,PTPRD,RORA,SHISA6,PLCB1,LOXL2,BPTF,PRKG1,RASGRP1,ELAVL4,NLRC5,STXBP6,MXI1,TTC28,MAGI2,NELL1,PLCL1,ABI1,TSHZ3,ASIC2,RALA,DOCK10,TRPM1,CACNG3,NECAB1,PRKD1,ATP8A1,TNFAIP8,BCL2L1,HDAC2,ETS1,MRPS27,TNN,RYR2,SEMA3D,BANP,TGFA,PRLR,TBX20,PTPRA,FAT3,OR11G2,MTMR2,KCNH5,ATF6,IL16,VCL,DEPTOR,BACH1,ATAT1,ROBO2,EWSR1,IFT81,ZMYND11,RGS6,SRGAP3,AKAP13,WDR41,NEDD9,MYRIP,ENPP1,UNC13C,PCP4,RIMS2,STAC,SCN8A,RAB27A,EYA4,RALGAPA1,L3MBTL3,DLGAP2,POMT2,HIVEP3,CLIP1,SEMA5A,CABLES1,PRDM15,OR4N2,BCL11A,DCC,ZNF112,CTNNA2,CHN1,ETV6,VPS13C,KCNJ15,PARD3,NRG1,CAST,FANK1,ZNF845,NPAS3,PRKCA,FMN2,SEMA3C,FANCB,CSF2RB,PC

			NT,ST18
GO:0120039	plasma membrane bounded cell projection morphogenesis	8.206233043717653e-23	CD44,SEMA4D,RPS6KA5,TAOK3,FBXO31,BCL2,NCAM1,GRIP1,TANC2,KALRN,EPB41L3,LAMA1,CDH11,NRXN1,IL1RAPL1,LAMA3,ZDHHC17,MYO9A,NTRK2,TIAM2,CNTN1,NUMB,UNC5D,SLIT2,ADAMTSL1,HECW1,KANK1,MACF1,TRIO,CCDC141,CNTN4,ITGA1,SLIT3,ROBO1,PAK3,CHODL,DSCAM,EPHA6,NTN1,IGF1R,NLGN1,CNTNAP2,MAP2,FLRT2,PTPRO,COBL,CUX1,ANK3,RELN,CDH4,TNR,CNTN6,APP,PAK1,LRRC4C,ALCAM,TIAM1,PHACTR1,DISC1,FYN,EPHA7,S100B,CNTN5,RIMS1,EPHB1,EXT1,EFNA5,TRPC5,PPFIA2,MARK2,LRP2,SEMA6D,NTF3,NTNG1,TMEM108,RAPGEF2,PRKCQ,KIRREL3,NRXN3,ATP8A2,UBE3A,AUTS2,EPHB2,PDLIM5,PRTG,CTNND2,NRP1,DAB1,SEMA3A,SEMA3E,DCLK1,CDH2,FSTL4,GLI3,DNM3,SYT1,PLXNA2,PTPRD,ELAVL4,ABI1,DOCK10,TNN,SEMA3D,VCL,ROBO2,RIMS2,SEMA5A,BCL11A,DCC,CTNNA2,CHN1,PARD3,SEMA3C
GO:0032501	multicellular organismal process	1.4311349599532275e-22	CD44,KCNMA1,ZHX3,APBB2,PTPRR,RTN1,PARN,SEMA4D,IGSF3,EVC,TEAD1,NFIA,RPS6KA5,TAOK3,PRKCB,A2M,SLC8A1,KCNC1,GPR55,SIAH3,CD38,FBXO31,PIWIL3,TRAPPC9,BCL2,MMP16,CAMTA1,COL18A1,THRB,DNAH11,RAG1,GRIK2,IGSF11,UNC13B,NCAM1,KDM4B,IL6R,ZBTB20,SCN11A,SORBS2,CASP5,KCNK10,ZNF568,NDRG2,GRIP1,APBA2,ASTN2,TANC2,CSMD3,DLGAP1,RCAN1,NTN4,JAK2,ABCG8,KALRN,USH2A,NEGR1,FGF12,CACNG2,BTBD9,FLI1,MEGF11,TRHDE,ZNF536,EPB41L3,LAMA1,CDH11,GRIN2A,NRXN1,ARID1B,IL1RAPL1,WDPCP,LAMA3,ADCYAP1R1,CTDP1,ADAMTS6,GRID1,RPS6KA2,SMOC2,ZDHHC17,KCNH1,CACNA1C,EXOC4,BBS9,CTNNA1,MYO9A,NTRK2,FOXN3,OCLN,AK8,THSD7A,NCAM2,TIAM2,MYT1L,TMPRSS3,SRGAP2B,SMARCA4,CNTN1,ZNRF3,MEOX2,ENPEP,NRG3,PTPRG,SLC24A4,SYNE1,FBXL17,GAS2,CACNB2,NUMB,MED15,MTPN,SOX6,MECOM,SYBU,TRPM3,VCAN,NHS,SHANK2,RAPGEF5,UNC5D,NREP,GABRA5,DOK5,DLG5,ASTN1,CHRM3,CPE,CALD1,ARHGAP24,SPRED2,SLIT2,MYLK3,ABCA5,ROR1,SLC4A4,ADAMTSL1,KAZN,ZNF675,DTNA,AKT3,CRB1,KCNE4,ALPK2,HECW1,RAP1GDS1,AFF3,LCE1F,ERBB4,KANK1,ATRX,DMRT1,CHST8,MACF1,MNAT1,TAF4B,RAP1A,TRIO,CTNNBL1,RAD51B,MYO3B,CHSY1,MYOM1,EXT2,OR4C46,ABL2,NOS1,ACACA,ABCG1,SPAG16,EML1,HTR2C,CCDC141,CNTN4,RNF17,SLC40A1,SLCO3A1,GABRR2,SLC9C1,TRAF3,SND1,HMCN1,ETS2,ITGA1,TCF12,HIRA,CORO2B,ITGA8,RBM19,RUNX1,ALDH1A2,GABRG1,ESRRG,PTGFR,CDH17,EYA1,KIAA1217,MORC3,P2RX6,HSF2BP,PLS1,ANK2,PLA2G4A,SLC1A2,ZBTB16,SLIT3,GRIN2B,PHC2,ROBO1,ANKRD11,PAK3,DGKB,GABRA2,MDM1,SMPX,OVOL2,BRINP1,MLLT3,CHODL,GABPA,DGKK,PRKCE,GLIS1,PSG9,ARHGEF11,PRKAA2,PACRG,BBS2,IL1RAPL2,MYOM2,MYO3A,GRIK1,SOX5,DSCAM,DGKI,ANKFN1,KDM4C,SDK1,SLC1A1,GRM5,EPHA6,NTN1,CA10,NR5A2,LDB2,IGF1R,NLGN1,DNAH9,SHISA9,OR9Q1,SHROOM3,JAM2,ALX4,CORIN,CNTNAP2,MAP2,CFTR,CAMK1D,FLRT2,NOS1AP,PTPRO,CD96,INSR,COBL,CATSPER2,CRTAC1,CUX1,ANK3,LHFPL3,TBCD,GPC6,RELN,TRPS1,ADAMTS5,FBXO32,MYO18B,CDH4,TNR,ADCY9,OCA2,CELF4,VAV3,CNTN6,APP,PUM1,CCDC88A,ARNT2,RNLS,SPOCK1,PLCE1,TACC2,ADAM12,PAK1,GNAL,MITF,IGF2BP3,CACNA2D1,HCN1,CHRM5,FRY,CXADR,EPS8,LRFN5,UTRN,TENM4,CECR2,GHR,RASGRF1,RIN2,PRDM16,RBFOX1,MEIS2,KIR2DL4,STARD13,KL,LRRC4C,ALCAM,PPP1R9A,ADAMTS16,LTBP1,SLAMF1,INO80D,CLSTN2,NEBL,RARB,MYH13,TCF4,FRYL,TIAM1,PBX1,PHACTR1,MLIP,SLC39A12,DISC1,FMN1,ARHGAP42,ZFPM2,PIEZO2,VSTM4,SVEP1,NTM,VTI1A,ASAP1,EDAR,EGF,PDGFD,FYN,KCND3,XRCC4,EPHA7,GRIA1,NAV2,STK3,COL19A1,MSR1,AP2B1,S100B,TOX,PCDH15,ESR1,GABRG3,SGCZ,GRM1,PDE4D,CNTN5,LRIG1,PRKACB,PDE3A,RIMS1,POR,DOCK4,CERS3,WWOX,PCSK2,FUT8,EPHB1,CREM,LSAMP,CTTNBP2,FHOD3,GREB1L,EFEMP1,ARMC2,AJAP1,IGSF21,ABCC9,HERC1,DOCK1,FAM126A,FLT1,EXT1,EFNA5,NXN,C14ORF39,HDAC4,STK36,KLHL1,TRPC5,AMFR,FTO,PPFIA2,ADAM28,AKAP6,ACSBG1,SORCS3,POU6F2,TENM3,LINGO2,OPCML,MARK2,ATF2,TUSC3,RBBP8,GRID2,ZNF423,LRP2,SEMA6D,NTF3,FER,SNRK,CAMK4,CELF2,NTNG1,MAPRE2,ISX,SGCD,TMEM108,GABRB3,GRM7,SLC39A8,RAPGEF2,NAV3,IMMP2L,GTF2I,ATXN1,PRKCQ,SSPN,KIRREL3,GABRG2,CPS1,PRKCH,NRXN3,RHPN2,KRT25

			,DLC1,KCND2,ATP8A2,SNTB1,SLC24A3,UBE3A,IDE,LOXHD1,APC,MACROD2,TTLL5,INO80,AUTS2,TFF1,EPHB2,PDLIM5,XYLT1,PRTG,NBN,ADAMTS18,CTNND2,COL22A1,SETD2,DOCK2,SDCCAG8,FLVCR1,NRP1,CDH13,MDGA2,RPGRIP1,DACH1,TRDN,SLC2A13,DAB1,RFTN1,ALK,LDLRAD4,SEMA3A,SEMA3E,MYEF2,DCLK1,KIF16B,NRIP1,CDH2,ARID5B,TENM2,TANC1,PAPPA,SERPINB7,SYCP1,ASB3,HDAC9,PIK3R3,MAP2K6,FSTL4,MTOR,RORB,GABRB1,FBLN1,FHL2,NCAPG2,RGS7,CD2AP,TTC39C,SLC44A1,SPRED1,SIPA1L3,ADAM10,GALC,PAPPA2,ABCB5,SPECC1,DPY19L2,CSMD1,SLC24A2,GLI3,NTRK3,RXFP1,FBN1,SGCG,HYDIN,CTNNA3,TBX15,BMPER,ZNF521,HMGA2,NSUN2,DNM3,SYT1,SYNDIG1,DPF3,SCAPER,NPHP4,PPP1R12B,PPARA,PLXNA2,PTPRD,RORA,MYH15,SHISA6,PLCB1,LOXL2,BPTF,PRKG1,RASGRP1,ELAVL4,MAGI2,NELL1,PLCL1,ABI1,TSHZ3,ASIC2,RALA,DOCK10,TRPM1,CACNG3,FNDC3A,NECAB1,PRKD1,ATP8A1,BCL2L1,HDAC2,ETS1,TNN,RYR2,SEMA3D,TGFA,PRLR,TBX20,FAT3,OR11G2,MTMR2,ATF6,EYS,IL16,VCL,ATAT1,ROBO2,IFT81,CDH23,AKAP13,NEDD9,ENPP1,PCP4,RIMS2,SLC5A1,STAC,SCN8A,RAB27A,EYA4,L3MBTL3,DLGAP2,SEMA5A,CABLES1,OR4N2,BCL11A,FREM1,DCC,CTNNA2,CHN1,ETV6,PARD3,NRG1,PRKCA,FMN2,SEMA3C,CSF2RB,TOP1
GO:0048812	neuron projection morphogenesis	1.4361700009989167e-22	SEMA4D,RPS6KA5,TAOK3,FBXO31,BCL2,NCAM1,GRIP1,TANC2,KALRN,EPB41L3,LAMA1,CDH11,NRXN1,IL1RAPL1,LAMA3,ZDHHC17,MYO9A,NTRK2,TIAM2,CNTN1,NUMB,UNC5D,SLIT2,ADAMTSL1,HECW1,MACF1,TRIO,CCDC141,CNTN4,ITGA1,SLIT3,ROBO1,PAK3,CHODL,DSCAM,EPHA6,NTN1,IGF1R,NLGN1,CNTNAP2,MAP2,FLRT2,PTPRO,COBL,CUX1,ANK3,RELN,CDH4,TNR,CNTN6,APP,PAK1,LRRC4C,ALCAM,TIAM1,PHACTR1,DISC1,FYN,EPHA7,S100B,CNTN5,RIMS1,EPHB1,EXT1,EFNA5,TRPC5,PPFIA2,MARK2,LRP2,SEMA6D,NTF3,NTNG1,TMEM108,RAPGEF2,PRKCQ,KIRREL3,NRXN3,ATP8A2,UBE3A,AUTS2,EPHB2,PDLIM5,PRTG,CTNND2,NRP1,DAB1,SEMA3A,SEMA3E,DCLK1,CDH2,FSTL4,GLI3,DNM3,SYT1,PLXNA2,PTPRD,ELAVL4,ABI1,DOCK10,TNN,SEMA3D,VCL,ROBO2,RIMS2,SEMA5A,BCL11A,DCC,CTNNA2,CHN1,PARD3,SEMA3C
GO:0050808	synapse organization	1.625415630662284e-22	APBB2,SEMA4D,NFIA,UNC13B,CDH8,TANC2,KALRN,NEGR1,CACNG2,NRXN1,IL1RAPL1,NTRK2,CACNB2,SYBU,SHANK2,DLG5,ERBB4,GPHN,GRIN2B,PAK3,DGKB,GABRA2,IL1RAPL2,DSCAM,SDK1,SLC1A1,GRM5,NTN1,IGF1R,NLGN1,FLRT2,NOS1AP,PTPRO,INSR,ANK3,GPC6,RELN,TNR,APP,LRFN5,LRRC4C,PDZRN3,CLSTN2,DISC1,FRMPD4,FYN,EPHA7,CNTN5,ERC2,CNKSR2,EPHB1,CTTNBP2,IGSF21,EFNA5,PPFIA2,LINGO2,GRID2,NTNG1,TMEM108,GABRB3,KIRREL3,GABRG2,NRXN3,UBE3A,EPHB2,ERC1,PDLIM5,CTNND2,NRP1,SEMA3E,CDH2,TANC1,ADAM10,LRFN2,NTRK3,ABHD17C,DNM3,SYNDIG1,PTPRD,SHISA6,ASIC2,DOCK10,MTMR2,ROBO2,NEDD9,UNC13C,CTNNA2,NRG1,CAST
GO:0032989	cellular component morphogenesis	1.6423457858392527e-22	CD44,SEMA4D,RPS6KA5,TAOK3,FBXO31,BCL2,NCAM1,GRIP1,TANC2,KALRN,EPB41L3,LAMA1,CDH11,NRXN1,IL1RAPL1,LAMA3,ZDHHC17,MYO9A,NTRK2,TIAM2,CNTN1,NUMB,UNC5D,PGM5,SLIT2,MYLK3,ADAMTSL1,HECW1,KANK1,MACF1,TRIO,CCDC141,CNTN4,ITGA1,ANK2,SLIT3,ROBO1,PAK3,CHODL,MYOM2,NUBPL,DSCAM,EPHA6,NTN1,IGF1R,NLGN1,CNTNAP2,MAP2,FLRT2,PTPRO,COBL,CUX1,ANK3,RELN,CDH4,TNR,CNTN6,APP,PAK1,TENM4,LRRC4C,ALCAM,NEBL,TIAM1,PHACTR1,DISC1,FYN,EPHA7,S100B,CNTN5,RIMS1,EPHB1,FHOD3,EXT1,EFNA5,TRPC5,PPFIA2,MARK2,LRP2,SEMA6D,NTF3,NTNG1,TMEM108,RAPGEF2,PRKCQ,KIRREL3,NRXN3,ATP8A2,UBE3A,AUTS2,EPHB2,PDLIM5,PRTG,CTNND2,PACSIN2,NRP1,DAB1,SEMA3A,SEMA3E,DCLK1,CDH2,FSTL4,GLI3,DNM3,SYT1,PLXNA2,PTPRD,ELAVL4,ABI1,DOCK10,BCL2L1,TNN,SEMA3D,MTMR2,VCL,ROBO2,AKAP13,RIMS2,SEMA5A,BCL11A,DCC,CTNNA2,CHN1,PARD3,SEMA3C
GO:0000904	cell morphogenesis involved	8.033467917334814e-22	SEMA4D,RPS6KA5,FBXO31,BCL2,COL18A1,NCAM1,TANC2,NTN4,KALRN,USH2A,LAMA1,PARVB,CDH11,NRXN1,IL1RAPL1,WDPCP,LAMA3,ZDHHC17,NTRK2,TIAM2,CNTN1,NUMB,UNC5D,ATRNL1,SLIT2,ADAMTSL1,HECW1,KANK1,MACF1,TRIO,CCDC141,CNTN4,ITGA8,PEAK1,FRMD6,PLS1,SLIT3,ROBO1,PAK3,CHODL,DSCAM,EPHA6,NTN1,IGF1R,NLGN1,MAP2,FLRT2,PTPRO,COBL,CUX1,ANK3,TBCD,RELN,CDH4,TNR,CNTN6,APP,PAK1,LRRC4C,ALCAM,TIAM1,PHACTR

	in differentiation		1,DISC1,FYN,EPHA7,S100B,PCDH15,CNTN5,EPHB1,DOCK1,EXT1,EFNA5,TRPC5,PPFIA2,MARK2,SEMA6D,FER,NTNG1,RAPGEF2,PRKCQ,NRXN3,ATP8A2,UBE3A,AUTS2,EPHB2,PDLIM5,PRTG,CTNND2,COL22A1,NRP1,DAB1,SEMA3A,SEMA3E,DCLK1,CDH2,FSTL4,FBLN1,SIPA1L3,FLNB,GLI3,DNM3,PLXNA2,PTPRD,ELAVL4,ABI1,DOCK10,TNN,SEMA3D,FAT3,VCL,ROBO2,CDH23,NEDD9,SEMA5A,BCL11A,DCC,CTNNA2,CHN1,PARD3,SEMA3C
GO:0048667	cell morphogenesis involved in neuron differentiation	1.4639044267434077e-21	SEMA4D,RPS6KA5,FBXO31,BCL2,NCAM1,TANC2,KALRN,LAMA1,CDH11,NRXN1,IL1RAPL1,WDPCP,LAMA3,ZDHHC17,NTRK2,TIAM2,CNTN1,NUMB,UNC5D,SLIT2,ADAMTSL1,HECW1,MACF1,TRIO,CCDC141,CNTN4,PLS1,SLIT3,ROBO1,PAK3,CHODL,DSCAM,EPHA6,NTN1,IGF1R,NLGN1,MAP2,FLRT2,PTPRO,COBL,CUX1,ANK3,TBCD,RELN,CDH4,TNR,CNTN6,APP,PAK1,LRRC4C,ALCAM,TIAM1,PHACTR1,DISC1,FYN,EPHA7,S100B,PCDH15,CNTN5,EPHB1,EXT1,EFNA5,TRPC5,PPFIA2,MARK2,SEMA6D,NTNG1,RAPGEF2,PRKCQ,NRXN3,ATP8A2,UBE3A,AUTS2,EPHB2,PDLIM5,PRTG,CTNND2,NRP1,DAB1,SEMA3A,SEMA3E,DCLK1,CDH2,FSTL4,GLI3,DNM3,PLXNA2,PTPRD,ELAVL4,ABI1,DOCK10,TNN,SEMA3D,VCL,ROBO2,CDH23,SEMA5A,BCL11A,DCC,CTNNA2,CHN1,PARD3,SEMA3C
GO:0099537	trans-synaptic signaling	5.649611262235817e-21	SYN3,PRKCB,CD38,GRIK2,IGSF11,UNC13B,CDH8,APBA2,DLGAP1,JAK2,FGF12,CACNG2,BTBD9,CDH11,GRIN2A,NRXN1,IL1RAPL1,GRID1,RPS6KA2,AMPH,EXOC4,NTRK2,NRG3,GRIK3,CACNB2,SHANK2,GABRA5,CHRM3,DTNA,RAP1A,NOS1,HTR2C,CACNA1E,CNTN4,GABRR2,GRIK4,GABRG1,SV2B,P2RX6,SLC1A2,GRIN2B,DGKB,GABRA2,FCHSD2,PRKCE,PTPRN2,GRIK1,DGKI,SLC1A1,GRM5,NLGN1,SHISA9,SV2C,RELN,RASGRF2,TNR,CELF4,APP,HCN1,CHRM5,RASGRF1,LRRC4C,PPP1R9A,ZZEF1,CLSTN2,SORCS2,DISC1,FYN,RIMBP2,GRIA1,S100B,GABRG3,GRM1,ERC2,RIMS1,MCTP2,EPHB1,SNAP29,EXT1,PLCB4,SORCS3,GRID2,NTF3,NTNG1,TMEM108,GABRB3,GRM7,RAPGEF2,GABRG2,NRXN3,NSG1,GABBR2,KCND2,EPHB2,ERC1,MCTP1,PACSIN2,CDH2,GABRB1,CADPS,LRFN2,SLC24A2,SYT1,DLG2,PTPRD,SHISA6,PLCB1,ELAVL4,PLCL1,TSHZ3,ASIC2,CACNG3,PTPRA,MTMR2,UNC13C,RIMS2,DLGAP2,DCC
GO:0007268	chemical synaptic transmission	2.4160510443437632e-20	SYN3,PRKCB,CD38,GRIK2,IGSF11,UNC13B,CDH8,APBA2,DLGAP1,JAK2,FGF12,CACNG2,BTBD9,CDH11,GRIN2A,NRXN1,GRID1,RPS6KA2,AMPH,EXOC4,NTRK2,NRG3,GRIK3,CACNB2,SHANK2,GABRA5,CHRM3,DTNA,RAP1A,HTR2C,CACNA1E,CNTN4,GABRR2,GRIK4,GABRG1,SV2B,P2RX6,SLC1A2,GRIN2B,DGKB,GABRA2,FCHSD2,PRKCE,PTPRN2,GRIK1,DGKI,SLC1A1,GRM5,NLGN1,SHISA9,SV2C,RELN,RASGRF2,TNR,CELF4,APP,HCN1,CHRM5,RASGRF1,LRRC4C,PPP1R9A,ZZEF1,CLSTN2,SORCS2,DISC1,FYN,RIMBP2,GRIA1,S100B,GABRG3,GRM1,ERC2,RIMS1,MCTP2,EPHB1,SNAP29,EXT1,PLCB4,SORCS3,GRID2,NTF3,NTNG1,TMEM108,GABRB3,GRM7,RAPGEF2,GABRG2,NRXN3,NSG1,GABBR2,KCND2,EPHB2,ERC1,MCTP1,PACSIN2,CDH2,GABRB1,CADPS,LRFN2,SLC24A2,SYT1,DLG2,PTPRD,SHISA6,PLCB1,ELAVL4,PLCL1,TSHZ3,ASIC2,CACNG3,PTPRA,MTMR2,UNC13C,RIMS2,DLGAP2,DCC
GO:0098916	anterograde trans-synaptic signaling	2.4160510443437632e-20	SYN3,PRKCB,CD38,GRIK2,IGSF11,UNC13B,CDH8,APBA2,DLGAP1,JAK2,FGF12,CACNG2,BTBD9,CDH11,GRIN2A,NRXN1,GRID1,RPS6KA2,AMPH,EXOC4,NTRK2,NRG3,GRIK3,CACNB2,SHANK2,GABRA5,CHRM3,DTNA,RAP1A,HTR2C,CACNA1E,CNTN4,GABRR2,GRIK4,GABRG1,SV2B,P2RX6,SLC1A2,GRIN2B,DGKB,GABRA2,FCHSD2,PRKCE,PTPRN2,GRIK1,DGKI,SLC1A1,GRM5,NLGN1,SHISA9,SV2C,RELN,RASGRF2,TNR,CELF4,APP,HCN1,CHRM5,RASGRF1,LRRC4C,PPP1R9A,ZZEF1,CLSTN2,SORCS2,DISC1,FYN,RIMBP2,GRIA1,S100B,GABRG3,GRM1,ERC2,RIMS1,MCTP2,EPHB1,SNAP29,EXT1,PLCB4,SORCS3,GRID2,NTF3,NTNG1,TMEM108,GABRB3,GRM7,RAPGEF2,GABRG2,NRXN3,NSG1,GABBR2,KCND2,EPHB2,ERC1,MCTP1,PACSIN2,CDH2,GABRB1,CADPS,LRFN2,SLC24A2,SYT1,DLG2,PTPRD,SHISA6,PLCB1,ELAVL4,PLCL1,TSHZ3,ASIC2,CACNG3,PTPRA,MTMR2,UNC13C,RIMS2,DLGAP2,DCC
GO:0099536	synaptic	2.934312467524761e-20	SYN3,PRKCB,CD38,GRIK2,IGSF11,UNC13B,CDH8,APBA2,DLGAP1,JAK2,FGF12,CACNG2,BTBD9,CDH11,GRIN2A,NRXN1,IL1RAPL1,GRID1,RPS6KA2,AMPH,EXOC4,NTRK2,NRG3,GRIK3,CACNB2,SHAN

	signaling		K2,GABRA5,CHRM3,DTNA,RAP1A,NOS1,HTR2C,CACNA1E,CNTN4,GABRR2,GRIK4,GABRG1,SV2B,P2RX6,SLC1A2,GRIN2B,DGKB,GABRA2,FCHSD2,PRKCE,PTPRN2,GRIK1,DGKI,SLC1A1,GRM5,NLGN1,SHISA9,SV2C,RELN,RASGRF2,TNR,CELF4,APP,HCN1,CHRM5,RASGRF1,LRRC4C,PPP1R9A,ZZEF1,CLSTN2,SORCS2,DISC1,FYN,RIMBP2,GRIA1,S100B,GABRG3,GRM1,ERC2,RIMS1,MCTP2,EPHB1,SNAP29,EXT1,PLCB4,SORCS3,GRID2,NTF3,NTNG1,TMEM108,GABRB3,GRM7,RAPGEF2,GABRG2,NRXN3,NSG1,GABBR2,KCND2,EPHB2,ERC1,MCTP1,PACSIN2,CDH2,GABRB1,CADPS,LRFN2,SLC24A2,SYT1,DLG2,PTPRD,SHISA6,PLCB1,ELAVL4,PLCL1,TSHZ3,ASIC2,CACNG3,PTPRA,MTMR2,UNC13C,RIMS2,DLGAP2,DCC,NRG1
GO:0007155	cell adhesion	1.9948467750521133e-19	CD44,SEMA4D,BCL2,COL18A1,RAG1,IGSF11,NCAM1,CDH8,ASTN2,NTN4,JAK2,TM9SF4,USH2A,NEGR1,NFAT5,MEGF11,LAMA1,PARVB,CDH11,NRXN1,ARID1B,IL1RAPL1,WDPCP,MAGI1,LAMA3,CTNNA1,ITGBL1,NCAM2,SMARCA4,CNTN1,PDZD2,VCAN,CNTNAP5,UNC5D,DLG5,CFDP1,PGM5,ASTN1,ATRNL1,ADAMTSL1,CRB1,KANK1,LPP,MACF1,DUSP22,ABL2,CCDC141,CNTN4,MUC16,HMCN1,ITGA1,CORO2B,ITGA8,RUNX1,CDH17,PEAK1,CDH18,SPON1,ZBTB16,ROBO1,EGFLAM,IGSF5,ITGB3BP,PRKCE,CNTN3,EDIL3,DSCAM,SDK1,NTN1,NLGN1,JAM2,CNTNAP2,FLRT2,PTPRO,PCDH9,CD96,CTNNAL1,ITGA9,ANK3,CDH12,TBCD,GPC6,RELN,CDH4,TNR,VAV3,CNTN6,APP,FBLN5,SPOCK1,ADAM12,CXADR,LRFN5,UTRN,TENM4,RIN2,FRMD5,PCDH7,LRRC4C,ALCAM,SLAMF1,CLSTN2,TIAM1,DISC1,FMN1,SVEP1,NTM,FYN,EPHA7,COL19A1,PCDH15,CNTN5,PCDH11X,EPHB1,LSAMP,AJAP1,IGSF21,DOCK1,TRPM7,EXT1,EFNA5,TLN2,PPFIA2,TENM3,OPCML,GRID2,FER,NTNG1,SLC39A8,PRKCQ,SSPN,KIRREL3,NRXN3,DLC1,APC,EPHB2,PDLIM5,PRTG,ADAMTS18,RGMB,CTNND2,PKP1,NRP1,CDH13,DAB1,SEMA3E,CADM2,CDH2,TENM2,FBLN1,CD2AP,ADAM10,PTPRT,GLI3,FBN1,CTNNA3,PTPRK,NPHP4,DLG2,PPARA,PLXNA2,PTPRD,LOXL2,PRKG1,RASGRP1,CDH9,STXBP6,CNTNAP3,FNDC3A,ETS1,TNN,PRLR,PTPRA,FAT3,VCL,ROBO2,CDH23,NEDD9,SEMA5A,FREM1,DCC,CTNNA2,PARD3B,PARD3,NRG1,PRKCA
GO:0023052	signaling	6.7218962948901985e-19	CD44,APBB2,PTPRR,ERG,PDE1C,SEMA4D,INIP,EVC,TEAD1,SYN3,RPS6KA5,TAOK3,PRKCB,SLC8A1,ANKRD6,GPR55,CD38,FBXO31,BCL2,CAMTA1,CHFR,THRB,CDC42EP3,GRIK2,IGSF11,SNX25,UNC13B,MAPK10,NCAM1,GNG12,IL6R,CDH8,SCN11A,SORBS2,SKAP2,CASP5,KCNK10,BRD4,GPR158,NDRG2,GRIP1,APBA2,TLK1,DLGAP1,RCAN1,NTN4,JAK2,BICD1,ABCG8,OTUD7A,TPTE2,KALRN,FGF12,CACNG2,BTBD9,NFAT5,TRHDE,ZNF536,LAMA1,CDH11,GRIN2A,MAST4,NRXN1,IL1RAPL1,WDPCP,MAGI1,LAMA3,ADCYAP1R1,GRID1,RPS6KA2,SMOC2,ZDHHC17,KCNH1,DCDC1,CACNA1C,AMPH,BMF,EXOC4,FAM83B,CTNNA1,MYO9A,NTRK2,FOXN3,NLK,ITGBL1,RASGEF1B,AFAP1,TIAM2,IQCJ-SCHIP1,SMARCA4,RNF152,CNTN1,ZNRF3,ENPEP,NRG3,PTPRG,MC2R,BTBD11,ELMO1,SLC24A4,CABIN1,FBXL17,GAS2,GRIK3,CACNB2,PDE10A,STXBP4,MECOM,STK32B,RGS12,SHANK2,RAPGEF5,KCTD8,UNC5D,NREP,GABRA5,DOK5,DLG5,ATRNL1,CHRM3,CPE,ARHGAP24,SPRED2,IGHV1OR15-9,SLIT2,PITPNC1,ROR1,GLP2R,ZNF675,CSNK2A1,DTNA,AKT3,CRB1,KCNE4,TRIM5,PSD3,ALPK2,HECW1,RAP1GDS1,ERBB4,KANK1,ATRX,DMRT1,BID,MACF1,RAP1A,TRIO,PTPRE,DUSP22,CHSY1,MYOM1,PSG8,EXT2,OR4C46,ABL2,MAP3K5,NOS1,RGS3,MAML2,RERG,HTR2C,CACNA1E,CNTN4,GABRR2,PIK3C3,TRAF3,FGD4,ITGA1,ITGA8,GRIK4,ALDH1A2,GABRG1,MAPK9,ESRRG,PTGFR,IGHV1OR21-1,CDH17,SV2B,EYA1,ANKS1B,P2RX6,AKAP10,ANK2,PLA2G4A,SLC1A2,SLIT3,GRIN2B,ROBO1,PAK3,DGKB,GARNL3,TNKS,NDFIP2,GABRA2,OVOL2,FCHSD2,SGMS1,ITPR2,MLLT3,LEMD3,RNF138,PRICKLE2,ITGB3BP,DGKK,PRKCE,PSG9,ARHGEF11,PRKAA2,BBS2,IL1RAPL2,PTPRN2,GRIK1,DSCAM,DGKI,RIN3,DNMBP,EFHB,KDM4C,SLC1A1,GRM5,EPHA6,NTN1,ARAP2,NR5A2,IGF1R,NLGN1,SHISA9,OR9Q1,CORIN,CNTNAP2,CFTR,FLRT2,NOS1AP,PTPRO,RBMS3,INSR,CTNNAL1,CLEC16A,ITGA9,ANK3,SV2C,GMDS,CNIH3,DOCK3,GPC6,RELN,RASGRF2,STK38,TNR,ADCY9,CELF4,DAPK1,VAV3,INPP5A,VRK1,CNTN6,APP,PUM1,CCDC88A,PLCE1,ADAM12,PAK1,GN

			AL,MITF,CACNA2D1,HCN1,PPP1R13B,CHRM5,CXADR,EPS8,GPC5,TENM4,GHR,RASGRF1,RIN2,PRDM16,USP7,KIR2DL4,STARD13,KL,LRRC4C,ALCAM,PPP1R9A,TMEM117,ZZEF1,LTBP1,SLAMF1,RGL1,CLSTN2,RARB,DIDO1,WDR12,TIAM1,SORCS2,SLC39A12,DISC1,RALGPS1,ARHGAP42,SVEP1,EDAR,EGF,PDGFD,FYN,FAM3B,KCND3,RIMBP2,EPHA7,FHIT,NSG2,GRIA1,TRABD2B,STK3,CNOT7,USP18,S100B,NET1,ESR1,ARHGAP12,GABRG3,PLCXD3,GRM1,PDE4D,ERC2,PRKACB,GNG2,PDE3A,RIMS1,POR,DOCK4,MCTP2,WWOX,HUNK,CNKSR2,FUT8,EPHB1,CREM,EFEMP1,ABCC9,SNAP29,GSG1L,DOCK1,FLT1,EXT1,EFNA5,NXN,CDC14B,HDAC4,STK36,AMFR,PLCB4,SH3BP5,AKAP6,SORCS3,TENM3,MARK2,ATF2,RBBP8,GRID2,ZNF423,LRP2,SEMA6D,RALGPS2,NTF3,FER,SNRK,CAMK4,NTNG1,MAPRE2,SGCD,TMEM108,RIC8B,GABRB3,TPTE,GRM7,RAPGEF2,MX1,PRKCQ,GABRG2,PRKCH,NRXN3,RHPN2,DLC1,NSG1,GABBR2,KCND2,UBE3A,GRIA4,IDE,APC,AUTS2,TFF1,EPHB2,ERC1,AGO3,MCTP1,MOB3B,NBN,ADAMTS18,RGMB,CTNND2,PACSIN2,PKP1,DOCK2,NRP1,CDH13,TRDN,DAB1,RFTN1,ALK,EVC2,LDLRAD4,SEMA3A,SEMA3E,MGAT5,DCLK1,MAGI3,KIF16B,CDH2,ARID5B,SIPA1L2,RCAN2,LRRC69,TENM2,ASB3,PIK3R3,MAP2K6,FSTL4,ARHGAP28,MTOR,STK38L,KSR1,RALGAPA2,RORB,GABRB1,FBLN1,BLM,SH3KBP1,FHL2,CADPS,NEU3,NCAPG2,RGS7,STK32A,CD2AP,SPRED1,SIPA1L3,ADAM10,LRFN2,FLNB,SCAI,PTPRT,TRERF1,SLC24A2,GLI3,NTRK3,RXFP1,FBN1,CTNNA3,RAPGEF4,BMPER,PDE1A,HMGA2,NSUN2,DEFA3,PTPRK,SORCS1,SYT1,APIP,NPHP4,DOCK9,DLG2,PPP1R12B,PPARA,PLXNA2,PTPRD,RORA,SHISA6,PLCB1,PRKG1,RASGRP1,ELAVL4,NLRC5,MAGI2,PLCL1,ABI1,TSHZ3,ASIC2,RALA,DOCK10,TRPM1,CACNG3,PRKD1,BCL2L1,HDAC2,TNN,RYR2,SEMA3D,TGFA,PRLR,TBX20,PTPRA,OR11G2,MTMR2,ATF6,IL16,DEPTOR,ROBO2,IFT81,ZMYND11,RGS6,SRGAP3,AKAP13,NEDD9,MYRIP,ENPP1,UNC13C,PCP4,RIMS2,STAC,SCN8A,EYA4,RALGAPA1,DLGAP2,SEMA5A,PRDM15,OR4N2,DCC,CHN1,PARD3,NRG1,PRKCA,FMN2,SEMA3C,CSF2RB,PCNT,ST18
GO:0007154	cell communication	1.0466865133937966e-18	CD44,APBB2,PTPRR,ERG,PDE1C,SEMA4D,INIP,ZFYVE1,EVC,TEAD1,SYN3,RPS6KA5,TAOK3,PRKCB,SLC8A1,ANKRD6,GPR55,DNAJC15,CD38,FBXO31,BCL2,CAMTA1,CHFR,THRB,CDC42EP3,GRIK2,IGSF11,SNX25,UNC13B,MAPK10,NCAM1,GNG12,IL6R,CDH8,SCN11A,SORBS2,SKAP2,CASP5,KCNK10,BRD4,GPR158,NDRG2,GRIP1,APBA2,TLK1,DLGAP1,RCAN1,NTN4,JAK2,BICD1,ABCG8,OTUD7A,TPTE2,KALRN,FGF12,CACNG2,BTBD9,NFAT5,TRHDE,ZNF536,LAMA1,CDH11,GRIN2A,MAST4,NRXN1,IL1RAPL1,WDPCP,MAGI1,LAMA3,ADCYAP1R1,GRID1,RPS6KA2,SMOC2,ZDHHC17,KCNH1,DCDC1,CACNA1C,AMPH,BMF,EXOC4,FAM83B,CTNNA1,MYO9A,NTRK2,FOXN3,NLK,ITGBL1,RASGEF1B,AFAP1,TIAM2,IQCJ-SCHIP1,SMARCA4,RNF152,CNTN1,ZNRF3,ENPEP,NRG3,PTPRG,MC2R,BTBD11,ELMO1,SLC24A4,CABIN1,FBXL17,GAS2,GRIK3,CACNB2,PDE10A,STXBP4,MECOM,STK32B,RGS12,SHANK2,RAPGEF5,KCTD8,UNC5D,NREP,GABRA5,DOK5,DLG5,ATRNL1,CHRM3,CPE,ARHGAP24,SPRED2,IGHV1OR15-9,SLIT2,PITPNC1,ROR1,GLP2R,ZNF675,CSNK2A1,DTNA,AKT3,CRB1,TRIM5,PSD3,ALPK2,HECW1,RAP1GDS1,ERBB4,KANK1,ATRX,DMRT1,BID,MACF1,RAP1A,TRIO,PTPRE,DUSP22,CHSY1,MYOM1,PSG8,EXT2,OR4C46,ABL2,MAP3K5,NOS1,RGS3,MAML2,RERG,HTR2C,CACNA1E,CNTN4,GABRR2,PIK3C3,TRAF3,FGD4,ITGA1,ITGA8,GRIK4,ALDH1A2,GABRG1,MAPK9,ESRRG,PTGFR,IGHV1OR21-1,CDH17,SV2B,EYA1,ANKS1B,P2RX6,AKAP10,ANK2,PLA2G4A,SLC1A2,SLIT3,GRIN2B,ROBO1,PAK3,DGKB,GARNL3,TNKS,NDFIP2,GABRA2,OVOL2,FCHSD2,SGMS1,ITPR2,MLLT3,LEMD3,RNF138,PRICKLE2,ITGB3BP,DGKK,PRKCE,PSG9,ARHGEF11,PRKAA2,BBS2,IL1RAPL2,PTPRN2,GRIK1,DSCAM,DGKI,RIN3,DNMBP,EFHB,KDM4C,SLC1A1,GRM5,EPHA6,NTN1,ARAP2,NR5A2,IGF1R,NLGN1,SHISA9,OR9Q1,CNTNAP2,CFTR,FLRT2,NOS1AP,PTPRO,RBMS3,INSR,CTNNAL1,CLEC16A,ITGA9,ANK3,SV2C,GMDS,CNIH3,DOCK3,GPC6,RELN,RASGRF2,STK38,TNR,ADCY9,CELF4,DAPK1,VAV3,INPP5A,VRK1,CNTN6,APP,PUM1,CCDC88A,PLCE1,ADAM12,PAK1,GNAL,MITF,CACNA2D1,HCN1,PPP1R13B,CHRM5,CXADR,EPS8,GPC5,TENM4,GHR,RASGRF1,RIN2,PRDM16,USP7,KIR2DL4,STARD13,KL,LRRC4

			C,ALCAM,PPP1R9A,TMEM117,ZZEF1,LTBP1,SLAMF1,RGL1,CLSTN2,RARB,DIDO1,MYH13,WDR12,TIAM1,SORCS2,SLC39A12,DISC1,RALGPS1,ARHGAP42,SVEP1,EDAR,EGF,PDGFD,FYN,FAM3B,RIMBP2,EPHA7,FHIT,NSG2,GRIA1,TRABD2B,STK3,CNOT7,USP18,S100B,NET1,ESR1,ARHGAP12,GABRG3,PLCXD3,GRM1,PDE4D,ERC2,PRKACB,GNG2,PDE3A,RIMS1,POR,DOCK4,MCTP2,WWOX,HUNK,CNKSR2,FUT8,EPHB1,CREM,EFEMP1,SNAP29,GSG1L,DOCK1,FLT1,EXT1,EFNA5,NXN,CDC14B,HDAC4,STK36,AMFR,PLCB4,SH3BP5,AKAP6,SORCS3,TENM3,MARK2,ATF2,RBBP8,GRID2,ZNF423,LRP2,SEMA6D,RALGPS2,NTF3,FER,SNRK,CAMK4,NTNG1,MAPRE2,SGCD,TMEM108,RIC8B,GABRB3,TPTE,GRM7,RAPGEF2,MX1,PRKCQ,GABRG2,PRKCH,NRXN3,RHPN2,DLC1,NSG1,GABBR2,KCND2,UBE3A,GRIA4,IDE,APC,AUTS2,TFF1,EPHB2,ERC1,AGO3,MCTP1,MOB3B,NBN,ADAMTS18,RGMB,CTNND2,PACSIN2,PKP1,DOCK2,NRP1,CDH13,TRDN,DAB1,RFTN1,SNTG1,ALK,EVC2,LDLRAD4,SEMA3A,SEMA3E,MGAT5,DCLK1,MAGI3,KIF16B,CDH2,ARID5B,SIPA1L2,RCAN2,LRRC69,TENM2,VPS41,ASB3,PIK3R3,MAP2K6,FSTL4,ARHGAP28,MTOR,STK38L,KSR1,RALGAPA2,RORB,GABRB1,FBLN1,BLM,SH3KBP1,FHL2,CADPS,NEU3,NCAPG2,RGS7,STK32A,CD2AP,SPRED1,SIPA1L3,ADAM10,LRFN2,FLNB,SCAI,PTPRT,TRERF1,SLC24A2,GLI3,NTRK3,RXFP1,FBN1,CHKA,CTNNA3,RAPGEF4,BMPER,PDE1A,HMGA2,NSUN2,DEFA3,PTPRK,SORCS1,SYT1,APIP,NPHP4,DOCK9,DLG2,PPP1R12B,PPARA,PLXNA2,PTPRD,RORA,SHISA6,PLCB1,PRKG1,RASGRP1,ELAVL4,NLRC5,MAGI2,PLCL1,ABI1,TSHZ3,ASIC2,RALA,DOCK10,TRPM1,CACNG3,PRKD1,BCL2L1,HDAC2,TNN,RYR2,SEMA3D,TGFA,PRLR,TBX20,PTPRA,OR11G2,MTMR2,ATF6,IL16,DEPTOR,ROBO2,IFT81,ZMYND11,RGS6,SRGAP3,AKAP13,NEDD9,MYRIP,ENPP1,UNC13C,PCP4,RIMS2,STAC,SCN8A,EYA4,RALGAPA1,DLGAP2,SEMA5A,PRDM15,OR4N2,FREM1,DCC,CHN1,PARD3,NRG1,PRKCA,FMN2,SEMA3C,CSF2RB,PCNT,ST18
GO:0010975	regulation of neuron projection development	5.179767157491692e-18	SEMA4D,CD38,FBXO31,TANC2,CSMD3,KALRN,NEGR1,NRXN1,IL1RAPL1,NTRK2,TIAM2,CNTN1,PTPRG,SLIT2,ROR1,HECW1,KANK1,MACF1,RAP1A,ABL2,ROBO1,PAK3,CHODL,DSCAM,NTN1,IGF1R,NLGN1,MAP2,CAMK1D,PTPRO,COBL,CUX1,RELN,CDH4,TNR,CCDC88A,SPOCK1,PAK1,LRRC4C,TIAM1,SLC39A12,DISC1,FYN,EPHA7,TOX,EFNA5,TRPC5,PPFIA2,TENM3,MARK2,GRID2,SEMA6D,NTNG1,RAPGEF2,ATP8A2,UBE3A,EPHB2,PDLIM5,NRP1,DAB1,ALK,SEMA3A,SEMA3E,CDH2,FSTL4,NTRK3,DNM3,PLXNA2,PTPRD,ELAVL4,MAGI2,PRKD1,HDAC2,TNN,SEMA3D,FAT3,ROBO2,SEMA5A,BCL11A,DCC,CTNNA2,CHN1,SEMA3C
GO:0051128	regulation of cellular component organization	6.7880480348993945e-18	CD44,C10ORF90,SCAF8,PARN,SEMA4D,TEAD1,DNAJC15,CD38,FBXO31,BCL2,CHFR,CDC42EP3,UNC13B,CDH8,ATP10A,GRIP1,TLK1,TANC2,CSMD3,BICD1,KALRN,NEGR1,BTBD9,EPB41L3,LAMA1,NRXN1,ARID1B,IL1RAPL1,WDPCP,CTDP1,RPS6KA2,BMF,MYO9A,NTRK2,OCLN,AFAP1,TIAM2,IQCJ-SCHIP1,SMARCA4,CNTN1,NRG3,PTPRG,NUMB,MTPN,PDE4DIP,SHANK2,DLG5,ARHGAP24,SLIT2,MYLK3,ROR1,CSNK2A1,ABCA13,HECW1,RAP1GDS1,KANK1,ATRX,DMRT1,BID,MACF1,MNAT1,RAP1A,DUSP22,ABL2,RERG,TBC1D5,CORO2B,RUNX1,MAPK9,CDH17,PEAK1,PLS1,SLIT3,GRIN2B,ROBO1,PAK3,DGKB,TNKS,MDM1,FCHSD2,MLLT3,CHODL,TMEM67,PRKCE,ARHGEF11,PRKAA2,IL1RAPL2,DSCAM,RIN3,NTN1,IGF1R,SPTB,SNX30,NLGN1,CNTNAP2,MAP2,CAMK1D,FLRT2,PTPRO,INSR,COBL,CLEC16A,MTRF1,CUX1,MORC2,TBCD,GPC6,RELN,CDH4,TNR,APP,FBLN5,CCDC88A,SPOCK1,PLCE1,PAK1,ADCK1,NSMCE2,EPS8,LRFN5,USP7,LRRC4C,ADAMTS16,SLAMF1,INO80D,CLSTN2,TIAM1,SLC39A12,DISC1,FMN1,ASAP1,FRMPD4,EGF,FYN,EPHA7,TRABD2B,SPIDR,NET1,TOX,ESR1,PDE3A,RIMS1,EPHB1,CTTNBP2,FHOD3,GSG1L,EFNA5,HDAC4,TRPC5,PPFIA2,AKAP6,TENM3,LINGO2,MARK2,GRID2,SEMA6D,NTF3,FER,NTNG1,MAPRE2,RAD51AP1,RAPGEF2,NAV3,PRKCQ,PRKCH,RHPN2,DLC1,ATP8A2,UBE3A,APC,INO80,AUTS2,EPHB2,SCAF4,PDLIM5,MCTP1,NBN,PACSIN2,SDCCAG8,NRP1,CDH13,DAB1,ALK,LDLRAD4,SEMA3A,SEMA3E,CDH2,TENM2,TANC1,VPS41,FSTL4,ARHGAP28,MTOR,STK38L,NEU3,CD2AP,ADAM10,LRFN2,PAPPA2,NTRK3,RAB31,VPS1

			3D,ABHD17C,ATF7IP,TBC1D4,DNM3,SYT1,SYNDIG1,DPF3,NPHP4,SACS,PPARA,PLXNA2,PTPRD,PLCB1,ELAVL4,STXBP6,MAGI2,ASIC2,RALA,PRKD1,ATP8A1,BCL2L1,HDAC2,TNN,SEMA3D,TGFA,TBX20,PTPRA,FAT3,MTMR2,VCL,ATAT1,ROBO2,AKAP13,NEDD9,ENPP1,RIMS2,L3MBTL3,CLIP1,SEMA5A,BCL11A,DCC,CTNNA2,CHN1,VPS13C,NRG1,SEMA3C
GO:0031344	regulation of cell projection organization	1.0089153924858561e-17	CD44,SEMA4D,CD38,FBXO31,CDC42EP3,GRIP1,TANC2,CSMD3,KALRN,NEGR1,NRXN1,IL1RAPL1,WDPCP,MYO9A,NTRK2,OCLN,TIAM2,CNTN1,PTPRG,ARHGAP24,SLIT2,ROR1,HECW1,KANK1,MACF1,RAP1A,ABL2,PLS1,GRIN2B,ROBO1,PAK3,CHODL,DSCAM,NTN1,IGF1R,NLGN1,MAP2,CAMK1D,PTPRO,COBL,CUX1,RELN,CDH4,TNR,CCDC88A,SPOCK1,PLCE1,PAK1,EPS8,LRRC4C,ADAMTS16,TIAM1,SLC39A12,DISC1,FYN,EPHA7,TOX,EFNA5,HDAC4,TRPC5,PPFIA2,TENM3,MARK2,GRID2,SEMA6D,FER,NTNG1,RAPGEF2,ATP8A2,UBE3A,APC,AUTS2,EPHB2,PDLIM5,SDCCAG8,NRP1,DAB1,ALK,SEMA3A,SEMA3E,CDH2,TENM2,FSTL4,MTOR,NTRK3,DNM3,PLXNA2,PTPRD,ELAVL4,MAGI2,RALA,PRKD1,HDAC2,TNN,SEMA3D,FAT3,ROBO2,NEDD9,SEMA5A,BCL11A,DCC,CTNNA2,CHN1,SEMA3C
GO:0048869	cellular developmental process	1.5482827560464953e-17	CD44,ZHX3,RTN1,ERG,SEMA4D,RPS6KA5,TAOK3,A2M,SLC8A1,GPR55,CD38,FBXO31,PIWIL3,TRAPPC9,BCL2,COL18A1,THRB,RAG1,NCAM1,IL6R,SORBS2,NDRG2,GRIP1,ASTN2,TANC2,CSMD3,RCAN1,NTN4,JAK2,KALRN,USH2A,NEGR1,FLI1,ZNF536,EPB41L3,LAMA1,PARVB,CDH11,ZBTB7C,GRIN2A,NRXN1,ARID1B,IL1RAPL1,WDPCP,LAMA3,ADCYAP1R1,CTDP1,RPS6KA2,ZDHHC17,KCNH1,PNPLA3,BBS9,CTNNA1,MYO9A,NTRK2,THSD7A,NCAM2,TIAM2,MYT1L,SMARCA4,CNTN1,PLEKHB2,NRG3,PTPRG,SYNE1,FBXL17,NUMB,MTPN,SOX6,MECOM,VCAN,NHS,PTGFRN,UNC5D,NREP,GABRA5,DOK5,DLG5,PGM5,ASTN1,ATRNL1,ARHGAP24,SPRED2,SLIT2,MYLK3,ABCA5,ROR1,ADAMTSL1,KAZN,ZNF675,CRB1,ALPK2,HECW1,LCE1F,ERBB4,KANK1,ATRX,DMRT1,MACF1,TAF4B,RAP1A,TRIO,CHSY1,EXT2,ABL2,MAP3K5,NOS1,ABCG1,SPAG16,EML1,HTR2C,CCDC141,CNTN4,RNF17,SLC9C1,SND1,ETS2,ITGA1,TCF12,HIRA,ITGA8,RUNX1,ALDH1A2,MAPK9,CDH17,PEAK1,EYA1,FRMD6,PLS1,ANK2,ZBTB16,SLIT3,ROBO1,PAK3,OVOL2,BRINP1,MLLT3,CHODL,GABPA,GLIS1,PSG9,PACRG,BBS2,MYOM2,NUBPL,SOX5,DSCAM,KDM4C,SDK1,SLC1A1,GRM5,EPHA6,NTN1,NR5A2,IGF1R,NLGN1,SHROOM3,JAM2,MSI2,CNTNAP2,MAP2,CFTR,CAMK1D,FLRT2,PTPRO,NHSL1,COBL,CATSPER2,CRTAC1,CUX1,ANK3,TBCD,RELN,TRPS1,ADAMTS5,MYO18B,CDH4,TNR,OCA2,CELF4,CNTN6,APP,PUM1,CCDC88A,SPOCK1,ADAM12,PAK1,MITF,HCN1,FRY,CXADR,TENM4,CECR2,GHR,RASGRF1,RIN2,PRDM16,RBFOX1,MEIS2,LRRC4C,ALCAM,PPP1R9A,SLAMF1,NEBL,RARB,TCF4,FRYL,TIAM1,PBX1,PHACTR1,SLC39A12,DISC1,ZFPM2,NTM,ASAP1,EDAR,FYN,EPHA7,NAV2,STK3,COL19A1,MSR1,S100B,TOX,PCDH15,ESR1,SGCZ,PDE4D,CNTN5,PDE3A,RIMS1,POR,CERS3,WWOX,EPHB1,CREM,FHOD3,EFEMP1,ARMC2,AJAP1,HERC1,DOCK1,FLT1,EXT1,EFNA5,NXN,C14ORF39,HDAC4,KLHL1,TRPC5,FTO,PPFIA2,AKAP6,POU6F2,TENM3,OPCML,MARK2,ATF2,GRID2,ZNF423,LRP2,SEMA6D,NTF3,FER,SNRK,CAMK4,NTNG1,SGCD,TMEM108,GRM7,RAPGEF2,NAV3,PRKCQ,KIRREL3,CPS1,PRKCH,NRXN3,ATP8A2,UBE3A,APC,AUTS2,TFF1,EPHB2,PDLIM5,PRTG,CTNND2,COL22A1,SETD2,PACSIN2,DOCK2,SDCCAG8,FLVCR1,NRP1,MDGA2,RPGRIP1,DAB1,ALK,LDLRAD4,SEMA3A,SEMA3E,MYEF2,DCLK1,CDH2,ARID5B,TENM2,TANC1,SYCP1,HDAC9,PIK3R3,MAP2K6,FSTL4,MTOR,RORB,GABRB1,FBLN1,FHL2,NCAPG2,SPRED1,SIPA1L3,FLNB,ABCB5,DPY19L2,GLI3,NTRK3,RXFP1,FBN1,HYDIN,TBX15,ZNF521,HMGA2,NSUN2,DNM3,SYT1,DPF3,NPHP4,PPARA,PLXNA2,PTPRD,RORA,PLCB1,LOXL2,PRKG1,RASGRP1,ELAVL4,DMBT1,MAGI2,NELL1,ABI1,DOCK10,FNDC3A,PRKD1,BCL2L1,HDAC2,ETS1,TNN,SEMA3D,PRLR,TBX20,FAT3,MTMR2,VCL,ATAT1,ROBO2,IFT81,CDH23,AKAP13,NEDD9,ENPP1,PCP4,RIMS2,RAB27A,EYA4,L3MBTL3,HIVEP3,SEMA5A,BCL11A,DCC,CTNNA2,CHN1,ETV6,PARD3,NRG1,PRKCA,FMN2,SEMA3C
GO:0120035	regulation of	1.6423465992151876e-17	CD44,SEMA4D,CD38,FBXO31,CDC42EP3,TANC2,CSMD3,KALRN,NEGR1,NRXN1,IL1RAPL1,WDPCP,NTRK2,OCLN,TIAM2,CNTN1,PTPRG,ARHGAP24,SLIT2,ROR1,HECW1,KANK1,MACF1,RAP1A,ABL2,PLS1,GRIN2B,ROBO1,PAK3,CHODL,DSCAM,NTN1,IGF1R,NLGN1,MAP2

	plasma membrane bounded cell projection organization		,CAMK1D,PTPRO,COBL,CUX1,RELN,CDH4,TNR,CCDC88A,SPOCK1,PLCE1,PAK1,EPS8,LRRC4C,ADAMTS16,TIAM1,SLC39A12,DISC1,FYN,EPHA7,TOX,EFNA5,HDAC4,TRPC5,PPFIA2,TENM3,MARK2,GRID2,SEMA6D,FER,NTNG1,RAPGEF2,ATP8A2,UBE3A,APC,AUTS2,EPHB2,PDLIM5,SDCCAG8,NRP1,DAB1,ALK,SEMA3A,SEMA3E,CDH2,TENM2,FSTL4,MTOR,NTRK3,DNM3,PLXNA2,PTPRD,ELAVL4,MAGI2,RALA,PRKD1,HDAC2,TNN,SEMA3D,FAT3,ROBO2,NEDD9,SEMA5A,BCL11A,DCC,CTNNA2,CHN1,SEMA3C
GO:0050804	modulation of chemical synaptic transmission	1.6962693054422702e-17	SYN3,PRKCB,CD38,GRIK2,IGSF11,APBA2,DLGAP1,JAK2,CACNG2,BTBD9,CDH11,GRIN2A,NRXN1,GRID1,NTRK2,NRG3,GRIK3,CACNB2,SHANK2,RAP1A,CNTN4,GRIK4,GRIN2B,DGKB,PRKCE,GRIK1,DGKI,SLC1A1,GRM5,NLGN1,SHISA9,RELN,RASGRF2,TNR,CELF4,APP,HCN1,RASGRF1,LRRC4C,PPP1R9A,ZZEF1,CLSTN2,SORCS2,DISC1,FYN,GRIA1,S100B,GRM1,ERC2,RIMS1,MCTP2,EPHB1,PLCB4,SORCS3,GRID2,NTF3,NTNG1,TMEM108,GRM7,RAPGEF2,NRXN3,NSG1,EPHB2,ERC1,MCTP1,PACSIN2,CDH2,LRFN2,SLC24A2,SYT1,PTPRD,SHISA6,PLCB1,ELAVL4,PLCL1,TSHZ3,CACNG3,PTPRA,MTMR2,UNC13C,RIMS2,DLGAP2,DCC
GO:0061564	axon development	1.883320783996041e-17	SEMA4D,RPS6KA5,BCL2,NCAM1,JAK2,KALRN,LAMA1,CDH11,NRXN1,LAMA3,ZDHHC17,CTNNA1,NTRK2,NCAM2,TIAM2,CNTN1,NUMB,UNC5D,NREP,SLIT2,ADAMTSL1,MACF1,TRIO,CCDC141,CNTN4,SLIT3,ROBO1,PAK3,CHODL,DSCAM,EPHA6,NTN1,IGF1R,MAP2,FLRT2,PTPRO,COBL,CRTAC1,ANK3,RELN,CDH4,TNR,CNTN6,APP,PAK1,LRRC4C,ALCAM,TIAM1,DISC1,FYN,EPHA7,S100B,CNTN5,EPHB1,EXT1,EFNA5,TRPC5,MARK2,SEMA6D,NTNG1,GRM7,PRKCQ,NRXN3,ATP8A2,AUTS2,EPHB2,PRTG,NRP1,DAB1,SEMA3A,SEMA3E,DCLK1,CDH2,FSTL4,GLI3,PLXNA2,TNN,SEMA3D,VCL,ROBO2,SEMA5A,BCL11A,DCC,CTNNA2,CHN1,PARD3,SEMA3C
GO:0099177	regulation of trans-synaptic signaling	1.96294310256885e-17	SYN3,PRKCB,CD38,GRIK2,IGSF11,APBA2,DLGAP1,JAK2,CACNG2,BTBD9,CDH11,GRIN2A,NRXN1,GRID1,NTRK2,NRG3,GRIK3,CACNB2,SHANK2,RAP1A,CNTN4,GRIK4,GRIN2B,DGKB,PRKCE,GRIK1,DGKI,SLC1A1,GRM5,NLGN1,SHISA9,RELN,RASGRF2,TNR,CELF4,APP,HCN1,RASGRF1,LRRC4C,PPP1R9A,ZZEF1,CLSTN2,SORCS2,DISC1,FYN,GRIA1,S100B,GRM1,ERC2,RIMS1,MCTP2,EPHB1,PLCB4,SORCS3,GRID2,NTF3,NTNG1,TMEM108,GRM7,RAPGEF2,NRXN3,NSG1,EPHB2,ERC1,MCTP1,PACSIN2,CDH2,LRFN2,SLC24A2,SYT1,PTPRD,SHISA6,PLCB1,ELAVL4,PLCL1,TSHZ3,CACNG3,PTPRA,MTMR2,UNC13C,RIMS2,DLGAP2,DCC
GO:0030154	cell differentiation	3.612571409660855e-17	ZHX3,RTN1,ERG,SEMA4D,RPS6KA5,TAOK3,A2M,SLC8A1,GPR55,CD38,FBXO31,PIWIL3,TRAPPC9,BCL2,COL18A1,THRB,RAG1,NCAM1,IL6R,SORBS2,NDRG2,GRIP1,ASTN2,TANC2,CSMD3,RCAN1,NTN4,JAK2,KALRN,USH2A,NEGR1,FLI1,ZNF536,EPB41L3,LAMA1,PARVB,CDH11,ZBTB7C,GRIN2A,NRXN1,ARID1B,IL1RAPL1,WDPCP,LAMA3,ADCYAP1R1,CTDP1,RPS6KA2,ZDHHC17,KCNH1,PNPLA3,BBS9,CTNNA1,MYO9A,NTRK2,THSD7A,NCAM2,TIAM2,MYT1L,SMARCA4,CNTN1,PLEKHB2,NRG3,PTPRG,SYNE1,FBXL17,NUMB,MTPN,SOX6,MECOM,VCAN,NHS,PTGFRN,UNC5D,NREP,GABRA5,DOK5,DLG5,PGM5,ASTN1,ATRNL1,ARHGAP24,SPRED2,SLIT2,MYLK3,ABCA5,ROR1,ADAMTSL1,KAZN,ZNF675,CRB1,ALPK2,HECW1,LCE1F,ERBB4,KANK1,ATRX,DMRT1,MACF1,TAF4B,RAP1A,TRIO,CHSY1,EXT2,ABL2,MAP3K5,NOS1,ABCG1,SPAG16,EML1,HTR2C,CCDC141,CNTN4,RNF17,SLC9C1,SND1,ETS2,ITGA1,TCF12,HIRA,ITGA8,RUNX1,ALDH1A2,MAPK9,CDH17,PEAK1,EYA1,FRMD6,PLS1,ANK2,ZBTB16,SLIT3,ROBO1,PAK3,OVOL2,BRINP1,MLLT3,CHODL,GABPA,GLIS1,PSG9,PACRG,BBS2,MYOM2,SOX5,DSCAM,KDM4C,SDK1,SLC1A1,GRM5,EPHA6,NTN1,NR5A2,IGF1R,NLGN1,SHROOM3,JAM2,MSI2,CNTNAP2,MAP2,CFTR,CAMK1D,FLRT2,PTPRO,NHSL1,COBL,CATSPER2

			,CRTAC1,CUX1,ANK3,TBCD,RELN,TRPS1,ADAMTS5,MYO18B,CDH4,TNR,OCA2,CELF4,CNTN6,APP,PUM1,CCDC88A,SPOCK1,ADAM12,PAK1,MITF,HCN1,FRY,CXADR,TENM4,CECR2,GHR,RASGRF1,RIN2,PRDM16,RBFOX1,MEIS2,LRRC4C,ALCAM,PPP1R9A,SLAMF1,NEBL,RARB,TCF4,FRYL,TIAM1,PBX1,PHACTR1,SLC39A12,DISC1,ZFPM2,NTM,ASAP1,EDAR,FYN,EPHA7,NAV2,STK3,COL19A1,MSR1,S100B,TOX,PCDH15,ESR1,SGCZ,PDE4D,CNTN5,PDE3A,RIMS1,POR,CERS3,WWOX,EPHB1,CREM,FHOD3,EFEMP1,ARMC2,AJAP1,HERC1,DOCK1,FLT1,EXT1,EFNA5,NXN,C14ORF39,HDAC4,KLHL1,TRPC5,FTO,PPFIA2,AKAP6,POU6F2,TENM3,OPCML,MARK2,ATF2,GRID2,ZNF423,LRP2,SEMA6D,NTF3,FER,SNRK,CAMK4,NTNG1,SGCD,TMEM108,GRM7,RAPGEF2,NAV3,PRKCQ,KIRREL3,CPS1,PRKCH,NRXN3,ATP8A2,UBE3A,APC,AUTS2,TFF1,EPHB2,PDLIM5,PRTG,CTNND2,COL22A1,SETD2,DOCK2,SDCCAG8,FLVCR1,NRP1,MDGA2,RPGRIP1,DAB1,ALK,LDLRAD4,SEMA3A,SEMA3E,MYEF2,DCLK1,CDH2,ARID5B,TENM2,TANC1,SYCP1,HDAC9,PIK3R3,MAP2K6,FSTL4,MTOR,RORB,GABRB1,FBLN1,FHL2,NCAPG2,SPRED1,SIPA1L3,FLNB,ABCB5,DPY19L2,GLI3,NTRK3,RXFP1,FBN1,HYDIN,TBX15,ZNF521,HMGA2,NSUN2,DNM3,SYT1,DPF3,NPHP4,PPARA,PLXNA2,PTPRD,RORA,PLCB1,LOXL2,PRKG1,RASGRP1,ELAVL4,DMBT1,MAGI2,NELL1,ABI1,DOCK10,FNDC3A,PRKD1,BCL2L1,HDAC2,ETS1,TNN,SEMA3D,PRLR,TBX20,FAT3,MTMR2,VCL,ATAT1,ROBO2,IFT81,CDH23,AKAP13,NEDD9,ENPP1,PCP4,RIMS2,RAB27A,EYA4,L3MBTL3,HIVEP3,SEMA5A,BCL11A,DCC,CTNNA2,CHN1,ETV6,PARD3,NRG1,PRKCA,FMN2,SEMA3C
GO:0065007	biological regulation	5.741685810707726e-17	CD44,SAMD4A,KCNMA1,C10ORF90,PKNOX2,ZHX3,APBB2,PTPRR,SCAF8,RTN1,ERG,PARN,PDE1C,SEMA4D,INIP,SLC12A8,MED13L,EVC,TEAD1,NFIA,SYN3,RPS6KA5,DHRS11,TAOK3,PRKCB,EIF4G3,A2M,SLC8A1,ANKRD6,KCNC1,GPR55,DNAJC15,SIAH3,CD38,ZNF257,FBXO31,PIWIL3,TRAPPC9,BCL2,MMP16,CAMTA1,SAMSN1,CHFR,THADA,COL18A1,TOX3,THRB,DNAH11,ZSCAN5C,RAG1,CDC42EP3,GRIK2,IGSF11,SNX25,UNC13B,MAPK10,NCAM1,KDM4B,GNG12,IL6R,KCNQ5,CDH8,ZBTB20,HEPHL1,SCN11A,ATP10A,SORBS2,SKAP2,HS1BP3,KCNJ6,CASP5,KCNK10,BRD4,GPR158,ZNF568,NDRG2,GRIP1,APBA2,TLK1,ASTN2,TANC2,CSMD3,DLGAP1,RCAN1,NTN4,JAK2,TM9SF4,BICD1,MIR17HG,ABCG8,OTUD7A,TPTE2,KALRN,USH2A,NEGR1,FGF12,CACNG2,BTBD9,NFAT5,FLI1,TRHDE,ZNF536,EPB41L3,LAMA1,PARVB,CDH11,ZBTB7C,GRIN2A,MAST4,NRXN1,NELL2,ARID1B,IL1RAPL1,WDPCP,MAGI1,LAMA3,ADCYAP1R1,CTDP1,DPP6,GRID1,RPS6KA2,SMOC2,ZDHHC17,KCNH1,DCDC1,CACNA1C,BMF,EXOC4,WDR70,PNPLA3,ANO4,FAM83B,CTNNA1,MYO9A,NTRK2,FOXN3,OCLN,NLK,ITGBL1,NBEA,RASGEF1B,AFAP1,TIAM2,MYT1L,TMPRSS3,SRGAP2B,IQCJ-SCHIP1,SMARCA4,RNF152,CNTN1,ZNRF3,PALMD,MEOX2,TTC39B,ENPEP,PLEKHB2,GLIS3,NRG3,PTPRG,MC2R,BTBD11,ELMO1,SLC24A4,CABIN1,FBXL17,GAS2,GRIK3,CACNB2,PDE10A,NUMB,STXBP4,MED15,MTPN,MT1HL1,SOX6,MECOM,TBC1D22A,PDE4DIP,STK32B,RGS12,SHANK2,RAPGEF5,UBE2E2,KCTD8,UNC5D,ZNF567,NREP,GABRA5,DOK5,DLG5,CFDP1,SMARCAD1,ZIM3,ATRNL1,CHRM3,CPE,ARHGAP24,SPRED2,IGHV1OR15-9,SLIT2,PITPNC1,MYLK3,ABCA5,ROR1,GLP2R,SLC4A4,ZNF675,CSNK2A1,DTNA,AKT3,CRB1,KMT2C,KCNE4,TRIM5,KCNS3,PSD3,ALPK2,ABCA13,HECW1,RAP1GDS1,AFF3,ERBB4,KANK1,GPHN,ATRX,DMRT1,CHST8,BID,MACF1,MNAT1,TAF4B,RAP1A,TRIO,CTNNBL1,RAD51B,PTPRE,DUSP22,CHSY1,MYOM1,PSG8,EXT2,ZSCAN30,OR4C46,ABL2,PSMA1,MAP3K5,NOS1,ACACA,ABCG1,RGS3,MAML2,RERG,HTR2C,NEK4,CACNA1E,CTIF,CNTN4,TBC1D5,SAMD13,SLC40A1,SLCO3A1,GABRR2,PIK3C3,SLC9C1,TRAF3,SND1,CHD6,FGD4,ETS2,ITGA1,TCF12,ZNF721,HIRA,CORO2B,ITGA8,GRIK4,RBM19,RUNX1,KIR3DL2,ALDH1A2,GABRG1,TSHZ2,MAPK9,ESRRG,PTGFR,IGHV1OR21-1,FAM171A1,ZNF595,CDH17,PEAK1,EYA1,MORC3,ANKS1B,P2RX6,AKAP10,SPOCK3,FRMD6,PLS1,SPON1,ANK2,PLA2G4A,SLC1A2,ZBTB16,SUPT3H,SLIT3,GRIN2B,ZNF518A,PHC2,ROBO1,ZNF578,EGFLAM,PAK3,DGKB,GARNL3,DPH6,EBF1,TNKS,KLF12,NDFIP2,GABRA2,MDM1,OVOL2,FCHSD2,SGMS1,ITPR2,BRINP1,MLLT3,BCL2L

			13,LEMD3,KHDRBS2,CPQ,RNF138,CHODL,GABPA,PRICKLE2,ITGB3BP,CACNA2D3,DGKK,TMEM67,PRKCE,GLIS1,PSG9,ARHGEF11,PRKAA2,PACRG,BBS2,IL1RAPL2,EDIL3,CDYL2,PTPRN2,HIVEP2,KCNH8,GRIK1,SOX5,DSCAM,DGKI,RIN3,ANKFN1,DNMBP,EFHB,KDM4C,ACSM2A,DCUN1D4,SDK1,SLC1A1,SLC12A1,GRM5,EPHA6,NTN1,ARAP2,NR5A2,LDB2,IGF1R,SPTB,SNX30,NLGN1,SHISA9,OR9Q1,SHROOM3,JAM2,ALX4,CORIN,CNTNAP2,MAP2,KCNIP4,CFTR,CAMK1D,FLRT2,MLLT10,NOS1AP,PTPRO,NKAIN2,CD96,RBMS3,INSR,COBL,CTNNAL1,CLEC16A,PHF20L1,ITGA9,MTRF1,CATSPER2,EGLN3,CUX1,ANK3,MORC2,GMDS,CNIH3,DOCK3,TBCD,GPC6,RELN,RASGRF2,TRPS1,ADAMTS5,STK38,AOAH,FBXO32,CDH4,TNR,ADCY9,DPP10,CELF4,DAPK1,VAV3,INPP5A,ZNF600,VRK1,ZNF678,CNTN6,CLIC6,ZNF420,APP,FBLN5,PUM1,CCDC88A,ARNT2,KCTD1,RNLS,SPOCK1,HPSE2,PLCE1,ADAM12,PAK1,ATP9B,GNAL,MITF,IGF2BP3,CACNA2D1,ADCK1,HCN1,PPP1R13B,CHRM5,NSMCE2,ZNF208,FRY,CXADR,EPS8,LRFN5,UTRN,GPC5,TENM4,PRR16,TSPAN13,GHR,DUX4,RIPK4,RASGRF1,RIN2,PRDM16,FRMD5,RNF217,USP7,RBFOX1,MEIS2,KIR2DL4,STARD13,SCP2,KL,LRRC4C,ALCAM,PPP1R9A,AVEN,TMEM117,ADAMTS16,TASP1,MICU1,ZZEF1,LTBP1,SLAMF1,RGL1,NKAIN3,BACE2,INO80D,CLSTN2,RARB,DIDO1,WDR12,TCF4,TIAM1,FOCAD,PBX1,PHACTR1,MLIP,SORCS2,PRIM2,SLC39A12,ASAP2,DISC1,FMN1,RALGPS1,ARHGAP42,ZFPM2,PIEZO2,VSTM4,SVEP1,ASAP1,PCBP3,FRMPD4,EDAR,EGF,PDGFD,FYN,FAM3B,KCND3,PRMT8,XRCC4,EPHA7,FHIT,NSG2,GRIA1,ZNF627,TRABD2B,SPIDR,NAV2,STK3,CNOT7,MSR1,PSIP1,AP2B1,USP18,S100B,NET1,TOX,PCDH15,ESR1,ARHGAP12,GABRG3,SGCZ,PLCXD3,KCNAB1,GRM1,PDE4D,ERC2,PRKACB,GNG2,PDE3A,RIMS1,POR,L3MBTL4,DOCK4,ATP6V1E1,FRMD4A,MCTP2,WWOX,PCSK2,HUNK,CNKSR2,FUT8,EPHB1,SSBP2,CREM,CTTNBP2,FHOD3,EFEMP1,TNRC6B,AJAP1,ABCC9,SNAP29,GSG1L,HERC1,DOCK1,PARP15,TRPM7,FLT1,EXT1,EFNA5,NXN,CDC14B,C14ORF39,HDAC4,ZNF717,STK36,TRPC5,AMFR,PLCB4,ATP9A,FTO,PPFIA2,SH3BP5,AKAP6,SORCS3,POU6F2,TENM3,LINGO2,MARK2,ATF2,PHACTR2,ZNF880,RBBP8,GRID2,ZNF423,LRP2,SEMA6D,ZNF573,C2,RALGPS2,NTF3,FER,SNRK,SUSD4,CAMK4,CELF2,TP53I11,NTNG1,MAPRE2,ARFGAP3,MICU2,ISX,RAD51AP1,SGCD,TMEM108,RIC8B,GABRB3,TPTE,GRM7,SLC39A8,RAPGEF2,NAV3,MX1,PLGRKT,PPA2,ZNF615,GTF2I,ATXN1,PRKCQ,GABRG2,CPS1,PRKCH,NRXN3,RHPN2,RABGAP1L,DLC1,NSG1,GABBR2,KCND2,ATP8A2,SLC24A3,UBE3A,GRIA4,IDE,APC,ZBTB25,INO80,AUTS2,TFF1,EPHB2,SCAF4,ERC1,ZNF850,PDLIM5,AGO3,C9,TMTC2,MCTP1,MOB3B,RYR3,NBAS,PRTG,NBN,ADAMTS18,RGMB,CTNND2,SETD2,PACSIN2,PKP1,DOCK2,NUP214,TRIM23,SDCCAG8,FLVCR1,NRP1,CDH13,RFC3,PHACTR3,ZNF879,DACH1,TRDN,SLC2A13,ZNF397,DAB1,RFTN1,ALK,EVC2,LDLRAD4,SEMA3A,SEMA3E,MGAT5,ATP13A3,MALRD1,MYEF2,DCLK1,MAGI3,KIF16B,NRIP1,CDH2,ARID5B,SIPA1L2,CCNG2,RCAN2,LRRC69,TENM2,TANC1,SERPINB7,EVI5,VPS41,SYCP1,ZNF407,ASB3,HDAC9,PIK3R3,MAP2K6,FSTL4,ARHGAP28,MTOR,STK38L,KSR1,RALGAPA2,RORB,GABRB1,FBLN1,SGSM1,ST8SIA1,BLM,SH3KBP1,FHL2,CADPS,NEU3,NCAPG2,RGS7,STK32A,CD2AP,ZFP30,USP25,SPRED1,SIPA1L3,ADAM10,DRAM1,PPP2R2C,KANSL1,LRFN2,FLNB,SCAI,PAPPA2,ABCB5,PTPRT,CSMD1,TRERF1,SLC24A2,GLI3,NTRK3,RXFP1,FBN1,RAB31,CTNNA3,VPS13D,ABHD17C,ZNF292,TBX15,RAPGEF4,BMPER,ANKRD31,ZNF521,PDE1A,TMPRSS2,ATF7IP,HMGA2,MX2,CREB5,NSUN2,DEFA3,PTPRK,SORCS1,TBC1D4,DNM3,SYT1,APIP,SYNDIG1,ASXL3,DPF3,NPHP4,DOCK9,PPP1R12B,SACS,PPARA,PLXNA2,PTPRD,RORA,SHISA6,SCARA5,PLCB1,LOXL2,BPTF,PRKG1,RASGRP1,ELAVL4,NLRC5,STXBP6,MXI1,TTC28,MAGI2,NELL1,STX12,PLCL1,ABI1,TSHZ3,ASIC2,RALA,DOCK10,TRPM1,CACNG3,NECAB1,PRKD1,ATP8A1,TNFAIP8,BCL2L1,HDAC2,ETS1,MRPS27,TNN,RYR2,SEMA3D,LDLRAD3,BANP,TGFA,PRLR,TBX20,PTPRA,FAT3,OR11G2,MTMR2,KCNH5,ATF6,IL16,VCL,DEPTOR,BACH1,ATAT1,ROBO2,EWSR1,IFT81,ZMYND11,CDH23,RGS6,SRGAP3,AKAP13,WDR41,NEDD9,MYRIP,ENPP1,UNC13C,PCP4,RIMS2,STAC,SCN8A,RAB27A,EYA4,RALGAPA1,L3MBTL3,DLGAP2,POMT2,HIVEP3,CLIP1,SEMA5A,CABLES1,PRDM15,OR4N2,BCL11A,AGAP1,DCC,ZNF112,CTNNA

			2,PVT1,CHN1,ETV6,VPS13C,KCNJ15,PARD3,NRG1,CAST,TBC1D9,FANK1,ZNF845,ATP10B,NPAS3,PRKCA,FMN2,SEMA3C,FANCB,CSF2RB,PCNT,PAH,ST18,TOP1
GO:0007409	axonogenesis	5.81453645767252e-17	SEMA4D,RPS6KA5,BCL2,NCAM1,KALRN,LAMA1,CDH11,NRXN1,LAMA3,ZDHHC17,NTRK2,TIAM2,CNTN1,NUMB,UNC5D,SLIT2,ADAMTSL1,MACF1,TRIO,CCDC141,CNTN4,SLIT3,ROBO1,PAK3,CHODL,DSCAM,EPHA6,NTN1,IGF1R,MAP2,FLRT2,PTPRO,COBL,ANK3,RELN,CDH4,TNR,CNTN6,APP,PAK1,LRRC4C,ALCAM,TIAM1,DISC1,FYN,EPHA7,S100B,CNTN5,EPHB1,EXT1,EFNA5,TRPC5,MARK2,SEMA6D,NTNG1,PRKCQ,NRXN3,ATP8A2,AUTS2,EPHB2,PRTG,NRP1,DAB1,SEMA3A,SEMA3E,DCLK1,CDH2,FSTL4,GLI3,PLXNA2,TNN,SEMA3D,VCL,ROBO2,SEMA5A,BCL11A,DCC,CTNNA2,CHN1,PARD3,SEMA3C
GO:0065008	regulation of biological quality	8.517457066082835e-17	SAMD4A,KCNMA1,C10ORF90,APBB2,PARN,SEMA4D,SLC12A8,SYN3,DHRS11,PRKCB,SLC8A1,KCNC1,GPR55,SIAH3,CD38,BCL2,CHFR,THADA,THRB,RAG1,CDC42EP3,GRIK2,IGSF11,UNC13B,CDH8,ZBTB20,HEPHL1,SCN11A,ATP10A,KCNK10,GRIP1,APBA2,TANC2,JAK2,TM9SF4,ABCG8,KALRN,USH2A,NEGR1,FGF12,CACNG2,BTBD9,FLI1,TRHDE,GRIN2A,NRXN1,NELL2,IL1RAPL1,ADCYAP1R1,GRID1,RPS6KA2,KCNH1,CACNA1C,PNPLA3,ANO4,NTRK2,OCLN,NLK,NBEA,TMPRSS3,SMARCA4,TTC39B,ENPEP,SLC24A4,GRIK3,CACNB2,NUMB,STXBP4,MTPN,MT1HL1,SHANK2,GABRA5,DLG5,CHRM3,CPE,SLIT2,MYLK3,ABCA5,SLC4A4,ZNF675,AKT3,CRB1,KCNE4,RAP1GDS1,ERBB4,KANK1,GPHN,CHST8,BID,MACF1,RAP1A,EXT2,ABL2,NOS1,ACACA,ABCG1,HTR2C,CNTN4,SLC40A1,GABRR2,SLC9C1,ITGA1,CORO2B,GRIK4,ALDH1A2,GABRG1,ESRRG,PTGFR,MORC3,P2RX6,PLS1,ANK2,PLA2G4A,SLC1A2,GRIN2B,PAK3,DGKB,GABRA2,FCHSD2,ITPR2,CPQ,DGKK,PRKCE,PRKAA2,BBS2,IL1RAPL2,PTPRN2,KCNH8,GRIK1,DSCAM,DGKI,RIN3,ACSM2A,SLC1A1,SLC12A1,GRM5,NTN1,NR5A2,LDB2,IGF1R,SPTB,NLGN1,SHISA9,JAM2,CORIN,MAP2,CFTR,FLRT2,NOS1AP,PTPRO,INSR,ANK3,GPC6,RELN,RASGRF2,ADAMTS5,CDH4,TNR,CELF4,VAV3,APP,PUM1,RNLS,PLCE1,ATP9B,IGF2BP3,CACNA2D1,ADCK1,HCN1,CXADR,EPS8,LRFN5,PRR16,GHR,RASGRF1,PRDM16,USP7,SCP2,KL,ADAMTS16,MICU1,ZZEF1,BACE2,CLSTN2,SORCS2,SLC39A12,DISC1,FMN1,ARHGAP42,PIEZO2,VSTM4,FRMPD4,FYN,FAM3B,KCND3,EPHA7,GRIA1,NAV2,STK3,CNOT7,AP2B1,S100B,PCDH15,ESR1,GABRG3,SGCZ,GRM1,PDE4D,ERC2,PRKACB,PDE3A,RIMS1,POR,DOCK4,MCTP2,PCSK2,EPHB1,CTTNBP2,FHOD3,TNRC6B,ABCC9,SNAP29,GSG1L,TRPM7,EXT1,EFNA5,TRPC5,ATP9A,FTO,PPFIA2,AKAP6,SORCS3,LINGO2,ATF2,GRID2,ZNF423,SEMA6D,FER,MICU2,SGCD,TMEM108,GABRB3,SLC39A8,RAPGEF2,PPA2,PRKCQ,GABRG2,CPS1,NRXN3,NSG1,KCND2,ATP8A2,SLC24A3,UBE3A,IDE,APC,TFF1,EPHB2,ERC1,PDLIM5,AGO3,TMTC2,MCTP1,RYR3,ADAMTS18,FLVCR1,NRP1,TRDN,ALK,SEMA3A,SEMA3E,ATP13A3,MALRD1,MYEF2,CDH2,TANC1,HDAC9,MAP2K6,FSTL4,ARHGAP28,MTOR,GABRB1,FBLN1,CADPS,NCAPG2,ADAM10,LRFN2,ABCB5,CSMD1,SLC24A2,NTRK3,CTNNA3,ABHD17C,RAPGEF4,ATF7IP,NSUN2,DNM3,SYT1,SYNDIG1,NPHP4,PPARA,PTPRD,RORA,SHISA6,SCARA5,PRKG1,ELAVL4,STX12,PLCL1,TSHZ3,ASIC2,DOCK10,TRPM1,CACNG3,PRKD1,ATP8A1,BCL2L1,ETS1,RYR2,SEMA3D,PRLR,MTMR2,KCNH5,VCL,DEPTOR,ROBO2,CDH23,NEDD9,MYRIP,ENPP1,UNC13C,RIMS2,SCN8A,RAB27A,L3MBTL3,SEMA5A,BCL11A,DCC,CTNNA2,NRG1,ATP10B,PRKCA,SEMA3C,PAH
GO:0010646	regulation of cell communication	4.1293350305836235e-16	CD44,PTPRR,SEMA4D,EVC,SYN3,TAOK3,PRKCB,SLC8A1,ANKRD6,GPR55,CD38,BCL2,CAMTA1,THRB,GRIK2,IGSF11,SNX25,UNC13B,NCAM1,IL6R,BRD4,NDRG2,APBA2,DLGAP1,RCAN1,JAK2,BICD1,OTUD7A,TPTE2,KALRN,FGF12,CACNG2,BTBD9,NFAT5,ZNF536,LAMA1,CDH11,GRIN2A,NRXN1,ADCYAP1R1,GRID1,SMOC2,ZDHHC17,CTNNA1,MYO9A,NTRK2,NLK,AFAP1,TIAM2,IQCJ-SCHIP1,SMARCA4,RNF152,ZNRF3,NRG3,SLC24A4,FBXL17,GAS2,GRIK3,CACNB2,PDE10A,STXBP4,MECOM,RGS12,SHANK2,KCTD8,NREP,DOK5,DLG5,ARHGAP24,SPRED2,SLIT2,ROR1,ZNF675,CSNK2A1,AKT3,TRIM5,PSD3,ALPK2,HECW1,RAP1GDS1,ERBB4,KANK1,DMRT1,BID,MACF1,RAP1A,TRIO,PTPRE,DUSP22,CHSY1,ABL2,MAP3K5,RGS3,HTR2C,CNTN4,TRAF3,FGD4,ITGA1,ITGA8,GRIK4,MAPK9,EYA1,ANK2,SLIT3,GRIN2B,ROBO1,PAK3,DGKB,GARNL3,TNKS,NDFIP2,OVOL2,SGMS1,MLLT3,LEMD3,PRKCE,ARHGEF11,PRKAA2

			,GRIK1,DGKI,DNMBP,EFHB,KDM4C,SLC1A1,GRM5,IGF1R,NLGN1,SHISA9,CFTR,NOS1AP,PTPRO,RBMS3,INSR,CLEC16A,ANK3,CNIH3,DOCK3,GPC6,RELN,RASGRF2,STK38,TNR,CELF4,DAPK1,VAV3,CNTN6,APP,PUM1,CCDC88A,PLCE1,PAK1,HCN1,CXADR,EPS8,GPC5,GHR,RASGRF1,PRDM16,USP7,STARD13,KL,LRRC4C,PPP1R9A,ZZEF1,LTBP1,SLAMF1,CLSTN2,TIAM1,SORCS2,DISC1,RALGPS1,ARHGAP42,EDAR,EGF,PDGFD,FYN,EPHA7,GRIA1,TRABD2B,STK3,CNOT7,USP18,S100B,NET1,ESR1,ARHGAP12,GRM1,PDE4D,ERC2,PRKACB,PDE3A,RIMS1,POR,MCTP2,WWOX,CNKSR2,EPHB1,GSG1L,FLT1,EFNA5,NXN,STK36,AMFR,PLCB4,AKAP6,SORCS3,GRID2,ZNF423,LRP2,RALGPS2,NTF3,FER,NTNG1,MAPRE2,TMEM108,RIC8B,TPTE,GRM7,RAPGEF2,PRKCQ,PRKCH,NRXN3,DLC1,NSG1,UBE3A,APC,AUTS2,EPHB2,ERC1,AGO3,MCTP1,MOB3B,CTNND2,PACSIN2,DOCK2,NRP1,CDH13,TRDN,DAB1,ALK,LDLRAD4,SEMA3A,SEMA3E,MGAT5,MAGI3,CDH2,SIPA1L2,MAP2K6,FSTL4,ARHGAP28,MTOR,KSR1,RALGAPA2,FBLN1,FHL2,NEU3,NCAPG2,RGS7,CD2AP,SPRED1,SIPA1L3,ADAM10,LRFN2,SCAI,PTPRT,SLC24A2,GLI3,NTRK3,FBN1,BMPER,SYT1,APIP,NPHP4,PPARA,PTPRD,RORA,SHISA6,PLCB1,RASGRP1,ELAVL4,NLRC5,MAGI2,PLCL1,TSHZ3,CACNG3,PRKD1,BCL2L1,HDAC2,TNN,RYR2,TGFA,PRLR,TBX20,PTPRA,MTMR2,ATF6,DEPTOR,ROBO2,IFT81,ZMYND11,RGS6,SRGAP3,AKAP13,MYRIP,ENPP1,UNC13C,RIMS2,EYA4,RALGAPA1,DLGAP2,SEMA5A,PRDM15,DCC,CHN1,NRG1,PRKCA
GO:0023051	regulation of signaling	1.2984168247683734e-15	CD44,PTPRR,SEMA4D,EVC,SYN3,TAOK3,PRKCB,SLC8A1,ANKRD6,GPR55,CD38,BCL2,CAMTA1,THRB,GRIK2,IGSF11,SNX25,UNC13B,NCAM1,IL6R,BRD4,NDRG2,APBA2,DLGAP1,RCAN1,JAK2,BICD1,OTUD7A,TPTE2,KALRN,FGF12,CACNG2,BTBD9,NFAT5,ZNF536,LAMA1,CDH11,GRIN2A,NRXN1,ADCYAP1R1,GRID1,SMOC2,ZDHHC17,CTNNA1,MYO9A,NTRK2,NLK,AFAP1,TIAM2,IQCJ-SCHIP1,SMARCA4,RNF152,ZNRF3,NRG3,SLC24A4,FBXL17,GAS2,GRIK3,CACNB2,PDE10A,STXBP4,MECOM,RGS12,SHANK2,KCTD8,NREP,DOK5,DLG5,ARHGAP24,SPRED2,SLIT2,ROR1,ZNF675,CSNK2A1,AKT3,TRIM5,PSD3,ALPK2,HECW1,RAP1GDS1,ERBB4,KANK1,DMRT1,BID,MACF1,RAP1A,TRIO,PTPRE,DUSP22,CHSY1,ABL2,MAP3K5,RGS3,HTR2C,CNTN4,TRAF3,FGD4,ITGA1,ITGA8,GRIK4,MAPK9,EYA1,ANK2,SLIT3,GRIN2B,ROBO1,PAK3,DGKB,GARNL3,TNKS,NDFIP2,OVOL2,SGMS1,MLLT3,LEMD3,PRKCE,ARHGEF11,PRKAA2,GRIK1,DGKI,DNMBP,EFHB,KDM4C,SLC1A1,GRM5,IGF1R,NLGN1,SHISA9,CORIN,CFTR,NOS1AP,PTPRO,RBMS3,INSR,CLEC16A,CNIH3,DOCK3,GPC6,RELN,RASGRF2,STK38,TNR,CELF4,DAPK1,VAV3,CNTN6,APP,PUM1,CCDC88A,PLCE1,PAK1,HCN1,EPS8,GPC5,GHR,RASGRF1,PRDM16,USP7,STARD13,KL,LRRC4C,PPP1R9A,ZZEF1,LTBP1,SLAMF1,CLSTN2,TIAM1,SORCS2,DISC1,RALGPS1,ARHGAP42,EDAR,EGF,PDGFD,FYN,EPHA7,GRIA1,TRABD2B,STK3,CNOT7,USP18,S100B,NET1,ESR1,ARHGAP12,GRM1,PDE4D,ERC2,PRKACB,PDE3A,RIMS1,POR,MCTP2,WWOX,CNKSR2,EPHB1,GSG1L,FLT1,EFNA5,NXN,STK36,AMFR,PLCB4,AKAP6,SORCS3,GRID2,ZNF423,LRP2,RALGPS2,NTF3,FER,NTNG1,MAPRE2,TMEM108,RIC8B,TPTE,GRM7,RAPGEF2,PRKCQ,PRKCH,NRXN3,DLC1,NSG1,UBE3A,APC,AUTS2,EPHB2,ERC1,AGO3,MCTP1,MOB3B,CTNND2,PACSIN2,DOCK2,NRP1,CDH13,DAB1,ALK,LDLRAD4,SEMA3A,SEMA3E,MGAT5,MAGI3,KIF16B,CDH2,SIPA1L2,MAP2K6,FSTL4,ARHGAP28,MTOR,KSR1,RALGAPA2,FBLN1,FHL2,NEU3,NCAPG2,RGS7,CD2AP,SPRED1,SIPA1L3,ADAM10,LRFN2,SCAI,PTPRT,SLC24A2,GLI3,NTRK3,FBN1,BMPER,SYT1,APIP,NPHP4,PPARA,PTPRD,RORA,SHISA6,PLCB1,RASGRP1,ELAVL4,NLRC5,MAGI2,PLCL1,TSHZ3,CACNG3,PRKD1,BCL2L1,HDAC2,TNN,RYR2,TGFA,PRLR,TBX20,PTPRA,MTMR2,ATF6,DEPTOR,ROBO2,IFT81,ZMYND11,RGS6,SRGAP3,AKAP13,MYRIP,ENPP1,UNC13C,RIMS2,EYA4,RALGAPA1,DLGAP2,SEMA5A,PRDM15,DCC,CHN1,NRG1,PRKCA
GO:0035556	intracellular signal	1.1004525050090167e-14	CD44,APBB2,PTPRR,SEMA4D,INIP,TEAD1,RPS6KA5,TAOK3,PRKCB,SLC8A1,ANKRD6,GPR55,FBXO31,BCL2,CAMTA1,CHFR,CDC42EP3,MAPK10,IL6R,BRD4,NDRG2,GRIP1,TLK1,RCAN1,JAK2,OTUD7A,TPTE2,KALRN,FGF12,NFAT5,GRIN2A,MAST4,NRXN1,ADCYAP1R1,RPS6KA2,ZDHHC17,KCNH1,DCDC1,CACNA1C,MYO9A,NTRK2,FOXN3,NLK,RASGEF1B,TIAM2,IQCJ-

	transduction		SCHIP1,RNF152,NRG3,ELMO1,SLC24A4,PDE10A,MECOM,STK32B,SHANK2,RAPGEF5,DOK5,DLG5,CHRM3,ARHGAP24,SPRED2,SLIT2,ROR1,ZNF675,AKT3,TRIM5,PSD3,RAP1GDS1,ERBB4,KANK1,ATRX,DMRT1,BID,RAP1A,TRIO,DUSP22,MYOM1,ABL2,MAP3K5,NOS1,RERG,HTR2C,PIK3C3,TRAF3,FGD4,ITGA1,MAPK9,PTGFR,ANK2,GRIN2B,ROBO1,PAK3,DGKB,GARNL3,NDFIP2,SGMS1,ITPR2,LEMD3,DGKK,PRKCE,ARHGEF11,PRKAA2,DGKI,DNMBP,EFHB,GRM5,NTN1,NR5A2,IGF1R,NLGN1,NOS1AP,INSR,CTNNAL1,CLEC16A,DOCK3,RELN,RASGRF2,STK38,ADCY9,DAPK1,VAV3,INPP5A,APP,PUM1,CCDC88A,PLCE1,PAK1,PPP1R13B,EPS8,GHR,RASGRF1,RIN2,USP7,STARD13,KL,PPP1R9A,TMEM117,SLAMF1,RGL1,TIAM1,DISC1,RALGPS1,ARHGAP42,EDAR,EGF,PDGFD,FYN,EPHA7,FHIT,STK3,S100B,NET1,ESR1,ARHGAP12,GRM1,PDE4D,PRKACB,PDE3A,DOCK4,MCTP2,WWOX,HUNK,CNKSR2,EPHB1,DOCK1,FLT1,CDC14B,HDAC4,STK36,PLCB4,SH3BP5,AKAP6,MARK2,ATF2,RBBP8,LRP2,RALGPS2,NTF3,FER,SNRK,CAMK4,MAPRE2,SGCD,TPTE,RAPGEF2,PRKCQ,PRKCH,DLC1,UBE3A,APC,AUTS2,EPHB2,ERC1,AGO3,MCTP1,MOB3B,NBN,DOCK2,NRP1,CDH13,DAB1,ALK,SEMA3A,SEMA3E,DCLK1,MAGI3,CDH2,SIPA1L2,RCAN2,ASB3,PIK3R3,MAP2K6,ARHGAP28,MTOR,STK38L,KSR1,RALGAPA2,FBLN1,BLM,FHL2,RGS7,STK32A,CD2AP,SPRED1,SIPA1L3,SCAI,NTRK3,RAPGEF4,BMPER,HMGA2,NSUN2,APIP,DOCK9,PPARA,RORA,PLCB1,PRKG1,RASGRP1,NLRC5,MAGI2,PLCL1,RALA,DOCK10,PRKD1,BCL2L1,RYR2,TGFA,DEPTOR,ZMYND11,RGS6,SRGAP3,AKAP13,RALGAPA1,SEMA5A,PRDM15,CHN1,NRG1,PRKCA,FMN2
GO:0051716	cellular response to stimulus	1.2191171450127982e-14	CD44,APBB2,PTPRR,ERG,PDE1C,SEMA4D,INIP,ZFYVE1,EVC,TEAD1,RPS6KA5,TAOK3,PRKCB,CPNE4,SLC8A1,ANKRD6,KCNC1,GPR55,DNAJC15,CD38,FBXO31,BCL2,CAMTA1,CHFR,THRB,CDC42EP3,GRIK2,IGSF11,SNX25,UNC13B,MAPK10,NCAM1,GNG12,IL6R,ZBTB20,SORBS2,SKAP2,CASP5,KCNK10,BRD4,GPR158,NDRG2,GRIP1,TLK1,DLGAP1,RCAN1,JAK2,BICD1,ABCG8,OTUD7A,TPTE2,KALRN,FGF12,CACNG2,NFAT5,TRHDE,ZNF536,LAMA1,GRIN2A,MAST4,NRXN1,ARID1B,IL1RAPL1,WDPCP,MAGI1,LAMA3,GBP4,ADCYAP1R1,GRID1,RPS6KA2,TDP1,SMOC2,ZDHHC17,KCNH1,DCDC1,CACNA1C,BMF,WDR70,PNPLA3,FAM83B,CTNNA1,MYO9A,NTRK2,FOXN3,NLK,ITGBL1,RASGEF1B,AFAP1,TIAM2,IQCJ-SCHIP1,SMARCA4,RNF152,CNTN1,ZNRF3,ENPEP,NRG3,PTPRG,MC2R,BTBD11,ELMO1,SLC24A4,CABIN1,FBXL17,GAS2,GRIK3,PDE10A,STXBP4,MTPN,MT1HL1,SOX6,MECOM,STK32B,RGS12,SHANK2,RAPGEF5,UBE2E2,KCTD8,CHCHD6,UNC5D,NREP,GABRA5,DOK5,DLG5,SMARCAD1,ATRNL1,CHRM3,CPE,ARHGAP24,SPRED2,IGHV1OR15-9,SLIT2,PITPNC1,MYLK3,ROR1,GLP2R,ZNF675,CSNK2A1,DTNA,AKT3,CRB1,TRIM5,PSD3,ALPK2,HECW1,RAP1GDS1,ERBB4,KANK1,ATRX,DMRT1,BID,MACF1,MNAT1,RAP1A,TRIO,RAD51B,PTPRE,DUSP22,CHSY1,MYOM1,PSG8,EXT2,OR4C46,ABL2,MAP3K5,NOS1,ARPP21,ACACA,ABCG1,RGS3,MAML2,RERG,HTR2C,NEK4,SLC40A1,GABRR2,PIK3C3,TRAF3,CHD6,FGD4,ITGA1,CORO2B,ITGA8,GRIK4,ALDH1A2,GABRG1,MAPK9,ESRRG,PTGFR,IGHV1OR21-1,CDH17,EYA1,MORC3,ANKS1B,P2RX6,HSF2BP,AKAP10,ANK2,PLA2G4A,SLC1A2,SUPT3H,SLIT3,GRIN2B,ROBO1,PAK3,DGKB,GARNL3,TNKS,NDFIP2,GABRA2,OVOL2,SGMS1,ITPR2,BRINP1,MLLT3,LEMD3,RNF138,GABPA,PRICKLE2,ITGB3BP,DGKK,TMEM67,PRKCE,PSG9,ARHGEF11,PRKAA2,PACRG,BBS2,IL1RAPL2,PTPRN2,GRIK1,SOX5,DSCAM,DGKI,RIN3,DNMBP,EFHB,KDM4C,SLC1A1,GRM5,EPHA6,NTN1,ARAP2,NR5A2,IGF1R,NLGN1,SHISA9,OR9Q1,SHROOM3,CFTR,CAMK1D,FLRT2,NOS1AP,PTPRO,MSRA,RBMS3,INSR,CTNNAL1,CLEC16A,ITGA9,EGLN3,ANK3,MORC2,GMDS,CNIH3,DOCK3,GPC6,RELN,RASGRF2,STK38,FBXO32,TNR,ADCY9,CELF4,DAPK1,VAV3,INPP5A,VRK1,CNTN6,ACSM2B,APP,FBLN5,PUM1,CCDC88A,PLCE1,ADAM12,PAK1,GNAL,MITF,CACNA2D1,HCN1,PPP1R13B,TOP3A,CHRM5,NSMCE2,CXADR,EPS8,GPC5,TENM4,GHR,RASGRF1,RIN2,PRDM16,USP7,KIR2DL4,STARD13,KL,ALCAM,PPP1R9A,TMEM117,MICU1,LTBP1,SLAMF1,RGL1,INO80D,RARB,DIDO1,MYH13,WDR12,TIAM1,SORCS2,SLC39A12,DISC1,RALGPS1,ARHGAP42,CHAF1A,PIEZO2,SVEP1,EDAR,EGF,PDGFD,FYN,FAM3B,XRCC4,EPHA7,F

			HIT,NSG2,GRIA1,TRABD2B,SPIDR,STK3,CNOT7,MSR1,USP18,S100B,NET1,ESR1,ARHGAP12,GABRG3,SEL1L2,PLCXD3,GRM1,PDE4D,PRKACB,GNG2,PDE3A,RIMS1,POR,DOCK4,MCTP2,WWOX,HUNK,CNKSR2,FUT8,EPHB1,CREM,EFEMP1,GSG1L,DOCK1,FLT1,EXT1,EFNA5,NXN,CDC14B,C14ORF39,HDAC4,STK36,AMFR,PLCB4,FTO,SH3BP5,AKAP6,SORCS3,TENM3,MARK2,ATF2,RBBP8,GRID2,ZNF423,LRP2,SEMA6D,RALGPS2,NTF3,FER,SNRK,GLDC,CAMK4,MAPRE2,RAD51AP1,SGCD,TMEM108,RIC8B,GABRB3,TPTE,GRM7,RAPGEF2,MX1,IMMP2L,PRKCQ,GABRG2,CPS1,PRKCH,NRXN3,RHPN2,DLC1,NSG1,GABBR2,KCND2,UBE3A,GRIA4,IDE,TPH2,APC,MACROD2,INO80,AUTS2,TFF1,EPHB2,ERC1,AGO3,MCTP1,MOB3B,RYR3,NBN,ADAMTS18,RGMB,CTNND2,SETD2,PKP1,DOCK2,NRP1,CDH13,RFC3,DAB1,RFTN1,ALK,EVC2,LDLRAD4,SEMA3A,SEMA3E,MGAT5,MYEF2,DCLK1,MAGI3,KIF16B,NRIP1,CDH2,ARID5B,SIPA1L2,RCAN2,LRRC69,TENM2,VPS41,SYCP1,ASB3,HDAC9,PIK3R3,MAP2K6,FSTL4,ARHGAP28,MTOR,STK38L,KSR1,RALGAPA2,RORB,GABRB1,FBLN1,ST8SIA1,BLM,FHL2,NEU3,NCAPG2,RGS7,STK32A,CD2AP,USP25,SPRED1,SIPA1L3,ADAM10,FLNB,SCAI,PTPRT,TRERF1,SLC24A2,GLI3,NTRK3,RXFP1,FBN1,CHKA,RAB31,RAPGEF4,BMPER,PDE1A,HMGA2,NSUN2,DEFA3,PTPRK,SORCS1,TBC1D4,SYT1,APIP,DPF3,NPHP4,DOCK9,DLG2,PPP1R12B,PPARA,PLXNA2,PTPRD,RORA,SHISA6,SCARA5,PLCB1,PRKG1,RASGRP1,ELAVL4,NLRC5,MAGI2,PLCL1,ABI1,PXDNL,ASIC2,RALA,DOCK10,TRPM1,CACNG3,PRKD1,BCL2L1,HDAC2,TNN,RYR2,SEMA3D,TGFA,PRLR,TBX20,PTPRA,OR11G2,MTMR2,ATF6,IL16,DEPTOR,BACH1,ROBO2,IFT81,ZMYND11,RGS6,SRGAP3,AKAP13,NEDD9,ENPP1,PCP4,RIMS2,STAC,EYA4,RALGAPA1,DLGAP2,SEMA5A,PRDM15,OR4N2,BCL11A,DCC,CHN1,VPS13C,PARD3,NRG1,PRKCA,FMN2,SEMA3C,FANCB,CSF2RB,PCNT,ST18
GO:0007165	signal transduction	1.7585164230371407e-14	CD44,APBB2,PTPRR,ERG,PDE1C,SEMA4D,INIP,EVC,TEAD1,RPS6KA5,TAOK3,PRKCB,SLC8A1,ANKRD6,GPR55,CD38,FBXO31,BCL2,CAMTA1,CHFR,THRB,CDC42EP3,GRIK2,IGSF11,SNX25,UNC13B,MAPK10,NCAM1,GNG12,IL6R,SORBS2,SKAP2,CASP5,KCNK10,BRD4,GPR158,NDRG2,GRIP1,TLK1,DLGAP1,RCAN1,JAK2,BICD1,ABCG8,OTUD7A,TPTE2,KALRN,FGF12,CACNG2,NFAT5,TRHDE,ZNF536,LAMA1,GRIN2A,MAST4,NRXN1,IL1RAPL1,WDPCP,MAGI1,LAMA3,ADCYAP1R1,GRID1,RPS6KA2,SMOC2,ZDHHC17,KCNH1,DCDC1,CACNA1C,BMF,FAM83B,CTNNA1,MYO9A,NTRK2,FOXN3,NLK,ITGBL1,RASGEF1B,AFAP1,TIAM2,IQCJ-SCHIP1,SMARCA4,RNF152,CNTN1,ZNRF3,ENPEP,NRG3,PTPRG,MC2R,BTBD11,ELMO1,SLC24A4,CABIN1,FBXL17,GAS2,GRIK3,PDE10A,STXBP4,MECOM,STK32B,RGS12,SHANK2,RAPGEF5,KCTD8,UNC5D,NREP,GABRA5,DOK5,DLG5,ATRNL1,CHRM3,CPE,ARHGAP24,SPRED2,IGHV1OR15-9,SLIT2,PITPNC1,ROR1,GLP2R,ZNF675,CSNK2A1,DTNA,AKT3,TRIM5,PSD3,ALPK2,HECW1,RAP1GDS1,ERBB4,KANK1,ATRX,DMRT1,BID,MACF1,RAP1A,TRIO,PTPRE,DUSP22,CHSY1,MYOM1,PSG8,EXT2,OR4C46,ABL2,MAP3K5,NOS1,RGS3,MAML2,RERG,HTR2C,GABRR2,PIK3C3,TRAF3,FGD4,ITGA1,ITGA8,GRIK4,ALDH1A2,GABRG1,MAPK9,ESRRG,PTGFR,IGHV1OR21-1,CDH17,EYA1,ANKS1B,P2RX6,AKAP10,ANK2,SLIT3,GRIN2B,ROBO1,PAK3,DGKB,GARNL3,TNKS,NDFIP2,GABRA2,OVOL2,SGMS1,ITPR2,MLLT3,LEMD3,RNF138,PRICKLE2,ITGB3BP,DGKK,PRKCE,PSG9,ARHGEF11,PRKAA2,BBS2,IL1RAPL2,GRIK1,DSCAM,DGKI,RIN3,DNMBP,EFHB,KDM4C,SLC1A1,GRM5,EPHA6,NTN1,ARAP2,NR5A2,IGF1R,NLGN1,SHISA9,OR9Q1,FLRT2,NOS1AP,PTPRO,RBMS3,INSR,CTNNAL1,CLEC16A,ITGA9,ANK3,GMDS,CNIH3,DOCK3,GPC6,RELN,RASGRF2,STK38,ADCY9,CELF4,DAPK1,VAV3,INPP5A,VRK1,CNTN6,APP,PUM1,CCDC88A,PLCE1,ADAM12,PAK1,GNAL,MITF,PPP1R13B,CHRM5,EPS8,GPC5,TENM4,GHR,RASGRF1,RIN2,PRDM16,USP7,KIR2DL4,STARD13,KL,ALCAM,PPP1R9A,TMEM117,LTBP1,SLAMF1,RGL1,RARB,DIDO1,WDR12,TIAM1,SORCS2,SLC39A12,DISC1,RALGPS1,ARHGAP42,SVEP1,EDAR,EGF,PDGFD,FYN,FAM3B,EPHA7,FHIT,NSG2,GRIA1,TRABD2B,STK3,CNOT7,USP18,S100B,NET1,ESR1,ARHGAP12,GABRG3,PLCXD3,GRM1,PDE4D,PRKACB,GNG2,PDE3A,RIMS1,POR,DOCK4,MCTP2,WWOX,HUNK,CNKSR2,FUT8,E

			PHB1,CREM,EFEMP1,GSG1L,DOCK1,FLT1,EXT1,EFNA5,NXN,CDC14B,HDAC4,STK36,AMFR,PLCB4,SH3BP5,AKAP6,SORCS3,TENM3,MARK2,ATF2,RBBP8,GRID2,ZNF423,LRP2,SEMA6D,RALGPS2,NTF3,FER,SNRK,CAMK4,MAPRE2,SGCD,TMEM108,RIC8B,GABRB3,TPTE,GRM7,RAPGEF2,MX1,PRKCQ,GABRG2,PRKCH,NRXN3,RHPN2,DLC1,NSG1,GABBR2,UBE3A,GRIA4,IDE,APC,AUTS2,TFF1,EPHB2,ERC1,AGO3,MCTP1,MOB3B,NBN,ADAMTS18,RGMB,CTNND2,PKP1,DOCK2,NRP1,CDH13,DAB1,RFTN1,ALK,EVC2,LDLRAD4,SEMA3A,SEMA3E,MGAT5,DCLK1,MAGI3,KIF16B,CDH2,ARID5B,SIPA1L2,RCAN2,LRRC69,TENM2,ASB3,PIK3R3,MAP2K6,FSTL4,ARHGAP28,MTOR,STK38L,KSR1,RALGAPA2,RORB,GABRB1,FBLN1,BLM,FHL2,NEU3,NCAPG2,RGS7,STK32A,CD2AP,SPRED1,SIPA1L3,ADAM10,FLNB,SCAI,PTPRT,TRERF1,SLC24A2,GLI3,NTRK3,RXFP1,FBN1,RAPGEF4,BMPER,PDE1A,HMGA2,NSUN2,DEFA3,PTPRK,SORCS1,APIP,NPHP4,DOCK9,PPP1R12B,PPARA,PLXNA2,PTPRD,RORA,SHISA6,PLCB1,PRKG1,RASGRP1,NLRC5,MAGI2,PLCL1,ABI1,ASIC2,RALA,DOCK10,TRPM1,CACNG3,PRKD1,BCL2L1,HDAC2,TNN,RYR2,SEMA3D,TGFA,PRLR,TBX20,PTPRA,OR11G2,MTMR2,ATF6,IL16,DEPTOR,ROBO2,IFT81,ZMYND11,RGS6,SRGAP3,AKAP13,NEDD9,ENPP1,PCP4,RIMS2,STAC,EYA4,RALGAPA1,DLGAP2,SEMA5A,PRDM15,OR4N2,DCC,CHN1,PARD3,NRG1,PRKCA,FMN2,SEMA3C,CSF2RB,PCNT,ST18
GO:0034329	cell junction assembly	3.2545595967897345e-14	SEMA4D,BCL2,CDH8,NEGR1,EPB41L3,CDH11,NRXN1,IL1RAPL1,WDPCP,CTNNA1,MYO9A,NTRK2,OCLN,SHANK2,DLG5,ERBB4,MACF1,RAP1A,DUSP22,CORO2B,PEAK1,CDH18,ANK2,GABRA2,IL1RAPL2,DSCAM,SDK1,NTN1,NLGN1,CNTNAP2,FLRT2,PTPRO,CDH12,TBCD,GPC6,APP,LRFN5,CLSTN2,FMN1,EPHA7,CNTN5,EPHB1,EFNA5,TLN2,LINGO2,GRID2,FER,GABRB3,RAPGEF2,KIRREL3,GABRG2,PRKCH,NRXN3,DLC1,APC,EPHB2,PDLIM5,CTNND2,PKP1,NRP1,CDH2,NTRK3,PTPRK,DNM3,SYNDIG1,NPHP4,PTPRD,CDH9,ASIC2,PTPRA,VCL,ROBO2,PARD3,NRG1,PRKCA
GO:0007267	cell-cell signaling	9.192089330293162e-14	SYN3,PRKCB,ANKRD6,CD38,GRIK2,IGSF11,UNC13B,CDH8,NDRG2,APBA2,DLGAP1,JAK2,KALRN,FGF12,CACNG2,BTBD9,TRHDE,CDH11,GRIN2A,NRXN1,IL1RAPL1,GRID1,RPS6KA2,CACNA1C,AMPH,EXOC4,NTRK2,NLK,SMARCA4,ZNRF3,ENPEP,NRG3,GRIK3,CACNB2,STXBP4,SHANK2,GABRA5,CHRM3,CPE,ROR1,CSNK2A1,DTNA,CRB1,ALPK2,HECW1,KANK1,MACF1,RAP1A,NOS1,HTR2C,CACNA1E,CNTN4,GABRR2,GRIK4,GABRG1,SV2B,P2RX6,ANK2,PLA2G4A,SLC1A2,GRIN2B,DGKB,TNKS,GABRA2,FCHSD2,MLLT3,RNF138,PRICKLE2,PRKCE,PSG9,PRKAA2,PTPRN2,GRIK1,DGKI,SLC1A1,GRM5,NLGN1,SHISA9,CFTR,PTPRO,RBMS3,SV2C,GPC6,RELN,RASGRF2,TNR,CELF4,APP,CCDC88A,MITF,CACNA2D1,HCN1,CHRM5,CXADR,GPC5,RASGRF1,LRRC4C,PPP1R9A,ZZEF1,LTBP1,CLSTN2,TIAM1,SORCS2,DISC1,EGF,FYN,FAM3B,RIMBP2,GRIA1,TRABD2B,STK3,S100B,GABRG3,GRM1,ERC2,RIMS1,MCTP2,WWOX,EPHB1,SNAP29,EXT1,EFNA5,NXN,AMFR,PLCB4,SORCS3,MARK2,GRID2,ZNF423,NTF3,NTNG1,TMEM108,GABRB3,GRM7,RAPGEF2,GABRG2,NRXN3,NSG1,GABBR2,KCND2,APC,EPHB2,ERC1,MCTP1,CTNND2,PACSIN2,NRP1,CDH2,MAP2K6,GABRB1,SH3KBP1,CADPS,ADAM10,LRFN2,SLC24A2,GLI3,RAPGEF4,HMGA2,SYT1,NPHP4,DLG2,PTPRD,SHISA6,PLCB1,ELAVL4,MAGI2,PLCL1,TSHZ3,ASIC2,CACNG3,TNN,RYR2,PTPRA,MTMR2,MYRIP,UNC13C,RIMS2,DLGAP2,SEMA5A,PRDM15,DCC,NRG1
GO:0050789	regulation of biological process	1.4226054388158902e-13	CD44,SAMD4A,KCNMA1,C10ORF90,PKNOX2,ZHX3,APBB2,PTPRR,SCAF8,RTN1,ERG,PARN,PDE1C,SEMA4D,INIP,MED13L,EVC,TEAD1,NFIA,SYN3,RPS6KA5,TAOK3,PRKCB,EIF4G3,A2M,SLC8A1,ANKRD6,KCNC1,GPR55,DNAJC15,SIAH3,CD38,ZNF257,FBXO31,PIWIL3,TRAPPC9,BCL2,CAMTA1,SAMSN1,CHFR,THADA,COL18A1,TOX3,THRB,DNAH11,ZSCAN5C,RAG1,CDC42EP3,GRIK2,IGSF11,SNX25,UNC13B,MAPK10,NCAM1,KDM4B,GNG12,IL6R,KCNQ5,CDH8,ZBTB20,SCN11A,ATP10A,SORBS2,SKAP2,HS1BP3,KCNJ6,CASP5,KCNK10,BRD4,GPR158,ZNF568,NDRG2,GRIP1,APBA2,TLK1,ASTN2,TANC2,CSMD3,DLGAP1,RCAN1,NTN4,JAK2,TM9SF4,BICD1,MIR17HG,ABCG8,OTUD7A,TPTE2,KALRN,USH2A,NEGR1,FGF12,CACNG2,BTBD9,NFAT5,FLI1,TRHDE,ZNF536,EPB41L3,LAMA1,PARVB,CDH11,ZBTB7C,GRIN2A,MAST4,NRXN1,ARID1B,IL1RAPL1,WDPCP,MAGI1,LAMA3,ADCYAP1R1,CTDP1,DPP6,GRID1,RPS6KA2,SMOC2,ZDHHC

			17,KCNH1,DCDC1,CACNA1C,BMF,EXOC4,WDR70,FAM83B,CTNNA1,MYO9A,NTRK2,FOXN3,OCLN,NLK,ITGBL1,RASGEF1B,AFAP1,TIAM2,MYT1L,SRGAP2B,IQCJ-SCHIP1,SMARCA4,RNF152,CNTN1,ZNRF3,PALMD,MEOX2,TTC39B,ENPEP,PLEKHB2,GLIS3,NRG3,PTPRG,MC2R,BTBD11,ELMO1,SLC24A4,CABIN1,FBXL17,GAS2,GRIK3,CACNB2,PDE10A,NUMB,STXBP4,MED15,MTPN,SOX6,MECOM,PDE4DIP,STK32B,RGS12,SHANK2,RAPGEF5,UBE2E2,KCTD8,UNC5D,ZNF567,NREP,GABRA5,DOK5,DLG5,CFDP1,SMARCAD1,ZIM3,ATRNL1,CHRM3,CPE,ARHGAP24,SPRED2,IGHV1OR15-9,SLIT2,PITPNC1,MYLK3,ABCA5,ROR1,GLP2R,SLC4A4,ZNF675,CSNK2A1,DTNA,AKT3,KMT2C,KCNE4,TRIM5,KCNS3,PSD3,ALPK2,ABCA13,HECW1,RAP1GDS1,AFF3,ERBB4,KANK1,ATRX,DMRT1,BID,MACF1,MNAT1,TAF4B,RAP1A,TRIO,CTNNBL1,RAD51B,PTPRE,DUSP22,CHSY1,MYOM1,PSG8,EXT2,ZSCAN30,OR4C46,ABL2,PSMA1,MAP3K5,NOS1,ABCG1,RGS3,MAML2,RERG,HTR2C,NEK4,CACNA1E,CTIF,CNTN4,TBC1D5,SAMD13,SLC40A1,SLCO3A1,GABRR2,PIK3C3,TRAF3,SND1,CHD6,FGD4,ETS2,ITGA1,TCF12,ZNF721,HIRA,CORO2B,ITGA8,GRIK4,RBM19,RUNX1,KIR3DL2,ALDH1A2,GABRG1,TSHZ2,MAPK9,ESRRG,PTGFR,IGHV1OR21-1,FAM171A1,ZNF595,CDH17,PEAK1,EYA1,MORC3,ANKS1B,P2RX6,AKAP10,SPOCK3,FRMD6,PLS1,SPON1,ANK2,PLA2G4A,SLC1A2,ZBTB16,SUPT3H,SLIT3,GRIN2B,ZNF518A,PHC2,ROBO1,ZNF578,EGFLAM,PAK3,DGKB,GARNL3,DPH6,EBF1,TNKS,KLF12,NDFIP2,GABRA2,MDM1,OVOL2,FCHSD2,SGMS1,ITPR2,BRINP1,MLLT3,BCL2L13,LEMD3,KHDRBS2,RNF138,CHODL,GABPA,PRICKLE2,ITGB3BP,CACNA2D3,DGKK,TMEM67,PRKCE,GLIS1,PSG9,ARHGEF11,PRKAA2,PACRG,BBS2,IL1RAPL2,EDIL3,CDYL2,HIVEP2,KCNH8,GRIK1,SOX5,DSCAM,DGKI,RIN3,ANKFN1,DNMBP,EFHB,KDM4C,DCUN1D4,SDK1,SLC1A1,GRM5,EPHA6,NTN1,ARAP2,NR5A2,LDB2,IGF1R,SPTB,SNX30,NLGN1,SHISA9,OR9Q1,SHROOM3,JAM2,ALX4,CORIN,CNTNAP2,MAP2,KCNIP4,CFTR,CAMK1D,FLRT2,MLLT10,NOS1AP,PTPRO,NKAIN2,CD96,RBMS3,INSR,COBL,CTNNAL1,CLEC16A,PHF20L1,ITGA9,MTRF1,CATSPER2,EGLN3,CUX1,ANK3,MORC2,GMDS,CNIH3,DOCK3,TBCD,GPC6,RELN,RASGRF2,TRPS1,ADAMTS5,STK38,AOAH,FBXO32,CDH4,TNR,ADCY9,DPP10,CELF4,DAPK1,VAV3,INPP5A,ZNF600,VRK1,ZNF678,CNTN6,CLIC6,ZNF420,APP,FBLN5,PUM1,CCDC88A,ARNT2,KCTD1,RNLS,SPOCK1,HPSE2,PLCE1,ADAM12,PAK1,GNAL,MITF,IGF2BP3,CACNA2D1,ADCK1,HCN1,PPP1R13B,CHRM5,NSMCE2,ZNF208,FRY,CXADR,EPS8,LRFN5,UTRN,GPC5,TENM4,PRR16,TSPAN13,GHR,DUX4,RIPK4,RASGRF1,RIN2,PRDM16,FRMD5,RNF217,USP7,RBFOX1,MEIS2,KIR2DL4,STARD13,SCP2,KL,LRRC4C,ALCAM,PPP1R9A,AVEN,TMEM117,ADAMTS16,TASP1,MICU1,ZZEF1,LTBP1,SLAMF1,RGL1,NKAIN3,BACE2,INO80D,CLSTN2,RARB,DIDO1,WDR12,TCF4,TIAM1,FOCAD,PBX1,PHACTR1,MLIP,SORCS2,PRIM2,SLC39A12,DISC1,FMN1,RALGPS1,ARHGAP42,ZFPM2,SVEP1,ASAP1,PCBP3,FRMPD4,EDAR,EGF,PDGFD,FYN,FAM3B,KCND3,EPHA7,FHIT,NSG2,GRIA1,ZNF627,TRABD2B,SPIDR,STK3,CNOT7,MSR1,PSIP1,USP18,S100B,NET1,TOX,ESR1,ARHGAP12,GABRG3,PLCXD3,KCNAB1,GRM1,PDE4D,ERC2,PRKACB,GNG2,PDE3A,RIMS1,POR,L3MBTL4,DOCK4,ATP6V1E1,FRMD4A,MCTP2,WWOX,HUNK,CNKSR2,FUT8,EPHB1,SSBP2,CREM,CTTNBP2,FHOD3,EFEMP1,TNRC6B,AJAP1,ABCC9,GSG1L,HERC1,DOCK1,PARP15,FLT1,EXT1,EFNA5,NXN,CDC14B,C14ORF39,HDAC4,ZNF717,STK36,TRPC5,AMFR,PLCB4,ATP9A,FTO,PPFIA2,SH3BP5,AKAP6,SORCS3,POU6F2,TENM3,LINGO2,MARK2,ATF2,ZNF880,RBBP8,GRID2,ZNF423,LRP2,SEMA6D,ZNF573,C2,RALGPS2,NTF3,FER,SNRK,SUSD4,CAMK4,CELF2,TP53I11,NTNG1,MAPRE2,ISX,RAD51AP1,SGCD,TMEM108,RIC8B,GABRB3,TPTE,GRM7,SLC39A8,RAPGEF2,NAV3,MX1,PLGRKT,ZNF615,GTF2I,ATXN1,PRKCQ,GABRG2,PRKCH,NRXN3,RHPN2,RABGAP1L,DLC1,NSG1,GABBR2,KCND2,ATP8A2,SLC24A3,UBE3A,GRIA4,IDE,APC,ZBTB25,INO80,AUTS2,TFF1,EPHB2,SCAF4,ERC1,ZNF850,PDLIM5,AGO3,C9,MCTP1,MOB3B,RYR3,NBAS,PRTG,NBN,ADAMTS18,RGMB,CTNND2,SETD2,PACSIN2,PKP1,DOCK2,NUP214,SDCCAG8,FLVCR1,NRP1,CDH13,RFC3,ZNF879,DACH1,TRDN,SLC2A13,ZNF397,DAB1,RFTN1,ALK,EVC2,LDLRAD4,SEMA3A,SE

			MA3E,MGAT5,MALRD1,MYEF2,DCLK1,MAGI3,KIF16B,NRIP1,CDH2,ARID5B,SIPA1L2,CCNG2,RCAN2,LRRC69,TENM2,TANC1,SERPINB7,VPS41,SYCP1,ZNF407,ASB3,HDAC9,PIK3R3,MAP2K6,FSTL4,ARHGAP28,MTOR,STK38L,KSR1,RALGAPA2,RORB,GABRB1,FBLN1,ST8SIA1,BLM,SH3KBP1,FHL2,CADPS,NEU3,NCAPG2,RGS7,STK32A,CD2AP,ZFP30,USP25,SPRED1,SIPA1L3,ADAM10,DRAM1,KANSL1,LRFN2,FLNB,SCAI,PAPPA2,PTPRT,TRERF1,SLC24A2,GLI3,NTRK3,RXFP1,FBN1,RAB31,CTNNA3,VPS13D,ABHD17C,ZNF292,TBX15,RAPGEF4,BMPER,ANKRD31,ZNF521,PDE1A,TMPRSS2,ATF7IP,HMGA2,MX2,CREB5,NSUN2,DEFA3,PTPRK,SORCS1,TBC1D4,DNM3,SYT1,APIP,SYNDIG1,ASXL3,DPF3,NPHP4,DOCK9,PPP1R12B,SACS,PPARA,PLXNA2,PTPRD,RORA,SHISA6,PLCB1,LOXL2,BPTF,PRKG1,RASGRP1,ELAVL4,NLRC5,STXBP6,MXI1,TTC28,MAGI2,NELL1,PLCL1,ABI1,TSHZ3,ASIC2,RALA,DOCK10,TRPM1,CACNG3,NECAB1,PRKD1,ATP8A1,TNFAIP8,BCL2L1,HDAC2,ETS1,MRPS27,TNN,RYR2,SEMA3D,LDLRAD3,BANP,TGFA,PRLR,TBX20,PTPRA,FAT3,OR11G2,MTMR2,KCNH5,ATF6,IL16,VCL,DEPTOR,BACH1,ATAT1,ROBO2,EWSR1,IFT81,ZMYND11,RGS6,SRGAP3,AKAP13,WDR41,NEDD9,MYRIP,ENPP1,UNC13C,PCP4,RIMS2,STAC,SCN8A,RAB27A,EYA4,RALGAPA1,L3MBTL3,DLGAP2,POMT2,HIVEP3,CLIP1,SEMA5A,CABLES1,PRDM15,OR4N2,BCL11A,DCC,ZNF112,CTNNA2,PVT1,CHN1,ETV6,VPS13C,KCNJ15,PARD3,NRG1,CAST,FANK1,ZNF845,NPAS3,PRKCA,FMN2,SEMA3C,FANCB,CSF2RB,PCNT,ST18,TOP1
GO:0050896	response to stimulus	2.7132269572563334e-13	CD44,KCNMA1,APBB2,PTPRR,ERG,PDE1C,SEMA4D,INIP,ZFYVE1,EVC,TEAD1,RPS6KA5,TAOK3,PRKCB,A2M,TRPM6,CPNE4,SLC8A1,ANKRD6,KCNC1,GPR55,DNAJC15,CD38,FBXO31,BCL2,CAMTA1,SAMSN1,CHFR,RSRC1,THRB,B3GALT5,RAG1,CDC42EP3,GRIK2,IGSF11,SNX25,UNC13B,MAPK10,NCAM1,GNG12,IL6R,CDH8,ZBTB20,SCN11A,SORBS2,SKAP2,GOT2,CASP5,KCNK10,BRD4,GPR158,NDRG2,GRIP1,TLK1,DLGAP1,RCAN1,JAK2,TM9SF4,BICD1,ABCG8,OTUD7A,TPTE2,KALRN,USH2A,FGF12,CACNG2,NFAT5,TRHDE,ZNF536,LAMA1,GRIN2A,MAST4,NRXN1,ARID1B,IL1RAPL1,WDPCP,MAGI1,LAMA3,GBP4,ADCYAP1R1,CTDP1,GRID1,RPS6KA2,TDP1,SMOC2,ZDHHC17,KCNH1,HLCS,DCDC1,CACNA1C,BMF,WDR70,PNPLA3,BBS9,FAM83B,CTNNA1,MYO9A,NTRK2,FOXN3,OCLN,NLK,ITGBL1,RASGEF1B,AFAP1,TIAM2,IQCJ-SCHIP1,SMARCA4,RNF152,CNTN1,ZNRF3,ENPEP,NRG3,PTPRG,MC2R,BTBD11,ELMO1,SLC24A4,CABIN1,FBXL17,GAS2,GRIK3,PDE10A,STXBP4,MTPN,MT1HL1,SOX6,MECOM,TRPM3,STK32B,RGS12,SHANK2,RAPGEF5,UBE2E2,KCTD8,CHCHD6,UNC5D,NREP,GABRA5,DOK5,DLG5,SMARCAD1,ATRNL1,CHRM3,CPE,ARHGAP24,SPRED2,IGHV1OR15-9,SLIT2,PITPNC1,MYLK3,ROR1,GLP2R,ADAMTSL1,ZNF675,CSNK2A1,DTNA,AKT3,CRB1,TRIM5,PSD3,ALPK2,HECW1,RAP1GDS1,AFF3,ERBB4,KANK1,GPHN,ATRX,DMRT1,BID,MACF1,MNAT1,RAP1A,TRIO,RAD51B,PTPRE,MYO3B,DUSP22,CHSY1,MYOM1,PSG8,EXT2,OR4C46,ABL2,PSMA1,MAP3K5,NOS1,ARPP21,ACACA,ABCG1,RGS3,MAML2,RERG,HTR2C,CCDC141,NEK4,CNTN4,TBC1D5,SLC40A1,GABRR2,PIK3C3,TRAF3,CHD6,HMCN1,FGD4,ITGA1,TCF12,CORO2B,ITGA8,GRIK4,KIR3DL2,ALDH1A2,GABRG1,MAPK9,ESRRG,PTGFR,IGHV1OR21-1,CDH17,EYA1,MORC3,ANKS1B,P2RX6,HSF2BP,AKAP10,ANK2,PLA2G4A,SLC1A2,SUPT3H,SLIT3,GRIN2B,ROBO1,PAK3,DGKB,GARNL3,TNKS,NDFIP2,GABRA2,OVOL2,SGMS1,ITPR2,BRINP1,MLLT3,LEMD3,RNF138,GABPA,PRICKLE2,ITGB3BP,DGKK,TMEM67,PRKCE,PSG9,ARHGEF11,PRKAA2,CD163,PACRG,BBS2,IL1RAPL2,PTPRN2,MYO3A,GRIK1,SOX5,DSCAM,DGKI,RIN3,REG4,ANKFN1,DNMBP,EFHB,KDM4C,SDK1,SLC1A1,GRM5,EPHA6,NTN1,ARAP2,NR5A2,IGF1R,NLGN1,SHISA9,OR9Q1,SHROOM3,CNTNAP2,CFTR,CAMK1D,FLRT2,NOS1AP,PTPRO,MSRA,CD96,RBMS3,INSR,CTNNAL1,CLEC16A,ITGA9,EGLN3,ANK3,MORC2,GMDS,CNIH3,DOCK3,GPC6,RELN,RASGRF2,ADAMTS5,STK38,AOAH,FBXO32,CDH4,TNR,ADCY9,CELF4,DAPK1,VAV3,INPP5A,VRK1,CNTN6,ACSM2B,APP,FBLN5,PUM1,CCDC88A,ARNT2,RNLS,PLCE1,ADAM12,PAK1,GNAL,MITF,CACNA2D1,HCN1,PPP1R13B,TOP3A,CHRM5,NSMCE2,CXADR,EPS8,LRFN5,GPC5,TENM4,GHR,RASGRF1,RIN2,PRDM16,USP7,MEIS2,KIR2DL4,S

			TARD13,KL,ALCAM,PPP1R9A,TMEM117,MICU1,LTBP1,SLAMF1,RGL1,INO80D,RARB,DIDO1,MYH13,WDR12,NAALADL2,TIAM1,MLIP,SORCS2,SLC39A12,DISC1,RALGPS1,ARHGAP42,CHAF1A,PIEZO2,SVEP1,EDAR,EGF,PDGFD,FYN,FAM3B,XRCC4,EPHA7,MAP7,FHIT,NSG2,GRIA1,TRABD2B,SPIDR,STK3,CNOT7,MSR1,PSIP1,USP18,S100B,NET1,PCDH15,ESR1,ARHGAP12,GABRG3,SEL1L2,PLCXD3,GRM1,PDE4D,CNTN5,PRKACB,GNG2,PDE3A,RIMS1,POR,DOCK4,MCTP2,WWOX,HUNK,CNKSR2,FUT8,EPHB1,CREM,EFEMP1,AJAP1,ABCC9,GSG1L,DOCK1,FLT1,EXT1,EFNA5,NXN,CDC14B,C14ORF39,HDAC4,STK36,AMFR,PLCB4,FTO,SH3BP5,AKAP6,ACSBG1,SORCS3,TENM3,MARK2,ATF2,RBBP8,GRID2,ZNF423,LRP2,SEMA6D,C2,RALGPS2,NTF3,FER,SNRK,GLDC,SUSD4,CAMK4,MAPRE2,RAD51AP1,SGCD,TMEM108,RIC8B,GABRB3,TPTE,GRM7,RAPGEF2,MX1,PLGRKT,IMMP2L,PRKCQ,GABRG2,CPS1,PRKCH,NRXN3,RHPN2,DLC1,NSG1,GABBR2,KCND2,ATP8A2,UBE3A,GRIA4,IDE,CERS6,TPH2,APC,MACROD2,INO80,AUTS2,TFF1,EPHB2,ERC1,AGO3,C9,MCTP1,MOB3B,RYR3,PRTG,NBN,ADAMTS18,RGMB,CTNND2,SETD2,PKP1,DOCK2,TRIM23,NRP1,CDH13,RFC3,RPGRIP1,DACH1,TRDN,DEFB116,DAB1,RFTN1,ALK,EVC2,LDLRAD4,SEMA3A,SEMA3E,MGAT5,MYEF2,DCLK1,MAGI3,KIF16B,NRIP1,CDH2,ARID5B,SIPA1L2,RCAN2,LRRC69,TENM2,TANC1,PAPPA,VPS41,SYCP1,ASB3,HDAC9,PIK3R3,MAP2K6,FSTL4,ARHGAP28,MTOR,STK38L,KSR1,RALGAPA2,RORB,GABRB1,FBLN1,ST8SIA1,BLM,FHL2,PSMB2,NEU3,NCAPG2,RGS7,KYNU,STK32A,CD2AP,USP25,SPRED1,SIPA1L3,ADAM10,FLNB,WDFY4,SCAI,PAPPA2,PTPRT,CSMD1,TRERF1,SLC24A2,GLI3,NTRK3,RXFP1,FBN1,CHKA,RAB31,PRB3,RAPGEF4,BMPER,PDE1A,HMGA2,MX2,NSUN2,DEFA3,PTPRK,SORCS1,TBC1D4,SYT1,APIP,DPF3,NPHP4,DOCK9,DLG2,PPP1R12B,PPARA,PLXNA2,PTPRD,RORA,SHISA6,SCARA5,PLCB1,LOXL2,PRKG1,RASGRP1,ELAVL4,NLRC5,DMBT1,MAGI2,PLCL1,ABI1,PXDNL,ASIC2,RALA,DOCK10,TRPM1,CACNG3,PRKD1,BCL2L1,HDAC2,ETS1,TNN,RYR2,SEMA3D,TGFA,PRLR,TBX20,PTPRA,OR11G2,MTMR2,ATF6,EYS,IL16,VCL,DEPTOR,BACH1,ROBO2,IFT81,OSCP1,ZMYND11,CDH23,RGS6,SRGAP3,AKAP13,WDR41,NEDD9,ENPP1,PCP4,RIMS2,SLC5A1,STAC,RAB27A,EYA4,RALGAPA1,DLGAP2,SEMA5A,PRDM15,OR4N2,BCL11A,DCC,CTNNA2,CHN1,VPS13C,PARD3,NRG1,PRKCA,FMN2,SEMA3C,FANCB,CSF2RB,PCNT,BCKDHB,ST18,TOP1
GO:0042391	regulation of membrane potential	5.706491773287466e-13	KCNMA1,SLC8A1,KCNC1,BCL2,GRIK2,IGSF11,UNC13B,SCN11A,KCNK10,FGF12,CACNG2,GRIN2A,NRXN1,GRID1,KCNH1,CACNA1C,NTRK2,SLC24A4,GRIK3,CACNB2,GABRA5,SLC4A4,KCNE4,BID,GABRR2,GRIK4,GABRG1,P2RX6,ANK2,GRIN2B,GABRA2,KCNH8,GRIK1,DGKI,GRM5,NLGN1,CFTR,NOS1AP,ANK3,RELN,CELF4,APP,CACNA2D1,HCN1,CXADR,PIEZO2,KCND3,GRIA1,GABRG3,GRM1,RIMS1,TRPC5,AKAP6,GRID2,TMEM108,GABRB3,SLC39A8,PPA2,GABRG2,KCND2,TRDN,MTOR,GABRB1,ABCB5,CTNNA3,ASIC2,BCL2L1,RYR2,MTMR2,KCNH5,RIMS2,SCN8A
GO:0050807	regulation of synapse organization	6.019442019142057e-13	SEMA4D,CDH8,TANC2,KALRN,NEGR1,NRXN1,IL1RAPL1,NTRK2,SHANK2,DLG5,GRIN2B,PAK3,DGKB,IL1RAPL2,NTN1,NLGN1,FLRT2,PTPRO,GPC6,RELN,APP,LRFN5,CLSTN2,DISC1,FRMPD4,FYN,EPHA7,EPHB1,CTTNBP2,EFNA5,PPFIA2,LINGO2,GRID2,UBE3A,EPHB2,PDLIM5,CDH2,TANC1,LRFN2,NTRK3,ABHD17C,DNM3,SYNDIG1,PTPRD,ASIC2,ROBO2,NEDD9,CTNNA2
GO:0003008	system process	1.2718226522997241e-12	KCNMA1,APBB2,SLC8A1,CD38,BCL2,CAMTA1,COL18A1,THRB,DNAH11,RAG1,GRIK2,IGSF11,UNC13B,SCN11A,SORBS2,KCNK10,DLGAP1,RCAN1,JAK2,ABCG8,KALRN,USH2A,FGF12,CACNG2,BTBD9,FLI1,TRHDE,GRIN2A,NRXN1,CTDP1,RPS6KA2,KCNH1,CACNA1C,BBS9,MYO9A,NTRK2,OCLN,NCAM2,TMPRSS3,ENPEP,SLC24A4,CACNB2,MTPN,TRPM3,SHANK2,GABRA5,CHRM3,CALD1,SLIT2,MYLK3,ROR1,SLC4A4,DTNA,CRB1,KCNE4,RAP1GDS1,MYO3B,MYOM1,EXT2,OR4C46,NOS1,SPAG16,HTR2C,SLCO3A1,GABRR2,HMCN1,ITGA1,CORO2B,ITGA8,GABRG1,EYA1,P2RX6,PLS1,ANK2,SLC1A2,GRIN2B,GABRA2,SMPX,BRINP1,ARHGEF11,BBS2,MYOM2,MYO3A,DGKI,ANK

			FN1,SLC1A1,GRM5,NLGN1,DNAH9,SHISA9,OR9Q1,JAM2,CORIN,CNTNAP2,NOS1AP,PTPRO,INSR,ANK3,LHFPL3,RELN,FBXO32,TNR,CELF4,APP,RNLS,PLCE1,GNAL,CACNA2D1,HCN1,CHRM5,CXADR,UTRN,TENM4,RASGRF1,RBFOX1,MEIS2,KL,ADAMTS16,MYH13,MLIP,ARHGAP42,PIEZO2,VSTM4,SVEP1,VTI1A,FYN,KCND3,GRIA1,NAV2,S100B,PCDH15,GABRG3,SGCZ,GRM1,PDE4D,CNTN5,LRIG1,PRKACB,PDE3A,RIMS1,DOCK4,EPHB1,EFEMP1,ABCC9,HERC1,EXT1,HDAC4,AMFR,FTO,AKAP6,SORCS3,POU6F2,TUSC3,GRID2,LRP2,NTF3,CAMK4,CELF2,SGCD,TMEM108,GABRB3,GRM7,IMMP2L,ATXN1,SSPN,GABRG2,CPS1,NRXN3,RHPN2,KCND2,ATP8A2,SNTB1,SLC24A3,UBE3A,LOXHD1,TFF1,EPHB2,PDLIM5,NBN,RPGRIP1,TRDN,SLC2A13,TANC1,ASB3,MAP2K6,MTOR,RORB,GABRB1,SLC44A1,SPECC1,CSMD1,SLC24A2,SGCG,CTNNA3,PPP1R12B,PPARA,SHISA6,PLCB1,PRKG1,ELAVL4,TSHZ3,ASIC2,TRPM1,CACNG3,ATP8A1,HDAC2,RYR2,TBX20,OR11G2,MTMR2,ATF6,EYS,CDH23,AKAP13,NEDD9,RIMS2,SLC5A1,STAC,SCN8A,EYA4,DLGAP2,OR4N2,CTNNA2,PARD3,PRKCA
GO:0031346	positive regulation of cell projection organization	1.7073523372863418e-12	SEMA4D,FBXO31,CDC42EP3,GRIP1,KALRN,NEGR1,NRXN1,IL1RAPL1,NTRK2,OCLN,TIAM2,CNTN1,SLIT2,ROR1,MACF1,RAP1A,ABL2,ROBO1,PAK3,CHODL,DSCAM,NTN1,IGF1R,NLGN1,CAMK1D,COBL,CUX1,RELN,CDH4,CCDC88A,PLCE1,EPS8,TIAM1,DISC1,FYN,TOX,EFNA5,HDAC4,TRPC5,TENM3,MARK2,RAPGEF2,ATP8A2,APC,AUTS2,EPHB2,NRP1,ALK,TENM2,MTOR,NTRK3,DNM3,PLXNA2,PTPRD,ELAVL4,MAGI2,RALA,PRKD1,TNN,ROBO2,NEDD9,SEMA5A,BCL11A
GO:0050803	regulation of synapse structure or activity	1.956242209995526e-12	SEMA4D,CDH8,TANC2,KALRN,NEGR1,NRXN1,IL1RAPL1,NTRK2,SHANK2,DLG5,GRIN2B,PAK3,DGKB,IL1RAPL2,NTN1,NLGN1,FLRT2,PTPRO,GPC6,RELN,APP,LRFN5,CLSTN2,DISC1,FRMPD4,FYN,EPHA7,EPHB1,CTTNBP2,EFNA5,PPFIA2,LINGO2,GRID2,UBE3A,EPHB2,PDLIM5,CDH2,TANC1,LRFN2,NTRK3,ABHD17C,DNM3,SYNDIG1,PTPRD,ASIC2,ROBO2,NEDD9,CTNNA2
GO:0016358	dendrite development	7.576567920984004e-12	SEMA4D,FBXO31,GRIP1,TANC2,CSMD3,KALRN,IL1RAPL1,DLG5,HECW1,PAK3,DSCAM,SDK1,NTN1,NLGN1,MAP2,CAMK1D,COBL,CUX1,RELN,APP,PHACTR1,DISC1,ASAP1,FYN,EPHB1,KLHL1,TRPC5,PPFIA2,RAPGEF2,UBE3A,EPHB2,PDLIM5,CTNND2,NRP1,DAB1,ALK,SEMA3A,DCLK1,FSTL4,DNM3,PTPRD,PRKG1,ELAVL4,ABI1,DOCK10,HDAC2,FAT3,BCL11A,DCC,CTNNA2
GO:0050793	regulation of developmental process	1.1657216127808094e-11	CD44,ZHX3,SEMA4D,PRKCB,SLC8A1,ANKRD6,GPR55,FBXO31,BCL2,RAG1,CDC42EP3,IL6R,ATP10A,GRIP1,TANC2,CSMD3,NTN4,JAK2,KALRN,USH2A,ZNF536,EPB41L3,LAMA1,PARVB,ZBTB7C,NRXN1,ARID1B,IL1RAPL1,WDPCP,LAMA3,CTDP1,SMOC2,CTNNA1,MYO9A,NTRK2,TIAM2,SMARCA4,ZNRF3,PALMD,PLEKHB2,GAS2,NUMB,MTPN,SOX6,NREP,DLG5,CFDP1,SPRED2,SLIT2,MYLK3,ABCA5,ROR1,ZNF675,AKT3,ALPK2,HECW1,ERBB4,KANK1,DMRT1,MACF1,RAP1A,TRIO,MAP3K5,ABCG1,HTR2C,CNTN4,FGD4,TCF12,RBM19,RUNX1,MAPK9,FAM171A1,EYA1,PLS1,ZBTB16,ROBO1,PAK3,OVOL2,BRINP1,MLLT3,CHODL,GABPA,PRICKLE2,GLIS1,PSG9,BBS2,SOX5,DSCAM,DNMBP,KDM4C,SDK1,GRM5,NTN1,IGF1R,NLGN1,SHROOM3,JAM2,MAP2,CFTR,CAMK1D,FLRT2,INSR,COBL,CUX1,GPC6,RELN,TRPS1,CDH4,TNR,CELF4,APP,ADAM12,PAK1,MITF,ADCK1,CXADR,EPS8,TENM4,GHR,RIN2,RBFOX1,MEIS2,KL,LRRC4C,INO80D,CLSTN2,RARB,TCF4,TIAM1,PBX1,SLC39A12,DISC1,ZFPM2,ASAP1,EGF,FYN,EPHA7,STK3,MSR1,TOX,ESR1,PDE3A,RIMS1,POR,EPHB1,EFEMP1,AJAP1,DOCK1,FLT1,EFNA5,HDAC4,TRPC5,FTO,PPFIA2,AKAP6,LINGO2,MARK2,ATF2,GRID2,LRP2,SEMA6D,NTF3,CAM

			K4,NTNG1,RAPGEF2,GTF2I,PRKCH,DLC1,ATP8A2,UBE3A,APC,INO80,EPHB2,PDLIM5,PRTG,FLVCR1,NRP1,DAB1,ALK,LDLRAD4,SEMA3A,SEMA3E,CDH2,HDAC9,FSTL4,MTOR,RORB,FBLN1,SH3KBP1,SPRED1,ADAM10,GLI3,NTRK3,FBN1,BMPER,HMGA2,NSUN2,DNM3,SYT1,SYNDIG1,DPF3,PPARA,PLXNA2,PTPRD,RORA,PLCB1,LOXL2,RASGRP1,ELAVL4,MAGI2,NELL1,ASIC2,RALA,PRKD1,BCL2L1,HDAC2,ETS1,TNN,SEMA3D,PRLR,TBX20,FAT3,MTMR2,VCL,ATAT1,ROBO2,NEDD9,ENPP1,PCP4,RIMS2,SEMA5A,BCL11A,DCC,CHN1,PARD3,NRG1,PRKCA,SEMA3C
GO:0048513	animal organ development	1.9449855962781347e-11	CD44,SEMA4D,IGSF3,EVC,TEAD1,NFIA,A2M,SLC8A1,ANKRD6,KCNC1,GPR55,TRAPPC9,BCL2,MMP16,COL18A1,THRB,DNAH11,RAG1,KDM4B,IL6R,SORBS2,CASP5,ZNF568,NDRG2,RCAN1,NTN4,JAK2,KALRN,USH2A,NEGR1,FGF12,FLI1,MEGF11,LAMA1,GRIN2A,NRXN1,ARID1B,WDPCP,LAMA3,CTDP1,ADAMTS6,RPS6KA2,CACNA1C,EXOC4,CTNNA1,NTRK2,FOXN3,AK8,SMARCA4,CNTN1,ZNRF3,MEOX2,ENPEP,NRG3,PTPRG,SLC24A4,FBXL17,GAS2,NUMB,MTPN,SOX6,MECOM,NHS,SHANK2,GABRA5,DLG5,ATRNL1,CPE,SPRED2,SLIT2,MYLK3,ROR1,KAZN,ZNF675,AKT3,CRB1,ALPK2,LCE1F,ERBB4,ATRX,DMRT1,MNAT1,MYO3B,CHSY1,EXT2,EML1,CCDC141,CNTN4,SLC40A1,TCF12,ITGA8,RUNX1,ALDH1A2,CDH17,EYA1,PLS1,ANK2,SLC1A2,ZBTB16,SLIT3,GRIN2B,ROBO1,ANKRD11,EGFLAM,MDM1,OVOL2,MLLT3,CHODL,GABPA,PRICKLE2,PSG9,BBS2,MYO3A,SOX5,DSCAM,SDK1,SLC1A1,NTN1,CA10,NR5A2,LDB2,IGF1R,WDR72,ALX4,CNTNAP2,CFTR,FLRT2,PTPRO,INSR,COBL,GPC6,RELN,TRPS1,ADAMTS5,MYO18B,TNR,CELF4,APP,ARNT2,PLCE1,TACC2,MITF,HCN1,CXADR,UTRN,TENM4,CECR2,GHR,RBFOX1,MEIS2,KL,ADAMTS16,SLAMF1,NEBL,RARB,TIAM1,PBX1,PHACTR1,DISC1,FMN1,ZFPM2,VSTM4,EDAR,EGF,PDGFD,FYN,EPHA7,STK3,COL19A1,AP2B1,TOX,PCDH15,ESR1,SGCZ,CNTN5,LRIG1,POR,CERS3,WWOX,EPHB1,CTTNBP2,FHOD3,GREB1L,EFEMP1,AJAP1,HERC1,DOCK1,FLT1,EXT1,HDAC4,STK36,KLHL1,FTO,AKAP6,ACSBG1,TENM3,ATF2,GRID2,ZNF423,LRP2,SEMA6D,FER,SNRK,CAMK4,NTNG1,SGCD,TMEM108,RAPGEF2,IMMP2L,ATXN1,KIRREL3,CPS1,PRKCH,KRT25,DLC1,ATP8A2,SLC24A3,UBE3A,MACROD2,TTLL5,EPHB2,PDLIM5,XYLT1,ADAMTS18,SETD2,DOCK2,FLVCR1,NRP1,MDGA2,RPGRIP1,DAB1,ALK,LDLRAD4,SEMA3A,SEMA3E,DCLK1,NRIP1,CDH2,ARID5B,SERPINB7,HDAC9,PIK3R3,MAP2K6,MTOR,RORB,FHL2,NCAPG2,RGS7,TTC39C,SPRED1,SIPA1L3,ADAM10,FLNB,PAPPA2,ABCB5,CSMD1,GLI3,NTRK3,RXFP1,FBN1,SGCG,HYDIN,TBX15,BMPER,HMGA2,NSUN2,SYT1,ASXL3,DPF3,SCAPER,NPHP4,PPARA,PLXNA2,RORA,MYH15,PLCB1,LOXL2,BPTF,PRKG1,RASGRP1,ELAVL4,MAGI2,NELL1,ABI1,DOCK10,FNDC3A,BCL2L1,HDAC2,ETS1,TNN,RYR2,SEMA3D,TGFA,PRLR,TBX20,FAT3,ATF6,ATAT1,ROBO2,CDH23,AKAP13,NEDD9,ENPP1,EYA4,L3MBTL3,HIVEP3,SEMA5A,FREM1,DCC,CTNNA2,ETV6,NRG1,PRKCA,SEMA3C
GO:0051179	localization	2.183582038410327e-11	KCNMA1,PARN,SLC12A8,ZFYVE1,SYN3,PRKCB,TRPM6,SLC8A1,KCNC1,DNAJC15,SIAH3,CD38,TRAPPC9,BCL2,THADA,RSRC1,DNAH11,SLC13A4,SAMM50,GRIK2,IGSF11,SNX25,UNC13B,KCNQ5,HEPHL1,SCN11A,ATP10A,GOT2,KCNJ6,KCNK10,ZDHHC11B,GPR158,TMEM241,GRIP1,APBA2,TLK1,ASTN2,TANC2,KIF4A,JAK2,TM9SF4,BICD1,LRP1B,ABCG8,KALRN,USH2A,FGF12,CACNG2,BTBD9,SLC44A5,EPB41L3,CDS2,GRIN2A,NRXN1,IL1RAPL1,WDPCP,NIPA2,SLC14A2,ADCYAP1R1,DPP6,PRELID2,GRID1,ZDHHC17,KCNH1,CACNA1C,AMPH,EXOC4,HEATR5A,ANO4,BBS9,CTNNA1,NTRK2,ENTHD1,OCLN,ABCA6,NBEA,TMPRSS3,CNTN1,TTC39B,ELMO1,SLC24A4,SYNE1,GRIK3,CACNB2,NUMB,STXBP4,ESYT2,SYBU,TRPM3,GABRA5,DLG5,CHRM3,CPE,IGHV1OR15-9,PITPNC1,CEP128,ABCA5,SLC4A4,CRB1,KCNE4,TRIM5,KCNS3,ABCA13,HECW1,RAP1GDS1,ERBB4,GPHN,ATRX,BID,MACF1,RAP1A,SLC15A5,MYOM1,EXT2,ABL2,NOS1,ABCG1,SPAG16,HTR2C,CCDC141,CACNA1E,TBC1D5,SLC40A1,SLCO3A1,GABRR2,PIK3C3,SLC9C1,SNAP25-AS1,CORO2B,ITGA8,GRIK4,GABRG1,MAPK9,IGHV1OR21-1,CDH17,SV2B,MORC3,ANKS1B,P2RX6,AKAP10,FRMD6,PLS1,ANK2,PLA2G4A,SLC1A2,ZBTB16,ANO2,GRIN2B,TNKS,NDFIP2,GABRA2,CSE1L,FCHSD2,ITPR2,TRAPPC10,EHBP1,CACNA2D3,PRKCE,PR

			KAA2,CD163,PACRG,BBS2,PTPRN2,KCNH8,GRIK1,DGKI,RIN3,ANKFN1,EFHB,TRAPPC8,SLC1A1,SLC12A1,GRM5,NTN1,IGF1R,WDR72,SNX30,NLGN1,DNAH9,SHISA9,SHROOM3,CORIN,MON2,CNTNAP2,MAP2,KCNIP4,CFTR,CAMK1D,NOS1AP,ZDHHC14,NKAIN2,INSR,CLEC16A,CATSPER2,CUX1,ANK3,SV2C,CNIH3,GPC6,RELN,RASGRF2,MFSD9,DPP10,OCA2,DAPK1,VAV3,VRK1,CLIC6,TTC7B,APP,FBLN5,CCDC88A,PAK1,ATP9B,IGF2BP3,CACNA2D1,HCN1,CHRM5,CXADR,UTRN,GPC5,CECR2,TSPAN13,GHR,RASGRF1,RIN2,USP7,RBFOX1,SCP2,MICU1,LTBP1,SLAMF1,NKAIN3,SLC25A21,FAM126B,SORCS2,SLC39A12,DISC1,OSBPL10,PIEZO2,SLC35F1,VTI1A,EGF,FYN,FAM3B,KCND3,IFT43,XRCC4,LRBA,MAP7,NSG2,GRIA1,SPIDR,STK3,ANO10,MSR1,AP2B1,ESR1,ARHGAP12,GABRG3,KCNN3,KCNAB1,GRM1,PDE4D,ERC2,RIMS1,ATP6V1E1,FRMD4A,MCTP2,PIGK,MIPEP,ABCC9,SNAP29,GSG1L,DOCK1,FAM126A,TRPM7,EXT1,EFNA5,ABCA10,STK36,TRPC5,ATP9A,FTO,PPFIA2,AKAP6,VPS37A,MARK2,ATF2,TUSC3,CCDC91,GRID2,ZNF423,LRP2,C2,NTF3,FER,MAPRE2,ARFGAP3,MICU2,SGCD,TMEM108,GABRB3,GRM7,SLC39A8,RAPGEF2,IMMP2L,ATXN1,GABRG2,NUDCD3,PRKCH,NRXN3,RABGAP1L,NSG1,KCND2,ATP8A2,SLC24A3,UBE3A,GRIA4,APC,EPHB2,STON1-GTF2A1L,SYT16,ERC1,MCTP1,RYR3,NBAS,SETD2,PACSIN2,DOCK2,NUP214,TRIM23,FLVCR1,NRP1,CDH13,TRDN,SLC2A13,DAB1,RFTN1,EXOC6B,ATP13A3,STOML1,DCLK1,KIF16B,NRIP1,CDH2,EVI5,VPS41,SYCP1,ASB3,MAP2K6,MTOR,GABRB1,SH3KBP1,CADPS,NEU3,RGS7,CD2AP,SLC44A1,AP5M1,ADAM10,TSPAN33,ABCB5,SLC24A2,GLI3,FBN1,CHKA,RAB31,VPS13D,ABHD17C,RAPGEF4,BMPER,TMPRSS2,TMPRSS15,MX2,NSUN2,PTPRK,SORCS1,TBC1D4,DNM3,SYT1,SYNDIG1,NPHP4,DLG2,PPARA,PLXNA2,SCFD2,SHISA6,SCARA5,LOXL2,RASGRP1,STXBP6,DMBT1,MAGI2,STX12,ASIC2,RALA,GNPTAB,TRPM1,CACNG3,PRKD1,ATP8A1,BCL2L1,MICAL3,RANBP17,RYR2,LDLRAD3,BANP,PRLR,MTMR2,KCNH5,TMEM163,IPO11,IL16,VCL,IFT81,OSCP1,CDH23,AKAP13,WDR41,NEDD9,MYRIP,SLC39A11,ENPP1,UNC13C,RIMS2,SLC5A1,STAC,SCN8A,RAB27A,AGAP1,PARD3B,VPS13C,KCNJ15,CEP112,PARD3,NRG1,SLC25A48,ATP10B,SLC35F4,FMN2,PCNT,OSBPL5
GO:0007411	axon guidance	2.3460480017578863e-11	SEMA4D,RPS6KA5,NCAM1,KALRN,LAMA1,NRXN1,LAMA3,CNTN1,UNC5D,SLIT2,ADAMTSL1,TRIO,CCDC141,CNTN4,SLIT3,ROBO1,DSCAM,EPHA6,NTN1,FLRT2,PTPRO,RELN,CDH4,TNR,CNTN6,APP,ALCAM,FYN,EPHA7,CNTN5,EPHB1,EXT1,EFNA5,SEMA6D,PRKCQ,NRXN3,EPHB2,PRTG,NRP1,SEMA3A,SEMA3E,GLI3,PLXNA2,SEMA3D,ROBO2,SEMA5A,DCC,CHN1,SEMA3C
GO:0097485	neuron projection guidance	2.7848485825461376e-11	SEMA4D,RPS6KA5,NCAM1,KALRN,LAMA1,NRXN1,LAMA3,CNTN1,UNC5D,SLIT2,ADAMTSL1,TRIO,CCDC141,CNTN4,SLIT3,ROBO1,DSCAM,EPHA6,NTN1,FLRT2,PTPRO,RELN,CDH4,TNR,CNTN6,APP,ALCAM,FYN,EPHA7,CNTN5,EPHB1,EXT1,EFNA5,SEMA6D,PRKCQ,NRXN3,EPHB2,PRTG,NRP1,SEMA3A,SEMA3E,GLI3,PLXNA2,SEMA3D,ROBO2,SEMA5A,DCC,CHN1,SEMA3C
GO:0051130	positive regulation of cellular component organization	8.516360922009528e-11	PARN,SEMA4D,FBXO31,CDC42EP3,UNC13B,ATP10A,GRIP1,BICD1,KALRN,NEGR1,NRXN1,IL1RAPL1,BMF,NTRK2,OCLN,TIAM2,CNTN1,PDE4DIP,DLG5,SLIT2,MYLK3,ROR1,ABCA13,ATRX,DMRT1,BID,MACF1,RAP1A,ABL2,TBC1D5,RUNX1,MAPK9,CDH17,ROBO1,PAK3,TNKS,FCHSD2,CHODL,PRKCE,DSCAM,NTN1,IGF1R,SNX30,NLGN1,CNTNAP2,CAMK1D,FLRT2,INSR,COBL,CUX1,MORC2,RELN,CDH4,APP,CCDC88A,PLCE1,PAK1,ADCK1,NSMCE2,EPS8,INO80D,CLSTN2,TIAM1,DISC1,FMN1,ASAP1,FRMPD4,EGF,FYN,TRABD2B,SPIDR,TOX,ESR1,EPHB1,EFNA5,HDAC4,TRPC5,TENM3,LINGO2,MARK2,GRID2,NTF3,FER,MAPRE2,RAD51AP1,RAPGEF2,NAV3,PRKCQ,ATP8A2,APC,INO80,AUTS2,EPHB2,NBN,NRP1,ALK,TENM2,MTOR,NTRK3,RAB31,VPS13D,ATF7IP,DNM3,SYT1,SYNDIG1,NPHP4,PLXNA2,PTPRD,PLCB1,ELAVL4,MAGI2,ASIC2,RALA,PRKD1,ATP8A1,TNN,TGFA,ATAT1,ROBO2,NEDD9,CLIP1,SEMA5A,BCL11A,NRG1
GO:0099173	postsynapse	2.378084847823599e-10	TANC2,KALRN,NRXN1,IL1RAPL1,SHANK2,GPHN,GRIN2B,PAK3,DGKB,IGF1R,NLGN1,NOS1AP,INSR,RELN,FRMPD4,FYN,EPHA7,CNKSR2,EPHB1,PPFIA2,GRID2,TMEM108,UBE3A,EPHB2,PDLIM5,CTNND2,NRP1,CDH2,TANC1,ADAM10,LRFN2,NTRK3,ABHD17C,DNM3,PT

	organization		PRD,SHISA6,DOCK10,MTMR2,NEDD9
GO:0050770	regulation of axonogenesis	3.351998236578787e-10	SEMA4D,NTRK2,TIAM2,SLIT2,MACF1,ROBO1,PAK3,CHODL,DSCAM,NTN1,MAP2,CDH4,TNR,PAK1,LRRC4C,TIAM1,DISC1,EPHA7,EFNA5,TRPC5,MARK2,SEMA6D,EPHB2,NRP1,DAB1,SEMA3A,SEMA3E,CDH2,FSTL4,PLXNA2,SEMA3D,ROBO2,SEMA5A,BCL11A,DCC,CHN1,SEMA3C
GO:0007417	central nervous system development	5.385691712317555e-10	SLC8A1,KCNC1,TRAPPC9,BCL2,KDM4B,CASP5,NDRG2,KALRN,NEGR1,CDH11,GRIN2A,NRXN1,CTNNA1,NTRK2,AK8,CNTN1,NRG3,PTPRG,NUMB,MTPN,SOX6,VCAN,SHANK2,GABRA5,DLG5,SLIT2,ROR1,AKT3,ERBB4,ATRX,CHST8,MNAT1,TRIO,EML1,CCDC141,CNTN4,ITGA8,ALDH1A2,SLC1A2,ZBTB16,GRIN2B,ROBO1,BBS2,IL1RAPL2,GRIK1,SLC1A1,CA10,IGF1R,CNTNAP2,MAP2,RELN,TNR,CNTN6,APP,ARNT2,SPOCK1,TACC2,TENM4,MEIS2,RARB,PBX1,PHACTR1,DISC1,EGF,FYN,EPHA7,NAV2,STK3,S100B,TOX,CNTN5,EPHB1,CTTNBP2,HERC1,EXT1,STK36,KLHL1,POU6F2,ATF2,GRID2,ZNF423,LRP2,SEMA6D,TMEM108,RAPGEF2,IMMP2L,ATXN1,KIRREL3,DLC1,UBE3A,MACROD2,EPHB2,SETD2,NRP1,MDGA2,DAB1,ALK,SEMA3A,SEMA3E,DCLK1,CDH2,MTOR,GABRB1,RGS7,GLI3,NTRK3,HYDIN,SYT1,PLXNA2,RORA,PLCB1,BPTF,PRKG1,ELAVL4,ASIC2,HDAC2,TBX20,ATAT1,ROBO2,SEMA5A,DCC,CTNNA2,NRG1
GO:0048523	negative regulation of cellular process	5.613615445022996e-10	CD44,SAMD4A,ZHX3,APBB2,PTPRR,SCAF8,RTN1,PARN,SEMA4D,INIP,RPS6KA5,TAOK3,PRKCB,SLC8A1,ANKRD6,GPR55,DNAJC15,SIAH3,CD38,FBXO31,BCL2,SAMSN1,CHFR,THADA,COL18A1,TOX3,THRB,RAG1,GRIK2,SNX25,KDM4B,ZBTB20,SKAP2,BRD4,ZNF568,NDRG2,ASTN2,RCAN1,JAK2,BICD1,OTUD7A,TPTE2,KALRN,USH2A,ZNF536,ZBTB7C,NRXN1,IL1RAPL1,ADCYAP1R1,CTDP1,RPS6KA2,CACNA1C,BMF,CTNNA1,NTRK2,FOXN3,OCLN,NLK,MYT1L,SRGAP2B,IQCJ-SCHIP1,SMARCA4,RNF152,ZNRF3,MEOX2,GLIS3,NRG3,PTPRG,SLC24A4,GRIK3,PDE10A,NUMB,MTPN,SOX6,MECOM,RGS12,SHANK2,GABRA5,DLG5,CFDP1,ARHGAP24,SPRED2,SLIT2,ABCA5,ZNF675,CSNK2A1,AKT3,KCNE4,ALPK2,HECW1,ERBB4,KANK1,ATRX,DMRT1,BID,MNAT1,RAP1A,TRIO,PTPRE,DUSP22,ABL2,NOS1,ABCG1,RGS3,RERG,HTR2C,CNTN4,SAMD13,SLC40A1,ETS2,ITGA1,HIRA,CORO2B,RUNX1,KIR3DL2,ALDH1A2,PTGFR,EYA1,MORC3,SPOCK3,SPON1,ANK2,ZBTB16,SLIT3,GRIN2B,PHC2,ROBO1,TNKS,KLF12,NDFIP2,MDM1,OVOL2,ITPR2,BRINP1,MLLT3,LEMD3,GABPA,TMEM67,PRKCE,GLIS1,PRKAA2,PACRG,CDYL2,DSCAM,DGKI,RIN3,KDM4C,SLC1A1,GRM5,NTN1,LDB2,IGF1R,SPTB,NLGN1,JAM2,MAP2,CAMK1D,PTPRO,RBMS3,CLEC16A,CUX1,ANK3,TBCD,TRPS1,STK38,TNR,CELF4,DAPK1,INPP5A,APP,PUM1,KCTD1,SPOCK1,PAK1,MITF,IGF2BP3,ADCK1,HCN1,PPP1R13B,CXADR,EPS8,LRFN5,GHR,DUX4,PRDM16,FRMD5,USP7,MEIS2,KIR2DL4,STARD13,AVEN,LTBP1,SLAMF1,BACE2,RARB,PBX1,MLIP,SORCS2,ARHGAP42,ZFPM2,PCBP3,FYN,EPHA7,FHIT,GRIA1,TRABD2B,STK3,CNOT7,USP18,ESR1,ARHGAP12,KCNAB1,PDE4D,PRKACB,PDE3A,POR,L3MBTL4,FRMD4A,WWOX,EPHB1,CREM,FHOD3,EFEMP1,TNRC6B,AJAP1,HERC1,PARP15,FLT1,EFNA5,NXN,CDC14B,HDAC4,TRPC5,AMFR,ATP9A,FTO,SH3BP5,AKAP6,SORCS3,ATF2,RBBP8,GRID2,ZNF423,LRP2,SEMA6D,NTF3,FER,TP53I11,TPTE,GRM7,RAPGEF2,NAV3,ATXN1,PRKCQ,PRKCH,RHPN2,DLC1,ATP8A2,UBE3A,GRIA4,APC,ZBTB25,TFF1,EPHB2,SCAF4,AGO3,MCTP1,RYR3,NBAS,PRTG,NBN,ADAMTS18,PACSIN2,PKP1,NRP1,CDH13,DACH1,TRDN,ZNF397,DAB1,LDLRAD4,SEMA3A,SEMA3E,MGAT5,MALRD1,DCLK1,NRIP1,CDH2,ARID5B,TENM2,HDAC9,PIK3R3,FSTL4,ARHGAP28,MTOR,RORB,FBLN1,BLM,FHL2,NEU3,NCAPG2,RGS7,CD2AP,USP25,SPRED1,ADAM10,SCAI,PTPRT,TRERF1,SLC24A2,GLI3,NTRK3,FBN1,ABHD17C,TBX15,BMPER,ATF7IP,HMGA2,NSUN2,PTPRK,TBC1D4,DNM3,APIP,DPF3,NPHP4,SACS,PPARA,PLXNA2,PTPRD,RORA,SHISA6,PLCB1,LOXL2,BPTF,PRKG1,ELAVL4,NLRC5,STXBP6,MXI1,MAGI2,NELL1,ABI1,TSHZ3,ASIC2,PRKD1,TNFAIP8,BCL2L1,HDAC2,ETS1,TNN,RYR2,SE

			MA3D,TGFA,PRLR,TBX20,FAT3,MTMR2,VCL,DEPTOR,BACH1,ROBO2,ZMYND11,RGS6,SRGAP3,WDR41,NEDD9,ENPP1,EYA4,L3MBTL3,SEMA5A,PRDM15,BCL11A,DCC,CTNNA2,ETV6,VPS13C,PARD3,NRG1,CAST,FANK1,PRKCA,FMN2,SEMA3C,FANCB,ST18
GO:0016477	cell migration	7.417027339748524e-10	CD44,PTPRR,SEMA4D,SLC8A1,FBXO31,BCL2,CDC42BPA,IL6R,ASTN2,NTN4,JAK2,USH2A,LAMA1,WDPCP,LAMA3,SMOC2,CTNNA1,NTRK2,ITGBL1,SRGAP2B,MEOX2,ENPEP,NRG3,PTPRG,ELMO1,NUMB,VCAN,UNC5D,DLG5,ASTN1,ATRNL1,ARHGAP24,SLIT2,AKT3,ERBB4,KANK1,DMRT1,MACF1,DUSP22,ABL2,CCDC141,ITGA1,PEAK1,ROBO1,PAK3,OVOL2,PSTPIP2,PRKCE,RIN3,NTN1,LDB2,IGF1R,JAM2,CAMK1D,FLRT2,PTPRO,INSR,ITGA9,GPC6,RELN,TNR,VAV3,APP,CCDC88A,SPOCK1,PAK1,MITF,CXADR,EPS8,GPC5,RIN2,FRMD5,STARD13,SLAMF1,TIAM1,PHACTR1,DISC1,VSTM4,EGF,PDGFD,FYN,NET1,DOCK4,FUT8,EPHB1,DOCK1,FLT1,EXT1,HDAC4,MARK2,SEMA6D,NTF3,FER,NTNG1,MAPRE2,RAPGEF2,NAV3,PRKCQ,KIRREL3,DLC1,APC,AUTS2,EPHB2,MCTP1,SETD2,DOCK2,SDCCAG8,NRP1,CDH13,DACH1,DAB1,LDLRAD4,SEMA3A,SEMA3E,MGAT5,DCLK1,CDH2,ARID5B,HDAC9,PIK3R3,MTOR,FBLN1,SH3KBP1,CD2AP,SPRED1,ADAM10,SCAI,PTPRT,GLI3,NTRK3,CTNNA3,BMPER,PTPRK,PLXNA2,PLCB1,LOXL2,PRKG1,MAGI2,DOCK10,PRKD1,ATP8A1,HDAC2,ETS1,TNN,SEMA3D,TBX20,FAT3,IL16,VCL,SRGAP3,NEDD9,SEMA5A,DCC,CTNNA2,NRG1,PRKCA,SEMA3C
GO:0007420	brain development	1.3120724944305458e-9	SLC8A1,KCNC1,TRAPPC9,BCL2,KDM4B,CASP5,NDRG2,NEGR1,GRIN2A,NRXN1,CTNNA1,NTRK2,AK8,CNTN1,NRG3,PTPRG,NUMB,MTPN,SOX6,SHANK2,GABRA5,DLG5,SLIT2,AKT3,ERBB4,ATRX,MNAT1,EML1,CCDC141,CNTN4,ITGA8,ALDH1A2,SLC1A2,GRIN2B,ROBO1,BBS2,SLC1A1,CA10,IGF1R,CNTNAP2,RELN,TNR,APP,ARNT2,TACC2,MEIS2,RARB,PBX1,PHACTR1,DISC1,EGF,FYN,EPHA7,TOX,CNTN5,EPHB1,CTTNBP2,HERC1,EXT1,STK36,KLHL1,ATF2,GRID2,ZNF423,LRP2,SEMA6D,TMEM108,RAPGEF2,IMMP2L,ATXN1,KIRREL3,DLC1,UBE3A,MACROD2,EPHB2,SETD2,NRP1,DAB1,ALK,SEMA3A,SEMA3E,DCLK1,CDH2,RGS7,GLI3,HYDIN,SYT1,PLXNA2,RORA,PLCB1,BPTF,PRKG1,ELAVL4,ATAT1,ROBO2,SEMA5A,CTNNA2,NRG1
GO:0060322	head development	1.4622389763805098e-9	SLC8A1,KCNC1,TRAPPC9,BCL2,KDM4B,CASP5,NDRG2,NEGR1,GRIN2A,NRXN1,CTNNA1,NTRK2,AK8,CNTN1,NRG3,PTPRG,NUMB,MTPN,SOX6,SHANK2,GABRA5,DLG5,SLIT2,AKT3,ERBB4,ATRX,MNAT1,EML1,CCDC141,CNTN4,ITGA8,ALDH1A2,SLC1A2,GRIN2B,ROBO1,ANKRD11,BBS2,SLC1A1,CA10,IGF1R,CNTNAP2,RELN,TNR,APP,ARNT2,TACC2,MEIS2,RARB,PBX1,PHACTR1,DISC1,EGF,FYN,EPHA7,TOX,CNTN5,EPHB1,CTTNBP2,HERC1,EXT1,STK36,KLHL1,ATF2,GRID2,ZNF423,LRP2,SEMA6D,DDX10,TMEM108,RAPGEF2,IMMP2L,ATXN1,KIRREL3,DLC1,UBE3A,MACROD2,EPHB2,SETD2,FLVCR1,NRP1,DAB1,ALK,SEMA3A,SEMA3E,DCLK1,CDH2,ARID5B,RGS7,GLI3,HYDIN,SYT1,PLXNA2,RORA,PLCB1,BPTF,PRKG1,ELAVL4,ATAT1,ROBO2,SEMA5A,CTNNA2,NRG1
GO:0034220	ion transmembrane transport	1.5612362903597418e-9	KCNMA1,SLC12A8,TRPM6,SLC8A1,KCNC1,BCL2,THADA,SLC13A4,GRIK2,KCNQ5,SCN11A,ATP10A,KCNJ6,KCNK10,FGF12,CACNG2,GRIN2A,NRXN1,NIPA2,ADCYAP1R1,DPP6,GRID1,ZDHHC17,KCNH1,CACNA1C,ANO4,SLC24A4,GRIK3,CACNB2,TRPM3,GABRA5,CHRM3,SLC4A4,KCNE4,KCNS3,HECW1,NOS1,HTR2C,CACNA1E,SLC40A1,GABRR2,SLC9C1,GRIK4,GABRG1,P2RX6,ANK2,SLC1A2,ANO2,GRIN2B,GABRA2,ITPR2,CACNA2D3,PRKCE,KCNH8,GRIK1,SLC1A1,SLC12A1,GRM5,NLGN1,SHISA9,KCNIP4,CFTR,NOS1AP,CATSPER2,ANK3,CNIH3,RELN,RASGRF2,DPP10,DAPK1,CLIC6,APP,CACNA2D1,HCN1,UTRN,TSPAN13,RASGRF1,MICU1,SLC25A21,SLC39A12,PIEZO2,FYN,KCND3,GRIA1,ANO10,GABRG3,KCNN3,KCNAB1,GRM1,PDE4D,ATP6V1E1,ABCC9,GSG1L,TRPM7,TRPC5,AKAP6,TUSC3,GRID2,LRP2,MICU2,GABRB3,SLC39A8,GABRG2,KCND2,SLC24A3,GRIA4,EPHB2,RYR3,TRDN,ATP13A3,GABRB1,RGS7,SLC24A2,SHISA6,SCARA5,ASIC2,TRPM1,CACNG3,PRKD1,ATP8A1,RYR2,KCNH5,TMEM163,SLC39A11,SLC5A1,STAC,SCN8A,KCNJ15
GO:0048870	cell motility	1.7955325307806966e-9	CD44,PTPRR,SEMA4D,SLC8A1,FBXO31,BCL2,DNAH11,CDC42BPA,IL6R,ASTN2,NTN4,JAK2,TPTE2,USH2A,LAMA1,WDPCP,LAMA3,SMOC2,CTNNA1,NTRK2,ITGBL1,SRGAP2B,MEOX2,ENPEP,NRG3,PTPRG,ELMO1,NUMB,VCAN,DNAH8,UNC5D,DLG5,ASTN1,ATRNL1,ARHGAP24,SLIT2,AKT3,ERBB4,KANK1,DMRT1,MACF1,DUSP22,ABL2,SP

			AG16,CCDC141,SLC9C1,ITGA1,PEAK1,SPOCK3,ROBO1,PAK3,OVOL2,PSTPIP2,PRKCE,BBS2,RIN3,NTN1,LDB2,IGF1R,JAM2,CAMK1D,FLRT2,PTPRO,INSR,ITGA9,CATSPER2,GPC6,RELN,TNR,VAV3,APP,CCDC88A,SPOCK1,PAK1,MITF,CXADR,EPS8,GPC5,RIN2,FRMD5,STARD13,SLAMF1,TIAM1,PHACTR1,DISC1,VSTM4,EGF,PDGFD,FYN,NET1,DOCK4,FUT8,EPHB1,ARMC2,DOCK1,FLT1,EXT1,HDAC4,MARK2,SEMA6D,NTF3,FER,NTNG1,MAPRE2,TPTE,RAPGEF2,NAV3,DNAH3,PRKCQ,KIRREL3,DLC1,APC,AUTS2,EPHB2,MCTP1,SETD2,DOCK2,SDCCAG8,NRP1,CDH13,DACH1,DAB1,LDLRAD4,SEMA3A,SEMA3E,DNAH6,MGAT5,DCLK1,CDH2,ARID5B,HDAC9,PIK3R3,MTOR,FBLN1,SH3KBP1,CD2AP,SPRED1,ADAM10,SCAI,PTPRT,GLI3,NTRK3,CTNNA3,BMPER,PTPRK,NPHP4,PLXNA2,PLCB1,LOXL2,PRKG1,MAGI2,DOCK10,PRKD1,ATP8A1,HDAC2,ETS1,TNN,SEMA3D,TBX20,FAT3,IL16,VCL,IFT81,SRGAP3,NEDD9,SEMA5A,DCC,CTNNA2,NRG1,PRKCA,SEMA3C
GO:0040011	locomotion	2.0379714867941387e-9	PTPRR,SEMA4D,RPS6KA5,SLC8A1,FBXO31,BCL2,NCAM1,IL6R,RCAN1,JAK2,KALRN,LAMA1,GRIN2A,NRXN1,WDPCP,LAMA3,SMOC2,CTNNA1,SRGAP2B,CNTN1,MEOX2,NRG3,PTPRG,NUMB,UNC5D,DLG5,SLIT2,ADAMTSL1,AKT3,ERBB4,KANK1,MACF1,TRIO,DUSP22,ABL2,CCDC141,CNTN4,ITGA1,SPOCK3,SLIT3,ROBO1,PAK3,PRKCE,BBS2,DSCAM,RIN3,GRM5,EPHA6,NTN1,LDB2,IGF1R,JAM2,CAMK1D,FLRT2,PTPRO,INSR,ITGA9,RELN,CDH4,TNR,VAV3,CNTN6,APP,PAK1,MITF,CXADR,RIN2,FRMD5,STARD13,ALCAM,SLAMF1,TIAM1,PHACTR1,EGF,PDGFD,FYN,EPHA7,CNTN5,DOCK4,EPHB1,DOCK1,FLT1,EXT1,EFNA5,HDAC4,SEMA6D,NTF3,FER,NTNG1,MAPRE2,RAPGEF2,NAV3,PLGRKT,PRKCQ,NRXN3,DLC1,APC,EPHB2,MCTP1,PRTG,DOCK2,NRP1,CDH13,DACH1,LDLRAD4,SEMA3A,SEMA3E,MGAT5,HDAC9,PIK3R3,MTOR,FBLN1,SPRED1,ADAM10,SCAI,PTPRT,GLI3,NTRK3,BMPER,PTPRK,PLXNA2,PLCB1,PRKG1,MAGI2,RALA,DOCK10,PRKD1,ATP8A1,HDAC2,ETS1,TNN,SEMA3D,IL16,VCL,ROBO2,SRGAP3,NEDD9,SEMA5A,DCC,CTNNA2,CHN1,NRG1,PRKCA,SEMA3C
GO:0055085	transmembrane transport	3.166995046899109e-9	KCNMA1,SLC12A8,PRKCB,TRPM6,SLC8A1,KCNC1,DNAJC15,BCL2,THADA,SLC13A4,GRIK2,KCNQ5,SCN11A,ATP10A,KCNJ6,KCNK10,TMEM241,ABCG8,FGF12,CACNG2,SLC44A5,GRIN2A,NRXN1,NIPA2,SLC14A2,ADCYAP1R1,DPP6,GRID1,ZDHHC17,KCNH1,CACNA1C,ANO4,OCLN,ABCA6,SLC24A4,GRIK3,CACNB2,STXBP4,TRPM3,GABRA5,CHRM3,ABCA5,SLC4A4,KCNE4,KCNS3,ABCA13,HECW1,SLC15A5,NOS1,ABCG1,HTR2C,CACNA1E,SLC40A1,SLCO3A1,GABRR2,SLC9C1,GRIK4,GABRG1,CDH17,SV2B,P2RX6,ANK2,SLC1A2,ANO2,GRIN2B,GABRA2,ITPR2,CACNA2D3,PRKCE,KCNH8,GRIK1,SLC1A1,SLC12A1,GRM5,NLGN1,SHISA9,KCNIP4,CFTR,NOS1AP,INSR,CATSPER2,ANK3,SV2C,CNIH3,RELN,RASGRF2,MFSD9,DPP10,OCA2,DAPK1,CLIC6,APP,CACNA2D1,HCN1,UTRN,TSPAN13,RASGRF1,MICU1,SLC25A21,SLC39A12,PIEZO2,SLC35F1,FYN,KCND3,GRIA1,ANO10,GABRG3,KCNN3,KCNAB1,GRM1,PDE4D,ATP6V1E1,ABCC9,GSG1L,TRPM7,ABCA10,TRPC5,AKAP6,TUSC3,GRID2,LRP2,MICU2,GABRB3,SLC39A8,GABRG2,KCND2,SLC24A3,GRIA4,EPHB2,RYR3,FLVCR1,TRDN,SLC2A13,ATP13A3,GABRB1,RGS7,SLC44A1,ABCB5,SLC24A2,SHISA6,SCARA5,ASIC2,TRPM1,CACNG3,PRKD1,ATP8A1,RYR2,KCNH5,TMEM163,OSCP1,SLC39A11,ENPP1,SLC5A1,STAC,SCN8A,KCNJ15,SLC25A48,SLC35F4
GO:0009966	regulation of signal transduction	4.617157053836016e-9	CD44,PTPRR,SEMA4D,EVC,TAOK3,PRKCB,ANKRD6,GPR55,BCL2,CAMTA1,THRB,IGSF11,SNX25,UNC13B,NCAM1,IL6R,BRD4,NDRG2,DLGAP1,RCAN1,JAK2,BICD1,OTUD7A,TPTE2,KALRN,CACNG2,NFAT5,ZNF536,LAMA1,GRIN2A,NRXN1,ADCYAP1R1,SMOC2,ZDHHC17,CTNNA1,MYO9A,NTRK2,NLK,AFAP1,TIAM2,IQCJ-SCHIP1,SMARCA4,RNF152,ZNRF3,SLC24A4,FBXL17,GAS2,PDE10A,MECOM,RGS12,SHANK2,KCTD8,NREP,DOK5,DLG5,ARHGAP24,SPRED2,SLIT2,ROR1,ZNF675,CSNK2A1,AKT3,TRIM5,PSD3,ALPK2,HECW1,RAP1GDS1,ERBB4,KANK1,DMRT1,BID,MACF1,RAP1A,TRIO,PTPRE,DUSP22,CHSY1,ABL2,MAP3K5,RGS3,HTR2C,TRAF3,FGD4,ITGA1,ITGA8,MAPK9,EYA1,SLIT3,GRIN2B,ROBO1,PAK3,GARNL3,TNKS,NDFIP2,OVOL2,SGMS1,MLLT3,LEMD3,PRKCE,ARHGEF11,PRKAA2,DGKI,DNMBP,EFHB,KDM4C,GRM5,IGF1R,NLGN1,SHISA9,NOS1AP,PTPRO,RBMS3,INSR,CLEC16A,CNIH3,DOCK3,GPC6,RELN

			,RASGRF2,STK38,CELF4,DAPK1,VAV3,CNTN6,APP,PUM1,CCDC88A,PLCE1,PAK1,EPS8,GPC5,GHR,RASGRF1,PRDM16,USP7,STARD13,KL,LTBP1,SLAMF1,TIAM1,DISC1,RALGPS1,ARHGAP42,EDAR,EGF,PDGFD,FYN,EPHA7,TRABD2B,STK3,CNOT7,USP18,S100B,NET1,ESR1,ARHGAP12,GRM1,PDE4D,PRKACB,PDE3A,RIMS1,POR,WWOX,CNKSR2,EPHB1,GSG1L,FLT1,NXN,STK36,AMFR,AKAP6,ZNF423,LRP2,RALGPS2,NTF3,FER,MAPRE2,TMEM108,RIC8B,TPTE,RAPGEF2,PRKCQ,PRKCH,DLC1,UBE3A,APC,AUTS2,EPHB2,AGO3,MOB3B,CTNND2,DOCK2,NRP1,CDH13,DAB1,ALK,LDLRAD4,SEMA3A,SEMA3E,MGAT5,MAGI3,CDH2,SIPA1L2,MAP2K6,FSTL4,ARHGAP28,MTOR,KSR1,RALGAPA2,FBLN1,FHL2,NEU3,NCAPG2,RGS7,CD2AP,SPRED1,SIPA1L3,ADAM10,SCAI,PTPRT,GLI3,NTRK3,FBN1,BMPER,APIP,NPHP4,PPARA,PTPRD,RORA,SHISA6,PLCB1,RASGRP1,NLRC5,MAGI2,CACNG3,PRKD1,BCL2L1,HDAC2,TNN,TGFA,PRLR,TBX20,MTMR2,ATF6,DEPTOR,ROBO2,IFT81,ZMYND11,RGS6,SRGAP3,AKAP13,ENPP1,RIMS2,EYA4,RALGAPA1,DLGAP2,SEMA5A,PRDM15,CHN1,NRG1,PRKCA
GO:0030029	actin filament-based process	5.658952952924201e-9	BCL2,CDC42BPA,CDC42EP3,SORBS2,JAK2,FGF12,EPB41L3,PARVB,CACNA1C,CTNNA1,THSD7A,ELMO1,GAS2,CACNB2,MTPN,PGM5,CALD1,SLIT2,MYLK3,KCNE4,RAP1GDS1,KANK1,ABL2,HMCN1,FGD4,CORO2B,FAM171A1,FRMD6,PLS1,ANK2,PAK3,FCHSD2,EHBP1,PSTPIP2,PRKCE,ARHGEF11,MYOM2,SPTB,SHROOM3,NOS1AP,COBL,CCDC88A,PAK1,CACNA2D1,CXADR,EPS8,UTRN,FRMD5,STARD13,PPP1R9A,NEBL,PHACTR1,FMN1,FRMPD4,KCND3,PCDH15,ARHGAP12,PDE4D,FHOD3,ABCC9,DIAPH3,TRPM7,EFNA5,KLHL1,PHACTR2,NTF3,FER,SGCD,RHPN2,DLC1,MPRIP,AUTS2,PDLIM5,FRMD3,PACSIN2,DOCK2,NRP1,PHACTR3,SEMA3E,ARHGAP28,MTOR,SH3KBP1,CD2AP,FLNB,SPECC1,NTRK3,CTNNA3,THSD7B,NPHP4,PRKG1,ABI1,RALA,MICAL3,RYR2,AKAP13,NEDD9,SEMA5A,CTNNA2,FMN2
GO:0022603	regulation of anatomical structure morphogenesis	6.0098718570856385e-9	CD44,SEMA4D,PRKCB,ANKRD6,FBXO31,CDC42EP3,ATP10A,GRIP1,TANC2,NTN4,KALRN,EPB41L3,PARVB,IL1RAPL1,WDPCP,SMOC2,MYO9A,NTRK2,TIAM2,ZNRF3,PALMD,GAS2,CFDP1,SLIT2,ROR1,AKT3,HECW1,KANK1,MACF1,FGD4,RUNX1,FAM171A1,ROBO1,PAK3,MLLT3,CHODL,PRICKLE2,DSCAM,DNMBP,NTN1,NLGN1,SHROOM3,MAP2,CUX1,GPC6,RELN,CDH4,TNR,ADAM12,PAK1,ADCK1,EPS8,TENM4,LRRC4C,TIAM1,SLC39A12,DISC1,EGF,FYN,EPHA7,ESR1,RIMS1,AJAP1,DOCK1,FLT1,EFNA5,TRPC5,PPFIA2,MARK2,ATF2,SEMA6D,NTNG1,RAPGEF2,GTF2I,DLC1,UBE3A,EPHB2,PDLIM5,NRP1,DAB1,SEMA3A,SEMA3E,CDH2,FSTL4,FBLN1,SH3KBP1,SPRED1,BMPER,HMGA2,DNM3,SYT1,PLXNA2,PTPRD,MAGI2,RALA,PRKD1,ETS1,TNN,SEMA3D,ROBO2,NEDD9,RIMS2,SEMA5A,BCL11A,DCC,CHN1,PRKCA,SEMA3C
GO:0007416	synapse assembly	7.257092643107974e-9	SEMA4D,NEGR1,NRXN1,IL1RAPL1,NTRK2,SHANK2,DLG5,ERBB4,GABRA2,IL1RAPL2,DSCAM,SDK1,NTN1,NLGN1,FLRT2,GPC6,APP,LRFN5,CLSTN2,EPHA7,CNTN5,EPHB1,EFNA5,LINGO2,GRID2,GABRB3,KIRREL3,GABRG2,NRXN3,EPHB2,PDLIM5,CDH2,NTRK3,DNM3,SYNDIG1,PTPRD,ASIC2,ROBO2,NRG1
GO:0007610	behavior	1.3051792987845306e-8	BCL2,THRB,DNAH11,RAG1,GRIK2,KCNK10,APBA2,RCAN1,KALRN,NEGR1,FGF12,BTBD9,GRIN2A,NRXN1,GRID1,NTRK2,CNTN1,SLC24A4,SHANK2,GABRA5,ASTN1,ABL2,HTR2C,ITGA8,SLC1A2,GRIN2B,BRINP1,PRKCE,BBS2,DSCAM,DGKI,ANKFN1,SDK1,SLC1A1,GRM5,NLGN1,CNTNAP2,INSR,RELN,TNR,APP,PUM1,EPS8,RASGRF1,MEIS2,FYN,GRIA1,NAV2,S100B,PCDH15,GRM1,EXT1,HDAC4,KLHL1,AMFR,SORCS3,NTF3,CAMK4,ATXN1,KIRREL3,GABRG2,NRXN3,KCND2,ATP8A2,UBE3A,EPHB2,DACH1,DAB1,ALK,TANC1,MTOR,SPECC1,CSMD1,GLI3,NPHP4,PPARA,PLCB1,ELAVL4,ATP8A1,HDAC2,CDH23,NEDD9,NRG1
GO:0060078	regulation of postsynaptic membr	1.838453859064167e-8	GRIK2,IGSF11,UNC13B,GRIN2A,NRXN1,GRID1,GRIK3,GABRA5,GABRR2,GRIK4,GABRG1,P2RX6,GRIN2B,GABRA2,GRIK1,DGKI,GRM5,NLGN1,RELN,CELF4,APP,GRIA1,GABRG3,GRM1,RIMS1,GRID2,TMEM108,GABRG2,KCND2,GABRB1,MTMR2,RIMS2

	ane potential		
GO:1901888	regulation of cell junction assembly	2.534083834799884e-8	SEMA4D,NEGR1,NRXN1,IL1RAPL1,WDPCP,NTRK2,DLG5,MACF1,RAP1A,DUSP22,PEAK1,IL1RAPL2,NTN1,NLGN1,CNTNAP2,FLRT2,GPC6,APP,LRFN5,CLSTN2,FMN1,EPHA7,EPHB1,EFNA5,LINGO2,GRID2,RAPGEF2,PRKCH,DLC1,EPHB2,PDLIM5,NRP1,NTRK3,SYNDIG1,NPHP4,PTPRD,ASIC2,PTPRA,VCL,ROBO2
GO:0007264	small GTPase mediated signal transduction	2.676861277863848e-8	GPR55,CDC42EP3,KALRN,ADCYAP1R1,MYO9A,RASGEF1B,TIAM2,ELMO1,RAPGEF5,ARHGAP24,SLIT2,PSD3,KANK1,RAP1A,TRIO,ABL2,RERG,FGD4,ROBO1,GARNL3,ARHGEF11,DGKI,DNMBP,NTN1,CTNNAL1,DOCK3,RELN,RASGRF2,VAV3,CCDC88A,PLCE1,EPS8,RASGRF1,RIN2,STARD13,RGL1,TIAM1,RALGPS1,ARHGAP42,NET1,ARHGAP12,DOCK4,DOCK1,RALGPS2,MAPRE2,RAPGEF2,DLC1,AUTS2,EPHB2,DOCK2,NRP1,CDH13,DAB1,SIPA1L2,ARHGAP28,KSR1,RALGAPA2,CD2AP,SIPA1L3,SCAI,RAPGEF4,DOCK9,RASGRP1,RALA,DOCK10,PRKD1,SRGAP3,AKAP13,RALGAPA1,CHN1,NRG1
GO:0048589	developmental growth	2.7237556538076303e-8	SEMA4D,EVC,BCL2,SORBS2,APBA2,CTDP1,GAS2,MTPN,PTGFRN,SLIT2,ERBB4,ATRX,MACF1,RAD51B,RUNX1,PLS1,SLC1A2,SLIT3,CPQ,BBS2,DSCAM,NTN1,MAP2,INSR,COBL,CDH4,TNR,APP,CXADR,TENM4,GHR,ALCAM,RARB,DISC1,FMN1,ZFPM2,EPHA7,STK3,PCDH15,ESR1,RIMS1,POR,EXT1,EFNA5,TRPC5,FTO,AKAP6,ATF2,SEMA6D,TMEM108,ATP8A2,UBE3A,AUTS2,PDLIM5,NBN,FLVCR1,NRP1,SEMA3A,SEMA3E,DCLK1,ARID5B,FSTL4,MTOR,NCAPG2,GLI3,SYT1,SCAPER,PPARA,PLCB1,MAGI2,TNN,SEMA3D,PRLR,TBX20,EYS,VCL,AKAP13,RIMS2,SEMA5A,BCL11A,DCC,NRG1,SEMA3C
GO:0035249	synaptic transmission, glutamatergic	3.107958401152773e-8	GRIK2,UNC13B,CDH8,CACNG2,GRIN2A,NRXN1,GRID1,GRIK3,GRIK4,GRIN2B,GRIK1,DGKI,GRM5,NLGN1,RELN,TNR,HCN1,DISC1,GRM1,EXT1,GRID2,GRM7,CDH2,SYT1,TSHZ3,CACNG3,UNC13C
GO:0065009	regulation of molecular function	3.8214553953293556e-8	CD44,PARN,SEMA4D,RPS6KA5,TAOK3,PRKCB,A2M,SLC8A1,KCNC1,GPR55,TRAPPC9,BCL2,MMP16,THADA,RAG1,MAPK10,IL6R,DLGAP1,RCAN1,JAK2,BICD1,KALRN,FGF12,CACNG2,ZBTB7C,GRIN2A,NRXN1,ADCYAP1R1,CACNA1C,MYO9A,NTRK2,RASGEF1B,TIAM2,SMARCA4,NRG3,ELMO1,SLC24A4,CABIN1,CACNB2,MTPN,TBC1D22A,RGS12,RAPGEF5,CHRM3,ARHGAP24,SPRED2,SLIT2,ROR1,ZNF675,CSNK2A1,KCNE4,TRIM5,PSD3,HECW1,RAP1GDS1,ERBB4,BID,MNAT1,RAP1A,TRIO,DUSP22,ABL2,MAP3K5,NOS1,RGS3,TBC1D5,SLCO3A1,TRAF3,FGD4,ITGA1,MAPK9,SPOCK3,SPON1,ANK2,GRIN2B,ROBO1,GARNL3,TNKS,NDFIP2,BCL2L13,PRKCE,ARHGEF11,DGKI,RIN3,DNMBP,EFHB,DCUN1D4,SLC1A1,GRM5,EPHA6,ARAP2,LDB2,IGF1R,NLGN1,SHISA9,MAP2,CFTR,CAMK1D,NOS1AP,PTPRO,INSR,EGLN3,ANK3,CNIH3,DOCK3,TBCD,RELN,RASGRF2,STK38,DAPK1,VAV3,APP,CCDC88A,SPOCK1,PLCE1,PAK1,CACNA2D1,HCN1,FRY,UTRN,GHR,RIPK4,RASGRF1,RIN2,USP7,STARD13,SLAMF1,RGL1,TIAM1,PBX1,PHACTR1,PRIM2,ASAP2,DISC1,RALGPS1,ARHGAP42,ASAP1,EGF,PDGFD,FYN,PRMT8,XRCC4,EPHA7,STK3,NET1,ESR1,ARHGAP12,KCNAB1,PDE4D,PDE3A,RIMS1,POR,DOCK4,EPHB1,ABCC9,GSG1L,HERC1,DOCK1,FLT1,EFNA5,CDC14B,C14ORF39,HDAC4,STK36,AMFR,SH3BP5,AKAP6,MARK2,ATF2,PHACTR2,RALGPS2,NTF3,FER,MAPRE2,ARFGAP3,RIC8B,GRM7,RAPGEF2,PRKCQ,PRKCH,RABGAP1L,DLC1,GABBR2,IDE,APC,EPHB2,ERC1,MOB3B,NBN,DOCK2,TRIM23,NRP1,RFC3,PHACTR3,TRDN,DAB1,ALK,MGAT5,A

			RID5B,SIPA1L2,CCNG2,RCAN2,SERPINB7,EVI5,HDAC9,PIK3R3,MAP2K6,ARHGAP28,MTOR,KSR1,RALGAPA2,FBLN1,SGSM1,BLM,NCAPG2,RGS7,SPRED1,SIPA1L3,PPP2R2C,PTPRT,GLI3,NTRK3,RXFP1,RAPGEF4,HMGA2,TBC1D4,DOCK9,PPP1R12B,PPARA,PLXNA2,SHISA6,PLCB1,PRKG1,RASGRP1,NLRC5,MAGI2,PLCL1,ABI1,DOCK10,CACNG3,PRKD1,TNFAIP8,HDAC2,RYR2,TGFA,PRLR,DEPTOR,RGS6,SRGAP3,AKAP13,WDR41,NEDD9,ENPP1,STAC,RALGAPA1,DLGAP2,AGAP1,CHN1,NRG1,CAST,TBC1D9,FANK1,ST18
GO:0034765	regulation of ion transmembrane transport	4.99755125133524e-8	KCNMA1,SLC8A1,KCNC1,BCL2,THADA,KCNQ5,SCN11A,KCNJ6,KCNK10,FGF12,CACNG2,GRIN2A,NRXN1,ADCYAP1R1,DPP6,KCNH1,CACNA1C,CACNB2,CHRM3,KCNE4,KCNS3,HECW1,NOS1,CACNA1E,ANK2,GRIN2B,CACNA2D3,PRKCE,KCNH8,GRM5,NLGN1,SHISA9,KCNIP4,CFTR,NOS1AP,CATSPER2,ANK3,CNIH3,RELN,RASGRF2,DPP10,DAPK1,CLIC6,APP,CACNA2D1,HCN1,UTRN,TSPAN13,RASGRF1,FYN,KCND3,KCNAB1,PDE4D,ABCC9,GSG1L,AKAP6,KCND2,EPHB2,TRDN,RGS7,SHISA6,ASIC2,CACNG3,PRKD1,RYR2,KCNH5,STAC,SCN8A,KCNJ15
GO:0009887	animal organ morphogenesis	6.188487667660966e-8	ANKRD6,BCL2,MMP16,COL18A1,THRB,DNAH11,NTN4,USH2A,FLI1,MEGF11,LAMA1,WDPCP,LAMA3,EXOC4,CTNNA1,NTRK2,FOXN3,ZNRF3,NRG3,SLC24A4,SOX6,DLG5,ATRNL1,CPE,SLIT2,ROR1,AKT3,CRB1,ALPK2,ERBB4,MYO3B,CHSY1,EXT2,SLC40A1,ITGA8,ALDH1A2,EYA1,PLS1,SLIT3,ROBO1,ANKRD11,EGFLAM,OVOL2,MLLT3,PRICKLE2,BBS2,MYO3A,SOX5,DSCAM,SDK1,SLC1A1,NTN1,NR5A2,WDR72,ALX4,CFTR,FLRT2,INSR,GPC6,ADAMTS5,HCN1,GHR,MEIS2,ADAMTS16,RARB,TIAM1,PBX1,FMN1,ZFPM2,EDAR,PCDH15,ESR1,LRIG1,POR,WWOX,EPHB1,GREB1L,EFEMP1,AJAP1,EXT1,TENM3,ATF2,LRP2,NTNG1,DLC1,ATP8A2,EPHB2,SETD2,FLVCR1,NRP1,RPGRIP1,CDH2,ARID5B,MTOR,RORB,FHL2,TTC39C,PAPPA2,CSMD1,GLI3,RXFP1,FBN1,TBX15,ASXL3,PPARA,MAGI2,HDAC2,RYR2,TGFA,TBX20,FAT3,ROBO2,CDH23,FREM1,CTNNA2,NRG1,SEMA3C
GO:0044093	positive regulation of molecular function	7.031516471036862e-8	CD44,PARN,SEMA4D,RPS6KA5,TAOK3,PRKCB,KCNC1,GPR55,TRAPPC9,BCL2,IL6R,JAK2,KALRN,CACNG2,GRIN2A,NRXN1,ADCYAP1R1,CACNA1C,MYO9A,NTRK2,RASGEF1B,TIAM2,SMARCA4,NRG3,CACNB2,MTPN,TBC1D22A,RAPGEF5,ARHGAP24,ROR1,TRIM5,RAP1GDS1,ERBB4,BID,MNAT1,RAP1A,ABL2,MAP3K5,NOS1,TBC1D5,SLCO3A1,ITGA1,SPON1,ANK2,GRIN2B,ROBO1,GARNL3,TNKS,BCL2L13,DCUN1D4,SLC1A1,GRM5,EPHA6,ARAP2,IGF1R,CFTR,CAMK1D,NOS1AP,INSR,EGLN3,ANK3,DOCK3,RELN,RASGRF2,DAPK1,VAV3,APP,CCDC88A,PAK1,CACNA2D1,GHR,RIPK4,RASGRF1,RGL1,TIAM1,PRIM2,ASAP2,RALGPS1,ARHGAP42,ASAP1,EGF,PDGFD,FYN,XRCC4,EPHA7,STK3,NET1,ESR1,POR,EPHB1,FLT1,EFNA5,CDC14B,HDAC4,STK36,AMFR,AKAP6,MARK2,ATF2,RALGPS2,NTF3,FER,MAPRE2,RAPGEF2,PRKCQ,PRKCH,RABGAP1L,DLC1,IDE,EPHB2,ERC1,MOB3B,NBN,NRP1,RFC3,TRDN,DAB1,ALK,ARID5B,SIPA1L2,EVI5,MAP2K6,MTOR,RALGAPA2,FBLN1,SGSM1,RGS7,SIPA1L3,NTRK3,RXFP1,RAPGEF4,HMGA2,TBC1D4,DOCK9,RASGRP1,MAGI2,PLCL1,ABI1,DOCK10,CACNG3,PRKD1,HDAC2,RYR2,TGFA,PRLR,RGS6,AKAP13,WDR41,NEDD9,STAC,RALGAPA1,CHN1,NRG1,TBC1D9,FANK1,ST18
GO:0098609	cell-cell adhesion	7.965403267760546e-8	CD44,SEMA4D,BCL2,RAG1,IGSF11,CDH8,ASTN2,JAK2,NEGR1,NFAT5,MEGF11,CDH11,NRXN1,ARID1B,IL1RAPL1,MAGI1,LAMA3,CTNNA1,NCAM2,SMARCA4,CNTN1,UNC5D,DLG5,ASTN1,CRB1,LPP,DUSP22,ABL2,CNTN4,HMCN1,ITGA1,ITGA8,RUNX1,CDH17,CDH18,ZBTB16,ROBO1,IGSF5,DSCAM,SDK1,NTN1,NLGN1,JAM2,PCDH9,ITGA9,ANK3,CDH12,GPC6,CDH4,TNR,CNTN6,CXADR,LRFN5,TENM4,PCDH7,LRRC4C,ALCAM,SLAMF1,CLSTN2,FYN,EPHA7,COL19A1,PCDH15,CNTN5,PCDH11X,IGSF21,EXT1,EFNA5,TLN2,TENM3,GRID2,FER,NTNG1,SLC39A8,PRKCQ,KIRREL3,NRXN3,PDLIM5,ADAMTS18,CTNND2,PKP1,CDH13,DAB1,CDH2,TENM2,CD2AP,PTPRT,GLI3,CTNNA3,NPHP4,DLG2,PPARA,PTPRD,PRKG1,RASGRP1,CDH9,STXBP6,FNDC3A,ETS1,FAT3,VCL,ROBO2,CDH23,DCC,CTNNA2,NRG1,PRKCA
GO:0048583	regulation	8.847276707293356e-8	CD44,PTPRR,SEMA4D,EVC,TAOK3,PRKCB,A2M,ANKRD6,GPR55,CD38,BCL2,CAMTA1,SAMSN1,THRB,RAG1,IGSF11,SNX25,UNC13B,M

	of response to stimulus		APK10,NCAM1,IL6R,CASP5,BRD4,NDRG2,DLGAP1,RCAN1,JAK2,BICD1,OTUD7A,TPTE2,KALRN,CACNG2,NFAT5,ZNF536,LAMA1,GRIN2A,NRXN1,ARID1B,ADCYAP1R1,CTDP1,SMOC2,ZDHHC17,CTNNA1,MYO9A,NTRK2,OCLN,NLK,AFAP1,TIAM2,IQCJ-SCHIP1,SMARCA4,RNF152,ZNRF3,SLC24A4,FBXL17,GAS2,PDE10A,MTPN,MECOM,RGS12,SHANK2,KCTD8,NREP,DOK5,DLG5,ARHGAP24,SPRED2,IGHV1OR15-9,SLIT2,ROR1,ZNF675,CSNK2A1,AKT3,TRIM5,PSD3,ALPK2,HECW1,RAP1GDS1,ERBB4,KANK1,DMRT1,BID,MACF1,RAP1A,TRIO,PTPRE,DUSP22,CHSY1,ABL2,PSMA1,MAP3K5,RGS3,HTR2C,NEK4,TRAF3,FGD4,ITGA1,CORO2B,ITGA8,MAPK9,IGHV1OR21-1,EYA1,PLA2G4A,SUPT3H,SLIT3,GRIN2B,ROBO1,PAK3,GARNL3,TNKS,NDFIP2,OVOL2,SGMS1,MLLT3,LEMD3,PRKCE,PSG9,ARHGEF11,PRKAA2,BBS2,DSCAM,DGKI,RIN3,DNMBP,EFHB,KDM4C,GRM5,IGF1R,NLGN1,SHISA9,CAMK1D,NOS1AP,PTPRO,CD96,RBMS3,INSR,CLEC16A,CNIH3,DOCK3,GPC6,RELN,RASGRF2,STK38,AOAH,FBXO32,TNR,CELF4,DAPK1,VAV3,CNTN6,APP,FBLN5,PUM1,CCDC88A,PLCE1,PAK1,EPS8,LRFN5,GPC5,GHR,RASGRF1,PRDM16,USP7,KIR2DL4,STARD13,KL,MICU1,LTBP1,SLAMF1,INO80D,TIAM1,MLIP,DISC1,RALGPS1,ARHGAP42,EDAR,EGF,PDGFD,FYN,EPHA7,TRABD2B,SPIDR,STK3,CNOT7,USP18,S100B,NET1,ESR1,ARHGAP12,GRM1,PDE4D,PRKACB,PDE3A,RIMS1,POR,WWOX,CNKSR2,FUT8,EPHB1,AJAP1,GSG1L,FLT1,NXN,HDAC4,STK36,AMFR,AKAP6,RBBP8,GRID2,ZNF423,LRP2,SEMA6D,C2,RALGPS2,NTF3,FER,SUSD4,MAPRE2,RAD51AP1,TMEM108,RIC8B,TPTE,RAPGEF2,PRKCQ,PRKCH,DLC1,UBE3A,APC,INO80,AUTS2,EPHB2,AGO3,C9,MCTP1,MOB3B,ADAMTS18,CTNND2,SETD2,DOCK2,NRP1,CDH13,DAB1,RFTN1,ALK,LDLRAD4,SEMA3A,SEMA3E,MGAT5,MAGI3,CDH2,SIPA1L2,MAP2K6,FSTL4,ARHGAP28,MTOR,KSR1,RALGAPA2,FBLN1,FHL2,NEU3,NCAPG2,RGS7,CD2AP,USP25,SPRED1,SIPA1L3,ADAM10,SCAI,PTPRT,GLI3,NTRK3,FBN1,BMPER,HMGA2,APIP,DPF3,NPHP4,PPARA,PTPRD,RORA,SHISA6,PLCB1,PRKG1,RASGRP1,NLRC5,MAGI2,CACNG3,PRKD1,BCL2L1,HDAC2,ETS1,TNN,SEMA3D,TGFA,PRLR,TBX20,MTMR2,ATF6,IL16,DEPTOR,ROBO2,IFT81,ZMYND11,RGS6,SRGAP3,AKAP13,WDR41,NEDD9,ENPP1,RIMS2,EYA4,RALGAPA1,DLGAP2,SEMA5A,PRDM15,CTNNA2,CHN1,VPS13C,NRG1,PRKCA,FMN2,SEMA3C,FANCB
GO:0051960	regulation of nervous system development	1.0619813411631149e-7	SEMA4D,FBXO31,KALRN,NRXN1,IL1RAPL1,CTNNA1,NTRK2,TIAM2,NUMB,DLG5,SLIT2,MACF1,ROBO1,PAK3,BRINP1,CHODL,DSCAM,GRM5,NTN1,NLGN1,JAM2,MAP2,FLRT2,CUX1,RELN,CDH4,TNR,TENM4,CLSTN2,TIAM1,DISC1,EPHA7,EPHB1,EFNA5,TRPC5,LINGO2,GRID2,LRP2,SEMA6D,RAPGEF2,PRKCH,EPHB2,PRTG,NRP1,DAB1,SEMA3A,SEMA3E,FSTL4,MTOR,GLI3,NTRK3,SYNDIG1,PLXNA2,PTPRD,ASIC2,HDAC2,SEMA3D,MTMR2,ROBO2,SEMA5A,BCL11A,DCC,PARD3,SEMA3C
GO:0048588	developmental cell growth	1.1954714937252826e-7	SEMA4D,SORBS2,CTDP1,SLIT2,MACF1,SLIT3,DSCAM,NTN1,MAP2,COBL,CDH4,TNR,APP,ALCAM,DISC1,EPHA7,RIMS1,EXT1,EFNA5,TRPC5,AKAP6,SEMA6D,TMEM108,AUTS2,PDLIM5,NRP1,SEMA3A,SEMA3E,DCLK1,FSTL4,SYT1,PPARA,TNN,SEMA3D,VCL,AKAP13,RIMS2,SEMA5A,BCL11A,DCC,SEMA3C
GO:0030036	actin cytoskeleton organization	1.2669084558949914e-7	BCL2,CDC42BPA,CDC42EP3,SORBS2,JAK2,EPB41L3,PARVB,CTNNA1,THSD7A,ELMO1,GAS2,MTPN,PGM5,CALD1,SLIT2,MYLK3,RAP1GDS1,KANK1,ABL2,HMCN1,FGD4,CORO2B,FAM171A1,FRMD6,PLS1,PAK3,FCHSD2,EHBP1,PSTPIP2,PRKCE,ARHGEF11,MYOM2,SPTB,SHROOM3,NOS1AP,COBL,CCDC88A,PAK1,CXADR,EPS8,UTRN,FRMD5,STARD13,PPP1R9A,NEBL,PHACTR1,FMN1,FRMPD4,PCDH15,ARHGAP12,FHOD3,DIAPH3,TRPM7,EFNA5,KLHL1,PHACTR2,NTF3,FER,RHPN2,DLC1,MPRIP,AUTS2,PDLIM5,FRMD3,PACSIN2,DOCK2,NRP1,PHACTR3,SEMA3E,ARHGAP28,MTOR,SH3KBP1,CD2AP,FLNB,SPECC1,NTRK3,CTNNA3,THSD7B,NPHP4,PRKG1,ABI1,RALA,MICAL3

			,AKAP13,NEDD9,SEMA5A,CTNNA2,FMN2
GO:0048522	positive regulation of cellular process	1.4915470456662957e-7	CD44,SAMD4A,KCNMA1,ZHX3,APBB2,SCAF8,ERG,PARN,SEMA4D,EVC,TEAD1,NFIA,RPS6KA5,TAOK3,PRKCB,SLC8A1,ANKRD6,KCNC1,GPR55,CD38,FBXO31,BCL2,CAMTA1,CHFR,TOX3,THRB,RAG1,CDC42EP3,GRIK2,IGSF11,UNC13B,IL6R,ZBTB20,ATP10A,BRD4,GRIP1,JAK2,TM9SF4,BICD1,KALRN,NEGR1,CACNG2,NFAT5,FLI1,LAMA1,ZBTB7C,GRIN2A,NRXN1,ARID1B,IL1RAPL1,MAGI1,ADCYAP1R1,RPS6KA2,SMOC2,ZDHHC17,BMF,CTNNA1,NTRK2,OCLN,TIAM2,IQCJ-SCHIP1,SMARCA4,RNF152,CNTN1,MEOX2,GLIS3,NRG3,CACNB2,NUMB,STXBP4,MED15,MTPN,SOX6,MECOM,PDE4DIP,SHANK2,UBE2E2,DOK5,DLG5,SPRED2,IGHV1OR15-9,SLIT2,MYLK3,ROR1,GLP2R,SLC4A4,CSNK2A1,AKT3,KMT2C,TRIM5,ABCA13,ERBB4,KANK1,ATRX,DMRT1,BID,MACF1,MNAT1,TAF4B,RAP1A,CTNNBL1,RAD51B,DUSP22,CHSY1,MYOM1,ABL2,MAP3K5,NOS1,ABCG1,MAML2,HTR2C,NEK4,CTIF,TBC1D5,SLC40A1,SLCO3A1,TRAF3,CHD6,ETS2,ITGA1,TCF12,ZNF721,CORO2B,ITGA8,RUNX1,ALDH1A2,MAPK9,ESRRG,PTGFR,IGHV1OR21-1,CDH17,EYA1,MORC3,PLS1,ANK2,PLA2G4A,SLC1A2,ZBTB16,SUPT3H,GRIN2B,ROBO1,EGFLAM,PAK3,TNKS,KLF12,NDFIP2,OVOL2,FCHSD2,BRINP1,MLLT3,CHODL,GABPA,PRKCE,GLIS1,ARHGEF11,PRKAA2,EDIL3,SOX5,DSCAM,DGKI,KDM4C,SLC1A1,GRM5,EPHA6,NTN1,NR5A2,LDB2,IGF1R,SNX30,NLGN1,JAM2,ALX4,CNTNAP2,MAP2,CFTR,CAMK1D,FLRT2,MLLT10,NOS1AP,INSR,COBL,CLEC16A,CUX1,ANK3,MORC2,DOCK3,RELN,RASGRF2,CDH4,TNR,DPP10,CELF4,DAPK1,VAV3,ZNF600,VRK1,CNTN6,APP,PUM1,CCDC88A,ARNT2,HPSE2,PLCE1,PAK1,MITF,CACNA2D1,ADCK1,NSMCE2,ZNF208,EPS8,UTRN,GPC5,TENM4,PRR16,GHR,DUX4,RASGRF1,RIN2,PRDM16,FRMD5,RNF217,USP7,MEIS2,KIR2DL4,SCP2,KL,TASP1,SLAMF1,INO80D,CLSTN2,RARB,TCF4,TIAM1,PBX1,MLIP,PRIM2,DISC1,FMN1,ZFPM2,ASAP1,FRMPD4,EDAR,EGF,PDGFD,FYN,EPHA7,TRABD2B,SPIDR,STK3,CNOT7,MSR1,PSIP1,S100B,NET1,TOX,ESR1,GRM1,PDE3A,RIMS1,POR,DOCK4,FRMD4A,WWOX,EPHB1,SSBP2,CREM,TNRC6B,DOCK1,FLT1,EFNA5,CDC14B,HDAC4,ZNF717,STK36,TRPC5,FTO,AKAP6,TENM3,LINGO2,MARK2,ATF2,RBBP8,GRID2,ZNF423,LRP2,SEMA6D,NTF3,FER,CAMK4,MAPRE2,RAD51AP1,TMEM108,RAPGEF2,NAV3,ZNF615,GTF2I,PRKCQ,PRKCH,DLC1,NSG1,ATP8A2,UBE3A,APC,INO80,AUTS2,EPHB2,ZNF850,AGO3,MOB3B,NBN,RGMB,NRP1,CDH13,RFC3,TRDN,SLC2A13,DAB1,ALK,SEMA3A,SEMA3E,MGAT5,NRIP1,CDH2,ARID5B,TENM2,SERPINB7,ZNF407,HDAC9,PIK3R3,MAP2K6,MTOR,KSR1,RORB,FBLN1,ST8SIA1,BLM,SH3KBP1,CADPS,NEU3,NCAPG2,RGS7,CD2AP,SPRED1,ADAM10,KANSL1,TRERF1,SLC24A2,GLI3,NTRK3,RAB31,VPS13D,ABHD17C,ZNF292,TBX15,BMPER,ANKRD31,ZNF521,ATF7IP,HMGA2,CREB5,DNM3,SYT1,SYNDIG1,ASXL3,DPF3,NPHP4,PPARA,PLXNA2,PTPRD,RORA,PLCB1,LOXL2,BPTF,RASGRP1,ELAVL4,NLRC5,MAGI2,NELL1,ABI1,TSHZ3,ASIC2,RALA,CACNG3,PRKD1,ATP8A1,TNFAIP8,BCL2L1,HDAC2,ETS1,MRPS27,TNN,RYR2,SEMA3D,BANP,TGFA,PRLR,TBX20,MTMR2,ATF6,BACH1,ATAT1,ROBO2,AKAP13,NEDD9,MYRIP,PCP4,RIMS2,STAC,RAB27A,EYA4,POMT2,HIVEP3,CLIP1,SEMA5A,PRDM15,BCL11A,ZNF112,ETV6,PARD3,NRG1,FANK1,ZNF845,NPAS3,PRKCA,FMN2,SEMA3C,FANCB,CSF2RB,PCNT,ST18
GO:0034762	regulation of transmembrane transport	2.061207891955606e-7	KCNMA1,PRKCB,SLC8A1,KCNC1,BCL2,THADA,KCNQ5,SCN11A,KCNJ6,KCNK10,FGF12,CACNG2,GRIN2A,NRXN1,ADCYAP1R1,DPP6,KCNH1,CACNA1C,OCLN,CACNB2,STXBP4,CHRM3,KCNE4,KCNS3,HECW1,NOS1,CACNA1E,ANK2,SLC1A2,GRIN2B,CACNA2D3,PRKCE,KCNH8,GRM5,NLGN1,SHISA9,KCNIP4,CFTR,NOS1AP,INSR,CATSPER2,ANK3,CNIH3,RELN,RASGRF2,DPP10,DAPK1,CLIC6,APP,CACNA2D1,HCN1,UTRN,TSPAN13,RASGRF1,FYN,KCND3,KCNAB1,PDE4D,ABCC9,GSG1L,AKAP6,KCND2,EPHB2,TRDN,RGS7,SHISA6,ASIC2,CACNG3,PRKD1,RYR2,KCNH5,ENPP1,STAC,SCN8A,KCNJ15
GO:0007010	cytoskeleton organ	2.2894262429484163e-7	C10ORF90,BCL2,CDC42BPA,CDC42EP3,SORBS2,KIF4A,JAK2,BICD1,EPB41L3,PARVB,MAST4,WDPCP,CCSER2,CTNNA1,OCLN,THSD7A,AFAP1,IQCJ-SCHIP1,LRRC49,ELMO1,GAS2,MTPN,PDE4DIP,DNAH8,PGM5,CALD

	ization		1,SLIT2,MYLK3,RAP1GDS1,KANK1,ATRX,MACF1,ABL2,SPAG16,EML1,HMCN1,FGD4,CORO2B,FAM171A1,FRMD6,PLS1,ANK2,PAK3,TNKS,MDM1,FCHSD2,EHBP1,PSTPIP2,TMEM67,PRKCE,ARHGEF11,PRKAA2,BBS2,MYOM2,ANKFN1,SPTB,NLGN1,SHROOM3,MAP2,NOS1AP,COBL,ANK3,TBCD,CCDC88A,PLCE1,TACC2,PAK1,CXADR,EPS8,UTRN,CECR2,FRMD5,STARD13,PPP1R9A,TTLL11,NEBL,PHACTR1,SLC39A12,DISC1,FMN1,FRMPD4,MAP7,PCDH15,ARHGAP12,FHOD3,ARMC2,DIAPH3,TRPM7,EFNA5,CDC14B,TLN2,STK36,KLHL1,MARK2,ATF2,PHACTR2,NTF3,FER,MAPRE2,NAV3,RHPN2,KRT25,DLC1,ATP8A2,MPRIP,APC,TTLL5,INO80,AUTS2,PDLIM5,FRMD3,SETD2,PACSIN2,PKP1,DOCK2,SDCCAG8,NRP1,PHACTR3,TRDN,SEMA3E,DCLK1,ARHGAP28,MTOR,SH3KBP1,CD2AP,SIPA1L3,FLNB,SPECC1,NTRK3,HYDIN,CTNNA3,THSD7B,NPHP4,PRKG1,ABI1,RALA,MICAL3,ATAT1,AKAP13,NEDD9,CLIP1,SEMA5A,CTNNA2,CEP44,PARD3B,PARD3,FMN2,PCNT
GO:0006810	transport	2.5709771229211975e-7	KCNMA1,SLC12A8,SYN3,PRKCB,TRPM6,SLC8A1,KCNC1,DNAJC15,SIAH3,CD38,TRAPPC9,BCL2,THADA,RSRC1,DNAH11,SLC13A4,SAMM50,GRIK2,SNX25,UNC13B,KCNQ5,HEPHL1,SCN11A,ATP10A,GOT2,KCNJ6,KCNK10,ZDHHC11B,TMEM241,GRIP1,APBA2,TLK1,ASTN2,TANC2,KIF4A,JAK2,TM9SF4,BICD1,LRP1B,ABCG8,KALRN,FGF12,CACNG2,BTBD9,SLC44A5,GRIN2A,NRXN1,IL1RAPL1,WDPCP,NIPA2,SLC14A2,ADCYAP1R1,DPP6,PRELID2,GRID1,ZDHHC17,KCNH1,CACNA1C,AMPH,EXOC4,HEATR5A,ANO4,BBS9,NTRK2,ENTHD1,OCLN,ABCA6,TMPRSS3,CNTN1,TTC39B,ELMO1,SLC24A4,GRIK3,CACNB2,NUMB,STXBP4,ESYT2,SYBU,TRPM3,GABRA5,CHRM3,CPE,IGHV1OR15-9,PITPNC1,ABCA5,SLC4A4,KCNE4,KCNS3,ABCA13,HECW1,RAP1GDS1,ERBB4,BID,MACF1,RAP1A,SLC15A5,MYOM1,EXT2,ABL2,NOS1,ABCG1,SPAG16,HTR2C,CACNA1E,TBC1D5,SLC40A1,SLCO3A1,GABRR2,PIK3C3,SLC9C1,SNAP25-AS1,GRIK4,GABRG1,IGHV1OR21-1,CDH17,SV2B,P2RX6,PLS1,ANK2,PLA2G4A,SLC1A2,ANO2,GRIN2B,TNKS,NDFIP2,GABRA2,CSE1L,FCHSD2,ITPR2,TRAPPC10,EHBP1,CACNA2D3,PRKCE,CD163,BBS2,PTPRN2,KCNH8,GRIK1,DGKI,RIN3,EFHB,TRAPPC8,SLC1A1,SLC12A1,GRM5,NTN1,IGF1R,SNX30,NLGN1,DNAH9,SHISA9,CORIN,MON2,MAP2,KCNIP4,CFTR,CAMK1D,NOS1AP,ZDHHC14,NKAIN2,INSR,CLEC16A,CATSPER2,CUX1,ANK3,SV2C,CNIH3,RELN,RASGRF2,MFSD9,DPP10,OCA2,DAPK1,VAV3,CLIC6,APP,FBLN5,CCDC88A,PAK1,ATP9B,IGF2BP3,CACNA2D1,HCN1,CHRM5,CXADR,UTRN,CECR2,TSPAN13,GHR,RASGRF1,RIN2,USP7,RBFOX1,SCP2,MICU1,SLAMF1,NKAIN3,SLC25A21,SORCS2,SLC39A12,OSBPL10,PIEZO2,SLC35F1,VTI1A,EGF,FYN,FAM3B,KCND3,IFT43,NSG2,GRIA1,STK3,ANO10,MSR1,AP2B1,ARHGAP12,GABRG3,KCNN3,KCNAB1,GRM1,PDE4D,ERC2,RIMS1,ATP6V1E1,FRMD4A,MCTP2,MIPEP,ABCC9,SNAP29,GSG1L,DOCK1,TRPM7,EXT1,EFNA5,ABCA10,STK36,TRPC5,ATP9A,PPFIA2,AKAP6,VPS37A,ATF2,TUSC3,CCDC91,GRID2,LRP2,C2,NTF3,FER,ARFGAP3,MICU2,TMEM108,GABRB3,GRM7,SLC39A8,IMMP2L,ATXN1,GABRG2,NRXN3,RABGAP1L,NSG1,KCND2,ATP8A2,SLC24A3,UBE3A,GRIA4,EPHB2,STON1-GTF2A1L,SYT16,ERC1,MCTP1,RYR3,NBAS,SETD2,PACSIN2,DOCK2,NUP214,TRIM23,FLVCR1,NRP1,CDH13,TRDN,SLC2A13,RFTN1,EXOC6B,ATP13A3,STOML1,DCLK1,KIF16B,CDH2,EVI5,VPS41,MAP2K6,GABRB1,SH3KBP1,CADPS,NEU3,RGS7,CD2AP,SLC44A1,AP5M1,ADAM10,ABCB5,SLC24A2,GLI3,CHKA,RAB31,VPS13D,RAPGEF4,TMPRSS2,TMPRSS15,MX2,NSUN2,SORCS1,TBC1D4,DNM3,SYT1,SYNDIG1,DLG2,PPARA,SCFD2,SHISA6,SCARA5,LOXL2,RASGRP1,STXBP6,DMBT1,MAGI2,STX12,ASIC2,RALA,GNPTAB,TRPM1,CACNG3,PRKD1,ATP8A1,BCL2L1,MICAL3,RANBP17,RYR2,LDLRAD3,PRLR,MTMR2,KCNH5,TMEM163,IPO11,IL16,IFT81,OSCP1,CDH23,AKAP13,WDR41,MYRIP,SLC39A11,ENPP1,UNC13C,RIMS2,SLC5A1,STAC,SCN8A,RAB27A,AGAP1,VPS13C,KCNJ15,PARD3,NRG1,SLC25A48,ATP10B,SLC35F4,FMN2,PCNT,OSBPL5
GO:0051239	regulation	3.243606165677337e-7	KCNMA1,PTPRR,SEMA4D,RPS6KA5,PRKCB,SLC8A1,GPR55,CD38,FBXO31,BCL2,THRB,RAG1,IGSF11,UNC13B,IL6R,ZBTB20,SCN11A,NDRG2,DLGAP1,NTN4,JAK2,ABCG8,KALRN,FGF12,LAMA1,GRIN2

	of multicellular organismal process		A,NRXN1,ARID1B,IL1RAPL1,WDPCP,LAMA3,CTDP1,SMOC2,CACNA1C,CTNNA1,NTRK2,TIAM2,SMARCA4,MEOX2,PTPRG,SLC24A4,CACNB2,NUMB,MTPN,SOX6,DLG5,CHRM3,SPRED2,SLIT2,ZNF675,AKT3,KCNE4,ALPK2,ERBB4,DMRT1,MACF1,CHSY1,NOS1,HTR2C,TRAF3,CORO2B,RBM19,RUNX1,ESRRG,PLS1,ANK2,ZBTB16,GRIN2B,ROBO1,PAK3,OVOL2,BRINP1,CHODL,GABPA,PRKCE,PSG9,BBS2,SOX5,DSCAM,SLC1A1,GRM5,NTN1,IGF1R,NLGN1,SHISA9,JAM2,CORIN,MAP2,CFTR,FLRT2,NOS1AP,PTPRO,CD96,INSR,CUX1,RELN,TRPS1,ADAMTS5,FBXO32,CDH4,TNR,CELF4,APP,RNLS,PLCE1,ADAM12,MITF,IGF2BP3,CACNA2D1,HCN1,CXADR,TENM4,GHR,RIN2,PRDM16,MEIS2,KIR2DL4,STARD13,KL,LTBP1,SLAMF1,INO80D,CLSTN2,RARB,TIAM1,PBX1,MLIP,SLC39A12,DISC1,ARHGAP42,ZFPM2,EGF,KCND3,EPHA7,STK3,TOX,ESR1,GRM1,PDE4D,PDE3A,RIMS1,POR,DOCK4,EPHB1,EFEMP1,AJAP1,ABCC9,DOCK1,FLT1,EFNA5,HDAC4,TRPC5,FTO,AKAP6,LINGO2,ATF2,GRID2,ZNF423,LRP2,SEMA6D,CAMK4,CELF2,MAPRE2,TMEM108,RAPGEF2,NAV3,GTF2I,PRKCQ,PRKCH,ATP8A2,APC,INO80,EPHB2,PRTG,ADAMTS18,SETD2,FLVCR1,NRP1,TRDN,DAB1,RFTN1,LDLRAD4,SEMA3A,SEMA3E,SERPINB7,ASB3,HDAC9,MAP2K6,FSTL4,MTOR,FBLN1,CD2AP,SPRED1,ADAM10,GLI3,NTRK3,FBN1,CTNNA3,BMPER,HMGA2,SYNDIG1,PPP1R12B,PPARA,PLXNA2,PTPRD,RORA,SHISA6,PLCB1,LOXL2,PRKG1,RASGRP1,NELL1,PLCL1,TSHZ3,ASIC2,PRKD1,HDAC2,ETS1,TNN,RYR2,SEMA3D,PRLR,TBX20,MTMR2,IL16,VCL,ROBO2,NEDD9,ENPP1,RIMS2,DLGAP2,SEMA5A,BCL11A,DCC,PARD3,NRG1,PRKCA,SEMA3C
GO:0007166	cell surface receptor signaling pathway	3.873429454982823e-7	CD44,PTPRR,SEMA4D,EVC,RPS6KA5,PRKCB,ANKRD6,CD38,BCL2,GRIK2,IGSF11,SNX25,UNC13B,MAPK10,NCAM1,IL6R,SORBS2,NDRG2,JAK2,KALRN,FGF12,LAMA1,GRIN2A,NRXN1,WDPCP,MAGI1,LAMA3,ADCYAP1R1,GRID1,SMOC2,ZDHHC17,FAM83B,CTNNA1,NTRK2,NLK,ITGBL1,SMARCA4,CNTN1,ZNRF3,NRG3,PTPRG,BTBD11,CABIN1,FBXL17,GAS2,GRIK3,STXBP4,UNC5D,NREP,DOK5,DLG5,CPE,SPRED2,IGHV1OR15-9,SLIT2,ROR1,GLP2R,ZNF675,CSNK2A1,TRIM5,ALPK2,HECW1,ERBB4,KANK1,DMRT1,BID,MACF1,RAP1A,TRIO,PTPRE,DUSP22,CHSY1,MAML2,TRAF3,ITGA1,ITGA8,GRIK4,MAPK9,IGHV1OR21-1,CDH17,EYA1,ANKS1B,P2RX6,SLIT3,GRIN2B,ROBO1,PAK3,TNKS,OVOL2,MLLT3,LEMD3,RNF138,PRICKLE2,PRKCE,PSG9,PRKAA2,BBS2,IL1RAPL2,GRIK1,DSCAM,DGKI,SLC1A1,GRM5,EPHA6,IGF1R,NLGN1,FLRT2,PTPRO,RBMS3,INSR,ITGA9,GMDS,GPC6,RELN,CELF4,DAPK1,VAV3,CNTN6,APP,CCDC88A,PLCE1,ADAM12,PAK1,MITF,GPC5,GHR,PRDM16,KL,LTBP1,SLAMF1,WDR12,TIAM1,DISC1,SVEP1,EGF,PDGFD,FYN,EPHA7,GRIA1,TRABD2B,STK3,CNOT7,USP18,GRM1,PDE4D,PRKACB,RIMS1,POR,WWOX,FUT8,EPHB1,EFEMP1,DOCK1,FLT1,EXT1,EFNA5,NXN,STK36,AMFR,MARK2,ATF2,GRID2,ZNF423,LRP2,SEMA6D,NTF3,FER,TMEM108,GRM7,RAPGEF2,MX1,PRKCQ,PRKCH,GRIA4,IDE,APC,EPHB2,ADAMTS18,RGMB,CTNND2,NRP1,CDH13,DAB1,RFTN1,ALK,EVC2,LDLRAD4,SEMA3A,SEMA3E,MGAT5,KIF16B,CDH2,ARID5B,PIK3R3,FSTL4,NEU3,SPRED1,ADAM10,PTPRT,GLI3,NTRK3,FBN1,BMPER,PTPRK,NPHP4,PPARA,PLXNA2,PTPRD,RORA,SHISA6,PLCB1,NLRC5,MAGI2,ABI1,TRPM1,PRKD1,BCL2L1,HDAC2,TNN,SEMA3D,TGFA,PRLR,TBX20,PTPRA,MTMR2,DEPTOR,ROBO2,IFT81,ZMYND11,NEDD9,ENPP1,RIMS2,EYA4,SEMA5A,PRDM15,DCC,CHN1,NRG1,PRKCA,SEMA3C,CSF2RB,ST18
GO:0051056	regulation of small GTPase mediated signal	4.052060046602118e-7	GPR55,KALRN,ADCYAP1R1,MYO9A,TIAM2,ARHGAP24,SLIT2,PSD3,KANK1,TRIO,ABL2,FGD4,ROBO1,GARNL3,ARHGEF11,DGKI,DNMBP,DOCK3,RELN,RASGRF2,VAV3,PLCE1,EPS8,RASGRF1,STARD13,TIAM1,RALGPS1,ARHGAP42,NET1,ARHGAP12,RALGPS2,MAPRE2,DLC1,AUTS2,EPHB2,DOCK2,NRP1,SIPA1L2,ARHGAP28,RALGAPA2,CD2AP,SIPA1L3,SCAI,RASGRP1,SRGAP3,AKAP13,RALGAPA1,CHN1,NRG1

	transduction		
GO:0051963	regulation of synapse assembly	4.825722129407593e-7	SEMA4D,NEGR1,NRXN1,IL1RAPL1,NTRK2,DLG5,IL1RAPL2,NTN1,NLGN1,FLRT2,GPC6,APP,LRFN5,CLSTN2,EPHA7,EPHB1,EFNA5,LINGO2,GRID2,EPHB2,PDLIM5,NTRK3,SYNDIG1,PTPRD,ASIC2,ROBO2
GO:0048813	dendrite morphogenesis	5.365335499919316e-7	SEMA4D,FBXO31,TANC2,KALRN,IL1RAPL1,HECW1,PAK3,DSCAM,NLGN1,MAP2,CUX1,RELN,PHACTR1,FYN,EPHB1,TRPC5,PPFIA2,RAPGEF2,UBE3A,EPHB2,PDLIM5,CTNND2,NRP1,SEMA3A,DCLK1,DNM3,PTPRD,ELAVL4,ABI1,DOCK10,CTNNA2
GO:0099175	regulation of postsynapse organization	5.683185549628624e-7	TANC2,KALRN,NRXN1,IL1RAPL1,GRIN2B,PAK3,DGKB,NLGN1,RELN,FYN,EPHA7,PPFIA2,GRID2,UBE3A,EPHB2,PDLIM5,CDH2,TANC1,LRFN2,NTRK3,ABHD17C,DNM3,PTPRD,NEDD9
GO:0098742	cell-cell adhesion via plasma-membrane adhesion molecules	7.141820748333678e-7	IGSF11,CDH8,CDH11,NRXN1,IL1RAPL1,UNC5D,CRB1,CNTN4,HMCN1,CDH17,CDH18,ROBO1,DSCAM,SDK1,NLGN1,PCDH9,CDH12,GPC6,CDH4,CNTN6,CXADR,LRFN5,TENM4,PCDH7,LRRC4C,ALCAM,CLSTN2,PCDH15,PCDH11X,IGSF21,EFNA5,TENM3,GRID2,NTNG1,KIREL3,CDH13,DAB1,CDH2,TENM2,PTPRT,PTPRD,CDH9,FAT3,ROBO2,CDH23,NRG1
GO:0043087	regulation of GTPase activity	9.423298738262362e-7	SEMA4D,KALRN,MYO9A,NTRK2,RASGEF1B,TIAM2,TBC1D22A,RAPGEF5,ARHGAP24,RAP1GDS1,RAP1A,TBC1D5,FGD4,GARNL3,DGKI,ARAP2,RASGRF2,VAV3,RASGRF1,RGL1,TIAM1,ASAP2,RALGPS1,ARHGAP42,ASAP1,NET1,ARHGAP12,EFNA5,RALGPS2,NTF3,MAPRE2,RAPGEF2,RABGAP1L,SIPA1L2,EVI5,MTOR,RALGAPA2,SGSM1,RGS7,SIPA1L3,NTRK3,RAPGEF4,TBC1D4,DOCK9,PLXNA2,PRKG1,RASGRP1,DOCK10,RGS6,WDR41,NEDD9,RALGAPA1,CHN1,TBC1D9
GO:0043547	positive regulation of GTPase activity	0.00000100744902179163 67	SEMA4D,KALRN,MYO9A,RASGEF1B,TIAM2,TBC1D22A,RAPGEF5,ARHGAP24,RAP1GDS1,RAP1A,TBC1D5,GARNL3,ARAP2,RASGRF2,RASGRF1,RGL1,TIAM1,ASAP2,RALGPS1,ARHGAP42,ASAP1,NET1,RALGPS2,NTF3,MAPRE2,RAPGEF2,RABGAP1L,SIPA1L2,EVI5,RALGAPA2,SGSM1,RGS7,SIPA1L3,NTRK3,RAPGEF4,TBC1D4,DOCK9,RASGRP1,DOCK10,RGS6,WDR41,NEDD9,RALGAPA1,CHN1,TBC1D9
GO:0007215	glutamate recep	0.000001351743403560851	GRIK2,GRIN2A,GRID1,GRIK3,GRIK4,GRIN2B,GRIK1,SLC1A1,GRM5,APP,FYN,GRIA1,GRM1,GRID2,GRM7,GRIA4,PLCB1,TRPM1

	tor signaling pathway		
GO:0051962	positive regulation of nervous system development	0.00000144261640970580 13	SEMA4D,FBXO31,KALRN,NRXN1,IL1RAPL1,NTRK2,TIAM2,NUMB,DLG5,SLIT2,MACF1,ROBO1,PAK3,CHODL,DSCAM,GRM5,NTN1,NLGN1,FLRT2,CUX1,RELN,CDH4,TENM4,CLSTN2,TIAM1,DISC1,EPHB1,EFNA5,TRPC5,LINGO2,GRID2,LRP2,PRKCH,EPHB2,NRP1,MTOR,GLI3,SYNDIG1,PLXNA2,PTPRD,ASIC2,HDAC2,ROBO2,SEMA5A,BCL11A
GO:0098655	cation transmembrane transport	0.00000146867825504953 83	KCNMA1,SLC12A8,TRPM6,SLC8A1,KCNC1,BCL2,THADA,KCNQ5,SCN11A,KCNJ6,KCNK10,FGF12,CACNG2,GRIN2A,NRXN1,NIPA2,ADCYAP1R1,DPP6,ZDHHC17,KCNH1,CACNA1C,SLC24A4,CACNB2,TRPM3,SLC4A4,KCNE4,KCNS3,HECW1,NOS1,HTR2C,CACNA1E,SLC40A1,SLC9C1,P2RX6,ANK2,SLC1A2,GRIN2B,ITPR2,CACNA2D3,PRKCE,KCNH8,SLC1A1,SLC12A1,NLGN1,SHISA9,KCNIP4,NOS1AP,CATSPER2,ANK3,CNIH3,RELN,RASGRF2,DPP10,DAPK1,APP,CACNA2D1,HCN1,UTRN,TSPAN13,RASGRF1,MICU1,SLC39A12,PIEZO2,FYN,KCND3,ANO10,KCNN3,KCNAB1,PDE4D,ATP6V1E1,ABCC9,GSG1L,TRPM7,TRPC5,AKAP6,TUSC3,MICU2,SLC39A8,KCND2,SLC24A3,EPHB2,RYR3,TRDN,ATP13A3,RGS7,SLC24A2,SHISA6,SCARA5,ASIC2,TRPM1,CACNG3,PRKD1,ATP8A1,RYR2,KCNH5,TMEM163,SLC39A11,SLC5A1,STAC,SCN8A,KCNJ15
GO:0051234	establishment of localization	0.00000148848800807778 17	KCNMA1,SLC12A8,SYN3,PRKCB,TRPM6,SLC8A1,KCNC1,DNAJC15,SIAH3,CD38,TRAPPC9,BCL2,THADA,RSRC1,DNAH11,SLC13A4,SAMM50,GRIK2,SNX25,UNC13B,KCNQ5,HEPHL1,SCN11A,ATP10A,GOT2,KCNJ6,KCNK10,ZDHHC11B,TMEM241,GRIP1,APBA2,TLK1,ASTN2,TANC2,KIF4A,JAK2,TM9SF4,BICD1,LRP1B,ABCG8,KALRN,USH2A,FGF12,CACNG2,BTBD9,SLC44A5,GRIN2A,NRXN1,IL1RAPL1,WDPCP,NIPA2,SLC14A2,ADCYAP1R1,DPP6,PRELID2,GRID1,ZDHHC17,KCNH1,CACNA1C,AMPH,EXOC4,HEATR5A,ANO4,BBS9,NTRK2,ENTHD1,OCLN,ABCA6,TMPRSS3,CNTN1,TTC39B,ELMO1,SLC24A4,GRIK3,CACNB2,NUMB,STXBP4,ESYT2,SYBU,TRPM3,GABRA5,CHRM3,CPE,IGHV1OR15-9,PITPNC1,ABCA5,SLC4A4,KCNE4,KCNS3,ABCA13,HECW1,RAP1GDS1,ERBB4,BID,MACF1,RAP1A,SLC15A5,MYOM1,EXT2,ABL2,NOS1,ABCG1,SPAG16,HTR2C,CACNA1E,TBC1D5,SLC40A1,SLCO3A1,GABRR2,PIK3C3,SLC9C1,SNAP25-AS1,CORO2B,ITGA8,GRIK4,GABRG1,IGHV1OR21-1,CDH17,SV2B,P2RX6,PLS1,ANK2,PLA2G4A,SLC1A2,ANO2,GRIN2B,TNKS,NDFIP2,GABRA2,CSE1L,FCHSD2,ITPR2,TRAPPC10,EHBP1,CACNA2D3,PRKCE,CD163,BBS2,PTPRN2,KCNH8,GRIK1,DGKI,RIN3,ANKFN1,EFHB,TRAPPC8,SLC1A1,SLC12A1,GRM5,NTN1,IGF1R,SNX30,NLGN1,DNAH9,SHISA9,CORIN,MON2,MAP2,KCNIP4,CFTR,CAMK1D,NOS1AP,ZDHHC14,NKAIN2,INSR,CLEC16A,CATSPER2,CUX1,ANK3,SV2C,CNIH3,RELN,RASGRF2,MFSD9,DPP10,OCA2,DAPK1,VAV3,CLIC6,APP,FBLN5,CCDC88A,PAK1,ATP9B,IGF2BP3,CACNA2D1,HCN1,CHRM5,CXADR,UTRN,CECR2,TSPAN13,GHR,RASGRF1,RIN2,USP7,RBFOX1,SCP2,MICU1,SLAMF1,NKAIN3,SLC25A21,SORCS2,SLC39A12,OSBPL10,PIEZO2,SLC35F1,VTI1A,EGF,FYN,FAM3B,KCND3,IFT43,NSG2,GRIA1,SPIDR,STK3,ANO10,MSR1,AP2B1,ARHGAP12,GABRG3,KCNN3,KCNAB1,GRM1,PDE4D,ERC2,RIMS1,ATP6V1E1,FRMD4A,MCTP2,MIPEP,ABCC9,SNAP29,GSG1L,DOCK1,TRPM7,EXT1,EFNA5,ABCA10,STK36,TRPC5,ATP9A,PPFIA2,AKAP6,VPS37A,ATF2,TUSC3,CCDC91,GRID2,LRP2,C2,NTF3,FER,ARFGAP3,MICU2,TMEM108,GABRB3,GRM7,SLC39A8,IMMP2L,ATX

			N1,GABRG2,NRXN3,RABGAP1L,NSG1,KCND2,ATP8A2,SLC24A3,UBE3A,GRIA4,EPHB2,STON1-GTF2A1L,SYT16,ERC1,MCTP1,RYR3,NBAS,SETD2,PACSIN2,DOCK2,NUP214,TRIM23,FLVCR1,NRP1,CDH13,TRDN,SLC2A13,RFTN1,EXOC6B,ATP13A3,STOML1,DCLK1,KIF16B,CDH2,EVI5,VPS41,MAP2K6,GABRB1,SH3KBP1,CADPS,NEU3,RGS7,CD2AP,SLC44A1,AP5M1,ADAM10,ABCB5,SLC24A2,GLI3,CHKA,RAB31,VPS13D,RAPGEF4,TMPRSS2,TMPRSS15,MX2,NSUN2,SORCS1,TBC1D4,DNM3,SYT1,SYNDIG1,DLG2,PPARA,SCFD2,SHISA6,SCARA5,LOXL2,RASGRP1,STXBP6,DMBT1,MAGI2,STX12,ASIC2,RALA,GNPTAB,TRPM1,CACNG3,PRKD1,ATP8A1,BCL2L1,MICAL3,RANBP17,RYR2,LDLRAD3,PRLR,MTMR2,KCNH5,TMEM163,IPO11,IL16,IFT81,OSCP1,CDH23,AKAP13,WDR41,MYRIP,SLC39A11,ENPP1,UNC13C,RIMS2,SLC5A1,STAC,SCN8A,RAB27A,AGAP1,PARD3B,VPS13C,KCNJ15,PARD3,NRG1,SLC25A48,ATP10B,SLC35F4,FMN2,PCNT,OSBPL5
GO:0043085	positive regulation of catalytic activity	0.00000166889979290590 44	CD44,PARN,SEMA4D,TAOK3,GPR55,BCL2,IL6R,JAK2,KALRN,GRIN2A,NRXN1,ADCYAP1R1,CACNA1C,MYO9A,NTRK2,RASGEF1B,TIAM2,NRG3,TBC1D22A,RAPGEF5,ARHGAP24,ROR1,RAP1GDS1,ERBB4,BID,MNAT1,RAP1A,ABL2,MAP3K5,NOS1,TBC1D5,ITGA1,GRIN2B,ROBO1,GARNL3,TNKS,BCL2L13,DCUN1D4,SLC1A1,GRM5,EPHA6,ARAP2,IGF1R,NOS1AP,INSR,EGLN3,DOCK3,RELN,RASGRF2,DAPK1,VAV3,APP,CCDC88A,PAK1,GHR,RASGRF1,RGL1,TIAM1,PRIM2,ASAP2,RALGPS1,ARHGAP42,ASAP1,EGF,PDGFD,FYN,XRCC4,EPHA7,STK3,NET1,ESR1,POR,EPHB1,FLT1,EFNA5,CDC14B,MARK2,RALGPS2,NTF3,MAPRE2,RAPGEF2,PRKCQ,RABGAP1L,DLC1,EPHB2,MOB3B,NBN,RFC3,DAB1,ALK,SIPA1L2,EVI5,MAP2K6,MTOR,RALGAPA2,FBLN1,SGSM1,RGS7,SIPA1L3,NTRK3,RXFP1,RAPGEF4,HMGA2,TBC1D4,DOCK9,RASGRP1,MAGI2,ABI1,DOCK10,PRKD1,TGFA,PRLR,RGS6,AKAP13,WDR41,NEDD9,RALGAPA1,CHN1,NRG1,TBC1D9,ST18
GO:0040007	growth	0.00000173472958857416 43	SEMA4D,EVC,TEAD1,CD38,BCL2,IGSF11,SORBS2,APBA2,EPB41L3,CTDP1,SMARCA4,NRG3,GAS2,MTPN,PTGFRN,SLIT2,CSNK2A1,ERBB4,ATRX,MACF1,RAD51B,RERG,RUNX1,PLS1,SLC1A2,SLIT3,CPQ,ARHGEF11,BBS2,DSCAM,NTN1,MAP2,INSR,COBL,CDH4,TNR,APP,FBLN5,SPOCK1,PLCE1,CXADR,TENM4,GHR,ALCAM,RARB,DISC1,FMN1,ZFPM2,EPHA7,STK3,NET1,PCDH15,ESR1,RIMS1,POR,EXT1,EFNA5,TRPC5,FTO,AKAP6,ATF2,SEMA6D,TMEM108,PRKCQ,ATP8A2,UBE3A,INO80,AUTS2,PDLIM5,NBN,FLVCR1,NRP1,RFTN1,SEMA3A,SEMA3E,DCLK1,ARID5B,FSTL4,MTOR,NCAPG2,ADAM10,PAPPA2,GLI3,HMGA2,SYT1,SCAPER,PPARA,PLCB1,MAGI2,BCL2L1,TNN,SEMA3D,PRLR,TBX20,EYS,VCL,AKAP13,NEDD9,ENPP1,RIMS2,SEMA5A,BCL11A,DCC,NRG1,SEMA3C
GO:0030334	regulation of cell migration	0.00000186299774602961 57	PTPRR,SEMA4D,SLC8A1,FBXO31,BCL2,IL6R,JAK2,LAMA1,WDPCP,LAMA3,SMOC2,SRGAP2B,MEOX2,NRG3,PTPRG,NUMB,UNC5D,DLG5,SLIT2,AKT3,ERBB4,KANK1,MACF1,DUSP22,ABL2,ROBO1,PAK3,PRKCE,RIN3,NTN1,LDB2,IGF1R,JAM2,CAMK1D,FLRT2,INSR,RELN,TNR,APP,PAK1,MITF,RIN2,FRMD5,STARD13,SLAMF1,TIAM1,PHACTR1,EGF,PDGFD,DOCK4,DOCK1,FLT1,HDAC4,SEMA6D,NTF3,FER,NTNG1,MAPRE2,RAPGEF2,NAV3,DLC1,APC,EPHB2,MCTP1,NRP1,CDH13,DACH1,LDLRAD4,SEMA3A,SEMA3E,MGAT5,HDAC9,PIK3R3,MTOR,FBLN1,SPRED1,ADAM10,SCAI,PTPRT,NTRK3,BMPER,PTPRK,PLXNA2,PLCB1,PRKG1,MAGI2,DOCK10,PRKD1,ATP8A1,HDAC2,ETS1,TNN,SEMA3D,VCL,SRGAP3,NEDD9,SEMA5A,CTNNA2,NRG1,PRKCA,SEMA3C
GO:0050919	negative chemotaxis	0.00000193360281644860 7	SEMA4D,NRG3,SLIT2,SLIT3,ROBO1,NTN1,FLRT2,EPHA7,EFNA5,SEMA6D,SEMA3A,SEMA3E,SEMA3D,ROBO2,SEMA5A,NRG1,SEMA3C
GO:0040012	regulation of locomotion	0.00000261648848334992	PTPRR,SEMA4D,SLC8A1,FBXO31,BCL2,IL6R,JAK2,LAMA1,WDPCP,LAMA3,SMOC2,CTNNA1,SRGAP2B,MEOX2,NRG3,PTPRG,NUMB,UNC5D,DLG5,SLIT2,AKT3,ERBB4,KANK1,MACF1,DUSP22,ABL2,SPOCK3,ROBO1,PAK3,PRKCE,BBS2,DSCAM,RIN3,GRM5,NTN1,LDB2,IGF1R,JAM2,CAMK1D,FLRT2,PTPRO,INSR,RELN,TNR,APP,PAK1,MITF,RIN2,FRMD5,STARD13,SLAMF1,TIAM1,PHACTR1,EGF,PDGFD,DOCK4,DOCK1,FLT1,HDAC4,SEMA6D,NTF3,FER,NTNG1,MAPRE2,R

			APGEF2,NAV3,DLC1,APC,EPHB2,MCTP1,NRP1,CDH13,DACH1,LDLRAD4,SEMA3A,SEMA3E,MGAT5,HDAC9,PIK3R3,MTOR,FBLN1,SPRED1,ADAM10,SCAI,PTPRT,NTRK3,BMPER,PTPRK,PLXNA2,PLCB1,PRKG1,MAGI2,DOCK10,PRKD1,ATP8A1,HDAC2,ETS1,TNN,SEMA3D,IL16,VCL,ROBO2,SRGAP3,NEDD9,SEMA5A,CTNNA2,NRG1,PRKCA,SEMA3C
GO:0009888	tissue development	0.00000295580575770977 65	CD44,SEMA4D,EVC,SLC8A1,ANKRD6,BCL2,COL18A1,THRB,IL6R,SORBS2,ASTN2,RCAN1,NTN4,JAK2,USH2A,LAMA1,WDPCP,LAMA3,CTDP1,EXOC4,MYO9A,ZNRF3,MEOX2,SLC24A4,FBXL17,MTPN,SOX6,DLG5,PGM5,ATRNL1,ARHGAP24,SPRED2,SLIT2,MYLK3,ROR1,KAZN,ALPK2,LCE1F,ERBB4,ATRX,DMRT1,RAP1A,RAD51B,CHSY1,EXT2,SLC40A1,ETS2,ITGA8,RUNX1,ALDH1A2,EYA1,FRMD6,PLS1,ZBTB16,ROBO1,EGFLAM,OVOL2,MLLT3,PRICKLE2,BBS2,SOX5,NTN1,NR5A2,LDB2,WDR72,SHROOM3,ALX4,CFTR,PTPRO,INSR,COBL,GPC6,TRPS1,MYO18B,PAK1,CXADR,TENM4,CECR2,GHR,RIPK4,RBFOX1,STARD13,KL,ADAMTS16,NEBL,RARB,TIAM1,PBX1,FMN1,ZFPM2,SVEP1,EDAR,EGF,PDGFD,EPHA7,STK3,COL19A1,TOX,PCDH15,ESR1,ARHGAP12,SGCZ,PDE4D,PRKACB,POR,CERS3,EPHB1,FHOD3,GREB1L,EFEMP1,AJAP1,EXT1,HDAC4,FTO,AKAP6,ATF2,LRP2,SEMA6D,FER,NTNG1,SGCD,RAPGEF2,CPS1,PRKCH,KRT25,DLC1,SLC24A3,PDLIM5,COL22A1,SETD2,NRP1,LDLRAD4,SEMA3A,SEMA3E,KIF16B,CDH2,ARID5B,SERPINB7,HDAC9,MTOR,FHL2,SPRED1,SIPA1L3,FLNB,CSMD1,GLI3,RXFP1,SGCG,HYDIN,BMPER,HMGA2,NSUN2,PPARA,PLXNA2,MYH15,PLCB1,LOXL2,BPTF,DMBT1,MAGI2,NELL1,ABI1,RALA,FNDC3A,HDAC2,TNN,RYR2,SEMA3D,PRLR,TBX20,VCL,ROBO2,CDH23,AKAP13,ENPP1,HIVEP3,SEMA5A,NRG1,SEMA3C
GO:0032879	regulation of localization	0.00000340647618651284 26	KCNMA1,PARN,PRKCB,SLC8A1,KCNC1,SIAH3,CD38,BCL2,THADA,UNC13B,KCNQ5,SCN11A,KCNJ6,KCNK10,APBA2,ASTN2,JAK2,TM9SF4,BICD1,ABCG8,KALRN,FGF12,CACNG2,BTBD9,GRIN2A,NRXN1,IL1RAPL1,WDPCP,ADCYAP1R1,DPP6,KCNH1,CACNA1C,CTNNA1,OCLN,CNTN1,TTC39B,CACNB2,NUMB,STXBP4,CHRM3,ABCA5,KCNE4,TRIM5,KCNS3,ABCA13,HECW1,RAP1GDS1,ERBB4,RAP1A,MYOM1,ABL2,NOS1,ABCG1,HTR2C,CACNA1E,TBC1D5,PIK3C3,CORO2B,PLS1,ANK2,PLA2G4A,SLC1A2,GRIN2B,NDFIP2,ITPR2,CACNA2D3,PRKCE,PRKAA2,KCNH8,DGKI,RIN3,EFHB,SLC1A1,GRM5,NLGN1,SHISA9,CORIN,MAP2,KCNIP4,CFTR,CAMK1D,NOS1AP,NKAIN2,INSR,CATSPER2,ANK3,CNIH3,GPC6,RELN,RASGRF2,DPP10,DAPK1,VRK1,CLIC6,APP,CCDC88A,CACNA2D1,HCN1,UTRN,GPC5,TSPAN13,RASGRF1,USP7,SCP2,SLAMF1,NKAIN3,EGF,FYN,KCND3,SPIDR,MSR1,KCNAB1,PDE4D,RIMS1,FRMD4A,MCTP2,ABCC9,GSG1L,EFNA5,ATP9A,FTO,AKAP6,C2,NTF3,FER,GRM7,PRKCH,NRXN3,RABGAP1L,KCND2,ATP8A2,APC,EPHB2,MCTP1,RYR3,SETD2,PACSIN2,DOCK2,NUP214,NRP1,CDH13,TRDN,DCLK1,CDH2,SYCP1,MAP2K6,CADPS,NEU3,RGS7,CD2AP,ADAM10,GLI3,RAB31,ABHD17C,RAPGEF4,BMPER,MX2,NSUN2,TBC1D4,DNM3,SYT1,PPARA,SHISA6,STXBP6,MAGI2,ASIC2,RALA,CACNG3,PRKD1,ATP8A1,BCL2L1,RYR2,MTMR2,KCNH5,IL16,VCL,WDR41,NEDD9,MYRIP,ENPP1,RIMS2,STAC,SCN8A,RAB27A,KCNJ15,NRG1,PCNT
GO:0003013	circulatory system process	0.00000366834343945079 4	KCNMA1,SLC8A1,CD38,THRB,JAK2,FGF12,FLI1,TRHDE,RPS6KA2,CACNA1C,OCLN,ENPEP,CACNB2,CHRM3,SLIT2,MYLK3,SLC4A4,KCNE4,RAP1GDS1,EXT2,NOS1,SLCO3A1,ITGA1,CORO2B,ANK2,SLC1A2,BBS2,SLC1A1,CORIN,NOS1AP,PTPRO,INSR,RNLS,CACNA2D1,HCN1,CXADR,KL,ADAMTS16,ARHGAP42,VSTM4,SVEP1,FYN,KCND3,NAV2,SGCZ,PDE4D,PDE3A,DOCK4,ABCC9,EXT1,HDAC4,LRP2,CELF2,SGCD,IMMP2L,CPS1,SLC24A3,TRDN,SLC2A13,ASB3,MAP2K6,MTOR,SLC44A1,SGCG,CTNNA3,PPARA,PRKG1,ASIC2,ATP8A1,RYR2,TBX20,AKAP13,SLC5A1
GO:0098660	inorganic ion transmembrane	0.00000493328394615758 35	KCNMA1,SLC12A8,TRPM6,SLC8A1,KCNC1,BCL2,THADA,SLC13A4,KCNQ5,SCN11A,KCNJ6,KCNK10,FGF12,CACNG2,GRIN2A,NIPA2,ADCYAP1R1,DPP6,ZDHHC17,KCNH1,CACNA1C,ANO4,SLC24A4,CACNB2,TRPM3,GABRA5,SLC4A4,KCNE4,KCNS3,HECW1,NOS1,HTR2C,CACNA1E,SLC40A1,GABRR2,SLC9C1,GABRG1,ANK2,ANO2,GRIN2B,GABRA2,ITPR2,CACNA2D3,PRKCE,KCNH8,SLC1A1,SLC12A1,GRM5,KCNIP4,CFTR,NOS1AP,CATSPER2,ANK3,DPP10,CLIC6,CACNA2D1,HCN1,UTRN,TSPAN13,MICU1,SLC39A12,FYN,KCND3,ANO10,GA

	transport		BRG3,KCNN3,KCNAB1,PDE4D,ATP6V1E1,ABCC9,TRPM7,TRPC5,AKAP6,TUSC3,MICU2,GABRB3,SLC39A8,GABRG2,KCND2,SLC24A3,RYR3,TRDN,GABRB1,RGS7,SLC24A2,SCARA5,ASIC2,TRPM1,CACNG3,PRKD1,RYR2,KCNH5,TMEM163,SLC39A11,SLC5A1,STAC,SCN8A,KCNJ15
GO:0006811	ion transport	0.00000506224745939847 04	KCNMA1,SLC12A8,PRKCB,TRPM6,SLC8A1,KCNC1,BCL2,THADA,SLC13A4,GRIK2,KCNQ5,HEPHL1,SCN11A,ATP10A,KCNJ6,KCNK10,FGF12,CACNG2,SLC44A5,GRIN2A,NRXN1,NIPA2,ADCYAP1R1,DPP6,GRID1,ZDHHC17,KCNH1,CACNA1C,ANO4,NTRK2,CNTN1,SLC24A4,GRIK3,CACNB2,TRPM3,GABRA5,CHRM3,SLC4A4,KCNE4,KCNS3,HECW1,NOS1,HTR2C,CACNA1E,SLC40A1,SLCO3A1,GABRR2,SLC9C1,GRIK4,GABRG1,P2RX6,ANK2,PLA2G4A,SLC1A2,ANO2,GRIN2B,NDFIP2,GABRA2,ITPR2,CACNA2D3,PRKCE,KCNH8,GRIK1,EFHB,SLC1A1,SLC12A1,GRM5,NLGN1,SHISA9,KCNIP4,CFTR,NOS1AP,NKAIN2,CATSPER2,ANK3,CNIH3,RELN,RASGRF2,DPP10,DAPK1,CLIC6,APP,CACNA2D1,HCN1,CHRM5,UTRN,TSPAN13,RASGRF1,MICU1,NKAIN3,SLC25A21,SLC39A12,PIEZO2,EGF,FYN,KCND3,GRIA1,ANO10,GABRG3,KCNN3,KCNAB1,GRM1,PDE4D,ATP6V1E1,ABCC9,GSG1L,TRPM7,TRPC5,AKAP6,TUSC3,GRID2,LRP2,MICU2,GABRB3,GRM7,SLC39A8,GABRG2,KCND2,SLC24A3,GRIA4,EPHB2,RYR3,FLVCR1,TRDN,ATP13A3,MAP2K6,GABRB1,RGS7,SLC44A1,SLC24A2,SYT1,SHISA6,SCARA5,ASIC2,TRPM1,CACNG3,PRKD1,ATP8A1,RYR2,KCNH5,TMEM163,IL16,CDH23,SLC39A11,ENPP1,SLC5A1,STAC,SCN8A,KCNJ15
GO:1902531	regulation of intracellular signal transduction	0.00000626188222489857 2	CD44,PTPRR,SEMA4D,TAOK3,PRKCB,ANKRD6,GPR55,BCL2,CAMTA1,IL6R,BRD4,NDRG2,RCAN1,JAK2,OTUD7A,TPTE2,KALRN,NFAT5,NRXN1,ADCYAP1R1,ZDHHC17,MYO9A,NTRK2,TIAM2,IQCJ-SCHIP1,RNF152,SLC24A4,PDE10A,MECOM,SHANK2,DOK5,DLG5,ARHGAP24,SPRED2,SLIT2,ROR1,ZNF675,AKT3,TRIM5,PSD3,RAP1GDS1,ERBB4,KANK1,BID,RAP1A,TRIO,DUSP22,ABL2,MAP3K5,HTR2C,TRAF3,FGD4,ITGA1,ROBO1,PAK3,GARNL3,NDFIP2,SGMS1,LEMD3,PRKCE,ARHGEF11,PRKAA2,DGKI,DNMBP,EFHB,GRM5,IGF1R,NLGN1,NOS1AP,INSR,CLEC16A,DOCK3,RELN,RASGRF2,STK38,VAV3,APP,PUM1,PLCE1,PAK1,EPS8,GHR,RASGRF1,USP7,STARD13,KL,SLAMF1,TIAM1,RALGPS1,ARHGAP42,EDAR,EGF,PDGFD,FYN,EPHA7,STK3,S100B,NET1,ESR1,ARHGAP12,GRM1,PDE4D,PDE3A,EPHB1,FLT1,AKAP6,LRP2,RALGPS2,NTF3,MAPRE2,TPTE,RAPGEF2,DLC1,UBE3A,AUTS2,EPHB2,AGO3,MOB3B,DOCK2,NRP1,CDH13,ALK,SEMA3A,SEMA3E,MAGI3,CDH2,SIPA1L2,MAP2K6,ARHGAP28,MTOR,KSR1,RALGAPA2,FBLN1,FHL2,CD2AP,SPRED1,SIPA1L3,SCAI,NTRK3,BMPER,APIP,PPARA,RORA,PLCB1,RASGRP1,MAGI2,PRKD1,BCL2L1,TGFA,DEPTOR,ZMYND11,SRGAP3,AKAP13,RALGAPA1,SEMA5A,PRDM15,CHN1,NRG1,PRKCA
GO:0048518	positive regulation of biological process	0.00000732131365646702 9	CD44,SAMD4A,KCNMA1,ZHX3,APBB2,SCAF8,ERG,PARN,SEMA4D,EVC,TEAD1,NFIA,RPS6KA5,TAOK3,PRKCB,A2M,SLC8A1,ANKRD6,KCNC1,GPR55,CD38,FBXO31,BCL2,CAMTA1,CHFR,TOX3,THRB,RAG1,CDC42EP3,GRIK2,IGSF11,UNC13B,IL6R,ZBTB20,ATP10A,BRD4,GRIP1,JAK2,TM9SF4,BICD1,KALRN,NEGR1,FGF12,CACNG2,NFAT5,FLI1,LAMA1,ZBTB7C,GRIN2A,NRXN1,ARID1B,IL1RAPL1,MAGI1,ADCYAP1R1,RPS6KA2,SMOC2,ZDHHC17,BMF,CTNNA1,NTRK2,OCLN,TIAM2,IQCJ-SCHIP1,SMARCA4,RNF152,CNTN1,MEOX2,GLIS3,NRG3,SLC24A4,CACNB2,NUMB,STXBP4,MED15,MTPN,SOX6,MECOM,PDE4DIP,SHANK2,UBE2E2,DOK5,DLG5,CHRM3,SPRED2,IGHV1OR15-9,SLIT2,MYLK3,ABCA5,ROR1,GLP2R,SLC4A4,CSNK2A1,AKT3,KMT2C,TRIM5,ABCA13,HECW1,ERBB4,KANK1,ATRX,DMRT1,BID,MACF1,MNAT1,TAF4B,RAP1A,CTNNBL1,RAD51B,DUSP22,CHSY1,MYOM1,ABL2,MAP3K5,NOS1,ABCG1,MAML2,HTR2C,NEK4,CTIF,TBC1D5,SLC40A1,SLCO3A1,TRAF3,CHD6,ETS2,ITGA1,TCF12,ZNF721,CORO2B,ITGA8,RBM19,RUNX1,ALDH1A2,MAPK9,ESRRG,PTGFR,IGHV1OR21-1,CDH17,EYA1,MORC3,PLS1,SPON1,ANK2,PLA2G4A,SLC1A2,ZBTB16,SUPT3H,GRIN2B,ROBO1,EGFLAM,PAK3,TNKS,KLF12,NDFIP2,OVOL2,FCHSD2,BRINP1,MLLT3,BCL2L13,CHODL,GABPA,PRKCE,GLIS1,PSG9,ARHGEF11,PRKAA2,BBS2,EDIL3,SOX5,DSCAM,DGKI,KDM4C,DCUN1D4,SLC1A1,GRM5,EPHA6,NTN1,NR5A2,LDB2,IGF1

			R,SNX30,NLGN1,JAM2,ALX4,CNTNAP2,MAP2,CFTR,CAMK1D,FLRT2,MLLT10,NOS1AP,RBMS3,INSR,COBL,CLEC16A,EGLN3,CUX1,ANK3,MORC2,DOCK3,RELN,RASGRF2,CDH4,TNR,DPP10,CELF4,DAPK1,VAV3,ZNF600,VRK1,CNTN6,APP,PUM1,CCDC88A,ARNT2,HPSE2,PLCE1,ADAM12,PAK1,MITF,IGF2BP3,CACNA2D1,ADCK1,NSMCE2,ZNF208,EPS8,UTRN,GPC5,TENM4,PRR16,GHR,DUX4,RASGRF1,RIN2,PRDM16,FRMD5,RNF217,USP7,MEIS2,KIR2DL4,SCP2,KL,TASP1,SLAMF1,INO80D,CLSTN2,RARB,TCF4,TIAM1,PBX1,MLIP,PRIM2,SLC39A12,DISC1,FMN1,ZFPM2,ASAP1,FRMPD4,EDAR,EGF,PDGFD,FYN,EPHA7,TRABD2B,SPIDR,STK3,CNOT7,MSR1,PSIP1,S100B,NET1,TOX,ESR1,GRM1,PDE4D,PDE3A,RIMS1,POR,DOCK4,FRMD4A,WWOX,EPHB1,SSBP2,CREM,TNRC6B,DOCK1,FLT1,EFNA5,CDC14B,HDAC4,ZNF717,STK36,TRPC5,FTO,AKAP6,TENM3,LINGO2,MARK2,ATF2,RBBP8,GRID2,ZNF423,LRP2,SEMA6D,C2,NTF3,FER,SUSD4,CAMK4,MAPRE2,RAD51AP1,TMEM108,RAPGEF2,NAV3,PLGRKT,ZNF615,GTF2I,PRKCQ,PRKCH,DLC1,NSG1,ATP8A2,SLC24A3,UBE3A,IDE,APC,INO80,AUTS2,EPHB2,ZNF850,AGO3,C9,MOB3B,NBAS,NBN,RGMB,SETD2,PKP1,DOCK2,NRP1,CDH13,RFC3,TRDN,SLC2A13,DAB1,RFTN1,ALK,SEMA3A,SEMA3E,MGAT5,NRIP1,CDH2,ARID5B,TENM2,SERPINB7,ZNF407,HDAC9,PIK3R3,MAP2K6,MTOR,KSR1,RORB,FBLN1,ST8SIA1,BLM,SH3KBP1,CADPS,NEU3,NCAPG2,RGS7,CD2AP,SPRED1,ADAM10,KANSL1,TRERF1,SLC24A2,GLI3,NTRK3,RAB31,VPS13D,ABHD17C,ZNF292,TBX15,BMPER,ANKRD31,ZNF521,TMPRSS2,ATF7IP,HMGA2,CREB5,DNM3,SYT1,SYNDIG1,ASXL3,DPF3,NPHP4,PPARA,PLXNA2,PTPRD,RORA,PLCB1,LOXL2,BPTF,RASGRP1,ELAVL4,NLRC5,MAGI2,NELL1,ABI1,TSHZ3,ASIC2,RALA,CACNG3,PRKD1,ATP8A1,TNFAIP8,BCL2L1,HDAC2,ETS1,MRPS27,TNN,RYR2,SEMA3D,BANP,TGFA,PRLR,TBX20,MTMR2,ATF6,IL16,BACH1,ATAT1,ROBO2,AKAP13,NEDD9,MYRIP,PCP4,RIMS2,STAC,RAB27A,EYA4,POMT2,HIVEP3,CLIP1,SEMA5A,PRDM15,BCL11A,ZNF112,PVT1,ETV6,PARD3,NRG1,FANK1,ZNF845,NPAS3,PRKCA,FMN2,SEMA3C,FANCB,CSF2RB,PCNT,ST18
GO:0006468	protein phosphorylation	0.0000073418518930211667	CD44,ERG,SEMA4D,RPS6KA5,TAOK3,PRKCB,TRPM6,SLC8A1,BCL2,SAMSN1,RSRC1,CDC42BPA,SNX25,MAPK10,IL6R,BRD4,TLK1,JAK2,KALRN,LAMA1,MAST4,NRXN1,RPS6KA2,NTRK2,OCLN,NLK,CNTN1,NRG3,STK32B,SPRED2,SLIT2,MYLK3,ROR1,ZNF675,CSNK2A1,AKT3,PHKB,ALPK2,ERBB4,MNAT1,TAF4B,RAP1A,TRIO,MYO3B,DUSP22,ABL2,MAP3K5,NOS1,NEK4,SLCO3A1,PIK3C3,MAPK9,PEAK1,MORC3,ROBO1,PAK3,TNKS,PRKCE,PRKAA2,MYO3A,SLC1A1,GRM5,EPHA6,IGF1R,CAMK1D,PTPRO,INSR,DOCK3,RELN,STK38,DAPK1,VRK1,APP,CCDC88A,PLCE1,PAK1,GHR,RIPK4,EGF,PDGFD,FYN,EPHA7,STK3,CNOT7,PDE4D,PRKACB,HUNK,EPHB1,EFEMP1,TRPM7,FLT1,EFNA5,STK36,TRPC5,SH3BP5,MARK2,ATF2,NTF3,FER,SNRK,CAMK4,RAPGEF2,PRKCQ,PRKCH,APC,EPHB2,ERC1,MOB3B,NBN,NRP1,DAB1,ALK,LDLRAD4,DCLK1,CCNG2,PIK3R3,MAP2K6,MTOR,STK38L,KSR1,FBLN1,BLM,NCAPG2,STK32A,SPRED1,ADAM10,PTPRT,NTRK3,CHKA,BMPER,HMGA2,PRKG1,RASGRP1,ABI1,PRKD1,HDAC2,TGFA,PRLR,PTPRA,DEPTOR,AKAP13,NEDD9,ENPP1,PARD3,NRG1,PRKCA,CSF2RB,TOP1
GO:2000145	regulation of cell motility	0.0000073564482187662227	PTPRR,SEMA4D,SLC8A1,FBXO31,BCL2,IL6R,JAK2,LAMA1,WDPCP,LAMA3,SMOC2,CTNNA1,SRGAP2B,MEOX2,NRG3,PTPRG,NUMB,UNC5D,DLG5,SLIT2,AKT3,ERBB4,KANK1,MACF1,DUSP22,ABL2,SPOCK3,ROBO1,PAK3,PRKCE,BBS2,RIN3,NTN1,LDB2,IGF1R,JAM2,CAMK1D,FLRT2,INSR,RELN,TNR,APP,PAK1,MITF,RIN2,FRMD5,STARD13,SLAMF1,TIAM1,PHACTR1,EGF,PDGFD,DOCK4,DOCK1,FLT1,HDAC4,SEMA6D,NTF3,FER,NTNG1,MAPRE2,RAPGEF2,NAV3,DLC1,APC,EPHB2,MCTP1,NRP1,CDH13,DACH1,LDLRAD4,SEMA3A,SEMA3E,MGAT5,HDAC9,PIK3R3,MTOR,FBLN1,SPRED1,ADAM10,SCAI,PTPRT,NTRK3,BMPER,PTPRK,PLXNA2,PLCB1,PRKG1,MAGI2,DOCK10,PRKD1,ATP8A1,HDAC2,ETS1,TNN,SEMA3D,VCL,SRGAP3,NEDD9,SEMA5A,CTNNA2,NRG1,PRKCA,SEMA3C
GO:0044057	regulation of syste	0.000007698443214335858	KCNMA1,SLC8A1,CD38,THRB,IGSF11,UNC13B,SCN11A,DLGAP1,JAK2,ABCG8,FGF12,GRIN2A,NRXN1,CTDP1,CACNA1C,CACNB2,MTPN,CHRM3,KCNE4,NOS1,HTR2C,CORO2B,ANK2,GRIN2B,SLC1A1,NLGN1,SHISA9,JAM2,CORIN,NOS1AP,PTPRO,RELN,FBXO32,TNR,CELF4,APP,RNLS,PLCE1,CACNA2D1,HCN1,CXADR,TENM4,MLIP,ARH

	m process		GAP42,KCND3,GRM1,PDE4D,RIMS1,DOCK4,ABCC9,HDAC4,FTO,AKAP6,CELF2,TMEM108,TRDN,ASB3,CTNNA3,PPP1R12B,PPARA,SHISA6,PRKG1,TSHZ3,ASIC2,RYR2,MTMR2,RIMS2,DLGAP2,PARD3,PRKCA
GO:0044087	regulation of cellular component biogenesis	0.000008776908517884329	C10ORF90,SEMA4D,DNAJC15,CDC42EP3,UNC13B,NEGR1,NRXN1,IL1RAPL1,WDPCP,BMF,NTRK2,OCLN,MTPN,PDE4DIP,DLG5,ARHGAP24,SLIT2,KANK1,BID,MACF1,RAP1A,DUSP22,CORO2B,MAPK9,CDH17,PEAK1,PAK3,MDM1,FCHSD2,PRKCE,PRKAA2,IL1RAPL2,NTN1,LDB2,SPTB,SNX30,NLGN1,CNTNAP2,MAP2,FLRT2,COBL,MORC2,TBCD,GPC6,APP,CCDC88A,PLCE1,PAK1,EPS8,LRFN5,ADAMTS16,CLSTN2,SLC39A12,FMN1,ASAP1,EPHA7,TRABD2B,SPIDR,ESR1,EPHB1,FHOD3,EFNA5,HDAC4,LINGO2,GRID2,FER,RAPGEF2,NAV3,PRKCH,RHPN2,DLC1,APC,AUTS2,EPHB2,PDLIM5,SDCCAG8,NRP1,LDLRAD4,TENM2,VPS41,ARHGAP28,MTOR,NTRK3,ATF7IP,DNM3,SYNDIG1,NPHP4,SACS,PTPRD,STXBP6,ASIC2,RALA,TBX20,PTPRA,VCL,ATAT1,ROBO2,CLIP1,BCL11A,NRG1,PRKCA
GO:0016310	phosphorylation	0.000009088575150701139	CD44,ERG,SEMA4D,RPS6KA5,TAOK3,PRKCB,TRPM6,SLC8A1,BCL2,SAMSN1,RSRC1,CDC42BPA,SNX25,MAPK10,IL6R,ZBTB20,BRD4,TLK1,JAK2,KALRN,LAMA1,MAST4,NRXN1,RPS6KA2,CKMT1B,NTRK2,OCLN,AK8,NLK,CNTN1,NRG3,STK32B,SPRED2,SLIT2,MYLK3,ROR1,SLC4A4,ZNF675,CSNK2A1,AKT3,PHKB,ALPK2,ERBB4,MNAT1,TAF4B,RAP1A,TRIO,MYO3B,DUSP22,ABL2,MAP3K5,NOS1,NEK4,SLCO3A1,PIK3C3,MAPK9,PEAK1,MORC3,AKAP10,ROBO1,PAK3,DGKB,TNKS,SGMS1,DGKK,PRKCE,PRKAA2,MYO3A,DSCAM,DGKI,NME7,SLC1A1,GRM5,EPHA6,LDB2,IGF1R,CAMK1D,PTPRO,INSR,DOCK3,RELN,STK38,DAPK1,VAV3,VRK1,APP,CCDC88A,PLCE1,PAK1,ADCK1,GHR,RIPK4,EGF,PDGFD,FYN,EPHA7,STK3,CNOT7,PDE4D,PRKACB,HUNK,EPHB1,EFEMP1,TRPM7,FLT1,EFNA5,HDAC4,STK36,TRPC5,SH3BP5,MARK2,ATF2,NTF3,FER,SNRK,CAMK4,RAPGEF2,PRKCQ,PRKCH,APC,EPHB2,ERC1,MOB3B,NBN,NRP1,DAB1,ALK,LDLRAD4,DCLK1,CCNG2,PIK3R3,MAP2K6,MTOR,STK38L,KSR1,FBLN1,BLM,SH3KBP1,NCAPG2,STK32A,SPRED1,ADAM10,PTPRT,NTRK3,CHKA,BMPER,HMGA2,PPARA,PRKG1,RASGRP1,NLRC5,MAGI2,ABI1,GNPTAB,PRKD1,ADK,HDAC2,TGFA,PRLR,PTPRA,DEPTOR,AKAP13,NEDD9,ENPP1,PARD3,NRG1,PRKCA,CSF2RB,TOP1
GO:0006935	chemotaxis	0.000010236456488778686	SEMA4D,RPS6KA5,NCAM1,IL6R,KALRN,LAMA1,NRXN1,LAMA3,SMOC2,CNTN1,NRG3,UNC5D,SLIT2,ADAMTSL1,TRIO,CCDC141,CNTN4,ITGA1,SLIT3,ROBO1,DSCAM,RIN3,EPHA6,NTN1,CAMK1D,FLRT2,PTPRO,ITGA9,RELN,CDH4,TNR,VAV3,CNTN6,APP,CXADR,ALCAM,SLAMF1,PDGFD,FYN,EPHA7,CNTN5,DOCK4,EPHB1,FLT1,EXT1,EFNA5,SEMA6D,NTF3,FER,PLGRKT,PRKCQ,NRXN3,EPHB2,PRTG,DOCK2,NRP1,CDH13,SEMA3A,SEMA3E,ADAM10,GLI3,NTRK3,PLXNA2,RALA,PRKD1,SEMA3D,IL16,ROBO2,NEDD9,SEMA5A,DCC,CHN1,NRG1,SEMA3C
GO:0031345	negative regulation of cell projection organization	0.00001083984559169229	SEMA4D,CD38,NRXN1,PTPRG,ARHGAP24,SLIT2,KANK1,GRIN2B,NTN1,NLGN1,MAP2,PTPRO,TNR,SPOCK1,FYN,EPHA7,TRPC5,SEMA6D,RAPGEF2,UBE3A,EPHB2,NRP1,DAB1,SEMA3A,SEMA3E,FSTL4,DNM3,HDAC2,SEMA3D,FAT3,SEMA5A,BCL11A,DCC,SEMA3C
GO:0042330	taxis	0.000011772362197277484	SEMA4D,RPS6KA5,NCAM1,IL6R,KALRN,LAMA1,NRXN1,LAMA3,SMOC2,CNTN1,NRG3,UNC5D,SLIT2,ADAMTSL1,TRIO,CCDC141,CNTN4,ITGA1,SLIT3,ROBO1,DSCAM,RIN3,EPHA6,NTN1,CAMK1D,FLRT2,PTPRO,ITGA9,RELN,CDH4,TNR,VAV3,CNTN6,APP,CXADR,ALCAM,SLAMF1,PDGFD,FYN,EPHA7,CNTN5,DOCK4,EPHB1,FLT1,EXT1,EFNA5,SEMA6D,NTF3,FER,PLGRKT,PRKCQ,NRXN3,EPHB2,PRTG,DOCK2,NRP1,CDH13,SEMA3A,SEMA3E,ADAM10,GLI3,NTRK3,PLXNA2,RALA,PRKD1,SEMA3D,IL16,ROBO2,NEDD9,SEMA5A,DCC,CHN1,NRG1,SEMA3C

GO:0010976	positive regulation of neuron projection development	0.0000151415952344345556	KALRN,NEGR1,NRXN1,NTRK2,CNTN1,ROR1,RAP1A,ABL2,PAK3,IGF1R,NLGN1,CAMK1D,COBL,RELN,DISC1,FYN,TOX,TENM3,MARK2,RAPGEF2,ATP8A2,EPHB2,NRP1,ALK,NTRK3,ELAVL4,MAGI2,PRKD1,TNN,BCL11A
GO:0008038	neuron recognition	0.0000169191278982355222	NCAM2,CNTN4,ROBO1,DSCAM,CNTNAP2,CRTAC1,CNTN6,APP,NTM,EXT1,OPCML,EPHB2,NRP1,TNN,ROBO2,SEMA5A
GO:0106027	neuron projection organization	0.0000188365953219945776	TANC2,KALRN,PLS1,GRIN2B,PAK3,IGF1R,NLGN1,INSR,RELN,APP,FYN,EPHB1,PPFIA2,UBE3A,EPHB2,PDLIM5,CTNND2,TANC1,DNM3,DOCK10,MTMR2,NEDD9
GO:0051345	positive regulation of hydrolase activity	0.000020954484135395555	SEMA4D,GPR55,JAK2,KALRN,GRIN2A,ADCYAP1R1,MYO9A,RASGEF1B,TIAM2,TBC1D22A,RAPGEF5,ARHGAP24,RAP1GDS1,BID,RAP1A,ABL2,MAP3K5,TBC1D5,ITGA1,GRIN2B,ROBO1,GARNL3,BCL2L13,SLC1A1,ARAP2,EGLN3,RASGRF2,DAPK1,APP,RASGRF1,RGL1,TIAM1,ASAP2,RALGPS1,ARHGAP42,ASAP1,FYN,NET1,ESR1,FLT1,RALGPS2,NTF3,MAPRE2,RAPGEF2,RABGAP1L,DLC1,SIPA1L2,EVI5,MTOR,RALGAPA2,FBLN1,SGSM1,RGS7,SIPA1L3,NTRK3,RAPGEF4,TBC1D4,DOCK9,RASGRP1,MAGI2,DOCK10,PRKD1,RGS6,WDR41,NEDD9,RALGAPA1,CHN1,TBC1D9,ST18
GO:0072359	circulatory system development	0.0000215921481194648744	PRKCB,SLC8A1,COL18A1,DNAH11,IL6R,SORBS2,FGF12,LAMA1,NRXN1,WDPCP,CTDP1,ADAMTS6,RPS6KA2,SMOC2,CACNA1C,NTRK2,THSD7A,MEOX2,ENPEP,SOX6,CPE,CALD1,ARHGAP24,SLIT2,MYLK3,AKT3,ALPK2,ERBB4,MNAT1,RAP1A,RUNX1,ALDH1A2,EYA1,ANK2,SLIT3,ROBO1,OVOL2,SLC1A1,IGF1R,FLRT2,INSR,MYO18B,VAV3,PLCE1,ADAM12,CXADR,TENM4,RIN2,STARD13,NEBL,RARB,SLC39A12,ZFPM2,VSTM4,SVEP1,EGF,PDGFD,STK3,AP2B1,SGCZ,EPHB1,FHOD3,GREB1L,FLT1,EXT1,NXN,AKAP6,ATF2,LRP2,SGCD,RAPGEF2,IMMP2L,GTF2I,NRXN3,DLC1,EPHB2,PDLIM5,COL22A1,SETD2,FLVCR1,NRP1,CDH13,SEMA3E,CDH2,SERPINB7,HDAC9,PIK3R3,MTOR,FHL2,SPRED1,ADAM10,GLI3,NTRK3,FBN1,SGCG,BMPER,HMGA2,PPARA,RORA,LOXL2,PRKD1,ETS1,TNN,RYR2,TGFA,TBX20,ROBO2,AKAP13,SEMA5A,NRG1,PRKCA,SEMA3C
GO:0010977	negative regulation of neuron projection development	0.0000227468685054988895	SEMA4D,CD38,PTPRG,SLIT2,KANK1,NTN1,NLGN1,MAP2,PTPRO,TNR,SPOCK1,EPHA7,SEMA6D,UBE3A,EPHB2,NRP1,DAB1,SEMA3A,SEMA3E,FSTL4,DNM3,HDAC2,SEMA3D,FAT3,SEMA5A,BCL11A,DCC,SEMA3C

GO:0051966	regulation of synaptic transmission, glutamatergic	0.00002310910327113776	GRIK2,CACNG2,GRIN2A,NRXN1,GRIK3,GRIN2B,GRIK1,DGKI,GRM5,NLGN1,RELN,TNR,HCN1,DISC1,GRM1,GRM7,CDH2,SYT1,TSHZ3,CACNG3
GO:0007611	learning or memory	0.00002540901910041243	DNAH11,RAG1,KCNK10,RCAN1,KALRN,BTBD9,GRIN2A,NRXN1,NTRK2,SHANK2,GABRA5,ITGA8,GRIN2B,BRINP1,DGKI,SLC1A1,GRM5,CNTNAP2,INSR,RELN,TNR,APP,RASGRF1,MEIS2,FYN,GRIA1,S100B,AMFR,SORCS3,NTF3,CAMK4,ATXN1,NRXN3,UBE3A,EPHB2,TANC1,SPECC1,CSMD1,PLCB1,ELAVL4,ATP8A1,NEDD9
GO:0030001	metal ion transport	0.000025518705619438668	KCNMA1,SLC12A8,PRKCB,TRPM6,SLC8A1,KCNC1,BCL2,THADA,SLC13A4,KCNQ5,HEPHL1,SCN11A,KCNJ6,KCNK10,FGF12,CACNG2,GRIN2A,NIPA2,ADCYAP1R1,DPP6,ZDHHC17,KCNH1,CACNA1C,CNTN1,SLC24A4,CACNB2,TRPM3,SLC4A4,KCNE4,KCNS3,HECW1,NOS1,HTR2C,CACNA1E,SLC40A1,SLC9C1,ANK2,GRIN2B,NDFIP2,ITPR2,CACNA2D3,PRKCE,KCNH8,EFHB,SLC1A1,SLC12A1,KCNIP4,NOS1AP,NKAIN2,CATSPER2,ANK3,DPP10,CACNA2D1,HCN1,UTRN,TSPAN13,MICU1,NKAIN3,SLC39A12,EGF,FYN,KCND3,KCNN3,KCNAB1,PDE4D,ABCC9,TRPM7,TRPC5,AKAP6,TUSC3,LRP2,MICU2,SLC39A8,KCND2,SLC24A3,RYR3,FLVCR1,TRDN,RGS7,SLC24A2,SCARA5,ASIC2,TRPM1,CACNG3,PRKD1,RYR2,KCNH5,TMEM163,IL16,CDH23,SLC39A11,SLC5A1,STAC,SCN8A,KCNJ15
GO:0051049	regulation of transport	0.00002652150915708233	KCNMA1,PRKCB,SLC8A1,KCNC1,SIAH3,CD38,BCL2,THADA,UNC13B,KCNQ5,SCN11A,KCNJ6,KCNK10,APBA2,JAK2,TM9SF4,BICD1,ABCG8,KALRN,FGF12,CACNG2,BTBD9,GRIN2A,NRXN1,IL1RAPL1,ADCYAP1R1,DPP6,KCNH1,CACNA1C,OCLN,CNTN1,TTC39B,CACNB2,NUMB,STXBP4,CHRM3,ABCA5,KCNE4,KCNS3,ABCA13,HECW1,RAP1GDS1,RAP1A,MYOM1,ABL2,NOS1,ABCG1,HTR2C,CACNA1E,TBC1D5,PIK3C3,ANK2,PLA2G4A,SLC1A2,GRIN2B,NDFIP2,CACNA2D3,PRKCE,KCNH8,DGKI,RIN3,EFHB,SLC1A1,GRM5,NLGN1,SHISA9,CORIN,MAP2,KCNIP4,CFTR,CAMK1D,NOS1AP,NKAIN2,INSR,CATSPER2,ANK3,CNIH3,RELN,RASGRF2,DPP10,DAPK1,CLIC6,APP,CACNA2D1,HCN1,UTRN,TSPAN13,RASGRF1,USP7,SCP2,SLAMF1,NKAIN3,EGF,FYN,KCND3,KCNAB1,PDE4D,RIMS1,FRMD4A,MCTP2,ABCC9,GSG1L,EFNA5,ATP9A,AKAP6,C2,NTF3,FER,GRM7,NRXN3,KCND2,ATP8A2,EPHB2,MCTP1,SETD2,PACSIN2,DOCK2,NUP214,NRP1,CDH13,TRDN,CDH2,MAP2K6,CADPS,NEU3,RGS7,CD2AP,GLI3,RAB31,RAPGEF4,MX2,NSUN2,TBC1D4,DNM3,SYT1,PPARA,SHISA6,STXBP6,MAGI2,ASIC2,RALA,CACNG3,PRKD1,ATP8A1,BCL2L1,RYR2,MTMR2,KCNH5,IL16,WDR41,MYRIP,ENPP1,RIMS2,STAC,SCN8A,RAB27A,KCNJ15,NRG1,PCNT
GO:0001764	neuron migration	0.000027188357300846656	FBXO31,ASTN2,NTRK2,NRG3,UNC5D,ASTN1,NTN1,FLRT2,RELN,SPOCK1,PHACTR1,DISC1,FYN,MARK2,NTNG1,RAPGEF2,KIRREL3,AUTS2,SDCCAG8,NRP1,DAB1,SEMA3A,SEMA3E,DCLK1,NTRK3,PRKG1,TNN,TBX20,FAT3,DCC,CTNNA2,NRG1
GO:0050767	regulation of neurogenesis	0.000027772277335050667	SEMA4D,FBXO31,KALRN,IL1RAPL1,CTNNA1,NTRK2,TIAM2,NUMB,SLIT2,MACF1,ROBO1,PAK3,BRINP1,CHODL,DSCAM,GRM5,NTN1,MAP2,CUX1,RELN,CDH4,TNR,TENM4,TIAM1,DISC1,EPHA7,EFNA5,TRPC5,LRP2,SEMA6D,RAPGEF2,PRKCH,EPHB2,PRTG,NRP1,DAB1,SEMA3A,SEMA3E,FSTL4,MTOR,GLI3,NTRK3,PLXNA2,PTPRD,HDAC2,SEMA3D,ROBO2,SEMA5A,BCL11A,DCC,SEMA3C
GO:0006812	cation transport	0.00003659183109543805	KCNMA1,SLC12A8,PRKCB,TRPM6,SLC8A1,KCNC1,BCL2,THADA,SLC13A4,KCNQ5,HEPHL1,SCN11A,KCNJ6,KCNK10,FGF12,CACNG2,SLC44A5,GRIN2A,NRXN1,NIPA2,ADCYAP1R1,DPP6,ZDHHC17,KCNH1,CACNA1C,CNTN1,SLC24A4,CACNB2,TRPM3,SLC4A4,KCNE4,KCN

			S3,HECW1,NOS1,HTR2C,CACNA1E,SLC40A1,SLC9C1,P2RX6,ANK2,SLC1A2,GRIN2B,NDFIP2,ITPR2,CACNA2D3,PRKCE,KCNH8,EFHB,SLC1A1,SLC12A1,NLGN1,SHISA9,KCNIP4,NOS1AP,NKAIN2,CATSPER2,ANK3,CNIH3,RELN,RASGRF2,DPP10,DAPK1,APP,CACNA2D1,HCN1,CHRM5,UTRN,TSPAN13,RASGRF1,MICU1,NKAIN3,SLC39A12,PIEZO2,EGF,FYN,KCND3,ANO10,KCNN3,KCNAB1,PDE4D,ATP6V1E1,ABCC9,GSG1L,TRPM7,TRPC5,AKAP6,TUSC3,LRP2,MICU2,SLC39A8,KCND2,SLC24A3,EPHB2,RYR3,FLVCR1,TRDN,ATP13A3,RGS7,SLC44A1,SLC24A2,SYT1,SHISA6,SCARA5,ASIC2,TRPM1,CACNG3,PRKD1,ATP8A1,RYR2,KCNH5,TMEM163,IL16,CDH23,SLC39A11,SLC5A1,STAC,SCN8A,KCNJ15
GO:0016049	cell growth	0.000036890012666203	SEMA4D,TEAD1,CD38,BCL2,SORBS2,EPB41L3,CTDP1,SMARCA4,NRG3,MTPN,SLIT2,CSNK2A1,MACF1,RERG,SLIT3,ARHGEF11,DSCAM,NTN1,MAP2,COBL,CDH4,TNR,APP,FBLN5,SPOCK1,PLCE1,ALCAM,DISC1,EPHA7,NET1,RIMS1,EXT1,EFNA5,TRPC5,AKAP6,SEMA6D,TMEM108,PRKCQ,INO80,AUTS2,PDLIM5,NRP1,SEMA3A,SEMA3E,DCLK1,FSTL4,MTOR,ADAM10,PAPPA2,SYT1,PPARA,TNN,SEMA3D,VCL,AKAP13,ENPP1,RIMS2,SEMA5A,BCL11A,DCC,NRG1,SEMA3C
GO:0060560	developmental growth involved in morphogenesis	0.0000370228170856777796	SEMA4D,SLIT2,MACF1,SLIT3,DSCAM,NTN1,MAP2,COBL,CDH4,TNR,APP,ALCAM,DISC1,FMN1,EPHA7,ESR1,RIMS1,EXT1,EFNA5,TRPC5,SEMA6D,TMEM108,AUTS2,NRP1,SEMA3A,SEMA3E,DCLK1,FSTL4,SYT1,MAGI2,TNN,SEMA3D,VCL,RIMS2,SEMA5A,BCL11A,DCC,SEMA3C
GO:0003012	muscle system process	0.0000381100486427302 44	KCNMA1,APBB2,SLC8A1,CD38,SORBS2,FGF12,CTDP1,CACNA1C,CACNB2,MTPN,CHRM3,CALD1,DTNA,KCNE4,RAP1GDS1,MYOM1,NOS1,P2RX6,ANK2,SMPX,ARHGEF11,BBS2,MYOM2,NOS1AP,FBXO32,PLCE1,CACNA2D1,UTRN,MYH13,MLIP,ARHGAP42,KCND3,PDE4D,DOCK4,ABCC9,HDAC4,AKAP6,SGCD,SSPN,ATP8A2,SNTB1,PDLIM5,TRDN,ASB3,MAP2K6,MTOR,CTNNA3,PPP1R12B,PPARA,PRKG1,HDAC2,RYR2,TBX20,AKAP13,STAC,PRKCA
GO:0048167	regulation of synaptic plasticity	0.00003885414032265373	CD38,GRIK2,IGSF11,GRIN2A,NTRK2,SHANK2,CNTN4,GRIN2B,DGKI,SLC1A1,GRM5,SHISA9,RELN,RASGRF2,TNR,APP,RASGRF1,ZZEF1,SORCS2,GRIA1,S100B,ERC2,RIMS1,SORCS3,GRID2,RAPGEF2,NSG1,EPHB2,ERC1,MCTP1,SLC24A2,SHISA6,TSHZ3,UNC13C,RIMS2
GO:0071495	cellular response to endogenous stimulus	0.0000395594905434610 13	CD44,PRKCB,SLC8A1,THRB,SNX25,JAK2,FGF12,NRXN1,ARID1B,SMOC2,PNPLA3,CTNNA1,NTRK2,NLK,SMARCA4,STXBP4,SOX6,NREP,CHRM3,SPRED2,SLIT2,GLP2R,RAP1GDS1,ERBB4,KANK1,RAP1A,PTPRE,DUSP22,EXT2,ACACA,HTR2C,ITGA8,ESRRG,PTGFR,SLC1A2,SLIT3,OVOL2,ITPR2,LEMD3,GABPA,PRKCE,PSG9,PRKAA2,BBS2,SOX5,KDM4C,SLC1A1,GRM5,NR5A2,IGF1R,CFTR,FLRT2,INSR,FBXO32,APP,PAK1,GNAL,CACNA2D1,HCN1,CHRM5,GHR,PRDM16,KL,LTBP1,RARB,PDGFD,FYN,NSG2,SPIDR,ESR1,PDE4D,GNG2,PDE3A,POR,WWOX,FUT8,EXT1,EFNA5,HDAC4,AKAP6,ATF2,ZNF423,LRP2,NTF3,FER,TMEM108,GABRB3,RAPGEF2,PRKCQ,GABRG2,CPS1,NSG1,UBE3A,IDE,APC,EPHB2,RYR3,RGMB,ALK,LDLRAD4,KIF16B,HDAC9,PIK3R3,MTOR,GABRB1,BLM,SPRED1,TRERF1,NTRK3,RXFP1,FBN1,RAB31,BMPER,DEFA3,PTPRK,TBC1D4,PPARA,PLCB1,ELAVL4,MAGI2,BCL2L1,HDAC2,RYR2,PRLR,TBX20,PTPRA,ROBO2,ENPP1,BCL11A
GO:0043269	regulation of ion	0.00004283089119544846	KCNMA1,SLC8A1,KCNC1,BCL2,THADA,KCNQ5,SCN11A,KCNJ6,KCNK10,FGF12,CACNG2,GRIN2A,NRXN1,ADCYAP1R1,DPP6,KCNH1,CACNA1C,CNTN1,CACNB2,CHRM3,KCNE4,KCNS3,HECW1,NOS1,CACNA1E,ANK2,PLA2G4A,GRIN2B,CACNA2D3,PRKCE,KCNH8,EFHB,GRM5,NLGN1,SHISA9,KCNIP4,CFTR,NOS1AP,NKAIN2,CATSPER2,ANK3

	transport		,CNIH3,RELN,RASGRF2,DPP10,DAPK1,CLIC6,APP,CACNA2D1,HCN1,UTRN,TSPAN13,RASGRF1,NKAIN3,EGF,FYN,KCND3,KCNAB1,PDE4D,ABCC9,GSG1L,AKAP6,GRM7,KCND2,EPHB2,TRDN,MAP2K6,RGS7,SYT1,SHISA6,ASIC2,CACNG3,PRKD1,RYR2,KCNH5,IL16,STAC,SCN8A,KCNJ15
GO:0009719	response to endogenous stimulus	0.00006425694000270094	CD44,PRKCB,A2M,SLC8A1,KCNC1,CD38,BCL2,THRB,SNX25,JAK2,FGF12,NRXN1,ARID1B,SMOC2,PNPLA3,CTNNA1,NTRK2,NLK,SMARCA4,SLC24A4,STXBP4,SOX6,NREP,CHRM3,SPRED2,SLIT2,GLP2R,RAP1GDS1,ERBB4,KANK1,RAP1A,PTPRE,DUSP22,EXT2,NOS1,ACACA,RERG,HTR2C,ITGA8,ESRRG,PTGFR,SLC1A2,SLIT3,OVOL2,ITPR2,LEMD3,GABPA,PRKCE,PSG9,PRKAA2,BBS2,SOX5,KDM4C,SLC1A1,GRM5,NR5A2,IGF1R,CFTR,FLRT2,INSR,FBXO32,APP,PAK1,GNAL,CACNA2D1,HCN1,CHRM5,GHR,PRDM16,KL,LTBP1,RARB,PDGFD,FYN,NSG2,SPIDR,ESR1,PDE4D,GNG2,PDE3A,POR,WWOX,FUT8,EXT1,EFNA5,HDAC4,AKAP6,ACSBG1,ATF2,ZNF423,LRP2,C2,NTF3,FER,TMEM108,GABRB3,RAPGEF2,PRKCQ,GABRG2,CPS1,NSG1,UBE3A,IDE,TPH2,APC,TFF1,EPHB2,RYR3,RGMB,CDH13,ALK,LDLRAD4,KIF16B,PAPPA,HDAC9,PIK3R3,MTOR,GABRB1,BLM,FHL2,SPRED1,TRERF1,GLI3,NTRK3,RXFP1,FBN1,RAB31,BMPER,DEFA3,PTPRK,TBC1D4,PPARA,PLCB1,ELAVL4,MAGI2,BCL2L1,HDAC2,RYR2,PRLR,TBX20,PTPRA,ROBO2,ENPP1,BCL11A,VPS13C,BCKDHB
GO:0097061	dendritic spine organization	0.00007521235173048459	TANC2,KALRN,GRIN2B,PAK3,IGF1R,NLGN1,INSR,RELN,FYN,EPHB1,PPFIA2,UBE3A,EPHB2,PDLIM5,CTNND2,TANC1,DNM3,DOCK10,MTMR2,NEDD9
GO:0040013	negative regulation of locomotion	0.0000870822631598776	PTPRR,SEMA4D,BCL2,CTNNA1,SRGAP2B,MEOX2,NRG3,PTPRG,DLG5,SLIT2,KANK1,DUSP22,SPOCK3,ROBO1,RIN3,GRM5,PTPRO,MITF,FRMD5,STARD13,SEMA6D,NAV3,DLC1,MCTP1,NRP1,DACH1,LDLRAD4,SEMA3A,SEMA3E,FBLN1,SPRED1,SCAI,PTPRT,PTPRK,PLCB1,PRKG1,MAGI2,HDAC2,TNN,SEMA3D,VCL,ROBO2,SRGAP3,NEDD9,SEMA5A,NRG1,SEMA3C
GO:0018193	peptidyl-amino acid modification	0.00009107084370003272	CD44,SEMA4D,RPS6KA5,PRKCB,BCL2,SAMSN1,CDC42BPA,KDM4B,IL6R,BRD4,ZDHHC11B,TLK1,JAK2,MAST4,NRXN1,AGBL1,RPS6KA2,ZDHHC17,NTRK2,NLK,CNTN1,STK32B,SPRED2,ROR1,CSNK2A1,AKT3,KMT2C,ERBB4,STT3A,ATRX,DUSP22,ABL2,NOS1,MAPK9,PEAK1,EYA1,MORC3,SPOCK3,SUPT3H,EGFLAM,DPH6,TNKS,MLLT3,PRKCE,PRKAA2,KDM4C,SLC1A1,GRM5,EPHA6,IGF1R,CAMK1D,NOS1AP,ZDHHC14,INSR,PHF20L1,EGLN3,DOCK3,RELN,STK38,VRK1,APP,PAK1,NSMCE2,GHR,TTLL11,EGF,PDGFD,FYN,PRMT8,EPHA7,CNOT7,PDE4D,POR,FUT8,EPHB1,EFEMP1,FLT1,EFNA5,HDAC4,TRPC5,SH3BP5,MARK2,ATF2,TUSC3,NTF3,FER,CAMK4,PRKCQ,PRKCH,TTLL5,AUTS2,EPHB2,SETD2,NRP1,ALK,MGAT5,DCLK1,HDAC9,MAP2K6,MTOR,STK38L,NCAPG2,STK32A,SPRED1,KANSL1,DPY19L2,NTRK3,CHKA,LOXL2,ABI1,GALNTL6,PRKD1,HDAC2,TGFA,PRLR,ATAT1,NEDD9,BCL11A,PARD3,NRG1,PRKCA,DPY19L1,TOP1
GO:0016043	cellular component organization	0.00010020880239026214	CD44,KCNMA1,C10ORF90,APBB2,SCAF8,PARN,SEMA4D,SLC12A8,ZFYVE1,TEAD1,NFIA,RPS6KA5,TAOK3,PRKCB,TRPM6,KCNC1,DNAJC15,SIAH3,CD38,FBXO31,TRAPPC9,BCL2,MMP16,CHFR,COL18A1,CDC42BPA,RAG1,SAMM50,CDC42EP3,UNC13B,NCAM1,KDM4B,CDH8,ATP10A,SORBS2,SKAP2,BRD4,GRIP1,TLK1,TANC2,KIF4A,CSMD3,NTN4,JAK2,TM9SF4,BICD1,ABCG8,KALRN,NEGR1,CACNG2,BTBD9,EPB41L3,LAMA1,PARVB,CDH11,CDS2,MAST4,NRXN1,ARID1B,IL1RAPL1,WDPCP,MAGI1,LAMA3,ADAMTS17,CTDP1,ADAMTS6,PRELID2,GOLGA8J,RPS6KA2,SMOC2,ZDHHC17,CCSER2,BMF,EXOC4,PNPLA3,ANO4,BBS9,CTNNA1,MYO9A,NTRK2,OCLN,THSD7A,AFAP1,NCAM2,TIAM2,IQCJ-SCHIP1,LRRC49,SMARCA4,CNTN1,NRG3,PTPRG,ELMO1,CABIN1,SYNE1,GAS2,CACNB2,NUMB,MED15,MTPN,ESYT2,MECOM,SYBU,PDE4DIP,TRPM3,DNAH8,SHANK2,PTGFRN,KCTD8,CHCHD6,UNC5D,NRE

			P,DLG5,CFDP1,PGM5,SMARCAD1,CALD1,ARHGAP24,IGHV1OR15-9,SLIT2,PITPNC1,MYLK3,ABCA5,ROR1,ADAMTSL1,CSNK2A1,AKT3,CRB1,KMT2C,KCNS3,ABCA13,HECW1,RAP1GDS1,ERBB4,KANK1,GPHN,ATRX,DMRT1,BID,MACF1,MNAT1,RAP1A,TRIO,RAD51B,TRMT61B,DUSP22,ABL2,ACACA,ABCG1,SPAG16,EML1,RERG,CCDC141,CNTN4,TBC1D5,PIK3C3,SNAP25-AS1,CHD6,HMCN1,FGD4,GOLGA6D,ITGA1,HIRA,CORO2B,ITGA8,RUNX1,ALDH1A2,MAPK9,IGHV1OR21-1,FAM171A1,CDH17,PEAK1,EYA1,ADAMTS19,CDH18,HSF2BP,FRMD6,PLS1,ANK2,SLC1A2,SLIT3,GRIN2B,ZNF518A,ROBO1,ANKRD30BL,EGFLAM,PAK3,DGKB,TNKS,GABRA2,MDM1,FCHSD2,MLLT3,TRAPPC10,LEMD3,CHODL,EHBP1,PSTPIP2,ITGB3BP,TMEM67,PRKCE,ARHGEF11,PRKAA2,BBS2,IL1RAPL2,CHCHD3,MYOM2,NUBPL,DSCAM,RIN3,ANKFN1,TRAPPC8,KDM4C,SDK1,SLC1A1,SLC12A1,GRM5,EPHA6,NTN1,IGF1R,SPTB,WDR72,SNX30,NLGN1,DNAH9,SHROOM3,CNTNAP2,MAP2,CAMK1D,FLRT2,NOS1AP,PTPRO,MATN2,INSR,COBL,MDN1,CLEC16A,MTRF1,CRTAC1,CUX1,ANK3,CDH12,MORC2,THSD4,TBCD,GPC6,RELN,ADAMTS5,ADAMTSL3,CDH4,TNR,CELF4,VAV3,VRK1,CNTN6,APP,FBLN5,CCDC88A,KCTD1,SPOCK1,HPSE2,PLCE1,TACC2,PAK1,ATP9B,MITF,ADCK1,HCN1,TOP3A,NSMCE2,FRY,CXADR,EPS8,LRFN5,UTRN,TENM4,CECR2,PRR16,RASGRF1,PRDM16,FRMD5,USP7,STARD13,SCP2,LRRC4C,ALCAM,PPP1R9A,PDZRN3,ADAMTS16,MICU1,SLAMF1,INO80D,CLSTN2,TTLL11,NEBL,TCF4,FRYL,TIAM1,PHACTR1,SLC39A12,DISC1,FMN1,CHAF1A,SVEP1,VTI1A,ASAP1,FRMPD4,COL23A1,EGF,FYN,KCND3,PRMT8,IFT43,LRBA,EPHA7,MAP7,NSG2,TRABD2B,SPIDR,NAV2,STK3,CNOT7,COL19A1,MSR1,PSIP1,S100B,NET1,TOX,PCDH15,ESR1,ARHGAP12,SGCZ,CNTN5,ERC2,PRKACB,PDE3A,RIMS1,L3MBTL4,CNKSR2,EPHB1,CTTNBP2,FHOD3,ARMC2,IGSF21,MIPEP,SNAP29,GSG1L,HERC1,DOCK1,DIAPH3,TRPM7,EXT1,EFNA5,CDC14B,TLN2,C14ORF39,HDAC4,STK36,KLHL1,TRPC5,ATP9A,PPFIA2,AKAP6,VPS37A,TENM3,LINGO2,MARK2,ATF2,PHACTR2,GRID2,ZNF423,LRP2,SEMA6D,NTF3,FER,TTC29,CELF2,NTNG1,MAPRE2,ARFGAP3,RAD51AP1,TMEM108,GABRB3,GRM7,SLC39A8,RAPGEF2,NAV3,IMMP2L,PRKCQ,KIRREL3,GABRG2,NUDCD3,PRKCH,NRXN3,RHPN2,KRT25,DLC1,NSG1,KCND2,ATP8A2,UBE3A,MPRIP,APC,TTLL5,INO80,AUTS2,EPHB2,SCAF4,ERC1,PDLIM5,AGO3,C9,MCTP1,RNU1-51P,RYR3,PRTG,NBN,ADAMTS18,CTNND2,FRMD3,COL22A1,SETD2,PACSIN2,PKP1,DOCK2,SDCCAG8,NRP1,CDH13,RFC3,PHACTR3,RPGRIP1,TRDN,DAB1,RFTN1,ALK,EXOC6B,LDLRAD4,SEMA3A,SEMA3E,DCLK1,CDH2,TENM2,TANC1,VPS41,SYCP1,HDAC9,FSTL4,ARHGAP28,MTOR,STK38L,FBLN1,BLM,SH3KBP1,NEU3,NCAPG2,CD2AP,TTC39C,CLVS2,SIPA1L3,ADAM10,TSPAN33,KANSL1,LRFN2,FLNB,PAPPA2,SPECC1,GLI3,NTRK3,RXFP1,HYDIN,CHKA,RAB31,CTNNA3,VPS13D,ABHD17C,ANKRD31,ATF7IP,HMGA2,THSD7B,PTPRK,TBC1D4,DNM3,SYT1,APIP,SYNDIG1,DPF3,NPHP4,SACS,PPARA,PLXNA2,PTPRD,SHISA6,SCARA5,PLCB1,LOXL2,BPTF,PRKG1,ELAVL4,CDH9,STXBP6,MAGI2,STX12,ABI1,ASIC2,RALA,DOCK10,GNPTAB,TRPM1,PRKD1,ATP8A1,BCL2L1,MICAL3,HDAC2,ETS1,TNN,SEMA3D,BANP,TGFA,PRLR,TBX20,PTPRA,FAT3,MTMR2,VCL,DEPTOR,ATAT1,ROBO2,IFT81,ZMYND11,CDH23,AKAP13,NEDD9,ENPP1,UNC13C,RIMS2,RAB27A,EYA4,L3MBTL3,CLIP1,SEMA5A,BCL11A,DCC,CTNNA2,CEP44,PVT1,PARD3B,CHN1,ETV6,VPS13C,PARD3,NRG1,CAST,ATP10B,PRKCA,FMN2,SEMA3C,PCNT,OSBPL5,TOP1
GO:0060284	regulation of cell development	0.00011205285157845214	SEMA4D,FBXO31,BCL2,KALRN,IL1RAPL1,CTNNA1,NTRK2,TIAM2,NUMB,SLIT2,KANK1,MACF1,ROBO1,PAK3,BRINP1,CHODL,DSCAM,GRM5,NTN1,MAP2,CUX1,RELN,CDH4,TNR,TENM4,TIAM1,DISC1,EPHA7,PDE3A,DOCK1,EFNA5,HDAC4,TRPC5,LRP2,SEMA6D,RAPGEF2,PRKCH,EPHB2,PRTG,NRP1,DAB1,SEMA3A,SEMA3E,HDAC9,FSTL4,MTOR,FBLN1,GLI3,NTRK3,FBN1,PLXNA2,PTPRD,PLCB1,HDAC2,SEMA3D,VCL,ROBO2,NEDD9,SEMA5A,BCL11A,DCC,SEMA3C
GO:0031589	cell-substrate	0.0001256670588895394	CD44,BCL2,NTN4,JAK2,USH2A,PARVB,WDPCP,ITGBL1,ATRNL1,KANK1,MACF1,DUSP22,ITGA1,CORO2B,ITGA8,PEAK1,EGFLAM,PRKCE,EDIL3,PTPRO,CD96,ITGA9,TBCD,FBLN5,SPOCK1,UTRN,RIN2,TIAM1,DISC1,FMN1,EPHB1,AJAP1,DOCK1,TRPM7,EFNA5,PPFIA

	adhesion		2,FER,NTNG1,DLC1,NRP1,CDH13,SEMA3E,FBLN1,PTPRK,TNN,PTPRA,VCL,NEDD9,FREM1
GO:0007167	enzyme-linked receptor protein signaling pathway	0.00013517075938737616	PTPRR,PRKCB,SNX25,JAK2,KALRN,FGF12,NRXN1,SMOC2,ZDHHC17,FAM83B,NTRK2,NLK,NRG3,PTPRG,BTBD11,STXBP4,NREP,DOK5,SPRED2,ROR1,ERBB4,KANK1,DMRT1,TRIO,PTPRE,DUSP22,ITGA1,ITGA8,ANKS1B,ROBO1,PAK3,OVOL2,LEMD3,PSG9,EPHA6,IGF1R,FLRT2,INSR,CCDC88A,PLCE1,PAK1,GHR,PRDM16,KL,LTBP1,TIAM1,SVEP1,EGF,PDGFD,FYN,EPHA7,FUT8,EPHB1,EFEMP1,FLT1,EXT1,EFNA5,ATF2,ZNF423,LRP2,NTF3,FER,TMEM108,RAPGEF2,PRKCQ,IDE,APC,EPHB2,RGMB,NRP1,CDH13,ALK,LDLRAD4,KIF16B,ARID5B,PIK3R3,FSTL4,NEU3,SPRED1,PTPRT,NTRK3,FBN1,BMPER,PTPRK,PPARA,PTPRD,PLCB1,MAGI2,ABI1,PRKD1,HDAC2,TGFA,PRLR,TBX20,PTPRA,NEDD9,ENPP1,CHN1,NRG1
GO:0035235	ionotropic glutamate receptor signaling pathway	0.00014293095967962463	GRIK2,GRIN2A,GRID1,GRIK3,GRIK4,GRIN2B,GRIK1,APP,GRIA1,GRID2,GRIA4
GO:1990806	ligand-gated ion channel signaling pathway	0.00014293095967962463	GRIK2,GRIN2A,GRID1,GRIK3,GRIK4,GRIN2B,GRIK1,APP,GRIA1,GRID2,GRIA4
GO:0007612	learning	0.00014742089142736023	RAG1,KALRN,GRIN2A,NRXN1,NTRK2,SHANK2,GABRA5,DGKI,SLC1A1,GRM5,CNTNAP2,INSR,RELN,TNR,APP,MEIS2,FYN,SORCS3,ATXN1,NRXN3,UBE3A,EPHB2,TANC1,SPECC1,CSMD1,PLCB1,ELAVL4,ATP8A1
GO:2000026	regulation of multicellular organismal development	0.00015769532661605065	SEMA4D,PRKCB,SLC8A1,GPR55,FBXO31,RAG1,KALRN,LAMA1,NRXN1,ARID1B,IL1RAPL1,WDPCP,LAMA3,CTDP1,SMOC2,CTNNA1,NTRK2,TIAM2,SMARCA4,NUMB,SOX6,DLG5,SPRED2,SLIT2,ZNF675,AKT3,ERBB4,MACF1,RBM19,RUNX1,ZBTB16,ROBO1,PAK3,OVOL2,BRINP1,CHODL,GABPA,PSG9,SOX5,DSCAM,GRM5,NTN1,NLGN1,JAM2,MAP2,CFTR,FLRT2,INSR,CUX1,RELN,TRPS1,CDH4,TNR,ADAM12,MITF,CXADR,TENM4,MEIS2,KL,INO80D,CLSTN2,RARB,TIAM1,SLC39A12,DISC1,ZFPM2,EGF,EPHA7,TOX,POR,EPHB1,EFEMP1,AJAP1,FLT1,EFNA5,TRPC5,AKAP6,LINGO2,ATF2,GRID2,LRP2,SEMA6D,CAMK4,RAPGEF2,GTF2I,PRKCH,INO80,EPHB2,PRTG,NRP1,DAB1,SEMA3A,SEMA3E,FSTL4,MTOR,SPRED1,ADAM10,GLI3,NTRK3,FBN1,BMPER,HMGA2,SYNDIG1,PPARA,PLXNA2,PTPRD,PLCB1,LOXL2,RASGRP1,NELL1,ASIC2,PRKD1,HDAC2,ETS1,TNN,SEMA3D,PRLR,TBX20,MTMR2,VCL,ROBO2,NEDD9,ENPP1,SEMA5A,BCL11A,DCC,PARD3,NRG1,PRKCA,SEMA3C
GO:0050772	positive regulation of axono	0.00021036493493207332	SEMA4D,NTRK2,TIAM2,SLIT2,MACF1,ROBO1,CHODL,DSCAM,NTN1,CDH4,TIAM1,DISC1,EFNA5,TRPC5,NRP1,PLXNA2,ROBO2,SEMA5A,BCL11A

	genesis		
GO:0006793	phosphorus metabolic process	0.00022911503643373605	CD44,PTPRR,ERG,SEMA4D,RPS6KA5,TAOK3,PRKCB,TRPM6,SLC8A1,CD38,BCL2,CAMTA1,SAMSN1,RSRC1,CDC42BPA,SNX25,DPYD,MAPK10,IL6R,ZBTB20,BRD4,TLK1,RCAN1,JAK2,TPTE2,KALRN,SLC44A5,LAMA1,CDS2,MAST4,NRXN1,ADCYAP1R1,CTDP1,RPS6KA2,CKMT1B,PNPLA3,NTRK2,OCLN,AK8,NLK,CNTN1,NRG3,PTPRG,PDE10A,STK32B,SPRED2,SLIT2,MYLK3,ROR1,SLC4A4,ZNF675,CSNK2A1,AKT3,PHKB,ALPK2,ERBB4,GPHN,MNAT1,TAF4B,RAP1A,TRIO,PTPRE,MYO3B,DUSP22,ABL2,MAP3K5,NOS1,ACACA,HTR2C,NEK4,SLCO3A1,PIK3C3,ITGA1,MAPK9,FAR2,PEAK1,EYA1,MORC3,AKAP10,PLA2G4A,ROBO1,PAK3,DGKB,TNKS,SGMS1,DGKK,PRKCE,PRKAA2,PTPRN2,MYO3A,DSCAM,DGKI,NME7,ACSM2A,SLC1A1,GRM5,EPHA6,LDB2,IGF1R,FAR1,CAMK1D,PTPRO,INSR,GMDS,DOCK3,RELN,STK38,ADCY9,DAPK1,VAV3,INPP5A,VRK1,ACSM2B,TTC7B,APP,CCDC88A,PLCE1,PAK1,ADCK1,CHRM5,GHR,RIPK4,SCP2,FAM126B,LIPI,OSBPL10,EGF,PDGFD,FYN,EPHA7,FHIT,STK3,CNOT7,PDE4D,PRKACB,PIGK,HUNK,FUT8,EPHB1,EFEMP1,PIGB,FAM126A,TRPM7,FLT1,EFNA5,CDC14B,HDAC4,STK36,TRPC5,SH3BP5,ACSBG1,MARK2,ATF2,NTF3,FER,SNRK,CAMK4,TPTE,RAPGEF2,PPA2,PRKCQ,CPS1,PRKCH,DLC1,PNPLA7,APC,MOCS2,EPHB2,ERC1,MOB3B,NBN,NRP1,DAB1,ALK,LDLRAD4,MGAT5,DCLK1,MAGI3,CCNG2,ELOVL7,PIK3R3,MAP2K6,MTOR,STK38L,KSR1,FBLN1,BLM,SH3KBP1,NCAPG2,KYNU,STK32A,SLC44A1,SPRED1,ADAM10,PPP2R2C,MTMR10,PTPRT,NTRK3,CHKA,BMPER,HMGA2,PTPRK,DLG2,PPARA,PTPRD,RORA,PLCB1,PRKG1,RASGRP1,NLRC5,MAGI2,ABI1,GNPTAB,PRKD1,ADK,HDAC2,TGFA,PRLR,PTPRA,MTMR2,DEPTOR,AKAP13,NEDD9,ENPP1,PARD3,NRG1,PRKCA,CSF2RB,OSBPL5,TOP1
GO:0007169	transmembrane receptor protein tyrosine kinase signaling pathway	0.00023593386246146305	PTPRR,PRKCB,JAK2,KALRN,FGF12,NRXN1,SMOC2,ZDHHC17,FAM83B,NTRK2,NRG3,PTPRG,STXBP4,DOK5,ROR1,ERBB4,KANK1,PTPRE,ITGA1,ANKS1B,ROBO1,PAK3,EPHA6,IGF1R,FLRT2,INSR,CCDC88A,PLCE1,PAK1,GHR,KL,TIAM1,SVEP1,EGF,PDGFD,FYN,EPHA7,EPHB1,EFEMP1,FLT1,EXT1,EFNA5,NTF3,FER,TMEM108,RAPGEF2,PRKCQ,IDE,APC,EPHB2,NRP1,CDH13,ALK,KIF16B,ARID5B,PIK3R3,FSTL4,NEU3,PTPRT,NTRK3,PLCB1,ABI1,PRKD1,TGFA,PRLR,PTPRA,NEDD9,ENPP1,CHN1,NRG1
GO:0045595	regulation of cell differentiation	0.00026722471036145823	ZHX3,SEMA4D,GPR55,FBXO31,BCL2,RAG1,IL6R,JAK2,KALRN,USH2A,ZNF536,LAMA1,ZBTB7C,ARID1B,IL1RAPL1,CTDP1,CTNNA1,NTRK2,TIAM2,SMARCA4,PLEKHB2,NUMB,SOX6,NREP,SPRED2,SLIT2,MYLK3,ABCA5,ZNF675,KANK1,MACF1,RAP1A,TRIO,MAP3K5,ABCG1,HTR2C,CNTN4,TCF12,RUNX1,MAPK9,EYA1,ZBTB16,ROBO1,PAK3,OVOL2,BRINP1,CHODL,GABPA,GLIS1,PSG9,SOX5,DSCAM,KDM4C,GRM5,NTN1,NLGN1,MAP2,CUX1,RELN,TRPS1,CDH4,TNR,APP,MITF,TENM4,GHR,RIN2,RBFOX1,MEIS2,RARB,TCF4,TIAM1,PBX1,DISC1,ZFPM2,EPHA7,STK3,MSR1,TOX,PDE3A,POR,EPHB1,EFEMP1,AJAP1,DOCK1,EFNA5,HDAC4,TRPC5,FTO,AKAP6,LRP2,SEMA6D,NTF3,CAMK4,RAPGEF2,PRKCH,APC,EPHB2,PRTG,NRP1,DAB1,ALK,LDLRAD4,SEMA3A,SEMA3E,HDAC9,FSTL4,MTOR,RORB,FBLN1,SPRED1,GLI3,NTRK3,FBN1,NSUN2,DPF3,PPARA,PLXNA2,PTPRD,RORA,PLCB1,LOXL2,RASGRP1,NELL1,PRKD1,HDAC2,ETS1,TNN,SEMA3D,PRLR,TBX20,VCL,ATAT1,ROBO2,NEDD9,ENPP1,PCP4,SEMA5A,BCL11A,DCC,NRG1,PRKCA,SEMA3C
GO:0006796	phosphate-conta	0.0002704145769230013	CD44,PTPRR,ERG,SEMA4D,RPS6KA5,TAOK3,PRKCB,TRPM6,SLC8A1,CD38,BCL2,CAMTA1,SAMSN1,RSRC1,CDC42BPA,SNX25,DPYD,MAPK10,IL6R,ZBTB20,BRD4,TLK1,RCAN1,JAK2,TPTE2,KALRN,SLC44A5,LAMA1,CDS2,MAST4,NRXN1,ADCYAP1R1,CTDP1,RPS6KA2,

	ining compound metabolic process		CKMT1B,PNPLA3,NTRK2,OCLN,AK8,NLK,CNTN1,NRG3,PTPRG,PDE10A,STK32B,SPRED2,SLIT2,MYLK3,ROR1,SLC4A4,ZNF675,CSNK2A1,AKT3,PHKB,ALPK2,ERBB4,GPHN,MNAT1,TAF4B,RAP1A,TRIO,PTPRE,MYO3B,DUSP22,ABL2,MAP3K5,NOS1,ACACA,HTR2C,NEK4,SLCO3A1,PIK3C3,ITGA1,MAPK9,FAR2,PEAK1,EYA1,MORC3,AKAP10,PLA2G4A,ROBO1,PAK3,DGKB,TNKS,SGMS1,DGKK,PRKCE,PRKAA2,PTPRN2,MYO3A,DSCAM,DGKI,NME7,ACSM2A,SLC1A1,GRM5,EPHA6,LDB2,IGF1R,FAR1,CAMK1D,PTPRO,INSR,DOCK3,RELN,STK38,ADCY9,DAPK1,VAV3,INPP5A,VRK1,ACSM2B,TTC7B,APP,CCDC88A,PLCE1,PAK1,ADCK1,CHRM5,GHR,RIPK4,SCP2,FAM126B,LIPI,OSBPL10,EGF,PDGFD,FYN,EPHA7,FHIT,STK3,CNOT7,PDE4D,PRKACB,PIGK,HUNK,EPHB1,EFEMP1,PIGB,FAM126A,TRPM7,FLT1,EFNA5,CDC14B,HDAC4,STK36,TRPC5,SH3BP5,ACSBG1,MARK2,ATF2,NTF3,FER,SNRK,CAMK4,TPTE,RAPGEF2,PPA2,PRKCQ,CPS1,PRKCH,DLC1,PNPLA7,APC,MOCS2,EPHB2,ERC1,MOB3B,NBN,NRP1,DAB1,ALK,LDLRAD4,MGAT5,DCLK1,MAGI3,CCNG2,ELOVL7,PIK3R3,MAP2K6,MTOR,STK38L,KSR1,FBLN1,BLM,SH3KBP1,NCAPG2,KYNU,STK32A,SLC44A1,SPRED1,ADAM10,PPP2R2C,MTMR10,PTPRT,NTRK3,CHKA,BMPER,HMGA2,PTPRK,DLG2,PPARA,PTPRD,RORA,PLCB1,PRKG1,RASGRP1,NLRC5,MAGI2,ABI1,GNPTAB,PRKD1,ADK,HDAC2,TGFA,PRLR,PTPRA,MTMR2,DEPTOR,AKAP13,NEDD9,ENPP1,PARD3,NRG1,PRKCA,CSF2RB,OSBPL5,TOP1
GO:0048638	regulation of developmental growth	0.00030244994244081207	SEMA4D,BCL2,CTDP1,ERBB4,MACF1,RUNX1,PLS1,BBS2,DSCAM,NTN1,MAP2,INSR,CDH4,TNR,APP,CXADR,GHR,DISC1,ZFPM2,EPHA7,STK3,RIMS1,EFNA5,TRPC5,FTO,AKAP6,SEMA6D,ATP8A2,FLVCR1,NRP1,SEMA3A,SEMA3E,FSTL4,SYT1,PPARA,PLCB1,SEMA3D,TBX20,RIMS2,SEMA5A,BCL11A,DCC,NRG1,SEMA3C
GO:0022604	regulation of cell morphogenesis	0.00032742905817638993	CD44,SEMA4D,FBXO31,CDC42EP3,ATP10A,GRIP1,KALRN,EPB41L3,PARVB,IL1RAPL1,WDPCP,MYO9A,PALMD,GAS2,CFDP1,KANK1,MACF1,FGD4,FAM171A1,PAK3,DNMBP,SHROOM3,CUX1,RELN,EPS8,FYN,RIMS1,DOCK1,EFNA5,MARK2,NTNG1,DLC1,EPHB2,NRP1,SEMA3E,FBLN1,SH3KBP1,SYT1,PLXNA2,PTPRD,NEDD9,RIMS2,BCL11A
GO:0050771	negative regulation of axonogenesis	0.00033019726309565264	SEMA4D,NTN1,MAP2,TNR,EPHA7,SEMA6D,EPHB2,NRP1,DAB1,SEMA3A,SEMA3E,FSTL4,SEMA3D,SEMA5A,BCL11A,DCC,SEMA3C
GO:0022898	regulation of transmembrane transporter activity	0.0003521620243626539	KCNC1,BCL2,THADA,FGF12,CACNG2,NRXN1,CACNB2,CHRM3,KCNE4,HECW1,NOS1,ANK2,PRKCE,GRM5,NLGN1,SHISA9,CFTR,NOS1AP,ANK3,CNIH3,RELN,RASGRF2,DAPK1,APP,CACNA2D1,HCN1,UTRN,RASGRF1,KCNAB1,PDE4D,ABCC9,GSG1L,AKAP6,EPHB2,TRDN,SHISA6,CACNG3,RYR2,STAC
GO:0050790	regulation of	0.0003569681845165647	CD44,PARN,SEMA4D,TAOK3,A2M,SLC8A1,GPR55,BCL2,MMP16,RAG1,IL6R,RCAN1,JAK2,BICD1,KALRN,GRIN2A,NRXN1,ADCYAP1R1,CACNA1C,MYO9A,NTRK2,RASGEF1B,TIAM2,NRG3,ELMO1,CABIN1,TBC1D22A,RGS12,RAPGEF5,ARHGAP24,SPRED2,SLIT2,ROR1,ZN

	catalytic activity		F675,CSNK2A1,PSD3,RAP1GDS1,ERBB4,BID,MNAT1,RAP1A,TRIO,DUSP22,ABL2,MAP3K5,NOS1,RGS3,TBC1D5,FGD4,ITGA1,SPOCK3,GRIN2B,ROBO1,GARNL3,TNKS,BCL2L13,PRKCE,ARHGEF11,DGKI,RIN3,DNMBP,DCUN1D4,SLC1A1,GRM5,EPHA6,ARAP2,LDB2,IGF1R,NOS1AP,PTPRO,INSR,EGLN3,DOCK3,TBCD,RELN,RASGRF2,STK38,DAPK1,VAV3,APP,CCDC88A,SPOCK1,PLCE1,PAK1,FRY,GHR,RASGRF1,RIN2,STARD13,SLAMF1,RGL1,TIAM1,PHACTR1,PRIM2,ASAP2,RALGPS1,ARHGAP42,ASAP1,EGF,PDGFD,FYN,XRCC4,EPHA7,STK3,NET1,ESR1,ARHGAP12,PDE3A,RIMS1,POR,DOCK4,EPHB1,HERC1,DOCK1,FLT1,EFNA5,CDC14B,SH3BP5,MARK2,PHACTR2,RALGPS2,NTF3,MAPRE2,ARFGAP3,RIC8B,GRM7,RAPGEF2,PRKCQ,RABGAP1L,DLC1,GABBR2,APC,EPHB2,MOB3B,NBN,DOCK2,TRIM23,NRP1,RFC3,PHACTR3,DAB1,ALK,MGAT5,SIPA1L2,CCNG2,RCAN2,SERPINB7,EVI5,HDAC9,PIK3R3,MAP2K6,ARHGAP28,MTOR,KSR1,RALGAPA2,FBLN1,SGSM1,BLM,NCAPG2,RGS7,SPRED1,SIPA1L3,PPP2R2C,PTPRT,NTRK3,RXFP1,RAPGEF4,HMGA2,TBC1D4,DOCK9,PPP1R12B,PLXNA2,PLCB1,PRKG1,RASGRP1,NLRC5,MAGI2,ABI1,DOCK10,PRKD1,TNFAIP8,TGFA,PRLR,DEPTOR,RGS6,SRGAP3,AKAP13,WDR41,NEDD9,RALGAPA1,AGAP1,CHN1,NRG1,CAST,TBC1D9,ST18
GO:0032412	regulation of ion transmembrane transporter activity	0.00037029057967441975	KCNC1,THADA,FGF12,CACNG2,NRXN1,CACNB2,CHRM3,KCNE4,HECW1,NOS1,ANK2,PRKCE,GRM5,NLGN1,SHISA9,CFTR,NOS1AP,ANK3,CNIH3,RELN,RASGRF2,DAPK1,APP,CACNA2D1,HCN1,UTRN,RASGRF1,KCNAB1,PDE4D,ABCC9,GSG1L,AKAP6,EPHB2,TRDN,SHISA6,CACNG3,RYR2,STAC
GO:0050769	positive regulation of neurogenesis	0.0004589236698642424	SEMA4D,FBXO31,KALRN,IL1RAPL1,NTRK2,TIAM2,NUMB,SLIT2,MACF1,ROBO1,PAK3,CHODL,DSCAM,GRM5,NTN1,CUX1,RELN,CDH4,TENM4,TIAM1,DISC1,EFNA5,TRPC5,LRP2,PRKCH,EPHB2,NRP1,MTOR,GLI3,PLXNA2,PTPRD,HDAC2,ROBO2,SEMA5A,BCL11A
GO:0008015	blood circulation	0.00047798471783645183	KCNMA1,SLC8A1,CD38,THRB,JAK2,FGF12,FLI1,TRHDE,RPS6KA2,CACNA1C,OCLN,ENPEP,CACNB2,CHRM3,SLIT2,MYLK3,KCNE4,RAP1GDS1,EXT2,NOS1,ITGA1,CORO2B,ANK2,BBS2,SLC1A1,CORIN,NOS1AP,PTPRO,RNLS,CACNA2D1,HCN1,CXADR,KL,ADAMTS16,ARHGAP42,VSTM4,KCND3,NAV2,SGCZ,PDE4D,PDE3A,DOCK4,ABCC9,EXT1,HDAC4,CELF2,SGCD,IMMP2L,CPS1,TRDN,ASB3,MAP2K6,MTOR,SGCG,CTNNA3,PPARA,PRKG1,ASIC2,RYR2,TBX20
GO:2001257	regulation of cation channel activity	0.0005086319930358956	KCNC1,FGF12,CACNG2,NRXN1,CACNB2,KCNE4,NOS1,ANK2,NLGN1,SHISA9,NOS1AP,ANK3,CNIH3,RELN,RASGRF2,DAPK1,APP,CACNA2D1,HCN1,RASGRF1,KCNAB1,PDE4D,ABCC9,GSG1L,AKAP6,EPHB2,TRDN,SHISA6,CACNG3,STAC
GO:1904062	regulation of catio	0.000533798537192781	SLC8A1,KCNC1,BCL2,THADA,FGF12,CACNG2,GRIN2A,NRXN1,ADCYAP1R1,DPP6,CACNA1C,CACNB2,KCNE4,HECW1,NOS1,ANK2,GRIN2B,PRKCE,NLGN1,SHISA9,KCNIP4,NOS1AP,ANK3,CNIH3,RELN,RASGRF2,DPP10,DAPK1,APP,CACNA2D1,HCN1,UTRN,TSPAN13,RASGRF1,FYN,KCNAB1,PDE4D,ABCC9,GSG1L,AKAP6,EPHB2,TRDN,RG

	n transmembrane transport		S7,SHISA6,CACNG3,PRKD1,RYR2,STAC
GO:0050890	cognition	0.0005642160406530876	DNAH11,RAG1,KCNK10,RCAN1,KALRN,BTBD9,GRIN2A,NRXN1,NTRK2,SHANK2,GABRA5,ITGA8,GRIN2B,BRINP1,DGKI,SLC1A1,GRM5,CNTNAP2,INSR,RELN,TNR,APP,RASGRF1,MEIS2,FYN,GRIA1,S100B,AMFR,SORCS3,TUSC3,NTF3,CAMK4,ATXN1,NRXN3,UBE3A,EPHB2,TANC1,SPECC1,CSMD1,PLCB1,ELAVL4,ATP8A1,NEDD9
GO:0032409	regulation of transporter activity	0.000652542921089627	KCNC1,BCL2,THADA,FGF12,CACNG2,NRXN1,CACNB2,CHRM3,KCNE4,HECW1,NOS1,ANK2,NDFIP2,PRKCE,GRM5,NLGN1,SHISA9,CFTR,NOS1AP,ANK3,CNIH3,RELN,RASGRF2,DAPK1,APP,CACNA2D1,HCN1,UTRN,RASGRF1,KCNAB1,PDE4D,ABCC9,GSG1L,AKAP6,EPHB2,TRDN,SHISA6,CACNG3,RYR2,STAC
GO:0050877	nervous system process	0.0007043159352103833	CAMTA1,COL18A1,THRB,DNAH11,RAG1,GRIK2,IGSF11,UNC13B,SCN11A,KCNK10,DLGAP1,RCAN1,KALRN,USH2A,FGF12,CACNG2,BTBD9,GRIN2A,NRXN1,RPS6KA2,KCNH1,BBS9,MYO9A,NTRK2,NCAM2,TMPRSS3,SLC24A4,CACNB2,TRPM3,SHANK2,GABRA5,ROR1,CRB1,MYO3B,OR4C46,SPAG16,HTR2C,GABRR2,HMCN1,ITGA8,GABRG1,EYA1,P2RX6,GRIN2B,GABRA2,BRINP1,BBS2,MYO3A,DGKI,ANKFN1,SLC1A1,GRM5,NLGN1,DNAH9,SHISA9,OR9Q1,JAM2,CNTNAP2,INSR,ANK3,LHFPL3,RELN,TNR,CELF4,APP,GNAL,CHRM5,TENM4,RASGRF1,RBFOX1,MEIS2,PIEZO2,VTI1A,FYN,GRIA1,NAV2,S100B,PCDH15,GABRG3,GRM1,CNTN5,LRIG1,RIMS1,EPHB1,EFEMP1,HERC1,AMFR,SORCS3,POU6F2,TUSC3,GRID2,LRP2,NTF3,CAMK4,TMEM108,GABRB3,GRM7,ATXN1,GABRG2,NRXN3,KCND2,ATP8A2,UBE3A,LOXHD1,EPHB2,NBN,RPGRIP1,TANC1,MTOR,RORB,GABRB1,SPECC1,CSMD1,SLC24A2,SHISA6,PLCB1,ELAVL4,TSHZ3,ASIC2,TRPM1,CACNG3,ATP8A1,OR11G2,MTMR2,ATF6,EYS,CDH23,NEDD9,RIMS2,STAC,SCN8A,EYA4,DLGAP2,OR4N2,CTNNA2,PARD3
GO:0007157	heterophilic cell-cell adhesion via plasma membrane cell adhesion molecules	0.0007680988840274947	NRXN1,IL1RAPL1,CRB1,HMCN1,NLGN1,CDH4,CXADR,TENM4,ALCAM,IGSF21,TENM3,GRID2,CDH2,TENM2,PTPRD
GO:0090066	regulation of anatomical structure	0.0008193494369769914	KCNMA1,SEMA4D,SLC12A8,SLC8A1,CD38,CDC42EP3,MTPN,CHRM3,SLIT2,AKT3,RAP1GDS1,KANK1,MACF1,EXT2,NOS1,ITGA1,PLS1,PAK3,FCHSD2,PRKCE,BBS2,DSCAM,RIN3,SLC12A1,NTN1,SPTB,MAP2,CDH4,TNR,VAV3,EPS8,PRR16,DISC1,FMN1,ARHGAP42,VSTM4,EPHA7,DOCK4,FHOD3,EXT1,EFNA5,TRPC5,SEMA6D,FER,CPS1,NRP1,SEMA3A,SEMA3E,FSTL4,ARHGAP28,MTOR,PRKG1,ASIC2,SEMA3D,DEPTOR,SEMA5A,BCL11A,DCC,SEMA3C

	size		
GO:0048519	negative regulation of biological process	0.0008636836456892352	CD44,SAMD4A,KCNMA1,ZHX3,APBB2,PTPRR,SCAF8,RTN1,PARN,SEMA4D,INIP,RPS6KA5,TAOK3,PRKCB,A2M,SLC8A1,ANKRD6,GPR55,DNAJC15,SIAH3,CD38,FBXO31,PIWIL3,BCL2,SAMSN1,CHFR,THADA,COL18A1,TOX3,THRB,RAG1,GRIK2,SNX25,KDM4B,IL6R,ZBTB20,SKAP2,BRD4,ZNF568,NDRG2,ASTN2,RCAN1,JAK2,BICD1,MIR17HG,ABCG8,OTUD7A,TPTE2,KALRN,USH2A,ZNF536,ZBTB7C,GRIN2A,NRXN1,IL1RAPL1,ADCYAP1R1,CTDP1,RPS6KA2,CACNA1C,BMF,CTNNA1,NTRK2,FOXN3,OCLN,NLK,MYT1L,SRGAP2B,IQCJ-SCHIP1,SMARCA4,RNF152,ZNRF3,MEOX2,TTC39B,GLIS3,NRG3,PTPRG,SLC24A4,GRIK3,PDE10A,NUMB,MTPN,SOX6,MECOM,RGS12,SHANK2,GABRA5,DLG5,CFDP1,ARHGAP24,SPRED2,SLIT2,ABCA5,ZNF675,CSNK2A1,AKT3,KCNE4,TRIM5,ALPK2,HECW1,RAP1GDS1,ERBB4,KANK1,ATRX,DMRT1,BID,MNAT1,RAP1A,TRIO,PTPRE,DUSP22,CHSY1,ABL2,PSMA1,NOS1,ABCG1,RGS3,RERG,HTR2C,CTIF,CNTN4,SAMD13,SLC40A1,SND1,ETS2,ITGA1,HIRA,CORO2B,RUNX1,KIR3DL2,ALDH1A2,PTGFR,EYA1,MORC3,SPOCK3,SPON1,ANK2,ZBTB16,SLIT3,GRIN2B,PHC2,ROBO1,TNKS,KLF12,NDFIP2,MDM1,OVOL2,ITPR2,BRINP1,MLLT3,LEMD3,GABPA,TMEM67,PRKCE,GLIS1,PSG9,PRKAA2,PACRG,BBS2,CDYL2,DSCAM,DGKI,RIN3,KDM4C,SLC1A1,GRM5,NTN1,LDB2,IGF1R,SPTB,NLGN1,JAM2,MAP2,CAMK1D,PTPRO,CD96,RBMS3,CLEC16A,CUX1,ANK3,MORC2,TBCD,TRPS1,ADAMTS5,STK38,AOAH,TNR,CELF4,DAPK1,INPP5A,APP,PUM1,KCTD1,RNLS,SPOCK1,PAK1,MITF,IGF2BP3,ADCK1,HCN1,PPP1R13B,FRY,CXADR,EPS8,LRFN5,GHR,DUX4,PRDM16,FRMD5,USP7,MEIS2,KIR2DL4,STARD13,AVEN,LTBP1,SLAMF1,BACE2,RARB,FOCAD,PBX1,MLIP,SORCS2,ARHGAP42,ZFPM2,ASAP1,PCBP3,EGF,FYN,EPHA7,FHIT,GRIA1,TRABD2B,STK3,CNOT7,MSR1,USP18,ESR1,ARHGAP12,KCNAB1,PDE4D,PRKACB,PDE3A,POR,L3MBTL4,DOCK4,FRMD4A,WWOX,EPHB1,CREM,FHOD3,EFEMP1,TNRC6B,AJAP1,HERC1,PARP15,FLT1,EFNA5,NXN,CDC14B,HDAC4,TRPC5,AMFR,ATP9A,FTO,SH3BP5,AKAP6,SORCS3,ATF2,RBBP8,GRID2,ZNF423,LRP2,SEMA6D,NTF3,FER,SUSD4,TP53I11,TPTE,GRM7,RAPGEF2,NAV3,MX1,GTF2I,ATXN1,PRKCQ,PRKCH,RHPN2,DLC1,ATP8A2,SLC24A3,UBE3A,GRIA4,APC,ZBTB25,TFF1,EPHB2,SCAF4,AGO3,MCTP1,RYR3,NBAS,PRTG,NBN,ADAMTS18,PACSIN2,PKP1,NRP1,CDH13,DACH1,TRDN,ZNF397,DAB1,ALK,LDLRAD4,SEMA3A,SEMA3E,MGAT5,MALRD1,MYEF2,DCLK1,NRIP1,CDH2,ARID5B,TENM2,SERPINB7,HDAC9,PIK3R3,MAP2K6,FSTL4,ARHGAP28,MTOR,RORB,FBLN1,BLM,FHL2,NEU3,NCAPG2,RGS7,CD2AP,USP25,SPRED1,ADAM10,SCAI,PTPRT,TRERF1,SLC24A2,GLI3,NTRK3,FBN1,ABHD17C,TBX15,BMPER,ATF7IP,HMGA2,NSUN2,PTPRK,TBC1D4,DNM3,APIP,ASXL3,DPF3,NPHP4,SACS,PPARA,PLXNA2,PTPRD,RORA,SHISA6,PLCB1,LOXL2,BPTF,PRKG1,ELAVL4,NLRC5,STXBP6,MXI1,MAGI2,NELL1,PLCL1,ABI1,TSHZ3,ASIC2,PRKD1,TNFAIP8,BCL2L1,HDAC2,ETS1,TNN,RYR2,SEMA3D,BANP,TGFA,PRLR,TBX20,FAT3,MTMR2,VCL,DEPTOR,BACH1,ROBO2,ZMYND11,RGS6,SRGAP3,WDR41,NEDD9,ENPP1,EYA4,L3MBTL3,SEMA5A,PRDM15,BCL11A,DCC,CTNNA2,PVT1,ETV6,VPS13C,PARD3,NRG1,CAST,FANK1,PRKCA,FMN2,SEMA3C,FANCB,ST18
GO:0035637	multicellular organismal signaling	0.0008792703222121363	SLC8A1,GRIK2,SCN11A,FGF12,CACNG2,CACNA1C,NTRK2,CACNB2,KCNE4,ANK2,CORIN,CNTNAP2,ANK3,TNR,CACNA2D1,HCN1,CHRM5,CXADR,KCND3,PDE4D,ABCC9,KCND2,TRDN,MTOR,CTNNA3,CACNG3,RYR2,SCN8A
GO:0006936	muscle contraction	0.0009312059739069467	KCNMA1,APBB2,SLC8A1,CD38,FGF12,CACNA1C,CACNB2,CHRM3,CALD1,DTNA,KCNE4,RAP1GDS1,MYOM1,NOS1,P2RX6,ANK2,SMPX,ARHGEF11,BBS2,MYOM2,NOS1AP,PLCE1,CACNA2D1,UTRN,MYH13,ARHGAP42,KCND3,PDE4D,DOCK4,ABCC9,HDAC4,SGCD,SSPN,ATP8A2,SNTB1,TRDN,ASB3,MAP2K6,MTOR,CTNNA3,PPP1R12B,PRKG1,RYR2,TBX20,STAC
GO:00	regul	0.000938588	KCNMA1,SEMA4D,SLC12A8,MTPN,AKT3,MACF1,DSCAM,SLC12A1,N

08361	ation of cell size	3404210717	TN1,MAP2,CDH4,TNR,VAV3,PRR16,DISC1,EPHA7,EFNA5,TRPC5,SEMA6D,NRP1,SEMA3A,SEMA3E,FSTL4,MTOR,SEMA3D,DEPTOR,SEMA5A,BCL11A,DCC,SEMA3C
GO:0071840	cellular component organization or biogenesis	0.0009479119870233036	CD44,KCNMA1,C10ORF90,APBB2,SCAF8,PARN,SEMA4D,SLC12A8,ZFYVE1,TEAD1,NFIA,RPS6KA5,TAOK3,PRKCB,TRPM6,KCNC1,DNAJC15,SIAH3,CD38,FBXO31,TRAPPC9,BCL2,MMP16,CHFR,COL18A1,CDC42BPA,RAG1,SAMM50,CDC42EP3,UNC13B,NCAM1,KDM4B,CDH8,ATP10A,SORBS2,SKAP2,BRD4,GRIP1,TLK1,TANC2,KIF4A,CSMD3,NTN4,JAK2,TM9SF4,BICD1,ABCG8,KALRN,NEGR1,CACNG2,BTBD9,EPB41L3,LAMA1,PARVB,CDH11,CDS2,MAST4,NRXN1,ARID1B,IL1RAPL1,WDPCP,MAGI1,LAMA3,ADAMTS17,CTDP1,ADAMTS6,PRELID2,GOLGA8J,RPS6KA2,SMOC2,ZDHHC17,CCSER2,BMF,EXOC4,PNPLA3,ANO4,BBS9,CTNNA1,MYO9A,NTRK2,OCLN,THSD7A,AFAP1,NCAM2,TIAM2,IQCJ-SCHIP1,LRRC49,SMARCA4,CNTN1,NRG3,PTPRG,ELMO1,CABIN1,SYNE1,GAS2,CACNB2,NUMB,MED15,MTPN,ESYT2,MECOM,SYBU,PDE4DIP,TRPM3,DNAH8,SHANK2,PTGFRN,KCTD8,CHCHD6,UNC5D,NREP,DLG5,CFDP1,PGM5,SMARCAD1,CALD1,ARHGAP24,IGHV1OR15-9,SLIT2,PITPNC1,MYLK3,ABCA5,ROR1,ADAMTSL1,CSNK2A1,AKT3,CRB1,KMT2C,KCNS3,ABCA13,HECW1,RAP1GDS1,ERBB4,KANK1,GPHN,ATRX,DMRT1,BID,MACF1,MNAT1,RAP1A,TRIO,RAD51B,TRMT61B,DUSP22,URB1,ABL2,ACACA,ABCG1,SPAG16,EML1,RERG,CCDC141,CNTN4,TBC1D5,PIK3C3,SNAP25-AS1,CHD6,HMCN1,FGD4,GOLGA6D,ITGA1,HIRA,CORO2B,ITGA8,RUNX1,ALDH1A2,MAPK9,IGHV1OR21-1,FAM171A1,CDH17,PEAK1,EYA1,ADAMTS19,CDH18,HSF2BP,FRMD6,PLS1,ANK2,SLC1A2,SLIT3,GRIN2B,ZNF518A,ROBO1,ANKRD30BL,EGFLAM,PAK3,DGKB,TNKS,GABRA2,MDM1,FCHSD2,MLLT3,TRAPPC10,LEMD3,CHODL,EHBP1,PSTPIP2,ITGB3BP,TMEM67,PRKCE,ARHGEF11,PRKAA2,BBS2,IL1RAPL2,CHCHD3,MYOM2,NUBPL,DSCAM,RIN3,ANKFN1,TRAPPC8,KDM4C,SDK1,SLC1A1,SLC12A1,GRM5,EPHA6,NTN1,LDB2,IGF1R,SPTB,WDR72,SNX30,NLGN1,DNAH9,SHROOM3,CNTNAP2,MAP2,CAMK1D,FLRT2,NOS1AP,PTPRO,MATN2,INSR,COBL,MDN1,CLEC16A,MTRF1,CRTAC1,CUX1,ANK3,CDH12,MORC2,THSD4,TBCD,GPC6,RELN,ADAMTS5,ADAMTSL3,CDH4,TNR,CELF4,VAV3,VRK1,CNTN6,APP,FBLN5,CCDC88A,KCTD1,SPOCK1,HPSE2,PLCE1,TACC2,PAK1,ATP9B,MITF,ADCK1,HCN1,TOP3A,NSMCE2,FRY,CXADR,EPS8,LRFN5,UTRN,TENM4,CECR2,PRR16,RASGRF1,PRDM16,FRMD5,USP7,STARD13,SCP2,LRRC4C,ALCAM,PPP1R9A,PDZRN3,ADAMTS16,MICU1,SLAMF1,INO80D,CLSTN2,TTLL11,NEBL,WDR12,TCF4,FRYL,TIAM1,PHACTR1,SLC39A12,DISC1,FMN1,CHAF1A,SVEP1,VTI1A,ASAP1,FRMPD4,COL23A1,EGF,FYN,KCND3,PRMT8,IFT43,LRBA,EPHA7,MAP7,NSG2,TRABD2B,SPIDR,NAV2,STK3,CNOT7,COL19A1,MSR1,PSIP1,S100B,NET1,TOX,PCDH15,ESR1,ARHGAP12,SGCZ,CNTN5,ERC2,PRKACB,PDE3A,RIMS1,L3MBTL4,CNKSR2,EPHB1,CTTNBP2,FHOD3,ARMC2,IGSF21,MIPEP,SNAP29,GSG1L,HERC1,DOCK1,DIAPH3,TRPM7,EXT1,EFNA5,CDC14B,TLN2,C14ORF39,HDAC4,STK36,KLHL1,TRPC5,MRM1,ATP9A,PPFIA2,AKAP6,VPS37A,TENM3,LINGO2,MARK2,ATF2,PHACTR2,GRID2,ZNF423,LRP2,SEMA6D,NTF3,FER,TTC29,CELF2,NTNG1,DDX10,MAPRE2,ARFGAP3,RAD51AP1,TMEM108,GABRB3,GRM7,SLC39A8,RAPGEF2,NAV3,IMMP2L,PRKCQ,KIRREL3,GABRG2,NUDCD3,PRKCH,NRXN3,RHPN2,KRT25,DLC1,NSG1,KCND2,ATP8A2,UBE3A,MPRIP,APC,TTLL5,INO80,AUTS2,EPHB2,SCAF4,ERC1,PDLIM5,AGO3,C9,MCTP1,RNU1-51P,RYR3,PRTG,NBN,ADAMTS18,CTNND2,FRMD3,COL22A1,SETD2,PACSIN2,PKP1,DOCK2,SDCCAG8,NRP1,CDH13,RFC3,PHACTR3,RPGRIP1,TRDN,DAB1,RFTN1,ALK,EXOC6B,LDLRAD4,SEMA3A,SEMA3E,DCLK1,CDH2,TENM2,TANC1,VPS41,SYCP1,HDAC9,FSTL4,ARHGAP28,MTOR,STK38L,FBLN1,BLM,SH3KBP1,NEU3,NCAPG2,CD2AP,TTC39C,CLVS2,SIPA1L3,ADAM10,TSPAN33,KANSL1,LRFN2,FLNB,PAPPA2,SPECC1,GLI3,NTRK3,RXFP1,HYDIN,CHKA,RAB31,CTNNA3,VPS13D,ABHD17C,ANKRD31,ATF7IP,HMGA2,THSD7B,PTPRK,TBC1D4,DNM3,SYT1,APIP,SYNDIG1,DPF3,NPHP4,SACS,PPARA,P

			LXNA2,PTPRD,SHISA6,SCARA5,PLCB1,LOXL2,BPTF,PRKG1,ELAVL4,CDH9,STXBP6,MAGI2,STX12,ABI1,ASIC2,RALA,DOCK10,GNPTAB,TRPM1,PRKD1,ATP8A1,BCL2L1,MICAL3,HDAC2,ETS1,TNN,SEMA3D,BANP,TGFA,PRLR,TBX20,PTPRA,FAT3,MTMR2,VCL,DEPTOR,ATAT1,ROBO2,IFT81,ZMYND11,CDH23,AKAP13,NEDD9,ENPP1,UNC13C,RIMS2,RAB27A,EYA4,L3MBTL3,CLIP1,SEMA5A,BCL11A,DCC,CTNNA2,CEP44,PVT1,PARD3B,CHN1,ETV6,VPS13C,PARD3,NRG1,CAST,ATP10B,PRKCA,FMN2,SEMA3C,PCNT,OSBPL5,TOP1
GO:0098662	inorganic cation transmembrane transport	0.0009521051716197967	KCNMA1,SLC12A8,TRPM6,SLC8A1,KCNC1,BCL2,THADA,KCNQ5,SCN11A,KCNJ6,KCNK10,FGF12,CACNG2,GRIN2A,NIPA2,ADCYAP1R1,DPP6,ZDHHC17,KCNH1,CACNA1C,SLC24A4,CACNB2,TRPM3,SLC4A4,KCNE4,KCNS3,HECW1,NOS1,HTR2C,CACNA1E,SLC40A1,SLC9C1,ANK2,GRIN2B,ITPR2,CACNA2D3,PRKCE,KCNH8,SLC1A1,SLC12A1,KCNIP4,NOS1AP,CATSPER2,ANK3,DPP10,CACNA2D1,HCN1,UTRN,TSPAN13,MICU1,SLC39A12,FYN,KCND3,KCNN3,KCNAB1,PDE4D,ATP6V1E1,ABCC9,TRPM7,TRPC5,AKAP6,TUSC3,MICU2,SLC39A8,KCND2,SLC24A3,RYR3,TRDN,RGS7,SLC24A2,SCARA5,ASIC2,TRPM1,CACNG3,PRKD1,RYR2,KCNH5,TMEM163,SLC39A11,SLC5A1,STAC,SCN8A,KCNJ15
GO:0007156	homophilic cell adhesion via plasma membrane adhesion molecules	0.001287385134566112	IGSF11,CDH8,CDH11,CNTN4,HMCN1,CDH17,CDH18,ROBO1,DSCAM,SDK1,PCDH9,CDH12,CDH4,CNTN6,PCDH7,CLSTN2,PCDH15,PCDH11X,IGSF21,TENM3,KIRREL3,CDH13,CDH2,PTPRT,CDH9,FAT3,ROBO2,CDH23
GO:0060998	regulation of dendritic spine development	0.0012916836924163884	TANC2,KALRN,DLG5,PAK3,SDK1,NLGN1,RELN,DISC1,ASAP1,PPFIA2,UBE3A,EPHB2,FSTL4,DNM3,HDAC2
GO:0007626	locomotory behavior	0.0013151586064768937	APBA2,RCAN1,KALRN,NEGR1,FGF12,BTBD9,CNTN1,SHANK2,ASTN1,HTR2C,PRKCE,DSCAM,ANKFN1,SLC1A1,GRM5,RELN,TNR,APP,PUM1,EPS8,NAV2,PCDH15,GRM1,KLHL1,KCND2,UBE3A,DAB1,ALK,MTOR,ELAVL4,CDH23,NRG1
GO:0048585	negative regulation of response to stimulus	0.0013950934655214413	CD44,PTPRR,SEMA4D,TAOK3,PRKCB,A2M,ANKRD6,BCL2,SAMSN1,SNX25,BRD4,NDRG2,RCAN1,BICD1,OTUD7A,ZNF536,NRXN1,CTNNA1,NLK,SMARCA4,RNF152,ZNRF3,SLC24A4,PDE10A,MECOM,RGS12,SHANK2,DLG5,ARHGAP24,SPRED2,SLIT2,ZNF675,CSNK2A1,ALPK2,HECW1,KANK1,BID,PTPRE,DUSP22,ABL2,PSMA1,RGS3,ITGA1,EYA1,SLIT3,ROBO1,OVOL2,MLLT3,LEMD3,PRKAA2,BBS2,RIN3,GRM5,IGF1R,PTPRO,CD96,RBMS3,STK38,AOAH,TNR,CELF4,LRFN5,PRDM16,KIR2DL4,LTBP1,SLAMF1,MLIP,ARHGAP42,FYN,TRABD2B,STK3,CNOT7,USP18,ESR1,ARHGAP12,PDE4D,PRKACB,PDE3A,WWOX,AJAP1,NXN,AMFR,GRID2,LRP2,SEMA6D,FER,SUSD4,PRKCQ,DLC1,APC,EPHB2,MCTP1,ADAMTS18,NRP1,DAB1,LDLRAD4,SEMA3A,SEMA3E,CDH2,FSTL4,MTOR,FBLN1,FHL2,RGS7,CD2AP,USP25,SPRED1,SCAI,PTPRT,GLI3,FBN1,BMPER,HMGA2,NPHP4,PPARA,PTPRD,RORA,SHISA6,PRKG1,NLRC5,MAGI2,BCL2L1,HDAC2,TNN,SEMA3D,TBX20,MTMR2,DEPTOR,ROBO2,ZMYND11,RGS6,WDR41,E

			NPP1,EYA4,SEMA5A,PRDM15,CTNNA2,VPS13C,NRG1,SEMA3C,FANCB
GO:0099601	regulation of neurotransmitter receptor activity	0.0015090479249697459	DLGAP1,CACNG2,NRXN1,NLGN1,SHISA9,CNIH3,RELN,RASGRF2,DAPK1,APP,RASGRF1,GSG1L,EPHB2,SHISA6,CACNG3,DLGAP2
GO:0051965	positive regulation of synapse assembly	0.0015090479249697459	SEMA4D,NRXN1,IL1RAPL1,NTRK2,DLG5,NLGN1,FLRT2,CLSTN2,EPHB1,EFNA5,LINGO2,GRID2,EPHB2,SYNDIG1,PTPRD,ASIC2
GO:0003015	heart process	0.0016687339656148818	SLC8A1,THRB,JAK2,FGF12,RPS6KA2,CACNA1C,CACNB2,KCNE4,RAP1GDS1,EXT2,NOS1,ANK2,SLC1A1,CORIN,NOS1AP,RNLS,CACNA2D1,HCN1,CXADR,FYN,KCND3,SGCZ,PDE4D,ABCC9,EXT1,HDAC4,CELF2,SGCD,TRDN,ASB3,MAP2K6,MTOR,SGCG,CTNNA3,RYR2,AKAP13
GO:0016192	vesicle-mediated transport	0.0017746966838272607	SYN3,PRKCB,TRAPPC9,UNC13B,GRIP1,APBA2,TM9SF4,BICD1,LRP1B,KALRN,CACNG2,BTBD9,NRXN1,IL1RAPL1,AMPH,EXOC4,HEATR5A,ENTHD1,TMPRSS3,ELMO1,CACNB2,NUMB,ESYT2,IGHV1OR15-9,ABCA13,MACF1,RAP1A,ABL2,TBC1D5,PIK3C3,IGHV1OR21-1,ANK2,FCHSD2,TRAPPC10,EHBP1,PRKCE,CD163,BBS2,DGKI,RIN3,TRAPPC8,SLC1A1,IGF1R,NLGN1,MON2,CFTR,CAMK1D,INSR,CLEC16A,CUX1,ANK3,CNIH3,VAV3,APP,PAK1,ATP9B,CECR2,GHR,RIN2,USP7,SLAMF1,VTI1A,EGF,FYN,NSG2,GRIA1,MSR1,AP2B1,ARHGAP12,ERC2,RIMS1,SNAP29,GSG1L,DOCK1,ATP9A,VPS37A,CCDC91,LRP2,C2,NTF3,FER,ARFGAP3,TMEM108,NRXN3,RABGAP1L,NSG1,UBE3A,EPHB2,STON1-GTF2A1L,SYT16,ERC1,MCTP1,NBAS,PACSIN2,DOCK2,TRIM23,NRP1,CDH13,EXOC6B,DCLK1,KIF16B,CDH2,EVI5,VPS41,SH3KBP1,CADPS,NEU3,CD2AP,AP5M1,RAB31,RAPGEF4,TMPRSS2,TMPRSS15,SORCS1,TBC1D4,DNM3,SYT1,SYNDIG1,SCFD2,SCARA5,LOXL2,RASGRP1,STXBP6,DMBT1,MAGI2,STX12,RALA,CACNG3,PRKD1,BCL2L1,MICAL3,LDLRAD3,MTMR2,WDR41,ENPP1,UNC13C,RIMS2,RAB27A,VPS13C,NRG1,FMN2,OSBPL5
GO:0009987	cellular process	0.0017960208134453813	CD44,SAMD4A,KCNMA1,C10ORF90,PKNOX2,ZHX3,APBB2,PTPRR,SCAF8,CPXM2,RTN1,ERG,PARN,PDE1C,SEMA4D,INIP,SLC12A8,MED13L,ZFYVE1,EVC,TEAD1,NFIA,SYN3,RPS6KA5,DHRS11,TAOK3,GADL1,PRKCB,EIF4G3,A2M,TRPM6,CPNE4,SLC8A1,ANKRD6,KCNC1,GPR55,DNAJC15,SIAH3,CD38,ZNF257,FBXO31,PIWIL3,TRAPPC9,BCL2,MMP16,CAMTA1,SAMSN1,CHFR,THADA,COL18A1,TOX3,RSRC1,THRB,DNAH11,ZSCAN5C,CDC42BPA,SLC13A4,B3GALT5,RAG1,SAMM50,CDC42EP3,GRIK2,IGSF11,SNX25,DPYD,UNC13B,MAPK10,NCAM1,KDM4B,GNG12,IL6R,KCNQ5,CDH8,ZBTB20,HEPHL1,SCN11A,ATP10A,SORBS2,SKAP2,HS1BP3,GOT2,KCNJ6,CASP5,KCNK10,BRD4,ZDHHC11B,GPR158,ZNF568,NDRG2,TMEM241,GRIP1,APBA2,TTC3,TLK1,ASTN2,TANC2,KIF4A,CSMD3,DLGAP1,RCAN1,NTN4,JAK2,TM9SF4,BICD1,LRP1B,ZBTB8OS,ABCG8,OTUD7A,TPTE2,KALRN,SUMF1,USH2A,NEGR1,FGF12,CACNG2,BTBD9,NFAT5,FLI1,SLC44A5,MEGF11,TRHDE,ZNF536,EPB41L3,LAMA1,PARVB,CDH11,ZBTB7C,CDS2,GRIN2A,MAST4,NRXN1,WDR26,DTWD2,NELL2,ARID1B,IL1RAPL1,WDPCP,NIPA2,MAGI1,LAMA3,SLC14A2,ADAMTS

			17,GBP4,ADCYAP1R1,ST6GALNAC3,CTDP1,ADAMTS6,DPP6,PRELID2,GOLGA8J,GRID1,RPS6KA2,TDP1,SMOC2,ZDHHC17,KCNH1,HLCS,ACSS3,DCDC1,CACNA1C,CCSER2,AMPH,BMF,EXOC4,HEATR5A,WDR70,CKMT1B,PNPLA3,ANO4,BBS9,FAM83B,CTNNA1,MYO9A,NTRK2,FOXN3,WDFY3,OCLN,AK8,NLK,ITGBL1,THSD7A,ABCA6,NBEA,RASGEF1B,AFAP1,NCAM2,TIAM2,MYT1L,TMPRSS3,SRGAP2B,IQCJ-SCHIP1,LRRC49,SMARCA4,RNF152,CNTN1,ZNRF3,MEOX2,ENPEP,PLEKHB2,GLIS3,NRG3,PTPRG,MC2R,BTBD11,ELMO1,SLC24A4,CABIN1,SYNE1,FBXL17,PDZD2,DNAH10,GAS2,GRIK3,CACNB2,PDE10A,NUMB,STXBP4,MED15,MTPN,MT1HL1,ESYT2,SOX6,MECOM,SYBU,PDE4DIP,TRPM3,STK32B,VCAN,DNAH8,NHS,CNTNAP5,RGS12,SHANK2,RAPGEF5,UBE2E2,PTGFRN,KCTD8,CHCHD6,UNC5D,EVA1A,ZNF567,NREP,GABRA5,DOK5,AGMO,DLG5,CFDP1,PGM5,SMARCAD1,ZIM3,ASTN1,AIFM3,ATRNL1,CHRM3,CPE,CALD1,AIG1,ARHGAP24,SPRED2,IGHV1OR15-9,SLIT2,PITPNC1,MYLK3,CEP128,ABCA5,ROR1,GLP2R,SLC4A4,ADAMTSL1,KAZN,ZNF675,CSNK2A1,DTNA,AKT3,CRB1,PHKB,KMT2C,KCNE4,TRIM5,KCNS3,CYP4B1,PSD3,ALPK2,ABCA13,HECW1,RAP1GDS1,AFF3,LCE1F,ERBB4,KANK1,STT3A,GPHN,LPP,ATRX,DMRT1,CHST8,BID,MACF1,MNAT1,TAF4B,RAP1A,TRIO,SLC15A5,CTNNBL1,RAD51B,TRMT61B,PTPRE,MYO3B,DUSP22,CHSY1,MYOM1,PSG8,EXT2,URB1,ZSCAN30,OR4C46,ABL2,PSMA1,MAP3K5,NOS1,ARPP21,ACACA,ABCG1,RGS3,MAML2,SPAG16,EML1,RERG,HTR2C,CCDC141,NEK4,CACNA1E,CTIF,CNTN4,TBC1D5,MUC16,CSTF3,SAMD13,RNF17,SLC40A1,SLCO3A1,GABRR2,PIK3C3,SLC9C1,TRAF3,SND1,SNAP25-AS1,CHD6,HMCN1,FGD4,ETS2,GOLGA6D,ITGA1,TCF12,ZNF721,HIRA,CORO2B,POC5,ITGA8,GRIK4,RBM19,RUNX1,KIR3DL2,ALDH1A2,GABRG1,TSHZ2,MAPK9,ESRRG,PTGFR,IGHV1OR21-1,FAR2,FAM171A1,ZNF595,CDH17,SV2B,PEAK1,EYA1,ADAMTS19,MORC3,ANKS1B,CDH18,P2RX6,HSF2BP,AKAP10,SPOCK3,FRMD6,PLS1,SPON1,ANK2,PLA2G4A,SLC1A2,ZBTB16,ANO2,SUPT3H,SLIT3,GRIN2B,ZNF518A,PHC2,ROBO1,ZNF578,ANKRD30BL,EGFLAM,PAK3,DGKB,GARNL3,DPH6,EBF1,TNKS,KLF12,NDFIP2,AQR,GABRA2,MDM1,OVOL2,CSE1L,PRUNE2,FCHSD2,SGMS1,ITPR2,BRINP1,MLLT3,BCL2L13,IGSF5,TRAPPC10,LEMD3,KHDRBS2,CPQ,RNF138,CHODL,EHBP1,GABPA,PRICKLE2,PSTPIP2,ITGB3BP,CACNA2D3,DGKK,TMEM67,PRKCE,CNTN3,MGAM,GLIS1,PSG9,ARHGEF11,PRKAA2,PACRG,BBS2,IL1RAPL2,CHCHD3,EDIL3,CDYL2,PTPRN2,MYOM2,MYO3A,USP31,UBE2R2,HIVEP2,KCNH8,GRIK1,NUBPL,SOX5,KIF6,DSCAM,DGKI,RIN3,ANKFN1,NME7,DNMBP,EFHB,TRAPPC8,KDM4C,ACSM2A,SDK1,SLC1A1,SLC12A1,GRM5,EPHA6,NTN1,CA10,ARAP2,NR5A2,LDB2,IGF1R,FAR1,SPTB,WDR72,SNX30,NLGN1,DNAH9,SHISA9,OR9Q1,SHROOM3,JAM2,SNRPN,ALX4,CORIN,MSI2,MON2,CNTNAP2,MAP2,KCNIP4,CFTR,CAMK1D,FLRT2,MLLT10,NOS1AP,PTPRO,ZDHHC14,MSRA,PCDH9,CD96,RBMS3,MATN2,NHSL1,INSR,COBL,MDN1,CTNNAL1,CLEC16A,PHF20L1,ME3,ITGA9,MTRF1,CATSPER2,CRTAC1,HS6ST3,EGLN3,CUX1,ANK3,CDH12,MORC2,SV2C,GMDS,CNIH3,DOCK3,THSD4,TBCD,GPC6,RELN,RASGRF2,TRPS1,HS3ST4,MFSD9,ADAMTS5,STK38,ADAMTSL3,SNHG14,AOAH,FBXO32,MYO18B,CDH4,TNR,ADCY9,DPP10,OCA2,CELF4,CDKAL1,DAPK1,VAV3,INPP5A,ZNF600,VRK1,ZNF678,CNTN6,CLIC6,ACSM2B,ZNF420,TTC7B,APP,FBLN5,PUM1,CCDC88A,ARNT2,KCTD1,SPOCK1,HPSE2,PLCE1,TACC2,ADAM12,PAK1,ATP9B,GNAL,MITF,IGF2BP3,CACNA2D1,ADCK1,HCN1,PPP1R13B,TOP3A,CHRM5,NSMCE2,ZNF208,FRY,CXADR,EPS8,LRFN5,UTRN,GPC5,TENM4,CECR2,PRR16,TSPAN13,GHR,DUX4,RIPK4,RASGRF1,RIN2,PRDM16,FRMD5,RNF217,USP7,RBFOX1,MEIS2,KIR2DL4,STARD13,PCDH7,SCP2,KL,LRRC4C,ALCAM,PPP1R9A,PDZRN3,AVEN,TMEM117,ADAMTS16,TASP1,MICU1,ZZEF1,LTBP1,SLAMF1,RGL1,BACE2,INO80D,SLC25A21,CLSTN2,MEG8,TTLL11,NEBL,RARB,DIDO1,MYH13,WDR12,TCF4,FRYL,TIAM1,PBX1,FAM126B,PHACTR1,MLIP,SORCS2,PRIM2,SLC39A12,LIPI,DISC1,OSBPL10,FMN1,RALGPS1,ARHGAP42,CHAF1A,ZFPM2,PIEZO2,SLC35F1,VSTM4,SVEP1,NTM,VTI1A,ASAP1,PCBP3,FRMPD4,COL23A1,EDAR,EGF,PDGFD,FYN,FAM3B,KCND3,RIMBP2,PRMT8,IFT43,XRCC4,LRBA,EPHA7,MAP7,FHIT,NSG2,GRIA1,ZN

			F627,TRABD2B,SPIDR,NAV2,STK3,ANO10,CNOT7,COL19A1,MSR1,PSIP1,AP2B1,USP18,S100B,NET1,TOX,PCDH15,ESR1,ARHGAP12,GABRG3,KCNN3,SGCZ,SEL1L2,PLCXD3,KCNAB1,GRM1,PDE4D,CNTN5,ERC2,PRKACB,GNG2,PDE3A,PCDH11X,RIMS1,POR,L3MBTL4,DOCK4,ATP6V1E1,FRMD4A,MCTP2,CERS3,PIGK,WWOX,PCSK2,HUNK,CNKSR2,FUT8,EPHB1,SSBP2,CREM,LSAMP,CTTNBP2,FHOD3,EFEMP1,ARMC2,TNRC6B,PIGB,AJAP1,IGSF21,MIPEP,ABCC9,SNAP29,GSG1L,HERC1,DOCK1,DIAPH3,PARP15,FAM126A,TRPM7,FLT1,EXT1,EFNA5,NXN,CDC14B,ABCA10,TLN2,C14ORF39,HDAC4,ZNF717,STK36,KLHL1,TRPC5,AMFR,PLCB4,MRM1,ATP9A,FTO,PPFIA2,SH3BP5,AKAP6,ACSBG1,SORCS3,VPS37A,POU6F2,TENM3,LINGO2,OPCML,MARK2,ATF2,TUSC3,PHACTR2,ZNF880,RBBP8,CCDC91,GRID2,ZNF423,LRP2,SEMA6D,ZNF573,C2,RALGPS2,NTF3,FER,SNRK,GLDC,TTC29,CAMK4,GALNT14,CELF2,TP53I11,PDXDC1,NTNG1,DDX10,FBXL7,MAPRE2,ARFGAP3,MICU2,ISX,RAD51AP1,SGCD,TMEM108,RIC8B,GABRB3,TPTE,GRM7,SLC39A8,RAPGEF2,NAV3,MX1,PPA2,IMMP2L,ZNF615,GTF2I,DNAH3,ATXN1,PRKCQ,SSPN,KIRREL3,GABRG2,NUDCD3,CPS1,PRKCH,NRXN3,RHPN2,RABGAP1L,KRT25,DLC1,PNPLA7,NSG1,GABBR2,KCND2,ATP8A2,SLC24A3,UBE3A,ORC4,MPRIP,GRIA4,IDE,CERS6,TPH2,APC,ZBTB25,MACROD2,TTLL5,INO80,TMTC1,MOCS2,AUTS2,TFF1,EPHB2,STON1-GTF2A1L,SCAF4,SYT16,ADARB2,ERC1,ZNF850,PDLIM5,XYLT1,AGO3,C9,TMTC2,MCTP1,RNU1-51P,MOB3B,RYR3,NBAS,PRTG,NBN,ADAMTS18,RGMB,CTNND2,FRMD3,COL22A1,SETD2,PACSIN2,PKP1,DOCK2,NUP214,TRIM23,SDCCAG8,FLVCR1,NRP1,CDH13,MDGA2,RFC3,PHACTR3,ZNF879,RPGRIP1,DACH1,TRDN,SLC2A13,ZNF397,DAB1,RFTN1,SNTG1,ALK,EXOC6B,EVC2,LDLRAD4,SEMA3A,SEMA3E,DNAH6,MGAT5,ATP13A3,CADM2,MALRD1,MYEF2,DCLK1,MAGI3,FAM135B,KIF16B,NRIP1,CDH2,ARID5B,SIPA1L2,CCNG2,RCAN2,LRRC69,TENM2,TANC1,SERPINB7,EVI5,VPS41,SYCP1,ZNF407,ASB3,HDAC9,ELOVL7,PIK3R3,MAP2K6,FSTL4,ARHGAP28,MTOR,STK38L,KSR1,RALGAPA2,RORB,GABRB1,FBLN1,ST8SIA1,BLM,SH3KBP1,FHL2,PSMB2,CADPS,NEU3,NCAPG2,RGS7,KYNU,STK32A,CD2AP,ZFP30,TTC39C,CLVS2,USP25,SLC44A1,SPRED1,AP5M1,SIPA1L3,ADAM10,GALC,MRPS22,DRAM1,TSPAN33,PPP2R2C,KANSL1,LRFN2,FLNB,WDFY4,SCAI,PAPPA2,ABCB5,SPECC1,DPY19L2,MTMR10,PTPRT,TRIM9,TRERF1,SLC24A2,GLI3,NTRK3,RXFP1,FBN1,HYDIN,CHKA,RAB31,CTNNA3,VPS13D,ABHD17C,ZNF292,TBX15,RAPGEF4,BMPER,ANKRD31,ZNF521,PDE1A,ATF7IP,HMGA2,MX2,CREB5,THSD7B,NSUN2,DEFA3,PTPRK,SORCS1,TBC1D4,DNM3,SYT1,APIP,SYNDIG1,ASXL3,DPF3,NPHP4,DOCK9,DLG2,PPP1R12B,SACS,PPARA,PLXNA2,SCFD2,PTPRD,RORA,SHISA6,SCARA5,PLCB1,LOXL2,BPTF,PRKG1,RASGRP1,ELAVL4,CDH9,NLRC5,STXBP6,CYP4Z1,DMBT1,MXI1,TTC28,MAGI2,NELL1,STX12,PLCL1,ABI1,GALNTL6,PXDNL,TSHZ3,ASIC2,RALA,DOCK10,GNPTAB,TRPM1,CACNG3,CNTNAP3,FNDC3A,NECAB1,PRKD1,ATP8A1,TNFAIP8,BCL2L1,MICAL3,ADK,HDAC2,RANBP17,ETS1,MRPS27,TNN,ST6GAL2,RYR2,SEMA3D,BANP,TGFA,PRLR,TBX20,PTPRA,FAT3,OR11G2,MTMR2,KCNH5,TMEM163,ATF6,IPO11,IL16,VCL,DEPTOR,BACH1,ATAT1,ROBO2,EWSR1,IFT81,OSCP1,ZMYND11,CDH23,RGS6,SRGAP3,AKAP13,WDR41,NEDD9,MYRIP,SLC39A11,ENPP1,UNC13C,PCP4,RIMS2,SLC5A1,STAC,SCN8A,RAB27A,EYA4,RALGAPA1,L3MBTL3,DLGAP2,POMT2,HIVEP3,CLIP1,SEMA5A,CABLES1,PRDM15,OR4N2,BCL11A,AGAP1,FREM1,DCC,ZNF112,CTNNA2,ATE1,CEP44,PVT1,PARD3B,CHN1,ETV6,VPS13C,KCNJ15,PARD3,NRG1,NPL,CAST,FANK1,ZNF845,SLC25A48,ATP10B,SLC35F4,NPAS3,PRKCA,FMN2,SEMA3C,FANCB,DPY19L1,CSF2RB,PCNT,BCKDHB,PAH,ST18,FRMD4B,OSBPL5,TOP1
GO:0018108	peptidyl-tyrosine phosphorylation	0.0018051860288991856	CD44,SEMA4D,SAMSN1,IL6R,JAK2,NTRK2,CNTN1,ROR1,ERBB4,DUSP22,ABL2,PEAK1,PRKCE,GRM5,EPHA6,IGF1R,INSR,DOCK3,RELN,APP,GHR,EGF,PDGFD,FYN,EPHA7,CNOT7,EPHB1,EFEMP1,FLT1,EFNA5,SH3BP5,NTF3,FER,EPHB2,NRP1,ALK,MAP2K6,MTOR,NCAPG2,NTRK3,CHKA,ABI1,HDAC2,TGFA,PRLR,NEDD9,NRG1

GO:1902284	neuron projection extension involved in neuron projection guidance	0.0018786473160058205	SEMA4D,SLIT2,SLIT3,DSCAM,ALCAM,SEMA6D,NRP1,SEMA3A,SEMA3E,SEMA3D,SEMA5A,SEMA3C
GO:0048846	axon extension involved in axon guidance	0.0018786473160058205	SEMA4D,SLIT2,SLIT3,DSCAM,ALCAM,SEMA6D,NRP1,SEMA3A,SEMA3E,SEMA3D,SEMA5A,SEMA3C
GO:0048675	axon extension	0.0019521746025241106	SEMA4D,SLIT2,MACF1,SLIT3,DSCAM,NTN1,MAP2,CDH4,TNR,ALCAM,DISC1,TRPC5,SEMA6D,AUTS2,NRP1,SEMA3A,SEMA3E,DCLK1,SEMA3D,VCL,SEMA5A,BCL11A,SEMA3C
GO:0018212	peptidyl-tyrosine modification	0.0021035540860819637	CD44,SEMA4D,SAMSN1,IL6R,JAK2,NTRK2,CNTN1,ROR1,ERBB4,DUSP22,ABL2,PEAK1,PRKCE,GRM5,EPHA6,IGF1R,INSR,DOCK3,RELN,APP,GHR,EGF,PDGFD,FYN,EPHA7,CNOT7,EPHB1,EFEMP1,FLT1,EFNA5,SH3BP5,NTF3,FER,EPHB2,NRP1,ALK,MAP2K6,MTOR,NCAPG2,NTRK3,CHKA,ABI1,HDAC2,TGFA,PRLR,NEDD9,NRG1
GO:0097120	receptor localization to synapse	0.0021173751785926597	GRIP1,CACNG2,NRXN1,GPHN,RAP1A,ANKS1B,NLGN1,GPC6,RELN,NSG1,ADAM10,DLG2,SHISA6,CACNG3,CEP112
GO:0048646	anatomical structure formation involved in morphogenesis	0.0021552381345268045	PRKCB,COL18A1,MEGF11,EPB41L3,NRXN1,LAMA3,SMOC2,KCNH1,EXOC4,THSD7A,CNTN1,MEOX2,ENPEP,NRG3,SLC24A4,MTPN,PTGFRN,PGM5,CALD1,ARHGAP24,SLIT2,MYLK3,AKT3,CRB1,EXT2,NOS1,SLC40A1,ETS2,ITGA8,RUNX1,ALDH1A2,EYA1,ANK2,ROBO1,OVOL2,GABPA,MYOM2,DSCAM,KDM4C,SDK1,SLC1A1,WDR72,SHROOM3,JAM2,CFTR,COBL,RELN,ADAMTS5,VAV3,ADAM12,TENM4,CECR2,STARD13,NEBL,SLC39A12,FMN1,VSTM4,EDAR,EGF,STK3,PRKACB,EPHB1,FHOD3,HERC1,FLT1,EXT1,ATF2,GRID2,LRP2,GTF2I,NRXN3,DLC1,ATP8A2,EPHB2,COL22A1,SETD2,SDCCAG8,NRP1,CDH13,SEMA3E,KIF16B,TANC1,HDAC9,PIK3R3,FHL2,SPRED1,GLI3,BMPER,HMGA2,PPARA,PLXNA2,RORA,LOXL2,ABI1,RALA,PRKD1,HDAC2,ETS1,TNN,TGFA,TBX20,FAT3,MTMR2,ROBO2,AKAP13,SEMA5A,PRKCA,SEMA3C
GO:1990138	neuron proje	0.0023743154085618026	SEMA4D,SLIT2,MACF1,SLIT3,DSCAM,NTN1,MAP2,CDH4,TNR,ALCAM,DISC1,RIMS1,TRPC5,SEMA6D,TMEM108,AUTS2,NRP1,SEMA3A,SEMA3E,DCLK1,SYT1,TNN,SEMA3D,VCL,RIMS2,SEMA5A,BCL11A,SEMA3C

	ction extension		
GO:0061387	regulation of extent of cell growth	0.003074144639618054	SEMA4D,MACF1,DSCAM,NTN1,MAP2,CDH4,TNR,DISC1,EPHA7,EFNA5,TRPC5,SEMA6D,NRP1,SEMA3A,SEMA3E,FSTL4,SEMA3D,SEMA5A,BCL11A,DCC,SEMA3C
GO:0051129	negative regulation of cellular component organization	0.0030784880826445183	SCAF8,SEMA4D,DNAJC15,CD38,NRXN1,RPS6KA2,IQCJ-SCHIP1,PTPRG,MTPN,ARHGAP24,SLIT2,KANK1,ATRX,DMRT1,MNAT1,DUSP22,CORO2B,GRIN2B,TNKS,MDM1,TMEM67,RIN3,NTN1,SPTB,NLGN1,MAP2,PTPRO,CLEC16A,TBCD,TNR,SPOCK1,ADCK1,EPS8,FYN,EPHA7,FHOD3,TRPC5,SEMA6D,RAPGEF2,NAV3,RHPN2,DLC1,UBE3A,APC,EPHB2,SCAF4,MCTP1,NBN,PACSIN2,NRP1,DAB1,LDLRAD4,SEMA3A,SEMA3E,FSTL4,ARHGAP28,NEU3,TBC1D4,DNM3,SACS,PRKD1,BCL2L1,HDAC2,SEMA3D,TBX20,FAT3,MTMR2,ROBO2,SEMA5A,BCL11A,DCC,CTNNA2,SEMA3C
GO:0031644	regulation of nervous system process	0.003110215284024939	IGSF11,UNC13B,SCN11A,DLGAP1,FGF12,GRIN2A,NRXN1,HTR2C,GRIN2B,NLGN1,SHISA9,JAM2,RELN,TNR,CELF4,APP,TENM4,GRM1,RIMS1,TMEM108,SHISA6,MTMR2,RIMS2,DLGAP2,PARD3
GO:0030900	forebrain development	0.003142491978402117	SLC8A1,KCNC1,TRAPPC9,NTRK2,NRG3,NUMB,SLIT2,ERBB4,ATRX,CCDC141,ALDH1A2,SLC1A2,ROBO1,BBS2,IGF1R,CNTNAP2,RELN,TNR,APP,TACC2,RARB,PHACTR1,DISC1,FYN,TOX,EXT1,LRP2,TMEM108,RAPGEF2,KIRREL3,DLC1,EPHB2,SETD2,NRP1,DAB1,ALK,SEMA3A,SEMA3E,DCLK1,CDH2,GLI3,PLCB1,PRKG1,ELAVL4,ATAT1,ROBO2,SEMA5A,NRG1
GO:0071417	cellular response to organonitrogen compound	0.0031716483837773155	PRKCB,SLC8A1,JAK2,ARID1B,PNPLA3,CTNNA1,NTRK2,STXBP4,CHRM3,SLIT2,GLP2R,RAP1GDS1,KANK1,RAP1A,PTPRE,HTR2C,SLC1A2,ITPR2,GABPA,SLC1A1,GRM5,IGF1R,CFTR,INSR,APP,GNAL,CACNA2D1,HCN1,CHRM5,GHR,KL,PDGFD,FYN,NSG2,SPIDR,PDE4D,GNG2,PDE3A,POR,AKAP6,ATF2,FER,GABRB3,RAPGEF2,PRKCQ,GABRG2,CPS1,NSG1,IDE,APC,EPHB2,RYR3,ALK,HDAC9,PIK3R3,MTOR,GABRB1,BLM,FBN1,RAB31,TBC1D4,PLCB1,BCL2L1,HDAC2,RYR2,PTPRA,ENPP1,BCL11A
GO:1905114	cell surface receptor signaling pathway	0.0032521953951728817	ANKRD6,GRIK2,IGSF11,UNC13B,NDRG2,GRIN2A,NRXN1,NLK,SMARCA4,ZNRF3,CPE,ROR1,CSNK2A1,ALPK2,HECW1,KANK1,MACF1,P2RX6,GRIN2B,TNKS,MLLT3,RNF138,PRICKLE2,PRKAA2,DGKI,NLGN1,PTPRO,RBMS3,GPC6,RELN,CELF4,APP,CCDC88A,MITF,GPC5,TIAM1,DISC1,EGF,TRABD2B,STK3,RIMS1,WWOX,EXT1,NXN,AMFR,MARK2,GRID2,ZNF423,TMEM108,APC,CTNND2,CDH2,GLI3,NPHP4,SHISA6,MAGI2,TNN,MTMR2,RIMS2,SEMA5A,PRDM15

	involved in cell-cell signaling		
GO:0051094	positive regulation of developmental process	0.0038095220734387677	ZHX3,SEMA4D,PRKCB,SLC8A1,FBXO31,BCL2,RAG1,IL6R,GRIP1,JAK2,KALRN,LAMA1,ZBTB7C,NRXN1,ARID1B,IL1RAPL1,SMOC2,NTRK2,TIAM2,SMARCA4,NUMB,SOX6,DLG5,SLIT2,MYLK3,AKT3,ERBB4,DMRT1,MACF1,RAP1A,MAP3K5,HTR2C,TCF12,RBM19,RUNX1,MAPK9,PLS1,ZBTB16,ROBO1,PAK3,OVOL2,BRINP1,CHODL,BBS2,SOX5,DSCAM,GRM5,NTN1,IGF1R,NLGN1,CFTR,FLRT2,INSR,COBL,CUX1,RELN,CDH4,ADAM12,TENM4,GHR,RIN2,KL,CLSTN2,TCF4,TIAM1,SLC39A12,DISC1,ZFPM2,EGF,STK3,MSR1,TOX,PDE3A,RIMS1,POR,EPHB1,DOCK1,FLT1,EFNA5,TRPC5,AKAP6,LINGO2,GRID2,LRP2,RAPGEF2,PRKCH,ATP8A2,EPHB2,NRP1,DAB1,ALK,MTOR,GLI3,BMPER,HMGA2,SYT1,SYNDIG1,DPF3,PLXNA2,PTPRD,PLCB1,LOXL2,RASGRP1,ELAVL4,NELL1,ASIC2,RALA,PRKD1,HDAC2,ETS1,TNN,TBX20,ROBO2,NEDD9,PCP4,RIMS2,SEMA5A,BCL11A,NRG1,PRKCA
GO:0007043	cell-cell junction assembly	0.004577407732796014	CDH8,EPB41L3,CDH11,CTNNA1,OCLN,DLG5,CDH18,ANK2,CNTNAP2,PTPRO,CDH12,TBCD,TLN2,FER,PRKCH,APC,EPHB2,CTNND2,PKP1,CDH2,NPHP4,CDH9,VCL,PARD3,PRKCA
GO:0060996	dendritic spine development	0.00462495984923291	TANC2,KALRN,DLG5,PAK3,SDK1,NLGN1,RELN,DISC1,ASAP1,EPHB1,PPFIA2,UBE3A,EPHB2,PDLIM5,CTNND2,FSTL4,DNM3,DOCK10,HDAC2
GO:0040008	regulation of growth	0.004794842303496823	SEMA4D,TEAD1,CD38,BCL2,IGSF11,EPB41L3,CTDP1,SMARCA4,NRG3,MTPN,SLIT2,CSNK2A1,ERBB4,MACF1,RERG,RUNX1,PLS1,SLIT3,ARHGEF11,BBS2,DSCAM,NTN1,MAP2,INSR,CDH4,TNR,APP,FBLN5,SPOCK1,PLCE1,CXADR,GHR,DISC1,ZFPM2,EPHA7,STK3,NET1,RIMS1,EFNA5,TRPC5,FTO,AKAP6,SEMA6D,PRKCQ,ATP8A2,INO80,FLVCR1,NRP1,RFTN1,SEMA3A,SEMA3E,FSTL4,MTOR,ADAM10,PAPPA2,HMGA2,SYT1,PPARA,PLCB1,BCL2L1,SEMA3D,TBX20,NEDD9,ENPP1,RIMS2,SEMA5A,BCL11A,DCC,NRG1,SEMA3C
GO:0007423	sensory organ development	0.004795384813977243	BCL2,THRB,USH2A,MEGF11,LAMA1,WDPCP,CACNA1C,NTRK2,SMARCA4,NHS,GABRA5,SPRED2,ROR1,CRB1,MYO3B,ITGA8,ALDH1A2,EYA1,PLS1,MDM1,MYO3A,DSCAM,SDK1,SLC1A1,NTN1,CELF4,MITF,HCN1,CECR2,MEIS2,RARB,PBX1,VSTM4,PCDH15,LRIG1,EPHB1,EFEMP1,FLT1,TENM3,ATP8A2,TTLL5,EPHB2,ADAMTS18,NRP1,RPGRIP1,DCLK1,RORB,TTC39C,SPRED1,SIPA1L3,ADAM10,ABCB5,GLI3,NTRK3,FBN1,BMPER,SCAPER,NPHP4,MYH15,HDAC2,FAT3,ATF6,CDH23,EYA4
GO:0031290	retinal ganglion cell axon guidance	0.005065093549011559	SLIT2,PTPRO,ALCAM,EPHA7,EPHB1,EFNA5,EPHB2,NRP1,ROBO2
GO:0007158	neuron cell-	0.0053058010384715365	ASTN2,NRXN1,NCAM2,ASTN1,CNTN4,NLGN1,TNR,NRXN3

	cell adhesion		
GO:0050773	regulation of dendrite development	0.0056568198875852705	SEMA4D,FBXO31,CSMD3,KALRN,IL1RAPL1,HECW1,PAK3,CAMK1D,COBL,CUX1,RELN,TRPC5,RAPGEF2,EPHB2,ALK,PTPRD,ELAVL4,FAT3,BCL11A,DCC
GO:0007214	gamma-aminobutyric acid signaling pathway	0.005886223800356885	GABRA5,GABRR2,GABRG1,GABRA2,GABRG3,GABRB3,GABRG2,GABBR2,GABRB1,PLCL1
GO:0010469	regulation of signaling receptor activity	0.0060109688166137334	DLGAP1,JAK2,BICD1,CACNG2,NRXN1,SLC24A4,NLGN1,SHISA9,CNIH3,RELN,RASGRF2,DAPK1,APP,RASGRF1,EGF,PDE4D,GSG1L,EPHB2,NRP1,NCAPG2,PPARA,SHISA6,CACNG3,HDAC2,TGFA,DLGAP2
GO:0044089	positive regulation of cellular component biogenesis	0.006185105945627232	SEMA4D,CDC42EP3,UNC13B,NRXN1,IL1RAPL1,BMF,NTRK2,OCLN,PDE4DIP,DLG5,BID,MAPK9,CDH17,FCHSD2,PRKCE,LDB2,SNX30,NLGN1,CNTNAP2,FLRT2,COBL,MORC2,CCDC88A,PLCE1,PAK1,EPS8,CLSTN2,FMN1,ASAP1,TRABD2B,SPIDR,ESR1,EPHB1,EFNA5,HDAC4,LINGO2,GRID2,FER,NAV3,APC,AUTS2,EPHB2,NRP1,TENM2,MTOR,ATF7IP,DNM3,SYNDIG1,NPHP4,PTPRD,ASIC2,RALA,ATAT1,CLIP1,NRG1,PRKCA
GO:0010648	negative regulation of cell communication	0.006605001455828056	CD44,PTPRR,TAOK3,PRKCB,ANKRD6,CD38,BCL2,GRIK2,SNX25,BRD4,NDRG2,RCAN1,BICD1,OTUD7A,KALRN,ZNF536,CTNNA1,NLK,SMARCA4,RNF152,ZNRF3,SLC24A4,GRIK3,PDE10A,MECOM,RGS12,SHANK2,DLG5,ARHGAP24,SPRED2,SLIT2,ZNF675,CSNK2A1,ALPK2,HECW1,KANK1,BID,RAP1A,PTPRE,DUSP22,ABL2,RGS3,ITGA1,EYA1,SLIT3,ROBO1,OVOL2,MLLT3,LEMD3,PRKAA2,DGKI,GRM5,IGF1R,PTPRO,RBMS3,STK38,TNR,CELF4,HCN1,PRDM16,LTBP1,SLAMF1,SORCS2,ARHGAP42,GRIA1,TRABD2B,STK3,CNOT7,USP18,ESR1,ARHGAP12,PDE4D,PRKACB,PDE3A,WWOX,NXN,AMFR,SORCS3,GRID2,LRP2,PRKCQ,DLC1,APC,EPHB2,NRP1,DAB1,LDLRAD4,CDH2,FSTL4,MTOR,FBLN1,FHL2,RGS7,CD2AP,SPRED1,SCAI,PTPRT,SLC24A2,GLI3,FBN1,BMPER,NPHP4,PPARA,PTPRD,RORA,SHISA6,NLRC5,MAGI2,BCL2L1,HDAC2,TNN,TBX20,MTMR2,DEPTOR,ZMYND11,RGS6,ENPP1,EYA4,PRDM15,NRG1
GO:0048639	positive regul	0.006768453578365107	SEMA4D,BCL2,ERBB4,MACF1,PLS1,BBS2,DSCAM,NTN1,INSR,CDH4,GHR,DISC1,ZFPM2,RIMS1,EFNA5,TRPC5,AKAP6,ATP8A2,NRP1,SYT1,PLCB1,TBX20,RIMS2,SEMA5A,BCL11A,NRG1

	ation of developmental growth		
GO:0001558	regulation of cell growth	0.006928586277388833	SEMA4D,TEAD1,CD38,BCL2,EPB41L3,CTDP1,SMARCA4,NRG3,MTPN,SLIT2,CSNK2A1,MACF1,RERG,SLIT3,ARHGEF11,DSCAM,NTN1,MAP2,CDH4,TNR,FBLN5,SPOCK1,PLCE1,DISC1,EPHA7,NET1,RIMS1,EFNA5,TRPC5,AKAP6,SEMA6D,PRKCQ,INO80,NRP1,SEMA3A,SEMA3E,FSTL4,MTOR,ADAM10,PAPPA2,SYT1,PPARA,SEMA3D,ENPP1,RIMS2,SEMA5A,BCL11A,DCC,NRG1,SEMA3C
GO:0014706	striated muscle tissue development	0.007087327900535896	SLC8A1,SORBS2,CTDP1,MTPN,SOX6,PGM5,MYLK3,ALPK2,ERBB4,RUNX1,ALDH1A2,EYA1,MYO18B,CXADR,TENM4,NEBL,RARB,ZFPM2,SGCZ,FHOD3,AKAP6,LRP2,SGCD,PDLIM5,MTOR,FHL2,SGCG,PPARA,RYR2,TBX20,AKAP13,NRG1,SEMA3C
GO:0051336	regulation of hydrolase activity	0.007243706156187095	CD44,SEMA4D,A2M,GPR55,RAG1,RCAN1,JAK2,BICD1,KALRN,GRIN2A,ADCYAP1R1,MYO9A,NTRK2,RASGEF1B,TIAM2,TBC1D22A,RAPGEF5,ARHGAP24,CSNK2A1,RAP1GDS1,BID,RAP1A,ABL2,MAP3K5,NOS1,TBC1D5,FGD4,ITGA1,SPOCK3,GRIN2B,ROBO1,GARNL3,BCL2L13,DGKI,SLC1A1,ARAP2,EGLN3,RASGRF2,DAPK1,VAV3,APP,SPOCK1,RASGRF1,RGL1,TIAM1,ASAP2,RALGPS1,ARHGAP42,ASAP1,FYN,EPHA7,NET1,ESR1,ARHGAP12,PDE3A,POR,FLT1,EFNA5,RALGPS2,NTF3,MAPRE2,RAPGEF2,RABGAP1L,DLC1,MGAT5,SIPA1L2,SERPINB7,EVI5,HDAC9,MTOR,RALGAPA2,FBLN1,SGSM1,RGS7,SIPA1L3,NTRK3,RAPGEF4,TBC1D4,DOCK9,PLXNA2,PRKG1,RASGRP1,MAGI2,DOCK10,PRKD1,TNFAIP8,RGS6,WDR41,NEDD9,RALGAPA1,CHN1,CAST,TBC1D9,ST18
GO:0090630	activation of GTPase activity	0.007595968484409961	TIAM2,TBC1D22A,ARHGAP24,TBC1D5,GARNL3,RASGRF1,TIAM1,ARHGAP42,NTF3,RABGAP1L,SIPA1L2,EVI5,RALGAPA2,SGSM1,SIPA1L3,NTRK3,TBC1D4,RASGRP1,NEDD9,RALGAPA1,TBC1D9
GO:0045216	cell-cell junction organization	0.0076943049692955266	CDH8,EPB41L3,CDH11,CTNNA1,OCLN,NUMB,DLG5,CDH18,ANK2,CNTNAP2,PTPRO,CDH12,TBCD,CXADR,SVEP1,EXT1,TLN2,FER,PRKCH,APC,EPHB2,CTNND2,PKP1,CDH2,ADAM10,NPHP4,CDH9,VCL,PARD3,PRKCA
GO:0023057	negative regulation of signaling	0.007905638442819884	CD44,PTPRR,TAOK3,PRKCB,ANKRD6,CD38,BCL2,GRIK2,SNX25,BRD4,NDRG2,RCAN1,BICD1,OTUD7A,KALRN,ZNF536,CTNNA1,NLK,SMARCA4,RNF152,ZNRF3,SLC24A4,GRIK3,PDE10A,MECOM,RGS12,SHANK2,DLG5,ARHGAP24,SPRED2,SLIT2,ZNF675,CSNK2A1,ALPK2,HECW1,KANK1,BID,RAP1A,PTPRE,DUSP22,ABL2,RGS3,ITGA1,EYA1,SLIT3,ROBO1,OVOL2,MLLT3,LEMD3,PRKAA2,DGKI,GRM5,IGF1R,PTPRO,RBMS3,STK38,TNR,CELF4,HCN1,PRDM16,LTBP1,SLAMF1,SORCS2,ARHGAP42,GRIA1,TRABD2B,STK3,CNOT7,USP18,ESR1,ARHGAP12,PDE4D,PRKACB,PDE3A,WWOX,NXN,AMFR,SORCS3,GRID2,LRP2,PRKCQ,DLC1,APC,EPHB2,NRP1,DAB1,LDLRAD4,CDH2,FSTL4,MTOR,FBLN1,FHL2,RGS7,CD2AP,SPRED1,SCAI,PTPRT,SLC24A2,GLI3,FBN1,BMPER,NPHP4,PPARA,PTPRD,RORA,SHISA

			6,NLRC5,MAGI2,BCL2L1,HDAC2,TNN,TBX20,MTMR2,DEPTOR,ZMYND11,RGS6,ENPP1,EYA4,PRDM15,NRG1
GO:1901699	cellular response to nitrogen compound	0.008130749789162919	PRKCB,SLC8A1,JAK2,ARID1B,PNPLA3,CTNNA1,NTRK2,STXBP4,CHRM3,SLIT2,GLP2R,RAP1GDS1,KANK1,ATRX,RAP1A,PTPRE,MAP3K5,HTR2C,SLC1A2,ITPR2,GABPA,SLC1A1,GRM5,IGF1R,CFTR,INSR,APP,GNAL,CACNA2D1,HCN1,CHRM5,GHR,KL,PDGFD,FYN,NSG2,GRIA1,SPIDR,PDE4D,GNG2,PDE3A,POR,AKAP6,ATF2,FER,GABRB3,RAPGEF2,PRKCQ,GABRG2,CPS1,NSG1,IDE,APC,EPHB2,RYR3,ALK,HDAC9,PIK3R3,MTOR,GABRB1,BLM,FBN1,RAB31,TBC1D4,PLCB1,BCL2L1,HDAC2,RYR2,PTPRA,ENPP1,BCL11A
GO:0071805	potassium ion transmembrane transport	0.008904055858258325	KCNMA1,SLC12A8,KCNC1,KCNQ5,KCNJ6,KCNK10,DPP6,KCNH1,SLC24A4,KCNE4,KCNS3,SLC9C1,ANK2,KCNH8,SLC12A1,KCNIP4,NOS1AP,ANK3,DPP10,HCN1,KCND3,KCNN3,KCNAB1,ABCC9,AKAP6,KCND2,SLC24A3,RGS7,SLC24A2,KCNH5,KCNJ15
GO:1901890	positive regulation of cell junction assembly	0.00896039507637462	SEMA4D,NRXN1,IL1RAPL1,NTRK2,DLG5,NLGN1,CNTNAP2,FLRT2,CLSTN2,FMN1,EPHB1,EFNA5,LINGO2,GRID2,EPHB2,NRP1,SYNDIG1,NPHP4,PTPRD,ASIC2
GO:0010720	positive regulation of cell development	0.009674577755211168	SEMA4D,FBXO31,BCL2,KALRN,IL1RAPL1,NTRK2,TIAM2,NUMB,SLIT2,MACF1,ROBO1,PAK3,CHODL,DSCAM,GRM5,NTN1,CUX1,RELN,CDH4,TENM4,TIAM1,DISC1,PDE3A,DOCK1,EFNA5,TRPC5,LRP2,PRKCH,EPHB2,NRP1,MTOR,GLI3,PLXNA2,PTPRD,HDAC2,ROBO2,NEDD9,SEMA5A,BCL11A
GO:0048640	negative regulation of developmental growth	0.010400333793186617	SEMA4D,CTDP1,BBS2,NTN1,MAP2,TNR,CXADR,EPHA7,STK3,SEMA6D,NRP1,SEMA3A,SEMA3E,FSTL4,PPARA,SEMA3D,SEMA5A,BCL11A,DCC,SEMA3C
GO:0055013	cardiac muscle cell development	0.011950251641009935	SLC8A1,SORBS2,CTDP1,MYLK3,ALPK2,MYO18B,CXADR,NEBL,FHOD3,AKAP6,SGCD,PDLIM5,MTOR,FHL2,PPARA,AKAP13

GO:0046545	development of primary female sexual characteristics	0.012046668434437996	A2M,BCL2,ADCYAP1R1,CTNNA1,GAS2,SLIT2,SLIT3,INSR,ZFPM2,ESR1,ACSBG1,IMMP2L,UBE3A,DACH1,NRIP1,ARID5B,CSMD1,SCAPER,BCL2L1,ROBO2
GO:0099072	regulation of postsynaptic membrane neurotransmitter receptor levels	0.012287951770181004	GRIP1,CACNG2,NUMB,GPHN,RAP1A,GPC6,EPS8,AP2B1,GSG1L,NSG1,ADAM10,DNM3,SHISA6,CACNG3,NRG1
GO:0060047	heart contraction	0.012337570873167078	SLC8A1,THRB,JAK2,FGF12,RPS6KA2,CACNA1C,CACNB2,KCNE4,EXT2,NOS1,ANK2,SLC1A1,CORIN,NOS1AP,RNLS,CACNA2D1,HCN1,CXADR,KCND3,SGCZ,PDE4D,ABCC9,EXT1,HDAC4,CELF2,SGCD,TRDN,ASB3,MAP2K6,MTOR,SGCG,CTNNA3,RYR2
GO:0002009	morphogenesis of an epithelium	0.013161224004967062	CD44,ANKRD6,BCL2,ASTN2,NTN4,LAMA1,WDPCP,LAMA3,MYO9A,ZNRF3,DLG5,ARHGAP24,SLIT2,ROR1,ERBB4,ALDH1A2,EYA1,OVOL2,MLLT3,PRICKLE2,NTN1,SHROOM3,COBL,GPC6,PAK1,CECR2,RIPK4,STARD13,ADAMTS16,TIAM1,PBX1,FMN1,EGF,EPHA7,STK3,PCDH15,ESR1,ARHGAP12,PRKACB,GREB1L,AJAP1,EXT1,LRP2,KRT25,DLC1,SETD2,NRP1,SEMA3E,MTOR,CSMD1,GLI3,MAGI2,RALA,RYR2,TBX20,VCL,SEMA3C
GO:0051961	negative regulation of nervous system development	0.013518450148743198	SEMA4D,CTNNA1,BRINP1,NTN1,MAP2,TNR,EPHA7,TRPC5,SEMA6D,RAPGEF2,EPHB2,PRTG,NRP1,DAB1,SEMA3A,SEMA3E,FSTL4,NTRK3,SEMA3D,ROBO2,SEMA5A,BCL11A,DCC,SEMA3C
GO:0098815	modulation of excitatory posts	0.014611917251826863	IGSF11,GRIN2A,NRXN1,GRIN2B,NLGN1,RELN,CELF4,APP,RIMS1,TMEM108,MTMR2,RIMS2

	ynaptic potential		
GO:0032535	regulation of cellular component size	0.01515371281368811	KCNMA1,SEMA4D,SLC12A8,CDC42EP3,MTPN,SLIT2,AKT3,KANK1,MACF1,PLS1,PAK3,FCHSD2,PRKCE,DSCAM,RIN3,SLC12A1,NTN1,SPTB,MAP2,CDH4,TNR,VAV3,EPS8,PRR16,DISC1,FMN1,EPHA7,FHOD3,EFNA5,TRPC5,SEMA6D,FER,NRP1,SEMA3A,SEMA3E,FSTL4,ARHGAP28,MTOR,SEMA3D,DEPTOR,SEMA5A,BCL11A,DCC,SEMA3C
GO:0036211	protein modification process	0.015158728724417944	CD44,C10ORF90,PTPRR,ERG,SEMA4D,RPS6KA5,TAOK3,PRKCB,TRPM6,SLC8A1,SIAH3,FBXO31,BCL2,CAMTA1,SAMSN1,CHFR,RSRC1,CDC42BPA,B3GALT5,RAG1,SNX25,MAPK10,KDM4B,IL6R,RNF182,BRD4,ZDHHC11B,TTC3,TLK1,RCAN1,JAK2,OTUD7A,TPTE2,KALRN,SUMF1,LAMA1,MAST4,NRXN1,AGBL1,ST6GALNAC3,CTDP1,RPS6KA2,ZDHHC17,HLCS,WDR70,NTRK2,OCLN,NLK,RNF152,CNTN1,ZNRF3,NRG3,PTPRG,FBXL17,MECOM,STK32B,UBE2E2,SMARCAD1,CHRM3,CPE,SPRED2,SLIT2,MYLK3,ROR1,ZNF675,CSNK2A1,AKT3,PHKB,KMT2C,TRIM5,ALPK2,HECW1,ERBB4,STT3A,GPHN,ATRX,MNAT1,TAF4B,RAP1A,TRIO,PTPRE,MYO3B,DUSP22,EXT2,ABL2,MAP3K5,NOS1,NEK4,SLCO3A1,PIK3C3,TRAF3,ITGA1,MAPK9,PEAK1,EYA1,MORC3,SPOCK3,ZBTB16,SUPT3H,ROBO1,EGFLAM,PAK3,DPH6,TNKS,NDFIP2,MLLT3,RNF138,PRKCE,PRKAA2,PTPRN2,MYO3A,USP31,UBE2R2,KDM4C,DCUN1D4,SLC1A1,GRM5,EPHA6,IGF1R,CAMK1D,NOS1AP,PTPRO,ZDHHC14,MSRA,INSR,PHF20L1,EGLN3,DOCK3,RELN,STK38,FBXO32,DAPK1,VRK1,APP,CCDC88A,PLCE1,PAK1,NSMCE2,FRY,GHR,RIPK4,PRDM16,RNF217,USP7,PDZRN3,ZZEF1,TTLL11,EGF,PDGFD,FYN,PRMT8,EPHA7,SENP8,TRABD2B,STK3,CNOT7,USP18,PDE4D,PRKACB,POR,PIGK,HUNK,FUT8,EPHB1,PARP8,EFEMP1,PIGB,HERC1,PARP15,TRPM7,FLT1,EXT1,EFNA5,NXN,CDC14B,HDAC4,STK36,TRPC5,AMFR,SH3BP5,MARK2,ATF2,TUSC3,NTF3,FER,SNRK,CAMK4,GALNT14,FBXL7,TPTE,SLC39A8,RAPGEF2,PRKCQ,PRKCH,DLC1,UBE3A,APC,MACROD2,TTLL5,TMTC1,AUTS2,EPHB2,ERC1,TMTC2,MOB3B,NBN,SETD2,TRIM23,NRP1,DAB1,ALK,LDLRAD4,MGAT5,DCLK1,CCNG2,ASB3,HDAC9,PIK3R3,MAP2K6,MTOR,STK38L,KSR1,FBLN1,ST8SIA1,BLM,NCAPG2,STK32A,USP25,SPRED1,ADAM10,PPP2R2C,KANSL1,TULP4,DPY19L2,PTPRT,TRIM9,TRERF1,NTRK3,CHKA,ABHD17C,BMPER,HMGA2,PTPRK,PTPRD,LOXL2,PRKG1,RASGRP1,MAGI2,ABI1,GALNTL6,PRKD1,HDAC2,ST6GAL2,TGFA,PRLR,PTPRA,MTMR2,DEPTOR,ATAT1,AKAP13,NEDD9,ENPP1,POMT2,BCL11A,ATE1,PARD3,NRG1,PRKCA,FANCB,DPY19L1,CSF2RB,TOP1
GO:0010243	response to organonitrogen compound	0.015423375655413013	PRKCB,SLC8A1,KCNC1,JAK2,GRIN2A,ARID1B,HLCS,PNPLA3,CTNNA1,NTRK2,SLC24A4,STXBP4,CHRM3,SLIT2,GLP2R,RAP1GDS1,KANK1,RAP1A,PTPRE,HTR2C,PIK3C3,P2RX6,SLC1A2,ITPR2,GABPA,TMEM67,PRKCE,SDK1,SLC1A1,GRM5,IGF1R,CFTR,INSR,APP,RNLS,GNAL,CACNA2D1,HCN1,CHRM5,GHR,KL,PDGFD,FYN,NSG2,SPIDR,SEL1L2,PDE4D,GNG2,PDE3A,POR,ABCC9,EXT1,AMFR,AKAP6,ATF2,FER,GLDC,GABRB3,RAPGEF2,PRKCQ,GABRG2,CPS1,NSG1,UBE3A,IDE,APC,TFF1,EPHB2,RYR3,CDH13,ALK,HDAC9,PIK3R3,MTOR,GABRB1,BLM,PSMB2,RGS7,KYNU,USP25,FBN1,RAB31,TBC1D4,PPARA,PLCB1,ELAVL4,BCL2L1,HDAC2,RYR2,PTPRA,ATF6,ENPP1,BCL11A,VPS13C,BCKDHB
GO:0043408	regulation of MAPK cascade	0.01553022668486957	CD44,PTPRR,TAOK3,ANKRD6,GPR55,IL6R,NDRG2,JAK2,NRXN1,ZDHHC17,NTRK2,MECOM,DOK5,SPRED2,ZNF675,TRIM5,RAP1GDS1,ERBB4,RAP1A,DUSP22,MAP3K5,HTR2C,TRAF3,ITGA1,ROBO1,PAK3,PRKCE,GRM5,IGF1R,INSR,STK38,APP,PLCE1,PAK1,GHR,KL,SLAMF1,EDAR,EGF,PDGFD,EPHA7,STK3,GRM1,EPHB1,FLT1,NTF3,RAPGEF2,EPHB2,NRP1,ALK,SEMA3A,MAGI3,CDH2,MAP2K6,KSR1,FBLN1,SPRED1,NTRK3,BMPER,APIP,PLCB1,RASGRP1,TGFA,ZMYND11,AKAP13,PRDM15,NRG1,PRKCA

GO:0048729	tissue morphogenesis	0.016937367181254326	CD44,ANKRD6,BCL2,ASTN2,NTN4,LAMA1,WDPCP,LAMA3,EXOC4,MYO9A,ZNRF3,DLG5,ARHGAP24,SLIT2,ROR1,ERBB4,EXT2,ITGA8,ALDH1A2,EYA1,ROBO1,OVOL2,MLLT3,PRICKLE2,NTN1,SHROOM3,COBL,GPC6,PAK1,CECR2,RIPK4,STARD13,ADAMTS16,TIAM1,PBX1,FMN1,ZFPM2,EGF,EPHA7,STK3,PCDH15,ESR1,ARHGAP12,PRKACB,GREB1L,AJAP1,EXT1,LRP2,KRT25,DLC1,SETD2,NRP1,SEMA3E,MTOR,CSMD1,GLI3,HMGA2,MAGI2,RALA,RYR2,TBX20,VCL,ROBO2,NRG1,SEMA3C
GO:0048013	ephrin receptor signaling pathway	0.017193830652475404	KALRN,ANKS1B,PAK3,EPHA6,PAK1,TIAM1,FYN,EPHA7,EPHB1,EFNA5,EPHB2,NTRK3,CHN1
GO:0010721	negative regulation of cell development	0.018096964134599273	SEMA4D,CTNNA1,KANK1,BRINP1,NTN1,MAP2,TNR,EPHA7,EFNA5,TRPC5,SEMA6D,RAPGEF2,EPHB2,PRTG,NRP1,DAB1,SEMA3A,SEMA3E,FSTL4,FBLN1,NTRK3,FBN1,SEMA3D,SEMA5A,BCL11A,DCC,SEMA3C
GO:0030517	negative regulation of axon extension	0.01887134911409489	SEMA4D,NTN1,MAP2,TNR,SEMA6D,NRP1,SEMA3A,SEMA3E,SEMA3D,SEMA5A,BCL11A,SEMA3C
GO:0007507	heart development	0.020035960602883093	SLC8A1,DNAH11,SORBS2,FGF12,CTDP1,ADAMTS6,RPS6KA2,CACNA1C,SOX6,CPE,SLIT2,MYLK3,ALPK2,ERBB4,MNAT1,RUNX1,ALDH1A2,EYA1,ANK2,SLIT3,ROBO1,OVOL2,IGF1R,FLRT2,INSR,MYO18B,PLCE1,CXADR,TENM4,NEBL,RARB,ZFPM2,STK3,AP2B1,SGCZ,FHOD3,GREB1L,EXT1,AKAP6,ATF2,LRP2,SGCD,DLC1,PDLIM5,SETD2,NRP1,HDAC9,MTOR,FHL2,SPRED1,GLI3,NTRK3,FBN1,SGCG,PPARA,RYR2,TBX20,ROBO2,AKAP13,NRG1,SEMA3C
GO:0048880	sensory system development	0.02004043025908226	BCL2,THRB,MEGF11,LAMA1,WDPCP,CACNA1C,NTRK2,SMARCA4,NHS,SPRED2,CRB1,ALDH1A2,MDM1,DSCAM,SDK1,SLC1A1,CELF4,MITF,HCN1,MEIS2,RARB,PBX1,VSTM4,EPHB1,EFEMP1,FLT1,TENM3,ATP8A2,TTLL5,EPHB2,ADAMTS18,NRP1,RPGRIP1,SEMA3A,DCLK1,RORB,SPRED1,SIPA1L3,ABCB5,GLI3,FBN1,SCAPER,NPHP4,MYH15,HDAC2,FAT3,ATF6
GO:0001654	eye development	0.022934640327953656	BCL2,THRB,MEGF11,LAMA1,WDPCP,CACNA1C,NTRK2,SMARCA4,NHS,SPRED2,CRB1,ALDH1A2,MDM1,DSCAM,SDK1,SLC1A1,CELF4,MITF,HCN1,MEIS2,RARB,PBX1,VSTM4,EPHB1,EFEMP1,FLT1,TENM3,ATP8A2,TTLL5,EPHB2,ADAMTS18,NRP1,RPGRIP1,DCLK1,RORB,SPRED1,SIPA1L3,ABCB5,GLI3,FBN1,SCAPER,NPHP4,MYH15,HDAC2,FAT3,ATF6
GO:0050768	negative regulation of neuro	0.024268363348262146	SEMA4D,CTNNA1,BRINP1,NTN1,MAP2,TNR,EPHA7,TRPC5,SEMA6D,RAPGEF2,EPHB2,PRTG,NRP1,DAB1,SEMA3A,SEMA3E,FSTL4,NTRK3,SEMA3D,SEMA5A,BCL11A,DCC,SEMA3C

	genesis		
GO:0019932	second-messenger-mediated signaling	0.024401484025134926	SLC8A1,CAMTA1,RCAN1,NFAT5,GRIN2A,ADCYAP1R1,CACNA1C,SLC24A4,PDE10A,CHRM3,NOS1,HTR2C,PTGFR,ANK2,GRIN2B,ITPR2,EFHB,GRM5,NR5A2,NOS1AP,INPP5A,PLCE1,PPP1R9A,PDE4D,PDE3A,MCTP2,HDAC4,AKAP6,SGCD,RAPGEF2,MCTP1,CDH13,RCAN2,MTOR,KSR1,FHL2,PRKG1,RYR2,NRG1
GO:0045927	positive regulation of growth	0.024567930456201505	SEMA4D,TEAD1,CD38,BCL2,MTPN,CSNK2A1,ERBB4,MACF1,PLS1,BBS2,DSCAM,NTN1,INSR,CDH4,GHR,DISC1,ZFPM2,RIMS1,EFNA5,TRPC5,AKAP6,ATP8A2,INO80,NRP1,RFTN1,MTOR,ADAM10,SYT1,PLCB1,TBX20,RIMS2,SEMA5A,BCL11A,NRG1
GO:2000311	regulation of AMPA receptor activity	0.025714911582076887	CACNG2,NRXN1,NLGN1,SHISA9,CNIH3,RELN,GSG1L,SHISA6,CACNG3
GO:0007265	Ras protein signal transduction	0.02588565974653477	GPR55,CDC42EP3,RASGEF1B,ELMO1,RAPGEF5,ARHGAP24,PSD3,KANK1,RAP1A,ABL2,RERG,ROBO1,ARHGEF11,DGKI,NTN1,CTNNAL1,RASGRF2,PLCE1,EPS8,RASGRF1,STARD13,RGL1,TIAM1,RALGPS1,ARHGAP42,NET1,RALGPS2,MAPRE2,RAPGEF2,DLC1,AUTS2,EPHB2,NRP1,CDH13,KSR1,SCAI,RAPGEF4,RASGRP1,RALA,PRKD1,AKAP13,NRG1
GO:0046660	female sex differentiation	0.02598088277770804	A2M,BCL2,ADCYAP1R1,CTNNA1,GAS2,SLIT2,SLIT3,INSR,ZFPM2,ESR1,ACSBG1,LRP2,IMMP2L,UBE3A,DACH1,NRIP1,ARID5B,CSMD1,SCAPER,BCL2L1,ROBO2
GO:0018209	peptidyl-serine modification	0.02691731052097304	CD44,RPS6KA5,PRKCB,BCL2,TLK1,MAST4,NRXN1,RPS6KA2,NTRK2,NLK,STK32B,CSNK2A1,AKT3,NOS1,MAPK9,MORC3,SPOCK3,EGFLAM,TNKS,PRKCE,SLC1A1,CAMK1D,STK38,VRK1,APP,PAK1,PDE4D,MARK2,NTF3,CAMK4,PRKCQ,PRKCH,DCLK1,MTOR,STK38L,STK32A,NTRK3,PRKD1,PRKCA,TOP1
GO:0018105	peptidyl-serine phosphorylation	0.027487870764118433	CD44,RPS6KA5,PRKCB,BCL2,TLK1,MAST4,NRXN1,RPS6KA2,NTRK2,NLK,STK32B,CSNK2A1,AKT3,NOS1,MAPK9,MORC3,TNKS,PRKCE,SLC1A1,CAMK1D,STK38,VRK1,APP,PAK1,PDE4D,MARK2,NTF3,CAMK4,PRKCQ,PRKCH,DCLK1,MTOR,STK38L,STK32A,NTRK3,PRKD1,PRKCA,TOP1
GO:0055006	cardiac cell devel	0.02907509169318759	SLC8A1,SORBS2,CTDP1,MYLK3,ALPK2,MYO18B,CXADR,NEBL,FHOD3,AKAP6,SGCD,PDLIM5,MTOR,FHL2,PPARA,AKAP13

	opment		
GO:0150063	visual system development	0.0297317426483145	BCL2,THRB,MEGF11,LAMA1,WDPCP,CACNA1C,NTRK2,SMARCA4,NHS,SPRED2,CRB1,ALDH1A2,MDM1,DSCAM,SDK1,SLC1A1,CELF4,MITF,HCN1,MEIS2,RARB,PBX1,VSTM4,EPHB1,EFEMP1,FLT1,TENM3,ATP8A2,TTLL5,EPHB2,ADAMTS18,NRP1,RPGRIP1,DCLK1,RORB,SPRED1,SIPA1L3,ABCB5,GLI3,FBN1,SCAPER,NPHP4,MYH15,HDAC2,FAT3,ATF6
GO:0051146	striated muscle cell differentiation	0.030653219848824503	SLC8A1,BCL2,SORBS2,RCAN1,CTDP1,KCNH1,MTPN,SOX6,PTGFRN,PGM5,MYLK3,ALPK2,NOS1,MYOM2,JAM2,ADAMTS5,MYO18B,ADAM12,CXADR,NEBL,RARB,FHOD3,HDAC4,AKAP6,SGCD,PDLIM5,MYEF2,CDH2,TANC1,HDAC9,MTOR,FHL2,PPARA,AKAP13,NRG1
GO:0060537	muscle tissue development	0.03169979502461116	SLC8A1,BCL2,SORBS2,RCAN1,CTDP1,MEOX2,MTPN,SOX6,PGM5,MYLK3,ALPK2,ERBB4,ITGA8,RUNX1,ALDH1A2,EYA1,MYO18B,CXADR,TENM4,RBFOX1,NEBL,RARB,ZFPM2,COL19A1,SGCZ,EPHB1,FHOD3,HDAC4,AKAP6,LRP2,SGCD,PDLIM5,HDAC9,MTOR,FHL2,FLNB,SGCG,PPARA,MYH15,TNN,RYR2,TBX20,AKAP13,HIVEP3,NRG1,SEMA3C
GO:0006813	potassium ion transport	0.032526977140880216	KCNMA1,SLC12A8,KCNC1,KCNQ5,KCNJ6,KCNK10,DPP6,KCNH1,SLC24A4,KCNE4,KCNS3,NOS1,SLC9C1,ANK2,KCNH8,SLC12A1,KCNIP4,NOS1AP,ANK3,DPP10,HCN1,KCND3,KCNN3,KCNAB1,ABCC9,AKAP6,KCND2,SLC24A3,RGS7,SLC24A2,KCNH5,KCNJ15
GO:0099560	synaptic membrane adhesion	0.03657588480278346	NRXN1,NLGN1,GPC6,LRFN5,LRRC4C,EFNA5,NTNG1,PTPRD,NRG1
GO:0050805	negative regulation of synaptic transmission	0.0380308239919109	CD38,GRIK2,GRIK3,SHANK2,RAP1A,DGKI,TNR,CELF4,HCN1,SORCS2,GRIA1,SORCS3,GRID2,SLC24A2,MTMR2
GO:2001222	regulation of neuron migration	0.03884169426198854	FBXO31,NRG3,UNC5D,FLRT2,RELN,PHACTR1,NTNG1,RAPGEF2,SEMA3A,TNN,CTNNA2,NRG1
GO:0055007	cardiac muscle	0.03885724162845732	SLC8A1,SORBS2,CTDP1,SOX6,MYLK3,ALPK2,MYO18B,CXADR,NEBL,RARB,FHOD3,AKAP6,SGCD,PDLIM5,MTOR,FHL2,PPARA,AKAP13,NRG1

	cell differentiation		
GO:0050806	positive regulation of synaptic transmission	0.04081791249295337	GRIK2,IGSF11,CACNG2,GRIN2A,NRXN1,NTRK2,CACNB2,SHANK2,GRIN2B,PRKCE,SLC1A1,NLGN1,RELN,RASGRF2,TNR,APP,CLSTN2,RIMS1,NSG1,EPHB2,SLC24A2,SYT1,TSHZ3,CACNG3,RIMS2
GO:0006996	organelle organization	0.045271543990990225	C10ORF90,PARN,ZFYVE1,PRKCB,DNAJC15,SIAH3,TRAPPC9,BCL2,CHFR,CDC42BPA,SAMM50,CDC42EP3,UNC13B,SORBS2,KIF4A,JAK2,BICD1,EPB41L3,PARVB,CDS2,MAST4,NRXN1,ARID1B,WDPCP,CTDP1,GOLGA8J,RPS6KA2,CCSER2,BMF,PNPLA3,BBS9,CTNNA1,OCLN,THSD7A,AFAP1,IQCJ-SCHIP1,LRRC49,SMARCA4,ELMO1,SYNE1,GAS2,MTPN,ESYT2,PDE4DIP,DNAH8,SHANK2,PTGFRN,CHCHD6,PGM5,SMARCAD1,CALD1,SLIT2,MYLK3,AKT3,RAP1GDS1,ERBB4,KANK1,ATRX,DMRT1,BID,MACF1,MNAT1,RAD51B,ABL2,SPAG16,EML1,PIK3C3,CHD6,HMCN1,FGD4,GOLGA6D,CORO2B,MAPK9,FAM171A1,HSF2BP,FRMD6,PLS1,ANK2,PAK3,TNKS,MDM1,FCHSD2,TRAPPC10,LEMD3,EHBP1,PSTPIP2,ITGB3BP,TMEM67,PRKCE,ARHGEF11,PRKAA2,BBS2,CHCHD3,MYOM2,NUBPL,ANKFN1,TRAPPC8,SPTB,SNX30,NLGN1,SHROOM3,MAP2,NOS1AP,INSR,COBL,MDN1,CLEC16A,ANK3,MORC2,TBCD,RELN,VAV3,VRK1,CCDC88A,PLCE1,TACC2,PAK1,ADCK1,TOP3A,NSMCE2,CXADR,EPS8,UTRN,CECR2,FRMD5,USP7,STARD13,PPP1R9A,ADAMTS16,SLAMF1,INO80D,TTLL11,NEBL,PHACTR1,SLC39A12,DISC1,FMN1,VTI1A,ASAP1,FRMPD4,EGF,IFT43,LRBA,MAP7,NAV2,CNOT7,PCDH15,ARHGAP12,PDE3A,FHOD3,ARMC2,MIPEP,SNAP29,DIAPH3,TRPM7,EXT1,EFNA5,CDC14B,TLN2,C14ORF39,STK36,KLHL1,VPS37A,MARK2,ATF2,PHACTR2,GRID2,ZNF423,NTF3,FER,TTC29,MAPRE2,ARFGAP3,RAD51AP1,TMEM108,NAV3,IMMP2L,PRKCQ,NUDCD3,RHPN2,KRT25,DLC1,ATP8A2,MPRIP,APC,TTLL5,INO80,AUTS2,PDLIM5,NBN,FRMD3,SETD2,PACSIN2,PKP1,DOCK2,SDCCAG8,NRP1,RFC3,PHACTR3,RPGRIP1,TRDN,SEMA3E,DCLK1,CDH2,VPS41,SYCP1,ARHGAP28,MTOR,BLM,SH3KBP1,NCAPG2,CD2AP,TTC39C,CLVS2,SIPA1L3,FLNB,SPECC1,NTRK3,HYDIN,CHKA,RAB31,CTNNA3,VPS13D,ANKRD31,ATF7IP,HMGA2,THSD7B,TBC1D4,SYT1,DPF3,NPHP4,PTPRD,PLCB1,PRKG1,STXBP6,STX12,ABI1,RALA,GNPTAB,PRKD1,BCL2L1,MICAL3,ETS1,TGFA,ATAT1,IFT81,AKAP13,NEDD9,UNC13C,RAB27A,CLIP1,SEMA5A,CTNNA2,CEP44,PARD3B,VPS13C,PARD3,ATP10B,PRKCA,FMN2,PCNT,TOP1
GO:0043412	macromolecule modification	0.045787650853022915	CD44,C10ORF90,PTPRR,ERG,PARN,SEMA4D,RPS6KA5,TAOK3,PRKCB,TRPM6,SLC8A1,SIAH3,FBXO31,BCL2,CAMTA1,SAMSN1,CHFR,THADA,RSRC1,CDC42BPA,B3GALT5,RAG1,SNX25,MAPK10,KDM4B,IL6R,RNF182,BRD4,ZDHHC11B,TTC3,TLK1,RCAN1,JAK2,OTUD7A,TPTE2,KALRN,SUMF1,LAMA1,MAST4,NRXN1,DTWD2,AGBL1,ST6GALNAC3,CTDP1,RPS6KA2,ZDHHC17,HLCS,WDR70,NTRK2,OCLN,NLK,RNF152,CNTN1,ZNRF3,NRG3,PTPRG,FBXL17,MECOM,STK32B,UBE2E2,SMARCAD1,CHRM3,CPE,SPRED2,SLIT2,MYLK3,ROR1,ZNF675,CSNK2A1,AKT3,PHKB,KMT2C,TRIM5,ALPK2,HECW1,ERBB4,ST3A,GPHN,ATRX,MNAT1,TAF4B,RAP1A,TRIO,TRMT61B,PTPRE,MYO3B,DUSP22,EXT2,ABL2,MAP3K5,NOS1,NEK4,SLCO3A1,PIK3C3,TRAF3,ITGA1,MAPK9,PEAK1,EYA1,MORC3,SPOCK3,ZBTB16,SUPT3H,ROBO1,EGFLAM,PAK3,DPH6,TNKS,NDFIP2,MLLT3,RNF138,PRKCE,PRKAA2,PTPRN2,MYO3A,USP31,UBE2R2,KDM4C,DCUN1D4,SLC1A1,GRM5,EPHA6,IGF1R,CAMK1D,NOS1AP,PTPRO,ZDHHC14,MSRA,INSR,PHF20L1,EGLN3,DOCK3,RELN,STK38,FBXO32,CDKAL1,DAPK1,VRK1,APP,CCDC88A,PLCE1,PAK1,NSMCE2,FRY,GHR,RIPK4

			,PRDM16,RNF217,USP7,PDZRN3,ZZEF1,TTLL11,EGF,PDGFD,FYN,PRMT8,EPHA7,SENP8,TRABD2B,STK3,CNOT7,USP18,TOX,PDE4D,PRKACB,POR,PIGK,HUNK,FUT8,EPHB1,PARP8,EFEMP1,PIGB,HERC1,PARP15,TRPM7,FLT1,EXT1,EFNA5,NXN,CDC14B,HDAC4,STK36,TRPC5,AMFR,MRM1,FTO,SH3BP5,MARK2,ATF2,TUSC3,NTF3,FER,SNRK,CAMK4,GALNT14,FBXL7,TPTE,SLC39A8,RAPGEF2,PRKCQ,PRKCH,DLC1,UBE3A,APC,MACROD2,TTLL5,TMTC1,AUTS2,EPHB2,ADARB2,ERC1,TMTC2,MOB3B,NBN,SETD2,TRIM23,NRP1,DAB1,ALK,LDLRAD4,MGAT5,DCLK1,CCNG2,ASB3,HDAC9,PIK3R3,MAP2K6,MTOR,STK38L,KSR1,FBLN1,ST8SIA1,BLM,NCAPG2,STK32A,USP25,SPRED1,ADAM10,PPP2R2C,KANSL1,TULP4,DPY19L2,PTPRT,TRIM9,TRERF1,NTRK3,CHKA,ABHD17C,BMPER,ATF7IP,HMGA2,NSUN2,PTPRK,PTPRD,LOXL2,PRKG1,RASGRP1,MAGI2,ABI1,GALNTL6,PRKD1,HDAC2,ST6GAL2,TGFA,PRLR,PTPRA,MTMR2,DEPTOR,ATAT1,AKAP13,NEDD9,ENPP1,POMT2,BCL11A,ATE1,PARD3,NRG1,PRKCA,FANCB,DPY19L1,CSF2RB,TOP1
GO:0035265	organ growth	0.047090486434941216	EVC,BCL2,SORBS2,CTDP1,ERBB4,RUNX1,CXADR,TENM4,RARB,ZFPM2,STK3,ESR1,POR,EXT1,AKAP6,ATF2,UBE3A,PDLIM5,FLVCR1,PPARA,PRLR,TBX20,AKAP13,NRG1
GO:2000146	negative regulation of cell motility	0.048419503548104766	PTPRR,BCL2,CTNNA1,SRGAP2B,MEOX2,NRG3,PTPRG,DLG5,SLIT2,KANK1,DUSP22,SPOCK3,ROBO1,RIN3,MITF,FRMD5,STARD13,SEMA6D,NAV3,DLC1,MCTP1,DACH1,LDLRAD4,FBLN1,SPRED1,SCAI,PTPRT,PTPRK,PLCB1,PRKG1,MAGI2,HDAC2,TNN,VCL,SRGAP3,NEDD9,NRG1

Table S6. GO associations with biological processes (GO Profiler) of 1307 rDNA-contacting genes associated with genes increase the number of contacts with rDNA clusters. The search was performed using g:Profiler (https://biit.cs.ut.ee/gprofiler/gost. Related to Figure 2B.

GO.ID	Description	padj	Genes
		**BP**	
GO:0050794	regulation of cellular process	2.0864298608533356e-13	*WWC1,GARNL3,MTOR,SMOC1,NSG1,LRP12,PLCB1,SPOCK1,ABCA13,ANKS1B,ZNF536,KSR1,BRINP3,SGCD,CACNA2D3,CNTN4,KCNH5,ZFPM2,TENM4,RIPOR2,RP1,ERC1,ODAD2,KCNMA1,FBN1,CDH8,DCDC1,RIMS1,PIK3C3,SPIRE1,TENM3,RARB,ENPEP,USH2A,MINAR1,CDC42EP3,RIMS2,ADGRE1,CDYL2,PJA2,BABAM2,ERBIN,RHPN2,CACNG2,NEGR1,MAP3K9,MYO3B,TCF4,ZNF573,NEK4,DOCK10,TSHZ2,EGFR,DENND1A,USP14,ANGPT1,CDK12,MACF1,PRKACB,NEK7,NCOR1,DOCK2,ZNF407,NEDD4,MAML2,NSMCE2,BTBD9,SOX6,PHACTR1,DKK2,DNAJC13,THRAP3,MAPKBP1,GABRB1,DGKI,C12ORF4,GRIA1,CAST,NEO1,CNTN6,SLC39A12,SLC8A3,TOM1L2,PAK1,EPHA7,CTNNAL1,NCOA7,RALGPS1,SPEN,RAPGEF2,ADGRB3,RUNX2,ARSB,GABRA6,TAOK3,CPEB4,PRICKLE2,LDB2,PUM3,PATJ,RPTOR,EPB41L3,COL4A2,PPP1R12B,ADAM10,IL1R1,APBB2,KDM1B,CACNB2,KLHL13,MTUS1,STAU2,TMC1,USP18,SEMA5A,SYT1,VCL,ARHGAP44,NTF3,ACER2,PARP15,AURKA,CFDP1,PYGO1,SLC8A1,SSBP2,SRGAP2C,ANKRD31,TAFA2,SRGAP2B,MAP4K4,BMPR1B,FMN2,HOMER2,RAB8B,PAK3,RFTN1,PDE1A,ZNF257,DIP2B,LARP1,ITPKB,RGS20,PDE10A,RAP1GDS1,HHAT,CLIC6,KICS2,SCP2,IFT57,INTS7,PRKCZ,BTLA,GRB10,MCPH1,CNST,RGS9,DEFA3,MBNL2,ABCA5,SENP6,EBF2,YAP1,PPM1L,RIPK4,RABGAP1L,USP25,ALCAM,PLG,PAPPA,PDGFD,ZNRF3,ITGBL1,UBE2O,GFRA1,SYCP1,NIPBL,EWSR1,MICU1,CORO2B,CARD18,CHD6,STK38,HRH4,SORCS3,MYLK3,KANSL1,MBNL1,A*

			TF6,ZNF684,CCNG2,TLK1,TPM1,LRRC38,BIRC6,KLF15,PPARA,SNX30,KCNS3,PPP6R3,SYNJ1,ADAMTS3,ARAP2,PTPRK,ARHGEF12,TRERF1,SEMA3C,DAPK1,SLC24A4,SEC14L1,VPS13C,STK32B,PHC3,MAGI1,ALPK2,DNAH11,JARID2,SCN2A,DNAJC15,GATAD2B,CPE,EVC2,IL34,TANC1,ZNF846,MELK,BBS2,RANBP3L,OR4F6,NKG7,USP8,PIAS1,BLK,EBF1,TNR,MXI1,OXR1,SDC2,GAS2,KCNH1,MRPS27,CREG1,DROSHA,APBB1IP,EIPR1,SLFN11,GLIS1,MORC1,MYO10,LATS2,GSG1L,ASPM,AP3B1,DENND2B,ATP11C,ZNF438,ABCB7,ZBTB16,MUSK,KIR3DL2,GNG7,SMARCAD1,SETDB2,PRKCE,FOXK2,SLMAP,ZNF718,USP33,CD44,RGS12,PTPRO,PRRC1,ABCC9,STXBP6,NSMAF,LNPEP,LIMD1,PEX14,SPRED2,RPS6KA3,PTPN2,PLXNA2,MCF2L,OR4F15,ATXN3,RIC3,ARHGEF7,ALG10B,ATP8A1,AMBRA1,KDM7A,OPRM1,FANCA,SEMA3E,RPRD1B,TMEM67,ABHD17C,TMOD2,MSH2,ZNF397,RELL1,HIPK3,EPN2,CLSPN,BICRAL,MOSMO,MNAT1,TMEM116,MDFIC,ANK3,HMGA2,BCL11B,VPS41,DOCK5,STK32A,LYPLA1,PLCE1,IL17RA,CRIM1,FUT9,PRR5L,VAV1,MYT1L,ZNF160,HLA-B,IQSEC1,CACNA1I,PDLIM5,BLM,NRK,MAGI3,INTS8,LIN54,ADCY10,BMP2,RC3H2,ATP9A,TRAK1,GFI1B,RIN3,BMP2K,SEMA3D,NETO2,NFATC2,SH3BP5,SLC23A2,ZNF106,MYOM1,TRAF3,ANKRD26,TTC21B,ZNF875,UIMC1,LRRFIP1,RAP1GAP,IKZF2,DRAXIN,ATF1,KCNH8,CGAS,GABRR2,CNKSR3,CASP5,VENTX,WDR12,KIF15,PRDM10,CUL1,BTAF1,ZNF618,FARP1,MOB1B,BBS4,MAPK8IP1,COL5A1,CFTR,ME2,UBASH3A,AHDC1,MRPL13,KITLG,YLPM1,GTF2I,TADA2A,ZNF208,NMD3,AKAP10,PTPRE,MTMR2,ZNF608,TBX20,SP110,AFAP1,WSB1,PRKCH,TG,IL6R,ALS2,ZNF627,OR51E1,TFDP1,HEMGN,KANK4,SNX25,TOX,PTPRB,PDE6A,SCN10A,USP7,ENPP3,PLAGL1,MESD,MOK,KIR2DL4,RALB,NPAS2,VCAM1,SEL1L,ARHGAP31,ZNF169,KIF11,DTX1,ZBTB33,ADA2,FANCL,DPYSL5,ZNF44,SUPT16H,BAZ1A,CUL5,OR7A17,NEK6,HECTD1,NMU,GAST,SNAI2,IGHV3-74,BID,SIAH2,RXRG,SP3,ERN2,ZNF879,MBTPS2,FLNB,TRIM58,TIAL1,ELF2,ZDHHC17,FYCO1,SH3GLB1,SAMHD1,IFT81,ENPP1,TP53I11,TMEM225,KCNC1,CSF1,GHRH,BCL2L1,CTDP1,ASB4,DHRS3,SMAD5,TCERG1,SLC40A1,PRAME,CIDEC,LPGAT1,MED1,CDC14B,CFH,SCML2,PRAMEF25,PTH,PRKAA2,CSF2RB,SOHLH1,PHF20L1,ABHD2,VSTM2A,PLA2G4A,CAMLG,COX7A2L,ZBTB7C,TEAD1,ANP32B,YBX3,AIMP1,FYB2,PCID2,ZNF234,CIBAR1,PBLD,FICD,CADM1,PEG10,NET1,ELOC,ANLN,SLC1A7,VSX1,FSTL1,SVEP1,MADD,HCRTR1,PATL1,ZNF287,ZNF449,PRSS2,CREBBP,MELTF,GORAB,SIAH3,NFKBIA,ABCC8,ZC3H15,RFC2,ZNF354C,ALX4,RTRAF,ZBTB21,NEDD9,OLFM4,ASS1,ADGRE3,SAR1A,PPP1R17,BTG3,ERLIN2,OTOP1,ZBTB49,EXOC1,HEPACAM,KRT6A,STOX2,AGO1,GID8,ELL2,FAM189A2,NDFIP2,NR2C1,CMTM7,GATAD1,MTPN,ABI1,ITGA4,OAZ2,ZSCAN30,POU1F1,UBE2J2,TM9SF4,OR6C75,ASB2,CEP120,ZSCAN5C,CYFIP2,HNRNPM,ASCC2,OR13C9,ARID3B,RXRA,ADGRB1,WNT7A,NDFIP1,MAP3K4,SERPINI2,FOXO6,ZNF112,ATP6V1C2,C16ORF72,MAGEL2,OR10H2,PDE2A,LRRC2,SDCBP,JPT2,NSMCE1,ZNF813,MLLT1,NCK1,SCAF8,FGR,C2,IFNAR1,RNF8,CYTH4,INTS13,DNMT3L,LHX9,WNT2B,OCLN,POSTN,CD101,AKAP11,DTHD1,MVB12B,CD5L,ANKRD6,SCGN,ASCL3,FEZ2,INIP,LAMB1,ZNF66,KIRREL1,PLCZ1,FCRLA,DIDO1,GPR55,NSUN2,TNFSF11,ZNF705G,PPM1F,ARL13B,SH2D3C,TRNAU1AP,ZFYVE28,TET1,ASB3,RAD9A,RP1L1,ZNF705D,ITGA1,POR,ZNF850,ZNF235,NSG2,B9D1,PRDM15,SRGAP3,MACROH2A1,TOGARAM1,CSNK1G1,ZNF705B,ATP6V0D2,SERPINB2,ATG5,UNK,FLRT2,OR2T2,BTBD10,TMEM25,NUDT21,DDX6,PPP1R13B,RFX2,PKNOX2
GO:0065007	biological regulation	1.3739950414517639e-9	WWC1,GARNL3,MTOR,SMOC1,NSG1,LRP12,TMPRSS2,PLCB1,SPOCK1,ABCA13,ANKS1B,ZNF536,KSR1,BRINP3,SGCD,CACNA2D3,CNTN4,KCNH5,ZFPM2,PIEZO2,TENM4,RIPOR2,RP1,ERC1,ODAD2,KCNMA1,FBN1,F13A1,CDH8,DCDC1,RIMS1,PIK3C3,SPIRE1,TENM3,RARB,NAV2,ENPEP,USH2A,MINAR1,CDC42E

			P3,RIMS2,ADGRE1,CDYL2,PJA2,BABAM2,ERBIN,RHPN2,PARVB,CACNG2,NEGR1,SUSD4,MAP3K9,MYO3B,RTN1,TCF4,ZNF573,NEK4,DOCK10,TSHZ2,EGFR,DENND1A,USP14,ANGPT1,CDK12,MACF1,PRKACB,NEK7,NCOR1,DOCK2,ZNF407,NEDD4,MAML2,NSMCE2,BTBD9,SOX6,PHACTR1,DKK2,DNAJC13,THRAP3,MAPKBP1,AOAH,GABRB1,DGKI,C12ORF4,GRIA1,CAST,NEO1,CNTN6,SLC39A12,SLC8A3,TOM1L2,TBC1D19,PAK1,EPHA7,CTNNAL1,NCOA7,RALGPS1,SPEN,RAPGEF2,ADGRB3,RUNX2,ARSB,GABRA6,CPS1,TAOK3,LDLRAD3,CPEB4,PRICKLE2,LDB2,PPP2R2B,PUM3,PATJ,RPTOR,EPB41L3,COL4A2,PPP1R12B,ADAM10,IL1R1,APBB2,PHACTR2,KDM1B,CACNB2,KLHL13,MTUS1,STAU2,TMC1,USP18,SEMA5A,SYT1,VCL,ARHGAP44,NTF3,ACER2,PARP15,AURKA,CFDP1,PYGO1,SLC8A1,SSBP2,SRGAP2C,ANKRD31,TAFA2,SERPINA6,SRGAP2B,MAP4K4,BMPR1B,FMN2,HOMER2,RAB8B,PAK3,RFTN1,PDE1A,ZNF257,DIP2B,LARP1,ITPKB,RGS20,PDE10A,RAP1GDS1,HHAT,RNLS,CLIC6,CHST8,KICS2,SCP2,IFT57,INTS7,PRKCZ,SPOP,BTLA,GRB10,MCPH1,CNST,RGS9,DEFA3,MBNL2,ABCA5,SENP6,EBF2,YAP1,PPM1L,RIPK4,RABGAP1L,USP25,ALCAM,PLG,PAPPA,PDGFD,ZNRF3,ITGBL1,UBE2O,GFRA1,SYCP1,NIPBL,EWSR1,MICU1,CORO2B,CARD18,CHD6,STK38,HRH4,SORCS3,MYLK3,KANSL1,MBNL1,ATF6,ZNF684,CCNG2,TLK1,TPM1,LRRC38,CORIN,BIRC6,KLF15,PPARA,SNX30,KCNS3,PPP6R3,SYNJ1,ADAMTS3,ARAP2,PTPRK,ARHGEF12,TRERF1,SEMA3C,DAPK1,SLC24A4,SEC14L1,VPS13C,STK32B,PHC3,MAGI1,ALPK2,DNAH11,JARID2,SCN2A,TBC1D9,RAB22A,DNAJC15,GATAD2B,CPE,EVC2,IL34,TANC1,ZNF846,MELK,BBS2,SLC9C1,RANBP3L,OR4F6,NKG7,USP8,PIAS1,BLK,EBF1,TNR,MXI1,OXR1,SDC2,GAS2,KCNH1,MRPS27,CREG1,DROSHA,APBB1IP,EIPR1,PSMF1,SLFN11,GLIS1,MORC1,MYO10,LATS2,GSG1L,ASPM,AP3B1,DENND2B,ATP11C,ZNF438,ABCB7,ZBTB16,MUSK,KIR3DL2,GNG7,SMARCAD1,SETDB2,PRKCE,FOXK2,ASAP2,SLMAP,ZNF718,USP33,CD44,RGS12,PTPRO,PRRC1,ABCC9,STXBP6,NSMAF,NLRP13,LNPEP,LIMD1,PEX14,SPRED2,RPS6KA3,PTPN2,PLXNA2,MCF2L,OR4F15,ATXN3,RIC3,ARHGEF7,ALG10B,ATP8A1,AMBRA1,KDM7A,OPRM1,FANCM,FANCA,SEMA3E,RPRD1B,TMEM67,ALPL,ABHD17C,TMOD2,MSH2,ZNF397,LUC7L,RELL1,HIPK3,EPN2,CLSPN,BICRAL,AFG3L2,MOSMO,MNAT1,TMEM116,MDFIC,ANK3,HMGA2,BCL11B,VPS41,DOCK5,F5,STK32A,LYPLA1,LINC01151,PLCE1,IL17RA,CRIM1,FUT9,PRR5L,VAV1,MYT1L,FBXO32,ZNF160,HLA-B,IQSEC1,CACNA1I,PDLIM5,BLM,NRK,MAGI3,INTS8,LIN54,ADCY10,STX12,BMP2,RC3H2,ATP9A,TRAK1,GFI1B,RIN3,BMP2K,SEMA3D,NETO2,POLR3A,NFATC2,TDRD7,SH3BP5,SLC23A2,ZNF106,MYOM1,TRAF3,ANKRD26,TTC21B,ZNF875,UIMC1,LRRFIP1,RAP1GAP,IKZF2,DRAXIN,ATF1,CCDC186,KCNH8,CGAS,GABRR2,CNKSR3,CASP5,VENTX,WDR12,KIF15,PRDM10,CUL1,BTAF1,ZNF618,FARP1,MOB1B,BBS4,MAPK8IP1,COL5A1,CFTR,ME2,TBC1D13,UBASH3A,AHDC1,MRPL13,KITLG,YLPM1,GTF2I,TADA2A,ZNF208,NMD3,AKAP10,PTPRE,MTMR2,ZNF608,SH3PXD2A,TBX20,SP110,AFAP1,WSB1,PRKCH,SLC12A1,TG,IL6R,ALS2,ZNF627,OR51E1,TFDP1,DHRS11,HEMGN,KANK4,SNX25,TOX,PTPRB,PDE6A,SCN10A,USP7,ENPP3,PLAGL1,MESD,MOK,KIR2DL4,RALB,NPAS2,DENND2C,VCAM1,SEL1L,ARHGAP31,TTC37,ZNF169,KIF11,DTX1,ZBTB33,ADA2,FANCL,DPYSL5,ZNF44,SUPT16H,BAZ1A,CUL5,OR7A17,NEK6,HECTD1,SHROOM3,XRCC4,NMU,GAST,SNAI2,IGHV3-74,BID,SIAH2,RXRG,SP3,ERN2,ZNF879,MBTPS2,FLNB,TRIM58,TIAL1,ELF2,ZDHHC17,FYCO1,SH3GLB1,XKR5,SAMHD1,IFT81,ENPP1,TP53I11,TMEM225,KCNC1,CSF1,GHRH,BCL2L1,MIR3142HG,CTDP1,ASB4,DHRS3,SMAD5,TCERG1,SLC40A1,PRAME,CIDEC,LPGAT1,MED1,CDC14B,CFH,SCML2,PRAMEF25,PTH,PRKAA2,CSF2RB,SOHLH1,PHF20L1,ABHD2,VSTM2A,PLA2G4A,CAMLG,COX7A2L,ZBTB7C,TEAD1,ANP32B,YBX3,AIMP1,FYB2,PCID2,ZNF234,CIBAR1,PBLD,FICD,CADM1,PEG10,NET1,ELOC,ANLN,SLC1A7,VSX1,FSTL1,SVEP1,MADD,HCRT

			R1,PATL1,ZNF287,ZNF449,PRSS2,CREBBP,MELTF,GORAB,SIAH3,TRPV5,NFKBIA,ABCC8,ZC3H15,RFC2,ZNF354C,ALX4,RTRAF,ZBTB21,NEDD9,OLFM4,ASS1,ADGRE3,SAR1A,PPP1R17,BTG3,ERLIN2,OTOP1,ZBTB49,EXOC1,HEPACAM,KRT6A,STOX2,AGO1,PDP2,GID8,ELL2,FAM189A2,NDFIP2,NR2C1,CMTM7,SLC6A11,GATAD1,MTPN,ABI1,ITGA4,OAZ2,PPME1,ZSCAN30,POU1F1,UBE2J2,TM9SF4,OR6C75,ASB2,CEP120,ZSCAN5C,CYFIP2,HNRNPM,ASCC2,OR13C9,ARID3B,RXRA,ADGRB1,WNT7A,NDFIP1,MAP3K4,TRIM43B,SERPINI2,TRIM43,FOXO6,ERI1,ZNF112,ATP6V1C2,C16ORF72,MAGEL2,OR10H2,PDE2A,LRRC2,SDCBP,DSG1,JPT2,NSMCE1,ZNF813,MLLT1,NCK1,FLVCR1,SCAF8,FGR,SNAP29,C2,IFNAR1,RNF8,CYTH4,INTS13,DNMT3L,LHX9,WNT2B,TNNI1,OCLN,POSTN,CD101,AKAP11,DTHD1,MVB12B,CD5L,ANKRD6,SCGN,ASCL3,FEZ2,INIP,LAMB1,MIR17HG,ZNF66,KIRREL1,PLCZ1,SLC9A5,FCRLA,DIDO1,GPR55,NSUN2,TNFSF11,ZNF705G,PPM1F,ARL13B,SH2D3C,PSME3IP1,TRNAU1AP,ZFYVE28,OPA3,TET1,ASB3,RAD9A,RP1L1,SPOPL,ZNF705D,IFT46,ITGA1,POR,ZNF850,ZNF235,NSG2,B9D1,PRDM15,SRGAP3,MACROH2A1,TOGARAM1,CSNK1G1,ZNF705B,ATP6V0D2,SERPINB2,ATG5,UNK,FLRT2,OR2T2,BTBD10,TMEM25,NUDT21,DDX6,PPP1R13B,RFX2,PKNOX2,SERPINB11
GO:0050789	regulation of biological process	2.2813341245005094e-8	*WWC1,GARNL3,MTOR,SMOC1,NSG1,LRP12,TMPRSS2,PLCB1,SPOCK1,ABCA13,ANKS1B,ZNF536,KSR1,BRINP3,SGCD,CACNA2D3,CNTN4,KCNH5,ZFPM2,TENM4,RIPOR2,RP1,ERC1,ODAD2,KCNMA1,FBN1,CDH8,DCDC1,RIMS1,PIK3C3,SPIRE1,TENM3,RARB,ENPEP,USH2A,MINAR1,CDC42EP3,RIMS2,ADGRE1,CDYL2,PJA2,BABAM2,ERBIN,RHPN2,PARVB,CACNG2,NEGR1,SUSD4,MAP3K9,MYO3B,RTN1,TCF4,ZNF573,NEK4,DOCK10,TSHZ2,EGFR,DENND1A,USP14,ANGPT1,CDK12,MACF1,PRKACB,NEK7,NCOR1,DOCK2,ZNF407,NEDD4,MAML2,NSMCE2,BTBD9,SOX6,PHACTR1,DKK2,DNAJC13,THRAP3,MAPKBP1,AOAH,GABRB1,DGKI,C12ORF4,GRIA1,CAST,NEO1,CNTN6,SLC39A12,SLC8A3,TOM1L2,PAK1,EPHA7,CTNNAL1,NCOA7,RALGPS1,SPEN,RAPGEF2,ADGRB3,RUNX2,ARSB,GABRA6,TAOK3,LDLRAD3,CPEB4,PRICKLE2,LDB2,PUM3,PATJ,RPTOR,EPB41L3,COL4A2,PPP1R12B,ADAM10,IL1R1,APBB2,KDM1B,CACNB2,KLHL13,MTUS1,STAU2,TMC1,USP18,SEMA5A,SYT1,VCL,ARHGAP44,NTF3,ACER2,PARP15,AURKA,CFDP1,PYGO1,SLC8A1,SSBP2,SRGAP2C,ANKRD31,TAFA2,SERPINA6,SRGAP2B,MAP4K4,BMPR1B,FMN2,HOMER2,RAB8B,PAK3,RFTN1,PDE1A,ZNF257,DIP2B,LARP1,ITPKB,RGS20,PDE10A,RAP1GDS1,HHAT,RNLS,CLIC6,KICS2,SCP2,IFT57,INTS7,PRKCZ,SPOP,BTLA,GRB10,MCPH1,CNST,RGS9,DEFA3,MBNL2,ABCA5,SENP6,EBF2,YAP1,PPM1L,RIPK4,RABGAP1L,USP25,ALCAM,PLG,PAPPA,PDGFD,ZNRF3,ITGBL1,UBE2O,GFRA1,SYCP1,NIPBL,EWSR1,MICU1,CORO2B,CARD18,CHD6,STK38,HRH4,SORCS3,MYLK3,KANSL1,MBNL1,ATF6,ZNF684,CCNG2,TLK1,TPM1,LRRC38,CORIN,BIRC6,KLF15,PPARA,SNX30,KCNS3,PPP6R3,SYNJ1,ADAMTS3,ARAP2,PTPRK,ARHGEF12,TRERF1,SEMA3C,DAPK1,SLC24A4,SEC14L1,VPS13C,STK32B,PHC3,MAGI1,ALPK2,DNAH11,JARID2,SCN2A,DNAJC15,GATAD2B,CPE,EVC2,IL34,TANC1,ZNF846,MELK,BBS2,RANBP3L,OR4F6,NKG7,USP8,PIAS1,BLK,EBF1,TNR,MXI1,OXR1,SDC2,GAS2,KCNH1,MRPS27,CREG1,DROSHA,APBB1IP,EIPR1,PSMF1,SLFN11,GLIS1,MORC1,MYO10,LATS2,GSG1L,ASPM,AP3B1,DENND2B,ATP11C,ZNF438,ABCB7,ZBTB16,MUSK,KIR3DL2,GNG7,SMARCAD1,SETDB2,PRKCE,FOXK2,SLMAP,ZNF718,USP33,CD44,RGS12,PTPRO,PRRC1,ABCC9,STXBP6,NSMAF,NLRP13,LNPEP,LIMD1,PEX14,SPRED2,RPS6KA3,PTPN2,PLXNA2,MCF2L,OR4F15,ATXN3,RIC3,ARHGEF7,ALG10B,ATP8A1,AMBRA1,KDM7A,OPRM1,FANCM,FANCA,SEMA3E,RPRD1B,TMEM67,ALPL,ABHD17C,TMOD2,MSH2,ZNF397,LUC7L,RELL1,HIPK3,EPN2,CLSPN,BICRAL,AFG3L2,MOSMO,MNAT1,TMEM116,MDFIC,ANK3,HMGA2,BCL11B,VPS41,DOCK5,STK32A,LYPLA1,LINC01151,PLCE1,IL17RA,CRIM1,FUT9,PRR5L,VAV1,MYT1L,FBXO32,ZNF160,HLA-*

			B,IQSEC1,CACNA1I,PDLIM5,BLM,NRK,MAGI3,INTS8,LIN54,ADCY10,BMP2,RC3H2,ATP9A,TRAK1,GFI1B,RIN3,BMP2K,SEMA3D,NETO2,POLR3A,NFATC2,TDRD7,SH3BP5,SLC23A2,ZNF106,MYOM1,TRAF3,ANKRD26,TTC21B,ZNF875,UIMC1,LRRFIP1,RAP1GAP,IKZF2,DRAXIN,ATF1,KCNH8,CGAS,GABRR2,CNKSR3,CASP5,VENTX,WDR12,KIF15,PRDM10,CUL1,BTAF1,ZNF618,FARP1,MOB1B,BBS4,MAPK8IP1,COL5A1,CFTR,ME2,UBASH3A,AHDC1,MRPL13,KITLG,YLPM1,GTF2I,TADA2A,ZNF208,NMD3,AKAP10,PTPRE,MTMR2,ZNF608,TBX20,SP110,AFAP1,WSB1,PRKCH,TG,IL6R,ALS2,ZNF627,OR51E1,TFDP1,HEMGN,KANK4,SNX25,TOX,PTPRB,PDE6A,SCN10A,USP7,ENPP3,PLAGL1,MESD,MOK,KIR2DL4,RALB,NPAS2,VCAM1,SEL1L,ARHGAP31,TTC37,ZNF169,KIF11,DTX1,ZBTB33,ADA2,FANCL,DPYSL5,ZNF44,SUPT16H,BAZ1A,CUL5,OR7A17,NEK6,HECTD1,SHROOM3,NMU,GAST,SNAI2,IGHV3-74,BID,SIAH2,RXRG,SP3,ERN2,ZNF879,MBTPS2,FLNB,TRIM58,TIAL1,ELF2,ZDHHC17,FYCO1,SH3GLB1,SAMHD1,IFT81,ENPP1,TP53I11,TMEM225,KCNC1,CSF1,GHRH,BCL2L1,MIR3142HG,CTDP1,ASB4,DHRS3,SMAD5,TCERG1,SLC40A1,PRAME,CIDEC,LPGAT1,MED1,CDC14B,CFH,SCML2,PRAMEF25,PTH,PRKAA2,CSF2RB,SOHLH1,PHF20L1,ABHD2,VSTM2A,PLA2G4A,CAMLG,COX7A2L,ZBTB7C,TEAD1,ANP32B,YBX3,AIMP1,FYB2,PCID2,ZNF234,CIBAR1,PBLD,FICD,CADM1,PEG10,NET1,ELOC,ANLN,SLC1A7,VSX1,FSTL1,SVEP1,MADD,HCRTR1,PATL1,ZNF287,ZNF449,PRSS2,CREBBP,MELTF,GORAB,SIAH3,NFKBIA,ABCC8,ZC3H15,RFC2,ZNF354C,ALX4,RTRAF,ZBTB21,NEDD9,OLFM4,ASS1,ADGRE3,SAR1A,PPP1R17,BTG3,ERLIN2,OTOP1,ZBTB49,EXOC1,HEPACAM,KRT6A,STOX2,AGO1,GID8,ELL2,FAM189A2,NDFIP2,NR2C1,CMTM7,GATAD1,MTPN,ABI1,ITGA4,OAZ2,ZSCAN30,POU1F1,UBE2J2,TM9SF4,OR6C75,ASB2,CEP120,ZSCAN5C,CYFIP2,HNRNPM,ASCC2,OR13C9,ARID3B,RXRA,ADGRB1,WNT7A,NDFIP1,MAP3K4,TRIM43B,SERPINI2,TRIM43,FOXO6,ERI1,ZNF112,ATP6V1C2,C16ORF72,MAGEL2,OR10H2,PDE2A,LRRC2,SDCBP,JPT2,NSMCE1,ZNF813,MLLT1,NCK1,FLVCR1,SCAF8,FGR,C2,IFNAR1,RNF8,CYTH4,INTS13,DNMT3L,LHX9,WNT2B,TNNI1,OCLN,POSTN,CD101,AKAP11,DTHD1,MVB12B,CD5L,ANKRD6,SCGN,ASCL3,FEZ2,INIP,LAMB1,MIR17HG,ZNF66,KIRREL1,PLCZ1,FCRLA,DIDO1,GPR55,NSUN2,TNFSF11,ZNF705G,PPM1F,ARL13B,SH2D3C,PSME3IP1,TRNAU1AP,ZFYVE28,OPA3,TET1,ASB3,RAD9A,RP1L1,SPOPL,ZNF705D,ITGA1,POR,ZNF850,ZNF235,NSG2,B9D1,PRDM15,SRGAP3,MACROH2A1,TOGARAM1,CSNK1G1,ZNF705B,ATP6V0D2,SERPINB2,ATG5,UNK,FLRT2,OR2T2,BTBD10,TMEM25,NUDT21,DDX6,PPP1R13B,RFX2,PKNOX2,SERPINB11
GO:0120036	plasma membrane bounded cell projection organization	9.626615646767171e-8	*LRRC49,MTOR,LRP12,SPOCK1,CNTN4,RIPOR2,RP1,ODAD2,RIMS1,TENM3,SPAG16,MINAR1,CDC42EP3,RIMS2,PARVB,NEGR1,MYO3B,DOCK10,MACF1,NEDD4,ARMC2,PHACTR1,NEO1,CNTN6,SLC39A12,PAK1,EPHA7,RAPGEF2,ADGRB3,ARSB,TAOK3,LRGUK,EPB41L3,STAU2,SEMA5A,SYT1,VCL,ARHGAP44,NTF3,AURKA,SRGAP2C,MAP4K4,BMPR1B,PAK3,DIP2B,IFT57,PRKCZ,KLHL1,YAP1,ALCAM,ABLIM1,GFRA1,TPM1,SEMA3C,TANC1,BBS2,BLK,TNR,SDC2,DNAH5,MYO10,USP33,CD44,PTPRO,PLXNA2,ARHGEF7,SEMA3E,TMEM67,ATL1,AFG3L2,CFAP61,ANK3,BCL11B,PLCE1,FUT9,IQSEC1,PDLIM5,NRK,DNAL1,SEMA3D,SLC23A2,TTC21B,B4GALT6,TSPAN2,RAP1GAP,DRAXIN,DNAH8,ATF1,DAW1,FARP1,BBS4,MTMR2,ALS2,TOX,CFAP74,DPYSL5,ZDHHC17,IFT81,FAM149B1,CDC14B,CIBAR1,ANLN,GORAB,NEDD9,ABI1,ITGA4,CEP120,CYFIP2,ADGRB1,WNT7A,FOXO6,SDCBP,NCK1,SNAP29,LHX9,OCLN,FEZ2,LAMB1,ARL13B,RP1L1,IFT46,ITGA1,B9D1,TOGARAM1,ATG5,FLRT2,RFX2,CCDC141*
GO:0030030	cell projection organizati	1.232228669096611e-7	*LRRC49,MTOR,LRP12,SPOCK1,CNTN4,RIPOR2,RP1,ODAD2,RIMS1,TENM3,SPAG16,MINAR1,CDC42EP3,RIMS2,PARVB,NEGR1,MYO3B,DOCK10,MACF1,NEDD4,ARMC2,PHACTR1,NEO1,CNTN6,SLC39A12,PAK1,EPHA7,RAPGEF2,ADGRB3,DEUP1,ARSB*

	on		,TAOK3,LRGUK,EPB41L3,STAU2,SEMA5A,SYT1,VCL,ARHGAP44,NTF3,AURKA,SRGAP2C,MAP4K4,BMPR1B,RAB8B,PAK3,DIP2B,IFT57,PRKCZ,KLHL1,YAP1,ALCAM,ABLIM1,GFRA1,TPM1,SEMA3C,TANC1,BBS2,BLK,TNR,SDC2,DNAH5,MYO10,USP33,CD44,PTPRO,PLXNA2,ARHGEF7,SEMA3E,TMEM67,ATL1,AFG3L2,CFAP61,ANK3,BCL11B,PLCE1,FUT9,IQSEC1,PDLIM5,NRK,DNAL1,SEMA3D,SLC23A2,TTC21B,B4GALT6,TSPAN2,RAP1GAP,DRAXIN,DNAH8,ATF1,DAW1,FARP1,BBS4,MTMR2,ALS2,TOX,CFAP74,DPYSL5,ZDHHC17,IFT81,FAM149B1,CDC14B,CIBAR1,ANLN,GORAB,NEDD9,ABI1,ITGA4,CEP120,CYFIP2,ADGRB1,WNT7A,FOXO6,SDCBP,NCK1,SNAP29,LHX9,OCLN,FEZ2,LAMB1,ARL13B,RP1L1,IFT46,ITGA1,B9D1,TOGARAM1,ATG5,FLRT2,RFX2,CCDC141
GO:0007275	multicellular organism development	4.2370911610607367e-7	MTOR,SMOC1,LRP12,PLCB1,NEBL,SPOCK1,ZNF536,BRINP3,SGCD,CNTN4,ZFPM2,TENM4,RIPOR2,RP1,ODAD2,FBN1,RIMS1,TENM3,RARB,NAV2,ENPEP,USH2A,MINAR1,RIMS2,ASTN1,NEGR1,MYO3B,RTN1,TCF4,DOCK10,EGFR,ANGPT1,MACF1,PRKACB,NEDD4,CRB1,SOX6,PHACTR1,DKK2,GABRB1,NEO1,CNTN6,SLC39A12,SLC8A3,PAK1,EPHA7,SPEN,RAPGEF2,ADGRB3,RUNX2,ARSB,CPS1,TAOK3,LDB2,EPB41L3,COL4A2,ADAM10,STAU2,TMC1,SEMA5A,SYT1,VCL,ARHGAP44,NTF3,AURKA,PYGO1,SLC8A1,SRGAP2C,SRGAP2B,MAP4K4,BMPR1B,PAK3,TTLL7,DIP2B,ITPKB,CHST8,IFT57,PRKCZ,KLHL1,MCPH1,ZSWIM6,YAP1,CADM2,ALCAM,PLG,PDGFD,ZNRF3,GFRA1,NIPBL,MYLK3,MBNL1,ATF6,TPM1,ANKRD11,BIRC6,KLF15,PPARA,SYNJ1,ADAMTS3,SF3B6,SEMA3C,SLC24A4,ALPK2,DNAH11,JARID2,SCN2A,CPE,IL34,BBS2,RANBP3L,LDB3,BLK,TNR,XIRP2,SDC2,GAS2,DROSHA,TTLL5,DNAH5,GALC,LATS2,ASPM,AP3B1,ATP11C,ZBTB16,MUSK,SETDB2,USP33,CD44,PTPRO,ALPK3,SPRED2,RPS6KA3,NHS,PTPN2,PLXNA2,ATXN3,ST8SIA6,ARHGEF7,AMBRA1,KDM7A,OPRM1,FANCA,SEMA3E,ALPL,TMOD2,MSH2,ATL1,EPN2,AFG3L2,MOSMO,MNAT1,ANK3,XYLT1,HMGA2,BCL11B,AK8,PLCE1,CRIM1,FUT9,MYT1L,HLA-B,IQSEC1,PDLIM5,NRK,SLC10A7,BMP2,RC3H2,TRAK1,GFI1B,BMP2K,RNF38,SEMA3D,NFATC2,TDRD7,SLC23A2,TTC21B,B4GALT6,TSPAN2,RAP1GAP,DRAXIN,ATF1,CASP5,DAW1,FARP1,BBS4,COL5A1,CFTR,AHDC1,KITLG,GTF2I,TADA2A,MTMR2,SH3PXD2A,TBX20,LGI2,PRKCH,TG,IL6R,ALS2,TOX,PTPRB,PDE6A,NPAS2,YIPF6,VCAM1,LRIG1,DTX1,DPYSL5,HECTD1,SHROOM3,XRCC4,SNAI2,SIAH2,RXRG,SP3,MBTPS2,ZDHHC17,SAMHD1,ENPP1,KCNC1,CSF1,GHRH,BCL2L1,CTDP1,ASB4,DHRS3,SMAD5,SYNJ2,SLC40A1,MED1,FAT1,PTH,TEAD1,ANP32B,YBX3,AIMP1,PCID2,CIBAR1,CADM1,VSX1,BPNT1,SVEP1,CREBBP,GORAB,SIAH3,NFKBIA,ABCC8,ALX4,NEDD9,ASS1,PPP1R17,OTOP1,STOX2,AGO1,SLC6A11,MTPN,ABI1,ITGA4,POU1F1,ASB2,CEP120,CYFIP2,ST8SIA4,RXRA,ADGRB1,WNT7A,NDFIP1,MAP3K4,FOXO6,PDE2A,SDCBP,NCK1,FLVCR1,FGR,RNF8,KIAA0319L,DNMT3L,LHX9,WNT2B,TNNI1,CD101,FEZ2,LAMB1,GPR55,NSUN2,TNFSF11,ARL13B,UGP2,TET1,RP1L1,ITGA1,POR,B9D1,MACROH2A1,ATG5,UNK,FLRT2,DDX6,CCDC141
GO:0050896	response to stimulus	4.567510757295763e-7	WWC1,GARNL3,MTOR,NSG1,LRP12,PLCB1,ANKS1B,ZNF536,KSR1,BRINP3,SGCD,CNTN4,PIEZO2,TENM4,RIPOR2,RP1,ERC1,KCNMA1,ARPP21,FBN1,F13A1,CDH8,DCDC1,RIMS1,PIK3C3,SPIRE1,TENM3,RARB,ENPEP,USH2A,MINAR1,CDC42EP3,RIMS2,ADGRE1,PJA2,BABAM2,ERBIN,RHPN2,CACNG2,GLYAT,SUSD4,MAP3K9,MYO3B,NEK4,DOCK10,EGFR,DENND1A,USP14,ANGPT1,MACF1,PRKACB,NEK7,NCOR1,HMCN2,DOCK2,UGT3A2,NEDD4,MAML2,CRB1,NSMCE2,SOX6,PSMB2,B3GALT5,DKK2,MAPKBP1,AOAH,NAT1,GABRB1,DGKI,C12ORF4,GRIA1,NEO1,CNTN6,SLC39A12,SLC8A3,TOM1L2,PAK1,EPHA7,CTNNAL1,NCOA7,GRAMD1B,RALGPS1,SPEN,RAPGEF2,ADGRB3,RUNX2,ARSB,GABRA6,CPS1,TAOK3,CPEB4,BCKDHB,PRICKLE2,PATJ,RPTOR,COL4A2,PPP1R12B,ADAM10,IL1R1,APBB2,MTUS1,STAU2,TMC1,USP18,SEMA5A,SYT1,VCL,ARHGAP44,NTF3,ACER2,AURKA,PYGO1,SLC8A1,TAFA2,MAP4K4,BMPR1B,FMN2,HOM

			ER2,HADHB,PAK3,RFTN1,PDE1A,LARP1,ITPKB,RGS20,PDE10A,RAP1GDS1,HHAT,RNLS,KICS2,CUBN,IFT57,INTS7,SUSD6,PRKCZ,BTLA,GRB10,MCPH1,FER1L6,RGS9,DEFA3,YAP1,PPM1L,ABCD3,SGTB,USP25,ALCAM,PLG,PAPPA,PDGFD,ZNRF3,ITGBL1,UBE2O,GFRA1,SYCP1,NIPBL,MICU1,CORO2B,CARD18,CHD6,STK38,HRH4,SORCS3,MYLK3,ATF6,TLK1,TPM1,BIRC6,KLF15,PPARA,SYNJ1,ADAMTS3,ARAP2,RSRC1,PTPRK,ARHGEF12,TRERF1,SEMA3C,DAPK1,SLC24A4,SEC14L1,VPS13C,STK32B,MAGI1,ALPK2,JARID2,SCN2A,DNAJC15,CPE,EVC2,IL34,TANC1,MELK,BBS2,OR4F6,NKG7,USP8,PIAS1,BLK,TNR,OXR1,GAS2,KCNH1,DROSHA,APBB1IP,SLFN11,MORC1,MYO10,LATS2,GSG1L,ASPM,AP3B1,DENND2B,MUSK,KIR3DL2,GNG7,SMARCAD1,PRKCE,FOXK2,USP33,CD44,RGS12,PTPRO,ABCC9,NSMAF,NLRP13,LNPEP,LIMD1,SPRED2,RPS6KA3,PTPN2,PLXNA2,MCF2L,OR4F15,ATXN3,ARHGEF7,AMBRA1,RFTN2,OPRM1,BIN2,FANCM,FANCA,CYBRD1,CNNM4,SEMA3E,TMEM67,ALPL,TMOD2,MSH2,IGLV2-14,RELL1,HIPK3,EPN2,CD163,CLSPN,AFG3L2,MOSMO,MNAT1,TMEM116,MDFIC,ANK3,HMGA2,BCL11B,VPS41,DOCK5,F5,STK32A,PLCE1,IL17RA,CRIM1,PRR5L,VAV1,FBXO32,HLA-B,IQSEC1,CACNA1I,BLM,NRK,MAGI3,ADCY10,BMP2,RC3H2,RIN3,BMP2K,SEMA3D,NETO2,POLR3A,NFATC2,SH3BP5,SLC23A2,ZNF106,MYOM1,TRAF3,PRG4,TTC21B,UIMC1,TSPAN2,RAP1GAP,DRAXIN,ATF1,CCDC186,CGAS,GABRR2,CNKSR3,CASP5,WDR12,CUL1,MOB1B,BBS4,MAPK8IP1,COL5A1,CFTR,UBASH3A,KITLG,AKAP10,PTPRE,MTMR2,TBX20,AFAP1,WSB1,TRPM6,PRKCH,TG,IL6R,ALS2,OR51E1,SNX25,OSCP1,PDE6A,MAP7,USP7,ENPP3,HAAO,MESD,MOK,KIR2DL4,RALB,NPAS2,VCAM1,SEL1L,ARHGAP31,GSTA3,DTX1,ZBTB33,ADA2,FANCL,DPYSL5,SLC13A5,SUPT16H,CUL5,OR7A17,NEK6,SHROOM3,XRCC4,NMU,GAST,SNAI2,IGHV3-74,BID,SIAH2,RXRG,ERN2,MBTPS2,FLNB,TRIM58,TIAL1,ZDHHC17,SH3GLB1,SAMHD1,IFT81,ENPP1,KCNC1,CSF1,GHRH,BCL2L1,CTDP1,ASB4,DHRS3,SMAD5,SLC40A1,PRAME,HADHA,MED1,IPCEF1,CDC14B,CFH,PTH,PRKAA2,CSF2RB,TRAV8-6,ABHD2,VSTM2A,PLA2G4A,REG4,CAMLG,TEAD1,YBX3,AIMP1,FYB2,PCID2,CIBAR1,PBLD,FICD,CADM1,PEG10,NET1,SLC1A7,VSX1,FSTL1,SVEP1,MADD,HCRTR1,PRSS2,CREBBP,GORAB,NFKBIA,ABCC8,MT1HL1,ZC3H15,RFC2,NEDD9,OLFM4,ASS1,ADGRE3,PPP1R17,ERLIN2,OTOP1,EXOC1,KRT6A,GID8,FAM189A2,NDFIP2,NR2C1,CMTM7,SLC6A11,MARCHF6,MTPN,ABI1,ITGA4,TOP3A,UBE2J2,TM9SF4,OR6C75,ASB2,CYFIP2,HNRNPM,ACACA,ASCC2,OR13C9,RXRA,ADGRB1,WNT7A,NDFIP1,MAP3K4,TRIM43B,TRIM43,ATP6V1C2,C16ORF72,OR10H2,PDE2A,LRRC2,SDCBP,DSG1,JPT2,NSMCE1,NCK1,FGR,C2,IFNAR1,RNF8,CYTH4,LHX9,WNT2B,TNNI1,OCLN,POSTN,CD101,AKAP11,DTHD1,MVB12B,ERP27,CD5L,ANKRD6,FEZ2,INIP,LAMB1,SCARA5,PLCZ1,FCRLA,DIDO1,GPR55,NSUN2,TNFSF11,PPM1F,ARL13B,SH2D3C,IGLV4-3,ZFYVE28,OPA3,TET1,ASB3,RAD9A,RP1L1,ITGA1,POR,NSG2,B9D1,PRDM15,SRGAP3,MACROH2A1,CSNK1G1,SERPINB2,ATG5,FLRT2,OR2T2,TMEM25,PPP1R13B,RFX2,CCDC141,MTREX
GO:0048731	system development	5.943159123485349e-7	MTOR,SMOC1,LRP12,PLCB1,NEBL,SPOCK1,ZNF536,BRINP3,SGCD,CNTN4,ZFPM2,TENM4,RIPOR2,RP1,ODAD2,FBN1,RIMS1,TENM3,RARB,NAV2,ENPEP,USH2A,MINAR1,RIMS2,ASTN1,NEGR1,RTN1,TCF4,DOCK10,EGFR,ANGPT1,MACF1,PRKACB,NEDD4,CRB1,SOX6,PHACTR1,GABRB1,NEO1,CNTN6,SLC39A12,SLC8A3,PAK1,EPHA7,SPEN,RAPGEF2,ADGRB3,RUNX2,ARSB,CPS1,TAOK3,LDB2,EPB41L3,COL4A2,ADAM10,STAU2,TMC1,SEMA5A,SYT1,VCL,ARHGAP44,NTF3,AURKA,PYGO1,SLC8A1,SRGAP2C,SRGAP2B,MAP4K4,BMPR1B,PAK3,TTLL7,DIP2B,CHST8,IFT57,PRKCZ,KLHL1,MCPH1,ZSWIM6,YAP1,CADM2,ALCAM,PLG,PDGFD,GFRA1,NIPBL,MYLK3,MBNL1,ATF6,TPM1,ANKRD11,KLF15,PPARA,SYNJ1,SEMA3C,SLC24A4,ALPK2,DNAH11,JARID2,SCN2A,CPE,IL34,BBS2,RANBP3L,LDB3,BLK,T

			NR,XIRP2,SDC2,GAS2,TTLL5,DNAH5,GALC,ASPM,AP3B1,ZBTB16,MUSK,SETDB2,USP33,CD44,PTPRO,ALPK3,SPRED2,RPS6KA3,NHS,PLXNA2,ATXN3,ARHGEF7,AMBRA1,KDM7A,OPRM1,FANCA,SEMA3E,ALPL,TMOD2,MSH2,ATL1,EPN2,AFG3L2,MOSMO,MNAT1,ANK3,XYLT1,HMGA2,BCL11B,AK8,PLCE1,CRIM1,FUT9,MYT1L,HLA-B,IQSEC1,PDLIM5,NRK,SLC10A7,BMP2,RC3H2,TRAK1,RNF38,SEMA3D,NFATC2,TDRD7,SLC23A2,TTC21B,B4GALT6,TSPAN2,RAP1GAP,DRAXIN,ATF1,CASP5,DAW1,FARP1,BBS4,COL5A1,KITLG,GTF2I,MTMR2,TBX20,LGI2,PRKCH,TG,IL6R,ALS2,TOX,PTPRB,PDE6A,NPAS2,YIPF6,VCAM1,LRIG1,DTX1,DPYSL5,HECTD1,SHROOM3,XRCC4,SNAI2,RXRG,SP3,MBTPS2,ZDHHC17,SAMHD1,KCNC1,CSF1,GHRH,BCL2L1,CTDP1,ASB4,DHRS3,SMAD5,SYNJ2,SLC40A1,MED1,FAT1,PTH,ANP32B,YBX3,AIMP1,PCID2,CADM1,VSX1,BPNT1,SVEP1,GORAB,ABCC8,ALX4,ASS1,PPP1R17,AGO1,SLC6A11,MTPN,ABI1,ITGA4,POU1F1,ASB2,CEP120,CYFIP2,ST8SIA4,ADGRB1,WNT7A,NDFIP1,FOXO6,PDE2A,SDCBP,NCK1,FLVCR1,FGR,RNF8,KIAA0319L,LHX9,WNT2B,TNNI1,FEZ2,LAMB1,TNFSF11,ARL13B,UGP2,RP1L1,ITGA1,POR,B9D1,ATG5,UNK,FLRT2,DDX6,CCDC141
GO:0030154	cell differentiation	6.962333610905045e-7	*MTOR,SMOC1,LRP12,PLCB1,NEBL,SPOCK1,ZNF536,BRINP3,SGCD,CNTN4,ZFPM2,TENM4,RIPOR2,RP1,FBN1,RIMS1,TENM3,RARB,NAV2,SPAG16,USH2A,MINAR1,RIMS2,ASTN1,NEGR1,RTN1,TCF4,OCA2,DOCK10,EGFR,ANGPT1,CDK12,MACF1,DOCK2,NEDD4,CRB1,SOX6,ARMC2,PHACTR1,DNAJC13,GABRB1,NEO1,CNTN6,SLC39A12,SLC8A3,PAK1,EPHA7,SPEN,RAPGEF2,ADGRB3,DEUP1,RUNX2,ARSB,CPS1,TAOK3,LRGUK,EPB41L3,COL4A2,STAU2,TMC1,SEMA5A,SYT1,VCL,ARHGAP44,NTF3,AURKA,PYGO1,SLC8A1,SRGAP2C,MAP4K4,BMPR1B,FMN2,PAK3,TTLL7,DIP2B,ITPKB,PRKCZ,KLHL1,ZSWIM6,ABCA5,EBF2,YAP1,ALCAM,PLG,GFRA1,SYCP1,NIPBL,RNF17,MYLK3,MBNL1,TPM1,KLF15,PPARA,SYNJ1,SEMA3C,ALPK2,JARID2,GATAD2B,IL34,TANC1,MELK,BBS2,SLC9C1,RANBP3L,LDB3,PIAS1,BLK,TNR,SDC2,KCNH1,DROSHA,GLIS1,MORC1,LATS2,ASPM,AP3B1,ATP11C,ZBTB16,MUSK,USP33,PTPRO,ALPK3,LIMD1,SPRED2,RPS6KA3,NHS,PTPN2,PLXNA2,ARHGEF7,AMBRA1,OPRM1,FANCA,SEMA3E,ALPL,TMOD2,MSH2,ATL1,BICRAL,AFG3L2,MOSMO,ANK3,HMGA2,BCL11B,DOCK5,CRIM1,FUT9,VAV1,MYT1L,ZNF160,HLA-B,IQSEC1,PDLIM5,NRK,BMP2,RC3H2,TRAK1,GFI1B,SEMA3D,NFATC2,TDRD7,SLC23A2,ANKRD26,TTC21B,B4GALT6,TSPAN2,RAP1GAP,DRAXIN,ATF1,NHSL1,FARP1,BBS4,COL5A1,CFTR,KITLG,MTMR2,SH3PXD2A,TBX20,PRKCH,IL6R,ALS2,HEMGN,TOX,PTPRB,TBATA,YIPF6,VCAM1,DTX1,DPYSL5,HECTD1,SHROOM3,SNAI2,RXRG,SP3,FLNB,TRIM58,TIAL1,ELF2,ZDHHC17,SLC22A14,KRT6B,IFT81,ENPP1,CSF1,BCL2L1,SPATA48,CTDP1,ASB4,SMAD5,PRAME,CABYR,MED1,FAT1,PRAMEF25,PTH,SDF4,SOHLH1,ABHD2,VSTM2A,ZBTB7C,ANP32B,YBX3,PCID2,CADM1,PEG10,ANLN,VSX1,FSTL1,ARL11,NFKBIA,ABCC8,NEDD9,KRT6A,NR2C1,CMTM7,MTPN,ABI1,ITGA4,BCAP29,ASB2,CEP120,DHTKD1,CYFIP2,KRT85,RXRA,ADGRB1,WNT7A,NDFIP1,MAP3K4,FOXO6,PDE2A,SDCBP,NCK1,FLVCR1,FGR,SPRR2D,RNF8,KIAA0319L,DNMT3L,LHX9,WNT2B,CD101,FEZ2,LAMB1,FCRLA,GPR55,NSUN2,TNFSF11,ARL13B,TET1,RP1L1,ITGA1,POR,B9D1,MACROH2A1,ATG5,UNK,FLRT2,NUDT21,DDX6,RFX2,CCDC141*
GO:0051716	cellular response to stimulus	7.211684044997644e-7	*WWC1,GARNL3,MTOR,NSG1,LRP12,PLCB1,ANKS1B,ZNF536,KSR1,BRINP3,SGCD,PIEZO2,TENM4,RIPOR2,RP1,ERC1,ARPP21,FBN1,DCDC1,RIMS1,PIK3C3,SPIRE1,TENM3,RARB,ENPEP,MINAR1,CDC42EP3,RIMS2,ADGRE1,PJA2,BABAM2,ERBIN,RHPN2,CACNG2,GLYAT,MAP3K9,NEK4,DOCK10,EGFR,DENND1A,USP14,ANGPT1,MACF1,PRKACB,NEK7,NCOR1,DOCK2,UGT3A2,NEDD4,MAML2,CRB1,NSMCE2,DKK2,MAPKBP1,NAT1,GABRB1,DGKI,GRIA1,NEO1,CNTN6,SLC39A12,SLC8A3,TOM1L2,PAK1,EPHA7,CTNNAL1,NCOA7,GRAMD1B,RALGPS1,SPEN,RAPG*

			EF2,ADGRB3,RUNX2,GABRA6,CPS1,TAOK3,CPEB4,PRICKLE2,PATJ,RPTOR,COL4A2,PPP1R12B,ADAM10,IL1R1,APBB2,MTUS1,STAU2,USP18,SEMA5A,SYT1,ARHGAP44,NTF3,ACER2,AURKA,PYGO1,SLC8A1,TAFA2,MAP4K4,BMPR1B,FMN2,HOMER2,HADHB,PAK3,RFTN1,PDE1A,LARP1,ITPKB,RGS20,PDE10A,RAP1GDS1,HHAT,KICS2,IFT57,INTS7,SUSD6,PRKCZ,BTLA,GRB10,RGS9,DEFA3,YAP1,PPM1L,SGTB,USP25,ALCAM,PAPPA,PDGFD,ZNRF3,ITGBL1,UBE2O,GFRA1,SYCP1,NIPBL,MICU1,CORO2B,CHD6,STK38,HRH4,SORCS3,MYLK3,ATF6,TLK1,TPM1,BIRC6,KLF15,PPARA,ADAMTS3,ARAP2,PTPRK,ARHGEF12,TRERF1,SEMA3C,DAPK1,SLC24A4,SEC14L1,VPS13C,STK32B,MAGI1,ALPK2,JARID2,SCN2A,DNAJC15,CPE,EVC2,IL34,MELK,BBS2,OR4F6,NKG7,USP8,PIAS1,BLK,TNR,OXR1,GAS2,KCNH1,APBB1IP,SLFN11,MYO10,LATS2,GSG1L,ASPM,AP3B1,DENND2B,MUSK,GNG7,SMARCAD1,PRKCE,USP33,CD44,RGS12,PTPRO,NSMAF,LNPEP,LIMD1,SPRED2,RPS6KA3,PTPN2,PLXNA2,MCF2L,OR4F15,ATXN3,ARHGEF7,AMBRA1,OPRM1,BIN2,FANCM,FANCA,SEMA3E,TMEM67,ALPL,TMOD2,MSH2,RELL1,HIPK3,EPN2,CLSPN,MOSMO,MNAT1,TMEM116,MDFIC,ANK3,HMGA2,VPS41,DOCK5,STK32A,PLCE1,IL17RA,CRIM1,PRR5L,VAV1,FBXO32,IQSEC1,CACNA1I,BLM,NRK,MAGI3,ADCY10,BMP2,RC3H2,RIN3,BMP2K,SEMA3D,NETO2,NFATC2,SH3BP5,SLC23A2,ZNF106,MYOM1,TRAF3,TTC21B,UIMC1,RAP1GAP,DRAXIN,ATF1,CCDC186,CGAS,GABRR2,CNKSR3,CASP5,WDR12,CUL1,MOB1B,BBS4,MAPK8IP1,CFTR,UBASH3A,KITLG,AKAP10,PTPRE,MTMR2,TBX20,AFAP1,WSB1,PRKCH,TG,IL6R,ALS2,OR51E1,SNX25,PDE6A,USP7,MESD,MOK,KIR2DL4,RALB,NPAS2,VCAM1,SEL1L,ARHGAP31,GSTA3,DTX1,ZBTB33,ADA2,FANCL,DPYSL5,SLC13A5,SUPT16H,CUL5,OR7A17,NEK6,SHROOM3,XRCC4,NMU,GAST,SNAI2,IGHV3-74,BID,SIAH2,RXRG,ERN2,MBTPS2,FLNB,TIAL1,ZDHHC17,SH3GLB1,SAMHD1,IFT81,ENPP1,KCNC1,CSF1,GHRH,BCL2L1,ASB4,DHRS3,SMAD5,SLC40A1,PRAME,MED1,IPCEF1,CDC14B,PTH,PRKAA2,CSF2RB,ABHD2,PLA2G4A,CAMLG,TEAD1,YBX3,AIMP1,FYB2,PCID2,CIBAR1,PBLD,FICD,CADM1,PEG10,NET1,SLC1A7,FSTL1,SVEP1,MADD,HCRTR1,CREBBP,GORAB,NFKBIA,ABCC8,MT1HL1,ZC3H15,RFC2,NEDD9,OLFM4,ASS1,ADGRE3,PPP1R17,ERLIN2,OTOP1,EXOC1,GID8,FAM189A2,NDFIP2,NR2C1,CMTM7,MARCHF6,MTPN,ABI1,ITGA4,TOP3A,UBE2J2,OR6C75,ASB2,CYFIP2,HNRNPM,ACACA,ASCC2,OR13C9,RXRA,ADGRB1,WNT7A,NDFIP1,MAP3K4,ATP6V1C2,C16ORF72,OR10H2,PDE2A,LRRC2,SDCBP,JPT2,NSMCE1,NCK1,FGR,IFNAR1,RNF8,CYTH4,WNT2B,POSTN,CD101,AKAP11,DTHD1,MVB12B,ERP27,ANKRD6,FEZ2,INIP,LAMB1,SCARA5,PLCZ1,FCRLA,DIDO1,GPR55,NSUN2,TNFSF11,PPM1F,ARL13B,SH2D3C,ZFYVE28,TET1,ASB3,RAD9A,RP1L1,ITGA1,POR,NSG2,B9D1,PRDM15,SRGAP3,CSNK1G1,ATG5,FLRT2,OR2T2,TMEM25,PPP1R13B,RFX2,MTREX
GO:0048869	cellular developmental process	8.122362928924366e-7	*MTOR,SMOC1,LRP12,PLCB1,NEBL,SPOCK1,ZNF536,BRINP3,SGCD,CNTN4,ZFPM2,TENM4,RIPOR2,RP1,FBN1,RIMS1,TENM3,RARB,NAV2,SPAG16,USH2A,MINAR1,RIMS2,ASTN1,NEGR1,RTN1,TCF4,OCA2,DOCK10,EGFR,ANGPT1,CDK12,MACF1,DOCK2,NEDD4,CRB1,SOX6,ARMC2,PHACTR1,DNAJC13,GABRB1,NEO1,CNTN6,SLC39A12,SLC8A3,PAK1,EPHA7,SPEN,RAPGEF2,ADGRB3,DEUP1,RUNX2,ARSB,CPS1,TAOK3,LRGUK,EPB41L3,COL4A2,STAU2,TMC1,SEMA5A,SYT1,VCL,ARHGAP44,NTF3,AURKA,PYGO1,SLC8A1,SRGAP2C,MAP4K4,BMPR1B,FMN2,PAK3,TTLL7,DIP2B,ITPKB,PRKCZ,KLHL1,ZSWIM6,ABCA5,EBF2,YAP1,ALCAM,PLG,GFRA1,SYCP1,NIPBL,RNF17,MYLK3,MBNL1,TPM1,KLF15,PPARA,SYNJ1,SEMA3C,ALPK2,JARID2,GATAD2B,IL34,TANC1,MELK,BBS2,SLC9C1,RANBP3L,LDB3,PIAS1,BLK,TNR,SDC2,KCNH1,DROSHA,GLIS1,MORC1,LATS2,ASPM,AP3B1,ATP11C,ZBTB16,MUSK,USP33,CD44,PTPRO,ALPK3,LIMD1,SPRED2,RPS6KA3,NHS,PTPN2,PLXNA2,ARHGEF7,AMBRA1,OPRM1,FANCA,SEMA3E,ALPL,TMOD2,MSH2,ATL1,BICRAL,AFG3L2,MOSMO,ANK3,HMGA2,BCL11B,DOCK5,CRIM1,F*

			UT9,VAV1,MYT1L,ZNF160,HLA-B,IQSEC1,PDLIM5,NRK,BMP2,RC3H2,TRAK1,GFI1B,SEMA3D,NFATC2,TDRD7,SLC23A2,ANKRD26,TTC21B,B4GALT6,TSPAN2,RAP1GAP,DRAXIN,ATF1,NHSL1,FARP1,BBS4,COL5A1,CFTR,KITLG,MTMR2,SH3PXD2A,TBX20,PRKCH,IL6R,ALS2,HEMGN,TOX,PTPRB,TBATA,YIPF6,VCAM1,DTX1,DPYSL5,HECTD1,SHROOM3,SNAI2,RXRG,SP3,FLNB,TRIM58,TIAL1,ELF2,ZDHHC17,SLC22A14,KRT6B,IFT81,ENPP1,CSF1,BCL2L1,SPATA48,CTDP1,ASB4,SMAD5,PRAME,CABYR,MED1,FAT1,PRAMEF25,PTH,SDF4,SOHLH1,ABHD2,VSTM2A,ZBTB7C,ANP32B,YBX3,PCID2,CADM1,PEG10,ANLN,VSX1,FSTL1,ARL11,NFKBIA,ABCC8,NEDD9,KRT6A,NR2C1,CMTM7,MTPN,ABI1,ITGA4,BCAP29,ASB2,CEP120,DHTKD1,CYFIP2,KRT85,RXRA,ADGRB1,WNT7A,NDFIP1,MAP3K4,FOXO6,PDE2A,SDCBP,NCK1,FLVCR1,FGR,SPRR2D,RNF8,KIAA0319L,DNMT3L,LHX9,WNT2B,CD101,FEZ2,LAMB1,FCRLA,GPR55,NSUN2,TNFSF11,ARL13B,TET1,RP1L1,ITGA1,POR,B9D1,MACROH2A1,ATG5,UNK,FLRT2,NUDT21,DDX6,RFX2,CCDC141
GO:0023052	signaling	0.0000010747297121753465	*WWC1,GARNL3,MTOR,NSG1,LRP12,PLCB1,ANKS1B,ZNF536,KSR1,SGCD,CNTN4,TENM4,RIPOR2,RP1,ERC1,FBN1,CDH8,DCDC1,RIMS1,PIK3C3,TENM3,RARB,ENPEP,MINAR1,CDC42EP3,RIMS2,ADGRE1,PJA2,BABAM2,SV2C,ERBIN,RHPN2,CACNG2,MAP3K9,DOCK10,EGFR,DENND1A,USP14,ANGPT1,MACF1,PRKACB,NCOR1,DOCK2,NEDD4,MAML2,CRB1,BTBD9,DKK2,MAPKBP1,GABRB1,DGKI,GRIA1,NEO1,CNTN6,SLC39A12,SLC8A3,TOM1L2,PAK1,EPHA7,CTNNAL1,RALGPS1,SPEN,RAPGEF2,ADGRB3,RUNX2,GABRA6,TAOK3,CPEB4,PRICKLE2,PATJ,RPTOR,COL4A2,PPP1R12B,ADAM10,IL1R1,APBB2,CACNB2,STAU2,USP18,SEMA5A,SYT1,ARHGAP44,NTF3,ACER2,AURKA,PYGO1,SLC8A1,TAFA2,MAP4K4,BMPR1B,FMN2,HOMER2,RAB8B,PAK3,RFTN1,PDE1A,LARP1,ITPKB,RGS20,PDE10A,RAP1GDS1,HHAT,KICS2,IFT57,INTS7,PRKCZ,BTLA,GRB10,RGS9,DEFA3,YAP1,PPM1L,ALCAM,PLG,PAPPA,PDGFD,ZNRF3,ITGBL1,UBE2O,GFRA1,STK38,HRH4,SORCS3,ATF6,TLK1,BIRC6,KLF15,PPARA,SYNJ1,ADAMTS3,ARAP2,PTPRK,ARHGEF12,TRERF1,SEMA3C,DAPK1,SLC24A4,SEC14L1,STK32B,MAGI1,ALPK2,SCN2A,AMPH,CPE,EVC2,IL34,MELK,BBS2,OR4F6,NKG7,USP8,PIAS1,BLK,TNR,GAS2,KCNH1,APBB1IP,EIPR1,MYO10,LATS2,GSG1L,ASPM,AP3B1,DENND2B,MUSK,GNG7,PRKCE,USP33,CD44,RGS12,PTPRO,NSMAF,LNPEP,LIMD1,SPRED2,RPS6KA3,PTPN2,PLXNA2,MCF2L,OR4F15,ATXN3,RIC3,ARHGEF7,OPRM1,FANCA,SEMA3E,TMOD2,MSH2,RELL1,HIPK3,EPN2,CLSPN,MOSMO,TMEM116,MDFIC,ANK3,HMGA2,DOCK5,STK32A,PLCE1,IL17RA,CRIM1,PRR5L,VAV1,IQSEC1,CACNA1I,BLM,NRK,MAGI3,ADCY10,BMP2,RC3H2,RIN3,BMP2K,SEMA3D,NETO2,NFATC2,SH3BP5,ZNF106,MYOM1,TRAF3,TTC21B,UIMC1,RAP1GAP,DRAXIN,ATF1,CCDC186,CGAS,GABRR2,CNKSR3,CASP5,WDR12,CUL1,FARP1,MOB1B,BBS4,MAPK8IP1,CFTR,UBASH3A,KITLG,AKAP10,PTPRE,MTMR2,TBX20,AFAP1,WSB1,PRKCH,TG,IL6R,ALS2,OR51E1,SNX25,PDE6A,SCN10A,USP7,MESD,MOK,KIR2DL4,RALB,VCAM1,SEL1L,ARHGAP31,DTX1,ZBTB33,ADA2,DPYSL5,CUL5,OR7A17,NEK6,NMU,GAST,SNAI2,IGHV3-74,BID,SIAH2,RXRG,ERN2,MBTPS2,FLNB,TIAL1,ZDHHC17,SAMHD1,IFT81,ENPP1,CSF1,GHRH,BCL2L1,ASB4,DHRS3,SMAD5,PRAME,MED1,CDC14B,FAT1,PTH,PRKAA2,CSF2RB,ABHD2,PLA2G4A,CAMLG,TEAD1,YBX3,AIMP1,FYB2,PCID2,CIBAR1,PBLD,FICD,CADM1,PEG10,NET1,SLC1A7,FSTL1,SVEP1,MADD,HCRTR1,CREBBP,GORAB,NFKBIA,ABCC8,ZC3H15,NEDD9,OLFM4,ADGRE3,PPP1R17,ERLIN2,OTOP1,EXOC1,GID8,FAM189A2,NDFIP2,NR2C1,CMTM7,ABI1,ITGA4,OR6C75,ASB2,CYFIP2,OR13C9,RXRA,ADGRB1,WNT7A,NDFIP1,MAP3K4,ATP6V1C2,C16ORF72,OR10H2,PDE2A,LRRC2,SDCBP,JPT2,NCK1,FGR,SNAP29,IFNAR1,CYTH4,WNT2B,POSTN,CD101,AKAP11,DTHD1,MVB12B,ANKRD6,SCGN,FEZ2,INIP,LAMB1,PLCZ1,FCRLA,DIDO1,GPR55,NSUN2,TNFSF11,PPM1F,ARL13B,SH2D3C*

			,ZFYVE28,TET1,ASB3,RAD9A,RP1L1,ITGA1,POR,NSG2,B9D1,PRDM15,SRGAP3,CSNK1G1,FLRT2,OR2T2,TMEM25,PPP1R13B
GO:0051179	localization	0.0000011936566781097806	WWC1,MICU2,MTOR,NSG1,LRP12,SLC25A21,TMPRSS2,ABCA13,ANKS1B,SGCD,CACNA2D3,KCNH5,SLC37A1,PIEZO2,RIPOR2,ERC1,SLC44A5,KCNMA1,FBN1,COG5,RIMS1,PIK3C3,SPIRE1,EXOC6B,SPAG16,TRAPPC8,USH2A,RIMS2,SV2C,ERBIN,FCHO2,CACNG2,MYO5C,OCA2,EGFR,DENND1A,ANGPT1,MACF1,DOCK2,NEDD4,CRB1,BTBD9,TUSC3,DNAJC13,GABRB1,DGKI,C12ORF4,GRIA1,SLC39A12,SLC8A3,TOM1L2,CEP128,PAK1,GRAMD1B,RAPGEF2,ARSB,GABRA6,CPS1,LDLRAD3,AGK,RANBP17,SLC44A1,EPB41L3,KIF4A,ADAM10,SLC7A2,CACNB2,STAU2,TMC1,SYT1,VCL,ARHGAP44,NTF3,AURKA,PYGO1,SLC8A1,FMN2,HOMER2,RAB8B,RFTN1,RAP1GDS1,CLIC6,KICS2,CUBN,SCP2,IFT57,PRKCZ,SPOP,GRB10,MCPH1,CNST,ABCA5,YAP1,VPS35L,ABCD3,RABGAP1L,SGTB,TRPC7,SLC45A4,UBE2O,SYCP1,NIPBL,NIPAL2,IPO11,MICU1,CORO2B,TLK1,LRRC38,ZDHHC14,CORIN,KLF15,PPARA,SNX30,KCNS3,SYNJ1,RSRC1,PTPRK,DAPK1,SLC24A4,SEC14L1,VPS13C,DNAH11,JARID2,SCN2A,RAB22A,DNAJC15,AMPH,CPE,BBS2,SLC9C1,RANBP3L,NKG7,USP8,SLC36A1,BLK,KCNH1,FHIP1A,EIPR1,DNAH5,MYO10,PLEKHA8,LATS2,GSG1L,ASPM,AP3B1,ATP11C,ABCB7,ZBTB16,MUSK,PRKCE,SLMAP,USP33,ABCC9,STXBP6,PEX14,PTPN2,PLXNA2,ATXN3,RIC3,SLC2A3,ARHGEF7,ALG10B,ATP8A1,RFTN2,OPRM1,BIN2,CYBRD1,CNNM4,ABHD17C,MSH2,EPN2,ABCA10,CD163,AFG3L2,MDFIC,ANK3,NIPA2,COG2,VPS41,LYPLA1,PRR5L,VPS37A,VAV1,CACNA1I,BHLHE40-AS1,SLC10A7,ADCY10,STX12,BMP2,ATP9A,TRAK1,RIN3,BMP2K,SLC15A5,NETO2,AP4E1,SLC23A2,MYOM1,PRG4,TTC21B,SNX8,CCDC186,KCNH8,SLC37A2,GABRR2,CNKSR3,DAW1,BBS4,LRRC8B,MAPK8IP1,CFTR,TBC1D13,NMD3,AKAP10,REPS1,MTMR2,HEPHL1,TRPM6,PRKCH,SLC12A1,TG,ALS2,SNX25,OSCP1,TSPAN33,SCN10A,MAP7,USP7,MON2,MESD,RALB,YIPF6,SEL1L,SLC13A5,NPIPA1,CUL5,HECTD1,SHROOM3,XRCC4,IGHV3-74,BID,OSBPL10,COX5A,TRIM58,ZDHHC17,FYCO1,SH3GLB1,SLC22A14,XKR5,IFT81,ENPP1,KCNC1,GHRH,BCL2L1,SYNJ2,SLC40A1,FAM149B1,CABYR,CIDEC,MED1,IPCEF1,ATG4B,PTH,PRKAA2,VSTM2A,PLA2G4A,SLC25A52,KIFC1,CAMLG,COX7A2L,ANP32B,AIMP1,LASP1,FYB2,PCID2,PEG10,SLC1A7,MELTF,ARL11,SIAH3,TRPV5,NFKBIA,ABCC8,RTRAF,NEDD9,SLC14A2,SAR1A,TRAPPC3,OTOP1,EXOC1,HEPACAM,FAM189A2,NDFIP2,SLC6A11,ITGA4,OAZ2,BCAP29,UBE2J2,TM9SF4,CEP120,STOML1,RXRA,ADGRB1,WNT7A,NDFIP1,ATP6V1C2,MAGEL2,SDCBP,JPT2,FLVCR1,FGR,SNAP29,C2,INTS13,RN7SL767P,PLEKHA3,OCLN,STON1-GTF2A1L,AKAP11,MFSD9,MVB12B,CD5L,SCARA5,HEATR5A,PLCZ1,SLC9A5,NSUN2,ANO10,TNFSF11,PPM1F,ARL13B,XPO7,ODR4,TMEM63C,SLC16A9,ASB3,IFT46,SLC14A1,NSG2,B9D1,MACROH2A1,CSNK1G1,ATP6V0D2,ATG5,NUP43,DDX6,CCDC141
GO:0007154	cell communication	0.0000013607603553342886	WWC1,GARNL3,MTOR,NSG1,LRP12,PLCB1,ANKS1B,ZNF536,KSR1,SGCD,CNTN4,TENM4,RIPOR2,RP1,ERC1,FBN1,CDH8,DCDC1,RIMS1,PIK3C3,TENM3,RARB,ENPEP,MINAR1,CDC42EP3,RIMS2,ADGRE1,PJA2,BABAM2,SV2C,ERBIN,RHPN2,CACNG2,MAP3K9,DOCK10,EGFR,DENND1A,USP14,ANGPT1,MACF1,PRKACB,NCOR1,DOCK2,NEDD4,MAML2,CRB1,BTBD9,DKK2,MAPKBP1,GABRB1,DGKI,GRIA1,NEO1,CNTN6,SLC39A12,SLC8A3,TOM1L2,PAK1,EPHA7,CTNNAL1,RALGPS1,SPEN,RAPGEF2,ADGRB3,RUNX2,GABRA6,TAOK3,CPEB4,PRICKLE2,PATJ,RPTOR,COL4A2,PPP1R12B,ADAM10,IL1R1,APBB2,CACNB2,STAU2,USP18,SEMA5A,SYT1,ARHGAP44,NTF3,ACER2,AURKA,PYGO1,SLC8A1,TAFA2,MAP4K4,BMPR1B,FMN2,HOMER2,RAB8B,PAK3,RFTN1,PDE1A,LARP1,ITPKB,RGS20,PDE10A,RAP1GDS1,HHAT,KICS2,IFT57,INTS7,PRKCZ,BTLA,GRB10,RGS9,DEFA3,YAP1,PPM1L,ALCAM,PLG,PAPPA,PDGFD,ZNRF3,ITGBL1,UB

			E2O,GFRA1,STK38,HRH4,SORCS3,ATF6,TLK1,BIRC6,KLF15,PPARA,SYNJ1,ADAMTS3,ARAP2,PTPRK,ARHGEF12,TRERF1,SEMA3C,DAPK1,SLC24A4,SEC14L1,STK32B,MAGI1,ALPK2,SCN2A,DNAJC15,AMPH,CPE,EVC2,IL34,MELK,BBS2,OR4F6,NKG7,USP8,PIAS1,BLK,TNR,GAS2,KCNH1,APBB1IP,EIPR1,MYO10,LATS2,GSG1L,ASPM,AP3B1,DENND2B,MUSK,GNG7,PRKCE,USP33,CD44,RGS12,PTPRO,NSMAF,LNPEP,LIMD1,SPRED2,RPS6KA3,PTPN2,PLXNA2,MCF2L,OR4F15,ATXN3,RIC3,ARHGEF7,AMBRA1,OPRM1,FANCA,SEMA3E,TMOD2,MSH2,RELL1,HIPK3,EPN2,CLSPN,MOSMO,TMEM116,MDFIC,ANK3,HMGA2,VPS41,DOCK5,STK32A,PLCE1,IL17RA,CRIM1,PRR5L,VAV1,IQSEC1,CACNA1I,BLM,NRK,MAGI3,ADCY10,BMP2,RC3H2,RIN3,BMP2K,SEMA3D,NETO2,NFATC2,SH3BP5,ZNF106,MYOM1,TRAF3,TTC21B,UIMC1,RAP1GAP,DRAXIN,ATF1,CCDC186,CGAS,GABRR2,CNKSR3,CASP5,WDR12,CUL1,FARP1,MOB1B,BBS4,MAPK8IP1,CFTR,UBASH3A,KITLG,AKAP10,PTPRE,MTMR2,TBX20,AFAP1,WSB1,PRKCH,TG,IL6R,ALS2,OR51E1,SNX25,PDE6A,SCN10A,USP7,MESD,MOK,KIR2DL4,RALB,VCAM1,SEL1L,ARHGAP31,DTX1,ZBTB33,ADA2,DPYSL5,CUL5,OR7A17,NEK6,NMU,GAST,SNAI2,IGHV3-74,BID,SIAH2,RXRG,ERN2,MBTPS2,FLNB,TIAL1,ZDHHC17,SH3GLB1,SAMHD1,IFT81,ENPP1,CSF1,GHRH,BCL2L1,ASB4,DHRS3,SMAD5,PRAME,MED1,CDC14B,FAT1,PTH,PRKAA2,CSF2RB,ABHD2,PLA2G4A,CAMLG,TEAD1,YBX3,AIMP1,FYB2,PCID2,CIBAR1,PBLD,FICD,CADM1,PEG10,NET1,SLC1A7,FSTL1,SVEP1,MADD,HCRTR1,CREBBP,GORAB,NFKBIA,ABCC8,ZC3H15,NEDD9,OLFM4,ADGRE3,PPP1R17,ERLIN2,OTOP1,EXOC1,GID8,FAM189A2,NDFIP2,NR2C1,CMTM7,ABI1,ITGA4,OR6C75,ASB2,CYFIP2,OR13C9,RXRA,ADGRB1,WNT7A,NDFIP1,MAP3K4,ATP6V1C2,C16ORF72,OR10H2,PDE2A,LRRC2,SDCBP,JPT2,NCK1,FGR,SNAP29,IFNAR1,CYTH4,WNT2B,POSTN,CD101,AKAP11,DTHD1,MVB12B,ANKRD6,SCGN,FEZ2,INIP,LAMB1,PLCZ1,FCRLA,DIDO1,GPR55,NSUN2,TNFSF11,PPM1F,ARL13B,SH2D3C,ZFYVE28,TET1,ASB3,RAD9A,RP1L1,ITGA1,POR,NSG2,B9D1,PRDM15,SRGAP3,CSNK1G1,ATG5,FLRT2,OR2T2,TMEM25,PPP1R13B
GO:0007399	nervous system development	0.000002745392228573166	MTOR,LRP12,PLCB1,SPOCK1,ZNF536,BRINP3,CNTN4,TENM4,RIPOR2,RP1,ODAD2,RIMS1,TENM3,RARB,NAV2,USH2A,MINAR1,RIMS2,ASTN1,NEGR1,RTN1,TCF4,DOCK10,EGFR,MACF1,PRKACB,NEDD4,CRB1,SOX6,PHACTR1,GABRB1,NEO1,CNTN6,SLC39A12,SLC8A3,PAK1,EPHA7,SPEN,RAPGEF2,ADGRB3,RUNX2,ARSB,TAOK3,LDB2,EPB41L3,STAU2,TMC1,SEMA5A,SYT1,VCL,ARHGAP44,NTF3,AURKA,SLC8A1,SRGAP2C,SRGAP2B,MAP4K4,BMPR1B,PAK3,TTLL7,DIP2B,CHST8,IFT57,PRKCZ,KLHL1,MCPH1,ZSWIM6,YAP1,CADM2,ALCAM,GFRA1,NIPBL,MBNL1,KLF15,SYNJ1,SEMA3C,SLC24A4,DNAH11,JARID2,SCN2A,IL34,BBS2,BLK,TNR,SDC2,DNAH5,GALC,ASPM,ZBTB16,MUSK,USP33,PTPRO,RPS6KA3,PLXNA2,ATXN3,ARHGEF7,AMBRA1,KDM7A,OPRM1,SEMA3E,TMOD2,ATL1,AFG3L2,MOSMO,MNAT1,ANK3,BCL11B,AK8,CRIM1,FUT9,MYT1L,IQSEC1,PDLIM5,NRK,BMP2,TRAK1,SEMA3D,SLC23A2,TTC21B,B4GALT6,TSPAN2,RAP1GAP,DRAXIN,ATF1,CASP5,FARP1,BBS4,MTMR2,TBX20,LGI2,PRKCH,TG,ALS2,TOX,PTPRB,NPAS2,VCAM1,LRIG1,DTX1,DPYSL5,HECTD1,SHROOM3,RXRG,ZDHHC17,KCNC1,CSF1,GHRH,SYNJ2,MED1,ANP32B,CADM1,VSX1,BPNT1,GORAB,ABCC8,PPP1R17,SLC6A11,MTPN,ABI1,ITGA4,POU1F1,CEP120,CYFIP2,ST8SIA4,ADGRB1,WNT7A,FOXO6,SDCBP,NCK1,KIAA0319L,LHX9,WNT2B,FEZ2,LAMB1,ARL13B,UGP2,RP1L1,ITGA1,UNK,FLRT2,DDX6,CCDC141
GO:0048856	anatomical structure development	0.0000033640997780029777	MTOR,SMOC1,LRP12,PLCB1,NEBL,SPOCK1,ZNF536,BRINP3,SGCD,CNTN4,ZFPM2,TENM4,RIPOR2,RP1,ODAD2,FBN1,CDH8,RIMS1,SPIRE1,TENM3,RARB,NAV2,ENPEP,SPAG16,USH2A,MINAR1,CDC42EP3,RIMS2,ASTN1,PARVB,NEGR1,MYO3B,RTN1,TCF4,OCA2,DOCK10,EGFR,ANGPT1,MACF1,PRKACB,DOCK2,NEDD4,CRB1,SOX6,ARMC2,PHACTR1,DKK2,GABRB1,NEO1,CNTN6,SLC39A12,SLC8A3,PAK1,EPHA7,SPEN,RAPGEF2,ADGR

			B3,DEUP1,RUNX2,ARSB,CPS1,TAOK3,PRICKLE2,LDB2,EPB41L3,COL4A2,ADAM10,STAU2,TMC1,SEMA5A,SYT1,VCL,ARHGAP44,NTF3,AURKA,CFDP1,PYGO1,SLC8A1,SRGAP2C,SRGAP2B,MAP4K4,BMPR1B,FMN2,PAK3,TTLL7,DIP2B,ITPKB,CHST8,IFT57,PRKCZ,KLHL1,MCPH1,ZSWIM6,EBF2,YAP1,RIPK4,CADM2,ALCAM,PLG,PDGFD,ZNRF3,ABLIM1,GFRA1,SYCP1,NIPBL,RNF17,MYLK3,MBNL1,ATF6,TPM1,ANKRD11,BIRC6,KLF15,PPARA,SYNJ1,ADAMTS3,SF3B6,SEMA3C,SLC24A4,ALPK2,DNAH11,JARID2,SCN2A,CPE,IL34,TANC1,MELK,BBS2,RANBP3L,LDB3,BLK,TNR,XIRP2,SDC2,GAS2,KCNH1,DROSHA,TTLL5,DNAH5,MYO10,GALC,LATS2,ASPM,AP3B1,ATP11C,ZBTB16,MUSK,SETDB2,USP33,CD44,PTPRO,ALPK3,COL5A3,LIMD1,SPRED2,RPS6KA3,NHS,PTPN2,PLXNA2,ATXN3,ST8SIA6,ARHGEF7,AMBRA1,KDM7A,OPRM1,FANCA,CNNM4,SEMA3E,ALPL,TMOD2,MSH2,ATL1,LUC7L,EPN2,AFG3L2,MOSMO,MNAT1,ANK3,XYLT1,HMGA2,BCL11B,DOCK5,AK8,PLCE1,CRIM1,FUT9,VAV1,MYT1L,ZNF160,HLA-B,IQSEC1,PDLIM5,NRK,SLC10A7,BMP2,RC3H2,TRAK1,GFI1B,BMP2K,RNF38,SEMA3D,NFATC2,TDRD7,SLC23A2,TTC21B,B4GALT6,TSPAN2,RAP1GAP,DRAXIN,ATF1,CASP5,CUL1,DAW1,FARP1,BBS4,COL5A1,CFTR,AHDC1,KITLG,GTF2I,TADA2A,MTMR2,SH3PXD2A,TBX20,LGI2,PRKCH,TG,IL6R,ALS2,TFDP1,TOX,PTPRB,PDE6A,SCN10A,MESD,NPAS2,YIPF6,VCAM1,LRIG1,DTX1,DPYSL5,HECTD1,SHROOM3,XRCC4,SNAI2,SIAH2,RXRG,SP3,MBTPS2,FLNB,TRIM58,TIAL1,ZDHHC17,SLC22A14,KRT6B,XKR5,SAMHD1,IFT81,ENPP1,KCNC1,CSF1,GHRH,BCL2L1,KRT25,CTDP1,ASB4,DHRS3,SMAD5,SYNJ2,SLC40A1,CABYR,MED1,ATG4B,FAT1,SCML2,PTH,SOHLH1,ABHD2,TEAD1,ANP32B,YBX3,AIMP1,PCID2,CIBAR1,CADM1,ANLN,VSX1,FSTL1,BPNT1,SVEP1,CREBBP,ARL11,GORAB,SIAH3,NFKBIA,ABCC8,ALX4,NEDD9,ASS1,PPP1R17,OTOP1,KRT6A,STOX2,AGO1,NR2C1,CMTM7,SLC6A11,MTPN,ABI1,ITGA4,POU1F1,ASB2,CEP120,DHTKD1,CYFIP2,KRT85,ST8SIA4,RXRA,ADGRB1,WNT7A,NDFIP1,MAP3K4,FOXO6,PDE2A,SDCBP,NCK1,FLVCR1,FGR,SPRR2D,RNF8,KIAA0319L,DNMT3L,LHX9,WNT2B,TNNI1,POSTN,CD101,ANKRD6,FEZ2,LAMB1,GPR55,NSUN2,TNFSF11,ARL13B,UGP2,TET1,RP1L1,DDX10,ITGA1,POR,B9D1,MACROH2A1,ATG5,UNK,FLRT2,NUDT21,DDX6,RFX2,CCDC141
GO:0048468	cell development	0.0000034562975566551235	MTOR,LRP12,PLCB1,NEBL,SPOCK1,SGCD,CNTN4,TENM4,RIPOR2,RP1,FBN1,RIMS1,TENM3,RARB,SPAG16,MINAR1,RIMS2,NEGR1,OCA2,DOCK10,ANGPT1,MACF1,DOCK2,NEDD4,CRB1,ARMC2,PHACTR1,GABRB1,NEO1,CNTN6,SLC39A12,SLC8A3,PAK1,EPHA7,SPEN,RAPGEF2,ADGRB3,RUNX2,ARSB,TAOK3,EPB41L3,STAU2,TMC1,SEMA5A,SYT1,VCL,ARHGAP44,NTF3,AURKA,PYGO1,SLC8A1,SRGAP2C,MAP4K4,BMPR1B,FMN2,PAK3,DIP2B,ITPKB,PRKCZ,KLHL1,YAP1,ALCAM,GFRA1,SYCP1,RNF17,MYLK3,TPM1,PPARA,SYNJ1,SEMA3C,ALPK2,IL34,MELK,BBS2,LDB3,BLK,TNR,SDC2,DROSHA,LATS2,ASPM,AP3B1,ATP11C,ZBTB16,USP33,PTPRO,ALPK3,LIMD1,PTPN2,PLXNA2,ARHGEF7,AMBRA1,OPRM1,FANCA,SEMA3E,TMOD2,MSH2,ATL1,AFG3L2,ANK3,BCL11B,FUT9,VAV1,MYT1L,ZNF160,HLA-B,IQSEC1,PDLIM5,NRK,BMP2,RC3H2,GFI1B,SEMA3D,NFATC2,TDRD7,SLC23A2,B4GALT6,TSPAN2,DRAXIN,ATF1,FARP1,BBS4,CFTR,KITLG,MTMR2,SH3PXD2A,TBX20,PRKCH,IL6R,ALS2,TOX,YIPF6,VCAM1,DTX1,DPYSL5,HECTD1,SHROOM3,SNAI2,SP3,FLNB,TRIM58,TIAL1,ZDHHC17,SLC22A14,IFT81,CSF1,BCL2L1,CTDP1,SMAD5,CABYR,MED1,FAT1,SOHLH1,ABHD2,YBX3,PCID2,ANLN,VSX1,ARL11,NFKBIA,ABCC8,NEDD9,CMTM7,ABI1,ITGA4,ASB2,DHTKD1,CYFIP2,ADGRB1,WNT7A,NDFIP1,FOXO6,PDE2A,NCK1,FLVCR1,RNF8,LHX9,WNT2B,CD101,FEZ2,LAMB1,GPR55,NSUN2,TNFSF11,ARL13B,RP1L1,ITGA1,ATG5,UNK,FLRT2,NUDT21,RFX2,CCDC141
GO:0007165	signal transducti	0.0000044039938387	WWC1,GARNL3,MTOR,NSG1,LRP12,PLCB1,ANKS1B,ZNF536,KSR1,SGCD,TENM4,RIPOR2,RP1,ERC1,FBN1,DCDC1,RIMS1,PIK3C3,TENM3,RARB,ENPEP,MINAR1,CDC42EP3,RIMS2,ADGR

	on	12315	*E1,PJA2,BABAM2,ERBIN,RHPN2,CACNG2,MAP3K9,DOCK10,EGFR,DENND1A,ANGPT1,MACF1,PRKACB,NCOR1,DOCK2,NEDD4,MAML2,DKK2,MAPKBP1,GABRB1,DGKI,GRIA1,NEO1,CNTN6,SLC39A12,SLC8A3,TOM1L2,PAK1,EPHA7,CTNNAL1,RALGPS1,SPEN,RAPGEF2,ADGRB3,RUNX2,GABRA6,TAOK3,CPEB4,PRICKLE2,PATJ,RPTOR,COL4A2,PPP1R12B,ADAM10,IL1R1,APBB2,USP18,SEMA5A,ARHGAP44,NTF3,ACER2,AURKA,PYGO1,SLC8A1,TAFA2,MAP4K4,BMPR1B,FMN2,HOMER2,PAK3,RFTN1,PDE1A,LARP1,ITPKB,RGS20,PDE10A,RAP1GDS1,HHAT,KICS2,IFT57,INTS7,PRKCZ,BTLA,GRB10,RGS9,DEFA3,YAP1,PPM1L,ALCAM,PAPPA,PDGFD,ZNRF3,ITGBL1,UBE2O,GFRA1,STK38,HRH4,SORCS3,ATF6,TLK1,BIRC6,KLF15,PPARA,ADAMTS3,ARAP2,PTPRK,ARHGEF12,TRERF1,SEMA3C,DAPK1,SLC24A4,SEC14L1,STK32B,MAGI1,ALPK2,SCN2A,CPE,EVC2,IL34,MELK,BBS2,OR4F6,NKG7,USP8,PIAS1,BLK,GAS2,KCNH1,APBB1IP,MYO10,LATS2,GSG1L,ASPM,AP3B1,DENND2B,MUSK,GNG7,PRKCE,USP33,CD44,RGS12,PTPRO,NSMAF,LNPEP,LIMD1,SPRED2,RPS6KA3,PTPN2,PLXNA2,MCF2L,OR4F15,ARHGEF7,OPRM1,FANCA,SEMA3E,TMOD2,MSH2,RELL1,HIPK3,EPN2,CLSPN,MOSMO,TMEM116,MDFIC,ANK3,HMGA2,DOCK5,STK32A,PLCE1,IL17RA,CRIM1,PRR5L,VAV1,IQSEC1,CACNA1I,BLM,NRK,MAGI3,ADCY10,BMP2,RC3H2,RIN3,BMP2K,SEMA3D,NETO2,NFATC2,SH3BP5,ZNF106,MYOM1,TRAF3,TTC21B,UIMC1,RAP1GAP,DRAXIN,ATF1,CGAS,GABRR2,CNKSR3,CASP5,WDR12,CUL1,MOB1B,BBS4,MAPK8IP1,UBASH3A,KITLG,AKAP10,PTPRE,MTMR2,TBX20,AFAP1,WSB1,PRKCH,TG,IL6R,ALS2,OR51E1,SNX25,PDE6A,USP7,MESD,MOK,KIR2DL4,RALB,VCAM1,SEL1L,ARHGAP31,DTX1,ZBTB33,ADA2,DPYSL5,CUL5,OR7A17,NEK6,NMU,GAST,SNAI2,IGHV3-74,BID,SIAH2,RXRG,ERN2,MBTPS2,FLNB,TIAL1,ZDHHC17,SAMHD1,IFT81,ENPP1,CSF1,GHRH,BCL2L1,ASB4,DHRS3,SMAD5,PRAME,MED1,CDC14B,PTH,PRKAA2,CSF2RB,ABHD2,CAMLG,TEAD1,YBX3,AIMP1,FYB2,PCID2,CIBAR1,PBLD,FICD,CADM1,PEG10,NET1,SLC1A7,FSTL1,SVEP1,MADD,HCRTR1,CREBBP,GORAB,NFKBIA,ZC3H15,NEDD9,OLFM4,ADGRE3,PPP1R17,ERLIN2,OTOP1,EXOC1,GID8,FAM189A2,NDFIP2,NR2C1,CMTM7,ABI1,ITGA4,OR6C75,ASB2,CYFIP2,OR13C9,RXRA,ADGRB1,WNT7A,NDFIP1,MAP3K4,ATP6V1C2,C16ORF72,OR10H2,PDE2A,LRRC2,SDCBP,JPT2,NCK1,FGR,IFNAR1,CYTH4,WNT2B,POSTN,CD101,AKAP11,DTHD1,MVB12B,ANKRD6,FEZ2,INIP,LAMB1,PLCZ1,FCRLA,DIDO1,GPR55,NSUN2,TNFSF11,PPM1F,ARL13B,SH2D3C,ZFYVE28,TET1,ASB3,RAD9A,RP1L1,ITGA1,POR,NSG2,B9D1,PRDM15,SRGAP3,CSNK1G1,FLRT2,OR2T2,TMEM25,PPP1R13B*
GO:0032501	multicellular organismal process	0.00000583626014632336	*WWC1,MTOR,SMOC1,LRP12,PLCB1,NEBL,SPOCK1,ZNF536,BRINP3,SGCD,CNTN4,ZFPM2,PIEZO2,TENM4,RIPOR2,RP1,ODAD2,KCNMA1,FBN1,F13A1,RIMS1,SPIRE1,TENM3,RARB,NAV2,ENPEP,SPAG16,USH2A,MINAR1,RIMS2,PJA2,ERBIN,RHPN2,ASTN1,CACNG2,NEGR1,MYO3B,RTN1,TCF4,OCA2,DOCK10,EGFR,ANGPT1,MACF1,PRKACB,NCOR1,DOCK2,NEDD4,CRB1,BTBD9,SOX6,ARMC2,TUSC3,PHACTR1,DKK2,DNAJC13,MAPKBP1,GABRB1,DGKI,C12ORF4,GRIA1,NEO1,CNTN6,SLC39A12,SLC8A3,PAK1,EPHA7,SPEN,RAPGEF2,ADGRB3,RUNX2,ARSB,GABRA6,CPS1,TAOK3,SLC44A1,LDB2,LRGUK,RPTOR,EPB41L3,COL4A2,PPP1R12B,ADAM10,IL1R1,APBB2,SLC7A2,KDM1B,CACNB2,STAU2,TMC1,SEMA5A,SYT1,VCL,ARHGAP44,NTF3,AURKA,PYGO1,SLC8A1,SRGAP2C,TAFA2,SRGAP2B,MAP4K4,BMPR1B,FMN2,HOMER2,RAB8B,PAK3,RFTN1,TTLL7,DIP2B,ITPKB,RAP1GDS1,RNLS,CHST8,CUBN,IFT57,PRKCZ,KLHL1,GRB10,MCPH1,ZSWIM6,RGS9,ABCA5,EBF2,YAP1,CADM2,ALCAM,PLG,PAPPA,PDGFD,ZNRF3,ABLIM1,GFRA1,SYCP1,NIPBL,RNF17,CORO2B,CARD18,SORCS3,MYLK3,MBNL1,ATF6,TPM1,CORIN,ANKRD11,BIRC6,KLF15,PPARA,SYNJ1,ADAMTS3,SF3B6,SEMA3C,SLC24A4,ALPK2,DNAH11,JARID2,SCN2A,CPE,IL34,TANC1,BBS2,SLC9C1,RANBP3L,OR4F6,NKG7,LDB3,BLK,TNR,XIRP2,OXR1,SDC2,GAS2,KCNH1,DROSHA,TTLL5,APBB1IP*

			,DNAH5,GLIS1,MORC1,GALC,LATS2,ASPM,AP3B1,ATP11C,ZBTB16,MUSK,SETDB2,PRKCE,SLMAP,USP33,CD44,PTPRO,ALPK3,ABCC9,LNPEP,LIMD1,SPRED2,RPS6KA3,NHS,PTPN2,PLXNA2,OR4F15,ATXN3,ST8SIA6,SLC2A3,ARHGEF7,ATP8A1,AMBRA1,KDM7A,OPRM1,FANCA,CNNM4,SEMA3E,ALPL,TMOD2,MSH2,ATL1,LUC7L,EPN2,BICRAL,AFG3L2,MOSMO,MNAT1,ANK3,XYLT1,HMGA2,BCL11B,DOCK5,F5,AK8,PLCE1,IL17RA,CRIM1,FUT9,VAV1,MYT1L,FBXO32,HLA-B,IQSEC1,CACNA1I,PDLIM5,NRK,SLC10A7,ADCY10,BMP2,RC3H2,TRAK1,GFI1B,BMP2K,RNF38,SEMA3D,POLR3A,NFATC2,TDRD7,SLC23A2,MYOM1,TRAF3,TTC21B,B4GALT6,TSPAN2,RAP1GAP,DRAXIN,ATF1,CGAS,GABRR2,CASP5,DAW1,MYL1,FARP1,BBS4,MAPK8IP1,COL5A1,CFTR,UBASH3A,AHDC1,KITLG,GTF2I,TADA2A,MTMR2,SH3PXD2A,TBX20,LGI2,PRKCH,TG,IL6R,ALS2,OR51E1,HEMGN,TOX,PTPRB,PDE6A,TBATA,SCN10A,ENPP3,MESD,KIR2DL4,NPAS2,YIPF6,VCAM1,LRIG1,DTX1,FANCL,DPYSL5,OR7A17,HECTD1,SHROOM3,XRCC4,NMU,SNAI2,IGHV3-74,BID,SIAH2,RXRG,SP3,MBTPS2,TRIM58,TIAL1,ZDHHC17,SLC22A14,KRT6B,SAMHD1,IFT81,ENPP1,KCNC1,CSF1,GHRH,BCL2L1,SPATA48,KRT25,CTDP1,ASB4,DHRS3,SMAD5,SYNJ2,SLC40A1,CABYR,MED1,FAT1,OTOG,PTH,PRKAA2,CSF2RB,SOHLH1,ABHD2,PLA2G4A,CAMLG,TEAD1,ANP32B,YBX3,AIMP1,PCID2,CIBAR1,PBLD,CADM1,SSPN,ANLN,ADAM28,SLC1A7,VSX1,FSTL1,BPNT1,SVEP1,HCRTR1,ZNF287,ZNF449,PRSS2,CREBBP,GORAB,SIAH3,TRPV5,NFKBIA,ABCC8,ALX4,NEDD9,ASS1,PPP1R17,OTOP1,KRT6A,STOX2,AGO1,CMTM7,SLC6A11,MTPN,ABI1,ITGA4,BCAP29,POU1F1,OR6C75,ASB2,CEP120,CYFIP2,ACACA,KRT85,ST8SIA4,OR13C9,RXRA,ADGRB1,WNT7A,NDFIP1,MAP3K4,FOXO6,OR10H2,PDE2A,SDCBP,DSG1,NCK1,FLVCR1,FGR,SPRR2D,RNF8,PPIP5K2,KIAA0319L,DNMT3L,LHX9,WNT2B,TNNI1,OCLN,CD101,AKAP11,FEZ2,LAMB1,KIRREL1,PLCZ1,GPR55,NSUN2,TNFSF11,PPM1F,ARL13B,TMEM63C,UGP2,OPA3,TET1,ASB3,RP1L1,ITGA1,POR,B9D1,MACROH2A1,SERPINB2,ATG5,UNK,FLRT2,OR2T2,TMEM25,DDX6,RFX2,CCDC141
GO:0032502	developmental process	0.00000616372889469349	WWC1,MTOR,SMOC1,LRP12,PLCB1,NEBL,SPOCK1,ZNF536,BRINP3,SGCD,CNTN4,ZFPM2,TENM4,RIPOR2,RP1,ODAD2,FBN1,CDH8,RIMS1,SPIRE1,TENM3,RARB,NAV2,ENPEP,SPAG16,USH2A,MINAR1,CDC42EP3,RIMS2,ASTN1,PARVB,NEGR1,MYO3B,RTN1,TCF4,OCA2,DOCK10,EGFR,ANGPT1,CDK12,MACF1,PRKACB,DOCK2,NEDD4,CRB1,SOX6,ARMC2,PHACTR1,DKK2,DNAJC13,GABRB1,NEO1,CNTN6,SLC39A12,SLC8A3,PAK1,EPHA7,SPEN,RAPGEF2,ADGRB3,DEUP1,RUNX2,ARSB,CPS1,TAOK3,PRICKLE2,LDB2,LRGUK,EPB41L3,COL4A2,ADAM10,STAU2,TMC1,SEMA5A,SYT1,VCL,ARHGAP44,NTF3,AURKA,CFDP1,PYGO1,SLC8A1,SRGAP2C,SRGAP2B,MAP4K4,BMPR1B,FMN2,PAK3,TTLL7,DIP2B,ITPKB,CHST8,IFT57,PRKCZ,KLHL1,MCPH1,ZSWIM6,ABCA5,EBF2,YAP1,RIPK4,CADM2,ALCAM,PLG,PDGFD,ZNRF3,ABLIM1,GFRA1,SYCP1,NIPBL,RNF17,MYLK3,MBNL1,ATF6,TPM1,ANKRD11,BIRC6,KLF15,PPARA,SYNJ1,ADAMTS3,SF3B6,SEMA3C,SLC24A4,ALPK2,DNAH11,JARID2,SCN2A,GATAD2B,CPE,IL34,TANC1,MELK,BBS2,SLC9C1,RANBP3L,LDB3,PIAS1,BLK,TNR,XIRP2,SDC2,GAS2,KCNH1,DROSHA,TTLL5,DNAH5,GLIS1,MORC1,MYO10,GALC,LATS2,ASPM,AP3B1,ATP11C,ZBTB16,MUSK,SETDB2,USP33,CD44,PTPRO,ALPK3,COL5A3,LIMD1,SPRED2,RPS6KA3,NHS,PTPN2,PLXNA2,ATXN3,ST8SIA6,ARHGEF7,AMBRA1,KDM7A,OPRM1,FANCA,CNNM4,SEMA3E,ALPL,TMOD2,MSH2,ATL1,LUC7L,EPN2,BICRAL,AFG3L2,MOSMO,MNAT1,ANK3,XYLT1,HMGA2,BCL11B,DOCK5,AK8,PLCE1,CRIM1,FUT9,VAV1,MYT1L,ZNF160,HLA-B,IQSEC1,PDLIM5,NRK,SLC10A7,ADCY10,BMP2,RC3H2,TRAK1,GFI1B,BMP2K,RNF38,SEMA3D,NFATC2,TDRD7,SLC23A2,ANKRD26,TTC21B,B4GALT6,TSPAN2,RAP1GAP,DRAXIN,ATF1,NHSL1,CASP5,CUL1,DAW1,FARP1,BBS4,COL5A1,CFTR,AHDC1,KITLG,GTF2I,TADA2A,MTMR2,SH3PXD2A,TBX20,LGI2,P

			RKCH,TG,IL6R,ALS2,TFDP1,HEMGN,TOX,PTPRB,PDE6A,TBATA,SCN10A,MESD,NPAS2,YIPF6,VCAM1,LRIG1,DTX1,DPYSL5,HECTD1,SHROOM3,XRCC4,SNAI2,SIAH2,RXRG,SP3,MBTPS2,FLNB,TRIM58,TIAL1,ELF2,ZDHHC17,SLC22A14,KRT6B,XKR5,SAMHD1,IFT81,ENPP1,KCNC1,CSF1,GHRH,BCL2L1,SPATA48,KRT25,CTDP1,ASB4,DHRS3,SMAD5,SYNJ2,SLC40A1,PRAME,CABYR,MED1,ATG4B,FAT1,SCML2,PRAMEF25,PTH,SDF4,SOHLH1,ABHD2,VSTM2A,ZBTB7C,TEAD1,ANP32B,YBX3,AIMP1,PCID2,CIBAR1,CADM1,PEG10,ANLN,ADAM28,VSX1,FSTL1,BPNT1,SVEP1,ZNF449,CREBBP,ARL11,GORAB,SIAH3,NFKBIA,ABCC8,ALX4,NEDD9,ASS1,PPP1R17,OTOP1,KRT6A,STOX2,AGO1,NR2C1,CMTM7,SLC6A11,MTPN,ABI1,ITGA4,BCAP29,POU1F1,ASB2,CEP120,DHTKD1,CYFIP2,KRT85,ST8SIA4,RXRA,ADGRB1,WNT7A,NDFIP1,MAP3K4,FOXO6,PDE2A,SDCBP,NCK1,FLVCR1,FGR,SPRR2D,RNF8,KIAA0319L,DNMT3L,LHX9,WNT2B,TNNI1,POSTN,CD101,ANKRD6,FEZ2,LAMB1,FCRLA,GPR55,NSUN2,TNFSF11,ARL13B,UGP2,TET1,RP1L1,DDX10,ITGA1,POR,B9D1,MACROH2A1,ATG5,UNK,FLRT2,NUDT21,DDX6,RFX2,CCDC141
GO:0048523	negative regulation of cellular process	0.0000205844207739 16217	*WWC1,MTOR,PLCB1,SPOCK1,ZNF536,BRINP3,CNTN4,ZFPM2,RIPOR2,FBN1,RARB,USH2A,MINAR1,CDYL2,BABAM2,ERBIN,RHPN2,EGFR,USP14,ANGPT1,CDK12,PRKACB,NCOR1,NEDD4,SOX6,DKK2,THRAP3,MAPKBP1,DGKI,GRIA1,CAST,SLC8A3,TOM1L2,PAK1,EPHA7,NCOA7,SPEN,RAPGEF2,RUNX2,TAOK3,CPEB4,LDB2,RPTOR,ADAM10,APBB2,KDM1B,STAU2,USP18,SEMA5A,VCL,ARHGAP44,NTF3,ACER2,PARP15,AURKA,CFDP1,SLC8A1,SRGAP2C,SRGAP2B,MAP4K4,BMPR1B,FMN2,HOMER2,DIP2B,LARP1,ITPKB,RGS20,PDE10A,KICS2,IFT57,INTS7,PRKCZ,GRB10,MCPH1,RGS9,ABCA5,YAP1,USP25,PLG,ZNRF3,UBE2O,NIPBL,CORO2B,STK38,SORCS3,ZNF684,TPM1,BIRC6,PPARA,PTPRK,TRERF1,SEMA3C,DAPK1,SLC24A4,SEC14L1,VPS13C,PHC3,ALPK2,JARID2,DNAJC15,GATAD2B,ZNF846,RANBP3L,PIAS1,BLK,TNR,MXI1,OXR1,CREG1,SLFN11,GLIS1,MORC1,LATS2,ASPM,ABCB7,ZBTB16,KIR3DL2,SETDB2,PRKCE,FOXK2,CD44,RGS12,PTPRO,STXBP6,LIMD1,PEX14,SPRED2,RPS6KA3,PTPN2,PLXNA2,ARHGEF7,AMBRA1,OPRM1,SEMA3E,TMEM67,ABHD17C,TMOD2,MSH2,ZNF397,HIPK3,EPN2,CLSPN,BICRAL,MOSMO,MNAT1,MDFIC,ANK3,HMGA2,BCL11B,LYPLA1,CRIM1,PRR5L,MYT1L,HLA-B,BLM,BMP2,RC3H2,ATP9A,GFI1B,RIN3,SEMA3D,NFATC2,SH3BP5,SLC23A2,ANKRD26,ZNF875,UIMC1,LRRFIP1,RAP1GAP,DRAXIN,CGAS,CNKSR3,BTAF1,BBS4,MAPK8IP1,COL5A1,UBASH3A,MRPL13,KITLG,PTPRE,MTMR2,ZNF608,TBX20,PRKCH,TFDP1,KANK4,SNX25,PTPRB,USP7,ENPP3,PLAGL1,KIR2DL4,NPAS2,ZNF169,DTX1,ZBTB33,DPYSL5,HECTD1,SNAI2,BID,SIAH2,SP3,ERN2,TIAL1,ELF2,SAMHD1,ENPP1,TP53I11,TMEM225,CSF1,BCL2L1,CTDP1,DHRS3,SMAD5,TCERG1,SLC40A1,PRAME,MED1,CDC14B,SCML2,PRAMEF25,PTH,PRKAA2,ABHD2,CAMLG,ZBTB7C,ANP32B,YBX3,AIMP1,PCID2,PBLD,CADM1,PEG10,FSTL1,PATL1,CREBBP,MELTF,SIAH3,NFKBIA,ABCC8,RTRAF,ZBTB21,NEDD9,ASS1,BTG3,ERLIN2,OTOP1,ZBTB49,AGO1,FAM189A2,NDFIP2,NR2C1,MTPN,ABI1,OAZ2,POU1F1,RXRA,ADGRB1,WNT7A,NDFIP1,C16ORF72,MAGEL2,PDE2A,SDCBP,MLLT1,NCK1,SCAF8,FGR,RNF8,DNMT3L,LHX9,OCLN,ANKRD6,ASCL3,FEZ2,INIP,KIRREL1,GPR55,NSUN2,TNFSF11,ZNF705G,PPM1F,ZFYVE28,TET1,RAD9A,ZNF705D,ITGA1,POR,PRDM15,SRGAP3,MACROH2A1,ZNF705B,SERPINB2,ATG5,UNK,BTBD10,DDX6,PPP1R13B*
GO:0022008	neurogenesis	0.0000217575467311 64253	*MTOR,LRP12,SPOCK1,ZNF536,BRINP3,CNTN4,TENM4,RIPOR2,RP1,RIMS1,TENM3,RARB,NAV2,USH2A,MINAR1,RIMS2,ASTN1,NEGR1,RTN1,TCF4,DOCK10,MACF1,NEDD4,CRB1,SOX6,PHACTR1,GABRB1,NEO1,CNTN6,SLC39A12,SLC8A3,PAK1,EPHA7,SPEN,RAPGEF2,ADGRB3,RUNX2,ARSB,TAOK3,EPB41L3,STAU2,TMC1,SEMA5A,SYT1,VCL,ARHGAP44,NTF3,AURKA,SRGAP2C,MAP4K4,BMPR1B,PAK3,DIP2B,PRKCZ,KLHL1,ZSWIM6,YAP1,ALCAM,GFRA1,NIPBL,KLF15,SYNJ1,SEMA3C,IL34,B*

			LK,TNR,SDC2,ASPM,USP33,PTPRO,PLXNA2,OPRM1,SEMA3E,ATL1,AFG3L2,MOSMO,ANK3,BCL11B,FUT9,MYT1L,IQSEC1,PDLIM5,NRK,BMP2,TRAK1,SEMA3D,SLC23A2,TTC21B,B4GALT6,TSPAN2,RAP1GAP,DRAXIN,ATF1,FARP1,BBS4,MTMR2,TBX20,PRKCH,ALS2,TOX,PTPRB,VCAM1,DTX1,DPYSL5,RXRG,ZDHHC17,CSF1,MED1,VSX1,ABCC8,MTPN,ABI1,ITGA4,CEP120,CYFIP2,ADGRB1,WNT7A,FOXO6,NCK1,KIAA0319L,LHX9,WNT2B,FEZ2,LAMB1,RP1L1,ITGA1,UNK,FLRT2,DDX6,CCDC141
GO:0048583	regulation of response to stimulus	0.00005749943985912488	*WWC1,GARNL3,MTOR,PLCB1,ZNF536,KSR1,RIPOR2,FBN1,RIMS1,SPIRE1,MINAR1,RIMS2,PJA2,BABAM2,ERBIN,CACNG2,SUSD4,NEK4,EGFR,DENND1A,USP14,ANGPT1,MACF1,PRKACB,NCOR1,DOCK2,NEDD4,DKK2,MAPKBP1,AOAH,DGKI,C12ORF4,NEO1,CNTN6,SLC8A3,PAK1,EPHA7,NCOA7,RALGPS1,RAPGEF2,RUNX2,TAOK3,RPTOR,ADAM10,IL1R1,MTUS1,USP18,SEMA5A,ARHGAP44,NTF3,AURKA,MAP4K4,BMPR1B,FMN2,HOMER2,PAK3,RFTN1,ITPKB,RGS20,PDE10A,RAP1GDS1,KICS2,PRKCZ,BTLA,GRB10,MCPH1,RGS9,YAP1,USP25,PLG,PDGFD,ZNRF3,UBE2O,MICU1,CORO2B,STK38,HRH4,ATF6,BIRC6,KLF15,PPARA,ADAMTS3,ARHGEF12,SEMA3C,DAPK1,SLC24A4,SEC14L1,VPS13C,ALPK2,JARID2,IL34,BBS2,NKG7,USP8,BLK,TNR,OXR1,GAS2,DROSHA,LATS2,GSG1L,ASPM,AP3B1,DENND2B,GNG7,PRKCE,USP33,CD44,RGS12,PTPRO,NLRP13,LIMD1,SPRED2,RPS6KA3,PTPN2,MCF2L,ATXN3,OPRM1,FANCA,SEMA3E,TMOD2,MSH2,RELL1,HIPK3,EPN2,MOSMO,MDFIC,HMGA2,PLCE1,IL17RA,CRIM1,PRR5L,VAV1,FBXO32,HLA-B,IQSEC1,NRK,MAGI3,BMP2,RC3H2,RIN3,BMP2K,SEMA3D,NETO2,NFATC2,TRAF3,TTC21B,UIMC1,RAP1GAP,DRAXIN,CGAS,CNKSR3,CASP5,BBS4,MAPK8IP1,UBASH3A,KITLG,PTPRE,MTMR2,TBX20,AFAP1,PRKCH,IL6R,ALS2,SNX25,USP7,ENPP3,KIR2DL4,RALB,NPAS2,ARHGAP31,DTX1,NEK6,SNAI2,IGHV3-74,BID,SIAH2,ERN2,MBTPS2,TIAL1,ZDHHC17,SAMHD1,IFT81,ENPP1,CSF1,GHRH,BCL2L1,CTDP1,DHRS3,PRAME,MED1,CFH,PTH,PRKAA2,PLA2G4A,YBX3,FYB2,PCID2,CIBAR1,PBLD,FICD,CADM1,PEG10,NET1,FSTL1,MADD,HCRTR1,CREBBP,GORAB,NFKBIA,ABCC8,NEDD9,OTOP1,GID8,FAM189A2,NDFIP2,NR2C1,MTPN,CYFIP2,RXRA,WNT7A,NDFIP1,MAP3K4,ATP6V1C2,C16ORF72,PDE2A,SDCBP,JPT2,NSMCE1,NCK1,FGR,C2,RNF8,CYTH4,TNNI1,OCLN,POSTN,MVB12B,CD5L,ANKRD6,LAMB1,GPR55,TNFSF11,PPM1F,ZFYVE28,TET1,RAD9A,ITGA1,POR,PRDM15,SRGAP3,MACROH2A1,CSNK1G1,SERPINB2,ATG5,TMEM25*
GO:0030182	neuron differentiation	0.00012693869115496697	*LRP12,SPOCK1,ZNF536,BRINP3,CNTN4,TENM4,RIPOR2,RP1,RIMS1,TENM3,USH2A,MINAR1,RIMS2,NEGR1,RTN1,TCF4,DOCK10,MACF1,NEDD4,CRB1,PHACTR1,GABRB1,NEO1,CNTN6,SLC39A12,PAK1,EPHA7,RAPGEF2,ADGRB3,RUNX2,ARSB,TAOK3,EPB41L3,STAU2,TMC1,SEMA5A,SYT1,VCL,ARHGAP44,NTF3,AURKA,SRGAP2C,MAP4K4,BMPR1B,PAK3,DIP2B,PRKCZ,KLHL1,ZSWIM6,ALCAM,GFRA1,SEMA3C,BLK,TNR,SDC2,ASPM,USP33,PTPRO,PLXNA2,SEMA3E,ATL1,AFG3L2,MOSMO,ANK3,BCL11B,FUT9,MYT1L,IQSEC1,PDLIM5,NRK,BMP2,SEMA3D,SLC23A2,TTC21B,B4GALT6,TSPAN2,RAP1GAP,DRAXIN,ATF1,FARP1,BBS4,MTMR2,TBX20,ALS2,TOX,VCAM1,DTX1,DPYSL5,RXRG,ZDHHC17,MED1,VSX1,MTPN,ABI1,ITGA4,CYFIP2,ADGRB1,WNT7A,FOXO6,NCK1,LHX9,WNT2B,FEZ2,LAMB1,RP1L1,ITGA1,UNK,FLRT2,DDX6,CCDC141*
GO:0036211	protein modification process	0.00017077832165452602	*MTOR,TMTC1,KSR1,ERC1,F13A1,PIK3C3,MINAR1,PJA2,BABAM2,MAP3K9,MYO3B,NEK4,EGFR,USP14,ANGPT1,CDK12,PRKACB,NEK7,NEDD4,NSMCE2,B3GALT5,TUSC3,SLC8A3,PAK1,EPHA7,NCOA7,RAPGEF2,TAOK3,RPTOR,ADAM10,KDM1B,KLHL13,USP18,NTF3,ACER2,PARP15,AURKA,SLC8A1,MAP4K4,BMPR1B,PAK3,TTLL7,DIP2B,ITPKB,HHAT,PRKCZ,SPOP,MAN2A2,ST8SIA5,SENP6,GALNT14,PPM1L,RIPK4,USP25,PDGFD,ZNRF3,XXYLT1,UBE2O,GFRA1,NIPBL,STK38,MYLK3,KANSL1,CCNG2,TLK1,ZDHHC14,BIRC6,KLF15,PPP6R3,RSRC1,PTPRK,TRERF1,PDZRN3,DAPK1,STK32B,ALPK2,GATAD2B,CPE,IL34*

			,MELK,USP8,PIAS1,UBE2R2,BLK,OXR1,TTLL5,EIPR1,ATE1,LATS2,AP3B1,ZBTB16,MUSK,SMARCAD1,SETDB2,PRKCE,USP33,CD44,PTPRO,ALPK3,PRRC1,LNPEP,SPRED2,RPS6KA3,PTPN2,ATXN3,ST8SIA6,ALG10B,AMBRA1,GALNT10,KDM7A,FANCM,FANCA,RPRD1B,ABHD17C,HIPK3,CLSPN,MNAT1,HMGA2,FOLH1,STK32A,LYPLA1,CWC27,PLCE1,FUT9,PRR5L,GXYLT2,FBXO32,BLM,NRK,BMP2,RC3H2,TRAK1,BMP2K,RNF38,SH3BP5,TRAF3,UIMC1,B4GALT6,CNKSR3,CUL1,DAW1,MOB1B,MAPK8IP1,KITLG,TADA2A,UBE2E1,PTPRE,MTMR2,WSB1,TRPM6,PRKCH,IL6R,ALS2,SNX25,PTPRB,USP7,MOK,RALB,DTX1,FANCL,CUL5,NEK6,HECTD1,SNAI2,SIAH2,PGAP4,ERN2,TRIM58,ZDHHC17,PTAR1,ENPP1,CSF1,PPIL6,EOGT,CTDP1,ASB4,SMAD5,PRAME,KLHL7,ATG4B,CDC14B,PRKAA2,CSF2RB,RNF182,PHF20L1,CAMLG,FICD,CADM1,ELOC,MADD,CREBBP,SIAH3,RTRAF,NEDD9,PCMTD2,PDP2,NDFIP2,MARCHF6,ABI1,PPME1,UBE2J2,ASB2,ST8SIA4,ADGRB1,NDFIP1,MAP3K4,TRIM43B,TRIM43,MAGEL2,SDCBP,NSMCE1,MLLT1,NCK1,FGR,RNF8,OCLN,KIRREL1,TNFSF11,DPY19L1,PPM1F,SH2D3C,ZFYVE28,STT3A,TET1,ASB3,SPOPL,ITGA1,POR,SENP8,USP49,MACROH2A1,CSNK1G1,CAMK1G,ATG5,TTLL11
GO:0050793	regulation of developmental process	0.0001891491531018646	*WWC1,MTOR,SMOC1,PLCB1,ZNF536,BRINP3,CNTN4,ZFPM2,TENM4,RIPOR2,FBN1,RIMS1,SPIRE1,RARB,USH2A,MINAR1,CDC42EP3,RIMS2,PARVB,TCF4,EGFR,CDK12,MACF1,NEDD4,SOX6,SLC39A12,PAK1,EPHA7,SPEN,RAPGEF2,ADGRB3,RUNX2,PRICKLE2,EPB41L3,COL4A2,ADAM10,STAU2,SEMA5A,SYT1,VCL,ARHGAP44,NTF3,AURKA,CFDP1,SLC8A1,SRGAP2C,BMPR1B,PAK3,DIP2B,ITPKB,PRKCZ,ABCA5,YAP1,ZNRF3,NIPBL,MYLK3,TPM1,PPARA,SYNJ1,SEMA3C,ALPK2,JARID2,GATAD2B,IL34,BBS2,RANBP3L,TNR,SDC2,GAS2,DROSHA,GLIS1,MYO10,LATS2,ASPM,AP3B1,ATP11C,ZBTB16,MUSK,CD44,LIMD1,SPRED2,RPS6KA3,PTPN2,PLXNA2,ARHGEF7,AMBRA1,OPRM1,FANCA,SEMA3E,MSH2,LUC7L,EPN2,BICRAL,AFG3L2,MOSMO,HMGA2,BCL11B,CRIM1,HLA-B,PDLIM5,BMP2,RC3H2,GFI1B,BMP2K,SEMA3D,NFATC2,SLC23A2,ANKRD26,RAP1GAP,DRAXIN,BBS4,COL5A1,CFTR,KITLG,GTF2I,TADA2A,MTMR2,TBX20,PRKCH,TG,IL6R,HEMGN,TOX,DTX1,DPYSL5,SHROOM3,SNAI2,RXRG,TRIM58,ENPP1,CSF1,GHRH,BCL2L1,CTDP1,ASB4,SMAD5,PRAME,MED1,PRAMEF25,PTH,VSTM2A,ZBTB7C,ANP32B,YBX3,PCID2,NFKBIA,ABCC8,NEDD9,AGO1,MTPN,RXRA,ADGRB1,WNT7A,NDFIP1,FOXO6,SDCBP,FLVCR1,FGR,WNT2B,CD101,ANKRD6,LAMB1,GPR55,NSUN2,TNFSF11,TET1,POR,MACROH2A1,FLRT2,NUDT21,DDX6*
GO:0048699	generation of neurons	0.0002772328347334582	*LRP12,SPOCK1,ZNF536,BRINP3,CNTN4,TENM4,RIPOR2,RP1,RIMS1,TENM3,USH2A,MINAR1,RIMS2,ASTN1,NEGR1,RTN1,TCF4,DOCK10,MACF1,NEDD4,CRB1,PHACTR1,GABRB1,NEO1,CNTN6,SLC39A12,PAK1,EPHA7,RAPGEF2,ADGRB3,RUNX2,ARSB,TAOK3,EPB41L3,STAU2,TMC1,SEMA5A,SYT1,VCL,ARHGAP44,NTF3,AURKA,SRGAP2C,MAP4K4,BMPR1B,PAK3,DIP2B,PRKCZ,KLHL1,ZSWIM6,ALCAM,GFRA1,NIPBL,SEMA3C,BLK,TNR,SDC2,ASPM,USP33,PTPRO,PLXNA2,SEMA3E,ATL1,AFG3L2,MOSMO,ANK3,BCL11B,FUT9,MYT1L,IQSEC1,PDLIM5,NRK,BMP2,SEMA3D,SLC23A2,TTC21B,B4GALT6,TSPAN2,RAP1GAP,DRAXIN,ATF1,FARP1,BBS4,MTMR2,TBX20,ALS2,TOX,VCAM1,DTX1,DPYSL5,RXRG,ZDHHC17,MED1,VSX1,MTPN,ABI1,ITGA4,CYFIP2,ADGRB1,WNT7A,FOXO6,NCK1,KIAA0319L,LHX9,WNT2B,FEZ2,LAMB1,RP1L1,ITGA1,UNK,FLRT2,DDX6,CCDC141*
GO:0000902	cell morphogenesis	0.00028595336331746463	*CNTN4,RIPOR2,CDH8,RIMS1,CDC42EP3,RIMS2,PARVB,DOCK10,EGFR,MACF1,NEDD4,CRB1,PHACTR1,NEO1,CNTN6,PAK1,EPHA7,RAPGEF2,ADGRB3,TAOK3,EPB41L3,STAU2,SEMA5A,SYT1,VCL,ARHGAP44,NTF3,AURKA,CFDP1,MAP4K4,BMPR1B,PAK3,DIP2B,PRKCZ,YAP1,ALCAM,TPM1,SEMA3C,TNR,SDC2,GAS2,MYO10,LATS2,AP3B1,USP33,CD44,PTPRO,LIMD1,PLXNA2,ARHGEF7,SEMA3E,ATL1,AFG3L2,ANK3,BCL11B,PDLIM5,NRK,SEMA3D,SLC23A2,B4GALT6,DRAXIN,FARP1,ALS2,DPYSL5,SHROOM3,FLNB,ZDHHC17,MED1,FAT1,ABI1,ITGA4,CYFI*

			P2,ADGRB1,WNT7A,FGR,LHX9,FEZ2,ARL13B,ITGA1,UNK,FLRT2,CCDC141
GO:0032989	cellular component morphogenesis	0.0002937769272867346	*NEBL,CNTN4,TENM4,RIMS1,RIMS2,DOCK10,MACF1,NEDD4,PHACTR1,NEO1,CNTN6,PAK1,EPHA7,RAPGEF2,ADGRB3,TAOK3,EPB41L3,STAU2,SEMA5A,SYT1,VCL,ARHGAP44,NTF3,AURKA,MAP4K4,BMPR1B,PAK3,DIP2B,PRKCZ,ALCAM,MYLK3,TPM1,SEMA3C,LDB3,TNR,SDC2,USP33,CD44,PTPRO,PLXNA2,ARHGEF7,SEMA3E,TMOD2,ATL1,AFG3L2,ANK3,BCL11B,PDLIM5,NRK,SEMA3D,SLC23A2,B4GALT6,DRAXIN,FARP1,MTMR2,ALS2,DPYSL5,ZDHHC17,BCL2L1,ABI1,ITGA4,CYFIP2,ADGRB1,WNT7A,LHX9,FEZ2,ITGA1,FLRT2,RFX2,CCDC141*
GO:0009653	anatomical structure morphogenesis	0.00035584826056229393	*MTOR,NEBL,SGCD,CNTN4,ZFPM2,TENM4,RIPOR2,RP1,FBN1,CDH8,RIMS1,SPIRE1,TENM3,RARB,ENPEP,USH2A,MINAR1,CDC42EP3,RIMS2,PARVB,MYO3B,DOCK10,EGFR,ANGPT1,MACF1,PRKACB,DOCK2,NEDD4,CRB1,SOX6,PHACTR1,NEO1,CNTN6,SLC39A12,PAK1,EPHA7,RAPGEF2,ADGRB3,RUNX2,TAOK3,PRICKLE2,EPB41L3,COL4A2,STAU2,SEMA5A,SYT1,VCL,ARHGAP44,NTF3,AURKA,CFDP1,MAP4K4,BMPR1B,PAK3,DIP2B,IFT57,PRKCZ,YAP1,RIPK4,ALCAM,ZNRF3,ABLIM1,NIPBL,MYLK3,MBNL1,TPM1,ANKRD11,PPARA,SF3B6,SEMA3C,SLC24A4,ALPK2,DNAH11,CPE,TANC1,BBS2,LDB3,TNR,XIRP2,SDC2,GAS2,KCNH1,MYO10,LATS2,AP3B1,ZBTB16,SETDB2,USP33,CD44,PTPRO,LIMD1,PLXNA2,ARHGEF7,CNNM4,SEMA3E,ALPL,TMOD2,ATL1,EPN2,AFG3L2,ANK3,HMGA2,BCL11B,DOCK5,PDLIM5,NRK,BMP2,SEMA3D,NFATC2,TDRD7,SLC23A2,TTC21B,B4GALT6,DRAXIN,CUL1,FARP1,BBS4,COL5A1,CFTR,AHDC1,GTF2I,MTMR2,SH3PXD2A,TBX20,ALS2,PTPRB,SCN10A,LRIG1,DPYSL5,HECTD1,SHROOM3,SNAI2,SP3,FLNB,ZDHHC17,CSF1,BCL2L1,KRT25,ASB4,DHRS3,SMAD5,SLC40A1,MED1,FAT1,SCML2,AIMP1,CIBAR1,VSX1,SVEP1,CREBBP,GORAB,ABCC8,ALX4,OTOP1,KRT6A,AGO1,MTPN,ABI1,ITGA4,ASB2,CYFIP2,ADGRB1,WNT7A,FLVCR1,FGR,LHX9,WNT2B,TNNI1,ANKRD6,FEZ2,LAMB1,TNFSF11,ARL13B,TET1,ITGA1,POR,B9D1,MACROH2A1,UNK,FLRT2,RFX2,CCDC141*
GO:0120039	plasma membrane bounded cell projection morphogenesis	0.00048082947154752314	*CNTN4,RIMS1,RIMS2,DOCK10,MACF1,NEDD4,PHACTR1,NEO1,CNTN6,PAK1,EPHA7,RAPGEF2,ADGRB3,TAOK3,EPB41L3,STAU2,SEMA5A,SYT1,VCL,ARHGAP44,NTF3,AURKA,MAP4K4,BMPR1B,PAK3,DIP2B,PRKCZ,ALCAM,SEMA3C,TNR,SDC2,USP33,CD44,PTPRO,PLXNA2,ARHGEF7,SEMA3E,ATL1,AFG3L2,ANK3,BCL11B,PDLIM5,NRK,SEMA3D,SLC23A2,B4GALT6,DRAXIN,FARP1,ALS2,DPYSL5,ZDHHC17,ABI1,ITGA4,CYFIP2,ADGRB1,WNT7A,LHX9,FEZ2,ITGA1,FLRT2,CCDC141*
GO:0048858	cell projection morphogenesis	0.0006261254021740971	*CNTN4,RIMS1,RIMS2,DOCK10,MACF1,NEDD4,PHACTR1,NEO1,CNTN6,PAK1,EPHA7,RAPGEF2,ADGRB3,TAOK3,EPB41L3,STAU2,SEMA5A,SYT1,VCL,ARHGAP44,NTF3,AURKA,MAP4K4,BMPR1B,PAK3,DIP2B,PRKCZ,ALCAM,SEMA3C,TNR,SDC2,USP33,CD44,PTPRO,PLXNA2,ARHGEF7,SEMA3E,ATL1,AFG3L2,ANK3,BCL11B,PDLIM5,NRK,SEMA3D,SLC23A2,B4GALT6,DRAXIN,FARP1,ALS2,DPYSL5,ZDHHC17,ABI1,ITGA4,CYFIP2,ADGRB1,WNT7A,LHX9,FEZ2,ITGA1,FLRT2,CCDC141*
GO:0035556	intracellular signal transduction	0.0006682023814466897	*WWC1,GARNL3,MTOR,PLCB1,KSR1,SGCD,RIPOR2,RP1,ERC1,DCDC1,PIK3C3,MINAR1,CDC42EP3,PJA2,BABAM2,ERBIN,MAP3K9,DOCK10,EGFR,DENND1A,ANGPT1,PRKACB,NCOR1,DOCK2,NEDD4,MAPKBP1,DGKI,PAK1,EPHA7,CTNNAL1,RALGPS1,RAPGEF2,TAOK3,PATJ,RPTOR,APBB2,SEMA5A,ARHGAP44,NTF3,ACER2,AURKA,SLC8A1,MAP4K4,FMN2,HOMER2,PAK3,LARP1,ITPKB,PDE10A,RAP1GDS1,KICS2,INTS7,PRKCZ,GRB10,RGS9,YAP1,PPM1L,PDGFD,STK38,HRH4,TLK1,PPARA,ARHGEF12,DAPK1,SLC24A4,STK32B,SCN2A,IL34,MELK,USP8,BLK,KCNH1,LATS2,DENND2B,PRKCE,CD44,LNPEP,LIMD1,SPRED2,RPS6KA3,PTPN2,MCF2L,ARHGEF7,OPRM1,SEMA3E,MSH2,RELL1,HIPK3,CLSPN,MDFIC,HMGA2,DOCK5,STK32A,PLCE1,PRR5L,VAV1,IQSEC1,BLM,NRK,MAGI3,ADCY10,BMP2,RC3H2,NFATC2,SH3BP5,MYOM1,TRAF3,UIMC1,RAP1GAP,ATF1,CGAS,CNKSR3,CUL1,MOB1B,MAPK8IP1,KITLG,WSB1,PRKCH,IL6R,*

			ALS2,USP7,MOK,RALB,VCAM1,ARHGAP31,ZBTB33,CUL5,NEK6,SNAI2,BID,SIAH2,ERN2,TIAL1,ZDHHC17,CSF1,BCL2L1,ASB4,SMAD5,CDC14B,PTH,PRKAA2,TEAD1,YBX3,PCID2,PBLD,NET1,MADD,HCRTR1,NFKBIA,PPP1R17,EXOC1,NDFIP2,ASB2,WNT7A,NDFIP1,MAP3K4,C16ORF72,PDE2A,SDCBP,JPT2,NCK1,FGR,CYTH4,AKAP11,ANKRD6,INIP,PLCZ1,GPR55,NSUN2,TNFSF11,PPM1F,SH2D3C,ASB3,RAD9A,RP1L1,ITGA1,PRDM15,SRGAP3,PPP1R13B
GO:0006810	transport	0.0007440132763410945	*WWC1,MICU2,NSG1,LRP12,SLC25A21,TMPRSS2,ABCA13,CACNA2D3,KCNH5,SLC37A1,PIEZO2,ERC1,SLC44A5,KCNMA1,COG5,RIMS1,PIK3C3,SPIRE1,EXOC6B,SPAG16,TRAPPC8,RIMS2,SV2C,ERBIN,FCHO2,CACNG2,MYO5C,OCA2,EGFR,DENND1A,ANGPT1,MACF1,DOCK2,NEDD4,BTBD9,TUSC3,DNAJC13,GABRB1,DGKI,C12ORF4,GRIA1,SLC39A12,SLC8A3,TOM1L2,PAK1,GRAMD1B,ARSB,GABRA6,LDLRAD3,AGK,RANBP17,SLC44A1,KIF4A,ADAM10,SLC7A2,CACNB2,STAU2,TMC1,SYT1,ARHGAP44,NTF3,SLC8A1,FMN2,HOMER2,RAB8B,RFTN1,RAP1GDS1,CLIC6,CUBN,SCP2,IFT57,PRKCZ,GRB10,CNST,ABCA5,VPS35L,ABCD3,RABGAP1L,SGTB,TRPC7,SLC45A4,UBE2O,NIPAL2,IPO11,MICU1,TLK1,LRRC38,ZDHHC14,CORIN,KLF15,PPARA,SNX30,KCNS3,SYNJ1,RSRC1,DAPK1,SLC24A4,SEC14L1,VPS13C,DNAH11,SCN2A,RAB22A,DNAJC15,AMPH,CPE,BBS2,SLC9C1,RANBP3L,NKG7,SLC36A1,BLK,KCNH1,EIPR1,DNAH5,MYO10,PLEKHA8,GSG1L,AP3B1,ATP11C,ABCB7,PRKCE,SLMAP,USP33,ABCC9,STXBP6,PEX14,SLC2A3,ARHGEF7,ALG10B,ATP8A1,RFTN2,OPRM1,BIN2,CYBRD1,CNNM4,EPN2,ABCA10,CD163,AFG3L2,MDFIC,ANK3,NIPA2,COG2,VPS41,LYPLA1,PRR5L,VPS37A,VAV1,CACNA1I,BHLHE40-AS1,SLC10A7,ADCY10,STX12,BMP2,ATP9A,TRAK1,RIN3,BMP2K,SLC15A5,NETO2,AP4E1,SLC23A2,MYOM1,PRG4,TTC21B,SNX8,CCDC186,KCNH8,SLC37A2,GABRR2,CNKSR3,DAW1,BBS4,LRRC8B,MAPK8IP1,CFTR,TBC1D13,NMD3,REPS1,MTMR2,HEPHL1,TRPM6,SLC12A1,TG,ALS2,SNX25,OSCP1,SCN10A,USP7,MON2,MESD,RALB,YIPF6,SEL1L,SLC13A5,NPIPA1,CUL5,IGHV3-74,BID,OSBPL10,COX5A,TRIM58,ZDHHC17,FYCO1,SH3GLB1,SLC22A14,XKR5,IFT81,ENPP1,KCNC1,GHRH,BCL2L1,SYNJ2,SLC40A1,CABYR,CIDEC,MED1,IPCEF1,ATG4B,PTH,PLA2G4A,SLC25A52,CAMLG,COX7A2L,ANP32B,AIMP1,LASP1,PCID2,PEG10,SLC1A7,MELTF,ARL11,SIAH3,TRPV5,NFKBIA,ABCC8,RTRAF,SLC14A2,SAR1A,TRAPPC3,OTOP1,EXOC1,FAM189A2,NDFIP2,SLC6A11,ITGA4,OAZ2,BCAP29,UBE2J2,TM9SF4,CEP120,STOML1,RXRA,ADGRB1,WNT7A,NDFIP1,ATP6V1C2,MAGEL2,SDCBP,JPT2,FLVCR1,FGR,SNAP29,C2,RN7SL767P,PLEKHA3,OCLN,STON1-GTF2A1L,MFSD9,MVB12B,CD5L,SCARA5,HEATR5A,PLCZ1,SLC9A5,NSUN2,ANO10,TNFSF11,PPM1F,XPO7,TMEM63C,SLC16A9,IFT46,SLC14A1,NSG2,CSNK1G1,ATP6V0D2,ATG5,NUP43,DDX6*
GO:0032990	cell part morphogenesis	0.0007658269034249816	*CNTN4,RIMS1,RIMS2,DOCK10,MACF1,NEDD4,PHACTR1,NEO1,CNTN6,PAK1,EPHA7,RAPGEF2,ADGRB3,TAOK3,EPB41L3,STAU2,SEMA5A,SYT1,VCL,ARHGAP44,NTF3,AURKA,MAP4K4,BMPR1B,PAK3,DIP2B,PRKCZ,ALCAM,SEMA3C,TNR,SDC2,USP33,CD44,PTPRO,PLXNA2,ARHGEF7,SEMA3E,ATL1,AFG3L2,ANK3,BCL11B,PDLIM5,NRK,SEMA3D,SLC23A2,B4GALT6,DRAXIN,FARP1,ALS2,DPYSL5,ZDHHC17,BCL2L1,ABI1,ITGA4,CYFIP2,ADGRB1,WNT7A,LHX9,FEZ2,ITGA1,FLRT2,CCDC141*
GO:0007010	cytoskeleton organization	0.0008827880245925006	*LRRC49,MTOR,NEBL,RIPOR2,RP1,ODAD2,SPIRE1,SPAG16,CDC42EP3,ERBIN,RHPN2,PARVB,MYO3B,MYO5C,MACF1,NEK7,NCOR1,DOCK2,DIAPH3,ARMC2,PHACTR1,SLC39A12,PAK1,DEUP1,LRGUK,EPB41L3,KIF4A,PHACTR2,STAU2,SEMA5A,ARHGAP44,NTF3,AURKA,SRGAP2C,CCSER2,FMN2,PAK3,TTLL7,RAP1GDS1,PRKCZ,KLHL1,MCPH1,SENP6,ABLIM1,CORO2B,MYLK3,TPM1,BBS2,LDB3,XIRP2,GAS2,TTLL5,DNAH5,ASPM,PRKCE,USP33,LIMD1,PEX14,ATXN3,ARHGEF7,SEMA3E,TMEM67,TMOD2,ANK3,PLCE1,IQSEC1,PDLIM5,NRK,DNAL1,TUBGCP3,D*

			NAH8,KIF15,DAW1,FARP1,GOLGA8B,BBS4,AFAP1,KANK4,MAP7,CFAP74,KIF11,NEK6,SHROOM3,FLNB,KRT6B,KRT25,CDC14B,FAT1,PRKAA2,KIFC1,TUBB6,ANLN,SGO1,NEDD9,KRT6A,MTPN,ABI1,ASB2,CEP120,CYFIP2,KRT85,MAGEL2,SDCBP,NCK1,INTS13,OCLN,AKAP11,KIRREL1,PSTPIP2,PPM1F,RP1L1,TOGARAM1,TTLL11
GO:0048812	neuron projection morphogenesis	0.0010092436490375019	CNTN4,RIMS1,RIMS2,DOCK10,MACF1,NEDD4,PHACTR1,NEO1,CNTN6,PAK1,EPHA7,RAPGEF2,ADGRB3,TAOK3,EPB41L3,STAU2,SEMA5A,SYT1,VCL,ARHGAP44,NTF3,AURKA,MAP4K4,BMPR1B,PAK3,DIP2B,PRKCZ,ALCAM,SEMA3C,TNR,SDC2,USP33,PTPRO,PLXNA2,SEMA3E,ATL1,AFG3L2,ANK3,BCL11B,PDLIM5,NRK,SEMA3D,SLC23A2,B4GALT6,DRAXIN,FARP1,ALS2,DPYSL5,ZDHHC17,ABI1,ITGA4,CYFIP2,ADGRB1,WNT7A,LHX9,FEZ2,ITGA1,FLRT2,CCDC141
GO:0048522	positive regulation of cellular process	0.0010387455555336998	WWC1,MTOR,NSG1,PLCB1,ABCA13,KSR1,BRINP3,ZFPM2,TENM4,RIPOR2,RP1,KCNMA1,RIMS1,SPIRE1,TENM3,RARB,CDC42EP3,RIMS2,PJA2,BABAM2,ERBIN,CACNG2,NEGR1,MAP3K9,MYO3B,TCF4,NEK4,EGFR,ANGPT1,CDK12,MACF1,NEK7,ZNF407,NEDD4,MAML2,NSMCE2,SOX6,DKK2,THRAP3,MAPKBP1,DGKI,NEO1,CNTN6,SLC8A3,PAK1,EPHA7,NCOA7,SPEN,RAPGEF2,ADGRB3,RUNX2,ARSB,TAOK3,LDB2,RPTOR,ADAM10,IL1R1,APBB2,KDM1B,CACNB2,STAU2,SEMA5A,SYT1,NTF3,ACER2,AURKA,PYGO1,SLC8A1,SSBP2,SRGAP2C,ANKRD31,MAP4K4,BMPR1B,FMN2,RAB8B,PAK3,LARP1,ITPKB,SCP2,PRKCZ,GRB10,CNST,EBF2,YAP1,PDGFD,UBE2O,GFRA1,NIPBL,CORO2B,CHD6,MYLK3,KANSL1,ATF6,TPM1,LRRC38,BIRC6,KLF15,PPARA,SNX30,SYNJ1,ADAMTS3,TRERF1,SEMA3C,DAPK1,MAGI1,GATAD2B,IL34,MELK,RANBP3L,USP8,PIAS1,BLK,TNR,MRPS27,APBB1IP,EIPR1,SLFN11,GLIS1,MYO10,LATS2,ASPM,AP3B1,DENND2B,ATP11C,ZNF438,ABCB7,ZBTB16,MUSK,SETDB2,PRKCE,FOXK2,CD44,PRRC1,NSMAF,SPRED2,RPS6KA3,PTPN2,PLXNA2,MCF2L,ATXN3,RIC3,ARHGEF7,ALG10B,ATP8A1,AMBRA1,KDM7A,OPRM1,SEMA3E,RPRD1B,ABHD17C,TMOD2,MSH2,RELL1,EPN2,CLSPN,BICRAL,MNAT1,MDFIC,ANK3,HMGA2,BCL11B,DOCK5,PLCE1,FUT9,PRR5L,VAV1,HLA-B,IQSEC1,CACNA1I,BLM,ADCY10,BMP2,RC3H2,GFI1B,BMP2K,SEMA3D,NFATC2,SLC23A2,MYOM1,TRAF3,TTC21B,UIMC1,ATF1,CGAS,CNKSR3,VENTX,PRDM10,MOB1B,BBS4,MAPK8IP1,CFTR,KITLG,GTF2I,TADA2A,ZNF208,NMD3,MTMR2,TBX20,PRKCH,IL6R,ALS2,TFDP1,TOX,USP7,PLAGL1,MESD,KIR2DL4,RALB,NPAS2,VCAM1,DTX1,SUPT16H,BAZ1A,NEK6,HECTD1,NMU,SNAI2,IGHV3-74,BID,RXRG,SP3,ERN2,MBTPS2,TRIM58,TIAL1,ELF2,ZDHHC17,FYCO1,SH3GLB1,KCNC1,CSF1,GHRH,BCL2L1,ASB4,SMAD5,TCERG1,SLC40A1,PRAME,LPGAT1,MED1,CDC14B,PRAMEF25,PTH,PRKAA2,CSF2RB,VSTM2A,PLA2G4A,ZBTB7C,TEAD1,ANP32B,YBX3,AIMP1,PCID2,CIBAR1,CADM1,NET1,ANLN,MADD,HCRTR1,PATL1,ZNF287,PRSS2,CREBBP,MELTF,GORAB,NFKBIA,ABCC8,RFC2,ALX4,RTRAF,NEDD9,OLFM4,ASS1,SAR1A,ZBTB49,EXOC1,KRT6A,STOX2,AGO1,GID8,ELL2,NDFIP2,NR2C1,MTPN,ABI1,ITGA4,OAZ2,POU1F1,UBE2J2,TM9SF4,CEP120,CYFIP2,ARID3B,RXRA,ADGRB1,WNT7A,NDFIP1,MAP3K4,ZNF112,ATP6V1C2,MAGEL2,SDCBP,NSMCE1,NCK1,SCAF8,FGR,IFNAR1,RNF8,DNMT3L,OCLN,CD101,CD5L,ANKRD6,SCGN,ASCL3,LAMB1,KIRREL1,GPR55,TNFSF11,PPM1F,SH2D3C,TET1,RAD9A,ITGA1,POR,ZNF850,PRDM15,MACROH2A1,TOGARAM1,CSNK1G1,FLRT2,BTBD10,NUDT21,RFX2
GO:0051234	establishment of localization	0.0011616755523986984	WWC1,MICU2,NSG1,LRP12,SLC25A21,TMPRSS2,ABCA13,CACNA2D3,KCNH5,SLC37A1,PIEZO2,RIPOR2,ERC1,SLC44A5,KCNMA1,COG5,RIMS1,PIK3C3,SPIRE1,EXOC6B,SPAG16,TRAPPC8,USH2A,RIMS2,SV2C,ERBIN,FCHO2,CACNG2,MYO5C,OCA2,EGFR,DENND1A,ANGPT1,MACF1,DOCK2,NEDD4,BTBD9,TUSC3,DNAJC13,GABRB1,DGKI,C12ORF4,GRIA1,SLC39A12,SLC8A3,TOM1L2,PAK1,GRAMD1B,ARSB,GABRA6,LDLRAD3,AGK,RANBP17,SLC44A1,KIF4A,ADAM10,SLC7A2,CACNB2,STAU2,TMC1,SYT1,ARHGAP44,NTF3,SLC8A1,FMN2,HOMER2,RAB8B,RF

			TN1,RAP1GDS1,CLIC6,CUBN,SCP2,IFT57,PRKCZ,GRB10,MCPH1,CNST,ABCA5,VPS35L,ABCD3,RABGAP1L,SGTB,TRPC7,SLC45A4,UBE2O,NIPBL,NIPAL2,IPO11,MICU1,CORO2B,TLK1,LRRC38,ZDHHC14,CORIN,KLF15,PPARA,SNX30,KCNS3,SYNJ1,RSRC1,DAPK1,SLC24A4,SEC14L1,VPS13C,DNAH11,SCN2A,RAB22A,DNAJC15,AMPH,CPE,BBS2,SLC9C1,RANBP3L,NKG7,SLC36A1,BLK,KCNH1,EIPR1,DNAH5,MYO10,PLEKHA8,GSG1L,AP3B1,ATP11C,ABCB7,PRKCE,SLMAP,USP33,ABCC9,STXBP6,PEX14,SLC2A3,ARHGEF7,ALG10B,ATP8A1,RFTN2,OPRM1,BIN2,CYBRD1,CNNM4,EPN2,ABCA10,CD163,AFG3L2,MDFIC,ANK3,NIPA2,COG2,VPS41,LYPLA1,PRR5L,VPS37A,VAV1,CACNA1I,BHLHE40-AS1,SLC10A7,ADCY10,STX12,BMP2,ATP9A,TRAK1,RIN3,BMP2K,SLC15A5,NETO2,AP4E1,SLC23A2,MYOM1,PRG4,TTC21B,SNX8,CCDC186,KCNH8,SLC37A2,GABRR2,CNKSR3,DAW1,BBS4,LRRC8B,MAPK8IP1,CFTR,TBC1D13,NMD3,REPS1,MTMR2,HEPHL1,TRPM6,SLC12A1,TG,ALS2,SNX25,OSCP1,SCN10A,USP7,MON2,MESD,RALB,YIPF6,SEL1L,SLC13A5,NPIPA1,CUL5,IGHV3-74,BID,OSBPL10,COX5A,TRIM58,ZDHHC17,FYCO1,SH3GLB1,SLC22A14,XKR5,IFT81,ENPP1,KCNC1,GHRH,BCL2L1,SYNJ2,SLC40A1,CABYR,CIDEC,MED1,IPCEF1,ATG4B,PTH,PLA2G4A,SLC25A52,KIFC1,CAMLG,COX7A2L,ANP32B,AIMP1,LASP1,PCID2,PEG10,SLC1A7,MELTF,ARL11,SIAH3,TRPV5,NFKBIA,ABCC8,RTRAF,SLC14A2,SAR1A,TRAPPC3,OTOP1,EXOC1,FAM189A2,NDFIP2,SLC6A11,ITGA4,OAZ2,BCAP29,UBE2J2,TM9SF4,CEP120,STOML1,RXRA,ADGRB1,WNT7A,NDFIP1,ATP6V1C2,MAGEL2,SDCBP,JPT2,FLVCR1,FGR,SNAP29,C2,RN7SL767P,PLEKHA3,OCLN,STON1-GTF2A1L,MFSD9,MVB12B,CD5L,SCARA5,HEATR5A,PLCZ1,SLC9A5,NSUN2,ANO10,TNFSF11,PPM1F,XPO7,TMEM63C,SLC16A9,IFT46,SLC14A1,NSG2,MACROH2A1,CSNK1G1,ATP6V0D2,ATG5,NUP43,DDX6
GO:0048666	neuron development	0.0012160107975043915	*LRP12,SPOCK1,CNTN4,TENM4,RIPOR2,RP1,RIMS1,TENM3,MINAR1,RIMS2,NEGR1,DOCK10,MACF1,NEDD4,CRB1,PHACTR1,GABRB1,NEO1,CNTN6,SLC39A12,PAK1,EPHA7,RAPGEF2,ADGRB3,ARSB,TAOK3,EPB41L3,STAU2,TMC1,SEMA5A,SYT1,VCL,ARHGAP44,NTF3,AURKA,SRGAP2C,MAP4K4,BMPR1B,PAK3,DIP2B,PRKCZ,KLHL1,ALCAM,GFRA1,SEMA3C,BLK,TNR,SDC2,USP33,PTPRO,PLXNA2,SEMA3E,ATL1,AFG3L2,ANK3,BCL11B,FUT9,MYT1L,IQSEC1,PDLIM5,NRK,SEMA3D,SLC23A2,B4GALT6,TSPAN2,DRAXIN,ATF1,FARP1,BBS4,MTMR2,ALS2,TOX,DPYSL5,ZDHHC17,VSX1,ABI1,ITGA4,CYFIP2,ADGRB1,WNT7A,FOXO6,NCK1,LHX9,FEZ2,LAMB1,RP1L1,ITGA1,UNK,FLRT2,CCDC141*
GO:0043412	macromolecule modification	0.0012580846054179367	*MTOR,TMTC1,KSR1,ERC1,F13A1,PIK3C3,MINAR1,PJA2,BABAM2,MAP3K9,MYO3B,NEK4,EGFR,USP14,ANGPT1,CDK12,PRKACB,NEK7,NEDD4,NSMCE2,B3GALT5,TUSC3,SLC8A3,PAK1,EPHA7,NCOA7,RAPGEF2,TAOK3,RPTOR,ADAM10,KDM1B,KLHL13,USP18,NTF3,ACER2,PARP15,AURKA,SLC8A1,DTWD2,MAP4K4,BMPR1B,PAK3,TTLL7,DIP2B,ITPKB,HHAT,PRKCZ,SPOP,MAN2A2,ST8SIA5,SENP6,GALNT14,PPM1L,RIPK4,USP25,PDGFD,ZNRF3,XXYLT1,UBE2O,GFRA1,NIPBL,STK38,MYLK3,KANSL1,CCNG2,TLK1,ZDHHC14,BIRC6,KLF15,PPP6R3,RSRC1,PTPRK,TRERF1,PDZRN3,DAPK1,STK32B,ALPK2,GATAD2B,CPE,IL34,MELK,USP8,PIAS1,UBE2R2,BLK,OXR1,TTLL5,EIPR1,ATE1,MORC1,LATS2,AP3B1,ZBTB16,MUSK,SMARCAD1,SETDB2,PRKCE,USP33,CD44,PTPRO,ALPK3,PRRC1,LNPEP,SPRED2,RPS6KA3,PTPN2,ATXN3,ST8SIA6,ALG10B,AMBRA1,GALNT10,KDM7A,FANCM,FANCA,RPRD1B,ABHD17C,HIPK3,CLSPN,MNAT1,HMGA2,FOLH1,STK32A,LYPLA1,CWC27,PLCE1,FUT9,PRR5L,GXYLT2,FBXO32,BLM,NRK,BMP2,RC3H2,TRAK1,BMP2K,RNF38,SH3BP5,TRAF3,UIMC1,B4GALT6,CNKSR3,CUL1,DAW1,MOB1B,MAPK8IP1,KITLG,TADA2A,UBE2E1,PTPRE,MTMR2,WSB1,TRPM6,PRKCH,IL6R,ALS2,SNX25,TOX,PTPRB,USP7,MOK,RALB,DTX1,FANCL,CUL5,NEK6,HECTD1,SNAI2,SIAH2,*

			PGAP4,ERN2,TRIM58,ZDHHC17,PTAR1,ENPP1,CSF1,PPIL6,EOGT,CTDP1,ASB4,SMAD5,PRAME,KLHL7,ATG4B,CDC14B,PRKAA2,CSF2RB,RNF182,PHF20L1,CAMLG,FICD,CADM1,ELOC,MADD,CREBBP,MRM1,SIAH3,RTRAF,NEDD9,PCMTD2,PDP2,NDFIP2,MARCHF6,ABI1,PPME1,UBE2J2,ASB2,ASCC2,ST8SIA4,ADGRB1,NDFIP1,MAP3K4,TRIM43B,TRIM43,MAGEL2,SDCBP,NSMCE1,MLLT1,NCK1,FGR,RNF8,DNMT3L,OCLN,KIRREL1,NSUN2,TNFSF11,DPY19L1,PPM1F,SH2D3C,ZFYVE28,STT3A,TET1,ASB3,SPOPL,ITGA1,POR,SENP8,USP49,MACROH2A1,CSNK1G1,CAMK1G,ATG5,TTLL11
GO:0031175	neuron projection development	0.001751893965180288	*LRP12,SPOCK1,CNTN4,RIPOR2,RIMS1,TENM3,MINAR1,RIMS2,NEGR1,DOCK10,MACF1,NEDD4,PHACTR1,NEO1,CNTN6,SLC39A12,PAK1,EPHA7,RAPGEF2,ADGRB3,ARSB,TAOK3,EPB41L3,STAU2,SEMA5A,SYT1,VCL,ARHGAP44,NTF3,AURKA,SRGAP2C,MAP4K4,BMPR1B,PAK3,DIP2B,PRKCZ,KLHL1,ALCAM,GFRA1,SEMA3C,BLK,TNR,SDC2,USP33,PTPRO,PLXNA2,SEMA3E,ATL1,AFG3L2,ANK3,BCL11B,FUT9,IQSEC1,PDLIM5,NRK,SEMA3D,SLC23A2,B4GALT6,TSPAN2,DRAXIN,ATF1,FARP1,BBS4,ALS2,TOX,DPYSL5,ZDHHC17,ABI1,ITGA4,CYFIP2,ADGRB1,WNT7A,FOXO6,NCK1,LHX9,FEZ2,LAMB1,ITGA1,FLRT2,CCDC141*
GO:0009966	regulation of signal transduction	0.0020847681704016865	*WWC1,GARNL3,MTOR,PLCB1,ZNF536,KSR1,RIPOR2,FBN1,RIMS1,MINAR1,RIMS2,PJA2,BABAM2,ERBIN,CACNG2,EGFR,DENND1A,ANGPT1,MACF1,PRKACB,NCOR1,DOCK2,NEDD4,DKK2,MAPKBP1,DGKI,NEO1,CNTN6,SLC8A3,PAK1,EPHA7,RALGPS1,RAPGEF2,RUNX2,TAOK3,RPTOR,ADAM10,IL1R1,USP18,SEMA5A,ARHGAP44,NTF3,AURKA,MAP4K4,BMPR1B,HOMER2,PAK3,ITPKB,RGS20,PDE10A,RAP1GDS1,KICS2,PRKCZ,GRB10,RGS9,YAP1,PDGFD,ZNRF3,UBE2O,STK38,HRH4,ATF6,BIRC6,KLF15,PPARA,ADAMTS3,ARHGEF12,DAPK1,SLC24A4,SEC14L1,ALPK2,IL34,USP8,BLK,GAS2,LATS2,GSG1L,ASPM,DENND2B,GNG7,PRKCE,USP33,CD44,RGS12,PTPRO,LIMD1,SPRED2,PTPN2,MCF2L,OPRM1,FANCA,SEMA3E,TMOD2,RELL1,HIPK3,EPN2,MOSMO,MDFIC,PLCE1,CRIM1,PRR5L,VAV1,IQSEC1,NRK,MAGI3,BMP2,RC3H2,BMP2K,NETO2,TRAF3,TTC21B,RAP1GAP,DRAXIN,CNKSR3,MAPK8IP1,UBASH3A,KITLG,PTPRE,MTMR2,TBX20,AFAP1,PRKCH,IL6R,ALS2,SNX25,USP7,RALB,ARHGAP31,DTX1,NEK6,SNAI2,BID,SIAH2,ERN2,TIAL1,ZDHHC17,SAMHD1,IFT81,ENPP1,CSF1,GHRH,BCL2L1,DHRS3,PRAME,MED1,PTH,PRKAA2,YBX3,PCID2,CIBAR1,PBLD,FICD,CADM1,PEG10,NET1,FSTL1,MADD,HCRTR1,CREBBP,GORAB,NFKBIA,OTOP1,GID8,FAM189A2,NDFIP2,NR2C1,CYFIP2,RXRA,WNT7A,NDFIP1,MAP3K4,ATP6V1C2,C16ORF72,PDE2A,SDCBP,JPT2,NCK1,FGR,CYTH4,POSTN,MVB12B,ANKRD6,LAMB1,GPR55,TNFSF11,ZFYVE28,TET1,RAD9A,ITGA1,POR,PRDM15,SRGAP3,CSNK1G1,TMEM25*
GO:0010646	regulation of cell communication	0.002249682218135826	*WWC1,GARNL3,MTOR,NSG1,PLCB1,ZNF536,KSR1,CNTN4,RIPOR2,ERC1,FBN1,RIMS1,MINAR1,RIMS2,PJA2,BABAM2,ERBIN,CACNG2,EGFR,DENND1A,ANGPT1,MACF1,PRKACB,NCOR1,DOCK2,NEDD4,BTBD9,DKK2,MAPKBP1,DGKI,GRIA1,NEO1,CNTN6,SLC8A3,PAK1,EPHA7,RALGPS1,RAPGEF2,RUNX2,TAOK3,RPTOR,ADAM10,IL1R1,CACNB2,STAU2,USP18,SEMA5A,SYT1,ARHGAP44,NTF3,AURKA,SLC8A1,MAP4K4,BMPR1B,HOMER2,RAB8B,PAK3,ITPKB,RGS20,PDE10A,RAP1GDS1,KICS2,PRKCZ,GRB10,RGS9,YAP1,PDGFD,ZNRF3,UBE2O,STK38,HRH4,SORCS3,ATF6,BIRC6,KLF15,PPARA,ADAMTS3,ARHGEF12,DAPK1,SLC24A4,SEC14L1,ALPK2,IL34,USP8,BLK,TNR,GAS2,EIPR1,LATS2,GSG1L,ASPM,DENND2B,GNG7,PRKCE,USP33,CD44,RGS12,PTPRO,LIMD1,SPRED2,PTPN2,MCF2L,ARHGEF7,OPRM1,FANCA,SEMA3E,TMOD2,RELL1,HIPK3,EPN2,MOSMO,MDFIC,ANK3,PLCE1,CRIM1,PRR5L,VAV1,IQSEC1,NRK,MAGI3,BMP2,RC3H2,BMP2K,NETO2,TRAF3,TTC21B,RAP1GAP,DRAXIN,CNKSR3,MAPK8IP1,CFTR,UBASH3A,KITLG,PTPRE,MTMR2,TBX20,AFAP1,PRKCH,IL6R,ALS2,SNX25,USP7,RALB,ARHGAP31,DTX1,NEK6,NMU,SNAI2,BID,SIAH2,ERN2,TIAL1,ZDHHC17,SAMHD1,IFT81,ENPP1,CSF1,GHRH,BCL2L1,DHRS3,PRAM*

			E,MED1,PTH,PRKAA2,YBX3,AIMP1,PCID2,CIBAR1,PBLD,FICD,CADM1,PEG10,NET1,FSTL1,MADD,HCRTR1,CREBBP,GORAB,NFKBIA,ABCC8,OTOP1,GID8,FAM189A2,NDFIP2,NR2C1,CYFIP2,RXRA,ADGRB1,WNT7A,NDFIP1,MAP3K4,ATP6V1C2,C16ORF72,PDE2A,SDCBP,JPT2,NCK1,FGR,CYTH4,POSTN,MVB12B,ANKRD6,SCGN,LAMB1,GPR55,TNFSF11,ZFYVE28,TET1,RAD9A,ITGA1,POR,PRDM15,SRGAP3,CSNK1G1,TMEM25
GO:0023051	regulation of signaling	0.004064219736681283	WWC1,GARNL3,MTOR,NSG1,PLCB1,ZNF536,KSR1,CNTN4,RIPOR2,ERC1,FBN1,RIMS1,MINAR1,RIMS2,PJA2,BABAM2,ERBIN,CACNG2,EGFR,DENND1A,ANGPT1,MACF1,PRKACB,NCOR1,DOCK2,NEDD4,BTBD9,DKK2,MAPKBP1,DGKI,GRIA1,NEO1,CNTN6,SLC8A3,PAK1,EPHA7,RALGPS1,RAPGEF2,RUNX2,TAOK3,RPTOR,ADAM10,IL1R1,CACNB2,STAU2,USP18,SEMA5A,SYT1,ARHGAP44,NTF3,AURKA,MAP4K4,BMPR1B,HOMER2,RAB8B,PAK3,ITPKB,RGS20,PDE10A,RAP1GDS1,KICS2,PRKCZ,GRB10,RGS9,YAP1,PDGFD,ZNRF3,UBE2O,STK38,HRH4,SORCS3,ATF6,BIRC6,KLF15,PPARA,ADAMTS3,ARHGEF12,DAPK1,SLC24A4,SEC14L1,ALPK2,IL34,USP8,BLK,TNR,GAS2,EIPR1,LATS2,GSG1L,ASPM,DENND2B,GNG7,PRKCE,USP33,CD44,RGS12,PTPRO,LIMD1,SPRED2,PTPN2,MCF2L,ARHGEF7,OPRM1,FANCA,SEMA3E,TMOD2,RELL1,HIPK3,EPN2,MOSMO,MDFIC,PLCE1,CRIM1,PRR5L,VAV1,IQSEC1,NRK,MAGI3,BMP2,RC3H2,BMP2K,NETO2,TRAF3,TTC21B,RAP1GAP,DRAXIN,CNKSR3,MAPK8IP1,CFTR,UBASH3A,KITLG,PTPRE,MTMR2,TBX20,AFAP1,PRKCH,IL6R,ALS2,SNX25,USP7,RALB,ARHGAP31,DTX1,NEK6,NMU,SNAI2,BID,SIAH2,ERN2,TIAL1,ZDHHC17,SAMHD1,IFT81,ENPP1,CSF1,GHRH,BCL2L1,DHRS3,PRAME,MED1,PTH,PRKAA2,YBX3,AIMP1,PCID2,CIBAR1,PBLD,FICD,CADM1,PEG10,NET1,FSTL1,MADD,HCRTR1,CREBBP,GORAB,NFKBIA,ABCC8,OTOP1,GID8,FAM189A2,NDFIP2,NR2C1,CYFIP2,RXRA,ADGRB1,WNT7A,NDFIP1,MAP3K4,ATP6V1C2,C16ORF72,PDE2A,SDCBP,JPT2,NCK1,FGR,CYTH4,POSTN,MVB12B,ANKRD6,SCGN,LAMB1,GPR55,TNFSF11,ZFYVE28,TET1,RAD9A,ITGA1,POR,PRDM15,SRGAP3,CSNK1G1,TMEM25
GO:0048518	positive regulation of biological process	0.004847366922259332	WWC1,MTOR,NSG1,TMPRSS2,PLCB1,ABCA13,KSR1,BRINP3,ZFPM2,TENM4,RIPOR2,RP1,KCNMA1,RIMS1,SPIRE1,TENM3,RARB,CDC42EP3,RIMS2,PJA2,BABAM2,ERBIN,CACNG2,NEGR1,SUSD4,MAP3K9,MYO3B,TCF4,NEK4,EGFR,ANGPT1,CDK12,MACF1,NEK7,NCOR1,DOCK2,ZNF407,NEDD4,MAML2,NSMCE2,SOX6,DKK2,THRAP3,MAPKBP1,DGKI,C12ORF4,NEO1,CNTN6,SLC39A12,SLC8A3,PAK1,EPHA7,NCOA7,SPEN,RAPGEF2,ADGRB3,RUNX2,ARSB,TAOK3,LDB2,RPTOR,ADAM10,IL1R1,APBB2,KDM1B,CACNB2,STAU2,SEMA5A,SYT1,NTF3,ACER2,AURKA,PYGO1,SLC8A1,SSBP2,SRGAP2C,ANKRD31,MAP4K4,BMPR1B,FMN2,RAB8B,PAK3,RFTN1,DIP2B,LARP1,ITPKB,SCP2,IFT57,PRKCZ,GRB10,CNST,ABCA5,EBF2,YAP1,PLG,PDGFD,UBE2O,GFRA1,NIPBL,CORO2B,CHD6,MYLK3,KANSL1,ATF6,TPM1,LRRC38,BIRC6,KLF15,PPARA,SNX30,SYNJ1,ADAMTS3,TRERF1,SEMA3C,DAPK1,SLC24A4,SEC14L1,MAGI1,GATAD2B,IL34,MELK,BBS2,RANBP3L,NKG7,USP8,PIAS1,BLK,TNR,MRPS27,DROSHA,APBB1IP,EIPR1,SLFN11,GLIS1,MYO10,LATS2,ASPM,AP3B1,DENND2B,ATP11C,ZNF438,ABCB7,ZBTB16,MUSK,SETDB2,PRKCE,FOXK2,CD44,PRRC1,NSMAF,LIMD1,SPRED2,RPS6KA3,PTPN2,PLXNA2,MCF2L,ATXN3,RIC3,ARHGEF7,ALG10B,ATP8A1,AMBRA1,KDM7A,OPRM1,FANCM,SEMA3E,RPRD1B,ALPL,ABHD17C,TMOD2,MSH2,RELL1,EPN2,CLSPN,BICRAL,MNAT1,MDFIC,ANK3,HMGA2,BCL11B,DOCK5,PLCE1,IL17RA,FUT9,PRR5L,VAV1,HLA-B,IQSEC1,CACNA1I,BLM,ADCY10,BMP2,RC3H2,GFI1B,BMP2K,SEMA3D,POLR3A,NFATC2,SLC23A2,MYOM1,TRAF3,TTC21B,UIMC1,ATF1,CGAS,CNKSR3,VENTX,PRDM10,MOB1B,BBS4,MAPK8IP1,CFTR,UBASH3A,KITLG,GTF2I,TADA2A,ZNF208,NMD3,MTMR2,TBX20,PRKCH,IL6R,ALS2,TFDP1,TOX,USP7,ENPP3,PLAGL1,MESD,KIR2DL4,RALB,NPAS2,VCAM1,DTX1,SUPT16H,BAZ1A,NEK6,HECTD1,NMU,SNAI2,IGHV3-74,BID,RXRG,SP3,ERN2,MBTPS2,TRIM58,TIAL1,ELF2,ZDH

			HC17,FYCO1,SH3GLB1,KCNC1,CSF1,GHRH,BCL2L1,ASB4,SMAD5,TCERG1,SLC40A1,PRAME,LPGAT1,MED1,CDC14B,CFH,PRAMEF25,PTH,PRKAA2,CSF2RB,VSTM2A,PLA2G4A,ZBTB7C,TEAD1,ANP32B,YBX3,AIMP1,FYB2,PCID2,CIBAR1,CADM1,NET1,ANLN,MADD,HCRTR1,PATL1,ZNF287,PRSS2,CREBBP,MELTF,GORAB,NFKBIA,ABCC8,RFC2,ALX4,RTRAF,NEDD9,OLFM4,ASS1,SAR1A,ZBTB49,EXOC1,KRT6A,STOX2,AGO1,GID8,ELL2,NDFIP2,NR2C1,MTPN,ABI1,ITGA4,OAZ2,POU1F1,UBE2J2,TM9SF4,CEP120,CYFIP2,ARID3B,RXRA,ADGRB1,WNT7A,NDFIP1,MAP3K4,FOXO6,ZNF112,ATP6V1C2,MAGEL2,PDE2A,SDCBP,NSMCE1,NCK1,SCAF8,FGR,C2,IFNAR1,RNF8,DNMT3L,WNT2B,OCLN,CD101,CD5L,ANKRD6,SCGN,ASCL3,LAMB1,KIRREL1,GPR55,TNFSF11,PPM1F,SH2D3C,TET1,RAD9A,ITGA1,POR,ZNF850,PRDM15,MACROH2A1,TOGARAM1,CSNK1G1,ATG5,FLRT2,BTBD10,NUDT21,RFX2
GO:0051172	negative regulation of nitrogen compound metabolic process	0.011993471850971275	*WWC1,PLCB1,SPOCK1,ZNF536,ZFPM2,RARB,MINAR1,CDYL2,RTN1,EGFR,USP14,ANGPT1,NCOR1,NEDD4,SOX6,THRAP3,CAST,SLC8A3,NCOA7,SPEN,RUNX2,CPEB4,LDB2,APBB2,KDM1B,NTF3,PARP15,SLC8A1,SERPINA6,FMN2,LARP1,PRKCZ,MCPH1,YAP1,USP25,UBE2O,NIPBL,CARD18,STK38,ZNF684,BIRC6,KLF15,PPARA,PTPRK,TRERF1,DAPK1,PHC3,JARID2,GATAD2B,ZNF846,PIAS1,MXI1,OXR1,CREG1,PSMF1,SLFN11,GLIS1,MORC1,LATS2,ZBTB16,SETDB2,PRKCE,FOXK2,CD44,PTPRO,LIMD1,PEX14,SPRED2,RPS6KA3,PTPN2,MSH2,ZNF397,HIPK3,MDFIC,HMGA2,CRIM1,PRR5L,MYT1L,BLM,BMP2,RC3H2,GFI1B,NFATC2,SH3BP5,ZNF875,UIMC1,LRRFIP1,CGAS,CNKSR3,BTAF1,MAPK8IP1,MRPL13,ZNF608,TBX20,SNX25,PTPRB,USP7,PLAGL1,ZNF169,ZBTB33,SNAI2,SIAH2,SP3,ERN2,ELF2,ENPP1,SMAD5,TCERG1,PRAME,MED1,SCML2,PRAMEF25,PRKAA2,CAMLG,YBX3,PCID2,CADM1,PATL1,CREBBP,RTRAF,ZBTB21,ZBTB49,AGO1,NR2C1,POU1F1,RXRA,ADGRB1,NDFIP1,SERPINI2,MAGEL2,PDE2A,SDCBP,MLLT1,NCK1,SCAF8,RNF8,DNMT3L,LHX9,OCLN,ASCL3,KIRREL1,NSUN2,TNFSF11,ZNF705G,PPM1F,PSME3IP1,ZFYVE28,TET1,SPOPL,ZNF705D,POR,MACROH2A1,ZNF705B,SERPINB2,ATG5,UNK,DDX6,SERPINB11*
GO:0065009	regulation of molecular function	0.013837459422381874	*GARNL3,MTOR,PLCB1,SPOCK1,KSR1,RIPOR2,ERC1,RIMS1,ERBIN,CACNG2,DOCK10,EGFR,DENND1A,USP14,ANGPT1,CDK12,NEK7,DOCK2,NEDD4,PHACTR1,DKK2,DGKI,CAST,SLC8A3,TBC1D19,PAK1,EPHA7,RALGPS1,RAPGEF2,ADGRB3,TAOK3,LDB2,PPP2R2B,PUM3,RPTOR,PPP1R12B,PHACTR2,CACNB2,ARHGAP44,NTF3,ACER2,AURKA,SLC8A1,SERPINA6,MAP4K4,BMPR1B,RGS20,RAP1GDS1,IFT57,PRKCZ,MCPH1,RGS9,EBF2,RIPK4,RABGAP1L,PDGFD,UBE2O,CARD18,STK38,CCNG2,TPM1,LRRC38,BIRC6,PPARA,PPP6R3,ARAP2,ARHGEF12,DAPK1,SLC24A4,TBC1D9,IL34,RANBP3L,BLK,PSMF1,LATS2,GSG1L,AP3B1,DENND2B,MUSK,PRKCE,ASAP2,SLMAP,USP33,CD44,RGS12,PTPRO,PRRC1,ABCC9,NSMAF,PEX14,SPRED2,RPS6KA3,PTPN2,PLXNA2,MCF2L,ARHGEF7,ALG10B,AMBRA1,OPRM1,FANCA,MSH2,HIPK3,CLSPN,MNAT1,ANK3,HMGA2,DOCK5,PLCE1,CRIM1,VAV1,IQSEC1,BLM,BMP2,RIN3,BMP2K,NETO2,SH3BP5,TRAF3,RAP1GAP,CNKSR3,BTAF1,ZNF618,FARP1,MOB1B,BBS4,MAPK8IP1,CFTR,TBC1D13,KITLG,NMD3,SH3PXD2A,PRKCH,IL6R,ALS2,TFDP1,PTPRB,USP7,RALB,DENND2C,ARHGAP31,XRCC4,BID,SIAH2,ERN2,MBTPS2,ENPP1,TMEM225,KCNC1,CSF1,CDC14B,PTH,ZBTB7C,ANP32B,PCID2,FICD,NET1,MADD,NFKBIA,ABCC8,ZC3H15,RFC2,RTRAF,NEDD9,PPP1R17,PDP2,NDFIP2,MTPN,ABI1,ITGA4,OAZ2,PPME1,CYFIP2,RXRA,NDFIP1,MAP3K4,SERPINI2,MLLT1,NCK1,FGR,CYTH4,DNMT3L,GPR55,TNFSF11,PPM1F,SH2D3C,PSME3IP1,ZFYVE28,ITGA1,POR,SRGAP3,MACROH2A1,SERPINB2,SERPINB11*
GO:1901564	organonitrogen compound metabolic	0.01433407834789898	*MTOR,NSG1,SLC25A21,TMPRSS2,TMTC1,PLCB1,SPOCK1,KSR1,ERC1,NME7,SLC44A5,F13A1,PIK3C3,ENPEP,MINAR1,PJA2,BABAM2,GLYAT,MAP3K9,MYO3B,CPA6,RTN1,NEK4,EGFR,USP14,ANGPT1,CDK12,PRKACB,NEK7,NCOR1,NEDD4,NSMCE2,BTBD9,PSMB2,B3GALT5,TUSC3,CAST,SLC8A3,PAK1,EPHA7,*

	process		*NCOA7,CHSY3,RAPGEF2,CPS1,TAOK3,LDLRAD3,CPEB4,AGK,BCKDHB,SLC44A1,PUM3,LRGUK,RPTOR,ADAM32,ADAM10,ADK,KDM1B,KLHL13,USP18,NTF3,ACER2,PARP15,AURKA,SLC8A1,SERPINA6,UPP2,MAP4K4,BMPR1B,FMN2,PAK3,TTLL7,DIP2B,LARP1,ITPKB,PDE10A,RAP1GDS1,HHAT,CHST8,CUBN,IFT57,PRKCZ,SPOP,MAN2A2,ST8SIA5,SENP6,GALNT14,PPM1L,RIPK4,SGTB,USP25,PLG,PAPPA,PDGFD,ZNRF3,XXYLT1,UBE2O,GFRA1,NIPBL,CARD18,STK38,MYLK3,KANSL1,ATF6,CCNG2,TLK1,MRPS22,ZDHHC14,CORIN,BIRC6,KLF15,PPARA,HS3ST2,PPP6R3,ADAMTS3,RSRC1,PTPRK,TRERF1,PDZRN3,DAPK1,FAR2,STK32B,ALPK2,GATAD2B,CPE,IL34,MELK,ASAH2B,USP8,PIAS1,UBE2R2,BLK,OLA1,AGPS,OXR1,MRPS27,TTLL5,EIPR1,PSMF1,ATE1,GALC,LATS2,AP3B1,ABCB7,ZBTB16,MUSK,SMARCAD1,SETDB2,PRKCE,FOXK2,USP33,CD44,PTPRO,ALPK3,PRRC1,NSMAF,LNPEP,SPRED2,RPS6KA3,PTPN2,ATXN3,ST8SIA6,ALG10B,AMBRA1,GALNT10,KDM7A,FANCM,FANCA,RPRD1B,TMEM67,ALPL,ABHD17C,HIPK3,CPXM2,CLSPN,AFG3L2,MNAT1,XYLT1,HMGA2,FOLH1,STK32A,LYPLA1,CWC27,PLCE1,IL17RA,CRIM1,FUT9,PRR5L,GXYLT2,VPS37A,FBXO32,BLM,NRK,SLC10A7,MAGI3,ADCY10,BMP2,RC3H2,TRAK1,WDR26,BMP2K,RNF38,SH3BP5,TRAF3,UIMC1,B4GALT6,CNKSR3,CASP5,CUL1,DAW1,MOB1B,MAPK8IP1,ME2,MRPL13,KITLG,TADA2A,UBE2E1,PTPRE,MTMR2,WSB1,TRPM6,PRKCH,TG,IL6R,ALS2,SNX25,PTPRB,TSPAN33,USP7,ENPP3,HAAO,FAH,MOK,RALB,VCAM1,SEL1L,GSTA3,DTX1,ADA2,FANCL,CUL5,NEK6,HECTD1,SNAI2,BID,SIAH2,OSBPL10,PGAP4,ERN2,MBTPS2,TRIM58,ZDHHC17,PTAR1,SAMHD1,ENPP1,MOCS2,PAMR1,UCK2,CSF1,PPIL6,EOGT,CTDP1,ASB4,SMAD5,PRAME,KLHL7,LPGAT1,MED1,ATG4B,CDC14B,CFH,NPL,HGD,PTH,PRKAA2,CSF2RB,GLYATL1,RNF182,PHF20L1,PLA2G4A,CAMLG,ANP32B,YBX3,AIMP1,PCID2,CYP4F22,FICD,CADM1,ELOC,ADAM28,BPNT1,MADD,PATL1,PRSS2,CREBBP,MELTF,SIAH3,NFKBIA,ZC3H15,RTRAF,NEDD9,NOXRED1,ASS1,CNDP2,ERLIN2,PCMTD2,ZBTB49,AGO1,PDP2,GID8,NDFIP2,MARCHF6,MTPN,ABI1,OAZ2,PPME1,UBE2J2,ASB2,DHTKD1,CYFIP2,ACACA,ASCC2,ST8SIA4,ADGRB1,WNT7A,NDFIP1,MAP3K4,TRIM43B,SERPINI2,TRIM43,MAGEL2,PDE2A,SDCBP,NSMCE1,MLLT1,NCK1,FLVCR1,FGR,C2,RNF8,OCLN,MVB12B,CD5L,KIRREL1,TNFSF11,DPY19L1,PPM1F,SH2D3C,PSME3IP1,TRNAU1AP,ZFYVE28,STT3A,SLC16A9,TET1,ASB3,OVCH2,SPOPL,ITGA1,POR,SENP8,USP49,MACROH2A1,CSNK1G1,PRSS51,CAMK1G,SERPINB2,ATG5,UNK,DDX6,TTLL11,SERPINB11*
GO:0048513	animal organ development	0.017811301284173853	*MTOR,SMOC1,PLCB1,NEBL,SGCD,CNTN4,ZFPM2,TENM4,RIPOR2,RP1,ODAD2,FBN1,TENM3,RARB,ENPEP,USH2A,NEGR1,MYO3B,EGFR,ANGPT1,CRB1,SOX6,PHACTR1,EPHA7,RAPGEF2,RUNX2,CPS1,PRICKLE2,LDB2,ADAM10,STAU2,TMC1,SEMA5A,SYT1,AURKA,PYGO1,SLC8A1,SRGAP2C,BMPR1B,IFT57,KLHL1,MCPH1,ZSWIM6,EBF2,YAP1,CADM2,PLG,PDGFD,ZNRF3,ABLIM1,GFRA1,NIPBL,MYLK3,ATF6,TPM1,ANKRD11,BIRC6,KLF15,PPARA,SYNJ1,SEMA3C,SLC24A4,ALPK2,DNAH11,JARID2,CPE,BBS2,RANBP3L,LDB3,TNR,XIRP2,GAS2,TTLL5,DNAH5,LATS2,ASPM,AP3B1,ZBTB16,SETDB2,CD44,PTPRO,ALPK3,COL5A3,SPRED2,NHS,PLXNA2,KDM7A,FANCA,CNNM4,SEMA3E,ALPL,MSH2,LUC7L,MNAT1,XYLT1,HMGA2,BCL11B,AK8,PLCE1,PDLIM5,SLC10A7,BMP2,RC3H2,BMP2K,RNF38,SEMA3D,TDRD7,TTC21B,TSPAN2,RAP1GAP,DRAXIN,CASP5,CUL1,DAW1,BBS4,COL5A1,CFTR,AHDC1,KITLG,TBX20,PRKCH,TG,IL6R,TOX,PDE6A,SCN10A,VCAM1,LRIG1,HECTD1,SNAI2,RXRG,SP3,MBTPS2,FLNB,KRT6B,ENPP1,KCNC1,CSF1,GHRH,BCL2L1,KRT25,CTDP1,DHRS3,SMAD5,SYNJ2,SLC40A1,MED1,ATG4B,FAT1,PTH,TEAD1,ANP32B,YBX3,PCID2,CADM1,VSX1,GORAB,ALX4,ASS1,OTOP1,KRT6A,STOX2,SLC6A11,MTPN,ABI1,POU1F1,ASB2,CEP120,KRT85,RXRA,ADGRB1,WNT7A,MAP3K4,PDE2A,SDCBP,FLVCR1,FGR,SPRR2D,DNMT3L,LHX9,WNT2B,TNNI1,ANKRD6,LAMB1,NSUN2,TNFSF11,ARL13B,UGP2,RP1L1,POR,B9D1,MACROH2A1,ATG5,FLRT2,CCDC141*

GO:0014706	striated muscle tissue development	0.019204680454221103	*MTOR,NEBL,SGCD,ZFPM2,TENM4,RARB,SOX6,SLC8A1,YAP1,MYLK3,TPM1,PPARA,SEMA3C,ALPK2,JARID2,XIRP2,ALPK3,LUC7L,PDLIM5,BMP2,TBX20,CTDP1,SMAD5,MED1,MTPN,ASB2,TNNI1,ATG5*
GO:0120031	plasma membrane bounded cell projection assembly	0.019355257515482793	*LRRC49,MTOR,RIPOR2,RP1,ODAD2,SPAG16,CDC42EP3,PARVB,MYO3B,ARMC2,RAPGEF2,LRGUK,STAU2,VCL,ARHGAP44,SRGAP2C,IFT57,YAP1,ABLIM1,BBS2,DNAH5,MYO10,PTPRO,ARHGEF7,TMEM67,PLCE1,DNAL1,TTC21B,RAP1GAP,DNAH8,DAW1,BBS4,CFAP74,IFT81,FAM149B1,CDC14B,CIBAR1,ANLN,GORAB,CEP120,SDCBP,NCK1,SNAP29,OCLN,ARL13B,RP1L1,IFT46,B9D1,TOGARAM1,ATG5,RFX2*
GO:0007166	cell surface receptor signaling pathway	0.019679105423894862	*PLCB1,ANKS1B,FBN1,RIMS1,RIMS2,ADGRE1,PJA2,ERBIN,EGFR,ANGPT1,MACF1,PRKACB,NEDD4,MAML2,DKK2,GABRB1,DGKI,GRIA1,NEO1,CNTN6,SLC8A3,PAK1,EPHA7,SPEN,RAPGEF2,ADGRB3,RUNX2,GABRA6,CPEB4,PRICKLE2,COL4A2,ADAM10,IL1R1,USP18,SEMA5A,NTF3,PYGO1,BMPR1B,HOMER2,PAK3,RFTN1,ITPKB,HHAT,IFT57,PRKCZ,BTLA,GRB10,YAP1,PPM1L,PAPPA,PDGFD,ZNRF3,ITGBL1,UBE2O,GFRA1,BIRC6,KLF15,PPARA,ADAMTS3,PTPRK,SEMA3C,DAPK1,MAGI1,ALPK2,CPE,EVC2,IL34,BBS2,USP8,PIAS1,BLK,GAS2,LATS2,ASPM,MUSK,PRKCE,CD44,PTPRO,LNPEP,LIMD1,SPRED2,PTPN2,PLXNA2,ARHGEF7,OPRM1,FANCA,SEMA3E,EPN2,MOSMO,MDFIC,PLCE1,IL17RA,CRIM1,VAV1,BMP2,RC3H2,BMP2K,SEMA3D,NFATC2,ZNF106,TRAF3,TTC21B,DRAXIN,GABRR2,WDR12,BBS4,UBASH3A,KITLG,PTPRE,MTMR2,TBX20,PRKCH,IL6R,SNX25,MESD,RALB,SEL1L,DTX1,ZBTB33,CUL5,SNAI2,IGHV3-74,BID,SIAH2,ZDHHC17,SAMHD1,IFT81,ENPP1,CSF1,GHRH,BCL2L1,SMAD5,MED1,PRKAA2,CSF2RB,CAMLG,FYB2,CIBAR1,PBLD,CADM1,PEG10,SLC1A7,FSTL1,SVEP1,MADD,CREBBP,GORAB,NFKBIA,ZC3H15,NEDD9,ADGRE3,OTOP1,GID8,FAM189A2,ABI1,ITGA4,CYFIP2,ADGRB1,WNT7A,ATP6V1C2,SDCBP,NCK1,FGR,IFNAR1,WNT2B,POSTN,CD101,MVB12B,ANKRD6,LAMB1,FCRLA,TNFSF11,ARL13B,ZFYVE28,TET1,ITGA1,POR,B9D1,PRDM15,CSNK1G1,FLRT2,TMEM25*
GO:0019538	protein metabolic process	0.023475391224244715	*MTOR,NSG1,TMPRSS2,TMTC1,PLCB1,SPOCK1,KSR1,ERC1,F13A1,PIK3C3,ENPEP,MINAR1,PJA2,BABAM2,MAP3K9,MYO3B,CPA6,RTN1,NEK4,EGFR,USP14,ANGPT1,CDK12,PRKACB,NEK7,NEDD4,NSMCE2,PSMB2,B3GALT5,TUSC3,CAST,SLC8A3,PAK1,EPHA7,NCOA7,CHSY3,RAPGEF2,TAOK3,LDLRAD3,CPEB4,PUM3,RPTOR,ADAM32,ADAM10,KDM1B,KLHL13,USP18,NTF3,ACER2,PARP15,AURKA,SLC8A1,SERPINA6,MAP4K4,BMPR1B,FMN2,PAK3,TTLL7,DIP2B,LARP1,ITPKB,RAP1GDS1,HHAT,CHST8,IFT57,PRKCZ,SPOP,MAN2A2,ST8SIA5,SENP6,GALNT14,PPM1L,RIPK4,SGTB,USP25,PLG,PAPPA,PDGFD,ZNRF3,XXYLT1,UBE2O,GFRA1,NIPBL,CARD18,STK38,MYLK3,KANSL1,ATF6,CCNG2,TLK1,MRPS22,ZDHHC14,CORIN,BIRC6,KLF15,PPARA,HS3ST2,PPP6R3,ADAMTS3,RSRC1,PTPRK,TRERF1,PDZRN3,DAPK1,STK32B,ALPK2,GATAD2B,CPE,IL34,MELK,USP8,PIAS1,UBE2R2,BLK,OXR1,MRPS27,TTLL5,EIPR1,PSMF1,ATE1,LATS2,AP3B1,ZBTB16,MUSK,SMARCAD1,SETDB2,PRKCE,USP33,CD44,PTPRO,ALPK3,PRRC1,LNPEP,SPRED2,RPS6KA3,PTPN2,ATXN3,ST8SIA6,ALG10B,AMBRA1,GALNT10,KDM7A,FANCM,FANCA,RPRD1B,TMEM67,ABHD17C,HIPK3,CPXM2,CLSPN,AFG3L2,MNAT1,XYLT1,HMGA2,FOLH1,STK32A,LYPLA1,CWC27,PLCE1,IL17RA,CRIM1,FUT9,PRR5L,GXYLT2,VPS37A,FBXO32,BLM,NRK,BMP2,RC3H2,TRAK1,WDR26,BMP2K,RNF38,SH3BP5,TRAF3,UIMC1,B4GALT6,CNKSR3,CASP5,CUL1,DAW1,MOB1B,MAPK8IP1,MRPL13,KITLG,TADA2A,UBE2E1,PTPRE,MTMR2,WSB1,TRPM6,PRKCH,IL6R,ALS2,SNX25,PTPRB,TSPAN33,USP7,MOK,RALB,SEL1L,DTX1,FANCL,CUL5,NEK6,HECTD1,SNAI2,BID,SIAH2,PGAP4,ERN2,MBTPS2,TRIM58,ZDHHC17,PTAR1,ENPP1,MOCS2,PAMR1,CSF1,PPIL6,EOGT,CTDP1,ASB4,SMAD5,PRAME,KLHL7,ATG4B,CDC14B,CFH,PRKAA2,CSF2RB,RNF182,PHF20L1,CAMLG,ANP32B,YBX3,AIMP1,PC*

			ID2,FICD,CADM1,ELOC,ADAM28,MADD,PATL1,PRSS2,CREBBP,MELTF,SIAH3,NFKBIA,ZC3H15,RTRAF,NEDD9,CNDP2,ERLIN2,PCMTD2,AGO1,PDP2,GID8,NDFIP2,MARCHF6,MTPN,ABI1,OAZ2,PPME1,UBE2J2,ASB2,CYFIP2,ACACA,ASCC2,ST8SIA4,ADGRB1,WNT7A,NDFIP1,MAP3K4,TRIM43B,SERPINI2,TRIM43,MAGEL2,SDCBP,NSMCE1,MLLT1,NCK1,FGR,C2,RNF8,OCLN,MVB12B,CD5L,KIRREL1,TNFSF11,DPY19L1,PPM1F,SH2D3C,PSME3IP1,TRNAU1AP,ZFYVE28,STT3A,TET1,ASB3,OVCH2,SPOPL,ITGA1,POR,SENP8,USP49,MACROH2A1,CSNK1G1,PRSS51,CAMK1G,SERPINB2,ATG5,UNK,DDX6,TTLL11,SERPINB11
GO:0030031	cell projection assembly	0.032557152992683684	LRRC49,MTOR,RIPOR2,RP1,ODAD2,SPAG16,CDC42EP3,PARVB,MYO3B,ARMC2,RAPGEF2,LRGUK,STAU2,VCL,ARHGAP44,SRGAP2C,IFT57,YAP1,ABLIM1,BBS2,DNAH5,MYO10,PTPRO,ARHGEF7,TMEM67,PLCE1,DNAL1,TTC21B,RAP1GAP,DNAH8,DAW1,BBS4,CFAP74,IFT81,FAM149B1,CDC14B,CIBAR1,ANLN,GORAB,CEP120,SDCBP,NCK1,SNAP29,OCLN,ARL13B,RP1L1,IFT46,B9D1,TOGARAM1,ATG5,RFX2
GO:0007017	microtubule-based process	0.033988872164939885	LRRC49,RIPOR2,RP1,ODAD2,SPIRE1,SPAG16,MACF1,NEK7,NCOR1,ARMC2,SLC39A12,PAK1,DEUP1,LRGUK,KIF4A,STAU2,AURKA,SRGAP2C,CCSER2,FMN2,TTLL7,IFT57,PRKCZ,MCPH1,SENP6,DNAH14,DNAH11,BBS2,SLC9C1,TTLL5,DNAH5,ASPM,AP3B1,USP33,PEX14,ATXN3,ARHGEF7,TMEM67,CFAP61,CACNA1I,ADCY10,TRAK1,DNAL1,TUBGCP3,TTC21B,DNAH8,KIF15,DAW1,GOLGA8B,BBS4,MAP7,CFAP74,KIF11,NEK6,TRIM58,FYCO1,SLC22A14,IFT81,KIF21B,CABYR,CDC14B,PRKAA2,KIFC1,TUBB6,SGO1,CEP120,INTS13,OCLN,DNAH10,RP1L1,IFT46,TOGARAM1,TTLL11
GO:0051128	regulation of cellular component organization	0.04313465406896168	MTOR,PLCB1,SPOCK1,ABCA13,RIPOR2,RP1,CDH8,RIMS1,SPIRE1,TENM3,MINAR1,CDC42EP3,RIMS2,RHPN2,NEGR1,MYO3B,EGFR,ANGPT1,MACF1,NEK7,NEDD4,NSMCE2,BTBD9,SLC39A12,TOM1L2,PAK1,EPHA7,RAPGEF2,ADGRB3,ARSB,RPTOR,EPB41L3,ADAM10,STAU2,SEMA5A,SYT1,VCL,ARHGAP44,NTF3,AURKA,SRGAP2C,MAP4K4,RAB8B,PAK3,DIP2B,RAP1GDS1,PRKCZ,MCPH1,SENP6,YAP1,CORO2B,MYLK3,TLK1,TPM1,PPARA,SNX30,SYNJ1,SEMA3C,VPS13C,DNAJC15,TANC1,TNR,SDC2,MYO10,GSG1L,ABCB7,MUSK,SETDB2,PRKCE,CD44,PTPRO,STXBP6,RPS6KA3,PLXNA2,ARHGEF7,ATP8A1,AMBRA1,SEMA3E,TMEM67,ABHD17C,TMOD2,MNAT1,VPS41,PLCE1,CRIM1,FUT9,IQSEC1,PDLIM5,BMP2,RIN3,BMP2K,SEMA3D,NFATC2,SLC23A2,RAP1GAP,DRAXIN,ATF1,KIF15,FARP1,BBS4,COL5A1,YLPM1,MTMR2,TBX20,AFAP1,PRKCH,KANK4,TOX,USP7,RALB,DPYSL5,BAZ1A,NEK6,SNAI2,BID,TRIM58,FYCO1,SH3GLB1,ENPP1,BCL2L1,CTDP1,PRKAA2,TEAD1,PCID2,NET1,ANLN,PRSS2,MELTF,ABCC8,NEDD9,SAR1A,MTPN,CEP120,CYFIP2,ADGRB1,WNT7A,MAP3K4,MAGEL2,PDE2A,SDCBP,NSMCE1,NCK1,SCAF8,OCLN,FEZ2,LAMB1,KIRREL1,PPM1F,TET1,MACROH2A1,TOGARAM1,ATG5,FLRT2
GO:0009987	cellular process	0.04393577722998982	WWC1,GARNL3,MICU2,LRRC49,MTOR,SMOC1,EBNA1BP2,NSG1,LRP12,SNHG14,SLC25A21,TMTC1,PLCB1,NEBL,SPOCK1,ABCA13,ANKS1B,ZNF536,KSR1,BRINP3,SGCD,CACNA2D3,CNTN4,KCNH5,SLC37A1,ZFPM2,PIEZO2,TENM4,RIPOR2,RP1,ERC1,NME7,SLC44A5,ODAD2,KCNMA1,ARPP21,FBN1,COG5,CDH8,DCDC1,PUDP,RIMS1,PIK3C3,SPIRE1,TENM3,RARB,EXOC6B,NAV2,ENPEP,SPAG16,TRAPPC8,USH2A,MINAR1,CDC42EP3,RIMS2,ADGRE1,CDYL2,PJA2,FAM135B,BABAM2,SV2C,ERBIN,RHPN2,ASTN1,FCHO2,PARVB,CACNG2,NEGR1,GLYAT,MAP3K9,MYO3B,MYO5C,RTN1,TCF4,ZNF573,OCA2,NEK4,DOCK10,TSHZ2,EGFR,DENND1A,USP14,ANGPT1,CDK12,MACF1,PRKACB,NEK7,NCOR1,HMCN2,DOCK2,DIAPH3,ZNF407,UGT3A2,NEDD4,MAML2,CRB1,NSMCE2,BTBD9,SOX6,PSMB2,ARMC2,B3GALT5,TUSC3,PHACTR1,DKK2,DNAJC13,CNTN3,THRAP3,MAPKBP1,AOAH,NAT1,GABRB1,DGKI,C12ORF4,GRIA1,CAST,NEO1,CNTN6,SLC39A12,SLC8A3,TOM1L2,CEP128,NELL2,PAK1,EPHA7,CTNNAL1,NCOA7,GRAMD1B,RALGPS1,SPEN,CHSY3,RAPGEF2,ADGRB3,DEUP1,RUNX2,ARSB,GABRA6,CPS1,TAOK3,CPEB4

			,AGK,BCKDHB,PRICKLE2,RANBP17,SLC44A1,LDB2,PPP2R2B,PUM3,PATJ,LRGUK,RPTOR,EPB41L3,KIF4A,COL4A2,ADAM32,PPP1R12B,ADAM10,IL1R1,APBB2,PHACTR2,SLC7A2,ADK,KDM1B,CACNB2,KLHL13,MTUS1,STAU2,TMC1,USP18,SEMA5A,SYT1,VCL,ARHGAP44,NTF3,ACER2,PARP15,AURKA,CFDP1,PYGO1,SLC8A1,SSBP2,SRGAP2C,DTWD2,ANKRD31,TAFA2,UPP2,CCSER2,SRGAP2B,MAP4K4,BMPR1B,FMN2,HOMER2,HADHB,RAB8B,PAK3,RFTN1,PDE1A,ZNF257,TTLL7,DIP2B,LARP1,ITPKB,RGS20,PDE10A,RAP1GDS1,HHAT,CLIC6,CHST8,KICS2,CUBN,SCP2,IFT57,INTS7,SUSD6,PRKCZ,KLHL1,SPOP,BTLA,MAN2A2,GRB10,MCPH1,ZSWIM6,FER1L6,ST8SIA5,CNST,RGS9,DEFA3,MBNL2,ABCA5,SENP6,GALNT14,LMNTD1,PDXDC1,EBF2,YAP1,PPM1L,RIPK4,VPS35L,CADM2,ABCD3,RABGAP1L,SGTB,DNAH14,TRPC7,USP25,ALCAM,PLG,PAPPA,PDGFD,ZNRF3,DNAJC21,CA5A,XXYLT1,ABLIM1,ITGBL1,UBE2O,GFRA1,SYCP1,NIPBL,RNF17,NIPAL2,PI4K2B,IPO11,EWSR1,MICU1,CORO2B,CARD18,CHD6,STK38,HRH4,SORCS3,MYLK3,KANSL1,MBNL1,ATF6,ZNF684,CCNG2,TLK1,TPM1,LRRC38,MRPS22,ZDHHC14,BIRC6,KLF15,PPARA,HS3ST2,SNX30,KCNS3,PPP6R3,SYNJ1,ADAMTS3,ARAP2,RSRC1,PTPRK,ARHGEF12,TRERF1,SF3B6,PDZRN3,SEMA3C,DAPK1,SLC24A4,SEC14L1,FAR2,VPS13C,STK32B,PHC3,MAGI1,ALPK2,DNAH11,JARID2,SCN2A,RAB22A,DNAJC15,AMPH,GATAD2B,CPE,PALS2,EVC2,IL34,TANC1,ZNF846,MELK,BBS2,SLC9C1,RANBP3L,OR4F6,NKG7,ASAH2B,USP8,LDB3,SLC36A1,PIAS1,UBE2R2,BLK,EBF1,TNR,OLA1,XIRP2,AGPS,MXI1,OXR1,SDC2,GAS2,KCNH1,MRPS27,FHIP1A,CREG1,DROSHA,TTLL5,APBB1IP,EIPR1,DNAH5,PSMF1,ATE1,SLFN11,GLIS1,ACSS3,MORC1,LYRM4,MYO10,PLEKHA8,GALC,LATS2,GSG1L,ASPM,AP3B1,DENND2B,COL6A5,ATP11C,ZNF438,ABCB7,ZBTB16,MUSK,KIR3DL2,GNG7,SMARCAD1,SETDB2,PRKCE,FOXK2,SLMAP,ZNF718,USP33,CD44,RGS12,PTPRO,ALPK3,PRRC1,ABCC9,STXBP6,COL5A3,NSMAF,LNPEP,LIMD1,PEX14,SPRED2,RPS6KA3,NHS,PTPN2,PLXNA2,POC5,MCF2L,OR4F15,ATXN3,ST8SIA6,RIC3,SLC2A3,ARHGEF7,ALG10B,ATP8A1,AMBRA1,GALNT10,KDM7A,OPRM1,BIN2,FANCM,FANCA,CYBRD1,CNNM4,SEMA3E,RPRD1B,TMEM67,RCL1,ALPL,ABHD17C,TMOD2,MSH2,COL6A6,ZNF397,ATL1,LUC7L,RELL1,HIPK3,EPN2,ABCA10,CLSPN,BICRAL,AFG3L2,MOSMO,MNAT1,TMEM116,MDFIC,CFAP61,ANK3,NIPA2,XYLT1,HMGA2,COG2,BCL11B,VPS41,FOLH1,DOCK5,STK32A,LYPLA1,AK8,LINC01151,CWC27,PLCE1,IL17RA,CRIM1,FUT9,PRR5L,GXYLT2,VPS37A,VAV1,MYT1L,FBXO32,ZNF160,HLA-B,IQSEC1,CACNA1I,PDLIM5,BHLHE40-AS1,EXD3,BLM,NRK,SLC10A7,MAGI3,INTS8,NAP1L4,LIN54,ADCY10,STX12,BMP2,RC3H2,ATP9A,TRAK1,WDR26,GFI1B,RIN3,BMP2K,DNAL1,SLC15A5,SEMA3D,NETO2,POLR3A,TUBGCP3,AP4E1,NFATC2,TDRD7,SH3BP5,SLC23A2,ZNF106,MYOM1,TRAF3,ANKRD26,TTC21B,ZNF875,UIMC1,B4GALT6,LRRFIP1,TSPAN2,PFKFB4,RAP1GAP,IKZF2,SNX8,DRAXIN,DNAH8,ATF1,CCDC186,KCNH8,CGAS,NHSL1,SLC37A2,GABRR2,CNKSR3,CASP5,VENTX,WDR12,KIF15,PRDM10,CUL1,BTAF1,DAW1,MYL1,ZNF618,FARP1,MOB1B,GOLGA8B,BBS4,LRRC8B,MAPK8IP1,CLVS2,COL5A1,CFTR,ME2,TBC1D13,UBASH3A,AHDC1,MRPL13,KITLG,YLPM1,GTF2I,TADA2A,ZNF208,NMD3,AKAP10,UBE2E1,PTPRE,REPS1,MTMR2,ZNF608,SH3PXD2A,TBX20,SP110,AFAP1,LGI2,HEPHL1,WSB1,TRPM6,PRKCH,SLC12A1,TG,IL6R,ALS2,ZNF627,OR51E1,TFDP1,HEMGN,KANK4,SNX25,OSCP1,TOX,PTPRB,PDE6A,TSPAN33,TBATA,SCN10A,RBMX2,MAP7,USP7,MON2,ENPP3,PLAGL1,HAAO,FAH,MESD,MOK,KIR2DL4,RALB,NPAS2,YIPF6,CFAP74,CA1,VCAM1,SEL1L,ARHGAP31,TTC37,GSTA3,ZNF169,KIF11,DTX1,ZBTB33,ADA2,FANCL,DPYSL5,SLC13A5,ZNF44,SUPT16H,BAZ1A,NPIPA1,CUL5,OR7A17,NEK6,HECTD1,SHROOM3,XRCC4,NMU,GAST,SNAI2,IGHV3-74,BID,SIAH2,OSBPL10,PGAP4,ZBTB8OS,COX5A,RXRG,SP3,ERN2,ZNF879,MBTPS2,FLNB,TRIM58,TIAL1,ELF2,ZDHHC1

			7,FYCO1,SH3GLB1,PTAR1,SLC22A14,KRT6B,XKR5,SAMHD1,IFT81,ENPP1,MOCS2,TP53I11,TMEM225,UCK2,KCNC1,CSF1,GHRH,PPIL6,EOGT,BCL2L1,SPATA48,KRT25,CTDP1,ASB4,DHRS3,SMAD5,KIF21B,SYNJ2,TCERG1,SLC40A1,PRAME,HADHA,FAM149B1,CABYR,CIDEC,LPGAT1,MED1,IPCEF1,ATG4B,CDC14B,CFH,NPL,FAT1,HGD,SCML2,PRAMEF25,HDHD5,PTH,SDF4,PRKAA2,CSF2RB,GLYATL1,QSOX2,SOHLH1,ERO1B,PHF20L1,ABHD2,VSTM2A,PLA2G4A,SLC25A52,KIFC1,CAMLG,COX7A2L,ZBTB7C,TEAD1,ANP32B,YBX3,AIMP1,LASP1,FYB2,PCID2,ZNF234,CYP4F22,CIBAR1,PBLD,FICD,CADM1,SSPN,PEG10,NET1,TUBB6,ELOC,ANLN,RNU6-1150P,SLC1A7,VSX1,FSTL1,BPNT1,SVEP1,MADD,HCRTR1,PATL1,ZNF287,ZNF449,PRSS2,CREBBP,MELTF,MRM1,ARL11,SGO1,GORAB,SIAH3,TRPV5,NFKBIA,ABCC8,MT1HL1,ZC3H15,RFC2,ZNF354C,ALX4,RTRAF,ZBTB21,NEDD9,OLFM4,SLC14A2,ATP1A1-AS1,NOXRED1,ASS1,ADGRE3,SAR1A,PPP1R17,BTG3,ERLIN2,TRAPPC3,OTOP1,PCMTD2,ZBTB49,EXOC1,HEPACAM,KRT6A,STOX2,AGO1,PDP2,GID8,ELL2,FAM189A2,NDFIP2,NR2C1,CMTM7,SLC6A11,MARCHF6,GATAD1,MTPN,ABI1,ITGA4,TOP3A,OAZ2,BCAP29,PPME1,ZSCAN30,POU1F1,UBE2J2,TM9SF4,OR6C75,ASB2,CEP120,DHTKD1,ZSCAN5C,CYFIP2,HNRNPM,ACACA,KRT85,ASCC2,ST8SIA4,OR13C9,ARID3B,RXRA,ADGRB1,WNT7A,ECHDC1,NDFIP1,MAP3K4,SERPINI2,FOXO6,ERI1,ZNF112,ATP6V1C2,C16ORF72,MAGEL2,OR10H2,PDE2A,LRRC2,SDCBP,DSG1,JPT2,NSMCE1,ZNF813,MLLT1,NCK1,FLVCR1,SCAF8,FGR,RNU6-1007P,SNAP29,C2,SPRR2D,IFNAR1,RNF8,PPIP5K2,CYTH4,INTS13,KIAA0319L,DNMT3L,KHDC4,LHX9,RN7SL767P,WNT2B,PLEKHA3,OCLN,POSTN,CD101,STON1-GTF2A1L,AKAP11,DTHD1,MFSD9,MVB12B,ERP27,CD5L,ANKRD6,SCGN,ASCL3,FEZ2,INIP,LAMB1,DNAH10,ZNF66,KIRREL1,SCARA5,HEATR5A,PSTPIP2,PLCZ1,SLC9A5,FCRLA,DIDO1,GPR55,NSUN2,ANO10,TNFSF11,DPY19L1,ZNF705G,PPM1F,ARL13B,XPO7,ODR4,TMEM63C,SH2D3C,TRNAU1AP,UGP2,ZFYVE28,STT3A,SLC16A9,TET1,ASB3,RAD9A,RP1L1,DDX10,SPOPL,ZNF705D,IFT46,ITGA1,POR,ZNF850,ZNF235,SLC14A1,USP49,NSG2,B9D1,PRDM15,SRGAP3,MACROH2A1,TOGARAM1,CSNK1G1,ZNF705B,ATP6V0D2,CAMK1G,SERPINB2,ATG5,UNK,FLRT2,NUP43,OR2T2,BTBD10,TMEM25,NUDT21,DDX6,PPP1R13B,RFX2,PKNOX2,TTLL11,PRPF18,RNU6-835P,CCDC141,MTREX
GO:0016310	phosphorylation	0.044065671557724055	MTOR,KSR1,ERC1,NME7,PIK3C3,MAP3K9,MYO3B,NEK4,EGFR,ANGPT1,CDK12,PRKACB,NEK7,NCOR1,DGKI,SLC8A3,PAK1,EPHA7,RAPGEF2,TAOK3,AGK,LDB2,LRGUK,RPTOR,ADAM10,ADK,NTF3,AURKA,SLC8A1,MAP4K4,BMPR1B,PAK3,ITPKB,PRKCZ,GRB10,MCPH1,RIPK4,PDGFD,GFRA1,PI4K2B,STK38,MYLK3,CCNG2,TLK1,BIRC6,PPARA,RSRC1,DAPK1,STK32B,ALPK2,IL34,MELK,BLK,AGPS,LATS2,MUSK,PRKCE,FOXK2,CD44,PTPRO,ALPK3,PRRC1,LIMD1,SPRED2,RPS6KA3,PTPN2,AMBRA1,HIPK3,CLSPN,MNAT1,HMGA2,STK32A,AK8,PLCE1,PRR5L,BLM,NRK,BMP2,BMP2K,SH3BP5,PFKFB4,CNKSR3,MOB1B,MAPK8IP1,KITLG,TADA2A,AKAP10,TRPM6,PRKCH,IL6R,ALS2,SNX25,PTPRB,MOK,RALB,NEK6,ERN2,ENPP1,UCK2,CSF1,SMAD5,PRKAA2,CSF2RB,CADM1,MADD,RTRAF,NEDD9,ABI1,PPME1,DHTKD1,MAP3K4,SDCBP,MLLT1,NCK1,FGR,PPIP5K2,OCLN,KIRREL1,TNFSF11,PPM1F,SH2D3C,ZFYVE28,MACROH2A1,CSNK1G1,CAMK1G,BTBD10

Table S7. GO associations with biological processes (GO Profiler) of 1200 rDNA-contacting genes associated with genes that decrease the number of contacts with rDNA clusters. The search was performed using g:Profiler (https://biit.cs.ut.ee/gprofiler/gost. Related to Figure 2C.

GO.ID	Description	padj	Genes
**BP**			
GO:0032502	developmental process	2.0167429786094905e-8	*FTO,BCAR3,TRAPPC9,MGA,LRRC4C,NOTCH2,IMMP2L,SCAPER,FREM1,MYO9A,TAFA5,ULK2,UNC13C,SVIL,CLTCL1,NUBPL,DLC1,ZDHHC21,RDX,STXBP1,RALA,IL1RAPL2,BCL2,PRDM16,ALDH1A2,ARHGAP26,ZEB1,AKR1C3,SDCCAG8,FGD4,SPRED1,MYO1E,PLPPR1,ALK,FOXJ2,CARMIL1,PAPPA2,RIN2,ANO6,MAP4,APC,ZMYM4,RBFOX3,PLPPR5,CRKL,ILDR2,SETD2,ARHGAP24,TNIK,SLC4A10,PTPRJ,MYOF,SND1,BCL11A,TMEM182,CECR2,CHSY1,FLI1,ATP2B2,RXFP1,C5,FLT1,EDAR,CABLES1,PRKD1,CHRM3,LRP2,FGF12,ONECUT1,SMYD3,HERC2,GRM7,RETREG1,LUZP1,SSBP3,TBCD,NEDD4L,ZHX3,ABCB5,DCLK1,GABRG2,HERPUD2,PTPRR,FIG4,CMIP,ABCD2,THSD7A,ARNT,TRPC5,NBN,RBM47,CALD1,SNTG2,DIP2A,MSH6,COL27A1,HECW1,PHF19,MRTFA,ESS2,FRYL,SHC4,BRINP1,ADAM22,CRISPLD2,KMT2E,NCAM1,GABPA,LCE1F,CHN1,ACSBG1,PAFAH1B1,EFEMP1,TLL1,DCAF1,ITGB8,NF2,CTNNA1,PPP1R9A,CDH7,MEIS2,MRTFB,PRTG,NR5A2,FOXJ3,PCDH11Y,AGO2,DYSF,ANK2,BRWD1,SYNE2,WNT9B,ANKS6,SMARCA4,CDH11,FABP7,CXADR,ATRX,PTPN12,HDAC4,SLC1A1,PRKAA1,CRTAC1,L3MBTL3,CAMK4,FGF10,CDHR3,TGM1,INO80D,RASGRF1,SYNE1,ZNF675,SH3GL3,NXN,FBN2,EGF,PDE3A,ADAMTS2,HTR2A,CYP4A11,DAZL,KREMEN1,MARK2,FHL2,IGF2BP3,ANKRD17,APBA2,CDKN2C,EVC,KNDC1,GFRA2,RBBP8,EMILIN2,MYOM2,CREM,MBP,TRPS1,TGFA,HIP1,NPHP4,PACSIN2,MTHFD1L,SNX3,BRCA2,CFAP97,STRN,PSG9,PTCD2,MSI2,PDE6C,HMGB1,FGF9,UST,CPAMD8,MDM1,ESRP1,ETS2,PLS1,NIN,SLAMF1,ETS1,SMARCC1,SLC9A4,SMOC2,ZNF431,MAP2,LAMC1,RRBP1,TDRD5,ATF2,HIRA,UMODL1,KPNA1,MYT1,MED27,SELENON,RB1CC1,ZNF541,JPH1,RACGAP1,ACO1,DMC1,CATSPERG,ITSN2,SOX30,PTGFRN,SYBU,SEC24D,CTSB,PLEKHB2,OVOL2,NTN1,HOXC13,CRACR2A,CASZ1,YTHDF3,COLQ,HDAC11,DDHD1,ASH1L,HOXC4,UFD1,PLPP4,CD9,CARD10,DZANK1,NDRG2,BMP5,HDGFL3,INO80,CNMD,VSTM4,MYCL,TNN,PSAP,MICALL2,KDM6A,ATRN,IL33,KL,CSDE1,LMX1A,IL10,TTC39C,MAP6,VASP,ETV6,PALMD,HIPK1,CACYBP,LMX1B,TWIST1,ALKAL2,ISX,CELSR2,PCNA,UFL1,ADAMTS5,SMTN,SMPD4,ITGA6,ATP2B1,GAP43,ADCY9,EXT2,MEOX2,GRXCR1,STAT1,BRMS1L,SHROOM2,ARMC6,PRAMEF2,IMPACT,ADCYAP1R1,NCAPG2,MYOCD,MEF2C,RBPMS2,S100B,PRDM13,RAB38,NECTIN1,DRC7,TOP1,LCE3D,EPHA4,EMP1,GABRA5,RSPH1,NUMB,MEGF10,IL17RD,FBXO31,PRKAB1,HS6ST1,MARK4,CDH5,NFKBID,ARHGAP12,CLDN18,CYFIP1,HOATZ,PCDH8,SEMA4D,FAT4,AKR1B1,WNT5B,AMFR,SANBR,DPY19L2,PDCL3,SPAG6,MYL12B,NLRP14,UNC45B,UHRF2,HDAC2,GON4L,TBX15,NCS1,ATP5PF,MAPK9,CRTAM,APELA,GPR137B,FAIM,NRIP1,SNRK,STK36,MB,RRAS2,CD38,VMP1,GNAS,SERPINB7,BMP7,TRAPPC6B,EHMT1*
GO:0050794	regulation of cellular process	5.6208964260267515e-8	*FTO,BCAR3,LONP2,TRAPPC9,MGA,LRRC4C,NOTCH2,ZNF236,MYO9A,TAFA5,ULK2,MX2,RFX7,NLK,UNC13C,SVIL,CLTCL1,DLC1,TNRC6B,DPP10,ZDHHC21,PTPRA,ITPR2,RDX,STXBP1,RALA,IL1RAPL2,BCL2,PRDM16,ALDH1A2,ARHGAP26,LRFN2,EPC2,ZEB1,AKR1C3,SDCCAG8,FGD4,SPRED1,MYO1E,PLPPR1,ALK,FOXJ2,CARMIL1,MCTP1,PAPPA2,RIN2,ANO6,ZNF880,EGLN3,MAP4,SPON1,APC,ZNF595,HHLA2,TSHZ3,RBFOX3,PLP*

			PR5,CRKL,ILDR2,SETD2,ARHGAP24,TNIK,SLC4A10,PTPRJ,ZNF280B,BACH1,RGS3,MTRF1,SND1,SCAI,BCL11A,FAM83F,TMEM182,SGMS1,GRIK3,CHSY1,FLI1,RPRD1A,RXFP1,C5,ZFAND6,FLT1,ZNF648,RFC3,RABEP1,ZNF382,TASP1,INVS,EDAR,CRACD,NUP214,CABLES1,PRKD1,TPTE2,CHRM3,PELI2,LRP2,FGF12,ONECUT1,TAFA4,BTBD11,CCL28,SMYD3,GRM7,SEPTIN9,RETREG1,TMEM117,THADA,SSBP3,RALGAPA1,CELF2,TBCD,NEDD4L,TRPM1,ZHX3,DCLK1,GABRG2,DOCK8,MAPRE2,ZNF600,NDUFAF2,CD2AP,ZNF723,PARN,HERPUD2,PTPRR,FIG4,KCNE4,HIVEP2,ABCD2,ARNT,KCNK10,RANBP2,TRPC5,UBE2E2,NBN,TAF15,MSH6,ARHGAP32,RAB27B,HECW1,PHF19,MRTFA,DUSP22,ZKSCAN5,SHC4,BRINP1,KCNJ1,HRH2,ADAM22,KMT2E,PCGF5,SYT10,PPP1R1C,ARHGEF17,ANKFY1,NCAM1,SLC16A1,GABPA,ZNF735,CHN1,GLP2R,LIMCH1,PAFAH1B1,EFEMP1,TM7SF3,DCAF1,ITGB8,STON2,VPS13D,NF2,CNKSR2,HIVEP1,CTNNA1,PPP1R9A,MOB3B,AKAP9,MEIS2,ERMP1,MRTFB,PRTG,RGL1,NR5A2,GRM1,FOXJ3,GABRG1,PCDH11Y,PPP2R5E,PLA2R1,AGO2,RIC8B,DYSF,ANK2,BRWD1,SYNE2,WNT9B,ZNF606,DUSP16,SMARCA4,CDH11,FABP7,TNRC6C,SPG21,CXADR,ATRX,NUAK1,PTPN12,HDAC4,SLC1A1,PRKAA1,ITGB3BP,L3MBTL3,NFAT5,GUCY1A2,TOX3,CAMK4,BAZ2A,CPSF3,FGF10,ZC3HAV1,TGM1,INO80D,CLIP1,RASGRF1,ZNF675,SH3GL3,NXN,WNK2,ESRRG,FBN2,EGF,P2RX6,PDE3A,SCG5,MTMR3,TRIM5,RFC1,CLEC16A,STK38L,HTR2A,DAZL,GTF2F2,KREMEN1,TAF3,MARK2,GCSAML,EBF3,ZNF33B,FHL2,ADGRA3,CNIH3,IGF2BP3,ANKRD17,APBA2,SLC2A13,CDKN2C,EVC,GRK3,KNDC1,SPSB4,NOS2,STK10,GFRA2,RBBP8,EMILIN2,CCND3,ZIM3,CREM,MBP,TRPS1,TGFA,HIP1,GSR,ATP6V1E1,UTP4,CAPN5,RUFY2,NPHP4,PACSIN2,SNX3,NAA35,BRCA2,ZBTB2,ASB7,STRN,OR9Q1,ZNF121,PSG9,CDC42BPB,SOGA1,PTCD2,RALGAPA2,ZC3H14,RANBP9,RESF1,TMEM161A,PDE6C,LEMD3,HMGB1,FGF9,UST,MDM1,ZNF567,ESRP1,ETS2,GEMIN5,DSTYK,PLS1,NIN,SLAMF1,ETS1,FAM83B,SMARCC1,SNX6,SMOC2,ZFYVE26,ZNF431,MAP2,LAMC1,NEK10,ATF2,HIRA,CYLD,UMODL1,PSG6,ITGA9,KPNA1,RGMB,ZZEF1,DNAJC7,MYT1,MED27,SELENON,RB1CC1,ZNF541,ZBED9,JPH1,LALBA,PKP1,RACGAP1,NLRC5,ACO1,CNOT6L,FBLN5,SLC4A4,ZFP90,COPS8,ZNF124,ITSN2,SOX30,ZNF780B,CTSB,SUMO3,SLC15A2,PLEKHB2,BZW1,OVOL2,NTN1,RRAGD,BANP,HOXC13,CRACR2A,CASZ1,BMF,YTHDF3,DEDD2,COLQ,DDHD1,SUMO2,HS1BP3,ZNF292,PDE4DIP,POGK,ASH1L,HOXC4,ABCA4,UFD1,TOM1,PLPP4,CD9,CARD10,RALGPS2,ANAPC1,NDRG2,BMP5,HDGFL3,SERPINB9,GRB14,INO80,IGHV2-70D,CLNS1A,CNMD,KCNK5,MYCL,TNN,PSAP,MICALL2,PCNT,IL33,GPRC5C,KL,RASGEF1C,CSDE1,LMX1A,IL10,OR1L6,SFPQ,RIOK1,DIRAS2,SKA1,LARP6,ITPRIP,MAP6,VASP,ETV6,RAB12,RPS12,MORC2,SREBF2,THNSL2,HIPK1,CISD1,ZNF518A,DGKK,CD70,CENPE,LMX1B,NGDN,TWIST1,ALKAL2,RPF2,ZBTB38,ISX,PTGS1,CELSR2,PCNA,UFL1,OR2T3,BRD4,SERBP1,NRBP1,ITGA6,ATP2B1,GAP43,IGHV1OR15-9,ADCY9,CNIH1,ZNF528,ZNF611,CIDEA,ARFGEF3,EXT2,MEOX2,SLC6A1,STAT1,BRMS1L,KCNJ18,PRAMEF2,IMPACT,PARK7,MED12L,ADCYAP1R1,NCAPG2,MYOCD,EFHB,MEF2C,ZNF613,RBPMS2,S100B,PRDM13,RAD51AP1,RAB38,DBF4B,NECTIN1,SPPL2B,ZBTB25,PASK,CWC22,ATP6V1B2,CXCL2,EPHA4,GABRA5,NUMB,ZBTB10,MEGF10,IL17RD,FBXO31,EXTL3,PRKAB1,MARK4,CDH5,NFKBID,ARHGAP12,CLDN18,APIP,CYFIP1,PCDH8,SEMA4D,MC2R,FAT4,IMPA2,AKR1B1,WNT5B,AM

			FR,NENF,POMT2,ZNF845,OR4L1,SAMD13,ICA1,TPTE,PDCL3,SRP9,CNKSR1,CHCHD2,CDC45,OR11G2,BUB1,SDE2,RBMS3,UHRF2,HDAC2,SLF1,GON4L,TBX15,NCS1,ALB,MAPK9,CRTAM,APELA,GPR137B,SPTB,MIDEAS,FAIM,ZNF615,RNF138,NRIP1,ZNF738,SNRK,TM9SF2,STK36,RRAS2,GNA14,CD38,VMP1,GNAS,SERPINB7,DHX29,BMP7,TNFAIP8,RNF217,CNOT7,IL20RB,NSD1,EHMT1
GO:0048856	anatomical structure development	1.1694466254793915e-7	*FTO,BCAR3,TRAPPC9,LRRC4C,NOTCH2,IMMP2L,SCAPER,FREM1,MYO9A,TAFA5,ULK2,SVIL,CLTCL1,NUBPL,DLC1,ZDHHC21,RDX,STXBP1,RALA,IL1RAPL2,BCL2,ALDH1A2,ARHGAP26,ZEB1,AKR1C3,SDCCAG8,FGD4,SPRED1,MYO1E,PLPPR1,ALK,FOXJ2,CARMIL1,PAPPA2,RIN2,ANO6,MAP4,APC,ZMYM4,RBFOX3,PLPPR5,CRKL,ILDR2,SETD2,ARHGAP24,TNIK,SLC4A10,PTPRJ,MYOF,BCL11A,TMEM182,CECR2,CHSY1,FLI1,ATP2B2,RXFP1,C5,FLT1,EDAR,CABLES1,PRKD1,CHRM3,LRP2,FGF12,ONECUT1,SMYD3,GRM7,LUZP1,SSBP3,TBCD,NEDD4L,ABCB5,DCLK1,GABRG2,PTPRR,FIG4,CMIP,ABCD2,THSD7A,ARNT,TRPC5,NBN,RBM47,CALD1,SNTG2,DIP2A,MSH6,COL27A1,HECW1,MRTFA,ESS2,FRYL,BRINP1,ADAM22,CRISPLD2,KMT2E,NCAM1,GABPA,LCE1F,CHN1,ACSBG1,PAFAH1B1,EFEMP1,TLL1,DCAF1,ITGB8,NF2,CTNNA1,PPP1R9A,CDH7,MEIS2,MRTFB,PRTG,NR5A2,AGO2,DYSF,ANK2,BRWD1,SYNE2,WNT9B,ANKS6,SMARCA4,CDH11,FABP7,CXADR,ATRX,PTPN12,HDAC4,SLC1A1,PRKAA1,CRTAC1,L3MBTL3,CAMK4,FGF10,CDHR3,TGM1,INO80D,RASGRF1,SYNE1,ZNF675,SH3GL3,NXN,FBN2,EGF,PDE3A,ADAMTS2,CYP4A11,DAZL,KREMEN1,MARK2,FHL2,IGF2BP3,ANKRD17,APBA2,CDKN2C,EVC,KNDC1,GFRA2,RBBP8,EMILIN2,MYOM2,MBP,TRPS1,TGFA,NPHP4,PACSIN2,MTHFD1L,SNX3,BRCA2,STRN,PSG9,PTCD2,MSI2,PDE6C,HMGB1,FGF9,UST,CPAMD8,MDM1,ESRP1,ETS2,PLS1,NIN,SLAMF1,ETS1,SMARCC1,SLC9A4,SMOC2,MAP2,LAMC1,TDRD5,ATF2,HIRA,UMODL1,KPNA1,MYT1,SELENON,RB1CC1,JPH1,RACGAP1,ACO1,DMC1,ITSN2,SOX30,PTGFRN,SYBU,SEC24D,CTSB,OVOL2,NTN1,HOXC13,CRACR2A,CASZ1,YTHDF3,COLQ,HDAC11,DDHD1,ASH1L,HOXC4,UFD1,PLPP4,CD9,CARD10,DZANK1,NDRG2,BMP5,HDGFL3,INO80,CNMD,VSTM4,MYCL,TNN,PSAP,MICALL2,KDM6A,ATRN,IL33,KL,CSDE1,LMX1A,IL10,TTC39C,MAP6,VASP,ETV6,PALMD,HIPK1,CACYBP,LMX1B,TWIST1,ALKAL2,ISX,CELSR2,PCNA,UFL1,ADAMTS5,SMTN,SMPD4,ITGA6,ATP2B1,GAP43,ADCY9,EXT2,MEOX2,GRXCR1,STAT1,SHROOM2,ARMC6,IMPACT,ADCYAP1R1,NCAPG2,MYOCD,MEF2C,RBPMS2,S100B,PRDM13,NECTIN1,DRC7,TOP1,LCE3D,EPHA4,EMP1,GABRA5,RSPH1,NUMB,MEGF10,IL17RD,FBXO31,PRKAB1,HS6ST1,MARK4,CDH5,NFKBID,ARHGAP12,CLDN18,CYFIP1,PCDH8,SEMA4D,FAT4,AKR1B1,WNT5B,SANBR,DPY19L2,PDCL3,SPAG6,MYL12B,UNC45B,HDAC2,GON4L,TBX15,NCS1,ATP5PF,CRTAM,APELA,GPR137B,FAIM,NRIP1,SNRK,STK36,MB,RRAS2,CD38,VMP1,GNAS,SERPINB7,BMP7,TRAPPC6B,EHMT1*
GO:0007275	multicellular organism development	2.5895630451827137e-7	*BCAR3,TRAPPC9,LRRC4C,NOTCH2,IMMP2L,SCAPER,FREM1,MYO9A,TAFA5,ULK2,DLC1,ZDHHC21,STXBP1,RALA,IL1RAPL2,BCL2,ALDH1A2,ARHGAP26,ZEB1,AKR1C3,SDCCAG8,SPRED1,MYO1E,PLPPR1,ALK,FOXJ2,PAPPA2,RIN2,ANO6,MAP4,APC,RBFOX3,PLPPR5,CRKL,SETD2,ARHGAP24,TNIK,SLC4A10,PTPRJ,BCL11A,CECR2,CHSY1,FLI1,ATP2B2,RXFP1,C5,FLT1,EDAR,CABLES1,PRKD1,CHRM3,LRP2,FGF12,ONECUT1,GRM7,LUZP1,SSBP3,TBCD,NEDD4L,ABCB5,DCLK1,GABRG2,PTPRR,FIG4,CMIP,ABCD2,THSD7A,ARNT,TRPC5,NBN,RBM47,CALD1,SNTG2,DIP2A,MSH6,COL27A1,HECW1*

			,ESS2,FRYL,BRINP1,ADAM22,CRISPLD2,KMT2E,NCAM1,GABPA,CHN1,ACSBG1,PAFAH1B1,EFEMP1,TLL1,DCAF1,ITGB8,NF2,CTNNA1,PPP1R9A,MEIS2,PRTG,NR5A2,AGO2,ANK2,SYNE2,WNT9B,ANKS6,SMARCA4,CDH11,FABP7,CXADR,ATRX,HDAC4,SLC1A1,CRTAC1,L3MBTL3,CAMK4,FGF10,INO80D,RASGRF1,ZNF675,SH3GL3,NXN,FBN2,EGF,ADAMTS2,CYP4A11,KREMEN1,MARK2,FHL2,IGF2BP3,ANKRD17,APBA2,CDKN2C,EVC,KNDC1,GFRA2,RBBP8,EMILIN2,MBP,TRPS1,TGFA,NPHP4,MTHFD1L,SNX3,BRCA2,STRN,PSG9,PTCD2,PDE6C,HMGB1,FGF9,UST,CPAMD8,MDM1,ESRP1,ETS2,PLS1,NIN,SLAMF1,ETS1,SMARCC1,SMOC2,MAP2,TDRD5,ATF2,HIRA,UMODL1,MYT1,SELENON,RB1CC1,RACGAP1,ACO1,DMC1,ITSN2,SYBU,SEC24D,OVOL2,NTN1,HOXC13,CRACR2A,CASZ1,YTHDF3,COLQ,HDAC11,ASH1L,HOXC4,UFD1,PLPP4,CD9,CARD10,DZANK1,NDRG2,BMP5,HDGFL3,INO80,CNMD,VSTM4,MYCL,TNN,PSAP,MICALL2,KDM6A,ATRN,IL33,KL,CSDE1,LMX1A,IL10,TTC39C,MAP6,VASP,ETV6,HIPK1,CACYBP,LMX1B,TWIST1,ALKAL2,ISX,CELSR2,PCNA,UFL1,SMPD4,ITGA6,ATP2B1,GAP43,ADCY9,EXT2,MEOX2,GRXCR1,STAT1,SHROOM2,ARMC6,IMPACT,ADCYAP1R1,NCAPG2,MYOCD,MEF2C,RBPMS2,S100B,PRDM13,NECTIN1,TOP1,EPHA4,GABRA5,NUMB,FBXO31,PRKAB1,HS6ST1,MARK4,CDH5,NFKBID,CLDN18,CYFIP1,PCDH8,SEMA4D,FAT4,AKR1B1,WNT5B,SANBR,PDCL3,SPAG6,UNC45B,HDAC2,GON4L,TBX15,NCS1,ATP5PF,CRTAM,APELA,GPR137B,FAIM,NRIP1,SNRK,STK36,MB,RRAS2,CD38,VMP1,GNAS,SERPINB7,BMP7,TRAPPC6B,EHMT1
GO:0048731	system development	0.0000017246278539398789	BCAR3,TRAPPC9,LRRC4C,NOTCH2,IMMP2L,SCAPER,FREM1,MYO9A,TAFA5,ULK2,DLC1,STXBP1,RALA,IL1RAPL2,BCL2,ALDH1A2,ARHGAP26,ZEB1,AKR1C3,SDCCAG8,SPRED1,MYO1E,PLPPR1,ALK,FOXJ2,PAPPA2,RIN2,MAP4,APC,RBFOX3,PLPPR5,CRKL,SETD2,ARHGAP24,TNIK,SLC4A10,PTPRJ,BCL11A,CECR2,CHSY1,FLI1,ATP2B2,RXFP1,C5,FLT1,EDAR,CABLES1,PRKD1,CHRM3,LRP2,FGF12,ONECUT1,GRM7,LUZP1,SSBP3,TBCD,NEDD4L,ABCB5,DCLK1,GABRG2,FIG4,ABCD2,THSD7A,ARNT,TRPC5,NBN,RBM47,CALD1,SNTG2,DIP2A,MSH6,COL27A1,HECW1,ESS2,FRYL,BRINP1,ADAM22,CRISPLD2,KMT2E,NCAM1,GABPA,CHN1,ACSBG1,PAFAH1B1,EFEMP1,TLL1,DCAF1,ITGB8,NF2,CTNNA1,PPP1R9A,MEIS2,PRTG,AGO2,ANK2,SYNE2,WNT9B,ANKS6,SMARCA4,CDH11,FABP7,CXADR,ATRX,HDAC4,SLC1A1,CRTAC1,L3MBTL3,CAMK4,FGF10,RASGRF1,ZNF675,SH3GL3,NXN,FBN2,EGF,ADAMTS2,CYP4A11,KREMEN1,MARK2,FHL2,IGF2BP3,ANKRD17,APBA2,CDKN2C,EVC,KNDC1,GFRA2,EMILIN2,MBP,TRPS1,TGFA,NPHP4,MTHFD1L,SNX3,BRCA2,STRN,PSG9,PTCD2,PDE6C,HMGB1,FGF9,UST,CPAMD8,MDM1,ESRP1,ETS2,PLS1,NIN,SLAMF1,ETS1,SMARCC1,SMOC2,MAP2,ATF2,UMODL1,MYT1,SELENON,RB1CC1,RACGAP1,DMC1,ITSN2,SYBU,OVOL2,NTN1,CRACR2A,CASZ1,COLQ,HDAC11,ASH1L,HOXC4,UFD1,CD9,CARD10,DZANK1,NDRG2,BMP5,HDGFL3,CNMD,VSTM4,MYCL,TNN,PSAP,MICALL2,KDM6A,ATRN,IL33,CSDE1,LMX1A,IL10,MAP6,VASP,ETV6,HIPK1,CACYBP,LMX1B,TWIST1,ALKAL2,ISX,CELSR2,PCNA,UFL1,ITGA6,ATP2B1,GAP43,EXT2,MEOX2,GRXCR1,STAT1,SHROOM2,ARMC6,IMPACT,NCAPG2,MYOCD,MEF2C,RBPMS2,S100B,PRDM13,NECTIN1,EPHA4,GABRA5,NUMB,FBXO31,HS6ST1,MARK4,CDH5,NFKBID,CLDN18,CYFIP1,SEMA4D,FAT4,AKR1B1,WNT5B,SANBR,PDCL3,SPAG6,UNC45B,HDAC2,GON4L,TBX15,NCS1,ATP5PF,CRTAM,APELA,GPR137B,FAIM,NRIP1,SNRK,STK36,MB,RRAS2,CD38,GNAS,SERPINB7,BMP7,TRAPPC6B

GO:0048869	cellular developmental process	0.00001749156392819301	*FTO,TRAPPC9,MGA,LRRC4C,NOTCH2,MYO9A,ULK2,NUBPL,ZDHHC21,RDX,STXBP1,BCL2,PRDM16,ALDH1A2,ZEB1,AKR1C3,SDCCAG8,SPRED1,MYO1E,ALK,FOXJ2,CARMIL1,RIN2,MAP4,APC,PLPPR5,CRKL,ILDR2,SETD2,ARHGAP24,TNIK,SLC4A10,PTPRJ,MYOF,SND1,BCL11A,TMEM182,CECR2,CHSY1,FLI1,ATP2B2,RXFP1,FLT1,EDAR,PRKD1,LRP2,ONECUT1,SMYD3,GRM7,RETREG1,SSBP3,TBCD,NEDD4L,ZHX3,ABCB5,DCLK1,FIG4,THSD7A,ARNT,TRPC5,RBM47,DIP2A,COL27A1,HECW1,PHF19,MRTFA,FRYL,SHC4,BRINP1,ADAM22,KMT2E,NCAM1,GABPA,LCE1F,CHN1,PAFAH1B1,EFEMP1,TLL1,DCAF1,ITGB8,NF2,CTNNA1,PPP1R9A,MEIS2,MRTFB,PRTG,NR5A2,FOXJ3,DYSF,ANK2,SYNE2,WNT9B,SMARCA4,CDH11,CXADR,ATRX,HDAC4,SLC1A1,CRTAC1,L3MBTL3,CAMK4,FGF10,TGM1,RASGRF1,SYNE1,ZNF675,SH3GL3,NXN,FBN2,PDE3A,HTR2A,DAZL,KREMEN1,MARK2,FHL2,ANKRD17,CDKN2C,KNDC1,MYOM2,CREM,MBP,TRPS1,HIP1,NPHP4,PACSIN2,SNX3,BRCA2,STRN,PSG9,PTCD2,MSI2,PDE6C,HMGB1,FGF9,UST,ESRP1,ETS2,PLS1,NIN,SLAMF1,ETS1,SMARCC1,SLC9A4,ZNF431,MAP2,LAMC1,RRBP1,TDRD5,ATF2,HIRA,MYT1,SELENON,ZNF541,RACGAP1,DMC1,CATSPERG,ITSN2,SOX30,PTGFRN,CTSB,PLEKHB2,OVOL2,NTN1,CRACR2A,CASZ1,HDAC11,CD9,DZANK1,NDRG2,BMP5,HDGFL3,CNMD,MYCL,TNN,PSAP,MICALL2,KDM6A,ATRN,IL33,LMX1A,IL10,MAP6,VASP,ETV6,HIPK1,LMX1B,TWIST1,ALKAL2,CELSR2,PCNA,UFL1,ADAMTS5,ITGA6,GAP43,EXT2,GRXCR1,STAT1,ARMC6,PRAMEF2,IMPACT,ADCYAP1R1,NCAPG2,MYOCD,MEF2C,RBPMS2,S100B,PRDM13,RAB38,NECTIN1,DRC7,LCE3D,EPHA4,GABRA5,RSPH1,NUMB,MEGF10,IL17RD,FBXO31,HS6ST1,CDH5,NFKBID,CLDN18,CYFIP1,SEMA4D,FAT4,AKR1B1,WNT5B,DPY19L2,SPAG6,NLRP14,UNC45B,UHRF2,HDAC2,GON4L,TBX15,NCS1,MAPK9,CRTAM,APELA,GPR137B,FAIM,SNRK,MB,RRAS2,CD38,BMP7*
GO:0030154	cell differentiation	0.000026321432572629996	*FTO,TRAPPC9,MGA,LRRC4C,NOTCH2,MYO9A,ULK2,ZDHHC21,RDX,STXBP1,BCL2,PRDM16,ALDH1A2,ZEB1,AKR1C3,SDCCAG8,SPRED1,MYO1E,ALK,FOXJ2,CARMIL1,RIN2,MAP4,APC,PLPPR5,CRKL,ILDR2,SETD2,ARHGAP24,TNIK,SLC4A10,PTPRJ,MYOF,SND1,BCL11A,TMEM182,CECR2,CHSY1,FLI1,ATP2B2,RXFP1,FLT1,EDAR,PRKD1,LRP2,ONECUT1,SMYD3,GRM7,RETREG1,SSBP3,TBCD,NEDD4L,ZHX3,ABCB5,DCLK1,FIG4,THSD7A,ARNT,TRPC5,RBM47,DIP2A,COL27A1,HECW1,PHF19,MRTFA,FRYL,SHC4,BRINP1,ADAM22,KMT2E,NCAM1,GABPA,LCE1F,CHN1,PAFAH1B1,EFEMP1,TLL1,DCAF1,ITGB8,NF2,CTNNA1,PPP1R9A,MEIS2,MRTFB,PRTG,NR5A2,FOXJ3,DYSF,ANK2,SYNE2,WNT9B,SMARCA4,CDH11,CXADR,ATRX,HDAC4,SLC1A1,CRTAC1,L3MBTL3,CAMK4,FGF10,TGM1,RASGRF1,SYNE1,ZNF675,SH3GL3,NXN,FBN2,PDE3A,HTR2A,DAZL,KREMEN1,MARK2,FHL2,ANKRD17,CDKN2C,KNDC1,MYOM2,CREM,MBP,TRPS1,HIP1,NPHP4,SNX3,BRCA2,STRN,PSG9,PTCD2,MSI2,PDE6C,HMGB1,FGF9,UST,ESRP1,ETS2,PLS1,NIN,SLAMF1,ETS1,SMARCC1,SLC9A4,ZNF431,MAP2,LAMC1,RRBP1,TDRD5,ATF2,HIRA,MYT1,SELENON,ZNF541,RACGAP1,DMC1,CATSPERG,ITSN2,SOX30,PTGFRN,CTSB,PLEKHB2,OVOL2,NTN1,CRACR2A,CASZ1,HDAC11,CD9,DZANK1,NDRG2,BMP5,HDGFL3,CNMD,MYCL,TNN,PSAP,MICALL2,KDM6A,ATRN,IL33,LMX1A,IL10,MAP6,VASP,ETV6,HIPK1,LMX1B,TWIST1,ALKAL2,CELSR2,PCNA,UFL1,ADAMTS5,ITGA6,GAP43,EXT2,GRXCR1,STAT1,ARMC6,PRAMEF2,IMPACT,ADCYAP1R1,NCAPG2,MYOCD,MEF2C,RBPMS2,S100B,PRDM13,RAB38,NECTIN1,DRC7,LCE3D,EPHA4,GABRA5,RSPH1,NUMB,MEGF10,IL17RD,FBXO31,HS6ST1,CDH5,NFKBID,C*

			LDN18,CYFIP1,SEMA4D,FAT4,AKR1B1,WNT5B,DPY19L2,SPAG6,NLRP14,UNC45B,UHRF2,HDAC2,GON4L,TBX15,NCS1,MAPK9,CRTAM,APELA,GPR137B,FAIM,SNRK,MB,RRAS2,CD38,BMP7
GO:0007399	nervous system development	0.00011167231795449553	TRAPPC9,LRRC4C,NOTCH2,IMMP2L,MYO9A,ULK2,DLC1,STXBP1,RALA,IL1RAPL2,BCL2,ALDH1A2,ARHGAP26,ZEB1,SDCCAG8,PLPPR1,ALK,MAP4,APC,RBFOX3,PLPPR5,CRKL,SETD2,TNIK,SLC4A10,BCL11A,CECR2,ATP2B2,CABLES1,PRKD1,CHRM3,LRP2,FGF12,GRM7,LUZP1,SSBP3,TBCD,NEDD4L,DCLK1,GABRG2,FIG4,ABCD2,TRPC5,SNTG2,DIP2A,HECW1,ESS2,FRYL,BRINP1,ADAM22,NCAM1,CHN1,ACSBG1,PAFAH1B1,NF2,CTNNA1,PPP1R9A,MEIS2,PRTG,ANK2,SYNE2,WNT9B,SMARCA4,CDH11,FABP7,ATRX,HDAC4,SLC1A1,CRTAC1,FGF10,RASGRF1,SH3GL3,EGF,KREMEN1,MARK2,IGF2BP3,APBA2,CDKN2C,KNDC1,GFRA2,MBP,NPHP4,MTHFD1L,SNX3,BRCA2,STRN,PDE6C,HMGB1,FGF9,UST,ESRP1,PLS1,NIN,SMARCC1,MAP2,ATF2,MYT1,RACGAP1,ITSN2,SYBU,OVOL2,NTN1,CASZ1,COLQ,HDAC11,CD9,DZANK1,NDRG2,BMP5,HDGFL3,MYCL,TNN,MICALL2,KDM6A,ATRN,IL33,LMX1A,MAP6,VASP,ETV6,HIPK1,LMX1B,TWIST1,ALKAL2,ISX,CELSR2,UFL1,ITGA6,ATP2B1,GAP43,GRXCR1,SHROOM2,IMPACT,MEF2C,S100B,PRDM13,NECTIN1,EPHA4,GABRA5,NUMB,FBXO31,HS6ST1,MARK4,CYFIP1,SEMA4D,FAT4,WNT5B,SPAG6,HDAC2,NCS1,ATP5PF,FAIM,STK36,RRAS2,CD38,BMP7,TRAPPC6B
GO:0009653	anatomical structure morphogenesis	0.00027158174772785534	BCAR3,LRRC4C,NOTCH2,FREM1,MYO9A,TAFA5,ULK2,CLTCL1,NUBPL,DLC1,RDX,STXBP1,RALA,BCL2,ALDH1A2,ZEB1,SDCCAG8,FGD4,SPRED1,MYO1E,FOXJ2,CARMIL1,PAPPA2,RIN2,ZMYM4,CRKL,SETD2,ARHGAP24,TNIK,SLC4A10,MYOF,BCL11A,TMEM182,CECR2,CHSY1,FLI1,RXFP1,C5,FLT1,EDAR,PRKD1,LRP2,ONECUT1,LUZP1,SSBP3,TBCD,NEDD4L,DCLK1,FIG4,THSD7A,TRPC5,CALD1,DIP2A,COL27A1,HECW1,MRTFA,FRYL,CRISPLD2,NCAM1,GABPA,CHN1,PAFAH1B1,EFEMP1,ITGB8,NF2,CTNNA1,CDH7,MEIS2,PRTG,NR5A2,AGO2,ANK2,BRWD1,WNT9B,CDH11,ATRX,SLC1A1,FGF10,CDHR3,FBN2,EGF,MARK2,FHL2,IGF2BP3,KNDC1,EMILIN2,MYOM2,MBP,TGFA,PACSIN2,MTHFD1L,PTCD2,PDE6C,FGF9,UST,ETS2,PLS1,NIN,ETS1,SMARCC1,SMOC2,MAP2,LAMC1,ATF2,HIRA,ITSN2,SOX30,PTGFRN,OVOL2,NTN1,HOXC13,DDHD1,ASH1L,HOXC4,CD9,CARD10,DZANK1,BMP5,CNMD,VSTM4,TNN,MICALL2,KDM6A,ATRN,LMX1A,IL10,TTC39C,MAP6,VASP,PALMD,HIPK1,TWIST1,CELSR2,ADAMTS5,ITGA6,GAP43,EXT2,MEOX2,GRXCR1,STAT1,SHROOM2,IMPACT,MYOCD,MEF2C,RBPMS2,S100B,NECTIN1,EPHA4,NUMB,FBXO31,HS6ST1,CDH5,ARHGAP12,CYFIP1,PCDH8,SEMA4D,FAT4,WNT5B,PDCL3,SPAG6,MYL12B,HDAC2,TBX15,APELA,GNAS,BMP7
GO:0023051	regulation of signaling	0.0008180579569025943	BCAR3,LRRC4C,NOTCH2,MYO9A,NLK,UNC13C,DLC1,PTPRA,RDX,STXBP1,BCL2,PRDM16,ARHGAP26,LRFN2,ZEB1,AKR1C3,FGD4,SPRED1,ALK,MCTP1,APC,TSHZ3,CRKL,ARHGAP24,TNIK,SLC4A10,PTPRJ,RGS3,SCAI,SGMS1,GRIK3,CHSY1,ATP2B2,ZFAND6,FLT1,INVS,EDAR,PRKD1,TPTE2,PELI2,LRP2,FGF12,ONECUT1,TAFA4,GRM7,RALGAPA1,DOCK8,MAPRE2,NDUFAF2,CD2AP,PTPRR,ARNT,ARHGAP32,HECW1,DUSP22,ARHGEF17,NCAM1,SLC16A1,CHN1,PAFAH1B1,TM7SF3,ITGB8,NF2,CNKSR2,CTNNA1,PPP1R9A,MOB3B,AKAP9,GRM1,PCDH11Y,PLA2R1,RIC8B,ANK2,DUSP16,SMARCA4,CDH11,NUAK1,PTPN12,SLC1A1,PRKAA1,NFAT5,GUCY1A2,FGF10,ZC3HAV1,RASGRF1,ZNF675,NXN,WNK2,FBN2,EGF,PDE3A,SCG5,TRIM5,CLEC16A,HTR2A,KREMEN1,GCSAML,FHL2,CNIH3,ANKRD17,APBA2,EVC,GRK3,

			NOS2,CCND3,TGFA,HIP1,NPHP4,PACSIN2,SNX3,BRCA2,RALGAPA2,RANBP9,TMEM161A,LEMD3,HMGB1,FGF9,DSTYK,SLAMF1,SNX6,SMOC2,LAMC1,NEK10,CYLD,KPNA1,ZZEF1,RB1CC1,RACGAP1,NLRC5,SOX30,SLC15A2,OVOL2,RRAGD,CRACR2A,YTHDF3,DEDD2,ASH1L,UFD1,RALGPS2,NDRG2,BMP5,GRB14,TNN,KL,IL10,SFPQ,ITPRIP,RPS12,SREBF2,HIPK1,TWIST1,ALKAL2,RPF2,UFL1,BRD4,ITGA6,ATP2B1,CIDEA,ARFGEF3,SLC6A1,STAT1,BRMS1L,PARK7,ADCYAP1R1,NCAPG2,MYOCD,EFHB,MEF2C,RBPMS2,S100B,SPPL2B,PASK,EPHA4,IL17RD,CDH5,ARHGAP12,APIP,CYFIP1,SEMA4D,WNT5B,AMFR,NENF,ICA1,TPTE,RBMS3,HDAC2,MAPK9,APELA,GPR137B,FAIM,STK36,CD38,GNAS,BMP7,CNOT7
GO:0010646	regulation of cell communication	0.0010133185663000787	*BCAR3,LRRC4C,NOTCH2,MYO9A,NLK,UNC13C,DLC1,PTPRA,RDX,STXBP1,BCL2,PRDM16,ARHGAP26,LRFN2,ZEB1,AKR1C3,FGD4,SPRED1,ALK,MCTP1,APC,TSHZ3,CRKL,ARHGAP24,TNIK,SLC4A10,PTPRJ,RGS3,SCAI,SGMS1,GRIK3,CHSY1,ZFAND6,FLT1,INVS,EDAR,PRKD1,TPTE2,PELI2,LRP2,FGF12,ONECUT1,TAFA4,GRM7,RALGAPA1,DOCK8,MAPRE2,NDUFAF2,CD2AP,PTPRR,ARNT,ARHGAP32,HECW1,DUSP22,ARHGEF17,NCAM1,SLC16A1,CHN1,PAFAH1B1,TM7SF3,ITGB8,NF2,CNKSR2,CTNNA1,PPP1R9A,MOB3B,AKAP9,GRM1,PCDH11Y,PLA2R1,RIC8B,ANK2,DUSP16,SMARCA4,CDH11,CXADR,NUAK1,PTPN12,SLC1A1,PRKAA1,NFAT5,GUCY1A2,FGF10,ZC3HAV1,RASGRF1,ZNF675,NXN,WNK2,FBN2,EGF,PDE3A,SCG5,TRIM5,CLEC16A,HTR2A,KREMEN1,GCSAML,FHL2,CNIH3,ANKRD17,APBA2,EVC,GRK3,NOS2,CCND3,TGFA,HIP1,NPHP4,PACSIN2,SNX3,BRCA2,RALGAPA2,RANBP9,TMEM161A,LEMD3,HMGB1,FGF9,DSTYK,SLAMF1,SNX6,SMOC2,LAMC1,NEK10,CYLD,KPNA1,ZZEF1,RB1CC1,RACGAP1,NLRC5,SOX30,SLC15A2,OVOL2,RRAGD,CRACR2A,YTHDF3,DEDD2,ASH1L,UFD1,RALGPS2,NDRG2,BMP5,GRB14,TNN,KL,IL10,SFPQ,ITPRIP,RPS12,SREBF2,HIPK1,TWIST1,ALKAL2,RPF2,UFL1,BRD4,ITGA6,CIDEA,ARFGEF3,SLC6A1,STAT1,BRMS1L,PARK7,ADCYAP1R1,NCAPG2,MYOCD,EFHB,MEF2C,RBPMS2,S100B,SPPL2B,PASK,EPHA4,IL17RD,CDH5,ARHGAP12,APIP,CYFIP1,SEMA4D,WNT5B,AMFR,NENF,ICA1,TPTE,RBMS3,HDAC2,MAPK9,APELA,GPR137B,FAIM,STK36,CD38,GNAS,BMP7,CNOT7*
GO:0050789	regulation of biological process	0.0014713848318905335	*FTO,BCAR3,LONP2,TRAPPC9,MGA,PVT1,LRRC4C,NOTCH2,ZNF236,MYO9A,TAFA5,ULK2,MX2,RFX7,NLK,UNC13C,SVIL,CLTCL1,DLC1,TNRC6B,DPP10,ZDHHC21,PTPRA,ITPR2,RDX,STXBP1,RALA,IL1RAPL2,BCL2,PRDM16,ALDH1A2,ARHGAP26,LRFN2,EPC2,ZEB1,AKR1C3,SDCCAG8,FGD4,SPRED1,MYO1E,PLPPR1,ALK,FOXJ2,CARMIL1,MCTP1,PAPPA2,RIN2,ANO6,ZNF880,EGLN3,MAP4,SPON1,APC,ZMYM4,ZNF595,HHLA2,TSHZ3,RBFOX3,PLPPR5,CRKL,ILDR2,SETD2,ARHGAP24,TNIK,SLC4A10,PTPRJ,ZNF280B,BACH1,RGS3,MTRF1,SND1,SCAI,BCL11A,FAM83F,TMEM182,SGMS1,GRIK3,CHSY1,FLI1,RPRD1A,ATP2B2,RXFP1,C5,ZFAND6,FLT1,ZNF648,RFC3,RABEP1,ZNF382,TASP1,INVS,EDAR,CRACD,NUP214,CABLES1,PRKD1,TPTE2,CHRM3,PELI2,LRP2,FGF12,ONECUT1,TAFA4,BTBD11,CCL28,SMYD3,GRM7,SEPTIN9,RETREG1,TMEM117,THADA,SSBP3,RALGAPA1,CELF2,TBCD,NEDD4L,TRPM1,ZHX3,DCLK1,GABRG2,DOCK8,MAPRE2,ZNF600,NDUFAF2,CD2AP,ZNF723,PARN,HERPUD2,PTPRR,FIG4,PLGRKT,KCNE4,HIVEP2,ABCD2,ARNT,KCNK10,RANBP2,TRPC5,UBE2E2,NBN,TAF15,DIP2A,MSH6,ARHGAP32,RAB27B,HECW1,PHF19,MRTFA,DUSP22,ZKSCAN5,SHC4,BRINP1,KCNJ1,HRH2,ITIH5,ADAM22,KMT2E,PCGF5,SYT10,PPP1R1C,ARHGEF17,MIR663AHG,ANKFY1,NCAM1,SLC*

			16A1,GABPA,ZNF735,CHN1,GLP2R,LIMCH1,PAFAH1B1,EFEMP1,TM7SF3,DCAF1,ITGB8,STON2,VPS13D,NF2,CNKSR2,HIVEP1,CTNNA1,PPP1R9A,MOB3B,AKAP9,MEIS2,ERMP1,MRTFB,PRTG,RGL1,NR5A2,GRM1,FOXJ3,GABRG1,PCDH11Y,PPP2R5E,PLA2R1,AGO2,RIC8B,DYSF,ANK2,BRWD1,SYNE2,WNT9B,ZNF606,CLPX,DUSP16,SMARCA4,CDH11,FABP7,TNRC6C,SPG21,CXADR,ATRX,NUAK1,PTPN12,HDAC4,SLC1A1,PRKAA1,ITGB3BP,L3MBTL3,NFAT5,GUCY1A2,TOX3,CAMK4,BAZ2A,CPSF3,FGF10,ZC3HAV1,TGM1,INO80D,CLIP1,RASGRF1,ZNF675,SH3GL3,NXN,WNK2,ESRRG,FBN2,EGF,P2RX6,PDE3A,SCG5,MTMR3,TRIM5,RFC1,CLEC16A,STK38L,HTR2A,CYP4A11,DAZL,GTF2F2,KREMEN1,TAF3,MARK2,GCSAML,EBF3,ZNF33B,FHL2,ADGRA3,CNIH3,IGF2BP3,ANKRD17,APBA2,SLC2A13,CDKN2C,EVC,GRK3,KNDC1,SPSB4,NOS2,STK10,GFRA2,RBBP8,EMILIN2,CCND3,ZIM3,CREM,MBP,TRPS1,TGFA,HIP1,GSR,ATP6V1E1,UTP4,CAPN5,RUFY2,NPHP4,PACSIN2,SNX3,NAA35,BRCA2,ZBTB2,ASB7,STRN,OR9Q1,ZNF121,PSG9,CDC42BPB,SOGA1,PTCD2,RALGAPA2,ZC3H14,RANBP9,RESF1,TMEM161A,PDE6C,LEMD3,HMGB1,FGF9,UST,CPAMD8,MDM1,ZNF567,ESRP1,ETS2,GEMIN5,DSTYK,PLS1,NIN,SLAMF1,ETS1,FAM83B,SMARCC1,SNX6,SMOC2,ZFYVE26,ZNF431,MAP2,LAMC1,NEK10,ATF2,HIRA,CYLD,UMODL1,PSG6,ITGA9,KPNA1,RGMB,ZZEF1,DNAJC7,MYT1,MED27,SELENON,RB1CC1,ZNF541,ZBED9,JPH1,LALBA,PKP1,RACGAP1,NLRC5,ACO1,CNOT6L,FBLN5,SLC4A4,ZFP90,COPS8,ZNF124,ITSN2,SOX30,ZNF780B,CTSB,SUMO3,SLC15A2,PLEKHB2,BZW1,OVOL2,NTN1,RRAGD,BANP,HOXC13,CRACR2A,CASZ1,BMF,YTHDF3,DEDD2,COLQ,DDHD1,SUMO2,HS1BP3,ZNF292,PDE4DIP,POGK,ASH1L,HOXC4,ABCA4,UFD1,TOM1,PLPP4,CD9,CARD10,RALGPS2,ANAPC1,NDRG2,BMP5,HDGFL3,SERPINB9,GRB14,INO80,IGHV2-70D,CLNS1A,CNMD,KCNK5,DCUN1D4,MYCL,TNN,PSAP,MICALL2,PCNT,KDM6A,ATRN,IL33,GPRC5C,KL,RASGEF1C,CSDE1,LMX1A,IL10,OR1L6,SFPQ,RIOK1,DIRAS2,SKA1,LARP6,ITPRIP,MAP6,VASP,ETV6,PALMD,RAB12,RPS12,MORC2,SREBF2,THNSL2,HIPK1,CISD1,ZNF518A,DGKK,CD70,CENPE,LMX1B,NGDN,TWIST1,ALKAL2,RPF2,ZBTB38,ISX,PTGS1,CELSR2,FH,PCNA,UFL1,ADAMTS5,OR2T3,BRD4,SERBP1,NRBP1,ITGA6,ATP2B1,GAP43,IGHV1OR15-9,ADCY9,CNIH1,ZNF528,ZNF611,UBAP2,CIDEA,ARFGEF3,EXT2,MEOX2,SLC6A1,STAT1,BRMS1L,KCNJ18,PRAMEF2,IMPACT,PARK7,MED12L,UBL7,ADCYAP1R1,NCAPG2,MYOCD,EFHB,MEF2C,ZNF613,RBPMS2,S100B,PRDM13,RAD51AP1,RAB38,DBF4B,NECTIN1,SPPL2B,ZBTB25,PASK,CWC22,ATP6V1B2,CXCL2,TOP1,EPHA4,GABRA5,NUMB,ZBTB10,MIR548H4,MEGF10,IL17RD,FBXO31,EXTL3,PRKAB1,MARK4,CDH5,NFKBID,ARHGAP12,CLDN18,APIP,CYFIP1,PCDH8,SEMA4D,SERPINB10,MC2R,FAT4,IMPA2,AKR1B1,C9,WNT5B,AMFR,NENF,SH2D1B,POMT2,ZNF845,OR4L1,SAMD13,ICA1,MTTP,TPTE,PDCL3,SRP9,CNKSR1,CHCHD2,CDC45,OR11G2,BUB1,MYL12B,SDE2,RBMS3,UHRF2,HDAC2,SLF1,GON4L,TBX15,NCS1,ALB,MAPK9,CRTAM,APELA,GPR137B,SPTB,MIDEAS,FAIM,ZNF615,RNF138,NRIP1,ZNF738,SNRK,TM9SF2,STK36,RRAS2,GNA14,CD38,VMP1,GNAS,SERPINB7,DHX29,BMP7,TNFAIP8,RNF217,CNOT7,IL20RB,NSD1,EHMT1
GO:0065007	biological regulation	0.0015309980797171415	*FTO,BCAR3,LONP2,TRAPPC9,UNC80,MGA,PVT1,LRRC4C,NOTCH2,ZNF236,MYO9A,TAFA5,ULK2,MX2,RFX7,NLK,UNC13C,SVIL,CLTCL1,DLC1,TNRC6B,DPP10,ZDHHC21,PTPRA,ITPR2,RDX,STXBP1,RALA,IL1RAPL2,BCL2,PRDM16,ALDH1A2,ARHGAP26,LRFN2,EPC2,ZEB*

			1,AKR1C3,SDCCAG8,FGD4,SPRED1,MYO1E,PLPPR1,ALK,FOXJ2,CARMIL1,MCTP1,PAPPA2,RIN2,ANO6,ZNF880,EGLN3,MAP4,SPON1,APC,ZMYM4,ZNF595,HHLA2,TSHZ3,RBFOX3,PLPPR5,CRKL,ILDR2,SETD2,ARHGAP24,TNIK,SLC4A10,PTPRJ,ZNF280B,BACH1,RGS3,MTRF1,SND1,SCAI,BCL11A,FAM83F,TMEM182,SGMS1,GRIK3,CHSY1,FLI1,RPRD1A,ATP2B2,RXFP1,C5,ZFAND6,CYP2C9,FLT1,ZNF648,RFC3,RABEP1,ZNF382,TASP1,INVS,EDAR,CRACD,NUP214,CABLES1,PRKD1,TPTE2,CHRM3,PELI2,LRP2,FGF12,ONECUT1,TAFA4,BTBD11,SYN2,CCL28,SMYD3,HERC2,GRM7,SEPTIN9,RETREG1,TMEM117,THADA,SSBP3,RALGAPA1,CELF2,TBCD,NEDD4L,TRPM1,ZHX3,ABCB5,DCLK1,GABRG2,DOCK8,MAPRE2,ZNF600,NDUFAF2,CD2AP,ZNF723,PARN,HERPUD2,PTPRR,FIG4,PLGRKT,KCNE4,HIVEP2,ABCD2,ARNT,KCNK10,RANBP2,TRPC5,UBE2E2,NBN,TAF15,DIP2A,MSH6,ARHGAP32,RAB27B,HECW1,PHF19,MRTFA,DUSP22,ZKSCAN5,SHC4,BRINP1,KCNJ1,HRH2,ITIH5,ADAM22,KMT2E,PCGF5,SYT10,PPP1R1C,ARHGEF17,MIR663AHG,ANKFY1,NCAM1,SLC16A1,GABPA,ZNF735,CHN1,GLP2R,LIMCH1,ECT2L,PAFAH1B1,EFEMP1,TM7SF3,DCAF1,ITGB8,STON2,VPS13D,NF2,CNKSR2,HIVEP1,CTNNA1,PPP1R9A,MOB3B,AKAP9,MEIS2,ERMP1,MRTFB,PRTG,RGL1,NR5A2,GRM1,FOXJ3,GABRG1,PCDH11Y,PPP2R5E,PLA2R1,AGO2,RIC8B,DYSF,ANK2,BRWD1,SYNE2,WNT9B,ZNF606,CLPX,DUSP16,SMARCA4,CDH11,FABP7,TNRC6C,SPG21,CXADR,ATRX,NUAK1,PTPN12,HDAC4,SLC1A1,PRKAA1,ITGB3BP,L3MBTL3,DMXL2,NFAT5,GUCY1A2,TOX3,CAMK4,BAZ2A,CPSF3,FGF10,ZC3HAV1,TGM1,INO80D,CLIP1,RASGRF1,PAH,ZNF675,SH3GL3,NXN,WNK2,ESRRG,FBN2,EGF,P2RX6,PDE3A,SCG5,MTMR3,TRIM5,RFC1,CLEC16A,STK38L,HTR2A,CYP4A11,DAZL,GTF2F2,PPP2R2C,KREMEN1,TAF3,MARK2,GCSAML,EBF3,ZNF33B,FHL2,ADGRA3,CNIH3,IGF2BP3,ANKRD17,APBA2,SLC2A13,CDKN2C,EVC,GRK3,KNDC1,SPSB4,NOS2,STK10,GFRA2,RBBP8,TMTC2,EMILIN2,CCND3,ZIM3,CREM,MBP,TRPS1,TRAPPC11,TGFA,HIP1,GSR,ATP6V1E1,UTP4,CAPN5,RUFY2,NPHP4,PACSIN2,SNX3,NAA35,BRCA2,ZBTB2,ASB7,STRN,OR9Q1,ZNF121,PSG9,CDC42BPB,SOGA1,PTCD2,RALGAPA2,ZC3H14,RANBP9,RESF1,TMEM161A,PDE6C,LEMD3,HMGB1,FGF9,UST,CPAMD8,MDM1,ZNF567,ESRP1,ETS2,UBAP2L,GEMIN5,DSTYK,PLS1,SLC39A6,NIN,SLAMF1,ETS1,FAM83B,SMARCC1,SNX6,SLC9A4,SMOC2,ZFYVE26,ZNF431,MAP2,PEX6,LAMC1,NEK10,ATF2,HIRA,CYLD,UMODL1,PSG6,ITGA9,KPNA1,RGMB,ZZEF1,DNAJC7,MYT1,MED27,SELENON,RB1CC1,ZNF541,ZBED9,JPH1,LALBA,PKP1,RACGAP1,NLRC5,ACO1,CNOT6L,FBLN5,SLC4A4,ZFP90,COPS8,ZNF124,ITSN2,SOX30,PPA2,ZNF780B,CTSB,SUMO3,SLC15A2,PLEKHB2,BZW1,OVOL2,NTN1,RRAGD,BANP,HOXC13,CRACR2A,CASZ1,BMF,YTHDF3,DEDD2,COLQ,DDHD1,SUMO2,HS1BP3,ZNF292,PDE4DIP,POGK,ASH1L,HOXC4,ABCA4,UFD1,TOM1,PLPP4,CD9,CARD10,RALGPS2,ANAPC1,NDRG2,BMP5,HDGFL3,SERPINB9,GRB14,INO80,IGHV2-70D,CLNS1A,CNMD,KCNK5,DCUN1D4,VSTM4,MYCL,TNN,PSAP,MICALL2,PCNT,KDM6A,ATRN,IL33,GPRC5C,PPP2R2A,KL,RASGEF1C,CSDE1,LMX1A,IL10,OR1L6,SFPQ,RIOK1,DIRAS2,SKA1,LARP6,ITPRIP,MAP6,VASP,ETV6,PALMD,RAB12,RPS12,MORC2,SREBF2,THNSL2,HIPK1,CISD1,ZNF518A,DGKK,CD70,CENPE,LMX1B,NGDN,TWIST1,ALKAL2,RPF2,ZBTB38,ISX,PTGS1,CELSR2,FH,PCNA,UFL1,ADAMTS5,OR2T3,BRD4,SERBP1,NRBP1,ITGA6,ATP2B1,GAP43,IARS2,IGHV1OR15-

			9,AGAP9,ADCY9,CNIH1,ZNF528,ZNF611,UBAP2,CIDEA,ARFGEF3,EXT2,MEOX2,SLC6A1,STAT1,BRMS1L,KCNJ18,PRAMEF2,IMPACT,PARK7,MED12L,UBL7,ADCYAP1R1,PLA2G12B,NCAPG2,MYOCD,EFHB,MEF2C,ZNF613,RBPMS2,S100B,PRDM13,RAD51AP1,RAB38,DBF4B,NECTIN1,SPPL2B,ZBTB25,PASK,CWC22,TRIM23,ATP6V1B2,CXCL2,TOP1,EPHA4,GABRA5,ACSM2A,NUMB,FRRS1,ZBTB10,MIR548H4,MEGF10,IL17RD,FBXO31,EXTL3,PRKAB1,MARK4,CDH5,NFKBID,ARHGAP12,CLDN18,APIP,CYFIP1,PCDH8,SEMA4D,SERPINB10,MC2R,FAT4,IMPA2,AKR1B1,C9,WNT5B,AMFR,NENF,SH2D1B,POMT2,ZNF845,OR4L1,SAMD13,ICA1,MTTP,TPTE,PDCL3,SRP9,CNKSR1,CHCHD2,HKDC1,CDC45,OR11G2,BUB1,MYL12B,SDE2,RBMS3,UHRF2,HDAC2,SLF1,GON4L,TBX15,NCS1,ALB,MAPK9,CRTAM,APELA,POTEJ,GPR137B,SPTB,MIDEAS,FAIM,ZNF615,RNF138,NRIP1,ZNF738,SNRK,ARFGAP3,TM9SF2,STK36,MB,RRAS2,GNA14,CD38,VMP1,GNAS,SERPINB7,DHX29,BMP7,TNFAIP8,RNF217,TRAPPC6B,CNOT7,IL20RB,NSD1,EHMT1
GO:0035556	intracellular signal transduction	0.0022717952454322466	*BCAR3,NOTCH2,MYO9A,NLK,DLC1,ITPR2,RDX,RALA,BCL2,ARHGAP26,AKR1C3,FGD4,SPRED1,ALK,MCTP1,RIN2,APC,CRKL,ARHGAP24,TNIK,PTPRJ,SCAI,SGMS1,ZFAND6,FLT1,EDAR,PRKD1,TPTE2,CHRM3,PELI2,LRP2,FGF12,TMEM117,RALGAPA1,DCLK1,DOCK8,MAPRE2,CD2AP,PTPRR,NBN,MSH6,ARHGAP32,DUSP22,SHC4,PPP1R1C,ARHGEF17,CHN1,PAFAH1B1,NF2,CNKSR2,PPP1R9A,MOB3B,RGL1,NR5A2,GRM1,PLA2R1,ANK2,DUSP16,ATRX,NUAK1,HDAC4,PRKAA1,NFAT5,GUCY1A2,CAMK4,FGF10,ZC3HAV1,RASGRF1,ZNF675,WNK2,EGF,PDE3A,TRIM5,CLEC16A,STK38L,HTR2A,MARK2,FHL2,ANKRD17,KNDC1,SPSB4,NOS2,RBBP8,MBP,TGFA,HIP1,BRCA2,ASB7,RALGAPA2,RANBP9,TMEM161A,LEMD3,HMGB1,DSTYK,SLAMF1,NEK10,ATF2,CYLD,RB1CC1,RACGAP1,NLRC5,COPS8,SLC15A2,NTN1,RRAGD,CRACR2A,DEDD2,ASH1L,UFD1,CARD10,RALGPS2,NDRG2,KL,RASGEF1C,SFPQ,RAB12,HIPK1,DGKK,TWIST1,ALKAL2,RPF2,UFL1,BRD4,NRBP1,ADCY9,ARFGEF3,STAT1,PARK7,ADCYAP1R1,EFHB,MEF2C,S100B,RAB38,PASK,EPHA4,FBXO31,MARK4,ARHGAP12,APIP,CYFIP1,SEMA4D,FAT4,NENF,TPTE,CNKSR1,CDC45,BUB1,SDE2,MAPK9,APELA,GPR137B,FAIM,SNRK,STK36,RRAS2,GNAS,BMP7*
GO:0120036	plasma membrane bounded cell projection organization	0.0023972948055687344	*LRRC4C,NOTCH2,MYO9A,ULK2,RDX,STXBP1,RALA,BCL2,SDCCAG8,FGD4,ALK,CARMIL1,ANO6,MAP4,APC,PLPPR5,CRKL,ARHGAP24,TNIK,BCL11A,CECR2,PRKD1,LRP2,ONECUT1,GRM7,SEPTIN9,NEDD4L,DCLK1,CD2AP,TTC29,FIG4,ABCD2,TRPC5,DIP2A,HECW1,FRYL,NCAM1,CHN1,PAFAH1B1,CTNNA1,PPP1R9A,PRTG,SYNE2,AIF1L,CDH11,HDAC4,CRTAC1,RASGRF1,IFT43,ABCC4,KREMEN1,MARK2,KNDC1,MBP,SNX3,STRN,HMGB1,NUDCD3,UST,PLS1,NIN,MAP2,CYLD,KIAA0753,ITSN2,NTN1,BMP5,HDGFL3,TNN,MICALL2,PCNT,LMX1A,TTC39C,MAP6,VASP,ALKAL2,CELSR2,ITGA6,GAP43,GRXCR1,IMPACT,MEF2C,S100B,NECTIN1,DRC7,EPHA4,EMP1,RSPH1,NUMB,FBXO31,MARK4,CYFIP1,HOATZ,SEMA4D,FAT4,SPAG6,HDAC2,NCS1,STK36,CD38,BMP7*
GO:0031175	neuron projection development	0.0027517124780565587	*LRRC4C,NOTCH2,MYO9A,ULK2,STXBP1,BCL2,ALK,MAP4,PLPPR5,CRKL,TNIK,BCL11A,CECR2,PRKD1,LRP2,GRM7,NEDD4L,DCLK1,FIG4,TRPC5,DIP2A,HECW1,FRYL,NCAM1,CHN1,PAFAH1B1,CTNNA1,PPP1R9A,PRTG,CDH11,CRTAC1,RASGRF1,KREMEN1,MARK2,KNDC1,MBP,SNX3,STRN,HMGB1,UST,PLS1,NIN,MAP2,ITSN2,NTN1,BMP5,HDGFL3,TNN,MICALL2,LMX1A,MAP6,VASP,ALKAL2,CELSR2,ITGA6,GAP43,GRXCR1,IMPACT,M*

			EF2C,S100B,NECTIN1,EPHA4,NUMB,FBXO31,CYFIP1,SEMA4D,FAT4,SPAG6,HDAC2,NCS1,CD38,BMP7
GO:0022008	neurogenesis	0.0037587736091569818	*TRAPPC9,LRRC4C,NOTCH2,MYO9A,ULK2,STXBP1,BCL2,ALDH1A2,ZEB1,SDCCAG8,ALK,MAP4,PLPPR5,CRKL,TNIK,SLC4A10,BCL11A,CECR2,ATP2B2,PRKD1,LRP2,GRM7,TBCD,NEDD4L,DCLK1,FIG4,TRPC5,DIP2A,HECW1,FRYL,BRINP1,ADAM22,NCAM1,CHN1,PAFAH1B1,NF2,CTNNA1,PPP1R9A,PRTG,SYNE2,WNT9B,CDH11,SLC1A1,CRTAC1,FGF10,RASGRF1,SH3GL3,KREMEN1,MARK2,CDKN2C,KNDC1,MBP,NPHP4,SNX3,STRN,PDE6C,HMGB1,UST,ESRP1,PLS1,NIN,MAP2,RACGAP1,ITSN2,NTN1,CASZ1,HDAC11,CD9,DZANK1,BMP5,HDGFL3,MYCL,TNN,MICALL2,IL33,LMX1A,MAP6,VASP,ETV6,HIPK1,LMX1B,TWIST1,ALKAL2,CELSR2,UFL1,ITGA6,GAP43,GRXCR1,IMPACT,MEF2C,S100B,PRDM13,NECTIN1,EPHA4,GABRA5,NUMB,FBXO31,HS6ST1,CYFIP1,SEMA4D,FAT4,WNT5B,SPAG6,HDAC2,NCS1,FAIM,RRAS2,CD38,BMP7*
GO:0030030	cell projection organization	0.004532180213776525	*LRRC4C,NOTCH2,MYO9A,ULK2,RDX,STXBP1,RALA,BCL2,SDCCAG8,FGD4,ALK,CARMIL1,ANO6,MAP4,APC,PLPPR5,CRKL,ARHGAP24,TNIK,BCL11A,CECR2,PRKD1,LRP2,ONECUT1,GRM7,SEPTIN9,NEDD4L,DCLK1,CD2AP,TTC29,FIG4,ABCD2,TRPC5,DIP2A,HECW1,FRYL,NCAM1,CHN1,PAFAH1B1,CTNNA1,PPP1R9A,PRTG,SYNE2,AIF1L,CDH11,HDAC4,CRTAC1,RASGRF1,IFT43,ABCC4,KREMEN1,MARK2,KNDC1,MBP,PACSIN2,SNX3,STRN,HMGB1,NUDCD3,UST,PLS1,NIN,MAP2,CYLD,KIAA0753,ITSN2,NTN1,BMP5,HDGFL3,TNN,MICALL2,PCNT,LMX1A,TTC39C,MAP6,VASP,ALKAL2,CELSR2,ITGA6,GAP43,GRXCR1,IMPACT,MEF2C,S100B,NECTIN1,DRC7,EPHA4,EMP1,RSPH1,NUMB,FBXO31,MARK4,CYFIP1,HOATZ,SEMA4D,FAT4,SPAG6,HDAC2,NCS1,STK36,CD38,BMP7*
GO:0001842	neural fold formation	0.005849283988301977	*CECR2,LUZP1,OVOL2,BMP5,BMP7*
GO:0032501	multicellular organismal process	0.006079506390014592	*FTO,BCAR3,TRAPPC9,LRRC4C,NOTCH2,IMMP2L,SCAPER,FREM1,MYO9A,TAFA5,ULK2,DLC1,ZDHHC21,STXBP1,RALA,IL1RAPL2,BCL2,PRDM16,ALDH1A2,ARHGAP26,ZEB1,AKR1C3,SDCCAG8,SPRED1,MYO1E,PLPPR1,ALK,FOXJ2,PAPPA2,RIN2,ANO6,MAP4,APC,HHLA2,TSHZ3,RBFOX3,PLPPR5,CRKL,ILDR2,SETD2,ARHGAP24,TNIK,SLC4A10,PTPRJ,MYOF,SND1,BCL11A,CECR2,CHSY1,FLI1,ATP2B2,RXFP1,C5,FLT1,EDAR,CRACD,CABLES1,PRKD1,CHRM3,LRP2,FGF12,ONECUT1,TAFA4,HERC2,GRM7,RETREG1,LUZP1,SSBP3,CELF2,TBCD,NEDD4L,TRPM1,ZHX3,ABCB5,DCLK1,GABRG2,MAPRE2,CD2AP,PARN,HERPUD2,PTPRR,FIG4,CMIP,LOXHD1,KCNE4,ABCD2,THSD7A,ARNT,KCNK10,TRPC5,NBN,RBM47,CALD1,SNTG2,DIP2A,MSH6,COL27A1,HECW1,PHF19,ESS2,FRYL,BRINP1,HRH2,ADAM22,CRISPLD2,KMT2E,SYT10,NCAM1,SLC16A1,GABPA,LCE1F,CHN1,ACSBG1,PAFAH1B1,EFEMP1,TLL1,DCAF1,ITGB8,NF2,CTNNA1,PPP1R9A,AKAP9,MEIS2,MRTFB,PRTG,NR5A2,GRM1,FOXJ3,GABRG1,PCDH11Y,PLA2R1,AGO2,ANK2,SYNE2,WNT9B,ANKS6,SMARCA4,CDH11,FABP7,CXADR,ATRX,HDAC4,SLC1A1,PRKAA1,CRTAC1,L3MBTL3,CAMK4,FGF10,ZC3HAV1,TGM1,INO80D,RASGRF1,SYNE1,ZNF675,SH3GL3,NXN,ESRRG,FBN2,EGF,P2RX6,PDE3A,ADAMTS2,PKHD1L1,ABCC4,HTR2A,CYP4A11,DAZL,KREMEN1,MARK2,FHL2,IGF2BP3,ANKRD17,APBA2,SLC2A13,CDKN2C,EVC,KNDC1,NOS2,GFRA2,RBBP8,EMILIN2,MYOM2,CREM,MBP,TRPS1,TGFA,NPHP4,MTHFD1L,SNX3,BRCA2,CFAP97,STRN,OR9Q1,PSG9,PTCD2,PDE6C,HMGB1,FGF9,UST,CPAMD8,MDM1,ESRP1,E*

			TS2,PLS1,NIN,SLAMF1,ETS1,SMARCC1,SLC9A4,SMOC2,MAP2,LAMC1,NEK10,RRBP1,TDRD5,ATF2,HIRA,CYLD,UMODL1,PSG6,MYT1,MED27,SELENON,RB1CC1,ZNF541,SMPX,RACGAP1,ACO1,DMC1,SLC4A4,CATSPERG,ITSN2,SOX30,SYBU,SEC24D,CTSB,SLC15A2,OVOL2,NTN1,HOXC13,CRACR2A,CASZ1,YTHDF3,COLQ,HDAC11,ASH1L,HOXC4,ABCA4,UFD1,PLPP4,CD9,CARD10,DZANK1,NDRG2,BMP5,HDGFL3,INO80,CNMD,VSTM4,MYCL,TNN,PSAP,MICALL2,KDM6A,ATRN,IL33,KL,CSDE1,LMX1A,IL10,OR1L6,TTC39C,MAP6,VASP,ETV6,HIPK1,DGKK,CACYBP,LMX1B,TWIST1,ALKAL2,ISX,PTGS1,CELSR2,FH,PCNA,UFL1,ADAMTS5,OR2T3,SMTN,SMPD4,ITGA6,ATP2B1,GAP43,ADCY9,CIDEA,EXT2,MEOX2,SLC6A1,GRXCR1,STAT1,BRMS1L,SHROOM2,ARMC6,IMPACT,PARK7,ADCYAP1R1,NCAPG2,MYOCD,MEF2C,RBPMS2,S100B,PRDM13,NECTIN1,PASK,DRC7,TOP1,LCE3D,EPHA4,GABRA5,RSPH1,NUMB,IL17RD,FBXO31,PRKAB1,HS6ST1,MARK4,CDH5,APOL2,NFKBID,CLDN18,CYFIP1,HOATZ,PCDH8,SEMA4D,FAT4,AKR1B1,WNT5B,AMFR,SLC26A2,OR4L1,SANBR,MTTP,DPY19L2,PDCL3,SPAG6,OR11G2,NLRP14,UNC45B,HDAC2,GON4L,TBX15,NCS1,ATP5PF,ALB,CRTAM,APELA,POTEJ,GPR137B,FAIM,NRIP1,SNRK,STK36,MB,RRAS2,CD38,VMP1,GNAS,SERPINB7,BMP7,TRAPPC6B,IL20RB,EHMT1
GO:0048666	neuron development	0.006280170127881384	*LRRC4C,NOTCH2,MYO9A,ULK2,STXBP1,BCL2,ALK,MAP4,PLPPR5,CRKL,TNIK,SLC4A10,BCL11A,CECR2,PRKD1,LRP2,GRM7,TBCD,NEDD4L,DCLK1,FIG4,TRPC5,DIP2A,HECW1,FRYL,NCAM1,CHN1,PAFAH1B1,CTNNA1,PPP1R9A,PRTG,CDH11,CRTAC1,RASGRF1,KREMEN1,MARK2,KNDC1,MBP,NPHP4,SNX3,STRN,PDE6C,HMGB1,UST,PLS1,NIN,MAP2,ITSN2,NTN1,DZANK1,BMP5,HDGFL3,TNN,MICALL2,LMX1A,MAP6,VASP,ALKAL2,CELSR2,ITGA6,GAP43,GRXCR1,IMPACT,MEF2C,S100B,NECTIN1,EPHA4,GABRA5,NUMB,FBXO31,HS6ST1,CYFIP1,SEMA4D,FAT4,SPAG6,HDAC2,NCS1,CD38,BMP7*
GO:0007154	cell communication	0.010208411590548052	*BCAR3,LRRC4C,NOTCH2,FREM1,MYO9A,TAFA5,ULK2,NLK,UNC13C,DLC1,ZDHHC21,PTPRA,ITPR2,RDX,STXBP1,RALA,IL1RAPL2,BCL2,PRDM16,ALDH1A2,ARHGAP26,LRFN2,ZEB1,AKR1C3,FGD4,SPRED1,MYO1E,PLPPR1,ALK,MCTP1,RIN2,ANO6,APC,HHLA2,TSHZ3,PLPPR5,CRKL,ILDR2,ARHGAP24,TNIK,SLC4A10,PTPRJ,RGS3,SCAI,FAM83F,SGMS1,GRIK3,CHSY1,RXFP1,C5,ZFAND6,FLT1,RABEP1,INVS,EDAR,PRKD1,TPTE2,CHRM3,PELI2,LRP2,FGF12,ONECUT1,TAFA4,BTBD11,SYN2,CCL28,GRM7,TMEM117,RALGAPA1,TRPM1,DCLK1,GABRG2,DOCK8,MAPRE2,NDUFAF2,CD2AP,HERPUD2,PTPRR,ARNT,KCNK10,NBN,MSH6,ARHGAP32,HECW1,DUSP22,SV2B,SHC4,HRH2,SYT10,PPP1R1C,ARHGEF17,NCAM1,SLC16A1,CHN1,GLP2R,PAFAH1B1,EFEMP1,TM7SF3,ITGB8,NF2,CNKSR2,HIVEP1,CTNNA1,PPP1R9A,MOB3B,AKAP9,ERMP1,RGL1,NR5A2,GRM1,GABRG1,PCDH11Y,PPP2R5E,PLA2R1,RIC8B,ANK2,WNT9B,DUSP16,SMARCA4,CDH11,SPG21,CXADR,ATRX,NUAK1,PTPN12,HDAC4,SLC1A1,PRKAA1,ITGB3BP,RIMBP2,NFAT5,GUCY1A2,CAMK4,FGF10,ZC3HAV1,RASGRF1,ZNF675,SH3GL3,NXN,WNK2,ESRRG,FBN2,EGF,P2RX6,PDE3A,SCG5,MTMR3,TRIM5,CLEC16A,STK38L,ABCC4,HTR2A,KREMEN1,MARK2,GCSAML,FHL2,ADGRA3,CNIH3,ANKRD17,APBA2,EVC,GRK3,KNDC1,SPSB4,NOS2,GFRA2,RBBP8,CCND3,CREM,MBP,TGFA,HIP1,CAPN5,NPHP4,PACSIN2,SNX3,BRCA2,ASB7,STRN,OR9Q1,PSG9,CDC42BPB,SOGA1,RALGAPA2,RANBP9,TMEM161A,PDE6C,LEMD3,HMGB1,FGF9,DSTYK,SLAMF1,FAM83B,SMARCC1,SNX6,SMOC2,LAMC1,NEK10,ATF2,CYLD,PSG6,ITGA9,KPNA1,RGMB,ZZEF1,RB1CC1,LALBA,PKP1,R*

			ACGAP1,NLRC5,COPS8,SOX30,SLC15A2,OVOL2,NTN1,CHKA,RRAGD,CRACR2A,BMF,YTHDF3,DEDD2,COLQ,ASH1L,ABCA4,UFD1,TOM1,PLPP4,CARD10,RALGPS2,NDRG2,BMP5,HDGFL3,GRB14,IGHV2-70D,TNN,PSAP,PCNT,IL33,GPRC5C,KL,RASGEF1C,IL10,OR1L6,SFPQ,DIRAS2,ITPRIP,RAB12,RPS12,SREBF2,THNSL2,HIPK1,DGKK,CD70,TWIST1,ALKAL2,RPF2,CELSR2,UFL1,OR2T3,BRD4,NRBP1,ITGA6,ATP2B1,GAP43,IGHV1OR15-9,ADCY9,CNIH1,CIDEA,ARFGEF3,EXT2,SLC6A1,STAT1,BRMS1L,IMPACT,PARK7,ADCYAP1R1,NCAPG2,MYOCD,EFHB,MEF2C,RBPMS2,S100B,RAB38,SPPL2B,PASK,CXCL2,EPHA4,GABRA5,IL17RD,FBXO31,PRKAB1,MARK4,CDH5,NFKBID,ARHGAP12,CLDN18,APIP,CYFIP1,PCDH8,SEMA4D,MC2R,FAT4,IMPA2,WNT5B,AMFR,NENF,ZFYVE1,OR4L1,ICA1,TPTE,CNKSR1,CDC45,OR11G2,BUB1,SDE2,RBMS3,HDAC2,ALB,MAPK9,APELA,GPR137B,FAIM,RNF138,SNRK,STK36,RRAS2,GNA14,CD38,GNAS,BMP7,CNOT7,IL20RB
GO:0030182	neuron differentiation	0.013150209196540513	*TRAPPC9,LRRC4C,NOTCH2,MYO9A,ULK2,STXBP1,BCL2,ALDH1A2,ZEB1,ALK,MAP4,PLPPR5,CRKL,TNIK,SLC4A10,BCL11A,CECR2,ATP2B2,PRKD1,LRP2,GRM7,TBCD,NEDD4L,DCLK1,FIG4,TRPC5,DIP2A,HECW1,FRYL,BRINP1,NCAM1,CHN1,PAFAH1B1,CTNNA1,PPP1R9A,PRTG,WNT9B,CDH11,CRTAC1,RASGRF1,SH3GL3,KREMEN1,MARK2,KNDC1,MBP,NPHP4,SNX3,STRN,PDE6C,HMGB1,UST,ESRP1,PLS1,NIN,MAP2,ITSN2,NTN1,CASZ1,DZANK1,BMP5,HDGFL3,MYCL,TNN,MICALL2,LMX1A,MAP6,VASP,HIPK1,LMX1B,ALKAL2,CELSR2,ITGA6,GAP43,GRXCR1,IMPACT,MEF2C,S100B,NECTIN1,EPHA4,GABRA5,NUMB,FBXO31,HS6ST1,CYFIP1,SEMA4D,FAT4,WNT5B,SPAG6,HDAC2,NCS1,CD38,BMP7*
GO:0051716	cellular response to stimulus	0.013168107819126317	*FTO,BCAR3,NOTCH2,IMMP2L,ZNF236,MYO9A,TAFA5,ULK2,NLK,DLC1,ZDHHC21,PTPRA,ITPR2,RDX,STXBP1,RALA,IL1RAPL2,BCL2,MYO5A,PRDM16,ALDH1A2,ARHGAP26,EPC2,ZEB1,AKR1C3,FGD4,SPRED1,MYO1E,PLPPR1,ALK,MCTP1,ERCC6L2,RIN2,ANO6,EGLN3,APC,HHLA2,PLPPR5,CRKL,SETD2,ARHGAP24,TNIK,PTPRJ,BACH1,RGS3,SCAI,BCL11A,FAM83F,SGMS1,GRIK3,CHSY1,RXFP1,C5,ZFAND6,CYP2C9,FLT1,RFC3,RABEP1,INVS,EDAR,PRKD1,TPTE2,CHRM3,ADSS2,PELI2,LRP2,FGF12,ONECUT1,TAFA4,BTBD11,CCL28,SMYD3,HERC2,GRM7,TMEM117,RALGAPA1,TRPM1,DCLK1,GABRG2,DOCK8,MAPRE2,NDUFAF2,CD2AP,HERPUD2,PTPRR,ECPAS,ARNT,KCNK10,UBE2E2,NBN,MSH6,ARHGAP32,HECW1,DUSP22,SEM1,SHC4,BRINP1,HRH2,SYT10,PPP1R1C,ARHGEF17,NCAM1,SLC16A1,GABPA,CHN1,GLP2R,PAFAH1B1,EFEMP1,TM7SF3,ITGB8,NF2,CNKSR2,HIVEP1,CTNNA1,PPP1R9A,MOB3B,AKAP9,ERMP1,RGL1,NR5A2,GRM1,GABRG1,PCDH11Y,PPP2R5E,PLA2R1,ACSM2B,WDHD1,RIC8B,ANK2,WNT9B,DUSP16,SMARCA4,EFTUD2,SPG21,CXADR,ATRX,NUAK1,PTPN12,HDAC4,SLC1A1,PRKAA1,ITGB3BP,NFAT5,GUCY1A2,CAMK4,FGF10,ZC3HAV1,INO80D,RASGRF1,ZNF675,SH3GL3,NXN,WNK2,ESRRG,FBN2,EGF,P2RX6,PDE3A,SCG5,MTMR3,TRIM5,RFC1,CLEC16A,STK38L,ABCC4,HTR2A,KREMEN1,MARK2,GCSAML,FHL2,ADGRA3,CNIH3,ANKRD17,EVC,GRK3,KNDC1,SPSB4,NOS2,GFRA2,RBBP8,GBP6,CCND3,CREM,MBP,TGFA,HIP1,GSR,CAPN5,NPHP4,SNX3,BRCA2,ASB7,STRN,OR9Q1,PSG9,CDC42BPB,SOGA1,RALGAPA2,RANBP9,TMEM161A,PDE6C,LEMD3,HMGB1,FGF9,DSTYK,SLAMF1,FAM83B,SMARCC1,SNX6,SMOC2,ZFYVE26,LAMC1,NEK10,ATF2,CYLD,UMODL1,PSG6,ITGA9,KPNA1,RGMB,DNAJC7,CHAF1A,SELENON,RB1CC1,USP43,LALBA,PKP1,RACGAP1,NLRC5,DMC1,FBLN5,COPS8,SOX30,CTSB,SLC15A2,OVOL2*

			,*NTN1,CHKA,RRAGD,CRACR2A,BMF,YTHDF3,DEDD2,ASH1L,ABCA4,UFD1,TOM1,PLPP4,CD9,CARD10,RALGPS2,NDRG2,BMP5,PWWP3A,HDGFL3,SERPINB9,GRB14,INO80,IGHV2-70D,TNN,PSAP,PCNT,IL33,GPRC5C,KL,RASGEF1C,IL10,OR1L6,SFPQ,DIRAS2,ITPRIP,RAB12,RPS12,MORC2,SREBF2,THNSL2,HIPK1,DGKK,CD70,CACYBP,TWIST1,ALKAL2,RPF2,ZBTB38,PTGS1,CELSR2,FH,TDP1,PCNA,UFL1,OR2T3,BRD4,SMPD4,NRBP1,ITGA6,ATP2B1,GAP43,IGHV1OR15-9,ADCY9,CNIH1,CIDEA,ARFGEF3,EXT2,STAT1,BRMS1L,SHROOM2,IMPACT,PARK7,UBL7,ADCYAP1R1,NCAPG2,MYOCD,EFHB,MEF2C,RBPMS2,S100B,RAD51AP1,RAB38,SPPL2B,PASK,CXCL2,EPHA4,GABRA5,IL17RD,FBXO31,PRKAB1,MARK4,CDH5,TPH2,NFKBID,ARHGAP12,CLDN18,APIP,CYFIP1,SEMA4D,MC2R,FAT4,IMPA2,AKR1B1,WNT5B,AMFR,NENF,ZFYVE1,OR4L1,TPTE,CNKSR1,CHCHD2,CDC45,OR11G2,BUB1,SDE2,RBMS3,HDAC2,SLF1,ALB,MAPK9,APELA,GPR137B,GBP4,FAIM,FAAP24,RNF138,NRIP1,SNRK,STK36,RRAS2,GNA14,CD38,GNAS,BMP7,CNOT7,IL20RB*
GO:0006996	organelle organization	0.015656980782319543	*LONP2,TRAPPC9,NOTCH2,IMMP2L,ULK2,UNC13C,SVIL,MICAL3,NUBPL,DLC1,RDX,STXBP1,RALA,BCL2,MYO5A,ARHGAP26,SDCCAG8,FGD4,MYO1E,CEP192,MICOS10,FOXJ2,CARMIL1,MAP4,APC,ZMYM4,SETD2,TNIK,GNPTAB,CECR2,ZFAND6,RFC3,CRACD,PRKD1,ONECUT1,SEPTIN9,RETREG1,TBCD,DCLK1,MAPRE2,NDUFAF2,CD2AP,PARN,TTC29,FIG4,FRMD3,ABCD2,THSD7A,NBN,CALD1,MRTFA,ARHGEF17,ANKFY1,SLC16A1,LIMCH1,PAFAH1B1,VPS13D,NF2,CTNNA1,PPP1R9A,AKAP9,FOXJ3,DYSF,ANK2,STAG2,BRWD1,SYNE2,AIF1L,SHOC1,SMARCA4,CXADR,ATRX,PRKAA1,ITGB3BP,BAZ2A,FGF10,UQCC1,INO80D,CLIP1,SYNE1,EGF,PDE3A,IFT43,MTMR3,RFC1,CLEC16A,ABCC4,GTF2F2,MARK2,MYOM2,TRAPPC11,TGFA,HIP1,NPHP4,PACSIN2,SNX3,BRCA2,CDC42BPB,PTCD2,RANBP9,RESF1,LEMD3,HMGB1,NUDCD3,CDS2,MDM1,UBAP2L,PLS1,NIN,SLAMF1,ETS1,SMARCC1,ZFYVE26,MAP2,PEX6,TDRD5,ATF2,NDUFAF6,CYLD,GOLGA6B,KIAA0753,CEP44,GOLGA6D,SELENON,RB1CC1,PKP1,RACGAP1,CNOT6L,DMC1,GOLGA6C,SOX30,PTGFRN,SEC24D,CHKA,BMF,YTHDF3,DDHD1,PDE4DIP,ESYT2,ANAPC1,HDGFL3,INO80,MICALL2,PCNT,CSDE1,SFPQ,TTC39C,TOP3B,SKA1,MAP6,VASP,MORC2,SREBF2,CENPE,RPF2,CELSR2,PCNA,UFL1,MIPEP,SMTN,SERBP1,SMPD4,GAP43,GOLGA8J,ARFGEF3,SHROOM2,PARK7,NCAPG2,CHAMP1,RAD51AP1,RAB38,DRC7,TOP1,RSPH1,MARK4,CDH5,ARHGAP12,CYFIP1,HOATZ,ZFYVE1,PDCL3,CHCHD2,SPAG6,CDC45,BUB1,SLF1,MAPK9,SPTB,ARFGAP3,STK36,VMP1,DHX29,BMP7,TRAPPC6B,CNOT7,SAMM50*
GO:0023052	signaling	0.016465463012106395	*BCAR3,LRRC4C,NOTCH2,MYO9A,TAFA5,ULK2,NLK,UNC13C,DLC1,ZDHHC21,PTPRA,ITPR2,RDX,STXBP1,RALA,IL1RAPL2,BCL2,PRDM16,ALDH1A2,ARHGAP26,LRFN2,ZEB1,AKR1C3,FGD4,SPRED1,MYO1E,PLPPR1,ALK,MCTP1,RIN2,ANO6,APC,HHLA2,TSHZ3,PLPPR5,CRKL,ILDR2,ARHGAP24,TNIK,SLC4A10,PTPRJ,RGS3,SCAI,FAM83F,SGMS1,GRIK3,CHSY1,ATP2B2,RXFP1,C5,ZFAND6,FLT1,RABEP1,INVS,EDAR,PRKD1,TPTE2,CHRM3,PELI2,LRP2,FGF12,ONECUT1,TAFA4,BTBD11,SYN2,CCL28,GRM7,TMEM117,RALGAPA1,TRPM1,DCLK1,GABRG2,DOCK8,MAPRE2,NDUFAF2,CD2AP,HERPUD2,PTPRR,KCNE4,ARNT,KCNK10,NBN,MSH6,ARHGAP32,HECW1,DUSP22,SV2B,SHC4,HRH2,SYT10,PPP1R1C,ARHGEF17,NCAM1,SLC16A1,CHN1,GLP2R,PAFAH1B1,EFEMP1,TM7SF3,ITGB8,NF2,CNKSR2,HIVEP1,CTNNA1,PPP1R9A,MOB3B,AKAP9,ERMP1,RGL1,NR5A2,GRM1*

			,GABRG1,PCDH11Y,PPP2R5E,PLA2R1,RIC8B,ANK2,WNT9B,DUSP16,SMARCA4,CDH11,SPG21,CXADR,ATRX,NUAK1,PTPN12,HDAC4,SLC1A1,PRKAA1,ITGB3BP,RIMBP2,NFAT5,GUCY1A2,CAMK4,FGF10,ZC3HAV1,RASGRF1,ZNF675,SH3GL3,NXN,WNK2,ESRRG,FBN2,EGF,P2RX6,PDE3A,SCG5,TRIM5,CLEC16A,STK38L,ABCC4,HTR2A,KREMEN1,MARK2,GCSAML,FHL2,ADGRA3,CNIH3,ANKRD17,APBA2,EVC,GRK3,KNDC1,SPSB4,NOS2,GFRA2,RBBP8,CCND3,CREM,MBP,TGFA,HIP1,CAPN5,NPHP4,PACSIN2,SNX3,BRCA2,ASB7,STRN,OR9Q1,PSG9,CDC42BPB,SOGA1,RALGAPA2,RANBP9,TMEM161A,PDE6C,LEMD3,HMGB1,FGF9,DSTYK,SLAMF1,FAM83B,SMARCC1,SNX6,SMOC2,LAMC1,NEK10,ATF2,CYLD,PSG6,ITGA9,KPNA1,RGMB,ZZEF1,RB1CC1,LALBA,PKP1,RACGAP1,NLRC5,COPS8,SOX30,SLC15A2,OVOL2,NTN1,RRAGD,CRACR2A,BMF,YTHDF3,DEDD2,COLQ,ASH1L,ABCA4,UFD1,TOM1,PLPP4,CARD10,RALGPS2,NDRG2,BMP5,HDGFL3,GRB14,IGHV2-70D,TNN,PSAP,PCNT,IL33,GPRC5C,KL,RASGEF1C,IL10,OR1L6,SFPQ,DIRAS2,ITPRIP,RAB12,RPS12,SREBF2,THNSL2,HIPK1,DGKK,CD70,TWIST1,ALKAL2,RPF2,CELSR2,UFL1,OR2T3,BRD4,NRBP1,ITGA6,ATP2B1,GAP43,IGHV1OR15-9,ADCY9,CNIH1,CIDEA,ARFGEF3,EXT2,SLC6A1,STAT1,BRMS1L,PARK7,ADCYAP1R1,NCAPG2,MYOCD,EFHB,MEF2C,RBPMS2,S100B,RAB38,SPPL2B,PASK,CXCL2,EPHA4,GABRA5,IL17RD,FBXO31,PRKAB1,MARK4,CDH5,NFKBID,ARHGAP12,CLDN18,APIP,CYFIP1,PCDH8,SEMA4D,MC2R,FAT4,IMPA2,WNT5B,AMFR,NENF,OR4L1,ICA1,TPTE,CNKSR1,CDC45,OR11G2,BUB1,SDE2,RBMS3,HDAC2,MAPK9,APELA,GPR137B,FAIM,RNF138,SNRK,STK36,RRAS2,GNA14,CD38,GNAS,BMP7,CNOT7,IL20RB
GO:0048468	cell development	0.01773230544209162	LRRC4C,NOTCH2,MYO9A,ULK2,ZDHHC21,RDX,STXBP1,BCL2,ALDH1A2,MYO1E,ALK,CARMIL1,MAP4,PLPPR5,CRKL,SETD2,TNIK,SLC4A10,MYOF,BCL11A,TMEM182,CECR2,CHSY1,FLI1,PRKD1,LRP2,ONECUT1,SMYD3,GRM7,TBCD,NEDD4L,DCLK1,FIG4,TRPC5,DIP2A,COL27A1,HECW1,FRYL,BRINP1,ADAM22,NCAM1,CHN1,PAFAH1B1,NF2,CTNNA1,PPP1R9A,PRTG,DYSF,ANK2,CDH11,CXADR,ATRX,HDAC4,CRTAC1,L3MBTL3,RASGRF1,PDE3A,DAZL,KREMEN1,MARK2,FHL2,KNDC1,MYOM2,MBP,NPHP4,SNX3,BRCA2,STRN,PTCD2,MSI2,PDE6C,HMGB1,UST,PLS1,NIN,SLC9A4,MAP2,LAMC1,TDRD5,SELENON,DMC1,ITSN2,SOX30,OVOL2,NTN1,HDAC11,DZANK1,BMP5,HDGFL3,TNN,MICALL2,ATRN,IL33,LMX1A,MAP6,VASP,TWIST1,ALKAL2,CELSR2,UFL1,ITGA6,GAP43,GRXCR1,IMPACT,MEF2C,S100B,NECTIN1,DRC7,EPHA4,GABRA5,RSPH1,NUMB,MEGF10,FBXO31,HS6ST1,CDH5,CLDN18,CYFIP1,SEMA4D,FAT4,AKR1B1,WNT5B,DPY19L2,SPAG6,HDAC2,NCS1,FAIM,CD38,BMP7
GO:0048699	generation of neurons	0.019351240251334764	TRAPPC9,LRRC4C,NOTCH2,MYO9A,ULK2,STXBP1,BCL2,ALDH1A2,ZEB1,SDCCAG8,ALK,MAP4,PLPPR5,CRKL,TNIK,SLC4A10,BCL11A,CECR2,ATP2B2,PRKD1,LRP2,GRM7,TBCD,NEDD4L,DCLK1,FIG4,TRPC5,DIP2A,HECW1,FRYL,BRINP1,NCAM1,CHN1,PAFAH1B1,CTNNA1,PPP1R9A,PRTG,WNT9B,CDH11,CRTAC1,RASGRF1,SH3GL3,KREMEN1,MARK2,KNDC1,MBP,NPHP4,SNX3,STRN,PDE6C,HMGB1,UST,ESRP1,PLS1,NIN,MAP2,RACGAP1,ITSN2,NTN1,CASZ1,DZANK1,BMP5,HDGFL3,MYCL,TNN,MICALL2,LMX1A,MAP6,VASP,HIPK1,LMX1B,TWIST1,ALKAL2,CELSR2,ITGA6,GAP43,GRXCR1,IMPACT,MEF2C,S100B,NECTIN1,EPHA4,GABRA5,NUMB,FBXO31,HS6ST1,CYFIP1,SEMA4D,FAT4,WNT5B,SPAG6,HDAC2,NCS1,CD38,BMP7

GO:0007423	sensory organ development	0.021028678741676195	*BCAR3,NOTCH2,SCAPER,BCL2,ALDH1A2,ZEB1,SPRED1,CECR2,ATP2B2,FLT1,ABCB5,DCLK1,PAFAH1B1,EFEMP1,NF2,MEIS2,WNT9B,SMARCA4,SLC1A1,FGF10,FBN2,NPHP4,PDE6C,FGF9,CPAMD8,MDM1,ESRP1,PLS1,NTN1,HOXC13,DZANK1,BMP5,VSTM4,MYCL,TTC39C,HIPK1,TWIST1,ATP2B1,GRXCR1,SHROOM2,NECTIN1,EPHA4,GABRA5,FAT4,WNT5B,UNC45B,HDAC2,BMP7*
GO:0031344	regulation of cell projection organization	0.024869152380703946	*LRRC4C,MYO9A,ULK2,RDX,RALA,SDCCAG8,ALK,CARMIL1,MAP4,APC,PLPPR5,CRKL,ARHGAP24,TNIK,BCL11A,PRKD1,SEPTIN9,NEDD4L,FIG4,TRPC5,HECW1,CHN1,PAFAH1B1,SYNE2,HDAC4,KREMEN1,MARK2,KNDC1,MBP,SNX3,UST,PLS1,NIN,MAP2,CYLD,NTN1,BMP5,TNN,MAP6,ALKAL2,ITGA6,GAP43,EPHA4,FBXO31,MARK4,CYFIP1,SEMA4D,HDAC2,NCS1,CD38,BMP7*
GO:0120035	regulation of plasma membrane bounded cell projection organization	0.026486009672339512	*LRRC4C,ULK2,RDX,RALA,SDCCAG8,ALK,CARMIL1,MAP4,APC,PLPPR5,CRKL,ARHGAP24,TNIK,BCL11A,PRKD1,SEPTIN9,NEDD4L,FIG4,TRPC5,HECW1,CHN1,PAFAH1B1,SYNE2,HDAC4,KREMEN1,MARK2,KNDC1,MBP,SNX3,UST,PLS1,NIN,MAP2,CYLD,NTN1,BMP5,TNN,MAP6,ALKAL2,ITGA6,GAP43,EPHA4,FBXO31,MARK4,CYFIP1,SEMA4D,HDAC2,NCS1,CD38,BMP7*
GO:0034330	cell junction organization	0.048591796621255096	*LRRC4C,MYO9A,UNC13C,DLC1,PTPRA,RDX,IL1RAPL2,BCL2,LRFN2,APC,CRKL,PTPRJ,TBCD,GABRG2,MAPRE2,DIP2A,DUSP22,LIMCH1,PAFAH1B1,NF2,CNKSR2,CTNNA1,CDH7,ANK2,CDH11,CXADR,SLC1A1,CDHR3,NPHP4,STRN,LAMC1,PKP1,SYBU,NTN1,COLQ,CD9,MICALL2,LMX1A,IL10,HIPK1,ITGA6,GAP43,SLC6A1,MEF2C,NECTIN1,EPHA4,NUMB,CDH5,CLDN18,CYFIP1,PCDH8,SEMA4D,VMP1*

Table S8. The 184 sets of co-expressed rDNA-contacting genes in differentiating K562 cells. The search was performed in https://maayanlab.cloud/Enrichr/enrich# for ARCHS4 TFs Coexp. The database presents the top-300 genes that are co-expressed with transcription factors. All 1485 genes specify the transcription factors and are co-expressed in different combinations.

Term	Overlap	Adjusted P-value	Genes
ZNF704 human tf ARCHS4 coexpression	126/299	4.551107476008999E-34	*ATP8A2;CTNND2;ZBTB20;SLC4A4;SLC8A1;MYLK3;HERC2;ZNF608;HERC1;AKT3;KIF21A;DIP2C;SCAPER;MAGI1;RBFOX1;RBFOX2;TMEM178B;CACNA2D1;TMOD2;MTUS1;FRMD4A;ANK3;TANC2;PYGO1;TANC1;WDFY3;ASTN2;DGKI;ASTN1;MACF1;SHC3;IGSF3;TNKS;KMT2C;PCDH15;ILDR2;RASAL2;CACNA1C;KALRN;NPAS3;FLRT2;FUT9;PLXNA2;SRGAP3;MPDZ;PLXNA4;BPTF;AUTS2;CADM2;NEBL;PTCH1;MICAL3;MYO5A;GRIN2B;CORO2B;DCLK1;PBX1;PTPRD;CCDC88A;ARHGAP32;SDK1;DLG2;NBEA;SPIRE1;FAT3;TCF4;ROBO2;RERE;DOCK3;TENM4;MAST2;GRIK3;ROBO1;AKAP11;TRIM2;MCF2L;PSD3;TMEM108;HYDIN2;DLGAP1;NPIPA1;NEO1;ADAMTS9;ARHGEF12;DST;NAV2;SEZ6L;MPPED1;LRRC7;MPPED2;ARHGEF7;RAPGEF5;PLCB1;DSCAML1;SHANK2;PPM1L;LUZP2;NRXN3;AKAP6;MIPOL1;STOX2;KIAA1328;HECTD4;CLVS2;CTNNA3;ATP9A;MAP4K4;ARNT2;ZNF462;FARP1;MYEF2;NTRK3;PCDH7;LSAMP;YLPM1;KIAA1549L;PDE4DIP;SORBS2;MYO9A;TTLL7;TJP1;PDE10A;APC;ASXL3;TTC3;RGS12*
PLXNA4 human tf ARCHS4	126/299	4.551107476008999E-34	*ATP8A2;FRMPD4;MYT1L;ANKRD36;CTNND2;RORB;FRY;SLC8A1;HS6ST3;RIMS1;CDH4;ZNF608;DPYSL5;AKT3;TNR;KIF21A;KIF21B;ANKS1B;SOX5;PPFIA2;ANKRD36C;RBFOX1;EPHA6;RBFOX2;KCNH5;TMEM178B;CACNA2D1;PR*

coexpression			KCE;TMOD2;FRMD4A;ANK3;TANC2;MAPK8IP1;SCN8A;HECW1;WDFY3;DGKI;ASTN1;MACF1;SHC3;CTTNBP2;STXBP1;ILDR2;KALRN;CACNA1E;NKAIN2;CTIF;DPP6;FLRT2;FUT9;ZNF704;PLXNA2;ST8SIA5;HIVEP2;SRGAP3;NDFIP1;ARPP21;SYT1;CADM2;TMEM132B;MYO5A;CORO2B;DCLK1;PBX1;SNAP91;PTPRD;CCDC88A;LRFN2;DLG2;DAB1;NBEA;SYNJ1;GNAQ;CNTN1;SPIRE1;FAT3;CCSER1;TCF4;SCN2A;SLC44A5;ROBO2;DOCK3;TENM4;PTPRO;AFF3;ROBO1;C4ORF50;GRM5;AKAP11;GRM7;TRIM2;PSD3;DLGAP1;ZNF385D;NEO1;FAM219A;SORCS3;AJAP1;CNKSR2;MPPED1;NAV3;LRRC7;ARHGEF7;PLCB1;PPM1L;NRXN3;MAPK8;HECTD4;CLVS2;NCAM1;CTNNA2;CSMD2;PAK3;CSMD1;ATP9A;OPCML;ARNT2;NTRK3;LSAMP;SYT16;KIAA1549L;TTLL7;APC;PPP2R2B;ASXL3;TTC3
SOX5 human tf ARCHS4 coexpression	119/299	2.1321254175670423E-29	ATP8A2;MYT1L;ANKRD36;CTNND2;RORB;SLC8A1;CDH7;SRGAP2C;GRIP1;AKT3;KIF21A;DIP2C;SRGAP2B;POTEB;PPFIA2;MAGI1;EPHA6;RBFOX2;KCNH5;TMEM178B;CACNA2D1;TMOD2;MAGI2;RFX3;KAZN;FRMD4A;ANK3;TANC2;TIAM2;ADGRB3;HECW1;KCNQ3;XPR1;DGKI;GRIA1;SHC3;CTTNBP2;ILDR2;NREP;KALRN;CACNA1E;FLRT2;FUT9;ZNF704;SRGAP3;MPDZ;PLXNA4;BPTF;BCL11B;SIAH3;TMEM132B;SLC4A10;MYO5A;CORO2B;DCLK1;ST18;PBX1;LRP1B;PTPRD;CCDC88A;FER;DLG2;DAB1;NBEA;SPIRE1;FAT3;TCF4;CNTN4;FGF12;LRP12;SLC44A5;ROBO2;CNTNAP2;DOCK3;TENM4;PTPRO;GRIK3;ELAVL4;GRIK2;FMN2;ROBO1;SLC22A14;NHSL1;ADAMTS3;GRM7;LRRTM4;TRIM2;TMEM108;PHACTR3;DLGAP1;ZNF385D;ERC1;NEO1;TTC37;LRRC49;MPPED1;NAV3;LRRC7;MPPED2;SLC24A2;PPM1L;NOL4;FGD4;MAPK8;NCAM1;PAK3;CSMD1;MYEF2;NTRK3;LSAMP;SYT16;KIAA1549L;PDE10A;NFIA;APC;NFIB;PPP2R2B;TTC3;SSBP2
ZNF483 human tf ARCHS4 coexpression	118/299	7.439628970064889E-29	ATP8A1;FRMPD4;MYT1L;DGKB;ZBTB20;RORB;SLC8A1;RIMS2;RIMS1;AKT3;SAMD12;PRKACB;ANKS1B;KCNH1;UNC13C;EPHA6;KCNH5;TMOD2;MAGI2;ANK3;SHISA9;PIAS2;GABRG1;ADGRB3;SCN8A;HECW1;WDPCP;WDFY3;ASTN2;DGKI;NECAB1;SLC1A2;KALRN;NALCN;GLB1L3;MTMR7;GRIN2A;DPP6;PDZD2;SNTG1;FUT9;DTNA;SYT1;CADM2;SLC4A10;MYO5A;GRIN2B;LRP1B;PTPRD;FAM135B;DLG2;NBEA;TMEM116;CNTN1;CPE;FAT3;SCN2A;GABRB3;SPAG16;CCDC122;DOCK3;RASGRF2;EFCAB6;RYR3;C4ORF50;UNC80;GRM5;AKAP11;LRRTM4;CA5A;TRIM2;ADAMTSL3;PSD3;HYDIN2;DLGAP1;DLGAP2;SLC2A13;OPRM1;KIF6;AJAP1;CNKSR2;DNM3;LRRC7;KCNMA1;RAPGEF5;PLCB1;SLC24A2;PPM1L;RGPD6;RGPD5;AKAP6;MIPOL1;RANBP3L;KIAA1328;SV2B;CLVS2;PAK3;CSMD1;ATP9B;GPR158;ATP9A;OPCML;RIC3;PCDH9;NEGR1;NTRK3;CADPS;SYT16;KIAA1549L;TTLL7;MAPK10;AGBL4;APC;PPP2R2B;PTPN4;ASB3;TNRC6B;HCN1
MYT1L human tf ARCHS4 coexpression	117/299	2.729558005106311E-28	ATP8A2;FRMPD4;ANKRD36;CTNND2;ZBTB20;RORB;RIMS2;RPH3A;RIMS1;AKT3;SH3GL2;ANKS1B;PPFIA2;RGS7;ANKRD36C;RBFOX1;RBFOX2;TMEM178B;TMOD2;MAGI2;ANK3;TANC2;ADGRB3;SCN8A;HECW1;KCNQ3;ASTN1;GRIA1;RTN1;SHC3;CTTNBP2;STXBP1;NTM;SLC1A2;RASAL2;NREP;NYAP2;KALRN;NALCN;CACNA1E;GRIN2A;DPP6;PGBD5;FUT9;CAMTA1;SRGAP3;PLXNA4;PTPRN2;DTNA;SYT1;CADM2;SLC4A10;MYO5A;GRIN2B;DCLK1;SNAP91;PTPRD;CCDC88A;RALYL;DLG2;NBEA;CNTN1;FAT3;TCF4;C8ORF34;GABRB3;DPP10;DOCK3;PTPRO;CELF4;ELAVL4;GRIK2;UNC80;GRM5;GRM7;TRIM2;MCF2L;PSD3;PHACTR3;DLGAP1;DLGAP2;GARNL3;LRRC49;SYN2;PGM2L1;AJAP1;CNKSR2;DNM3;MPPED1;LRRC7;PLCB1;SLC24A2;PPM1L;NRXN3;AKAP6;STOX2;SV2B;CLVS2;NCAM1;CTNNA2;PAK3;PAK5;ATP9A;OPCML;GABBR2;ARNT2;KCNJ6;NTRK3;SYT16;KIAA1549L;ATP2B2;TTLL7;MAPK10;APC;PPP2R2C;PPP2R2B;TTC3

SETBP1 human tf ARCHS4 coexpression	112/299	3.7167301841993506E-25	CPNE4;MYT1L;ANKRD36;CTNND2;ZBTB20;GRIP1;ZNF608;DACH1;CDH2;DPYSL5;AKT3;KIF21A;SOX6;SRGAP2B;SOX5;PPFIA2;MAGI1;ANKRD36C;WSB1;RBFOX2;TMEM178B;CACNA2D1;MAGI2;RFX3;FRMD4A;PYGO1;IFT81;ADGRB3;DOK5;ASTN2;TOX;XPR1;ASTN1;GRIA1;TNKS;PDE1A;ILDR2;RASAL2;NREP;KALRN;NPAS3;NKAIN3;SNTG1;FUT9;ZNF704;PLXNA2;SRGAP3;LRRC4C;MPDZ;PLXNA4;AUTS2;ST8SIA1;ST8SIA2;CORO2B;DCLK1;PBX1;PTPRD;CCDC88A;LRFN5;DLG2;NBEA;RNF182;ZNF536;FAT3;TCF4;SLC44A5;ROBO2;DRAXIN;TENM4;ELAVL4;BICD1;SYNE2;ROBO1;GRM7;LRRTM4;TRIM2;RALGPS1;GARNL3;ADGRV1;KCND3;LRRC49;TCF12;LRRC7;MPPED2;NRXN3;NOL4;MIPOL1;FGD4;STOX2;GNG2;NCAM1;CTNNA2;CSMD2;PAK3;PAK5;ZNF462;FARP1;NTRK2;MBD5;MYEF2;NTRK3;LSAMP;YLPM1;MAPK10;NFIA;APC;FABP7;ASXL3;TTC3;YPEL1;SSBP2;TNRC6B
SORBS2 human tf ARCHS4 coexpression	108/299	9.116971763335655E-23	FHOD3;MYT1L;SLC8A1;MYLK3;CDH2;DPYSL5;ZSCAN30;AKT3;LONP2;KIF21A;LARGE1;MAGI1;EPHA7;MLIP;RBFOX2;CACNA2D1;MAGI2;MTUS2;FRMD4A;ANK3;UNC5D;TANC2;TIAM2;TOM1L2;KCNQ3;SLC27A6;ALPK3;WDFY3;MXRA7;ALPK2;XPR1;DGKI;MACF1;RASAL2;CACNA1C;NREP;KALRN;LPP;ZNF704;PLXNA2;SRGAP3;MPDZ;CADM1;SPHKAP;AUTS2;NEBL;ST8SIA2;ST18;SNAP91;PTPRD;DLG2;RCAN2;PDE3A;TCF4;ROBO2;RYR2;MYOM1;TENM4;ELAVL4;PTPRM;LDB3;SIPA1L2;PTPRG;RASGEF1B;TRIM2;MYO18B;PSD3;PGM5;RALGPS1;DST;DCC;LRRC49;ENAH;LRRC7;NOS1AP;SHANK2;PRKAA2;AKAP6;STOX2;TBX20;NHS;CTNNA3;NCAM1;CSMD3;PAK3;PDLIM5;CORIN;CLVS1;ZNF462;FARP1;MYEF2;NTRK3;CADPS;SYT16;PDE4DIP;PPP2R3A;KIAA0232;CDC42BPA;MAPK10;PDE10A;NFIA;NFIB;KLHL7;ASXL3;TTC3;YPEL1;TACC2;HCN1
SATB2 human tf ARCHS4 coexpression	103/299	5.3826378970828905E-20	ZFYVE9;FRMPD4;MYT1L;ANKRD36;SLC8A1;EPS8;CDH4;KIF21A;ANKS1B;SOX5;PPFIA2;RGS7;ANKRD36C;KCNH5;TMEM178B;CACNA2D1;TMOD2;MAGI2;FRMD4A;ANK3;SHISA9;EML1;TANC2;TIAM2;GAREM1;ULK2;ASTN2;DGKI;SHC3;CTTNBP2;TNKS;RASAL2;KALRN;NKAIN2;FLRT2;FUT9;ZNF704;PLXNA2;ANKRD20A5P;ST8SIA5;PLXNA4;ARPP21;CADM2;MYO5A;GRIN2B;CORO2B;DCLK1;PTPRD;CCDC88A;DLG2;DAB1;NBEA;SYNJ1;SPIRE1;FAT3;CCSER1;CNTN3;TCF4;SCN2A;RGL1;SLC44A5;ROBO2;SETD2;DOCK3;TENM4;PTPRO;NHSL1;GRM7;TRIM2;TMEM108;PHACTR3;HYDIN2;DLGAP1;DLGAP2;NEO1;SLC2A13;COBL;FAM126B;ARID1B;ITFG1;DNM3;MMP16;MPPED1;NAV3;LRRC7;SHANK2;LUZP2;RGPD6;ATP10B;MIPOL1;MAPK8;NCAM1;PAK5;ATP9A;NTRK3;KIAA1549L;LHFPL3;C1ORF21;TTLL7;APC;ASXL3;PTPN4;CCDC171
PBX1 human tf ARCHS4 coexpression	103/299	5.3826378970828905E-20	ATP8A2;MYT1L;CTNND2;KLHL32;ZBTB20;ADARB2;RPS6KA5;ZNF608;CDH2;DPYSL5;PEG10;KIF21A;KIF21B;EPHB2;LARGE1;MAGI1;RBFOX2;TMEM178B;CACNA2D1;MAGI2;FRMD4A;ANK3;SHISA9;FOXP2;TANC2;PYGO1;WDPCP;WDFY3;ASTN2;XPR1;DGKI;IGSF3;CHRNA7;NTM;CACNA1C;NREP;KALRN;NPAS3;PGBD5;ZNF704;CAMTA1;SRGAP3;ASIC2;ZNF423;MPDZ;PLXNA4;CADM1;AUTS2;ST8SIA2;TMEM132B;NETO2;GRIN2B;DCLK1;PTPRD;CCDC88A;NBEA;GNAQ;FAT3;GABRB3;ROBO2;DRAXIN;DOCK3;TENM4;PTPRO;CELF4;ELAVL4;ROBO1;TRIM2;ADAMTSL3;ZNF385D;NEO1;LRRC49;GFRA1;NAV2;SORCS3;PGM2L1;NAALADL2;AJAP1;LRRC7;PPP1R12B;PPM1L;MIPOL1;STOX2;CECR2;GTF2IP1;KIAA1328;CTNNA2;PAK3;CSMD1;PAK5;ATP9A;ZNF462;FARP1;MBD5;KCNJ6;MYEF2;NTRK3;LSAMP;MAPK10;NEDD4;TTC3;ASB4;ADGRL2
RORB human tf ARCHS4 coexpression	103/299	5.3826378970828905E-20	ATP8A2;FRMPD4;MYT1L;CTNND2;SLC8A1;RIMS2;RPH3A;RIMS1;PRKACB;SH3GL2;ANKS1B;PPFIA2;KCNH1;RGS7;UNC13C;RBFOX1;KCNH5;TMOD2;ANK3;GABRG2;GABRG1;ADGRB3;SCN8A;HECW1;DGKI;ASTN1;NECAB1;SHC3;STXBP1;SLC1A2;KALRN;NALCN;GRIN2A;DPP6;PGBD5;FUT

			9;ST8SIA5;CAMTA1;SRGAP3;PLXNA4;NDFIP1;DTNA;SYT1;CADM2;SLC4A10;MYO5A;GRIN2B;DCLK1;SNAP91;PTPRD;DLG2;SYNJ1;CNTN1;SCN2A;GABRB3;PTPRT;DOCK3;KCNC1;OTUD7A;UNC80;TRIM9;GRM5;AKAP11;TRIM2;PSD3;DLGAP1;DLGAP2;MYRIP;SYN2;AJAP1;CNKSR2;DNM3;MPPED1;LRRC7;RAPGEF5;PLCB1;RAPGEF4;SLC24A2;PPM1L;KIAA0513;SV2B;CLVS2;NCAM1;CTNNA2;PDE6A;PAK3;ATP9A;WASF3;OPCML;GABBR2;ARNT2;ATRNL1;NTRK3;CADPS;LSAMP;SYT16;KIAA1549L;ATP2B2;TTLL7;APC;PPP2R2C;PPP2R2B;HCN1
MEIS2 human tf ARCHS4 coexpression	101/299	5.978116687893467E-19	ERO1B;MYT1L;CTNND2;ZBTB20;SLC8A1;HS6ST3;SCGN;CDH4;ZNF608;DACH1;KIF21A;KIF21B;RGS8;SOX5;MAGI1;RBFOX2;KCNH5;TMEM178B;CACNA2D1;TMOD2;MAGI2;MTUS2;FRMD4A;ANK3;SHISA9;EML1;TANC2;WDPCP;ASTN2;DGKI;CTTNBP2;RASAL2;NREP;NYAP2;KALRN;FLRT2;FUT9;ZNF704;PLXNA2;STXBP6;SRGAP3;PLXNA4;ZFHX3;PTPRN2;CADM1;AUTS2;NEBL;BTBD9;GRIN2B;LRP1B;PTPRD;RALYL;PLCXD3;FGF14;NBEA;RNF182;SPIRE1;CPE;LHX9;GABRB3;ROBO2;DRAXIN;TENM4;ELAVL4;GLI3;ROBO1;UNC80;GRM7;TRIM2;MCF2L;PHACTR3;HYDIN2;ABCC8;NAV3;LRRC7;CNKSR3;MPPED2;MYO3A;RGPD5;NOL4;MIPOL1;GNG2;NCAM1;CSMD3;PAK3;MBD5;MYEF2;ANKRD30BL;NTRK3;CADPS;LSAMP;SYT16;MAPK10;MEIS1;PDE10A;APC;PPP2R2B;NEDD4;ASXL3;TTC3;ASB3
TRIM23 human tf ARCHS4 coexpression	101/299	5.978116687893467E-19	ATP8A2;ATP8A1;FRMPD4;MYT1L;DGKB;CTNND2;RPH3A;SYNPR;AKT3;KIF21A;PRKACB;SH3GL2;ANKS1B;PPFIA2;KCNH1;RBFOX1;CACNA2D1;TMOD2;CACNA2D3;SCAMP1;GABRG2;GABRG1;ADGRB3;SCN8A;FAR1;KCNQ5;WDFY3;ASTN1;NECAB1;RTN1;STXBP1;SLC1A2;NALCN;GRIN2A;FUT9;NDFIP1;DTNA;SYT1;CADM2;SLC4A10;MYO5A;BTBD10;CORO2B;DCLK1;SNAP91;PTPRD;DLG2;FGF14;NBEA;SYNJ1;RCAN2;CNTN1;SCN2A;CDK14;FGF12;CPEB4;GABRB3;DPP10;RNF11;DOCK3;TRIM9;GRM5;AKAP11;TRIM2;PSD3;DLGAP1;EDIL3;KCND2;PDE4D;SYN2;PJA2;CNKSR2;DNM3;RAPGEF2;RAPGEF5;PLCB1;RAPGEF4;SLC24A2;PPM1L;NRXN3;AKAP6;KIAA0513;SV2B;CLVS2;SPOCK1;NCAM1;CTNNA2;NCAM2;GPR158;ATP9A;OPCML;ARNT2;CA10;ATRNL1;SYT16;KIAA1549L;ATP2B2;TTLL7;APC;PPP2R2C;PPP2R2B
TCF4 human tf ARCHS4 coexpression	100/299	1.9691704253345607E-18	MYT1L;ANKRD36;SLC8A1;SRGAP2C;ZNF608;ZSCAN30;AKT3;KIF21A;SCAPER;SRGAP2B;ANKS1B;PPFIA2;MAGI1;ANKRD36C;RBFOX2;MAGI2;RFX3;SHISA9;TIAM2;ADGRB3;DOK5;KCNQ3;ASTN2;GRIA1;CTTNBP2;NTM;PCDH15;RASAL2;NREP;NYAP2;KALRN;NPAS3;NKAIN2;FLRT2;SNTG1;FUT9;ZNF704;ST8SIA5;SRGAP3;PLXNA4;GRIA4;ST8SIA2;CORO2B;DCLK1;PTPRD;CCDC88A;FER;DLG2;NBEA;ZNF536;FAT3;CCSER1;SCN2A;ROBO2;TENM4;CHD9;PTPRO;ELAVL4;SLC35F1;AFF3;BICD1;NHSL1;GRM7;LRRTM4;TRIM2;DLGAP1;TCF12;FAM126B;ZDHHC17;ARID1B;MMP16;MPPED1;NAV3;IL1RAPL2;MPPED2;VSTM2A;DSCAML1;CRB1;NRXN3;MIPOL1;STOX2;CLVS2;NCAM1;PAK5;RFTN2;ZNF462;MBD5;MYEF2;NTRK3;KIAA1549L;TTLL7;NFIA;APC;NFIB;KLHL7;ASXL3;TTC3;PTPN4;SSBP2;ASB3
KIAA1549 human tf ARCHS4 coexpression	99/299	5.997659546927141E-18	ATP8A2;MYT1L;CTNND2;CDH4;ZNF608;CDH2;DPYSL5;AKT3;KIF21A;RGS8;ANKS1B;PPFIA2;MAGI1;RBFOX2;TMEM178B;CACNA2D1;MAGI2;RFX3;FRMD4A;ANK3;SHISA9;TANC2;IFT81;HECW1;KCNQ3;XPR1;SHC3;IGSF3;CHRNA7;NTM;RASAL2;NREP;KALRN;CACNA1E;NPAS3;PHF21B;FUT9;ZNF704;PLXNA2;CAMTA1;SRGAP3;MPDZ;PLXNA4;AUTS2;ST8SIA2;PTCH1;NETO2;GRIN2B;DCLK1;PBX1;PTPRD;CCDC88A;DLG2;NBEA;DLG5;FAT3;TCF4;SCN2A;GABRB3;PTPRT;ROBO2;DRAXIN;TENM3;TENM4;PTPRO;CELF4;ELAVL4;GRIK2;ROBO1;GRM7;TRIM2;ADAMTSL3;TMEM108;DCC;LRRC49;HUNK;PGM2L1;NAV3;LRRC7;ARHGEF7;AKAP6;STOX2;MAPK8;GNG2;NCAM1;CSMD2;PAK3;P

			AK5;ARNT2;ZNF462;FARP1;MBD5;MYEF2;NTRK3;LSAMP;KIAA1549L;APC;ZNF618;ASXL3
SLC4A10 human tf ARCHS4 coexpression	99/299	5.997659546927141E-18	*ATP8A1;FRMPD4;MYT1L;DGKB;LDLRAD4;SLC8A1;HS6ST3;RPH3A;SYNPR;SAMD12;PRKACB;SH3GL2;ANKS1B;PPFIA2;KCNH1;RGS7;UNC13C;RBFOX1;PRKCE;TMOD2;GABRG2;GABRG1;PCP4;ADGRB3;SCN8A;KCNQ3;NGEF;NECAB1;SHC3;STXBP1;SLC1A2;KALRN;NALCN;GRIN2A;DPP6;PGBD5;PTPRN2;NDFIP1;SYT1;CADM2;MYO5A;GRIN2B;CORO2B;DCLK1;SNAP91;LRP1B;DLG2;SYNJ1;RCAN2;CNTN1;CPE;SCN2A;FGF12;GABRB3;DOCK3;RASGRF2;UNC80;TRIM9;GRM5;AKAP11;TRIM2;PSD3;DLGAP1;KCND3;SLC2A13;MYRIP;SYN2;PJA2;AJAP1;CNKSR2;DNM3;LRRC7;KCNMA1;RAPGEF5;PLCB1;RAPGEF4;SLC24A2;KIAA0513;SV2B;CLVS2;CTNNA2;NCAM2;GPR158;ATP9A;OPCML;GABBR2;ARNT2;CA10;ATRNL1;NTRK3;KCNIP4;SYT16;KIAA1549L;ATP2B2;TTLL7;MAPK10;PPP2R2C;PPP2R2B;HCN1*
MACF1 human tf ARCHS4 coexpression	98/299	1.945964858218302E-17	*TRIO;MAML2;ATP8A1;RORA;FRY;DOCK10;PTAR1;HERC2;HERC1;MPRIP;PIEZO2;SACS;ANKFY1;DIP2B;MAP3K5;MBNL1;FNDC3B;TANC2;TANC1;WDFY3;UTRN;NOTCH2;KMT2C;ITPR2;IQGAP1;CACNA1C;LPP;PCNX1;ATXN1;ZNF704;HIVEP2;CREBBP;LRBA;DENND4C;EXOC6B;MICAL3;MYO5A;LNPEP;DNAJC13;ARHGAP26;SMARCA2;MED13L;PTPRB;DMXL2;FAT1;CCSER2;DOCK4;DOCK9;DOCK8;RASGRF2;PTPRM;LIMD1;LYST;MYSM1;SYNE2;AKAP13;NIPBL;C16ORF72;AKAP11;ZNF407;KIF13A;RBM33;ARHGEF12;ITGA4;DST;VPS13C;ARAP2;VPS13B;URB1;ARID1B;NCOR1;PEAK1;BIRC6;PPP1R12B;DOCK2;DOCK1;KDM7A;NFAT5;WDR26;HERC2P2;BTAF1;KIAA1328;HECTD1;HECTD4;ZNF106;ARFGEF1;USP24;SPEN;MON2;TRAPPC10;MGA;ERBIN;YLPM1;ADAM32;MYO9A;MTOR;TJP1;SLMAP*
ASH1L human tf ARCHS4 coexpression	97/299	5.894800713503227E-17	*PATJ;TRIO;ATP8A1;ANKRD36;RORA;FRY;HERC2;HERC1;AKT3;DIP2B;TMOD2;TANC2;WDPCP;WDFY3;ASTN2;UTRN;MACF1;KMT2C;ITPR2;CACNA1C;KALRN;CACNA1E;LPP;PCNX1;PDZD2;ZNF704;TRPM7;HIVEP2;BPTF;CREBBP;LRBA;DENND4C;MICAL3;MYO5A;LNPEP;ARHGAP26;SMARCA2;PHC3;GRIN2B;MED13L;ARHGAP32;DMXL2;CDK12;SETD2;DOCK3;DOCK9;RASGRF2;LYST;MYSM1;SYNE2;UNC80;AKAP13;NIPBL;C16ORF72;AKAP11;ADAMTSL3;ZNF407;PSD3;ERC1;RBM33;ARHGEF12;KCND2;DST;VPS13C;ARAP2;VPS13B;ARID1B;NCOR1;PEAK1;BIRC6;RAPGEF5;PLCB1;NFAT5;MIPOL1;BTAF1;KIAA1328;HECTD1;HECTD4;ATP9B;ARFGEF1;USP24;SPEN;MBD5;MON2;MGA;ZNF804B;ERBIN;YLPM1;ADAM32;PDE4DIP;MYO9A;GATAD2B;TTLL7;APC;KANSL1;NEDD4;SLMAP*
NPAS3 human tf ARCHS4 coexpression	97/299	5.894800713503227E-17	*APP;CTNND2;ZBTB20;CDH4;DACH1;CDH2;DPYSL5;PEG10;AKT3;KIF21A;SOX6;SRGAP2B;ANKS1B;MAGI1;TMEM178B;TMOD2;MAGI2;RFX3;KAZN;FRMD4A;PYGO1;IFT81;ADGRB3;TNIK;ASTN1;GRIA1;SHC3;NTM;ILDR2;NREP;DPP6;NKAIN3;CDH20;FUT9;CAMTA1;SRGAP3;MPDZ;NDFIP1;DTNA;CADM1;AUTS2;CADM2;ST8SIA1;PXDNL;NETO2;CORO2B;DCLK1;PBX1;PTPRD;CCDC88A;SDK1;CNTN1;FAT3;TCF4;SPAG16;DPP10;DRAXIN;TENM3;GRIK4;SLC35F1;FMN2;SIPA1L2;GRM3;TRIM9;GRM5;TRIM2;TMEM108;ADGRV1;KCND3;LRRC49;SEZ6L;PARD3B;NRG3;MPPED2;LUZP2;STOX2;GNG2;TMEM67;LRIG1;NCAM1;CTNNA2;PAK3;JAM2;ATP9A;ARNT2;ZNF462;NTRK2;MYEF2;NTRK3;KIAA1549L;TTLL7;MAPK10;APC;PPP2R2B;FABP7;SMOC1;TTC3*
FAM171B human tf ARCHS4 coexpression	95/299	6.190821108679311E-16	*GABRB3;SPAG16;ROBO2;SEMA5A;DPP10;ERO1B;FRMPD4;DGKB;SLC35F1;PTPRG;TIAL1;PCMTD2;SRGAP2C;SYNPR;TRIM9;GRM5;NHSL1;LRRTM4;TRIM2;TLK1;KIF21A;ZNF568;SAMD12;PRKACB;DLGAP2;EDIL3;SRGAP2B;ANKS1B;PCMTD1;PPFIA2;EPHA7;WSB1;SLC2A13;MAGI2;RFX3;SEZ6L;GABRG2;PJA2;CNKSR2;ADGRB3;LRRC7;MPPED2;KCNQ3;PLCB1;SLC24A2;NECAB1;NLGN1;SAR1A;CTTN*

			BP2;ATL1;NRXN1;BTF3L4;RAP1GDS1;EFNA5;NOL4;NALCN;SDCBP;GRIN2A;SNTG1;FUT9;NCAM2;GPR158;AKAIN1;RFTN2;MYEF2;CADM1;SYT1;CADM2;INSR;NTRK3;CADPS;SLC4A10;SYT16;CDC42BPA;OXR1;SNAP91;TTLL7;PTPRD;IGSF11;FGF14;NFIA;NBEA;APC;NFIB;PPP2R2B;TTC3;CNTN1;CPE;TCF4;ASB4;SCN2A;PTPN4;SSBP2;FGF12;CPEB4
PKNOX2 human tf ARCHS4 coexpression	94/299	1.912843891850123E-15	*ROBO2;DOCK3;TENM4;CTNND2;PTPRO;GRIK3;RORB;ADARB2;MYLK3;ROBO1;RIMS1;RPS6KA5;ZNF608;GRM7;CA5A;TRIM2;ADAMTSL3;MCF2L;PSD3;TNR;DLGAP1;KIF21B;ZNF385D;NEO1;SOX5;PPFIA2;RBFOX1;EPHA6;RBFOX2;KCNH5;TMEM178B;CACNA2D1;TMOD2;FRMD4A;ABCC9;ANK3;SORCS3;SHISA9;KLF15;TANC2;AJAP1;MPPED1;NAV3;LRRC7;PEAK1;CNKSR3;WDPCP;DGKI;SHC3;STXBP1;ILDR2;KALRN;DPP6;KIAA1328;FLRT2;FUT9;ZNF704;HECTD4;NCAM1;SRGAP3;PAK3;CSMD1;ATP9B;PLXNA4;ATP9A;ARNT2;FARP1;ZFHX3;AUTS2;NEGR1;ANKRD30BL;TMEM132B;NTRK3;MICAL3;LSAMP;YLPM1;KIAA1549L;MYO5A;GRIN2B;DCLK1;PBX1;PTPRD;CCDC88A;DLG2;DAB1;PDE10A;NBEA;APC;PPP2R2B;NEDD4;FAT3;RGS12;ASB3;FBXL7*
NFIA human tf ARCHS4 coexpression	92/299	1.838184372535877E-14	*ROBO2;CNTNAP2;TENM4;MYT1L;ANKRD36;CLCN3P1;PTPRO;GRIK3;ELAVL4;SLC35F1;GRIK2;EFCAB6;BICD1;SYNE2;PTPRG;CDH7;SRGAP2C;NHSL1;ADAMTS3;RASGEF1B;LRRTM4;AKT3;TMEM108;PHACTR3;KIF21A;SRGAP2B;SOX5;GARNL3;PPFIA2;RGS7;EPHA7;ZNF160;LRRC49;MAGI2;RFX3;KAZN;CDKAL1;FRMD4B;ENAH;ADGRB3;LRRC7;HECW1;MPPED2;KCNQ3;RAPGEF2;ZFPM2;ZNF234;XPR1;SHANK2;GRIA1;SLC24A2;CRB1;CTTNBP2;BTF3L4;NYAP2;NOL4;NPAS3;SNTG1;PLXNA2;SRGAP3;JAM2;PAK5;OPCML;RFTN2;ZNF462;MYEF2;BCL11B;ST8SIA1;INSR;NTRK3;SYT16;SORBS2;PPP2R3A;KIAA0232;CORO2B;NBEAP1;ST18;PTPRD;CCDC88A;DAB1;NBEA;APC;PPP2R2B;AGO1;TTC3;FAT3;CNTN3;TCF4;ASB4;FGF12;LRP12;HCN1*
NFAT5 human tf ARCHS4 coexpression	91/299	4.989038209990635E-14	*DIDO1;DOCK5;SETD2;TRIO;MAML2;DOCK9;KIAA1671;PTPRM;RORA;LIMD1;MYSM1;SYNE2;AKAP13;NIPBL;HERC2;C16ORF72;AKAP11;HERC1;MPRIP;SH3PXD2A;ADAMTSL3;ZNF407;KIF13A;RBM33;ARHGEF11;MBNL1;MBNL2;ARHGEF12;DST;VPS13C;FNDC3B;ARAP2;VPS13B;ARID1B;TANC2;PATL1;TANC1;NCOR1;PEAK1;WDPCP;WDFY3;BIRC6;ASTN2;UTRN;KDM7A;NOTCH2;MACF1;WDR26;KMT2C;ITPR2;IQGAP1;NLK;LPP;MIPOL1;ATXN3;PCNX1;SEC14L1;ATXN1;KIAA1328;HECTD1;HECTD4;HIVEP2;ATP9B;ARFGEF1;USP24;SPEN;FARP1;CREBBP;MON2;LRBA;TRAPPC10;DENND4C;MICAL3;ERBIN;YLPM1;ADAM32;LNPEP;ARHGAP26;SMARCA2;PHC3;MYO9A;GATAD2B;MED13L;TJP1;KANSL1;NEDD4;SLMAP;AGO2;DMXL2;FAT1;CDK12*
AFF3 human tf ARCHS4 coexpression	91/299	4.989038209990635E-14	*PTPRT;DPP10;CCDC122;MYT1L;ANKRD36;CTNND2;DPY19L2P2;LDLRAD4;EFCAB6;FRY;SLC8A1;CEP128;UNC80;POTEKP;CCDC91;GRM7;ZSCAN30;PPIP5K2;TRIM2;ADAMTSL3;AKT3;PRKACB;ANKS1B;PPFIA2;POTEC;ANKRD36C;KCND2;PRKCB;PRKCE;TMOD2;MAGI2;VPS13B;TBC1D9;FRMD4B;SHISA9;ARID1B;GAREM1;MPPED1;BANK1;LRRC7;HECW1;KCNQ3;WDPCP;ASTN2;ZMYND11;ANKRD36B;BLK;GRIA1;AKAP6;NREP;LRP2;KALRN;MIPOL1;NPAS3;KIAA1328;GNG7;HECTD4;CLVS2;ANKRD36BP2;NCAM1;SRGAP3;ATP9B;PLXNA4;NTRK2;MBD5;CEP112;AUTS2;SYT1;CADM2;ST8SIA2;NTRK3;SETDB2;MYO5A;ADAM32;PARP15;GRIN2B;CORO2B;DCLK1;TTLL7;PTPRD;CCDC88A;DLG2;SYNJ1;TTC3;NEK10;YPEL1;TCF4;SSBP2;CDK14;ASB3;TNRC6B*
POGZ human tf ARCHS4 coexpressi	91/299	4.989038209990635E-14	*GABRB3;RERE;CNTNAP2;ANKRD36;CHD9;GADL1;AFF3;SYNE2;IGF1R;TIAL1;DTWD2;UNC80;HERC2;ZNF608;LIPI;TRIM2;ADAMTSL3;MCF2L;AKT3;TLK1;KIF21A;NPIPA1;NEO1;SRGAP2B;POTEC;MAGI1;ANKRD36C;HFM1;VPS1*

on			3B;NAV2;SHISA9;SEZ6L;ARID1B;FOXP2;TANC2;NAV3;PEAK1;WDPCP;FAM193A;ASTN2;XPR1;SHANK2;MACF1;NFAT5;TNKS;KMT2C;NRXN1;RGPD6;RGPD5;KALRN;FAM214A;MIPOL1;ATXN3;KIAA1328;ZNF704;CECR7;HECTD4;GSE1;TRPM7;MPDZ;BPTF;SPEN;ZNF462;FARP1;CREBBP;MYEF2;AUTS2;MGA;RANBP17;CADPS;MICAL3;YLPM1;ADAM32;CDC42BPA;PHC3;GATAD2B;PTPRD;MLLT10;NFIA;NBEA;KANSL1;NFIB;AGO1;DLG5;AGO2;TTC3;TCF4;ASB4;EIF4G3;ASB3;TNRC6B
MBNL2 human tf ARCHS4 coexpression	90/299	1.462083056152094E-13	RNF11;ATP8A1;FRMPD4;DOCK9;PTPRM;RORA;PTPRK;RPH3A;SYNPR;RPS6KA3;EFR3A;AKAP11;UBL3;TRIM2;KIF13A;PSD3;PRKACB;EDIL3;SH3GL2;PCMTD1;KCNH1;CAST;RBFOX1;ARHGEF12;DST;PRKCE;SLC2A13;TMOD2;MTUS1;ARAP2;COBL;MYRIP;SYN2;GABRG2;GABRG1;DNM3;TOM1L2;TANC1;KCNMA1;WDFY3;PLCB1;NGEF;RAPGEF4;SLC24A2;NECAB1;NFAT5;STXBP1;SEL1L;SLC1A2;NALCN;RAP1GAP;KIAA0513;GRIN2A;ABLIM1;PDZD2;SV2B;SPOCK3;SPOCK1;CTNNA3;GPR158;ATP9A;OPCML;NDFIP2;ABCA5;CADM2;ATRNL1;SAMD4A;CRIM1;SLC4A10;SYT16;KIAA1549L;MYO5A;PDE4DIP;ATP2B2;SYNJ2;GNG12;ELL2;SNAP91;TTLL7;TJP1;ARHGAP32;PTPRB;DLG2;SYNJ1;PPP2R2C;RCAN2;CNTN1;CPE;SCN2A;CPEB4
PLXNA2 human tf ARCHS4 coexpression	89/299	4.2232694123581743E-13	APP;DOCK4;TENM4;MYT1L;DOCK9;CTNND2;MAST2;GRIK3;PTPRM;ECE1;SIPA1L2;ROBO1;CDH4;MPRIP;DPYSL5;PEG10;TRIM2;MCF2L;AKT3;PHACTR3;KIF21B;EPHB2;NEO1;PPFIA2;MAGI1;RBFOX1;RBFOX2;TMEM178B;CACNA2D1;TMOD2;AFAP1;KAZN;FRMD4A;FAM219A;NAV2;TANC2;MAPK8IP1;MPPED1;COL4A2;LRRC7;HECW2;SCN8A;KCNQ3;WDFY3;TNIK;XPR1;GRIA1;SHC3;IGSF3;STXBP1;NREP;KALRN;FAM171A1;CTIF;STOX2;GNG2;ZNF704;HECTD4;EPB41L3;GTF2IP4;MVB12B;NCAM1;CTNNA2;SRGAP3;PAK5;PLXNA4;ATP9A;MAP4K4;ARNT2;NDFIP1;AUTS2;SYT1;ST8SIA2;NTRK3;KIAA1549L;NETO2;CORO2B;DCLK1;SNAP91;SGSM1;PTPRD;SDK1;PTPRB;APC;NFIB;DLG5;TTC3;SPIRE1;APBA2
SOX6 human tf ARCHS4 coexpression	86/299	9.646149406694208E-12	SLC44A5;SPAG16;ROBO2;SEMA5A;OSCP1;CCDC122;CHD9;GADL1;ZBTB20;SLC35F1;EFCAB6;RYR3;MYLK3;UNC80;SRGAP2C;NHSL1;DPYSL5;ZSCAN30;TRIM2;ADAMTSL3;SNAPC3;TNR;NOS1;DENND2C;WSB1;TMEM178B;LRRC49;TCF12;MAGI2;RFX3;FRMD4A;ABCC9;PIAS2;EPN2;MMP16;IFT81;ADGRB3;ASTN2;ZFPM2;ZNF234;ASTN1;CRB1;TNKS;LUZP2;PDE1A;PCDH15;NRXN3;TPTE2P2;ILDR2;NOL4;MIPOL1;NPAS3;FGD4;GNG2;KIAA1328;SNTG1;FUT9;ANKRD36BP2;SRGAP3;JAM2;PAK5;GRIA4;GABRA2;RFTN2;SPECC1;ATF7IP;RIC3;ZNF462;MBD5;ANKRD26;SLC14A2;NTRK3;LSAMP;PDE4DIP;LHFPL3;ARHGAP28;DCDC1;MAPK10;CCDC88A;APC;KLHL7;TTC3;TCF4;ASB4;ASB3;CPEB4
ZNF236 human tf ARCHS4 coexpression	86/299	9.646149406694208E-12	RERE;DIDO1;SETD2;USP33;ZBTB21;SMG1P5;RORA;LYST;SYNE2;AKAP13;NIPBL;PTAR1;C16ORF72;AKAP11;HERC1;PPIP5K2;ADAMTSL3;ZNF407;RTTN;ERC1;POTEC;RBM33;MBNL1;USP7;DST;VPS13C;ARAP2;VPS13B;ARID1B;FAM153A;WDFY3;BIRC6;ASTN2;UTRN;KDM7A;CFAP61;MACF1;NFAT5;WDR26;ROCK1;KMT2C;RNF38;RGPD6;RGPD8;LPP;MIPOL1;FGD4;PCNX1;SCAF8;GRK3;ATXN1;KIAA1328;HECTD1;HECTD4;ADAMTS17;ANKRD36BP2;HIVEP2;ATP9B;BPTF;LRRC37A3;ARFGEF1;SPEN;USP25;CREBBP;MBD5;MON2;LRBA;TRAPPC10;MGA;DENND4C;ERBIN;YLPM1;ADAM32;LNPEP;DNAJC13;ARHGAP26;SMARCA2;MYO9A;MED13L;ARHGAP32;TTLL5;DMXL2;APLF;CDK12;EIF4G3;TNRC6B
ST18 human tf ARCHS4 coexpression	85/299	2.4522380337575943E-11	PCSK2;GABRB3;ROBO2;ERO1B;MYT1L;DGKB;ELAVL4;GRIK2;EFCAB6;BICD1;CDH7;RIMS2;UNC80;DPYSL5;RASGEF1B;TRIM2;MCF2L;AKT3;HYDIN2;KIF21A;EDIL3;SH3GL2;ANKS1B;EPHA7;KCND3;DCC;LRRC49;MAGI2;MTUS2;EBF2;CDKAL1;SEZ6L;SHISA6;HECW1;KCNQ3;XPR1;SL

			C24A2;CRB1;PPM1L;TNKS;STXBP1;ATL1;BTF3L4;NRXN3;AKAP6;NREP;NYAP2;KALRN;NOL4;NALCN;MTMR7;DPP6;SNTG1;CLVS2;NCAM1;CTNNA2;CSMD3;SRGAP3;PAK3;MBD5;PTPRN2;MYEF2;CADM1;PCDH9;NTRK3;CADPS;SLC4A10;SYT16;KLHL3;SORBS2;SNAP91;TTLL7;PTPRD;MAPK10;CCDC88A;PLCXD3;NFIB;ASXL3;TTC3;CNTN1;CPE;APBA2;ASB3;CPEB4;HCN1
ZFPM2 human tf ARCHS4 coexpression	85/299	2.4522380337575943E-11	*SLC44A5;OSCP1;CCDC122;CNTNAP2;MYT1L;DPY19L2P2;GADL1;GRIK2;EFCAB6;MYLK3;GPHN;RPH3A;UNC80;SYNPR;ADAMTS3;LRRTM4;TRIM2;ADAMTSL3;ZNF407;AKT3;PSD3;DPF3;DLGAP1;NOS1;ERC1;SOX6;DIP2C;SRGAP2B;SH3GL2;PPFIA2;POTEC;UNC13C;RBFOX1;KCND3;CACNA2D1;TMOD2;KAZN;SHISA9;FAM126A;SYN2;CNKSR2;LRRC7;HECW1;KCNQ3;PLCB1;SHANK2;GRIA1;NECAB1;NLGN1;SHC3;STXBP1;KALRN;MIPOL1;STOX2;GRIN2A;HECTD1;SV2B;CECR7;CLVS2;RFPL3S;OPCML;SLC14A2;SPHKAP;GABRA6;SYT1;CADM2;ST8SIA1;INSR;NTRK3;KIAA1549L;ADAM32;PDE4DIP;ATP2B2;MYO9A;SNAP91;TTLL7;FER;NFIA;NFIB;NEDD4;TTC3;SPIRE1;SCN2A;LRP12;CPEB4*
ZNF385D human tf ARCHS4 coexpression	85/299	2.4522380337575943E-11	*ROBO2;DPP10;CCDC122;ATP8A2;MYT1L;ANKRD36;C2ORF88;MAP3K7CL;CELF4;SLC8A1;MYLK3;RIMS2;UNC80;GRM5;GRM7;CA5A;TRIM2;ADAMTSL3;PHACTR3;DLGAP1;ANKS1B;SOX5;GARNL3;KCNH5;LRRC49;TMOD2;MAGI2;ABCC9;ANK3;SHISA9;AJAP1;CNKSR2;FRMD3;DNM3;LRRC7;HECW1;ASTN2;PLCB1;ZNF397;SLC24A2;PPM1L;ATL1;NTM;KALRN;CACNA1E;MIPOL1;GRIN2A;DPP6;KIAA1328;FLRT2;PGBD5;FUT9;CLVS2;CAMTA1;NCAM1;CSMD3;PAK3;ASIC2;MORC1;CSMD1;PLXNA4;GABBR2;RIC3;SYT1;NEGR1;CADM2;NTRK3;LSAMP;SLC4A10;SYT16;ADAM32;GRIN2B;DCLK1;PBX1;SNAP91;TTLL7;CCDC88A;AGBL4;GFI1B;DLG2;PDE10A;PPP2R2B;GNAQ;SCN2A;ASB3*
FOXG1 human tf ARCHS4 coexpression	84/299	6.576674901778489E-11	*SLC44A5;ROBO2;TENM4;MYT1L;ANKRD36;CTNND2;GRIK3;ELAVL4;RORB;BICD1;GLI3;SYNE2;GLI2;TTC28;CDH4;NHSL1;CDH2;DPYSL5;LRRTM4;TRIM2;AKT3;LUC7L;KIF21A;SOX6;SOX5;PPFIA2;RGS7;ANKRD36C;RBFOX2;LRRC49;MAGI2;RFX3;EML1;ENAH;C12ORF4;GAREM1;MPPED1;IFT81;LRRC7;HECW1;MPPED2;KCNQ3;AMPH;XPR1;ASTN1;GRIA1;CRB1;PHLPP1;SHC3;CTTNBP2;TNKS;NTM;NREP;KALRN;PHF21B;STOX2;FUT9;ZNF704;NCAM1;CTNNA2;SRGAP3;MPDZ;PAK5;PLXNA4;MYEF2;ST8SIA1;ST8SIA2;NTRK3;CORO2B;ST18;TTLL7;PTPRD;NELL2;CCDC88A;DLG2;DAB1;NFIA;NBEA;APC;NFIB;KLHL7;TTC3;RNF182;TCF4*
POU3F2 human tf ARCHS4 coexpression	83/299	1.7363996020301883E-10	*SLC44A5;ROBO2;DRAXIN;TENM4;MYT1L;CTNND2;ELAVL4;LDLRAD3;ROBO1;GRM3;CDH4;DACH1;CDH2;DPYSL5;TRIM2;DNER;KIF21A;EPHB2;MAGI1;ANKRD36C;WSB1;ADGRV1;TMEM178B;DCC;LRRC49;TCF12;HUNK;RFX3;FRMD4A;MPPED1;ADGRB3;LRRC7;TNKS;CHRNA7;NTM;ILDR2;NREP;KALRN;NPAS3;PHF21B;STOX2;GTF2IP1;NKAIN3;GNG2;ZNF704;PLXNA2;CAMTA1;ZSWIM6;NCAM1;CTNNA2;SRGAP3;PAK3;JAM2;PAK5;PLXNA4;ATP9A;MAP4K4;ARNT2;MYEF2;ST8SIA1;ST8SIA2;PTCH1;NTRK3;NETO2;CORO2B;DCLK1;PBX1;ENOX1;TTLL7;PTPRD;MAPK10;NELL2;CCDC88A;FABP7;ASXL3;TTC3;YPEL1;RNF182;FAT3;TCF4;RGS12;APBA2;ADGRL2*
CUX2 human tf ARCHS4 coexpression	82/299	4.037540535965851E-10	*SLC44A5;RERE;DOCK3;TENM4;FRMPD4;MYT1L;ANKRD36;CTNND2;SLC8A1;RPH3A;CDH4;ZNF608;GRM7;DPYSL5;TRIM2;MCF2L;PHACTR3;KIF21A;DLGAP1;KIF21B;NEO1;PPFIA2;ANKRD36C;RBFOX1;RBFOX2;KCNH5;TMEM178B;CACNA2D1;TMOD2;ANK3;MYRIP;SYN2;MAPK8IP1;MPPED1;LRRC7;SCN8A;ARHGEF7;DSCAML1;NGEF;SHANK2;ASTN1;SHC3;CTTNBP2;STXBP1;KALRN;CTIF;SV2B;FUT9;ZNF704;HECTD4;PLXNA2;CLVS2;NCAM1;CTNNA2;SRGAP3;PAK5;ATP9A;GABBR2;ARNT2;AUTS2;SYT1;CADM2;NTRK3;KIAA1549L;MYO5A;ATP2B2;GRIN2B;CORO2B;DCLK*

			1;PBX1;SNAP91;PTPRD;DLG2;NBEA;APC;PPP2R2C;PPP2R2B;ASXL3;FAT3;TCF4;SCN2A;APBA2
GLIS3 human tf ARCHS4 coexpression	82/299	4.037540535965851E-10	SPAG16;APP;NRP1;ERO1B;TRIO;TENM3;MAML2;TUSC3;WWC1;PTPRM;ZBTB20;ECE1;FMN2;SLC4A4;IGF1R;SCGN;C1ORF127;ALCAM;CDH2;PEG10;HYDIN;TEAD1;NEO1;DST;IL1R1;ABCC8;ANXA4;FNDC3B;AFAP1;RFX3;ARID5B;FNDC3A;NAV2;STON1-GTF2A1L;TANC2;PARD3B;EVC;HEATR5A;KCNMA1;MYO3A;MXRA7;DOCK1;CD44;CHST3;RAI14;SDC2;ARHGEF28;DNAH5;SEL1L;DNAH6;RGPD6;FSTL1;SLC7A2;THSD4;NPAS3;ERICH5;ARSJ;SNX9;TSPAN3;MPDZ;ARSB;MAP4K4;NTRK2;STARD13;VCAM1;NEK6;PXDNL;LAMB1;GNG12;CDC42BPA;ELL2;TJP1;IGSF11;MOB1B;SDK1;RCAN1;PLCXD3;TTC6;COL5A1;DLG5;STT3A;CPE
ZNF536 human tf ARCHS4 coexpression	82/299	4.037540535965851E-10	MYT1L;DGKB;CTNND2;PTPRO;GRIK3;ELAVL4;GPHN;ROBO1;GRM3;DPYSL5;TRIM2;DNER;PSD3;PHACTR3;KIF21A;DLGAP1;WSB1;RBFOX2;KCND3;TMEM178A;LRRC49;TMOD2;RFX3;ANK3;DNM3;NRG3;ADGRB3;LRRC7;HECW1;MPPED2;AMPH;RAPGEF5;TOX;ASTN1;GRIA1;RTN1;STXBP1;PDE1A;ATL1;BTF3L4;NRXN3;ILDR2;AKAP6;NREP;KALRN;NOL4;HDAC9;STOX2;MAPK8;GNG2;CLVS2;APBB2;NCAM1;CTNNA2;SRGAP3;PAK5;GRIA4;GABRA2;ATF7IP;ARNT2;ZNF462;MYEF2;AUTS2;SYT1;PCDH9;ST8SIA2;LSAMP;DCLK1;PBX1;ENOX1;TTLL7;PTPRD;MAPK10;NELL2;CCDC88A;LRFN5;APC;KLHL7;TTC3;FAT3;TCF4;APBA2
ZNF638 human tf ARCHS4 coexpression	82/299	4.037540535965851E-10	GABRB1;DOCK3;ATP8A1;DOCK9;CTNND2;DPY19L2P2;OSBPL10;UNC80;PTAR1;HERC2;C16ORF72;AKAP11;HERC1;ZSCAN30;PPIP5K2;TRIM2;SACS;DIP2B;EDIL3;PPFIA2;KCNH1;UNC13C;VWFP1;ARHGEF12;DST;VPS13C;TMOD2;VPS13B;WDR72;ANK3;UNC5D;ZDHHC17;ARID1B;PIAS2;DNM3;HEATR5A;HECW2;WDFY3;BIRC6;RAPGEF5;PLCB1;ASTN1;SLC24A2;MACF1;NFAT5;TNKS;KMT2C;HERC2P3;AKAP6;ZDHHC21;KALRN;MIPOL1;PCNX1;RANBP3L;KIAA1328;HECTD1;FUT9;HECTD4;SPOCK3;TRPM7;ATP9B;BPTF;MBD5;MON2;PCDH9;CADM2;ST8SIA1;ATRNL1;LRBA;MGA;NTRK3;SYT16;PHC3;MYO9A;LRP1B;TJP1;DLG2;POLR3A;APC;FAT3;PTPN4;ASB3
HELZ human tf ARCHS4 coexpression	82/299	4.037540535965851E-10	SETD2;USP32;DOCK8;SMG1P2;RORA;LYST;MYSM1;SYNE2;DOCK10;AKAP13;NIPBL;PTAR1;C16ORF72;HERC1;ZNF407;THSD7A;RBM33;MBNL1;PRKCH;ARHGEF12;ITGA4;DST;VPS13C;ARAP2;ADAM10;VPS13B;ARID1B;PARP8;INPP4B;WDFY3;BIRC6;PIK3C3;UTRN;DOCK2;MCTP2;KDM7A;MACF1;NFAT5;WDR26;ROCK1;KMT2C;ITPR2;LPP;RASGRP1;PCNX1;SCAF8;BTAF1;KIAA1328;HECTD1;HECTD4;TRPM7;HIVEP2;BPTF;MAP2K6;ARFGEF1;USP24;SPEN;CREBBP;RABGAP1L;MBD5;MON2;KDM4C;ARPP21;LRBA;TRAPPC10;MGA;DENND4C;ERBIN;YLPM1;ADAM32;LNPEP;DNAJC13;SMARCA2;PHC3;MYO9A;GATAD2B;MED13L;KANSL1;SLMAP;SP3;CDK12;UNC79
ZMAT3 human tf ARCHS4 coexpression	81/299	1.0098578168621822E-9	SEMA5A;APP;RNF11;TRIO;ANKRD33B;PTPRM;ECE1;ANTXR1;SLC8A1;GALNT10;ADAMTSL1;TRIM9;SPRED1;AKAP11;SH3PXD2A;RPS6KA2;PSD3;SACS;TEAD1;PAMR1;EDIL3;KCNH1;ARHGEF12;DST;TMOD2;FNDC3B;AFAP1;FRMD6;EVC;COL4A2;HECW2;KCNMA1;CDC42EP3;WDFY3;MXRA7;DOCK1;DGKI;CHST3;DDR2;MACF1;NFAT5;SHC3;ROCK1;TWIST2;TMTC1;NLK;FSTL1;TRHDE;LTBP1;CTIF;ATXN1;ARSJ;SNX25;MGAT5;CLVS2;SPOCK1;ATP9A;MAP4K4;NTRK2;STARD13;EGLN3;CADM2;NEK6;RFTN1;NEK7;SAMD4A;CRIM1;SYT16;MYO5A;CYBRD1;LAMB1;GNG12;DCLK1;EXT1;ARHGAP31;COL5A1;FAT1;ITGBL1;SPIRE1;RGL1;FBN1
PBRM1 human tf ARCHS4 coexpressi	81/299	1.0098578168621822E-9	SLC44A5;SETD2;TENM4;ANKRD36;CHD9;ELAVL4;BICD1;SYNE2;PTPRG;ROBO1;VN1R7P;GRIP1;NIPBL;ZNF608;HERC1;ZNF407;SACS;SMARCAD1;THSD7A;GTF2I;RBFOX2;DCC;LRRC49;VPS13C;VPS13B;FRMD4A;ZDHHC17;ARID1B;PARD3B;ASPM;NCOR1;MMP16;RFX7;WDFY3;BIRC6;

on			PIK3C3;UTRN;XPR1;MACF1;ROCK1;STAU2;TNKS;KMT2C;IREB2;RASAL2;NREP;PHF21B;MAPK8;KIAA1328;ZNF704;ZSWIM6;NCAM1;PAK3;MAP4K4;BPTF;ARFGEF1;USP24;ATF7IP;ZNF462;MBD5;MYEF2;BCL11B;AUTS2;ST8SIA2;MGA;YLPM1;SYT14;PPP2R3A;MYO9A;PBX1;GATAD2B;MED13L;PLEKHA8;PTPRD;FER;APC;KANSL1;ASXL3;TTC3;TCF4;EIF4G3
POU3F3 human tf ARCHS4 coexpression	80/299	2.4316801562333346E-9	DRAXIN;TENM4;MYT1L;CTNND2;ELAVL4;ZBTB20;ROBO1;TRIM9;CDH4;ZNF608;CDH2;DPYSL5;PEG10;TRIM2;AKT3;DNER;KIF21A;KIF21B;ERC2;PPFIA2;RBFOX2;TMEM178B;TMOD2;RFX3;KAZN;FRMD4A;ANK3;SORCS3;MPPED1;LRRC7;ADGRB1;TNIK;ASTN1;GRIA1;SHC3;STAU2;ILDR2;NREP;KALRN;NPAS3;PHF21B;FAM171A1;STOX2;GTF2IP1;GNG2;CDH20;FUT9;ZNF704;PLXNA2;CAMTA1;NCAM1;CTNNA2;SRGAP3;ZNF423;PAK5;PLXNA4;ATP9A;ARNT2;NTRK2;NDFIP1;MYEF2;AUTS2;ST8SIA2;NTRK3;GRIN2B;CORO2B;DCLK1;TTLL7;PTPRD;MAPK10;CCDC88A;DLG2;APC;SMOC1;TTC3;ZNF536;FAT3;TCF4;RGS12;APBA2
NCOA1 human tf ARCHS4 coexpression	80/299	2.4316801562333346E-9	ATP8A1;CPQ;DOCK8;DYSF;RORA;FRY;LYST;RYR3;SYNE2;UNC80;SENP6;AKAP13;GLT1D1;RASSF2;AKAP11;HERC1;ADGRE3;KIF21B;DIP2B;ANKS1B;MAP3K5;RBFOX1;MBNL1;PRKCB;VPS13C;TMOD2;ARAP2;VPS13B;ANK3;FAM126B;IPCEF1;IL17RA;PARP8;CNKSR2;ITPKB;MADD;RAPGEF2;WDFY3;PLCB1;UTRN;DOCK2;MCTP2;KDM7A;SLC24A2;MACF1;MTMR3;KMT2C;AOAH;KALRN;KIAA0513;GRIN2A;PCNX1;ATXN1;HECTD4;MAPK1;HIVEP2;LYN;GABBR2;CREBBP;IQSEC1;PLCL1;ERBIN;MYO5A;PDE4DIP;LNPEP;ATP2B2;FOXN3;ARHGAP26;SMARCA2;PHC3;GRIN2B;MYO9A;MED13L;DLG2;SYNJ1;APC;GNAQ;DMXL2;FAT3;PTPN4
ZMAT4 human tf ARCHS4 coexpression	80/299	2.4316801562333346E-9	GABRB3;DPP10;DOCK3;ATP8A1;FRMPD4;MYT1L;DGKB;CTNND2;OTUD7A;CELF4;ZBTB20;GRIK1;C4ORF50;GRM5;CDH2;PEG10;TRIM2;DNER;KIF21A;DLGAP1;KCNH1;POU1F1;TMEM178B;TMEM178A;KCNH8;TMOD2;PGM2L1;FOXP2;PJA2;AJAP1;ADGRB3;DOK5;SCN8A;HECW1;TNIK;DGKI;CDH18;MAGEL2;KHDRBS2;NLGN1;RTN1;SHC3;BTF3L4;RNF8;KLHL13;NREP;MTMR7;CECR2;GRIN2A;DPP6;FUT9;CLVS2;CAMTA1;DPH6;NCAM1;STXBP6;PAK3;ATP9A;GRIA4;ARNT2;ZNF462;SYT1;PCDH9;CADM2;ATRNL1;TMEM132B;NTRK3;PCDH7;PBX3;LSAMP;KLHL1;ESRRG;DCLK1;TTLL7;PTPRD;IGSF11;RALYL;PPP2R2B;SCN2A;ADGRL2
SMAD4 human tf ARCHS4 coexpression	79/299	5.543374746019774E-9	PATJ;TENM4;ZFYVE9;XYLT1;PTPRM;MYSM1;SYNE2;PTPRG;AKAP13;NIPBL;PTAR1;HERC2;ZNF608;C16ORF72;AKAP11;HERC1;KIF13A;SACS;DIP2B;TEAD1;NEO1;GTF2I;UNC13B;CADPS2;ARHGEF12;DST;VPS13C;ADAM10;VPS13B;ELF2;WDFY3;BIRC6;UTRN;DOCK1;KDM7A;NOTCH2;MACF1;NFAT5;WDR26;KMT2C;IREB2;ZDHHC21;LPP;CECR2;PCNX1;SCAF8;BTAF1;HECTD1;HECTD4;MPDZ;BPTF;ARFGEF1;USP24;SVIL;CREBBP;MON2;LRBA;TRAPPC10;MGA;DENND4C;ERBIN;LNPEP;DNAJC13;FOXN3;PUM1;PTPN13;MYO9A;MTOR;MED13L;PLEKHA8;TJP1;ITCH;SLMAP;AGO2;SP3;FAT1;PKN2;ADGRL2;FGF10
ZNF540 human tf ARCHS4 coexpression	79/299	5.543374746019774E-9	PCSK2;ZNRF2P2;MYT1L;DGKB;ELAVL4;FRG1HP;CDH8;UNC80;SCGN;DPYSL5;TRIM2;RNF17;KIF21A;RALGPS1;ANKS1B;RGS7;ZNF287;EPHA7;TANGO6;WSB1;LRRC49;TMOD2;MAGI2;RFX3;SEZ6L;GABRG2;ITFG1;PJA2;ADGRB3;LRRC7;HECW1;MAPRE2;ZNF675;CDH18;ASTN1;ZNF112;GRIA1;STX12;MAGEL2;RTN1;STOML1;STXBP1;ATL1;NRXN3;RGPD5;NREP;NYAP2;NOL4;MTMR7;RIC8B;GNG2;SNTG1;SPOCK3;CTNNA2;CSMD3;SRGAP3;PAK3;GRIA4;CCDC178;PTPRN2;CADM1;CADM2;ST8SIA2;LSAMP;SLC4A10;SYT16;SGCZ;MAPK10;AGBL4;LRFN5;APC;ASXL3;TTC3;CNTN1;PARGP1;CNTN4;SCN2A;FSIP1;SSBP2

ZNF407 human tf ARCHS4 coexpression	79/299	5.543374746019774E-9	*DOCK5;SETD2;ATP8A1;DOCK8;SMG1P2;SMG1P5;GRIK2;FRY;BACH1;LYST;SYNE2;DOCK10;AKAP13;NIPBL;HERC2;C16ORF72;PPIP5K2;DIP2B;JAK2;PCMTD1;MAP3K5;RBM33;MBNL1;ITGA4;DST;VPS13C;ARAP2;VPS13B;PARP8;PHF20L1;NCOR1;DPYD;RELL1;WDFY3;BIRC6;ZFPM2;UTRN;DOCK2;MCTP2;KDM7A;NOTCH2;MACF1;NFAT5;MTMR3;WDR26;ROCK1;KMT2C;ITPR2;IQGAP1;PCNX1;SCAF8;KIAA1328;HECTD1;HECTD4;HIVEP2;ATP9B;ARFGEF1;USP24;USP25;CREBBP;MON2;KDM4C;LRBA;TRAPPC10;MGA;DENND4C;ERBIN;LNPEP;DNAJC13;ARHGAP26;SMARCA2;MYO9A;MED13L;KANSL1;DMXL2;SP3;CCSER1;CDK12;KIAA0825*
ZNF804A human tf ARCHS4 coexpression	79/299	5.543374746019774E-9	*ROBO2;DRAXIN;PPP1R17;TENM4;MYT1L;ANKRD36;CHD9;CTNND2;PTPRO;ELAVL4;CDH9;CMIP;RPH3A;CDH4;GRM7;DPYSL5;ZSCAN30;TRIM2;DNER;DLGAP1;ZNF385D;PRKACB;ZNF521;ANKS1B;CALN1;SOX5;PPFIA2;RBFOX2;KCNH5;TMEM178B;DCC;CACNA2D1;LRRC49;TMOD2;SORCS1;ANK3;UNC5D;ZDHHC17;TIAM2;LRRC7;IL1RAPL2;CDC42EP3;IL1RAPL1;DSCAML1;NLGN1;AKAP6;NREP;KALRN;NKAIN2;NEU3;STOX2;GRK3;MAPK8;FUT9;PLXNA2;CLVS2;ZSWIM6;NCAM1;CTNNA2;SRGAP3;ZSWIM5;PAK5;PLXNA4;CLVS1;NTRK3;GRIN2B;DCLK1;SNAP91;PTPRD;NELL2;CCDC88A;FER;DLG2;NFIA;APC;PPP2R2B;COL5A3;TTC3;LRP12*
CHD9 human tf ARCHS4 coexpression	79/299	5.543374746019774E-9	*DPP10;ANKRD36;USP33;FRG1HP;ZBTB20;EFCAB6;SYNE2;SENP6;SRGAP2C;CNST;PPIP5K2;LRRTM4;TRIM2;ADAMTSL3;AKT3;DLEU1;KIF21A;SOX6;SCAPER;SRGAP2B;GABPA;ANKS1B;ANKRD36C;WSB1;DST;GRID1;LRRC49;KCNH8;VPS13C;MAGI2;VPS13B;ARID1B;PIAS2;PCCA;IFT81;PEAK1;MPPED2;ZNF438;USP41;ASTN2;ZNF675;ANKRD36B;SDCCAG8;CRB1;STXBP4;KMT2C;RGPD6;RGPD5;RASAL2;MIPOL1;FGGY;KIAA1328;SNTG1;TTC21B;ANKRD36BP2;PAK3;LRRC4C;ATP9B;JAM2;BPTF;PRELID2;MBD5;ATP8B4;SLC14A2;MYEF2;CDC42BPA;DCDC1;MAPK10;CCDC88A;FER;APC;NEDD4;TTC3;CEP83;TCF4;SSBP2;ASB3;ZNF354C;TNRC6B*
SOX9 human tf ARCHS4 coexpression	78/299	1.2325312265316335E-8	*APP;TENM4;CTNND2;WWC1;ZBTB20;SLC35F1;PTPRK;SLC4A4;GLI3;CMIP;KIF15;SPRED2;GRM5;CDH2;TRIM2;KIF21A;EPHB2;NEO1;SRGAP2B;ANKRD6;MAGI1;COL27A1;ADGRV1;TMEM178B;TCF12;RFX3;SLC6A11;SEZ6L;SORCS2;PARD3B;EPN2;PYGO1;ASPM;ADGRB3;PARD3;MPPED2;CHST3;CREB5;NFAT5;PHLPP1;SHC3;NTM;ILDR2;ASAP2;NPAS2;NPAS3;STOX2;RELN;ZNRF3;ZNF704;LRIG1;APBB2;SRGAP3;MPDZ;JAM2;MAP4K4;RFTN2;ARNT2;ZNF462;FARP1;NTRK2;DTNA;MYEF2;AUTS2;SGTB;PTCH1;HMGA2;BTBD9;CORO2B;PTK2;ARHGAP32;NFIA;FABP7;SMOC1;DLG5;FAT1;RNF182;SPIRE1*
ZNF91 human tf ARCHS4 coexpression	78/299	1.2325312265316335E-8	*SLC44A5;ROBO2;SETD2;MYT1L;ANKRD36;CHD9;PTPRO;ELAVL4;ZBTB20;SMG1P5;EFCAB6;UNC80;GRM7;PPIP5K2;TRIM2;AKT3;TMEM108;TLK1;KIF21A;SOX6;PCMTD1;EPHA6;WSB1;DST;LRRC49;SEMA6D;TMOD2;MAGI2;RFX3;ANK3;FAM126B;TANC2;PJA2;NAV3;LRRC7;ASTN2;XPR1;ANKRD36B;TNKS;NRXN3;AKAP6;RASAL2;NREP;NYAP2;KALRN;NOL4;MIPOL1;FGD4;MAPK8;GNG2;SNTG1;FUT9;ZNF627;SRGAP3;PAK3;PLXNA4;MBD5;NTRK3;RANBP17;SYT16;GRIN2B;PTPRD;MAPK10;CCDC88A;DLG2;NBEA;APC;KLHL7;ASXL3;TTC3;FAT3;CCSER1;TCF4;PTPN4;SSBP2;ASB3;CPEB4;TNRC6B*
ZNF25 human tf ARCHS4 coexpression	78/299	1.2325312265316335E-8	*GABRB1;ATP8A1;MYT1L;CTNND2;ELAVL4;TRIM9;DPYSL5;TRIM2;DNER;PSD3;DLGAP1;PRKACB;RALGPS1;PPFIA2;RBFOX1;WSB1;RBFOX2;TMEM178B;TMEM178A;CACNA2D1;TMOD2;FRMD4B;SYN2;GABRG2;PGM2L1;PJA2;DNM3;ADGRB3;ULK2;AMPH;ZMYND11;VSTM2A;ASTN1;GRIA1;RTN1;STXBP1;ATL1;NRXN3;AKAP6;NREP;PRKCZ;KALRN;NALCN;DPP6;GNG2;SV2B;CDH20;FUT9;CLVS2;NCAM1;CTNNA2;PAK3;ATP9A;OPCML;GABRA2;ARNT2;NDFIP1;SY*

			T1;CADM2;ST8SIA2;PBX3;SLC4A10;SYT16;ATP2B2;DCLK1;SNAP91;TTLL7;MAPK10;DLG2;APC;PPP2R2B;RCAN2;TTC3;YPEL1;CNTN1;SCN2A;SSBP2;APBA2
ZNF532 human tf ARCHS4 coexpression	78/299	1.2325312265316335E-8	*GABRB3;DRAXIN;TENM3;TENM4;CTNND2;GRIK3;ELAVL4;PSIP1;BICD1;PTPRG;GLI2;ROBO1;IGF1R;ZNF608;CDH2;DPYSL5;TRIM2;AKT3;DIP2C;LARGE1;NEO1;MAGI1;WSB1;RBFOX2;TMEM178B;DCC;CACNA2D1;RFX3;FRMD4A;NAV2;ENAH;ADGRB3;LRRC7;ASTN1;SHC3;CTTNBP2;TNKS;ILDR2;AKAP6;ADCY2;RASAL2;NREP;KALRN;NPAS3;FAM171A1;STOX2;CECR2;ZNF704;LRIG1;NCAM1;CTNNA2;SRGAP3;CSMD2;ZNF423;MPDZ;WASF3;ARNT2;ZNF462;FARP1;MYEF2;AUTS2;PTCH1;NTRK3;YLPM1;PTPN13;CORO2B;DCLK1;PBX1;PTK2;GULP1;TJP1;PTPRD;KLHL7;DLG5;TTC3;FAT3;TCF4;ADGRL2*
TUB human tf ARCHS4 coexpression	78/299	1.2325312265316335E-8	*GABRB3;APP;CNTNAP2;GALNT13;ATP8A2;DOCK3;MYT1L;CTNND2;RPH3A;TRIM9;GRM5;DPYSL5;TRIM2;MCF2L;AKT3;PSD3;TNR;KIF21A;DLGAP1;SH3GL2;PPFIA2;RBFOX2;TMEM178B;TMEM178A;TMOD2;KAZN;FAM219A;SEZ6L;SYN2;MAPK8IP1;LRRC7;SCN8A;DSCAML1;SHANK2;ASTN1;RTN1;SHC3;CTTNBP2;TNKS;STXBP1;NTM;NREP;KALRN;NOL4;CTIF;STOX2;SV2B;CLVS2;CAMTA1;NCAM1;CTNNA2;ATP9A;WASF3;OPCML;ARNT2;NDFIP1;MYEF2;SYT1;CADM2;NTRK3;LSAMP;KIAA1549L;ATP2B2;CORO2B;DCLK1;SNAP91;PTPRD;MAPK10;DLG2;NBEA;APC;PPP2R2C;PPP2R2B;RCAN2;TTC3;CNTN1;SCN2A;APBA2*
DZIP1 human tf ARCHS4 coexpression	78/299	1.2325312265316335E-8	*APP;SH3GL3;TENM3;TENM4;CPNE4;CTNND2;FMN2;SIPA1L2;PTPRG;ROBO1;ZNF608;CDH2;TRIM2;AKT3;PSD3;SACS;KIF21A;TEAD1;NEO1;MAGI1;RBFOX2;TMEM178B;DST;TMOD2;EML1;ZDHHC17;MAPK8IP1;ENAH;PYGO1;DOK5;PEAK1;MXRA7;DOCK1;RAI14;RABGAP1;SHC3;RGPD6;ILDR2;FSTL1;EHBP1;NPAS3;FAM171A1;CTIF;STOX2;FLRT2;ZNF704;NCAM1;CTNNA2;SRGAP3;ZNF423;GPC6;MPDZ;ATP9A;WASF3;ARNT2;FARP1;MYEF2;AUTS2;NTRK3;RDX;KIAA1549L;HMGA2;PTPN13;CDC42BPA;MYO9A;CORO2B;DCLK1;PLEKHA8;TTLL7;PTPRD;CCDC88A;TTLL5;FER;SPIRE1;FAT3;EIF4G3;ADGRL2;TBATA*
ETV1 human tf ARCHS4 coexpression	77/299	2.556655119321648E-8	*GABRB3;PPP1R17;GALNT13;DOCK3;MTCL1;MEGF11;CTNND2;LDLRAD3;RORB;PCMTD2;RPH3A;SRGAP2C;TRIM9;TRIM2;MCF2L;DNER;PSD3;TNR;DLGAP1;SRGAP2B;SH3GL2;MAGI1;CADPS2;KCND2;KCND3;TMEM178A;DCC;SEMA6D;TCF12;TMOD2;RFX3;SEZ6L;SYN2;PIAS2;MAPK8IP1;EPN2;CNKSR2;TIAM1;ADGRB3;ADGRB1;ASTN1;NLGN1;PHLPP1;SHC3;STXBP1;BTF3L4;ILDR2;DPP6;SV2B;SNTG1;FUT9;LRIG1;CLVS2;SPOCK1;GPR158;SLC15A5;WASF3;GRIA4;BBS2;RFTN2;ARNT2;PTPRN2;DTNA;MYEF2;GABRA6;SYT1;CADM2;ATP2B2;CORO2B;SNAP91;IGSF11;MAPK10;APC;SMOC1;CNTN1;SPIRE1;APBA2*
NFIB human tf ARCHS4 coexpression	77/299	2.556655119321648E-8	*SLC44A5;CNTNAP3;CNTNAP2;ATP8A2;MYT1L;GRIK3;GRIK2;BICD1;CDH7;SRGAP2C;NHSL1;ADAMTS3;LRRTM4;AKT3;TMEM108;PHACTR3;HYDIN2;THSD7A;SRGAP2B;SH3GL2;SOX5;GARNL3;PPFIA2;RGS7;EPHA7;ZNF160;KAZN;PRKCA;EML1;TIAM2;ADGRB3;LRRC7;MPPED2;KCNQ3;RAPGEF2;ZFPM2;ZNF234;XPR1;SHANK2;GRIA1;SLC24A2;CTTNBP2;TNKS;EFNA5;NOL4;CACNA1E;PLXNA2;ANKRD20A5P;PAK3;OPCML;CA10;MYEF2;BCL11B;ARPP21;INSR;NTRK3;SLC4A10;SORBS2;SYT14;PPP2R3A;CORO2B;ST18;SNAP91;SGSM1;CCDC88A;NELL1;DLG2;DAB1;SLCO3A1;NBEA;ASXL3;TTC3;TCF4;ASB4;FGF12;LRP12;UNC79*
PBX3 human tf ARCHS4 coexpression	77/299	2.556655119321648E-8	*DPP10;DRAXIN;TENM3;ATP8A1;TUSC3;GRIK1;GRIK2;BICD1;CDH8;PTPRG;ROBO1;DACH1;CDH2;DPYSL5;PEG10;TRIM2;DNER;DIRC3;ZNF521;RALGPS1;MAGI1;RBFOX2;TMEM178B;DCC;CACNA2D1;LRRC49;MAGI2;EBF1;EBF2;GFRA1;PGM2L1;FOXP2;PJA2;ADGRB3;NME7;LRRC7;DOK5;TOX;XPR1;CNTNAP5;KHDRBS2;RTN1;GREB1L;KLHL13;NREP;KALRN;STOX2;MAPK8;GNG2;ZMAT4;MVB12B;NC*

			AM1;CTNNA2;CSMD3;PAK3;PAK5;ATF7IP;CA10;NDFIP1;MYEF2;CNTN5;AUTS2;ST8SIA2;KLHL1;DCLK1;ARHGAP24;ENOX1;PTPRD;MAPK10;RALYL;FER;KLHL7;ASXL3;TTC3;KIAA1958;SSBP2;APBA2
ZBTB38 human tf ARCHS4 coexpression	77/299	2.556655119321648E-8	LPGAT1;TRIO;ANKRD33B;DOCK9;PTPRM;EFCAB14;PTPRK;IKZF2;LIMD1;GALNT10;EPS8;AKAP13;EFR3A;AKAP11;SH3PXD2A;KIF13A;SAMD12;TEAD1;CAST;MBNL1;MBNL2;ARHGEF12;DST;IL1R1;ANXA4;VPS13C;FNDC3B;KIAA1217;FRMD6;EVC;TRAF3;KCNMA1;CMPK1;ITGA8;MET;DDR2;MACF1;NFAT5;ARHGEF28;SEL1L;TMTC1;IQGAP1;LPP;ATXN1;IGLV2-14;ARSJ;SNX25;MGAT5;BTLA;SPOCK1;SNX9;HIVEP2;STARD13;NEK6;RFTN1;NEK7;SAMD4A;ERBIN;CRIM1;CYBRD1;CABLES1;LNPEP;DNAJC13;LAMB1;SYNJ2;GNG12;SMARCA2;ELL2;EXT1;MYO1D;MYO1E;PPFIBP1;DPY19L1;SLMAP;FAT1;ATP13A3;FBN1
ZKSCAN1 human tf ARCHS4 coexpression	77/299	2.556655119321648E-8	PCSK2;SPAG16;CCDC122;ERO1B;ANKRD36;TUSC3;CHD9;ELAVL4;ZBTB20;PCMTD2;UNC80;RPS6KA5;PPIP5K2;CA5A;TRIM2;ADAMTSL3;GATAD1;LONP2;LUC7L;KIF21A;SOX6;MAGI1;ANKRD36C;WSB1;TCF12;MAGI2;HUNK;RFX3;COBL;ARID1B;TANC2;EPN2;PLCB4;EVC;IFT81;PEAK1;CNKSR3;WDPCP;ASTN2;ANKRD36B;ZNF397;STXBP4;RGPD5;RASAL2;HERC2P9;NOL4;NALCN;LPP;MIPOL1;NPAS3;FGD4;KIAA1328;SRGAP3;PAK3;ZNF462;FARP1;MBD5;SLC14A2;PTPRN2;MYEF2;CADM1;CADPS;C1ORF21;SORBS2;PUM1;ST18;DCDC1;CCDC88A;TMEM116;NEDD4;TTC3;CPE;ASB4;RGS12;ASB3;XKR6;TNRC6B
TBX20 human tf ARCHS4 coexpression	77/299	2.556655119321648E-8	FHOD3;COL18A1;RYR2;MYOM1;PTPRM;LDB3;SIPA1L2;SLC8A1;MYLK3;AKAP13;ARHGAP42;CDH2;KIF13A;MYO18B;DPF3;PGM5;TEAD1;ADAMTS9;PDK1;MLIP;CERS6;TPM1;MTUS2;TOM1L2;TANC1;COL4A2;COL21A1;SLC27A6;ALPK3;MXRA7;ALPK2;TLN2;PPP1R12B;DGKI;ITGA9;YAP1;FBN2;PRKAA2;TMTC1;CACNA1C;LTBP1;THSD4;ABLIM1;INPP5A;MGAT5;TNNI1;CTNNA3;SLIT3;B4GALNT3;PDLIM5;MPDZ;CORIN;PLXNA4;SVIL;SPHKAP;CNTN5;NEBL;PCDH7;NEK7;EXOC6B;MICAL3;SAMD4A;FAM189A2;PDE4DIP;SORBS2;PXDNL;LAMB1;MCC;PTPN13;TJP1;PLCXD3;BMP2;COL5A1;DLC1;SLMAP;PDE3A;TACC2
CAMTA1 human tf ARCHS4 coexpression	77/299	2.556655119321648E-8	GABRB3;PTPRT;ATP8A2;DOCK3;MYT1L;CTNND2;CELF4;ELAVL4;RPH3A;RIMS1;GRM5;TRIM2;PSD3;KIF21A;DLGAP1;ZNF385D;ANKS1B;PPFIA2;RBFOX1;TMEM178B;TMEM178A;TMOD2;KAZN;ANK3;SYN2;GABRG2;PGM2L1;AJAP1;CNKSR2;DNM3;SCN8A;HECW1;NOS1AP;ASTN1;SLC24A2;RTN1;SHC3;STXBP1;ATL1;NTM;NRXN3;KALRN;STOX2;GRIN2A;DPP6;PGBD5;SV2B;FUT9;CLVS2;NCAM1;CTNNA2;SRGAP3;PAK3;PAK5;ATP9A;WASF3;OPCML;GABBR2;ARNT2;SYT1;CADM2;NTRK3;LSAMP;KIAA1549L;ATP2B2;DCLK1;PBX1;SNAP91;TTLL7;PTPRD;MAPK10;RALYL;DLG2;PPP2R2C;PPP2R2B;RCAN2;SCN2A
AFF4 human tf ARCHS4 coexpression	77/299	2.556655119321648E-8	APP;TRIO;ZFYVE9;PTPRM;PTPRK;CMIP;ALCAM;AKAP11;HERC1;MPRIP;SH3PXD2A;KIF13A;SACS;ANKFY1;DIP2B;ERC1;TEAD1;NEO1;GTF2I;UNC13B;ARHGEF11;ARHGEF12;DST;IL1R1;VPS13C;FNDC3B;AFAP1;FNDC3A;RC3H2;TANC2;TANC1;WDFY3;BIRC6;UTRN;DOCK1;YAP1;NOTCH2;MACF1;NFAT5;RABGAP1;WDR26;ABHD2;KMT2C;LPP;PCNX1;SCAF8;MAPK8;HECTD1;HECTD4;ABL1;FLNB;HIVEP2;ATP9A;MAP4K4;USP24;SPEN;CREBBP;TRAPPC10;EXOC6B;SAMD4A;ERBIN;DNAJC13;LAMB1;AP2B1;SYNJ2;PUM1;GNG12;MTOR;MED13L;PLEKHA8;EXT1;TJP1;SLMAP;DLG5;FAT1;CDK12;ATP13A3
RAPGEF5 human tf ARCHS4 coexpression	77/299	2.556655119321648E-8	DOCK4;DOCK3;ATP8A1;FRMPD4;DOCK9;CTNND2;RASGRF2;OTUD7A;RPH3A;SYNPR;GRM5;AKAP11;TRIM2;MCF2L;PSD3;DLGAP1;EDIL3;CALN1;KCNH1;CADPS2;RBFOX1;MBNL2;DST;PRKCE;TMOD2;MTUS1;COBL;MYRIP;SYN2;MAPK8IP1;GABRG1;AJAP1;CNKSR2;DNM3;SCN8A;WDFY3;PLCB1;NGEF;RAPGEF4;SLC24A2;NECAB1;SHC3;STXBP1;

			SLC1A2;PRKCZ;KALRN;NALCN;RAP1GAP;KIAA0513;GRIN2A;SV2B;EPB41L3;SPOCK3;CLVS2;SPOCK1;GPR158;ATP9A;OPCML;ARNT2;DTNA;SYT1;CADM2;ATRNL1;KIAA1549L;MYO5A;ATP2B2;SNAP91;LRP1B;ETNPPL;TTLL7;PTPRD;ARHGAP32;DLG2;SYNJ1;PPP2R2C;CNTN1;SCN2A
RGS7 human tf ARCHS4 coexpression	77/299	2.556655119321648E-8	PCSK2;DOCK3;KCNC1;FRMPD4;MYT1L;CTNND2;HS6ST3;RPH3A;UNC80;SYNPR;GRM5;TRIM2;MCF2L;PSD3;DLGAP1;PRKACB;SH3GL2;PPFIA2;KCNH1;UNC13C;RBFOX1;PRKCE;TMOD2;MYRIP;SEZ6L;SYN2;GABRG2;MAPK8IP1;AJAP1;CNKSR2;DNM3;MPPED1;SCN8A;KCNMA1;PLCB1;NGEF;RAPGEF4;SLC24A2;NECAB1;RTN1;SHC3;STXBP1;SLC1A2;PRKCZ;KALRN;NALCN;RAP1GAP;KIAA0513;GRIN2A;DPP6;PGBD5;SV2B;SPOCK3;CLVS2;GPR158;ATP9A;OPCML;GABBR2;ARNT2;PTPRN2;NDFIP1;SYT1;CADM2;CADPS;SLC4A10;KIAA1549L;MYO5A;ATP2B2;CORO2B;SNAP91;DLG2;SYNJ1;PPP2R2C;RCAN2;CNTN1;CPE;SCN2A
DACH2 human tf ARCHS4 coexpression	76/299	5.989632969160491E-8	GABRB3;MYT1L;PTPRO;CELF4;ELAVL4;CDH8;SYNPR;C1ORF127;GRM7;DPYSL5;TRIM2;KIF21A;ZNF385D;ANKS1B;PPFIA2;EPHA6;CACNA2D1;LRRC49;TMOD2;MAGI2;EBF1;MTUS2;SEZ6L;PGM2L1;NAV3;NRG3;IFT81;ADGRB3;LRRC7;CDH18;ASTN1;ZNF112;NECAB1;RTN1;NRXN3;ZNF66;NREP;NOL4;NALCN;CACNA1E;FGD4;STOX2;DPP6;GNG2;SUSD4;CAMTA1;SUSD6;NCAM1;CTNNA2;SRGAP3;PAK3;PAK5;CADM1;SYT1;PCDH9;CADM2;LSAMP;SLC4A10;GRIN2B;USH2A;SNAP91;DCDC1;MAPK10;NELL2;RALYL;DLG2;FGF14;NBEA;PPP2R2B;ASXL3;TTC3;CNTN1;FAT3;GALNTL6;SCN2A;SSBP2
THRB human tf ARCHS4 coexpression	76/299	5.989632969160491E-8	PTPRT;DOCK3;MAST4;FRMPD4;CHRM5;C12ORF42;RPH3A;UNC80;SYNPR;GRM5;ADAMTSL3;PSD3;DLGAP1;ANKS1B;KCNH1;UNC13C;RBFOX1;KCNH5;ARHGEF12;KCND3;PRKCE;TMOD2;KCTD1;ANK3;ELOVL7;SYN2;CNKSR2;DNM3;SCN8A;PPARA;PLCB1;VSTM2A;NGEF;RAPGEF4;SLC24A2;STOML1;HEPACAM;STXBP1;SLC1A2;AKAP6;KALRN;NALCN;KIAA0513;GRIN2A;DPP6;PCNX2;RIC8B;PGBD5;SV2B;ST8SIA5;CAMTA1;GPC5;ATP9A;OPCML;GABBR2;ARNT2;OSBPL6;SYT1;NEGR1;CADM2;ATRNL1;SLC4A10;KIAA1549L;FBXL17;ATP2B2;SNAP91;ETNPPL;TTC39B;TTLL7;ARHGAP32;DLG2;PPP2R2C;RCAN2;SCN2A;OSBPL1A;HCN1
ZFHX3 human tf ARCHS4 coexpression	75/299	1.3406515125423934E-7	RYR2;PATJ;DOCK4;TRIO;DOCK9;CELF4;KIAA1671;PTPRM;SIPA1L3;RYR3;RPTOR;C4ORF50;UNC80;HERC2;RPS6KA5;HERC1;MPRIP;ADAMTSL3;MCF2L;PSD3;GAST;ERC1;VWFP1;ARHGEF12;DST;ABCC9;ANK3;URB1;SHISA9;KLF15;TANC2;TANC1;NAV3;PEAK1;HECW2;WDPCP;WDFY3;BIRC6;RAPGEF5;PPARA;DGKI;NOTCH2;MACF1;NFAT5;KMT2C;CACNA1C;CACNA1E;LPP;PDZD2;KIAA1328;HECTD4;PLXNA2;GSG1L;ABL2;FLNB;NCAM1;CSMD1;ATP9B;ATP9A;ARNT2;FARP1;ANKRD30BL;NTRK3;MICAL3;NSG1;GRIN2B;MYO9A;HS3ST4;PBX1;TJP1;ARHGAP32;GNAL;PDE10A;NEDD4;FAT3
ZEB2 human tf ARCHS4 coexpression	75/299	1.3406515125423934E-7	DOCK4;PID1;MCTP1;ANKRD36;MEGF11;DOCK8;ANKRD20A1;FRY;BACH1;LYST;SLC8A1;FAM107B;DOCK10;AKAP13;GRM5;RASSF2;HERC1;GRM7;TRIM2;TMEM108;LUC7L;KIF21B;DAPK1;GRID1;VPS13C;PDE4D;TMOD2;ARAP2;VPS13B;FAM126B;ZDHHC17;RUNX1;PHF20L1;DNM3;MPPED1;LRRC7;DPYD;KCNQ3;RAPGEF2;WDFY3;COL6A6;DOCK2;MACF1;RABGAP1;WDR26;CTTNBP2;KMT2C;AOAH;NR2C1;GNG2;NCAM1;PLXNA4;MYEF2;NTRK3;SYT16;MYO5A;RAB27A;SYT14;FOXN3;PPP2R3A;PLXDC2;ARHGAP26;SMARCA2;GRIN2B;MED13L;PTPRD;CCDC88A;PTPRE;DLG2;DAB1;APC;NFIB;DMXL2;CCSER1;TCF4
ZNF462 human tf ARCHS4 coexpressi	75/299	1.3406515125423934E-7	GABRB3;ROBO2;DRAXIN;TENM4;CHD6;ZBTB20;PSIP1;BICD1;IGF1R;TTC28;RIMS2;ZNF608;CDH2;DPYSL5;TRIM2;ADAMTSL3;KIF21A;JARID2;PPFIA2;MAGI1;RBFOX2;ADGRV1;LRRC49;HUNK;RFX3;ANK3;NAV2;ARID1B;NAALADL2;PIAS2;ENAH;PYGO1;NCOR1;IFT81;ZNF397;BTF

on			*3L4;ILDR2;RASAL2;NREP;KALRN;PHF21B;STOX2;CECR2;ZNF704;CAMTA1;APBB2;NCAM1;SRGAP3;ZNF423;MPDZ;PAK5;BPTF;FARP1;MYEF2;CADM1;AUTS2;ST8SIA2;PTCH1;YLPM1;PBX1;GULP1;TJP1;PTPRD;CCDC88A;AGO1;KLHL7;TTC3;YPEL1;ZNF536;FAT3;TCF4;RGS12;KIAA1958;ADGRL2;TNRC6B*
NCOA2 human tf ARCHS4 coexpression	75/299	1.3406515125423934E-7	*RERE;DIDO1;RNF11;LPGAT1;ATP8A1;USP32;DOCK8;EFCAB14;FRY;BACH1;SIPA1L3;LYST;SYNE2;AKAP13;EFR3A;GLT1D1;NIPBL;RASSF2;C16ORF72;HERC1;ZNF407;ADGRE3;DIP2B;JAK2;IL6R;MAP3K5;RBM33;MBNL1;ARHGEF12;VPS13C;ARAP2;VPS13B;IL17RA;PARP8;DPYD;WDFY3;BIRC6;UTRN;DOCK2;MCTP2;KDM7A;NOTCH2;MACF1;NFAT5;MTMR3;WDR26;ABHD2;KMT2C;RNF38;ITPR2;CSF2RB;IQGAP1;PCNX1;SCAF8;HECTD1;HECTD4;HIVEP2;LYN;ARFGEF1;USP24;CREBBP;MGAM;IQSEC1;LRBA;TRAPPC10;DENND4C;ERBIN;LNPEP;DNAJC13;ARHGAP26;SMARCA2;MED13L;DMXL2;SP3;CDK12*
FOXP2 human tf ARCHS4 coexpression	74/299	3.04000285494918E-7	*DPP10;DRAXIN;MYT1L;TUSC3;CELF4;ELAVL4;ZBTB20;GRIK1;GRIK2;EFCAB6;PTPRG;RIMS1;DACH1;ZNF280B;DNER;KIF21A;RALGPS1;ANKS1B;ZNF684;TMEM178B;DCC;LRRC49;EBF1;MTUS2;ANK3;ARID1B;PGM2L1;SHISA6;ADGRB3;DOK5;AMPH;ALG10B;CDH18;KHDRBS2;RTN1;TSHZ3;CHRNA7;TSHZ2;ZNF66;KLHL13;NREP;NYAP2;ADAM29;DPP6;PDCD6IPP2;GNG2;ZMAT4;CAMTA1;NCAM1;CTNNA2;CSMD3;PAK3;VAT1L;CLVS1;CNTN5;AUTS2;NEGR1;ST8SIA1;ST8SIA2;RANBP17;CADPS;PBX3;KLHL1;PBX1;ENOX1;PTPRD;CCDC88A;RALYL;FER;ASXL3;TTC3;ZNF74;LRP12;LHX9*
MGA human tf ARCHS4 coexpression	74/299	3.04000285494918E-7	*LIN54;PATJ;SETD2;SMG1P2;ZBTB20;MYSM1;SYNE2;OSBPL10;IGF1R;AKAP13;NIPBL;PTAR1;HERC2;C16ORF72;HERC1;ADAMTSL3;PPP6R3;SACS;ERC1;USP8;AQR;ARHGEF12;ESCO1;DST;VPS13C;VPS13B;ARID1B;PIAS2;NCOR1;PEAK1;WDPCP;WDFY3;PIK3C3;ASTN2;UTRN;MACF1;NFAT5;RABGAP1;KMT2C;IREB2;RGPD5;ITPR2;THADA;UBR1;MIPOL1;PCNX1;SCAF8;BTAF1;KIAA1328;HECTD1;TRPM7;MPDZ;ATP9B;BPTF;SPECC1;ARFGEF1;USP24;MBD5;LRBA;DENND4C;ERBIN;ADAM32;LNPEP;PHC3;MYO9A;PTK2;MED13L;TJP1;KANSL1;AGO1;NEDD4;CDK12;ASB3;TNRC6B*
MEF2C human tf ARCHS4 coexpression	73/299	6.776943140783708E-7	*MYOM1;MYLK2;ATP8A1;MYT1L;LDB3;LRRC2;SLC8A1;GRM5;AKAP11;SGCD;HERC1;TRIM2;PEBP4;MYO18B;DLGAP1;PRKACB;SH3GL2;SGCG;RBFOX1;DSCAM;PRKCB;CACNA2D1;PRKCE;TMOD2;TRDN;DNM3;MMP16;MPPED1;MYL1;LRRC7;SCN8A;ALPK3;KCNQ5;DGKI;PRKAA2;PPM1L;TRAK1;KALRN;GRIN2A;SV2B;FUT9;NRAP;SYNDIG1;TNNI1;XIRP2;ZNF106;CTNNA3;NCAM1;HIVEP2;PLXNA4;ATP9A;OPCML;SVIL;SYT1;CADM2;AGL;SYT16;KIAA1549L;MYO5A;PDE4DIP;ATP2B2;GRIN2B;DCLK1;SNAP91;TTLL7;PTPRD;DLG2;AGBL1;APC;TCF4;SCN2A;ASB2;FGF12*
ELK4 human tf ARCHS4 coexpression	73/299	6.776943140783708E-7	*PATJ;MAML2;DOCK9;DOCK8;CEP120;EFCAB14;RORA;LIMD1;MYSM1;SYNE2;DOCK10;AKAP13;POTEM;NIPBL;PTAR1;HERC1;LRRFIP1;RBM33;CAST;MBNL1;PRKCH;ARHGEF12;ITGA4;VPS13C;ARAP2;VPS13B;TC2N;ARID1B;RUNX1;PARP8;INPP4B;PACS1;BIRC6;DOCK2;MCTP2;KDM7A;NOTCH2;MACF1;NFAT5;WDR26;DNAH8;ABHD2;KMT2C;ITPR2;IQGAP1;LPP;RASGRP1;PCNX1;KIAA1328;HECTD1;HECTD4;TRPM7;HIVEP2;PDLIM5;ARFGEF1;USP24;OR51E1;CREBBP;LRBA;TRAPPC10;DENND4C;ERBIN;LNPEP;FOXN3;SMARCA2;PHC3;PARP15;GATAD2B;MED13L;SLMAP;AGO2;SP3;CDK12*
AFF1 human tf ARCHS4 coexpression	72/299	1.4849784821845063E-6	*RERE;DIDO1;ATP8A1;KDM1B;DOCK9;DOCK8;CHD6;KIAA1671;EFCAB14;LIMD1;SIPA1L3;MYSM1;YBX3;AKAP13;FYCO1;NIPBL;HERC1;ZNF407;KIF13A;LRRFIP1;RBM33;CAST;MBNL1;ARHGEF12;ITGA4;VPS13C;VPS13B;RUNX1;KIAA1217;BMP2K;WDFY3;BIRC6;UTRN;DOCK2;MCTP2;KDM7A;FKBP5;MACF1;NFAT5;MTMR3;WDR26;ABHD2;KM*

			T2C;DNAH5;ITPR2;IQGAP1;AMBRA1;PCNX1;SCAF8;LARP1;HECTD1;HECTD4;FLNB;HIVEP2;ARFGEF1;USP24;SPEN;CREBBP;LRBA;TRAPPC10;DENND4C;ERBIN;XPO7;LNPEP;DNAJC13;FOXN3;SMARCA2;MTOR;MED13L;DIAPH1;SP3;CDK12
BDP1 human tf ARCHS4 coexpression	72/299	1.4849784821845063E-6	TCERG1;SETD2;ZFYVE9;ANKRD36;USP33;CWC27;MYSM1;SYNE2;AKAP13;NIPBL;HERC2;NHSL1;HERC1;ADAMTSL3;SACS;PPFIA2;POTEC;ANKRD36C;TTC37;ARHGEF12;ESCO1;DST;VPS13C;VPS13B;ARID1B;SUPT3H;NCOR1;LRRC7;PEAK1;WDPCP;WDFY3;ZNF678;BIRC6;ASTN2;ZNF236;UTRN;ANKRD36B;MACF1;NFAT5;TBC1D19;ROCK1;KMT2C;RGPD6;MIPOL1;KIAA1328;TTC21B;ANKRD20A5P;ANKRD36BP2;ATP9B;BPTF;ARFGEF1;USP24;SPEN;USP25;MBD5;LRBA;MGA;ERBIN;YLPM1;ADAM32;MYO9A;CCDC88A;NBEA;APC;NEDD4;FAT3;CCSER1;TCF4;CDK12;PTPN4;TNRC6B;CCDC171
SOX4 human tf ARCHS4 coexpression	71/299	3.0418558031735515E-6	SLC44A5;ROBO2;CNTNAP2;DRAXIN;MYT1L;CLCN3P1;PTPRO;ELAVL4;SLC35F1;GRIK2;GPHN;SRGAP2C;NHSL1;DPYSL5;LRRTM4;TMEM108;KIF21A;SOX6;SRGAP2B;MAGI1;EPHA7;WSB1;LRRC49;TCF12;ENAH;IFT81;LRRC7;MPPED2;ZNF234;XPR1;GRIA1;CRB1;RTN1;CTTNBP2;ATL1;CHRNA7;BTF3L4;NRXN3;NREP;FGD4;GNG2;SNTG1;SRGAP3;PAK3;JAM2;PAK5;GRIA4;RFTN2;ZNF462;CADM1;AUTS2;SIAH3;INSR;ST8SIA2;RANBP17;CORO2B;ST18;PBX1;MAPK10;CCDC88A;NFIA;NBEA;APC;NFIB;KLHL7;ASXL3;TTC3;ZNF536;TCF4;SSBP2;LRP12
SOX11 human tf ARCHS4 coexpression	71/299	3.0418558031735515E-6	SLC44A5;DRAXIN;MYT1L;ANKRD36;PTPRO;ELAVL4;MSANTD4;GPHN;NHSL1;CDH2;DPYSL5;TRIM2;SNAPC3;LUC7L;KIF21A;WSB1;RBFOX2;DCC;LRRC49;MAGI2;RFX3;ENAH;IFT81;LRRC7;MPPED2;ZNF675;XPR1;GRIA1;CRB1;RTN1;STAU2;ATL1;NTM;BTF3L4;NRXN3;AKAP6;NREP;NOL4;PHF21B;FGD4;GNG2;ZNF627;NCAM1;CTNNA2;SRGAP3;PAK3;PAK5;CLVS1;ATF7IP;RIC3;ZNF462;MON2;MYEF2;CADM1;AUTS2;ST8SIA2;ST18;MAPK10;NELL2;CCDC88A;NFIA;NFIB;AGO1;KLHL7;ASXL3;TTC3;YPEL1;ZNF536;TCF4;SSBP2;LRP12
ZNF365 human tf ARCHS4 coexpression	71/299	3.0418558031735515E-6	DOCK3;KCNC1;FRMPD4;OTUD7A;RPH3A;UNC80;SYNPR;GRM5;AKAP11;MCF2L;PSD3;DLGAP1;PRKACB;SH3GL2;CALN1;KCNH1;RBFOX1;TMEM178A;PRKCE;TMOD2;MYRIP;SYN2;GABRG2;MAPK8IP1;GABRG1;AJAP1;CNKSR2;DNM3;MPPED1;SCN8A;KCNMA1;RAPGEF5;PLCB1;NGEF;ASTN1;RAPGEF4;SLC24A2;NECAB1;SHC3;STXBP1;SLC1A2;PRKCZ;NALCN;RAP1GAP;KIAA0513;GRIN2A;CLVS2;SPOCK1;GPR158;ATP9A;OPCML;GABBR2;ARNT2;GABRA6;SYT1;CADM2;ATRNL1;SLC4A10;SYT16;KIAA1549L;MYO5A;ATP2B2;SNAP91;TTLL7;DLG2;SYNJ1;PPP2R2C;RCAN2;CNTN1;SCN2A;HCN1
BNC2 human tf ARCHS4 coexpression	71/299	3.0418558031735515E-6	NRP1;PTPRQ;COL14A1;CHRM5;EFCAB6;GXYLT2;C12ORF42;ANTXR1;PTPRG;EPS8;ADAMTSL1;TUBB6;ARHGAP42;SGCD;HMCN2;PHACTR2;TEAD1;PRKG1;ADAMTS6;POSTN;MUSK;DST;FNDC3B;ARID5B;NAV2;EML1;FAM126A;TBC1D1;FRMD6;ELF2;EVC;HEATR5A;NAV3;PEAK1;CDH13;ALPK2;TLN2;PPP1R12B;DOCK1;VCL;DDR2;RAI14;MACF1;SAR1A;FSTL1;LPP;LTBP1;GLIS1;ARSJ;PDGFC;CHSY3;PDLIM5;STARD13;EYA4;CRIM1;CYBRD1;ARHGAP28;LAMB1;GNG12;FBXO32;CDC42BPA;TJP1;PPFIBP1;COL5A1;DLC1;SLMAP;SSPN;ITGBL1;SNAI2;FBXL7;FBN1
TOX3 human tf ARCHS4 coexpression	71/299	3.0418558031735515E-6	ROBO2;DRAXIN;TENM4;ELAVL4;SLC35F1;SRGAP2C;ZNF608;NHSL1;DACH1;CDH2;DPYSL5;TRIM2;TMEM108;KIF21A;SOX6;EPHB2;SRGAP2B;MAGI1;WSB1;TMEM178B;LRRC49;MAGI3;TCF12;MAGI2;RFX3;GAREM1;CFDP1;IFT81;MPPED2;PDZRN3;ZNF234;CRB1;CHRNA7;ILDR2;NREP;NPAS3;FGD4;STOX2;GTF2IP1;NKAIN3;ZNRF3;SNTG1;ZNF704;ZNF627;SRGAP3;PAK3;JAM2;BPTF;RFTN2;FOXB1;ZNF462;FARP1;PTPRN2;MYEF2;AUTS2;PTCH1;RANBP17;LSAMP;ST18;PBX1;PTPRD;IGSF11;CCDC88A;NFIA

			;TTC3;YPEL1;FAT3;TCF4;KIAA1958;BMPR1B;TNRC6B
NCOR1 human tf ARCHS4 coexpression	71/299	3.0418558031735515E-6	SETD2;DOCK8;DPY19L2P2;GADL1;SYNE2;TTC28;UNC80;AKAP13;NIPBL;HERC2;HERC1;CA5A;ADAMTSL3;ZNF407;ERC1;GTF2I;MAP3K5;RBM33;USP8;MBNL1;ARHGEF12;VPS13C;ANK3;NBPF1;ARID1B;ZDHHC14;PEAK1;CNKSR3;WDPCP;WDFY3;BIRC6;ASTN2;UTRN;MACF1;NFAT5;KMT2C;ITPR2;ATP10B;LPP;MIPOL1;ATXN3;PCNX1;KIAA1328;HECTD4;ANKRD36BP2;ATP9B;BPTF;SPECC1;ARFGEF1;USP24;SPEN;ZNF462;CREBBP;RABGAP1L;MBD5;LRBA;MGA;DENND4C;YLPM1;ADAM32;LNPEP;PHC3;PARP15;MYO9A;MED13L;ARHGAP32;KANSL1;NEDD4;CDK12;ASB3;TNRC6B
MEF2A human tf ARCHS4 coexpression	70/299	6.151475741225199E-6	ATF1;RYR2;MYOM1;DOCK4;DOCK9;EFCAB14;LDB3;FRY;SLC8A1;AKAP13;FYCO1;EFR3A;ALCAM;SGCD;HERC1;PEBP4;KIF13A;MYO18B;PGM5;SGCG;SH3GLB1;MBNL1;MLIP;MBNL2;SFMBT2;VPS13C;TRDN;TOM1L2;ZNF717;MYL1;KCNMA1;BMP2K;ALPK3;KCNQ5;WDFY3;ALPK2;TLN2;PPP1R12B;MACF1;RABGAP1;ITPR2;CACNA1C;TRAK1;BCL2L13;ABLIM1;SCFD2;NRAP;TBX20;TNNI1;XIRP2;ZNF106;CTNNA3;HIVEP2;PDLIM5;SVIL;NEBL;EFL1;NEK7;SAMD4A;PDE4DIP;LNPEP;LHFPL2;PLXDC2;FBXO32;SMARCA2;MOB1B;ARHGAP31;ANKRD18A;RGL1;ASB2
TEAD1 human tf ARCHS4 coexpression	70/299	6.151475741225199E-6	TRIO;TENM3;MAST2;PTPRM;PTPRK;ANTXR1;FYCO1;TUBB6;SGCD;MPRIP;SH3PXD2A;KIF13A;MYO18B;SACS;PRKG1;ARHGEF12;DST;FNDC3B;AFAP1;NAV2;FRMD6;EVC;COL4A2;ALPK3;MXRA7;ALPK2;DOCK1;MET;VCL;CHST3;DDR2;RAI14;YAP1;NOTCH2;SEMA3C;NTN4;TMTC1;FSTL1;LTBP1;HECTD1;ARSJ;NRAP;ABL1;TNNI1;XIRP2;ZNF106;FLNB;PDLIM5;MPDZ;CTNNAL1;SVIL;NEK7;EYA4;SAMD4A;CRIM1;LAMB1;AP2B1;GNG12;FBXO32;EXT1;TJP1;ANLN;PPFIBP1;DPY19L1;COL5A1;DLC1;DLG5;FAT1;SNAI2;FBN1
ZNF436 human tf ARCHS4 coexpression	70/299	6.151475741225199E-6	SEMA5A;DRAXIN;THSD7B;SLC35F1;BICD1;PTPRG;ROBO1;SRGAP2C;TRIM9;ADAMTS3;CDH2;DPYSL5;TRIM2;AKT3;DNER;SACS;KIF21A;ERC1;SOX6;SRGAP2B;MAGI1;RBFOX2;DST;SEMA6D;FOXP2;TANC2;PJA2;ADGRB3;COL21A1;ZMYND11;VSTM2A;CHST3;EXOC1;GRIA1;MACF1;MAGEL2;SHC3;IGSF3;TNKS;ZBTB49;NREP;NPAS3;GNG2;ZNF704;MVB12B;APBB2;NCAM1;TSPAN3;JAM2;PAK5;VAT1L;RFTN2;NDFIP1;ST8SIA2;NTRK3;SORBS2;NETO2;AP2B1;CORO2B;DCLK1;PTPRD;DLG2;APC;RCAN2;TTC3;TRPV5;SPIRE1;TCF4;FBXL7;LRP12
ZNF248 human tf ARCHS4 coexpression	70/299	6.151475741225199E-6	MYT1L;PTPRO;ELAVL4;GRM3;DPYSL5;TRIM2;AKT3;DNER;HMCN2;KIF21A;RALGPS1;ANKS1B;PPFIA2;PRMT8;WSB1;RBFOX2;LRRC49;TMOD2;OPRM1;SEZ6L;PJA2;ADGRB3;LRRC7;PPP1R12B;MAPRE2;ANKRD36B;ASTN1;ZNF112;GRIA1;KHDRBS2;RTN1;TNKS;STXBP1;NRXN3;AKAP6;CACNA1C;NREP;NYAP2;CACNA1E;LPP;STOX2;MAPK8;GNG2;CLVS2;NCAM1;CTNNA2;SRGAP3;PAK3;GRIA4;GABRA2;CADM1;SYT1;ST8SIA2;LSAMP;DCLK1;PBX1;ENOX1;TTLL7;PTPRD;MAPK10;CCDC88A;DLG2;APC;KLHL7;SLMAP;TTC3;SCN2A;KIAA1958;SSBP2;APBA2
ID4 human tf ARCHS4 coexpression	70/299	6.151475741225199E-6	TENM4;CTNND2;GRIK4;ZBTB20;FMN2;SIPA1L2;SYNE2;GRM3;TRIM9;UBL3;DACH1;CDH2;TRIM2;KIF21A;NEO1;MAGI1;ADGRV1;TMEM178B;KCND3;LRRC49;RFX3;KAZN;SEZ6L;PARD3B;PYGO1;ANKFN1;IFT81;ADGRB3;PARD3;ZNF678;DGKI;CHST3;MAGEL2;PHLPP1;SHC3;NTM;ILDR2;APCDD1;NPAS3;STOX2;GTF2IP1;ZNF704;LRIG1;GPC5;NCAM1;TSPAN3;SRGAP3;SLIT2;MPDZ;JAM2;ARNT2;NTRK2;MBD5;NDFIP1;DTNA;MYEF2;VCAM1;AUTS2;ST8SIA1;PTCH1;PCDH7;FAM189A2;DCLK1;PBX1;PTPRD;TTC6;FABP7;SMOC1;SPIRE1;FAT3
BAZ2B human tf ARCHS4	70/299	6.151475741225199E-6	DPP10;CCDC122;ANKRD36;CHD9;USP33;GADL1;ELAVL4;FRG1HP;ZBTB20;EFCAB6;MYLK3;SYNE2;UNC80;DACH1;ZSCAN30;PPIP5K2;CA5A;ADAMTSL3;GATAD1;SCAPER;POTEC;MAGI1;ANKRD36C;WSB1;LRRC49;KCNH8;MAGI2;

coexpression			RFX3;VPS13B;FAM126B;SHISA9;ARID1B;PCCA;IFT81;PEAK1;WDPCP;WDFY3;ASTN2;ANKRD36B;SDCCAG8;CRB1;KMT2C;RGPD5;MIPOL1;STK3;FGD4;FGGY;KIAA1328;SNTG1;ANKRD36BP2;CSMD3;SRGAP3;LRRC4C;ATP9B;BPTF;RFTN2;MBD5;MYEF2;RANBP17;ADAM32;ARHGAP28;ST18;DCDC1;MAPK10;CCDC88A;FER;NEDD4;TTC3;ASB3;TNRC6B
PAX8 human tf ARCHS4 coexpression	69/299	1.2675352153749261E-5	COL18A1;PATJ;ATP8A1;DOCK9;CPQ;KIAA1671;PTPRM;ZBTB20;SIPA1L3;SYNE2;PTPRG;AKAP13;DEPTOR;MPRIP;MECOM;ENPP3;ADAMTS9;GTF2I;CUBN;ARHGEF12;COL23A1;ARAP2;WDR72;KIAA1217;TANC1;GOLGA8B;FNDC1;WDFY3;BIRC6;UTRN;DOCK1;MET;DGKI;FKBP5;ITGA9;YAP1;NOTCH2;MACF1;NFAT5;SEMA3D;SDC2;ARHGEF28;GLIS3;LRP2;PRDM11;RAP1GAP;THSD4;PKHD1;HECTD1;FLNB;ZBTB7C;FARP1;STARD13;HOMER2;LRBA;ZBTB16;SLC12A1;ZNF804B;FAM189A2;CYBRD1;SORBS2;LAMB1;MYO9A;PTPRB;ARHGAP31;MYO5B;GGT3P;ATP13A3;ADGRL2
ZFP28 human tf ARCHS4 coexpression	69/299	1.2675352153749261E-5	CYFIP2;GABRB3;SPAG16;DOCK3;FRMPD4;ANKRD36;CELF4;ZBTB20;ZNF44;UNC80;TRIM9;DACH1;DPYSL5;PEG10;AKT3;HYDIN2;BBS9;RGS7;GUSBP1;EPHA6;RBFOX2;ABCC8;PDE4D;PRKCA;ABCC9;SCAMP1;ARID1B;FOXP2;PJA2;CACNB2;LRRC7;MYO3A;AMPH;CNTNAP5;STX12;PPM1L;TNKS;NTM;NOL4;NALCN;SLC7A2;MTMR7;SDCBP;GRIN2A;FUT9;ZMAT4;NCAM1;CSMD2;ATP9A;OPCML;PTPRN2;NTRK3;LSAMP;FAM189A2;SLC4A10;MYO5A;CDC42BPA;GRIN2B;PBX1;PTK2;PTPRD;TMEM232;FER;NBEA;APC;GSTA3;RCAN2;TTC3;CPEB4
FMNL2 human tf ARCHS4 coexpression	69/299	1.2675352153749261E-5	APP;DOCK4;DOCK3;ATP8A1;CTNND2;TRIM9;SPRED1;AKAP11;MPRIP;TRIM2;MCF2L;PSD3;TNR;DLGAP1;DIP2B;EDIL3;KCNH1;RBFOX1;DST;TMOD2;MTUS1;KAZN;FAM219A;SYN2;MAPK8IP1;GABRG1;DNM3;SCN8A;WDFY3;TLN2;RAPGEF5;NGEF;ASTN1;RAPGEF4;SLC24A2;SHC3;PDE1C;STXBP1;SLC1A2;FAM171A1;CTIF;SV2B;SPOCK3;CLVS2;SPOCK1;NCAM1;GPR158;ATP9A;WASF3;MAP4K4;OPCML;ARNT2;DTNA;SYT1;CADM2;EXOC6B;KIAA1549L;MYO5A;ATP2B2;CORO2B;SNAP91;ETNPPL;TTLL7;TJP1;PTPRD;DLG2;PPP2R2C;SCN2A;OSBPL1A
TCF12 human tf ARCHS4 coexpression	68/299	2.5667347466297275E-5	MAML2;LDLRAD3;SLC35F1;KIF15;ROBO1;SRGAP2C;TRIM9;CDH2;SACS;TNR;SMARCAD1;SOX6;ZNF521;SRGAP2B;GTF2I;MAGI1;WSB1;ADGRV1;DST;DCC;SEMA6D;RFX3;ENAH;ASPM;IFT81;ADGRB3;TOX;ZNF112;CREB5;CRB1;STXBP4;TNKS;BTF3L4;ILDR2;KLHL13;NREP;NPAS3;STOX2;NKAIN3;SNTG1;ZNF704;LRIG1;APBB2;TSPAN3;SRGAP3;MPDZ;JAM2;SLC15A5;GRIA4;BBS2;RFTN2;ATF7IP;NTRK2;MYEF2;RANBP17;PUM1;CORO2B;ST18;PTPRD;IGSF11;MAPK10;CCDC88A;APC;FABP7;SMOC1;FAT3;TCF4;KIAA1958
ZBTB37 human tf ARCHS4 coexpression	68/299	2.5667347466297275E-5	CCDC122;ANKRD36;DOCK8;USP33;GADL1;FRY;LYST;MYSM1;MYLK3;SYNE2;AKAP13;NIPBL;PTAR1;RPS6KA5;C16ORF72;HERC1;ZSCAN30;CA5A;ADAMTSL3;LONP2;POTEC;VPS13C;VPS13B;ABCC9;SHISA9;ARID1B;PARP8;PHF20L1;PEAK1;CNKSR3;WDPCP;WDFY3;BIRC6;PIK3C3;ASTN2;UTRN;MCTP2;KDM7A;MACF1;NFAT5;KMT2C;RGPD6;RGPD5;LPP;MIPOL1;ATXN3;PCNX1;KIAA1328;ANKRD36BP2;TRPM7;ATP9B;BPTF;RABGAP1L;MBD5;INSR;MGA;ERBIN;ADAM32;LNPEP;ARHGAP28;PHC3;PARP15;KANSL1;NEDD4;DMXL2;PTPN4;ASB3;TNRC6B
ZNF292 human tf ARCHS4 coexpression	68/299	2.5667347466297275E-5	SLC44A5;TCERG1;CCDC122;ANKRD36;CHD9;ELAVL4;EFCAB6;SYNE2;GRM7;ZSCAN30;PPIP5K2;TRIM2;ADAMTSL3;KIF21A;ANKRD36C;WSB1;HFM1;DCC;LRRC49;VPS13C;MAGI2;RFX3;VPS13B;FAM126B;ARID1B;IFT81;LRRC7;HECW1;MPPED2;WDPCP;PIK3C3;ASTN2;ANKRD36B;CHRNA7;KMT2C;RGPD6;RGPD5;NREP;CACNA1E;MTMR7;MIPOL1;MAPK8;GNG2;KIAA1328;SNTG1;ANKRD36BP2;SRGAP3;CSMD2;PAK3;ATP9B;PAK5;BPTF;RIC3;ZNF462;MBD5

			;MYEF2;ADAM32;DCDC1;CCDC88A;NBEA;KLHL7;NEDD4;ASXL3;TTC3;TCF4;SSBP2;ASB3;TNRC6B
PGR human tf ARCHS4 coexpression	67/299	4.809324016235369E-5	PCSK2;ERO1B;PATJ;ZBTB20;ECE1;RORB;SERPINA6;AFF3;IGF1R;POTEKP;SCGN;PPP6R3;TMED3;LONP2;PLCE1;PAMR1;ADAMTS9;VAV3;VWFP1;ARHGEF12;DST;VPS13C;MAGI2;VPS13B;TBC1D9;PRLR;CACNB2;ALDH1A2;PPP1R12B;FREM1;DOCK1;MACF1;SEMA3C;KMT2C;PRICKLE2;LRP2;FAM214A;LPP;SLC7A2;THSD4;TTR;ZNF704;GSE1;FLNB;AKAIN1;ARFGEF1;STARD13;ABCA5;ANKRD30A;LRBA;PTCH1;LAMB1;TDRD5;CDC42BPA;ELL2;ESR1;MPP7;MED13L;MOB1B;SDK1;PLCXD3;DLG5;MYO5C;VPS41;NEK10;CPE;CNTN4
HIVEP1 human tf ARCHS4 coexpression	67/299	4.809324016235369E-5	ANKRD33B;MAML2;KDM1B;IKZF2;BACH1;MYSM1;DOCK10;AKAP13;NIPBL;RPS6KA5;C16ORF72;HERC1;ADAMTSL3;ZNF407;RNF111;STPG2;KSR1;SFMBT2;VPS13C;FNDC3B;ARAP2;VPS13B;ARID1B;TRAF3;PEAK1;WDPCP;WDFY3;BIRC6;UTRN;DOCK2;WDFY4;KDM7A;MACF1;NFAT5;MTMR3;WDR26;KMT2C;ITPR2;IQGAP1;AMBRA1;PCNX1;SCAF8;BTAF1;ATXN1;KIAA1328;NLRP4;BTLA;TRPM7;SUSD6;HIVEP2;ATP9B;LYN;USP24;SPEN;CREBBP;LRBA;TRAPPC10;MGA;LNPEP;DNAJC13;SMARCA2;PARP15;GATAD2B;MED13L;NLRP13;DMXL2;CDK12
KLF12 human tf ARCHS4 coexpression	67/299	4.809324016235369E-5	CYFIP2;ABCD2;MAML2;DOCK8;CEP120;RORA;LYST;SYNE2;DOCK10;AKAP13;RASSF2;HERC1;TRIM2;SACS;UBASH3A;KIF21B;RBM33;CD96;MBNL1;RBFOX2;PRKCH;ITGA4;CACNA2D1;VPS13C;TCF12;ARAP2;RFX3;VPS13B;TC2N;IPCEF1;ZDHHC17;PARP8;PHF20L1;ITPKB;GOLGA8B;DPYD;BIRC6;TNIK;UTRN;DOCK2;KDM7A;MACF1;TNKS;KMT2C;NREP;RASGRP1;CACNA1I;STOX2;PCNX1;GNG2;NCAM1;FYN;HIVEP2;CREBBP;BCL11B;TRAPPC10;ERBIN;MYO5A;LNPEP;DCLK1;CCDC88A;APC;KANSL1;TTC3;CCSER2;TCF4;PTPN4
ZNF510 human tf ARCHS4 coexpression	67/299	4.809324016235369E-5	DIDO1;LPGAT1;TRIO;ATP8A1;ZFYVE9;DOCK9;USP32;RASGRF2;CEP120;ZBTB21;NIPBL;PTAR1;GRM5;HERC2;ZNF608;AKAP11;HERC1;SACS;DIP2B;GTF2I;MED1;WDHD1;CD96;ARHGEF12;DST;PRKCE;VPS13C;TMOD2;VPS13B;WDFY3;BIRC6;TNIK;RAPGEF5;UTRN;DGKI;MACF1;MTPN;KMT2C;PCNX1;SCAF8;BTAF1;HECTD1;ZNF704;HECTD4;GTF2IP4;HIVEP2;ATP9A;ARFGEF1;USP24;SPEN;KDM4C;LRBA;TRAPPC10;MGA;ERBIN;MYO5A;LNPEP;DNAJC13;ESRRG;AP2B1;SMARCA2;MTOR;MED13L;APC;SP3;FAT3;CDK12
ZNF827 human tf ARCHS4 coexpression	67/299	4.809324016235369E-5	DPP10;DRAXIN;MYT1L;CHD9;SYCP1;CTNND2;ZBTB20;BICD1;PTPRG;IGF1R;ZNF608;NHSL1;DACH1;CDH2;SCN11A;ADAMTSL3;KIF21A;STK32A;PPFIA2;MAGI1;TMEM178B;CACNA2D1;LRRC49;HUNK;RFX3;FRMD4A;ZDHHC17;ARID1B;ENAH;PEAK1;ASTN2;ZNF236;ARHGEF7;TNKS;CHRNA7;ILDR2;NREP;MIPOL1;NPAS3;PHF21B;STOX2;PDCD6IPP2;SNTG1;FUT9;NCAM1;SRGAP3;JAM2;BPTF;ZNF462;FARP1;MYEF2;AUTS2;NTRK3;RANBP17;CADPS;SORBS2;DCLK1;PBX1;PTPRD;CCDC88A;NFIA;PPP2R2B;ASXL3;TTC3;RNF182;TCF4;TNRC6B
MYSM1 human tf ARCHS4 coexpression	67/299	4.809324016235369E-5	TCERG1;DIDO1;SETD2;LPGAT1;SMG1P2;RORA;SYNE2;AKAP13;NIPBL;PTAR1;C16ORF72;HERC1;JARID2;RBM33;MBNL1;USP7;VPS13C;ARAP2;VPS13B;TC2N;ARID1B;PARP8;MSH6;GOLGA8B;FAR1;WDFY3;BIRC6;UTRN;KDM7A;MACF1;NFAT5;WDR26;ROCK1;KMT2C;IREB2;IQGAP1;LPP;PCNX1;SCAF8;LARP1;BTAF1;HECTD1;HECTD4;TRPM7;MDN1;BPTF;ARFGEF1;USP24;SPEN;KDM4C;LRBA;TRAPPC10;MGA;ERBIN;LNPEP;PHC3;SCAF4;MTOR;MED13L;TJP1;MLLT10;KANSL1;AGO2;DMXL2;SP3;PKN2;CDK12
GATAD2B human tf ARCHS4 coexpressi	67/299	4.809324016235369E-5	CYFIP2;RERE;SETD2;DOCK8;LDLRAD4;CMIP;SYNE2;IGF1R;AKAP13;NIPBL;AKAP11;HERC1;AKT3;KIF21B;ERC1;RBM33;CAMK1D;TMEM178B;PRKCB;SFMBT2;ANK3;ARID1B;TANC2;AJAP1;UBE2R2;RFX7;PACS1;MADD;FAM193A;TNIK;ARHGEF7;MACF1;NFAT5;WDR26;ANP32A;KMT2C

on			;ANKRD11;KALRN;KIAA1328;HECTD4;GSE1;DROSHA;TRPM7;NCAM1;HIVEP2;PAK5;BPTF;SPEN;CREBBP;KDM4B;AUTS2;IQSEC1;TRAPPC10;PTCH1;MICAL3;LSAMP;YLPM1;LNPEP;SMARCA2;SCAF4;MED13L;DLG2;NBEA;KANSL1;AGO2;CDK12;TNRC6B
CHD7 human tf ARCHS4 coexpression	67/299	4.809324016235369E-5	TCERG1;DRAXIN;TENM4;ELAVL4;PSIP1;GLI3;SYNE2;KIF15;PTPRG;ZNF608;NHSL1;DACH1;CDH2;DPYSL5;ADAMTSL3;SMARCAD1;KIF21A;SOX6;SRGAP2B;MAGI1;WSB1;DCC;ANKRD20A11P;LRRC49;MAGI3;TCF12;RFX3;ENAH;MMP16;IFT81;CCDC150;UBE2Q2P1;CRB1;PPM1L;TNKS;BTF3L4;GREB1L;ILDR2;NREP;KALRN;PHF21B;FGD4;STOX2;CECR2;KIAA1328;SRGAP3;MPDZ;BPTF;ATF7IP;ZNF462;FARP1;MYEF2;AUTS2;ST8SIA2;RANBP17;ST18;MAPK10;CCDC88A;NFIA;KANSL1;AGO1;KLHL7;TTC3;YPEL1;TCF4;KIAA1958;ASB3
CHD2 human tf ARCHS4 coexpression	67/299	4.809324016235369E-5	RERE;SETD2;DOCK8;SMG1P2;RORA;LYST;SYNE2;DOCK10;AKAP13;NIPBL;C16ORF72;HERC1;ADAMTSL3;ZNF407;LRRFIP1;RBM33;MBNL1;PRKCH;ITGA4;VPS13C;ARAP2;VPS13B;ARID1B;PARP8;GOLGA8B;NCOR1;PEAK1;FAM153A;BIRC6;UTRN;DOCK2;MCTP2;KDM7A;MACF1;NFAT5;WDR26;KMT2C;RGPD6;RGPD5;RGPD8;PCNX1;BTAF1;KIAA1328;HECTD4;TRPM7;HIVEP2;ATP9B;BPTF;LRRC37A3;USP24;SPEN;CREBBP;MBD5;KDM4C;LRBA;TRAPPC10;MGA;YLPM1;LNPEP;ARHGAP26;SMARCA2;PHC3;PARP15;MED13L;KANSL1;DMXL2;CDK12
ZFHX4 human tf ARCHS4 coexpression	66/299	9.241681330475861E-5	ROBO2;DRAXIN;PTPRO;CELF4;ELAVL4;SLC35F1;GRIK2;ZNF608;DACH1;CDH2;DPYSL5;DNER;KIF21A;ZNF521;ANKS1B;GARNL3;PPFIA2;KIRREL3;MAGI1;WSB1;TMEM178B;DCC;LRRC49;KCNH8;MAGI2;EBF1;RFX3;FRMD4A;ANK3;PGM2L1;FOXP2;IFT81;AMPH;ZNF397;CHRNA7;GREB1L;TPTE2P2;NREP;EFNA5;PHF21B;FGD4;STOX2;GTF2IP1;NCAM1;CTNNA2;SRGAP3;PAK3;ZNF423;LINGO2;ZNF462;MYEF2;AUTS2;NEGR1;ST8SIA1;ST8SIA2;NTRK3;PBX3;LSAMP;PBX1;MAPK10;CCDC88A;PPP2R2B;TTC3;FAT3;KIAA1958;LHX9
KLF7 human tf ARCHS4 coexpression	66/299	9.241681330475861E-5	ROBO2;DRAXIN;MYT1L;PTPRO;CELF4;ELAVL4;GRIK1;GRIK2;BICD1;GPHN;GRIP1;ZNF608;DPYSL5;TRIM2;AKT3;DNER;ZNF449;KIF21A;RALGPS1;PPFIA2;WSB1;RBFOX2;DCC;RC3H1;RFX3;ANK3;ZDHHC17;PGM2L1;LRRC7;ALPK3;XPR1;EXOC1;DEFB103A;GRIA1;RTN1;ATL1;RASAL2;NREP;KALRN;STOX2;GNG2;ZNF627;MYH13;TNNI1;APBB2;NCAM1;CTNNA2;SRGAP3;PAK3;PAK5;MBD5;OSBPL6;CADM1;AUTS2;ST8SIA2;MYO5A;UBE2G1;SYNJ2;DCLK1;ST18;CCDC88A;TTC3;YPEL1;TCF4;FSIP1;FBXL7
ZNF608 human tf ARCHS4 coexpression	66/299	9.241681330475861E-5	GABRB3;RERE;SH3GL3;DRAXIN;TENM3;TENM4;CTNND2;ELAVL4;ROBO1;CDH4;DACH1;CDH2;DPYSL5;PEG10;TRIM2;HYDIN2;NEO1;PPFIA2;MAGI1;WSB1;RBFOX2;TMEM178B;RFX3;FRMD4A;TNKS;GREB1L;ILDR2;NREP;KALRN;PHF21B;STOX2;CECR2;FLRT2;ZNF704;HECTD4;NCAM1;CTNNA2;SRGAP3;PAK3;ZNF423;MPDZ;PLXNA4;MAP4K4;BPTF;LRRC37A3;ZNF462;AUTS2;ST8SIA2;YLPM1;ESRRG;PTPN13;DCLK1;PBX1;PTPRD;CCDC88A;SDK1;NBEA;APC;ZNF618;ASXL3;TTC3;TCF4;KIAA1958;EIF4G3;ADGRL2;TBATA
ZBTB41 human tf ARCHS4 coexpression	66/299	9.241681330475861E-5	PCSK2;ROBO2;ERO1B;TUSC3;PCMTD2;TRIM9;SCGN;ZSCAN30;TRIM2;PPP6R3;LONP2;PCMTD1;TTC37;WSB1;DST;CACNA2D1;SLC2A13;LRRC49;VPS13C;RFX3;FNDC3A;SEZ6L;PJA2;CACNB2;MMP16;MYO3A;PIGK;FICD;SAR1A;STXBP4;TNKS;BTF3L4;UBR1;NOL4;NALCN;NR2C1;TTR;TTC21B;SYNDIG1;PAK3;EVI5;MON2;PTPRN2;NDFIP1;ABCA5;CADPS;SYT16;CDC42BPA;ELL2;OXR1;ST18;MOB1B;MAPK10;ITCH;NBEA;APC;ASXL3;TTC3;VPS41;CNTN1;CPE;CNTN4;SCN2A;PTPN4;LRP12;CPEB4
ZNF521 human tf	66/299	9.241681330475861E-5	ZNF573;CHRM3;DRAXIN;TENM3;WNT2B;TENM4;CTNND2;CELF4;LDLRAD3;CDH9;CDH8;TTC28;ZNF608;DACH1;CDH2;DPYSL5;PSD3;CALN1;WSB1;RBFOX2;DCC;LRRC49;T

ARCHS4 coexpression			CF12;RFX3;EML1;PYGO1;IFT81;ST6GALNAC3;ASTN1;ZNF397;KHDRBS2;CHRNA7;GREB1L;ILDR2;NREP;HERC2P9;NPAS3;STOX2;CAMTA1;NCAM1;TSPAN3;CTNNA2;SRGAP3;SLIT2;ZNF423;MPDZ;TRPM3;JAM2;WASF3;TMEM132C;MYEF2;AUTS2;ANGPT1;PBX3;PBX1;PTPRD;MAPK10;NELL2;MEIS1;ZNF618;COL5A3;TTC3;YPEL1;KIAA1958;SSBP2;ADGRL2
HIVEP2 human tf ARCHS4 coexpression	65/299	1.7619210894916884E-4	CYFIP2;MAML2;ATP8A1;FRMPD4;DOCK9;DOCK8;RASGRF2;RORA;DOCK10;RPH3A;AKAP13;AKAP11;HERC1;MPRIP;PSD3;DLGAP1;MAP3K5;KCNH1;ARHGEF11;RBFOX1;MBNL1;PRKCH;PRKCB;PRKCE;VPS13C;TMOD2;ARAP2;IPCEF1;SYN2;ITPKB;PACS1;MADD;KCNQ5;WDFY3;RAPGEF5;UTRN;DOCK2;NGEF;KDM7A;MACF1;NFAT5;SLC1A2;IQGAP1;RASGRP1;KIAA0513;CACNA1I;GRIN2A;PCNX1;ABLIM1;ATXN1;SV2B;HECTD4;CREBBP;BCL11B;IQSEC1;ERBIN;MYO5A;PDE4DIP;LNPEP;ATP2B2;ARHGAP26;SMARCA2;GRIN2B;SYNJ1;PPP2R2C
ZHX1 human tf ARCHS4 coexpression	65/299	1.7619210894916884E-4	PCSK2;LIN54;ERO1B;ABCD3;ZFYVE9;CPQ;USP32;LAMC3;RAB22A;SCGN;EFR3A;AKAP11;TRIM2;PSD3;ANKRD31;LONP2;BCAP29;KIF21A;DIP2B;SOX6;DIP2C;SCAPER;PCMTD1;PPFIA2;MBNL2;ARHGEF12;MUSK;DST;ARL15;ZNF160;FNDC3A;ITFG1;TANC2;PJA2;CMPK1;WDFY3;ARHGEF7;MICU1;ZMYND11;STX12;ZDHHC21;SLC30A10;NOL4;SNTG2;HECTD1;FCHO2;GSE1;MAPK1;ATP9A;MON2;NDFIP1;ABCA5;ERBIN;SYT16;CDC42BPA;ELL2;MOB1B;ARHGAP32;SYNJ1;APC;TTC3;CNTN1;CCSER2;SPIRE1;CPE
ZKSCAN2 human tf ARCHS4 coexpression	65/299	1.7619210894916884E-4	TENM4;ZFYVE9;ZBTB21;ELAVL4;PSIP1;KIF11;BICD1;TXNDC16;GLI3;PTPRG;ROBO1;ZNF608;CDH2;DPYSL5;TRIM2;AKT3;DNER;SACS;SMARCAD1;KIF21A;NEO1;MAGI1;SUPT16H;WSB1;RBFOX2;CSNK2A1;DCC;CACNA2D1;RFX3;NBPF1;MSH6;ADGRB3;DOK5;MPPED2;ZNF678;ST6GALNAC3;GREB1L;NREP;EHBP1;PHF21B;STOX2;FUT9;ZNF704;CLVS2;DROSHA;NCAM1;RPRD1A;SRGAP3;MPDZ;VAT1L;BPTF;ZNF462;FARP1;MYEF2;AUTS2;ST8SIA2;YLPM1;PUM1;BTBD10;PBX1;PLEKHA8;PTPRD;CCDC88A;FAT3;ADGRL2
ZFP1 human tf ARCHS4 coexpression	65/299	1.7619210894916884E-4	ELAVL4;GRIK2;BICD1;TPGS2;ROBO1;CDH2;DPYSL5;LRRTM4;TRIM2;DNER;PHACTR3;KIF21A;PPFIA2;RGS7;WSB1;RBFOX2;RFX3;FOXP2;PYGO1;FRMD5;IFT81;ADGRB3;DOK5;MPPED2;IL1RAPL1;XPR1;MAGEL2;NLGN1;RTN1;STAU2;TNKS;BTF3L4;NRXN3;NREP;NOL4;MAPK8;GNG2;FUT9;ZMAT4;CLVS2;CTNNA2;SRGAP3;PAK5;OPCML;CLVS1;ATF7IP;CADM2;ST8SIA2;RANBP17;PBX3;LSAMP;KLHL1;ESRRG;BTBD10;PTPRD;MAPK10;CCDC88A;NFIA;NBEA;APC;NFIB;KLHL7;TTC3;ZNF536;MDGA2
ARX human tf ARCHS4 coexpression	64/299	3.2166063352786627E-4	PCSK2;ROBO2;ERO1B;TUSC3;PTPRO;ELAVL4;HS6ST3;SCGN;GRM7;DPYSL5;TRIM2;ZNF846;PLCE1;SOX6;RGS7;ANKRD36C;PACRG;KCND3;LRRC49;MAGI2;HUNK;CACNB2;MMP16;MPPED2;VSTM2A;PDE1A;BTF3L4;NRXN3;RGPD5;NREP;NOL4;SLC7A2;MTMR7;NPAS3;GNG2;TTR;SNTG1;NCAM1;PAK3;CORIN;AKAIN1;PRELID2;RIC3;ANKRD26;PTPRN2;MYEF2;CADPS;POU6F2;ELL2;ST18;DCDC1;MOB1B;MAPK10;CCDC88A;KLHL7;ASXL3;TTC3;CNTN1;ZNF536;CPE;TCF4;CNTN4;FGF12;XKR5
RFX3 human tf ARCHS4 coexpression	64/299	3.2166063352786627E-4	FANK1;SPAG16;OSCP1;ELAVL4;EFCAB6;TRIM9;ZNF608;DACH1;CDH2;DPYSL5;LRRTM4;TRIM2;SNAPC3;HYDIN;KIF21A;MAGI1;WSB1;ADGRV1;DCC;LRRC49;TCF12;IFT81;LRRC7;ASTN1;CFAP61;GRIA1;DNAH3;TNKS;DNAH6;GREB1L;ILDR2;AKAP6;DNAH9;NREP;NOL4;NPAS3;STOX2;GNG2;FUT9;TMEM67;TSPAN3;CTNNA2;CSMD3;SRGAP3;SLIT2;PAK3;ZNF462;NTRK2;MYEF2;AUTS2;CFAP70;VWA3B;DCLK1;PTPRD;TMEM232;MAPK10;NELL2;CCDC88A;APC;FABP7;TTC3;FAT3;SSBP2;PPIL6
NEUROD6 human tf	64/299	3.2166063352786627E-4	TCERG1;CNTNAP2;ZNRF2P2;MYT1L;PTPRO;GRIK3;RORB;BICD1;SRGAP2C;SLC22A14;NHSL1;ADAMTS3;TRIM2;AKT3;TMEM108;PHACTR3;SOX5;RGS7;EPHA7;RBFOX2;CA

ARCHS4 coexpression			*MLG;KAZN;EML1;MPPED1;LRRC7;HECW1;KCNQ3;RAPGEF2;ZFPM2;XPR1;SHANK2;LINC00643;GRIA1;SLC24A2;CTTNBP2;NREP;NYAP2;NOL4;CACNA1E;AP5M1;PCBP3;PAK5;OPCML;CLVS1;OSBPL6;BCL11B;SIAH3;NTRK3;SORBS2;CORO2B;ST18;PTK2;SNAP91;NELL2;DAB1;NFIA;NBEA;APC;NFIB;PPP2R2B;TTC3;TCF4;SSBP2;LRP12*
ETV5 human tf ARCHS4 coexpression	64/299	3.2166063352786627E-4	*SEMA5A;GLDC;LDLRAD3;FMN1;KIF11;KIF15;PTPRG;EPS8;GRM3;SRGAP2C;SPRED2;TUBB6;ARHGAP42;SPRED1;NHSL1;PSMD2;NUF2;BCAP29;HMCN1;STK32A;TEAD1;SRGAP2B;SUPT16H;CSNK2A1;KCND3;TCF12;FNDC3B;RC3H1;MITF;SEZ6L;EPN2;ENAH;TANC1;MELK;FRMD6;DOCK1;CHST3;EXOC1;RAI14;MTMR2;SHC3;ILDR2;ASAP2;C10ORF90;SDCBP;MGAT5;ARNT2;STARD13;EFL1;MYO5A;HMGA2;UBE2G1;NETO2;SYNJ2;GNG12;CDC42BPA;CORO2B;NDC80;ANLN;PPFIBP1;DPY19L1;FABP7;SPIRE1;FBXL7*
FOXE1 human tf ARCHS4 coexpression	64/299	3.2166063352786627E-4	*DOCK5;PATJ;ATP8A1;DOCK9;CPQ;GALNT18;KIAA1671;PTPRM;SYNE2;AKAP13;MPRIP;KIF13A;GTF2I;KRT6A;CAST;ARHGEF12;COL23A1;RIPK4;ARAP2;WDR72;KIAA1217;TOM1L2;TANC1;GOLGA8B;FNDC1;BIRC6;MXRA7;UTRN;DOCK1;MET;DGKI;FKBP5;ITGA9;YAP1;NOTCH2;MACF1;SEMA3D;SDC2;ARHGEF28;GLIS3;NTN4;LRP2;RAP1GAP;THSD4;HECTD1;ABL1;FLNB;CDH26;CTNNAL1;ZBTB7C;STARD13;ZBTB16;ZNF804B;FAM189A2;CYBRD1;SORBS2;MYO9A;LRP1B;ARHGAP31;TG;MYO5B;GGT3P;ATP13A3;FBN1*
ZNF300 human tf ARCHS4 coexpression	64/299	3.2166063352786627E-4	*SLC44A5;DRAXIN;USP33;PTPRO;ELAVL4;GRIK2;TXNDC16;CDH8;TPGS2;ROBO1;GRIP1;NHSL1;CDH2;GRM7;DPYSL5;PPIP5K2;TRIM2;PHACTR3;SMARCAD1;KIF21A;PPFIA2;WSB1;RBFOX2;ARL15;LRRC49;TCF12;MAGI2;RFX3;ITFG1;PGM2L1;IFT81;LRRC7;PDZRN4;XPR1;GRIA1;RTN1;STAU2;BTF3L4;NRXN3;NREP;NOL4;STOX2;MAPK8;GNG2;ZNF627;NCAM1;CTNNA2;PAK5;MYEF2;CADM1;ST8SIA2;ZNF804B;PBX3;LHFPL3;PTPRD;MAPK10;CCDC88A;RALYL;APC;KLHL7;TTC3;CCSER1;TCF4;SSBP2*
TOX human tf ARCHS4 coexpression	64/299	3.2166063352786627E-4	*ROBO2;DRAXIN;ELAVL4;GRIK2;CDH8;DACH1;DPYSL5;TRIM2;SH3GL2;GARNL3;WSB1;LRRC49;TCF12;MAGI2;RFX3;ZDHHC17;IFT81;ADGRB3;MAPRE2;XPR1;ANKRD36B;ASTN1;CRB1;MAGEL2;KHDRBS2;RTN1;PDE1A;BTF3L4;NRXN3;NREP;NOL4;NKAIN3;PDCD6IPP2;GNG2;SNTG1;FUT9;DPH6;NCAM1;TSPAN3;CTNNA2;SRGAP3;PAK3;JAM2;GRIA4;GABRA2;ATF7IP;CA10;MYEF2;ST8SIA1;ST8SIA2;PBX3;SLC4A10;MAPK10;NELL2;AGBL4;LRFN5;RALYL;APC;KLHL7;TTC3;ZNF536;SSBP2;FGF12;LRP12*
DACH1 human tf ARCHS4 coexpression	63/299	5.92140279591383E-4	*ROBO2;DRAXIN;TENM4;CTNND2;CELF4;ELAVL4;RIMS2;GRIP1;CDH4;ZNF608;CDH2;DPYSL5;EPHB2;ZNF521;PPFIA2;DCC;LRRC49;KCNH8;MAGI3;EBF2;RFX3;PGM2L1;HECW1;PRR16;CNTNAP5;PPM1L;CHRNA7;TSHZ2;GREB1L;NREP;KALRN;NPAS3;PHF21B;STOX2;GTF2IP1;PDCD6IPP2;NCAM1;CTNNA2;SRGAP3;PAK3;ZNF423;LRRC4C;JAM2;CLVS1;ZNF462;PTPRN2;MYEF2;ST8SIA1;ST8SIA2;CADPS;PBX3;ST18;PBX1;PTPRD;MAPK10;CCDC88A;ZNF618;AGO1;ASXL3;TTC3;KIAA1958;VSX1;LHX9*
ARNT2 human tf ARCHS4 coexpression	63/299	5.92140279591383E-4	*DOCK3;FRMPD4;MYT1L;CTNND2;RPH3A;TRIM9;GRM5;TRIM2;MCF2L;PSD3;DLGAP1;KCNH1;RBFOX1;TMEM178A;TMOD2;FAM219A;SYN2;MAPK8IP1;GABRG1;AJAP1;CNKSR2;MPPED1;SCN8A;ADGRB1;RAPGEF5;NGEF;ASTN1;RAPGEF4;SHC3;STXBP1;SLC1A2;KALRN;RAP1GAP;KIAA0513;CTIF;GRIN2A;PGBD5;SV2B;GNG7;NCAM1;CTNNA2;GPR158;ATP9A;WASF3;OPCML;GABBR2;NDFIP1;DTNA;SYT1;CADM2;NTRK3;KIAA1549L;MYO5A;ATP2B2;CORO2B;DCLK1;SNAP91;DLG2;PPP2R2C;RCAN2;CNTN1;SCN2A;APBA2*
ZNF354B human tf ARCHS4	63/299	5.92140279591383E-4	*DIDO1;DOCK5;TRIO;DPY19L2P2;BACH1;ANTXR1;RPTOR;AKAP13;POTEM;PTAR1;HERC2;C16ORF72;HERC1;ZNF407;SACS;ARHGEF12;VPS13C;PDE4D;VPS13B;URB1;PEAK1;WDPCP;RELL1;WDFY3;BIRC6;UTRN;MCTP2;NOTCH2;*

coexpression			*MACF1;NFAT5;NBAS;KMT2C;ITPR2;LPP;ATXN3;PCNX1;KIAA1328;HECTD1;HECTD4;TRPM7;ATP9B;USP24;ZFHX3;ST8SIA1;LRBA;TRAPPC10;MGA;MOCOS;MICAL3;ERBIN;FANCA;MTOR;MED13L;TMPRSS15;MYO1E;NEDD4;SLMAP;AGO2;DMXL2;SP3;FAT1;CDK12;KIAA0825*
SCAPER human tf ARCHS4 coexpression	63/299	5.92140279591383E-4	*PCSK2;ROBO2;DPP10;ERO1B;MYT1L;ANKRD36;CHD9;FRG1HP;EFCAB6;RIMS2;UNC80;GRM7;ZSCAN30;LRRTM4;ADAMTSL3;KIF21A;ZNF568;ANKS1B;ANKRD36C;TMEM178B;MAGI2;OPRM1;SEZ6L;ARID1B;DNM3;LRRC7;ASTN2;ANKRD36B;SDCCAG8;LUZP2;PCDH15;RGPD6;RGPD5;ILDR2;RASAL2;MTMR7;MIPOL1;AK9;SNTG2;TTR;SNTG1;ZNF704;ANKRD20A5P;ANKRD36BP2;NCAM1;SRGAP3;PAK3;CORIN;BPTF;RIC3;DCDC1;TTLL7;MAPK10;CCDC88A;FER;NBEA;ASXL3;TTC3;TCF4;CNTN4;PTPN4;ASB3;CCDC171*
RARB human tf ARCHS4 coexpression	62/299	0.0010536650874352492	*SEMA5A;RYR2;BNC2;ZFYVE9;SYCP1;PTPRM;SLC2A3;LDB2;SLC8A1;ROBO1;ARHGAP42;CDH2;RGS8;DIRC3;RNF152;TEAD1;ADAMTS9;PRKG1;KCNH1;POSTN;MUSK;TPM1;WDR72;DKK2;CNKSR2;TANC1;AGPS;ALPK2;TLN2;ST6GALNAC3;FREM1;DOCK1;KANK4;TRABD2B;DGKI;DDR2;RAI14;FBN2;FOCAD;SAR1A;SEMA3D;SEMA3E;RANBP3L;FLRT2;ERBB4;ALX4;ZNF423;RXRG;MPDZ;STARD13;CNTN5;NEBL;TXNRD2;PCDH7;LAMB1;GNG12;MCC;PTPN13;ARHGAP24;TJP1;DLC1;SNAI2*
EBF3 human tf ARCHS4 coexpression	62/299	0.0010536650874352492	*APP;DRAXIN;MYT1L;CTNND2;CELF4;ELAVL4;XYLT1;SLC6A3;ROBO1;ZNF608;DACH1;DPYSL5;PEG10;DNER;KIF21A;KIF21B;EPHB2;NEO1;CHST8;RBFOX2;DCC;CACNA2D1;EBF1;AFAP1;EBF2;GFRA1;AJAP1;ELF2;PDZRN4;PPM1L;NTM;NREP;NYAP2;KALRN;NPAS3;PHF21B;STOX2;GTF2IP1;GNG2;NCAM1;CTNNA2;SRGAP3;ZNF423;ATP9A;MAP4K4;KCNJ6;NDFIP1;MYEF2;AUTS2;ST8SIA2;PLCL1;PBX3;LSAMP;DCLK1;PBX1;ENOX1;PTPRD;CCDC88A;ZNF618;ZNF536;FAT3;APBA2*
NR3C1 human tf ARCHS4 coexpression	62/299	0.0010536650874352492	*PI4K2B;TRIO;ANKRD33B;ATP8A1;SH3KBP1;DOCK8;EFCAB14;IKZF2;BACH1;LYST;FAM107B;DOCK10;AKAP13;EFR3A;HERC1;KIF13A;DIP2B;LRRFIP1;MAP3K5;CAST;CD96;MBNL1;ITGA4;PRKCB;VPS13C;ARAP2;APBB1IP;TRAF3;DPYD;AGPS;BIRC6;UTRN;DOCK2;WDFY4;KDM7A;NOTCH2;WDR26;ITPR2;IQGAP1;SAMSN1;PCNX1;BTAF1;RAP1A;ATXN1;BTLA;SUSD6;HIVEP2;LYN;USP24;TRAPPC10;NEK6;RFTN1;NEK7;ERBIN;LNPEP;DNAJC13;SMARCA2;MED13L;MYO1E;ARHGAP31;SP3;ATP13A3*
RREB1 human tf ARCHS4 coexpression	62/299	0.0010536650874352492	*DIDO1;PATJ;DOCK8;KIAA1671;LIMD1;SIPA1L3;CABIN1;RPTOR;AKAP13;HERC2;HERC1;ZNF407;LRRFIP1;MAP3K5;RBM33;NUP214;VAV3;MBNL1;ITGA4;SFMBT2;VPS13C;FNDC3B;VPS13B;URB1;ARID1B;RUNX1;NCOR1;BMP2K;WDPCP;BIRC6;PPARA;UTRN;DOCK2;WDFY4;MACF1;NFAT5;CTDP1;KMT2C;ITPR2;LPP;PCNX1;KIAA1328;HECTD1;CUX1;TRPM7;ATP9B;MDN1;USP24;SPEN;CREBBP;ATP8B4;LRBA;TRAPPC10;MGA;LNPEP;SMARCA2;PHC3;PARP15;MED13L;DIAPH1;AGO2;CDK12*
ZNF81 human tf ARCHS4 coexpression	62/299	0.0010536650874352492	*MCTP1;THSD7B;ATP8A1;DOCK8;USP33;LYST;MYSM1;RAB22A;AKAP13;NIPBL;RASSF2;C16ORF72;HERC1;JAK2;TRPC7;MBNL1;VPS13C;VPS13B;FAM126B;PJA2;PHF20L1;KCNQ3;WDFY3;BIRC6;UTRN;DOCK2;MCTP2;KDM7A;CNTNAP5;MACF1;NFAT5;NLGN1;KMT2C;THADA;UBR1;FGD4;PCNX1;MAPK8;BTAF1;CECR7;FCHO2;ANKRD36BP2;TRPM7;ST8SIA6;BPTF;AKAIN1;ARFGEF1;USP24;RABGAP1L;MBD5;FANCM;LRBA;MGA;PBX3;KLHL1;ERBIN;SYT16;LNPEP;SMARCA2;PHC3;DMXL2;CCDC171*
ZC3H6 human tf ARCHS4 coexpression	62/299	0.0010536650874352492	*CCDC122;CRYBB2P1;MYT1L;ANKRD36;DPY19L2P2;GADL1;ZBTB20;EFCAB6;MYLK3;RIMS2;UNC80;CCDC91;RPS6KA5;HERC1;ZSCAN30;CA5A;ADAMTSL3;MACROD2;NOS1;ANKS1B;PCMTD1;POTEC;USP8;ANKRD36C;MAGI2;VPS13B;ANK3;FAM126B;SHISA9;ARID1B;NCOR1;LRRC7;PEAK1;CNKSR3;WDPCP;ASTN2;ANKRD36B;SDCCAG8;RGPD5;M*

			IPOL1;STK3;FGD4;DPP6;KIAA1328;ANKRD36BP2;SRGAP3;ATP9B;LRRC37A3;MBD5;SLC14A2;NTRK3;ADAM32;ARHGAP28;MYO9A;DCDC1;TTC39B;TTLL7;TMEM116;NEDD4;TTC3;ASB3;TNRC6B
RC3H2 human tf ARCHS4 coexpression	61/299	0.0018988661313926161	*TRIO;FRY;PITPNC1;SYNE2;CEP128;C16ORF72;AKAP11;HERC1;TRIM2;PSD3;SACS;DIP2B;JAK2;ACTR2;USP7;ARHGEF12;KSR1;DST;NSUN2;VPS13C;PDE4D;FRMD4B;TANC2;HADHB;TBC1D5;FAR1;WDFY3;BIRC6;ZMYND11;DGKI;DTHD1;MACF1;MTPN;NFAT5;RABGAP1;WDR26;ASAP2;AMBRA1;FAM214A;PCNX1;MAPK8;LARP1;ATXN1;HECTD1;HIVEP2;ATP9A;ARFGEF1;USP24;LRBA;TRAPPC10;MGA;ERBIN;MYO5A;LNPEP;TRAPPC8;DNAJC13;KIAA0232;MED13L;APC;SP3;FAT1*
ZNF781 human tf ARCHS4 coexpression	61/299	0.0018988661313926161	*SLC44A5;ROBO2;MYT1L;PTPRO;CHRM5;ELAVL4;SLC35F1;C12ORF40;EFCAB6;PTPRG;SRGAP2C;SYNPR;GRM7;TRIM2;KIF21A;SOX6;SRGAP2B;BBS4;ANKS1B;ANKRD36C;WSB1;LRRC49;TMOD2;MAGI2;SEZ6L;TIAM2;MMP16;LRRC7;VSTM2A;CRB1;ATL1;BTF3L4;NREP;NOL4;GNG2;SNTG1;SRGAP3;JAM2;PAK5;GRIA4;CLVS1;RFTN2;ATF7IP;SIAH3;ST8SIA2;RANBP17;SORBS2;NBEA;APC;NFIB;TMEM116;KLHL7;ASXL3;TTC3;YPEL1;ZNF536;TCF4;SSBP2;KIAA0825;ASB3;XKR5*
HIF1A human tf ARCHS4 coexpression	60/299	0.0033310251820726443	*NRP1;DOCK5;TRIO;C2CD2;ECE1;PTPRK;BACH1;GALNT10;TUBB6;ALCAM;C16ORF72;TEAD1;ADAMTS9;CAST;IL1R1;FNDC3B;AFAP1;ADAM10;FRMD6;LATS2;DPYD;CDC42EP3;DOCK1;MET;CD44;VCL;CHST3;DDR2;RAI14;YAP1;NOTCH2;GRAMD1B;WDR26;SEMA3C;NTN4;IQGAP1;FSTL1;LTBP1;PCNX1;DRAM1;ARSJ;FAM180A;ABL2;SNX9;FLNB;CTNNAL1;NEK7;CRIM1;DNAJC13;LAMB1;SYNJ2;GNG12;EXT1;MYO1E;COL5A1;FAT1;ITGBL1;SNAI2;ATP13A3;FBN1*
NR2C2 human tf ARCHS4 coexpression	60/299	0.0033310251820726443	*RERE;DIDO1;TRIO;DOCK8;LIMD1;MYSM1;CABIN1;RPTOR;AKAP13;NIPBL;PTAR1;HERC2;HERC1;MPRIP;NPIPA1;DIP2B;GTF2I;RBM33;ARHGEF11;MBNL1;ARHGEF12;PRKCB;VPS13C;VPS13B;URB1;PACS1;MADD;WDFY3;BIRC6;UTRN;DOCK2;KDM7A;NOTCH2;MACF1;NFAT5;WDR26;KMT2C;HERC2P2;PCNX1;BTAF1;HECTD1;MAN2A2;HECTD4;MAPK1;HIVEP2;USP24;SPEN;CREBBP;MON2;LRBA;TRAPPC10;MICAL3;ERBIN;LNPEP;SMARCA2;MTOR;MED13L;DIAPH1;SP3;CDK12*
POU3F4 human tf ARCHS4 coexpression	60/299	0.0033310251820726443	*DRAXIN;CHD9;CTNND2;ELAVL4;MSANTD4;DACH1;CDH2;DPYSL5;KIF21A;EPHB2;WSB1;RBFOX2;ADGRV1;CAMK1D;LRRC49;KCNH8;TCF12;RFX3;KCTD1;GAB4;PCCA;ADGRB3;MPPED2;GRIA1;CHRNA7;NTM;GREB1L;NREP;NPAS3;PHF21B;STOX2;MAPK8;NKAIN3;GNG2;MVB12B;CAMTA1;NCAM1;CTNNA2;SRGAP3;ZNF423;LRRC4C;JAM2;FOXB1;ZNF462;NTRK2;MYEF2;AUTS2;ST8SIA2;PTCH1;PLCL1;PBX3;PBX1;ENOX1;PTPRD;MAPK10;CCDC88A;FABP7;TTC3;FAT3;TCF4*
CLOCK human tf ARCHS4 coexpression	59/299	0.0053780379169078	*PATJ;RNF11;ZFYVE9;KDM1B;OSBPL10;SRGAP2C;EFR3A;PPP6R3;LONP2;TEAD1;UNC13C;ARHGEF12;DST;SLC2A13;FNDC3B;VPS13B;TANC2;KIAA1217;PEAK1;FEZ2;PLIN2;WDFY3;CD44;NFAT5;PRKAA2;KMT2C;RGPD6;PRICKLE2;ASAP1;RASAL2;FAM214A;LPP;AURKA;NLRP8;HECTD1;ARSJ;NLRP4;TRPM7;MAP4K3;ARFGEF1;ABCA5;MGA;DENND4C;KIAA1549L;DNAJC13;CDC42BPA;ELL2;MYO9A;PTK2;EXT1;TJP1;MOB1B;NLRP13;PPFIBP1;FGF14;SYNJ1;SLMAP;SPIRE1;CPEB4*
DMTF1 human tf ARCHS4 coexpression	59/299	0.0053780379169078	*ANKRD36;CHD9;USP33;SMG1P5;C12ORF40;MYSM1;BICD1;RYR3;SYNE2;PCMTD2;SENP6;AKAP13;NIPBL;C16ORF72;HERC1;ZSCAN30;PPIP5K2;LUC7L;ANKRD36C;VPS13C;VPS13B;FAM126B;ARID1B;GOLGA8B;BIRC6;ASTN2;ZMYND11;MCTP2;ALG10B;CREB5;MACF1;TNKS;KMT2C;RGPD5;HERC2P9;MIPOL1;SCAF8;BTAF1;KIAA1328;ATP9B;BPTF;USP24;ATF7IP;MBD5;RANBP17;ADAM32;LNPEP;PHC3;PARP15;MLLT10;FER;APC;KANSL1;CEP83;PKN2;*

			TCF4;PTPN4;ASB3;TNRC6B
EBF1 human tf ARCHS4 coexpression	59/299	0.0053780379169078	*DRAXIN;PTPRO;ELAVL4;BICD1;PTPRG;ROBO1;ZNF608;DACH1;DPYSL5;DNER;ANKS1B;PPFIA2;TRPC7;RBFOX2;DCC;CACNA2D1;LRRC49;EBF2;UBE2E2;ANK3;ARID1B;PGM2L1;FOXP2;MMP16;NAV3;HECW1;ARHGEF7;CNTNAP5;RTN1;CHRNA7;GREB1L;NREP;KALRN;PHF21B;FGD4;STOX2;NKAIN3;GNG2;NCAM1;SRGAP3;CADM1;AUTS2;PCDH9;ST8SIA2;PLCL1;PBX3;POU6F2;KLHL1;YLPM1;ST18;PBX1;NELL2;CCDC88A;RALYL;TTC3;YPEL1;GALNTL6;CDK14;LHX9*
HEY1 human tf ARCHS4 coexpression	59/299	0.0053780379169078	*APP;TENM3;GALNT13;TENM4;CTNND2;GRIK4;SIPA1L2;ROBO1;TRIM9;DACH1;CDH2;PEG10;AKT3;TNR;KIF21A;ADGRV1;TMEM178B;TCF12;KAZN;FAM219A;SEZ6L;MAPK8IP1;PARD3B;EPN2;COL4A2;ADGRB3;TNIK;DSCAML1;ASTN1;GRIA1;SHC3;NTM;NPAS3;FAM171A1;STOX2;CDH20;PLXNA2;LRIG1;NCAM1;FYN;TSPAN3;SRGAP3;SLIT2;MPDZ;JAM2;ATP9A;MAP4K4;ARNT2;NTRK2;NDFIP1;DTNA;MYEF2;AUTS2;LHFPL3;PXDNL;CORO2B;DCLK1;FABP7;SMOC1*
NR1D2 human tf ARCHS4 coexpression	59/299	0.0053780379169078	*MYOM1;MYLK2;RNF11;ATP8A1;ZFYVE9;DOCK9;RORA;LDB3;FYCO1;EFR3A;AKAP11;UBL3;SGCD;HERC1;MPRIP;PEBP4;KIF13A;MYO18B;PSD3;TEAD1;CAST;RBFOX1;MBNL2;ARHGEF12;DST;CACNA2D1;VPS13C;MTUS1;TRDN;INPP4B;MYL1;ALPK3;KCNQ5;WDFY3;PRKAA2;RGPD6;ABLIM1;HECTD1;NRAP;XIRP2;MAPK1;ZNF106;CTNNA3;HIVEP2;USP24;SVIL;ABCA5;AGL;NEK7;SAMD4A;ERBIN;FBXL17;PDE4DIP;LNPEP;ATP2B2;FBXO32;KCNS3;SLMAP;OSBPL1A*
MITF human tf ARCHS4 coexpression	59/299	0.0053780379169078	*MYOM1;PIGN;GADL1;PTPRM;FMN1;LDB3;LYST;EPS8;FYCO1;SPRED1;SGCD;CRTAC1;PEBP4;CHCHD6;KIF13A;MYO18B;PPP6R3;PHACTR1;PGM5;HMCN1;STK32A;TEAD1;MLIP;PDE4D;ADAM10;ANO2;TRDN;FRMD3;INPP4B;BACE2;PPP1R12B;CD44;OCA2;MTMR2;CFH;ABCB5;NPL;C10ORF90;SDCBP;CREG1;NRAP;XIRP2;ZNF106;RXRG;SVIL;PCDH7;SAMD4A;MYO5A;PDE4DIP;CABLES1;LHFPL2;GNG12;FBXO32;IGSF11;ARHGAP31;SLMAP;PDE3A;SNAI2;CCDC171*
ZSCAN23 human tf ARCHS4 coexpression	59/299	0.0053780379169078	*SPAG16;ELAVL4;CWC27;PTPRG;ANKRD30BP2;CDH2;DPYSL5;TRIM2;SOX6;ZNF521;MAGI1;WDHD1;ANKRD36C;ZNF880;EPHA7;GUSBP1;WSB1;RBFOX2;DCC;LRRC49;TCF12;RFX3;FAM126A;IFT81;ADGRB3;MPPED2;ANKRD36B;KHDRBS2;STXBP4;TNKS;BTF3L4;GREB1L;ZNF66;NREP;NR2C1;HDAC9;GNG2;ARSJ;ZNF627;CSMD3;SRGAP3;SLIT2;MPDZ;JAM2;PAK5;RFTN2;ATF7IP;ZNF462;FANCM;ST8SIA2;RANBP17;POU6F2;PTK2;MAPK10;APC;KLHL7;TTC3;TCF4;SSBP2*
ZNF33A human tf ARCHS4 coexpression	59/299	0.0053780379169078	*SPAG16;PATJ;ANKRD36;USP33;ELAVL4;RORB;BICD1;SYNE2;RIMS2;PPIP5K2;LRRTM4;ADAMTSL3;DNER;SCAPER;BBS4;ANKRD36C;DCC;LRRC49;KCNH8;VPS13C;VPS13B;ANK3;ZDHHC17;ARID1B;PGM2L1;PARP8;ZNF675;ANKRD36B;KDM7A;TNKS;KMT2C;NREP;NOL4;GLB1L3;MTMR7;GTF2IP1;ANKRD36BP2;PAK3;EVI5;BPTF;CLVS1;ATF7IP;RIC3;MBD5;MYEF2;ST8SIA2;PBX3;PHC3;MAPK10;CCDC88A;CLCN5;PPP2R2B;NEDD4;TTC3;CCSER1;KIAA1958;SSBP2;KIAA0825;TNRC6B*
ZNF460 human tf ARCHS4 coexpression	59/299	0.0053780379169078	*SMG1P2;SMG1P5;MYSM1;CDH8;C16ORF72;HERC1;CDH2;ZSCAN30;TRIM2;ZNF407;PPP6R3;SACS;WSB1;ESCO1;DST;VPS13C;PDE4D;RFX3;VPS13B;ZDHHC17;TANC2;PJA2;LRRC7;WDFY3;ZNF678;BIRC6;PIK3C3;ZNF236;UTRN;ZNF112;MACF1;NFAT5;FOCAD;TNKS;KMT2C;BTF3L4;RGPD5;RASAL2;RGPD2;FGD4;BTAF1;HECTD1;FUT9;ZNF704;ZNF627;ATP9B;GRIA4;GABRA2;MBD5;MON2;ST8SIA2;LRBA;MGA;RANBP17;LRFN5;APC;TTC3;PARGP1;TNRC6B*
FOXJ3	59/299	0.005378037	*RERE;DIDO1;SETD2;DPY19L2P3;KDM1B;MAST2;CEP120*

human tf ARCHS4 coexpression		9169078	;EFCAB14;MYSM1;RPS6KA3;AKAP13;NIPBL;PTAR1;HERC2;HERC1;MPRIP;QSOX2;NEO1;RBM33;TRPC7;MBNL1;CACNA2D1;TANC2;PHF20L1;RFX7;BIRC6;PABPC1;MACF1;NFAT5;MTMR3;WDR26;KMT2C;HERC2P2;AMBRA1;MAPK8;LARP1;BTAF1;HECTD1;HECTD4;HIVEP2;USP24;SPEN;CREBBP;TRAPPC10;ERBIN;YLPM1;LNPEP;AP2B1;FBXO32;SMARCA2;GATAD2B;SCAF4;MTOR;MED13L;DIAPH1;KANSL1;AGO2;PKN2;CDK12
NPAS4 human tf ARCHS4 coexpression	59/299	0.0053780379169078	PCSK2;GABRB3;PTPRT;ERO1B;DOCK3;MYT1L;TUSC3;CELF4;RIMS2;IQCJ-SCHIP1;UNC80;SCGN;HYDIN2;ERC1;RGS9;ANKS1B;ABCC8;LRRC49;MAGI2;FOXP2;CACNB2;LRRC7;ZNF236;DSCAML1;LINC00643;STOML1;CTTNBP2;STXBP1;ATL1;CACNA1C;NREP;KALRN;NOL4;SLC7A2;MTMR7;DPP6;UNK;PDZD2;TTR;HECTD4;SUSD4;PAK3;ATP9A;LINGO2;ZNF462;PTPRN2;CADPS;POU6F2;SYT16;ELL2;GRIN2B;ST18;MOB1B;NELL1;MEIS1;PPP2R2B;ASXL3;CPE;CNIH3
ZC3H11A human tf ARCHS4 coexpression	59/299	0.0053780379169078	DIDO1;SETD2;LPGAT1;USP32;MYSM1;RPS6KA3;AKAP13;EFR3A;NIPBL;PTAR1;C16ORF72;HERC1;KIF13A;DIP2B;GABPA;PCMTD1;GTF2I;CAST;MBNL1;USP7;ARHGEF12;VPS13C;VPS13B;WDFY3;BIRC6;UTRN;DOCK1;KDM7A;NOTCH2;MACF1;MTPN;NFAT5;MTMR3;WDR26;ROCK1;IQGAP1;PCNX1;SCAF8;ABLIM1;BTAF1;HECTD1;TRPM7;USP24;SPEN;CREBBP;EGLN3;HOMER2;LRBA;SIAH2;TRAPPC10;ERBIN;LNPEP;DNAJC13;SMARCA2;MTOR;MED13L;SLMAP;SP3;PKN2
ISL1 human tf ARCHS4 coexpression	58/299	0.008848914530534845	PCSK2;SPAG16;ERO1B;TUSC3;SLC35F4;HS6ST3;RIMS2;SCGN;C1ORF127;ACOXL;FAM3B;PLCE1;TLK1;SAMD12;SCAPER;RGS9;RGS7;ABCC8;SLC2A13;MTUS2;FNDC3A;ZDHHC14;CACNB2;CDC42EP3;MYO3A;GAS2;USP41;LINC00643;RGPD6;USH1C;RGPD5;NOL4;SLC7A2;MTMR7;TTR;SUSD4;TSPAN2;SUSD6;AKAIN1;KCNJ6;PTPRN2;ABCA5;CADPS;POU6F2;PARVB;CDC42BPA;ELL2;USH2A;ST18;MOB1B;OCLN;PLCXD3;FGF14;ASXL3;CNTN1;CPE;CNTN4;CPEB4
PRRX1 human tf ARCHS4 coexpression	58/299	0.008848914530534845	NRP1;TRIO;COL14A1;CPXM2;PTPRM;ANTXR1;ADAMTSL1;FYCO1;SGCD;SH3PXD2A;RPS6KA2;KIF13A;SVEP1;TEAD1;PAMR1;PRKG1;POSTN;DST;IL1R1;FNDC3B;AFAP1;FRMD6;EVC;FNDC1;MXRA7;ALPK2;PDZRN3;DOCK1;DDR2;YAP1;NOTCH2;CEMIP;TWIST2;TMTC1;FSTL1;LTBP1;CTIF;ABL1;FAM180A;SNX9;SLIT3;SVIL;NEK7;SAMD4A;CYBRD1;PDE4DIP;DNAJC13;LAMB1;GNG12;FBXO32;EXT1;SMOC2;PPFIBP1;COL5A1;DLC1;FAT1;ITGBL1;SNAI2
RCOR3 human tf ARCHS4 coexpression	58/299	0.008848914530534845	PCSK2;SPAG16;ERO1B;LPGAT1;ATP8A1;CHD9;ZBTB20;ZNF44;EFCAB6;FRY;MYLK3;IGF1R;PCMTD2;SENP6;SCGN;CNST;HERC1;PPIP5K2;FBXO3;TLK1;PCMTD1;ABCC8;VPS13C;COMMD10;VPS13B;OPRM1;ARID1B;MYO3A;USP41;ASTN2;ZNF236;KMT2C;RGPD6;RGPD5;RGPD8;ZDHHC21;FAM214A;GRK3;TTR;ANKRD36BP2;USP24;RABGAP1L;ABCA5;PHKB;PARVB;SMARCA2;PHC3;ELL2;MOB1B;OCLN;PLCXD3;CCSER2;CPE;ASB4;ZNF354C;CPEB4;TNRC6B;CCDC171
ZNF385B human tf ARCHS4 coexpression	58/299	0.008848914530534845	RERE;DOCK3;ATP8A1;CHD6;SIPA1L3;IGF1R;RPH3A;UNC80;GRM5;ZNF608;MCF2L;PSD3;DLGAP1;PPFIA2;UNC13B;CADPS2;RBFOX1;ARHGEF12;PRKCE;TMOD2;COBL;MYRIP;SYN2;KIAA1217;TOM1L2;SCN8A;ARHGEF7;RAPGEF5;UTRN;NGEF;RAPGEF4;STXBP1;DNAH5;TMPRSS2;NLK;PRKCZ;RAP1GAP;KIAA0513;SV2B;HECTD4;CLVS2;STXBP6;GPR158;ATP9A;WASF3;OPCML;ZNF462;GABRA6;SYT1;HOMER2;CADM2;IQSEC1;ATP2B2;SNAP91;ARHGAP32;DLG2;PPP2R2C;HCN1
ZNF84 human tf ARCHS4 coexpressi	58/299	0.008848914530534845	ANKRD36;USP33;MSANTD4;GPHN;CDH2;DPYSL5;TRIM2;KIF21A;ANKS1B;PDK1;MAGI1;ANKRD36C;WSB1;HFM1;DST;LRRC49;RFX3;ITFG1;ENAH;IFT81;ADGRB3;LRRC7;ZNF234;ANKRD36B;ZNF397;CRB1;STAU2;TNKS;BTF3L4;NREP;CACNA1E;NR2C1;STOX2;GNG2;CTNNA2;SRGAP3;

on			PAK3;PAK5;BBS2;ZNF462;MBD5;MYEF2;ST8SIA2;TMEM132B;RANBP17;LSAMP;ENOX1;PTPRD;MAPK10;CCDC88A;NBEA;APC;KLHL7;TTC3;TCF4;PTPN4;SSBP2;CCDC171
SHPRH human tf ARCHS4 coexpression	58/299	0.008848914530534845	CCDC122;ANKRD36;USP33;DPY19L2P2;GADL1;EFCAB6;AFF3;MYLK3;CEP128;UNC80;CCDC91;HERC1;ZSCAN30;PPIP5K2;LRRTM4;ADAMTSL3;PCMTD1;POTEC;ANKRD36C;MAGI2;VPS13B;FRMD4B;OPRM1;SHISA9;ARID1B;GAREM1;PEAK1;WDPCP;ASTN2;ZMYND11;L3MBTL4;ANKRD36B;RGPD5;ATP10B;RASAL2;GLB1L3;MIPOL1;STK3;ATXN3;KIAA1328;CECR7;ANKRD36BP2;CSMD3;ATP9B;MBD5;CEP112;SLC14A2;RANBP17;ADAM32;ARHGAP28;PARP15;CCDC88A;NEDD4;TCF4;ASB4;ASB3;TNRC6B;CCDC171
YOD1 human tf ARCHS4 coexpression	58/299	0.008848914530534845	DOCK5;RNF11;DOCK8;SMG1P2;C12ORF42;BACH1;LYST;YBX3;AKAP13;NIPBL;PTAR1;RASSF2;CCDC91;HERC1;JAK2;RBM33;MBNL1;ITGA4;SP110;PRKCB;VPS13C;HAUS6;PARP8;ALDH1A2;DPYD;BMP2K;TRIM58;RELL1;BIRC6;UTRN;DOCK2;MCTP2;KDM7A;HEMGN;MACF1;WDR26;ROCK1;IQGAP1;PRAMENP;ADAM28;MXI1;MAPK1;RPRD1B;ASCC2;USP24;SIAH2;SETDB2;ERBIN;XPO7;LNPEP;ARHGAP26;ARHGAP24;MED13L;DIAPH1;SP3;UBE2O;CPEB4;BCL2L1
TSC22D1 human tf ARCHS4 coexpression	57/299	0.01421038216820919	GABRB1;MAP3K7CL;TPGS2;SYNPR;TRIM9;DPYSL5;TRIM2;MCF2L;DLGAP1;PRKACB;SH3GL2;ZNF287;WSB1;RBFOX2;TMOD2;SYN2;GABRG2;ITFG1;PJA2;FRMD3;DNM3;ADGRB3;SLC27A6;VSTM2A;MAPRE2;ASTN1;ZNF112;GRIA1;RTN1;STXBP1;PDE1A;ATL1;BTF3L4;NRXN3;NREP;PRKCZ;GNAI1;GNG2;NCAM1;TSPAN3;CTNNA2;GRIA4;GABRA2;ARNT2;NDFIP1;DTNA;SYT1;CADM2;OXR1;SNAP91;MAPK10;MEIS1;PPP2R2B;RCAN2;CNTN1;SCN2A;SSBP2
PRDM2 human tf ARCHS4 coexpression	57/299	0.01421038216820919	CYFIP2;RERE;SETD2;ANKRD33B;DOCK8;AFF3;SYNE2;FAM107B;FCRLA;DOCK10;AKAP13;NIPBL;C16ORF72;AKAP11;HERC1;RBM33;MBNL1;PRKCB;VPS13C;ARAP2;VPS13B;IPCEF1;FCHSD2;BANK1;PACS1;BIRC6;UTRN;DOCK2;WDFY4;KDM7A;BLK;NFAT5;WDR26;ROCK1;KMT2C;RASGRP1;PCNX1;HECTD4;BTLA;HIVEP2;BPTF;LYN;SPEN;CREBBP;RABGAP1L;BCL11B;IQSEC1;TRAPPC10;YLPM1;LNPEP;SMARCA2;PARP15;GATAD2B;SCAF4;MED13L;ARHGAP32;KANSL1
PAX3 human tf ARCHS4 coexpression	57/299	0.01421038216820919	TENM3;WNT2B;DENND1A;ROBO1;ZNF608;NHSL1;DACH1;CDH2;PEG10;EPHB2;ZNF521;ANKRD6;WSB1;ADGRV1;DST;TCF12;RFX3;PLA2G4A;PARD3B;ENAH;ANKFN1;IFT81;ADGRB3;ATF6;GRIA1;MTMR2;STXBP4;GREB1L;GLIS3;RASAL2;NR2C1;OAZ2;NPAS3;STOX2;GNG2;PLXNA2;NHS;TSPAN3;CSMD3;SRGAP3;ZNF423;LRRC4C;MPDZ;BBS2;NTRK2;MYEF2;AUTS2;ST8SIA1;RANBP17;PXDNL;PHKB;PTPN13;TMEM232;MAPK10;TTC3;FAT3;BMPR1B
RFX7 human tf ARCHS4 coexpression	57/299	0.01421038216820919	LIN54;DIDO1;SETD2;ATP8A1;PSIP1;MYSM1;NIPBL;HERC1;GNPTAB;SACS;SMARCAD1;QSOX2;GTF2I;RBM33;AQR;ITGA4;TANC2;RUNX1;FCHSD2;AGPS;GCSAML;BIRC6;PABPC1;MCTP2;MACF1;NFAT5;ANP32A;TNKS;ITPR2;SCAF8;LARP1;BTAF1;HECTD1;FUT9;HECTD4;BPTF;USP24;SPEN;CREBBP;TRAPPC10;YLPM1;XPO7;LNPEP;FOXN3;PUM1;GATAD2B;SCAF4;MED13L;PLEKHA8;MLLT10;APC;KANSL1;AGO2;SP3;CDK12;PTPN4;EIF4G3
SIM1 human tf ARCHS4 coexpression	57/299	0.01421038216820919	PCSK2;SEMA5A;MYOM1;ERO1B;PTPRQ;SLC35F4;ZBTB20;LRRC2;SCGN;FYCO1;C1ORF127;SGCD;MYO18B;SLC16A9;PCMTD1;MLIP;IL1R1;ABCC8;SLC2A13;ABCC9;TRDN;ABCA10;PLCB4;NAV3;MYO3A;GAS2;ALPK3;PRKAA2;STXBP4;GLIS3;NOL4;NALCN;SLC7A2;TTR;NRAP;SUSD4;XIRP2;TSPAN2;STXBP6;AKAIN1;PTPRN2;ABCA5;CNTN5;CADPS;PDE4DIP;PARVB;BTBD9;CDC42BPA;ELL2;MOB1B;OCLN;PLCXD3;AGBL1;ASXL3;CPE;CNTN4;CPEB4
MBNL3 human tf ARCHS4	57/299	0.01421038216820919	DOCK5;RNF11;LPGAT1;DOCK8;PCSK6;SERPINA6;BACH1;LYST;SYNE2;RPS6KA3;AKAP13;NIPBL;RASSF2;HERC1;ADGRE3;JAK2;IL6R;RBM33;ABCG8;MBNL1;COL27A1;I

coexpression			*TGA4;VPS13C;F5;PARP8;DPYD;TRIM58;BIRC6;UTRN;DOCK2;MCTP2;KDM7A;HEMGN;MTPN;WDR26;ITPR2;IQGAP1;C2;C5;PCNX1;RELN;HECTD1;MXI1;ASCC2;A2M;LYN;USP24;RABGAP1L;SIAH2;TRAPPC10;ERBIN;LNPEP;ARHGAP26;DIAPH1;SP3;SNTB1;BCL2L1*
ZCCHC11 human tf ARCHS4 coexpression	57/299	0.01421038216820919	*CCDC122;DRAXIN;CRYBB2P1;ANKRD36;CHD9;USP33;ELAVL4;EFCAB6;SYNE2;NIPBL;ZNF608;ZSCAN30;PPIP5K2;CA5A;ADAMTSL3;SCAPER;ZNF521;ANKRD36C;WSB1;DCC;ZNF160;LRRC49;MAGI2;RFX3;VPS13B;IFT81;NME7;WDPCP;ASTN2;ANKRD36B;ZNF397;GREB1L;TPTE2P2;RGPD5;NREP;NR2C1;MIPOL1;AK9;STK3;KIAA1328;ANKRD36BP2;SRGAP3;ATP9B;BPTF;RIC3;MBD5;ST8SIA1;MGA;ARHGAP28;CCDC88A;KANSL1;TMEM116;KLHL7;NEDD4;TTC3;ASB3;TNRC6B*
ZC3H13 human tf ARCHS4 coexpression	57/299	0.01421038216820919	*GABRB3;TCERG1;DRAXIN;TENM3;CTNND2;CELF4;ELAVL4;PSIP1;CWC27;TXNDC16;ROBO1;RIMS2;NIPBL;ZNF608;DPYSL5;ADAMTSL3;CHCHD6;LUC7L;KIF21A;ANKS1B;PPFIA2;MAGI1;RBFOX2;KCNH8;FRMD4A;ANK3;PGM2L1;PEAK1;ROR1;TNIK;ANKRD36B;MTMR2;ANKRD11;CWF19L2;NREP;NPAS3;PHF21B;FAM171A1;STOX2;ZNF704;CAMTA1;CLSPN;SRGAP3;PAK3;MAP4K4;BPTF;ZNF462;MYEF2;AUTS2;YLPM1;DCLK1;PTPRD;CCDC88A;ZNF618;TTC3;KIAA1958;EIF4G3*
RORA human tf ARCHS4 coexpression	56/299	0.02202597445180155	*DOCK9;DOCK8;FRY;LYST;SYNE2;DOCK10;AKAP13;HERC1;KIF13A;PCMTD1;CERS3;MBNL1;GRID2;PRKCH;MBNL2;ITGA4;VPS13C;ARAP2;VPS13B;TC2N;IPCEF1;PARP8;INPP4B;PCP4;CARD18;PRKCQ;BIRC6;ASTN2;UTRN;KDM7A;MACF1;NFAT5;RASGRP1;CACNA1I;PCNX1;ABLIM1;ATXN1;HIVEP2;KDM4C;BCL11B;ABCA5;KCNIP4;ERBIN;ADAM32;LNPEP;FOXN3;ARHGAP26;SMARCA2;PHC3;PARP15;MPP7;TTC39B;NEDD4;CCSER2;PTPN4;CPEB4*
ATF2 human tf ARCHS4 coexpression	56/299	0.02202597445180155	*GABRB3;ELAVL4;BACH1;PCMTD2;DPYSL5;TRIM2;FBXO3;KIF21A;PRKACB;PCMTD1;WSB1;DCC;VPS13C;RFX3;ZDHHC17;RUNX2;PGM2L1;PJA2;PIAS1;PYGO1;LATS2;MDFIC;LRRC7;NAA35;ZNF678;TOX;GRIA1;STX12;RTN1;STAU2;TNKS;ATL1;BTF3L4;NREP;NOL4;GNAI1;MAPK8;GNG2;SNTG1;CTNNA2;PAK3;CADM1;SYT1;ST8SIA2;SYT16;PUM1;OXR1;PTPRD;MAPK10;SYNJ1;APC;KLHL7;TTC3;CNTN1;TCF4;SSBP2*
TSHZ3 human tf ARCHS4 coexpression	56/299	0.02202597445180155	*NRP1;DOCK4;BNC2;PTPRO;ANTXR1;ADAMTSL1;SLC22A14;GLT1D1;SH3PXD2A;DIP2C;EPHB2;PAMR1;ARHGEF11;RBFOX1;IL1R1;AFAP1;MYRIP;PGM2L1;FOXP2;TANC2;FRMD6;EVC;KCNMA1;CDC42EP3;WDFY3;MXRA7;PDZRN3;DDR2;CEMIP;TNKS;NTM;TWIST2;ASAP1;FSTL1;CACNA1E;LTBP1;CTIF;RANBP3L;FLRT2;SH3BP5;SPOCK1;CSMD2;MAP4K4;CLVS1;CA10;CADM1;STAC;SIAH3;EXT1;PLCXD3;GNAL;COL5A1;ITGBL1;SNAI2;LRP12;FBN1*
POU2F1 human tf ARCHS4 coexpression	56/299	0.02202597445180155	*GABRB3;DRAXIN;PSIP1;MYSM1;GLI2;ZNF608;ADAMTSL3;FLVCR1;JARID2;MAGI1;WDHD1;ADGRV1;LRRC49;ARID1B;SGO1;MSH6;MSH2;ROR1;CCDC150;FAM72B;ZNF397;TNKS;IREB2;GREB1L;GLB1L3;MIPOL1;PHF21B;FGD4;ATXN3;STOX2;GTF2IP1;RIC8B;KIAA1328;CLSPN;SRGAP3;ZNF423;BPTF;ZNF462;MYEF2;AUTS2;PTCH1;MGA;YLPM1;ARHGAP28;ARID3B;PBX1;GATAD2B;GULP1;MLLT10;CCDC88A;KANSL1;NEDD4;FAT3;KIAA1958;ASB3;TNRC6B*
IRX1 human tf ARCHS4 coexpression	56/299	0.02202597445180155	*APP;DRAXIN;TENM2;TENM3;CTNND2;SIPA1L2;CDH4;ZNF608;DACH1;CDH2;DPYSL5;PEG10;TRIM2;KIF21A;ANKRD20A7P;EPHB2;TMEM178B;EBF2;RFX3;KAZN;FRMD4A;FAM219A;SORCS2;CFDP1;TNIK;CHST3;GRIA1;IGSF3;GREB1L;MLLT1;NPAS3;PHF21B;STOX2;GTF2IP1;GNG2;CDH20;PLXNA2;NCAM1;SRGAP3;ZNF423;MAP4K4;TMEM132C;NTRK2;NDFIP1;MYEF2;AUTS2;PTCH1;YLPM1;PXDNL;ESRRG;NETO2;DCLK1;PTPRD;SDK1;ZNF618;TTC3*
LCORL	56/299	0.022025974	*SPAG16;CNTNAP2;ANKRD36;USP33;ELAVL4;NHSL1;PPI*

human tf ARCHS4 coexpression		45180155	*P5K2;LRRTM4;TRIM2;SNAPC3;SMARCAD1;KIF21A;SOX6;ANKRD36C;WSB1;LRRC49;TCF12;MAGI2;RFX3;ZDHHC17;PYGO1;IFT81;NME7;LRRC7;ZNF678;ASTN2;XPR1;ANKRD36B;STXBP4;STAU2;ATL1;BTF3L4;NREP;NOL4;MTMR7;MIPOL1;MAPK8;GNG2;ATP9B;ATF7IP;MBD5;MYEF2;RANBP17;ST18;PTPRD;CCDC88A;LRFN5;FER;NBEA;APC;KLHL7;ASXL3;TTC3;PARGP1;CNTN4;ASB3*
LCOR human tf ARCHS4 coexpression	56/299	0.02202597445180155	*MYT1L;ANKRD36;GALNT18;CLCN3P1;ELAVL4;FRG1HP;C12ORF40;C16ORF72;NHSL1;LRRTM4;TRIM2;PDK1;ANKRD36C;WSB1;MAGI2;RFX3;PRKCA;OPRM1;ARID1B;ENAH;FRMD5;IFT81;LRRC7;BIRC6;ASTN2;ALG10B;XPR1;ANKRD36B;MCPH1;UBE2Q2P1;CUL5;KMT2C;BTF3L4;RGPD6;TPTE2P2;RGPD5;NREP;NOL4;MIPOL1;NKAIN2;PAK5;MBD5;MYEF2;INSR;MGA;PHC3;KITLG;DAB1;NBEA;KLHL7;TTC3;TCF4;ASB4;ASB3;XKR5;TNRC6B*
ESRRG human tf ARCHS4 coexpression	56/299	0.02202597445180155	*TENM2;PID1;TENM3;FRMPD4;ANKRD36;AP4E1;GRIK1;GRIK2;MYLK3;GRM5;ZNF608;DNER;KIF21A;NPIPA1;DIP2C;RNF111;MED1;PRMT8;ARHGEF12;LMNTD1;EML1;KIF6;ZDHHC14;PIAS2;KCTD8;MPPED2;CDH18;IGSF3;STAU2;RGPD6;RGPD4;PAK1;NKAIN3;FLRT2;PGBD5;SNTG1;ZMAT4;DNAJC21;NCAM1;CTNNA2;PAK3;ATP9A;MBD5;SYT1;PCDH9;NEGR1;CADM2;ST8SIA2;NTRK3;BTBD11;PTPRD;CCDC88A;NEDD4;TTC3;ZNF578;SCN2A*
EN2 human tf ARCHS4 coexpression	56/299	0.02202597445180155	*BTG3;TENM3;TENM4;CTNND2;PTPRO;CELF4;ELAVL4;ZBTB20;KIF15;DACH1;CDH2;DPYSL5;PEG10;PSD3;EPHB2;ERC2;TMEM178B;GRID1;TCF12;KAZN;GFRA1;FRMD4A;SHISA6;IL1RAPL2;GRIA1;NTM;NF1P9;ILDR2;NREP;ABHD17C;NPAS3;STOX2;GTF2IP1;NKAIN3;SNTG1;CAMTA1;NCAM1;SRGAP3;ZNF423;ATP9A;NTRK2;MYEF2;AUTS2;PTCH1;PLCL1;NETO2;DCLK1;PBX1;PTPRD;LRFN2;SDK1;TTLL5;FABP7;RNF182;ZNF536;FAT3*
NEUROG2 human tf ARCHS4 coexpression	56/299	0.02202597445180155	*APOOP5;ELAVL4;GLI3;ZNF608;SGCD;SH3PXD2A;AKT3;KIF13A;SOX6;EPHB2;PAMR1;NEO1;PRKG1;PRMT8;RBFOX2;TTC33;ANKRD20A11P;AFAP1;ZDHHC14;MPPED1;UBE2R2;HECW2;HECW1;RELL1;ROR2;TSHZ3;SEMA3A;CDCA8;OR9Q1;TMTC1;ZNF66;CARM1P1;GLIS1;IGSF21;GTF2IP1;ARSJ;ZNF106;STXBP6;SRGAP3;EVI5;PAK5;MAP4K4;ZNF462;ANKRD26;AUTS2;EYA4;ARHGAP28;FOXN3;PUM1;MCC;CPHL1P;ST18;CCDC88A;NFIA;CCNG2;TTC3*
CHD1 human tf ARCHS4 coexpression	56/299	0.02202597445180155	*TCERG1;SETD2;DOCK8;SMG1P2;BACH1;LYST;MYSM1;SYNE2;KIF15;AKAP13;NIPBL;PTAR1;C16ORF72;HERC1;PPP6R3;LRRFIP1;JARID2;ZNF367;RBM33;ANKRD36C;AQR;MBNL1;USP7;ESCO1;ITGA4;VPS13C;VPS13B;HAUS6;BAZ1A;PARP8;PHF20L1;ASPM;BIRC6;UTRN;DOCK2;KDM7A;WDR26;ROCK1;KMT2C;RGPD8;PCNX1;SCAF8;BTAF1;BPTF;ARFGEF1;USP24;SPEN;TRAPPC10;MGA;ERBIN;MED13L;KANSL1;DMXL2;SP3;PKN2;CDK12*
SALL1 human tf ARCHS4 coexpression	55/299	0.03340394542430667	*GABRB3;TENM4;GLDC;CTNND2;PSIP1;LDLRAD3;SLC35F1;KIF15;PTPRG;GLI2;ROBO1;SPRED1;CDH2;NUF2;NEO1;SRGAP2B;MAGI1;WDHD1;SUPT16H;ADGRV1;SEMA6D;TCF12;HAUS6;NAV2;SGO1;ASPM;PHLPP1;ILDR2;STOX2;CECR2;GTF2IP4;LRIG1;ZNF423;SRGAP2;MPDZ;JAM2;WASF3;ZNF462;FARP1;NTRK2;MYEF2;AUTS2;PTCH1;HMGA2;ARID3B;PTPN13;CORO2B;GULP1;TJP1;PTPRD;FABP7;KIF4A;FAT3;TCF4;ADGRL2*
ATF6 human tf ARCHS4 coexpression	55/299	0.03340394542430667	*NRP1;DOCK5;PATJ;SETD2;IDE;SRGAP2C;GABRR2;VN1R7P;LONP2;PHACTR2;DIP2B;CAST;DST;VPS13C;FNDC3B;ADAM10;ARID5B;AP3B1;FNDC3A;ENAH;HEATR5A;EOGT;PIGK;BMP2K;RELL1;WDFY3;BIRC6;IFNAR1;WDR26;SAR1A;ROCK1;ARHGEF28;SEL1L;ANKRD19P;ASAP1;IQGAP1;SLC7A2;HECTD1;FCHO2;STARD13;CADM1;MYO10;NUBPL;MGA;DENND4C;ERBIN;LNPEP;DNAJC13;GNG12;CDC42BPA;PHC3;ELL2;ITCH;GSTA3;ATP13A3*
MXI1 human tf ARCHS4	55/299	0.03340394542430667	*DOCK5;RNF11;CTNND2;DOCK8;RORA;FRY;LYST;YBX3;SYNE2;TRIM9;AKAP13;NIPBL;CA1;RASSF2;HERC1;ADGR*

coexpression			E3;UIMC1;SOX6;JAK2;RBM33;MBNL1;ARHGEF12;TANGO2;VPS13C;ARAP2;KAZN;VPS13B;PARP8;TRIM58;WDFY3;UTRN;MCTP2;KDM7A;HEMGN;WDR26;PHLPP1;RAP1GAP;KIAA0513;MAN2A2;HECTD4;ZSWIM6;ASCC2;JAZF1;SPECC1;NDFIP1;SIAH2;SUSD1;ERBIN;XPO7;LNPEP;ARHGAP26;TTLL7;UBE2O;CPEB4;BCL2L1
JAZF1 human tf ARCHS4 coexpression	55/299	0.03340394542430667	RNF11;CTNND2;CELF4;FRY;LYST;HS6ST3;SYNPR;SCGN;GLT1D1;CA1;RASSF2;ADGRE3;TANGO2;TMOD2;IPCEF1;SYN2;ZDHHC17;PGM2L1;IL17RA;PIGK;TRIM58;VSTM2A;KDM7A;HEMGN;MTMR3;WDR26;RTN1;STXBP1;ATL1;PRDM11;NALCN;KIAA0513;GNG2;MXI1;ASCC2;CTNNA2;PAK3;OPCML;LYN;PTPRN2;NDFIP1;SYT1;SIAH3;IQSEC1;SYT16;FAM135B;SYNJ1;APC;RCAN2;TRPV5;CNTN1;CPE;SCN2A;SSBP2;CPEB4
SOX1 human tf ARCHS4 coexpression	55/299	0.03340394542430667	DRAXIN;TENM3;CTNND2;PSIP1;GLI3;GLI2;ROBO1;CDH4;ZNF608;DACH1;CDH2;DPYSL5;PEG10;TRIM2;KIF21A;ZNF521;MAGI1;WSB1;TMEM178B;RFX3;FRMD4A;CFDP1;IFT81;MPPED2;GRIA1;CRB1;SHC3;NRXN3;ILDR2;NREP;NPAS3;PHF21B;STOX2;RIC8B;GNG2;CDH20;SRGAP3;ZNF423;NTRK2;MYEF2;AUTS2;ST8SIA2;NETO2;DCLK1;PTPRD;MAPK10;SDK1;LYRM4;ZNF618;FABP7;SMOC1;TTC3;TCF4;RGS12;APBA2
ZFP37 human tf ARCHS4 coexpression	55/299	0.03340394542430667	MYT1L;PTPRO;ELAVL4;CDH8;NHSL1;GRM7;DPYSL5;LRRTM4;AKT3;TMEM108;PHACTR3;KIF21A;ERC1;DIP2C;RALGPS1;WSB1;RBFOX2;LRRC49;MAGI3;HUNK;ZDHHC17;PGM2L1;LRRC7;KCNQ3;AMPH;XPR1;ANKRD36B;GRIA1;KHDRBS2;NLGN1;RTN1;ATL1;RASAL2;NREP;UBR1;NYAP2;NOL4;FGD4;GNG2;SNTG1;CTNNA2;PAK3;MPDZ;PAK5;GABRA2;MAPK10;CCDC88A;ASAH2B;APC;PPP2R2B;KLHL7;ASXL3;TTC3;TCF4;SSBP2
SALL3 human tf ARCHS4 coexpression	55/299	0.03340394542430667	GABRB3;GRIK3;PSIP1;LDLRAD3;SLC35F1;KIF15;GLI2;ZNF608;NHSL1;CDH2;NUF2;JARID2;ZNF521;SRGAP2B;MAGI1;WDHD1;SUPT16H;ADGRV1;SEMA6D;TCF12;NAV2;SEZ6L;SGO1;ASPM;MSH2;CNKSR3;BLM;PHLPP1;LUZP2;ILDR2;NPAS3;STOX2;CECR2;UGP2;ZNF704;CLSPN;CSMD2;ZNF423;JAM2;BPTF;BARD1;RFTN2;ZNF462;NTRK2;MYEF2;AUTS2;CCDC138;PTCH1;WNT7A;CORO2B;NDC80;GULP1;FABP7;AGO2;FAT3
ZBTB20 human tf ARCHS4 coexpression	55/299	0.03340394542430667	CCDC122;PATJ;MYT1L;CELF4;KLHL33;MYLK3;UNC80;RPS6KA5;CA5A;ADAMTSL3;SLC16A7;ERC2;USP8;ANKRD36C;MAGI2;ABCC9;SORCS3;SHISA9;ARID1B;NAALADL2;PIAS2;MYO3B;PEAK1;CNKSR3;WDPCP;ASTN2;PPARA;ANKRD36B;LINC00643;SDCCAG8;ZNF397;NMD3;LPP;MIPOL1;DPP6;PCNX2;KIAA1328;FLRT2;ZNF704;PAK3;MBD5;KCNJ6;CADM1;NEGR1;HOMER2;NUBPL;KCNJ15;BTBD9;PHC3;NEDD4;RGS12;CEACAM22P;ASB3;C8ORF34;TNRC6B
SMARCA1 human tf ARCHS4 coexpression	55/299	0.03340394542430667	PCSK2;TENM3;ABCD3;C2CD2;TUSC3;PTPRK;LDB2;PTPRG;ROBO1;IGF1R;ARHGAP42;CDH2;PEG10;TEAD1;ADAMTS9;MAGI1;RBFOX2;COL27A1;CSNK2A1;FNDC3B;F5;ENAH;FREM1;DOCK1;CHST3;RAI14;YAP1;GRAMD1B;ACSS3;SDC2;SEMA3A;FSTL1;EHBP1;RELN;UGP2;TTR;GPC6;MPDZ;MAP4K4;FARP1;MYEF2;RDX;HMGA2;LAMB1;PTPN13;CDC42BPA;PTK2;PLEKHA8;TJP1;SDK1;DLG5;FAT1;CPE;EIF4G3;ADGRL2
EEA1 human tf ARCHS4 coexpression	55/299	0.03340394542430667	CHD9;RORA;GXYLT2;GLI3;PCMTD2;SRGAP2C;CCDC91;NHSL1;PPIP5K2;ZNF208;PPP6R3;PHACTR2;TLK1;ERC1;METTL15;PCMTD1;HSDL2;MBNL2;DST;VPS13C;ADAM10;ENAH;MMP16;KIF16B;WDFY3;BIRC6;DGKI;DDR2;MCPH1;RABGAP1;SAR1A;CTTNBP2;KMT2C;RASAL2;ERBB4;EVI5;SEC23B;ARSB;PAK5;BPTF;OSBPL6;SIAH3;INSR;MGA;RDX;DENND4C;SAMD4A;GNG12;TJP1;OCLN;ITCH;NBEA;SPIRE1;CNIH3;ZNF354C
CHD6 human tf ARCHS4	55/299	0.03340394542430667	RERE;DIDO1;PATJ;TRIO;SIPA1L3;SYNE2;IGF1R;HERC2;ZNF608;C16ORF72;HERC1;MPRIP;FBXO3;GTF2I;ARH

coexpression			*GEF12;VPS13C;VPS13B;ARID1B;NAALADL2;INIP;KIAA1217;PEAK1;WDFY3;BIRC6;ARHGEF7;UTRN;MACF1;NFAT5;CCDC186;KMT2C;DNAH5;KALRN;LARP1;KIAA1328;HECTD1;ZNF704;HECTD4;STK38;GSE1;FLNB;BPTF;SPEN;ZNF462;CREBBP;GLYATL2;LRBA;PTCH1;MGA;YLPM1;MED13L;TJP1;ARHGAP32;PDP2;KANSL1;CDK12*
FBN1 human tf ARCHS4 coexpression	55/299	0.03340394542430667	*NRP1;TRIO;COL14A1;PTPRM;ANTXR1;PRSS23;GALNT10;ADAMTSL1;TUBB6;SGCD;SH3PXD2A;RPS6KA2;SVEP1;TEAD1;PAMR1;POSTN;IL1R1;FNDC3B;AFAP1;FRMD6;EVC;COL4A2;CDH13;MXRA7;ALPK2;DOCK1;VCL;DDR2;RAI14;YAP1;NOTCH2;CEMIP;SEMA3C;TWIST2;NTN4;ATP10A;LTBP1;GLIS1;CTIF;ARSJ;ABL1;FAM180A;SLIT3;CHSY3;NEK7;CRIM1;CYBRD1;LAMB1;GNG12;EXT1;COL5A1;DLC1;FAT1;ITGBL1;SNAI2*

Table S9. The results of RNA-Seq data in initial and differentiating K562 cells. baseMean—mean of counts divided by the size factors for the counts for both conditions. log2FoldChange—the log2 value of the fold change. lfcSE presents the standard error of the log2FoldChange. Stat is the Wald statistic: the log2 fold change divided by lfcSE, which is compared to a standard normal distribution to generate a two-tailed *p*-value. padj—adjusted *p*-values. Excel file attached separately. Related to Figure 3A.

Table S10. Venn diagram showing the intersections of upregulated genes (p-value < 0.05) with lists of genes that reveal an increase or decrease in rDNA contacts. Related to Figure 3B.

Names	total	elements
4C-increased-1309 upregulated-1285	74	*THRAP3 FOXK2 TAFA2 USP14 LIMD1 DIDO1 WDR12 PHACTR1 OPA3 UIMC1 GUSBP1 TLK1 CREBBP FAM193A MIR17HG SUPT16H NIPA2 LRRFIP1 CTDP1 HECTD1 BIRC6 PHACTR2 DNAJC21 INTS13 DDX10 CLSPN PPP6R3 GUCD1 PPIP5K2 EBNA1BP2 SNX8 NSMAF GSE1 ABLIM1 UCK2 RPTOR STT3A ECHDC1 ACACA MAN2A2 CDK12 AFG3L2 CCDC138 MTOR LARP1 PSMB2 ANKRD11 SPEN NAP1L4 KANSL1 CRIM1 MTREX GID8 LINC00861 CUL1 NSUN2 LINC01128 GRB10 MLLT1 SDCBP MBNL1 ABI1 MED1 HNRNPM MACROH2A1 JPT2 MSH2 BAZ1A EWSR1 ELL2 PRAME NUP43 EOGT ANP32B*
4C-decreased-1200 upregulated-1285	78	*IGF2BP3 MRPL45 HERC2 ARMC6 ASH1L GOT2 BRD4 CHAF1A CHAMP1 NSD1 AGO2 KIAA0753 ANKRD17 IBA57 SREBF2 RESF1 MAPK1IP1L ZC3H14 ZNF33B BRCA2 SSBP3 LCLAT1 MS4A4A SMARCA4 CWC22 ZNF121 DHX29 TM9SF3 CFAP97 ANKRD33B UBAP2 APC GEMIN5 KTN1 HMGB1 MEF2C SETD2 MRPS35 UTP4 SMARCC1 NUP214 ECPAS SFPQ URB1 STAG2 PAFAH1B1 CSDE1 ZBTB2 EFTUD2 NRIP1 STON2 DNAJC7 ZC3HAV1 CPSF3 SNRPD1 SUMO3 BZW1 PCNA AQR RNF138 ZCCHC14 ADSS2 LINC00923 MGA NIN PPIL2 SPTB UBAP2L ZFYVE26 BACH1 OXNAD1 ZNF431 TBCD KCNK5 SERBP1 RANBP2 PEPD PCNT*
4C-increased-1309	1233	*FSTL1 CD44 PLCE1 SLMAP FAM219A FARP1 KCNMA1 PKNOX2 ARHGAP5-AS1 APBB2 ZNF684 KLHL13 GLT1D1 ABCB7 COX7A2L FYB2 RNF38 AHDC1 LINC02073 CUBN SLC10A7 LDB3 PAK1 SCAF8 SLC6A11 B9D1 CPXM2 RTN1 KIRREL1 LINC01467 PEBP4 PPP1R13B FGR TOP3A EPN2 LINC01491 LINC02664 INIP NSMCE2 TTC37 ZNF208 RCL1 PRSS51 SLC25A52 OAZ2 TEAD1 ZNF718 DKK2 GLRA4 OR7A17 DHRS11 PCAT19 CCDC34 TAOK3 DNAJC27-AS1 PUM3 ZNF66 LINC02563 SCYGR8 LRGUK TENM4 TRPM6 FAAHP1 LINC01479 CCDC106 SLC8A1 SMIM35 ANLN ARHGEF26-AS1 SLC1A7 LGI2 ANKRD6 KCNC1 ADGRE3 GPR55 DNAJC15 STAU2 PGAP4 SIAH3 RIPK4 TMEM63C ZNF257 TIAL1 ST8SIA4 HYDIN2 ADGRE4P IGLV3-2 USP7 CD82 KIR2DL4 FAM167B ARL13B SCP2 TMEM156 ABHD2 ALCAM DENND1A SEC14L1 ERLIN2 ATP11C PDZRN3 SF3B6 ADAM32 RSRC1 DNAH11 ASS1 ZSCAN5C NIPBL CEP120 B3GALT5 CDC42EP3 MICU1 FAM204A SNX25 MYO5C SDC2 OTX2-AS1 ATF1 SLC25A21 SLC22A14 SAMD12-AS1 RBPJP6 IL6R C12orf4 TTLL11 LINC01579 NEBL RARB CCDC18 CDH8 AGPS TRPV5 HEPHL1 PJA2 KLHL7 TCF4 ECM1P1 GACAT3 RPRD1B AURKA LINC01145 PTPN2 AP3B1 PBLD GORAB ERICH5 BCAP29 TTC21B PSMF1 SLC5A4-AS1 SH3PXD2A NEK6 SLC39A12 MOB1B*

		OR13C9 ASAP2 SEMA3F-AS1 YIPF6 TMEM25 OSBPL10 RIC3 CASP5 EXOC1 RNF182 RALGPS1 UPP2 PHF21B ZFPM2 PIEZO2 FAM66A BCRP2 SVEP1 FANCA DEUP1 ZNF354C LINC02325 LRRC2 ANKRD26 RGS20 MIR3118-2 PDGFD CNDP2 HCRTR1 RELL1 LINC02176 BRINP3 LINC01237 KIF4A XRCC4 OVCH2 COP1P1 EPHA7 MAP7 TM9SF4 SENP8 SUSD6 NSG2 ZBTB8OS GRIA1 ZNF627 IFNAR1 KIAA0319L USH2A LHFPL6 OPN3 NEGR1 NAV2 XXYLT1 CACNG2 ANO10 BTBD9 SPATA48 CA5A FAM72D USP18 NET1 TTC28-AS1 BMP2 TOX KIAA0232 LINC02011 SRGAP2C TMOD2 SLC44A5 FAM107B LUZP2 BTBD10 SH2D3C MELK RBPJP2 LRIG1 YPEL1 PRKACB ZNF536 RIMS1 GXYLT2 HIPK3 EPB41L3 WSB1 TMEM225 POR LINC00896 PARVB MORC1 OR10H2 ZBTB7C SCGB1D5P KANK4 GAST SGO1 WDR26 SAR1A SLC37A1 BCL11B LINC01814 DTWD2 LINC01213 NELL2 TSPAN2 MAGI1 SLC14A2 LINC02668 OR52B3P ASAH2B RALB MOSMO KRTAP19-10P BLK PPP1R17 PIAS1 PTCSC3 LINC02180 SSBP2 ZNF705CP GALNT10 FHIP2A CFAP74 ZNF846 HAGLR ZNF287 ARMC2 FER1L6 ALS2 CYTH4 COMMD8 ZNF608 PTGER4P2 TBATA ATL1 SERPINB11 ZDHHC17 KCNH1 ABCC9 SNAP29 QSOX2 GSG1L MCF2L LINC01098 ACSS3 BBOX1-AS1 ABCD3 DCDC1 NPAS2 DIAPH3 CCSER2 INTS7 AMPH ASB4 TMEM178B COL5A3 COL4A2 PARP15 ME2 GRAMD1B SIAH2 POLR3A ZNF44 LINC00536 HEATR5A ADA2 PRKCZ BPNT1 F13A1 ALG10B CDC14B GIPC2 RNU6-1007P KDM7A CYP4F22 ABCA10 LINC01182 KLHL1 SP110 LINC01566 PRSS2 MRM1 ZNF705D ATP9A POSTN OLFM4 SAMHD1 OCLN AK8 SDF4 ITGBL1 TNFSF11 SPOP EFHD2 EGFR MYEOV ADAM28 MRPL13 B4GALNT3 AFAP1 DPY19L2P1 SH3BP5 SLC49A4 FANCM NEO1 MELTF MXRA7 MYT1L SRGAP2B SLC37A2 ATP1A1-AS1 SORCS3 PDP2 VPS37A LRRC49 ERP27 ZNRF3 ZBTB21 BBS4 TENM3 ITPKB ENPEP TUSC3 IPCEF1 RTRAF NF1P9 OR4K6P BIN2 LINC00877 TCERG1 UBE2O VENTX DIRC3 PLCZ1 CPEB4 COL6A5 ZFYVE28 NCOR1 LINC02213 PRDM10 EBF2 C16orf72 USP33 ERBIN RNY4 SLC24A4 ZNF573 MBTPS2 KHDC4 C2 NTF3 OR6C75 ZNF705G LINC01684 PI4K2B USP41 RBMX2 FAM72A IL17RA SOX1-OT SUSD4 PTH GALNT14 RAB22A H2ACP1 FAM66C ZNF160 LINC00466 HADHB NSMCE1 DNAH10 GAS2 PDE10A CACNB2 REPS1 MAP3K4 TP53I11 PDXDC1 MTPN MT1HL1 LINC02646 GNG7 VSTM2A RUNX2 ZNF804B LRP12 LRRC8B CSNK1G1 ZNF169 MICU2 SOX6 JAZF1-AS1 SGCD RC3H2 LINC01492 RNU6-374P TNNI1 RAC1P3 SP3 STK32B PLG ZNF106 LINC01020 NR2C1 SLFN11 ADAMTS3 ERO1B DNAH8 NHS LINC02505 CABYR LINC01476 ANK3-DT RGS12 RAPGEF2 ZNF438 GTF2I NCK1 SOHLH1 LINC01192 CDV3P1 C15orf32 PUDP KDM1B SSPN HS3ST2 F5 PATJ FAT1 NUP50-DT CPS1 MESD PRKCH TRAK1 TET1 FAM66B RHPN2 ANKRD30A RABGAP1L KRT25 NMU DENND2B LINC00603 HADHA CFDP1 LINC00944 SMARCAD1 MIR3118-3 FNDC3B ADAMTS9-AS2 ASTN1 GNG12-AS1 GFRA1 NSG1 RMST ANKRD20A8P MAPKBP1 CPE TDRD7 RNF8 LY86-AS1 LINC02613 PYGO1 LINC01723 NFKBIA TEX29 DNAL1 TRAPPC3 CD101 PFKFB4 TMEM132D HMCN2 FHIP1A EFCAB8 LINC01204 SPRED2 SCN10A HSDL2 MYLK3 NCOA7 ANKRD18A ZNF350-AS1 CEP128 ZC3H15 LINC01135 ANKRD7 ABCA5 PTCSC2 CIB4 ABCC8 TTLL5 AKAP11 NOXRED1 TMTC1 TTC33 MOCS2 NRK NAT1 KICS2 CYBRD1 MCPH1 MINAR1 EIPR1 STON1-GTF2A1L BMP2K LINC02543 CYFIP2 APOOP5 CCDC126 BABAM2 MSANTD4 CRB1 IL1R1 OTOG HEPACAM USP8 NUDT21 XPO7 ARSJ KCNS3 ENPP3 ZNF235 ERC1 LINC02006 VWA3B ZNF850 ALPL PDLIM5 MAP3K9 XYLT1 BTAF1 PDCD6IPP2 ALPK2 LINC02660 ABCA13 HNRNPCP9 RFX2 MAPK8IP1 ADGRB1 SLC66A1L LYPLAL1-DT ADGRE1 RAP1GDS1 FOXO1B TET1P1 ST8SIA5 TBC1D19 ZNRF2P2 PDE6A CHST8 BID COX5A MACF1 MDFIC MNAT1 PCMTD2 MBNL2 DNAH14 STARD4-AS1 ERI1 TC2N TUBGCP3 BTLA LGALS9DP SLC15A5 HCP5 AMBRA1 CLEC20A NETO2 DOCK2 SERPINA6 ASCL3 PTPRE IFT46 FAM66D NUP210L LINC02063 TSPAN3 ALPK3 LINC02465 FLVCR1 SLC9A5 MUSK EPDR1 MYO3B LMNTD1 RAB8B LINC00583 MYOM1 ZSCAN30 MTCO2P3 LINC00469 RNU6-835P RXRA CGAS ARHGEF7 SLC23A2 LIN54 LINC01649 ARPP21 ARL11 MAML2 SPAG16 ADAM5 TRIM43B ZNF879 ARHGEF12 LYPLA1 LNPEP DDX39BP1 LINC02198 UNC93B3 RPS3AP6 POU1F1 ZNF397 KIAA1958 CARD18 LINC00623 NEDD4 RFTN1 CCDC141 NEK4 RSU1P1 PEX14 CFAP61 FYCO1 LPGAT1 CNTN4 FOLH1 HRH4 SPRR2D LRRC38 EXOC6B EVC2 CNKSR3 USP49 DRAXIN SEMA3E CSF1 CEACAM22P LINC02109 LINC00511 SLC8A3 TRNAU1AP LINC02145 RNF17 HAS2-AS1 KIF11 LINC02400 SLC2A3 FAM72B PRAMEF26 SLC40A1 PHC3 STOML1 CADM2 SDR42E1 LINC01581 FANCL SH3GLB1 GABRR2 RAP1GAP PIK3C3 OTULINL RAD9A SLC9C1 SCML2 SPOPL MAGI3 LINC00701 TRAF3 MPPED1 CCDC122 CHD6 FAM135B TMEM273 MORN1 CCDC186 CFH PAXIP1-AS2 LINC01695 PTPRB INTS8 LINC01412 ITGA1 VN1R7P MARCHF6 CCNG2 ATG4B CIBAR1 ODR4 GAGE13 TANC1 CORO2B PAPPA DHX40 KIFC1 POC5 IGHVII-65-1 TRPC7 SYCP1 VPS41 DTX1 MYO10 GFI1B ZNF407 MIR3118-4 ASB3 TENM3-AS1 KIR3DL2 GAGE12J TRIM43 FOXO6 TSHZ2 C1orf21 TOM1L2 STOX2 FAR2 BICRAL PLAGL1 NEK7 NKG7 CNN2P12 NLRP13 COG2 RPL5P35 ERN2 CYP2C58P TLNRD1 SERPINB2 KSR1 AOX3P LINC01322 GABRB1 ANKS1B RP1 LUC7L AKAP10 TTLL7 EFCAB14 SLC16A9 WWC1 PLEKHA3 RSRP1 BLM PLA2G4A SENP6

		GATAD1 ZBTB16 DDX39AP1 UNK B4GALT6 ATG5 LINC01938 SGTB TRIM60P19 ZNF875 XKR5 SCGN SYNJ2 STK32A ARSB IL34 ZNF234 CLVS2 DIO2-AS1 SIGLEC29P DPYSL5 CWC27 AGK USP25 ASCC2 SLC44A1 CNNM4 ADAM10 ATXN3 GALC MRPS22 TMC1 PLD5 OXR1 PAK3 CAMLG TSPAN33 GARNL3 RNU6-1150P NPIPA1 TPM1 CES1P2 CIDEC EBF1 DHTKD1 OBI1-AS1 FLNB OR2T2 MADD PCID2 LINC00667 NDFIP2 DUX4L45 ZSWIM6 MYL1 ANKRD36BP2 CAMK1G DSG1 C1orf87 LINC02327 FAM30A PDZPH1P ERICH3 TRERF1 CENPBD1P1 TADA2A RPL15P2 LRRC9 LINC00158 FBN1 NIPAL2 MTUS1 GABRA6 PTPRVP DHRS3 PRRC1 PANTR1 LASP1 VPS35L ABHD17C KRT6A C21orf91-OT1 GGT4P DROSHA ZNF813 ANKRD31 PALS2 ADCY10 FAM149B1 PDE1A TMPRSS2 LINC02165 PTAR1 PRICKLE2 CMAHP ANGPT1 TRIM58 HMGA2 HHAT KLHL32 CHASERR PSTPIP2 MVB12B C4orf50 LINC02253 UBASH3A CACNA2D3 SEL1L ELOC SLC7A2 TMEM67 BTF3L4 MIR3936HG ZNF618 ITGA4 CPA6 AGO1 PRKCE DEFA3 GLYATL1 RBPJP5 PTPRK MIR3142HG CNTN3 GLIS1 ODAD2 ARHGAP31 RXRG ETNPPL KIR2DS4 PRKAA2 CD163 ARMC3 BBS2 SYT1 OR4F15 LATS2 IKZF2 PPP1R12B APCDD1L-DT PDE2A DNMT3L ASPM RFC2 PPARA PLXNA2 KLHL33 DENND2C LINC01602 TBC1D13 CDYL2 SCARA5 PRG4 SCGB1D1 RANBP3L MARK2P12 TG ADGRB3 HGD SLC36A1 PLCB1 UBE2R2 KCNH8 ATP6V0CP3 LAMB1 ANKRD19P RPS10P7 CUL5 HOMER2 DGKI RIN3 LINC01221 REG4 LINC01151 DIP2B CD5L LINC01524 TBC1D30 IGLV2-14 NME7 STXBP6 TFDP1 FAM66E ZBTB33 MXI1 ZNF876P PPME1 TRAPPC8 OR4R3P STX12 LINC02291 FUT9 MOK GARS1-DT CREG1 LINC02488 SLC12A1 SCN2A MAPKAPK5P1 WNT7A TUBB6 ZNF449 DOCK10 FAM27C PAMR1 DDX6 SPIRE1 TMEM71 COG5 AIMP1 UBE2E1 ARAP2 LDB2 LINC02149 SNX30 ATP8A1 BCL2L1 IQSEC1 LINC02008 JARID2 LINC02236 SHROOM3 ADK PRR5L ALX4 CORIN FAM241A LINC00838 RANBP17 WNT2B MRPS27 PPM1L CPHL1P LRRC37A3 TRIM43CP PRPF18 SMOC1 GSTA3 EXD3 SETDB2 FAH MON2 OR51E1 TMEM116 TOGARAM1 SLC45A4 ZNF705B ELF2 SEMA3D LDLRAD3 GLYAT KIF15 CFTR VSX1 TBX20 FLRT2 NFATC2 NALCN-AS1 PRAMEF25 KRTAP20-4 ARHGAP44 MTMR2 ZNF970P KCNH5 FAM189A2 CADM1 KLHL29 PTPRO ARID3B OPRM1 ACER2 ZDHHC14 RGS9 YLPM1 ATF6 ITFG1 IPO11 VCL SLC13A5 GLB1L3 ABCA9-AS1 DOCK5 VCAM1 C7orf31 LINC02511 LINC01818 ATP6V1C2 MAGEL2 IFT81 NHSL1 OSCP1 PLEKHA8 SGO1-AS1 DTHD1 SRGAP3 IGHVIII-13-1 HAAO CTNNAL1 CIBAR1-DT CYP2A7P1 ATP6V0D2 SYNJ1 PHF20L1 HLA-B KLF15 PPP2R2B NEDD9 HDHD5 IL12A-AS1 ANKRD66 ENPP1 SDR42E2 LYRM4 CCDC150 DNM1P47 LINC02099 HEMGN RIMS2 UGP2 RP1L1 PPM1F OR4K8P EFCAB6-AS1 CHSY3 YBX3 YAP1 ARL15 ANK3 BMPR1B LINC01173 FCRLA LINC01937 SEMA5A C1orf127 PRDM15 KITLG KRT6B SV2C DAW1 COL5A1 IGHV3-74 IFT57 LINC01426 RIPOR2 MAP4K4 LINC02899 ZNF112 FICD KIF21B FEZ2 ATE1 PEG10 INHBA-AS1 HSPD1P3 NMD3 OLA1 GATAD2B VPS13C ANKRD55 XIRP2 KRT85 SLC14A1 CA1 C5orf52 FAM72C MFSD9 SERPINI2 STK38 APBB1IP NPL CAST TBC1D9 FBXO32 AOAH SNHG14 TSBP1-AS1 SMG1P4 SNAI2 ZBTB49 FAM90A28P PHF2P2 AP4E1 DLEU1 NDFIP1 TNR FAM180A LHX9 LINC02074 OCA2 ST8SIA6 DAPK1 MSANTD2 FCHO2 RFTN2 TRAV8-6 COL6A6 RN7SL767P DNAH5 CNTN6 CLIC6 LINC02406 FMN2 BHLHE40-AS1 SEMA3C VAV1 LINC00895 CACNA1I BTG3 DPY19L1 CSF2RB CMTM7 RPS6KA3 SMAD5 DNAJC13 BCKDHB PPIL6 IGLV3-30 CNST PATL1 UBE2J2 ASB2 OTOP1 PSME3IP1 OR4F6 GHRH UGT3A2 GOLGA8B RNLS IGLV4-3 SPOCK1 LINC02315 NF1P6
4C-decreased-1200	1122	LRRC37A5P CCDC116 ZHX3 APOL2 RPS4XP6 PDCL3 PTPRR HERPUD2 CRTAM SLC52A1 UBE2G1 PELI2 TPI1P1 NOS2 MIR548H4 ZEB1 LINC01708 FAT4 PARN SEMA4D SLC15A2 RN7SL483P WSCD1 MIR4435-2HG KNDC1 LINC02355 ZFYVE1 EVC HERC2P2 LINC00670 DMC1 CXADR SPPL2B C9orf43 DIP2A NBPF21P OR7E19P RIOK1 GGT2 UNC45B LUZP1 LINC01346 BVES-AS1 ZNF723 LINC01138 CECR2 LINC01782 SMYD3 GNAS DYSF NPM1P2 CD38 SERPINB9 LINC01876 PGBD5 LAIR1 MSH6 RASGRF1 HECTD2 FBXO31 C12orf40 LINC01163 GMPR RIN2 PRDM16 FRRS1 PDE6C RNF217 TRAPPC9 LINC00323 MEIS2 CARMIL1 PCDH7 BCL2 KL LRRC4C LINC02558 RACGAP1 MIR3667HG THADA NUP37 PPP1R9A SAA3P TMEM117 KCNK15-AS1 TOX3 TDRD5 TASP1 SNX6 POTEG GOLGA6L3 SAMM50 ZZEF1 FRA10AC1 HHLA2 NCF4-AS1 C3orf52 SLAMF1 UQCC1 RGL1 ATP5PBP3 SHOC1 LINC00841 FAAP24 INO80D KDM6A MED27 NCAM1 PDYN-AS1 GDAP1L1 LINC02096 LINC01358 UFL1 EPHA4 LINC01967 PLA2R1 LYSMD2 NGDN H1-9P ADAMTS2 GTSF1L WARS2-AS1 CCDC172 FRYL LINC01828 GPRC5C COLCA1 CPAMD8 RNU6-929P GPR1-AS UBL7 LINC02675 CALM1P2 RBM6 MEGF10 IGHV2-70D HS1BP3 LIPI LINC01445 RAB38 DZANK1 CLTCL1 NUAK1 PCAT1 KCNK10 ZNF738 BLOC1S5-TXNDC5 SLC16A1-AS1 VMP1 NENF HRH2 VSTM4 ATP6AP1L RNU2-47P RDX SNTG2 CTSB SVIL NDRG2 APBA2 TTC3 COL23A1 NEDD4L EDAR C5 EGF LINC00960 ATP2B2 HDGFL3 RPL37P3 CCNYL3 ABCC12 PARK7 DSTYK RIMBP2 ZNF271P IFT43 ADAMTS19-AS1 SNRPC C2orf69P4 DRC7 DSE POTEJ LINC01427 MYCL LINC02256 TPTE2 SPAG6 BMP7 PDE4DIPP1 GALNT2 FGF12 EPHX4 CYP2C9 CNOT7 CASC9 IMPACT ITGA6 HHIPL1 NOTCH2 IMPA2 ZFP90 S100B ARHGAP12 USP43 KCNN3 FKBP5 NFAT5 FLI1 ANAPC1

		GRM1 LINC02147 ARHGAP26 ITGB8 GAP43 LINC02422 OR4K3 PRDM13 PDE3A PTPN4 BCAR3 MAP6 APOL1 CDH11 SETBP1 AIF1L CDS2 ZNF780B LINC01900 ATP6V1E1 LINC01993 LMX1A AGBL1 RSPH3 DEFT1P2 LINC02439 ZFAND6 GBP4 CNKSR2 PSMA5 DPH6-DT GFRA2 NEK10 ADCYAP1R1 SNX29 MAGI2-AS3 ZNF611 STAT1 SAMD5 MROH5 SLC44A3-AS1 MYOCD CREM GOLGA8J XKR3 UBE2QL1 PLPPR5 TDP1 AVL9 EFEMP1 TNRC6B WBP2P1 LINC02542 SYN2 PTCD2 MYO1E SMOC2 MIPEP NCSTNP1 HDAC2-AS2 HLCS FH RWDD2B PLPP4 STK10 PWRN4 CCDC102B SDS GSR CCDC162P LINC01571 FIG4 SOGA1 ARHGAP32 BMF NECTIN1 FLT1 RB1CC1 ZNF528 LINC01222 LALBA NXN LINC00375 FOXJ3 CENPE CKMT1B MYL12B RSPH14 IL17RD ANKMY1 HCG22 APELA UBN1 PLA2G12B FAM83B HDAC4 CTNNA1 STK36 GNAI2P1 FAM102A NCOR1P1 TRPC5 MYO9A TMEM182 IL10 LINC02305 AMFR LIFR-AS1 C19orf18 FTO SLC6A1 EPC2 DMXL2 SEM1 SEMA6A-AS2 MOGAT3 TMEM236 NLK THSD7A CXCL2 GOLGA6B LINC00334 CARD10 ACSBG1 GCSAML DNPEP TRAPPC11 HOXC4 IGHV3-62 NECTIN4 CNMD LINC01309 UFD1 LINC00299 BAZ2B HERC2P3 CRACD NGF-AS1 AGL PALMD HS6ST1 MARK2 MEOX2 SFI1 ZBED9 CTSE ATF2 PLEKHB2 FAIM ZNF72P RPRD1A ZNF880 PPP2R2A ESS2 PHAF1 ZNF541 RBBP8 AK6P2 CKMT1A RFC1 NSUN6 LINC02174 CDC45 MC2R AKR1B1 BTBD11 LRP2 LINC02087 HERC2P9 SYNE1 MTHFD1L RALGPS2 HOATZ SNRK ABCD1P4 EXTL3 EHMT1 TTC29 FBXO47 KRT89P ZNF735 ZBTB10 PKHD1L1 SLC9B1P4 PLPPR1 CEP192 SLC26A2 CAMK4 GUSBP11 CLPX OR7H1P ROCR ANKRD20A9P HDAC11 SLC9A4 ANKRD20A17P GRIK3 GRXCR1 NUMB STPG2 MIDEAS TM9SF2 CD70 CELF2 SH2D1B ZNF606 ESYT2 HEATR6 ARFGAP3 MAPRE2 FGF10 TAPT1-AS1 KRT18P59 ISX RAD51AP1 POTEM SYBU SMTN LINC01035 PDE4DIP SCG3 ESRP1 RIC8B TAF3 ZMYM4 TPTE FRG1JP MED12L GRM7 ZDHHC21 BRMS1L DDHD1 ICA1 PLEKHD1 CDH7 EMILIN2 TLDC2 CYCSP39 HORMAD2-AS1 VASP PLGRKT UBE2E2 UNC80 SDE2 PTGFRN PPA2 ILDR2 IMMP2L ZNF615 SPSB4 SPSB1 GABRG2 ZNF567 ITCH NUDCD3 MBP S100PBP TANGO6 GABRA5 CELSR2 CDKN2C STXBP1 SLC46A3 PTPRJ DLC1 PNPLA7 SELENON RPS3AP4 CNIH1 FRG1BP C2orf69P3 LINC00114 ZIM3 MANBA DUX4L2 CHRM3 ECT2L UST MIR663AHG CALD1 LINC01543 AIG1 ERICH1 DEDD2 TYW1 TAF15 ALB ARHGAP24 JPH1 ANKRD20A3P EFR3A HTR2A TPH2 N4BP2L1 IGHV1OR15-9 TPTE2P6 EIF4BP3 LOXHD1 MDS2 GOLGA8G LINC01622 GALNT1 MARCHF1 OR4L1 ZBTB25 INO80 RBM15-AS1 HSD17B14 KRT16P6 COL27A1 GLP2R CHCHD2 BMP5 SLC4A4 ZNF675 LINC02366 GOLGA6L17P SYT16 CRYBG1 TWIST1 HACD2 LINC02653 LINC02061 RPL15P3 TRIM77BP ERCC6L2 PASK PHKB RUFY2 SLC16A1 RANBP9 FAM245A MRTFB LINC01344 KCNE4 TRIM5 LINC02228 KRTAP21-3 LINC01801 LINC01320 LYPLAL1 THNSL2 BRWD1 COLQ TMEM54 PPIP5K1 C9 TMTC2 HECW1 MCTP1 RNU1-51P MOB3B ACTR3C LCE1F ARPC3P2 MAP4 TSPAN11 MTND1P17 HIVEP1 ATRX TNIK KRT18P55 OR1L6 NBN PRTG OR2T7 SLC17A1 SEC24D RGMB KMT2E WNK2 FRMD3 RBFOX3 SDAD1P2 PWWP3A ITIH5 PACSIN2 TRGJ1 HOXC13 PKP1 SYNE2 GTF2IP6 MIR181A1HG TRMT61B TRIM23 FABP7 SDCCAG8 GK LINC00355 DUSP22 KCNJ18 CHSY1 RFC3 MAB21L3 SMPD4 EXT2 PTPN12 GPR137B ZYG11A LINC00434 LINC02424 TOP3B MPPE1 RGS3 UBA6-DT ANKRD26P1 CMIP DEFB116 SLC2A13 FBN2 YTHDF3 SPATA17 SYT10 ZBTB38 LINC02380 CYFIP1 ALK DOCK8 GON4L GREB1 ARNT LINC01340 LINC00929 CSTF3 ZNF648 LINC02058 SAMD13 DNAH6 ARFGEF3 TMCO5A UHRF2 EPCAM-DT DCLK1 DEFT1P RNF215 ANKRD28 GRK3 SND1 IFNG-AS1 SNAP25-AS1 ZFAND4 LINC01664 FGD4 ETS2 LINC00649 ZNF382 NDUFAF6 GOLGA6D ERMP1 CATSPERG ARL4C ITSN2 LINC01917 KTN1-AS1 HIRA OSER1-DT SERPINB7 ANKS6 TMEM74 PRKAA1 RASGEF1C TAFA4 ALDH1A2 GABRG1 MTTP POGK CROT MAPK9 ESRRG FBXW2 LINC02458 LINC02652 LINC01229 FOXJ2 AGAP9 ABCC4 MYT1 FRG1HP ABCD2 ZNF595 EMP1 TMEM171 LNCAROD RRAS2 SV2B FAM110A NRBP1 SEC14L3 STK38L GTF2F2 RALGAPA2 FAM245B ADAMTS19 ZNF236 RAB27B SOX30 LINC01337 MYOF P2RX6 PLS1 UNC79 RSPH1 SPON1 ANK2 SH3GL3 CFHR4 INVS FHL2 NCAPG2 LPCAT2 LNP1 TPTE2P5 PHF19 ADAMTS14 ZNF518A LINC02191 IGLV3-31 KYNU DCAF1 ZCCHC7 CD2AP TTC39C LINC02680 ZNF124 EBF3 TAFA5 NFKBID SPRED1 MYO5A CIDEA LINC01924 PPP2R2C PWRN1 LINGO1 LINC01706 SEPTIN9 SMPDL3A LRFN2 ACO1 WDFY4 SCAI PAPPA2 LTN1 TINAG NCOR1P3 DIRAS2 ABCB5 HEATR4 SLC5A9 KLHL4 DPY19L2 MDM1 RRBP1 SMPX OVOL2 CD9 MTMR10 EIF3F CDHR3 CSE1L NEK2P2 TOM1 FAM118A PRUNE2 SGMS1 ANKRD24 COL25A1 RBPMS2 ITPR2 CYP4A11 BRINP1 IGLV2-34 MTND2P8 RPL23AP7 GRB14 LARP6 RXFP1 IGSF5 LONP2 CCDC192 LEMD3 PEX6 CHKA BUB1 INTS4P1 VPS13D KRTAP21-2 ZNF292 SPIN1 TBX15 PRB3 TTC3P1 KPNA1 SKA1 CCND3 LAMC1 SRP9 SCGB2B2 GABPA SMIM11B DSG1-AS1 PCNX1 DUXAP10 LINC02091 NAA35 ATP5PF RPF2 UBAP1L MX2 AGGF1P10 MTCO1P1 ITGB3BP ZNF831 PRAMEF2 ENTPD5 DGKK SLC4A10 LINC00844 TSSC2 LIX1-AS1 TGM1 AGAP14P MGAM FTLP13 ZDHHC18 LINC01310 PSG9 FAM183A UHRF1BP1L IL1RAPL2 APIP MUC19 SCAPER IKBIP NPHP4 LINC02141 OR8B9P CBLIF ATP6V1B2 KCNJ1 LRRTM4 CLCA4 PSAP LINC01877 MYOM2

		SLC5A12 NCS1 ONECUT1 PCDH11Y METAP1D USP31 HIVEP2 SUMO2 OR5AQ1P MICOS10 XPNPEP1 GLYATL3 NUBPL PCGF5 FGF7P1 NLRC5 TUBBP9 LINC00363 NDUFAF2 GUCY1A2 KREMEN1 UMODL1 LINC01189 MB LINC00383 WDR25 SOD1P2 CYP4Z1 LCE3D TNPO3 EFHB ACSM2A FGF9 DCUN1D4 DAZL RPL23AP53 SLC1A1 HPCAL1 SPG21 ANKFY1 LIMCH1 ZKSCAN5 CCDC195 TSHZ3 TMCO4 LINC02098 RALA GMDS-DT BAZ2A MIR548A1HG RPS12 GNPTAB TRPM1 NTN1 COPS8 SACM1L AKAP9 C1QTNF3-AMACR AP4S1 NR5A2 PRKD1 TRAV8-1 TNFAIP8 RBM47 TMEM260 GBP6 SANBR DBF4B OR9Q1 CCL28 FBXO41 MICAL3 SNRPN HDAC2 MSI2 MRTFA LRRC7 LINC00221 ETS1 TNN CASZ1 PCDH8 LINC01673 WDHD1 MIR155HG MAMDC2-AS1 RABEP1 ST6GAL2 LINC00930 NUTM2HP SLX4IP PTCHD4 BANP ALKAL2 MAP2 YME1L1P1 TGFA SNX3 LINC01422 ZNF280B PTPRA CEACAM7 TNRC6C TMEM161A CYLD DEFB108B OR11G2 LINC02451 WSCD2 UBE3D GLIDR ZNF402P CAPN5 CERS3-AS1 RPL23AP87 GOLGA6C ABCA4 ZFAND3 LINC01718 THUMPD2 C6orf118 FAM83F CNKSR1 RAB12 TMEM163 PCDH9 LINC02235 DIPK1A SLF1 EXOC1L RBMS3 POTEH GNA14 INMT-MINDY4 LINC01992 SRFBP1 COPB1 MATN2 TM7SF3 IL33 EFCAB2 MARK4 CRACR2A CLEC16A LINC01901 OR2T3 SERPINB10 PROX1-AS1 ACTN1-DT ARHGEF17 ME3 PSG6 CACYBP ITGA9 PPP1R1C ULK2 IARS2 UNC13C CDC42BPB HNRNPH3P1 TMEM44-AS1 LINC00298 FAM217B MTRF1 FAM25G SHROOM2 OVCH1 CISD1 C16orf95 CRTAC1 EGLN3 PRKAB1 RALGAPA1 LMX1B LINC01748 L3MBTL3 RRAGD IGKV2OR22-3 POMT2 MICALL2 ST13 LINC02245 CLIP1 MARCHF8 SHC4 C2orf42 CABLES1 MORC2 ZNF613 TLL1 DEFA8P FRMD6-AS2 DUSP16 BCL11A AKR1C3 PPP2R5E ASB7 FREM1 IL20RB ADAM22 RABL2A PTGS1 LINC02582 LINC01811 CNIH3 RPF1 TRIT1 CEPT1 WNT5B CEP44 HKDC1 CLNS1A EPS15L1 HIP1 PVT1 CHN1 ETV6 WNT9B RFX7 TRPS1 SKINT1L IGKV3OR22-2 ADAMTS5 NF2 STRN CRISPLD2 NPM1P1 ANTXRLP1 MTMR3 ZNF845 RN7SL250P FAM25C CRKL ITPRIP H2BC15 ADGRA3 SCG5 CLDN18 LINC00581 ADCY9 DPP10 TUBB2BP1 GALNT16 CDH5 ANO6 ZNF600 HIPK1 A2MP1 PGPEP1 ACSM2B TTC7B ATP2B1 IFI44 RETREG1 NLRP14 NPHP3-AS1 PAH FAM138E ATRN FBLN5 CNOT6L TRAPPC6B TOP1 ZNHIT6 SLC39A6 WDSUB1 ANKRD20A7P
upregulated-1285	1133	*TFIP11 UTP20 ERAL1 H2AZ2 TKT LRRC41 SLC25A3 IRAK1 G3BP1 NUP155 DDB1 RNU6-322P DAZAP1 EP300 MITF DDX5 IMP4 CLUH ZNF131 HROB MSH3 NOSTRIN LYAR SPN ARF6 IPO9 MED13L MRPL1 NOC4L MNS1 NAT10 FADS1 PCYT2 FAHD1 ARHGAP21 ACTR8 ENSG00000261770 STK25 SLK BSN NASP NOL8 BAZ1B GRPEL1 LMNB2 SDAD1 SLC25A46 INTS6 CLTA CEP350 RBM10 BCLAF1 TRA2B RAD23B STK17A CHRAC1 NEFH CCNY SSU72 TRUB2 IP6K1 SRSF6 ZNF598 POLE3 HSPD1 DUS3L BOP1 POLR1E C1orf216 CCAR1 EP400 GPN2 UBR3 TUBB KIAA0100 HNRNPR GCLM HBZ ZNF75A ADI1 ZNF239 SAMSN1 SON PTP4A2 TRAM1 PSMD3 RHEB RAB35 OTUD6B NPM1 TSR3 XRN2 FUS CELF1 ABRAXAS2 SEC23IP CNOT1 RCSD1 DDX18 PPP2R5A CCT8 EIF3M SRP72 ZNF24 DDX49 CAPN1 TRIP12 BTF3 ATAD3B IKZF3 PRXL2C SETMAR EZR DYNC1H1 TMEM33 ABCF2 WDR6 ITFG2 DHX16 MIS18BP1 KIF2A HGH1 HSPA8 MRPL15 KCNQ5 DHX15 NEU1 WASHC5 SPRY2 LTBR NOP58 TBC1D9B SF3A3 FUBP1 HCFC1 AHNAK TRMT6 DHFR EIF4A3 ATP6V1C1 PRPF3 ALYREF UBE2N FAM83H ENSG00000286122 LINC02393 PPM1H NFYC SSRP1 STRIP1 ASXL2 CCDC6 MTHFD1 CYP3A5 FTH1 IER3 PITX1 IL17D GRWD1 JUND PHB2 LIN28B TNF MRTO4 ERMAP DDX39A C1QBP TIMM17A ASAP1 PDSS1 HNRNPA0 AMMECR1 TCP1 BRCC3 TRAM2 KLHL21 EIF3D ZNF586 SET NAB2 FAM120A AURKAIP1 POLR1A TAF9 CRCP BICD1 CORO1C FTH1P16 JRK RANBP1 FDFT1 SQLE EIF5A WBP11 ZNF614 MCM10 TMEM69 TMEM185B GABRE HNRNPK ELF1 ICE1 SAFB2 BEGAIN OXA1L TRIM35 USP11 PRKDC PSIP1 EXOSC3 ADD1 NOL11 E2F4 IPO5 ACLY IK SURF4 NACA GPX4 CDK4 PRMT1 TEX10 CEBPZ MCM5 CLN6 CSNK2A2 SRRT RBM3 RABGGTB CUL3 NFATC3 KMT2B TSPYL5 MIX23 FASTKD2 HRAS RABL6 NR2F2-AS1 DHCR7 NUTM2B-AS1 SLC19A1 AHCTF1 VAT1 APEX1 CAVIN2 MRPL11 MYC UBE2L3 NAA15 ENSG00000279348 TOMM22 MUS81 ARID1B YES1 VCP ABT1 ARID2 SMG1 RAVER1 RCC1 SRSF3 CALM2 ENSG00000287905 WDR82 FUT8 ZNF74 RBMXL1 STMN1 NRROS ENSG00000282386 CWC25 MT-RNR1 NR2F2 PES1 MS4A3 ENSG00000288271 TRIM24 PTDSS1 ENSG00000253853 CHTOP ENSG00000276742 ZFX HMGCR URB2 YBX1 STK24 NCOA5 H2BC12 BUB3 CTR9 CDC27 MCCC1 VPS35 VAC14 HNRNPH1 OR10Z1 CITED2 KPNA4 PFAS NUCKS1 HNRNPD U2AF2 YWHAG TRMT1 KMT2D ZMYND19 EIF5B WDR70 UTP25 LMNB1 MAF1 MT-RNR2 TFAM PUM2 PIK3C2B HHEX SRSF7 GTF2H1 VKORC1L1 ELOA PPP5C RNASEH2C PCLAF ACP5 WDR33 PRMT5 MAPK1 MPHOSPH10 CHD7 HNRNPA2B1 RNF126 UTP18 SBF1 API5 POLR1B PPP3R1 RBM45 FAM117A SH3YL1 RBM14 SF3A1 CAPNS1 EIF3A DANCR SEC24B LHX4 LINS1 CYB5B TNPO1 EEF1D RRP1 TFAP4 SNX9 ANKRD36C WDR74 MCM7 POLDIP2 RIOK2 STAG1 DHX38 BTBD1 ABCF1 CDT1 CFL1 LRP8 MCOLN3 TGFBRAP1 GPATCH3 PSMG1 TMEM43 EML4 PRPF8 SSB SKI TIMM23 ENSG00000289474 CTPS1 NUDC EIF3J EMP3 SYNCRIP DNAJC8 STAR JADE2 FARSA TRNT1 TXNRD1 TRMO STIP1 SART1 MTDH SPTA1 HSP90AB1 CCT3 MVK NOP14*

		NCL GVINP1 GSPT1 CDK7 COPS3 HCG18 TMEM97 MCM2 MCM4 DCAF13 ARPC4 TOMM70 ENSG00000286680 TARDBP TRIM28 DDX21 FKBP15 PWP1 COA7 METAP2 PAN3 ALG8 RIOX1 MED15 SETD1B CDC37 PPP2CA POLR2A ODC1 ZNF26 PPRC1 HAT1 POLE DNTTIP2 ENO1 SBDS DDX1 AZIN1 HAND2-AS1 RNPS1 KIF1A NOL7 BCL7B PPP6C TOMM5 PSMC5 CBX3 ANKRD13A SETD1A SYPL1 FAM71F2 SLC39A10 ENSG00000227706 ZNF512B ATP6V0A1 KIAA1586 BRD2 SLC7A1 TIRAP USP36 NONO ZC3H4 MAGOH ELAVL1 AGPAT5 CSTB SNHG6 VPS72 TCF20 SRM PPM1G AKAP8 XRCC5 CERT1 CUTALP NFKB1 FOSB H3-3B PCBP2 DUS1L MFAP1 ZNF789 TOMM40 VGF ADNP IGF2R RBM15B ENSG00000268362 POM121C LARP4 ZMPSTE24 HMGCS1 SNRNP200 RAB10 TOP2B PKP3 DNM1L ZNF252P EXOC7 DEK PSPC1 UPF2 ALMS1 CERS6 BEND3 UBTF GYG1 PHB R3HDM1 RSF1 P2RY11 KCTD3 GAPDH IFRD2 RRP15 RSL1D1 SBNO1 ADNP2 RBM25 B4GALT5 TPR BICRA MCMBP NAA11 WDR3 PROSER2-AS1 TNPO2 MTCH2 BACH2 PPARGC1B ACTG1 PTDSS2 FAM13B CCDC78 HNRNPU SCAP NEMP1 DDX56 SRRM2 PEBP1 HNRNPA1 ATXN1-AS1 DDX20 KAT7 UTP15 MYBBP1A CCT6A TCOF1 SF3B3 PIM2 CPNE7 BAIAP2 INSIG1 GPR75 TEX15 ARID1A MBD1 RBM48 ARL8B STARD7 TRMT61A ZBTB40 NCLN CHST3 MT-TL1 RYBP MAEA NCR3LG1 C8orf82 SAFB S1PR3 TRMT2A RTL10 LBR CBFA2T3 RNASEH1 IQGAP2 MYB CDC25A XRCC2 MMS19 PTGER3 ENSG00000271971 KIF26B NBAS EIF4EBP2 DHCR24 LINC01963 ATP6V0D1 TAF4B AFF1 MTA2 SLTM TBC1D14 AXIN1 MALT1 POLR1C ENSG00000288884 ENSG00000285730 TFDP2 NDST1 NOSIP SNHG4 GOLM1 PELP1 LINC00645 KCTD15 C22orf46 EIF3B SMARCD1 TPP2 MED29 FASN METTL8 PCBP1-AS1 PSMA3 ASCC3 MECP2 H4C8 CLDN11 TMEM18 VPS26A FUBP3 CLCN6 SQSTM1 TMEM127 RALY ZNF274 ZNF581 DAP3 H2AW PHF3 DCBLD2 DVL2 BTG1 PSMC2 CAMSAP1-DT PCYT1A UBQLN4 RAP1GAP2 EZH2 ATP11A TMEM223 SLC25A5 ENSG00000271781 PI4KA PRPF19 LSM14A TRIR C19orf25 PQBP1 SMARCB1 CHEK1 LRRC58 WDR43 ATP13A3 KIF5B CCT2 NUP153 MACO1 CLPTM1 XRCC6 DHDDS AGPAT3 ABO KCNH2 LETM1 DHX33 CHD3 TEX261 LINC00958 SNRPA GATAD2A ARHGEF2 CASP8 N4BP2 DHX30 ENSG00000177788 CPSF7 PRDX1 CASC3 SNU13 FBRSL1 BAG1 DSG2 HSPA4 ENSG00000266976 MMAB RRP9 SMC1A PSMG2 GNB1L C11orf58 RBM19 RUNX1 CASD1 FADS2 MT-CYB MYO16 EGR1 SLC12A2 PDCD7 GRSF1 EXOSC9 ZFP36L2 PBRM1 FTL VAPA TMPO PPP1CC PAXIP1 YY1 SLBP OXCT1 LEPR AASDHPPT MCM3 ANAPC7 DELE1 DDX42 FES YJU2 PITHD1 RPUSD1 FAF1 CDK6 RRP36 CSK MRRF RAPGEF1 SUB1 PRPF6 NAA20 ZNF587B SMARCA5 TIMM10B SF3A2 ARPP19 RIF1 COPS2 H4C5 NAA50 MRFAP1L1 LMO2 RBBP4 PDPR CSNK1G2 DOLPP1 RPL22 SCD SLC38A2 WDR36 CCDC86 MYH10 SPIN4 THOC1 STXBP5 ENSG00000176349 DNAJA1 NOP16 PCNX4 GFM1 RAD51C AP5M1 ENSG00000272341 ADO DPYS TGFBR1 GBP2 LRPPRC RRS1 TICRR TULP4 PRRC2C HNRNPUL1 ECSIT QRFP DCAF7 CLCN7 POLR3E SLC12A9 TXNL1 THG1L SPECC1 PPIA PPP1R10 VDAC1 TJP1 MED6 TPRN DHX37 ARFGAP2 AATF PRMT6 CENPN TAF4 RETREG2 NXF1 RRP1B DKC1 GART SLC24A2 THOP1 TFRC DNAJB6 DHX34 DDX46 AK2 ZNF787 PRPF38B KAT6A EPRS1 ILF3 MLLT3 PLK4 KEAP1 MED16 POLR3C NUP50 DRG1 BEX4 MRPS30 GAB2 MT-ND1 SLITRK6 CTCF ATAD3A TRIP13 CLTC ZNF521 RPRD2 SRSF8 PTBP1 USP37 RAN MCCC2 HEATR1 UFC1 NSDHL KDM3B POLR2D LSS ETF1 GAR1 EIF4G1 HNRNPC ZC3H18 SF1 HSP90AA1 CASP3 FTH1P7 LRRC47 RANBP3 PPAN YWHAB DHX9 BAG6 EIF4B OR2AT4 KIFC3 STX3 PHF5A NCAPH WDR81 ZNF45 AP5Z1 PTMA NAPA SNHG3 GNL3L UHRF1BP1 SGPP2 BRD9 PRKAR2B ST3GAL2 PGAM5 EEF2 RPIA CHCHD3 RBM12 RFWD3 ENSG00000234160 NUP98 SNHG20 NIP7 RNF220 PRPF4 ENSG00000255099 ZC3H7B UROD COMMD4 SACS NT5C3A NIFK WDR46 CAPZA1 DNAJA2 ZNF512 CSTF2 LYN ZFP91 AHSA1 TFB2M FRMD8 NUP188 NOP56 RAI1 MT-TF BPTF TASOR2 DENR TXK DNMT1 ENSG00000284024 FAM136A FOXRED2 ENSG00000261342 AIFM2 CYP20A1 AAMP CAPRIN1 NLN HNRNPF RREB1 NQO1 HSPA9 SRSF10 RBM42 UTP3 ENSG00000279669 NUP160 HNRNPDL DOK3 FOS EMD CMPK1 PUS7 ZNF451-AS1 XPO1 GNAQ LINC02434 PDZD8 PABPC4 ACAT2 SRSF1 IDI1 GLUL DIAPH1 ZDHHC5 RRM2 ELOVL6 C19orf48 HNRNPAB SLC20A1 SLC30A10 FEN1 PPIF IPO7 PCM1 NOB1 STRBP GTF3C4 NAV1 PATZ1 LINC00342 ABCF3 ELOF1 NCBP1 PLAGL2 PDCL3P4 CTSL SNHG17 SMG5 ANK1 EDC4 GLYR1 PPP4R3A UBE4B TRIM44 CTDSP1 TRRAP LRWD1 AP3D1 ATP6V1G1 LRRC59 KHSRP LYL1 DDX54 MED28 BAP1 KMT2A ZEB2 GDI2 HIF1AN WTAP ACSF3 TMEM201 JMJD1C STK35 CCNH SURF6 MLLT10 RRM1 PA2G4 RRP12 MRPS2 NSRP1 RILP HSPH1 THUMPD1 ACTB MSRA UBA2 CMBL SRSF2 DDX51 TCF3 SERPINE1 WDR5 NVL RANBP10 PSMD1 TIMM44 PRPF38A SPART-AS1 RNF40 NOL9 SLC29A2 RBMX CDC123 CIZ1 MDN1 BCCIP DYRK1A SLC9A3-AS1 SLC38A1 ARHGAP6 PAICS DNAJB12 PDS5A CRK NCAPH2 TCF7L2 PKM DDX23 ARHGDIA RBM8A CDV3 ZFR HNRNPA3 QSER1 NOLC1 CANX CUL4A ENSG00000286064 MCM6 PABPC1 TMX2 SNRPD3 TSR1 ENSG00000247934 CCT5 MCRIP2 FBXO45 RPUSD4 SNRPB ULK3 ENC1 PNO1 SS18L1 CNPPD1 GTF3C6 SREK1 SF3B4 PSMC3 UBP1 SAE1 ZNF282 CERS2 HDGF SUPT6H SETX WWP2 ZNF326 PAF1

		TRMT10C GMPS EIF4G2 DLAT CDC5L KPNB1 KHDRBS1 NORAD NFILZ PSME3 RELN ST7 METTL3 HMGA1 SUPT7L PARD3 ATP2A2 POM121 PNN DDX3X NUP62 HNRNPL PGD UBE2Q1 SMG9 CUTA NDC1 WAC CEROX1 EIF3G AMD1 PRMT7 MAD2L2 MAT2A URM1 PAK2 RBL1 CENPF IGF2BP1 ZNRF1 GNL2 SNX17 DCLRE1C UBC HBG1 HBA2 PUM1 RAB7A ZNF622 ATP5MC3 MTCYBP18 HBA1

Table S11. Venn diagram showing the intersections of downregulated genes (*p*-value < 0.05) with lists of genes that reveal an increase or decrease in rDNA contacts. Related to Figure 3D.

Names	total	elements
4C-increased-1307 downregulated-1147 0,05	62	*TTC37 TEAD1 TIAL1 ASS1 IL6R ASAP2 SUSD6 USP18 TMOD2 YPEL1 WSB1 WDR26 MAGI1 GALNT10 ZNF608 BPNT1 KDM7A BBS4 CPEB4 COL6A5 ZFYVE28 GAS2 TP53I11 GNG7 ERO1B RAPGEF2 FNDC3B PFKFB4 MYLK3 NCOA7 AKAP11 TTC33 MAP3K9 MBNL2 DNAH14 ERI1 ARHGEF12 LYPLA1 ZNF397 SEMA3E TMEM273 CFH INTS8 PAPPA CNN2P12 KSR1 LUC7L TTLL7 TSPAN33 GARNL3 DHTKD1 FLNB PRRC1 PTAR1 LAMB1 GARS1-DT TMEM116 ATF6 PLEKHA8 HLA-B KIF21B CMTM7*
4C-decreased-1200 downregulated-1147 0,05	54	*SPPL2B DIP2A SMYD3 DYSF FRRS1 RNF217 FRA10AC1 UFL1 VSTM4 CTSB NDRG2 HDGFL3 DSTYK IMPA2 APOL1 AIF1L RSPH3 STK10 CCDC162P BMF FLT1 RB1CC1 AMFR DMXL2 CELF2 SYBU SMTN PDE4DIP SCG3 EMILIN2 CELSR2 PTPRJ SELENON AIG1 PHKB TMEM54 SEC24D EXT2 P2RX6 ADAMTS14 KYNU ITPR2 RXFP1 VPS13D CCND3 PSAP HIVEP2 CYLD CAPN5 DIPK1A COPB1 EGLN3 PTGS1 HIP1*
4C-increased-1307	1245	*FSTL1 CD44 PLCE1 SLMAP FAM219A FARP1 KCNMA1 PKNOX2 ARHGAP5-AS1 APBB2 ZNF684 KLHL13 GLT1D1 ABCB7 COX7A2L FYB2 RNF38 AHDC1 LINC02073 CUBN SLC10A7 LDB3 PAK1 SCAF8 SLC6A11 B9D1 CPXM2 RTN1 KIRREL1 LINC01467 PEBP4 PPP1R13B FGR TOP3A EPN2 LINC01491 LINC02664 THRAP3 INIP NSMCE2 ZNF208 RCL1 PRSS51 SLC25A52 OAZ2 ZNF718 DKK2 GLRA4 OR7A17 DHRS11 PCAT19 CCDC34 TAOK3 DNAJC27-AS1 PUM3 ZNF66 LINC02563 SCYGR8 LRGUK TENM4 TRPM6 FAAHP1 LINC01479 FOXK2 CCDC106 SLC8A1 SMIM35 ANLN ARHGEF26-AS1 SLC1A7 LGI2 ANKRD6 KCNC1 ADGRE3 GPR55 DNAJC15 TAFA2 STAU2 PGAP4 SIAH3 RIPK4 TMEM63C ZNF257 USP14 ST8SIA4 HYDIN2 ADGRE4P IGLV3-2 USP7 CD82 KIR2DL4 FAM167B ARL13B SCP2 TMEM156 ABHD2 ALCAM DENND1A SEC14L1 ERLIN2 ATP11C PDZRN3 SF3B6 LIMD1 ADAM32 RSRC1 DNAH11 ZSCAN5C NIPBL CEP120 B3GALT5 CDC42EP3 MICU1 FAM204A SNX25 MYO5C SDC2 OTX2-AS1 ATF1 SLC25A21 SLC22A14 SAMD12-AS1 RBPJP6 C12orf4 TTLL11 LINC01579 NEBL DIDO1 RARB CCDC18 CDH8 AGPS TRPV5 HEPHL1 WDR12 PJA2 KLHL7 TCF4 ECM1P1 GACAT3 RPRD1B AURKA LINC01145 PTPN2 AP3B1 PBLD GORAB ERICH5 BCAP29 PHACTR1 TTC21B PSMF1 SLC5A4-AS1 SH3PXD2A NEK6 OPA3 SLC39A12 MOB1B OR13C9 UIMC1 SEMA3F-AS1 YIPF6 TMEM25 OSBPL10 RIC3 CASP5 EXOC1 RNF182 RALGPS1 UPP2 PHF21B GUSBP1 ZFPM2 PIEZO2 FAM66A BCRP2 SVEP1 FANCA DEUP1 ZNF354C LINC02325 LRRC2 ANKRD26 RGS20 MIR3118-2 PDGFD CNDP2 HCRTR1 TLK1 CREBBP RELL1 LINC02176 BRINP3 LINC01237 KIF4A XRCC4 OVCH2 FAM193A COP1P1 EPHA7 MAP7 TM9SF4 SENP8 NSG2 ZBTB8OS MIR17HG GRIA1 ZNF627 IFNAR1 KIAA0319L USH2A LHFPL6 OPN3 NEGR1 NAV2 XXYLT1 CACNG2 ANO10 BTBD9 SPATA48 CA5A FAM72D NET1 TTC28-AS1 BMP2 TOX KIAA0232 LINC02011 SRGAP2C SLC44A5 FAM107B LUZP2 BTBD10 SH2D3C MELK RBPJP2 LRIG1 PRKACB ZNF536 RIMS1 GXYLT2 HIPK3 EPB41L3 TMEM225 POR LINC00896 PARVB MORC1 OR10H2 ZBTB7C SCGB1D5P KANK4 GAST SGO1 SAR1A SLC37A1 SUPT16H BCL11B LINC01814 DTWD2 LINC01213 NELL2 TSPAN2 NIPA2 SLC14A2 LINC02668 OR52B3P ASAH2B RALB MOSMO KRTAP19-10P BLK PPP1R17 PIAS1 PTCSC3 LRRFIP1 LINC02180 CTDP1 SSBP2 ZNF705CP FHIP2A CFAP74 ZNF846 HAGLR ZNF287 ARMC2 FER1L6 ALS2 CYTH4 COMMD8 PTGER4P2 TBATA ATL1 SERPINB11 ZDHHC17 KCNH1 ABCC9 SNAP29 QSOX2 GSG1L MCF2L LINC01098 ACSS3 BBOX1-AS1 ABCD3 DCDC1 NPAS2 DIAPH3 CCSER2 INTS7 AMPH ASB4 TMEM178B COL5A3 COL4A2 PARP15 ME2 GRAMD1B SIAH2 POLR3A ZNF44 LINC00536 HEATR5A ADA2 PRKCZ F13A1 ALG10B CDC14B GIPC2 RNU6-1007P HECTD1 CYP4F22 ABCA10 LINC01182 KLHL1 SP110 LINC01566 PRSS2 MRM1 ZNF705D ATP9A POSTN BIRC6 OLFM4 SAMHD1 OCLN AK8 SDF4 ITGBL1 TNFSF11 SPOP EFHD2 EGFR MYEOV ADAM28 MRPL13 B4GALNT3 AFAP1 DPY19L2P1*

		SH3BP5 SLC49A4 FANCM NEO1 MELTF MXRA7 MYT1L SRGAP2B SLC37A2 ATP1A1-AS1 SORCS3 PDP2 VPS37A LRRC49 ERP27 ZNRF3 ZBTB21 TENM3 ITPKB ENPEP TUSC3 IPCEF1 RTRAF NF1P9 OR4K6P BIN2 LINC00877 TCERG1 PHACTR2 UBE2O VENTX DIRC3 PLCZ1 NCOR1 LINC02213 PRDM10 EBF2 DNAJC21 C16orf72 USP33 ERBIN RNY4 SLC24A4 ZNF573 MBTPS2 KHDC4 C2 NTF3 OR6C75 ZNF705G LINC01684 PI4K2B USP41 RBMX2 FAM72A IL17RA SOX1-OT SUSD4 PTH GALNT14 RAB22A H2ACP1 FAM66C ZNF160 LINC00466 HADHB NSMCE1 DNAH10 PDE10A CACNB2 REPS1 MAP3K4 PDXDC1 MTPN MT1HL1 LINC02646 INTS13 VSTM2A RUNX2 DDX10 ZNF804B LRP12 LRRC8B CSNK1G1 ZNF169 MICU2 SOX6 JAZF1-AS1 SGCD RC3H2 LINC01492 RNU6-374P TNNI1 RAC1P3 SP3 STK32B PLG ZNF106 LINC01020 NR2C1 SLFN11 ADAMTS3 DNAH8 NHS LINC02505 CABYR LINC01476 ANK3-DT CLSPN RGS12 PPP6R3 ZNF438 GUCD1 PPIP5K2 GTF2I NCK1 SOHLH1 LINC01192 CDV3P1 C15orf32 PUDP KDM1B SSPN HS3ST2 F5 PATJ FAT1 NUP50-DT CPS1 MESD PRKCH EBNA1BP2 TRAK1 TET1 FAM66B RHPN2 ANKRD30A RABGAP1L KRT25 NMU DENND2B LINC00603 SNX8 HADHA CFDP1 LINC00944 SMARCAD1 MIR3118-3 ADAMTS9-AS2 ASTN1 GNG12-AS1 GFRA1 NSG1 RMST ANKRD20A8P MAPKBP1 CPE TDRD7 RNF8 LY86-AS1 LINC02613 NSMAF PYGO1 LINC01723 NFKBIA TEX29 DNAL1 TRAPPC3 CD101 TMEM132D GSE1 HMCN2 FHIP1A EFCAB8 LINC01204 SPRED2 SCN10A HSDL2 ANKRD18A ZNF350-AS1 CEP128 ZC3H15 LINC01135 ANKRD7 ABCA5 PTCSC2 CIB4 ABCC8 TTLL5 NOXRED1 TMTC1 MOCS2 NRK NAT1 KICS2 CYBRD1 MCPH1 MINAR1 EIPR1 STON1-GTF2A1L BMP2K LINC02543 CYFIP2 APOOP5 CCDC126 BABAM2 MSANTD4 CRB1 IL1R1 OTOG HEPACAM USP8 NUDT21 XPO7 ARSJ KCNS3 ENPP3 ZNF235 ERC1 LINC02006 VWA3B ZNF850 ALPL PDLIM5 ABLIM1 XYLT1 BTAF1 PDCD6IPP2 ALPK2 LINC02660 ABCA13 HNRNPCP9 RFX2 MAPK8IP1 ADGRB1 SLC66A1L LYPLAL1-DT ADGRE1 UCK2 RAP1GDS1 FOXO1B TET1P1 ST8SIA5 TBC1D19 RPTOR ZNRF2P2 STT3A PDE6A CHST8 BID COX5A MACF1 MDFIC MNAT1 PCMTD2 STARD4-AS1 TC2N TUBGCP3 BTLA LGALS9DP SLC15A5 ECHDC1 HCP5 AMBRA1 CLEC20A NETO2 DOCK2 SERPINA6 ASCL3 PTPRE IFT46 FAM66D NUP210L LINC02063 TSPAN3 ALPK3 LINC02465 FLVCR1 SLC9A5 MUSK EPDR1 MYO3B LMNTD1 RAB8B LINC00583 MYOM1 ZSCAN30 MTCO2P3 LINC00469 RNU6-835P RXRA CGAS ARHGEF7 SLC23A2 LIN54 LINC01649 ARPP21 ACACA ARL11 MAML2 SPAG16 ADAM5 TRIM43B ZNF879 MAN2A2 LNPEP DDX39BP1 LINC02198 UNC93B3 RPS3AP6 CDK12 POU1F1 KIAA1958 CARD18 LINC00623 NEDD4 RFTN1 CCDC141 NEK4 RSU1P1 PEX14 CFAP61 FYCO1 LPGAT1 CNTN4 FOLH1 HRH4 SPRR2D LRRC38 EXOC6B EVC2 AFG3L2 CNKSR3 USP49 DRAXIN CSF1 CEACAM22P LINC02109 LINC00511 SLC8A3 TRNAU1AP LINC02145 RNF17 HAS2-AS1 KIF11 LINC02400 SLC2A3 FAM72B PRAMEF26 SLC40A1 PHC3 STOML1 CADM2 SDR42E1 LINC01581 FANCL SH3GLB1 GABRR2 RAP1GAP PIK3C3 OTULINL RAD9A SLC9C1 SCML2 SPOPL MAGI3 LINC00701 TRAF3 MPPED1 CCDC122 CHD6 FAM135B MORN1 CCDC186 PAXIP1-AS2 LINC01695 PTPRB LINC01412 ITGA1 VN1R7P MARCHF6 CCNG2 ATG4B CIBAR1 ODR4 GAGE13 TANC1 CORO2B DHX40 KIFC1 POC5 IGHVII-65-1 TRPC7 SYCP1 VPS41 DTX1 MYO10 GFI1B ZNF407 MIR3118-4 ASB3 TENM3-AS1 KIR3DL2 GAGE12J TRIM43 FOXO6 TSHZ2 C1orf21 TOM1L2 STOX2 FAR2 BICRAL PLAGL1 NEK7 NKG7 NLRP13 COG2 CCDC138 MTOR RPL5P35 ERN2 CYP2C58P TLNRD1 SERPINB2 AOX3P LINC01322 GABRB1 ANKS1B RP1 AKAP10 EFCAB14 SLC16A9 WWC1 PLEKHA3 RSRP1 BLM PLA2G4A SENP6 LARP1 GATAD1 ZBTB16 PSMB2 DDX39AP1 UNK B4GALT6 ATG5 LINC01938 SGTB TRIM60P19 ZNF875 XKR5 SCGN SYNJ2 STK32A ARSB IL34 ZNF234 CLVS2 DIO2-AS1 SIGLEC29P DPYSL5 CWC27 AGK USP25 ANKRD11 ASCC2 SLC44A1 CNNM4 ADAM10 ATXN3 SPEN GALC NAP1L4 MRPS22 TMC1 PLD5 OXR1 PAK3 CAMLG KANSL1 RNU6-1150P NPIPA1 TPM1 CES1P2 CIDEC EBF1 CRIM1 OBI1-AS1 OR2T2 MADD PCID2 LINC00667 NDFIP2 DUX4L45 ZSWIM6 MYL1 ANKRD36BP2 MTREX CAMK1G DSG1 C1orf87 LINC02327 FAM30A PDZPH1P ERICH3 TRERF1 CENPBD1P1 GID8 TADA2A RPL15P2 LRRC9 LINC00158 FBN1 NIPAL2 MTUS1 GABRA6 PTPRVP DHRS3 PANTR1 LASP1 VPS35L ABHD17C KRT6A C21orf91-OT1 GGT4P DROSHA ZNF813 ANKRD31 PALS2 ADCY10 FAM149B1 PDE1A TMPRSS2 LINC02165 PRICKLE2 CMAHP ANGPT1 TRIM58 HMGA2 HHAT KLHL32 CHASERR PSTPIP2 LINC00861 MVB12B C4orf50 LINC02253 UBASH3A CACNA2D3 SEL1L ELOC CUL1 SLC7A2 TMEM67 BTF3L4 MIR3936HG ZNF618 ITGA4 CPA6 AGO1 NSUN2 PRKCE DEFA3 GLYATL1 RBPJP5 PTPRK MIR3142HG CNTN3 GLIS1 ODAD2 ARHGAP31 RXRG ETNPPL KIR2DS4 PRKAA2 CD163 ARMC3 BBS2 SYT1 LINC01128 GRB10 OR4F15 LATS2 IKZF2 PPP1R12B APCDD1L-DT PDE2A DNMT3L ASPM RFC2 PPARA PLXNA2 KLHL33 DENND2C LINC01602 TBC1D13 CDYL2 SCARA5 PRG4 SCGB1D1 MLLT1 RANBP3L MARK2P12

		TG ADGRB3 HGD SLC36A1 PLCB1 UBE2R2 KCNH8 ATP6V0CP3 SDCBP ANKRD19P RPS10P7 CUL5 HOMER2 DGKI RIN3 LINC01221 REG4 LINC01151 DIP2B CD5L LINC01524 TBC1D30 IGLV2-14 NME7 STXBP6 TFDP1 FAM66E ZBTB33 MXI1 ZNF876P PPME1 TRAPPC8 OR4R3P MBNL1 STX12 ABI1 LINC02291 FUT9 MOK CREG1 LINC02488 SLC12A1 SCN2A MAPKAPK5P1 WNT7A TUBB6 ZNF449 DOCK10 FAM27C MED1 PAMR1 DDX6 HNRNPM SPIRE1 TMEM71 COG5 AIMP1 UBE2E1 ARAP2 LDB2 LINC02149 SNX30 ATP8A1 BCL2L1 IQSEC1 LINC02008 JARID2 LINC02236 SHROOM3 ADK PRR5L ALX4 CORIN MACROH2A1 FAM241A LINC00838 RANBP17 WNT2B MRPS27 PPM1L CPHL1P LRRC37A3 TRIM43CP PRPF18 SMOC1 GSTA3 EXD3 SETDB2 FAH MON2 OR51E1 TOGARAM1 SLC45A4 ZNF705B ELF2 SEMA3D LDLRAD3 GLYAT KIF15 JPT2 CFTR VSX1 TBX20 FLRT2 NFATC2 MSH2 NALCN-AS1 PRAMEF25 KRTAP20-4 ARHGAP44 MTMR2 ZNF970P KCNH5 FAM189A2 BAZ1A CADM1 KLHL29 PTPRO ARID3B OPRM1 ACER2 ZDHHC14 RGS9 YLPM1 ITFG1 IPO11 VCL SLC13A5 GLB1L3 ABCA9-AS1 DOCK5 VCAM1 C7orf31 LINC02511 LINC01818 ATP6V1C2 EWSR1 MAGEL2 IFT81 NHSL1 OSCP1 SGO1-AS1 DTHD1 SRGAP3 IGHVIII-13-1 HAAO CTNNAL1 CIBAR1-DT CYP2A7P1 ATP6V0D2 SYNJ1 PHF20L1 KLF15 PPP2R2B NEDD9 HDHD5 IL12A-AS1 ANKRD66 ENPP1 SDR42E2 LYRM4 CCDC150 DNM1P47 LINC02099 HEMGN RIMS2 UGP2 RP1L1 PPM1F OR4K8P EFCAB6-AS1 CHSY3 YBX3 YAP1 ARL15 ANK3 BMPR1B LINC01173 FCRLA LINC01937 SEMA5A C1orf127 PRDM15 KITLG KRT6B SV2C ELL2 DAW1 COL5A1 IGHV3-74 IFT57 LINC01426 RIPOR2 MAP4K4 LINC02899 ZNF112 FICD FEZ2 ATE1 PEG10 INHBA-AS1 PRAME HSPD1P3 NUP43 NMD3 OLA1 GATAD2B VPS13C ANKRD55 XIRP2 KRT85 SLC14A1 CA1 C5orf52 FAM72C MFSD9 EOGT SERPINI2 STK38 APBB1IP NPL CAST TBC1D9 FBXO32 AOAH SNHG14 TSBP1-AS1 SMG1P4 SNAI2 ZBTB49 ANP32B FAM90A28P PHF2P2 AP4E1 DLEU1 NDFIP1 TNR FAM180A LHX9 LINC02074 OCA2 ST8SIA6 DAPK1 MSANTD2 FCHO2 RFTN2 TRAV8-6 COL6A6 RN7SL767P DNAH5 CNTN6 CLIC6 LINC02406 FMN2 BHLHE40-AS1 SEMA3C VAV1 LINC00895 CACNA1I BTG3 DPY19L1 CSF2RB RPS6KA3 SMAD5 DNAJC13 BCKDHB PPIL6 IGLV3-30 CNST PATL1 UBE2J2 ASB2 OTOP1 PSME3IP1 OR4F6 GHRH UGT3A2 GOLGA8B RNLS IGLV4-3 SPOCK1 LINC02315 NF1P6
4C-decreased-1200	1146	LRRC37A5P CCDC116 ZHX3 APOL2 RPS4XP6 PDCL3 PTPRR HERPUD2 CRTAM SLC52A1 UBE2G1 PELI2 TPI1P1 NOS2 IGF2BP3 MIR548H4 ZEB1 LINC01708 FAT4 PARN SEMA4D SLC15A2 MRPL45 RN7SL483P WSCD1 MIR4435-2HG KNDC1 LINC02355 ZFYVE1 EVC HERC2P2 LINC00670 DMC1 CXADR C9orf43 NBPF21P OR7E19P RIOK1 HERC2 GGT2 UNC45B LUZP1 LINC01346 BVES-AS1 ZNF723 LINC01138 CECR2 LINC01782 GNAS NPM1P2 CD38 SERPINB9 LINC01876 PGBD5 LAIR1 MSH6 RASGRF1 HECTD2 FBXO31 C12orf40 LINC01163 GMPR RIN2 PRDM16 PDE6C TRAPPC9 LINC00323 MEIS2 CARMIL1 PCDH7 BCL2 KL LRRC4C LINC02558 RACGAP1 MIR3667HG THADA NUP37 PPP1R9A SAA3P TMEM117 KCNK15-AS1 TOX3 TDRD5 TASP1 SNX6 POTEG GOLGA6L3 SAMM50 ZZEF1 HHLA2 NCF4-AS1 C3orf52 SLAMF1 UQCC1 RGL1 ATP5PBP3 SHOC1 LINC00841 FAAP24 INO80D KDM6A MED27 NCAM1 PDYN-AS1 GDAP1L1 LINC02096 LINC01358 EPHA4 LINC01967 PLA2R1 LYSMD2 NGDN H1-9P ADAMTS2 GTSF1L WARS2-AS1 CCDC172 FRYL LINC01828 GPRC5C COLCA1 CPAMD8 RNU6-929P GPR1-AS UBL7 ARMC6 LINC02675 ASH1L CALM1P2 RBM6 MEGF10 IGHV2-70D HS1BP3 LIPI LINC01445 GOT2 RAB38 DZANK1 CLTCL1 NUAK1 PCAT1 KCNK10 ZNF738 BLOC1S5-TXNDC5 BRD4 SLC16A1-AS1 VMP1 CHAF1A NENF HRH2 ATP6AP1L RNU2-47P RDX SNTG2 SVIL APBA2 TTC3 CHAMP1 COL23A1 NSD1 NEDD4L EDAR C5 EGF LINC00960 ATP2B2 RPL37P3 CCNYL3 AGO2 ABCC12 PARK7 RIMBP2 ZNF271P IFT43 ADAMTS19-AS1 SNRPC C2orf69P4 DRC7 DSE POTEJ LINC01427 MYCL LINC02256 TPTE2 SPAG6 BMP7 PDE4DIPP1 GALNT2 KIAA0753 FGF12 ANKRD17 EPHX4 CYP2C9 CNOT7 CASC9 IMPACT ITGA6 HHIPL1 NOTCH2 ZFP90 S100B ARHGAP12 USP43 KCNN3 FKBP5 NFAT5 FLI1 ANAPC1 GRM1 IBA57 LINC02147 ARHGAP26 ITGB8 GAP43 LINC02422 OR4K3 PRDM13 PDE3A PTPN4 BCAR3 MAP6 SREBF2 CDH11 SETBP1 CDS2 ZNF780B LINC01900 ATP6V1E1 LINC01993 LMX1A AGBL1 DEFT1P2 LINC02439 ZFAND6 GBP4 CNKSR2 PSMA5 RESF1 MAPK1IP1L DPH6-DT GFRA2 NEK10 ADCYAP1R1 SNX29 MAGI2-AS3 ZNF611 STAT1 SAMD5 ZC3H14 MROH5 SLC44A3-AS1 MYOCD CREM GOLGA8J XKR3 UBE2QL1 PLPPR5 TDP1 AVL9 EFEMP1 TNRC6B WBP2P1 ZNF33B LINC02542 SYN2 PTCD2 MYO1E SMOC2 MIPEP NCSTNP1 HDAC2-AS2 HLCS FH RWDD2B PLPP4 PWRN4 CCDC102B SDS GSR LINC01571 FIG4 SOGA1 ARHGAP32 NECTIN1 ZNF528 LINC01222 LALBA NXN LINC00375 FOXJ3 CENPE CKMT1B MYL12B RSPH14 IL17RD BRCA2 ANKMY1 HCG22 APELA UBN1 SSBP3 PLA2G12B FAM83B HDAC4 CTNNA1 STK36 GNAI2P1 FAM102A NCOR1P1 TRPC5 MYO9A TMEM182 IL10 LINC02305 LCLAT1 LIFR-AS1 C19orf18 FTO

		SLC6A1 EPC2 SEM1 SEMA6A-AS2 MOGAT3 MS4A4A TMEM236 NLK THSD7A CXCL2 GOLGA6B LINC00334 CARD10 ACSBG1 GCSAML DNPEP TRAPPC11 HOXC4 IGHV3-62 NECTIN4 CNMD LINC01309 UFD1 SMARCA4 LINC00299 BAZ2B HERC2P3 CRACD NGF-AS1 AGL PALMD HS6ST1 MARK2 MEOX2 SFI1 ZBED9 CTSE ATF2 PLEKHB2 FAIM ZNF72P RPRD1A ZNF880 PPP2R2A ESS2 PHAF1 ZNF541 RBBP8 AK6P2 CKMT1A RFC1 NSUN6 LINC02174 CDC45 MC2R AKR1B1 BTBD11 CWC22 LRP2 LINC02087 ZNF121 HERC2P9 SYNE1 MTHFD1L RALGPS2 HOATZ SNRK ABCD1P4 EXTL3 EHMT1 TTC29 FBXO47 KRT89P ZNF735 ZBTB10 PKHD1L1 SLC9B1P4 PLPPR1 CEP192 SLC26A2 CAMK4 GUSBP11 CLPX OR7H1P ROCR ANKRD20A9P HDAC11 SLC9A4 DHX29 ANKRD20A17P GRIK3 GRXCR1 NUMB STPG2 MIDEAS TM9SF2 CD70 SH2D1B ZNF606 ESYT2 HEATR6 ARFGAP3 MAPRE2 FGF10 TAPT1-AS1 KRT18P59 ISX RAD51AP1 POTEM LINC01035 ESRP1 RIC8B TAF3 ZMYM4 TPTE FRG1JP MED12L GRM7 ZDHHC21 BRMS1L TM9SF3 DDHD1 ICA1 PLEKHD1 CDH7 TLDC2 CYCSP39 HORMAD2-AS1 VASP PLGRKT UBE2E2 UNC80 SDE2 PTGFRN PPA2 ILDR2 IMMP2L ZNF615 SPSB4 SPSB1 GABRG2 ZNF567 ITCH NUDCD3 MBP S100PBP TANGO6 GABRA5 CDKN2C CFAP97 STXBP1 SLC46A3 DLC1 ANKRD33B PNPLA7 RPS3AP4 CNIH1 FRG1BP C2orf69P3 LINC00114 ZIM3 MANBA DUX4L2 CHRM3 ECT2L UST MIR663AHG CALD1 LINC01543 ERICH1 DEDD2 TYW1 TAF15 ALB ARHGAP24 JPH1 ANKRD20A3P EFR3A HTR2A UBAP2 TPH2 N4BP2L1 IGHV1OR15-9 TPTE2P6 EIF4BP3 LOXHD1 APC MDS2 GOLGA8G LINC01622 GALNT1 MARCHF1 OR4L1 ZBTB25 INO80 RBM15-AS1 HSD17B14 KRT16P6 COL27A1 GLP2R CHCHD2 BMP5 SLC4A4 ZNF675 LINC02366 GOLGA6L17P SYT16 CRYBG1 TWIST1 HACD2 LINC02653 LINC02061 GEMIN5 RPL15P3 TRIM77BP KTN1 ERCC6L2 PASK RUFY2 SLC16A1 RANBP9 FAM245A MRTFB LINC01344 KCNE4 TRIM5 LINC02228 KRTAP21-3 LINC01801 LINC01320 LYPLAL1 THNSL2 BRWD1 COLQ PPIP5K1 C9 TMTC2 HECW1 HMGB1 MEF2C MCTP1 RNU1-51P MOB3B ACTR3C LCE1F ARPC3P2 MAP4 TSPAN11 MTND1P17 HIVEP1 ATRX TNIK KRT18P55 OR1L6 NBN PRTG OR2T7 SLC17A1 RGMB KMT2E WNK2 FRMD3 SETD2 RBFOX3 MRPS35 SDAD1P2 PWWP3A ITIH5 UTP4 PACSIN2 TRGJ1 HOXC13 PKP1 SMARCC1 SYNE2 GTF2IP6 MIR181A1HG TRMT61B NUP214 TRIM23 FABP7 SDCCAG8 GK LINC00355 DUSP22 KCNJ18 ECPAS CHSY1 RFC3 MAB21L3 SFPQ SMPD4 URB1 PTPN12 GPR137B ZYG11A LINC00434 LINC02424 TOP3B MPPE1 STAG2 RGS3 UBA6-DT ANKRD26P1 CMIP DEFB116 SLC2A13 FBN2 YTHDF3 SPATA17 SYT10 ZBTB38 PAFAH1B1 LINC02380 CYFIP1 ALK DOCK8 GON4L GREB1 ARNT LINC01340 LINC00929 CSTF3 ZNF648 LINC02058 SAMD13 DNAH6 ARFGEF3 TMCO5A UHRF2 EPCAM-DT CSDE1 DCLK1 DEFT1P RNF215 ANKRD28 GRK3 ZBTB2 SND1 IFNG-AS1 SNAP25-AS1 ZFAND4 LINC01664 FGD4 EFTUD2 NRIP1 ETS2 LINC00649 ZNF382 NDUFAF6 GOLGA6D ERMP1 CATSPERG ARL4C ITSN2 LINC01917 KTN1-AS1 HIRA OSER1-DT SERPINB7 ANKS6 TMEM74 PRKAA1 RASGEF1C TAFA4 ALDH1A2 GABRG1 MTTP POGK CROT MAPK9 ESRRG FBXW2 STON2 LINC02458 LINC02652 LINC01229 FOXJ2 AGAP9 ABCC4 MYT1 FRG1HP ABCD2 DNAJC7 ZNF595 EMP1 TMEM171 ZC3HAV1 LNCAROD RRAS2 SV2B FAM110A NRBP1 SEC14L3 STK38L GTF2F2 RALGAPA2 FAM245B ADAMTS19 ZNF236 RAB27B SOX30 LINC01337 MYOF CPSF3 PLS1 UNC79 RSPH1 SPON1 ANK2 SH3GL3 CFHR4 INVS FHL2 SNRPD1 NCAPG2 LPCAT2 SUMO3 LNP1 BZW1 PCNA TPTE2P5 PHF19 ZNF518A LINC02191 IGLV3-31 DCAF1 ZCCHC7 CD2AP TTC39C LINC02680 ZNF124 EBF3 TAFA5 NFKBID SPRED1 MYO5A CIDEA LINC01924 PPP2R2C PWRN1 LINGO1 LINC01706 SEPTIN9 SMPDL3A LRFN2 ACO1 WDFY4 SCAI PAPPA2 LTN1 TINAG NCOR1P3 DIRAS2 AQR ABCB5 HEATR4 SLC5A9 KLHL4 DPY19L2 MDM1 RRBP1 SMPX OVOL2 CD9 MTMR10 EIF3F CDHR3 CSE1L NEK2P2 TOM1 FAM118A PRUNE2 SGMS1 ANKRD24 COL25A1 RBPMS2 CYP4A11 BRINP1 IGLV2-34 MTND2P8 RPL23AP7 GRB14 LARP6 IGSF5 LONP2 CCDC192 LEMD3 PEX6 CHKA BUB1 RNF138 INTS4P1 KRTAP21-2 ZNF292 SPIN1 TBX15 PRB3 TTC3P1 KPNA1 SKA1 LAMC1 SRP9 SCGB2B2 GABPA SMIM11B DSG1-AS1 PCNX1 DUXAP10 LINC02091 NAA35 ATP5PF RPF2 UBAP1L ZCCHC14 MX2 AGGF1P10 MTCO1P1 ITGB3BP ZNF831 PRAMEF2 ENTPD5 DGKK SLC4A10 LINC00844 TSSC2 LIX1-AS1 TGM1 AGAP14P MGAM FTLP13 ZDHHC18 LINC01310 PSG9 FAM183A UHRF1BP1L IL1RAPL2 APIP MUC19 SCAPER ADSS2 IKBIP NPHP4 LINC02141 OR8B9P CBLIF ATP6V1B2 KCNJ1 LRRTM4 CLCA4 LINC01877 MYOM2 SLC5A12 NCS1 ONECUT1 PCDH11Y LINC00923 METAP1D USP31 SUMO2 OR5AQ1P MICOS10 XPNPEP1 GLYATL3 NUBPL PCGF5 FGF7P1 NLRC5 TUBBP9 LINC00363 NDUFAF2 GUCY1A2 KREMEN1 UMODL1 LINC01189 MB LINC00383 WDR25 SOD1P2 CYP4Z1 MGA LCE3D TNPO3 EFHB ACSM2A FGF9 DCUN1D4 DAZL NIN RPL23AP53 SLC1A1 HPCAL1 SPG21 ANKFY1 LIMCH1 ZKSCAN5 CCDC195 TSHZ3 TMCO4 LINC02098 RALA GMDS-DT BAZ2A MIR548A1HG RPS12 GNPTAB TRPM1 NTN1 COPS8 SACM1L AKAP9 C1QTNF3-AMACR PPIL2 AP4S1

	1031	*NR5A2 PRKD1 SPTB TRAV8-1 TNFAIP8 RBM47 TMEM260 GBP6 SANBR DBF4B OR9Q1 CCL28 FBXO41 MICAL3 SNRPN HDAC2 MSI2 MRTFA LRRC7 LINC00221 ETS1 TNN UBAP2L CASZ1 PCDH8 LINC01673 WDHD1 MIR155HG MAMDC2-AS1 RABEP1 ST6GAL2 LINC00930 NUTM2HP SLX4IP PTCHD4 BANP ALKAL2 MAP2 YME1L1P1 TGFA ZFYVE26 SNX3 LINC01422 ZNF280B PTPRA CEACAM7 TNRC6C TMEM161A DEFB108B OR11G2 LINC02451 WSCD2 UBE3D GLIDR ZNF402P CERS3-AS1 RPL23AP87 GOLGA6C ABCA4 ZFAND3 LINC01718 THUMPD2 C6orf118 FAM83F CNKSR1 RAB12 TMEM163 PCDH9 LINC02235 SLF1 EXOC1L BACH1 RBMS3 POTEH GNA14 OXNAD1 INMT-MINDY4 LINC01992 SRFBP1 MATN2 TM7SF3 IL33 EFCAB2 MARK4 CRACR2A CLEC16A LINC01901 OR2T3 SERPINB10 PROX1-AS1 ACTN1-DT ARHGEF17 ME3 PSG6 ZNF431 CACYBP ITGA9 PPP1R1C ULK2 IARS2 UNC13C CDC42BPB HNRNPH3P1 TMEM44-AS1 LINC00298 FAM217B MTRF1 FAM25G SHROOM2 OVCH1 CISD1 C16orf95 CRTAC1 PRKAB1 RALGAPA1 LMX1B LINC01748 L3MBTL3 RRAGD IGKV2OR22-3 POMT2 MICALL2 ST13 LINC02245 CLIP1 MARCHF8 SHC4 C2orf42 CABLES1 MORC2 ZNF613 TLL1 DEFA8P FRMD6-AS2 DUSP16 BCL11A AKR1C3 PPP2R5E ASB7 FREM1 IL20RB ADAM22 RABL2A LINC02582 LINC01811 CNIH3 RPF1 TRIT1 TBCD CEPT1 WNT5B KCNK5 CEP44 HKDC1 CLNS1A EPS15L1 PVT1 CHN1 ETV6 WNT9B RFX7 TRPS1 SERBP1 SKINT1L IGKV3OR22-2 RANBP2 ADAMTS5 NF2 STRN CRISPLD2 NPM1P1 ANTXRLP1 MTMR3 ZNF845 RN7SL250P FAM25C CRKL ITPRIP H2BC15 ADGRA3 SCG5 CLDN18 LINC00581 ADCY9 DPP10 TUBB2BP1 GALNT16 CDH5 ANO6 ZNF600 HIPK1 A2MP1 PGPEP1 ACSM2B TTC7B PEPD ATP2B1 IFI44 RETREG1 NLRP14 PCNT NPHP3-AS1 PAH FAM138E ATRN FBLN5 CNOT6L TRAPPC6B TOP1 ZNHIT6 SLC39A6 WDSUB1 ANKRD20A7P*
downregulated-1147 0,05	1031	*GRAP2 ITGA5 BTG2 CYP26A1 RTKN2 IGF1 LMAN1 NTRK1 UBAC1 GDF15 ASNSP1 TRIB3 REEP6 CCDC88B LINC02864 STK16 XK HEPH BBC3 CASTOR2 PCOLCE TSC22D1 LHFPL2 EPS8 ITM2B UTRN TRIQK ACSBG2 SNX16 GPC5 GTPBP1 PDIA3 CA11 ALDOC NECTIN2 ZNF83 SEL1L3 ZCRB1 GDPD5 ENSG00000213963 OXLD1 DDB2 ANO5 LINC02267 LTBP4 TSPAN13 STARD5 MT-ND4L VWA5A NAT8L TNFRSF9 ENSG00000224271 GTF3C3 DMTN AP3M2 LINC00656 CORO2A APOE C3 KLHL36 BTN3A1 DNM1 HYOU1 SLC16A5 KBTBD3 SORT1 LLGL2 NFKB2 PGGHG MYO15B IFT140 GMPPA MLF1 SLC6A6 DUSP5 ID1 COL18A1 ELAPOR2 A4GALT BCAM PCK2 ADAM19 THRB MMP15 ZBTB11 EXOC2 GUCA1B LSM4 POMT1 NUCB1 TLCD2 DDHD2 RYR1 NUDT12 ZNF467 MAN2B1 FGFR3 HES7 EPHA1-AS1 TKTL1 TM6SF1 TFAP2B AKNA NBR1 PKHD1 INPP5J FAM234A SELENOP UBE2L6 TMED9 MYORG TDRKH TLE2 LONRF2 GIPR AHCYL1 STON1 YIPF2 FN1 RBPMS PTPRH SHFL GGA1 DGKD SERTAD2 CYSTM1 IGSF8 ZNF275 ACAD11 GAA ENTR1 ACSF2 BTK TJP3 CLCA1 AURKB HID1 ZC3H6 GABBR1 PAX8-AS1 DEF8 MANF PLA2G6 CCDC113 PRR36 CEBPD GPR158 CALR4P WARS1 ADCY3 TMEM241 PHYKPL GRHL1 LARGE2 WDR91 HAGH TMED7 YPEL5 ITGB1 PI15 TAB2 CPD BRSK1 PECR KRT8 SYDE2 UCA1 SVIP PCAT14 TMPRSS4 GABPB1-AS1 ACADVL ISCU GLI1 HBP1 PHGDH MCFD2 EPS15 IL13RA1 SH3BGRL2 ENO2 B2M ADAMTSL4 EHD2 MAP1A AGER SAT2 SNTA1 TCFL5 CLIP2 WNK4 VPS16 C1GALT1 CD93 KDM6B RRAS PCDH15 PCED1A PPDPF TMEM106C BAIAP3 IL15RA H1-10 SCFD1 ZKSCAN1 RABAC1 ADGRE2 ESRRB EPSTI1 BSDC1 POFUT2 RASGEF1A CTCFL XYLT2 TMEM263 BCL6 TPD52L1 PROS1 FGFR4 CRAT NYAP1 VEGFA PIK3CD DMBX1 TGFB1 ABHD18 PSEN2 CHST2 SNHG32 NTS ULBP1 PIGK ZDHHC8P1 PLOD2 KLF9 RPL27 LAMA3 SLC30A2 KIF1B GPD1L B4GALT4 PSRC1 ALAS1 PDK4 SMIM1 ENSG00000286403 CPZ PTTG1 LINC02416 ZSWIM4 TARBP1 BLVRB GTPBP2 ATP2A3 SMARCA1 PLXND1 SMURF2 ACER3 UCP2 ARHGAP8 RFK DNAJB11 CACNA2D2 SLC22A5 DOCK6 SLC25A42 FMNL1 PTPRC EEF1A2 FAM83A HEXD GPR155 RAB24 SPEG IQGAP3 SHC2 INPPL1 TMCO6 MAP3K14-AS1 GSEC RTCA-AS1 DCTN4 HNRNPCP4 CNN2 DBP PPP1R13L LRRC63 GFPT1 FCGR2A MC4R MAN1A1 STARD8 FNDC4 PCED1B OCEL1 MKNK2 BATF2 SLC29A4 NIBAN2 UXT-AS1 LINC02432 PALM PARP12 SLC22A23 C6orf89 LDLRAP1 TUBB2B SLC31A1 AFDN-DT IRAK2 MFGE8 SH3BP2 SH3TC2 AGRN RTN2 TSKU RHOT1P2 APBB3 GPSM1 GADD45A TMEM70 LRP1 SH3PXD2B MMP14 SERPINI1 PPP2R5B PTPRF NIPSNAP2 NDRG1 CPXM1 MRPL23-AS1 ERFE ABCB9 TRAK2 ENSG00000249631 LGALS3 PDLIM4 CLSTN3 NFE2L3 STAT5B ENSG00000253154 ENSG00000286488 SLC22A31 ARL6IP5 TMEM50B DBNDD1 C11orf80 SCN1B ACVR1 PLEKHH1 GOLGA2 F2R LPCAT4 THBS1 ACSL1 ASMTL CMAS AFDN UBE2H SLC6A8 PDE7A NFE2L1 GEMIN2 PAAF1 C2CD2L GSDMB GAL3ST4 FUCA1 MACC1 SOCS1 BHLHE40 KIAA0895 ROBO3 SLC17A6-DT LRSAM1 PLXNB2 INHBE NUP107 SLC25A36 RAB11FIP1 SDC3 NR2F6 ZNF396 CAMTA2 HIP1R PLEKHA2 ENO3 ZNF204P ENSG00000271382 STX10 TSPAN18 MCF2 MAN1B1 P4HA1 TMEM243 FECH LRRC20 ANKRD20A11P IL10RA VCAN PCYOX1L ARG2 FBXL16 GDE1 MAP4K2 LAMB2 PDIA6*

		TGFBR3 ZMYM2 DYNC2LI1 CDKN1A IL32 RPS6KA1 CUL7 FAM214B ERMN CCDC71L HRC LRG1 ENSG00000236393 GUK1 MAF ENSG00000229191 HOOK1 GTF2E2 MAPK8IP3 CRYL1 PRKACA IFIH1 MVB12A IDH1 ZKSCAN8P1 ANO8 NUDT4 MICAL1 SWI5 SEMA3F HAX1 NEK9 NHLH1 PIDD1 CD200R1 INPP4A PTPRU ATP8B3 ST6GAL1 SIAE SKIL ALAS2 ST3GAL5 SDF2L1 KLF13 RNF11 DLK1 COL11A1 LTK YARS1 RAB18 RPL29P14 AMPD2 PITPNC1 SEMA6B PLP1 MLLT11 LINC00663 ENGASE PYCR2 KCNJ11 SEC24A IFITM1 GAS6 KRBA1 THUMPD3-AS1 MT2A MYO1D SFXN5 FLT4 SESTD1 UBR5-DT RHOBTB1 LINC02384 CHPF SMAP2 CALCOCO1 MAP7D1 DEPP1 COL1A2 DTNA AMIGO2 RELL2 PROCR ZNF697 EHHADH LAPTM4B ITGB5 C4orf46 REEP2 ARFGAP1 SMAD6 RHBDD1 FAM171A2 PVR ZBED5 TMEM63A FNBP1 PITPNM1 ANKZF1 DUSP8 MLXIP KHNYN RCN3 PRCP YIF1B WASL NFE2 ADD3 PTEN MAST2 ANXA1 GTF2A1 ARVCF DENND11 ID3 NBEAL1 TIA1 HLTF POLI FAM234B GPR180 POLG2 PTK2B UNC5B LINC01419 MFSD6 CHID1 PTPDC1 USP20 PPM1J CALB1 B4GALNT4 MDGA2 PKD1 CCDC50 RGS16 CRYZL1 SMIM3 TGM2 TCAF1 LYRM1 NFATC4 FHIP2B PDLIM7 STX2 SLC44A2 COG6 CD55 ACAT1 AARS1 SMAD3 ATF3 TRDN CARD11 APOL6 RTN4R SEPTIN6 OSTF1 CAMSAP3 TEX19 CTIF TBC1D5 SOCS2-AS1 LINC00173 THOC2 LGALS1 COL15A1 CASP10 NPAS1 DACT3 MINK1 HLA-E BTN3A2 SYT5 SEMA3A R3HDM4 TRIM3 MGAT5 RTN3 ULK1 ZNF175 TMEM143 MRAP2 MYO5B COPG1 CD36 ZNF117 CENPH TTYH3 ZNF692 IDH2 EFHD1 COX6A1P2 PAX9 GALNT5 NIPSNAP1 TNNI3 RNF145 GDI1 TSC22D3 CCM2 ARID5B FLNC TMCC2 HACE1 NOTUM SPTAN1 ATP7A COLGALT2 ENSG00000283125 PAN2 RAB26 DCLRE1B NDUFB4 BCL9L MAPT HSPA4L TTBK2 RNF103 CXCL8 KHK HDAC9 WIPF3 LITAF NLGN2 DLG3 MSI1 CC2D1A ZCCHC24 PIM1 PCLO THBD SERINC2 ADGRL1 HECW2 IARS1 TENM1 BNIP3 PRR14L CCNL2 CDC42SE1 CBLB MIIP AP2M1 OAS3 ANXA6 TSPYL2 VBP1 MORF4L2 NEMP2 PRKAB2 MAP3K8 DLG4 STOM GSN ENSG00000264112 MAPRE3 RIMS3 ENSG00000286750 RHBDD2 LMOD1 PDGFB CALR RGL3 ITGAV RTN4RL2 ITPKA ANGEL1 BTBD2 RHAG PTK7 PHKA1 HELZ2 ENSG00000255347 RAB6B DENND4B SLC25A29 ENSG00000204745 ENSG00000258274 ROBO1 APAF1 TRPT1 TNK2 TBC1D20 KIF5A CCSAP ENSG00000287737 LINC01033 RNF187 MRC2 AGPAT4 SFMBT2 ACVR1C SETD5 GALNT12 SALL2 ENSG00000279164 SH3GLB2 SNX2 ABI3BP ATP1B2 SEMA7A GABARAPL1 ATM ODF2L KLF12 GTF2H4 ARRB1 ITM2C NSD3 USP45 AHSA2P LINC02863 MAN2A1 OBSCN PPIB RBL2 CD46 ENSG00000286980 C4orf33 HJV RPH3AL G0S2 SLC4A7 PLEKHH2 SEC22B TTC7A SLC17A7 ENSG00000273901 MEIS3 MUC4 BRPF3-AS1 SLC35F3 ARRDC4 RAB31 ENSG00000257086 ZBED8 ANKRD9 AZU1 LAMA5 SPATS2 KPNA5 KMT5C EHBP1 ENSG00000257337 TXNIP NIM1K TMEM41B CXorf38 CORO6 EPHX2 LPAR2 MARCHF2 FBXO44 FAM131A PSME1 CHAC1 MAST1 ZMYM1 MTATP6P1 ADGRA2 LINC02772 ARHGEF40 ZNF558 TRPM4 ZP3 RNF213 ACSS2 PDIA5 KIAA0040 KCTD20 FLNC-AS1 ACE CLDN12 TPD52 EML2 TUBE1 WDR31 SLC37A4 CREB3L2 WBP1L IVNS1ABP OGT ABCG2 RHPN1 P4HA2 FUT1 SERGEF PTPRS COTL1 TMEM9 PEX2 SESN3 WDR11 DAB2 MARCKS RBCK1 SERPINH1 SH2D3A ZDHHC8 VWDE CD59 IL2RA CD24 NFASC DDIT4 BST2 ATP1A2 OPTN HERPUD1 SCPEP1 ENSG00000285108 KLF7 DMKN DNASE2 LGMN MSH5 MYRF PYGL SNPH STARD10 EPB41L2 SOX5 GOLM2 ETFDH PCSK4 ENSG00000225528 KCNAB2 CCDC18-AS1 GALK2 MTURN TSPO RAB3B SEMA6C ERBB3 SERPINB1 TCAF2 GGT7 TUT7 CALU HSPA5 WIPI1 DENND3 PAFAH2 TP53INP1 LDAF1 FADS3 VPS28 ULBP2 ANAPC16 EPHX1 NCBP2 GPSM3 ENSG00000259953 ADGRL3 SIL1 MICAL2 CPNE3 CAPRIN2 TNFRSF1B LGALS9 RBM22 STK4 HMGCL FGFR1OP2 TRIM38 ENSG00000272941 CA2 CTBS DCP2 FGD1 PIGS HSP90B1 ANKRD29 GTDC1 MXD3 NIT1 TNFRSF10B TCIRG1 PAPSS1 SIDT2 GRN ACAD10 LINC01278 SMIM14 EDEM1 SEMA4G METTL25B RIPOR3 DZIP3 TTLL3 ERP44 ITM2A ETAA1 MTCL1 NRIP3 GPC2 PAIP2B ACBD4 CDKN1C NIPA1 CLYBL CYP26B1 PRSS16 GNB5 C3orf18 EIF2AK1 RAVER2 SOCS2 PLPPR2 BRWD3 CR2 IFNGR1 MIR223HG PLEKHH3 TMOD1 NT5C2 PDK1 PPP1R16B ALS2CL SLC45A3 MEG3 REEP4 AMOTL1 DRAP1 PLCD3 TMEM30A OGA MORN4 SMPDL3B FAM227A SETD7 GSTO1 ACSL6 CORO7 RGS10 ZMIZ1 TKFC CCND2 QSOX1 ANKIB1 ABCA7 PLD3 CUEDC1 ENSG00000237643 PLEKHA4 STARD9 HEMK1 FDXR PAPLN NES CDCP1 RAB27A TXNRD3 CITED4 CUX1 UNC119 MALAT1 LRP4 MLXIPL ELP1 BCL3 HIVEP3 ITGB3 RUSC1-AS1 MIF4GD PPP1R14C YIPF5 CERCAM F11R TDO2 GIPC1 FAM193B CEMIP2 MIR22HG SPARC FASTKD1 PPFIA4 CTSZ LMNA NDUFA10 CCDC92 KLF10 PPP1R18 PANK4 SLC2A1-DT C18orf54 MAGED2 PDIA4 RFLNB ADAM15 LNCSRLR SLC4A11 PGM3 SESN2 TENT5A ZNF133 CISH CYP4F29P PXK WHRN NCOA4 RPS6KC1 SELENBP1 TMED4 MROH1 SUSD1 ICAM5 TUBB4A MBOAT2 MYBPHL GPAT3 TUBB1 NPTXR MNX1 PHF21A LINC01630 HDAC6 CYP2R1 TNNT1 MISP3 DCAF8 CTSC

Table S12. Top-20 GO associations with molecular function (MF), biological process (BP), or cellular component (CC) of 1285 upregulated genes. The search was performed in GO Profiler (https://biit.cs.ut.ee/gprofiler/gost

GO.ID	Description	padj	Genes
**MF**			
GO:0003676	nucleic acid binding	4.044611317588401e-59	*NOP14,SNRPB,MITF,JMJD1C,MIS18BP1,DAP3,RBM48,PPAN,MSH3,CHTOP,ERAL1,ZNF274,SEC23IP,PRPF38B,KMT2B,RNASEH1,SREK1,MACROH2A1,BRD9,FBRSL1,MUS81,NOL11,MTA2,RAD51C,UTP3,CWC22,MTREX,PHF5A,NACA,POLR1C,MED6,SRP72,METTL3,EZH2,TCF20,DCLRE1C,NOC4L,MFAP1,LRRFIP1,GPATCH3,H4C5,SMG5,BRD4,RRP9,MRPL1,ZNF581,ZNF74,MRPL11,AHCTF1,RPUSD4,DENR,HROB,MRPS30,MED1,NVL,HNRNPL,WDR46,MCM10,EFTUD2,CHRAC1,KMT2D,TAF4B,PQBP1,AIFM2,PSPC1,CEP350,POLDIP2,ZBTB40,DHX38,ADNP2,NOP16,ARID2,DDX23,PATZ1,TRNT1,RNPS1,LHX4,ZNF512B,RBL1,RPUSD1,RBM45,NOSIP,HNRNPH1,ZNF512,DNMT1,LYL1,PPP5C,BAP1,JRK,SUB1,DHX16,TOP2B,LYAR,LRWD1,EIF4EBP2,CCT6A,KAT7,CLTC,EEF2,FUBP3,IMP4,BCCIP,FAM120A,RNF40,NONO,MEF2C,TCF3,MAF1,CLSPN,NOL9,PRPF6,ZC3H4,PEBP1,ZNF26,ZNF24,GSPT1,GRSF1,NXF1,ZC3HAV1,HNRNPA3,MAPK1,EEF1D,POLR1E,ABT1,R3HDM1,CERS2,DHX33,NCOA5,DHX15,UIMC1,PUM1,MTOR,DHX30,UTP18,IKZF3,CPSF3,SPEN,GOT2,SMARCA5,SF3A2,H2AW,EIF3D,ZNF45,TFAP4,NIFK,HNRNPA0,ZNF131,SBNO1,ZNF431,NAP1L4,DHFR,MTDH,HSPA9,TCF7L2,ZNF239,SRRM2,MRPL15,ASCC3,UBE2L3,THOC1,SETMAR,RPTOR,PPP1R10,MRPS35,TMEM18,CSTF2,TNF,RAVER1,EIF4B,SAMSN1,WDR33,NIP7,IGF2BP3,NOL7,ELL2,PUM2,H2AZ2,WDR3,MT-TL1,SSBP3,SNRPA,ZNF586,BACH1,DDX10,RBM10,SETX,NFILZ,TRMT2A,APEX1,BPTF,NAA15,ARID1A,PPP1CC,ZC3H14,SRSF10,AKAP8,SBDS,RCC1,C1QBP,VPS72,ZNF587B,SRSF8,PRPF4,KTN1,PRMT5,PCBP2,ADD1,TRMT6,RBMX,BAZ1A,MLLT3,RAD23B,TAF4,RBM14,RBM8A,KDM3B,BOP1,ZNF326,API5,FEN1,ZNF282,TFDP2,GTF3C6,SLBP,NUCKS1,UTP25,TMPO,PRPF38A,SSB,GNL3L,DNTTIP2,IBA57,RNU6-322P,NSRP1,MT-TF,TFIP11,STRBP,EIF3G,DHX29,BRCA2,EIF3M,RBM42,ZFX,ZC3H7B,SNRPD1,PAN3,ZC3H18,FASTKD2,TCOF1,XRCC2,ZNF75A,SMARCB1,TRIR,CREBBP,EXOSC3,WBP11,EPRS1,LSM14A,NUP98,HNRNPUL1,CNOT1,ASH1L,LRRC59,GEMIN5,PLAGL2,BTF3,POLR1B,DDX42,ELOF1,WDR6,NFYC,SF1,PELP1,ZCCHC14,XRN2,EZR,TRUB2,DDX20,DDX51,ELOA,TAF9,YWHAG,ELAVL1,UTP15,VCP,RYBP,SAFB2,DHX34,PSIP1,CSTB,DCAF13,RBM12,STIP1,PNO1,POLR3C,THG1L,RBM25,SETD1A,RRP12,SMC1A,AGO2,E2F4,G3BP1,GNL2,RRP36,MCM6,AQR,CLCN6,DDX18,ADNP,NFKB1,UBTF,ZNF622,CHD7,RBM15B,HNRNPA1,GAR1,RRS1,PPARGC1B,MAGOH,PBRM1,CTCF,HHEX,ANKRD17,HNRNPR,ELF1,ZNF614,NSD1,RSL1D1,CCT3,H3-3B,RNF138,SETD1B,PKM,FOXK2,HMGB1,BZW1,CANX,YY1,NOL8,MLLT10,PUS7,NAT10,TRMT10C,METAP2,SNU13,SRSF6,EP400,SART1,CEBPZ,MECP2,CSDE1,MBNL1,SKI,DDX54,TARDBP,LARP4,XRCC6,PNN,RP*

			L22,PCNA,MBD1,UPF2,DIAPH1,DDX3X,LBR,HDGF,ZNF789,EIF4G2,MCM2,JUND,AATF,ZMPSTE24,UBAP2,SLC25A5,SLTM,URB1,DNAJC21,UBP1,POLE,EXOSC9,POLR2A,CCDC86,DYNC1H1,TSR1,RBM19,XPO1,PPIA,PRPF3,TPR,SRSF7,UTP20,ZFP36L2,SF3B4,PPRC1,EBNA1BP2,ZFR,TFB2M,UBE2N,NRIP1,MRTO4,GFM1,MYBBP1A,DDB1,BACH2,PITX1,GTF3C4,LRRC47,ASXL2,TCP1,RALY,ENO1,CCT5,FUBP1,POLE3,CDC5L,CHD3,NUP153,RAN,BEND3,UBC,ZNF787,TFAM,MSH2,ZBTB2,DIDO1,GLYR1,KMT2A,MCM3,NOSTRIN,GRWD1,H4C8,CERS6,PRDX1,TNPO1,GATAD2A,DKC1,DDX1,H2BC12,THUMPD1,EWSR1,DDX49,CASC3,SSRP1,ARID1B,KPNB1,CPSF7,RRP1,ZNF33B,HNRNPAB,KAT6A,USP36,RBBP4,SAFB,NCBP1,HEATR1,HSP90AB1,EIF3B,DUS3L,PA2G4,HNRNPK,SMG1,DHX37,SMARCA4,EIF3J,PRKDC,ZNF121,TRA2B,PRMT1,HNRNPC,SF3A3,SUPT16H,HMGA1,FARSA,CELF1,RBMXL1,XRCC5,DDX5,UTP4,DEK,LMNB1,DDX46,EIF4A3,SURF6,DDX39A,DDX56,CIZ1,CDT1,UBAP2L,NOP56,HNRNPDL,KHDRBS1,HNRNPD,LRPPRC,EIF5A,SUPT6H,RANBP2,DNAJB6,NOLC1,SNRNP200,SREBF2,ABCF1,MGA,NSUN2,HSPD1,YBX1,LIN28B,EIF5B,RBM3,SF3A1,MPHOSPH10,GDI2,PES1,MYH10,IPO5,RREB1,EP300,DAZAP1,ALYREF,RRP1B,CLUH,FOSB,SRRT,PABPC1,PRRC2C,SERBP1,AHNAK,PRPF8,SF3B3,THRAP3,HNRNPF,NPM1,ETF1,SON,WDR43,TFRC,RUNX1,NQO1,EIF4G1,TRIM28,U2AF2,NR2F2,BCLAF1,SRSF2,HSP90AA1,NFATC3,ZNF521,CAPRIN1,TRMT1,MCM4,TRIM24,NOP58,HNRNPA2B1,LARP1,PTBP1,CCAR1,DDX21,SFPQ,PABPC4,HNRNPM,POLR1A,KCNH2,SRSF3,EIF3A,MCM7,SMARCC1,MYC,SET,IGF2BP1,FOS,MYB,KHSRP,ZEB2,FUS,ILF3,FASN,ACTB,DHX9,HNRNPU,MCM5,NCL,EGR1,HSPA8
GO:0005515	protein binding	2.8669471371796823e-55	HRAS,NOP14,CENPN,TXK,ACSF3,DSG2,SNRPB,TMEM127,MITF,C1ORF216,PPP6C,JMJD1C,MIS18BP1,DAP3,RBM48,MSH3,TRIP13,CHTOP,ERAL1,ZNF274,SEC23IP,ABRAXAS2,DNAJA2,GYG1,KMT2B,AFF1,MMAB,RNASEH1,SUPT7L,SREK1,MACROH2A1,BRD9,MED16,FADS2,MUS81,NOL11,CCDC138,BICRA,KCNK5,MTA2,TIRAP,RAD51C,NRROS,AAMP,UTP3,CWC22,CPNE7,BEGAIN,UBE2Q1,NEU1,DCAF7,MTREX,OTUD6B,RIOX1,PHF5A,QRFP,NACA,POLR1C,MED6,SRP72,TRIM35,CBX3,PHB,METTL3,EZH2,SNX17,TOMM70,TCF20,FES,RAB10,DCLRE1C,NOC4L,RFWD3,MFAP1,ULK3,DNM1L,PAXIP1,LRRFIP1,GPATCH3,RHEB,ASAP1,INTS6,PPP6R3,H4C5,FUT8,NCAPH2,SMG5,IFRD2,BRD4,STT3A,DHDDS,VKORC1L1,RRP9,MRPL1,ZNF581,SPIN4,SH3YL1,MRPL11,RPUSD4,CHCHD3,DENR,RNASEH2C,HROB,ANKRD13A,CTDP1,NUP155,MED1,NVL,POM121C,KIF26B,SRM,HNRNPL,MYO16,PWP1,ATP11A,WDR46,MCM10,OXA1L,EFTUD2,CHRAC1,EML4,CRCP,GPN2,RANBP3,FRMD8,KMT2D,STAG2,CASP8,TAF4B,PSMA3,PQBP1,PSMD3,AIFM2,PSPC1,CEP350,MAD2L2,MRRF,POLDIP2,PSMC3,ZBTB40,NAA11,TIMM44,DHX38,ADNP2,NT5C3A,MS4A4A,FBXO45,ARID2,DDX23,PATZ1,TRIM44,TRNT1,MNS1,RNPS1,MTCH2,ALMS1,LHX4,ZNF512B,RBL1,PPP3R1,TPRN,SLC25A46,SYPL1,HIF1AN,USP11,RPUSD1,SS18L1,RBM45,MED29,NOSIP,PAFAH1B1,STAG1,HNRNPH1,USP14,RAB7A,LIMD1,ZNF512,DNMT1,LYL1,NAA20,BICD1,PPP5C,BAP1,JRK,DUS1L,ANAPC7,SUB1,GTF2H1,DHX16,BUB3,GAB2,TOP2B,LYAR,LRWD1,THOP1,CCNH,NAB2,EXOC7,EIF4EBP2,CCT6A,KAT7,TPP2,CLTC,EEF2,AFG3L2,MSRA,AURKAIP1,WDR5,CCT8,KIF5B,TMEM43,RTL10,FUBP3,IMP4,BCCIP,SPRY2,DLAT,NOB1,RNF40,CUL3,ARPP19,CMPK1,NONO,MEF2C,TCF3,NUP1

			60,MAF1,GID8,CLSPN,NOL9,MVK,NBAS,PLK4,CCNY,CRIM1,SLK,ZFYVE26,PRPF6,ZC3H4,TMEM33,PEBP1,ZNF26,ZNF24,GSPT1,CLPTM1,PEPD,GRSF1,NXF1,UBQLN4,ACTR8,FAHD1,PSMC5,AGPAT3,STK35,TIMM23,ZC3HAV1,CAPZA1,HNRNPA3,MAPK1,STAR,ZNRF1,EEF1D,CASP3,POLR1E,ANKRD36C,ATP2A2,IPO9,PKP3,ABCF3,ABT1,TULP4,SURF4,PCLAF,WDR36,R3HDM1,HBG1,PDSS1,CERS2,DHX33,STRIP1,PGAM5,NSMAF,LETM1,CTR9,NCOA5,TBCD,DHX15,UIMC1,PUM1,NEMP1,MTOR,DHX30,UHRF1BP1,RPRD2,UTP18,IKZF3,KANSL1,CPSF3,SPEN,LCLAT1,VDAC1,SMG9,SMARCA5,SNX8,CWC25,SF3A2,H2AW,EIF3D,RSF1,TFAP4,NAA50,KCTD3,PTP4A2,CLDN11,DELE1,PRMT6,TSPYL5,KPNA4,NIFK,TBC1D9B,FAF1,HNRNPA0,BAG6,EMD,ZNF131,PCYT1A,SBNO1,TASOR2,FKBP15,NAP1L4,DHFR,MTDH,BAIAP2,COMMD4,HSPA9,MCCC1,TCF7L2,ZNF239,SRRM2,NSDHL,MRPL15,PRAME,PCNX4,ASCC3,UROD,UBE2L3,THOC1,PSMC2,MCMBP,SETMAR,RPTOR,PPIL2,PPP1R10,HECTD1,TMEM18,CSTF2,TNF,RAVER1,GRPEL1,RABGGTB,EIF4B,SAMSN1,WDR33,NIP7,PPP2R5A,TRMT61A,IGF2BP3,NOL7,ANKRD33B,MED28,ELL2,FAM71F2,PUM2,H2AZ2,HAT1,MRFAP1L1,MCRIP2,CLTA,WDR3,SSBP3,GUCD1,SNRPA,BACH1,PDZD8,RBM10,CERT1,TRAM2,SETX,TRMT2A,AP3D1,NAPA,PSMG2,STK24,APEX1,BPTF,ATP6V1G1,UFC1,EDC4,ICE1,RIOK2,NAA15,ARID1A,NUP43,AMMECR1,TRAM1,SPECC1,ADO,PPP1CC,KCNQ5,CAVIN2,ZC3H14,CDC27,HERC2,RAB35,SRSF10,CTSL,AKAP8,SBDS,RCC1,C1QBP,VPS72,PPM1H,CRK,TICRR,MED15,EOGT,STK25,CAPNS1,SRSF8,WDR74,PAK2,ZMYND19,ARHGAP21,PRPF4,KTN1,PRMT5,SNX9,PCBP2,GNB1L,ADD1,TRMT6,RBMX,RNF126,BAZ1A,DPYS,GPX4,YJU2,WDR70,MCCC2,SLC25A3,MLLT3,ALG8,RAD23B,VAT1,TAF4,RBM14,RBM8A,ARHGAP6,BOP1,GOLM1,LRP8,LRRC58,SETD2,ZNF326,API5,INTS13,RPIA,UCK2,BTBD1,OXNAD1,TUBB,FEN1,CHAF1A,TRMO,DYRK1A,TFDP2,WDR12,FAM193A,GTF3C6,SLBP,CUL4A,DNAJC8,JADE2,NUCKS1,SDCBP,PSMD1,UTP25,AMD1,TMPO,NUP50,PRPF38A,SSB,CDC37,ECHDC1,AGPAT5,GNL3L,DNTTIP2,CYB5B,IBA57,RILP,NSRP1,TFIP11,KCTD15,STRBP,PSMB2,WAC,EIF3G,ADI1,BSN,DHX29,ARHGEF2,IL17D,BRCA2,RANBP1,POLR2D,ZDHHC5,EIF3M,WTAP,COPS2,NIN,BCL7B,RBM42,ZC3H7B,ACLY,SNRPD1,PAN3,PAF1,ZC3H18,BIRC6,ERMAP,FASTKD2,RABL6,PHB2,TCOF1,LRRC41,TOMM22,RAP1GAP2,GRB10,XRCC2,PDS5A,BRCC3,ZNF75A,SMARCB1,TRIR,CREBBP,EXOSC3,WBP11,KIFC3,AHSA1,EPRS1,COPS3,UBE4B,INSIG1,LSM14A,ABLIM1,TMX2,NUP98,HNRNPUL1,CNOT1,CUTA,LYN,ASH1L,LRRC59,GEMIN5,PHACTR2,PLAGL2,APC,BTF3,POLR1B,JPT2,DDX42,CAPN1,ELOF1,WDR6,NFYC,SF1,PELP1,ZCCHC14,SLC12A2,XRN2,TMEM201,EZR,DDX20,URM1,MMS19,ELOA,TAF9,ACAT2,YWHAG,ELAVL1,UTP15,VCP,DNAJB12,WASHC5,RYBP,SAFB2,GBP2,MAPK1IP1L,KIAA0100,DHX34,TMEM69,PSIP1,CSTB,CSNK1G2,DCAF13,BEX4,RBM12,STIP1,CDK4,DVL2,PNO1,POLR3C,THG1L,RBM25,SETD1A,SCAP,WDR82,SMC1A,AGO2,E2F4,DCBLD2,TJP1,VPS26A,FOXRED2,G3BP1,PCM1,RNF220,TBC1D14,SAE1,MCM6,AQR,DHCR7,ARL8B,DDX18,IER3,IGF2R,ATP6V0D1,ADNP,UBA2,NFKB1,UBTF,ZNF622,ARHGDIA,BRD2,CHD7,RBM15B,DOK3,USP37,HNRNPA1,GAR1,RRS1,PPARGC1B,MAGOH,PBRM1,CTCF,RANBP10,HHEX,ANKRD17,HNRNPR,RRM2,RRM1,PI4KA,SEC24B,ELF1,ZNF614,NSD1,RSL1D1,CCT3,CDK7,H3-3B,RNF138,SETD1B,PPIF,PKM,FOXK2,KIF2A,HMGB

			1,MAEA,BZW1,SMARCD1,GNAQ,CANX,YY1,NOL8,MLLT10,IRAK1,PUS7,C19ORF25,PIK3C2B,IP6K1,NAT10,TRMT10C,METAP2,SNU13,SRSF6,EP400,AASDHPPT,MTHFD1,ADSS2,SART1,LEPR,MECP2,CSDE1,PARD3,MBNL1,SKI,PRKAR2B,TKT,DDX54,AZIN1,CCDC6,NCR3LG1,N4BP2,TARDBP,LARP4,XRCC6,PNN,RPL22,ABI1,KLHL21,PCNA,CDC25A,MBD1,CSNK2A2,UPF2,DIAPH1,DDX3X,KIAA0753,IQGAP2,IDI1,TNPO2,SACS,LBR,HDGF,NCAPH,ZNF789,VPS35,ATP5MC3,EIF4G2,MCM2,JUND,CFL1,HCFC1,CHEK1,AATF,CLN6,ECSIT,ZMPSTE24,UBAP2,PRMT7,OXCT1,RETREG2,SLC25A5,ARPC4,SLTM,CYP3A5,S1PR3,VAC14,POM121,CHAMP1,DNAJC21,UBP1,POLE,TRRAP,PPIP5K2,NUDC,EXOSC9,CSK,CCDC86,DNAJC7,DNAJA1,SUMO3,NUP62,DYNC1H1,TSR1,SLC19A1,XPO1,PPIA,PRPF3,IK,CLCN7,PHACTR1,SERPINE1,TPR,ENC1,SRSF7,UTP20,TGFBRAP1,ZFP36L2,SF3B4,SLC38A2,EBNA1BP2,ZFR,KEAP1,UBE2N,BAZ1B,NRIP1,MACO1,MRTO4,VAPA,TLK1,TEX10,GFM1,MYBBP1A,DDB1,BACH2,PSMG1,PITX1,GTF3C4,LRRC47,NASP,ASXL2,TCP1,SLITRK6,EMP3,CENPF,YWHAB,RALY,ENO1,FAM83H,CCT5,FUBP1,POLE3,CDK6,CDC5L,CHD3,ST3GAL2,NUP153,RAN,BEND3,UBC,WDR81,TFAM,HSPA4,AK2,PPP2CA,SSU72,MSH2,CDK12,ZBTB2,GLYR1,KMT2A,MCM3,NOSTRIN,GRWD1,TOMM40,PAICS,H4C8,SLC30A10,CERS6,ARMC6,PRDX1,TNPO1,GATAD2A,DKC1,DDX1,H2BC12,STX3,CUL1,LTBR,THUMPD1,CTPS1,EWSR1,DDX49,CASC3,SSRP1,ARID1B,KPNB1,CPSF7,RRP1,SPTB,TXNRD1,ZNF33B,ATP6V0A1,HNRNPAB,KAT6A,ATAD3A,ARF6,USP36,RBBP4,CNPPD1,SAFB,NCBP1,HEATR1,CCT2,HSP90AB1,EIF3B,RCSD1,DUS3L,PA2G4,HNRNPK,SMG1,DHX37,FDFT1,PRPF19,SMARCA4,EIF3J,PRKDC,YES1,ZNF121,TRA2B,PRMT1,HNRNPC,MALT1,WWP2,SF3A3,SUPT16H,HMGA1,FARSA,CELF1,NCLN,XRCC5,DDX5,PSME3,TMEM97,ANK1,UTP4,DEK,LMO2,LMNB1,DDX46,LSS,EIF4A3,NDST1,NUP214,TRIP12,IPO7,ACTG1,SURF6,DDX39A,DDX56,SLC38A1,CIZ1,CDT1,UBAP2L,NOP56,HNRNPDL,CORO1C,KHDRBS1,HNRNPD,RAPGEF1,LRPPRC,EIF5A,FAM136A,CTDSP1,SLC7A1,SUPT6H,RANBP2,DNAJB6,NOLC1,GSE1,SNRNP200,SREBF2,CBFA2T3,STXBP5,ABCF1,ACACA,TAFA2,MGA,AP5Z1,HSPD1,PIM2,GAPDH,YBX1,LIN28B,EIF5B,PCYT2,ATP6V1C1,RBM3,STARD7,SF3A1,LMNB2,AXIN1,MS4A3,MPHOSPH10,GDI2,PES1,ANP32B,MYH10,IPO5,HSPH1,EP300,TGFBR1,DAZAP1,PCNT,STMN1,RRP1B,FOSB,PPM1G,STON2,SRRT,PABPC1,PRRC2C,SERBP1,ST7,AHNAK,PRPF8,COA7,SF3B3,PTMA,FTL,THRAP3,MLLT1,ELOVL6,HNRNPF,MT-ND1,NPM1,RIF1,CITED2,ETF1,SON,WDR43,TFRC,RUNX1,NQO1,EIF4G1,TRIM28,NEFH,U2AF2,NR2F2,BCLAF1,ECPAS,SRSF2,HSP90AA1,MDN1,NFATC3,ZNF521,CAPRIN1,TRMT1,SQLE,MCM4,TRIM24,NOP58,RESF1,HNRNPA2B1,KIF1A,SPN,BAG1,LARP1,SQSTM1,HBZ,PTBP1,CCAR1,MAT2A,DDX21,HMGCS1,SFPQ,SCD,PABPC4,HNRNPM,POLR1A,KCNH2,SRSF3,ANKRD11,EIF3A,MCM7,SMARCC1,MYC,SET,VGF,BTG1,IGF2BP1,FOS,GCLM,HMGCR,SPTA1,MYB,ODC1,KHSRP,ZEB2,RELN,FUS,ILF3,FASN,GLUL,ACTB,DHX9,HNRNPU,MCM5,NCL,EGR1,DHCR24,HSPA8,FTH1
GO:0003723	RNA binding	3.0604824045501514e-55	NOP14,SNRPB,DAP3,RBM48,PPAN,CHTOP,ERAL1,SEC23IP,PRPF38B,RNASEH1,SREK1,FBRSL1,NOL11,UTP3,CWC22,MTREX,PHF5A,SRP72,METTL3,EZH2,TCF20,NOC4L,MFAP1,LRRFIP1,H4C5,SMG5,RRP9,MRPL1,ZNF74,MRPL11,RPUSD4,DENR,MRPS30,NVL,HNRNPL,WDR46,EFTUD2,PSPC1,DHX38,NOP16,DDX23,T

			RNT1,RNPS1,RPUSD1,RBM45,NOSIP,HNRNPH1,DNMT1,PPP5C,JRK,SUB1,DHX16,LYAR,CCT6A,CLTC,EEF2,FUBP3,IMP4,BCCIP,FAM120A,RNF40,NONO,NOL9,PRPF6,ZC3H4,PEBP1,GSPT1,GRSF1,NXF1,ZC3HAV1,HNRNPA3,ABT1,R3HDM1,DHX33,NCOA5,DHX15,PUM1,DHX30,UTP18,CPSF3,SPEN,GOT2,SF3A2,EIF3D,NIFK,HNRNPA0,NAP1L4,DHFR,MTDH,HSPA9,ZNF239,SRRM2,MRPL15,ASCC3,UBE2L3,THOC1,PPP1R10,MRPS35,CSTF2,RAVER1,EIF4B,SAMSN1,WDR33,NIP7,IGF2BP3,NOL7,PUM2,WDR3,MT-TL1,SNRPA,DDX10,RBM10,SETX,TRMT2A,APEX1,NAA15,PPP1CC,ZC3H14,SRSF10,AKAP8,SBDS,C1QBP,SRSF8,PRPF4,KTN1,PCBP2,ADD1,TRMT6,RBMX,RBM14,RBM8A,BOP1,ZNF326,API5,SLBP,NUCKS1,UTP25,PRPF38A,SSB,GNL3L,DNTTIP2,IBA57,RNU6-322P,NSRP1,MT-TF,STRBP,EIF3G,DHX29,RBM42,ZC3H7B,SNRPD1,PAN3,ZC3H18,FASTKD2,TCOF1,TRIR,EXOSC3,WBP11,EPRS1,LSM14A,NUP98,HNRNPUL1,CNOT1,LRRC59,GEMIN5,BTF3,DDX42,WDR6,SF1,PELP1,XRN2,EZR,TRUB2,DDX20,DDX51,YWHAG,ELAVL1,UTP15,VCP,SAFB2,DHX34,PSIP1,CSTB,DCAF13,RBM12,STIP1,PNO1,THG1L,RBM25,SETD1A,RRP12,SMC1A,AGO2,G3BP1,GNL2,RRP36,AQR,CLCN6,DDX18,UBTF,ZNF622,RBM15B,HNRNPA1,GAR1,RRS1,PPARGC1B,MAGOH,ANKRD17,HNRNPR,RSL1D1,CCT3,SETD1B,PKM,HMGB1,BZW1,CANX,YY1,NOL8,PUS7,NAT10,TRMT10C,METAP2,SNU13,SRSF6,SART1,CEBPZ,MECP2,CSDE1,MBNL1,DDX54,TARDBP,LARP4,XRCC6,PNN,RPL22,UPF2,DIAPH1,DDX3X,LBR,HDGF,EIF4G2,AATF,UBAP2,SLC25A5,SLTM,URB1,DNAJC21,EXOSC9,CCDC86,DYNC1H1,TSR1,RBM19,XPO1,PPIA,PRPF3,TPR,SRSF7,UTP20,ZFP36L2,SF3B4,PPRC1,EBNA1BP2,ZFR,TFB2M,UBE2N,MRTO4,GFM1,MYBBP1A,LRRC47,TCP1,RALY,ENO1,CCT5,FUBP1,CDC5L,CHD3,RAN,UBC,TFAM,DIDO1,GRWD1,H4C8,PRDX1,TNPO1,DKC1,DDX1,THUMPD1,EWSR1,DDX49,CASC3,SSRP1,KPNB1,CPSF7,RRP1,HNRNPAB,USP36,SAFB,NCBP1,HEATR1,HSP90AB1,EIF3B,DUS3L,PA2G4,HNRNPK,SMG1,DHX37,SMARCA4,PRKDC,TRA2B,PRMT1,HNRNPC,SF3A3,SUPT16H,HMGA1,FARSA,CELF1,RBMXL1,XRCC5,DDX5,UTP4,DEK,DDX46,EIF4A3,SURF6,DDX39A,DDX56,UBAP2L,NOP56,HNRNPDL,KHDRBS1,HNRNPD,LRPPRC,EIF5A,SUPT6H,RANBP2,NOLC1,SNRNP200,ABCF1,NSUN2,HSPD1,YBX1,LIN28B,EIF5B,RBM3,SF3A1,MPHOSPH10,GDI2,PES1,MYH10,IPO5,EP300,DAZAP1,ALYREF,RRP1B,CLUH,SRRT,PABPC1,PRRC2C,SERBP1,AHNAK,PRPF8,SF3B3,THRAP3,HNRNPF,NPM1,ETF1,SON,WDR43,TFRC,NQO1,EIF4G1,TRIM28,U2AF2,BCLAF1,SRSF2,HSP90AA1,CAPRIN1,TRMT1,NOP58,HNRNPA2B1,LARP1,PTBP1,CCAR1,DDX21,SFPQ,PABPC4,HNRNPM,SRSF3,EIF3A,MYC,IGF2BP1,KHSRP,FUS,ILF3,FASN,DHX9,HNRNPU,NCL,HSPA8
GO:1901363	heterocyclic compound binding	3.820583544160709e-54	HRAS,NOP14,TXK,ACSF3,SNRPB,MITF,JMJD1C,MIS18BP1,DAP3,RBM48,PPAN,MSH3,TRIP13,CHTOP,ERAL1,ZNF274,SEC23IP,DNAJA2,PRPF38B,KMT2B,MMAB,RNASEH1,SREK1,MACROH2A1,BRD9,FBRSL1,MUS81,NOL11,MTA2,RAD51C,UTP3,CWC22,UBE2Q1,MTREX,PHF5A,NACA,POLR1C,MED6,SRP72,METTL3,EZH2,TCF20,FES,RAB10,DCLRE1C,NOC4L,MFAP1,ULK3,DNM1L,LRRFIP1,GPATCH3,RHEB,H4C5,SMG5,BRD4,RRP9,MRPL1,ZNF581,ZNF74,MRPL11,AHCTF1,RPUSD4,DENR,HROB,MRPS30,MED1,NVL,KIF26B,HNRNPL,MYO16,ATP11A,WDR46,MCM10,EFTUD2,CHRAC1,CRCP,GPN2,KMT2D,TAF4B,PQBP1,AIFM2,PSPC1,CEP350,POLDIP2,PSMC3,ZBTB40,TIMM44,DHX38,ADNP

			2,GART,NT5C3A,NOP16,ARID2,DDX23,PATZ1,TRNT1,RNPS1,LHX4,ZNF512B,RBL1,RPUSD1,RBM45,NOSIP,HNRNPH1,RAB7A,ZNF512,DNMT1,LYL1,PPP5C,BAP1,JRK,DUS1L,SUB1,DHX16,TOP2B,LYAR,LRWD1,EIF4EBP2,CCT6A,KAT7,CLTC,EEF2,AFG3L2,CCT8,KIF5B,FUBP3,IMP4,BCCIP,FAM120A,RNF40,CMPK1,NONO,MEF2C,TCF3,MAF1,CLSPN,NOL9,MVK,PLK4,SLK,PRPF6,ZC3H4,PEBP1,ZNF26,ZNF24,GSPT1,GRSF1,NXF1,ACTR8,PSMC5,STK35,ZC3HAV1,HNRNPA3,MAPK1,EEF1D,POLR1E,ATP2A2,ABCF3,ABT1,R3HDM1,HBG1,CERS2,DHX33,NCOA5,DHX15,UIMC1,PUM1,MTOR,DHX30,UTP18,IKZF3,CPSF3,SPEN,GOT2,SMARCA5,SF3A2,H2AW,EIF3D,ZNF45,TFAP4,NIFK,HNRNPA0,ZNF131,SBNO1,ZNF431,NAP1L4,DHFR,MTDH,HSPA9,MCCC1,TCF7L2,ZNF239,SRRM2,MRPL15,GMPS,ASCC3,UBE2L3,THOC1,PSMC2,SETMAR,RPTOR,PPP1R10,MRPS35,TMEM18,CSTF2,TNF,RAVER1,GRPEL1,EIF4B,SAMSN1,WDR33,NIP7,IGF2BP3,NOL7,ELL2,PUM2,H2AZ2,WDR3,MT-TL1,SSBP3,SNRPA,ZNF586,BACH1,DDX10,RBM10,SETX,NFILZ,TRMT2A,STK24,APEX1,BPTF,RIOK2,NAA15,ARID1A,PPP1CC,ZC3H14,RAB35,SRSF10,AKAP8,SBDS,RCC1,C1QBP,VPS72,ZNF587B,STK25,SRSF8,PAK2,PRPF4,KTN1,PRMT5,PCBP2,ADD1,TRMT6,RBMX,BAZ1A,MCCC2,MLLT3,RAD23B,TAF4,RBM14,RBM8A,KDM3B,BOP1,ZNF326,API5,UCK2,TUBB,FEN1,ZNF282,DYRK1A,TFDP2,GTF3C6,SLBP,NUCKS1,UTP25,TMPO,PRPF38A,SSB,GNL3L,DNTTIP2,CYB5B,IBA57,RNU6-322P,NSRP1,MT-TF,TFIP11,STRBP,EIF3G,DHX29,BRCA2,POLR2D,EIF3M,NIN,RBM42,ZFX,ZC3H7B,ACLY,SNRPD1,PAN3,ZC3H18,FASTKD2,RABL6,TCOF1,XRCC2,ZNF75A,SMARCB1,TRIR,CREBBP,EXOSC3,WBP11,KIFC3,EPRS1,LSM14A,NUP98,HNRNPUL1,CNOT1,LYN,ASH1L,LRRC59,GEMIN5,PLAGL2,BTF3,POLR1B,DDX42,ELOF1,WDR6,NFYC,SF1,PELP1,ZCCHC14,XRN2,EZR,TRUB2,DDX20,DDX51,ELOA,TAF9,YWHAG,ELAVL1,UTP15,VCP,RYBP,SAFB2,GBP2,DHX34,PSIP1,CSTB,CSNK1G2,DCAF13,RBM12,STIP1,CDK4,PNO1,POLR3C,THG1L,RBM25,SETD1A,RRP12,SMC1A,AGO2,E2F4,FOXRED2,G3BP1,GNL2,RRP36,MCM6,AQR,DHCR7,CLCN6,ARL8B,DDX18,ADNP,UBA2,NFKB1,UBTF,ZNF622,CHD7,RBM15B,HNRNPA1,GAR1,RRS1,PPARGC1B,MAGOH,PBRM1,CTCF,HHEX,ANKRD17,HNRNPR,RRM1,PI4KA,ELF1,ZNF614,NSD1,RSL1D1,CCT3,CDK7,H3-3B,RNF138,SETD1B,PKM,FOXK2,KIF2A,HMGB1,ATAD3B,BZW1,GNAQ,CANX,YY1,NOL8,MLLT10,IRAK1,PUS7,PIK3C2B,IP6K1,NAT10,TRMT10C,METAP2,SNU13,SRSF6,EP400,MTHFD1,ADSS2,SART1,CEBPZ,MECP2,CSDE1,MBNL1,SKI,PRKAR2B,TKT,DDX54,N4BP2,TARDBP,LARP4,XRCC6,PNN,RPL22,PCNA,MBD1,CSNK2A2,UPF2,DIAPH1,DDX3X,LBR,HDGF,ZNF789,EIF4G2,MCM2,JUND,PFAS,CHEK1,AATF,ZMPSTE24,UBAP2,SLC25A5,SLTM,CYP3A5,URB1,DNAJC21,UBP1,POLE,PPIP5K2,EXOSC9,CSK,POLR2A,CCDC86,DNAJA1,DYNC1H1,TSR1,RBM19,SLC19A1,XPO1,PPIA,PRPF3,CLCN7,TPR,SRSF7,UTP20,ZFP36L2,SF3B4,STK17A,PPRC1,EBNA1BP2,ZFR,TFB2M,UBE2N,BAZ1B,NRIP1,MRTO4,TLK1,GFM1,MYBBP1A,DDB1,BACH2,PITX1,GTF3C4,LRRC47,ASXL2,TCP1,RALY,ENO1,CCT5,FUBP1,POLE3,CDK6,CDC5L,CHD3,NUP153,RAN,BEND3,UBC,ZNF787,TFAM,HSPA4,AK2,MSH2,CDK12,ZBTB2,DIDO1,GLYR1,KMT2A,MCM3,NOSTRIN,GRWD1,PAICS,H4C8,CERS6,PRDX1,TNPO1,GATAD2A,DKC1,DDX1,H2BC12,THUMPD1,CTPS1,EWSR1,DDX49,CASC3,SSRP1,ARID1B,KPNB1,CPSF7,RRP1,TXNRD1,

			ZNF33B,HNRNPAB,KAT6A,ATAD3A,ARF6,USP36,RBBP4,SAFB,NCBP1,HEATR1,CCT2,HSP90AB1,EIF3B,DUS3L,PA2G4,HNRNPK,SMG1,DHX37,SMARCA4,CYP20A1,EIF3J,PRKDC,YES1,ZNF121,TRA2B,PRMT1,HNRNPC,SF3A3,SUPT16H,HMGA1,FARSA,CELF1,RBMXL1,XRCC5,DDX5,UTP4,DEK,LMNB1,DDX46,EIF4A3,ACTG1,SURF6,DDX39A,DDX56,CIZ1,CDT1,UBAP2L,NOP56,HNRNPDL,KHDRBS1,HNRNPD,LRPPRC,EIF5A,SUPT6H,RANBP2,DNAJB6,NOLC1,SNRNP200,SREBF2,ABCF1,ACACA,MGA,NSUN2,HSPD1,PIM2,GAPDH,YBX1,LIN28B,EIF5B,RBM3,PGD,SF3A1,MPHOSPH10,GDI2,PES1,MYH10,IPO5,RREB1,HSPH1,EP300,TGFBR1,DAZAP1,ALYREF,RRP1B,CLUH,FOSB,SRRT,PABPC1,PRRC2C,SERBP1,ATP13A3,AHNAK,PRPF8,SF3B3,THRAP3,HNRNPF,NPM1,ETF1,SON,WDR43,TFRC,RUNX1,NQO1,EIF4G1,TRIM28,U2AF2,NR2F2,BCLAF1,SRSF2,HSP90AA1,MDN1,NFATC3,ZNF521,CAPRIN1,TRMT1,SQLE,MCM4,TRIM24,NOP58,HNRNPA2B1,KIF1A,BAG1,LARP1,HBZ,PTBP1,CCAR1,MAT2A,DDX21,SFPQ,PABPC4,HNRNPM,POLR1A,KCNH2,SRSF3,EIF3A,MCM7,SMARCC1,MYC,SET,IGF2BP1,FOS,HMGCR,MYB,KHSRP,ZEB2,FUS,ILF3,FASN,GLUL,ACTB,DHX9,HNRNPU,MCM5,NCL,EGR1,DHCR24,HSPA8
GO:0097159	organic cyclic compound binding	6.396976601369362e-54	*HRAS,NOP14,TXK,ACSF3,SNRPB,MITF,JMJD1C,MIS18BP1,DAP3,RBM48,PPAN,MSH3,TRIP13,CHTOP,ERAL1,ZNF274,SEC23IP,DNAJA2,PRPF38B,KMT2B,MMAB,RNASEH1,SREK1,MACROH2A1,BRD9,FBRSL1,MUS81,NOL11,MTA2,RAD51C,UTP3,CWC22,UBE2Q1,MTREX,PHF5A,NACA,POLR1C,MED6,SRP72,METTL3,EZH2,TCF20,FES,RAB10,DCLRE1C,NOC4L,MFAP1,ULK3,DNM1L,LRRFIP1,GPATCH3,RHEB,H4C5,SMG5,BRD4,RRP9,MRPL1,ZNF581,ZNF74,MRPL11,AHCTF1,RPUSD4,DENR,HROB,MRPS30,MED1,NVL,KIF26B,HNRNPL,MYO16,ATP11A,WDR46,MCM10,EFTUD2,CHRAC1,CRCP,GPN2,KMT2D,TAF4B,PQBP1,AIFM2,PSPC1,CEP350,POLDIP2,PSMC3,ZBTB40,TIMM44,DHX38,ADNP2,GART,NT5C3A,NOP16,ARID2,DDX23,PATZ1,TRNT1,RNPS1,LHX4,ZNF512B,RBL1,RPUSD1,RBM45,NOSIP,HNRNPH1,RAB7A,ZNF512,DNMT1,LYL1,PPP5C,BAP1,JRK,DUS1L,SUB1,DHX16,TOP2B,LYAR,LRWD1,EIF4EBP2,CCT6A,KAT7,CLTC,EEF2,AFG3L2,CCT8,KIF5B,FUBP3,IMP4,BCCIP,FAM120A,RNF40,CMPK1,NONO,MEF2C,TCF3,MAF1,CLSPN,NOL9,MVK,PLK4,SLK,PRPF6,ZC3H4,PEBP1,ZNF26,ZNF24,GSPT1,GRSF1,NXF1,ACTR8,PSMC5,STK35,ZC3HAV1,HNRNPA3,MAPK1,STAR,EEF1D,POLR1E,ATP2A2,ABCF3,ABT1,R3HDM1,HBG1,CERS2,DHX33,NCOA5,DHX15,UIMC1,PUM1,MTOR,DHX30,UTP18,IKZF3,CPSF3,SPEN,GOT2,VDAC1,SMARCA5,SF3A2,H2AW,EIF3D,ZNF45,TFAP4,NIFK,HNRNPA0,ZNF131,SBNO1,ZNF431,NAP1L4,DHFR,MTDH,HSPA9,MCCC1,TCF7L2,ZNF239,SRRM2,MRPL15,GMPS,ASCC3,UBE2L3,THOC1,PSMC2,SETMAR,RPTOR,PPP1R10,MRPS35,TMEM18,CSTF2,TNF,RAVER1,GRPEL1,EIF4B,SAMSN1,WDR33,NIP7,IGF2BP3,NOL7,ELL2,PUM2,H2AZ2,WDR3,MT-TL1,SSBP3,SNRPA,ZNF586,BACH1,DDX10,RBM10,SETX,NFILZ,TRMT2A,STK24,APEX1,BPTF,RIOK2,NAA15,ARID1A,PPP1CC,ZC3H14,RAB35,SRSF10,AKAP8,SBDS,RCC1,C1QBP,VPS72,ZNF587B,STK25,SRSF8,PAK2,PRPF4,KTN1,PRMT5,PCBP2,ADD1,TRMT6,RBMX,BAZ1A,MCCC2,MLLT3,RAD23B,TAF4,RBM14,RBM8A,KDM3B,BOP1,ZNF326,API5,UCK2,TUBB,FEN1,ZNF282,DYRK1A,TFDP2,GTF3C6,SLBP,NUCKS1,UTP25,TMPO,PRPF38A,SSB,GNL3L,DNTTIP2,CYB5B,IBA57,RNU6-322P,NSRP1,MT-TF,TFIP11,STRBP,EIF3G,DHX29,BRCA2,POLR2D,E*

			IF3M,NIN,RBM42,ZFX,ZC3H7B,ACLY,SNRPD1,PAN3,ZC3H18,FASTKD2,RABL6,TCOF1,XRCC2,ZNF75A,SMARCB1,TRIR,CREBBP,EXOSC3,WBP11,KIFC3,EPRS1,INSIG1,LSM14A,NUP98,HNRNPUL1,CNOT1,LYN,ASH1L,LRRC59,GEMIN5,PLAGL2,BTF3,POLR1B,DDX42,ELOF1,WDR6,NFYC,SF1,PELP1,ZCCHC14,XRN2,EZR,TRUB2,DDX20,DDX51,ELOA,TAF9,YWHAG,ELAVL1,UTP15,VCP,RYBP,SAFB2,GBP2,DHX34,PSIP1,CSTB,CSNK1G2,DCAF13,RBM12,STIP1,CDK4,PNO1,POLR3C,THG1L,RBM25,SETD1A,RRP12,SCAP,SMC1A,AGO2,E2F4,FOXRED2,G3BP1,GNL2,RRP36,MCM6,AQR,DHCR7,CLCN6,ARL8B,DDX18,ADNP,UBA2,NFKB1,UBTF,ZNF622,CHD7,RBM15B,HNRNPA1,GAR1,RRS1,PPARGC1B,MAGOH,PBRM1,CTCF,HHEX,ANKRD17,HNRNPR,RRM1,PI4KA,ELF1,ZNF614,NSD1,RSL1D1,CCT3,CDK7,H3-3B,RNF138,SETD1B,PKM,FOXK2,KIF2A,HMGB1,ATAD3B,BZW1,GNAQ,CANX,YY1,NOL8,MLLT10,IRAK1,PUS7,PIK3C2B,IP6K1,NAT10,TRMT10C,METAP2,SNU13,SRSF6,EP400,MTHFD1,ADSS2,SART1,CEBPZ,MECP2,CSDE1,MBNL1,SKI,PRKAR2B,TKT,DDX54,N4BP2,TARDBP,LARP4,XRCC6,PNN,RPL22,PCNA,MBD1,CSNK2A2,UPF2,DIAPH1,DDX3X,LBR,HDGF,ZNF789,EIF4G2,MCM2,JUND,PFAS,CHEK1,AATF,ZMPSTE24,UBAP2,SLC25A5,SLTM,CYP3A5,URB1,DNAJC21,UBP1,POLE,PPIP5K2,EXOSC9,CSK,POLR2A,CCDC86,DNAJA1,DYNC1H1,TSR1,RBM19,SLC19A1,XPO1,PPIA,PRPF3,CLCN7,TPR,SRSF7,UTP20,ZFP36L2,SF3B4,STK17A,PPRC1,EBNA1BP2,ZFR,TFB2M,UBE2N,BAZ1B,NRIP1,MRTO4,TLK1,GFM1,MYBBP1A,DDB1,BACH2,PITX1,GTF3C4,LRRC47,ASXL2,TCP1,RALY,ENO1,CCT5,FUBP1,POLE3,CDK6,CDC5L,CHD3,NUP153,RAN,BEND3,UBC,ZNF787,TFAM,HSPA4,AK2,MSH2,CDK12,ZBTB2,DIDO1,GLYR1,KMT2A,MCM3,NOSTRIN,GRWD1,PAICS,H4C8,CERS6,PRDX1,TNPO1,GATAD2A,DKC1,DDX1,H2BC12,THUMPD1,CTPS1,EWSR1,DDX49,CASC3,SSRP1,ARID1B,KPNB1,CPSF7,RRP1,TXNRD1,ZNF33B,HNRNPAB,KAT6A,ATAD3A,ARF6,USP36,RBBP4,SAFB,NCBP1,HEATR1,CCT2,HSP90AB1,EIF3B,DUS3L,PA2G4,HNRNPK,SMG1,DHX37,SMARCA4,CYP20A1,EIF3J,PRKDC,YES1,ZNF121,TRA2B,PRMT1,HNRNPC,SF3A3,SUPT16H,HMGA1,FARSA,CELF1,RBMXL1,XRCC5,DDX5,UTP4,DEK,LMNB1,DDX46,EIF4A3,ACTG1,SURF6,DDX39A,DDX56,CIZ1,CDT1,UBAP2L,NOP56,HNRNPDL,KHDRBS1,HNRNPD,LRPPRC,EIF5A,SUPT6H,RANBP2,DNAJB6,NOLC1,SNRNP200,SREBF2,ABCF1,ACACA,MGA,NSUN2,HSPD1,PIM2,GAPDH,YBX1,LIN28B,EIF5B,RBM3,PGD,SF3A1,MPHOSPH10,GDI2,PES1,MYH10,IPO5,RREB1,HSPH1,EP300,TGFBR1,DAZAP1,ALYREF,RRP1B,CLUH,FOSB,SRRT,PABPC1,PRRC2C,SERBP1,ATP13A3,AHNAK,PRPF8,SF3B3,THRAP3,HNRNPF,NPM1,ETF1,SON,WDR43,TFRC,RUNX1,NQO1,EIF4G1,TRIM28,U2AF2,NR2F2,BCLAF1,SRSF2,HSP90AA1,MDN1,NFATC3,ZNF521,CAPRIN1,TRMT1,SQLE,MCM4,TRIM24,NOP58,HNRNPA2B1,KIF1A,BAG1,LARP1,HBZ,PTBP1,CCAR1,MAT2A,DDX21,SFPQ,PABPC4,HNRNPM,POLR1A,KCNH2,SRSF3,EIF3A,MCM7,SMARCC1,MYC,SET,IGF2BP1,FOS,HMGCR,MYB,KHSRP,ZEB2,FUS,ILF3,FASN,GLUL,ACTB,DHX9,HNRNPU,MCM5,NCL,EGR1,DHCR24,HSPA8
GO:0003729	mRNA binding	7.544244813888879e-34	*CHTOP,METTL3,DENR,HNRNPL,RNPS1,JRK,FUBP3,RNF40,GRSF1,NXF1,HNRNPA3,DHX33,PUM1,SPEN,EIF3D,HNRNPA0,DHFR,SRRM2,CSTF2,RAVER1,IGF2BP3,PUM2,MT-TL1,C1QBP,PCBP2,RBMX,RBM14,RBM8A,SLBP,SSB,NSRP1,MT-*

			TF,LSM14A,NUP98,GEMIN5,SF1,ELAVL1,RBM25,AGO2,G3BP1,AQR,CLCN6,RBM15B,HNRNPA1,HNRNPR,RSL1D1,PKM,SRSF6,MECP2,TARDBP,LARP4,DDX3X,EIF4G2,EXOSC9,TPR,ZFP36L2,CCT5,FUBP1,CASC3,CPSF7,HNRNPAB,NCBP1,HNRNPK,TRA2B,HNRNPC,CELF1,DDX5,EIF4A3,KHDRBS1,HNRNPD,LRPPRC,YBX1,LIN28B,RBM3,MYH10,DAZAP1,CLUH,PABPC1,SERBP1,ETF1,EIF4G1,BCLAF1,HNRNPA2B1,LARP1,PTBP1,PABPC4,HNRNPM,EIF3A,MYC,IGF2BP1,KHSRP,FUS,ILF3,DHX9,HNRNPU,NCL
GO:0003682	chromatin binding	5.204156114683076e-26	*MITF,JMJD1C,ZNF274,MACROH2A1,MTA2,CBX3,EZH2,NCAPH2,BRD4,MED1,STAG2,PATZ1,RBL1,STAG1,DNMT1,BAP1,GTF2H1,TOP2B,LRWD1,NONO,ZC3H4,PCLAF,NCOA5,DHX30,IKZF3,SMARCA5,PRMT6,TSPYL5,SBNO1,ZNF431,NAP1L4,MCMBP,APEX1,ARID1A,RCC1,TICRR,RBMX,MLLT3,KDM3B,CHAF1A,NUCKS1,WAC,ZFX,PAF1,SMARCB1,CREBBP,NUP98,ASH1L,PELP1,WDR82,SMC1A,E2F4,ADNP,NFKB1,UBTF,BRD2,CHD7,PBRM1,CTCF,ANKRD17,NSD1,H3-3B,SMARCD1,YY1,MLLT10,EP400,MECP2,PCNA,NCAPH,HCFC1,POLE,NUP62,TPR,ASXL2,CENPF,POLE3,CHD3,RAN,TFAM,MSH2,GLYR1,KMT2A,GRWD1,DDX1,SSRP1,ARID1B,RBBP4,SAFB,SMARCA4,HNRNPC,SUPT16H,HMGA1,DDX5,CDT1,HNRNPD,SUPT6H,YBX1,EP300,NPM1,CITED2,TRIM28,TRIM24,SFPQ,POLR1A,SMARCC1,SET,FOS,FUS,ACTB,DHX9,HNRNPU,EGR1*
GO:0003677	DNA binding	2.244036499431702e-23	*MITF,JMJD1C,MIS18BP1,MSH3,CHTOP,ZNF274,KMT2B,MACROH2A1,MUS81,MTA2,RAD51C,PHF5A,NACA,POLR1C,MED6,EZH2,TCF20,DCLRE1C,LRRFIP1,H4C5,SMG5,BRD4,ZNF581,ZNF74,AHCTF1,HROB,MED1,HNRNPL,MCM10,CHRAC1,KMT2D,TAF4B,PQBP1,AIFM2,CEP350,POLDIP2,ZBTB40,ADNP2,ARID2,PATZ1,LHX4,ZNF512B,RBL1,ZNF512,DNMT1,LYL1,BAP1,JRK,SUB1,TOP2B,LYAR,LRWD1,KAT7,FUBP3,NONO,MEF2C,TCF3,MAF1,CLSPN,ZNF26,ZNF24,MAPK1,EEF1D,POLR1E,ABT1,CERS2,DHX33,UIMC1,MTOR,IKZF3,SPEN,SMARCA5,H2AW,ZNF45,TFAP4,ZNF131,SBNO1,ZNF431,TCF7L2,ZNF239,THOC1,SETMAR,RPTOR,PPP1R10,TMEM18,TNF,ELL2,H2AZ2,SSBP3,SNRPA,ZNF586,BACH1,SETX,NFILZ,APEX1,BPTF,ARID1A,AKAP8,RCC1,VPS72,ZNF587B,PRMT5,PCBP2,RBMX,BAZ1A,MLLT3,RAD23B,TAF4,KDM3B,ZNF326,FEN1,ZNF282,TFDP2,GTF3C6,NUCKS1,TMPO,SSB,STRBP,BRCA2,ZFX,XRCC2,ZNF75A,SMARCB1,CREBBP,WBP11,LSM14A,ASH1L,PLAGL2,POLR1B,NFYC,XRN2,DDX20,TAF9,RYBP,SAFB2,PSIP1,POLR3C,SMC1A,AGO2,E2F4,G3BP1,MCM6,ADNP,NFKB1,UBTF,CHD7,HNRNPA1,PBRM1,CTCF,HHEX,ELF1,ZNF614,NSD1,H3-3B,RNF138,FOXK2,HMGB1,YY1,MLLT10,EP400,MECP2,SKI,TARDBP,XRCC6,PNN,PCNA,MBD1,UPF2,DDX3X,LBR,HDGF,ZNF789,MCM2,JUND,ZMPSTE24,SLTM,UBP1,POLE,POLR2A,ZFR,NRIP1,MYBBP1A,DDB1,BACH2,PITX1,GTF3C4,ASXL2,ENO1,FUBP1,POLE3,CDC5L,CHD3,NUP153,BEND3,ZNF787,TFAM,MSH2,ZBTB2,GLYR1,KMT2A,MCM3,NOSTRIN,GRWD1,H4C8,CERS6,GATAD2A,DDX1,H2BC12,SSRP1,ARID1B,ZNF33B,HNRNPAB,KAT6A,RBBP4,SAFB,HNRNPK,SMG1,SMARCA4,PRKDC,ZNF121,PRMT1,HNRNPC,HMGA1,XRCC5,DEK,LMNB1,SURF6,CDT1,HNRNPDL,KHDRBS1,HNRNPD,LRPPRC,SUPT6H,DNAJB6,SREBF2,MGA,HSPD1,YBX1,LIN28B,RREB1,EP300,FOSB,SRRT,THRAP3,NPM1,SON,RUNX1,TRIM28,NR2F2,BCLAF1,NFATC3,ZNF521,MCM4,TRIM24,HNRNPA2B1,PTBP1,CCAR1,SFPQ,POLR1A,KCNH2,MCM7,SMARCC1,MYC,SET,FOS,MYB,KHSRP,ZEB2,FUS,ILF3,ACTB,DHX9,HNRNPU,MCM5,NCL,EGR1*

GO:0140640	catalytic activity, acting on a nucleic acid	3.729442806839756e-18	*MSH3,RNASEH1,MUS81,RAD51C,MTREX,POLR1C,METTL3,DCLRE1C,CHRAC1,CRCP,DHX38,NT5C3A,DDX23,TRNT1,DNMT1,DUS1L,SUB1,DHX16,TOP2B,TSR3,NOB1,DHX33,DHX15,DHX30,CPSF3,SMARCA5,RSF1,ASCC3,SETMAR,TRMT61A,DDX10,SETX,TRMT2A,METTL8,APEX1,BPTF,FEN1,TRMO,DHX29,PAN3,XRCC2,EXOSC3,EPRS1,CNOT1,POLR1B,DDX42,XRN2,DDX20,DDX51,DHX34,POLR3C,THG1L,AGO2,G3BP1,MCM6,AQR,DDX18,CHD7,CDK7,TRMT10C,EP400,DDX54,N4BP2,XRCC6,PCNA,DDX3X,MCM2,POLE,EXOSC9,POLR2A,TFB2M,LRRC47,POLE3,CHD3,MSH2,MCM3,DKC1,DDX1,DDX49,RBBP4,DUS3L,DHX37,SMARCA4,HMGA1,FARSA,XRCC5,DDX5,DDX46,EIF4A3,DDX39A,DDX56,SNRNP200,NSUN2,TRMT1,MCM4,DDX21,POLR1A,MCM7,DHX9,MCM5*
GO:0140657	ATP-dependent activity	3.762752166632928e-18	*ACSF3,MSH3,TRIP13,RAD51C,MTREX,NVL,KIF26B,ATP11A,PSMC3,DHX38,DDX23,SUB1,DHX16,TOP2B,CCT6A,AFG3L2,CCT8,KIF5B,PSMC5,ATP2A2,DHX33,DHX15,DHX30,SMARCA5,RSF1,HSPA9,ASCC3,PSMC2,DDX10,SETX,BPTF,ATP6V1G1,DHX29,XRCC2,KIFC3,DDX42,DDX20,DDX51,VCP,DHX34,SMC1A,G3BP1,SAE1,MCM6,AQR,DDX18,ATP6V0D1,UBA2,CHD7,CCT3,CDK7,KIF2A,ATAD3B,EP400,DDX54,XRCC6,DDX3X,MCM2,DYNC1H1,TCP1,CCT5,CHD3,HSPA4,MSH2,MCM3,DDX1,DDX49,ATP6V0A1,ATAD3A,RBBP4,CCT2,HSP90AB1,DHX37,SMARCA4,XRCC5,DDX5,DDX46,EIF4A3,DDX39A,DDX56,SNRNP200,HSPD1,ATP6V1C1,MYH10,HSPH1,ATP13A3,HSP90AA1,MDN1,MCM4,KIF1A,DDX21,MCM7,DHX9,MCM5,HSPA8*
GO:0016887	ATP hydrolysis activity	2.0089993144624836e-17	*TRIP13,MTREX,NVL,ATP11A,PSMC3,DHX38,DDX23,DHX16,CCT6A,AFG3L2,CCT8,KIF5B,PSMC5,ATP2A2,DHX33,DHX15,DHX30,SMARCA5,HSPA9,ASCC3,PSMC2,DDX10,ATP6V1G1,DHX29,DDX42,DDX20,DDX51,VCP,DHX34,SMC1A,G3BP1,MCM6,AQR,DDX18,CHD7,CCT3,KIF2A,ATAD3B,DDX54,DDX3X,TCP1,CCT5,CHD3,MSH2,MCM3,DDX1,DDX49,ATAD3A,CCT2,HSP90AB1,DHX37,SMARCA4,DDX5,DDX46,EIF4A3,DDX39A,DDX56,SNRNP200,HSPD1,ATP13A3,HSP90AA1,MDN1,MCM4,KIF1A,DDX21,MCM7,DHX9,MCM5,HSPA8*
GO:0004386	helicase activity	1.6301182781795197e-16	*MTREX,DHX38,DDX23,SUB1,DHX16,DHX33,DHX15,DHX30,SMARCA5,ASCC3,DDX10,SETX,DHX29,DDX42,DDX20,DDX51,DHX34,G3BP1,MCM6,AQR,DDX18,CHD7,EP400,DDX54,XRCC6,DDX3X,MCM2,CHD3,MCM3,DDX1,DDX49,DHX37,SMARCA4,XRCC5,DDX5,DDX46,EIF4A3,DDX39A,DDX56,SNRNP200,MCM4,DDX21,MCM7,DHX9,MCM5*
GO:0008134	transcription factor binding	3.0595870923560274e-16	*JMJD1C,MED16,MTA2,MED6,CBX3,EZH2,CTDP1,MED1,TAF4B,MAD2L2,HIF1AN,USP11,SUB1,GTF2H1,LYAR,MEF2C,TCF3,PRPF6,PSMC5,EEF1D,POLR1E,DHX33,MTOR,SPEN,FAF1,MTDH,BAIAP2,TCF7L2,PRAME,RPTOR,APEX1,ARID1A,AKAP8,C1QBP,ADD1,RAD23B,TAF4,TFDP2,NUCKS1,PHB2,SMARCB1,CREBBP,CNOT1,DDX20,MMS19,TAF9,PSIP1,DCAF13,SETD1A,PPARGC1B,CTCF,HHEX,NSD1,HMGB1,YY1,SKI,DDX54,PCNA,DDX3X,HDGF,JUND,HCFC1,EXOSC9,DNAJA1,KEAP1,NRIP1,MYBBP1A,PITX1,CENPF,ENO1,TFAM,KAT6A,SMARCA4,PRKDC,WWP2,HMGA1,DDX5,LMO2,TRIP12,NOLC1,EP300,PTMA,THRAP3,HNRNPF,NPM1,CITED2,TRIM24,NOP58,MYC,FOS,ACTB,DHX9,HNRNPU,NCL*
GO:0003712	transcription coregulator activity	1.2164672374882798e-15	*JMJD1C,TRIP13,SUPT7L,MED16,MTA2,NACA,MED6,PHB,EZH2,BRD4,MED1,KMT2D,PQBP1,SS18L1,MED29,LIMD1,SUB1,NAB2,KAT7,PRPF6,ABT1,NCOA5,SPEN,RSF1,MTDH,UBE2L3,SSBP3,APEX1,ARID1A,C1QBP,MED15,PRMT5,RBM14,KDM3B,DYRK1A,NUCKS1,COPS2,MED13L,SMARCB1,CREBBP,NUP98,SF1,MMS19*

			,TAF9,RYBP,PSIP1,PPARGC1B,NSD1,HMGB1,SMARCD1,CEBPZ,MECP2,DDX54,HDGF,HCFC1,TRRAP,PPRC1,TFB2M,NRIP1,MYBBP1A,RALY,ENO1,DDX1,EWSR1,ARID1B,KAT6A,PA2G4,SMARCA4,HMGA1,LMO2,CBFA2T3,EP300,RRP1B,THRAP3,NPM1,CITED2,TRIM28,BCLAF1,SRSF2,TRIM24,CCAR1,SMARCC1,BTG1,FUS,DHX9,HNRNPU
GO:0000166	nucleotide binding	3.127047203682427e-15	*HRAS,TXK,ACSF3,DAP3,MSH3,TRIP13,CHTOP,ERAL1,DNAJA2,MMAB,RAD51C,UBE2Q1,MTREX,FES,RAB10,ULK3,DNM1L,RHEB,NVL,KIF26B,MYO16,ATP11A,EFTUD2,CRCP,GPN2,AIFM2,PSMC3,TIMM44,DHX38,GART,NT5C3A,DDX23,TRNT1,LHX4,RAB7A,DNMT1,PPP5C,DUS1L,DHX16,TOP2B,LRWD1,CCT6A,EEF2,AFG3L2,CCT8,KIF5B,CMPK1,NOL9,MVK,PLK4,SLK,PEBP1,GSPT1,ACTR8,PSMC5,STK35,MAPK1,ATP2A2,ABCF3,DHX33,DHX15,MTOR,DHX30,SMARCA5,DHFR,HSPA9,MCCC1,GMPS,ASCC3,UBE2L3,PSMC2,GRPEL1,DDX10,SETX,STK24,RIOK2,RAB35,STK25,PAK2,PRMT5,MCCC2,UCK2,TUBB,DYRK1A,GNL3L,DHX29,POLR2D,NIN,ACLY,PAN3,RABL6,XRCC2,KIFC3,EPRS1,LYN,DDX42,DDX20,DDX51,VCP,GBP2,DHX34,CSNK1G2,CDK4,THG1L,SMC1A,FOXRED2,G3BP1,GNL2,MCM6,AQR,DHCR7,CLCN6,ARL8B,DDX18,UBA2,CHD7,RRM1,PI4KA,CCT3,CDK7,PKM,KIF2A,ATAD3B,GNAQ,IRAK1,PIK3C2B,IP6K1,NAT10,EP400,MTHFD1,ADSS2,MECP2,PRKAR2B,DDX54,N4BP2,XRCC6,MBD1,CSNK2A2,DDX3X,LBR,HDGF,MCM2,PFAS,CHEK1,POLE,PPIP5K2,CSK,DNAJA1,DYNC1H1,TSR1,SLC19A1,CLCN7,STK17A,UBE2N,BAZ1B,TLK1,GFM1,TCP1,CCT5,CDK6,CHD3,RAN,HSPA4,AK2,MSH2,CDK12,GLYR1,MCM3,PAICS,DDX1,CTPS1,DDX49,TXNRD1,ATAD3A,ARF6,CCT2,HSP90AB1,DUS3L,SMG1,DHX37,SMARCA4,PRKDC,YES1,PRMT1,FARSA,XRCC5,DDX5,DDX46,EIF4A3,ACTG1,DDX39A,DDX56,NOLC1,SNRNP200,ABCF1,ACACA,HSPD1,PIM2,GAPDH,EIF5B,PGD,MYH10,HSPH1,TGFBR1,ATP13A3,THRAP3,RUNX1,EIF4G1,HSP90AA1,MDN1,SQLE,MCM4,KIF1A,BAG1,MAT2A,DDX21,MCM7,HMGCR,GLUL,ACTB,DHX9,HNRNPU,MCM5,DHCR24,HSPA8*
GO:1901265	nucleoside phosphate binding	3.302441829774877e-15	*HRAS,TXK,ACSF3,DAP3,MSH3,TRIP13,CHTOP,ERAL1,DNAJA2,MMAB,RAD51C,UBE2Q1,MTREX,FES,RAB10,ULK3,DNM1L,RHEB,NVL,KIF26B,MYO16,ATP11A,EFTUD2,CRCP,GPN2,AIFM2,PSMC3,TIMM44,DHX38,GART,NT5C3A,DDX23,TRNT1,LHX4,RAB7A,DNMT1,PPP5C,DUS1L,DHX16,TOP2B,LRWD1,CCT6A,EEF2,AFG3L2,CCT8,KIF5B,CMPK1,NOL9,MVK,PLK4,SLK,PEBP1,GSPT1,ACTR8,PSMC5,STK35,MAPK1,ATP2A2,ABCF3,DHX33,DHX15,MTOR,DHX30,SMARCA5,DHFR,HSPA9,MCCC1,GMPS,ASCC3,UBE2L3,PSMC2,GRPEL1,DDX10,SETX,STK24,RIOK2,RAB35,STK25,PAK2,PRMT5,MCCC2,UCK2,TUBB,DYRK1A,GNL3L,DHX29,POLR2D,NIN,ACLY,PAN3,RABL6,XRCC2,KIFC3,EPRS1,LYN,DDX42,DDX20,DDX51,VCP,GBP2,DHX34,CSNK1G2,CDK4,THG1L,SMC1A,FOXRED2,G3BP1,GNL2,MCM6,AQR,DHCR7,CLCN6,ARL8B,DDX18,UBA2,CHD7,RRM1,PI4KA,CCT3,CDK7,PKM,KIF2A,ATAD3B,GNAQ,IRAK1,PIK3C2B,IP6K1,NAT10,EP400,MTHFD1,ADSS2,MECP2,PRKAR2B,DDX54,N4BP2,XRCC6,MBD1,CSNK2A2,DDX3X,LBR,HDGF,MCM2,PFAS,CHEK1,POLE,PPIP5K2,CSK,DNAJA1,DYNC1H1,TSR1,SLC19A1,CLCN7,STK17A,UBE2N,BAZ1B,TLK1,GFM1,TCP1,CCT5,CDK6,CHD3,RAN,HSPA4,AK2,MSH2,CDK12,GLYR1,MCM3,PAICS,DDX1,CTPS1,DDX49,TXNRD1,ATAD3A,ARF6,CCT2,HSP90AB1,DUS3L,SMG1,DHX37,SMARCA4,PRKDC,YES1,PRMT1,FARSA,XRCC5,DDX5,DDX46,EIF4A3,ACTG1,DDX39A,DDX56,NOLC1,SNRNP200,AB*

			CF1,ACACA,HSPD1,PIM2,GAPDH,EIF5B,PGD,MYH10,HSPH1,TGFBR1,ATP13A3,THRAP3,RUNX1,EIF4G1,HSP90AA1,MDN1,SQLE,MCM4,KIF1A,BAG1,MAT2A,DDX21,MCM7,HMGCR,GLUL,ACTB,DHX9,HNRNPU,MCM5,DHCR24,HSPA8
GO:0043021	ribonucleoprotein complex binding	1.4257583737685725e-14	*SNRPB,ERAL1,SRP72,EZH2,NVL,OXA1L,PQBP1,MRRF,EEF2,PRPF6,DHX33,LETM1,MTOR,BAG6,EIF4B,SNRPA,NAA15,SBDS,C1QBP,PRMT5,TMEM223,BOP1,WDR12,DHX29,SNRPD1,GEMIN5,ZNF622,DDX3X,PRMT7,NCLN,DDX5,EIF4A3,EIF5A,NOLC1,ABCF1,RBM3,PES1,SERBP1,NPM1,ETF1,LARP1,DHX9,HNRNPU*
GO:0019899	enzyme binding	2.1855488246916012e-14	*NOP14,TMEM127,MSH3,MACROH2A1,MTA2,TIRAP,CBX3,PHB,DNM1L,RHEB,PPP6R3,SMG5,BRD4,SH3YL1,CHCHD3,MYO16,MCM10,RANBP3,CASP8,PSMA3,MAD2L2,PATZ1,PPP3R1,TPRN,PAFAH1B1,RAB7A,BICD1,ANAPC7,GAB2,TOP2B,CLTC,EEF2,SPRY2,RNF40,CUL3,ARPP19,MEF2C,TCF3,MAF1,CCNY,ZFYVE26,PEBP1,MAPK1,CASP3,ATP2A2,IPO9,PKP3,STRIP1,CTR9,UHRF1BP1,RPRD2,IKZF3,KANSL1,VDAC1,TFAP4,FAF1,HNRNPA0,BAG6,HSPA9,TCF7L2,UBE2L3,SETMAR,RPTOR,PPP1R10,TNF,RABGGTB,PPP2R5A,CLTA,ATP6V1G1,PPP1CC,CAVIN2,CDC27,HERC2,AKAP8,RCC1,C1QBP,CRK,PAK2,SNX9,PCBP2,WDR70,ARHGAP6,ZNF326,TUBB,CUL4A,PSMD1,CDC37,RILP,WAC,ARHGEF2,BRCA2,RANBP1,NIN,PAF1,EPRS1,UBE4B,HNRNPUL1,CUTA,LYN,APC,ELOF1,PELP1,SLC12A2,EZR,DDX20,MMS19,TAF9,YWHAG,ELAVL1,VCP,CSTB,BEX4,DVL2,AGO2,TBC1D14,SAE1,IGF2R,UBA2,USP37,RNF138,HMGB1,IRAK1,PUS7,NAT10,SNU13,SKI,PRKAR2B,PCNA,CDC25A,DIAPH1,IQGAP2,TNPO2,MCM2,JUND,AATF,SLC25A5,ARPC4,CSK,DNAJA1,SUMO3,XPO1,PHACTR1,SERPINE1,TPR,UBE2N,NRIP1,TCP1,YWHAB,ENO1,FAM83H,UBC,MSH2,CDK12,TNPO1,CUL1,LTBR,CASC3,KPNB1,ATP6V0A1,ARF6,RBBP4,CNPPD1,CCT2,HSP90AB1,PA2G4,SMARCA4,PRKDC,YES1,PRMT1,MALT1,HMGA1,XRCC5,ANK1,LMNB1,IPO7,ACTG1,CDT1,NOP56,CORO1C,KHDRBS1,HNRNPD,LRPPRC,RANBP2,HSPD1,YBX1,AXIN1,GDI2,ANP32B,IPO5,NPM1,CITED2,WDR43,TFRC,TRIM28,NEFH,U2AF2,SRSF2,HSP90AA1,NOP58,RESF1,BAG1,SQSTM1,SFPQ,KCNH2,SRSF3,EIF3A,BTG1,GCLM,ACTB,DHX9,HNRNPU,NCL,EGR1,DHCR24,HSPA8*
GO:0003724	RNA helicase activity	4.95583457347062e-14	*MTREX,DHX38,DDX23,DHX16,DHX33,DHX15,DHX30,DDX10,DHX29,DDX42,DDX20,DDX51,DHX34,G3BP1,AQR,DDX18,DDX54,DDX3X,DDX1,DDX49,DHX37,DDX5,DDX46,EIF4A3,DDX39A,DDX56,SNRNP200,DDX21,DHX9*
GO:0005524	ATP binding	7.582381189180597e-14	*TXK,ACSF3,MSH3,TRIP13,DNAJA2,MMAB,RAD51C,UBE2Q1,MTREX,FES,ULK3,NVL,KIF26B,MYO16,ATP11A,PSMC3,TIMM44,DHX38,GART,DDX23,TRNT1,PPP5C,DHX16,TOP2B,CCT6A,AFG3L2,CCT8,KIF5B,CMPK1,NOL9,MVK,PLK4,SLK,PEBP1,ACTR8,PSMC5,STK35,MAPK1,ATP2A2,ABCF3,DHX33,DHX15,MTOR,DHX30,SMARCA5,HSPA9,MCCC1,GMPS,ASCC3,UBE2L3,PSMC2,DDX10,SETX,STK24,RIOK2,STK25,PAK2,MCCC2,UCK2,DYRK1A,DHX29,ACLY,PAN3,XRCC2,KIFC3,EPRS1,LYN,DDX42,DDX20,DDX51,VCP,DHX34,CSNK1G2,CDK4,THG1L,SMC1A,G3BP1,MCM6,AQR,CLCN6,DDX18,UBA2,CHD7,RRM1,PI4KA,CCT3,CDK7,PKM,KIF2A,ATAD3B,IRAK1,PIK3C2B,IP6K1,NAT10,EP400,MTHFD1,DDX54,N4BP2,XRCC6,CSNK2A2,DDX3X,MCM2,PFAS,CHEK1,PPIP5K2,CSK,DNAJA1,DYNC1H1,CLCN7,STK17A,UBE2N,BAZ1B,TLK1,TCP1,CCT5,CDK6,CHD3,HSPA4,AK2,MSH2,CDK12,MCM3,PAICS,DDX1,CTPS1,DDX49,ATAD3A,CCT2,HSP90AB1,SMG1,DHX37,SMARCA4,PRKDC,YES1,FARSA,XRCC5,DDX5,*

			DDX46,EIF4A3,ACTG1,DDX39A,DDX56,NOLC1,SNRNP200,ABCF1,ACACA,HSPD1,PIM2,MYH10,HSPH1,TGFBR1,ATP13A3,THRAP3,RUNX1,EIF4G1,HSP90AA1,MDN1,MCM4,KIF1A,MAT2A,DDX21,MCM7,GLUL,ACTB,DHX9,HNRNPU,MCM5,HSPA8
**BP**			
GO:0044260	cellular macromolecule metabolic process	8.282456461504237e-57	*SNRPB,DAP3,MSH3,TRIP13,CHTOP,ZNF274,GYG1,KMT2B,RNASEH1,SUPT7L,MACROH2A1,MUS81,MTA2,TIRAP,RAD51C,MTREX,OTUD6B,NACA,PHB,METTL3,EZH2,DCLRE1C,RFWD3,PAXIP1,PPP6R3,FUT8,SMG5,BRD4,STT3A,DHDDS,MRPL1,MRPL11,RPUSD4,DENR,CDC123,RNASEH2C,HROB,MRPS30,NVL,HNRNPL,PWP1,DOLPP1,MCM10,CHRAC1,KMT2D,PSMA3,PSMD3,MAD2L2,MRRF,POLDIP2,PSMC3,FBXO45,ARID2,RNPS1,USP11,USP14,DNMT1,PPP5C,BAP1,ANAPC7,SUB1,GTF2H1,TOP2B,LRWD1,EIF4EBP2,CCT6A,KAT7,EEF2,AURKAIP1,WDR5,CCT8,BCCIP,RNF40,CUL3,NONO,UBR3,TCF3,TEX15,GID8,CLSPN,NBAS,ZFYVE26,ZC3H4,GSPT1,GRSF1,UBQLN4,ACTR8,PSMC5,ZC3HAV1,MAPK1,ZNRF1,EEF1D,PKP3,PCLAF,CERS2,DHX33,CTR9,UIMC1,PUM1,MTOR,KANSL1,SMG9,SMARCA5,H2AW,EIF3D,PRMT6,FAF1,HNRNPA0,BAG6,DHFR,TCF7L2,MRPL15,PRAME,ASCC3,UBE2L3,THOC1,PSMC2,MCMBP,SETMAR,PPP1R10,MRPS35,HECTD1,TNF,EIF4B,WDR33,TRMT61A,IGF2BP3,PUM2,MT-TL1,BACH1,RBM10,SETX,TRMT2A,METTL8,APEX1,EDC4,ARID1A,PPP1CC,ZC3H14,B4GALT5,CDC27,HERC2,CTSL,C1QBP,VPS72,TICRR,EOGT,PRMT5,PCBP2,ADD1,TRMT6,RNF126,BAZ1A,WDR70,ALG8,RAD23B,TAF4,RBM8A,SETD2,FEN1,CHAF1A,TRMO,SLBP,CUL4A,JADE2,NUCKS1,SDCBP,PSMD1,SSB,GNL3L,MT-TF,TFIP11,PSMB2,WAC,EIF3G,DHX29,BRCA2,POLR2D,ZDHHC5,EIF3M,WTAP,BCL7B,SNRPD1,PAN3,PAF1,ZC3H18,FASTKD2,TCOF1,XRCC2,PDS5A,BRCC3,SMARCB1,TRIR,CREBBP,EXOSC3,EPRS1,COPS3,UBE4B,LSM14A,CNOT1,ASH1L,GEMIN5,APC,CAPN1,ELOF1,WDR6,NFYC,XRN2,TRUB2,MMS19,ELOA,TAF9,ELAVL1,VCP,DNAJB12,RYBP,DHX34,MRPS2,SETD1A,WDR82,SMC1A,AGO2,FOXRED2,MCM6,IER3,RBM15B,USP37,HNRNPA1,GAR1,MAGOH,PBRM1,CTCF,ANKRD17,RRM2,RRM1,NSD1,CCT3,CDK7,RNF138,SETD1B,PKM,HMGB1,MAEA,BZW1,SMARCD1,CANX,YY1,MLLT10,PUS7,NAT10,TRMT10C,EP400,LEPR,MECP2,CSDE1,N4BP2,TARDBP,LARP4,XRCC6,RPL22,PCNA,CSNK2A2,UPF2,DDX3X,EIF4G2,MCM2,HCFC1,CHEK1,AATF,CLN6,ZMPSTE24,PRMT7,POLE,TRRAP,EXOSC9,XPO1,TPR,ENC1,ZFP36L2,KEAP1,TFB2M,UBE2N,MRTO4,GFM1,DDB1,LRRC47,NASP,CHST3,TCP1,CENPF,CCT5,POLE3,CDC5L,ST3GAL2,RAN,BEND3,UBC,WDR81,PPP2CA,MSH2,MAN2A2,KMT2A,MCM3,GRWD1,ZNF598,GATAD2A,DKC1,DDX1,CUL1,DDX49,CASC3,SSRP1,ARID1B,HNRNPAB,USP36,RBBP4,NCBP1,CCT2,HSP90AB1,EIF3B,DUS3L,PA2G4,SMG1,PRPF19,SMARCA4,EIF3J,PRKDC,PRMT1,HNRNPC,WWP2,SUPT16H,HMGA1,FARSA,CELF1,XRCC5,DDX5,DEK,EIF4A3,NDST1,TRIP12,CIZ1,CDT1,KHDRBS1,HNRNPD,LRPPRC,EIF5A,SUPT6H,NOLC1,CBFA2T3,ABCF1,NSUN2,AP5Z1,HSPD1,GAPDH,YBX1,LIN28B,EIF5B,RBM3,AXIN1,PABPC1,SERBP1,SF3B3,THRAP3,NPM1,RIF1,ETF1,TFRC,EIF4G1,TRIM28,BCLAF1,ECPAS,HSP90AA1,CAPRIN1,TRMT1,MCM4,HNRNPA2B1,LARP1,SQSTM1,SFPQ,PABPC4,HNRNPM,EIF3A,MCM7,SMARCC1,MYC,SET,BTG1,IGF2BP1,FOS,MYB,KHSRP,FUS,ILF3,GLUL,ACTB,DHX9,HNRNPU,MCM5,NCL*

GO:0019219	regulation of nucleobase-containing compound metabolic process	1.3663776749014435e-50	*HRAS,TXK,MITF,JMJD1C,MSH3,ZNF274,KMT2B,SUPT7L,MACROH2A1,BRD9,MED16,NOL11,BICRA,MTA2,TIRAP,CWC22,RIOX1,PHF5A,NACA,MED6,PRXL2C,CBX3,PHB,METTL3,EZH2,TCF20,DNM1L,PAXIP1,LRRFIP1,GPATCH3,INTS6,SMG5,BRD4,ZNF581,SPIN4,ZNF74,CTDP1,MED1,NVL,HNRNPL,PWP1,CHRAC1,KMT2D,TAF4B,PQBP1,PSPC1,CEP350,MAD2L2,PSMC3,ZBTB40,ADNP2,ARID2,PATZ1,TRIM44,RNPS1,LHX4,ZNF512B,RBL1,PPP3R1,HIF1AN,SS18L1,MED29,HNRNPH1,USP14,LIMD1,DNMT1,LYL1,BAP1,SUB1,GTF2H1,TOP2B,LYAR,CCNH,NAB2,CCT6A,KAT7,WDR5,CCT8,FUBP3,CUL3,NONO,MEF2C,TCF3,TEX15,MAF1,NBAS,PRPF6,ZC3H4,ZNF26,ZNF24,GRSF1,UBQLN4,ACTR8,PSMC5,ZC3HAV1,MAPK1,PKP3,ABT1,DHX33,CTR9,NCOA5,FADS1,ZFP91,UIMC1,PUM1,MTOR,IKZF3,KANSL1,SPEN,SMARCA5,ZNF45,RSF1,TFAP4,PRMT6,FAF1,HNRNPA0,ZNF131,SBNO1,ZNF431,MTDH,TCF7L2,ZNF239,PRAME,UBE2L3,THOC1,SETMAR,RPTOR,PPP1R10,TNF,RAVER1,IGF2BP3,MED28,ELL2,PUM2,SSBP3,SNRPA,ZNF586,BACH1,RBM10,SETX,NFILZ,AP3D1,APEX1,BPTF,ICE1,RIOK2,NAA15,ARID1A,PITHD1,ZC3H14,SRSF10,C1QBP,VPS72,ZNF587B,CRK,TICRR,MED15,PRMT5,PCBP2,RBMX,BAZ1A,WDR70,MLLT3,TAF4,RBM14,RBM8A,KDM3B,LRP8,SETD2,ZNF326,INTS13,ZNF282,DYRK1A,TFDP2,CUL4A,JADE2,NUCKS1,SDCBP,GNL3L,NSRP1,TFIP11,WAC,ARHGEF2,BRCA2,POLR2D,WTAP,COPS2,BCL7B,RBM42,ZFX,PAN3,MED13L,PAF1,ZC3H18,FASTKD2,PHB2,PDS5A,BRCC3,ZNF75A,SMARCB1,CREBBP,EXOSC3,NUP98,CNOT1,ASH1L,PLAGL2,NFYC,SF1,PELP1,EZR,DDX20,MMS19,ELOA,TAF9,ELAVL1,UTP15,VCP,RYBP,SAFB2,DHX34,PSIP1,RBM12,CDK4,DVL2,POLR3C,RBM25,WDR82,AGO2,E2F4,RNF220,MCM6,IER3,ADNP,NFKB1,UBTF,BRD2,CHD7,RBM15B,USP37,HNRNPA1,PPARGC1B,MAGOH,PBRM1,CTCF,HHEX,ANKRD17,ELF1,ZNF614,NSD1,CCT3,CDK7,FOXK2,HMGB1,SMARCD1,YY1,MLLT10,IRAK1,NAT10,SRSF6,EP400,CEBPZ,MECP2,CSDE1,MBNL1,SKI,DDX54,TARDBP,XRCC6,PCNA,MBD1,DDX3X,HDGF,ZNF789,EIF4G2,MCM2,JUND,HCFC1,CHEK1,AATF,ZMPSTE24,SLTM,UBP1,TRRAP,EXOSC9,NUP62,PPIA,TPR,SRSF7,TGFBRAP1,ZFP36L2,SF3B4,PPRC1,SLC38A2,KEAP1,TFB2M,UBE2N,BAZ1B,NRIP1,MYBBP1A,BACH2,PITX1,ASXL2,TCP1,CENPF,RALY,ENO1,CCT5,FUBP1,POLE3,CDK6,CDC5L,CHD3,BEND3,ZNF787,TFAM,PPP2CA,SSU72,MSH2,CDK12,ZBTB2,GLYR1,KMT2A,MCM3,NOSTRIN,GATAD2A,DKC1,EWSR1,DDX49,CASC3,ARID1B,CPSF7,ZNF33B,HNRNPAB,KAT6A,USP36,RBBP4,SAFB,NCBP1,HEATR1,CCT2,HSP90AB1,PA2G4,HNRNPK,SMG1,PRPF19,SMARCA4,PRKDC,YES1,ZNF121,TRA2B,HNRNPC,MALT1,WWP2,SUPT16H,HMGA1,CELF1,RBMXL1,XRCC5,DDX5,UTP4,DEK,LMO2,EIF4A3,TRIP12,CIZ1,CDT1,KHDRBS1,HNRNPD,LRPPRC,EIF5A,CTDSP1,SUPT6H,DNAJB6,NOLC1,SREBF2,CBFA2T3,MGA,NSUN2,PIM2,YBX1,LIN28B,RBM3,AXIN1,RREB1,EP300,TGFBR1,DAZAP1,RRP1B,FOSB,SRRT,PABPC1,SERBP1,AHNAK,SF3B3,PTMA,THRAP3,MLLT1,HNRNPF,NPM1,RIF1,CITED2,SON,WDR43,TFRC,RUNX1,TRIM28,U2AF2,NR2F2,BCLAF1,SRSF2,HSP90AA1,NFATC3,ZNF521,MCM4,TRIM24,RESF1,HNRNPA2B1,LARP1,SQSTM1,PTBP1,CCAR1,DDX21,SFPQ,PABPC4,HNRNPM,KCNH2,SRSF3,MCM7,SMARCC1,MYC,SET,BTG1,IGF2BP1,FOS,MYB,KHSRP,ZEB2,RELN,FUS,ILF3,ACTB,DHX9,HNRNPU,MCM5,NCL,EGR1,HSPA8*
GO:0051171	regulatio	3.645982953	*HRAS,TXK,MITF,JMJD1C,MSH3,CHTOP,ZNF274,KMT2B,SUPT7L,MACROH2A1,BRD9,MED16,NOL11,BICRA*

	n of nitrogen compound metabolic process	961369e-48	,MTA2,TIRAP,CWC22,OTUD6B,RIOX1,PHF5A,NACA,MED6,PRXL2C,CBX3,PHB,METTL3,EZH2,TCF20,DNM1L,PAXIP1,LRRFIP1,GPATCH3,INTS6,PPP6R3,SMG5,BRD4,ZNF581,SPIN4,ZNF74,RPUSD4,CDC123,CTDP1,MED1,NVL,HNRNPL,PWP1,CHRAC1,KMT2D,CASP8,TAF4B,PSMA3,PQBP1,PSMD3,PSPC1,CEP350,MAD2L2,PSMC3,ZBTB40,ADNP2,ARID2,PATZ1,TRIM44,RNPS1,LHX4,ZNF512B,RBL1,PPP3R1,HIF1AN,SS18L1,MED29,HNRNPH1,USP14,RAB7A,LIMD1,DNMT1,LYL1,BAP1,SUB1,GTF2H1,BUB3,TOP2B,LYAR,SLC39A10,CCNH,NAB2,EIF4EBP2,CCT6A,KAT7,CLTC,EEF2,AURKAIP1,WDR5,CCT8,FUBP3,BCCIP,SPRY2,RNF40,CUL3,ARPP19,NONO,MEF2C,UBR3,TCF3,TEX15,MAF1,CLSPN,NBAS,CCNY,CRIM1,PRPF6,ZC3H4,PEBP1,ZNF26,ZNF24,GSPT1,GRSF1,UBQLN4,ACTR8,PSMC5,ZC3HAV1,MAPK1,CASP3,PKP3,ABT1,DHX33,CTR9,NCOA5,FADS1,ZFP91,UIMC1,PUM1,MTOR,IKZF3,KANSL1,SPEN,SMARCA5,EIF3D,ZNF45,RSF1,TFAP4,DELE1,PRMT6,TSPYL5,FAF1,HNRNPA0,BAG6,ZNF131,SBNO1,ZNF431,DHFR,MTDH,TCF7L2,ZNF239,PRAME,UBE2L3,THOC1,PSMC2,SETMAR,RPTOR,PPP1R10,HECTD1,TNF,RAVER1,EIF4B,SAMSN1,PPP2R5A,IGF2BP3,MED28,ELL2,PUM2,SSBP3,SNRPA,ZNF586,BACH1,RBM10,SETX,NFILZ,AP3D1,METTL8,APEX1,BPTF,ICE1,RIOK2,NAA15,ARID1A,PITHD1,ZC3H14,SRSF10,AKAP8,C1QBP,VPS72,ZNF587B,CRK,TICRR,MED15,PAK2,PRMT5,SNX9,PCBP2,RBMX,BAZ1A,WDR70,MLLT3,RAD23B,TAF4,RBM14,RBM8A,KDM3B,LRP8,SETD2,ZNF326,INTS13,ZNF282,DYRK1A,TFDP2,CUL4A,JADE2,NUCKS1,SDCBP,PSMD1,CDC37,GNL3L,RILP,NSRP1,TFIP11,WAC,DHX29,ARHGEF2,IL17D,BRCA2,POLR2D,WTAP,COPS2,BCL7B,RBM42,ZFX,PAN3,MED13L,PAF1,ZC3H18,BIRC6,FASTKD2,PHB2,TCOF1,PDS5A,BRCC3,ZNF75A,SMARCB1,CREBBP,EXOSC3,EPRS1,COPS3,LSM14A,NUP98,CNOT1,LYN,ASH1L,GEMIN5,PLAGL2,APC,NFYC,SF1,PELP1,EZR,TRUB2,DDX20,MMS19,ELOA,TAF9,YWHAG,ELAVL1,UTP15,VCP,RYBP,SAFB2,DHX34,PSIP1,CSTB,BEX4,RBM12,CDK4,DVL2,POLR3C,RBM25,WDR82,AGO2,E2F4,RNF220,SAE1,MCM6,IER3,ADNP,UBA2,NFKB1,UBTF,BRD2,CHD7,RBM15B,USP37,HNRNPA1,PPARGC1B,MAGOH,PBRM1,CTCF,HHEX,ANKRD17,ELF1,ZNF614,NSD1,CCT3,CDK7,PKM,FOXK2,HMGB1,BZW1,SMARCD1,GNAQ,YY1,MLLT10,IRAK1,PUS7,NAT10,TRMT10C,SRSF6,EP400,CEBPZ,LEPR,MECP2,CSDE1,PARD3,MBNL1,SKI,PRKAR2B,DDX54,AZIN1,TARDBP,LARP4,XRCC6,ABI1,PCNA,CDC25A,MBD1,CSNK2A2,DDX3X,HDGF,ZNF789,VPS35,EIF4G2,MCM2,JUND,HCFC1,CHEK1,AATF,CLN6,ZMPSTE24,SLTM,UBP1,TRRAP,EXOSC9,CSK,DNAJA1,NUP62,XPO1,PPIA,SERPINE1,TPR,ENC1,SRSF7,TGFBRAP1,ZFP36L2,SF3B4,PPRC1,SLC38A2,KEAP1,TFB2M,UBE2N,BAZ1B,NRIP1,MYBBP1A,DDB1,BACH2,PITX1,ASXL2,TCP1,CENPF,YWHAB,RALY,ENO1,CCT5,FUBP1,POLE3,CDK6,CDC5L,CHD3,BEND3,ZNF787,TFAM,PPP2CA,SSU72,MSH2,CDK12,ZBTB2,GLYR1,KMT2A,MCM3,NOSTRIN,GATAD2A,DKC1,DDX1,EWSR1,DDX49,CASC3,ARID1B,CPSF7,ZNF33B,HNRNPAB,KAT6A,USP36,RBBP4,CNPPD1,SAFB,NCBP1,HEATR1,CCT2,HSP90AB1,EIF3B,DUS3L,PA2G4,HNRNPK,SMG1,PRPF19,SMARCA4,PRKDC,YES1,ZNF121,TRA2B,PRMT1,HNRNPC,MALT1,WWP2,SUPT16H,HMGA1,CELF1,RBMXL1,XRCC5,DDX5,PSME3,UTP4,DEK,LMO2,EIF4A3,TRIP12,IPO7,CIZ1,CDT1,CORO1C,KHDRBS1,HNRNPD,LRPPRC,EIF5A,CTDSP1,SUPT6H,DNAJB6,NOLC1,SREBF2,CBFA2T3,ABCF1,MGA,NSUN2,HSPD1,PIM2,GAPDH,YBX1,LIN28B

			,EIF5B,RBM3,AXIN1,ANP32B,IPO5,RREB1,EP300,TGFBR1,DAZAP1,RRP1B,FOSB,SRRT,PABPC1,SERBP1,AHNAK,SF3B3,PTMA,THRAP3,MLLT1,HNRNPF,NPM1,RIF1,CITED2,ETF1,SON,WDR43,TFRC,RUNX1,NQO1,EIF4G1,TRIM28,ACP5,U2AF2,NR2F2,BCLAF1,SRSF2,HSP90AA1,NFATC3,ZNF521,CAPRIN1,MCM4,TRIM24,RESF1,HNRNPA2B1,LARP1,SQSTM1,PTBP1,CCAR1,DDX21,SFPQ,PABPC4,HNRNPM,KCNH2,SRSF3,MCM7,SMARCC1,MYC,SET,BTG1,IGF2BP1,FOS,HMGCR,MYB,ODC1,KHSRP,ZEB2,RELN,FUS,ILF3,ACTB,DHX9,HNRNPU,MCM5,NCL,EGR1,DHCR24,HSPA8
GO:0031323	regulation of cellular metabolic process	1.3828894735545929e-47	HRAS,TXK,MITF,JMJD1C,MSH3,CHTOP,ZNF274,KMT2B,SUPT7L,MACROH2A1,BRD9,MED16,NOL11,BICRA,MTA2,TIRAP,CWC22,OTUD6B,RIOX1,PHF5A,NACA,MED6,PRXL2C,CBX3,PHB,METTL3,EZH2,TCF20,DNM1L,PAXIP1,LRRFIP1,GPATCH3,RHEB,INTS6,PPP6R3,SMG5,BRD4,ZNF581,SPIN4,ZNF74,RPUSD4,CDC123,CTDP1,MED1,NVL,HNRNPL,NLN,PWP1,CHRAC1,KMT2D,TAF4B,PQBP1,PSPC1,CEP350,MAD2L2,POLDIP2,PSMC3,ZBTB40,ADNP2,ARID2,PATZ1,TRIM44,RNPS1,LHX4,ZNF512B,RBL1,PPP3R1,HIF1AN,SS18L1,MED29,HNRNPH1,USP14,LIMD1,DNMT1,LYL1,BAP1,SUB1,GTF2H1,TOP2B,LYAR,SLC39A10,CCNH,NAB2,EXOC7,EIF4EBP2,CCT6A,KAT7,EEF2,WDR5,CCT8,FUBP3,BCCIP,SPRY2,CUL3,ARPP19,NONO,MEF2C,TCF3,TEX15,MAF1,CLSPN,NBAS,CCNY,PRPF6,ZC3H4,ZNF26,ZNF24,GSPT1,GRSF1,UBQLN4,ACTR8,PSMC5,ZC3HAV1,MAPK1,STAR,CASP3,PKP3,ABT1,DHX33,CTR9,NCOA5,FADS1,ZFP91,UIMC1,PUM1,MTOR,IKZF3,KANSL1,SPEN,VDAC1,SMARCA5,EIF3D,ZNF45,RSF1,TFAP4,PRMT6,FAF1,HNRNPA0,BAG6,ZNF131,SBNO1,ZNF431,DHFR,MTDH,TCF7L2,ZNF239,PRAME,UBE2L3,THOC1,SETMAR,RPTOR,PPP1R10,HECTD1,TNF,RAVER1,EIF4B,SAMSN1,PPP2R5A,IGF2BP3,MED28,ELL2,PUM2,SSBP3,SNRPA,ZNF586,BACH1,RBM10,SETX,NFILZ,AP3D1,METTL8,APEX1,BPTF,ATP6V1G1,ICE1,RIOK2,NAA15,ARID1A,PITHD1,ZC3H14,SRSF10,C1QBP,VPS72,ZNF587B,CRK,TICRR,MED15,CAPNS1,PAK2,PRMT5,SNX9,PCBP2,RBMX,BAZ1A,WDR70,MLLT3,RAD23B,TAF4,RBM14,RBM8A,KDM3B,LRP8,SETD2,ZNF326,INTS13,ZNF282,DYRK1A,TFDP2,CUL4A,JADE2,NUCKS1,SDCBP,CDC37,GNL3L,NSRP1,TFIP11,WAC,DHX29,ARHGEF2,BRCA2,POLR2D,WTAP,COPS2,BCL7B,RBM42,ZFX,PAN3,MED13L,PAF1,ZC3H18,FASTKD2,PHB2,TCOF1,GRB10,PDS5A,BRCC3,ZNF75A,SMARCB1,CREBBP,EXOSC3,EPRS1,INSIG1,LSM14A,NUP98,CNOT1,LYN,ASH1L,GEMIN5,PLAGL2,APC,CAPN1,WDR6,NFYC,SF1,PELP1,EZR,TRUB2,DDX20,MMS19,ELOA,TAF9,YWHAG,ELAVL1,UTP15,VCP,RYBP,SAFB2,DHX34,PSIP1,RBM12,CDK4,DVL2,POLR3C,RBM25,SCAP,WDR82,AGO2,E2F4,VPS26A,RNF220,TBC1D14,MCM6,IER3,ATP6V0D1,ADNP,NFKB1,UBTF,ZNF622,BRD2,CHD7,RBM15B,USP37,HNRNPA1,PPARGC1B,MAGOH,PBRM1,CTCF,HHEX,ANKRD17,ELF1,ZNF614,NSD1,RSL1D1,CCT3,CDK7,PPIF,PKM,FOXK2,HMGB1,BZW1,SMARCD1,GNAQ,YY1,MLLT10,IRAK1,PUS7,NAT10,TRMT10C,SRSF6,EP400,CEBPZ,LEPR,MECP2,CSDE1,PARD3,MBNL1,SKI,PRKAR2B,DDX54,TARDBP,LARP4,XRCC6,ABI1,PCNA,CDC25A,MBD1,CSNK2A2,DDX3X,HDGF,ZNF789,VPS35,EIF4G2,MCM2,JUND,HCFC1,CHEK1,AATF,ZMPSTE24,SLC25A5,SLTM,VAC14,UBP1,TRRAP,EXOSC9,CSK,DNAJA1,NUP62,XPO1,PPIA,TPR,ENC1,SRSF7,TGFBRAP1,ZFP36L2,SF3B4,STK17A,PPRC1,SLC38A2,KEAP1,TFB2M,UBE2N,BAZ1B,NRIP1,MYBBP1A,DDB1,BACH2,PITX1,ASXL2,TCP1,CENPF,YWHAB,RALY,ENO1

			,CCT5,FUBP1,POLE3,CDK6,CDC5L,CHD3,BEND3,WDR81,ZNF787,TFAM,PPP2CA,SSU72,MSH2,CDK12,ZBTB2,GLYR1,KMT2A,MCM3,NOSTRIN,SLC30A10,GATAD2A,DKC1,DDX1,EWSR1,DDX49,CASC3,ARID1B,CPSF7,ZNF33B,ATP6V0A1,HNRNPAB,KAT6A,USP36,RBBP4,CNPPD1,SAFB,NCBP1,HEATR1,CCT2,HSP90AB1,EIF3B,DUS3L,PA2G4,HNRNPK,SMG1,PRPF19,SMARCA4,PRKDC,YES1,ZNF121,TRA2B,PRMT1,HNRNPC,MALT1,WWP2,SUPT16H,HMGA1,CELF1,RBMXL1,XRCC5,DDX5,UTP4,DEK,LMO2,EIF4A3,TRIP12,IPO7,CIZ1,CDT1,CORO1C,KHDRBS1,HNRNPD,LRPPRC,EIF5A,CTDSP1,SUPT6H,RANBP2,DNAJB6,NOLC1,SREBF2,CBFA2T3,ABCF1,MGA,NSUN2,PIM2,GAPDH,YBX1,LIN28B,EIF5B,ATP6V1C1,RBM3,AXIN1,IPO5,RREB1,EP300,TGFBR1,DAZAP1,RRP1B,FOSB,SRRT,PABPC1,SERBP1,AHNAK,SF3B3,PTMA,THRAP3,MLLT1,HNRNPF,NPM1,RIF1,CITED2,ETF1,SON,WDR43,TFRC,RUNX1,EIF4G1,TRIM28,ACP5,U2AF2,NR2F2,BCLAF1,SRSF2,HSP90AA1,NFATC3,ZNF521,CAPRIN1,MCM4,TRIM24,RESF1,HNRNPA2B1,LARP1,SQSTM1,PTBP1,CCAR1,DDX21,SFPQ,PABPC4,HNRNPM,KCNH2,SRSF3,MCM7,SMARCC1,MYC,SET,BTG1,IGF2BP1,FOS,HMGCR,MYB,KHSRP,ZEB2,RELN,FUS,ILF3,ACTB,DHX9,HNRNPU,MCM5,NCL,EGR1,HSPA8
GO:0080090	regulation of primary metabolic process	2.662849153270026e-47	HRAS,TXK,MITF,JMJD1C,MSH3,CHTOP,ZNF274,KMT2B,SUPT7L,MACROH2A1,BRD9,MED16,NOL11,BICRA,MTA2,TIRAP,CWC22,OTUD6B,RIOX1,PHF5A,NACA,MED6,PRXL2C,CBX3,PHB,METTL3,EZH2,TCF20,DNM1L,PAXIP1,LRRFIP1,GPATCH3,INTS6,PPP6R3,SMG5,BRD4,ZNF581,SPIN4,ZNF74,RPUSD4,CDC123,CTDP1,MED1,NVL,HNRNPL,NLN,PWP1,CHRAC1,KMT2D,CASP8,TAF4B,PSMA3,PQBP1,PSMD3,PSPC1,CEP350,MAD2L2,PSMC3,ZBTB40,ADNP2,ARID2,PATZ1,TRIM44,RNPS1,LHX4,ZNF512B,RBL1,PPP3R1,HIF1AN,SS18L1,MED29,HNRNPH1,USP14,RAB7A,LIMD1,DNMT1,LYL1,BAP1,SUB1,GTF2H1,BUB3,TOP2B,LYAR,SLC39A10,CCNH,NAB2,EIF4EBP2,CCT6A,KAT7,CLTC,EEF2,AURKAIP1,WDR5,CCT8,FUBP3,BCCIP,SPRY2,RNF40,CUL3,ARPP19,NONO,MEF2C,UBR3,TCF3,TEX15,MAF1,CLSPN,NBAS,CCNY,CRIM1,PRPF6,ZC3H4,PEBP1,ZNF26,ZNF24,GSPT1,GRSF1,UBQLN4,ACTR8,PSMC5,ZC3HAV1,MAPK1,STAR,CASP3,PKP3,ABT1,CERS2,DHX33,CTR9,NCOA5,FADS1,ZFP91,UIMC1,PUM1,MTOR,IKZF3,KANSL1,SPEN,SMARCA5,EIF3D,ZNF45,RSF1,TFAP4,DELE1,PRMT6,TSPYL5,FAF1,HNRNPA0,BAG6,ZNF131,SBNO1,ZNF431,DHFR,MTDH,TCF7L2,ZNF239,PRAME,UBE2L3,THOC1,PSMC2,SETMAR,RPTOR,PPP1R10,HECTD1,TNF,RAVER1,EIF4B,SAMSN1,PPP2R5A,IGF2BP3,MED28,ELL2,PUM2,SSBP3,SNRPA,ZNF586,BACH1,RBM10,SETX,NFILZ,AP3D1,METTL8,APEX1,BPTF,ICE1,RIOK2,NAA15,ARID1A,PITHD1,ZC3H14,SRSF10,AKAP8,C1QBP,VPS72,ZNF587B,CRK,TICRR,MED15,PAK2,PRMT5,SNX9,PCBP2,RBMX,BAZ1A,WDR70,MLLT3,OPA3,RAD23B,TAF4,RBM14,RBM8A,KDM3B,GOLM1,LRP8,SETD2,ZNF326,INTS13,ZNF282,DYRK1A,TFDP2,CUL4A,JADE2,NUCKS1,SDCBP,PSMD1,CDC37,GNL3L,RILP,NSRP1,TFIP11,WAC,DHX29,ARHGEF2,IL17D,BRCA2,POLR2D,WTAP,COPS2,BCL7B,RBM42,ZFX,PAN3,MED13L,PAF1,ZC3H18,BIRC6,FASTKD2,PHB2,TCOF1,PDS5A,BRCC3,ZNF75A,SMARCB1,CREBBP,EXOSC3,EPRS1,COPS3,INSIG1,LSM14A,NUP98,CNOT1,LYN,ASH1L,GEMIN5,PLAGL2,APC,NFYC,SF1,PELP1,EZR,TRUB2,DDX20,MMS19,ELOA,TAF9,YWHAG,ELAVL1,UTP15,VCP,RYBP,SAFB2,DHX34,PSIP1,CSTB,BEX4,RBM12,CDK4,DVL2,POLR3C,RBM25,SCAP,WDR82,AGO2,E2F4

			,RNF220,SAE1,MCM6,DHCR7,IER3,ADNP,UBA2,NFKB1,UBTF,BRD2,CHD7,RBM15B,USP37,HNRNPA1,PPARGC1B,MAGOH,PBRM1,CTCF,HHEX,ANKRD17,ELF1,ZNF614,NSD1,CCT3,CDK7,PKM,FOXK2,HMGB1,BZW1,SMARCD1,GNAQ,YY1,MLLT10,IRAK1,PUS7,NAT10,TRMT10C,SRSF6,EP400,CEBPZ,LEPR,MECP2,CSDE1,PARD3,MBNL1,SKI,PRKAR2B,DDX54,AZIN1,TARDBP,LARP4,XRCC6,ABI1,PCNA,CDC25A,MBD1,CSNK2A2,DDX3X,HDGF,ZNF789,VPS35,EIF4G2,MCM2,JUND,HCFC1,CHEK1,AATF,CLN6,ZMPSTE24,SLTM,UBP1,TRRAP,EXOSC9,CSK,DNAJA1,NUP62,XPO1,PPIA,SERPINE1,TPR,ENC1,SRSF7,TGFBRAP1,ZFP36L2,SF3B4,PPRC1,SLC38A2,KEAP1,TFB2M,UBE2N,BAZ1B,NRIP1,MYBBP1A,DDB1,BACH2,PITX1,ASXL2,TCP1,CENPF,YWHAB,RALY,ENO1,CCT5,FUBP1,POLE3,CDK6,CDC5L,CHD3,BEND3,ZNF787,TFAM,PPP2CA,SSU72,MSH2,CDK12,ZBTB2,GLYR1,KMT2A,MCM3,NOSTRIN,GATAD2A,DKC1,DDX1,EWSR1,DDX49,CASC3,ARID1B,CPSF7,ZNF33B,HNRNPAB,KAT6A,USP36,RBBP4,CNPPD1,SAFB,NCBP1,HEATR1,CCT2,HSP90AB1,EIF3B,DUS3L,PA2G4,HNRNPK,SMG1,PRPF19,SMARCA4,PRKDC,YES1,ZNF121,TRA2B,PRMT1,HNRNPC,MALT1,WWP2,SUPT16H,HMGA1,CELF1,RBMXL1,XRCC5,DDX5,PSME3,UTP4,DEK,LMO2,EIF4A3,TRIP12,IPO7,CIZ1,CDT1,CORO1C,KHDRBS1,HNRNPD,LRPPRC,EIF5A,CTDSP1,SUPT6H,RANBP2,DNAJB6,NOLC1,SREBF2,CBFA2T3,ABCF1,MGA,NSUN2,HSPD1,PIM2,GAPDH,YBX1,LIN28B,EIF5B,RBM3,AXIN1,ANP32B,IPO5,RREB1,EP300,TGFBR1,DAZAP1,RRP1B,FOSB,SRRT,PABPC1,SERBP1,AHNAK,SF3B3,PTMA,THRAP3,MLLT1,HNRNPF,NPM1,RIF1,CITED2,ETF1,SON,WDR43,TFRC,RUNX1,NQO1,EIF4G1,TRIM28,U2AF2,NR2F2,BCLAF1,SRSF2,HSP90AA1,NFATC3,ZNF521,CAPRIN1,MCM4,TRIM24,RESF1,HNRNPA2B1,LARP1,SQSTM1,PTBP1,CCAR1,DDX21,SFPQ,PABPC4,HNRNPM,KCNH2,SRSF3,MCM7,SMARCC1,MYC,SET,BTG1,IGF2BP1,FOS,HMGCR,MYB,ODC1,KHSRP,ZEB2,RELN,FUS,ILF3,ACTB,DHX9,HNRNPU,MCM5,NCL,EGR1,DHCR24,HSPA8*
GO:0034660	ncRNA metabolic process	3.9518264366669384e-47	*HRAS,NOP14,PPAN,MACROH2A1,NOL11,UTP3,MTREX,METTL3,NOC4L,INTS6,RRP9,MRPL1,RPUSD4,NUP155,NVL,PWP1,WDR46,TRNT1,ZNF512B,RPUSD1,DUS1L,LYAR,TSR3,IMP4,NOB1,NOL9,ZC3H4,GRSF1,POLR1E,ABT1,WDR36,PUM1,MTOR,UTP18,NIFK,TNF,TRMT61A,ELL2,PUM2,WDR3,DDX10,TRMT2A,METTL8,ICE1,RIOK2,SBDS,WDR74,TRMT6,BOP1,INTS13,TRMO,WDR12,GTF3C6,UTP25,SSB,ZC3H7B,TCOF1,SMARCB1,TRIR,EXOSC3,WBP11,EPRS1,POLR1B,WDR6,PELP1,XRN2,TRUB2,URM1,DDX51,UTP15,DCAF13,THG1L,RRP12,WDR82,AGO2,RRP36,DDX18,NFKB1,CHD7,GAR1,RRS1,RSL1D1,CDK7,YY1,NOL8,PUS7,NAT10,TRMT10C,SNU13,SART1,RRP15,DDX54,DDX3X,ZMPSTE24,URB1,EXOSC9,TSR1,UTP20,EBNA1BP2,TFB2M,MRTO4,GTF3C4,LRRC47,DKC1,DDX1,THUMPD1,DDX49,RRP1,USP36,NCBP1,HEATR1,DUS3L,PA2G4,DHX37,SMARCA4,PRKDC,FARSA,DDX5,UTP4,EIF4A3,DDX56,NOP56,NOLC1,SREBF2,NSUN2,LIN28B,MPHOSPH10,PES1,RRP1B,SRRT,WDR43,MDN1,NFATC3,TRMT1,NOP58,HNRNPA2B1,DDX21,SRSF3,MYC,FOS,MYB,KHSRP,NCL,EGR1*
GO:0042254	ribosome biogenesis	4.738824971283475e-47	*NOP14,PPAN,ERAL1,NOL11,UTP3,MTREX,NOC4L,RRP9,MRPL1,RPUSD4,NVL,PWP1,WDR46,NOP16,RPUSD1,LYAR,TSR3,IMP4,NOB1,NOL9,ABT1,WDR36,SDAD1,DHX30,UTP18,NIFK,NIP7,WDR3,DDX10,RIOK2,SBDS,C1QBP,WDR74,BOP1,WDR12,URB2,CUL4A,UTP25,GNL3L,DHX29,FASTKD2,EXOSC3,WBP11,PELP1,XRN2,DDX51,UTP15,DCAF13,MRPS2,RRP12,GNL2,RR*

			P36,DDX18,ZNF622,CHD7,GAR1,RRS1,RSL1D1,NOL8,NAT10,SNU13,SART1,RRP15,DDX54,DDX3X,AATF,URB1,EXOSC9,TSR1,XPO1,UTP20,EBNA1BP2,TFB2M,MRTO4,MYBBP1A,RAN,GRWD1,DKC1,DDX49,RRP1,USP36,HEATR1,PA2G4,DHX37,PRKDC,XRCC5,UTP4,EIF4A3,SURF6,DDX56,NOP56,NOLC1,MPHOSPH10,PES1,RRP1B,NPM1,WDR43,MDN1,NOP58,DDX21,GLUL
GO:0044271	cellular nitrogen compound biosynthetic process	7.22575707518893e-47	*HRAS,TXK,ACSF3,MITF,JMJD1C,DAP3,TRIP13,ZNF274,KMT2B,SUPT7L,MACROH2A1,BRD9,MED16,NOL11,BICRA,MTA2,TIRAP,POLR3E,OTUD6B,RIOX1,PHF5A,NACA,POLR1C,MED6,CBX3,PHB,METTL3,EZH2,TCF20,PAXIP1,LRRFIP1,GPATCH3,INTS6,SMG5,BRD4,PTDSS2,MRPL1,ZNF581,SPIN4,ZNF74,MRPL11,RPUSD4,DENR,CDC123,HROB,MRPS30,CTDP1,MED1,NVL,SRM,HNRNPL,PWP1,CHRAC1,CRCP,KMT2D,TAF4B,PQBP1,PSPC1,CEP350,MAD2L2,MRRF,POLDIP2,PSMC3,ZBTB40,ADNP2,GART,ARID2,PATZ1,TRIM44,RNPS1,LHX4,ZNF512B,RBL1,PPP3R1,HIF1AN,SS18L1,MED29,USP14,LIMD1,DNMT1,LYL1,PPP5C,BAP1,SUB1,GTF2H1,TOP2B,LYAR,CCNH,NAB2,EIF4EBP2,CCT6A,KAT7,EEF2,AURKAIP1,WDR5,CCT8,FUBP3,DLAT,CUL3,CMPK1,NONO,MEF2C,TCF3,MAF1,PRPF6,ZC3H4,ZNF26,ZNF24,GSPT1,ACTR8,PSMC5,MAPK1,EEF1D,POLR1E,ABT1,PCLAF,CERS2,DHX33,CTR9,NCOA5,FADS1,ZFP91,UIMC1,PUM1,MTOR,IKZF3,KANSL1,SPEN,SMARCA5,EIF3D,ZNF45,RSF1,TFAP4,PRMT6,NIFK,FAF1,ZNF131,SBNO1,ZNF431,DHFR,MTDH,TCF7L2,ZNF239,MRPL15,PRAME,GMPS,ASCC3,UROD,UBE2L3,THOC1,RPTOR,MRPS35,TNF,EIF4B,IGF2BP3,MED28,ELL2,PUM2,MT-TL1,SSBP3,ZNF586,BACH1,RBM10,SETX,NFILZ,AP3D1,METTL8,APEX1,BPTF,ICE1,NAA15,ARID1A,PITHD1,B4GALT5,SRSF10,C1QBP,VPS72,ZNF587B,CRK,MED15,PRMT5,PCBP2,RBMX,BAZ1A,MLLT3,TAF4,RBM14,RBM8A,KDM3B,LRP8,SETD2,ZNF326,INTS13,UCK2,ZNF282,DYRK1A,PHF3,TFDP2,GTF3C6,SLBP,JADE2,NUCKS1,SDCBP,AMD1,GNL3L,CYB5B,IBA57,MT-TF,WAC,EIF3G,DHX29,ARHGEF2,BRCA2,POLR2D,EIF3M,COPS2,BCL7B,ZFX,ACLY,PAN3,MED13L,PAF1,FASTKD2,PHB2,TCOF1,ZNF75A,SMARCB1,CREBBP,EXOSC3,EPRS1,LSM14A,NUP98,CNOT1,ASH1L,GEMIN5,PLAGL2,POLR1B,ELOF1,NFYC,SF1,PELP1,XRN2,EZR,TRUB2,DDX20,MMS19,ELOA,TAF9,ELAVL1,UTP15,VCP,RYBP,SAFB2,PSIP1,CDK4,DVL2,POLR3C,MRPS2,WDR82,AGO2,E2F4,RNF220,ADNP,NFKB1,UBTF,BRD2,CHD7,HNRNPA1,GAR1,PPARGC1B,MAGOH,PBRM1,CTCF,HHEX,RRM2,RRM1,ELF1,ZNF614,NSD1,CCT3,CDK7,PKM,FOXK2,HMGB1,BZW1,SMARCD1,YY1,MLLT10,IRAK1,PUS7,NAT10,TRMT10C,EP400,MTHFD1,ADSS2,CEBPZ,MECP2,CSDE1,SKI,DDX54,AZIN1,TARDBP,LARP4,XRCC6,RPL22,PCNA,MBD1,DDX3X,HDGF,ZNF789,VPS35,ATP5MC3,EIF4G2,JUND,PFAS,HCFC1,CHEK1,AATF,ZMPSTE24,SLTM,UBP1,POLE,TRRAP,EXOSC9,POLR2A,NUP62,PPIA,TPR,ENC1,TGFBRAP1,ZFP36L2,PPRC1,KEAP1,TFB2M,UBE2N,BAZ1B,NRIP1,VAPA,GFM1,MYBBP1A,BACH2,PITX1,GTF3C4,LRRC47,ASXL2,TCP1,CENPF,RALY,ENO1,CCT5,FUBP1,POLE3,CDK6,CDC5L,CHD3,ST3GAL2,BEND3,ZNF787,TFAM,AK2,PPP2CA,SSU72,CDK12,ZBTB2,DIDO1,GLYR1,KMT2A,NOSTRIN,PAICS,ZNF598,CERS6,GATAD2A,DKC1,DDX1,CTPS1,EWSR1,CASC3,ARID1B,ZNF33B,HNRNPAB,KAT6A,RBBP4,SAFB,NCBP1,HEATR1,CCT2,HSP90AB1,EIF3B,DUS3L,PA2G4,HNRNPK,SMARCA4,EIF3J,PRKDC,YES1,ZNF121,HNRNPC,MALT1,WWP2,SUPT16H,HMGA1,FARSA,CELF1,XRCC*

			5,DDX5,UTP4,DEK,LMO2,EIF4A3,DDX56,KHDRBS1,HNRNPD,LRPPRC,EIF5A,CTDSP1,SUPT6H,DNAJB6,NOLC1,SREBF2,CBFA2T3,ABCF1,ACACA,MGA,PIM2,GAPDH,YBX1,EIF5B,RBM3,AXIN1,RREB1,EP300,TGFBR1,RRP1B,FOSB,SRRT,PABPC1,SF3B3,PTMA,THRAP3,MLLT1,ELOVL6,MT-ND1,NPM1,RIF1,CITED2,ETF1,WDR43,TFRC,RUNX1,NQO1,EIF4G1,TRIM28,ACP5,NR2F2,BCLAF1,SRSF2,HSP90AA1,NFATC3,ZNF521,CAPRIN1,TRIM24,RESF1,HNRNPA2B1,LARP1,SQSTM1,CCAR1,DDX21,SFPQ,PABPC4,POLR1A,KCNH2,EIF3A,SMARCC1,MYC,SET,BTG1,IGF2BP1,FOS,GCLM,SPTA1,MYB,ODC1,KHSRP,ZEB2,RELN,FUS,ILF3,ACTB,DHX9,HNRNPU,NCL,EGR1,HSPA8
GO:1901576	organic substance biosynthetic process	2.0323421997065126e-44	*HRAS,TXK,ACSF3,MITF,JMJD1C,DAP3,TRIP13,ZNF274,GYG1,KMT2B,SUPT7L,MACROH2A1,BRD9,MED16,FADS2,NOL11,BICRA,MTA2,TIRAP,POLR3E,OTUD6B,RIOX1,PHF5A,NACA,POLR1C,MED6,CBX3,PHB,METTL3,EZH2,TCF20,PAXIP1,LRRFIP1,GPATCH3,INTS6,FUT8,SMG5,BRD4,STT3A,PTDSS2,DHDDS,MRPL1,ZNF581,SPIN4,ZNF74,SH3YL1,MRPL11,RPUSD4,DENR,CDC123,HROB,MRPS30,CTDP1,MED1,NVL,SRM,HNRNPL,NLN,PWP1,DOLPP1,CHRAC1,CRCP,KMT2D,TAF4B,PQBP1,PSPC1,CEP350,MAD2L2,MRRF,POLDIP2,PSMC3,ZBTB40,ADNP2,GART,ARID2,PATZ1,TRIM44,RNPS1,LHX4,ZNF512B,RBL1,PPP3R1,HIF1AN,SS18L1,MED29,USP14,LIMD1,DNMT1,LYL1,PPP5C,BAP1,SUB1,GTF2H1,TOP2B,LYAR,CCNH,NAB2,EIF4EBP2,CCT6A,KAT7,CLTC,EEF2,AURKAIP1,WDR5,CCT8,FUBP3,DLAT,CUL3,ARPP19,CMPK1,NONO,MEF2C,TCF3,MAF1,MVK,PRPF6,ZC3H4,ZNF26,ZNF24,GSPT1,ACTR8,PSMC5,AGPAT3,MAPK1,STAR,EEF1D,POLR1E,ABT1,PCLAF,PDSS1,CERS2,DHX33,CTR9,NCOA5,FADS1,ZFP91,UIMC1,PUM1,MTOR,IKZF3,KANSL1,SPEN,LCLAT1,GOT2,SMARCA5,EIF3D,ZNF45,RSF1,TFAP4,PRMT6,NIFK,FAF1,ZNF131,PCYT1A,SBNO1,ZNF431,DHFR,MTDH,TCF7L2,ZNF239,NSDHL,MRPL15,PRAME,GMPS,ASCC3,UROD,UBE2L3,THOC1,RPTOR,MRPS35,TNF,EIF4B,IGF2BP3,MED28,ELL2,PUM2,MT-TL1,SSBP3,ZNF586,BACH1,RBM10,SETX,NFILZ,AP3D1,METTL8,APEX1,BPTF,ICE1,NAA15,ARID1A,PITHD1,B4GALT5,SRSF10,C1QBP,VPS72,ZNF587B,CRK,MED15,EOGT,PRMT5,PCBP2,PTDSS1,RBMX,BAZ1A,GPX4,MLLT3,ALG8,TAF4,RBM14,RBM8A,KDM3B,LRP8,SETD2,ZNF326,INTS13,UCK2,ZNF282,DYRK1A,PHF3,TFDP2,GTF3C6,SLBP,JADE2,NUCKS1,SDCBP,AMD1,AGPAT5,GNL3L,IBA57,MT-TF,WAC,EIF3G,ADI1,DHX29,ARHGEF2,BRCA2,POLR2D,ZDHHC5,EIF3M,COPS2,BCL7B,ZFX,ACLY,PAN3,MED13L,PAF1,FASTKD2,PHB2,TCOF1,ZNF75A,SMARCB1,CREBBP,EXOSC3,EPRS1,INSIG1,LSM14A,NUP98,CNOT1,ASH1L,GEMIN5,PLAGL2,POLR1B,ELOF1,NFYC,SF1,PELP1,XRN2,EZR,TRUB2,DDX20,MMS19,SGPP2,ELOA,TAF9,ELAVL1,UTP15,VCP,RYBP,SAFB2,PSIP1,CSNK1G2,CDK4,DVL2,POLR3C,MRPS2,SCAP,WDR82,AGO2,E2F4,RNF220,DHCR7,ADNP,NFKB1,UBTF,BRD2,CHD7,HNRNPA1,GAR1,PPARGC1B,MAGOH,PBRM1,CTCF,HHEX,RRM2,RRM1,PI4KA,ELF1,ZNF614,NSD1,CCT3,CDK7,PKM,FOXK2,HMGB1,BZW1,SMARCD1,YY1,MLLT10,IRAK1,PUS7,PIK3C2B,IP6K1,NAT10,TRMT10C,EP400,AASDHPPT,MTHFD1,ADSS2,CEBPZ,LEPR,MECP2,CSDE1,SKI,TKT,DDX54,AZIN1,TARDBP,LARP4,XRCC6,RPL22,PCNA,MBD1,DDX3X,IDI1,LBR,HDGF,ZNF789,VPS35,ATP5MC3,EIF4G2,JUND,PFAS,HCFC1,CHEK1,AATF,ZMPSTE24,SLTM,VAC*

			14,UBP1,POLE,TRRAP,PPIP5K2,EXOSC9,POLR2A,NUP62,PPIA,TPR,ENC1,TGFBRAP1,ZFP36L2,PPRC1,KEAP1,TFB2M,UBE2N,BAZ1B,NRIP1,VAPA,GFM1,MYBBP1A,DDB1,BACH2,PITX1,GTF3C4,LRRC47,ASXL2,CHST3,TCP1,CENPF,RALY,ENO1,CCT5,FUBP1,POLE3,CDK6,CDC5L,CHD3,ST3GAL2,BEND3,ZNF787,TFAM,AK2,PPP2CA,SSU72,CDK12,ZBTB2,DIDO1,GLYR1,MAN2A2,KMT2A,NOSTRIN,PAICS,ZNF598,CERS6,GATAD2A,DKC1,DDX1,CTPS1,EWSR1,CASC3,ARID1B,ZNF33B,HNRNPAB,KAT6A,RBBP4,SAFB,NCBP1,HEATR1,CCT2,HSP90AB1,EIF3B,DUS3L,PA2G4,HNRNPK,SMG1,FDFT1,PRPF19,SMARCA4,EIF3J,PRKDC,YES1,ZNF121,PRMT1,HNRNPC,MALT1,WWP2,SUPT16H,HMGA1,FARSA,CELF1,XRCC5,DDX5,UTP4,DEK,LMO2,LSS,EIF4A3,NDST1,DDX56,SLC38A1,KHDRBS1,HNRNPD,LRPPRC,EIF5A,CTDSP1,SUPT6H,RANBP2,DNAJB6,NOLC1,SREBF2,CBFA2T3,ABCF1,ACACA,MGA,PIM2,GAPDH,YBX1,EIF5B,PCYT2,RBM3,PGD,AXIN1,RREB1,HSPH1,EP300,TGFBR1,RRP1B,FOSB,SRRT,PABPC1,SF3B3,PTMA,THRAP3,MLLT1,ELOVL6,MT-ND1,NPM1,RIF1,CITED2,ETF1,WDR43,TFRC,RUNX1,EIF4G1,TRIM28,NR2F2,BCLAF1,SRSF2,HSP90AA1,NFATC3,ZNF521,CAPRIN1,SQLE,TRIM24,RESF1,HNRNPA2B1,LARP1,SQSTM1,CCAR1,MAT2A,DDX21,HMGCS1,SFPQ,SCD,PABPC4,POLR1A,KCNH2,EIF3A,SMARCC1,MYC,SET,BTG1,IGF2BP1,FOS,GCLM,HMGCR,SPTA1,MYB,ODC1,KHSRP,ZEB2,RELN,FUS,ILF3,FASN,GLUL,ACTB,DHX9,HNRNPU,NCL,EGR1,DHCR24,HSPA8
GO:0009058	biosynthetic process	3.2624366353004245e-44	*HRAS,TXK,ACSF3,MITF,JMJD1C,DAP3,TRIP13,ZNF274,GYG1,KMT2B,SUPT7L,MACROH2A1,BRD9,MED16,FADS2,NOL11,BICRA,MTA2,TIRAP,POLR3E,OTUD6B,RIOX1,PHF5A,NACA,POLR1C,MED6,CBX3,PHB,METTL3,EZH2,TCF20,PAXIP1,LRRFIP1,GPATCH3,INTS6,FUT8,SMG5,BRD4,STT3A,PTDSS2,DHDDS,MRPL1,ZNF581,SPIN4,ZNF74,SH3YL1,MRPL11,RPUSD4,DENR,CDC123,HROB,MRPS30,CTDP1,MED1,NVL,SRM,HNRNPL,NLN,PWP1,DOLPP1,CHRAC1,CRCP,KMT2D,TAF4B,PQBP1,PSPC1,CEP350,MAD2L2,MRRF,POLDIP2,PSMC3,ZBTB40,ADNP2,GART,ARID2,PATZ1,TRIM44,RNPS1,LHX4,ZNF512B,RBL1,PPP3R1,HIF1AN,SS18L1,MED29,USP14,LIMD1,DNMT1,LYL1,PPP5C,BAP1,SUB1,GTF2H1,TOP2B,LYAR,CCNH,NAB2,EIF4EBP2,CCT6A,KAT7,CLTC,EEF2,AURKAIP1,WDR5,CCT8,FUBP3,DLAT,CUL3,ARPP19,CMPK1,NONO,MEF2C,TCF3,MAF1,MVK,PRPF6,ZC3H4,ZNF26,ZNF24,GSPT1,ACTR8,PSMC5,AGPAT3,MAPK1,STAR,EEF1D,POLR1E,ABT1,PCLAF,PDSS1,CERS2,DHX33,CTR9,NCOA5,FADS1,ZFP91,UIMC1,PUM1,MTOR,IKZF3,KANSL1,SPEN,LCLAT1,GOT2,SMARCA5,EIF3D,ZNF45,RSF1,TFAP4,PRMT6,NIFK,FAF1,ZNF131,PCYT1A,SBNO1,ZNF431,DHFR,MTDH,TCF7L2,ZNF239,NSDHL,MRPL15,PRAME,GMPS,ASCC3,UROD,UBE2L3,THOC1,RPTOR,MRPS35,TNF,EIF4B,IGF2BP3,MED28,ELL2,PUM2,MT-TL1,SSBP3,ZNF586,BACH1,RBM10,TRAM2,SETX,NFILZ,AP3D1,METTL8,APEX1,BPTF,ICE1,NAA15,ARID1A,PITHD1,B4GALT5,SRSF10,C1QBP,VPS72,ZNF587B,CRK,MED15,EOGT,PRMT5,PCBP2,PTDSS1,RBMX,BAZ1A,GPX4,MLLT3,ALG8,TAF4,RBM14,RBM8A,KDM3B,LRP8,SETD2,ZNF326,INTS13,UCK2,ZNF282,DYRK1A,PHF3,TFDP2,GTF3C6,SLBP,JADE2,NUCKS1,SDCBP,AMD1,AGPAT5,GNL3L,CYB5B,IBA57,MT-TF,WAC,EIF3G,ADI1,DHX29,ARHGEF2,BRCA2,POLR2D,ZDHHC5,EIF3M,COPS2,BCL7B,ZFX,ACLY,PAN3,MED13L,PAF1,FASTKD2,PHB2,TCOF1,ZNF75A,SMAR*

			CB1,CREBBP,EXOSC3,EPRS1,INSIG1,LSM14A,NUP98,CNOT1,ASH1L,GEMIN5,PLAGL2,POLR1B,ELOF1,NFYC,SF1,PELP1,XRN2,EZR,TRUB2,DDX20,MMS19,SGPP2,ELOA,TAF9,ELAVL1,UTP15,VCP,RYBP,SAFB2,PSIP1,CSNK1G2,CDK4,DVL2,POLR3C,MRPS2,SCAP,WDR82,AGO2,E2F4,RNF220,DHCR7,ADNP,NFKB1,UBTF,BRD2,CHD7,HNRNPA1,GAR1,PPARGC1B,MAGOH,PBRM1,CTCF,HHEX,RRM2,RRM1,PI4KA,ELF1,ZNF614,NSD1,CCT3,CDK7,PKM,FOXK2,HMGB1,BZW1,SMARCD1,YY1,MLLT10,IRAK1,PUS7,PIK3C2B,IP6K1,NAT10,TRMT10C,EP400,AASDHPPT,MTHFD1,ADSS2,CEBPZ,LEPR,MECP2,CSDE1,SKI,TKT,DDX54,AZIN1,TARDBP,LARP4,XRCC6,RPL22,PCNA,MBD1,DDX3X,IDI1,LBR,HDGF,ZNF789,VPS35,ATP5MC3,EIF4G2,JUND,PFAS,HCFC1,CHEK1,AATF,ZMPSTE24,SLTM,VAC14,UBP1,POLE,TRRAP,PPIP5K2,EXOSC9,POLR2A,NUP62,PPIA,TPR,ENC1,TGFBRAP1,ZFP36L2,PPRC1,KEAP1,TFB2M,UBE2N,BAZ1B,NRIP1,VAPA,GFM1,MYBBP1A,DDB1,BACH2,PITX1,GTF3C4,LRRC47,ASXL2,CHST3,TCP1,CENPF,RALY,ENO1,CCT5,FUBP1,POLE3,CDK6,CDC5L,CHD3,ST3GAL2,BEND3,ZNF787,TFAM,AK2,PPP2CA,SSU72,CDK12,ZBTB2,DIDO1,GLYR1,MAN2A2,KMT2A,NOSTRIN,PAICS,ZNF598,SLC30A10,CERS6,GATAD2A,DKC1,DDX1,CTPS1,EWSR1,CASC3,ARID1B,ZNF33B,HNRNPAB,KAT6A,RBBP4,SAFB,NCBP1,HEATR1,CCT2,HSP90AB1,EIF3B,DUS3L,PA2G4,HNRNPK,SMG1,FDFT1,PRPF19,SMARCA4,EIF3J,PRKDC,YES1,ZNF121,PRMT1,HNRNPC,MALT1,WWP2,SUPT16H,HMGA1,FARSA,CELF1,XRCC5,DDX5,UTP4,DEK,LMO2,LSS,EIF4A3,NDST1,DDX56,SLC38A1,KHDRBS1,HNRNPD,LRPPRC,EIF5A,CTDSP1,SUPT6H,RANBP2,DNAJB6,NOLC1,SREBF2,CBFA2T3,ABCF1,ACACA,MGA,PIM2,GAPDH,YBX1,EIF5B,PCYT2,RBM3,PGD,AXIN1,RREB1,HSPH1,EP300,TGFBR1,RRP1B,FOSB,SRRT,PABPC1,SF3B3,PTMA,THRAP3,MLLT1,ELOVL6,MT-ND1,NPM1,RIF1,CITED2,ETF1,WDR43,TFRC,RUNX1,NQO1,EIF4G1,TRIM28,ACP5,NR2F2,BCLAF1,SRSF2,HSP90AA1,NFATC3,ZNF521,CAPRIN1,SQLE,TRIM24,RESF1,HNRNPA2B1,LARP1,SQSTM1,CCAR1,MAT2A,DDX21,HMGCS1,SFPQ,SCD,PABPC4,POLR1A,KCNH2,EIF3A,SMARCC1,MYC,SET,BTG1,IGF2BP1,FOS,GCLM,HMGCR,SPTA1,MYB,ODC1,KHSRP,ZEB2,RELN,FUS,ILF3,FASN,GLUL,ACTB,DHX9,HNRNPU,NCL,EGR1,DHCR24,HSPA8*
GO:0044249	cellular biosynthetic process	7.774314040210125e-43	*HRAS,TXK,ACSF3,MITF,JMJD1C,DAP3,TRIP13,ZNF274,GYG1,KMT2B,SUPT7L,MACROH2A1,BRD9,MED16,FADS2,NOL11,BICRA,MTA2,TIRAP,POLR3E,OTUD6B,RIOX1,PHF5A,NACA,POLR1C,MED6,CBX3,PHB,METTL3,EZH2,TCF20,PAXIP1,LRRFIP1,GPATCH3,INTS6,FUT8,SMG5,BRD4,STT3A,PTDSS2,DHDDS,MRPL1,ZNF581,SPIN4,ZNF74,SH3YL1,MRPL11,RPUSD4,DENR,CDC123,HROB,MRPS30,CTDP1,MED1,NVL,SRM,HNRNPL,PWP1,DOLPP1,CHRAC1,CRCP,KMT2D,TAF4B,PQBP1,PSPC1,CEP350,MAD2L2,MRRF,POLDIP2,PSMC3,ZBTB40,ADNP2,GART,ARID2,PATZ1,TRIM44,RNPS1,LHX4,ZNF512B,RBL1,PPP3R1,HIF1AN,SS18L1,MED29,USP14,LIMD1,DNMT1,LYL1,PPP5C,BAP1,SUB1,GTF2H1,TOP2B,LYAR,CCNH,NAB2,EIF4EBP2,CCT6A,KAT7,EEF2,AURKAIP1,WDR5,CCT8,FUBP3,DLAT,CUL3,ARPP19,CMPK1,NONO,MEF2C,TCF3,MAF1,MVK,PRPF6,ZC3H4,ZNF26,ZNF24,GSPT1,ACTR8,PSMC5,AGPAT3,MAPK1,STAR,EEF1D,POLR1E,ABT1,PCLAF,PDSS1,CERS2,DHX33,CTR9,NCOA5,FADS1,ZFP91,UIMC1,PUM1,MTOR,IKZF3,KANSL1,SPEN,LCLAT1,GOT2,SMARCA5,EIF3D,ZNF45,RSF1,TFAP4,PRM*

			T6,NIFK,FAF1,ZNF131,PCYT1A,SBNO1,ZNF431,DHFR,MTDH,TCF7L2,ZNF239,MRPL15,PRAME,GMPS,ASCC3,UROD,UBE2L3,THOC1,RPTOR,MRPS35,TNF,EIF4B,IGF2BP3,MED28,ELL2,PUM2,MT-TL1,SSBP3,ZNF586,BACH1,RBM10,SETX,NFILZ,AP3D1,METTL8,APEX1,BPTF,ICE1,NAA15,ARID1A,PITHD1,B4GALT5,SRSF10,C1QBP,VPS72,ZNF587B,CRK,MED15,EOGT,PRMT5,PCBP2,PTDSS1,RBMX,BAZ1A,GPX4,MLLT3,ALG8,TAF4,RBM14,RBM8A,KDM3B,LRP8,SETD2,ZNF326,INTS13,UCK2,ZNF282,DYRK1A,PHF3,TFDP2,GTF3C6,SLBP,JADE2,NUCKS1,SDCBP,AMD1,AGPAT5,GNL3L,CYB5B,IBA57,MT-TF,WAC,EIF3G,ADI1,DHX29,ARHGEF2,BRCA2,POLR2D,ZDHHC5,EIF3M,COPS2,BCL7B,ZFX,ACLY,PAN3,MED13L,PAF1,FASTKD2,PHB2,TCOF1,ZNF75A,SMARCB1,CREBBP,EXOSC3,EPRS1,INSIG1,LSM14A,NUP98,CNOT1,ASH1L,GEMIN5,PLAGL2,POLR1B,ELOF1,NFYC,SF1,PELP1,XRN2,EZR,TRUB2,DDX20,MMS19,SGPP2,ELOA,TAF9,ELAVL1,UTP15,VCP,RYBP,SAFB2,PSIP1,CSNK1G2,CDK4,DVL2,POLR3C,MRPS2,SCAP,WDR82,AGO2,E2F4,RNF220,ADNP,NFKB1,UBTF,BRD2,CHD7,HNRNPA1,GAR1,PPARGC1B,MAGOH,PBRM1,CTCF,HHEX,RRM2,RRM1,PI4KA,ELF1,ZNF614,NSD1,CCT3,CDK7,PKM,FOXK2,HMGB1,BZW1,SMARCD1,YY1,MLLT10,IRAK1,PUS7,PIK3C2B,IP6K1,NAT10,TRMT10C,EP400,AASDHPPT,MTHFD1,ADSS2,CEBPZ,LEPR,MECP2,CSDE1,SKI,TKT,DDX54,AZIN1,TARDBP,LARP4,XRCC6,RPL22,PCNA,MBD1,DDX3X,IDI1,HDGF,ZNF789,VPS35,ATP5MC3,EIF4G2,JUND,PFAS,HCFC1,CHEK1,AATF,ZMPSTE24,SLTM,VAC14,UBP1,POLE,TRRAP,EXOSC9,POLR2A,NUP62,PPIA,TPR,ENC1,TGFBRAP1,ZFP36L2,PPRC1,KEAP1,TFB2M,UBE2N,BAZ1B,NRIP1,VAPA,GFM1,MYBBP1A,DDB1,BACH2,PITX1,GTF3C4,LRRC47,ASXL2,CHST3,TCP1,CENPF,RALY,ENO1,CCT5,FUBP1,POLE3,CDK6,CDC5L,CHD3,ST3GAL2,BEND3,ZNF787,TFAM,AK2,PPP2CA,SSU72,CDK12,ZBTB2,DIDO1,GLYR1,MAN2A2,KMT2A,NOSTRIN,PAICS,ZNF598,CERS6,GATAD2A,DKC1,DDX1,CTPS1,EWSR1,CASC3,ARID1B,ZNF33B,HNRNPAB,KAT6A,RBBP4,SAFB,NCBP1,HEATR1,CCT2,HSP90AB1,EIF3B,DUS3L,PA2G4,HNRNPK,SMG1,SMARCA4,EIF3J,PRKDC,YES1,ZNF121,PRMT1,HNRNPC,MALT1,WWP2,SUPT16H,HMGA1,FARSA,CELF1,XRCC5,DDX5,UTP4,DEK,LMO2,LSS,EIF4A3,NDST1,DDX56,SLC38A1,KHDRBS1,HNRNPD,LRPPRC,EIF5A,CTDSP1,SUPT6H,DNAJB6,NOLC1,SREBF2,CBFA2T3,ABCF1,ACACA,MGA,PIM2,GAPDH,YBX1,EIF5B,PCYT2,RBM3,AXIN1,RREB1,EP300,TGFBR1,RRP1B,FOSB,SRRT,PABPC1,SF3B3,PTMA,THRAP3,MLLT1,ELOVL6,MT-ND1,NPM1,RIF1,CITED2,ETF1,WDR43,TFRC,RUNX1,NQO1,EIF4G1,TRIM28,ACP5,NR2F2,BCLAF1,SRSF2,HSP90AA1,NFATC3,ZNF521,CAPRIN1,TRIM24,RESF1,HNRNPA2B1,LARP1,SQSTM1,CCAR1,MAT2A,DDX21,HMGCS1,SFPQ,SCD,PABPC4,POLR1A,KCNH2,EIF3A,SMARCC1,MYC,SET,BTG1,IGF2BP1,FOS,GCLM,HMGCR,SPTA1,MYB,ODC1,KHSRP,ZEB2,RELN,FUS,ILF3,FASN,GLUL,ACTB,DHX9,HNRNPU,NCL,EGR1,HSPA8
GO:0051252	regulation of RNA metabolic process	1.048050740301161e-41	*HRAS,TXK,MITF,JMJD1C,ZNF274,KMT2B,SUPT7L,MACROH2A1,BRD9,MED16,NOL11,BICRA,MTA2,TIRAP,CWC22,RIOX1,PHF5A,NACA,MED6,CBX3,PHB,METTL3,EZH2,TCF20,PAXIP1,LRRFIP1,GPATCH3,INTS6,BRD4,ZNF581,SPIN4,ZNF74,CTDP1,MED1,HNRNPL,PWP1,KMT2D,TAF4B,PQBP1,PSPC1,CEP350,MAD2L2,PSMC3,ZBTB40,ADNP2,ARID2,PATZ1,TRIM44,RNPS1,LHX4,ZNF512B,RBL1,PPP3R1,HIF1AN,SS18L1*

			,MED29,HNRNPH1,USP14,LIMD1,DNMT1,LYL1,BAP1,SUB1,GTF2H1,TOP2B,LYAR,CCNH,NAB2,KAT7,WDR5,FUBP3,CUL3,NONO,MEF2C,TCF3,MAF1,NBAS,PRPF6,ZC3H4,ZNF26,ZNF24,GRSF1,ACTR8,PSMC5,ZC3HAV1,PKP3,ABT1,DHX33,CTR9,NCOA5,FADS1,ZFP91,UIMC1,PUM1,MTOR,IKZF3,KANSL1,SPEN,SMARCA5,ZNF45,RSF1,TFAP4,PRMT6,FAF1,HNRNPA0,ZNF131,SBNO1,ZNF431,MTDH,TCF7L2,ZNF239,PRAME,UBE2L3,THOC1,RPTOR,TNF,RAVER1,IGF2BP3,MED28,ELL2,PUM2,SSBP3,SNRPA,ZNF586,BACH1,RBM10,SETX,NFILZ,AP3D1,APEX1,BPTF,ICE1,RIOK2,NAA15,ARID1A,PITHD1,ZC3H14,SRSF10,C1QBP,VPS72,ZNF587B,CRK,MED15,PRMT5,PCBP2,RBMX,BAZ1A,MLLT3,TAF4,RBM14,RBM8A,KDM3B,LRP8,SETD2,ZNF326,INTS13,ZNF282,DYRK1A,TFDP2,JADE2,NUCKS1,SDCBP,NSRP1,WAC,ARHGEF2,BRCA2,POLR2D,WTAP,COPS2,BCL7B,RBM42,ZFX,PAN3,MED13L,PAF1,ZC3H18,FASTKD2,PHB2,ZNF75A,SMARCB1,CREBBP,EXOSC3,NUP98,CNOT1,ASH1L,PLAGL2,NFYC,SF1,PELP1,EZR,DDX20,MMS19,ELOA,TAF9,ELAVL1,UTP15,RYBP,SAFB2,DHX34,PSIP1,RBM12,CDK4,DVL2,POLR3C,RBM25,WDR82,AGO2,E2F4,RNF220,ADNP,NFKB1,UBTF,BRD2,CHD7,RBM15B,HNRNPA1,PPARGC1B,MAGOH,PBRM1,CTCF,HHEX,ELF1,ZNF614,NSD1,CDK7,FOXK2,HMGB1,SMARCD1,YY1,MLLT10,IRAK1,SRSF6,EP400,CEBPZ,MECP2,CSDE1,MBNL1,SKI,DDX54,TARDBP,XRCC6,PCNA,MBD1,DDX3X,HDGF,ZNF789,EIF4G2,JUND,HCFC1,CHEK1,AATF,ZMPSTE24,SLTM,UBP1,TRRAP,EXOSC9,NUP62,PPIA,TPR,SRSF7,TGFBRAP1,ZFP36L2,SF3B4,PPRC1,SLC38A2,KEAP1,TFB2M,UBE2N,BAZ1B,NRIP1,MYBBP1A,BACH2,PITX1,ASXL2,CENPF,RALY,ENO1,FUBP1,CDK6,CDC5L,CHD3,BEND3,ZNF787,TFAM,PPP2CA,SSU72,CDK12,ZBTB2,GLYR1,KMT2A,NOSTRIN,GATAD2A,DKC1,EWSR1,DDX49,CASC3,ARID1B,CPSF7,ZNF33B,HNRNPAB,KAT6A,USP36,RBBP4,SAFB,NCBP1,HEATR1,PA2G4,HNRNPK,PRPF19,SMARCA4,PRKDC,YES1,ZNF121,TRA2B,HNRNPC,MALT1,WWP2,SUPT16H,HMGA1,CELF1,RBMXL1,XRCC5,DDX5,UTP4,DEK,LMO2,EIF4A3,KHDRBS1,HNRNPD,LRPPRC,EIF5A,CTDSP1,SUPT6H,DNAJB6,NOLC1,SREBF2,CBFA2T3,MGA,NSUN2,PIM2,YBX1,LIN28B,RBM3,AXIN1,RREB1,EP300,TGFBR1,DAZAP1,RRP1B,FOSB,SRRT,PABPC1,SERBP1,AHNAK,SF3B3,PTMA,THRAP3,MLLT1,HNRNPF,NPM1,RIF1,CITED2,SON,WDR43,TFRC,RUNX1,TRIM28,U2AF2,NR2F2,BCLAF1,SRSF2,NFATC3,ZNF521,TRIM24,RESF1,HNRNPA2B1,LARP1,SQSTM1,PTBP1,CCAR1,DDX21,SFPQ,PABPC4,HNRNPM,KCNH2,SRSF3,SMARCC1,MYC,SET,BTG1,IGF2BP1,FOS,MYB,KHSRP,ZEB2,RELN,FUS,ILF3,ACTB,DHX9,HNRNPU,NCL,EGR1,HSPA8
GO:0009059	macromolecule biosynthetic process	1.8891374905976274e-41	HRAS,TXK,MITF,JMJD1C,DAP3,TRIP13,ZNF274,GYG1,KMT2B,SUPT7L,MACROH2A1,BRD9,MED16,NOL11,BICRA,MTA2,TIRAP,POLR3E,OTUD6B,RIOX1,PHF5A,NACA,POLR1C,MED6,CBX3,PHB,METTL3,EZH2,TCF20,PAXIP1,LRRFIP1,GPATCH3,INTS6,FUT8,SMG5,BRD4,STT3A,DHDDS,MRPL1,ZNF581,SPIN4,ZNF74,MRPL11,RPUSD4,DENR,CDC123,HROB,MRPS30,CTDP1,MED1,NVL,HNRNPL,PWP1,DOLPP1,CHRAC1,CRCP,KMT2D,TAF4B,PQBP1,PSPC1,CEP350,MAD2L2,MRRF,POLDIP2,PSMC3,ZBTB40,ADNP2,ARID2,PATZ1,TRIM44,RNPS1,LHX4,ZNF512B,RBL1,PPP3R1,HIF1AN,SS18L1,MED29,USP14,LIMD1,DNMT1,LYL1,PPP5C,BAP1,SUB1,GTF2H1,TOP2B,LYAR,CCNH,NAB2,EIF4EBP2,CCT6A,KAT7,CLTC,EEF2,AURKAIP1,WDR5,CCT8,FUBP3,CUL3,NONO,MEF2C,TCF3,MAF1,PRPF6,ZC3H4,ZNF26,ZNF24,GSPT1,ACTR8,PSMC5,MAPK1

			,EEF1D,POLR1E,ABT1,PCLAF,CERS2,DHX33,CTR9,NCOA5,FADS1,ZFP91,UIMC1,PUM1,MTOR,IKZF3,KANSL1,SPEN,SMARCA5,EIF3D,ZNF45,RSF1,TFAP4,PRMT6,NIFK,FAF1,ZNF131,SBNO1,ZNF431,DHFR,MTDH,TCF7L2,ZNF239,MRPL15,PRAME,ASCC3,UBE2L3,THOC1,RPTOR,MRPS35,TNF,EIF4B,IGF2BP3,MED28,ELL2,PUM2,MT-TL1,SSBP3,ZNF586,BACH1,RBM10,SETX,NFILZ,AP3D1,METTL8,APEX1,BPTF,ICE1,NAA15,ARID1A,PITHD1,B4GALT5,SRSF10,C1QBP,VPS72,ZNF587B,CRK,MED15,EOGT,PRMT5,PCBP2,RBMX,BAZ1A,MLLT3,ALG8,TAF4,RBM14,RBM8A,KDM3B,LRP8,SETD2,ZNF326,INTS13,ZNF282,DYRK1A,PHF3,TFDP2,GTF3C6,SLBP,JADE2,NUCKS1,SDCBP,GNL3L,MT-TF,WAC,EIF3G,DHX29,ARHGEF2,BRCA2,POLR2D,ZDHHC5,EIF3M,COPS2,BCL7B,ZFX,PAN3,MED13L,PAF1,FASTKD2,PHB2,TCOF1,ZNF75A,SMARCB1,CREBBP,EXOSC3,EPRS1,LSM14A,NUP98,CNOT1,ASH1L,GEMIN5,PLAGL2,POLR1B,ELOF1,NFYC,SF1,PELP1,XRN2,EZR,TRUB2,DDX20,MMS19,ELOA,TAF9,ELAVL1,UTP15,VCP,RYBP,SAFB2,PSIP1,CDK4,DVL2,POLR3C,MRPS2,WDR82,AGO2,E2F4,RNF220,ADNP,NFKB1,UBTF,BRD2,CHD7,HNRNPA1,GAR1,PPARGC1B,MAGOH,PBRM1,CTCF,HHEX,RRM1,ELF1,ZNF614,NSD1,CCT3,CDK7,PKM,FOXK2,HMGB1,BZW1,SMARCD1,YY1,MLLT10,IRAK1,PUS7,NAT10,TRMT10C,EP400,CEBPZ,MECP2,CSDE1,SKI,DDX54,TARDBP,LARP4,XRCC6,RPL22,PCNA,MBD1,DDX3X,HDGF,ZNF789,EIF4G2,JUND,HCFC1,CHEK1,AATF,ZMPSTE24,SLTM,UBP1,POLE,TRRAP,EXOSC9,POLR2A,NUP62,PPIA,TPR,ENC1,TGFBRAP1,ZFP36L2,PPRC1,KEAP1,TFB2M,UBE2N,BAZ1B,NRIP1,GFM1,MYBBP1A,BACH2,PITX1,GTF3C4,LRRC47,ASXL2,CHST3,TCP1,CENPF,RALY,ENO1,CCT5,FUBP1,POLE3,CDK6,CDC5L,CHD3,ST3GAL2,BEND3,ZNF787,TFAM,PPP2CA,SSU72,CDK12,ZBTB2,DIDO1,GLYR1,MAN2A2,KMT2A,NOSTRIN,ZNF598,GATAD2A,DKC1,DDX1,EWSR1,CASC3,ARID1B,ZNF33B,HNRNPAB,KAT6A,RBBP4,SAFB,NCBP1,HEATR1,CCT2,HSP90AB1,EIF3B,DUS3L,PA2G4,HNRNPK,SMARCA4,EIF3J,PRKDC,YES1,ZNF121,PRMT1,HNRNPC,MALT1,WWP2,SUPT16H,HMGA1,FARSA,CELF1,XRCC5,DDX5,UTP4,DEK,LMO2,EIF4A3,NDST1,DDX56,KHDRBS1,HNRNPD,LRPPRC,EIF5A,CTDSP1,SUPT6H,DNAJB6,NOLC1,SREBF2,CBFA2T3,ABCF1,MGA,PIM2,GAPDH,YBX1,EIF5B,RBM3,AXIN1,RREB1,HSPH1,EP300,TGFBR1,RRP1B,FOSB,SRRT,PABPC1,SF3B3,PTMA,THRAP3,MLLT1,NPM1,RIF1,CITED2,ETF1,WDR43,TFRC,RUNX1,EIF4G1,TRIM28,NR2F2,BCLAF1,SRSF2,HSP90AA1,NFATC3,ZNF521,CAPRIN1,TRIM24,RESF1,HNRNPA2B1,LARP1,SQSTM1,CCAR1,DDX21,SFPQ,PABPC4,POLR1A,KCNH2,EIF3A,SMARCC1,MYC,SET,BTG1,IGF2BP1,FOS,MYB,KHSRP,ZEB2,RELN,FUS,ILF3,GLUL,ACTB,DHX9,HNRNPU,NCL,EGR1,HSPA8
GO:0034641	cellular nitrogen compound metabolic process	3.112118402543911e-41	HRAS,NOP14,TXK,ACSF3,SNRPB,MITF,JMJD1C,DAP3,PPAN,MSH3,TRIP13,ZNF274,PRPF38B,KMT2B,RNASEH1,SUPT7L,SREK1,MACROH2A1,BRD9,MED16,MUS81,NOL11,BICRA,MTA2,SNHG6,TIRAP,RAD51C,UTP3,CWC22,POLR3E,MTREX,OTUD6B,RIOX1,PHF5A,NACA,POLR1C,MED6,PRXL2C,CBX3,PHB,METTL3,EZH2,TCF20,DCLRE1C,NOC4L,RFWD3,MFAP1,DNM1L,PAXIP1,LRRFIP1,GPATCH3,INTS6,FUT8,SMG5,BRD4,PTDSS2,RRP9,MRPL1,ZNF581,SPIN4,ZNF74,MRPL11,RPUSD4,DENR,CDC123,RNASEH2C,HROB,MRPS30,CTDP1,NUP155,MED1,NVL,SRM,HNRNPL,PWP1,WDR46,MCM10,EFTUD2,CHRAC1,CRCP,KMT2D,TAF4B,PQBP1,PSPC1,CEP350,MAD2L2,MRRF,POLDIP2,PSMC3,

			ZBTB40,DHX38,ADNP2,GART,NT5C3A,ARID2,DDX23,PATZ1,TRIM44,TRNT1,RNPS1,LHX4,ZNF512B,RBL1,PPP3R1,HIF1AN,RPUSD1,SS18L1,MED29,HNRNPH1,USP14,LIMD1,DNMT1,LYL1,BICD1,PPP5C,BAP1,DUS1L,SUB1,GTF2H1,DHX16,TOP2B,LYAR,LRWD1,CCNH,NAB2,EIF4EBP2,CCT6A,KAT7,TSR3,EEF2,AURKAIP1,SNHG20,WDR5,CCT8,FUBP3,IMP4,SNHG17,BCCIP,DLAT,NOB1,CUL3,CMPK1,NONO,MEF2C,TCF3,TEX15,MAF1,CLSPN,NOL9,MVK,NBAS,ZFYVE26,PRPF6,ZC3H4,ZNF26,ZNF24,GSPT1,GRSF1,UBQLN4,ACTR8,PSMC5,ZC3HAV1,HNRNPA3,MAPK1,EEF1D,POLR1E,PKP3,ABT1,PCLAF,WDR36,CERS2,DHX33,CTR9,NCOA5,FADS1,ZFP91,DHX15,UIMC1,PUM1,MTOR,RPRD2,UTP18,IKZF3,KANSL1,CPSF3,SPEN,SMG9,SMARCA5,CWC25,SF3A2,H2AW,EIF3D,ZNF45,RSF1,TFAP4,PRMT6,NIFK,FAF1,HNRNPA0,ZNF131,SBNO1,ZNF431,DHFR,MTDH,TCF7L2,ZNF239,SRRM2,MRPL15,PRAME,GMPS,ASCC3,UROD,UBE2L3,THOC1,MCMBP,SETMAR,RPTOR,PPP1R10,MRPS35,CSTF2,TNF,RAVER1,EIF4B,WDR33,TRMT61A,IGF2BP3,PDCD7,MED28,ELL2,PUM2,WDR3,MT-TL1,SSBP3,SNRPA,ZNF586,BACH1,DDX10,RBM10,SETX,NFILZ,TRMT2A,AP3D1,METTL8,APEX1,BPTF,EDC4,ICE1,RIOK2,NAA15,ARID1A,PITHD1,ZC3H14,B4GALT5,HERC2,SRSF10,SBDS,C1QBP,VPS72,ZNF587B,CRK,TICRR,MED15,SRSF8,WDR74,PRPF4,PRMT5,PCBP2,TRMT6,RBMX,BAZ1A,DPYS,YJU2,WDR70,MCCC2,MLLT3,RAD23B,TAF4,RBM14,RBM8A,KDM3B,BOP1,LRP8,SETD2,ZNF326,INTS13,RPIA,UCK2,FEN1,CHAF1A,TRMO,ZNF282,DYRK1A,PHF3,TFDP2,WDR12,GTF3C6,SLBP,CUL4A,JADE2,NUCKS1,SDCBP,UTP25,AMD1,PRPF38A,SSB,GNL3L,DNTTIP2,CYB5B,IBA57,RNU6-322P,NSRP1,MT-TF,TFIP11,WAC,EIF3G,DHX29,ARHGEF2,BRCA2,POLR2D,EIF3M,WTAP,COPS2,BCL7B,RBM42,ZFX,ZC3H7B,ACLY,SNRPD1,PAN3,MED13L,PAF1,ZC3H18,FASTKD2,PHB2,TCOF1,XRCC2,PDS5A,BRCC3,ZNF75A,SMARCB1,TRIR,CREBBP,EXOSC3,WBP11,EPRS1,LSM14A,NUP98,HNRNPUL1,CNOT1,ASH1L,GEMIN5,PLAGL2,POLR1B,ELOF1,WDR6,NFYC,SF1,PELP1,XRN2,EZR,TRUB2,DDX20,URM1,DDX51,MMS19,ELOA,TAF9,ELAVL1,UTP15,VCP,RYBP,SAFB2,DHX34,PSIP1,DCAF13,RBM12,CDK4,DVL2,POLR3C,THG1L,MRPS2,RBM25,RRP12,WDR82,SMC1A,AGO2,E2F4,G3BP1,RNF220,RRP36,MCM6,AQR,DDX18,IER3,ADNP,NFKB1,UBTF,BRD2,CHD7,RBM15B,USP37,HNRNPA1,GAR1,RRS1,PPARGC1B,MAGOH,PBRM1,CTCF,HHEX,ANKRD17,HNRNPR,RRM2,RRM1,ELF1,ZNF614,NSD1,RSL1D1,CCT3,CDK7,RNF138,PKM,FOXK2,HMGB1,BZW1,SMARCD1,YY1,NOL8,MLLT10,IRAK1,PUS7,NAT10,TRMT10C,SNU13,SRSF6,EP400,AASDHPPT,MTHFD1,ADSS2,SART1,CEBPZ,MECP2,CSDE1,MBNL1,SKI,TKT,RRP15,DDX54,AZIN1,N4BP2,TARDBP,LARP4,XRCC6,PNN,RPL22,PCNA,MBD1,UPF2,DDX3X,HDGF,ZNF789,VPS35,ATP5MC3,EIF4G2,MCM2,JUND,PFAS,HCFC1,CHEK1,AATF,ZMPSTE24,PRMT7,SLTM,CYP3A5,URB1,UBP1,POLE,TRRAP,EXOSC9,POLR2A,NUP62,TSR1,RBM19,PPIA,PRPF3,IK,TPR,ENC1,SRSF7,UTP20,TGFBRAP1,ZFP36L2,SF3B4,PPRC1,SLC38A2,EBNA1BP2,KEAP1,TFB2M,UBE2N,BAZ1B,NRIP1,MRTO4,VAPA,GFM1,MYBBP1A,DDB1,BACH2,PITX1,GTF3C4,LRRC47,NASP,ASXL2,TCP1,CENPF,RALY,ENO1,CCT5,FUBP1,POLE3,CDK6,CDC5L,CHD3,ST3GAL2,RAN,BEND3,C19ORF48,ZNF787,TFAM,AK2,PPP2CA,SSU72,MSH2,CDK12,ZBTB2,DIDO1,GLYR1,KMT2A,MCM3,NOSTRIN,GRWD1,PAICS,ZNF598,CERS6,SNHG3,GATAD2A,DK

			C1,DDX1,THUMPD1,CTPS1,EWSR1,DDX49,CASC3,SSRP1,ARID1B,CPSF7,RRP1,ZNF33B,HNRNPAB,KAT6A,USP36,RBBP4,SAFB,NCBP1,HEATR1,CCT2,HSP90AB1,EIF3B,DUS3L,PA2G4,HNRNPK,SMG1,DHX37,PRPF19,SMARCA4,EIF3J,PRKDC,YES1,ZNF121,TRA2B,PRMT1,HNRNPC,MALT1,WWP2,SF3A3,SUPT16H,HMGA1,FARSA,CELF1,RBMXL1,XRCC5,DDX5,UTP4,DEK,LMO2,DDX46,EIF4A3,TRIP12,DDX39A,DDX56,CIZ1,CDT1,NOP56,HNRNPDL,KHDRBS1,HNRNPD,LRPPRC,EIF5A,CTDSP1,SUPT6H,DNAJB6,NOLC1,SNRNP200,SREBF2,CBFA2T3,ABCF1,DANCR,ACACA,MGA,NSUN2,AP5Z1,HSPD1,PIM2,GAPDH,YBX1,LIN28B,EIF5B,PCYT2,RBM3,PGD,SF3A1,AXIN1,MPHOSPH10,PES1,RREB1,EP300,TGFBR1,DAZAP1,ALYREF,RRP1B,FOSB,SRRT,PABPC1,SERBP1,AHNAK,PRPF8,SF3B3,PTMA,THRAP3,MLLT1,ELOVL6,HNRNPF,MT-ND1,NPM1,RIF1,CITED2,ETF1,SON,WDR43,TFRC,RUNX1,NQO1,EIF4G1,TRIM28,ACP5,U2AF2,NR2F2,BCLAF1,SRSF2,HSP90AA1,MDN1,NFATC3,ZNF521,CAPRIN1,TRMT1,MCM4,TRIM24,NOP58,RESF1,HNRNPA2B1,LARP1,SQSTM1,PTBP1,CCAR1,DDX21,HMGCS1,SFPQ,PABPC4,HNRNPM,POLR1A,KCNH2,SRSF3,EIF3A,MCM7,SMARCC1,MYC,SET,BTG1,IGF2BP1,FOS,GCLM,HMGCR,SPTA1,MYB,ODC1,KHSRP,ZEB2,RELN,FUS,ILF3,ACTB,DHX9,HNRNPU,MCM5,NCL,EGR1,HSPA8
GO:0016072	rRNA metabolic process	4.659727176400823e-41	*NOP14,PPAN,MACROH2A1,NOL11,UTP3,MTREX,NOC4L,RRP9,MRPL1,RPUSD4,NVL,PWP1,WDR46,RPUSD1,LYAR,TSR3,IMP4,NOB1,NOL9,POLR1E,ABT1,WDR36,MTOR,UTP18,NIFK,WDR3,DDX10,RIOK2,SBDS,WDR74,BOP1,WDR12,GTF3C6,UTP25,TCOF1,SMARCB1,TRIR,EXOSC3,WBP11,POLR1B,PELP1,XRN2,DDX51,UTP15,DCAF13,RRP12,RRP36,DDX18,CHD7,GAR1,RRS1,RSL1D1,NOL8,NAT10,SNU13,SART1,RRP15,DDX54,URB1,EXOSC9,TSR1,UTP20,EBNA1BP2,TFB2M,MRTO4,GTF3C4,DKC1,DDX49,RRP1,USP36,HEATR1,PA2G4,DHX37,SMARCA4,PRKDC,UTP4,EIF4A3,DDX56,NOP56,NOLC1,MPHOSPH10,PES1,RRP1B,WDR43,MDN1,NOP58,DDX21,NCL*
GO:0034470	ncRNA processing	3.0324239187311426e-40	*NOP14,PPAN,NOL11,UTP3,MTREX,METTL3,NOC4L,INTS6,RRP9,MRPL1,RPUSD4,NUP155,NVL,PWP1,WDR46,TRNT1,RPUSD1,DUS1L,LYAR,TSR3,IMP4,NOB1,NOL9,GRSF1,ABT1,WDR36,PUM1,UTP18,NIFK,TNF,TRMT61A,PUM2,WDR3,DDX10,TRMT2A,METTL8,RIOK2,SBDS,WDR74,TRMT6,BOP1,INTS13,TRMO,WDR12,UTP25,SSB,ZC3H7B,EXOSC3,WBP11,WDR6,PELP1,XRN2,TRUB2,URM1,DDX51,UTP15,DCAF13,THG1L,RRP12,AGO2,RRP36,DDX18,CHD7,GAR1,RRS1,RSL1D1,NOL8,PUS7,NAT10,TRMT10C,SNU13,SART1,RRP15,DDX54,DDX3X,ZMPSTE24,URB1,EXOSC9,TSR1,UTP20,EBNA1BP2,TFB2M,MRTO4,DKC1,DDX1,THUMPD1,DDX49,RRP1,USP36,NCBP1,HEATR1,DUS3L,PA2G4,DHX37,PRKDC,DDX5,UTP4,EIF4A3,DDX56,NOP56,NOLC1,NSUN2,LIN28B,MPHOSPH10,PES1,RRP1B,SRRT,WDR43,MDN1,TRMT1,NOP58,HNRNPA2B1,DDX21,SRSF3*
GO:0044237	cellular metabolic process	6.044757627314364e-39	*HRAS,NOP14,TXK,ACSF3,SNRPB,MITF,PPP6C,JMJD1C,DAP3,PPAN,MSH3,TRIP13,CHTOP,ZNF274,GYG1,PRPF38B,KMT2B,MMAB,RNASEH1,SUPT7L,SREK1,MACROH2A1,BRD9,MED16,FADS2,MUS81,NOL11,BICRA,MTA2,SNHG6,TIRAP,RAD51C,NRROS,UTP3,CWC22,BEGAIN,POLR3E,NEU1,MTREX,OTUD6B,RIOX1,PHF5A,NACA,POLR1C,MED6,PRXL2C,CBX3,PHB,METTL3,EZH2,TCF20,FES,DCLRE1C,NOC4L,RFWD3,MFAP1,ULK3,DNM1L,PAXIP1,LRRFIP1,GPATCH3,RHEB,INTS6,PPP6R3,FUT8,SMG5,BRD4,STT3A,PTDSS2,DHDD*

			S,VKORC1L1,RRP9,MRPL1,ZNF581,SPIN4,ZNF74,SH3YL1,MRPL11,RPUSD4,CMBL,DENR,CDC123,RNASEH2C,HROB,MRPS30,CTDP1,NUP155,MED1,NVL,SRM,HNRNPL,NLN,PWP1,DOLPP1,WDR46,MCM10,OXA1L,EFTUD2,CHRAC1,CRCP,KMT2D,TAF4B,PSMA3,PQBP1,PSMD3,AIFM2,PSPC1,CEP350,MAD2L2,MRRF,POLDIP2,PSMC3,ZBTB40,DHX38,ADNP2,GART,NT5C3A,FBXO45,ARID2,DDX23,PATZ1,TRIM44,TRNT1,RNPS1,LHX4,ZNF512B,RBL1,PPP3R1,SLC25A46,HIF1AN,USP11,RPUSD1,SS18L1,MED29,PAFAH1B1,HNRNPH1,USP14,RAB7A,LIMD1,DNMT1,LYL1,BICD1,PPP5C,BAP1,DUS1L,ANAPC7,SUB1,GTF2H1,DHX16,TOP2B,LYAR,LRWD1,SLC39A10,CCNH,NAB2,EXOC7,EIF4EBP2,CCT6A,KAT7,CLTC,TSR3,EEF2,MSRA,AURKAIP1,SNHG20,WDR5,CCT8,FUBP3,IMP4,SNHG17,BCCIP,SPRY2,DLAT,NOB1,SBF1,RNF40,CUL3,ARPP19,CMPK1,NONO,MEF2C,UBR3,TCF3,TEX15,MAF1,GID8,CLSPN,NOL9,MVK,NBAS,PLK4,CCNY,SLK,ZFYVE26,PRPF6,ZC3H4,ZNF26,ZNF24,GSPT1,PEPD,GRSF1,UBQLN4,ACTR8,PSMC5,AGPAT3,STK35,ZC3HAV1,HNRNPA3,MAPK1,STAR,ZNRF1,EEF1D,CASP3,POLR1E,ATP2A2,PKP3,ABT1,PCLAF,WDR36,HBG1,PDSS1,CERS2,DHX33,PGAM5,NSMAF,CTR9,NCOA5,FADS1,ZFP91,DHX15,UIMC1,PUM1,MTOR,PDPR,RPRD2,UTP18,IKZF3,KANSL1,CPSF3,SPEN,LCLAT1,GOT2,VDAC1,SMG9,SMARCA5,CWC25,SF3A2,H2AW,EIF3D,ZNF45,RSF1,TFAP4,PTP4A2,PRMT6,NIFK,FAF1,HNRNPA0,BAG6,ZNF131,PCYT1A,SBNO1,ZNF431,DHFR,MTDH,HSPA9,MCCC1,TCF7L2,ZNF239,SRRM2,MRPL15,PRAME,GMPS,ASCC3,UROD,UBE2L3,THOC1,PSMC2,MCMBP,SETMAR,RPTOR,PPP1R10,MRPS35,HECTD1,CSTF2,TNF,RAVER1,RABGGTB,EIF4B,PPP4R3A,SAMSN1,WDR33,PPP2R5A,TRMT61A,IGF2BP3,PDCD7,MED28,ELL2,PUM2,WDR3,MT-TL1,SSBP3,SNRPA,ZNF586,BACH1,DDX10,RBM10,CERT1,SETX,NFILZ,TRMT2A,AP3D1,STK24,METTL8,APEX1,BPTF,ATP6V1G1,UFC1,EDC4,ICE1,RIOK2,NAA15,ARID1A,PITHD1,PPP1CC,ZC3H14,B4GALT5,CDC27,HERC2,SRSF10,CTSL,SBDS,C1QBP,VPS72,PPM1H,ZNF587B,CRK,TICRR,MED15,EOGT,STK25,CAPNS1,SRSF8,WDR74,PAK2,PRPF4,PRMT5,SNX9,PCBP2,PTDSS1,ADD1,TRMT6,RBMX,RNF126,BAZ1A,DPYS,GPX4,YJU2,WDR70,MCCC2,MLLT3,ALG8,RAD23B,TAF4,RBM14,RBM8A,KDM3B,BOP1,LRP8,SETD2,ZNF326,INTS13,RPIA,UCK2,FEN1,CHAF1A,TRMO,ZNF282,DYRK1A,PHF3,TFDP2,WDR12,GTF3C6,SLBP,CUL4A,JADE2,NUCKS1,SDCBP,PSMD1,UTP25,AMD1,PRPF38A,SSB,CDC37,ECHDC1,AGPAT5,GNL3L,DNTTIP2,CYB5B,IBA57,RNU6-322P,NSRP1,MT-TF,TFIP11,PSMB2,WAC,EIF3G,ADI1,DHX29,ARHGEF2,BRCA2,POLR2D,ZDHHC5,EIF3M,WTAP,COPS2,BCL7B,RBM42,ZFX,ZC3H7B,ACLY,SNRPD1,PAN3,MED13L,PAF1,ZC3H18,BIRC6,FASTKD2,PHB2,TCOF1,GRB10,XRCC2,PDS5A,BRCC3,ZNF75A,SMARCB1,TRIR,CREBBP,EXOSC3,WBP11,EPRS1,COPS3,UBE4B,INSIG1,LSM14A,NUP98,HNRNPUL1,CNOT1,LYN,ASH1L,GEMIN5,PLAGL2,APC,POLR1B,CAPN1,ELOF1,WDR6,NFYC,SF1,PELP1,XRN2,EZR,TRUB2,DDX20,URM1,DDX51,MMS19,SGPP2,ELOA,TAF9,ACAT2,YWHAG,ELAVL1,UTP15,VCP,DNAJB12,RYBP,SAFB2,DHX34,PSIP1,CSNK1G2,DCAF13,RBM12,CDK4,DVL2,POLR3C,THG1L,MRPS2,RBM25,SETD1A,RRP12,SCAP,WDR82,SMC1A,AGO2,E2F4,VPS26A,FOXRED2,G3BP1,RNF220,TBC1D14,RRP36,MCM6,AQR,ARL8B,DDX18,IER3,ATP6V0D1,ADNP,NFKB1,UBTF,ZNF622,BRD2,CHD7,RBM15B,USP37,HNRNPA1,GAR1,RRS1,PPARGC1B,MAGO

			H,PBRM1,CTCF,HHEX,ANKRD17,HNRNPR,RRM2,RRM1,PI4KA,ELF1,ZNF614,NSD1,RSL1D1,CCT3,CDK7,RNF138,SETD1B,PPIF,PKM,FOXK2,HMGB1,MAEA,BZW1,SMARCD1,GNAQ,CANX,YY1,NOL8,MLLT10,IRAK1,PUS7,PIK3C2B,IP6K1,NAT10,TRMT10C,SNU13,SRSF6,EP400,AASDHPPT,MTHFD1,ADSS2,SART1,CEBPZ,LEPR,MECP2,CSDE1,PARD3,MBNL1,SKI,PRKAR2B,TKT,RRP15,DDX54,AZIN1,N4BP2,TARDBP,LARP4,XRCC6,PNN,RPL22,ABI1,PCNA,CDC25A,MBD1,CSNK2A2,UPF2,DDX3X,IDI1,HDGF,ZNF789,VPS35,ATP5MC3,EIF4G2,MCM2,JUND,PFAS,HCFC1,CHEK1,AATF,CLN6,ZMPSTE24,PRMT7,OXCT1,RETREG2,SLC25A5,SLTM,CYP3A5,URB1,VAC14,UBP1,POLE,TRRAP,PPIP5K2,EXOSC9,CSK,POLR2A,DNAJA1,NUP62,TSR1,RBM19,SLC19A1,XPO1,PPIA,PRPF3,IK,SERPINE1,TPR,ENC1,SRSF7,UTP20,TGFBRAP1,ZFP36L2,SF3B4,STK17A,PPRC1,SLC38A2,EBNA1BP2,KEAP1,TFB2M,UBE2N,BAZ1B,NRIP1,MRTO4,VAPA,TLK1,GFM1,MYBBP1A,DDB1,BACH2,PITX1,GTF3C4,LRRC47,NASP,ASXL2,CHST3,TCP1,CENPF,YWHAB,RALY,ENO1,CCT5,FUBP1,POLE3,CDK6,CDC5L,CHD3,ST3GAL2,RAN,BEND3,C19ORF48,UBC,WDR81,ZNF787,TFAM,AK2,PPP2CA,SSU72,MSH2,CDK12,ZBTB2,DIDO1,GLYR1,MAN2A2,KMT2A,MCM3,NOSTRIN,GRWD1,PAICS,ZNF598,SLC30A10,CERS6,PRDX1,SNHG3,GATAD2A,DKC1,DDX1,CUL1,THUMPD1,CTPS1,EWSR1,DDX49,CASC3,SSRP1,ARID1B,CPSF7,RRP1,ZNF33B,ATP6V0A1,HNRNPAB,KAT6A,USP36,RBBP4,CNPPD1,SAFB,NCBP1,HEATR1,CCT2,HSP90AB1,EIF3B,DUS3L,PA2G4,HNRNPK,SMG1,DHX37,FDFT1,PRPF19,SMARCA4,EIF3J,PRKDC,YES1,ZNF121,TRA2B,PRMT1,HNRNPC,MALT1,WWP2,SF3A3,SUPT16H,HMGA1,FARSA,CELF1,RBMXL1,XRCC5,DDX5,UTP4,DEK,LMO2,DDX46,LSS,EIF4A3,NDST1,TRIP12,IPO7,DDX39A,DDX56,SLC38A1,CIZ1,CDT1,NOP56,HNRNPDL,CORO1C,KHDRBS1,HNRNPD,LRPPRC,EIF5A,CTDSP1,SUPT6H,RANBP2,DNAJB6,NOLC1,SNRNP200,SREBF2,CBFA2T3,ABCF1,DANCR,ACACA,MGA,NSUN2,AP5Z1,HSPD1,PIM2,GAPDH,YBX1,LIN28B,EIF5B,PCYT2,ATP6V1C1,RBM3,PGD,SF3A1,AXIN1,MPHOSPH10,PES1,IPO5,RREB1,EP300,TGFBR1,DAZAP1,ALYREF,RRP1B,FOSB,PPM1G,SRRT,PABPC1,SERBP1,AHNAK,PRPF8,SF3B3,PTMA,THRAP3,MLLT1,ELOVL6,HNRNPF,MT-ND1,NPM1,RIF1,CITED2,ETF1,SON,WDR43,TFRC,RUNX1,NQO1,EIF4G1,TRIM28,ACP5,U2AF2,NR2F2,BCLAF1,ECPAS,SRSF2,HSP90AA1,MDN1,NFATC3,ZNF521,CAPRIN1,TRMT1,SQLE,MCM4,TRIM24,NOP58,RESF1,HNRNPA2B1,LARP1,SQSTM1,HBZ,PTBP1,CCAR1,MAT2A,DDX21,HMGCS1,SFPQ,SCD,PABPC4,HNRNPM,POLR1A,KCNH2,SRSF3,EIF3A,MCM7,SMARCC1,MYC,SET,VGF,BTG1,MT-CYB,IGF2BP1,FOS,GCLM,HMGCR,SPTA1,MYB,ODC1,KHSRP,ZEB2,RELN,FUS,ILF3,FASN,GLUL,ACTB,DHX9,HNRNPU,SLC20A1,MCM5,NCL,EGR1,HSPA8
GO:0045935	positive regulation of nucleobase-containing compound metabolic process	1.9429071964258657e-38	HRAS,TXK,MITF,KMT2B,SUPT7L,MED16,NOL11,BICRA,MTA2,PHF5A,NACA,MED6,PRXL2C,PHB,METTL3,TCF20,PAXIP1,GPATCH3,BRD4,MED1,NVL,PWP1,KMT2D,TAF4B,PQBP1,MAD2L2,PSMC3,ARID2,PATZ1,TRIM44,LHX4,PPP3R1,SS18L1,MED29,LYL1,SUB1,GTF2H1,TOP2B,LYAR,CCT6A,KAT7,WDR5,CCT8,FUBP3,MEF2C,TCF3,PRPF6,ZNF24,GRSF1,ACTR8,PSMC5,ZC3HAV1,MAPK1,ABT1,DHX33,UIMC1,PUM1,MTOR,IKZF3,KANSL1,SMARCA5,RSF1,TFAP4,FAF1,ZNF131,MTDH,TCF7L2,UBE2L3,THOC1,SETMAR,RPTOR,PPP1R10,TNF,MED28,ELL2,SSBP3,BACH1,SETX,AP3D1,APEX1,BPTF,ICE1,RIOK2,NAA15,ARID1A,PITHD

			1,VPS72,MED15,PRMT5,PCBP2,RBMX,BAZ1A,MLLT3,TAF4,RBM14,DYRK1A,TFDP2,NUCKS1,WAC,ARHGEF2,BRCA2,POLR2D,BCL7B,ZFX,PAN3,PAF1,ZC3H18,BRCC3,SMARCB1,CREBBP,EXOSC3,NUP98,CNOT1,ASH1L,PLAGL2,NFYC,PELP1,MMS19,TAF9,UTP15,VCP,RYBP,PSIP1,DVL2,AGO2,E2F4,NFKB1,UBTF,CHD7,HNRNPA1,PPARGC1B,PBRM1,CTCF,HHEX,ELF1,NSD1,CCT3,CDK7,FOXK2,HMGB1,SMARCD1,YY1,MLLT10,EP400,CEBPZ,MECP2,CSDE1,SKI,TARDBP,XRCC6,PCNA,DDX3X,HDGF,JUND,HCFC1,AATF,UBP1,TRRAP,EXOSC9,NUP62,ZFP36L2,SF3B4,PPRC1,SLC38A2,UBE2N,BAZ1B,NRIP1,MYBBP1A,PITX1,ASXL2,TCP1,ENO1,CCT5,CDC5L,CHD3,BEND3,TFAM,MSH2,CDK12,GLYR1,KMT2A,GATAD2A,DKC1,ARID1B,ZNF33B,HNRNPAB,KAT6A,RBBP4,NCBP1,HEATR1,CCT2,HSP90AB1,HNRNPK,PRPF19,SMARCA4,PRKDC,YES1,TRA2B,WWP2,SUPT16H,HMGA1,CELF1,RBMXL1,XRCC5,DDX5,DEK,LMO2,CIZ1,CDT1,HNRNPD,EIF5A,SUPT6H,NOLC1,SREBF2,MGA,PIM2,YBX1,LIN28B,RBM3,AXIN1,RREB1,EP300,TGFBR1,DAZAP1,RRP1B,FOSB,PABPC1,SF3B3,PTMA,THRAP3,NPM1,RIF1,CITED2,WDR43,TFRC,RUNX1,TRIM28,U2AF2,NR2F2,BCLAF1,HSP90AA1,NFATC3,ZNF521,TRIM24,HNRNPA2B1,SQSTM1,CCAR1,DDX21,SFPQ,KCNH2,SMARCC1,MYC,IGF2BP1,FOS,MYB,KHSRP,ZEB2,FUS,ILF3,ACTB,DHX9,HNRNPU,NCL,EGR1,HSPA8
GO:0010556	regulation of macromolecule biosynthetic process	3.9651577078188344e-38	*HRAS,TXK,MITF,JMJD1C,ZNF274,KMT2B,SUPT7L,MACROH2A1,BRD9,MED16,NOL11,BICRA,MTA2,TIRAP,OTUD6B,RIOX1,PHF5A,NACA,MED6,CBX3,PHB,METTL3,EZH2,TCF20,PAXIP1,LRRFIP1,GPATCH3,INTS6,SMG5,BRD4,ZNF581,SPIN4,ZNF74,RPUSD4,CDC123,CTDP1,MED1,NVL,HNRNPL,PWP1,KMT2D,TAF4B,PQBP1,PSPC1,CEP350,MAD2L2,PSMC3,ZBTB40,ADNP2,ARID2,PATZ1,TRIM44,LHX4,ZNF512B,RBL1,PPP3R1,HIF1AN,SS18L1,MED29,USP14,LIMD1,DNMT1,LYL1,BAP1,SUB1,GTF2H1,TOP2B,LYAR,CCNH,NAB2,EIF4EBP2,CCT6A,KAT7,CLTC,EEF2,WDR5,CCT8,FUBP3,CUL3,NONO,MEF2C,TCF3,MAF1,PRPF6,ZC3H4,ZNF26,ZNF24,GSPT1,ACTR8,PSMC5,MAPK1,ABT1,DHX33,CTR9,NCOA5,FADS1,ZFP91,UIMC1,PUM1,MTOR,IKZF3,KANSL1,SPEN,SMARCA5,EIF3D,ZNF45,RSF1,TFAP4,PRMT6,FAF1,ZNF131,SBNO1,ZNF431,DHFR,MTDH,TCF7L2,ZNF239,PRAME,UBE2L3,THOC1,RPTOR,TNF,EIF4B,IGF2BP3,MED28,ELL2,PUM2,SSBP3,ZNF586,BACH1,RBM10,SETX,NFILZ,AP3D1,METTL8,APEX1,BPTF,ICE1,NAA15,ARID1A,PITHD1,SRSF10,C1QBP,VPS72,ZNF587B,CRK,MED15,PRMT5,PCBP2,RBMX,BAZ1A,MLLT3,TAF4,RBM14,RBM8A,KDM3B,LRP8,SETD2,ZNF326,INTS13,ZNF282,DYRK1A,TFDP2,JADE2,NUCKS1,SDCBP,GNL3L,WAC,DHX29,ARHGEF2,BRCA2,POLR2D,COPS2,BCL7B,ZFX,PAN3,MED13L,PAF1,FASTKD2,PHB2,TCOF1,ZNF75A,SMARCB1,CREBBP,EXOSC3,EPRS1,LSM14A,NUP98,CNOT1,ASH1L,GEMIN5,PLAGL2,NFYC,SF1,PELP1,EZR,TRUB2,DDX20,MMS19,ELOA,TAF9,ELAVL1,UTP15,RYBP,SAFB2,PSIP1,CDK4,DVL2,POLR3C,WDR82,AGO2,E2F4,RNF220,ADNP,NFKB1,UBTF,BRD2,CHD7,HNRNPA1,PPARGC1B,MAGOH,PBRM1,CTCF,HHEX,ELF1,ZNF614,NSD1,CCT3,CDK7,PKM,FOXK2,HMGB1,BZW1,SMARCD1,YY1,MLLT10,IRAK1,PUS7,NAT10,TRMT10C,EP400,CEBPZ,MECP2,CSDE1,SKI,DDX54,TARDBP,LARP4,XRCC6,PCNA,MBD1,DDX3X,HDGF,ZNF789,EIF4G2,JUND,HCFC1,CHEK1,AATF,ZMPSTE24,SLTM,UBP1,TRRAP,EXOSC9,NUP62,PPIA,TPR,ENC1,TGFBRAP1,ZFP36L2,PPRC1,KEAP1,TFB2M,UBE2N,BAZ1B,NRIP1,MYBBP1A,BACH2,PITX1,ASXL2,TCP1,CENP*

			F,RALY,ENO1,CCT5,FUBP1,CDK6,CDC5L,CHD3,BEND3,ZNF787,TFAM,PPP2CA,SSU72,CDK12,ZBTB2,GLYR1,KMT2A,NOSTRIN,GATAD2A,DKC1,DDX1,EWSR1,CASC3,ARID1B,ZNF33B,HNRNPAB,KAT6A,RBBP4,SAFB,NCBP1,HEATR1,CCT2,HSP90AB1,EIF3B,DUS3L,PA2G4,HNRNPK,SMARCA4,PRKDC,YES1,ZNF121,PRMT1,HNRNPC,MALT1,WWP2,SUPT16H,HMGA1,CELF1,XRCC5,DDX5,UTP4,DEK,LMO2,EIF4A3,KHDRBS1,HNRNPD,LRPPRC,EIF5A,CTDSP1,SUPT6H,DNAJB6,NOLC1,SREBF2,CBFA2T3,ABCF1,MGA,PIM2,GAPDH,YBX1,EIF5B,RBM3,AXIN1,RREB1,HSPH1,EP300,TGFBR1,RRP1B,FOSB,SRRT,PABPC1,SF3B3,PTMA,THRAP3,MLLT1,NPM1,RIF1,CITED2,ETF1,WDR43,TFRC,RUNX1,EIF4G1,TRIM28,NR2F2,BCLAF1,SRSF2,HSP90AA1,NFATC3,ZNF521,CAPRIN1,TRIM24,RESF1,HNRNPA2B1,LARP1,SQSTM1,CCAR1,DDX21,SFPQ,KCNH2,SMARCC1,MYC,SET,BTG1,IGF2BP1,FOS,MYB,KHSRP,ZEB2,RELN,FUS,ILF3,ACTB,DHX9,HNRNPU,NCL,EGR1,HSPA8
GO:1903311	regulation of mRNA metabolic process	9.149991572233825e-38	*TIRAP,CWC22,METTL3,HNRNPL,RNPS1,NBAS,ZC3HAV1,PKP3,PUM1,HNRNPA0,RAVER1,IGF2BP3,PUM2,SNRPA,RBM10,APEX1,ZC3H14,SRSF10,C1QBP,PRMT5,RBMX,RBM8A,DYRK1A,NSRP1,POLR2D,WTAP,RBM42,PAN3,PAF1,FASTKD2,EXOSC3,NUP98,CNOT1,SF1,ELAVL1,SAFB2,DHX34,RBM25,AGO2,RBM15B,HNRNPA1,MAGOH,SRSF6,CSDE1,MBNL1,TARDBP,SLTM,EXOSC9,SRSF7,ZFP36L2,SF3B4,CASC3,CPSF7,HNRNPAB,SAFB,NCBP1,HNRNPK,PRPF19,TRA2B,HNRNPC,CELF1,RBMXL1,DDX5,EIF4A3,KHDRBS1,HNRNPD,SUPT6H,YBX1,RBM3,DAZAP1,PABPC1,SERBP1,THRAP3,NPM1,SON,U2AF2,SRSF2,HNRNPA2B1,LARP1,PTBP1,PABPC4,HNRNPM,SRSF3,IGF2BP1,KHSRP,FUS,DHX9,HNRNPU,NCL,HSPA8*
**CC**			
GO:0005654	nucleoplasm	8.600361741950438e-141	*HRAS,NOP14,CENPN,ACSF3,SNRPB,MITF,PPP6C,JMJD1C,MIS18BP1,DAP3,RBM48,MSH3,CHTOP,KMT2B,AFF1,SUPT7L,SREK1,MACROH2A1,BRD9,MED16,MUS81,NOL11,ITFG2,MTA2,RAD51C,UTP3,CWC22,POLR3E,DCAF7,MTREX,RIOX1,PHF5A,POLR1C,MED6,CBX3,PHB,METTL3,EZH2,TCF20,DCLRE1C,NOC4L,RFWD3,MFAP1,PAXIP1,GPATCH3,INTS6,PPP6R3,H4C5,NCAPH2,BRD4,TIMM17A,RRP9,SPIN4,ZNF74,AHCTF1,RPUSD4,CTDP1,MED1,NVL,POM121C,HNRNPL,MYO16,WDR46,MCM10,EFTUD2,CRCP,RANBP3,FRMD8,KMT2D,STAG2,CASP8,TAF4B,PSMA3,PQBP1,PSMD3,PSPC1,MAD2L2,PSMC3,NAA11,DHX38,NT5C3A,NOP16,ARID2,DDX23,PATZ1,TRNT1,RNPS1,ZNF512B,RBL1,PPP3R1,HIF1AN,USP11,SS18L1,RBM45,MED29,NOSIP,STAG1,HNRNPH1,LIMD1,DNMT1,LYL1,PPP5C,BAP1,ANAPC7,SUB1,GTF2H1,DHX16,BUB3,TOP2B,LYAR,LRWD1,CCNH,NAB2,KAT7,TPP2,MSRA,AURKAIP1,WDR5,CCT8,FUBP3,IMP4,BCCIP,NOB1,SBF1,RNF40,CUL3,ARPP19,CMPK1,NONO,MEF2C,TCF3,MAF1,GID8,CLSPN,NOL9,PRPF6,ZC3H4,ZNF24,GRSF1,NXF1,UBQLN4,ACTR8,FAHD1,PSMC5,STK35,HNRNPA3,MAPK1,EEF1D,CASP3,POLR1E,PKP3,PCLAF,DHX33,CTR9,NCOA5,ZFP91,SDAD1,DHX15,UIMC1,PUM1,MTOR,RPRD2,UTP18,IKZF3,KANSL1,CPSF3,SPEN,SMARCA5,CWC25,SF3A2,ZNF45,RSF1,TFAP4,PRMT6,KPNA4,NIFK,FAF1,HNRNPA0,BAG6,EMD,ZNF131,TASOR2,MTDH,BAIAP2,TCF7L2,SRRM2,PRAME,ASCC3,UROD,UBE2L3,THOC1,PSMC2,MCMBP,RPTOR,PPIL2,PPP1R10,HECTD1,CSTF2,GRPEL1,PPP4R3A,WDR33,NIP7,TRMT61A,PDCD7,MED28,ELL2,HAT1,WDR3,SNRPA,BACH1,RBM10,CERT1,SETX,STK24,APEX1,BPTF,ED*

			C4,ICE1,RIOK2,NAA15,ARID1A,NUP43,AMMECR1,SPECC1,PPP1CC,CAVIN2,ZC3H14,CDC27,HERC2,SRSF10,AKAP8,SBDS,RCC1,VPS72,PPM1H,TICRR,MED15,SRSF8,WDR74,PRPF4,PRMT5,PCBP2,ADD1,TRMT6,RBMX,RNF126,MLLT3,RAD23B,TAF4,RBM14,RBM8A,KDM3B,BOP1,SETD2,ZNF326,API5,INTS13,BTBD1,FEN1,DYRK1A,TFDP2,WDR12,GTF3C6,SLBP,CUL4A,DNAJC8,JADE2,NUCKS1,SDCBP,PSMD1,UTP25,NUP50,PRPF38A,GNL3L,DNTTIP2,NSRP1,TFIP11,PSMB2,WAC,BRCA2,POLR2D,WTAP,COPS2,NIN,RBM42,ZFX,ACLY,SNRPD1,PAF1,ZC3H18,TCOF1,XRCC2,PDS5A,BRCC3,SMARCB1,CREBBP,EXOSC3,WBP11,COPS3,NUP98,HNRNPUL1,ASH1L,GEMIN5,APC,POLR1B,DDX42,ELOF1,NFYC,SF1,PELP1,XRN2,DDX20,MMS19,ELOA,TAF9,ELAVL1,UTP15,VCP,WASHC5,RYBP,SAFB2,GBP2,PSIP1,DCAF13,BEX4,RBM12,CDK4,DVL2,PNO1,POLR3C,RBM25,SETD1A,WDR82,SMC1A,AGO2,E2F4,PCM1,TBC1D14,SAE1,RRP36,MCM6,AQR,UBA2,NFKB1,UBTF,ZNF622,BRD2,CHD7,RBM15B,USP37,HNRNPA1,GAR1,RRS1,PPARGC1B,MAGOH,PBRM1,CTCF,ANKRD17,HNRNPR,ELF1,NSD1,CDK7,H3-3B,SETD1B,FOXK2,KIF2A,HMGB1,MAEA,SMARCD1,YY1,MLLT10,IRAK1,PIK3C2B,IP6K1,NAT10,TRMT10C,SNU13,SRSF6,EP400,SART1,MECP2,MBNL1,SKI,TKT,DDX54,TARDBP,XRCC6,PNN,PCNA,CDC25A,MBD1,CSNK2A2,DDX3X,HDGF,NCAPH,ZNF789,MCM2,JUND,HCFC1,CHEK1,AATF,ECSIT,PRMT7,SLTM,POM121,CHAMP1,UBP1,POLE,TRRAP,NUDC,EXOSC9,CCDC86,DNAJC7,SUMO3,NUP62,TSR1,RBM19,XPO1,PRPF3,IK,TPR,ENC1,SRSF7,UTP20,SF3B4,STK17A,PPRC1,KEAP1,UBE2N,BAZ1B,NRIP1,TLK1,TEX10,MYBBP1A,DDB1,BACH2,PSMG1,GTF3C4,NASP,ASXL2,CENPF,FUBP1,POLE3,CDK6,CDC5L,CHD3,NUP153,RAN,BEND3,UBC,SSU72,MSH2,CDK12,ZBTB2,GLYR1,KMT2A,MCM3,GRWD1,H4C8,GATAD2A,DKC1,DDX1,H2BC12,CUL1,THUMPD1,EWSR1,DDX49,CASC3,SSRP1,ARID1B,KPNB1,CPSF7,TXNRD1,ATP6V0A1,HNRNPAB,KAT6A,USP36,RBBP4,SAFB,NCBP1,HEATR1,HSP90AB1,HNRNPK,SMG1,DHX37,PRPF19,SMARCA4,PRKDC,TRA2B,PRMT1,HNRNPC,SF3A3,SUPT16H,HMGA1,CELF1,XRCC5,DDX5,PSME3,UTP4,DEK,LMO2,LMNB1,DDX46,EIF4A3,NUP214,TRIP12,IPO7,SURF6,DDX39A,CIZ1,CDT1,NOP56,HNRNPDL,KHDRBS1,HNRNPD,LRPPRC,CTDSP1,SUPT6H,RANBP2,DNAJB6,NOLC1,SNRNP200,SREBF2,CBFA2T3,ABCF1,MGA,NSUN2,AP5Z1,YBX1,LIN28B,RBM3,SF3A1,MPHOSPH10,PES1,ANP32B,RREB1,HSPH1,EP300,DAZAP1,ALYREF,RRP1B,FOSB,PPM1G,SRRT,PRPF8,COA7,SF3B3,PTMA,THRAP3,MLLT1,HNRNPF,NPM1,RIF1,CITED2,SON,WDR43,RUNX1,TRIM28,U2AF2,NR2F2,BCLAF1,ECPAS,SRSF2,HSP90AA1,MDN1,NFATC3,ZNF521,TRMT1,MCM4,TRIM24,NOP58,HNRNPA2B1,SPN,BAG1,SQSTM1,PTBP1,CCAR1,DDX21,SFPQ,HNRNPM,POLR1A,SRSF3,ANKRD11,EIF3A,MCM7,SMARCC1,MYC,SET,BTG1,IGF2BP1,FOS,MYB,KHSRP,ZEB2,FUS,ILF3,ACTB,DHX9,HNRNPU,MCM5,NCL,EGR1,HSPA8
GO:0031981	nuclear lumen	1.5924688755699682e-113	*HRAS,NOP14,CENPN,ACSF3,SNRPB,MITF,PPP6C,JMJD1C,MIS18BP1,DAP3,RBM48,PPAN,MSH3,CHTOP,ZNF274,KMT2B,AFF1,SUPT7L,SREK1,MACROH2A1,BRD9,MED16,MUS81,NOL11,ITFG2,MTA2,SNHG6,RAD51C,UTP3,CWC22,POLR3E,DCAF7,MTREX,RIOX1,PHF5A,POLR1C,MED6,CBX3,PHB,METTL3,EZH2,TCF20,DCLRE1C,NOC4L,RFWD3,MFAP1,PAXIP1,GPATCH3,INTS6,PPP6R3,H4C5,NCAPH2,BRD4,TIMM17A,RRP9,SPIN4,ZNF74,AHCTF1,RPUSD4,CTDP1,MED1,NVL,POM121C,HNRNPL,MYO16,PWP1,WDR46,MCM10,EFTUD*

			2,CHRAC1,CRCP,RANBP3,FRMD8,KMT2D,STAG2,CASP8,TAF4B,PSMA3,PQBP1,PSMD3,PSPC1,MAD2L2,PSMC3,NAA11,TIMM44,DHX38,NT5C3A,NOP16,ARID2,DDX23,PATZ1,TRNT1,RNPS1,ZNF512B,RBL1,PPP3R1,HIF1AN,USP11,SS18L1,RBM45,MED29,NOSIP,STAG1,HNRNPH1,LIMD1,DNMT1,LYL1,PPP5C,BAP1,ANAPC7,SUB1,GTF2H1,DHX16,BUB3,TOP2B,LYAR,LRWD1,CCNH,NAB2,KAT7,TPP2,MSRA,AURKAIP1,SNHG20,WDR5,CCT8,FUBP3,IMP4,SNHG17,BCCIP,NOB1,SBF1,RNF40,CUL3,ARPP19,CMPK1,NONO,MEF2C,TCF3,MAF1,GID8,CLSPN,NOL9,PLK4,PRPF6,ZC3H4,ZNF24,GRSF1,NXF1,UBQLN4,ACTR8,FAHD1,PSMC5,STK35,HNRNPA3,MAPK1,EEF1D,CASP3,POLR1E,PKP3,ABT1,PCLAF,WDR36,DHX33,CTR9,NCOA5,ZFP91,SDAD1,DHX15,UIMC1,PUM1,MTOR,RPRD2,UTP18,IKZF3,KANSL1,CPSF3,SPEN,SMARCA5,CWC25,SF3A2,ZNF45,RSF1,TFAP4,NAA50,PRMT6,KPNA4,NIFK,FAF1,HNRNPA0,BAG6,EMD,ZNF131,TASOR2,MTDH,BAIAP2,HSPA9,TCF7L2,SRRM2,PRAME,ASCC3,UROD,SLC29A2,UBE2L3,THOC1,PSMC2,MCMBP,SETMAR,RPTOR,PPIL2,PPP1R10,HECTD1,CSTF2,GRPEL1,PPP4R3A,WDR33,NIP7,TRMT61A,NOL7,PDCD7,MED28,ELL2,HAT1,WDR3,SNRPA,BACH1,RBM10,CERT1,SETX,STK24,APEX1,BPTF,EDC4,ICE1,RIOK2,NAA15,ARID1A,NUP43,AMMECR1,SPECC1,PPP1CC,CAVIN2,ZC3H14,CDC27,HERC2,SRSF10,AKAP8,SBDS,RCC1,C1QBP,VPS72,PPM1H,TICRR,MED15,SRSF8,WDR74,PRPF4,PRMT5,PCBP2,ADD1,TRMT6,RBMX,RNF126,BAZ1A,MLLT3,RAD23B,TAF4,RBM14,RBM8A,KDM3B,BOP1,SETD2,ZNF326,API5,INTS13,BTBD1,FEN1,DYRK1A,TFDP2,WDR12,URB2,GTF3C6,SLBP,CUL4A,DNAJC8,JADE2,NUCKS1,SDCBP,PSMD1,UTP25,NUP50,PRPF38A,AGPAT5,GNL3L,DNTTIP2,NSRP1,TFIP11,PSMB2,WAC,BRCA2,POLR2D,WTAP,COPS2,NIN,RBM42,ZFX,ACLY,SNRPD1,PAF1,ZC3H18,PHB2,TCOF1,XRCC2,PDS5A,BRCC3,SMARCB1,CREBBP,EXOSC3,WBP11,COPS3,NUP98,HNRNPUL1,ASH1L,GEMIN5,APC,POLR1B,DDX42,ELOF1,NFYC,SF1,PELP1,XRN2,EZR,DDX20,DDX51,MMS19,ELOA,TAF9,ELAVL1,UTP15,VCP,WASHC5,RYBP,SAFB2,GBP2,PSIP1,CSTB,DCAF13,BEX4,RBM12,CDK4,DVL2,PNO1,POLR3C,RBM25,SETD1A,RRP12,WDR82,SMC1A,AGO2,E2F4,PCM1,RNF220,TBC1D14,GNL2,SAE1,RRP36,MCM6,AQR,DDX18,UBA2,NFKB1,UBTF,ZNF622,BRD2,CHD7,RBM15B,USP37,HNRNPA1,GAR1,RRS1,PPARGC1B,MAGOH,PBRM1,CTCF,ANKRD17,HNRNPR,ELF1,NSD1,RSL1D1,CDK7,H3-3B,SETD1B,FOXK2,KIF2A,HMGB1,MAEA,SMARCD1,YY1,NOL8,MLLT10,IRAK1,PIK3C2B,IP6K1,NAT10,TRMT10C,SNU13,SRSF6,EP400,SART1,MECP2,MBNL1,SKI,TKT,DDX54,TARDBP,XRCC6,PNN,PCNA,CDC25A,MBD1,CSNK2A2,DDX3X,HDGF,NCAPH,ZNF789,MCM2,JUND,CFL1,HCFC1,CHEK1,AATF,CLN6,ECSIT,PRMT7,SLTM,URB1,POM121,CHAMP1,DNAJC21,UBP1,POLE,TRRAP,NUDC,EXOSC9,POLR2A,CCDC86,DNAJC7,SUMO3,NUP62,TSR1,RBM19,XPO1,PRPF3,IK,TPR,ENC1,SRSF7,UTP20,SF3B4,STK17A,PPRC1,EBNA1BP2,KEAP1,UBE2N,BAZ1B,NRIP1,MRTO4,TLK1,TEX10,MYBBP1A,DDB1,BACH2,PSMG1,GTF3C4,NASP,ASXL2,TCP1,CENPF,FUBP1,POLE3,CDK6,CDC5L,CHD3,NUP153,RAN,BEND3,C19ORF48,UBC,SSU72,MSH2,CDK12,ZBTB2,GLYR1,KMT2A,MCM3,GRWD1,H4C8,SNHG3,GATAD2A,DKC1,DDX1,H2BC12,CUL1,THUMPD1,EWSR1,DDX49,CASC3,SSRP1,ARID1B,KPNB1,CPSF7,RRP1,TXNRD1,ATP6V0A1,HNRNPAB,KAT6A,USP36,RBBP4,SAFB,NCBP1,HEATR1,HSP90AB1,PA2G4,HNRNPK,SMG1,DHX37,PRPF19,SMARCA4,PRKDC,TRA2B,P

			RMT1,HNRNPC,MALT1,SF3A3,SUPT16H,HMGA1,CELF1,XRCC5,DDX5,PSME3,UTP4,DEK,LMO2,LMNB1,DDX46,EIF4A3,NUP214,TRIP12,IPO7,SURF6,DDX39A,DDX56,CIZ1,CDT1,NOP56,HNRNPDL,KHDRBS1,HNRNPD,LRPPRC,CTDSP1,SUPT6H,RANBP2,DNAJB6,NOLC1,SNRNP200,SREBF2,CBFA2T3,ABCF1,DANCR,ACACA,MGA,NSUN2,AP5Z1,YBX1,LIN28B,RBM3,SF3A1,LMNB2,MPHOSPH10,PES1,ANP32B,IPO5,RREB1,HSPH1,EP300,DAZAP1,ALYREF,RRP1B,FOSB,PPM1G,STON2,SRRT,PRPF8,COA7,SF3B3,PTMA,THRAP3,MLLT1,HNRNPF,NPM1,RIF1,CITED2,SON,WDR43,RUNX1,TRIM28,U2AF2,NR2F2,BCLAF1,ECPAS,SRSF2,HSP90AA1,MDN1,NFATC3,ZNF521,TRMT1,MCM4,TRIM24,NOP58,HNRNPA2B1,SPN,BAG1,SQSTM1,PTBP1,CCAR1,DDX21,SFPQ,SCD,HNRNPM,POLR1A,SRSF3,ANKRD11,EIF3A,MCM7,SMARCC1,MYC,SET,BTG1,IGF2BP1,FOS,MYB,KHSRP,ZEB2,FUS,ILF3,ACTB,DHX9,HNRNPU,MCM5,NCL,EGR1,HSPA8
GO:0031974	membrane-enclosed lumen	1.514657470535515e-106	*HRAS,NOP14,CENPN,ACSF3,SNRPB,MITF,PPP6C,JMJD1C,MIS18BP1,DAP3,RBM48,PPAN,MSH3,CHTOP,ERAL1,ZNF274,GYG1,KMT2B,AFF1,MMAB,SUPT7L,SREK1,MACROH2A1,BRD9,MED16,MUS81,NOL11,ITFG2,MTA2,SNHG6,RAD51C,MIX23,UTP3,CWC22,POLR3E,NEU1,DCAF7,MTREX,RIOX1,PHF5A,POLR1C,MED6,CBX3,PHB,METTL3,EZH2,TCF20,DCLRE1C,NOC4L,RFWD3,MFAP1,PAXIP1,GPATCH3,INTS6,PPP6R3,H4C5,NCAPH2,BRD4,TIMM17A,RRP9,MRPL1,SPIN4,ZNF74,MRPL11,AHCTF1,RPUSD4,MRPS30,CTDP1,MED1,NVL,POM121C,HNRNPL,NLN,MYO16,PWP1,WDR46,MCM10,OXA1L,EFTUD2,CHRAC1,CRCP,RANBP3,FRMD8,KMT2D,STAG2,CASP8,TAF4B,PSMA3,PQBP1,PSMD3,PSPC1,MAD2L2,MRRF,POLDIP2,PSMC3,NAA11,TIMM44,DHX38,NT5C3A,NOP16,ARID2,DDX23,PATZ1,TRNT1,RNPS1,ZNF512B,RBL1,PPP3R1,HIF1AN,USP11,SS18L1,RBM45,MED29,NOSIP,STAG1,HNRNPH1,LIMD1,DNMT1,LYL1,PPP5C,BAP1,ANAPC7,SUB1,GTF2H1,DHX16,BUB3,TOP2B,LYAR,LRWD1,THOP1,CCNH,NAB2,KAT7,TPP2,EEF2,MSRA,AURKAIP1,SNHG20,WDR5,CCT8,TMEM43,FUBP3,IMP4,SNHG17,BCCIP,DLAT,NOB1,SBF1,RNF40,CUL3,ARPP19,CMPK1,NONO,MEF2C,TCF3,MAF1,GID8,CLSPN,NOL9,PLK4,PRPF6,ZC3H4,ZNF24,GRSF1,NXF1,UBQLN4,ACTR8,FAHD1,PSMC5,STK35,TIMM23,HNRNPA3,MAPK1,STAR,EEF1D,CASP3,POLR1E,PKP3,ABT1,PCLAF,WDR36,PDSS1,DHX33,CTR9,NCOA5,ZFP91,SDAD1,DHX15,UIMC1,PUM1,MTOR,DHX30,PDPR,RPRD2,UTP18,IKZF3,KANSL1,CPSF3,SPEN,GOT2,VDAC1,SMARCA5,CWC25,SF3A2,ZNF45,RSF1,TFAP4,NAA50,PRMT6,KPNA4,NIFK,FAF1,HNRNPA0,BAG6,EMD,ZNF131,TASOR2,MTDH,BAIAP2,HSPA9,MCCC1,TCF7L2,SRRM2,MRPL15,PRAME,ASCC3,UROD,SLC29A2,UBE2L3,THOC1,PSMC2,MCMBP,SETMAR,RPTOR,PPIL2,PPP1R10,MRPS35,HECTD1,CSTF2,GRPEL1,PPP4R3A,WDR33,NIP7,TRMT61A,NOL7,PDCD7,MED28,ELL2,HAT1,WDR3,SNRPA,BACH1,RBM10,CERT1,SETX,STK24,APEX1,BPTF,EDC4,ICE1,RIOK2,NAA15,ARID1A,NUP43,AMMECR1,SPECC1,PPP1CC,CAVIN2,ZC3H14,CDC27,HERC2,SRSF10,CTSL,AKAP8,SBDS,RCC1,C1QBP,VPS72,PPM1H,TICRR,MED15,EOGT,SRSF8,WDR74,PRPF4,KTN1,PRMT5,PCBP2,ADD1,TRMT6,RBMX,RNF126,BAZ1A,MCCC2,MLLT3,RAD23B,VAT1,TAF4,RBM14,RBM8A,KDM3B,BOP1,GOLM1,SETD2,ZNF326,API5,INTS13,BTBD1,TUBB,FEN1,DYRK1A,TFDP2,WDR12,URB2,GTF3C6,SLBP,CUL4A,DNAJC8,JADE2,NUCKS1,SDCBP,PSMD1,UTP25,NUP50,PRPF38A,AGPAT5,GNL3L,DNTTIP2,IBA57,NSRP1,TFIP11,PSMB2,WAC,BRCA2,POLR2*

			D,WTAP,COPS2,NIN,RBM42,ZFX,ACLY,SNRPD1,PAF1,ZC3H18,FASTKD2,PHB2,TCOF1,XRCC2,PDS5A,BRCC3,SMARCB1,CREBBP,EXOSC3,WBP11,COPS3,NUP98,HNRNPUL1,LYN,ASH1L,LRRC59,GEMIN5,APC,POLR1B,DDX42,CAPN1,ELOF1,NFYC,SF1,PELP1,XRN2,EZR,TRUB2,DDX20,DDX51,MMS19,ELOA,TAF9,ELAVL1,UTP15,VCP,WASHC5,RYBP,SAFB2,GBP2,PSIP1,CSTB,DCAF13,BEX4,RBM12,CDK4,DVL2,PNO1,POLR3C,MRPS2,RBM25,SETD1A,RRP12,WDR82,SMC1A,AGO2,E2F4,FOXRED2,PCM1,RNF220,TBC1D14,GNL2,SAE1,RRP36,MCM6,AQR,DDX18,IGF2R,UBA2,NFKB1,UBTF,ZNF622,BRD2,CHD7,RBM15B,USP37,HNRNPA1,GAR1,RRS1,PPARGC1B,MAGOH,PBRM1,CTCF,ANKRD17,HNRNPR,ELF1,NSD1,RSL1D1,CDK7,H3-3B,SETD1B,PPIF,PKM,FOXK2,KIF2A,HMGB1,MAEA,SMARCD1,CANX,YY1,NOL8,MLLT10,IRAK1,PIK3C2B,IP6K1,NAT10,TRMT10C,SNU13,SRSF6,EP400,SART1,MECP2,MBNL1,SKI,TKT,DDX54,TARDBP,XRCC6,PNN,PCNA,CDC25A,MBD1,CSNK2A2,DDX3X,HDGF,NCAPH,ZNF789,MCM2,JUND,CFL1,HCFC1,CHEK1,AATF,CLN6,ECSIT,PRMT7,OXCT1,SLC25A5,SLTM,URB1,POM121,CHAMP1,DNAJC21,UBP1,POLE,TRRAP,NUDC,EXOSC9,POLR2A,CCDC86,DNAJC7,SUMO3,NUP62,DYNC1H1,TSR1,RBM19,XPO1,PPIA,PRPF3,IK,SERPINE1,TPR,ENC1,SRSF7,UTP20,SF3B4,STK17A,PPRC1,EBNA1BP2,KEAP1,TFB2M,UBE2N,BAZ1B,NRIP1,MRTO4,TLK1,TEX10,GFM1,MYBBP1A,DDB1,BACH2,PSMG1,GTF3C4,NASP,ASXL2,TCP1,CENPF,FUBP1,POLE3,CDK6,CDC5L,CHD3,NUP153,RAN,BEND3,C19ORF48,UBC,TFAM,AK2,SSU72,MSH2,CDK12,ZBTB2,GLYR1,KMT2A,MCM3,GRWD1,H4C8,SNHG3,GATAD2A,DKC1,DDX1,H2BC12,CUL1,THUMPD1,EWSR1,DDX49,CASC3,SSRP1,ARID1B,KPNB1,CPSF7,RRP1,TXNRD1,ATP6V0A1,HNRNPAB,KAT6A,ATAD3A,USP36,RBBP4,SAFB,NCBP1,HEATR1,CCT2,HSP90AB1,PA2G4,HNRNPK,SMG1,DHX37,PRPF19,SMARCA4,PRKDC,TRA2B,PRMT1,HNRNPC,MALT1,SF3A3,SUPT16H,HMGA1,CELF1,XRCC5,DDX5,PSME3,UTP4,DEK,LMO2,LMNB1,DDX46,EIF4A3,NUP214,TRIP12,IPO7,SURF6,DDX39A,DDX56,CIZ1,CDT1,NOP56,HNRNPDL,KHDRBS1,HNRNPD,LRPPRC,CTDSP1,SUPT6H,RANBP2,DNAJB6,NOLC1,SNRNP200,SREBF2,CBFA2T3,ABCF1,DANCR,ACACA,MGA,NSUN2,AP5Z1,HSPD1,YBX1,LIN28B,RBM3,SF3A1,LMNB2,MPHOSPH10,GDI2,PES1,ANP32B,IPO5,RREB1,HSPH1,EP300,DAZAP1,ALYREF,RRP1B,FOSB,PPM1G,STON2,SRRT,PRPF8,COA7,SF3B3,PTMA,FTL,THRAP3,MLLT1,HNRNPF,NPM1,RIF1,CITED2,SON,WDR43,RUNX1,TRIM28,U2AF2,NR2F2,BCLAF1,ECPAS,SRSF2,HSP90AA1,MDN1,NFATC3,ZNF521,TRMT1,MCM4,TRIM24,NOP58,HNRNPA2B1,SPN,BAG1,SQSTM1,PTBP1,CCAR1,DDX21,SFPQ,SCD,HNRNPM,POLR1A,SRSF3,ANKRD11,EIF3A,MCM7,SMARCC1,MYC,SET,VGF,BTG1,IGF2BP1,FOS,MYB,KHSRP,ZEB2,FUS,ILF3,ACTB,DHX9,HNRNPU,MCM5,NCL,EGR1,HSPA8,FTH1
GO:0070013	intracellular organelle lumen	1.514657470535515e-106	*HRAS,NOP14,CENPN,ACSF3,SNRPB,MITF,PPP6C,JMJD1C,MIS18BP1,DAP3,RBM48,PPAN,MSH3,CHTOP,ERAL1,ZNF274,GYG1,KMT2B,AFF1,MMAB,SUPT7L,SREK1,MACROH2A1,BRD9,MED16,MUS81,NOL11,ITFG2,MTA2,SNHG6,RAD51C,MIX23,UTP3,CWC22,POLR3E,NEU1,DCAF7,MTREX,RIOX1,PHF5A,POLR1C,MED6,CBX3,PHB,METTL3,EZH2,TCF20,DCLRE1C,NOC4L,RFWD3,MFAP1,PAXIP1,GPATCH3,INTS6,PPP6R3,H4C5,NCAPH2,BRD4,TIMM17A,RRP9,MRPL1,SPIN4,ZNF74,MRPL11,AHCTF1,RPUSD4,MRPS30,CTDP1,MED1,NVL,POM121C,HNRNPL,NLN,MYO16,PWP1,WDR46,MC*

			M10,OXA1L,EFTUD2,CHRAC1,CRCP,RANBP3,FRMD8,KMT2D,STAG2,CASP8,TAF4B,PSMA3,PQBP1,PSMD3,PSPC1,MAD2L2,MRRF,POLDIP2,PSMC3,NAA11,TIMM44,DHX38,NT5C3A,NOP16,ARID2,DDX23,PATZ1,TRNT1,RNPS1,ZNF512B,RBL1,PPP3R1,HIF1AN,USP11,SS18L1,RBM45,MED29,NOSIP,STAG1,HNRNPH1,LIMD1,DNMT1,LYL1,PPP5C,BAP1,ANAPC7,SUB1,GTF2H1,DHX16,BUB3,TOP2B,LYAR,LRWD1,THOP1,CCNH,NAB2,KAT7,TPP2,EEF2,MSRA,AURKAIP1,SNHG20,WDR5,CCT8,TMEM43,FUBP3,IMP4,SNHG17,BCCIP,DLAT,NOB1,SBF1,RNF40,CUL3,ARPP19,CMPK1,NONO,MEF2C,TCF3,MAF1,GID8,CLSPN,NOL9,PLK4,PRPF6,ZC3H4,ZNF24,GRSF1,NXF1,UBQLN4,ACTR8,FAHD1,PSMC5,STK35,TIMM23,HNRNPA3,MAPK1,STAR,EEF1D,CASP3,POLR1E,PKP3,ABT1,PCLAF,WDR36,PDSS1,DHX33,CTR9,NCOA5,ZFP91,SDAD1,DHX15,UIMC1,PUM1,MTOR,DHX30,PDPR,RPRD2,UTP18,IKZF3,KANSL1,CPSF3,SPEN,GOT2,VDAC1,SMARCA5,CWC25,SF3A2,ZNF45,RSF1,TFAP4,NAA50,PRMT6,KPNA4,NIFK,FAF1,HNRNPA0,BAG6,EMD,ZNF131,TASOR2,MTDH,BAIAP2,HSPA9,MCCC1,TCF7L2,SRRM2,MRPL15,PRAME,ASCC3,UROD,SLC29A2,UBE2L3,THOC1,PSMC2,MCMBP,SETMAR,RPTOR,PPIL2,PPP1R10,MRPS35,HECTD1,CSTF2,GRPEL1,PPP4R3A,WDR33,NIP7,TRMT61A,NOL7,PDCD7,MED28,ELL2,HAT1,WDR3,SNRPA,BACH1,RBM10,CERT1,SETX,STK24,APEX1,BPTF,EDC4,ICE1,RIOK2,NAA15,ARID1A,NUP43,AMMECR1,SPECC1,PPP1CC,CAVIN2,ZC3H14,CDC27,HERC2,SRSF10,CTSL,AKAP8,SBDS,RCC1,C1QBP,VPS72,PPM1H,TICRR,MED15,EOGT,SRSF8,WDR74,PRPF4,KTN1,PRMT5,PCBP2,ADD1,TRMT6,RBMX,RNF126,BAZ1A,MCCC2,MLLT3,RAD23B,VAT1,TAF4,RBM14,RBM8A,KDM3B,BOP1,GOLM1,SETD2,ZNF326,API5,INTS13,BTBD1,TUBB,FEN1,DYRK1A,TFDP2,WDR12,URB2,GTF3C6,SLBP,CUL4A,DNAJC8,JADE2,NUCKS1,SDCBP,PSMD1,UTP25,NUP50,PRPF38A,AGPAT5,GNL3L,DNTTIP2,IBA57,NSRP1,TFIP11,PSMB2,WAC,BRCA2,POLR2D,WTAP,COPS2,NIN,RBM42,ZFX,ACLY,SNRPD1,PAF1,ZC3H18,FASTKD2,PHB2,TCOF1,XRCC2,PDS5A,BRCC3,SMARCB1,CREBBP,EXOSC3,WBP11,COPS3,NUP98,HNRNPUL1,LYN,ASH1L,LRRC59,GEMIN5,APC,POLR1B,DDX42,CAPN1,ELOF1,NFYC,SF1,PELP1,XRN2,EZR,TRUB2,DDX20,DDX51,MMS19,ELOA,TAF9,ELAVL1,UTP15,VCP,WASHC5,RYBP,SAFB2,GBP2,PSIP1,CSTB,DCAF13,BEX4,RBM12,CDK4,DVL2,PNO1,POLR3C,MRPS2,RBM25,SETD1A,RRP12,WDR82,SMC1A,AGO2,E2F4,FOXRED2,PCM1,RNF220,TBC1D14,GNL2,SAE1,RRP36,MCM6,AQR,DDX18,IGF2R,UBA2,NFKB1,UBTF,ZNF622,BRD2,CHD7,RBM15B,USP37,HNRNPA1,GAR1,RRS1,PPARGC1B,MAGOH,PBRM1,CTCF,ANKRD17,HNRNPR,ELF1,NSD1,RSL1D1,CDK7,H3-3B,SETD1B,PPIF,PKM,FOXK2,KIF2A,HMGB1,MAEA,SMARCD1,CANX,YY1,NOL8,MLLT10,IRAK1,PIK3C2B,IP6K1,NAT10,TRMT10C,SNU13,SRSF6,EP400,SART1,MECP2,MBNL1,SKI,TKT,DDX54,TARDBP,XRCC6,PNN,PCNA,CDC25A,MBD1,CSNK2A2,DDX3X,HDGF,NCAPH,ZNF789,MCM2,JUND,CFL1,HCFC1,CHEK1,AATF,CLN6,ECSIT,PRMT7,OXCT1,SLC25A5,SLTM,URB1,POM121,CHAMP1,DNAJC21,UBP1,POLE,TRRAP,NUDC,EXOSC9,POLR2A,CCDC86,DNAJC7,SUMO3,NUP62,DYNC1H1,TSR1,RBM19,XPO1,PPIA,PRPF3,IK,SERPINE1,TPR,ENC1,SRSF7,UTP20,SF3B4,STK17A,PPRC1,EBNA1BP2,KEAP1,TFB2M,UBE2N,BAZ1B,NRIP1,MRTO4,TLK1,TEX10,GFM1,MYBBP1A,DDB1,BACH2,PSMG1,GTF3C4,NASP,ASXL2,TCP1,CENPF,FUBP1,POLE3,CDK6,CDC5L,CHD3,NUP153,RAN,BEND3,C19ORF

			48,UBC,TFAM,AK2,SSU72,MSH2,CDK12,ZBTB2,GLYR1,KMT2A,MCM3,GRWD1,H4C8,SNHG3,GATAD2A,DKC1,DDX1,H2BC12,CUL1,THUMPD1,EWSR1,DDX49,CASC3,SSRP1,ARID1B,KPNB1,CPSF7,RRP1,TXNRD1,ATP6V0A1,HNRNPAB,KAT6A,ATAD3A,USP36,RBBP4,SAFB,NCBP1,HEATR1,CCT2,HSP90AB1,PA2G4,HNRNPK,SMG1,DHX37,PRPF19,SMARCA4,PRKDC,TRA2B,PRMT1,HNRNPC,MALT1,SF3A3,SUPT16H,HMGA1,CELF1,XRCC5,DDX5,PSME3,UTP4,DEK,LMO2,LMNB1,DDX46,EIF4A3,NUP214,TRIP12,IPO7,SURF6,DDX39A,DDX56,CIZ1,CDT1,NOP56,HNRNPDL,KHDRBS1,HNRNPD,LRPPRC,CTDSP1,SUPT6H,RANBP2,DNAJB6,NOLC1,SNRNP200,SREBF2,CBFA2T3,ABCF1,DANCR,ACACA,MGA,NSUN2,AP5Z1,HSPD1,YBX1,LIN28B,RBM3,SF3A1,LMNB2,MPHOSPH10,GDI2,PES1,ANP32B,IPO5,RREB1,HSPH1,EP300,DAZAP1,ALYREF,RRP1B,FOSB,PPM1G,STON2,SRRT,PRPF8,COA7,SF3B3,PTMA,FTL,THRAP3,MLLT1,HNRNPF,NPM1,RIF1,CITED2,SON,WDR43,RUNX1,TRIM28,U2AF2,NR2F2,BCLAF1,ECPAS,SRSF2,HSP90AA1,MDN1,NFATC3,ZNF521,TRMT1,MCM4,TRIM24,NOP58,HNRNPA2B1,SPN,BAG1,SQSTM1,PTBP1,CCAR1,DDX21,SFPQ,SCD,HNRNPM,POLR1A,SRSF3,ANKRD11,EIF3A,MCM7,SMARCC1,MYC,SET,VGF,BTG1,IGF2BP1,FOS,MYB,KHSRP,ZEB2,FUS,ILF3,ACTB,DHX9,HNRNPU,MCM5,NCL,EGR1,HSPA8,FTH1
GO:0043233	organelle lumen	1.514657470535515e-106	HRAS,NOP14,CENPN,ACSF3,SNRPB,MITF,PPP6C,JMJD1C,MIS18BP1,DAP3,RBM48,PPAN,MSH3,CHTOP,ERAL1,ZNF274,GYG1,KMT2B,AFF1,MMAB,SUPT7L,SREK1,MACROH2A1,BRD9,MED16,MUS81,NOL11,ITFG2,MTA2,SNHG6,RAD51C,MIX23,UTP3,CWC22,POLR3E,NEU1,DCAF7,MTREX,RIOX1,PHF5A,POLR1C,MED6,CBX3,PHB,METTL3,EZH2,TCF20,DCLRE1C,NOC4L,RFWD3,MFAP1,PAXIP1,GPATCH3,INTS6,PPP6R3,H4C5,NCAPH2,BRD4,TIMM17A,RRP9,MRPL1,SPIN4,ZNF74,MRPL11,AHCTF1,RPUSD4,MRPS30,CTDP1,MED1,NVL,POM121C,HNRNPL,NLN,MYO16,PWP1,WDR46,MCM10,OXA1L,EFTUD2,CHRAC1,CRCP,RANBP3,FRMD8,KMT2D,STAG2,CASP8,TAF4B,PSMA3,PQBP1,PSMD3,PSPC1,MAD2L2,MRRF,POLDIP2,PSMC3,NAA11,TIMM44,DHX38,NT5C3A,NOP16,ARID2,DDX23,PATZ1,TRNT1,RNPS1,ZNF512B,RBL1,PPP3R1,HIF1AN,USP11,SS18L1,RBM45,MED29,NOSIP,STAG1,HNRNPH1,LIMD1,DNMT1,LYL1,PPP5C,BAP1,ANAPC7,SUB1,GTF2H1,DHX16,BUB3,TOP2B,LYAR,LRWD1,THOP1,CCNH,NAB2,KAT7,TPP2,EEF2,MSRA,AURKAIP1,SNHG20,WDR5,CCT8,TMEM43,FUBP3,IMP4,SNHG17,BCCIP,DLAT,NOB1,SBF1,RNF40,CUL3,ARPP19,CMPK1,NONO,MEF2C,TCF3,MAF1,GID8,CLSPN,NOL9,PLK4,PRPF6,ZC3H4,ZNF24,GRSF1,NXF1,UBQLN4,ACTR8,FAHD1,PSMC5,STK35,TIMM23,HNRNPA3,MAPK1,STAR,EEF1D,CASP3,POLR1E,PKP3,ABT1,PCLAF,WDR36,PDSS1,DHX33,CTR9,NCOA5,ZFP91,SDAD1,DHX15,UIMC1,PUM1,MTOR,DHX30,PDPR,RPRD2,UTP18,IKZF3,KANSL1,CPSF3,SPEN,GOT2,VDAC1,SMARCA5,CWC25,SF3A2,ZNF45,RSF1,TFAP4,NAA50,PRMT6,KPNA4,NIFK,FAF1,HNRNPA0,BAG6,EMD,ZNF131,TASOR2,MTDH,BAIAP2,HSPA9,MCCC1,TCF7L2,SRRM2,MRPL15,PRAME,ASCC3,UROD,SLC29A2,UBE2L3,THOC1,PSMC2,MCMBP,SETMAR,RPTOR,PPIL2,PPP1R10,MRPS35,HECTD1,CSTF2,GRPEL1,PPP4R3A,WDR33,NIP7,TRMT61A,NOL7,PDCD7,MED28,ELL2,HAT1,WDR3,SNRPA,BACH1,RBM10,CERT1,SETX,STK24,APEX1,BPTF,EDC4,ICE1,RIOK2,NAA15,ARID1A,NUP43,AMMECR1,SPECC1,PPP1CC,CAVIN2,ZC3H14,CDC27,HERC2,SRSF10,CTSL,AKAP8,SBDS,RCC1,C1QBP,VPS72,PPM1H

			,TICRR,MED15,EOGT,SRSF8,WDR74,PRPF4,KTN1,PRMT5,PCBP2,ADD1,TRMT6,RBMX,RNF126,BAZ1A,MCCC2,MLLT3,RAD23B,VAT1,TAF4,RBM14,RBM8A,KDM3B,BOP1,GOLM1,SETD2,ZNF326,API5,INTS13,BTBD1,TUBB,FEN1,DYRK1A,TFDP2,WDR12,URB2,GTF3C6,SLBP,CUL4A,DNAJC8,JADE2,NUCKS1,SDCBP,PSMD1,UTP25,NUP50,PRPF38A,AGPAT5,GNL3L,DNTTIP2,IBA57,NSRP1,TFIP11,PSMB2,WAC,BRCA2,POLR2D,WTAP,COPS2,NIN,RBM42,ZFX,ACLY,SNRPD1,PAF1,ZC3H18,FASTKD2,PHB2,TCOF1,XRCC2,PDS5A,BRCC3,SMARCB1,CREBBP,EXOSC3,WBP11,COPS3,NUP98,HNRNPUL1,LYN,ASH1L,LRRC59,GEMIN5,APC,POLR1B,DDX42,CAPN1,ELOF1,NFYC,SF1,PELP1,XRN2,EZR,TRUB2,DDX20,DDX51,MMS19,ELOA,TAF9,ELAVL1,UTP15,VCP,WASHC5,RYBP,SAFB2,GBP2,PSIP1,CSTB,DCAF13,BEX4,RBM12,CDK4,DVL2,PNO1,POLR3C,MRPS2,RBM25,SETD1A,RRP12,WDR82,SMC1A,AGO2,E2F4,FOXRED2,PCM1,RNF220,TBC1D14,GNL2,SAE1,RRP36,MCM6,AQR,DDX18,IGF2R,UBA2,NFKB1,UBTF,ZNF622,BRD2,CHD7,RBM15B,USP37,HNRNPA1,GAR1,RRS1,PPARGC1B,MAGOH,PBRM1,CTCF,ANKRD17,HNRNPR,ELF1,NSD1,RSL1D1,CDK7,H3-3B,SETD1B,PPIF,PKM,FOXK2,KIF2A,HMGB1,MAEA,SMARCD1,CANX,YY1,NOL8,MLLT10,IRAK1,PIK3C2B,IP6K1,NAT10,TRMT10C,SNU13,SRSF6,EP400,SART1,MECP2,MBNL1,SKI,TKT,DDX54,TARDBP,XRCC6,PNN,PCNA,CDC25A,MBD1,CSNK2A2,DDX3X,HDGF,NCAPH,ZNF789,MCM2,JUND,CFL1,HCFC1,CHEK1,AATF,CLN6,ECSIT,PRMT7,OXCT1,SLC25A5,SLTM,URB1,POM121,CHAMP1,DNAJC21,UBP1,POLE,TRRAP,NUDC,EXOSC9,POLR2A,CCDC86,DNAJC7,SUMO3,NUP62,DYNC1H1,TSR1,RBM19,XPO1,PPIA,PRPF3,IK,SERPINE1,TPR,ENC1,SRSF7,UTP20,SF3B4,STK17A,PPRC1,EBNA1BP2,KEAP1,TFB2M,UBE2N,BAZ1B,NRIP1,MRTO4,TLK1,TEX10,GFM1,MYBBP1A,DDB1,BACH2,PSMG1,GTF3C4,NASP,ASXL2,TCP1,CENPF,FUBP1,POLE3,CDK6,CDC5L,CHD3,NUP153,RAN,BEND3,C19ORF48,UBC,TFAM,AK2,SSU72,MSH2,CDK12,ZBTB2,GLYR1,KMT2A,MCM3,GRWD1,H4C8,SNHG3,GATAD2A,DKC1,DDX1,H2BC12,CUL1,THUMPD1,EWSR1,DDX49,CASC3,SSRP1,ARID1B,KPNB1,CPSF7,RRP1,TXNRD1,ATP6V0A1,HNRNPAB,KAT6A,ATAD3A,USP36,RBBP4,SAFB,NCBP1,HEATR1,CCT2,HSP90AB1,PA2G4,HNRNPK,SMG1,DHX37,PRPF19,SMARCA4,PRKDC,TRA2B,PRMT1,HNRNPC,MALT1,SF3A3,SUPT16H,HMGA1,CELF1,XRCC5,DDX5,PSME3,UTP4,DEK,LMO2,LMNB1,DDX46,EIF4A3,NUP214,TRIP12,IPO7,SURF6,DDX39A,DDX56,CIZ1,CDT1,NOP56,HNRNPDL,KHDRBS1,HNRNPD,LRPPRC,CTDSP1,SUPT6H,RANBP2,DNAJB6,NOLC1,SNRNP200,SREBF2,CBFA2T3,ABCF1,DANCR,ACACA,MGA,NSUN2,AP5Z1,HSPD1,YBX1,LIN28B,RBM3,SF3A1,LMNB2,MPHOSPH10,GDI2,PES1,ANP32B,IPO5,RREB1,HSPH1,EP300,DAZAP1,ALYREF,RRP1B,FOSB,PPM1G,STON2,SRRT,PRPF8,COA7,SF3B3,PTMA,FTL,THRAP3,MLLT1,HNRNPF,NPM1,RIF1,CITED2,SON,WDR43,RUNX1,TRIM28,U2AF2,NR2F2,BCLAF1,ECPAS,SRSF2,HSP90AA1,MDN1,NFATC3,ZNF521,TRMT1,MCM4,TRIM24,NOP58,HNRNPA2B1,SPN,BAG1,SQSTM1,PTBP1,CCAR1,DDX21,SFPQ,SCD,HNRNPM,POLR1A,SRSF3,ANKRD11,EIF3A,MCM7,SMARCC1,MYC,SET,VGF,BTG1,IGF2BP1,FOS,MYB,KHSRP,ZEB2,FUS,ILF3,ACTB,DHX9,HNRNPU,MCM5,NCL,EGR1,HSPA8,FTH1
GO:0005634	nucleus	3.816971580108217e-70	HRAS,NOP14,CENPN,TXK,ACSF3,SNRPB,MITF,PPP6C,JMJD1C,MIS18BP1,DAP3,RBM48,PPAN,MSH3,TRIP13,CHTOP,ZNF274,ABRAXAS2,PRPF38B,KMT2B,AF

			F1,SUPT7L,SREK1,MACROH2A1,BRD9,MED16,MUS81,NOL11,ITFG2,BICRA,MTA2,SNHG6,RAD51C,UTP3,CWC22,CPNE7,POLR3E,UBE2Q1,DCAF7,MTREX,RIOX1,PHF5A,NACA,POLR1C,MED6,TRIM35,CBX3,PHB,METTL3,EZH2,TCF20,DCLRE1C,NOC4L,RFWD3,MFAP1,PAXIP1,LRRFIP1,GPATCH3,RHEB,INTS6,PPP6R3,H4C5,NCAPH2,SMG5,IFRD2,BRD4,TIMM17A,RRP9,ZNF581,SPIN4,ZNF74,AHCTF1,RPUSD4,CHCHD3,RNASEH2C,HROB,CTDP1,NUP155,MED1,NVL,POM121C,HNRNPL,MYO16,PWP1,WDR46,MCM10,EFTUD2,CHRAC1,CRCP,RANBP3,FRMD8,KMT2D,STAG2,CASP8,TAF4B,PSMA3,PQBP1,PSMD3,AIFM2,PSPC1,CEP350,MAD2L2,POLDIP2,PSMC3,ZBTB40,NAA11,TIMM44,DHX38,ADNP2,NT5C3A,NOP16,ARID2,DDX23,PATZ1,TRNT1,MNS1,RNPS1,MTCH2,LHX4,ZNF512B,RBL1,PPP3R1,HIF1AN,USP11,SS18L1,RBM45,MED29,NOSIP,PAFAH1B1,STAG1,HNRNPH1,LIMD1,ZNF512,DNMT1,LYL1,NAA20,PPP5C,BAP1,JRK,ANAPC7,SUB1,GTF2H1,DHX16,BUB3,TOP2B,LYAR,LRWD1,CCNH,NAB2,KAT7,TPP2,EEF2,MSRA,AURKAIP1,SNHG20,WDR5,CCT8,TMEM43,FUBP3,IMP4,SNHG17,BCCIP,SPRY2,FAM120A,NOB1,SBF1,RNF40,CUL3,ARPP19,CMPK1,NONO,MEF2C,TCF3,NUP160,TEX15,MAF1,GID8,CLSPN,NOL9,PLK4,CCNY,PRPF6,ZC3H4,TMEM33,PEBP1,ZNF26,ZNF24,GRSF1,NXF1,UBQLN4,ACTR8,FAHD1,PSMC5,AGPAT3,STK35,ZC3HAV1,HNRNPA3,MAPK1,EEF1D,CASP3,POLR1E,IPO9,PKP3,ABT1,SURF4,PCLAF,WDR36,CERS2,DHX33,STRIP1,CTR9,NCOA5,ZFP91,SDAD1,DHX15,UIMC1,PUM1,NEMP1,MTOR,RPRD2,UTP18,IKZF3,KANSL1,CPSF3,SPEN,VDAC1,SMARCA5,CWC25,SF3A2,H2AW,ZNF45,RSF1,TFAP4,NAA50,PTP4A2,PRMT6,TSPYL5,KPNA4,NIFK,FAF1,HNRNPA0,BAG6,EMD,ZNF131,PCYT1A,SBNO1,TASOR2,ZNF431,NAP1L4,MTDH,BAIAP2,COMMD4,HSPA9,TCF7L2,ZNF239,SRRM2,PRAME,ASCC3,UROD,SLC29A2,UBE2L3,THOC1,PSMC2,MCMBP,SETMAR,RPTOR,PPIL2,PPP1R10,HECTD1,TMEM18,CSTF2,RAVER1,GRPEL1,PPP4R3A,SAMSN1,WDR33,NIP7,PPP2R5A,TRMT61A,IGF2BP3,NOL7,PDCD7,MED28,ELL2,PUM2,H2AZ2,HAT1,MCRIP2,WDR3,SSBP3,SNRPA,ZNF586,BACH1,DDX10,RBM10,CERT1,SETX,NFILZ,PSMG2,STK24,METTL8,APEX1,BPTF,EDC4,ICE1,RIOK2,NAA15,ARID1A,NUP43,AMMECR1,PITHD1,SPECC1,PPP1CC,CAVIN2,ZC3H14,CDC27,HERC2,SRSF10,CTSL,AKAP8,SBDS,RCC1,C1QBP,VPS72,PPM1H,ZNF587B,CRK,TICRR,MED15,SRSF8,WDR74,PAK2,PRPF4,PRMT5,PCBP2,ADD1,TRMT6,RBMX,RNF126,BAZ1A,GPX4,YJU2,WDR70,MLLT3,RAD23B,TAF4,RBM14,RBM8A,KDM3B,BOP1,SETD2,ZNF326,API5,INTS13,BTBD1,TUBB,FEN1,CHAF1A,ZNF282,DYRK1A,PHF3,TFDP2,WDR12,URB2,GTF3C6,SLBP,CUL4A,DNAJC8,JADE2,NUCKS1,SDCBP,PSMD1,UTP25,TMPO,NUP188,NUP50,PRPF38A,SSB,AGPAT5,GNL3L,DNTTIP2,RNU6-322P,NSRP1,TFIP11,KCTD15,STRBP,PSMB2,WAC,ADI1,BSN,BRCA2,RANBP1,POLR2D,WTAP,COPS2,NIN,BCL7B,RBM42,ZFX,ZC3H7B,ACLY,SNRPD1,MED13L,PAF1,ZC3H18,BIRC6,RABL6,PHB2,TCOF1,LRRC41,RAP1GAP2,XRCC2,PDS5A,BRCC3,ZNF75A,SMARCB1,CREBBP,EXOSC3,WBP11,COPS3,UBE4B,NUP98,HNRNPUL1,CNOT1,LYN,ASH1L,LRRC59,GEMIN5,PLAGL2,APC,BTF3,POLR1B,JPT2,DDX42,ELOF1,WDR6,NFYC,SF1,PELP1,XRN2,TMEM201,EZR,DDX20,URM1,DDX51,MMS19,ELOA,TAF9,ELAVL1,UTP15,VCP,DNAJB12,WASHC5,RYBP,SAFB2,GBP2,PSIP1,CSTB,CSNK1G2,DCAF13,BEX4,RBM12,STIP1,CDK4,DVL2,PNO1,POLR3C,RBM25,SETD1A,RRP12,WDR82,SMC1A,AGO2

			,E2F4,TJP1,G3BP1,PCM1,RNF220,TBC1D14,GNL2,SAE1,RRP36,MCM6,AQR,DHCR7,DDX18,IER3,IGF2R,ADNP,UBA2,NFKB1,UBTF,ZNF622,ARHGDIA,BRD2,CHD7,RBM15B,USP37,HNRNPA1,GAR1,RRS1,PPARGC1B,MAGOH,PBRM1,CTCF,RANBP10,HHEX,ANKRD17,HNRNPR,RRM2,ELF1,ZNF614,NSD1,RSL1D1,CDK7,H3-3B,RNF138,SETD1B,PKM,FOXK2,KIF2A,HMGB1,MAEA,SMARCD1,GNAQ,YY1,NOL8,MLLT10,IRAK1,PUS7,PIK3C2B,IP6K1,NAT10,TRMT10C,SNU13,SRSF6,EP400,SART1,CEBPZ,MECP2,MBNL1,SKI,TKT,DDX54,AZIN1,TARDBP,XRCC6,PNN,RPL22,ABI1,PCNA,CDC25A,MBD1,CSNK2A2,UPF2,DIAPH1,DDX3X,TNPO2,SACS,LBR,HDGF,NDC1,NCAPH,ZNF789,MCM2,JUND,CFL1,HCFC1,CHEK1,AATF,CLN6,ECSIT,ZMPSTE24,UBAP2,PRMT7,SLC25A5,ARPC4,SLTM,URB1,POM121,CHAMP1,DNAJC21,UBP1,POLE,TRRAP,NUDC,EXOSC9,POLR2A,CCDC86,DNAJC7,DNAJA1,SUMO3,NUP62,TSR1,RBM19,XPO1,PPIA,PRPF3,IK,PHACTR1,TPR,ENC1,SRSF7,UTP20,ZFP36L2,SF3B4,STK17A,PPRC1,EBNA1BP2,ZFR,KEAP1,UBE2N,BAZ1B,NRIP1,MACO1,MRTO4,VAPA,TLK1,TEX10,MYBBP1A,DDB1,BACH2,PSMG1,PITX1,GTF3C4,NASP,ASXL2,TCP1,CENPF,RALY,ENO1,FUBP1,POLE3,CDK6,CDC5L,CHD3,NUP153,RAN,BEND3,C19ORF48,UBC,ZNF787,TFAM,HSPA4,PPP2CA,SSU72,MSH2,CDK12,ZBTB2,DIDO1,GLYR1,KMT2A,MCM3,NOSTRIN,GRWD1,H4C8,PRDX1,TNPO1,SNHG3,GATAD2A,DKC1,DDX1,H2BC12,STX3,CUL1,THUMPD1,EWSR1,DDX49,CASC3,SSRP1,ARID1B,KPNB1,CPSF7,RRP1,TXNRD1,ZNF33B,ATP6V0A1,HNRNPAB,KAT6A,USP36,RBBP4,CNPPD1,SAFB,NCBP1,HEATR1,HSP90AB1,PA2G4,HNRNPK,SMG1,TXNL1,DHX37,PRPF19,SMARCA4,PRKDC,ZNF121,TRA2B,PRMT1,HNRNPC,MALT1,WWP2,SF3A3,SUPT16H,HMGA1,CELF1,RBMXL1,XRCC5,DDX5,PSME3,TMEM97,UTP4,DEK,LMO2,LMNB1,DDX46,EIF4A3,NUP214,TRIP12,IPO7,ACTG1,SURF6,DDX39A,DDX56,CIZ1,CDT1,UBAP2L,NOP56,HNRNPDL,KHDRBS1,HNRNPD,LRPPRC,EIF5A,CTDSP1,SUPT6H,RANBP2,DNAJB6,NOLC1,SNRNP200,SREBF2,CBFA2T3,ABCF1,DANCR,ACACA,TAFA2,MGA,NSUN2,AP5Z1,GAPDH,YBX1,LIN28B,RBM3,PGD,SF3A1,LMNB2,AXIN1,MPHOSPH10,PES1,ANP32B,MYH10,IPO5,RREB1,HSPH1,EP300,TGFBR1,DAZAP1,ALYREF,RRP1B,FOSB,PPM1G,STON2,SRRT,PABPC1,SERBP1,AHNAK,PRPF8,COA7,SF3B3,PTMA,THRAP3,MLLT1,HNRNPF,NPM1,RIF1,CITED2,SON,WDR43,TFRC,RUNX1,EIF4G1,TRIM28,U2AF2,NR2F2,BCLAF1,ECPAS,SRSF2,HSP90AA1,MDN1,NFATC3,ZNF521,TRMT1,MCM4,TRIM24,NOP58,RESF1,HNRNPA2B1,SPN,BAG1,SQSTM1,PTBP1,CCAR1,DDX21,SFPQ,PTGER3,SCD,PABPC4,HNRNPM,POLR1A,SRSF3,ANKRD11,EIF3A,MCM7,SMARCC1,MYC,SET,BTG1,IGF2BP1,FOS,MYB,KHSRP,ZEB2,FUS,ILF3,GLUL,ACTB,DHX9,HNRNPU,MCM5,NCL,EGR1,DHCR24,HSPA8,FTH1
GO:0043231	intracellular membrane-bounded organelle	2.1156696610029647e-60	*HRAS,NOP14,CENPN,TXK,ACSF3,DSG2,SNRPB,TMEM127,MITF,PPP6C,JMJD1C,MIS18BP1,DAP3,RBM48,TOMM5,PPAN,MSH3,TRIP13,CHTOP,ERAL1,ZNF274,SEC23IP,ABRAXAS2,GYG1,PRPF38B,KMT2B,AFF1,MMAB,SUPT7L,AP5M1,SREK1,MACROH2A1,BRD9,MED16,FADS2,MUS81,NOL11,ITFG2,BICRA,MTA2,SNHG6,TIRAP,RAD51C,NRROS,MIX23,UTP3,CWC22,CPNE7,POLR3E,UBE2Q1,NEU1,DCAF7,MTREX,RIOX1,PHF5A,NACA,POLR1C,MED6,SRP72,TRIM35,CBX3,PHB,METTL3,EZH2,SNX17,TOMM70,TCF20,FES,CCDC78,RAB10,DCLRE1C,NOC4L,RFWD3,MFAP1,ULK3,DNM1L,PAXIP1,LRRFIP1,GPATCH3,RHEB,INTS6,PPP6R3,H*

			4C5,FUT8,NCAPH2,SMG5,IFRD2,BRD4,STT3A,PTDSS2,DHDDS,VKORC1L1,TIMM17A,RRP9,MRPL1,ZNF581,SPIN4,ZNF74,MRPL11,AHCTF1,RPUSD4,CHCHD3,RNASEH2C,HROB,ANKRD13A,MRPS30,CTDP1,NUP155,MED1,NVL,POM121C,HNRNPL,NLN,MYO16,PWP1,ATP11A,DOLPP1,WDR46,MCM10,OXA1L,EFTUD2,CHRAC1,CRCP,RANBP3,FRMD8,KMT2D,STAG2,CASP8,TAF4B,PSMA3,PQBP1,PSMD3,AIFM2,PSPC1,CEP350,MAD2L2,MRRF,POLDIP2,PSMC3,ZBTB40,NAA11,TIMM44,DHX38,ADNP2,NT5C3A,MS4A4A,NOP16,ARID2,DDX23,PATZ1,TRNT1,MNS1,RNPS1,MTCH2,LHX4,ZNF512B,RBL1,PPP3R1,SLC25A46,SYPL1,HIF1AN,USP11,SS18L1,RBM45,MED29,NOSIP,PAFAH1B1,STAG1,HNRNPH1,USP14,RAB7A,LIMD1,ZNF512,DNMT1,LYL1,NAA20,BICD1,PPP5C,BAP1,JRK,ANAPC7,SUB1,GTF2H1,DHX16,BUB3,TOP2B,LYAR,LRWD1,THOP1,CCNH,NAB2,KAT7,TPP2,CLTC,EEF2,AFG3L2,MSRA,AURKAIP1,SNHG20,NIPA2,WDR5,CCT8,KIF5B,TMEM43,RTL10,FUBP3,IMP4,SNHG17,BCCIP,SPRY2,DLAT,FAM120A,NOB1,SBF1,RNF40,CUL3,ARPP19,CMPK1,NONO,MEF2C,TCF3,NUP160,TEX15,MAF1,GID8,CLSPN,NOL9,MVK,NBAS,PLK4,CCNY,ZFYVE26,PRPF6,ZC3H4,TMEM33,PEBP1,ZNF26,ZNF24,GRSF1,NXF1,UBQLN4,ACTR8,FAHD1,PSMC5,AGPAT3,STK35,TIMM23,ZC3HAV1,HNRNPA3,MAPK1,STAR,ZNRF1,EEF1D,CASP3,POLR1E,ATP2A2,IPO9,PKP3,ABT1,SURF4,PCLAF,WDR36,PDSS1,CERS2,DHX33,STRIP1,PGAM5,LETM1,CTR9,NCOA5,FADS1,ZFP91,SDAD1,DHX15,UIMC1,PUM1,NEMP1,MTOR,DHX30,PDPR,RPRD2,UTP18,IKZF3,KANSL1,CPSF3,SPEN,LCLAT1,GOT2,VDAC1,SMARCA5,SNX8,CWC25,SF3A2,H2AW,ZNF45,RSF1,TFAP4,NAA50,PTP4A2,DELE1,PRMT6,TSPYL5,KPNA4,NIFK,FAF1,HNRNPA0,BAG6,EMD,ZNF131,PCYT1A,SBNO1,TASOR2,ZNF431,FKBP15,NAP1L4,DHFR,MTDH,MCOLN3,BAIAP2,COMMD4,HSPA9,MCCC1,TCF7L2,ZNF239,SRRM2,NSDHL,MRPL15,PRAME,ASCC3,UROD,SLC29A2,UBE2L3,THOC1,PSMC2,MCMBP,SETMAR,RPTOR,MRPL45,PPIL2,PPP1R10,MRPS35,HECTD1,TMEM18,CSTF2,TNF,RAVER1,GRPEL1,PPP4R3A,SAMSN1,WDR33,NIP7,PPP2R5A,TRMT61A,IGF2BP3,NOL7,PDCD7,MED28,ELL2,FAM71F2,PUM2,H2AZ2,HAT1,MCRIP2,CLTA,WDR3,SSBP3,SNRPA,ZNF586,BACH1,DDX10,PDZD8,RBM10,CERT1,SETX,NFILZ,AP3D1,PSMG2,STK24,METTL8,APEX1,BPTF,ATP6V1G1,EDC4,ICE1,RIOK2,NAA15,ARID1A,NUP43,AMMECR1,TRAM1,PITHD1,SPECC1,PPP1CC,CAVIN2,ZC3H14,B4GALT5,CDC27,HERC2,RAB35,SRSF10,CTSL,AKAP8,SBDS,RCC1,C1QBP,VPS72,PPM1H,ZNF587B,CRK,TICRR,MED15,EOGT,STK25,SRSF8,WDR74,PAK2,ARHGAP21,PRPF4,KTN1,PRMT5,SNX9,PCBP2,PTDSS1,ADD1,TRMT6,RBMX,RNF126,TMEM223,BAZ1A,GPX4,YJU2,WDR70,MCCC2,SLC25A3,MLLT3,OPA3,ALG8,RAD23B,VAT1,TAF4,RBM14,RBM8A,KDM3B,BOP1,GOLM1,SETD2,ZNF326,API5,INTS13,RPIA,BTBD1,TUBB,FEN1,CHAF1A,ZNF282,DYRK1A,PHF3,TFDP2,CASD1,WDR12,URB2,GTF3C6,SLBP,CUL4A,DNAJC8,JADE2,NUCKS1,SDCBP,PSMD1,UTP25,TMPO,NUP188,NUP50,PRPF38A,SSB,AGPAT5,GNL3L,DNTTIP2,CYB5B,IBA57,RILP,RNU6-322P,NSRP1,TFIP11,KCTD15,STRBP,PSMB2,WAC,ADI1,BSN,ARHGEF2,BRCA2,RANBP1,POLR2D,ZDHHC5,WTAP,COPS2,NIN,BCL7B,RBM42,ZFX,ZC3H7B,ACLY,SNRPD1,MED13L,PAF1,ZC3H18,BIRC6,ERMAP,FASTKD2,RABL6,PHB2,TCOF1,LRRC41,TOMM22,RAP1GAP2,XRCC2,PDS5A,BRCC3,ZNF75A,SMARCB1,CREBBP,EXOSC3,WBP11,KIFC3,AHSA1,COPS3,UBE4B,INS

			IG1,TMX2,NUP98,HNRNPUL1,CNOT1,LYN,ASH1L,LRRC59,GEMIN5,PHACTR2,PLAGL2,APC,BTF3,POLR1B,JPT2,DDX42,CAPN1,ELOF1,WDR6,NFYC,SF1,PELP1,SLC12A2,XRN2,TMEM201,EZR,TRUB2,DDX20,URM1,DDX51,MMS19,SGPP2,ELOA,TAF9,ACAT2,ELAVL1,UTP15,VCP,DNAJB12,WASHC5,RYBP,SAFB2,GBP2,PSIP1,CSTB,CSNK1G2,DCAF13,BEX4,RBM12,STIP1,CDK4,DVL2,PNO1,POLR3C,THG1L,MRPS2,RBM25,SETD1A,RRP12,SCAP,WDR82,SMC1A,AGO2,E2F4,TJP1,VPS26A,FOXRED2,G3BP1,PCM1,RNF220,TBC1D14,GNL2,SAE1,RRP36,MCM6,AQR,DHCR7,CLCN6,ARL8B,DDX18,IER3,IGF2R,ATP6V0D1,ADNP,UBA2,NFKB1,UBTF,ZNF622,ARHGDIA,BRD2,CHD7,RBM15B,DOK3,USP37,HNRNPA1,GAR1,RRS1,PPARGC1B,MAGOH,PBRM1,CTCF,RANBP10,HHEX,ANKRD17,HNRNPR,RRM2,RRM1,PI4KA,SEC24B,ELF1,ZNF614,NSD1,RSL1D1,CDK7,H3-3B,RNF138,SETD1B,PPIF,PKM,FOXK2,KIF2A,HMGB1,ATAD3B,MAEA,SMARCD1,GNAQ,CANX,YY1,NOL8,MLLT10,IRAK1,PUS7,PIK3C2B,IP6K1,NAT10,TRMT10C,SNU13,SRSF6,EP400,MTHFD1,ADSS2,SART1,CEBPZ,MECP2,CSDE1,MBNL1,SKI,TKT,DDX54,AZIN1,TARDBP,XRCC6,PNN,RPL22,ABI1,PCNA,CDC25A,MBD1,CSNK2A2,UPF2,DIAPH1,DDX3X,IQGAP2,IDI1,TNPO2,SACS,LBR,HDGF,NDC1,NCAPH,ZNF789,VPS35,ATP5MC3,MCM2,JUND,CFL1,HCFC1,CHEK1,AATF,CLN6,ECSIT,ZMPSTE24,UBAP2,PRMT7,OXCT1,RETREG2,SLC25A5,ARPC4,SLTM,CYP3A5,URB1,VAC14,POM121,CHAMP1,DNAJC21,UBP1,POLE,TRRAP,NUDC,EXOSC9,POLR2A,CCDC86,DNAJC7,DNAJA1,TEX261,SUMO3,NUP62,DYNC1H1,TSR1,RBM19,XPO1,PPIA,PRPF3,IK,CLCN7,PHACTR1,SERPINE1,TPR,ENC1,SRSF7,UTP20,TGFBRAP1,ZFP36L2,SF3B4,STK17A,PPRC1,EBNA1BP2,ZFR,ARFGAP2,KEAP1,TFB2M,UBE2N,BAZ1B,NRIP1,MACO1,MRTO4,VAPA,TLK1,TEX10,GFM1,MYBBP1A,DDB1,BACH2,PSMG1,PITX1,GTF3C4,NASP,ASXL2,CHST3,TCP1,CENPF,YWHAB,RALY,ENO1,FUBP1,POLE3,CDK6,CDC5L,CHD3,ST3GAL2,NUP153,RAN,BEND3,C19ORF48,UBC,WDR81,ZNF787,TFAM,HSPA4,AK2,PPP2CA,SSU72,MSH2,CDK12,ZBTB2,DIDO1,GLYR1,MAN2A2,KMT2A,MCM3,NOSTRIN,GRWD1,TOMM40,H4C8,SLC30A10,CERS6,PRDX1,TNPO1,SNHG3,GATAD2A,DKC1,DDX1,H2BC12,STX3,CUL1,LTBR,THUMPD1,EWSR1,DDX49,CASC3,SSRP1,ARID1B,KPNB1,CPSF7,RRP1,TXNRD1,ZNF33B,ATP6V0A1,HNRNPAB,KAT6A,ATAD3A,ARF6,USP36,RBBP4,CNPPD1,SAFB,NCBP1,HEATR1,CCT2,HSP90AB1,PA2G4,HNRNPK,SMG1,TXNL1,DHX37,FDFT1,PRPF19,SMARCA4,PRKDC,YES1,ZNF121,TRA2B,PRMT1,HNRNPC,MALT1,WWP2,SF3A3,SUPT16H,HMGA1,CELF1,NCLN,RBMXL1,XRCC5,DDX5,PSME3,TMEM97,ANK1,UTP4,DEK,LMO2,LMNB1,DDX46,LSS,EIF4A3,NDST1,NUP214,TRIP12,IPO7,ACTG1,SURF6,DDX39A,DDX56,CIZ1,CDT1,UBAP2L,NOP56,HNRNPDL,CORO1C,KHDRBS1,HNRNPD,RAPGEF1,LRPPRC,EIF5A,CTDSP1,SUPT6H,RANBP2,DNAJB6,NOLC1,SNRNP200,SREBF2,CBFA2T3,STXBP5,ABCF1,DANCR,ACACA,TAFA2,MGA,NSUN2,AP5Z1,HSPD1,GAPDH,YBX1,LIN28B,EIF5B,PCYT2,ATP6V1C1,RBM3,STARD7,PGD,SF3A1,LMNB2,AXIN1,MS4A3,MPHOSPH10,GDI2,PES1,ANP32B,MYH10,IPO5,RREB1,HSPH1,EP300,TGFBR1,DAZAP1,ALYREF,RRP1B,FOSB,PPM1G,STON2,SRRT,PABPC1,SERBP1,ATP13A3,AHNAK,PRPF8,COA7,SF3B3,PTMA,FTL,THRAP3,MLLT1,ELOVL6,HNRNPF,MT-ND1,NPM1,RIF1,CITED2,SON,WDR43,TFRC,RUNX1,EIF4G1,TRIM28,ACP5,U2AF2,NR2F2,BCLAF1,ECPA

			S,SRSF2,HSP90AA1,MDN1,NFATC3,ZNF521,TRMT1,SQLE,MCM4,TRIM24,NOP58,RESF1,HNRNPA2B1,KIF1A,SPN,BAG1,SQSTM1,PTBP1,CCAR1,DDX21,SFPQ,PTGER3,SCD,PABPC4,HNRNPM,POLR1A,SRSF3,ANKRD11,EIF3A,MCM7,SMARCC1,MYC,SET,VGF,BTG1,MT-CYB,IGF2BP1,FOS,HMGCR,MYB,KHSRP,ZEB2,FUS,ILF3,FASN,GLUL,ACTB,DHX9,HNRNPU,MCM5,NCL,EGR1,DHCR24,HSPA8,FTH1
GO:0005622	intracellular anatomical structure	2.728636610854794e-54	*HRAS,NOP14,CENPN,TXK,ACSF3,DSG2,SNRPB,TMEM127,MITF,PPP6C,JMJD1C,MIS18BP1,DAP3,RBM48,TOMM5,PPAN,MSH3,TRIP13,CHTOP,ERAL1,ZNF274,SEC23IP,ABRAXAS2,DNAJA2,GYG1,PRPF38B,KMT2B,AFF1,MMAB,RNASEH1,SUPT7L,AP5M1,SREK1,MACROH2A1,BRD9,MED16,FADS2,MUS81,NOL11,ITFG2,BICRA,MTA2,SNHG6,TIRAP,RAD51C,NRROS,AAMP,MIX23,UTP3,CWC22,CPNE7,BEGAIN,POLR3E,UBE2Q1,NEU1,DCAF7,MTREX,OTUD6B,RIOX1,PHF5A,NACA,POLR1C,MED6,SRP72,TRIM35,CBX3,PHB,METTL3,EZH2,SNX17,TOMM70,TCF20,FES,CCDC78,RAB10,DCLRE1C,NOC4L,RFWD3,MFAP1,ULK3,DNM1L,PAXIP1,LRRFIP1,GPATCH3,RHEB,ASAP1,INTS6,PPP6R3,H4C5,FUT8,NCAPH2,SMG5,IFRD2,BRD4,STT3A,PTDSS2,DHDDS,VKORC1L1,TIMM17A,RRP9,MRPL1,ZNF581,SPIN4,ZNF74,MRPL11,AHCTF1,RPUSD4,CMBL,CHCHD3,CDC123,RNASEH2C,HROB,ANKRD13A,MRPS30,CTDP1,NUP155,MED1,NVL,POM121C,KIF26B,SRM,HNRNPL,NLN,MYO16,PWP1,ATP11A,DOLPP1,WDR46,MCM10,OXA1L,EFTUD2,CHRAC1,EML4,CRCP,RANBP3,FRMD8,KMT2D,STAG2,CASP8,TAF4B,PSMA3,PQBP1,PSMD3,AIFM2,PSPC1,CEP350,MAD2L2,MRRF,POLDIP2,PSMC3,ZBTB40,NAA11,TIMM44,DHX38,ADNP2,GART,NT5C3A,MS4A4A,NOP16,FBXO45,ARID2,DDX23,PATZ1,TRIM44,TRNT1,MNS1,RNPS1,MTCH2,ALMS1,LHX4,ZNF512B,RBL1,PPP3R1,SLC25A46,SYPL1,HIF1AN,USP11,SS18L1,RBM45,MED29,NOSIP,PAFAH1B1,STAG1,HNRNPH1,USP14,RAB7A,LIMD1,ZNF512,DNMT1,LYL1,NAA20,BICD1,PPP5C,BAP1,JRK,ANAPC7,SUB1,GTF2H1,DHX16,BUB3,GAB2,TOP2B,LYAR,LRWD1,THOP1,CCNH,NAB2,EXOC7,EIF4EBP2,CCT6A,KAT7,TPP2,CLTC,TSR3,EEF2,AFG3L2,MSRA,AURKAIP1,SNHG20,NIPA2,WDR5,CCT8,KIF5B,FAM13B,TMEM43,RTL10,FUBP3,IMP4,SNHG17,BCCIP,SPRY2,DLAT,FAM120A,NOB1,SBF1,RNF40,CUL3,ARPP19,CMPK1,NONO,MEF2C,UBR3,TCF3,NUP160,TEX15,MAF1,GID8,CLSPN,NOL9,MVK,NBAS,PLK4,CCNY,SLK,ZFYVE26,PRPF6,ZC3H4,TMEM33,PEBP1,ZNF26,ZNF24,GSPT1,GRSF1,NXF1,UBQLN4,ACTR8,FAHD1,PSMC5,AGPAT3,STK35,TIMM23,ZC3HAV1,CAPZA1,HNRNPA3,MAPK1,STAR,ZNRF1,EEF1D,CASP3,POLR1E,ATP2A2,IPO9,PKP3,ABT1,TULP4,SURF4,PCLAF,WDR36,HBG1,PDSS1,CERS2,DHX33,STRIP1,PGAM5,NSMAF,LETM1,CTR9,NCOA5,FADS1,ZFP91,SDAD1,TBCD,DHX15,UIMC1,PUM1,NEMP1,MTOR,DHX30,PDPR,RPRD2,UTP18,IKZF3,KANSL1,CPSF3,SPEN,LCLAT1,GOT2,VDAC1,SMG9,SMARCA5,SNX8,CWC25,SF3A2,H2AW,EIF3D,ZNF45,RSF1,TFAP4,NAA50,PTP4A2,CLDN11,DELE1,PRMT6,TSPYL5,KPNA4,NIFK,FAF1,HNRNPA0,BAG6,EMD,ZNF131,PCYT1A,SBNO1,TASOR2,ZNF431,FKBP15,NAP1L4,DHFR,MTDH,MCOLN3,BAIAP2,COMMD4,HSPA9,MCCC1,TCF7L2,ZNF239,SRRM2,NSDHL,MRPL15,PRAME,GMPS,ASCC3,UROD,SLC29A2,UBE2L3,THOC1,PSMC2,MCMBP,SETMAR,RPTOR,MRPL45,PPIL2,PPP1R10,MRPS35,HECTD1,TMEM18,CSTF2,TNF,RAVER1,GRPEL1,RABGGTB,EIF4B,PPP4R3A,SAMSN1,WDR33,NIP7,PPP2R5A,TRMT61A,IGF2BP3,NOL*

			7,PDCD7,MED28,ELL2,FAM71F2,PUM2,H2AZ2,HAT1,MCRIP2,CLTA,WDR3,SSBP3,SNRPA,ZNF586,BACH1,DDX10,PDZD8,RBM10,CERT1,SETX,NFILZ,TRMT2A,AP3D1,NAPA,PSMG2,STK24,METTL8,APEX1,BPTF,ATP6V1G1,EDC4,ICE1,RIOK2,NAA15,ARID1A,NUP43,AMMECR1,TRAM1,PITHD1,SPECC1,ADO,PPP1CC,KCNQ5,CAVIN2,ZC3H14,B4GALT5,CDC27,HERC2,RAB35,SRSF10,CTSL,AKAP8,SBDS,RCC1,C1QBP,VPS72,PPM1H,ZNF587B,CRK,TICRR,MED15,EOGT,STK25,CAPNS1,SRSF8,WDR74,PAK2,ZMYND19,ARHGAP21,PRPF4,KTN1,PRMT5,SNX9,PCBP2,PTDSS1,ADD1,TRMT6,RBMX,RNF126,TMEM223,BAZ1A,DPYS,GPX4,YJU2,WDR70,MCCC2,SLC25A3,MLLT3,OPA3,ALG8,RAD23B,VAT1,TAF4,RBM14,RBM8A,ARHGAP6,KDM3B,BOP1,GOLM1,LRP8,SETD2,ZNF326,API5,INTS13,RPIA,UCK2,BTBD1,TUBB,FEN1,CHAF1A,ZNF282,DYRK1A,PHF3,TFDP2,CASD1,WDR12,URB2,GTF3C6,SLBP,CUL4A,DNAJC8,JADE2,NUCKS1,SDCBP,PSMD1,UTP25,AMD1,TMPO,NUP188,NUP50,PRPF38A,SSB,CDC37,ECHDC1,AGPAT5,GNL3L,DNTTIP2,CYB5B,IBA57,RILP,RNU6-322P,NSRP1,TFIP11,KCTD15,STRBP,PSMB2,WAC,EIF3G,ADI1,BSN,DHX29,ARHGEF2,BRCA2,RANBP1,POLR2D,ZDHHC5,EIF3M,WTAP,COPS2,NIN,BCL7B,RBM42,ZFX,ZC3H7B,ACLY,SNRPD1,PAN3,MED13L,PAF1,ZC3H18,BIRC6,ERMAP,FASTKD2,RABL6,PHB2,TCOF1,LRRC41,TOMM22,RAP1GAP2,GRB10,XRCC2,PDS5A,BRCC3,ZNF75A,SMARCB1,CREBBP,EXOSC3,WBP11,KIFC3,AHSA1,EPRS1,COPS3,UBE4B,INSIG1,LSM14A,ABLIM1,TMX2,NUP98,HNRNPUL1,CNOT1,LYN,ASH1L,LRRC59,GEMIN5,PHACTR2,PLAGL2,APC,BTF3,POLR1B,JPT2,DDX42,CAPN1,ELOF1,WDR6,NFYC,SF1,PELP1,SLC12A2,XRN2,TMEM201,EZR,TRUB2,DDX20,URM1,DDX51,MMS19,SGPP2,ELOA,TAF9,ACAT2,YWHAG,ELAVL1,UTP15,VCP,DNAJB12,WASHC5,RYBP,SAFB2,GBP2,PSIP1,CSTB,CSNK1G2,DCAF13,BEX4,RBM12,STIP1,CDK4,DVL2,PNO1,POLR3C,THG1L,MRPS2,RBM25,SETD1A,RRP12,SCAP,WDR82,SMC1A,AGO2,E2F4,TJP1,VPS26A,FOXRED2,G3BP1,PCM1,RNF220,TBC1D14,GNL2,SAE1,RRP36,MCM6,AQR,DHCR7,CLCN6,ARL8B,DDX18,IER3,QSER1,IGF2R,ATP6V0D1,ADNP,UBA2,NFKB1,UBTF,ZNF622,ARHGDIA,BRD2,CHD7,RBM15B,DOK3,USP37,HNRNPA1,GAR1,RRS1,PPARGC1B,MAGOH,PBRM1,CTCF,RANBP10,HHEX,ANKRD17,HNRNPR,RRM2,RRM1,PI4KA,SEC24B,ELF1,ZNF614,NSD1,RSL1D1,CCT3,CDK7,H3-3B,RNF138,SETD1B,PPIF,PKM,FOXK2,KIF2A,HMGB1,ATAD3B,MAEA,BZW1,SMARCD1,GNAQ,CANX,YY1,NOL8,MLLT10,IRAK1,PUS7,PIK3C2B,IP6K1,NAT10,TRMT10C,METAP2,SNU13,SRSF6,EP400,AASDHPPT,MTHFD1,ADSS2,SART1,CEBPZ,MECP2,CSDE1,PARD3,MBNL1,SKI,PRKAR2B,TKT,DDX54,AZIN1,CCDC6,N4BP2,TARDBP,LARP4,XRCC6,PNN,RPL22,ABI1,KLHL21,PCNA,CDC25A,MBD1,CSNK2A2,UPF2,DIAPH1,DDX3X,KIAA0753,IQGAP2,IDI1,TNPO2,SACS,LBR,HDGF,NDC1,NCAPH,ZNF789,VPS35,ATP5MC3,EIF4G2,MCM2,JUND,CFL1,PFAS,HCFC1,CHEK1,AATF,CLN6,ECSIT,ZMPSTE24,UBAP2,PRMT7,OXCT1,RETREG2,SLC25A5,ARPC4,SLTM,CYP3A5,URB1,S1PR3,VAC14,POM121,CHAMP1,DNAJC21,UBP1,POLE,TRRAP,PPIP5K2,NUDC,EXOSC9,CSK,POLR2A,CCDC86,DNAJC7,DNAJA1,TEX261,SUMO3,NUP62,DYNC1H1,TSR1,RBM19,XPO1,PPIA,PRPF3,IK,CLCN7,PHACTR1,SERPINE1,TPR,ENC1,SRSF7,UTP20,TGFBRAP1,ZFP36L2,SF3B4,STK17A,PPRC1,SLC38A2,EBNA1BP2,ZFR,ARFGAP2,KEAP1,TFB2M,UBE2N,BAZ1B,NRIP1,MACO1,M

			RTO4,VAPA,TLK1,TEX10,GFM1,MYBBP1A,DDB1,BACH2,PSMG1,PITX1,GTF3C4,NASP,ASXL2,CHST3,TCP1,CENPF,YWHAB,RALY,ENO1,FAM83H,CCT5,FUBP1,POLE3,CDK6,CDC5L,CHD3,ST3GAL2,NUP153,RAN,BEND3,C19ORF48,UBC,WDR81,ZNF787,TFAM,HSPA4,AK2,PPP2CA,SSU72,MSH2,CDK12,ZBTB2,DIDO1,GLYR1,MAN2A2,KMT2A,MCM3,NOSTRIN,GRWD1,TOMM40,PAICS,H4C8,SLC30A10,CERS6,ARMC6,PRDX1,TNPO1,SNHG3,GATAD2A,DKC1,DDX1,H2BC12,STX3,CUL1,LTBR,THUMPD1,CTPS1,EWSR1,DDX49,CASC3,SSRP1,ARID1B,KPNB1,CPSF7,RRP1,SPTB,TXNRD1,ZNF33B,ATP6V0A1,HNRNPAB,KAT6A,ATAD3A,ARF6,USP36,RBBP4,MT-RNR2,CNPPD1,SAFB,NCBP1,HEATR1,CCT2,HSP90AB1,EIF3B,RCSD1,PA2G4,HNRNPK,SMG1,TXNL1,DHX37,FDFT1,PRPF19,SMARCA4,EIF3J,PRKDC,YES1,ZNF121,TRA2B,PRMT1,HNRNPC,MALT1,WWP2,SF3A3,SUPT16H,HMGA1,FARSA,CELF1,NCLN,RBMXL1,XRCC5,DDX5,PSME3,TMEM97,ANK1,UTP4,DEK,LMO2,LMNB1,DDX46,LSS,EIF4A3,NDST1,NUP214,TRIP12,IPO7,ACTG1,SURF6,DDX39A,DDX56,CIZ1,CDT1,UBAP2L,NOP56,HNRNPDL,CORO1C,KHDRBS1,HNRNPD,RAPGEF1,LRPPRC,EIF5A,FAM136A,CTDSP1,SUPT6H,RANBP2,DNAJB6,NOLC1,SNRNP200,SREBF2,CBFA2T3,STXBP5,ABCF1,DANCR,ACACA,TAFA2,MGA,NSUN2,AP5Z1,HSPD1,PIM2,GAPDH,YBX1,LIN28B,EIF5B,PCYT2,ATP6V1C1,RBM3,STARD7,PGD,SF3A1,LMNB2,AXIN1,MS4A3,MPHOSPH10,GDI2,PES1,ANP32B,MYH10,IPO5,RREB1,HSPH1,EP300,TGFBR1,DAZAP1,ALYREF,PCNT,STMN1,RRP1B,CLUH,FOSB,PPM1G,STON2,SRRT,PABPC1,PRRC2C,SERBP1,ATP13A3,AHNAK,PRPF8,COA7,SF3B3,PTMA,FTL,THRAP3,MLLT1,ELOVL6,HNRNPF,MT-ND1,NPM1,RIF1,CITED2,ETF1,SON,CDV3,WDR43,TFRC,RUNX1,NQO1,EIF4G1,TRIM28,ACP5,NEFH,U2AF2,NR2F2,BCLAF1,ECPAS,SRSF2,HSP90AA1,MDN1,NFATC3,ZNF521,CAPRIN1,TRMT1,SQLE,MCM4,TRIM24,NOP58,RESF1,HNRNPA2B1,KIF1A,SPN,BAG1,LARP1,SQSTM1,HBZ,PTBP1,CCAR1,MAT2A,DDX21,HMGCS1,SFPQ,PTGER3,SCD,PABPC4,HNRNPM,NAV1,POLR1A,KCNH2,SRSF3,ANKRD11,EIF3A,MCM7,SMARCC1,MYC,SET,VGF,BTG1,MT-CYB,IGF2BP1,FOS,GCLM,HMGCR,SPTA1,MYB,ODC1,KHSRP,ZEB2,RELN,FUS,ILF3,FASN,GLUL,ACTB,DHX9,MT-RNR1,HNRNPU,MCM5,NCL,EGR1,DHCR24,HSPA8,FTH1
GO:0043227	membrane-bounded organelle	6.633879674865536e-52	*HRAS,NOP14,CENPN,TXK,ACSF3,DSG2,SNRPB,TMEM127,MITF,PPP6C,JMJD1C,MIS18BP1,DAP3,RBM48,TOMM5,PPAN,MSH3,TRIP13,CHTOP,ERAL1,ZNF274,SEC23IP,ABRAXAS2,DNAJA2,GYG1,PRPF38B,KMT2B,AFF1,MMAB,SUPT7L,AP5M1,SREK1,MACROH2A1,BRD9,MED16,FADS2,MUS81,NOL11,ITFG2,BICRA,MTA2,SNHG6,TIRAP,RAD51C,NRROS,MIX23,UTP3,CWC22,CPNE7,POLR3E,UBE2Q1,NEU1,DCAF7,MTREX,RIOX1,PHF5A,NACA,POLR1C,MED6,SRP72,TRIM35,CBX3,PHB,METTL3,EZH2,SNX17,TOMM70,TCF20,FES,CCDC78,RAB10,DCLRE1C,NOC4L,RFWD3,MFAP1,ULK3,DNM1L,PAXIP1,LRRFIP1,GPATCH3,RHEB,INTS6,PPP6R3,H4C5,FUT8,NCAPH2,SMG5,IFRD2,BRD4,STT3A,PTDSS2,DHDDS,VKORC1L1,TIMM17A,RRP9,MRPL1,ZNF581,SPIN4,ZNF74,MRPL11,AHCTF1,RPUSD4,CMBL,CHCHD3,RNASEH2C,HROB,ANKRD13A,MRPS30,CTDP1,NUP155,MED1,NVL,POM121C,HNRNPL,NLN,MYO16,PWP1,ATP11A,DOLPP1,WDR46,MCM10,OXA1L,EFTUD2,CHRAC1,CRCP,RANBP3,FRMD8,KMT2D,STAG*

			2,CASP8,TAF4B,PSMA3,PQBP1,PSMD3,AIFM2,PSPC1,CEP350,MAD2L2,MRRF,POLDIP2,PSMC3,ZBTB40,NAA11,TIMM44,DHX38,ADNP2,GART,NT5C3A,MS4A4A,NOP16,ARID2,DDX23,PATZ1,TRNT1,MNS1,RNPS1,MTCH2,ALMS1,LHX4,ZNF512B,RBL1,PPP3R1,SLC25A46,SYPL1,HIF1AN,USP11,SS18L1,RBM45,MED29,NOSIP,PAFAH1B1,STAG1,HNRNPH1,USP14,RAB7A,LIMD1,ZNF512,DNMT1,LYL1,NAA20,BICD1,PPP5C,BAP1,JRK,ANAPC7,SUB1,GTF2H1,DHX16,BUB3,TOP2B,LYAR,LRWD1,THOP1,CCNH,NAB2,CCT6A,KAT7,TPP2,CLTC,EEF2,AFG3L2,MSRA,AURKAIP1,SNHG20,NIPA2,WDR5,CCT8,KIF5B,TMEM43,RTL10,FUBP3,IMP4,SNHG17,BCCIP,SPRY2,DLAT,FAM120A,NOB1,SBF1,RNF40,CUL3,ARPP19,CMPK1,NONO,MEF2C,TCF3,NUP160,TEX15,MAF1,GID8,CLSPN,NOL9,MVK,NBAS,PLK4,CCNY,SLK,ZFYVE26,PRPF6,ZC3H4,TMEM33,PEBP1,ZNF26,ZNF24,PEPD,GRSF1,NXF1,UBQLN4,ACTR8,FAHD1,PSMC5,AGPAT3,STK35,TIMM23,ZC3HAV1,CAPZA1,HNRNPA3,MAPK1,STAR,ZNRF1,EEF1D,CASP3,POLR1E,ATP2A2,IPO9,PKP3,ABT1,SURF4,PCLAF,WDR36,PDSS1,CERS2,DHX33,STRIP1,PGAM5,LETM1,CTR9,NCOA5,FADS1,ZFP91,SDAD1,DHX15,UIMC1,PUM1,NEMP1,MTOR,DHX30,PDPR,RPRD2,UTP18,IKZF3,KANSL1,CPSF3,SPEN,LCLAT1,GOT2,VDAC1,SMARCA5,SNX8,CWC25,SF3A2,H2AW,ZNF45,RSF1,TFAP4,NAA50,PTP4A2,DELE1,PRMT6,TSPYL5,KPNA4,NIFK,FAF1,HNRNPA0,BAG6,EMD,ZNF131,PCYT1A,SBNO1,TASOR2,ZNF431,FKBP15,NAP1L4,DHFR,MTDH,MCOLN3,BAIAP2,COMMD4,HSPA9,MCCC1,TCF7L2,ZNF239,SRRM2,NSDHL,MRPL15,PRAME,ASCC3,UROD,SLC29A2,UBE2L3,THOC1,PSMC2,MCMBP,SETMAR,RPTOR,MRPL45,PPIL2,PPP1R10,MRPS35,HECTD1,TMEM18,CSTF2,TNF,RAVER1,GRPEL1,PPP4R3A,SAMSN1,WDR33,NIP7,PPP2R5A,TRMT61A,IGF2BP3,NOL7,PDCD7,MED28,ELL2,FAM71F2,PUM2,H2AZ2,HAT1,MCRIP2,CLTA,WDR3,SSBP3,SNRPA,ZNF586,BACH1,DDX10,PDZD8,RBM10,CERT1,SETX,NFILZ,AP3D1,NAPA,PSMG2,STK24,METTL8,APEX1,BPTF,ATP6V1G1,UFC1,EDC4,ICE1,RIOK2,NAA15,ARID1A,NUP43,AMMECR1,TRAM1,PITHD1,SPECC1,PPP1CC,CAVIN2,ZC3H14,B4GALT5,CDC27,HERC2,RAB35,SRSF10,CTSL,AKAP8,SBDS,RCC1,C1QBP,VPS72,PPM1H,ZNF587B,CRK,TICRR,MED15,EOGT,STK25,CAPNS1,SRSF8,WDR74,PAK2,ARHGAP21,PRPF4,KTN1,PRMT5,SNX9,PCBP2,PTDSS1,ADD1,TRMT6,RBMX,RNF126,TMEM223,BAZ1A,DPYS,GPX4,YJU2,WDR70,MCCC2,SLC25A3,MLLT3,OPA3,ALG8,RAD23B,VAT1,TAF4,RBM14,RBM8A,KDM3B,BOP1,GOLM1,SETD2,ZNF326,API5,INTS13,RPIA,BTBD1,TUBB,FEN1,CHAF1A,ZNF282,DYRK1A,PHF3,TFDP2,CASD1,WDR12,URB2,GTF3C6,SLBP,CUL4A,DNAJC8,JADE2,NUCKS1,SDCBP,PSMD1,UTP25,TMPO,NUP188,NUP50,PRPF38A,SSB,CDC37,AGPAT5,GNL3L,DNTTIP2,CYB5B,IBA57,RILP,RNU6-322P,NSRP1,TFIP11,KCTD15,STRBP,PSMB2,WAC,ADI1,BSN,ARHGEF2,BRCA2,RANBP1,POLR2D,ZDHHC5,WTAP,COPS2,NIN,BCL7B,RBM42,ZFX,ZC3H7B,ACLY,SNRPD1,SLC12A9,MED13L,PAF1,ZC3H18,BIRC6,ERMAP,FASTKD2,RABL6,PHB2,TCOF1,LRRC41,TOMM22,RAP1GAP2,XRCC2,PDS5A,BRCC3,ZNF75A,SMARCB1,CREBBP,EXOSC3,WBP11,KIFC3,AHSA1,COPS3,UBE4B,INSIG1,TMX2,NUP98,HNRNPUL1,CNOT1,CUTA,LYN,ASH1L,LRRC59,GEMIN5,PHACTR2,PLAGL2,APC,BTF3,POLR1B,JPT2,DDX42,CAPN1,ELOF1,WDR6,NFYC,SF1,PELP1,SLC12A2,XRN2,TMEM201,EZR,TRUB2,DDX20,URM1,DDX51,MMS19,SGPP2,ELOA,TAF9

			,ACAT2,YWHAG,ELAVL1,UTP15,VCP,DNAJB12,WASHC5,RYBP,SAFB2,GBP2,PSIP1,CSTB,CSNK1G2,DCAF13,BEX4,RBM12,STIP1,CDK4,DVL2,PNO1,POLR3C,THG1L,MRPS2,RBM25,SETD1A,RRP12,SCAP,WDR82,SMC1A,AGO2,E2F4,TJP1,VPS26A,FOXRED2,G3BP1,PCM1,RNF220,TBC1D14,GNL2,SAE1,RRP36,MCM6,AQR,DHCR7,CLCN6,ARL8B,DDX18,IER3,IGF2R,ATP6V0D1,ADNP,UBA2,NFKB1,UBTF,ZNF622,ARHGDIA,BRD2,CHD7,RBM15B,DOK3,USP37,HNRNPA1,GAR1,RRS1,PPARGC1B,MAGOH,PBRM1,CTCF,RANBP10,HHEX,ANKRD17,HNRNPR,RRM2,RRM1,PI4KA,SEC24B,ELF1,ZNF614,NSD1,RSL1D1,CCT3,CDK7,H3-3B,RNF138,SETD1B,PPIF,PKM,FOXK2,KIF2A,HMGB1,ATAD3B,MAEA,SMARCD1,GNAQ,CANX,YY1,NOL8,MLLT10,IRAK1,PUS7,PIK3C2B,IP6K1,NAT10,TRMT10C,SNU13,SRSF6,EP400,AASDHPPT,MTHFD1,ADSS2,SART1,CEBPZ,MECP2,CSDE1,MBNL1,SKI,PRKAR2B,TKT,DDX54,AZIN1,TARDBP,XRCC6,PNN,RPL22,ABI1,PCNA,CDC25A,MBD1,CSNK2A2,UPF2,DIAPH1,DDX3X,IQGAP2,IDI1,TNPO2,SACS,LBR,HDGF,NDC1,NCAPH,ZNF789,VPS35,ATP5MC3,MCM2,JUND,CFL1,PFAS,HCFC1,CHEK1,AATF,CLN6,ECSIT,ZMPSTE24,UBAP2,PRMT7,OXCT1,RETREG2,SLC25A5,ARPC4,SLTM,CYP3A5,URB1,VAC14,POM121,CHAMP1,DNAJC21,UBP1,POLE,TRRAP,NUDC,EXOSC9,CSK,POLR2A,CCDC86,DNAJC7,DNAJA1,TEX261,SUMO3,NUP62,DYNC1H1,TSR1,RBM19,XPO1,PPIA,PRPF3,IK,CLCN7,PHACTR1,SERPINE1,TPR,ENC1,SRSF7,UTP20,TGFBRAP1,ZFP36L2,SF3B4,STK17A,PPRC1,EBNA1BP2,ZFR,ARFGAP2,KEAP1,TFB2M,UBE2N,BAZ1B,NRIP1,MACO1,MRTO4,VAPA,TLK1,TEX10,GFM1,MYBBP1A,DDB1,BACH2,PSMG1,PITX1,GTF3C4,NASP,ASXL2,CHST3,TCP1,CENPF,YWHAB,RALY,ENO1,CCT5,FUBP1,POLE3,CDK6,CDC5L,CHD3,ST3GAL2,NUP153,RAN,BEND3,C19ORF48,UBC,WDR81,ZNF787,TFAM,HSPA4,AK2,PPP2CA,SSU72,MSH2,CDK12,ZBTB2,DIDO1,GLYR1,MAN2A2,KMT2A,MCM3,NOSTRIN,GRWD1,TOMM40,PAICS,H4C8,SLC30A10,CERS6,PRDX1,TNPO1,SNHG3,GATAD2A,DKC1,DDX1,H2BC12,STX3,CUL1,LTBR,THUMPD1,EWSR1,DDX49,CASC3,SSRP1,ARID1B,KPNB1,CPSF7,RRP1,TXNRD1,ZNF33B,ATP6V0A1,HNRNPAB,KAT6A,ATAD3A,ARF6,USP36,RBBP4,CNPPD1,SAFB,NCBP1,HEATR1,CCT2,HSP90AB1,EIF3B,PA2G4,HNRNPK,SMG1,TXNL1,DHX37,FDFT1,PRPF19,SMARCA4,PRKDC,YES1,ZNF121,TRA2B,PRMT1,HNRNPC,MALT1,WWP2,SF3A3,SUPT16H,HMGA1,CELF1,NCLN,RBMXL1,XRCC5,DDX5,PSME3,TMEM97,ANK1,UTP4,DEK,LMO2,LMNB1,DDX46,LSS,EIF4A3,NDST1,NUP214,TRIP12,IPO7,ACTG1,SURF6,DDX39A,DDX56,SLC38A1,CIZ1,CDT1,UBAP2L,NOP56,HNRNPDL,CORO1C,KHDRBS1,HNRNPD,RAPGEF1,LRPPRC,EIF5A,CTDSP1,SUPT6H,RANBP2,DNAJB6,NOLC1,SNRNP200,SREBF2,CBFA2T3,STXBP5,ABCF1,DANCR,ACACA,TAFA2,MGA,NSUN2,AP5Z1,HSPD1,GAPDH,YBX1,LIN28B,EIF5B,PCYT2,ATP6V1C1,RBM3,STARD7,PGD,SF3A1,LMNB2,AXIN1,MS4A3,MPHOSPH10,GDI2,PES1,ANP32B,MYH10,IPO5,RREB1,HSPH1,EP300,TGFBR1,DAZAP1,ALYREF,STMN1,RRP1B,FOSB,PPM1G,STON2,SRRT,PABPC1,SERBP1,ATP13A3,AHNAK,PRPF8,COA7,SF3B3,PTMA,FTL,THRAP3,MLLT1,ELOVL6,HNRNPF,MT-ND1,NPM1,RIF1,CITED2,SON,WDR43,TFRC,RUNX1,EIF4G1,TRIM28,ACP5,U2AF2,NR2F2,BCLAF1,ECPAS,SRSF2,HSP90AA1,MDN1,NFATC3,ZNF521,TRMT1,SQLE,MCM4,TRIM24,NOP58,RESF1,HNRNPA2B1,KIF1A,SPN,BAG1,SQSTM1,HBZ,PTBP1,CCAR1,DDX21,SFPQ,PTGER3,SCD,PABPC4,HNRNPM,POLR1A,SRSF3,

			ANKRD11,EIF3A,MCM7,SMARCC1,MYC,SET,VGF,BTG1,MT-CYB,IGF2BP1,FOS,HMGCR,MYB,KHSRP,ZEB2,FUS,ILF3,FASN,GLUL,ACTB,DHX9,HNRNPU,MCM5,NCL,EGR1,DHCR24,HSPA8,FTH1
GO:0043229	intracellular organelle	1.4110597771612032e-51	*HRAS,NOP14,CENPN,TXK,ACSF3,DSG2,SNRPB,TMEM127,MITF,PPP6C,JMJD1C,MIS18BP1,DAP3,RBM48,TOMM5,PPAN,MSH3,TRIP13,CHTOP,ERAL1,ZNF274,SEC23IP,ABRAXAS2,GYG1,PRPF38B,KMT2B,AFF1,MMAB,SUPT7L,AP5M1,SREK1,MACROH2A1,BRD9,MED16,FADS2,MUS81,NOL11,ITFG2,BICRA,MTA2,SNHG6,TIRAP,RAD51C,NRROS,AAMP,MIX23,UTP3,CWC22,CPNE7,POLR3E,UBE2Q1,NEU1,DCAF7,MTREX,RIOX1,PHF5A,NACA,POLR1C,MED6,SRP72,TRIM35,CBX3,PHB,METTL3,EZH2,SNX17,TOMM70,TCF20,FES,CCDC78,RAB10,DCLRE1C,NOC4L,RFWD3,MFAP1,ULK3,DNM1L,PAXIP1,LRRFIP1,GPATCH3,RHEB,ASAP1,INTS6,PPP6R3,H4C5,FUT8,NCAPH2,SMG5,IFRD2,BRD4,STT3A,PTDSS2,DHDDS,VKORC1L1,TIMM17A,RRP9,MRPL1,ZNF581,SPIN4,ZNF74,MRPL11,AHCTF1,RPUSD4,CHCHD3,RNASEH2C,HROB,ANKRD13A,MRPS30,CTDP1,NUP155,MED1,NVL,POM121C,KIF26B,HNRNPL,NLN,MYO16,PWP1,ATP11A,DOLPP1,WDR46,MCM10,OXA1L,EFTUD2,CHRAC1,EML4,CRCP,RANBP3,FRMD8,KMT2D,STAG2,CASP8,TAF4B,PSMA3,PQBP1,PSMD3,AIFM2,PSPC1,CEP350,MAD2L2,MRRF,POLDIP2,PSMC3,ZBTB40,NAA11,TIMM44,DHX38,ADNP2,NT5C3A,MS4A4A,NOP16,ARID2,DDX23,PATZ1,TRNT1,MNS1,RNPS1,MTCH2,ALMS1,LHX4,ZNF512B,RBL1,PPP3R1,SLC25A46,SYPL1,HIF1AN,USP11,SS18L1,RBM45,MED29,NOSIP,PAFAH1B1,STAG1,HNRNPH1,USP14,RAB7A,LIMD1,ZNF512,DNMT1,LYL1,NAA20,BICD1,PPP5C,BAP1,JRK,ANAPC7,SUB1,GTF2H1,DHX16,BUB3,TOP2B,LYAR,LRWD1,THOP1,CCNH,NAB2,EXOC7,CCT6A,KAT7,TPP2,CLTC,EEF2,AFG3L2,MSRA,AURKAIP1,SNHG20,NIPA2,WDR5,CCT8,KIF5B,TMEM43,RTL10,FUBP3,IMP4,SNHG17,BCCIP,SPRY2,DLAT,FAM120A,NOB1,SBF1,RNF40,CUL3,ARPP19,CMPK1,NONO,MEF2C,TCF3,NUP160,TEX15,MAF1,GID8,CLSPN,NOL9,MVK,NBAS,PLK4,CCNY,ZFYVE26,PRPF6,ZC3H4,TMEM33,PEBP1,ZNF26,ZNF24,GRSF1,NXF1,UBQLN4,ACTR8,FAHD1,PSMC5,AGPAT3,STK35,TIMM23,ZC3HAV1,CAPZA1,HNRNPA3,MAPK1,STAR,ZNRF1,EEF1D,CASP3,POLR1E,ATP2A2,IPO9,PKP3,ABT1,SURF4,PCLAF,WDR36,PDSS1,CERS2,DHX33,STRIP1,PGAM5,LETM1,CTR9,NCOA5,FADS1,ZFP91,SDAD1,TBCD,DHX15,UIMC1,PUM1,NEMP1,MTOR,DHX30,PDPR,RPRD2,UTP18,IKZF3,KANSL1,CPSF3,SPEN,LCLAT1,GOT2,VDAC1,SMARCA5,SNX8,CWC25,SF3A2,H2AW,ZNF45,RSF1,TFAP4,NAA50,PTP4A2,CLDN11,DELE1,PRMT6,TSPYL5,KPNA4,NIFK,FAF1,HNRNPA0,BAG6,EMD,ZNF131,PCYT1A,SBNO1,TASOR2,ZNF431,FKBP15,NAP1L4,DHFR,MTDH,MCOLN3,BAIAP2,COMMD4,HSPA9,MCCC1,TCF7L2,ZNF239,SRRM2,NSDHL,MRPL15,PRAME,ASCC3,UROD,SLC29A2,UBE2L3,THOC1,PSMC2,MCMBP,SETMAR,RPTOR,MRPL45,PPIL2,PPP1R10,MRPS35,HECTD1,TMEM18,CSTF2,TNF,RAVER1,GRPEL1,PPP4R3A,SAMSN1,WDR33,NIP7,PPP2R5A,TRMT61A,IGF2BP3,NOL7,PDCD7,MED28,ELL2,FAM71F2,PUM2,H2AZ2,HAT1,MCRIP2,CLTA,WDR3,SSBP3,SNRPA,ZNF586,BACH1,DDX10,PDZD8,RBM10,CERT1,SETX,NFILZ,AP3D1,PSMG2,STK24,METTL8,APEX1,BPTF,ATP6V1G1,EDC4,ICE1,RIOK2,NAA15,ARID1A,NUP43,AMMECR1,TRAM1,PITHD1,SPECC1,PPP1CC,CAVIN2,ZC3H14,B4GALT5,CDC27,HERC2,RAB35,SRSF10,CTSL,AKAP8,SBDS,RCC1,C1QBP,VPS72,PPM1H*

			,ZNF587B,CRK,TICRR,MED15,EOGT,STK25,SRSF8,WDR74,PAK2,ARHGAP21,PRPF4,KTN1,PRMT5,SNX9,PCBP2,PTDSS1,ADD1,TRMT6,RBMX,RNF126,TMEM223,BAZ1A,GPX4,YJU2,WDR70,MCCC2,SLC25A3,MLLT3,OPA3,ALG8,RAD23B,VAT1,TAF4,RBM14,RBM8A,ARHGAP6,KDM3B,BOP1,GOLM1,LRP8,SETD2,ZNF326,API5,INTS13,RPIA,BTBD1,TUBB,FEN1,CHAF1A,ZNF282,DYRK1A,PHF3,TFDP2,CASD1,WDR12,URB2,GTF3C6,SLBP,CUL4A,DNAJC8,JADE2,NUCKS1,SDCBP,PSMD1,UTP25,TMPO,NUP188,NUP50,PRPF38A,SSB,AGPAT5,GNL3L,DNTTIP2,CYB5B,IBA57,RILP,RNU6-322P,NSRP1,TFIP11,KCTD15,STRBP,PSMB2,WAC,ADI1,BSN,DHX29,ARHGEF2,BRCA2,RANBP1,POLR2D,ZDHHC5,WTAP,COPS2,NIN,BCL7B,RBM42,ZFX,ZC3H7B,ACLY,SNRPD1,PAN3,MED13L,PAF1,ZC3H18,BIRC6,ERMAP,FASTKD2,RABL6,PHB2,TCOF1,LRRC41,TOMM22,RAP1GAP2,XRCC2,PDS5A,BRCC3,ZNF75A,SMARCB1,CREBBP,EXOSC3,WBP11,KIFC3,AHSA1,COPS3,UBE4B,INSIG1,LSM14A,ABLIM1,TMX2,NUP98,HNRNPUL1,CNOT1,LYN,ASH1L,LRRC59,GEMIN5,PHACTR2,PLAGL2,APC,BTF3,POLR1B,JPT2,DDX42,CAPN1,ELOF1,WDR6,NFYC,SF1,PELP1,SLC12A2,XRN2,TMEM201,EZR,TRUB2,DDX20,URM1,DDX51,MMS19,SGPP2,ELOA,TAF9,ACAT2,ELAVL1,UTP15,VCP,DNAJB12,WASHC5,RYBP,SAFB2,GBP2,PSIP1,CSTB,CSNK1G2,DCAF13,BEX4,RBM12,STIP1,CDK4,DVL2,PNO1,POLR3C,THG1L,MRPS2,RBM25,SETD1A,RRP12,SCAP,WDR82,SMC1A,AGO2,E2F4,TJP1,VPS26A,FOXRED2,G3BP1,PCM1,RNF220,TBC1D14,GNL2,SAE1,RRP36,MCM6,AQR,DHCR7,CLCN6,ARL8B,DDX18,IER3,QSER1,IGF2R,ATP6V0D1,ADNP,UBA2,NFKB1,UBTF,ZNF622,ARHGDIA,BRD2,CHD7,RBM15B,DOK3,USP37,HNRNPA1,GAR1,RRS1,PPARGC1B,MAGOH,PBRM1,CTCF,RANBP10,HHEX,ANKRD17,HNRNPR,RRM2,RRM1,PI4KA,SEC24B,ELF1,ZNF614,NSD1,RSL1D1,CCT3,CDK7,H3-3B,RNF138,SETD1B,PPIF,PKM,FOXK2,KIF2A,HMGB1,ATAD3B,MAEA,SMARCD1,GNAQ,CANX,YY1,NOL8,MLLT10,IRAK1,PUS7,PIK3C2B,IP6K1,NAT10,TRMT10C,SNU13,SRSF6,EP400,MTHFD1,ADSS2,SART1,CEBPZ,MECP2,CSDE1,PARD3,MBNL1,SKI,PRKAR2B,TKT,DDX54,AZIN1,CCDC6,TARDBP,LARP4,XRCC6,PNN,RPL22,ABI1,KLHL21,PCNA,CDC25A,MBD1,CSNK2A2,UPF2,DIAPH1,DDX3X,KIAA0753,IQGAP2,IDI1,TNPO2,SACS,LBR,HDGF,NDC1,NCAPH,ZNF789,VPS35,ATP5MC3,MCM2,JUND,CFL1,HCFC1,CHEK1,AATF,CLN6,ECSIT,ZMPSTE24,UBAP2,PRMT7,OXCT1,RETREG2,SLC25A5,ARPC4,SLTM,CYP3A5,URB1,VAC14,POM121,CHAMP1,DNAJC21,UBP1,POLE,TRRAP,NUDC,EXOSC9,POLR2A,CCDC86,DNAJC7,DNAJA1,TEX261,SUMO3,NUP62,DYNC1H1,TSR1,RBM19,XPO1,PPIA,PRPF3,IK,CLCN7,PHACTR1,SERPINE1,TPR,ENC1,SRSF7,UTP20,TGFBRAP1,ZFP36L2,SF3B4,STK17A,PPRC1,EBNA1BP2,ZFR,ARFGAP2,KEAP1,TFB2M,UBE2N,BAZ1B,NRIP1,MACO1,MRTO4,VAPA,TLK1,TEX10,GFM1,MYBBP1A,DDB1,BACH2,PSMG1,PITX1,GTF3C4,NASP,ASXL2,CHST3,TCP1,CENPF,YWHAB,RALY,ENO1,FAM83H,CCT5,FUBP1,POLE3,CDK6,CDC5L,CHD3,ST3GAL2,NUP153,RAN,BEND3,C19ORF48,UBC,WDR81,ZNF787,TFAM,HSPA4,AK2,PPP2CA,SSU72,MSH2,CDK12,ZBTB2,DIDO1,GLYR1,MAN2A2,KMT2A,MCM3,NOSTRIN,GRWD1,TOMM40,H4C8,SLC30A10,CERS6,PRDX1,TNPO1,SNHG3,GATAD2A,DKC1,DDX1,H2BC12,STX3,CUL1,LTBR,THUMPD1,EWSR1,DDX49,CASC3,SSRP1,ARID1B,KPNB1,CPSF7,RRP1,SPTB,TXNRD1,ZN

			F33B,ATP6V0A1,HNRNPAB,KAT6A,ATAD3A,ARF6,USP36,RBBP4,MT-RNR2,CNPPD1,SAFB,NCBP1,HEATR1,CCT2,HSP90AB1,EIF3B,RCSD1,PA2G4,HNRNPK,SMG1,TXNL1,DHX37,FDFT1,PRPF19,SMARCA4,PRKDC,YES1,ZNF121,TRA2B,PRMT1,HNRNPC,MALT1,WWP2,SF3A3,SUPT16H,HMGA1,CELF1,NCLN,RBMXL1,XRCC5,DDX5,PSME3,TMEM97,ANK1,UTP4,DEK,LMO2,LMNB1,DDX46,LSS,EIF4A3,NDST1,NUP214,TRIP12,IPO7,ACTG1,SURF6,DDX39A,DDX56,CIZ1,CDT1,UBAP2L,NOP56,HNRNPDL,CORO1C,KHDRBS1,HNRNPD,RAPGEF1,LRPPRC,EIF5A,CTDSP1,SUPT6H,RANBP2,DNAJB6,NOLC1,SNRNP200,SREBF2,CBFA2T3,STXBP5,ABCF1,DANCR,ACACA,TAFA2,MGA,NSUN2,AP5Z1,HSPD1,GAPDH,YBX1,LIN28B,EIF5B,PCYT2,ATP6V1C1,RBM3,STARD7,PGD,SF3A1,LMNB2,AXIN1,MS4A3,MPHOSPH10,GDI2,PES1,ANP32B,MYH10,IPO5,RREB1,HSPH1,EP300,TGFBR1,DAZAP1,ALYREF,PCNT,STMN1,RRP1B,FOSB,PPM1G,STON2,SRRT,PABPC1,PRRC2C,SERBP1,ATP13A3,AHNAK,PRPF8,COA7,SF3B3,PTMA,FTL,THRAP3,MLLT1,ELOVL6,HNRNPF,MT-ND1,NPM1,RIF1,CITED2,SON,WDR43,TFRC,RUNX1,EIF4G1,TRIM28,ACP5,NEFH,U2AF2,NR2F2,BCLAF1,ECPAS,SRSF2,HSP90AA1,MDN1,NFATC3,ZNF521,CAPRIN1,TRMT1,SQLE,MCM4,TRIM24,NOP58,RESF1,HNRNPA2B1,KIF1A,SPN,BAG1,LARP1,SQSTM1,PTBP1,CCAR1,DDX21,SFPQ,PTGER3,SCD,PABPC4,HNRNPM,NAV1,POLR1A,SRSF3,ANKRD11,EIF3A,MCM7,SMARCC1,MYC,SET,VGF,BTG1,MT-CYB,IGF2BP1,FOS,HMGCR,SPTA1,MYB,KHSRP,ZEB2,FUS,ILF3,FASN,GLUL,ACTB,DHX9,MT-RNR1,HNRNPU,MCM5,NCL,EGR1,DHCR24,HSPA8,FTH1
GO:1902494	catalytic complex	4.1848905931753614e-47	*HRAS,SNRPB,JMJD1C,KMT2B,SUPT7L,BRD9,MUS81,BICRA,MTA2,RAD51C,CWC22,POLR3E,DCAF7,MTREX,POLR1C,METTL3,EZH2,DCLRE1C,PAXIP1,SMG5,ST T3A,DHDDS,RNASEH2C,NVL,EFTUD2,CHRAC1,CRCP,KMT2D,CASP8,TAF4B,PSMA3,PSMD3,MAD2L2,PSMC3,NAA11,DHX38,FBXO45,ARID2,DDX23,PPP3R1,SS18L1,PAFAH1B1,HNRNPH1,USP14,NAA20,ANAPC7,GTF2H1,CCNH,KAT7,AFG3L2,WDR5,BCCIP,DLAT,RNF40,CUL3,UBR3,GID8,CCNY,PRPF6,UBQLN4,ACTR8,PSMC5,HNRNPA3,POLR1E,PDSS1,CTR9,PDPR,RPRD2,KANSL1,SMARCA5,CWC25,SF3A2,RSF1,NAA50,MCCC1,SRRM2,PRAME,ASCC3,UBE2L3,PSMC2,PPIL2,PPP1R10,RABGGTB,PPP4R3A,PPP2R5A,TRMT61A,BPTF,ATP6V1G1,NAA15,ARID1A,PPP1CC,CDC27,VPS72,CAPNS1,WDR74,PRMT5,TRMT6,RBMX,BAZ1A,YJU2,MCC2,MLLT3,RAD23B,TAF4,RBM8A,KDM3B,CUL4A,JADE2,PSMD1,GNL3L,CYB5B,TFIP11,PSMB2,BRCA2,POLR2D,WTAP,BCL7B,SNRPD1,PAF1,BRCC3,SMARCB1,CREBBP,EXOSC3,EPRS1,UBE4B,ASH1L,APC,POLR1B,PELP1,MMS19,TAF9,VCP,DCAF13,CDK4,POLR3C,THG1L,SETD1A,WDR82,AGO2,SAE1,AQR,ATP6V0D1,UBA2,RBM15B,HNRNPA1,GAR1,MAGOH,PBRM1,RANBP10,HNRNPR,RRM2,RRM1,CDK7,SETD1B,PPIF,MAEA,SMARCD1,GNAQ,YY1,PIK3C2B,NAT10,TRMT10C,EP400,SART1,PARD3,PRKAR2B,XRCC6,PNN,KLHL21,PCNA,CSNK2A2,ATP5MC3,HCFC1,VAC14,POLE,TRRAP,EXOSC9,POLR2A,DYNC1H1,TPR,ENC1,KEAP1,UBE2N,BAZ1B,NRIP1,TEX10,MYBBP1A,DDB1,RALY,ENO1,POLE3,CDK6,CDC5L,CHD3,PPP2CA,CDK12,KMT2A,MCM3,GATAD2A,DKC1,DDX1,CUL1,CASC3,ARID1B,ATP6V0A1,HNRNPAB,KAT6A,RBBP4,CNPPD1,HNRNPK,TXNL1,PRPF19,SMARCA4,PRKDC,PRMT1,HNRNPC,WWP2,SF3A3,FARSA,XRCC5,DDX5,PSME3,DEK,EIF4A3,*

			RANBP2,NOLC1,SNRNP200,MGA,ATP6V1C1,SF3A1,AXIN1,EP300,TGFBR1,PABPC1,PRPF8,SF3B3,MLLT1,ELOVL6,HNRNPF,MT-ND1,ECPAS,HNRNPA2B1,MAT2A,DDX21,HNRNPM,POLR1A,SMARCC1,MT-CYB,GCLM,KHSRP,ACTB,DHX9,HNRNPU
GO:0043226	organelle	6.2476559181553e-47	*HRAS,NOP14,CENPN,TXK,ACSF3,DSG2,SNRPB,TMEM127,MITF,PPP6C,JMJD1C,MIS18BP1,DAP3,RBM48,TOMM5,PPAN,MSH3,TRIP13,CHTOP,ERAL1,ZNF274,SEC23IP,ABRAXAS2,DNAJA2,GYG1,PRPF38B,KMT2B,AFF1,MMAB,SUPT7L,AP5M1,SREK1,MACROH2A1,BRD9,MED16,FADS2,MUS81,NOL11,ITFG2,BICRA,MTA2,SNHG6,TIRAP,RAD51C,NRROS,AAMP,MIX23,UTP3,CWC22,CPNE7,POLR3E,UBE2Q1,NEU1,DCAF7,MTREX,RIOX1,PHF5A,NACA,POLR1C,MED6,SRP72,TRIM35,CBX3,PHB,METTL3,EZH2,SNX17,TOMM70,TCF20,FES,CCDC78,RAB10,DCLRE1C,NOC4L,RFWD3,MFAP1,ULK3,DNM1L,PAXIP1,LRRFIP1,GPATCH3,RHEB,ASAP1,INTS6,PPP6R3,H4C5,FUT8,NCAPH2,SMG5,IFRD2,BRD4,STT3A,PTDSS2,DHDDS,VKORC1L1,TIMM17A,RRP9,MRPL1,ZNF581,SPIN4,ZNF74,MRPL11,AHCTF1,RPUSD4,CMBL,CHCHD3,RNASEH2C,HROB,ANKRD13A,MRPS30,CTDP1,NUP155,MED1,NVL,POM121C,KIF26B,HNRNPL,NLN,MYO16,PWP1,ATP11A,DOLPP1,WDR46,MCM10,OXA1L,EFTUD2,CHRAC1,EML4,CRCP,RANBP3,FRMD8,KMT2D,STAG2,CASP8,TAF4B,PSMA3,PQBP1,PSMD3,AIFM2,PSPC1,CEP350,MAD2L2,MRRF,POLDIP2,PSMC3,ZBTB40,NAA11,TIMM44,DHX38,ADNP2,GART,NT5C3A,MS4A4A,NOP16,FBXO45,ARID2,DDX23,PATZ1,TRNT1,MNS1,RNPS1,MTCH2,ALMS1,LHX4,ZNF512B,RBL1,PPP3R1,TPRN,SLC25A46,SYPL1,HIF1AN,USP11,SS18L1,RBM45,MED29,NOSIP,PAFAH1B1,STAG1,HNRNPH1,USP14,RAB7A,LIMD1,ZNF512,DNMT1,LYL1,NAA20,BICD1,PPP5C,BAP1,JRK,ANAPC7,SUB1,GTF2H1,DHX16,BUB3,TOP2B,LYAR,LRWD1,THOP1,CCNH,NAB2,EXOC7,CCT6A,KAT7,TPP2,CLTC,EEF2,AFG3L2,MSRA,AURKAIP1,SNHG20,NIPA2,WDR5,CCT8,KIF5B,TMEM43,RTL10,FUBP3,IMP4,SNHG17,BCCIP,SPRY2,DLAT,FAM120A,NOB1,SBF1,RNF40,CUL3,ARPP19,CMPK1,NONO,MEF2C,TCF3,NUP160,TEX15,MAF1,GID8,CLSPN,NOL9,MVK,NBAS,PLK4,CCNY,SLK,ZFYVE26,PRPF6,ZC3H4,TMEM33,PEBP1,ZNF26,ZNF24,PEPD,GRSF1,NXF1,UBQLN4,ACTR8,FAHD1,PSMC5,AGPAT3,STK35,TIMM23,ZC3HAV1,CAPZA1,HNRNPA3,MAPK1,STAR,ZNRF1,EEF1D,CASP3,POLR1E,ATP2A2,IPO9,PKP3,ABT1,SURF4,PCLAF,WDR36,PDSS1,CERS2,DHX33,STRIP1,PGAM5,LETM1,CTR9,NCOA5,FADS1,ZFP91,SDAD1,TBCD,DHX15,UIMC1,PUM1,NEMP1,MTOR,DHX30,PDPR,RPRD2,UTP18,IKZF3,KANSL1,CPSF3,SPEN,LCLAT1,GOT2,VDAC1,SMARCA5,SNX8,CWC25,SF3A2,H2AW,ZNF45,RSF1,TFAP4,NAA50,PTP4A2,CLDN11,DELE1,PRMT6,TSPYL5,KPNA4,NIFK,FAF1,HNRNPA0,BAG6,EMD,ZNF131,PCYT1A,SBNO1,TASOR2,ZNF431,FKBP15,NAP1L4,DHFR,MTDH,MCOLN3,BAIAP2,COMMD4,HSPA9,MCCC1,TCF7L2,ZNF239,SRRM2,NSDHL,MRPL15,PRAME,ASCC3,UROD,SLC29A2,UBE2L3,THOC1,PSMC2,MCMBP,SETMAR,RPTOR,MRPL45,PPIL2,PPP1R10,MRPS35,HECTD1,TMEM18,CSTF2,TNF,RAVER1,GRPEL1,PPP4R3A,SAMSN1,WDR33,NIP7,PPP2R5A,TRMT61A,IGF2BP3,NOL7,PDCD7,MED28,ELL2,FAM71F2,PUM2,H2AZ2,HAT1,MCRIP2,CLTA,WDR3,SSBP3,SNRPA,ZNF586,BACH1,DDX10,PDZD8,RBM10,CERT1,SETX,NFILZ,AP3D1,NAPA,PSMG2,STK24,METTL8,APEX1,BPTF,ATP6V1G1,UFC1,EDC4,ICE1,RIOK2,NAA15,ARID1A,NUP43,AMMECR1,TRAM1,PITHD1,SPECC1,PP*

			P1CC,CAVIN2,ZC3H14,B4GALT5,CDC27,HERC2,RAB35,SRSF10,CTSL,AKAP8,SBDS,RCC1,C1QBP,VPS72,PPM1H,ZNF587B,CRK,TICRR,MED15,EOGT,STK25,CAPNS1,SRSF8,WDR74,PAK2,ARHGAP21,PRPF4,KTN1,PRMT5,SNX9,PCBP2,PTDSS1,ADD1,TRMT6,RBMX,RNF126,TMEM223,BAZ1A,DPYS,GPX4,YJU2,WDR70,MCCC2,SLC25A3,MLLT3,OPA3,ALG8,RAD23B,VAT1,TAF4,RBM14,RBM8A,ARHGAP6,KDM3B,BOP1,GOLM1,LRP8,SETD2,ZNF326,API5,INTS13,RPIA,BTBD1,TUBB,FEN1,CHAF1A,ZNF282,DYRK1A,PHF3,TFDP2,CASD1,WDR12,URB2,GTF3C6,SLBP,CUL4A,DNAJC8,JADE2,NUCKS1,SDCBP,PSMD1,UTP25,TMPO,NUP188,NUP50,PRPF38A,SSB,CDC37,AGPAT5,GNL3L,DNTTIP2,CYB5B,IBA57,RILP,RNU6-322P,NSRP1,TFIP11,KCTD15,STRBP,PSMB2,WAC,ADI1,BSN,DHX29,ARHGEF2,BRCA2,RANBP1,POLR2D,ZDHHC5,WTAP,COPS2,NIN,BCL7B,RBM42,ZFX,ZC3H7B,ACLY,SNRPD1,SLC12A9,PAN3,MED13L,PAF1,ZC3H18,BIRC6,ERMAP,FASTKD2,RABL6,PHB2,TCOF1,LRRC41,TOMM22,RAP1GAP2,XRCC2,PDS5A,BRCC3,ZNF75A,SMARCB1,CREBBP,EXOSC3,WBP11,KIFC3,AHSA1,COPS3,UBE4B,INSIG1,LSM14A,ABLIM1,TMX2,NUP98,HNRNPUL1,CNOT1,CUTA,LYN,ASH1L,LRRC59,GEMIN5,PHACTR2,PLAGL2,APC,BTF3,POLR1B,JPT2,DDX42,CAPN1,ELOF1,WDR6,NFYC,SF1,PELP1,SLC12A2,XRN2,TMEM201,EZR,TRUB2,DDX20,URM1,DDX51,MMS19,SGPP2,ELOA,TAF9,ACAT2,YWHAG,ELAVL1,UTP15,VCP,DNAJB12,WASHC5,RYBP,SAFB2,GBP2,PSIP1,CSTB,CSNK1G2,DCAF13,BEX4,RBM12,STIP1,CDK4,DVL2,PNO1,POLR3C,THG1L,MRPS2,RBM25,SETD1A,RRP12,SCAP,WDR82,SMC1A,AGO2,E2F4,TJP1,VPS26A,FOXRED2,G3BP1,PCM1,RNF220,TBC1D14,GNL2,SAE1,RRP36,MCM6,AQR,DHCR7,CLCN6,ARL8B,DDX18,IER3,QSER1,IGF2R,ATP6V0D1,ADNP,UBA2,NFKB1,UBTF,ZNF622,ARHGDIA,BRD2,CHD7,RBM15B,DOK3,USP37,HNRNPA1,GAR1,RRS1,PPARGC1B,MAGOH,PBRM1,CTCF,RANBP10,HHEX,ANKRD17,HNRNPR,RRM2,RRM1,PI4KA,SEC24B,ELF1,ZNF614,NSD1,RSL1D1,CCT3,CDK7,H3-3B,RNF138,SETD1B,PPIF,PKM,FOXK2,KIF2A,HMGB1,ATAD3B,MAEA,SMARCD1,GNAQ,CANX,YY1,NOL8,MLLT10,IRAK1,PUS7,PIK3C2B,IP6K1,NAT10,TRMT10C,SNU13,SRSF6,EP400,AASDHPPT,MTHFD1,ADSS2,SART1,CEBPZ,MECP2,CSDE1,PARD3,MBNL1,SKI,PRKAR2B,TKT,DDX54,AZIN1,CCDC6,TARDBP,LARP4,XRCC6,PNN,RPL22,ABI1,KLHL21,PCNA,CDC25A,MBD1,CSNK2A2,UPF2,DIAPH1,DDX3X,KIAA0753,IQGAP2,IDI1,TNPO2,SACS,LBR,HDGF,NDC1,NCAPH,ZNF789,VPS35,ATP5MC3,MCM2,JUND,CFL1,PFAS,HCFC1,CHEK1,AATF,CLN6,ECSIT,ZMPSTE24,UBAP2,PRMT7,OXCT1,RETREG2,SLC25A5,ARPC4,SLTM,CYP3A5,URB1,VAC14,POM121,CHAMP1,DNAJC21,UBP1,POLE,TRRAP,NUDC,EXOSC9,CSK,POLR2A,CCDC86,DNAJC7,DNAJA1,TEX261,SUMO3,NUP62,DYNC1H1,TSR1,RBM19,XPO1,PPIA,PRPF3,IK,CLCN7,PHACTR1,SERPINE1,TPR,ENC1,SRSF7,UTP20,TGFBRAP1,ZFP36L2,SF3B4,STK17A,PPRC1,EBNA1BP2,ZFR,ARFGAP2,KEAP1,TFB2M,UBE2N,BAZ1B,NRIP1,MACO1,MRTO4,VAPA,TLK1,TEX10,GFM1,MYBBP1A,DDB1,BACH2,PSMG1,PITX1,GTF3C4,NASP,ASXL2,CHST3,TCP1,CENPF,YWHAB,RALY,ENO1,FAM83H,CCT5,FUBP1,POLE3,CDK6,CDC5L,CHD3,ST3GAL2,NUP153,RAN,BEND3,C19ORF48,UBC,WDR81,ZNF787,TFAM,HSPA4,AK2,PPP2CA,SSU72,MSH2,CDK12,ZBTB2,DIDO1,GLYR1,MAN2A2,KMT2A,MCM3,NOSTRIN,GRWD1,TOMM40,PAICS,H4C8,SLC30A10,CERS6,PRDX1,TNPO1,SNHG3,GA

			TAD2A,DKC1,DDX1,H2BC12,STX3,CUL1,LTBR,THUMPD1,EWSR1,DDX49,CASC3,SSRP1,ARID1B,KPNB1,CPSF7,RRP1,SPTB,TXNRD1,ZNF33B,ATP6V0A1,HNRNPAB,KAT6A,ATAD3A,ARF6,USP36,RBBP4,MT-RNR2,CNPPD1,SAFB,NCBP1,HEATR1,CCT2,HSP90AB1,EIF3B,RCSD1,PA2G4,HNRNPK,SMG1,TXNL1,DHX37,FDFT1,PRPF19,SMARCA4,PRKDC,YES1,ZNF121,TRA2B,PRMT1,HNRNPC,MALT1,WWP2,SF3A3,SUPT16H,HMGA1,CELF1,NCLN,RBMXL1,XRCC5,DDX5,PSME3,TMEM97,ANK1,UTP4,DEK,LMO2,LMNB1,DDX46,LSS,EIF4A3,NDST1,NUP214,TRIP12,IPO7,ACTG1,SURF6,DDX39A,DDX56,SLC38A1,CIZ1,CDT1,UBAP2L,NOP56,HNRNPDL,CORO1C,KHDRBS1,HNRNPD,RAPGEF1,LRPPRC,EIF5A,CTDSP1,SUPT6H,RANBP2,DNAJB6,NOLC1,SNRNP200,SREBF2,CBFA2T3,STXBP5,ABCF1,DANCR,ACACA,TAFA2,MGA,NSUN2,AP5Z1,HSPD1,GAPDH,YBX1,LIN28B,EIF5B,PCYT2,ATP6V1C1,RBM3,STARD7,PGD,SF3A1,LMNB2,AXIN1,MS4A3,MPHOSPH10,GDI2,PES1,ANP32B,MYH10,IPO5,RREB1,HSPH1,EP300,TGFBR1,DAZAP1,ALYREF,PCNT,STMN1,RRP1B,FOSB,PPM1G,STON2,SRRT,PABPC1,PRRC2C,SERBP1,ATP13A3,AHNAK,PRPF8,COA7,SF3B3,PTMA,FTL,THRAP3,MLLT1,ELOVL6,HNRNPF,MT-ND1,NPM1,RIF1,CITED2,SON,WDR43,TFRC,RUNX1,EIF4G1,TRIM28,ACP5,NEFH,U2AF2,NR2F2,BCLAF1,ECPAS,SRSF2,HSP90AA1,MDN1,NFATC3,ZNF521,CAPRIN1,TRMT1,SQLE,MCM4,TRIM24,NOP58,RESF1,HNRNPA2B1,KIF1A,SPN,BAG1,LARP1,SQSTM1,HBZ,PTBP1,CCAR1,DDX21,SFPQ,PTGER3,SCD,PABPC4,HNRNPM,NAV1,POLR1A,SRSF3,ANKRD11,EIF3A,MCM7,SMARCC1,MYC,SET,VGF,BTG1,MT-CYB,IGF2BP1,FOS,HMGCR,SPTA1,MYB,KHSRP,ZEB2,FUS,ILF3,FASN,GLUL,ACTB,DHX9,MT-RNR1,HNRNPU,MCM5,NCL,EGR1,DHCR24,HSPA8,FTH1
GO:0043232	intracellular non-membrane-bounded organelle	7.633612574 48226e-47	*NOP14,CENPN,MITF,JMJD1C,MIS18BP1,DAP3,PPAN,TRIP13,CHTOP,ZNF274,ABRAXAS2,SUPT7L,MACROH2A1,BRD9,MUS81,NOL11,BICRA,MTA2,SNHG6,RAD51C,AAMP,UTP3,MTREX,RIOX1,POLR1C,CBX3,EZH2,FES,CCDC78,RAB10,NOC4L,RFWD3,MFAP1,DNM1L,PAXIP1,LRRFIP1,ASAP1,INTS6,H4C5,NCAPH2,BRD4,RRP9,MRPL1,ZNF74,MRPL11,AHCTF1,RPUSD4,HROB,MRPS30,CTDP1,MED1,NVL,KIF26B,HNRNPL,MYO16,PWP1,WDR46,MCM10,CHRAC1,EML4,FRMD8,STAG2,CASP8,TAF4B,PQBP1,AIFM2,PSPC1,CEP350,MAD2L2,POLDIP2,PSMC3,NAA11,TIMM44,ADNP2,NOP16,ARID2,DDX23,MNS1,ALMS1,LHX4,RBL1,USP11,SS18L1,PAFAH1B1,STAG1,RAB7A,LIMD1,DNMT1,LYL1,BICD1,ANAPC7,SUB1,BUB3,TOP2B,LYAR,LRWD1,EXOC7,CCT6A,KAT7,CLTC,MSRA,AURKAIP1,SNHG20,WDR5,CCT8,KIF5B,FUBP3,IMP4,SNHG17,BCCIP,SPRY2,RNF40,CUL3,CMPK1,NONO,MEF2C,TCF3,NUP160,MAF1,NOL9,PLK4,ZFYVE26,ZC3H4,GRSF1,NXF1,UBQLN4,ACTR8,STK35,CAPZA1,HNRNPA3,MAPK1,EEF1D,POLR1E,ABT1,PCLAF,WDR36,DHX33,CTR9,NCOA5,ZFP91,SDAD1,TBCD,DHX15,PUM1,DHX30,UTP18,KANSL1,VDAC1,SMARCA5,H2AW,RSF1,TFAP4,NAA50,CLDN11,PRMT6,TSPYL5,NIFK,EMD,ZNF131,FKBP15,NAP1L4,MTDH,BAIAP2,HSPA9,TCF7L2,NSDHL,MRPL15,PRAME,SLC29A2,THOC1,PSMC2,SETMAR,RPTOR,PPP1R10,MRPS35,PPP4R3A,WDR33,NIP7,PPP2R5A,IGF2BP3,NOL7,MED28,PUM2,H2AZ2,HAT1,MCRIP2,CLTA,WDR3,BACH1,SETX,STK24,APEX1,BPTF,EDC4,ICE1,ARID1A,NUP43,SPECC1,PPP1CC,ZC3H14,CDC27,HERC2,AKAP8,SBDS,RCC1,C1QBP,VPS72,CRK,WDR74,ARHGAP21,PRMT5,SNX9,ADD1,RBMX,BAZ*

			1A,WDR70,MLLT3,TAF4,RBM14,ARHGAP6,KDM3B,BOP1,LRP8,SETD2,BTBD1,TUBB,FEN1,CHAF1A,DYRK1A,TFDP2,WDR12,URB2,SLBP,JADE2,NUCKS1,SDCBP,PSMD1,UTP25,TMPO,SSB,AGPAT5,GNL3L,DNTTIP2,RILP,TFIP11,STRBP,BSN,DHX29,ARHGEF2,BRCA2,RANBP1,POLR2D,NIN,BCL7B,ZFX,PAN3,BIRC6,FASTKD2,RABL6,PHB2,TCOF1,RAP1GAP2,XRCC2,PDS5A,BRCC3,SMARCB1,CREBBP,EXOSC3,KIFC3,LSM14A,ABLIM1,NUP98,CNOT1,LYN,ASH1L,LRRC59,APC,BTF3,POLR1B,NFYC,SF1,PELP1,XRN2,TMEM201,EZR,TRUB2,DDX20,DDX51,MMS19,ELOA,TAF9,ELAVL1,UTP15,VCP,PSIP1,CSTB,DCAF13,BEX4,STIP1,CDK4,PNO1,MRPS2,SETD1A,RRP12,WDR82,SMC1A,AGO2,E2F4,TJP1,G3BP1,PCM1,GNL2,RRP36,MCM6,ARL8B,DDX18,QSER1,ATP6V0D1,ADNP,NFKB1,UBTF,ZNF622,ARHGDIA,BRD2,CHD7,HNRNPA1,GAR1,RRS1,PBRM1,CTCF,HHEX,ANKRD17,ELF1,NSD1,RSL1D1,CCT3,CDK7,H3-3B,RNF138,SETD1B,FOXK2,KIF2A,HMGB1,MAEA,SMARCD1,CANX,YY1,NOL8,IRAK1,IP6K1,NAT10,TRMT10C,SNU13,EP400,MECP2,CSDE1,PARD3,MBNL1,SKI,PRKAR2B,DDX54,CCDC6,TARDBP,LARP4,XRCC6,PNN,RPL22,ABI1,KLHL21,PCNA,MBD1,CSNK2A2,UPF2,DIAPH1,DDX3X,KIAA0753,IQGAP2,NDC1,NCAPH,MCM2,JUND,CFL1,HCFC1,CHEK1,AATF,CLN6,UBAP2,PRMT7,SLC25A5,ARPC4,URB1,CHAMP1,DNAJC21,UBP1,POLE,TRRAP,NUDC,EXOSC9,POLR2A,CCDC86,DNAJC7,DNAJA1,SUMO3,NUP62,DYNC1H1,TSR1,RBM19,XPO1,IK,TPR,ENC1,UTP20,EBNA1BP2,ZFR,KEAP1,TFB2M,UBE2N,BAZ1B,NRIP1,MRTO4,VAPA,TEX10,MYBBP1A,DDB1,BACH2,PITX1,NASP,TCP1,CENPF,ENO1,FAM83H,CCT5,POLE3,CDK6,CDC5L,CHD3,NUP153,RAN,BEND3,C19ORF48,TFAM,PPP2CA,MSH2,DIDO1,GLYR1,MCM3,NOSTRIN,GRWD1,H4C8,SNHG3,GATAD2A,DKC1,DDX1,H2BC12,EWSR1,CASC3,SSRP1,ARID1B,KPNB1,RRP1,SPTB,TXNRD1,HNRNPAB,KAT6A,ATAD3A,USP36,RBBP4,MT-RNR2,HEATR1,CCT2,HSP90AB1,EIF3B,RCSD1,PA2G4,HNRNPK,SMG1,DHX37,PRPF19,SMARCA4,PRKDC,YES1,HNRNPC,MALT1,SUPT16H,HMGA1,CELF1,XRCC5,DDX5,ANK1,UTP4,DEK,LMNB1,DDX46,LSS,EIF4A3,ACTG1,SURF6,DDX56,CDT1,UBAP2L,NOP56,CORO1C,LRPPRC,DNAJB6,NOLC1,SREBF2,CBFA2T3,ABCF1,DANCR,ACACA,MGA,NSUN2,GAPDH,YBX1,LIN28B,RBM3,LMNB2,AXIN1,MPHOSPH10,PES1,ANP32B,MYH10,IPO5,RREB1,HSPH1,EP300,PCNT,STMN1,RRP1B,FOSB,STON2,PABPC1,PRRC2C,AHNAK,SF3B3,MLLT1,NPM1,RIF1,CITED2,WDR43,RUNX1,EIF4G1,TRIM28,NEFH,ECPAS,SRSF2,MDN1,NFATC3,CAPRIN1,MCM4,TRIM24,NOP58,RESF1,HNRNPA2B1,KIF1A,LARP1,SQSTM1,PTBP1,DDX21,SFPQ,SCD,PABPC4,HNRNPM,NAV1,POLR1A,EIF3A,MCM7,SMARCC1,MYC,SET,IGF2BP1,FOS,SPTA1,KHSRP,ZEB2,FUS,ILF3,ACTB,DHX9,MT-RNR1,HNRNPU,MCM5,NCL,EGR1,DHCR24,HSPA8
GO:0043228	non-membrane-bounded organelle	8.063434871350523e-47	*NOP14,CENPN,MITF,JMJD1C,MIS18BP1,DAP3,PPAN,TRIP13,CHTOP,ZNF274,ABRAXAS2,SUPT7L,MACROH2A1,BRD9,MUS81,NOL11,BICRA,MTA2,SNHG6,RAD51C,AAMP,UTP3,MTREX,RIOX1,POLR1C,CBX3,EZH2,FES,CCDC78,RAB10,NOC4L,RFWD3,MFAP1,DNM1L,PAXIP1,LRRFIP1,ASAP1,INTS6,H4C5,NCAPH2,BRD4,RRP9,MRPL1,ZNF74,MRPL11,AHCTF1,RPUSD4,HROB,MRPS30,CTDP1,MED1,NVL,KIF26B,HNRNPL,MYO16,PWP1,WDR46,MCM10,CHRAC1,EML4,FRMD8,STAG2,CASP8,TAF4B,PQBP1,AIFM2,PSPC1,CEP350,MAD2L2,POLDIP2,PSMC3,NAA11,TIMM44,ADNP2,NOP16*

			,ARID2,DDX23,MNS1,ALMS1,LHX4,RBL1,USP11,SS18L1,PAFAH1B1,STAG1,RAB7A,LIMD1,DNMT1,LYL1,BICD1,ANAPC7,SUB1,BUB3,TOP2B,LYAR,LRWD1,EXOC7,CCT6A,KAT7,CLTC,MSRA,AURKAIP1,SNHG20,WDR5,CCT8,KIF5B,FUBP3,IMP4,SNHG17,BCCIP,SPRY2,RNF40,CUL3,CMPK1,NONO,MEF2C,TCF3,NUP160,MAF1,NOL9,PLK4,ZFYVE26,ZC3H4,GRSF1,NXF1,UBQLN4,ACTR8,STK35,CAPZA1,HNRNPA3,MAPK1,EEF1D,POLR1E,ABT1,PCLAF,WDR36,DHX33,CTR9,NCOA5,ZFP91,SDAD1,TBCD,DHX15,PUM1,DHX30,UTP18,KANSL1,VDAC1,SMARCA5,H2AW,RSF1,TFAP4,NAA50,CLDN11,PRMT6,TSPYL5,NIFK,EMD,ZNF131,FKBP15,NAP1L4,MTDH,BAIAP2,HSPA9,TCF7L2,NSDHL,MRPL15,PRAME,SLC29A2,THOC1,PSMC2,SETMAR,RPTOR,PPP1R10,MRPS35,PPP4R3A,WDR33,NIP7,PPP2R5A,IGF2BP3,NOL7,MED28,PUM2,H2AZ2,HAT1,MCRIP2,CLTA,WDR3,BACH1,SETX,STK24,APEX1,BPTF,EDC4,ICE1,ARID1A,NUP43,SPECC1,PPP1CC,ZC3H14,CDC27,HERC2,AKAP8,SBDS,RCC1,C1QBP,VPS72,CRK,WDR74,ARHGAP21,PRMT5,SNX9,ADD1,RBMX,BAZ1A,WDR70,MLLT3,TAF4,RBM14,ARHGAP6,KDM3B,BOP1,LRP8,SETD2,BTBD1,TUBB,FEN1,CHAF1A,DYRK1A,TFDP2,WDR12,URB2,SLBP,JADE2,NUCKS1,SDCBP,PSMD1,UTP25,TMPO,SSB,AGPAT5,GNL3L,DNTTIP2,RILP,TFIP11,STRBP,BSN,DHX29,ARHGEF2,BRCA2,RANBP1,POLR2D,NIN,BCL7B,ZFX,PAN3,BIRC6,FASTKD2,RABL6,PHB2,TCOF1,RAP1GAP2,XRCC2,PDS5A,BRCC3,SMARCB1,CREBBP,EXOSC3,KIFC3,LSM14A,ABLIM1,NUP98,CNOT1,LYN,ASH1L,LRRC59,APC,BTF3,POLR1B,NFYC,SF1,PELP1,XRN2,TMEM201,EZR,TRUB2,DDX20,DDX51,MMS19,ELOA,TAF9,ELAVL1,UTP15,VCP,PSIP1,CSTB,DCAF13,BEX4,STIP1,CDK4,PNO1,MRPS2,SETD1A,RRP12,WDR82,SMC1A,AGO2,E2F4,TJP1,G3BP1,PCM1,GNL2,RRP36,MCM6,ARL8B,DDX18,QSER1,ATP6V0D1,ADNP,NFKB1,UBTF,ZNF622,ARHGDIA,BRD2,CHD7,HNRNPA1,GAR1,RRS1,PBRM1,CTCF,HHEX,ANKRD17,ELF1,NSD1,RSL1D1,CCT3,CDK7,H3-3B,RNF138,SETD1B,FOXK2,KIF2A,HMGB1,MAEA,SMARCD1,CANX,YY1,NOL8,IRAK1,IP6K1,NAT10,TRMT10C,SNU13,EP400,MECP2,CSDE1,PARD3,MBNL1,SKI,PRKAR2B,DDX54,CCDC6,TARDBP,LARP4,XRCC6,PNN,RPL22,ABI1,KLHL21,PCNA,MBD1,CSNK2A2,UPF2,DIAPH1,DDX3X,KIAA0753,IQGAP2,NDC1,NCAPH,MCM2,JUND,CFL1,HCFC1,CHEK1,AATF,CLN6,UBAP2,PRMT7,SLC25A5,ARPC4,URB1,CHAMP1,DNAJC21,UBP1,POLE,TRRAP,NUDC,EXOSC9,POLR2A,CCDC86,DNAJC7,DNAJA1,SUMO3,NUP62,DYNC1H1,TSR1,RBM19,XPO1,IK,TPR,ENC1,UTP20,EBNA1BP2,ZFR,KEAP1,TFB2M,UBE2N,BAZ1B,NRIP1,MRTO4,VAPA,TEX10,MYBBP1A,DDB1,BACH2,PITX1,NASP,TCP1,CENPF,ENO1,FAM83H,CCT5,POLE3,CDK6,CDC5L,CHD3,NUP153,RAN,BEND3,C19ORF48,TFAM,PPP2CA,MSH2,DIDO1,GLYR1,MCM3,NOSTRIN,GRWD1,H4C8,SNHG3,GATAD2A,DKC1,DDX1,H2BC12,EWSR1,CASC3,SSRP1,ARID1B,KPNB1,RRP1,SPTB,TXNRD1,HNRNPAB,KAT6A,ATAD3A,USP36,RBBP4,MT-RNR2,HEATR1,CCT2,HSP90AB1,EIF3B,RCSD1,PA2G4,HNRNPK,SMG1,DHX37,PRPF19,SMARCA4,PRKDC,YES1,HNRNPC,MALT1,SUPT16H,HMGA1,CELF1,XRCC5,DDX5,ANK1,UTP4,DEK,LMNB1,DDX46,LSS,EIF4A3,ACTG1,SURF6,DDX56,CDT1,UBAP2L,NOP56,CORO1C,LRPPRC,DNAJB6,NOLC1,SREBF2,CBFA2T3,ABCF1,DANCR,ACACA,MGA,NSUN2,GAPDH,YBX1,LIN28B,RBM3,LMNB2,AXIN1,MPHOSPH10,PES1,ANP32B,MYH10,IPO5,RREB1,HSPH1,EP300,PCNT,STMN1,RRP1B,

			FOSB,STON2,PABPC1,PRRC2C,AHNAK,SF3B3,MLLT1,NPM1,RIF1,CITED2,WDR43,RUNX1,EIF4G1,TRIM28,NEFH,ECPAS,SRSF2,MDN1,NFATC3,CAPRIN1,MCM4,TRIM24,NOP58,RESF1,HNRNPA2B1,KIF1A,LARP1,SQSTM1,PTBP1,DDX21,SFPQ,SCD,PABPC4,HNRNPM,NAV1,POLR1A,EIF3A,MCM7,SMARCC1,MYC,SET,IGF2BP1,FOS,SPTA1,KHSRP,ZEB2,FUS,ILF3,ACTB,DHX9,MT-RNR1,HNRNPU,MCM5,NCL,EGR1,DHCR24,HSPA8
GO:0005829	cytosol	6.849257879661888e-42	*HRAS,CENPN,SNRPB,PPP6C,ERAL1,SEC23IP,ABRAXAS2,DNAJA2,GYG1,AP5M1,ITFG2,TIRAP,RAD51C,AAMP,CWC22,POLR3E,UBE2Q1,DCAF7,POLR1C,SRP72,SNX17,FES,RAB10,DNM1L,LRRFIP1,GPATCH3,RHEB,ASAP1,PPP6R3,SMG5,MRPL1,SPIN4,AHCTF1,CMBL,NUP155,SRM,HNRNPL,NLN,MYO16,EFTUD2,EML4,CRCP,FRMD8,STAG2,CASP8,PSMA3,PSMD3,AIFM2,MAD2L2,PSMC3,NAA11,GART,NT5C3A,FBXO45,RNPS1,ALMS1,PPP3R1,HIF1AN,USP11,SS18L1,NOSIP,PAFAH1B1,STAG1,HNRNPH1,USP14,RAB7A,LIMD1,NAA20,BICD1,PPP5C,BAP1,ANAPC7,BUB3,GAB2,TOP2B,THOP1,EXOC7,CCT6A,KAT7,TPP2,CLTC,TSR3,EEF2,MSRA,CCT8,KIF5B,FAM13B,BCCIP,SPRY2,FAM120A,NOB1,SBF1,RNF40,CUL3,CMPK1,MEF2C,NUP160,MAF1,GID8,MVK,NBAS,PLK4,SLK,ZC3H4,PEBP1,GSPT1,NXF1,UBQLN4,FAHD1,PSMC5,ZC3HAV1,CAPZA1,MAPK1,ZNRF1,EEF1D,CASP3,IPO9,TULP4,SURF4,HBG1,DHX33,STRIP1,NSMAF,PUM1,MTOR,DHX30,IKZF3,LCLAT1,SMG9,SNX8,EIF3D,NAA50,PTP4A2,DELE1,KPNA4,FAF1,BAG6,EMD,PCYT1A,TASOR2,DHFR,BAIAP2,COMMD4,MCCC1,GMPS,ASCC3,UROD,UBE2L3,PSMC2,MCMBP,RPTOR,RABGGTB,EIF4B,PPP4R3A,SAMSN1,NIP7,PPP2R5A,IGF2BP3,PUM2,CLTA,BACH1,CERT1,TRMT2A,NAPA,PSMG2,STK24,ATP6V1G1,EDC4,RIOK2,NAA15,NUP43,SPECC1,ADO,PPP1CC,CAVIN2,CDC27,HERC2,RAB35,SRSF10,SBDS,C1QBP,CRK,TICRR,CAPNS1,SRSF8,PAK2,ARHGAP21,PRMT5,SNX9,PCBP2,ADD1,RNF126,DPYS,GPX4,MCCC2,MLLT3,RAD23B,TAF4,RBM8A,ARHGAP6,RPIA,UCK2,BTBD1,TUBB,SLBP,DNAJC8,SDCBP,PSMD1,AMD1,NUP188,CDC37,ECHDC1,GNL3L,RILP,PSMB2,EIF3G,ADI1,DHX29,ARHGEF2,BRCA2,RANBP1,POLR2D,EIF3M,COPS2,ACLY,SNRPD1,PAN3,BIRC6,ERMAP,RABL6,TCOF1,LRRC41,RAP1GAP2,GRB10,PDS5A,BRCC3,CREBBP,EXOSC3,WBP11,AHSA1,EPRS1,COPS3,LSM14A,NUP98,CNOT1,LYN,GEMIN5,APC,BTF3,POLR1B,JPT2,DDX42,CAPN1,WDR6,SLC12A2,EZR,DDX20,URM1,MMS19,ACAT2,YWHAG,ELAVL1,VCP,WASHC5,GBP2,PSIP1,CSTB,CSNK1G2,DCAF13,BEX4,STIP1,CDK4,DVL2,PNO1,POLR3C,THG1L,RRP12,SMC1A,AGO2,TJP1,VPS26A,G3BP1,PCM1,TBC1D14,ARL8B,IER3,NFKB1,ZNF622,ARHGDIA,USP37,PPARGC1B,MAGOH,RANBP10,RRM2,RRM1,PI4KA,SEC24B,CCT3,CDK7,PKM,KIF2A,MLLT10,IRAK1,PIK3C2B,IP6K1,METAP2,AASDHPPT,MTHFD1,ADSS2,SART1,MECP2,CSDE1,PARD3,MBNL1,PRKAR2B,TKT,AZIN1,CCDC6,N4BP2,LARP4,XRCC6,RPL22,ABI1,KLHL21,CDC25A,CSNK2A2,UPF2,DIAPH1,DDX3X,KIAA0753,IQGAP2,IDI1,NCAPH,VPS35,EIF4G2,CFL1,PFAS,CHEK1,ECSIT,PRMT7,ARPC4,VAC14,DNAJC21,UBP1,PPIP5K2,NUDC,EXOSC9,CSK,DNAJC7,DNAJA1,DYNC1H1,TSR1,XPO1,PPIA,PRPF3,PHACTR1,ARFGAP2,KEAP1,UBE2N,NRIP1,VAPA,BACH2,PSMG1,TCP1,CENPF,YWHAB,ENO1,CCT5,CDK6,NUP153,RAN,UBC,WDR81,TFAM,HSPA4,PPP2CA,SSU72,GLYR1,KMT2A,GRWD1,TOMM40,PAICS,ARMC6,PRDX1,TNPO1,DDX1,H2BC12,CUL1,CTPS1,CASC3,ARID1B,KPNB1,SPTB,TXNRD1,ATP6V0A1,KAT*

			6A,ARF6,USP36,RBBP4,NCBP1,CCT2,HSP90AB1,EIF3B,SMG1,TXNL1,EIF3J,PRKDC,YES1,PRMT1,HNRNPC,MALT1,WWP2,HMGA1,FARSA,XRCC5,DDX5,PSME3,ANK1,EIF4A3,NUP214,TRIP12,IPO7,ACTG1,DDX56,HNRNPDL,KHDRBS1,HNRNPD,RAPGEF1,EIF5A,RANBP2,DNAJB6,SREBF2,STXBP5,ABCF1,ACACA,HSPD1,GAPDH,YBX1,LIN28B,EIF5B,ATP6V1C1,PGD,AXIN1,GDI2,PES1,MYH10,HSPH1,EP300,DAZAP1,PCNT,STMN1,RRP1B,STON2,PABPC1,PRRC2C,SERBP1,AHNAK,PTMA,FTL,MLLT1,HNRNPF,NPM1,ETF1,CDV3,NQO1,EIF4G1,ACP5,NR2F2,SRSF2,HSP90AA1,MDN1,NFATC3,CAPRIN1,TRIM24,NOP58,BAG1,LARP1,SQSTM1,HBZ,MAT2A,DDX21,HMGCS1,SFPQ,PABPC4,ANKRD11,EIF3A,MCM7,SET,IGF2BP1,FOS,GCLM,SPTA1,MYB,ODC1,KHSRP,ZEB2,FASN,GLUL,ACTB,DHX9,HNRNPU,MCM5,DHCR24,HSPA8,FTH1
GO:0032991	protein-containing complex	5.635927156769828e-41	*HRAS,NOP14,SNRPB,MITF,JMJD1C,DAP3,TOMM5,PPAN,MSH3,CHTOP,ABRAXAS2,PRPF38B,KMT2B,AFF1,SUPT7L,AP5M1,SREK1,MACROH2A1,BRD9,MED16,MUS81,NOL11,ITFG2,BICRA,MTA2,RAD51C,UTP3,CWC22,POLR3E,DCAF7,MTREX,OTUD6B,PHF5A,NACA,POLR1C,MED6,SRP72,CBX3,PHB,METTL3,EZH2,SNX17,TOMM70,RAB10,DCLRE1C,NOC4L,MFAP1,DNM1L,PAXIP1,RHEB,INTS6,H4C5,NCAPH2,SMG5,STT3A,DHDDS,TIMM17A,RRP9,MRPL1,MRPL11,AHCTF1,CHCHD3,RNASEH2C,MRPS30,CTDP1,NUP155,MED1,NVL,POM121C,KIF26B,HNRNPL,MYO16,ATP11A,WDR46,MCM10,OXA1L,EFTUD2,CHRAC1,CRCP,RANBP3,KMT2D,STAG2,CASP8,TAF4B,PSMA3,PSMD3,MAD2L2,PSMC3,NAA11,TIMM44,DHX38,FBXO45,ARID2,DDX23,RNPS1,RBL1,PPP3R1,SS18L1,RBM45,MED29,PAFAH1B1,STAG1,HNRNPH1,USP14,RAB7A,LIMD1,LYL1,NAA20,PPP5C,BAP1,JRK,ANAPC7,SUB1,GTF2H1,DHX16,BUB3,TOP2B,LRWD1,CCNH,EXOC7,CCT6A,KAT7,CLTC,EEF2,AFG3L2,AURKAIP1,WDR5,CCT8,KIF5B,FUBP3,IMP4,BCCIP,DLAT,NOB1,RNF40,CUL3,NONO,MEF2C,UBR3,TCF3,NUP160,GID8,NBAS,PLK4,CCNY,PRPF6,GSPT1,GRSF1,NXF1,UBQLN4,MIR17HG,ACTR8,PSMC5,TIMM23,CAPZA1,HNRNPA3,EEF1D,CASP3,POLR1E,ATP2A2,PKP3,ABT1,WDR36,HBG1,PDSS1,DHX33,CTR9,DHX15,UIMC1,MTOR,PDPR,RPRD2,UTP18,KANSL1,CPSF3,SPEN,VDAC1,SMARCA5,SNX8,CWC25,SF3A2,H2AW,EIF3D,RSF1,TFAP4,NAA50,KPNA4,FAF1,HNRNPA0,BAG6,HSPA9,MCCC1,TCF7L2,SRRM2,MRPL15,PRAME,ASCC3,UBE2L3,THOC1,PSMC2,MCMBP,RPTOR,PPIL2,PPP1R10,MRPS35,CSTF2,GRPEL1,RABGGTB,EIF4B,PPP4R3A,WDR33,NIP7,PPP2R5A,TRMT61A,PDCD7,MED28,ELL2,H2AZ2,HAT1,CLTA,WDR3,SSBP3,SNRPA,BACH1,RBM10,CERT1,AP3D1,NAPA,PSMG2,APEX1,BPTF,ATP6V1G1,ICE1,RIOK2,NAA15,ARID1A,NUP43,SPECC1,PPP1CC,KCNQ5,ZC3H14,CDC27,RCC1,VPS72,CRK,MED15,CAPNS1,WDR74,PRPF4,PRMT5,PCBP2,ADD1,TRMT6,RBMX,BAZ1A,GPX4,YJU2,MCCC2,MLLT3,RAD23B,TAF4,RBM14,RBM8A,KDM3B,BOP1,LRP8,ZNF326,API5,INTS13,BTBD1,TUBB,FEN1,CHAF1A,DYRK1A,TFDP2,WDR12,GTF3C6,SLBP,CUL4A,JADE2,SDCBP,PSMD1,UTP25,NUP188,NUP50,PRPF38A,SSB,CDC37,GNL3L,CYB5B,RILP,RNU6-322P,NSRP1,TFIP11,PSMB2,WAC,EIF3G,DHX29,ARHGEF2,BRCA2,RANBP1,POLR2D,EIF3M,WTAP,COPS2,NIN,BCL7B,RBM42,SNRPD1,PAN3,MED13L,PAF1,ZC3H18,PHB2,TOMM22,GRB10,XRCC2,BRCC3,SMARCB1,CREBBP,EXOSC3,WBP11,KIFC3,EPRS1,COPS3,UBE4B,INSIG1,LSM14A,NUP98,HNRNPUL1,CNOT1,LYN,ASH1L,GEMIN5,APC,BTF3,POLR1B,ELOF1,WDR6,N*

			FYC,SF1,PELP1,EZR,DDX20,MMS19,ELOA,TAF9,ELAVL1,VCP,WASHC5,RYBP,DCAF13,RBM12,STIP1,CDK4,POLR3C,THG1L,MRPS2,SETD1A,SCAP,WDR82,SMC1A,AGO2,E2F4,TJP1,VPS26A,G3BP1,PCM1,RNF220,SAE1,RRP36,MCM6,AQR,IGF2R,ATP6V0D1,ADNP,UBA2,NFKB1,ZNF622,RBM15B,HNRNPA1,GAR1,RRS1,PPARGC1B,MAGOH,PBRM1,RANBP10,HHEX,HNRNPR,RRM2,RRM1,SEC24B,RSL1D1,CCT3,CDK7,H3-3B,SETD1B,PPIF,KIF2A,HMGB1,MAEA,SMARCD1,GNAQ,CANX,YY1,MLLT10,IRAK1,PIK3C2B,NAT10,TRMT10C,SNU13,EP400,SART1,CEBPZ,LEPR,CSDE1,PARD3,SKI,PRKAR2B,RRP15,LARP4,XRCC6,PNN,RPL22,ABI1,KLHL21,PCNA,CSNK2A2,UPF2,DDX3X,HDGF,NDC1,NCAPH,VPS35,ATP5MC3,EIF4G2,MCM2,JUND,HCFC1,CHEK1,AATF,ZMPSTE24,SLC25A5,ARPC4,VAC14,POM121,POLE,TRRAP,EXOSC9,POLR2A,NUP62,DYNC1H1,TSR1,XPO1,PPIA,PRPF3,IK,CLCN7,SERPINE1,TPR,ENC1,UTP20,TGFBRAP1,ZFP36L2,SF3B4,GABRE,EBNA1BP2,KEAP1,UBE2N,BAZ1B,NRIP1,MRTO4,TEX10,MYBBP1A,DDB1,BACH2,PSMG1,PITX1,GTF3C4,NASP,ASXL2,TCP1,CENPF,RALY,ENO1,CCT5,POLE3,CDK6,CDC5L,CHD3,NUP153,RAN,TFAM,PPP2CA,SSU72,MSH2,CDK12,GLYR1,KMT2A,MCM3,GRWD1,TOMM40,H4C8,GATAD2A,DKC1,DDX1,H2BC12,STX3,CUL1,CASC3,SSRP1,ARID1B,KPNB1,CPSF7,RRP1,SPTB,ATP6V0A1,HNRNPAB,KAT6A,RBBP4,CNPPD1,NCBP1,HEATR1,CCT2,HSP90AB1,EIF3B,PA2G4,HNRNPK,SMG1,TXNL1,PRPF19,SMARCA4,EIF3J,PRKDC,TRA2B,PRMT1,HNRNPC,MALT1,WWP2,SF3A3,SUPT16H,HMGA1,FARSA,CELF1,NCLN,RBMXL1,XRCC5,DDX5,PSME3,UTP4,DEK,LMO2,DDX46,EIF4A3,NUP214,IPO7,ACTG1,UBAP2L,NOP56,HNRNPDL,CORO1C,KHDRBS1,HNRNPD,RAPGEF1,LRPPRC,EIF5A,SLC7A1,SUPT6H,RANBP2,NOLC1,SNRNP200,SREBF2,STXBP5,ABCF1,MGA,AP5Z1,HSPD1,GAPDH,YBX1,ATP6V1C1,RBM3,SF3A1,AXIN1,MPHOSPH10,PES1,MYH10,IPO5,HSPH1,EP300,TGFBR1,DAZAP1,RRP1B,SRRT,PABPC1,AHNAK,PRPF8,SF3B3,FTL,THRAP3,MLLT1,ELOVL6,HNRNPF,MT-ND1,NPM1,CITED2,ETF1,TFRC,RUNX1,EIF4G1,TRIM28,U2AF2,BCLAF1,ECPAS,SRSF2,HSP90AA1,MDN1,NFATC3,MCM4,NOP58,RESF1,HNRNPA2B1,KIF1A,LARP1,HBZ,MAT2A,DDX21,SFPQ,PABPC4,HNRNPM,POLR1A,KCNH2,EIF3A,MCM7,SMARCC1,MYC,SET,MT-CYB,IGF2BP1,FOS,GCLM,SPTA1,MYB,KHSRP,RELN,ILF3,GLUL,ACTB,DHX9,HNRNPU,MCM5,NCL,HSPA8,FTH1
GO:0005694	chromosome	1.1434197020245962e-38	*CENPN,MITF,JMJD1C,MIS18BP1,TRIP13,SUPT7L,MACROH2A1,BRD9,MUS81,BICRA,MTA2,RAD51C,CBX3,EZH2,RFWD3,PAXIP1,H4C5,NCAPH2,BRD4,AHCTF1,HROB,HNRNPL,PWP1,MCM10,CHRAC1,STAG2,MAD2L2,ADNP2,ARID2,DDX23,LHX4,RBL1,USP11,SS18L1,PAFAH1B1,STAG1,DNMT1,LYL1,ANAPC7,BUB3,TOP2B,LRWD1,KAT7,WDR5,RNF40,NONO,MEF2C,TCF3,NUP160,PLK4,ZC3H4,UBQLN4,ACTR8,CTR9,KANSL1,SMARCA5,H2AW,RSF1,TFAP4,TSPYL5,NIFK,NAP1L4,TCF7L2,PRAME,THOC1,SETMAR,PPP1R10,PPP2R5A,NOL7,H2AZ2,HAT1,BACH1,SETX,APEX1,BPTF,ICE1,ARID1A,NUP43,PPP1CC,AKAP8,RCC1,VPS72,PRMT5,RBMX,BAZ1A,WDR70,MLLT3,TAF4,KDM3B,BOP1,SETD2,FEN1,CHAF1A,TFDP2,JADE2,NUCKS1,TMPO,SSB,DNTTIP2,TFIP11,BRCA2,BCL7B,ZFX,XRCC2,PDS5A,SMARCB1,CREBBP,EXOSC3,NUP98,ASH1L,APC,POLR1B,NFYC,PELP1,ELOA,TAF9,VCP,PSIP1,CDK4,SETD1A,WDR82,SMC1A,E2F4,MCM6,DDX18,QSER1,ADNP,NFKB1,BRD2,CHD7,GAR1,RRS1,PBRM1,CTCF*

			,HHEX,ANKRD17,ELF1,NSD1,RSL1D1,H3-3B,RNF138,SETD1B,FOXK2,HMGB1,SMARCD1,YY1,NOL8,NAT10,EP400,MECP2,XRCC6,PCNA,MBD1,CSNK2A2,NCAPH,MCM2,JUND,HCFC1,CHEK1,ARPC4,CHAMP1,UBP1,POLE,TRRAP,EXOSC9,POLR2A,CCDC86,SUMO3,RBM19,XPO1,IK,TPR,ENC1,EBNA1BP2,ZFR,BAZ1B,NRIP1,MYBBP1A,DDB1,BACH2,PITX1,NASP,TCP1,CENPF,POLE3,CDC5L,CHD3,RAN,BEND3,PPP2CA,MSH2,GLYR1,MCM3,GRWD1,H4C8,GATAD2A,H2BC12,SSRP1,ARID1B,RRP1,KAT6A,RBBP4,HNRNPK,PRPF19,SMARCA4,PRKDC,HNRNPC,SUPT16H,HMGA1,XRCC5,UTP4,DEK,SURF6,CDT1,LRPPRC,SREBF2,MGA,MPHOSPH10,PES1,EP300,RRP1B,FOSB,SF3B3,MLLT1,RIF1,CITED2,WDR43,RUNX1,TRIM28,SRSF2,NFATC3,MCM4,TRIM24,HNRNPA2B1,DDX21,SFPQ,POLR1A,MCM7,SMARCC1,MYC,SET,FOS,ZEB2,ACTB,DHX9,HNRNPU,MCM5,NCL,EGR1
GO:0005681	spliceosomal complex	1.4482077991152633e-37	SNRPB,PRPF38B,SREK1,CWC22,MTREX,PHF5A,MFAP1,RHEB,EFTUD2,DHX38,DDX23,HNRNPH1,DHX16,PRPF6,HNRNPA3,DHX15,CWC25,SF3A2,SRRM2,PPIL2,PDCD7,SNRPA,PRPF4,RBMX,YJU2,RBM8A,API5,PRPF38A,TFIP11,WAC,SNRPD1,WBP11,SF1,AQR,HNRNPA1,MAGOH,HNRNPR,SNU13,SART1,PNN,PRPF3,IK,SF3B4,RALY,CDC5L,CASC3,HNRNPK,PRPF19,TRA2B,HNRNPC,SF3A3,RBMXL1,DDX5,DDX46,EIF4A3,HNRNPDL,SNRNP200,YBX1,RBM3,SF3A1,PABPC1,PRPF8,SF3B3,HNRNPF,U2AF2,SRSF2,HNRNPA2B1,HNRNPM,HNRNPU,NCL,HSPA8
GO:0005737	cytoplasm	2.1817181438564374e-32	HRAS,CENPN,TXK,ACSF3,SNRPB,TMEM127,MITF,PPP6C,DAP3,TOMM5,CHTOP,ERAL1,ZNF274,SEC23IP,ABRAXAS2,DNAJA2,GYG1,MMAB,RNASEH1,AP5M1,FADS2,ITFG2,TIRAP,RAD51C,NRROS,AAMP,MIX23,CWC22,CPNE7,BEGAIN,POLR3E,UBE2Q1,NEU1,DCAF7,OTUD6B,NACA,POLR1C,SRP72,TRIM35,PHB,METTL3,EZH2,SNX17,TOMM70,FES,CCDC78,RAB10,DCLRE1C,RFWD3,ULK3,DNM1L,LRRFIP1,GPATCH3,RHEB,ASAP1,PPP6R3,FUT8,SMG5,STT3A,PTDSS2,DHDDS,VKORC1L1,TIMM17A,MRPL1,SPIN4,MRPL11,AHCTF1,RPUSD4,CMBL,CHCHD3,CDC123,ANKRD13A,MRPS30,CTDP1,NUP155,POM121C,KIF26B,SRM,HNRNPL,NLN,MYO16,PWP1,ATP11A,DOLPP1,OXA1L,EFTUD2,EML4,CRCP,RANBP3,FRMD8,STAG2,CASP8,TAF4B,PSMA3,PQBP1,PSMD3,AIFM2,PSPC1,CEP350,MAD2L2,MRRF,POLDIP2,PSMC3,NAA11,TIMM44,GART,NT5C3A,MS4A4A,FBXO45,TRIM44,TRNT1,MNS1,RNPS1,MTCH2,ALMS1,PPP3R1,SLC25A46,SYPL1,HIF1AN,USP11,SS18L1,RBM45,NOSIP,PAFAH1B1,STAG1,HNRNPH1,USP14,RAB7A,LIMD1,LYL1,NAA20,BICD1,PPP5C,BAP1,JRK,ANAPC7,BUB3,GAB2,TOP2B,LYAR,LRWD1,THOP1,EXOC7,EIF4EBP2,CCT6A,KAT7,TPP2,CLTC,TSR3,EEF2,AFG3L2,MSRA,AURKAIP1,NIPA2,CCT8,KIF5B,FAM13B,TMEM43,RTL10,FUBP3,BCCIP,SPRY2,DLAT,FAM120A,NOB1,SBF1,RNF40,CUL3,ARPP19,CMPK1,MEF2C,UBR3,TCF3,NUP160,TEX15,MAF1,GID8,CLSPN,MVK,NBAS,PLK4,CCNY,SLK,ZFYVE26,ZC3H4,TMEM33,PEBP1,GSPT1,GRSF1,NXF1,UBQLN4,FAHD1,PSMC5,AGPAT3,STK35,TIMM23,ZC3HAV1,CAPZA1,HNRNPA3,MAPK1,STAR,ZNRF1,EEF1D,CASP3,ATP2A2,IPO9,PKP3,TULP4,SURF4,PCLAF,HBG1,PDSS1,CERS2,DHX33,STRIP1,PGAM5,NSMAF,LETM1,FADS1,TBCD,PUM1,MTOR,DHX30,PDPR,IKZF3,LCLAT1,GOT2,VDAC1,SMG9,SNX8,EIF3D,TFAP4,NAA50,PTP4A2,CLDN11,DELE1,KPNA4,NIFK,FAF1,BAG6,EMD,PCYT1A,TASOR2,FKBP15,NAP1L4,DHFR,MTDH,MCOLN3,BAIAP2,COMMD4,HSPA9,MCCC1,NSDHL,MRPL15,PRAME,GMPS,ASCC3,UROD,UBE2L3,THOC1,PSMC2

			,MCMBP,RPTOR,MRPL45,PPIL2,PPP1R10,MRPS35,TMEM18,TNF,RAVER1,GRPEL1,RABGGTB,EIF4B,PPP4R3A,SAMSN1,NIP7,PPP2R5A,IGF2BP3,NOL7,MED28,FAM71F2,PUM2,HAT1,MCRIP2,CLTA,BACH1,PDZD8,CERT1,SETX,TRMT2A,AP3D1,NAPA,PSMG2,STK24,METTL8,APEX1,BPTF,ATP6V1G1,EDC4,RIOK2,NAA15,NUP43,AMMECR1,TRAM1,PITHD1,SPECC1,ADO,PPP1CC,KCNQ5,CAVIN2,ZC3H14,B4GALT5,CDC27,HERC2,RAB35,SRSF10,CTSL,AKAP8,SBDS,RCC1,C1QBP,PPM1H,CRK,TICRR,MED15,EOGT,STK25,CAPNS1,SRSF8,PAK2,ZMYND19,ARHGAP21,KTN1,PRMT5,SNX9,PCBP2,PTDSS1,ADD1,RNF126,TMEM223,DPYS,GPX4,MCCC2,SLC25A3,MLLT3,OPA3,ALG8,RAD23B,VAT1,TAF4,RBM14,RBM8A,ARHGAP6,GOLM1,API5,INTS13,RPIA,UCK2,BTBD1,TUBB,FEN1,DYRK1A,CASD1,SLBP,DNAJC8,NUCKS1,SDCBP,PSMD1,AMD1,TMPO,NUP188,SSB,CDC37,ECHDC1,AGPAT5,GNL3L,CYB5B,IBA57,RILP,TFIP11,STRBP,PSMB2,EIF3G,ADI1,BSN,DHX29,ARHGEF2,BRCA2,RANBP1,POLR2D,ZDHHC5,EIF3M,WTAP,COPS2,NIN,RBM42,ACLY,SNRPD1,PAN3,PAF1,BIRC6,ERMAP,FASTKD2,RABL6,PHB2,TCOF1,LRRC41,TOMM22,RAP1GAP2,GRB10,XRCC2,PDS5A,BRCC3,CREBBP,EXOSC3,WBP11,KIFC3,AHSA1,EPRS1,COPS3,UBE4B,INSIG1,LSM14A,ABLIM1,TMX2,NUP98,CNOT1,LYN,ASH1L,LRRC59,GEMIN5,PHACTR2,APC,BTF3,POLR1B,JPT2,DDX42,CAPN1,WDR6,PELP1,SLC12A2,TMEM201,EZR,TRUB2,DDX20,URM1,MMS19,SGPP2,ACAT2,YWHAG,ELAVL1,UTP15,VCP,DNAJB12,WASHC5,RYBP,SAFB2,GBP2,PSIP1,CSTB,CSNK1G2,DCAF13,BEX4,STIP1,CDK4,DVL2,PNO1,POLR3C,THG1L,MRPS2,RBM25,RRP12,SCAP,WDR82,SMC1A,AGO2,E2F4,TJP1,VPS26A,FOXRED2,G3BP1,PCM1,RNF220,TBC1D14,SAE1,DHCR7,CLCN6,ARL8B,IER3,IGF2R,ATP6V0D1,ADNP,UBA2,NFKB1,ZNF622,ARHGDIA,BRD2,DOK3,USP37,HNRNPA1,RRS1,PPARGC1B,MAGOH,RANBP10,HHEX,ANKRD17,HNRNPR,RRM2,RRM1,PI4KA,SEC24B,CCT3,CDK7,PPIF,PKM,FOXK2,KIF2A,HMGB1,ATAD3B,MAEA,BZW1,GNAQ,CANX,YY1,MLLT10,IRAK1,PIK3C2B,IP6K1,TRMT10C,METAP2,AASDHPPT,MTHFD1,ADSS2,SART1,MECP2,CSDE1,PARD3,MBNL1,SKI,PRKAR2B,TKT,DDX54,AZIN1,CCDC6,N4BP2,TARDBP,LARP4,XRCC6,RPL22,ABI1,KLHL21,CDC25A,CSNK2A2,UPF2,DIAPH1,DDX3X,KIAA0753,IQGAP2,IDI1,TNPO2,SACS,LBR,HDGF,NDC1,NCAPH,VPS35,ATP5MC3,EIF4G2,MCM2,CFL1,PFAS,HCFC1,CHEK1,AATF,CLN6,ECSIT,ZMPSTE24,UBAP2,PRMT7,OXCT1,RETREG2,SLC25A5,ARPC4,CYP3A5,S1PR3,VAC14,POM121,CHAMP1,DNAJC21,UBP1,TRRAP,PPIP5K2,NUDC,EXOSC9,CSK,DNAJC7,DNAJA1,TEX261,SUMO3,NUP62,DYNC1H1,TSR1,RBM19,XPO1,PPIA,PRPF3,IK,CLCN7,PHACTR1,SERPINE1,TPR,ENC1,SRSF7,UTP20,TGFBRAP1,ZFP36L2,SLC38A2,ZFR,ARFGAP2,KEAP1,TFB2M,UBE2N,NRIP1,MACO1,MRTO4,VAPA,TEX10,GFM1,MYBBP1A,DDB1,BACH2,PSMG1,PITX1,GTF3C4,NASP,CHST3,TCP1,CENPF,YWHAB,ENO1,FAM83H,CCT5,FUBP1,CDK6,CDC5L,CHD3,ST3GAL2,NUP153,RAN,UBC,WDR81,TFAM,HSPA4,AK2,PPP2CA,SSU72,DIDO1,GLYR1,MAN2A2,KMT2A,MCM3,NOSTRIN,GRWD1,TOMM40,PAICS,SLC30A10,CERS6,ARMC6,PRDX1,TNPO1,DKC1,DDX1,H2BC12,STX3,CUL1,LTBR,CTPS1,EWSR1,CASC3,ARID1B,KPNB1,CPSF7,SPTB,TXNRD1,ATP6V0A1,HNRNPAB,KAT6A,ATAD3A,ARF6,USP36,RBBP4,NCBP1,HEATR1,CCT2,HSP90AB1,EIF3B,PA2G4,HNRNPK,SMG1,TXNL1,DHX37,FDFT1,PRPF19,EIF3J,PRKDC,YES1,PRMT1,HNRNPC,MALT1,WWP2,HMGA1,FARSA,CELF1,NCLN,XRCC

			5,DDX5,PSME3,TMEM97,ANK1,DEK,LSS,EIF4A3,NDST1,NUP214,TRIP12,IPO7,ACTG1,DDX39A,DDX56,UBAP2L,NOP56,HNRNPDL,CORO1C,KHDRBS1,HNRNPD,RAPGEF1,LRPPRC,EIF5A,FAM136A,RANBP2,DNAJB6,NOLC1,SREBF2,CBFA2T3,STXBP5,ABCF1,ACACA,TAFA2,NSUN2,AP5Z1,HSPD1,PIM2,GAPDH,YBX1,LIN28B,EIF5B,PCYT2,ATP6V1C1,RBM3,STARD7,PGD,AXIN1,MS4A3,GDI2,PES1,ANP32B,MYH10,IPO5,RREB1,HSPH1,EP300,TGFBR1,DAZAP1,PCNT,STMN1,RRP1B,CLUH,PPM1G,STON2,SRRT,PABPC1,PRRC2C,SERBP1,ATP13A3,AHNAK,COA7,PTMA,FTL,MLLT1,ELOVL6,HNRNPF,MT-ND1,NPM1,RIF1,ETF1,CDV3,TFRC,NQO1,EIF4G1,ACP5,NEFH,NR2F2,BCLAF1,ECPAS,SRSF2,HSP90AA1,MDN1,NFATC3,CAPRIN1,SQLE,TRIM24,NOP58,HNRNPA2B1,KIF1A,BAG1,LARP1,SQSTM1,HBZ,CCAR1,MAT2A,DDX21,HMGCS1,SFPQ,SCD,PABPC4,HNRNPM,NAV1,KCNH2,SRSF3,ANKRD11,EIF3A,MCM7,SMARCC1,MYC,SET,VGF,BTG1,MT-CYB,IGF2BP1,FOS,GCLM,HMGCR,SPTA1,MYB,ODC1,KHSRP,ZEB2,RELN,FUS,ILF3,FASN,GLUL,ACTB,DHX9,HNRNPU,MCM5,NCL,EGR1,DHCR24,HSPA8,FTH1
GO:0016604	nuclear body	3.6926907951841867e-28	*CHTOP,SREK1,CWC22,DCAF7,MTREX,PHF5A,METTL3,TCF20,RFWD3,EFTUD2,PQBP1,PSPC1,NT5C3A,RNPS1,STAG1,TPP2,SBF1,NONO,MEF2C,PRPF6,NXF1,STK35,CTR9,DHX15,UIMC1,MTOR,CWC25,SF3A2,MTDH,TCF7L2,SRRM2,ASCC3,THOC1,PPP1R10,HECTD1,CSTF2,PPP4R3A,RBM10,SETX,APEX1,ICE1,NAA15,NUP43,PPP1CC,ZC3H14,SRSF10,VPS72,SRSF8,PRPF4,ADD1,RBM14,RBM8A,API5,INTS13,DYRK1A,GTF3C6,NSRP1,TFIP11,WAC,POLR2D,WTAP,ZC3H18,CREBBP,NUP98,GEMIN5,DDX42,SF1,DDX20,SAFB2,DVL2,RBM25,SETD1A,BRD2,RBM15B,GAR1,MAGOH,SETD1B,KIF2A,SRSF6,EP400,SART1,SKI,TKT,PNN,PCNA,MBD1,SLTM,CHAMP1,SUMO3,RBM19,XPO1,PRPF3,IK,SRSF7,STK17A,NRIP1,CDC5L,CHD3,CDK12,GATAD2A,DKC1,DDX1,EWSR1,CASC3,ATP6V0A1,KAT6A,USP36,PRPF19,SF3A3,DDX5,DDX46,EIF4A3,TRIP12,DDX39A,CDT1,NOLC1,AP5Z1,SF3A1,RREB1,ALYREF,SRRT,PRPF8,THRAP3,NPM1,RIF1,SON,U2AF2,BCLAF1,SRSF2,NOP58,HNRNPA2B1,SPN,SQSTM1,SFPQ,HNRNPM,SRSF3,DHX9,HNRNPU*

Table S13. Top-20 GO associations with molecular function (MF), biological process (BP), or cellular component (CC) of 1147 downregulated genes. The search was performed in GO Profiler (https://biit.cs.ut.ee/gprofiler/gost

GO.ID	Description	padj	Genes
GO:0005515	protein binding	2.655052739633818e-19	*HSPA5,FN1,TXNIP,LAMA3,ARRDC4,CISH,KSR1,HECW2,ADAMTSL4,FCGR2A,DDIT4,PTK2B,GDF15,PIM1,ELAPOR2,FLNC,ANXA1,CA2,LTBP4,HSP90B1,KIF21B,CARD11,ANKRD29,CAPRIN2,PLD3,ST6GAL1,NFASC,AIG1,UNC5B,REEP6,MAF,LAMB1,CLSTN3,LAPTM4B,KLF10,SCG3,IL10RA,WSB1,HDAC9,LRP1,EEF1A2,CCNL2,TSC22D1,RGS10,GADD45A,TGFB1,EPHX2,ANXA6,ABI3BP,ATM,ARG2,PLEKHH2,IVNS1ABP,F11R,NIPSNAP1,RAB6B,UBE2L6,FLNB,EPB41L2,ASS1,EML2,TGM2,COL6A5,MKNK2,HERPUD1,PPP1R13L,CD36,AIF1L,PTGS1,TMCC2,DYSF,APOL1,AKAP11,MA*

			RCKS,ANKZF1,ZNF692,PAPPA,CHAC1,MCFD2,VEGFA,GMPPA,ERBB3,OPTN,PTK7,USP20,ITGAV,DMBX1,MRC2,TNFRSF9,CTSZ,TEAD1,GTPBP2,ID3,ADD3,ATP2A3,FAM193B,CALB1,ERMN,ZMYM2,SERPINH1,LMAN1,KIAA0040,BTG2,TMOD1,SEMA6B,KDM6B,RIPOR3,IL2RA,BLVRB,RYR1,ADAM19,SEC24D,REEP2,HYOU1,CD46,BCL6,NHLH1,TTLL7,STARD9,FAM83A,F2R,RIMS3,SEPTIN6,RAB11FIP1,SOCS2,MLXIP,MICAL2,MYO1D,LGALS1,APOE,LAMA5,P4HA1,PTPDC1,GOLGA2,BHLHE40,HRC,ULBP1,FNBP1,ITGA5,PDIA4,TP53INP1,LLGL2,TRDN,ATF3,PYCR2,MFGE8,CALU,IL15RA,CD24,RFK,RHOBTB1,PALM,MLXIPL,MMP15,BST2,GRAP2,ABCG2,ALAS1,KRT8,SESN3,APOL6,RAB3B,NFATC4,GIPR,ID1,CTCFL,CPZ,RAB27A,RHPN1,IGF1,PYGL,UBE2H,TLE2,NUDT4,HELZ2,ICAM5,MAPRE3,MIIP,ITGB1,MAGI1,PITPNM1,NLGN2,CTSC,FGFR3,SMIM3,TFAP2B,G0S2,NTS,WHRN,INHBE,MICAL1,USP45,NECTIN2,MUC4,PLEKHA4,BRSK1,TM6SF1,SMAD3,AP2M1,EDEM1,POLI,B2M,PSME1,LYPLA1,MYRF,PROS1,PRKAB2,SVIP,HDAC6,ARHGEF12,SDC3,ULBP2,SEMA7A,ATF6,TMEM70,GDI1,PCK2,NUDT12,TMEM63A,RBL2,GPC2,ROBO1,CDKN1A,ACE,UBAC1,TMEM241,TBC1D5,ACVR1C,PCLO,EXT2,ZNF397,AFDN,SDF2L1,MFSD6,CNN2,DMTN,CYP26B1,PXK,AMOTL1,CXCL8,CDKN1C,SEMA3F,LARGE2,CDC42SE1,TRPM4,BAIAP3,BCAM,TNNT1,KCNAB2,ZMYM1,ACSF2,PTPRU,PTPRH,SKIL,POMT1,GDPD5,PAN2,KDM7A,PECR,BTN3A1,TMEM106C,SPEG,COLGALT2,PLXND1,DOCK6,NDRG2,HES7,PCED1B,PRKACA,EIF2AK1,MYBPHL,PSRC1,ADCY3,MIF4GD,GABBR1,AGRN,HLTF,PTPRS,FASTKD1,WIPI1,ATP7A,MT2A,NDRG1,TEX19,YPEL5,SESTD1,SHC2,NSD3,CAMTA2,DNAH14,MSI1,STAT5B,SH2D3A,NUP107,PROCR,CYP2R1,IFIH1,TGFBR3,FBXL16,SMARCA1,ULK1,GLI1,PTEN,TDO2,LRSAM1,CYSTM1,ITM2C,MTURN,RAB18,PDLIM7,TMPRSS4,KHNYN,KMT5C,ZNF275,CUL7,GSN,PKHD1,ZNF697,NFE2,ALAS2,DRAP1,PPP1R18,BTK,CTIF,ADGRL1,COL18A1,CDCP1,SMIM14,GABARAPL1,XK,ALS2CL,TIA1,SH3PXD2B,THOC2,LRP4,TTC37,SELENBP1,INTS8,EPSTI1,LUC7L,ANKRD9,RGS16,IFNGR1,RNF217,CMTM7,TDRKH,LGALS9,CAMSAP3,MINK1,RNF103,DMKN,SH3GLB2,NFE2L1,ETFDH,COTL1,SLC31A1,SEMA3A,SLC22A5,HACE1,ALDOC,SPPL2B,PITPNC1,P4HA2,SLC25A42,DDHD2,CALR,BTBD2,RHBDD1,AMFR,ITM2A,TSPO,AURKB,FLT1,AMPD2,TMED9,MAGED2,PTPRJ,IGSF8,PTPRF,PDIA6,LRRC20,MORF4L2,SYBU,ARL6IP5,NEK9,MACC1,PAPSS1,YIF1B,TJP3,NES,AGPAT4,INPPL1,BCL3,TRIM38,HID1,OBSCN,ARHGAP8,KYNU,MARCHF2,CXORF38,LSM4,ITGB5,CORO7,DLG4,STK10,ESRRB,UCP2,DHTKD1,CCDC113,MSH5,SLC22A23,C4ORF46,CEBPD,RCN3,NR2F6,LDAF1,MLLT11,PDLIM4,ACBD4,IFITM1,TMOD2,WNK4,COPB1,CYLD,SYT5,TCIRG1,HLA-E,SIL1,GUK1,RBM22,AHCYL1,RHAG,ANKIB1,NCOA7,ADGRA2,MROH1,TUBB2B,SUSD6,HIP1,ERFE,KIF1B,SPARC,LONRF2,FGFR1OP2,CC2D1A,PLA2G6,THBS1,CCND2,IL32,RRAS,TMEM9,CTSB,NFKB2,SH3TC2,TRPT1,CALCOCO1,SMAP2,DACT3,TRIM3,ZBTB11,PDE4DIP,DMXL2,IFT140,SEC22B,WDR26,PDK4,COPG1,MAP3K9,LMOD1,PIDD1,ZNF175,TKFC,PPIB,TCAF1,SERPINB1,LRG1,CLDN12,CASTOR2,KLF13,PAAF1,GPC5,MANF,PHF21A,SPTAN1,TPD52L1,DENND3,EPHX1,CORO6,VWA5A,FNDC4,MAN2A1,RTN3,NPAS1,PAIP2B,ENO2,NCBP2,CASP10,TUBB4A,RNF187,HAX1,PDIA5,SOCS1,MBNL2,CFH,HIVEP3,TMEM30A,ITPKA,SELENON,ACVR1,PLEKHA2,CCDC92,ITGB3,SMIM1,SNX16,CORO2A,MEIS3,MVB12A,NUCB1,NCOA4,MYO15B,PCSK4,BRWD3,PTTG1,FAM214B,AGER,NIPSNAP2,MAP3K8,DGKD,SORT1,SAT2,ACADVL,LITAF,AZU1,PTPRC,DAB2,TMCO6,IDH1,GGA1,NBEAL1,IL6R,TMEM243,DIP2A,EHBP1,NT5C2,PSAP,VWDE,SEMA6C,NFE2L3,POLG2,SLC4A11,ASAP2,PHKA1,H1-

			10,CERCAM,CCND3,PIK3CD,OAS3,EXOC2,RPS6KC1,DYNC2LI1,TSC22D3,REEP4,TSPYL2,SMYD3,AP3M2,HIP1R,ITPR2,ARFGAP1,SETD5,ASMTL,ARRB1,TNFRSF10B,TMEM50B,C4ORF33,BATF2,FECH,SMPDL3B,FBXO44,OSTF1,COG6,LRRC63,CD200R1,HJV,LTK,MMP14,OGT,MAP1A,TRIB3,PLP1,CLIP2,CHID1,GAS6,MYO5B,ADGRL3,PPFIA4,NTRK1,VCAN,RXFP1,LPAR2,GNG7,LGALS3,TAB2,WARS1,PHKB,CRYL1,LMNA,CCDC50,IARS1,BSDC1,GDE1,PLXNB2,IL13RA1,FNDC3B,STX10,CEMIP2,VSTM4,CD55,PPM1J,YIPF5,RELL2,ZNF558,ITM2B,ABCB9,GTF2E2,LDLRAP1,NBR1,RGL3,CENPH,IQGAP3,MRAP2,TIAL1,ARVCF,PDIA3,KIF5A,GGT7,PIGS,AMIGO2,KCNJ11,RBCK1,HAGH,DNM1,EGLN3,DLK1,ADGRE2,PDGFB,MAST2,NIBAN2,PCOLCE,HDGFL3,KPNA5,GSTO1,STK16,DBP,CPEB4,GTF2H4,BBS4,ELP1,SOX5,STX2,LAMB2,SFXN5,ERP44,EMILIN2,DCLRE1B,GPSM3,PPP2R5B,AKNA,ERI1,VPS28,DIPK1A,OGA,ISCU,SESN2,ATP1A2,C6ORF89,BMF,YIPF2,TSKU,SMTN,SMURF2,TNNI3,NRIP3,TUT7,CPNE3,GAS2,ZFYVE28,HEMK1,FLT4,MCF2,BTN3A2,KLF12,RBPMS,WIPF3,MTCL1,C3,ANO5,DCAF8,TMEM143,ZCRB1,RAB26,SLC4A7,FRA10AC1,PLPPR2,PFKFB4,TSPAN33,HOOK1,DZIP3,CCM2,RABAC1,RTN4RL2,YARS1,SCN1B,NAT8L,UNC119,ZMIZ1,RPH3AL,TTC33,SNPH,ANGEL1,MAST1,GUCA1B,ROBO3,COL1A2,STARD10,INPP5J,SALL2,BNIP3,KHK,PIGK,HLA-B,ETAA1,ACSL6,ZNF396,PHYKPL,KLHL36,GNB5,WASL,STOM,TCAF2,TCFL5,PEX2,SNTA1,THRB,SFMBT2,FGD1,FGFR4,MAPK8IP3,SEMA4G,SHFL,GIPC1,MLF1,GTF2A1,ARID5B,ZP3,PKD1,C1GALT1,HBP1,MAPT,SNX2,TBC1D20,GEMIN2,MC4R,FMNL1,SMAD6,MORN4,APAF1,GRN,EHD2,WBP1L,CCDC88B,APBB3,RAB24,DUSP5,NOTUM,SEMA3E,SLC30A2,TMEM41B,MDGA2,ZKSCAN1,TENM1,EPS8,RNF11,WDR91,WDR31,ACAT1,WDR11,LGMN,DNAJB11,EHHADH,ERO1B,CBLB,BBC3,PCED1A,SCFD1,PDK1,BCL9L,PRRC1,DLG3,ZNF133,DTNA,ZNF83,TPD52,RHBDD2,SEC24A,C2CD2L,PRCP,GTPBP1,TENT5A,RTN4R,SH3BGRL2,NIPA1,RFLNB,TMEM54,SETD7,RAB31,STK4,TRAK2,PVR,UTRN,STON1,RPL27,IMPA2,FADS3,CD93,B4GALT4,GALNT10,RTN2,ZBED8,SERGEF,SLC16A5,TNFRSF1B,CR2,UFL1,GRHL1,RB1CC1,PAX9,ACAD11,TTC7A,RAPGEF2,FUCA1,INPP4A,C18ORF54,ZNF467,CUEDC1,TNK2,GPSM1,DCP2,PPP1R16B,EPS15,ATP1B2,LHFPL2,DEF8,RPS6KA1,GTF3C3,MAP4K2,VPS16,DCTN4,GPD1L,KBTBD3,THBD,SH3BP2,VBP1,CCSAP,ENTR1,SERTAD2,DDB2,NYAP1,TTBK2,MXD3,USP18,ANAPC16,IRAK2,ADAM15
GO:0008092	cytoskeletal protein binding	3.02506949085435e-7	*FLNC,KIF21B,CLSTN3,ANXA6,PLEKHH2,RAB6B,FLNB,EPB41L2,EML2,AIF1L,MARCKS,ADD3,ERMN,TMOD1,REEP2,TTLL7,STARD9,MICAL2,MYO1D,APOE,GOLGA2,LLGL2,RAB3B,RAB27A,MAPRE3,ITGB1,MAGI1,MICAL1,BRSK1,HDAC6,AFDN,CNN2,DMTN,PXK,TNNT1,PSRC1,NDRG1,GLI1,PDLIM7,GSN,PPP1R18,GABARAPL1,CAMSAP3,COTL1,ALDOC,SYBU,INPPL1,OBSCN,CORO7,DLG4,PDLIM4,TMOD2,RHAG,HIP1,KIF1B,LMOD1,SPTAN1,CORO6,CORO2A,MYO15B,PTPRC,REEP4,HIP1R,MAP1A,CLIP2,MYO5B,IQGAP3,KIF5A,KCNJ11,DNM1,MAST2,HDGFL3,BBS4,SMTN,TNNI3,GAS2,WIPF3,MTCL1,HOOK1,RPH3AL,MAST1,WASL,SNTA1,MAPK8IP3,GIPC1,MAPT,FMNL1,CCDC88B,EPS8,LGMN,RFLNB,TRAK2,UTRN,VPS16,VBP1,CCSAP,TTBK2*
GO:0003824	catalytic activity	0.000008073409883835456	*HSPA5,KSR1,HECW2,ADAMTS14,CPXM1,PTK2B,PIM1,AARS1,RNF213,CA2,LTBP4,HSP90B1,KIF21B,CARD11,PLD3,GFPT1,ST6GAL1,AIG1,WSB1,HDAC9,EEF1A2,RGS10,EPHX2,ATM,ARG2,RAB6B,UBE2L6,ASS1,TGM2,MKNK2,CRAT,PTGS1,PAPPA,CHAC1,FUT1,GMPPA,ERBB3,GALNT12,PTK7,USP20,CTSZ,GTPBP2,ATP2A3,KDM6B,BLVRB,ADAM19,TKTL1,TTLL7,SEPTIN6,CMAS,MICAL2,P4HA1,PTPDC1,DSTYK,PDIA4,ENO3,PYCR2,RFK,RHOBTB1,MMP15,PTAR1,B4GALNT4,ALAS1,LPCAT4,SESN3,IDH2,RAB3B,CPZ,RA*

			B27A,PYGL,UBE2H,NUDT4,HELZ2,CTSC,FGFR3,MICAL1,USP45,GAA,MBOAT2,BRSK1,PLOD2,EDEM1,POLI,LYPLA1,MYRF,PRKAB2,HDAC6,PGGHG,PCK2,NUDT12,CDKN1A,ACE,ATP8B3,ACVR1C,EXT2,CYP26B1,PXK,LARGE2,MYLK3,ABCA7,ACAD10,KCNAB2,ACSF2,PTPRU,PTPRH,POMT1,GDPD5,PAN2,KDM7A,PECR,SPEG,HMGCL,PGM3,COLGALT2,CPD,CRYZL1,PCED1B,PRKACA,EIF2AK1,ADCY3,HLTF,PTPRS,ATP7A,NSD3,DNAH14,DUSP8,DNASE2,CYP2R1,IFIH1,TGFBR3,SMARCA1,ULK1,PTEN,TDO2,LRSAM1,RAB18,TMPRSS4,KHNYN,KMT5C,ALAS2,BTK,SELENBP1,RGS16,RNF217,MINK1,RNF103,ETFDH,NDUFA10,HACE1,ALDOC,QSOX1,CLYBL,SPPL2B,P4HA2,SLC25A42,DDHD2,RHBDD1,AMFR,AURKB,FLT1,AMPD2,PTPRJ,PTPRF,PDIA6,PRSS16,NEK9,PAPSS1,METTL25B,AGPAT4,INPPL1,TRIM38,OBSCN,POFUT2,CHST2,KYNU,MARCHF2,STK10,DHTKD1,MSH5,CYP26A1,WNK4,CYLD,GUK1,AHCYL1,ANKIB1,TUBB2B,KIF1B,LONRF2,PLA2G6,RRAS,CTSB,TRPT1,TRIM3,PDK4,MAP3K9,PIDD1,NDUFB4,TKFC,PPIB,XYLT2,EPHX1,NIT1,MAN2A1,ENO2,HEXD,CASP10,TUBB4A,RNF187,PDIA5,ITPKA,SELENON,ACSS2,ACVR1,TARBP1,ITGB3,CA11,PCSK4,MAP3K8,DGKD,ACER3,SAT2,ACADVL,AZU1,PAPLN,PTPRC,IDH1,GTDC1,DIP2A,NT5C2,POLG2,PHKA1,CCND3,PIK3CD,OAS3,GALNT5,RPS6KC1,SMYD3,SETD5,ASMTL,MAN1B1,FECH,SMPDL3B,ENGASE,FBXO44,HJV,LTK,MMP14,OGT,MAN2B1,TRIB3,ZDHHC8,NTRK1,CHPF,WARS1,CRYL1,IARS1,GDE1,CEMIP2,PPM1J,CAPN5,FDXR,PCYOX1L,PDIA3,KIF5A,PAFAH2,SCPEP1,GGT7,RBCK1,HAGH,DNM1,EGLN3,MAST2,GSTO1,STK16,GALK2,ERP44,FRRS1,DCLRE1B,ERI1,OGA,SESN2,ATP1A2,SIAE,SMURF2,NRIP3,TUT7,CLCA1,CPNE3,HEMK1,FLT4,PANK4,PHGDH,RAB26,FRA10AC1,PLPPR2,ACSL1,PFKFB4,DZIP3,MAN1A1,YARS1,PLCD3,NAT8L,ANGEL1,MAST1,INPP5J,KHK,PIGK,ACSL6,PHYKPL,GNB5,CTBS,FGFR4,GPAT3,C1GALT1,GAL3ST4,A4GALT,PARP12,EHD2,RAB24,DUSP5,NOTUM,RNF11,ACAT1,LGMN,EHHADH,ERO1B,ST3GAL5,RNF145,CBLB,PCED1A,PDK1,MGAT5,PDE7A,MT-ND4L,RHBDD2,PRCP,GTPBP1,TENT5A,TUBB1,SETD7,RAB31,STK4,IMPA2,FADS3,B4GALT4,TTLL3,GALNT10,SERGEF,BPNT1,UFL1,ACAD11,FUCA1,INPP4A,TNK2,ACSBG2,DCP2,MYORG,NIM1K,RPS6KA1,MAP4K2,GPD1L,DDB2,TTBK2,USP18,IRAK2,TXNRD3,HEPH,ADAM15
GO:0044877	protein-containing complex binding	0.000021090283502000077	*HSPA5,FN1,LAMA3,FCGR2A,PTK2B,PIM1,FLNC,LTBP4,LAMB1,LRP1,TGFB1,ANXA6,ABI3BP,ATM,F11R,FLNB,EPB41L2,CD36,AIF1L,MARCKS,ITGAV,MRC2,ADD3,ERMN,SERPINH1,TMOD1,MYO1D,APOE,LAMA5,ITGA5,MFGE8,KRT8,ID1,IGF1,ICAM5,ITGB1,MICAL1,SMAD3,B2M,HDAC6,SEMA7A,TMEM70,CDKN1A,TBC1D5,AFDN,DMTN,CDKN1C,IFIH1,TGFBR3,SMARCA1,ULK1,PTEN,GSN,CAMSAP3,COTL1,CALR,AMFR,PTPRF,NES,SIDT2,ITGB5,CORO7,DLG4,P2RX6,HLA-E,HIP1,SPARC,THBS1,RRAS,CTSB,PPIB,SPTAN1,CORO6,ENO2,ACVR1,ITGB3,CORO2A,AGER,SORT1,PTPRC,H1-10,HIP1R,MMP14,PLP1,MYO5B,LGALS3,RELL2,LDLRAP1,IQGAP3,PDGFB,PCOLCE,CPEB4,LAMB2,DCLRE1B,ERI1,VPS28,SESN2,TNNI3,GAS2,SALL2,SHFL,MAPT,SNX2,FMNL1,SCFD1,RTN4R,UTRN,CD93,VPS16,DDB2,ADAM15*
GO:0003779	actin binding	0.00002824821369260495	*FLNC,ANXA6,PLEKHH2,FLNB,EPB41L2,AIF1L,MARCKS,ADD3,ERMN,TMOD1,MICAL2,MYO1D,ITGB1,MICAL1,HDAC6,AFDN,CNN2,DMTN,PXK,PDLIM7,GSN,PPP1R18,CAMSAP3,COTL1,INPPL1,CORO7,PDLIM4,TMOD2,HIP1,LMOD1,SPTAN1,CORO6,CORO2A,MYO15B,HIP1R,MAP1A,MYO5B,IQGAP3,SMTN,TNNI3,GAS2,WIPF3,HOOK1,WASL,SNTA1,GIPC1,MAPT,FMNL1,EPS8,UTRN,VPS16*
GO:0043168	anion binding	0.0000476523896214494	*HSPA5,KSR1,PTK2B,PIM1,AARS1,RNF213,HSP90B1,KIF21B,EEF1A2,ANXA6,ATM,RAB6B,UBE2L6,ASS1,TGM2,MKNK2,ERBB3,PTK7,GTPBP2,ATP2A3,RYR1,HYOU1,TKTL1,*

			TTLL7,STARD9,SEPTIN6,MICAL2,MYO1D,P4HA1,DSTYK,RFK,RHOBTB1,ABCG2,ALAS1,RAB3B,RAB27A,PYGL,UBE2H,HELZ2,MAGI1,PITPNM1,CTSC,FGFR3,MICAL1,PLEKHA4,BRSK1,PLOD2,PCK2,ACE,ATP8B3,ACVR1C,CYP26B1,PXK,TRPM4,MYLK3,ABCA7,ACAD10,ACSF2,SPEG,PRKACA,EIF2AK1,ADCY3,AGRN,HLTF,WIPI1,ATP7A,SESTD1,DNAH14,IFIH1,SMARCA1,ULK1,ITM2C,RAB18,GSN,ALAS2,BTK,SH3PXD2B,MINK1,ETFDH,SLC22A5,QSOX1,PITPNC1,P4HA2,AURKB,FLT1,PLEKHA8,NEK9,PAPSS1,OBSCN,KYNU,STK10,DHTKD1,MSH5,CYP26A1,P2RX6,WNK4,SYT5,GUK1,TUBB2B,HIP1,KIF1B,RRAS,PDK4,MAP3K9,TKFC,TUBB4A,ITPKA,ACSS2,ACVR1,PLEKHA2,MYO15B,MAP3K8,DGKD,ACADVL,NT5C2,PSAP,PIK3CD,OAS3,RPS6KC1,GSDMB,HIP1R,ITPR2,LTK,OGT,TRIB3,MYO5B,NTRK1,WARS1,CRYL1,IARS1,ITM2B,ABCB9,LDLRAP1,KIF5A,KCNJ11,DNM1,EGLN3,MAST2,STK16,GALK2,ATP1A2,FLT4,PANK4,RAB26,ACSL1,PFKFB4,HSPA4L,YARS1,MAST1,TUBE1,KHK,ACSL6,PHYKPL,FGFR4,STARD5,GAL3ST4,APAF1,EHD2,RAB24,ACAT1,EHHADH,ERO1B,PDK1,GTPBP1,RTN4R,TUBB1,RAB31,STK4,TTLL3,ACAD11,RAPGEF2,TNK2,ACSBG2,NIM1K,RPS6KA1,MAP4K2,TTBK2,IRAK2,TXNRD3
GO:0043167	ion binding	0.00005235368824438149	*HSPA5,KSR1,ADAMTS14,CPXM1,PTK2B,PIM1,AARS1,ANXA1,RNF213,CA2,LTBP4,HSP90B1,KIF21B,CAPRIN2,CLSTN3,KLF10,HDAC9,LRP1,EEF1A2,EPHX2,ANXA6,ATM,ARG2,RAB6B,UBE2L6,ASS1,TGM2,MKNK2,AIF1L,PTGS1,DYSF,ANKZF1,ZNF692,PAPPA,MCFD2,EFHD1,ERBB3,GALNT12,OPTN,PTK7,USP20,ITGAV,GTPBP2,ATP2A3,CALB1,ZMYM2,LMAN1,KDM6B,RYR1,ADAM19,SEC24D,HYOU1,TKTL1,BCL6,TTLL7,STARD9,SEPTIN6,MICAL2,CELSR2,MYO1D,APOE,P4HA1,HRC,DSTYK,ITGA5,ENO3,CALU,RFK,RHOBTB1,MMP15,ABCG2,ALAS1,IDH2,COL11A1,RAB3B,CTCFL,CPZ,RAB27A,PYGL,UBE2H,NUDT4,HELZ2,ITGB1,MAGI1,PITPNM1,CTSC,FGFR3,MICAL1,USP45,PLEKHA4,BRSK1,PLOD2,SMAD3,EDEM1,POLI,PROS1,HDAC6,PCK2,NUDT12,CDKN1A,ACE,ATP8B3,ACVR1C,PCLO,EXT2,ZNF397,CYP26B1,PXK,LARGE2,TRPM4,BAIAP3,MYLK3,ABCA7,ACAD10,ZMYM1,ACSF2,POMT1,PAN2,KDM7A,SPEG,HMGCL,PGM3,CPD,PRKACA,EIF2AK1,ADCY3,AGRN,ZSWIM4,HLTF,WIPI1,ATP7A,MT2A,YPEL5,SESTD1,NSD3,DNAH14,CYP2R1,IFIH1,SMARCA1,ULK1,GLI1,TDO2,LRSAM1,NPTXR,ITM2C,RAB18,PDLIM7,KMT5C,ZNF275,GSN,ZNF697,ALAS2,BTK,COL18A1,SH3PXD2B,LRP4,ZC3H6,KLF9,RNF217,MINK1,RNF103,ETFDH,SLC22A5,QSOX1,CLYBL,PITPNC1,P4HA2,DDHD2,CALR,AMFR,AURKB,FLT1,AMPD2,PLEKHA8,NEK9,PAPSS1,TRIM38,OBSCN,KYNU,MARCHF2,STK10,PCDH15,ESRRB,DHTKD1,MSH5,RCN3,NR2F6,PDLIM4,CYP26A1,P2RX6,WNK4,CYLD,SYT5,GUK1,RBM22,ANKIB1,ZBED5,TUBB2B,HIP1,KIF1B,SPARC,LONRF2,THBS1,RRAS,CALCOCO1,SMAP2,TRIM3,KLF7,ZBTB11,PDK4,SUSD1,MAP3K9,ZNF175,TKFC,KLF13,PHF21A,SPTAN1,XYLT2,MAN2A1,ENO2,TUBB4A,RNF187,MBNL2,HIVEP3,ITPKA,SELENON,ACSS2,ACVR1,PLEKHA2,CA11,NUCB1,MYO15B,ZCCHC24,MAP3K8,DGKD,ACER3,SAT2,ACADVL,LITAF,IDH1,NT5C2,PSAP,VWDE,ASAP2,PIK3CD,OAS3,GALNT5,RPS6KC1,GSDMB,SMYD3,HIP1R,ITPR2,ARFGAP1,MAN1B1,FECH,SMPDL3B,LTK,MMP14,OGT,MAN2B1,TRIB3,GAS6,MYO5B,ADGRL3,NTRK1,VCAN,CHPF,RXFP1,TAB2,WARS1,CRYL1,IARS1,GDE1,CEMIP2,ZNF558,ITM2B,ABCB9,LDLRAP1,NBR1,KIF5A,KCNJ11,RBCK1,HAGH,DNM1,EGLN3,DLK1,ADGRE2,MAST2,STK16,GALK2,CPEB4,FRRS1,ERI1,ISCU,ATP1A2,TNNI3,TUT7,CLCA1,CPNE3,ZFYVE28,FLT4,PANK4,KLF12,ZCRB1,RAB26,ACSL1,PFKFB4,ZNF608,DZIP3,MAN1A1,HSPA4L,YARS1,PLCD3,ZMIZ1,RPH3AL,HIVEP2,MAST1,GUCA1B,COL1A2,SALL2,TUBE1,KHK,ACSL6,ZNF396,PHYKPL,PEX2,THRB,FGD1,FGFR4,STARD5,ZNF117,C1GALT1,GAL3ST4,PARP12,SMAD6,APAF1,EHD2,RAB24,ZK*

			SCAN1,RNF11,ACAT1,YPEL1,EHHADH,ERO1B,RNF145,CBLB,CACNA2D2,PDK1,MGAT5,PDE7A,ZNF133,DTNA,ZNF83,TPD52,SEC24A,GTPBP1,RTN4R,TUBB1,RAB31,STK4,UTRN,IMPA2,CD93,B4GALT4,TTLL3,GALNT10,BPNT1,ACAD11,RAPGEF2,ZNF467,TNK2,ACSBG2,DCP2,EPS15,DEF8,NIM1K,RPS6KA1,MAP4K2,THBD,TTBK2,IRAK2,TXNRD3,HEPH,ADAM15
GO:0140096	catalytic activity, acting on a protein	0.000101233925112449	*KSR1,HECW2,ADAMTS14,CPXM1,PTK2B,PIM1,RNF213,LTBP4,WSB1,HDAC9,ATM,UBE2L6,TGM2,MKNK2,PAPPA,ERBB3,GALNT12,PTK7,USP20,CTSZ,KDM6B,ADAM19,TTLL7,P4HA1,PTPDC1,DSTYK,PDIA4,MMP15,PTAR1,B4GALNT4,CPZ,UBE2H,CTSC,FGFR3,USP45,BRSK1,PLOD2,LYPLA1,MYRF,PRKAB2,HDAC6,PGGHG,CDKN1A,ACE,ACVR1C,PXK,MYLK3,PTPRU,PTPRH,POMT1,KDM7A,SPEG,COLGALT2,CPD,PRKACA,EIF2AK1,HLTF,PTPRS,NSD3,DUSP8,TGFBR3,ULK1,PTEN,LRSAM1,TMPRSS4,KMT5C,BTK,RNF217,MINK1,RNF103,HACE1,QSOX1,SPPL2B,P4HA2,RHBDD1,AMFR,AURKB,FLT1,PTPRJ,PTPRF,PDIA6,PRSS16,NEK9,TRIM38,OBSCN,POFUT2,MARCHF2,STK10,WNK4,CYLD,ANKIB1,LONRF2,CTSB,TRIM3,PDK4,MAP3K9,PIDD1,PPIB,XYLT2,CASP10,RNF187,PDIA5,ITPKA,ACVR1,ITGB3,PCSK4,MAP3K8,AZU1,PAPLN,PTPRC,PHKA1,CCND3,GALNT5,RPS6KC1,SMYD3,SETD5,FBXO44,HJV,LTK,MMP14,OGT,TRIB3,ZDHHC8,NTRK1,PPM1J,CAPN5,PDIA3,SCPEP1,GGT7,RBCK1,EGLN3,MAST2,STK16,ERP44,SMURF2,NRIP3,CLCA1,CPNE3,HEMK1,FLT4,DZIP3,MAST1,PIGK,FGFR4,C1GALT1,PARP12,DUSP5,RNF11,LGMN,ERO1B,RNF145,CBLB,PDK1,MGAT5,RHBDD2,PRCP,SETD7,STK4,TTLL3,GALNT10,SERGEF,UFL1,TNK2,NIM1K,RPS6KA1,MAP4K2,DDB2,TTBK2,USP18,IRAK2,TXNRD3,ADAM15*
GO:0042802	identical protein binding	0.0001689906771428019	*FN1,ADAMTSL4,GDF15,ST6GAL1,MAF,GADD45A,TGFB1,EPHX2,ANXA6,ATM,F11R,FLNB,ASS1,PPP1R13L,MARCKS,VEGFA,ERBB3,OPTN,DMBX1,GTPBP2,BCL6,FAM83A,APOE,P4HA1,GOLGA2,BHLHE40,FNBP1,ATF3,BST2,ABCG2,ALAS1,ID1,PYGL,MAPRE3,NLGN2,CTSC,FGFR3,SMIM3,TFAP2B,WHRN,NECTIN2,PLEKHA4,SMAD3,B2M,MYRF,SDC3,ATF6,ROBO1,ZNF397,AMOTL1,EIF2AK1,MIF4GD,MSI1,STAT5B,CYP2R1,IFIH1,ULK1,PTEN,TDO2,NFE2,DRAP1,BTK,ALS2CL,LRP4,LUC7L,SH3GLB2,NFE2L1,SLC31A1,SPPL2B,AMFR,AMPD2,PAPSS1,TRIM38,KYNU,STK10,CEBPD,PDLIM4,SIL1,AHCYL1,HIP1,ERFE,FGFR1OP2,PLA2G6,THBS1,DACT3,TRIM3,MAP3K9,CLDN12,CASTOR2,TPD52L1,MAN2A1,ENO2,CFH,ACVR1,CCDC92,ITGB3,SMIM1,SNX16,AGER,DGKD,SAT2,ACADVL,LITAF,IDH1,IL6R,NT5C2,PSAP,POLG2,CERCAM,HIP1R,FECH,NTRK1,WARS1,CRYL1,LMNA,ABCB9,MRAP2,PDIA3,RBCK1,DNM1,PDGFB,STX2,DCLRE1B,OGA,ISCU,SMURF2,FLT4,RBPMS,MTCL1,HOOK1,RABAC1,COL1A2,BNIP3,ACSL6,ZNF396,PHYKPL,STOM,SHFL,GIPC1,ZP3,MAPT,SNX2,SMAD6,APAF1,EHD2,SLC30A2,TENM1,ACAT1,TPD52,STK4,IMPA2,CR2,GRHL1,TNK2,GPD1L,IRAK2*
GO:0016773	phosphotransferase activity, alcohol group as acceptor	0.0003799101324081344	*KSR1,PTK2B,PIM1,LTBP4,ATM,MKNK2,ERBB3,PTK7,DSTYK,RFK,FGFR3,BRSK1,PRKAB2,CDKN1A,ACVR1C,PXK,MYLK3,SPEG,PRKACA,EIF2AK1,TGFBR3,ULK1,BTK,MINK1,AURKB,FLT1,NEK9,PAPSS1,OBSCN,STK10,WNK4,TRPT1,PDK4,MAP3K9,TKFC,ITPKA,ACVR1,MAP3K8,DGKD,NT5C2,PHKA1,CCND3,PIK3CD,RPS6KC1,HJV,LTK,TRIB3,NTRK1,MAST2,STK16,GALK2,CPNE3,FLT4,PANK4,PFKFB4,MAST1,KHK,FGFR4,PDK1,STK4,TNK2,NIM1K,RPS6KA1,MAP4K2,TTBK2,IRAK2*
BP			
GO:0050794	regulation of	3.949424296615564e-19	*HSPA5,FN1,TXNIP,LAMA3,CISH,KSR1,HECW2,ADAMTSL4,FCGR2A,DDIT4,PTK2B,GDF15,PIM1,ELAPOR2,AARS1,A*

	cellular process		NXA1,RNF213,CA2,LTBP4,HSP90B1,TSPAN13,CARD11,CAPRIN2,MEG3,ST6GAL1,UNC5B,REEP6,MAF,LAMB1,CLSTN3,LAPTM4B,KLF10,IL10RA,WSB1,HDAC9,LRP1,EEF1A2,CCNL2,TSC22D1,RGS10,GADD45A,TGFB1,ANXA6,ABI3BP,SLC6A6,ATM,ARG2,PLEKHH2,IVNS1ABP,F11R,FLNB,EPB41L2,ASS1,EML2,TGM2,MKNK2,HERPUD1,PPP1R13L,CD36,PTGS1,DYSF,AKAP11,ZNF692,PAPPA,CHAC1,VEGFA,FUT1,EFHD1,ERBB3,OPTN,PTK7,USP20,ITGAV,DMBX1,TNFRSF9,CTSZ,TEAD1,ID3,ADD3,ATP2A3,CALB1,ERMN,ZMYM2,LMAN1,BTG2,TMOD1,SEMA6B,KDM6B,IL2RA,RYR1,ADAM19,REEP2,HYOU1,CD46,BCL6,NHLH1,FAM83A,F2R,RIMS3,RAB11FIP1,SOCS2,MLXIP,MICAL2,CELSR2,LGALS1,APOE,LAMA5,PTPDC1,GOLGA2,BHLHE40,HRC,DSTYK,FNBP1,ITGA5,DEPP1,TP53INP1,LLGL2,TRDN,ATF3,MFGE8,IL15RA,CD24,RASGEF1A,RHOBTB1,PLEKHH3,PALM,MLXIPL,BST2,STARD8,GRAP2,GPR155,KRT8,SESN3,RAB3B,NFATC4,GIPR,ID1,CTCFL,CPZ,RAB27A,RHPN1,IGF1,TLE2,NUDT4,HELZ2,MAPRE3,MIIP,ITGB1,MAGI1,PITPNM1,MALAT1,NLGN2,CTSC,FGFR3,VPS13D,TFAP2B,PPP1R14C,G0S2,NTS,INHBE,MICAL1,NECTIN2,MUC4,PLEKHA4,CREB3L2,MBOAT2,BRSK1,SMAD3,AP2M1,EDEM1,B2M,PSME1,LYPLA1,MYRF,PRKAB2,SVIP,HDAC6,ARHGEF12,SEMA7A,ATF6,GDI1,PCK2,RBL2,GPC2,ROBO1,CDKN1A,ACE,TBC1D5,ACVR1C,PCLO,EXT2,ZNF397,AFDN,SDF2L1,CNN2,DMTN,CYP26B1,PXK,AMOTL1,CXCL8,CDKN1C,SEMA3F,CDC42SE1,TRPM4,BAIAP3,MYLK3,ABCA7,BCAM,KCNAB2,PTPRU,SLC44A2,PTPRH,SKIL,POMT1,GDPD5,PAN2,KDM7A,BTN3A1,SPEG,PLXND1,DOCK6,NDRG2,HES7,PRKACA,EIF2AK1,PSRC1,ADCY3,MIF4GD,GABBR1,AGRN,HLTF,PTPRS,FASTKD1,WIPI1,SERPINI1,ATP7A,NDRG1,TEX19,SESTD1,SHC2,NSD3,CAMTA2,MSI1,STAT5B,SH2D3A,DUSP8,NUP107,IFIH1,TGFBR3,SMARCA1,ULK1,GLI1,PTEN,LRSAM1,NPTXR,ITM2C,MTURN,RAB18,PDLIM7,KMT5C,ZNF275,CUL7,GSN,PKHD1,ZNF697,NFE2,DRAP1,BTK,CTIF,ADGRL1,COL18A1,XK,TIA1,LRP4,INTS8,ZC3H6,KRBA1,RGS16,KLF9,IFNGR1,RNF217,CMTM7,LGALS9,CAMSAP3,MINK1,NFE2L1,COTL1,SEMA3A,HACE1,QSOX1,CLYBL,SPPL2B,PITPNC1,DDHD2,CALR,RHBDD1,AMFR,ITM2A,TSPO,AURKB,FLT1,TMED9,GPR180,MAGED2,PTPRJ,PTPRF,MORF4L2,ARL6IP5,MACC1,NES,INPPL1,BCL3,TRIM38,OBSCN,POFUT2,SIDT2,ARHGAP8,CHST2,MNX1,ITGB5,CORO7,DLG4,STK10,ESRRB,UCP2,CEBPD,RCN3,NR2F6,MLLT11,IFITM1,TMOD2,CYP26A1,P2RX6,WNK4,CYLD,SYT5,TCIRG1,HLA-E,RBM22,AHCYL1,ANKIB1,NCOA7,ADGRA2,TUBB2B,HIP1,ERFE,SPARC,CC2D1A,PLA2G6,SLC17A7,THBS1,CCND2,IL32,RRAS,TMEM9,CTSB,NFKB2,SH3TC2,TRPT1,CALCOCO1,DACT3,KLF7,ZBTB11,PDE4DIP,IFT140,SEC22B,PDK4,MAP3K9,LMOD1,PIDD1,ZNF175,TKFC,TCAF1,LRG1,CASTOR2,KLF13,CCDC71L,GPC5,MANF,PHF21A,SPTAN1,TPD52L1,DENND3,MAN2A1,NPAS1,PAIP2B,ENO2,NCBP2,CASP10,TUBB4A,RNF187,HAX1,SOCS1,MBNL2,CFH,DBNDD1,HIVEP3,TMEM30A,ITPKA,SELENON,ACVR1,TARBP1,ITGB3,CORO2A,MEIS3,MVB12A,NUCB1,NCOA4,BRWD3,PTTG1,AGER,NIPSNAP2,MAP3K8,DGKD,ACER3,SORT1,ACADVL,LITAF,AZU1,PTPRC,DAB2,DENND4B,IDH1,IL6R,PSAP,SEMA6C,NFE2L3,POLG2,SLC4A11,H1-10,CCND3,PIK3CD,OAS3,RPS6KC1,DYNC2LI1,TSC22D3,TSPYL2,SMYD3,HIP1R,ITPR2,ARFGAP1,SETD5,ARRB1,TNFRSF10B,BATF2,FECH,RAVER2,SMPDL3B,OSTF1,CD200R1,HJV,LTK,MMP14,OGT,MAP1A,TRIB3,PLP1,GAS6,ZDHHC8,ADGRL3,NTRK1,RXFP1,GPR158,LPAR2,GNG7,LGALS3,TAB2,TMEM116,WARS1,LMNA,PLXNB2,IL13RA1,STX10,CD55,YIPF5,RELL2,ZNF558,CAPN5,ITM2B,LDLRAP1,NBR1,RGL3,IQGAP3,MRAP2,TIAL1,PDIA3,AMIGO2,KCNJ11,RBCK1,DNM1,EGLN3,DLK1,ADGRE2,PDGFB,MAST2,NIB

			AN2,HDGFL3,GSTO1,STK16,DBP,CPEB4,GTF2H4,BBS4,ELP1,SOX5,STX2,LAMB2,EMILIN2,GPSM3,PPP2R5B,AKNA,VPS28,CELF2,ISCU,SESN2,ATP1A2,C6ORF89,BMF,TSKU,SMURF2,TNNI3,TUT7,CPNE3,GAS2,ZFYVE28,FLT4,MCF2,BTN3A2,KLF12,RBPMS,MTCL1,C3,GARNL3,RAB26,PLPPR2,ACSL1,TMED4,ODF2L,ZNF608,CCM2,RTN4RL2,SCN1B,PLCD3,UNC119,ZMIZ1,RPH3AL,HIVEP2,MAST1,GUCA1B,KCTD20,COL1A2,INPP5J,SALL2,ARHGEF40,BNIP3,KHK,HLA-B,ETAA1,ZNF396,GNB5,WASL,STOM,TCAF2,TCFL5,PEX2,SNTA1,THRB,SFMBT2,FGD1,FGFR4,MAPK8IP3,SEMA4G,SHFL,GIPC1,MLF1,RTKN2,GTF2A1,ARID5B,ZNF117,ZP3,GPAT3,PKD1,HBP1,CUX1,MAPT,TBC1D20,CITED4,MC4R,SMAD6,SLC45A3,APAF1,GRN,EHD2,WBP1L,CCDC88B,APBB3,DUSP5,NOTUM,SEMA3E,SLC30A2,ZKSCAN1,TENM1,EPS8,WDR91,WDR11,LGMN,DNAJB11,CBLB,BBC3,TP53I11,CACNA2D2,SCFD1,PDK1,BCL9L,PRRC1,MGAT5,PDE7A,DLG3,ZNF133,DTNA,ZNF83,RHBDD2,SEC24A,C2CD2L,PRCP,GTPBP1,TENT5A,RTN4R,RFLNB,SETD7,RAB31,STK4,PVR,UTRN,STON1,COL15A1,IMPA2,RTN2,SERGEF,TNFRSF1B,CR2,SYDE2,UFL1,GRHL1,RB1CC1,PAX9,RAPGEF2,INPP4A,C18ORF54,ZNF467,TNK2,GPSM1,DCP2,PPP1R16B,MYORG,ATP1B2,DEF8,NIM1K,RPS6KA1,MAP4K2,VPS16,GPD1L,THBD,SH3BP2,VBP1,CCSAP,ENTR1,SERTAD2,NYAP1,TTBK2,MXD3,USP18,ANAPC16,IRAK2,ADAM15
GO:0048856	anatomical structure development	8.853877170428204e-18	HSPA5,FN1,TXNIP,LAMA3,HECW2,ADAMTSL4,DDIT4,PTK2B,GDF15,PIM1,ELAPOR2,AARS1,FLNC,ANXA1,RNF213,CA2,CARD11,CAPRIN2,PLD3,NFASC,UNC5B,MAF,LAMB1,CLSTN3,KLF10,HDAC9,LRP1,GADD45A,TGFB1,ANXA6,ABI3BP,ATM,ARG2,F11R,RAB6B,FLNB,ASS1,TGM2,MKNK2,PPP1R13L,CD36,DYSF,MARCKS,CHAC1,VEGFA,FUT1,EFHD1,ERBB3,PTK7,ITGAV,DMBX1,TNFRSF9,CTSZ,TEAD1,ID3,CALB1,ERMN,SERPINH1,BTG2,TMOD1,SEMA6B,KDM6B,IL2RA,RYR1,ADAM19,SEC24D,CD46,BCL6,NHLH1,TTLL7,F2R,SOCS2,MICAL2,CELSR2,LGALS1,APOE,LAMA5,BHLHE40,ITGA5,ATF3,ENO3,MFGE8,IL15RA,CD24,RHOBTB1,PALM,MLXIPL,MMP15,ALAS1,KRT8,COL11A1,NFATC4,GIPR,ID1,IGF1,TLE2,ITGB1,PITPNM1,NLGN2,CTSC,FGFR3,TFAP2B,WHRN,USP45,NECTIN2,GAA,PLEKHA4,CREB3L2,MBOAT2,BRSK1,SMAD3,B2M,MYRF,HDAC6,SEMA7A,ATF6,GDI1,PCK2,GPC2,ROBO1,CDKN1A,ACE,ACVR1C,PCLO,EXT2,AFDN,CNN2,DMTN,CYP26B1,AMOTL1,CXCL8,CDKN1C,SEMA3F,CDC42SE1,TRPM4,MYLK3,TNNT1,PTPRU,SKIL,GDPD5,KDM7A,SPEG,PGM3,PLXND1,NDRG2,HES7,PRKACA,EIF2AK1,MYBPHL,AGRN,HLTF,PTPRS,SERPINI1,ATP7A,NDRG1,TEX19,MSI1,STAT5B,NUP107,DNASE2,TGFBR3,SMARCA1,ULK1,GLI1,PTEN,ITM2C,MTURN,RAB18,PDLIM7,KMT5C,CUL7,GSN,PKHD1,ALAS2,BTK,ADGRL1,COL18A1,SMIM14,XK,SH3PXD2B,THOC2,LRP4,LUC7L,IFNGR1,CMTM7,TDRKH,LGALS9,CAMSAP3,MINK1,RNF103,NFE2L1,SEMA3A,ALDOC,DDHD2,CALR,BTBD2,ITM2A,TSPO,AURKB,FLT1,PTPRJ,IGSF8,PTPRF,SELENOP,SYBU,PAPSS1,TJP3,NES,INPPL1,BCL3,OBSCN,POFUT2,SIDT2,MNX1,ITGB5,DLG4,PCDH15,ESRRB,UCP2,DHTKD1,SLC37A4,CEBPD,RCN3,NR2F6,PDLIM4,TMOD2,CYP26A1,WNK4,TCIRG1,HLA-E,RHAG,ADGRA2,TUBB2B,SPARC,SLC17A7,THBS1,RRAS,CTSB,NFKB2,SH3TC2,DACT3,TRIM3,KLF7,IFT140,LMOD1,PPIB,LRG1,KLF13,MANF,MAN2A1,RTN3,NPAS1,HAX1,SOCS1,HIVEP3,TMEM30A,ITPKA,SELENON,ACVR1,ITGB3,MEIS3,NCOA4,PCSK4,BRWD3,AGER,ACER3,SORT1,ACADVL,AZU1,PTPRC,DAB2,IDH1,IL6R,DIP2A,PSAP,VWDE,SEMA6C,POLG2,PIK3CD,DYNC2LI1,SMYD3,HIP1R,SETD5,BATF2,FECH,LTK,MMP14,OGT,MAP1A,PLP1,GAS6,ADGRL3,NTRK1,VCAN,RXFP1,LGALS3,TAB2,WARS1,LMNA,PLXNB2,CEMIP2,VSTM4,ITM2B,NBR1,IQGAP3,TIAL1,KIF5A,

			AMIGO2,PDGFB,NIBAN2,HDGFL3,BBS4,SOX5,STX2,LAMB2,EMILIN2,PPP2R5B,AKNA,ATP1A2,TSKU,SMTN,SMURF2,TNNI3,TUT7,GAS2,FLT4,MCF2,C3,PHGDH,RAB26,SLC4A7,HOOK1,CCM2,RTN4RL2,SCN1B,PLCD3,UNC119,ZMIZ1,SNPH,MAST1,ROBO3,COL1A2,INPP5J,SALL2,BNIP3,HLA-B,ACSL6,WASL,THRB,FGD1,MAPK8IP3,SEMA4G,MLF1,RTKN2,ARID5B,ZP3,PKD1,C1GALT1,CUX1,MAPT,SNX2,TBC1D20,FMNL1,SMAD6,SLC45A3,APAF1,GRN,EHD2,WBP1L,DUSP5,NOTUM,SEMA3E,TMEM41B,MDGA2,TENM1,EPS8,ACAT1,WDR11,DNAJB11,CBLB,SCFD1,PPDPF,BCL9L,DLG3,TPD52,PRCP,TENT5A,RTN4R,RFLNB,STK4,TRAK2,UTRN,COL15A1,BPNT1,TNFRSF1B,CR2,UFL1,GRHL1,RB1CC1,PAX9,TTC7A,RAPGEF2,GPSM1,PPP1R16B,MYORG,ATP1B2,LHFPL2,RPS6KA1,CCSAP,NYAP1,TTBK2,ADAM15
GO:0032502	developmental process	2.914064808888199e-16	*HSPA5,FN1,TXNIP,LAMA3,HECW2,ADAMTSL4,DDIT4,PTK2B,GDF15,PIM1,ELAPOR2,AARS1,FLNC,ANXA1,RNF213,CA2,CARD11,CAPRIN2,PLD3,NFASC,UNC5B,MAF,LAMB1,CLSTN3,KLF10,HDAC9,LRP1,GADD45A,TGFB1,ANXA6,ABI3BP,SLC6A6,ATM,ARG2,F11R,RAB6B,FLNB,ASS1,TGM2,MKNK2,PPP1R13L,CD36,DYSF,MARCKS,CHAC1,VEGFA,FUT1,EFHD1,ERBB3,PTK7,ITGAV,DMBX1,MRC2,TNFRSF9,CTSZ,TEAD1,ID3,CALB1,ERMN,SERPINH1,BTG2,TMOD1,SEMA6B,KDM6B,IL2RA,RYR1,ADAM19,SEC24D,CD46,BCL6,NHLH1,TTLL7,F2R,SEPTIN6,SOCS2,MICAL2,CELSR2,LGALS1,APOE,LAMA5,BHLHE40,ITGA5,TP53INP1,ATF3,ENO3,MFGE8,IL15RA,CD24,RHOBTB1,PALM,MLXIPL,MMP15,ALAS1,KRT8,COL11A1,NFATC4,GIPR,ID1,RAB27A,IGF1,TLE2,ITGB1,PITPNM1,NLGN2,CTSC,FGFR3,TFAP2B,WHRN,USP45,NECTIN2,GAA,PLEKHA4,CREB3L2,MBOAT2,BRSK1,SMAD3,B2M,MYRF,HDAC6,SEMA7A,ATF6,GDI1,PCK2,RBL2,GPC2,ROBO1,CDKN1A,ACE,ACVR1C,PCLO,EXT2,AFDN,CNN2,DMTN,CYP26B1,AMOTL1,CXCL8,CDKN1C,SEMA3F,CDC42SE1,TRPM4,BAIAP3,MYLK3,TNNT1,PTPRU,SKIL,GDPD5,KDM7A,SPEG,PGM3,PLXND1,NDRG2,HES7,PRKACA,EIF2AK1,MYBPHL,GABBR1,AGRN,HLTF,PTPRS,SERPINI1,ATP7A,NDRG1,TEX19,MSI1,STAT5B,NUP107,DNASE2,TGFBR3,SMARCA1,ULK1,GLI1,PTEN,ITM2C,MTURN,RAB18,PDLIM7,KMT5C,CUL7,GSN,PKHD1,ALAS2,BTK,ADGRL1,COL18A1,SMIM14,XK,SH3PXD2B,THOC2,LRP4,LUC7L,IFNGR1,CMTM7,TDRKH,LGALS9,CAMSAP3,MINK1,RNF103,NFE2L1,SEMA3A,ALDOC,DDHD2,CALR,BTBD2,RHBDD1,AMFR,ITM2A,TSPO,AURKB,FLT1,PTPRJ,IGSF8,PTPRF,SELENOP,SYBU,PAPSS1,TJP3,NES,INPPL1,BCL3,OBSCN,POFUT2,SIDT2,MNX1,ITGB5,DLG4,PCDH15,ESRRB,UCP2,DHTKD1,SLC37A4,CEBPD,RCN3,NR2F6,PDLIM4,IFITM1,TMOD2,CYP26A1,WNK4,TCIRG1,HLA-E,RHAG,ADGRA2,TUBB2B,HIP1,SPARC,SLC17A7,THBS1,RRAS,CTSB,NFKB2,SH3TC2,DACT3,TRIM3,KLF7,IFT140,LMOD1,PPIB,LRG1,KLF13,CCDC71L,MANF,MAN2A1,RTN3,NPAS1,HAX1,SOCS1,HIVEP3,TMEM30A,ITPKA,SELENON,ACVR1,ITGB3,MEIS3,NCOA4,PCSK4,BRWD3,PTTG1,AGER,ACER3,SORT1,ACADVL,AZU1,PTPRC,DAB2,IDH1,IL6R,DIP2A,PSAP,VWDE,SEMA6C,POLG2,SLC4A11,PIK3CD,DYNC2LI1,SMYD3,HIP1R,SETD5,BATF2,FECH,LTK,MMP14,OGT,MAP1A,TRIB3,PLP1,GAS6,ADGRL3,NTRK1,VCAN,RXFP1,LGALS3,TAB2,WARS1,LMNA,IARS1,PLXNB2,CEMIP2,VSTM4,ITM2B,NBR1,IQGAP3,TIAL1,KIF5A,AMIGO2,DLK1,PDGFB,MAST2,NIBAN2,HDGFL3,BBS4,SOX5,STX2,LAMB2,EMILIN2,PPP2R5B,AKNA,ATP1A2,TSKU,SMTN,SMURF2,TNNI3,TUT7,GAS2,FLT4,MCF2,WIPF3,C3,PHGDH,RAB26,SLC4A7,HOOK1,CCM2,RTN4RL2,SCN1B,PLCD3,UNC119,ZMIZ1,SNPH,MAST1,ROBO3,COL1A2,INPP5J,SALL2,BNIP3,HLA-B,ACSL6,WASL,TCFL5,THRB,FGD1,MAPK8IP3,SEMA4G,MLF1,RTKN2,ARID5B,ZP3,PKD1,C1GALT1,CUX1,MAPT,SN*

			X2,TBC1D20,FMNL1,SMAD6,SLC45A3,APAF1,GRN,EHD2,WBP1L,DUSP5,NOTUM,SEMA3E,TMEM41B,MDGA2,TENM1,EPS8,ACAT1,WDR11,LGMN,DNAJB11,CBLB,SCFD1,PPDPF,BCL9L,DLG3,TPD52,PRCP,TENT5A,RTN4R,RFLNB,STK4,TRAK2,UTRN,COL15A1,BPNT1,TNFRSF1B,CR2,UFL1,GRHL1,RB1CC1,PAX9,TTC7A,RAPGEF2,TNK2,GPSM1,ACSBG2,PPP1R16B,MYORG,ATP1B2,LHFPL2,RPS6KA1,CCSAP,NYAP1,TTBK2,TXNRD3,ADAM15
GO:0048869	cellular developmental process	1.4567302385469244e-15	*HSPA5,FN1,TXNIP,LAMA3,HECW2,ADAMTSL4,DDIT4,PTK2B,GDF15,PIM1,FLNC,ANXA1,CARD11,CAPRIN2,PLD3,NFASC,UNC5B,MAF,LAMB1,KLF10,HDAC9,LRP1,TGFB1,ANXA6,ABI3BP,SLC6A6,ATM,F11R,RAB6B,FLNB,TGM2,MKNK2,PPP1R13L,CD36,DYSF,MARCKS,CHAC1,VEGFA,EFHD1,ERBB3,PTK7,ITGAV,MRC2,TNFRSF9,ID3,SERPINH1,BTG2,TMOD1,SEMA6B,KDM6B,IL2RA,RYR1,CD46,BCL6,NHLH1,TTLL7,SEPTIN6,SOCS2,CELSR2,LGALS1,APOE,LAMA5,BHLHE40,ITGA5,TP53INP1,ATF3,IL15RA,CD24,MMP15,ALAS1,KRT8,COL11A1,NFATC4,ID1,RAB27A,IGF1,ITGB1,NLGN2,FGFR3,TFAP2B,WHRN,NECTIN2,CREB3L2,MBOAT2,BRSK1,SMAD3,B2M,MYRF,HDAC6,SEMA7A,GDI1,PCK2,RBL2,GPC2,ROBO1,ACE,ACVR1C,EXT2,AFDN,CNN2,DMTN,CYP26B1,CDKN1C,SEMA3F,TRPM4,MYLK3,TNNT1,PTPRU,SKIL,GDPD5,SPEG,PGM3,PLXND1,NDRG2,HES7,PRKACA,EIF2AK1,GABBR1,AGRN,HLTF,PTPRS,SERPINI1,ATP7A,NDRG1,TEX19,MSI1,STAT5B,DNASE2,TGFBR3,SMARCA1,ULK1,GLI1,PTEN,ITM2C,MTURN,PDLIM7,CUL7,PKHD1,ALAS2,BTK,COL18A1,XK,SH3PXD2B,THOC2,LRP4,IFNGR1,CMTM7,TDRKH,LGALS9,CAMSAP3,MINK1,SEMA3A,ALDOC,CALR,BTBD2,RHBDD1,ITM2A,TSPO,FLT1,PTPRJ,PTPRF,TJP3,NES,BCL3,OBSCN,POFUT2,SIDT2,MNX1,ITGB5,DLG4,PCDH15,ESRRB,DHTKD1,SLC37A4,CEBPD,NR2F6,IFITM1,TMOD2,TCIRG1,RHAG,ADGRA2,TUBB2B,HIP1,RRAS,CTSB,NFKB2,SH3TC2,DACT3,KLF7,IFT140,LMOD1,LRG1,KLF13,CCDC71L,MANF,MAN2A1,RTN3,HAX1,SOCS1,HIVEP3,TMEM30A,ITPKA,SELENON,ACVR1,ITGB3,PCSK4,AGER,SORT1,ACADVL,AZU1,PTPRC,DAB2,IL6R,DIP2A,PSAP,SEMA6C,POLG2,SLC4A11,PIK3CD,SMYD3,BATF2,FECH,LTK,MMP14,OGT,MAP1A,TRIB3,PLP1,GAS6,ADGRL3,NTRK1,VCAN,RXFP1,LGALS3,LMNA,IARS1,PLXNB2,NBR1,TIAL1,KIF5A,DLK1,PDGFB,MAST2,NIBAN2,HDGFL3,BBS4,SOX5,STX2,LAMB2,PPP2R5B,AKNA,TSKU,TNNI3,TUT7,FLT4,MCF2,WIPF3,C3,PHGDH,SLC4A7,HOOK1,CCM2,RTN4RL2,SCN1B,ZMIZ1,SNPH,ROBO3,INPP5J,BNIP3,HLA-B,ACSL6,WASL,TCFL5,THRB,MAPK8IP3,SEMA4G,MLF1,RTKN2,ARID5B,ZP3,C1GALT1,CUX1,MAPT,SNX2,TBC1D20,SMAD6,SLC45A3,APAF1,GRN,EHD2,WBP1L,SEMA3E,MDGA2,TENM1,DNAJB11,PPDPF,BCL9L,TPD52,TENT5A,RTN4R,RFLNB,STK4,TRAK2,COL15A1,TNFRSF1B,CR2,UFL1,GRHL1,TTC7A,RAPGEF2,TNK2,GPSM1,ACSBG2,PPP1R16B,MYORG,ATP1B2,RPS6KA1,NYAP1,TXNRD3,ADAM15*
GO:0050789	regulation of biological process	1.762785007925035e-15	*HSPA5,FN1,TXNIP,LAMA3,ARRDC4,CISH,KSR1,HECW2,ADAMTSL4,FCGR2A,DDIT4,PTK2B,GDF15,PIM1,ELAPOR2,AARS1,ANXA1,RNF213,CA2,LTBP4,HSP90B1,TSPAN13,CARD11,CAPRIN2,PLD3,MEG3,GFPT1,ST6GAL1,UNC5B,REEP6,MAF,LAMB1,CLSTN3,LAPTM4B,KLF10,IL10RA,WSB1,HDAC9,LRP1,EEF1A2,CCNL2,TSC22D1,RGS10,GADD45A,TGFB1,EPHX2,ANXA6,ABI3BP,SLC6A6,ATM,ARG2,PLEKHH2,IVNS1ABP,F11R,FLNB,EPB41L2,ASS1,EML2,TGM2,MKNK2,HERPUD1,PPP1R13L,CD36,PTGS1,DYSF,AKAP11,ZNF692,PAPPA,CHAC1,VEGFA,FUT1,EFHD1,ERBB3,OPTN,PTK7,USP20,ITGAV,DMBX1,TNFRSF9,CTSZ,TEAD1,ID3,ADD3,ATP2A3,CALB1,ERMN,ZMYM2,SERPINH1,LMAN1,BTG2,TMOD1,SEMA6B,KDM6B,IL2RA,RYR1,ADAM19,REEP2,HYOU1,CD46,BCL6,NHLH1,FAM83A,F2R,RIMS3,RAB11FIP1,SOCS2,MLXIP,MICAL2,CELSR2,LGALS1,APOE,LAMA*

			5,PTPDC1,GOLGA2,BHLHE40,HRC,DSTYK,FNBP1,ITGA5,DEPP1,TP53INP1,LLGL2,TRDN,ATF3,MFGE8,IL15RA,CD24,RASGEF1A,RHOBTB1,PLEKHH3,PALM,MLXIPL,BST2,STARD8,GRAP2,GPR155,KRT8,SESN3,RAB3B,NFATC4,GIPR,ID1,CTCFL,CPZ,RAB27A,RHPN1,IGF1,TLE2,NUDT4,HELZ2,MAPRE3,MIIP,ITGB1,MAGI1,PITPNM1,MALAT1,NLGN2,CTSC,FGFR3,VPS13D,TFAP2B,PPP1R14C,G0S2,NTS,WHRN,INHBE,MICAL1,NECTIN2,MUC4,GAA,PLEKHA4,CREB3L2,MBOAT2,BRSK1,SMAD3,AP2M1,EDEM1,B2M,PSME1,LYPLA1,MYRF,PROS1,PRKAB2,SVIP,HDAC6,ARHGEF12,SEMA7A,UCA1,ATF6,GDI1,PCK2,NUDT12,RBL2,GPC2,ROBO1,CDKN1A,ACE,TBC1D5,ACVR1C,PCLO,EXT2,ZNF397,AFDN,SDF2L1,CNN2,DMTN,CYP26B1,PXK,AMOTL1,CXCL8,CDKN1C,SEMA3F,CDC42SE1,TRPM4,BAIAP3,MYLK3,ABCA7,BCAM,TNNT1,KCNAB2,PTPRU,SLC44A2,PTPRH,SKIL,POMT1,GDPD5,PAN2,KDM7A,BTN3A1,SPEG,PLXND1,DOCK6,NDRG2,HES7,PRKACA,EIF2AK1,PSRC1,ADCY3,MIF4GD,GABBR1,AGRN,HLTF,PTPRS,FASTKD1,WIPI1,SERPINI1,ATP7A,MT2A,NDRG1,TEX19,SESTD1,SHC2,NSD3,CAMTA2,MSI1,STAT5B,SH2D3A,DUSP8,NUP107,PROCR,DNASE2,IFIH1,TGFBR3,SMARCA1,ULK1,GLI1,PTEN,LRSAM1,NPTXR,ITM2C,MTURN,RAB18,PDLIM7,TMPRSS4,KMT5C,ZNF275,CUL7,GSN,PKHD1,ZNF697,NFE2,DRAP1,BTK,CTIF,ADGRL1,COL18A1,XK,TIA1,LRP4,TTC37,INTS8,ZC3H6,KRBA1,LUC7L,RGS16,KLF9,IFNGR1,RNF217,CMTM7,TDRKH,LGALS9,CAMSAP3,MINK1,NFE2L1,COTL1,SEMA3A,SLC22A5,HACE1,QSOX1,CLYBL,SPPL2B,PITPNC1,DDHD2,CALR,RHBDD1,AMFR,ITM2A,TSPO,AURKB,FLT1,TMED9,GPR180,MAGED2,PTPRJ,PTPRF,SELENOP,MORF4L2,ARL6IP5,MACC1,NES,INPPL1,BCL3,TRIM38,OBSCN,POFUT2,SIDT2,ARHGAP8,CHST2,MARCHF2,LSM4,MNX1,ITGB5,CORO7,DLG4,STK10,ESRRB,UCP2,SLC37A4,CEBPD,RCN3,NR2F6,MLLT11,IFITM1,TMOD2,CYP26A1,P2RX6,WNK4,CYLD,SYT5,TCIRG1,HLA-E,RBM22,AHCYL1,ANKIB1,NCOA7,ADGRA2,TUBB2B,HIP1,ERFE,SPARC,CC2D1A,PLA2G6,SLC17A7,THBS1,CCND2,IL32,RRAS,TMEM9,CTSB,NFKB2,SH3TC2,TRPT1,CALCOCO1,DACT3,KLF7,ZBTB11,PDE4DIP,IFT140,SEC22B,PDK4,MAP3K9,LMOD1,PIDD1,ZNF175,TKFC,PPIB,TCAF1,SERPINB1,LRG1,CASTOR2,KLF13,CCDC71L,GPC5,MANF,PHF21A,SPTAN1,TPD52L1,DENND3,FNDC4,MAN2A1,RTN3,NPAS1,PAIP2B,ENO2,NCBP2,CASP10,TUBB4A,RNF187,HAX1,SOCS1,MBNL2,CFH,DBNDD1,HIVEP3,TMEM30A,ITPKA,SELENON,ACVR1,CCDC92,TARBP1,ITGB3,CORO2A,MEIS3,MVB12A,NUCB1,NCOA4,BRWD3,PTTG1,AGER,NIPSNAP2,MAP3K8,DGKD,ACER3,SORT1,ACADVL,LITAF,AZU1,PAPLN,PTPRC,DAB2,DENND4B,IDH1,GGA1,IL6R,DIP2A,PSAP,SEMA6C,NFE2L3,POLG2,SLC4A11,H1-10,CCND3,PIK3CD,OAS3,EXOC2,RPS6KC1,DYNC2LI1,TSC22D3,TSPYL2,SMYD3,HIP1R,ITPR2,ARFGAP1,SETD5,ARRB1,TNFRSF10B,BATF2,FECH,RAVER2,SMPDL3B,OSTF1,CD200R1,HJV,LTK,MMP14,OGT,MAP1A,TRIB3,PLP1,CHID1,GAS6,ZDHHC8,ADGRL3,NTRK1,RXFP1,GPR158,LPAR2,GNG7,LGALS3,TAB2,TMEM116,WARS1,LMNA,PLXNB2,IL13RA1,STX10,CEMIP2,CD55,YIPF5,RELL2,ZNF558,CAPN5,ITM2B,LDLRAP1,NBR1,RGL3,IQGAP3,MRAP2,TIAL1,PDIA3,GGT7,AMIGO2,KCNJ11,RBCK1,DNM1,EGLN3,DLK1,ADGRE2,PDGFB,MAST2,NIBAN2,PCOLCE,HDGFL3,GSTO1,STK16,DBP,CPEB4,GTF2H4,BBS4,ELP1,SOX5,STX2,LAMB2,PI15,EMILIN2,GPSM3,PPP2R5B,AKNA,ERI1,VPS28,CELF2,ISCU,SESN2,ATP1A2,C6ORF89,BMF,TSKU,SIAE,SMURF2,TNNI3,TUT7,CPNE3,GAS2,ZFYVE28,FLT4,MCF2,BTN3A2,KLF12,RBPMS,MTCL1,C3,PHGDH,GARNL3,RAB26,PLPPR2,ACSL1,TMED4,ODF2L,ZNF608,CCM2,RTN4RL2,SCN1B,PLCD3,UNC119,ZMIZ1,RPH3AL,HIVEP2,MAST1,GUCA1B,KCTD20,COL1A2,INPP5J,SALL2,ARHGEF40,B

			NIP3,KHK,HLA-B,ETAA1,ZNF396,GNB5,WASL,STOM,TCAF2,TCFL5,PEX2,SNTA1,THRB,SFMBT2,FGD1,FGFR4,MAPK8IP3,SEMA4G,SHFL,GIPC1,MLF1,RTKN2,GTF2A1,ARID5B,ZNF117,ZP3,GPAT3,PKD1,HBP1,CUX1,MAPT,TBC1D20,CITED4,MC4R,FMNL1,SMAD6,SLC45A3,APAF1,GRN,EHD2,WBP1L,CCDC88B,APBB3,DUSP5,NOTUM,SEMA3E,SLC30A2,ZKSCAN1,TENM1,EPS8,WDR91,WDR11,LGMN,DNAJB11,CBLB,BBC3,TP53I11,CACNA2D2,SCFD1,PDK1,BCL9L,PRRC1,MGAT5,PDE7A,DLG3,ZNF133,DTNA,ZNF83,RHBDD2,SEC24A,C2CD2L,PRCP,GTPBP1,TENT5A,RTN4R,RFLNB,SETD7,RAB31,STK4,PVR,UTRN,STON1,COL15A1,IMPA2,RTN2,SERGEF,TNFRSF1B,CR2,SYDE2,UFL1,GRHL1,RB1CC1,PAX9,RAPGEF2,INPP4A,C18ORF54,ZNF467,TNK2,GPSM1,DCP2,PPP1R16B,MYORG,ATP1B2,LHFPL2,DEF8,NIM1K,RPS6KA1,MAP4K2,VPS16,GPD1L,THBD,SH3BP2,VBP1,CCSAP,ENTR1,SERTAD2,NYAP1,TTBK2,MXD3,USP18,ANAPC16,IRAK2,ADAM15
GO:0050896	response to stimulus	2.0023684986661803e-15	*HSPA5,FN1,TXNIP,LAMA3,CISH,KSR1,FCGR2A,DDIT4,PTK2B,GDF15,PIM1,ELAPOR2,ANXA1,RNF213,CA2,LTBP4,HSP90B1,CARD11,CAPRIN2,PLD3,ST6GAL1,NFASC,UNC5B,REEP6,MAF,LAMB1,KLF10,IL10RA,WSB1,HDAC9,LRP1,RGS10,GADD45A,TGFB1,EPHX2,ANXA6,ABI3BP,ATM,ARG2,IVNS1ABP,F11R,FLNB,ASS1,TGM2,MKNK2,HERPUD1,CD36,PTGS1,DYSF,APOL1,AKAP11,MARCKS,ANKZF1,PAPPA,CHAC1,VEGFA,EFHD1,ERBB3,OPTN,PTK7,USP20,ITGAV,TEAD1,ID3,ATP2A3,CALB1,SERPINH1,LMAN1,BTG2,SEMA6B,KDM6B,IL2RA,RYR1,HYOU1,CD46,BCL6,FAM83A,F2R,SOCS2,CELSR2,LGALS1,APOE,LAMA5,PTPDC1,BHLHE40,HRC,DSTYK,ULBP1,FNBP1,ITGA5,PDIA4,TP53INP1,LLGL2,TRDN,ATF3,PYCR2,IL15RA,CD24,RASGEF1A,RHOBTB1,PLEKHH3,PALM,MLXIPL,MMP15,BST2,STARD8,GRAP2,ABCG2,ALAS1,GPR155,KRT8,SESN3,COL11A1,NFATC4,GIPR,ID1,CPZ,RAB27A,RHPN1,IGF1,PYGL,TLE2,NUDT4,ITGB1,MAGI1,PITPNM1,NLGN2,CTSC,FGFR3,TFAP2B,G0S2,NTS,WHRN,INHBE,MICAL1,USP45,NECTIN2,MUC4,PLEKHA4,CREB3L2,BRSK1,PLOD2,SMAD3,EDEM1,POLI,B2M,PROS1,PRKAB2,SVIP,HDAC6,ARHGEF12,ULBP2,SEMA7A,ATF6,GDI1,PCK2,GPC2,ROBO1,CDKN1A,ACE,TBC1D5,ACVR1C,PCLO,EXT2,AFDN,SDF2L1,CNN2,DMTN,CYP26B1,PXK,AMOTL1,CXCL8,CDKN1C,SEMA3F,CDC42SE1,TRPM4,BAIAP3,MYLK3,ABCA7,BCAM,TNNT1,PTPRU,SLC44A2,SKIL,BTN3A1,PLXND1,DOCK6,NDRG2,HES7,PRKACA,EIF2AK1,ADCY3,GABBR1,AGRN,HLTF,PTPRS,WIPI1,ATP7A,MT2A,NDRG1,SHC2,CAMTA2,MSI1,STAT5B,SH2D3A,DUSP8,PROCR,DNASE2,CYP2R1,IFIH1,TGFBR3,ULK1,GLI1,PTEN,TDO2,LRSAM1,NPTXR,ITM2C,MTURN,RAB18,TMPRSS4,KMT5C,GSN,PKHD1,NFE2,ALAS2,BTK,ADGRL1,GABARAPL1,LRP4,RGS16,KLF9,IFNGR1,CMTM7,LGALS9,MINK1,RNF103,NFE2L1,ETFDH,COTL1,SEMA3A,SLC22A5,HACE1,SPPL2B,PITPNC1,DDHD2,CALR,RHBDD1,AMFR,ITM2A,TSPO,AURKB,FLT1,GPR180,PTPRJ,PTPRF,SELENOP,PDIA6,MORF4L2,ARL6IP5,MACC1,INPPL1,BCL3,TRIM38,OBSCN,SIDT2,ARHGAP8,CHST2,KYNU,MARCHF2,ITGB5,CORO7,DLG4,PCDH15,ESRRB,UCP2,SLC37A4,MSH5,CEBPD,RCN3,NR2F6,MLLT11,IFITM1,TMOD2,CYP26A1,P2RX6,WNK4,CYLD,SYT5,TCIRG1,HLA-E,GUK1,RBM22,AHCYL1,NCOA7,ADGRA2,TUBB2B,SUSD6,HIP1,ERFE,FGFR1OP2,CC2D1A,PLA2G6,THBS1,CCND2,IL32,RRAS,TMEM9,CTSB,NFKB2,SH3TC2,SWI5,CALCOCO1,DACT3,KLF7,IFT140,PDK4,MAP3K9,PIDD1,ZNF175,NDUFB4,TKFC,PPIB,LRG1,CASTOR2,GPC5,MANF,TPD52L1,DENND3,EPHX1,FNDC4,NPAS1,ENO2,CASP10,RNF187,HAX1,SOCS1,CFH,ITPKA,SELENON,ACVR1,CCDC92,ITGB3,CORO2A,MEIS3,MVB12A,NUCB1,NCOA4,PTTG1,AGER,MAP3K8,DGKD,ACER3,SORT1,ACADVL,LITAF,AZU1,PTPRC,D*

			AB2,DENND4B,IDH1,IL6R,PSAP,SEMA6C,NFE2L3,POLG2,SLC4A11,CCND3,PIK3CD,OAS3,RPS6KC1,GSDMB,TSC22D3,TSPYL2,SMYD3,HIP1R,ITPR2,ARFGAP1,ARRB1,TNFRSF10B,BATF2,MAN1B1,FECH,SMPDL3B,FBXO44,OSTF1,CD200R1,HJV,LTK,MMP14,OGT,TRIB3,PLP1,CHID1,GAS6,ADGRL3,NTRK1,RXFP1,GPR158,LPAR2,GNG7,LGALS3,TAB2,TMEM116,LMNA,PLXNB2,IL13RA1,CD55,RELL2,CAPN5,LDLRAP1,NBR1,RGL3,IQGAP3,MRAP2,TIAL1,PDIA3,KIF5A,PAFAH2,GGT7,KCNJ11,RBCK1,EGLN3,DLK1,ADGRE2,PDGFB,MAST2,NIBAN2,HDGFL3,GSTO1,STK16,CPEB4,GTF2H4,BBS4,SOX5,STX2,LAMB2,ERP44,EMILIN2,DCLRE1B,GPSM3,PPP2R5B,AKNA,SESN2,ATP1A2,C6ORF89,BMF,TSKU,SMURF2,TNNI3,CLCA1,CPNE3,GAS2,ZFYVE28,FLT4,MCF2,BTN3A2,RBPMS,C3,GARNL3,SLC4A7,PLPPR2,ACSL1,TMED4,CCM2,MAN1A1,HSPA4L,RTN4RL2,SCN1B,PLCD3,UNC119,ZMIZ1,RPH3AL,MAST1,GUCA1B,ROBO3,COL1A2,ARHGEF40,BNIP3,KHK,HLA-B,ETAA1,GNB5,WASL,THRB,FGD1,FGFR4,MAPK8IP3,SEMA4G,SHFL,GIPC1,MLF1,RTKN2,ARID5B,ZP3,GPAT3,PKD1,HBP1,MAPT,CITED4,MC4R,SMAD6,MORN4,APAF1,GRN,WBP1L,CCDC88B,DUSP5,NOTUM,SEMA3E,SLC30A2,TENM1,EPS8,ACAT1,WDR11,LGMN,CBLB,BBC3,SCFD1,PDK1,BCL9L,MGAT5,PDE7A,DTNA,RHBDD2,C2CD2L,PRCP,GTPBP1,TENT5A,RTN4R,SETD7,RAB31,STK4,PVR,STON1,COL15A1,RPL27,IMPA2,SERGEF,TNFRSF1B,CR2,SYDE2,UFL1,RB1CC1,PAX9,RAPGEF2,INPP4A,TNK2,PPP1R16B,MYORG,NIM1K,RPS6KA1,MAP4K2,GPD1L,THBD,SH3BP2,DDB2,NYAP1,TTBK2,USP18,IRAK2,TXNRD3,ADAM15
GO:0065007	biological regulation	3.117291731752886e-15	HSPA5,FN1,TXNIP,LAMA3,ARRDC4,CISH,KSR1,HECW2,ADAMTSL4,FCGR2A,DDIT4,PTK2B,GDF15,PIM1,ELAPOR2,AARS1,ANXA1,RNF213,CA2,LTBP4,HSP90B1,TSPAN13,CARD11,CAPRIN2,PLD3,MEG3,GFPT1,ST6GAL1,UNC5B,REEP6,MAF,LAMB1,CLSTN3,LAPTM4B,KLF10,IL10RA,WSB1,HDAC9,LRP1,EEF1A2,CCNL2,TSC22D1,RGS10,GADD45A,TGFB1,EPHX2,ANXA6,ABI3BP,SLC6A6,ATM,ARG2,PLEKHH2,IVNS1ABP,F11R,FLNB,EPB41L2,ASS1,EML2,TGM2,MKNK2,HERPUD1,PPP1R13L,CD36,PTGS1,DYSF,AKAP11,ZNF692,PAPPA,CHAC1,VEGFA,FUT1,EFHD1,ERBB3,OPTN,PTK7,USP20,ITGAV,DMBX1,TNFRSF9,CTSZ,TEAD1,ID3,ADD3,ATP2A3,CALB1,ERMN,ZMYM2,SERPINH1,LMAN1,BTG2,TMOD1,SEMA6B,KDM6B,IL2RA,RYR1,ADAM19,REEP2,HYOU1,CD46,BCL6,NHLH1,FAM83A,F2R,RIMS3,RAB11FIP1,SOCS2,MLXIP,MICAL2,CELSR2,LGALS1,APOE,LAMA5,PTPDC1,GOLGA2,BHLHE40,SLC29A4,HRC,DSTYK,FNBP1,ITGA5,DEPP1,TP53INP1,LLGL2,TRDN,ATF3,MFGE8,IL15RA,CD24,RASGEF1A,RFK,RHOBTB1,PLEKHH3,PALM,MLXIPL,MMP15,BST2,STARD8,GRAP2,GPR155,KRT8,SESN3,RAB3B,NFATC4,GIPR,ID1,CTCFL,CPZ,RAB27A,RHPN1,IGF1,TLE2,NUDT4,HELZ2,MAPRE3,MIIP,ITGB1,MAGI1,PITPNM1,MALAT1,NLGN2,CTSC,FGFR3,VPS13D,TFAP2B,PPP1R14C,G0S2,NTS,WHRN,INHBE,MICAL1,NECTIN2,MUC4,GAA,PLEKHA4,CREB3L2,MBOAT2,BRSK1,SMAD3,AP2M1,EDEM1,B2M,PSME1,LYPLA1,MYRF,PROS1,PRKAB2,SVIP,HDAC6,ARHGEF12,SEMA7A,UCA1,ATF6,GDI1,PCK2,NUDT12,RBL2,GPC2,ROBO1,CDKN1A,ACE,ATP8B3,TBC1D5,ACVR1C,PCLO,EXT2,ZNF397,AFDN,SDF2L1,CNN2,DMTN,CYP26B1,PXK,AMOTL1,CXCL8,CDKN1C,SEMA3F,CDC42SE1,TRPM4,BAIAP3,MYLK3,ABCA7,BCAM,TNNT1,KCNAB2,PTPRU,SLC44A2,PTPRH,SKIL,POMT1,GDPD5,PAN2,KDM7A,BTN3A1,SPEG,PLXND1,DOCK6,NDRG2,HES7,PRKACA,EIF2AK1,PSRC1,ADCY3,MIF4GD,GABBR1,AGRN,HLTF,PTPRS,FASTKD1,WIPI1,SERPINI1,ATP7A,MT2A,NDRG1,TEX19,SESTD1,SHC2,NSD3,CAMTA2,MSI1,STAT5B,SH2D3A,DUSP8,NUP107,PROCR,DNASE2,IFIH1,TGFBR3,SMARCA1,ULK1,GLI1,PTEN,LRSAM1,NPTXR,ITM2C,MTURN,RAB18,PDLIM7,TMPRSS4,KMT5C,ZNF275,CUL7,GSN,PKHD1,ZN

			F697,NFE2,DRAP1,BTK,CTIF,ADGRL1,COL18A1,XK,ALS2CL,TIA1,SH3PXD2B,LRP4,TTC37,INTS8,ZC3H6,KRBA1,LUC7L,RGS16,KLF9,IFNGR1,RNF217,CMTM7,TDRKH,LGALS9,CAMSAP3,MINK1,NFE2L1,COTL1,SEMA3A,SLC22A5,HACE1,QSOX1,CLYBL,SPPL2B,PITPNC1,DDHD2,CALR,RHBDD1,AMFR,ITM2A,TSPO,AURKB,FLT1,TMED9,GPR180,MAGED2,PTPRJ,PTPRF,SELENOP,MORF4L2,ARL6IP5,MACC1,TJP3,NES,INPPL1,BCL3,TRIM38,OBSCN,POFUT2,SIDT2,ARHGAP8,CHST2,MARCHF2,LSM4,MNX1,ITGB5,CORO7,DLG4,STK10,ESRRB,UCP2,SLC37A4,CEBPD,RCN3,NR2F6,MLLT11,IFITM1,TMOD2,CYP26A1,P2RX6,WNK4,CYLD,SYT5,TCIRG1,HLA-E,RBM22,AHCYL1,ANKIB1,NCOA7,ADGRA2,TUBB2B,HIP1,ERFE,SPARC,CC2D1A,PLA2G6,SLC17A7,THBS1,CCND2,IL32,RRAS,TMEM9,CTSB,NFKB2,SH3TC2,TRPT1,CALCOCO1,SMAP2,DACT3,KLF7,ZBTB11,PDE4DIP,DMXL2,IFT140,SEC22B,PDK4,MAP3K9,LMOD1,PIDD1,ZNF175,TKFC,PPIB,TCAF1,SERPINB1,LRG1,CASTOR2,KLF13,CCDC71L,GPC5,MANF,PHF21A,SPTAN1,TPD52L1,DENND3,FNDC4,MAN2A1,RTN3,NPAS1,PAIP2B,ENO2,NCBP2,CASP10,TUBB4A,RNF187,HAX1,SOCS1,MBNL2,CFH,DBNDD1,HIVEP3,TMEM30A,ITPKA,SELENON,ACVR1,CCDC92,TARBP1,ITGB3,CORO2A,MEIS3,MVB12A,NUCB1,NCOA4,PCSK4,BRWD3,PTTG1,AGER,NIPSNAP2,MAP3K8,DGKD,ACER3,SORT1,ACADVL,LITAF,AZU1,PAPLN,PTPRC,DAB2,DENND4B,IDH1,GGA1,IL6R,DIP2A,PSAP,SEMA6C,NFE2L3,POLG2,SLC4A11,ASAP2,H1-10,CCND3,PIK3CD,OAS3,EXOC2,RPS6KC1,DYNC2LI1,TSC22D3,TSPYL2,SMYD3,HIP1R,ITPR2,ARFGAP1,SETD5,ARRB1,TNFRSF10B,BATF2,FECH,RAVER2,SMPDL3B,OSTF1,CD200R1,HJV,LTK,MMP14,OGT,MAP1A,TRIB3,PLP1,CHID1,GAS6,ZDHHC8,MYO5B,ADGRL3,NTRK1,RXFP1,GPR158,DENND11,LPAR2,GNG7,LGALS3,TAB2,TMEM116,WARS1,LMNA,IARS1,PLXNB2,IL13RA1,STX10,CEMIP2,VSTM4,CD55,YIPF5,RELL2,ZNF558,CAPN5,ITM2B,LDLRAP1,NBR1,RGL3,IQGAP3,MRAP2,TIAL1,PDIA3,PAFAH2,SCPEP1,GGT7,AMIGO2,KCNJ11,RBCK1,DNM1,EGLN3,DLK1,ADGRE2,PDGFB,MAST2,NIBAN2,PCOLCE,HDGFL3,GSTO1,STK16,DBP,CPEB4,GTF2H4,BBS4,ELP1,SOX5,STX2,LAMB2,PI15,EMILIN2,GPSM3,PPP2R5B,AKNA,ERI1,VPS28,CELF2,SLC25A36,ISCU,SESN2,ATP1A2,C6ORF89,BMF,TSKU,SIAE,SMURF2,TNNI3,TUT7,CPNE3,GAS2,ZFYVE28,FLT4,MCF2,BTN3A2,KLF12,RBPMS,MTCL1,C3,PHGDH,GARNL3,RAB26,SLC4A7,PLPPR2,ACSL1,TMED4,ODF2L,ZNF608,CCM2,RTN4RL2,SCN1B,PLCD3,UNC119,ZMIZ1,RPH3AL,HIVEP2,SNPH,MAST1,GUCA1B,KCTD20,COL1A2,INPP5J,SALL2,ARHGEF40,BNIP3,KHK,HLA-B,ETAA1,ZNF396,GNB5,WASL,STOM,TCAF2,TCFL5,PEX2,SNTA1,THRB,SFMBT2,FGD1,FGFR4,MAPK8IP3,SEMA4G,SHFL,GIPC1,MLF1,RTKN2,GTF2A1,ARID5B,ZNF117,ZP3,GPAT3,PKD1,HBP1,CUX1,MAPT,TBC1D20,CITED4,GEMIN2,MC4R,FMNL1,SMAD6,SLC45A3,APAF1,GRN,EHD2,WBP1L,CCDC88B,APBB3,DUSP5,NOTUM,SEMA3E,SLC30A2,TMEM41B,ZKSCAN1,TENM1,EPS8,WDR91,WDR11,LGMN,DNAJB11,CBLB,BBC3,TP53I11,CACNA2D2,SCFD1,PDK1,BCL9L,PRRC1,MGAT5,PDE7A,DLG3,ZNF133,DTNA,ZNF83,RHBDD2,SEC24A,C2CD2L,PRCP,GTPBP1,TENT5A,RTN4R,RFLNB,SETD7,RAB31,STK4,PVR,UTRN,STON1,COL15A1,TLCD2,IMPA2,RTN2,SERGEF,TNFRSF1B,CR2,SYDE2,UFL1,GRHL1,RB1CC1,PAX9,RAPGEF2,INPP4A,C18ORF54,ZNF467,TNK2,GPSM1,DCP2,PPP1R16B,MYORG,ATP1B2,LHFPL2,DEF8,NIM1K,RPS6KA1,MAP4K2,VPS16,GPD1L,THBD,SH3BP2,VBP1,CCSAP,ENTR1,SERTAD2,NYAP1,TTBK2,MXD3,USP18,ANAPC16,IRAK2,ADAM15
GO:00301	cell	4.1124090924	*HSPA5,FN1,TXNIP,LAMA3,HECW2,ADAMTSL4,DDIT4,PTK2B,GDF15,PIM1,FLNC,ANXA1,CARD11,CAPRIN2,PLD3,N*

54	differentiation	03464e-15	*FASC,UNC5B,MAF,LAMB1,KLF10,HDAC9,LRP1,TGFB1,ANXA6,ABI3BP,SLC6A6,ATM,F11R,RAB6B,FLNB,TGM2,MKNK2,PPP1R13L,CD36,DYSF,MARCKS,CHAC1,VEGFA,EFHD1,ERBB3,PTK7,ITGAV,MRC2,TNFRSF9,ID3,SERPINH1,BTG2,TMOD1,SEMA6B,KDM6B,IL2RA,RYR1,CD46,BCL6,NHLH1,TTLL7,SEPTIN6,SOCS2,CELSR2,LGALS1,APOE,LAMA5,BHLHE40,ITGA5,TP53INP1,ATF3,IL15RA,CD24,MMP15,ALAS1,KRT8,COL11A1,NFATC4,ID1,RAB27A,IGF1,ITGB1,NLGN2,FGFR3,TFAP2B,WHRN,NECTIN2,CREB3L2,MBOAT2,BRSK1,SMAD3,B2M,MYRF,HDAC6,SEMA7A,GDI1,PCK2,RBL2,GPC2,ROBO1,ACE,ACVR1C,EXT2,AFDN,CNN2,DMTN,CYP26B1,CDKN1C,SEMA3F,TRPM4,MYLK3,TNNT1,PTPRU,SKIL,GDPD5,SPEG,PGM3,PLXND1,NDRG2,HES7,PRKACA,EIF2AK1,GABBR1,AGRN,HLTF,PTPRS,SERPINI1,ATP7A,NDRG1,TEX19,MSI1,STAT5B,DNASE2,TGFBR3,SMARCA1,ULK1,GLI1,PTEN,ITM2C,MTURN,PDLIM7,CUL7,PKHD1,ALAS2,BTK,COL18A1,XK,SH3PXD2B,THOC2,LRP4,IFNGR1,CMTM7,TDRKH,LGALS9,CAMSAP3,MINK1,SEMA3A,ALDOC,CALR,BTBD2,RHBDD1,ITM2A,TSPO,FLT1,PTPRJ,PTPRF,TJP3,BCL3,OBSCN,POFUT2,SIDT2,MNX1,ITGB5,DLG4,PCDH15,ESRRB,DHTKD1,SLC37A4,CEBPD,NR2F6,IFITM1,TMOD2,TCIRG1,RHAG,ADGRA2,TUBB2B,HIP1,RRAS,CTSB,NFKB2,SH3TC2,DACT3,KLF7,IFT140,LMOD1,LRG1,KLF13,CCDC71L,MANF,MAN2A1,RTN3,HAX1,SOCS1,HIVEP3,TMEM30A,ITPKA,SELENON,ACVR1,ITGB3,PCSK4,AGER,SORT1,ACADVL,AZU1,PTPRC,DAB2,IL6R,DIP2A,PSAP,SEMA6C,SLC4A11,PIK3CD,SMYD3,BATF2,FECH,LTK,MMP14,OGT,MAP1A,TRIB3,PLP1,GAS6,ADGRL3,NTRK1,VCAN,RXFP1,LGALS3,LMNA,IARS1,PLXNB2,NBR1,TIAL1,KIF5A,DLK1,PDGFB,MAST2,NIBAN2,HDGFL3,BBS4,SOX5,STX2,LAMB2,PPP2R5B,AKNA,TSKU,TNNI3,TUT7,FLT4,MCF2,WIPF3,C3,PHGDH,SLC4A7,HOOK1,CCM2,RTN4RL2,SCN1B,ZMIZ1,SNPH,ROBO3,INPP5J,BNIP3,HLA-B,ACSL6,WASL,TCFL5,THRB,MAPK8IP3,SEMA4G,MLF1,RTKN2,ARID5B,ZP3,C1GALT1,CUX1,MAPT,TBC1D20,SMAD6,SLC45A3,APAF1,GRN,EHD2,WBP1L,SEMA3E,MDGA2,TENM1,DNAJB11,PPDPF,BCL9L,TPD52,TENT5A,RTN4R,RFLNB,STK4,TRAK2,COL15A1,TNFRSF1B,CR2,UFL1,GRHL1,TTC7A,RAPGEF2,TNK2,GPSM1,ACSBG2,PPP1R16B,MYORG,ATP1B2,RPS6KA1,NYAP1,TXNRD3,ADAM15*
GO:0048731	system development	1.3099091970582402e-14	*HSPA5,FN1,LAMA3,HECW2,DDIT4,PTK2B,PIM1,ELAPOR2,AARS1,ANXA1,RNF213,CAPRIN2,NFASC,UNC5B,MAF,LAMB1,CLSTN3,HDAC9,LRP1,GADD45A,TGFB1,ANXA6,ABI3BP,ATM,ARG2,RAB6B,ASS1,TGM2,PPP1R13L,MARCKS,CHAC1,VEGFA,FUT1,EFHD1,ERBB3,PTK7,ITGAV,DMBX1,CTSZ,ID3,CALB1,SERPINH1,BTG2,TMOD1,SEMA6B,KDM6B,IL2RA,RYR1,BCL6,NHLH1,TTLL7,SOCS2,MICAL2,CELSR2,APOE,LAMA5,BHLHE40,ITGA5,ENO3,MFGE8,CD24,PALM,COL11A1,NFATC4,GIPR,ID1,IGF1,ITGB1,PITPNM1,NLGN2,CTSC,FGFR3,TFAP2B,WHRN,USP45,GAA,CREB3L2,MBOAT2,BRSK1,SMAD3,B2M,MYRF,HDAC6,SEMA7A,ATF6,GDI1,PCK2,GPC2,ROBO1,CDKN1A,ACE,ACVR1C,PCLO,EXT2,CYP26B1,AMOTL1,CXCL8,CDKN1C,SEMA3F,MYLK3,SKIL,GDPD5,KDM7A,PLXND1,NDRG2,HES7,PRKACA,AGRN,HLTF,PTPRS,SERPINI1,ATP7A,NDRG1,TEX19,MSI1,STAT5B,NUP107,TGFBR3,SMARCA1,ULK1,GLI1,PTEN,ITM2C,RAB18,PDLIM7,KMT5C,CUL7,GSN,PKHD1,ADGRL1,COL18A1,XK,SH3PXD2B,THOC2,LRP4,IFNGR1,LGALS9,CAMSAP3,MINK1,RNF103,SEMA3A,CALR,BTBD2,TSPO,FLT1,IGSF8,PTPRF,SELENOP,SYBU,PAPSS1,NES,INPPL1,BCL3,SIDT2,MNX1,DLG4,PCDH15,UCP2,RCN3,NR2F6,PDLIM4,TMOD2,CYP26A1,WNK4,TCIRG1,HLA-E,ADGRA2,TUBB2B,SPARC,SLC17A7,THBS1,RRAS,NFKB2,SH3TC2,TRIM3,KLF7,IFT140,PPIB,LRG1,MANF,MAN2A1,RTN3,NPAS1,TMEM30A,ITPKA,SELENON,ACVR1,ITGB3,MEIS3,NCOA4,AGER,ACER3,AZU1,PTPRC,DAB2,IDH1,I*

			L6R,DIP2A,PSAP,SEMA6C,PIK3CD,HIP1R,SETD5,LTK,MMP14,MAP1A,PLP1,GAS6,ADGRL3,NTRK1,VCAN,RXFP1,TAB2,WARS1,LMNA,PLXNB2,CEMIP2,VSTM4,ITM2B,KIF5A,AMIGO2,PDGFB,NIBAN2,HDGFL3,BBS4,SOX5,LAMB2,EMILIN2,PPP2R5B,AKNA,ATP1A2,TSKU,TNNI3,GAS2,FLT4,MCF2,C3,PHGDH,RAB26,SLC4A7,CCM2,RTN4RL2,SCN1B,PLCD3,UNC119,ZMIZ1,SNPH,MAST1,ROBO3,COL1A2,INPP5J,SALL2,BNIP3,HLA-B,ACSL6,WASL,THRB,MAPK8IP3,SEMA4G,ARID5B,ZP3,PKD1,C1GALT1,CUX1,MAPT,TBC1D20,SMAD6,SLC45A3,APAF1,GRN,NOTUM,SEMA3E,TMEM41B,MDGA2,TENM1,ACAT1,WDR11,DNAJB11,CBLB,PRCP,RTN4R,RFLNB,STK4,TRAK2,COL15A1,BPNT1,TNFRSF1B,UFL1,RB1CC1,RAPGEF2,GPSM1,PPP1R16B,ATP1B2,RPS6KA1,NYAP1,TTBK2,ADAM15
GO:0048518	positive regulation of biological process	3.137304948029567e-14	*HSPA5,FN1,TXNIP,ARRDC4,KSR1,ADAMTSL4,DDIT4,PTK2B,GDF15,PIM1,ELAPOR2,ANXA1,CA2,CARD11,CAPRIN2,ST6GAL1,UNC5B,MAF,LAMB1,CLSTN3,KLF10,IL10RA,HDAC9,LRP1,EEF1A2,TSC22D1,GADD45A,TGFB1,EPHX2,ABI3BP,SLC6A6,ATM,ARG2,F11R,EPB41L2,ASS1,TGM2,HERPUD1,PPP1R13L,CD36,VEGFA,FUT1,ERBB3,OPTN,PTK7,ITGAV,TEAD1,ID3,ATP2A3,CALB1,LMAN1,BTG2,SEMA6B,KDM6B,IL2RA,ADAM19,CD46,BCL6,NHLH1,F2R,RIMS3,SOCS2,MLXIP,MICAL2,LGALS1,APOE,GOLGA2,HRC,DSTYK,ITGA5,TP53INP1,TRDN,ATF3,MFGE8,IL15RA,CD24,RASGEF1A,PALM,MLXIPL,BST2,SESN3,RAB3B,NFATC4,GIPR,ID1,CTCFL,RAB27A,IGF1,HELZ2,MAPRE3,ITGB1,MAGI1,MALAT1,NLGN2,CTSC,FGFR3,VPS13D,TFAP2B,G0S2,NTS,WHRN,INHBE,NECTIN2,PLEKHA4,CREB3L2,SMAD3,AP2M1,EDEM1,B2M,PSME1,MYRF,PRKAB2,SVIP,HDAC6,SEMA7A,ATF6,GDI1,PCK2,GPC2,ROBO1,CDKN1A,ACE,TBC1D5,ACVR1C,AFDN,CNN2,DMTN,CYP26B1,CXCL8,CDKN1C,SEMA3F,TRPM4,BAIAP3,MYLK3,ABCA7,PTPRU,SLC44A2,SKIL,POMT1,GDPD5,PAN2,KDM7A,BTN3A1,PLXND1,PRKACA,PSRC1,MIF4GD,GABBR1,AGRN,HLTF,PTPRS,WIPI1,SERPINI1,ATP7A,SHC2,NSD3,CAMTA2,STAT5B,SH2D3A,IFIH1,TGFBR3,SMARCA1,ULK1,GLI1,PTEN,LRSAM1,ITM2C,MTURN,PDLIM7,TMPRSS4,KMT5C,CUL7,GSN,PKHD1,NFE2,DRAP1,BTK,CTIF,ADGRL1,LRP4,IFNGR1,RNF217,LGALS9,MINK1,SEMA3A,SLC22A5,CLYBL,DDHD2,CALR,RHBDD1,TSPO,AURKB,FLT1,TMED9,PTPRJ,MORF4L2,ARL6IP5,MACC1,NES,BCL3,TRIM38,POFUT2,ARHGAP8,CHST2,MARCHF2,CORO7,DLG4,ESRRB,UCP2,CEBPD,RCN3,MLLT11,IFITM1,TMOD2,WNK4,CYLD,TCIRG1,HLA-E,RBM22,AHCYL1,ANKIB1,NCOA7,ADGRA2,TUBB2B,HIP1,ERFE,SPARC,CC2D1A,PLA2G6,THBS1,CCND2,RRAS,TMEM9,NFKB2,CALCOCO1,KLF7,PDE4DIP,SEC22B,MAP3K9,LMOD1,PIDD1,ZNF175,TKFC,PPIB,TCAF1,LRG1,KLF13,CCDC71L,GPC5,TPD52L1,MAN2A1,NCBP2,CASP10,RNF187,HAX1,SOCS1,CFH,HIVEP3,TMEM30A,ITPKA,SELENON,ACVR1,ITGB3,MEIS3,NCOA4,AGER,NIPSNAP2,MAP3K8,DGKD,ACER3,LITAF,AZU1,PTPRC,DAB2,GGA1,IL6R,DIP2A,SEMA6C,POLG2,CCND3,PIK3CD,OAS3,SMYD3,HIP1R,ARRB1,TNFRSF10B,SMPDL3B,HJV,LTK,MMP14,OGT,MAP1A,TRIB3,PLP1,GAS6,ZDHHC8,NTRK1,LPAR2,LGALS3,TAB2,WARS1,LMNA,PLXNB2,IL13RA1,CD55,RELL2,LDLRAP1,IQGAP3,MRAP2,TIAL1,PDIA3,AMIGO2,RBCK1,EGLN3,ADGRE2,PDGFB,NIBAN2,PCOLCE,GSTO1,STK16,DBP,BBS4,SOX5,LAMB2,EMILIN2,GPSM3,PPP2R5B,AKNA,VPS28,ISCU,SESN2,ATP1A2,C6ORF89,BMF,TSKU,SMURF2,TUT7,CPNE3,FLT4,BTN3A2,KLF12,RBPMS,MTCL1,C3,ACSL1,TMED4,SCN1B,UNC119,ZMIZ1,RPH3AL,GUCA1B,KCTD20,SALL2,BNIP3,HLA-B,ETAA1,WASL,STOM,TCAF2,THRB,FGFR4,MAPK8IP3,SEMA4G,GIPC1,RTKN2,GTF2A1,ARID5B,ZP3,PKD1,CUX1,MAPT,TBC1D20,CITED4,MC4R,SMAD6,SLC45A3,APAF1,GR*

			N,EHD2,WBP1L,CCDC88B,APBB3,SEMA3E,SLC30A2,TENM1,EPS8,LGMN,CBLB,BBC3,BCL9L,PRRC1,MGAT5,DLG3,SEC24A,C2CD2L,GTPBP1,TENT5A,RTN4R,RAB31,STK4,PVR,UTRN,TNFRSF1B,CR2,UFL1,GRHL1,RB1CC1,PAX9,RAPGEF2,TNK2,GPSM1,DCP2,PPP1R16B,MYORG,ATP1B2,LHFPL2,DEF8,RPS6KA1,MAP4K2,GPD1L,THBD,ENTR1,SERTAD2,IRAK2
GO:0050920	regulation of chemotaxis	0.048599062309295976	*PTK2B,ST6GAL1,VEGFA,SEMA6B,SMAD3,HDAC6,SEMA7A,ROBO1,CXCL8,SEMA3F,LGALS9,SEMA3A,CALR,ADGRA2,TUBB2B,THBS1,AGER,AZU1,IL6R,SEMA6C,GAS6,PDGFB,GPSM3,SEMA4G,SEMA3E,LGMN*
CC			
GO:0005737	cytoplasm	9.52511254351904e-51	*HSPA5,FN1,TXNIP,LAMA3,ARRDC4,CISH,KSR1,HECW2,ADAMTSL4,FCGR2A,DDIT4,PTK2B,GDF15,PIM1,AARS1,FLNC,ANXA1,RNF213,CA2,HSP90B1,KIF21B,CARD11,CAPRIN2,PLD3,GFPT1,ST6GAL1,NFASC,REEP6,MAF,LAMB1,CLSTN3,LAPTM4B,SCG3,IL10RA,WSB1,HDAC9,LRP1,EEF1A2,TSC22D1,RGS10,GADD45A,TGFB1,EPHX2,ANXA6,ATM,ARG2,PLEKHH2,IVNS1ABP,NIPSNAP1,RAB6B,UBE2L6,FLNB,EPB41L2,ASS1,EML2,TGM2,MKNK2,HERPUD1,PPP1R13L,CD36,AIF1L,CRAT,PTGS1,TMCC2,DYSF,APOL1,AKAP11,MARCKS,ANKZF1,CHAC1,MCFD2,VEGFA,FUT1,EFHD1,GMPPA,GALNT12,OPTN,USP20,ITGAV,CTSZ,GTPBP2,ID3,ADD3,ATP2A3,FAM193B,CALB1,ERMN,ZMYM2,SERPINH1,LMAN1,BTG2,TMOD1,BLVRB,RYR1,ADAM19,SEC24D,REEP2,HYOU1,TKTL1,CD46,BCL6,TTLL7,STARD9,FAM83A,F2R,RIMS3,SEPTIN6,RAB11FIP1,SOCS2,MLXIP,MICAL2,CELSR2,MYO1D,LGALS1,APOE,P4HA1,PTPDC1,GOLGA2,BHLHE40,HRC,DSTYK,ULBP1,FNBP1,ITGA5,DEPP1,MAP7D1,PDIA4,TP53INP1,LLGL2,TRDN,ENO3,PYCR2,MFGE8,CALU,IL15RA,RASGEF1A,RFK,RHOBTB1,PALM,MLXIPL,PTAR1,BST2,B4GALNT4,GRAP2,ABCG2,ALAS1,LPCAT4,KRT8,SESN3,IDH2,APOL6,COL11A1,RAB3B,NFATC4,ID1,CTCFL,RAB27A,RHPN1,IGF1,PYGL,UBE2H,NUDT4,HELZ2,MAPRE3,ITGB1,MAGI1,PITPNM1,NLGN2,CTSC,FGFR3,PPP1R14C,G0S2,NTS,WHRN,MICAL1,USP45,MUC4,GAA,PLEKHA4,CREB3L2,MBOAT2,BRSK1,PLOD2,TM6SF1,SMAD3,AP2M1,EDEM1,POLI,B2M,PSME1,LYPLA1,MYRF,PROS1,PRKAB2,SVIP,HDAC6,PGGHG,ARHGEF12,SDC3,ULBP2,ATF6,TMEM70,GDI1,PCK2,NUDT12,TMEM63A,RBL2,GPC2,ROBO1,CDKN1A,ACE,ATP8B3,UBAC1,TMEM241,TBC1D5,PCLO,EXT2,ZNF397,AFDN,SDF2L1,CNN2,DMTN,CYP26B1,PXK,AMOTL1,CDKN1C,LARGE2,CDC42SE1,TRPM4,BAIAP3,MYLK3,ABCA7,ACAD10,TNNT1,KCNAB2,ACSF2,SLC44A2,PTPRH,SKIL,POMT1,GDPD5,PAN2,PECR,TMEM106C,HMGCL,PGM3,COLGALT2,DOCK6,CRYZL1,NDRG2,PRKACA,EIF2AK1,MYBPHL,PSRC1,ADCY3,MIF4GD,GABBR1,AGRN,HLTF,PTPRS,FASTKD1,WIPI1,SERPINI1,ATP7A,MT2A,NDRG1,TEX19,YPEL5,SHC2,DNAH14,MSI1,STAT5B,DUSP8,NUP107,PROCR,DNASE2,CYP2R1,IFIH1,TGFBR3,FBXL16,ULK1,GLI1,PTEN,TDO2,LRSAM1,CYSTM1,ITM2C,MTURN,RAB18,PDLIM7,TMPRSS4,KHNYN,CUL7,GSN,PKHD1,NFE2,ALAS2,PPP1R18,BTK,CTIF,COL18A1,SMIM14,GABARAPL1,XK,ALS2CL,TIA1,SH3PXD2B,TTC37,SELENBP1,ANKRD9,RGS16,KLF9,RNF217,TDRKH,LGALS9,CAMSAP3,MINK1,RNF103,SH3GLB2,NFE2L1,ETFDH,COTL1,SLC31A1,NDUFA10,SLC22A5,HACE1,ALDOC,QSOX1,CLYBL,SPPL2B,PITPNC1,P4HA2,SLC25A42,DDHD2,CALR,BTBD2,RHBDD1,AMFR,ITM2A,TSPO,AURKB,FLT1,AMPD2,TMED9,MAGED2,PTPRJ,SELENOP,PDIA6,PRSS16,PLEKHA8,SYBU,ARL6IP5,NEK9,MACC1,PAPSS1,YIF1B,NES,AGPAT4,INPPL1,BCL3,TRIM38,HID1,OBSCN,POFUT2,SIDT2,ARHGAP8,CHST2,KYNU,MARCHF2,LSM4,MNX1,ITGB5,CORO7,DLG4,STK10,PCDH15,ESRRB,UCP2,DHTKD1,CCDC113,SLC37A4,C4ORF46,RCN3,LDAF1,MLLT11,PDLIM4,ACBD4,IFITM1,TMOD2,C*

			YP26A1,SLC25A29,P2RX6,WNK4,COPB1,CYLD,SYT5,TCIRG1,HLA-E,SIL1,GUK1,RBM22,AHCYL1,ANKIB1,MROH1,TUBB2B,HIP1,KIF1B,SPARC,LONRF2,FGFR1OP2,CC2D1A,PLA2G6,SLC17A7,THBS1,CCND2,IL32,TMEM9,CTSB,NFKB2,SH3TC2,CALCOCO1,SMAP2,DACT3,TRIM3,KLF7,PDE4DIP,DMXL2,IFT140,SEC22B,WDR26,PDK4,COPG1,LMOD1,PIDD1,ZNF175,NDUFB4,TKFC,PPIB,SERPINB1,LRG1,CASTOR2,GPC5,MANF,SPTAN1,XYLT2,TPD52L1,DENND3,EPHX1,FNDC4,NIT1,MAN2A1,RTN3,PAIP2B,ENO2,NCBP2,HEXD,CASP10,TUBB4A,RNF187,HAX1,PDIA5,SOCS1,MBNL2,DBNDD1,HIVEP3,TMEM30A,ITPKA,SELENON,ACSS2,CCDC92,ITGB3,SNX16,MVB12A,NUCB1,NCOA4,MYO15B,PCSK4,PTTG1,NIPSNAP2,MAP3K8,DGKD,ACER3,SORT1,SAT2,ACADVL,LITAF,AZU1,PTPRC,DAB2,DENND4B,IDH1,GGA1,NBEAL1,DIP2A,EHBP1,NT5C2,PSAP,SEMA6C,POLG2,ASAP2,PHKA1,CERCAM,CCND3,PIK3CD,OAS3,EXOC2,GALNT5,RPS6KC1,GSDMB,DYNC2LI1,TSC22D3,REEP4,TSPYL2,SMYD3,AP3M2,OCEL1,HIP1R,ITPR2,ARFGAP1,ASMTL,ARRB1,TMEM50B,MAN1B1,FECH,TRIQK,RAVER2,ENGASE,FBXO44,OSTF1,COG6,MMP14,OGT,MAP1A,MAN2B1,TRIB3,CLIP2,CHID1,GAS6,ZDHHC8,MYO5B,PPFIA4,NTRK1,VCAN,CHPF,DENND11,LPAR2,LGALS3,TAB2,WARS1,PHKB,CRYL1,LMNA,CCDC50,IARS1,BSDC1,GDE1,STX10,CEMIP2,CD55,YIPF5,CAPN5,ITM2B,ABCB9,GTF2E2,FDXR,LDLRAP1,NBR1,RGL3,CENPH,IQGAP3,MRAP2,PCYOX1L,TIAL1,ARVCF,PDIA3,KIF5A,PAFAH2,SCPEP1,PIGS,SPATS2,RBCK1,HAGH,DNM1,EGLN3,DLK1,PDGFB,MAST2,NIBAN2,HDGFL3,KPNA5,GSTO1,STK16,GALK2,CPEB4,FAM234B,BBS4,ELP1,STX2,LAMB2,SFXN5,ERP44,DCLRE1B,GPSM3,PPP2R5B,AKNA,ERI1,VPS28,DIPK1A,OGA,CELF2,SLC25A36,ISCU,SESN2,ATP1A2,C6ORF89,TMED7,BMF,YIPF2,SIAE,SMTN,SMURF2,TNNI3,TUT7,CLCA1,CPNE3,GAS2,ZFYVE28,HEMK1,FLT4,PANK4,MCF2,KLF12,RBPMS,WIPF3,MTCL1,C3,ANO5,PHGDH,DCAF8,TMEM143,GARNL3,ZCRB1,RAB26,ACSL1,PFKFB4,TSPAN33,HOOK1,TMED4,ODF2L,DZIP3,CCM2,RABAC1,MAN1A1,HSPA4L,YARS1,PLCD3,NAT8L,UNC119,ZMIZ1,RPH3AL,SNPH,ANGEL1,MAST1,KCTD20,COL1A2,STARD10,INPP5J,TUBE1,ARHGEF40,BNIP3,KHK,PIGK,HLA-B,ETAA1,ACSL6,ZNF396,PHYKPL,GNB5,WASL,STOM,GOLM2,PEX2,SNTA1,CTBS,SFMBT2,FGD1,FGFR4,MAPK8IP3,SHFL,STARD5,GIPC1,MLF1,RTKN2,GTF2A1,GPAT3,PKD1,C1GALT1,GAL3ST4,A4GALT,CUX1,MAPT,SNX2,TBC1D20,CITED4,GEMIN2,MC4R,FMNL1,SMAD6,MORN4,APAF1,GRN,EHD2,CCDC88B,APBB3,RAB24,DUSP5,NOTUM,SLC30A2,TMEM41B,TENM1,EPS8,RNF11,WDR91,ACAT1,WDR11,LGMN,DNAJB11,EHHADH,ERO1B,ST3GAL5,RNF145,CBLB,BBC3,SCFD1,PDK1,PRRC1,MGAT5,PDE7A,DLG3,MT-ND4L,DTNA,TPD52,RHBDD2,SEC24A,C2CD2L,PRCP,GTPBP1,TENT5A,RTN4R,NIPA1,RFLNB,TUBB1,RAB31,STK4,TRAK2,PVR,UTRN,STON1,COL15A1,RPL27,IMPA2,FADS3,CD93,B4GALT4,TTLL3,GALNT10,RTN2,SERGEF,BPNT1,TNFRSF1B,SYDE2,UFL1,RB1CC1,ACAD11,TTC7A,RAPGEF2,FUCA1,INPP4A,ANO8,TNK2,GPSM1,ACSBG2,DCP2,PPP1R16B,EPS15,MYORG,ATP1B2,LHFPL2,RPS6KA1,MAP4K2,VPS16,DCTN4,GPD1L,THBD,VBP1,CCSAP,ENTR1,SERTAD2,TTBK2,USP18,ANAPC16,IRAK2,TXNRD3,HEPH,RSPH3,ADAM15*
GO:0012505	endomembrane system	1.749245117720525e-33	*HSPA5,FN1,LAMA3,ARRDC4,KSR1,ADAMTSL4,FCGR2A,GDF15,ANXA1,HSP90B1,PLD3,ST6GAL1,NFASC,AIG1,REEP6,LAMB1,CLSTN3,LAPTM4B,SCG3,LRP1,TGFB1,ANXA6,RAB6B,ASS1,TGM2,HERPUD1,CD36,CRAT,PTGS1,TMCC2,DYSF,APOL1,CHAC1,MCFD2,VEGFA,FUT1,GALNT12,OPTN,ITGAV,CTSZ,GTPBP2,ATP2A3,SERPINH1,LMAN1,RYR1,ADAM19,SEC24D,REEP2,HYOU1,CD46,BCL6,F2R,SEPTIN6*

			,*RAB11FIP1,MYO1D,LGALS1,APOE,P4HA1,GOLGA2,HRC,ULBP1,FNBP1,ITGA5,PDIA4,TRDN,MFGE8,CALU,IL15RA,RHOBTB1,BST2,B4GALNT4,GRAP2,LPCAT4,COL11A1,RAB3B,ID1,RAB27A,IGF1,PYGL,ITGB1,PITPNM1,CTSC,FGFR3,NTS,MUC4,GAA,CREB3L2,MBOAT2,BRSK1,PLOD2,SMAD3,AP2M1,EDEM1,B2M,LYPLA1,MYRF,PROS1,SVIP,HDAC6,SDC3,ULBP2,ATF6,GDI1,TMEM63A,GPC2,ACE,ATP8B3,UBAC1,TMEM241,TBC1D5,EXT2,SDF2L1,CNN2,DMTN,CYP26B1,LARGE2,TRPM4,BAIAP3,ABCA7,KCNAB2,SLC44A2,SKIL,POMT1,GDPD5,TMEM106C,COLGALT2,NDRG2,PRKACA,ADCY3,MIF4GD,GABBR1,AGRN,PTPRS,WIPI1,SERPINI1,ATP7A,NDRG1,YPEL5,NUP107,CYP2R1,ULK1,CYSTM1,ITM2C,RAB18,TMPRSS4,CUL7,GSN,PKHD1,COL18A1,SMIM14,GABARAPL1,XK,MINK1,RNF103,NFE2L1,COTL1,SLC31A1,HACE1,ALDOC,QSOX1,SPPL2B,P4HA2,DDHD2,CALR,RHBDD1,AMFR,ITM2A,TSPO,FLT1,TMED9,MAGED2,PTPRJ,SELENOP,PDIA6,PRSS16,PLEKHA8,SYBU,ARL6IP5,YIF1B,AGPAT4,INPPL1,HID1,POFUT2,CHST2,MARCHF2,CORO7,DLG4,STK10,SLC37A4,RCN3,LDAF1,PDLIM4,CYP26A1,COPB1,SYT5,TCIRG1,HLA-E,SIL1,AHCYL1,HIP1,KIF1B,SPARC,SLC17A7,THBS1,CCND2,TMEM9,CTSB,SH3TC2,TRIM3,PDE4DIP,DMXL2,SEC22B,COPG1,PIDD1,PPIB,SERPINB1,LRG1,GPC5,MANF,SPTAN1,XYLT2,EPHX1,FNDC4,MAN2A1,RTN3,HAX1,PDIA5,TMEM30A,SELENON,ITGB3,SNX16,MVB12A,NUCB1,PCSK4,DGKD,ACER3,SORT1,LITAF,AZU1,PTPRC,DAB2,DENND4B,IDH1,GGA1,EHBP1,PSAP,ASAP2,CERCAM,GALNT5,RPS6KC1,DYNC2LI1,REEP4,AP3M2,HIP1R,ITPR2,ARFGAP1,ARRB1,TMEM50B,MAN1B1,TRIQK,OSTF1,COG6,MMP14,MAN2B1,CHID1,GAS6,ZDHHC8,MYO5B,NTRK1,VCAN,CHPF,LGALS3,TAB2,LMNA,STX10,CD55,YIPF5,ITM2B,ABCB9,LDLRAP1,NBR1,MRAP2,PCYOX1L,PDIA3,PAFAH2,PIGS,PDGFB,STK16,CPEB4,FAM234B,STX2,LAMB2,ERP44,VPS28,DIPK1A,ATP1A2,C6ORF89,TMED7,BMF,YIPF2,CLCA1,CPNE3,ZFYVE28,NEMP2,C3,ANO5,RAB26,ACSL1,TSPAN33,TMED4,RABAC1,MAN1A1,NAT8L,RPH3AL,ANGEL1,COL1A2,BNIP3,PIGK,HLA-B,ACSL6,STOM,GOLM2,FGD1,FGFR4,MAPK8IP3,GIPC1,GPAT3,PKD1,C1GALT1,GAL3ST4,A4GALT,CUX1,SNX2,TBC1D20,SMAD6,APAF1,GRN,EHD2,CCDC88B,RAB24,NOTUM,SLC30A2,TMEM41B,TENM1,RNF11,WDR91,WDR11,LGMN,DNAJB11,ERO1B,ST3GAL5,RNF145,BBC3,SCFD1,PRRC1,MGAT5,TPD52,RHBDD2,SEC24A,C2CD2L,PRCP,RTN4R,SH3BGRL2,NIPA1,RAB31,TRAK2,STON1,COL15A1,RPL27,FADS3,CD93,B4GALT4,GALNT10,RTN2,TNFRSF1B,UFL1,RB1CC1,RAPGEF2,FUCA1,INPP4A,ANO8,TNK2,GPSM1,EPS15,MYORG,LHFPL2,GTF3C3,MAP4K2,VPS16,ENTR1,IRAK2,TXNRD3,ADAM15*
GO:0031982	vesicle	1.445498078836667e-28	*HSPA5,FN1,LAMA3,ARRDC4,FCGR2A,GDF15,AARS1,ANXA1,CA2,HSP90B1,KIF21B,CARD11,PLD3,GFPT1,NFASC,REEP6,LAMB1,LAPTM4B,SCG3,LRP1,TGFB1,EPHX2,ANXA6,ATM,F11R,RAB6B,FLNB,EPB41L2,ASS1,TGM2,CD36,AIF1L,PTGS1,DYSF,MARCKS,MCFD2,VEGFA,GMPPA,OPTN,ITGAV,CTSZ,GTPBP2,CALB1,ERMN,LMAN1,BTG2,BLVRB,RYR1,SEC24D,HYOU1,CD46,F2R,SEPTIN6,RAB11FIP1,MYO1D,LGALS1,APOE,LAMA5,GOLGA2,FNBP1,ITGA5,PDIA4,TP53INP1,ENO3,MFGE8,CALU,IL15RA,RHOBTB1,PALM,BST2,GRAP2,GPR155,KRT8,IDH2,RAB3B,RAB27A,IGF1,PYGL,ITGB1,CTSC,FGFR3,VPS13D,NTS,NECTIN2,MUC4,GAA,BRSK1,PLOD2,AP2M1,B2M,PSME1,LYPLA1,PROS1,SVIP,HDAC6,ARHGEF12,TMEM63A,RBL2,ACE,ATP8B3,UBAC1,TBC1D5,PCLO,EXT2,CNN2,DMTN,AMOTL1,BAIAP3,ABCA7,BCAM,KCNAB2,SLC44A2,SKIL,POMT1,CPD,NDRG2,PRKACA,GABBR1,AGRN,PTPRS,WIPI1,SERPINI1,ATP7A,NDRG1,YPEL5,PROCR,DNASE2,TGFBR3,ULK1,SERINC2,CYSTM1,ITM2C,RAB18,TMPRSS4,GSN,PKHD1,BTK,COL18A1*

			,GABARAPL1,ALS2CL,SELENBP1,ANKRD9,MINK1,COTL1,SLC31A1,SLC22A5,ALDOC,QSOX1,SPPL2B,DDHD2,CALR,TSPO,FLT1,TMED9,MAGED2,PTPRJ,IGSF8,PTPRF,SELENOP,PDIA6,PRSS16,SYBU,YIF1B,HID1,MARCHF2,ITGB5,CORO7,DLG4,STK10,PDLIM4,COPB1,SYT5,TCIRG1,HLA-E,AHCYL1,TTYH3,HIP1,KIF1B,SPARC,CC2D1A,SLC17A7,THBS1,RRAS,TMEM9,CTSB,SH3TC2,TRIM3,DMXL2,SEC22B,COPG1,TKFC,PPIB,SERPINB1,LRG1,SPTAN1,DENND3,MAN2A1,ENO2,HEXD,TUBB4A,HAX1,SOCS1,CFH,TMEM30A,FAM234A,ITGB3,SNX16,MVB12A,NUCB1,PCSK4,DGKD,SORT1,SAT2,LITAF,AZU1,PTPRC,DAB2,DENND4B,IDH1,GGA1,EHBP1,PSAP,SLC4A11,RPS6KC1,AP3M2,OCEL1,HIP1R,ITPR2,ARRB1,MAN1B1,SMPDL3B,OSTF1,MMP14,MAN2B1,CHID1,GAS6,MYO5B,NTRK1,LPAR2,GNG7,LGALS3,TAB2,WARS1,CRYL1,IARS1,GDE1,PLXNB2,STX10,CEMIP2,CD55,YIPF5,CAPN5,ITM2B,LDLRAP1,NBR1,PCYOX1L,PDIA3,SCPEP1,DNM1,PDGFB,NIBAN2,PCOLCE,GSTO1,STK16,STX2,LAMB2,PI15,ERP44,VPS28,ATP1A2,TMED7,BMF,YIPF2,SIAE,CLCA1,CPNE3,ZFYVE28,C3,ANO5,PHGDH,RAB26,TMED4,RABAC1,MAN1A1,RTN4RL2,RPH3AL,COL1A2,KHK,HLA-B,WASL,STOM,FGFR4,MAPK8IP3,GIPC1,PKD1,GAL3ST4,SNX2,FMNL1,APAF1,GRN,EHD2,RAB24,SLC30A2,EPS8,RNF11,WDR91,ACAT1,WDR11,LGMN,SCFD1,MGAT5,SEC24A,PRCP,RTN4R,NIPA1,TUBB1,RAB31,TRAK2,UTRN,STON1,COL15A1,RPL27,CD93,TNFRSF1B,CR2,RAPGEF2,FUCA1,INPP4A,TNK2,EPS15,LHFPL2,VPS16,GPD1L,ENTR1,IRAK2,ADAM15*
GO:0031410	cytoplasmic vesicle	2.243832165765238e-24	*HSPA5,FN1,ARRDC4,FCGR2A,ANXA1,HSP90B1,KIF21B,PLD3,NFASC,REEP6,LAPTM4B,SCG3,LRP1,TGFB1,ANXA6,ATM,RAB6B,FLNB,CD36,DYSF,MCFD2,VEGFA,OPTN,ITGAV,CTSZ,GTPBP2,LMAN1,RYR1,SEC24D,HYOU1,CD46,F2R,SEPTIN6,RAB11FIP1,MYO1D,APOE,GOLGA2,FNBP1,ITGA5,PDIA4,TP53INP1,MFGE8,CALU,IL15RA,RHOBTB1,PALM,BST2,GRAP2,RAB3B,RAB27A,IGF1,PYGL,ITGB1,CTSC,FGFR3,NTS,GAA,BRSK1,AP2M1,B2M,PROS1,SVIP,HDAC6,TMEM63A,ACE,ATP8B3,TBC1D5,CNN2,DMTN,AMOTL1,BAIAP3,ABCA7,KCNAB2,SLC44A2,SKIL,POMT1,PRKACA,GABBR1,PTPRS,WIPI1,SERPINI1,ATP7A,NDRG1,YPEL5,ULK1,CYSTM1,RAB18,TMPRSS4,GSN,BTK,GABARAPL1,ALS2CL,ANKRD9,COTL1,SLC31A1,ALDOC,QSOX1,SPPL2B,DDHD2,CALR,FLT1,TMED9,MAGED2,PTPRJ,SELENOP,PDIA6,PRSS16,SYBU,YIF1B,MARCHF2,ITGB5,CORO7,DLG4,STK10,PDLIM4,COPB1,SYT5,TCIRG1,HLA-E,HIP1,KIF1B,SPARC,SLC17A7,THBS1,TMEM9,CTSB,SH3TC2,TRIM3,DMXL2,SEC22B,COPG1,PPIB,SERPINB1,LRG1,SPTAN1,DENND3,HAX1,SOCS1,TMEM30A,ITGB3,SNX16,MVB12A,PCSK4,DGKD,SORT1,LITAF,AZU1,PTPRC,DAB2,DENND4B,IDH1,GGA1,EHBP1,PSAP,RPS6KC1,AP3M2,OCEL1,HIP1R,ITPR2,ARRB1,MAN1B1,OSTF1,MMP14,MAN2B1,CHID1,GAS6,MYO5B,NTRK1,LPAR2,LGALS3,TAB2,GDE1,CD55,YIPF5,ITM2B,LDLRAP1,NBR1,PCYOX1L,PDIA3,PDGFB,STK16,STX2,ERP44,VPS28,ATP1A2,TMED7,BMF,YIPF2,CLCA1,CPNE3,ZFYVE28,C3,RAB26,TMED4,RABAC1,MAN1A1,RPH3AL,HLA-B,WASL,STOM,FGFR4,MAPK8IP3,GIPC1,PKD1,SNX2,FMNL1,APAF1,GRN,EHD2,RAB24,SLC30A2,RNF11,WDR91,WDR11,LGMN,SCFD1,SEC24A,PRCP,NIPA1,RAB31,TRAK2,STON1,CD93,TNFRSF1B,RAPGEF2,FUCA1,INPP4A,TNK2,EPS15,LHFPL2,VPS16,ENTR1,IRAK2,ADAM15*
GO:0097708	intracellular vesicle	2.518111169437426e-24	*HSPA5,FN1,ARRDC4,FCGR2A,ANXA1,HSP90B1,KIF21B,PLD3,NFASC,REEP6,LAPTM4B,SCG3,LRP1,TGFB1,ANXA6,ATM,RAB6B,FLNB,CD36,DYSF,MCFD2,VEGFA,OPTN,ITGAV,CTSZ,GTPBP2,LMAN1,RYR1,SEC24D,HYOU1,CD46,F2R,SEPTIN6,RAB11FIP1,MYO1D,APOE,GOLGA2,FNBP1,ITGA5,PDIA4,TP53INP1,MFGE8,CALU,IL15RA,RHOBTB1,PA*

			LM,BST2,GRAP2,RAB3B,RAB27A,IGF1,PYGL,ITGB1,CTSC,FGFR3,NTS,GAA,BRSK1,AP2M1,B2M,PROS1,SVIP,HDAC6,TMEM63A,ACE,ATP8B3,TBC1D5,CNN2,DMTN,AMOTL1,BAIAP3,ABCA7,KCNAB2,SLC44A2,SKIL,POMT1,PRKACA,GABBR1,PTPRS,WIPI1,SERPINI1,ATP7A,NDRG1,YPEL5,ULK1,CYSTM1,RAB18,TMPRSS4,GSN,BTK,GABARAPL1,ALS2CL,ANKRD9,COTL1,SLC31A1,ALDOC,QSOX1,SPPL2B,DDHD2,CALR,FLT1,TMED9,MAGED2,PTPRJ,SELENOP,PDIA6,PRSS16,SYBU,YIF1B,MARCHF2,ITGB5,CORO7,DLG4,STK10,PDLIM4,COPB1,SYT5,TCIRG1,HLA-E,HIP1,KIF1B,SPARC,SLC17A7,THBS1,TMEM9,CTSB,SH3TC2,TRIM3,DMXL2,SEC22B,COPG1,PPIB,SERPINB1,LRG1,SPTAN1,DENND3,HAX1,SOCS1,TMEM30A,ITGB3,SNX16,MVB12A,PCSK4,DGKD,SORT1,LITAF,AZU1,PTPRC,DAB2,DENND4B,IDH1,GGA1,EHBP1,PSAP,RPS6KC1,AP3M2,OCEL1,HIP1R,ITPR2,ARRB1,MAN1B1,OSTF1,MMP14,MAN2B1,CHID1,GAS6,MYO5B,NTRK1,LPAR2,LGALS3,TAB2,GDE1,CD55,YIPF5,ITM2B,LDLRAP1,NBR1,PCYOX1L,PDIA3,PDGFB,STK16,STX2,ERP44,VPS28,ATP1A2,TMED7,BMF,YIPF2,CLCA1,CPNE3,ZFYVE28,C3,RAB26,TMED4,RABAC1,MAN1A1,RPH3AL,HLA-B,WASL,STOM,FGFR4,MAPK8IP3,GIPC1,PKD1,SNX2,FMNL1,APAF1,GRN,EHD2,RAB24,SLC30A2,RNF11,WDR91,WDR11,LGMN,SCFD1,SEC24A,PRCP,NIPA1,RAB31,TRAK2,STON1,CD93,TNFRSF1B,RAPGEF2,FUCA1,INPP4A,TNK2,EPS15,LHFPL2,VPS16,ENTR1,IRAK2,ADAM15
GO:0016020	membrane	4.354142577657899e-24	*HSPA5,FN1,LAMA3,ARRDC4,CISH,KSR1,FCGR2A,PTK2B,PIM1,ELAPOR2,AARS1,FLNC,ANXA1,RNF213,CA2,HSP90B1,TSPAN13,CARD11,ANKRD29,CAPRIN2,PLD3,ST6GAL1,NFASC,AIG1,UNC5B,REEP6,LAMB1,CLSTN3,LAPTM4B,SCG3,IL10RA,TMEM273,LRP1,EEF1A2,RGS10,ANXA6,SLC6A6,PLEKHH2,F11R,NIPSNAP1,RAB6B,FLNB,EPB41L2,ASS1,EML2,TGM2,HERPUD1,CD36,AIF1L,CRAT,PTGS1,TMCC2,DYSF,AKAP11,MARCKS,ANKZF1,MCFD2,VEGFA,FUT1,EFHD1,ERBB3,GALNT12,OPTN,PTK7,ITGAV,MRC2,TNFRSF9,CTSZ,ADD3,ATP2A3,SERPINH1,LMAN1,KIAA0040,TMOD1,SEMA6B,IL2RA,BLVRB,RYR1,ADAM19,SEC24D,REEP2,HYOU1,CD46,F2R,RIMS3,SEPTIN6,RAB11FIP1,SOCS2,MLXIP,CMAS,CELSR2,MYO1D,APOE,P4HA1,GOLGA2,SLC29A4,HRC,DSTYK,ULBP1,FNBP1,ITGA5,LLGL2,TRDN,ENO3,MFGE8,CALU,IL15RA,CD24,RASGEF1A,RHOBTB1,TSPAN18,PALM,MMP15,BST2,B4GALNT4,GRAP2,ABCG2,ALAS1,LPCAT4,GPR155,KRT8,APOL6,RAB3B,GIPR,TMEM263,RAB27A,IGF1,HELZ2,ICAM5,ITGB1,MAGI1,PITPNM1,NLGN2,CTSC,FGFR3,SMIM3,VPS13D,PPP1R14C,WHRN,MICAL1,NECTIN2,MUC4,GAA,PLEKHA4,CREB3L2,MBOAT2,PLOD2,TM6SF1,SMAD3,AP2M1,EDEM1,B2M,LYPLA1,MYRF,PROS1,SVIP,HDAC6,ARHGEF12,SDC3,ULBP2,SEMA7A,ATF6,TMEM70,TMEM63A,GPC2,ROBO1,ACE,ATP8B3,UBAC1,TMEM241,TBC1D5,ACVR1C,PCLO,EXT2,ZNF397,AFDN,SDF2L1,MFSD6,CNN2,DMTN,CYP26B1,PXK,AMOTL1,LARGE2,CDC42SE1,TRPM4,BAIAP3,ABCA7,BCAM,KCNAB2,PTPRU,SLC44A2,PTPRH,POMT1,GDPD5,PECR,BTN3A1,TMEM106C,CPD,PLXND1,NDRG2,PRKACA,ADCY3,GABBR1,AGRN,HLTF,PTPRS,WIPI1,ATP7A,NDRG1,SEL1L3,SESTD1,SHC2,NUP107,PROCR,CYP2R1,TGFBR3,ULK1,SERINC2,PTEN,LRSAM1,CYSTM1,NPTXR,ITM2C,RAB18,TMPRSS4,GSN,PKHD1,ALAS2,BTK,ADGRL1,CDCP1,SMIM14,GABARAPL1,XK,LRP4,SELENBP1,RGS16,KLF9,IFNGR1,RNF217,CMTM7,TDRKH,RNF103,SH3GLB2,NFE2L1,ETFDH,COTL1,SLC31A1,NDUFA10,SLC22A5,HACE1,QSOX1,CLYBL,SPPL2B,SLC25A42,DDHD2,CALR,RHBDD1,AMFR,ITM2A,TSPO,FLT1,TMED9,GPR180,MAGED2,PTPRJ,IGSF8,PTPRF,SELENOP,PDIA6,PRSS16,PLEKHA8,MORF4L2,SYBU,ARL6IP5,YIF1B,METTL25B,TJP3,AGPAT4,INPPL1,BCL3,HID1,OBSCN,POFUT2,SIDT2,CHST2,MARCHF2,LSM4,ITGB5,CORO7,DLG4,*

			STK10,PCDH15,UCP2,SLC37A4,SLC22A23,SLC35F3,LDAF1,PDLIM4,ACBD4,IFITM1,CYP26A1,SLC25A29,P2RX6,WNK4,COPB1,CYLD,SYT5,TCIRG1,HLA-E,AHCYL1,RHAG,TTYH3,ADGRA2,SUSD6,HIP1,KIF1B,SPARC,CC2D1A,PLA2G6,SLC17A7,THBS1,CCND2,IL32,RRAS,TMEM9,CTSB,SH3TC2,DMXL2,SEC22B,COPG1,SUSD1,LMOD1,NDUFB4,PPIB,TCAF1,SERPINB1,LRG1,CLDN12,FAM171A2,PAAF1,GPC5,SPTAN1,XYLT2,EPHX1,FNDC4,MAN2A1,RTN3,ENO2,CASP10,SLC22A31,HAX1,PDIA5,SOCS1,TMEM30A,SELENON,FAM234A,ACVR1,ITGB3,SMIM1,CA11,SNX16,MVB12A,NUCB1,PCSK4,AGER,NIPSNAP2,DGKD,ACER3,SORT1,ACADVL,LITAF,AZU1,PTPRC,DAB2,TMCO6,GGA1,NBEAL1,IL6R,TMEM243,DIP2A,EHBP1,PSAP,SEMA6C,SLC4A11,ASAP2,PHKA1,CERCAM,PIK3CD,OAS3,EXOC2,GALNT5,RPS6KC1,GSDMB,REEP4,AP3M2,OCEL1,HIP1R,ITPR2,ARFGAP1,ARRB1,TNFRSF10B,TMEM50B,MAN1B1,FECH,TRIQK,SMPDL3B,COG6,LRRC63,CD200R1,HJV,LTK,MMP14,OGT,TRIB3,PLP1,CHID1,ZDHHC8,MYO5B,ADGRL3,NTRK1,VCAN,CHPF,RXFP1,GPR158,LPAR2,GNG7,LGALS3,TAB2,TMEM116,PHKB,LMNA,IARS1,GDE1,PLXNB2,IL13RA1,FNDC3B,STX10,CEMIP2,VSTM4,CD55,YIPF5,RELL2,ITM2B,ABCB9,FDXR,LDLRAP1,NBR1,RGL3,IQGAP3,MRAP2,PCYOX1L,ARVCF,PDIA3,KIF5A,PAFAH2,GGT7,PIGS,AMIGO2,KCNJ11,DNM1,DLK1,ADGRE2,PDGFB,MAST2,NIBAN2,STK16,FAM234B,BBS4,STX2,SFXN5,ERP44,FRRS1,GPSM3,AKNA,VPS28,DIPK1A,OGA,SLC25A36,SLC6A8,ATP1A2,C6ORF89,TMED7,BMF,YIPF2,SMURF2,CLCA1,CPNE3,GAS2,ZFYVE28,FLT4,NEMP2,MCF2,BTN3A2,MTCL1,C3,ANO5,TMEM143,RAB26,SLC4A7,PLPPR2,ACSL1,TSPAN33,HOOK1,TMED4,RABAC1,MAN1A1,RTN4RL2,SCN1B,PLCD3,NAT8L,RPH3AL,SNPH,MAST1,GUCA1B,KCTD20,ROBO3,STARD10,INPP5J,ARHGEF40,BNIP3,PIGK,HLA-B,ACSL6,GNB5,WASL,STOM,GOLM2,TCAF2,PEX2,SNTA1,FGFR4,MAPK8IP3,SEMA4G,GIPC1,RTKN2,ZP3,GPAT3,PKD1,C1GALT1,GAL3ST4,A4GALT,CUX1,MAPT,SNX2,TBC1D20,MC4R,FMNL1,SLC45A3,GRN,EHD2,WBP1L,CCDC88B,APBB3,RAB24,SLC30A2,TMEM41B,MDGA2,TENM1,EPS8,WDR91,WDR11,DNAJB11,ERO1B,ST3GAL5,RNF145,CBLB,BBC3,TP53I11,CACNA2D2,SCFD1,MGAT5,DLG3,MT-ND4L,C3ORF18,DTNA,RHBDD2,SEC24A,C2CD2L,PRCP,GTPBP1,RTN4R,SH3BGRL2,NIPA1,TMEM54,RAB31,TRAK2,PVR,UTRN,STON1,TLCD2,RPL27,FADS3,CD93,B4GALT4,GALNT10,RTN2,SLC16A5,TNFRSF1B,CR2,SYDE2,UFL1,RB1CC1,ACAD11,TTC7A,RAPGEF2,INPP4A,ANO8,TNK2,GPSM1,ACSBG2,PPP1R16B,EPS15,MYORG,ATP1B2,LHFPL2,GTF3C3,MAP4K2,VPS16,GPD1L,THBD,ENTR1,IRAK2,HEPH,ADAM15
GO:0031090	organelle membrane	1.040554824358374e-18	*HSPA5,KSR1,FCGR2A,ANXA1,HSP90B1,PLD3,ST6GAL1,NFASC,REEP6,CLSTN3,LAPTM4B,SCG3,LRP1,EEF1A2,ANXA6,RAB6B,ASS1,HERPUD1,CD36,CRAT,PTGS1,TMCC2,DYSF,MCFD2,FUT1,EFHD1,GALNT12,OPTN,ITGAV,CTSZ,ATP2A3,LMAN1,RYR1,SEC24D,REEP2,CD46,RAB11FIP1,MLXIP,APOE,GOLGA2,HRC,TRDN,MFGE8,CALU,IL15RA,RHOBTB1,BST2,B4GALNT4,ABCG2,ALAS1,LPCAT4,RAB27A,ITGB1,PITPNM1,CTSC,GAA,CREB3L2,MBOAT2,PLOD2,TM6SF1,SMAD3,AP2M1,EDEM1,B2M,LYPLA1,MYRF,PROS1,SVIP,ATF6,TMEM70,TMEM63A,ROBO1,ATP8B3,TBC1D5,EXT2,DMTN,CYP26B1,LARGE2,BAIAP3,ABCA7,KCNAB2,SLC44A2,POMT1,PECR,TMEM106C,ADCY3,GABBR1,PTPRS,WIPI1,ATP7A,NDRG1,NUP107,CYP2R1,ULK1,CYSTM1,ITM2C,RAB18,ALAS2,SMIM14,GABARAPL1,XK,RNF103,NFE2L1,ETFDH,NDUFA10,HACE1,QSOX1,SPPL2B,SLC25A42,CALR,RHBDD1,AMFR,TSPO,TMED9,PTPRJ,PDIA6,PLEKHA8,SYBU,ARL6IP5,YIF1B,AGPAT4,HID1,POFUT2,SIDT2,CHST2,MARCHF2,CORO7,DLG4,STK10,UCP2,SLC37A4,LDAF1,PDLIM4,IFITM1,CYP26A1,SLC25A29,COPB1,SYT5,TCI*

			RG1,HLA-E,AHCYL1,HIP1,KIF1B,SPARC,SLC17A7,CCND2,TMEM9,DMXL2,SEC22B,COPG1,NDUFB4,XYLT2,EPHX1,MAN2A1,RTN3,HAX1,PDIA5,TMEM30A,SELENON,ITGB3,SNX16,MVB12A,PCSK4,NIPSNAP2,ACER3,SORT1,ACADVL,LITAF,AZU1,PTPRC,DAB2,GGA1,PSAP,SLC4A11,ASAP2,GALNT5,REEP4,AP3M2,HIP1R,ITPR2,ARFGAP1,ARRB1,TMEM50B,MAN1B1,FECH,TRIQK,COG6,OGT,ZDHHC8,MYO5B,NTRK1,CHPF,LGALS3,TAB2,LMNA,GDE1,STX10,CD55,YIPF5,ITM2B,ABCB9,FDXR,LDLRAP1,NBR1,MRAP2,PDIA3,PAFAH2,PIGS,PDGFB,BBS4,SFXN5,ERP44,VPS28,DIPK1A,SLC25A36,ATP1A2,C6ORF89,TMED7,BMF,YIPF2,CLCA1,CPNE3,ZFYVE28,NEMP2,ANO5,RAB26,ACSL1,TMED4,MAN1A1,NAT8L,RPH3AL,GUCA1B,BNIP3,PIGK,HLA-B,ACSL6,WASL,STOM,PEX2,MAPK8IP3,GIPC1,GPAT3,PKD1,C1GALT1,GAL3ST4,A4GALT,CUX1,SNX2,TBC1D20,GRN,EHD2,RAB24,SLC30A2,TMEM41B,WDR91,WDR11,ERO1B,ST3GAL5,RNF145,BBC3,SCFD1,MGAT5,MT-ND4L,RHBDD2,SEC24A,C2CD2L,PRCP,SH3BGRL2,RAB31,RPL27,FADS3,CD93,B4GALT4,GALNT10,RTN2,TNFRSF1B,UFL1,RB1CC1,ACAD11,INPP4A,TNK2,GPSM1,EPS15,MYORG,LHFPL2,GTF3C3,MAP4K2,VPS16,THBD,IRAK2
GO:0098588	bounding membrane of organelle	1.095806023926822e-17	*FCGR2A,ANXA1,PLD3,ST6GAL1,NFASC,REEP6,CLSTN3,LAPTM4B,SCG3,LRP1,EEF1A2,ANXA6,RAB6B,ASS1,CD36,MCFD2,FUT1,GALNT12,OPTN,ITGAV,CTSZ,ATP2A3,LMAN1,RYR1,SEC24D,CD46,RAB11FIP1,MLXIP,APOE,GOLGA2,HRC,TRDN,MFGE8,IL15RA,RHOBTB1,BST2,RAB27A,ITGB1,CTSC,GAA,PLOD2,TM6SF1,AP2M1,B2M,PROS1,SVIP,ATF6,TMEM63A,ROBO1,ATP8B3,TBC1D5,EXT2,DMTN,LARGE2,BAIAP3,ABCA7,KCNAB2,SLC44A2,PECR,ADCY3,PTPRS,WIPI1,ATP7A,NDRG1,ULK1,CYSTM1,ITM2C,RAB18,GABARAPL1,HACE1,QSOX1,SPPL2B,CALR,TSPO,TMED9,PTPRJ,PLEKHA8,SYBU,YIF1B,AGPAT4,HID1,SIDT2,CHST2,MARCHF2,CORO7,DLG4,STK10,PDLIM4,IFITM1,COPB1,SYT5,TCIRG1,HLA-E,HIP1,SPARC,SLC17A7,TMEM9,DMXL2,SEC22B,COPG1,XYLT2,MAN2A1,RTN3,HAX1,TMEM30A,ITGB3,SNX16,MVB12A,PCSK4,NIPSNAP2,ACER3,SORT1,LITAF,AZU1,PTPRC,DAB2,GGA1,PSAP,GALNT5,HIP1R,ITPR2,ARFGAP1,ARRB1,TMEM50B,COG6,ZDHHC8,NTRK1,CHPF,LGALS3,TAB2,STX10,CD55,ITM2B,ABCB9,LDLRAP1,NBR1,PDIA3,PDGFB,BBS4,VPS28,C6ORF89,TMED7,BMF,YIPF2,CLCA1,CPNE3,ZFYVE28,RAB26,ACSL1,MAN1A1,RPH3AL,BNIP3,HLA-B,ACSL6,WASL,STOM,PEX2,MAPK8IP3,PKD1,C1GALT1,A4GALT,CUX1,SNX2,TBC1D20,GRN,EHD2,RAB24,SLC30A2,WDR91,WDR11,ST3GAL5,BBC3,SCFD1,MGAT5,RHBDD2,SEC24A,PRCP,RAB31,RPL27,CD93,B4GALT4,GALNT10,RTN2,TNFRSF1B,RB1CC1,INPP4A,GPSM1,EPS15,LHFPL2,MAP4K2,VPS16,THBD,IRAK2*
GO:1903561	extracellular vesicle	5.612932659294167e-16	*HSPA5,FN1,LAMA3,ARRDC4,GDF15,AARS1,ANXA1,CA2,HSP90B1,CARD11,PLD3,GFPT1,LAMB1,EPHX2,ANXA6,F11R,FLNB,EPB41L2,ASS1,TGM2,AIF1L,PTGS1,DYSF,MARCKS,GMPPA,ITGAV,CTSZ,CALB1,ERMN,LMAN1,BTG2,BLVRB,RYR1,HYOU1,CD46,MYO1D,LGALS1,APOE,LAMA5,ENO3,MFGE8,BST2,GPR155,KRT8,IDH2,RAB3B,RAB27A,PYGL,ITGB1,CTSC,VPS13D,NECTIN2,MUC4,GAA,PLOD2,AP2M1,B2M,PSME1,LYPLA1,PROS1,SVIP,ARHGEF12,TMEM63A,RBL2,ACE,UBAC1,PCLO,EXT2,BCAM,SLC44A2,CPD,NDRG2,PRKACA,AGRN,PTPRS,SERPINI1,NDRG1,PROCR,DNASE2,TGFBR3,SERINC2,CYSTM1,ITM2C,GSN,PKHD1,COL18A1,SELENBP1,MINK1,COTL1,SLC22A5,ALDOC,QSOX1,CALR,TSPO,TMED9,PTPRJ,IGSF8,PTPRF,SELENOP,PDIA6,HID1,ITGB5,STK10,HLA-E,AHCYL1,TTYH3,CC2D1A,THBS1,RRAS,CTSB,TKFC,PPIB,SERPINB1,LRG1,SPTAN1,MAN2A1,ENO2,HEXD,TUBB4A*

			,CFH,FAM234A,ITGB3,MVB12A,NUCB1,SAT2,AZU1,PTPRC,IDH1,PSAP,MAN1B1,SMPDL3B,MAN2B1,CHID1,GAS6,MYO5B,GNG7,LGALS3,WARS1,CRYL1,IARS1,PLXNB2,CEMIP2,CD55,CAPN5,ITM2B,PDIA3,SCPEP1,DNM1,NIBAN2,PCOLCE,GSTO1,LAMB2,PI15,ERP44,VPS28,ATP1A2,SIAE,CPNE3,C3,PHGDH,MAN1A1,RTN4RL2,COL1A2,KHK,HLA-B,WASL,STOM,GIPC1,PKD1,GAL3ST4,FMNL1,APAF1,GRN,EHD2,EPS8,RNF11,ACAT1,LGMN,MGAT5,PRCP,RTN4R,TUBB1,UTRN,COL15A1,RPL27,CR2,FUCA1,GPD1L,ADAM15
GO:0065010	extracellular membrane-bounded organelle	5.901196968151801e-16	HSPA5,FN1,LAMA3,ARRDC4,GDF15,AARS1,ANXA1,CA2,HSP90B1,CARD11,PLD3,GFPT1,LAMB1,EPHX2,ANXA6,F11R,FLNB,EPB41L2,ASS1,TGM2,AIF1L,PTGS1,DYSF,MARCKS,GMPPA,ITGAV,CTSZ,CALB1,ERMN,LMAN1,BTG2,BLVRB,RYR1,HYOU1,CD46,MYO1D,LGALS1,APOE,LAMA5,ENO3,MFGE8,BST2,GPR155,KRT8,IDH2,RAB3B,RAB27A,PYGL,ITGB1,CTSC,VPS13D,NECTIN2,MUC4,GAA,PLOD2,AP2M1,B2M,PSME1,LYPLA1,PROS1,SVIP,ARHGEF12,TMEM63A,RBL2,ACE,UBAC1,PCLO,EXT2,BCAM,SLC44A2,CPD,NDRG2,PRKACA,AGRN,PTPRS,SERPINI1,NDRG1,PROCR,DNASE2,TGFBR3,SERINC2,CYSTM1,ITM2C,GSN,PKHD1,COL18A1,SELENBP1,MINK1,COTL1,SLC22A5,ALDOC,QSOX1,CALR,TSPO,TMED9,PTPRJ,IGSF8,PTPRF,SELENOP,PDIA6,HID1,ITGB5,STK10,HLA-E,AHCYL1,TTYH3,CC2D1A,THBS1,RRAS,CTSB,TKFC,PPIB,SERPINB1,LRG1,SPTAN1,MAN2A1,ENO2,HEXD,TUBB4A,CFH,FAM234A,ITGB3,MVB12A,NUCB1,SAT2,AZU1,PTPRC,IDH1,PSAP,MAN1B1,SMPDL3B,MAN2B1,CHID1,GAS6,MYO5B,GNG7,LGALS3,WARS1,CRYL1,IARS1,PLXNB2,CEMIP2,CD55,CAPN5,ITM2B,PDIA3,SCPEP1,DNM1,NIBAN2,PCOLCE,GSTO1,LAMB2,PI15,ERP44,VPS28,ATP1A2,SIAE,CPNE3,C3,PHGDH,MAN1A1,RTN4RL2,COL1A2,KHK,HLA-B,WASL,STOM,GIPC1,PKD1,GAL3ST4,FMNL1,APAF1,GRN,EHD2,EPS8,RNF11,ACAT1,LGMN,MGAT5,PRCP,RTN4R,TUBB1,UTRN,COL15A1,RPL27,CR2,FUCA1,GPD1L,ADAM15

Table S14. Venn diagram showing the intersections of upregulated genes with lists of genes that reveal an increase or decrease in rDNA contacts. Related to Figure 3B.

Names	total	elements
4C-increased-1309 upregulated-1285	74	*THRAP3 FOXK2 TAFA2 USP14 LIMD1 DIDO1 WDR12 PHACTR1 OPA3 UIMC1 GUSBP1 TLK1 CREBBP FAM193A MIR17HG SUPT16H NIPA2 LRRFIP1 CTDP1 HECTD1 BIRC6 PHACTR2 DNAJC21 INTS13 DDX10 CLSPN PPP6R3 GUCD1 PPIP5K2 EBNA1BP2 SNX8 NSMAF GSE1 ABLIM1 UCK2 RPTOR STT3A ECHDC1 ACACA MAN2A2 CDK12 AFG3L2 CCDC138 MTOR LARP1 PSMB2 ANKRD11 SPEN NAP1L4 KANSL1 CRIM1 MTREX GID8 LINC00861 CUL1 NSUN2 LINC01128 GRB10 MLLT1 SDCBP MBNL1 ABI1 MED1 HNRNPM MACROH2A1 JPT2 MSH2 BAZ1A EWSR1 ELL2 PRAME NUP43 EOGT ANP32B*
4C-decreased-1200 upregulated-1285	78	*IGF2BP3 MRPL45 HERC2 ARMC6 ASH1L GOT2 BRD4 CHAF1A CHAMP1 NSD1 AGO2 KIAA0753 ANKRD17 IBA57 SREBF2 RESF1 MAPK1IP1L ZC3H14 ZNF33B BRCA2 SSBP3 LCLAT1 MS4A4A SMARCA4 CWC22 ZNF121 DHX29 TM9SF3 CFAP97 ANKRD33B UBAP2 APC GEMIN5 KTN1 HMGB1 MEF2C SETD2 MRPS35 UTP4 SMARCC1 NUP214 ECPAS SFPQ URB1 STAG2 PAFAH1B1 CSDE1 ZBTB2 EFTUD2 NRIP1 STON2 DNAJC7 ZC3HAV1 CPSF3 SNRPD1 SUMO3 BZW1 PCNA AQR RNF138 ZCCHC14 ADSS2 LINC00923 MGA NIN PPIL2 SPTB UBAP2L ZFYVE26 BACH1 OXNAD1 ZNF431 TBCD KCNK5 SERBP1 RANBP2 PEPD PCNT*
4C-increased-1309	1233	*FSTL1 CD44 PLCE1 SLMAP FAM219A FARP1 KCNMA1 PKNOX2 ARHGAP5-AS1 APBB2 ZNF684 KLHL13 GLT1D1 ABCB7 COX7A2L FYB2 RNF38 AHDC1 LINC02073 CUBN SLC10A7 LDB3 PAK1 SCAF8 SLC6A11 B9D1 CPXM2 RTN1 KIRREL1 LINC01467 PEBP4 PPP1R13B FGR TOP3A EPN2 LINC01491 LINC02664 INIP NSMCE2 TTC37 ZNF208 RCL1 PRSS51 SLC25A52 OAZ2 TEAD1 ZNF718 DKK2 GLRA4 OR7A17 DHRS11 PCAT19 CCDC34 TAOK3 DNAJC27-AS1 PUM3 ZNF66 LINC02563 SCYGR8 LRGUK TENM4 TRPM6 FAAHP1 LINC01479 CCDC106 SLC8A1 SMIM35 ANLN ARHGEF26-AS1 SLC1A7 LGI2 ANKRD6 KCNC1 ADGRE3 GPR55 DNAJC15 STAU2 PGAP4 SIAH3 RIPK4 TMEM63C ZNF257 TIAL1*

		ST8SIA4 HYDIN2 ADGRE4P IGLV3-2 USP7 CD82 KIR2DL4 FAM167B ARL13B SCP2 TMEM156 ABHD2 ALCAM DENND1A SEC14L1 ERLIN2 ATP11C PDZRN3 SF3B6 ADAM32 RSRC1 DNAH11 ASS1 ZSCAN5C NIPBL CEP120 B3GALT5 CDC42EP3 MICU1 FAM204A SNX25 MYO5C SDC2 OTX2-AS1 ATF1 SLC25A21 SLC22A14 SAMD12-AS1 RBPJP6 IL6R C12orf4 TTLL11 LINC01579 NEBL RARB CCDC18 CDH8 AGPS TRPV5 HEPHL1 PJA2 KLHL7 TCF4 ECM1P1 GACAT3 RPRD1B AURKA LINC01145 PTPN2 AP3B1 PBLD GORAB ERICH5 BCAP29 TTC21B PSMF1 SLC5A4-AS1 SH3PXD2A NEK6 SLC39A12 MOB1B OR13C9 ASAP2 SEMA3F-AS1 YIPF6 TMEM25 OSBPL10 RIC3 CASP5 EXOC1 RNF182 RALGPS1 UPP2 PHF21B ZFPM2 PIEZO2 FAM66A BCRP2 SVEP1 FANCA DEUP1 ZNF354C LINC02325 LRRC2 ANKRD26 RGS20 MIR3118-2 PDGFD CNDP2 HCRTR1 RELL1 LINC02176 BRINP3 LINC01237 KIF4A XRCC4 OVCH2 COP1P1 EPHA7 MAP7 TM9SF4 SENP8 SUSD6 NSG2 ZBTB8OS GRIA1 ZNF627 IFNAR1 KIAA0319L USH2A LHFPL6 OPN3 NEGR1 NAV2 XXYLT1 CACNG2 ANO10 BTBD9 SPATA48 CA5A FAM72D USP18 NET1 TTC28-AS1 BMP2 TOX KIAA0232 LINC02011 SRGAP2C TMOD2 SLC44A5 FAM107B LUZP2 BTBD10 SH2D3C MELK RBPJP2 LRIG1 YPEL1 PRKACB ZNF536 RIMS1 GXYLT2 HIPK3 EPB41L3 WSB1 TMEM225 POR LINC00896 PARVB MORC1 OR10H2 ZBTB7C SCGB1D5P KANK4 GAST SGO1 WDR26 SAR1A SLC37A1 BCL11B LINC01814 DTWD2 LINC01213 NELL2 TSPAN2 MAGI1 SLC14A2 LINC02668 OR52B3P ASAH2B RALB MOSMO KRTAP19-10P BLK PPP1R17 PIAS1 PTCSC3 LINC02180 SSBP2 ZNF705CP GALNT10 FHIP2A CFAP74 ZNF846 HAGLR ZNF287 ARMC2 FER1L6 ALS2 CYTH4 COMMD8 ZNF608 PTGER4P2 TBATA ATL1 SERPINB11 ZDHHC17 KCNH1 ABCC9 SNAP29 QSOX2 GSG1L MCF2L LINC01098 ACSS3 BBOX1-AS1 ABCD3 DCDC1 NPAS2 DIAPH3 CCSER2 INTS7 AMPH ASB4 TMEM178B COL5A3 COL4A2 PARP15 ME2 GRAMD1B SIAH2 POLR3A ZNF44 LINC00536 HEATR5A ADA2 PRKCZ BPNT1 F13A1 ALG10B CDC14B GIPC2 RNU6-1007P KDM7A CYP4F22 ABCA10 LINC01182 KLHL1 SP110 LINC01566 PRSS2 MRM1 ZNF705D ATP9A POSTN OLFM4 SAMHD1 OCLN AK8 SDF4 ITGBL1 TNFSF11 SPOP EFHD2 EGFR MYEOV ADAM28 MRPL13 B4GALNT3 AFAP1 DPY19L2P1 SH3BP5 SLC49A4 FANCM NEO1 MELTF MXRA7 MYT1L SRGAP2B SLC37A2 ATP1A1-AS1 SORCS3 PDP2 VPS37A LRRC49 ERP27 ZNRF3 ZBTB21 BBS4 TENM3 ITPKB ENPEP TUSC3 IPCEF1 RTRAF NF1P9 OR4K6P BIN2 LINC00877 TCERG1 UBE2O VENTX DIRC3 PLCZ1 CPEB4 COL6A5 ZFYVE28 NCOR1 LINC02213 PRDM10 EBF2 C16orf72 USP33 ERBIN RNY4 SLC24A4 ZNF573 MBTPS2 KHDC4 C2 NTF3 OR6C75 ZNF705G LINC01684 PI4K2B USP41 RBMX2 FAM72A IL17RA SOX1-OT SUSD4 PTH GALNT14 RAB22A H2ACP1 FAM66C ZNF160 LINC00466 HADHB NSMCE1 DNAH10 GAS2 PDE10A CACNB2 REPS1 MAP3K4 TP53I11 PDXDC1 MTPN MT1HL1 LINC02646 GNG7 VSTM2A RUNX2 ZNF804B LRP12 LRRC8B CSNK1G1 ZNF169 MICU2 SOX6 JAZF1-AS1 SGCD RC3H2 LINC01492 RNU6-374P TNNI1 RAC1P3 SP3 STK32B PLG ZNF106 LINC01020 NR2C1 SLFN11 ADAMTS3 ERO1B DNAH8 NHS LINC02505 CABYR LINC01476 ANK3-DT RGS12 RAPGEF2 ZNF438 GTF2I NCK1 SOHLH1 LINC01192 CDV3P1 C15orf32 PUDP KDM1B SSPN HS3ST2 F5 PATJ FAT1 NUP50-DT CPS1 MESD PRKCH TRAK1 TET1 FAM66B RHPN2 ANKRD30A RABGAP1L KRT25 NMU DENND2B LINC00603 HADHA CFDP1 LINC00944 SMARCAD1 MIR3118-3 FNDC3B ADAMTS9-AS2 ASTN1 GNG12-AS1 GFRA1 NSG1 RMST ANKRD20A8P MAPKBP1 CPE TDRD7 RNF8 LY86-AS1 LINC02613 PYGO1 LINC01723 NFKBIA TEX29 DNAL1 TRAPPC3 CD101 PFKFB4 TMEM132D HMCN2 FHIP1A EFCAB8 LINC01204 SPRED2 SCN10A HSDL2 MYLK3 NCOA7 ANKRD18A ZNF350-AS1 CEP128 ZC3H15 LINC01135 ANKRD7 ABCA5 PTCSC2 CIB4 ABCC8 TTLL5 AKAP11 NOXRED1 TMTC1 TTC33 MOCS2 NRK NAT1 KICS2 CYBRD1 MCPH1 MINAR1 EIPR1 STON1-GTF2A1L BMP2K LINC02543 CYFIP2 APOOP5 CCDC126 BABAM2 MSANTD4 CRB1 IL1R1 OTOG HEPACAM USP8 NUDT21 XPO7 ARSJ KCNS3 ENPP3 ZNF235 ERC1 LINC02006 VWA3B ZNF850 ALPL PDLIM5 MAP3K9 XYLT1 BTAF1 PDCD6IPP2 ALPK2 LINC02660 ABCA13 HNRNPCP9 RFX2 MAPK8IP1 ADGRB1 SLC66A1L LYPLAL1-DT ADGRE1 RAP1GDS1 FOXO1B TET1P1 ST8SIA5 TBC1D19 ZNRF2P2 PDE6A CHST8 BID COX5A MACF1 MDFIC MNAT1 PCMTD2 MBNL2 DNAH14 STARD4-AS1 ERI1 TC2N TUBGCP3 BTLA LGALS9DP SLC15A5 HCP5 AMBRA1 CLEC20A NETO2 DOCK2 SERPINA6 ASCL3 PTPRE IFT46 FAM66D NUP210L LINC02063 TSPAN3 ALPK3 LINC02465 FLVCR1 SLC9A5 MUSK EPDR1 MYO3B LMNTD1 RAB8B LINC00583 MYOM1 ZSCAN30 MTCO2P3 LINC00469 RNU6-835P RXRA CGAS ARHGEF7 SLC23A2 LIN54 LINC01649 ARPP21 ARL11 MAML2 SPAG16 ADAM5 TRIM43B ZNF879 ARHGEF12 LYPLA1 LNPEP DDX39BP1 LINC02198 UNC93B3 RPS3AP6 POU1F1 ZNF397 KIAA1958 CARD18 LINC00623 NEDD4 RFTN1 CCDC141 NEK4 RSU1P1 PEX14 CFAP61 FYCO1 LPGAT1 CNTN4 FOLH1 HRH4 SPRR2D LRRC38 EXOC6B EVC2 CNKSR3 USP49 DRAXIN SEMA3E CSF1 CEACAM22P LINC02109 LINC00511 SLC8A3 TRNAU1AP LINC02145 RNF17 HAS2-AS1 KIF11 LINC02400 SLC2A3 FAM72B PRAMEF26 SLC40A1 PHC3 STOML1 CADM2 SDR42E1 LINC01581 FANCL SH3GLB1 GABRR2 RAP1GAP PIK3C3 OTULINL RAD9A SLC9C1 SCML2 SPOPL MAGI3 LINC00701 TRAF3 MPPED1 CCDC122 CHD6 FAM135B

		TMEM273 MORN1 CCDC186 CFH PAXIP1-AS2 LINC01695 PTPRB INTS8 LINC01412 ITGA1 VN1R7P MARCHF6 CCNG2 ATG4B CIBAR1 ODR4 GAGE13 TANC1 CORO2B PAPPA DHX40 KIFC1 POC5 IGHVII-65-1 TRPC7 SYCP1 VPS41 DTX1 MYO10 GFI1B ZNF407 MIR3118-4 ASB3 TENM3-AS1 KIR3DL2 GAGE12J TRIM43 FOXO6 TSHZ2 C1orf21 TOM1L2 STOX2 FAR2 BICRAL PLAGL1 NEK7 NKG7 CNN2P12 NLRP13 COG2 RPL5P35 ERN2 CYP2C58P TLNRD1 SERPINB2 KSR1 AOX3P LINC01322 GABRB1 ANKS1B RP1 LUC7L AKAP10 TTLL7 EFCAB14 SLC16A9 WWC1 PLEKHA3 RSRP1 BLM PLA2G4A SENP6 GATAD1 ZBTB16 DDX39AP1 UNK B4GALT6 ATG5 LINC01938 SGTB TRIM60P19 ZNF875 XKR5 SCGN SYNJ2 STK32A ARSB IL34 ZNF234 CLVS2 DIO2-AS1 SIGLEC29P DPYSL5 CWC27 AGK USP25 ASCC2 SLC44A1 CNNM4 ADAM10 ATXN3 GALC MRPS22 TMC1 PLD5 OXR1 PAK3 CAMLG TSPAN33 GARNL3 RNU6-1150P NPIPA1 TPM1 CES1P2 CIDEC EBF1 DHTKD1 OBI1-AS1 FLNB OR2T2 MADD PCID2 LINC00667 NDFIP2 DUX4L45 ZSWIM6 MYL1 ANKRD36BP2 CAMK1G DSG1 C1orf87 LINC02327 FAM30A PDZPH1P ERICH3 TRERF1 CENPBD1P1 TADA2A RPL15P2 LRRC9 LINC00158 FBN1 NIPAL2 MTUS1 GABRA6 PTPRVP DHRS3 PRRC1 PANTR1 LASP1 VPS35L ABHD17C KRT6A C21orf91-OT1 GGT4P DROSHA ZNF813 ANKRD31 PALS2 ADCY10 FAM149B1 PDE1A TMPRSS2 LINC02165 PTAR1 PRICKLE2 CMAHP ANGPT1 TRIM58 HMGA2 HHAT KLHL32 CHASERR PSTPIP2 MVB12B C4orf50 LINC02253 UBASH3A CACNA2D3 SEL1L ELOC SLC7A2 TMEM67 BTF3L4 MIR3936HG ZNF618 ITGA4 CPA6 AGO1 PRKCE DEFA3 GLYATL1 RBPJP5 PTPRK MIR3142HG CNTN3 GLIS1 ODAD2 ARHGAP31 RXRG ETNPPL KIR2DS4 PRKAA2 CD163 ARMC3 BBS2 SYT1 OR4F15 LATS2 IKZF2 PPP1R12B APCDD1L-DT PDE2A DNMT3L ASPM RFC2 PPARA PLXNA2 KLHL33 DENND2C LINC01602 TBC1D13 CDYL2 SCARA5 PRG4 SCGB1D1 RANBP3L MARK2P12 TG ADGRB3 HGD SLC36A1 PLCB1 UBE2R2 KCNH8 ATP6V0CP3 LAMB1 ANKRD19P RPS10P7 CUL5 HOMER2 DGKI RIN3 LINC01221 REG4 LINC01151 DIP2B CD5L LINC01524 TBC1D30 IGLV2-14 NME7 STXBP6 TFDP1 FAM66E ZBTB33 MXI1 ZNF876P PPME1 TRAPPC8 OR4R3P STX12 LINC02291 FUT9 MOK GARS1-DT CREG1 LINC02488 SLC12A1 SCN2A MAPKAPK5P1 WNT7A TUBB6 ZNF449 DOCK10 FAM27C PAMR1 DDX6 SPIRE1 TMEM71 COG5 AIMP1 UBE2E1 ARAP2 LDB2 LINC02149 SNX30 ATP8A1 BCL2L1 IQSEC1 LINC02008 JARID2 LINC02236 SHROOM3 ADK PRR5L ALX4 CORIN FAM241A LINC00838 RANBP17 WNT2B MRPS27 PPM1L CPHL1P LRRC37A3 TRIM43CP PRPF18 SMOC1 GSTA3 EXD3 SETDB2 FAH MON2 OR51E1 TMEM116 TOGARAM1 SLC45A4 ZNF705B ELF2 SEMA3D LDLRAD3 GLYAT KIF15 CFTR VSX1 TBX20 FLRT2 NFATC2 NALCN-AS1 PRAMEF25 KRTAP20-4 ARHGAP44 MTMR2 ZNF970P KCNH5 FAM189A2 CADM1 KLHL29 PTPRO ARID3B OPRM1 ACER2 ZDHHC14 RGS9 YLPM1 ATF6 ITFG1 IPO11 VCL SLC13A5 GLB1L3 ABCA9-AS1 DOCK5 VCAM1 C7orf31 LINC02511 LINC01818 ATP6V1C2 MAGEL2 IFT81 NHSL1 OSCP1 PLEKHA8 SGO1-AS1 DTHD1 SRGAP3 IGHVIII-13-1 HAAO CTNNAL1 CIBAR1-DT CYP2A7P1 ATP6V0D2 SYNJ1 PHF20L1 HLA-B KLF15 PPP2R2B NEDD9 HDHD5 IL12A-AS1 ANKRD66 ENPP1 SDR42E2 LYRM4 CCDC150 DNM1P47 LINC02099 HEMGN RIMS2 UGP2 RP1L1 PPM1F OR4K8P EFCAB6-AS1 CHSY3 YBX3 YAP1 ARL15 ANK3 BMPR1B LINC01173 FCRLA LINC01937 SEMA5A C1orf127 PRDM15 KITLG KRT6B SV2C DAW1 COL5A1 IGHV3-74 IFT57 LINC01426 RIPOR2 MAP4K4 LINC02899 ZNF112 FICD KIF21B FEZ2 ATE1 PEG10 INHBA-AS1 HSPD1P3 NMD3 OLA1 GATAD2B VPS13C ANKRD55 XIRP2 KRT85 SLC14A1 CA1 C5orf52 FAM72C MFSD9 SERPINI2 STK38 APBB1IP NPL CAST TBC1D9 FBXO32 AOAH SNHG14 TSBP1-AS1 SMG1P4 SNAI2 ZBTB49 FAM90A28P PHF2P2 AP4E1 DLEU1 NDFIP1 TNR FAM180A LHX9 LINC02074 OCA2 ST8SIA6 DAPK1 MSANTD2 FCHO2 RFTN2 TRAV8-6 COL6A6 RN7SL767P DNAH5 CNTN6 CLIC6 LINC02406 FMN2 BHLHE40-AS1 SEMA3C VAV1 LINC00895 CACNA1I BTG3 DPY19L1 CSF2RB CMTM7 RPS6KA3 SMAD5 DNAJC13 BCKDHB PPIL6 IGLV3-30 CNST PATL1 UBE2J2 ASB2 OTOP1 PSME3IP1 OR4F6 GHRH UGT3A2 GOLGA8B RNLS IGLV4-3 SPOCK1 LINC02315 NF1P6
4C-decreased-1200	1122	*LRRC37A5P CCDC116 ZHX3 APOL2 RPS4XP6 PDCL3 PTPRR HERPUD2 CRTAM SLC52A1 UBE2G1 PELI2 TPI1P1 NOS2 MIR548H4 ZEB1 LINC01708 FAT4 PARN SEMA4D SLC15A2 RN7SL483P WSCD1 MIR4435-2HG KNDC1 LINC02355 ZFYVE1 EVC HERC2P2 LINC00670 DMC1 CXADR SPPL2B C9orf43 DIP2A NBPF21P OR7E19P RIOK1 GGT2 UNC45B LUZP1 LINC01346 BVES-AS1 ZNF723 LINC01138 CECR2 LINC01782 SMYD3 GNAS DYSF NPM1P2 CD38 SERPINB9 LINC01876 PGBD5 LAIR1 MSH6 RASGRF1 HECTD2 FBXO31 C12orf40 LINC01163 GMPR RIN2 PRDM16 FRRS1 PDE6C RNF217 TRAPPC9 LINC00323 MEIS2 CARMIL1 PCDH7 BCL2 KL LRRC4C LINC02558 RACGAP1 MIR3667HG THADA NUP37 PPP1R9A SAA3P TMEM117 KCNK15-AS1 TOX3 TDRD5 TASP1 SNX6 POTEG GOLGA6L3 SAMM50 ZZEF1 FRA10AC1 HHLA2 NCF4-AS1 C3orf52 SLAMF1 UQCC1 RGL1 ATP5PBP3 SHOC1 LINC00841 FAAP24 INO80D KDM6A MED27 NCAM1 PDYN-AS1 GDAP1L1 LINC02096 LINC01358 UFL1 EPHA4 LINC01967 PLA2R1 LYSMD2 NGDN H1-9P ADAMTS2 GTSF1L WARS2-AS1 CCDC172 FRYL LINC01828 GPRC5C COLCA1 CPAMD8 RNU6-929P GPR1-AS UBL7 LINC02675 CALM1P2 RBM6 MEGF10 IGHV2-70D HS1BP3 LIPI LINC01445*

		RAB38 DZANK1 CLTCL1 NUAK1 PCAT1 KCNK10 ZNF738 BLOC1S5-TXNDC5 SLC16A1-AS1 VMP1 NENF HRH2 VSTM4 ATP6AP1L RNU2-47P RDX SNTG2 CTSB SVIL NDRG2 APBA2 TTC3 COL23A1 NEDD4L EDAR C5 EGF LINC00960 ATP2B2 HDGFL3 RPL37P3 CCNYL3 ABCC12 PARK7 DSTYK RIMBP2 ZNF271P IFT43 ADAMTS19-AS1 SNRPC C2orf69P4 DRC7 DSE POTEJ LINC01427 MYCL LINC02256 TPTE2 SPAG6 BMP7 PDE4DIPP1 GALNT2 FGF12 EPHX4 CYP2C9 CNOT7 CASC9 IMPACT ITGA6 HHIPL1 NOTCH2 IMPA2 ZFP90 S100B ARHGAP12 USP43 KCNN3 FKBP5 NFAT5 FLI1 ANAPC1 GRM1 LINC02147 ARHGAP26 ITGB8 GAP43 LINC02422 OR4K3 PRDM13 PDE3A PTPN4 BCAR3 MAP6 APOL1 CDH11 SETBP1 AIF1L CDS2 ZNF780B LINC01900 ATP6V1E1 LINC01993 LMX1A AGBL1 RSPH3 DEFT1P2 LINC02439 ZFAND6 GBP4 CNKSR2 PSMA5 DPH6-DT GFRA2 NEK10 ADCYAP1R1 SNX29 MAGI2-AS3 ZNF611 STAT1 SAMD5 MROH5 SLC44A3-AS1 MYOCD CREM GOLGA8J XKR3 UBE2QL1 PLPPR5 TDP1 AVL9 EFEMP1 TNRC6B WBP2P1 LINC02542 SYN2 PTCD2 MYO1E SMOC2 MIPEP NCSTNP1 HDAC2-AS2 HLCS FH RWDD2B PLPP4 STK10 PWRN4 CCDC102B SDS GSR CCDC162P LINC01571 FIG4 SOGA1 ARHGAP32 BMF NECTIN1 FLT1 RB1CC1 ZNF528 LINC01222 LALBA NXN LINC00375 FOXJ3 CENPE CKMT1B MYL12B RSPH14 IL17RD ANKMY1 HCG22 APELA UBN1 PLA2G12B FAM83B HDAC4 CTNNA1 STK36 GNAI2P1 FAM102A NCOR1P1 TRPC5 MYO9A TMEM182 IL10 LINC02305 AMFR LIFR-AS1 C19orf18 FTO SLC6A1 EPC2 DMXL2 SEM1 SEMA6A-AS2 MOGAT3 TMEM236 NLK THSD7A CXCL2 GOLGA6B LINC00334 CARD10 ACSBG1 GCSAML DNPEP TRAPPC11 HOXC4 IGHV3-62 NECTIN4 CNMD LINC01309 UFD1 LINC00299 BAZ2B HERC2P3 CRACD NGF-AS1 AGL PALMD HS6ST1 MARK2 MEOX2 SFI1 ZBED9 CTSE ATF2 PLEKHB2 FAIM ZNF72P RPRD1A ZNF880 PPP2R2A ESS2 PHAF1 ZNF541 RBBP8 AK6P2 CKMT1A RFC1 NSUN6 LINC02174 CDC45 MC2R AKR1B1 BTBD11 LRP2 LINC02087 HERC2P9 SYNE1 MTHFD1L RALGPS2 HOATZ SNRK ABCD1P4 EXTL3 EHMT1 TTC29 FBXO47 KRT89P ZNF735 ZBTB10 PKHD1L1 SLC9B1P4 PLPPR1 CEP192 SLC26A2 CAMK4 GUSBP11 CLPX OR7H1P ROCR ANKRD20A9P HDAC11 SLC9A4 ANKRD20A17P GRIK3 GRXCR1 NUMB STPG2 MIDEAS TM9SF2 CD70 CELF2 SH2D1B ZNF606 ESYT2 HEATR6 ARFGAP3 MAPRE2 FGF10 TAPT1-AS1 KRT18P59 ISX RAD51AP1 POTEM SYBU SMTN LINC01035 PDE4DIP SCG3 ESRP1 RIC8B TAF3 ZMYM4 TPTE FRG1JP MED12L GRM7 ZDHHC21 BRMS1L DDHD1 ICA1 PLEKHD1 CDH7 EMILIN2 TLDC2 CYCSP39 HORMAD2-AS1 VASP PLGRKT UBE2E2 UNC80 SDE2 PTGFRN PPA2 ILDR2 IMMP2L ZNF615 SPSB4 SPSB1 GABRG2 ZNF567 ITCH NUDCD3 MBP S100PBP TANGO6 GABRA5 CELSR2 CDKN2C STXBP1 SLC46A3 PTPRJ DLC1 PNPLA7 SELENON RPS3AP4 CNIH1 FRG1BP C2orf69P3 LINC00114 ZIM3 MANBA DUX4L2 CHRM3 ECT2L UST MIR663AHG CALD1 LINC01543 AIG1 ERICH1 DEDD2 TYW1 TAF15 ALB ARHGAP24 JPH1 ANKRD20A3P EFR3A HTR2A TPH2 N4BP2L1 IGHV1OR15-9 TPTE2P6 EIF4BP3 LOXHD1 MDS2 GOLGA8G LINC01622 GALNT1 MARCHF1 OR4L1 ZBTB25 INO80 RBM15-AS1 HSD17B14 KRT16P6 COL27A1 GLP2R CHCHD2 BMP5 SLC4A4 ZNF675 LINC02366 GOLGA6L17P SYT16 CRYBG1 TWIST1 HACD2 LINC02653 LINC02061 RPL15P3 TRIM77BP ERCC6L2 PASK PHKB RUFY2 SLC16A1 RANBP9 FAM245A MRTFB LINC01344 KCNE4 TRIM5 LINC02228 KRTAP21-3 LINC01801 LINC01320 LYPLAL1 THNSL2 BRWD1 COLQ TMEM54 PPIP5K1 C9 TMTC2 HECW1 MCTP1 RNU1-51P MOB3B ACTR3C LCE1F ARPC3P2 MAP4 TSPAN11 MTND1P17 HIVEP1 ATRX TNIK KRT18P55 OR1L6 NBN PRTG OR2T7 SLC17A1 SEC24D RGMB KMT2E WNK2 FRMD3 RBFOX3 SDAD1P2 PWWP3A ITIH5 PACSIN2 TRGJ1 HOXC13 PKP1 SYNE2 GTF2IP6 MIR181A1HG TRMT61B TRIM23 FABP7 SDCCAG8 GK LINC00355 DUSP22 KCNJ18 CHSY1 RFC3 MAB21L3 SMPD4 EXT2 PTPN12 GPR137B ZYG11A LINC00434 LINC02424 TOP3B MPPE1 RGS3 UBA6-DT ANKRD26P1 CMIP DEFB116 SLC2A13 FBN2 YTHDF3 SPATA17 SYT10 ZBTB38 LINC02380 CYFIP1 ALK DOCK8 GON4L GREB1 ARNT LINC01340 LINC00929 CSTF3 ZNF648 LINC02058 SAMD13 DNAH6 ARFGEF3 TMCO5A UHRF2 EPCAM-DT DCLK1 DEFT1P RNF215 ANKRD28 GRK3 SND1 IFNG-AS1 SNAP25-AS1 ZFAND4 LINC01664 FGD4 ETS2 LINC00649 ZNF382 NDUFAF6 GOLGA6D ERMP1 CATSPERG ARL4C ITSN2 LINC01917 KTN1-AS1 HIRA OSER1-DT SERPINB7 ANKS6 TMEM74 PRKAA1 RASGEF1C TAFA4 ALDH1A2 GABRG1 MTTP POGK CROT MAPK9 ESRRG FBXW2 LINC02458 LINC02652 LINC01229 FOXJ2 AGAP9 ABCC4 MYT1 FRG1HP ABCD2 ZNF595 EMP1 TMEM171 LNCAROD RRAS2 SV2B FAM110A NRBP1 SEC14L3 STK38L GTF2F2 RALGAPA2 FAM245B ADAMTS19 ZNF236 RAB27B SOX30 LINC01337 MYOF P2RX6 PLS1 UNC79 RSPH1 SPON1 ANK2 SH3GL3 CFHR4 INVS FHL2 NCAPG2 LPCAT2 LNP1 TPTE2P5 PHF19 ADAMTS14 ZNF518A LINC02191 IGLV3-31 KYNU DCAF1 ZCCHC7 CD2AP TTC39C LINC02680 ZNF124 EBF3 TAFA5 NFKBID SPRED1 MYO5A CIDEA LINC01924 PPP2R2C PWRN1 LINGO1 LINC01706 SEPTIN9 SMPDL3A LRFN2 ACO1 WDFY4 SCAI PAPPA2 LTN1 TINAG NCOR1P3 DIRAS2 ABCB5 HEATR4 SLC5A9 KLHL4 DPY19L2 MDM1 RRBP1 SMPX OVOL2 CD9 MTMR10 EIF3F CDHR3 CSE1L NEK2P2 TOM1 FAM118A PRUNE2 SGMS1 ANKRD24 COL25A1 RBPMS2 ITPR2 CYP4A11 BRINP1 IGLV2-34 MTND2P8 RPL23AP7

		GRB14 LARP6 RXFP1 IGSF5 LONP2 CCDC192 LEMD3 PEX6 CHKA BUB1 INTS4P1 VPS13D KRTAP21-2 ZNF292 SPIN1 TBX15 PRB3 TTC3P1 KPNA1 SKA1 CCND3 LAMC1 SRP9 SCGB2B2 GABPA SMIM11B DSG1-AS1 PCNX1 DUXAP10 LINC02091 NAA35 ATP5PF RPF2 UBAP1L MX2 AGGF1P10 MTCO1P1 ITGB3BP ZNF831 PRAMEF2 ENTPD5 DGKK SLC4A10 LINC00844 TSSC2 LIX1-AS1 TGM1 AGAP14P MGAM FTLP13 ZDHHC18 LINC01310 PSG9 FAM183A UHRF1BP1L IL1RAPL2 APIP MUC19 SCAPER IKBIP NPHP4 LINC02141 OR8B9P CBLIF ATP6V1B2 KCNJ1 LRRTM4 CLCA4 PSAP LINC01877 MYOM2 SLC5A12 NCS1 ONECUT1 PCDH11Y METAP1D USP31 HIVEP2 SUMO2 OR5AQ1P MICOS10 XPNPEP1 GLYATL3 NUBPL PCGF5 FGF7P1 NLRC5 TUBBP9 LINC00363 NDUFAF2 GUCY1A2 KREMEN1 UMODL1 LINC01189 MB LINC00383 WDR25 SOD1P2 CYP4Z1 LCE3D TNPO3 EFHB ACSM2A FGF9 DCUN1D4 DAZL RPL23AP53 SLC1A1 HPCAL1 SPG21 ANKFY1 LIMCH1 ZKSCAN5 CCDC195 TSHZ3 TMCO4 LINC02098 RALA GMDS-DT BAZ2A MIR548A1HG RPS12 GNPTAB TRPM1 NTN1 COPS8 SACM1L AKAP9 C1QTNF3-AMACR AP4S1 NR5A2 PRKD1 TRAV8-1 TNFAIP8 RBM47 TMEM260 GBP6 SANBR DBF4B OR9Q1 CCL28 FBXO41 MICAL3 SNRPN HDAC2 MSI2 MRTFA LRRC7 LINC00221 ETS1 TNN CASZ1 PCDH8 LINC01673 WDHD1 MIR155HG MAMDC2-AS1 RABEP1 ST6GAL2 LINC00930 NUTM2HP SLX4IP PTCHD4 BANP ALKAL2 MAP2 YME1L1P1 TGFA SNX3 LINC01422 ZNF280B PTPRA CEACAM7 TNRC6C TMEM161A CYLD DEFB108B OR11G2 LINC02451 WSCD2 UBE3D GLIDR ZNF402P CAPN5 CERS3-AS1 RPL23AP87 GOLGA6C ABCA4 ZFAND3 LINC01718 THUMPD2 C6orf118 FAM83F CNKSR1 RAB12 TMEM163 PCDH9 LINC02235 DIPK1A SLF1 EXOC1L RBMS3 POTEH GNA14 INMT-MINDY4 LINC01992 SRFBP1 COPB1 MATN2 TM7SF3 IL33 EFCAB2 MARK4 CRACR2A CLEC16A LINC01901 OR2T3 SERPINB10 PROX1-AS1 ACTN1-DT ARHGEF17 ME3 PSG6 CACYBP ITGA9 PPP1R1C ULK2 IARS2 UNC13C CDC42BPB HNRNPH3P1 TMEM44-AS1 LINC00298 FAM217B MTRF1 FAM25G SHROOM2 OVCH1 CISD1 C16orf95 CRTAC1 EGLN3 PRKAB1 RALGAPA1 LMX1B LINC01748 L3MBTL3 RRAGD IGKV2OR22-3 POMT2 MICALL2 ST13 LINC02245 CLIP1 MARCHF8 SHC4 C2orf42 CABLES1 MORC2 ZNF613 TLL1 DEFA8P FRMD6-AS2 DUSP16 BCL11A AKR1C3 PPP2R5E ASB7 FREM1 IL20RB ADAM22 RABL2A PTGS1 LINC02582 LINC01811 CNIH3 RPF1 TRIT1 CEPT1 WNT5B CEP44 HKDC1 CLNS1A EPS15L1 HIP1 PVT1 CHN1 ETV6 WNT9B RFX7 TRPS1 SKINT1L IGKV3OR22-2 ADAMTS5 NF2 STRN CRISPLD2 NPM1P1 ANTXRLP1 MTMR3 ZNF845 RN7SL250P FAM25C CRKL ITPRIP H2BC15 ADGRA3 SCG5 CLDN18 LINC00581 ADCY9 DPP10 TUBB2BP1 GALNT16 CDH5 ANO6 ZNF600 HIPK1 A2MP1 PGPEP1 ACSM2B TTC7B ATP2B1 IFI44 RETREG1 NLRP14 NPHP3-AS1 PAH FAM138E ATRN FBLN5 CNOT6L TRAPPC6B TOP1 ZNHIT6 SLC39A6 WDSUB1 ANKRD20A7P
upregulated-1285	1133	*TFIP11 UTP20 ERAL1 H2AZ2 TKT LRRC41 SLC25A3 IRAK1 G3BP1 NUP155 DDB1 RNU6-322P DAZAP1 EP300 MITF DDX5 IMP4 CLUH ZNF131 HROB MSH3 NOSTRIN LYAR SPN ARF6 IPO9 MED13L MRPL1 NOC4L MNS1 NAT10 FADS1 PCYT2 FAHD1 ARHGAP21 ACTR8 ENSG00000261770 STK25 SLK BSN NASP NOL8 BAZ1B GRPEL1 LMNB2 SDAD1 SLC25A46 INTS6 CLTA CEP350 RBM10 BCLAF1 TRA2B RAD23B STK17A CHRAC1 NEFH CCNY SSU72 TRUB2 IP6K1 SRSF6 ZNF598 POLE3 HSPD1 DUS3L BOP1 POLR1E C1orf216 CCAR1 EP400 GPN2 UBR3 TUBB KIAA0100 HNRNPR GCLM HBZ ZNF75A ADI1 ZNF239 SAMSN1 SON PTP4A2 TRAM1 PSMD3 RHEB RAB35 OTUD6B NPM1 TSR3 XRN2 FUS CELF1 ABRAXAS2 SEC23IP CNOT1 RCSD1 DDX18 PPP2R5A CCT8 EIF3M SRP72 ZNF24 DDX49 CAPN1 TRIP12 BTF3 ATAD3B IKZF3 PRXL2C SETMAR EZR DYNC1H1 TMEM33 ABCF2 WDR6 ITFG2 DHX16 MIS18BP1 KIF2A HGH1 HSPA8 MRPL15 KCNQ5 DHX15 NEU1 WASHC5 SPRY2 LTBR NOP58 TBC1D9B SF3A3 FUBP1 HCFC1 AHNAK TRMT6 DHFR EIF4A3 ATP6V1C1 PRPF3 ALYREF UBE2N FAM83H ENSG00000286122 LINC02393 PPM1H NFYC SSRP1 STRIP1 ASXL2 CCDC6 MTHFD1 CYP3A5 FTH1 IER3 PITX1 IL17D GRWD1 JUND PHB2 LIN28B TNF MRTO4 ERMAP DDX39A C1QBP TIMM17A ASAP1 PDSS1 HNRNPA0 AMMECR1 TCP1 BRCC3 TRAM2 KLHL21 EIF3D ZNF586 SET NAB2 FAM120A AURKAIP1 POLR1A TAF9 CRCP BICD1 CORO1C FTH1P16 JRK RANBP1 FDFT1 SQLE EIF5A WBP11 ZNF614 MCM10 TMEM69 TMEM185B GABRE HNRNPK ELF1 ICE1 SAFB2 BEGAIN OXA1L TRIM35 USP11 PRKDC PSIP1 EXOSC3 ADD1 NOL11 E2F4 IPO5 ACLY IK SURF4 NACA GPX4 CDK4 PRMT1 TEX10 CEBPZ MCM5 CLN6 CSNK2A2 SRRT RBM3 RABGGTB CUL3 NFATC3 KMT2B TSPYL5 MIX23 FASTKD2 HRAS RABL6 NR2F2-AS1 DHCR7 NUTM2B-AS1 SLC19A1 AHCTF1 VAT1 APEX1 CAVIN2 MRPL11 MYC UBE2L3 NAA15 ENSG00000279348 TOMM22 MUS81 ARID1B YES1 VCP ABT1 ARID2 SMG1 RAVER1 RCC1 SRSF3 CALM2 ENSG00000287905 WDR82 FUT8 ZNF74 RBMXL1 STMN1 NRROS ENSG00000282386 CWC25 MT-RNR1 NR2F2 PES1 MS4A3 ENSG00000288271 TRIM24 PTDSS1 ENSG00000253853 CHTOP ENSG00000276742 ZFX HMGCR URB2 YBX1 STK24 NCOA5 H2BC12 BUB3 CTR9 CDC27 MCCC1 VPS35 VAC14 HNRNPH1 OR10Z1 CITED2 KPNA4 PFAS NUCKS1 HNRNPD U2AF2 YWHAG TRMT1 KMT2D ZMYND19 EIF5B WDR70 UTP25 LMNB1 MAF1 MT-RNR2 TFAM PUM2 PIK3C2B HHEX SRSF7 GTF2H1 VKORC1L1*

		ELOA PPP5C RNASEH2C PCLAF ACP5 WDR33 PRMT5 MAPK1 MPHOSPH10 CHD7 HNRNPA2B1 RNF126 UTP18 SBF1 API5 POLR1B PPP3R1 RBM45 FAM117A SH3YL1 RBM14 SF3A1 CAPNS1 EIF3A DANCR SEC24B LHX4 LINS1 CYB5B TNPO1 EEF1D RRP1 TFAP4 SNX9 ANKRD36C WDR74 MCM7 POLDIP2 RIOK2 STAG1 DHX38 BTBD1 ABCF1 CDT1 CFL1 LRP8 MCOLN3 TGFBRAP1 GPATCH3 PSMG1 TMEM43 EML4 PRPF8 SSB SKI TIMM23 ENSG00000289474 CTPS1 NUDC EIF3J EMP3 SYNCRIP DNAJC8 STAR JADE2 FARSA TRNT1 TXNRD1 TRMO STIP1 SART1 MTDH SPTA1 HSP90AB1 CCT3 MVK NOP14 NCL GVINP1 GSPT1 CDK7 COPS3 HCG18 TMEM97 MCM2 MCM4 DCAF13 ARPC4 TOMM70 ENSG00000286680 TARDBP TRIM28 DDX21 FKBP15 PWP1 COA7 METAP2 PAN3 ALG8 RIOX1 MED15 SETD1B CDC37 PPP2CA POLR2A ODC1 ZNF26 PPRC1 HAT1 POLE DNTTIP2 ENO1 SBDS DDX1 AZIN1 HAND2-AS1 RNPS1 KIF1A NOL7 BCL7B PPP6C TOMM5 PSMC5 CBX3 ANKRD13A SETD1A SYPL1 FAM71F2 SLC39A10 ENSG00000227706 ZNF512B ATP6V0A1 KIAA1586 BRD2 SLC7A1 TIRAP USP36 NONO ZC3H4 MAGOH ELAVL1 AGPAT5 CSTB SNHG6 VPS72 TCF20 SRM PPM1G AKAP8 XRCC5 CERT1 CUTALP NFKB1 FOSB H3-3B PCBP2 DUS1L MFAP1 ZNF789 TOMM40 VGF ADNP IGF2R RBM15B ENSG00000268362 POM121C LARP4 ZMPSTE24 HMGCS1 SNRNP200 RAB10 TOP2B PKP3 DNM1L ZNF252P EXOC7 DEK PSPC1 UPF2 ALMS1 CERS6 BEND3 UBTF GYG1 PHB R3HDM1 RSF1 P2RY11 KCTD3 GAPDH IFRD2 RRP15 RSL1D1 SBNO1 ADNP2 RBM25 B4GALT5 TPR BICRA MCMBP NAA11 WDR3 PROSER2-AS1 TNPO2 MTCH2 BACH2 PPARGC1B ACTG1 PTDSS2 FAM13B CCDC78 HNRNPU SCAP NEMP1 DDX56 SRRM2 PEBP1 HNRNPA1 ATXN1-AS1 DDX20 KAT7 UTP15 MYBBP1A CCT6A TCOF1 SF3B3 PIM2 CPNE7 BAIAP2 INSIG1 GPR75 TEX15 ARID1A MBD1 RBM48 ARL8B STARD7 TRMT61A ZBTB40 NCLN CHST3 MT-TL1 RYBP MAEA NCR3LG1 C8orf82 SAFB S1PR3 TRMT2A RTL10 LBR CBFA2T3 RNASEH1 IQGAP2 MYB CDC25A XRCC2 MMS19 PTGER3 ENSG00000271971 KIF26B NBAS EIF4EBP2 DHCR24 LINC01963 ATP6V0D1 TAF4B AFF1 MTA2 SLTM TBC1D14 AXIN1 MALT1 POLR1C ENSG00000288884 ENSG00000285730 TFDP2 NDST1 NOSIP SNHG4 GOLM1 PELP1 LINC00645 KCTD15 C22orf46 EIF3B SMARCD1 TPP2 MED29 FASN METTL8 PCBP1-AS1 PSMA3 ASCC3 MECP2 H4C8 CLDN11 TMEM18 VPS26A FUBP3 CLCN6 SQSTM1 TMEM127 RALY ZNF274 ZNF581 DAP3 H2AW PHF3 DCBLD2 DVL2 BTG1 PSMC2 CAMSAP1-DT PCYT1A UBQLN4 RAP1GAP2 EZH2 ATP11A TMEM223 SLC25A5 ENSG00000271781 PI4KA PRPF19 LSM14A TRIR C19orf25 PQBP1 SMARCB1 CHEK1 LRRC58 WDR43 ATP13A3 KIF5B CCT2 NUP153 MACO1 CLPTM1 XRCC6 DHDDS AGPAT3 ABO KCNH2 LETM1 DHX33 CHD3 TEX261 LINC00958 SNRPA GATAD2A ARHGEF2 CASP8 N4BP2 DHX30 ENSG00000177788 CPSF7 PRDX1 CASC3 SNU13 FBRSL1 BAG1 DSG2 HSPA4 ENSG00000266976 MMAB RRP9 SMC1A PSMG2 GNB1L C11orf58 RBM19 RUNX1 CASD1 FADS2 MT-CYB MYO16 EGR1 SLC12A2 PDCD7 GRSF1 EXOSC9 ZFP36L2 PBRM1 FTL VAPA TMPO PPP1CC PAXIP1 YY1 SLBP OXCT1 LEPR AASDHPPT MCM3 ANAPC7 DELE1 DDX42 FES YJU2 PITHD1 RPUSD1 FAF1 CDK6 RRP36 CSK MRRF RAPGEF1 SUB1 PRPF6 NAA20 ZNF587B SMARCA5 TIMM10B SF3A2 ARPP19 RIF1 COPS2 H4C5 NAA50 MRFAP1L1 LMO2 RBBP4 PDPR CSNK1G2 DOLPP1 RPL22 SCD SLC38A2 WDR36 CCDC86 MYH10 SPIN4 THOC1 STXBP5 ENSG00000176349 DNAJA1 NOP16 PCNX4 GFM1 RAD51C AP5M1 ENSG00000272341 ADO DPYS TGFBR1 GBP2 LRPPRC RRS1 TICRR TULP4 PRRC2C HNRNPUL1 ECSIT QRFP DCAF7 CLCN7 POLR3E SLC12A9 TXNL1 THG1L SPECC1 PPIA PPP1R10 VDAC1 TJP1 MED6 TPRN DHX37 ARFGAP2 AATF PRMT6 CENPN TAF4 RETREG2 NXF1 RRP1B DKC1 GART SLC24A2 THOP1 TFRC DNAJB6 DHX34 DDX46 AK2 ZNF787 PRPF38B KAT6A EPRS1 ILF3 MLLT3 PLK4 KEAP1 MED16 POLR3C NUP50 DRG1 BEX4 MRPS30 GAB2 MT-ND1 SLITRK6 CTCF ATAD3A TRIP13 CLTC ZNF521 RPRD2 SRSF8 PTBP1 USP37 RAN MCCC2 HEATR1 UFC1 NSDHL KDM3B POLR2D LSS ETF1 GAR1 EIF4G1 HNRNPC ZC3H18 SF1 HSP90AA1 CASP3 FTH1P7 LRRC47 RANBP3 PPAN YWHAB DHX9 BAG6 EIF4B OR2AT4 KIFC3 STX3 PHF5A NCAPH WDR81 ZNF45 AP5Z1 PTMA NAPA SNHG3 GNL3L UHRF1BP1 SGPP2 BRD9 PRKAR2B ST3GAL2 PGAM5 EEF2 RPIA CHCHD3 RBM12 RFWD3 ENSG00000234160 NUP98 SNHG20 NIP7 RNF220 PRPF4 ENSG00000255099 ZC3H7B UROD COMMD4 SACS NT5C3A NIFK WDR46 CAPZA1 DNAJA2 ZNF512 CSTF2 LYN ZFP91 AHSA1 TFB2M FRMD8 NUP188 NOP56 RAI1 MT-TF BPTF TASOR2 DENR TXK DNMT1 ENSG00000284024 FAM136A FOXRED2 ENSG00000261342 AIFM2 CYP20A1 AAMP CAPRIN1 NLN HNRNPF RREB1 NQO1 HSPA9 SRSF10 RBM42 UTP3 ENSG00000279669 NUP160 HNRNPDL DOK3 FOS EMD CMPK1 PUS7 ZNF451-AS1 XPO1 GNAQ LINC02434 PDZD8 PABPC4 ACAT2 SRSF1 IDI1 GLUL DIAPH1 ZDHHC5 RRM2 ELOVL6 C19orf48 HNRNPAB SLC20A1 SLC30A10 FEN1 PPIF IPO7 PCM1 NOB1 STRBP GTF3C4 NAV1 PATZ1 LINC00342 ABCF3 ELOF1 NCBP1 PLAGL2 PDCL3P4 CTSL SNHG17 SMG5 ANK1 EDC4 GLYR1 PPP4R3A UBE4B TRIM44 CTDSP1 TRRAP LRWD1 AP3D1 ATP6V1G1 LRRC59 KHSRP LYL1 DDX54 MED28 BAP1 KMT2A ZEB2 GDI2 HIF1AN WTAP ACSF3 TMEM201 JMJD1C STK35 CCNH SURF6 MLLT10 RRM1 PA2G4 RRP12 MRPS2 NSRP1 RILP HSPH1

		THUMPD1 ACTB MSRA UBA2 CMBL SRSF2 DDX51 TCF3 SERPINE1 WDR5 NVL RANBP10 PSMD1 TIMM44 PRPF38A SPART-AS1 RNF40 NOL9 SLC29A2 RBMX CDC123 CIZ1 MDN1 BCCIP DYRK1A SLC9A3-AS1 SLC38A1 ARHGAP6 PAICS DNAJB12 PDS5A CRK NCAPH2 TCF7L2 PKM DDX23 ARHGDIA RBM8A CDV3 ZFR HNRNPA3 QSER1 NOLC1 CANX CUL4A ENSG00000286064 MCM6 PABPC1 TMX2 SNRPD3 TSR1 ENSG00000247934 CCT5 MCRIP2 FBXO45 RPUSD4 SNRPB ULK3 ENC1 PNO1 SS18L1 CNPPD1 GTF3C6 SREK1 SF3B4 PSMC3 UBP1 SAE1 ZNF282 CERS2 HDGF SUPT6H SETX WWP2 ZNF326 PAF1 TRMT10C GMPS EIF4G2 DLAT CDC5L KPNB1 KHDRBS1 NORAD NFILZ PSME3 RELN ST7 METTL3 HMGA1 SUPT7L PARD3 ATP2A2 POM121 PNN DDX3X NUP62 HNRNPL PGD UBE2Q1 SMG9 CUTA NDC1 WAC CEROX1 EIF3G AMD1 PRMT7 MAD2L2 MAT2A URM1 PAK2 RBL1 CENPF IGF2BP1 ZNRF1 GNL2 SNX17 DCLRE1C UBC HBG1 HBA2 PUM1 RAB7A ZNF622 ATP5MC3 MTCYBP18 HBA1

Table S15. Venn diagram showing the intersections of downregulated genes with lists of genes that reveal an increase or decrease in rDNA contacts. Related to Figure 3D.

Names	total	elements
4C-increased-1307 downregulated-1147 0,05	62	*TTC37 TEAD1 TIAL1 ASS1 IL6R ASAP2 SUSD6 USP18 TMOD2 YPEL1 WSB1 WDR26 MAGI1 GALNT10 ZNF608 BPNT1 KDM7A BBS4 CPEB4 COL6A5 ZFYVE28 GAS2 TP53I11 GNG7 ERO1B RAPGEF2 FNDC3B PFKFB4 MYLK3 NCOA7 AKAP11 TTC33 MAP3K9 MBNL2 DNAH14 ERI1 ARHGEF12 LYPLA1 ZNF397 SEMA3E TMEM273 CFH INTS8 PAPPA CNN2P12 KSR1 LUC7L TTLL7 TSPAN33 GARNL3 DHTKD1 FLNB PRRC1 PTAR1 LAMB1 GARS1-DT TMEM116 ATF6 PLEKHA8 HLA-B KIF21B CMTM7*
4C-decreased-1200 downregulated-1147 0,05	54	*SPPL2B DIP2A SMYD3 DYSF FRRS1 RNF217 FRA10AC1 UFL1 VSTM4 CTSB NDRG2 HDGFL3 DSTYK IMPA2 APOL1 AIF1L RSPH3 STK10 CCDC162P BMF FLT1 RB1CC1 AMFR DMXL2 CELF2 SYBU SMTN PDE4DIP SCG3 EMILIN2 CELSR2 PTPRJ SELENON AIG1 PHKB TMEM54 SEC24D EXT2 P2RX6 ADAMTS14 KYNU ITPR2 RXFP1 VPS13D CCND3 PSAP HIVEP2 CYLD CAPN5 DIPK1A COPB1 EGLN3 PTGS1 HIP1*
4C-increased-1307	1245	*FSTL1 CD44 PLCE1 SLMAP FAM219A FARP1 KCNMA1 PKNOX2 ARHGAP5-AS1 APBB2 ZNF684 KLHL13 GLT1D1 ABCB7 COX7A2L FYB2 RNF38 AHDC1 LINC02073 CUBN SLC10A7 LDB3 PAK1 SCAF8 SLC6A11 B9D1 CPXM2 RTN1 KIRREL1 LINC01467 PEBP4 PPP1R13B FGR TOP3A EPN2 LINC01491 LINC02664 THRAP3 INIP NSMCE2 ZNF208 RCL1 PRSS51 SLC25A52 OAZ2 ZNF718 DKK2 GLRA4 OR7A17 DHRS11 PCAT19 CCDC34 TAOK3 DNAJC27-AS1 PUM3 ZNF66 LINC02563 SCYGR8 LRGUK TENM4 TRPM6 FAAHP1 LINC01479 FOXK2 CCDC106 SLC8A1 SMIM35 ANLN ARHGEF26-AS1 SLC1A7 LGI2 ANKRD6 KCNC1 ADGRE3 GPR55 DNAJC15 TAFA2 STAU2 PGAP4 SIAH3 RIPK4 TMEM63C ZNF257 USP14 ST8SIA4 HYDIN2 ADGRE4P IGLV3-2 USP7 CD82 KIR2DL4 FAM167B ARL13B SCP2 TMEM156 ABHD2 ALCAM DENND1A SEC14L1 ERLIN2 ATP11C PDZRN3 SF3B6 LIMD1 ADAM32 RSRC1 DNAH11 ZSCAN5C NIPBL CEP120 B3GALT5 CDC42EP3 MICU1 FAM204A SNX25 MYO5C SDC2 OTX2-AS1 ATF1 SLC25A21 SLC22A14 SAMD12-AS1 RBPJP6 C12orf4 TTLL11 LINC01579 NEBL DIDO1 RARB CCDC18 CDH8 AGPS TRPV5 HEPHL1 WDR12 PJA2 KLHL7 TCF4 ECM1P1 GACAT3 RPRD1B AURKA LINC01145 PTPN2 AP3B1 PBLD GORAB ERICH5 BCAP29 PHACTR1 TTC21B PSMF1 SLC5A4-AS1 SH3PXD2A NEK6 OPA3 SLC39A12 MOB1B OR13C9 UIMC1 SEMA3F-AS1 YIPF6 TMEM25 OSBPL10 RIC3 CASP5 EXOC1 RNF182 RALGPS1 UPP2 PHF21B GUSBP1 ZFPM2 PIEZO2 FAM66A BCRP2 SVEP1 FANCA DEUP1 ZNF354C LINC02325 LRRC2 ANKRD26 RGS20 MIR3118-2 PDGFD CNDP2 HCRTR1 TLK1 CREBBP RELL1 LINC02176 BRINP3 LINC01237 KIF4A XRCC4 OVCH2 FAM193A COP1P1 EPHA7 MAP7 TM9SF4 SENP8 NSG2 ZBTB8OS MIR17HG GRIA1 ZNF627 IFNAR1 KIAA0319L USH2A LHFPL6 OPN3 NEGR1 NAV2 XXYLT1 CACNG2 ANO10 BTBD9 SPATA48 CA5A FAM72D NET1 TTC28-AS1 BMP2 TOX KIAA0232 LINC02011 SRGAP2C SLC44A5 FAM107B LUZP2 BTBD10 SH2D3C MELK RBPJP2 LRIG1 PRKACB ZNF536 RIMS1 GXYLT2 HIPK3 EPB41L3 TMEM225 POR LINC00896 PARVB MORC1 OR10H2 ZBTB7C SCGB1D5P KANK4 GAST SGO1 SAR1A SLC37A1 SUPT16H BCL11B LINC01814 DTWD2 LINC01213 NELL2 TSPAN2 NIPA2 SLC14A2 LINC02668 OR52B3P ASAH2B RALB MOSMO KRTAP19-10P BLK PPP1R17 PIAS1 PTCSC3 LRRFIP1 LINC02180 CTDP1 SSBP2 ZNF705CP FHIP2A CFAP74 ZNF846 HAGLR ZNF287 ARMC2 FER1L6 ALS2 CYTH4 COMMD8 PTGER4P2*

		TBATA ATL1 SERPINB11 ZDHHC17 KCNH1 ABCC9 SNAP29 QSOX2 GSG1L MCF2L LINC01098 ACSS3 BBOX1-AS1 ABCD3 DCDC1 NPAS2 DIAPH3 CCSER2 INTS7 AMPH ASB4 TMEM178B COL5A3 COL4A2 PARP15 ME2 GRAMD1B SIAH2 POLR3A ZNF44 LINC00536 HEATR5A ADA2 PRKCZ F13A1 ALG10B CDC14B GIPC2 RNU6-1007P HECTD1 CYP4F22 ABCA10 LINC01182 KLHL1 SP110 LINC01566 PRSS2 MRM1 ZNF705D ATP9A POSTN BIRC6 OLFM4 SAMHD1 OCLN AK8 SDF4 ITGBL1 TNFSF11 SPOP EFHD2 EGFR MYEOV ADAM28 MRPL13 B4GALNT3 AFAP1 DPY19L2P1 SH3BP5 SLC49A4 FANCM NEO1 MELTF MXRA7 MYT1L SRGAP2B SLC37A2 ATP1A1-AS1 SORCS3 PDP2 VPS37A LRRC49 ERP27 ZNRF3 ZBTB21 TENM3 ITPKB ENPEP TUSC3 IPCEF1 RTRAF NF1P9 OR4K6P BIN2 LINC00877 TCERG1 PHACTR2 UBE2O VENTX DIRC3 PLCZ1 NCOR1 LINC02213 PRDM10 EBF2 DNAJC21 C16orf72 USP33 ERBIN RNY4 SLC24A4 ZNF573 MBTPS2 KHDC4 C2 NTF3 OR6C75 ZNF705G LINC01684 PI4K2B USP41 RBMX2 FAM72A IL17RA SOX1-OT SUSD4 PTH GALNT14 RAB22A H2ACP1 FAM66C ZNF160 LINC00466 HADHB NSMCE1 DNAH10 PDE10A CACNB2 REPS1 MAP3K4 PDXDC1 MTPN MT1HL1 LINC02646 INTS13 VSTM2A RUNX2 DDX10 ZNF804B LRP12 LRRC8B CSNK1G1 ZNF169 MICU2 SOX6 JAZF1-AS1 SGCD RC3H2 LINC01492 RNU6-374P TNNI1 RAC1P3 SP3 STK32B PLG ZNF106 LINC01020 NR2C1 SLFN11 ADAMTS3 DNAH8 NHS LINC02505 CABYR LINC01476 ANK3-DT CLSPN RGS12 PPP6R3 ZNF438 GUCD1 PPIP5K2 GTF2I NCK1 SOHLH1 LINC01192 CDV3P1 C15orf32 PUDP KDM1B SSPN HS3ST2 F5 PATJ FAT1 NUP50-DT CPS1 MESD PRKCH EBNA1BP2 TRAK1 TET1 FAM66B RHPN2 ANKRD30A RABGAP1L KRT25 NMU DENND2B LINC00603 SNX8 HADHA CFDP1 LINC00944 SMARCAD1 MIR3118-3 ADAMTS9-AS2 ASTN1 GNG12-AS1 GFRA1 NSG1 RMST ANKRD20A8P MAPKBP1 CPE TDRD7 RNF8 LY86-AS1 LINC02613 NSMAF PYGO1 LINC01723 NFKBIA TEX29 DNAL1 TRAPPC3 CD101 TMEM132D GSE1 HMCN2 FHIP1A EFCAB8 LINC01204 SPRED2 SCN10A HSDL2 ANKRD18A ZNF350-AS1 CEP128 ZC3H15 LINC01135 ANKRD7 ABCA5 PTCSC2 CIB4 ABCC8 TTLL5 NOXRED1 TMTC1 MOCS2 NRK NAT1 KICS2 CYBRD1 MCPH1 MINAR1 EIPR1 STON1-GTF2A1L BMP2K LINC02543 CYFIP2 APOOP5 CCDC126 BABAM2 MSANTD4 CRB1 IL1R1 OTOG HEPACAM USP8 NUDT21 XPO7 ARSJ KCNS3 ENPP3 ZNF235 ERC1 LINC02006 VWA3B ZNF850 ALPL PDLIM5 ABLIM1 XYLT1 BTAF1 PDCD6IPP2 ALPK2 LINC02660 ABCA13 HNRNPCP9 RFX2 MAPK8IP1 ADGRB1 SLC66A1L LYPLAL1-DT ADGRE1 UCK2 RAP1GDS1 FOXO1B TET1P1 ST8SIA5 TBC1D19 RPTOR ZNRF2P2 STT3A PDE6A CHST8 BID COX5A MACF1 MDFIC MNAT1 PCMTD2 STARD4-AS1 TC2N TUBGCP3 BTLA LGALS9DP SLC15A5 ECHDC1 HCP5 AMBRA1 CLEC20A NETO2 DOCK2 SERPINA6 ASCL3 PTPRE IFT46 FAM66D NUP210L LINC02063 TSPAN3 ALPK3 LINC02465 FLVCR1 SLC9A5 MUSK EPDR1 MYO3B LMNTD1 RAB8B LINC00583 MYOM1 ZSCAN30 MTCO2P3 LINC00469 RNU6-835P RXRA CGAS ARHGEF7 SLC23A2 LIN54 LINC01649 ARPP21 ACACA ARL11 MAML2 SPAG16 ADAM5 TRIM43B ZNF879 MAN2A2 LNPEP DDX39BP1 LINC02198 UNC93B3 RPS3AP6 CDK12 POU1F1 KIAA1958 CARD18 LINC00623 NEDD4 RFTN1 CCDC141 NEK4 RSU1P1 PEX14 CFAP61 FYCO1 LPGAT1 CNTN4 FOLH1 HRH4 SPRR2D LRRC38 EXOC6B EVC2 AFG3L2 CNKSR3 USP49 DRAXIN CSF1 CEACAM22P LINC02109 LINC00511 SLC8A3 TRNAU1AP LINC02145 RNF17 HAS2-AS1 KIF11 LINC02400 SLC2A3 FAM72B PRAMEF26 SLC40A1 PHC3 STOML1 CADM2 SDR42E1 LINC01581 FANCL SH3GLB1 GABRR2 RAP1GAP PIK3C3 OTULINL RAD9A SLC9C1 SCML2 SPOPL MAGI3 LINC00701 TRAF3 MPPED1 CCDC122 CHD6 FAM135B MORN1 CCDC186 PAXIP1-AS2 LINC01695 PTPRB LINC01412 ITGA1 VN1R7P MARCHF6 CCNG2 ATG4B CIBAR1 ODR4 GAGE13 TANC1 CORO2B DHX40 KIFC1 POC5 IGHVII-65-1 TRPC7 SYCP1 VPS41 DTX1 MYO10 GFI1B ZNF407 MIR3118-4 ASB3 TENM3-AS1 KIR3DL2 GAGE12J TRIM43 FOXO6 TSHZ2 C1orf21 TOM1L2 STOX2 FAR2 BICRAL PLAGL1 NEK7 NKG7 NLRP13 COG2 CCDC138 MTOR RPL5P35 ERN2 CYP2C58P TLNRD1 SERPINB2 AOX3P LINC01322 GABRB1 ANKS1B RP1 AKAP10 EFCAB14 SLC16A9 WWC1 PLEKHA3 RSRP1 BLM PLA2G4A SENP6 LARP1 GATAD1 ZBTB16 PSMB2 DDX39AP1 UNK B4GALT6 ATG5 LINC01938 SGTB TRIM60P19 ZNF875 XKR5 SCGN SYNJ2 STK32A ARSB IL34 ZNF234 CLVS2 DIO2-AS1 SIGLEC29P DPYSL5 CWC27 AGK USP25 ANKRD11 ASCC2 SLC44A1 CNNM4 ADAM10 ATXN3 SPEN GALC NAP1L4 MRPS22 TMC1 PLD5 OXR1 PAK3 CAMLG KANSL1 RNU6-1150P NPIPA1 TPM1 CES1P2 CIDEC EBF1 CRIM1 OBI1-AS1 OR2T2 MADD PCID2 LINC00667 NDFIP2 DUX4L45 ZSWIM6 MYL1 ANKRD36BP2 MTREX CAMK1G DSG1 C1orf87 LINC02327 FAM30A PDZPH1P ERICH3 TRERF1 CENPBD1P1 GID8 TADA2A RPL15P2 LRRC9 LINC00158 FBN1 NIPAL2 MTUS1 GABRA6 PTPRVP DHRS3 PANTR1 LASP1 VPS35L ABHD17C KRT6A C21orf91-OT1 GGT4P DROSHA ZNF813 ANKRD31 PALS2 ADCY10 FAM149B1 PDE1A TMPRSS2 LINC02165 PRICKLE2 CMAHP ANGPT1 TRIM58

		HMGA2 HHAT KLHL32 CHASERR PSTPIP2 LINC00861 MVB12B C4orf50 LINC02253 UBASH3A CACNA2D3 SEL1L ELOC CUL1 SLC7A2 TMEM67 BTF3L4 MIR3936HG ZNF618 ITGA4 CPA6 AGO1 NSUN2 PRKCE DEFA3 GLYATL1 RBPJP5 PTPRK MIR3142HG CNTN3 GLIS1 ODAD2 ARHGAP31 RXRG ETNPPL KIR2DS4 PRKAA2 CD163 ARMC3 BBS2 SYT1 LINC01128 GRB10 OR4F15 LATS2 IKZF2 PPP1R12B APCDD1L-DT PDE2A DNMT3L ASPM RFC2 PPARA PLXNA2 KLHL33 DENND2C LINC01602 TBC1D13 CDYL2 SCARA5 PRG4 SCGB1D1 MLLT1 RANBP3L MARK2P12 TG ADGRB3 HGD SLC36A1 PLCB1 UBE2R2 KCNH8 ATP6V0CP3 SDCBP ANKRD19P RPS10P7 CUL5 HOMER2 DGKI RIN3 LINC01221 REG4 LINC01151 DIP2B CD5L LINC01524 TBC1D30 IGLV2-14 NME7 STXBP6 TFDP1 FAM66E ZBTB33 MXI1 ZNF876P PPME1 TRAPPC8 OR4R3P MBNL1 STX12 ABI1 LINC02291 FUT9 MOK CREG1 LINC02488 SLC12A1 SCN2A MAPKAPK5P1 WNT7A TUBB6 ZNF449 DOCK10 FAM27C MED1 PAMR1 DDX6 HNRNPM SPIRE1 TMEM71 COG5 AIMP1 UBE2E1 ARAP2 LDB2 LINC02149 SNX30 ATP8A1 BCL2L1 IQSEC1 LINC02008 JARID2 LINC02236 SHROOM3 ADK PRR5L ALX4 CORIN MACROH2A1 FAM241A LINC00838 RANBP17 WNT2B MRPS27 PPM1L CPHL1P LRRC37A3 TRIM43CP PRPF18 SMOC1 GSTA3 EXD3 SETDB2 FAH MON2 OR51E1 TOGARAM1 SLC45A4 ZNF705B ELF2 SEMA3D LDLRAD3 GLYAT KIF15 JPT2 CFTR VSX1 TBX20 FLRT2 NFATC2 MSH2 NALCN-AS1 PRAMEF25 KRTAP20-4 ARHGAP44 MTMR2 ZNF970P KCNH5 FAM189A2 BAZ1A CADM1 KLHL29 PTPRO ARID3B OPRM1 ACER2 ZDHHC14 RGS9 YLPM1 ITFG1 IPO11 VCL SLC13A5 GLB1L3 ABCA9-AS1 DOCK5 VCAM1 C7orf31 LINC02511 LINC01818 ATP6V1C2 EWSR1 MAGEL2 IFT81 NHSL1 OSCP1 SGO1-AS1 DTHD1 SRGAP3 IGHVIII-13-1 HAAO CTNNAL1 CIBAR1-DT CYP2A7P1 ATP6V0D2 SYNJ1 PHF20L1 KLF15 PPP2R2B NEDD9 HDHD5 IL12A-AS1 ANKRD66 ENPP1 SDR42E2 LYRM4 CCDC150 DNM1P47 LINC02099 HEMGN RIMS2 UGP2 RP1L1 PPM1F OR4K8P EFCAB6-AS1 CHSY3 YBX3 YAP1 ARL15 ANK3 BMPR1B LINC01173 FCRLA LINC01937 SEMA5A C1orf127 PRDM15 KITLG KRT6B SV2C ELL2 DAW1 COL5A1 IGHV3-74 IFT57 LINC01426 RIPOR2 MAP4K4 LINC02899 ZNF112 FICD FEZ2 ATE1 PEG10 INHBA-AS1 PRAME HSPD1P3 NUP43 NMD3 OLA1 GATAD2B VPS13C ANKRD55 XIRP2 KRT85 SLC14A1 CA1 C5orf52 FAM72C MFSD9 EOGT SERPINI2 STK38 APBB1IP NPL CAST TBC1D9 FBXO32 AOAH SNHG14 TSBP1-AS1 SMG1P4 SNAI2 ZBTB49 ANP32B FAM90A28P PHF2P2 AP4E1 DLEU1 NDFIP1 TNR FAM180A LHX9 LINC02074 OCA2 ST8SIA6 DAPK1 MSANTD2 FCHO2 RFTN2 TRAV8-6 COL6A6 RN7SL767P DNAH5 CNTN6 CLIC6 LINC02406 FMN2 BHLHE40-AS1 SEMA3C VAV1 LINC00895 CACNA1I BTG3 DPY19L1 CSF2RB RPS6KA3 SMAD5 DNAJC13 BCKDHB PPIL6 IGLV3-30 CNST PATL1 UBE2J2 ASB2 OTOP1 PSME3IP1 OR4F6 GHRH UGT3A2 GOLGA8B RNLS IGLV4-3 SPOCK1 LINC02315 NF1P6
4C-decreased-1200	1146	*LRRC37A5P CCDC116 ZHX3 APOL2 RPS4XP6 PDCL3 PTPRR HERPUD2 CRTAM SLC52A1 UBE2G1 PELI2 TPI1P1 NOS2 IGF2BP3 MIR548H4 ZEB1 LINC01708 FAT4 PARN SEMA4D SLC15A2 MRPL45 RN7SL483P WSCD1 MIR4435-2HG KNDC1 LINC02355 ZFYVE1 EVC HERC2P2 LINC00670 DMC1 CXADR C9orf43 NBPF21P OR7E19P RIOK1 HERC2 GGT2 UNC45B LUZP1 LINC01346 BVES-AS1 ZNF723 LINC01138 CECR2 LINC01782 GNAS NPM1P2 CD38 SERPINB9 LINC01876 PGBD5 LAIR1 MSH6 RASGRF1 HECTD2 FBXO31 C12orf40 LINC01163 GMPR RIN2 PRDM16 PDE6C TRAPPC9 LINC00323 MEIS2 CARMIL1 PCDH7 BCL2 KL LRRC4C LINC02558 RACGAP1 MIR3667HG THADA NUP37 PPP1R9A SAA3P TMEM117 KCNK15-AS1 TOX3 TDRD5 TASP1 SNX6 POTEG GOLGA6L3 SAMM50 ZZEF1 HHLA2 NCF4-AS1 C3orf52 SLAMF1 UQCC1 RGL1 ATP5PBP3 SHOC1 LINC00841 FAAP24 INO80D KDM6A MED27 NCAM1 PDYN-AS1 GDAP1L1 LINC02096 LINC01358 EPHA4 LINC01967 PLA2R1 LYSMD2 NGDN H1-9P ADAMTS2 GTSF1L WARS2-AS1 CCDC172 FRYL LINC01828 GPRC5C COLCA1 CPAMD8 RNU6-929P GPR1-AS UBL7 ARMC6 LINC02675 ASH1L CALM1P2 RBM6 MEGF10 IGHV2-70D HS1BP3 LIPI LINC01445 GOT2 RAB38 DZANK1 CLTCL1 NUAK1 PCAT1 KCNK10 ZNF738 BLOC1S5-TXNDC5 BRD4 SLC16A1-AS1 VMP1 CHAF1A NENF HRH2 ATP6AP1L RNU2-47P RDX SNTG2 SVIL APBA2 TTC3 CHAMP1 COL23A1 NSD1 NEDD4L EDAR C5 EGF LINC00960 ATP2B2 RPL37P3 CCNYL3 AGO2 ABCC12 PARK7 RIMBP2 ZNF271P IFT43 ADAMTS19-AS1 SNRPC C2orf69P4 DRC7 DSE POTEJ LINC01427 MYCL LINC02256 TPTE2 SPAG6 BMP7 PDE4DIPP1 GALNT2 KIAA0753 FGF12 ANKRD17 EPHX4 CYP2C9 CNOT7 CASC9 IMPACT ITGA6 HHIPL1 NOTCH2 ZFP90 S100B ARHGAP12 USP43 KCNN3 FKBP5 NFAT5 FLI1 ANAPC1 GRM1 IBA57 LINC02147 ARHGAP26 ITGB8 GAP43 LINC02422 OR4K3 PRDM13 PDE3A PTPN4 BCAR3 MAP6 SREBF2 CDH11 SETBP1 CDS2 ZNF780B LINC01900 ATP6V1E1 LINC01993 LMX1A AGBL1 DEFT1P2 LINC02439 ZFAND6 GBP4 CNKSR2 PSMA5 RESF1 MAPK1IP1L DPH6-DT GFRA2 NEK10 ADCYAP1R1 SNX29 MAGI2-AS3 ZNF611 STAT1 SAMD5 ZC3H14*

		MROH5 SLC44A3-AS1 MYOCD CREM GOLGA8J XKR3 UBE2QL1 PLPPR5 TDP1 AVL9 EFEMP1 TNRC6B WBP2P1 ZNF33B LINC02542 SYN2 PTCD2 MYO1E SMOC2 MIPEP NCSTNP1 HDAC2-AS2 HLCS FH RWDD2B PLPP4 PWRN4 CCDC102B SDS GSR LINC01571 FIG4 SOGA1 ARHGAP32 NECTIN1 ZNF528 LINC01222 LALBA NXN LINC00375 FOXJ3 CENPE CKMT1B MYL12B RSPH14 IL17RD BRCA2 ANKMY1 HCG22 APELA UBN1 SSBP3 PLA2G12B FAM83B HDAC4 CTNNA1 STK36 GNAI2P1 FAM102A NCOR1P1 TRPC5 MYO9A TMEM182 IL10 LINC02305 LCLAT1 LIFR-AS1 C19orf18 FTO SLC6A1 EPC2 SEM1 SEMA6A-AS2 MOGAT3 MS4A4A TMEM236 NLK THSD7A CXCL2 GOLGA6B LINC00334 CARD10 ACSBG1 GCSAML DNPEP TRAPPC11 HOXC4 IGHV3-62 NECTIN4 CNMD LINC01309 UFD1 SMARCA4 LINC00299 BAZ2B HERC2P3 CRACD NGF-AS1 AGL PALMD HS6ST1 MARK2 MEOX2 SFI1 ZBED9 CTSE ATF2 PLEKHB2 FAIM ZNF72P RPRD1A ZNF880 PPP2R2A ESS2 PHAF1 ZNF541 RBBP8 AK6P2 CKMT1A RFC1 NSUN6 LINC02174 CDC45 MC2R AKR1B1 BTBD11 CWC22 LRP2 LINC02087 ZNF121 HERC2P9 SYNE1 MTHFD1L RALGPS2 HOATZ SNRK ABCD1P4 EXTL3 EHMT1 TTC29 FBXO47 KRT89P ZNF735 ZBTB10 PKHD1L1 SLC9B1P4 PLPPR1 CEP192 SLC26A2 CAMK4 GUSBP11 CLPX OR7H1P ROCR ANKRD20A9P HDAC11 SLC9A4 DHX29 ANKRD20A17P GRIK3 GRXCR1 NUMB STPG2 MIDEAS TM9SF2 CD70 SH2D1B ZNF606 ESYT2 HEATR6 ARFGAP3 MAPRE2 FGF10 TAPT1-AS1 KRT18P59 ISX RAD51AP1 POTEM LINC01035 ESRP1 RIC8B TAF3 ZMYM4 TPTE FRG1JP MED12L GRM7 ZDHHC21 BRMS1L TM9SF3 DDHD1 ICA1 PLEKHD1 CDH7 TLDC2 CYCSP39 HORMAD2-AS1 VASP PLGRKT UBE2E2 UNC80 SDE2 PTGFRN PPA2 ILDR2 IMMP2L ZNF615 SPSB4 SPSB1 GABRG2 ZNF567 ITCH NUDCD3 MBP S100PBP TANGO6 GABRA5 CDKN2C CFAP97 STXBP1 SLC46A3 DLC1 ANKRD33B PNPLA7 RPS3AP4 CNIH1 FRG1BP C2orf69P3 LINC00114 ZIM3 MANBA DUX4L2 CHRM3 ECT2L UST MIR663AHG CALD1 LINC01543 ERICH1 DEDD2 TYW1 TAF15 ALB ARHGAP24 JPH1 ANKRD20A3P EFR3A HTR2A UBAP2 TPH2 N4BP2L1 IGHV1OR15-9 TPTE2P6 EIF4BP3 LOXHD1 APC MDS2 GOLGA8G LINC01622 GALNT1 MARCHF1 OR4L1 ZBTB25 INO80 RBM15-AS1 HSD17B14 KRT16P6 COL27A1 GLP2R CHCHD2 BMP5 SLC4A4 ZNF675 LINC02366 GOLGA6L17P SYT16 CRYBG1 TWIST1 HACD2 LINC02653 LINC02061 GEMIN5 RPL15P3 TRIM77BP KTN1 ERCC6L2 PASK RUFY2 SLC16A1 RANBP9 FAM245A MRTFB LINC01344 KCNE4 TRIM5 LINC02228 KRTAP21-3 LINC01801 LINC01320 LYPLAL1 THNSL2 BRWD1 COLQ PPIP5K1 C9 TMTC2 HECW1 HMGB1 MEF2C MCTP1 RNU1-51P MOB3B ACTR3C LCE1F ARPC3P2 MAP4 TSPAN11 MTND1P17 HIVEP1 ATRX TNIK KRT18P55 OR1L6 NBN PRTG OR2T7 SLC17A1 RGMB KMT2E WNK2 FRMD3 SETD2 RBFOX3 MRPS35 SDAD1P2 PWWP3A ITIH5 UTP4 PACSIN2 TRGJ1 HOXC13 PKP1 SMARCC1 SYNE2 GTF2IP6 MIR181A1HG TRMT61B NUP214 TRIM23 FABP7 SDCCAG8 GK LINC00355 DUSP22 KCNJ18 ECPAS CHSY1 RFC3 MAB21L3 SFPQ SMPD4 URB1 PTPN12 GPR137B ZYG11A LINC00434 LINC02424 TOP3B MPPE1 STAG2 RGS3 UBA6-DT ANKRD26P1 CMIP DEFB116 SLC2A13 FBN2 YTHDF3 SPATA17 SYT10 ZBTB38 PAFAH1B1 LINC02380 CYFIP1 ALK DOCK8 GON4L GREB1 ARNT LINC01340 LINC00929 CSTF3 ZNF648 LINC02058 SAMD13 DNAH6 ARFGEF3 TMCO5A UHRF2 EPCAM-DT CSDE1 DCLK1 DEFT1P RNF215 ANKRD28 GRK3 ZBTB2 SND1 IFNG-AS1 SNAP25-AS1 ZFAND4 LINC01664 FGD4 EFTUD2 NRIP1 ETS2 LINC00649 ZNF382 NDUFAF6 GOLGA6D ERMP1 CATSPERG ARL4C ITSN2 LINC01917 KTN1-AS1 HIRA OSER1-DT SERPINB7 ANKS6 TMEM74 PRKAA1 RASGEF1C TAFA4 ALDH1A2 GABRG1 MTTP POGK CROT MAPK9 ESRRG FBXW2 STON2 LINC02458 LINC02652 LINC01229 FOXJ2 AGAP9 ABCC4 MYT1 FRG1HP ABCD2 DNAJC7 ZNF595 EMP1 TMEM171 ZC3HAV1 LNCAROD RRAS2 SV2B FAM110A NRBP1 SEC14L3 STK38L GTF2F2 RALGAPA2 FAM245B ADAMTS19 ZNF236 RAB27B SOX30 LINC01337 MYOF CPSF3 PLS1 UNC79 RSPH1 SPON1 ANK2 SH3GL3 CFHR4 INVS FHL2 SNRPD1 NCAPG2 LPCAT2 SUMO3 LNP1 BZW1 PCNA TPTE2P5 PHF19 ZNF518A LINC02191 IGLV3-31 DCAF1 ZCCHC7 CD2AP TTC39C LINC02680 ZNF124 EBF3 TAFA5 NFKBID SPRED1 MYO5A CIDEA LINC01924 PPP2R2C PWRN1 LINGO1 LINC01706 SEPTIN9 SMPDL3A LRFN2 ACO1 WDFY4 SCAI PAPPA2 LTN1 TINAG NCOR1P3 DIRAS2 AQR ABCB5 HEATR4 SLC5A9 KLHL4 DPY19L2 MDM1 RRBP1 SMPX OVOL2 CD9 MTMR10 EIF3F CDHR3 CSE1L NEK2P2 TOM1 FAM118A PRUNE2 SGMS1 ANKRD24 COL25A1 RBPMS2 CYP4A11 BRINP1 IGLV2-34 MTND2P8 RPL23AP7 GRB14 LARP6 IGSF5 LONP2 CCDC192 LEMD3 PEX6 CHKA BUB1 RNF138 INTS4P1 KRTAP21-2 ZNF292 SPIN1 TBX15 PRB3 TTC3P1 KPNA1 SKA1 LAMC1 SRP9 SCGB2B2 GABPA SMIM11B DSG1-AS1 PCNX1 DUXAP10 LINC02091 NAA35 ATP5PF RPF2 UBAP1L ZCCHC14 MX2 AGGF1P10 MTCO1P1 ITGB3BP ZNF831 PRAMEF2 ENTPD5 DGKK SLC4A10 LINC00844 TSSC2 LIX1-AS1 TGM1 AGAP14P MGAM FTLP13 ZDHHC18 LINC01310 PSG9 FAM183A UHRF1BP1L IL1RAPL2 APIP MUC19 SCAPER ADSS2 IKBIP NPHP4 LINC02141 OR8B9P CBLIF ATP6V1B2 KCNJ1 LRRTM4 CLCA4

		LINC01877 MYOM2 SLC5A12 NCS1 ONECUT1 PCDH11Y LINC00923 METAP1D USP31 SUMO2 OR5AQ1P MICOS10 XPNPEP1 GLYATL3 NUBPL PCGF5 FGF7P1 NLRC5 TUBBP9 LINC00363 NDUFAF2 GUCY1A2 KREMEN1 UMODL1 LINC01189 MB LINC00383 WDR25 SOD1P2 CYP4Z1 MGA LCE3D TNPO3 EFHB ACSM2A FGF9 DCUN1D4 DAZL NIN RPL23AP53 SLC1A1 HPCAL1 SPG21 ANKFY1 LIMCH1 ZKSCAN5 CCDC195 TSHZ3 TMCO4 LINC02098 RALA GMDS-DT BAZ2A MIR548A1HG RPS12 GNPTAB TRPM1 NTN1 COPS8 SACM1L AKAP9 C1QTNF3-AMACR PPIL2 AP4S1 NR5A2 PRKD1 SPTB TRAV8-1 TNFAIP8 RBM47 TMEM260 GBP6 SANBR DBF4B OR9Q1 CCL28 FBXO41 MICAL3 SNRPN HDAC2 MSI2 MRTFA LRRC7 LINC00221 ETS1 TNN UBAP2L CASZ1 PCDH8 LINC01673 WDHD1 MIR155HG MAMDC2-AS1 RABEP1 ST6GAL2 LINC00930 NUTM2HP SLX4IP PTCHD4 BANP ALKAL2 MAP2 YME1L1P1 TGFA ZFYVE26 SNX3 LINC01422 ZNF280B PTPRA CEACAM7 TNRC6C TMEM161A DEFB108B OR11G2 LINC02451 WSCD2 UBE3D GLIDR ZNF402P CERS3-AS1 RPL23AP87 GOLGA6C ABCA4 ZFAND3 LINC01718 THUMPD2 C6orf118 FAM83F CNKSR1 RAB12 TMEM163 PCDH9 LINC02235 SLF1 EXOC1L BACH1 RBMS3 POTEH GNA14 OXNAD1 INMT-MINDY4 LINC01992 SRFBP1 MATN2 TM7SF3 IL33 EFCAB2 MARK4 CRACR2A CLEC16A LINC01901 OR2T3 SERPINB10 PROX1-AS1 ACTN1-DT ARHGEF17 ME3 PSG6 ZNF431 CACYBP ITGA9 PPP1R1C ULK2 IARS2 UNC13C CDC42BPB HNRNPH3P1 TMEM44-AS1 LINC00298 FAM217B MTRF1 FAM25G SHROOM2 OVCH1 CISD1 C16orf95 CRTAC1 PRKAB1 RALGAPA1 LMX1B LINC01748 L3MBTL3 RRAGD IGKV2OR22-3 POMT2 MICALL2 ST13 LINC02245 CLIP1 MARCHF8 SHC4 C2orf42 CABLES1 MORC2 ZNF613 TLL1 DEFA8P FRMD6-AS2 DUSP16 BCL11A AKR1C3 PPP2R5E ASB7 FREM1 IL20RB ADAM22 RABL2A LINC02582 LINC01811 CNIH3 RPF1 TRIT1 TBCD CEPT1 WNT5B KCNK5 CEP44 HKDC1 CLNS1A EPS15L1 PVT1 CHN1 ETV6 WNT9B RFX7 TRPS1 SERBP1 SKINT1L IGKV3OR22-2 RANBP2 ADAMTS5 NF2 STRN CRISPLD2 NPM1P1 ANTXRLP1 MTMR3 ZNF845 RN7SL250P FAM25C CRKL ITPRIP H2BC15 ADGRA3 SCG5 CLDN18 LINC00581 ADCY9 DPP10 TUBB2BP1 GALNT16 CDH5 ANO6 ZNF600 HIPK1 A2MP1 PGPEP1 ACSM2B TTC7B PEPD ATP2B1 IFI44 RETREG1 NLRP14 PCNT NPHP3-AS1 PAH FAM138E ATRN FBLN5 CNOT6L TRAPPC6B TOP1 ZNHIT6 SLC39A6 WDSUB1 ANKRD20A7P
downregulated-1147 0,05	1031	*GRAP2 ITGA5 BTG2 CYP26A1 RTKN2 IGF1 LMAN1 NTRK1 UBAC1 GDF15 ASNSP1 TRIB3 REEP6 CCDC88B LINC02864 STK16 XK HEPH BBC3 CASTOR2 PCOLCE TSC22D1 LHFPL2 EPS8 ITM2B UTRN TRIQK ACSBG2 SNX16 GPC5 GTPBP1 PDIA3 CA11 ALDOC NECTIN2 ZNF83 SEL1L3 ZCRB1 GDPD5 ENSG00000213963 OXLD1 DDB2 ANO5 LINC02267 LTBP4 TSPAN13 STARD5 MT-ND4L VWA5A NAT8L TNFRSF9 ENSG00000224271 GTF3C3 DMTN AP3M2 LINC00656 CORO2A APOE C3 KLHL36 BTN3A1 DNM1 HYOU1 SLC16A5 KBTBD3 SORT1 LLGL2 NFKB2 PGGHG MYO15B IFT140 GMPPA MLF1 SLC6A6 DUSP5 ID1 COL18A1 ELAPOR2 A4GALT BCAM PCK2 ADAM19 THRB MMP15 ZBTB11 EXOC2 GUCA1B LSM4 POMT1 NUCB1 TLCD2 DDHD2 RYR1 NUDT12 ZNF467 MAN2B1 FGFR3 HES7 EPHA1-AS1 TKTL1 TM6SF1 TFAP2B AKNA NBR1 PKHD1 INPP5J FAM234A SELENOP UBE2L6 TMED9 MYORG TDRKH TLE2 LONRF2 GIPR AHCYL1 STON1 YIPF2 FN1 RBPMS PTPRH SHFL GGA1 DGKD SERTAD2 CYSTM1 IGSF8 ZNF275 ACAD11 GAA ENTR1 ACSF2 BTK TJP3 CLCA1 AURKB HID1 ZC3H6 GABBR1 PAX8-AS1 DEF8 MANF PLA2G6 CCDC113 PRR36 CEBPD GPR158 CALR4P WARS1 ADCY3 TMEM241 PHYKPL GRHL1 LARGE2 WDR91 HAGH TMED7 YPEL5 ITGB1 PI15 TAB2 CPD BRSK1 PECR KRT8 SYDE2 UCA1 SVIP PCAT14 TMPRSS4 GABPB1-AS1 ACADVL ISCU GLI1 HBP1 PHGDH MCFD2 EPS15 IL13RA1 SH3BGRL2 ENO2 B2M ADAMTSL4 EHD2 MAP1A AGER SAT2 SNTA1 TCFL5 CLIP2 WNK4 VPS16 C1GALT1 CD93 KDM6B RRAS PCDH15 PCED1A PPDPF TMEM106C BAIAP3 IL15RA H1-10 SCFD1 ZKSCAN1 RABAC1 ADGRE2 ESRRB EPSTI1 BSDC1 POFUT2 RASGEF1A CTCFL XYLT2 TMEM263 BCL6 TPD52L1 PROS1 FGFR4 CRAT NYAP1 VEGFA PIK3CD DMBX1 TGFB1 ABHD18 PSEN2 CHST2 SNHG32 NTS ULBP1 PIGK ZDHHC8P1 PLOD2 KLF9 RPL27 LAMA3 SLC30A2 KIF1B GPD1L B4GALT4 PSRC1 ALAS1 PDK4 SMIM1 ENSG00000286403 CPZ PTTG1 LINC02416 ZSWIM4 TARBP1 BLVRB GTPBP2 ATP2A3 SMARCA1 PLXND1 SMURF2 ACER3 UCP2 ARHGAP8 RFK DNAJB11 CACNA2D2 SLC22A5 DOCK6 SLC25A42 FMNL1 PTPRC EEF1A2 FAM83A HEXD GPR155 RAB24 SPEG IQGAP3 SHC2 INPPL1 TMCO6 MAP3K14-AS1 GSEC RTCA-AS1 DCTN4 HNRNPCP4 CNN2 DBP PPP1R13L LRRC63 GFPT1 FCGR2A MC4R MAN1A1 STARD8 FNDC4 PCED1B OCEL1 MKNK2 BATF2 SLC29A4 NIBAN2 UXT-AS1 LINC02432 PALM PARP12 SLC22A23 C6orf89 LDLRAP1 TUBB2B SLC31A1 AFDN-DT IRAK2 MFGE8 SH3BP2 SH3TC2 AGRN RTN2 TSKU RHOT1P2 APBB3 GPSM1 GADD45A TMEM70 LRP1 SH3PXD2B MMP14 SERPINI1 PPP2R5B PTPRF NIPSNAP2 NDRG1 CPXM1 MRPL23-AS1 ERFE ABCB9 TRAK2 ENSG00000249631 LGALS3 PDLIM4 CLSTN3 NFE2L3 STAT5B ENSG00000253154*

		ENSG00000286488 SLC22A31 ARL6IP5 TMEM50B DBNDD1 C11orf80 SCN1B ACVR1 PLEKHH1 GOLGA2 F2R LPCAT4 THBS1 ACSL1 ASMTL CMAS AFDN UBE2H SLC6A8 PDE7A NFE2L1 GEMIN2 PAAF1 C2CD2L GSDMB GAL3ST4 FUCA1 MACC1 SOCS1 BHLHE40 KIAA0895 ROBO3 SLC17A6-DT LRSAM1 PLXNB2 INHBE NUP107 SLC25A36 RAB11FIP1 SDC3 NR2F6 ZNF396 CAMTA2 HIP1R PLEKHA2 ENO3 ZNF204P ENSG00000271382 STX10 TSPAN18 MCF2 MAN1B1 P4HA1 TMEM243 FECH LRRC20 ANKRD20A11P IL10RA VCAN PCYOX1L ARG2 FBXL16 GDE1 MAP4K2 LAMB2 PDIA6 TGFBR3 ZMYM2 DYNC2LI1 CDKN1A IL32 RPS6KA1 CUL7 FAM214B ERMN CCDC71L HRC LRG1 ENSG00000236393 GUK1 MAF ENSG00000229191 HOOK1 GTF2E2 MAPK8IP3 CRYL1 PRKACA IFIH1 MVB12A IDH1 ZKSCAN8P1 ANO8 NUDT4 MICAL1 SWI5 SEMA3F HAX1 NEK9 NHLH1 PIDD1 CD200R1 INPP4A PTPRU ATP8B3 ST6GAL1 SIAE SKIL ALAS2 ST3GAL5 SDF2L1 KLF13 RNF11 DLK1 COL11A1 LTK YARS1 RAB18 RPL29P14 AMPD2 PITPNC1 SEMA6B PLP1 MLLT11 LINC00663 ENGASE PYCR2 KCNJ11 SEC24A IFITM1 GAS6 KRBA1 THUMPD3-AS1 MT2A MYO1D SFXN5 FLT4 SESTD1 UBR5-DT RHOBTB1 LINC02384 CHPF SMAP2 CALCOCO1 MAP7D1 DEPP1 COL1A2 DTNA AMIGO2 RELL2 PROCR ZNF697 EHHADH LAPTM4B ITGB5 C4orf46 REEP2 ARFGAP1 SMAD6 RHBDD1 FAM171A2 PVR ZBED5 TMEM63A FNBP1 PITPNM1 ANKZF1 DUSP8 MLXIP KHNYN RCN3 PRCP YIF1B WASL NFE2 ADD3 PTEN MAST2 ANXA1 GTF2A1 ARVCF DENND11 ID3 NBEAL1 TIA1 HLTF POLI FAM234B GPR180 POLG2 PTK2B UNC5B LINC01419 MFSD6 CHID1 PTPDC1 USP20 PPM1J CALB1 B4GALNT4 MDGA2 PKD1 CCDC50 RGS16 CRYZL1 SMIM3 TGM2 TCAF1 LYRM1 NFATC4 FHIP2B PDLIM7 STX2 SLC44A2 COG6 CD55 ACAT1 AARS1 SMAD3 ATF3 TRDN CARD11 APOL6 RTN4R SEPTIN6 OSTF1 CAMSAP3 TEX19 CTIF TBC1D5 SOCS2-AS1 LINC00173 THOC2 LGALS1 COL15A1 CASP10 NPAS1 DACT3 MINK1 HLA-E BTN3A2 SYT5 SEMA3A R3HDM4 TRIM3 MGAT5 RTN3 ULK1 ZNF175 TMEM143 MRAP2 MYO5B COPG1 CD36 ZNF117 CENPH TTYH3 ZNF692 IDH2 EFHD1 COX6A1P2 PAX9 GALNT5 NIPSNAP1 TNNI3 RNF145 GDI1 TSC22D3 CCM2 ARID5B FLNC TMCC2 HACE1 NOTUM SPTAN1 ATP7A COLGALT2 ENSG00000283125 PAN2 RAB26 DCLRE1B NDUFB4 BCL9L MAPT HSPA4L TTBK2 RNF103 CXCL8 KHK HDAC9 WIPF3 LITAF NLGN2 DLG3 MSI1 CC2D1A ZCCHC24 PIM1 PCLO THBD SERINC2 ADGRL1 HECW2 IARS1 TENM1 BNIP3 PRR14L CCNL2 CDC42SE1 CBLB MIIP AP2M1 OAS3 ANXA6 TSPYL2 VBP1 MORF4L2 NEMP2 PRKAB2 MAP3K8 DLG4 STOM GSN ENSG00000264112 MAPRE3 RIMS3 ENSG00000286750 RHBDD2 LMOD1 PDGFB CALR RGL3 ITGAV RTN4RL2 ITPKA ANGEL1 BTBD2 RHAG PTK7 PHKA1 HELZ2 ENSG00000255347 RAB6B DENND4B SLC25A29 ENSG00000204745 ENSG00000258274 ROBO1 APAF1 TRPT1 TNK2 TBC1D20 KIF5A CCSAP ENSG00000287737 LINC01033 RNF187 MRC2 AGPAT4 SFMBT2 ACVR1C SETD5 GALNT12 SALL2 ENSG00000279164 SH3GLB2 SNX2 ABI3BP ATP1B2 SEMA7A GABARAPL1 ATM ODF2L KLF12 GTF2H4 ARRB1 ITM2C NSD3 USP45 AHSA2P LINC02863 MAN2A1 OBSCN PPIB RBL2 CD46 ENSG00000286980 C4orf33 HJV RPH3AL G0S2 SLC4A7 PLEKHH2 SEC22B TTC7A SLC17A7 ENSG00000273901 MEIS3 MUC4 BRPF3-AS1 SLC35F3 ARRDC4 RAB31 ENSG00000257086 ZBED8 ANKRD9 AZU1 LAMA5 SPATS2 KPNA5 KMT5C EHBP1 ENSG00000257337 TXNIP NIM1K TMEM41B CXorf38 CORO6 EPHX2 LPAR2 MARCHF2 FBXO44 FAM131A PSME1 CHAC1 MAST1 ZMYM1 MTATP6P1 ADGRA2 LINC02772 ARHGEF40 ZNF558 TRPM4 ZP3 RNF213 ACSS2 PDIA5 KIAA0040 KCTD20 FLNC-AS1 ACE CLDN12 TPD52 EML2 TUBE1 WDR31 SLC37A4 CREB3L2 WBP1L IVNS1ABP OGT ABCG2 RHPN1 P4HA2 FUT1 SERGEF PTPRS COTL1 TMEM9 PEX2 SESN3 WDR11 DAB2 MARCKS RBCK1 SERPINH1 SH2D3A ZDHHC8 VWDE CD59 IL2RA CD24 NFASC DDIT4 BST2 ATP1A2 OPTN HERPUD1 SCPEP1 ENSG00000285108 KLF7 DMKN DNASE2 LGMN MSH5 MYRF PYGL SNPH STARD10 EPB41L2 SOX5 GOLM2 ETFDH PCSK4 ENSG00000225528 KCNAB2 CCDC18-AS1 GALK2 MTURN TSPO RAB3B SEMA6C ERBB3 SERPINB1 TCAF2 GGT7 TUT7 CALU HSPA5 WIPI1 DENND3 PAFAH2 TP53INP1 LDAF1 FADS3 VPS28 ULBP2 ANAPC16 EPHX1 NCBP2 GPSM3 ENSG00000259953 ADGRL3 SIL1 MICAL2 CPNE3 CAPRIN2 TNFRSF1B LGALS9 RBM22 STK4 HMGCL FGFR1OP2 TRIM38 ENSG00000272941 CA2 CTBS DCP2 FGD1 PIGS HSP90B1 ANKRD29 GTDC1 MXD3 NIT1 TNFRSF10B TCIRG1 PAPSS1 SIDT2 GRN ACAD10 LINC01278 SMIM14 EDEM1 SEMA4G METTL25B RIPOR3 DZIP3 TTLL3 ERP44 ITM2A ETAA1 MTCL1 NRIP3 GPC2 PAIP2B ACBD4 CDKN1C NIPA1 CLYBL CYP26B1 PRSS16 GNB5 C3orf18 EIF2AK1 RAVER2 SOCS2 PLPPR2 BRWD3 CR2 IFNGR1 MIR223HG PLEKHH3 TMOD1 NT5C2 PDK1 PPP1R16B ALS2CL SLC45A3 MEG3 REEP4 AMOTL1 DRAP1 PLCD3 TMEM30A OGA MORN4 SMPDL3B FAM227A SETD7 GSTO1 ACSL6 CORO7 RGS10 ZMIZ1 TKFC CCND2 QSOX1 ANKIB1 ABCA7 PLD3 CUEDC1 ENSG00000237643 PLEKHA4 STARD9 HEMK1 FDXR PAPLN NES CDCP1 RAB27A TXNRD3 CITED4 CUX1 UNC119 MALAT1 LRP4

		MLXIPL ELP1 BCL3 HIVEP3 ITGB3 RUSC1-AS1 MIF4GD PPP1R14C YIPF5 CERCAM F11R TDO2 GIPC1 FAM193B CEMIP2 MIR22HG SPARC FASTKD1 PPFIA4 CTSZ LMNA NDUFA10 CCDC92 KLF10 PPP1R18 PANK4 SLC2A1-DT C18orf54 MAGED2 PDIA4 RFLNB ADAM15 LNCSRLR SLC4A11 PGM3 SESN2 TENT5A ZNF133 CISH CYP4F29P PXK WHRN NCOA4 RPS6KC1 SELENBP1 TMED4 MROH1 SUSD1 ICAM5 TUBB4A MBOAT2 MYBPHL GPAT3 TUBB1 NPTXR MNX1 PHF21A LINC01630 HDAC6 CYP2R1 TNNT1 MISP3 DCAF8 CTSC

Table S16. Expression levels (TPMs) of genes possessing the most frequent DSBs in HEK293T cells (mapped in hg9). Excel file attached separately. Related to Figure 6.

Figure S1

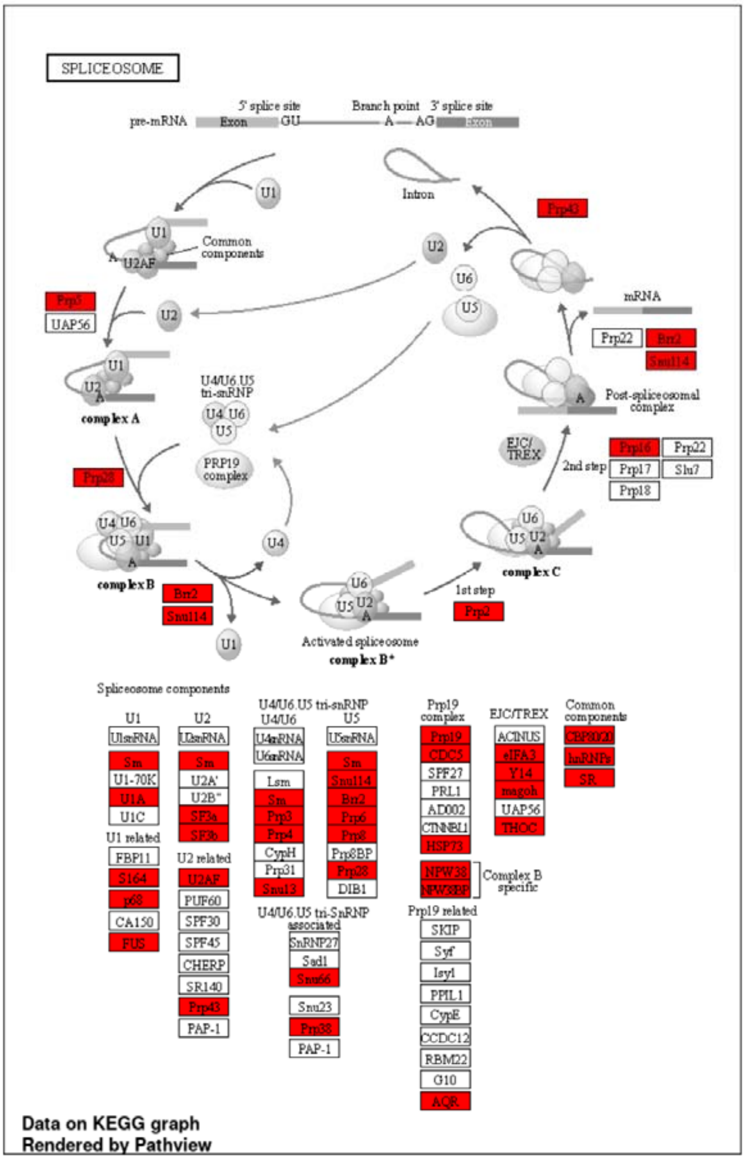

Figure S1. Analysis of 1285 upregulated genes in the course of hemin-induced differentiation genes in the KEGG database. The search was performed in the ShinyGO 0.77 database (http://bioinformatics.sdstate.edu/go/). Genes presented in red correspond to upregulated genes.
